# Supplementary figures and images for: Novel mechanistic insights into the role of Mer2 as the keystone of meiotic DNA break formation (part 1 of 2)
Source: eLife. 2021 Dec 24;10:e72330. doi: 10.7554/eLife.72330 (PMC8848140; doi:10.7554/eLife.72330)

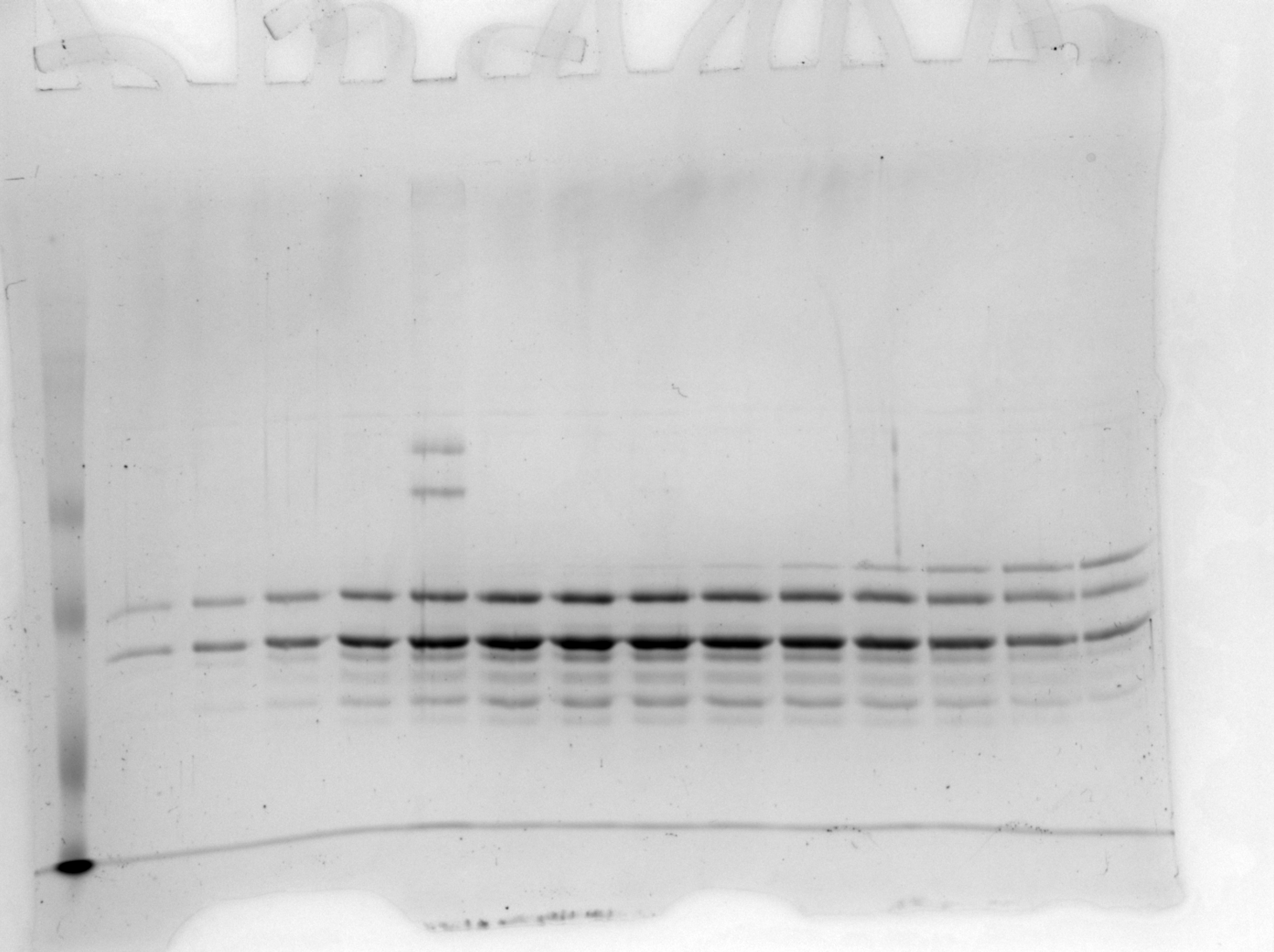

Supplement: Figure 1—source data 1. [file elife-72330-fig1-data1.zip › Figure 1-source data/Gel_SEC_Spp1-Mer2.tif]

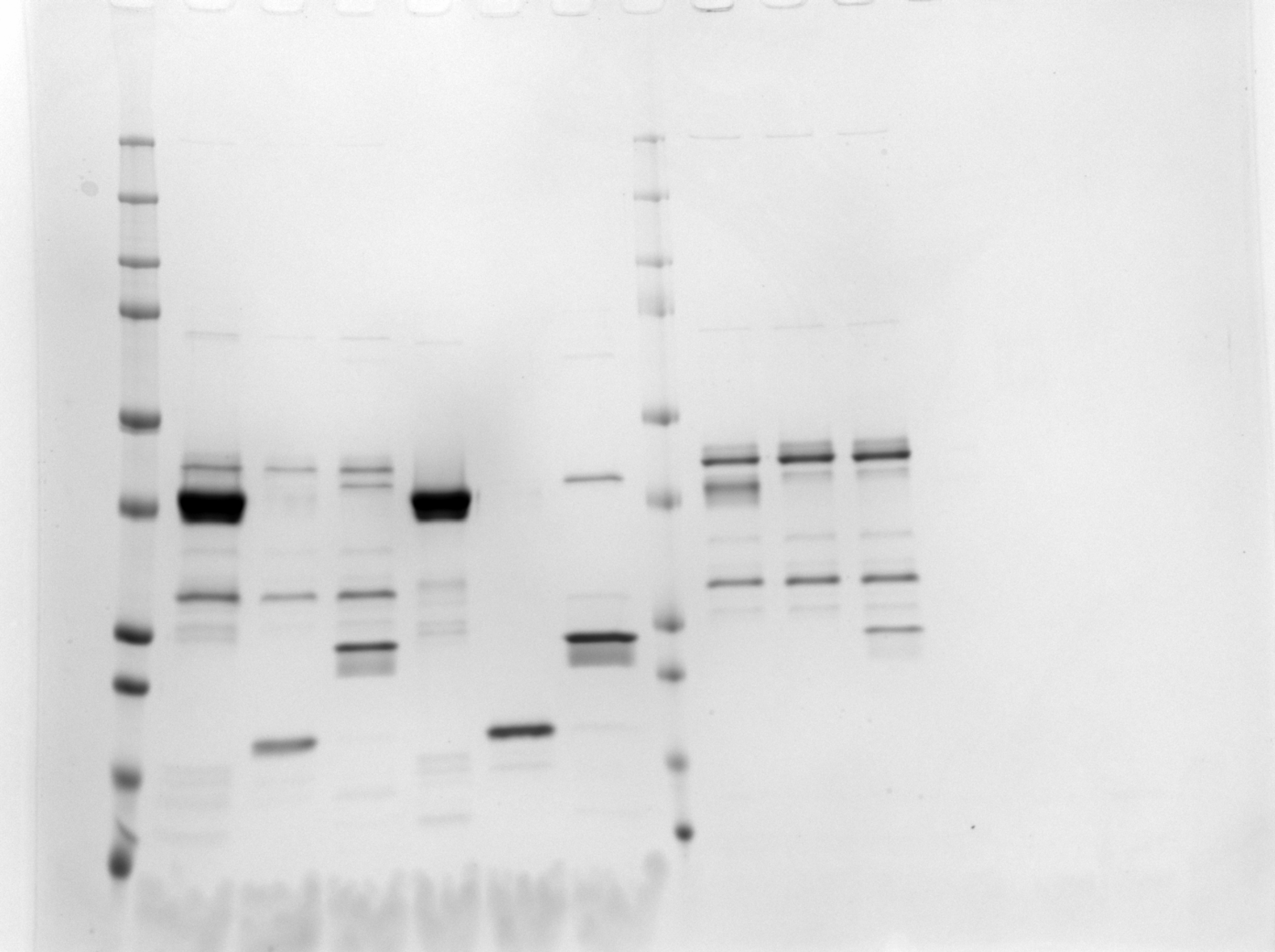

Supplement: Figure 1—figure supplement 2—source data 1. [file elife-72330-fig1-figsupp2-data1.zip › Figure 1-Sup2-source data/Gel_Pulldown_Mer2+Spp1.tif]

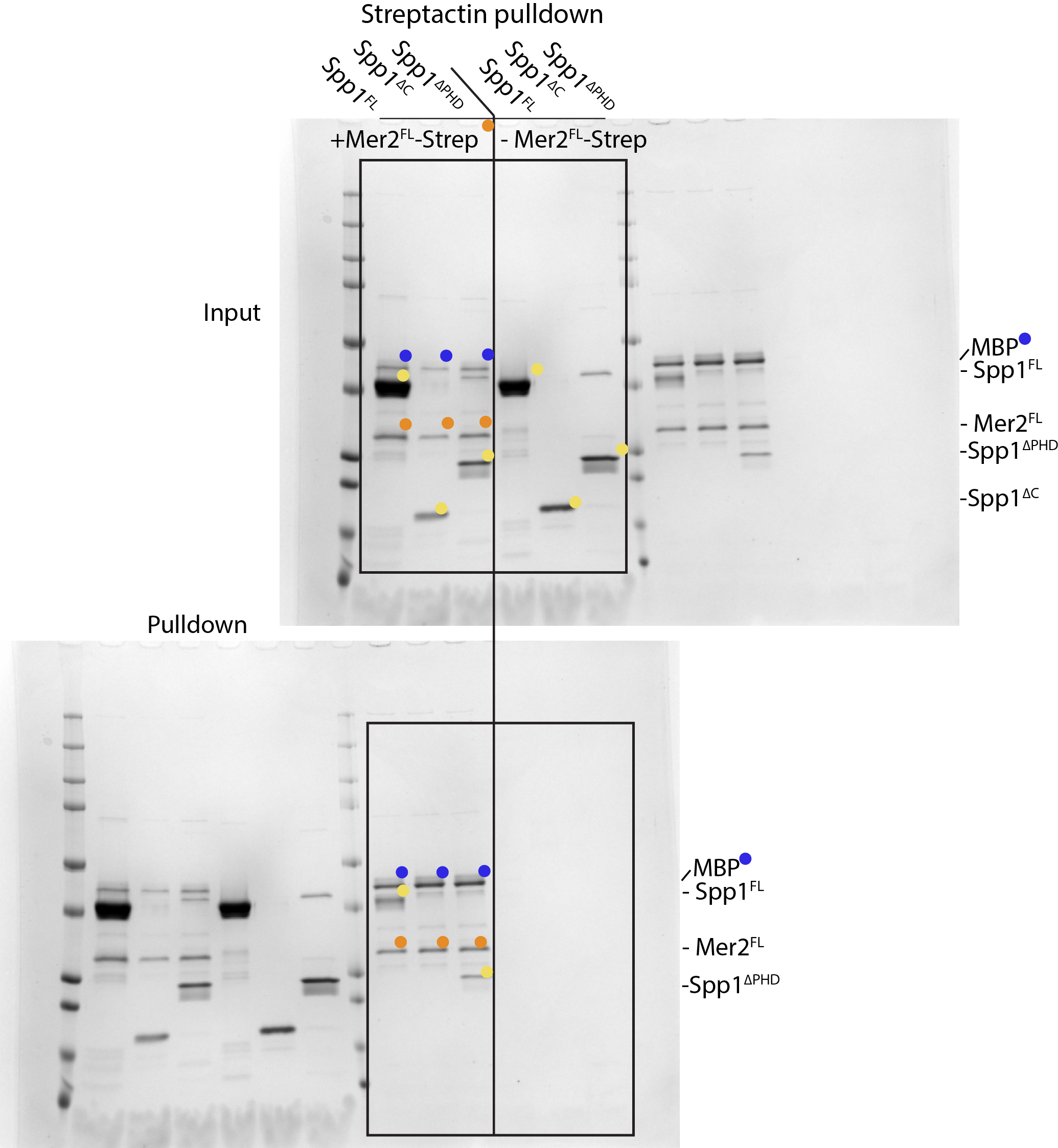

Supplement: Figure 1—figure supplement 2—source data 1. [file elife-72330-fig1-figsupp2-data1.zip › Figure 1-Sup2-source data/Pulldown_Spp1+Mer2.png]

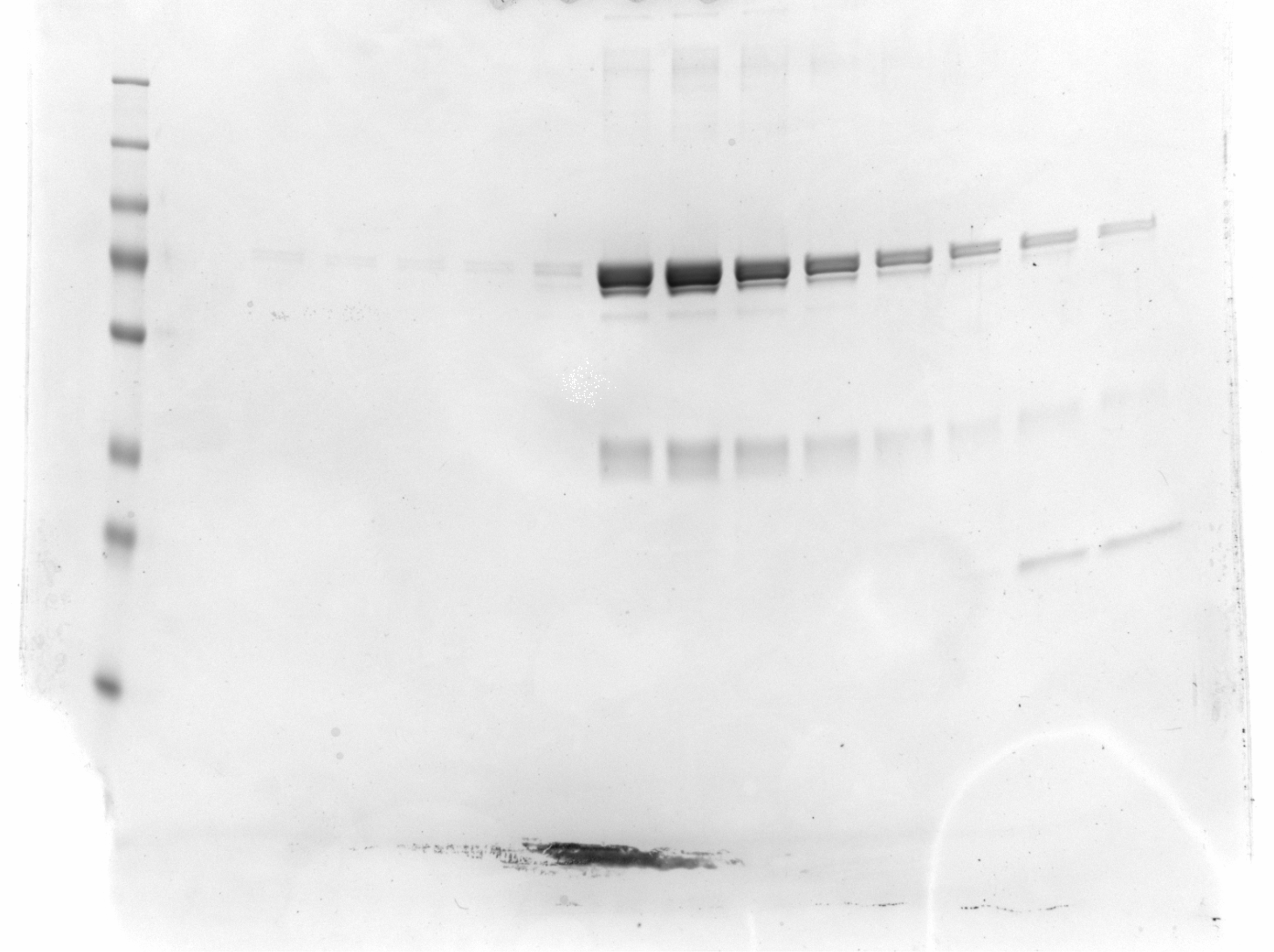

Supplement: Figure 2—source data 1. [file elife-72330-fig2-data1.zip › Figure 2-source data/Gel_SEC_Spp1+Mer2.tif]

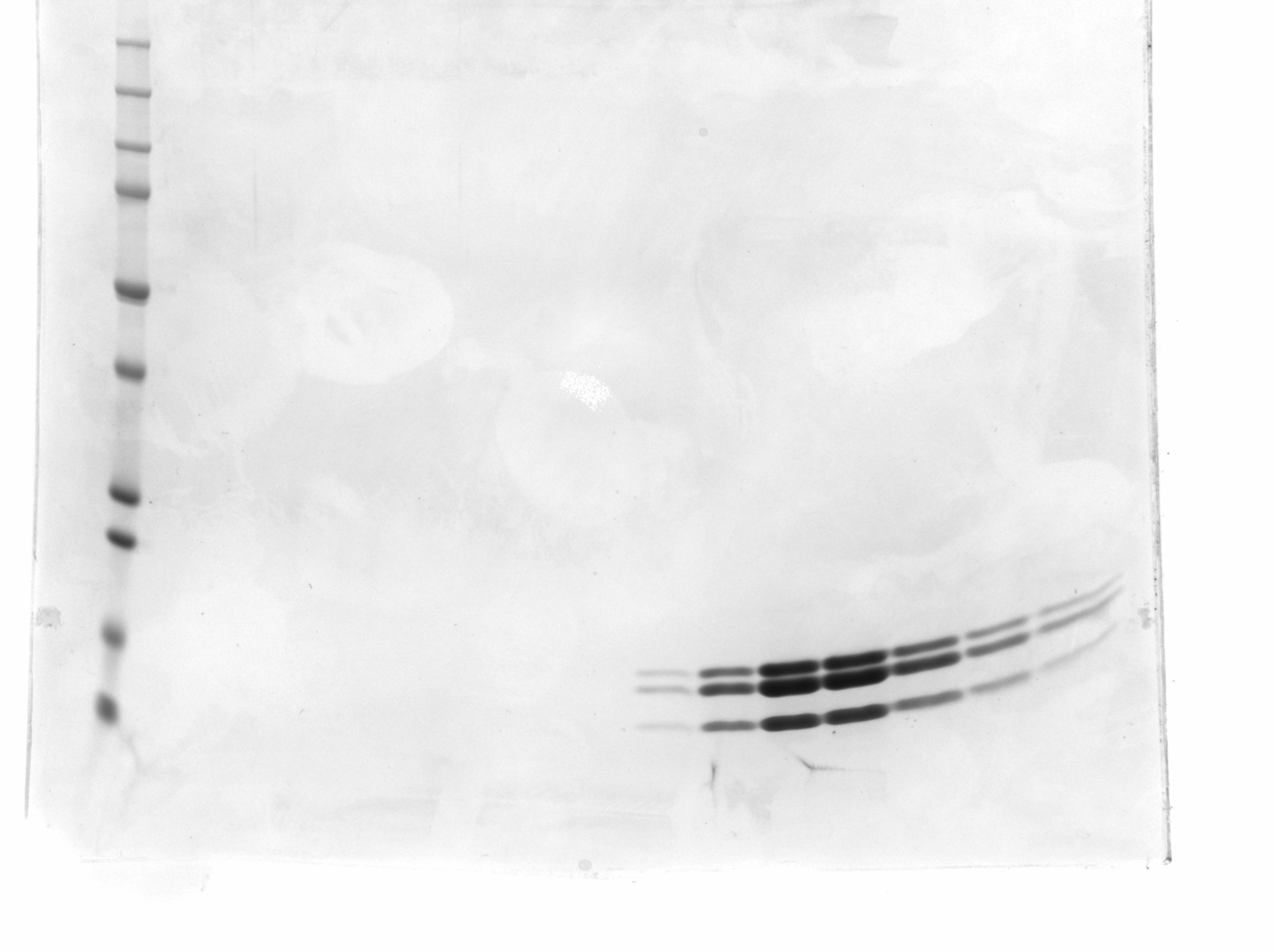

Supplement: Figure 2—source data 1. [file elife-72330-fig2-data1.zip › Figure 2-source data/Gel_SEC_MN.tif]

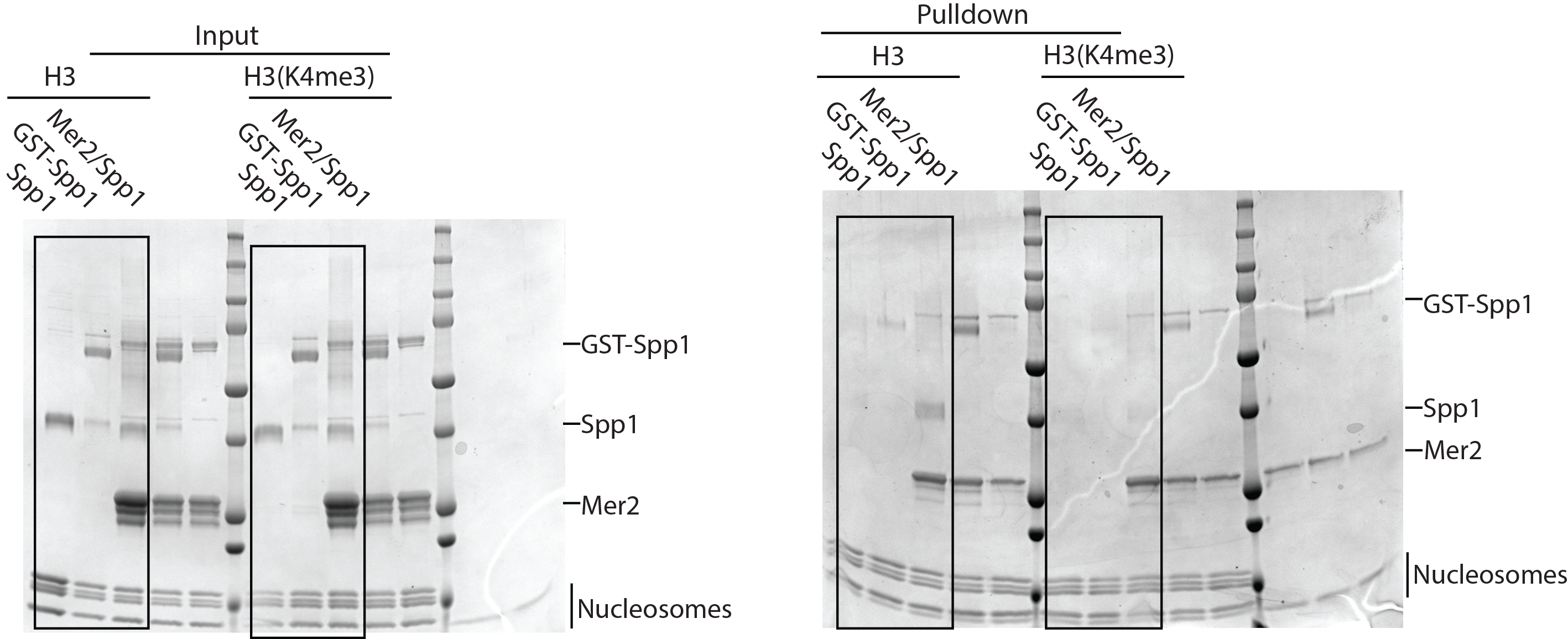

Supplement: Figure 2—source data 1. [file elife-72330-fig2-data1.zip › Figure 2-source data/Pulldown_MN+Spp1+Mer2.png]

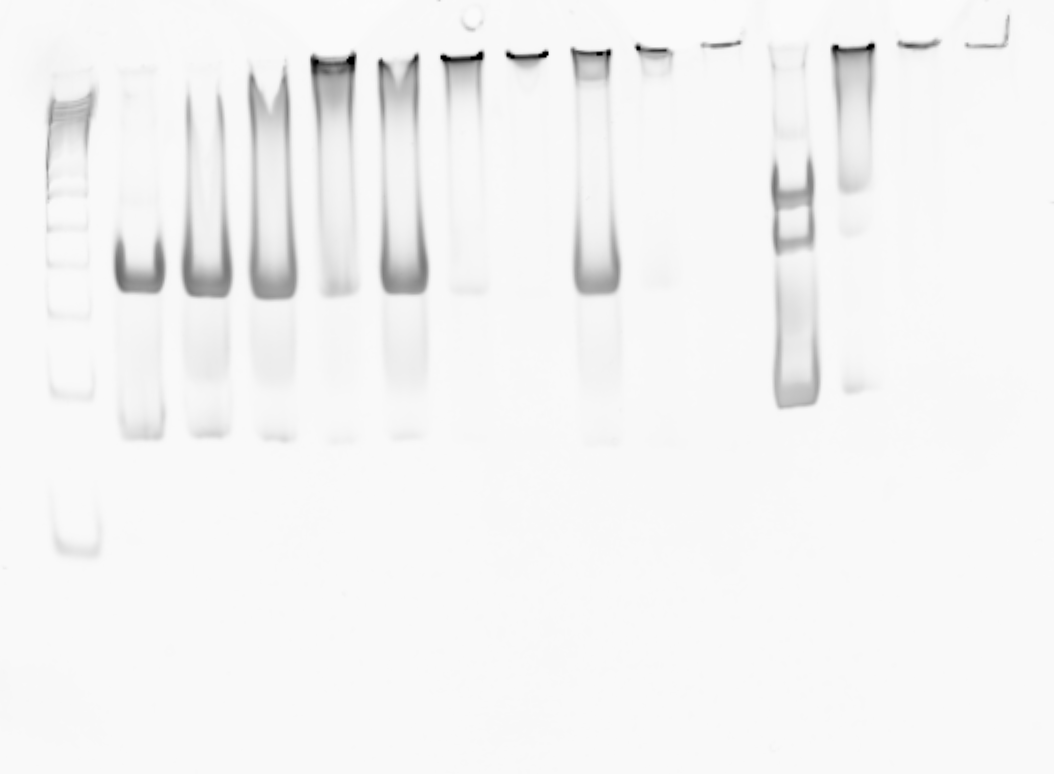

Supplement: Figure 2—source data 1. [file elife-72330-fig2-data1.zip › Figure 2-source data/Gel_EMSA.tif]

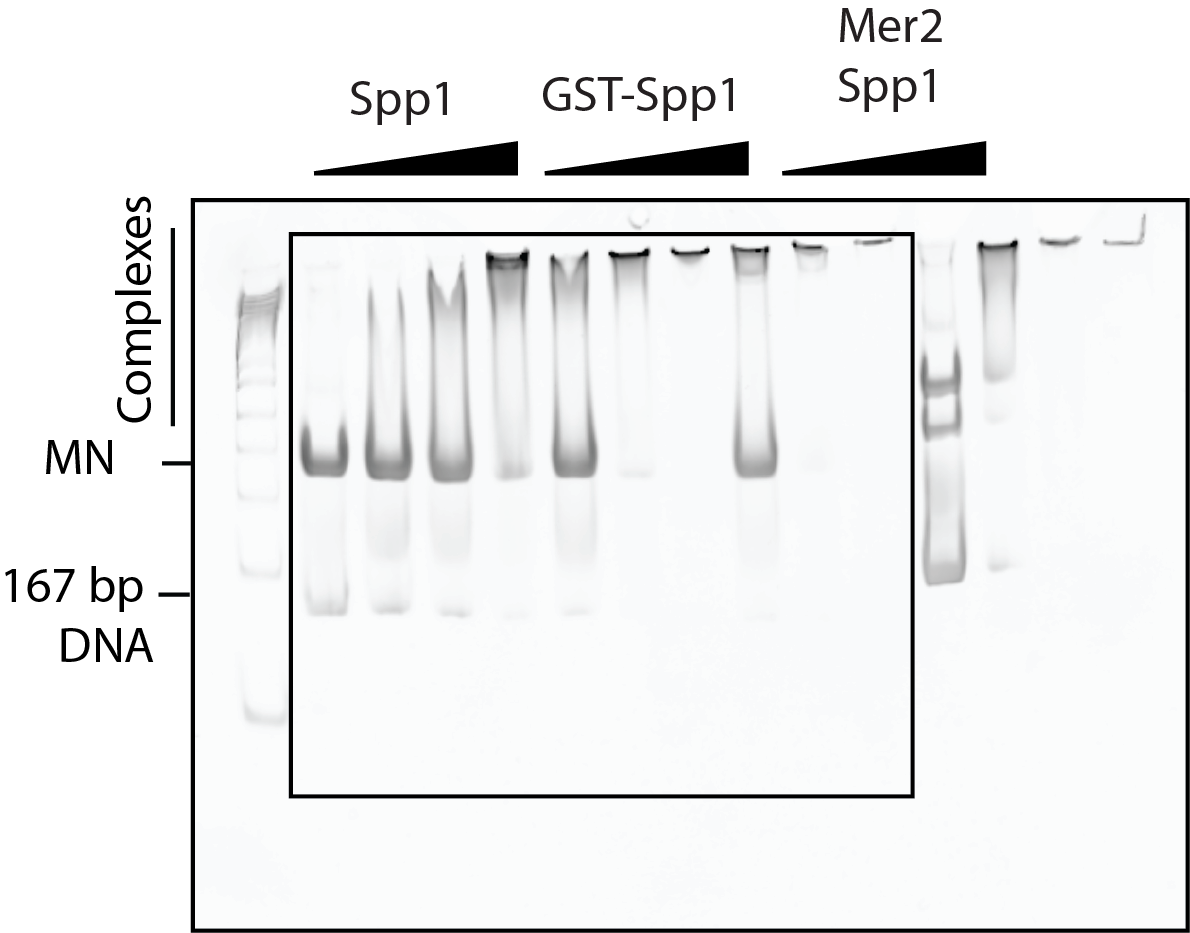

Supplement: Figure 2—source data 1. [file elife-72330-fig2-data1.zip › Figure 2-source data/EMSA_MN+Spp1.png]

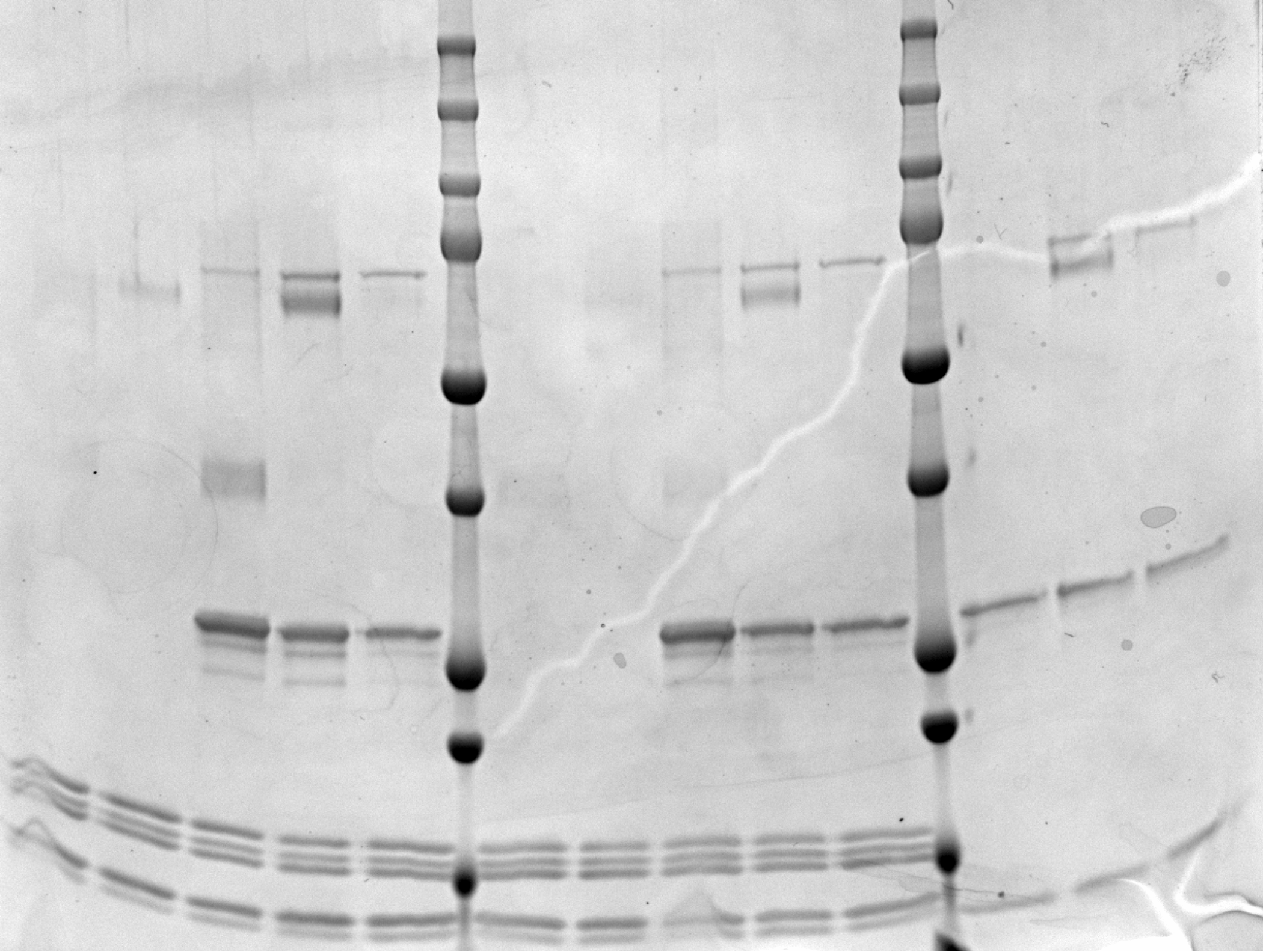

Supplement: Figure 2—source data 1. [file elife-72330-fig2-data1.zip › Figure 2-source data/Gel_MN_pulldown_elution.tif]

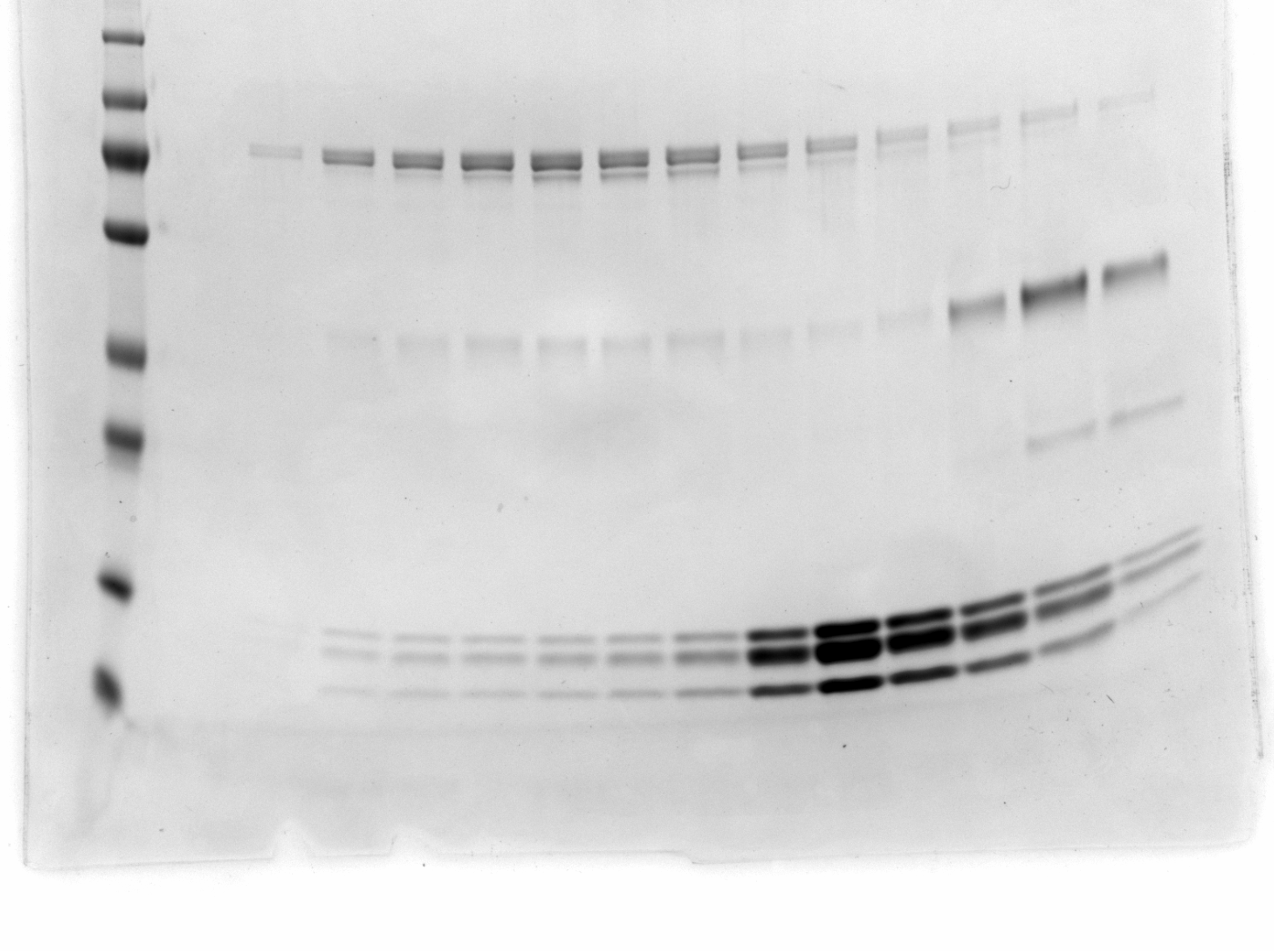

Supplement: Figure 2—source data 1. [file elife-72330-fig2-data1.zip › Figure 2-source data/Gel_SEC_MN+Spp1+Mer2.tif]

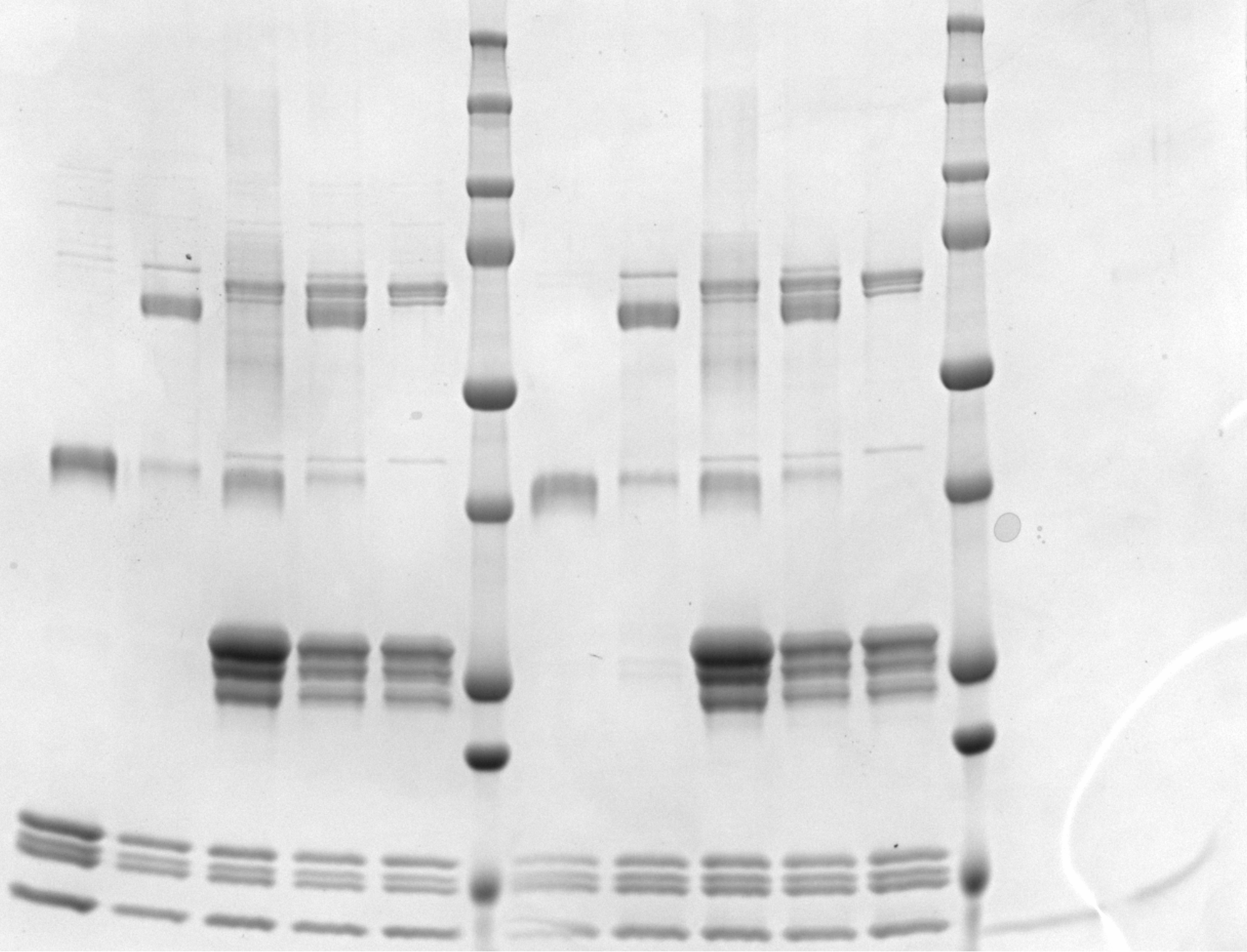

Supplement: Figure 2—source data 1. [file elife-72330-fig2-data1.zip › Figure 2-source data/Gel_MN_pulldown_input.tif]

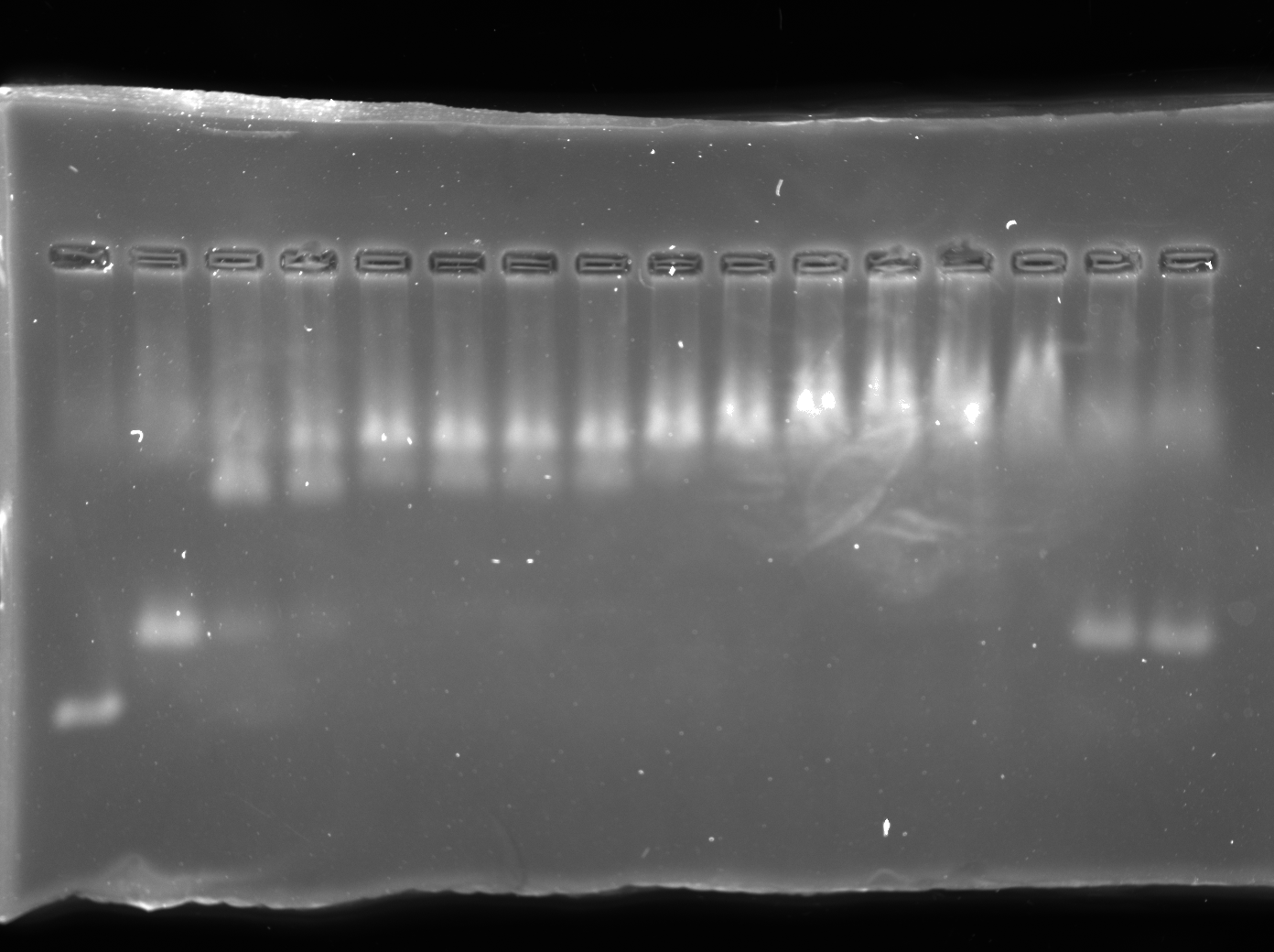

Supplement: Figure 3—source data 1. [file elife-72330-fig3-data1.zip › Figure 3-source data/EMSA_Mer2+MN(WT)-167_1.tif]

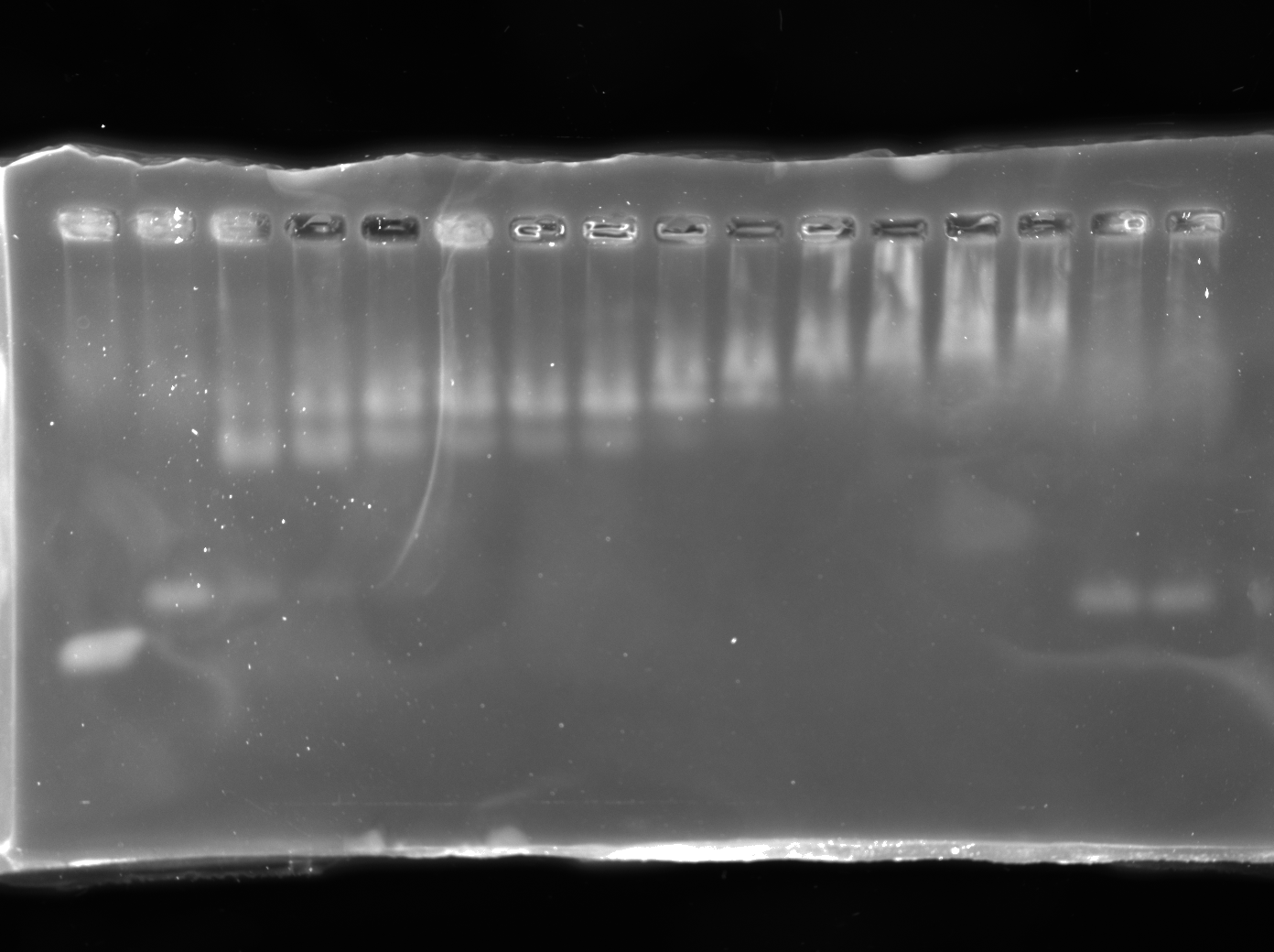

Supplement: Figure 3—source data 1. [file elife-72330-fig3-data1.zip › Figure 3-source data/EMSA_Mer2+MN(WT)-167_2.tif]

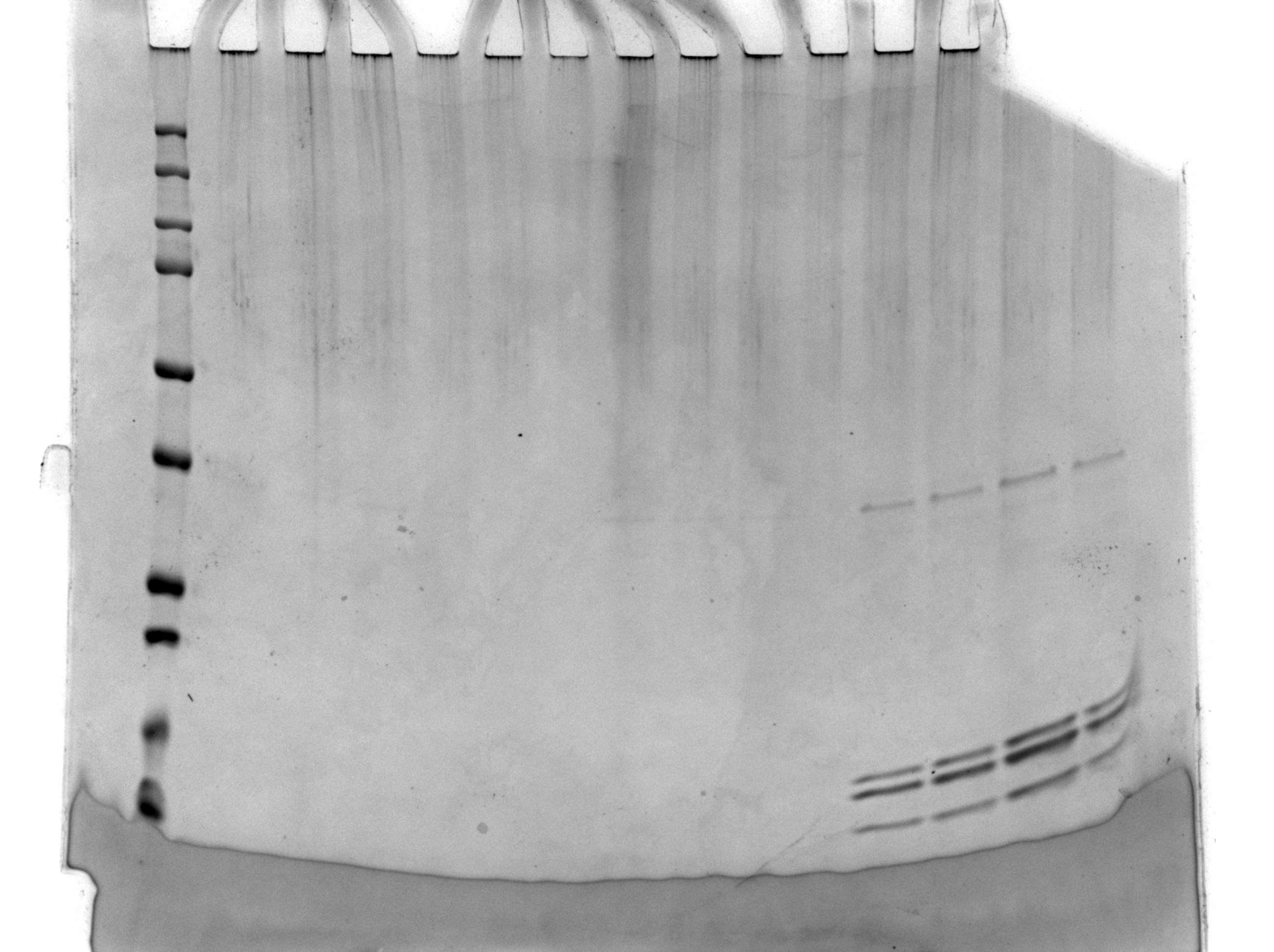

Supplement: Figure 3—source data 1. [file elife-72330-fig3-data1.zip › Figure 3-source data/Gel_SEC_MN.tif]

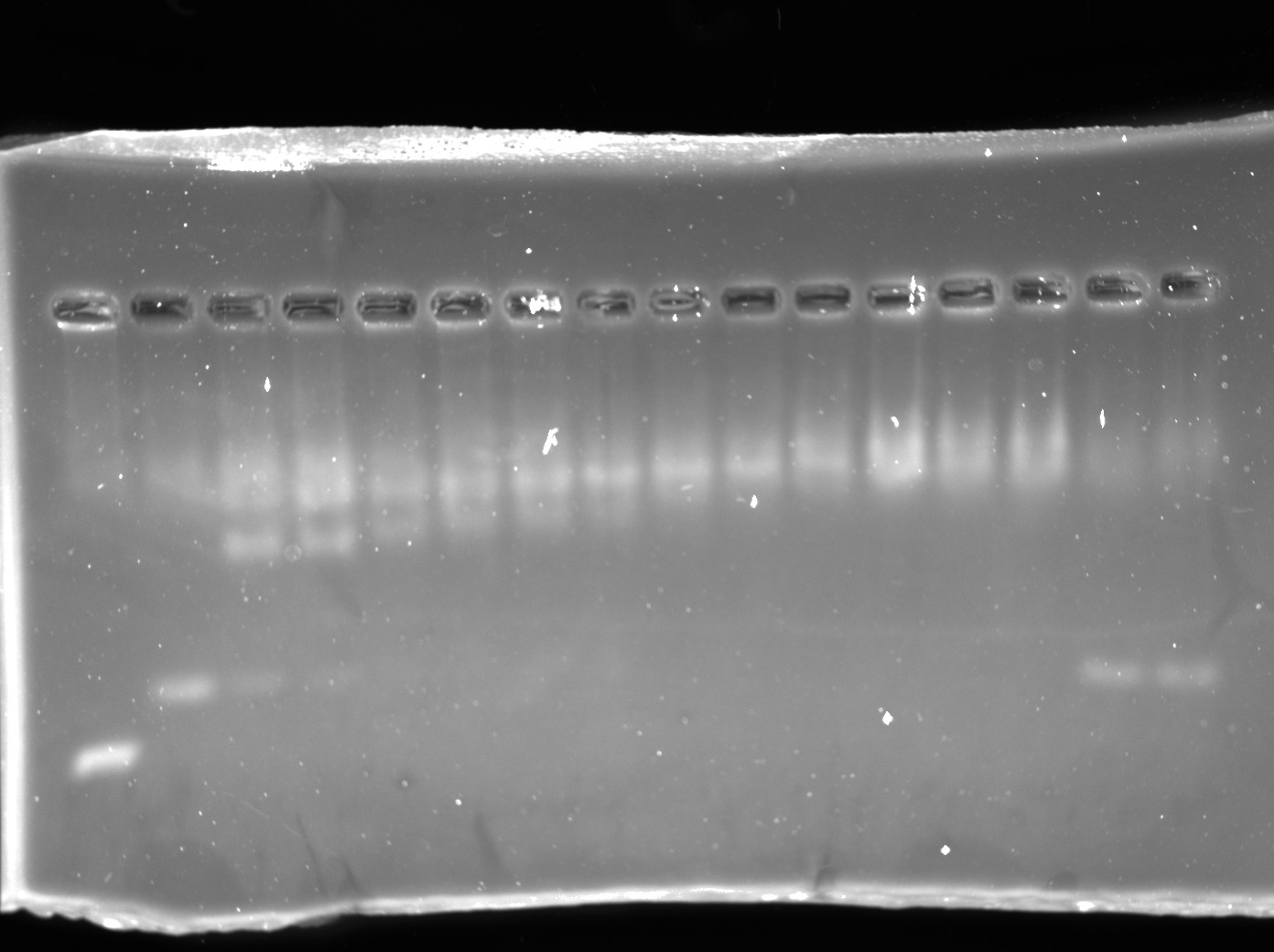

Supplement: Figure 3—source data 1. [file elife-72330-fig3-data1.zip › Figure 3-source data/EMSA_Mer2+MN(WT)-167_3.tif]

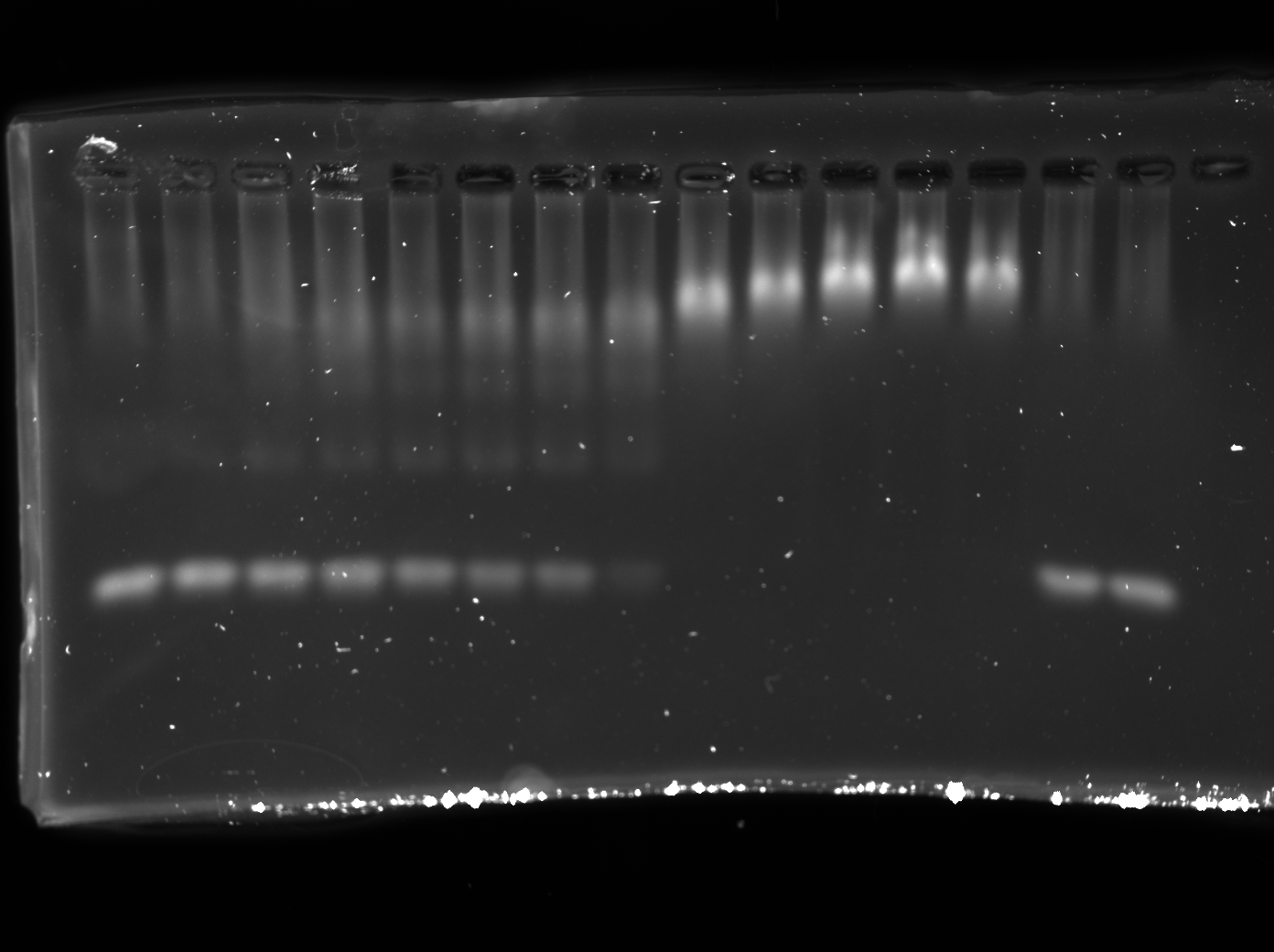

Supplement: Figure 3—source data 1. [file elife-72330-fig3-data1.zip › Figure 3-source data/EMSA_Mer2+DNA_3.tif]

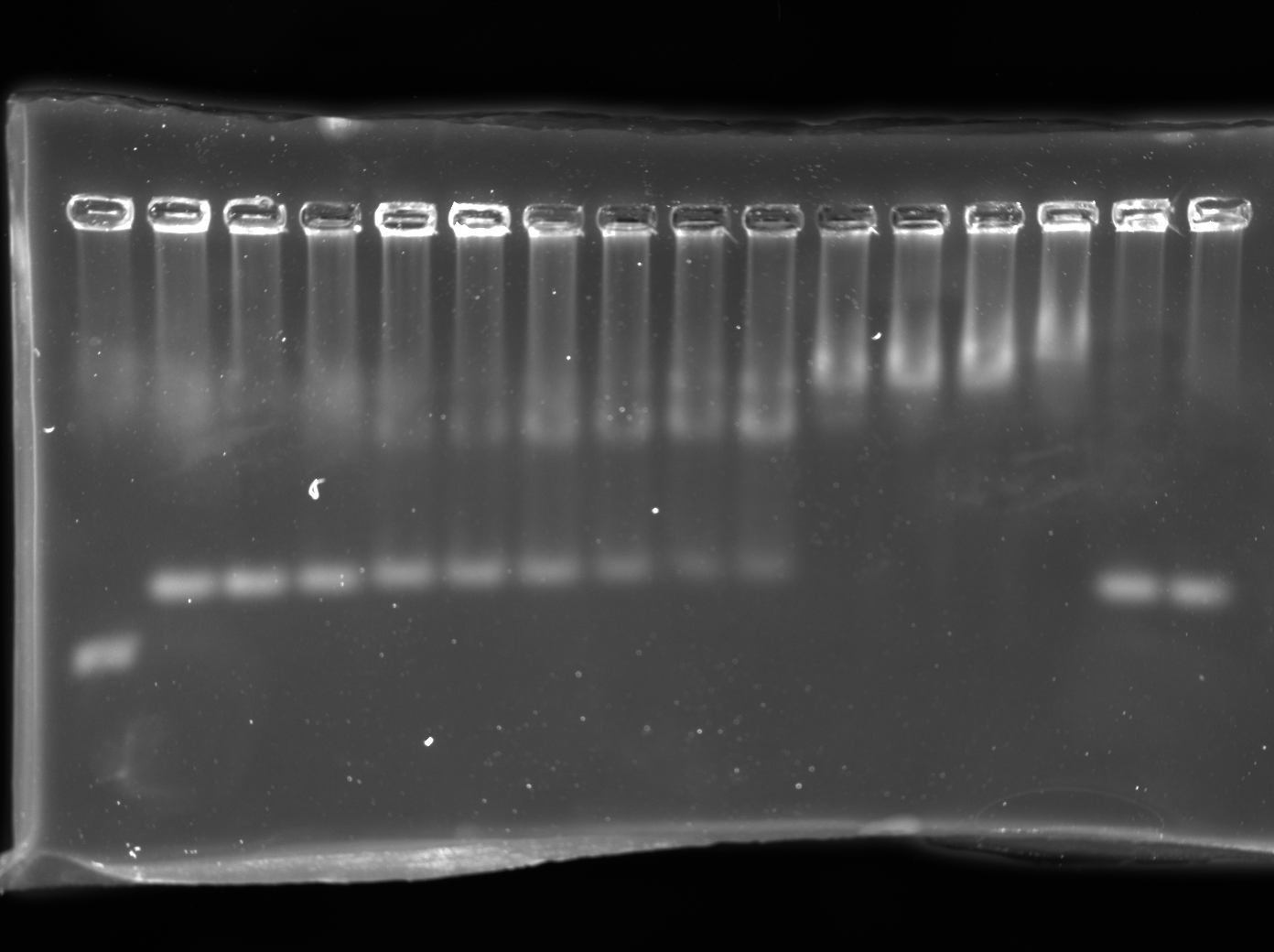

Supplement: Figure 3—source data 1. [file elife-72330-fig3-data1.zip › Figure 3-source data/EMSA_Mer2+MN(WT)-147_2.tif]

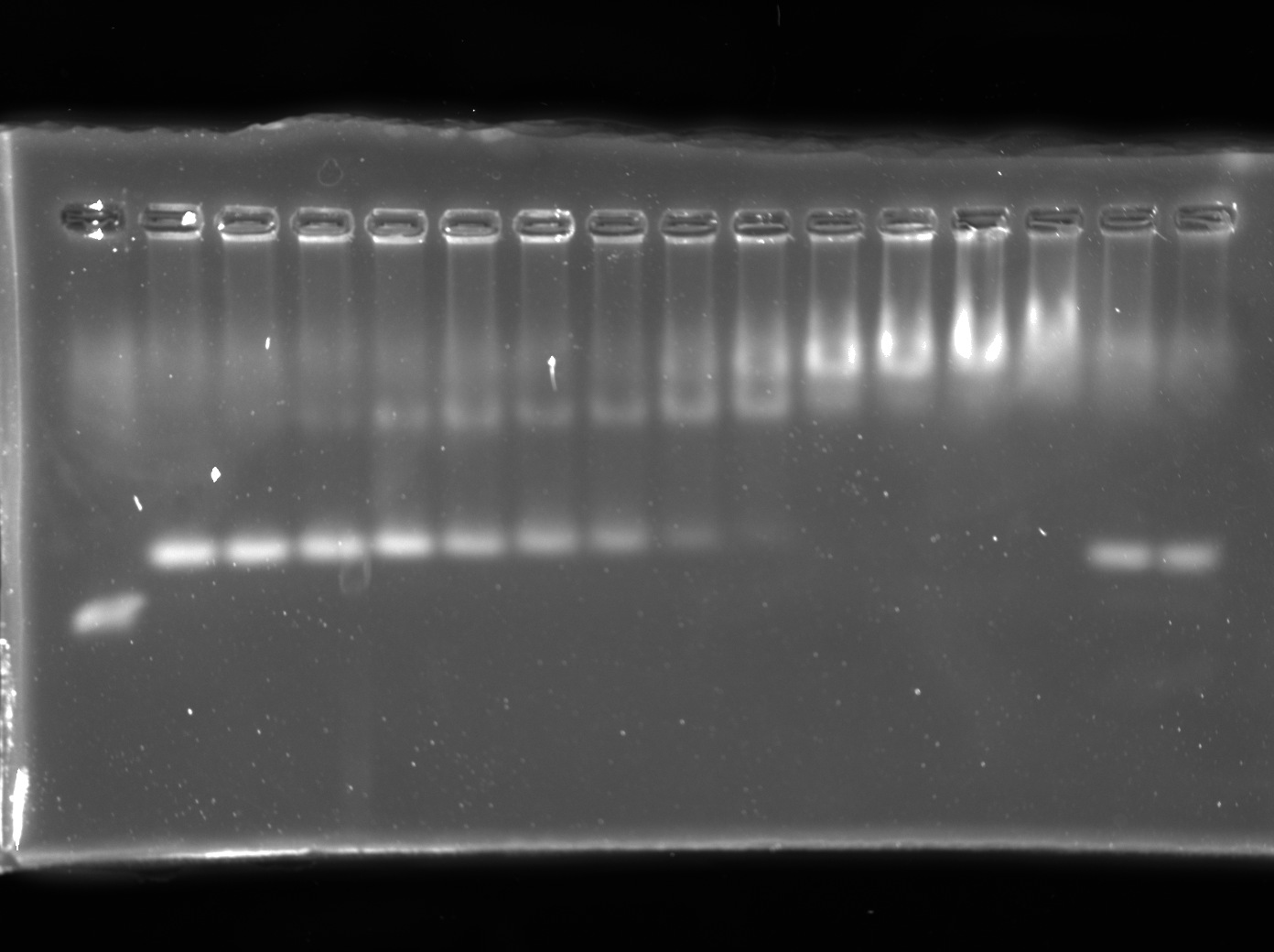

Supplement: Figure 3—source data 1. [file elife-72330-fig3-data1.zip › Figure 3-source data/EMSA_Mer2+MN(WT)-147_3.tif]

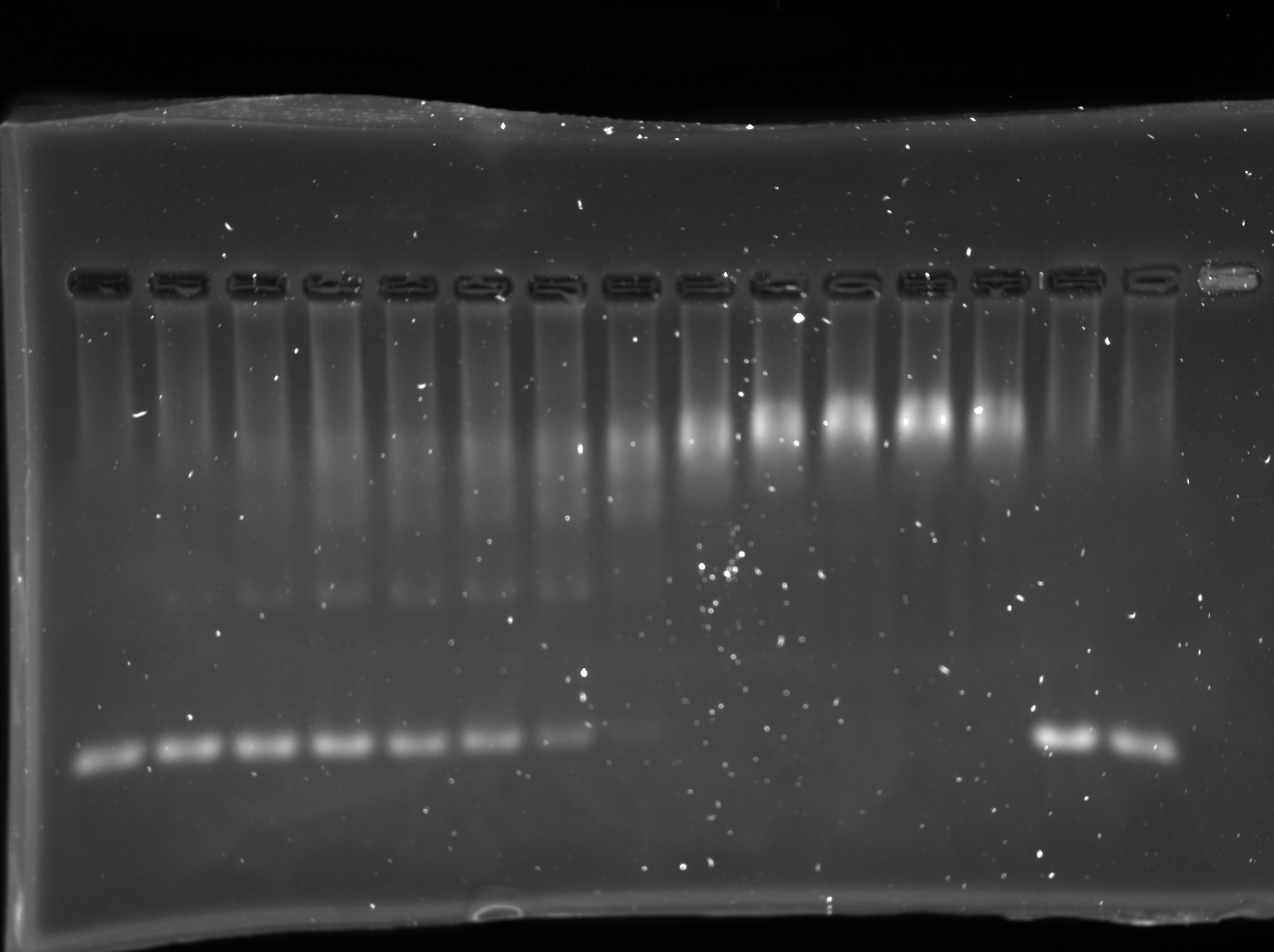

Supplement: Figure 3—source data 1. [file elife-72330-fig3-data1.zip › Figure 3-source data/EMSA_Mer2+DNA_2.tif]

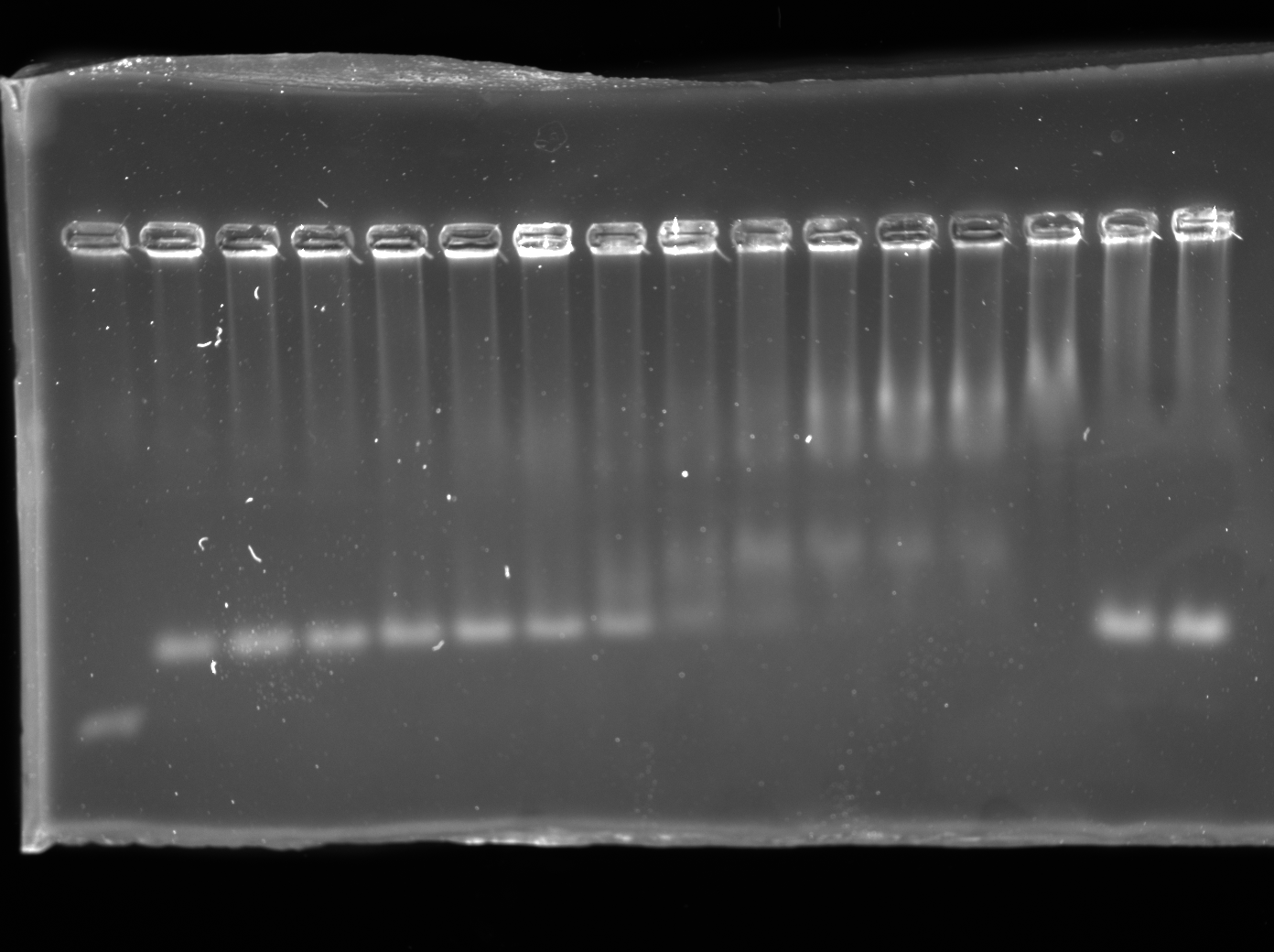

Supplement: Figure 3—source data 1. [file elife-72330-fig3-data1.zip › Figure 3-source data/EMSA_Mer2+MN(WT)-147_1.tif]

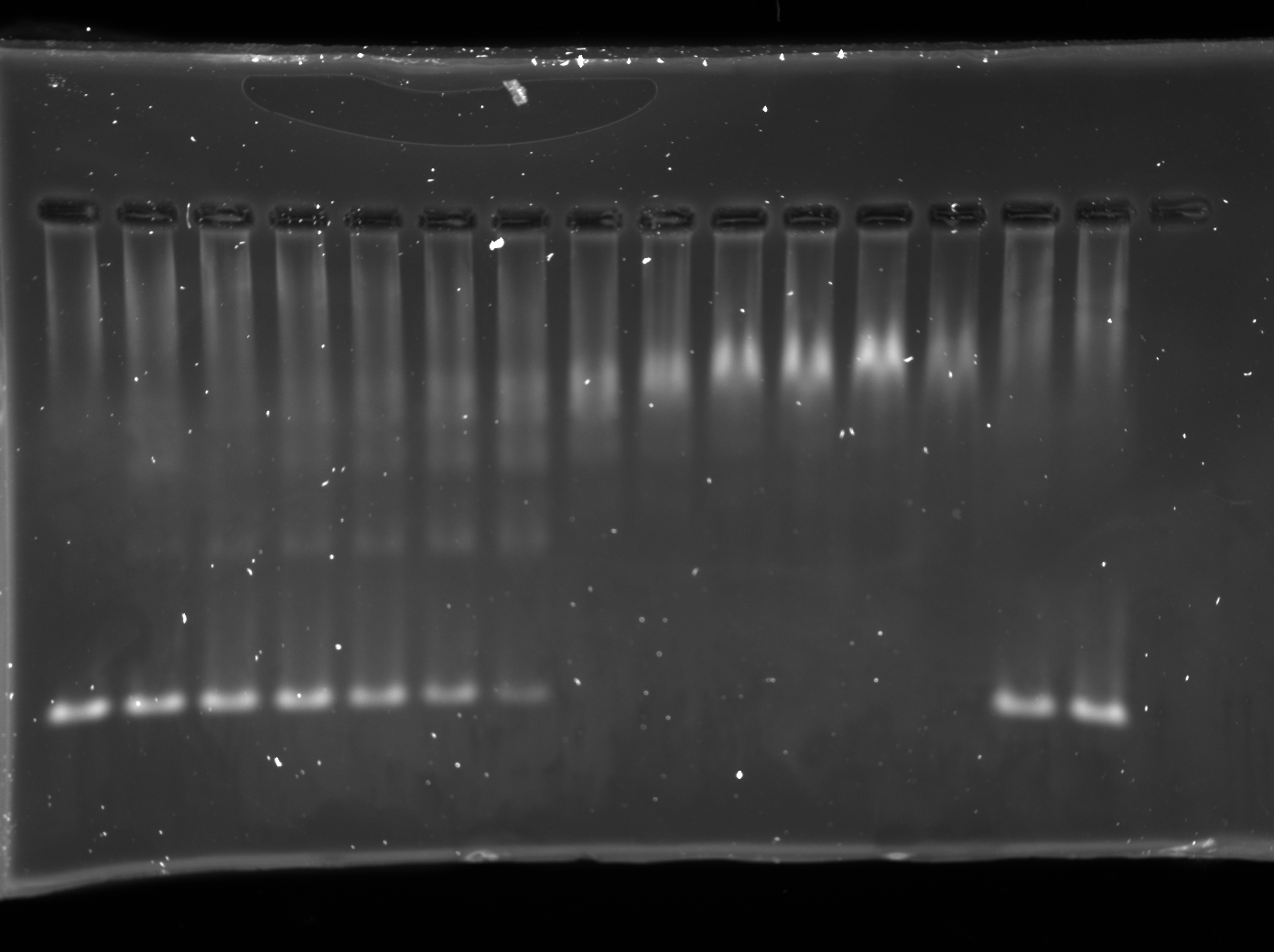

Supplement: Figure 3—source data 1. [file elife-72330-fig3-data1.zip › Figure 3-source data/EMSA_Mer2+DNA_1.tif]

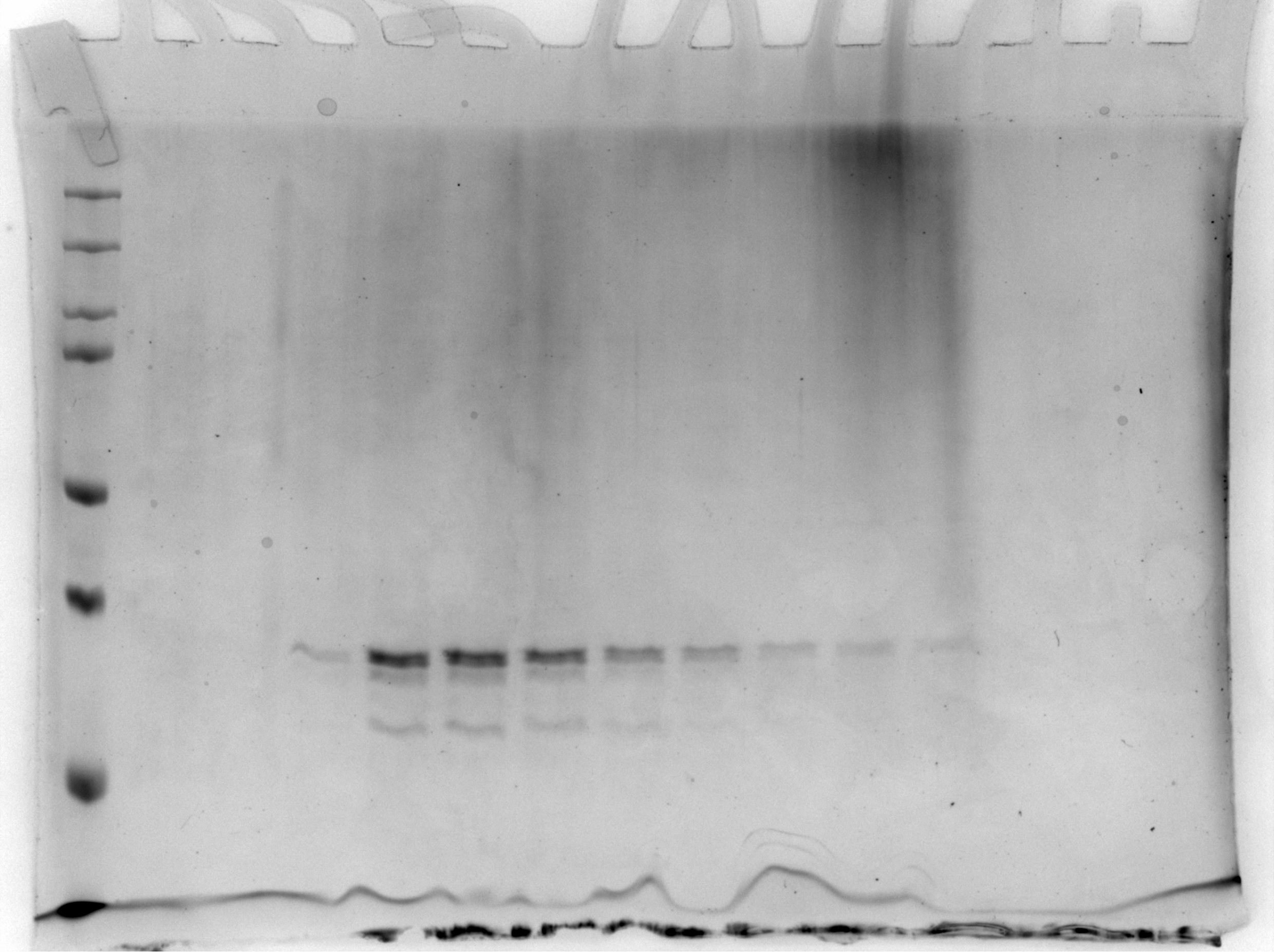

Supplement: Figure 3—source data 1. [file elife-72330-fig3-data1.zip › Figure 3-source data/Gel_SEC_Mer2.tif]

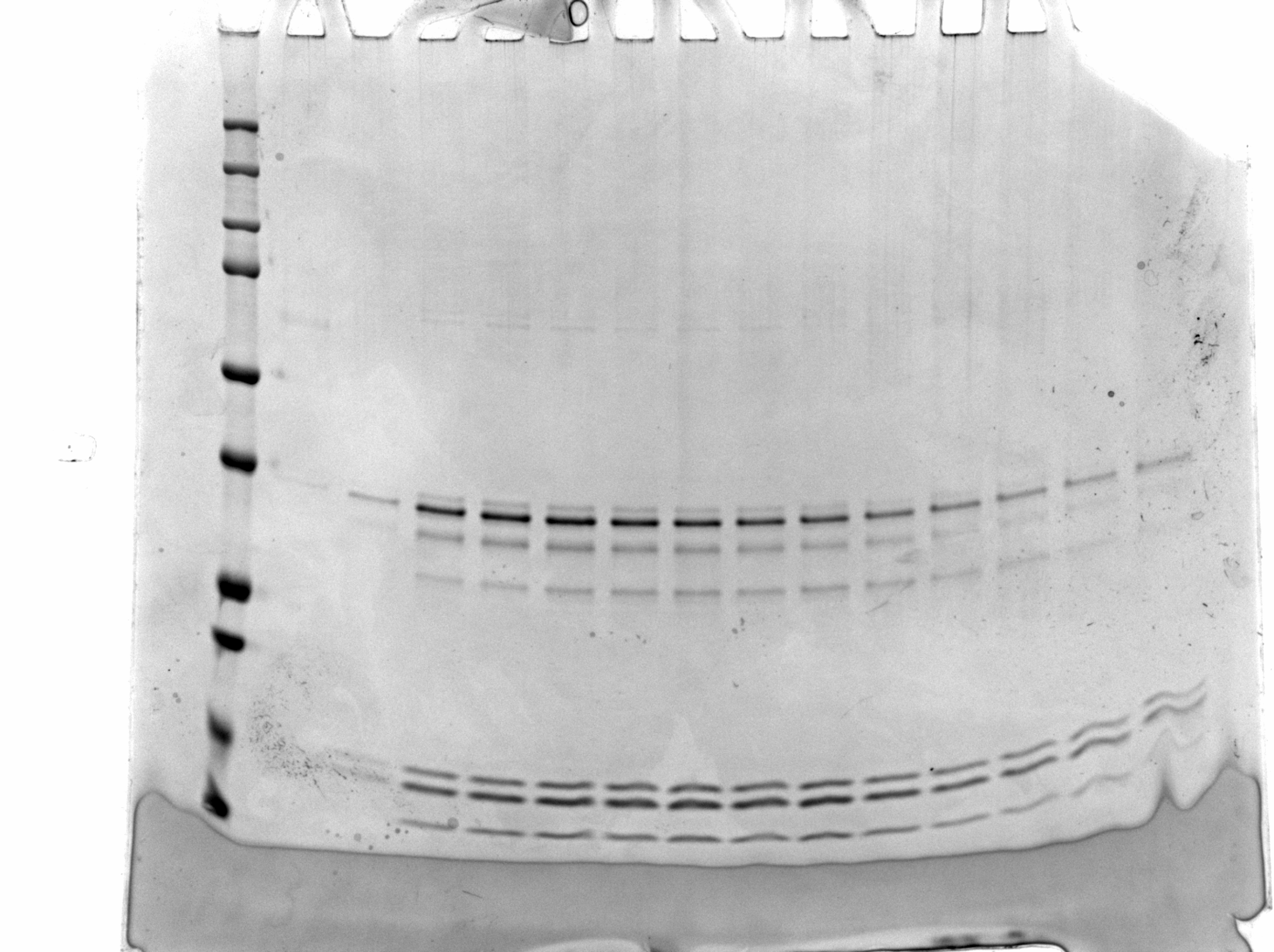

Supplement: Figure 3—source data 1. [file elife-72330-fig3-data1.zip › Figure 3-source data/Gel_SEC_MN+Mer2.tif]

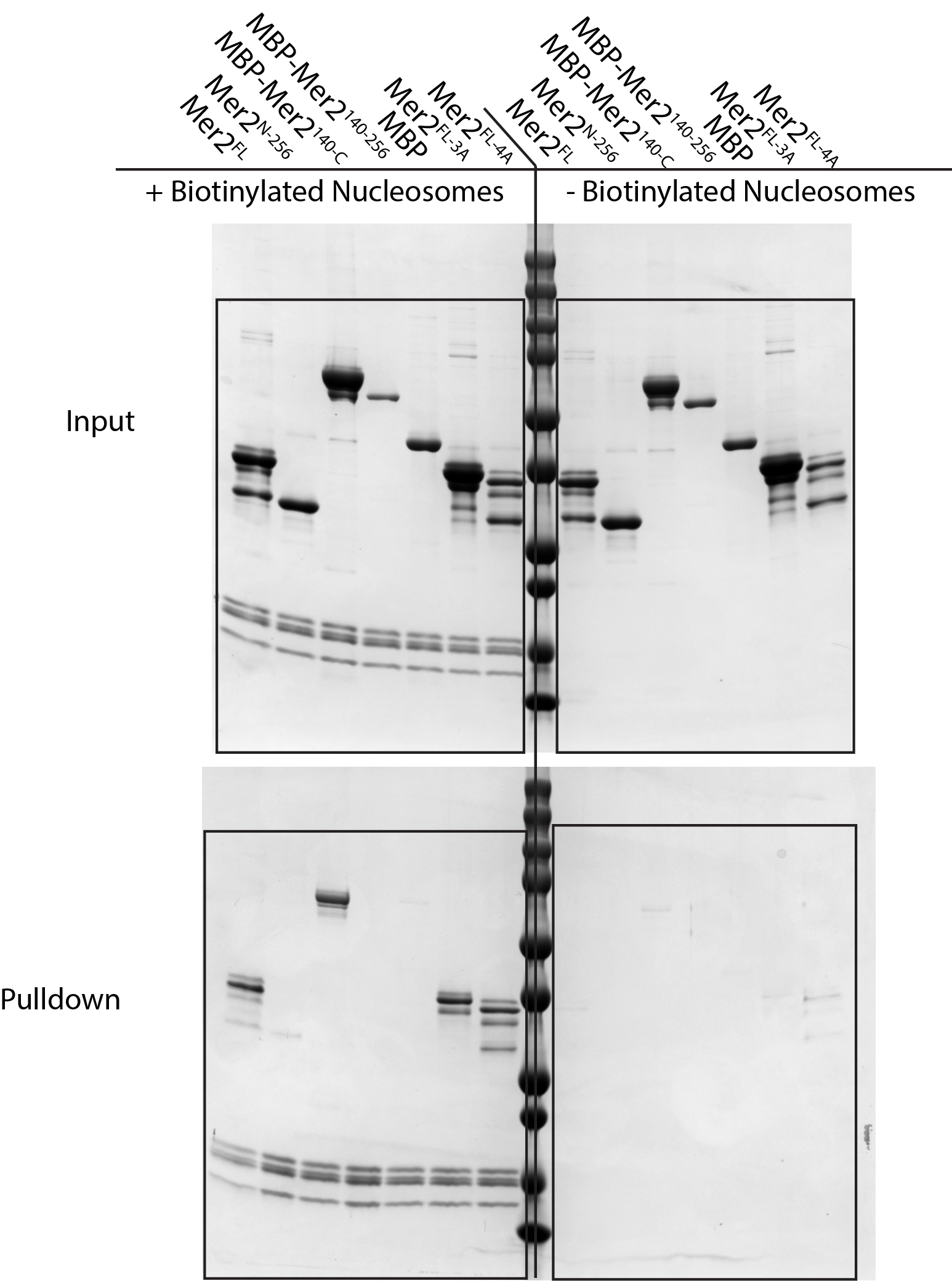

Supplement: Figure 3—source data 1. [file elife-72330-fig3-data1.zip › Figure 3-source data/Pulldown_Mer2+MN.png]

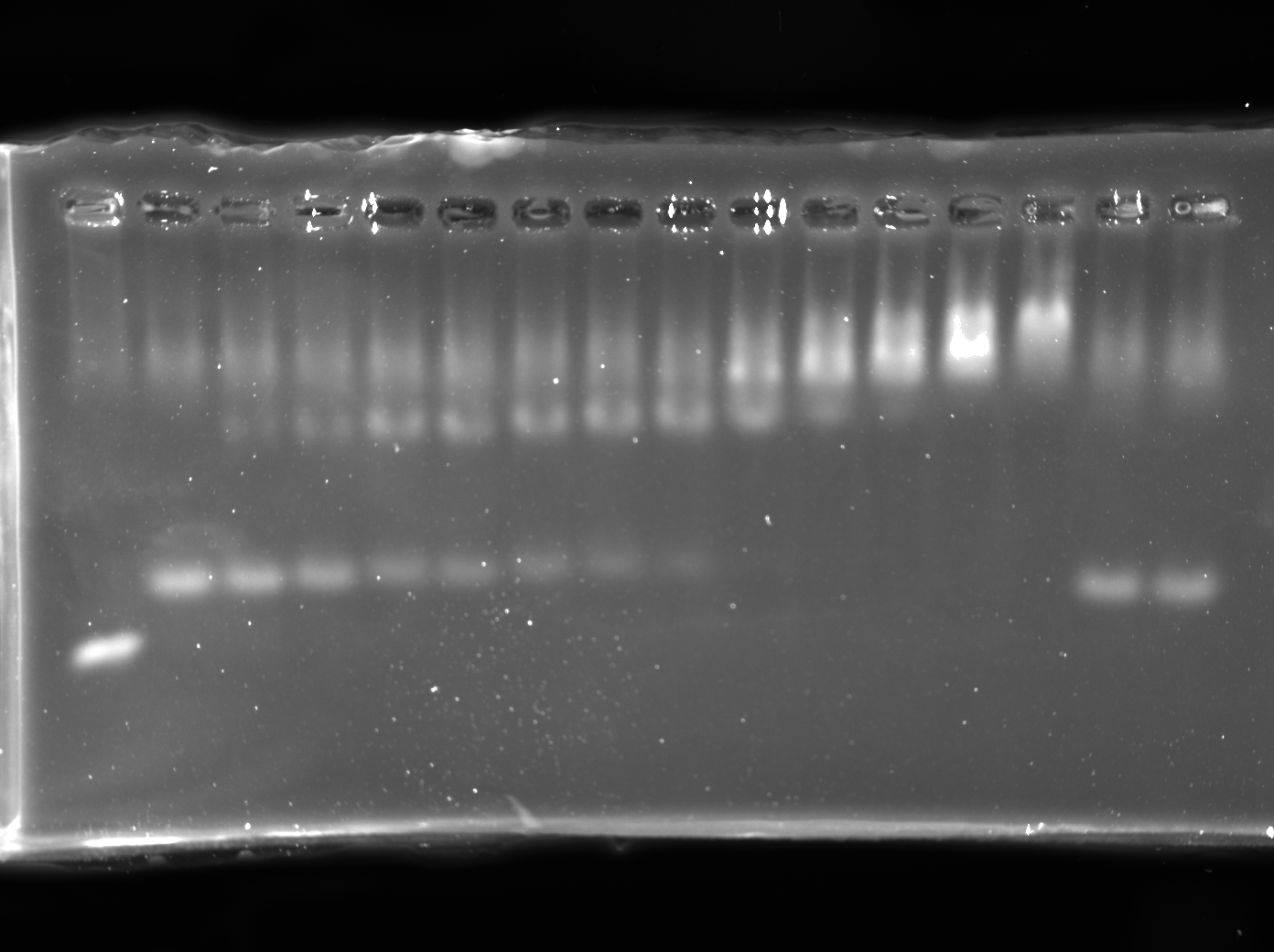

Supplement: Figure 3—figure supplement 2—source data 1. [file elife-72330-fig3-figsupp2-data1.zip › Figure 3-Sup2- source data/EMSA_Mer2_FL+MN(ac-patch)-147_1.tif]

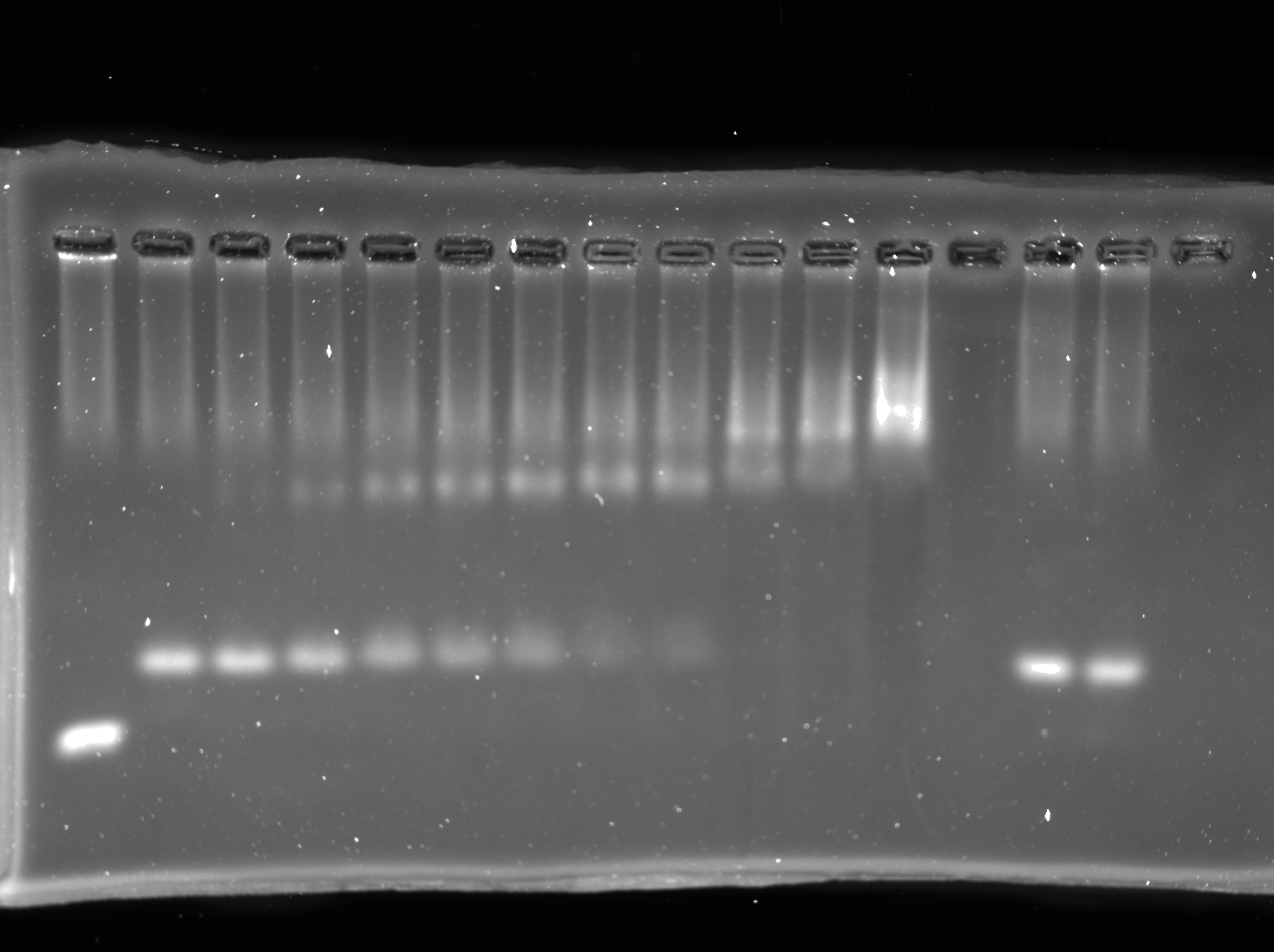

Supplement: Figure 3—figure supplement 2—source data 1. [file elife-72330-fig3-figsupp2-data1.zip › Figure 3-Sup2- source data/EMSA_Mer2_FL+MN(ac-patch)-147_2.tif]

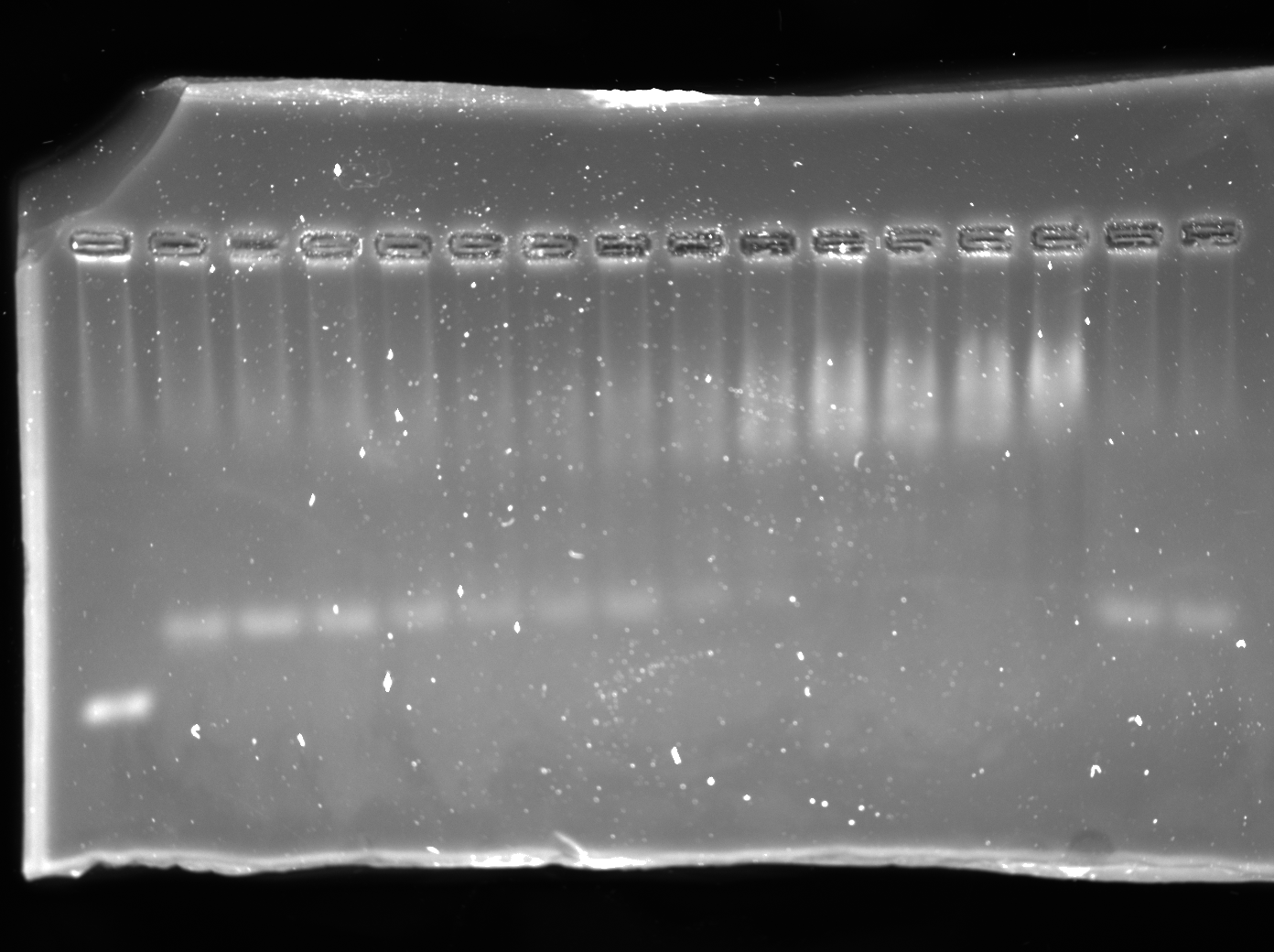

Supplement: Figure 3—figure supplement 2—source data 1. [file elife-72330-fig3-figsupp2-data1.zip › Figure 3-Sup2- source data/EMSA_Mer2_FL+MN(ac-patch)-147_3.tif]

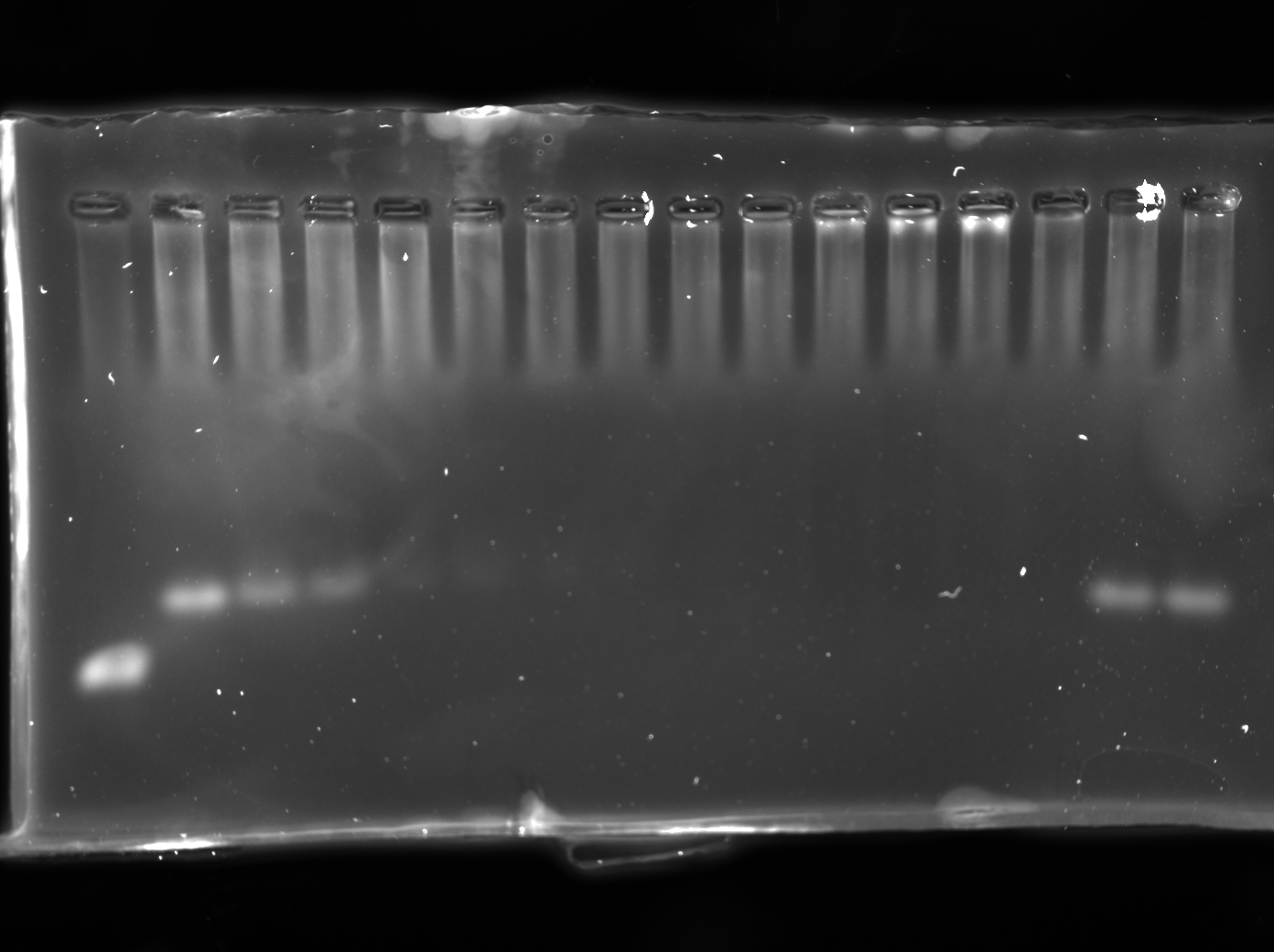

Supplement: Figure 3—figure supplement 2—source data 1. [file elife-72330-fig3-figsupp2-data1.zip › Figure 3-Sup2- source data/EMSA_Mer2_N-256+MN(WT)-167_1.tif]

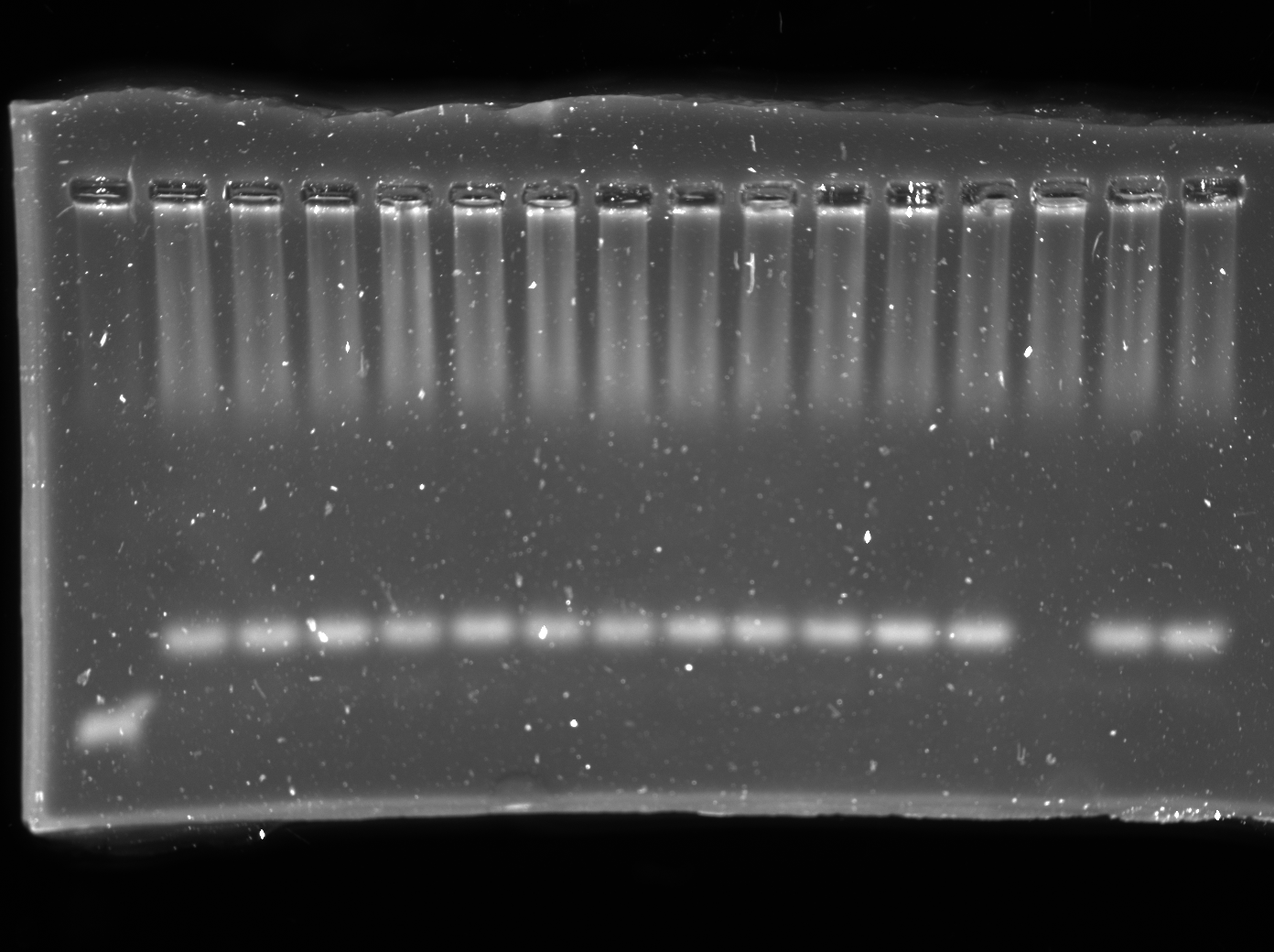

Supplement: Figure 3—figure supplement 2—source data 1. [file elife-72330-fig3-figsupp2-data1.zip › Figure 3-Sup2- source data/EMSA_Mer2_140-256+MN(WT)-167.tif]

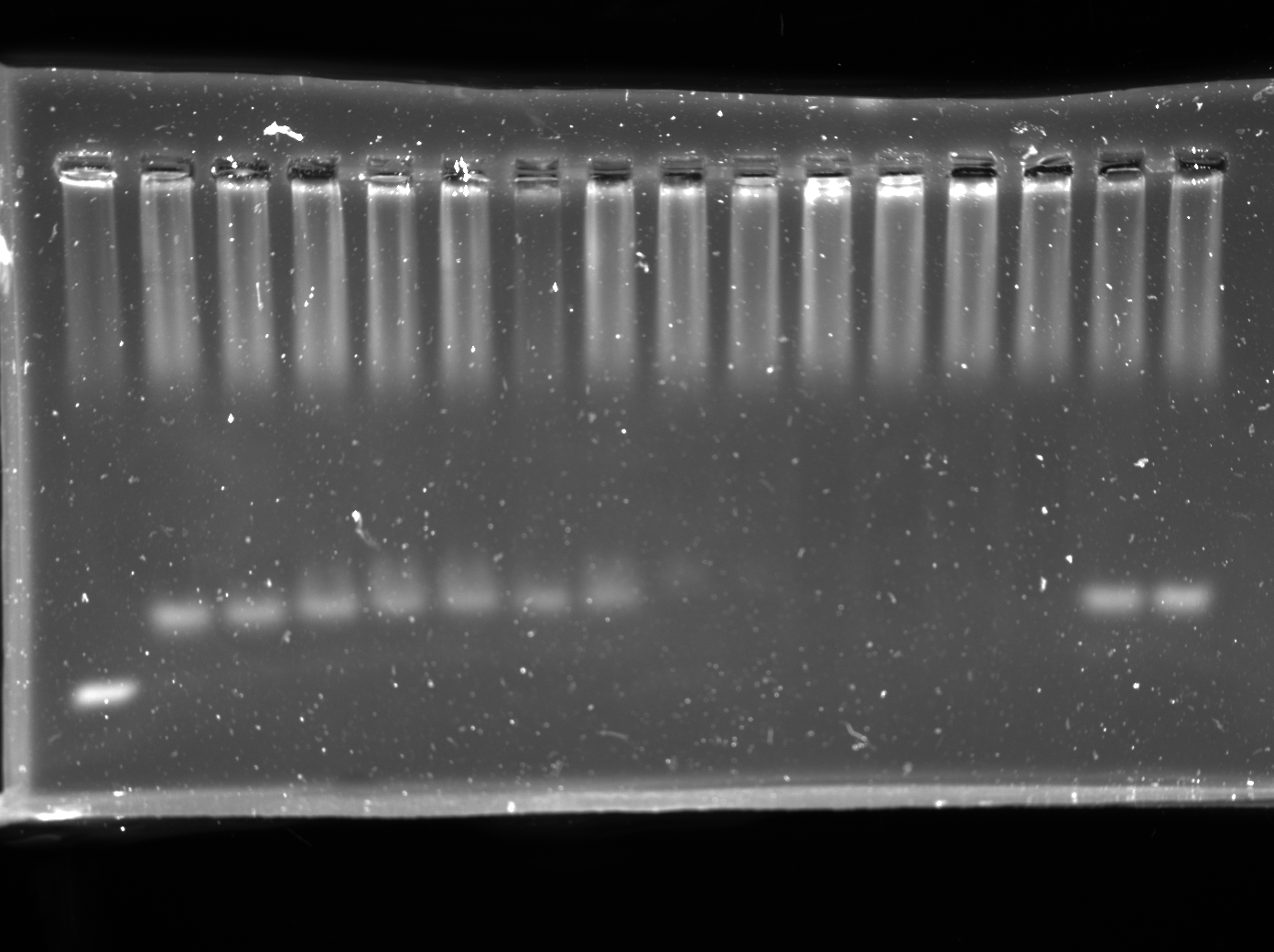

Supplement: Figure 3—figure supplement 2—source data 1. [file elife-72330-fig3-figsupp2-data1.zip › Figure 3-Sup2- source data/EMSA_Mer2_140-C+MN(WT)-167_4.tif]

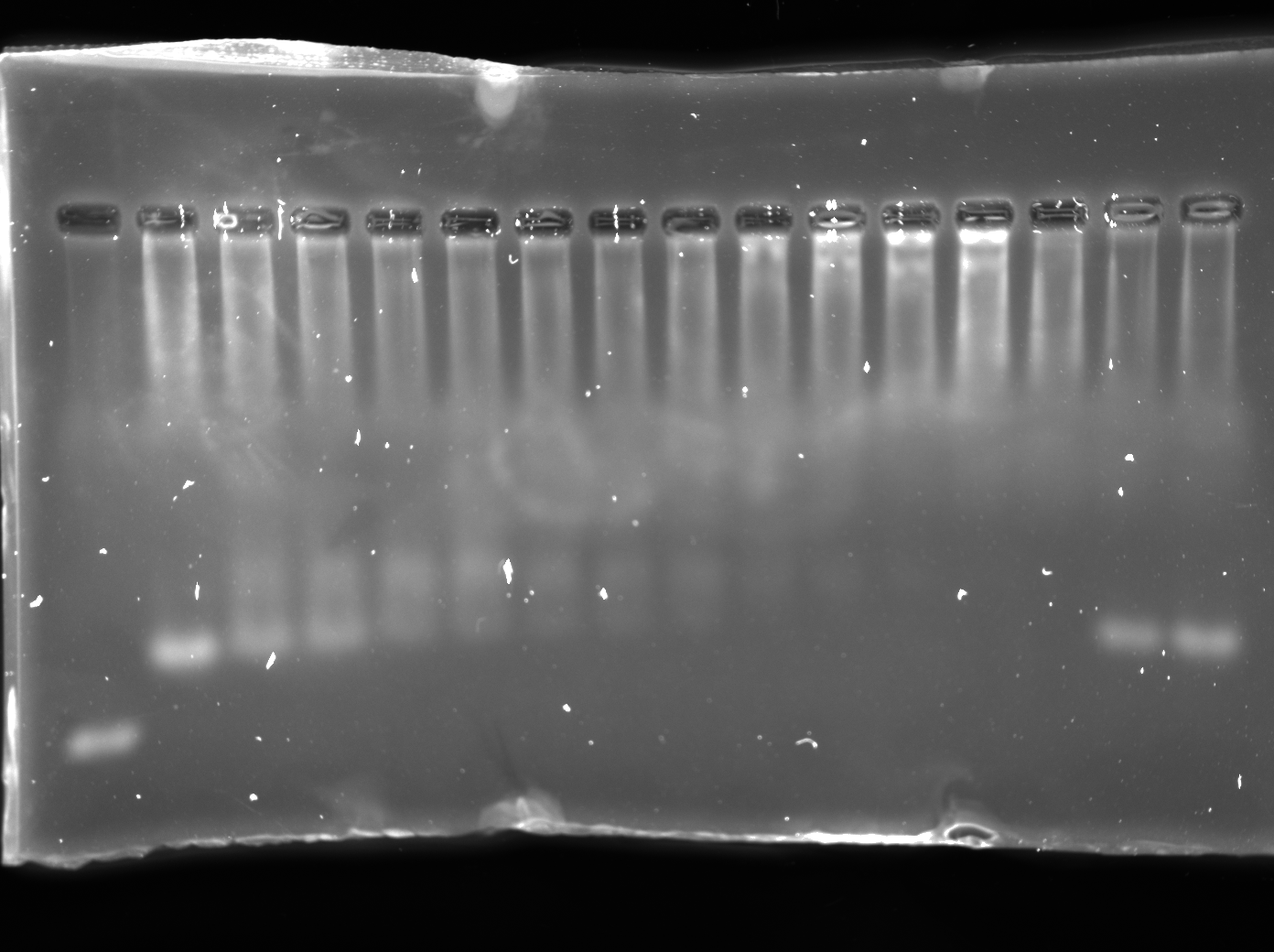

Supplement: Figure 3—figure supplement 2—source data 1. [file elife-72330-fig3-figsupp2-data1.zip › Figure 3-Sup2- source data/EMSA_Mer2_N-256+MN(WT)-167_2.tif]

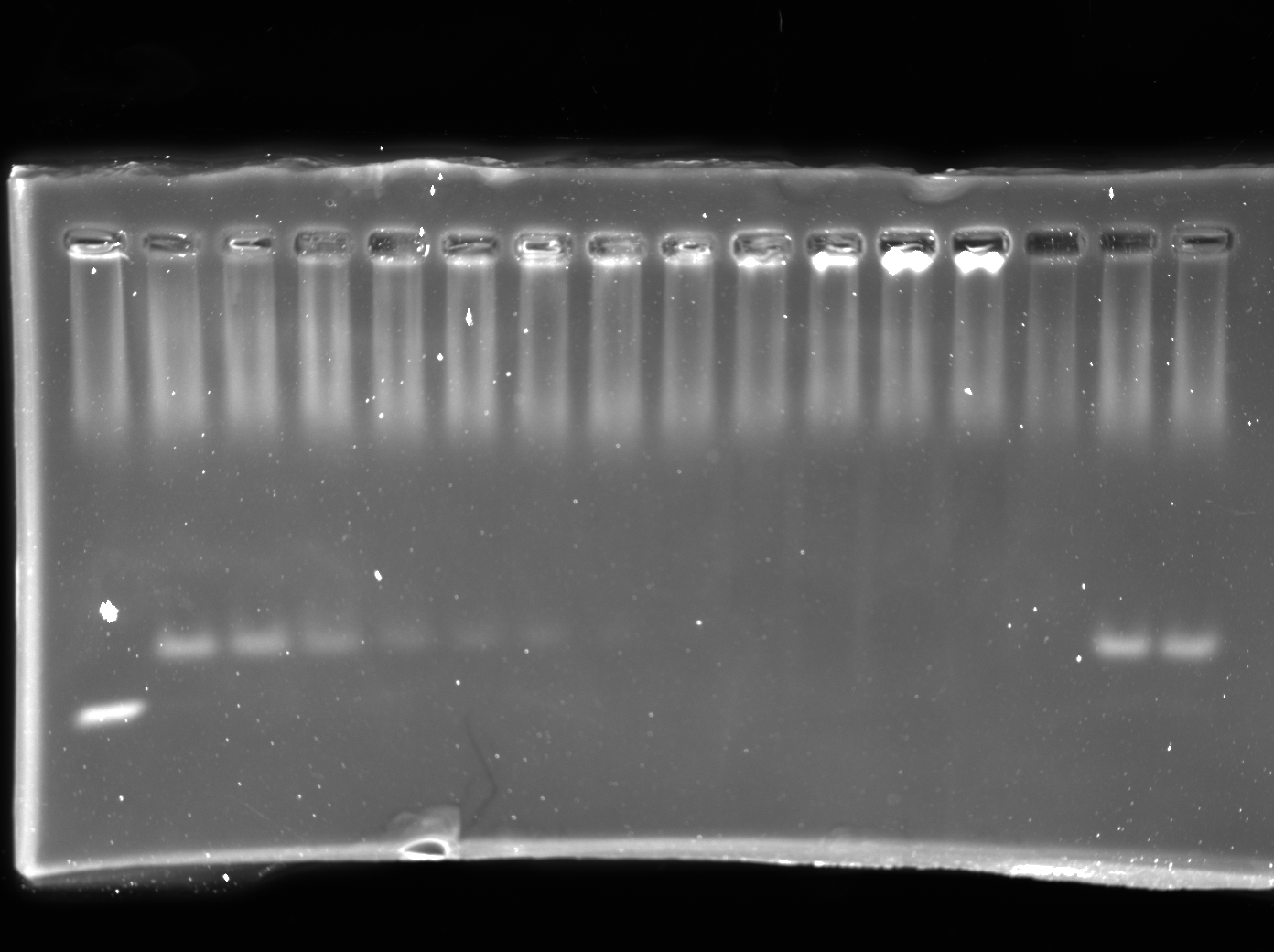

Supplement: Figure 3—figure supplement 2—source data 1. [file elife-72330-fig3-figsupp2-data1.zip › Figure 3-Sup2- source data/EMSA_Mer2_N-256+MN(WT)-167_3.tif]

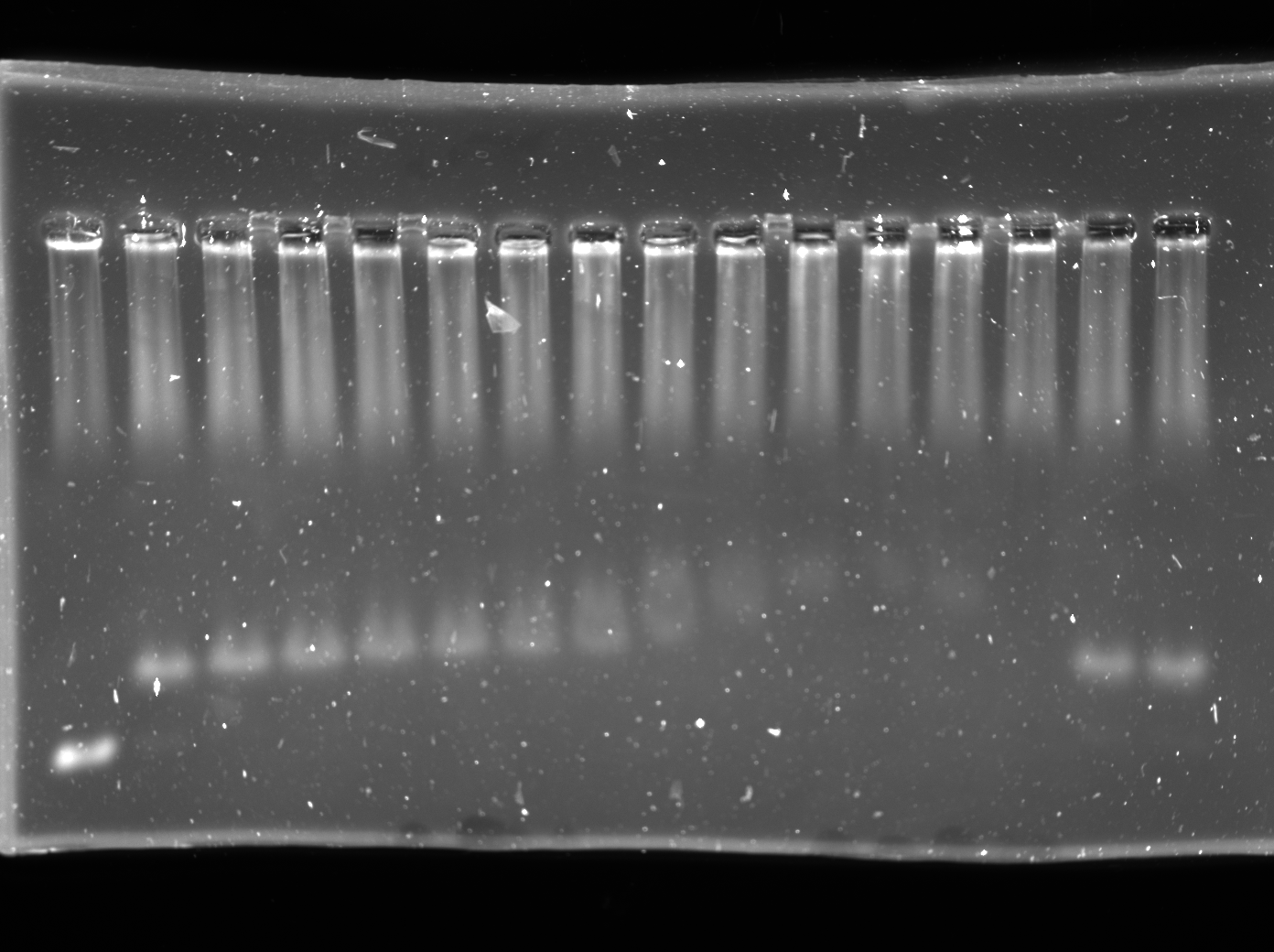

Supplement: Figure 3—figure supplement 2—source data 1. [file elife-72330-fig3-figsupp2-data1.zip › Figure 3-Sup2- source data/EMSA_Mer2_140-C+MN(WT)-167_3.tif]

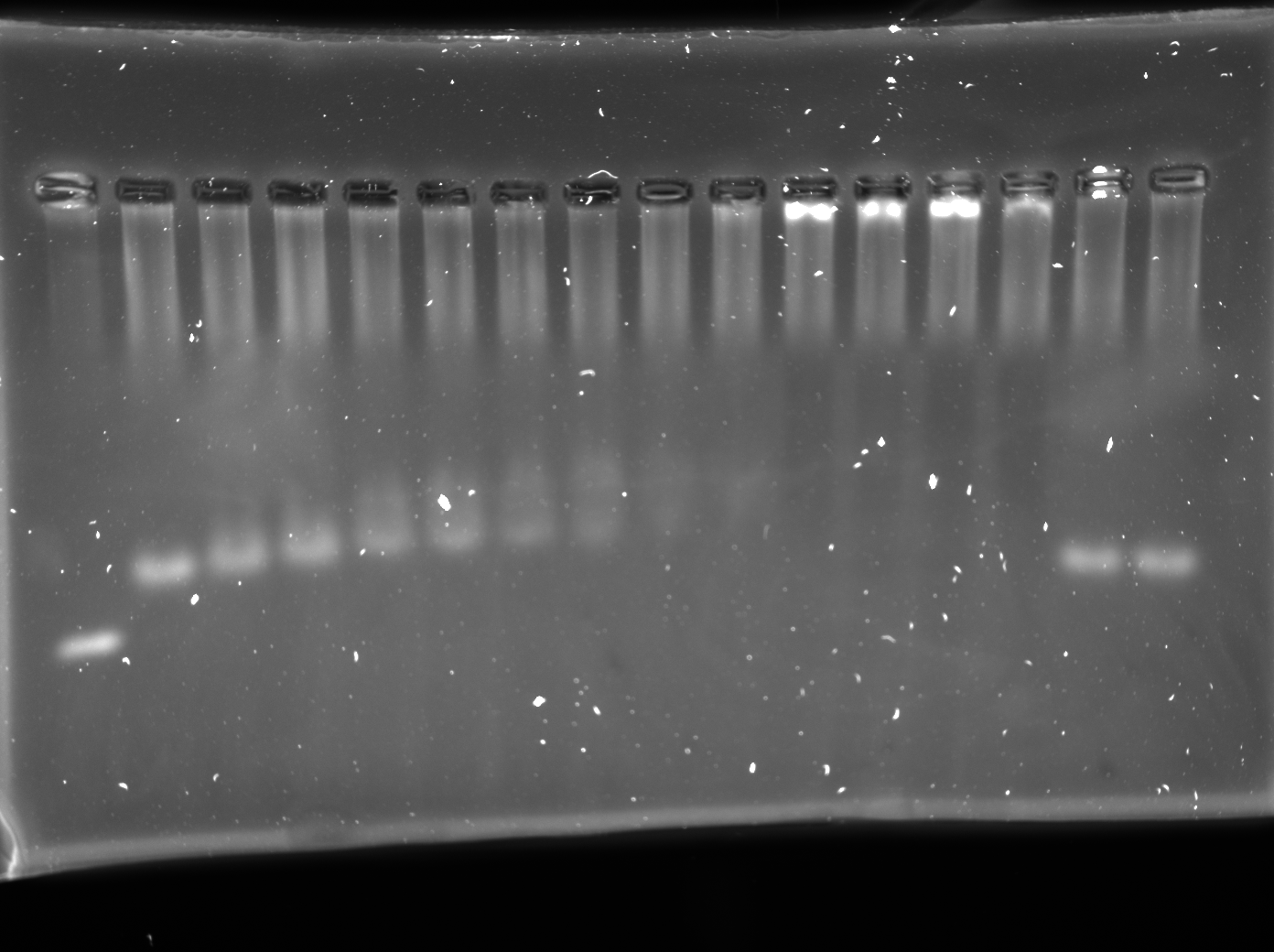

Supplement: Figure 3—figure supplement 2—source data 1. [file elife-72330-fig3-figsupp2-data1.zip › Figure 3-Sup2- source data/EMSA_Mer2_140-C+MN(WT)-167_2.tif]

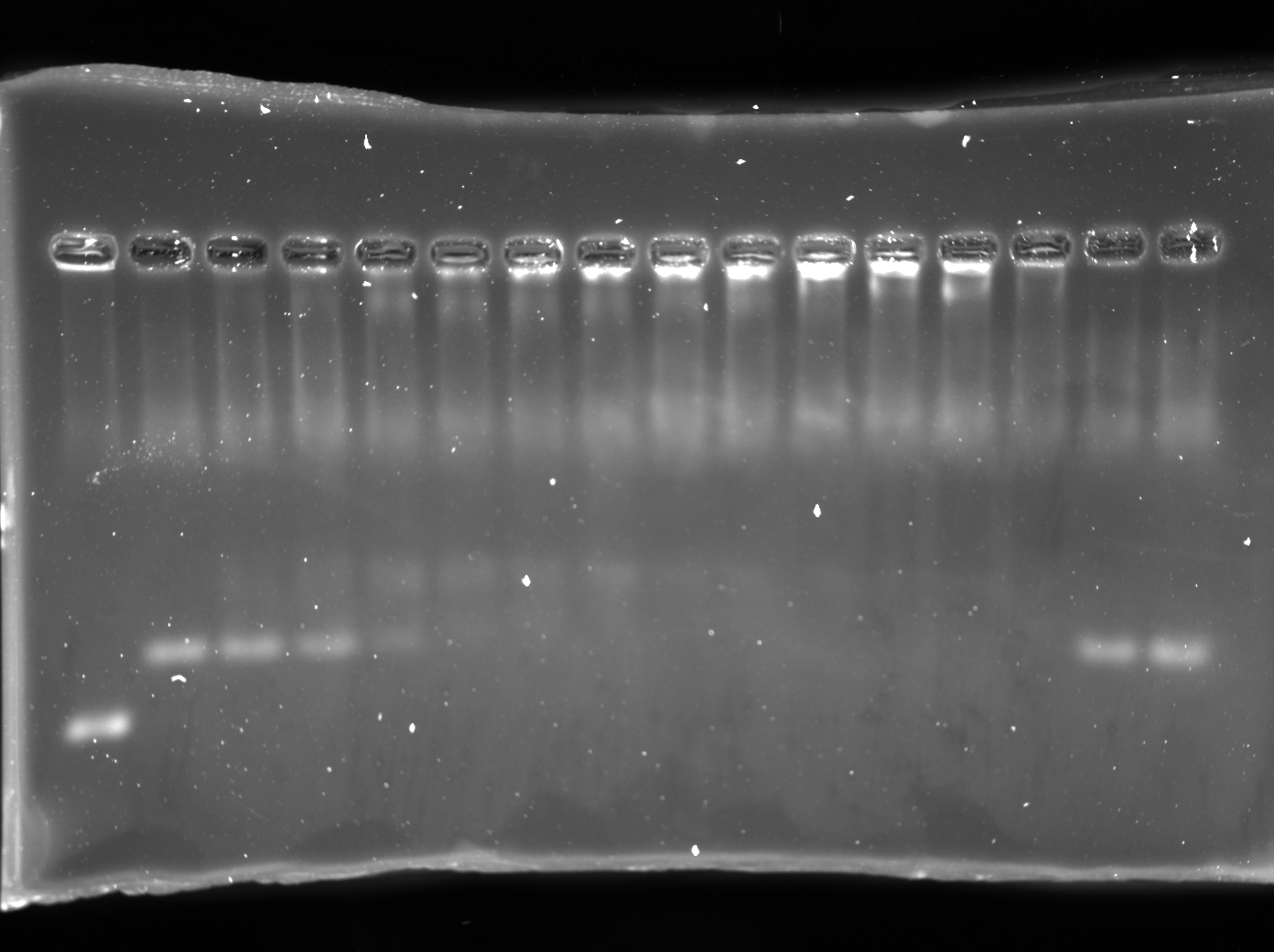

Supplement: Figure 3—figure supplement 2—source data 1. [file elife-72330-fig3-figsupp2-data1.zip › Figure 3-Sup2- source data/EMSA_Mer2_N-256+MN(WT)-167_4.tif]

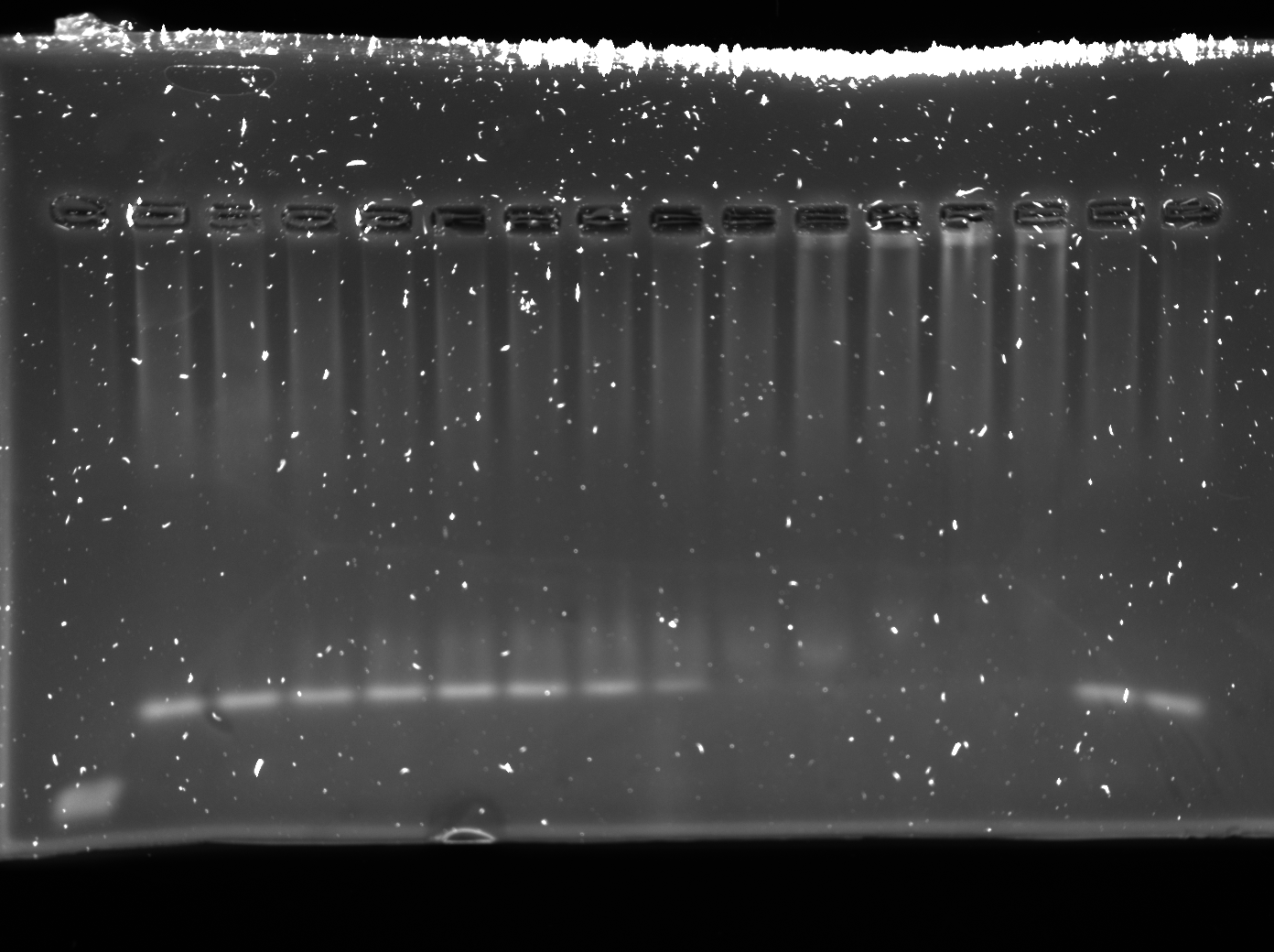

Supplement: Figure 3—figure supplement 2—source data 1. [file elife-72330-fig3-figsupp2-data1.zip › Figure 3-Sup2- source data/EMSA_Mer2_140-C+MN(WT)-167_1.tif]

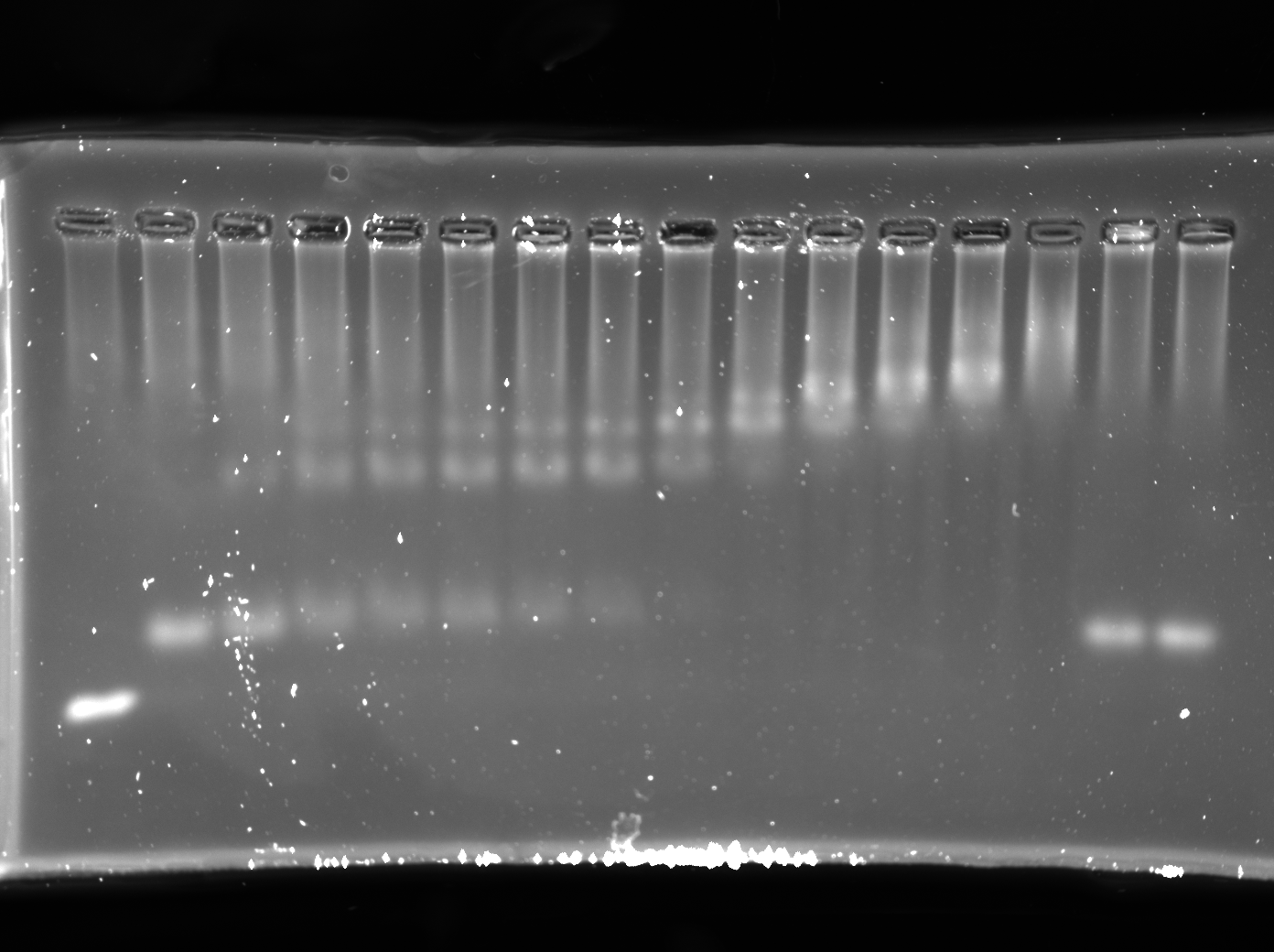

Supplement: Figure 3—figure supplement 2—source data 1. [file elife-72330-fig3-figsupp2-data1.zip › Figure 3-Sup2- source data/EMSA_Mer2_3A+MN(WT)-167_1.tif]

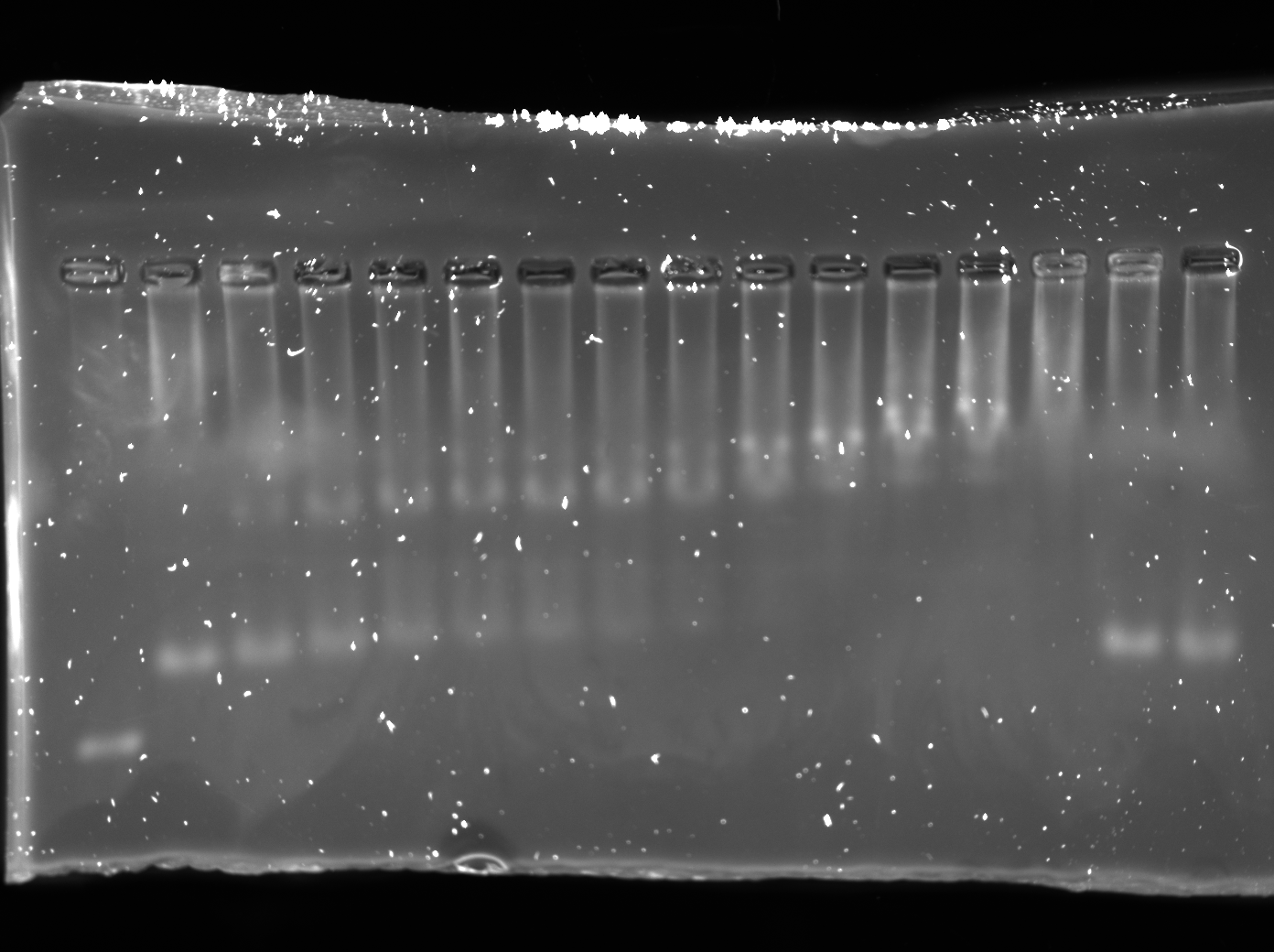

Supplement: Figure 3—figure supplement 2—source data 1. [file elife-72330-fig3-figsupp2-data1.zip › Figure 3-Sup2- source data/EMSA_Mer2_3A+MN(WT)-167_3.tif]

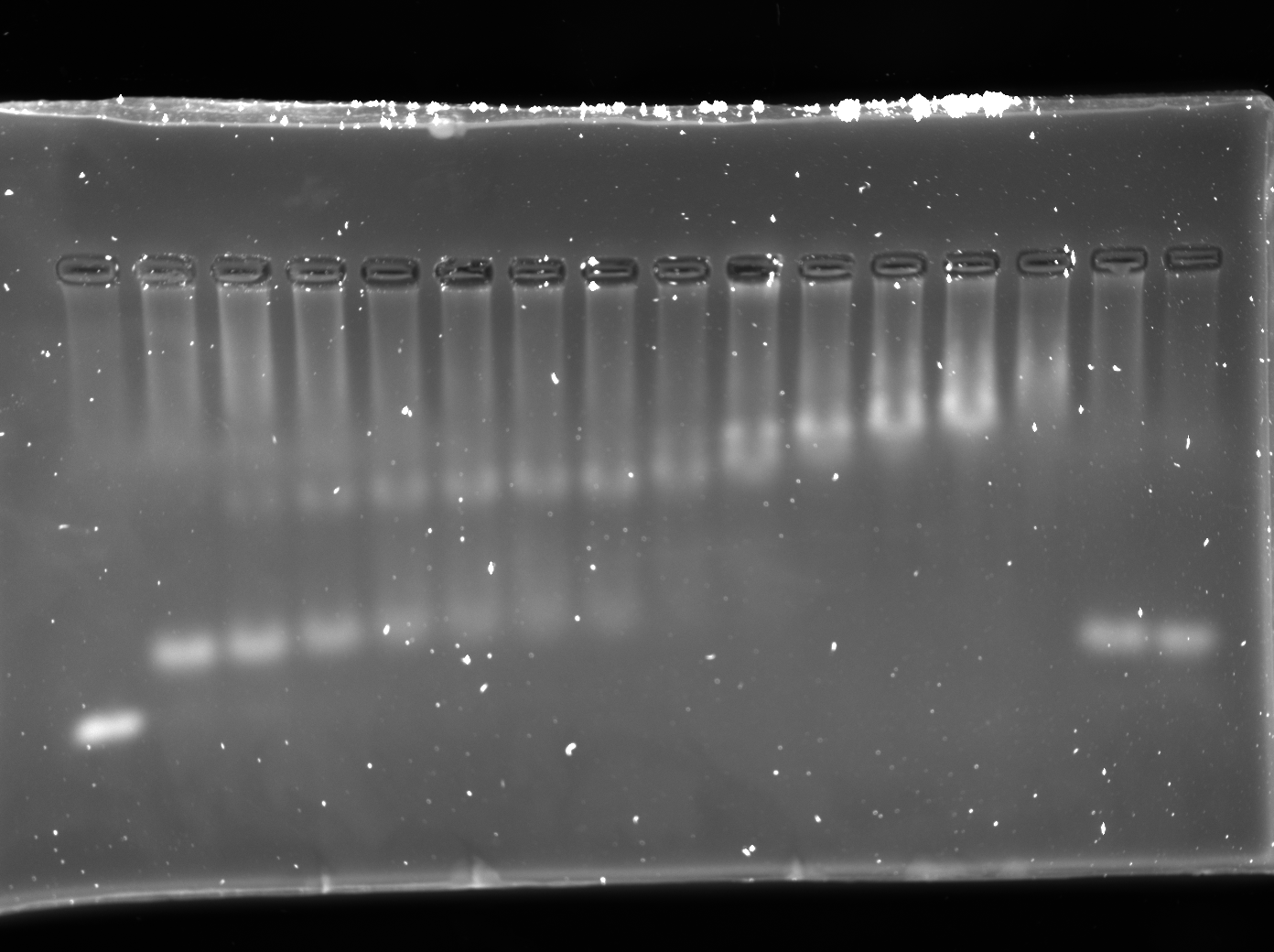

Supplement: Figure 3—figure supplement 2—source data 1. [file elife-72330-fig3-figsupp2-data1.zip › Figure 3-Sup2- source data/EMSA_Mer2_3A+MN(WT)-167_2.tif]

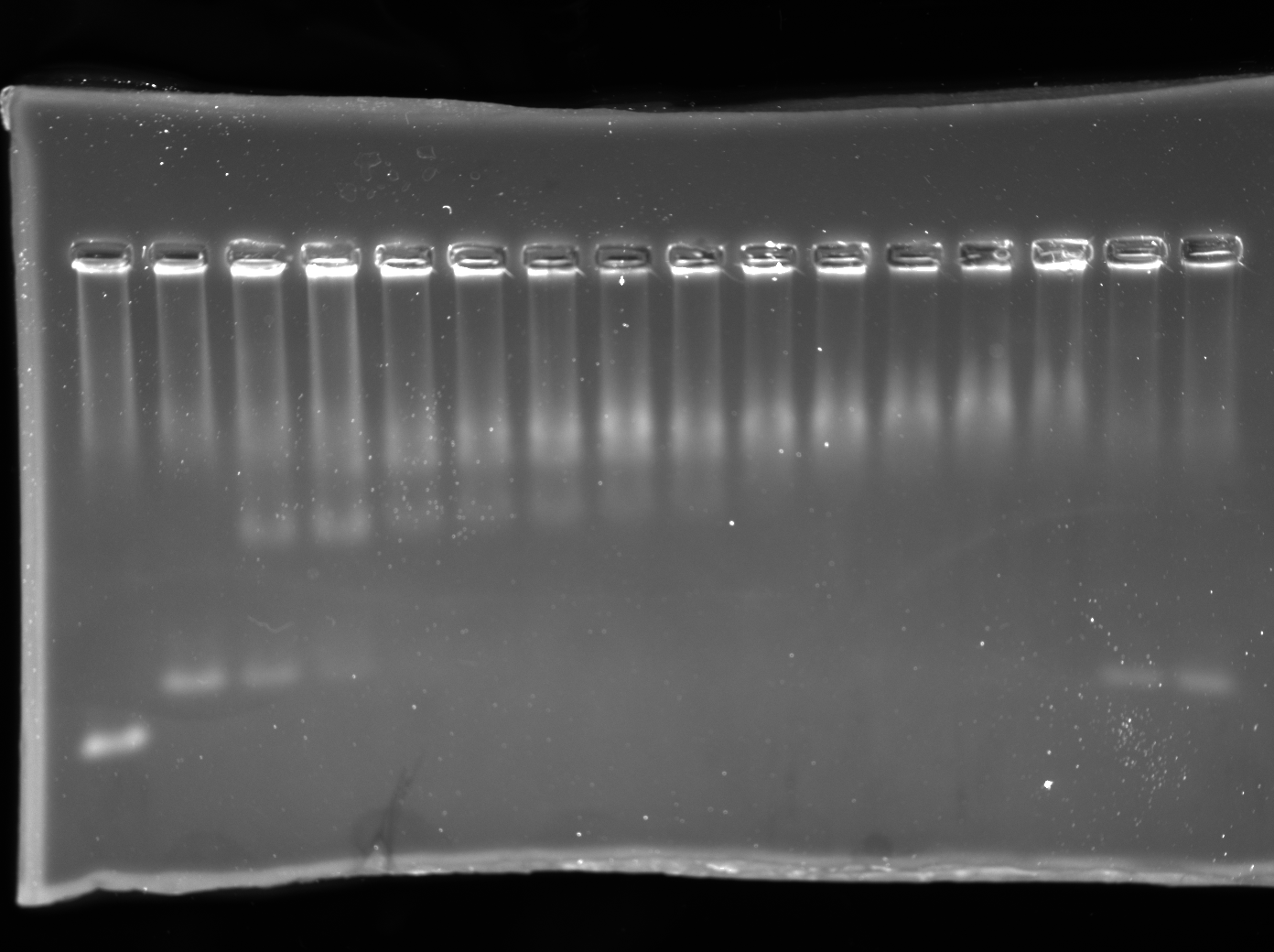

Supplement: Figure 3—figure supplement 2—source data 1. [file elife-72330-fig3-figsupp2-data1.zip › Figure 3-Sup2- source data/EMSA_Mer2_FL+MN(tailless)-147_2.tif]

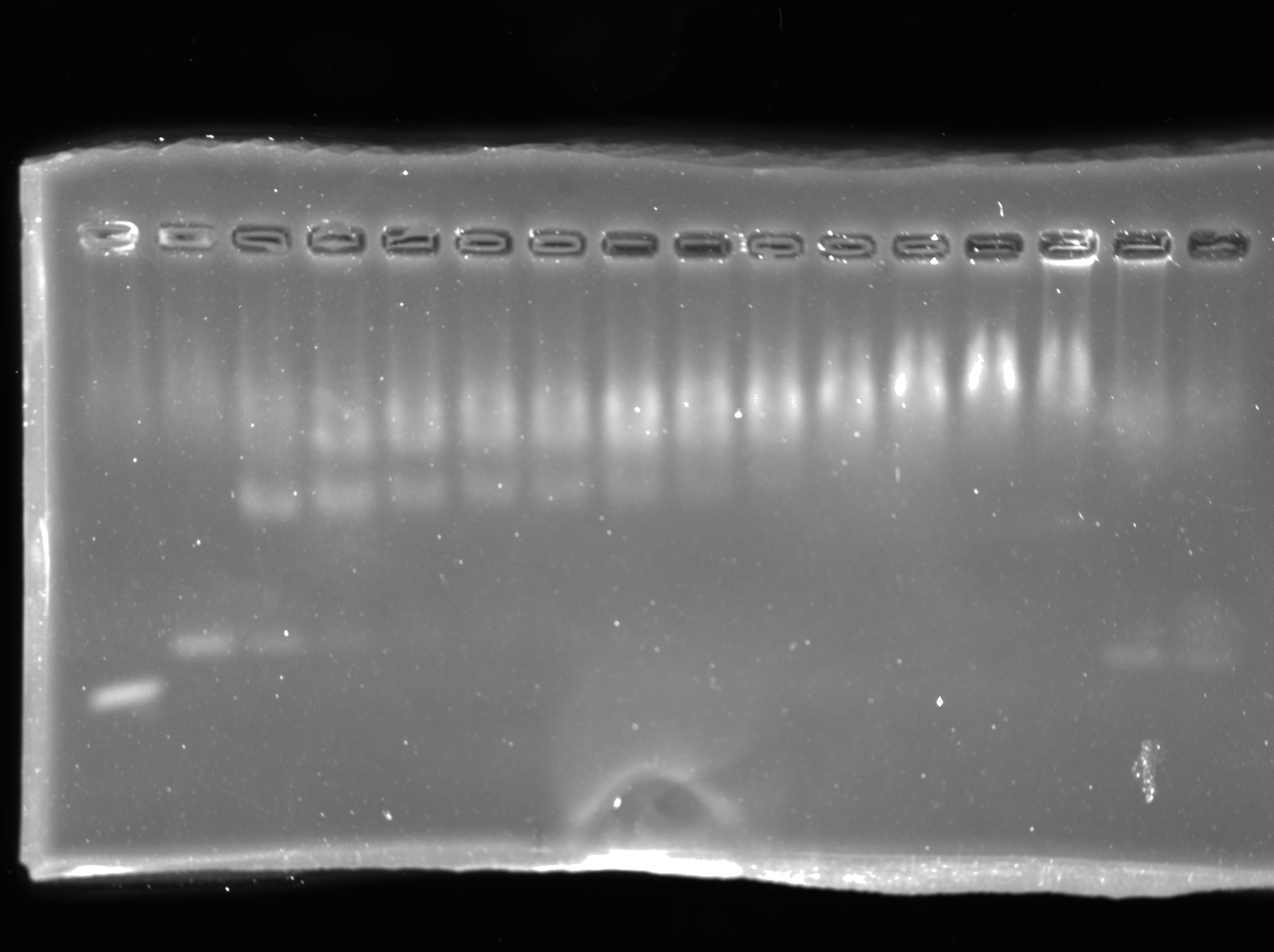

Supplement: Figure 3—figure supplement 2—source data 1. [file elife-72330-fig3-figsupp2-data1.zip › Figure 3-Sup2- source data/EMSA_Mer2_FL+MN(tailless)-147_3.tif]

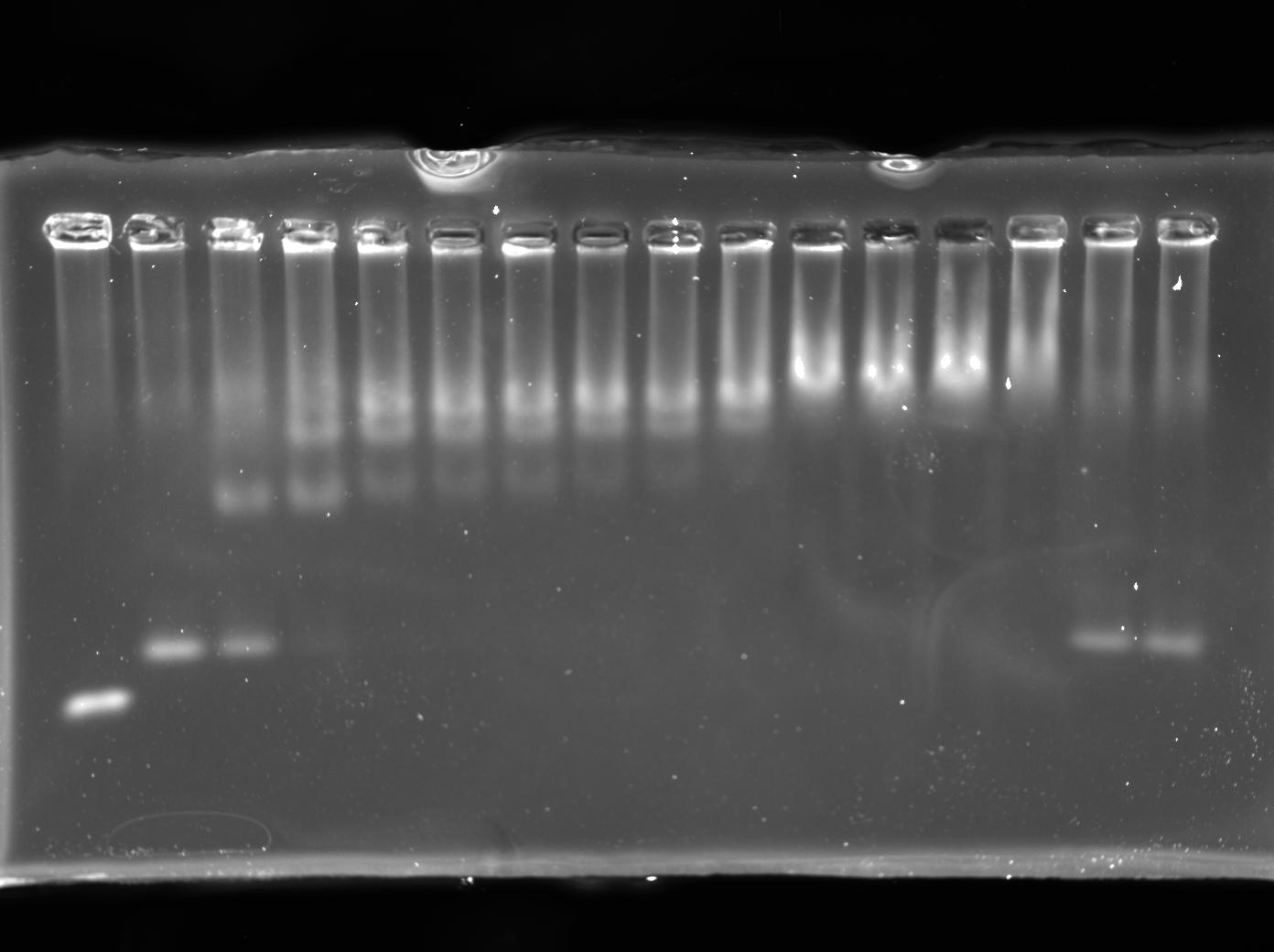

Supplement: Figure 3—figure supplement 2—source data 1. [file elife-72330-fig3-figsupp2-data1.zip › Figure 3-Sup2- source data/EMSA_Mer2_FL+MN(tailless)-147_1.tif]

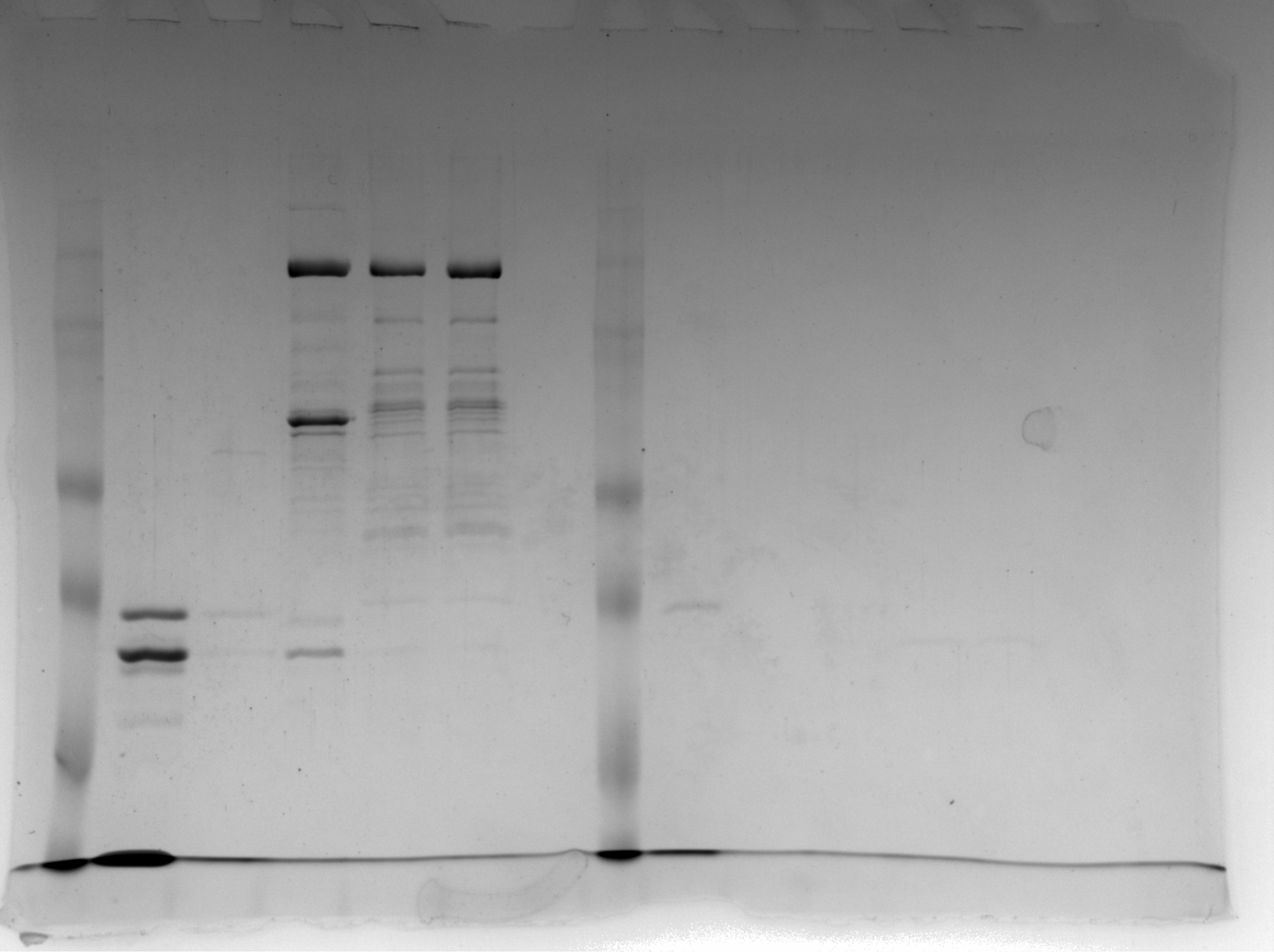

Supplement: Figure 4—source data 1. [file elife-72330-fig4-data1.zip › Figure 4-source data/Gel_Pulldown_Red1-pulldowns_elution.tif]

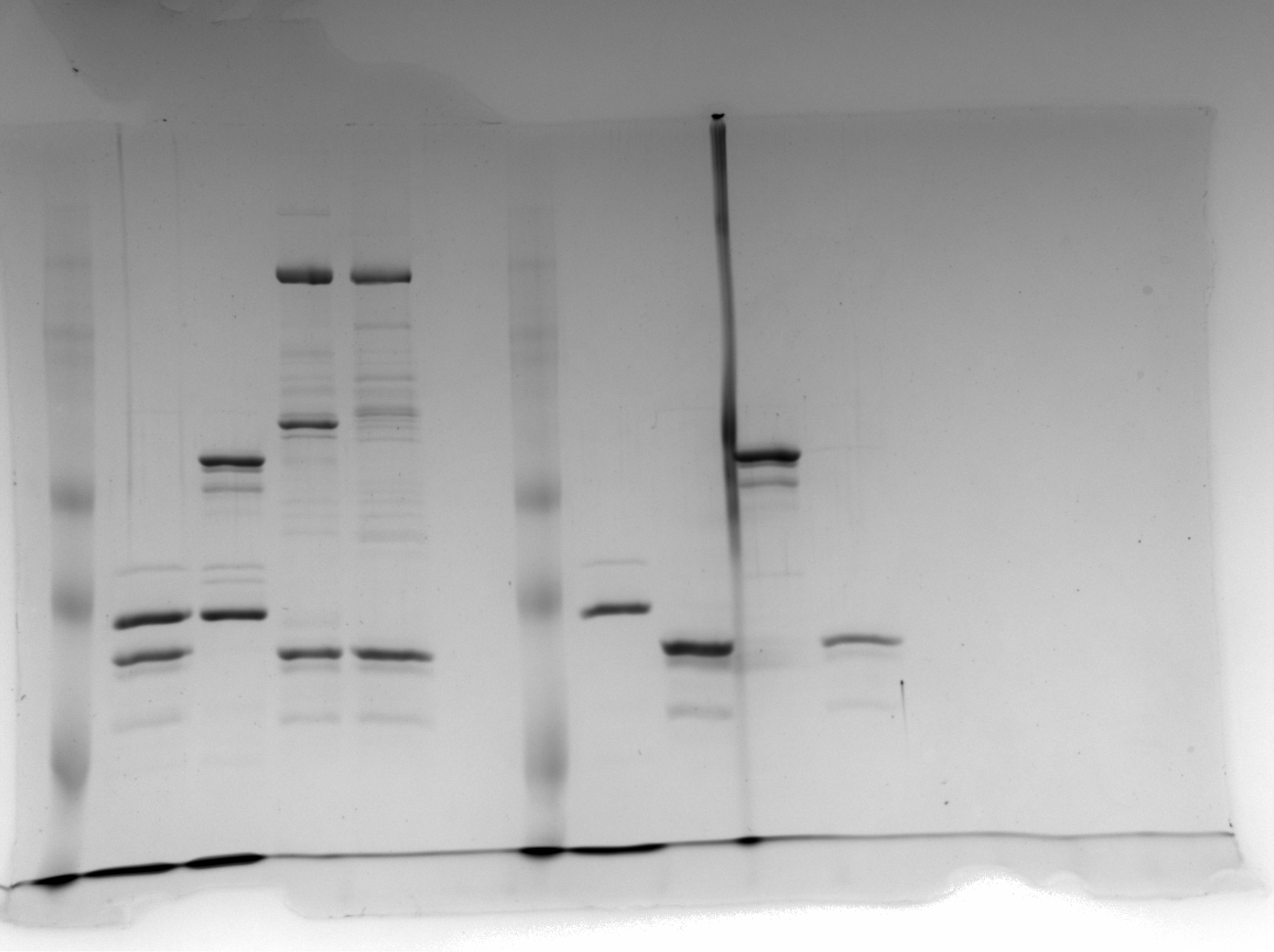

Supplement: Figure 4—source data 1. [file elife-72330-fig4-data1.zip › Figure 4-source data/Gel_Pulldown_Red1-pulldowns_input.tif]

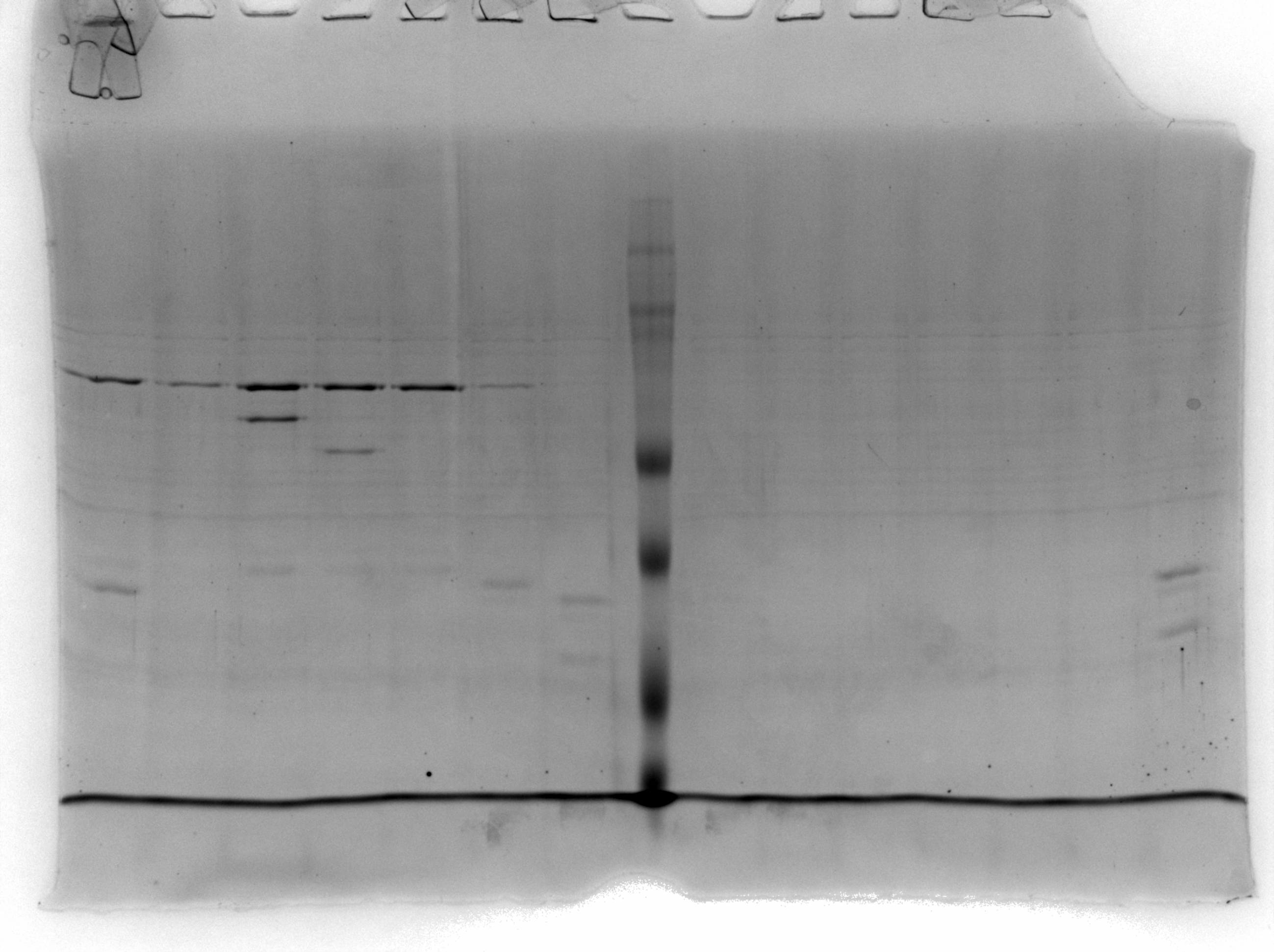

Supplement: Figure 4—source data 1. [file elife-72330-fig4-data1.zip › Figure 4-source data/Gel_Pulldown_Hop1_K593A+Mer2_elution.tif]

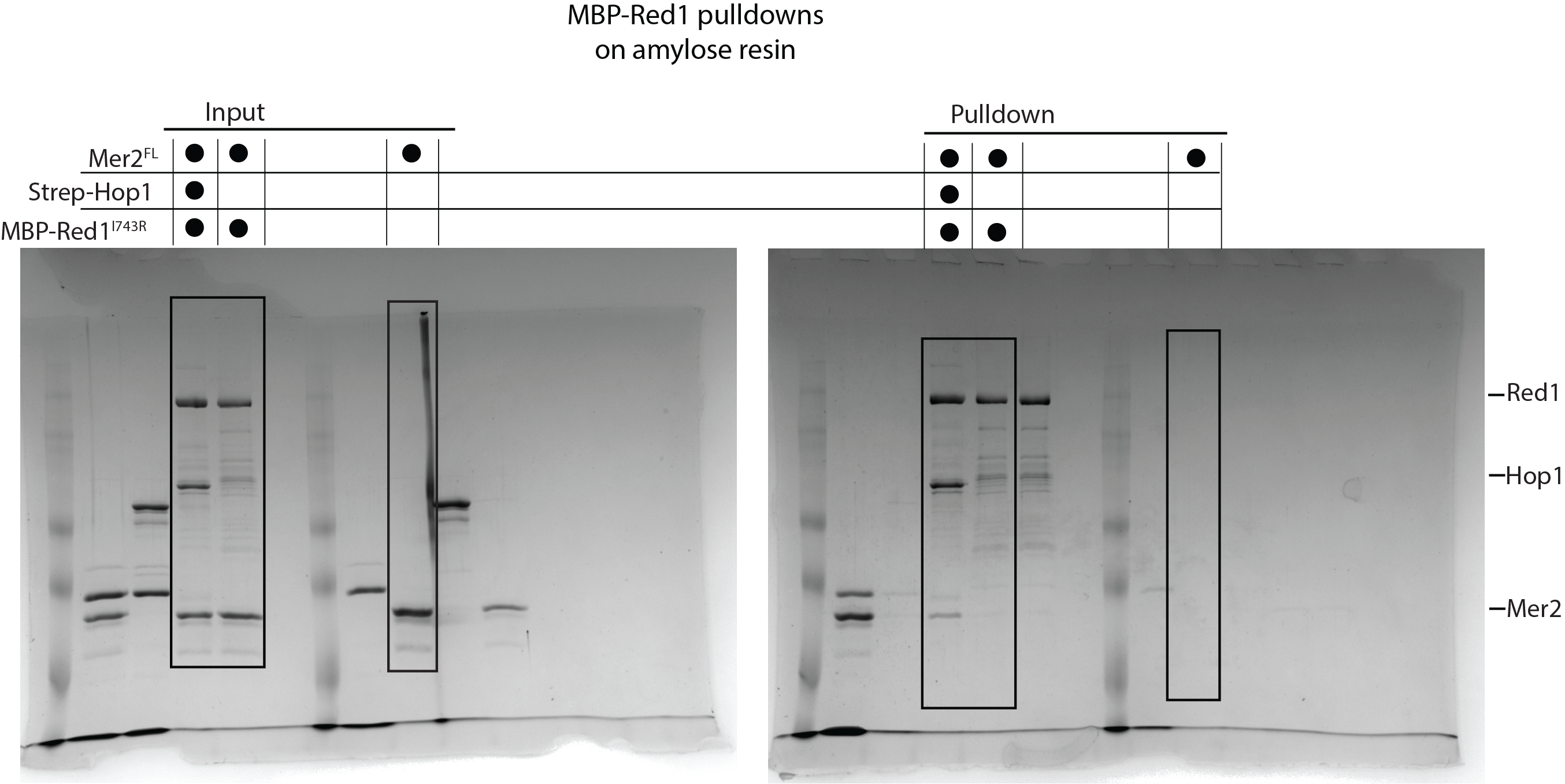

Supplement: Figure 4—source data 1. [file elife-72330-fig4-data1.zip › Figure 4-source data/Pulldown_Red1-pulldowns.png]

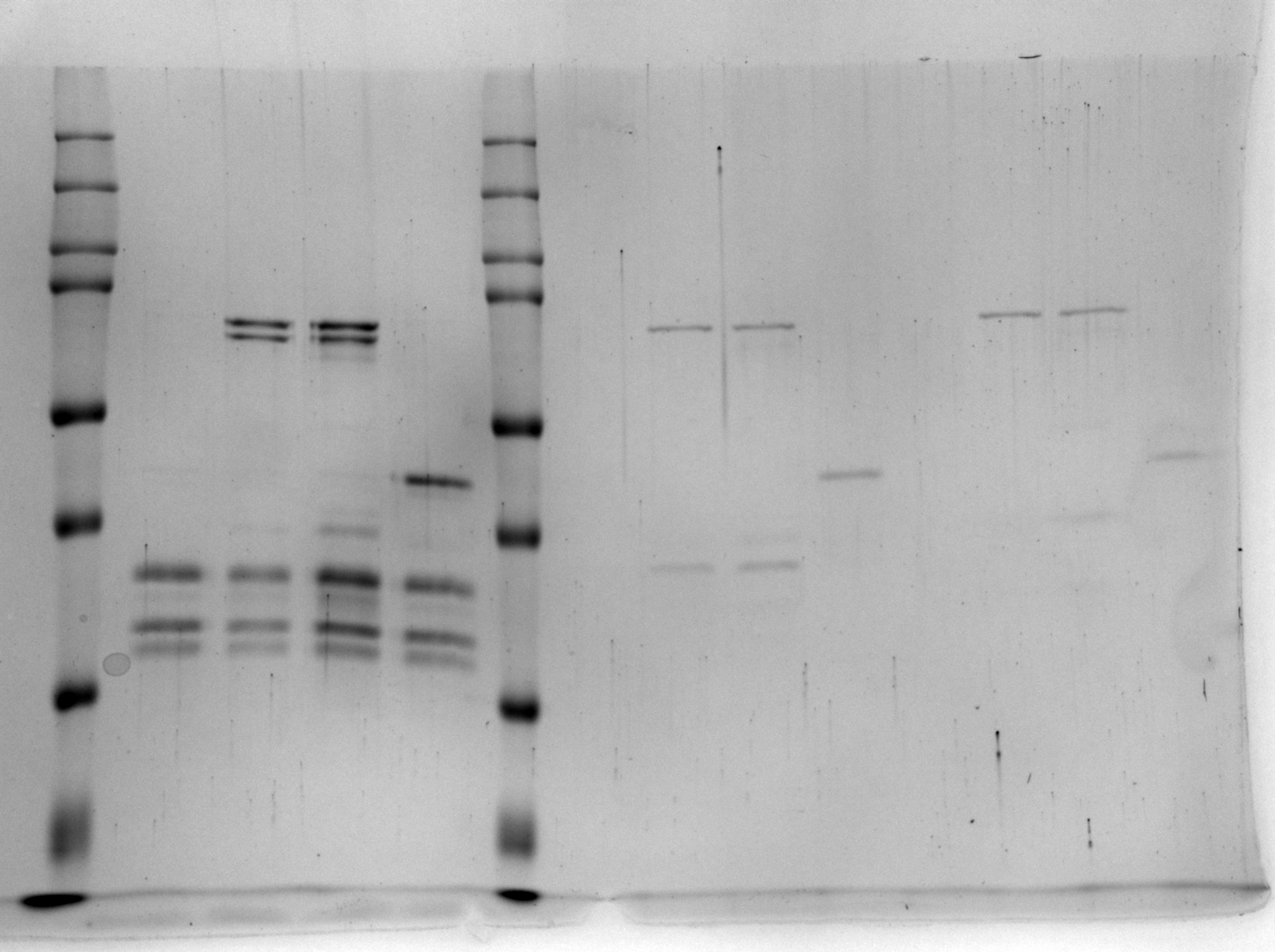

Supplement: Figure 4—source data 1. [file elife-72330-fig4-data1.zip › Figure 4-source data/Gel_Pulldown_Mer2+Hop1_6.tif]

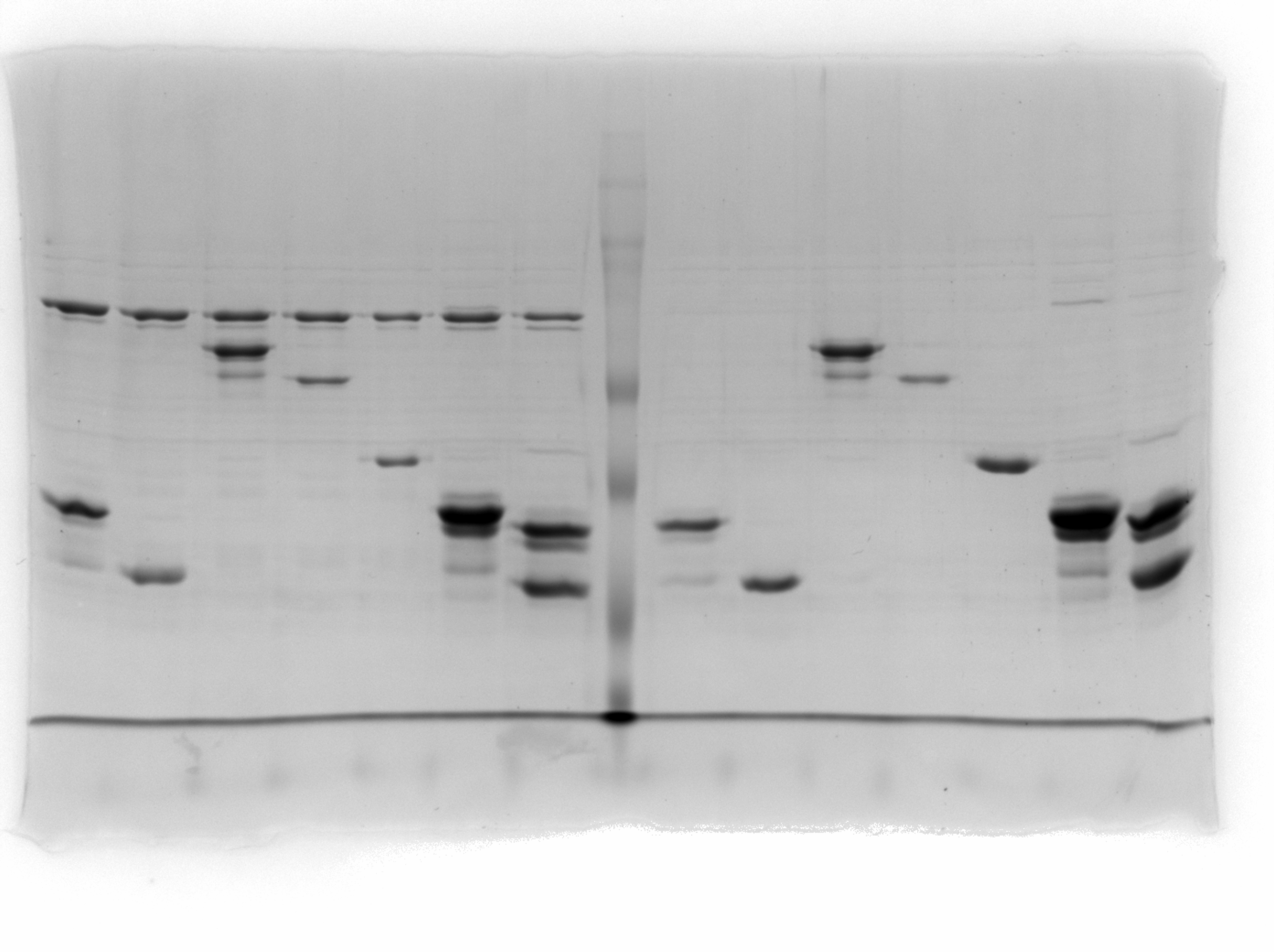

Supplement: Figure 4—source data 1. [file elife-72330-fig4-data1.zip › Figure 4-source data/Gel_Pulldown_Hop1_K593A+Mer2_input.tif]

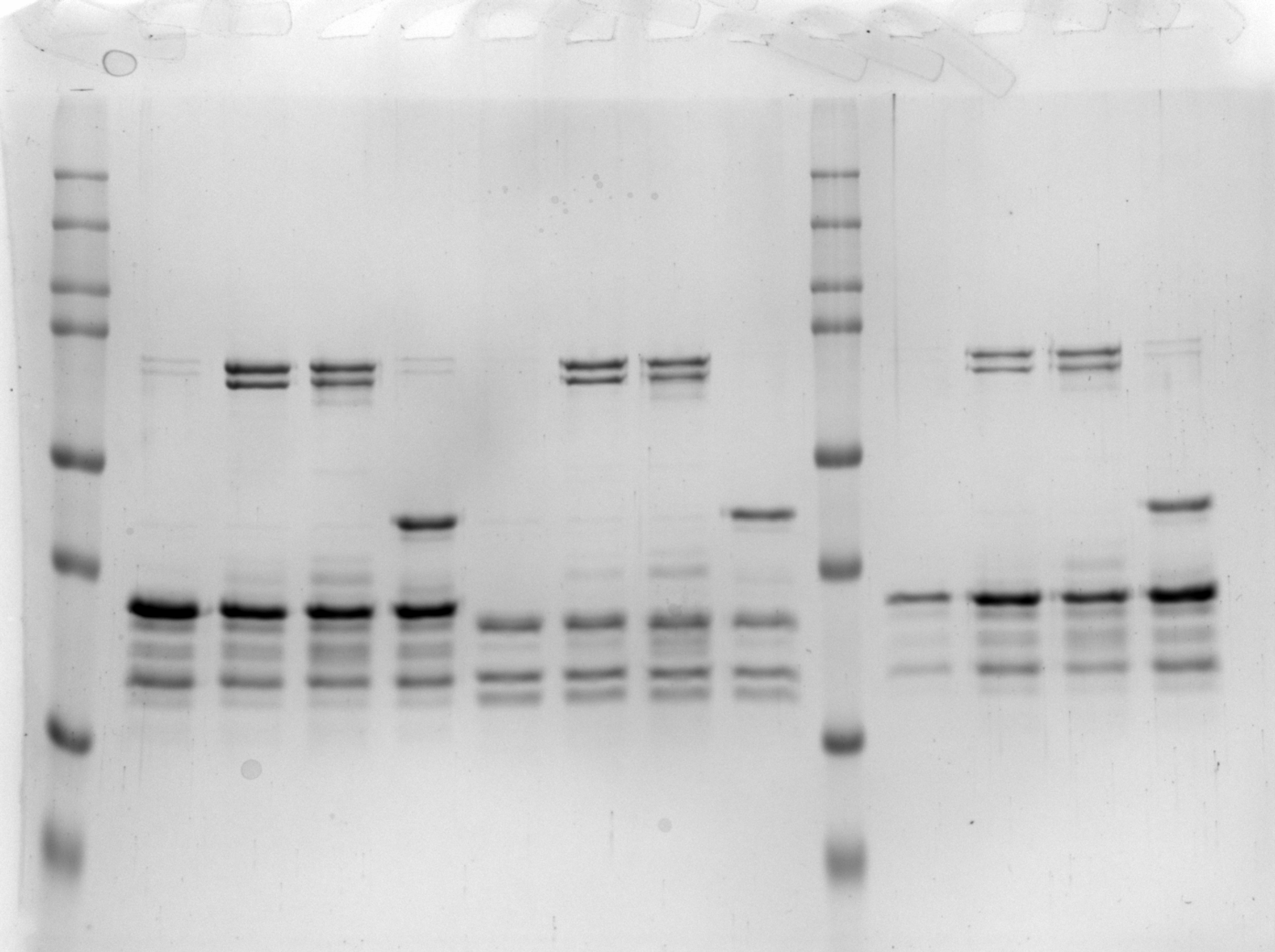

Supplement: Figure 4—source data 1. [file elife-72330-fig4-data1.zip › Figure 4-source data/Gel_Pulldown_Mer2+Hop1_5.tif]

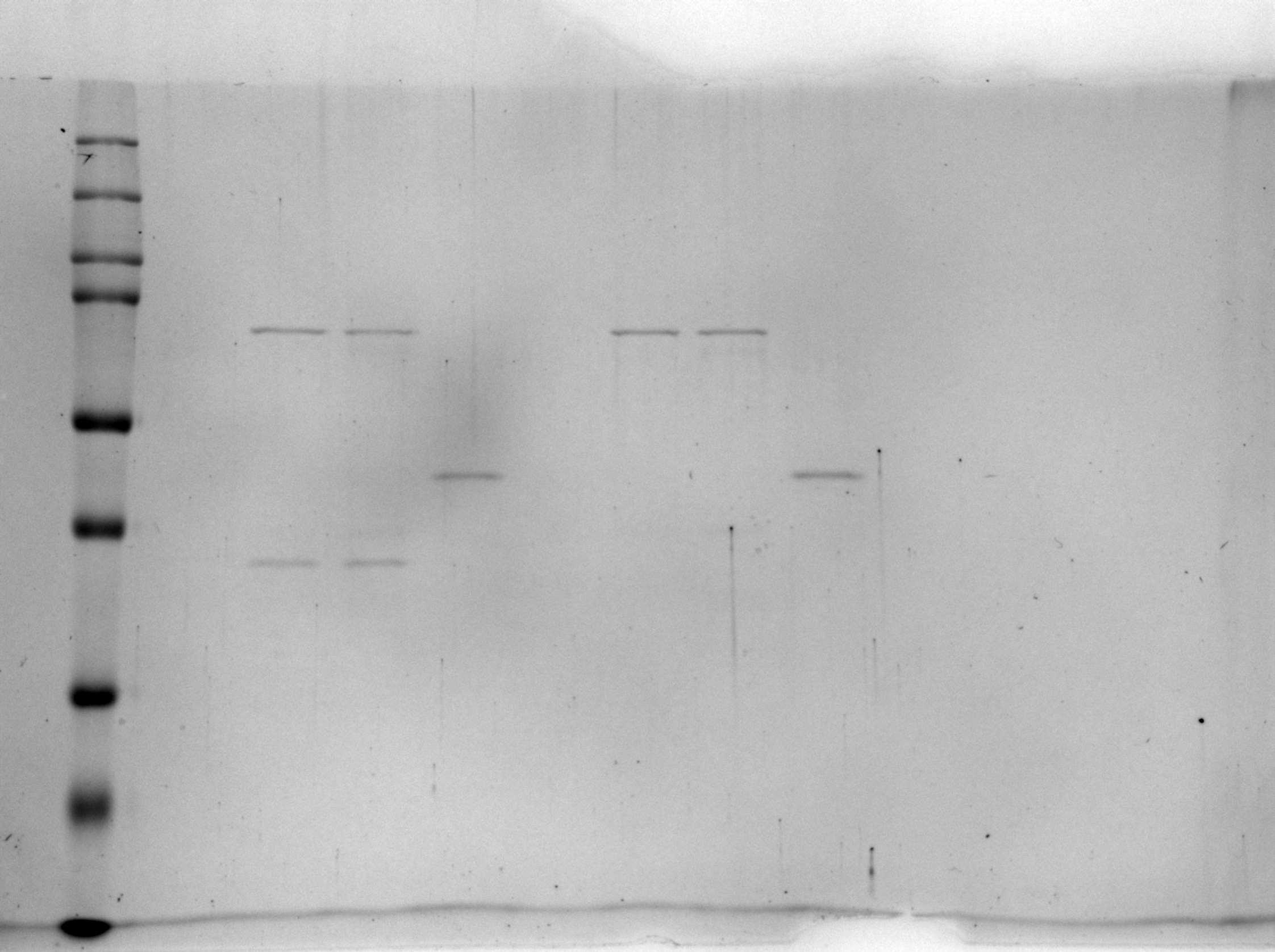

Supplement: Figure 4—source data 1. [file elife-72330-fig4-data1.zip › Figure 4-source data/Gel_Pulldown_Mer2+Hop1_4.tif]

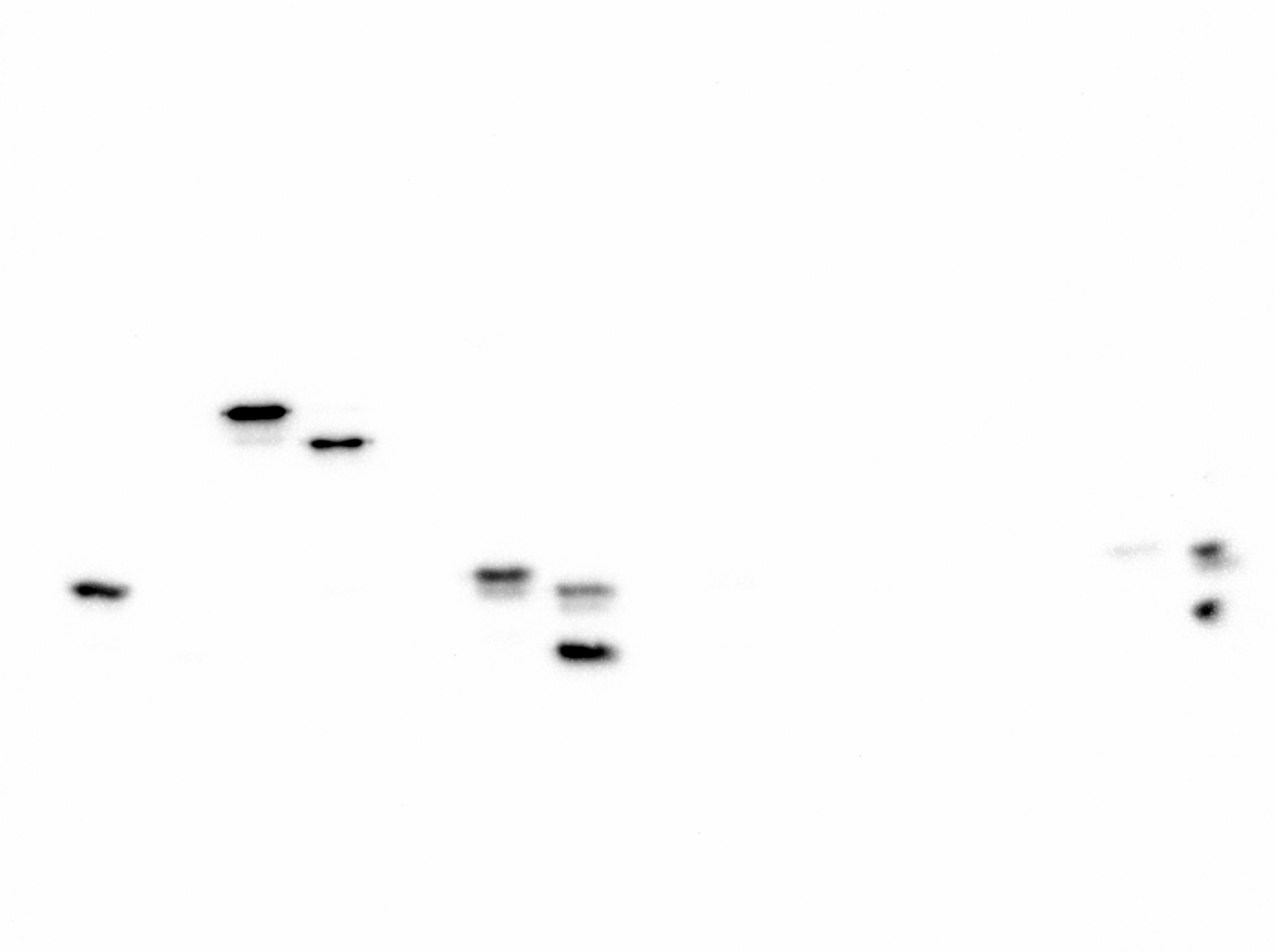

Supplement: Figure 4—source data 1. [file elife-72330-fig4-data1.zip › Figure 4-source data/Western_Pulldown_Hop1_K593A+Mer2.tif]

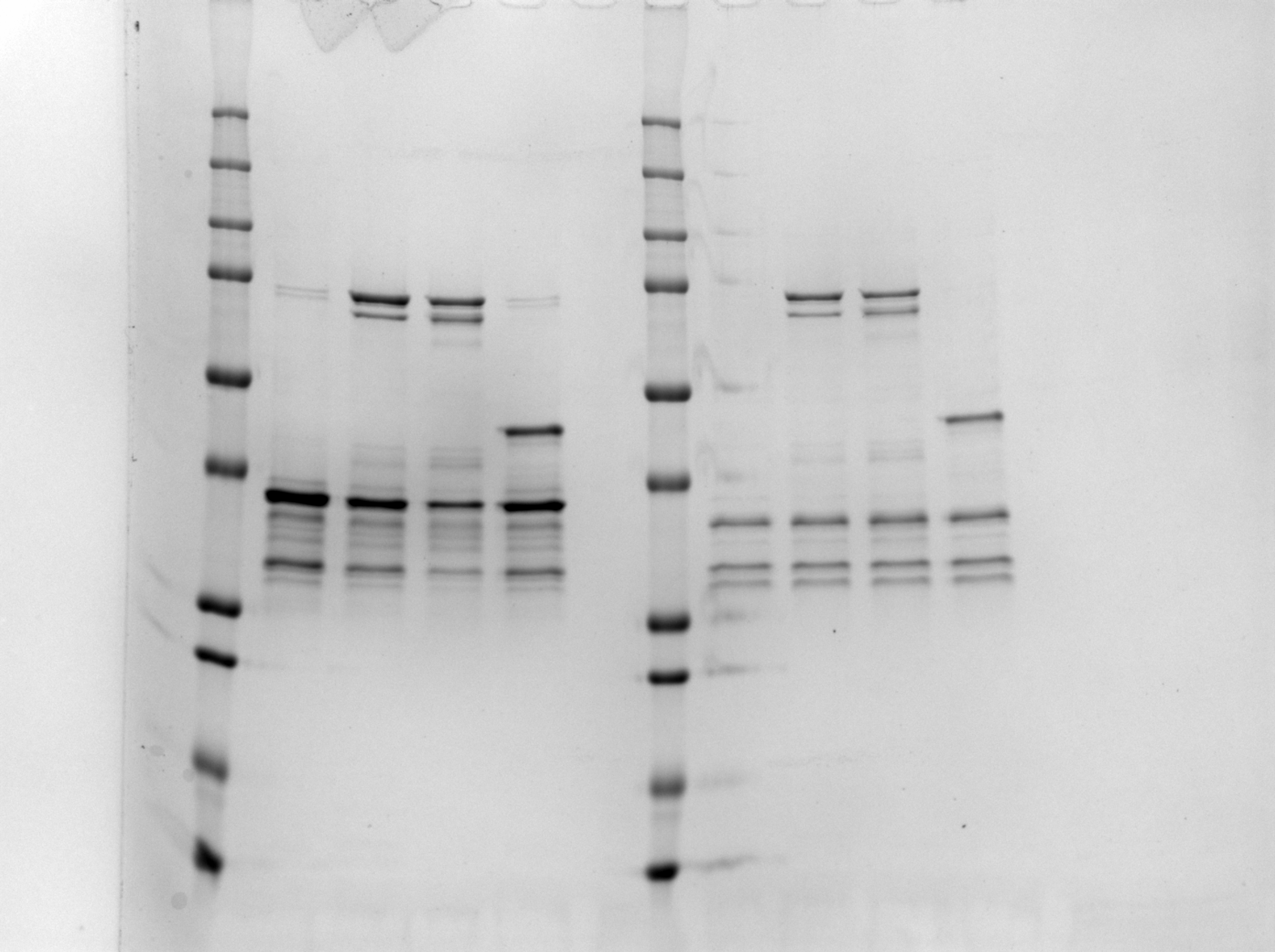

Supplement: Figure 4—source data 1. [file elife-72330-fig4-data1.zip › Figure 4-source data/Gel_Pulldown_Mer2+Hop1_1.tif]

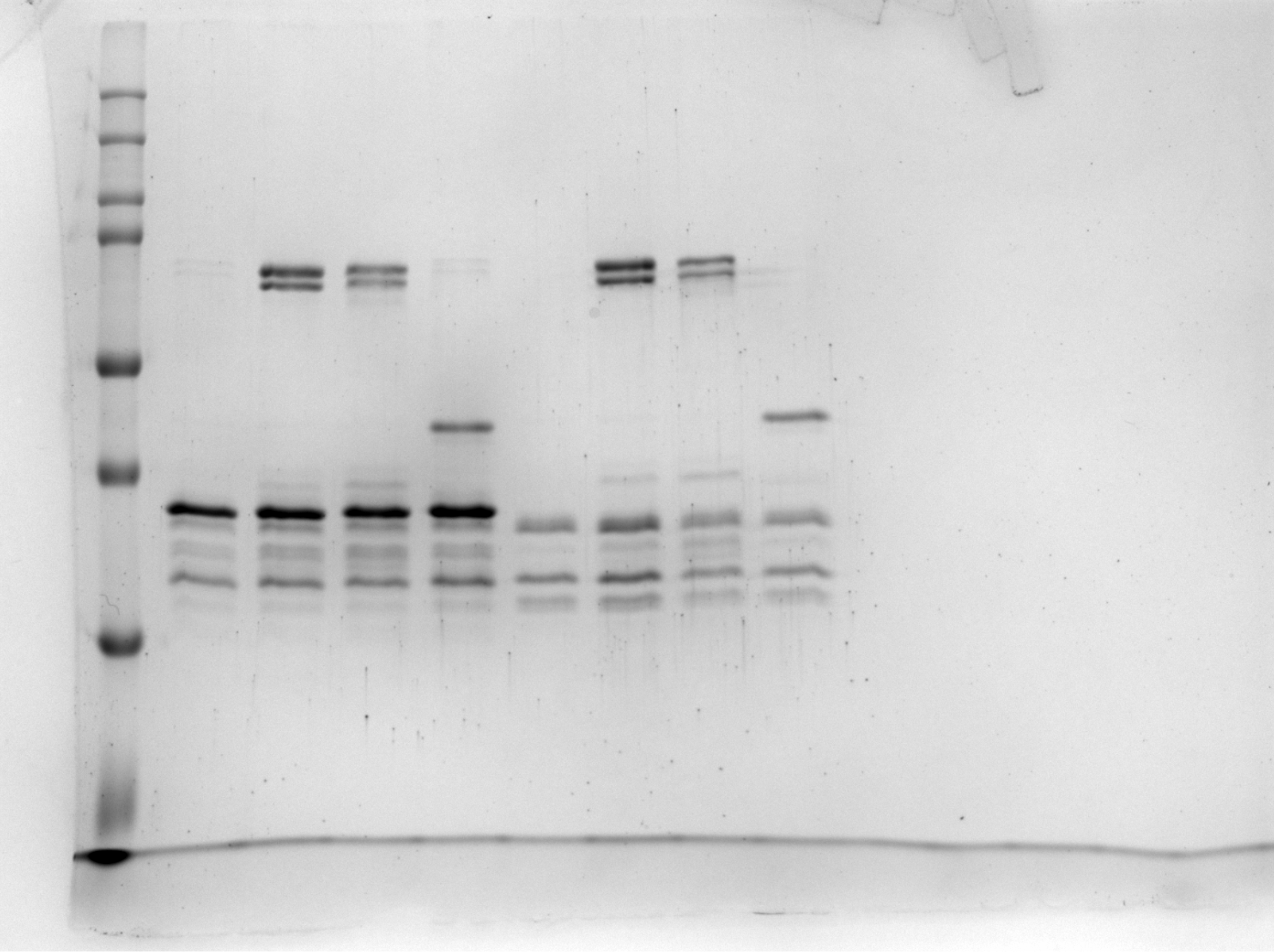

Supplement: Figure 4—source data 1. [file elife-72330-fig4-data1.zip › Figure 4-source data/Gel_Pulldown_Mer2+Hop1_3.tif]

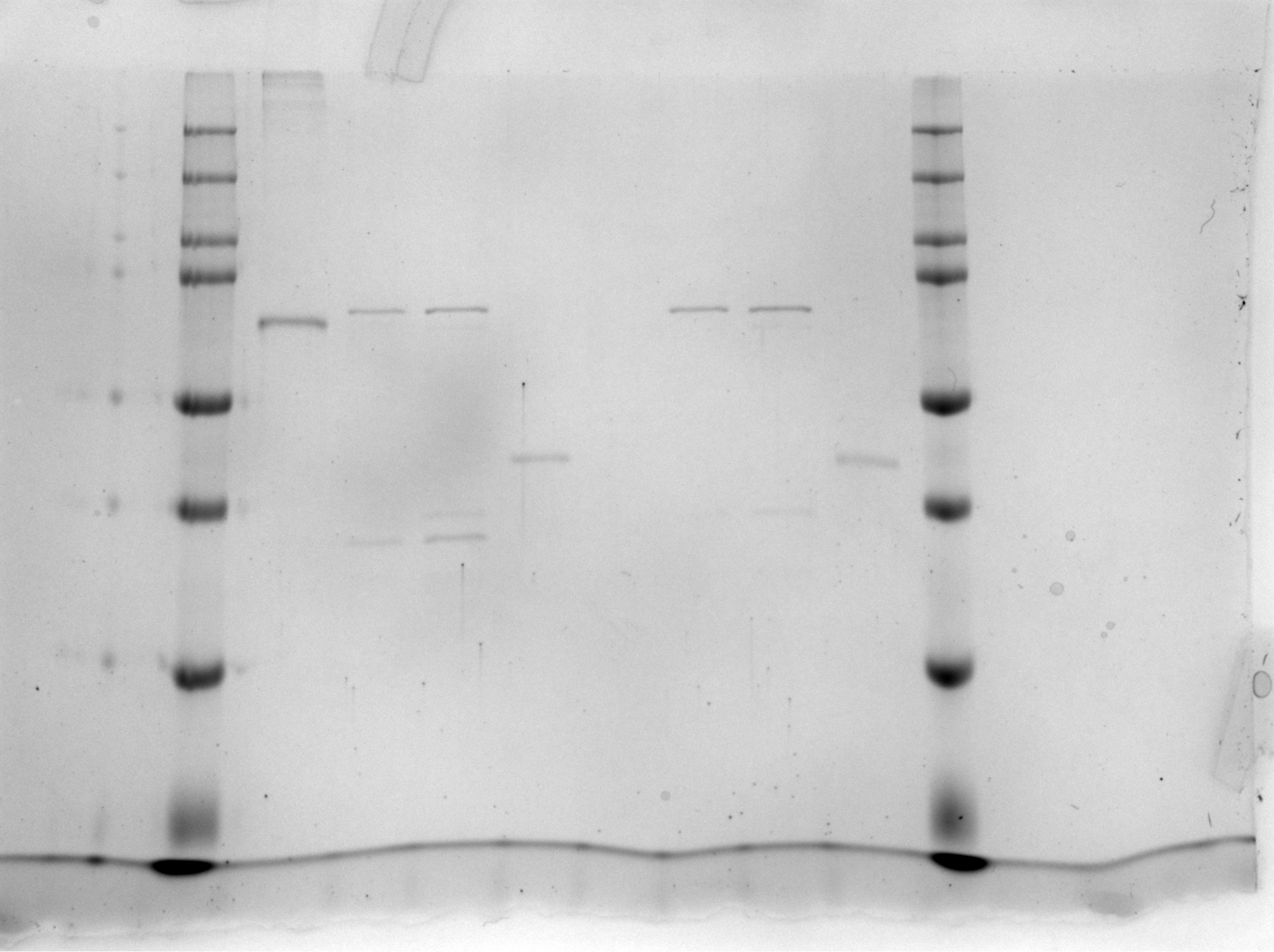

Supplement: Figure 4—source data 1. [file elife-72330-fig4-data1.zip › Figure 4-source data/Gel_Pulldown_Mer2+Hop1_2.tif]

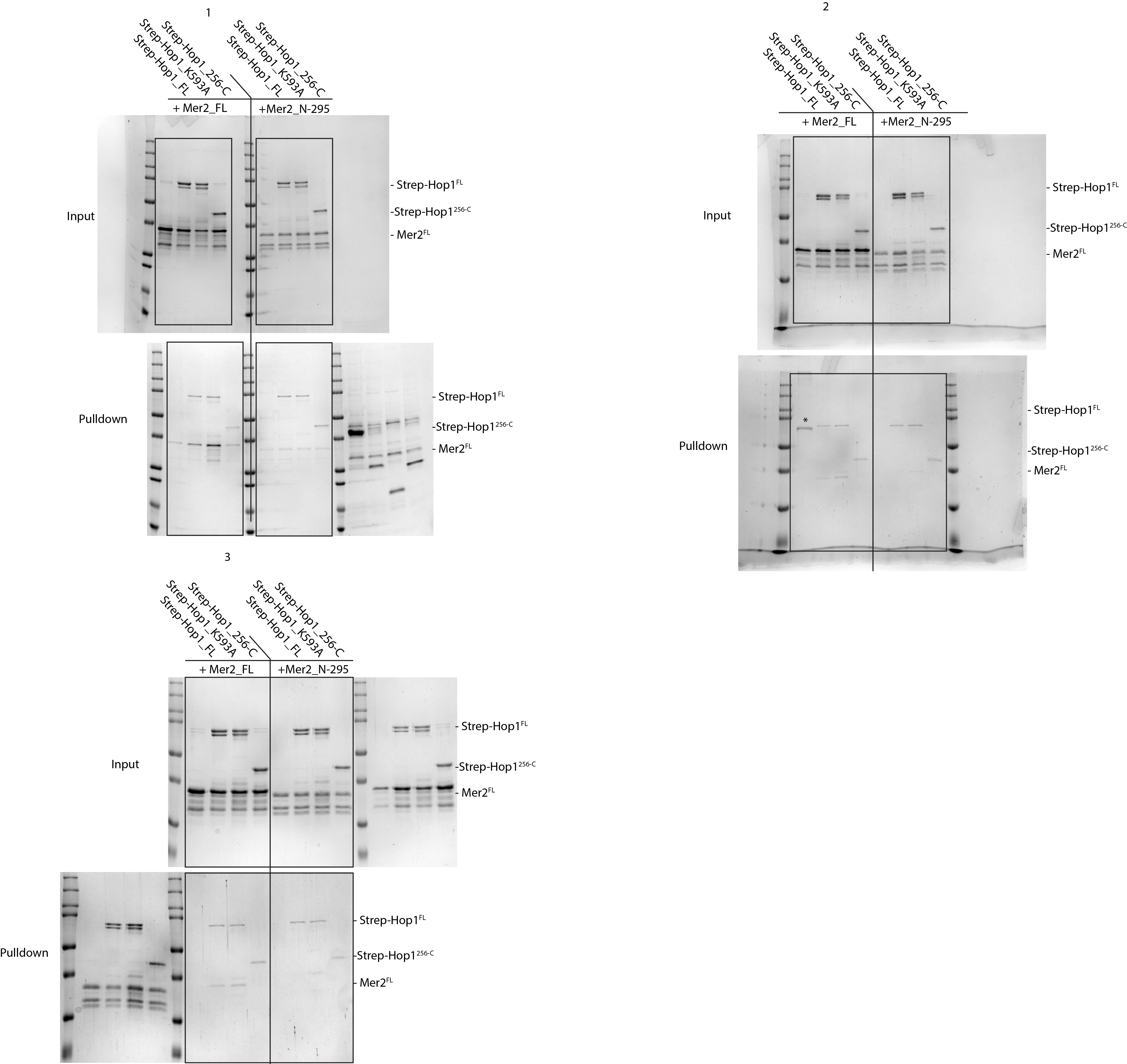

Supplement: Figure 4—source data 1. [file elife-72330-fig4-data1.zip › Figure 4-source data/Pulldown_Hop1+Mer2.png]

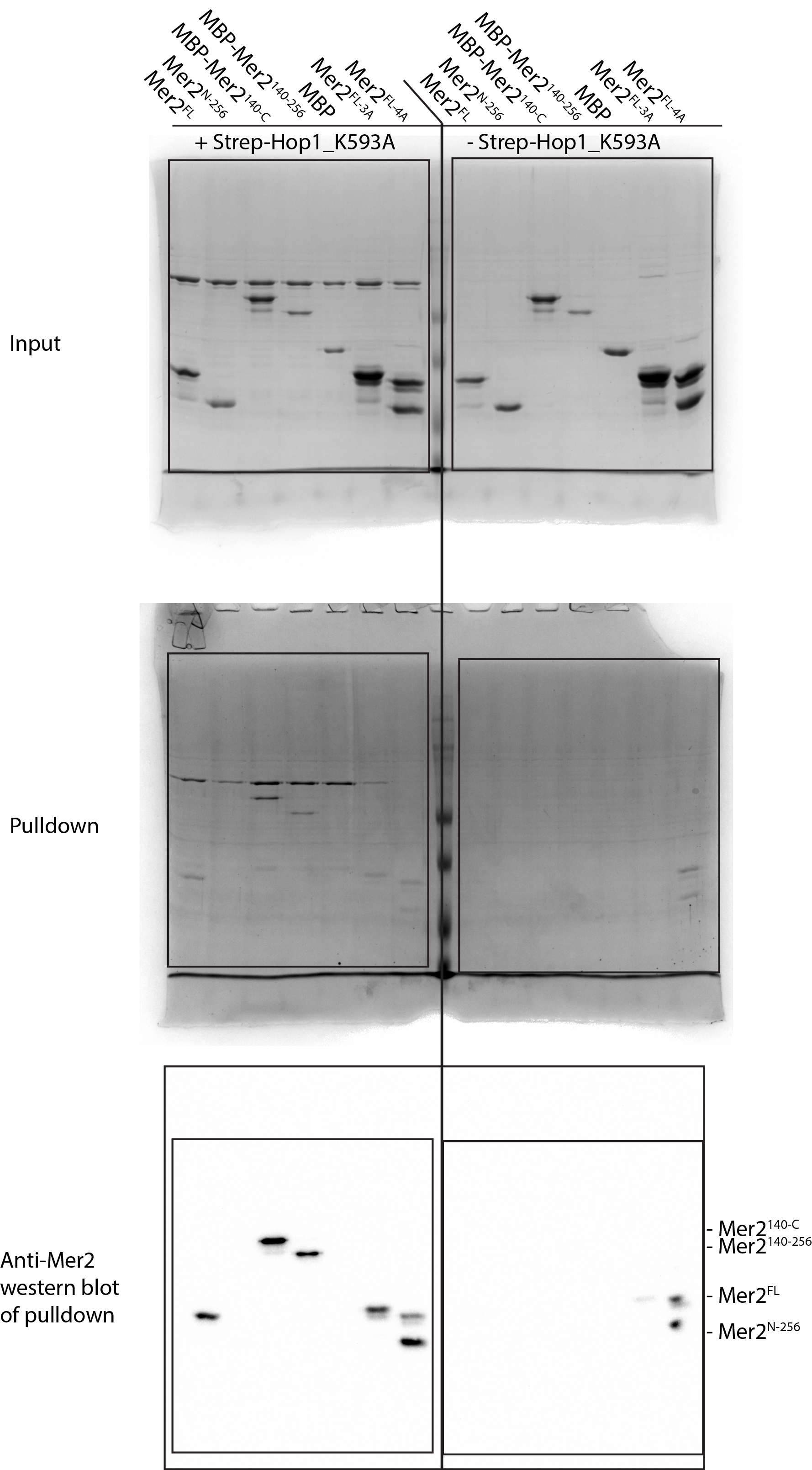

Supplement: Figure 4—source data 1. [file elife-72330-fig4-data1.zip › Figure 4-source data/Pulldown_Hop1_K593A+Mer2.png]

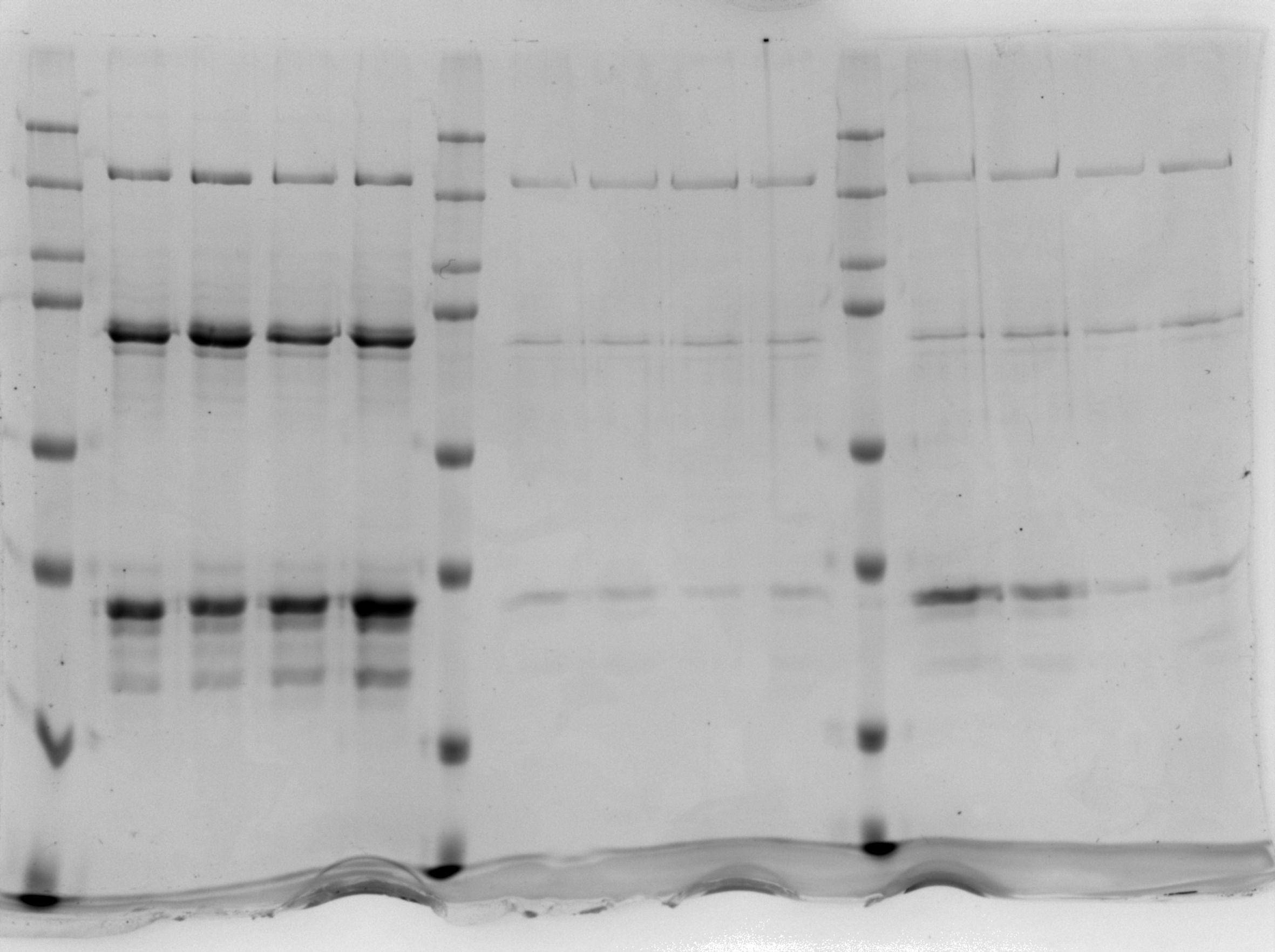

Supplement: Figure 4—figure supplement 1—source data 1. [file elife-72330-fig4-figsupp1-data1.zip › Figure 4-Sup1-source data/Gel_Pulldown_Red1+Hop1+Mer2.tif]

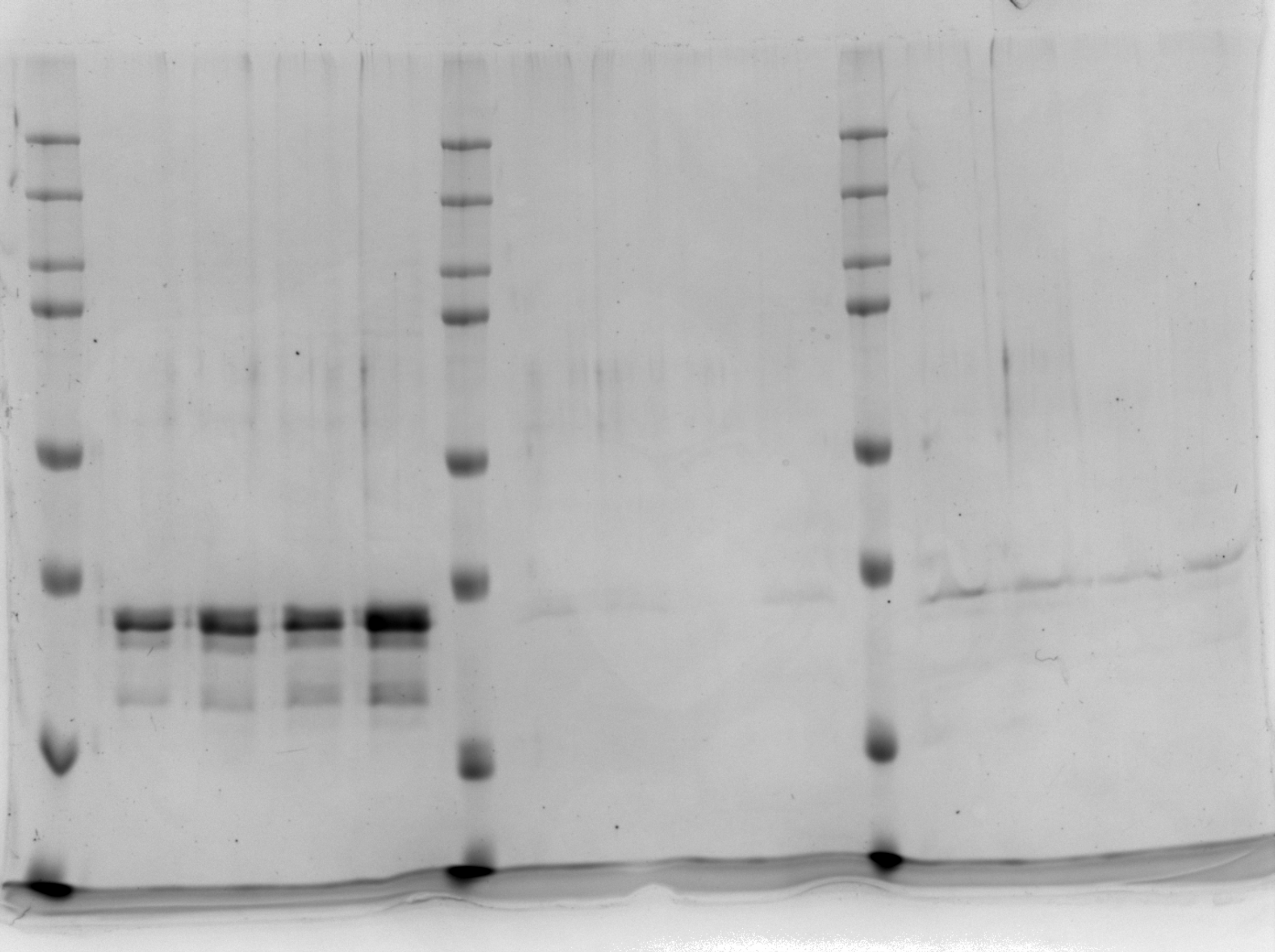

Supplement: Figure 4—figure supplement 1—source data 1. [file elife-72330-fig4-figsupp1-data1.zip › Figure 4-Sup1-source data/Gel_Pulldown_Red1+Hop1+Mer2_control.tif]

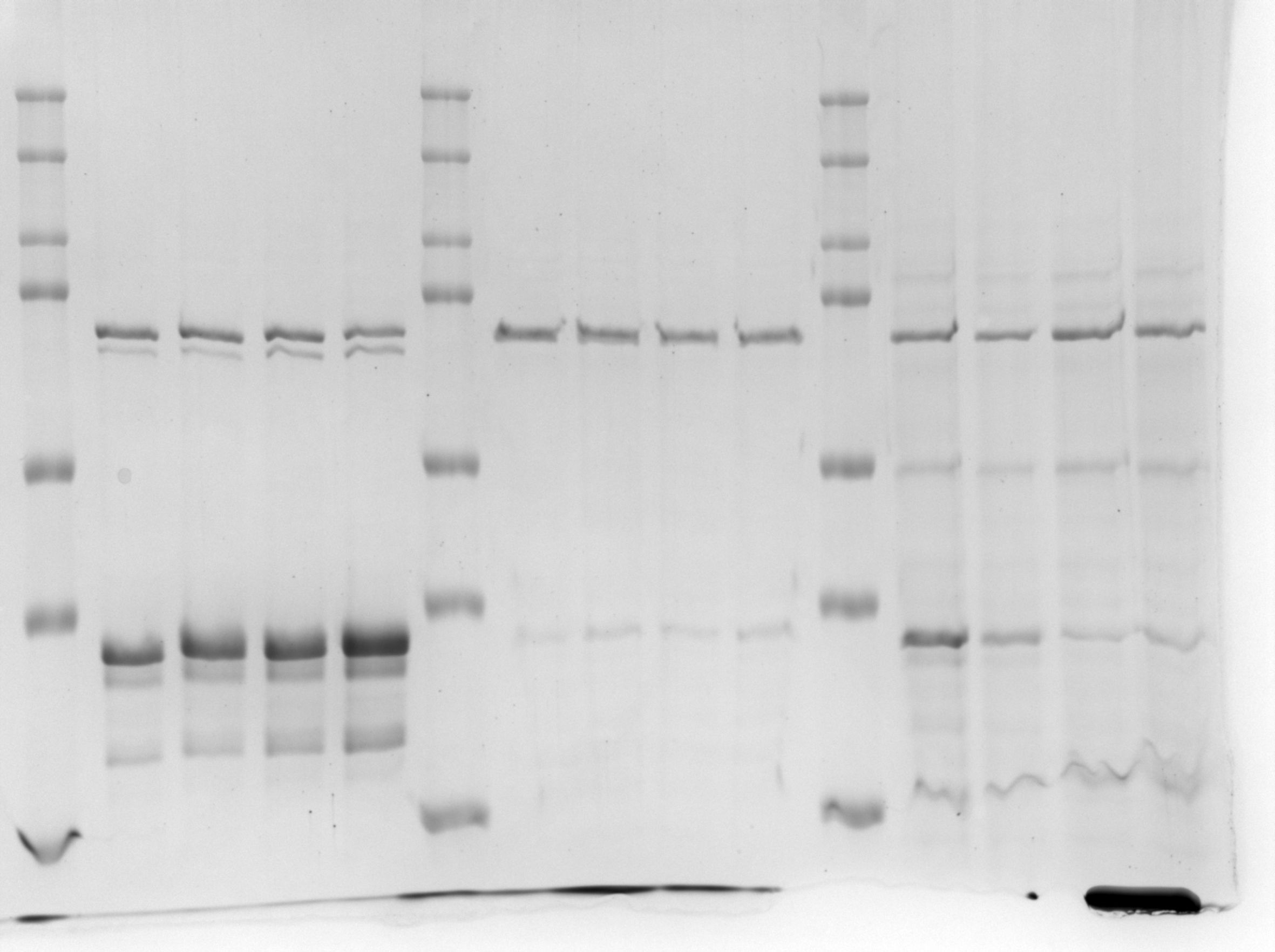

Supplement: Figure 4—figure supplement 1—source data 1. [file elife-72330-fig4-figsupp1-data1.zip › Figure 4-Sup1-source data/Gel_Pulldown_Hop1+Mer2.tif]

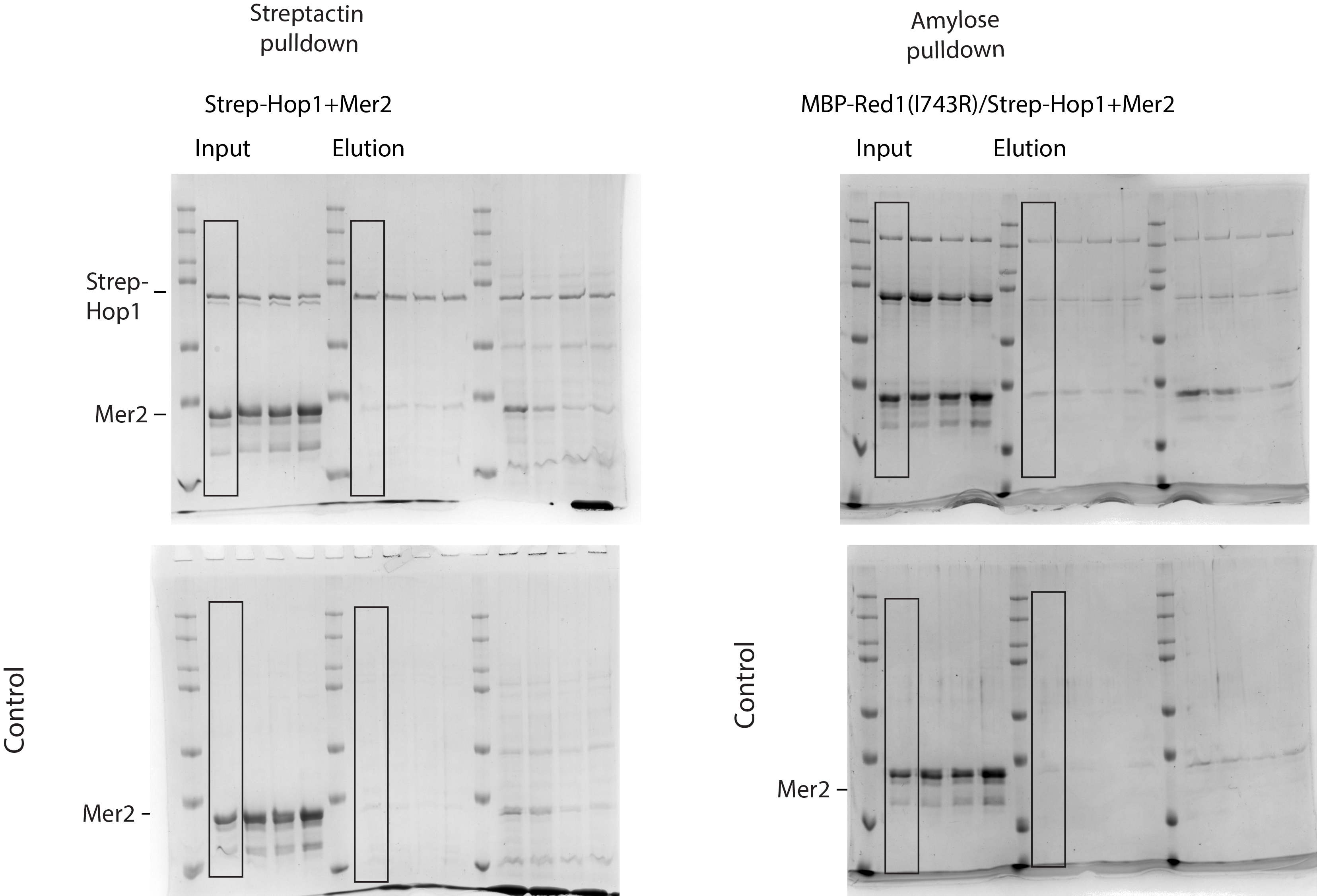

Supplement: Figure 4—figure supplement 1—source data 1. [file elife-72330-fig4-figsupp1-data1.zip › Figure 4-Sup1-source data/Pulldown_Red1+Hop1+Mer2.png]

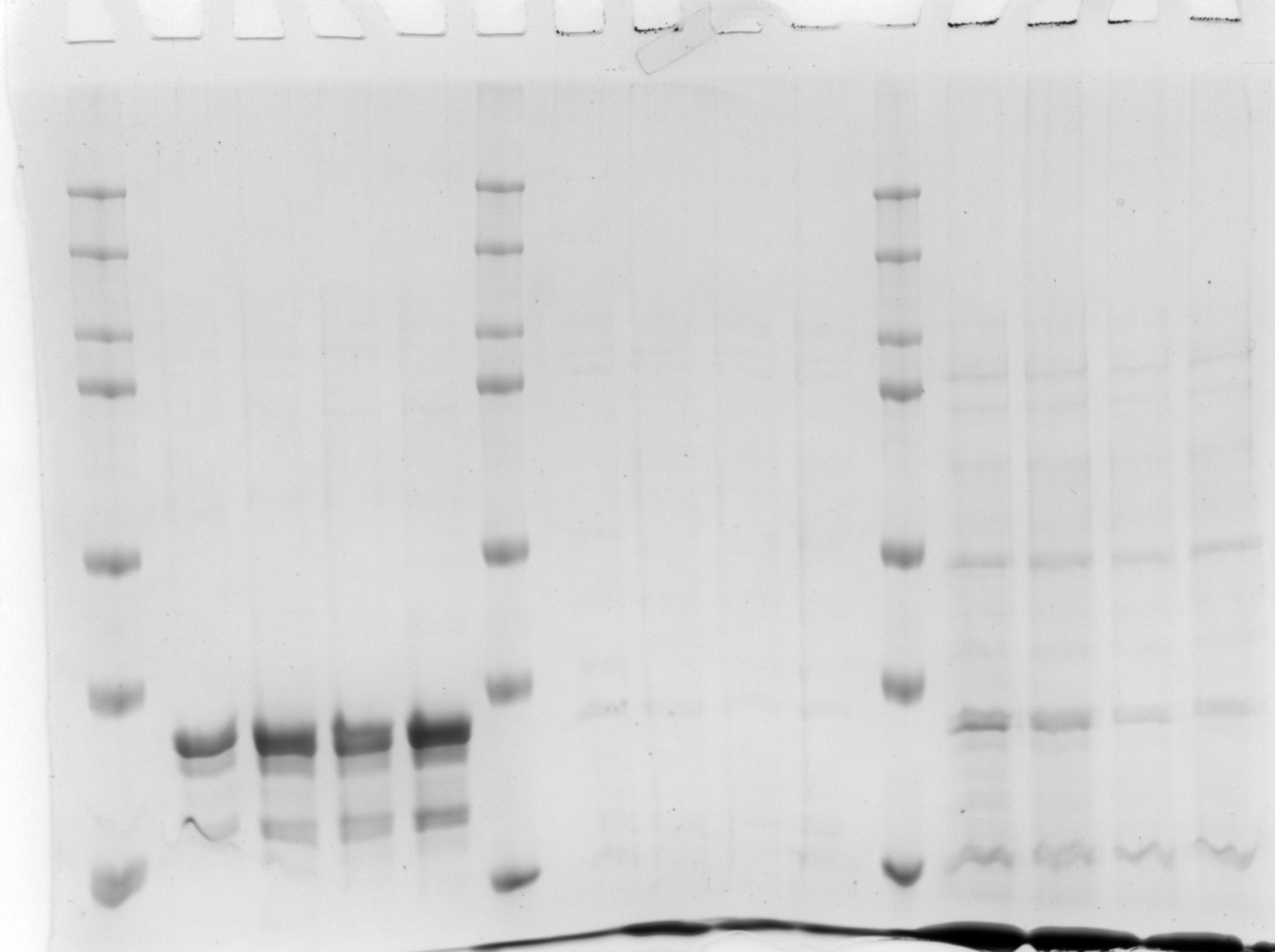

Supplement: Figure 4—figure supplement 1—source data 1. [file elife-72330-fig4-figsupp1-data1.zip › Figure 4-Sup1-source data/Gel_Pulldown_Hop1+Mer2_control.tif]

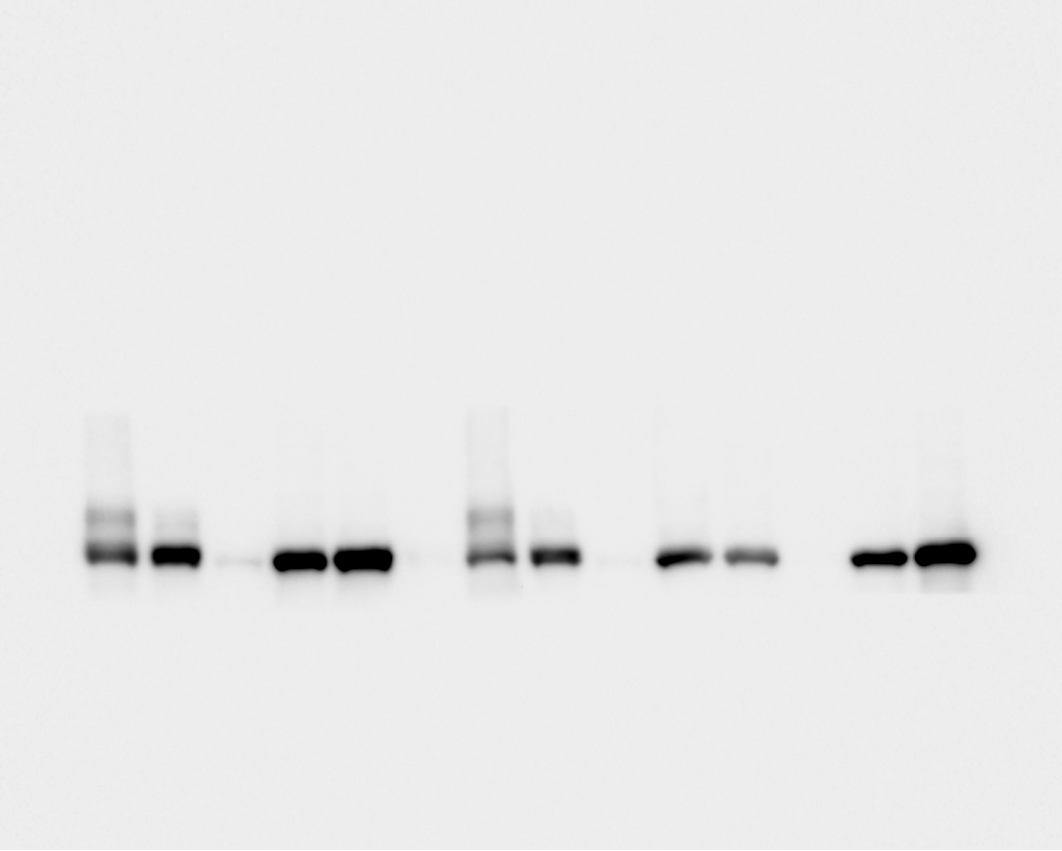

Supplement: Figure 5—source data 1. [file elife-72330-fig5-data1.zip › Figure 5 Source data/Hop1 blot.tif]

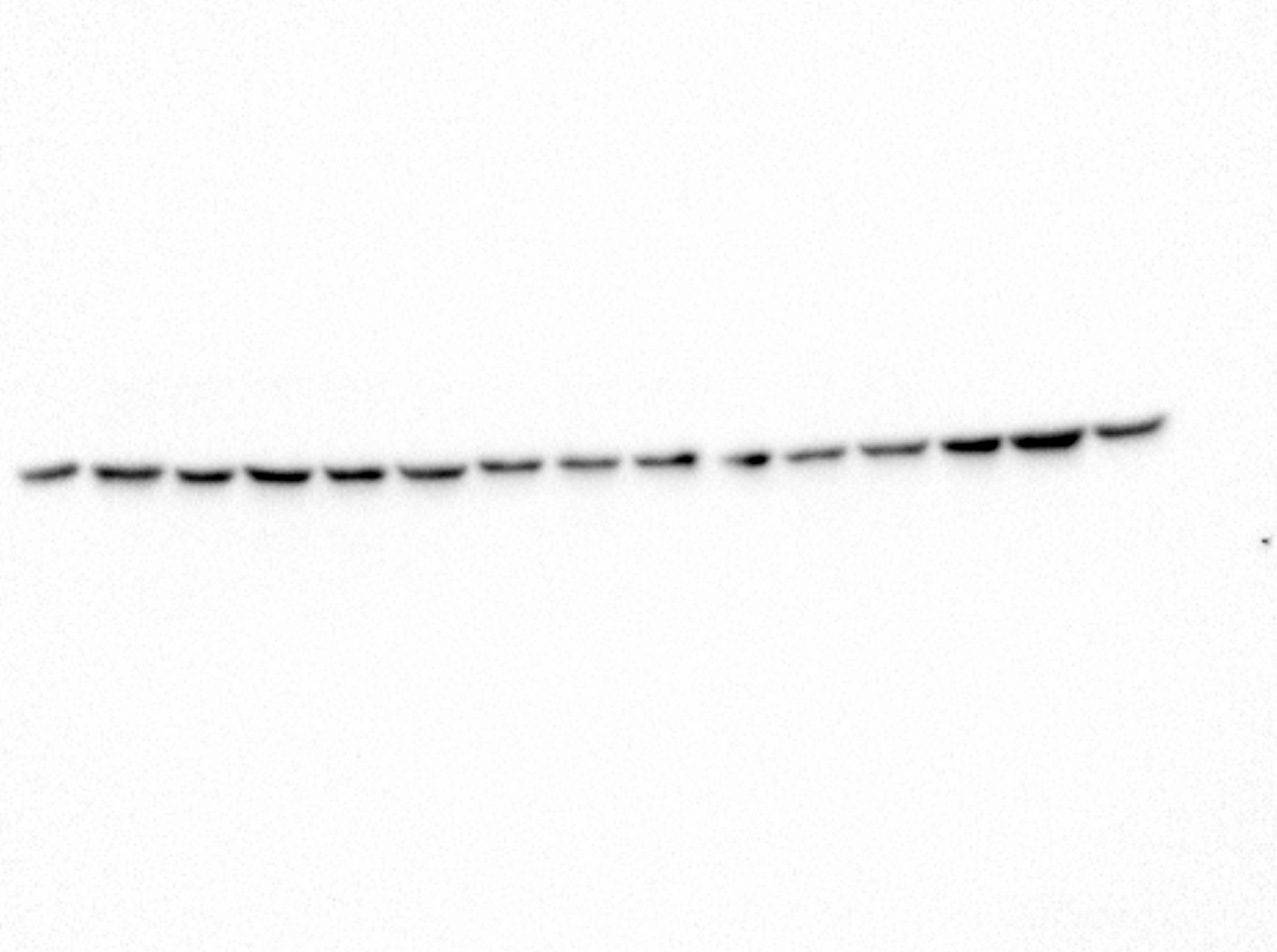

Supplement: Figure 5—source data 1. [file elife-72330-fig5-data1.zip › Figure 5 Source data/Pgk1 blot.tif]

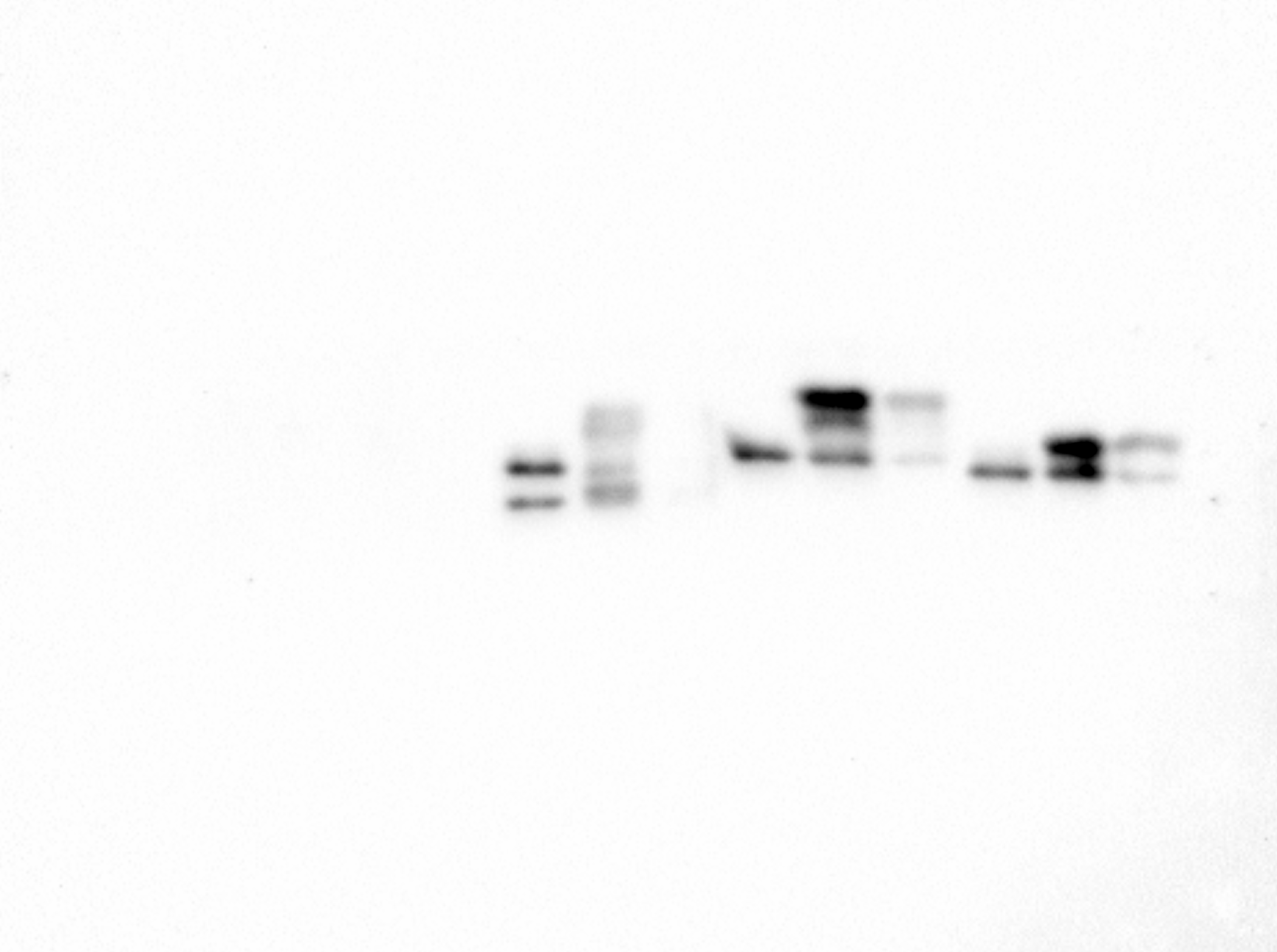

Supplement: Figure 5—source data 1. [file elife-72330-fig5-data1.zip › Figure 5 Source data/HA-Mer2 blot.tif]

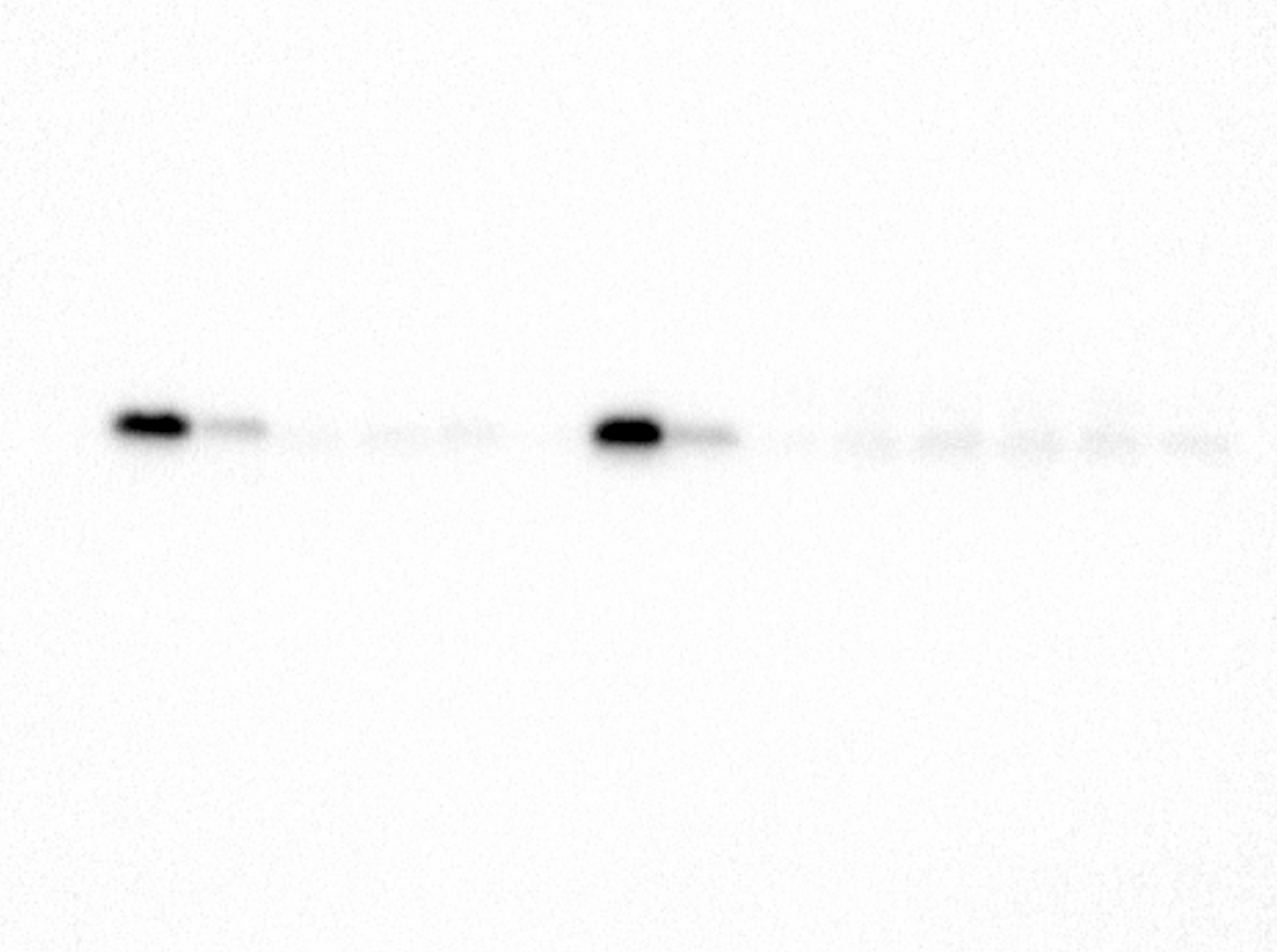

Supplement: Figure 5—source data 1. [file elife-72330-fig5-data1.zip › Figure 5 Source data/H3t11.tif]

**Rousova et al. paper 2021**

**Figures with unedited blots:**


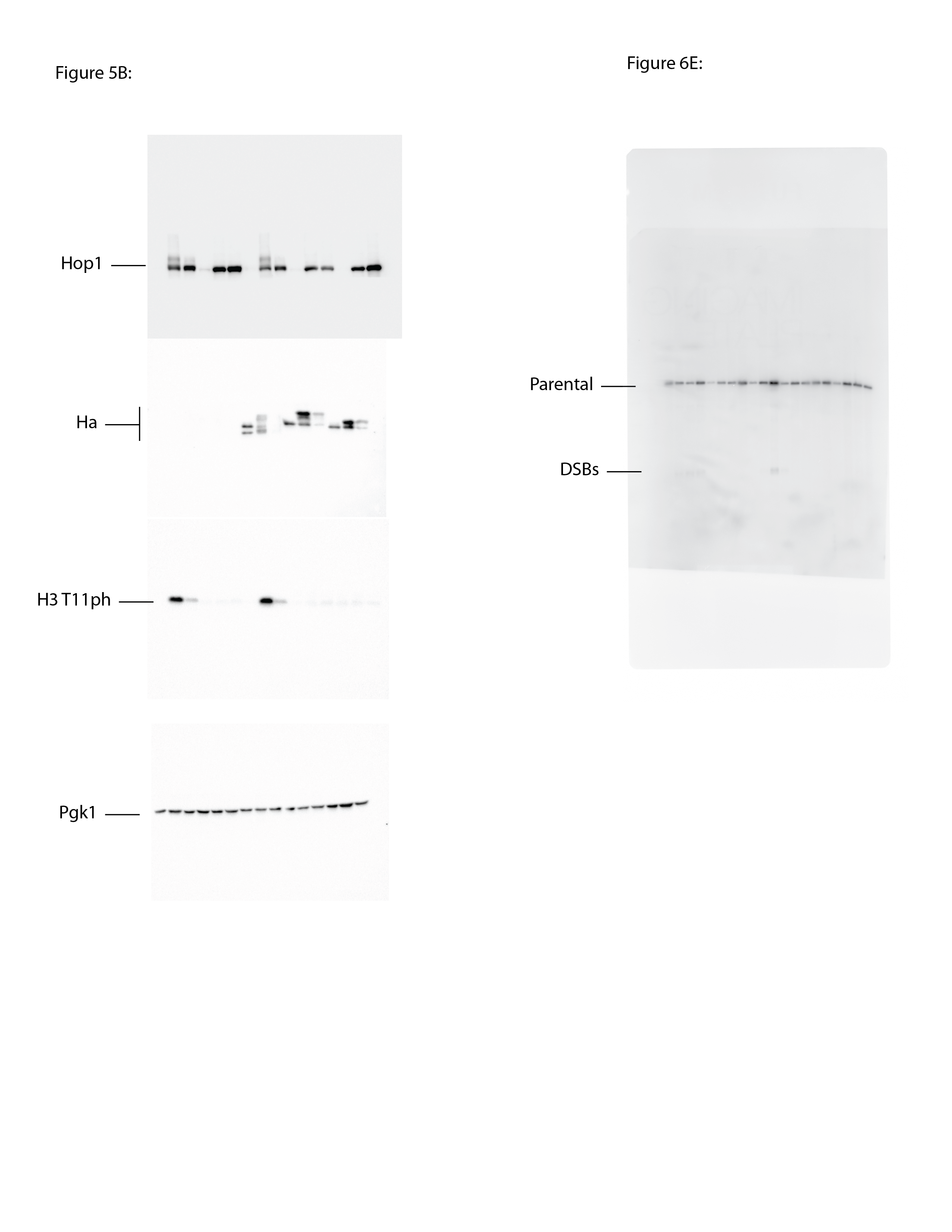


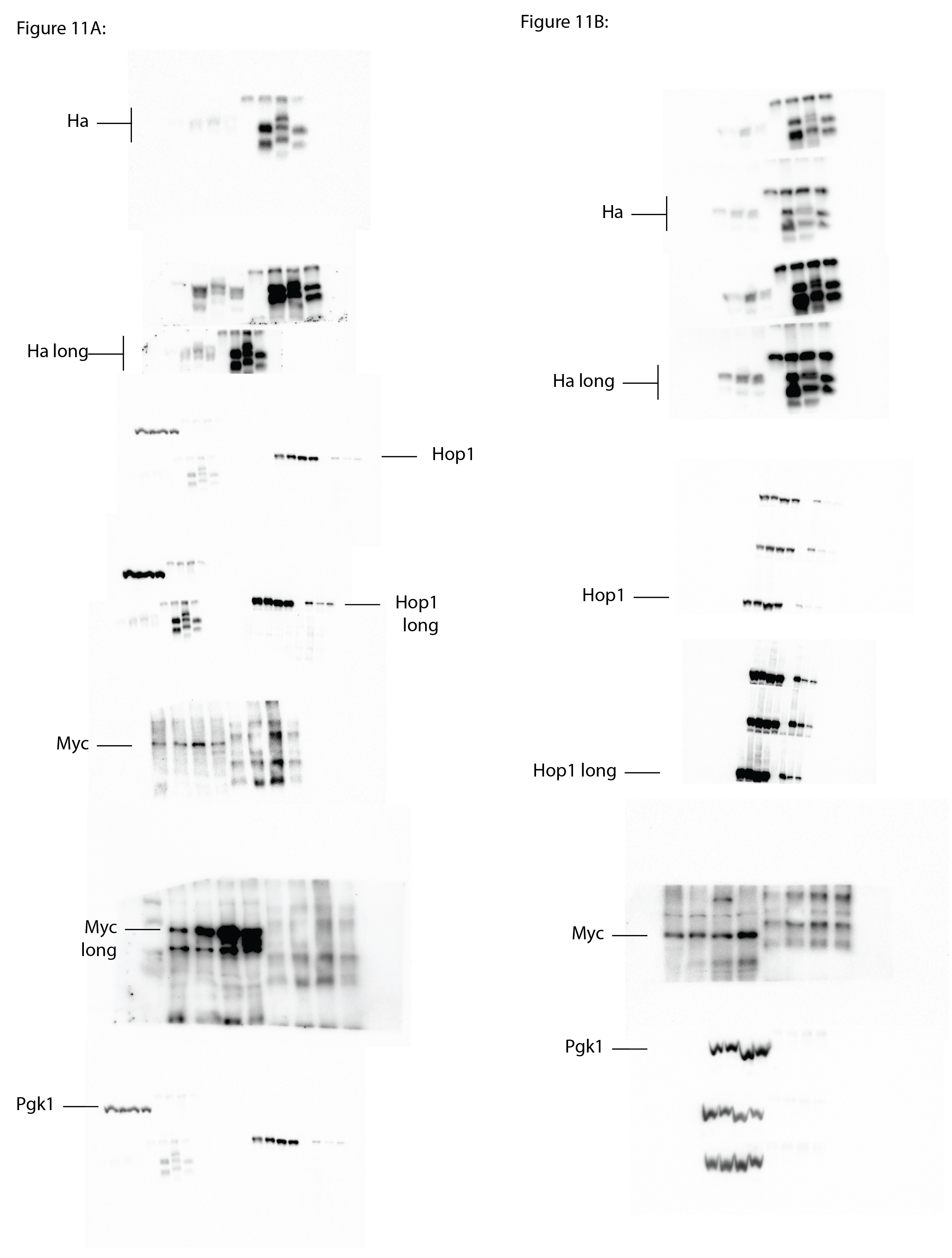

Supplement: Figure 5—source data 1. [file elife-72330-fig5-data1.zip › Figure 5 Source data/Rousova et al paper unedited figure blots.docx]

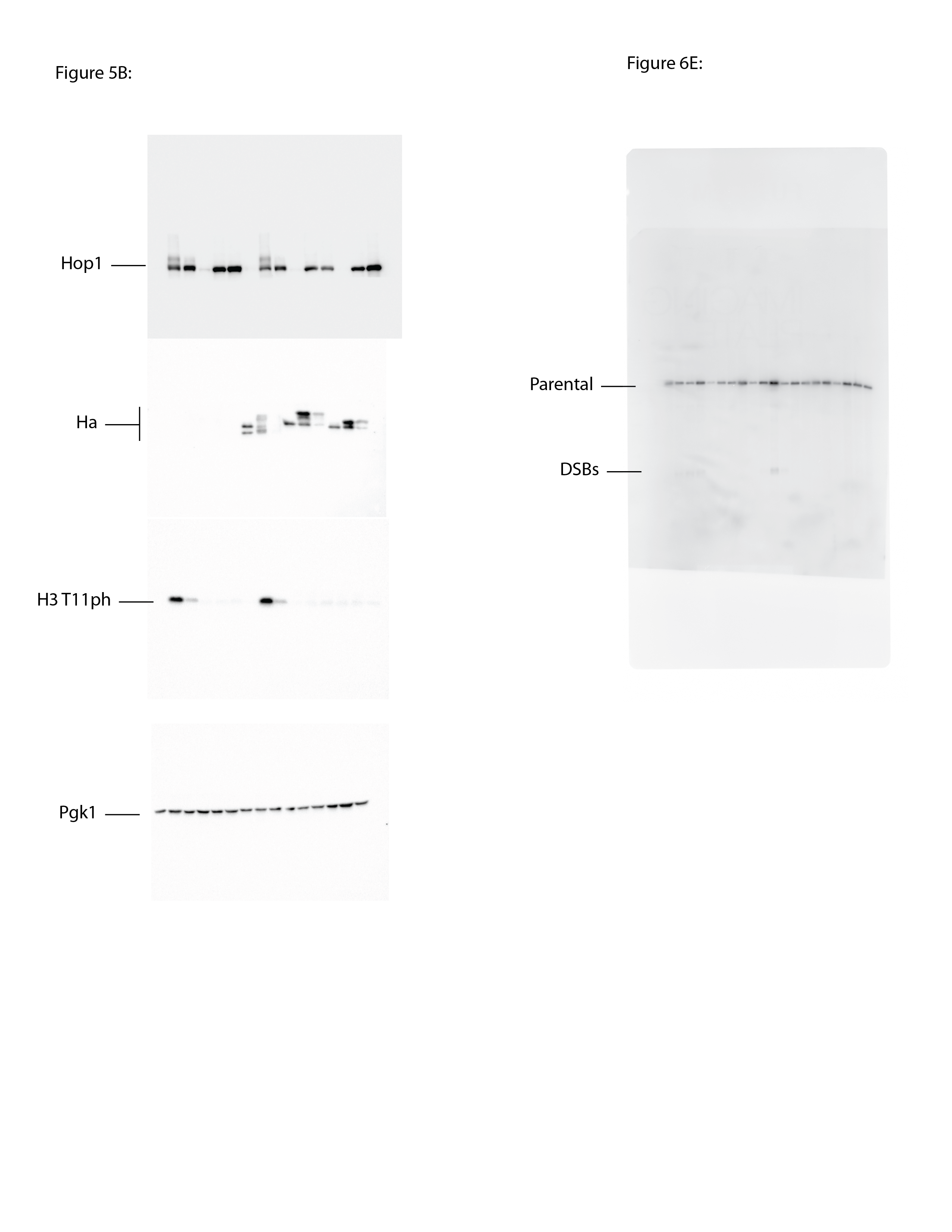

Supplement: Figure 5—source data 1. [file elife-72330-fig5-data1.zip › Figure 5 Source data/all figures raw files 02 atb01-01.png]

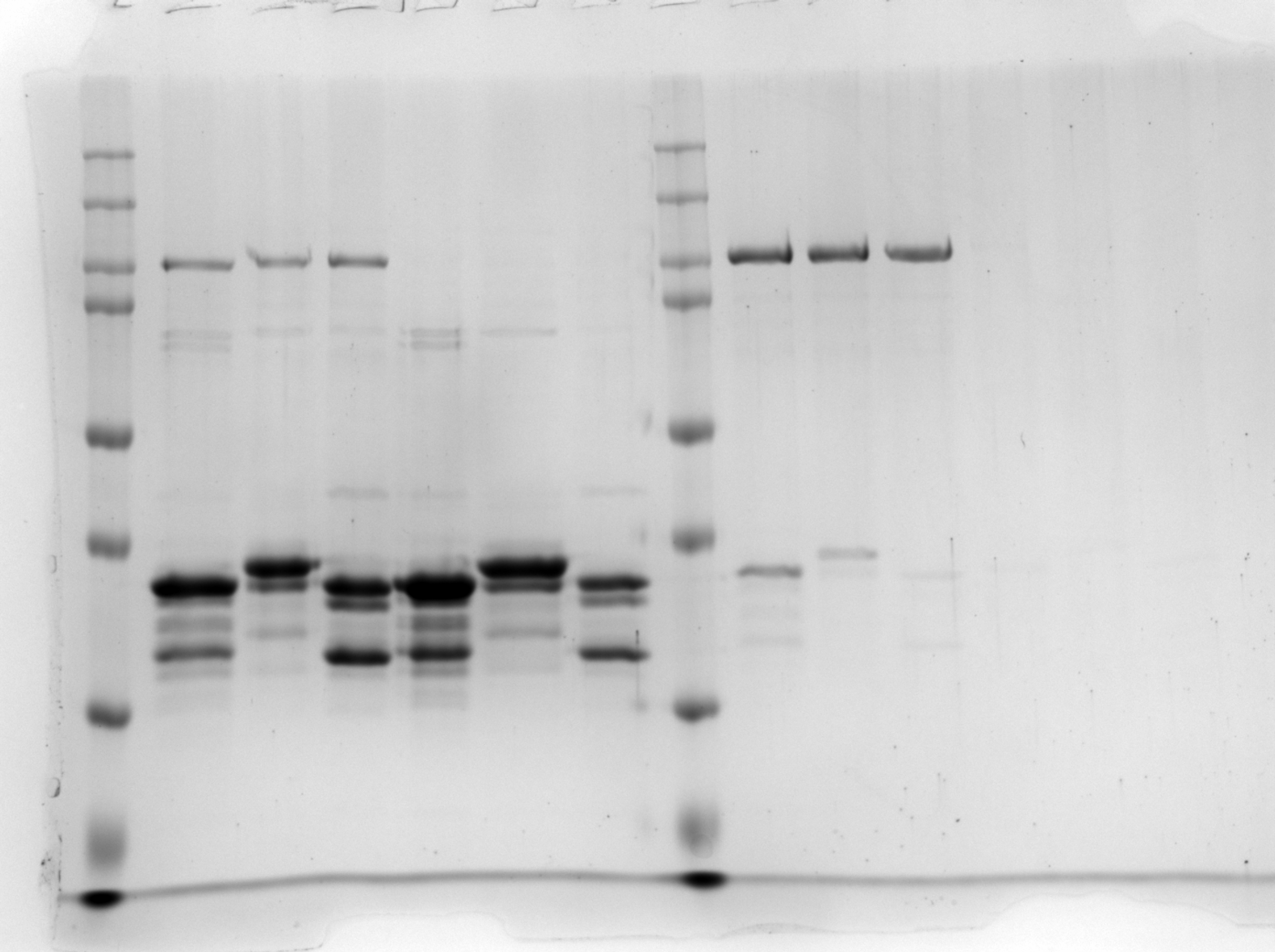

Supplement: Figure 6—source data 1. [file elife-72330-fig6-data1.zip › Figure 6 -source data 1/Gel_Pulldown_Mer2+Mre11_1.tif]

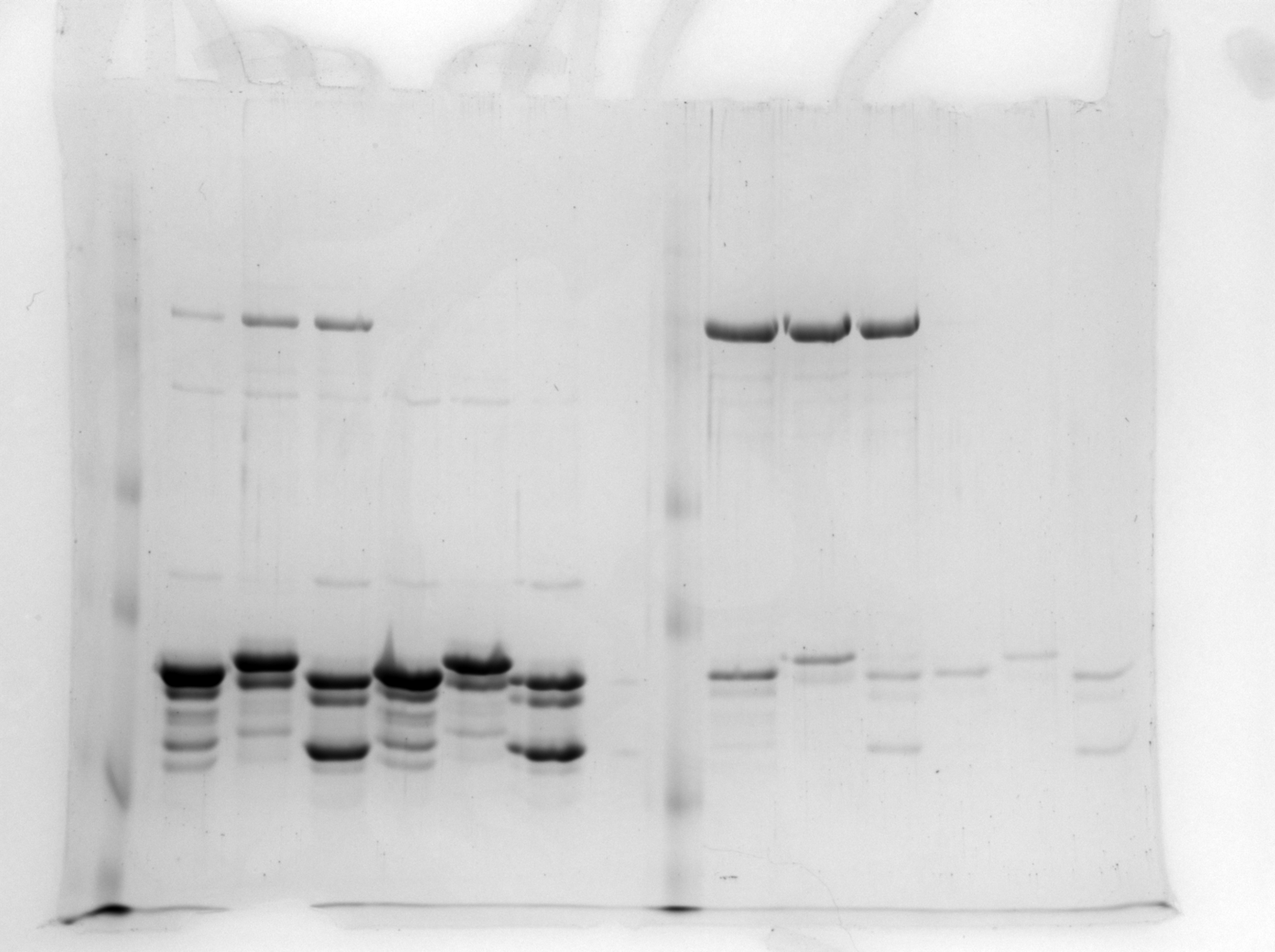

Supplement: Figure 6—source data 1. [file elife-72330-fig6-data1.zip › Figure 6 -source data 1/Gel_Pulldown_Mer2+Mre11_2.tif]

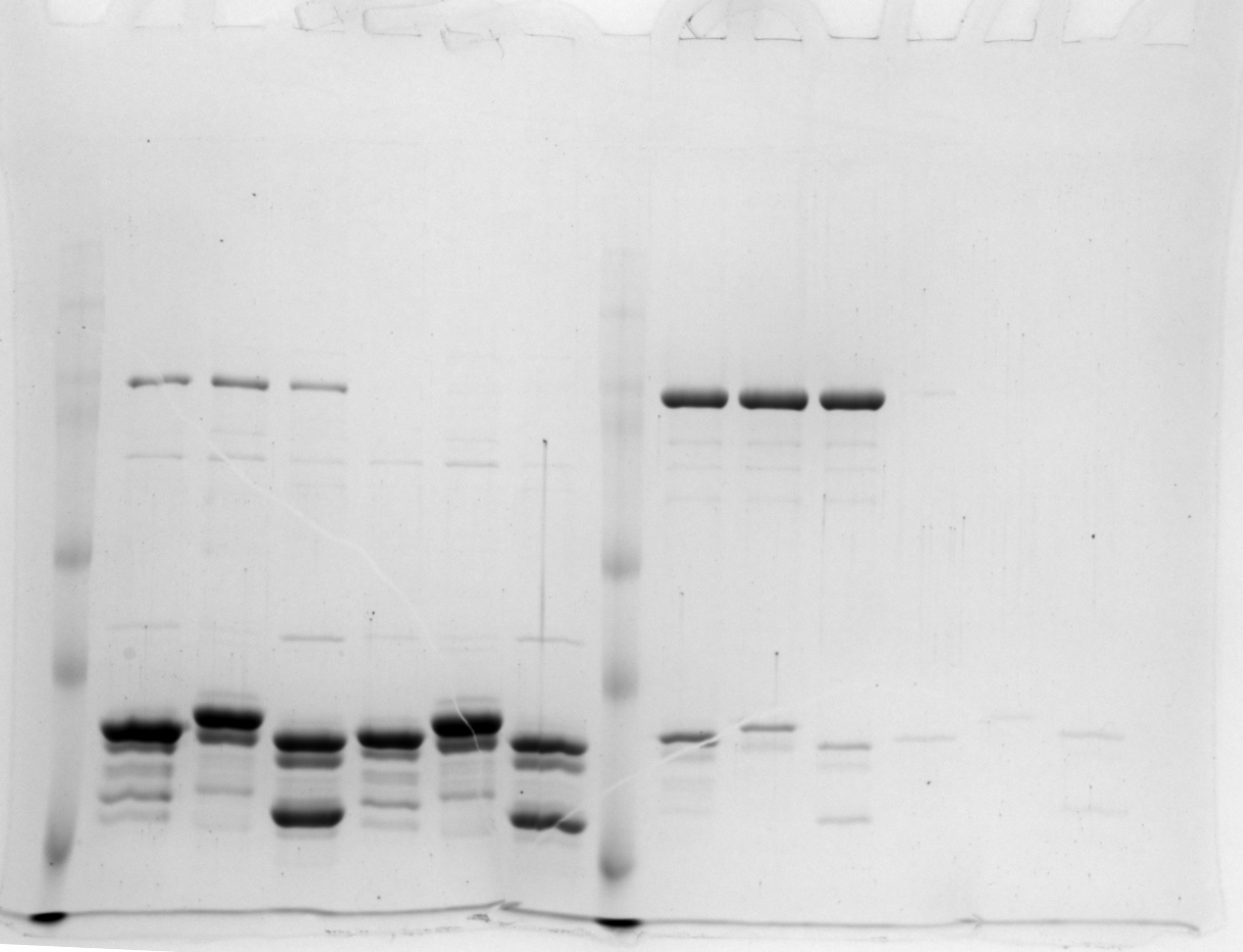

Supplement: Figure 6—source data 1. [file elife-72330-fig6-data1.zip › Figure 6 -source data 1/Gel_Pulldown_Mer2+Mre11_3.tif]

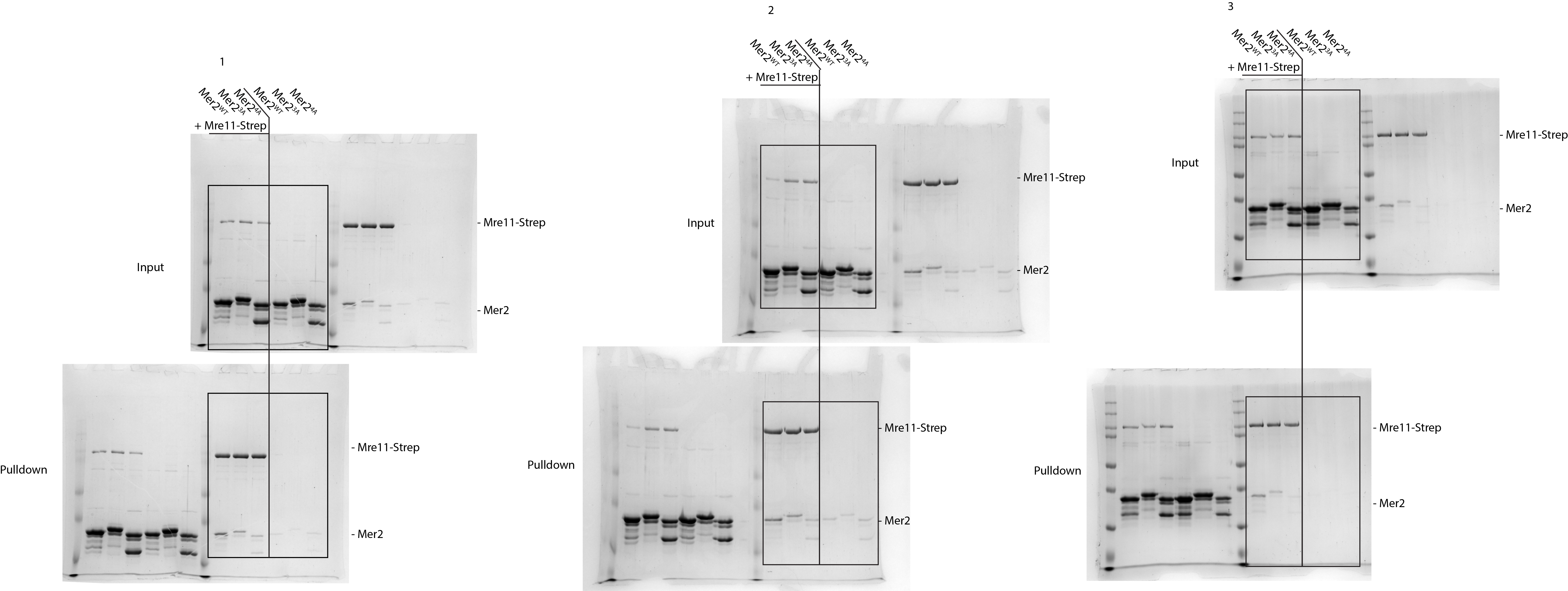

Supplement: Figure 6—source data 1. [file elife-72330-fig6-data1.zip › Figure 6 -source data 1/Pulldown_Mer2+Mre11.png]

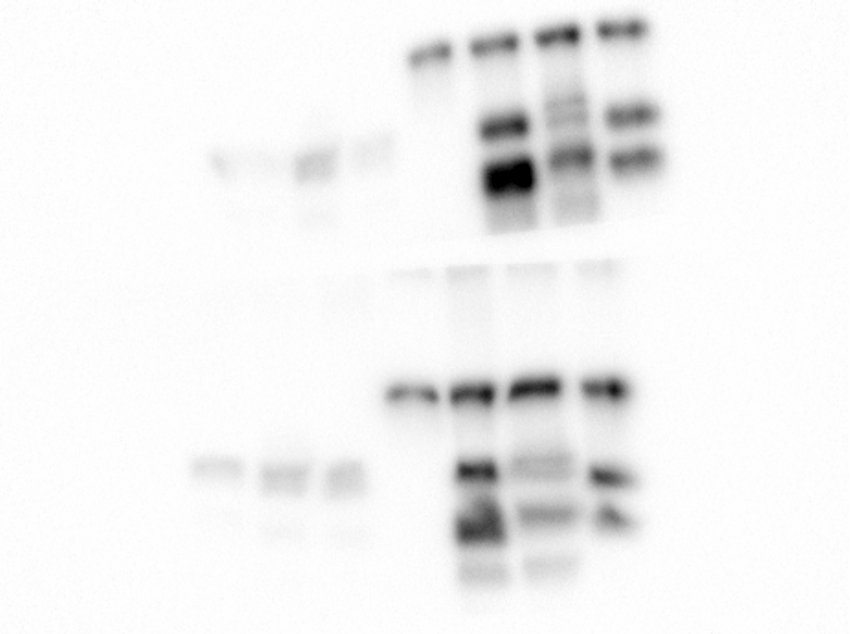

Supplement: Figure 6—figure supplement 2—source data 1. [file elife-72330-fig6-figsupp2-data1.zip › Figure 6 supplement 2 source data/Fig 6 Supp 2B/ha low.tif]

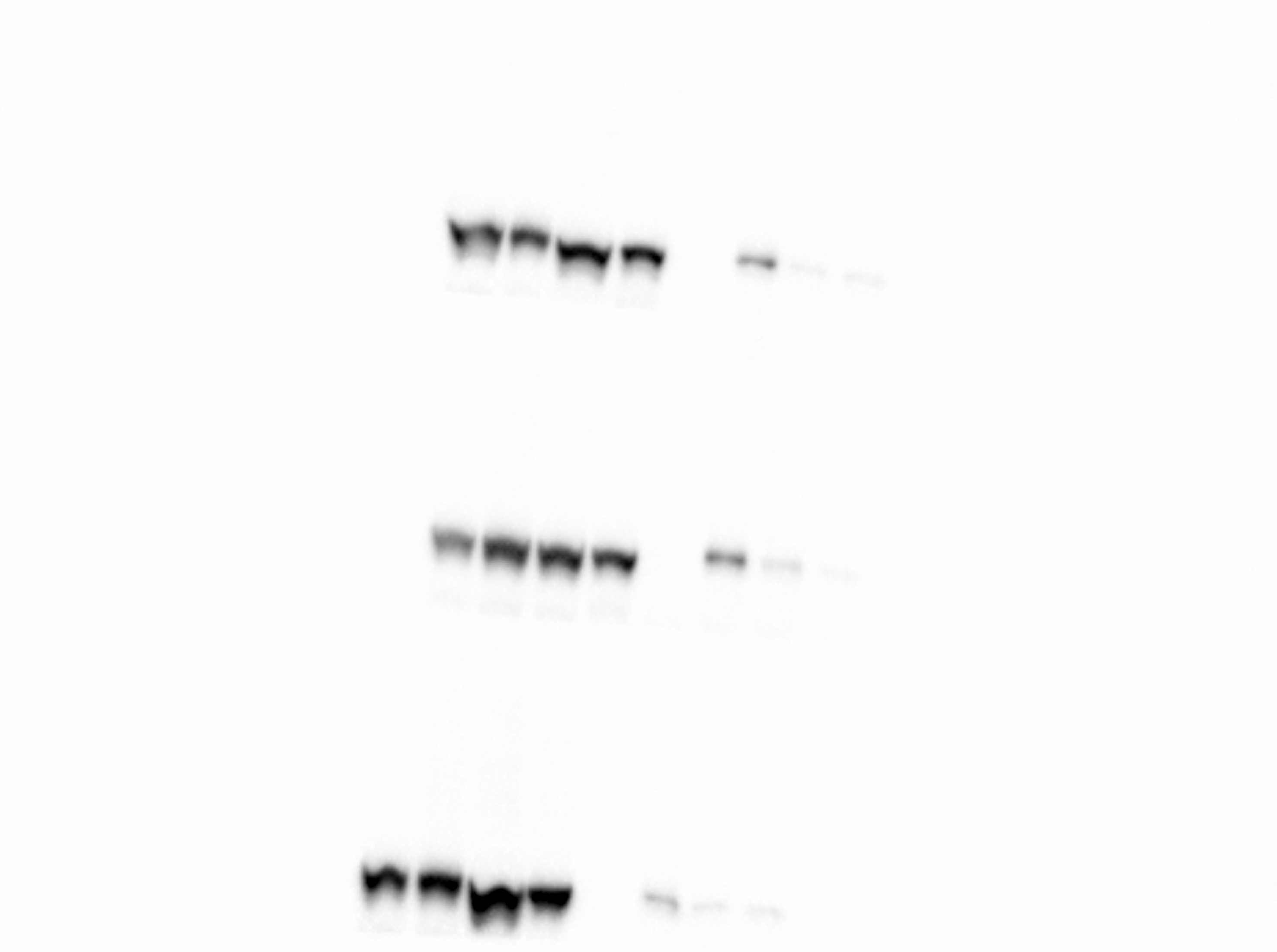

Supplement: Figure 6—figure supplement 2—source data 1. [file elife-72330-fig6-figsupp2-data1.zip › Figure 6 supplement 2 source data/Fig 6 Supp 2B/hop1 .tif]

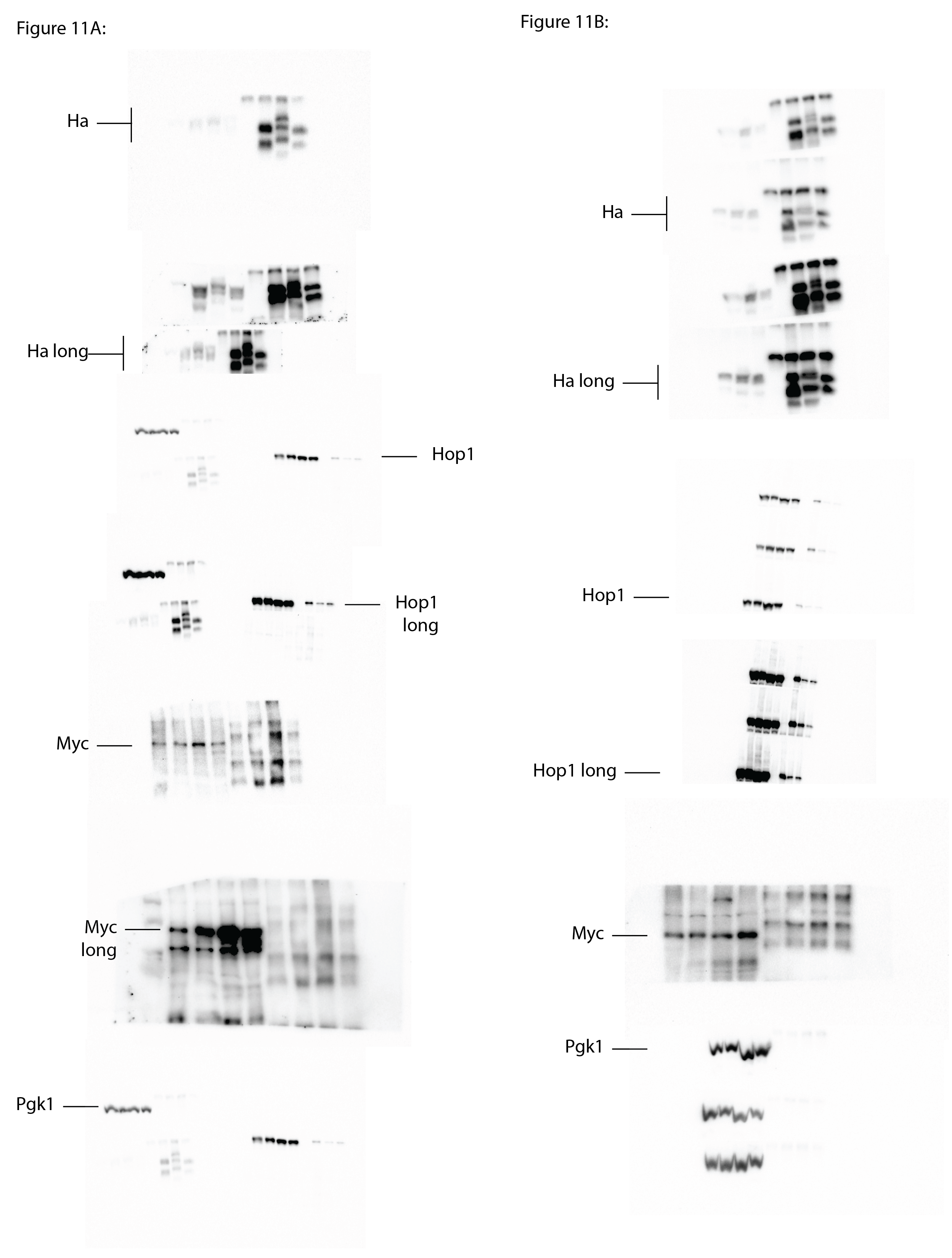

Supplement: Figure 6—figure supplement 2—source data 1. [file elife-72330-fig6-figsupp2-data1.zip › Figure 6 supplement 2 source data/Fig 6 Supp 2B/all figures raw files 02 abt02-02.png]

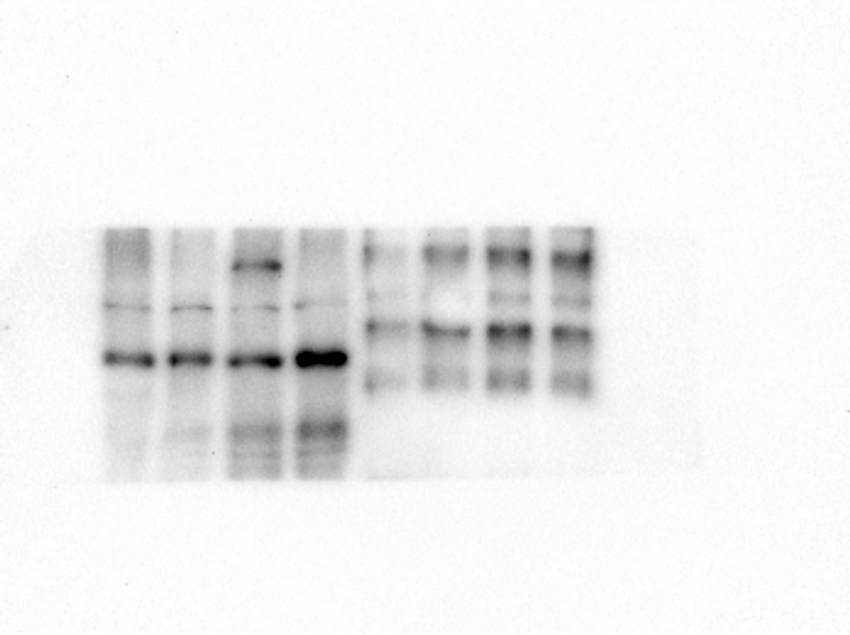

Supplement: Figure 6—figure supplement 2—source data 1. [file elife-72330-fig6-figsupp2-data1.zip › Figure 6 supplement 2 source data/Fig 6 Supp 2B/myc low.tif]

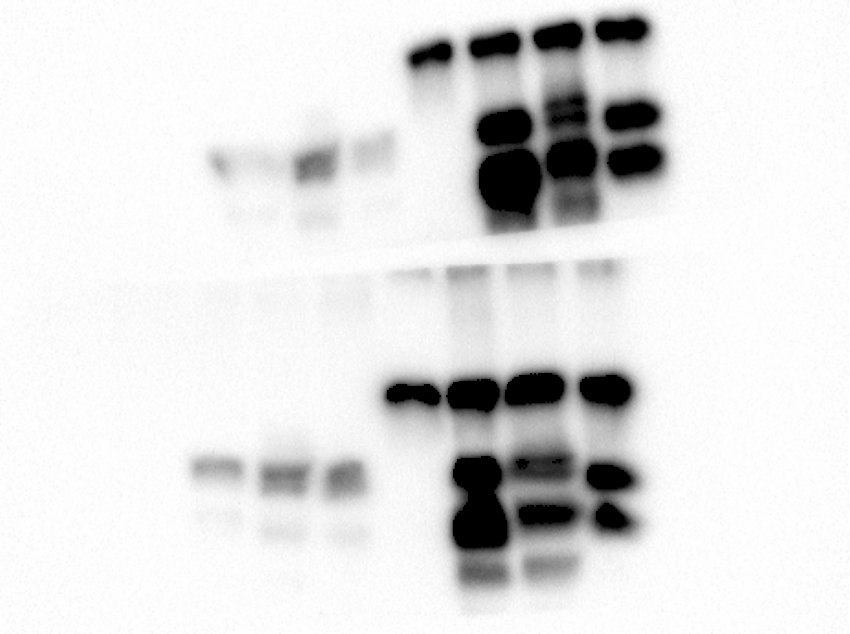

Supplement: Figure 6—figure supplement 2—source data 1. [file elife-72330-fig6-figsupp2-data1.zip › Figure 6 supplement 2 source data/Fig 6 Supp 2B/Ha high.tif]

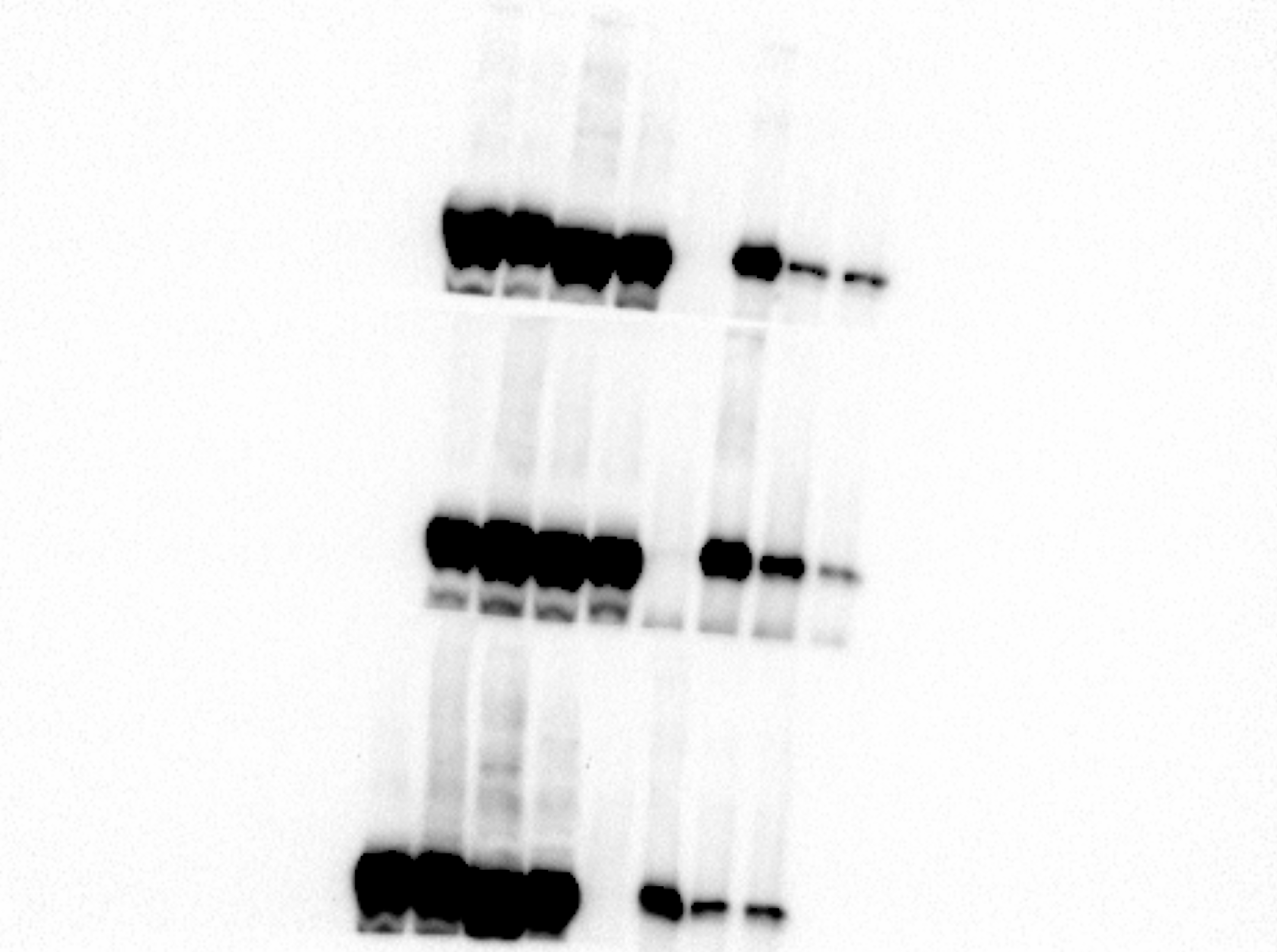

Supplement: Figure 6—figure supplement 2—source data 1. [file elife-72330-fig6-figsupp2-data1.zip › Figure 6 supplement 2 source data/Fig 6 Supp 2B/hop1 high.tif]

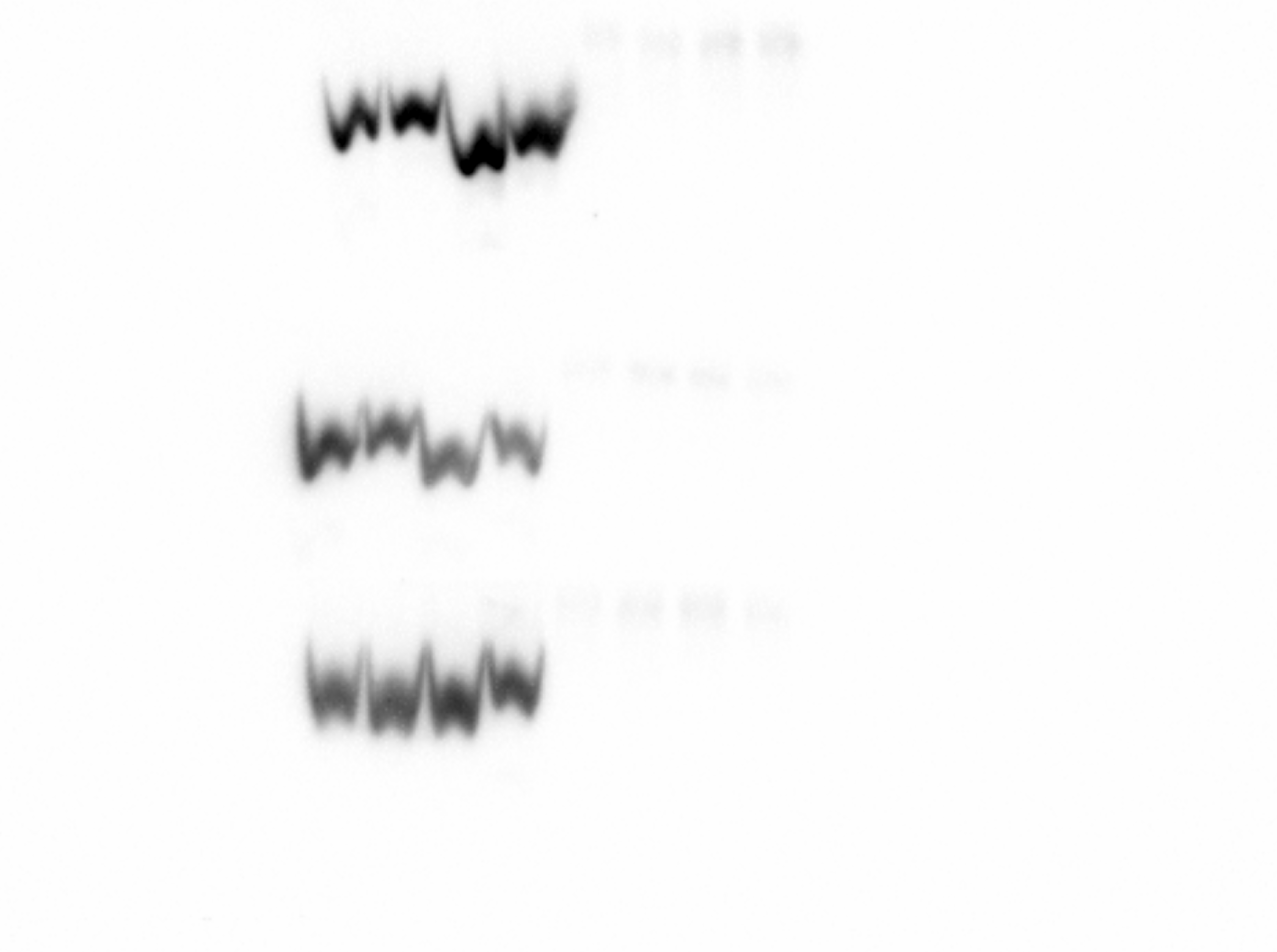

Supplement: Figure 6—figure supplement 2—source data 1. [file elife-72330-fig6-figsupp2-data1.zip › Figure 6 supplement 2 source data/Fig 6 Supp 2B/pgk.tif]

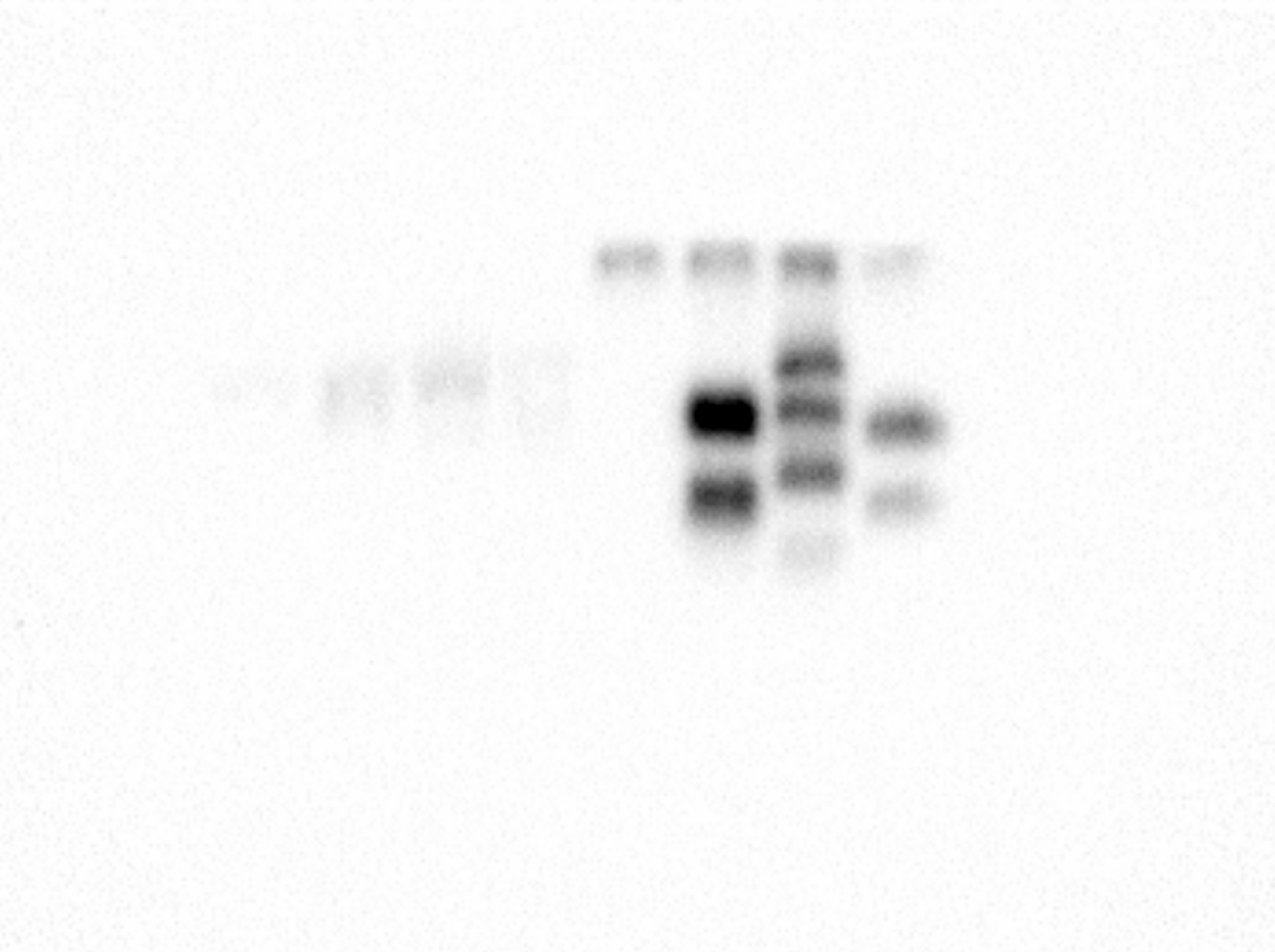

Supplement: Figure 6—figure supplement 2—source data 1. [file elife-72330-fig6-figsupp2-data1.zip › Figure 6 supplement 2 source data/Figure 6 Supp 2 A/Ha blot.tif]

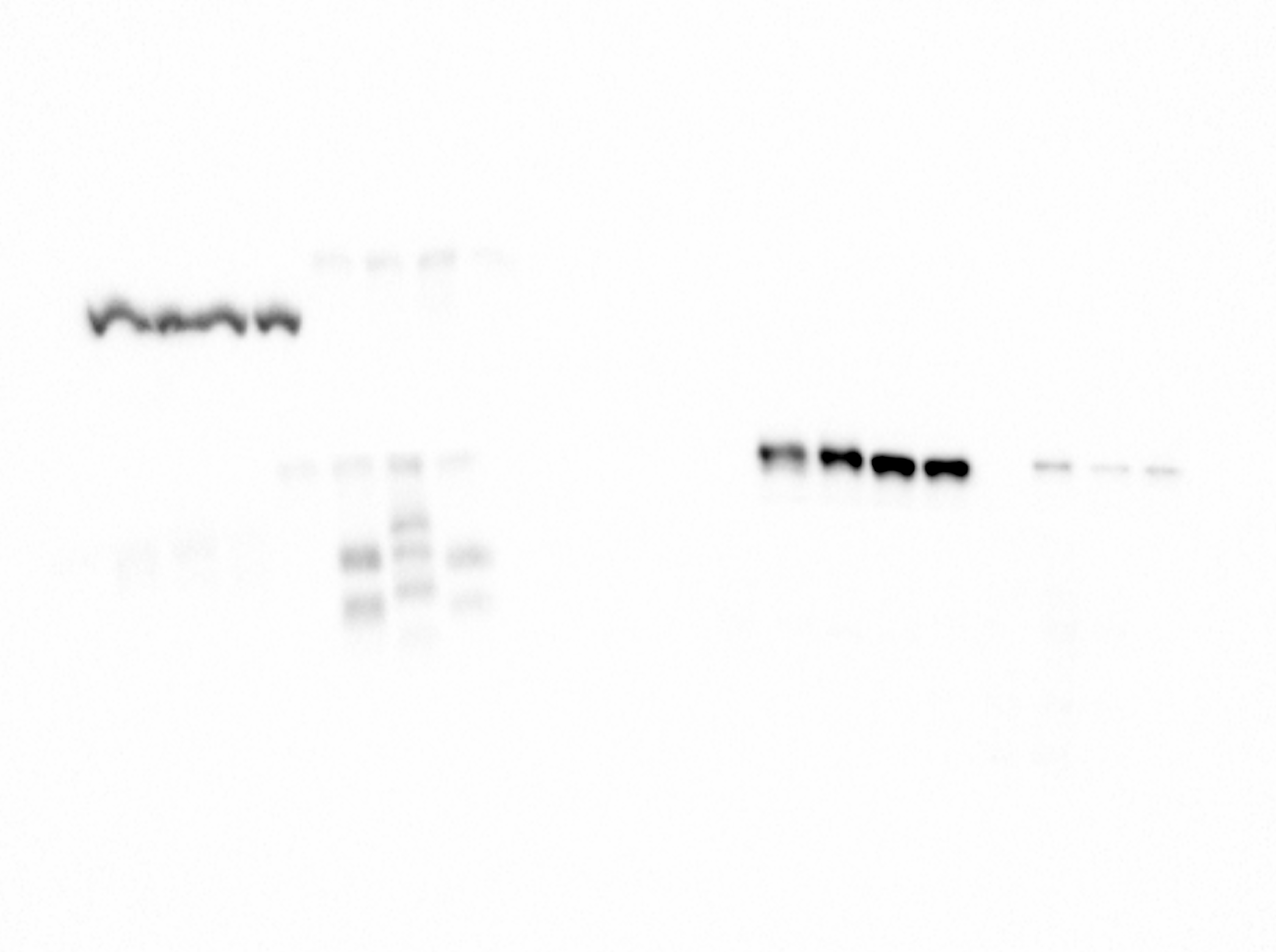

Supplement: Figure 6—figure supplement 2—source data 1. [file elife-72330-fig6-figsupp2-data1.zip › Figure 6 supplement 2 source data/Figure 6 Supp 2 A/hop1 blot.tif]

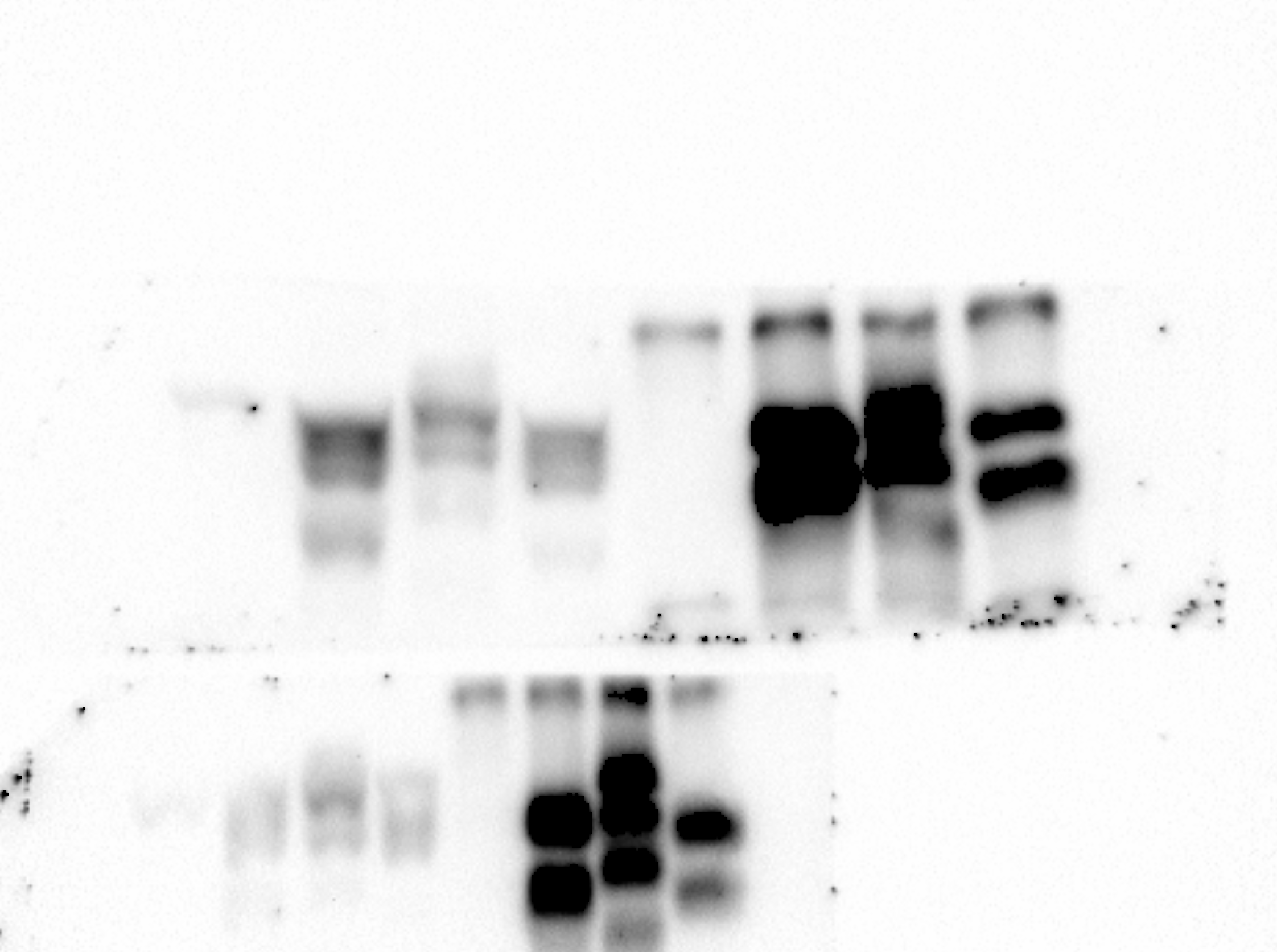

Supplement: Figure 6—figure supplement 2—source data 1. [file elife-72330-fig6-figsupp2-data1.zip › Figure 6 supplement 2 source data/Figure 6 Supp 2 A/Ha high.tif]

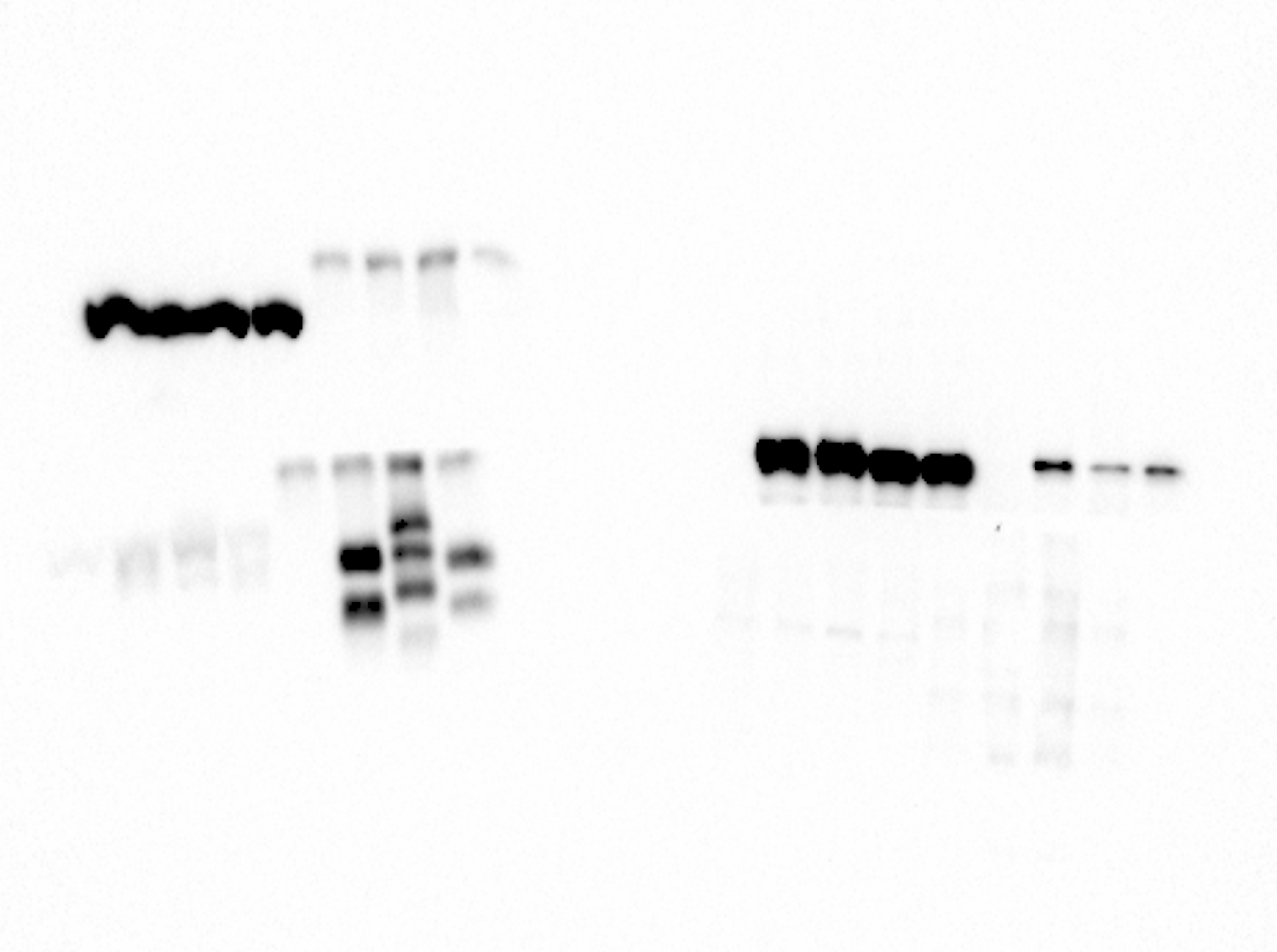

Supplement: Figure 6—figure supplement 2—source data 1. [file elife-72330-fig6-figsupp2-data1.zip › Figure 6 supplement 2 source data/Figure 6 Supp 2 A/hop1 high.tif]

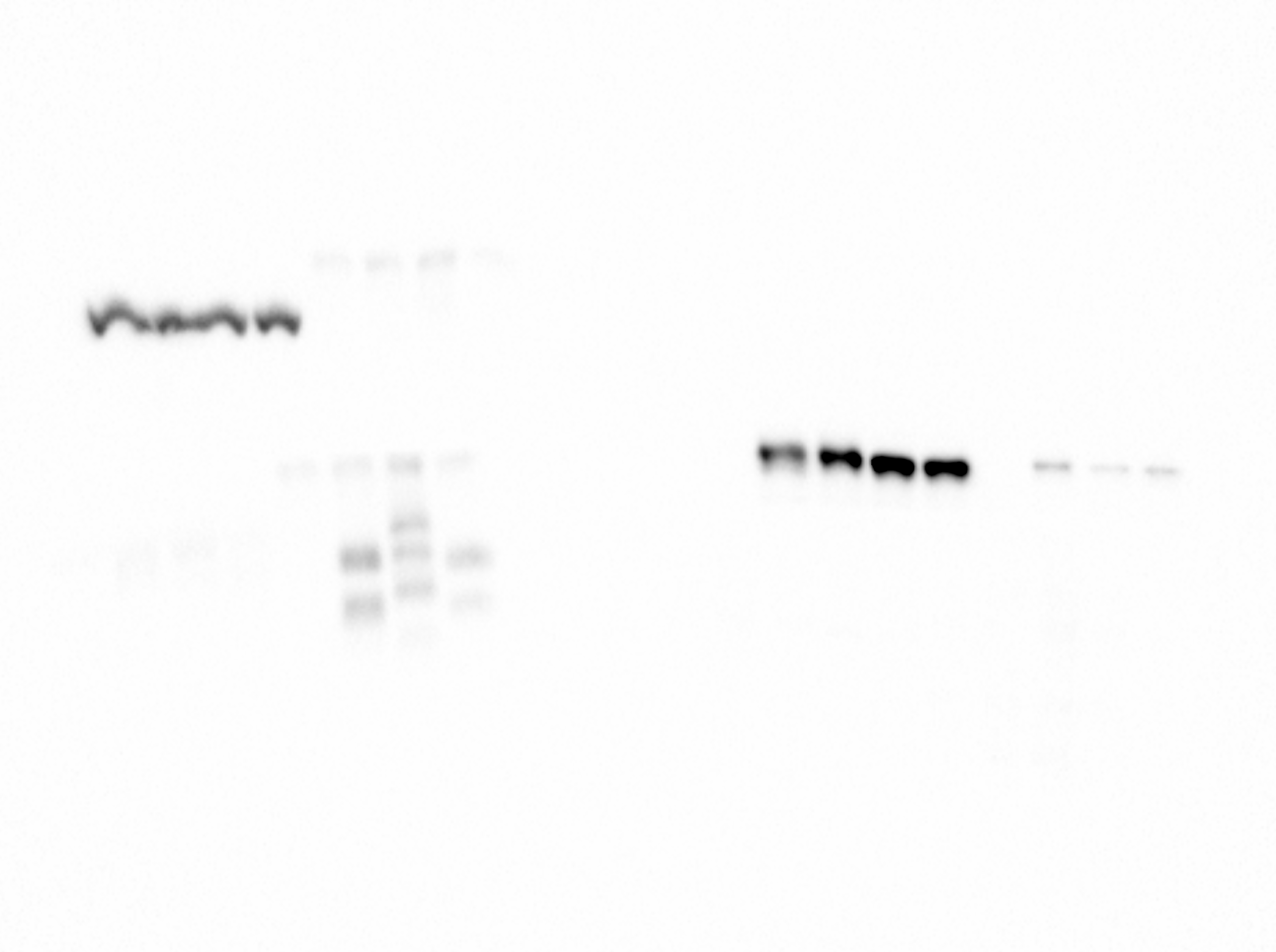

Supplement: Figure 6—figure supplement 2—source data 1. [file elife-72330-fig6-figsupp2-data1.zip › Figure 6 supplement 2 source data/Figure 6 Supp 2 A/Pgk.tif]

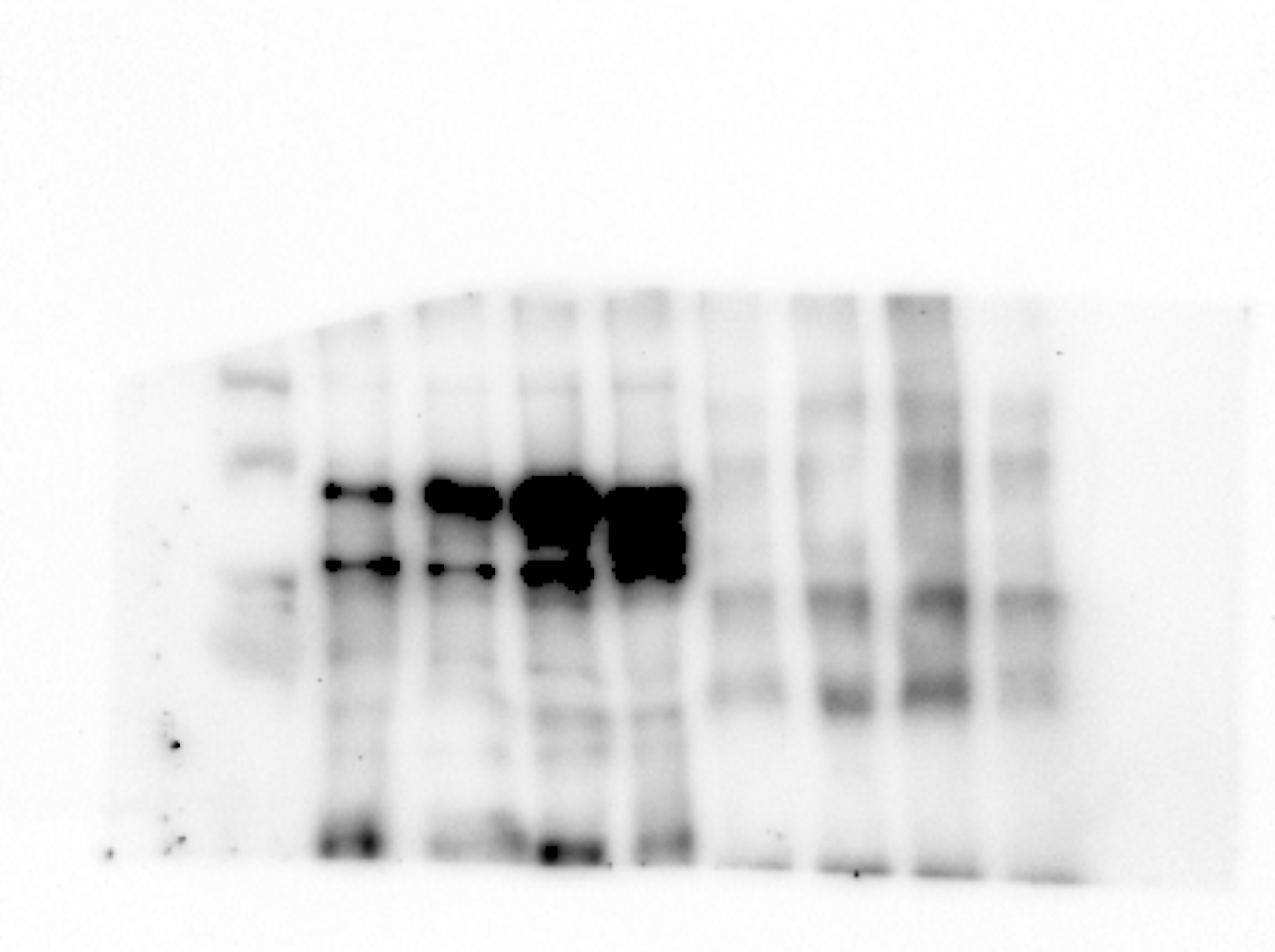

Supplement: Figure 6—figure supplement 2—source data 1. [file elife-72330-fig6-figsupp2-data1.zip › Figure 6 supplement 2 source data/Figure 6 Supp 2 A/myc high.tif]

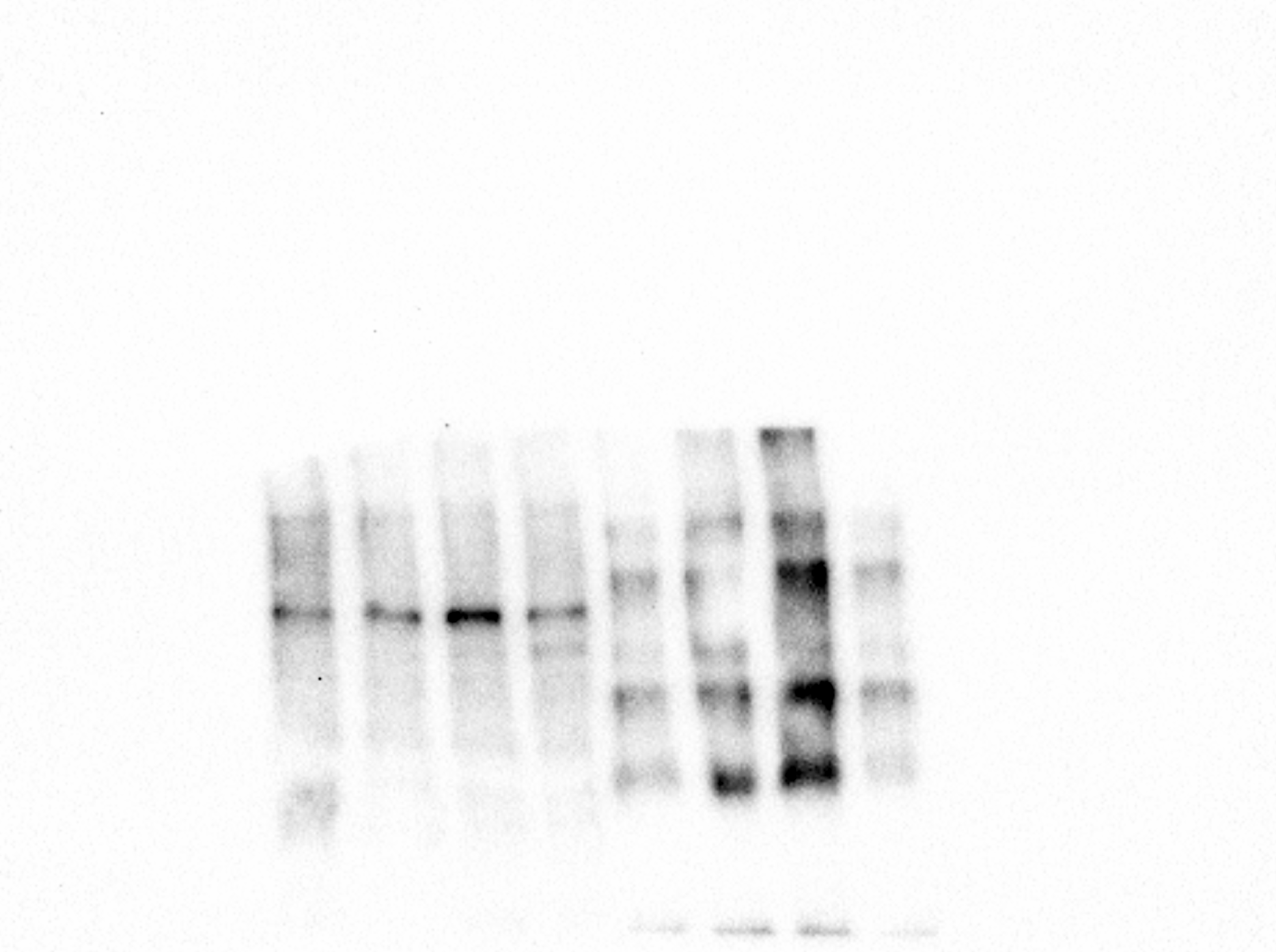

Supplement: Figure 6—figure supplement 2—source data 1. [file elife-72330-fig6-figsupp2-data1.zip › Figure 6 supplement 2 source data/Figure 6 Supp 2 A/myc blot.tif]

# kmeans clustering

gene

log2.ratio

6

4

2

0

1

2

3

4

5

6

7

8

9

10

WT

MUT

Control

condition

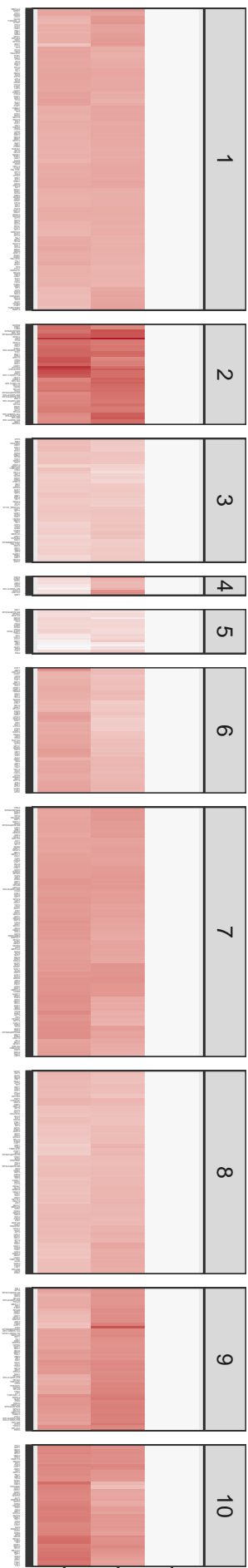

Supplement: Source data 2. [file elife-72330-data2.zip › data_analysis_results_V1/Clustering_heatmap_hits_kmeans_10_cluster_741_proteins_V1.pdf]

hierarchical clustering

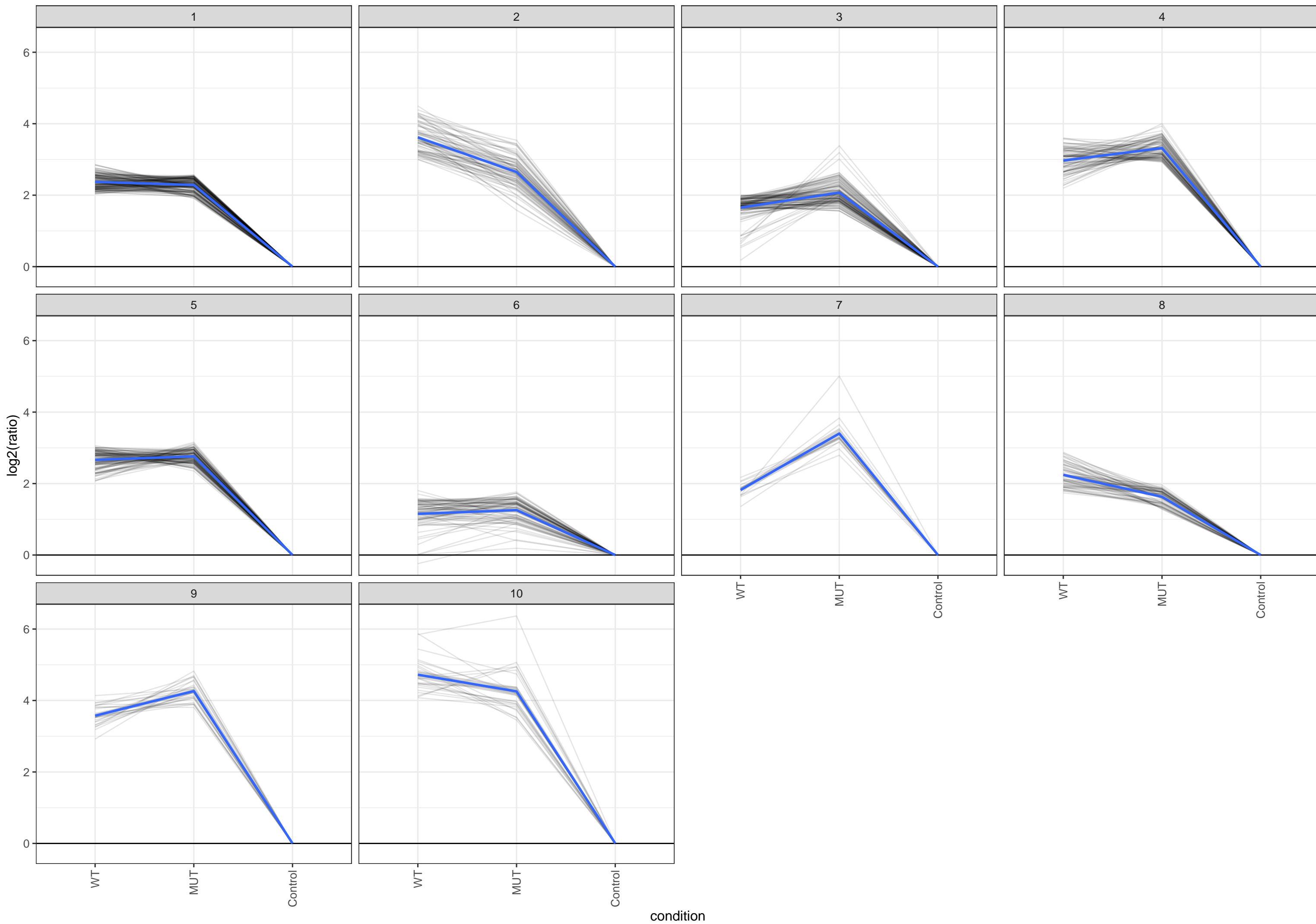

Supplement: Source data 2. [file elife-72330-data2.zip › data_analysis_results_V1/Clustering_line_plot_hclust_10_cluster_741_proteins_V1.pdf]

kmeans clustering

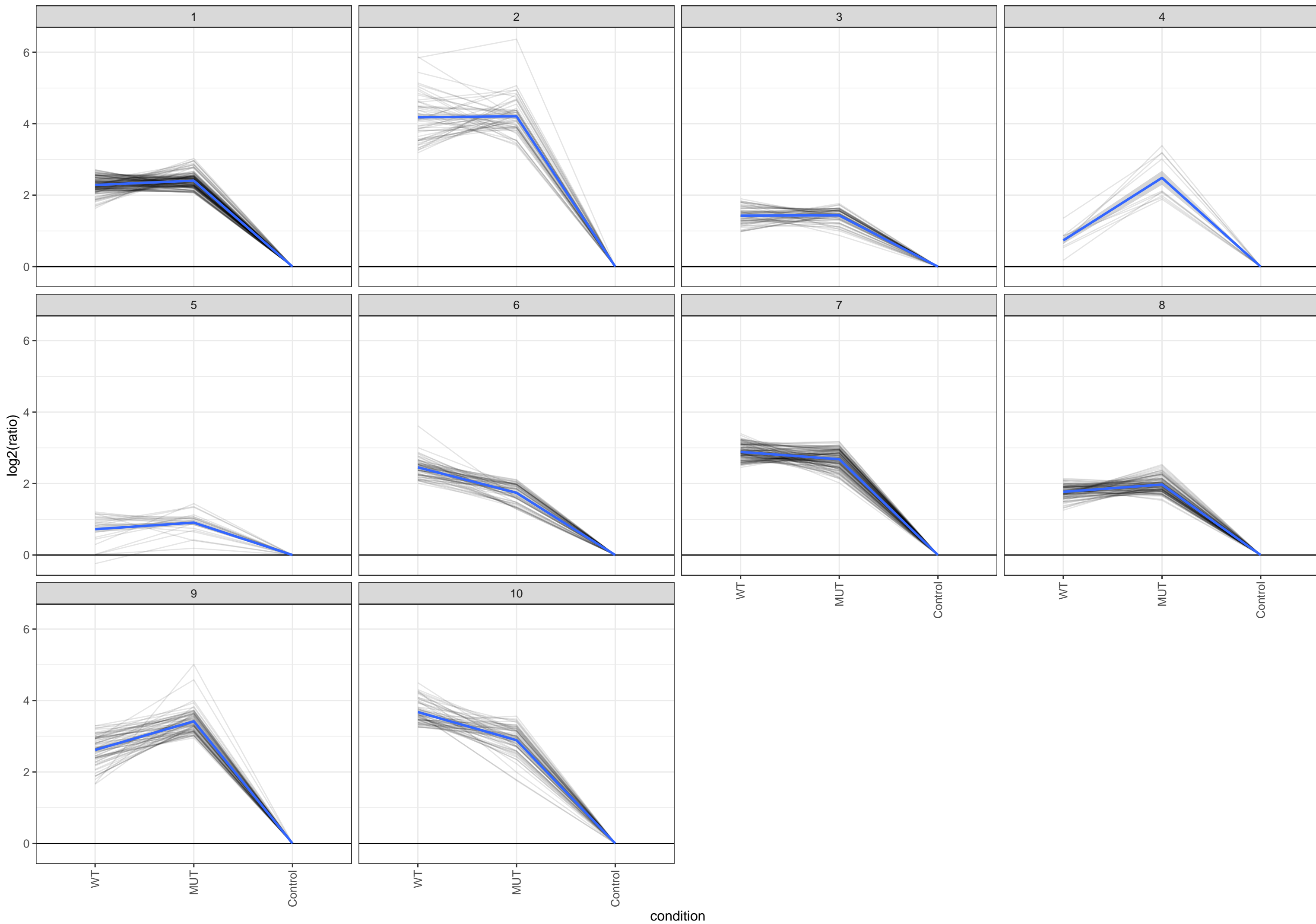

Supplement: Source data 2. [file elife-72330-data2.zip › data_analysis_results_V1/Clustering_line_plot_kmeans_10_cluster_741_proteins_V1.pdf]

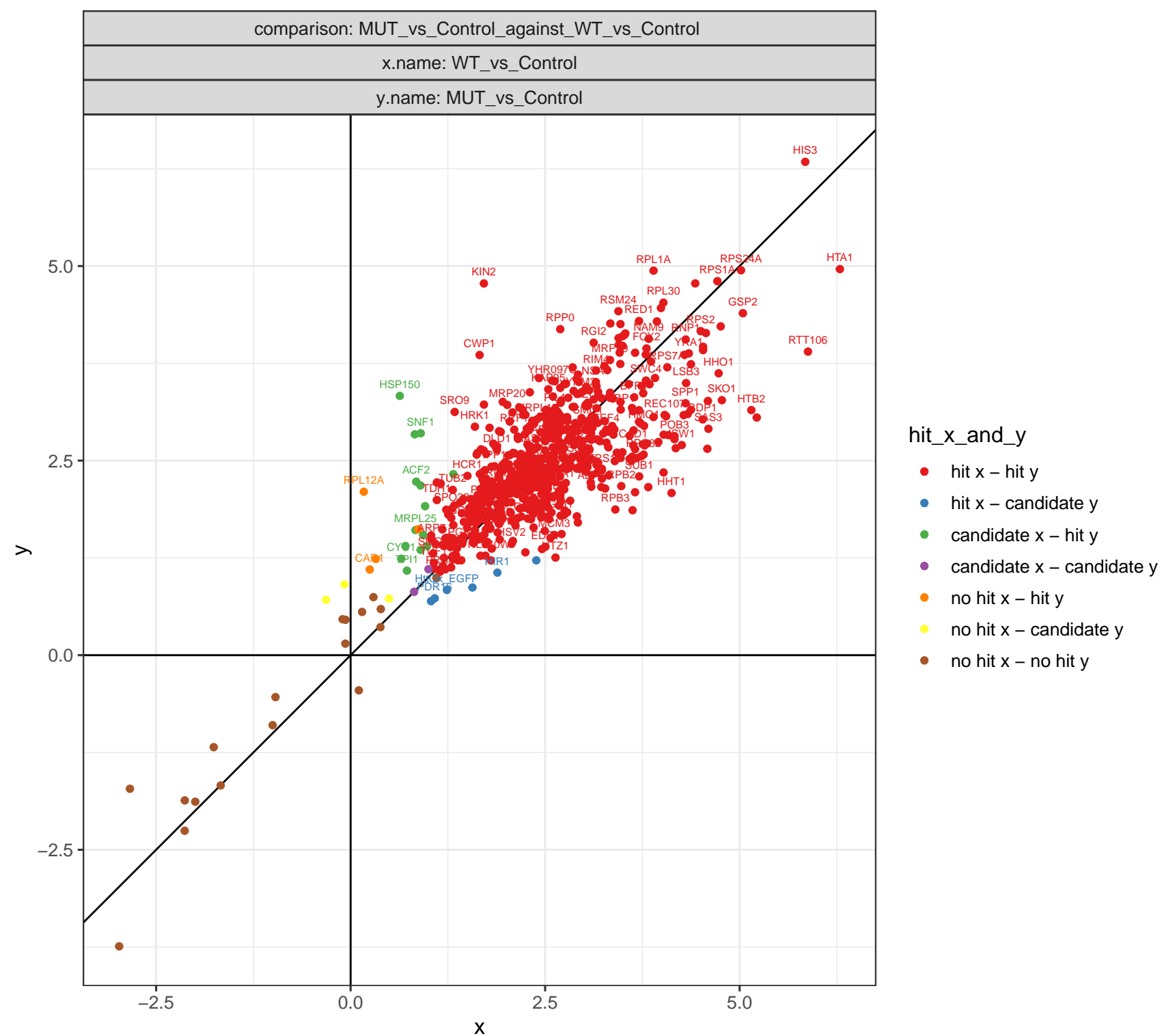

Supplement: Source data 2. [file elife-72330-data2.zip › data_analysis_results_V1/Fold_change_correlation_alt_hit_class_V1.pdf]

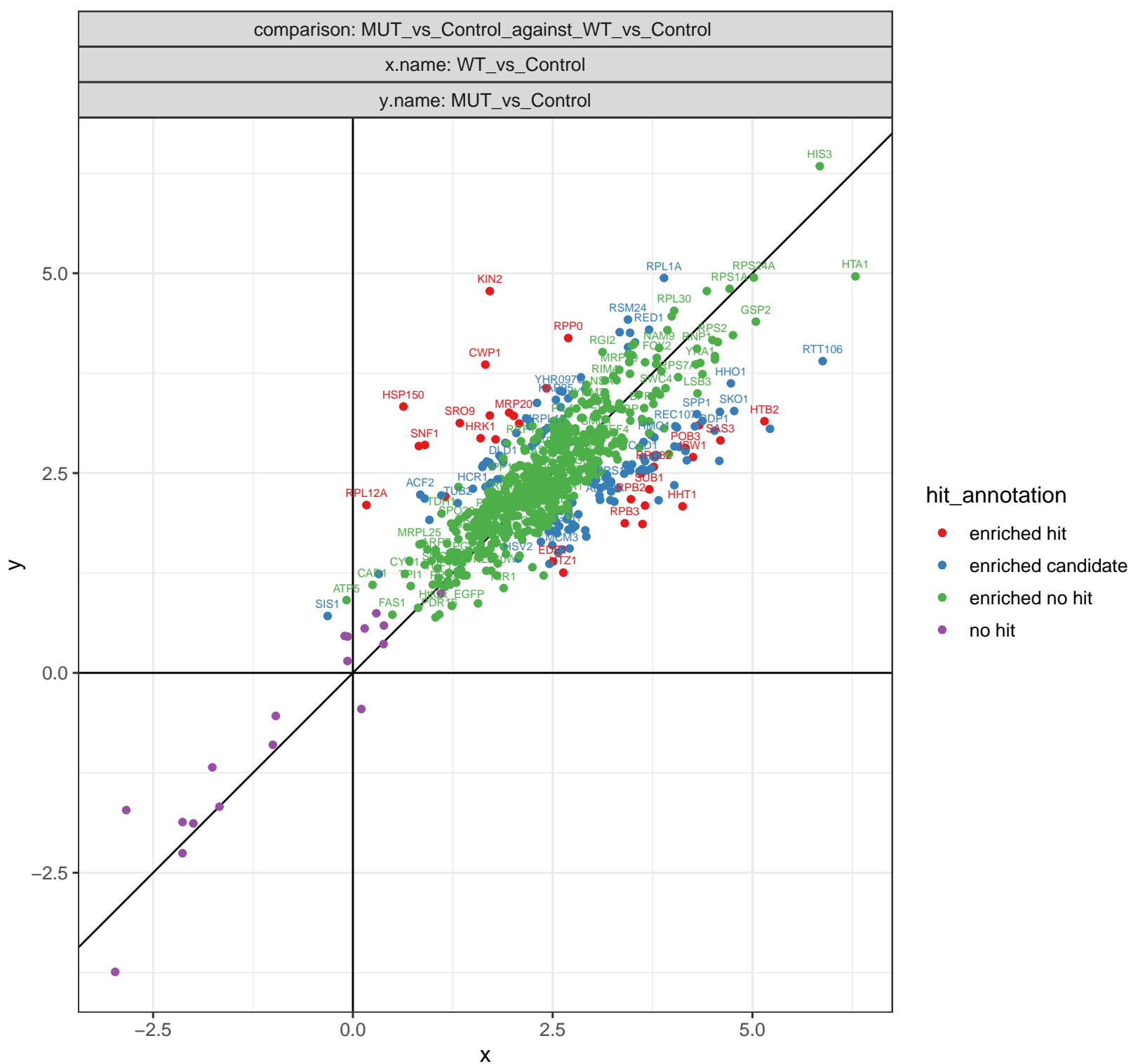

Supplement: Source data 2. [file elife-72330-data2.zip › data_analysis_results_V1/Fold_change_correlation_V1.pdf]

gene

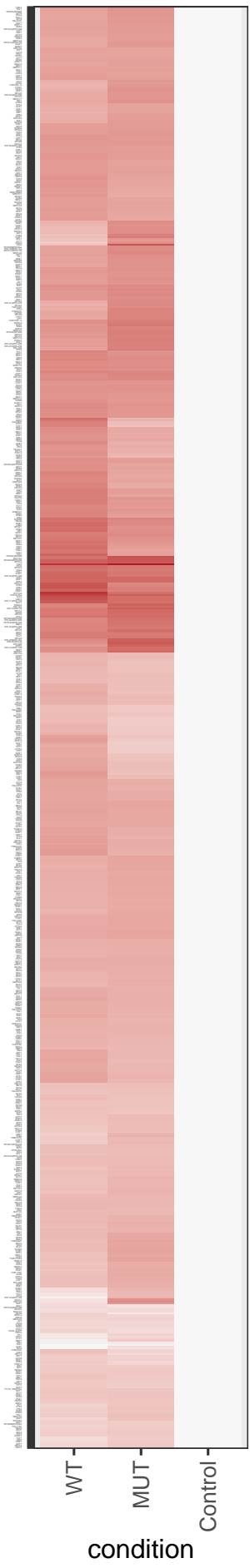

log2.ratio

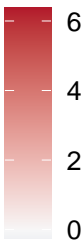

Supplement: Source data 2. [file elife-72330-data2.zip › data_analysis_results_V1/Heatmap_hits_741_proteins_V1.pdf]

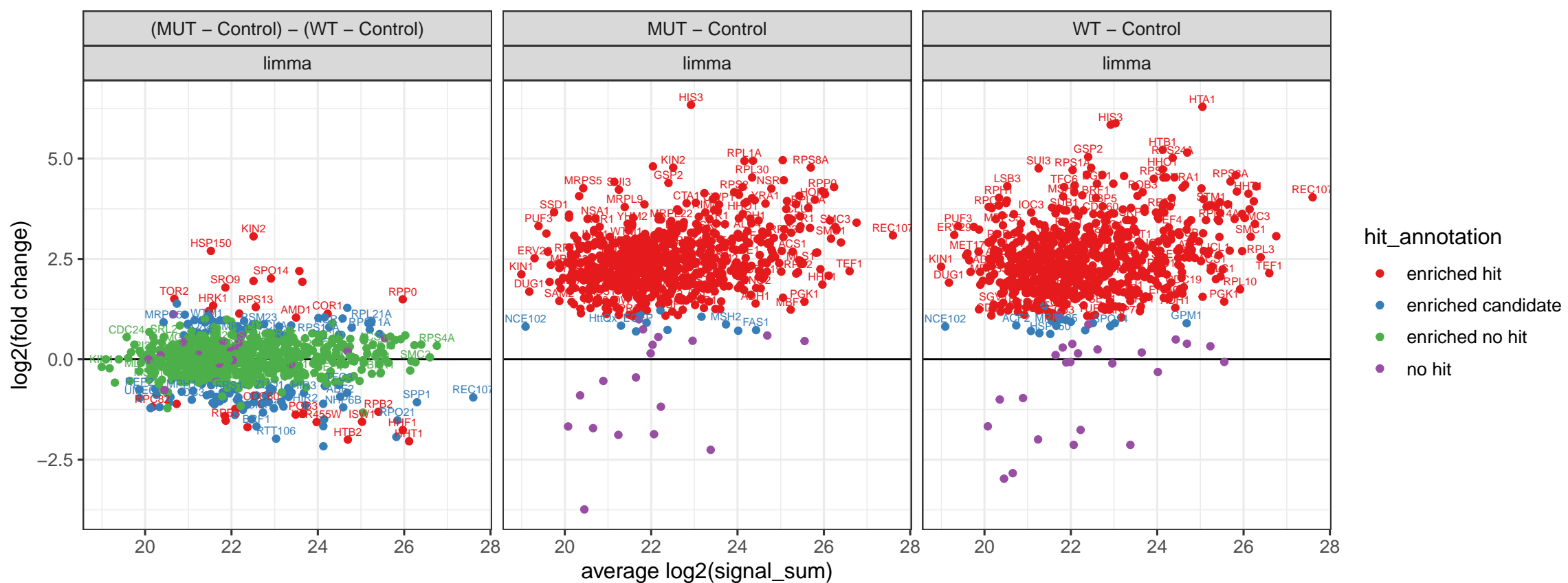

Supplement: Source data 2. [file elife-72330-data2.zip › data_analysis_results_V1/MA_plot_V1.pdf]

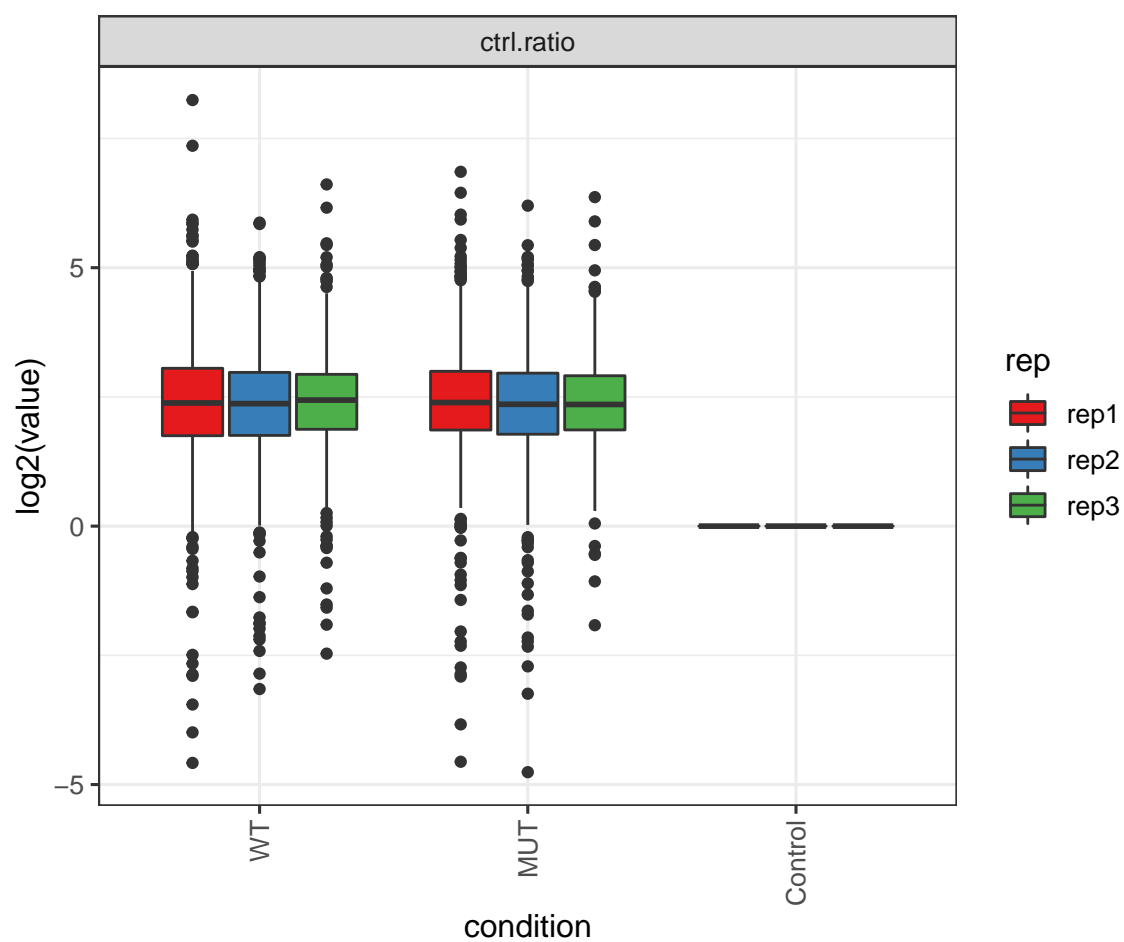

Supplement: Source data 2. [file elife-72330-data2.zip › data_analysis_results_V1/Normalization_overview_ratios_V1.pdf]

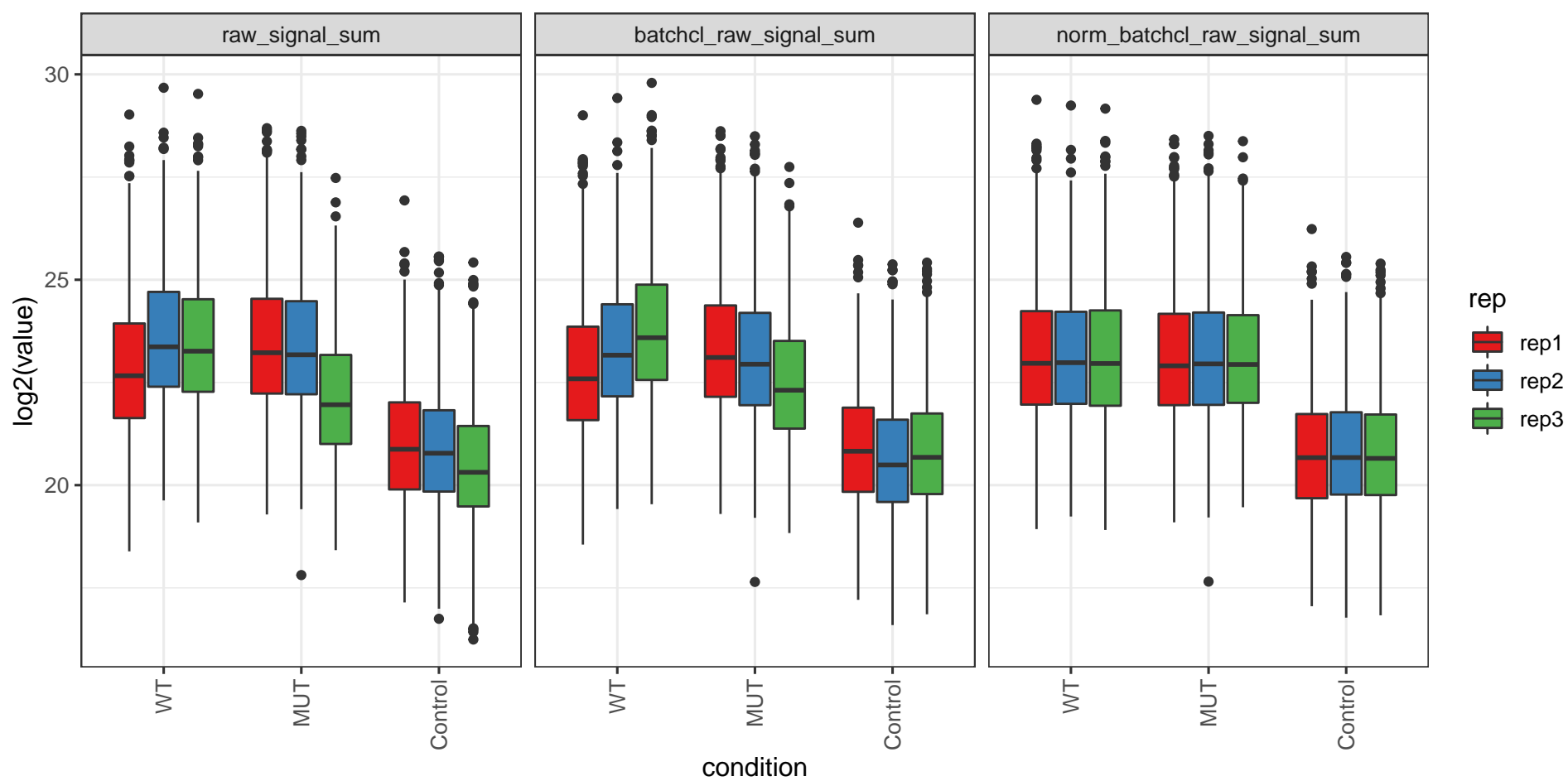

Supplement: Source data 2. [file elife-72330-data2.zip › data_analysis_results_V1/Normalization_overview_V1.pdf]

batchcl\_raw\_data

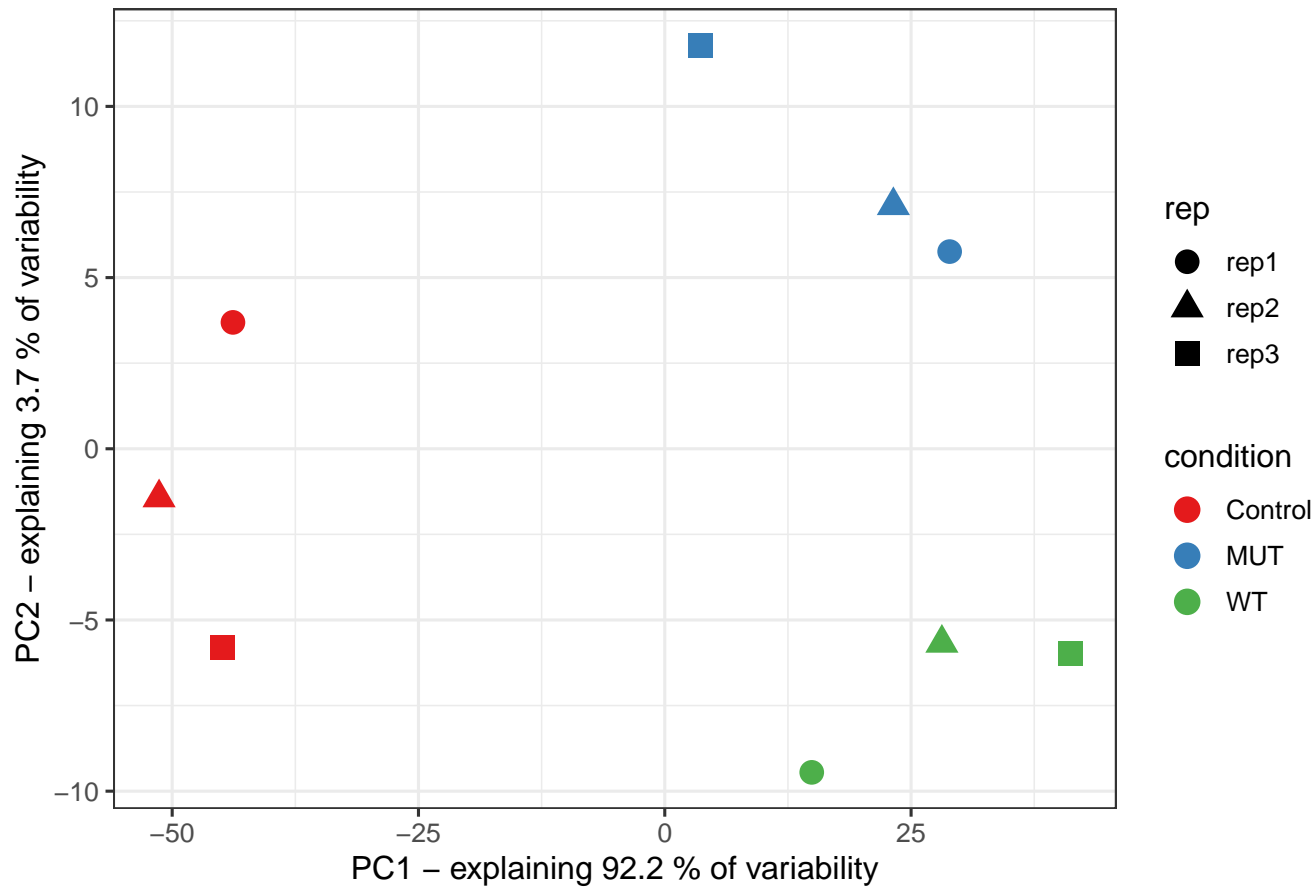

Supplement: Source data 2. [file elife-72330-data2.zip › data_analysis_results_V1/PCA_batchcl_raw_data_V1.pdf]

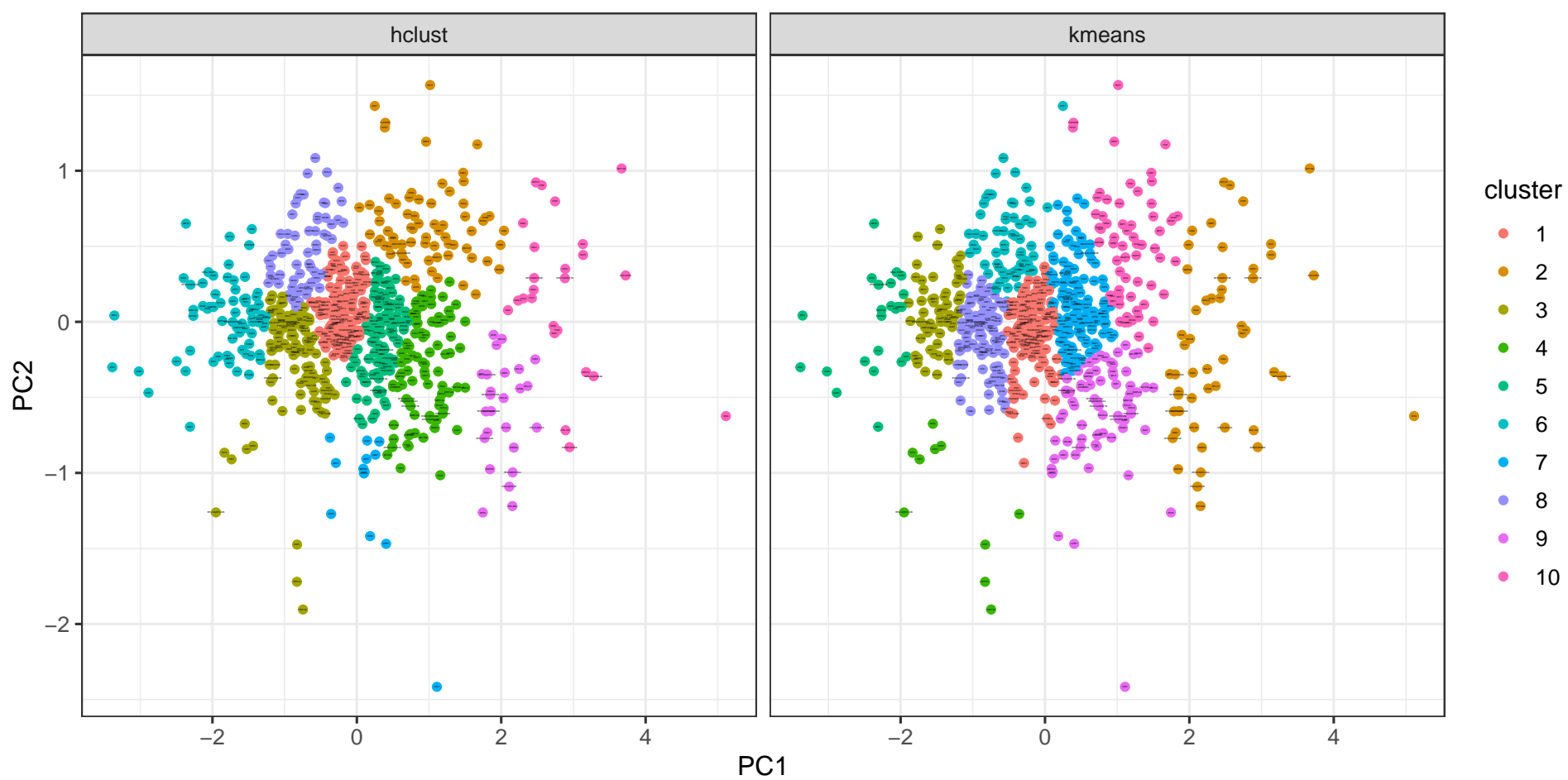

Supplement: Source data 2. [file elife-72330-data2.zip › data_analysis_results_V1/PCA_clustering_data_10_cluster_741_proteins_V1.pdf]

# PCA clustering data

PC3 – explaining 0 % of variability

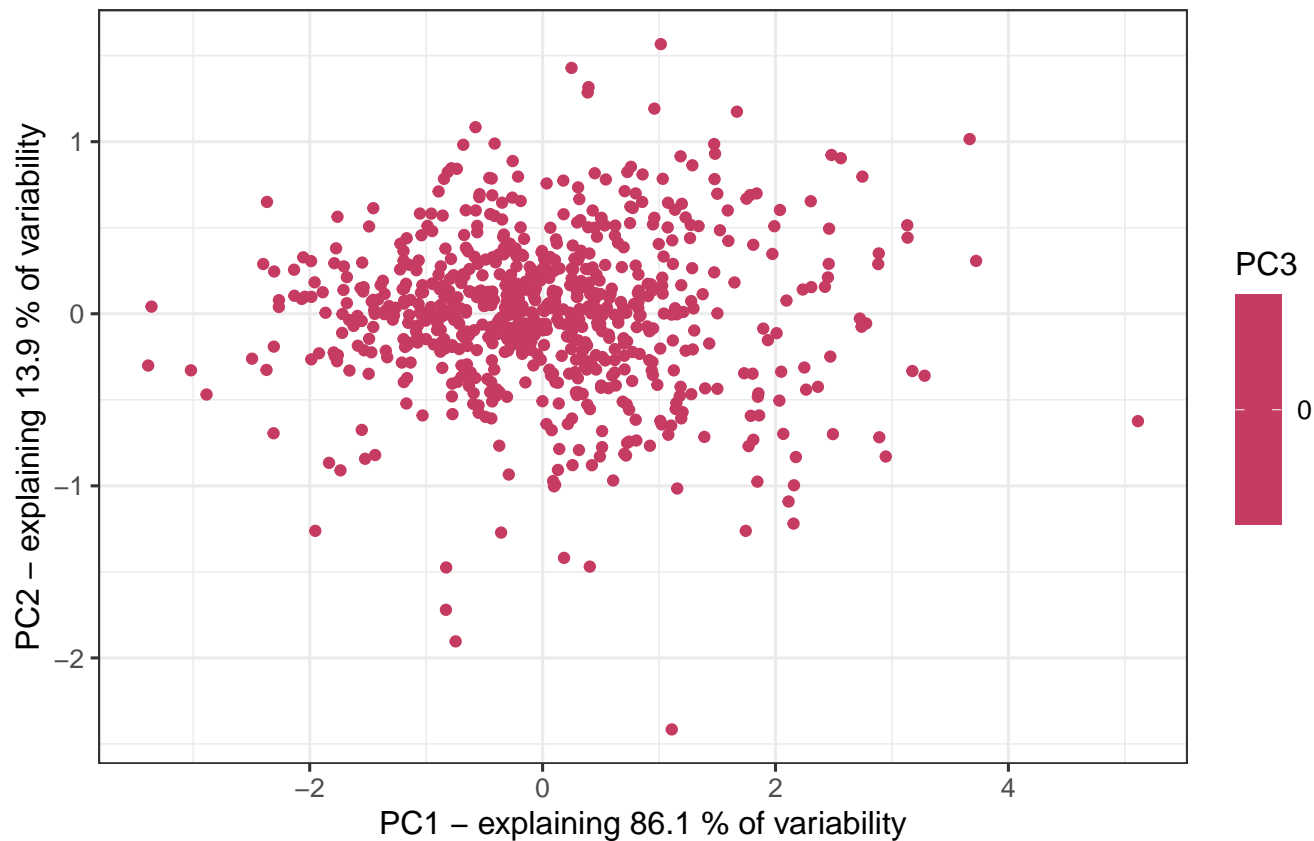

Supplement: Source data 2. [file elife-72330-data2.zip › data_analysis_results_V1/PCA_clustering_data_V1.pdf]

ctrl.ratio\_data

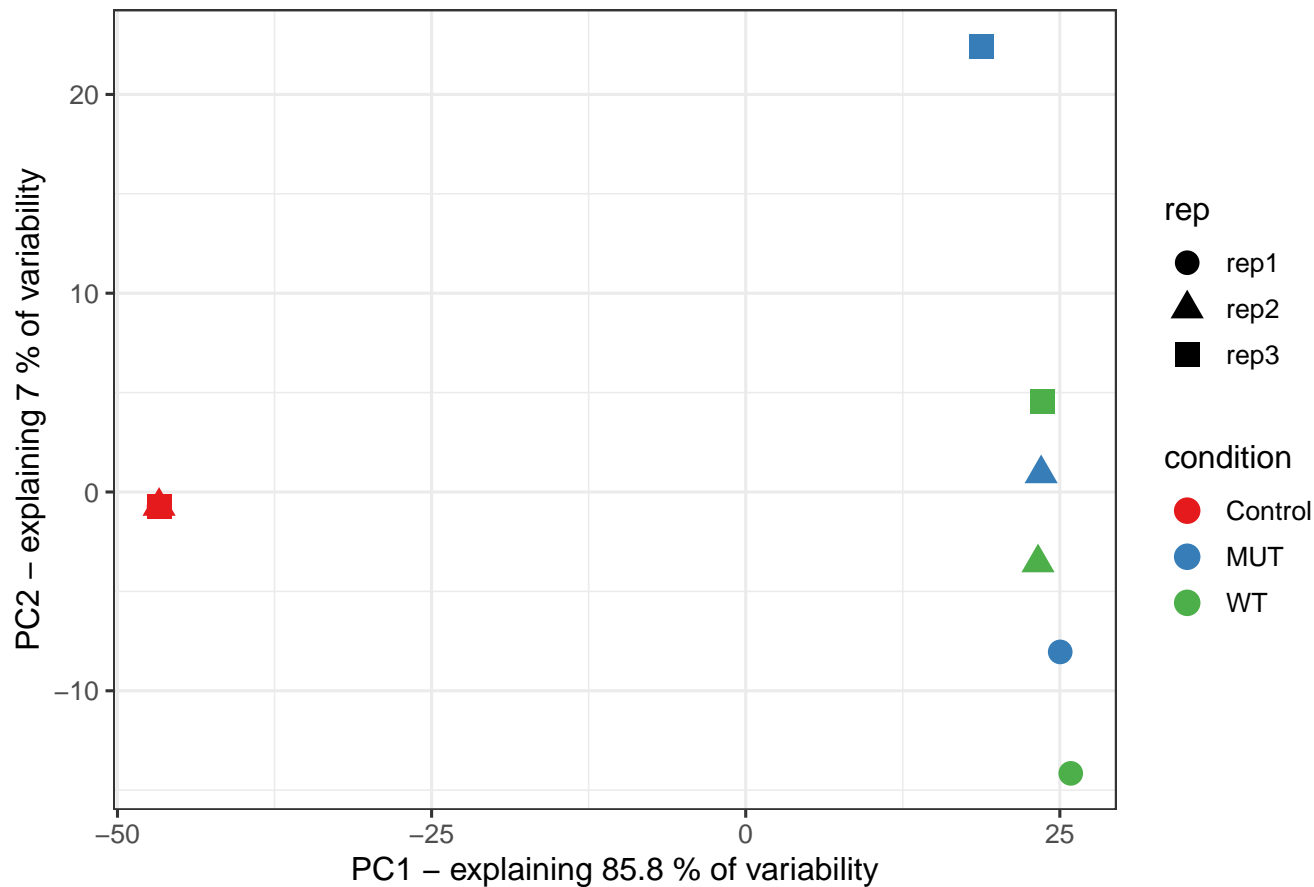

Supplement: Source data 2. [file elife-72330-data2.zip › data_analysis_results_V1/PCA_ctrl.ratio_data_V1.pdf]

norm\_batchcl\_raw\_data

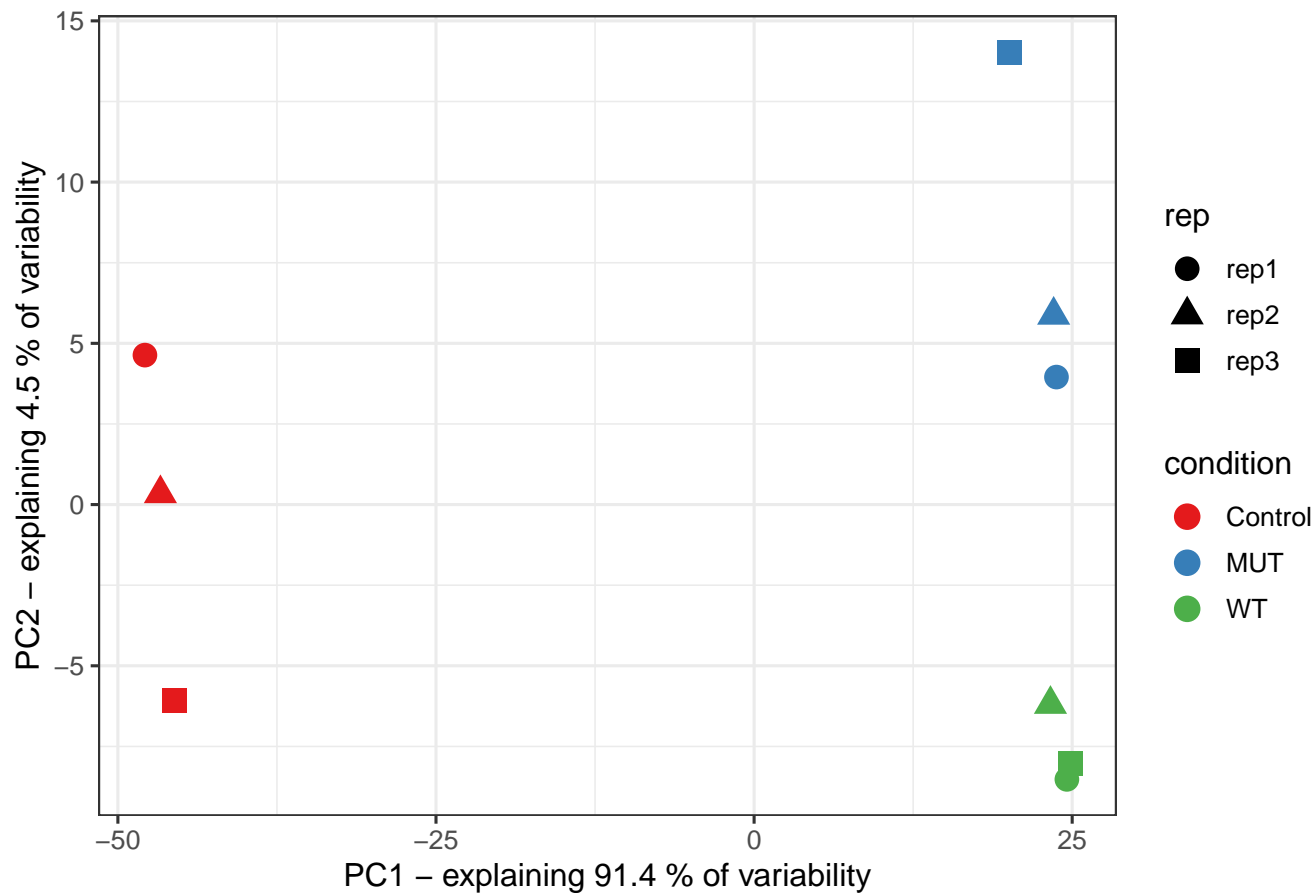

Supplement: Source data 2. [file elife-72330-data2.zip › data_analysis_results_V1/PCA_norm_batchcl_raw_data_V1.pdf]

raw\_data

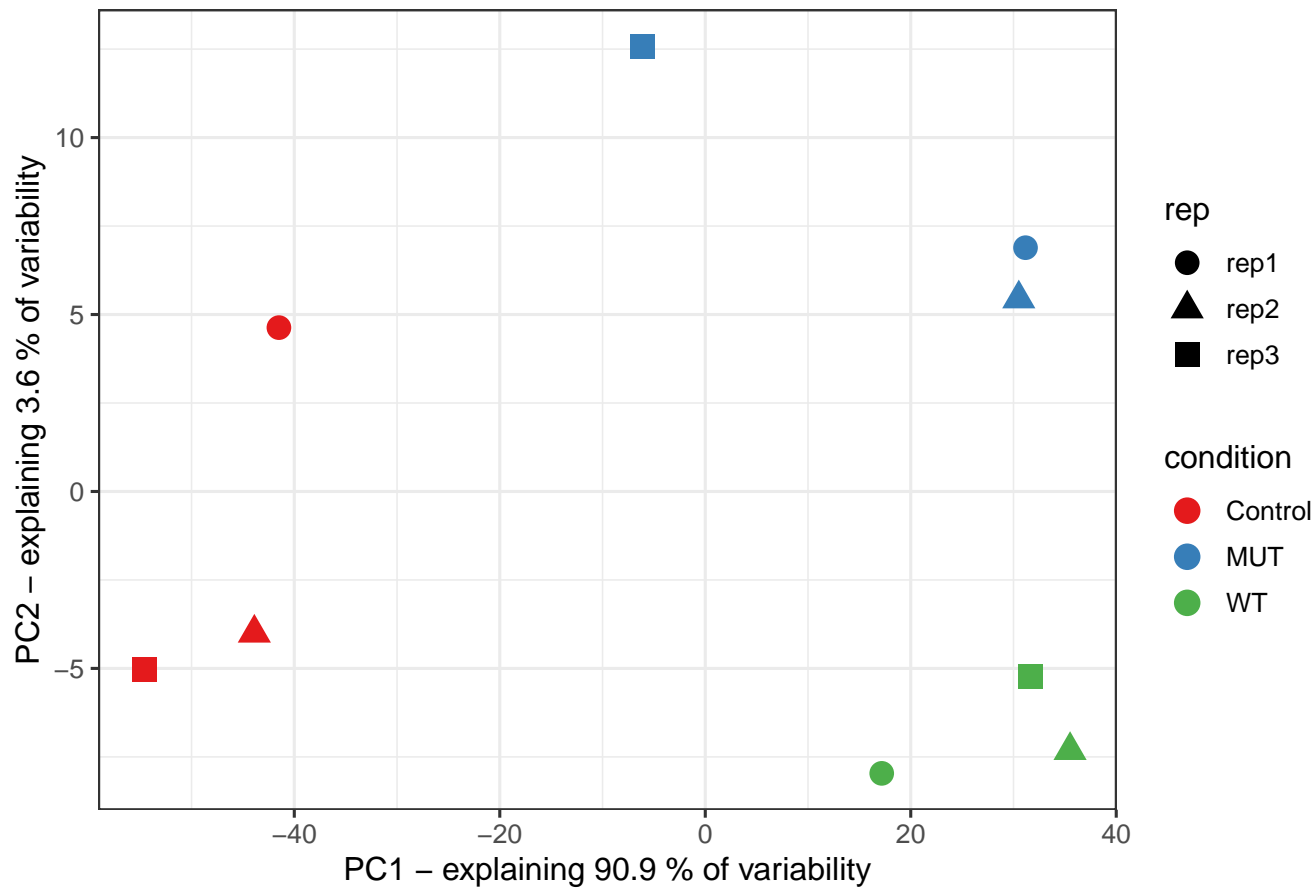

Supplement: Source data 2. [file elife-72330-data2.zip › data_analysis_results_V1/PCA_raw_data_V1.pdf]
