# Supplementary material for: Novel mechanistic insights into the role of Mer2 as the keystone of meiotic DNA break formation
Source: eLife. 2021 Dec 24;10:e72330. doi: 10.7554/eLife.72330 (PMC8848140; doi:10.7554/eLife.72330)
Supplement: Source data 2. [file elife-72330-data2.zip › VeronikaAltmannova_P1787_V1.pdf]

Veronika Altmannova - P1787

Version 1

Frank Stein

2021-09-01

This analysis pipeline is partly based on an analysis workflow developed by Bernd Klaus.

R setup

Defining a working directory

We defined the working directory for the analysis. This directory should contain the metadata file and the tab delimited text files containing the data.

```
setwd("~/home/path to the right file location")
```

Loading packages

```
library(vsn)
library(limma)
library(MSnbase)
library(gplots)
library(fdrtool)
library(biobroom)
library(tidyverse)
```

Defining some functions and parameters

```
customPlot <- list(
  theme_bw(base_size = 12),
  scale_fill_brewer(palette = "Set1"),
  scale_colour_brewer(palette = "Set1")
)
script.version <- "V1"
dir_save <- paste0("data_analysis_results_", script.version)
if (!dir.exists(dir_save))
{
  dir.create(dir_save)
}
```

Loading data and annotating experimental conditions

```
conditions <- read.delim("metadata.csv", sep = ",")
files <- file.path(unique(conditions$file))
```

```r
files <- files[file.exists(files)]
data <- NULL
for (i in seq_along(files))
{
  print(files[i])
  x <- read_delim(file.path(files[i]), delim = "    ")
  names(x) <- make.names(names(x))
  #keep only proteins with at least two unique peptide matches
  x <- x %>%
    dplyr::filter(qupm >= 2)
  #remove keratines
  x <- x %>%
    dplyr::filter(!grepl("[K, k][R, r][T, t][0-9]", gene_name))
  #remove known contaminants
  x <- x %>%
    dplyr::filter(!grepl("#+", protein_id))
  x$file <- files[i]
  keepnames <- as.character(subset(conditions, file == files[i])$col.name)
  x <- x[, c("gene_name", "protein_id", "description", "qupm", "top3", "file", keepnames)]
  x <- x %>%
    group_by(gene_name, protein_id, description, qupm, top3, file) %>%
    gather(key = "col.name", value = "value", keepnames)
  data <- bind_rows(data, x)
  rm(x, keepnames)
}
```

```
## [1] "fractionated_merged_results_20210825_1637_proteins.txt"
```

```r
rm(i, files)
data <- left_join(data, conditions)
data <- subset(data, value > 0)
data$gene_name[is.na(data$gene_name)] <- data$protein_id[is.na(data$gene_name)]
```

Protein identification overview

```r
sub <- unique(data[, c("gene_name", "file", "rep")])
sub$found <- 1
sub$file_old <- sub$file
sub$file <- paste("file", as.numeric(factor(sub$file)))
print(unique(with(sub, paste(file, "-", file_old))))
```

```
## [1] "file 1 - fractionated_merged_results_20210825_1637_proteins.txt"
```

```r
sub$file_old <- NULL
sub <- unique(sub)
ggplot(data = sub, aes(file, fill = rep)) +
  geom_bar(position = position_dodge()) +
  customPlot +
  theme(axis.text.x = element_text(angle = 45, hjust = 1, vjust = 1))
```

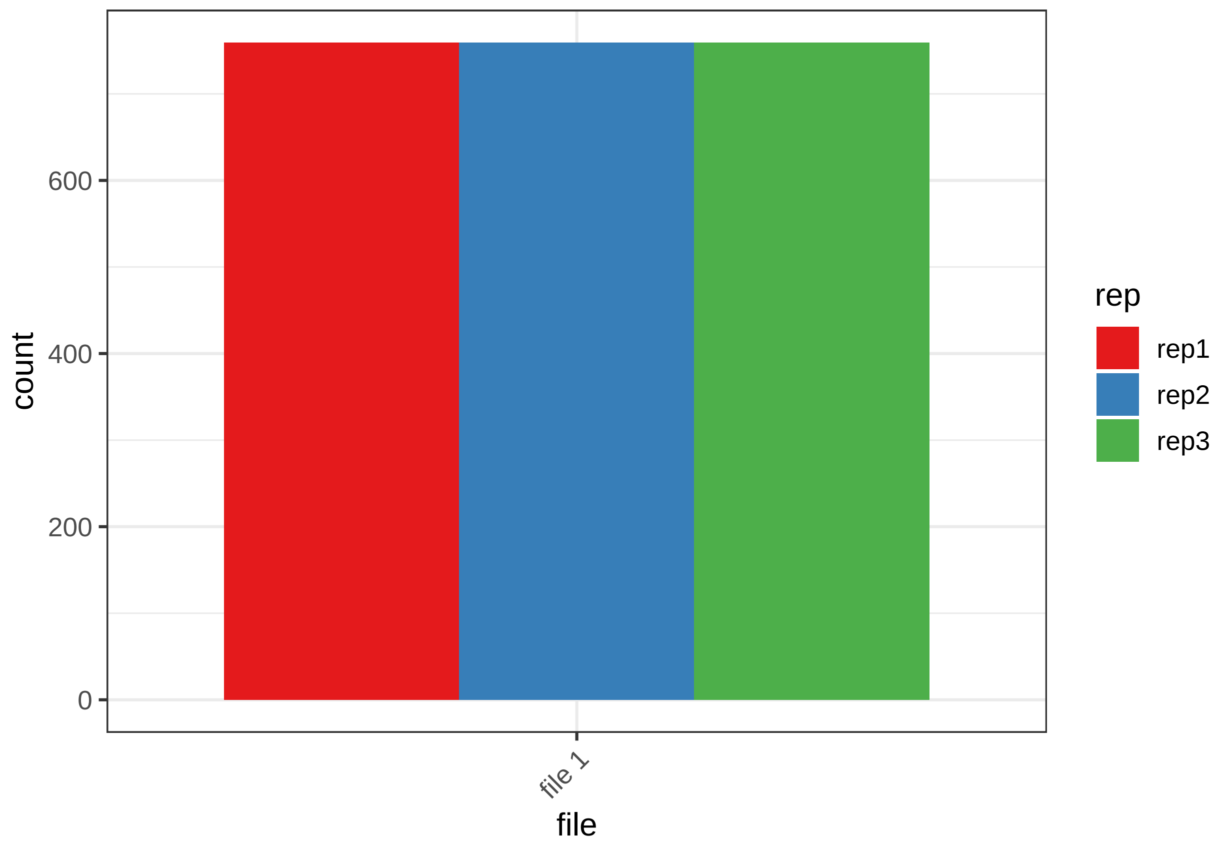


```
with(sub, table(file, rep))
```

```
##         rep
## file     rep1 rep2 rep3
##   file 1  759  759  759
```

```
sub_i <- data %>%
  group_by(gene_name) %>%
  dplyr::select(gene_name, rep, file) %>%
  unique() %>%
  summarise(found.in.files = n())
ggplot(data = sub_i, aes(found.in.files)) +
  geom_bar(position = position_dodge()) +
  customPlot +
  xlab("no of identifications per file")
```

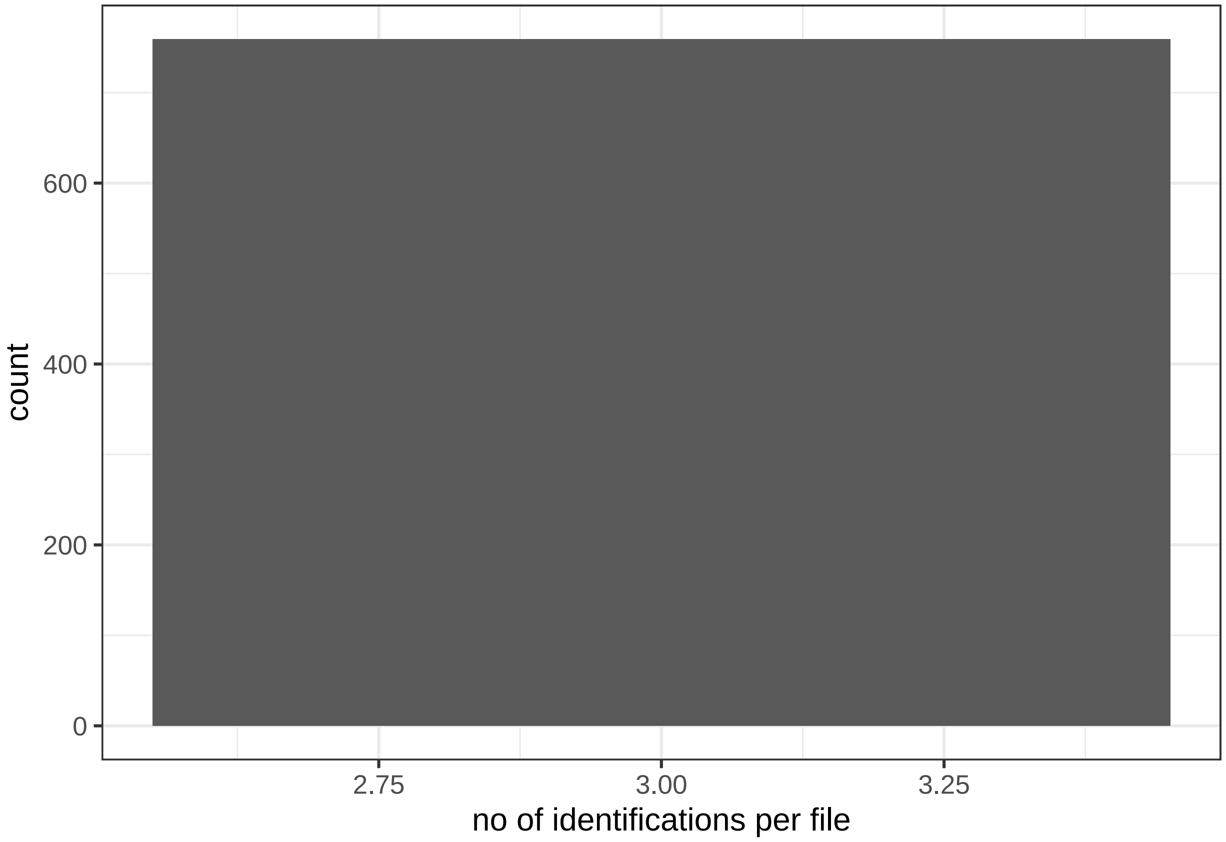


```
table(sub_i$found.in.files)
```

```
##
##   3
## 759
```

```
rm(sub_i)
rm(sub)
```

Identification and removal of duplicated gene names

```
dup.data <- data %>%
  ungroup() %>%
  dplyr::select(gene_name, protein_id, file, qupm) %>%
  unique() %>%
  group_by(file, gene_name) %>%
  dplyr::filter(n() > 1)
if (nrow(dup.data) > 0)
{
  write.csv(dup.data, file.path(dir_save, paste0("Duplicates_", script.version, ".csv")), row.names = F
}
dups <- unique(dup.data$gene_name)
if (length(dups) > 0)
{
  print("Duplicates were identified:")
  print(dups)
```

```r
  print("Removing genes with smaller numbers of quantified peptides.")
  for (dup in dups)
  {
    for (filen in unique(subset(dup.data, gene_name == dup)$file))
    {
      sub <- subset(data, gene_name == dup & file == filen)
      data <- subset(data, paste(gene_name, file) != paste(dup, filen))
      sub <- subset(sub, qupm == max(sub$qupm, na.rm = TRUE))
      data <- bind_rows(data, sub)
      rm(sub)
    }
    rm(filen)
  }
  rm(dup)
}
dup.data <- data %>%
  ungroup() %>%
  dplyr::select(gene_name, protein_id, file, qupm) %>%
  unique() %>%
  group_by(file, gene_name) %>%
  dplyr::filter(n() > 1)
dups <- unique(dup.data$gene_name)
if (length(dups) > 0)
{
  print("Duplicates:")
  print(dups)
  print("Removing genes with the least overlap of protein ids in other ms experiments")
  for (dup in dups)
  {
    sub <- subset(data, gene_name %in% dup)
    IDs <- unlist(strsplit(sub$protein_id, split = "[|]"))
    rm(sub)
    for (filen in unique(subset(dup.data, gene_name == dup)$file))
    {
      sub <- subset(data, gene_name == dup & file == filen)
      data <- subset(data, paste(gene_name, file) != paste(dup, filen))
      sub$ID.count <- 0
      for (ID in unique(sub$protein_id))
      {
        sub$ID.count[sub$protein_id == ID] <- length(which(IDs %in% unlist(strsplit(ID, split = "[|]"))
      }
      sub <- subset(sub, ID.count == max(sub$ID.count, na.rm = TRUE))
      sub$ID.count <- NULL
      data <- bind_rows(data, sub)
      rm(sub)
    }
    rm(filen, IDs)
  }
  rm(dup)
}
rm(dups, dup.data)
```

Transforming long data to wide data

```
ggplot(data = data, aes(condition, log2(value), fill = rep)) +
  geom_boxplot() +
  ylab("log2(signal_sum)") +
  customPlot +
  theme(axis.text.x = element_text(angle = 45, hjust = 1, vjust = 1))
```

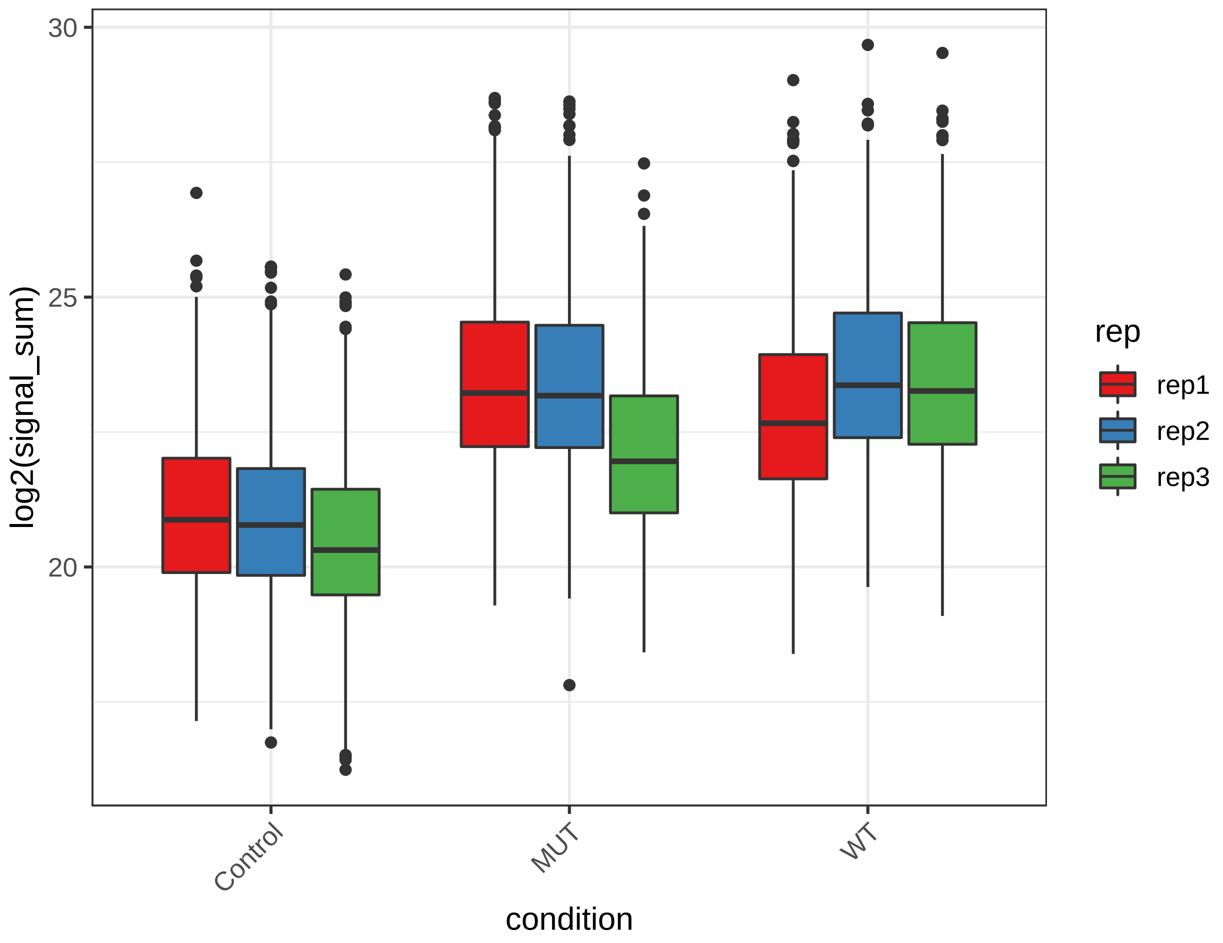


```
cdata <- data %>%
  group_by(gene_name) %>%
  mutate(key = paste("signal_sum", condition, rep, sep = "_")) %>%
  dplyr::select(gene_name, key, value) %>%
  group_by(gene_name, key) %>%
  summarise(value = sum(value, na.rm = TRUE)) %>%
  spread(key = key, value = value)
#found.in.files
fdata <- data %>%
  group_by(gene_name) %>%
  dplyr::select(gene_name, file) %>%
  unique() %>%
  summarise(found.in.files = n())
fdata_i <- data %>%
  mutate(file2 = make.names(file)) %>%
```

```
  group_by(gene_name, file2) %>%
  dplyr::select(gene_name, file, file2) %>%
  unique() %>%
  summarise(found.in.file = n()) %>%
  mutate(file2 = paste0("found.in.file_", file2)) %>%
  spread(key = file2, value = found.in.file)
fdata <- left_join(fdata, fdata_i)
#found.in.conditions
fdata_i <- data %>%
  group_by(gene_name) %>%
  dplyr::select(gene_name, condition) %>%
  unique() %>%
  summarise(found.in.conditions = n())
fdata <- left_join(fdata, fdata_i)
#found.in.reps
fdata_i <- data %>%
  group_by(gene_name) %>%
  dplyr::select(gene_name, rep) %>%
  unique() %>%
  summarise(found.in.reps = n())
fdata <- left_join(fdata, fdata_i)
#qupm overview
fdata_i <- data %>%
  group_by(gene_name) %>%
  dplyr::select(gene_name, qupm) %>%
  unique() %>%
  summarise(max.qupm = max(qupm, na.rm = TRUE))
fdata <- left_join(fdata, fdata_i)
#abundance overview
fdata_i <- data %>%
  group_by(gene_name) %>%
  dplyr::select(gene_name, top3) %>%
  unique() %>%
  summarise(average.top3 = mean(top3, na.rm = TRUE))
fdata <- left_join(fdata, fdata_i)
id_data <- data %>%
  ungroup() %>%
  dplyr::select(gene_name, protein_id, description) %>%
  unique()
dups <- id_data %>%
  group_by(gene_name) %>%
  summarize(n = n()) %>%
  dplyr::filter(n > 1) %>%
  dplyr::select(gene_name)
if (nrow(dups) > 0)
{
  dups <- dups$gene_name
  dup.data <- subset(id_data, gene_name %in% dups)
  id_data <- subset(id_data, !gene_name %in% dups)
  combine_ids <- function(ids)
  {
    ids <- as.character(ids)
    return(paste(
```

```
    sort(unique(unlist(strsplit(ids, split = "[|]")))), collapse = "|"))
  }
  dup.data <- dup.data %>%
    group_by(gene_name) %>%
    summarise(protein_id = combine_ids(protein_id),
              description = combine_ids(description))
  id_data <- bind_rows(id_data, dup.data)
  rm(dup.data, combine_ids)
}
rm(dups)
fdata <- left_join(id_data, fdata)
rm(id_data)
fdata <- left_join(fdata, fdata_i)
cdata <- left_join(fdata, cdata)
rm(fdata_i, fdata)
```

Filter data

Only proteins that were quantified with two unique peptide matches are kept for the analysis. Proteins were filtered according to these condition already when loading the data. Moreover, only proteins were kept if they were quantified in at least 2/3 of the replicates.

```
dim(cdata)
```

```
## [1] 759  18
```

```
min.found.in.files <- 1
min.qupm <- 2
cdata <- cdata %>%
  dplyr::filter(found.in.files >= min.found.in.files, max.qupm >= min.qupm)
dim(cdata)
```

```
## [1] 759  18
```

```
rm(min.found.in.files, min.qupm)
```

Building an expression set object

```
# Constructing assay data
raw_data <- cdata %>%
  dplyr::select(starts_with("signal_sum")) %>%
  as.data.frame()
rownames(raw_data) <- cdata$gene_name
names(raw_data) <- gsub("signal_sum_", "", names(raw_data))

# Constructing metadata
conditions_i <- data.frame(ID = names(raw_data))
conditions_i <- left_join(conditions_i, conditions)

# Constructing fdata
fdata <- cdata %>%
  dplyr::select(-starts_with("signal_sum")) %>%
  as.data.frame()
```

```r
# Defining ID columns
rownames(fdata) <- fdata$gene_name
rownames(conditions_i) <- conditions_i$ID
colnames(raw_data) <- conditions_i$ID

# Log2-transformation of raw_ signal_sums
raw_data_m <- log2(as.matrix(raw_data))
raw_data_m[is.infinite((raw_data_m))] <- NA
raw_data_m[is.na((raw_data_m))] <- NA

# Creating an expression set
raw_dataE <- ExpressionSet(assayData = raw_data_m,
                           phenoData = AnnotatedDataFrame(conditions_i),
                           featureData = AnnotatedDataFrame(fdata))
validObject(raw_dataE)
```

```
## [1] TRUE
```

```r
rm(raw_data, raw_data_m, conditions_i, fdata)
```

Batch effect removal

```r
batchcl_raw_dataE <- raw_dataE
exprs(batchcl_raw_dataE) <- removeBatchEffect(exprs(batchcl_raw_dataE),
      batch = as.character(pData(batchcl_raw_dataE)$rep),
      design = model.matrix(~0 + as.character(pData(batchcl_raw_dataE)$condition)))
```

Data normalization

The vsn package from Wolfgang Huber is used to apply a variance stabilization normalization method on the log2 raw data.

```r
norm_batchcl_raw_dataE <- batchcl_raw_dataE
for (ng in unique(conditions$norm_group))
{
  print(ng)
  vsn.fit <- vsn2(2^exprs(norm_batchcl_raw_dataE[, norm_batchcl_raw_dataE$norm_group == ng]))
  norm_batchcl_raw_dataE_i <-
    predict(vsn.fit, 2^exprs(norm_batchcl_raw_dataE[ , norm_batchcl_raw_dataE$norm_group == ng]))
  for (cn in colnames(norm_batchcl_raw_dataE_i))
  {
    exprs(norm_batchcl_raw_dataE)[ , cn] <- norm_batchcl_raw_dataE_i[, cn]
  }
  rm(cn, norm_batchcl_raw_dataE_i)
  sdplot.object <- meanSdPlot(vsn.fit, plot = FALSE)
  print(sdplot.object$gg + ggtitle(ng))
  rm(vsn.fit, sdplot.object, vsn.fit)
}
```

```
## [1] "Mer2"
```

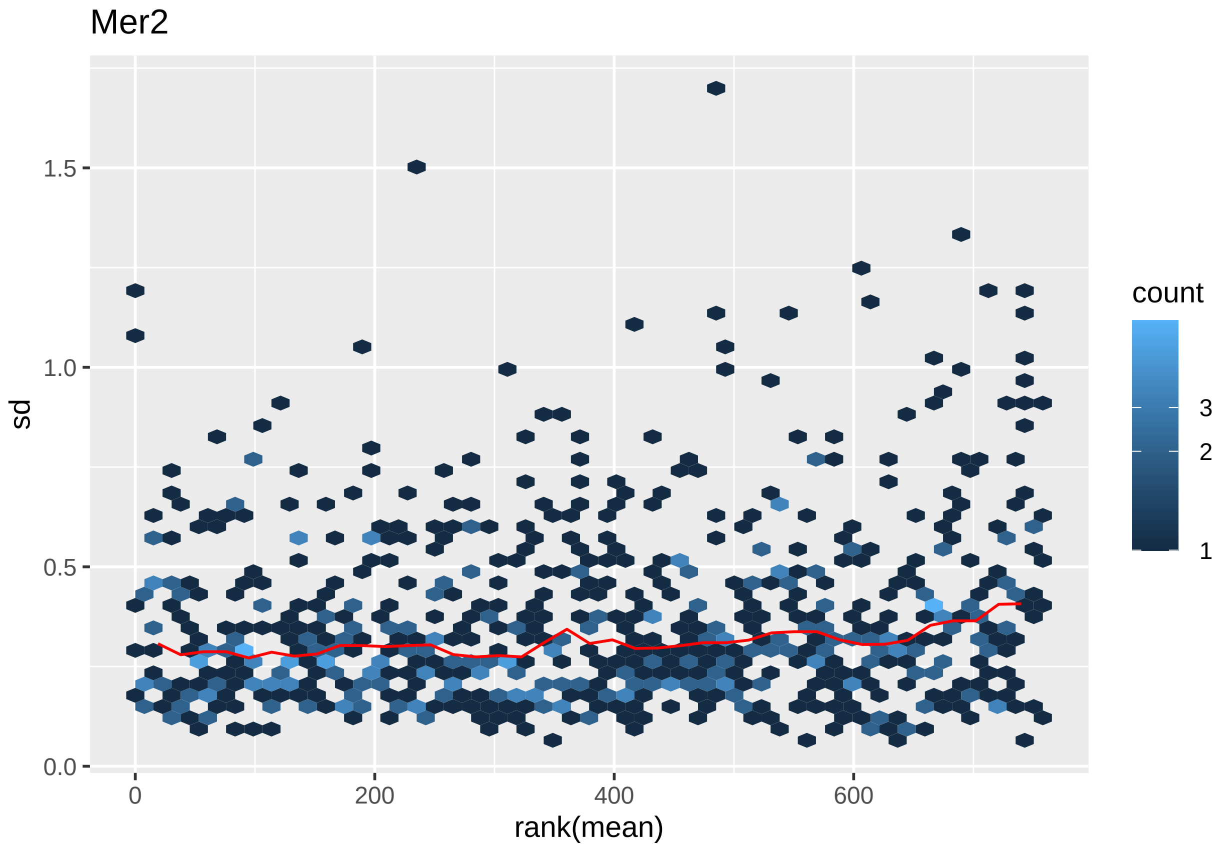


```
## [1] "control"
```

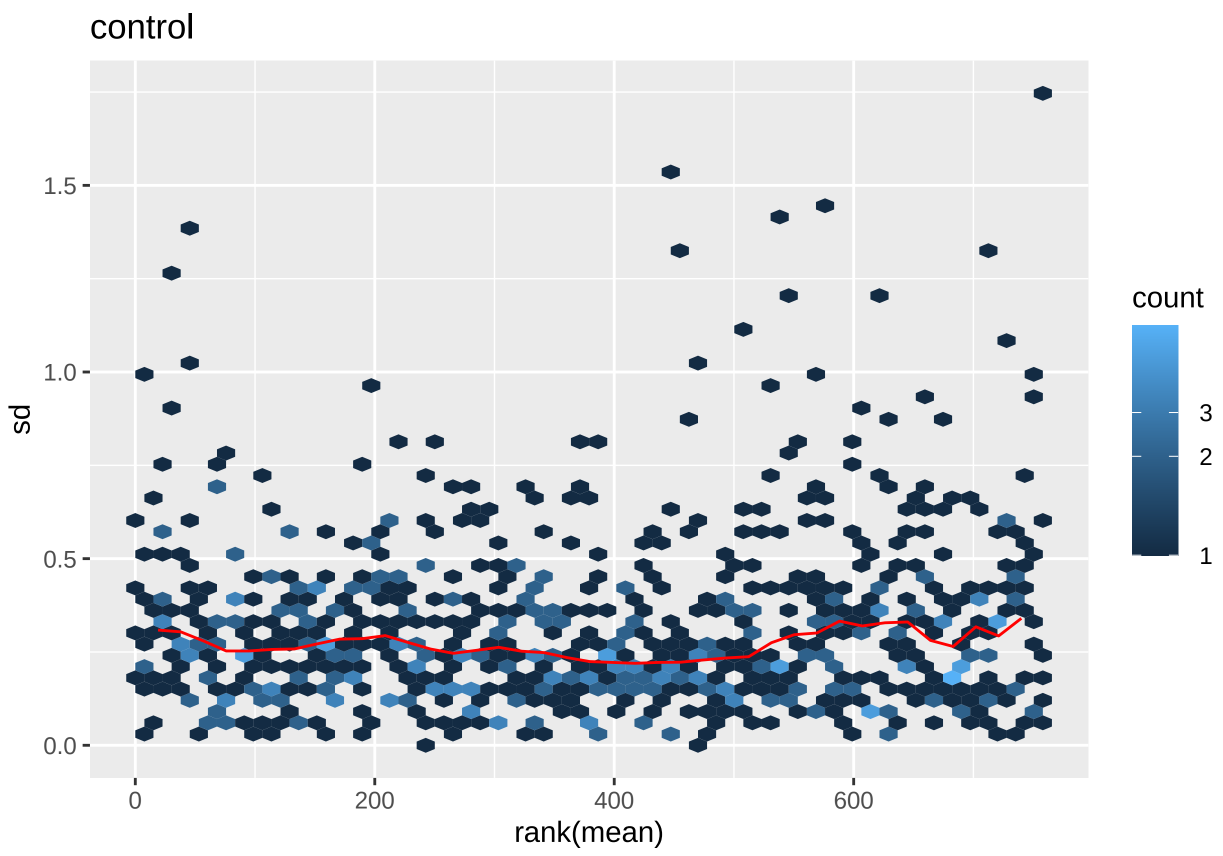


```
rm(ng)
```

Calculation of ctrl-fold changes

```
fc.data <- as.data.frame(2^exprs(norm_batchcl_raw_dataE))
for (i in seq_along(conditions$ID))
{
  fc.data[, paste0(conditions$ID[i], ".ctrl.ratio")] <-
    fc.data[, as.character(conditions$ID[i])] /
    fc.data[, as.character(conditions$ctrl.ID[i])]
}
rm(i)
fc.data <- fc.data %>%
  dplyr::select(ends_with(".ctrl.ratio"))
fc.data$gene_name <- rownames(fc.data)
fc.data <- fc.data %>%
  dplyr::select(ends_with(".ctrl.ratio"))
ctrl.ratio_dataE <- norm_batchcl_raw_dataE
names(fc.data) <- gsub(".ctrl.ratio", "", names(fc.data))
fc.data <- fc.data[ , colnames(ctrl.ratio_dataE)]
exprs(ctrl.ratio_dataE) <- fc.data %>%
  as.matrix() %>%
  log2()
rm(fc.data)
```

PCA - principal component analysis

```r
# PCA of raw_data
raw_PCA_m <- t(na.omit(exprs(raw_dataE)))
if (length(raw_PCA_m) > 50)
{
  raw_PCA <- prcomp(raw_PCA_m, scale = FALSE)
  perc_var <-
    round(100 * raw_PCA$sdev ^ 2 /
            sum(raw_PCA$sdev ^ 2), 1)
  PCA_raw_data <-
    data.frame(PC1 = raw_PCA$x[, 1],
               PC2 = raw_PCA$x[, 2],
               rep = pData(raw_dataE)$rep,
               condition = pData(raw_dataE)$condition)
  print(ggplot(data = PCA_raw_data, aes(PC1, PC2)) +
          geom_point(aes(color = condition, shape = rep), size = 4) +
          guides(size = FALSE) +
          customPlot +
          ggtitle("raw_data") +
          xlab(paste("PC1 - explaining", perc_var[1], "% of variability")) +
          ylab(paste("PC2 - explaining", perc_var[2], "% of variability")))
  ggsave(file.path(dir_save, paste0("PCA_raw_data_", script.version, ".pdf")),
         width = 7, height = 5)
  rm(raw_PCA, perc_var, PCA_raw_data)
}
```

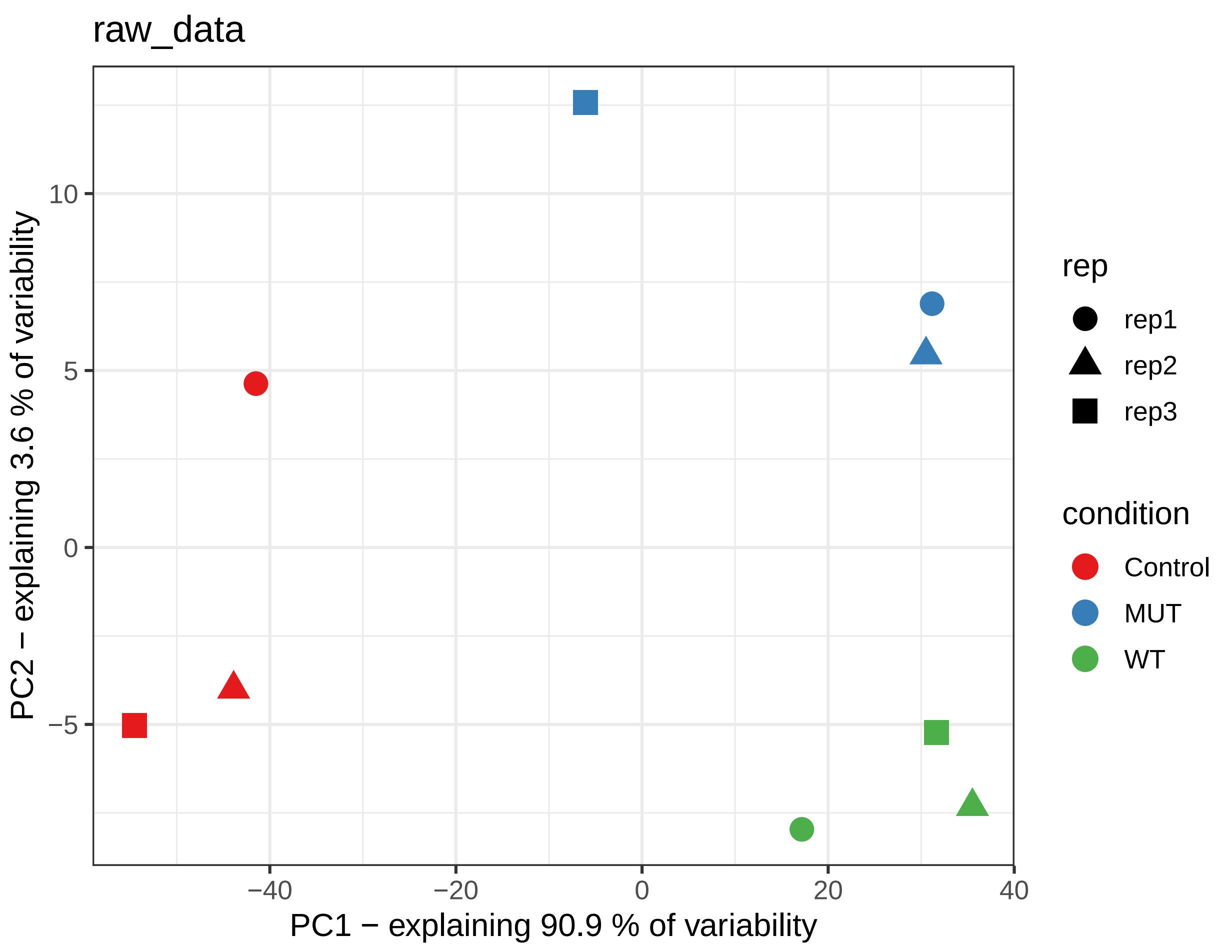


```
rm(raw_PCA_m)

# PCA of batchcl_raw_data
batchcl_raw_PCA_m <- t(na.omit(exprs(batchcl_raw_dataE)))
if (length(batchcl_raw_PCA_m) > 50)
{
  batchcl_raw_PCA <- prcomp(batchcl_raw_PCA_m, scale = FALSE)
  perc_var <-
    round(100 * batchcl_raw_PCA$sdev ^ 2 /
            sum(batchcl_raw_PCA$sdev ^ 2), 1)
  PCA_batchcl_raw_data <-
    data.frame(PC1 = batchcl_raw_PCA$x[, 1],
               PC2 = batchcl_raw_PCA$x[, 2],
               rep = pData(batchcl_raw_dataE)$rep,
               condition = pData(batchcl_raw_dataE)$condition)
  print(ggplot(data = PCA_batchcl_raw_data, aes(PC1, PC2)) +
          geom_point(aes(color = condition, shape = rep), size = 4) +
          guides(size = FALSE) +
          customPlot +
          ggtitle("batchcl_raw_data") +
          xlab(paste("PC1 - explaining", perc_var[1], "% of variability")) +
          ylab(paste("PC2 - explaining", perc_var[2], "% of variability")))
  ggsave(file.path(dir_save, paste0("PCA_batchcl_raw_data_", script.version, ".pdf")),
```

```
         width = 7, height = 5)
  rm(batchcl_raw_PCA, perc_var, PCA_batchcl_raw_data)
}
```

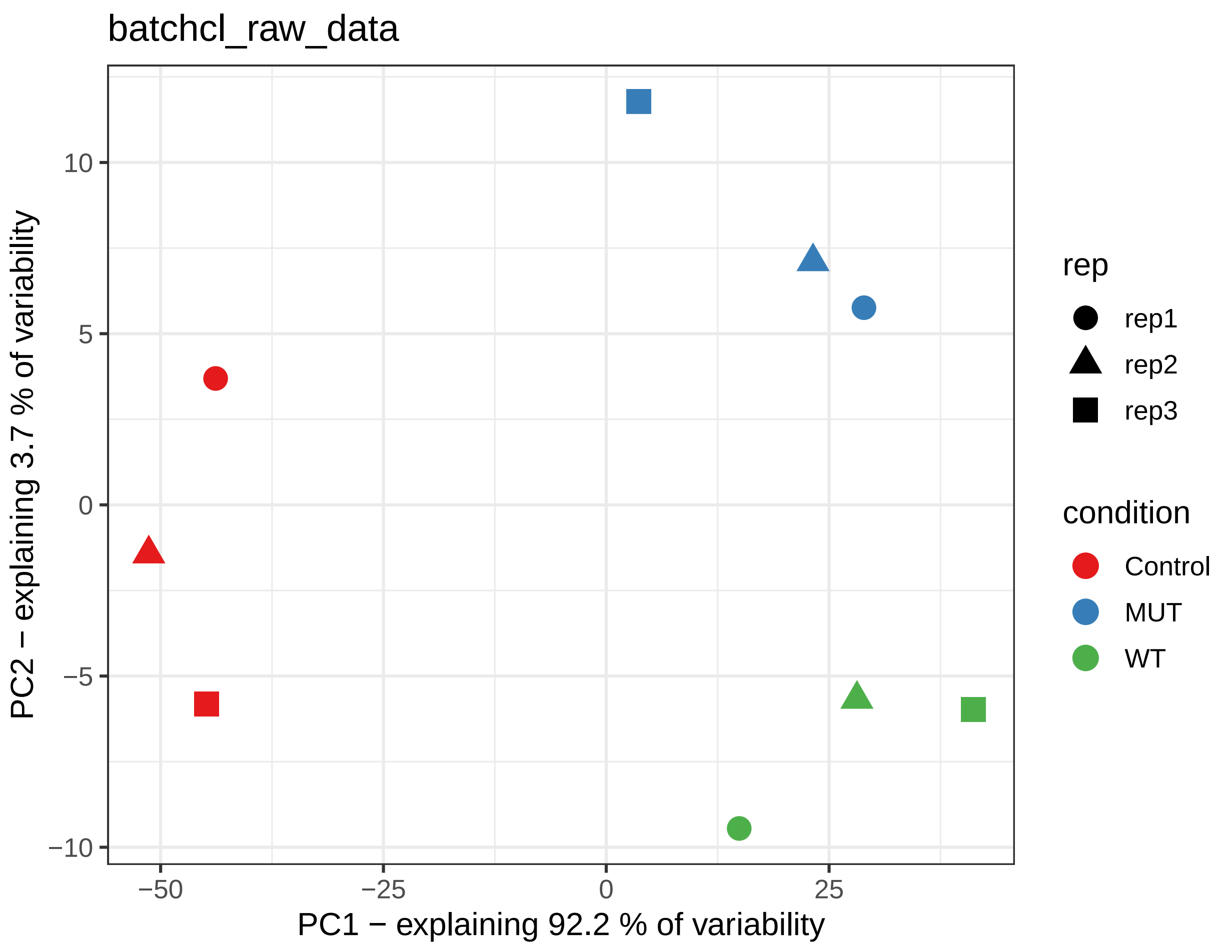


```
rm(batchcl_raw_PCA_m)

# PCA of norm_batchcl_raw_data
norm_batchcl_raw_PCA_m <- t(na.omit(exprs(norm_batchcl_raw_dataE)))
if (length(norm_batchcl_raw_PCA_m) > 50)
{
  norm_batchcl_raw_PCA <- prcomp(norm_batchcl_raw_PCA_m, scale = FALSE)
  perc_var <-
    round(100 * norm_batchcl_raw_PCA$sdev ^ 2 /
            sum(norm_batchcl_raw_PCA$sdev ^ 2), 1)
  PCA_norm_batchcl_raw_data <-
    data.frame(PC1 = norm_batchcl_raw_PCA$x[, 1],
               PC2 = norm_batchcl_raw_PCA$x[, 2],
               rep = pData(norm_batchcl_raw_dataE)$rep,
               condition = pData(norm_batchcl_raw_dataE)$condition)
  print(ggplot(data = PCA_norm_batchcl_raw_data, aes(PC1, PC2)) +
          geom_point(aes(color = condition, shape = rep), size = 4) +
          guides(size = FALSE) +
          customPlot +
```

```
        ggtitle("norm_batchcl_raw_data") +
        xlab(paste("PC1 - explaining", perc_var[1], "% of variability")) +
        ylab(paste("PC2 - explaining", perc_var[2], "% of variability")))
  ggsave(file.path(dir_save, paste0("PCA_norm_batchcl_raw_data_", script.version, ".pdf")),
         width = 7, height = 5)
  rm(norm_batchcl_raw_PCA, perc_var, PCA_norm_batchcl_raw_data)
}
```

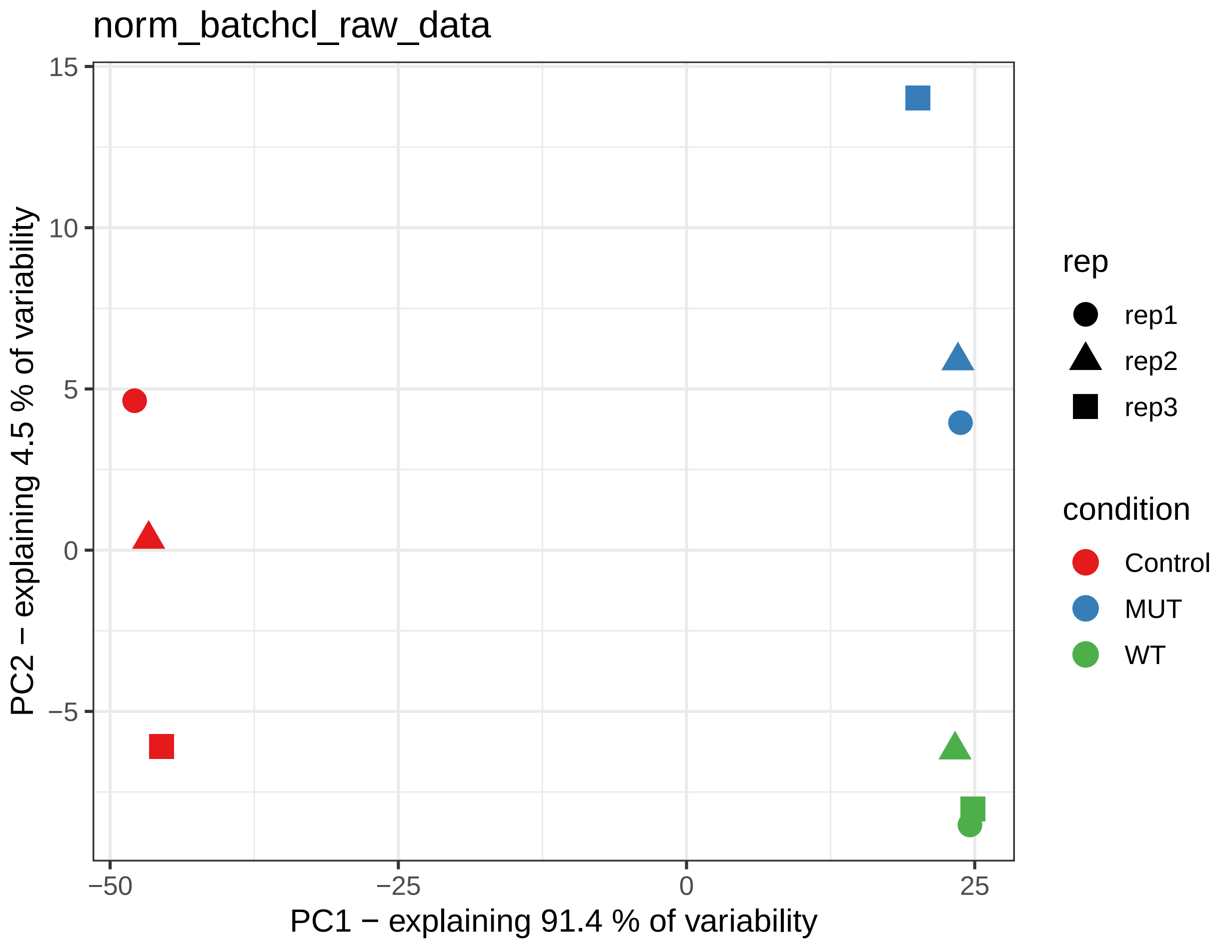


```
rm(norm_batchcl_raw_PCA_m)

# PCA of ctrl.ratio_data
ctrl.ratio_PCA_m <- t(na.omit(exprs(ctrl.ratio_dataE)))
if (length(ctrl.ratio_PCA_m) > 50)
{
  ctrl.ratio_PCA <- prcomp(ctrl.ratio_PCA_m, scale = FALSE)
  perc_var <-
    round(100 * ctrl.ratio_PCA$sdev ^ 2 /
            sum(ctrl.ratio_PCA$sdev ^ 2), 1)
  PCA_ctrl.ratio_data <-
    data.frame(PC1 = ctrl.ratio_PCA$x[, 1],
               PC2 = ctrl.ratio_PCA$x[, 2],
               rep = pData(ctrl.ratio_dataE)$rep,
               condition = pData(ctrl.ratio_dataE)$condition)
```

```
  print(ggplot(data = PCA_ctrl.ratio_data, aes(PC1, PC2)) +
          geom_point(aes(color = condition, shape = rep), size = 4) +
          guides(size = FALSE) +
          customPlot +
          ggtitle("ctrl.ratio_data") +
          xlab(paste("PC1 - explaining", perc_var[1], "% of variability")) +
          ylab(paste("PC2 - explaining", perc_var[2], "% of variability")))
  ggsave(file.path(dir_save, paste0("PCA_ctrl.ratio_data_", script.version, ".pdf")),
         width = 7, height = 5)
  rm(ctrl.ratio_PCA, perc_var, PCA_ctrl.ratio_data)
}
```

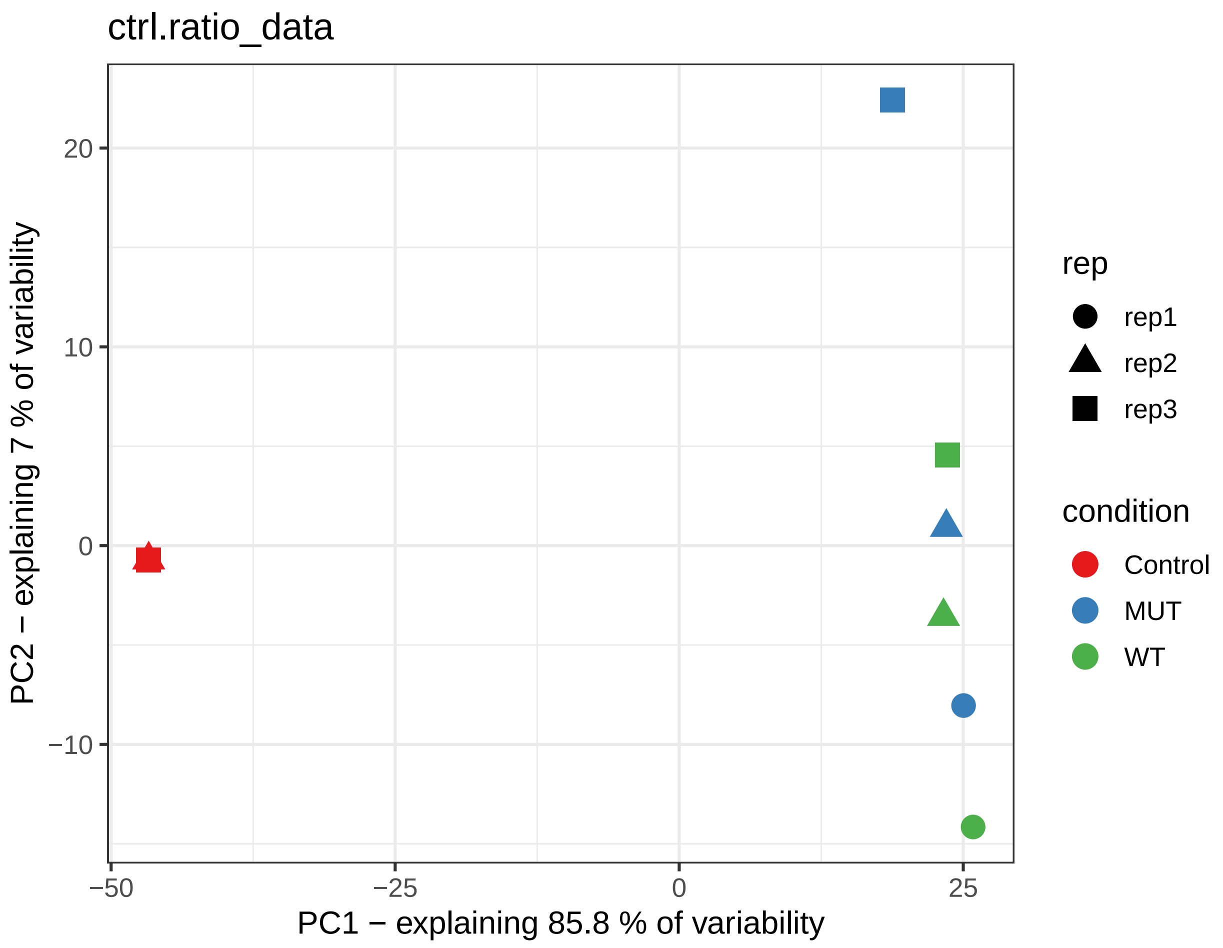


```
rm(ctrl.ratio_PCA_m)
```

Merge modified data back into 'cdata'

```
cdata_i <- as.data.frame(2^exprs(batchcl_raw_dataE))
names(cdata_i) <- paste0("batchcl_raw_signal_sum_", names(cdata_i))
cdata_i$gene_name <- rownames(cdata_i)
cdata <- left_join(cdata, cdata_i)
rm(cdata_i)
```

```
cdata_i <- as.data.frame(2^exprs(norm_batchcl_raw_dataE))
names(cdata_i) <- paste0("norm_batchcl_raw_signal_sum_", names(cdata_i))
cdata_i$gene_name <- rownames(cdata_i)
cdata <- left_join(cdata, cdata_i)
rm(cdata_i)

cdata_i <- as.data.frame(2^exprs(ctrl.ratio_dataE))
names(cdata_i) <- paste0("ctrl.ratio_", names(cdata_i))
cdata_i$gene_name <- rownames(cdata_i)
cdata <- left_join(cdata, cdata_i)
rm(cdata_i)

write.csv(cdata, file = file.path(dir_save, paste0("Full_dataset_", script.version, ".csv")), row.names
```

Create tidy data

```
mdata <- NULL

mdata_i <- tidy(raw_dataE)
mdata_i <- mdata_i %>%
  mutate(value = 2 ^ value)
names(mdata_i)[1] <- "gene_name"
names(mdata_i)[2] <- "ID"
mdata_i <- left_join(mdata_i, conditions)
mdata_i$measurement <- "raw_signal_sum"
mdata <- bind_rows(mdata, mdata_i)
rm(mdata_i)

mdata_i <- tidy(batchcl_raw_dataE)
mdata_i <- mdata_i %>%
  mutate(value = 2 ^ value)
names(mdata_i)[1] <- "gene_name"
names(mdata_i)[2] <- "ID"
mdata_i <- left_join(mdata_i, conditions)
mdata_i$measurement <- "batchcl_raw_signal_sum"
mdata <- bind_rows(mdata, mdata_i)
rm(mdata_i)

mdata_i <- tidy(norm_batchcl_raw_dataE)
mdata_i <- mdata_i %>%
  mutate(value = 2 ^ value)
names(mdata_i)[1] <- "gene_name"
names(mdata_i)[2] <- "ID"
mdata_i <- left_join(mdata_i, conditions)
mdata_i$measurement <- "norm_batchcl_raw_signal_sum"
mdata <- bind_rows(mdata, mdata_i)
rm(mdata_i)

mdata_i <- tidy(ctrl.ratio_dataE)
mdata_i <- mdata_i %>%
  mutate(value = 2 ^ value)
names(mdata_i)[1] <- "gene_name"
```

```
names(mdata_i)[2] <- "ID"
mdata_i <- left_join(mdata_i, conditions)
mdata_i$measurement <- "ctrl.ratio"
mdata <- bind_rows(mdata, mdata_i)
rm(mdata_i)

mdata$condition <- factor(mdata$condition, ordered = TRUE, levels = c("WT", "MUT", "Control"))
mdata$measurement <- factor(mdata$measurement, ordered = TRUE, levels = c("raw_signal_sum", "batchcl_ra
```

Data transformation overview

```
ggplot(data = subset(mdata, !grepl("ratio", measurement)), aes(condition, log2(value))) +
  geom_boxplot(aes(fill = rep)) +
  customPlot +
  theme(axis.text.x = element_text(angle = 90, hjust = 1, vjust = 0.5)) +
  facet_grid(. ~ measurement)
```

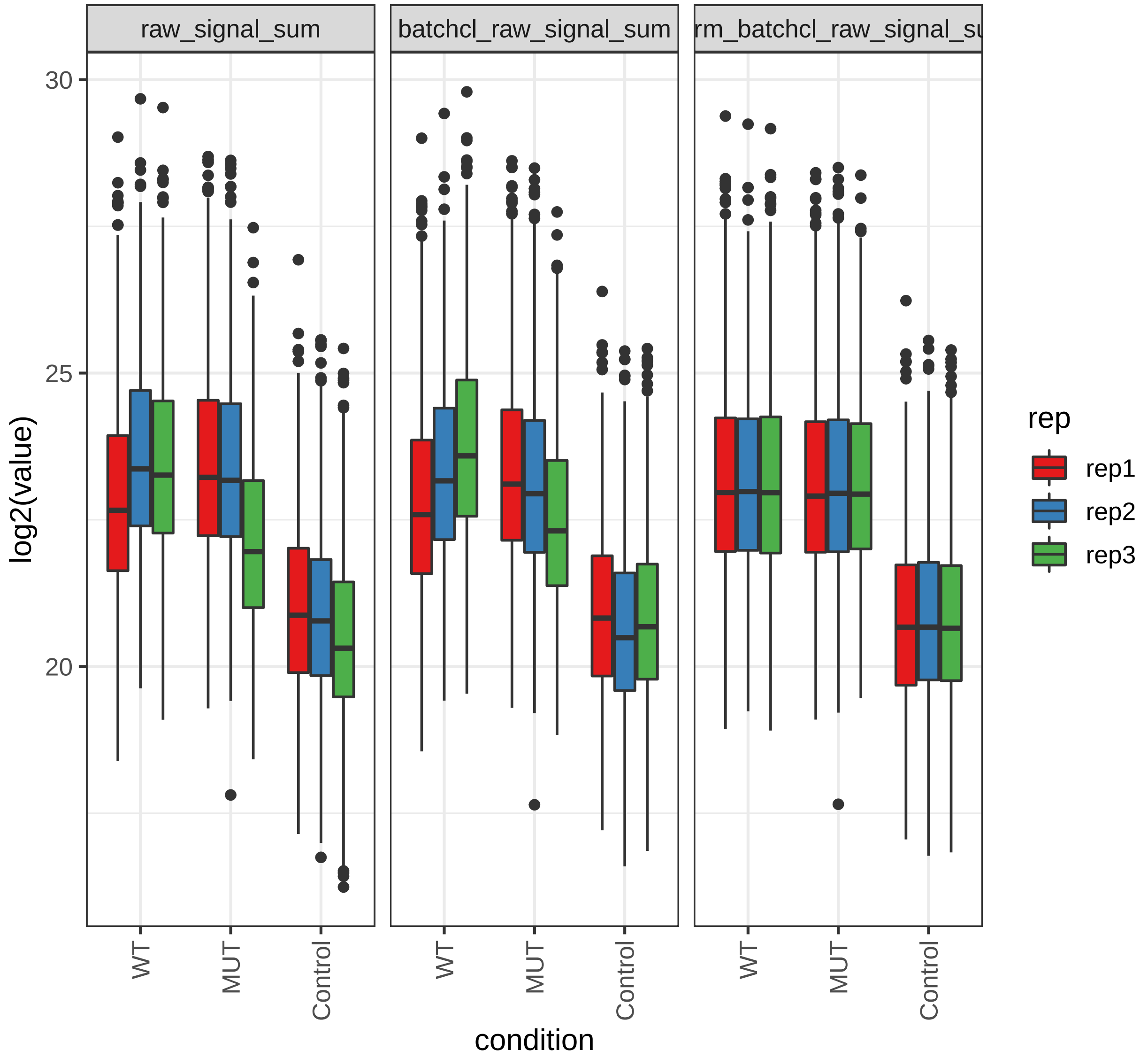


```
ggsave(file.path(dir_save, paste0("Normalization_overview_", script.version, ".pdf")), width = 10, heig

ggplot(data = subset(mdata, grepl("ratio", measurement)), aes(condition, log2(value))) +
  geom_boxplot(aes(fill = rep)) +
  customPlot +
  theme(axis.text.x = element_text(angle = 90, hjust = 1, vjust = 0.5)) +
  facet_grid(. ~ measurement, scale = "free")
```

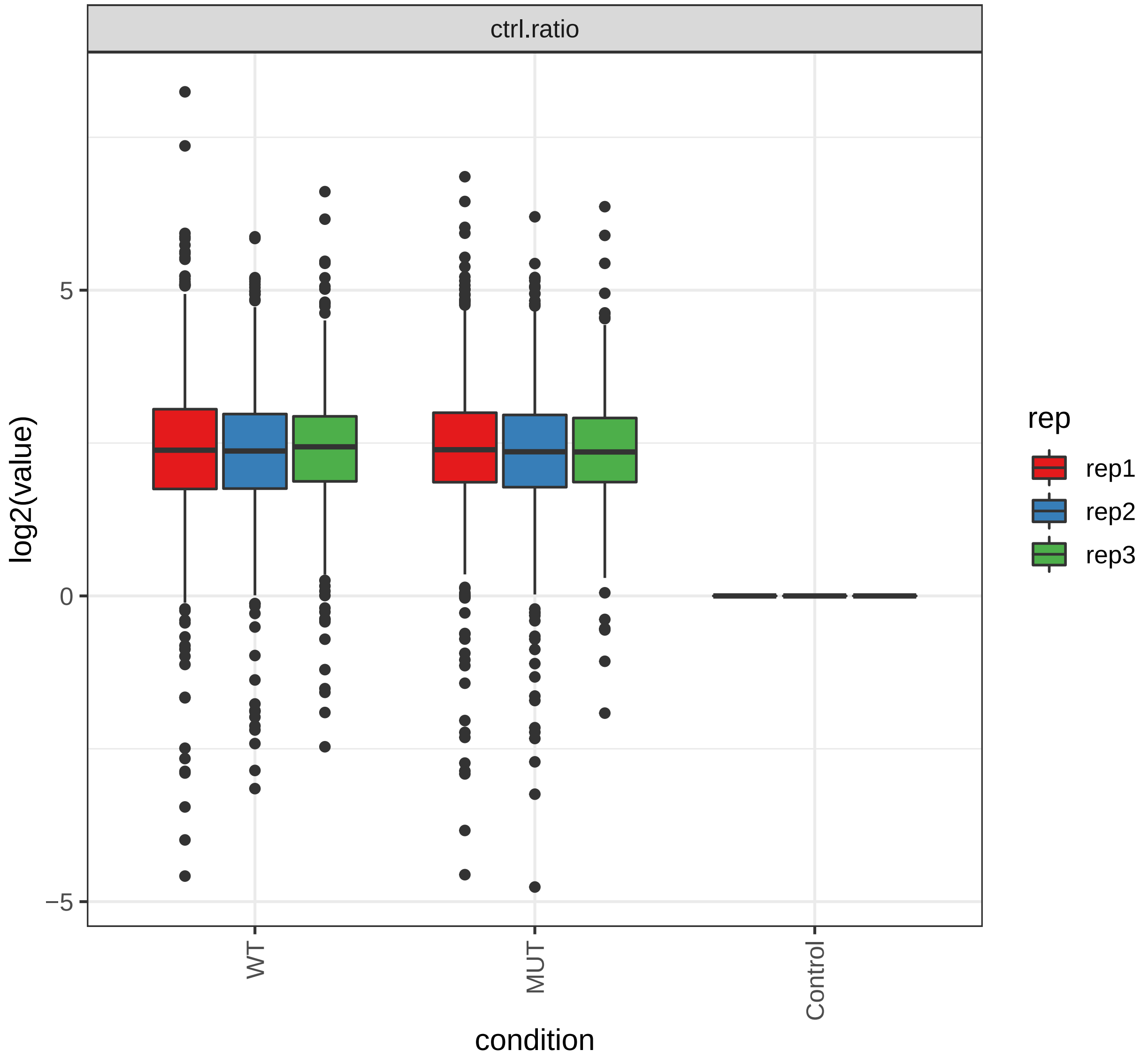


```
ggsave(file.path(dir_save, paste0("Normalization_overview_ratios_", script.version, ".pdf")), width = 6
```

LIMMA analysis

A protein is considered a 'hit', if the false discovery rate is smaller 0.05 and a fold change of at least 2-fold is observed. A protein is considered a 'candidate', if the false discovery rate is smaller 0.2 and a fold change of at least 1.5-fold is observed.

Initialize limma results data

```
limma_results <- NULL
```

Run limma analysis

```
limma_data <- norm_batchcl_raw_dataE
limma_weights <- exprs(raw_dataE)
```

```r
comparison.list <- c("WT - Control", "MUT - Control", "(MUT - Control) - (WT - Control)")
limma_weights <- ifelse(is.na(limma_weights), 0.05, 1)
limma.cond <- factor(pData(limma_data)$condition, ordered = FALSE)
limma.rep <- factor(pData(limma_data)$rep, ordered = FALSE)
contrast.matrix <- model.matrix( ~  0 + limma.cond + limma.rep)
colnames(contrast.matrix) <- gsub("limma.cond", "", colnames(contrast.matrix))

limma.object <- eBayes(
  contrasts.fit(
    lmFit(limma_data, design = contrast.matrix, weights = limma_weights),
    makeContrasts(contrasts = comparison.list, levels = contrast.matrix)
  )
)
for (comparison in comparison.list)
{
  limma_results_i <- limma::topTable(limma.object,
                         sort.by = "t",
                         coef = comparison,
                         number = Inf)
  names(limma_results_i)[grep("P.Value", names(limma_results_i))] <- "pvalue.limma"
  names(limma_results_i)[grep("adj.P.Val", names(limma_results_i))] <- "fdr.limma"
  limma_results_i <- subset(limma_results_i, !is.na(logFC))
  fdr_res <- NULL
  try(fdr_res <- fdrtool(limma_results_i$t, plot = FALSE, verbose = FALSE))
  if (!is.null(fdr_res))
  {
    limma_results_i$pvalue.fdrtool <- fdr_res$pval
    limma_results_i$qval.fdrtool <- fdr_res$qval
    limma_results_i$lfdr.fdrtool <- fdr_res$lfdr
  }else
  {
    limma_results_i$pvalue.fdrtool <- 1
    limma_results_i$qval.fdrtool <- 1
    limma_results_i$lfdr.fdrtool <- 1
  }
  limma_results_i$comparison <- comparison
  limma_results <- bind_rows(limma_results, limma_results_i)
  rm(limma_results_i, fdr_res)
}
rm(comparison.list, comparison, contrast.matrix, limma.cond, limma.rep, limma_data, limma_weights, limm
```

Fdr statistics comparison limma vs fdrtool

```r
ggplot(data = limma_results, aes(abs(t))) +
  geom_line(aes(y = fdr.limma, colour = "limma - adj.P.Val")) +
  geom_line(aes(y = qval.fdrtool, colour = "fdrtool - qval")) +
  geom_point(aes(y = fdr.limma, colour = "limma - adj.P.Val")) +
  geom_point(aes(y = qval.fdrtool, colour = "fdrtool - qval")) +
  facet_wrap( ~ comparison, scale = "free_x") +
  ylab("fdr") +
  customPlot
```

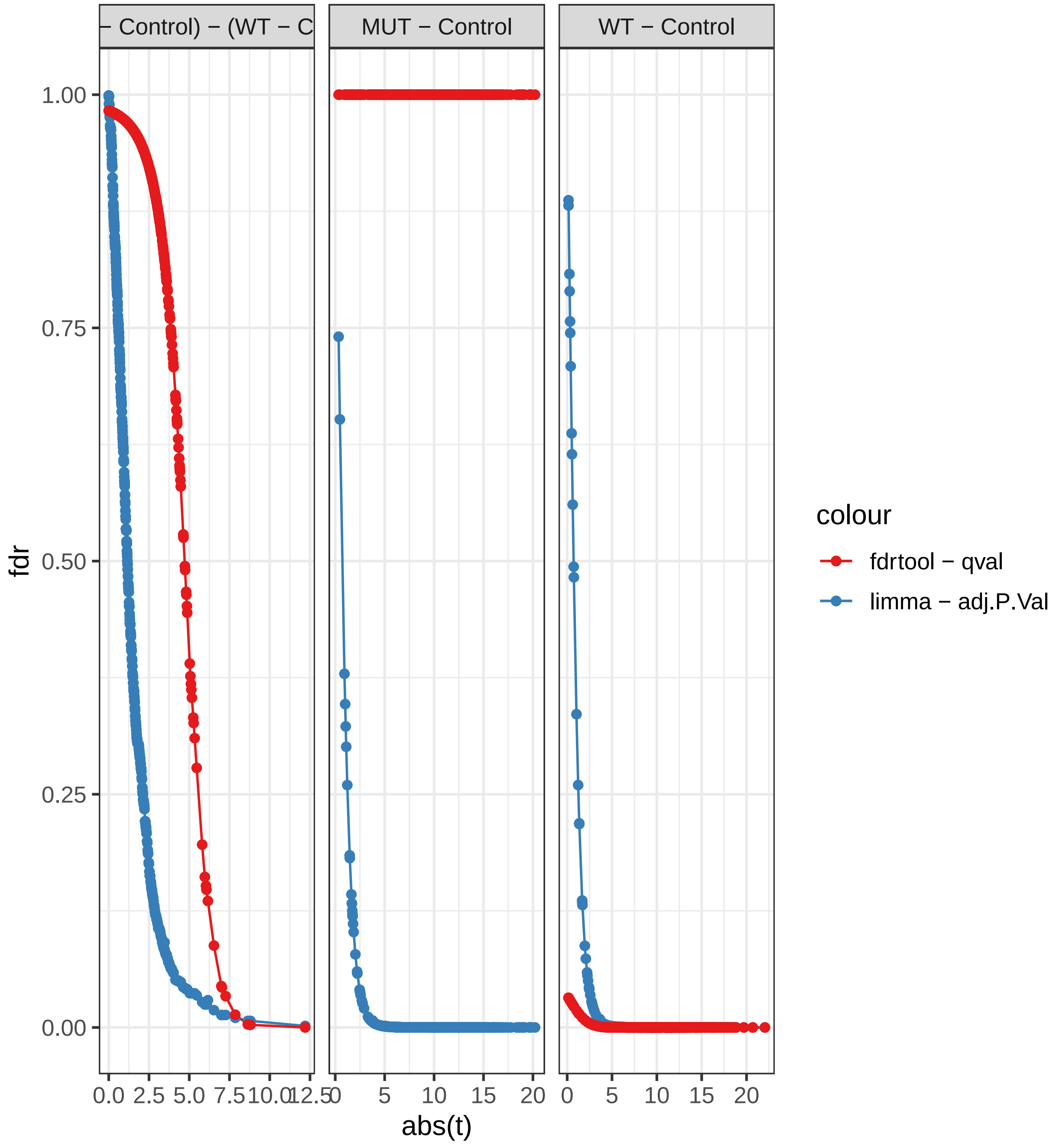


```
ggsave(file.path(dir_save, paste0("t_vs_fdr_limma_vs_fdrtool_", script.version, ".pdf")), width = 12, he

ggplot(data = limma_results) +
  geom_histogram(aes(pvalue.limma, alpha = 0.5, fill = "limma"), bins = 40) +
  geom_histogram(aes(pvalue.fdrtool, alpha = 0.5, fill = "fdrtool"), bins = 40) +
  guides(alpha = FALSE) +
  xlab("p-value") +
  facet_wrap( ~ comparison, scale = "free_y") +
  coord_cartesian(xlim = c(0, 1)) +
  customPlot
```

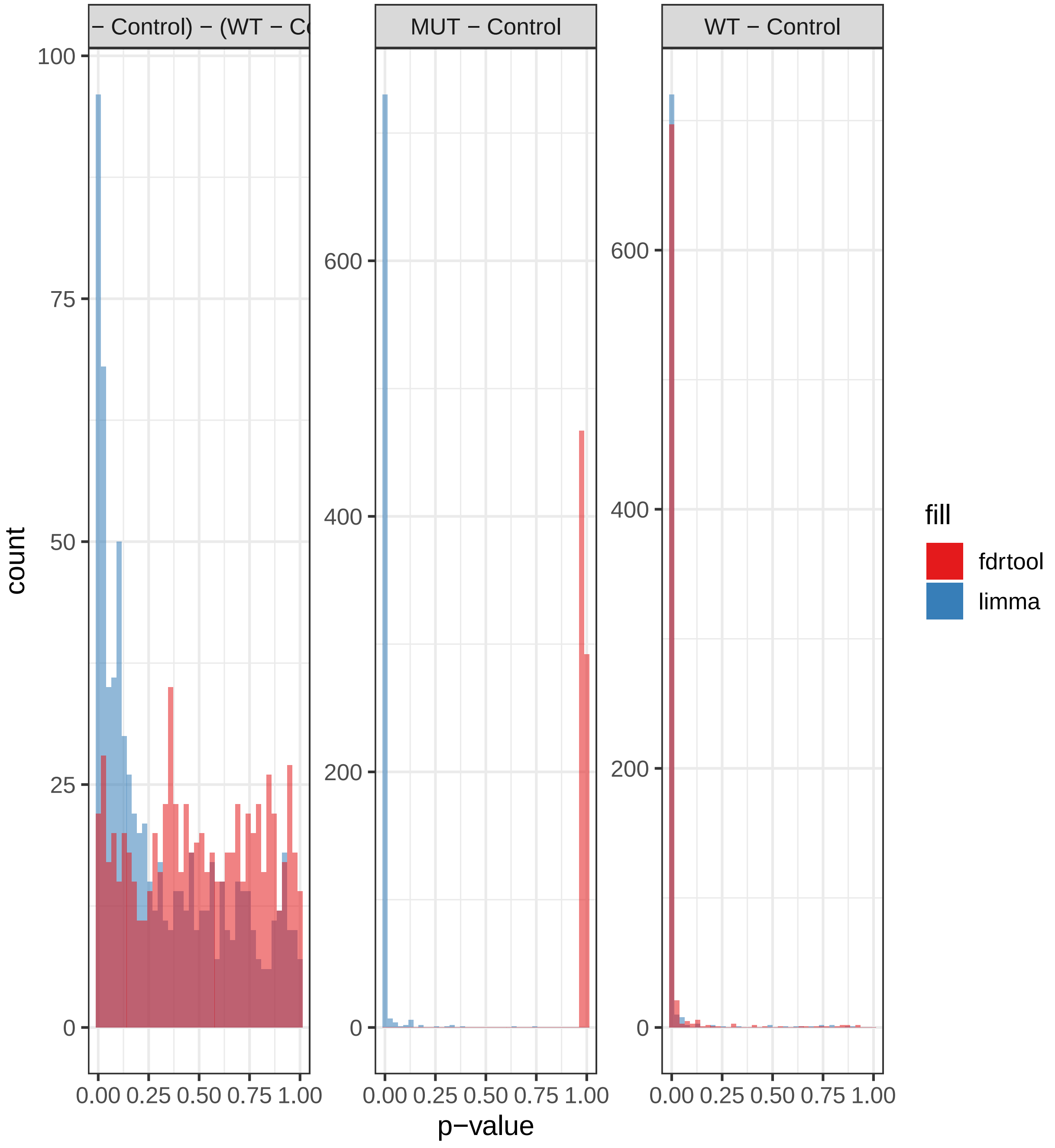


```
ggsave(file.path(dir_save, paste0("p-value_histogram_limma_vs_fdrtool_", script.version, ".pdf")), widt
```

Hit annotation

```
limma_results$hit_annotation_method <- NA
fdr_hit_threshold <- 0.05
fdr_candidate_threshold = 0.2
fc_hit_threshold <- 2
fc_candidate_threshold <- 1.5
limma_results$pvalue <- NA
```

```r
limma_results$fdr <- NA
for (comparison in unique(limma_results$comparison))
{
  limma_hits <-
    nrow(limma_results[limma_results$comparison == comparison &
                         limma_results$fdr.limma <= fdr_hit_threshold, ])
  fdrtool_hits <-
    nrow(limma_results[limma_results$comparison == comparison &
                         limma_results$qval.fdrtool <= fdr_hit_threshold, ])
  if (limma_hits >= fdrtool_hits)
  {
    limma_results$hit_annotation_method[limma_results$comparison == comparison] <-
      "limma"
  }
  if (fdrtool_hits > limma_hits)
  {
    limma_results$hit_annotation_method[limma_results$comparison == comparison] <-
      "fdrtool"
  }
  rm(limma_hits, fdrtool_hits)
}
rm(comparison)
table(limma_results$hit_annotation_method)
```

```
##
## fdrtool   limma
##     759    1518
```

```r
limma_results$hit_annotation_method <- "limma"

limma_results$pvalue[limma_results$hit_annotation_method == "limma"] <-
  limma_results$pvalue.limma[limma_results$hit_annotation_method == "limma"]
limma_results$fdr[limma_results$hit_annotation_method == "limma"] <-
  limma_results$fdr.limma[limma_results$hit_annotation_method == "limma"]

limma_results$pvalue[limma_results$hit_annotation_method == "fdrtool"] <-
  limma_results$pvalue.fdrtool[limma_results$hit_annotation_method == "fdrtool"]
limma_results$fdr[limma_results$hit_annotation_method == "fdrtool"] <-
  limma_results$qval.fdrtool[limma_results$hit_annotation_method == "fdrtool"]

limma_results$hit <-
  with(limma_results, ifelse(fdr <= fdr_hit_threshold & abs(logFC) >=
                               log2(fc_hit_threshold), TRUE, FALSE))
limma_results$hit_annotation <-
  with(limma_results,
       ifelse(fdr <= fdr_hit_threshold & abs(logFC) >=
                log2(fc_hit_threshold), "hit",
              ifelse(fdr <= fdr_candidate_threshold & abs(logFC) >=
                       log2(fc_candidate_threshold), "candidate", "no hit")))
limma_results$hit_annotation <-
  factor(limma_results$hit_annotation,
         ordered = TRUE,
         levels = c("hit", "candidate", "no hit"))
```

```r
# Keep only enriched hits
ctrl.comparisons <- c("WT - Control", "MUT - Control")
limma_results$hit[
  with(limma_results, comparison %in% ctrl.comparisons &
         hit & logFC < 0)
] <- FALSE
limma_results$hit_annotation[
  with(limma_results, comparison %in% ctrl.comparisons &
         hit_annotation %in% c("hit", "candidate") &
         logFC < 0)
] <- "no hit"

limma_results$hit_annotation <- as.character(limma_results$hit_annotation)
limma_results$hit_annotation.orig <- limma_results$hit_annotation
for (comparison in unique(limma_results$comparison))
{
  comparison.orig <- comparison
  comparison.list_i <- gsub("[(,)]", "",
                            unlist(base::strsplit(comparison, split = " - ")))
  comparison.list_i <- comparison.list_i[!comparison.list_i %in% conditions$ctrl]
  enriched <- NULL
  for (comparison_i in comparison.list_i)
  {
    to_compare <- grep(paste0("^", comparison_i, " -"), ctrl.comparisons, value = TRUE)
    enriched <- append(enriched,
                       as.character(subset(limma_results,
                                           comparison == to_compare &
                                             hit_annotation.orig %in%
                                             c("hit", "candidate"))$gene_name))
  }
  rm(comparison.list_i, comparison_i, to_compare)
  enriched <- unique(enriched)
  limma_results$hit_annotation[
    limma_results$comparison == comparison.orig & limma_results$gene_name %in% enriched
    ] <-
    paste("enriched", limma_results$hit_annotation[
      limma_results$comparison == comparison.orig & limma_results$gene_name %in% enriched
      ])
}
rm(comparison, ctrl.comparisons, comparison.orig)
limma_results$hit_annotation.orig <- NULL
limma_results$hit_annotation <- factor(limma_results$hit_annotation,
                                       ordered = TRUE,
                                       levels = c("enriched hit",
                                                  "hit",
                                                  "enriched candidate",
                                                  "candidate",
                                                  "enriched no hit",
                                                  "no hit"))

with(limma_results, table(comparison, hit_annotation))
```

```
##                                      hit_annotation
## comparison                            enriched hit hit enriched candidate
```

```
##   (MUT - Control) - (WT - Control)           31   0                 120
##   MUT - Control                             730   0                  11
##   WT - Control                              718   0                  16
##                                     hit_annotation
## comparison                            candidate enriched no hit no hit
##   (MUT - Control) - (WT - Control)          0             590     18
##   MUT - Control                             0               0     18
##   WT - Control                              0               0     25
```

Volcano-plot

```
ggplot(data = limma_results, aes(logFC, -log10(pvalue), colour = hit_annotation)) +
  geom_vline(aes(xintercept = 0)) +
  geom_point() +
  geom_text(aes(label = gsub("[|].+", "", gene_name)),
            data = subset(limma_results, hit_annotation != "no hit"),
            vjust = 0, nudge_y = 0.1, size = 2, check_overlap = TRUE) +
  facet_wrap( ~ comparison + hit_annotation_method) +
  xlab("log2(fold change)") +
  customPlot
```

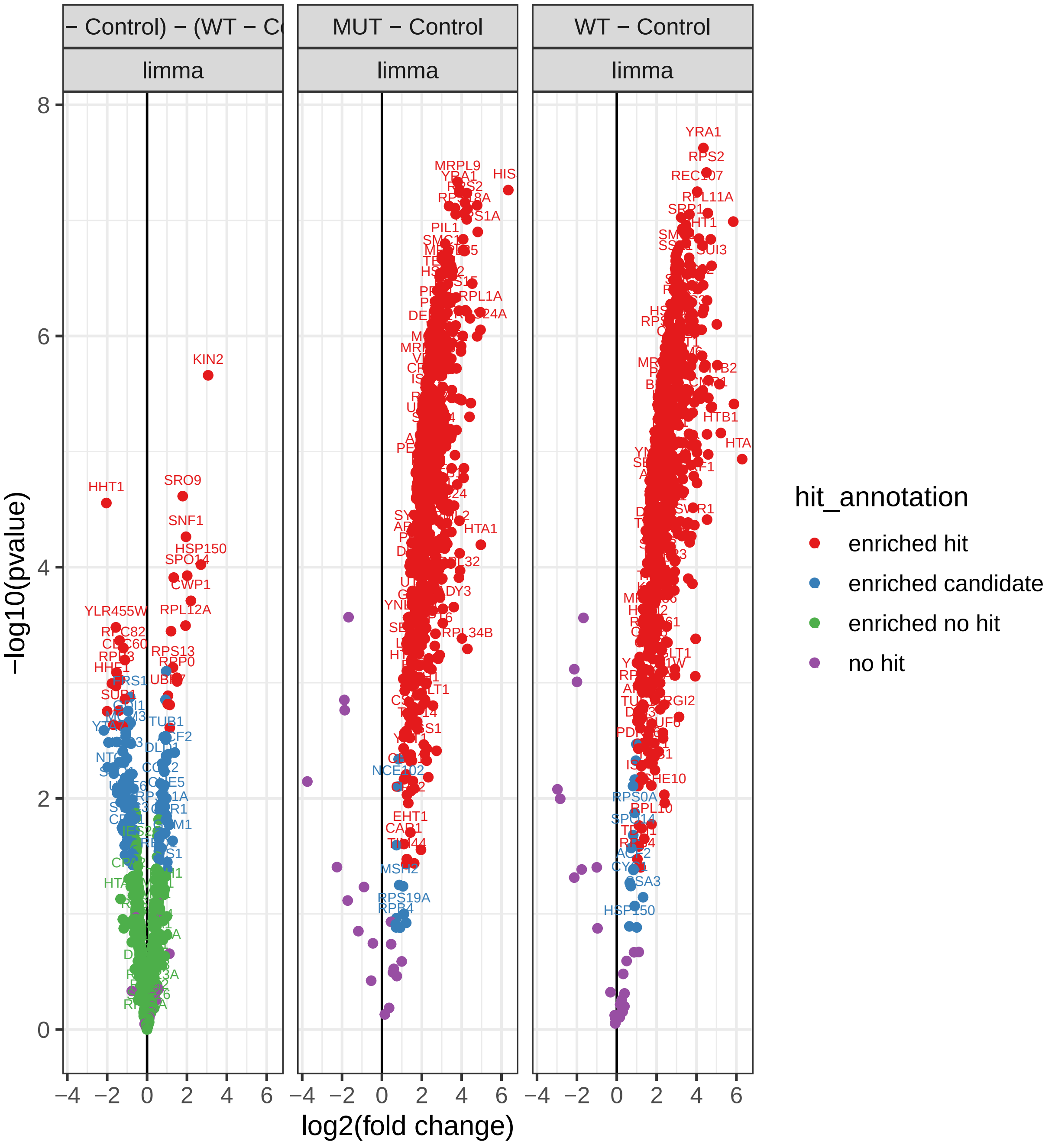


```
ggsave(file.path(dir_save, paste0("Volcano_plot_", script.version, ".pdf")), width = 12, height = 4.5)
```

MA-plot

```
ggplot(data = limma_results, aes(AveExpr, logFC, colour = hit_annotation)) +
  geom_hline(aes(yintercept = 0)) +
  geom_point() +
  geom_text(aes(label = gsub("[|].+", "", gene_name)),
            data = subset(limma_results, hit_annotation != "no hit"),
            vjust = 0, nudge_y = 0.1, size = 2, check_overlap = TRUE) +
```

```r
  facet_wrap( ~ comparison + hit_annotation_method) +
  xlab("average log2(signal_sum)") +
  ylab("log2(fold change)") +
  customPlot
```

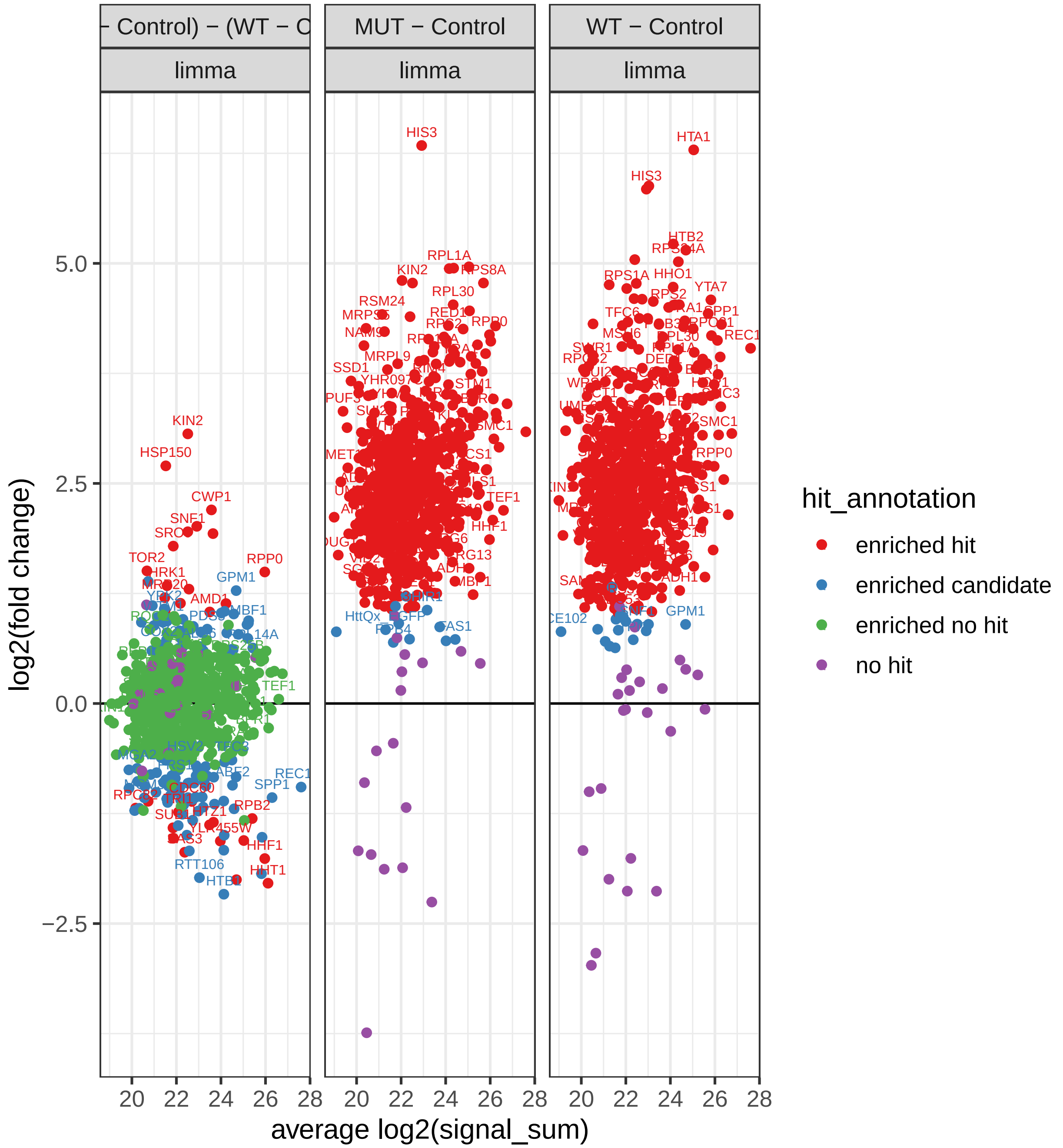


```r
ggsave(file.path(dir_save, paste0("MA_plot_", script.version, ".pdf")), width = 12, height = 4.5)
```

##top3-plot

```r
limma_results$top3 <- limma_results$average.top3
ggplot(data = limma_results, aes(top3, logFC, colour = hit_annotation)) +
  geom_hline(aes(yintercept = 0)) +
```

```
geom_point() +
geom_text(aes(label = gsub("[|].+", "", gene_name)),
          data = subset(limma_results, hit_annotation != "no hit"),
          vjust = 0, nudge_y = 0.1, size = 2, check_overlap = TRUE) +
facet_wrap( ~ comparison + hit_annotation_method) +
xlab("average(top3)") +
ylab("log2(fold change)") +
customPlot
```

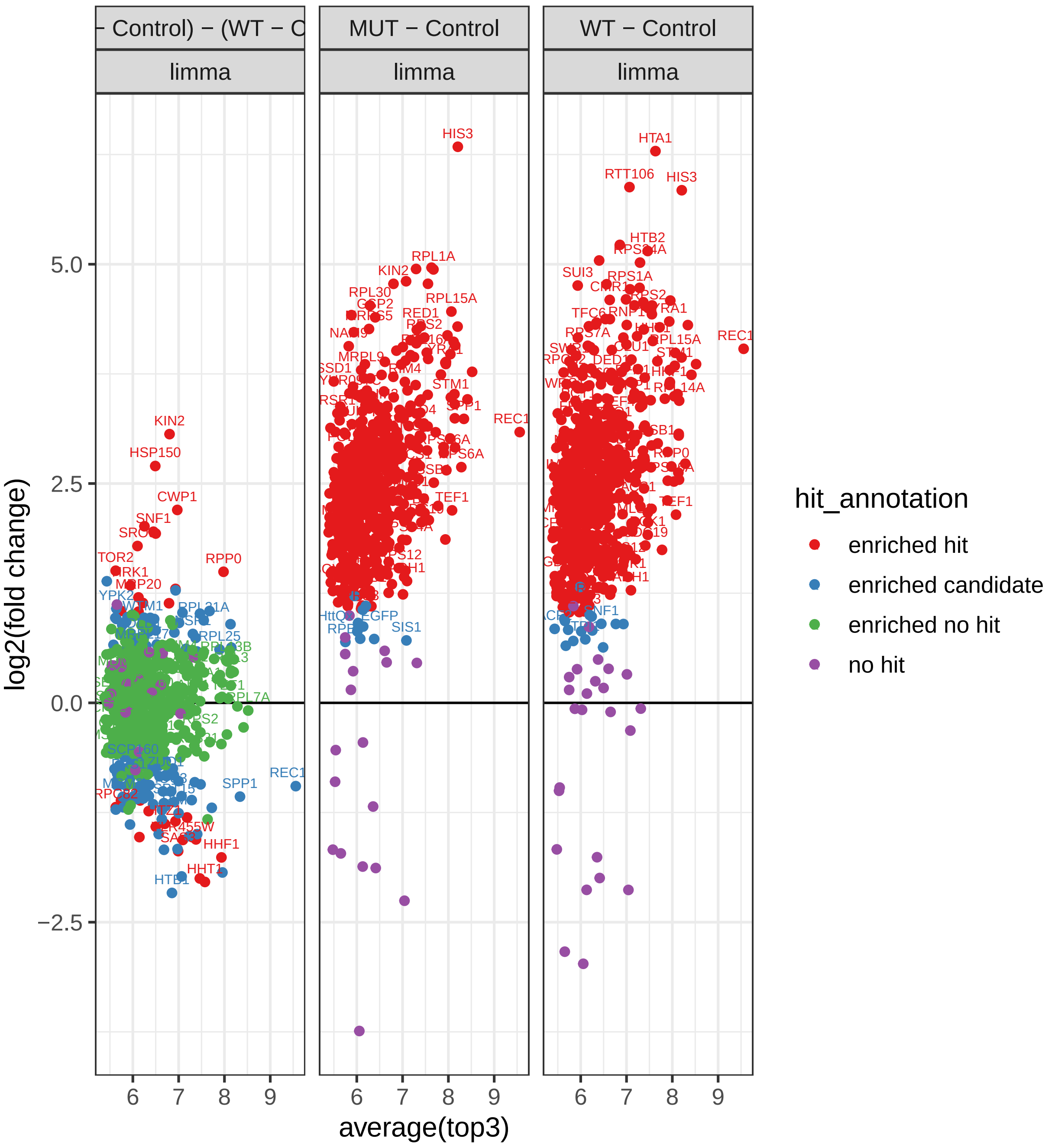

```r
ggsave(file.path(dir_save, paste0("top3_plot_", script.version, ".pdf")), width = 12, height = 4.5)
```

Fold change correlations

```r
FC.correlation.list <- list(
  structure(
    c("(MUT - Control) - (WT - Control)", "MUT - Control", "WT - Control"),
    .Names = c("MUT_vs_Control_against_WT_vs_Control ", "MUT_vs_Control", "WT_vs_Control")
  )
)
fc.cor.data <- NULL
for (i in seq_along(FC.correlation.list))
{
  FC.correlation.list_i <- FC.correlation.list[[i]]
  fc.cor.data_x <- limma_results %>%
    group_by(gene_name) %>%
    dplyr::filter(comparison == FC.correlation.list_i[3]) %>%
    mutate(x.name = names(FC.correlation.list_i)[3], x = logFC, x.hit = hit_annotation) %>%
    dplyr::select(gene_name, x, x.name, x.hit)
  fc.cor.data_y <- limma_results %>%
    group_by(gene_name) %>%
    dplyr::filter(comparison == FC.correlation.list_i[2]) %>%
    mutate(y.name = names(FC.correlation.list_i)[2], y = logFC, y.hit = hit_annotation) %>%
    dplyr::select(gene_name, y, y.name, y.hit)
  fc.cor.data_i <- left_join(fc.cor.data_x, fc.cor.data_y)
  fc.cor.data_hit <- limma_results %>%
    group_by(gene_name) %>%
    dplyr::filter(comparison == FC.correlation.list_i[1]) %>%
    mutate(comparison = names(FC.correlation.list_i)[1]) %>%
    dplyr::select(gene_name, comparison, hit_annotation)
  fc.cor.data_i <- left_join(fc.cor.data_i, fc.cor.data_hit)
  rm(fc.cor.data_x, fc.cor.data_y, fc.cor.data_hit)
  fc.cor.data <- bind_rows(fc.cor.data, fc.cor.data_i)
  rm(fc.cor.data_i)
}
fc.cor.data$hit_x_and_y <- paste(fc.cor.data$x.hit, "x -", fc.cor.data$y.hit, "y")
fc.cor.data$hit_x_and_y <- gsub("enriched ", "", fc.cor.data$hit_x_and_y)
fc.cor.data$hit_x_and_y <- factor(fc.cor.data$hit_x_and_y, ordered = TRUE,
                                  levels = c("hit x - hit y",
                                             "hit x - candidate y",
                                             "candidate x - hit y",
                                             "candidate x - candidate y",
                                             "hit x - no hit y",
                                             "no hit x - hit y",
                                             "candidate x - no hit y",
                                             "no hit x - candidate y",
                                             "no hit x - no hit y"))
no.of.comparisons <- with(fc.cor.data, length(unique(paste(comparison))))
gr.width <- 4 + ceiling(sqrt(no.of.comparisons)) * 5
gr.height <- 3 + floor(sqrt(no.of.comparisons)) * 5
ggplot(data = fc.cor.data, aes(x, y, colour = hit_annotation)) +
  geom_vline(aes(xintercept = 0)) +
  geom_hline(aes(yintercept = 0)) +
```

```
  geom_abline() +
  coord_fixed(ratio = 1) +
  geom_point() +
  geom_text(aes(label = gsub("[|].+", "", gene_name)),
            data = subset(fc.cor.data, hit_annotation != "no hit"),
            vjust = 0, nudge_y = 0.1, size = 2, check_overlap = TRUE) +
  facet_wrap( ~ comparison + x.name + y.name, labeller = "label_both") +
  customPlot
```

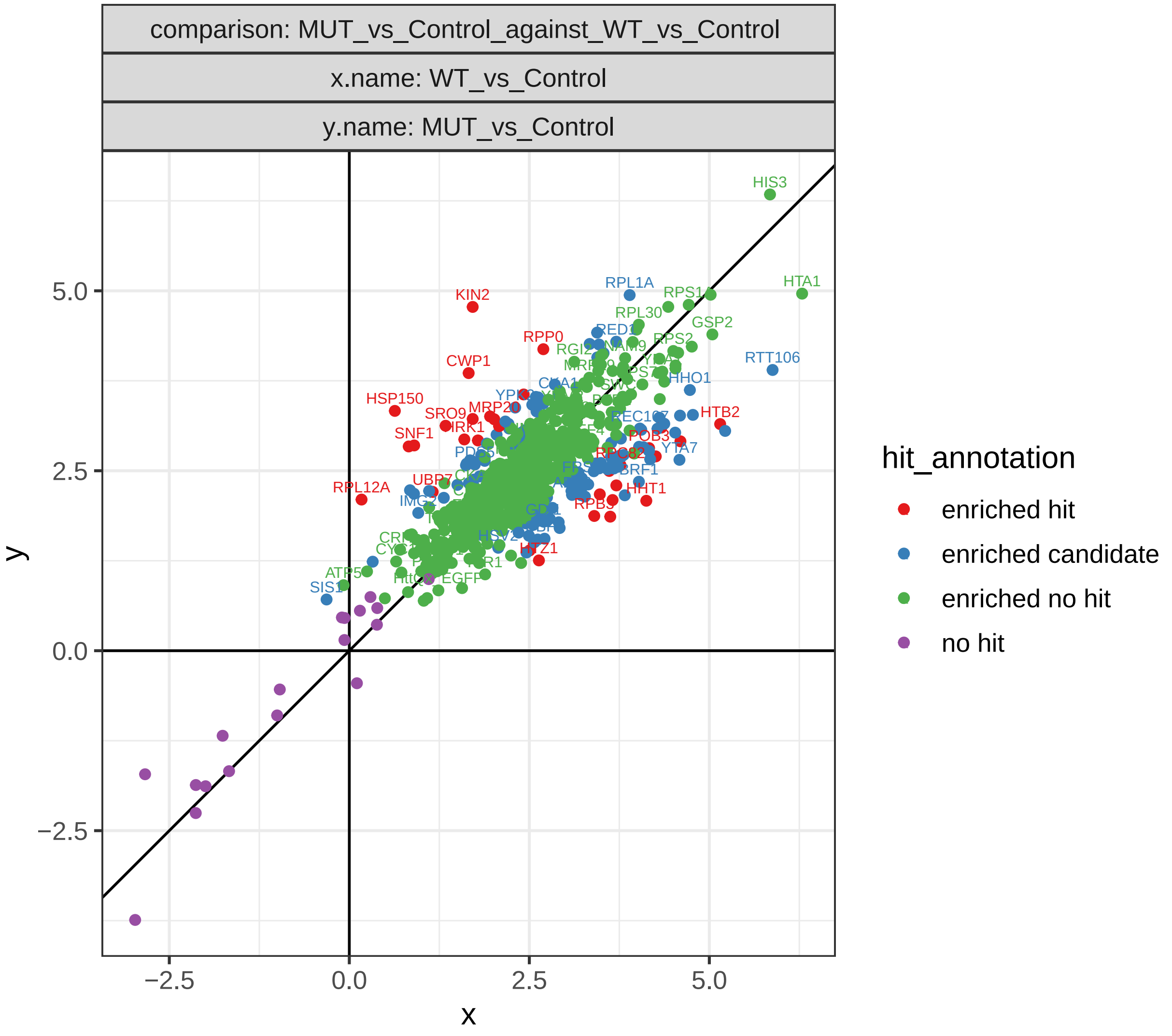


```
ggsave(file.path(dir_save, paste0("Fold_change_correlation_", script.version, ".pdf")), width = gr.widt
ggplot(data = fc.cor.data, aes(x, y, colour = hit_x_and_y)) +
```

```
geom_vline(aes(xintercept = 0)) +
geom_hline(aes(yintercept = 0)) +
geom_abline() +
coord_fixed(ratio = 1) +
geom_point() +
geom_text(aes(label = gsub("[|].+", "", gene_name)),
          data = subset(fc.cor.data, x.hit %in% c("enriched hit") | y.hit %in% c("enriched hit")),
          vjust = 0, nudge_y = 0.1, size = 2, check_overlap = TRUE) +
facet_wrap( ~ comparison + x.name + y.name, labeller = "label_both") +
customPlot
```

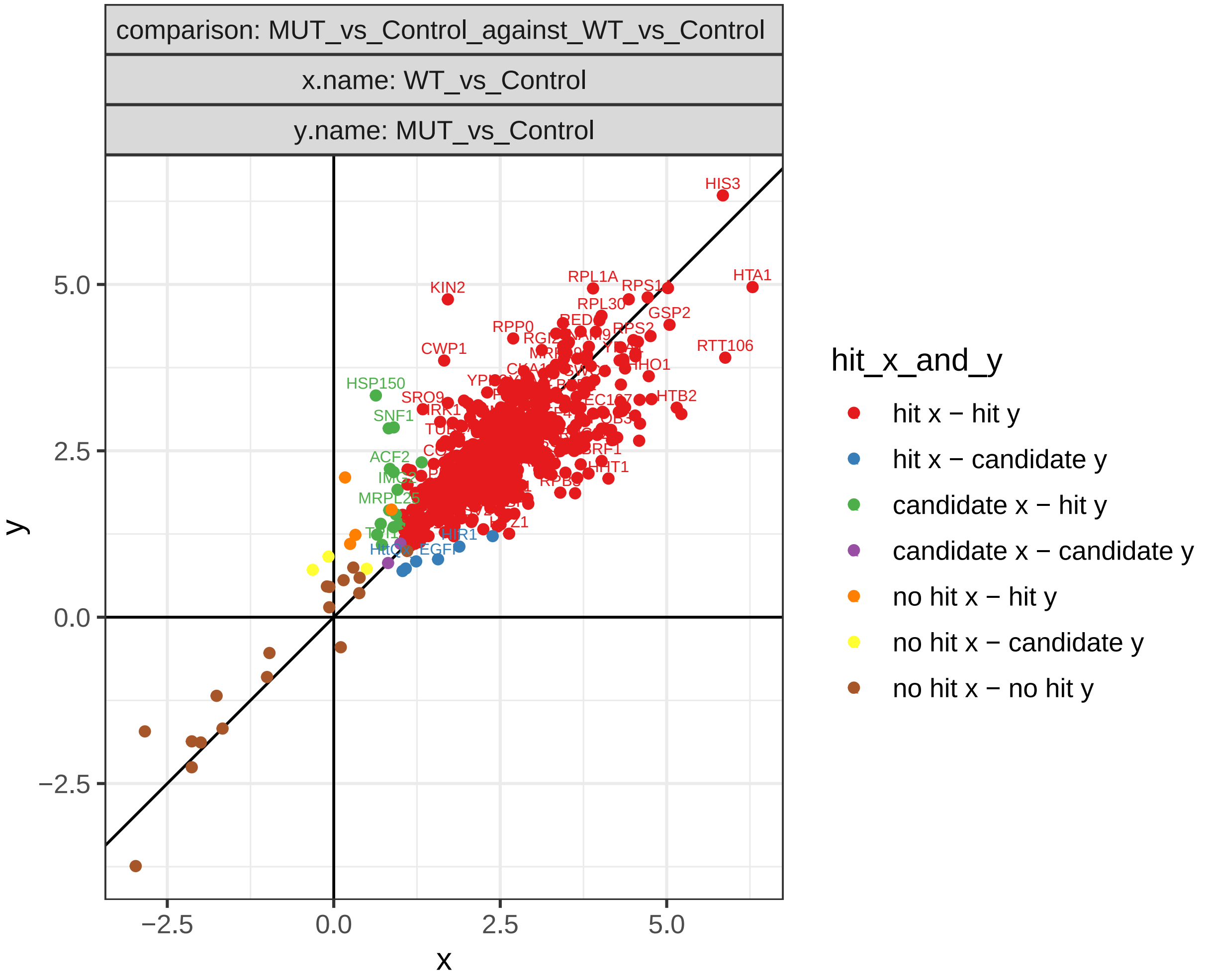

```
ggsave(file.path(dir_save, paste0("Fold_change_correlation_alt_hit_class_", script.version, ".pdf")), wi
write.csv(fc.cor.data, file = file.path(dir_save, paste0("Fold_change_correlation_data_", script.versior
rm(FC.correlation.list, gr.width, gr.height)
```

Save limma results

```
write.csv(limma_results, file.path(dir_save, paste0("Limma_results_", script.version, ".csv")), row.name
```

Clustering of data

Preparation of cluster data

```
hits <- unique(subset(limma_results, hit_annotation %in% c("enriched hit", "enriched candidate"))$gene_r
m_clust.data <- subset(mdata, measurement == "ctrl.ratio" & gene_name %in% hits) %>%
  group_by(gene_name, condition) %>%
  summarise(median.value = median(value, na.rm = TRUE))
clust.data_m <- m_clust.data %>%
  mutate(key = paste(condition, sep = "_")) %>%
  ungroup() %>%
  dplyr::select(gene_name, key, median.value) %>%
  group_by(gene_name) %>%
  spread(key = key, value = median.value) %>%
  as.data.frame()
  write.csv(clust.data_m, file.path(dir_save, paste0("Clustering_data_", nrow(clust.data_m), "_proteins_
rownames(clust.data_m) <- clust.data_m$gene_name
m_clust.data <- m_clust.data %>%
  dplyr::filter(gene_name %in% clust.data_m$gene_name)
clust.data_m$gene_name <- NULL
clust.data_m <- clust.data_m %>%
  as.matrix() %>%
  log2()
clust.data_m[is.na(clust.data_m)] <- 0
```

PCA of clustering data

```
clust.data_PCA <- prcomp(clust.data_m, scale = FALSE)
perc_var <-
  round(100 * clust.data_PCA$sdev ^ 2 /
          sum(clust.data_PCA$sdev ^ 2), 1)
PCA_clust.data <-
  data.frame(PC1 = clust.data_PCA$x[, 1],
             PC2 = clust.data_PCA$x[, 2],
             PC3 = clust.data_PCA$x[, 3])
ggplot(data = PCA_clust.data, aes(PC1, PC2, colour = PC3)) +
  geom_point() +
  theme_bw(base_size = 12) +
  scale_colour_gradientn(colours = c("black", "#377eb8", "#984ea3",
                                     "#e41a1c", "#ff7f00", "#ffff33")) +
  ggtitle("PCA clustering data",
          subtitle = paste("PC3 - explaining", perc_var[3], "% of variability")) +
  xlab(paste("PC1 - explaining", perc_var[1], "% of variability")) +
```

```
ylab(paste("PC2 - explaining", perc_var[2], "% of variability"))
```

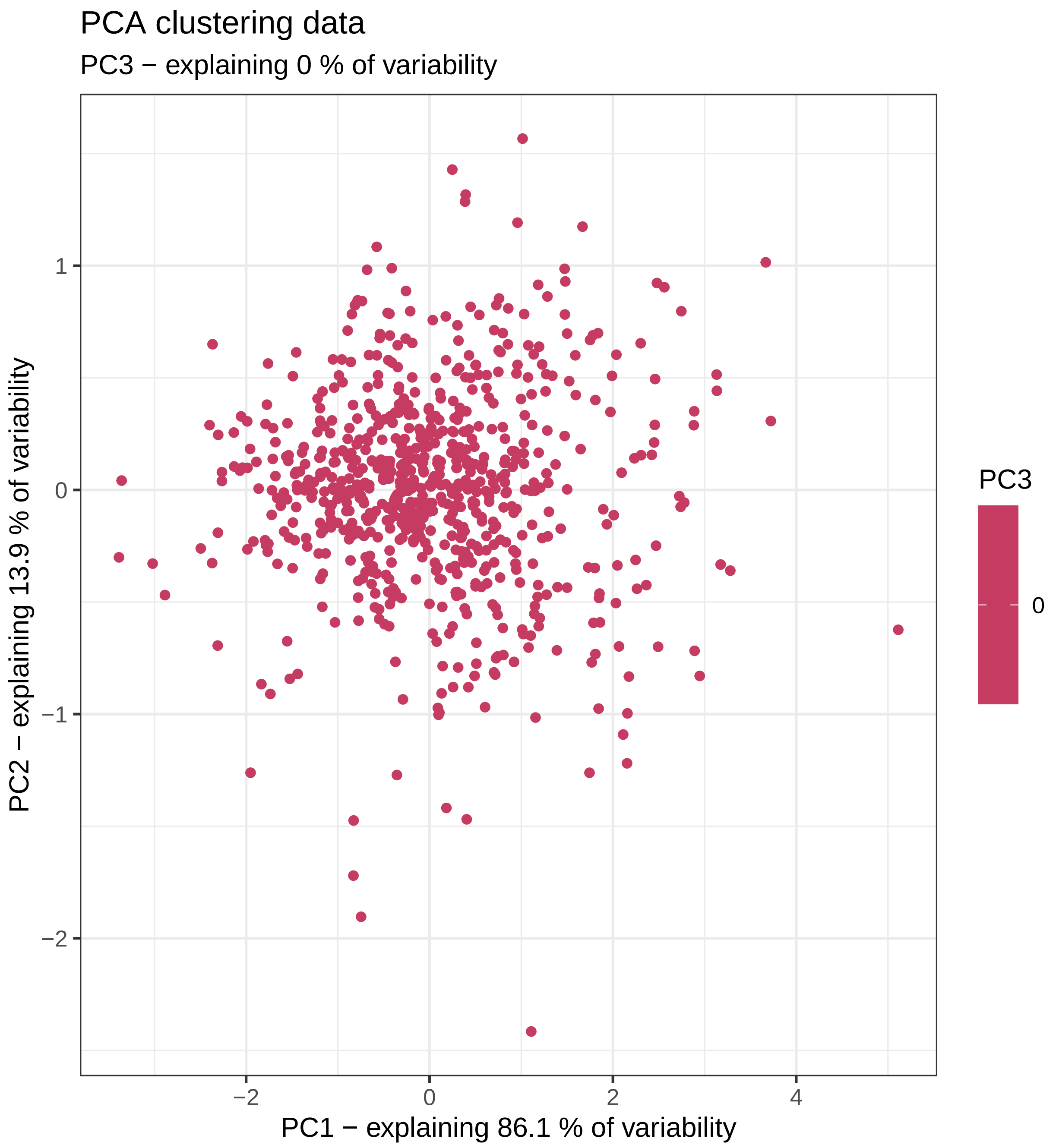


```
ggsave(file.path(dir_save, paste0("PCA_clustering_data_", script.version, ".pdf")),
       width = 7, height = 5)
rm(clust.data_PCA, perc_var)
```

Set number of clusters

```
cluster.number <- ceiling(sqrt(sqrt(nrow(clust.data_m)) * ncol(clust.data_m)))
```

K-means clustering

```
# plot within groups sum of squares for different cluster numbers
no <- ceiling(nrow(clust.data_m) / 4)
wss <- (no - 1) * sum(apply(clust.data_m, 2, var))
for (i in 2:(no - 1)) wss[i] <- sum(kmeans(clust.data_m,
                                           centers = i)$withinss)
plot(1:(no - 1), wss, type = "b", xlab = "number of Clusters",
     ylab = "within groups sum of squares")
abline(v = cluster.number, col = "red")
```

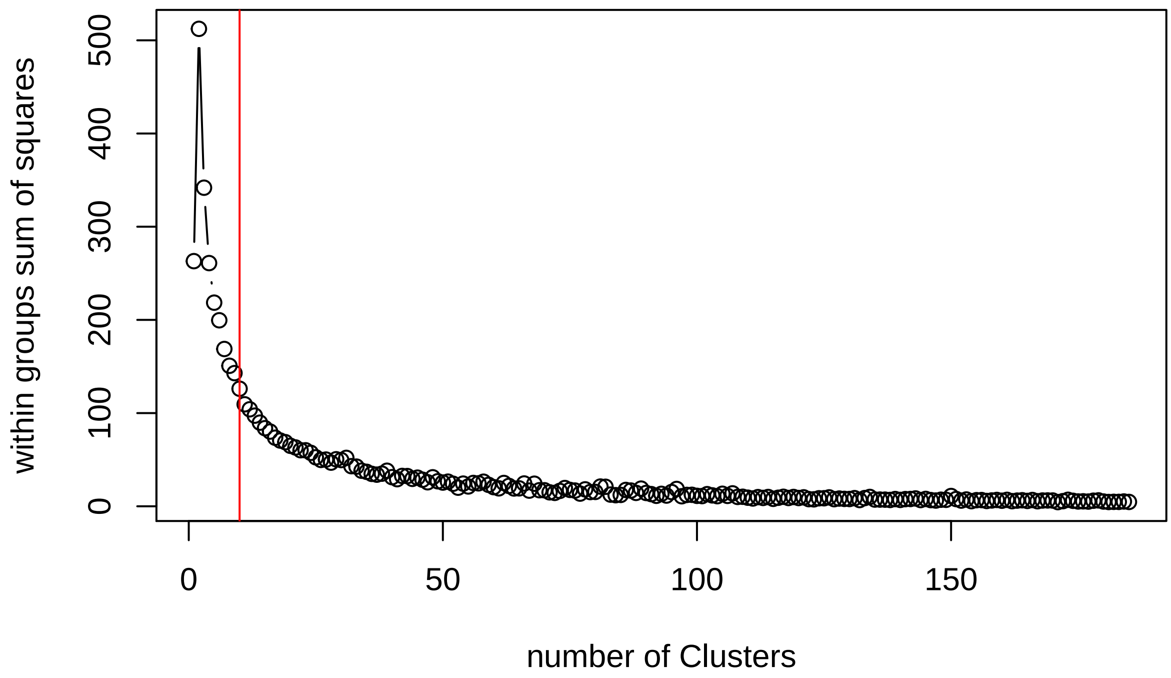


```
kmeans.fit <- kmeans(clust.data_m, cluster.number)
cluster.groups <- data.frame(gene_name = names(kmeans.fit$cluster),
                             kmeans.cluster.group = kmeans.fit$cluster)
rm(wss, kmeans.fit, no)
```

Hierarchical clustering

```
d <- dist(clust.data_m, method = "euclidean")
hclust.fit <- hclust(d, method = "ward.D2")
cluster.groups_hclust <-
  data.frame(gene_name = names(cutree(hclust.fit, k = cluster.number)),
             hclust.cluster.group = cutree(hclust.fit, k = cluster.number))
cluster.groups <- left_join(cluster.groups, cluster.groups_hclust)
rm(cluster.groups_hclust)
m_clust.data <- left_join(m_clust.data, cluster.groups)
```

```r
m_clust.data$gene_name <- factor(m_clust.data$gene_name, ordered = TRUE,
                                  levels = hclust.fit$labels[hclust.fit$order])
rm(d, hclust.fit)
```

Plot clustering results

```r
gr.width <- 2.5 + ncol(clust.data_m) * 0.1 + max(nchar(rownames(clust.data_m))) * 0.008
gr.height <- 2.5 + nrow(clust.data_m) * 0.01
ggplot(data = m_clust.data, aes(condition, gene_name)) +
  geom_tile(aes(fill = log2(median.value))) +
  scale_fill_gradient2(low = "#2166ac", high = "#b2182b", midpoint = 0,
                       mid = "#f7f7f7", name = "log2.ratio") +
  theme_bw(base_size = 12) +
  theme(axis.text.x = element_text(angle = 90, hjust = 1, vjust = 0.5),
        axis.text.y = element_text(size = 1)) +
  ylab("gene")
```

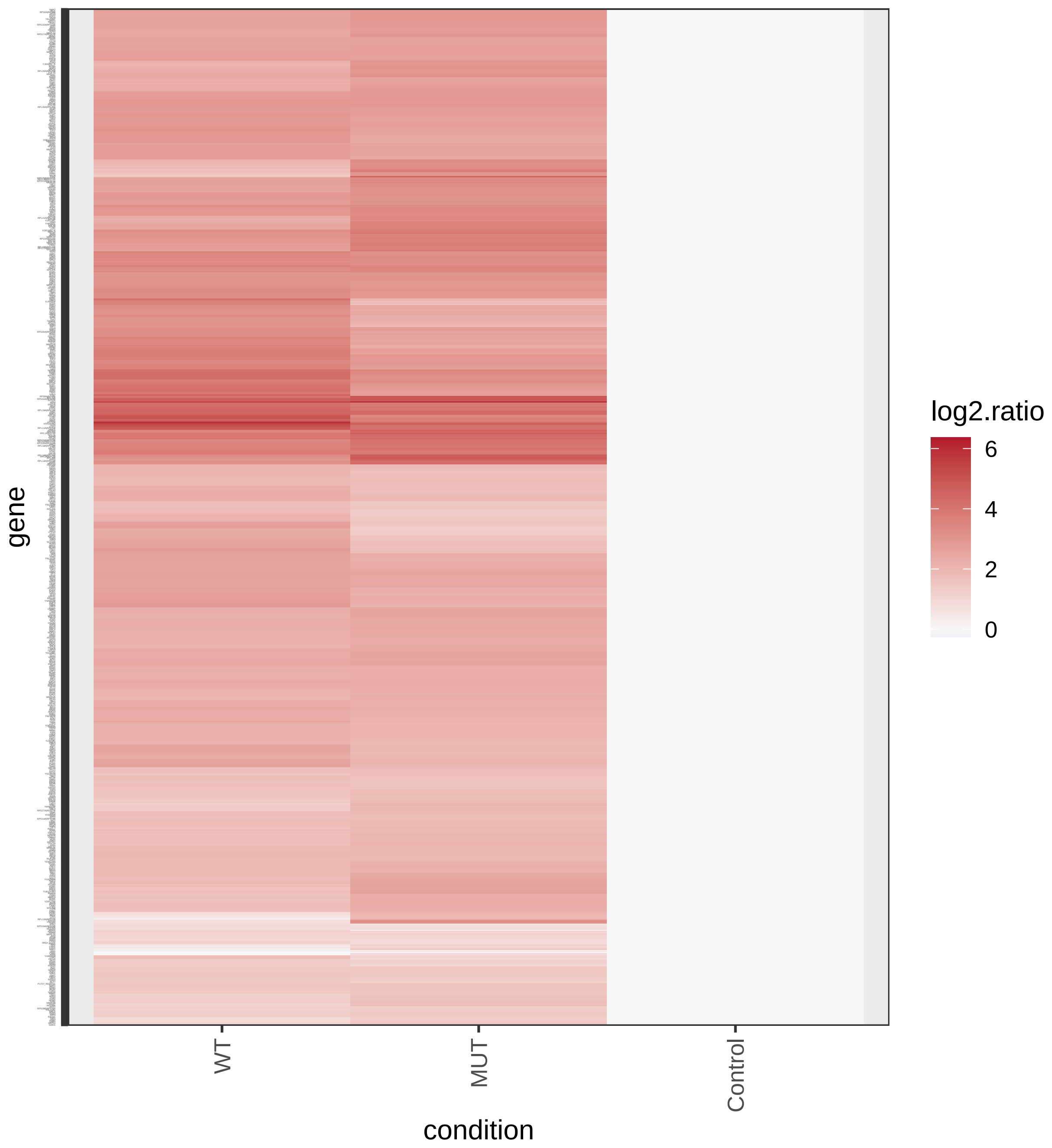


```r
ggsave(file.path(dir_save, paste0("Heatmap_hits_", nrow(clust.data_m), "_proteins_", script.version, ".

# Clustering heatmaps
ggplot(data = cluster.groups, aes(hclust.cluster.group, kmeans.cluster.group)) +
  geom_abline(colour = "grey") +
  geom_jitter(width = 0.2, height = 0.2, alpha = 0.3) +
  scale_x_continuous(breaks = 1:cluster.number) +
  scale_y_continuous(breaks = 1:cluster.number) +
  customPlot +
  theme(panel.grid.minor = element_blank())
```

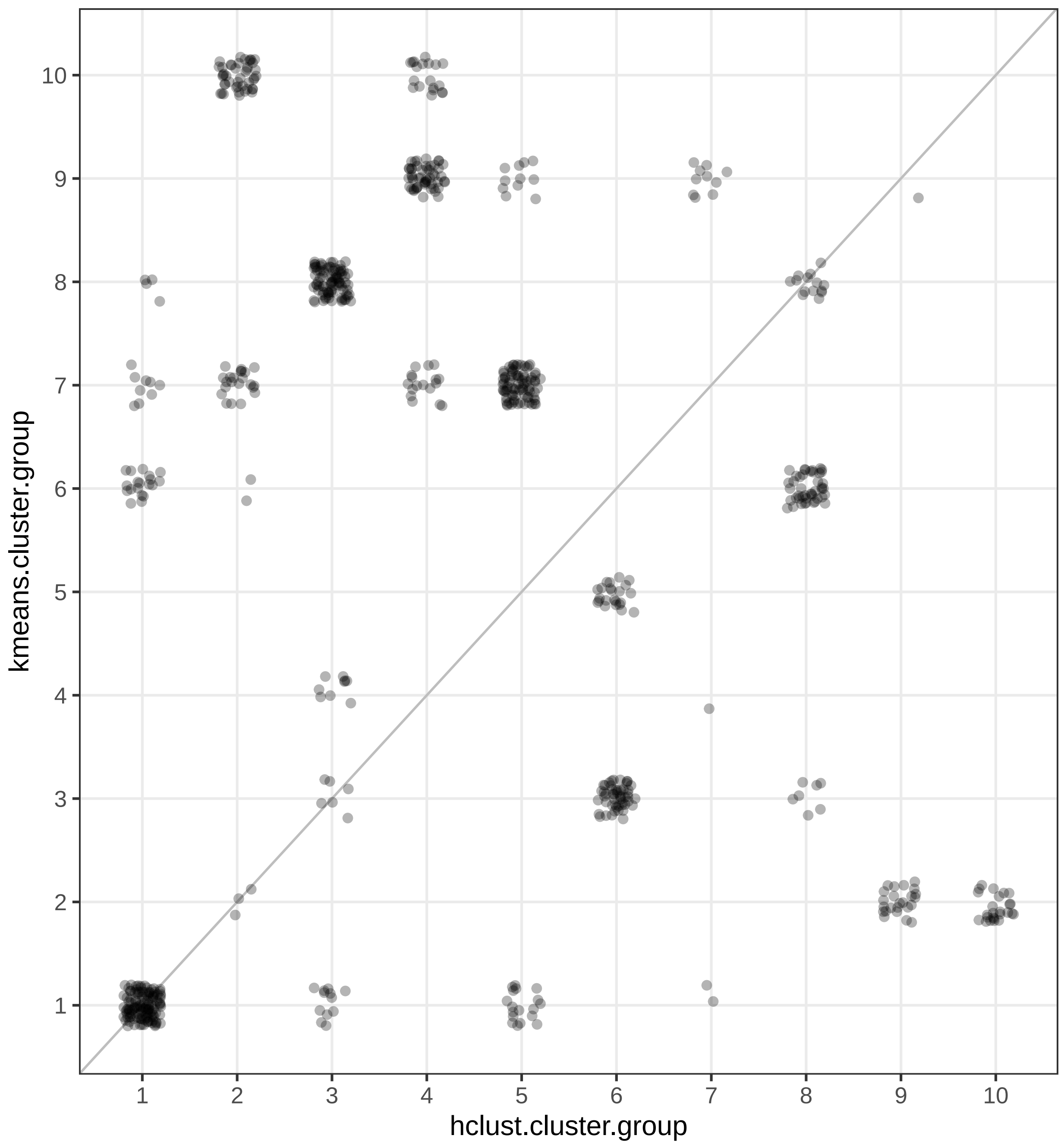


```r
# PCA with cluster results
m_cluster.groups <- cluster.groups %>%
 group_by(gene_name) %>%
 gather(-gene_name, key = "cluster.group", value = "cluster") %>%
 mutate(cluster.group = gsub(".cluster.group", "", cluster.group))
PCA_clust.data$gene_name <- rownames(PCA_clust.data)
PCA_clust.data <- left_join(PCA_clust.data, m_cluster.groups)
PCA_clust.data$cluster <- factor(PCA_clust.data$cluster)
ggplot(data = PCA_clust.data, aes(PC1, PC2, colour = cluster)) +
  geom_point() +
  theme_bw(base_size = 12) +
```

```
  facet_wrap(~ cluster.group) +
  geom_text(aes(label = gene_name), size = 0.2, colour = "black")
```

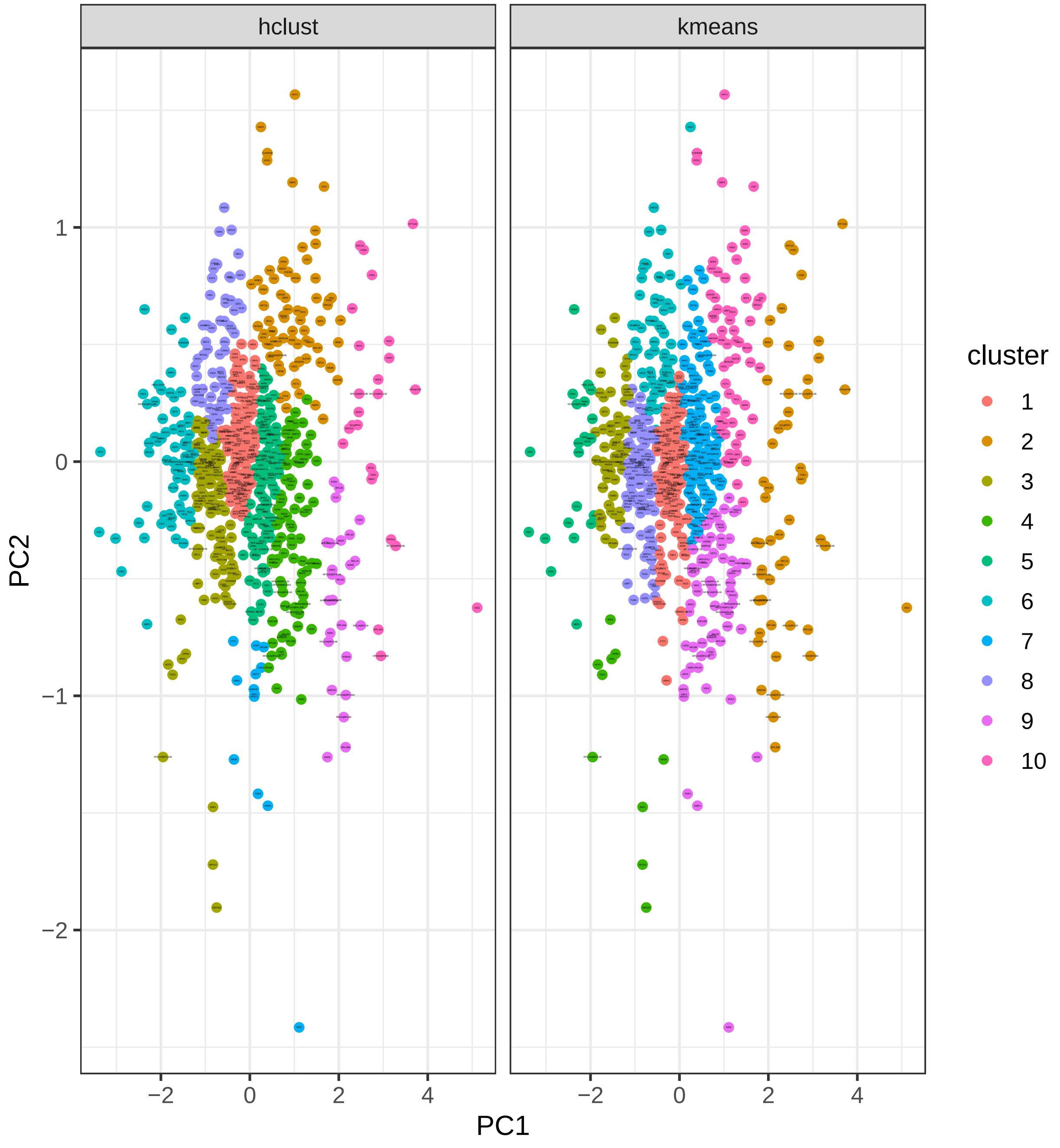


```
ggsave(file.path(dir_save, paste0("PCA_clustering_data_", cluster.number, "_cluster_", nrow(clust.data_m
       width = 10, height = 5)
gr.height <- 2.5 + nrow(clust.data_m) * 0.01 + cluster.number * 0.1
ggplot(data = m_clust.data, aes(condition, gene_name)) +
  geom_tile(aes(fill = log2(median.value))) +
  scale_fill_gradient2(low = "#2166ac", high = "#b2182b", midpoint = 0,
                       mid = "#f7f7f7", name = "log2.ratio") +
  facet_grid(hclust.cluster.group ~ ., scale = "free", space = "free") +
```

```r
  theme_bw(base_size = 12) +
  theme(axis.text.x = element_text(angle = 90, hjust = 1, vjust = 0.5),
        axis.text.y = element_text(size = 1)) +
  ylab("gene") +
  ggtitle("hierarchical clustering")
```

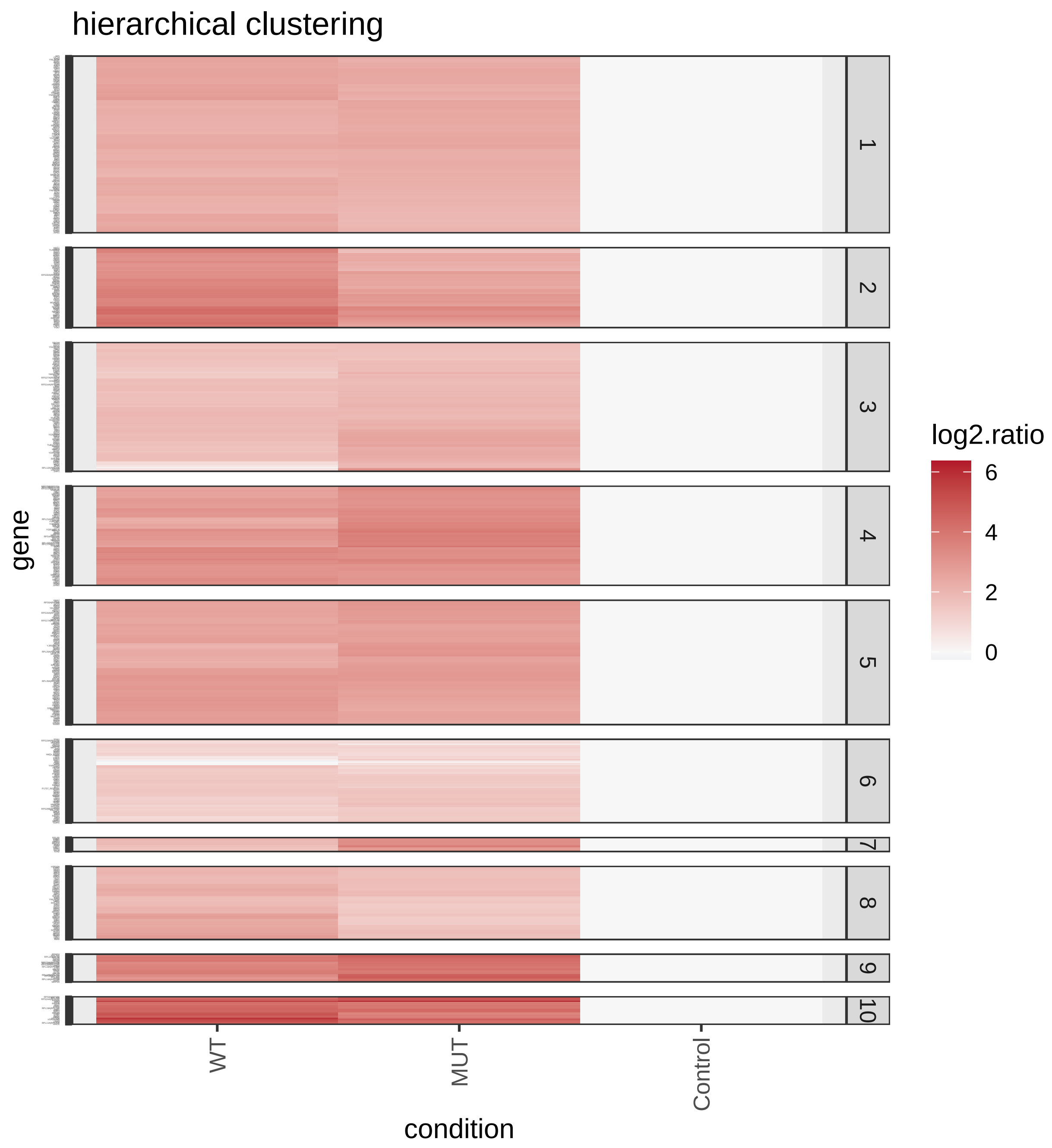


```r
ggsave(file.path(dir_save, paste0("Clustering_heatmap_hits_hclust_", cluster.number, "_cluster_", nrow(
       width = gr.width, height = gr.height)

ggplot(data = m_clust.data, aes(condition, gene_name)) +
  geom_tile(aes(fill = log2(median.value))) +
```

```
scale_fill_gradient2(low = "#2166ac", high = "#b2182b", midpoint = 0,
                     mid = "#f7f7f7", name = "log2.ratio") +
facet_grid(kmeans.cluster.group ~ ., scale = "free", space = "free") +
theme_bw(base_size = 12) +
theme(axis.text.x = element_text(angle = 90, hjust = 1, vjust = 0.5),
      axis.text.y = element_text(size = 1)) +
ylab("gene") +
ggtitle("kmeans clustering")
```

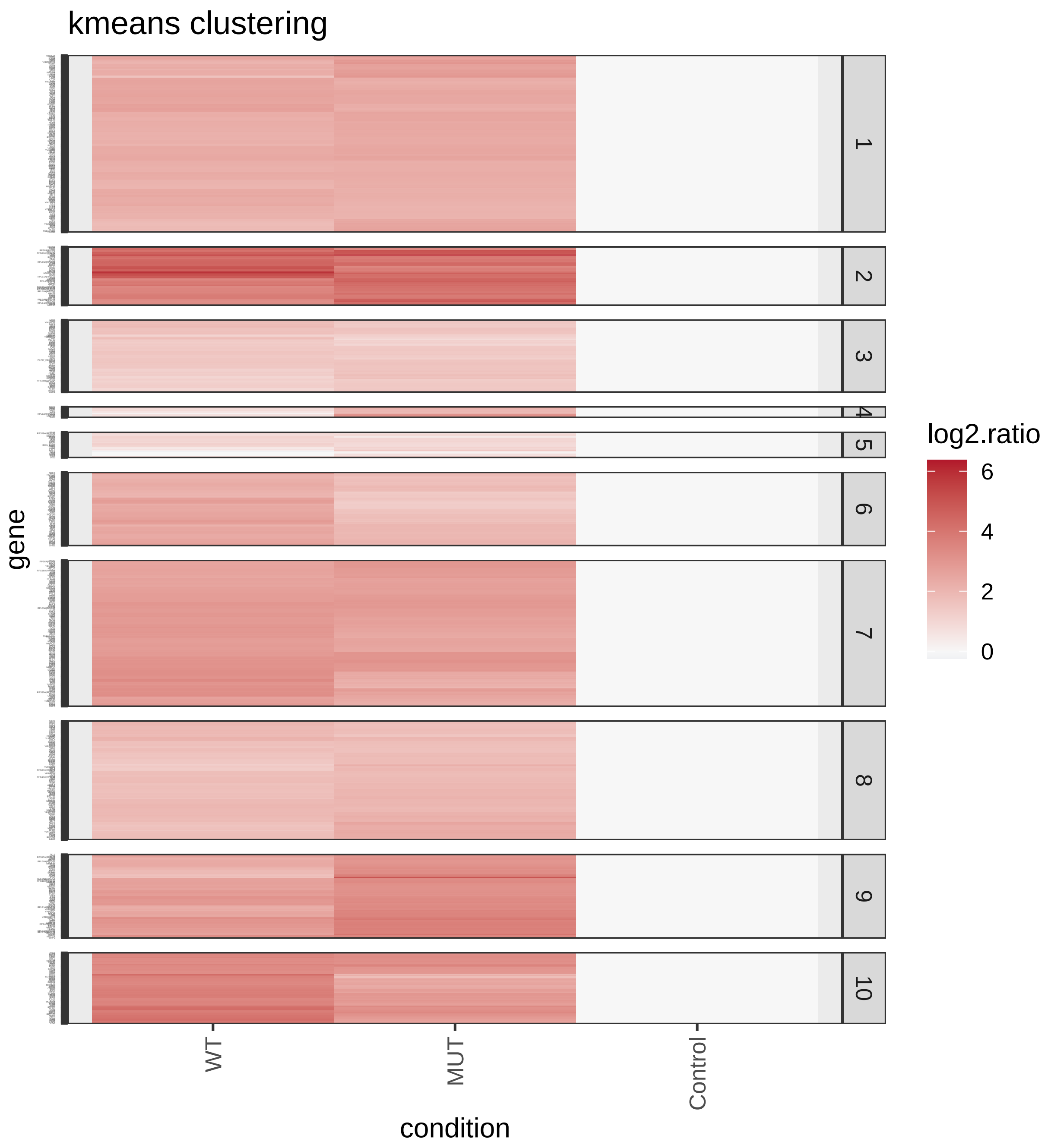


```
ggsave(file.path(dir_save, paste0("Clustering_heatmap_hits_kmeans_", cluster.number, "_cluster_", nrow(
       width = gr.width, height = gr.height)
```

```r
# Line plots
gr.width <- 4 + ceiling(sqrt(cluster.number)) * 3
gr.height <- 2.5 + floor(sqrt(cluster.number)) * 3
ggplot(data = m_clust.data, aes(condition, log2(median.value))) +
  geom_hline(yintercept = 0) +
  geom_line(aes(group = paste(gene_name, hclust.cluster.group)), alpha = 0.1) +
  geom_smooth(fun.data = "mean_se", stat = "summary", aes(group = paste(hclust.cluster.group))) +
  facet_wrap(~ hclust.cluster.group) +
  customPlot +
  theme(axis.text.x = element_text(angle = 90, hjust = 1, vjust = 0.5)) +
  ggtitle("hierarchical clustering") +
  ylab("log2(ratio)")
```

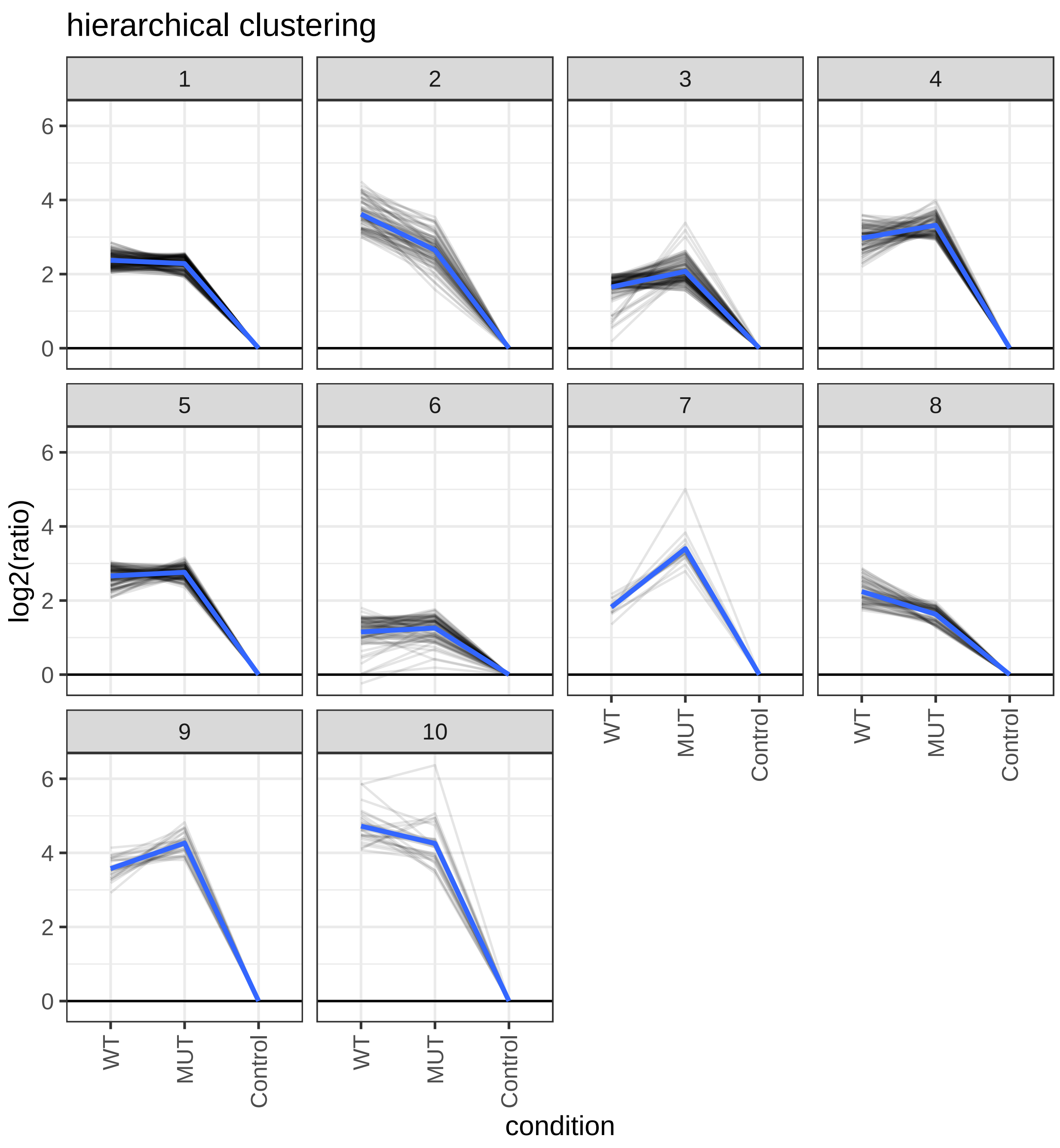


```r
ggsave(file.path(dir_save, paste0("Clustering_line_plot_hclust_", cluster.number, "_cluster_", nrow(clu
       width = gr.width, height = gr.height)

ggplot(data = m_clust.data, aes(condition, log2(median.value))) +
  geom_hline(yintercept = 0) +
  geom_line(aes(group = paste(gene_name, kmeans.cluster.group)), alpha = 0.1) +
  geom_smooth(fun.data = "mean_se", stat = "summary", aes(group = paste(kmeans.cluster.group))) +
  facet_wrap(~ kmeans.cluster.group) +
  customPlot +
  theme(axis.text.x = element_text(angle = 90, hjust = 1, vjust = 0.5)) +
  ggtitle("kmeans clustering") +
```

```r
  ylab("log2(ratio)")
```

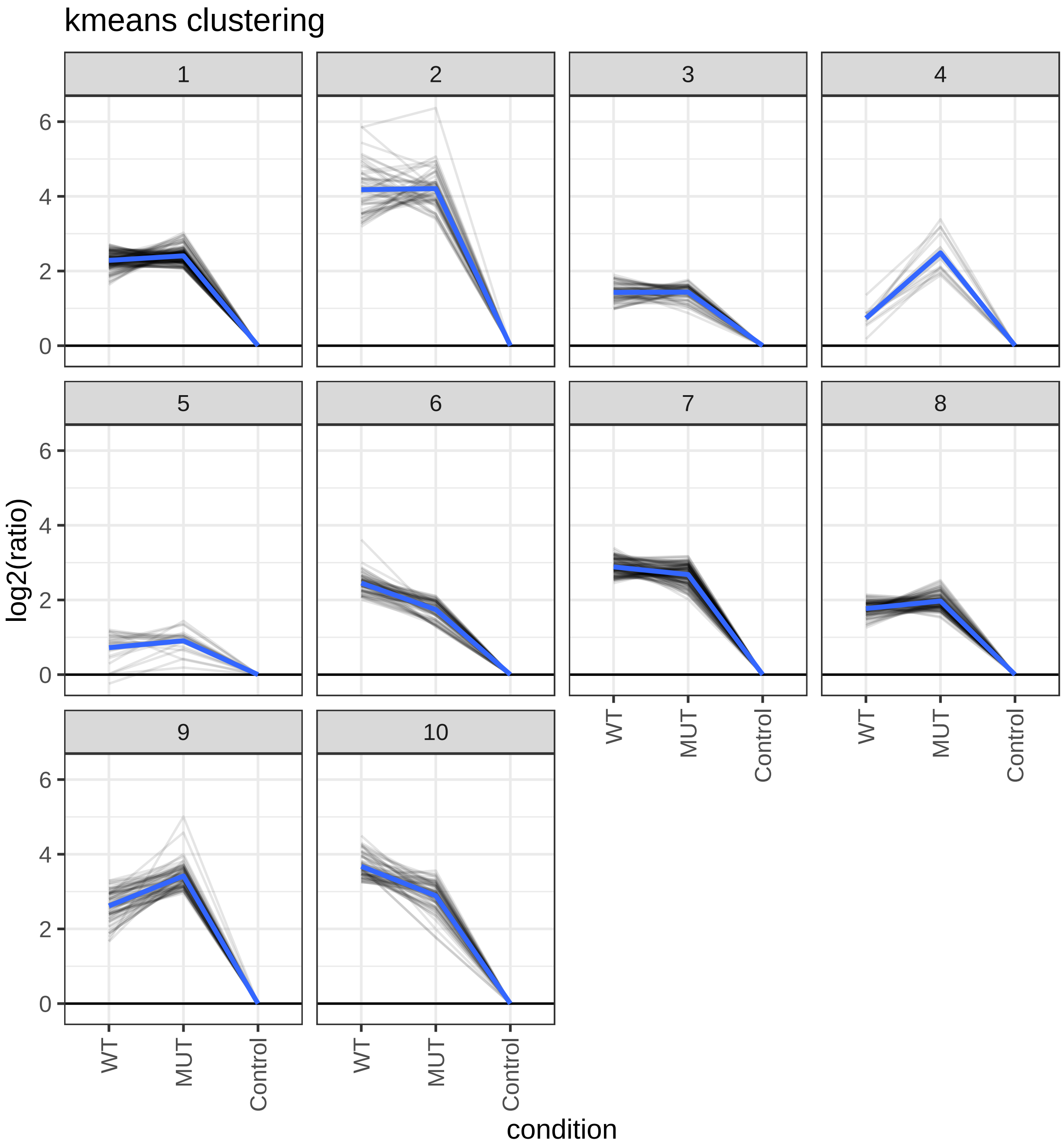


```r
ggsave(file.path(dir_save, paste0("Clustering_line_plot_kmeans_", cluster.number, "_cluster_", nrow(clu
       width = gr.width, height = gr.height)
write.csv(cluster.groups, file.path(dir_save, paste0("Cluster_results_",cluster.number,"cluster_", nrow
rm(gr.width, gr.height)
```

Save R workspace

```
save.image(file.path(dir_save, paste0("Workspace_", script.version, ".RData")))
# load(file.path(dir_save, paste0("Workspace_", script.version, ".RData")))
```

Save Session information

```
sessionInfo()
```

```
## R version 4.0.4 (2021-02-15)
## Platform: x86_64-w64-mingw32/x64 (64-bit)
## Running under: Windows 10 x64 (build 17763)
##
## Matrix products: default
##
## locale:
## [1] LC_COLLATE=English_United States.1252
## [2] LC_CTYPE=English_United States.1252
## [3] LC_MONETARY=English_United States.1252
## [4] LC_NUMERIC=C
## [5] LC_TIME=English_United States.1252
##
## attached base packages:
## [1] stats4    parallel  stats     graphics  grDevices utils     datasets
## [8] methods   base
##
## other attached packages:
##  [1] forcats_0.5.1       stringr_1.4.0       dplyr_1.0.4
##  [4] purrr_0.3.4         readr_1.4.0         tidyr_1.1.2
##  [7] tibble_3.0.6        ggplot2_3.3.3       tidyverse_1.3.0
## [10] biobroom_1.22.0     broom_0.7.5         fdrtool_1.2.16
## [13] gplots_3.1.1        MSnbase_2.15.7      ProtGenerics_1.22.0
## [16] S4Vectors_0.28.1    mzR_2.24.1          Rcpp_1.0.6
## [19] limma_3.46.0        vsn_3.58.0          Biobase_2.50.0
## [22] BiocGenerics_0.36.0
##
## loaded via a namespace (and not attached):
##  [1] bitops_1.0-6          fs_1.5.0              lubridate_1.7.9.2
##  [4] RColorBrewer_1.1-2    doParallel_1.0.16     httr_1.4.2
##  [7] tools_4.0.4           backports_1.2.1       utf8_1.1.4
## [10] R6_2.5.0              affyio_1.60.0         KernSmooth_2.23-18
## [13] DBI_1.1.1             colorspace_2.0-0      withr_2.4.1
## [16] tidyselect_1.1.0      compiler_4.0.4        preprocessCore_1.52.1
## [19] cli_2.3.1             rvest_0.3.6           xml2_1.3.2
## [22] labeling_0.4.2        caTools_1.18.1        scales_1.1.1
## [25] hexbin_1.28.2         affy_1.68.0           digest_0.6.27
## [28] rmarkdown_2.7         pkgconfig_2.0.3       htmltools_0.5.1.1
## [31] highr_0.8             dbplyr_2.1.0          rlang_0.4.10
## [34] readxl_1.3.1          rstudioapi_0.13       impute_1.64.0
## [37] farver_2.0.3          generics_0.1.0        jsonlite_1.7.2
## [40] mzID_1.28.0           BiocParallel_1.24.1   gtools_3.8.2
## [43] magrittr_2.0.1        MALDIquant_1.19.3     munsell_0.5.0
## [46] fansi_0.4.2           lifecycle_1.0.0       stringi_1.5.3
```

```
## [49] yaml_2.2.1           MASS_7.3-53.1        zlibbioc_1.36.0
## [52] plyr_1.8.6           grid_4.0.4           crayon_1.4.1
## [55] lattice_0.20-41      haven_2.3.1          hms_1.0.0
## [58] knitr_1.31           pillar_1.5.0         codetools_0.2-18
## [61] reprex_1.0.0         XML_3.99-0.5         glue_1.4.2
## [64] evaluate_0.14        pcaMethods_1.82.0    BiocManager_1.30.10
## [67] modelr_0.1.8         vctrs_0.3.6          foreach_1.5.1
## [70] cellranger_1.1.0     gtable_0.3.0         assertthat_0.2.1
## [73] xfun_0.21            ncdf4_1.17           iterators_1.0.13
## [76] IRanges_2.24.1       ellipsis_0.3.1
```

Plot individual proteins

```
f.name <- file.path(dir_save, paste0("all_proteins_", script.version, ".pdf"))
pdf(width = 6, height = 14, file = f.name)
genes <- unique(cdata$gene_name)
genes <- sort(genes)
pb <- txtProgressBar(min = 0, max = length(genes), style = 3)
for (i in seq_along(genes))
{
  gene <- genes[i]
  setTxtProgressBar(pb, i)
  mdata_i <- subset(mdata, gene_name == gene)
  cdata_i <- subset(cdata, gene_name == gene)
  if (nrow(cdata_i) == 1 & nrow(mdata_i) > 0)
  {
    titlestring <- paste(cdata_i$gene_name[1], "-", cdata_i$protein_id[1])
    try(
      print(
        ggplot(data = mdata_i, aes(condition, log2(value), fill = condition)) +
          geom_boxplot() +
          geom_point(aes(shape = rep)) +
          facet_grid(measurement ~ ., space = "free_x", scale = "free") +
          customPlot +
          theme(axis.text.x = element_text(angle = 90, hjust = 1, vjust = 0.5)) +
          ggtitle(titlestring, subtitle = cdata_i$description[1])
      )
    )
    rm(titlestring)
  }
  rm(mdata_i, cdata_i)
}
dev.off()
close(pb)

f.name <- file.path(dir_save, paste0("hit_proteins_", script.version, ".pdf"))
pdf(width = 6, height = 14, file = f.name)
genes <- unique(subset(limma_results, hit)$gene_name)
genes <- sort(genes)
pb <- txtProgressBar(min = 0, max = length(genes), style = 3)
for (i in seq_along(genes))
{
  gene <- genes[i]
```

```r
  setTxtProgressBar(pb, i)
  mdata_i <- subset(mdata, gene_name == gene)
  cdata_i <- subset(cdata, gene_name == gene)
  if (nrow(cdata_i) == 1 & nrow(mdata_i) > 0)
  {
    titlestring <- paste(cdata_i$gene_name[1], "-", cdata_i$protein_id[1])
    try(
      print(
        ggplot(data = mdata_i, aes(condition, log2(value), fill = condition)) +
          geom_boxplot() +
          geom_point(aes(shape = rep)) +
          facet_grid(measurement ~ ., space = "free_x", scale = "free") +
          customPlot +
          theme(axis.text.x = element_text(angle = 90, hjust = 1, vjust = 0.5)) +
          ggtitle(titlestring, subtitle = cdata_i$description[1])
      )
    )
    rm(titlestring)
  }
  rm(mdata_i, cdata_i)
}
dev.off()
close(pb)
```
