# Supplementary figures and images for: Novel mechanistic insights into the role of Mer2 as the keystone of meiotic DNA break formation (part 2 of 2)
Source: eLife. 2021 Dec 24;10:e72330. doi: 10.7554/eLife.72330 (PMC8848140; doi:10.7554/eLife.72330)

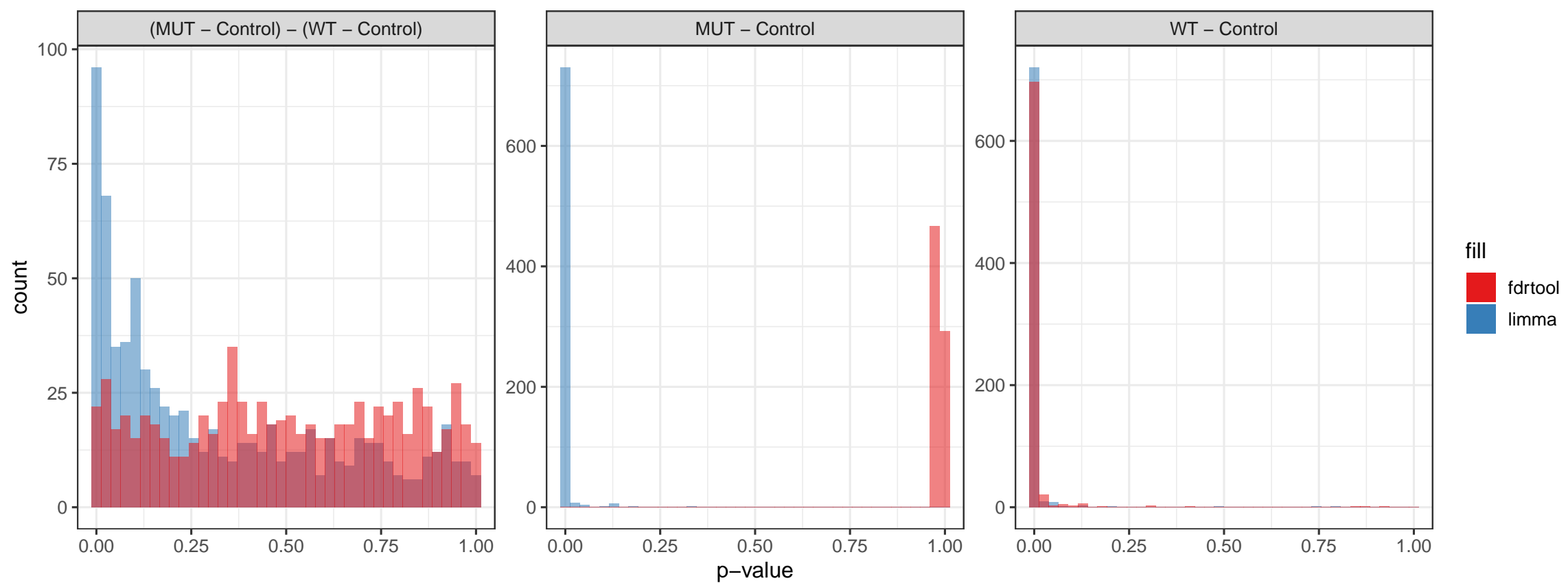

Supplement: Source data 2. [file elife-72330-data2.zip › data_analysis_results_V1/p-value_histogram_limma_vs_fdrtool_V1.pdf]

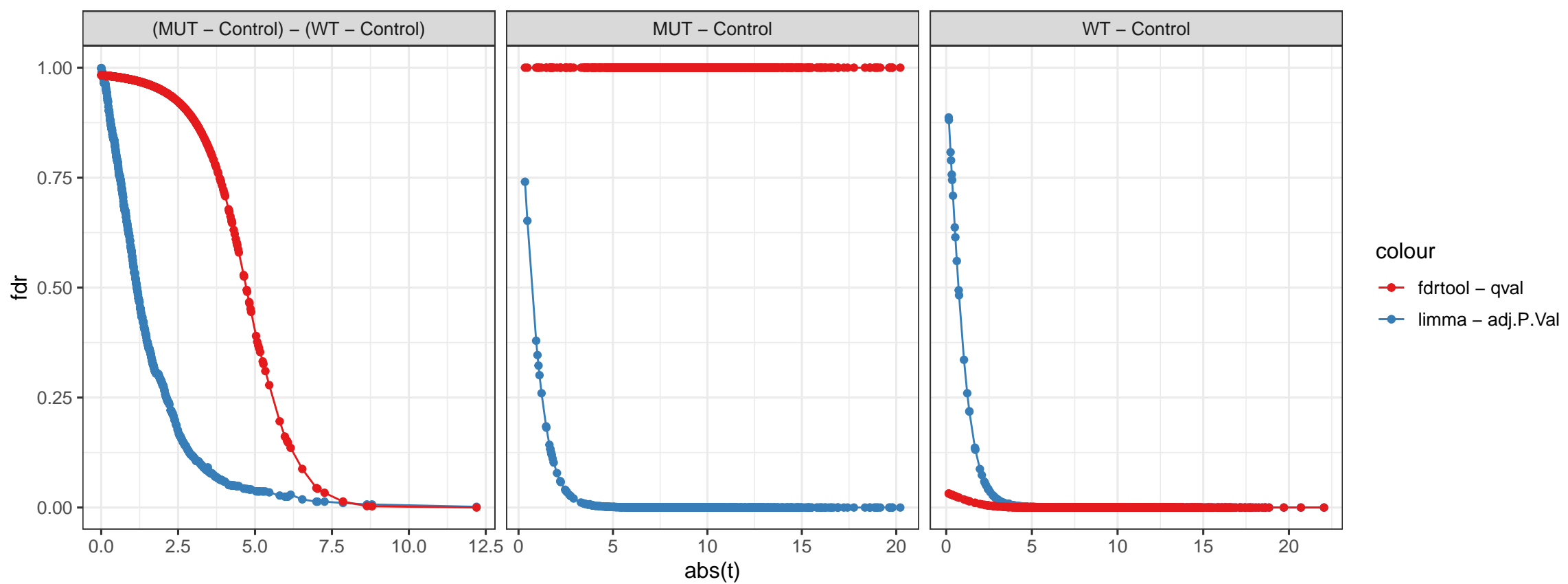

Supplement: Source data 2. [file elife-72330-data2.zip › data_analysis_results_V1/t_vs_fdr_limma_vs_fdrtool_V1.pdf]

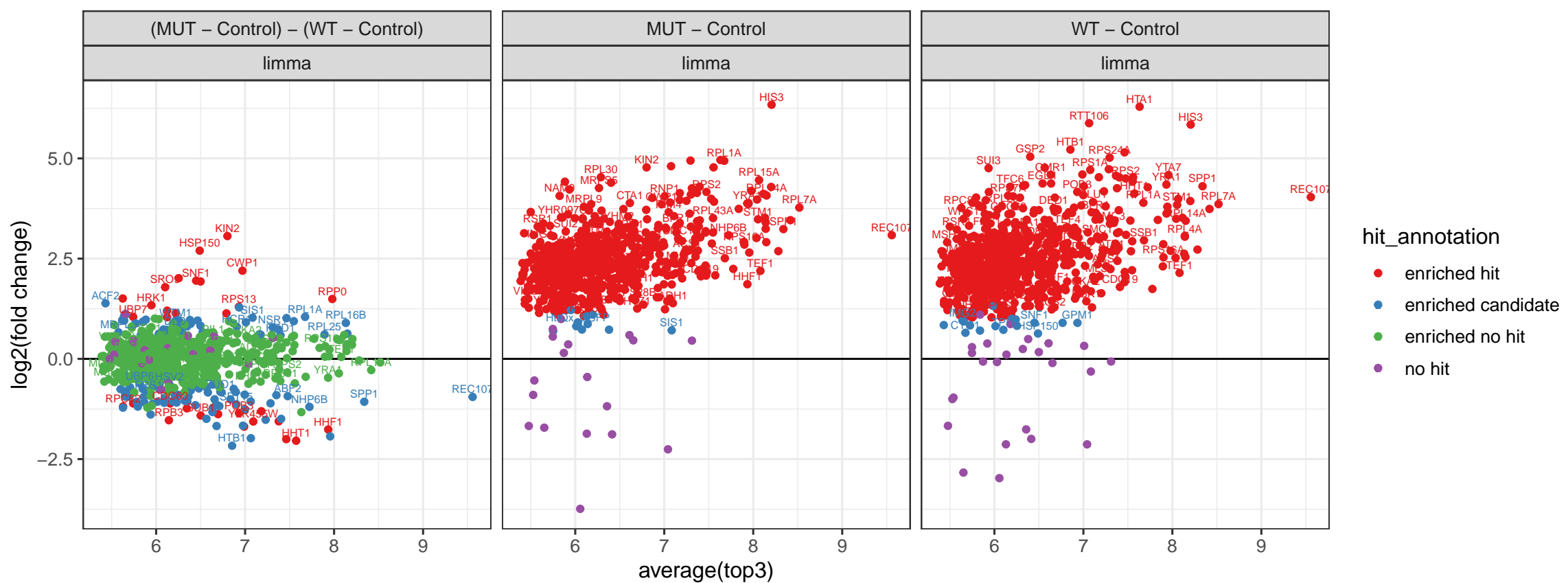

Supplement: Source data 2. [file elife-72330-data2.zip › data_analysis_results_V1/top3_plot_V1.pdf]

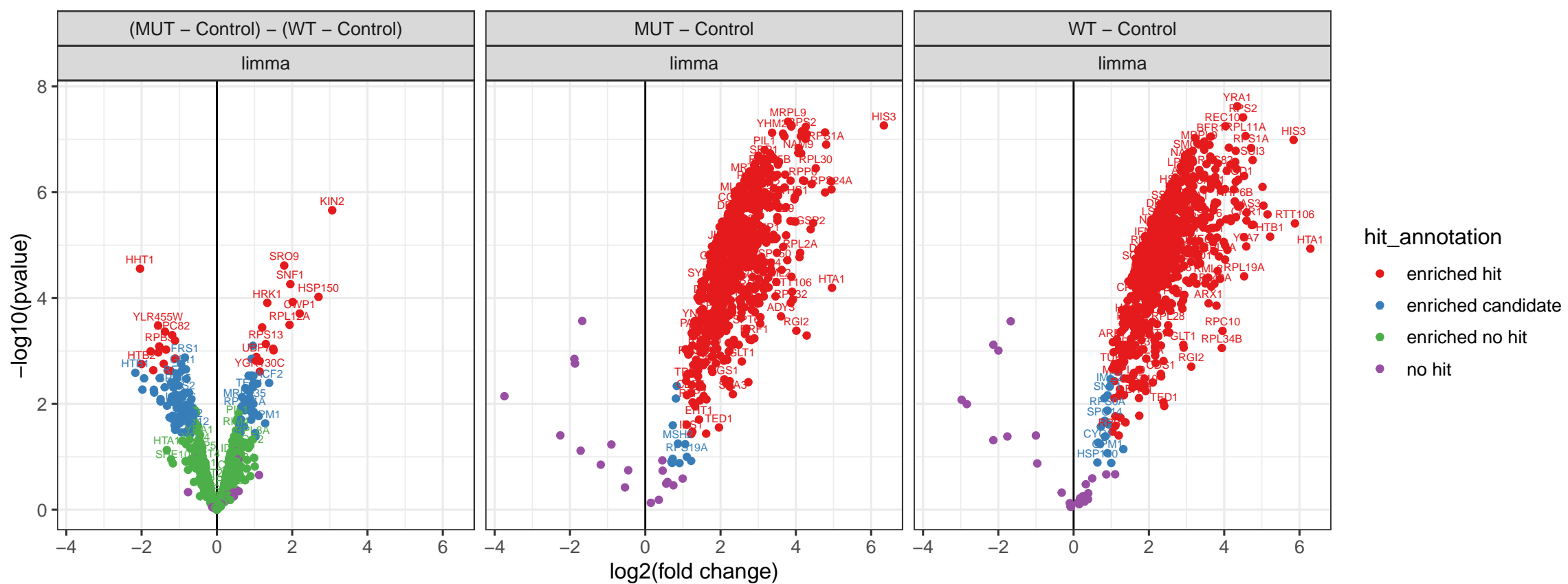

Supplement: Source data 2. [file elife-72330-data2.zip › data_analysis_results_V1/Volcano_plot_V1.pdf]
