# Supplementary material for: Novel mechanistic insights into the role of Mer2 as the keystone of meiotic DNA break formation
Source: eLife. 2021 Dec 24;10:e72330. doi: 10.7554/eLife.72330 (PMC8848140; doi:10.7554/eLife.72330)
Supplement: Source data 2. [file all_proteins_V1.pdf › data_analysis_results_V1/all_proteins_V1.pdf]

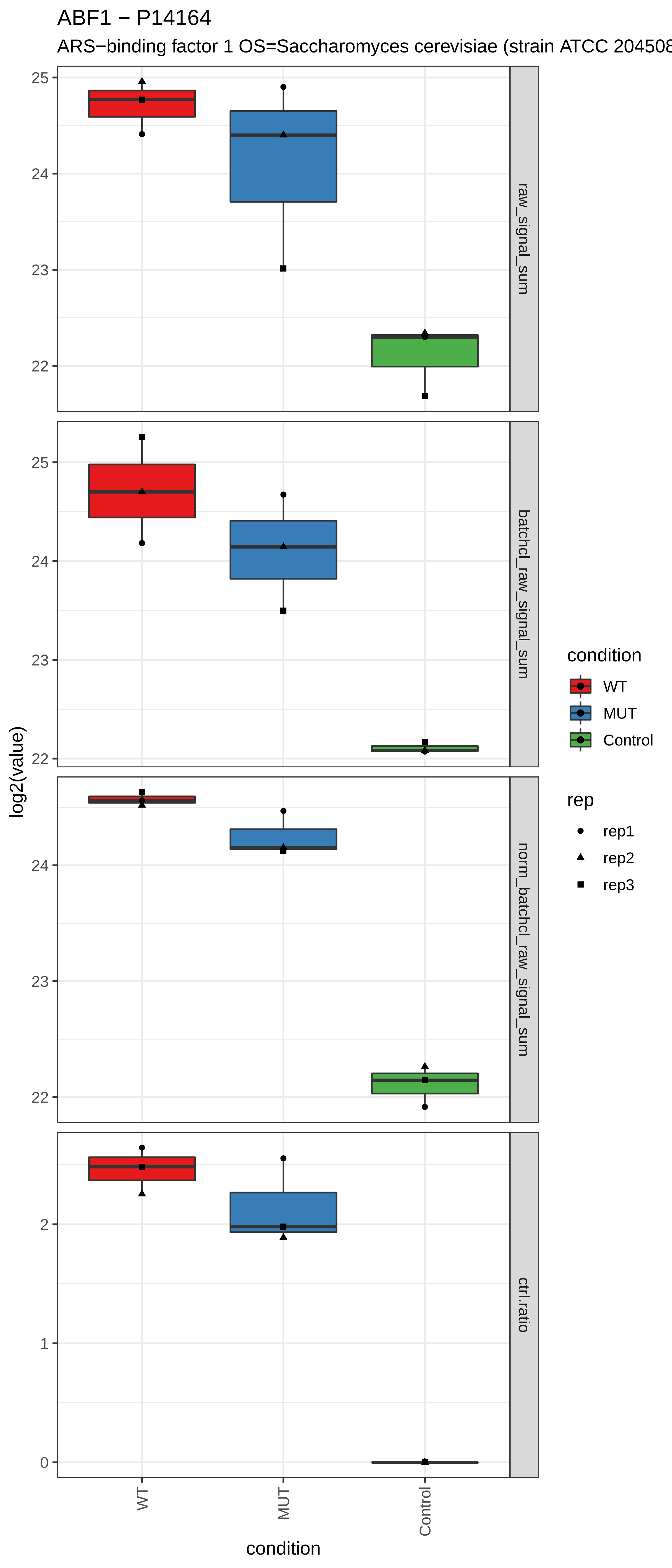
ABF1 − P14164
ARS−binding factor 1 OS=Saccharomyces cerevisiae (strain ATCC 204508
raw_signal_sum
batchcl_raw_signal_sum
norm_batchcl_raw_signal_sum
ctrl.ratio
log2(value)
condition
WT
MUT
Control
rep
rep1
rep2
rep3
25
24
23
22
2
1
0

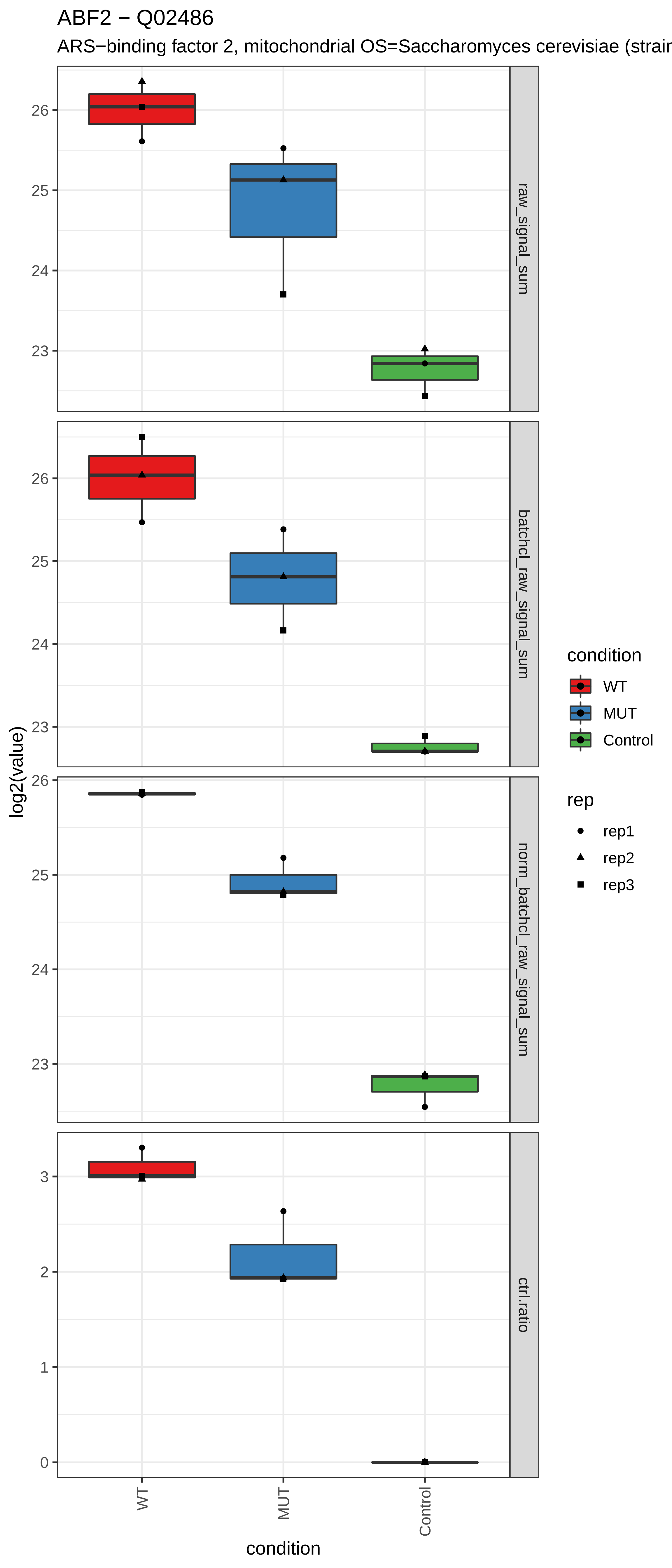

ABF2 − Q02486
ARS−binding factor 2, mitochondrial OS=Saccharomyces cerevisiae (strain
log2(value)
raw_signal_sum
batchcl_raw_signal_sum
norm_batchcl_raw_signal_sum
ctrl.ratio
26
25
24
23
3
2
1
0
WT
MUT
Control
condition
condition
WT
MUT
Control
rep
rep1
rep2
rep3

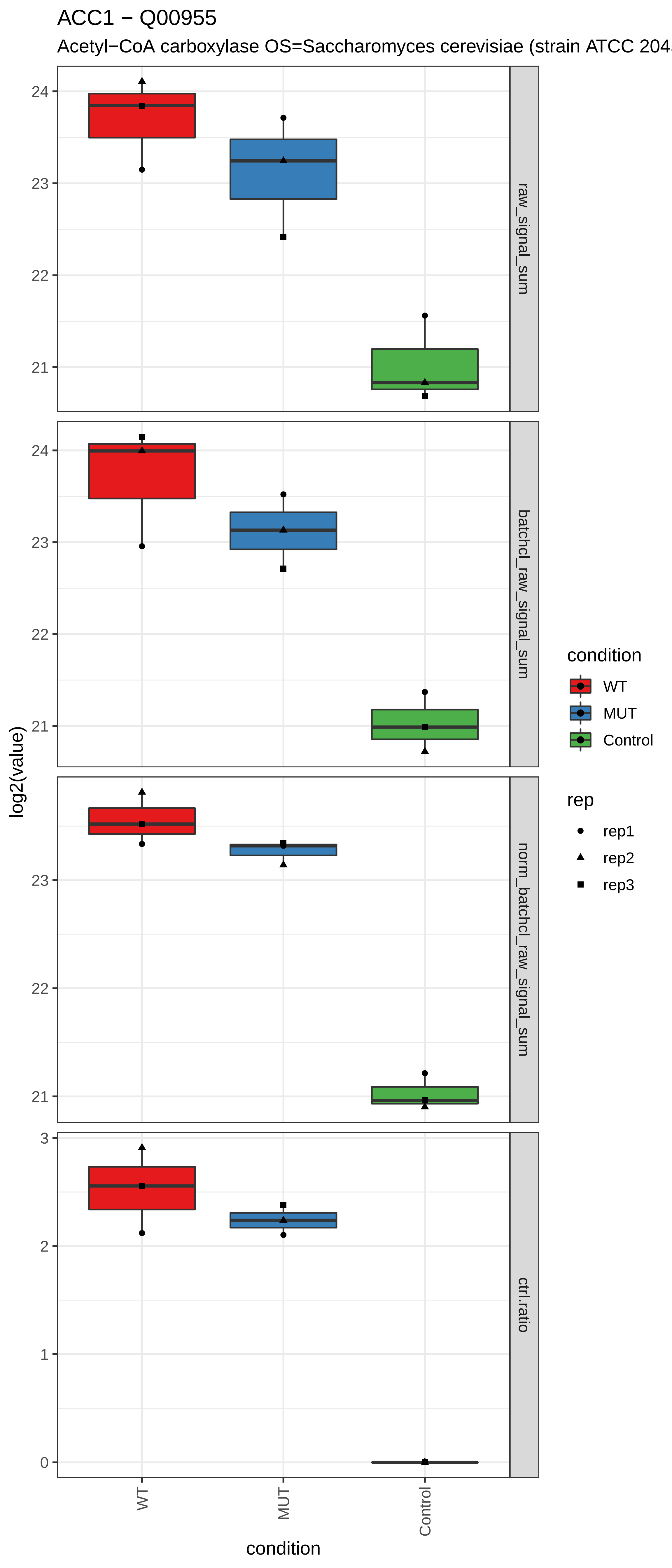
ACC1 − Q00955
Acetyl−CoA carboxylase OS=Saccharomyces cerevisiae (strain ATCC 204
log2(value)
raw_signal_sum
batchcl_raw_signal_sum
norm_batchcl_raw_signal_sum
ctrl.ratio
24
23
22
21
3
2
1
0
WT
MUT
Control
condition
condition
WT
MUT
Control
rep
rep1
rep2
rep3

ACF2 − Q12168

Endo−1,3(4)−beta−glucanase 2 OS=Saccharomyces cerevisiae (strain ATC

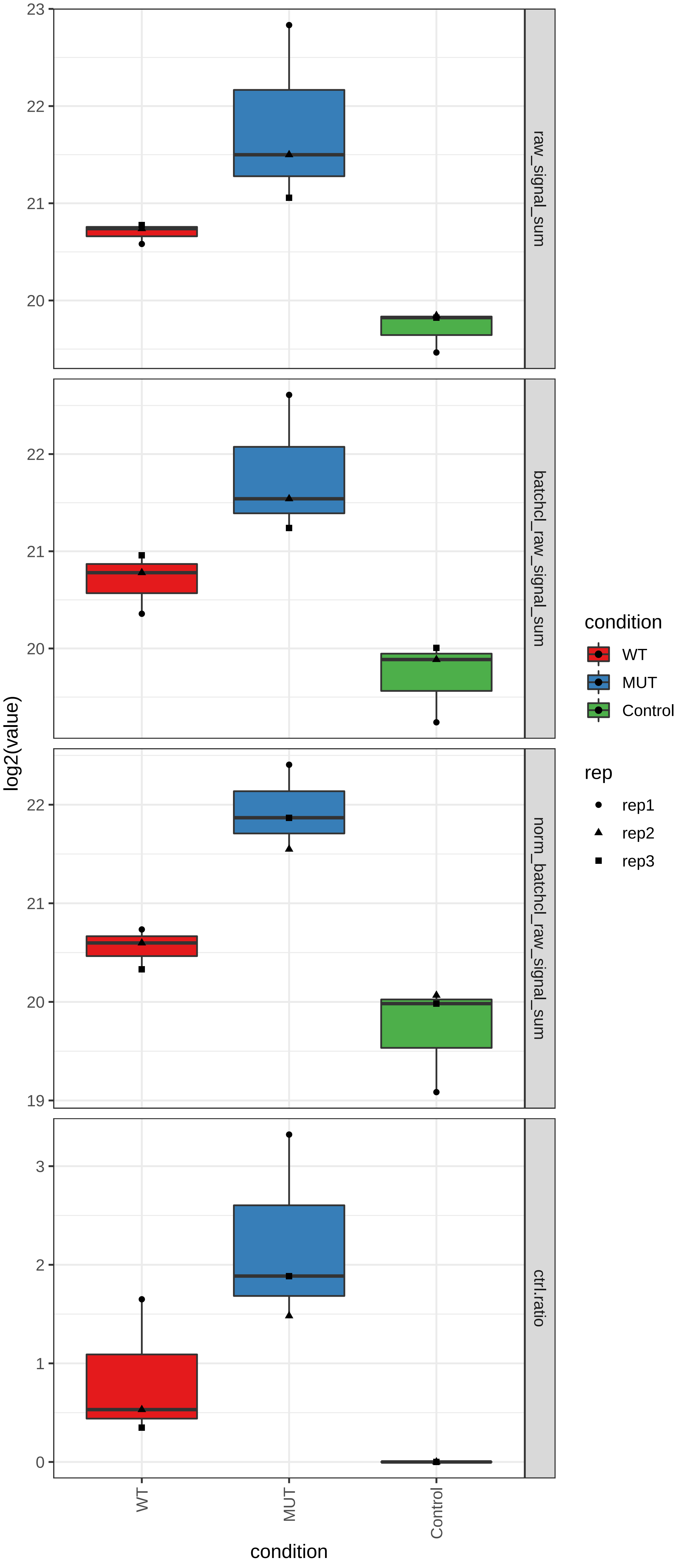

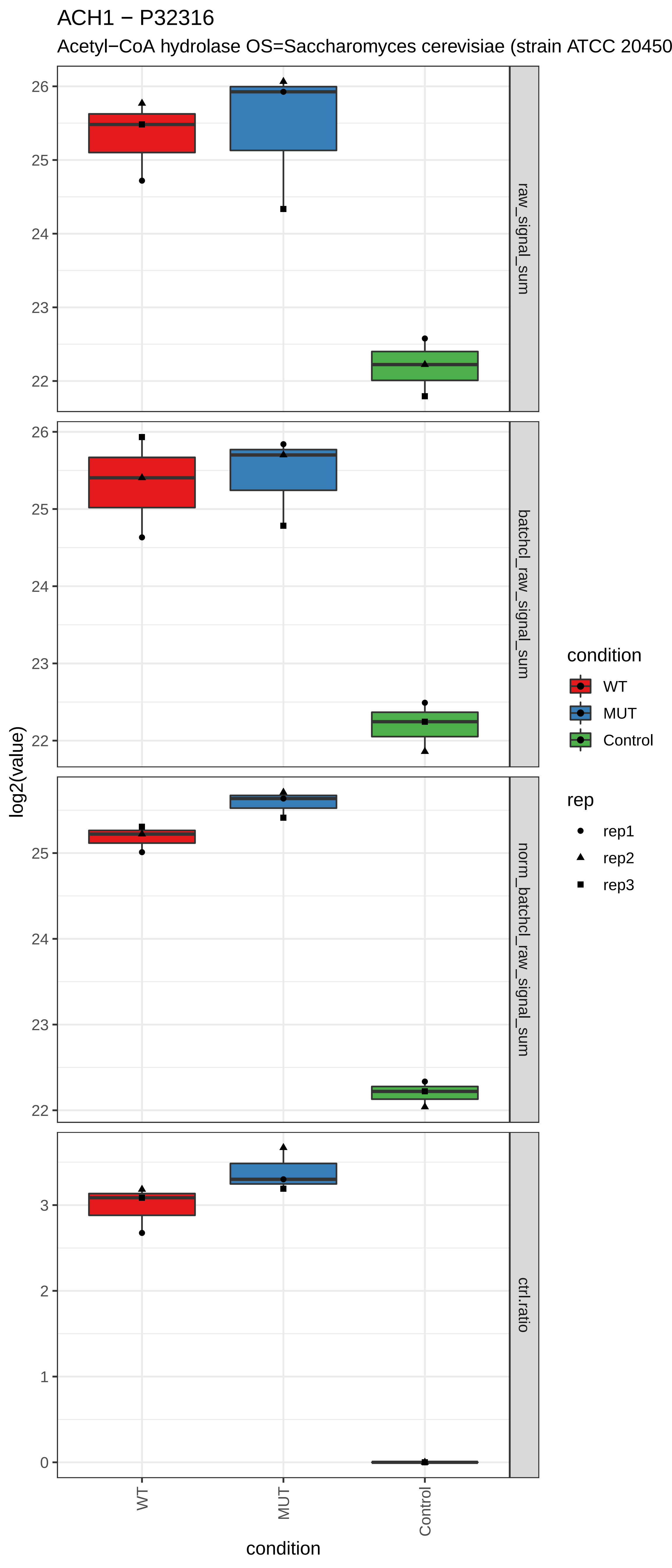

ACH1 − P32316
Acetyl−CoA hydrolase OS=Saccharomyces cerevisiae (strain ATCC 20450
raw_signal_sum
batchcl_raw_signal_sum
norm_batchcl_raw_signal_sum
ctrl.ratio
log2(value)
condition
WT
MUT
Control
rep
rep1
rep2
rep3

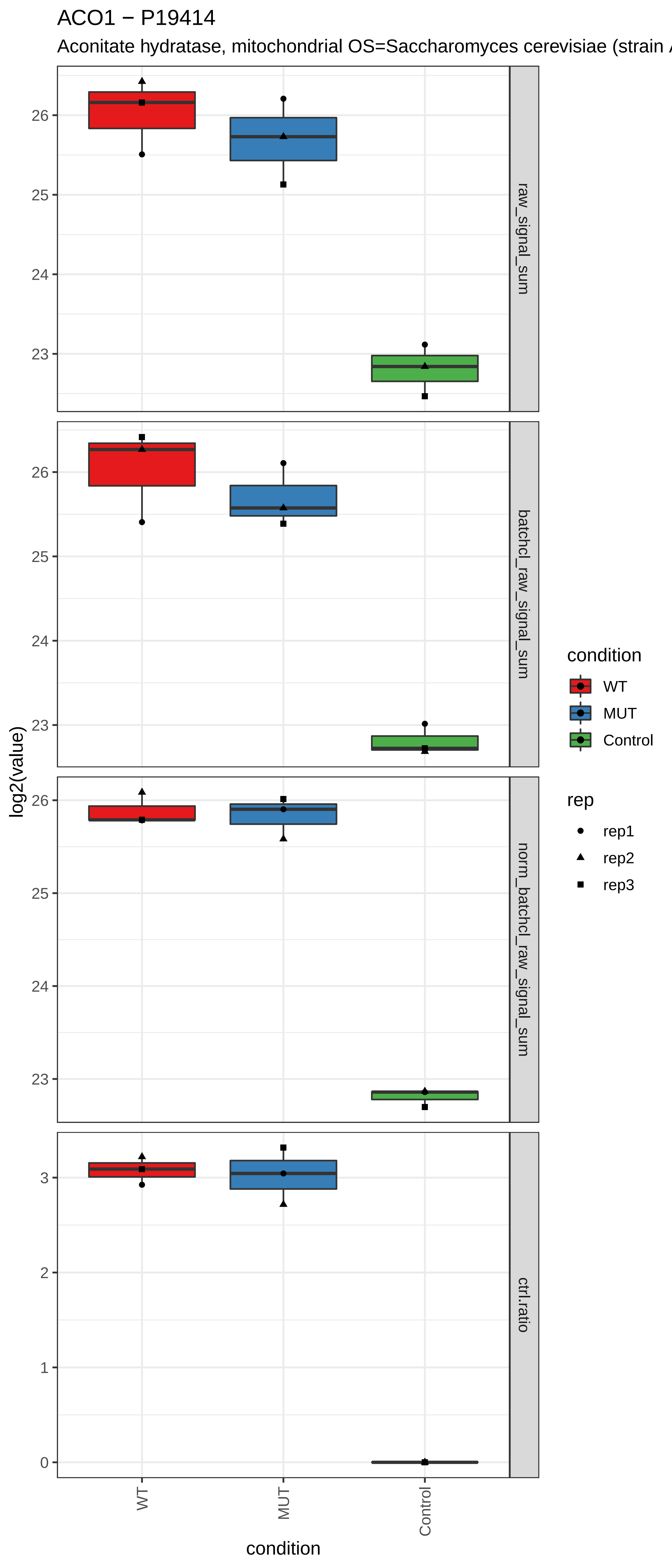

ACO1 − P19414
Aconitate hydratase, mitochondrial OS=Saccharomyces cerevisiae (strain
raw_signal_sum
batchcl_raw_signal_sum
norm_batchcl_raw_signal_sum
ctrl.ratio
log2(value)
26
25
24
23
3
2
1
0
WT
MUT
Control
condition
condition
WT
MUT
Control
rep
rep1
rep2
rep3

ACS1 − Q01574

Acetyl−coenzyme A synthetase 1 OS=Saccharomyces cerevisiae (strain

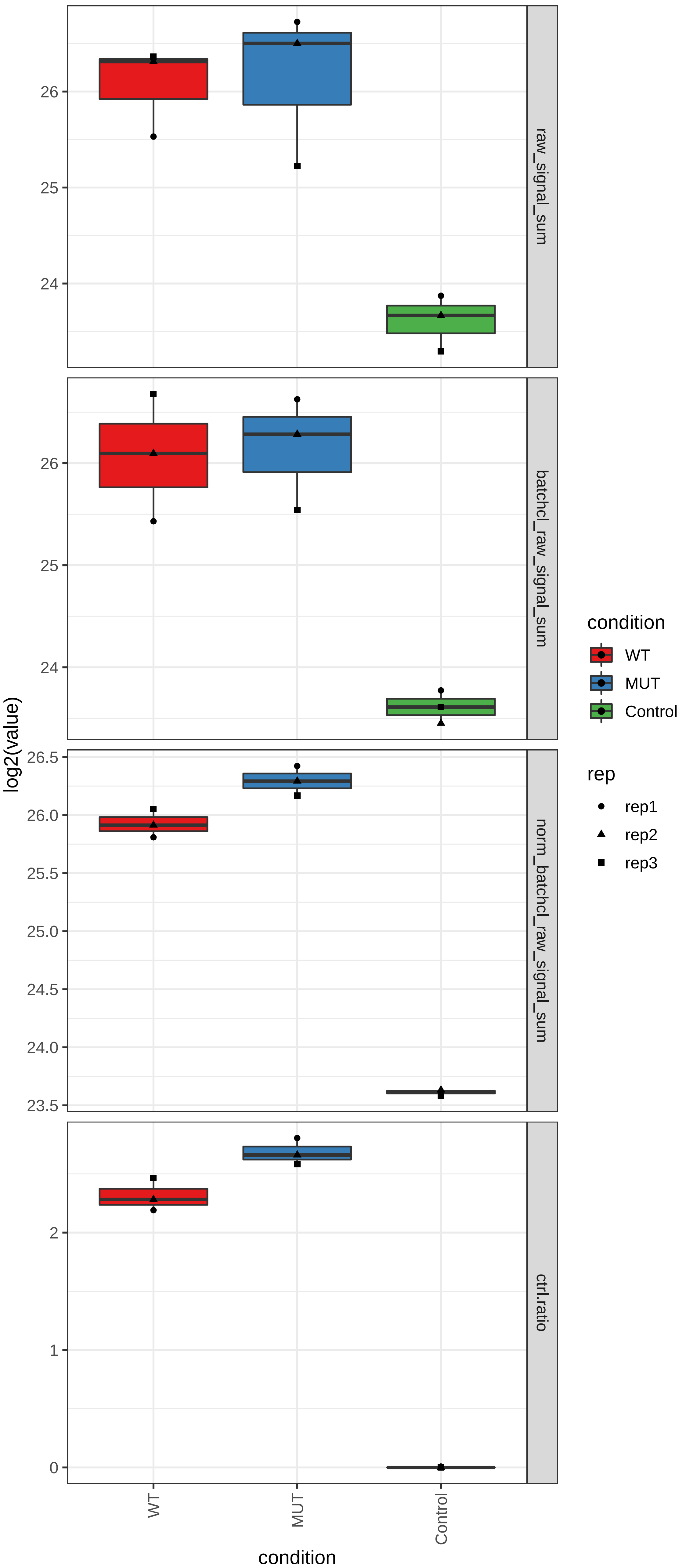

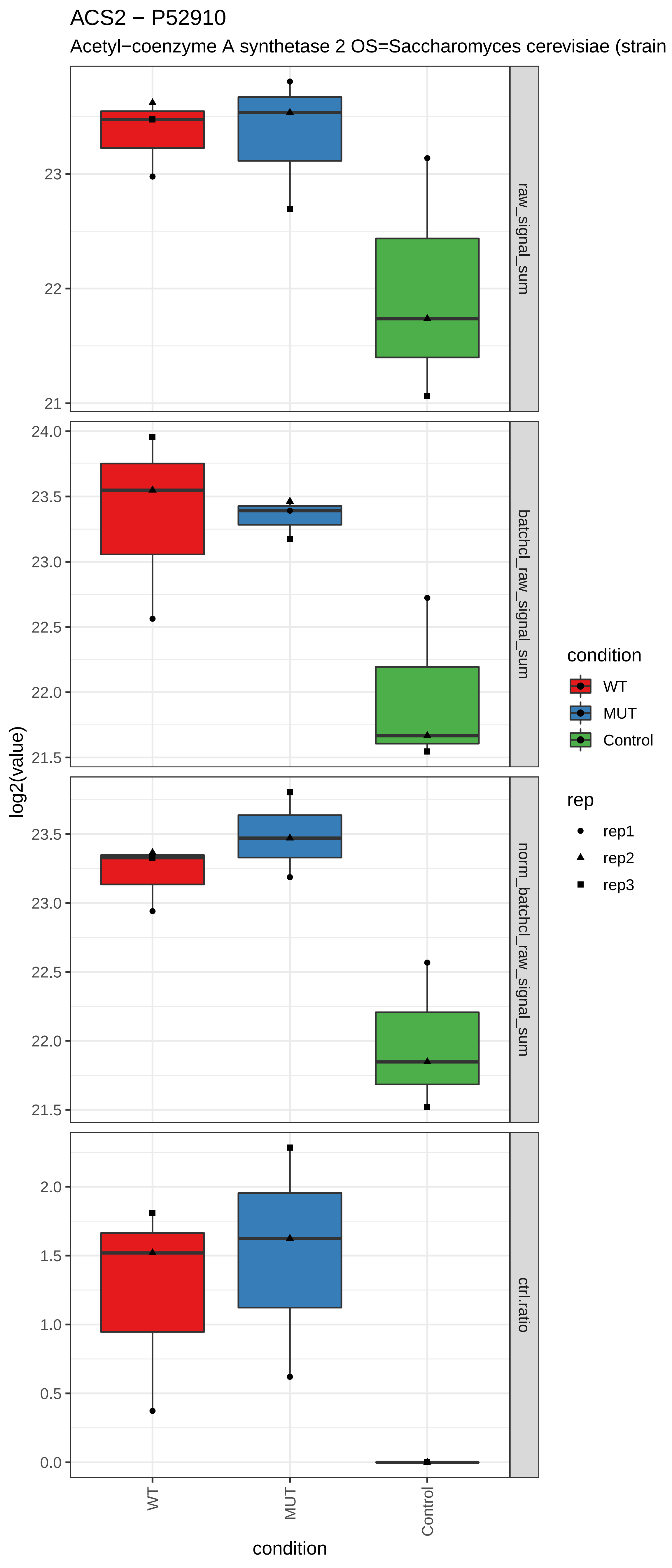
ACS2 − P52910
Acetyl−coenzyme A synthetase 2 OS=Saccharomyces cerevisiae (strain
raw_signal_sum
batchcl_raw_signal_sum
norm_batchcl_raw_signal_sum
ctrl.ratio
log2(value)
condition
WT
MUT
Control
rep
rep1
rep2
rep3
23
22
21
24.0
23.5
23.0
22.5
22.0
21.5
2.0
1.5
1.0
0.5
0.0

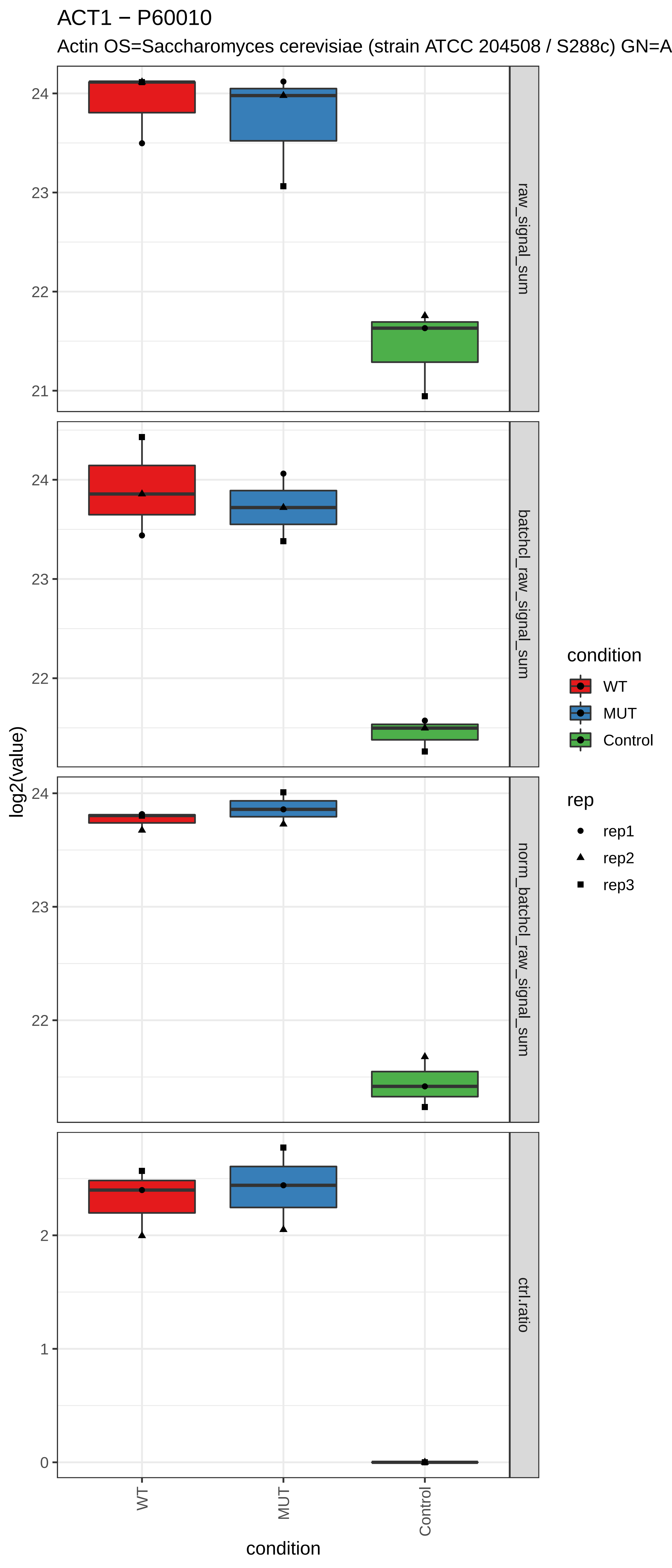
ACT1 − P60010
Actin OS=Saccharomyces cerevisiae (strain ATCC 204508 / S288c) GN=A
raw_signal_sum
batchcl_raw_signal_sum
norm_batchcl_raw_signal_sum
ctrl.ratio
log2(value)
condition
WT
MUT
Control
rep
rep1
rep2
rep3

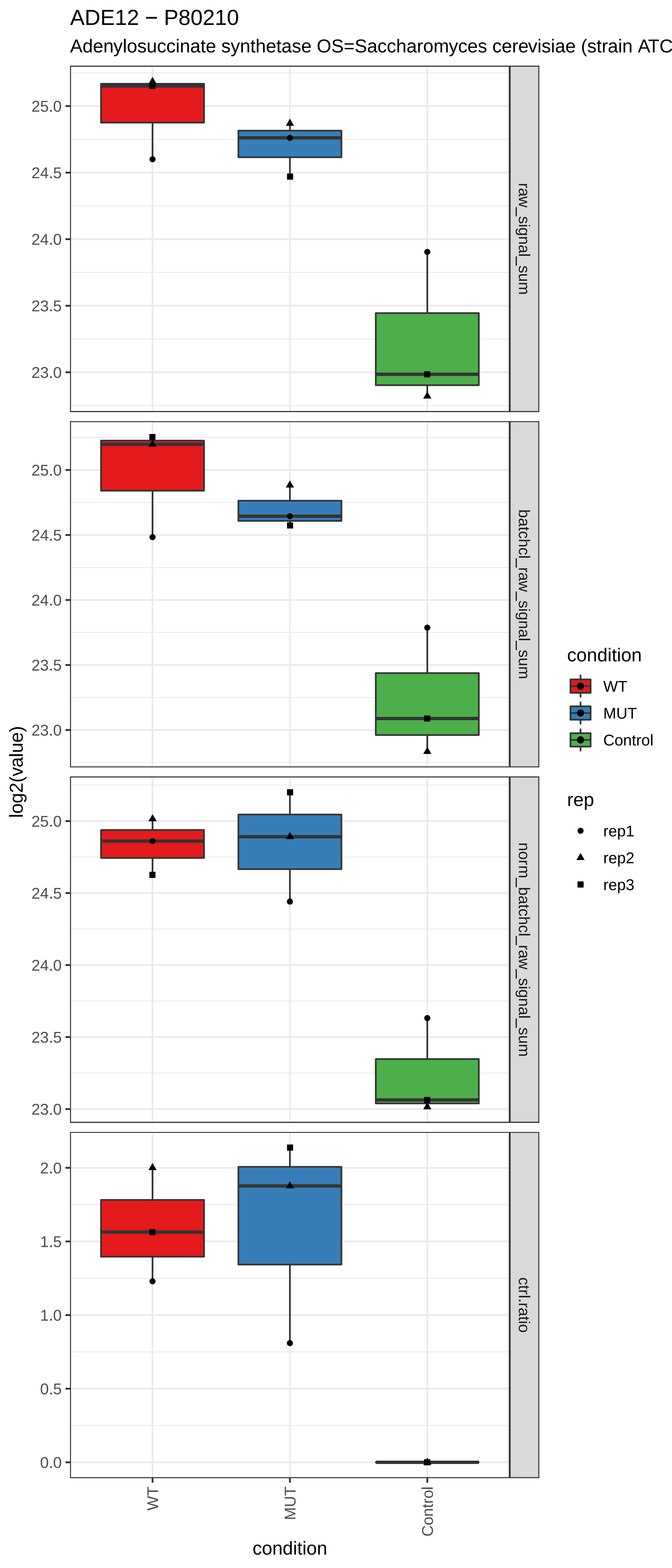

ADE12 − P80210
Adenylosuccinate synthetase OS=Saccharomyces cerevisiae (strain ATC
raw_signal_sum
batchcl_raw_signal_sum
norm_batchcl_raw_signal_sum
ctrl.ratio
log2(value)
condition
WT
MUT
Control
rep
rep1
rep2
rep3
25.0
24.5
24.0
23.5
23.0
2.0
1.5
1.0
0.5
0.0

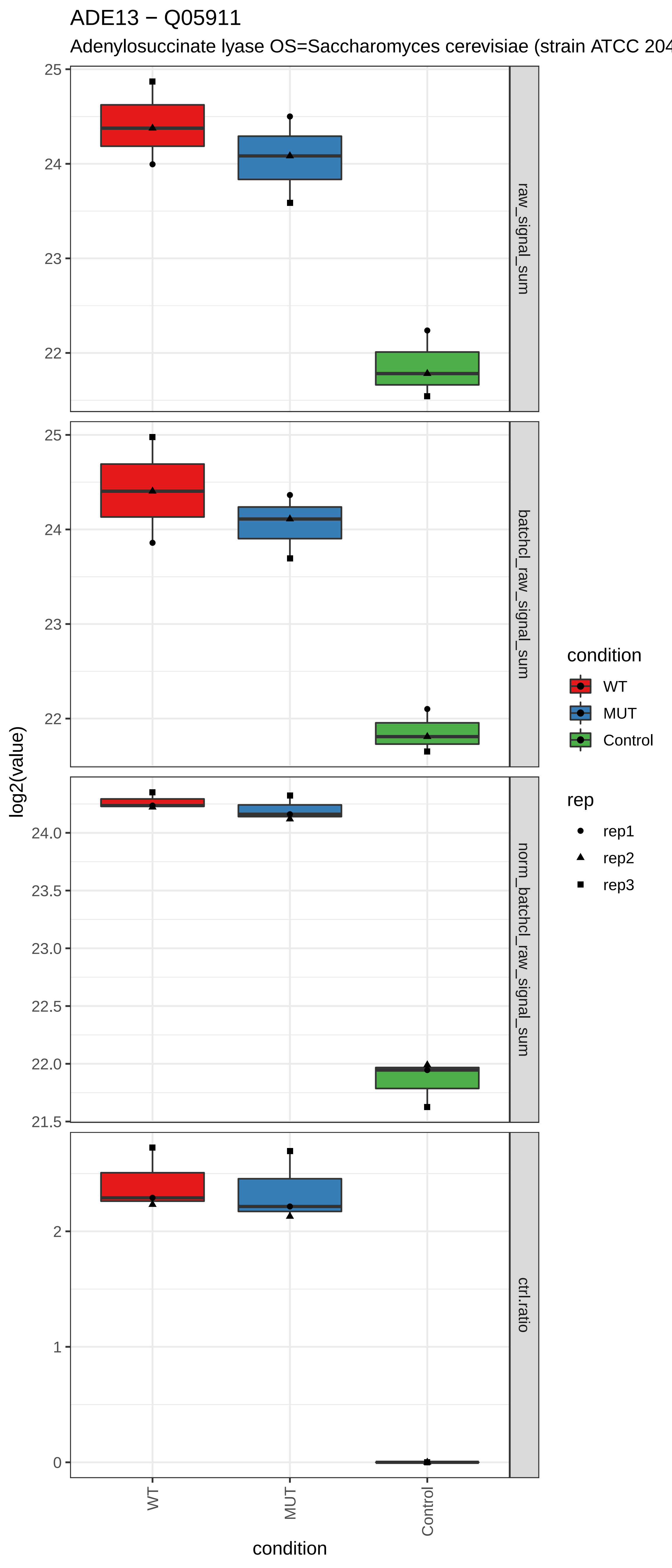
ADE13 − Q05911
Adenylosuccinate lyase OS=Saccharomyces cerevisiae (strain ATCC 204
raw_signal_sum
batchcl_raw_signal_sum
norm_batchcl_raw_signal_sum
ctrl.ratio
log2(value)
condition
WT
MUT
Control
rep
rep1
rep2
rep3
25
24
23
22
24.0
23.5
23.0
22.5
22.0
21.5
2
1
0

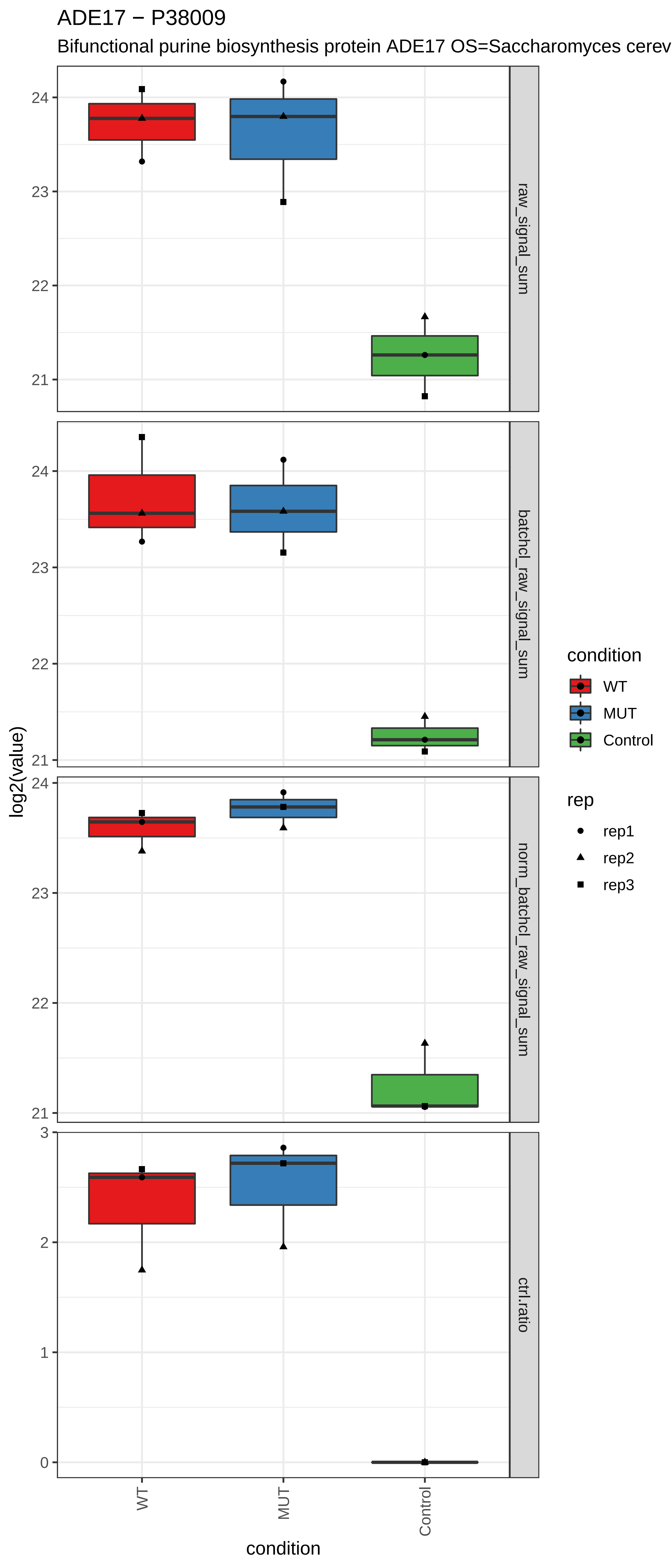

ADE17 − P38009
Bifunctional purine biosynthesis protein ADE17 OS=Saccharomyces cerev
raw_signal_sum
batchcl_raw_signal_sum
norm_batchcl_raw_signal_sum
ctrl.ratio
log2(value)
condition
WT
MUT
Control
rep
rep1
rep2
rep3

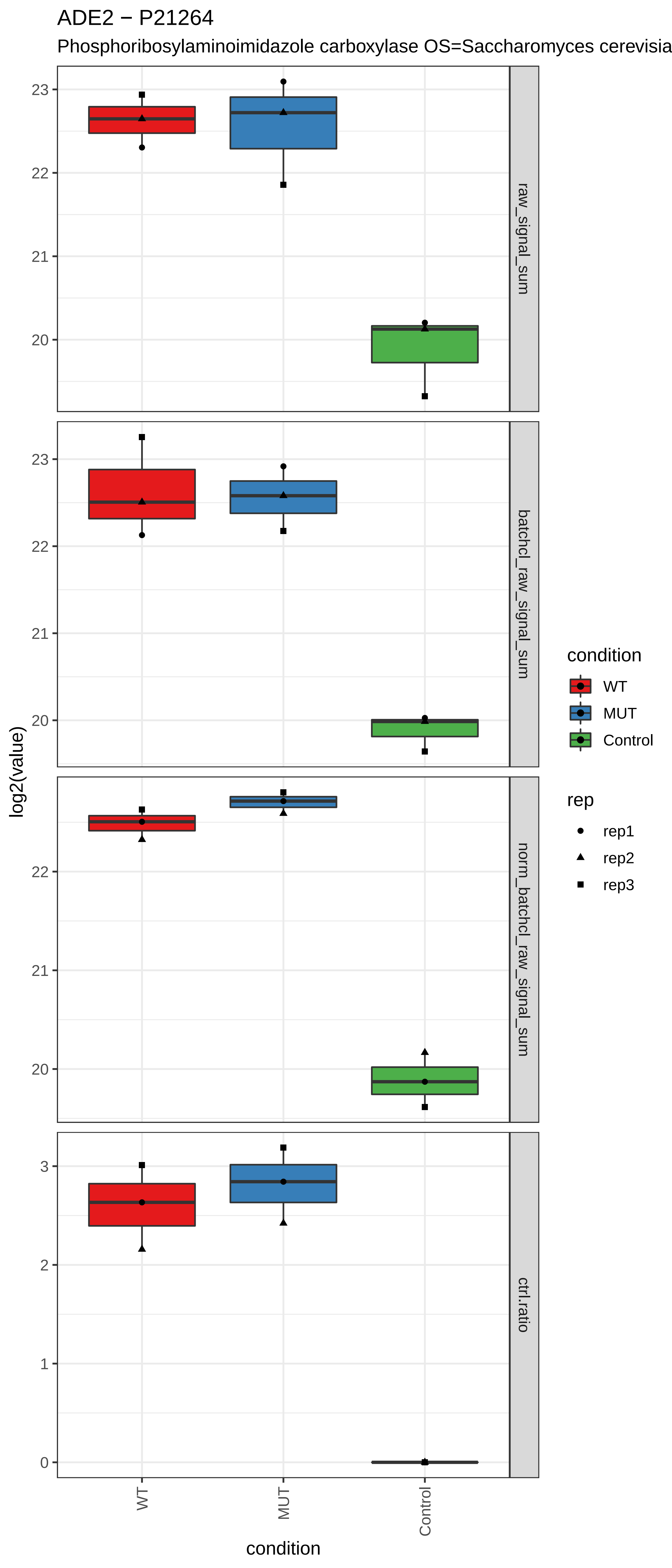
ADE2 − P21264
Phosphoribosylaminoimidazole carboxylase OS=Saccharomyces cerevisia
raw_signal_sum
batchcl_raw_signal_sum
norm_batchcl_raw_signal_sum
ctrl.ratio
log2(value)
condition
WT
MUT
Control
rep
rep1
rep2
rep3
23
22
21
20
3
2
1
0

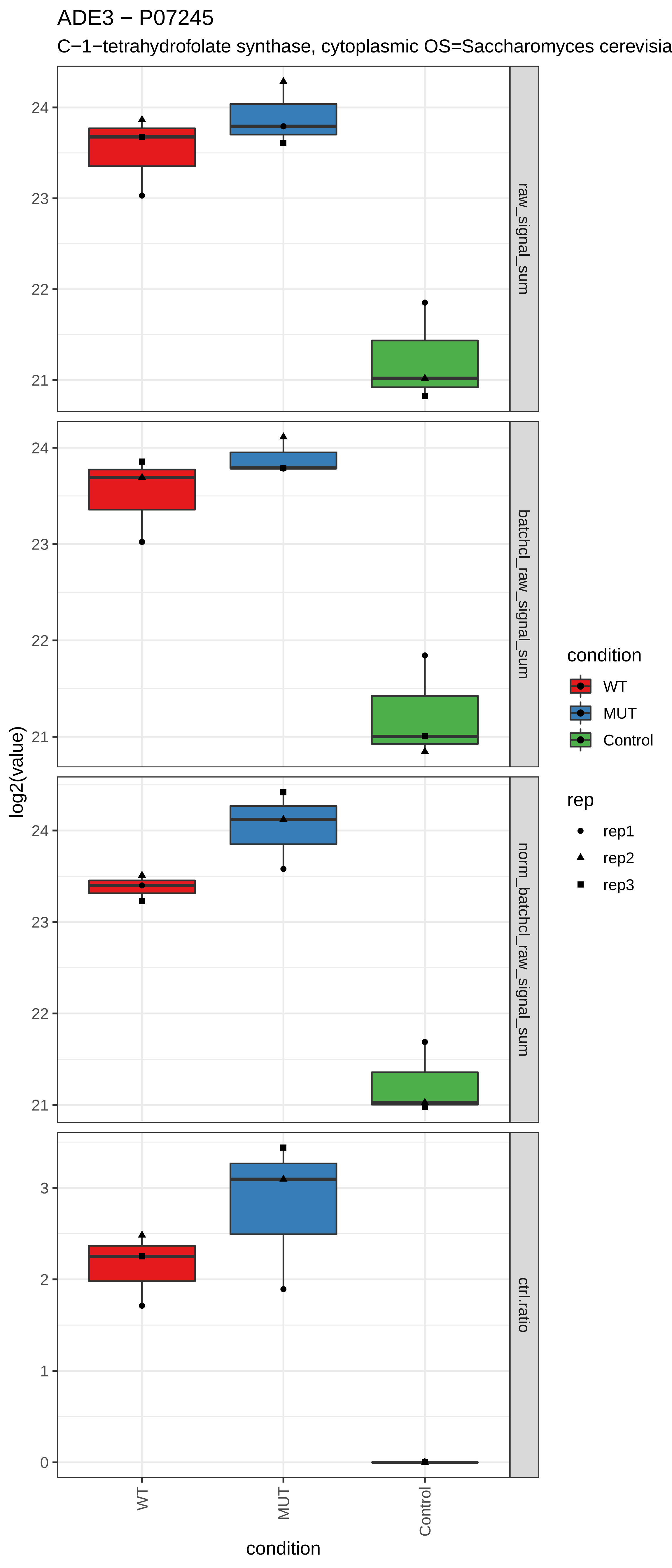
ADE3 − P07245
C−1−tetrahydrofolate synthase, cytoplasmic OS=Saccharomyces cerevisia
raw_signal_sum
batchcl_raw_signal_sum
norm_batchcl_raw_signal_sum
ctrl.ratio
log2(value)
condition
WT
MUT
Control
rep
rep1
rep2
rep3
24
23
22
21
3
2
1
0

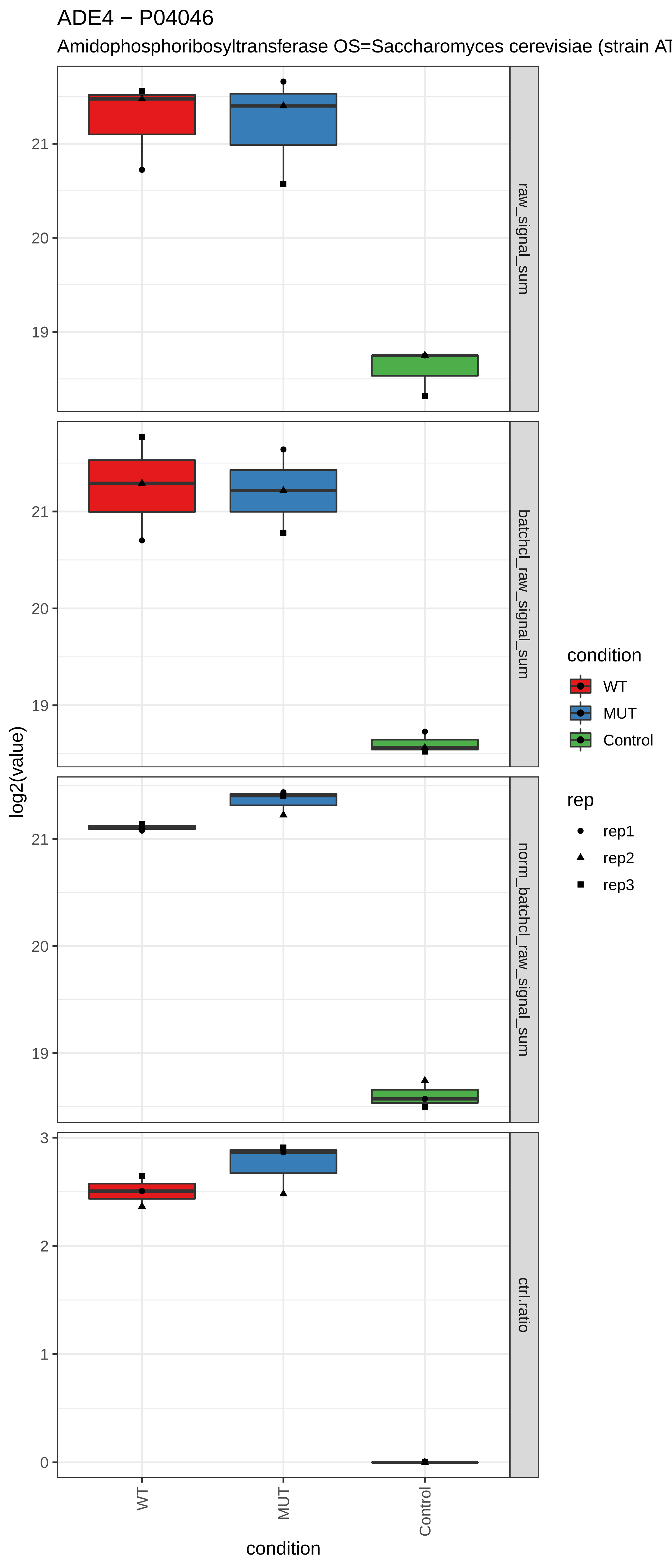
ADE4 − P04046
Amidophosphoribosyltransferase OS=Saccharomyces cerevisiae (strain AT
raw_signal_sum
batchcl_raw_signal_sum
norm_batchcl_raw_signal_sum
ctrl.ratio
log2(value)
condition
WT
MUT
Control
rep
rep1
rep2
rep3

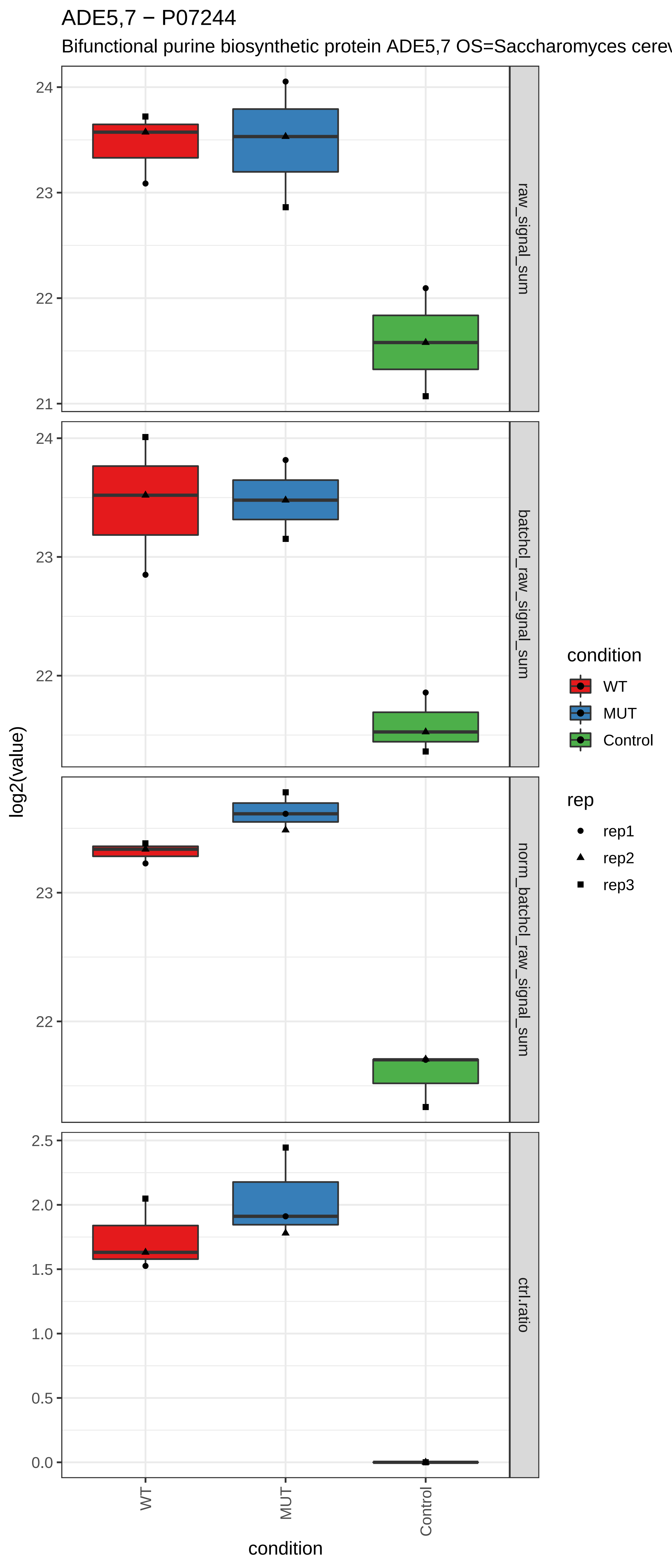
ADE5,7 − P07244
Bifunctional purine biosynthetic protein ADE5,7 OS=Saccharomyces cerev
raw_signal_sum
batchcl_raw_signal_sum
norm_batchcl_raw_signal_sum
ctrl.ratio
log2(value)
24
23
22
21
2.5
2.0
1.5
1.0
0.5
0.0
WT
MUT
Control
condition
condition
WT
MUT
Control
rep
rep1
rep2
rep3

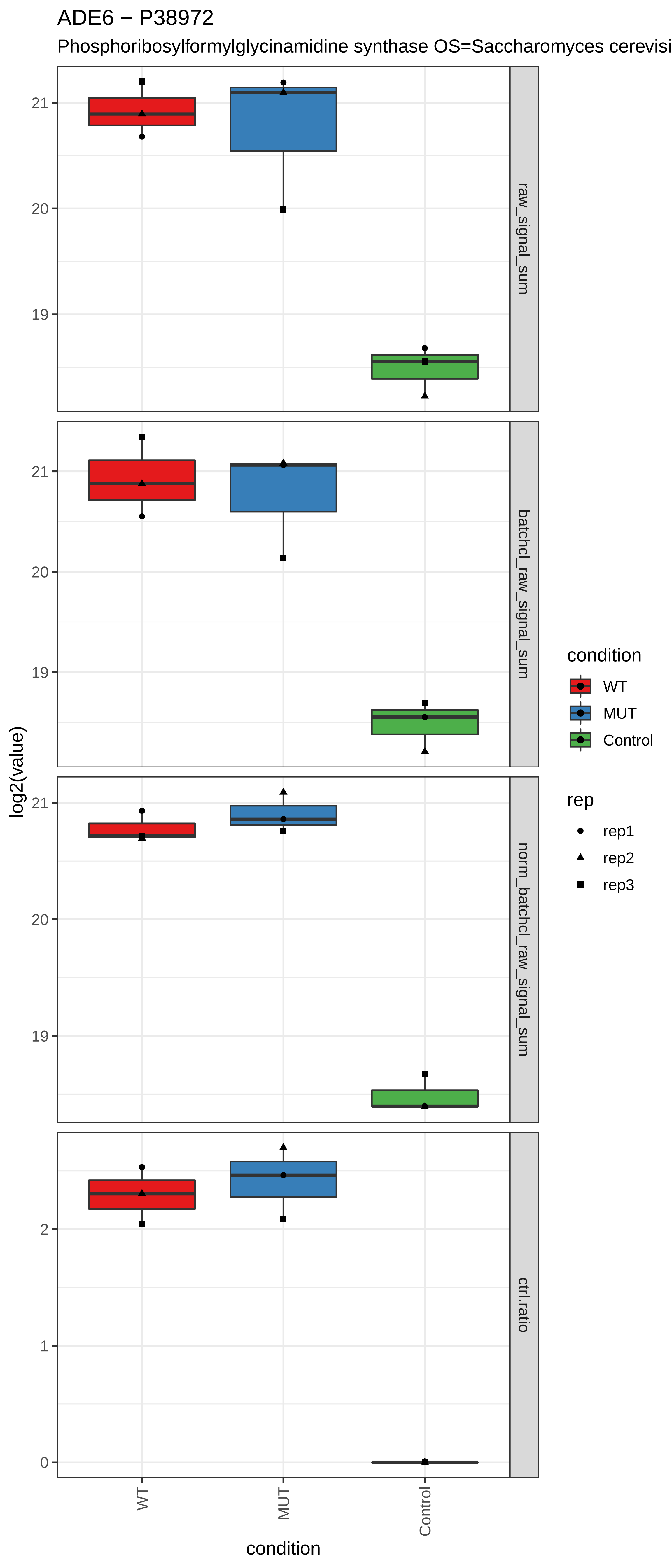
ADE6 − P38972
Phosphoribosylformylglycinamidine synthase OS=Saccharomyces cerevisi
raw_signal_sum
batchcl_raw_signal_sum
norm_batchcl_raw_signal_sum
ctrl.ratio
log2(value)
condition
WT
MUT
Control
rep
rep1
rep2
rep3
21
20
19
2
1
0

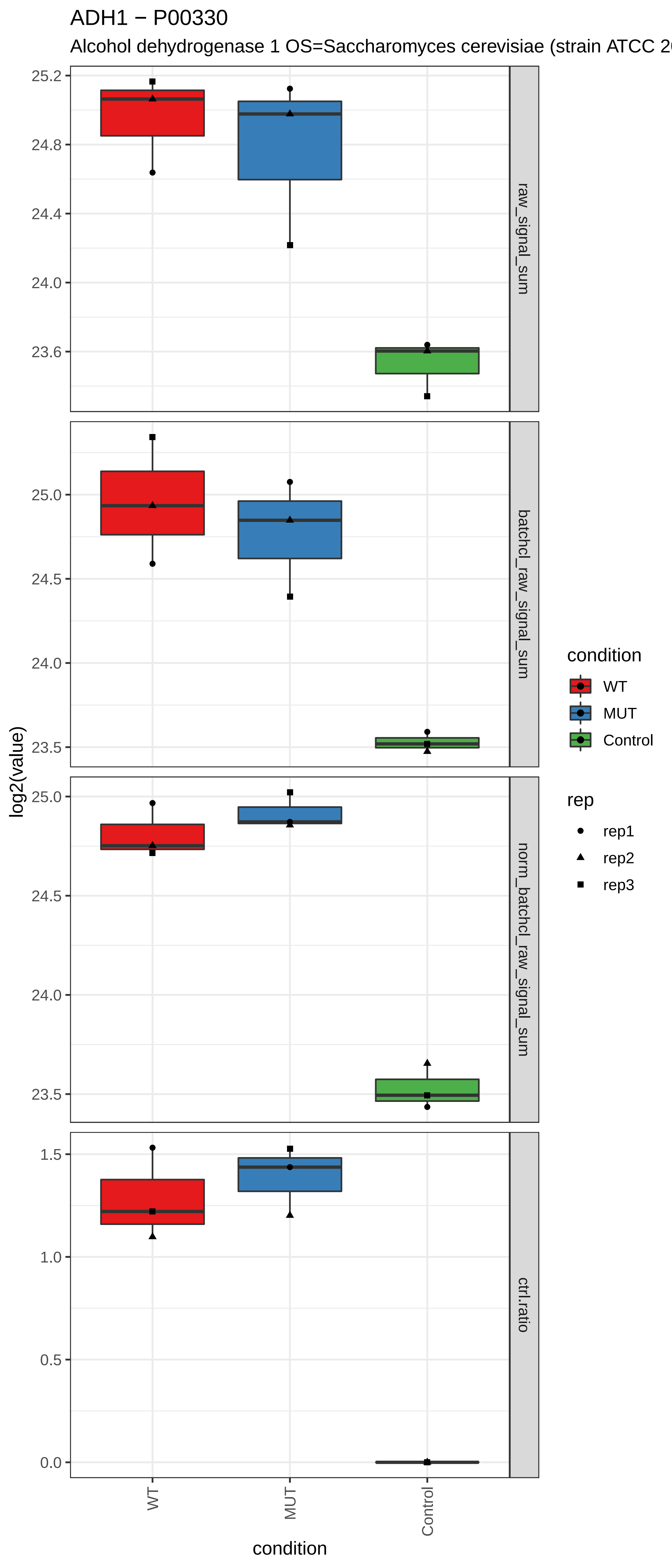

ADH1 − P00330
Alcohol dehydrogenase 1 OS=Saccharomyces cerevisiae (strain ATCC 2
log2(value)
raw_signal_sum
batchcl_raw_signal_sum
norm_batchcl_raw_signal_sum
ctrl.ratio
25.2
24.8
24.4
24.0
23.6
25.0
24.5
24.0
23.5
25.0
24.5
24.0
23.5
1.5
1.0
0.5
0.0
WT
MUT
Control
condition
condition
WT
MUT
Control
rep
rep1
rep2
rep3

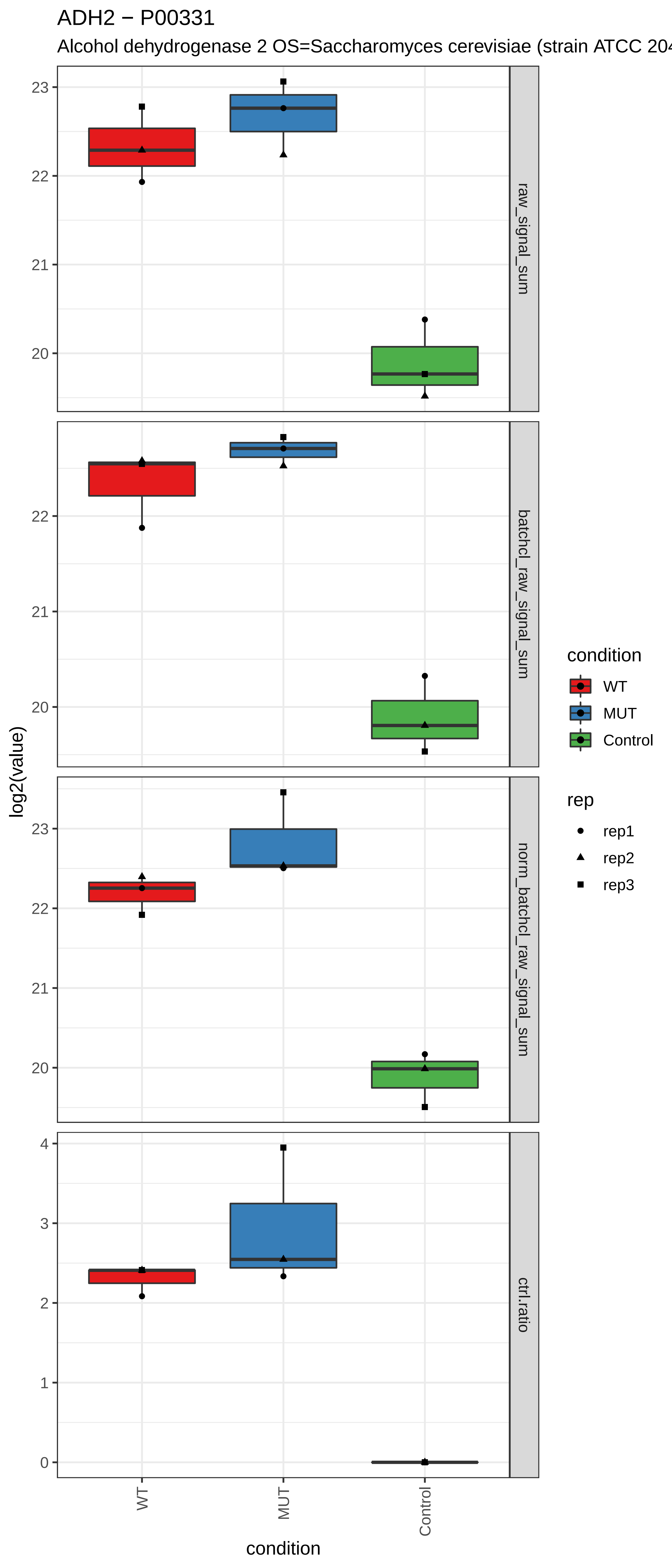
ADH2 − P00331
Alcohol dehydrogenase 2 OS=Saccharomyces cerevisiae (strain ATCC 204
raw_signal_sum
batchcl_raw_signal_sum
norm_batchcl_raw_signal_sum
ctrl.ratio
log2(value)
condition
WT
MUT
Control
rep
rep1
rep2
rep3
23
22
21
20
4
3
2
1
0

ADH3 − P07246

Alcohol dehydrogenase 3, mitochondrial OS=Saccharomyces cerevisiae (s

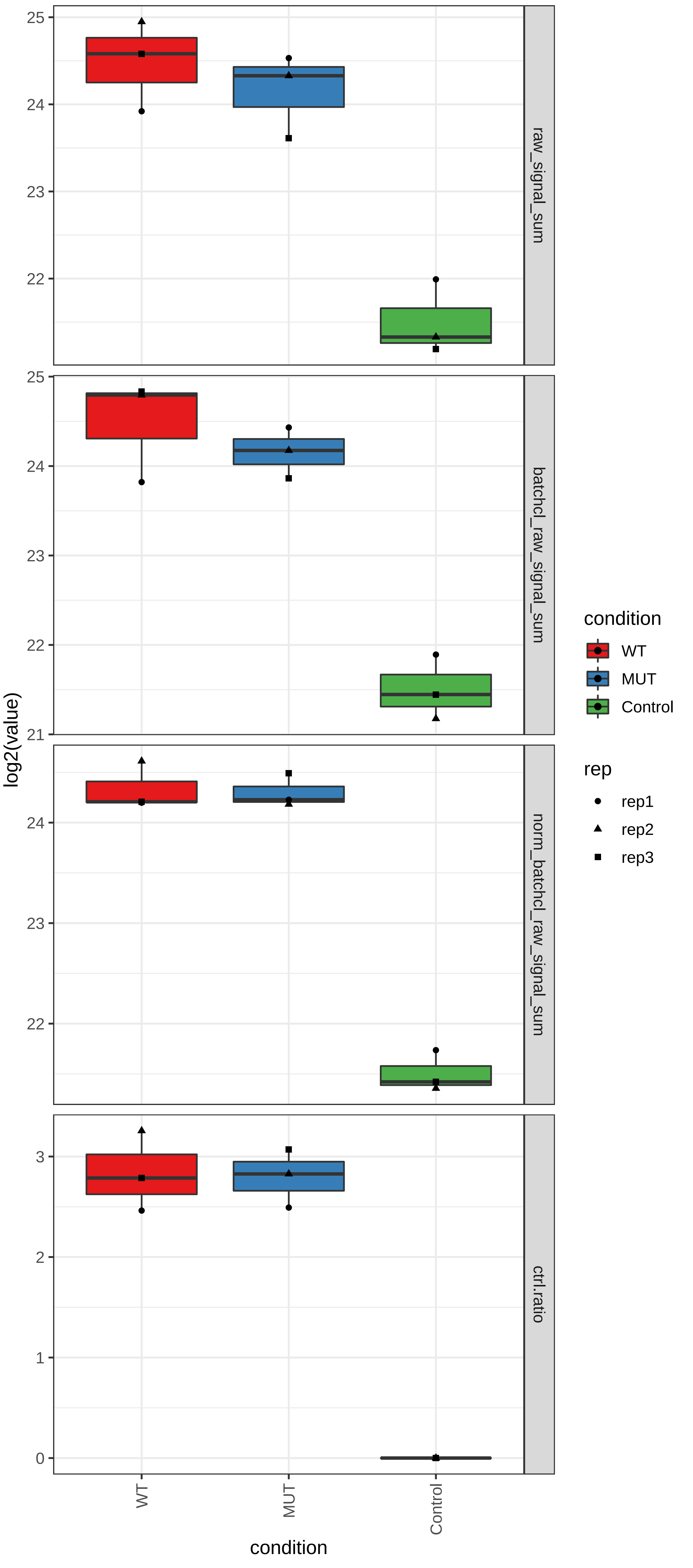

ADO1 − P47143

Adenosine kinase OS=Saccharomyces cerevisiae (strain ATCC 204508 / S

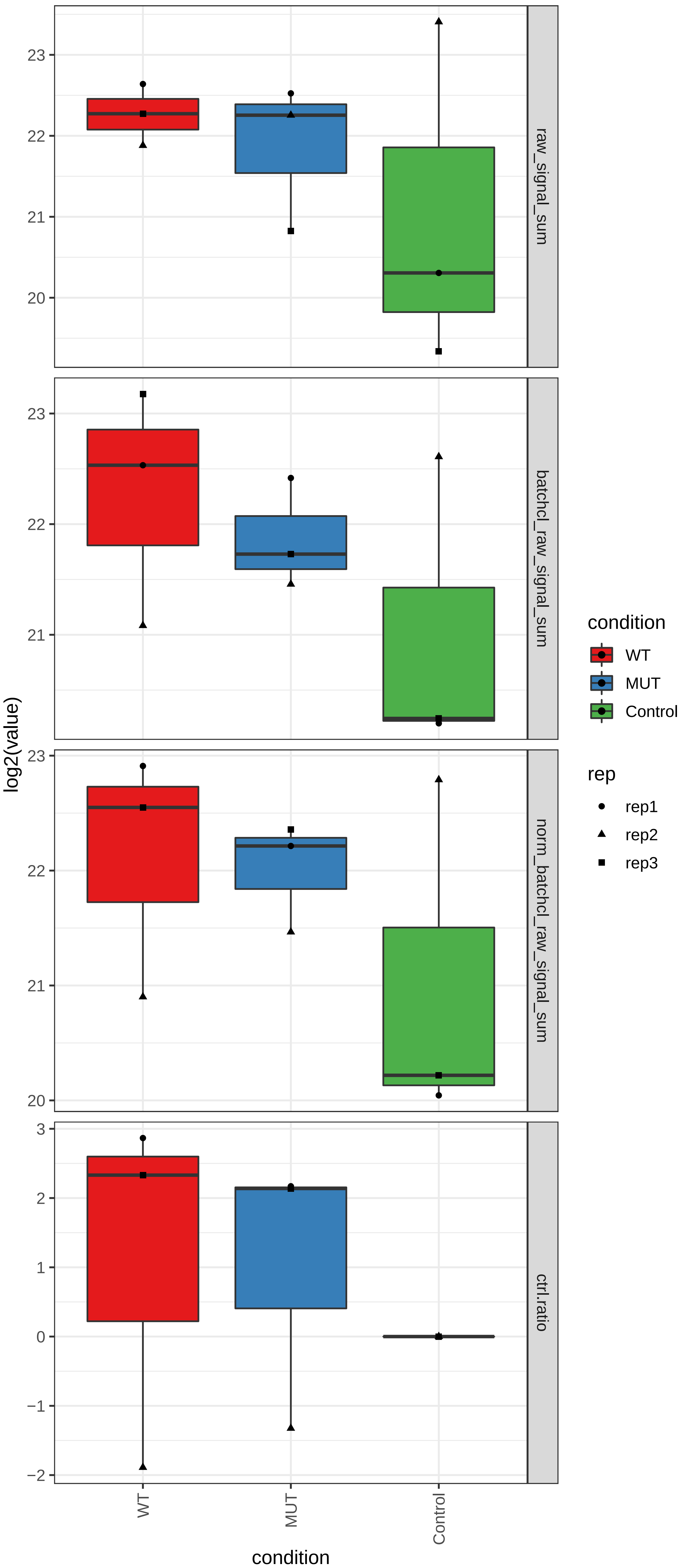

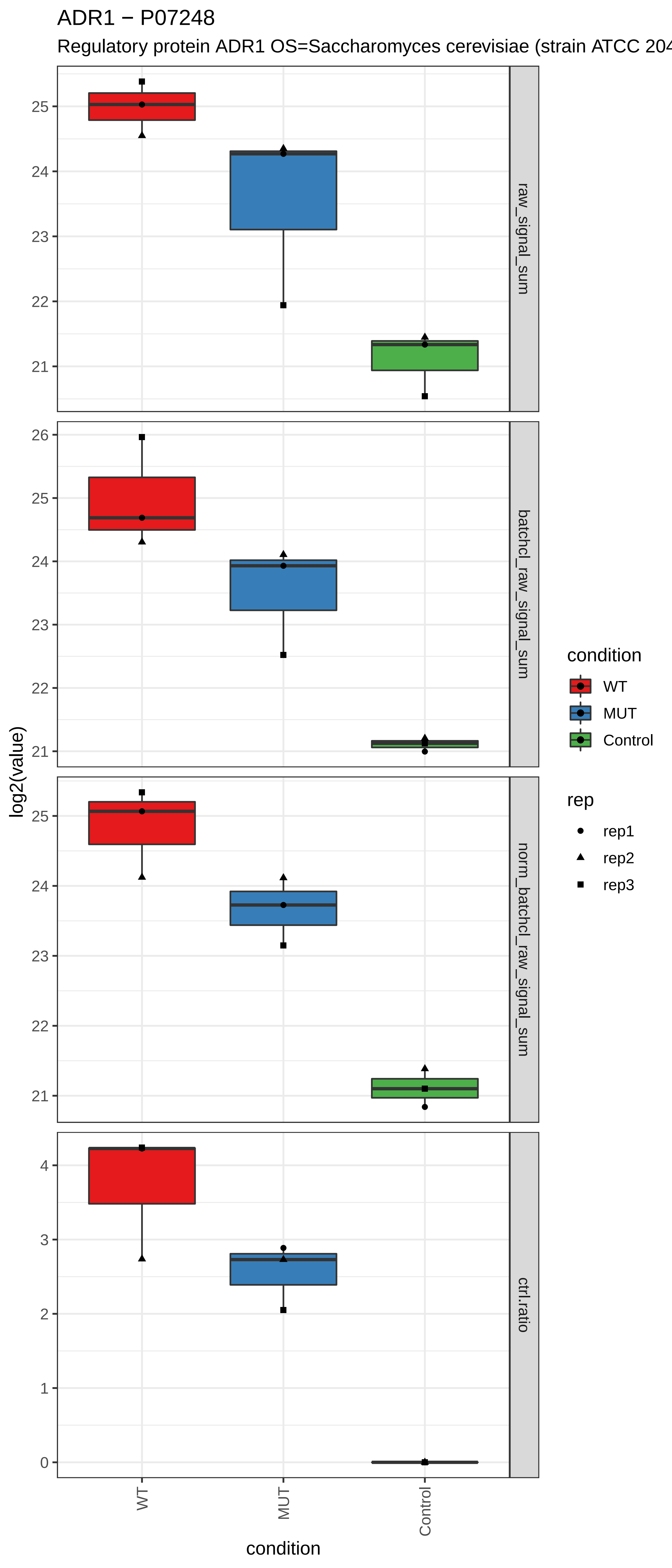
ADR1 − P07248
Regulatory protein ADR1 OS=Saccharomyces cerevisiae (strain ATCC 204
raw_signal_sum
batchcl_raw_signal_sum
norm_batchcl_raw_signal_sum
ctrl.ratio
log2(value)
condition
WT
MUT
Control
rep
rep1
rep2
rep3

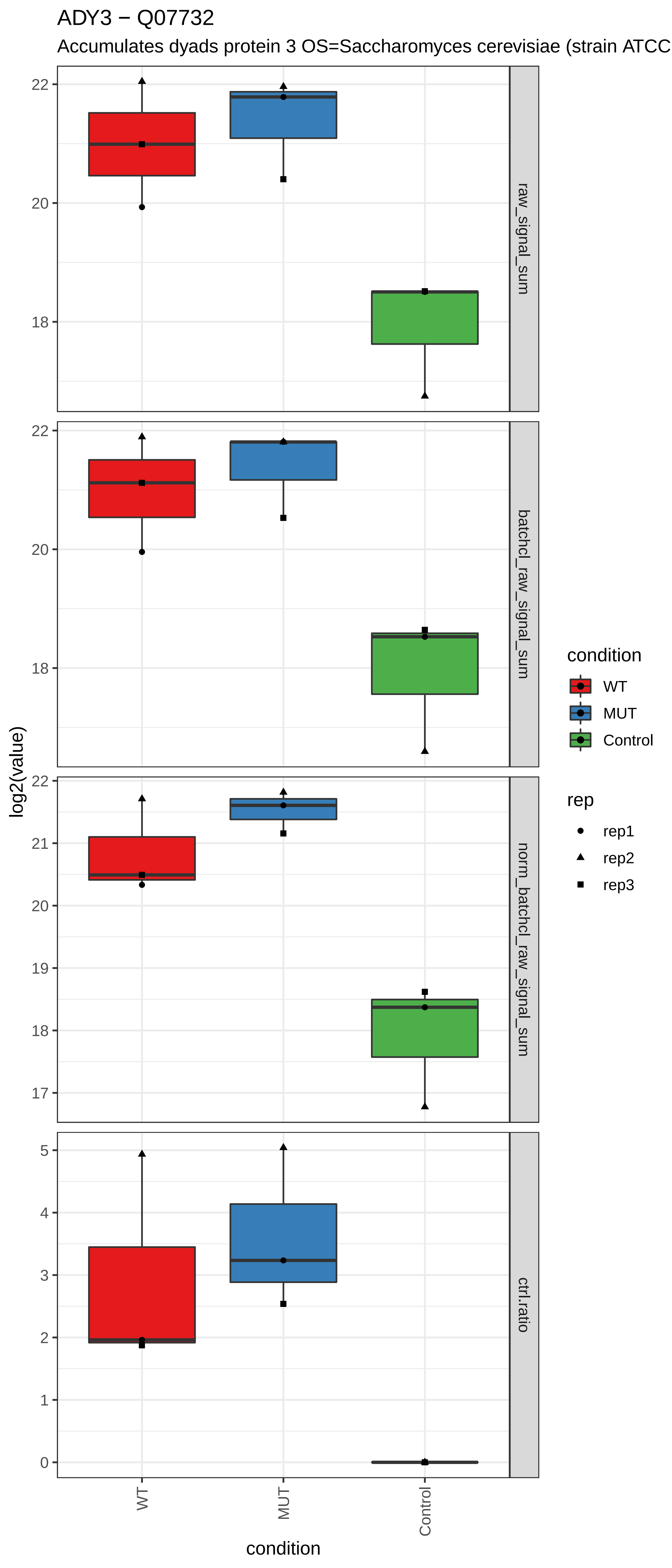

ADY3 − Q07732
Accumulates dyads protein 3 OS=Saccharomyces cerevisiae (strain ATCC
raw_signal_sum
batchcl_raw_signal_sum
norm_batchcl_raw_signal_sum
ctrl.ratio
log2(value)
condition
WT
MUT
Control
rep
rep1
rep2
rep3
22
20
18
21
19
17
5
4
3
2
1
0

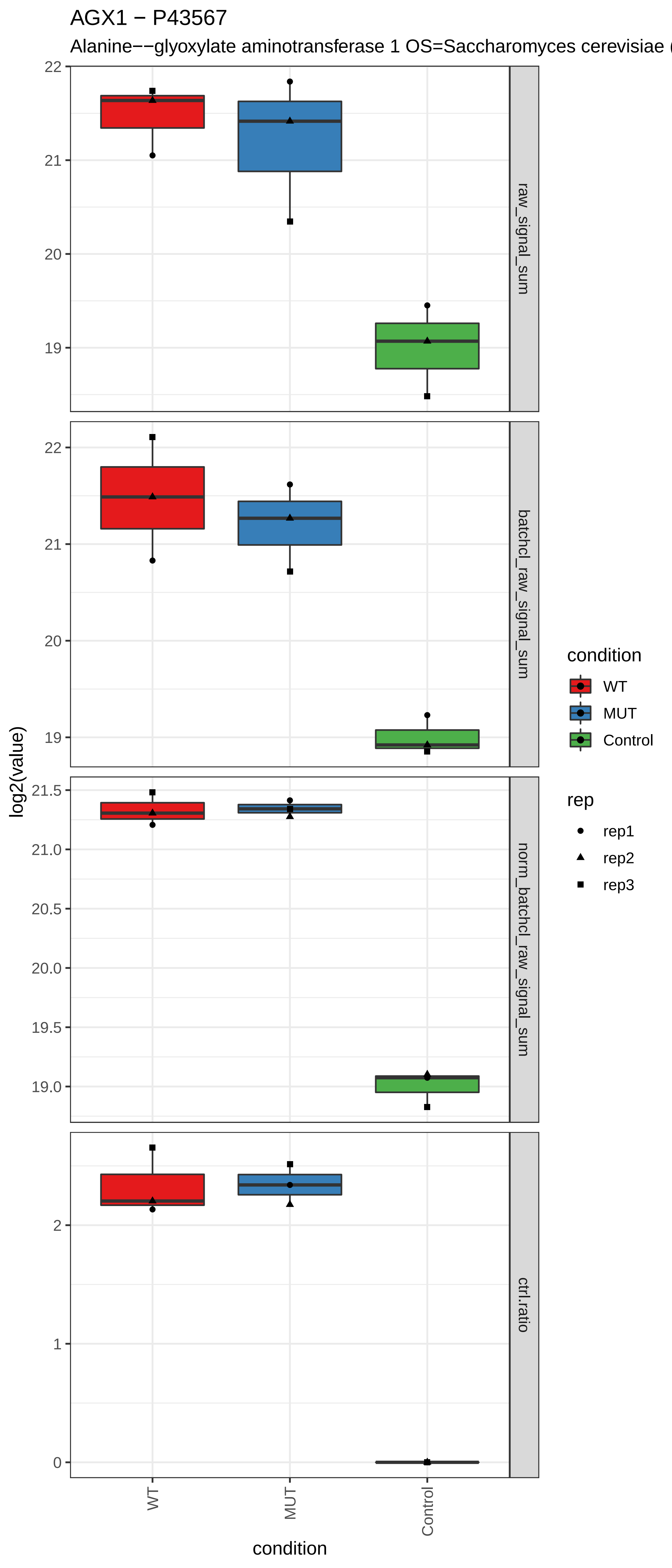

AGX1 − P43567
Alanine−−glyoxylate aminotransferase 1 OS=Saccharomyces cerevisiae (
raw_signal_sum
batchcl_raw_signal_sum
norm_batchcl_raw_signal_sum
ctrl.ratio
log2(value)
condition
WT
MUT
Control
rep
rep1
rep2
rep3

AIM45 − Q12480

Probable electron transfer flavoprotein subunit alpha, mitochondrial OS=S

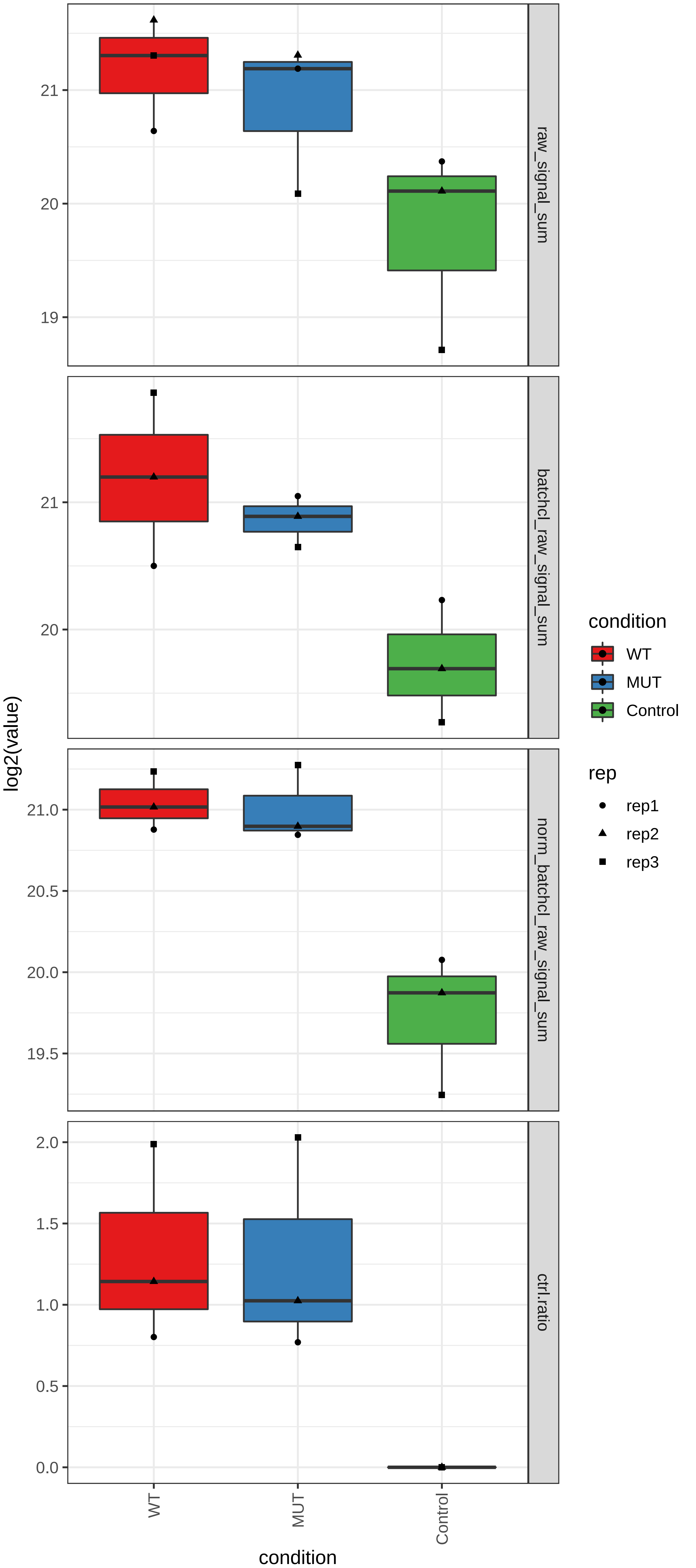

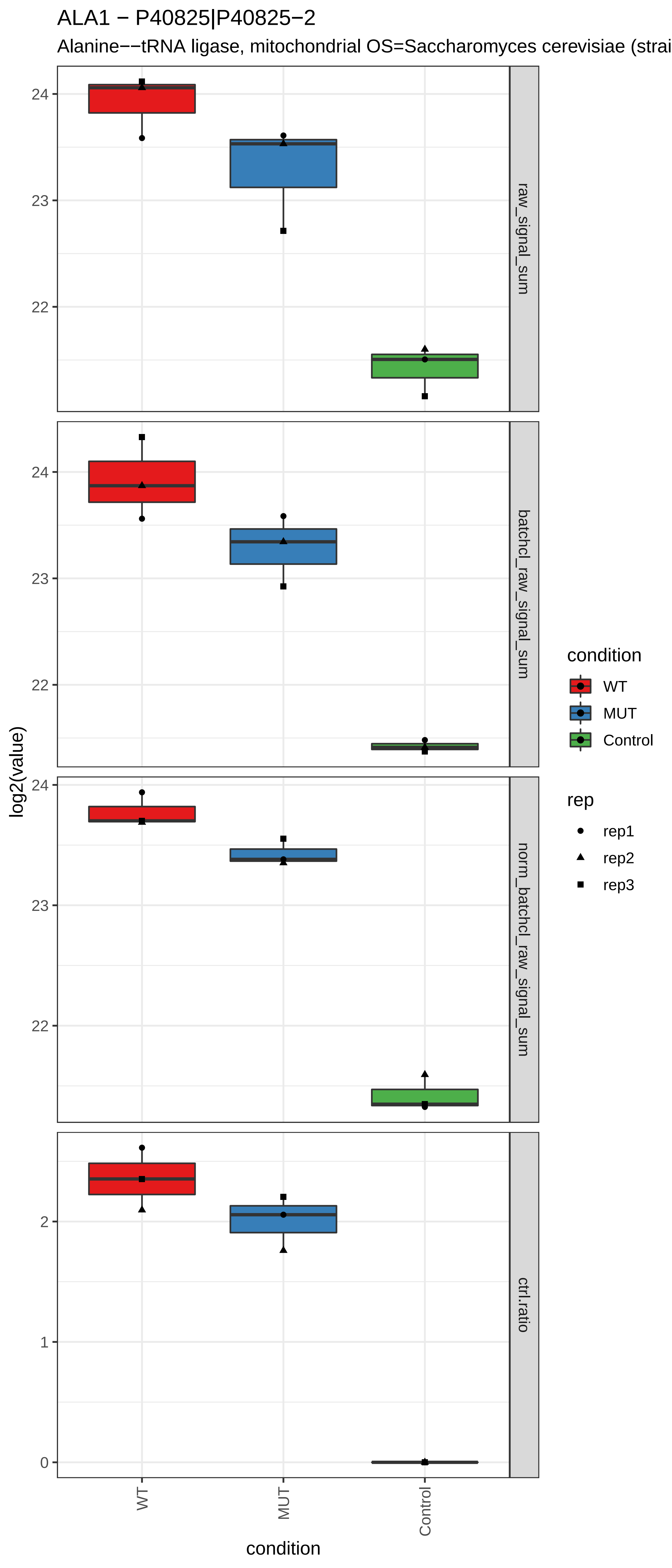
ALA1 − P40825|P40825−2
Alanine−−tRNA ligase, mitochondrial OS=Saccharomyces cerevisiae (strai
raw_signal_sum
batchcl_raw_signal_sum
norm_batchcl_raw_signal_sum
ctrl.ratio
log2(value)
condition
WT
MUT
Control
rep
rep1
rep2
rep3
24
23
22
2
1
0

ALD4 − P46367

Potassium−activated aldehyde dehydrogenase, mitochondrial OS=Sacchar

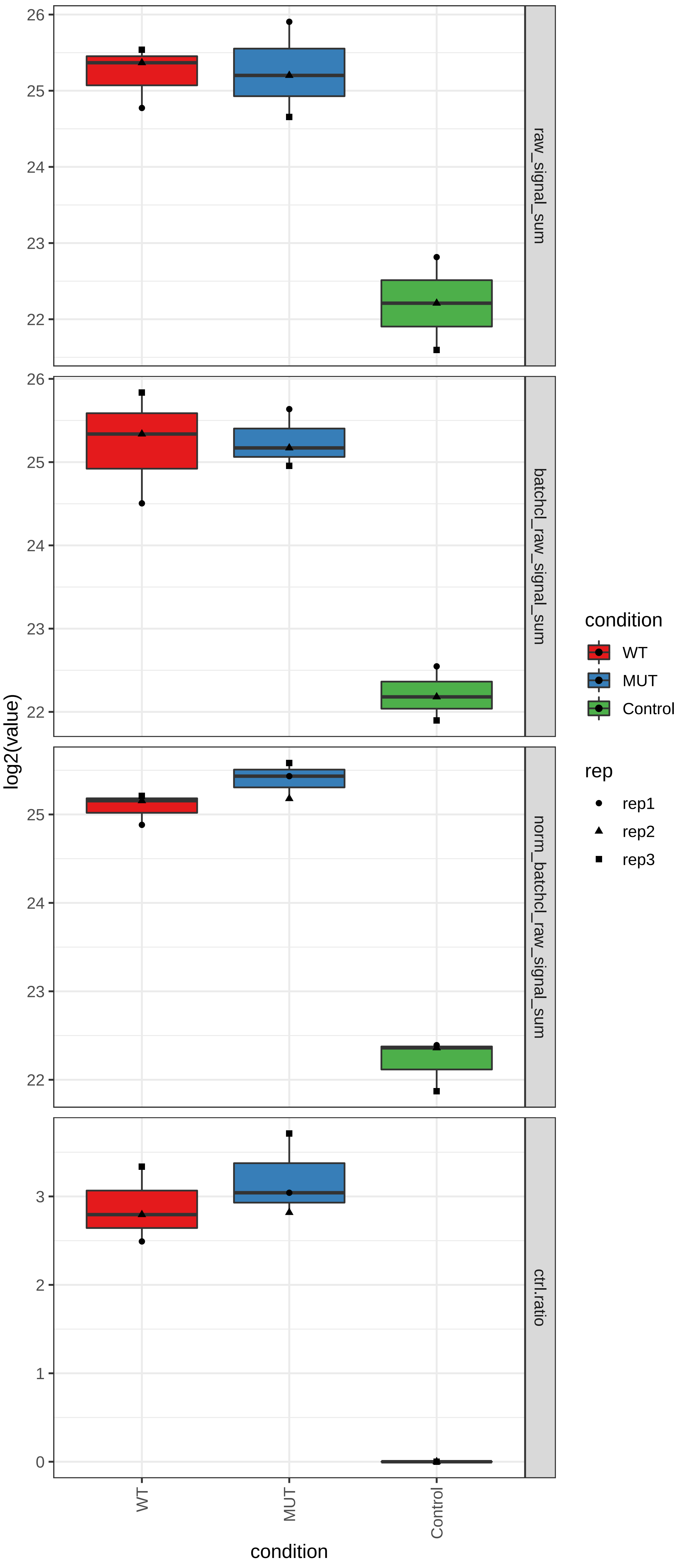

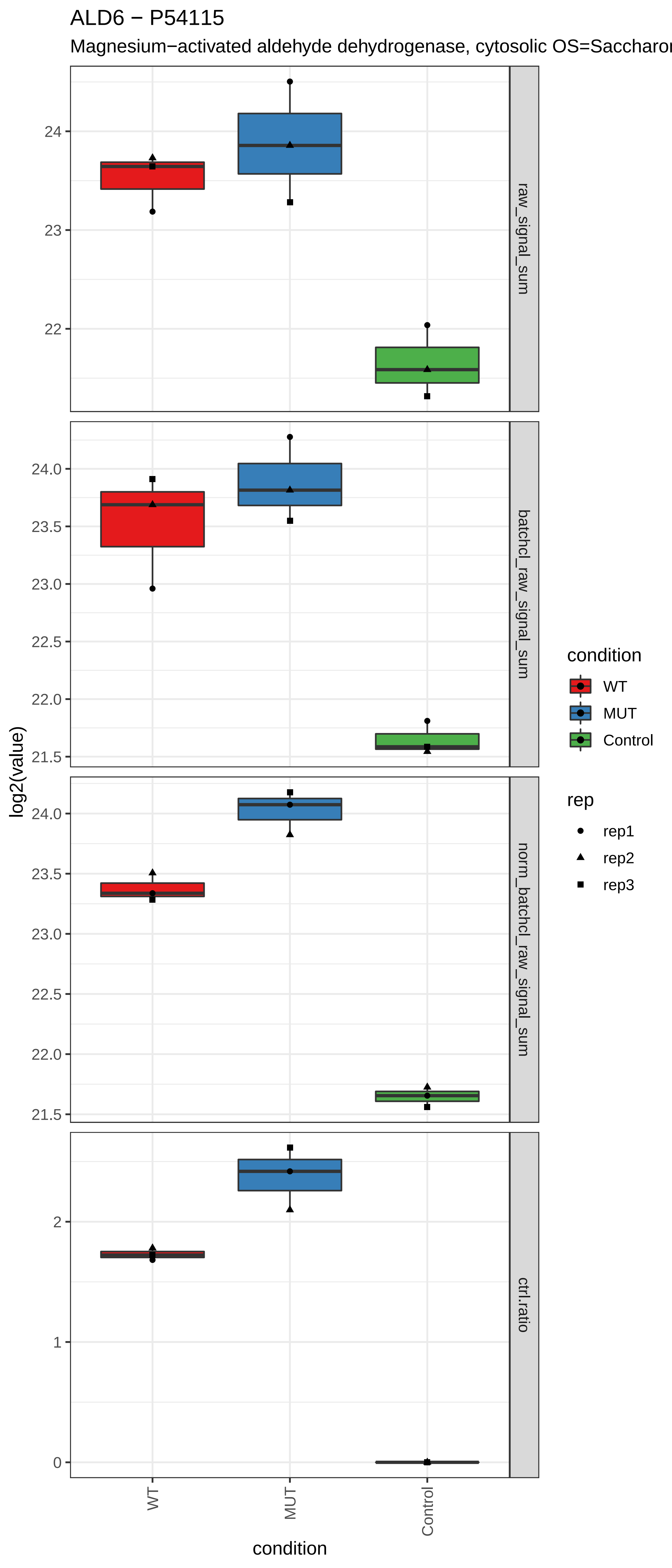
ALD6 − P54115
Magnesium−activated aldehyde dehydrogenase, cytosolic OS=Saccharo
raw_signal_sum
batchcl_raw_signal_sum
norm_batchcl_raw_signal_sum
ctrl.ratio
log2(value)
condition
WT
MUT
Control
rep
rep1
rep2
rep3
24
23
22
24.0
23.5
23.0
22.5
22.0
21.5
2
1
0

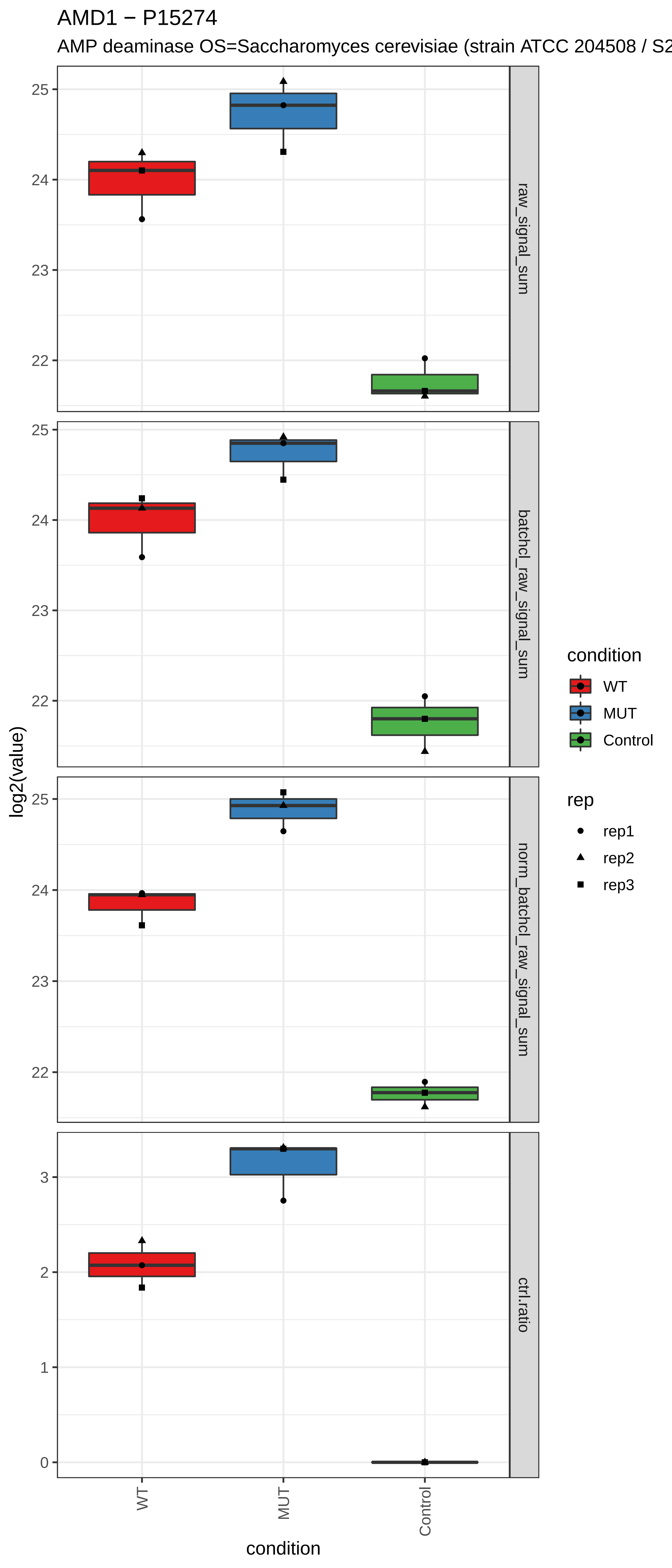
AMD1 − P15274
AMP deaminase OS=Saccharomyces cerevisiae (strain ATCC 204508 / S2
raw_signal_sum
batchcl_raw_signal_sum
norm_batchcl_raw_signal_sum
ctrl.ratio
log2(value)
condition
WT
MUT
Control
condition
WT
MUT
Control
rep
rep1
rep2
rep3

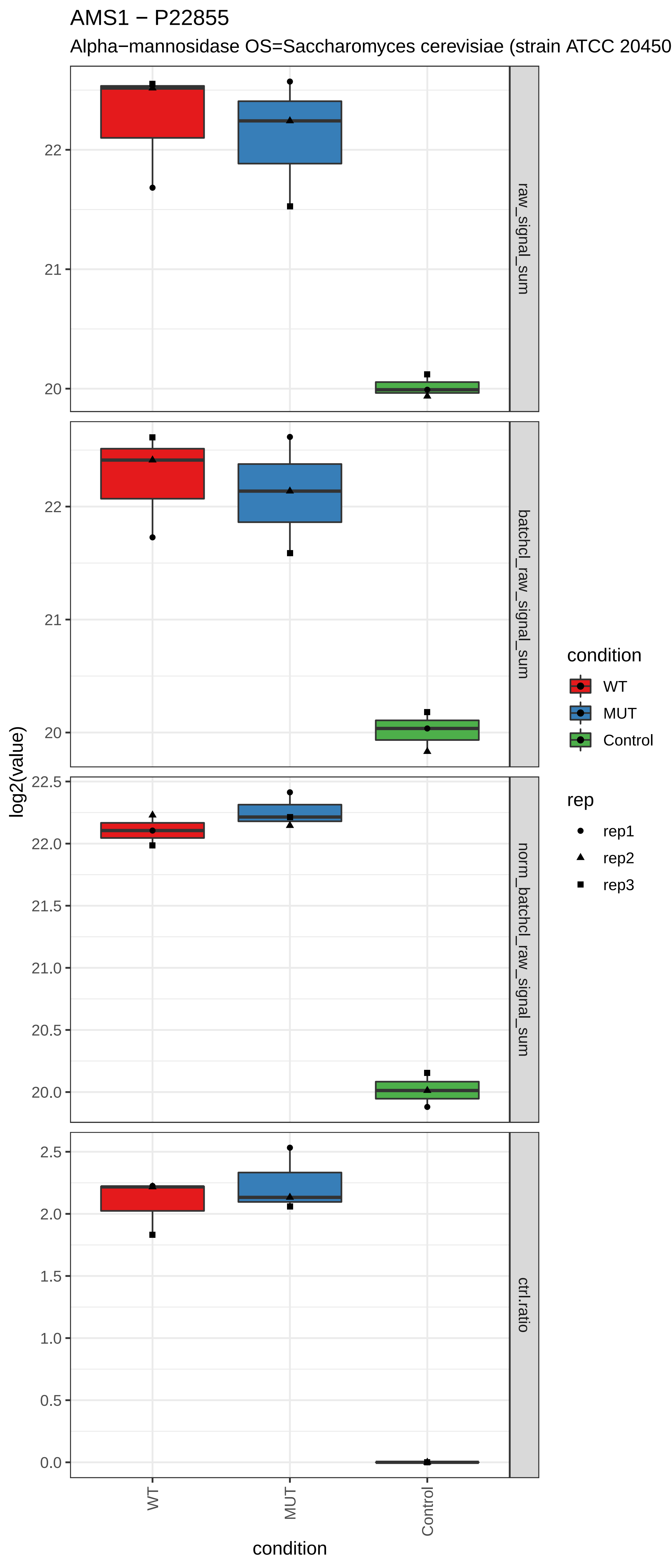

AMS1 − P22855
Alpha−mannosidase OS=Saccharomyces cerevisiae (strain ATCC 20450
raw_signal_sum
batchcl_raw_signal_sum
norm_batchcl_raw_signal_sum
ctrl.ratio
log2(value)
condition
WT
MUT
Control
rep
rep1
rep2
rep3

APE2 − P32454

Aminopeptidase 2, mitochondrial OS=Saccharomyces cerevisiae (strain AT

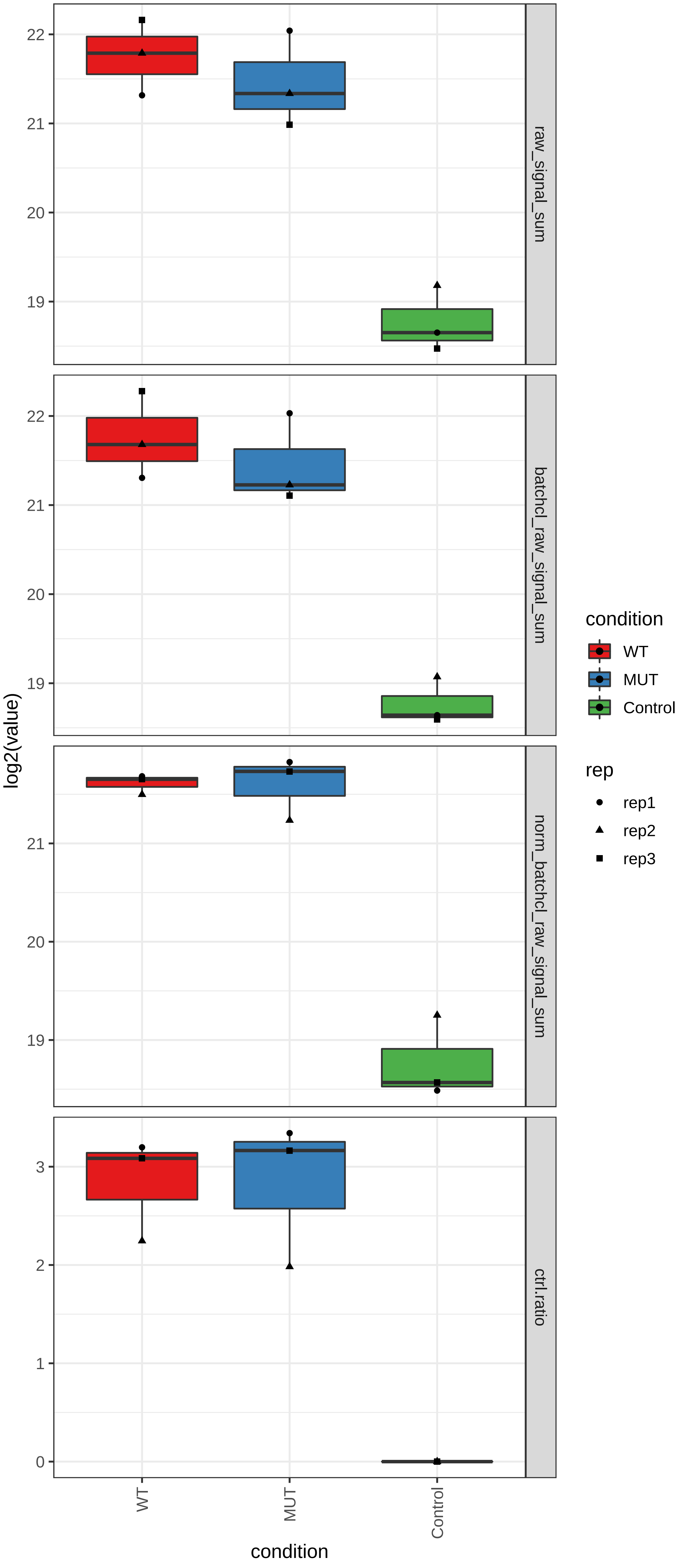

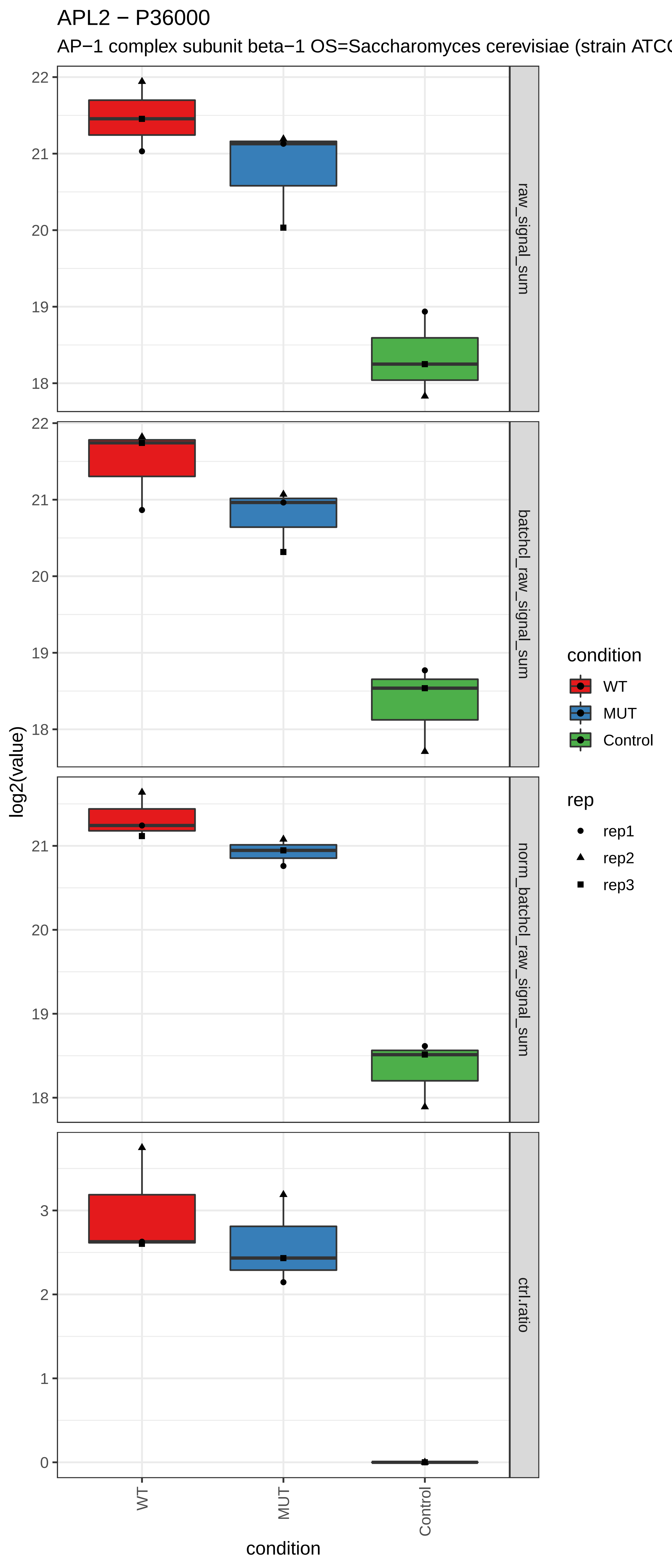

APL2 − P36000
AP−1 complex subunit beta−1 OS=Saccharomyces cerevisiae (strain ATCC
raw_signal_sum
batchcl_raw_signal_sum
norm_batchcl_raw_signal_sum
ctrl.ratio
log2(value)
condition
WT
MUT
Control
rep
rep1
rep2
rep3
22
21
20
19
18
3
2
1
0

APN1 − P22936

DNA−(apurinic or apyrimidinic site) lyase 1 OS=Saccharomyces cerevisiae

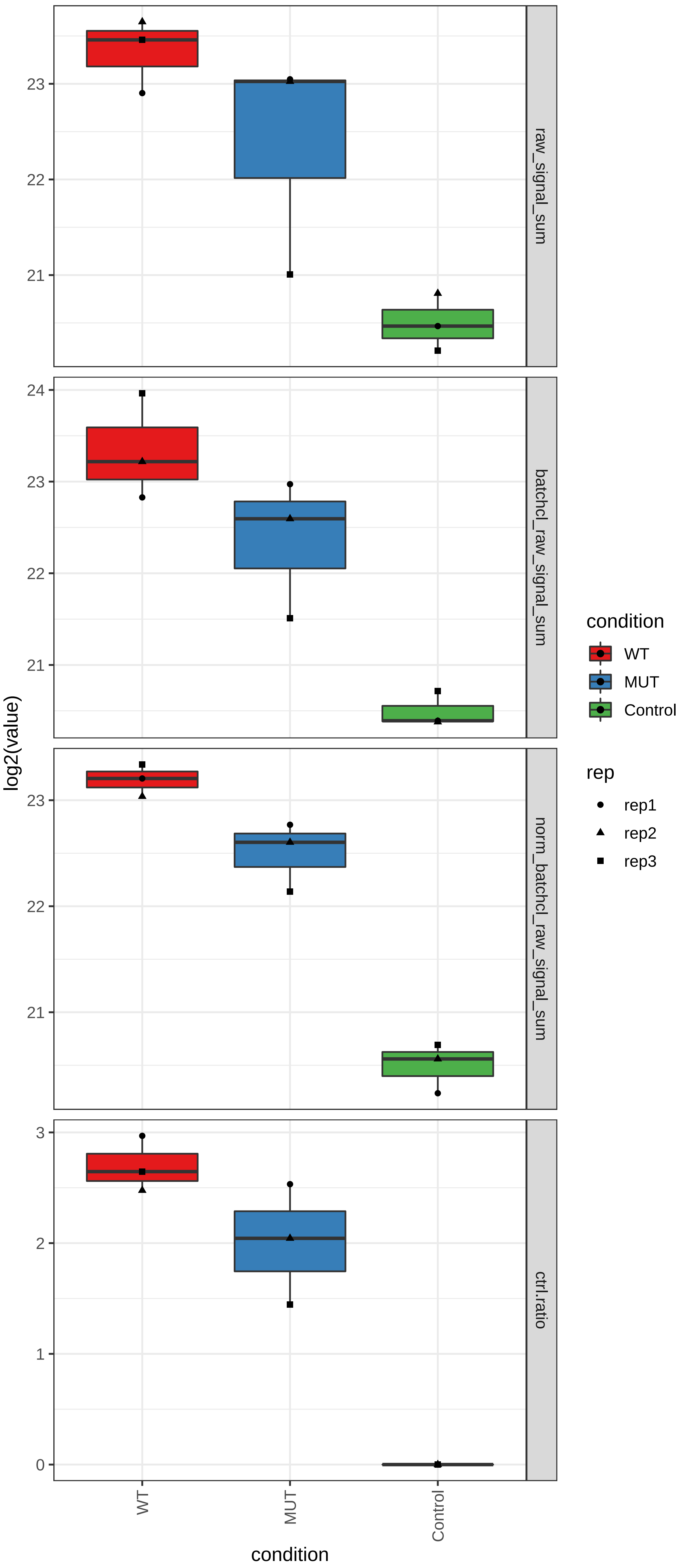

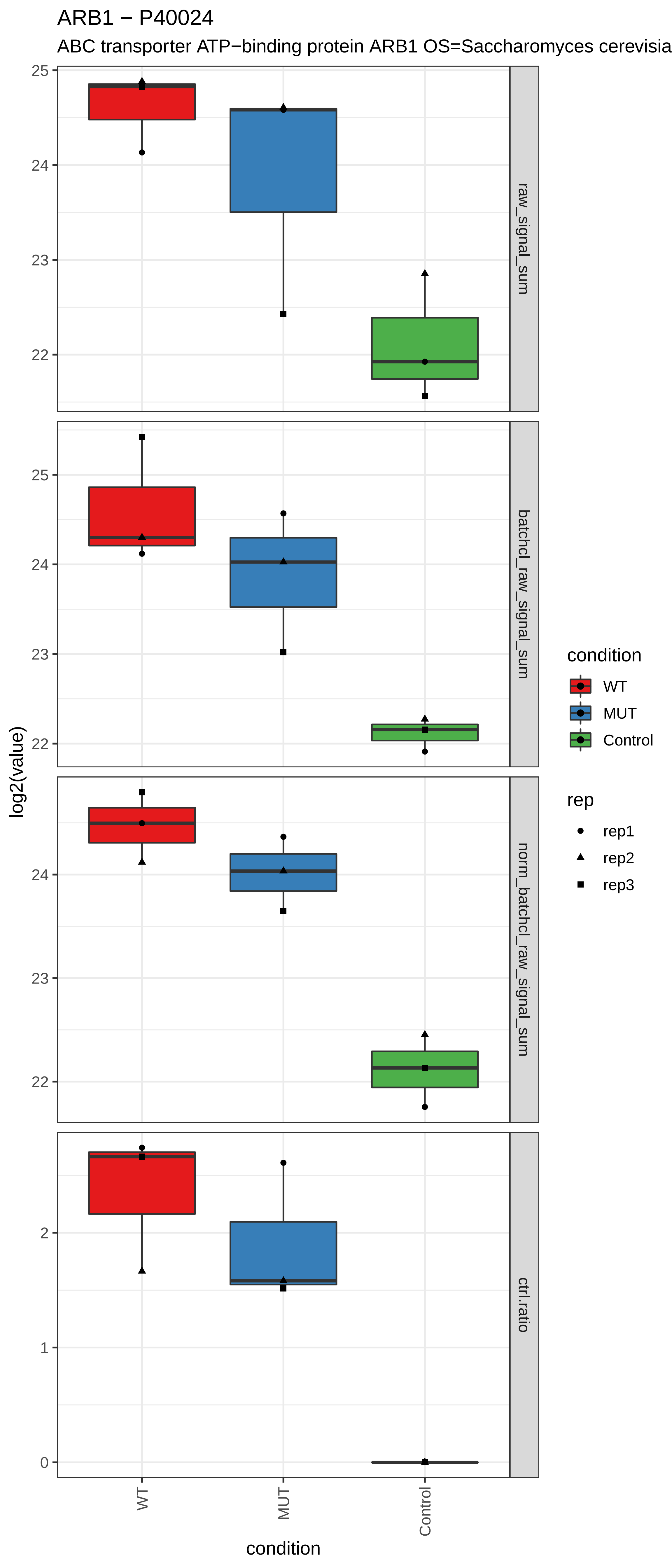

ARB1 − P40024
ABC transporter ATP−binding protein ARB1 OS=Saccharomyces cerevisia
log2(value)
raw_signal_sum
batchcl_raw_signal_sum
norm_batchcl_raw_signal_sum
ctrl.ratio
condition
WT
MUT
Control
rep
rep1
rep2
rep3
25
24
23
22
2
1
0

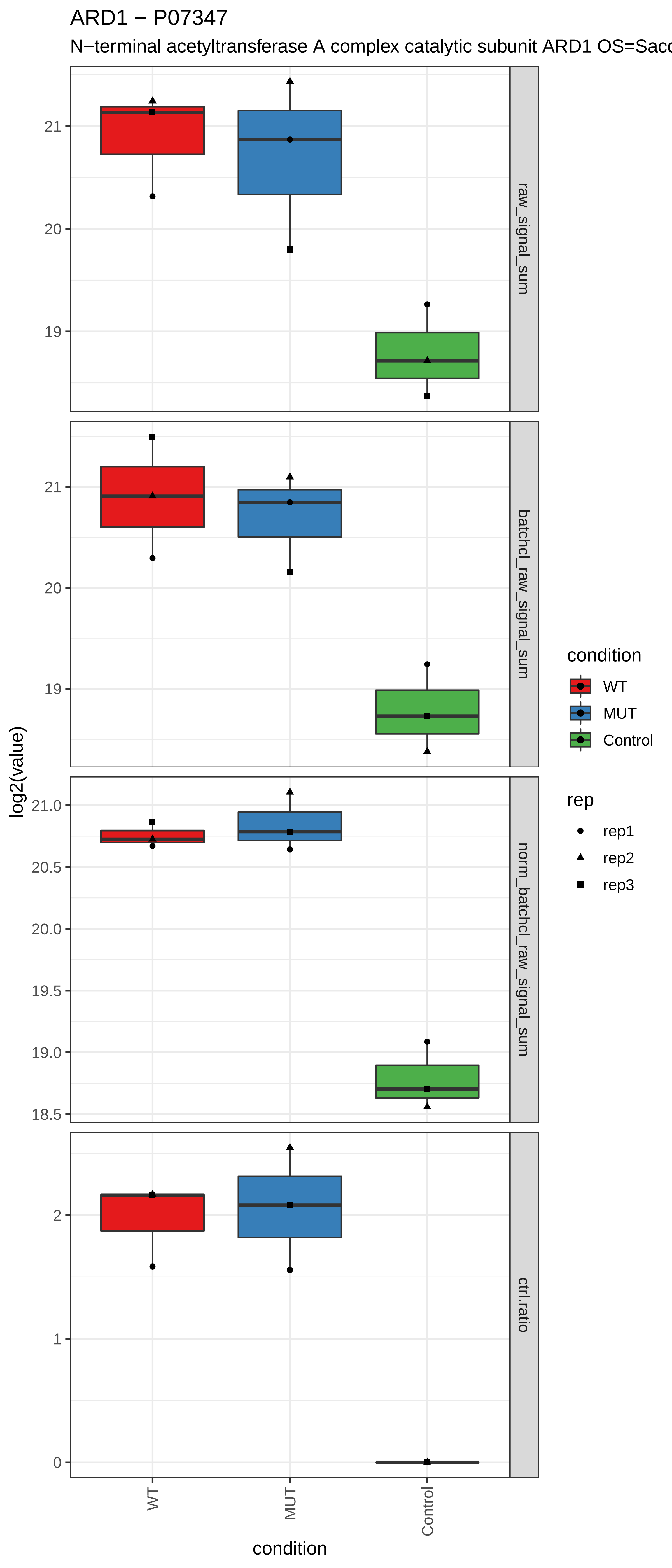

ARD1 − P07347
N−terminal acetyltransferase A complex catalytic subunit ARD1 OS=Sacc
raw_signal_sum
batchcl_raw_signal_sum
norm_batchcl_raw_signal_sum
ctrl.ratio
log2(value)
condition
WT
MUT
Control
rep
rep1
rep2
rep3

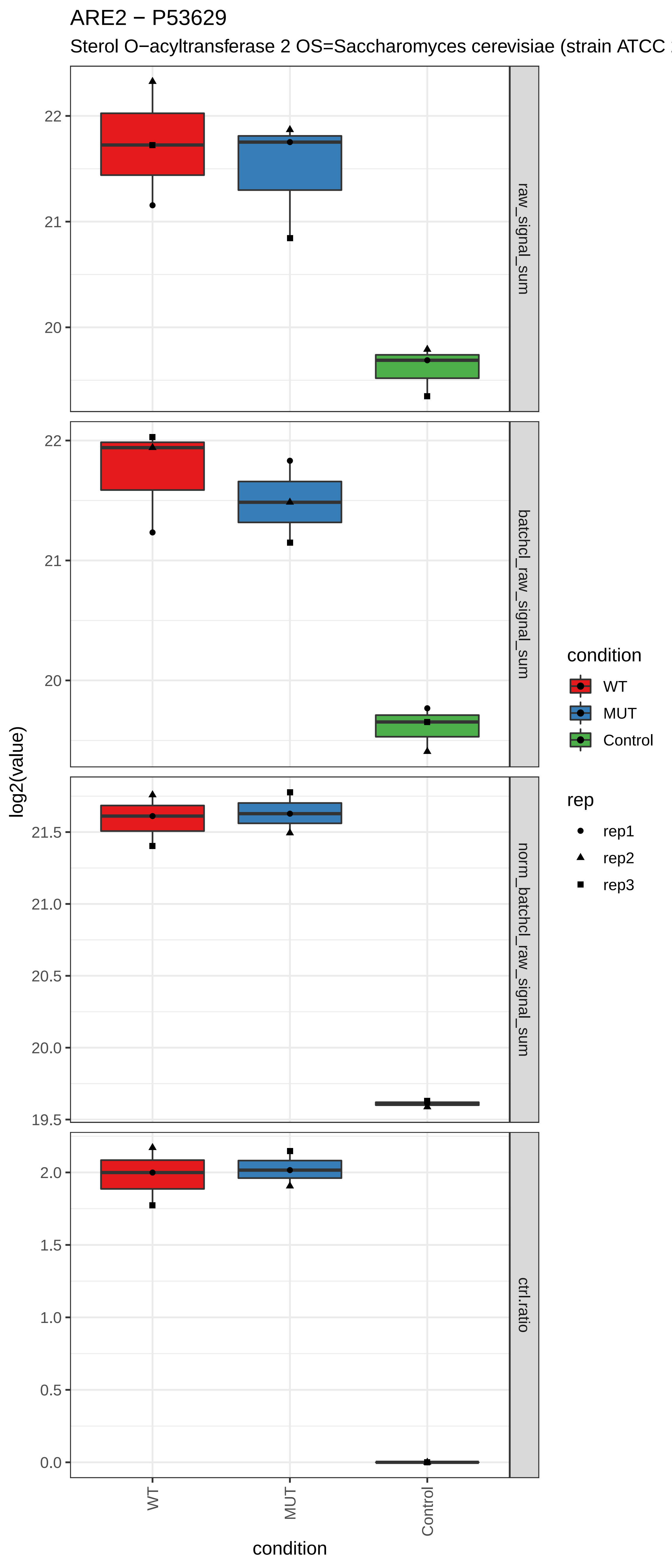

ARE2 − P53629
Sterol O−acyltransferase 2 OS=Saccharomyces cerevisiae (strain ATCC
raw_signal_sum
batchcl_raw_signal_sum
norm_batchcl_raw_signal_sum
ctrl.ratio
log2(value)
condition
WT
MUT
Control
rep
rep1
rep2
rep3

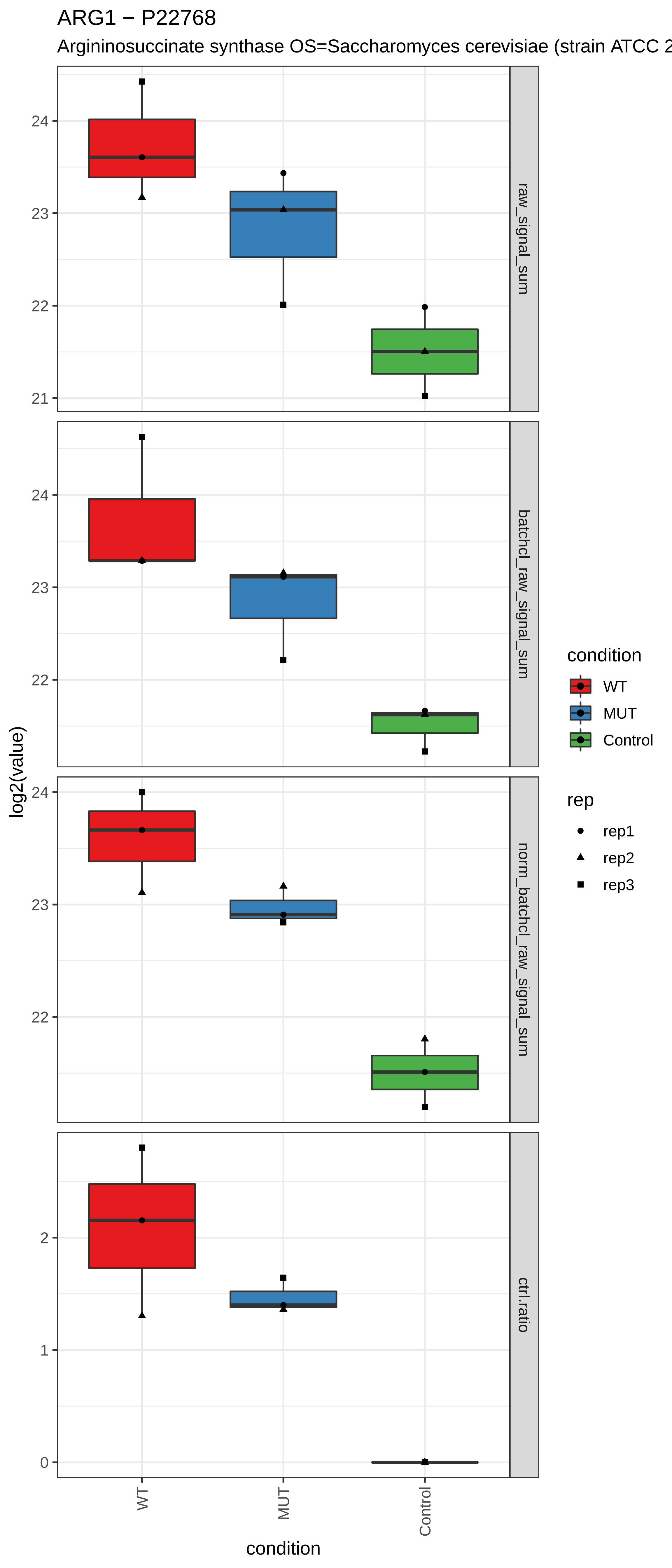

ARG1 − P22768
Argininosuccinate synthase OS=Saccharomyces cerevisiae (strain ATCC 2
raw_signal_sum
batchcl_raw_signal_sum
norm_batchcl_raw_signal_sum
ctrl.ratio
log2(value)
condition
WT
MUT
Control
rep
rep1
rep2
rep3

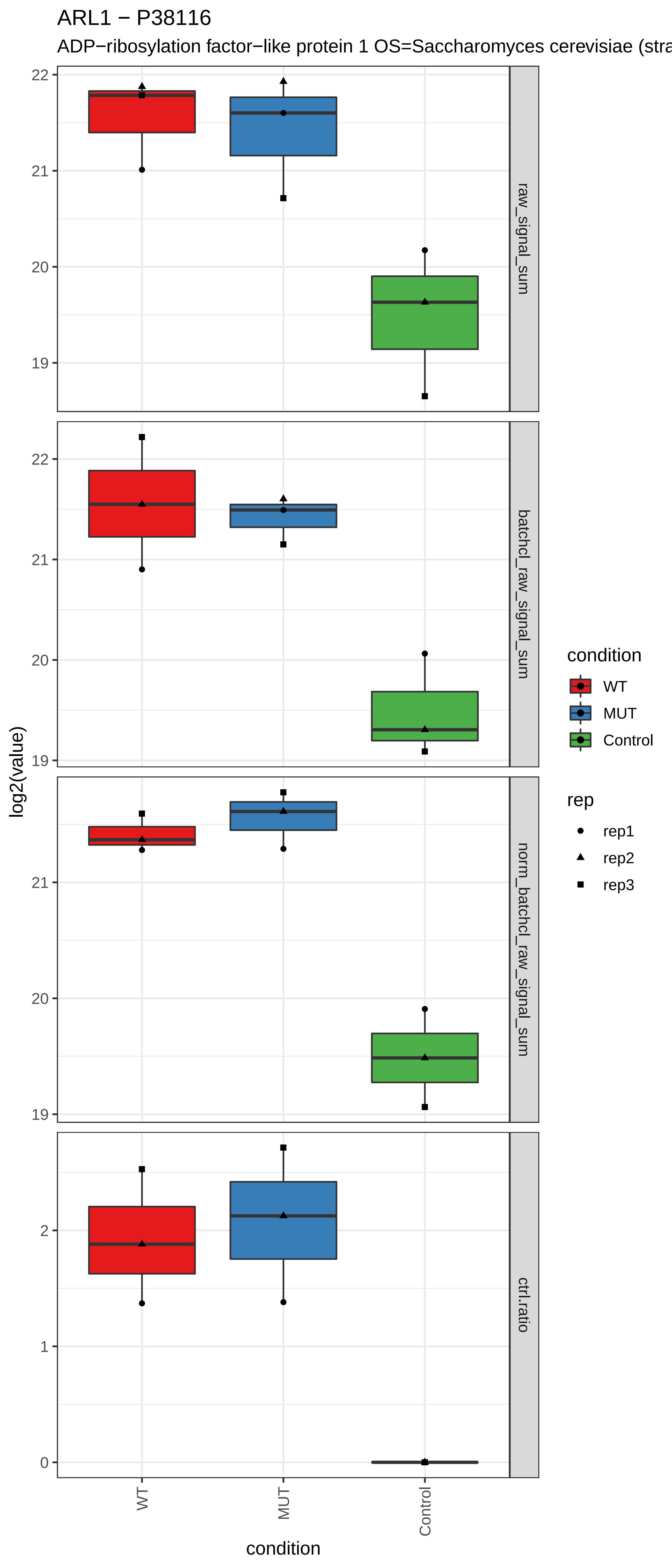
ARL1 − P38116
ADP−ribosylation factor−like protein 1 OS=Saccharomyces cerevisiae (stra
raw_signal_sum
batchcl_raw_signal_sum
norm_batchcl_raw_signal_sum
ctrl.ratio
log2(value)
condition
WT
MUT
Control
rep
rep1
rep2
rep3

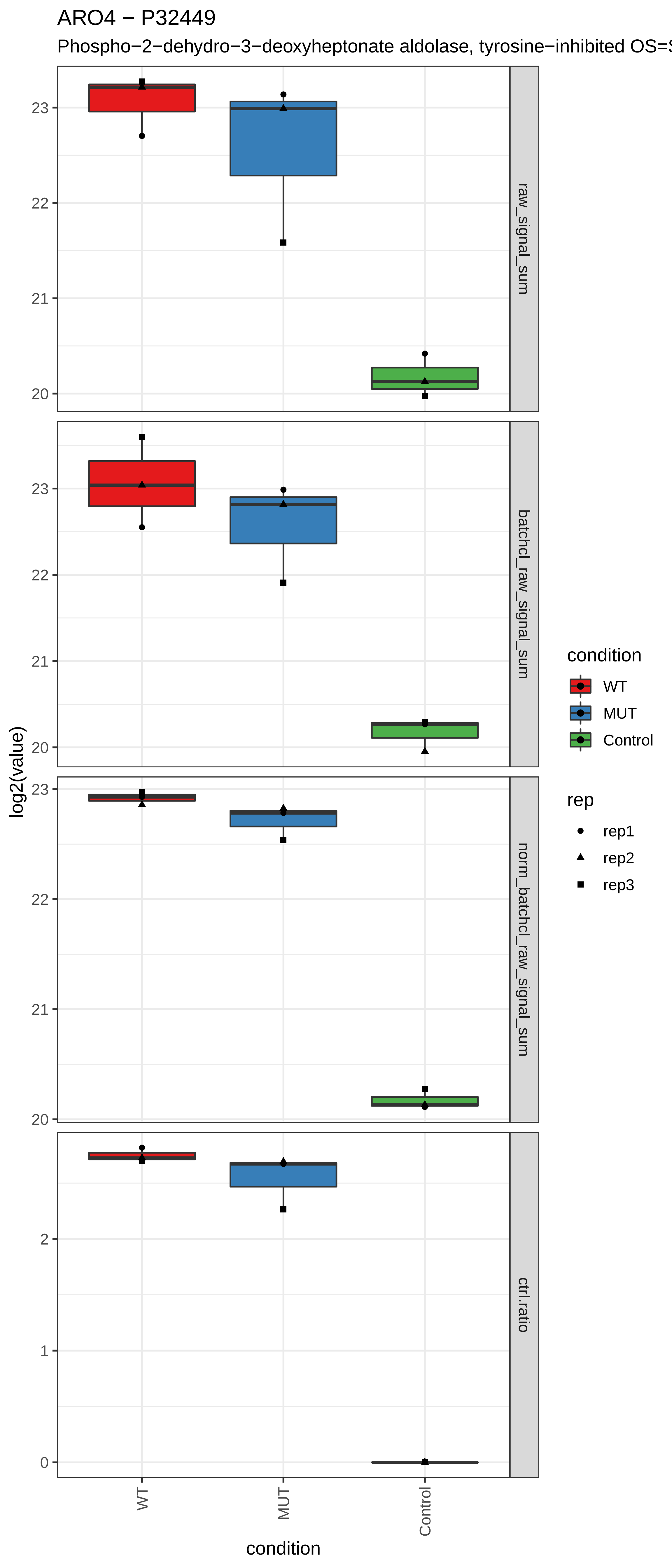

ARO4 − P32449
Phospho−2−dehydro−3−deoxyheptonate aldolase, tyrosine−inhibited OS=S
raw_signal_sum
batchcl_raw_signal_sum
norm_batchcl_raw_signal_sum
ctrl.ratio
log2(value)
condition
WT
MUT
Control
rep
rep1
rep2
rep3

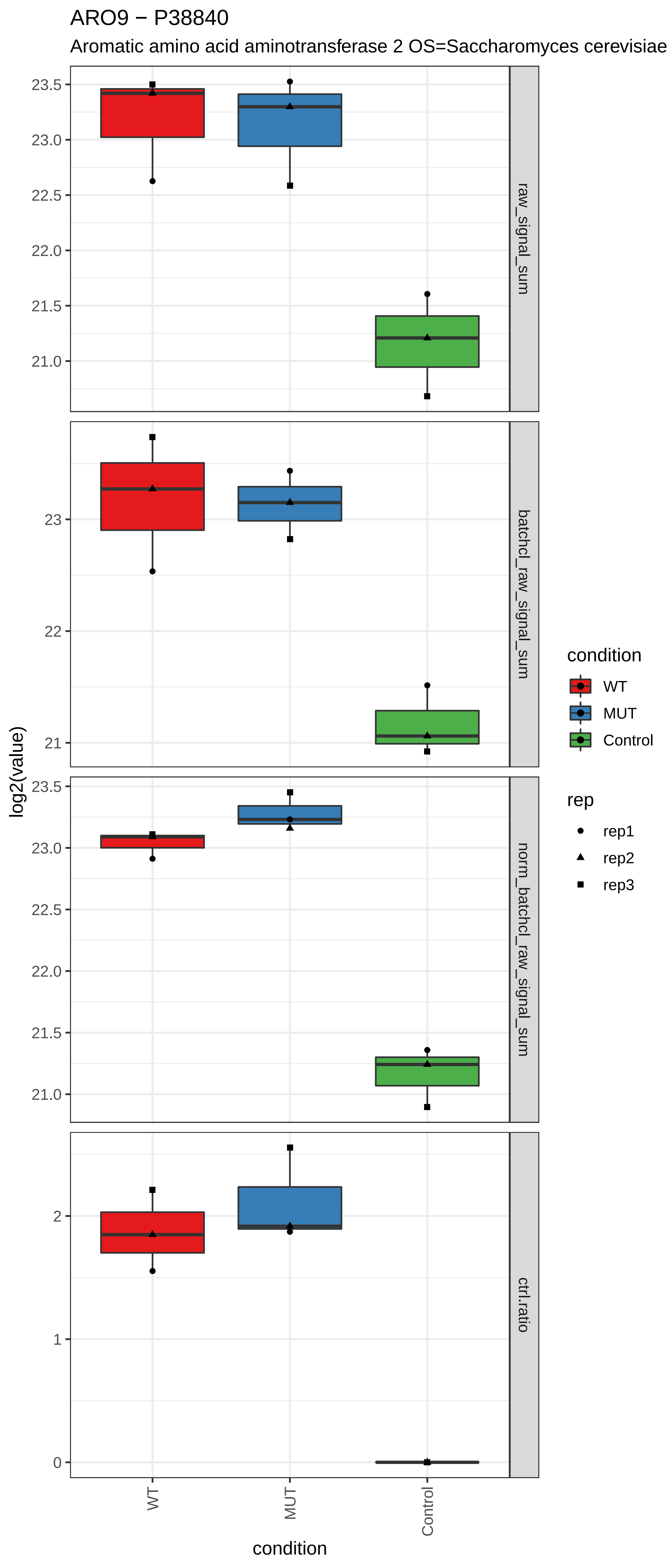
ARO9 − P38840
Aromatic amino acid aminotransferase 2 OS=Saccharomyces cerevisiae
raw_signal_sum
batchcl_raw_signal_sum
norm_batchcl_raw_signal_sum
ctrl.ratio
log2(value)
condition
WT
MUT
Control
rep
rep1
rep2
rep3
23.5
23.0
22.5
22.0
21.5
21.0
23
22
21
2
1
0

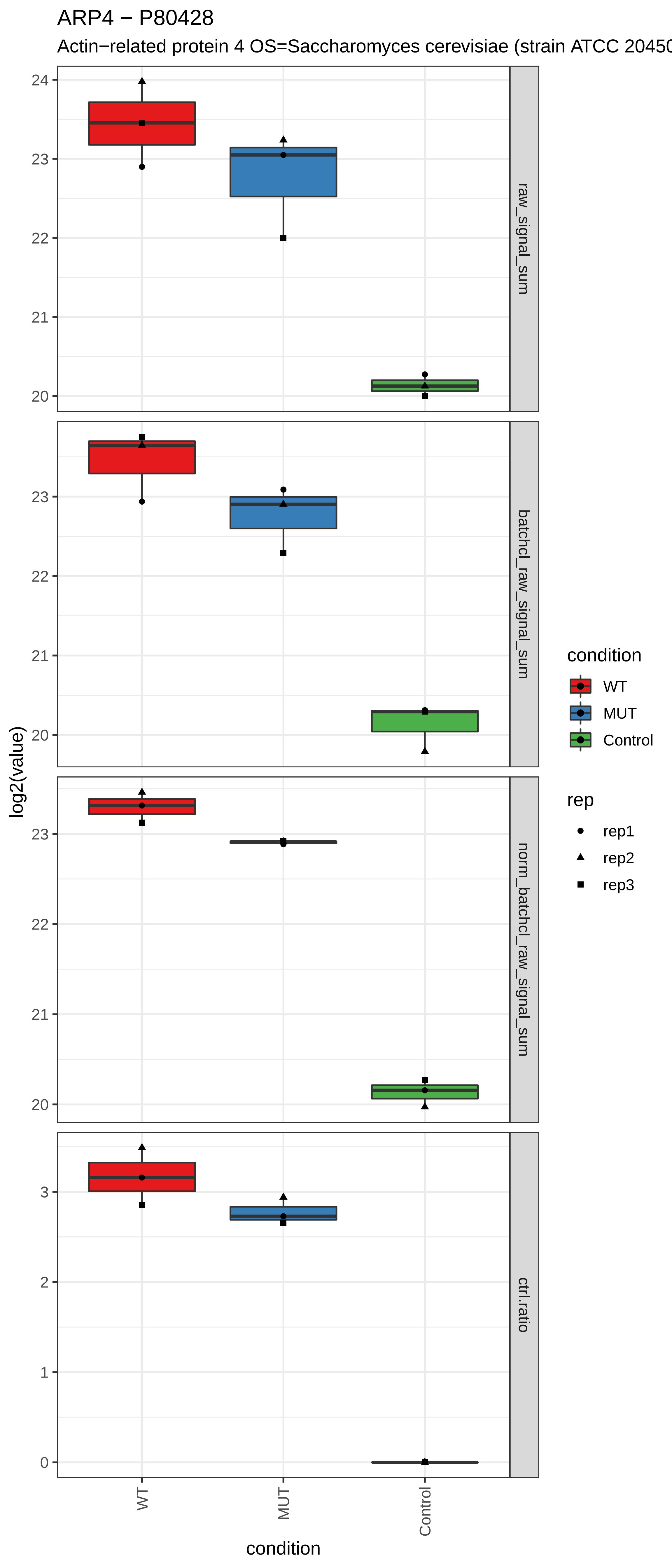
ARP4 − P80428
Actin−related protein 4 OS=Saccharomyces cerevisiae (strain ATCC 20450
raw_signal_sum
batchcl_raw_signal_sum
norm_batchcl_raw_signal_sum
ctrl.ratio
log2(value)
condition
WT
MUT
Control
rep
rep1
rep2
rep3
24
23
22
21
20
3
2
1
0

ARP7 − Q12406

Actin−related protein 7 OS=Saccharomyces cerevisiae (strain ATCC 2045

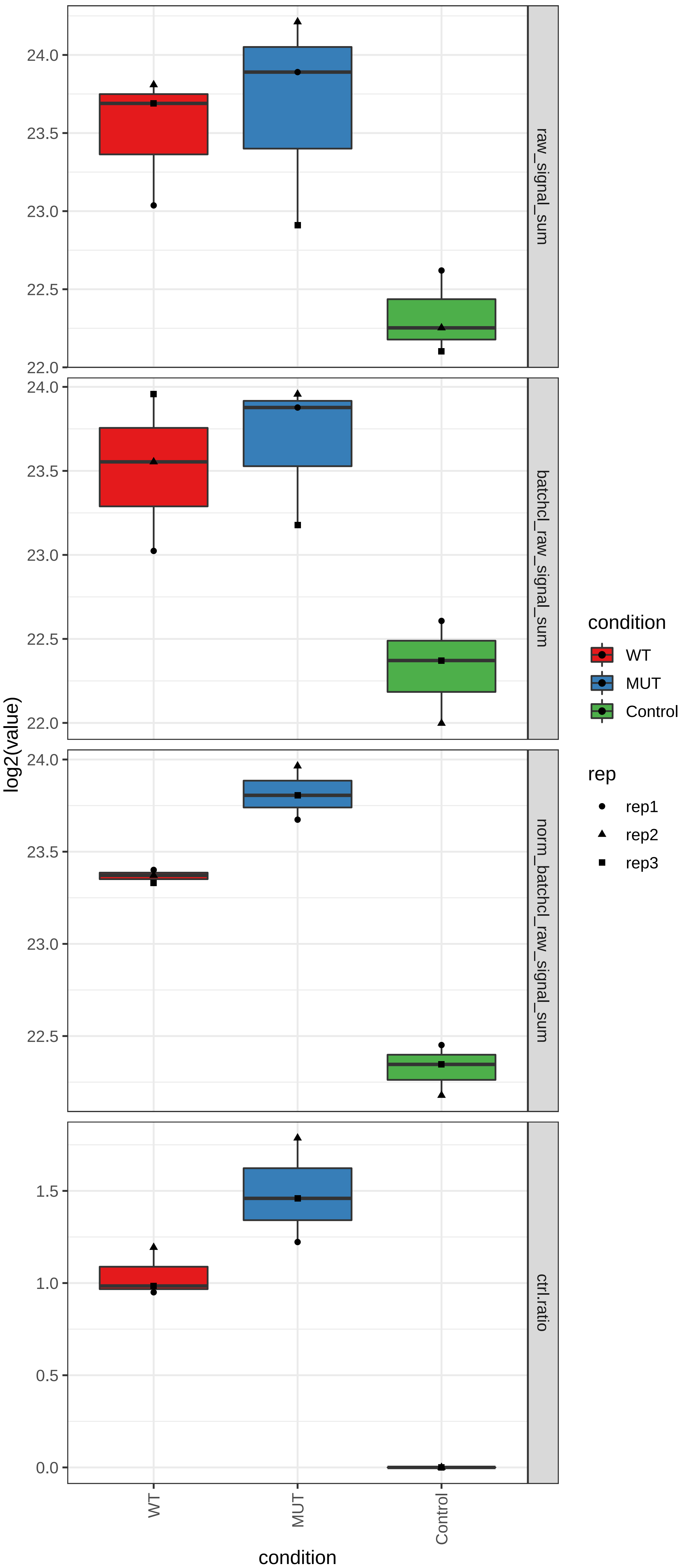

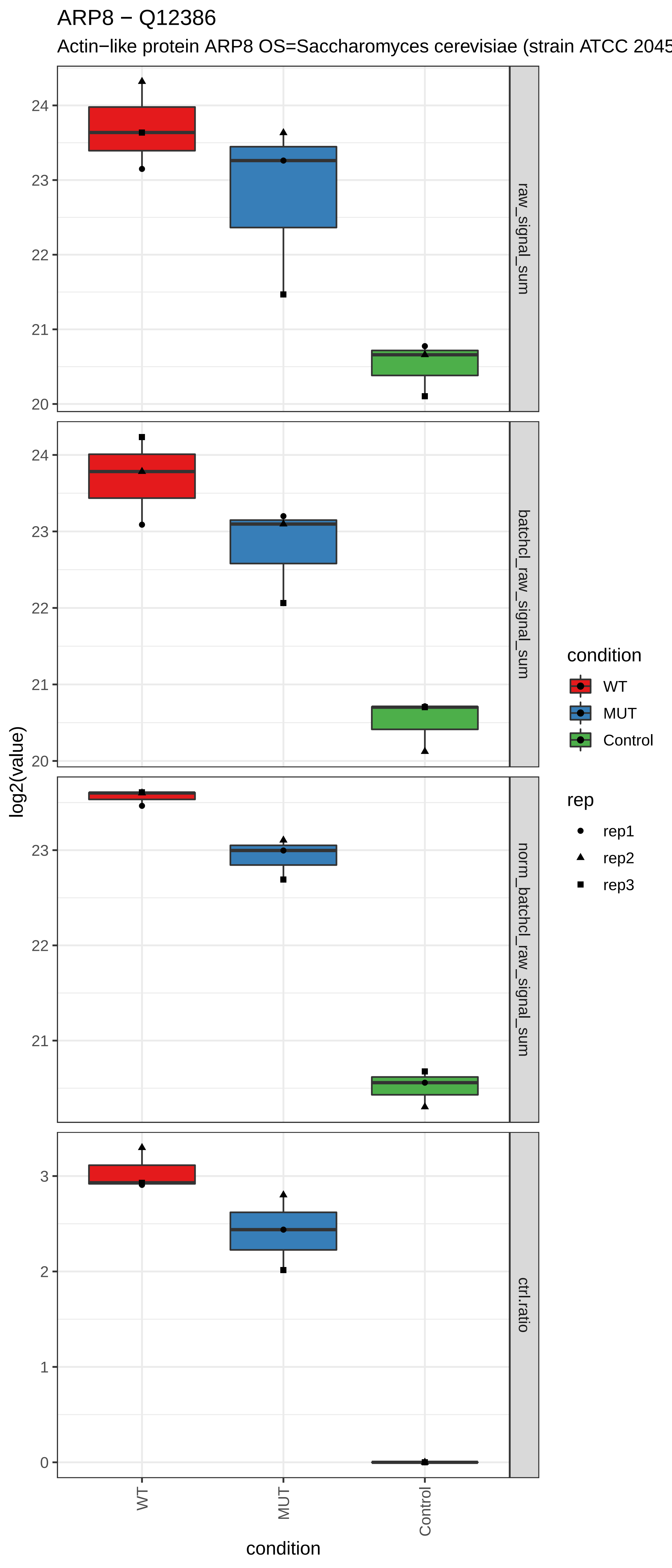

ARP8 − Q12386
Actin−like protein ARP8 OS=Saccharomyces cerevisiae (strain ATCC 2045
raw_signal_sum
batchcl_raw_signal_sum
norm_batchcl_raw_signal_sum
ctrl.ratio
log2(value)
condition
WT
MUT
Control
rep
rep1
rep2
rep3

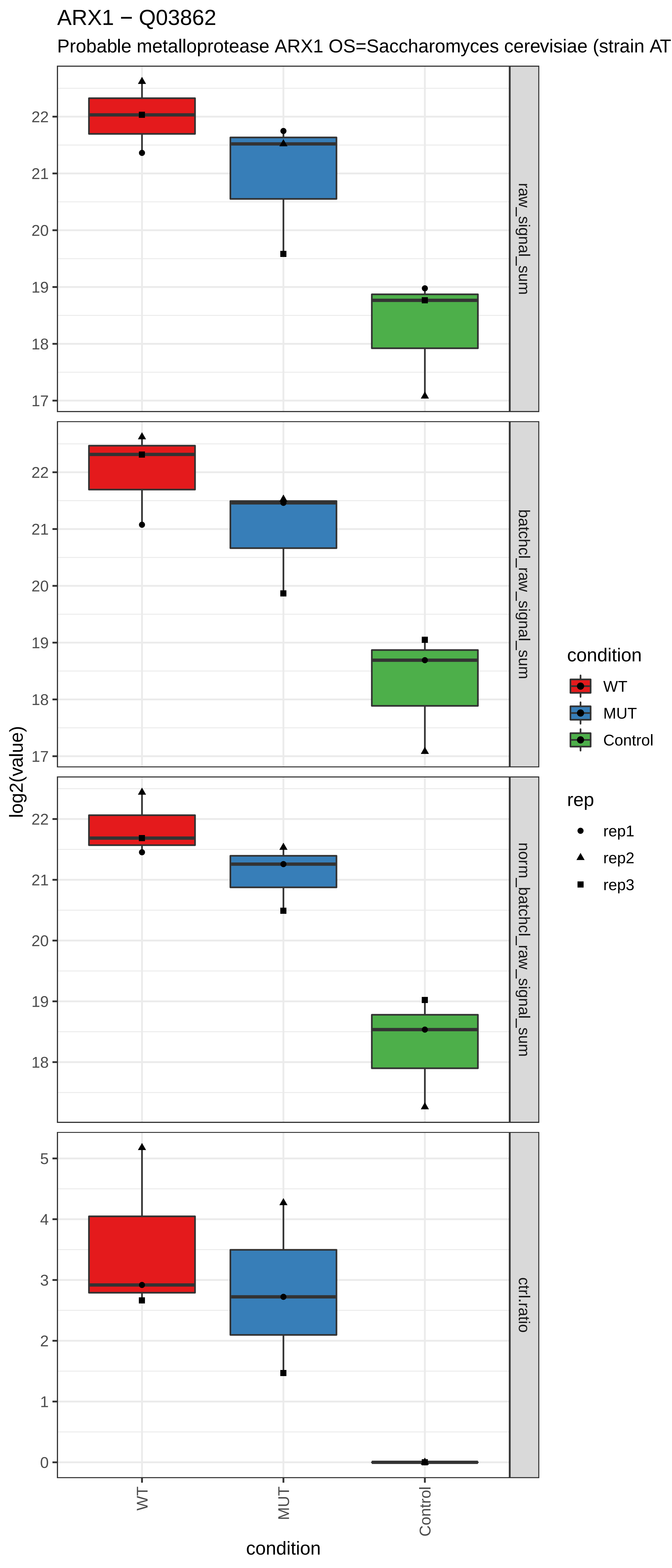

ARX1 − Q03862
Probable metalloprotease ARX1 OS=Saccharomyces cerevisiae (strain AT
raw_signal_sum
batchcl_raw_signal_sum
norm_batchcl_raw_signal_sum
ctrl.ratio
log2(value)
condition
WT
MUT
Control
rep
rep1
rep2
rep3

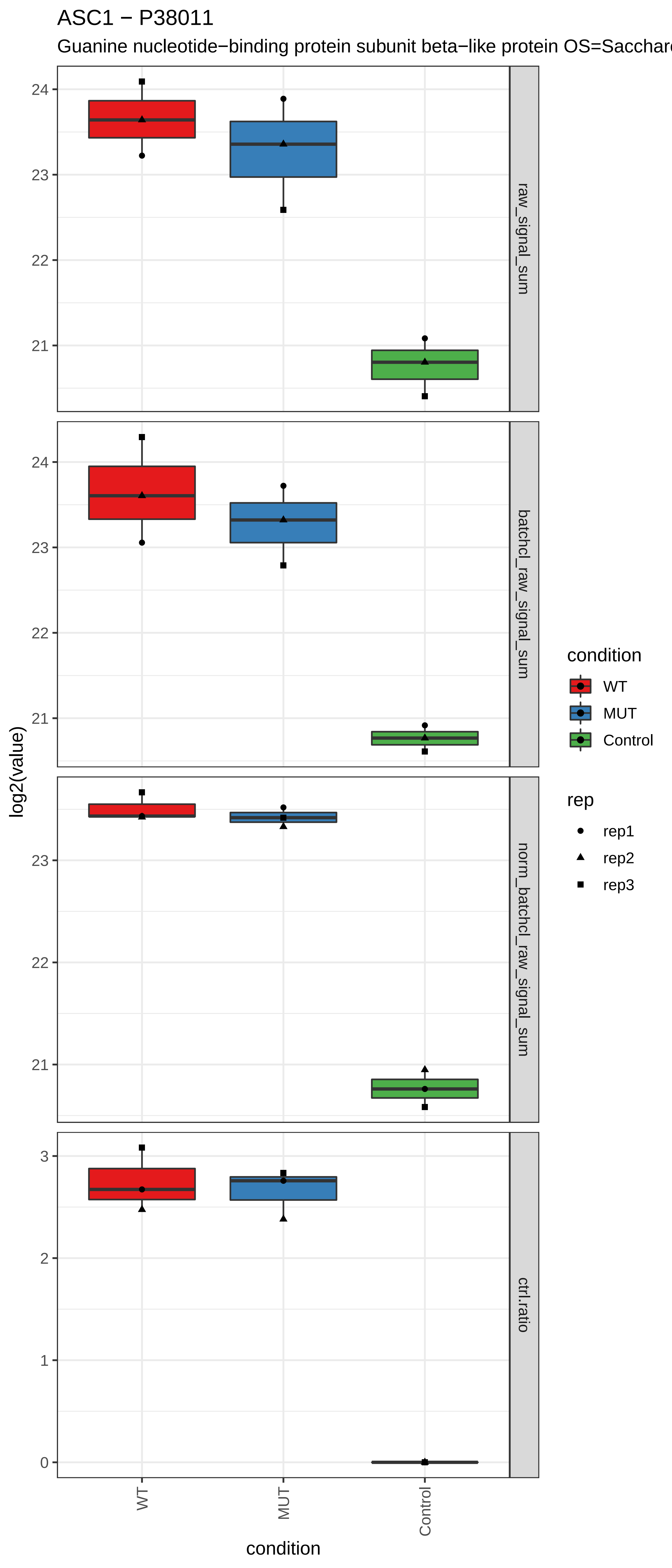

ASC1 − P38011
Guanine nucleotide−binding protein subunit beta−like protein OS=Sacchar
log2(value)
raw_signal_sum
batchcl_raw_signal_sum
norm_batchcl_raw_signal_sum
ctrl.ratio
condition
WT
MUT
Control
rep
rep1
rep2
rep3

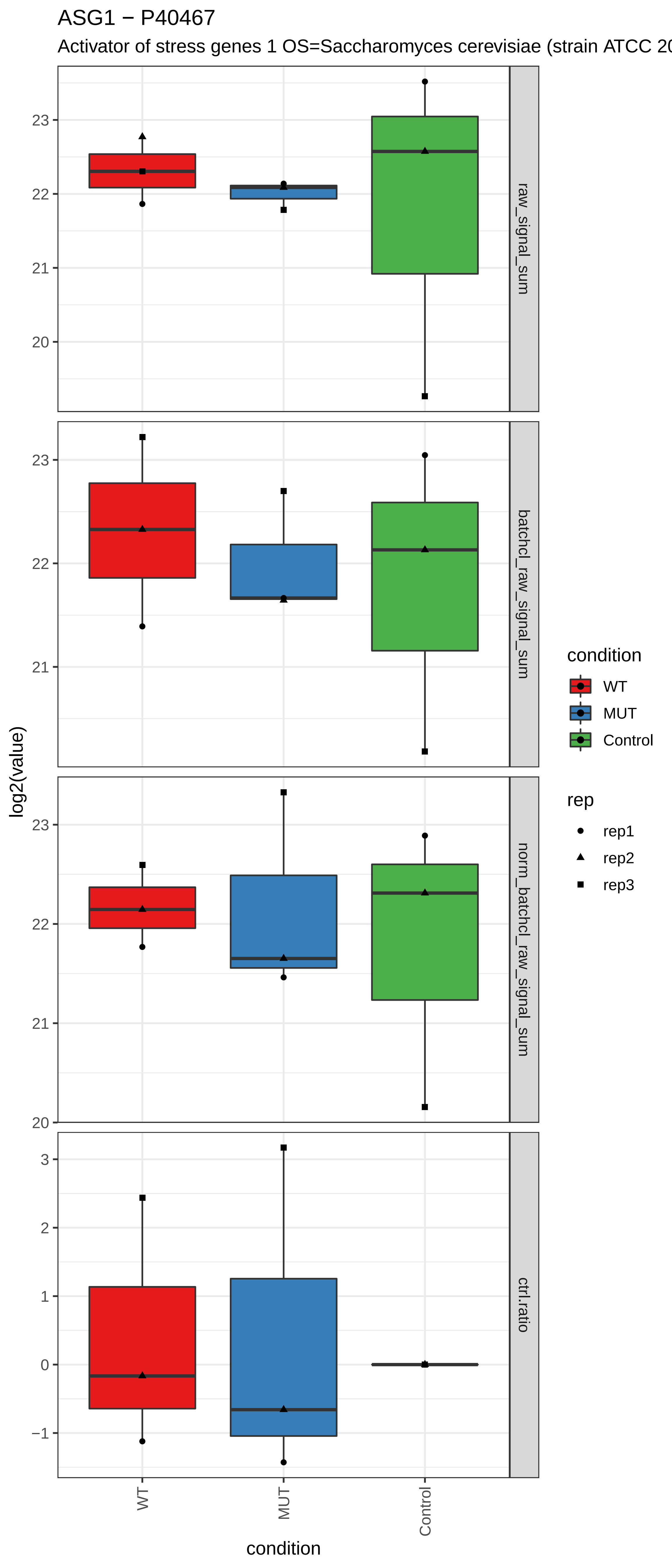
ASG1 − P40467
Activator of stress genes 1 OS=Saccharomyces cerevisiae (strain ATCC 20
raw_signal_sum
batchcl_raw_signal_sum
norm_batchcl_raw_signal_sum
ctrl.ratio
log2(value)
condition
WT
MUT
Control
rep
rep1
rep2
rep3
23
22
21
20
3
2
1
0
−1

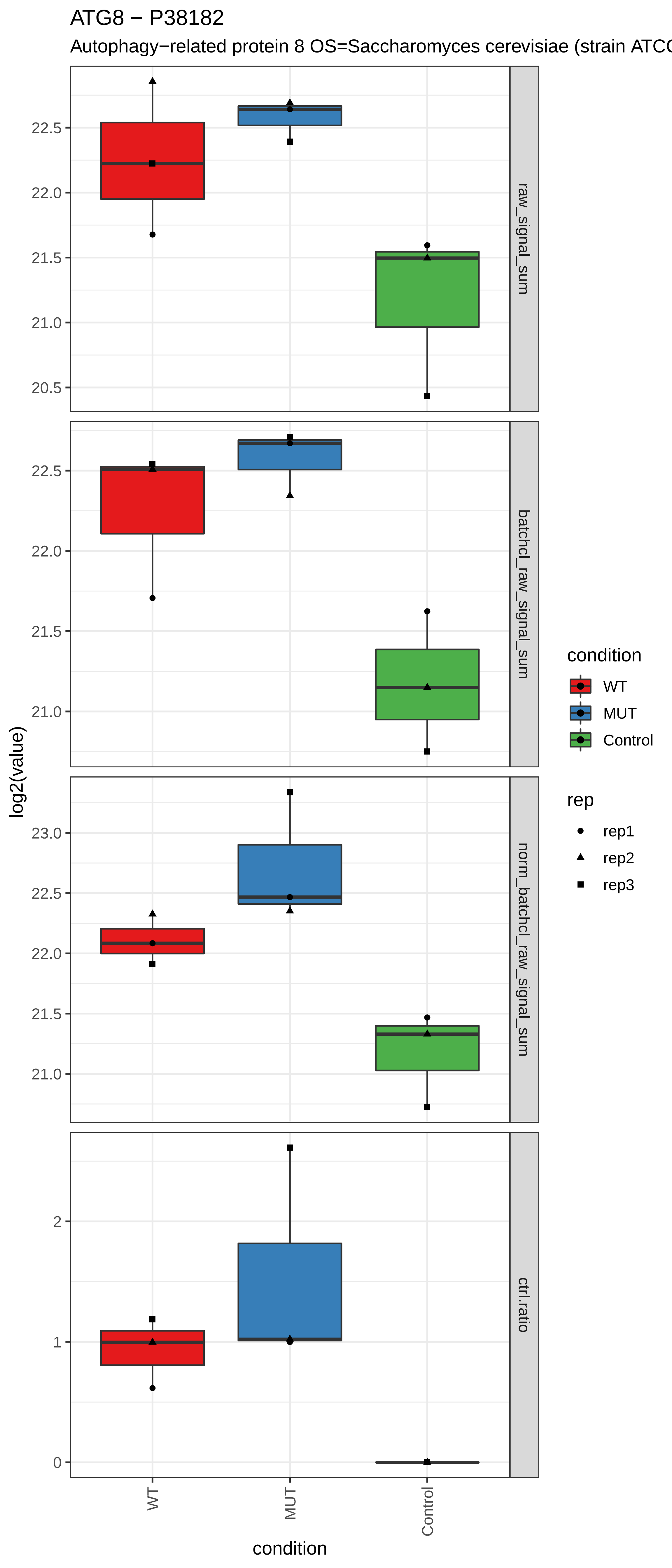

ATG8 − P38182
Autophagy−related protein 8 OS=Saccharomyces cerevisiae (strain ATCC
raw_signal_sum
batchcl_raw_signal_sum
norm_batchcl_raw_signal_sum
ctrl.ratio
log2(value)
condition
WT
MUT
Control
rep
rep1
rep2
rep3
22.5
22.0
21.5
21.0
20.5
23.0
2
1
0

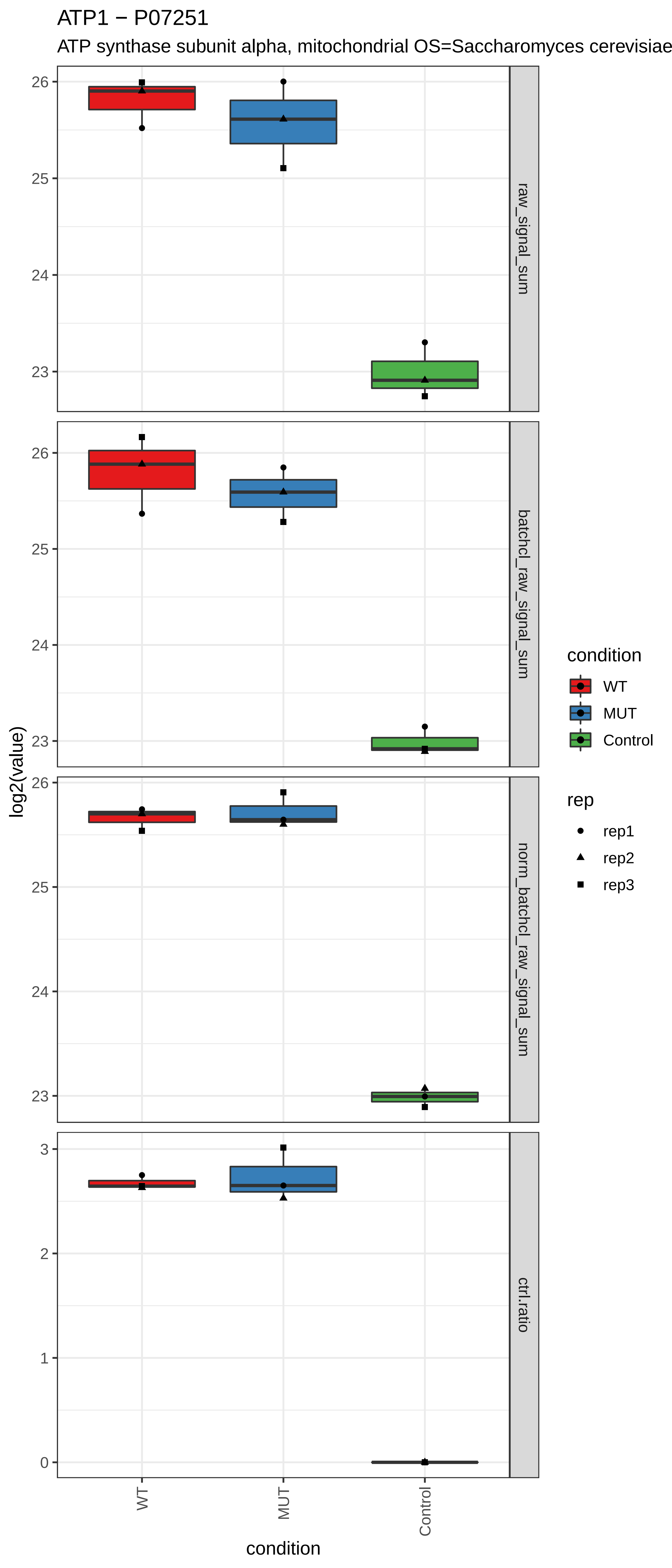

ATP1 − P07251
ATP synthase subunit alpha, mitochondrial OS=Saccharomyces cerevisiae
raw_signal_sum
batchcl_raw_signal_sum
norm_batchcl_raw_signal_sum
ctrl.ratio
log2(value)
condition
WT
MUT
Control
rep
rep1
rep2
rep3

ATP2 − P00830

ATP synthase subunit beta, mitochondrial OS=Saccharomyces cerevisiae (

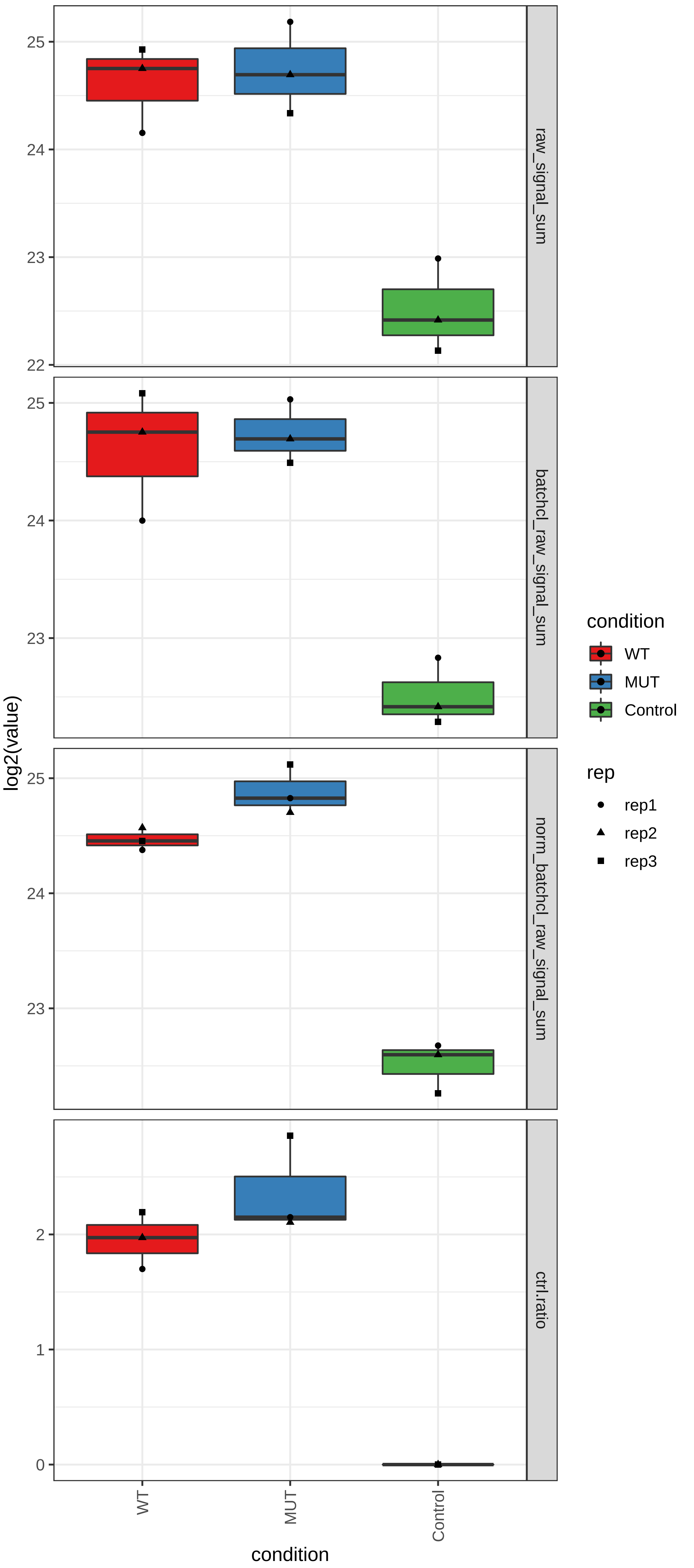

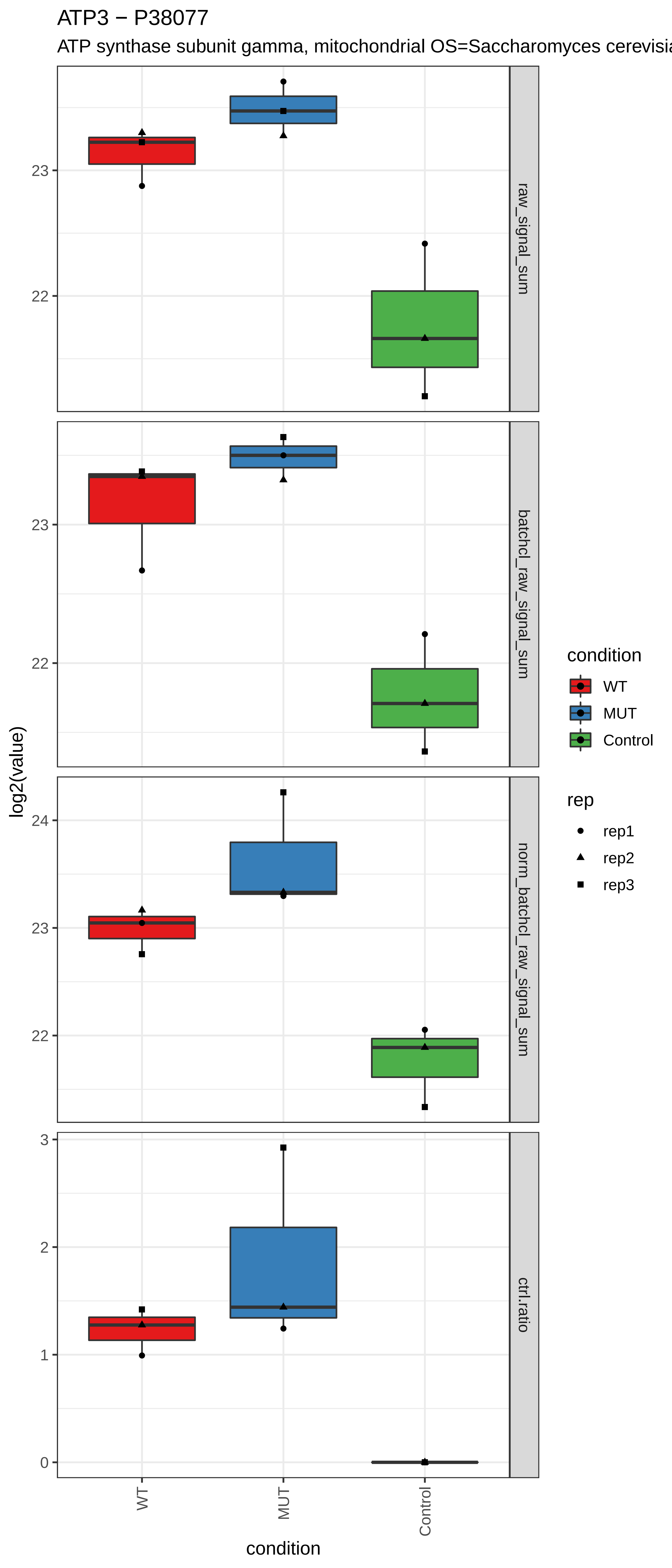

ATP3 − P38077
ATP synthase subunit gamma, mitochondrial OS=Saccharomyces cerevisia
log2(value)
raw_signal_sum
batchcl_raw_signal_sum
norm_batchcl_raw_signal_sum
ctrl.ratio
23
22
24
3
2
1
0
WT
MUT
Control
condition
condition
WT
MUT
Control
rep
rep1
rep2
rep3

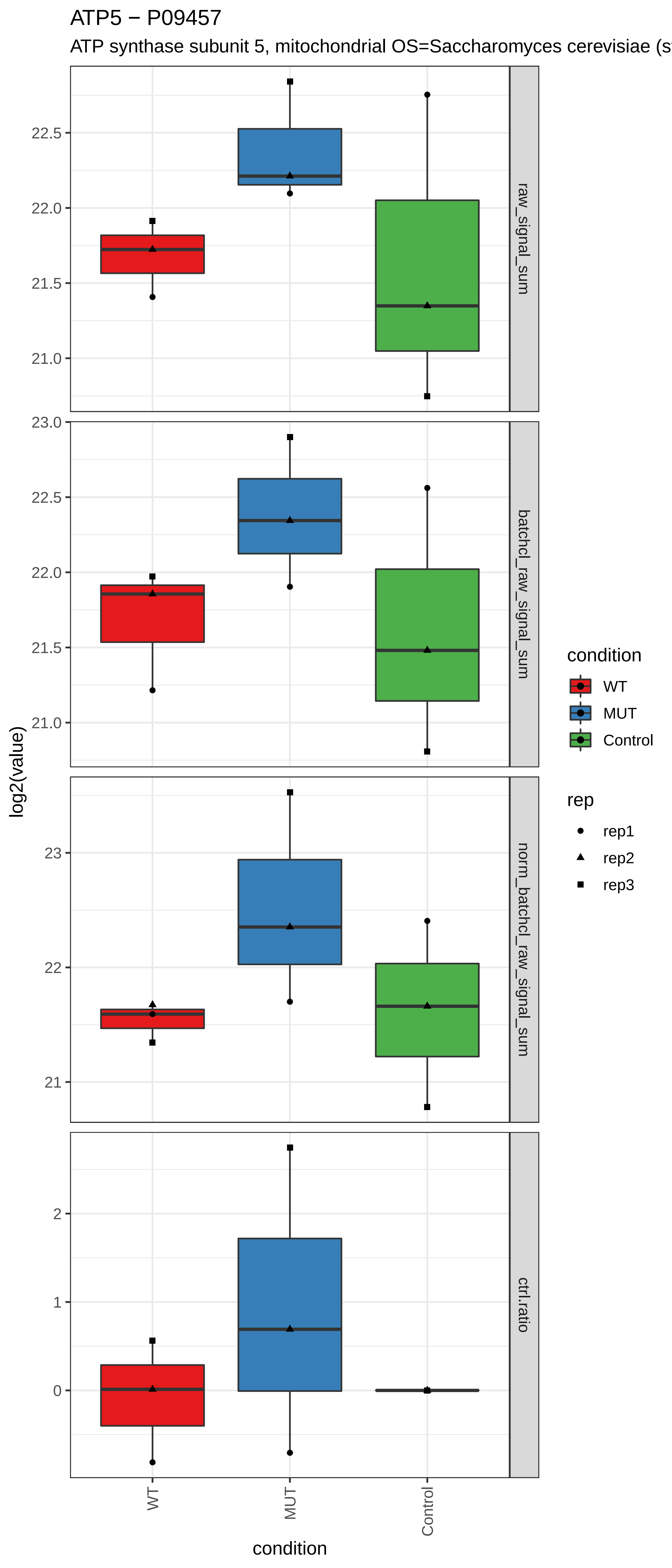
ATP5 − P09457
ATP synthase subunit 5, mitochondrial OS=Saccharomyces cerevisiae (s
raw_signal_sum
batchcl_raw_signal_sum
norm_batchcl_raw_signal_sum
ctrl.ratio
log2(value)
condition
WT
MUT
Control
rep
rep1
rep2
rep3
22.5
22.0
21.5
21.0
23.0
23
22
21
2
1
0

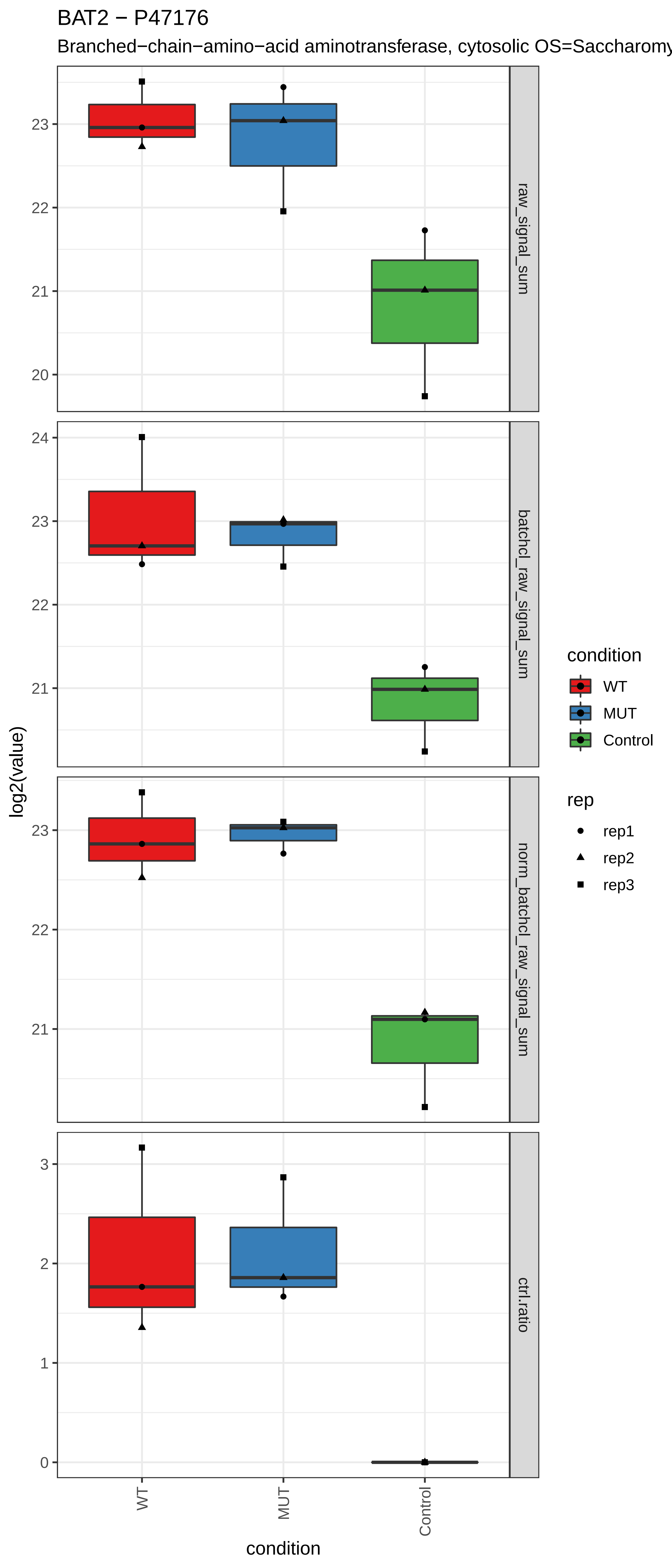

BAT2 − P47176
Branched−chain−amino−acid aminotransferase, cytosolic OS=Saccharomy
raw_signal_sum
batchcl_raw_signal_sum
norm_batchcl_raw_signal_sum
ctrl.ratio
log2(value)
condition
WT
MUT
Control
rep
rep1
rep2
rep3

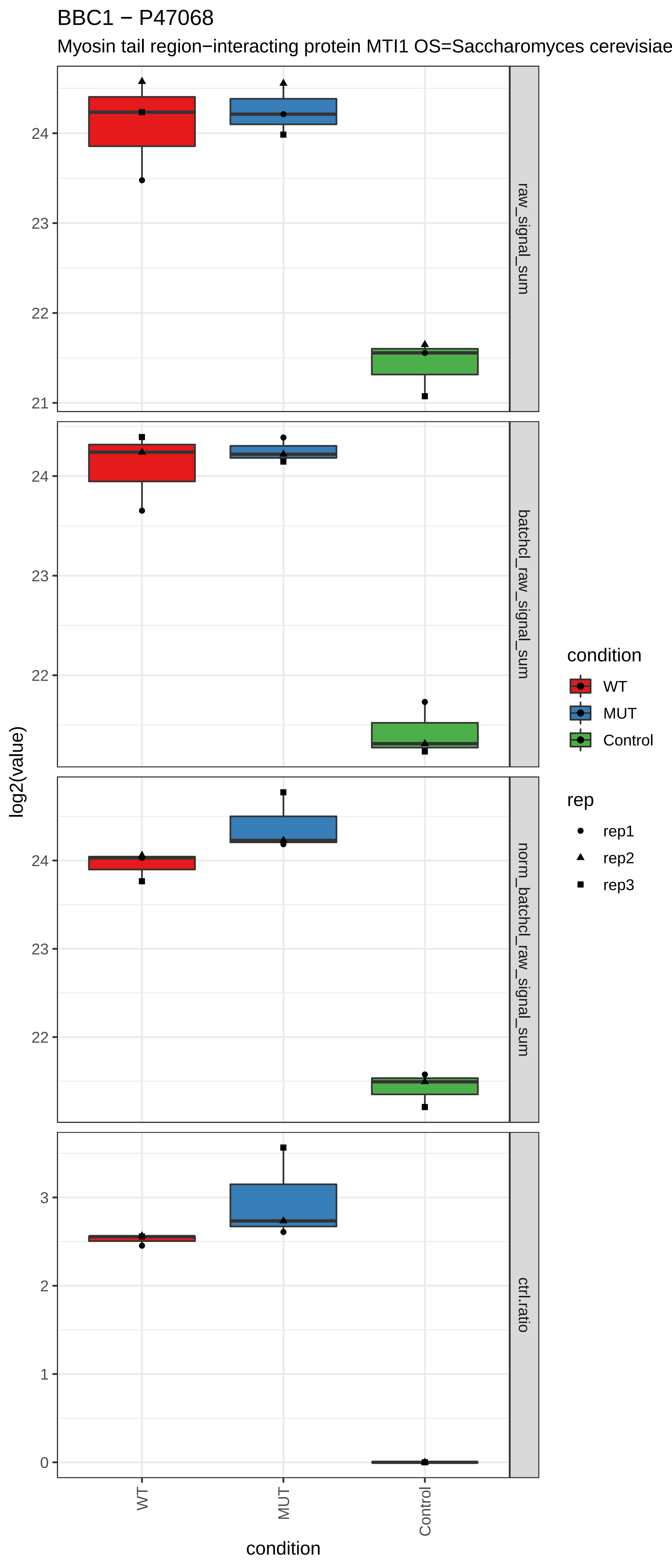

BBC1 − P47068
Myosin tail region−interacting protein MTI1 OS=Saccharomyces cerevisiae
raw_signal_sum
batchcl_raw_signal_sum
norm_batchcl_raw_signal_sum
ctrl.ratio
log2(value)
condition
WT
MUT
Control
rep
rep1
rep2
rep3
24
23
22
21
3
2
1
0

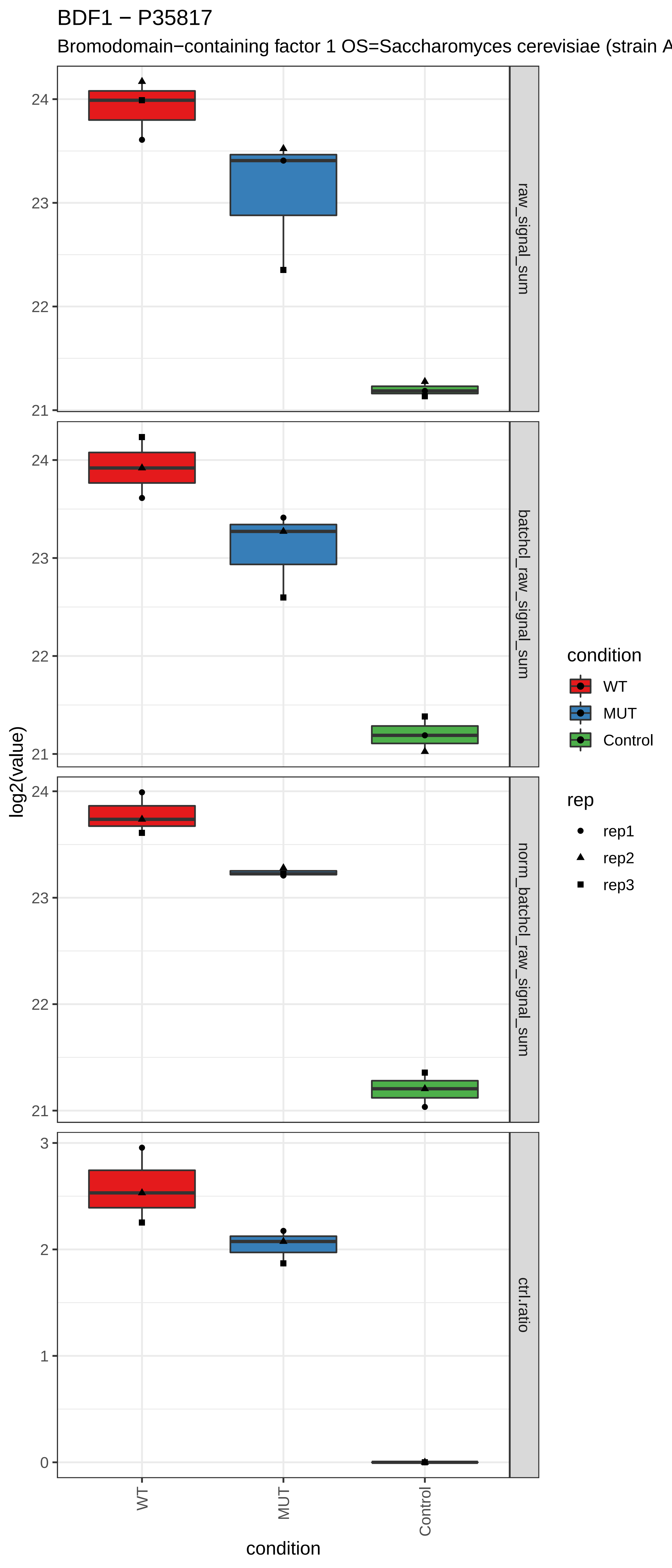
BDF1 − P35817
Bromodomain−containing factor 1 OS=Saccharomyces cerevisiae (strain A
raw_signal_sum
batchcl_raw_signal_sum
norm_batchcl_raw_signal_sum
ctrl.ratio
log2(value)
condition
WT
MUT
Control
rep
rep1
rep2
rep3

BDH1 − P39714

(R,R)−butanediol dehydrogenase OS=Saccharomyces cerevisiae (strain

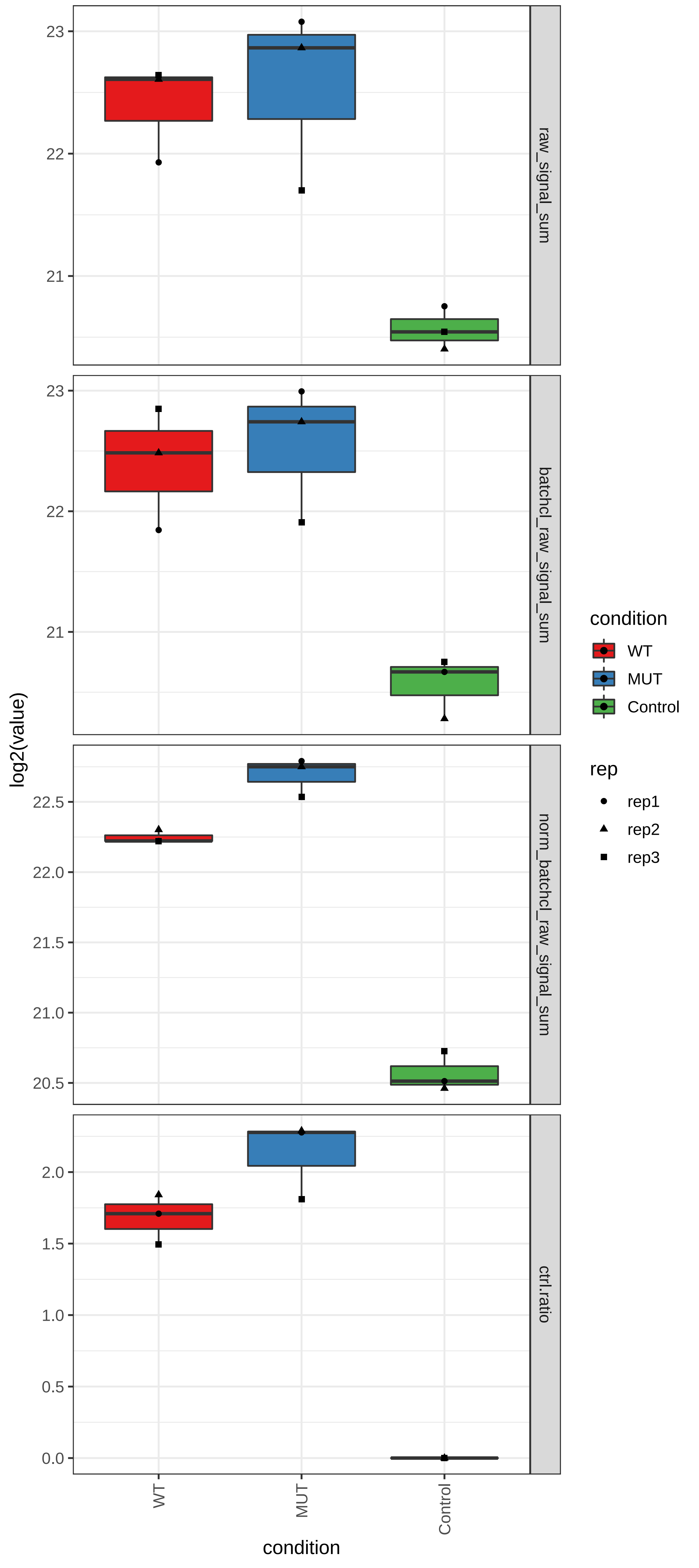

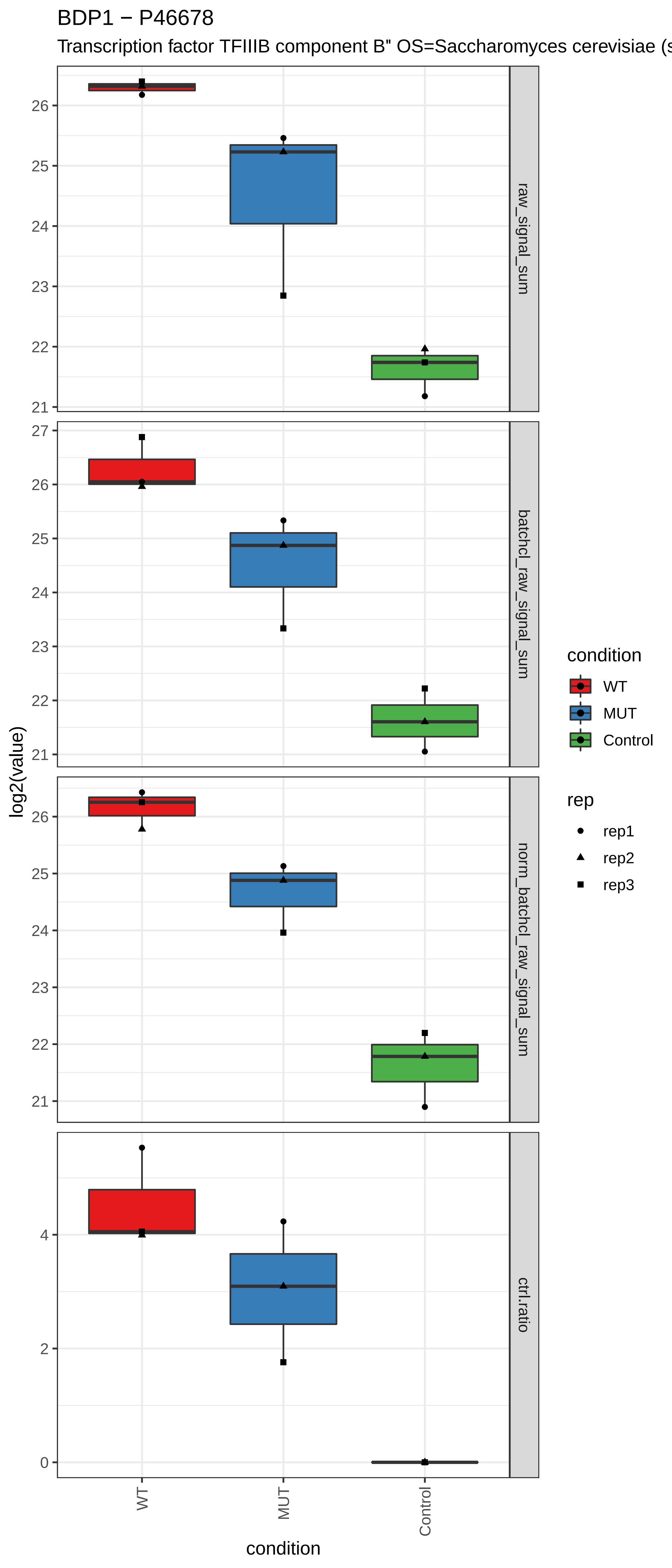
BDP1 − P46678
Transcription factor TFIIIB component B" OS=Saccharomyces cerevisiae (s
raw_signal_sum
batchcl_raw_signal_sum
norm_batchcl_raw_signal_sum
ctrl.ratio
log2(value)
condition
WT
MUT
Control
rep
rep1
rep2
rep3
26
25
24
23
22
21
27
4
2
0

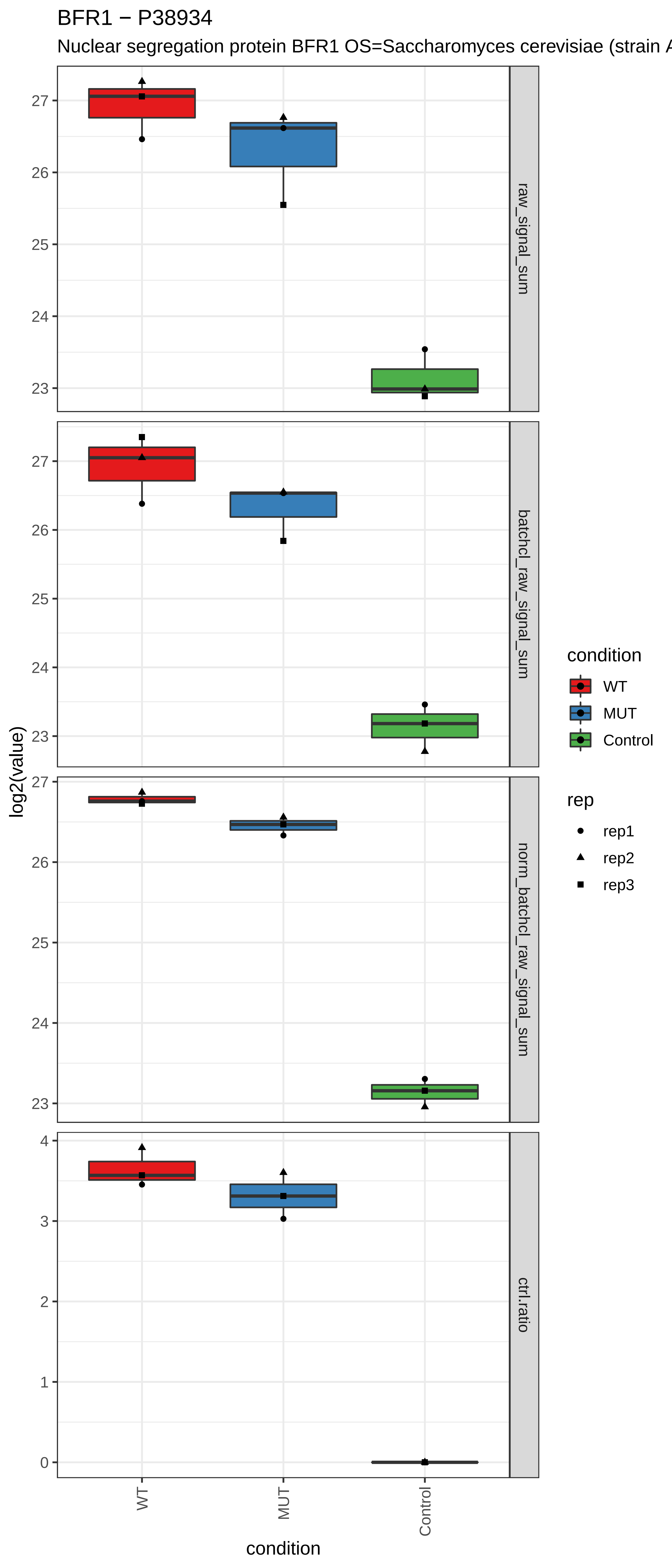
BFR1 − P38934
Nuclear segregation protein BFR1 OS=Saccharomyces cerevisiae (strain A
raw_signal_sum
batchcl_raw_signal_sum
norm_batchcl_raw_signal_sum
ctrl.ratio
log2(value)
condition
WT
MUT
Control
rep
rep1
rep2
rep3
27
26
25
24
23
4
3
2
1
0

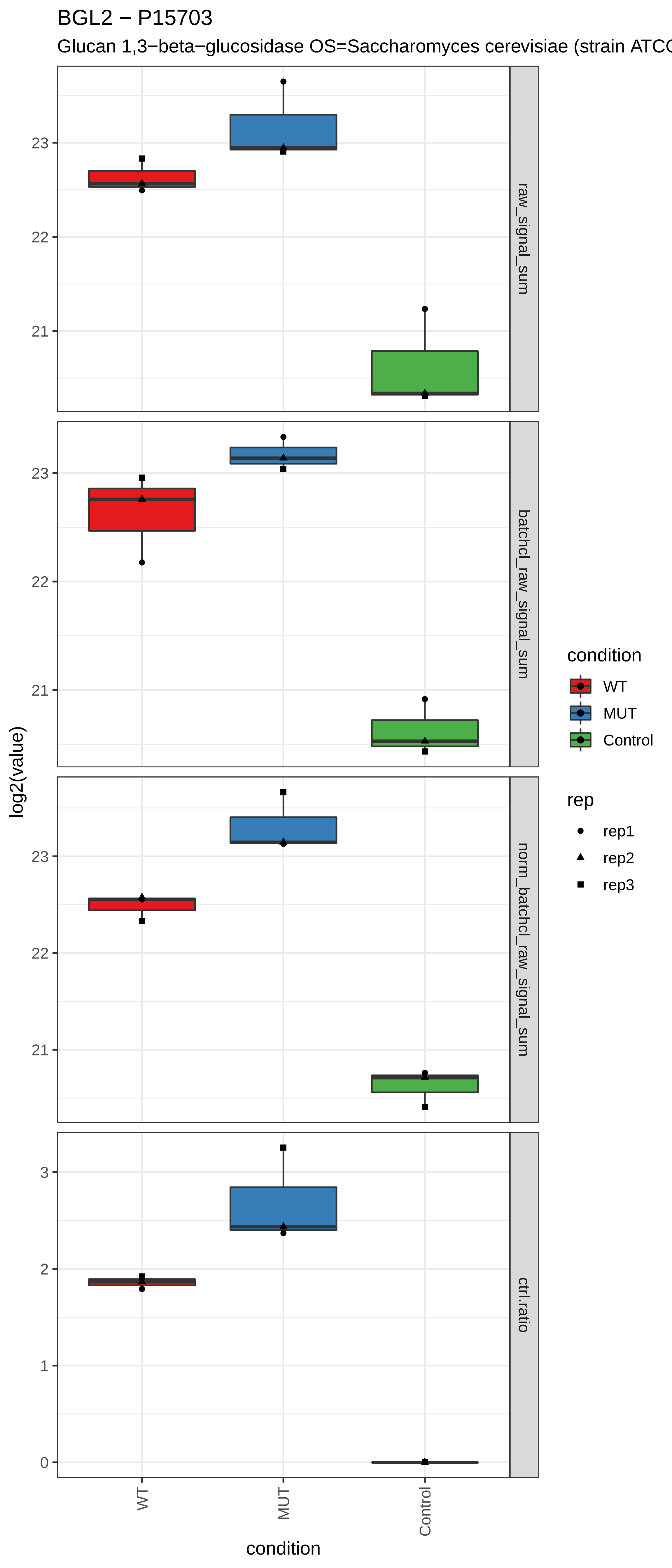
BGL2 − P15703
Glucan 1,3−beta−glucosidase OS=Saccharomyces cerevisiae (strain ATCC
log2(value)
raw_signal_sum
batchcl_raw_signal_sum
norm_batchcl_raw_signal_sum
ctrl.ratio
23
22
21
3
2
1
0
WT
MUT
Control
condition
condition
WT
MUT
Control
rep
rep1
rep2
rep3

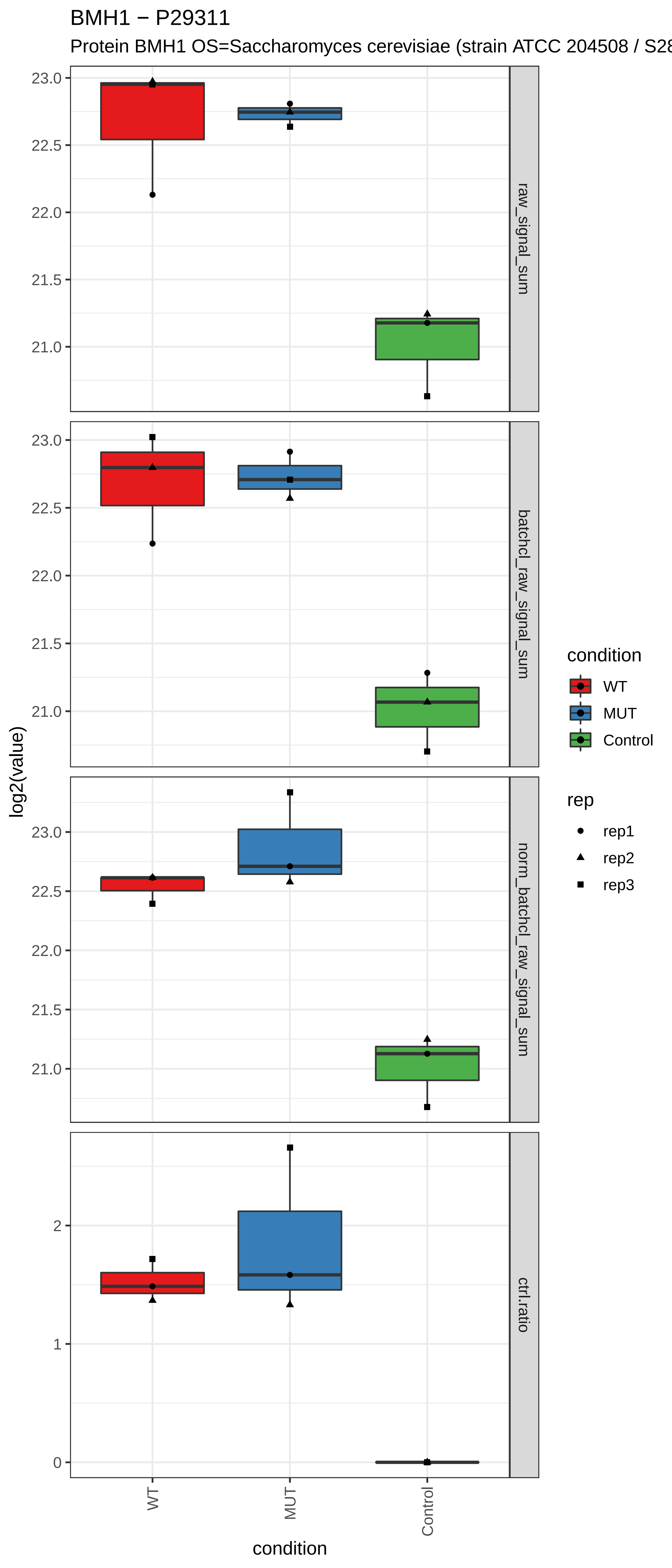

BMH1 − P29311
Protein BMH1 OS=Saccharomyces cerevisiae (strain ATCC 204508 / S28
raw_signal_sum
batchcl_raw_signal_sum
norm_batchcl_raw_signal_sum
ctrl.ratio
23.0
22.5
22.0
21.5
21.0
2
1
0
log2(value)
WT
MUT
Control
condition
condition
WT
MUT
Control
rep
rep1
rep2
rep3

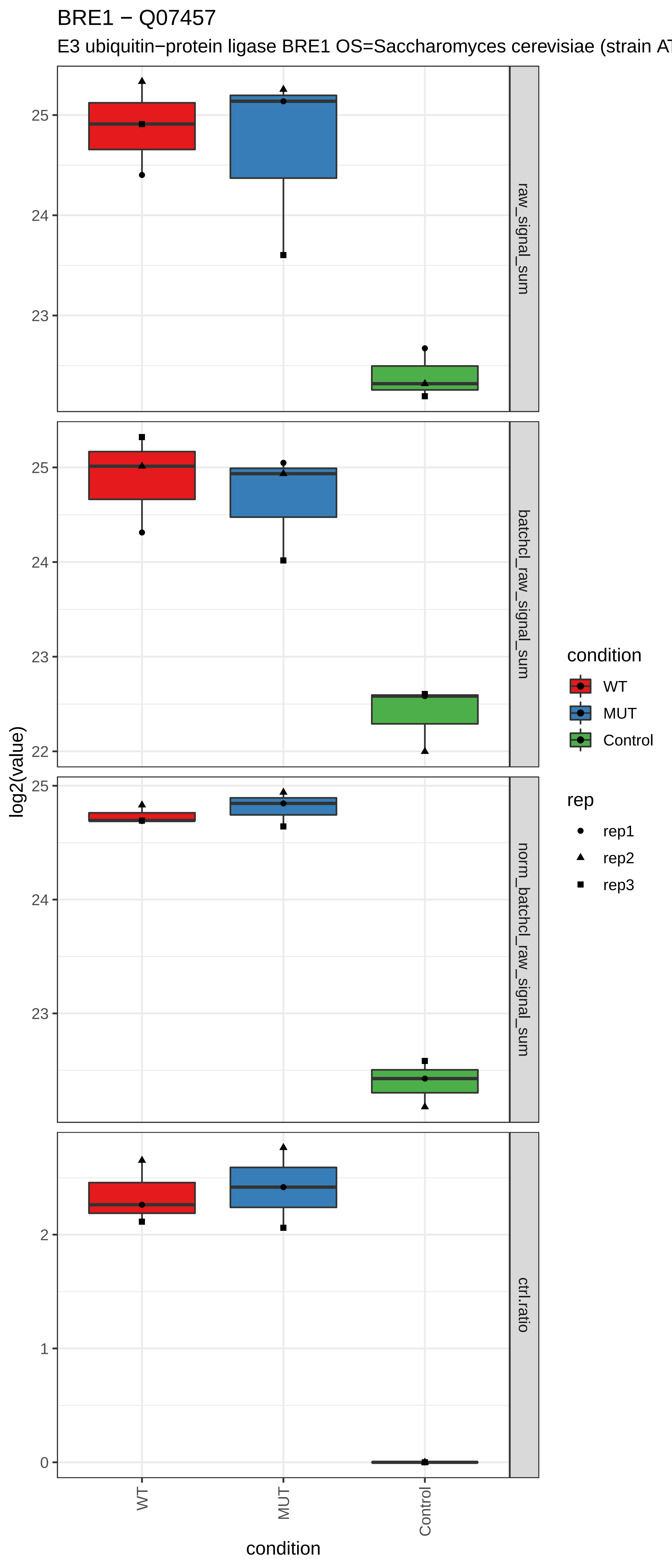
BRE1 − Q07457
E3 ubiquitin−protein ligase BRE1 OS=Saccharomyces cerevisiae (strain AT
raw_signal_sum
batchcl_raw_signal_sum
norm_batchcl_raw_signal_sum
ctrl.ratio
log2(value)
condition
WT
MUT
Control
rep
rep1
rep2
rep3
25
24
23
22
2
1
0

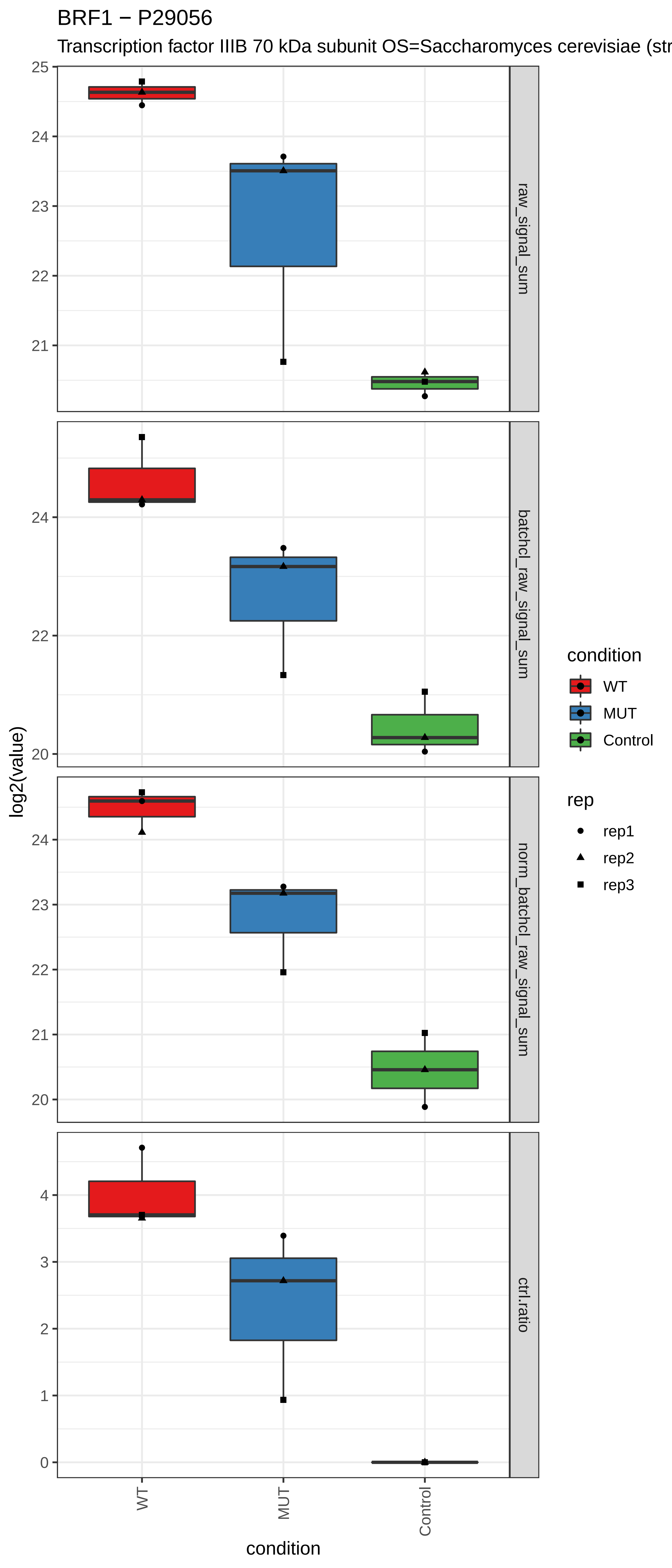
BRF1 − P29056
Transcription factor IIIB 70 kDa subunit OS=Saccharomyces cerevisiae (str
raw_signal_sum
batchcl_raw_signal_sum
norm_batchcl_raw_signal_sum
ctrl.ratio
log2(value)
condition
WT
MUT
Control
rep
rep1
rep2
rep3

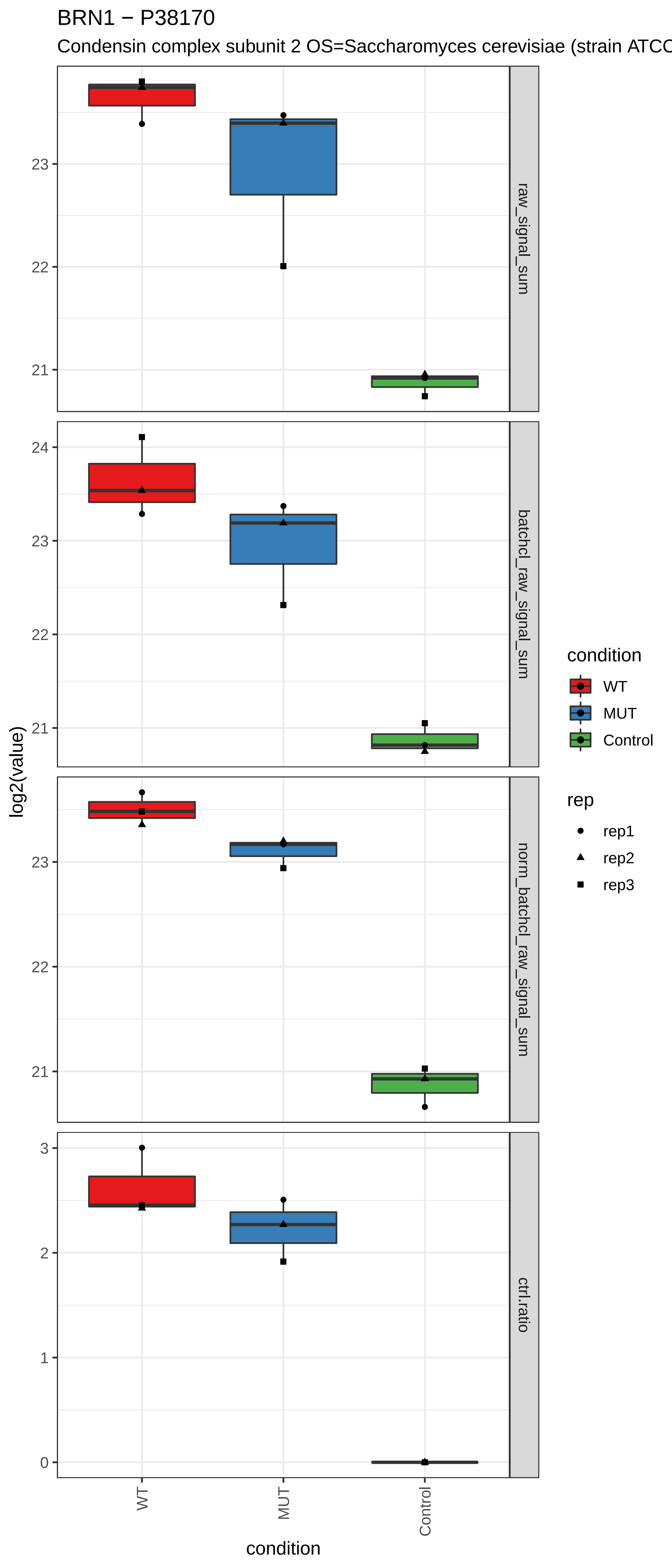
BRN1 − P38170
Condensin complex subunit 2 OS=Saccharomyces cerevisiae (strain ATCC
raw_signal_sum
batchcl_raw_signal_sum
norm_batchcl_raw_signal_sum
ctrl.ratio
log2(value)
condition
WT
MUT
Control
rep
rep1
rep2
rep3
23
22
21
24
3
2
1
0

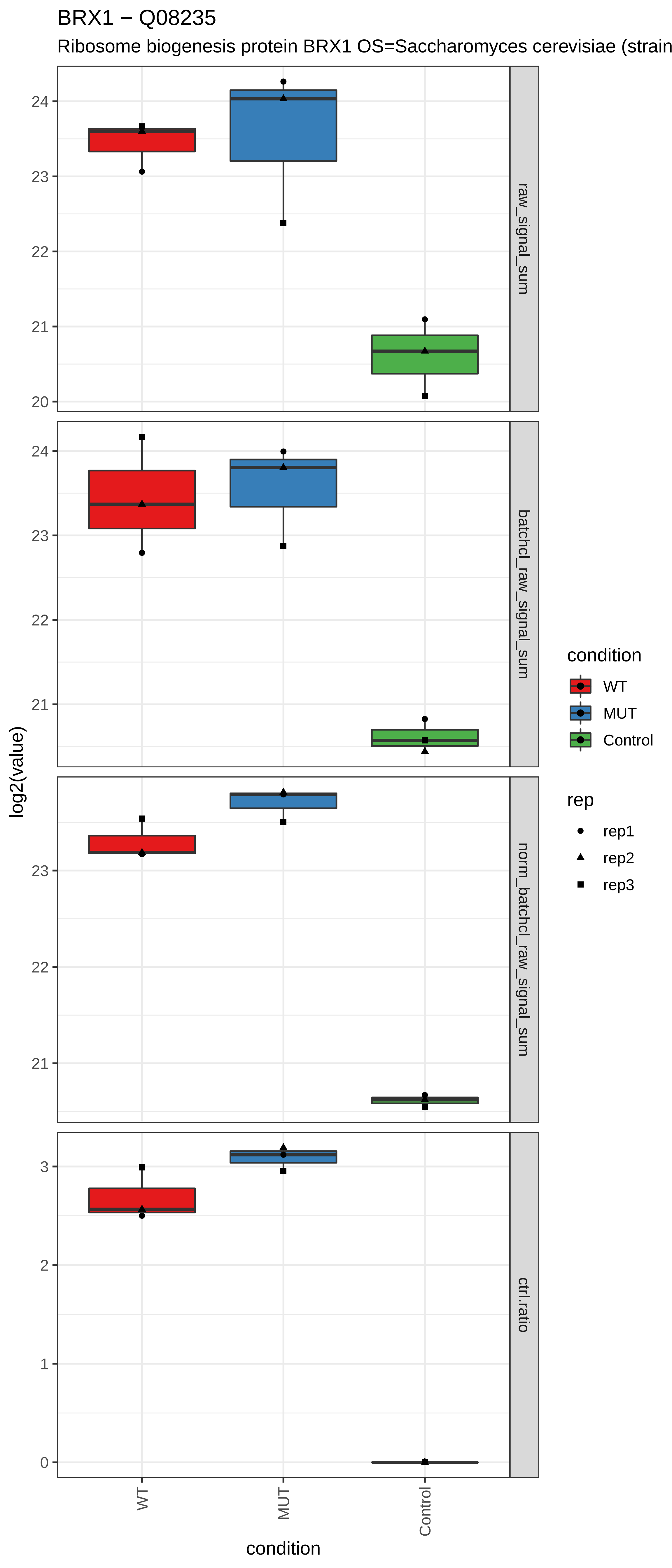
BRX1 − Q08235
Ribosome biogenesis protein BRX1 OS=Saccharomyces cerevisiae (strain
raw_signal_sum
batchcl_raw_signal_sum
norm_batchcl_raw_signal_sum
ctrl.ratio
log2(value)
condition
WT
MUT
Control
rep
rep1
rep2
rep3
24
23
22
21
20
3
2
1
0

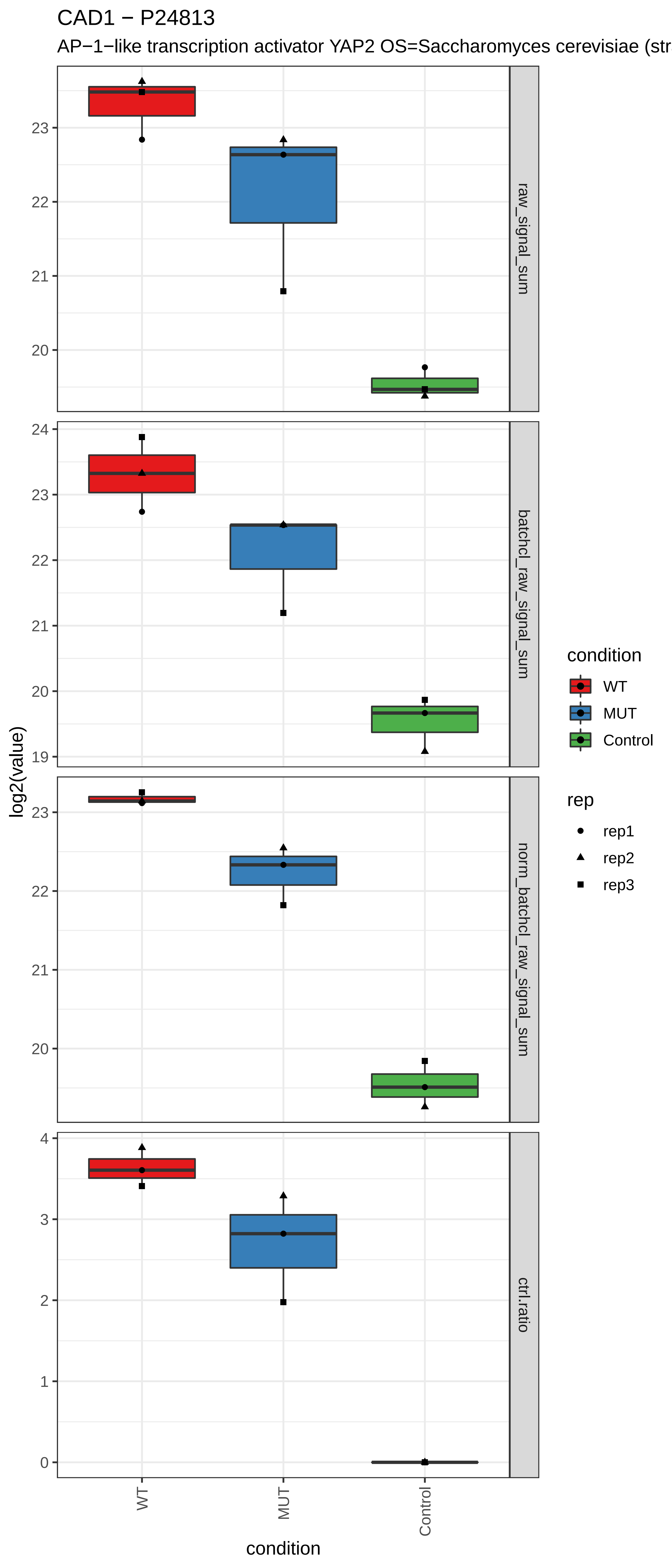

CAD1 − P24813
AP−1−like transcription activator YAP2 OS=Saccharomyces cerevisiae (str
raw_signal_sum
batchcl_raw_signal_sum
norm_batchcl_raw_signal_sum
ctrl.ratio
log2(value)
condition
WT
MUT
Control
rep
rep1
rep2
rep3

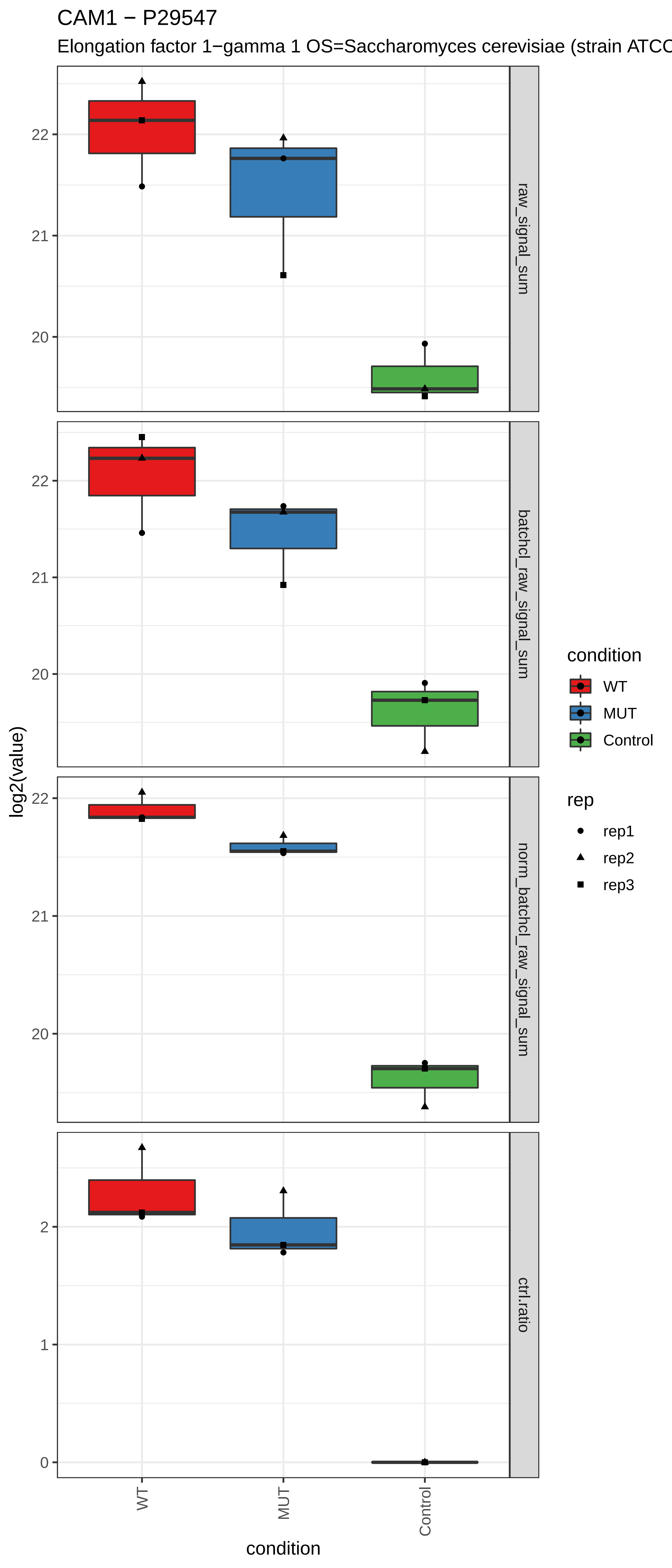

CAM1 − P29547
Elongation factor 1−gamma 1 OS=Saccharomyces cerevisiae (strain ATCC
raw_signal_sum
batchcl_raw_signal_sum
norm_batchcl_raw_signal_sum
ctrl.ratio
log2(value)
condition
WT
MUT
Control
rep
rep1
rep2
rep3
22
21
20
2
1
0

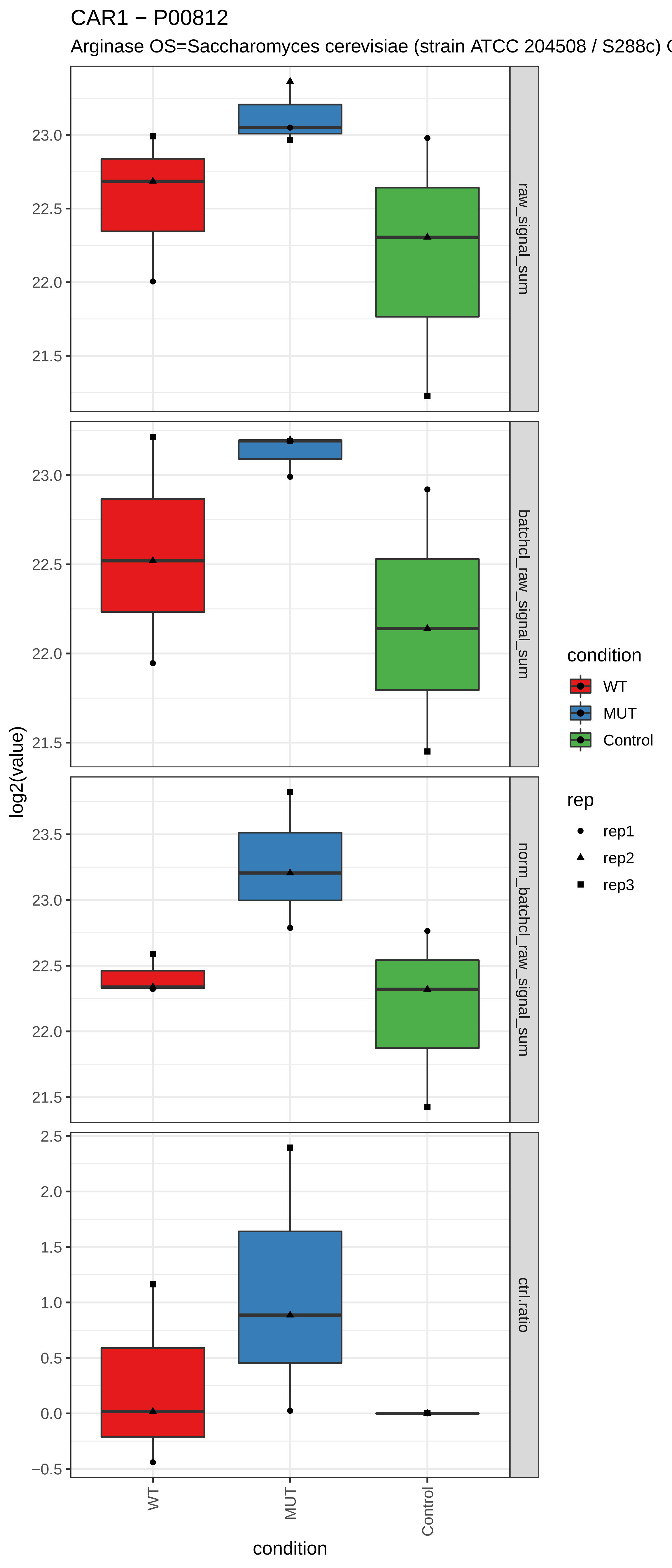

CAR1 − P00812
Arginase OS=Saccharomyces cerevisiae (strain ATCC 204508 / S288c) G
raw_signal_sum
batchcl_raw_signal_sum
norm_batchcl_raw_signal_sum
ctrl.ratio
log2(value)
condition
WT
MUT
Control
rep
rep1
rep2
rep3

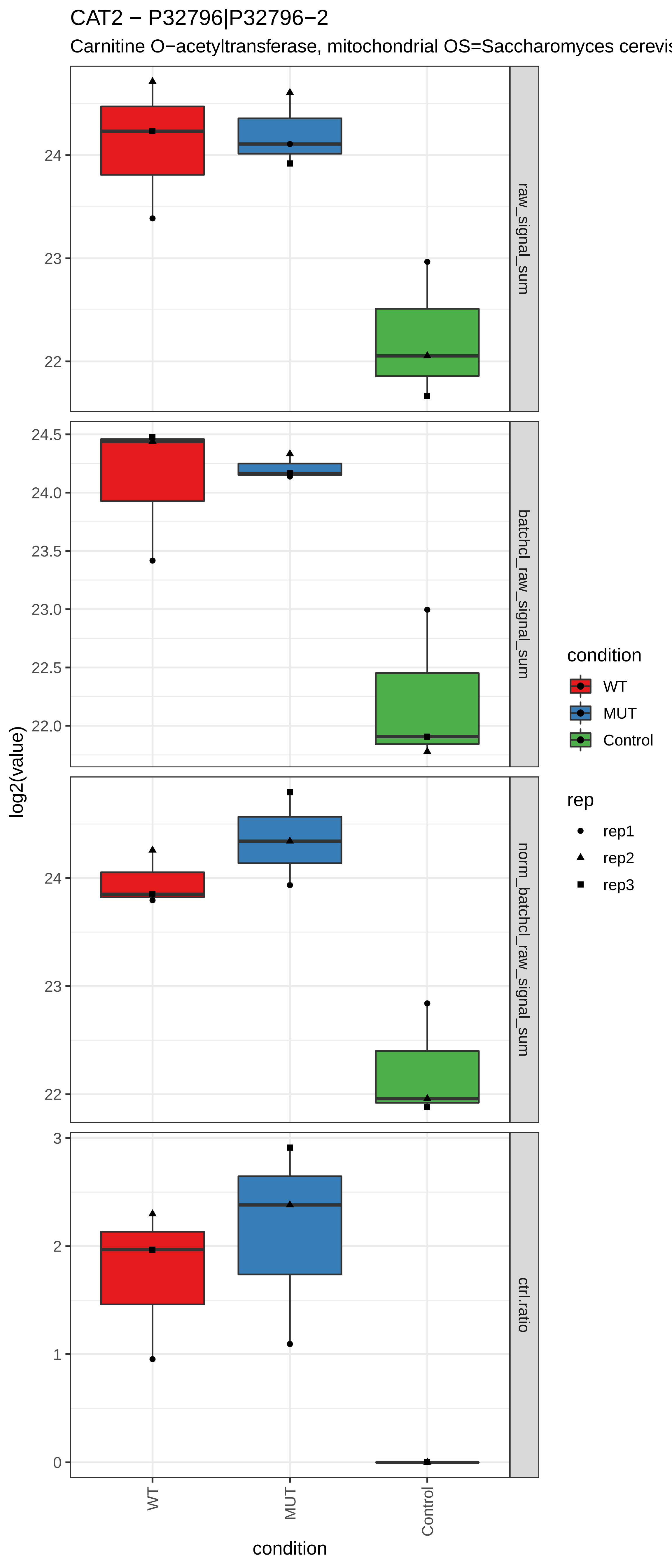

CAT2 − P32796|P32796−2
Carnitine O−acetyltransferase, mitochondrial OS=Saccharomyces cerevis
raw_signal_sum
batchcl_raw_signal_sum
norm_batchcl_raw_signal_sum
ctrl.ratio
log2(value)
condition
WT
MUT
Control
rep
rep1
rep2
rep3
24
23
22
24.5
24.0
23.5
23.0
22.5
22.0
3
2
1
0

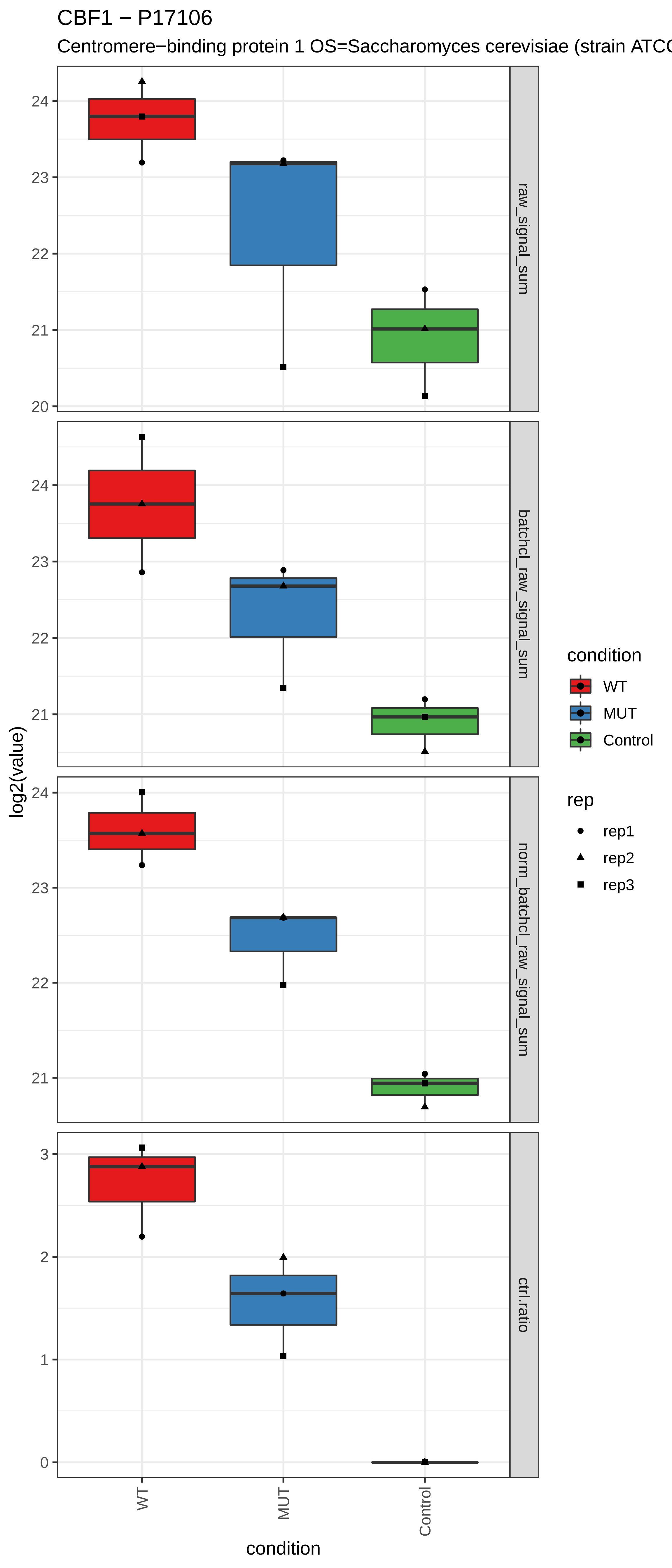

CBF1 − P17106
Centromere−binding protein 1 OS=Saccharomyces cerevisiae (strain ATCC
raw_signal_sum
batchcl_raw_signal_sum
norm_batchcl_raw_signal_sum
ctrl.ratio
log2(value)
condition
WT
MUT
Control
rep
rep1
rep2
rep3

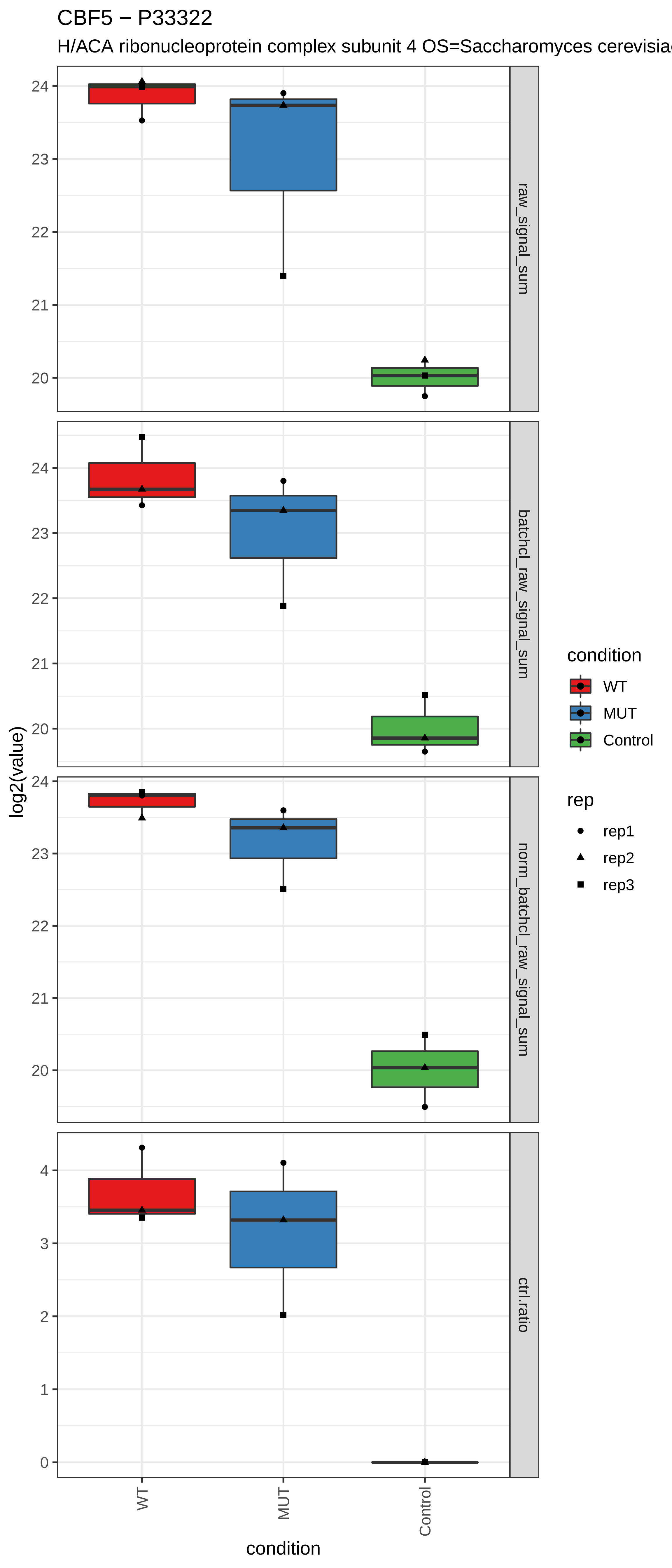
CBF5 − P33322
H/ACA ribonucleoprotein complex subunit 4 OS=Saccharomyces cerevisia
raw_signal_sum
batchcl_raw_signal_sum
norm_batchcl_raw_signal_sum
ctrl.ratio
log2(value)
condition
WT
MUT
Control
rep
rep1
rep2
rep3

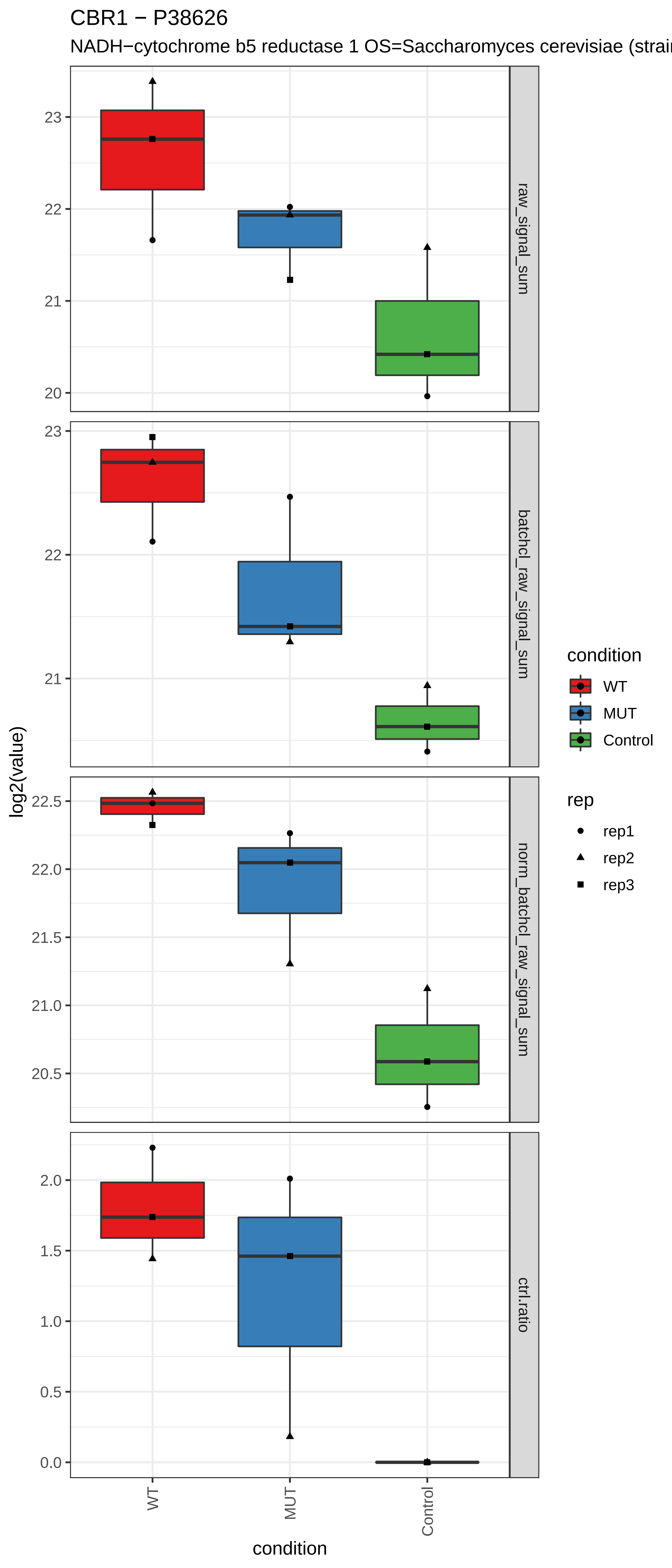

CBR1 − P38626
NADH−cytochrome b5 reductase 1 OS=Saccharomyces cerevisiae (strai
raw_signal_sum
batchcl_raw_signal_sum
norm_batchcl_raw_signal_sum
ctrl.ratio
log2(value)
condition
WT
MUT
Control
rep
rep1
rep2
rep3

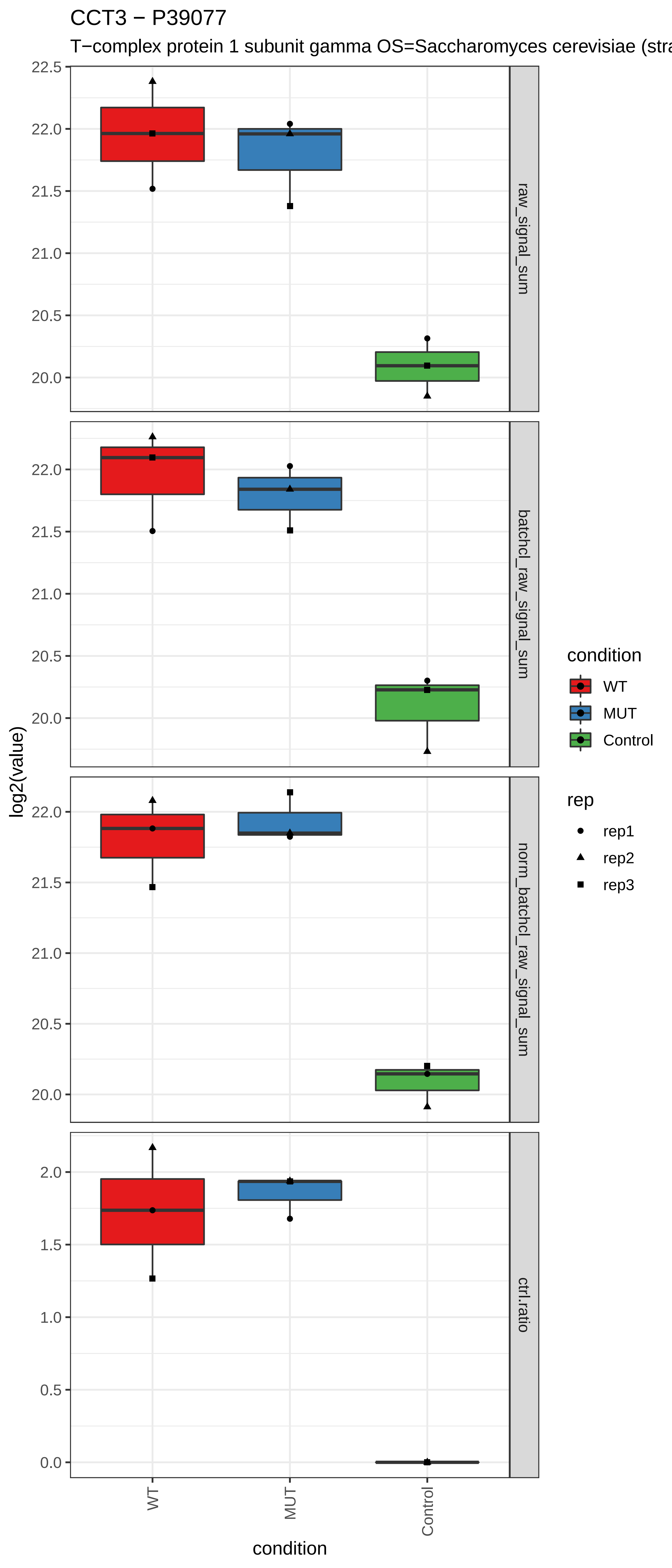

CCT3 − P39077
T−complex protein 1 subunit gamma OS=Saccharomyces cerevisiae (stra
raw_signal_sum
batchcl_raw_signal_sum
norm_batchcl_raw_signal_sum
ctrl.ratio
log2(value)
condition
WT
MUT
Control
rep
rep1
rep2
rep3

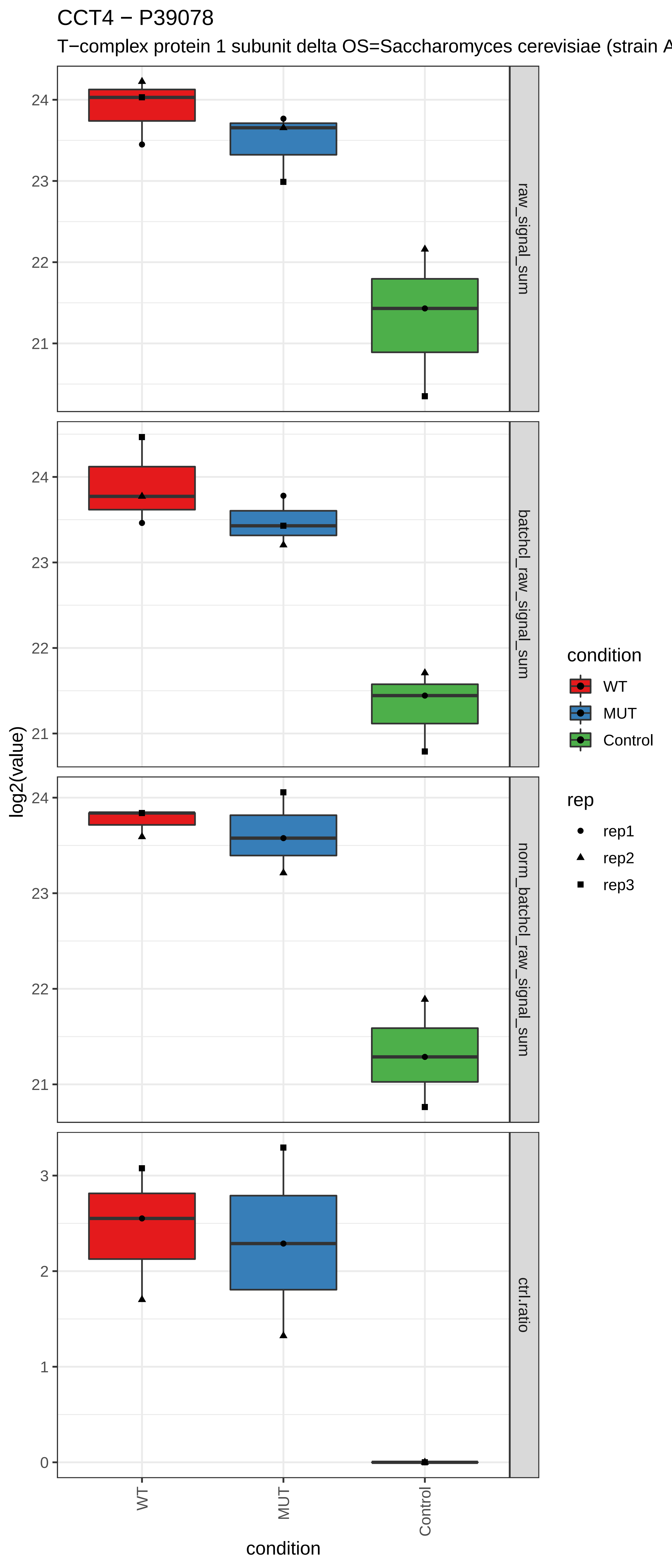
CCT4 − P39078
T−complex protein 1 subunit delta OS=Saccharomyces cerevisiae (strain A
raw_signal_sum
batchcl_raw_signal_sum
norm_batchcl_raw_signal_sum
ctrl.ratio
log2(value)
condition
WT
MUT
Control
rep
rep1
rep2
rep3

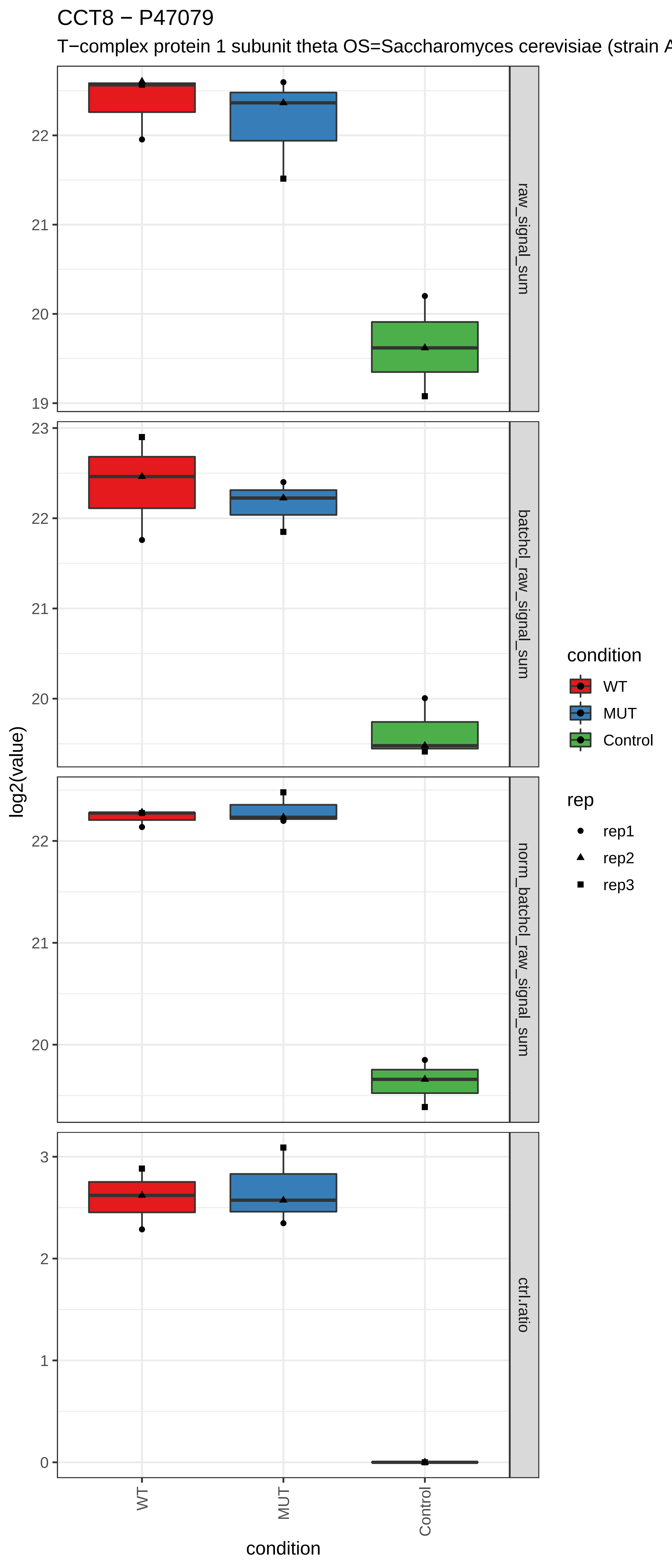

CCT8 − P47079
T−complex protein 1 subunit theta OS=Saccharomyces cerevisiae (strain A
raw_signal_sum
batchcl_raw_signal_sum
norm_batchcl_raw_signal_sum
ctrl.ratio
log2(value)
condition
WT
MUT
Control
rep
rep1
rep2
rep3

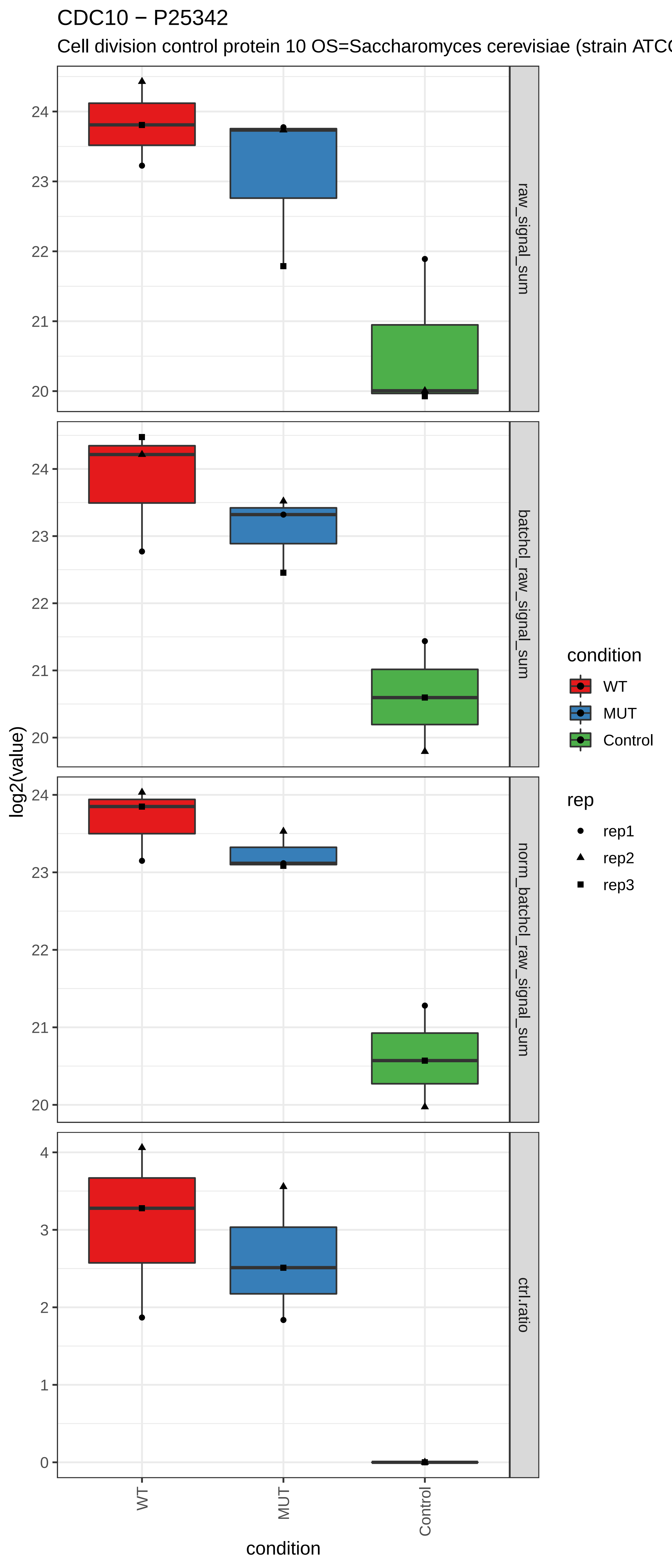
CDC10 − P25342
Cell division control protein 10 OS=Saccharomyces cerevisiae (strain ATCC
raw_signal_sum
batchcl_raw_signal_sum
norm_batchcl_raw_signal_sum
ctrl.ratio
log2(value)
condition
WT
MUT
Control
rep
rep1
rep2
rep3

CDC12 − P32468

Cell division control protein 12 OS=Saccharomyces cerevisiae (strain ATCC

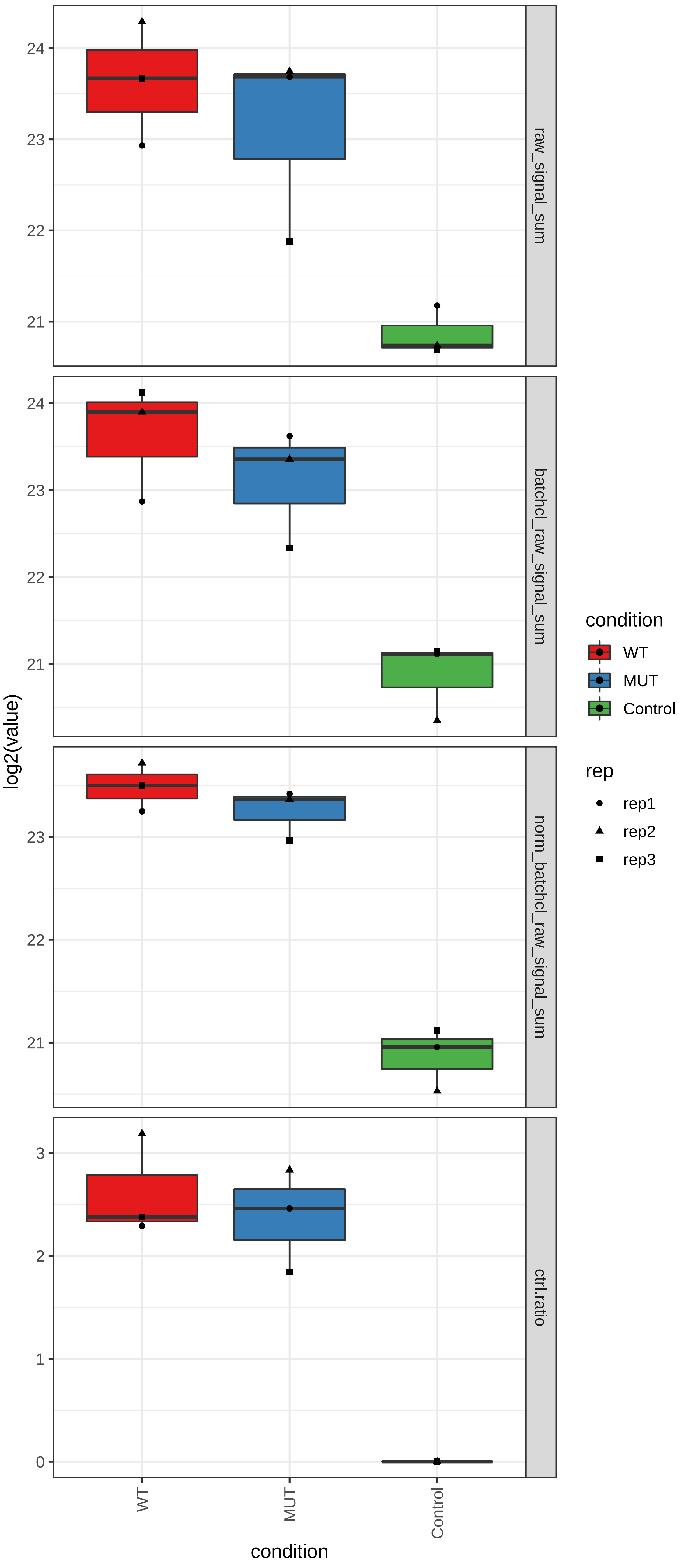

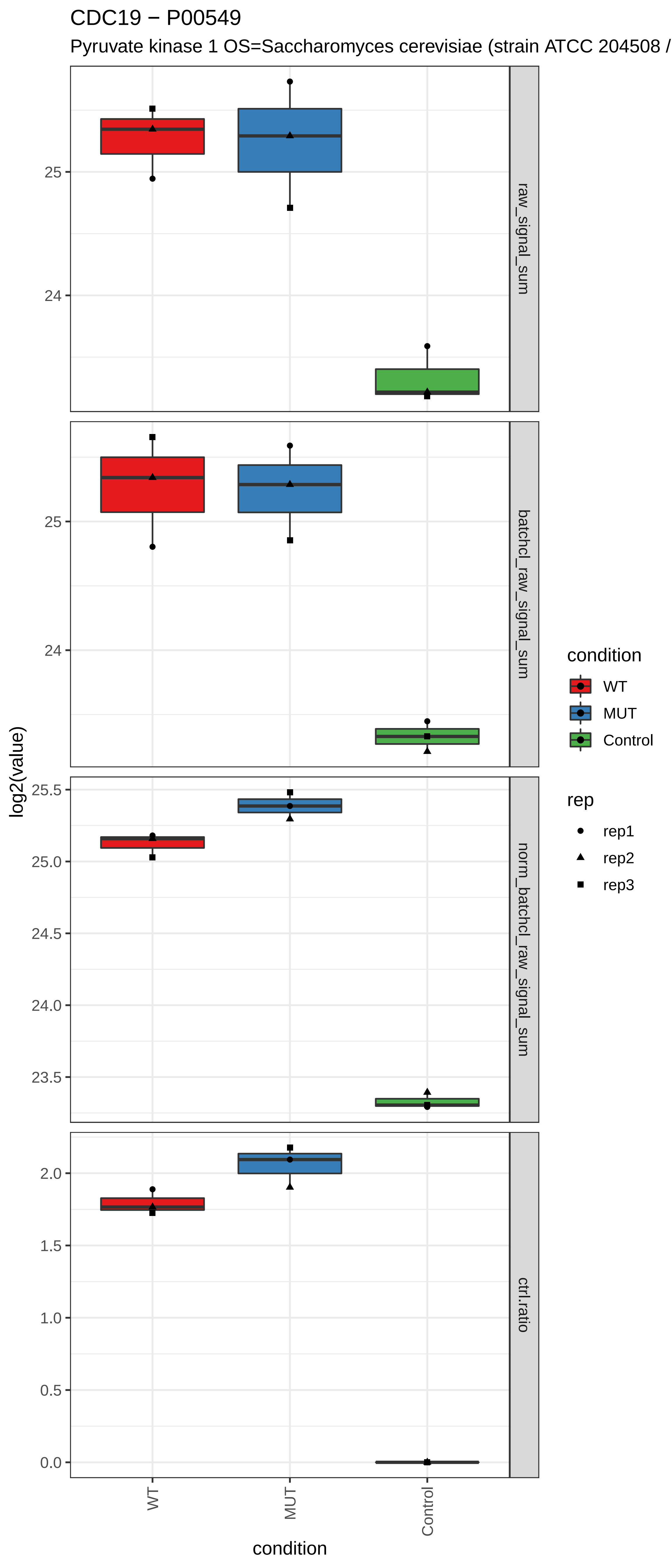

CDC19 − P00549
Pyruvate kinase 1 OS=Saccharomyces cerevisiae (strain ATCC 204508 /
raw_signal_sum
batchcl_raw_signal_sum
norm_batchcl_raw_signal_sum
ctrl.ratio
log2(value)
condition
WT
MUT
Control
rep
rep1
rep2
rep3
25
24
25.5
25.0
24.5
24.0
23.5
2.0
1.5
1.0
0.5
0.0

CDC24 − P11433

Cell division control protein 24 OS=Saccharomyces cerevisiae (strain ATCC

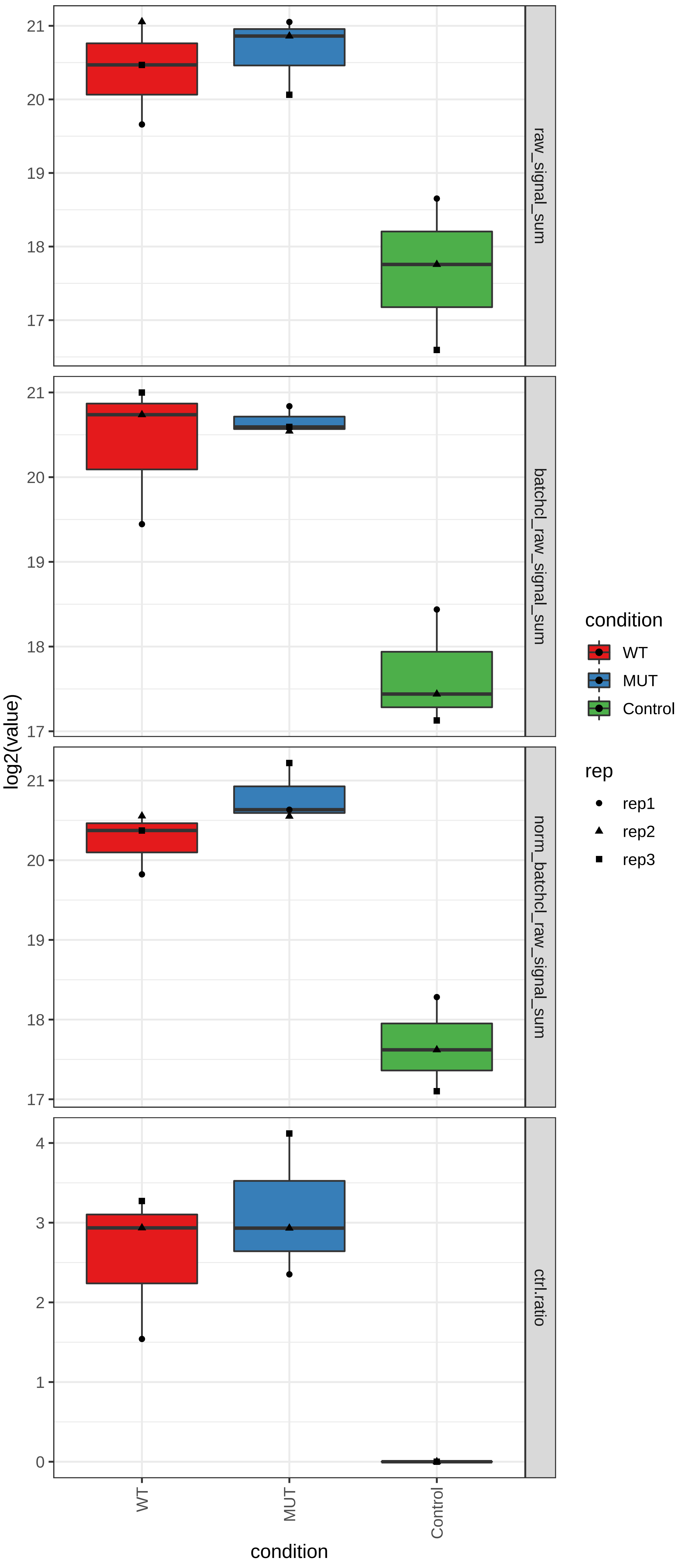

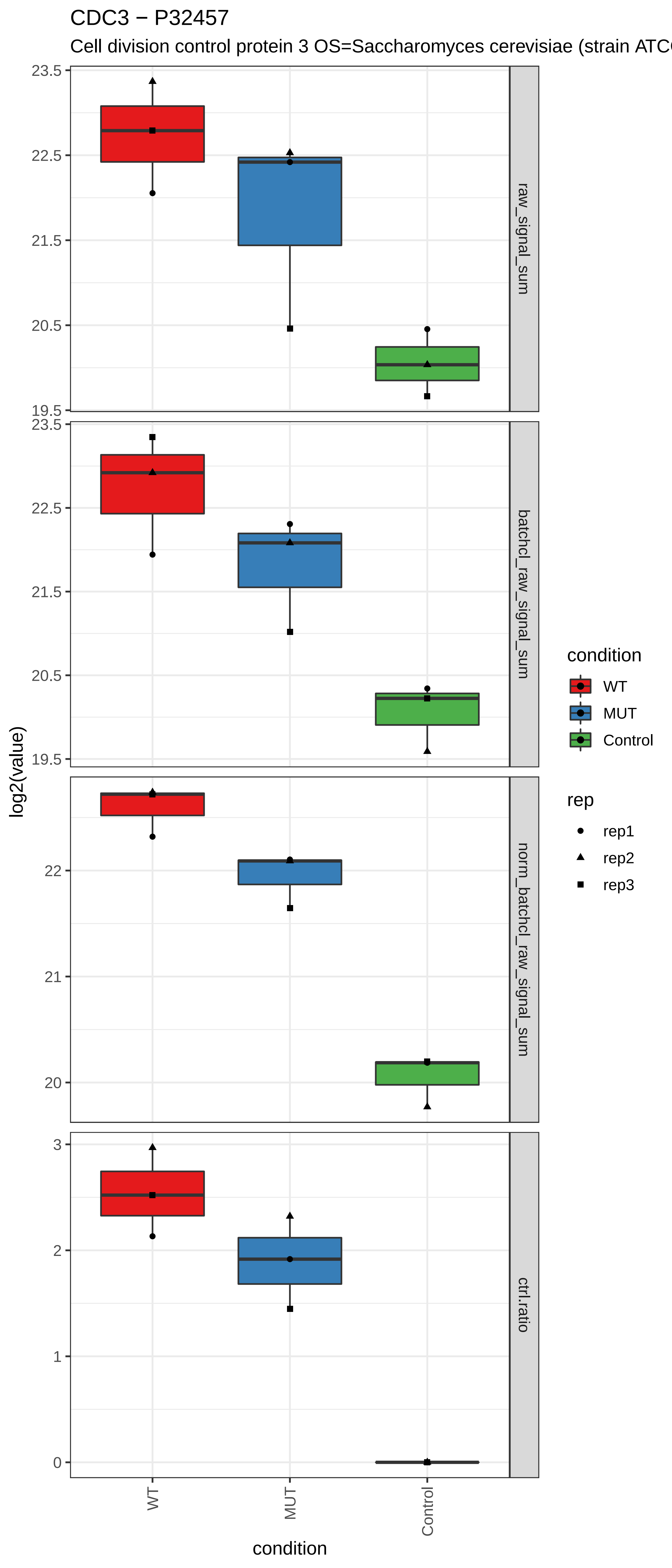
CDC3 − P32457
Cell division control protein 3 OS=Saccharomyces cerevisiae (strain ATC
raw_signal_sum
batchcl_raw_signal_sum
norm_batchcl_raw_signal_sum
ctrl.ratio
log2(value)
condition
WT
MUT
Control
rep
rep1
rep2
rep3
23.5
22.5
21.5
20.5
19.5
22
21
20
3
2
1
0

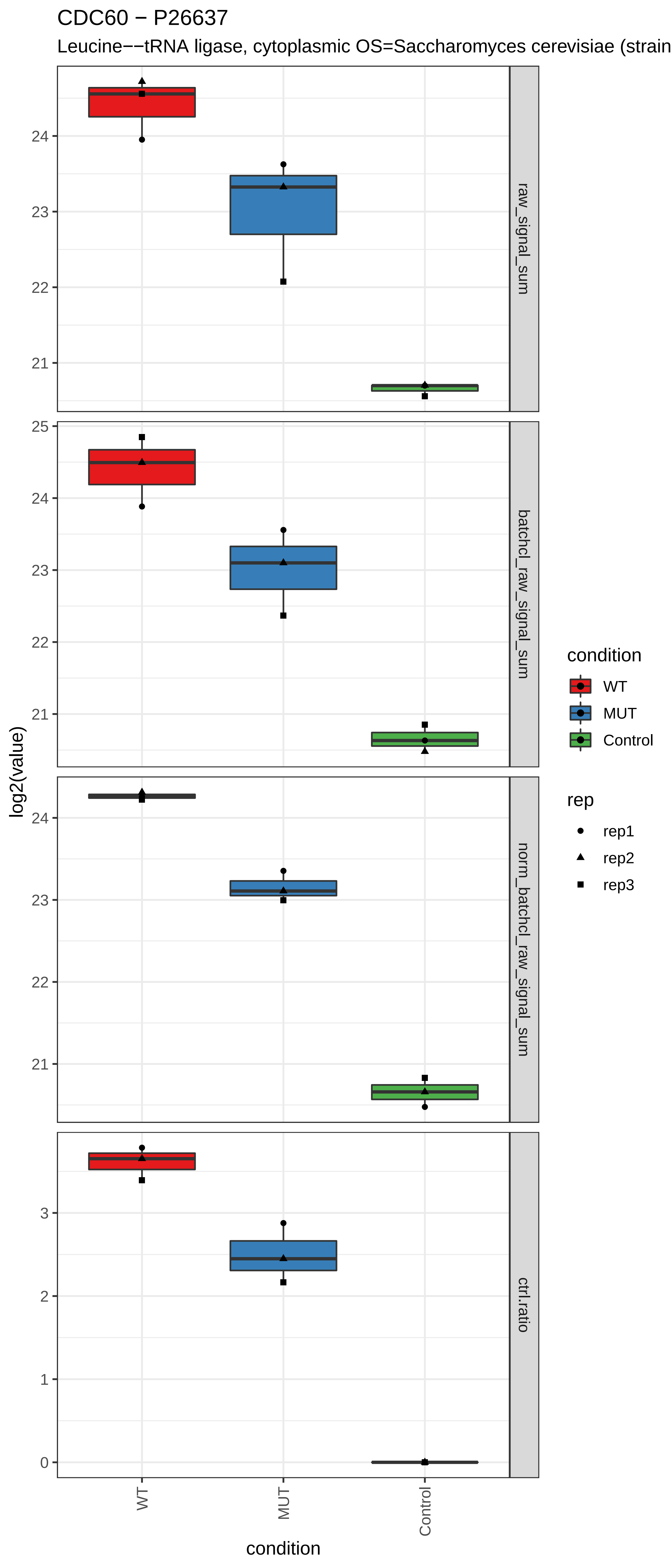
CDC60 − P26637
Leucine−−tRNA ligase, cytoplasmic OS=Saccharomyces cerevisiae (strain
raw_signal_sum
batchcl_raw_signal_sum
norm_batchcl_raw_signal_sum
ctrl.ratio
log2(value)
condition
WT
MUT
Control
rep
rep1
rep2
rep3
24
23
22
21
25
3
2
1
0

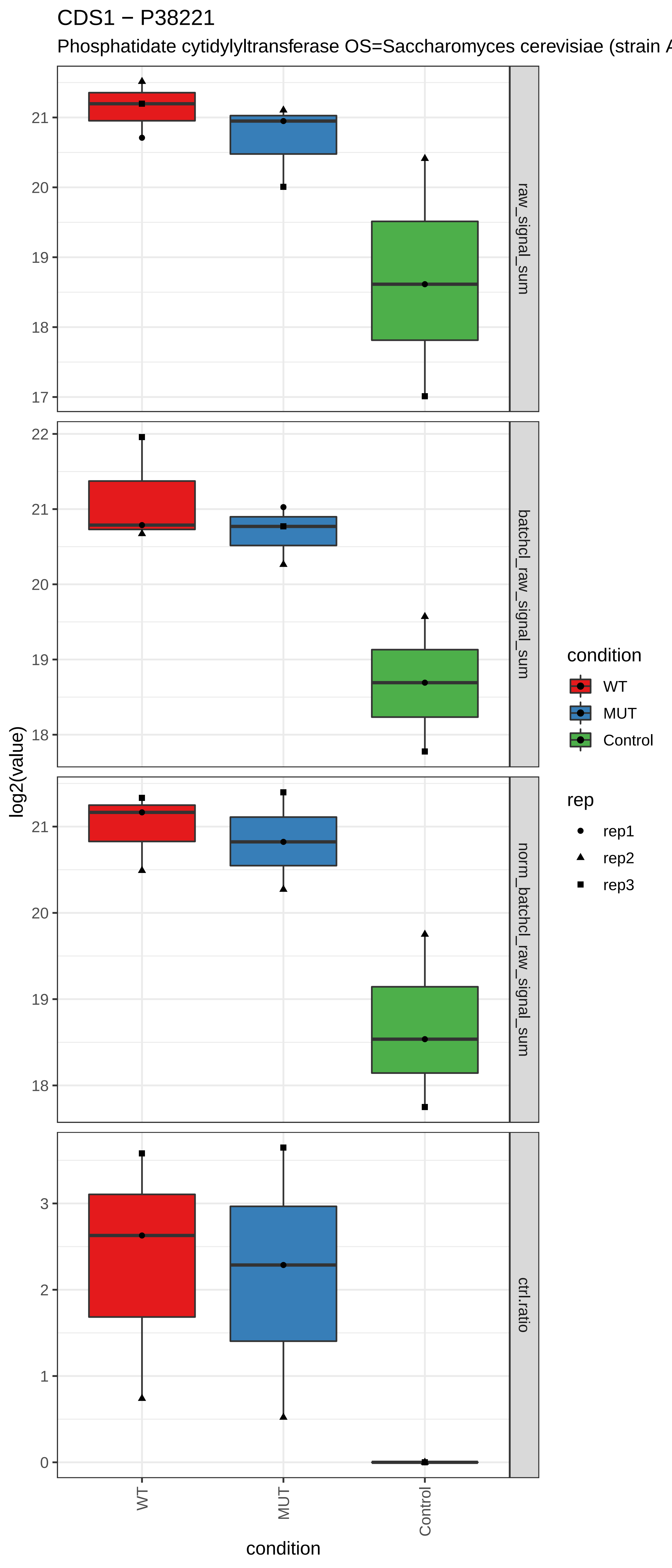

CDS1 − P38221
Phosphatidate cytidylyltransferase OS=Saccharomyces cerevisiae (strain A
raw_signal_sum
batchcl_raw_signal_sum
norm_batchcl_raw_signal_sum
ctrl.ratio
log2(value)
condition
WT
MUT
Control
rep
rep1
rep2
rep3

CFT2 − Q12102

Cleavage factor two protein 2 OS=Saccharomyces cerevisiae (strain ATC

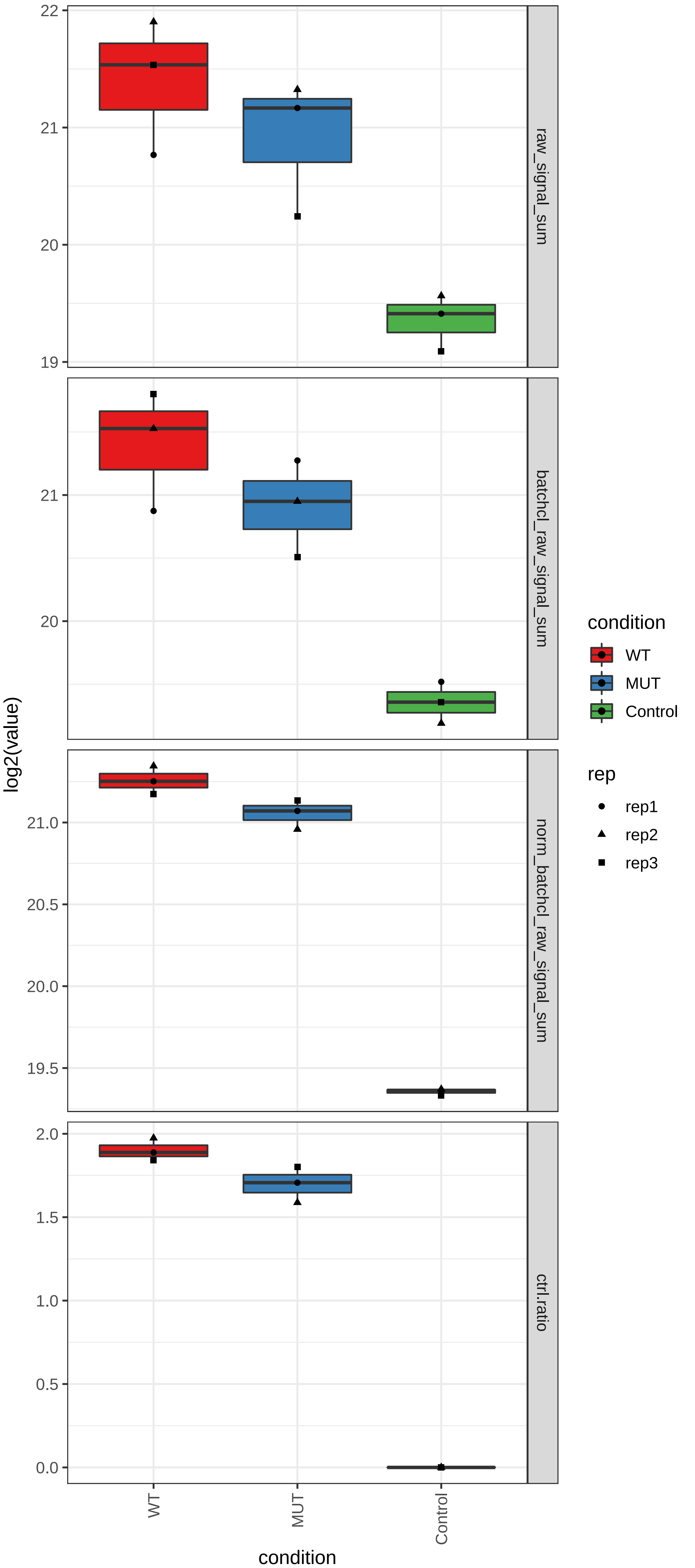

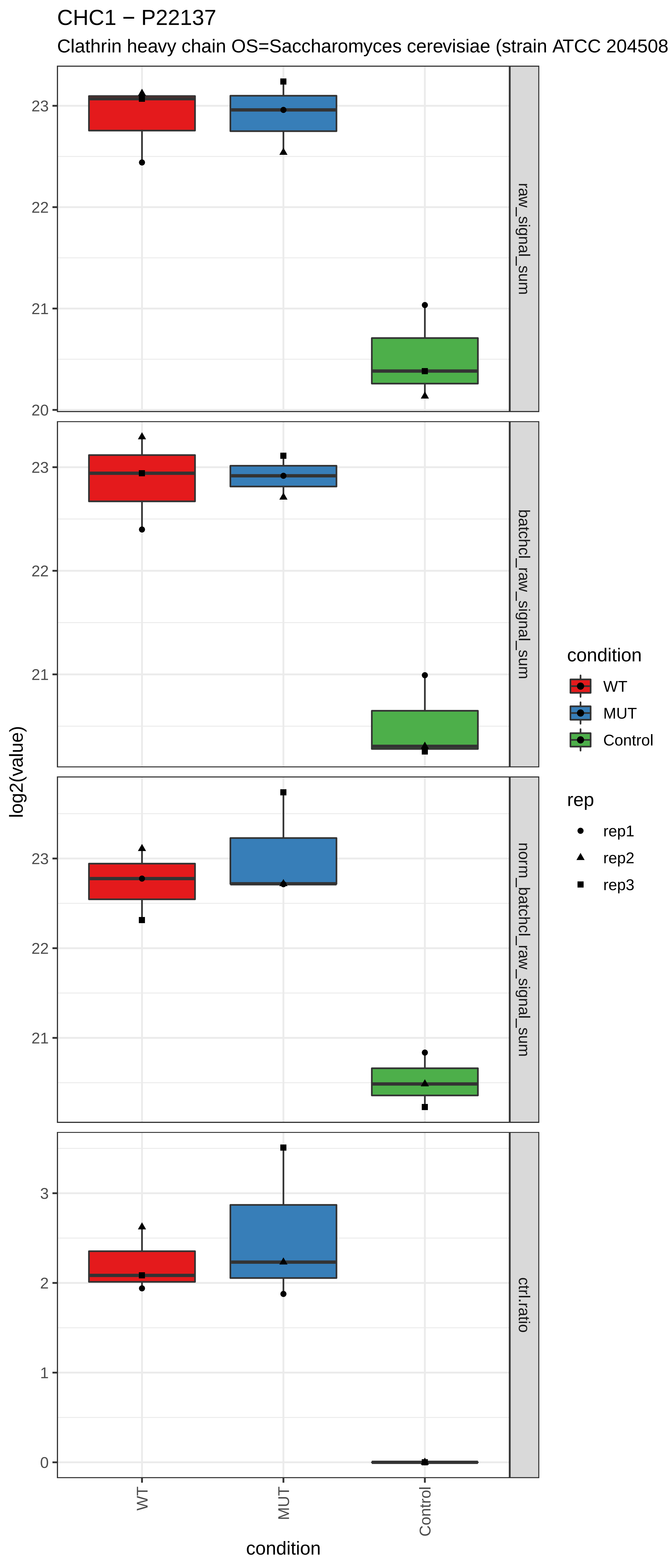
CHC1 − P22137
Clathrin heavy chain OS=Saccharomyces cerevisiae (strain ATCC 204508
raw_signal_sum
batchcl_raw_signal_sum
norm_batchcl_raw_signal_sum
ctrl.ratio
log2(value)
condition
WT
MUT
Control
rep
rep1
rep2
rep3
20
21
22
23
0
1
2
3

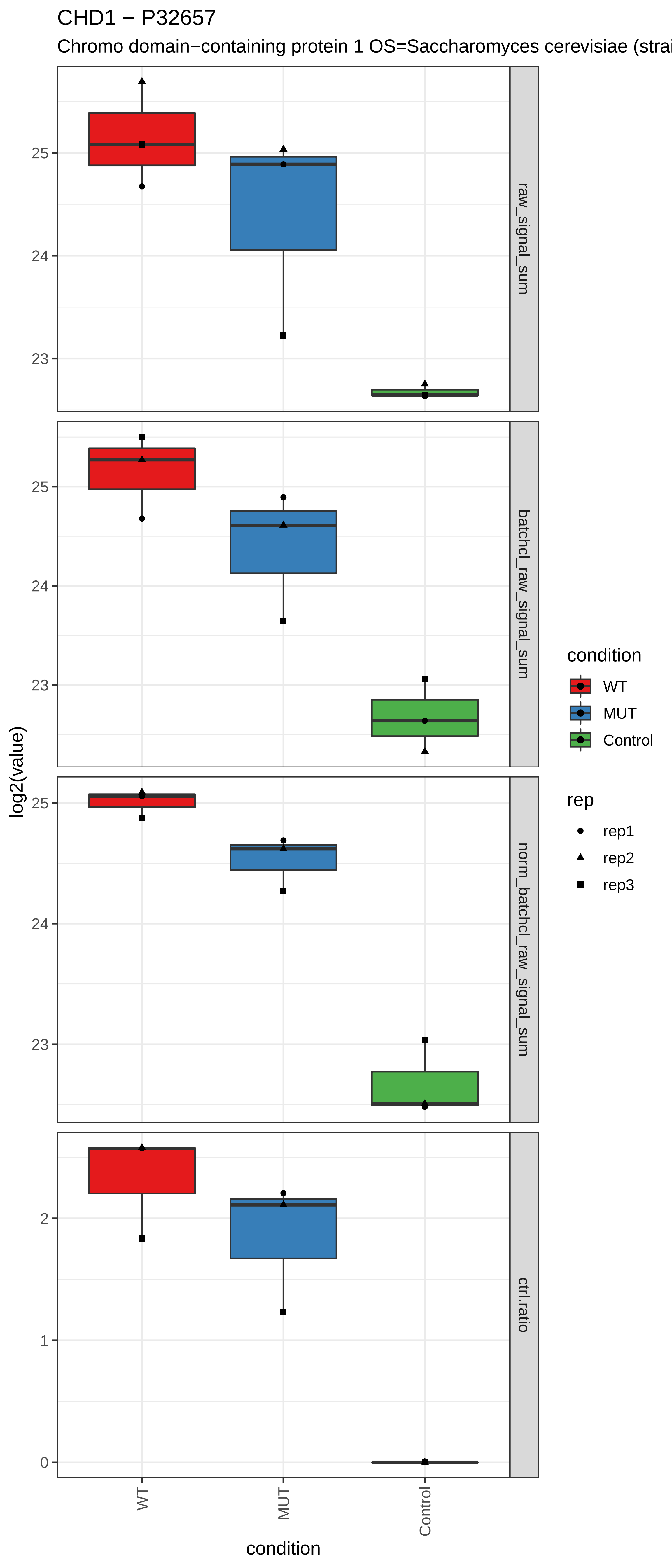

CHD1 − P32657
Chromo domain−containing protein 1 OS=Saccharomyces cerevisiae (strai
raw_signal_sum
batchcl_raw_signal_sum
norm_batchcl_raw_signal_sum
ctrl.ratio
log2(value)
condition
WT
MUT
Control
rep
rep1
rep2
rep3
25
24
23
2
1
0

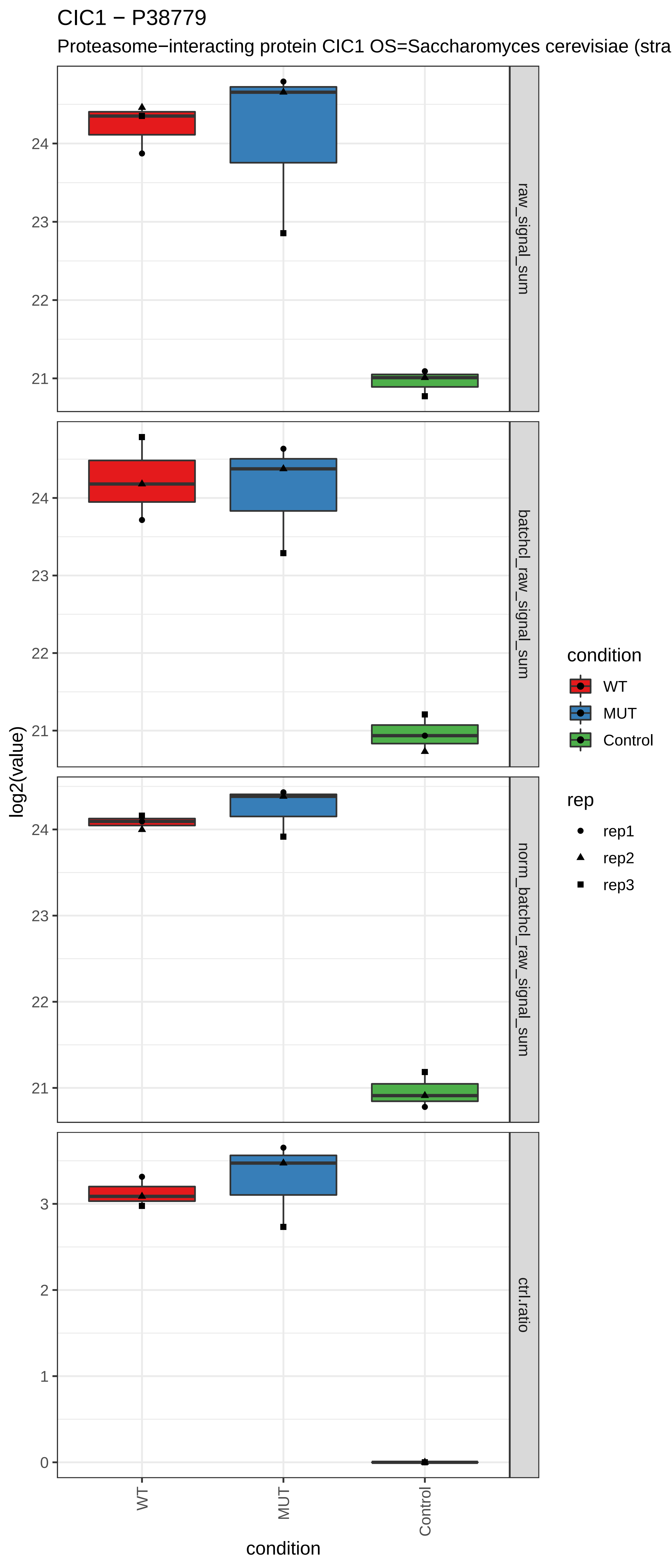
CIC1 − P38779
Proteasome−interacting protein CIC1 OS=Saccharomyces cerevisiae (stra
raw_signal_sum
batchcl_raw_signal_sum
norm_batchcl_raw_signal_sum
ctrl.ratio
log2(value)
condition
WT
MUT
Control
rep
rep1
rep2
rep3
24
23
22
21
3
2
1
0

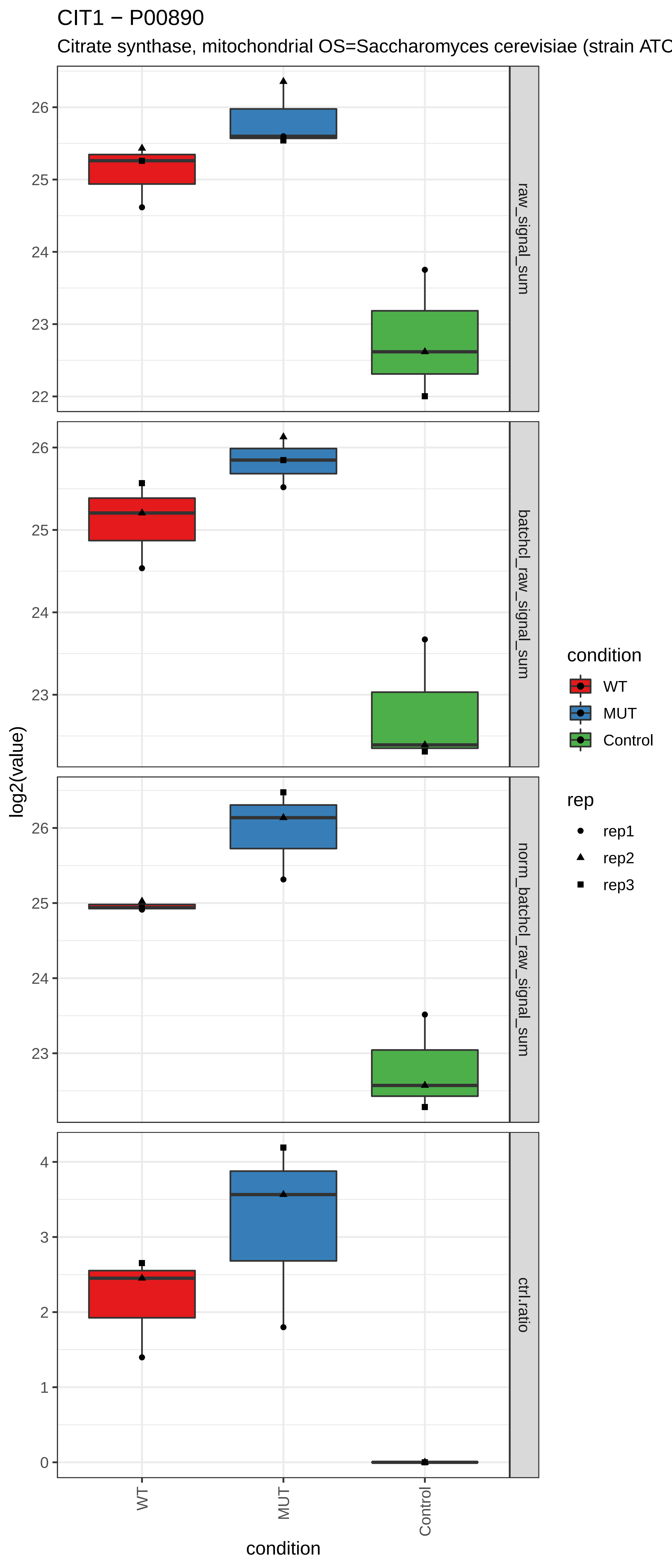

CIT1 − P00890
Citrate synthase, mitochondrial OS=Saccharomyces cerevisiae (strain ATC
raw_signal_sum
batchcl_raw_signal_sum
norm_batchcl_raw_signal_sum
ctrl.ratio
log2(value)
condition
WT
MUT
Control
rep
rep1
rep2
rep3
26
25
24
23
22
4
3
2
1
0

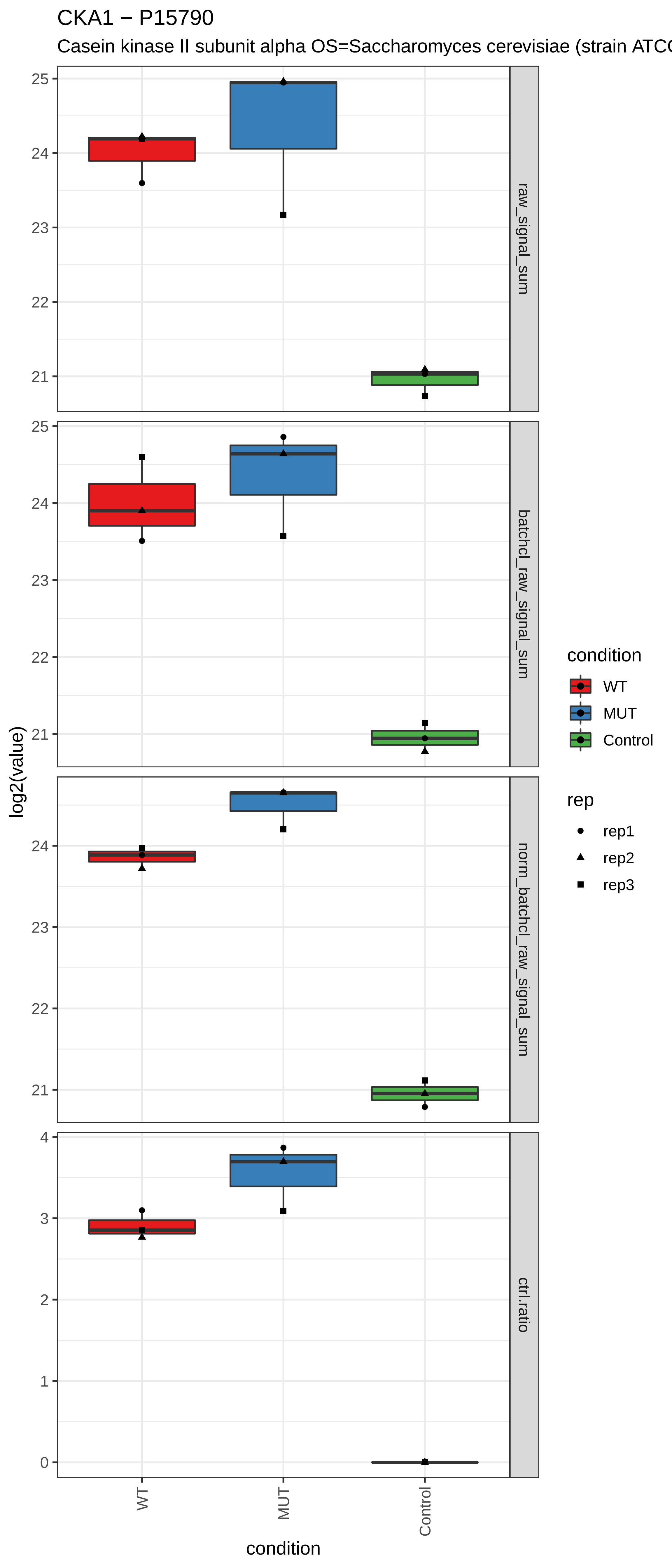

CKA1 − P15790
Casein kinase II subunit alpha OS=Saccharomyces cerevisiae (strain ATCC
raw_signal_sum
batchcl_raw_signal_sum
norm_batchcl_raw_signal_sum
ctrl.ratio
log2(value)
condition
WT
MUT
Control
rep
rep1
rep2
rep3

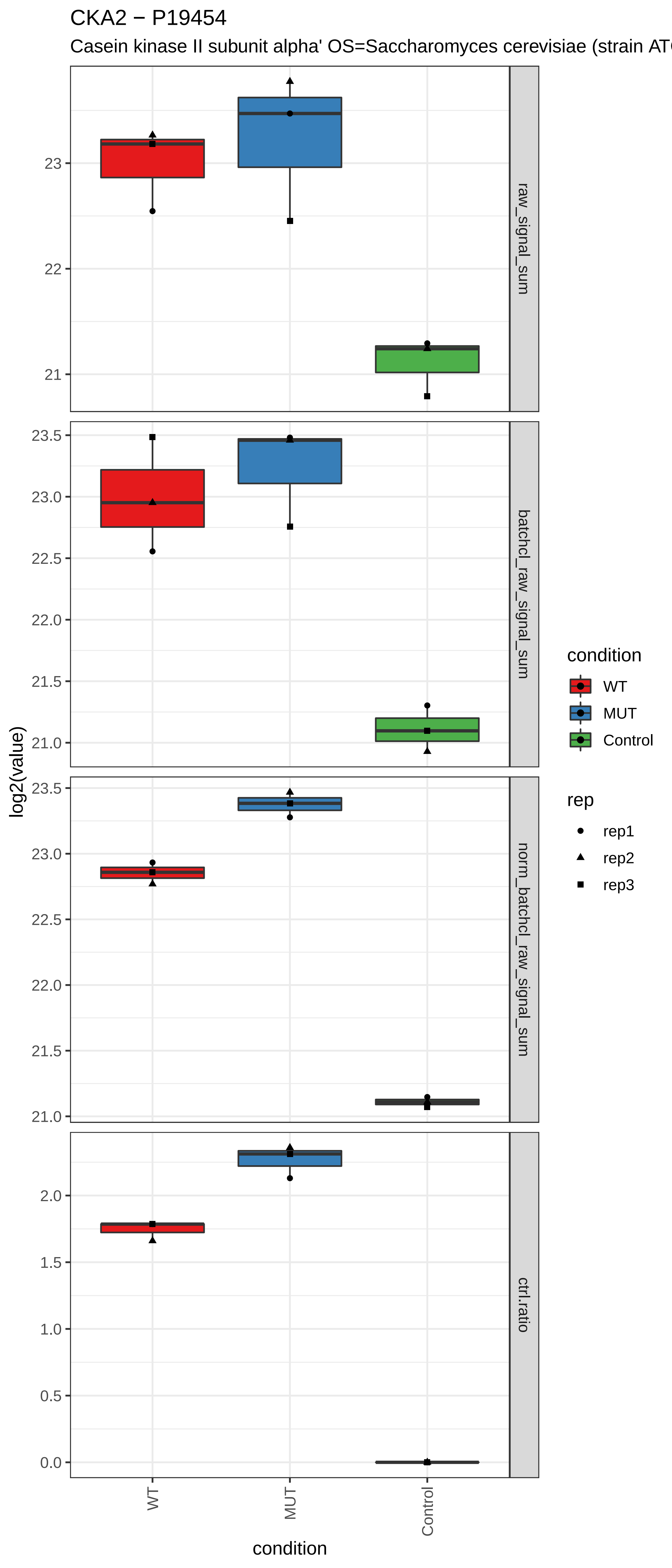

CKA2 − P19454
Casein kinase II subunit alpha' OS=Saccharomyces cerevisiae (strain AT
raw_signal_sum
batchcl_raw_signal_sum
norm_batchcl_raw_signal_sum
ctrl.ratio
log2(value)
condition
WT
MUT
Control
rep
rep1
rep2
rep3
23
22
21
23.5
23.0
22.5
22.0
21.5
21.0
2.0
1.5
1.0
0.5
0.0

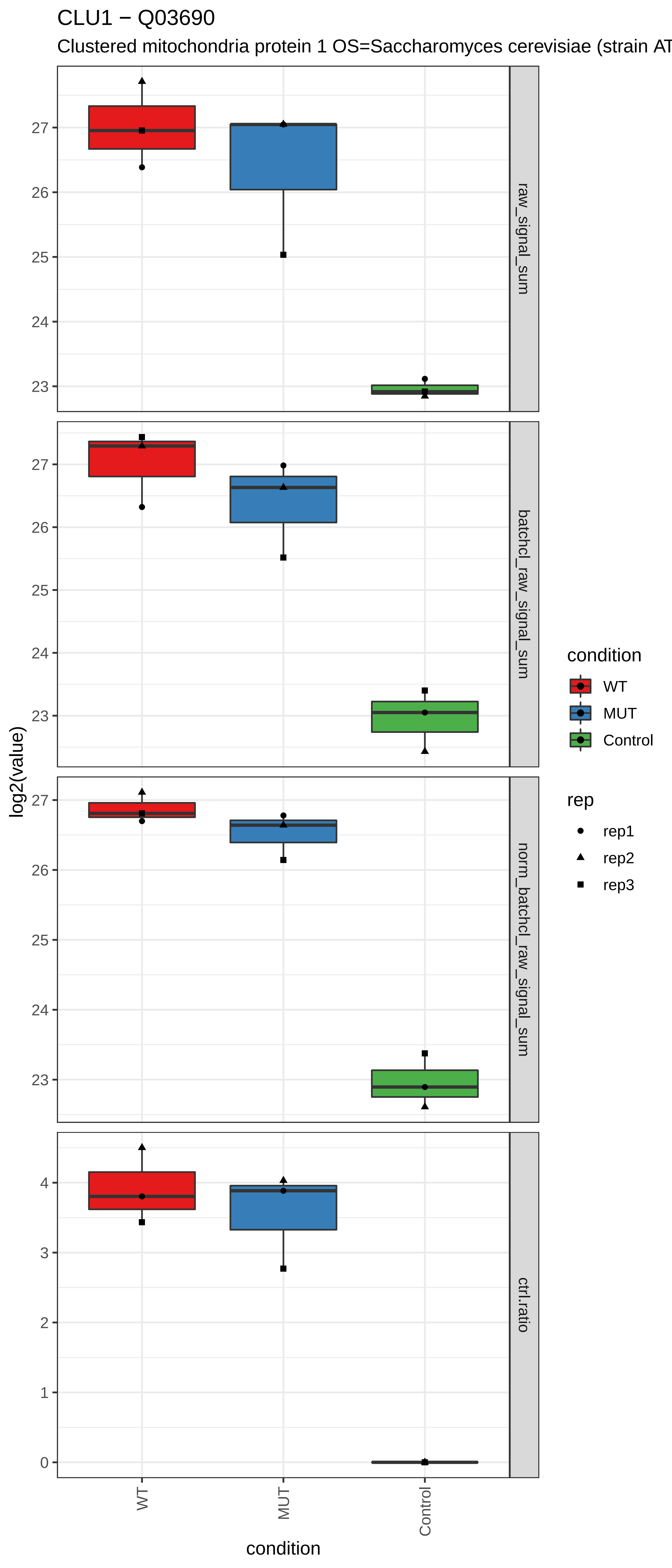
CLU1 − Q03690
Clustered mitochondria protein 1 OS=Saccharomyces cerevisiae (strain AT
raw_signal_sum
batchcl_raw_signal_sum
norm_batchcl_raw_signal_sum
ctrl.ratio
log2(value)
condition
WT
MUT
Control
rep
rep1
rep2
rep3

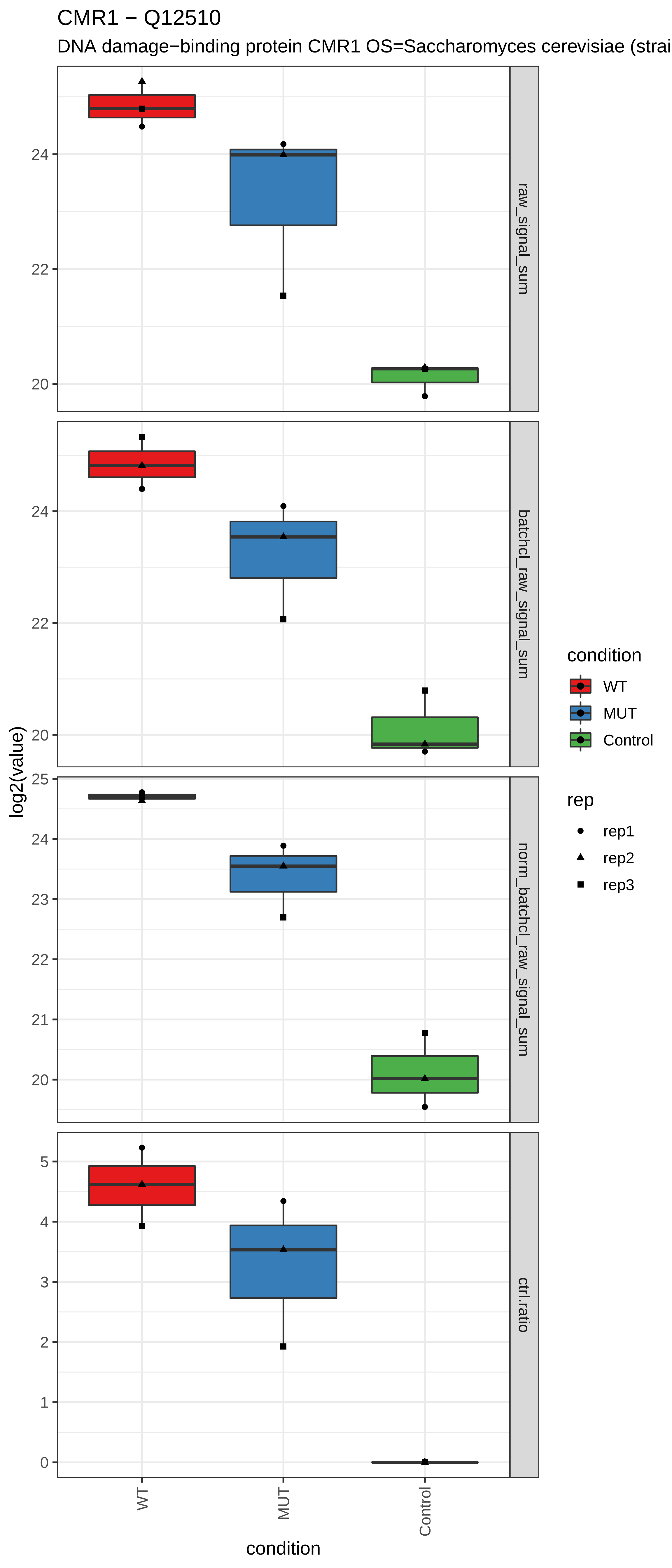

CMR1 − Q12510
DNA damage−binding protein CMR1 OS=Saccharomyces cerevisiae (strai
raw_signal_sum
batchcl_raw_signal_sum
norm_batchcl_raw_signal_sum
ctrl.ratio
log2(value)
condition
WT
MUT
Control
rep
rep1
rep2
rep3
24
22
20
25
23
21
5
4
3
2
1
0

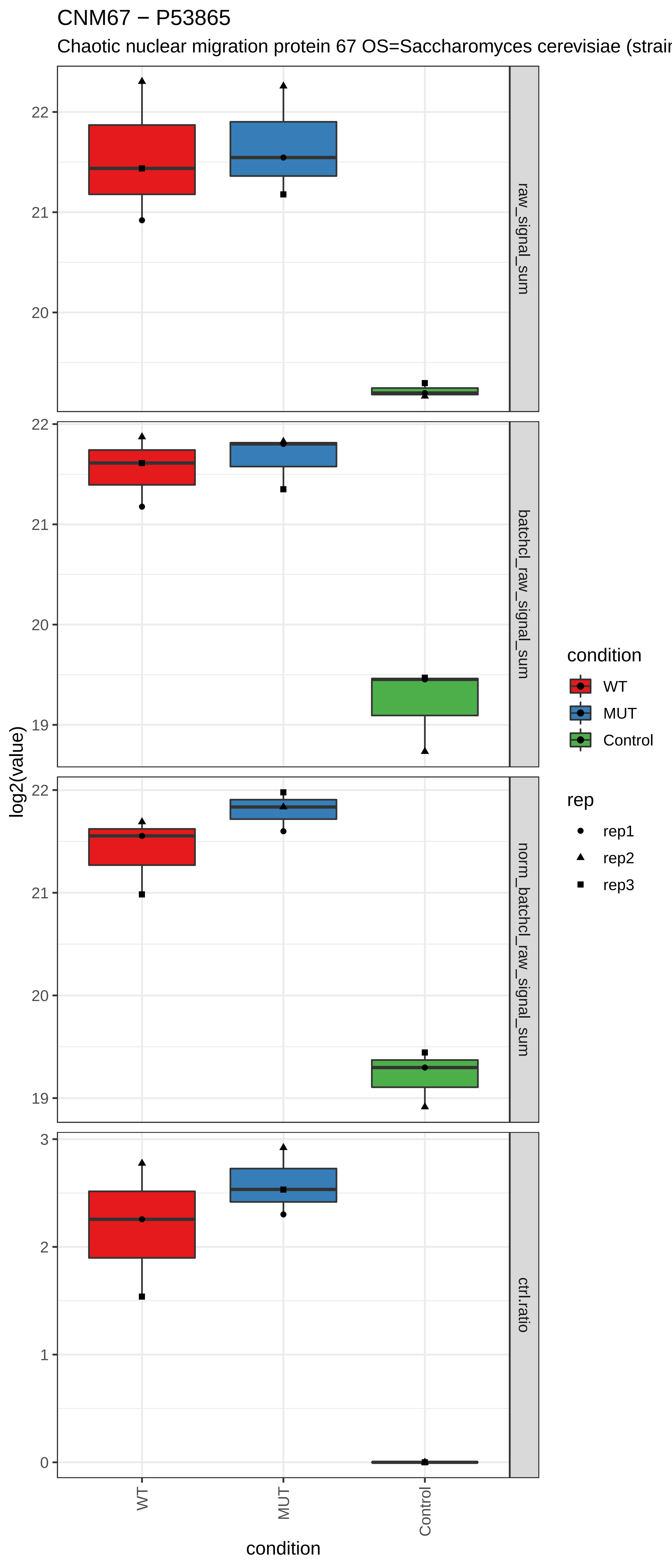
CNM67 − P53865
Chaotic nuclear migration protein 67 OS=Saccharomyces cerevisiae (strain
raw_signal_sum
batchcl_raw_signal_sum
norm_batchcl_raw_signal_sum
ctrl.ratio
log2(value)
condition
WT
MUT
Control
rep
rep1
rep2
rep3
22
21
20
19
3
2
1
0

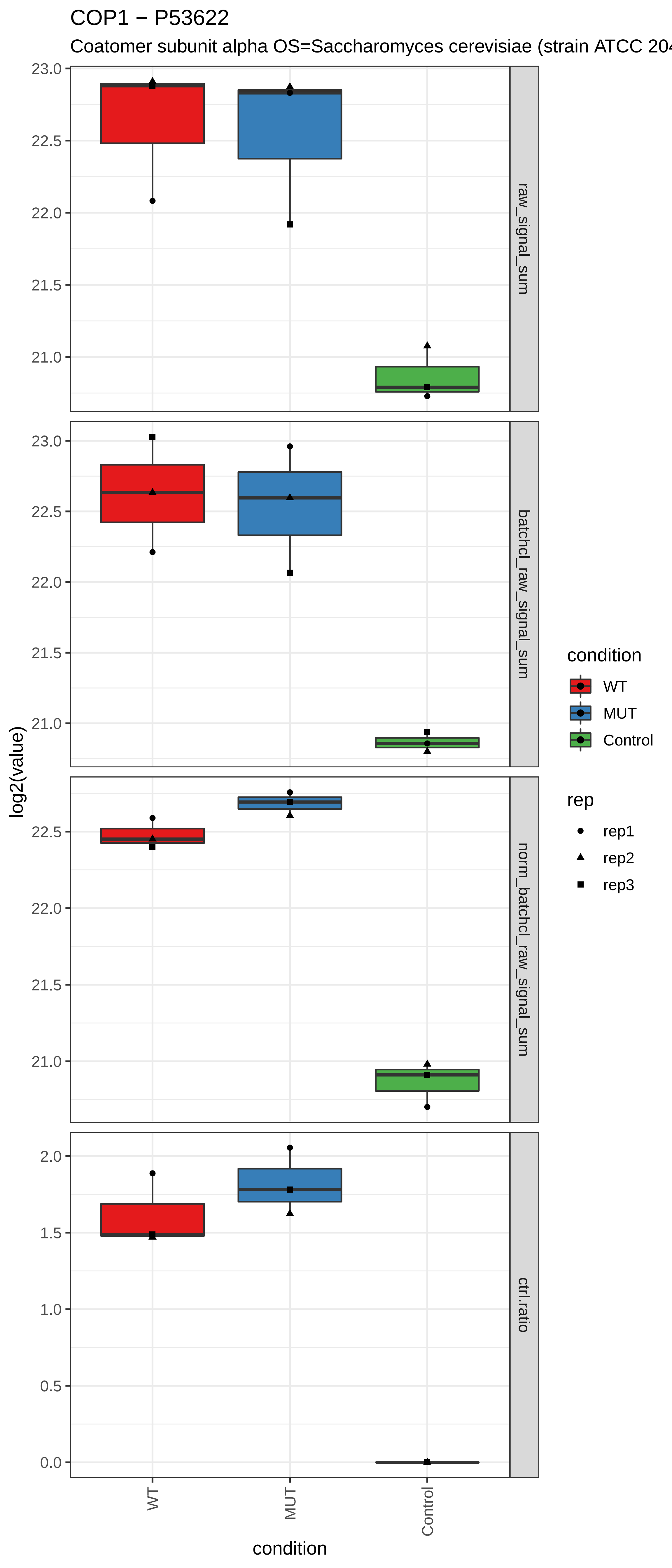

COP1 − P53622
Coatomer subunit alpha OS=Saccharomyces cerevisiae (strain ATCC 204
raw_signal_sum
batchcl_raw_signal_sum
norm_batchcl_raw_signal_sum
ctrl.ratio
log2(value)
condition
WT
MUT
Control
rep
rep1
rep2
rep3
23.0
22.5
22.0
21.5
21.0
2.0
1.5
1.0
0.5
0.0

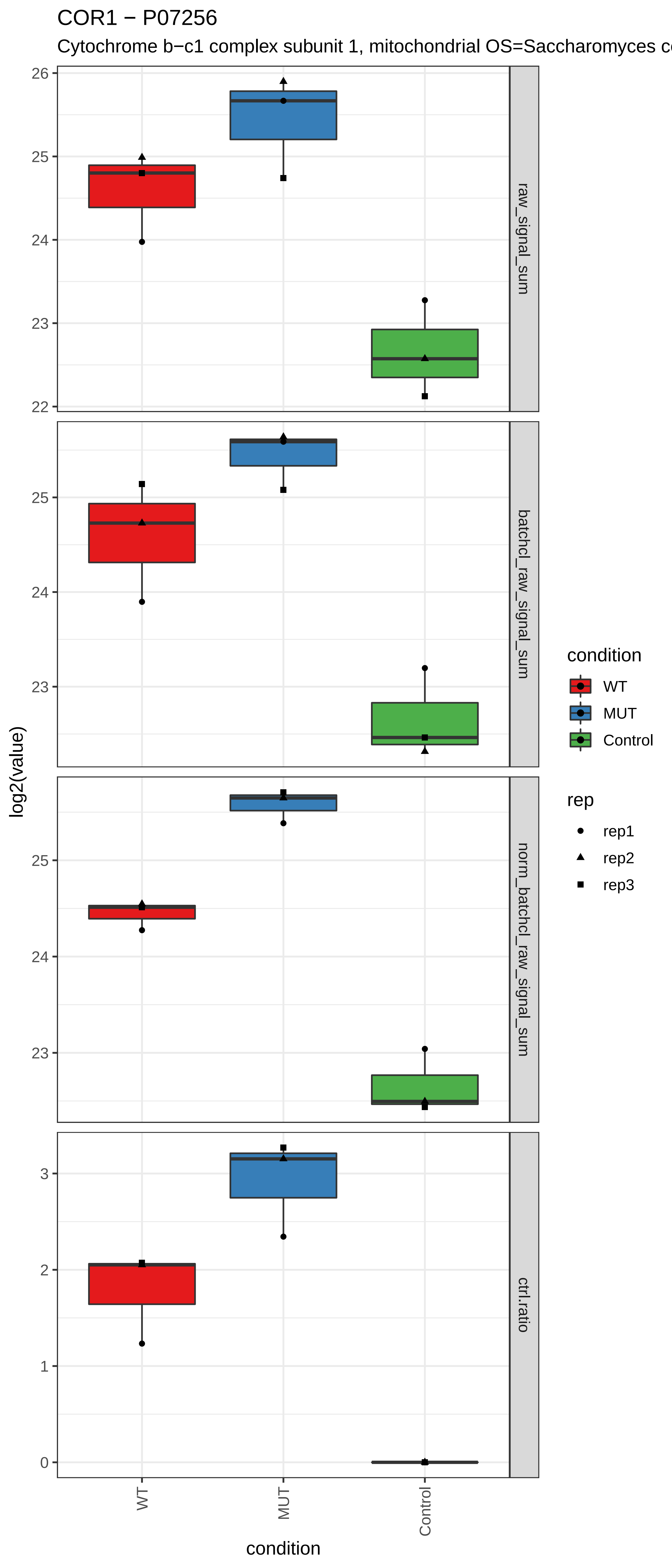
COR1 − P07256
Cytochrome b−c1 complex subunit 1, mitochondrial OS=Saccharomyces c
raw_signal_sum
batchcl_raw_signal_sum
norm_batchcl_raw_signal_sum
ctrl.ratio
log2(value)
condition
WT
MUT
Control
rep
rep1
rep2
rep3

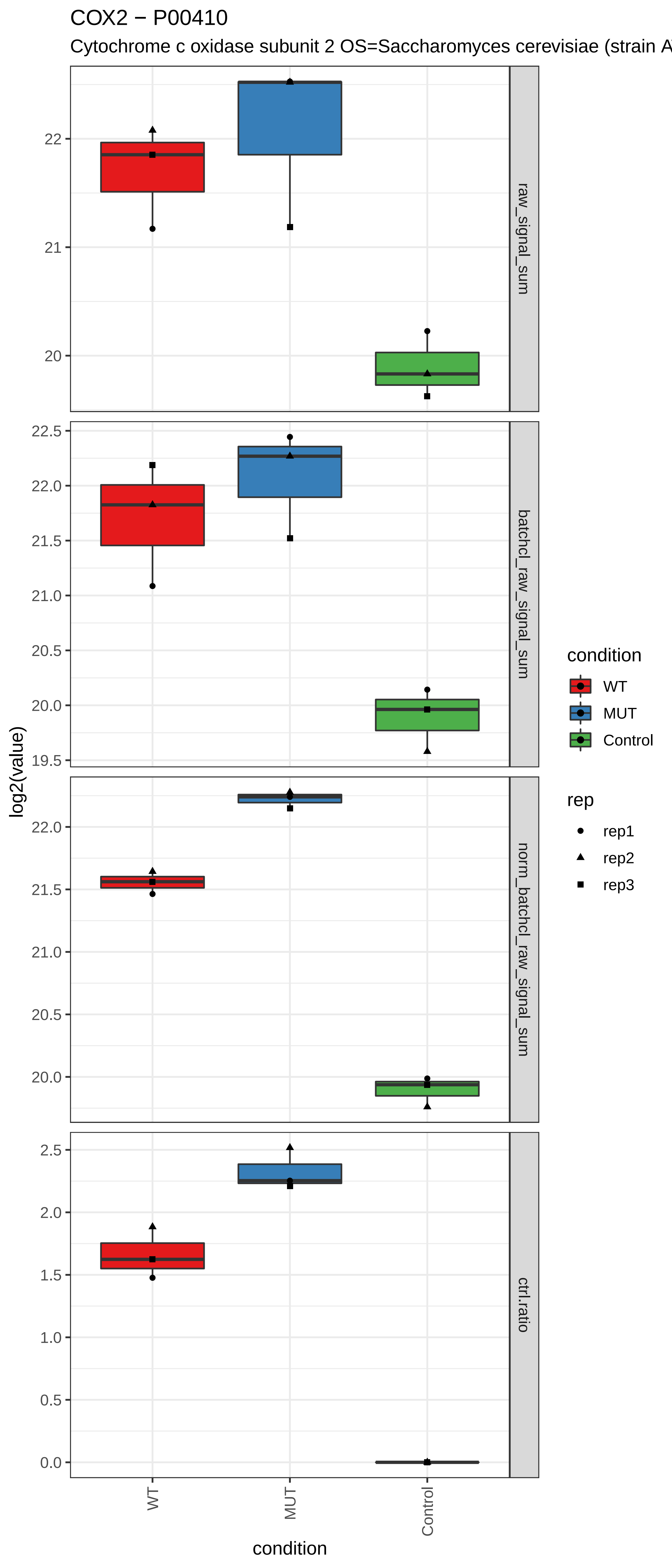
COX2 − P00410
Cytochrome c oxidase subunit 2 OS=Saccharomyces cerevisiae (strain A
raw_signal_sum
batchcl_raw_signal_sum
norm_batchcl_raw_signal_sum
ctrl.ratio
log2(value)
condition
WT
MUT
Control
rep
rep1
rep2
rep3
22
21
20
22.5
22.0
21.5
21.0
20.5
20.0
19.5
2.5
2.0
1.5
1.0
0.5
0.0

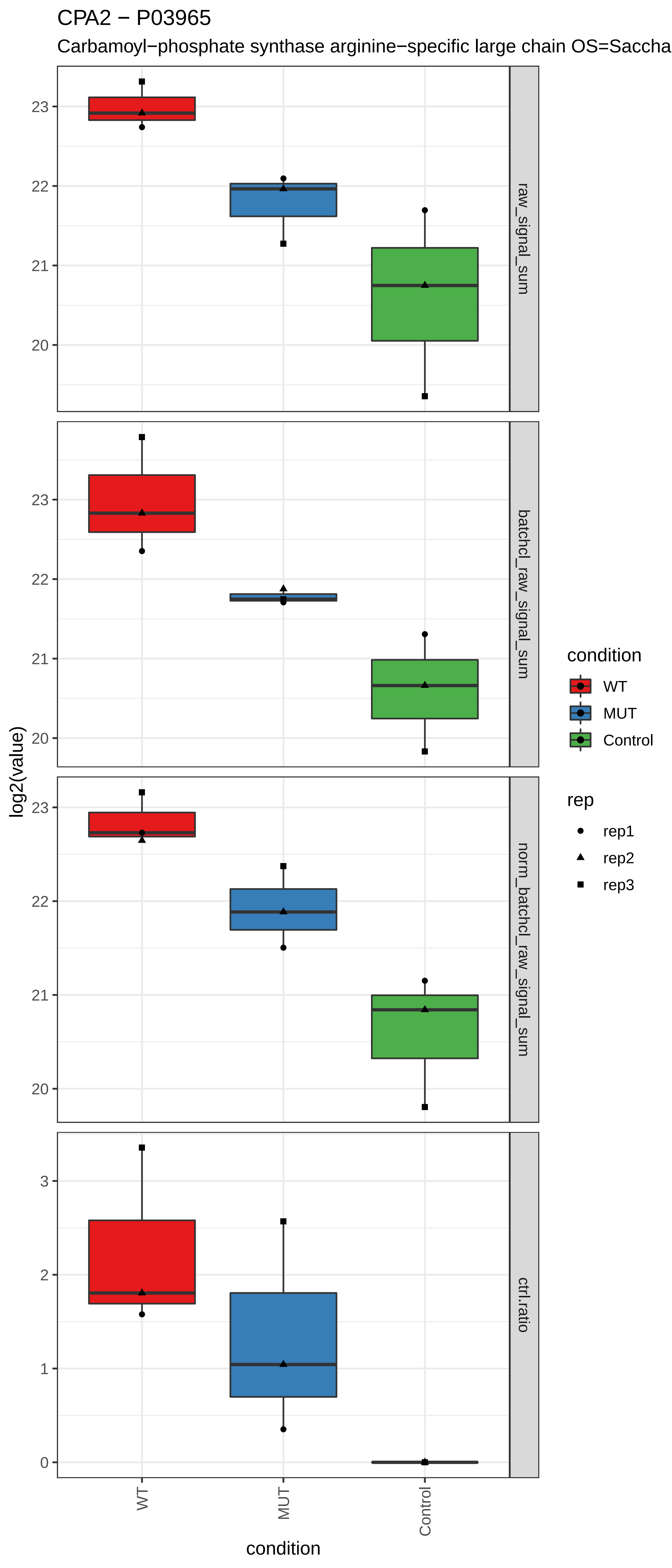

CPA2 − P03965
Carbamoyl−phosphate synthase arginine−specific large chain OS=Saccha
raw_signal_sum
batchcl_raw_signal_sum
norm_batchcl_raw_signal_sum
ctrl.ratio
log2(value)
condition
WT
MUT
Control
rep
rep1
rep2
rep3

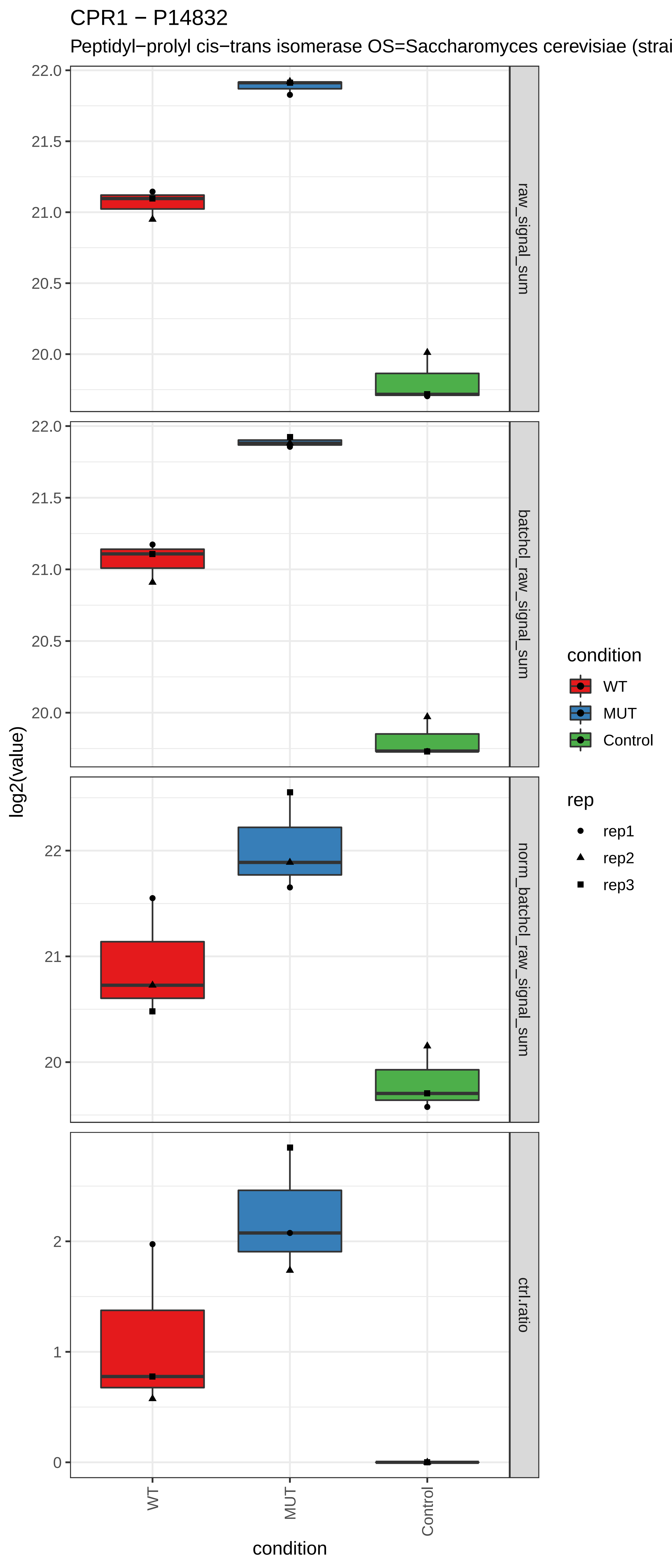
CPR1 − P14832
Peptidyl−prolyl cis−trans isomerase OS=Saccharomyces cerevisiae (strai
raw_signal_sum
batchcl_raw_signal_sum
norm_batchcl_raw_signal_sum
ctrl.ratio
22.0
21.5
21.0
20.5
20.0
22
21
20
2
1
0
log2(value)
WT
MUT
Control
condition
rep
rep1
rep2
rep3

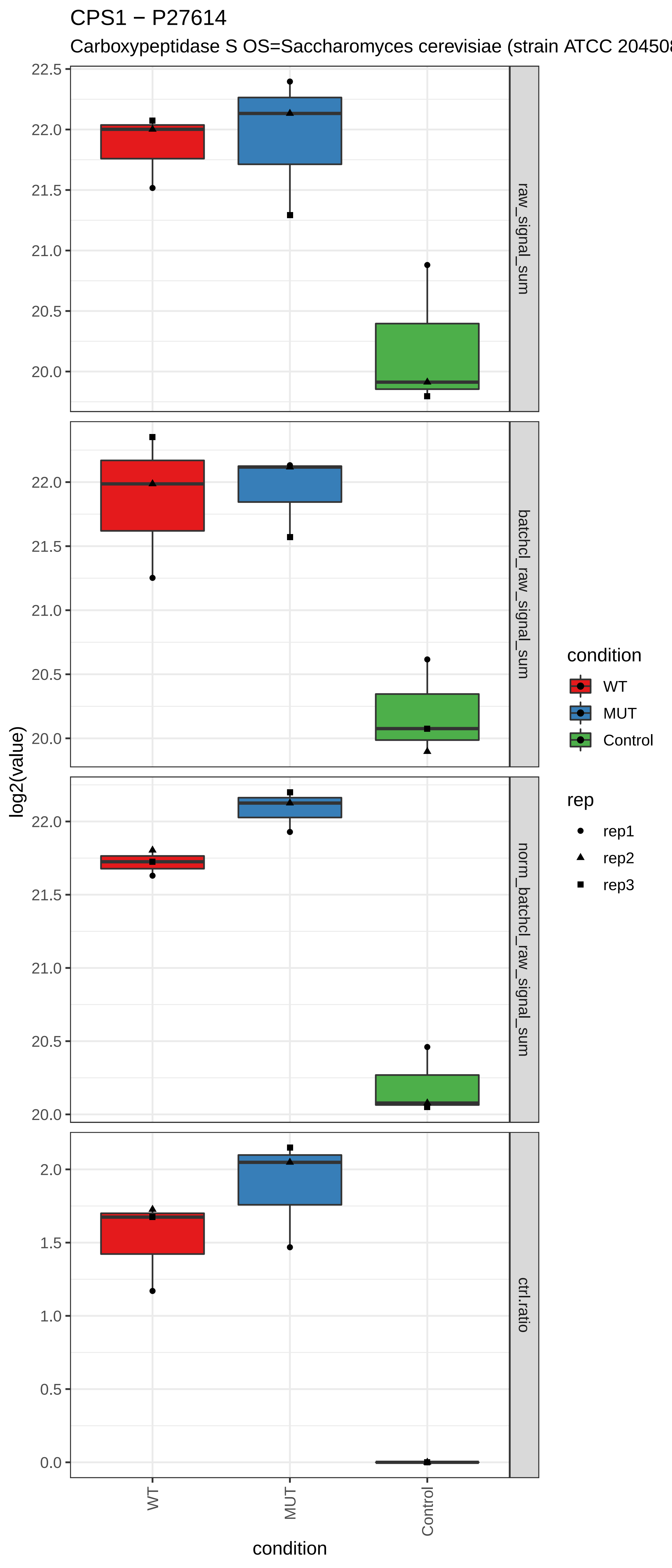

CPS1 − P27614
Carboxypeptidase S OS=Saccharomyces cerevisiae (strain ATCC 20450
22.5
22.0
21.5
21.0
20.5
20.0
2.0
1.5
1.0
0.5
0.0
raw_signal_sum
batchcl_raw_signal_sum
norm_batchcl_raw_signal_sum
ctrl.ratio
log2(value)
condition
WT
MUT
Control
rep
rep1
rep2
rep3

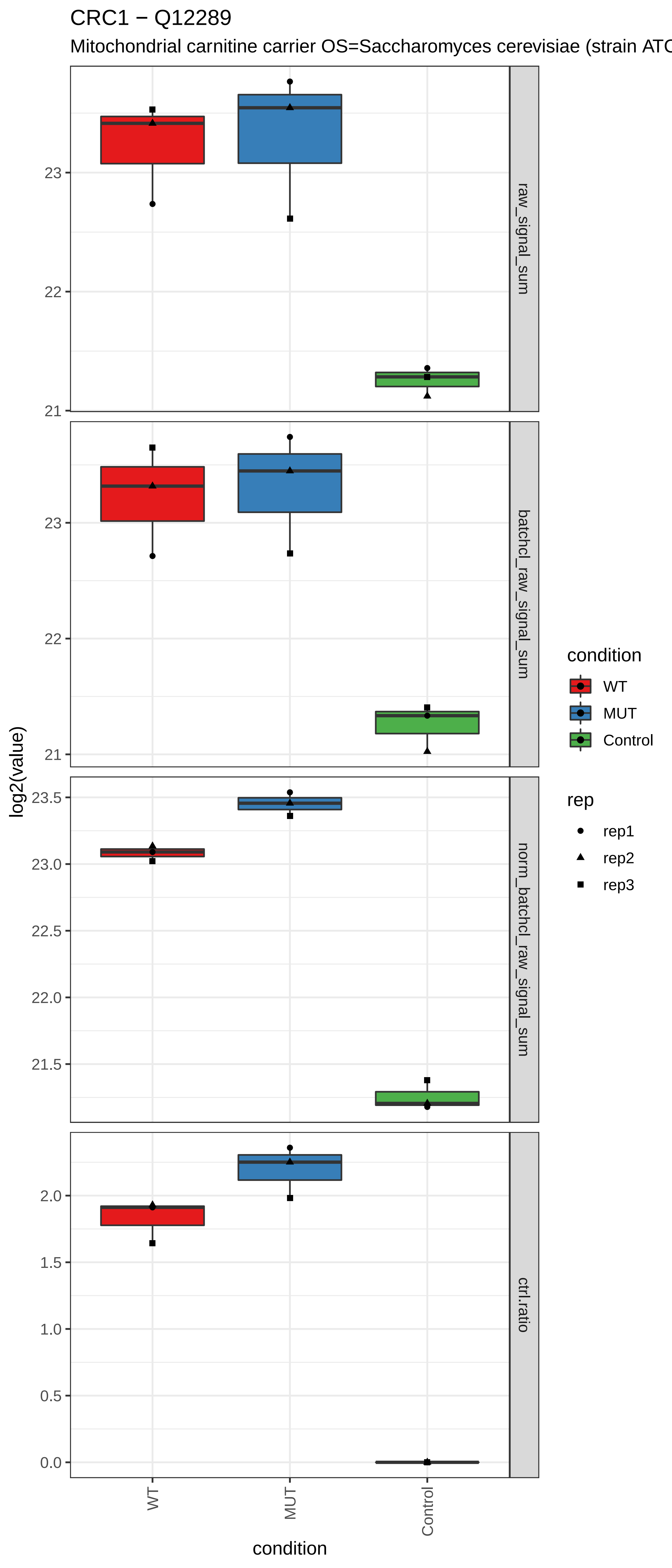
CRC1 − Q12289
Mitochondrial carnitine carrier OS=Saccharomyces cerevisiae (strain ATC
raw_signal_sum
batchcl_raw_signal_sum
norm_batchcl_raw_signal_sum
ctrl.ratio
log2(value)
condition
WT
MUT
Control
rep
rep1
rep2
rep3
23
22
21
23.5
23.0
22.5
22.0
21.5
2.0
1.5
1.0
0.5
0.0

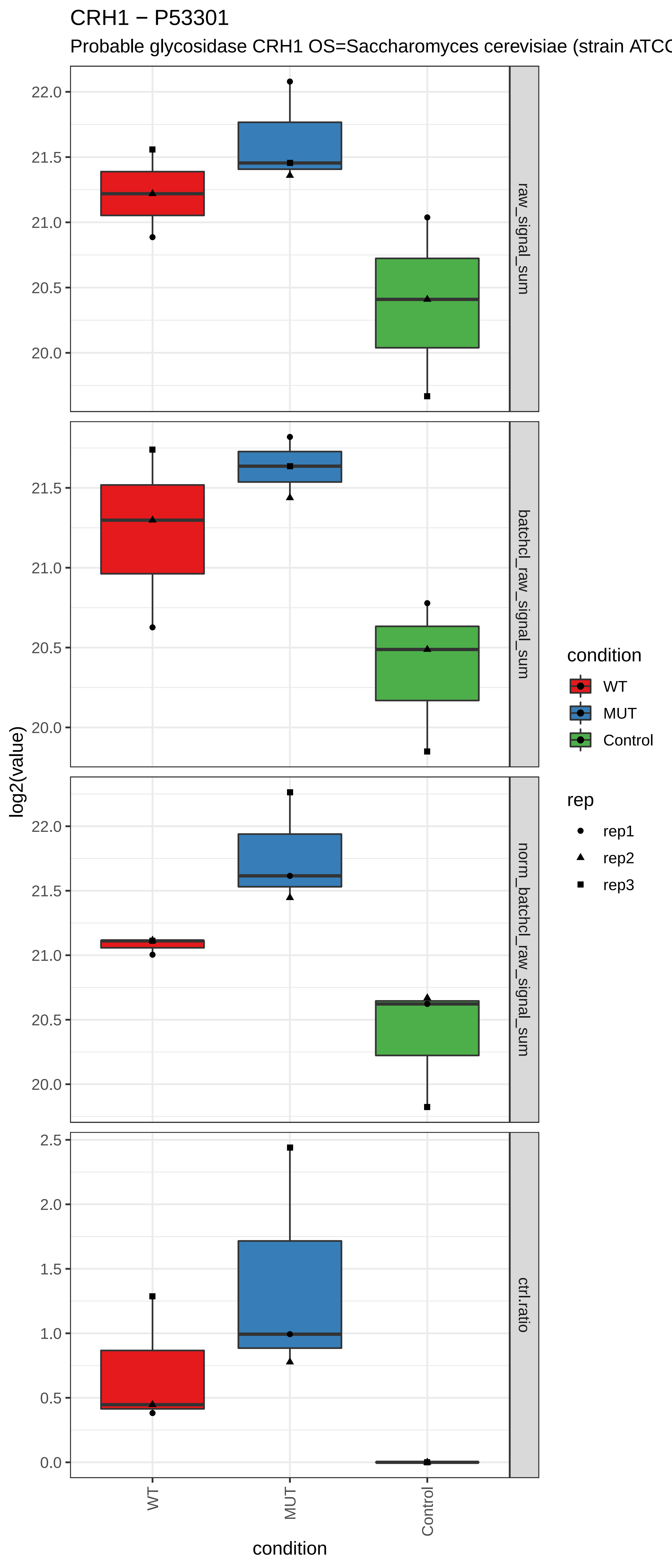
CRH1 − P53301
Probable glycosidase CRH1 OS=Saccharomyces cerevisiae (strain ATCC
raw_signal_sum
batchcl_raw_signal_sum
norm_batchcl_raw_signal_sum
ctrl.ratio
log2(value)
condition
WT
MUT
Control
rep
rep1
rep2
rep3
22.0
21.5
21.0
20.5
20.0
2.5
2.0
1.5
1.0
0.5
0.0

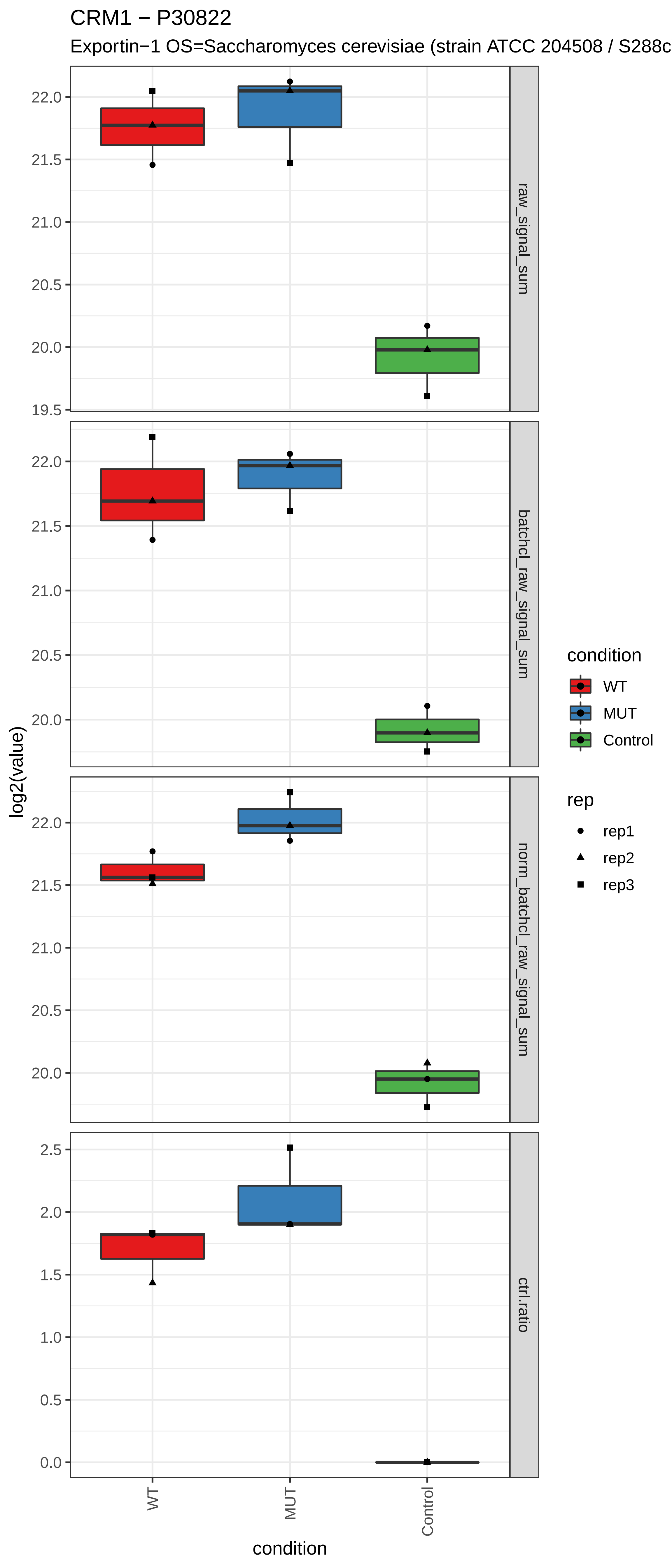
CRM1 − P30822
Exportin−1 OS=Saccharomyces cerevisiae (strain ATCC 204508 / S288c)
raw_signal_sum
batchcl_raw_signal_sum
norm_batchcl_raw_signal_sum
ctrl.ratio
log2(value)
condition
WT
MUT
Control
rep
rep1
rep2
rep3
22.0
21.5
21.0
20.5
20.0
19.5
2.5
2.0
1.5
1.0
0.5
0.0

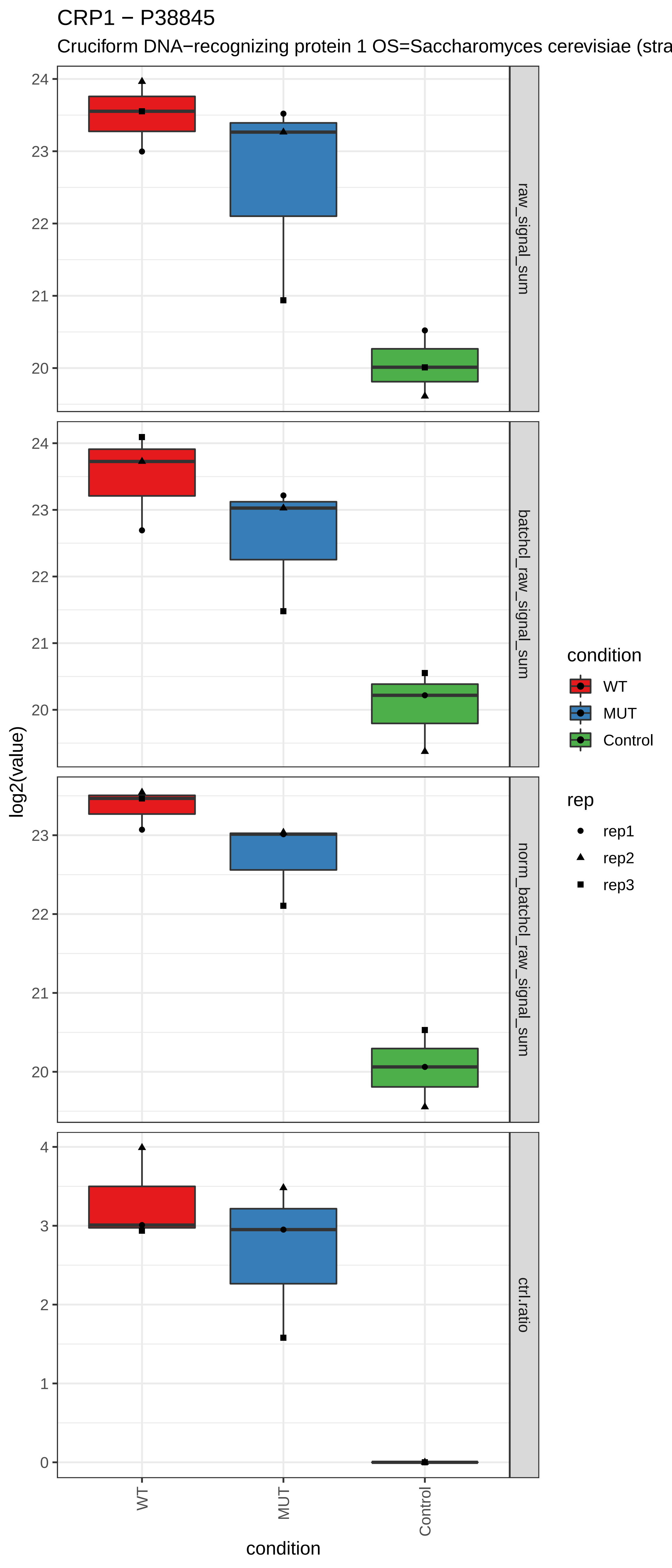
CRP1 − P38845
Cruciform DNA−recognizing protein 1 OS=Saccharomyces cerevisiae (stra
raw_signal_sum
batchcl_raw_signal_sum
norm_batchcl_raw_signal_sum
ctrl.ratio
log2(value)
condition
WT
MUT
Control
rep
rep1
rep2
rep3
24
23
22
21
20
4
3
2
1
0

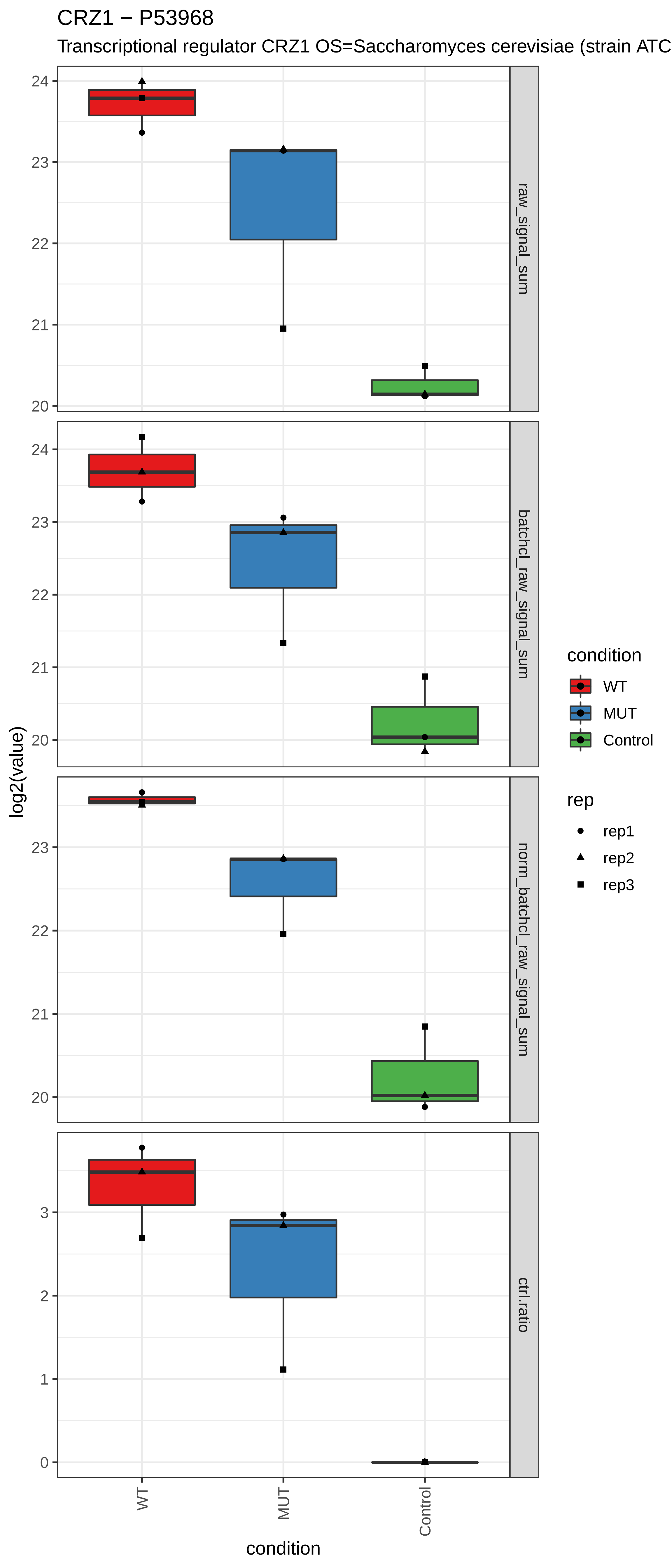

CRZ1 − P53968
Transcriptional regulator CRZ1 OS=Saccharomyces cerevisiae (strain ATC
raw_signal_sum
batchcl_raw_signal_sum
norm_batchcl_raw_signal_sum
ctrl.ratio
log2(value)
condition
WT
MUT
Control
rep
rep1
rep2
rep3
24
23
22
21
20
3
2
1
0

CSE4 − P36012

Histone H3−like centromeric protein CSE4 OS=Saccharomyces cerevisiae

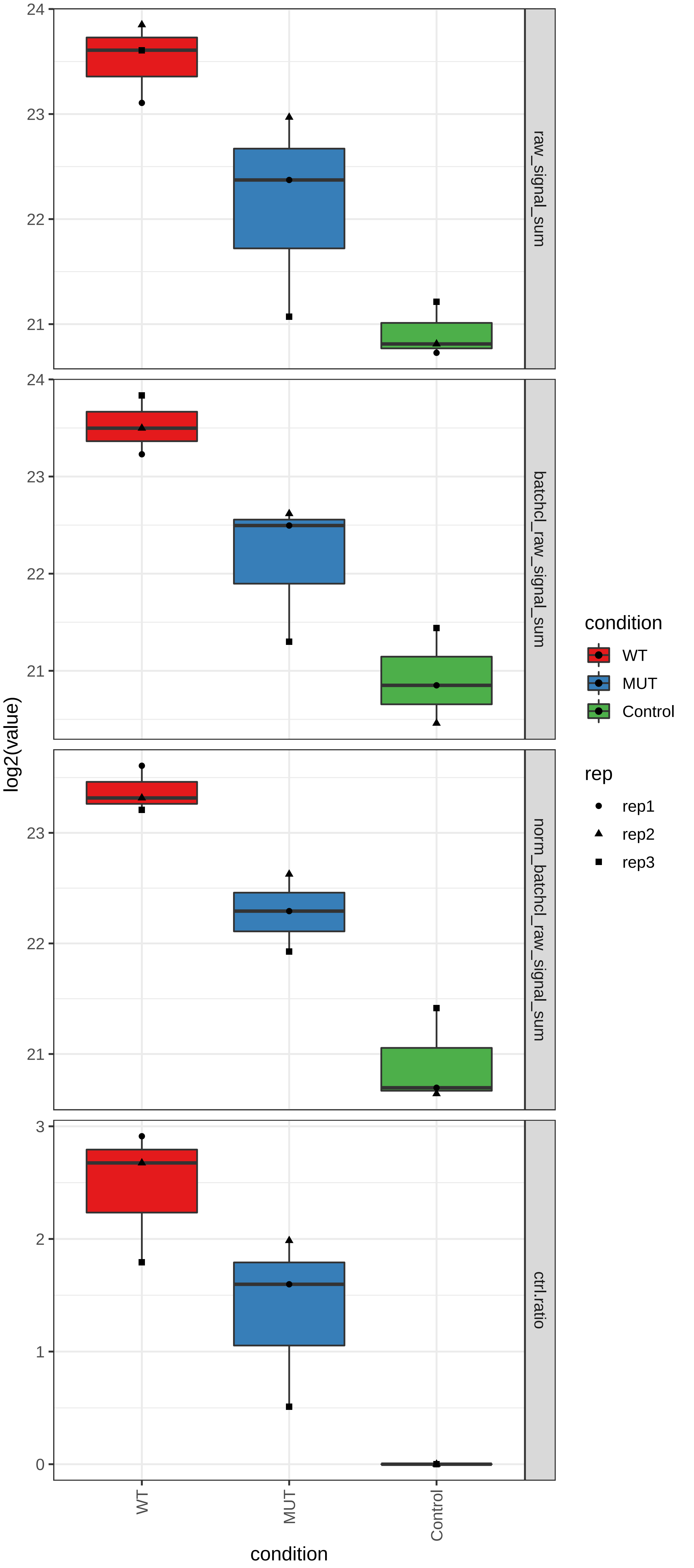

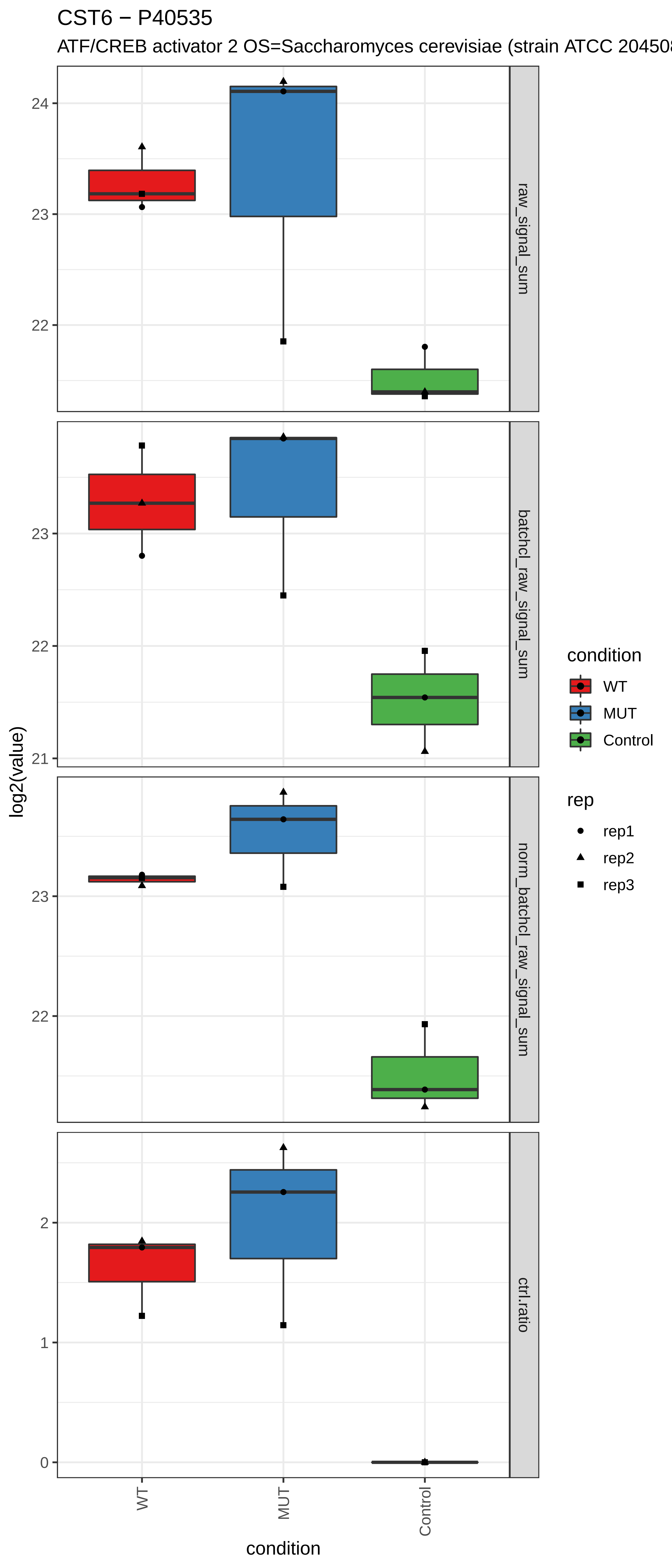

CST6 − P40535
ATF/CREB activator 2 OS=Saccharomyces cerevisiae (strain ATCC 20450
log2(value)
raw_signal_sum
batchcl_raw_signal_sum
norm_batchcl_raw_signal_sum
ctrl.ratio
24
23
22
21
2
1
0
WT
MUT
Control
condition
condition
WT
MUT
Control
rep
rep1
rep2
rep3

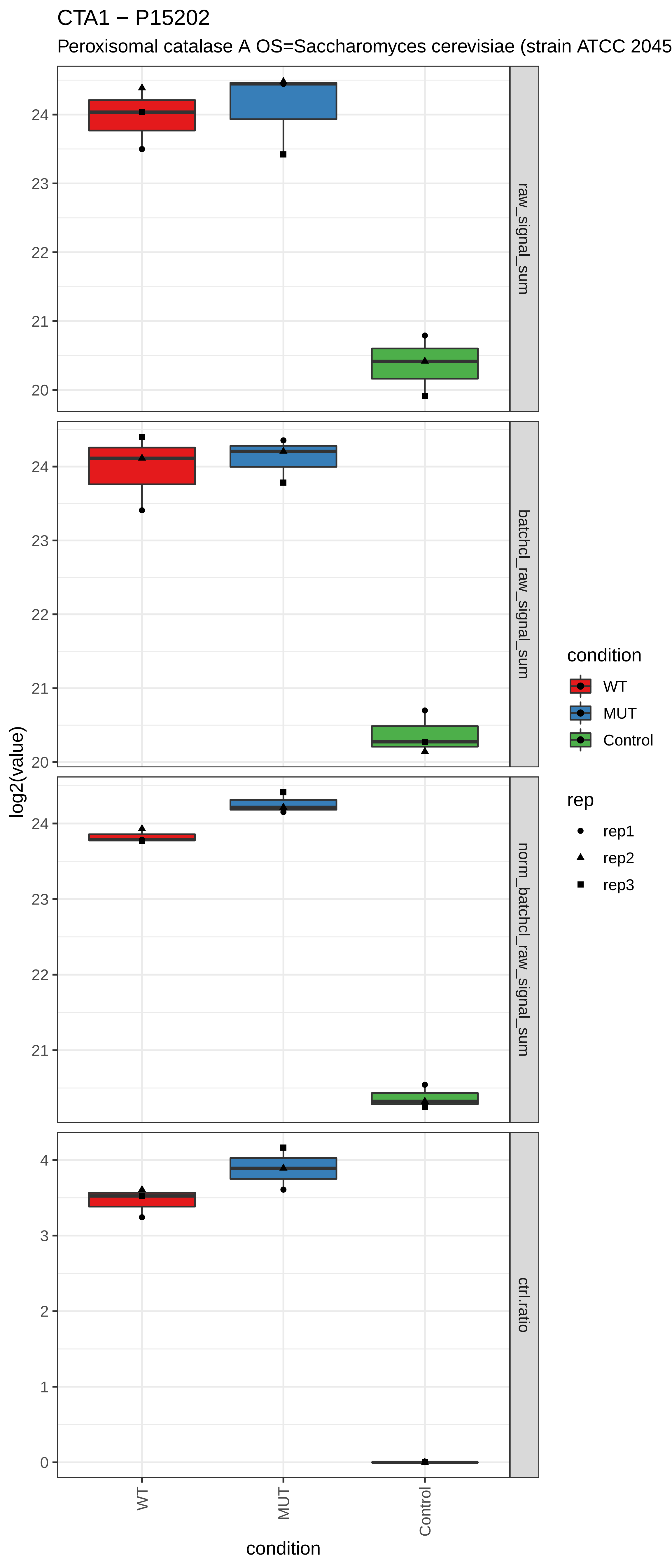
CTA1 − P15202
Peroxisomal catalase A OS=Saccharomyces cerevisiae (strain ATCC 2045
raw_signal_sum
batchcl_raw_signal_sum
norm_batchcl_raw_signal_sum
ctrl.ratio
log2(value)
condition
WT
MUT
Control
rep
rep1
rep2
rep3

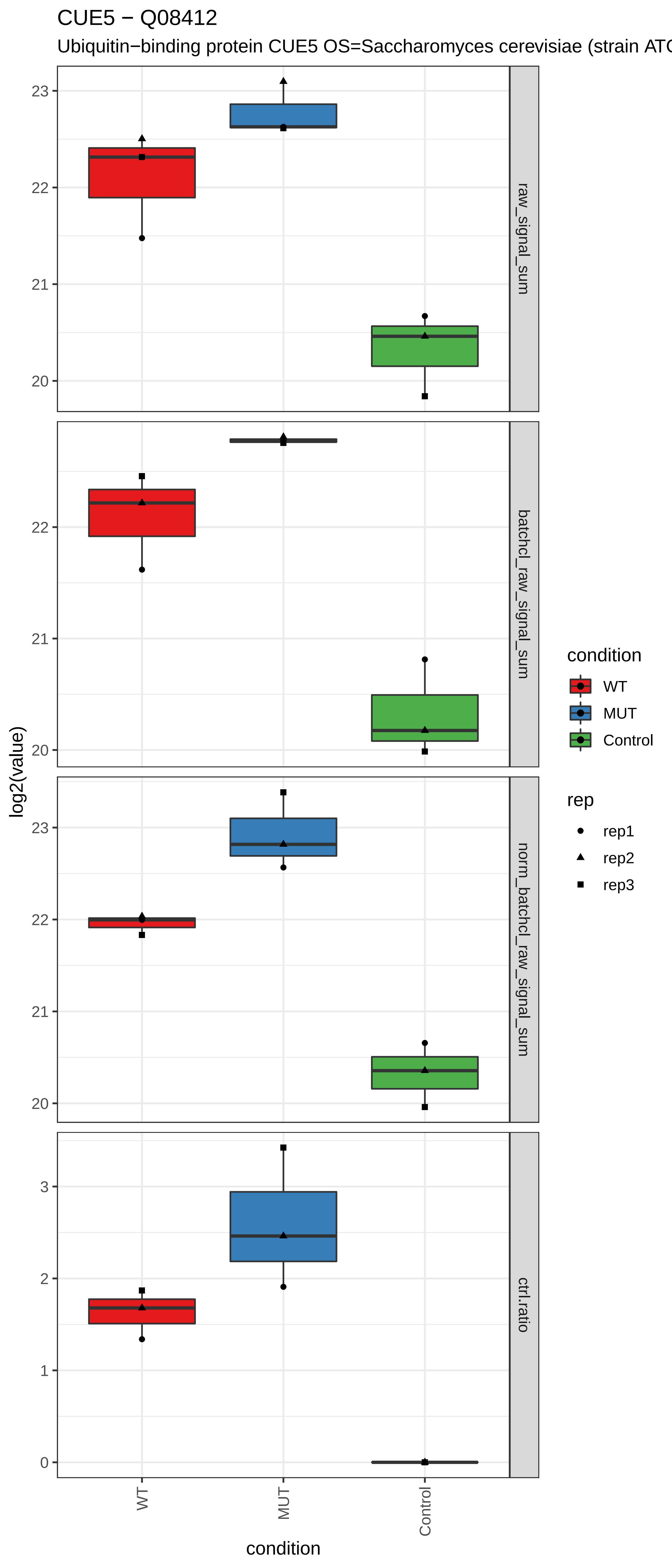

CUE5 − Q08412
Ubiquitin−binding protein CUE5 OS=Saccharomyces cerevisiae (strain ATC
raw_signal_sum
batchcl_raw_signal_sum
norm_batchcl_raw_signal_sum
ctrl.ratio
log2(value)
condition
WT
MUT
Control
rep
rep1
rep2
rep3

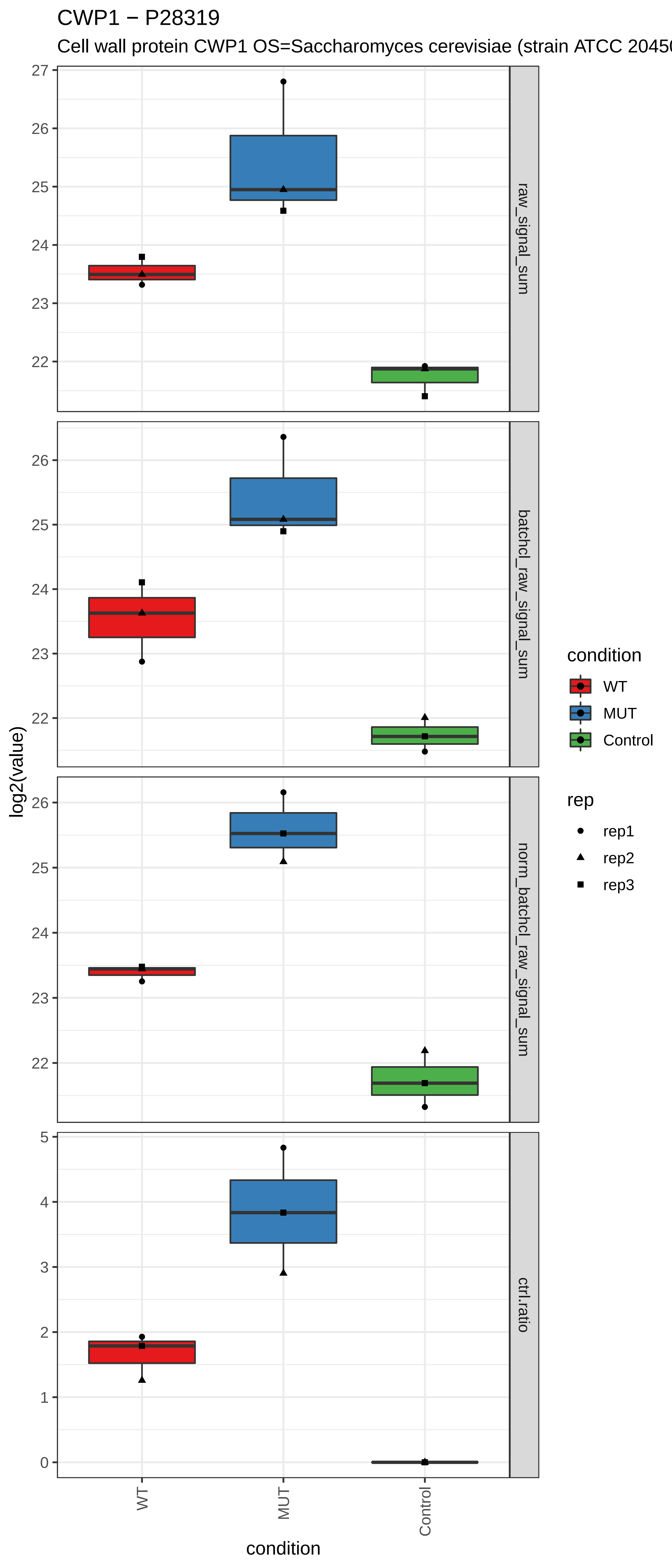
CWP1 − P28319
Cell wall protein CWP1 OS=Saccharomyces cerevisiae (strain ATCC 20450
raw_signal_sum
batchcl_raw_signal_sum
norm_batchcl_raw_signal_sum
ctrl.ratio
log2(value)
condition
WT
MUT
Control
rep
rep1
rep2
rep3

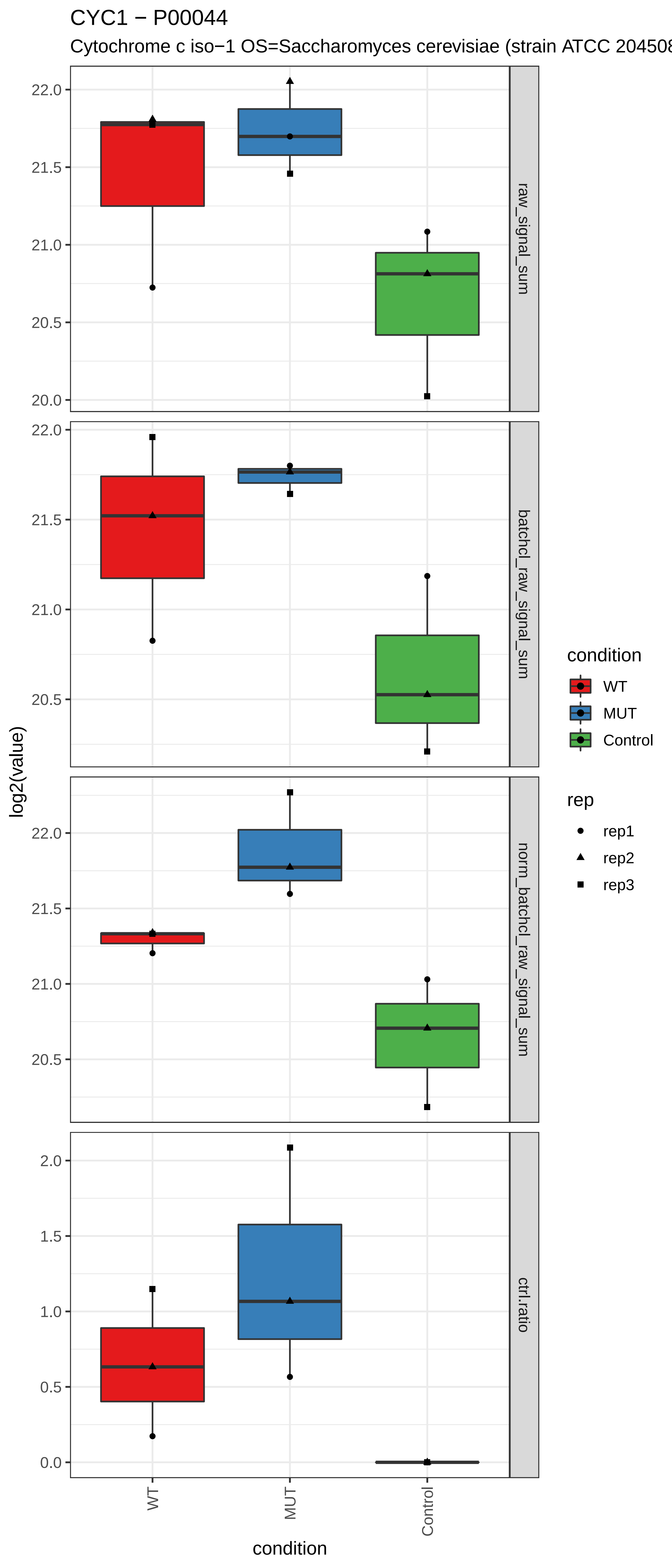

CYC1 − P00044
Cytochrome c iso−1 OS=Saccharomyces cerevisiae (strain ATCC 204508
raw_signal_sum
batchcl_raw_signal_sum
norm_batchcl_raw_signal_sum
ctrl.ratio
log2(value)
condition
WT
MUT
Control
rep
rep1
rep2
rep3
22.0
21.5
21.0
20.5
20.0
2.0
1.5
1.0
0.5
0.0

CYT1 − P07143

Cytochrome c1, heme protein, mitochondrial OS=Saccharomyces cerevisia

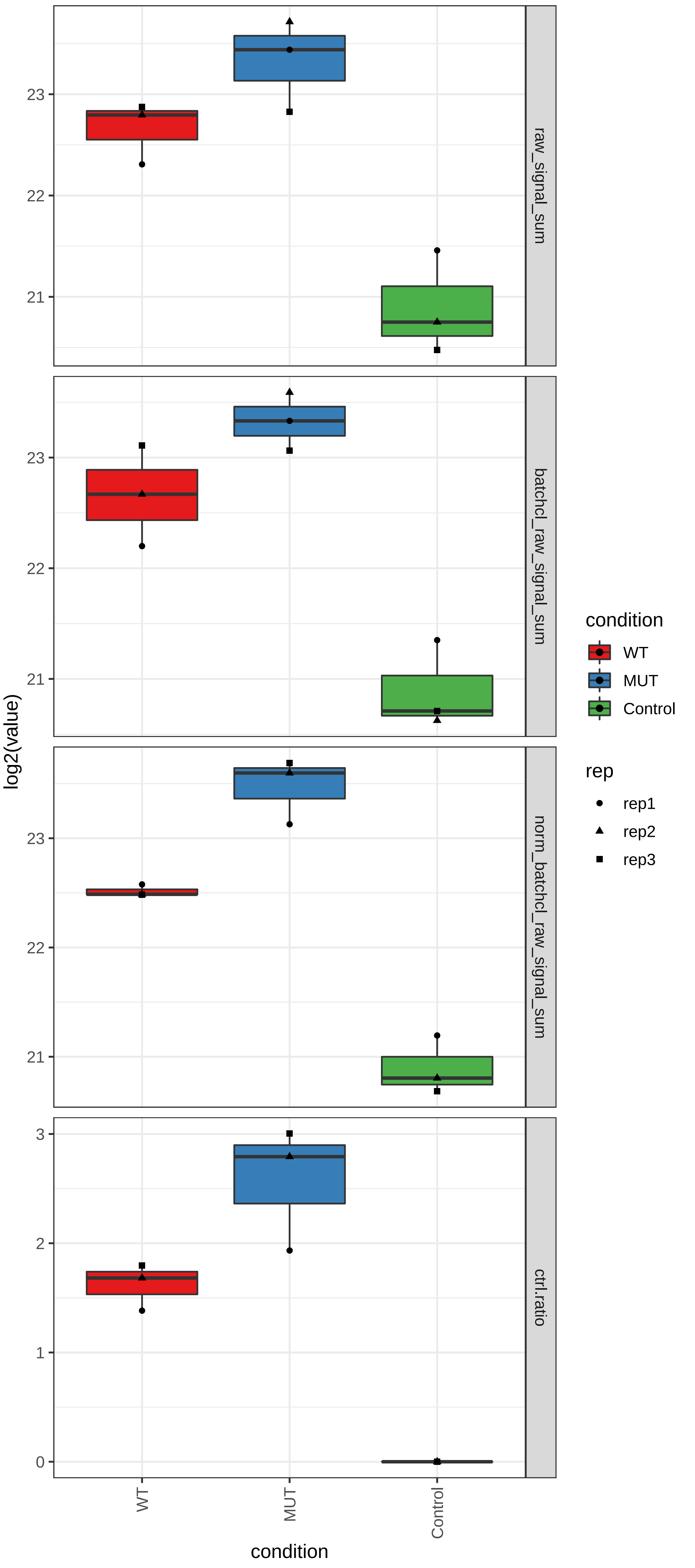

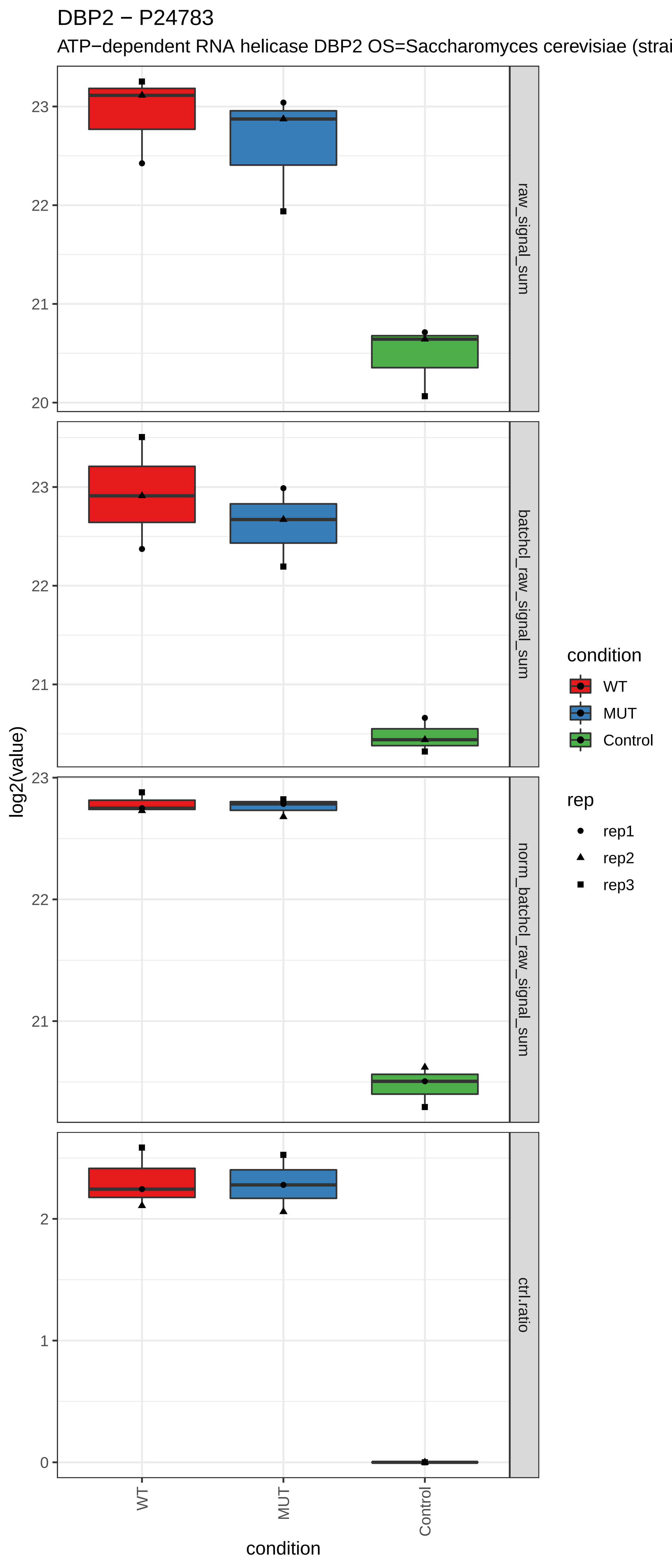
DBP2 − P24783
ATP−dependent RNA helicase DBP2 OS=Saccharomyces cerevisiae (strai
raw_signal_sum
batchcl_raw_signal_sum
norm_batchcl_raw_signal_sum
ctrl.ratio
log2(value)
condition
WT
MUT
Control
rep
rep1
rep2
rep3
23
22
21
20
2
1
0

DBP3 − P20447

ATP−dependent RNA helicase DBP3 OS=Saccharomyces cerevisiae (strai

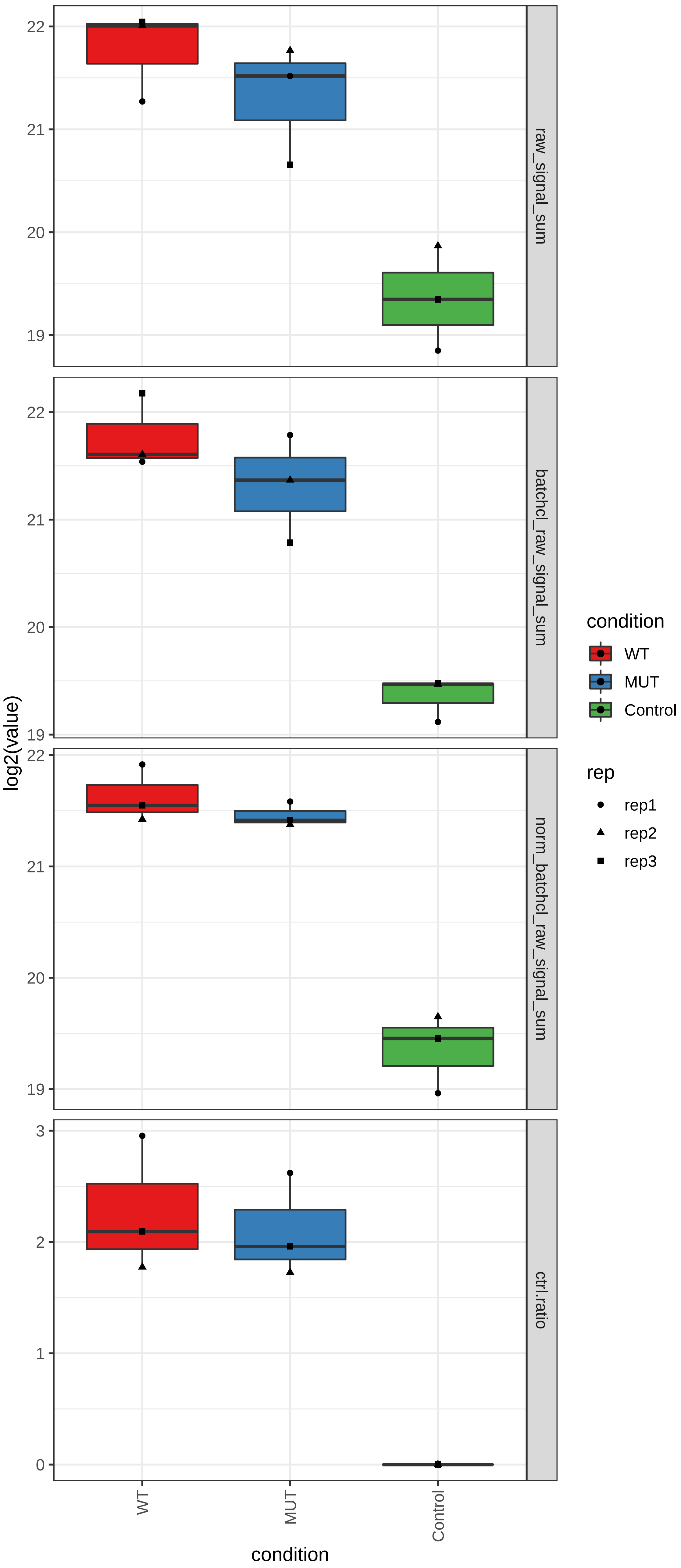

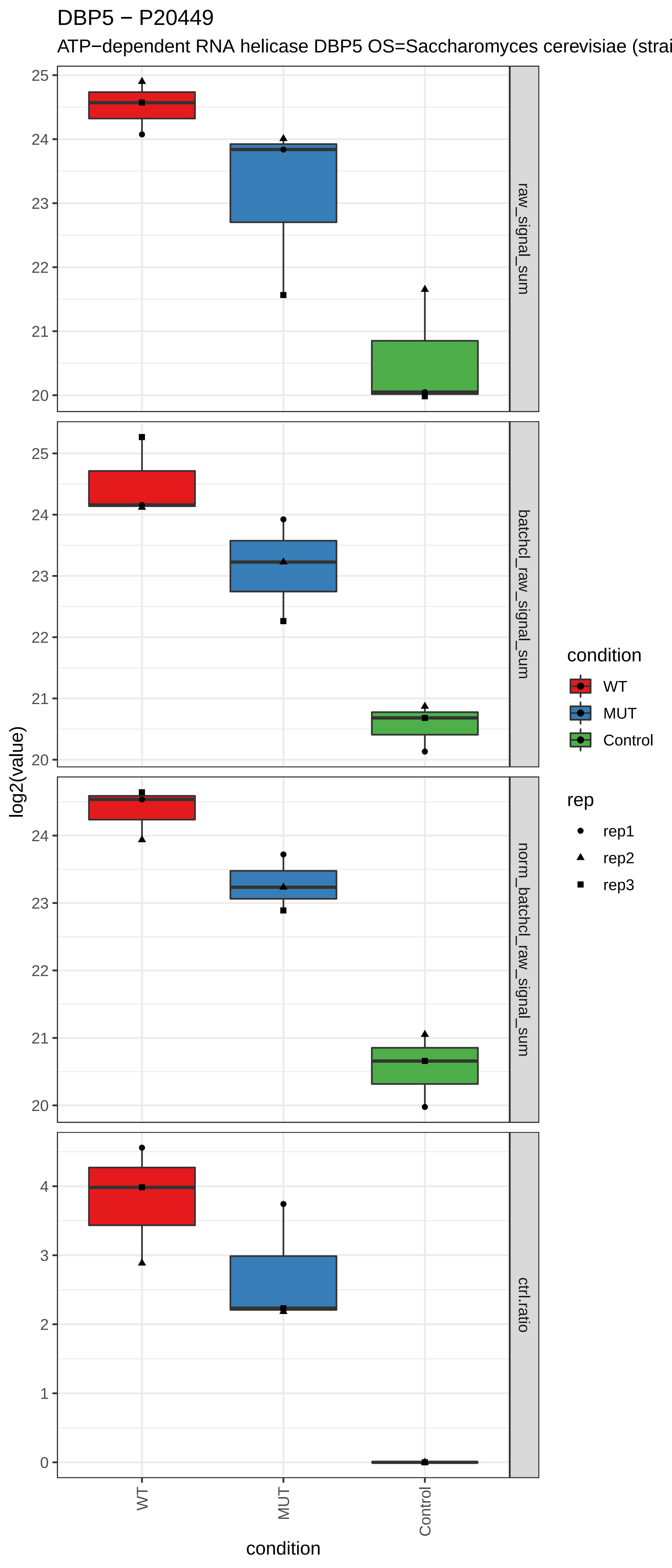

DBP5 − P20449
ATP−dependent RNA helicase DBP5 OS=Saccharomyces cerevisiae (strai
raw_signal_sum
batchcl_raw_signal_sum
norm_batchcl_raw_signal_sum
ctrl.ratio
log2(value)
condition
WT
MUT
Control
rep
rep1
rep2
rep3

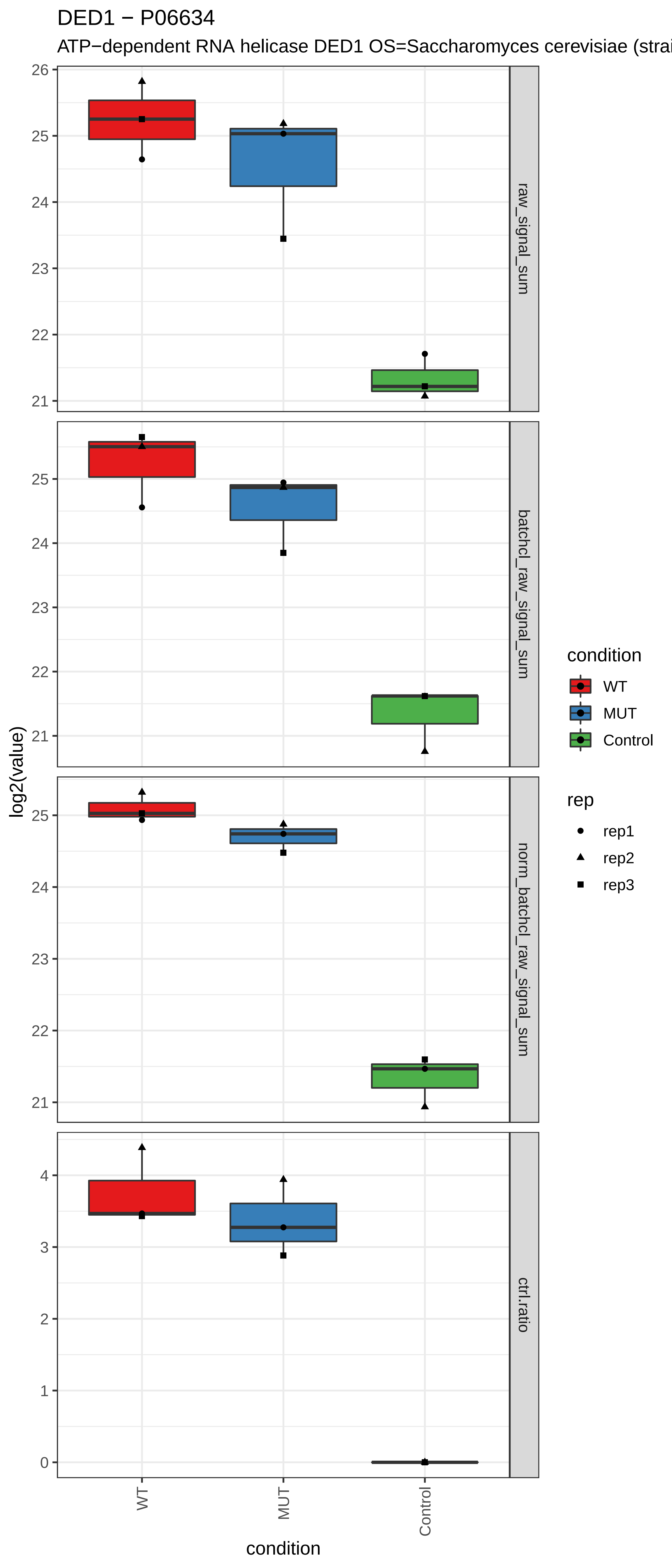
DED1 − P06634
ATP−dependent RNA helicase DED1 OS=Saccharomyces cerevisiae (strai
raw_signal_sum
batchcl_raw_signal_sum
norm_batchcl_raw_signal_sum
ctrl.ratio
log2(value)
condition
WT
MUT
Control
rep
rep1
rep2
rep3
26
25
24
23
22
21
4
3
2
1
0

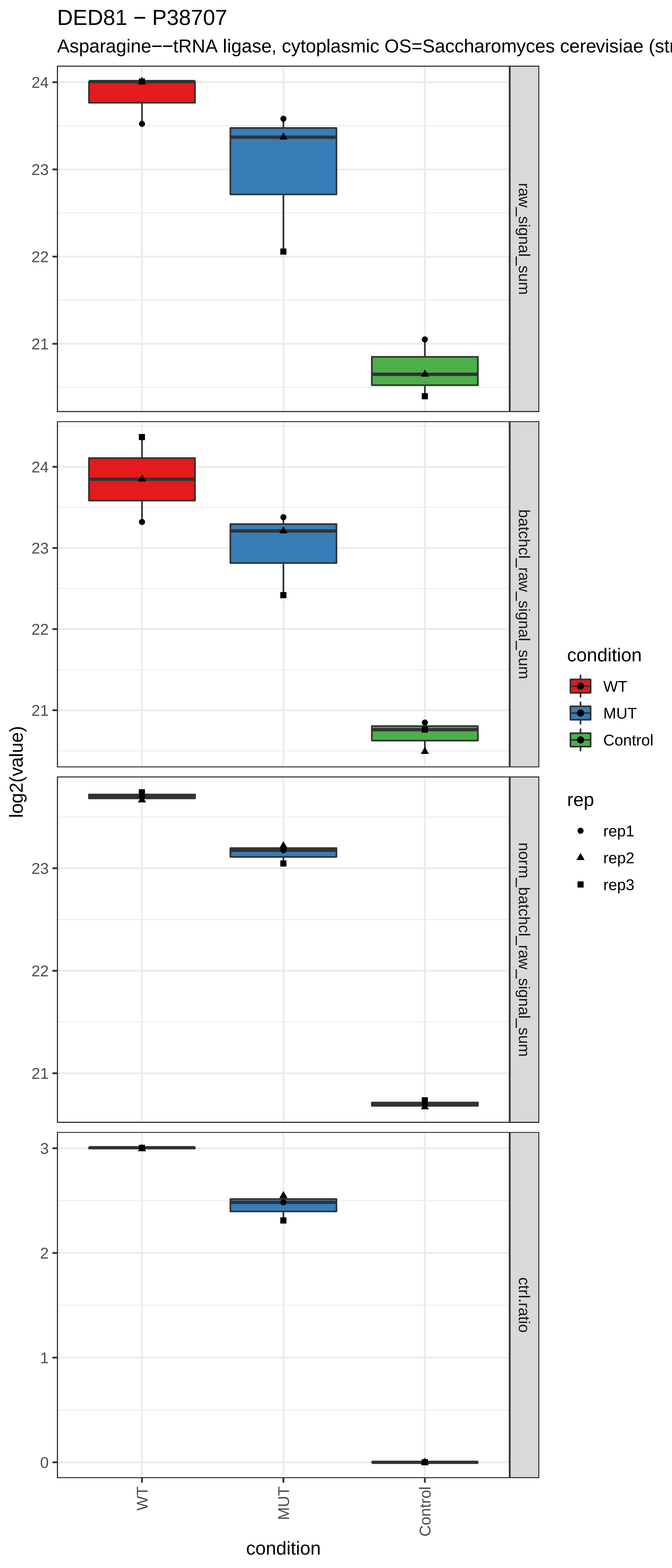

DED81 − P38707
Asparagine−−tRNA ligase, cytoplasmic OS=Saccharomyces cerevisiae (str
raw_signal_sum
batchcl_raw_signal_sum
norm_batchcl_raw_signal_sum
ctrl.ratio
log2(value)
condition
WT
MUT
Control
rep
rep1
rep2
rep3

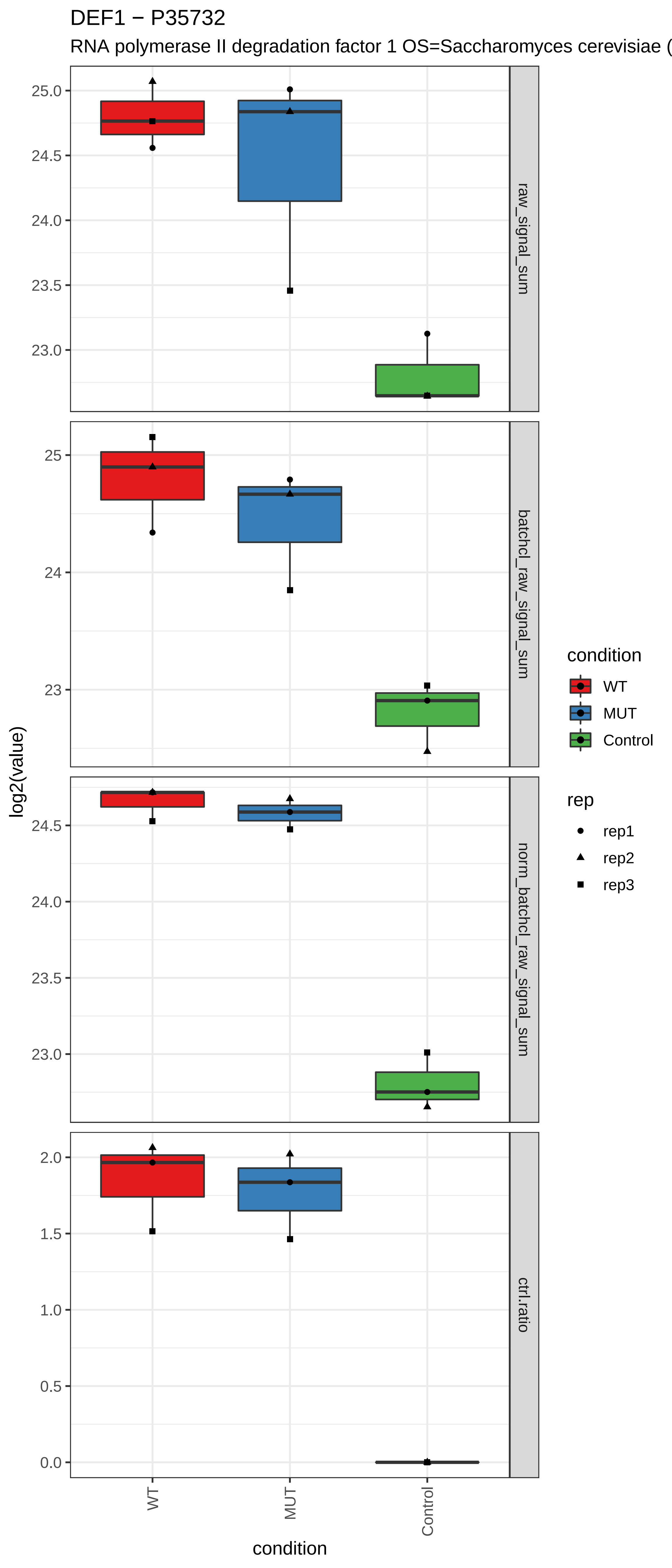

DEF1 − P35732
RNA polymerase II degradation factor 1 OS=Saccharomyces cerevisiae (
raw_signal_sum
batchcl_raw_signal_sum
norm_batchcl_raw_signal_sum
ctrl.ratio
log2(value)
condition
WT
MUT
Control
rep
rep1
rep2
rep3

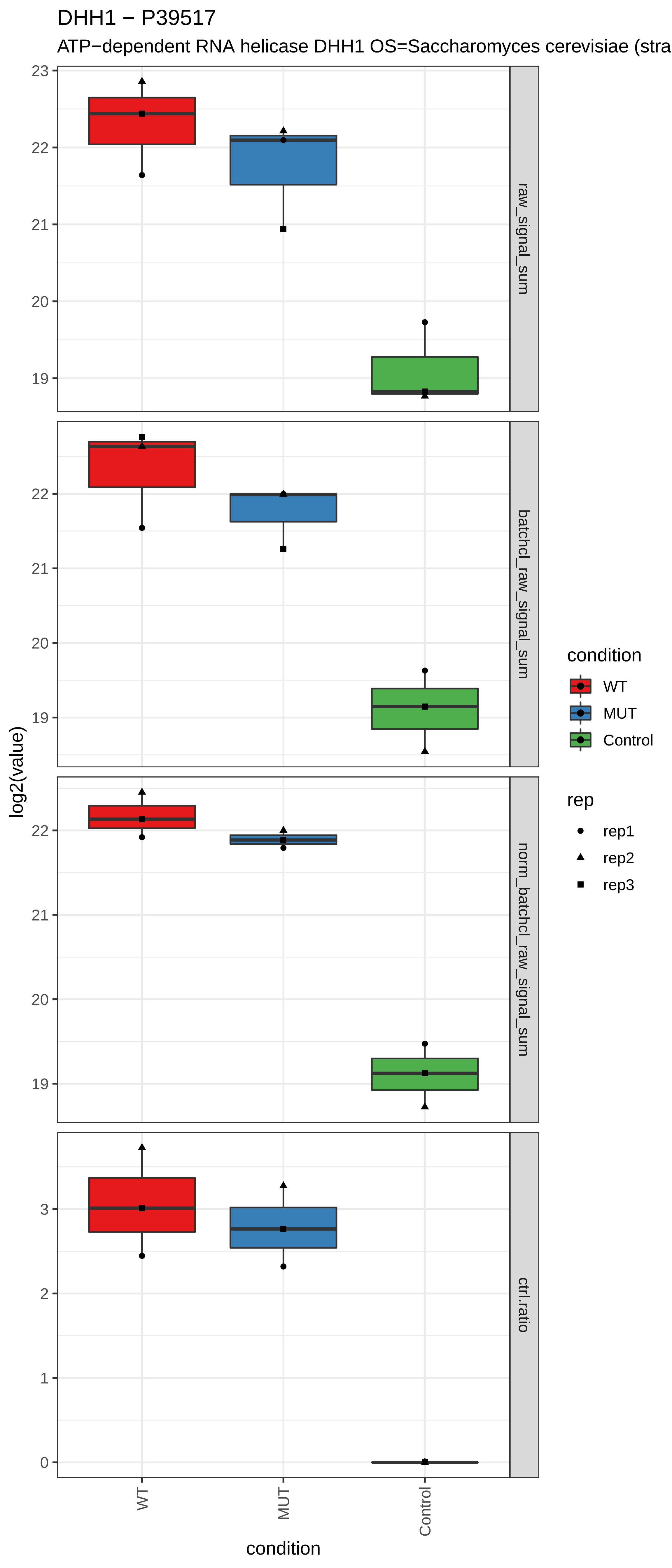

DHH1 − P39517
ATP−dependent RNA helicase DHH1 OS=Saccharomyces cerevisiae (stra
raw_signal_sum
batchcl_raw_signal_sum
norm_batchcl_raw_signal_sum
ctrl.ratio
log2(value)
condition
WT
MUT
Control
rep
rep1
rep2
rep3

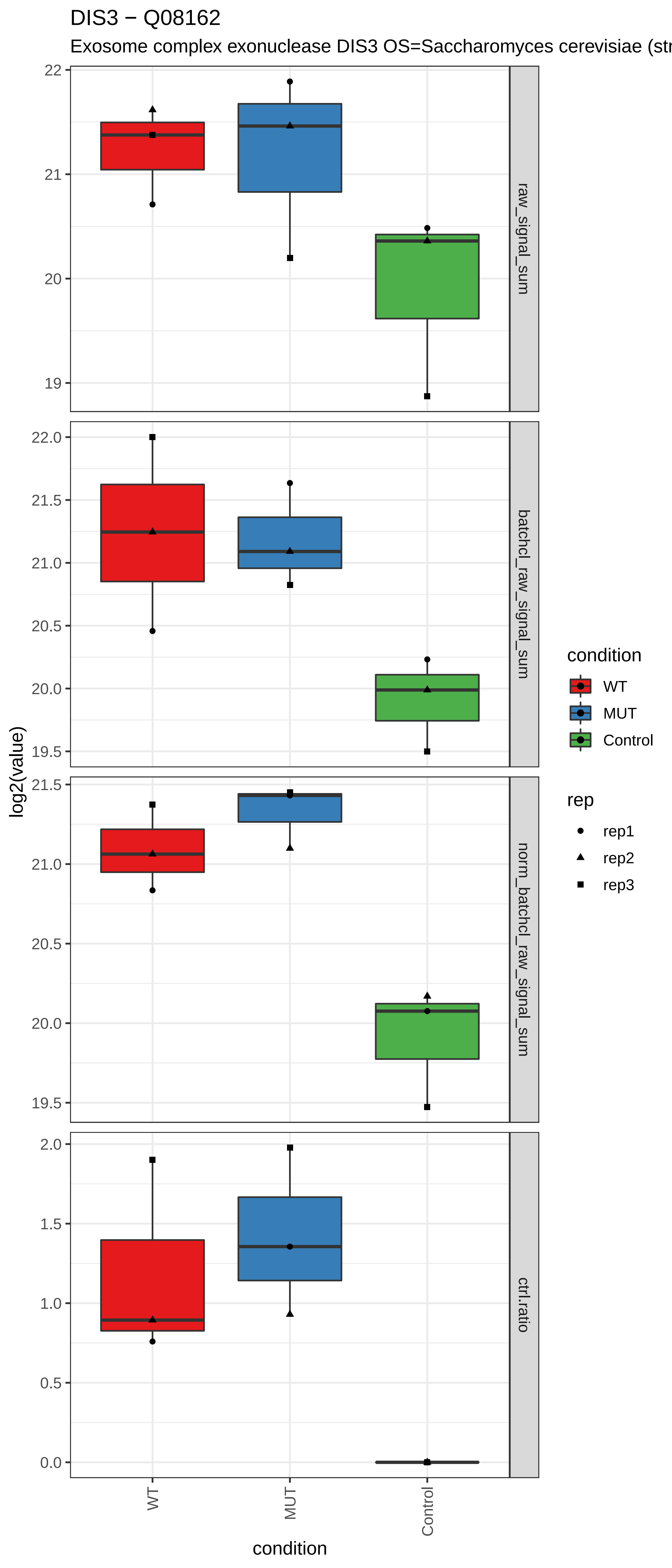

DIS3 − Q08162
Exosome complex exonuclease DIS3 OS=Saccharomyces cerevisiae (str
raw_signal_sum
batchcl_raw_signal_sum
norm_batchcl_raw_signal_sum
ctrl.ratio
log2(value)
condition
WT
MUT
Control
rep
rep1
rep2
rep3

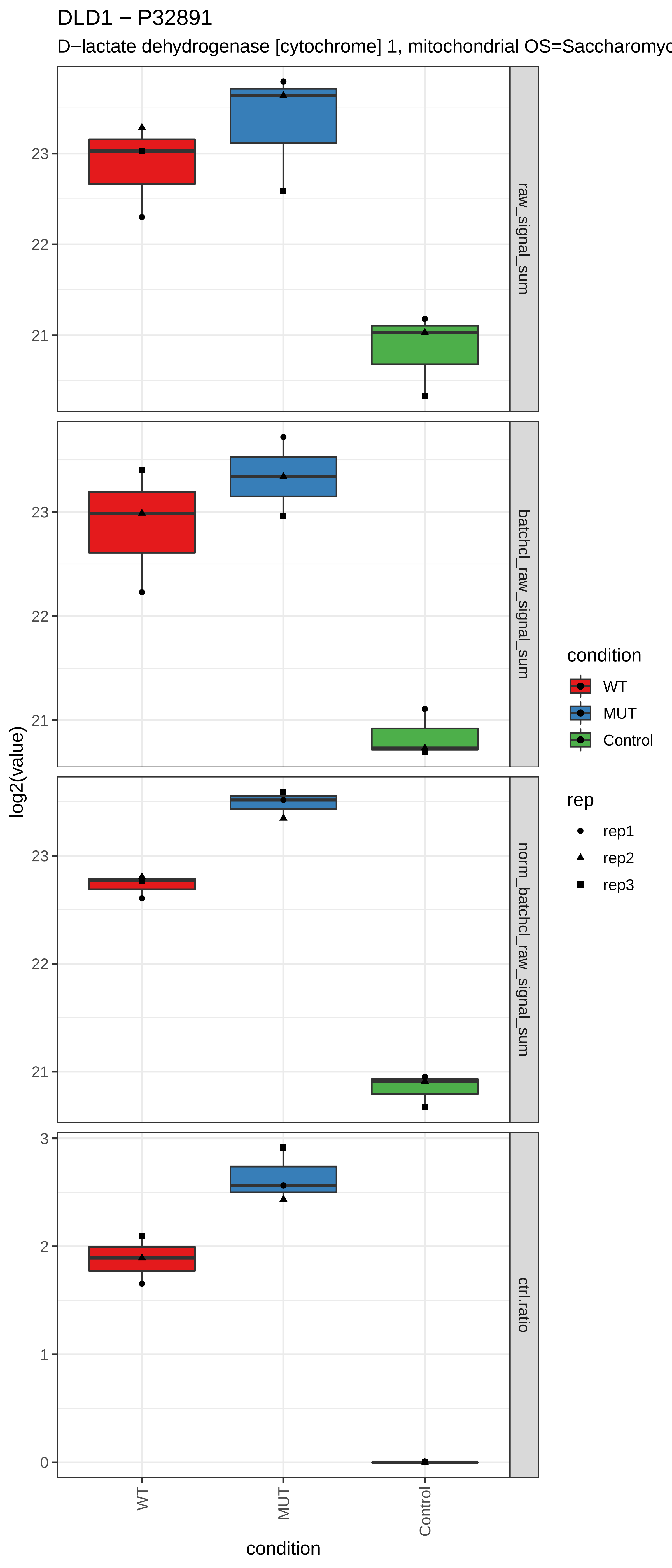
DLD1 − P32891
D−lactate dehydrogenase [cytochrome] 1, mitochondrial OS=Saccharomyc
raw_signal_sum
batchcl_raw_signal_sum
norm_batchcl_raw_signal_sum
ctrl.ratio
log2(value)
condition
WT
MUT
Control
rep
rep1
rep2
rep3

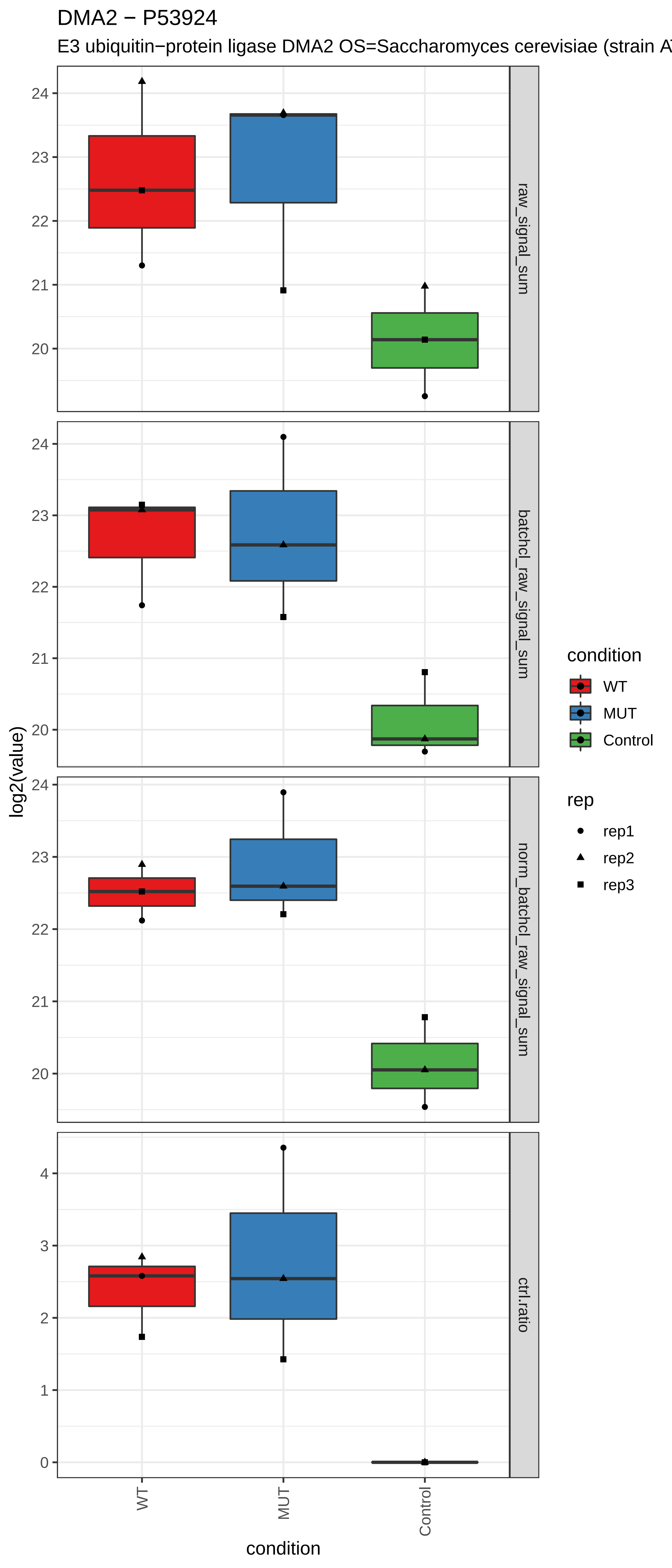

DMA2 − P53924
E3 ubiquitin−protein ligase DMA2 OS=Saccharomyces cerevisiae (strain A
raw_signal_sum
batchcl_raw_signal_sum
norm_batchcl_raw_signal_sum
ctrl.ratio
log2(value)
condition
WT
MUT
Control
rep
rep1
rep2
rep3

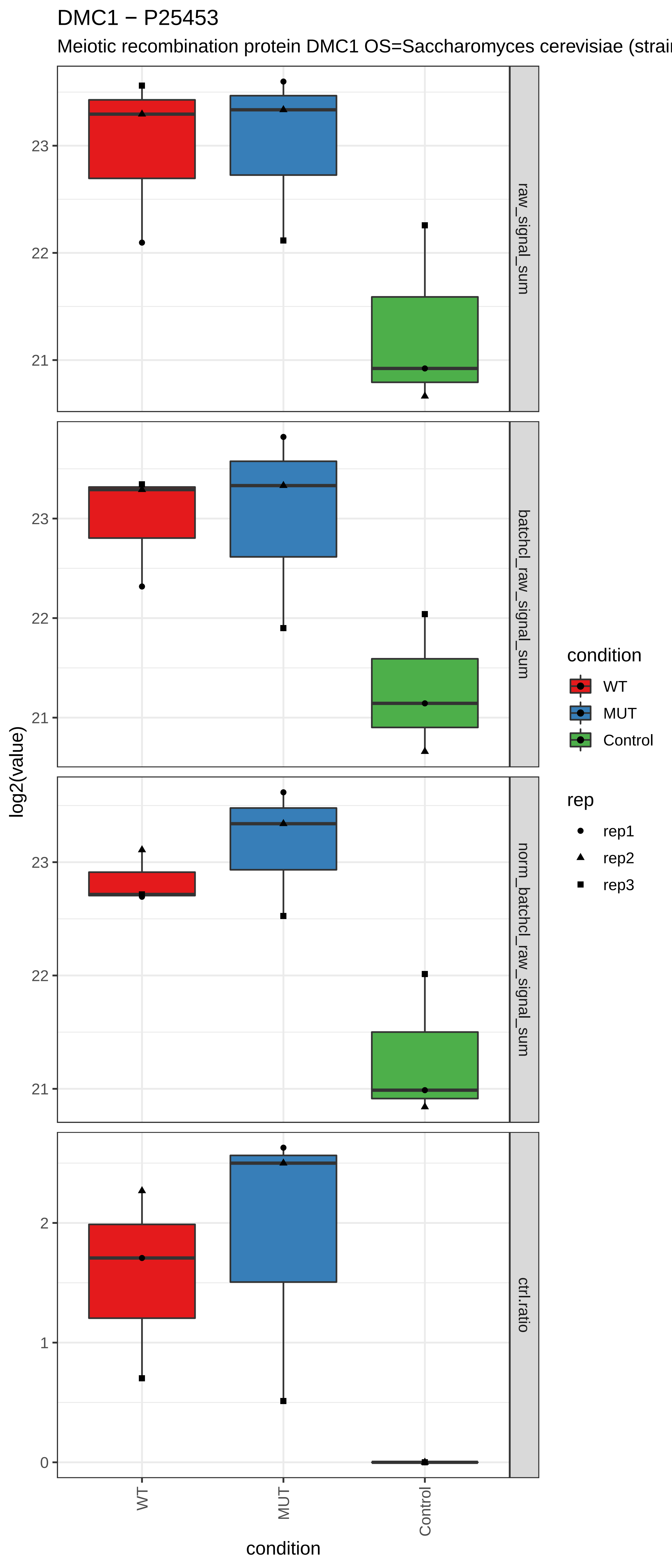
DMC1 − P25453
Meiotic recombination protein DMC1 OS=Saccharomyces cerevisiae (strai
raw_signal_sum
batchcl_raw_signal_sum
norm_batchcl_raw_signal_sum
ctrl.ratio
log2(value)
condition
WT
MUT
Control
rep
rep1
rep2
rep3
23
22
21
2
1
0

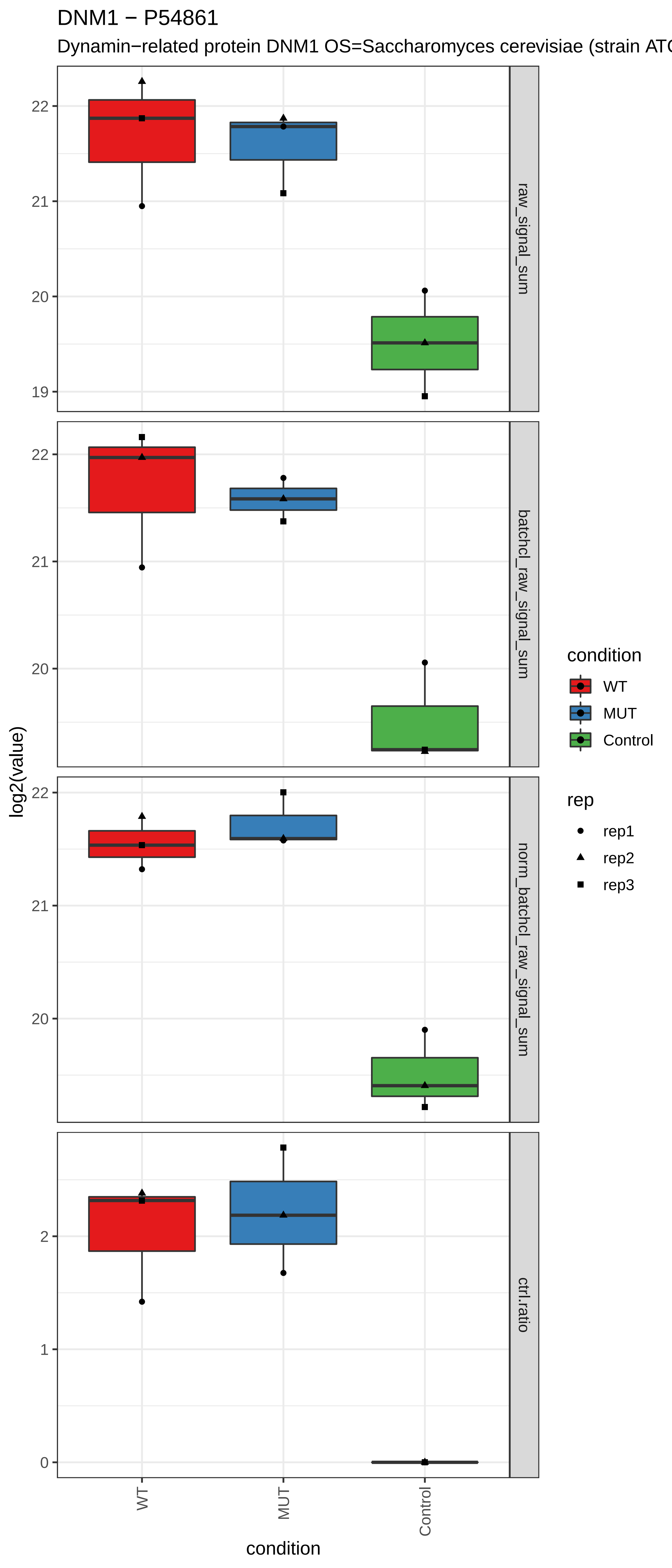
DNM1 − P54861
Dynamin−related protein DNM1 OS=Saccharomyces cerevisiae (strain ATC
raw_signal_sum
batchcl_raw_signal_sum
norm_batchcl_raw_signal_sum
ctrl.ratio
log2(value)
condition
WT
MUT
Control
rep
rep1
rep2
rep3
22
21
20
19
2
1
0

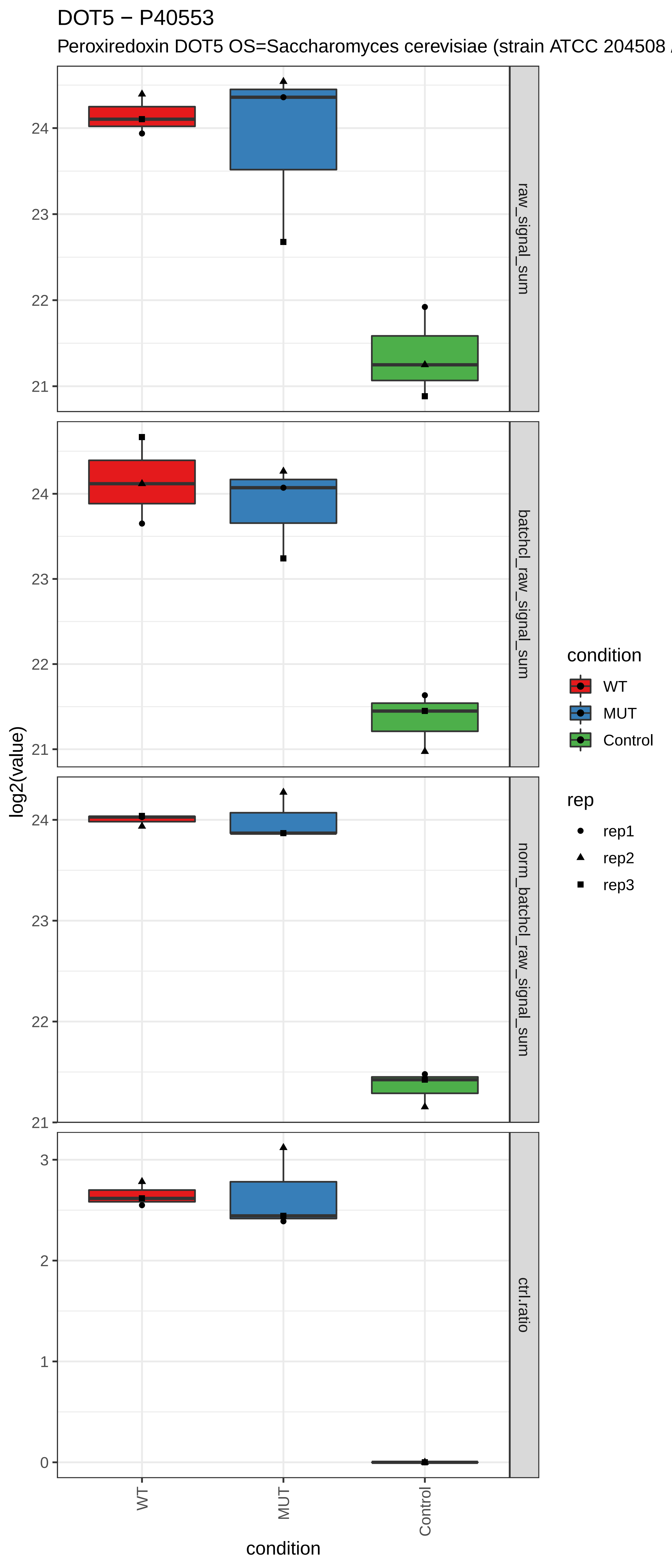

DOT5 − P40553
Peroxiredoxin DOT5 OS=Saccharomyces cerevisiae (strain ATCC 204508 /
raw_signal_sum
batchcl_raw_signal_sum
norm_batchcl_raw_signal_sum
ctrl.ratio
log2(value)
condition
WT
MUT
Control
rep
rep1
rep2
rep3

DPM1 − P14020

Dolichol−phosphate mannosyltransferase OS=Saccharomyces cerevisiae (

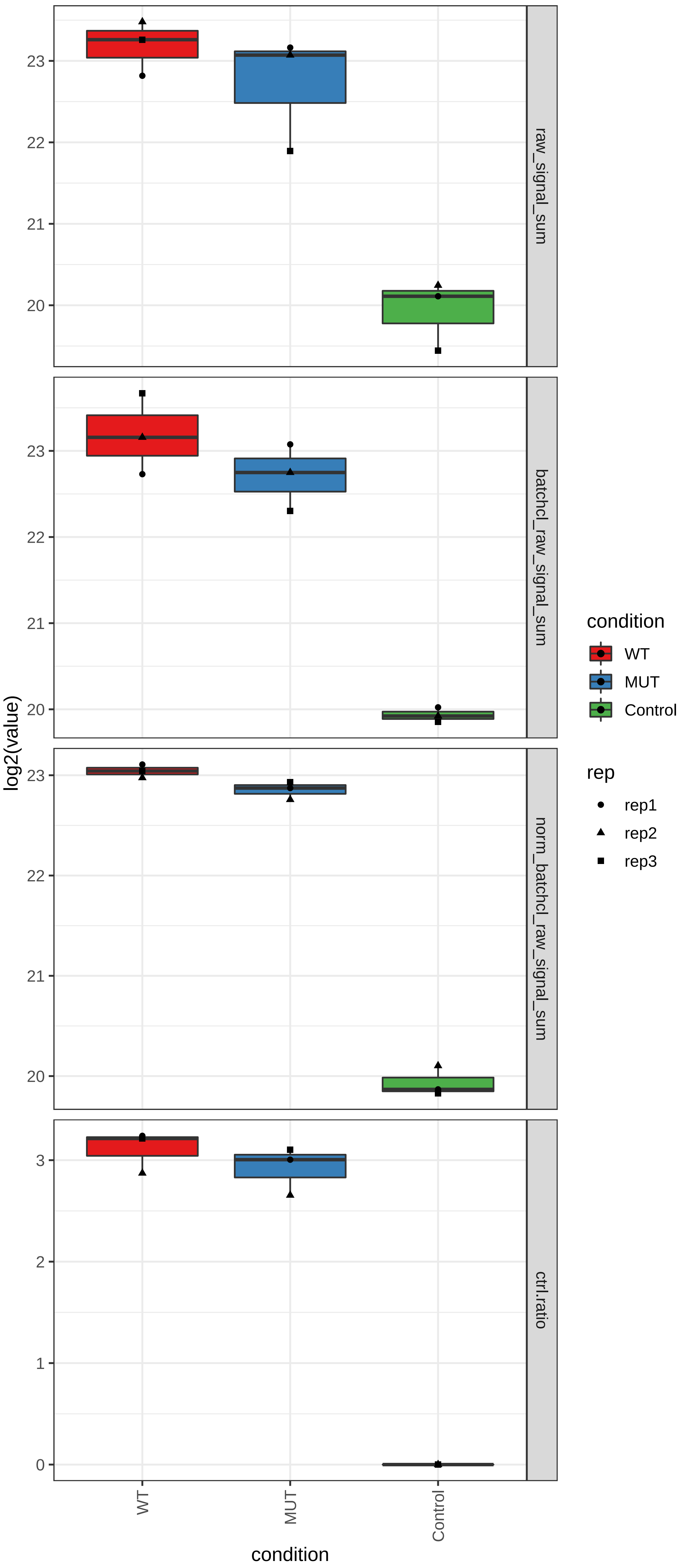

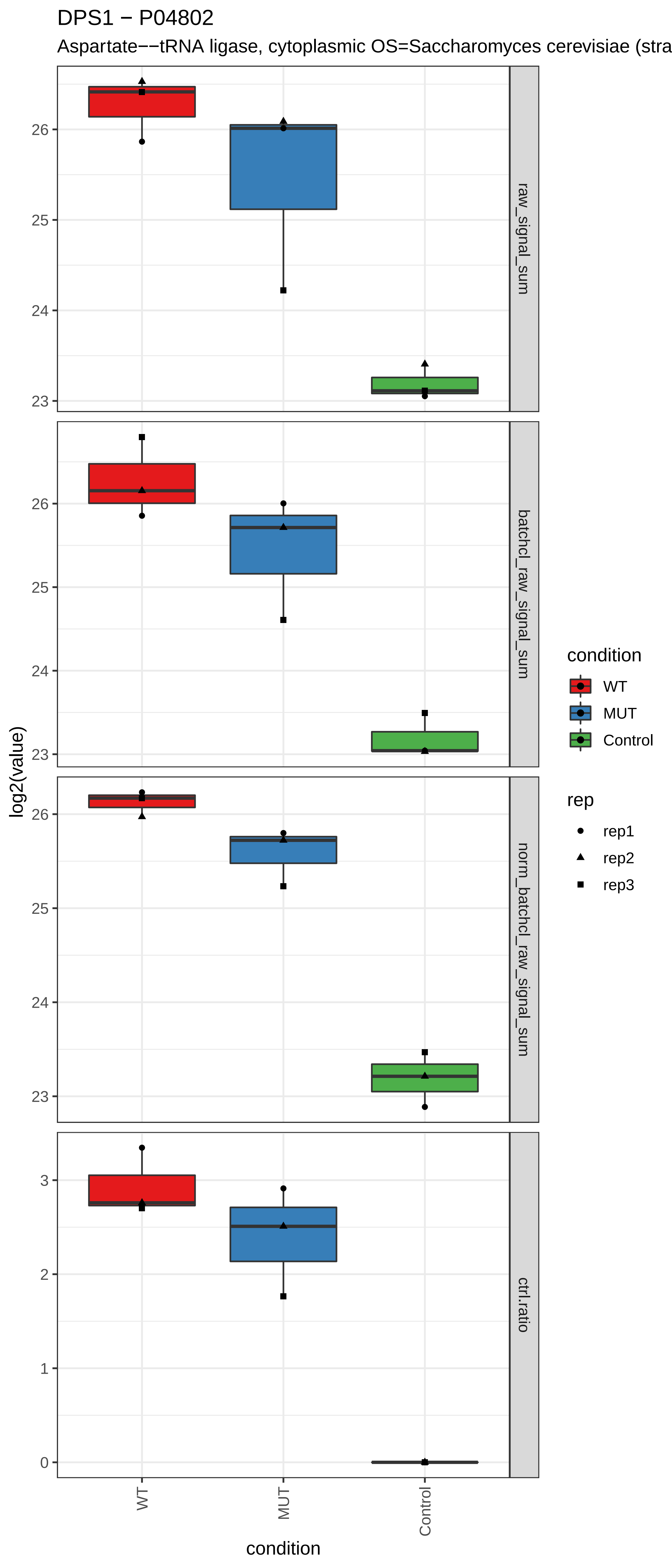
DPS1 − P04802
Aspartate−−tRNA ligase, cytoplasmic OS=Saccharomyces cerevisiae (stra
raw_signal_sum
batchcl_raw_signal_sum
norm_batchcl_raw_signal_sum
ctrl.ratio
log2(value)
condition
WT
MUT
Control
rep
rep1
rep2
rep3
26
25
24
23
3
2
1
0
WT
MUT
Control
condition

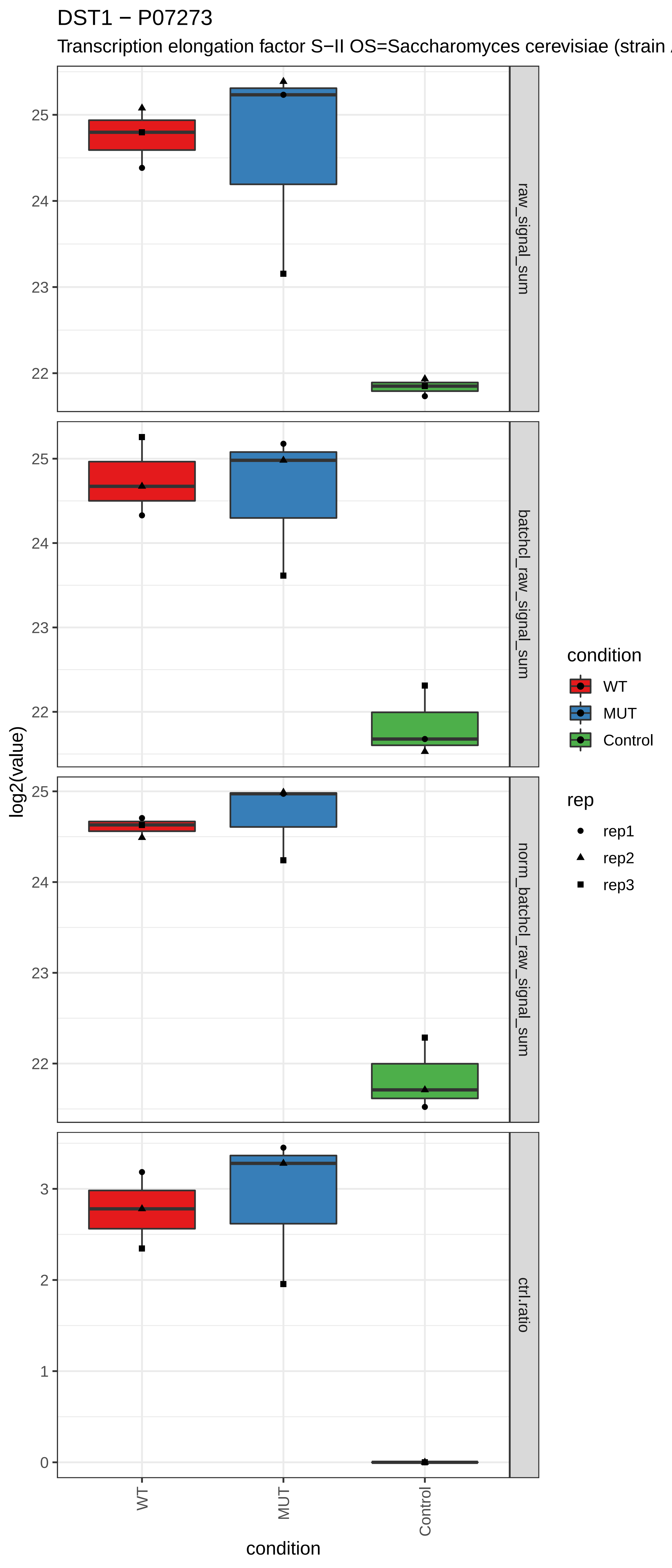
DST1 − P07273
Transcription elongation factor S−II OS=Saccharomyces cerevisiae (strain
raw_signal_sum
batchcl_raw_signal_sum
norm_batchcl_raw_signal_sum
ctrl.ratio
log2(value)
condition
WT
MUT
Control
rep
rep1
rep2
rep3
25
24
23
22
3
2
1
0

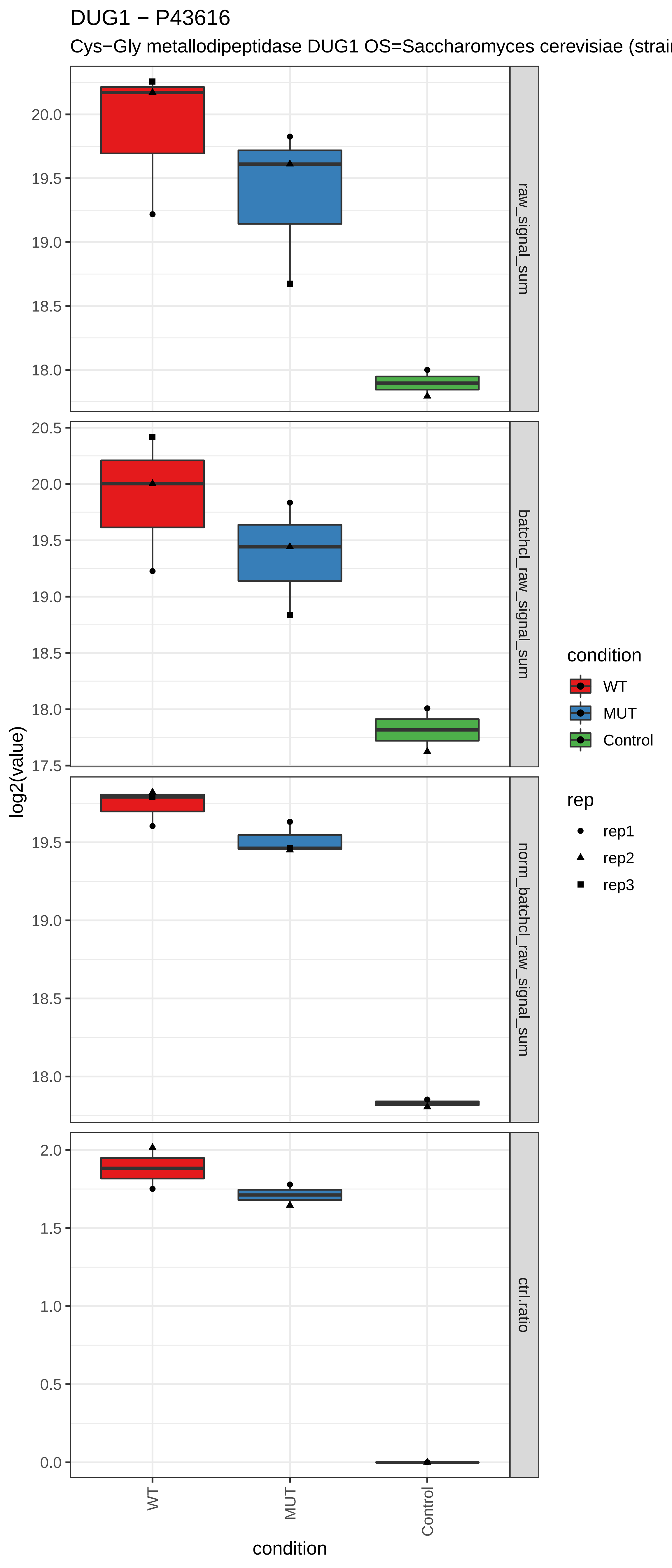

DUG1 − P43616
Cys−Gly metallodipeptidase DUG1 OS=Saccharomyces cerevisiae (strai
raw_signal_sum
batchcl_raw_signal_sum
norm_batchcl_raw_signal_sum
ctrl.ratio
log2(value)
condition
WT
MUT
Control
rep
rep1
rep2
rep3
20.0
19.5
19.0
18.5
18.0
20.5
17.5
2.0
1.5
1.0
0.5
0.0

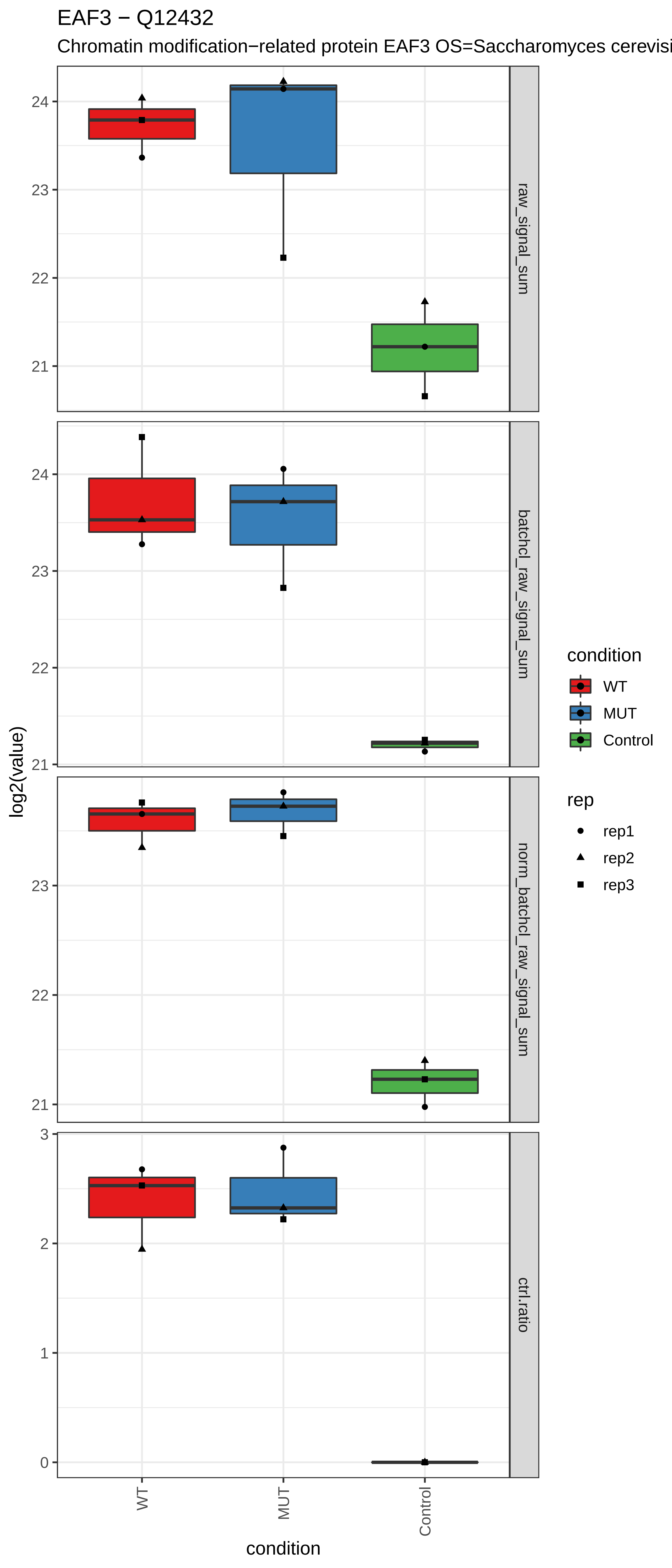

EAF3 − Q12432
Chromatin modification−related protein EAF3 OS=Saccharomyces cerevisi
raw_signal_sum
batchcl_raw_signal_sum
norm_batchcl_raw_signal_sum
ctrl.ratio
log2(value)
condition
WT
MUT
Control
rep
rep1
rep2
rep3
24
23
22
21
3
2
1
0

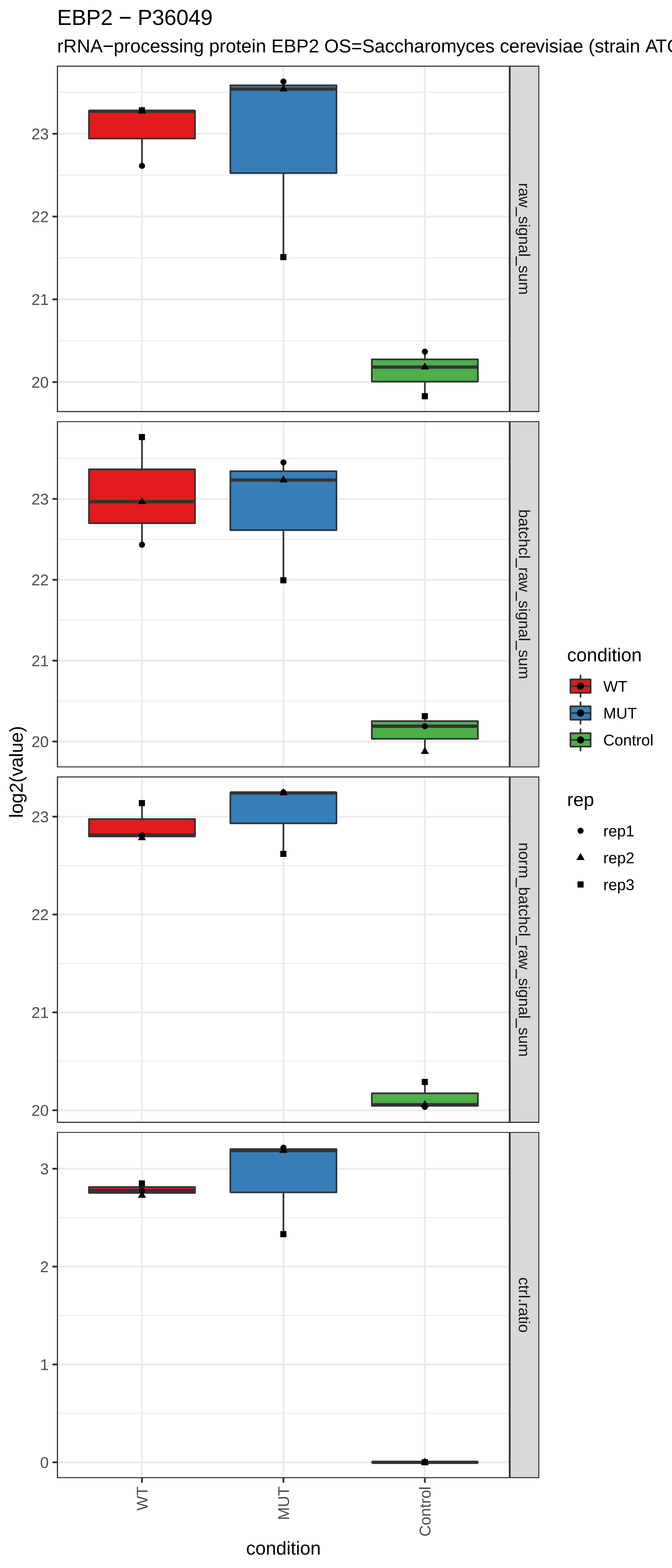
EBP2 − P36049
rRNA−processing protein EBP2 OS=Saccharomyces cerevisiae (strain ATC
raw_signal_sum
batchcl_raw_signal_sum
norm_batchcl_raw_signal_sum
ctrl.ratio
log2(value)
condition
WT
MUT
Control
rep
rep1
rep2
rep3
20
21
22
23
0
1
2
3

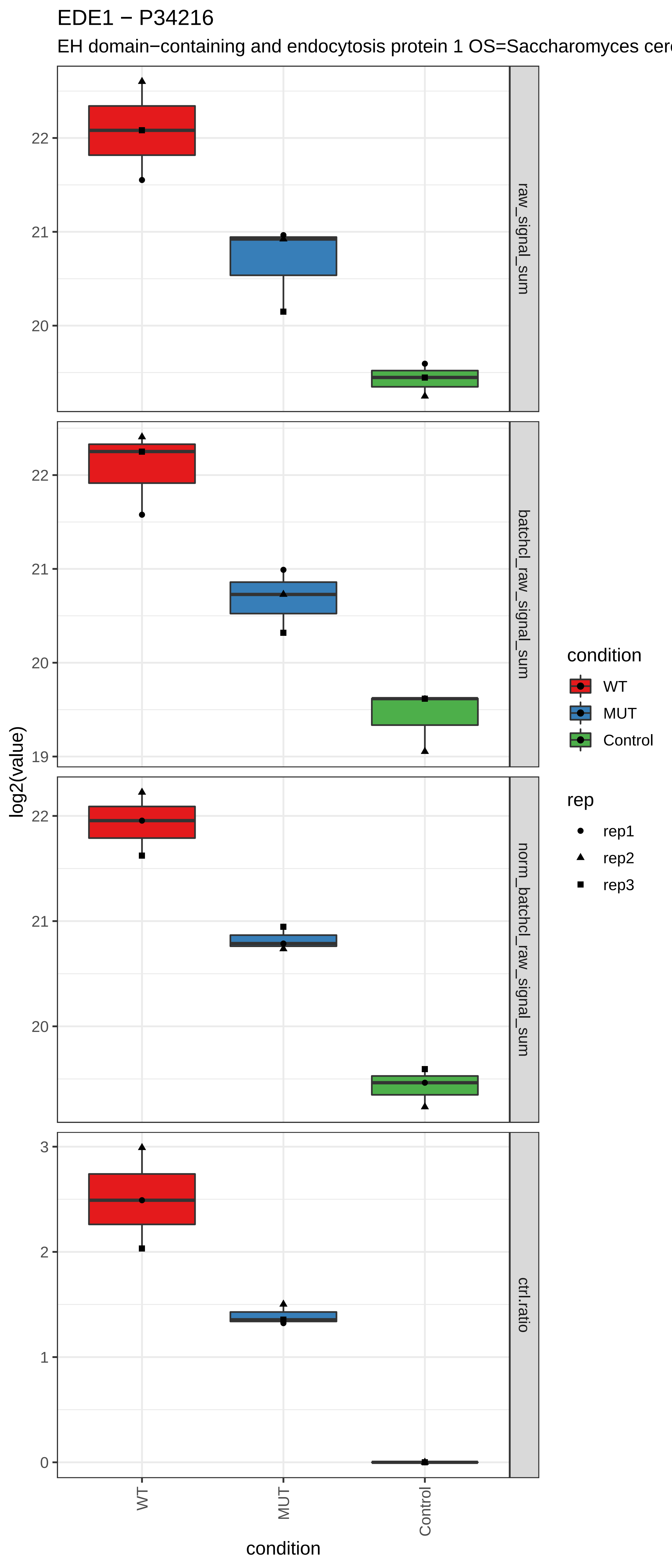

EDE1 − P34216
EH domain−containing and endocytosis protein 1 OS=Saccharomyces cere
raw_signal_sum
batchcl_raw_signal_sum
norm_batchcl_raw_signal_sum
ctrl.ratio
log2(value)
condition
WT
MUT
Control
rep
rep1
rep2
rep3
22
21
20
19
3
2
1
0

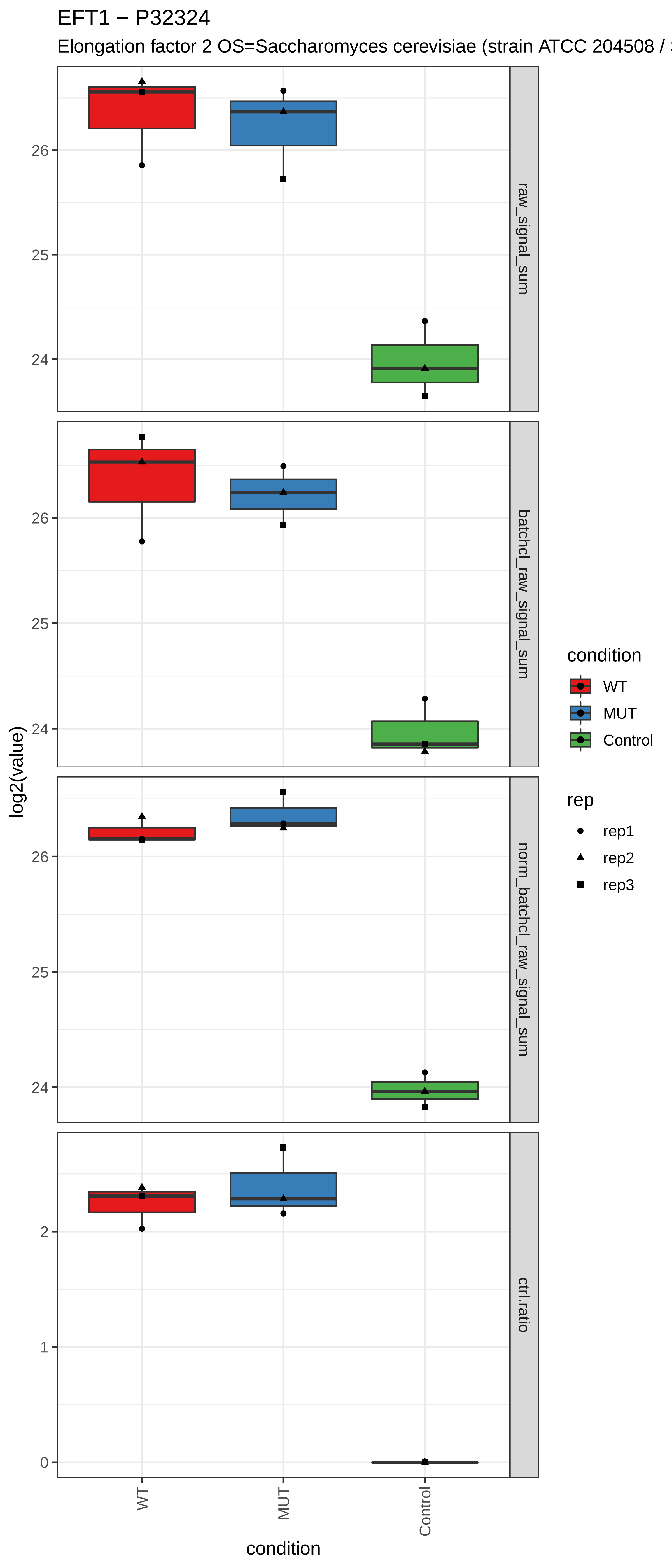
EFT1 − P32324
Elongation factor 2 OS=Saccharomyces cerevisiae (strain ATCC 204508 /
raw_signal_sum
batchcl_raw_signal_sum
norm_batchcl_raw_signal_sum
ctrl.ratio
log2(value)
condition
WT
MUT
Control
rep
rep1
rep2
rep3
26
25
24
2
1
0

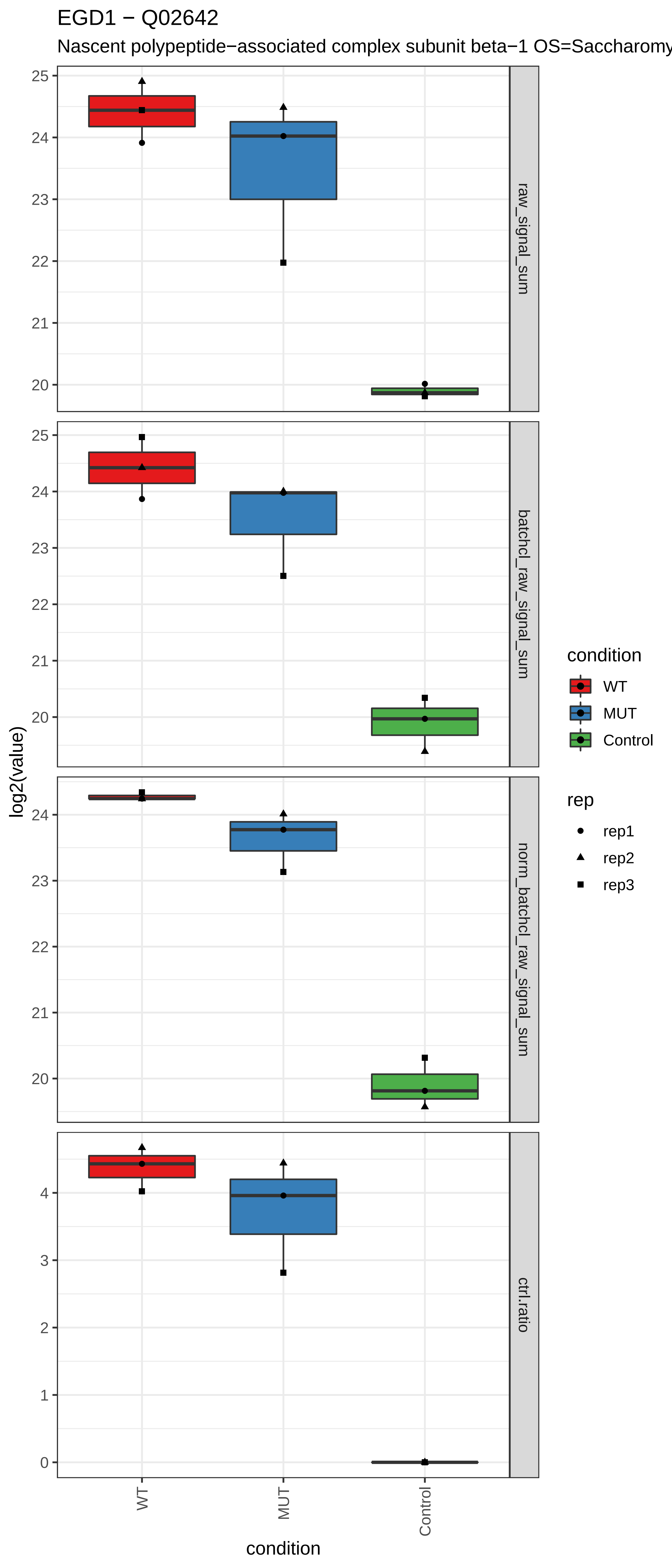

EGD1 − Q02642
Nascent polypeptide−associated complex subunit beta−1 OS=Saccharomy
raw_signal_sum
batchcl_raw_signal_sum
norm_batchcl_raw_signal_sum
ctrl.ratio
log2(value)
condition
WT
MUT
Control
rep
rep1
rep2
rep3

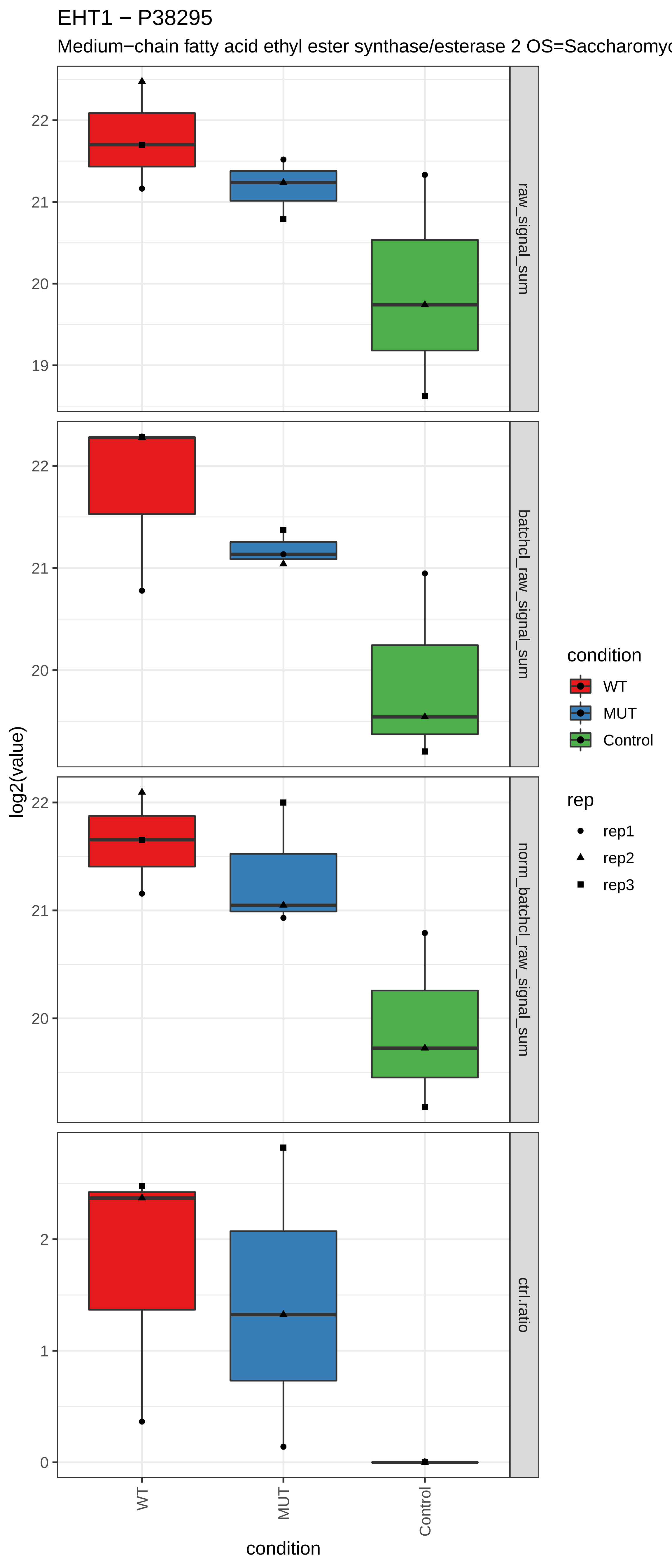
EHT1 − P38295
Medium−chain fatty acid ethyl ester synthase/esterase 2 OS=Saccharomyc
raw_signal_sum
batchcl_raw_signal_sum
norm_batchcl_raw_signal_sum
ctrl.ratio
log2(value)
condition
WT
MUT
Control
rep
rep1
rep2
rep3
22
21
20
19
2
1
0

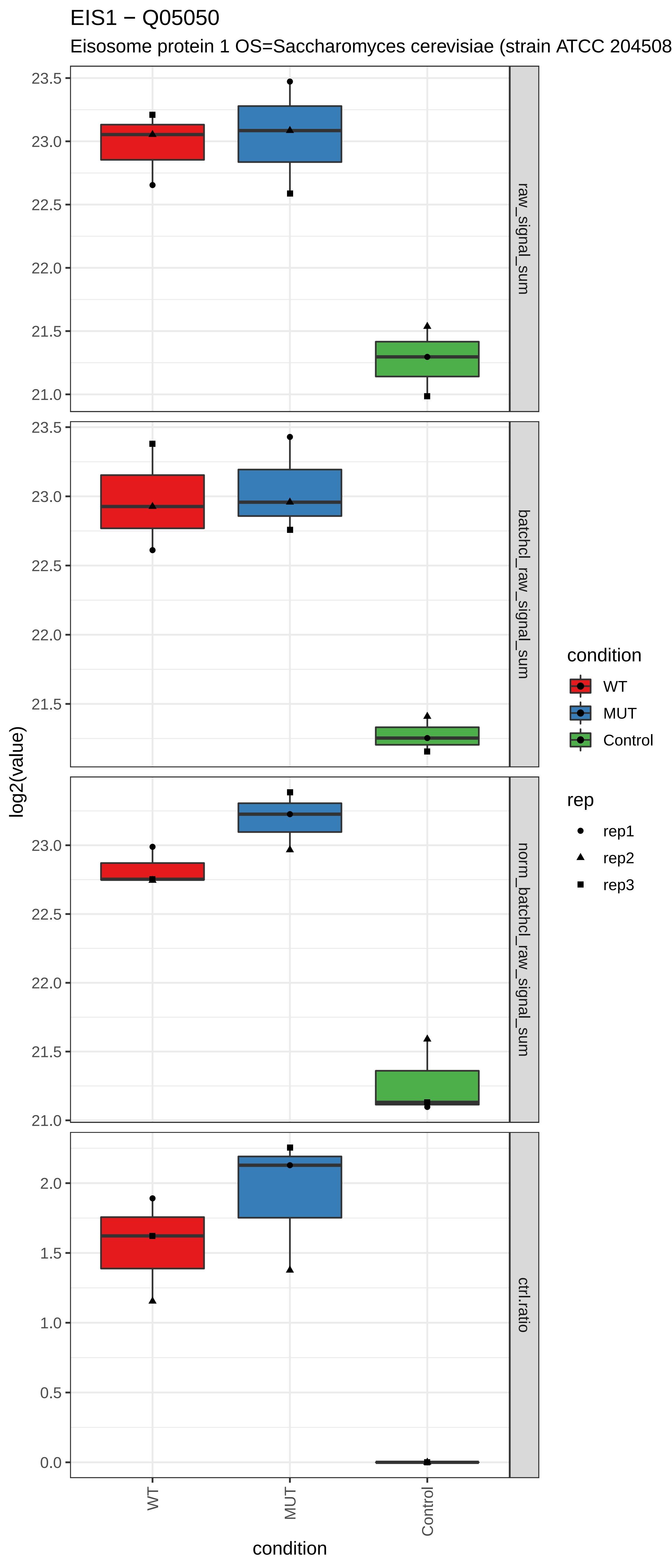

EIS1 − Q05050
Eisosome protein 1 OS=Saccharomyces cerevisiae (strain ATCC 204508
raw_signal_sum
batchcl_raw_signal_sum
norm_batchcl_raw_signal_sum
ctrl.ratio
log2(value)
condition
WT
MUT
Control
rep
rep1
rep2
rep3

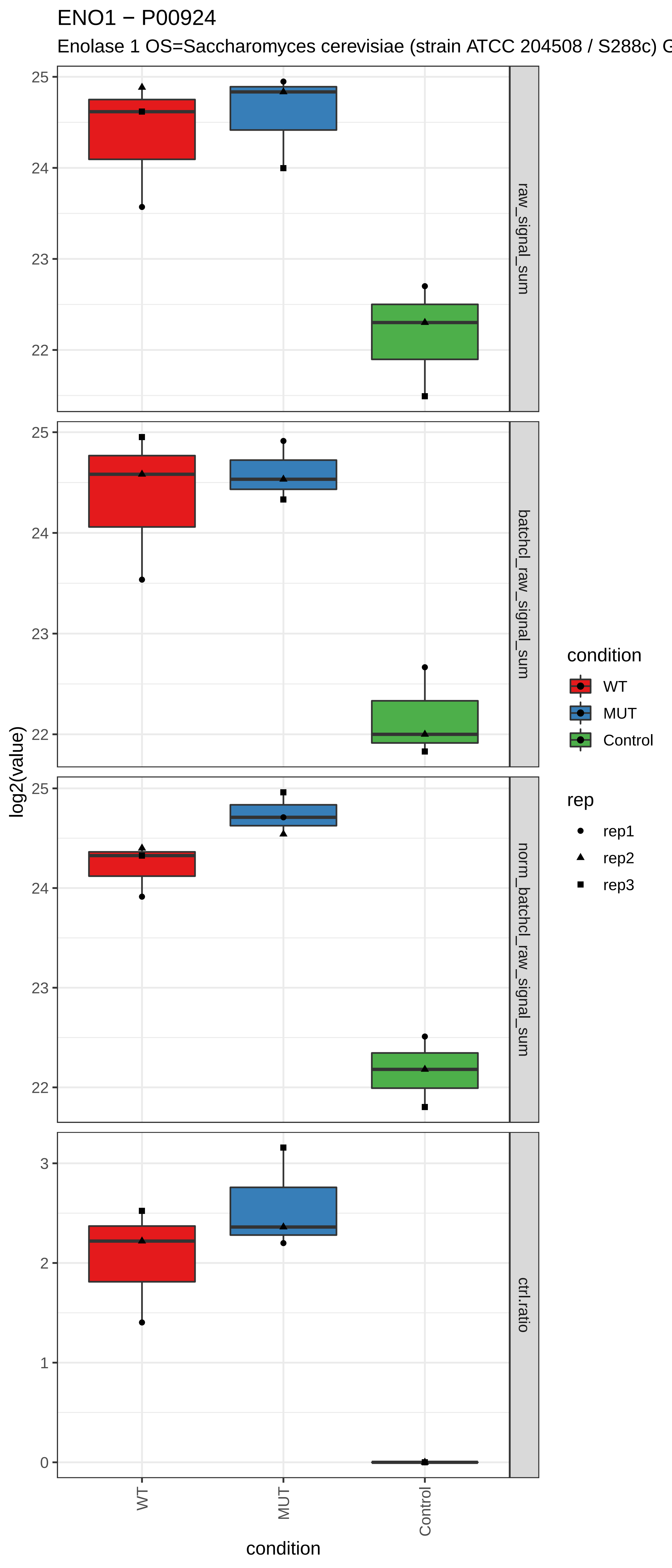

ENO1 − P00924
Enolase 1 OS=Saccharomyces cerevisiae (strain ATCC 204508 / S288c) G
raw_signal_sum
batchcl_raw_signal_sum
norm_batchcl_raw_signal_sum
ctrl.ratio
log2(value)
condition
WT
MUT
Control
rep
rep1
rep2
rep3
25
24
23
22
3
2
1
0

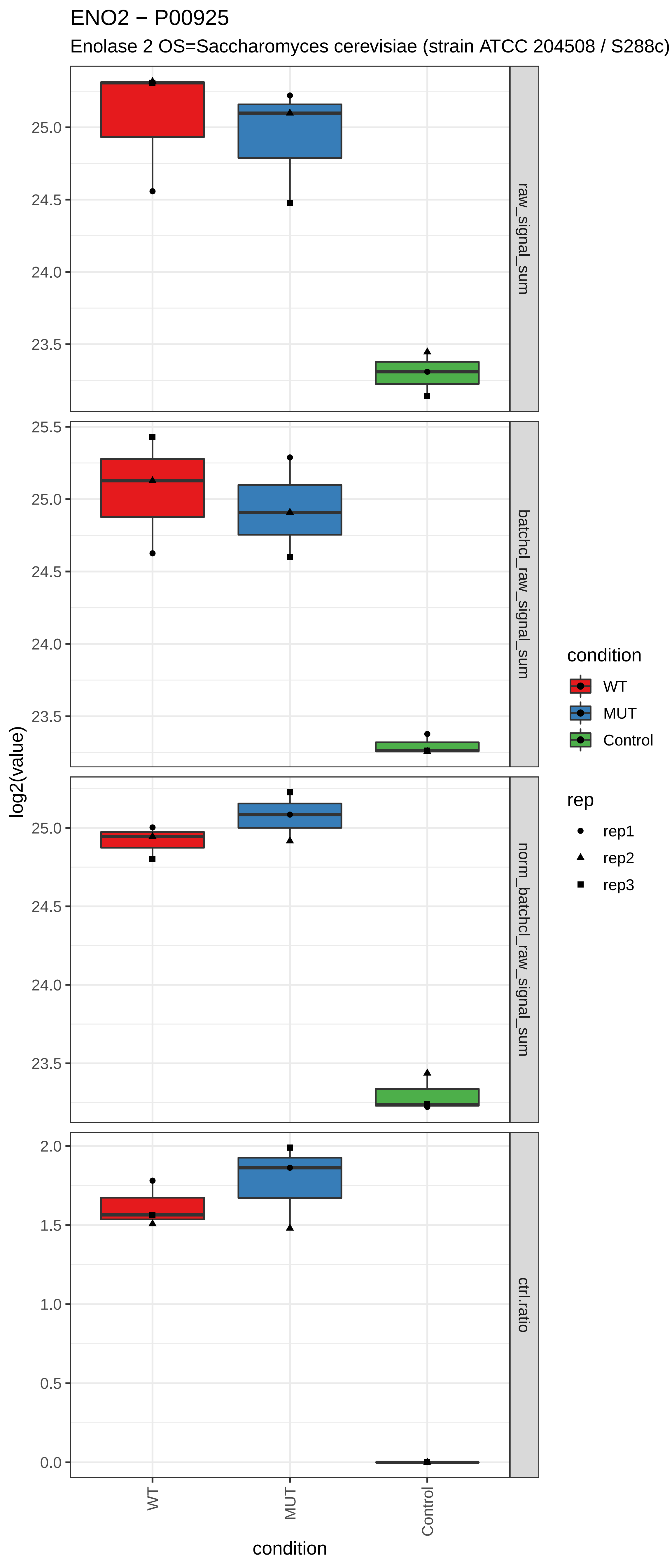

ENO2 − P00925
Enolase 2 OS=Saccharomyces cerevisiae (strain ATCC 204508 / S288c)
raw_signal_sum
batchcl_raw_signal_sum
norm_batchcl_raw_signal_sum
ctrl.ratio
25.0
24.5
24.0
23.5
25.5
2.0
1.5
1.0
0.5
0.0
log2(value)
WT
MUT
Control
condition
rep
rep1
rep2
rep3

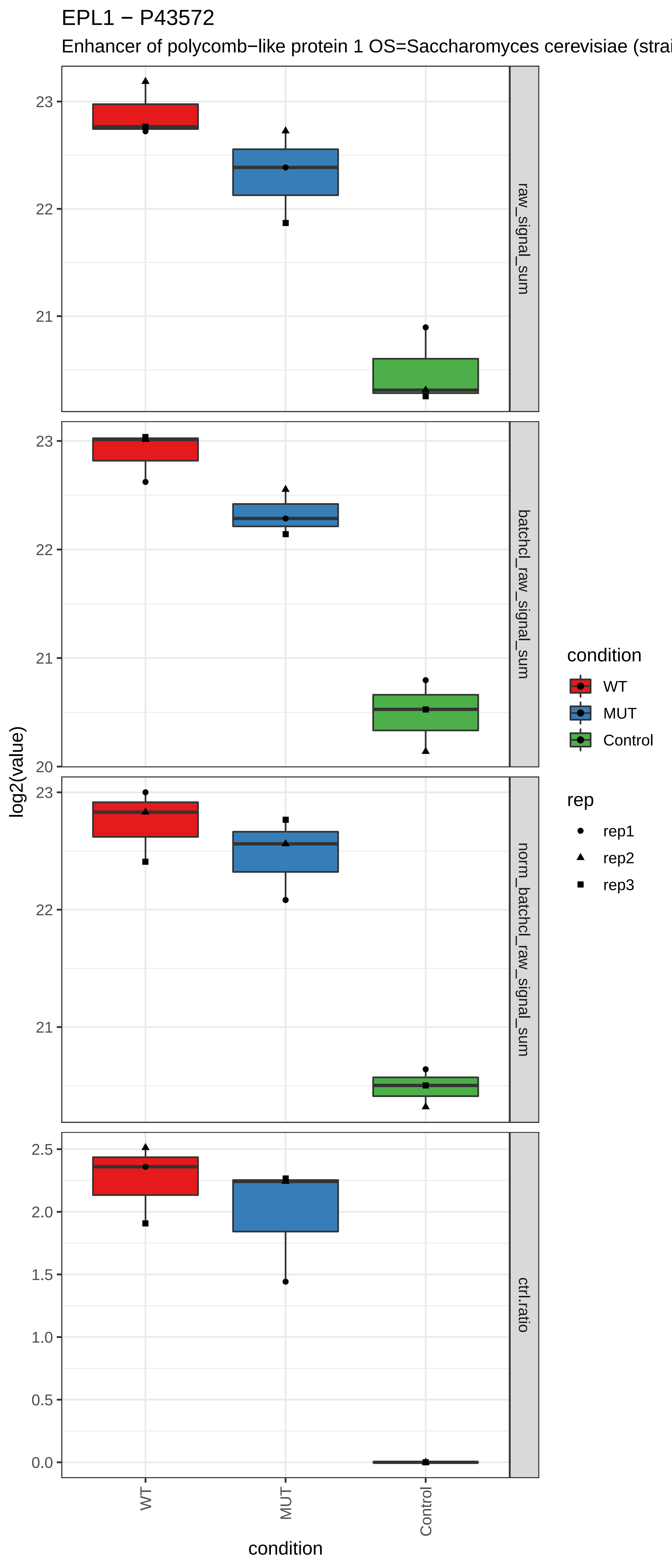

EPL1 − P43572
Enhancer of polycomb−like protein 1 OS=Saccharomyces cerevisiae (strai
raw_signal_sum
batchcl_raw_signal_sum
norm_batchcl_raw_signal_sum
ctrl.ratio
log2(value)
condition
WT
MUT
Control
rep
rep1
rep2
rep3

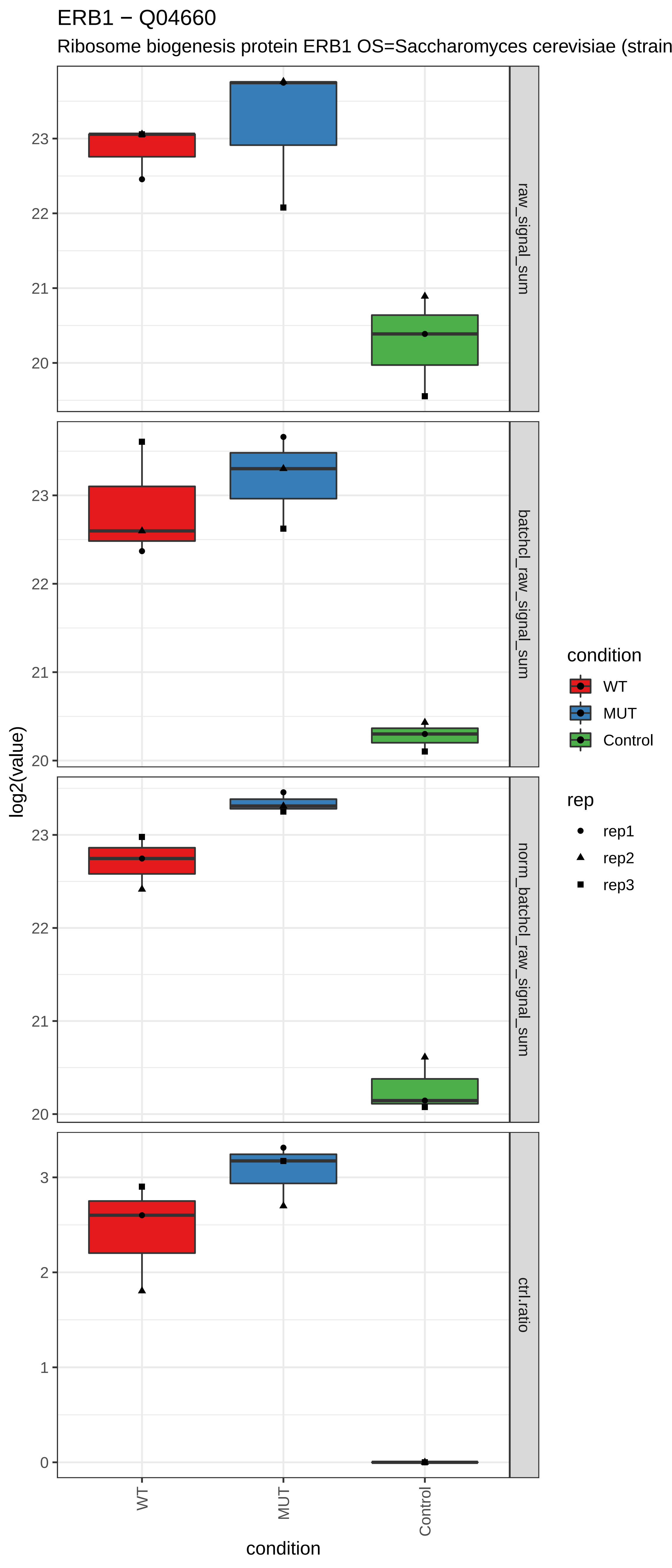

ERB1 − Q04660
Ribosome biogenesis protein ERB1 OS=Saccharomyces cerevisiae (strain
raw_signal_sum
batchcl_raw_signal_sum
norm_batchcl_raw_signal_sum
ctrl.ratio
log2(value)
condition
WT
MUT
Control
condition
WT
MUT
Control
rep
rep1
rep2
rep3

ERG10 − P41338

Acetyl−CoA acetyltransferase OS=Saccharomyces cerevisiae (strain ATCC

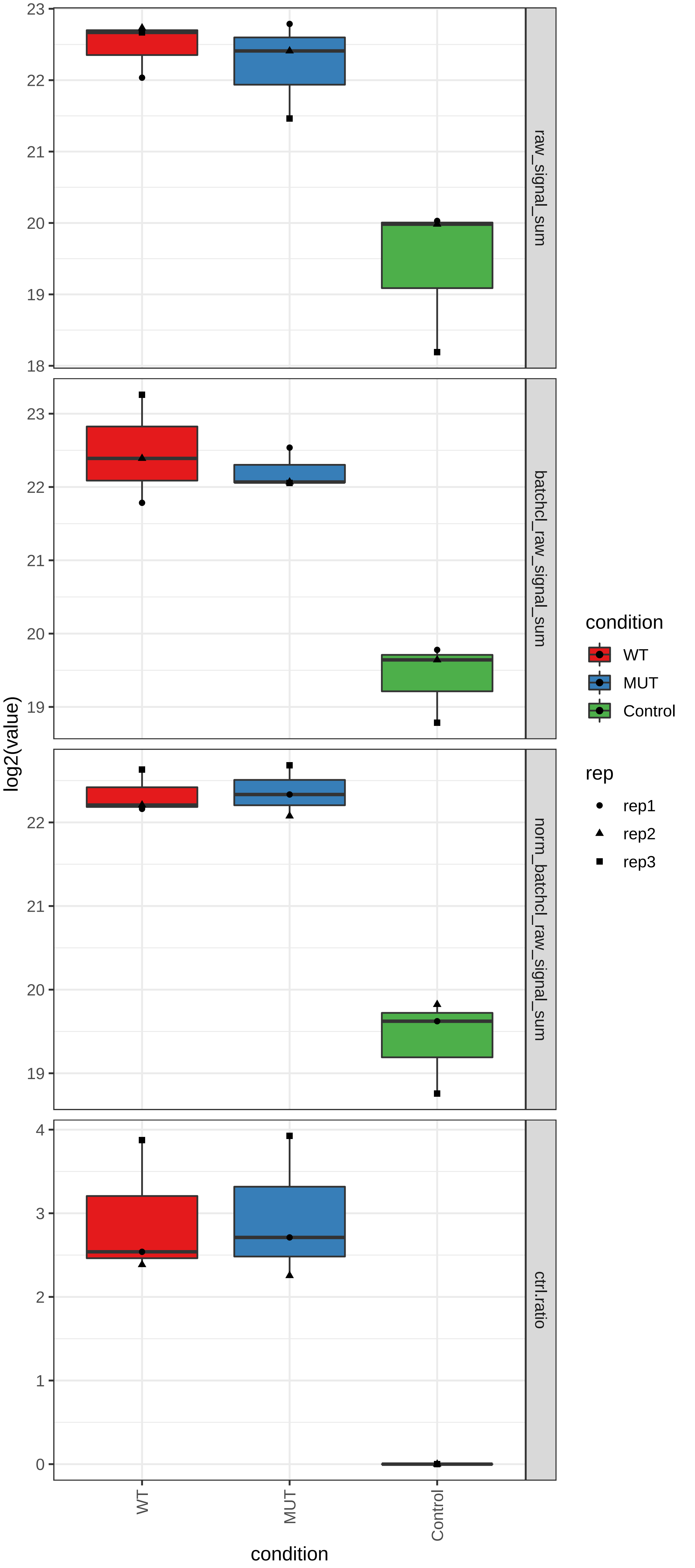

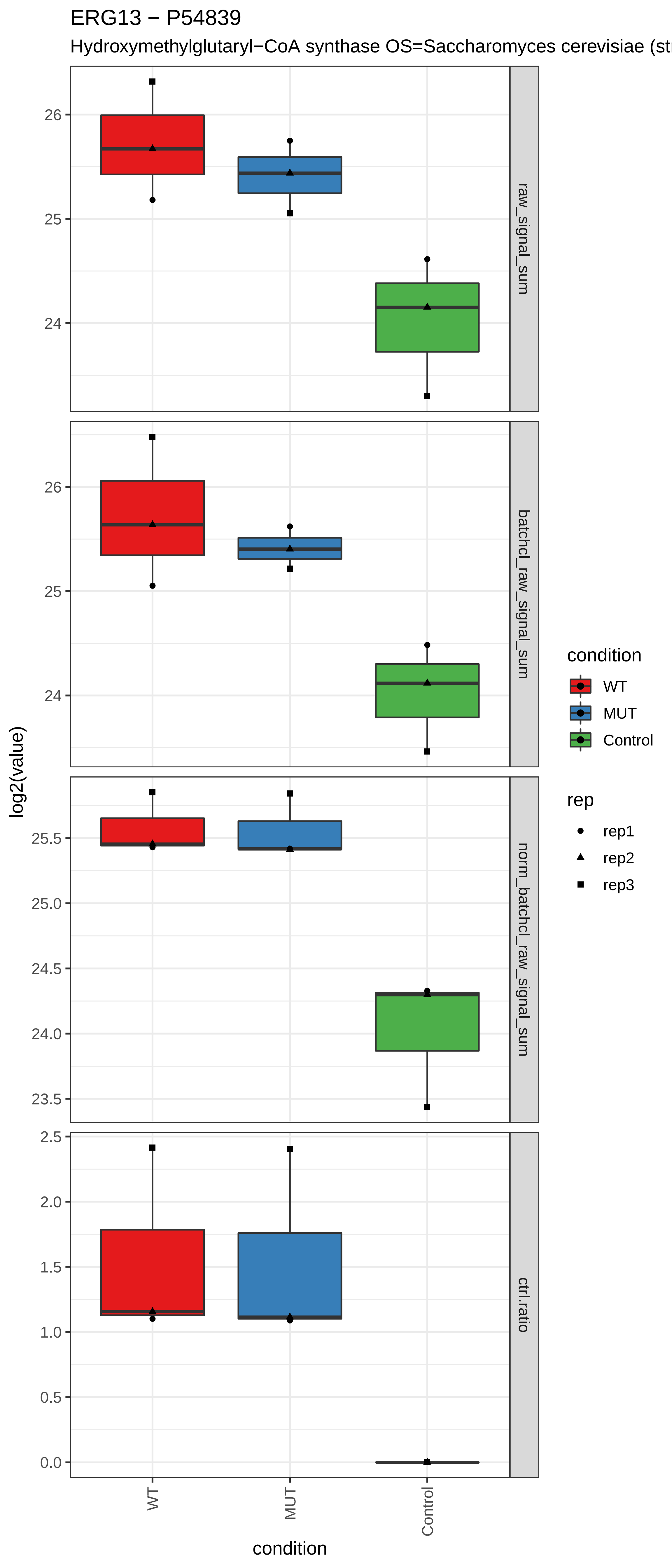
ERG13 − P54839
Hydroxymethylglutaryl−CoA synthase OS=Saccharomyces cerevisiae (st
raw_signal_sum
batchcl_raw_signal_sum
norm_batchcl_raw_signal_sum
ctrl.ratio
log2(value)
condition
WT
MUT
Control
rep
rep1
rep2
rep3
26
25
24
25.5
25.0
24.5
24.0
23.5
2.5
2.0
1.5
1.0
0.5
0.0

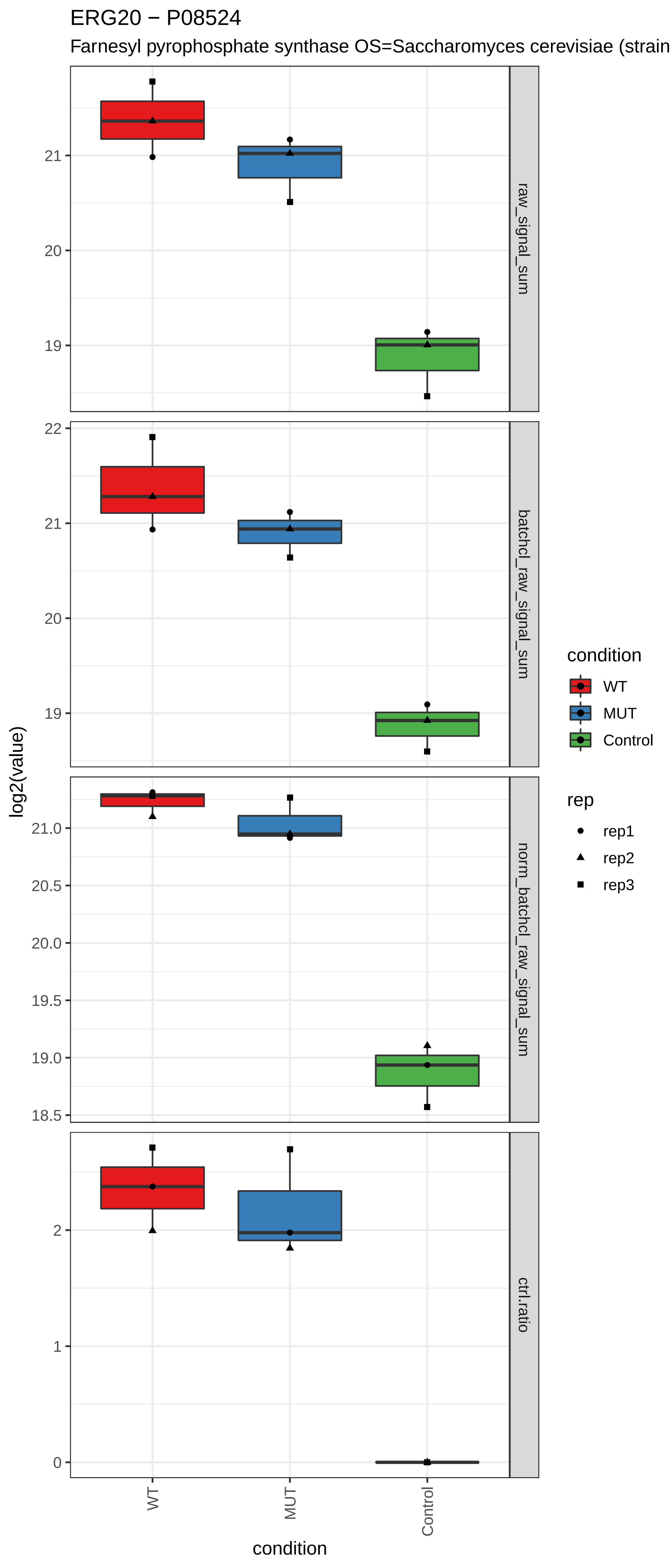

ERG20 − P08524
Farnesyl pyrophosphate synthase OS=Saccharomyces cerevisiae (strain
raw_signal_sum
batchcl_raw_signal_sum
norm_batchcl_raw_signal_sum
ctrl.ratio
log2(value)
condition
WT
MUT
Control
rep
rep1
rep2
rep3

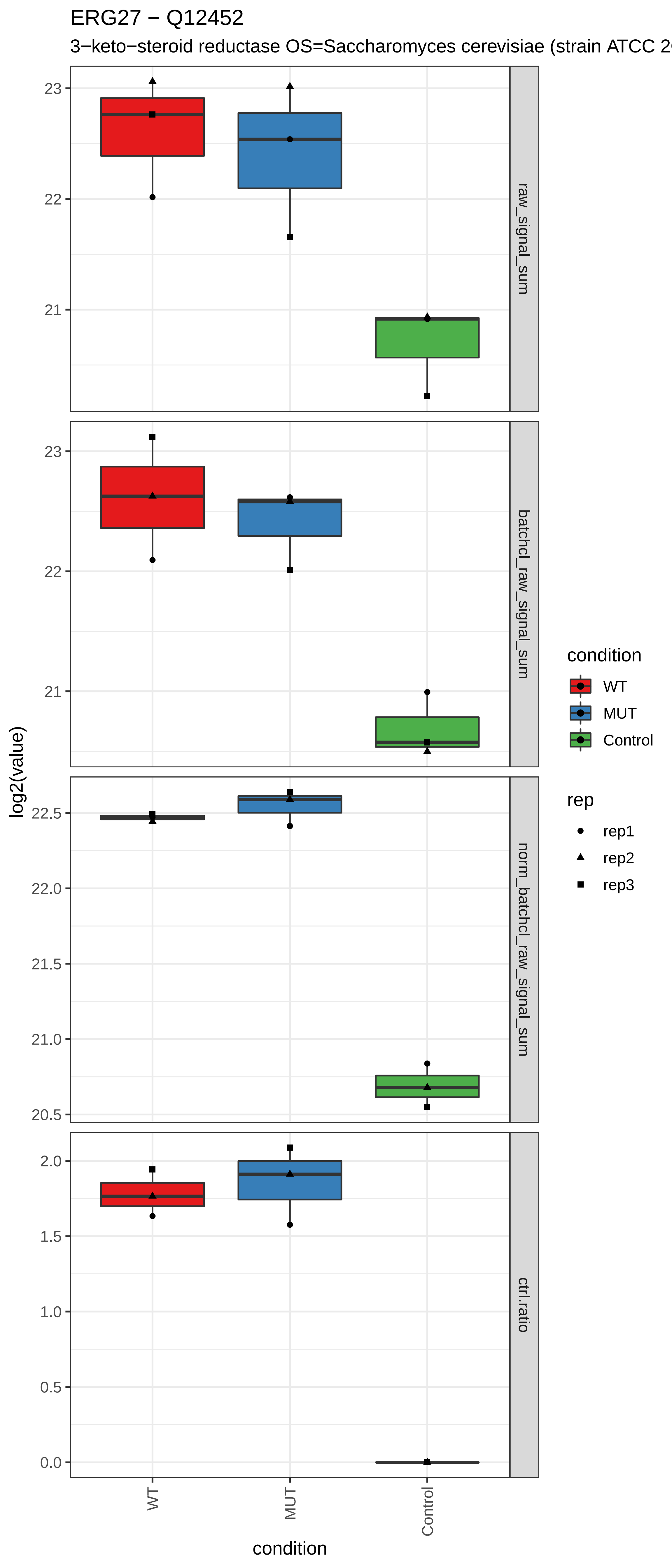

ERG27 − Q12452
3−keto−steroid reductase OS=Saccharomyces cerevisiae (strain ATCC 2
raw_signal_sum
batchcl_raw_signal_sum
norm_batchcl_raw_signal_sum
ctrl.ratio
log2(value)
condition
WT
MUT
Control
rep
rep1
rep2
rep3

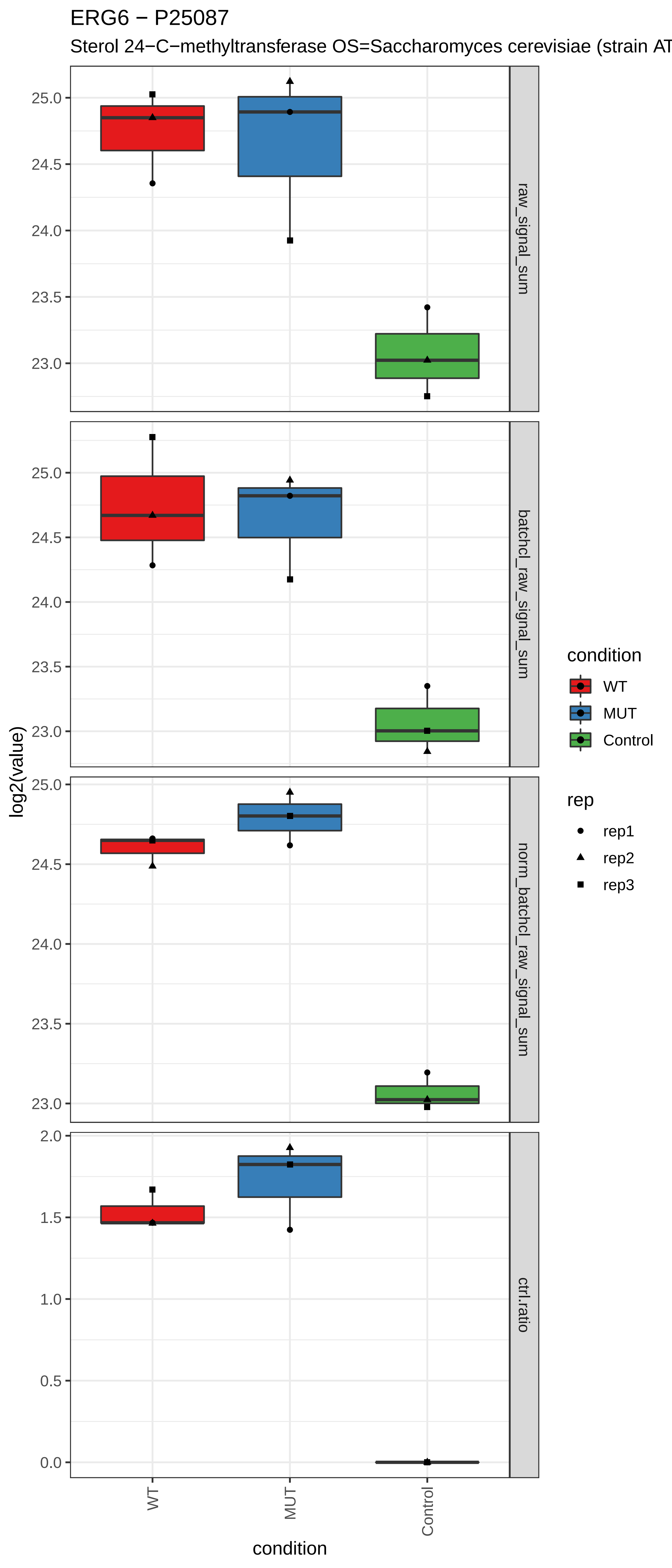

ERG6 − P25087
Sterol 24−C−methyltransferase OS=Saccharomyces cerevisiae (strain AT
raw_signal_sum
batchcl_raw_signal_sum
norm_batchcl_raw_signal_sum
ctrl.ratio
log2(value)
condition
WT
MUT
Control
rep
rep1
rep2
rep3
25.0
24.5
24.0
23.5
23.0
2.0
1.5
1.0
0.5
0.0

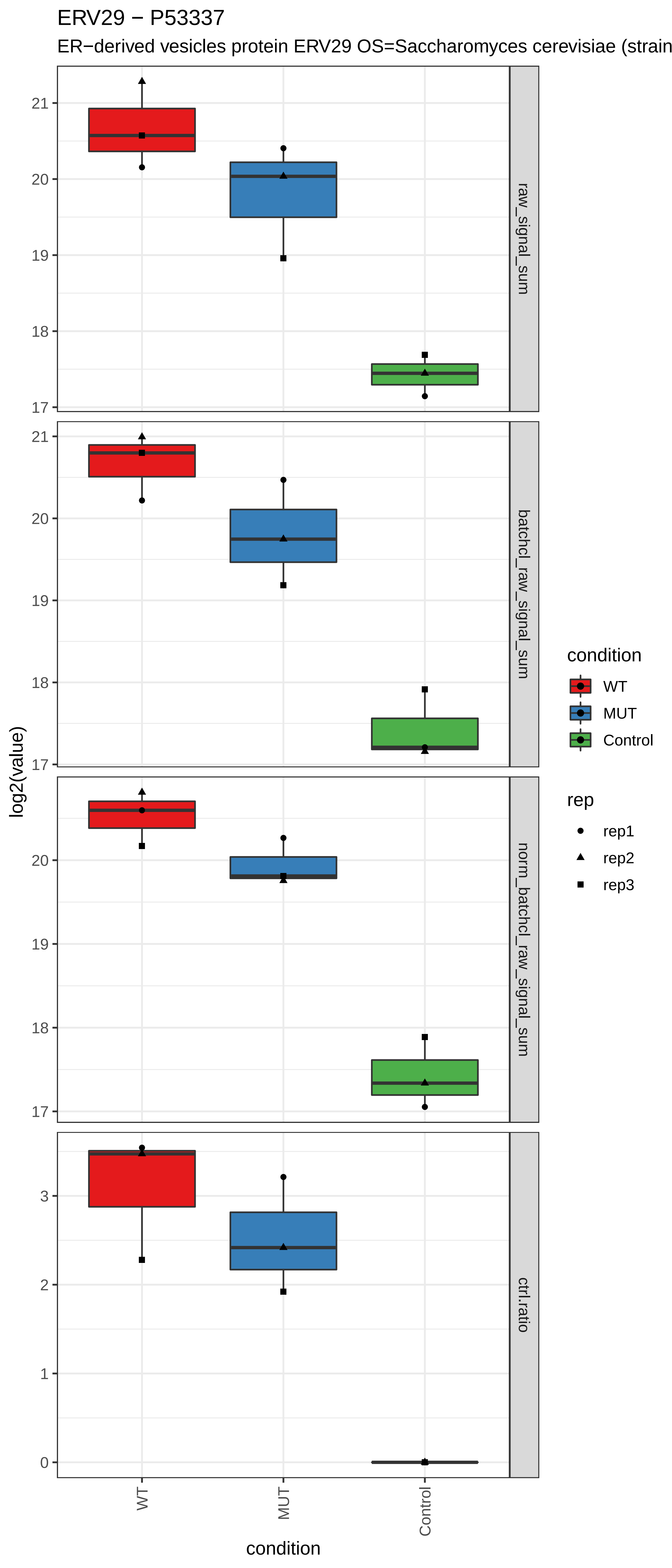

ERV29 − P53337
ER−derived vesicles protein ERV29 OS=Saccharomyces cerevisiae (strain
raw_signal_sum
batchcl_raw_signal_sum
norm_batchcl_raw_signal_sum
ctrl.ratio
log2(value)
condition
WT
MUT
Control
rep
rep1
rep2
rep3

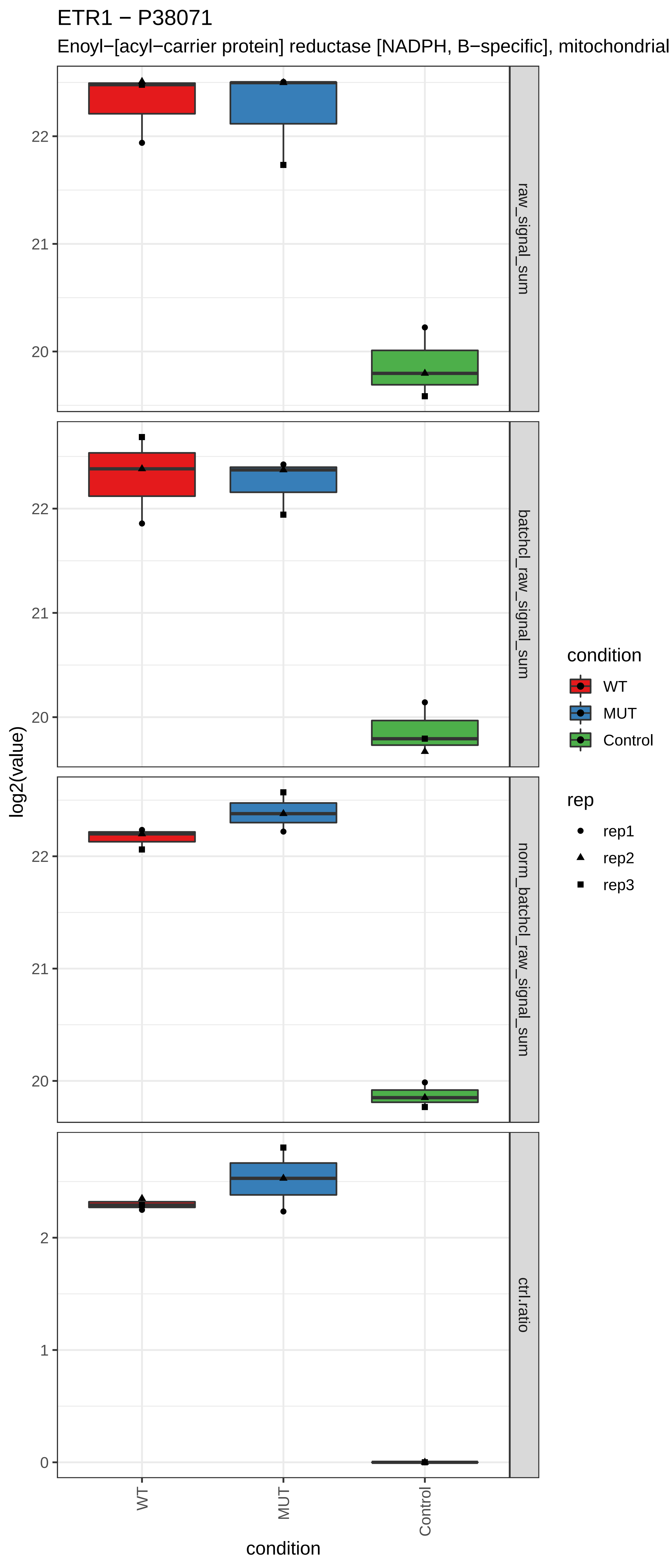

ETR1 − P38071
Enoyl−[acyl−carrier protein] reductase [NADPH, B−specific], mitochondrial
log2(value)
22
21
20
22
21
20
22
21
20
2
1
0
raw_signal_sum
batchcl_raw_signal_sum
norm_batchcl_raw_signal_sum
ctrl.ratio
WT
MUT
Control
condition
condition
WT
MUT
Control
rep
rep1
rep2
rep3

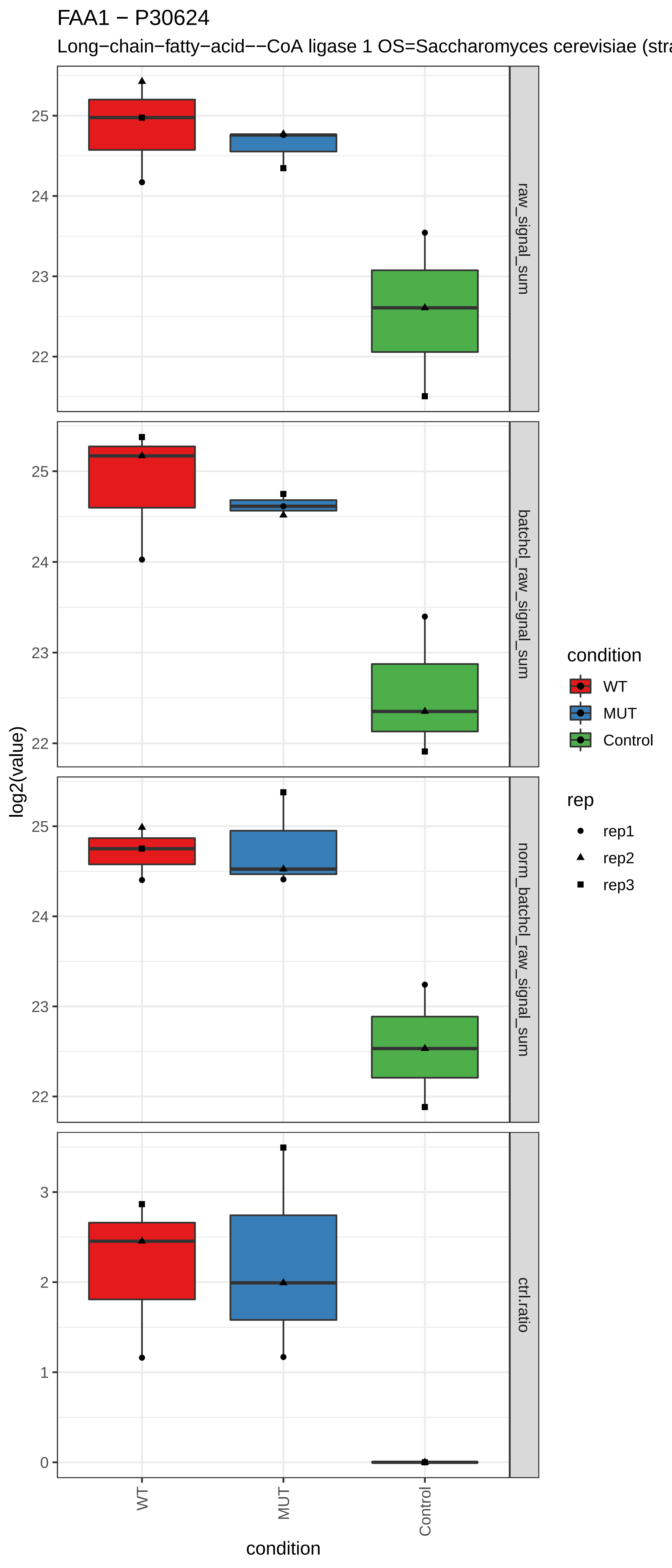
FAA1 − P30624
Long−chain−fatty−acid−−CoA ligase 1 OS=Saccharomyces cerevisiae (stra
raw_signal_sum
batchcl_raw_signal_sum
norm_batchcl_raw_signal_sum
ctrl.ratio
log2(value)
condition
WT
MUT
Control
rep
rep1
rep2
rep3
25
24
23
22
3
2
1
0

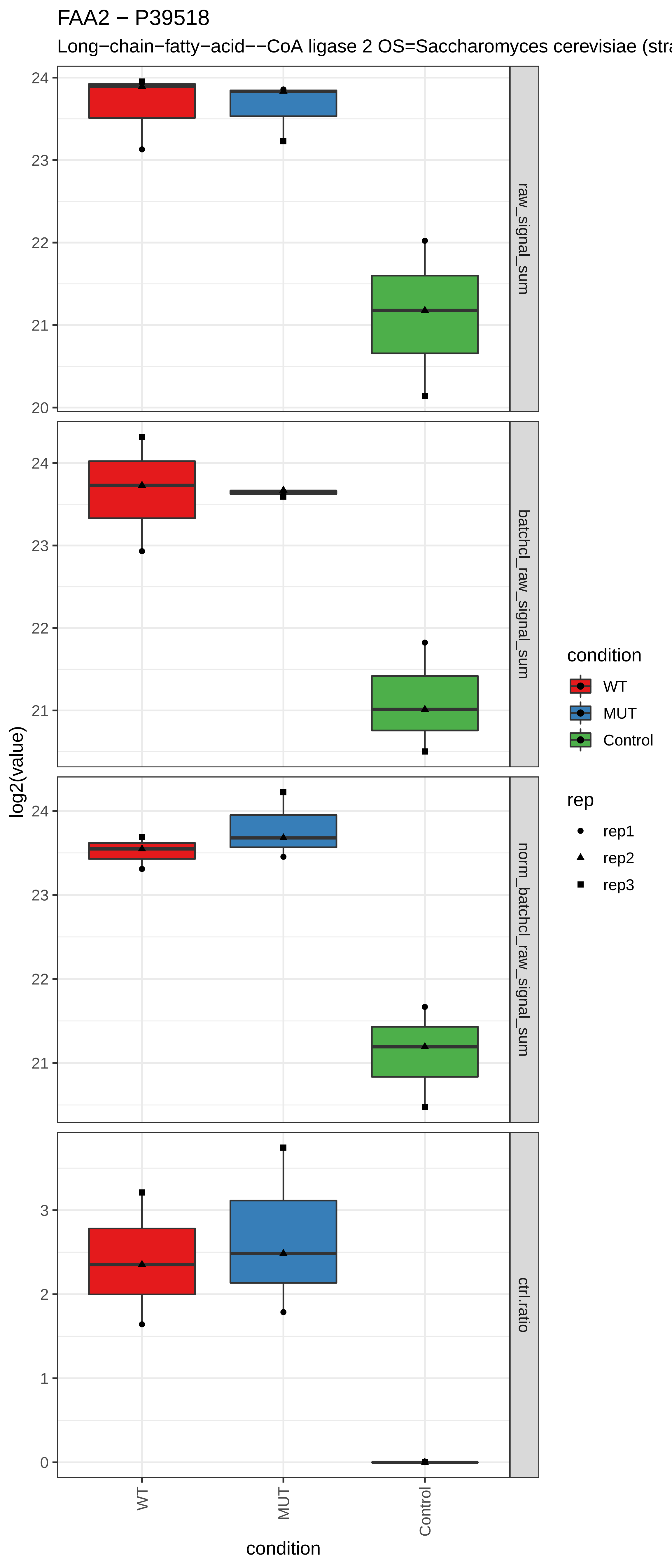

FAA2 − P39518
Long−chain−fatty−acid−−CoA ligase 2 OS=Saccharomyces cerevisiae (stra
raw_signal_sum
batchcl_raw_signal_sum
norm_batchcl_raw_signal_sum
ctrl.ratio
log2(value)
condition
WT
MUT
Control
rep
rep1
rep2
rep3

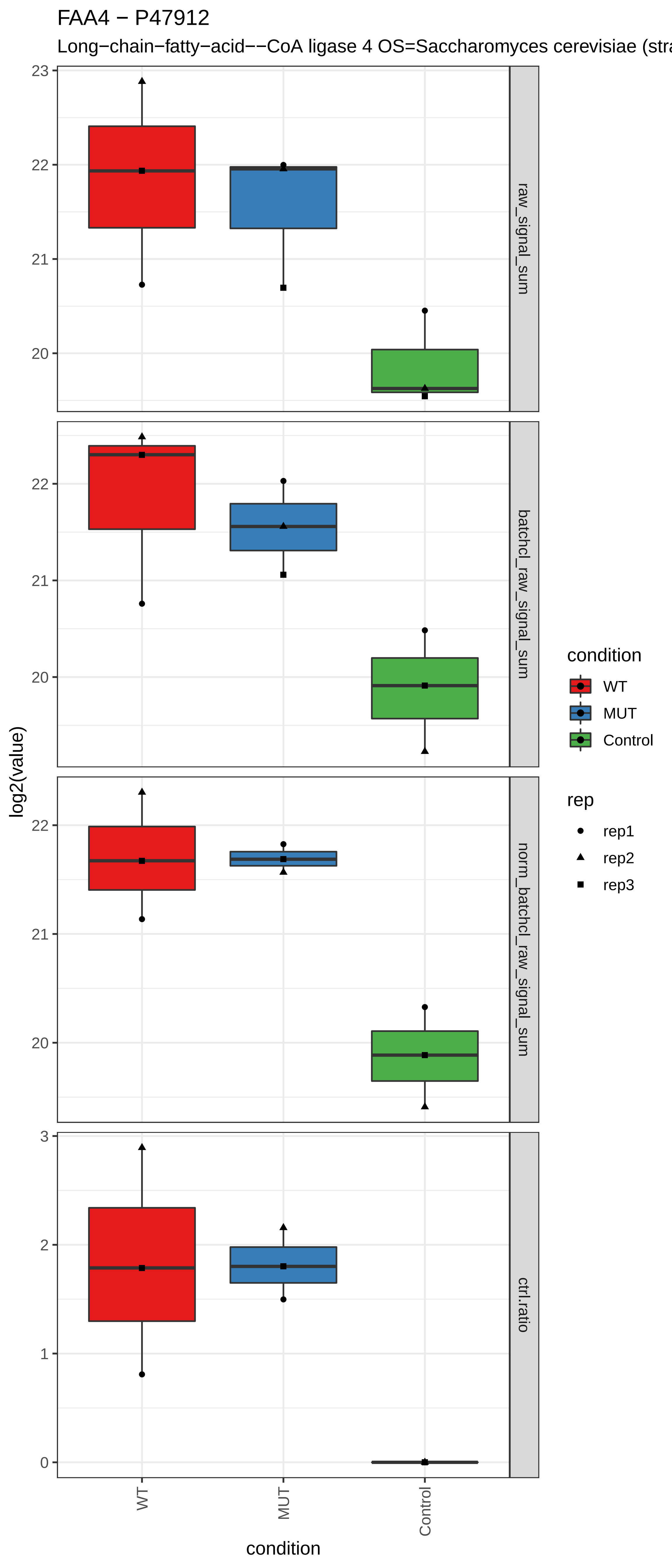

FAA4 − P47912
Long−chain−fatty−acid−−CoA ligase 4 OS=Saccharomyces cerevisiae (stra
raw_signal_sum
batchcl_raw_signal_sum
norm_batchcl_raw_signal_sum
ctrl.ratio
log2(value)
condition
WT
MUT
Control
rep
rep1
rep2
rep3

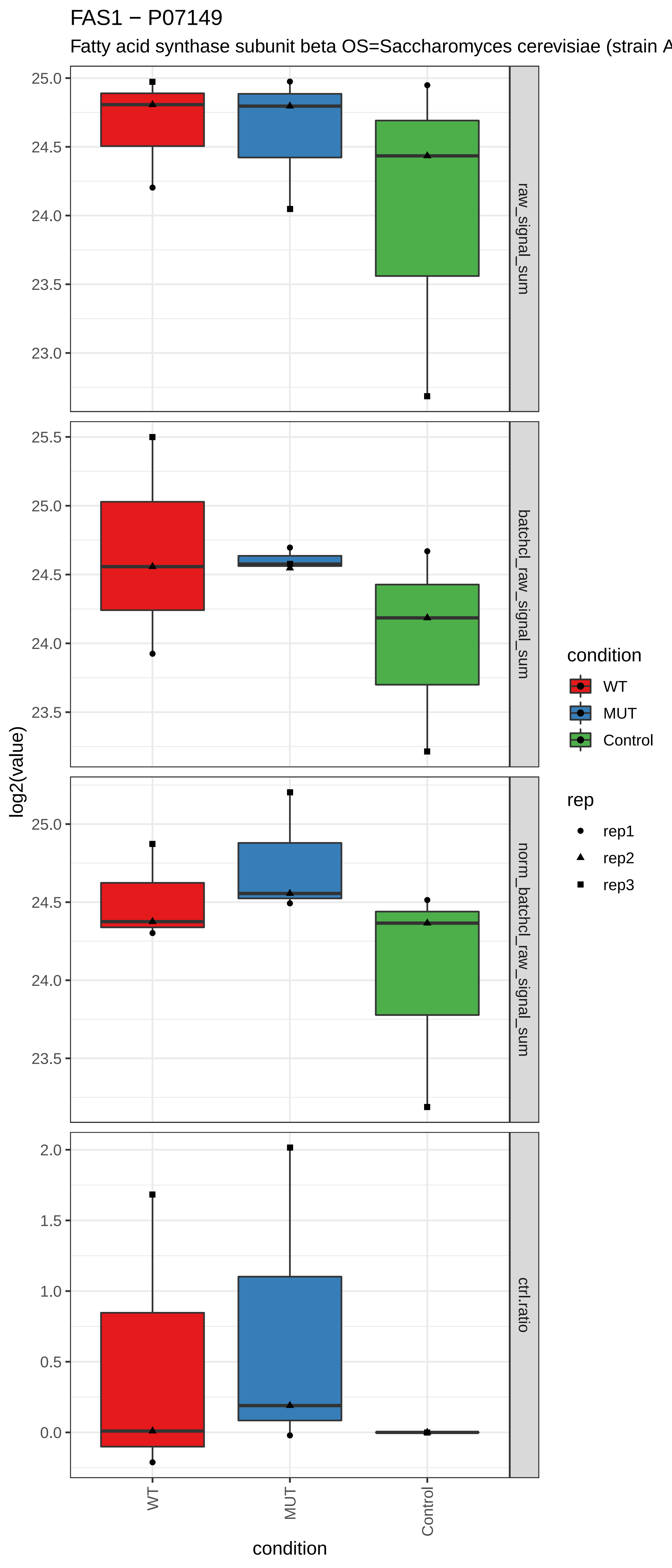
FAS1 − P07149
Fatty acid synthase subunit beta OS=Saccharomyces cerevisiae (strain A
raw_signal_sum
batchcl_raw_signal_sum
norm_batchcl_raw_signal_sum
ctrl.ratio
log2(value)
condition
WT
MUT
Control
rep
rep1
rep2
rep3
25.0
24.5
24.0
23.5
23.0
25.5
2.0
1.5
1.0
0.5
0.0

FAS2 − P19097

Fatty acid synthase subunit alpha OS=Saccharomyces cerevisiae (strain A

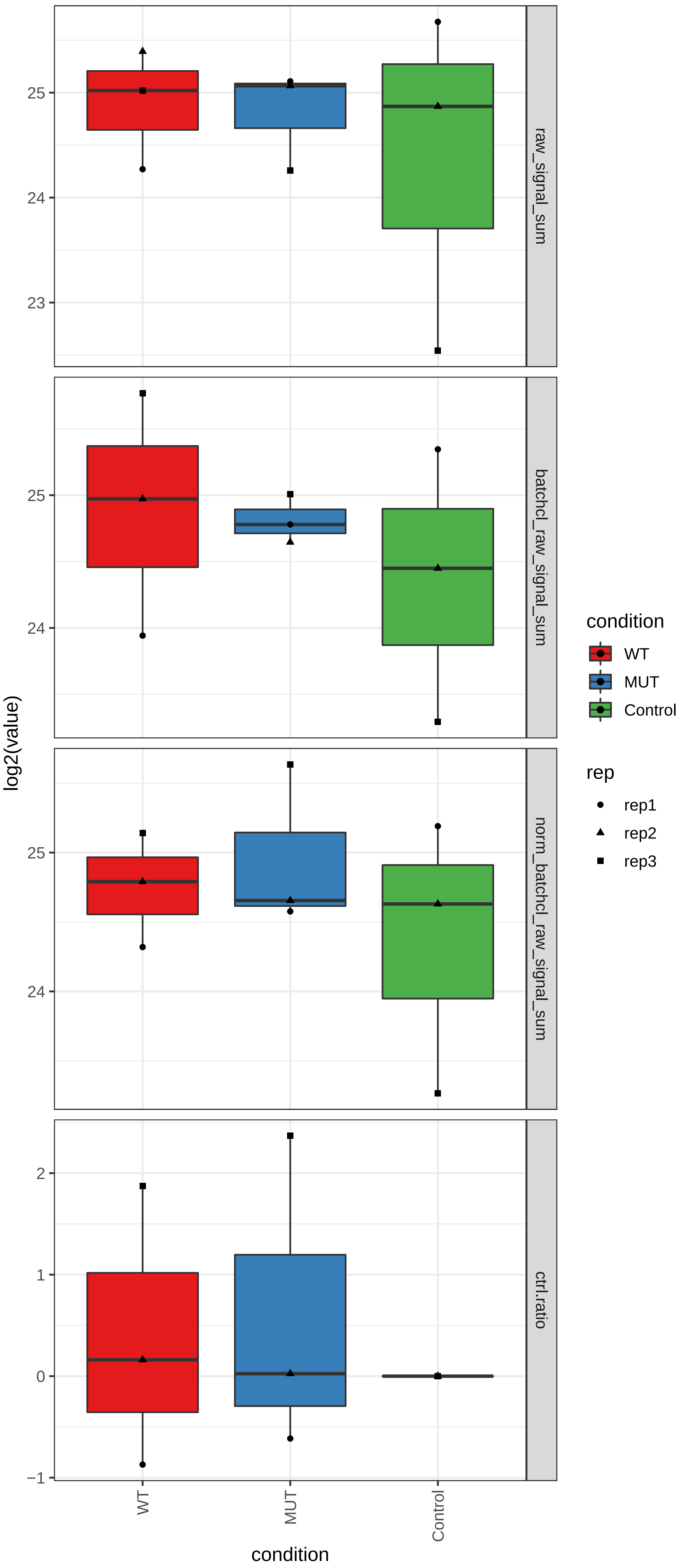

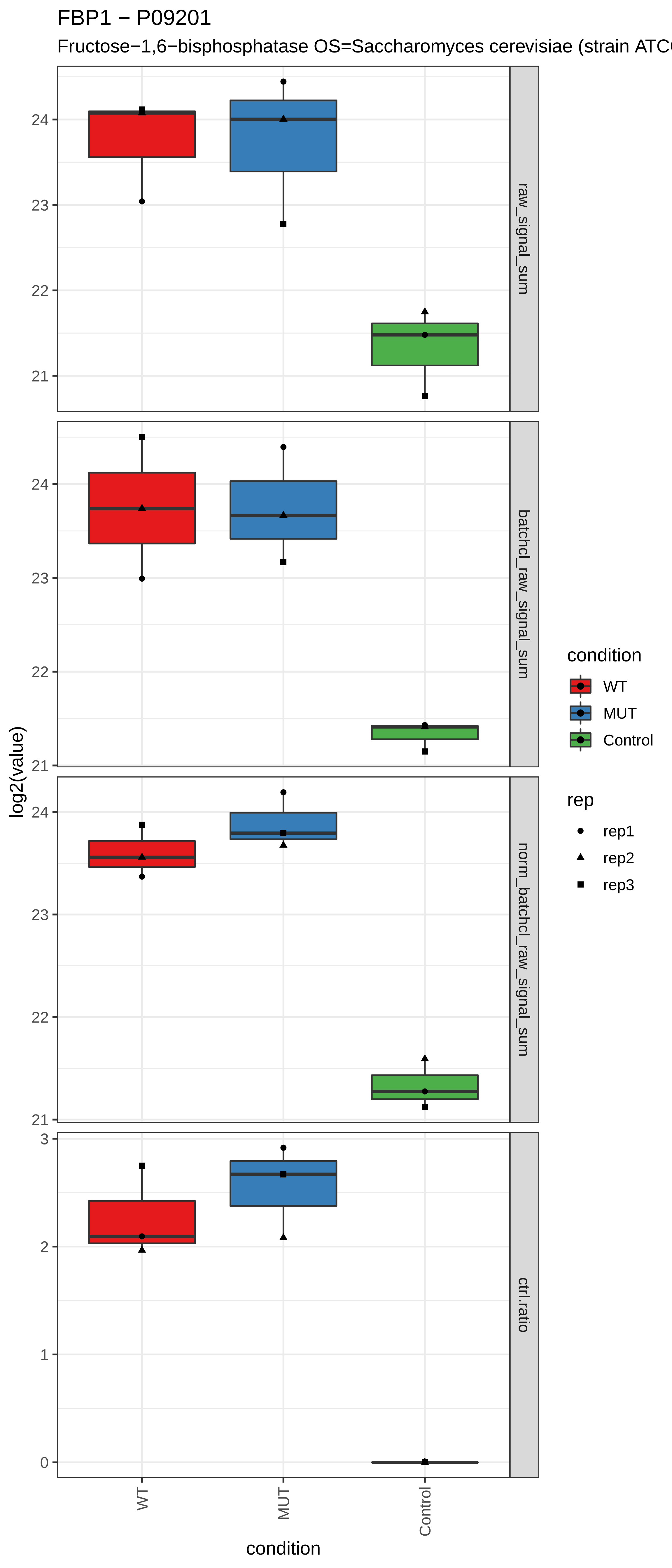

FBP1 − P09201
Fructose−1,6−bisphosphatase OS=Saccharomyces cerevisiae (strain ATCC
raw_signal_sum
batchcl_raw_signal_sum
norm_batchcl_raw_signal_sum
ctrl.ratio
log2(value)
condition
WT
MUT
Control
rep
rep1
rep2
rep3
24
23
22
21
3
2
1
0

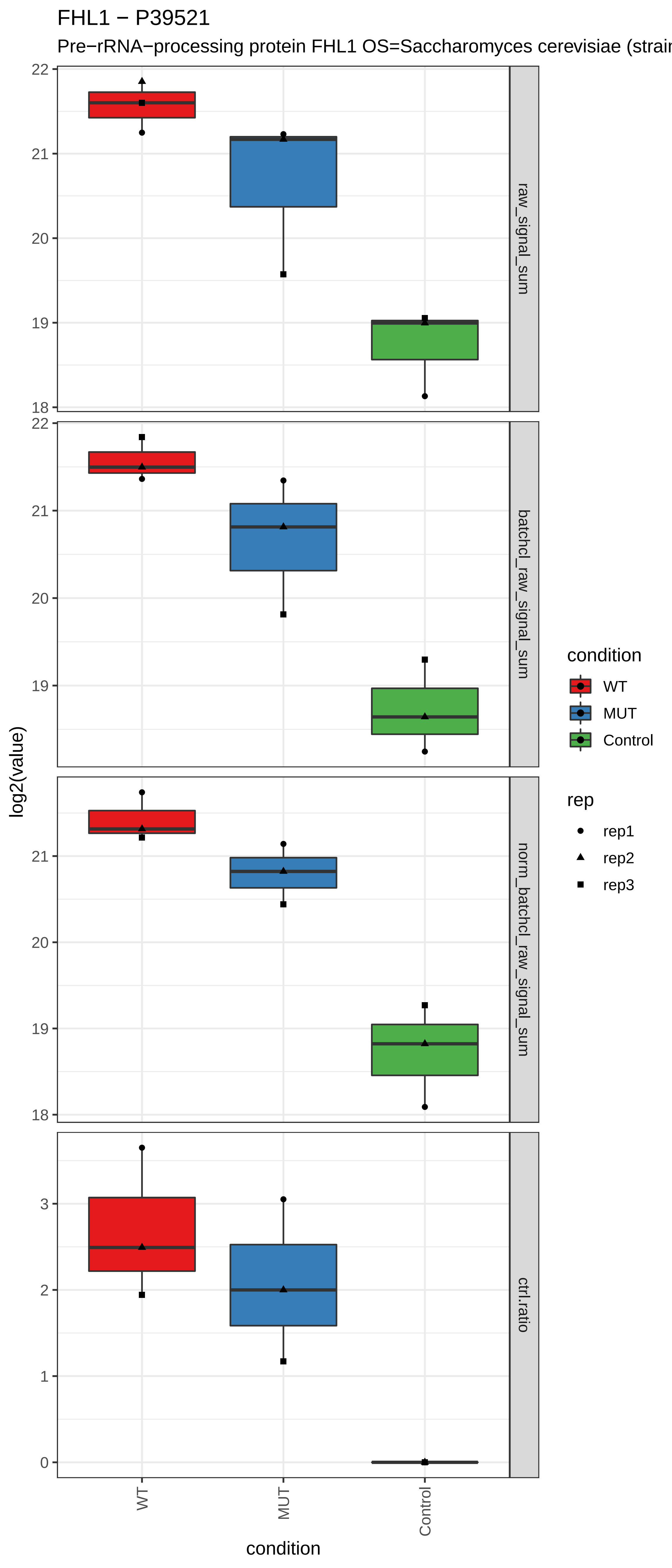

FHL1 − P39521
Pre−rRNA−processing protein FHL1 OS=Saccharomyces cerevisiae (strain
raw_signal_sum
batchcl_raw_signal_sum
norm_batchcl_raw_signal_sum
ctrl.ratio
log2(value)
condition
WT
MUT
Control
rep
rep1
rep2
rep3
22
21
20
19
18
3
2
1
0

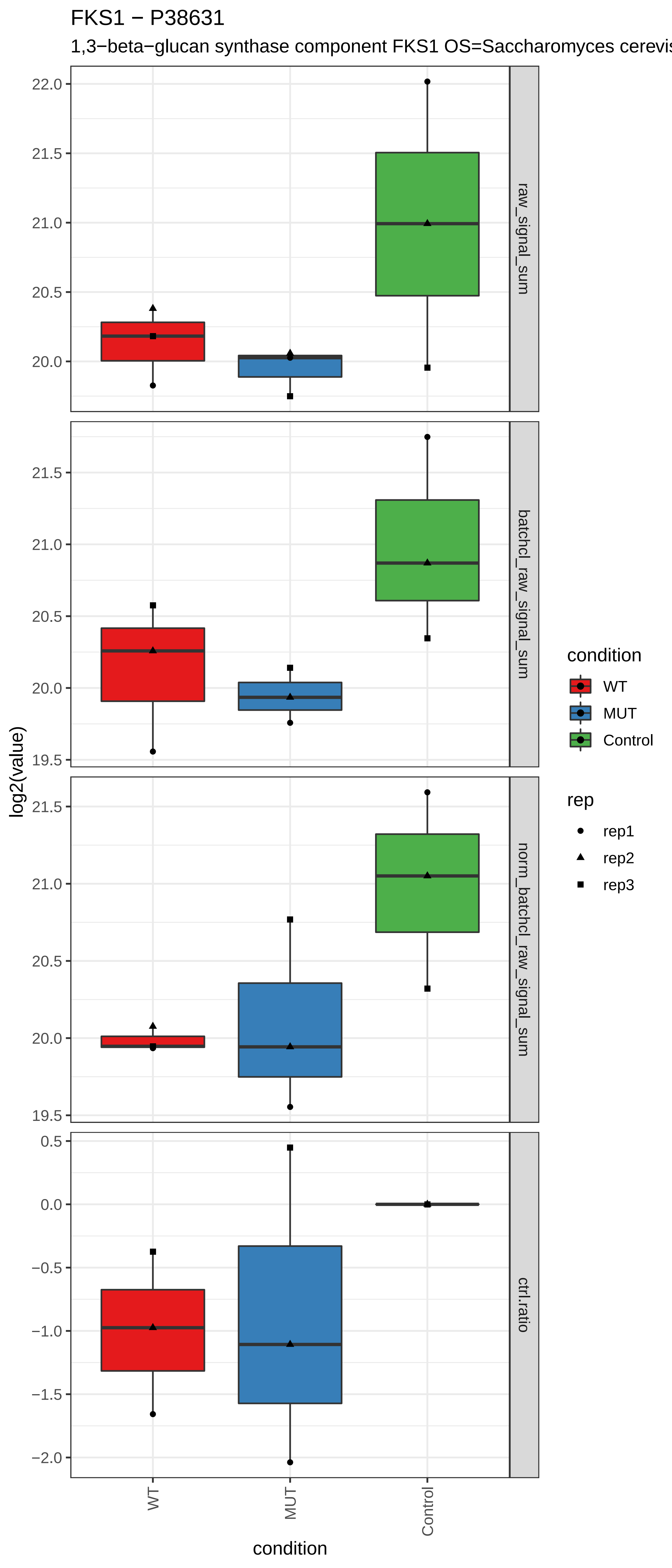

FKS1 − P38631
1,3−beta−glucan synthase component FKS1 OS=Saccharomyces cerevis
log2(value)
raw_signal_sum
batchcl_raw_signal_sum
norm_batchcl_raw_signal_sum
ctrl.ratio
22.0
21.5
21.0
20.5
20.0
19.5
0.5
0.0
−0.5
−1.0
−1.5
−2.0
WT
MUT
Control
condition
condition
WT
MUT
Control
rep
rep1
rep2
rep3

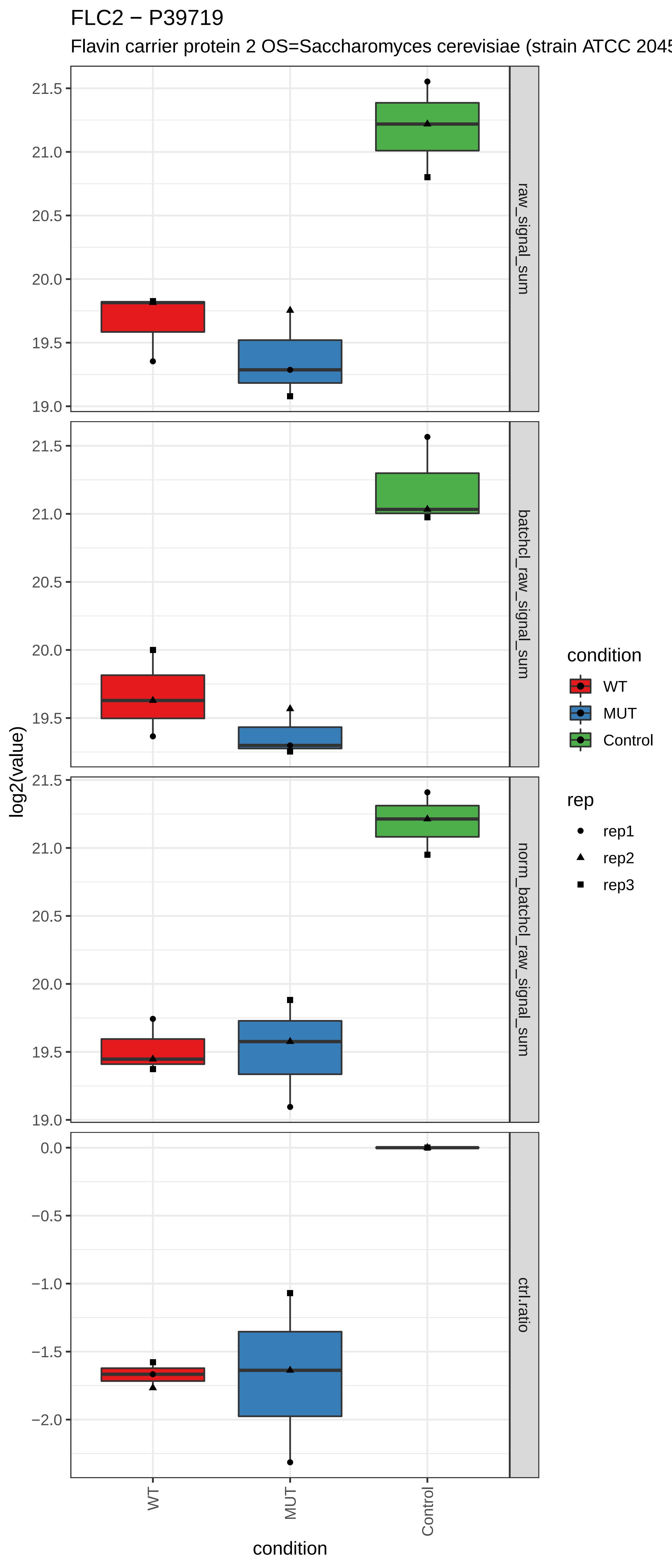

FLC2 − P39719
Flavin carrier protein 2 OS=Saccharomyces cerevisiae (strain ATCC 2045
raw_signal_sum
batchcl_raw_signal_sum
norm_batchcl_raw_signal_sum
ctrl.ratio
log2(value)
condition
WT
MUT
Control
rep
rep1
rep2
rep3

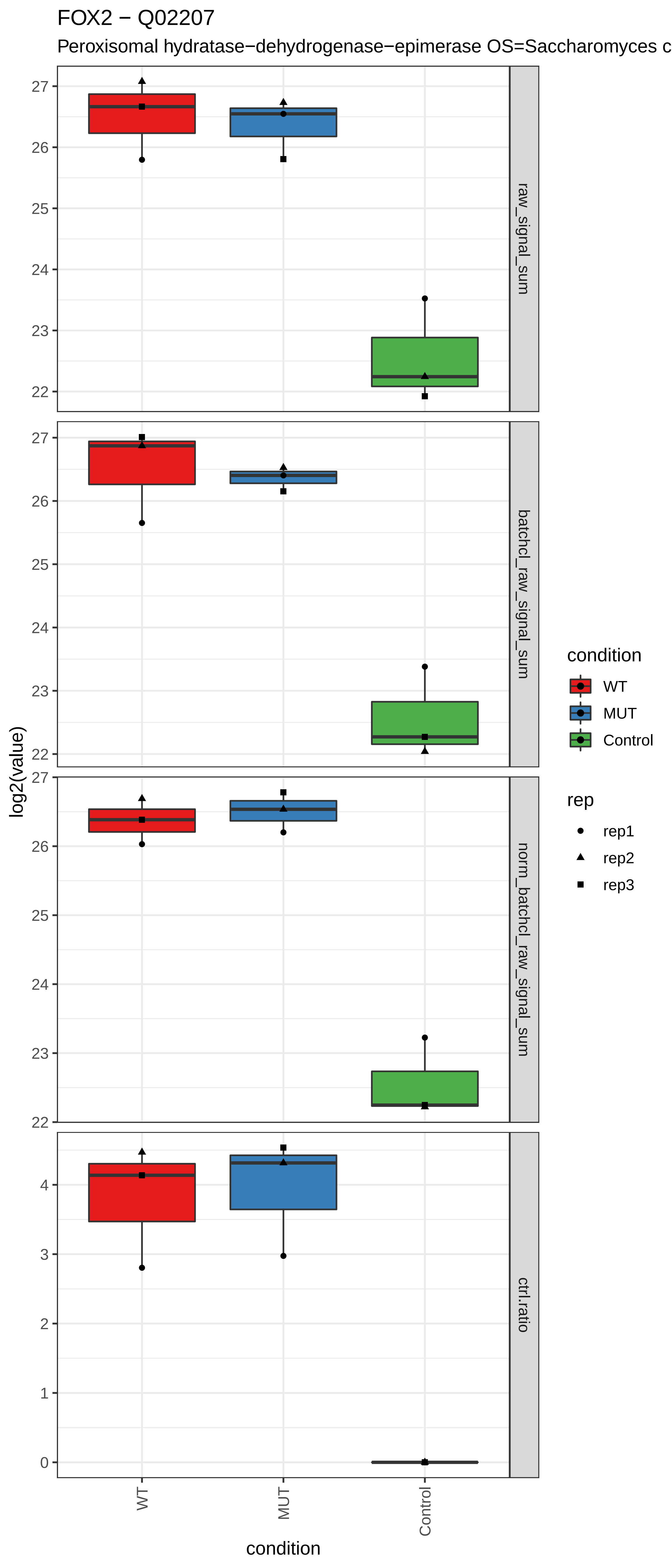

FOX2 − Q02207
Peroxisomal hydratase−dehydrogenase−epimerase OS=Saccharomyces c
raw_signal_sum
batchcl_raw_signal_sum
norm_batchcl_raw_signal_sum
ctrl.ratio
log2(value)
condition
WT
MUT
Control
rep
rep1
rep2
rep3

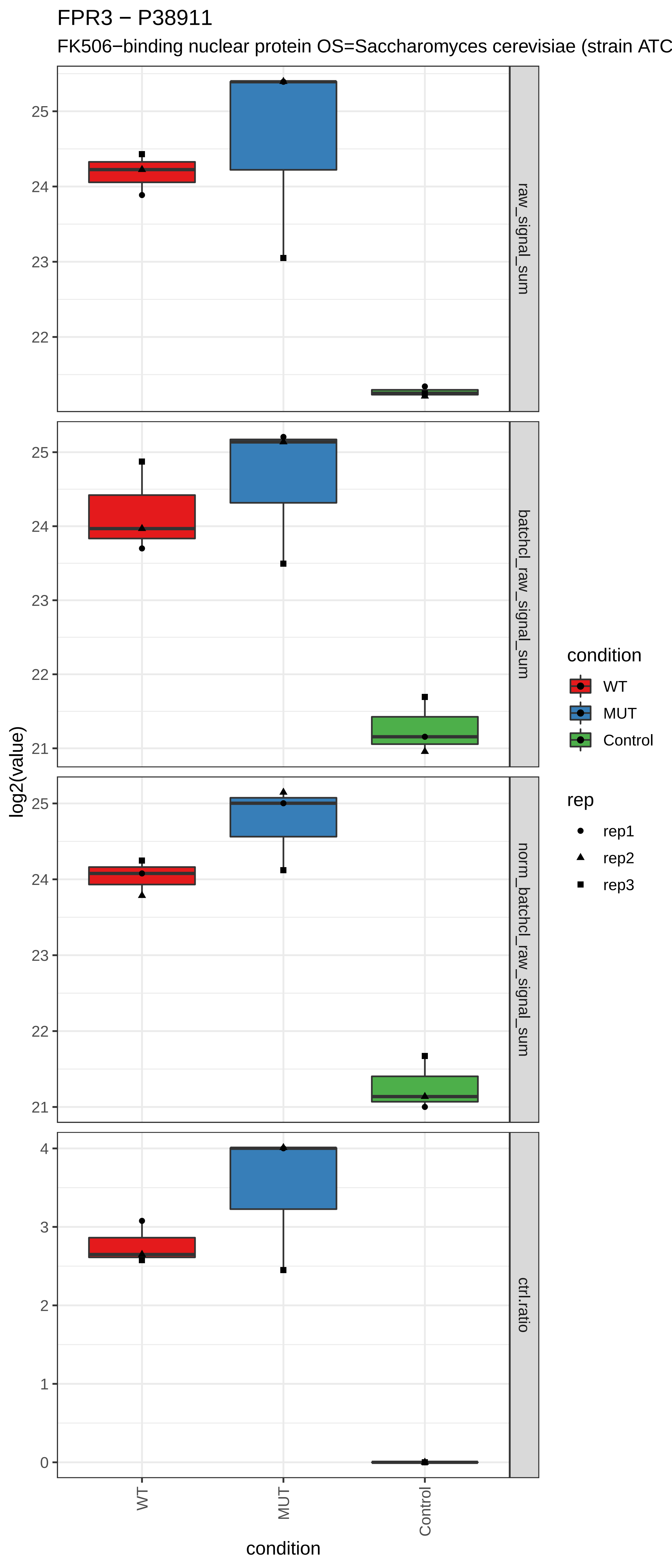

FPR3 − P38911
FK506−binding nuclear protein OS=Saccharomyces cerevisiae (strain ATC
raw_signal_sum
batchcl_raw_signal_sum
norm_batchcl_raw_signal_sum
ctrl.ratio
log2(value)
condition
WT
MUT
Control
rep
rep1
rep2
rep3

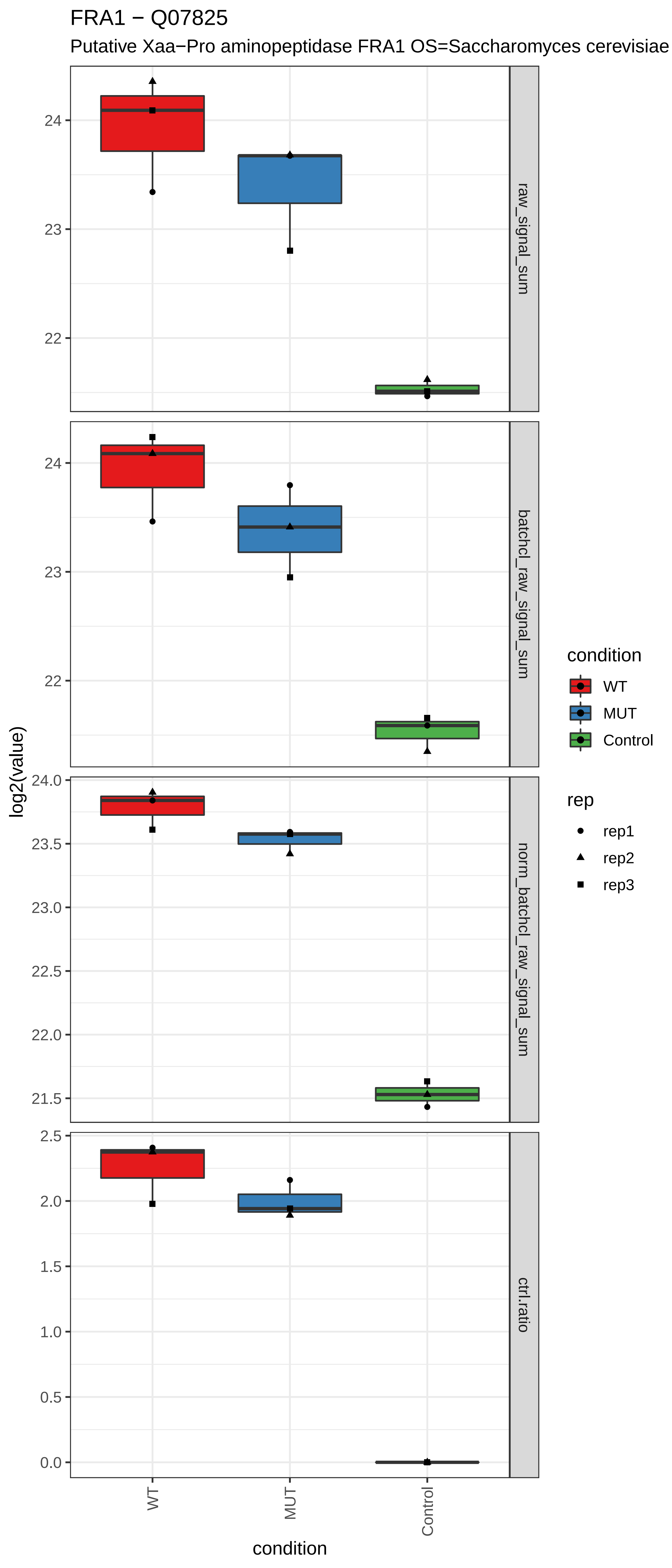

FRA1 − Q07825
Putative Xaa−Pro aminopeptidase FRA1 OS=Saccharomyces cerevisiae
log2(value)
raw_signal_sum
batchcl_raw_signal_sum
norm_batchcl_raw_signal_sum
ctrl.ratio
24
23
22
24.0
23.5
23.0
22.5
22.0
21.5
2.5
2.0
1.5
1.0
0.5
0.0
WT
MUT
Control
condition
condition
WT
MUT
Control
rep
rep1
rep2
rep3

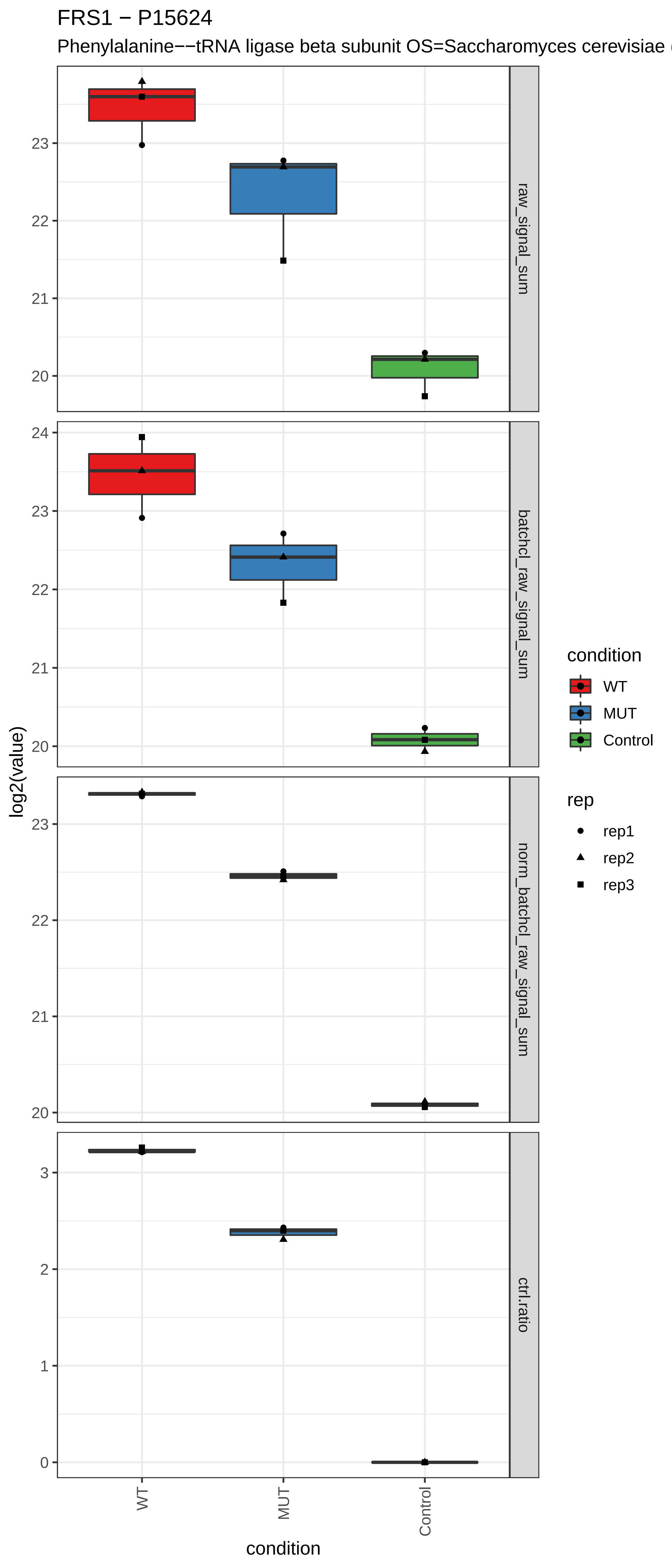

FRS1 − P15624
Phenylalanine−−tRNA ligase beta subunit OS=Saccharomyces cerevisiae
raw_signal_sum
batchcl_raw_signal_sum
norm_batchcl_raw_signal_sum
ctrl.ratio
log2(value)
condition
WT
MUT
Control
rep
rep1
rep2
rep3

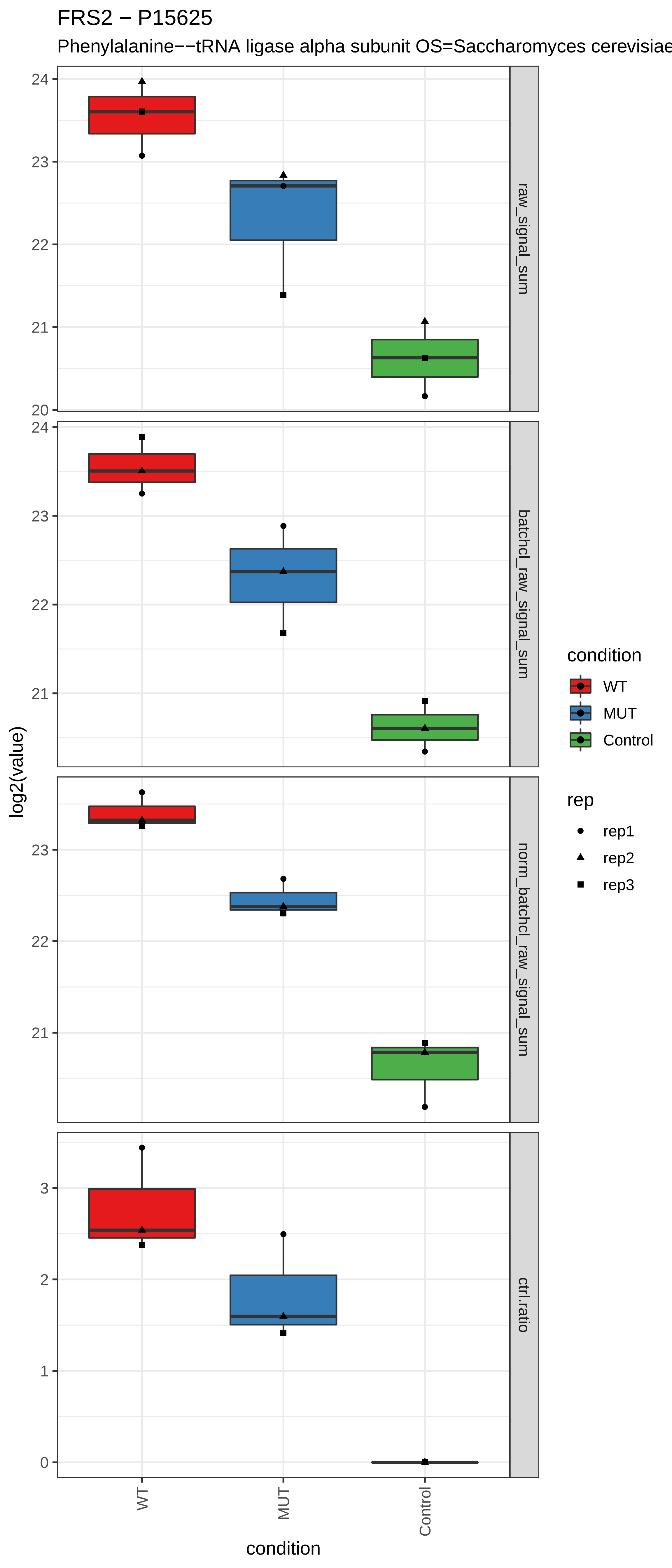
FRS2 − P15625
Phenylalanine−−tRNA ligase alpha subunit OS=Saccharomyces cerevisiae
raw_signal_sum
batchcl_raw_signal_sum
norm_batchcl_raw_signal_sum
ctrl.ratio
log2(value)
condition
WT
MUT
Control
rep
rep1
rep2
rep3
24
23
22
21
20
3
2
1
0

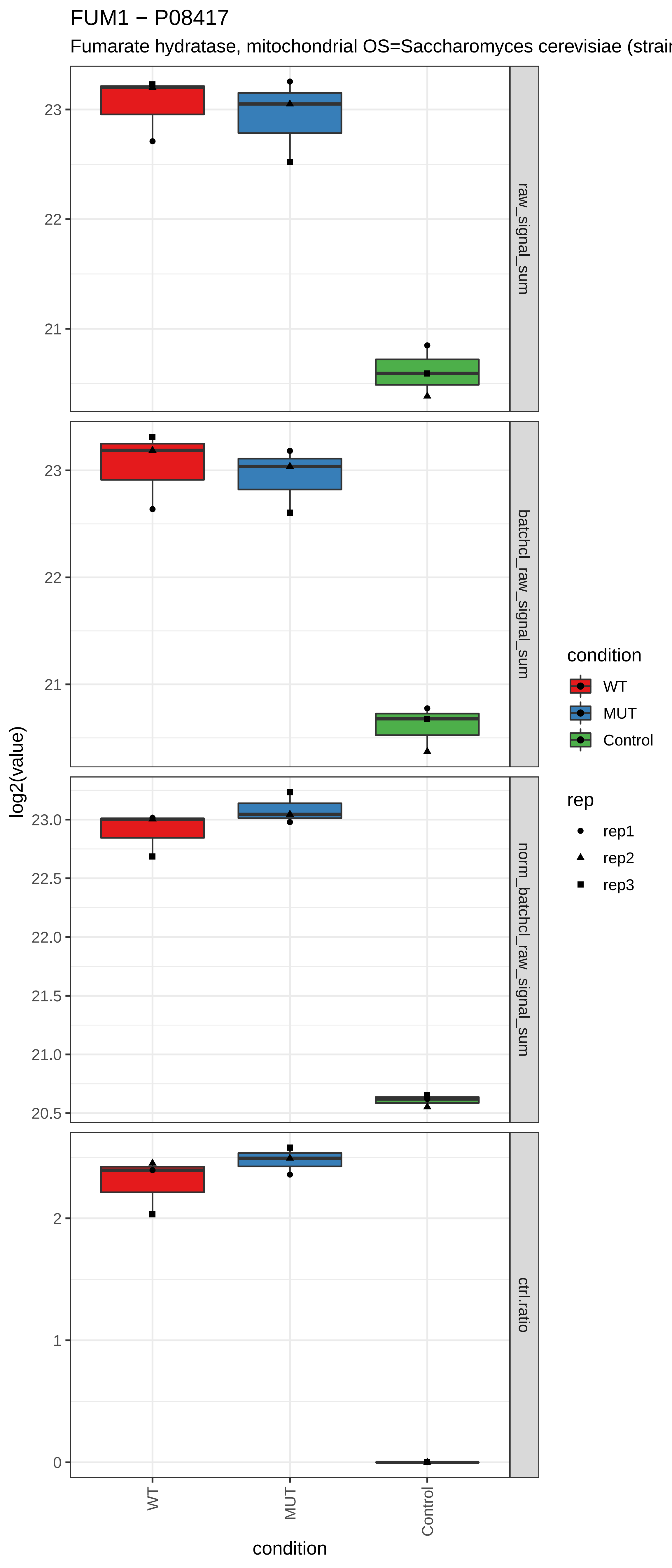
FUM1 − P08417
Fumarate hydratase, mitochondrial OS=Saccharomyces cerevisiae (strain
raw_signal_sum
batchcl_raw_signal_sum
norm_batchcl_raw_signal_sum
ctrl.ratio
log2(value)
condition
WT
MUT
Control
rep
rep1
rep2
rep3
23
22
21
23.0
22.5
22.0
21.5
21.0
20.5
2
1
0

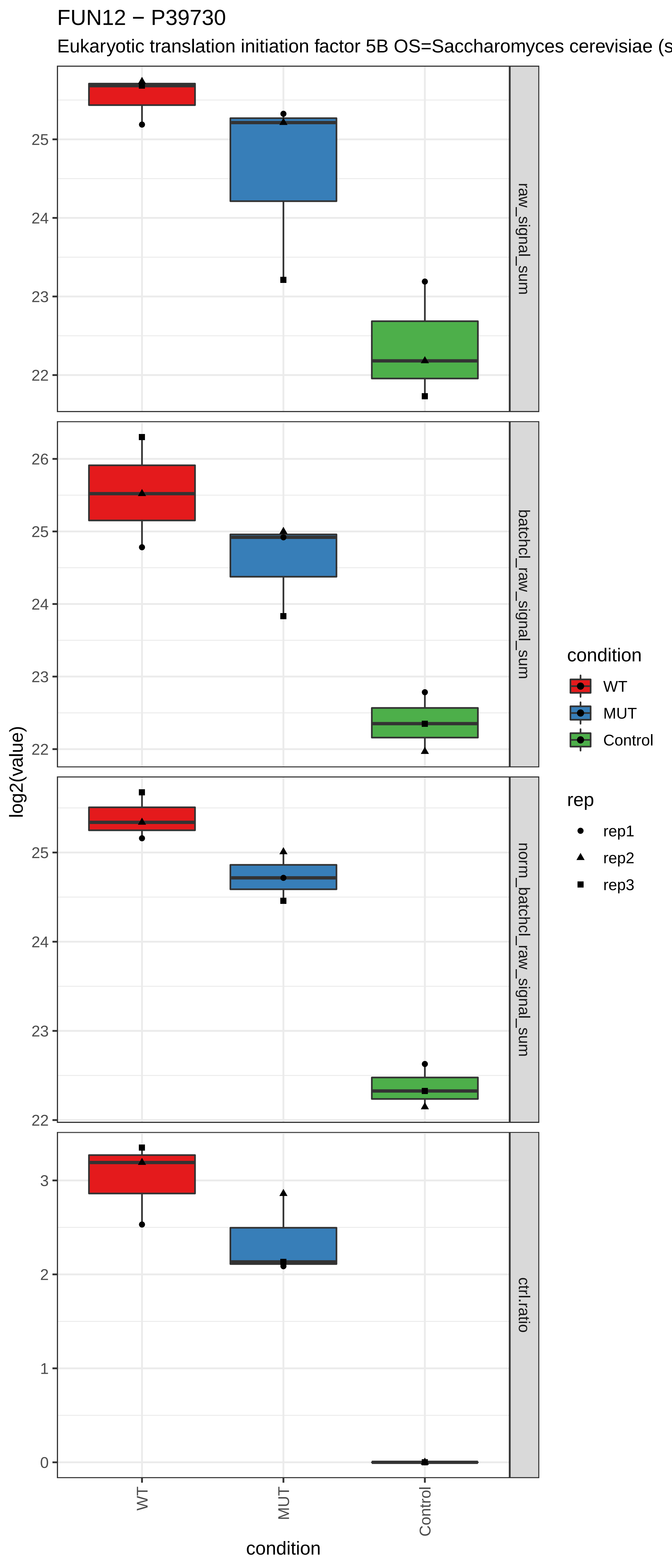

FUN12 − P39730
Eukaryotic translation initiation factor 5B OS=Saccharomyces cerevisiae (s
raw_signal_sum
batchcl_raw_signal_sum
norm_batchcl_raw_signal_sum
ctrl.ratio
log2(value)
condition
WT
MUT
Control
rep
rep1
rep2
rep3

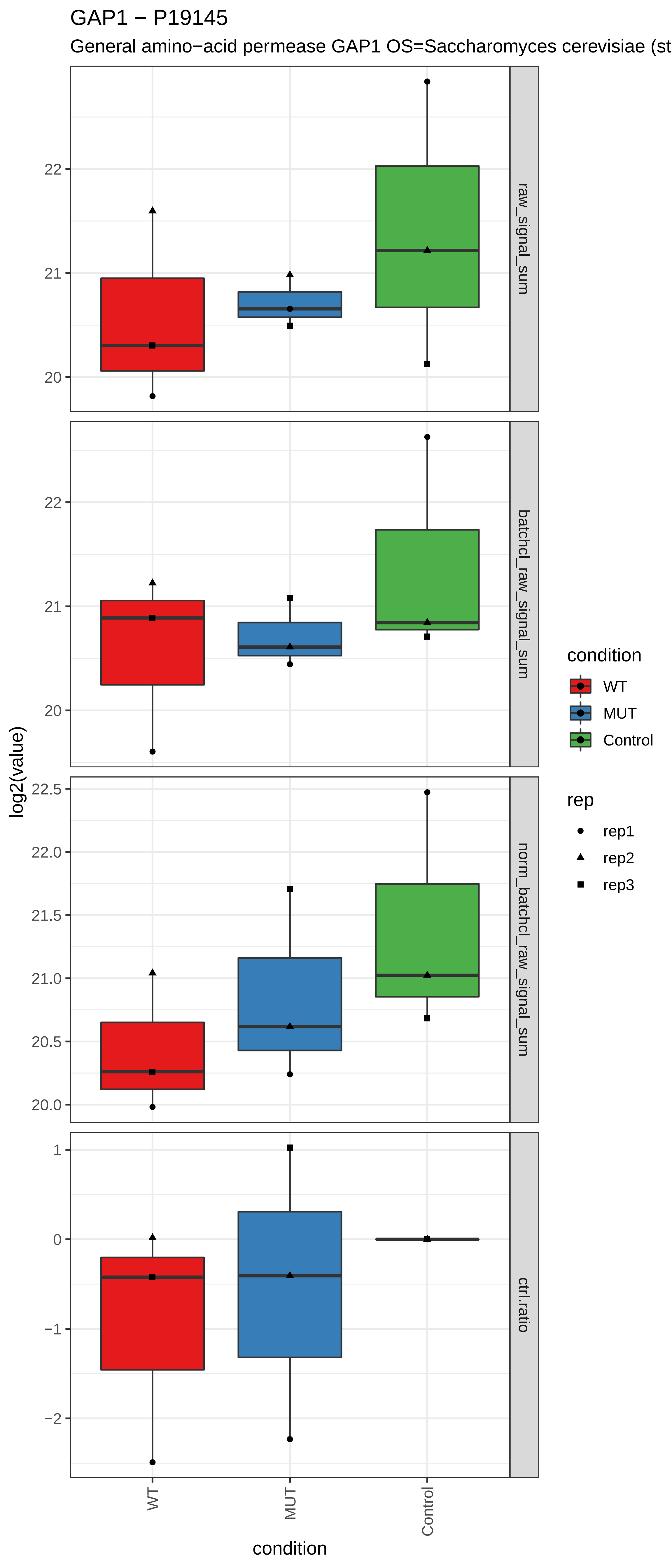
GAP1 − P19145
General amino−acid permease GAP1 OS=Saccharomyces cerevisiae (st
raw_signal_sum
batchcl_raw_signal_sum
norm_batchcl_raw_signal_sum
ctrl.ratio
log2(value)
condition
WT
MUT
Control
rep
rep1
rep2
rep3
22
21
20
22.5
22.0
21.5
21.0
20.5
20.0
1
0
−1
−2

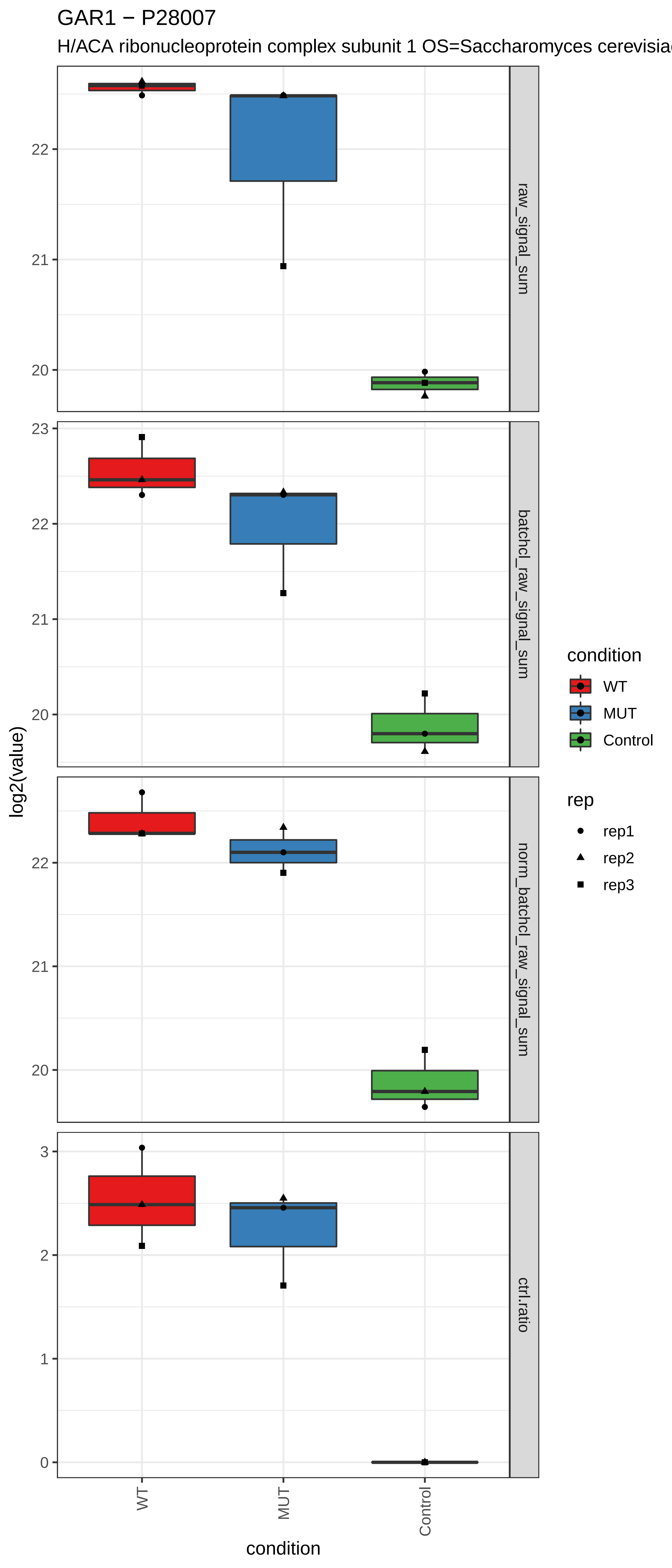
GAR1 − P28007
H/ACA ribonucleoprotein complex subunit 1 OS=Saccharomyces cerevisiae
log2(value)
raw_signal_sum
batchcl_raw_signal_sum
norm_batchcl_raw_signal_sum
ctrl.ratio
22
21
20
23
22
21
20
22
21
20
3
2
1
0
WT
MUT
Control
condition
condition
WT
MUT
Control
rep
rep1
rep2
rep3

GAS1 − P22146

1,3−beta−glucanosyltransferase GAS1 OS=Saccharomyces cerevisiae (str

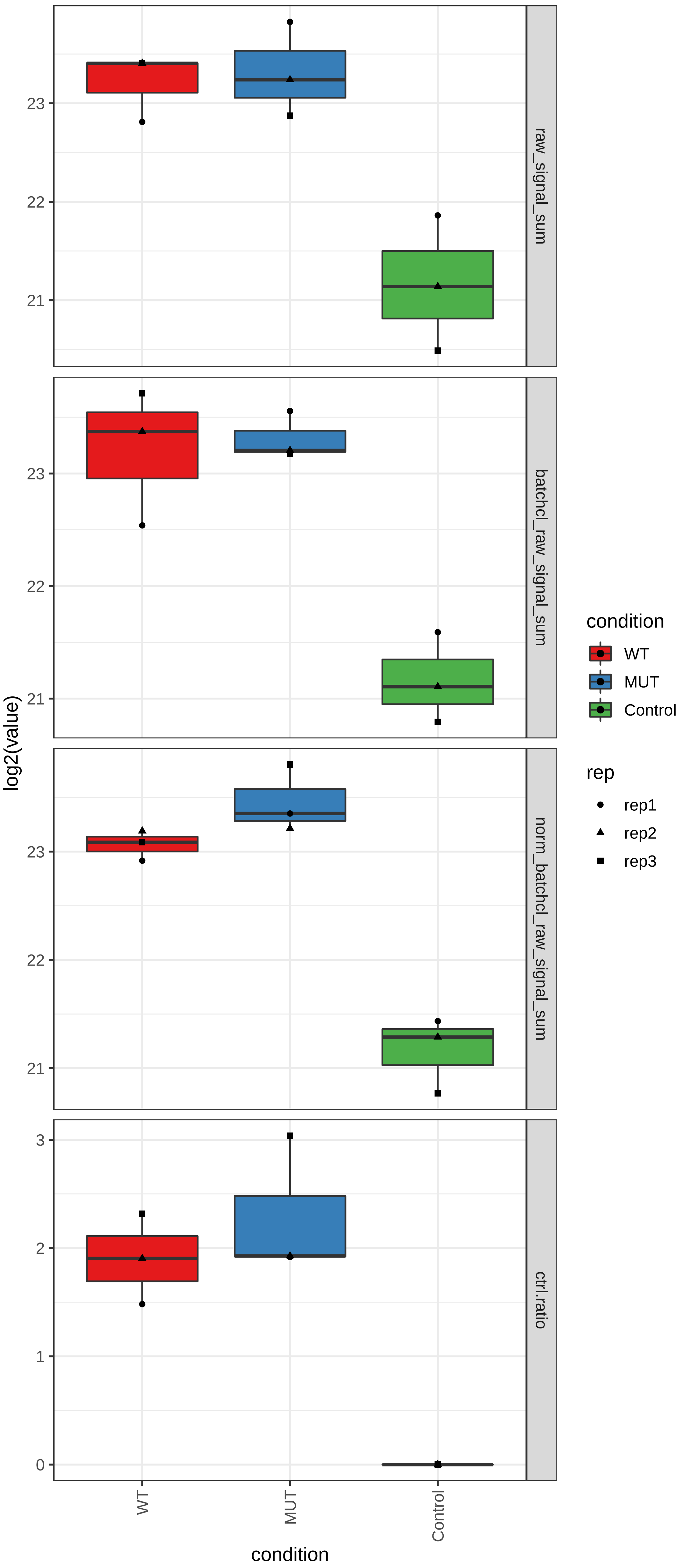

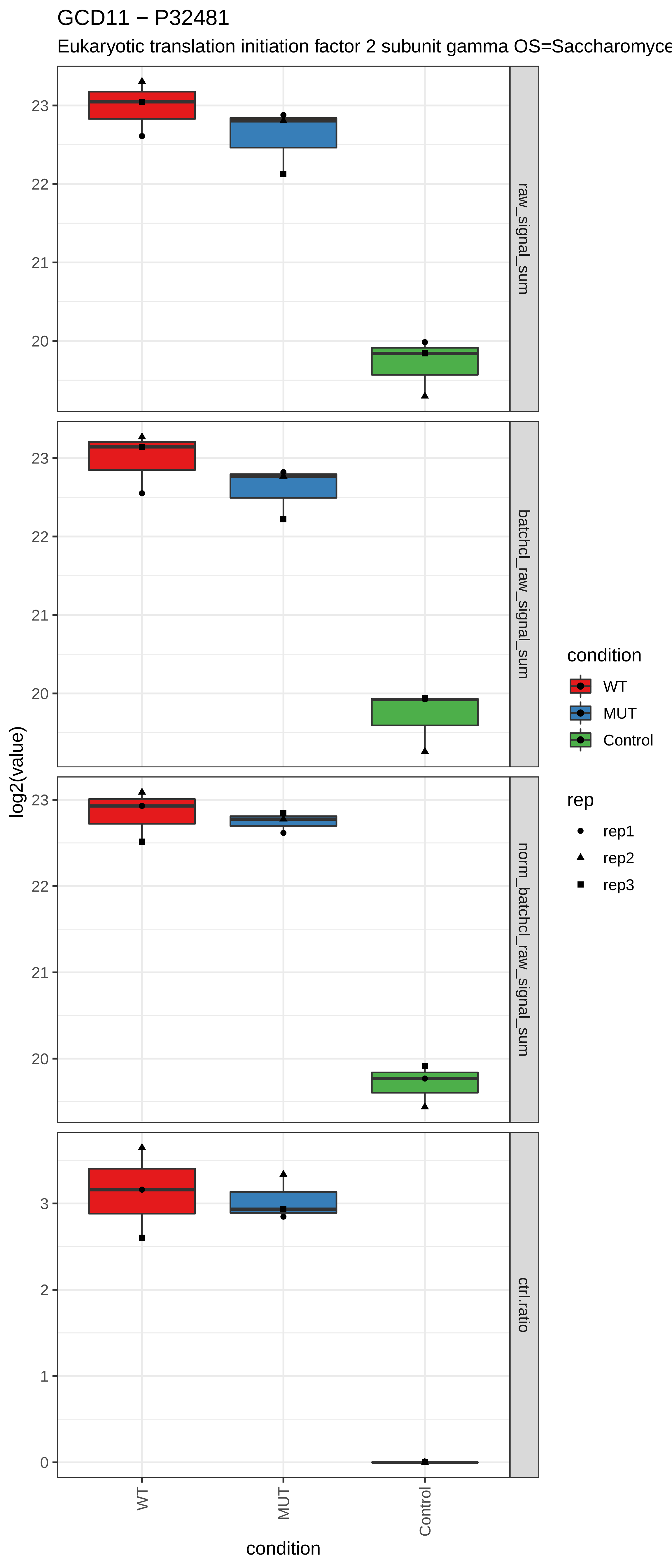
GCD11 − P32481
Eukaryotic translation initiation factor 2 subunit gamma OS=Saccharomyce
raw_signal_sum
batchcl_raw_signal_sum
norm_batchcl_raw_signal_sum
ctrl.ratio
log2(value)
condition
WT
MUT
Control
rep
rep1
rep2
rep3
23
22
21
20
3
2
1
0

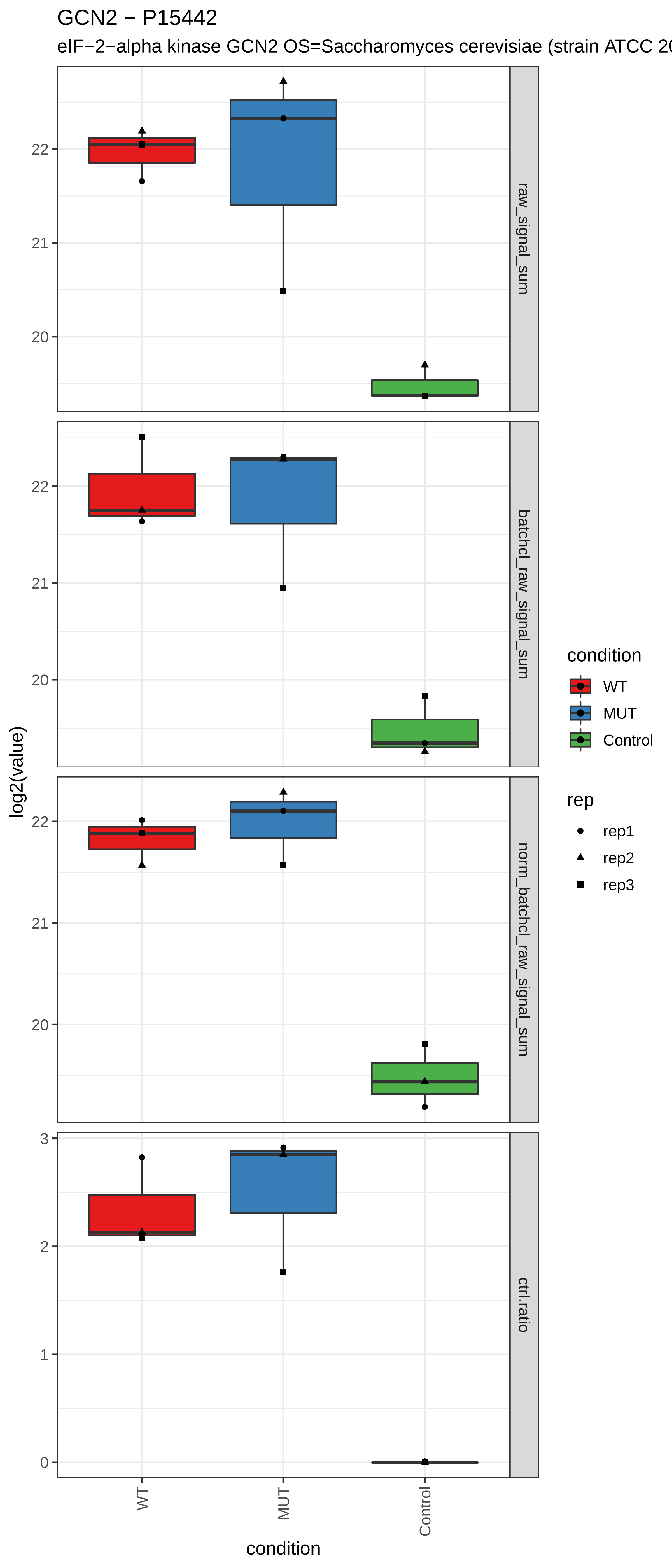
GCN2 − P15442
eIF−2−alpha kinase GCN2 OS=Saccharomyces cerevisiae (strain ATCC 2
raw_signal_sum
batchcl_raw_signal_sum
norm_batchcl_raw_signal_sum
ctrl.ratio
log2(value)
condition
WT
MUT
Control
rep
rep1
rep2
rep3
22
21
20
3
2
1
0

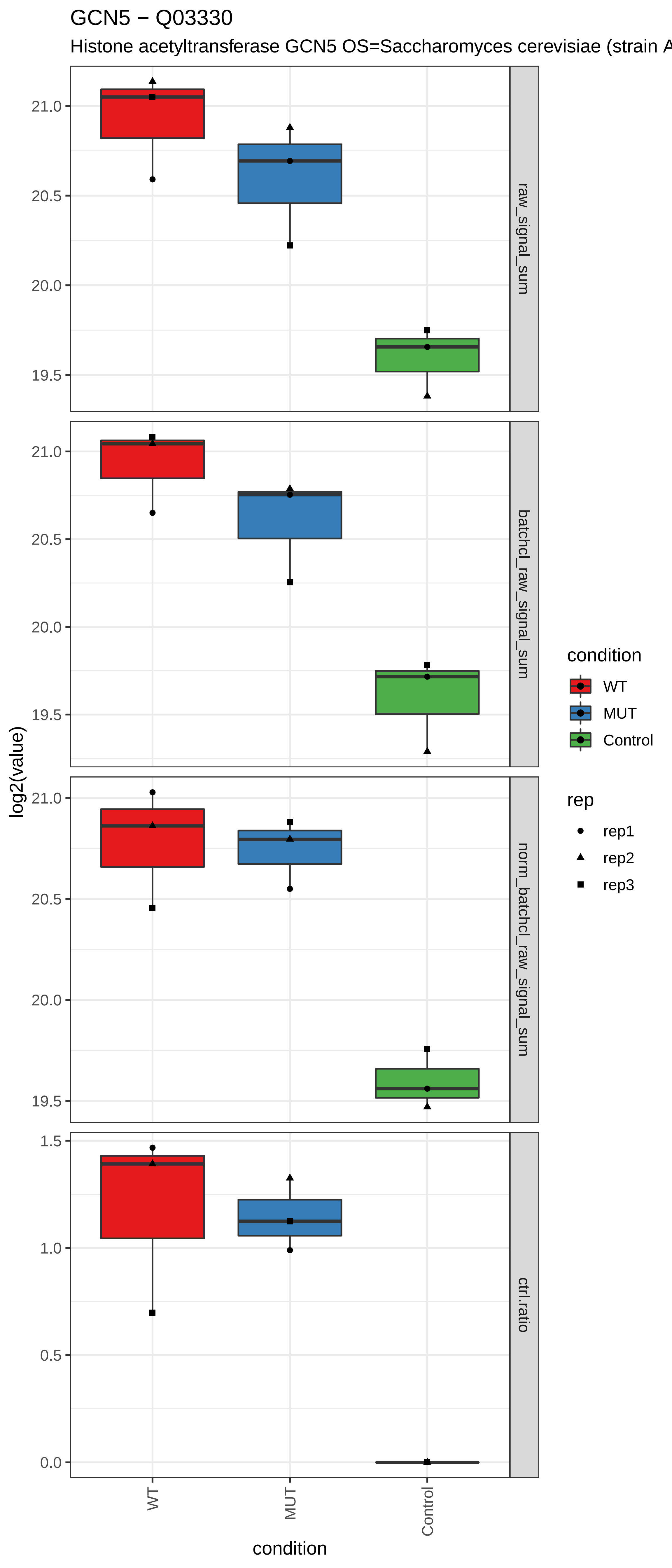

GCN5 − Q03330
Histone acetyltransferase GCN5 OS=Saccharomyces cerevisiae (strain A
raw_signal_sum
batchcl_raw_signal_sum
norm_batchcl_raw_signal_sum
ctrl.ratio
21.0
20.5
20.0
19.5
1.5
1.0
0.5
0.0
log2(value)
WT
MUT
Control
condition
rep
rep1
rep2
rep3

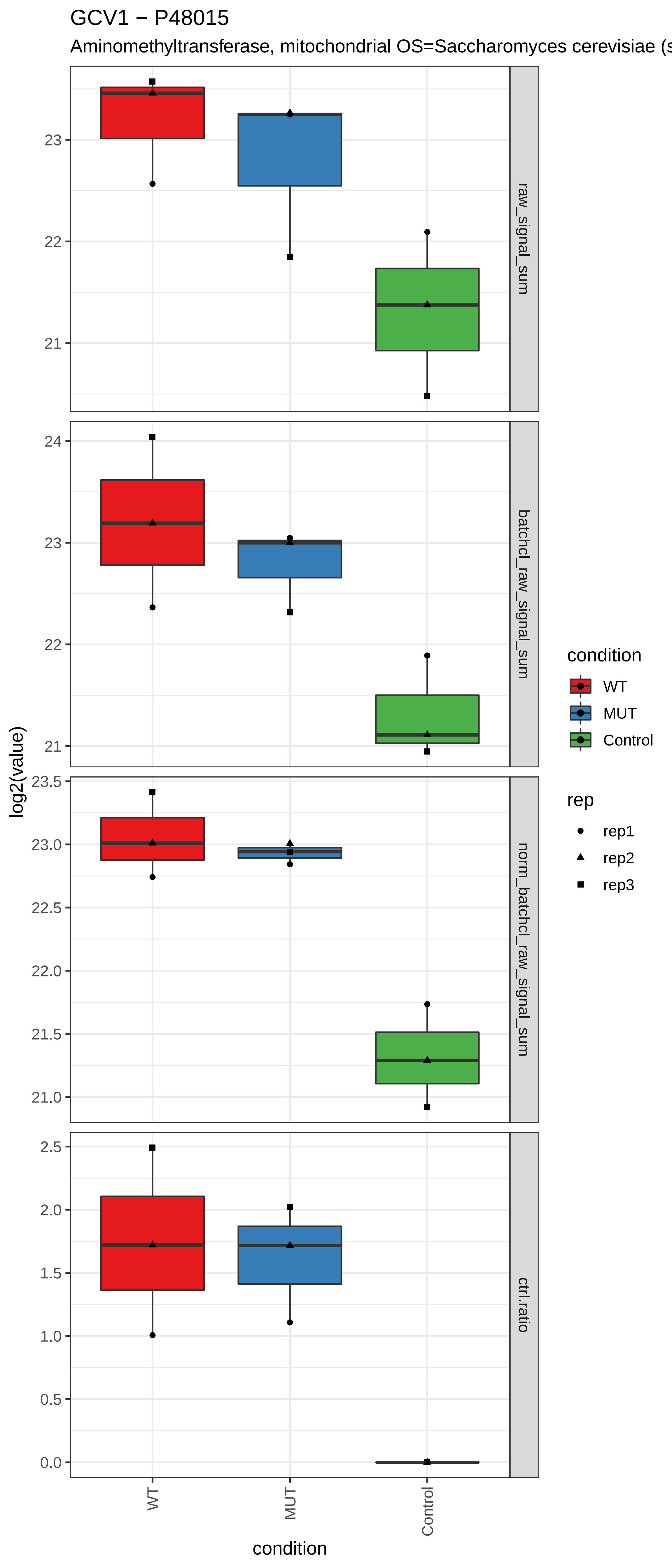

GCV1 − P48015
Aminomethyltransferase, mitochondrial OS=Saccharomyces cerevisiae (s
raw_signal_sum
batchcl_raw_signal_sum
norm_batchcl_raw_signal_sum
ctrl.ratio
log2(value)
condition
WT
MUT
Control
rep
rep1
rep2
rep3

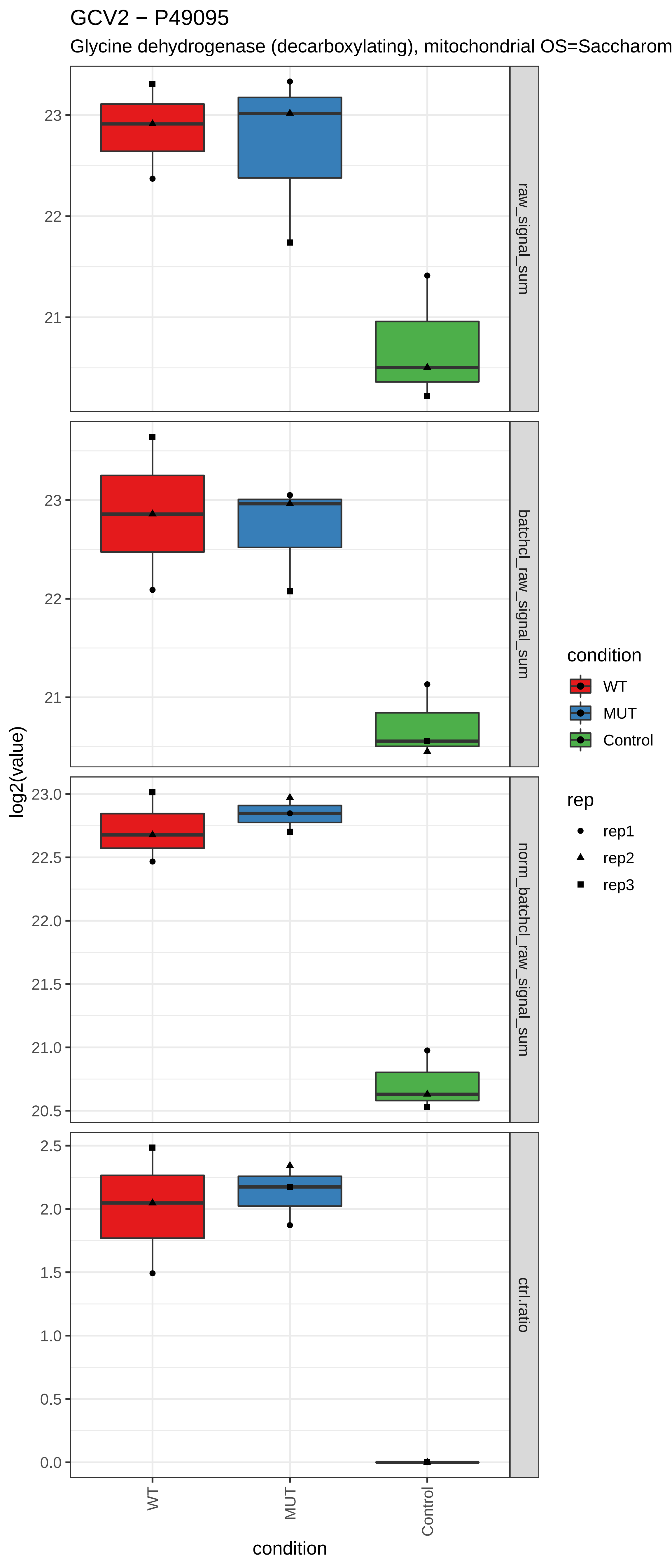
GCV2 − P49095
Glycine dehydrogenase (decarboxylating), mitochondrial OS=Saccharom
raw_signal_sum
batchcl_raw_signal_sum
norm_batchcl_raw_signal_sum
ctrl.ratio
log2(value)
condition
WT
MUT
Control
rep
rep1
rep2
rep3

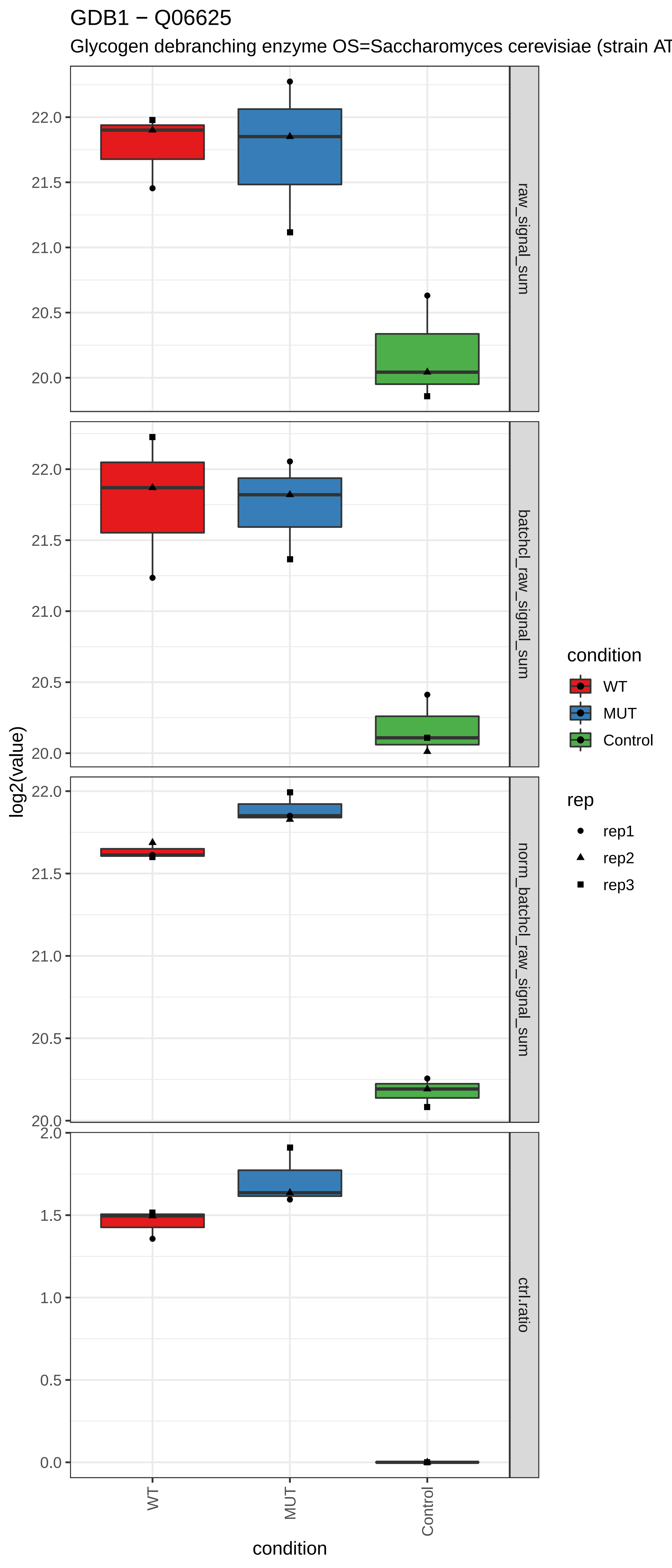

GDB1 − Q06625
Glycogen debranching enzyme OS=Saccharomyces cerevisiae (strain AT
raw_signal_sum
batchcl_raw_signal_sum
norm_batchcl_raw_signal_sum
ctrl.ratio
log2(value)
condition
WT
MUT
Control
rep
rep1
rep2
rep3
22.0
21.5
21.0
20.5
20.0
2.0
1.5
1.0
0.5
0.0

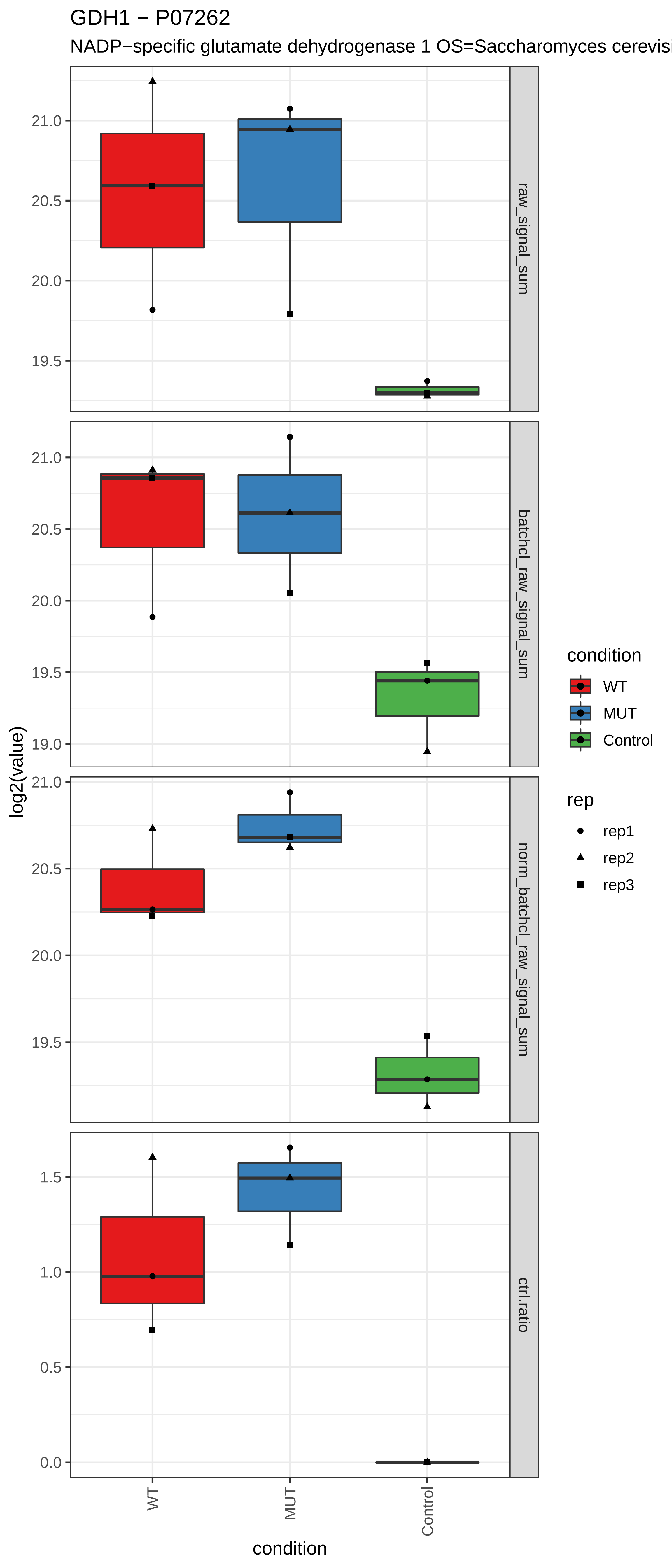

GDH1 − P07262
NADP−specific glutamate dehydrogenase 1 OS=Saccharomyces cerevisi
log2(value)
raw_signal_sum
batchcl_raw_signal_sum
norm_batchcl_raw_signal_sum
ctrl.ratio
21.0
20.5
20.0
19.5
19.0
1.5
1.0
0.5
0.0
WT
MUT
Control
condition
condition
WT
MUT
Control
rep
rep1
rep2
rep3

GDH2 − P33327

NAD−specific glutamate dehydrogenase OS=Saccharomyces cerevisiae (s

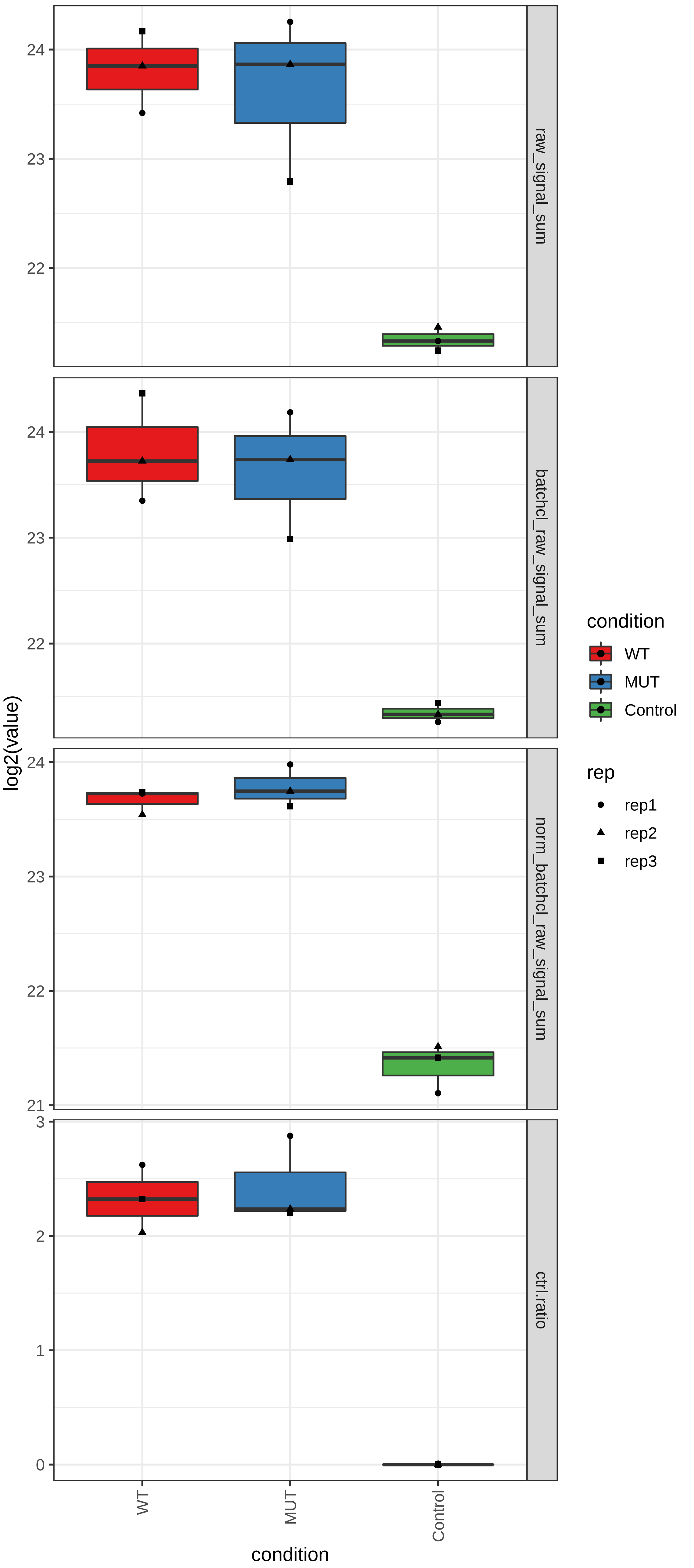

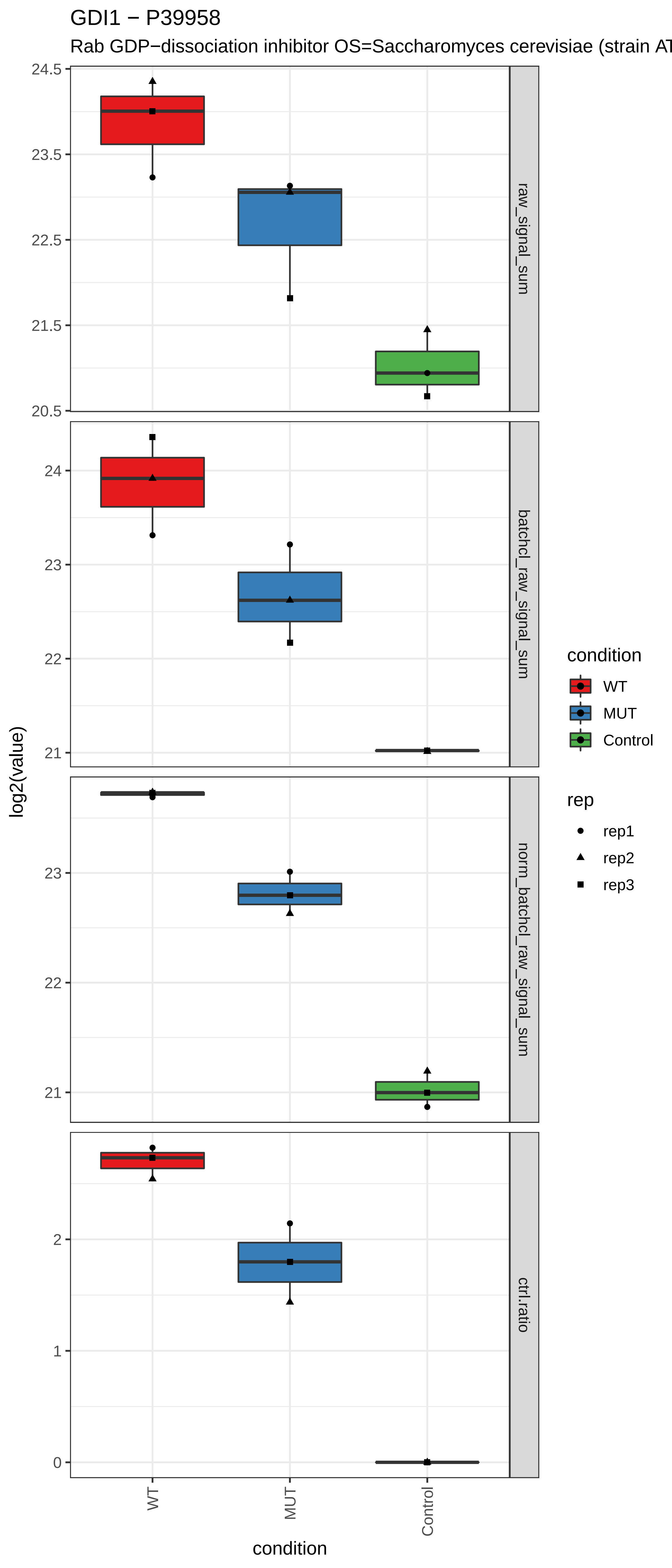
GDI1 − P39958
Rab GDP−dissociation inhibitor OS=Saccharomyces cerevisiae (strain AT
log2(value)
raw_signal_sum
batchcl_raw_signal_sum
norm_batchcl_raw_signal_sum
ctrl.ratio
condition
WT
MUT
Control
rep
rep1
rep2
rep3

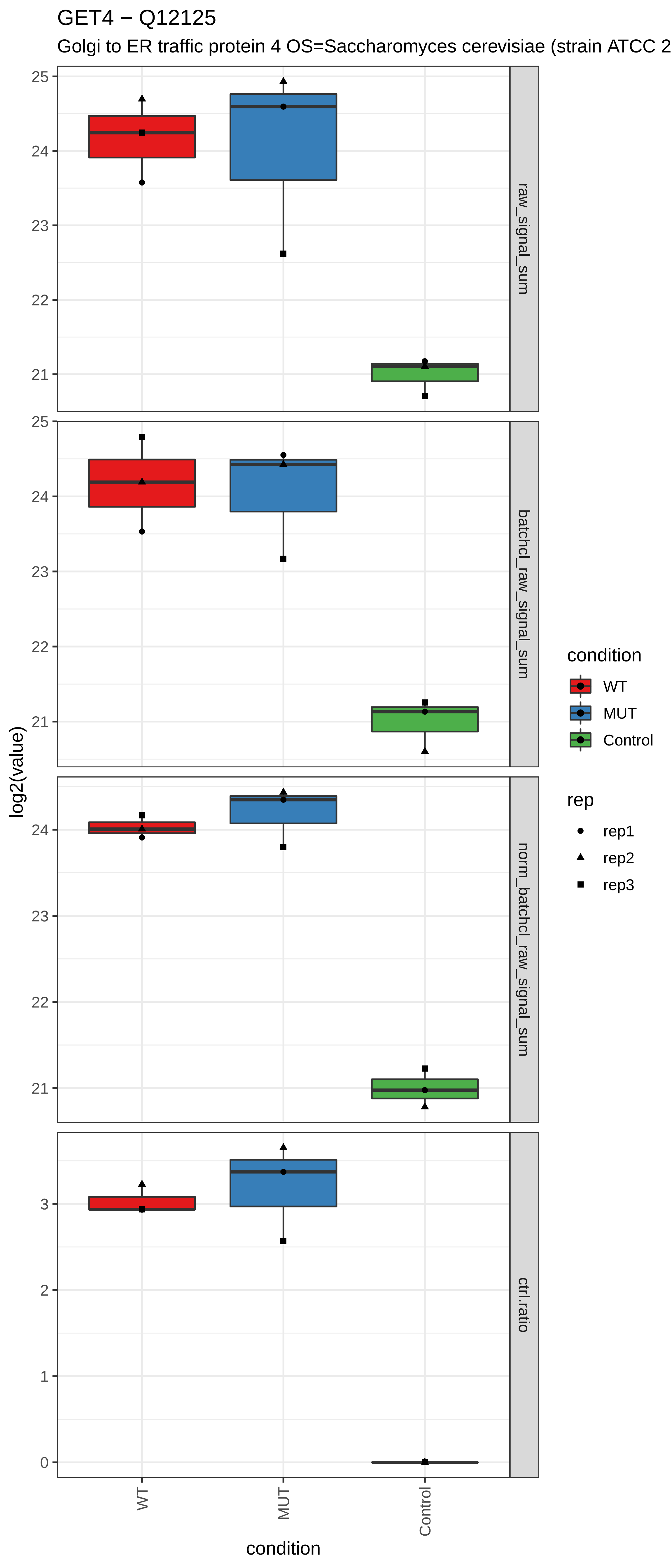

GET4 − Q12125
Golgi to ER traffic protein 4 OS=Saccharomyces cerevisiae (strain ATCC 2
raw_signal_sum
batchcl_raw_signal_sum
norm_batchcl_raw_signal_sum
ctrl.ratio
log2(value)
condition
WT
MUT
Control
rep
rep1
rep2
rep3

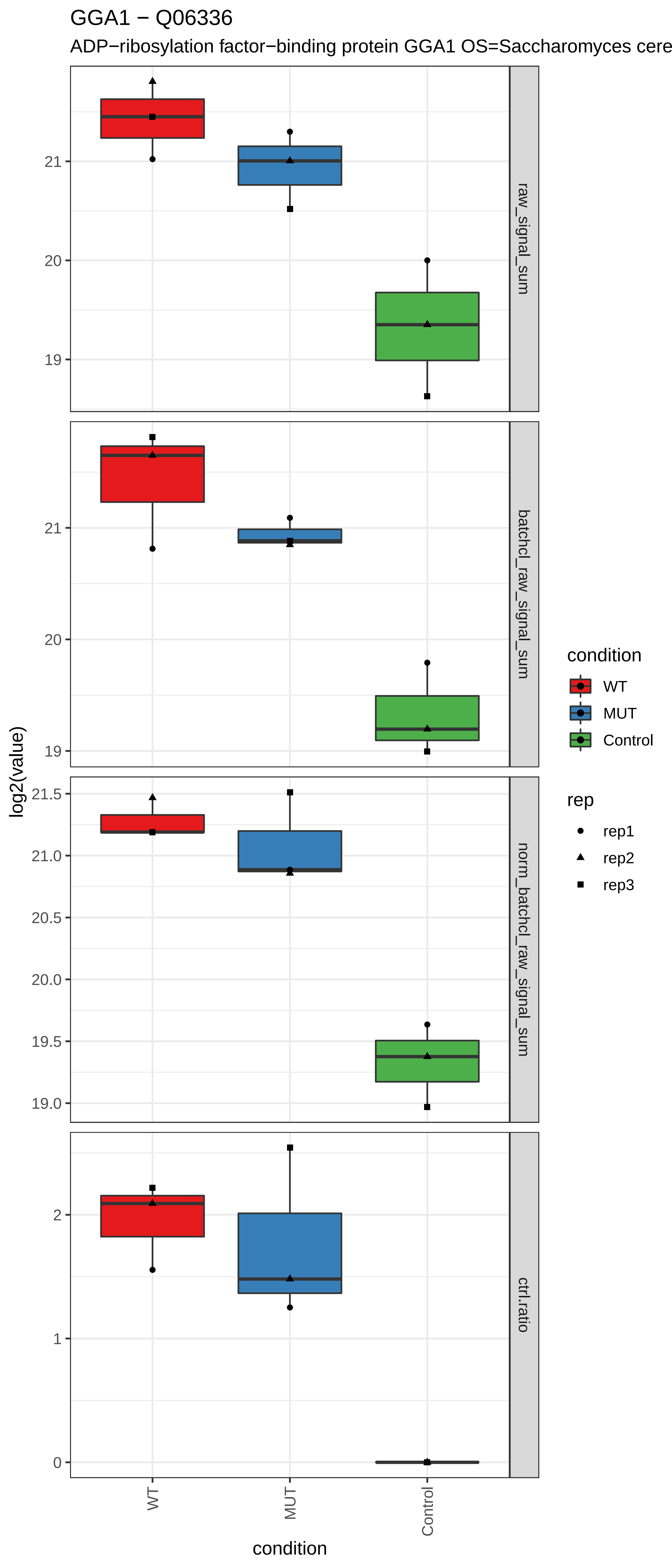
GGA1 − Q06336
ADP−ribosylation factor−binding protein GGA1 OS=Saccharomyces cere
raw_signal_sum
batchcl_raw_signal_sum
norm_batchcl_raw_signal_sum
ctrl.ratio
log2(value)
condition
WT
MUT
Control
rep
rep1
rep2
rep3
21
20
19
21.5
21.0
20.5
20.0
19.5
19.0
2
1
0

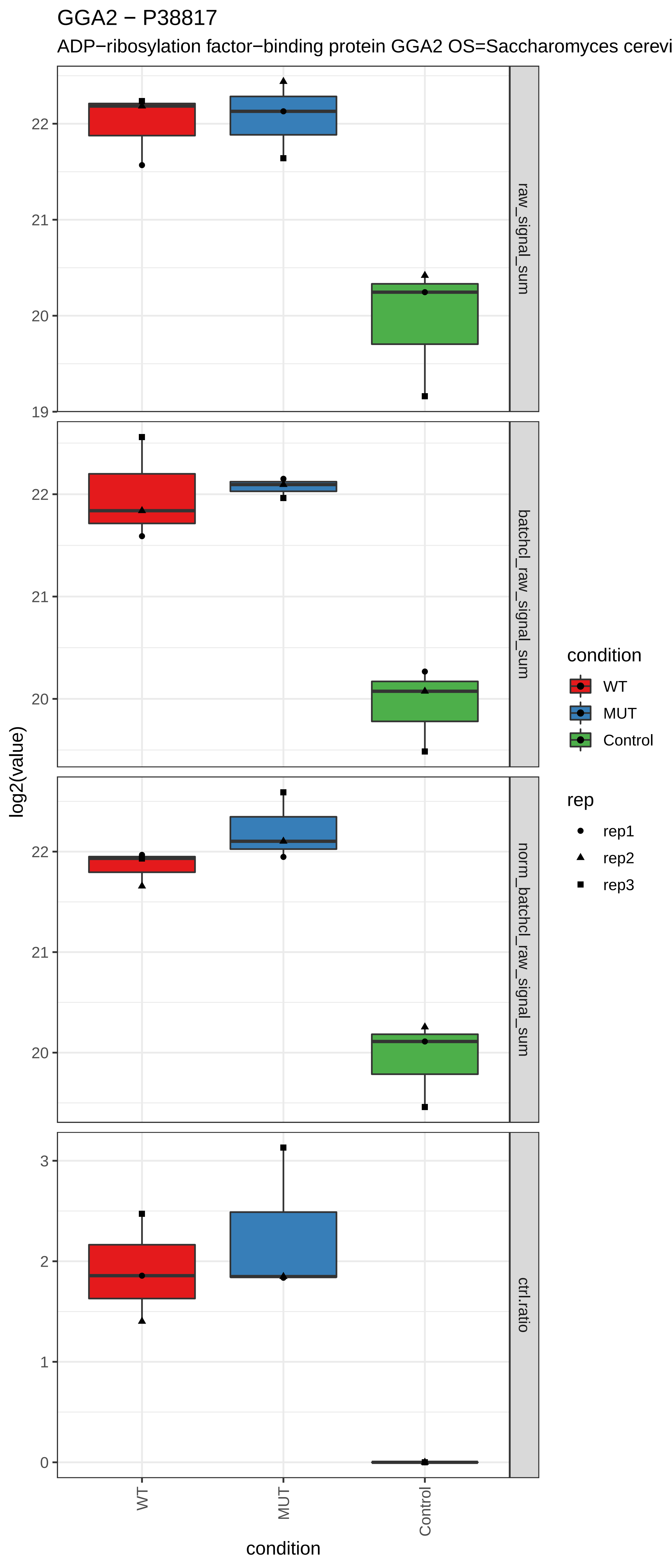

GGA2 − P38817
ADP−ribosylation factor−binding protein GGA2 OS=Saccharomyces cerevi
raw_signal_sum
batchcl_raw_signal_sum
norm_batchcl_raw_signal_sum
ctrl.ratio
log2(value)
condition
WT
MUT
Control
rep
rep1
rep2
rep3

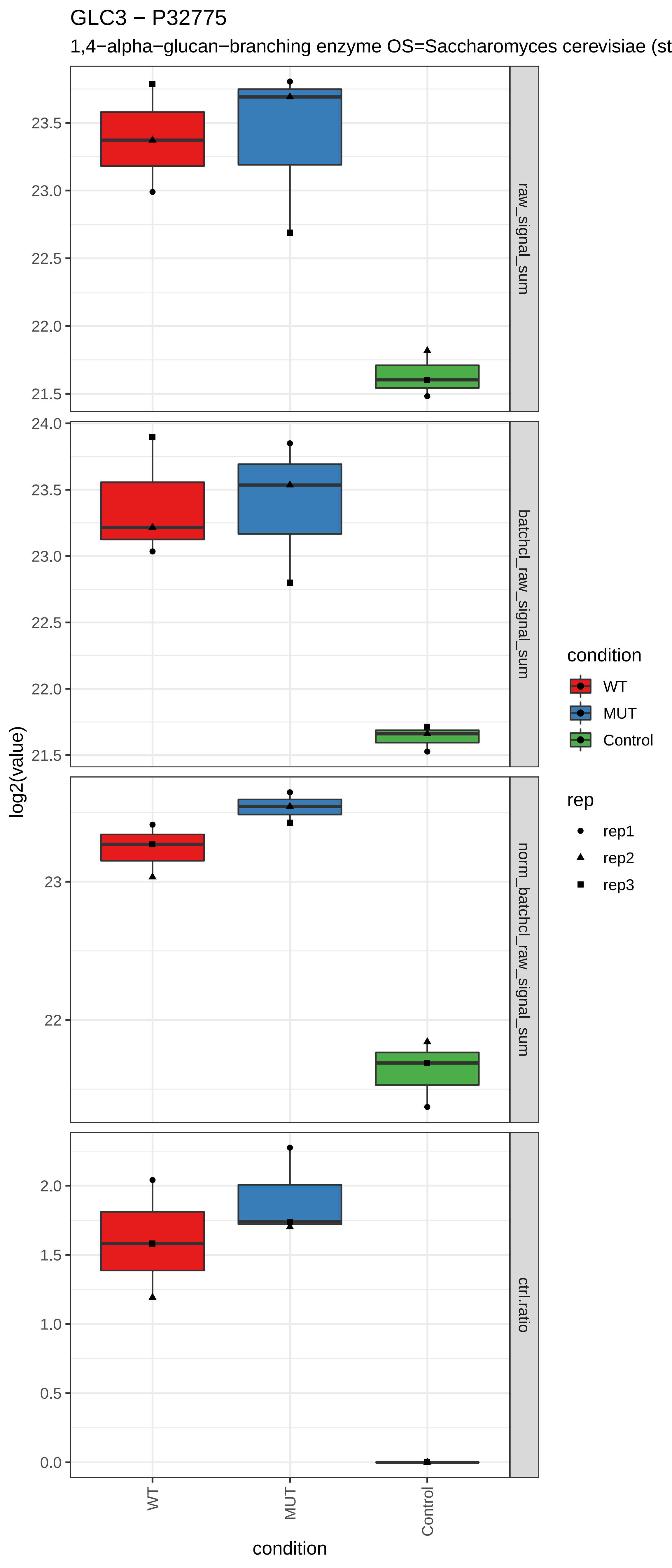
GLC3 − P32775
1,4−alpha−glucan−branching enzyme OS=Saccharomyces cerevisiae (st
raw_signal_sum
batchcl_raw_signal_sum
norm_batchcl_raw_signal_sum
ctrl.ratio
log2(value)
condition
WT
MUT
Control
rep
rep1
rep2
rep3
23.5
23.0
22.5
22.0
21.5
24.0
23
22
2.0
1.5
1.0
0.5
0.0

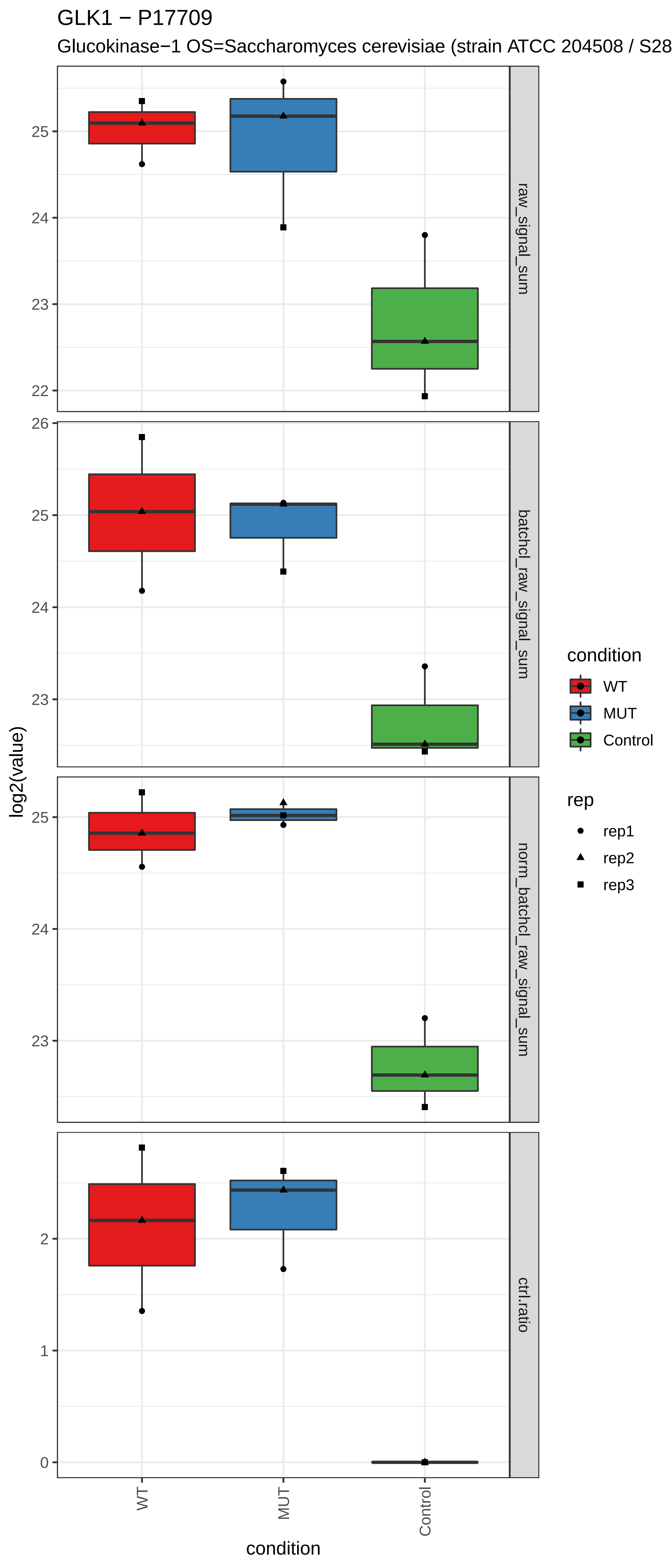
GLK1 − P17709
Glucokinase−1 OS=Saccharomyces cerevisiae (strain ATCC 204508 / S28
raw_signal_sum
batchcl_raw_signal_sum
norm_batchcl_raw_signal_sum
ctrl.ratio
log2(value)
condition
WT
MUT
Control
rep
rep1
rep2
rep3
26
25
24
23
22
2
1
0

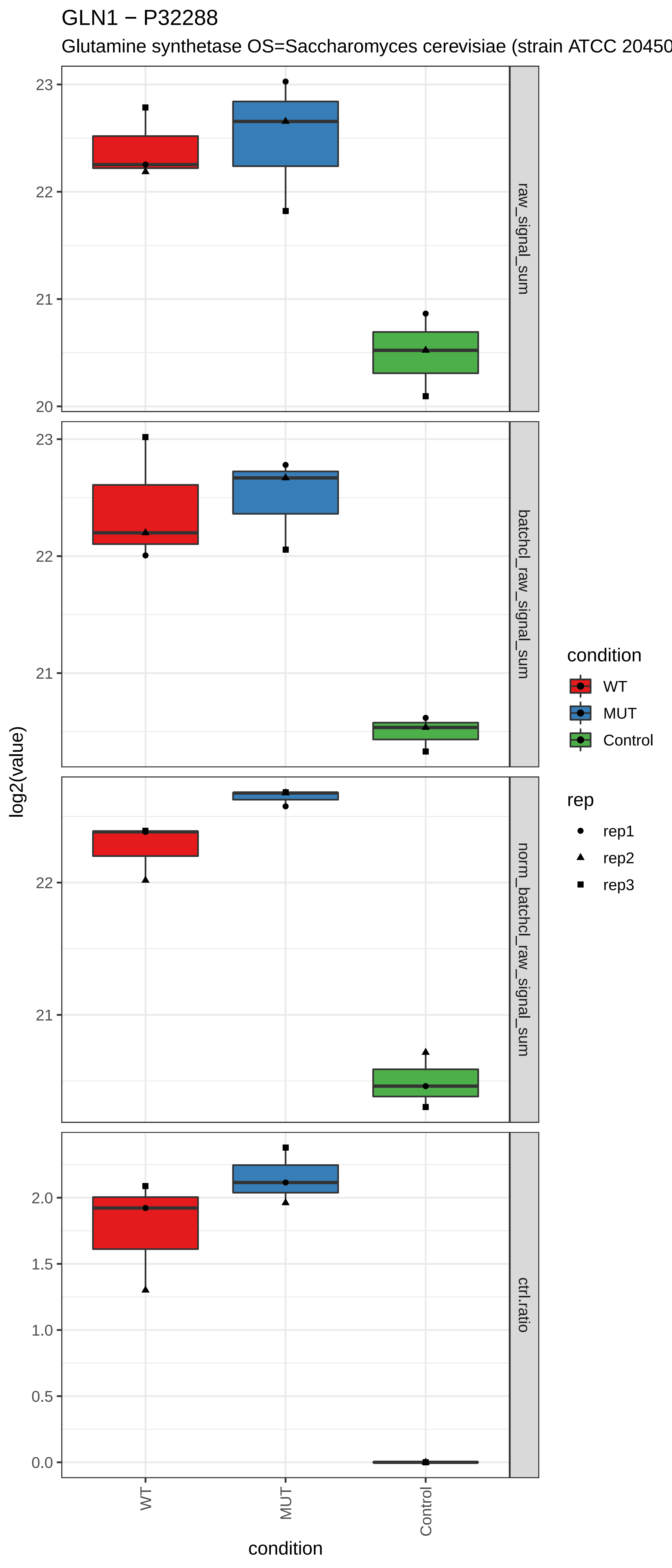

GLN1 − P32288
Glutamine synthetase OS=Saccharomyces cerevisiae (strain ATCC 20450
log2(value)
raw_signal_sum
batchcl_raw_signal_sum
norm_batchcl_raw_signal_sum
ctrl.ratio
23
22
21
20
2.0
1.5
1.0
0.5
0.0
WT
MUT
Control
condition
condition
WT
MUT
Control
rep
rep1
rep2
rep3

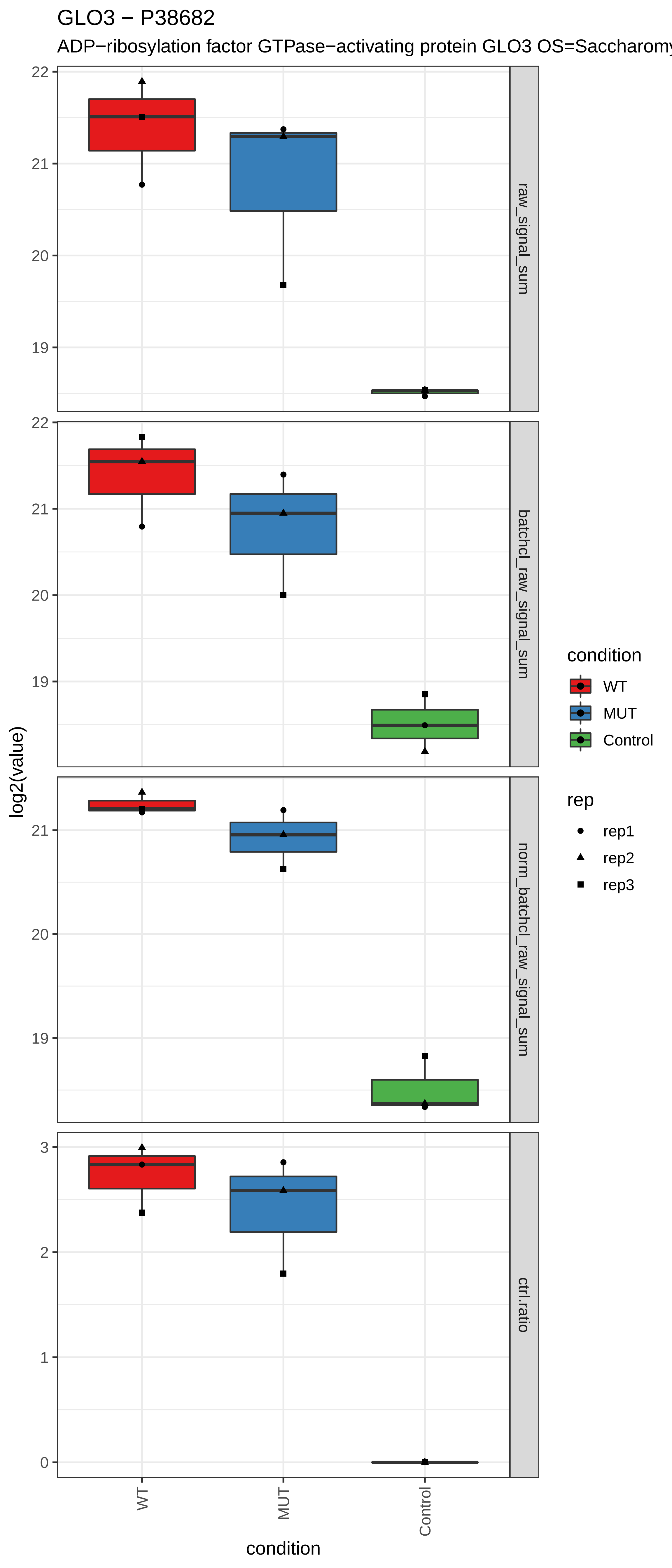

GLO3 − P38682
ADP−ribosylation factor GTPase−activating protein GLO3 OS=Saccharomy
raw_signal_sum
batchcl_raw_signal_sum
norm_batchcl_raw_signal_sum
ctrl.ratio
log2(value)
condition
WT
MUT
Control
rep
rep1
rep2
rep3

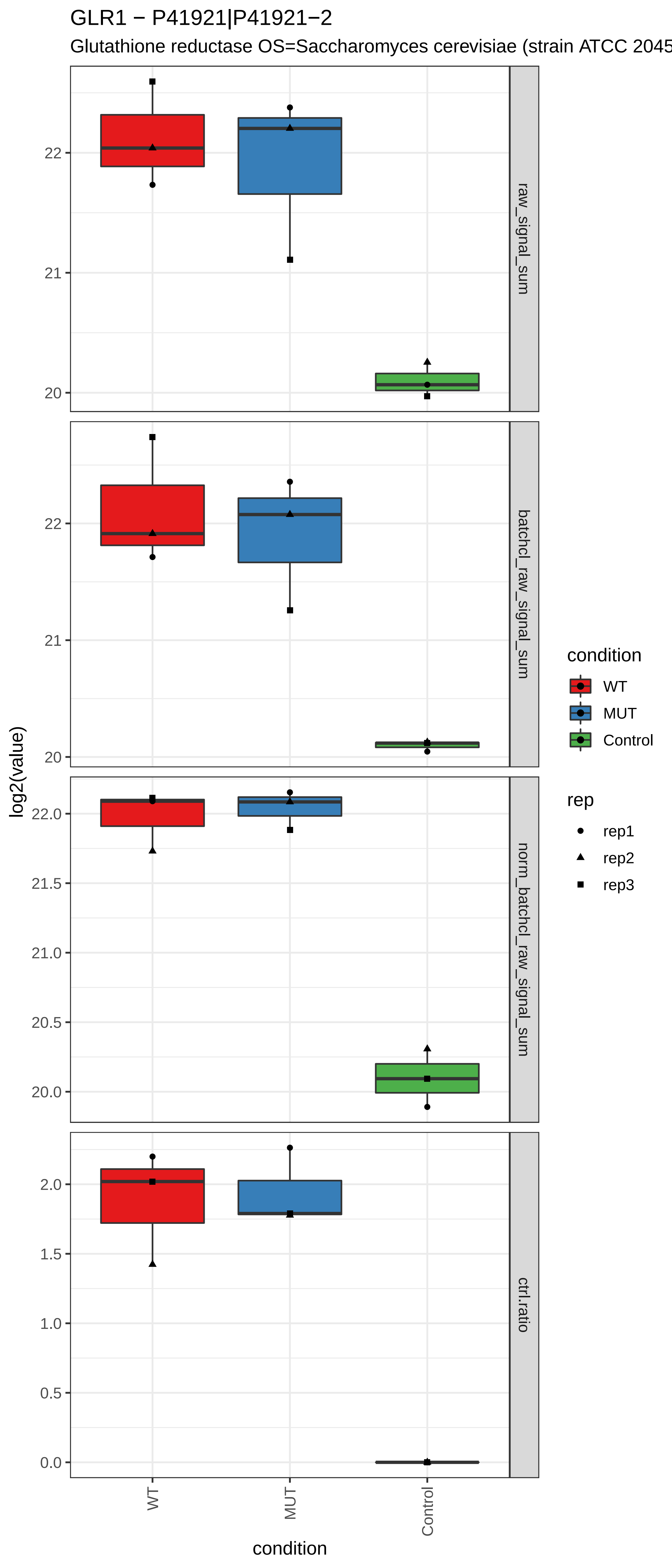
GLR1 − P41921|P41921−2
Glutathione reductase OS=Saccharomyces cerevisiae (strain ATCC 2045
raw_signal_sum
batchcl_raw_signal_sum
norm_batchcl_raw_signal_sum
ctrl.ratio
log2(value)
condition
WT
MUT
Control
rep
rep1
rep2
rep3
22
21
20
22.0
21.5
21.0
20.5
20.0
2.0
1.5
1.0
0.5
0.0

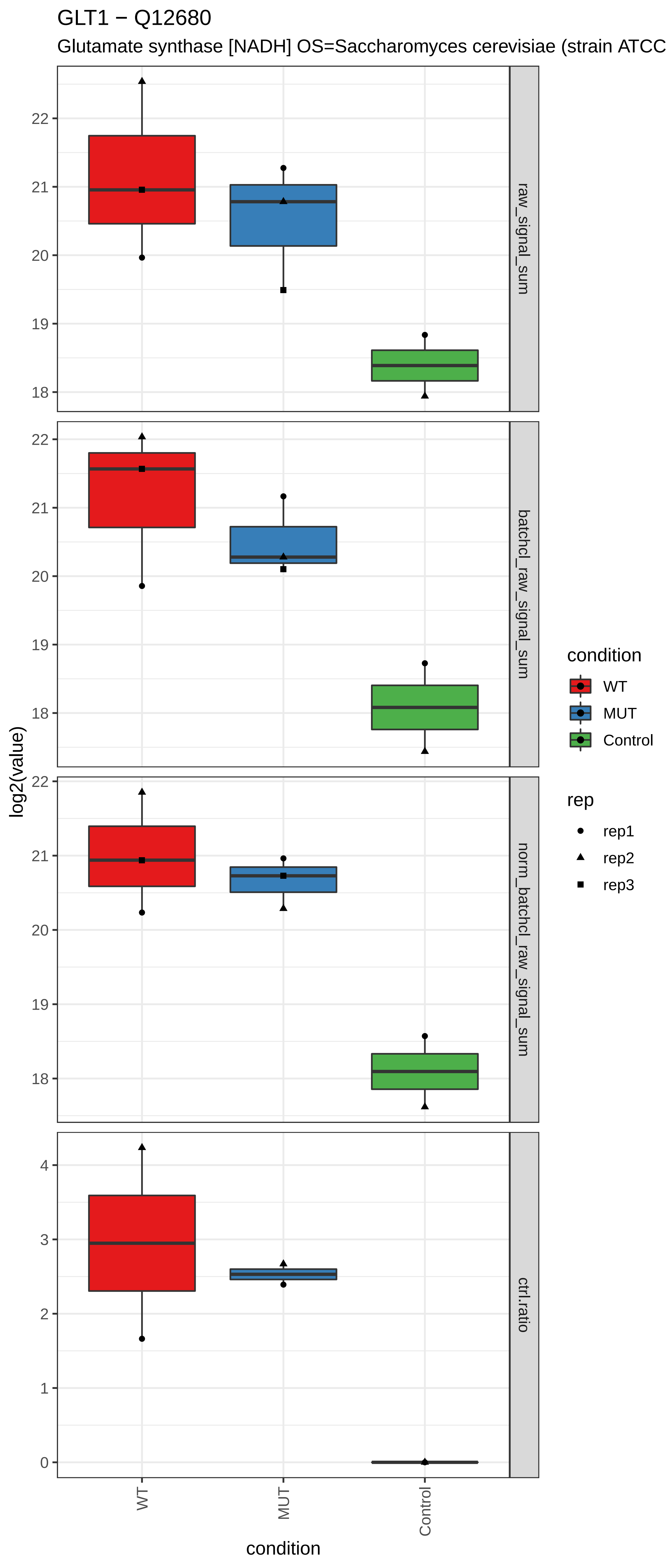

GLT1 − Q12680
Glutamate synthase [NADH] OS=Saccharomyces cerevisiae (strain ATCC
raw_signal_sum
batchcl_raw_signal_sum
norm_batchcl_raw_signal_sum
ctrl.ratio
log2(value)
condition
WT
MUT
Control
rep
rep1
rep2
rep3
22
21
20
19
18
4
3
2
1
0

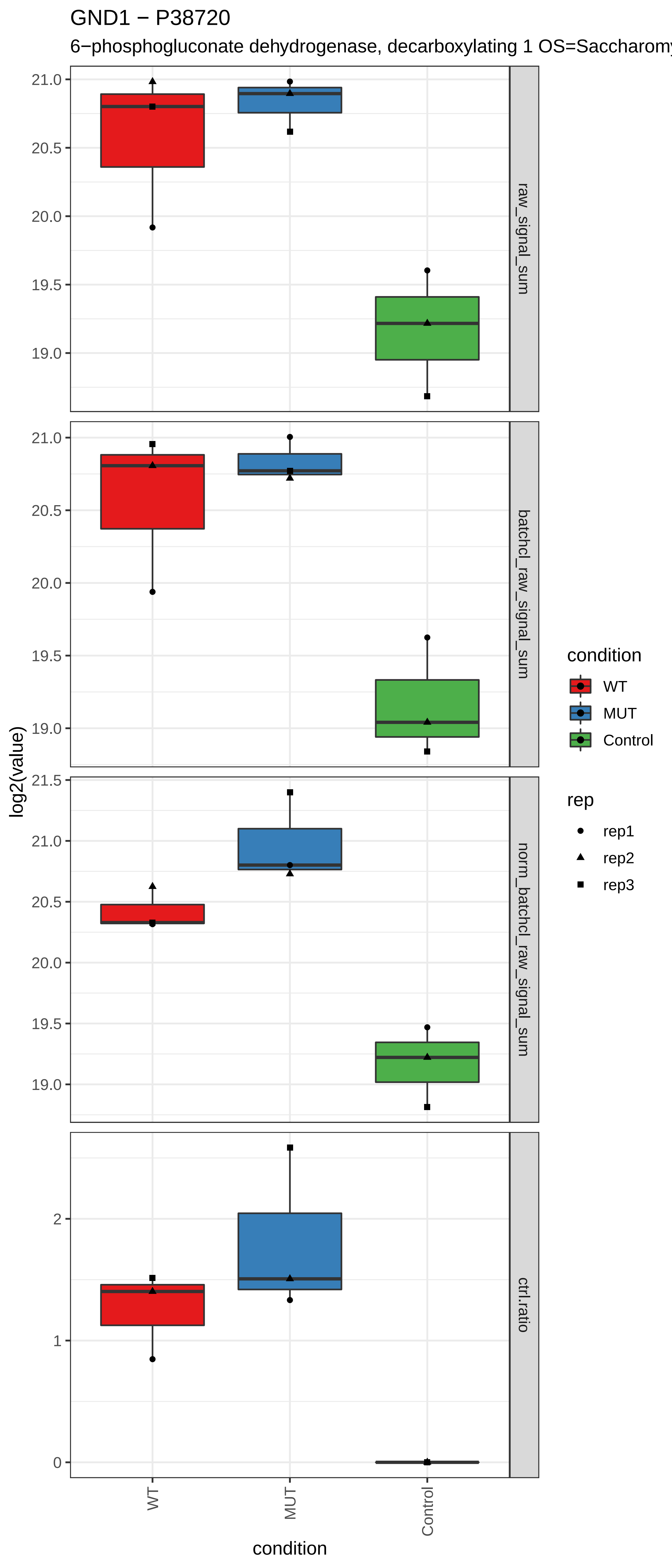
GND1 − P38720
6−phosphogluconate dehydrogenase, decarboxylating 1 OS=Saccharomy
raw_signal_sum
batchcl_raw_signal_sum
norm_batchcl_raw_signal_sum
ctrl.ratio
log2(value)
condition
WT
MUT
Control
rep
rep1
rep2
rep3
21.0
20.5
20.0
19.5
19.0
21.5
2
1
0

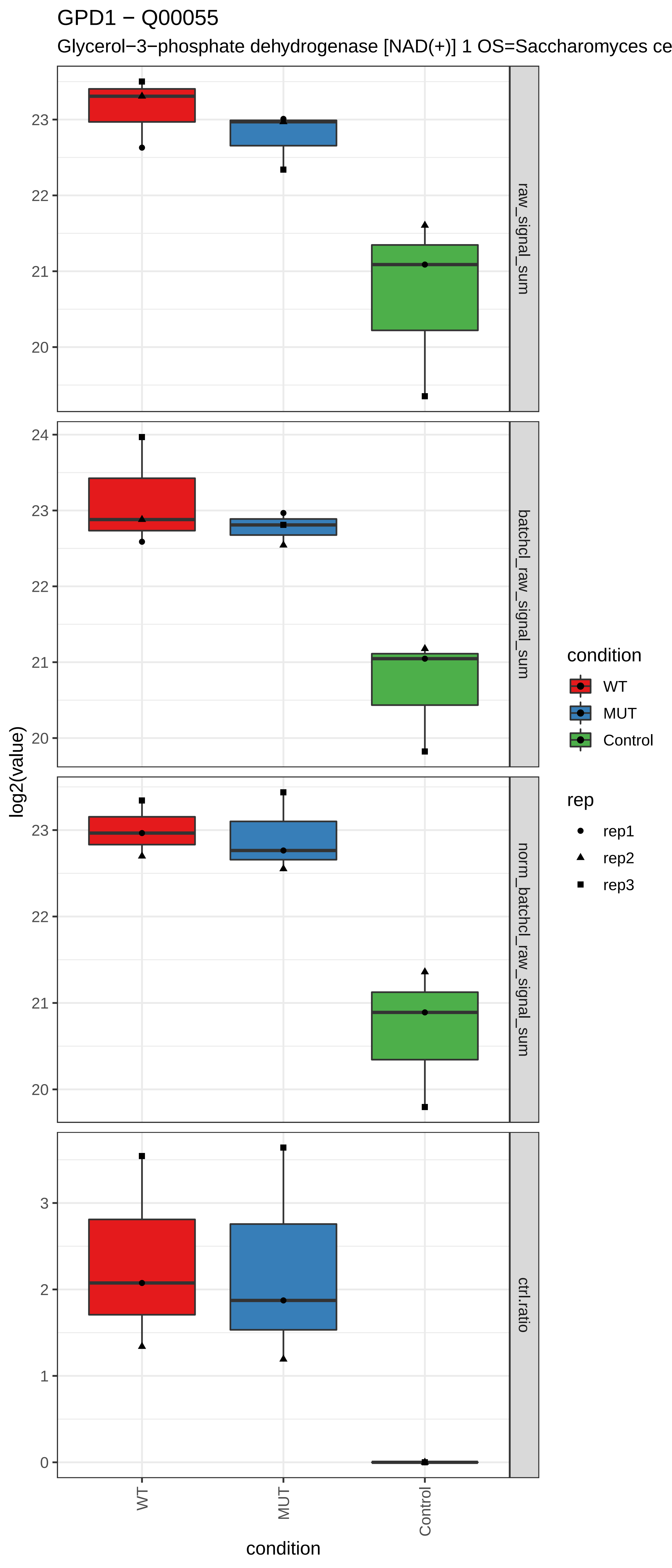

GPD1 − Q00055
Glycerol−3−phosphate dehydrogenase [NAD(+)] 1 OS=Saccharomyces ce
raw_signal_sum
batchcl_raw_signal_sum
norm_batchcl_raw_signal_sum
ctrl.ratio
log2(value)
condition
WT
MUT
Control
rep
rep1
rep2
rep3

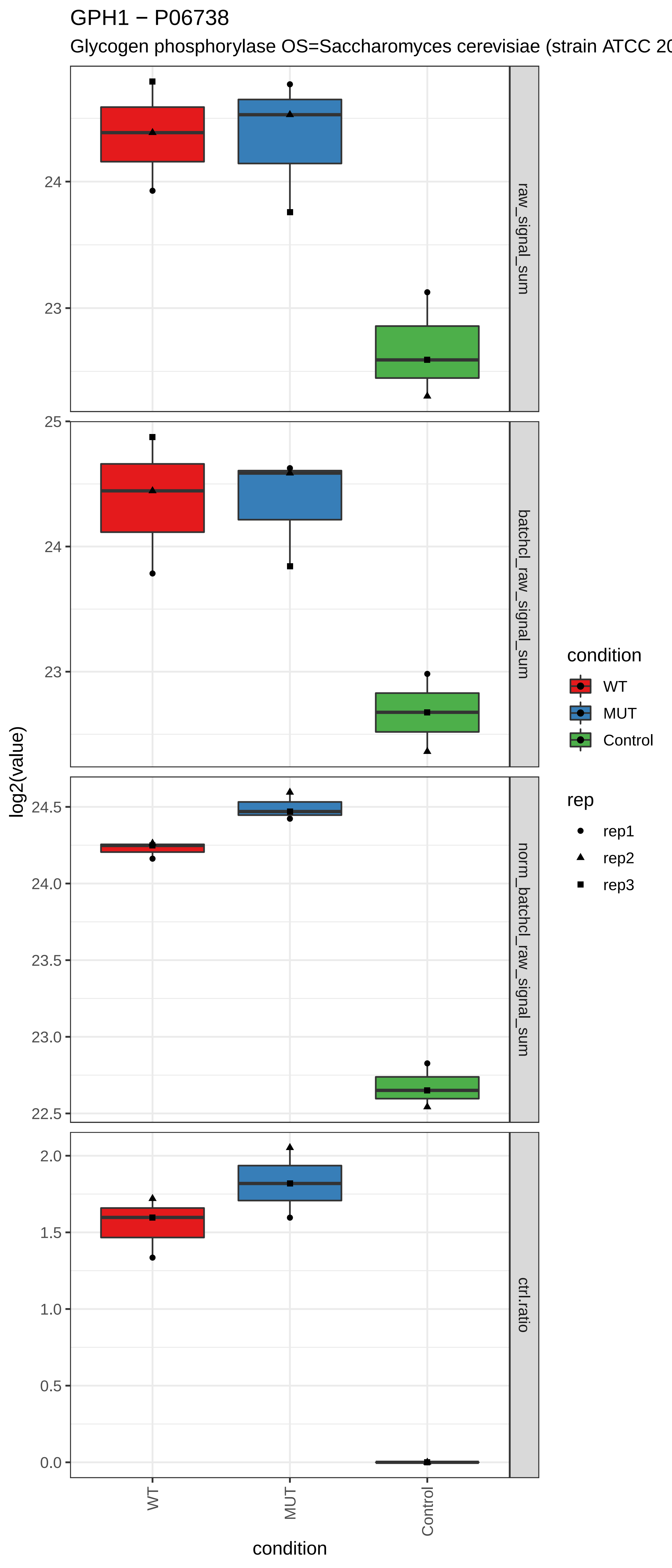

GPH1 − P06738
Glycogen phosphorylase OS=Saccharomyces cerevisiae (strain ATCC 20
raw_signal_sum
batchcl_raw_signal_sum
norm_batchcl_raw_signal_sum
ctrl.ratio
24
23
25
24
23
24.5
24.0
23.5
23.0
22.5
2.0
1.5
1.0
0.5
0.0
log2(value)
WT
MUT
Control
condition
condition
WT
MUT
Control
rep
rep1
rep2
rep3

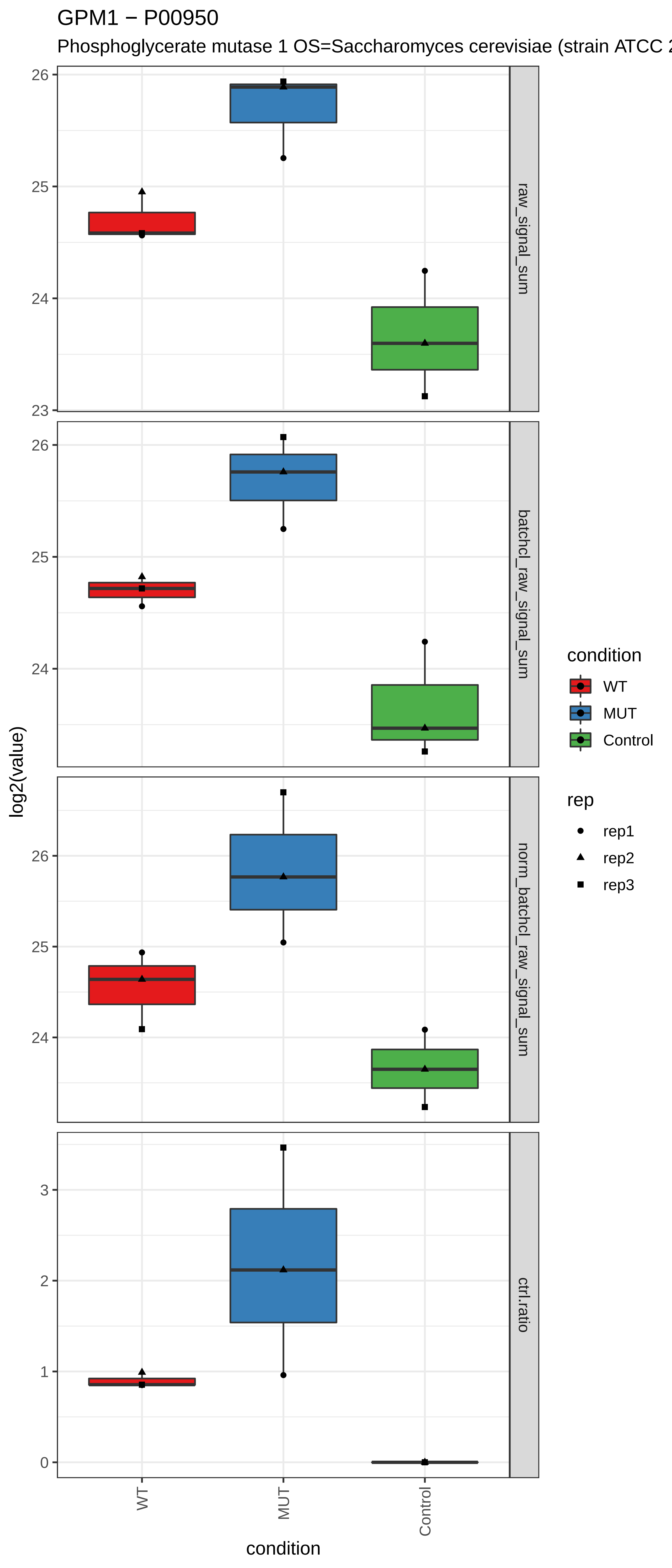
GPM1 − P00950
Phosphoglycerate mutase 1 OS=Saccharomyces cerevisiae (strain ATCC 2
raw_signal_sum
batchcl_raw_signal_sum
norm_batchcl_raw_signal_sum
ctrl.ratio
log2(value)
condition
WT
MUT
Control
rep
rep1
rep2
rep3

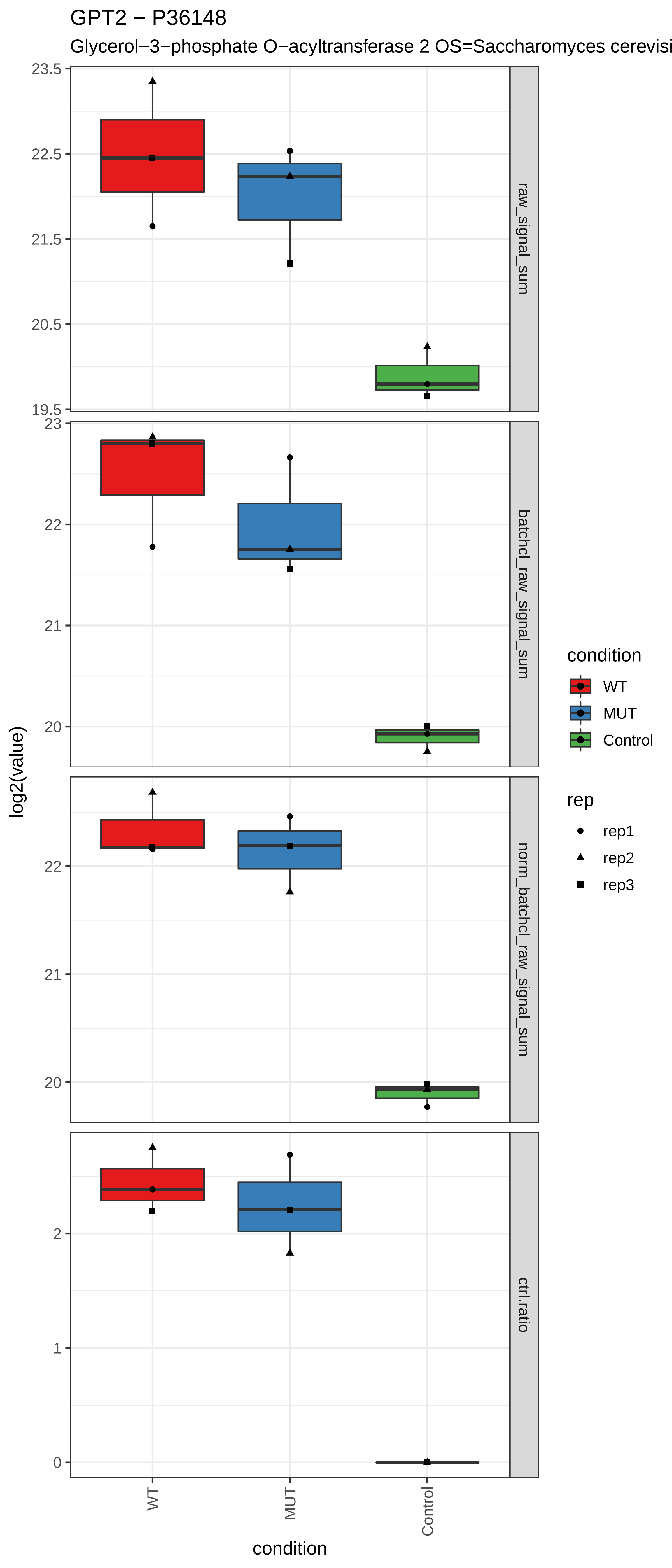
GPT2 − P36148
Glycerol−3−phosphate O−acyltransferase 2 OS=Saccharomyces cerevisi
raw_signal_sum
batchcl_raw_signal_sum
norm_batchcl_raw_signal_sum
ctrl.ratio
log2(value)
condition
WT
MUT
Control
rep
rep1
rep2
rep3
23.5
22.5
21.5
20.5
19.5
23
22
21
20
1
0
2

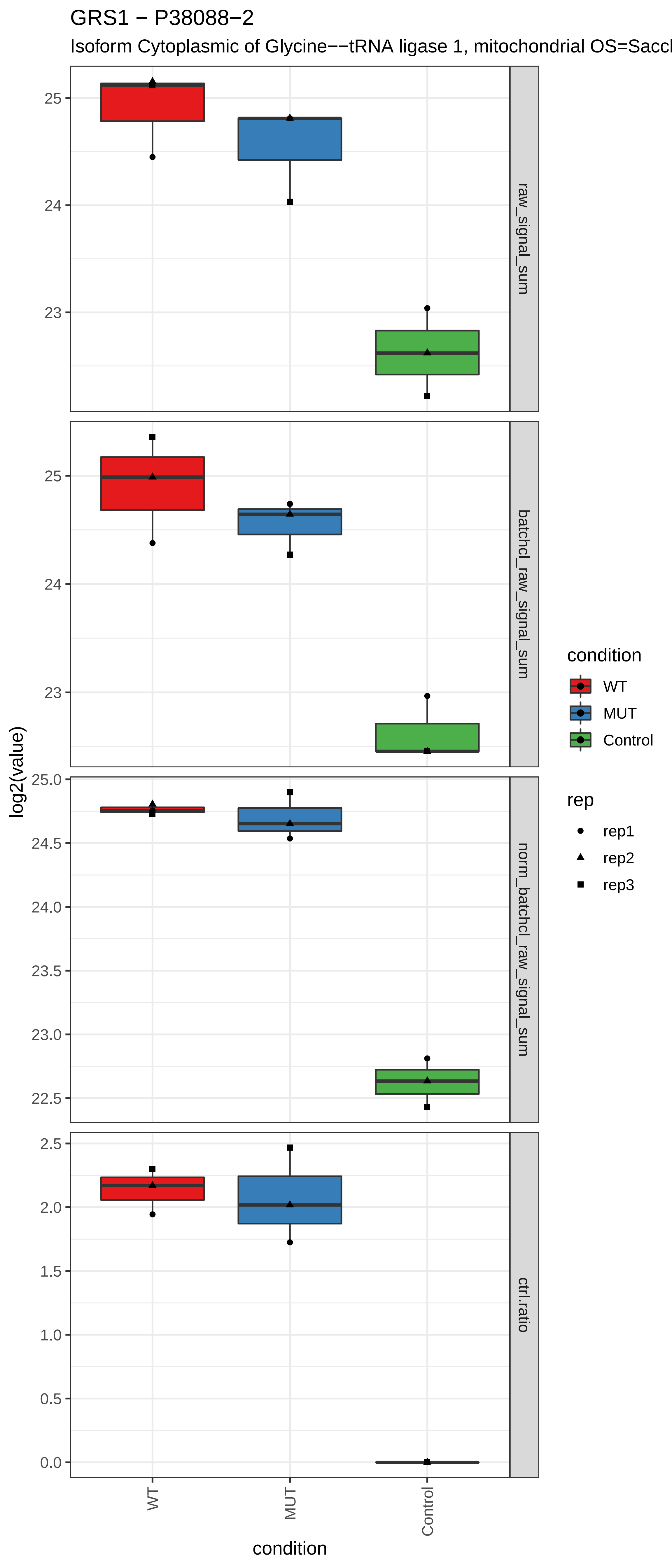
GRS1 − P38088−2
Isoform Cytoplasmic of Glycine−−tRNA ligase 1, mitochondrial OS=Saccl
raw_signal_sum
batchcl_raw_signal_sum
norm_batchcl_raw_signal_sum
ctrl.ratio
log2(value)
condition
WT
MUT
Control
rep
rep1
rep2
rep3
25
24
23
25.0
24.5
24.0
23.5
23.0
22.5
2.5
2.0
1.5
1.0
0.5
0.0

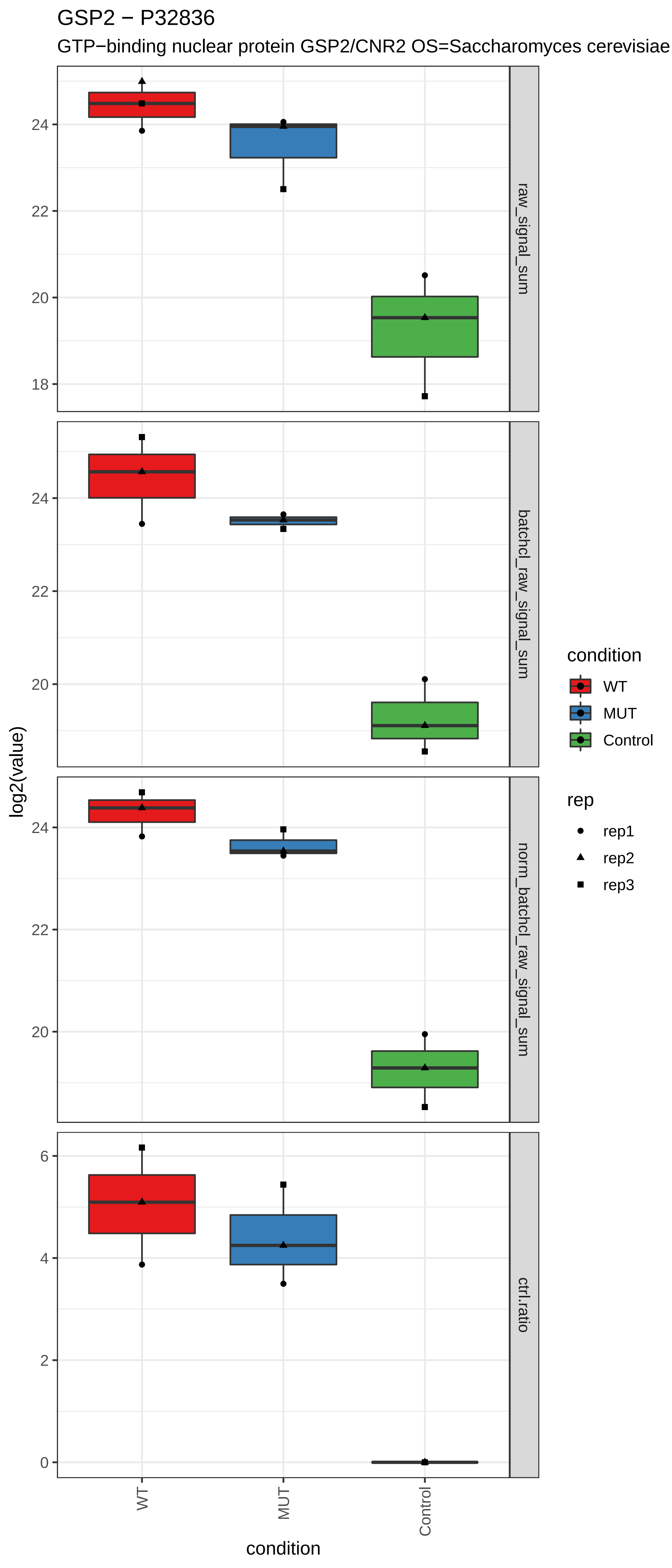
GSP2 − P32836
GTP−binding nuclear protein GSP2/CNR2 OS=Saccharomyces cerevisiae
raw_signal_sum
batchcl_raw_signal_sum
norm_batchcl_raw_signal_sum
ctrl.ratio
log2(value)
condition
WT
MUT
Control
rep
rep1
rep2
rep3
24
22
20
18
6
4
2
0

GSY1 − P23337

Glycogen [starch] synthase isoform 1 OS=Saccharomyces cerevisiae (stra

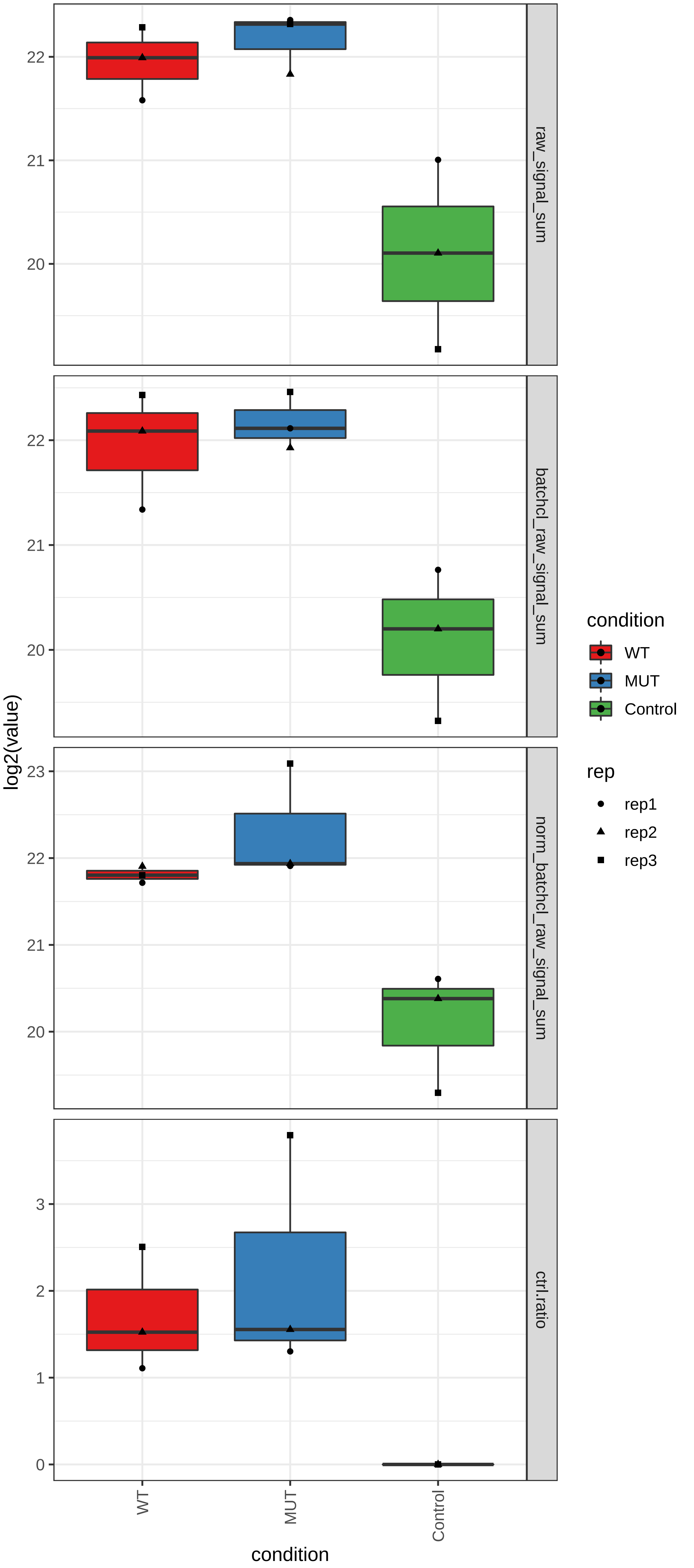

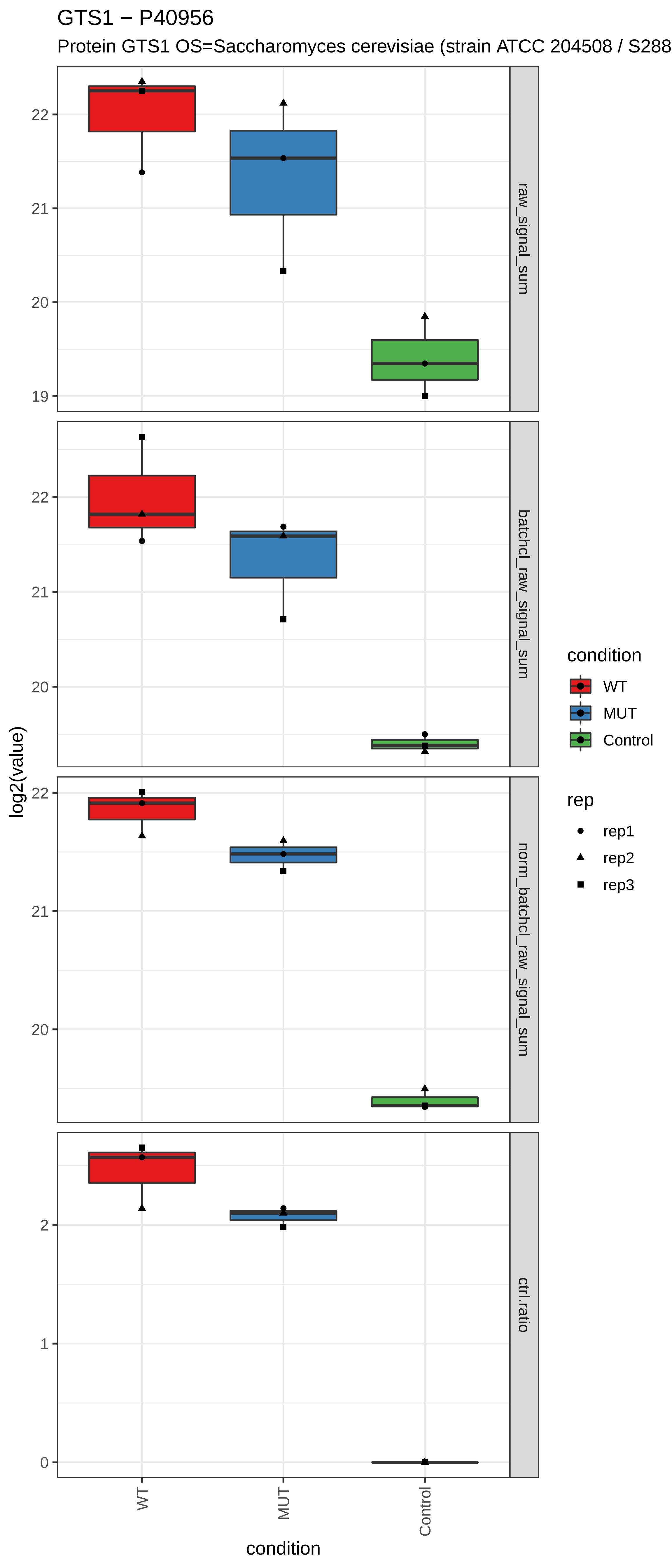

GTS1 − P40956
Protein GTS1 OS=Saccharomyces cerevisiae (strain ATCC 204508 / S288
raw_signal_sum
batchcl_raw_signal_sum
norm_batchcl_raw_signal_sum
ctrl.ratio
log2(value)
condition
WT
MUT
Control
rep
rep1
rep2
rep3
22
21
20
19
0
1
2

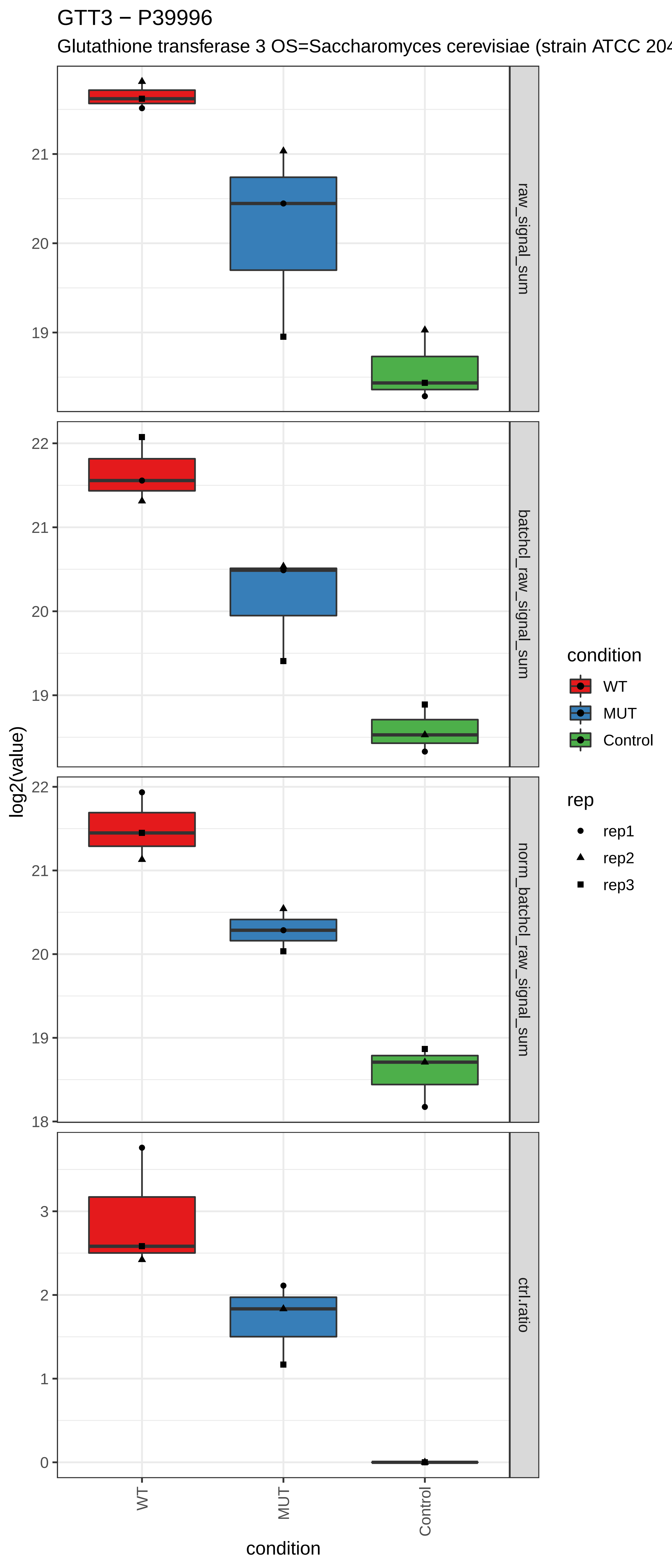

GTT3 − P39996
Glutathione transferase 3 OS=Saccharomyces cerevisiae (strain ATCC 204
raw_signal_sum
batchcl_raw_signal_sum
norm_batchcl_raw_signal_sum
ctrl.ratio
log2(value)
condition
WT
MUT
Control
rep
rep1
rep2
rep3

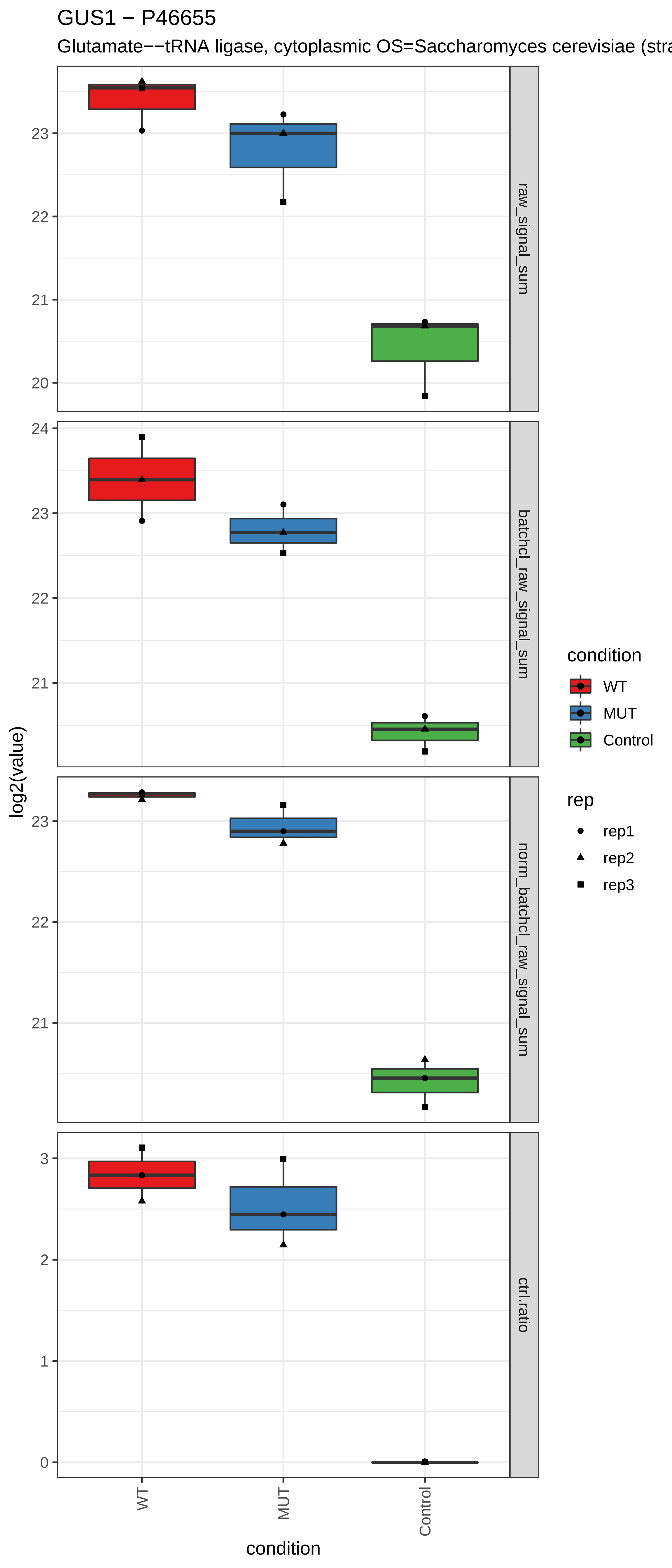

GUS1 − P46655
Glutamate−−tRNA ligase, cytoplasmic OS=Saccharomyces cerevisiae (stra
raw_signal_sum
batchcl_raw_signal_sum
norm_batchcl_raw_signal_sum
ctrl.ratio
log2(value)
condition
WT
MUT
Control
rep
rep1
rep2
rep3
20
21
22
23
24
0
1
2
3

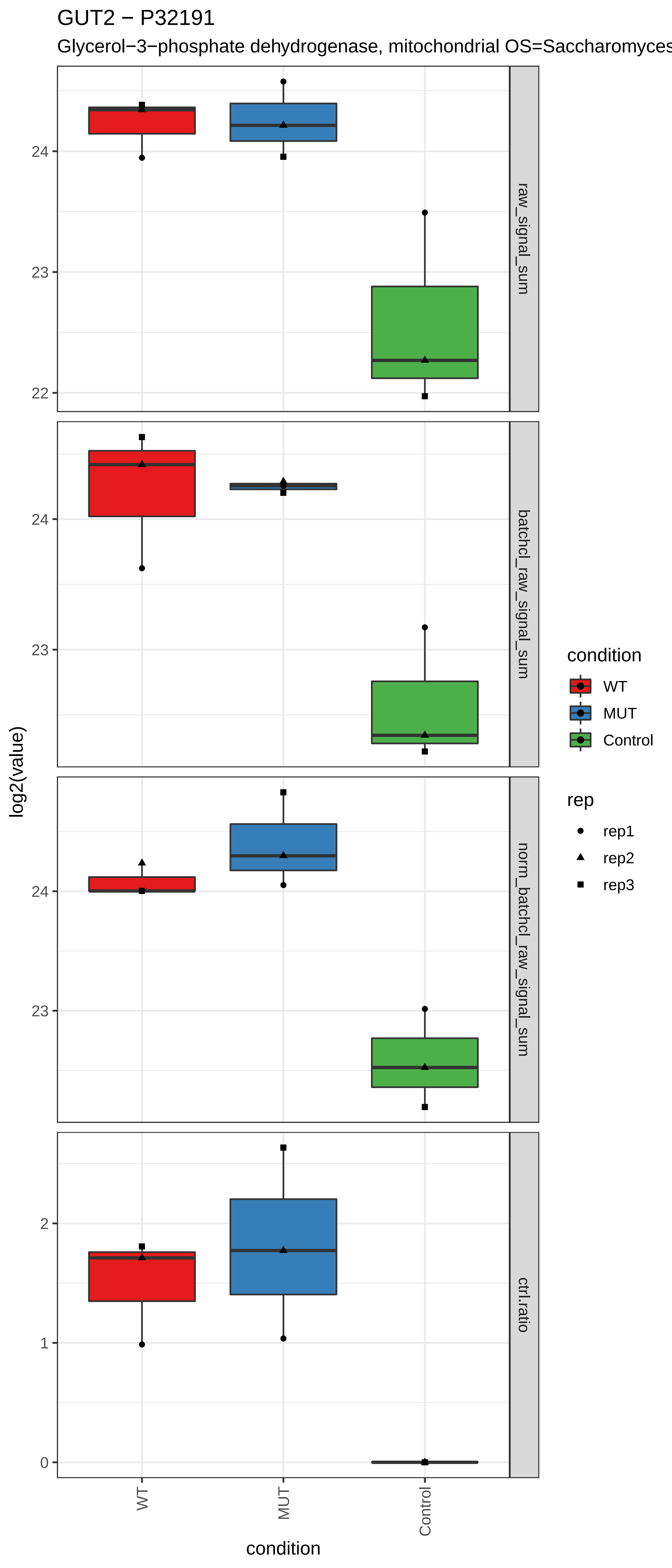

GUT2 − P32191
Glycerol−3−phosphate dehydrogenase, mitochondrial OS=Saccharomyces
raw_signal_sum
batchcl_raw_signal_sum
norm_batchcl_raw_signal_sum
ctrl.ratio
log2(value)
condition
WT
MUT
Control
rep
rep1
rep2
rep3
24
23
22
2
1
0

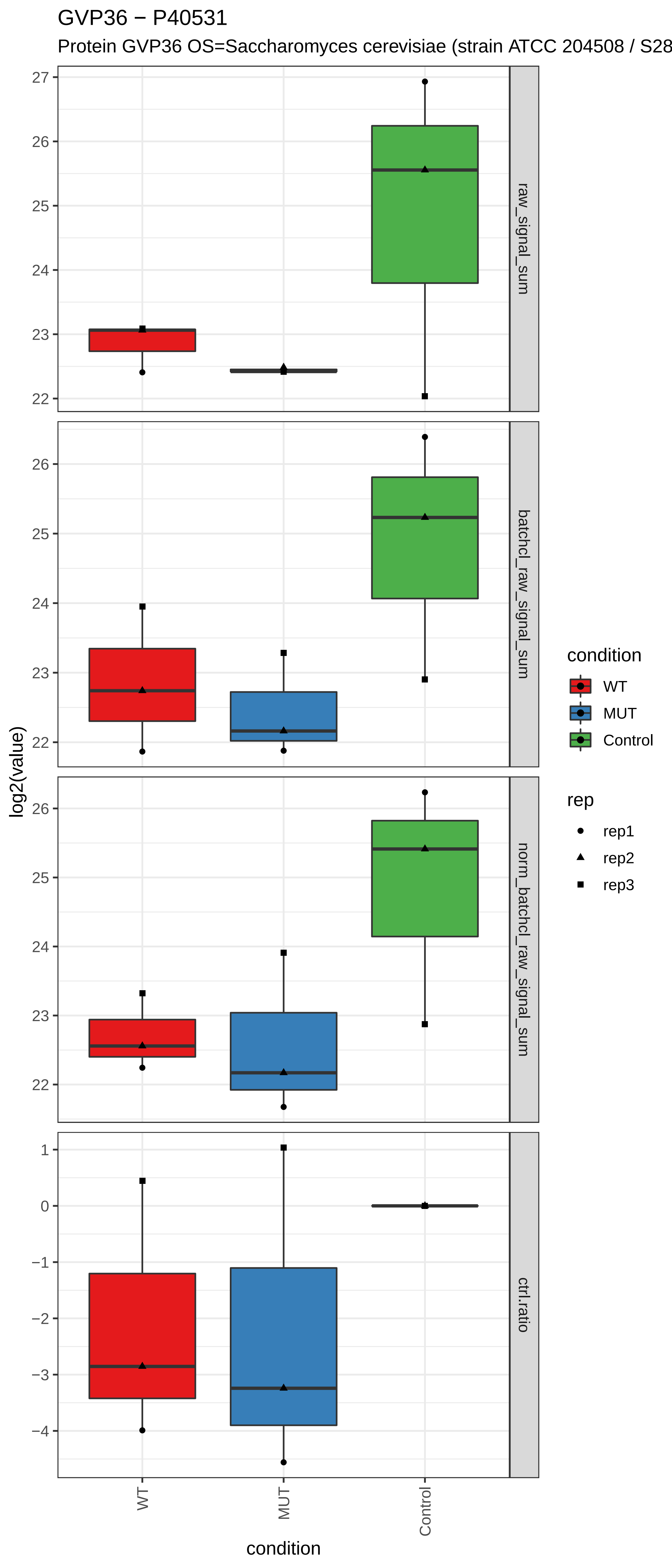

GVP36 − P40531
Protein GVP36 OS=Saccharomyces cerevisiae (strain ATCC 204508 / S28
raw_signal_sum
batchcl_raw_signal_sum
norm_batchcl_raw_signal_sum
ctrl.ratio
log2(value)
condition
WT
MUT
Control
rep
rep1
rep2
rep3

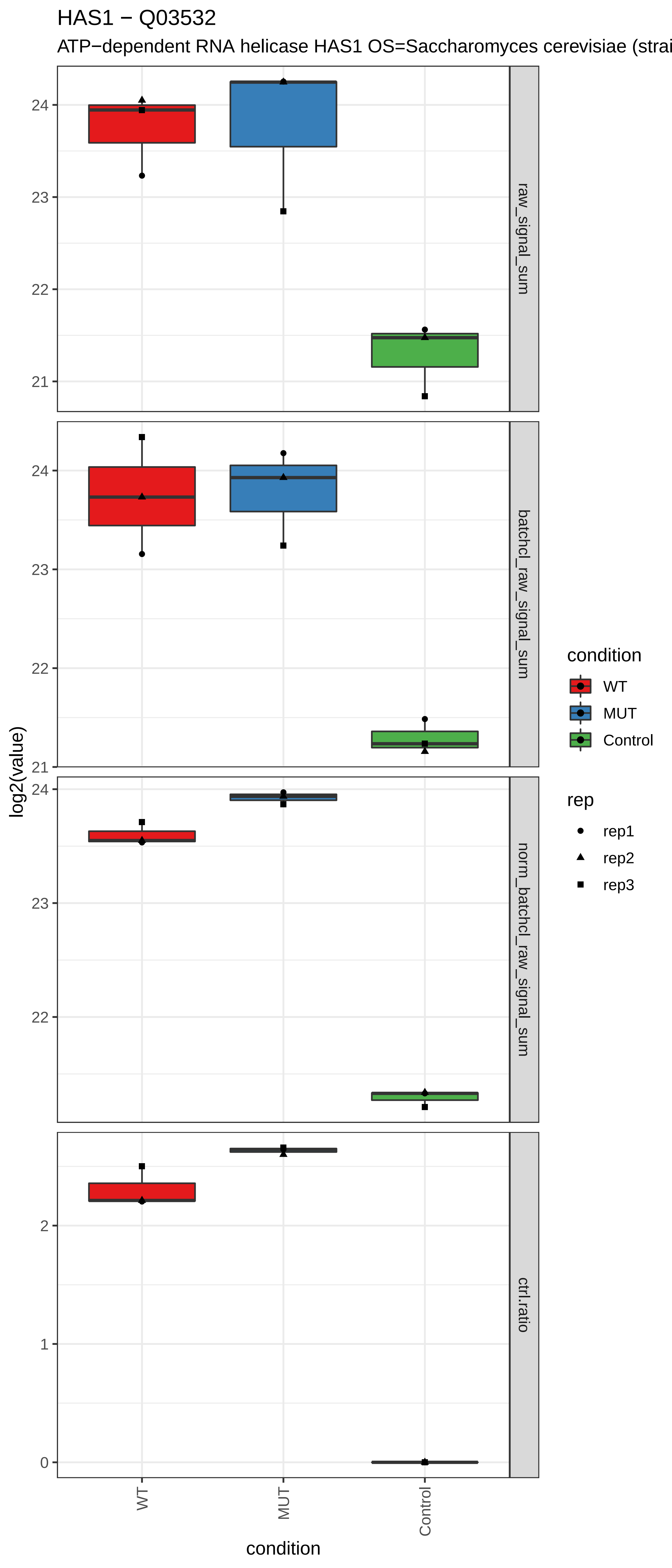

HAS1 − Q03532
ATP−dependent RNA helicase HAS1 OS=Saccharomyces cerevisiae (strai
raw_signal_sum
batchcl_raw_signal_sum
norm_batchcl_raw_signal_sum
ctrl.ratio
log2(value)
condition
WT
MUT
Control
rep
rep1
rep2
rep3
24
23
22
21
2
1
0

HCR1 − Q05775

Eukaryotic translation initiation factor 3 subunit J OS=Saccharomyces cer

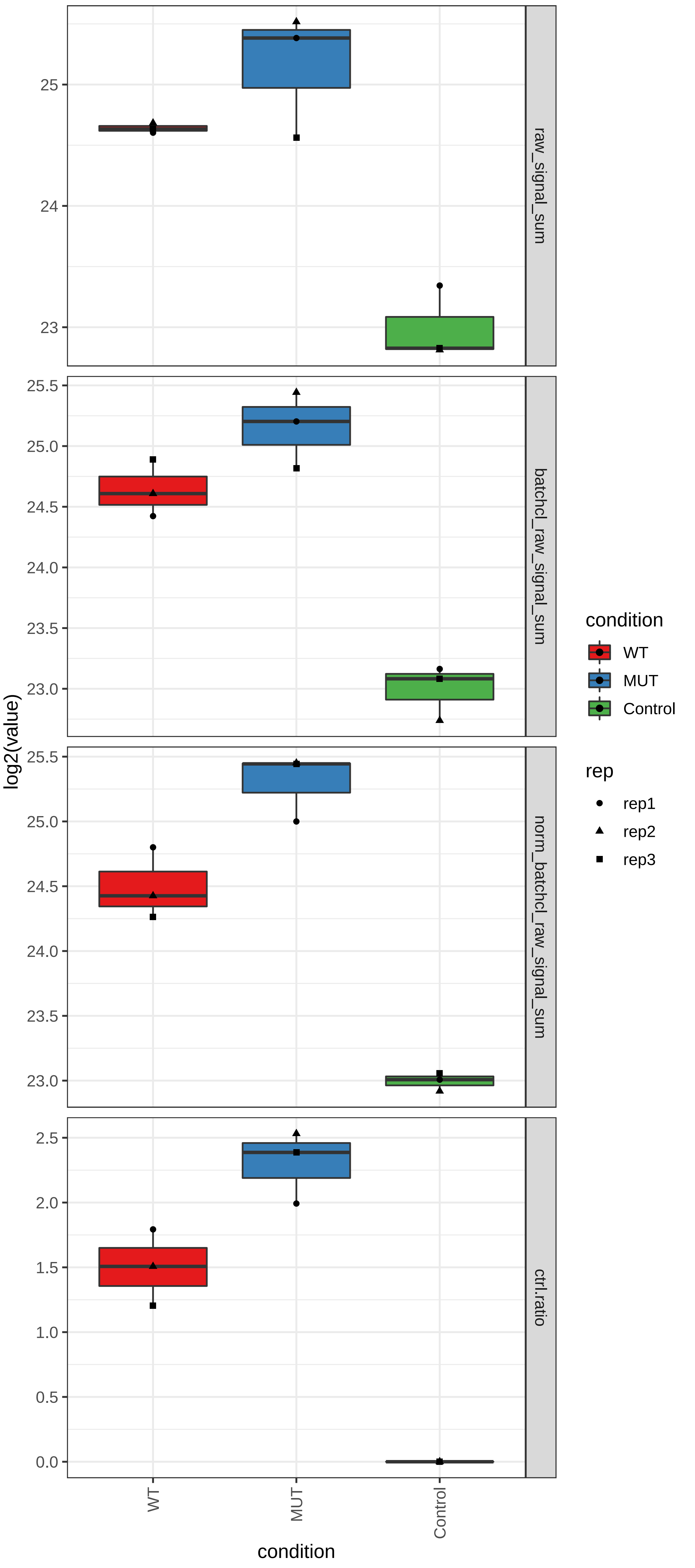

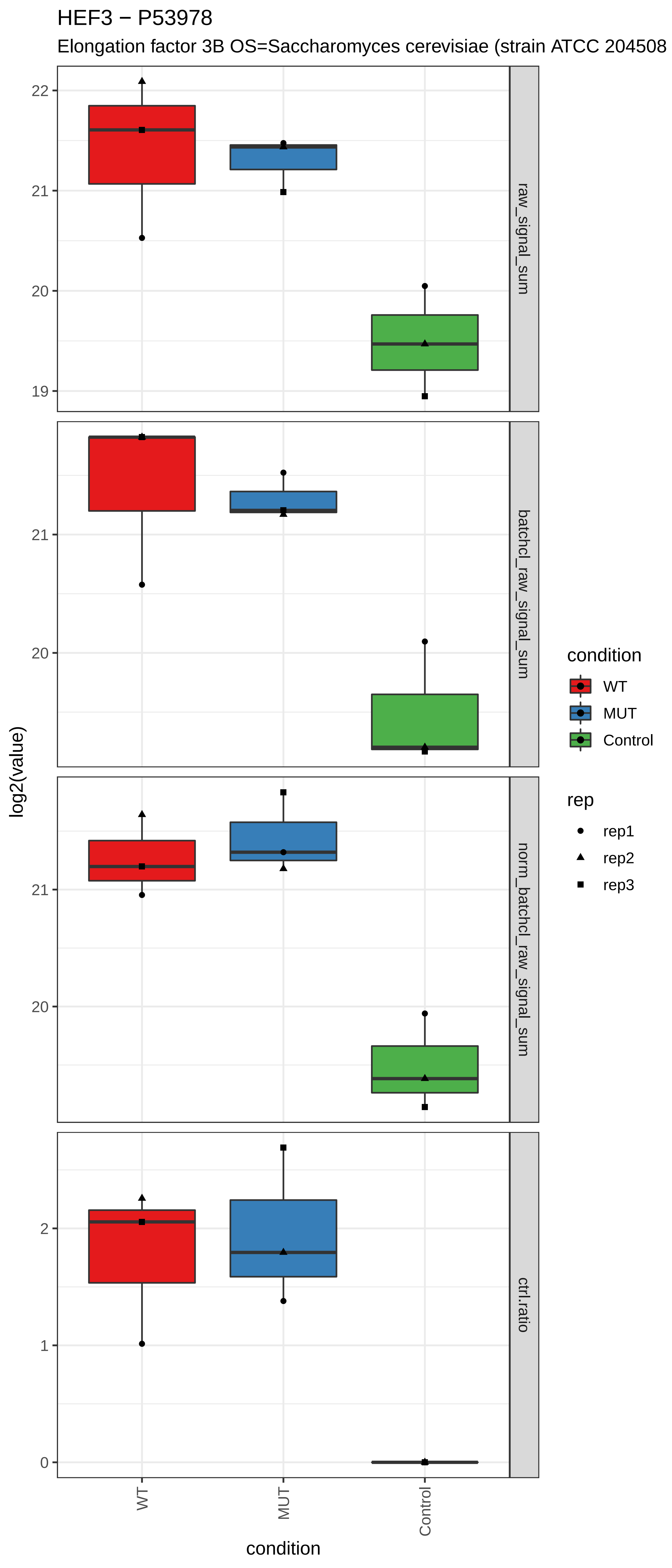

HEF3 − P53978
Elongation factor 3B OS=Saccharomyces cerevisiae (strain ATCC 204508
raw_signal_sum
batchcl_raw_signal_sum
norm_batchcl_raw_signal_sum
ctrl.ratio
log2(value)
condition
WT
MUT
Control
rep
rep1
rep2
rep3
22
21
20
19
0
1
2

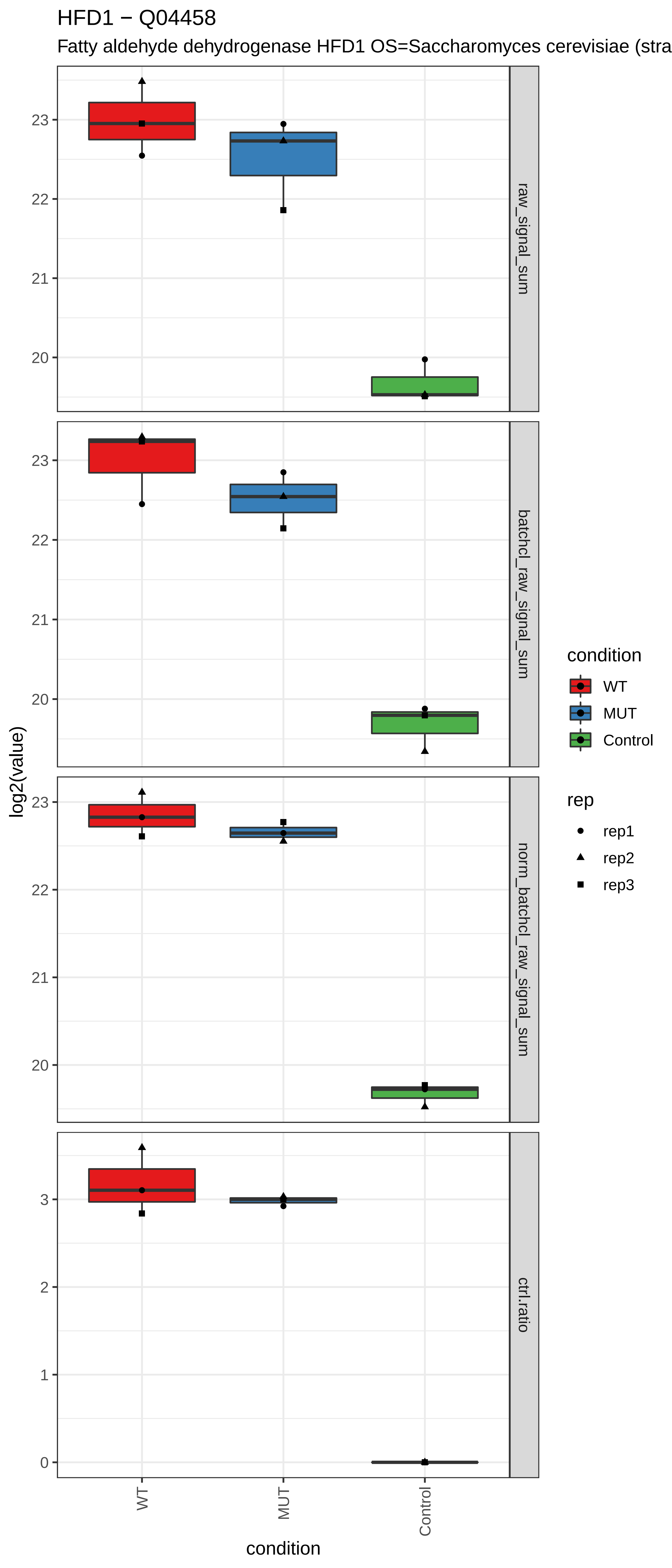

HFD1 − Q04458
Fatty aldehyde dehydrogenase HFD1 OS=Saccharomyces cerevisiae (stra
raw_signal_sum
batchcl_raw_signal_sum
norm_batchcl_raw_signal_sum
ctrl.ratio
log2(value)
23
22
21
20
3
2
1
0
WT
MUT
Control
condition
condition
WT
MUT
Control
rep
rep1
rep2
rep3

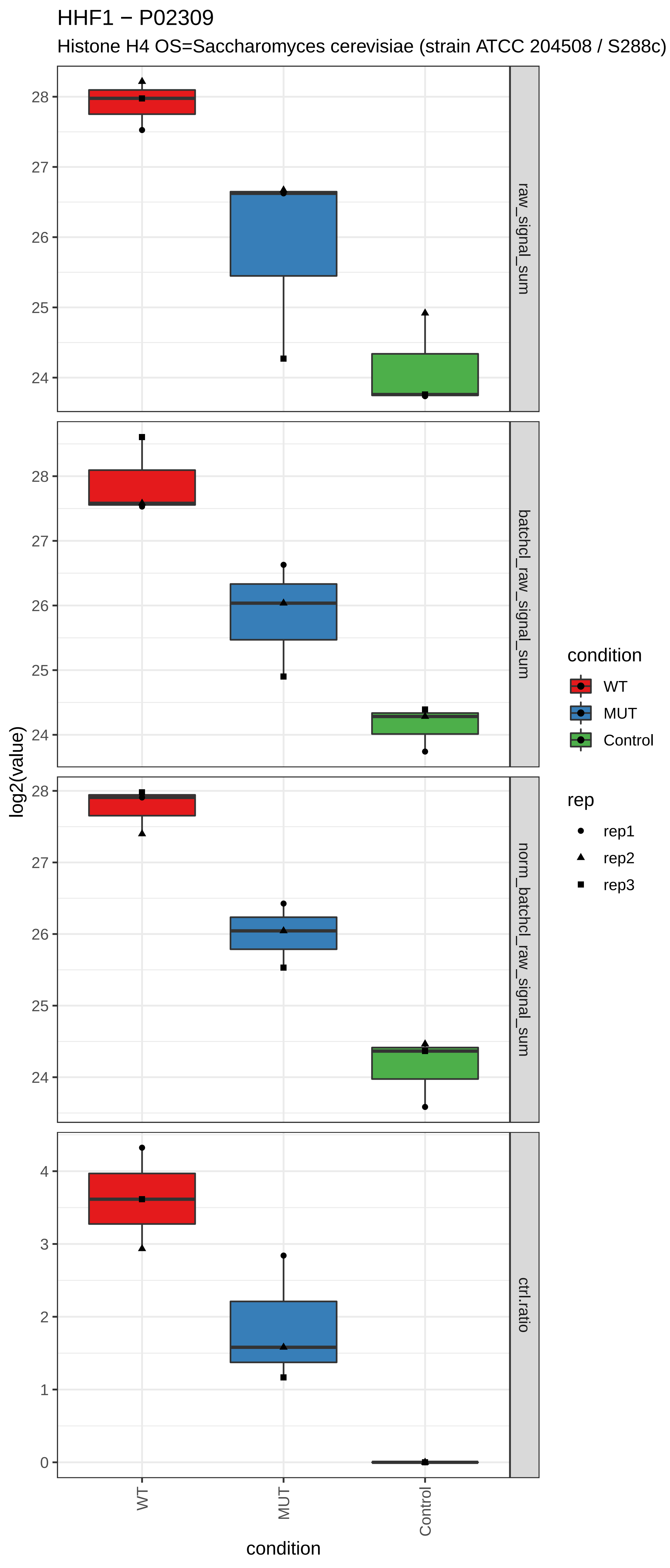
HHF1 − P02309
Histone H4 OS=Saccharomyces cerevisiae (strain ATCC 204508 / S288c)
raw_signal_sum
batchcl_raw_signal_sum
norm_batchcl_raw_signal_sum
ctrl.ratio
log2(value)
condition
WT
MUT
Control
rep
rep1
rep2
rep3

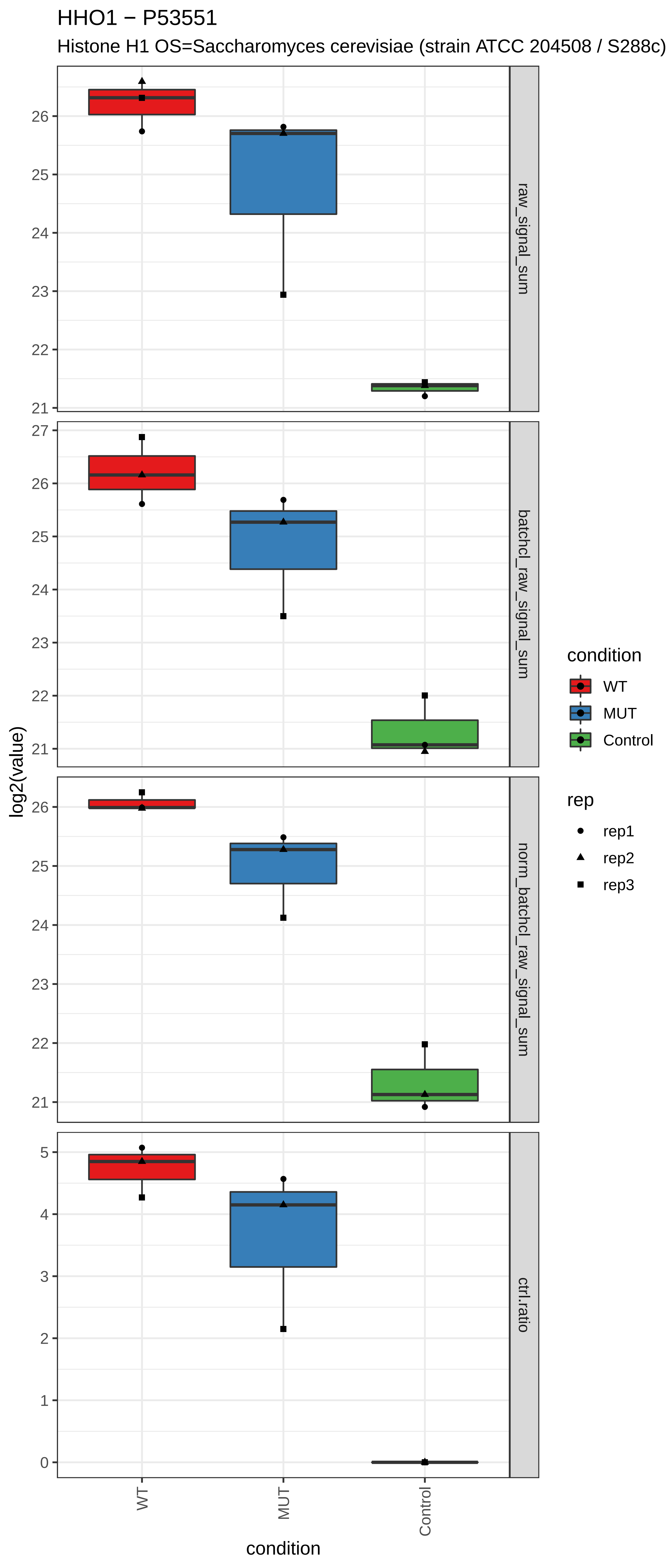
HHO1 − P53551
Histone H1 OS=Saccharomyces cerevisiae (strain ATCC 204508 / S288c)
raw_signal_sum
batchcl_raw_signal_sum
norm_batchcl_raw_signal_sum
ctrl.ratio
log2(value)
condition
WT
MUT
Control
rep
rep1
rep2
rep3

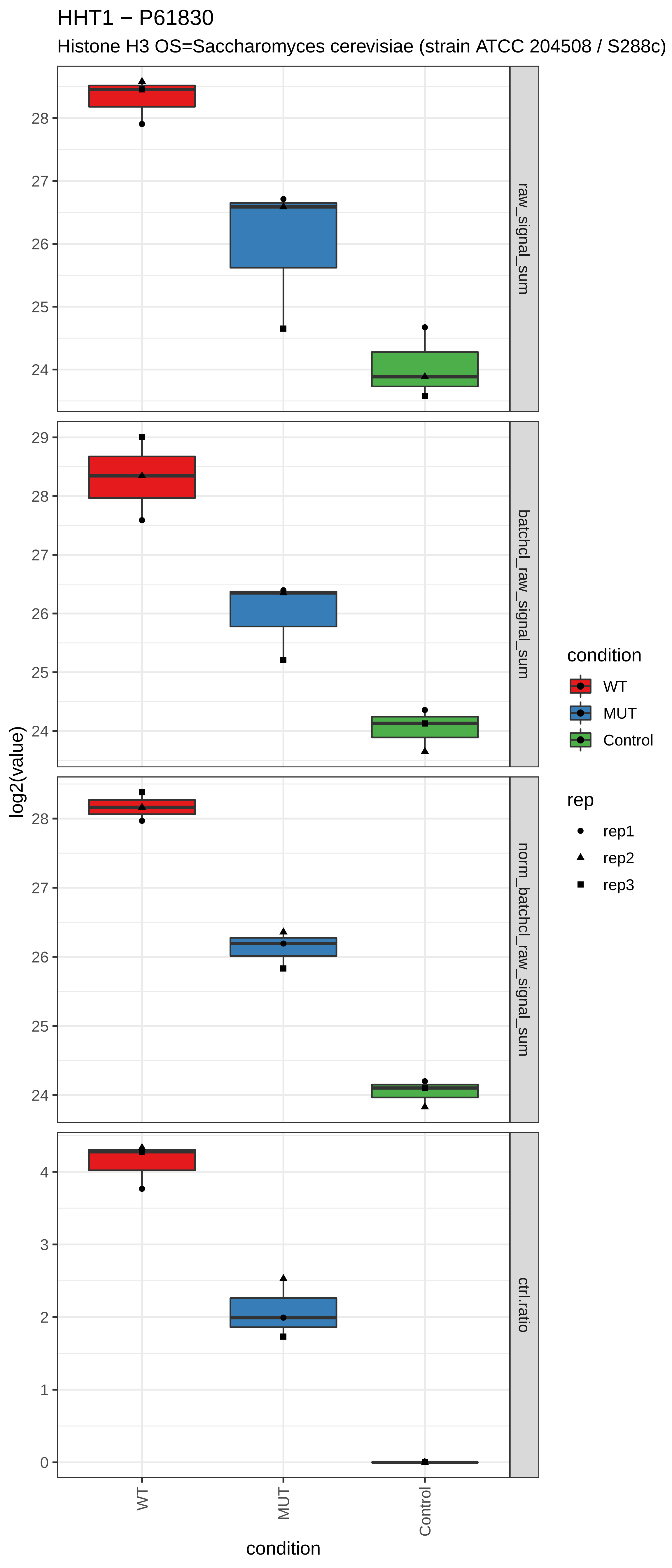

HHT1 − P61830
Histone H3 OS=Saccharomyces cerevisiae (strain ATCC 204508 / S288c)
raw_signal_sum
batchcl_raw_signal_sum
norm_batchcl_raw_signal_sum
ctrl.ratio
log2(value)
condition
WT
MUT
Control
rep
rep1
rep2
rep3

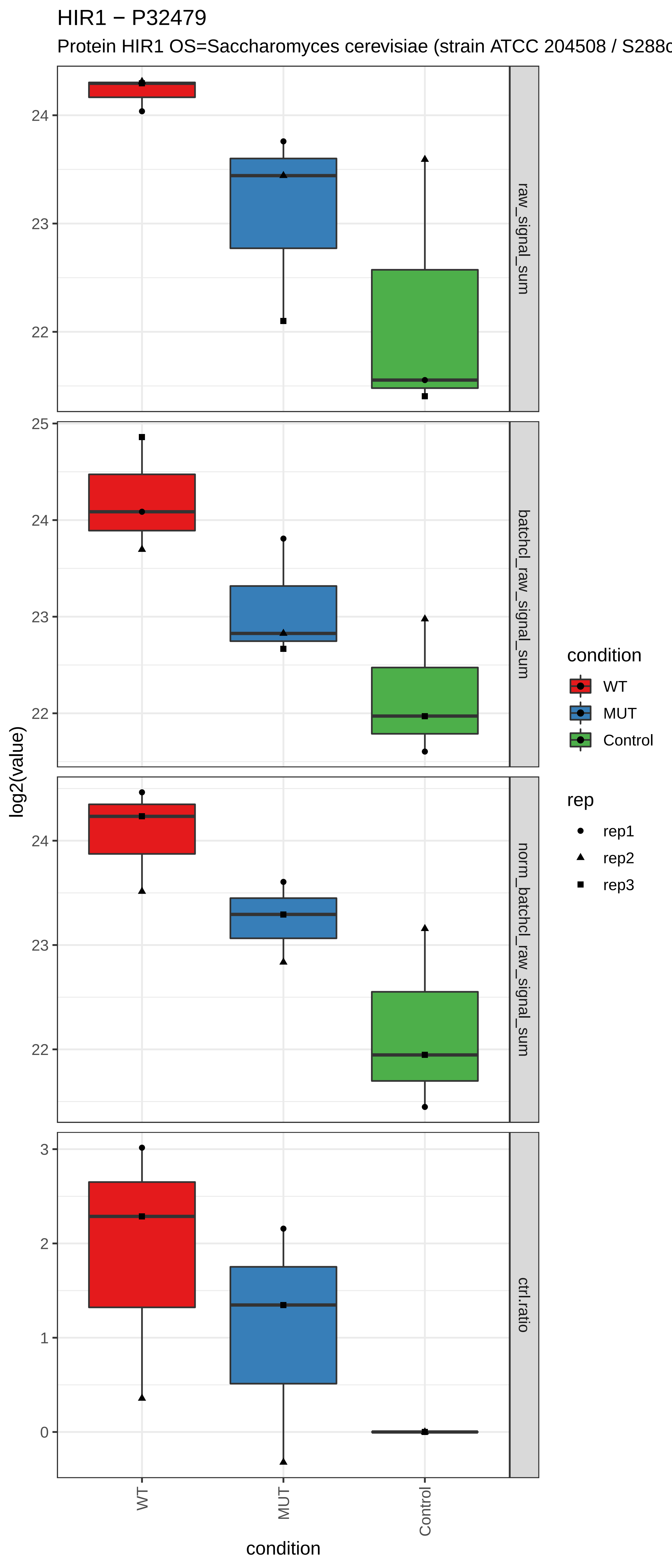

HIR1 − P32479
Protein HIR1 OS=Saccharomyces cerevisiae (strain ATCC 204508 / S288c
raw_signal_sum
batchcl_raw_signal_sum
norm_batchcl_raw_signal_sum
ctrl.ratio
log2(value)
condition
WT
MUT
Control
rep
rep1
rep2
rep3

HIR2 − P32480

Protein HIR2 OS=Saccharomyces cerevisiae (strain ATCC 204508 / S288c

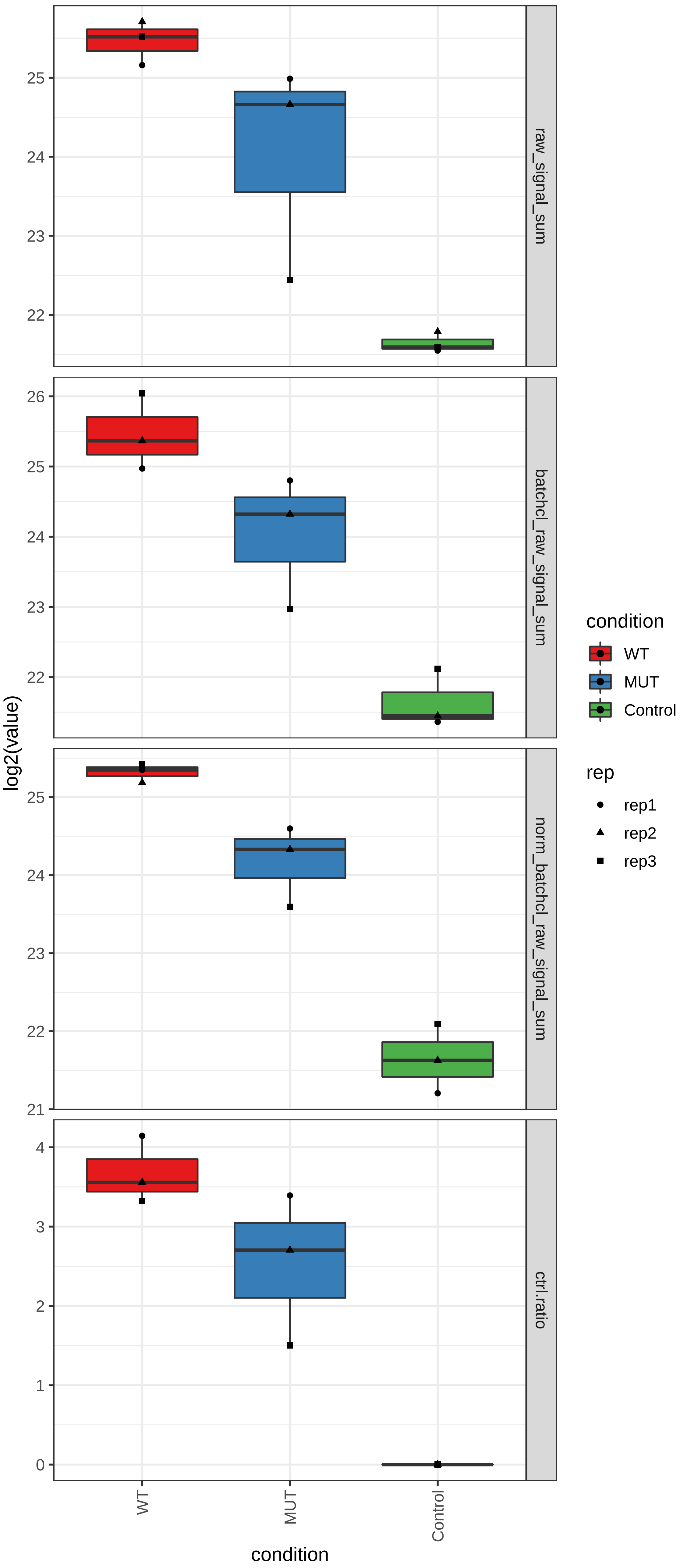

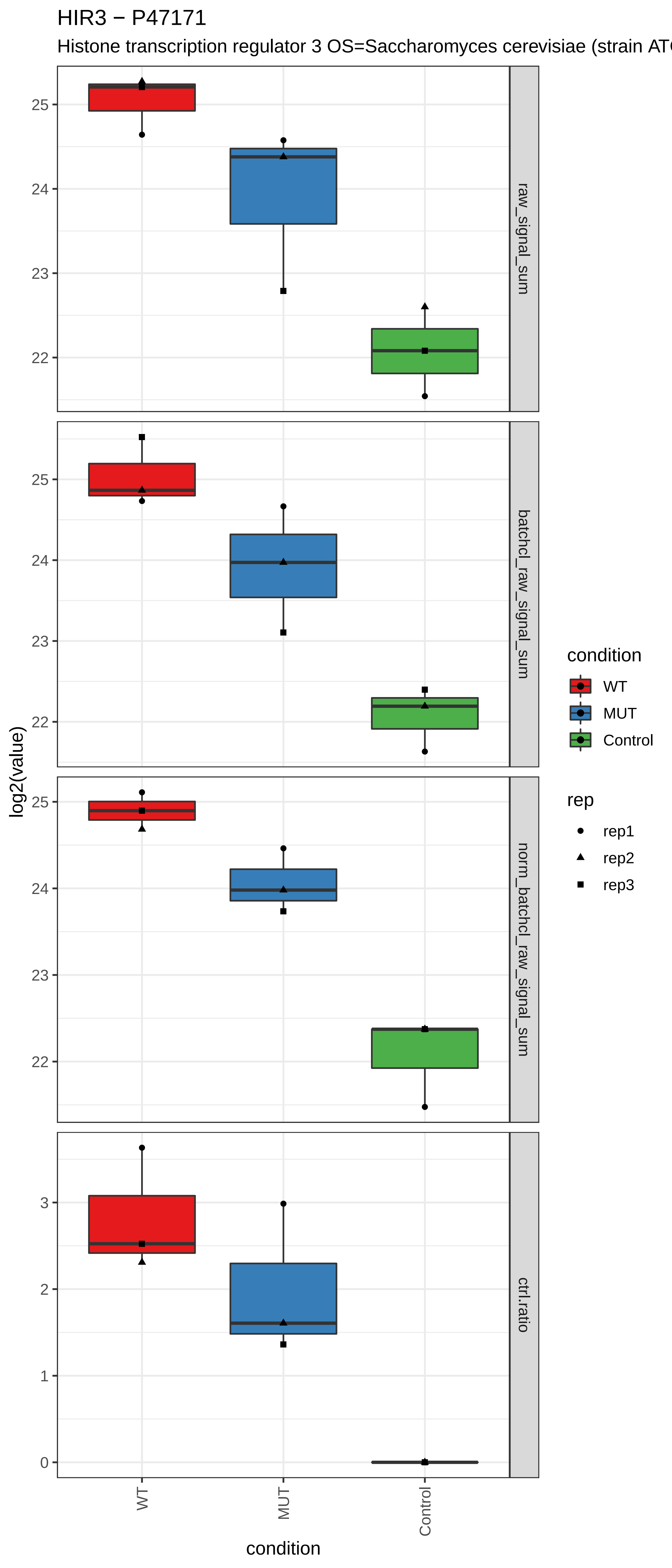

HIR3 − P47171
Histone transcription regulator 3 OS=Saccharomyces cerevisiae (strain ATC
raw_signal_sum
batchcl_raw_signal_sum
norm_batchcl_raw_signal_sum
ctrl.ratio
log2(value)
condition
WT
MUT
Control
rep
rep1
rep2
rep3

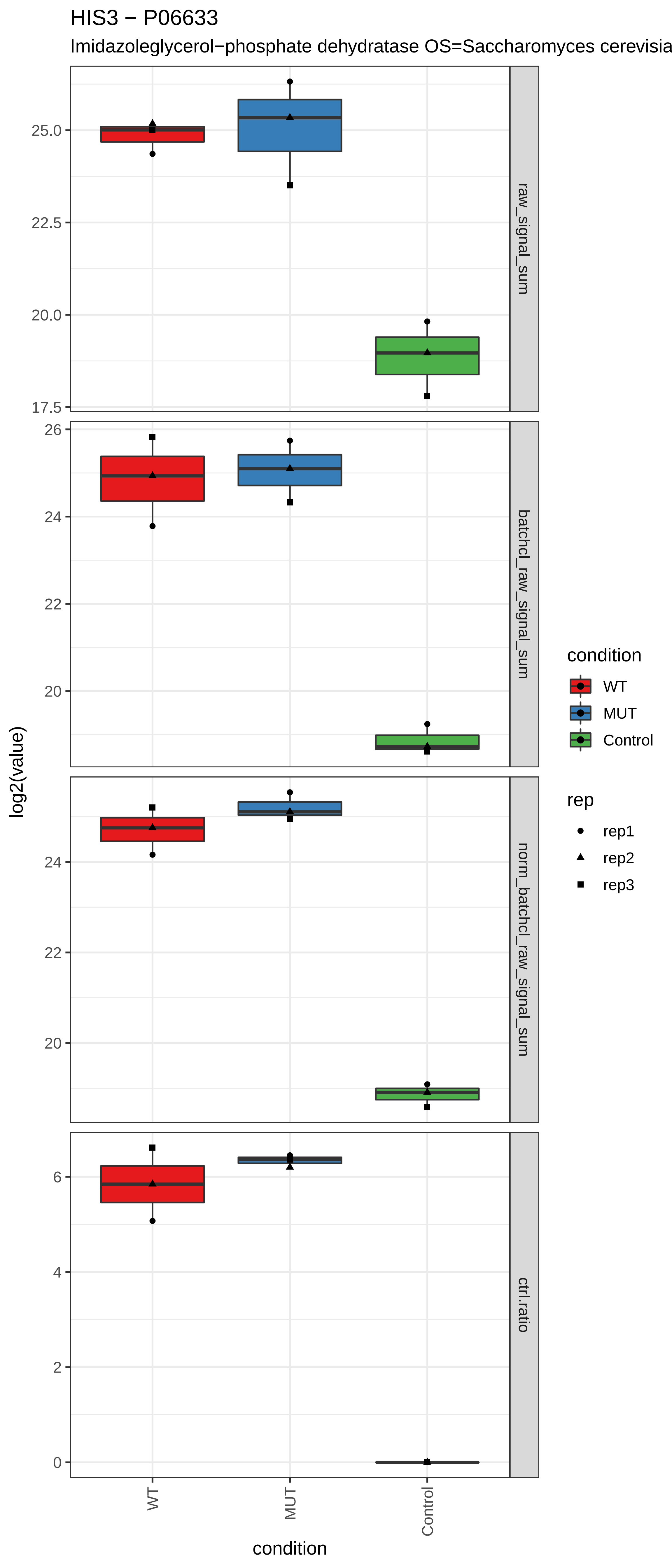

HIS3 − P06633
Imidazoleglycerol−phosphate dehydratase OS=Saccharomyces cerevisia
raw_signal_sum
batchcl_raw_signal_sum
norm_batchcl_raw_signal_sum
ctrl.ratio
25.0
22.5
20.0
17.5
26
24
22
20
24
22
20
6
4
2
0
log2(value)
WT
MUT
Control
condition
condition
WT
MUT
Control
rep
rep1
rep2
rep3

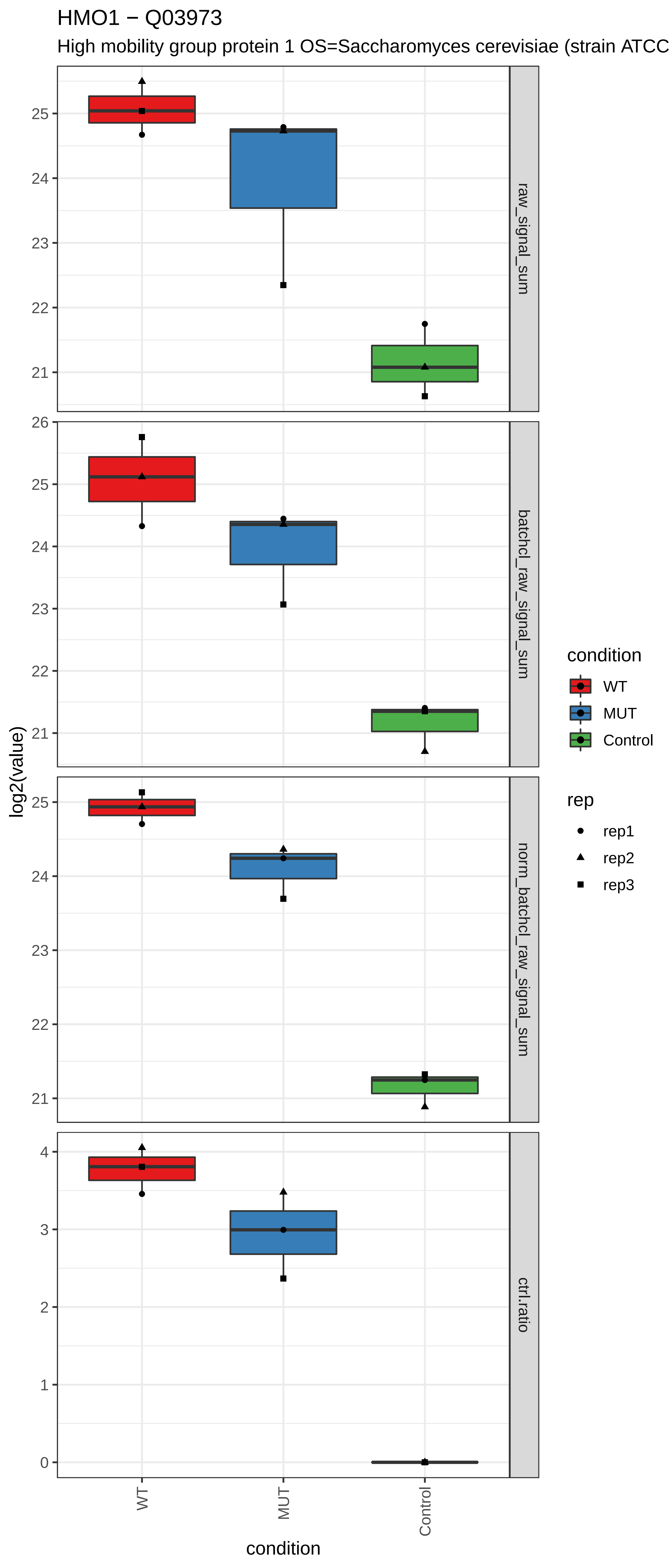

HMO1 − Q03973
High mobility group protein 1 OS=Saccharomyces cerevisiae (strain ATCC
raw_signal_sum
batchcl_raw_signal_sum
norm_batchcl_raw_signal_sum
ctrl.ratio
log2(value)
condition
WT
MUT
Control
rep
rep1
rep2
rep3

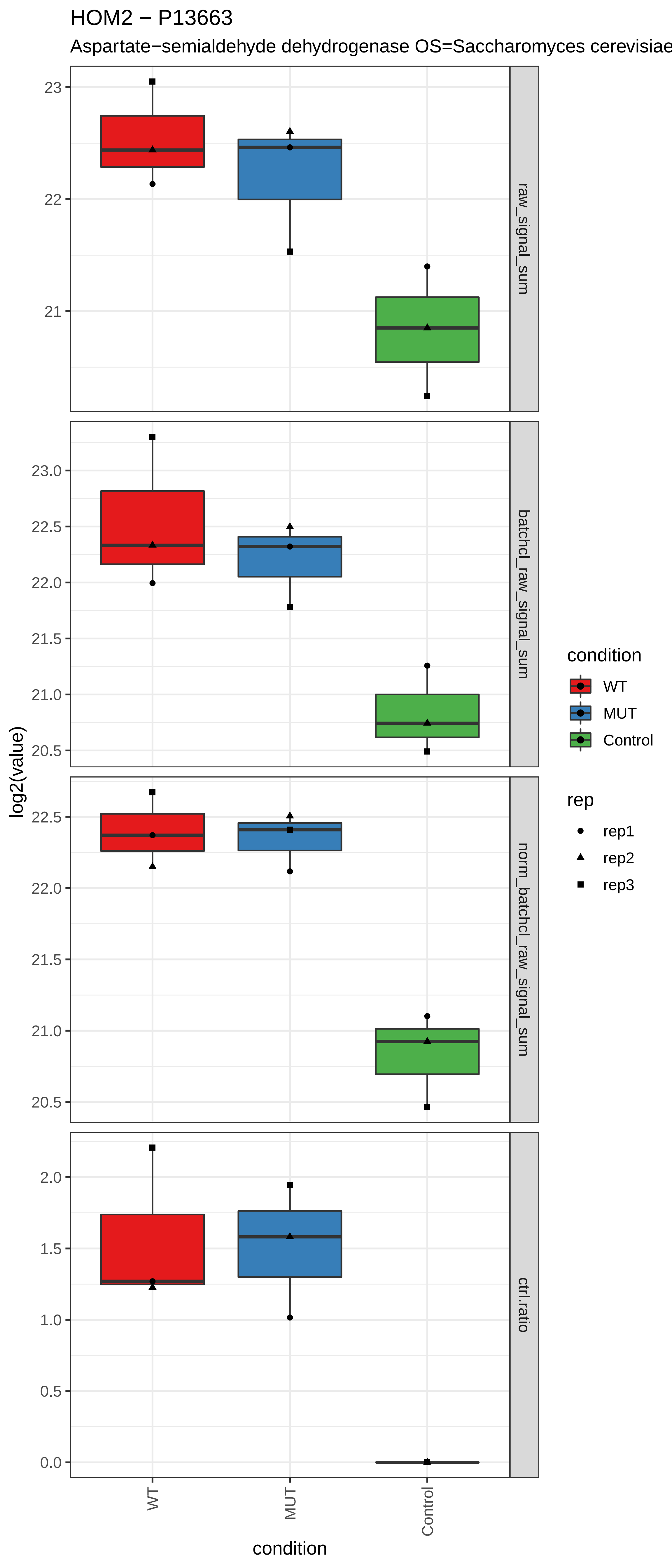

HOM2 − P13663
Aspartate−semialdehyde dehydrogenase OS=Saccharomyces cerevisiae
raw_signal_sum
batchcl_raw_signal_sum
norm_batchcl_raw_signal_sum
ctrl.ratio
log2(value)
condition
WT
MUT
Control
rep
rep1
rep2
rep3
23
22
21
23.0
22.5
22.0
21.5
21.0
20.5
2.0
1.5
1.0
0.5
0.0

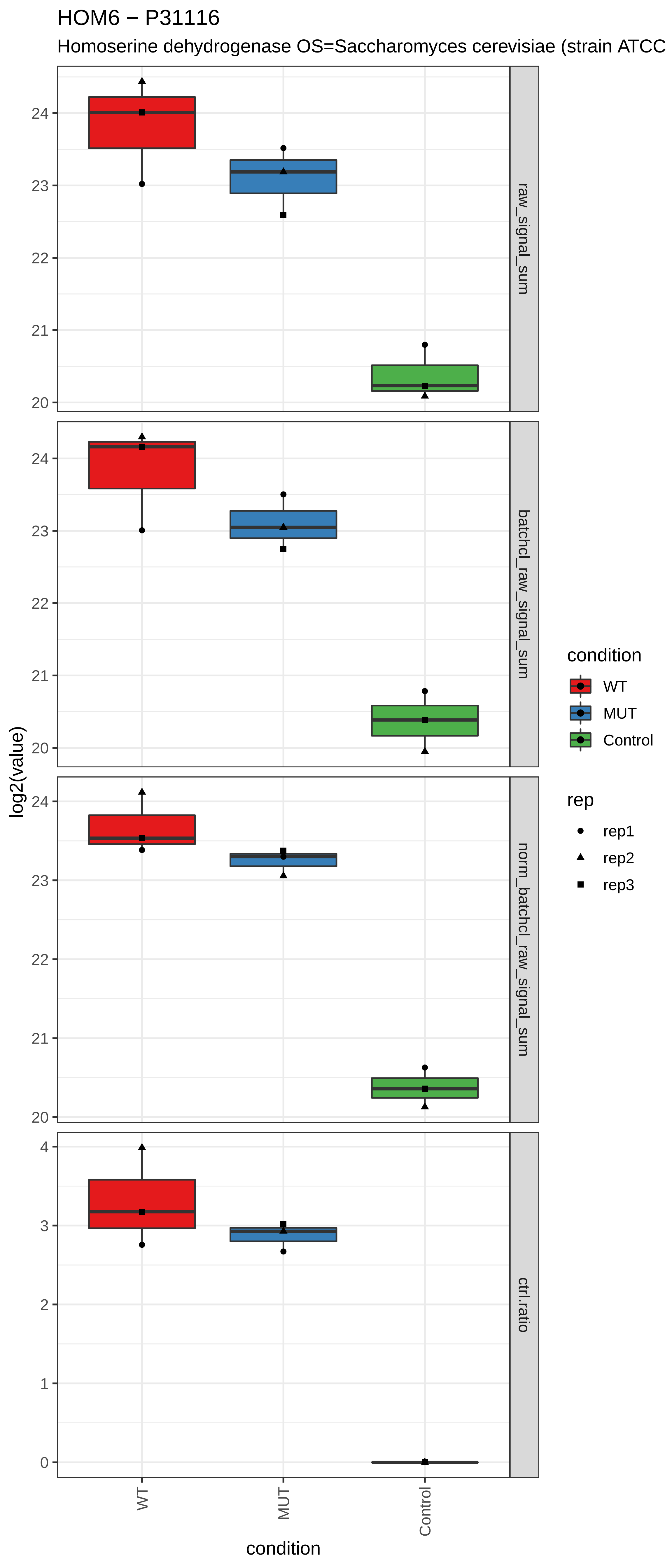

HOM6 − P31116
Homoserine dehydrogenase OS=Saccharomyces cerevisiae (strain ATCC
raw_signal_sum
batchcl_raw_signal_sum
norm_batchcl_raw_signal_sum
ctrl.ratio
log2(value)
condition
WT
MUT
Control
rep
rep1
rep2
rep3

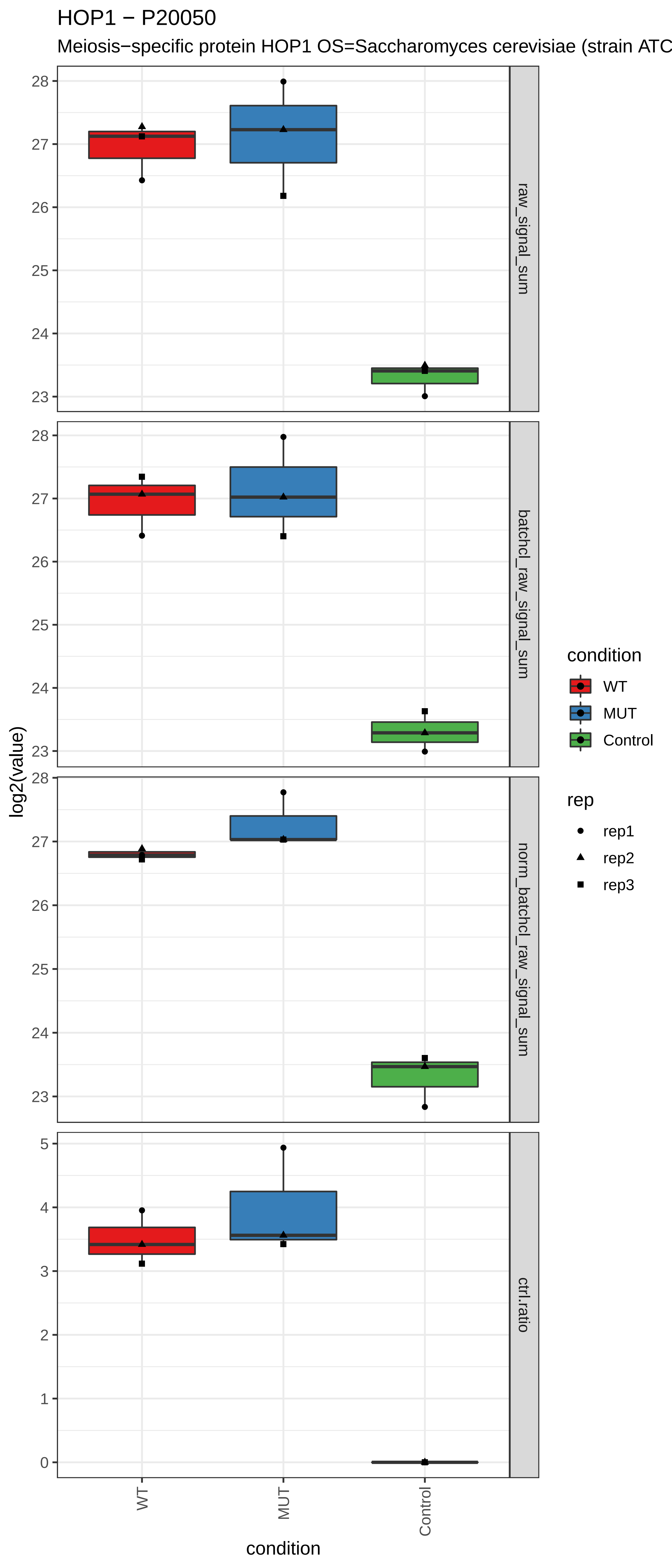
HOP1 − P20050
Meiosis−specific protein HOP1 OS=Saccharomyces cerevisiae (strain ATC
log2(value)
raw_signal_sum
batchcl_raw_signal_sum
norm_batchcl_raw_signal_sum
ctrl.ratio
28
27
26
25
24
23
5
4
3
2
1
0
WT
MUT
Control
condition
condition
WT
MUT
Control
rep
rep1
rep2
rep3

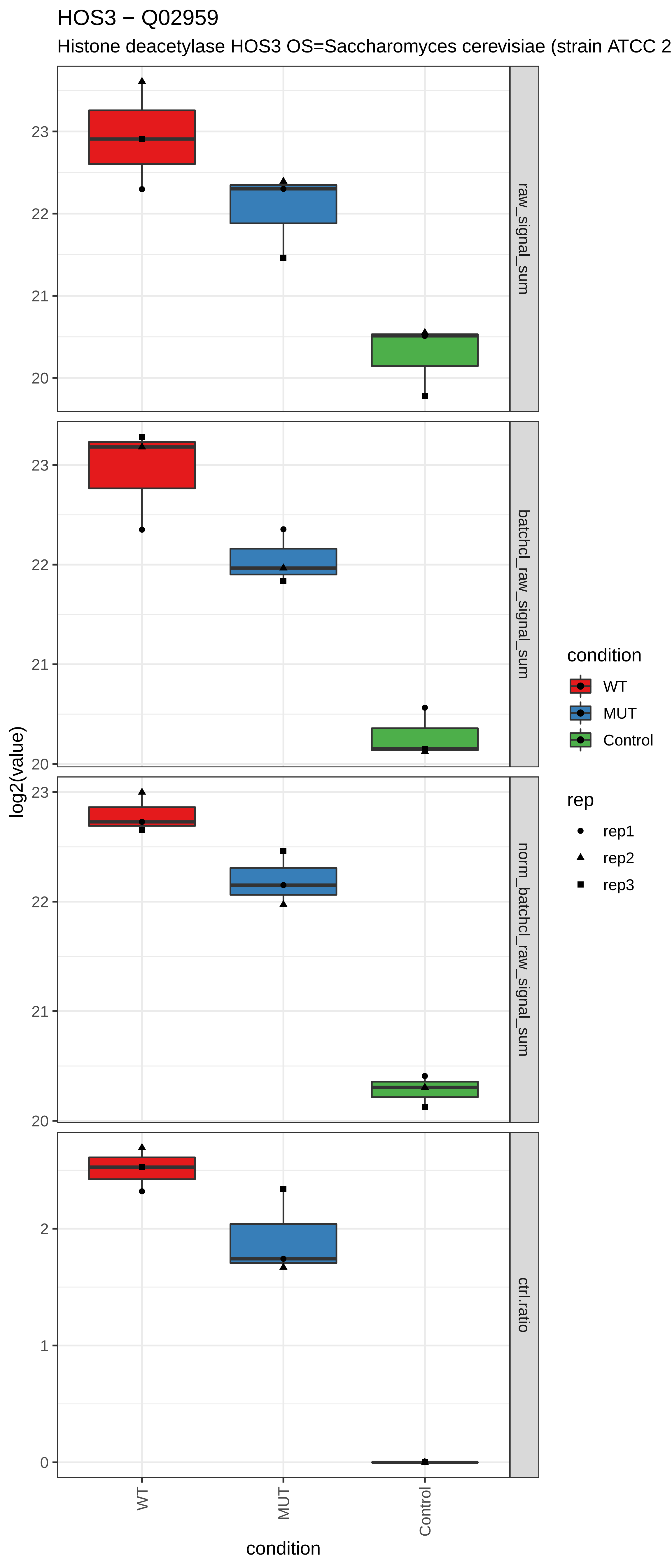
HOS3 − Q02959
Histone deacetylase HOS3 OS=Saccharomyces cerevisiae (strain ATCC 2
raw_signal_sum
batchcl_raw_signal_sum
norm_batchcl_raw_signal_sum
ctrl.ratio
log2(value)
condition
WT
MUT
Control
rep
rep1
rep2
rep3

HPC2 − Q01448

Histone promoter control protein 2 OS=Saccharomyces cerevisiae (strain A

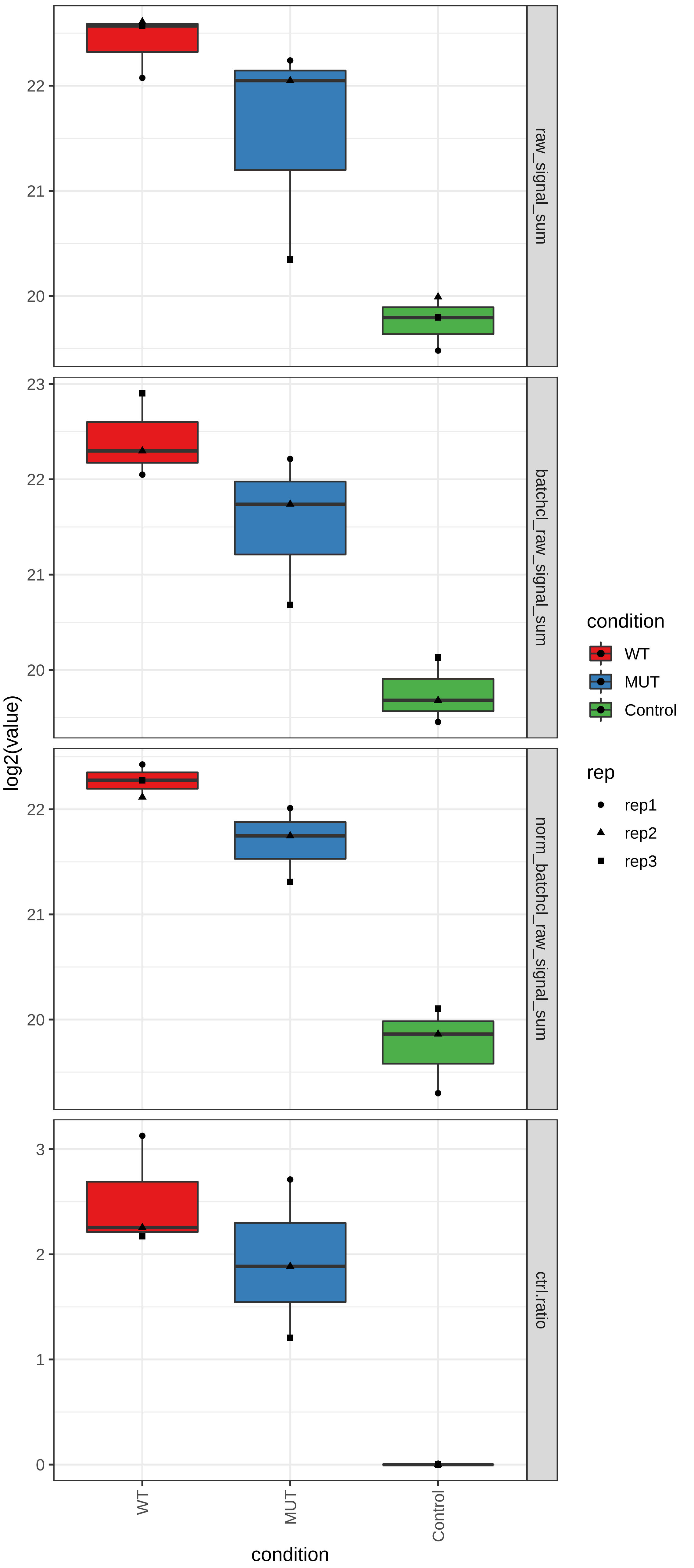

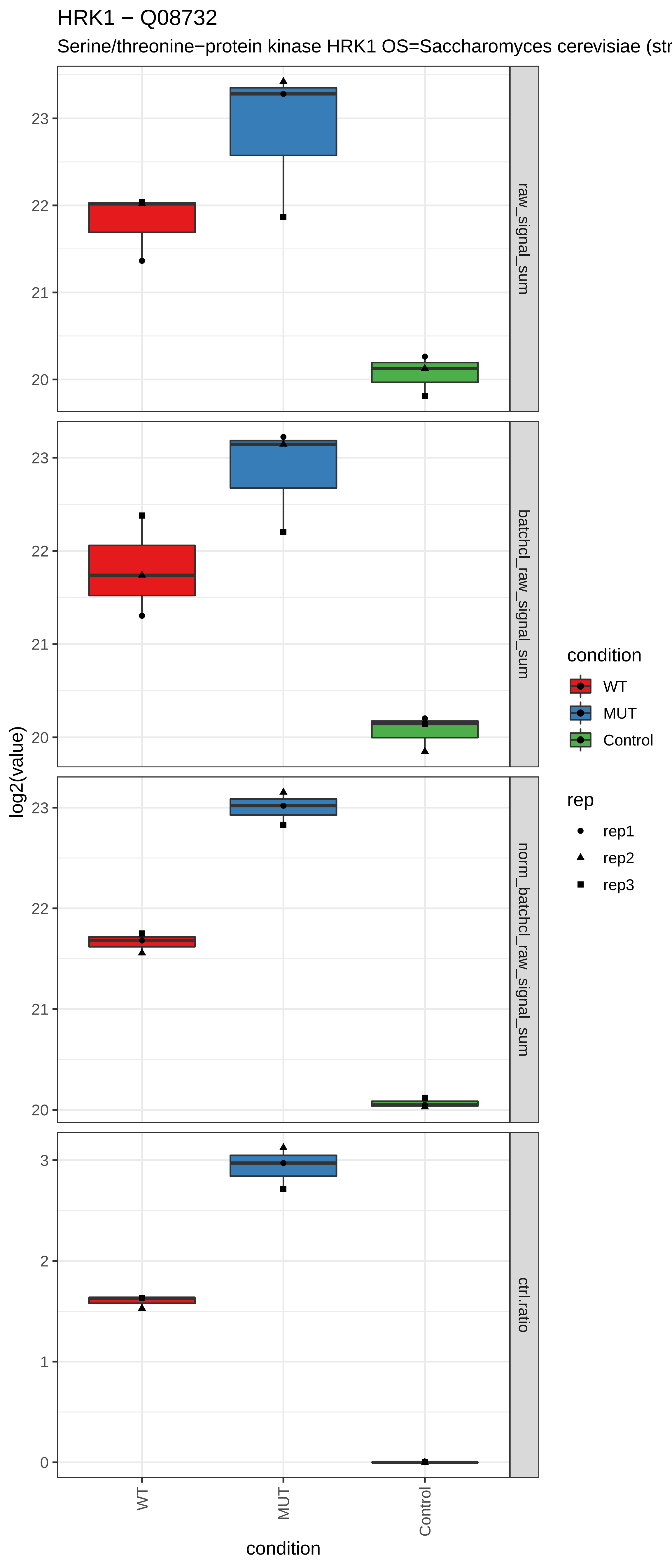

HRK1 − Q08732
Serine/threonine−protein kinase HRK1 OS=Saccharomyces cerevisiae (str
raw_signal_sum
batchcl_raw_signal_sum
norm_batchcl_raw_signal_sum
ctrl.ratio
log2(value)
condition
WT
MUT
Control
rep
rep1
rep2
rep3

HRR25 − P29295

Casein kinase I homolog HRR25 OS=Saccharomyces cerevisiae (strain AT

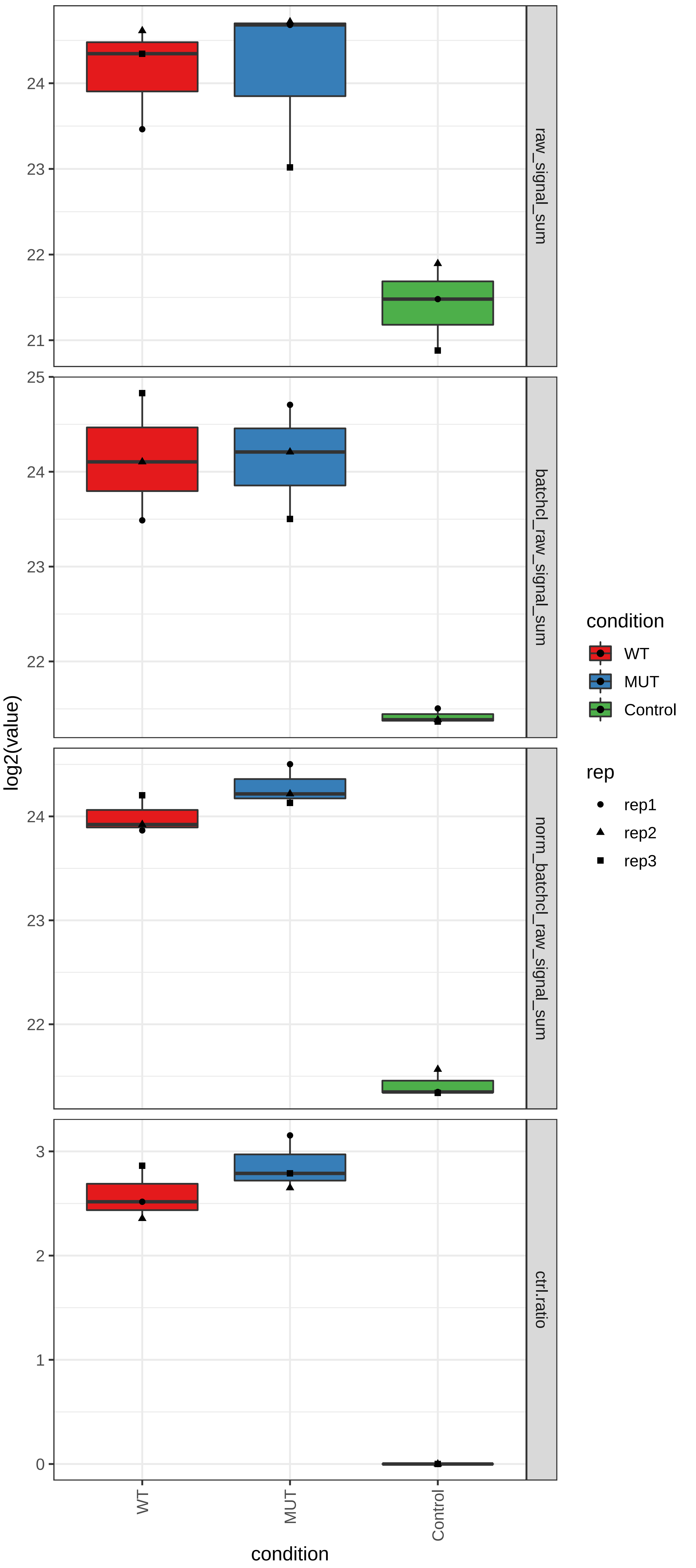

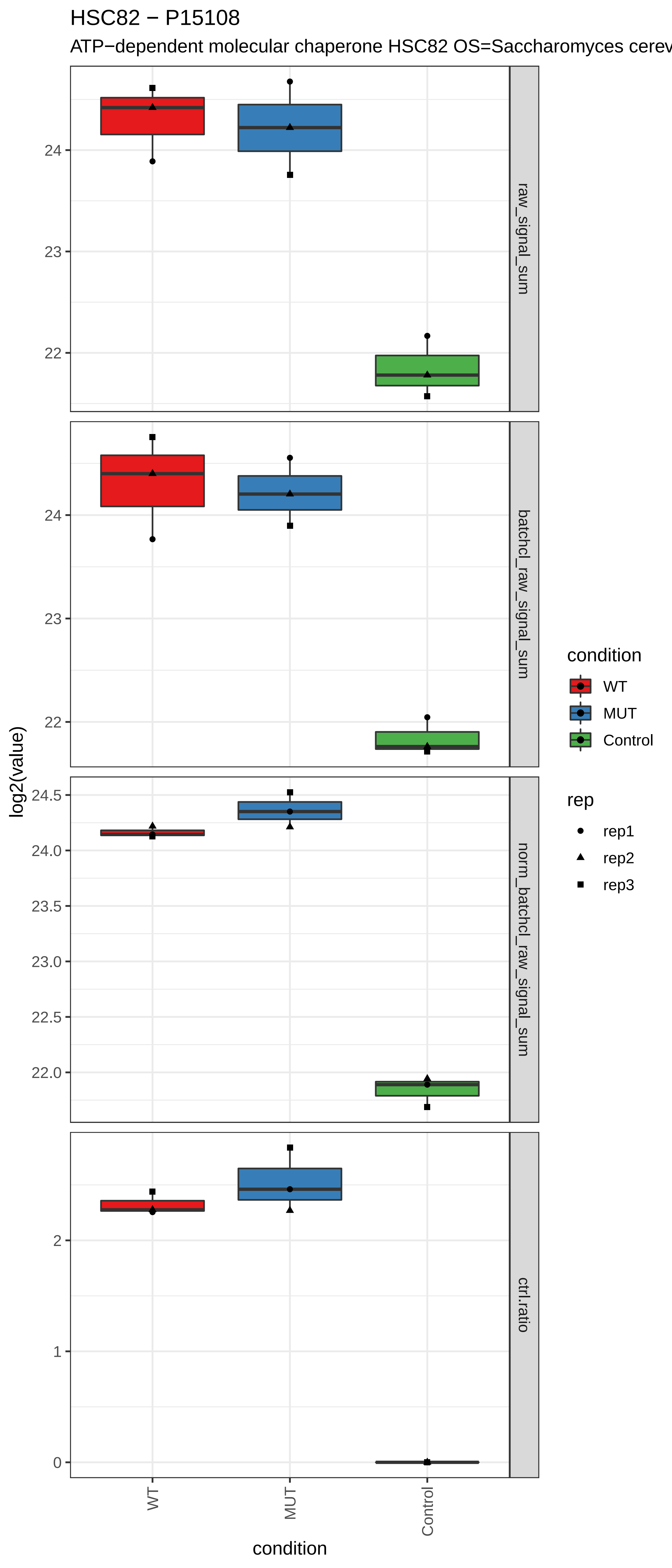
HSC82 − P15108
ATP−dependent molecular chaperone HSC82 OS=Saccharomyces cerev
raw_signal_sum
batchcl_raw_signal_sum
norm_batchcl_raw_signal_sum
ctrl.ratio
log2(value)
condition
WT
MUT
Control
rep
rep1
rep2
rep3
24
23
22
24.5
24.0
23.5
23.0
22.5
22.0
2
1
0

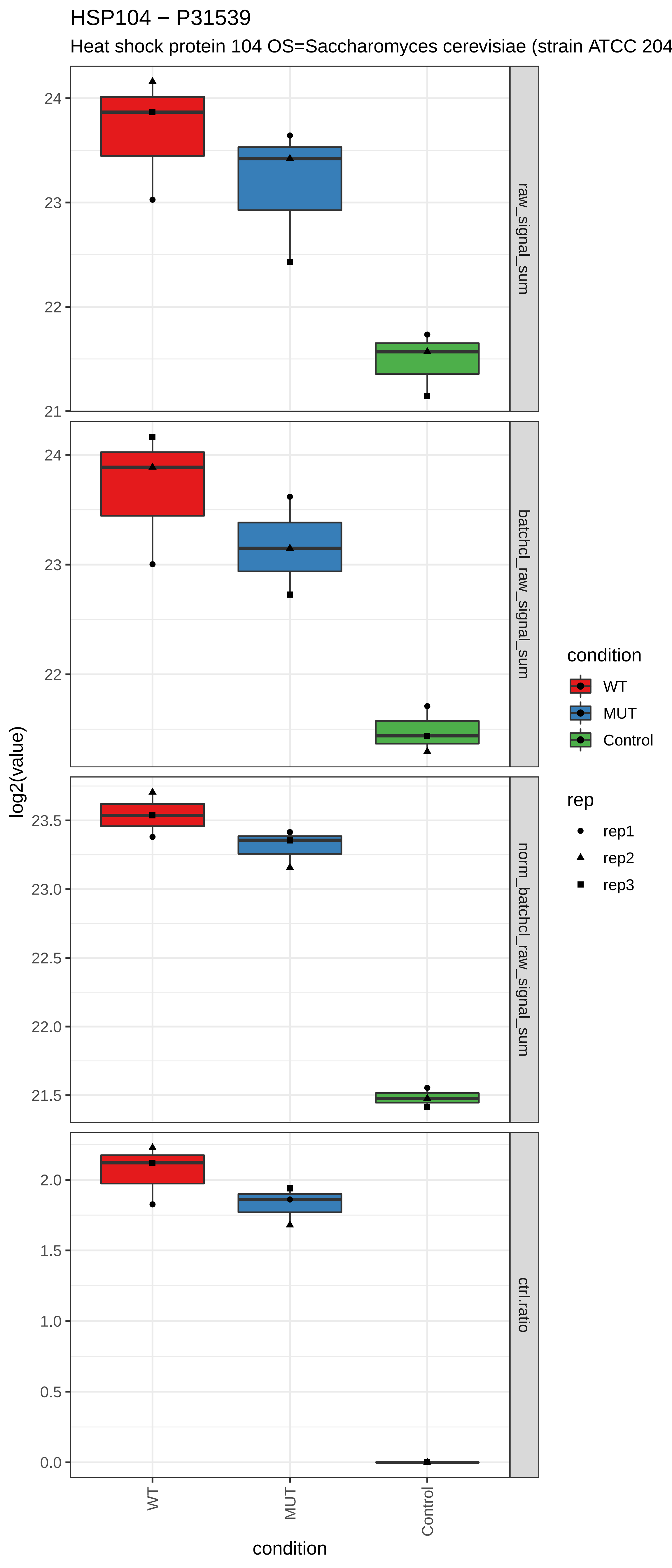

HSP104 − P31539
Heat shock protein 104 OS=Saccharomyces cerevisiae (strain ATCC 204
raw_signal_sum
batchcl_raw_signal_sum
norm_batchcl_raw_signal_sum
ctrl.ratio
log2(value)
condition
WT
MUT
Control
rep
rep1
rep2
rep3

HSP150 − P32478

Cell wall mannoprotein HSP150 OS=Saccharomyces cerevisiae (strain ATC

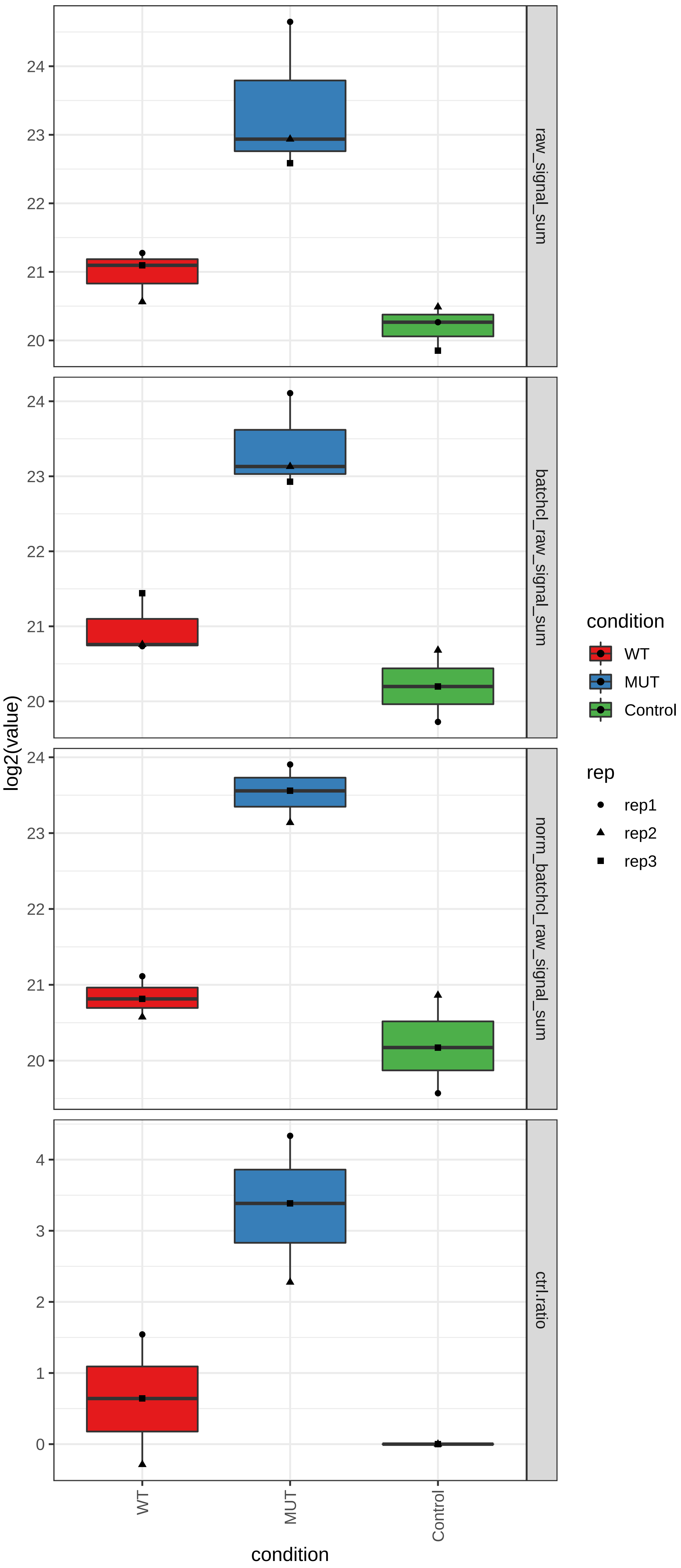

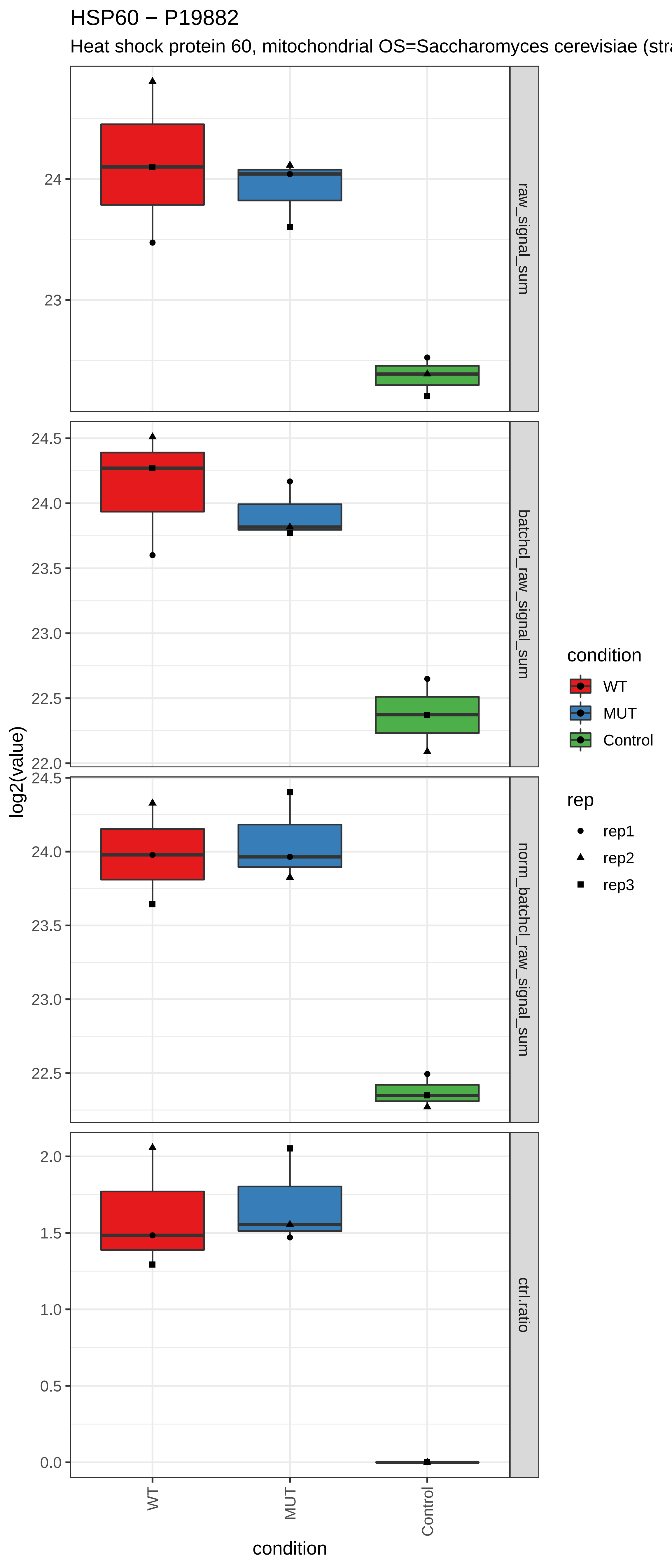

HSP60 − P19882
Heat shock protein 60, mitochondrial OS=Saccharomyces cerevisiae (stra
raw_signal_sum
batchcl_raw_signal_sum
norm_batchcl_raw_signal_sum
ctrl.ratio
log2(value)
condition
WT
MUT
Control
rep
rep1
rep2
rep3
24
23
24.5
24.0
23.5
23.0
22.5
22.0
2.0
1.5
1.0
0.5
0.0

HSP82 − P02829

ATP−dependent molecular chaperone HSP82 OS=Saccharomyces cerevis

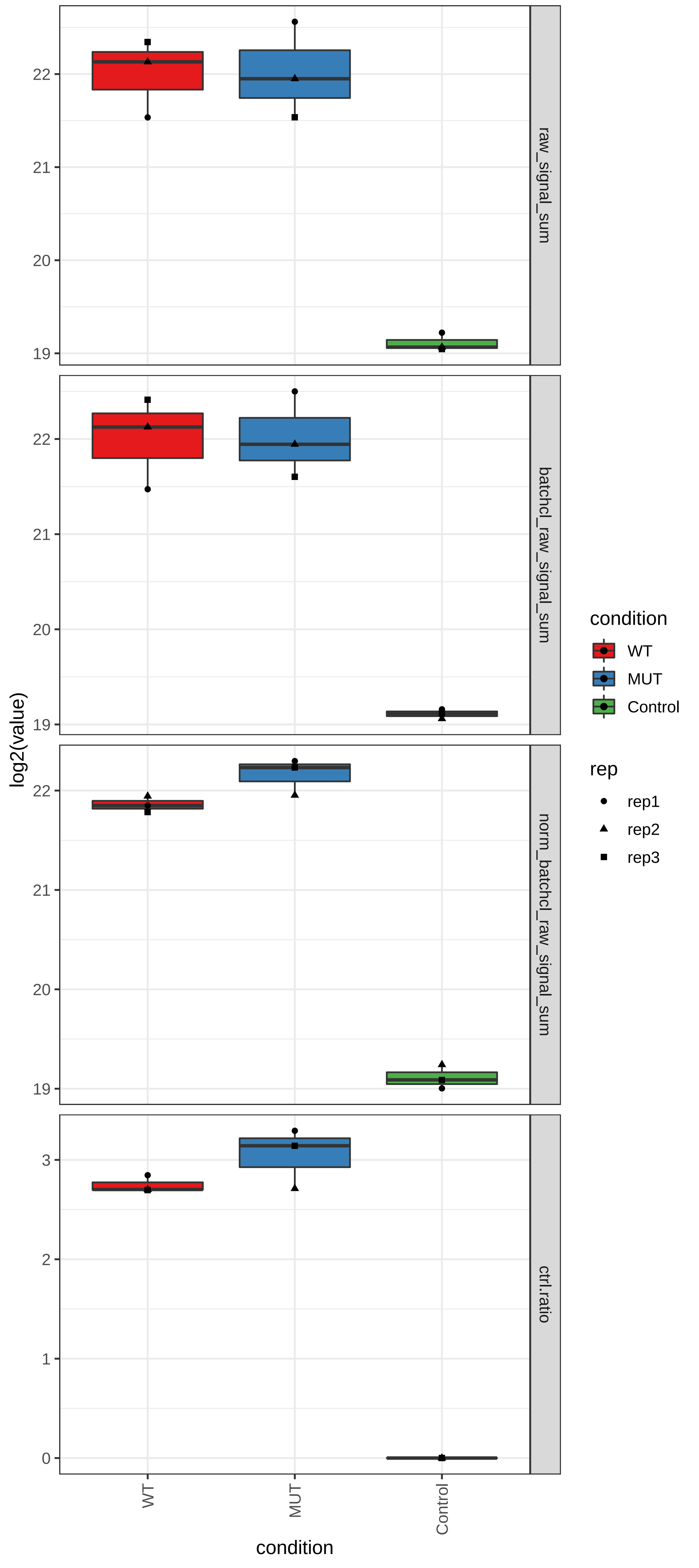

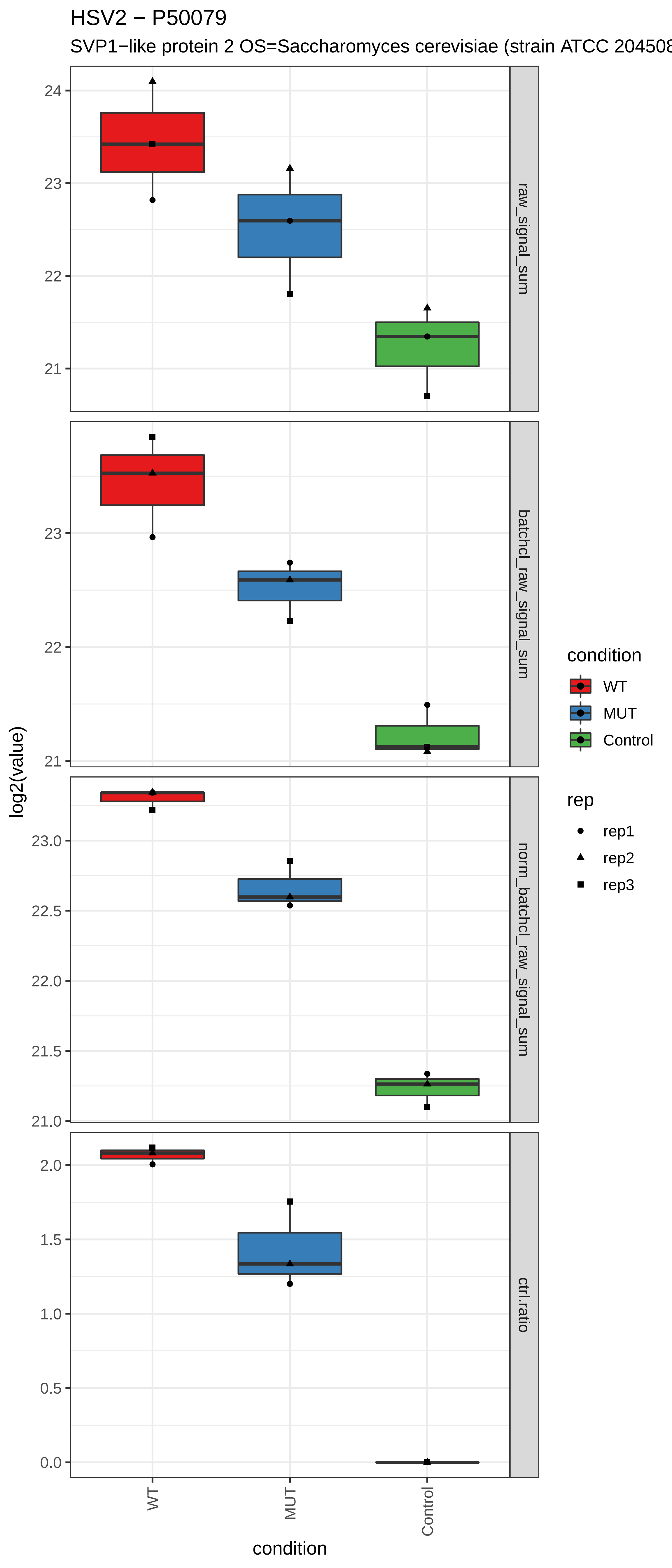
HSV2 − P50079
SVP1−like protein 2 OS=Saccharomyces cerevisiae (strain ATCC 204508
raw_signal_sum
batchcl_raw_signal_sum
norm_batchcl_raw_signal_sum
ctrl.ratio
log2(value)
condition
WT
MUT
Control
rep
rep1
rep2
rep3
24
23
22
21
23.0
22.5
22.0
21.5
21.0
2.0
1.5
1.0
0.5
0.0

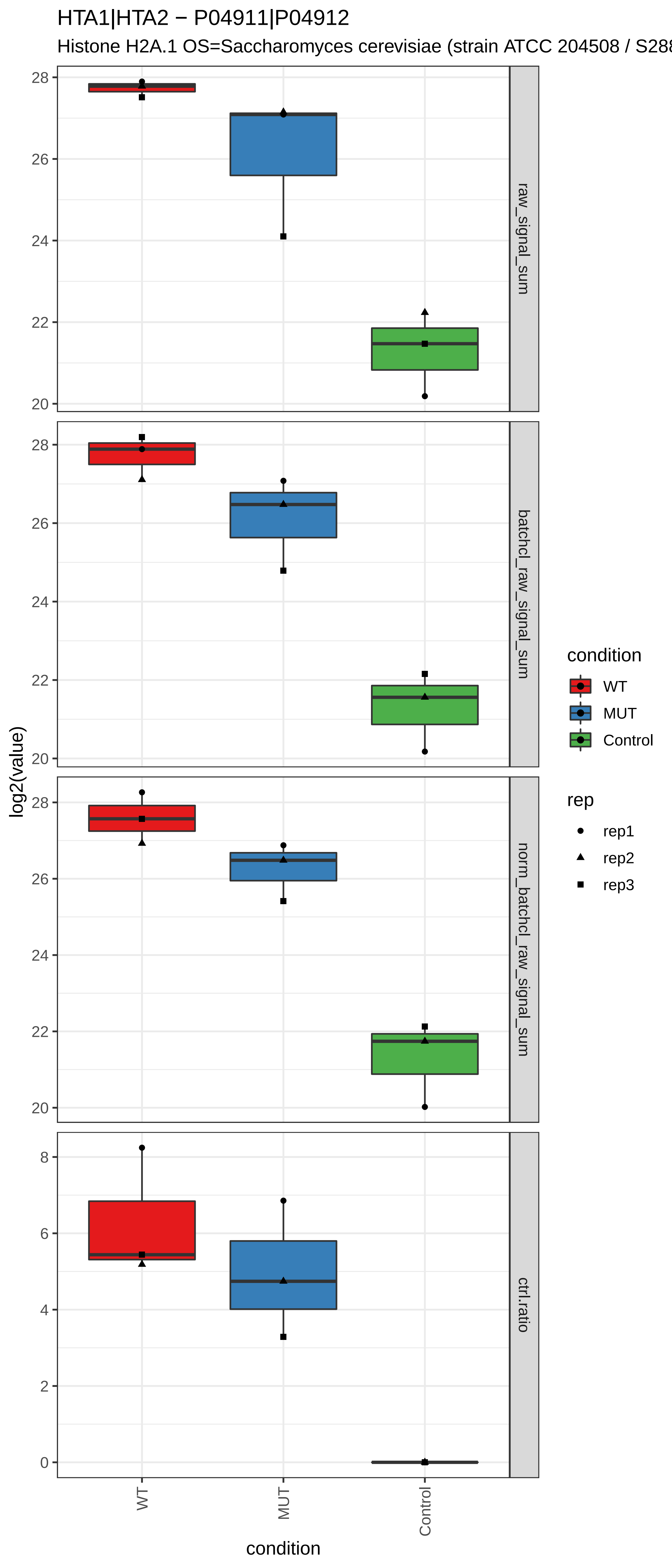
HTA1|HTA2 − P04911|P04912
Histone H2A.1 OS=Saccharomyces cerevisiae (strain ATCC 204508 / S288
raw_signal_sum
batchcl_raw_signal_sum
norm_batchcl_raw_signal_sum
ctrl.ratio
log2(value)
condition
WT
MUT
Control
rep
rep1
rep2
rep3
28
26
24
22
20
8
6
4
2
0

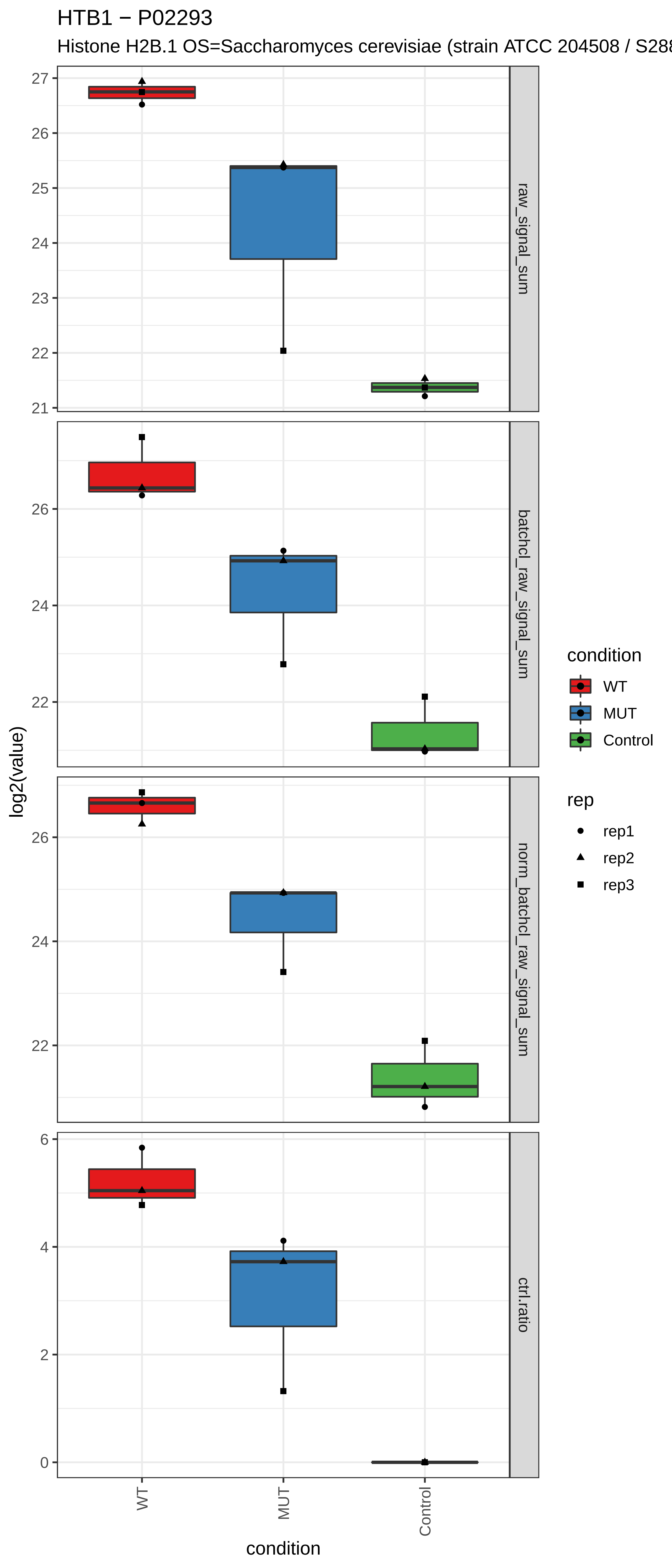
HTB1 − P02293
Histone H2B.1 OS=Saccharomyces cerevisiae (strain ATCC 204508 / S288
raw_signal_sum
batchcl_raw_signal_sum
norm_batchcl_raw_signal_sum
ctrl.ratio
log2(value)
condition
WT
MUT
Control
rep
rep1
rep2
rep3

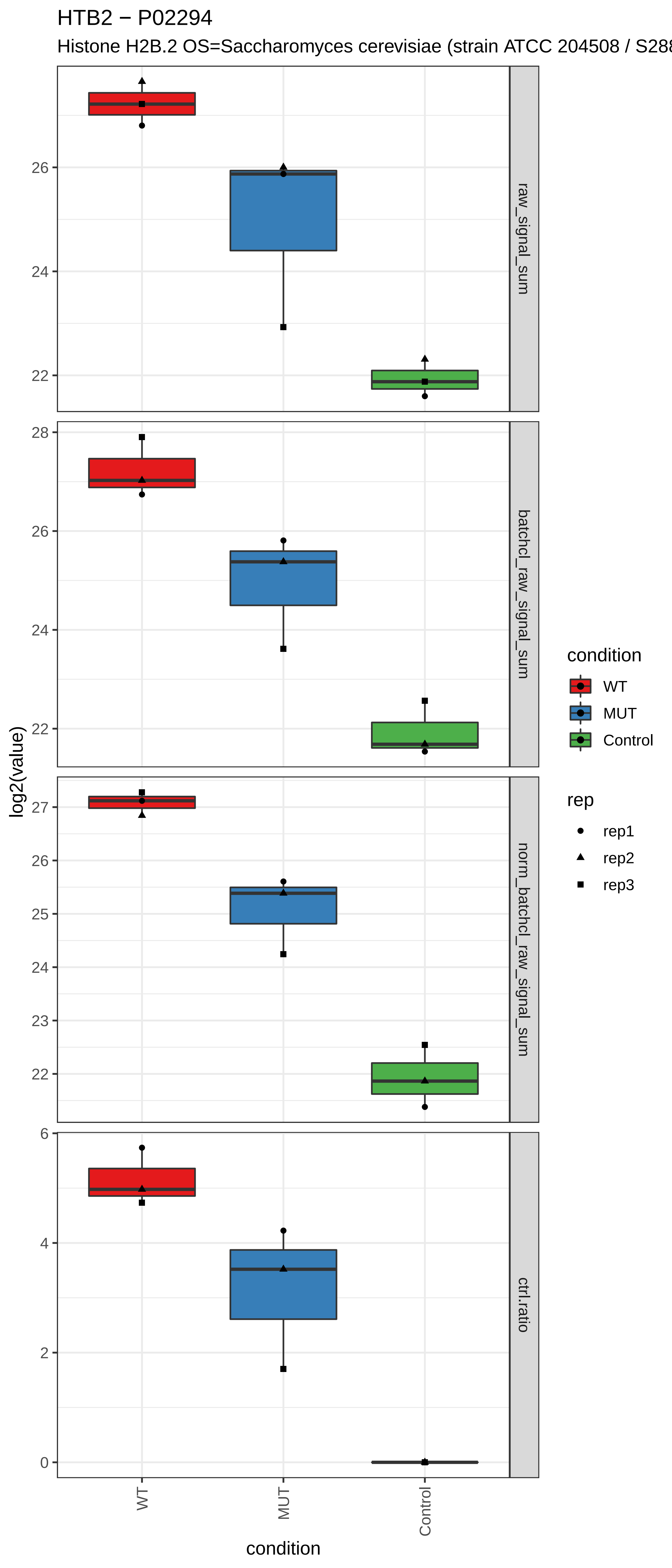
HTB2 − P02294
Histone H2B.2 OS=Saccharomyces cerevisiae (strain ATCC 204508 / S288
raw_signal_sum
batchcl_raw_signal_sum
norm_batchcl_raw_signal_sum
ctrl.ratio
log2(value)
condition
WT
MUT
Control
rep
rep1
rep2
rep3

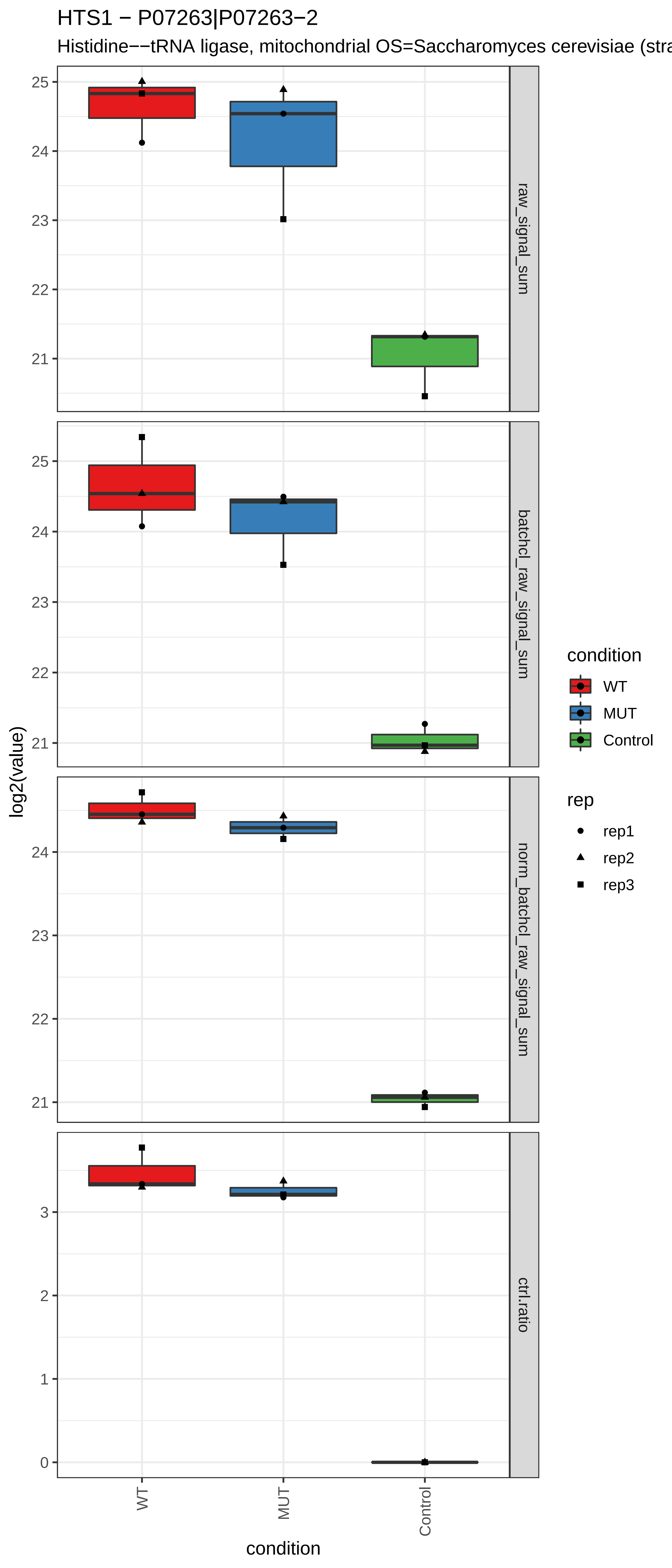

HTS1 − P07263|P07263−2
Histidine−−tRNA ligase, mitochondrial OS=Saccharomyces cerevisiae (stra
raw_signal_sum
batchcl_raw_signal_sum
norm_batchcl_raw_signal_sum
ctrl.ratio
log2(value)
condition
WT
MUT
Control
rep
rep1
rep2
rep3
25
24
23
22
21
3
2
1
0

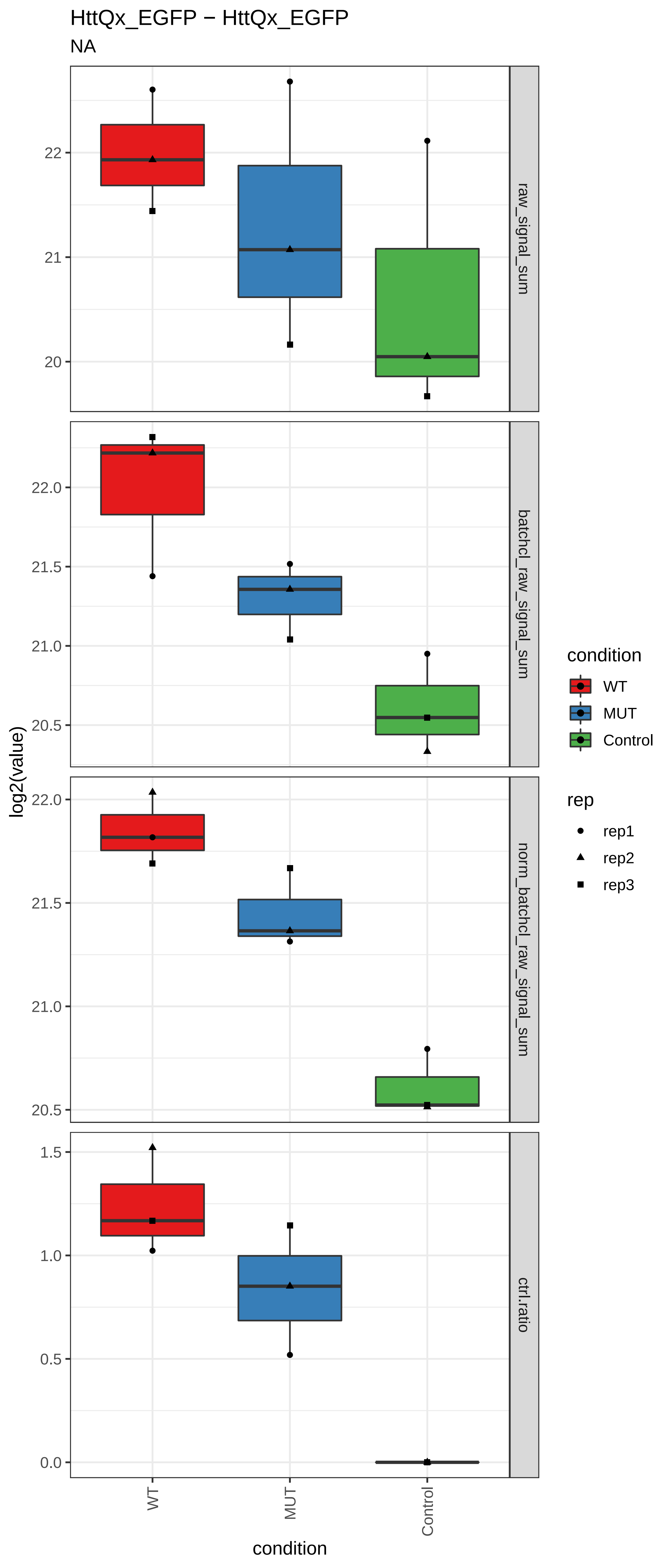

HttQx_EGFP − HttQx_EGFP
NA
raw_signal_sum
batchcl_raw_signal_sum
norm_batchcl_raw_signal_sum
ctrl.ratio
log2(value)
condition
WT
MUT
Control
rep
rep1
rep2
rep3
22
21
20
22.0
21.5
21.0
20.5
1.5
1.0
0.5
0.0

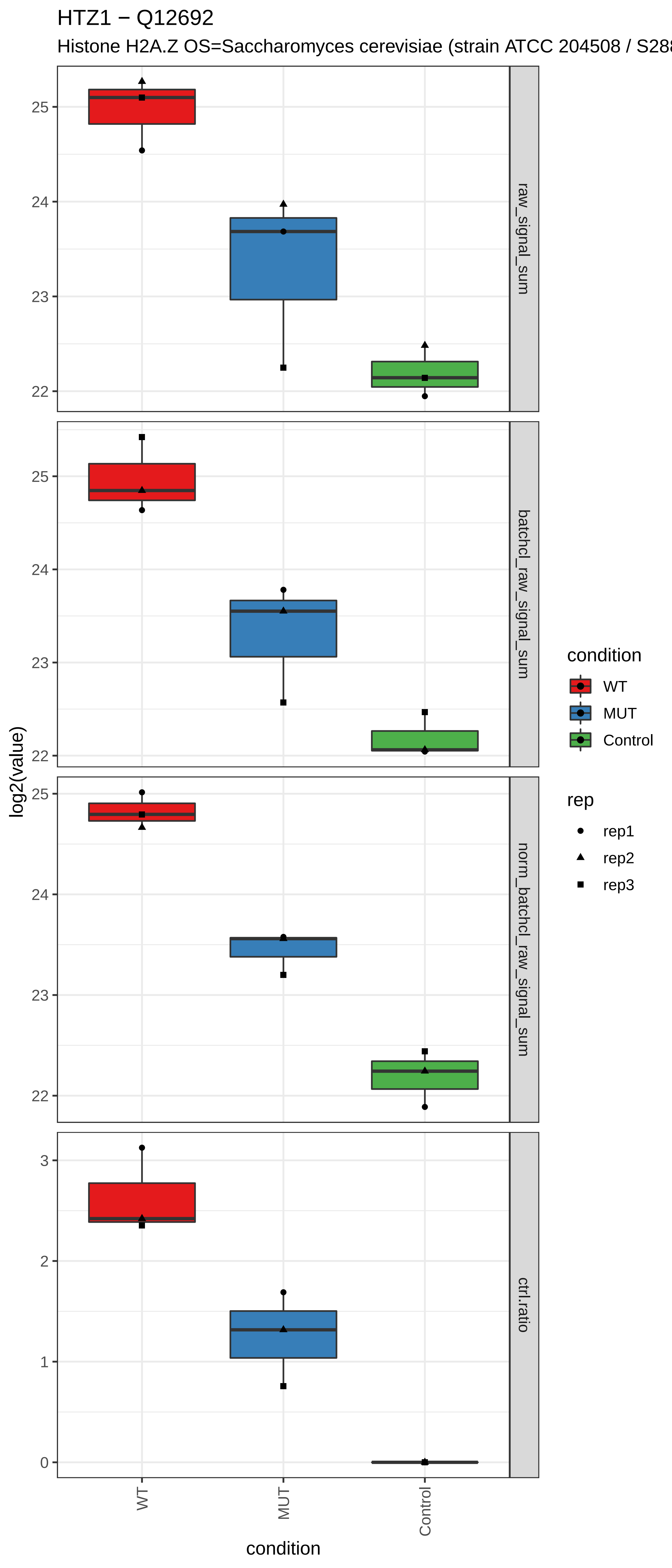

HTZ1 − Q12692
Histone H2A.Z OS=Saccharomyces cerevisiae (strain ATCC 204508 / S288
raw_signal_sum
batchcl_raw_signal_sum
norm_batchcl_raw_signal_sum
ctrl.ratio
log2(value)
condition
WT
MUT
Control
rep
rep1
rep2
rep3

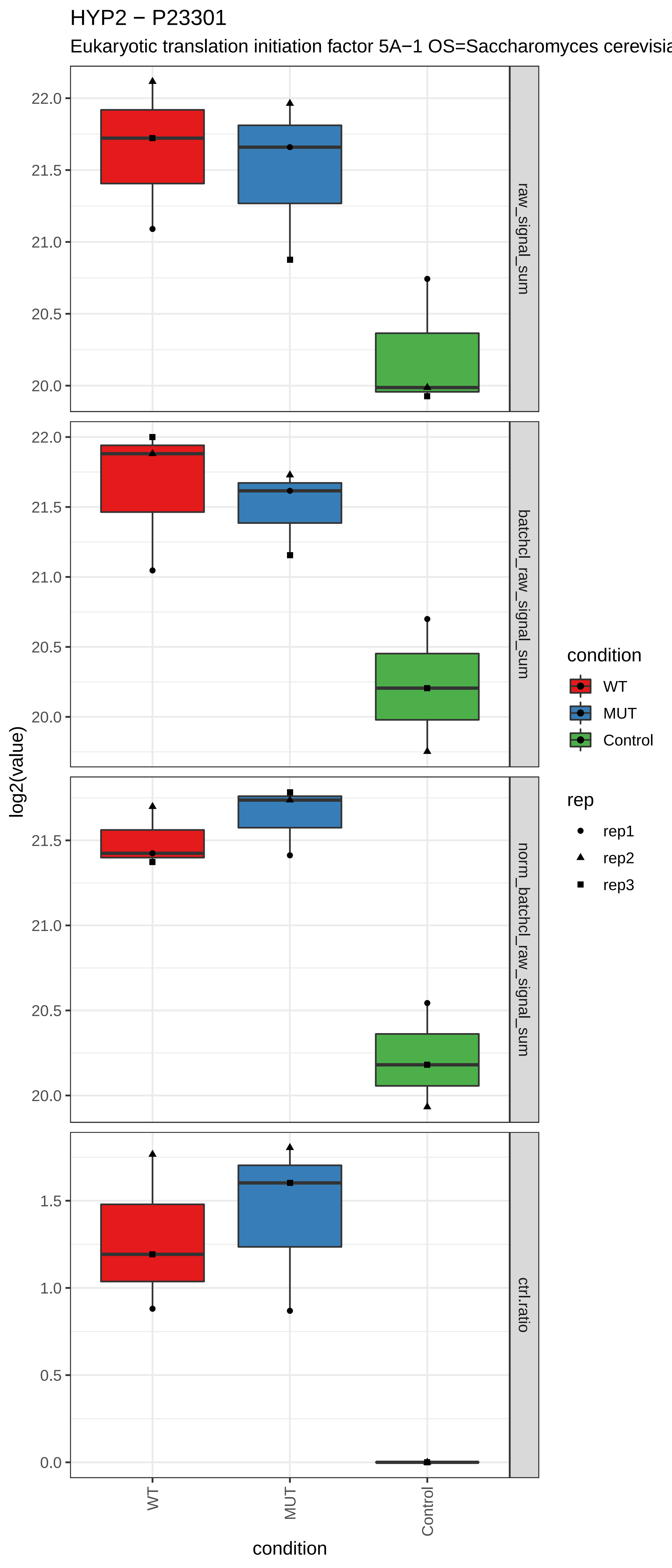
HYP2 − P23301
Eukaryotic translation initiation factor 5A−1 OS=Saccharomyces cerevisia
raw_signal_sum
batchcl_raw_signal_sum
norm_batchcl_raw_signal_sum
ctrl.ratio
log2(value)
condition
WT
MUT
Control
rep
rep1
rep2
rep3
22.0
21.5
21.0
20.5
20.0
1.5
1.0
0.5
0.0

ICL1 − P28240

Isocitrate lyase OS=Saccharomyces cerevisiae (strain ATCC 204508 / S28

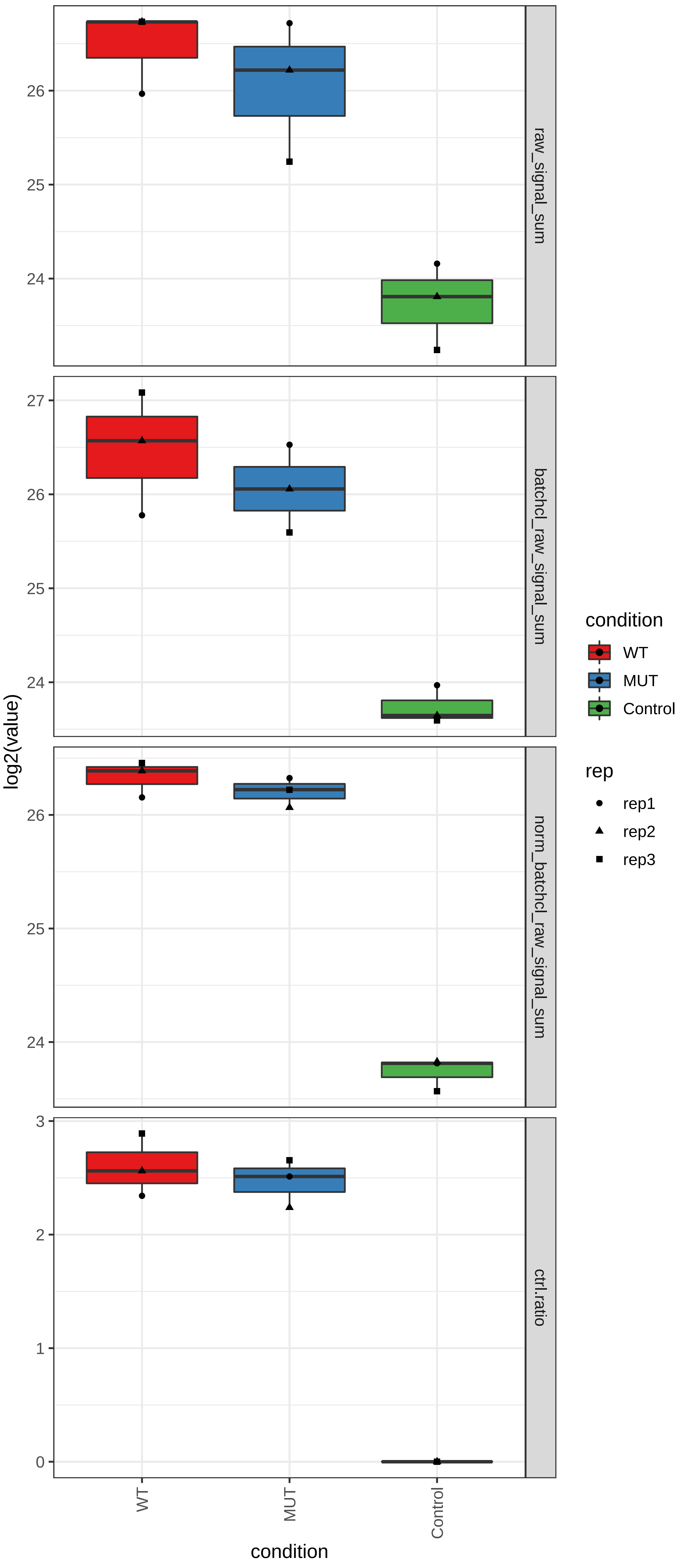

IDH1 − P28834

Isocitrate dehydrogenase [NAD] subunit 1, mitochondrial OS=Saccharom

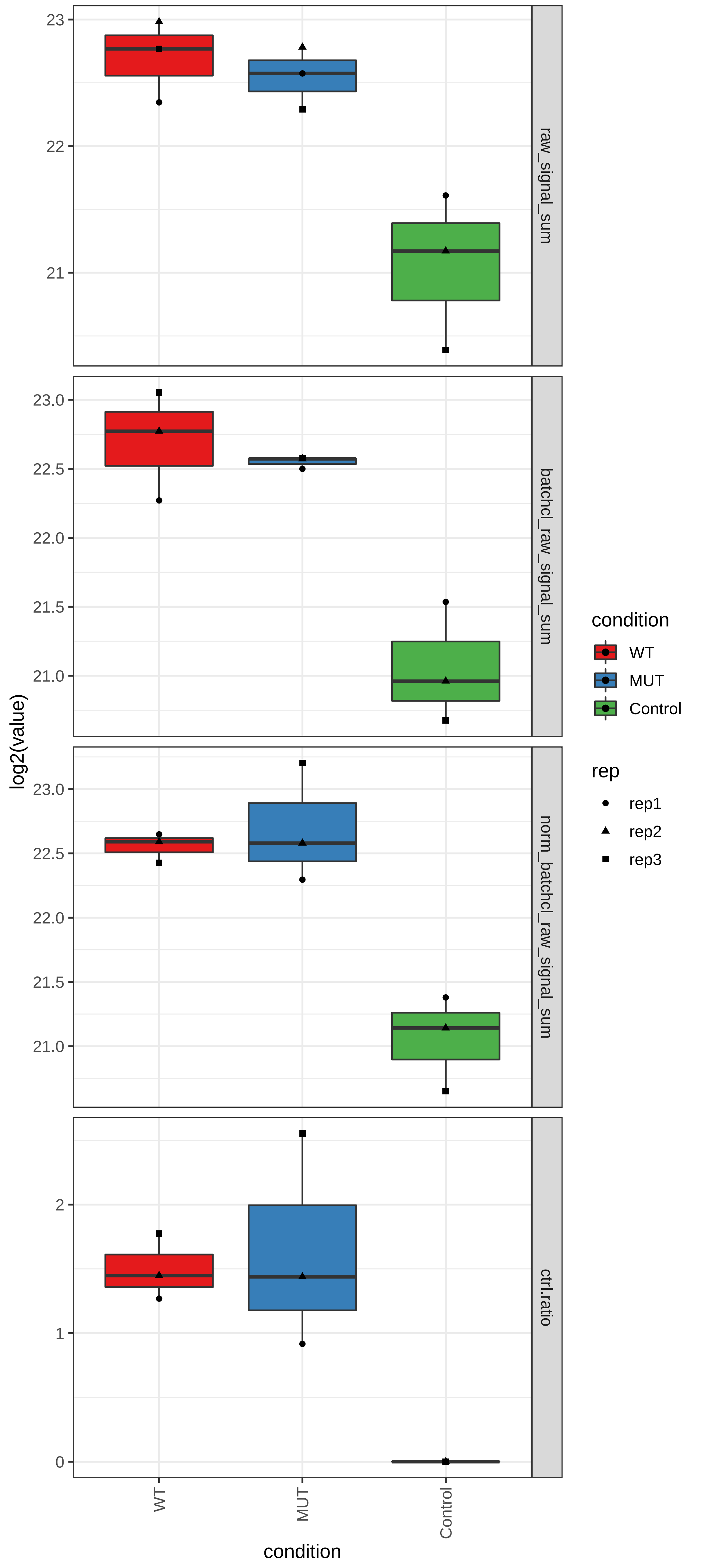

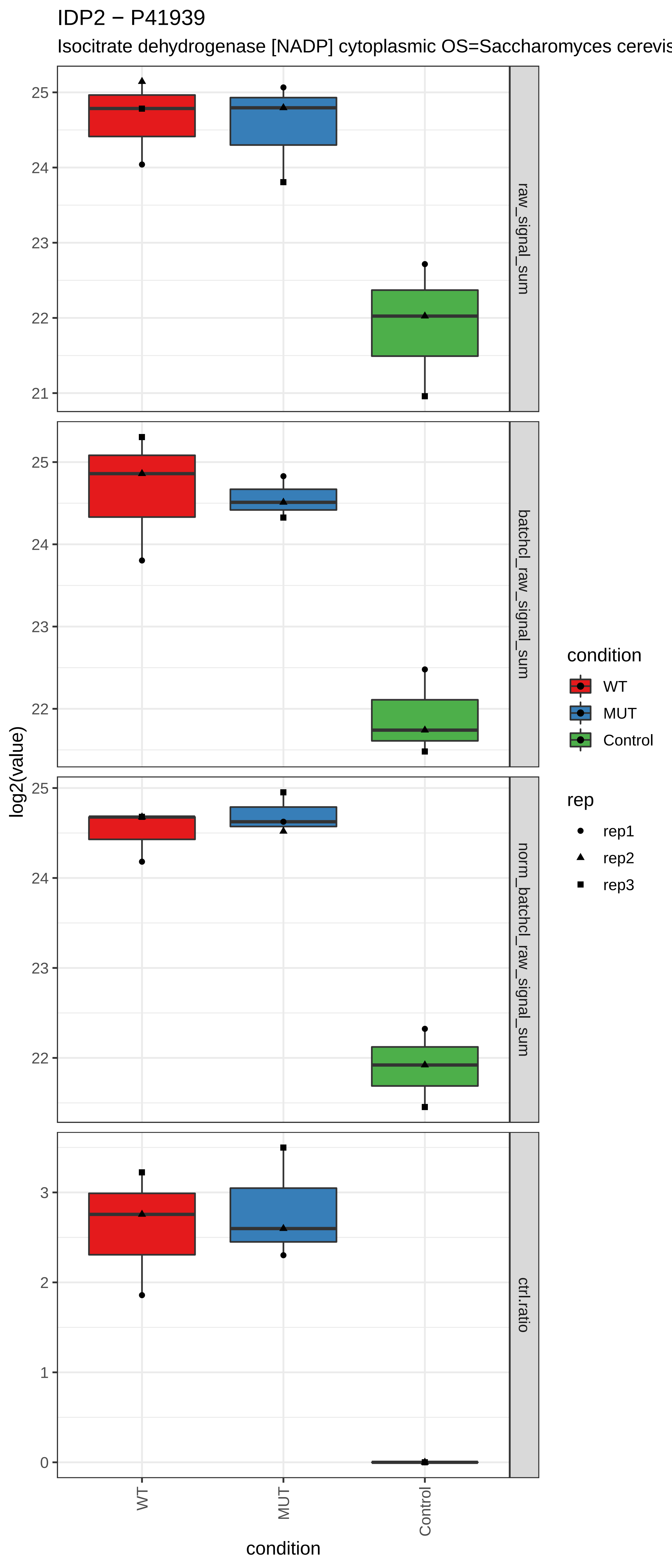

IDP2 − P41939
Isocitrate dehydrogenase [NADP] cytoplasmic OS=Saccharomyces cerevis
raw_signal_sum
batchcl_raw_signal_sum
norm_batchcl_raw_signal_sum
ctrl.ratio
log2(value)
condition
WT
MUT
Control
rep
rep1
rep2
rep3
25
24
23
22
21
3
2
1
0

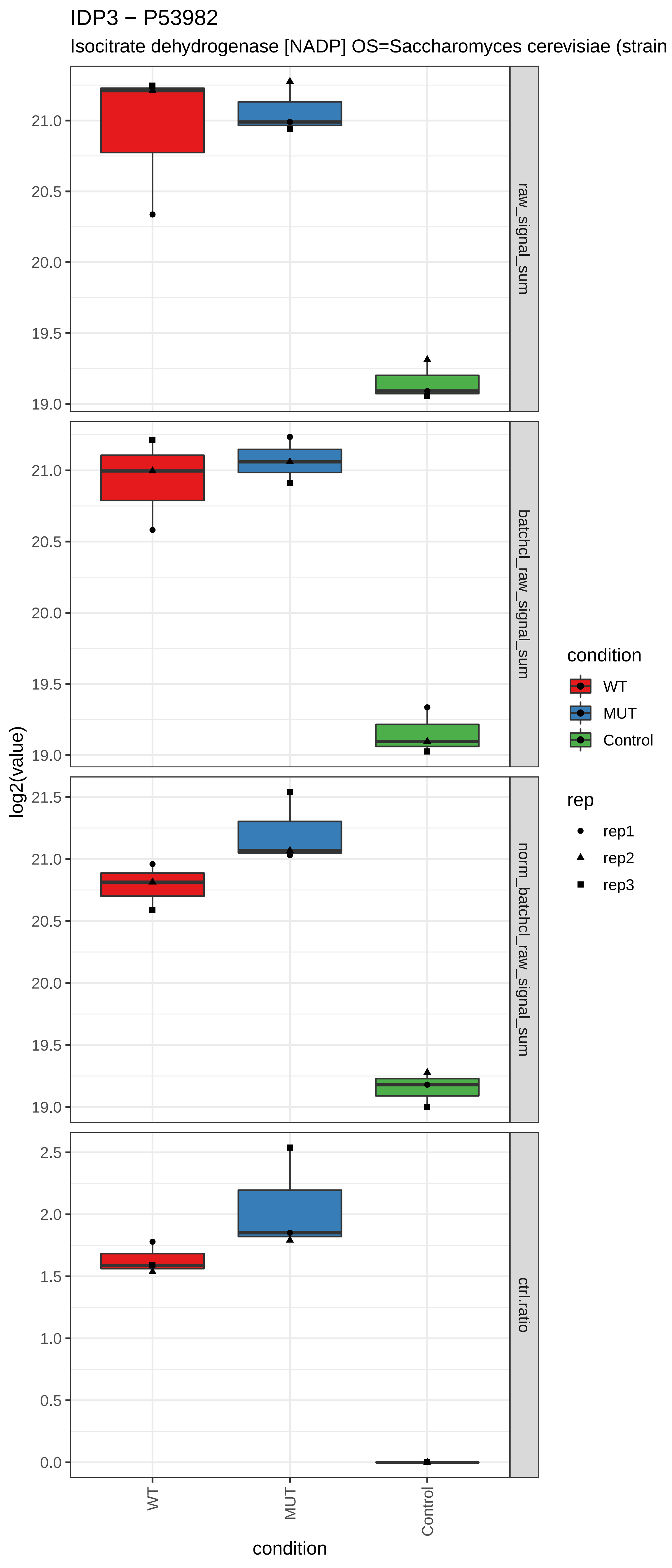

IDP3 − P53982
Isocitrate dehydrogenase [NADP] OS=Saccharomyces cerevisiae (strain
raw_signal_sum
batchcl_raw_signal_sum
norm_batchcl_raw_signal_sum
ctrl.ratio
log2(value)
condition
WT
MUT
Control
rep
rep1
rep2
rep3

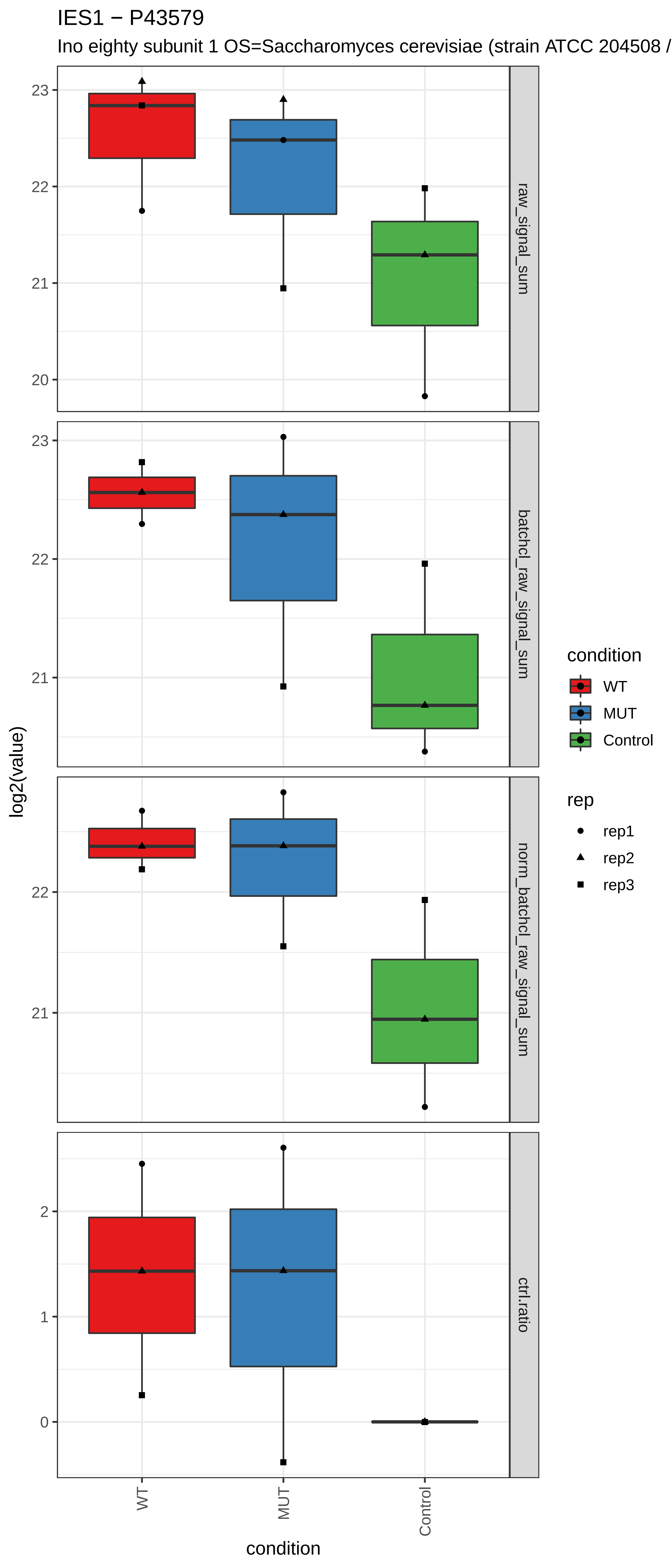

IES1 − P43579
Ino eighty subunit 1 OS=Saccharomyces cerevisiae (strain ATCC 204508 /
raw_signal_sum
batchcl_raw_signal_sum
norm_batchcl_raw_signal_sum
ctrl.ratio
log2(value)
condition
WT
MUT
Control
rep
rep1
rep2
rep3

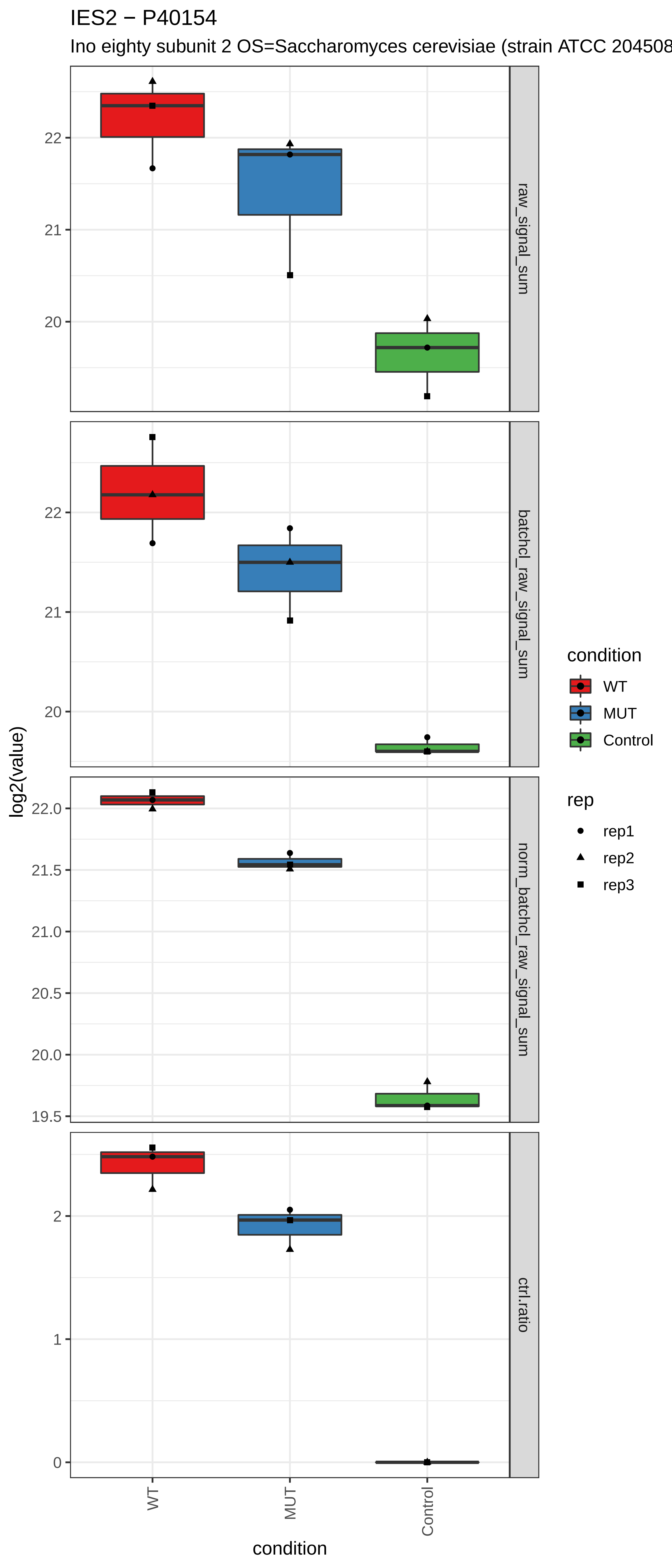

IES2 − P40154
Ino eighty subunit 2 OS=Saccharomyces cerevisiae (strain ATCC 204508
raw_signal_sum
batchcl_raw_signal_sum
norm_batchcl_raw_signal_sum
ctrl.ratio
log2(value)
22
21
20
22.0
21.5
21.0
20.5
20.0
19.5
2
1
0
WT
MUT
Control
condition
condition
WT
MUT
Control
rep
rep1
rep2
rep3

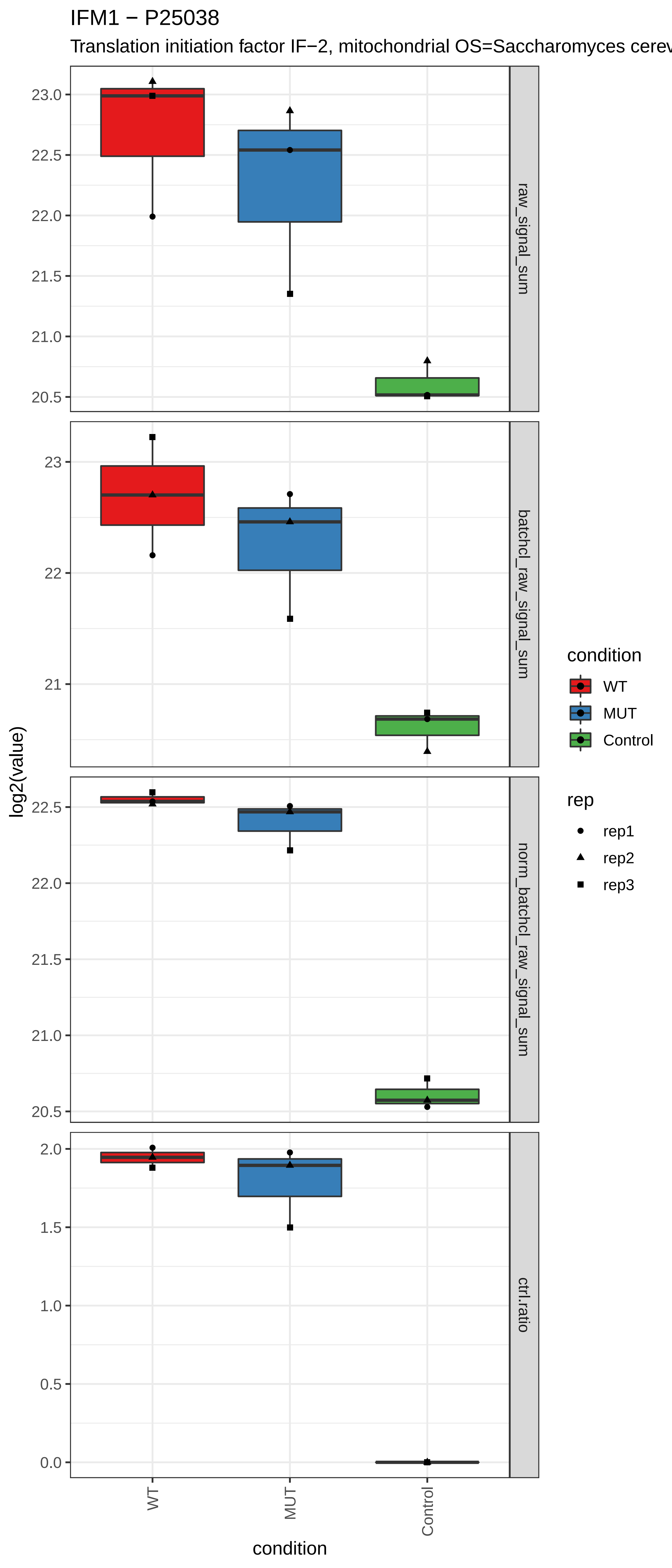

IFM1 − P25038
Translation initiation factor IF−2, mitochondrial OS=Saccharomyces cerev
raw_signal_sum
batchcl_raw_signal_sum
norm_batchcl_raw_signal_sum
ctrl.ratio
log2(value)
condition
WT
MUT
Control
rep
rep1
rep2
rep3
23.0
22.5
22.0
21.5
21.0
20.5
23
22
21
2.0
1.5
1.0
0.5
0.0

ILV2 − P07342

Acetolactate synthase catalytic subunit, mitochondrial OS=Saccharomyce

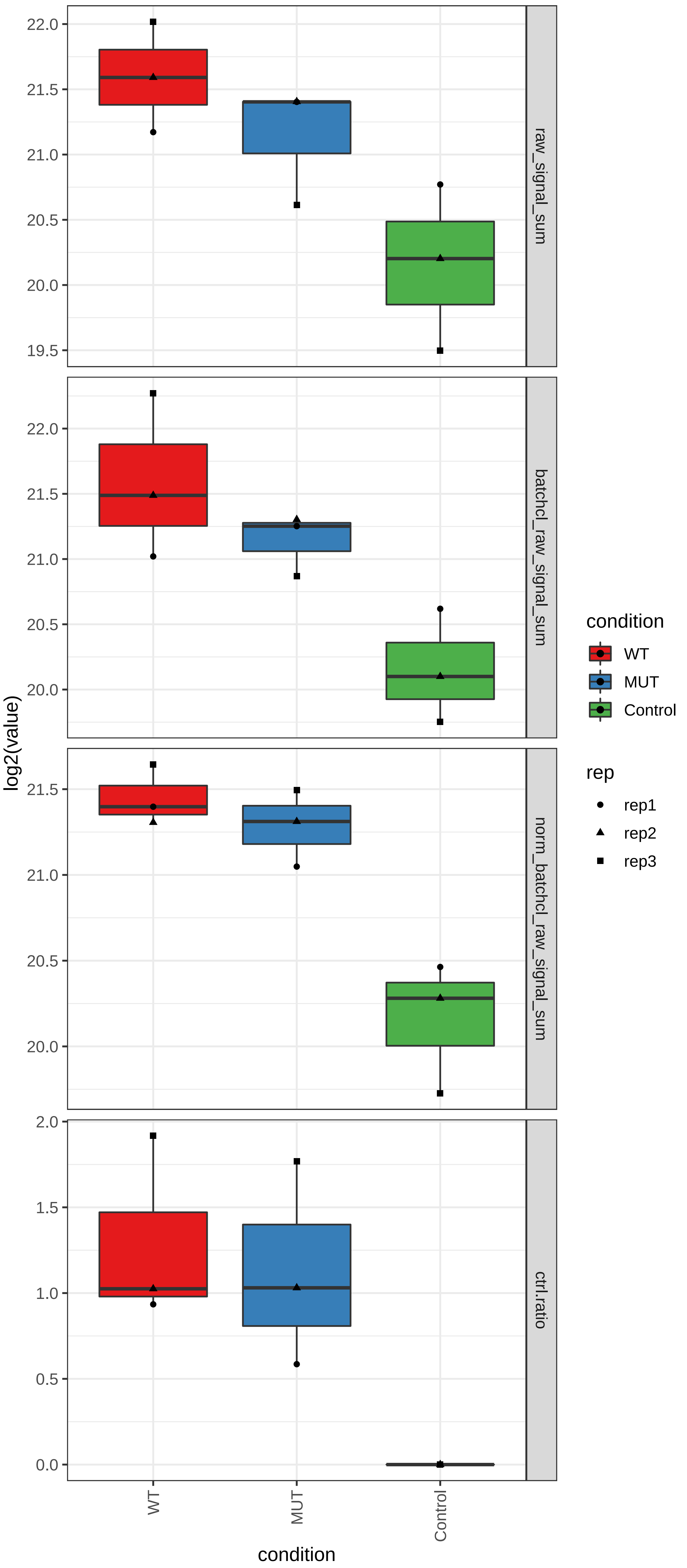

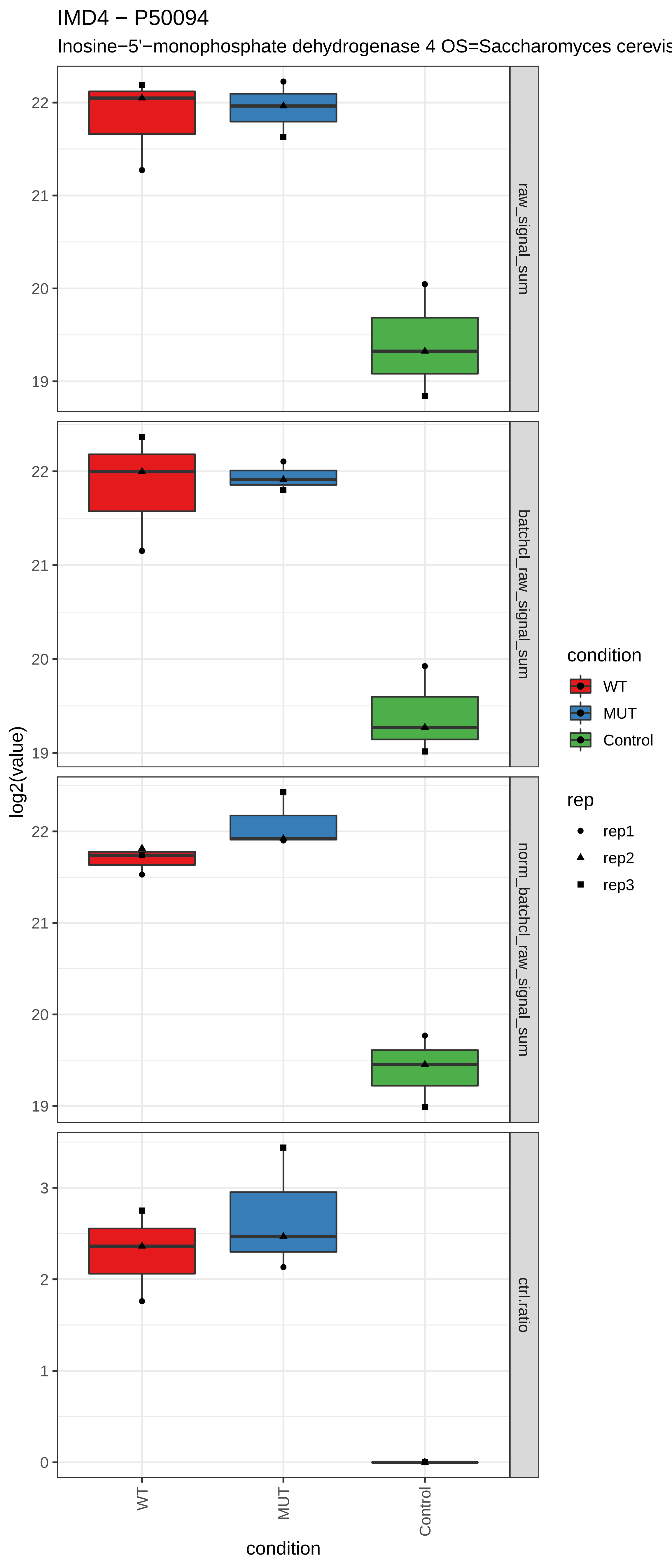
IMD4 − P50094
Inosine−5'−monophosphate dehydrogenase 4 OS=Saccharomyces cerevis
raw_signal_sum
batchcl_raw_signal_sum
norm_batchcl_raw_signal_sum
ctrl.ratio
log2(value)
condition
WT
MUT
Control
rep
rep1
rep2
rep3
19
20
21
22
0
1
2
3

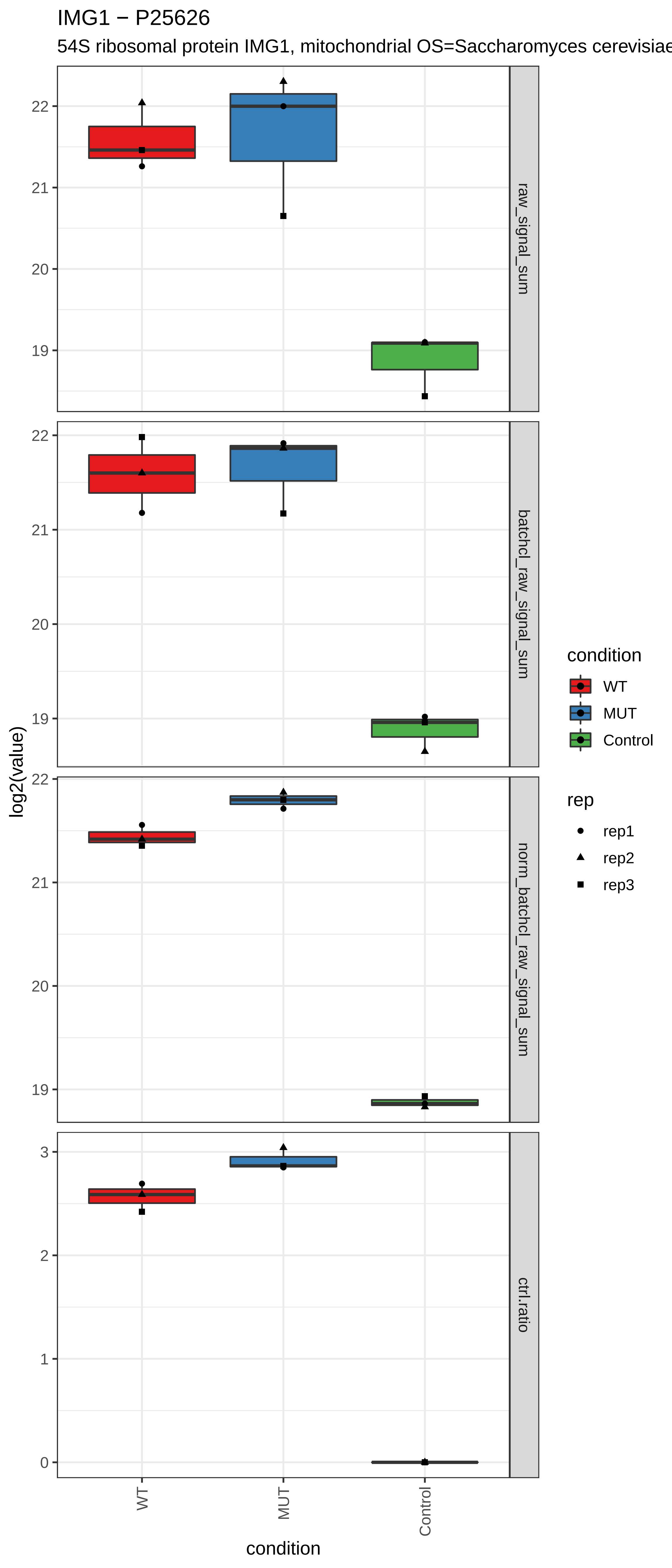

IMG1 − P25626
54S ribosomal protein IMG1, mitochondrial OS=Saccharomyces cerevisiae
raw_signal_sum
batchcl_raw_signal_sum
norm_batchcl_raw_signal_sum
ctrl.ratio
log2(value)
condition
WT
MUT
Control
rep
rep1
rep2
rep3
22
21
20
19
3
2
1
0

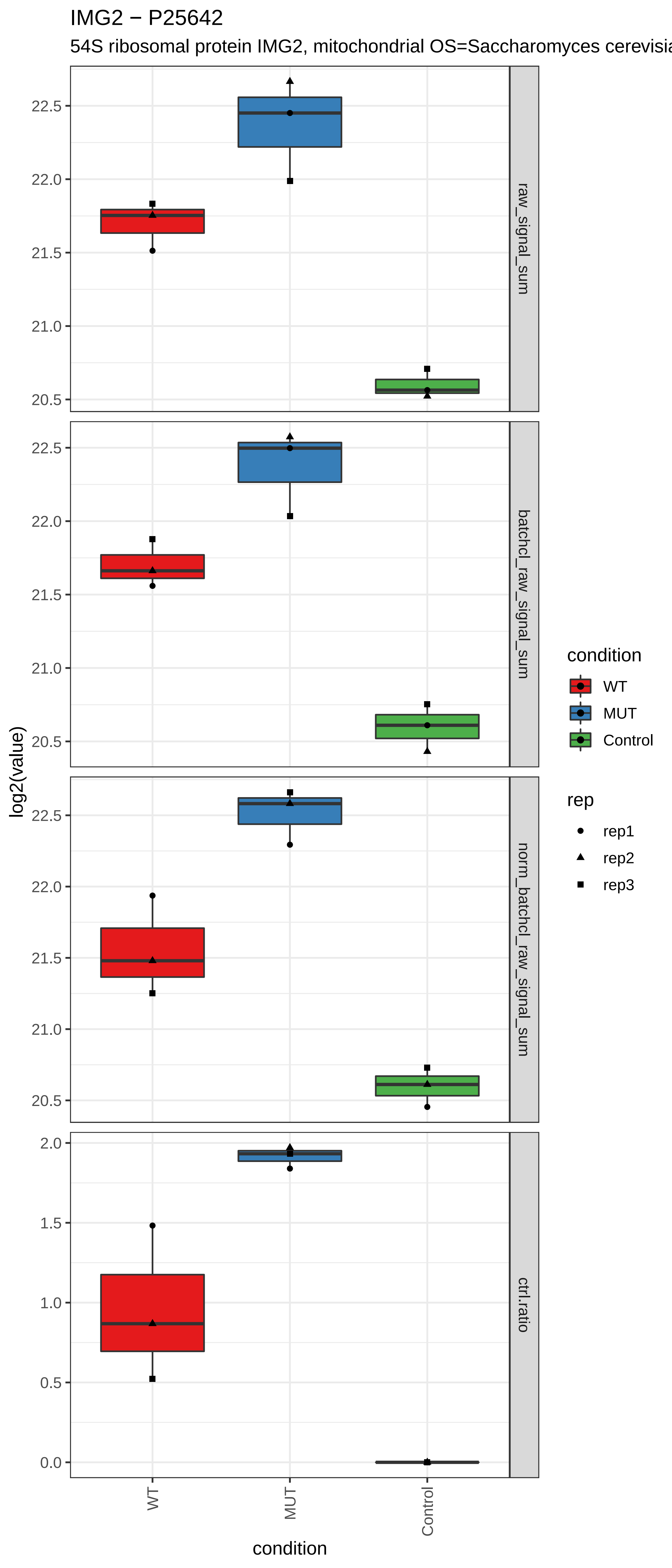
IMG2 − P25642
54S ribosomal protein IMG2, mitochondrial OS=Saccharomyces cerevisia
raw_signal_sum
batchcl_raw_signal_sum
norm_batchcl_raw_signal_sum
ctrl.ratio
log2(value)
condition
WT
MUT
Control
rep
rep1
rep2
rep3
22.5
22.0
21.5
21.0
20.5
2.0
1.5
1.0
0.5
0.0

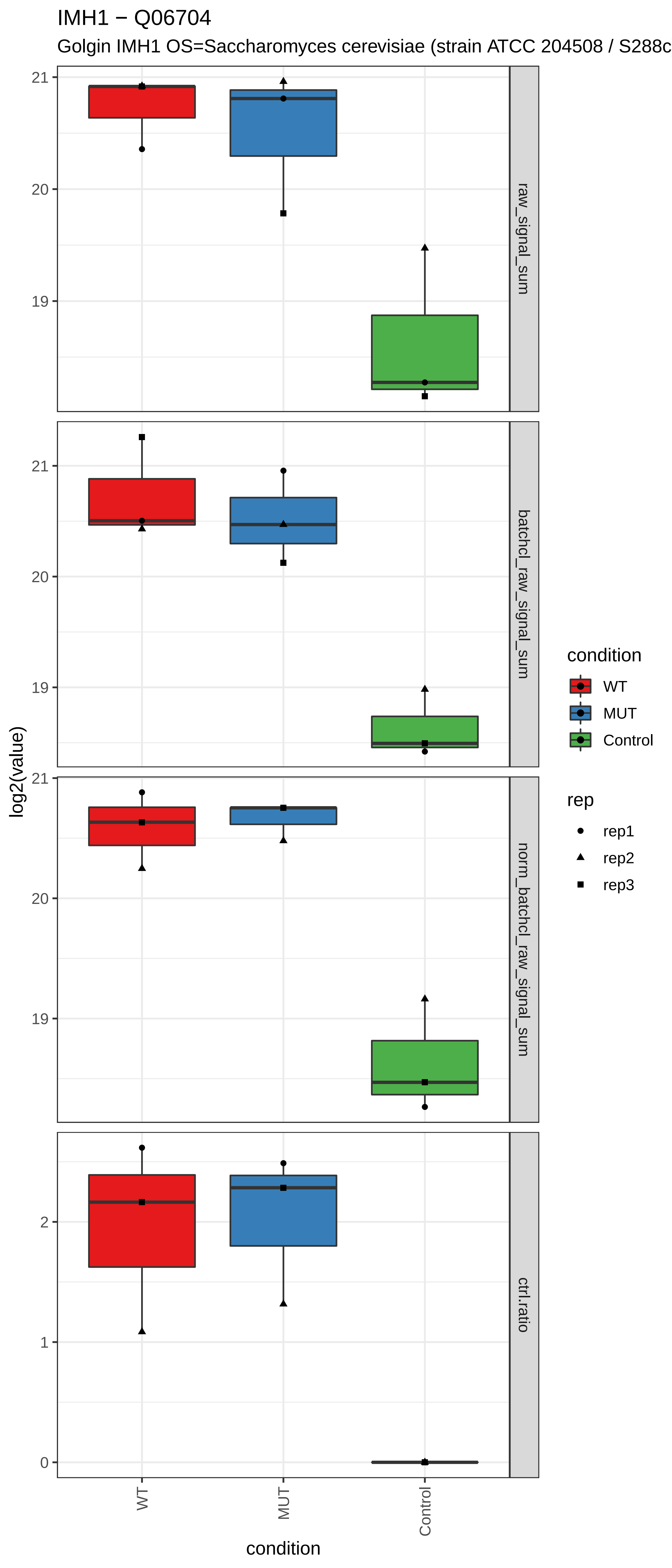

IMH1 − Q06704
Golgin IMH1 OS=Saccharomyces cerevisiae (strain ATCC 204508 / S288c
raw_signal_sum
batchcl_raw_signal_sum
norm_batchcl_raw_signal_sum
ctrl.ratio
log2(value)
condition
WT
MUT
Control
rep
rep1
rep2
rep3
21
20
19
2
1
0

INO1 − P11986

Inositol−3−phosphate synthase OS=Saccharomyces cerevisiae (strain ATC

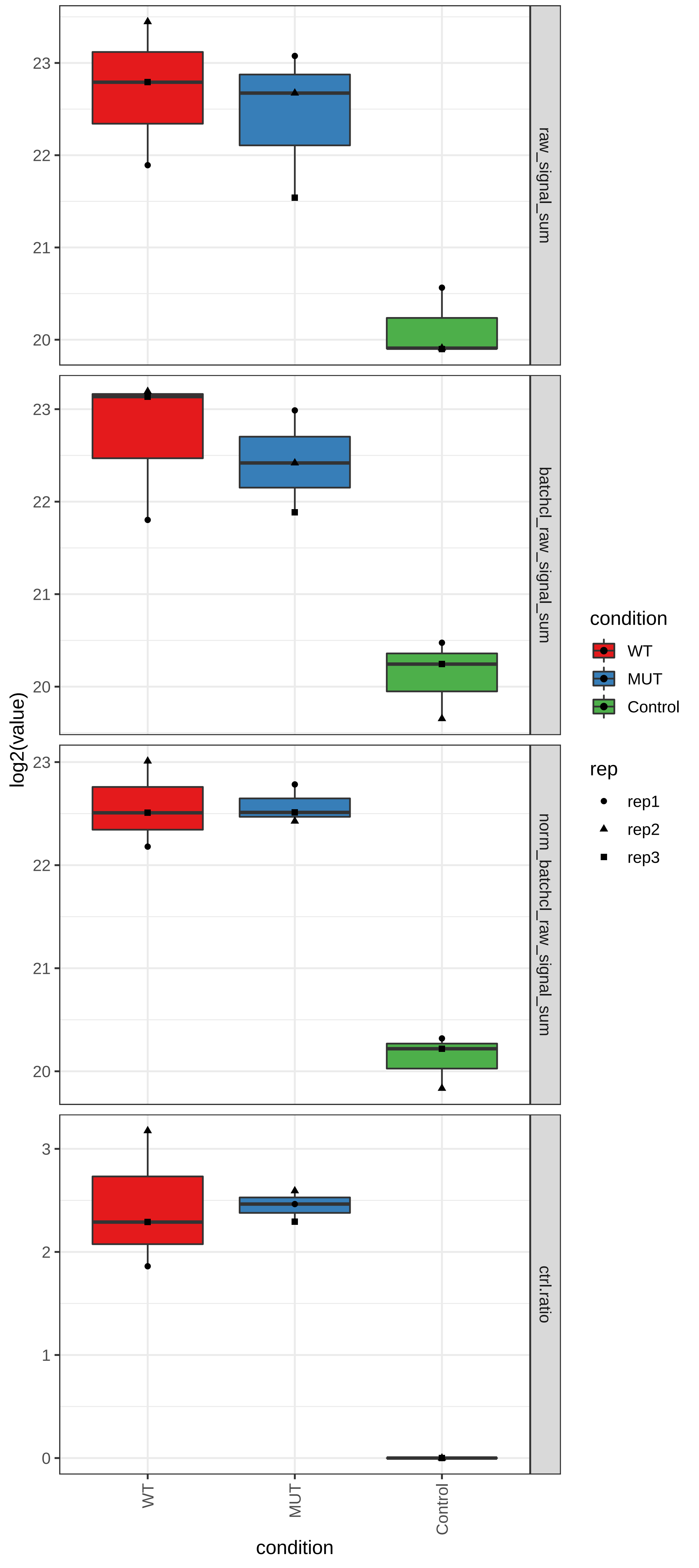

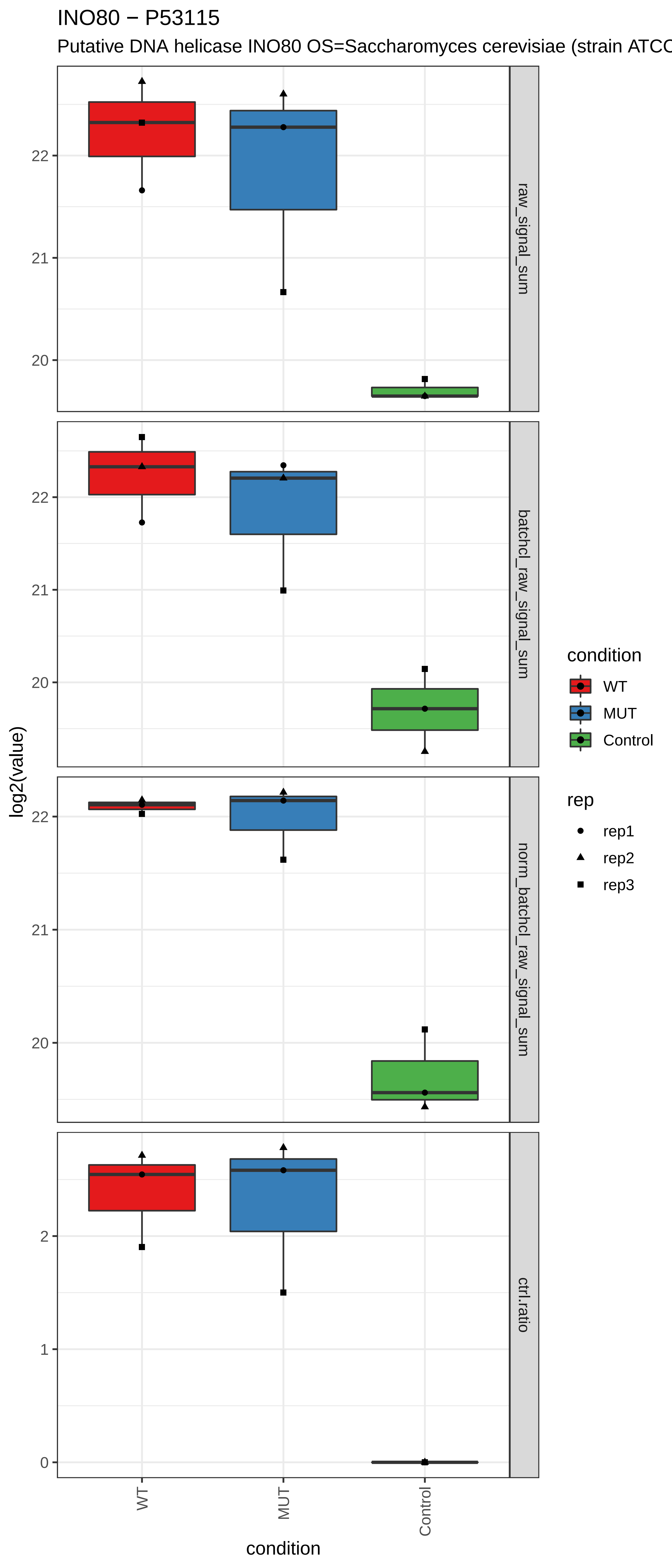

INO80 − P53115
Putative DNA helicase INO80 OS=Saccharomyces cerevisiae (strain ATCC
raw_signal_sum
batchcl_raw_signal_sum
norm_batchcl_raw_signal_sum
ctrl.ratio
log2(value)
22
21
20
2
1
0
WT
MUT
Control
condition
condition
WT
MUT
Control
rep
rep1
rep2
rep3

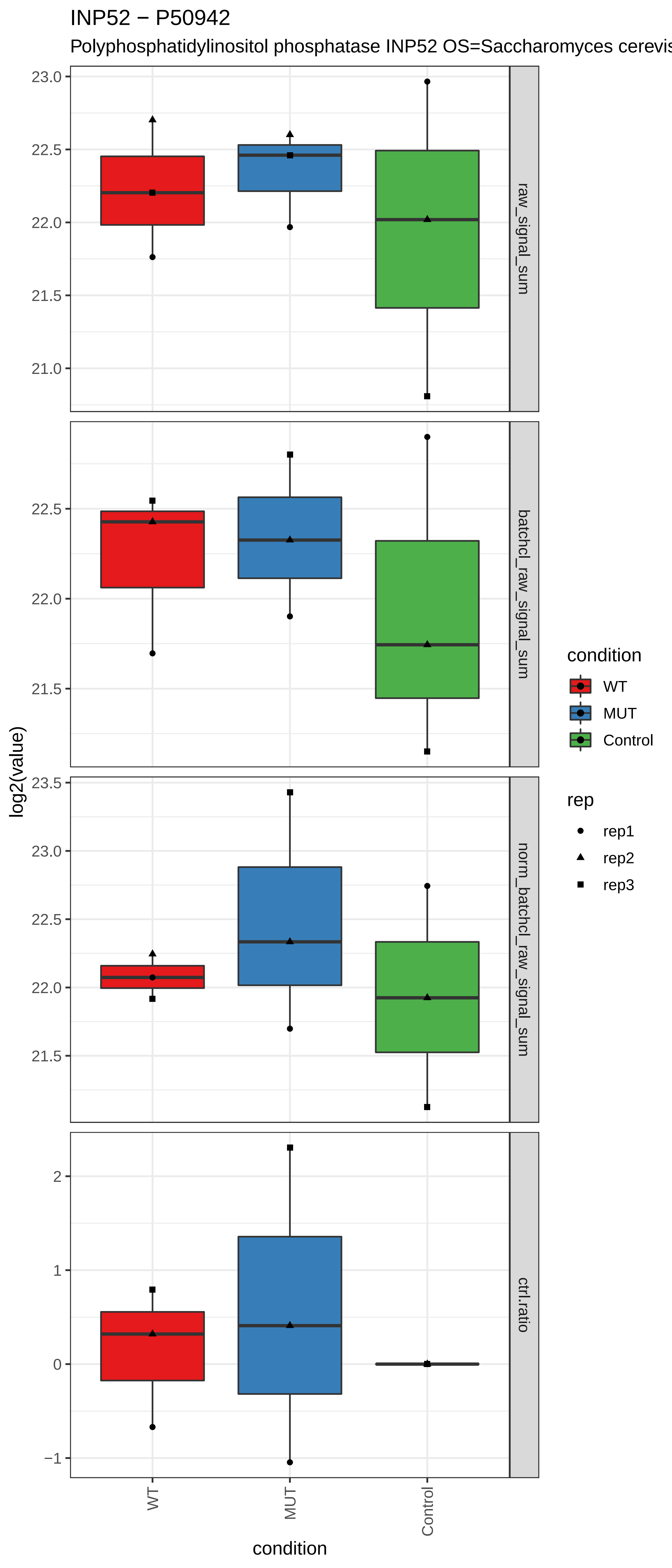

INP52 − P50942
Polyphosphatidylinositol phosphatase INP52 OS=Saccharomyces cerevis
raw_signal_sum
batchcl_raw_signal_sum
norm_batchcl_raw_signal_sum
ctrl.ratio
log2(value)
condition
WT
MUT
Control
rep
rep1
rep2
rep3

IOC2 − Q12072

ISWI one complex protein 2 OS=Saccharomyces cerevisiae (strain ATCC 2

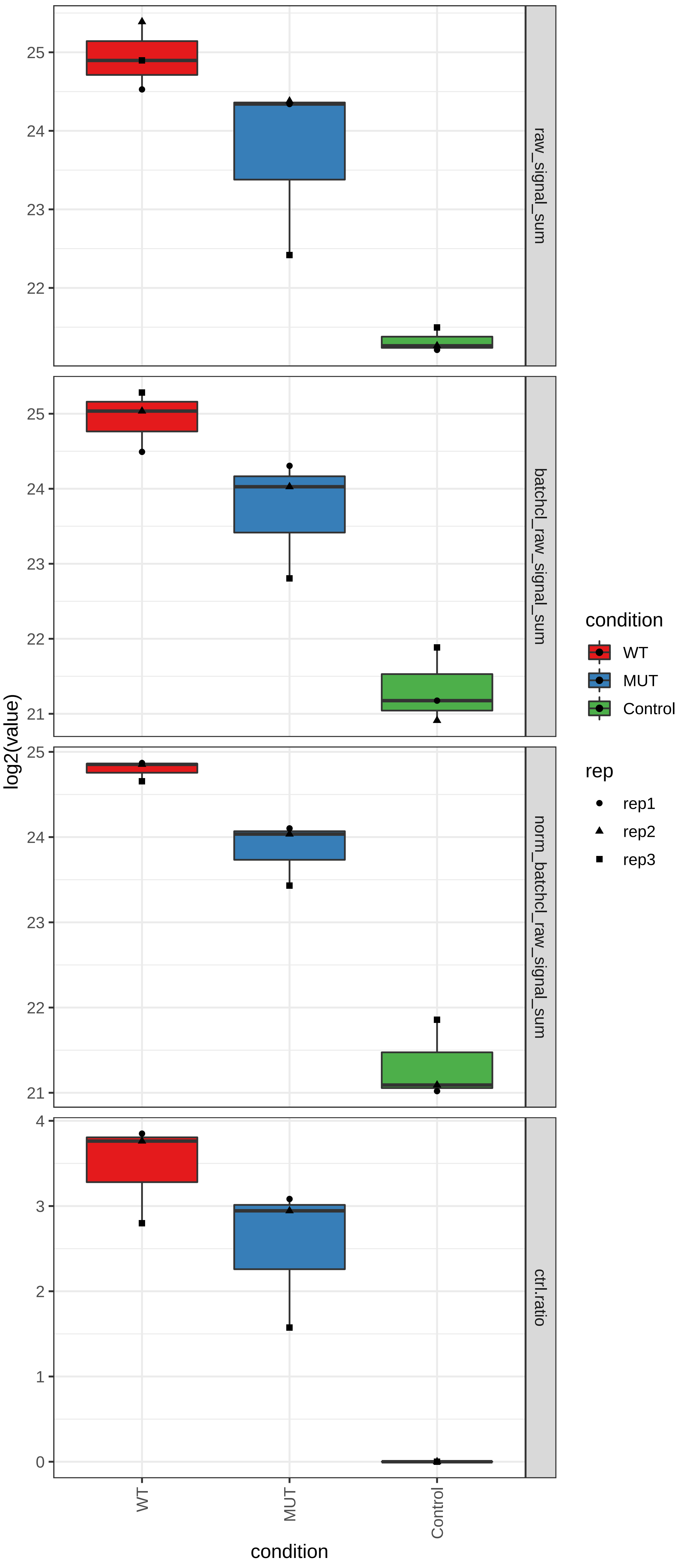

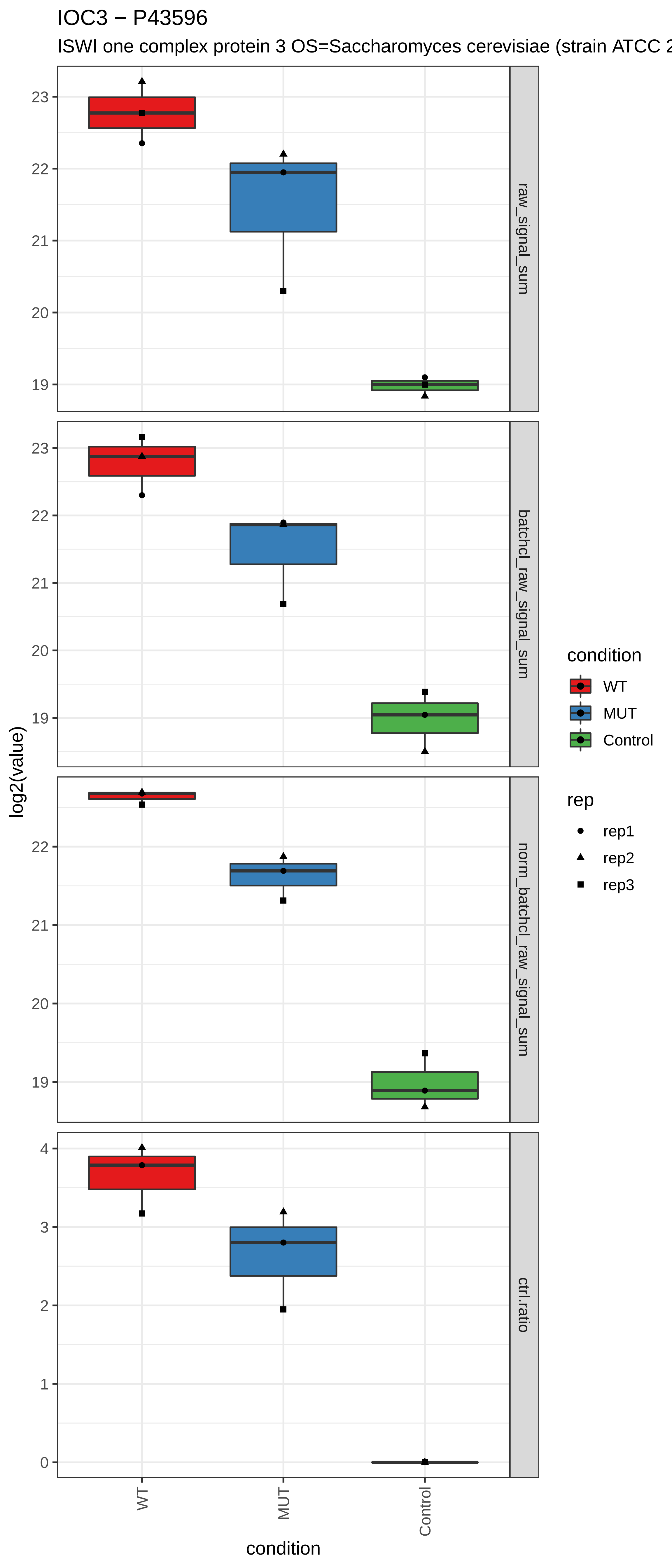

IOC3 − P43596
ISWI one complex protein 3 OS=Saccharomyces cerevisiae (strain ATCC 2
log2(value)
raw_signal_sum
batchcl_raw_signal_sum
norm_batchcl_raw_signal_sum
ctrl.ratio
condition
WT
MUT
Control
rep
rep1
rep2
rep3

IPI3 − P53877

Pre−rRNA−processing protein IPI3 OS=Saccharomyces cerevisiae (strain A

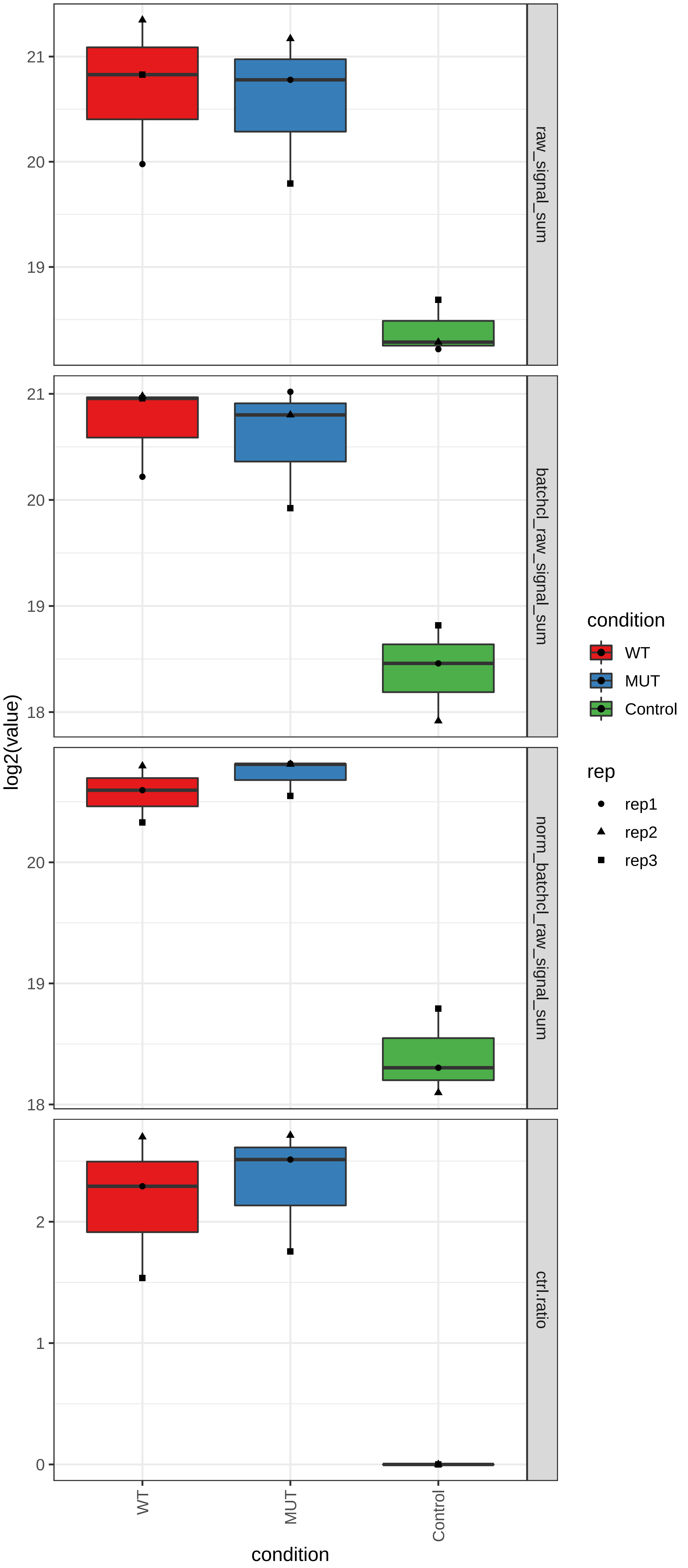

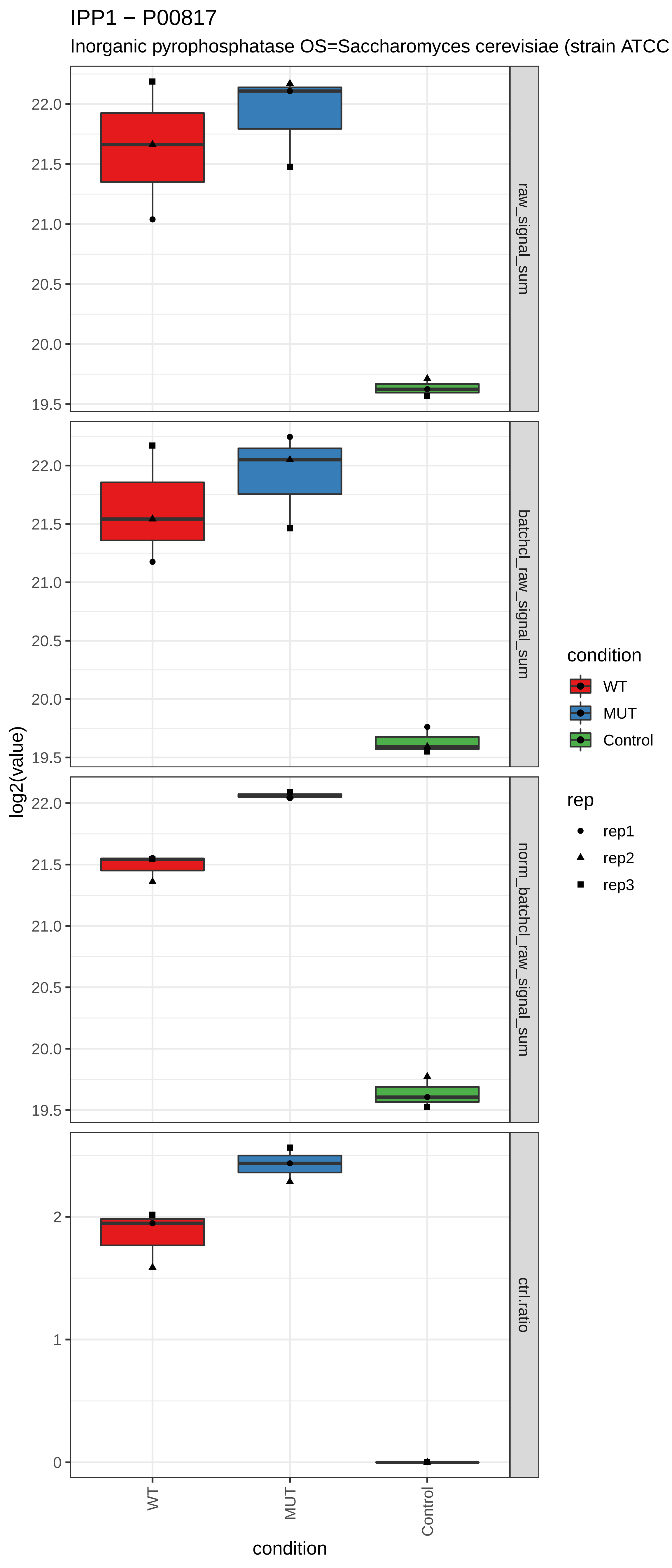

IPP1 − P00817
Inorganic pyrophosphatase OS=Saccharomyces cerevisiae (strain ATCC
raw_signal_sum
batchcl_raw_signal_sum
norm_batchcl_raw_signal_sum
ctrl.ratio
log2(value)
condition
WT
MUT
Control
rep
rep1
rep2
rep3

IRR1 − P40541

Cohesin subunit SCC3 OS=Saccharomyces cerevisiae (strain ATCC 20450

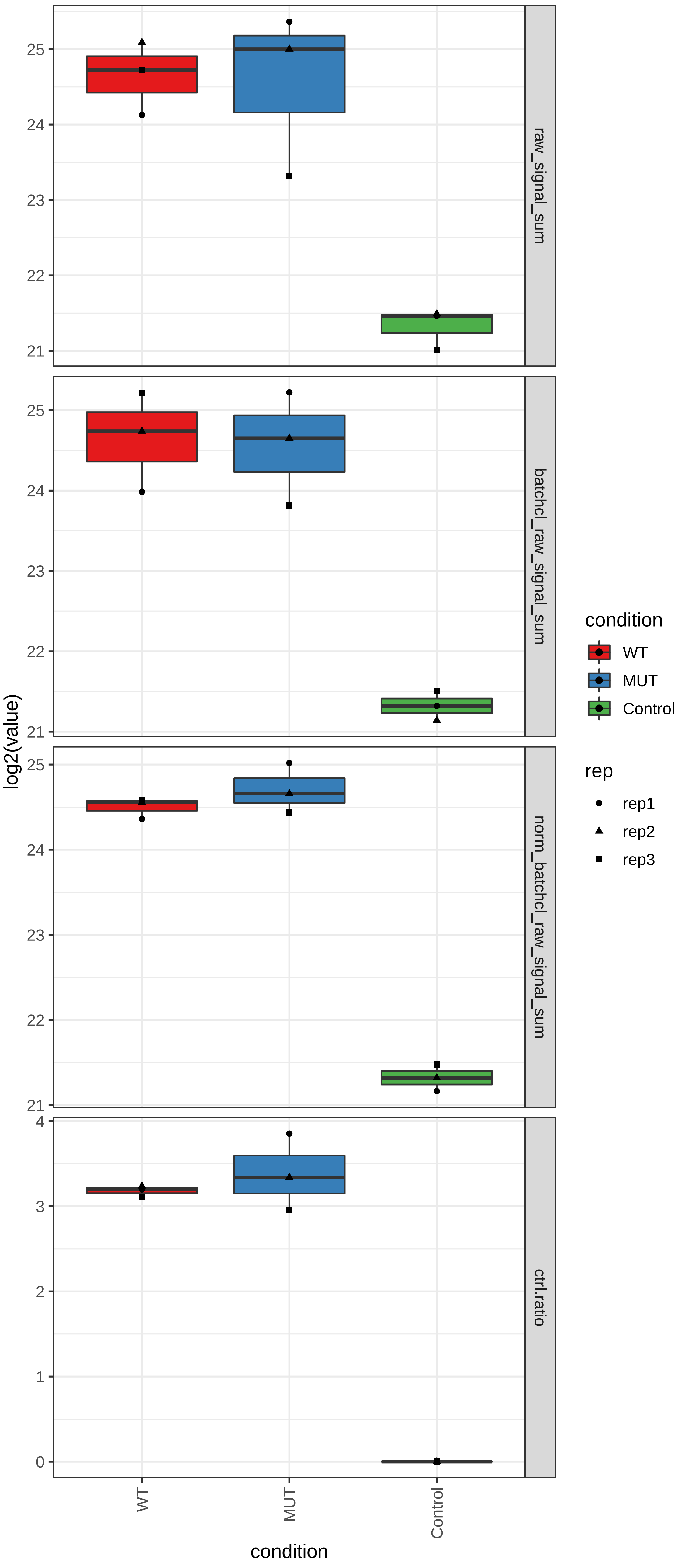

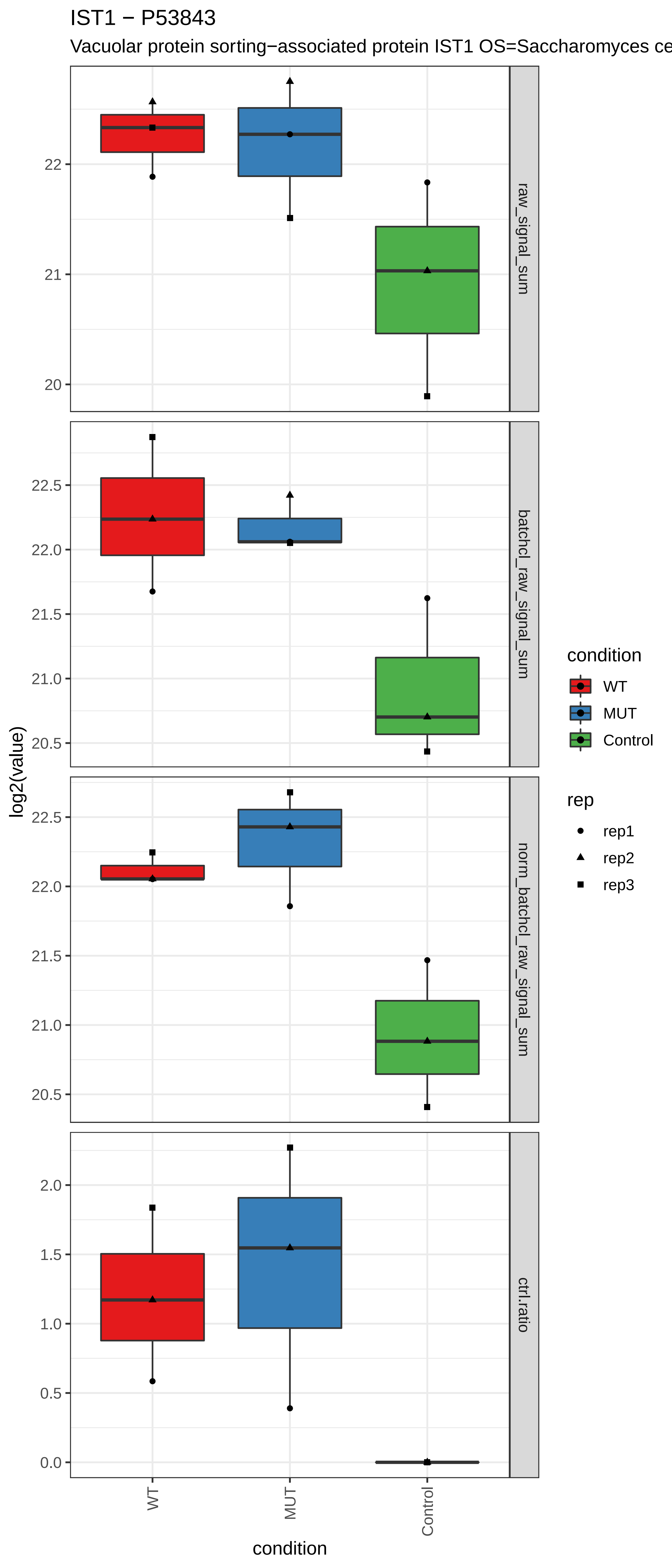
IST1 − P53843
Vacuolar protein sorting−associated protein IST1 OS=Saccharomyces ce
raw_signal_sum
batchcl_raw_signal_sum
norm_batchcl_raw_signal_sum
ctrl.ratio
log2(value)
condition
WT
MUT
Control
rep
rep1
rep2
rep3

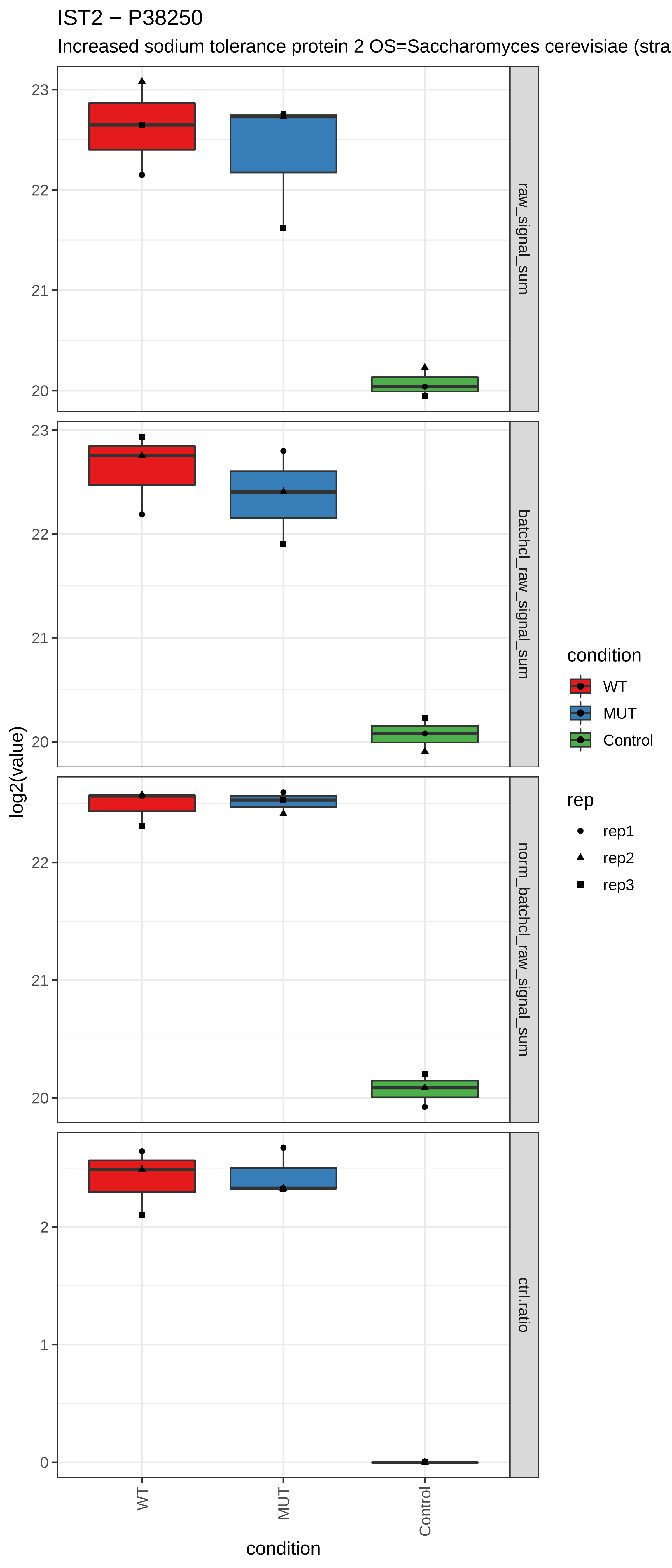

IST2 − P38250
Increased sodium tolerance protein 2 OS=Saccharomyces cerevisiae (strai
raw_signal_sum
batchcl_raw_signal_sum
norm_batchcl_raw_signal_sum
ctrl.ratio
log2(value)
condition
WT
MUT
Control
rep
rep1
rep2
rep3
23
22
21
20
2
1
0

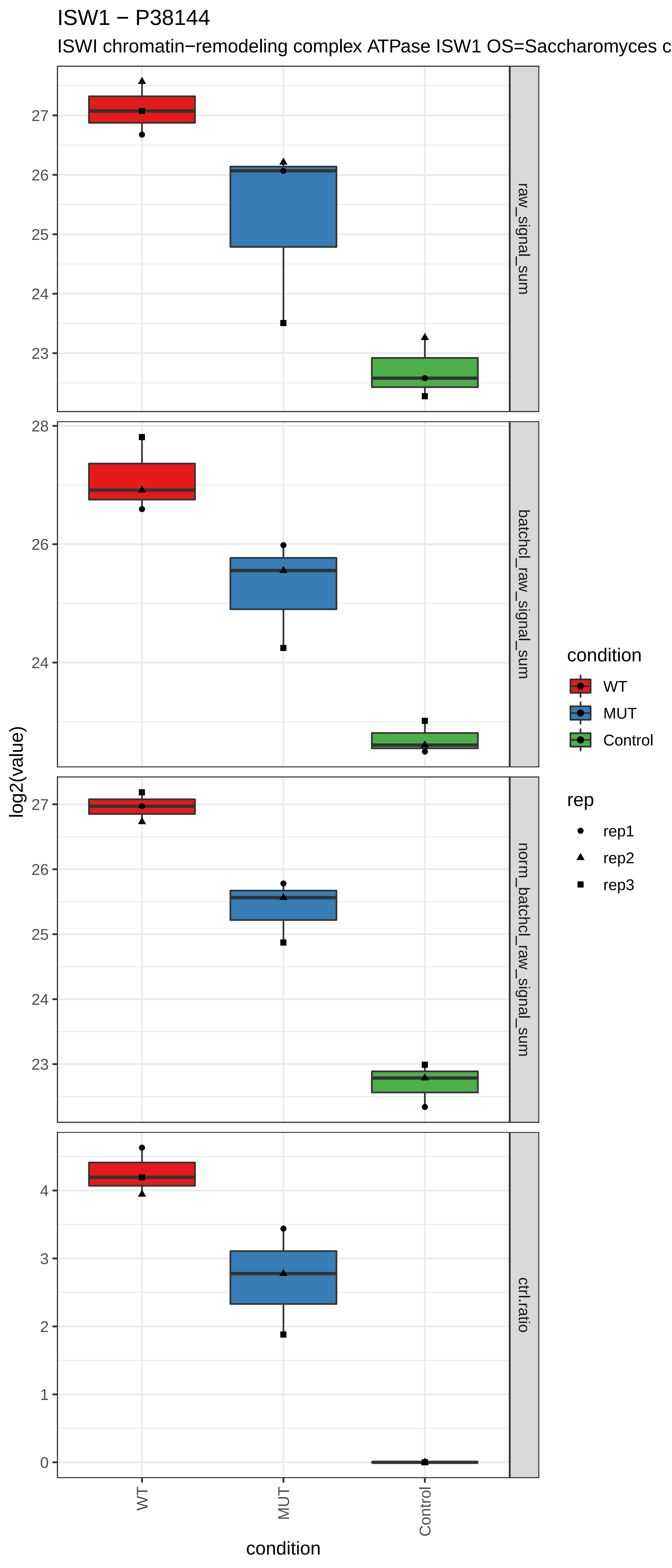

ISW1 − P38144
ISWI chromatin−remodeling complex ATPase ISW1 OS=Saccharomyces c
raw_signal_sum
batchcl_raw_signal_sum
norm_batchcl_raw_signal_sum
ctrl.ratio
log2(value)
condition
WT
MUT
Control
rep
rep1
rep2
rep3

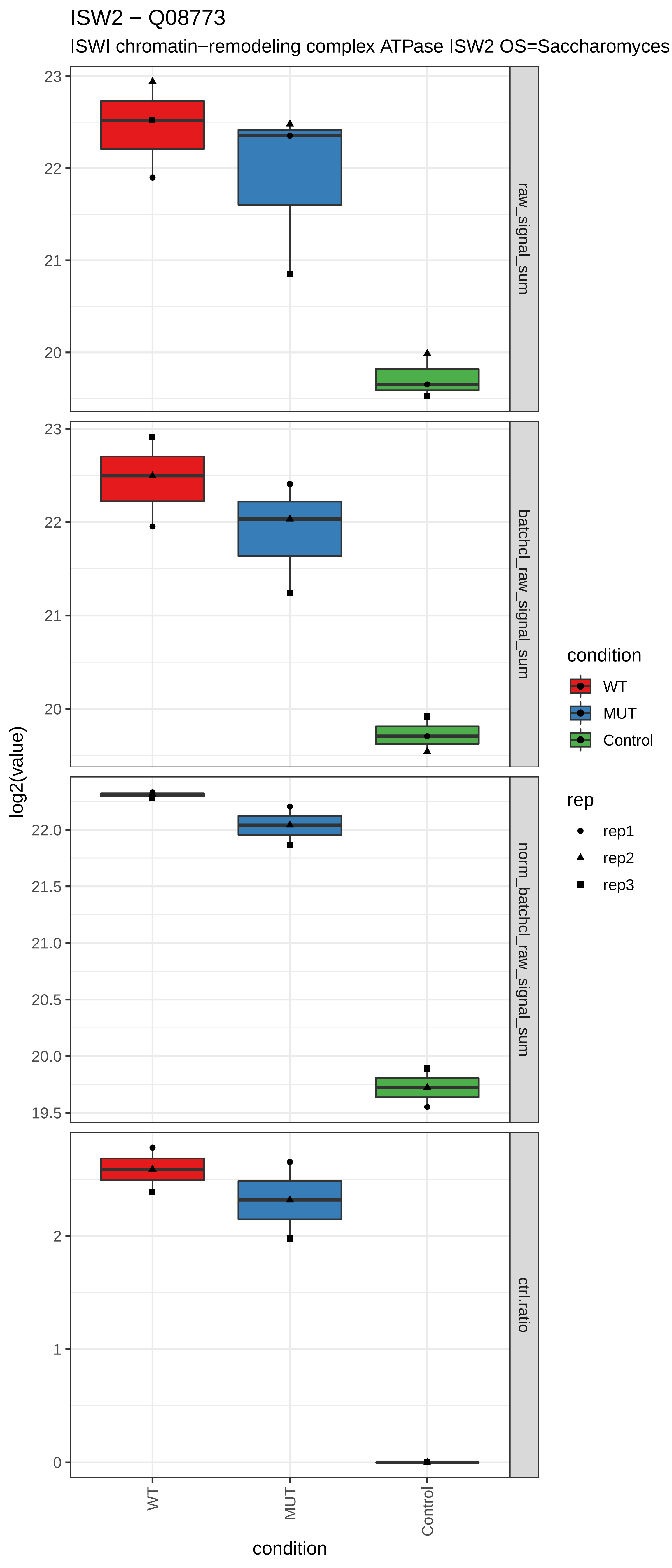
ISW2 − Q08773
ISWI chromatin−remodeling complex ATPase ISW2 OS=Saccharomyces
raw_signal_sum
batchcl_raw_signal_sum
norm_batchcl_raw_signal_sum
ctrl.ratio
log2(value)
condition
WT
MUT
Control
rep
rep1
rep2
rep3

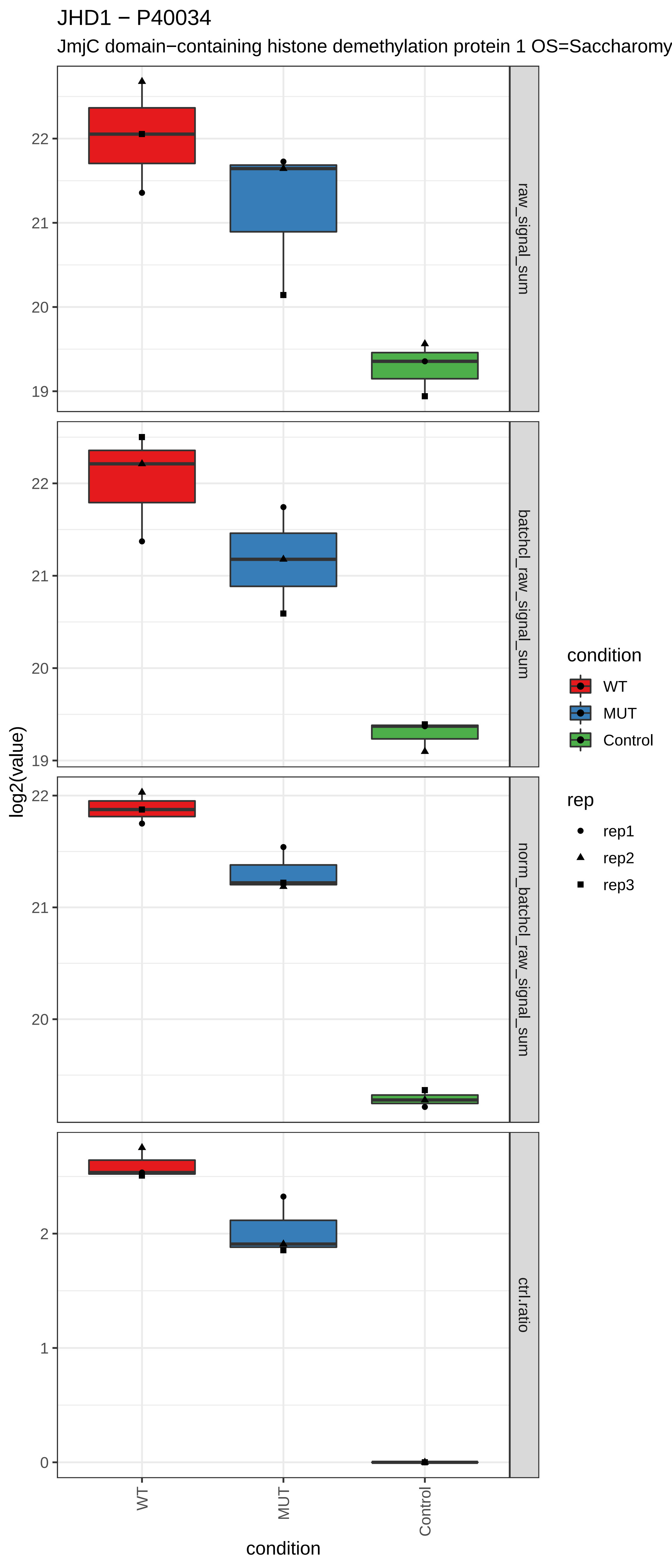

JHD1 − P40034
JmjC domain−containing histone demethylation protein 1 OS=Saccharomy
raw_signal_sum
batchcl_raw_signal_sum
norm_batchcl_raw_signal_sum
ctrl.ratio
log2(value)
condition
WT
MUT
Control
rep
rep1
rep2
rep3

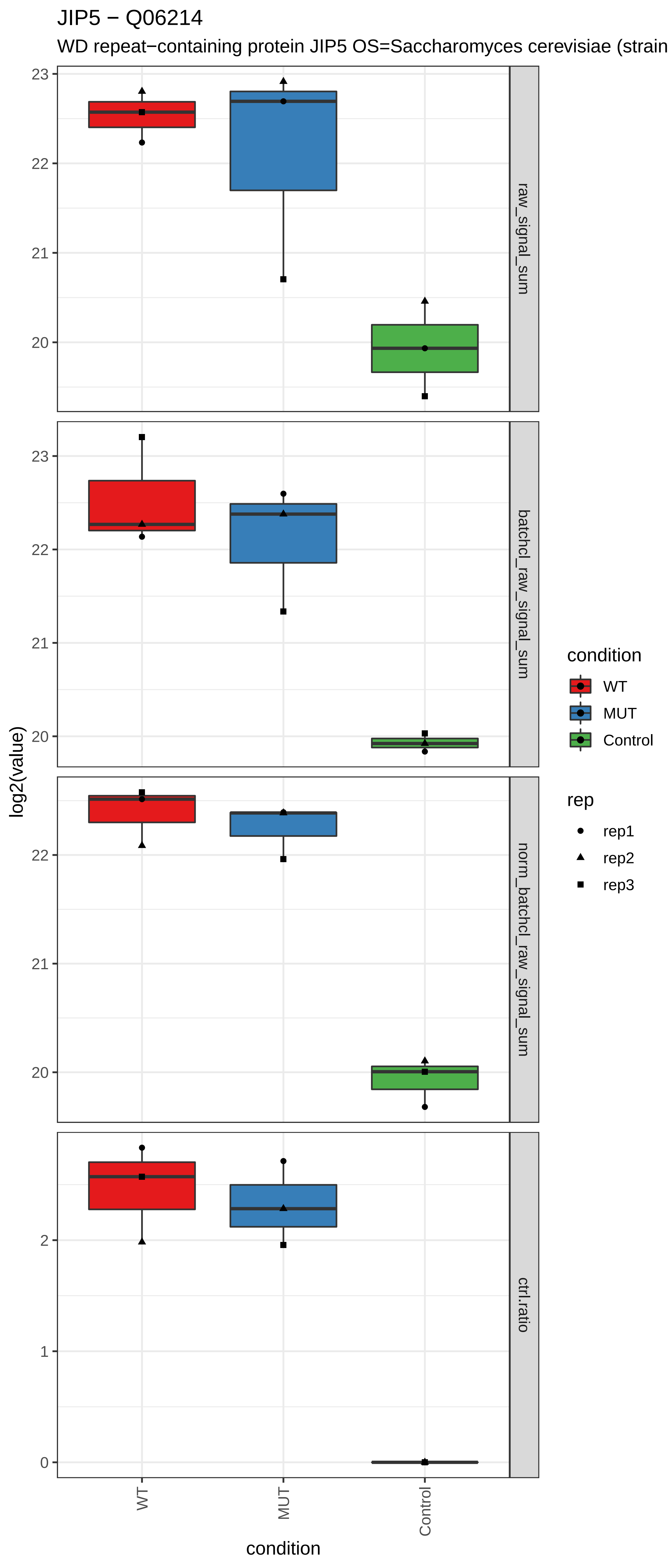
JIP5 − Q06214
WD repeat−containing protein JIP5 OS=Saccharomyces cerevisiae (strain
raw_signal_sum
batchcl_raw_signal_sum
norm_batchcl_raw_signal_sum
ctrl.ratio
log2(value)
condition
WT
MUT
Control
rep
rep1
rep2
rep3

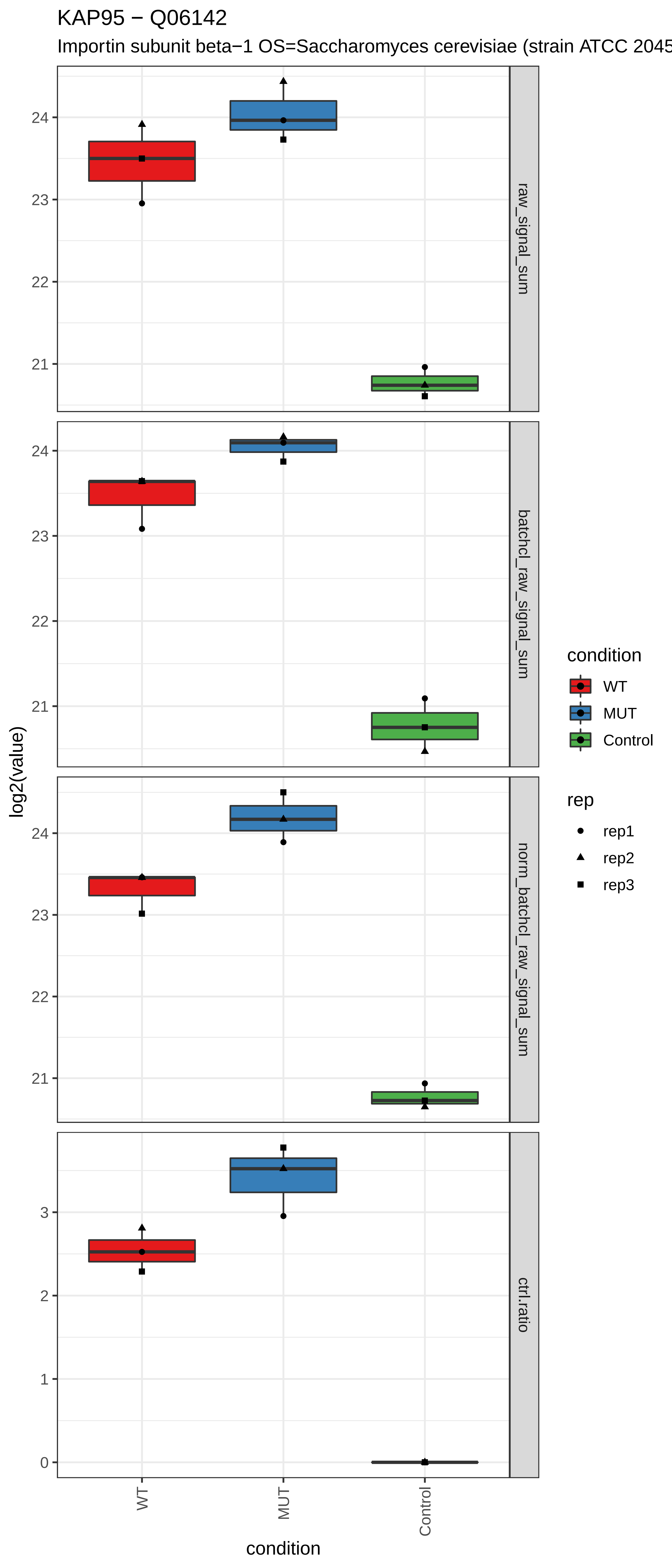

KAP95 − Q06142
Importin subunit beta−1 OS=Saccharomyces cerevisiae (strain ATCC 2045
raw_signal_sum
batchcl_raw_signal_sum
norm_batchcl_raw_signal_sum
ctrl.ratio
log2(value)
24
23
22
21
3
2
1
0
WT
MUT
Control
condition
condition
WT
MUT
Control
rep
rep1
rep2
rep3

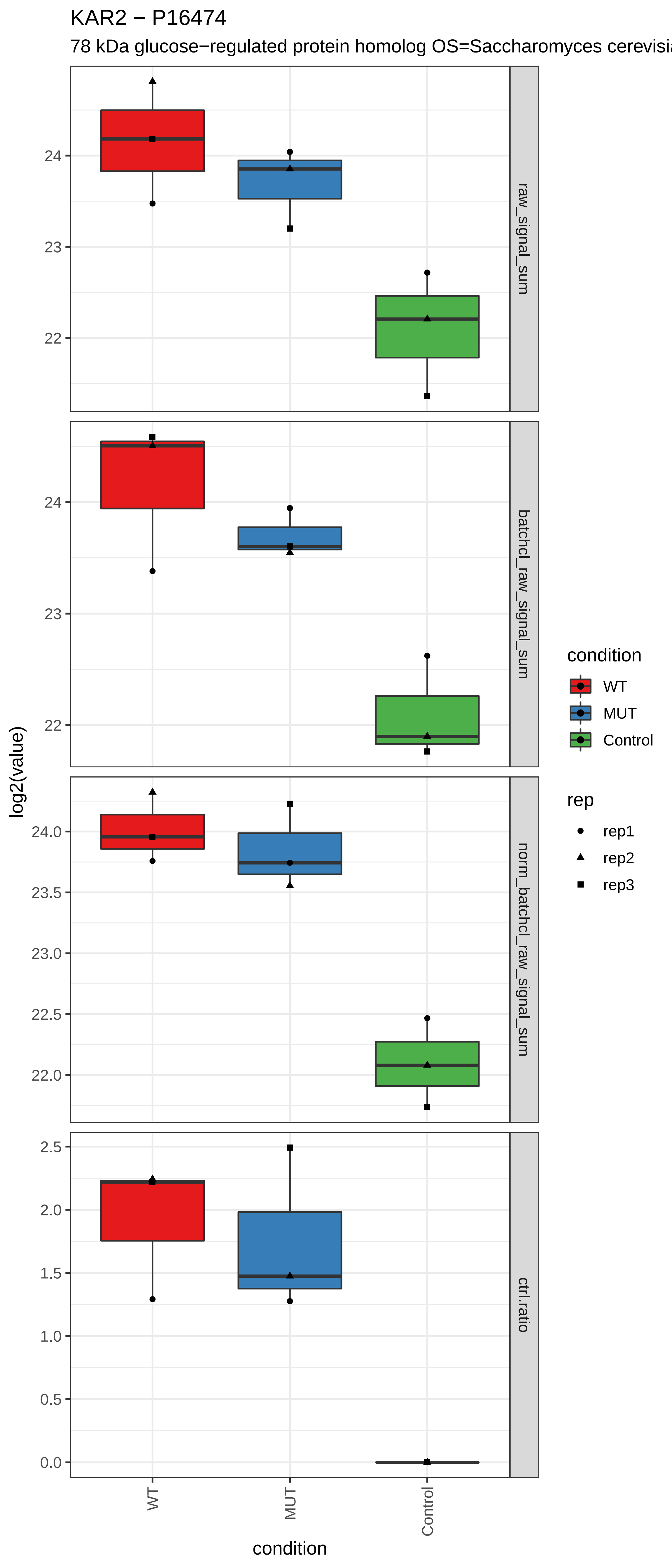
KAR2 − P16474
78 kDa glucose−regulated protein homolog OS=Saccharomyces cerevisia
raw_signal_sum
batchcl_raw_signal_sum
norm_batchcl_raw_signal_sum
ctrl.ratio
log2(value)
condition
WT
MUT
Control
rep
rep1
rep2
rep3

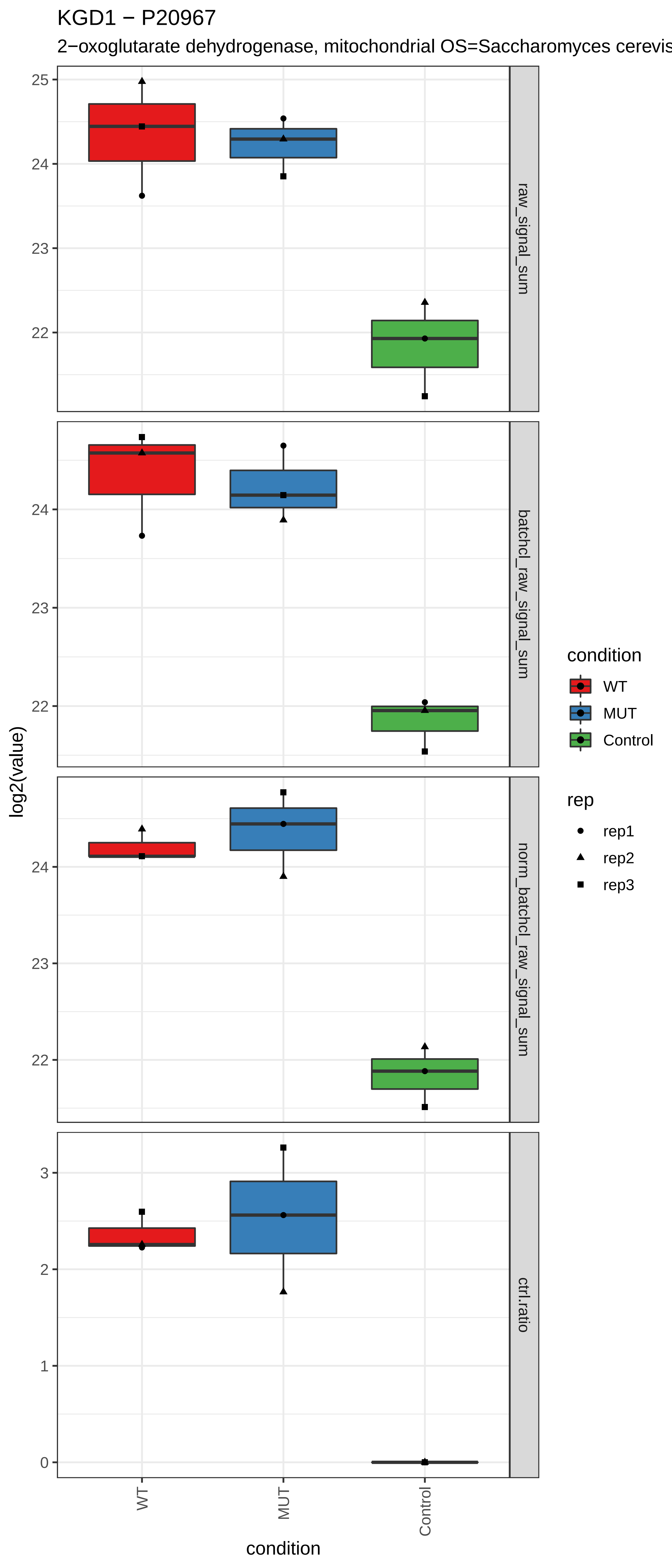

KGD1 − P20967
2−oxoglutarate dehydrogenase, mitochondrial OS=Saccharomyces cerevis
raw_signal_sum
batchcl_raw_signal_sum
norm_batchcl_raw_signal_sum
ctrl.ratio
log2(value)
25
24
23
22
3
2
1
0
WT
MUT
Control
condition
rep
rep1
rep2
rep3

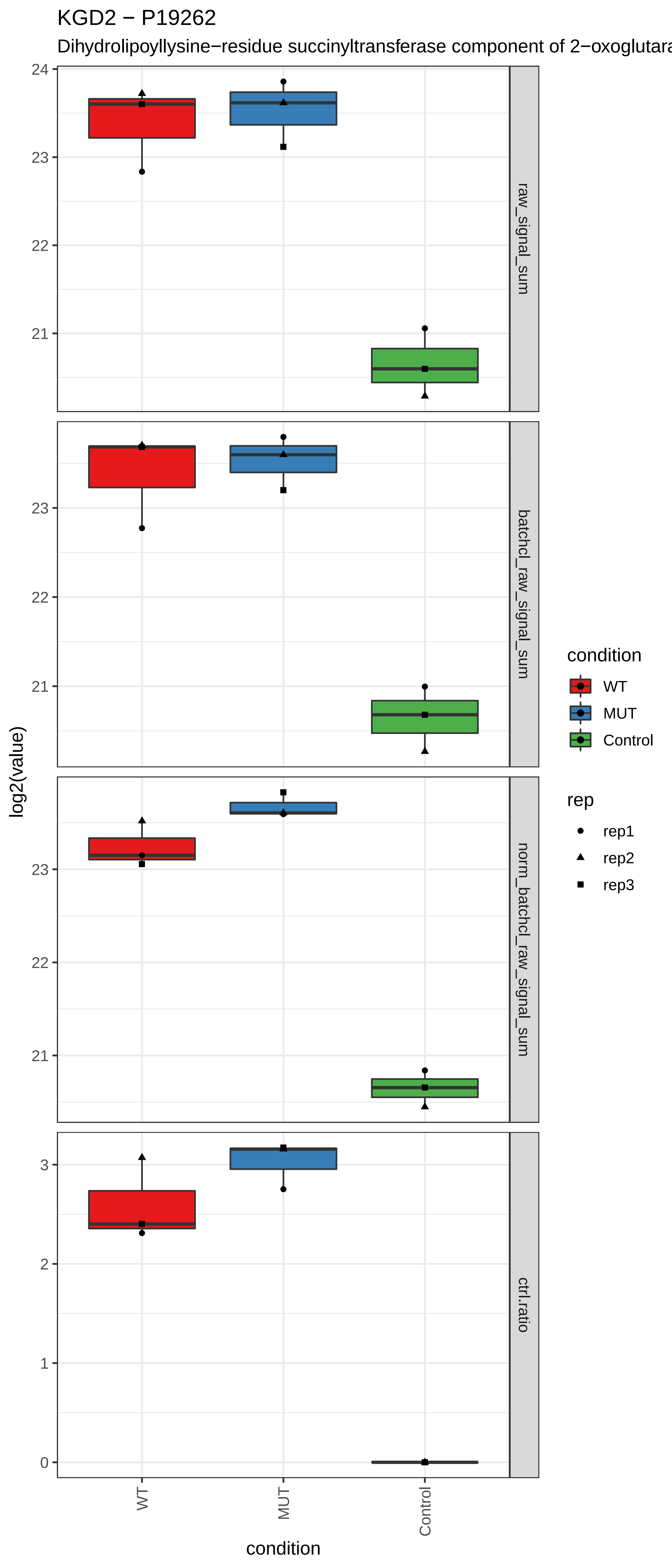

KGD2 − P19262
Dihydrolipoyllysine−residue succinyltransferase component of 2−oxoglutara
raw_signal_sum
batchcl_raw_signal_sum
norm_batchcl_raw_signal_sum
ctrl.ratio
log2(value)
condition
WT
MUT
Control
rep
rep1
rep2
rep3

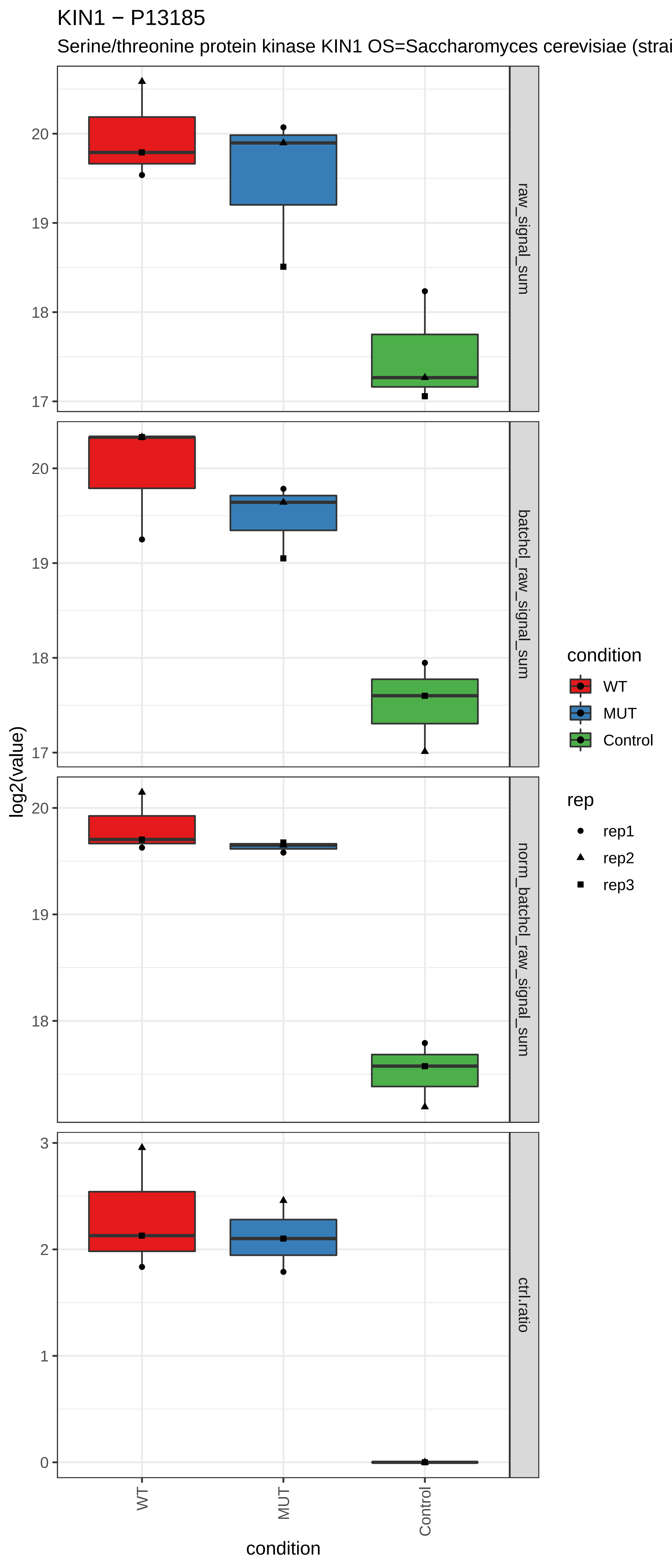

KIN1 − P13185
Serine/threonine protein kinase KIN1 OS=Saccharomyces cerevisiae (strai
raw_signal_sum
batchcl_raw_signal_sum
norm_batchcl_raw_signal_sum
ctrl.ratio
log2(value)
condition
WT
MUT
Control
rep
rep1
rep2
rep3

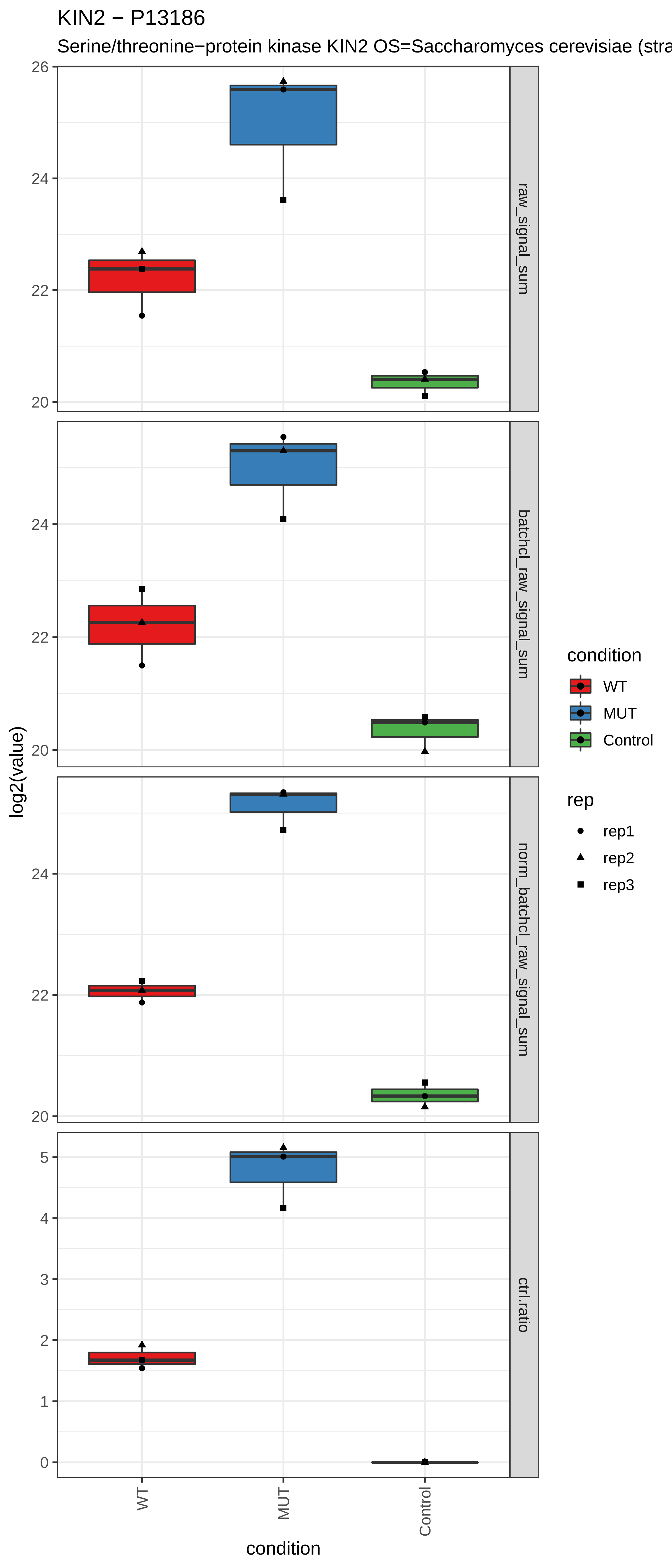
KIN2 − P13186
Serine/threonine−protein kinase KIN2 OS=Saccharomyces cerevisiae (stra
raw_signal_sum
batchcl_raw_signal_sum
norm_batchcl_raw_signal_sum
ctrl.ratio
log2(value)
condition
WT
MUT
Control
rep
rep1
rep2
rep3
26
24
22
20
5
4
3
2
1
0

KNS1 − P32350

Dual specificity protein kinase KNS1 OS=Saccharomyces cerevisiae (strain

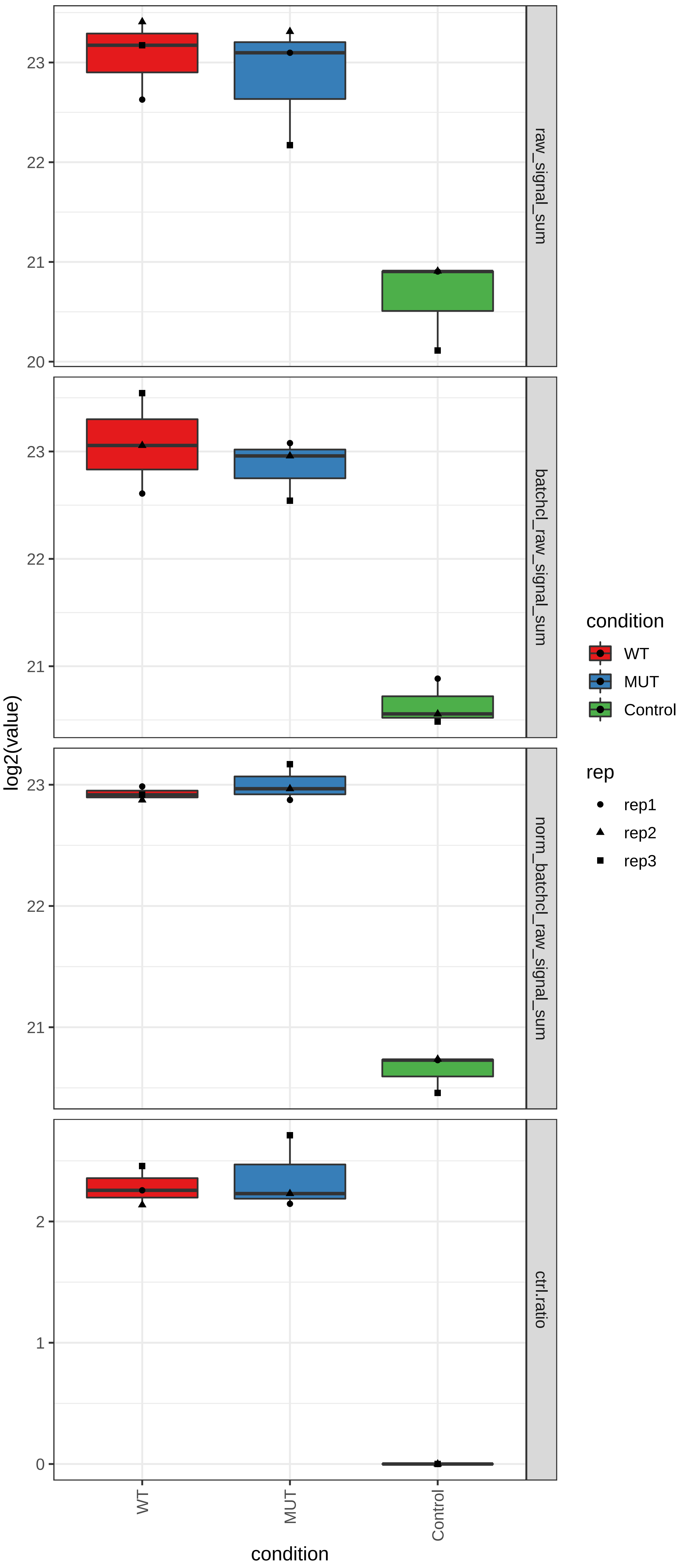

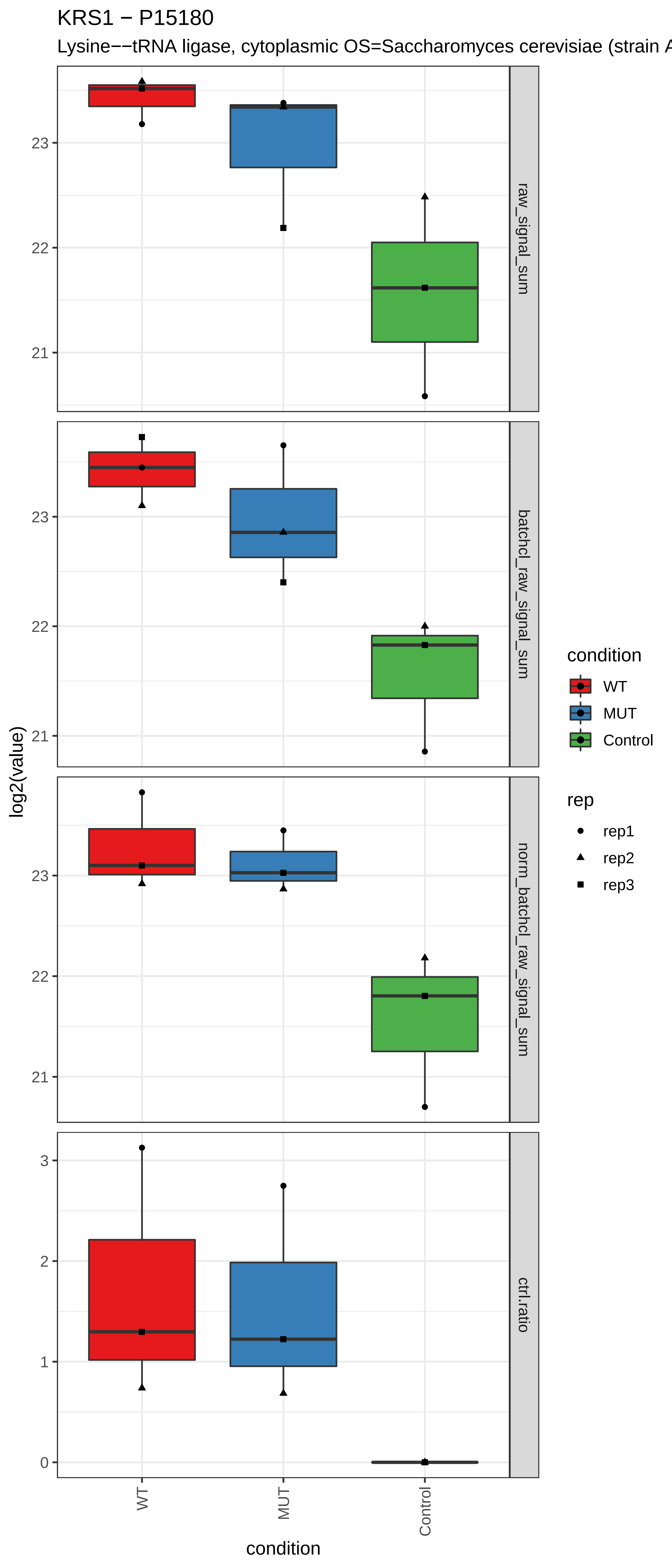

KRS1 − P15180
Lysine−−tRNA ligase, cytoplasmic OS=Saccharomyces cerevisiae (strain A
raw_signal_sum
batchcl_raw_signal_sum
norm_batchcl_raw_signal_sum
ctrl.ratio
log2(value)
condition
WT
MUT
Control
rep
rep1
rep2
rep3

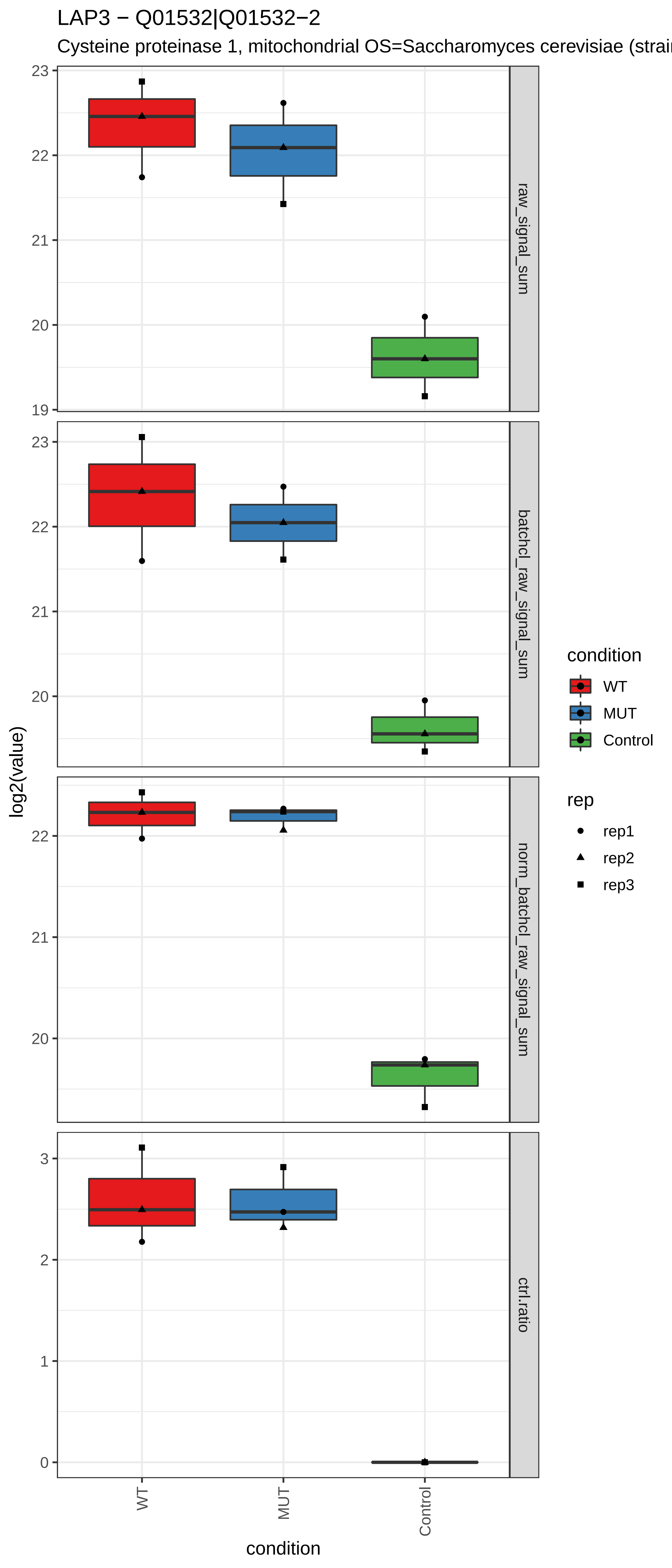

LAP3 − Q01532|Q01532−2
Cysteine proteinase 1, mitochondrial OS=Saccharomyces cerevisiae (strai
raw_signal_sum
batchcl_raw_signal_sum
norm_batchcl_raw_signal_sum
ctrl.ratio
log2(value)
condition
WT
MUT
Control
rep
rep1
rep2
rep3

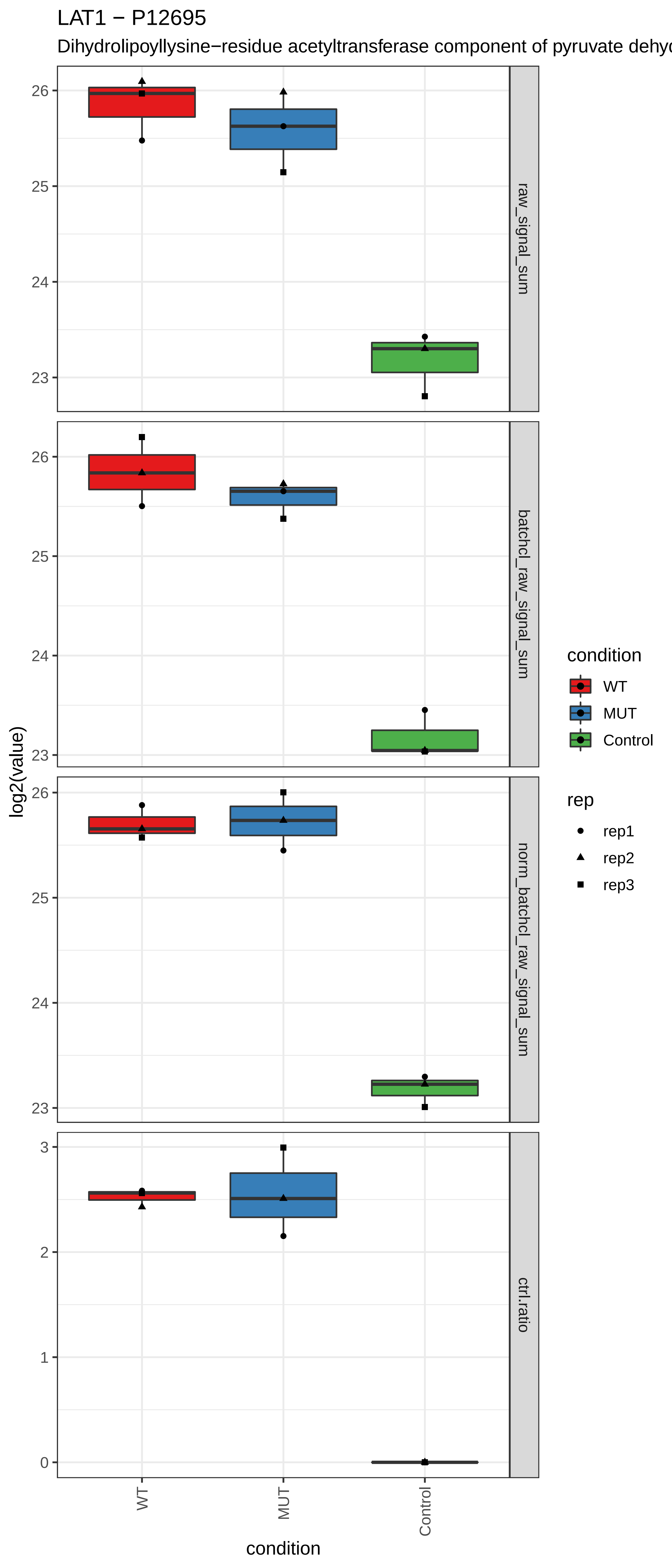
LAT1 − P12695
Dihydrolipoyllysine−residue acetyltransferase component of pyruvate dehyd
raw_signal_sum
batchcl_raw_signal_sum
norm_batchcl_raw_signal_sum
ctrl.ratio
log2(value)
condition
WT
MUT
Control
rep
rep1
rep2
rep3
26
25
24
23
3
2
1
0

LEU4 − P06208|P06208−2

2−isopropylmalate synthase OS=Saccharomyces cerevisiae (strain ATCC 2

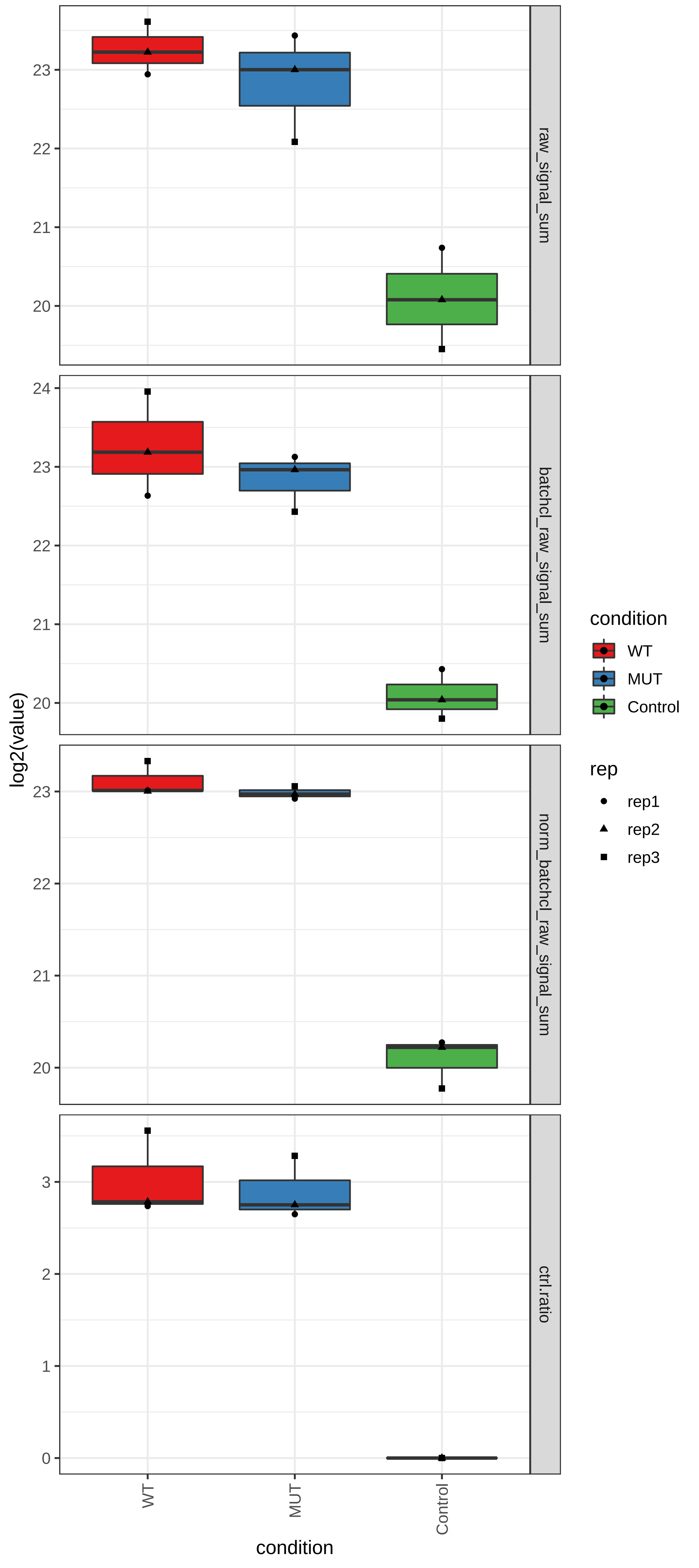

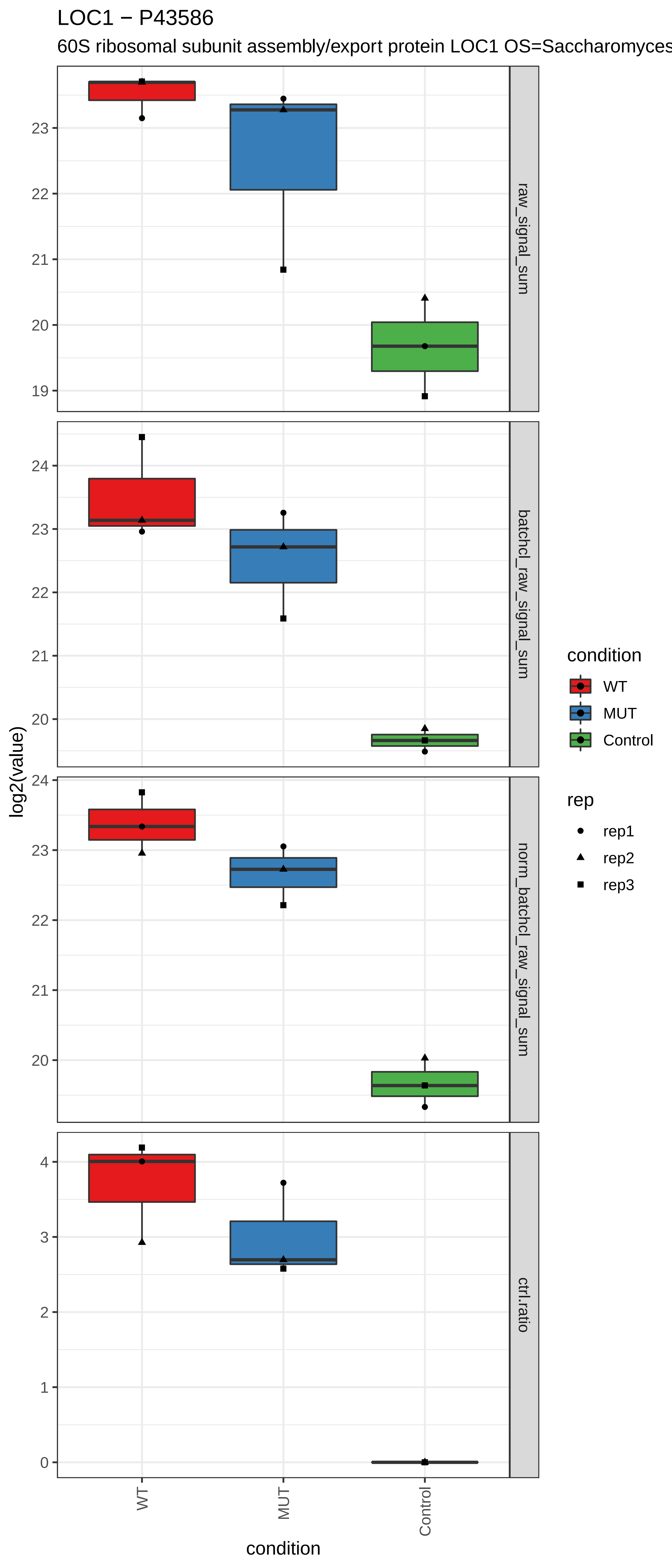

LOC1 − P43586
60S ribosomal subunit assembly/export protein LOC1 OS=Saccharomyces
raw_signal_sum
batchcl_raw_signal_sum
norm_batchcl_raw_signal_sum
ctrl.ratio
log2(value)
condition
WT
MUT
Control
rep
rep1
rep2
rep3

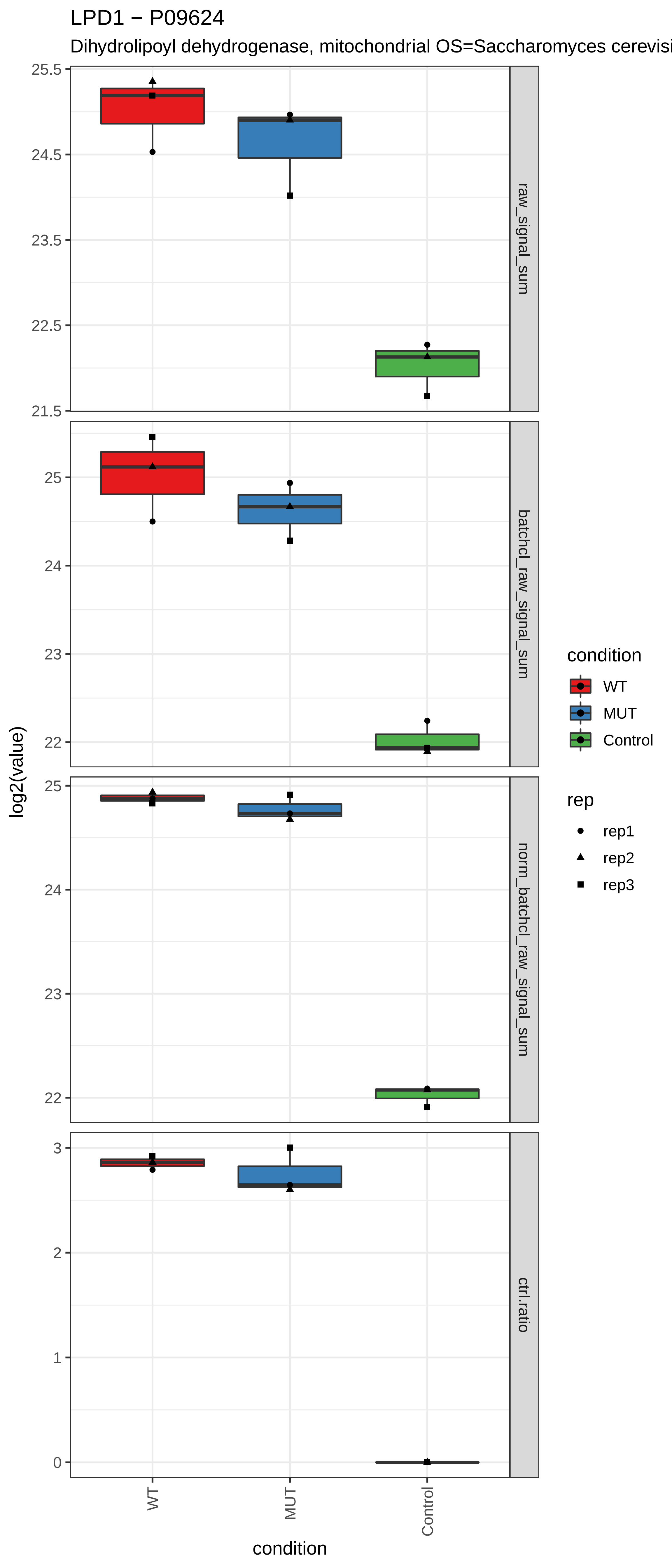
LPD1 − P09624
Dihydrolipoyl dehydrogenase, mitochondrial OS=Saccharomyces cerevisi
raw_signal_sum
batchcl_raw_signal_sum
norm_batchcl_raw_signal_sum
ctrl.ratio
log2(value)
condition
WT
MUT
Control
rep
rep1
rep2
rep3
25.5
24.5
23.5
22.5
21.5
25
24
23
22
3
2
1
0

LPX1 − Q12405

Peroxisomal membrane protein LPX1 OS=Saccharomyces cerevisiae (stra

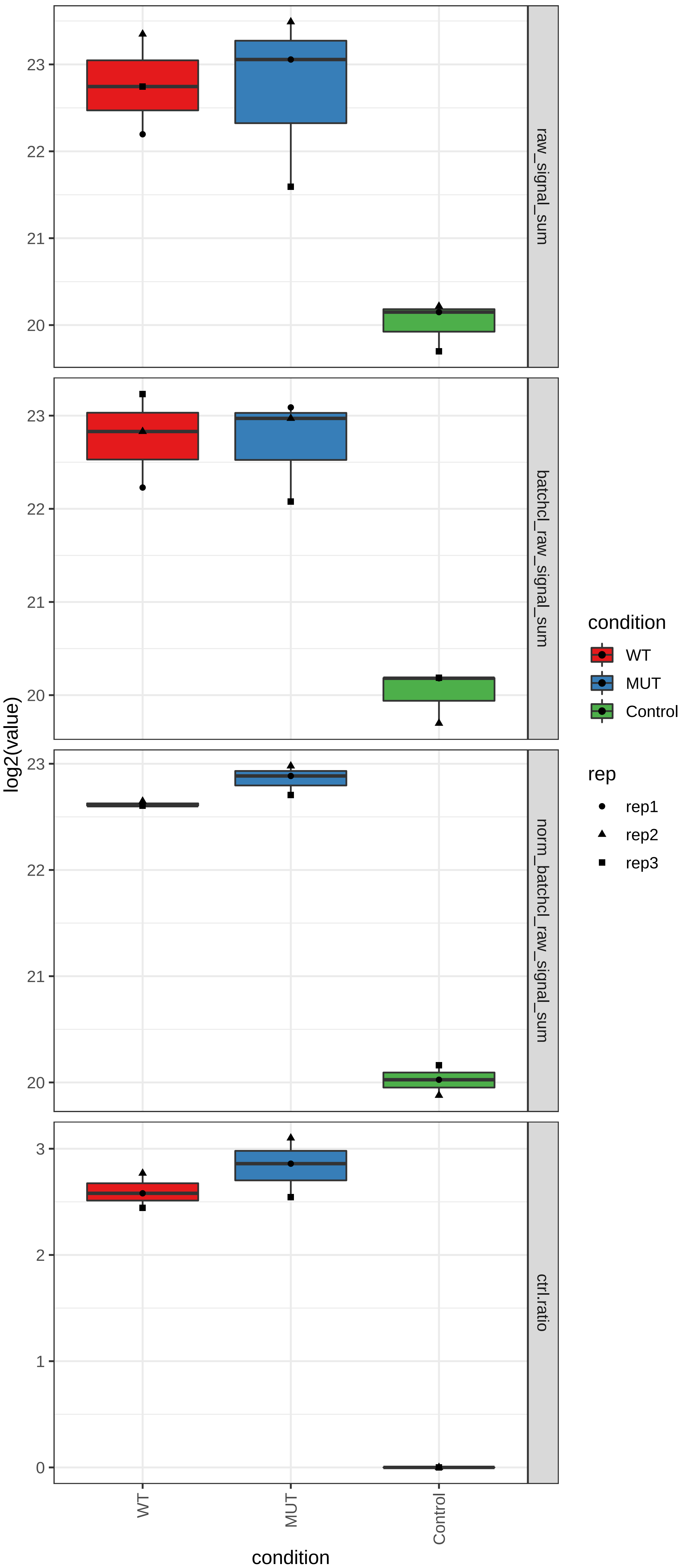

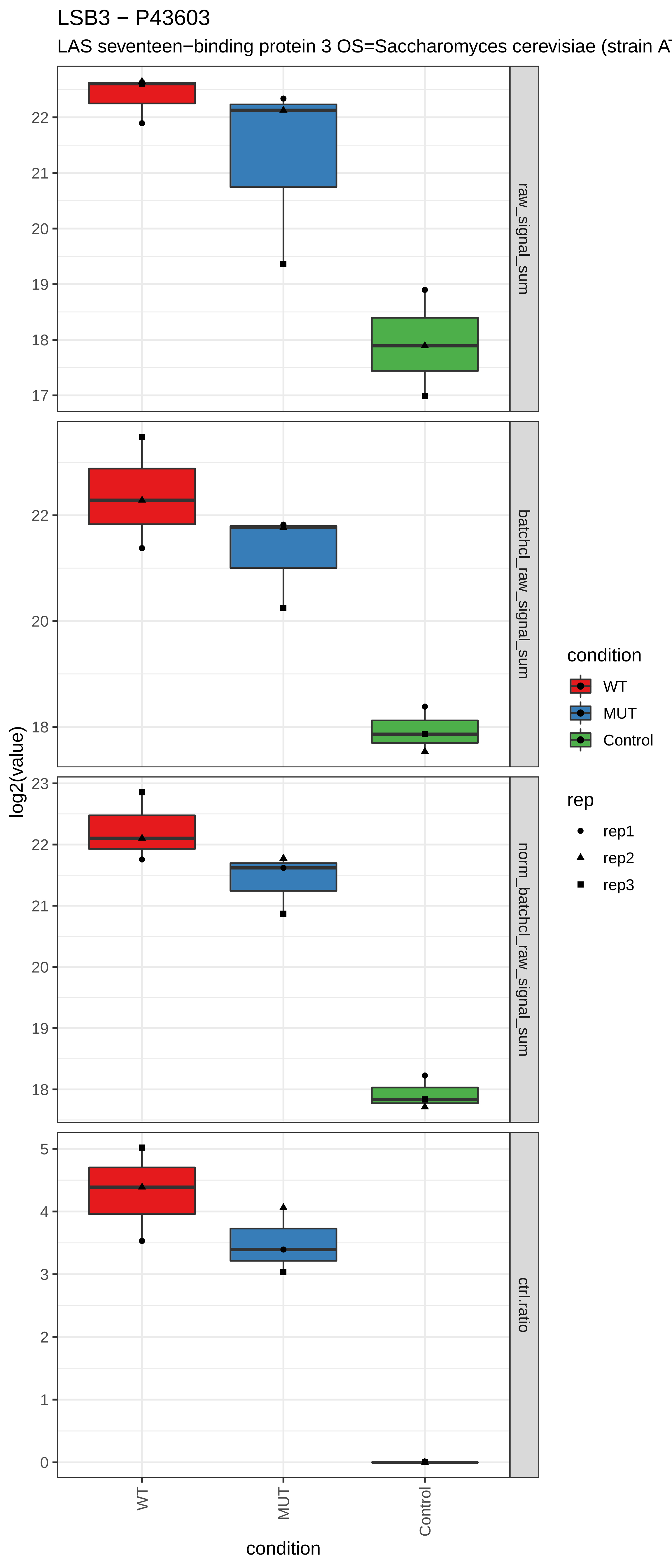

LSB3 − P43603
LAS seventeen−binding protein 3 OS=Saccharomyces cerevisiae (strain A
raw_signal_sum
batchcl_raw_signal_sum
norm_batchcl_raw_signal_sum
ctrl.ratio
log2(value)
condition
WT
MUT
Control
rep
rep1
rep2
rep3

LSC1 − P53598

Succinyl−CoA ligase [ADP−forming] subunit alpha, mitochondrial OS=Sa

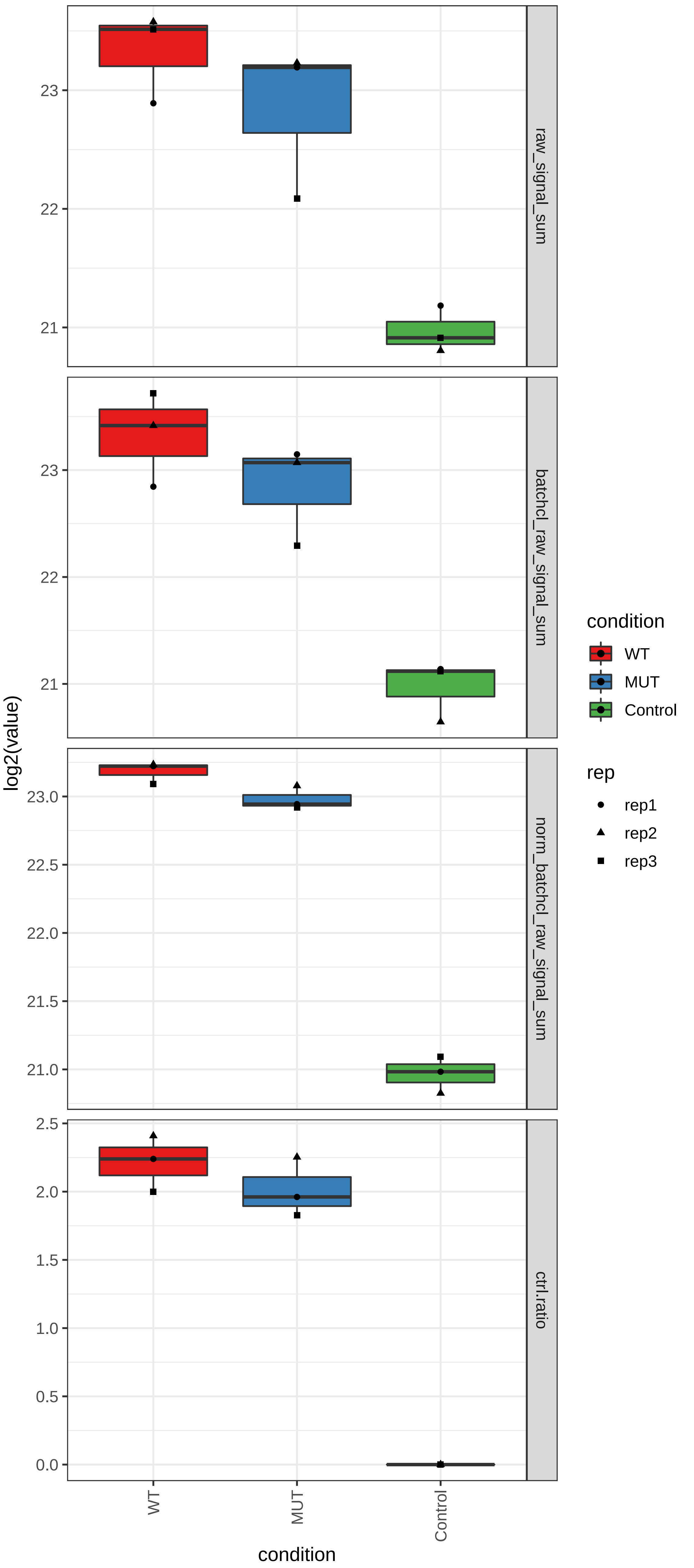

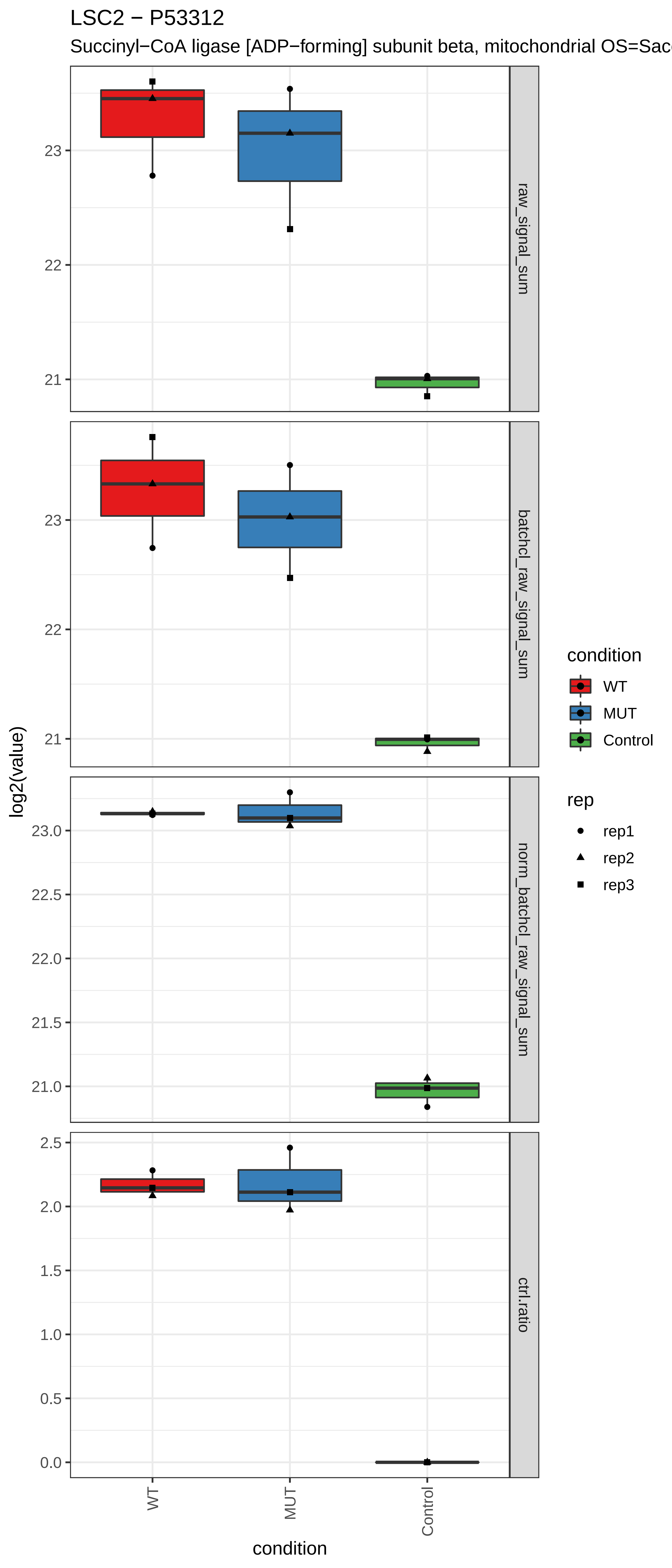

LSC2 − P53312
Succinyl−CoA ligase [ADP−forming] subunit beta, mitochondrial OS=Sac
raw_signal_sum
batchcl_raw_signal_sum
norm_batchcl_raw_signal_sum
ctrl.ratio
log2(value)
condition
WT
MUT
Control
rep
rep1
rep2
rep3
23
22
21
23.0
22.5
22.0
21.5
21.0
2.5
2.0
1.5
1.0
0.5
0.0

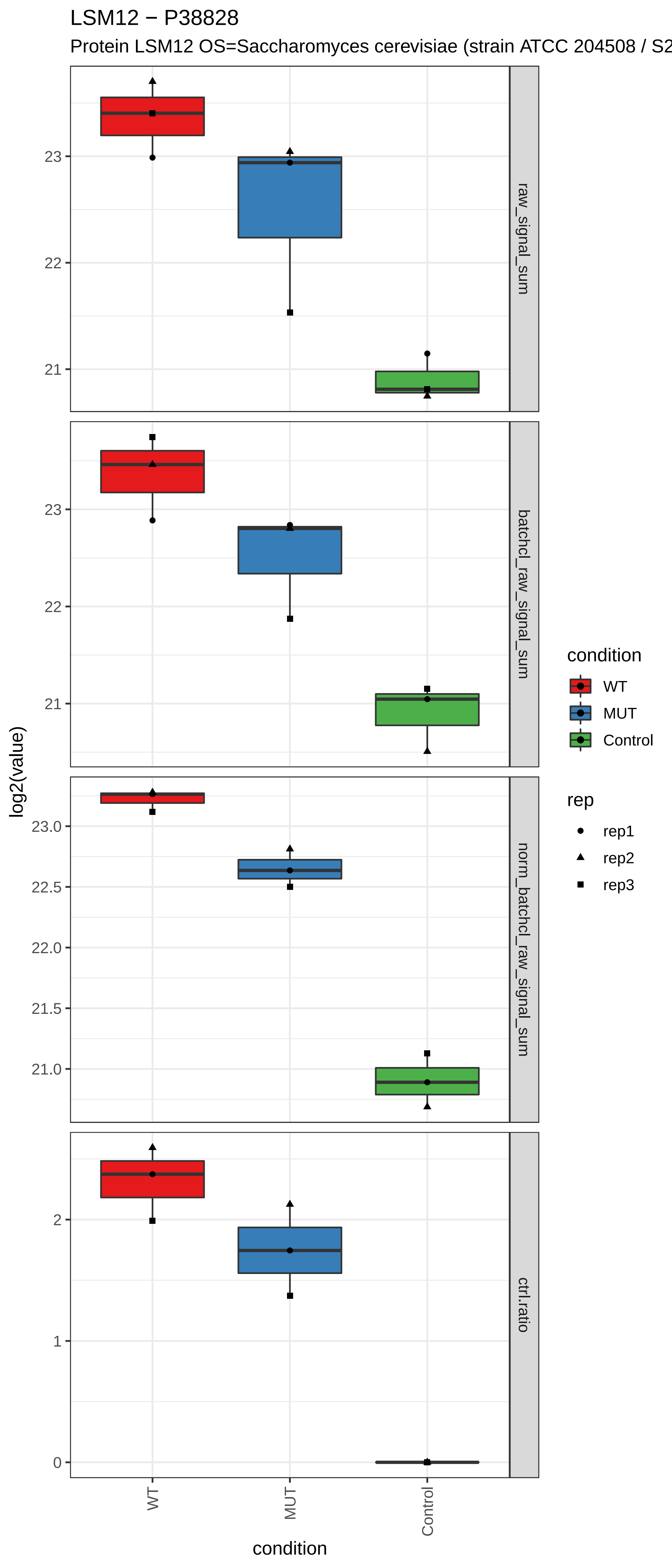
LSM12 − P38828
Protein LSM12 OS=Saccharomyces cerevisiae (strain ATCC 204508 / S2
raw_signal_sum
batchcl_raw_signal_sum
norm_batchcl_raw_signal_sum
ctrl.ratio
log2(value)
condition
WT
MUT
Control
rep
rep1
rep2
rep3
23
22
21
23.0
22.5
22.0
21.5
21.0
2
1
0

LSP1 − Q12230

Sphingolipid long chain base−responsive protein LSP1 OS=Saccharomyc

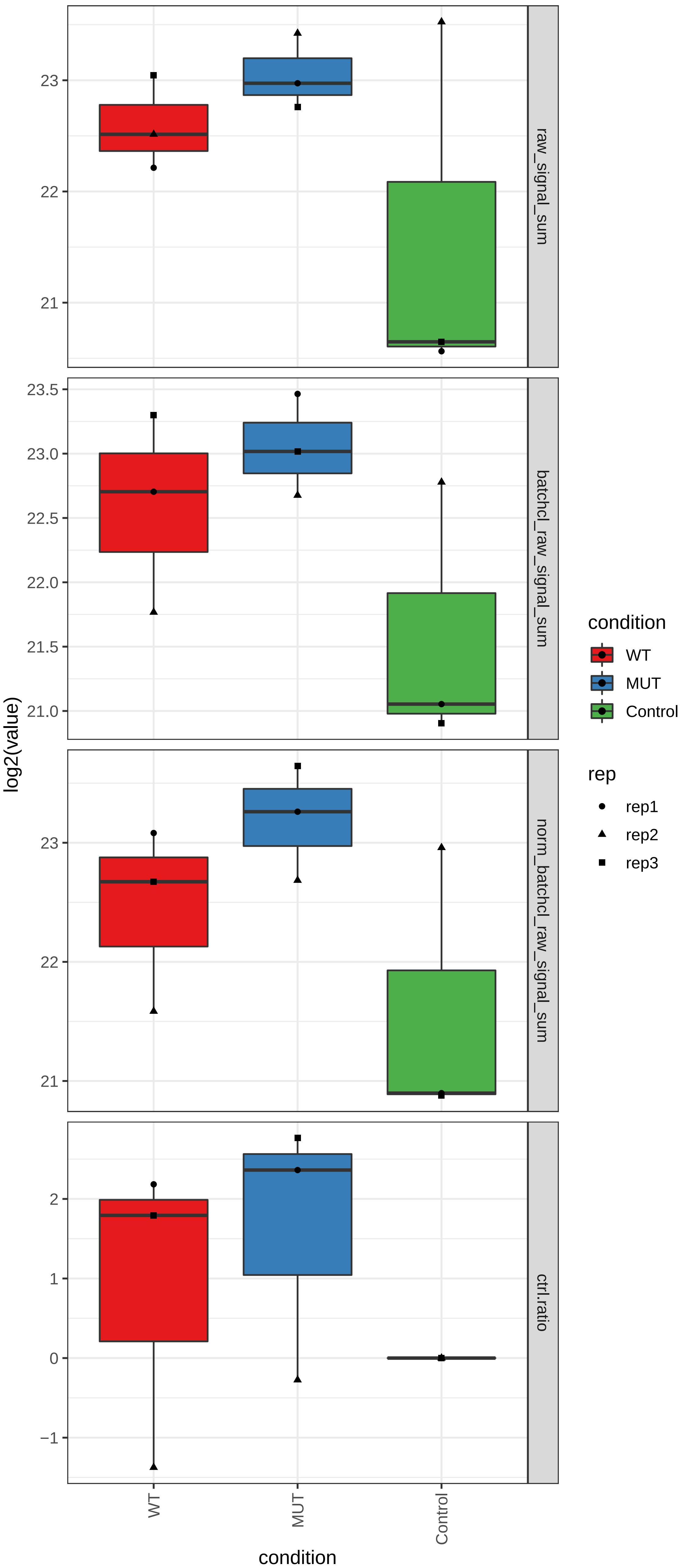

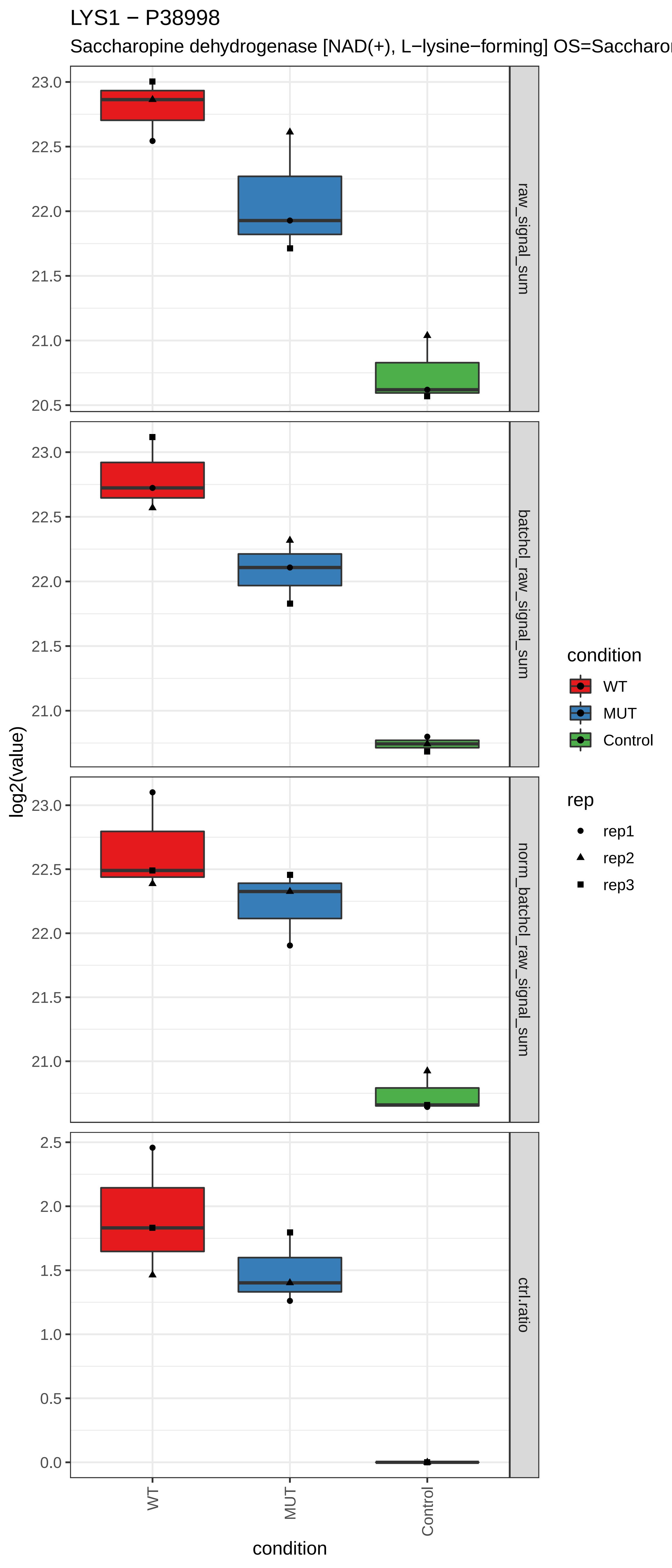

LYS1 − P38998
Saccharopine dehydrogenase [NAD(+), L−lysine−forming] OS=Saccharor
raw_signal_sum
batchcl_raw_signal_sum
norm_batchcl_raw_signal_sum
ctrl.ratio
log2(value)
condition
WT
MUT
Control
rep
rep1
rep2
rep3

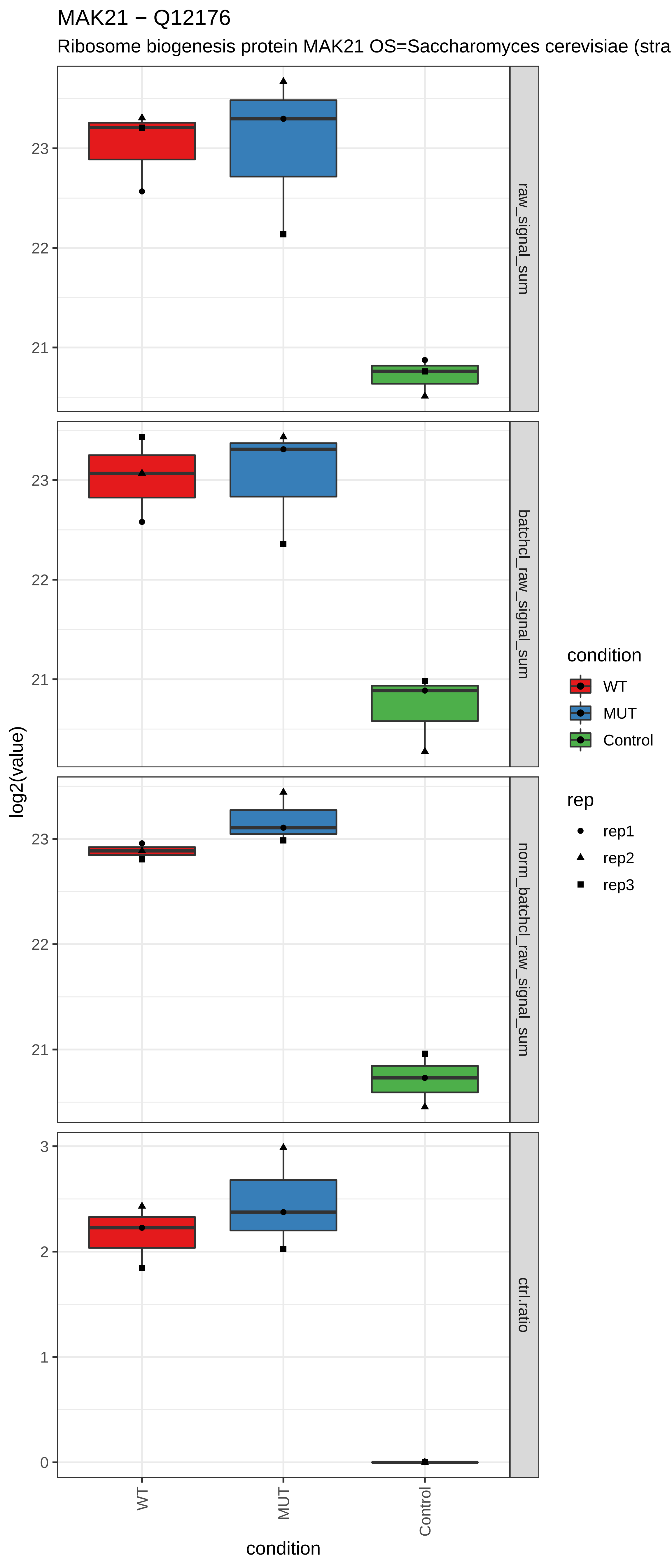
MAK21 − Q12176
Ribosome biogenesis protein MAK21 OS=Saccharomyces cerevisiae (stra
raw_signal_sum
batchcl_raw_signal_sum
norm_batchcl_raw_signal_sum
ctrl.ratio
log2(value)
condition
WT
MUT
Control
rep
rep1
rep2
rep3
23
22
21
3
2
1
0

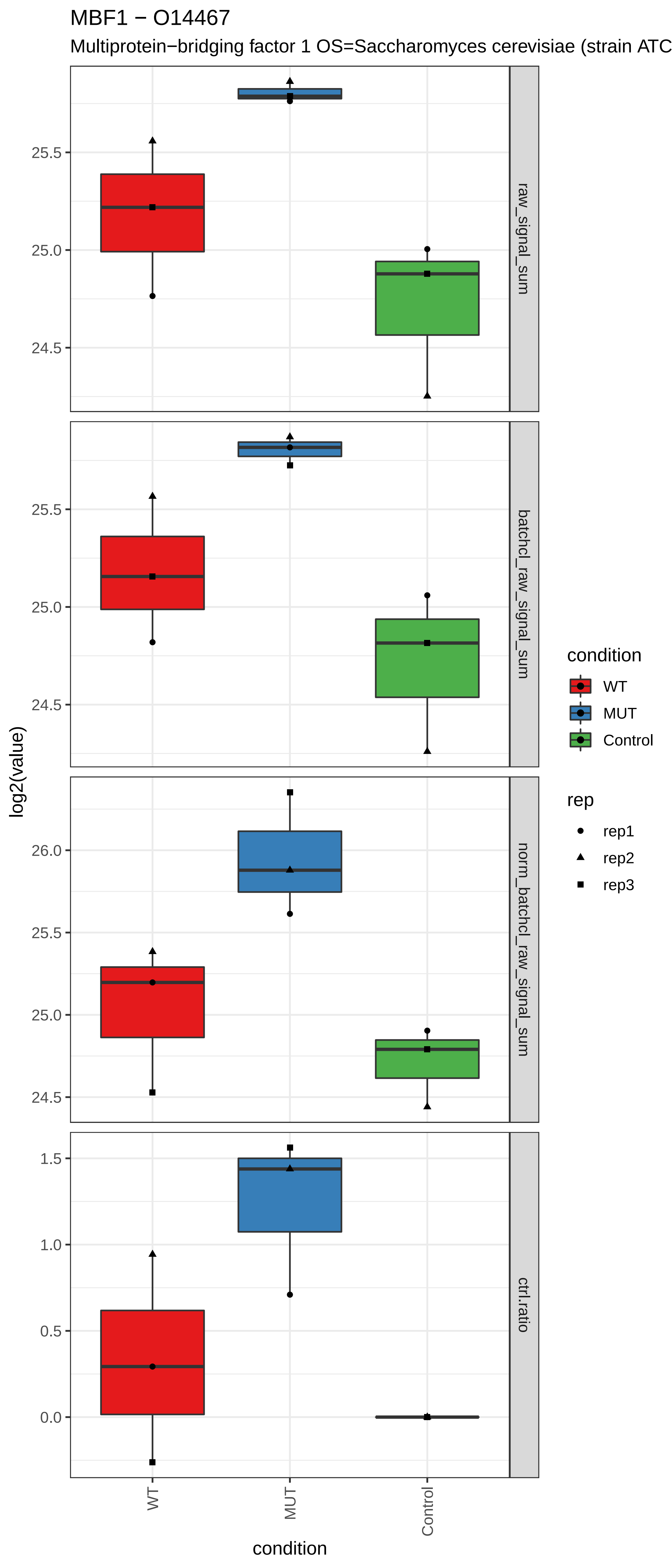
MBF1 − O14467
Multiprotein−bridging factor 1 OS=Saccharomyces cerevisiae (strain ATC
raw_signal_sum
batchcl_raw_signal_sum
norm_batchcl_raw_signal_sum
ctrl.ratio
25.5
25.0
24.5
26.0
1.5
1.0
0.5
0.0
log2(value)
WT
MUT
Control
condition
rep
rep1
rep2
rep3

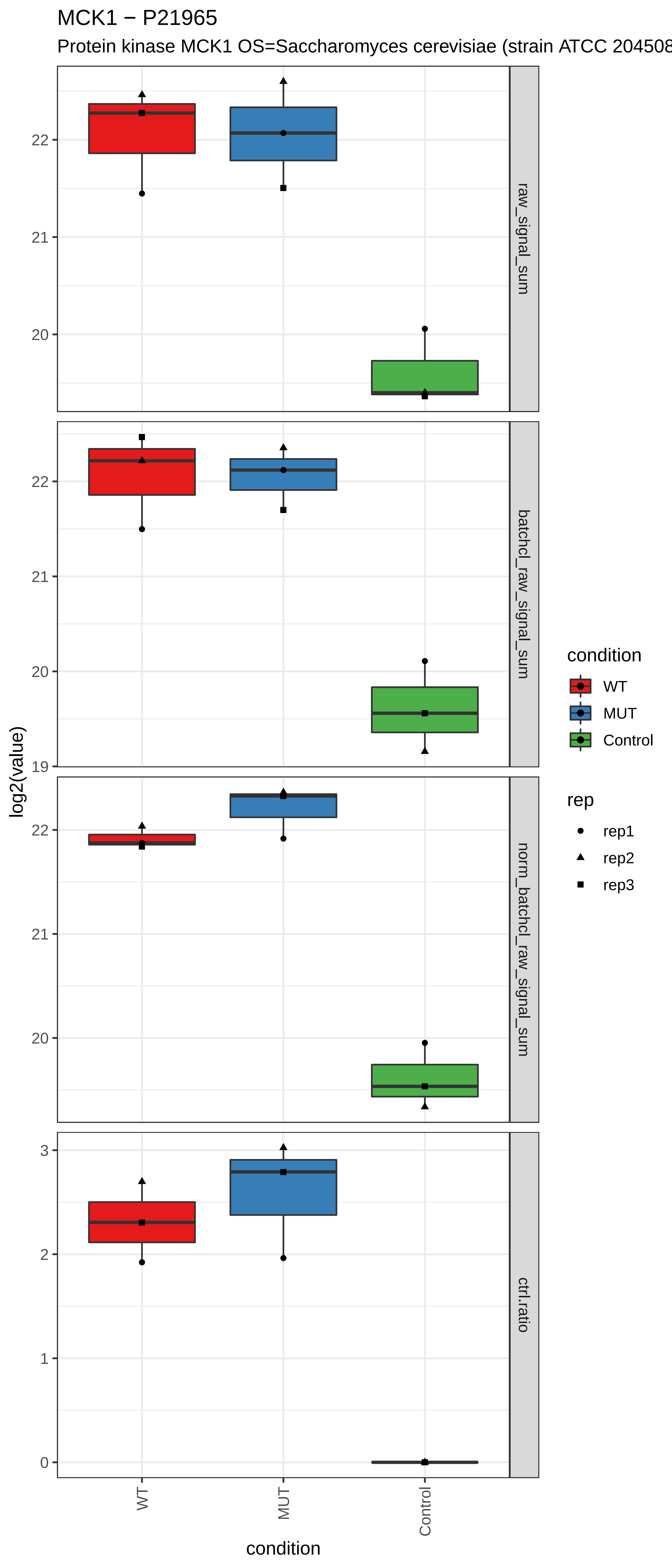
MCK1 − P21965
Protein kinase MCK1 OS=Saccharomyces cerevisiae (strain ATCC 204508
raw_signal_sum
batchcl_raw_signal_sum
norm_batchcl_raw_signal_sum
ctrl.ratio
log2(value)
condition
WT
MUT
Control
rep
rep1
rep2
rep3
22
21
20
19
3
2
1
0

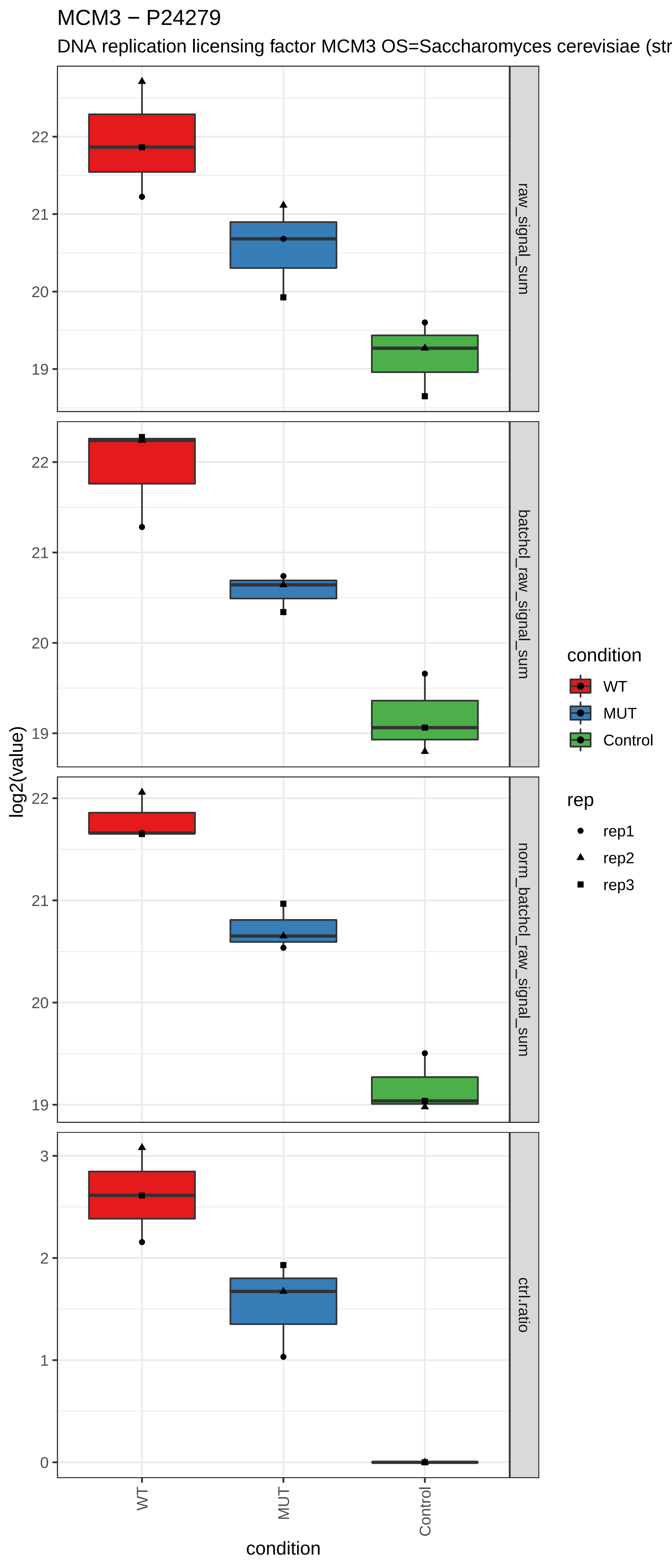

MCM3 − P24279
DNA replication licensing factor MCM3 OS=Saccharomyces cerevisiae (str
raw_signal_sum
batchcl_raw_signal_sum
norm_batchcl_raw_signal_sum
ctrl.ratio
log2(value)
condition
WT
MUT
Control
rep
rep1
rep2
rep3
22
21
20
19
3
2
1
0

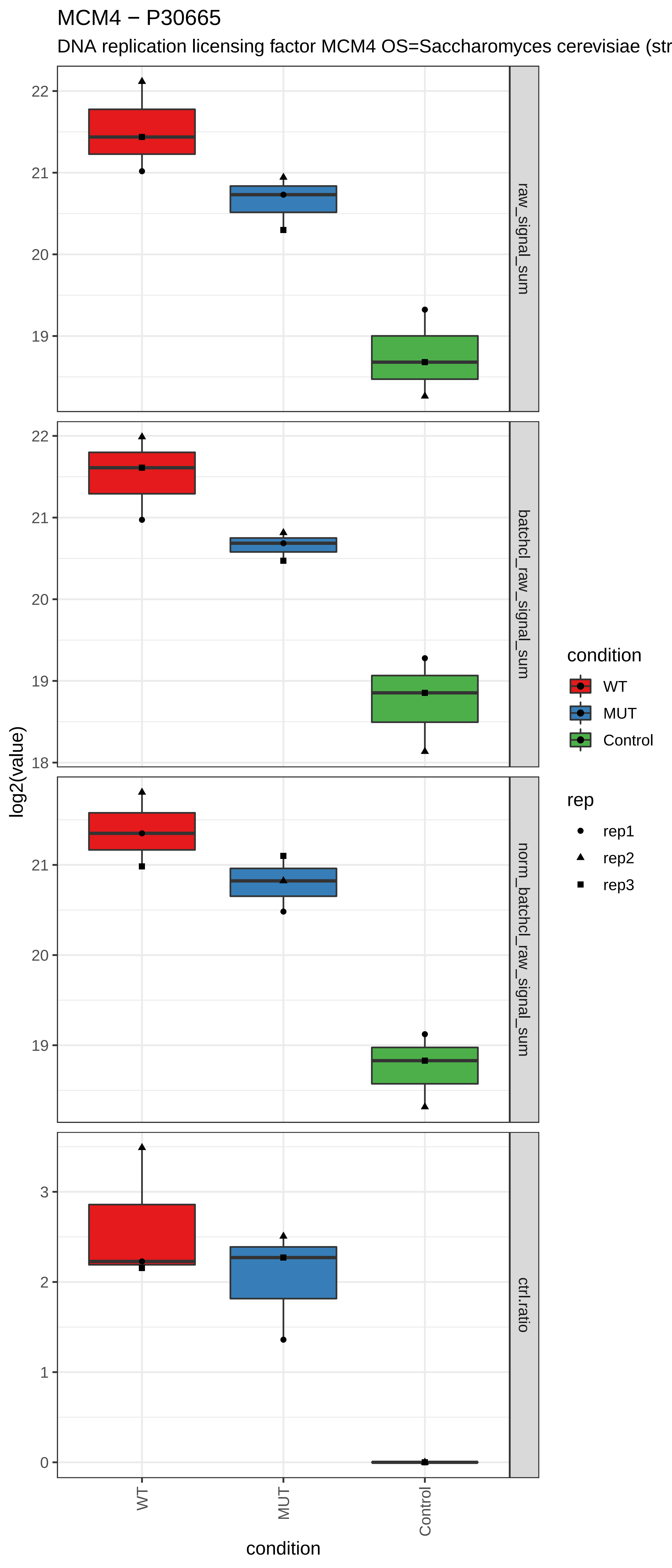
MCM4 − P30665
DNA replication licensing factor MCM4 OS=Saccharomyces cerevisiae (str
raw_signal_sum
batchcl_raw_signal_sum
norm_batchcl_raw_signal_sum
ctrl.ratio
log2(value)
condition
WT
MUT
Control
rep
rep1
rep2
rep3
22
21
20
19
18
3
2
1
0

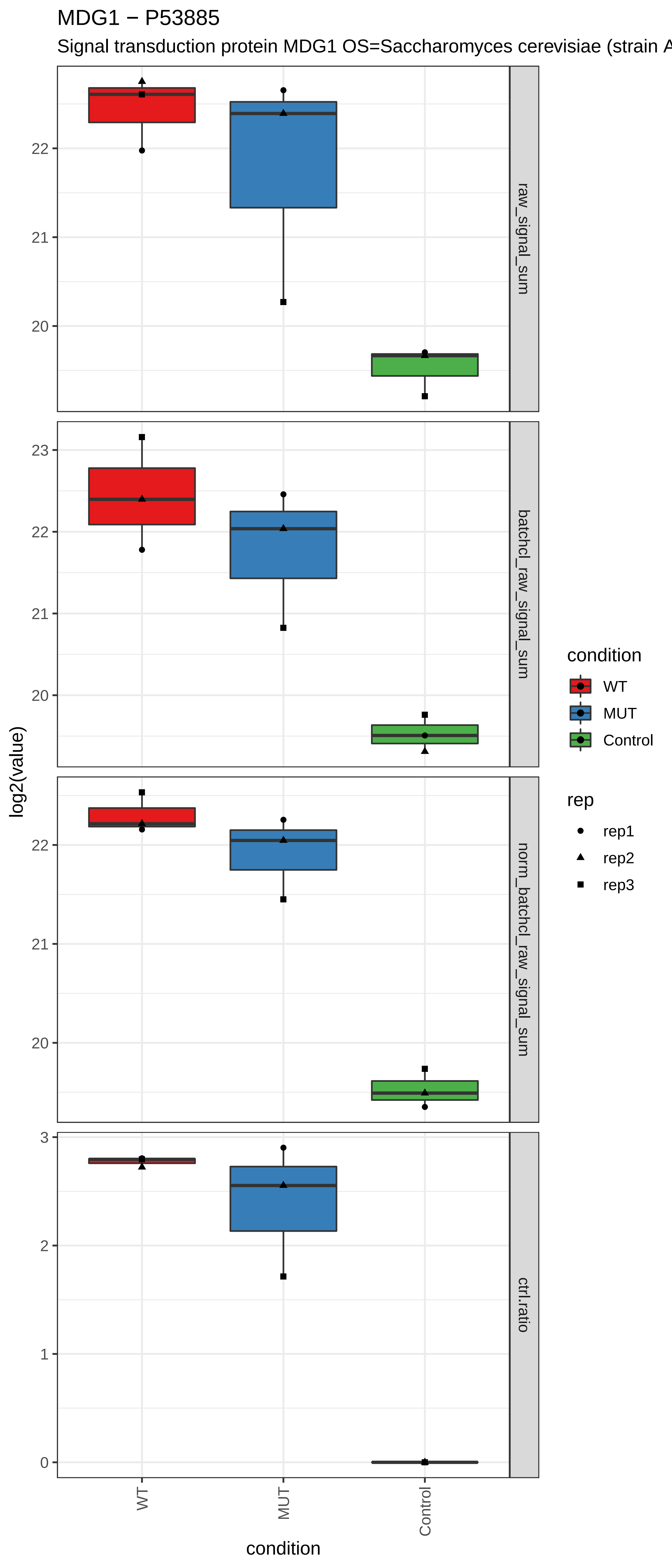

MDG1 − P53885
Signal transduction protein MDG1 OS=Saccharomyces cerevisiae (strain A
log2(value)
raw_signal_sum
batchcl_raw_signal_sum
norm_batchcl_raw_signal_sum
ctrl.ratio
condition
WT
MUT
Control
rep
rep1
rep2
rep3

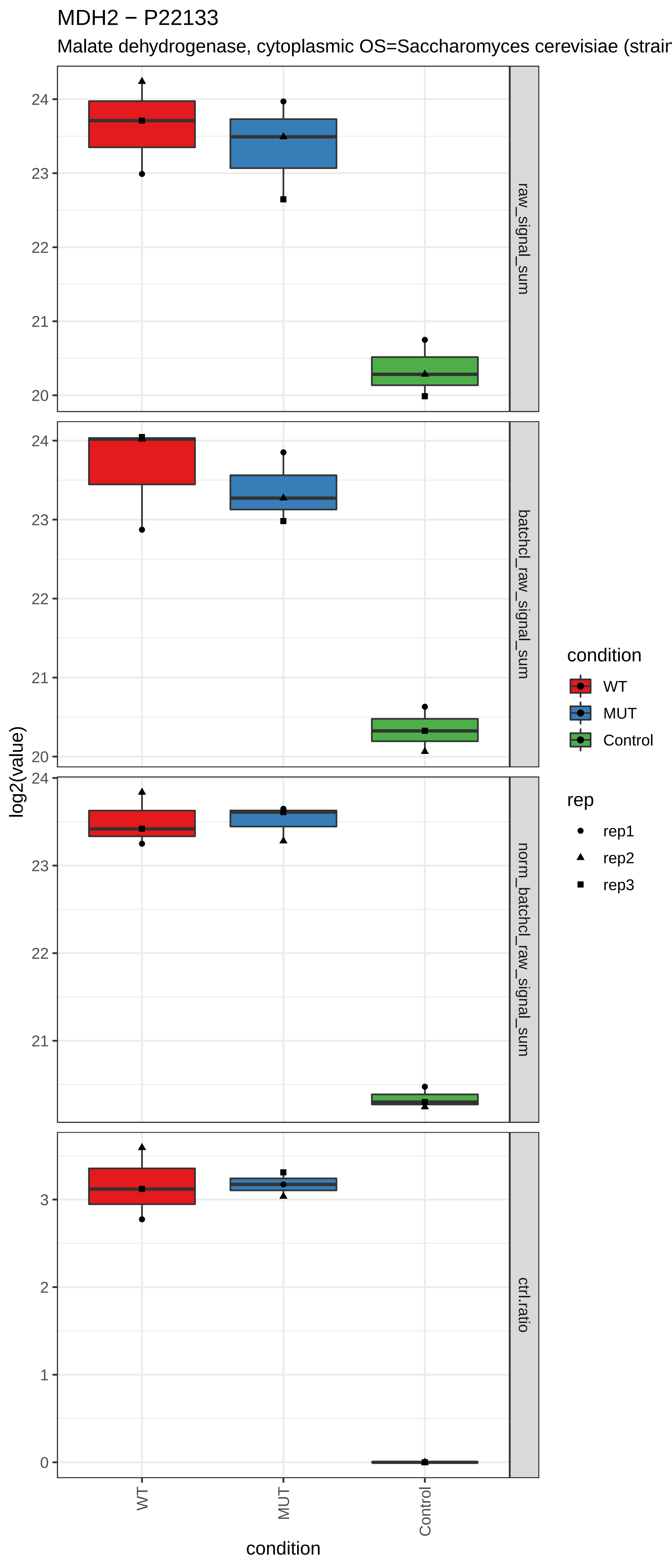
MDH2 − P22133
Malate dehydrogenase, cytoplasmic OS=Saccharomyces cerevisiae (strain
raw_signal_sum
batchcl_raw_signal_sum
norm_batchcl_raw_signal_sum
ctrl.ratio
log2(value)
condition
WT
MUT
Control
rep
rep1
rep2
rep3

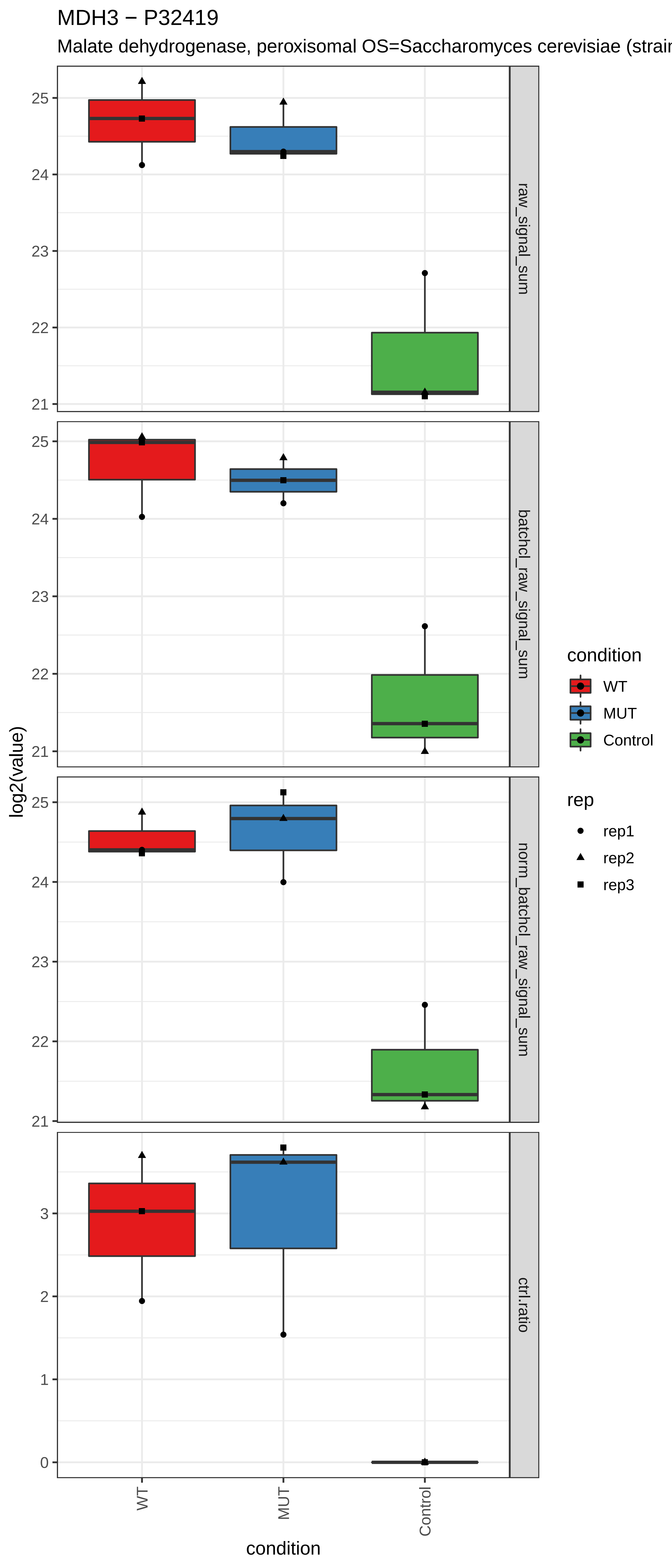
MDH3 − P32419
Malate dehydrogenase, peroxisomal OS=Saccharomyces cerevisiae (strain
raw_signal_sum
batchcl_raw_signal_sum
norm_batchcl_raw_signal_sum
ctrl.ratio
log2(value)
condition
WT
MUT
Control
rep
rep1
rep2
rep3
25
24
23
22
21
3
2
1
0

MDJ1 − P35191

DnaJ homolog 1, mitochondrial OS=Saccharomyces cerevisiae (strain ATC

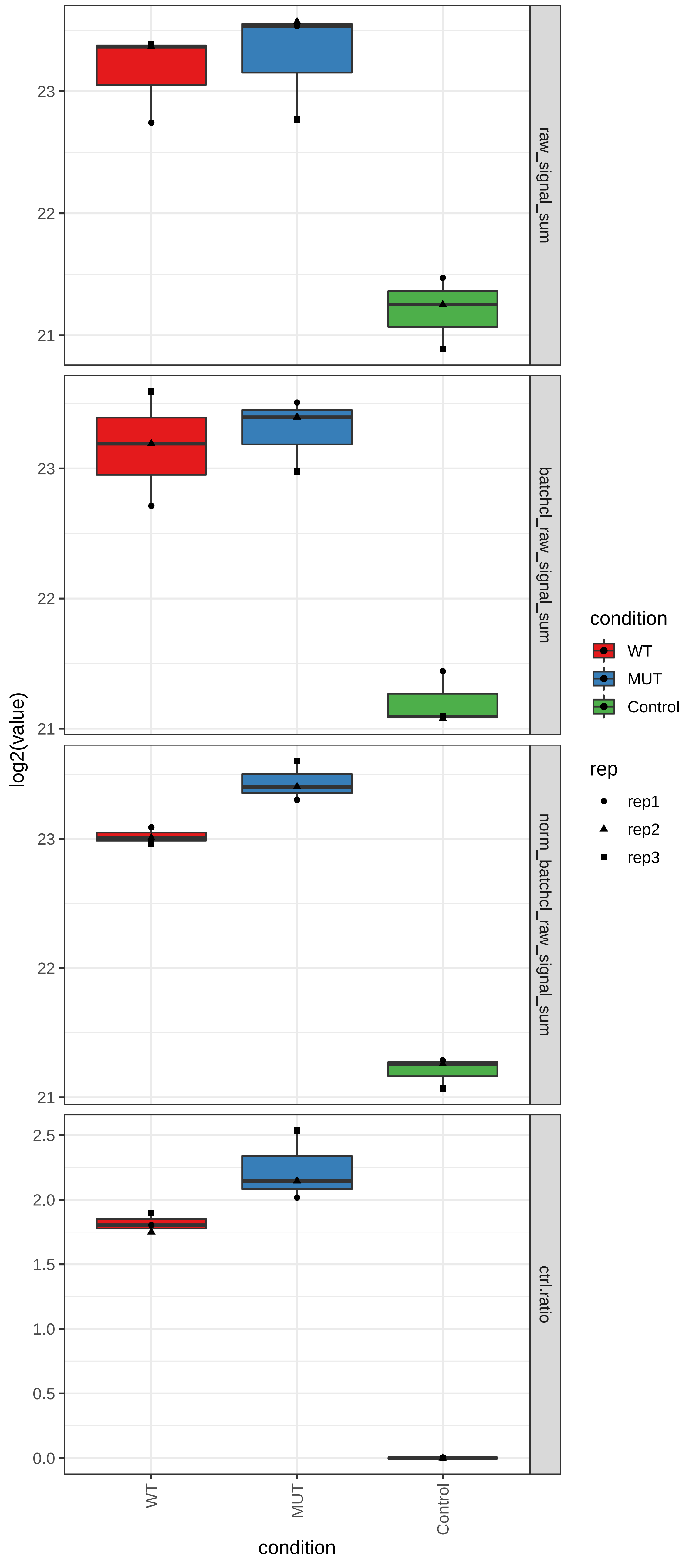

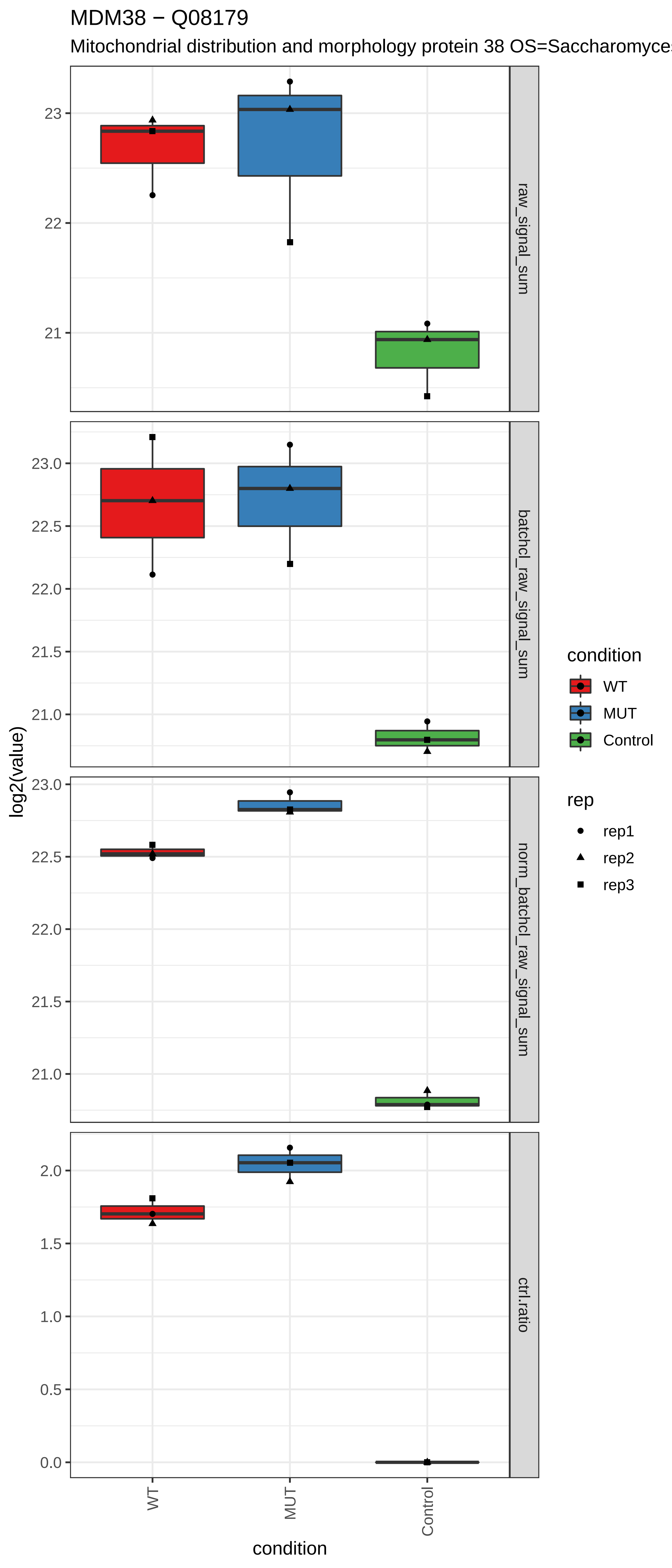

MDM38 − Q08179
Mitochondrial distribution and morphology protein 38 OS=Saccharomyces
raw_signal_sum
batchcl_raw_signal_sum
norm_batchcl_raw_signal_sum
ctrl.ratio
log2(value)
condition
WT
MUT
Control
rep
rep1
rep2
rep3
23
22
21
23.0
22.5
22.0
21.5
21.0
2.0
1.5
1.0
0.5
0.0

MET17 − P06106

Homocysteine/cysteine synthase OS=Saccharomyces cerevisiae (strain AT

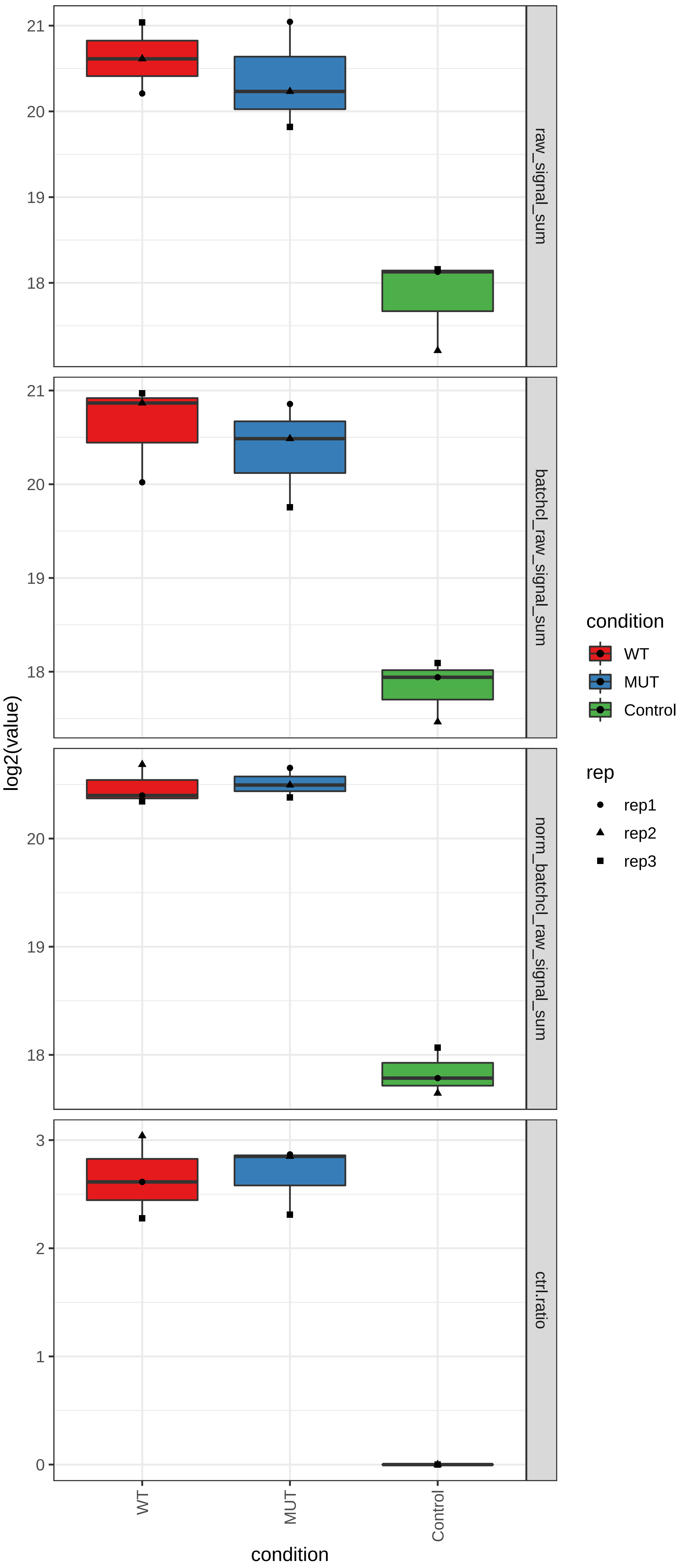

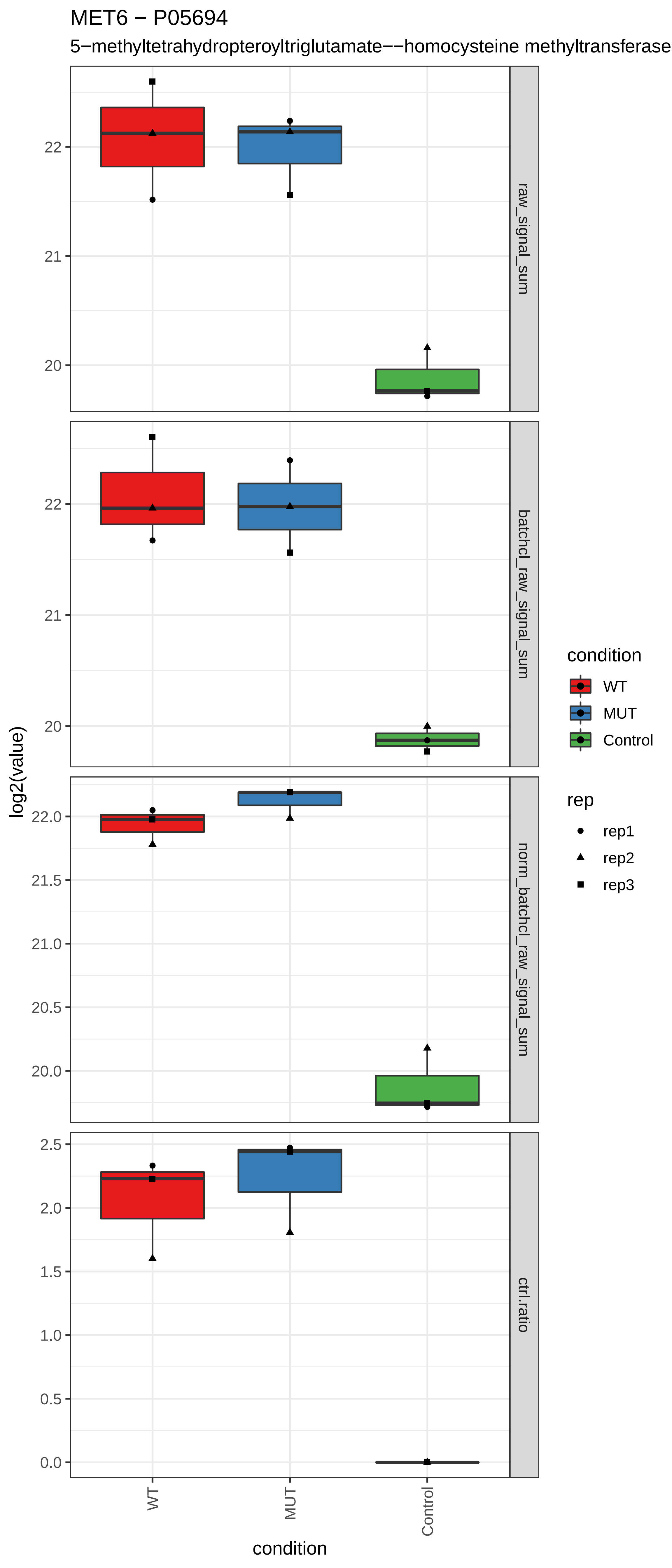
MET6 − P05694
5−methyltetrahydropteroyltriglutamate−−homocysteine methyltransferase
log2(value)
condition
WT
MUT
Control
raw_signal_sum
batchcl_raw_signal_sum
norm_batchcl_raw_signal_sum
ctrl.ratio
condition
WT
MUT
Control
rep
rep1
rep2
rep3

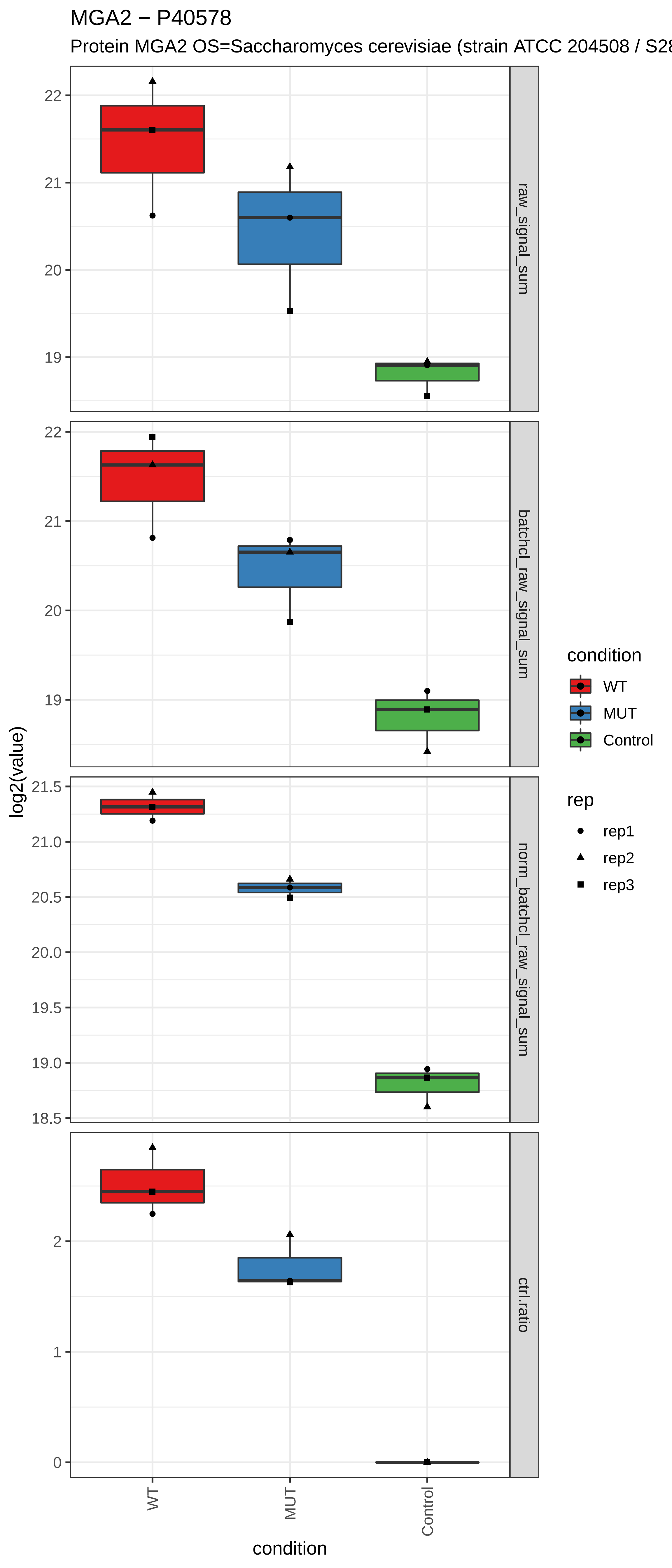
MGA2 − P40578
Protein MGA2 OS=Saccharomyces cerevisiae (strain ATCC 204508 / S28
raw_signal_sum
batchcl_raw_signal_sum
norm_batchcl_raw_signal_sum
ctrl.ratio
log2(value)
condition
WT
MUT
Control
rep
rep1
rep2
rep3
22
21
20
19
21.5
21.0
20.5
20.0
19.5
19.0
18.5
2
1
0

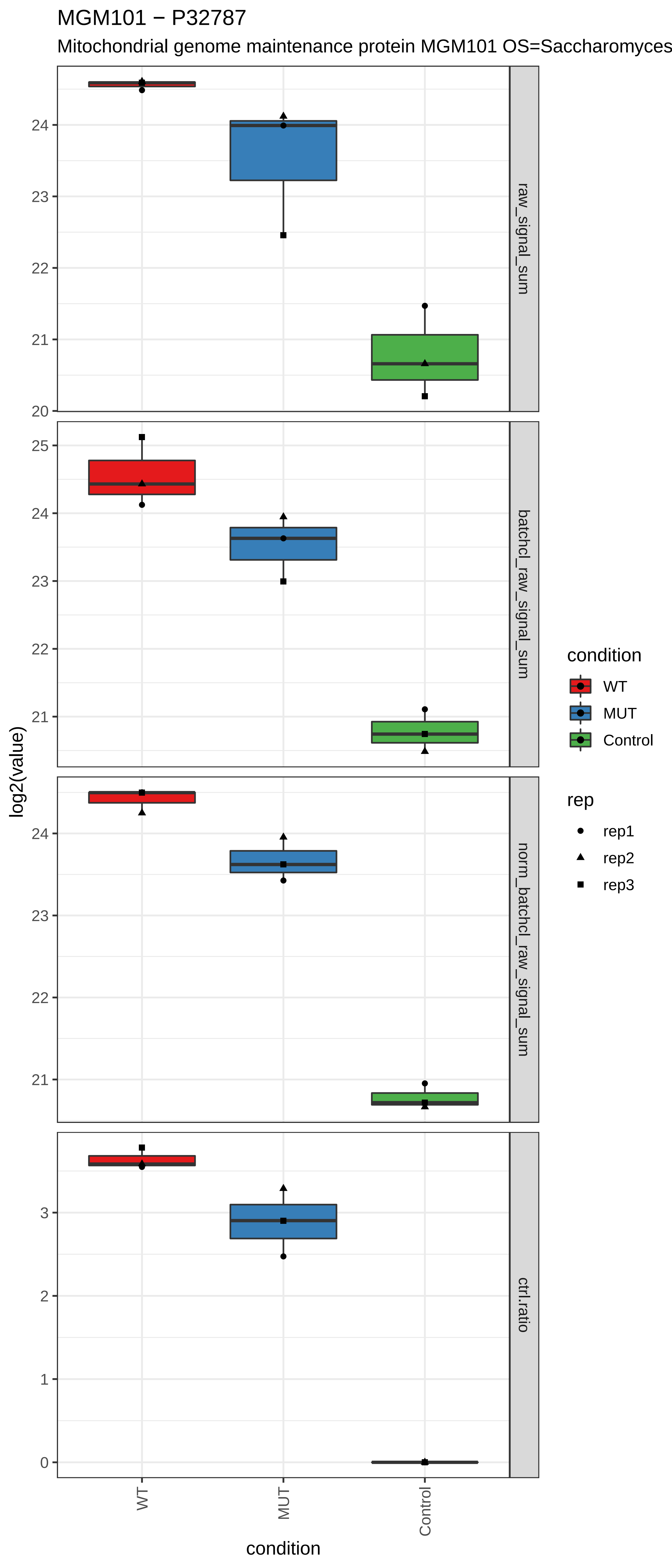

MGM101 − P32787
Mitochondrial genome maintenance protein MGM101 OS=Saccharomyces
raw_signal_sum
batchcl_raw_signal_sum
norm_batchcl_raw_signal_sum
ctrl.ratio
log2(value)
condition
WT
MUT
Control
rep
rep1
rep2
rep3

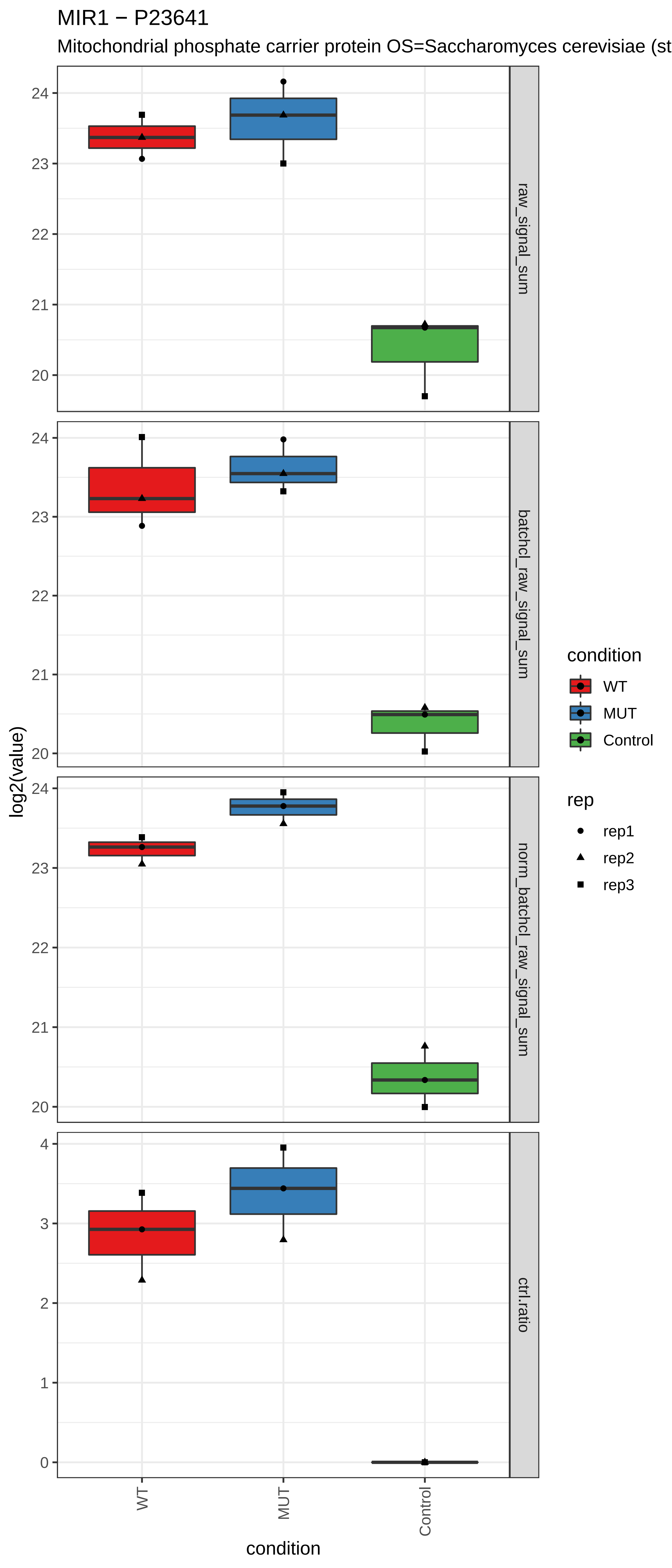
MIR1 − P23641
Mitochondrial phosphate carrier protein OS=Saccharomyces cerevisiae (st
raw_signal_sum
batchcl_raw_signal_sum
norm_batchcl_raw_signal_sum
ctrl.ratio
log2(value)
condition
WT
MUT
Control
rep
rep1
rep2
rep3
24
23
22
21
20
4
3
2
1
0

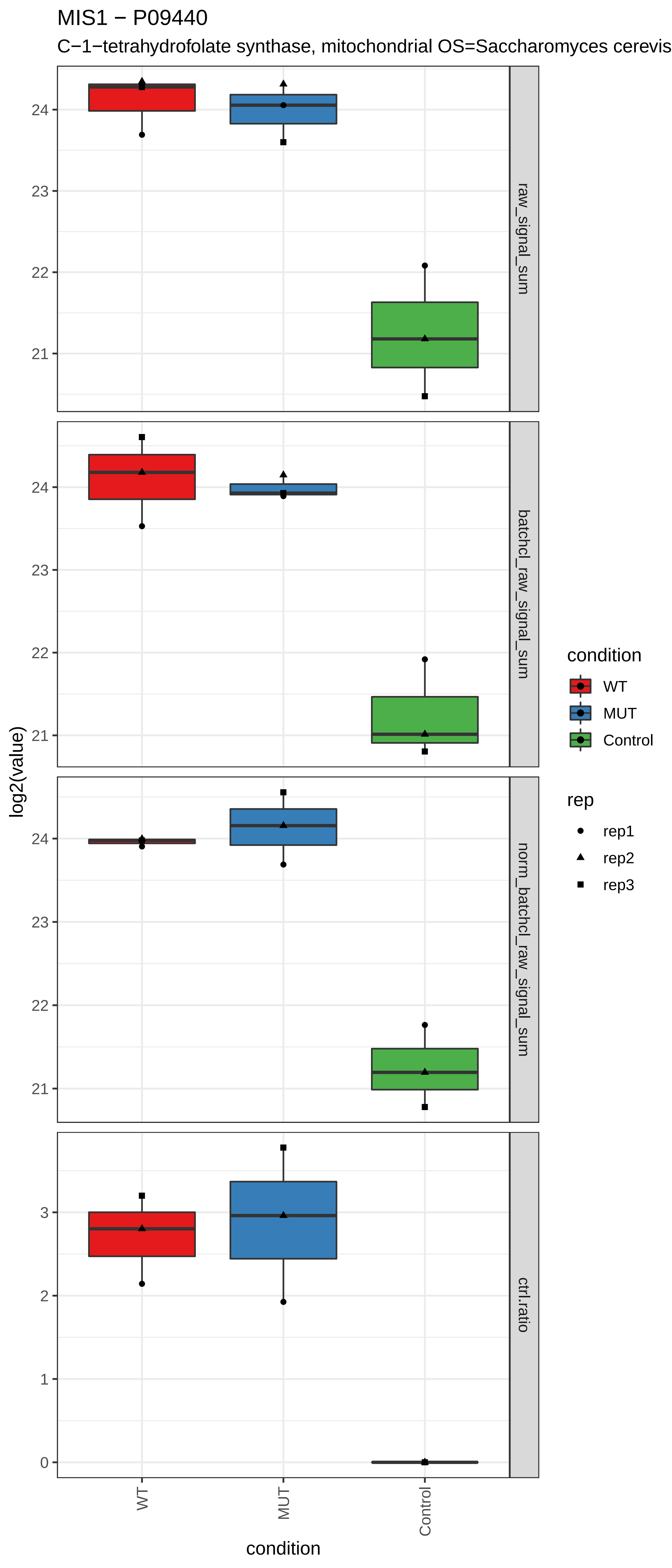

MIS1 − P09440
C−1−tetrahydrofolate synthase, mitochondrial OS=Saccharomyces cerevis
raw_signal_sum
batchcl_raw_signal_sum
norm_batchcl_raw_signal_sum
ctrl.ratio
log2(value)
condition
WT
MUT
Control
rep
rep1
rep2
rep3
24
23
22
21
3
2
1
0

MKT1 − P40850

Protein MKT1 OS=Saccharomyces cerevisiae (strain ATCC 204508 / S288

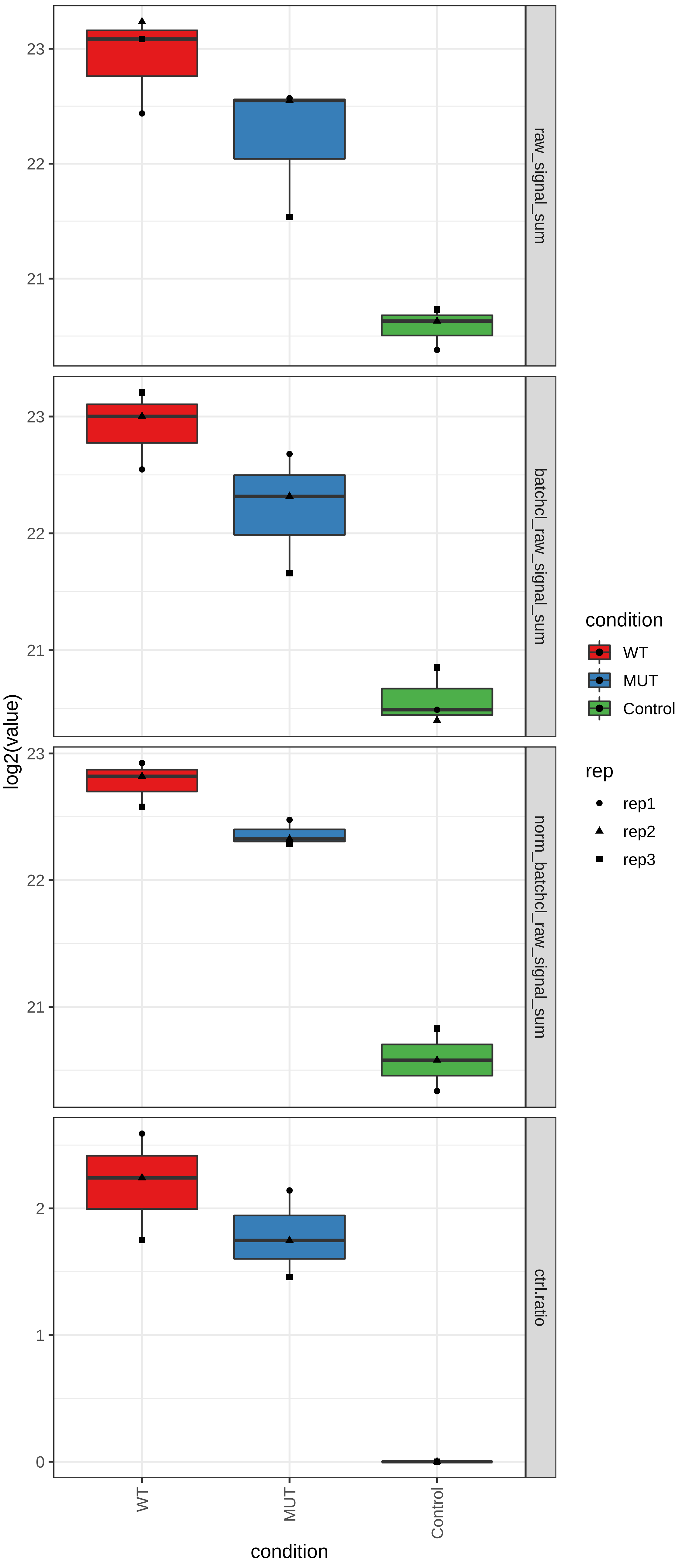

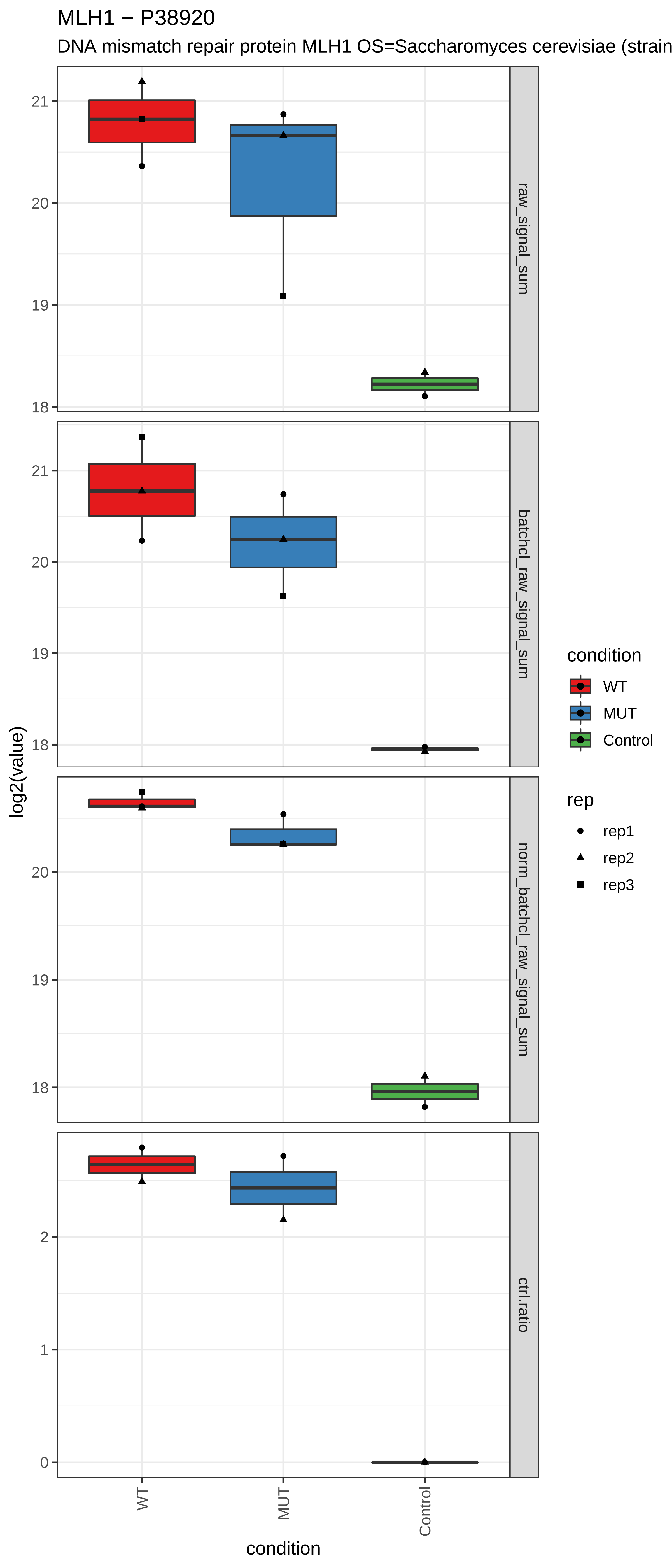

MLH1 − P38920
DNA mismatch repair protein MLH1 OS=Saccharomyces cerevisiae (strain
raw_signal_sum
batchcl_raw_signal_sum
norm_batchcl_raw_signal_sum
ctrl.ratio
log2(value)
condition
WT
MUT
Control
rep
rep1
rep2
rep3

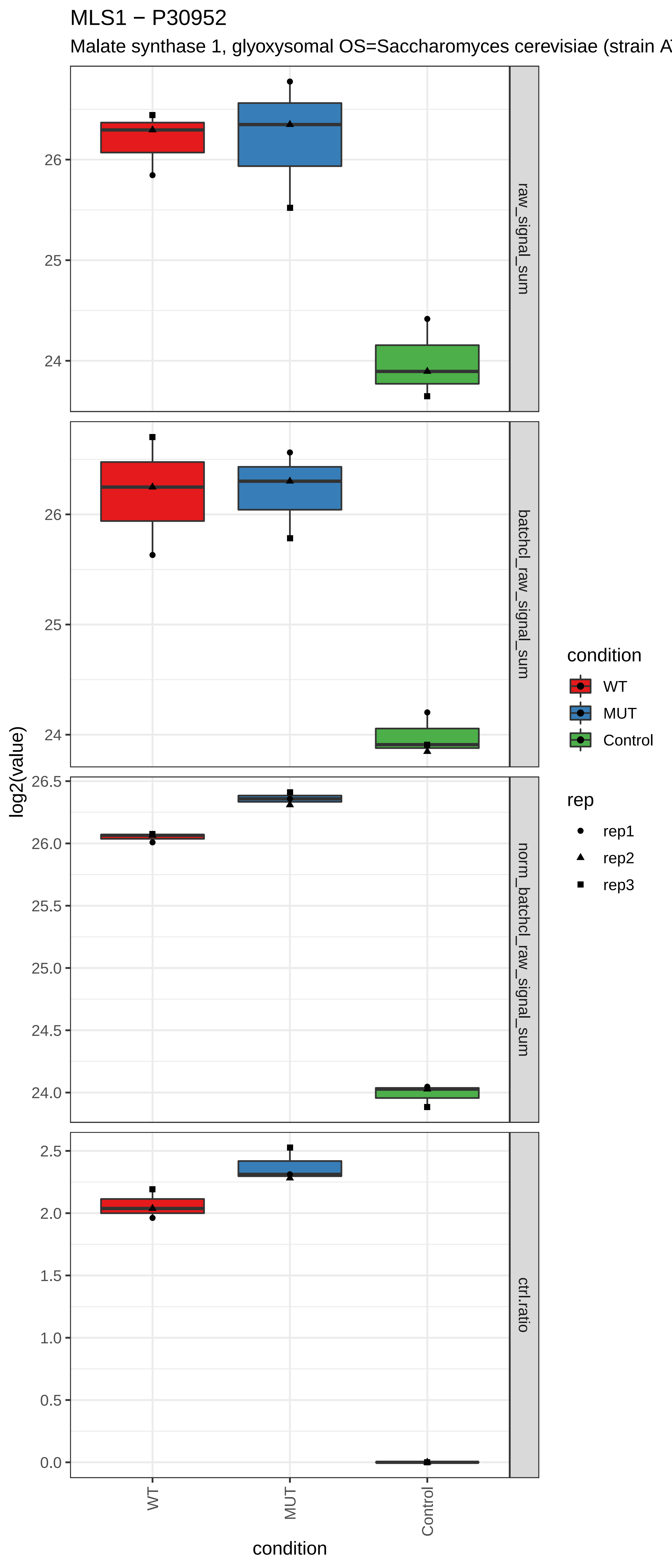

MLS1 − P30952
Malate synthase 1, glyoxysomal OS=Saccharomyces cerevisiae (strain A
log2(value)
raw_signal_sum
batchcl_raw_signal_sum
norm_batchcl_raw_signal_sum
ctrl.ratio
26
25
24
26.5
26.0
25.5
25.0
24.5
24.0
2.5
2.0
1.5
1.0
0.5
0.0
WT
MUT
Control
condition
condition
WT
MUT
Control
rep
rep1
rep2
rep3

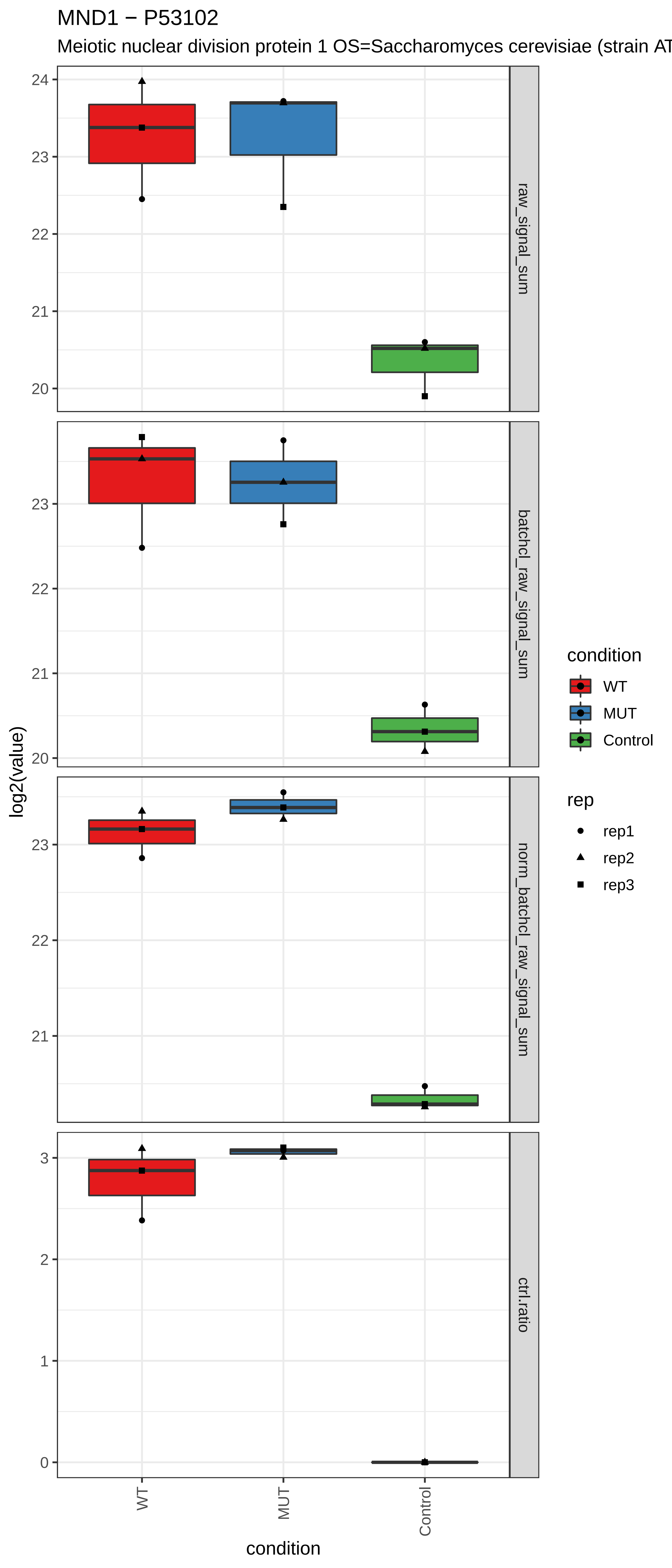
MND1 − P53102
Meiotic nuclear division protein 1 OS=Saccharomyces cerevisiae (strain AT
raw_signal_sum
batchcl_raw_signal_sum
norm_batchcl_raw_signal_sum
ctrl.ratio
log2(value)
condition
WT
MUT
Control
rep
rep1
rep2
rep3
24
23
22
21
20
3
2
1
0

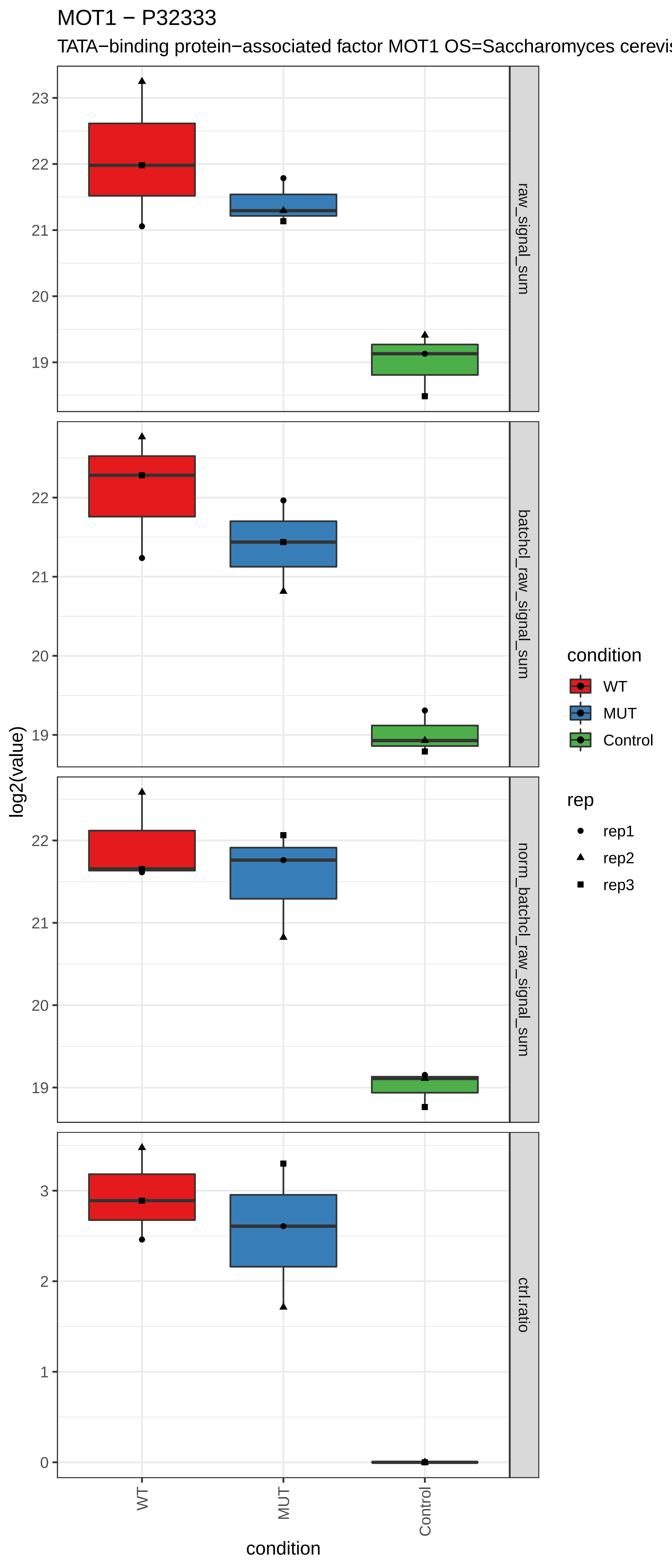
MOT1 − P32333
TATA−binding protein−associated factor MOT1 OS=Saccharomyces cerevis
raw_signal_sum
batchcl_raw_signal_sum
norm_batchcl_raw_signal_sum
ctrl.ratio
log2(value)
condition
WT
MUT
Control
rep
rep1
rep2
rep3

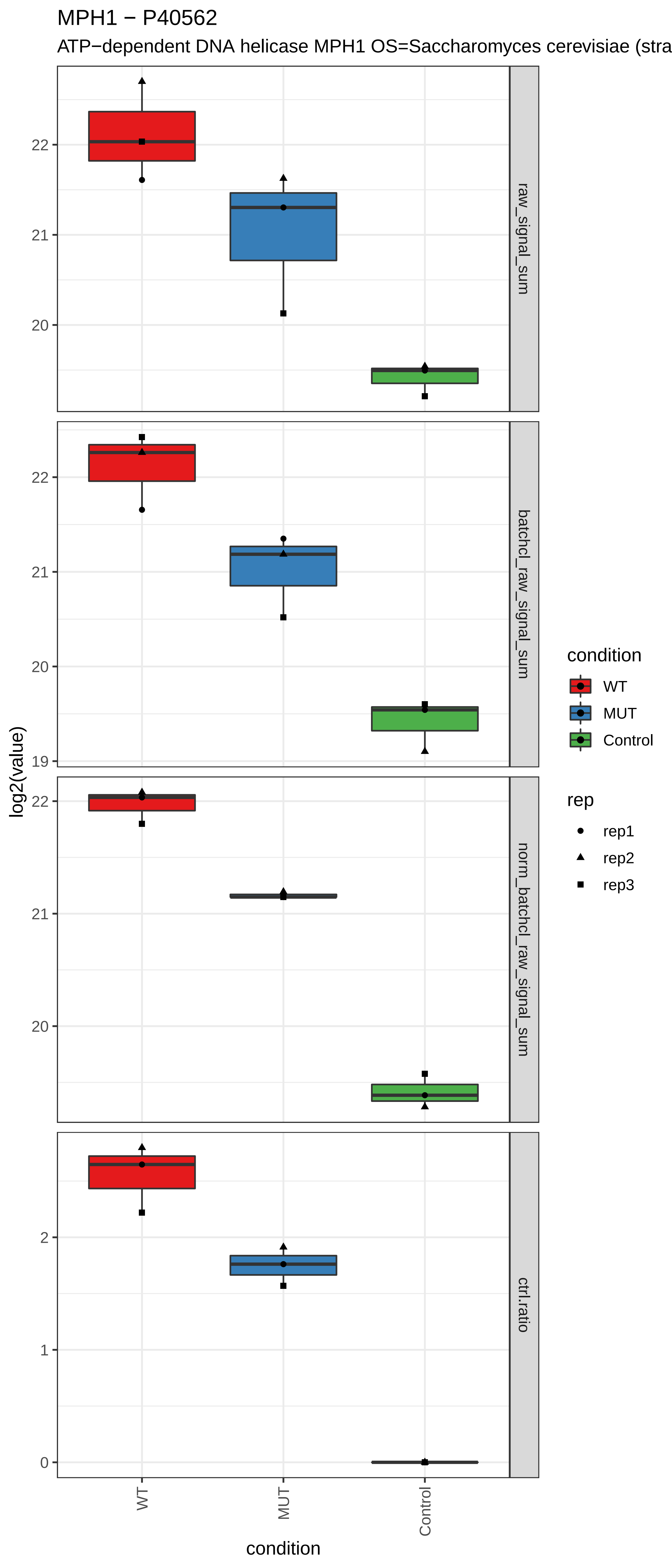

MPH1 − P40562
ATP−dependent DNA helicase MPH1 OS=Saccharomyces cerevisiae (stra
raw_signal_sum
batchcl_raw_signal_sum
norm_batchcl_raw_signal_sum
ctrl.ratio
log2(value)
condition
WT
MUT
Control
rep
rep1
rep2
rep3
22
21
20
19
2
1
0

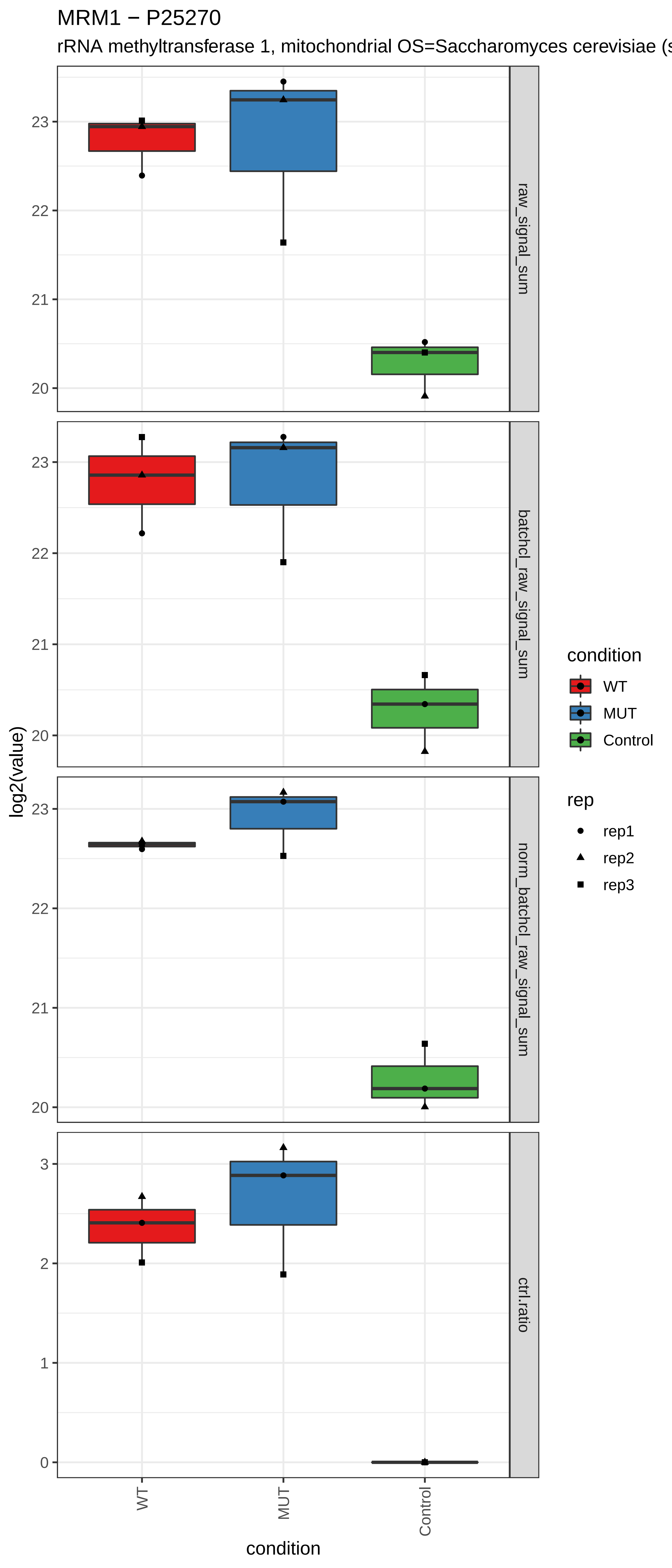
MRM1 − P25270
rRNA methyltransferase 1, mitochondrial OS=Saccharomyces cerevisiae (s
raw_signal_sum
batchcl_raw_signal_sum
norm_batchcl_raw_signal_sum
ctrl.ratio
log2(value)
condition
WT
MUT
Control
condition
WT
MUT
Control
rep
rep1
rep2
rep3

MRP20 − P32387

54S ribosomal protein L41, mitochondrial OS=Saccharomyces cerevisiae (

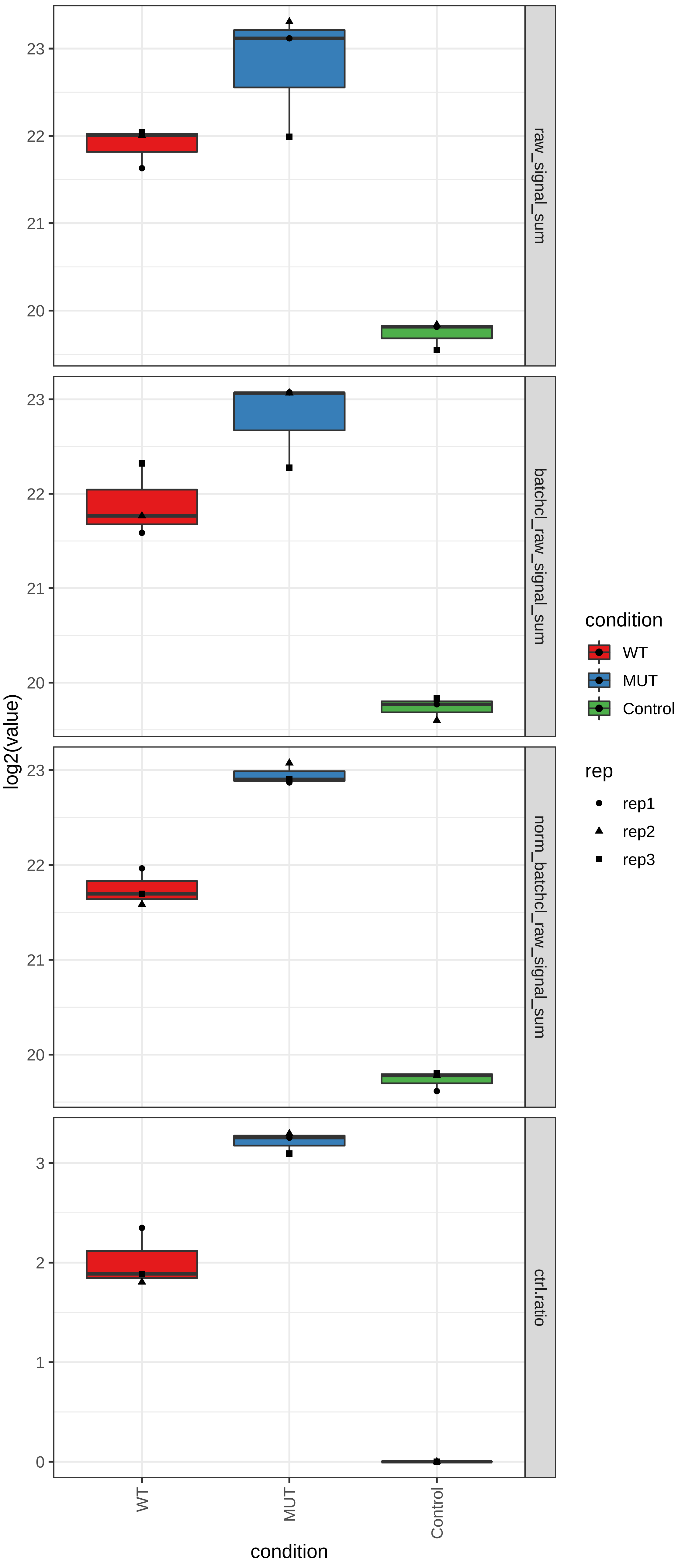

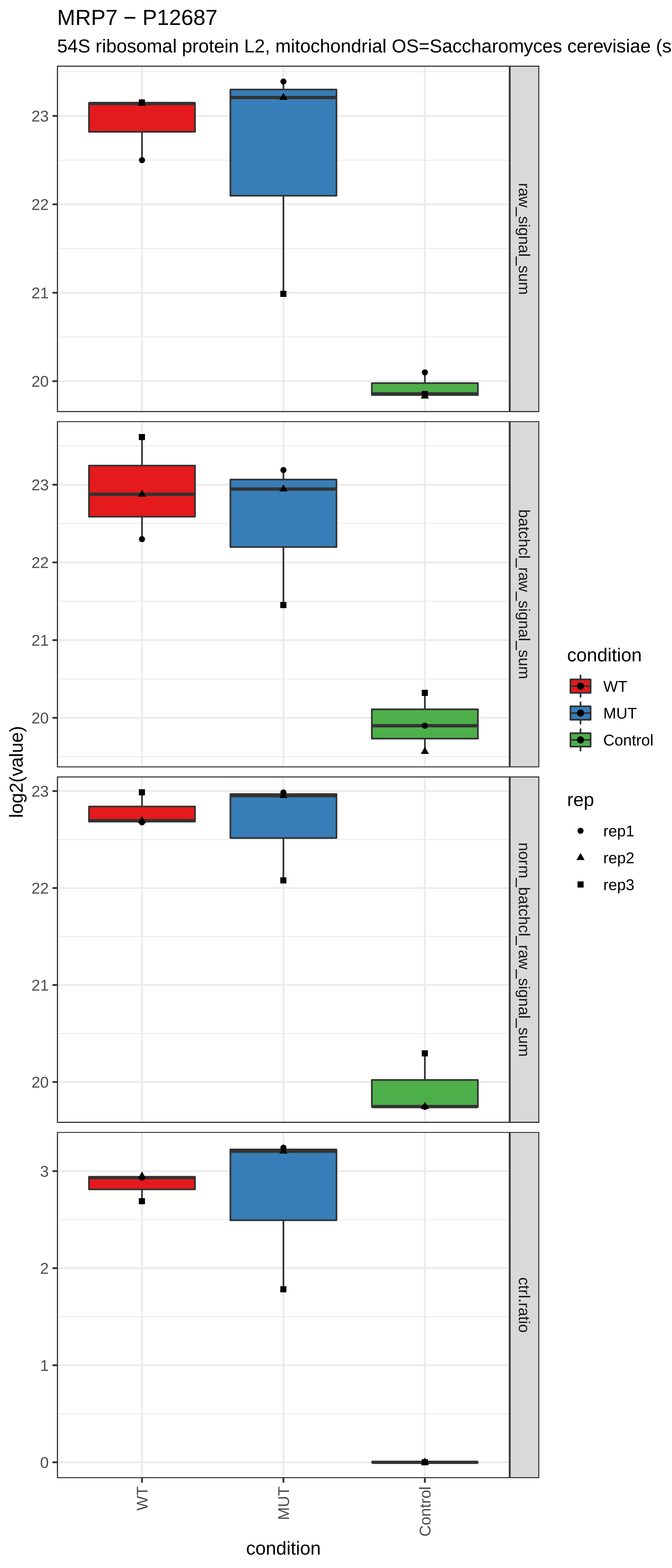

MRP7 − P12687
54S ribosomal protein L2, mitochondrial OS=Saccharomyces cerevisiae (s
raw_signal_sum
batchcl_raw_signal_sum
norm_batchcl_raw_signal_sum
ctrl.ratio
log2(value)
condition
WT
MUT
Control
rep
rep1
rep2
rep3
23
22
21
20
3
2
1
0

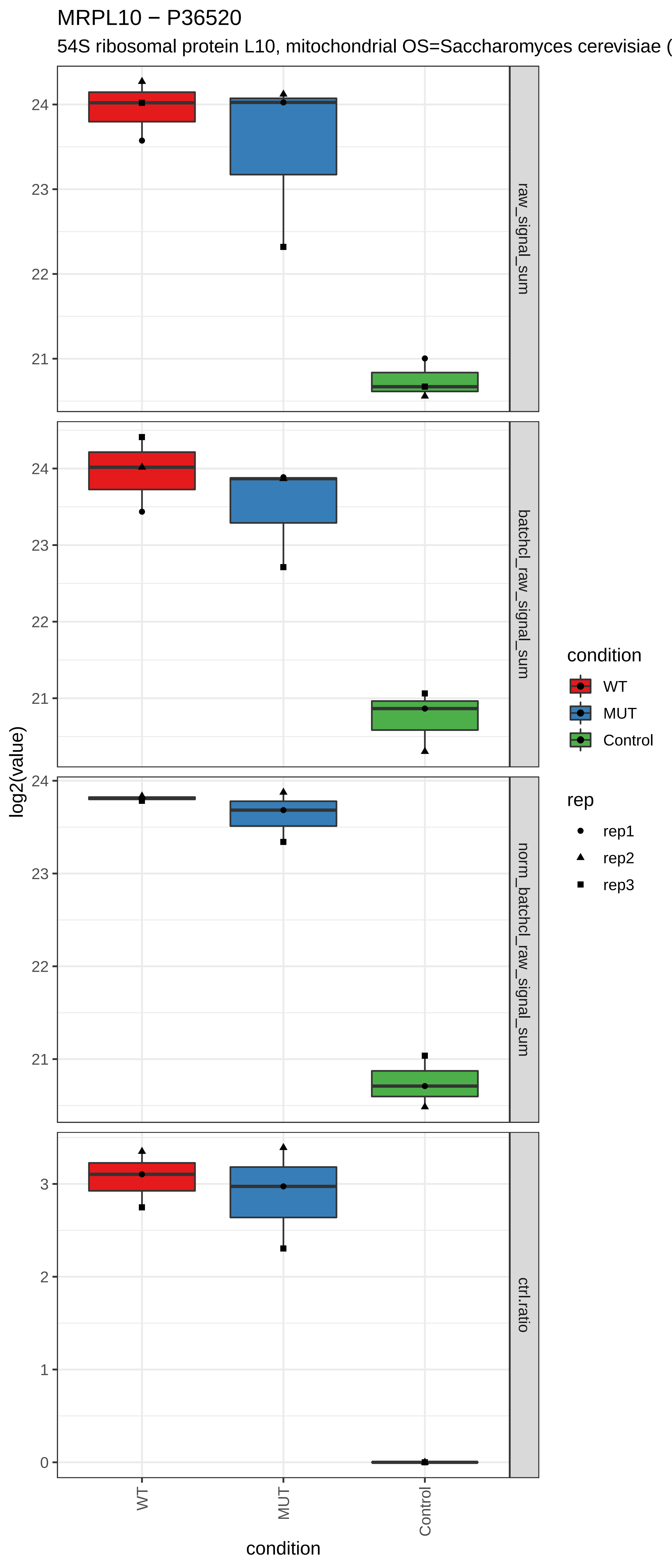

MRPL10 − P36520
54S ribosomal protein L10, mitochondrial OS=Saccharomyces cerevisiae (
raw_signal_sum
batchcl_raw_signal_sum
norm_batchcl_raw_signal_sum
ctrl.ratio
log2(value)
condition
WT
MUT
Control
rep
rep1
rep2
rep3
24
23
22
21
3
2
1
0

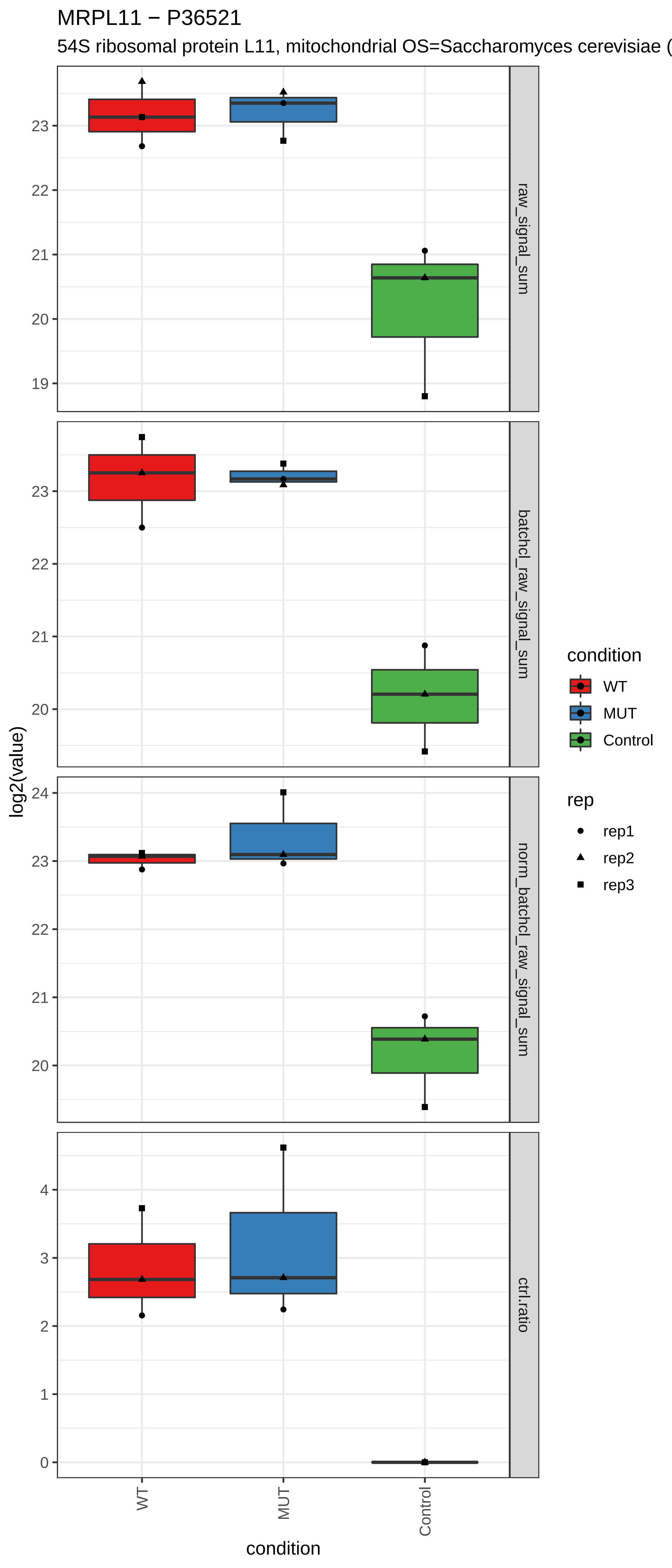
MRPL11 − P36521
54S ribosomal protein L11, mitochondrial OS=Saccharomyces cerevisiae (
raw_signal_sum
batchcl_raw_signal_sum
norm_batchcl_raw_signal_sum
ctrl.ratio
log2(value)
condition
WT
MUT
Control
rep
rep1
rep2
rep3
19
20
21
22
23
24
0
1
2
3
4

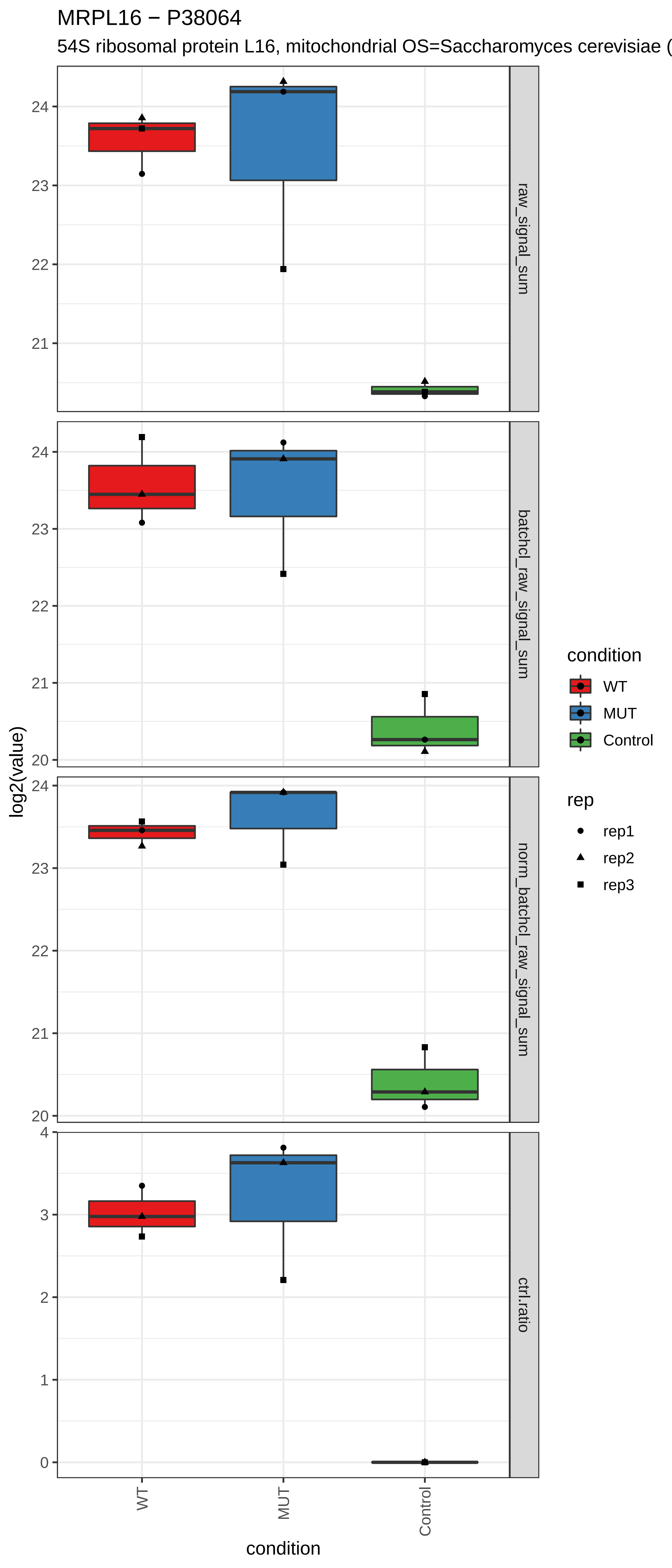

MRPL16 − P38064
54S ribosomal protein L16, mitochondrial OS=Saccharomyces cerevisiae (
raw_signal_sum
batchcl_raw_signal_sum
norm_batchcl_raw_signal_sum
ctrl.ratio
log2(value)
condition
WT
MUT
Control
rep
rep1
rep2
rep3

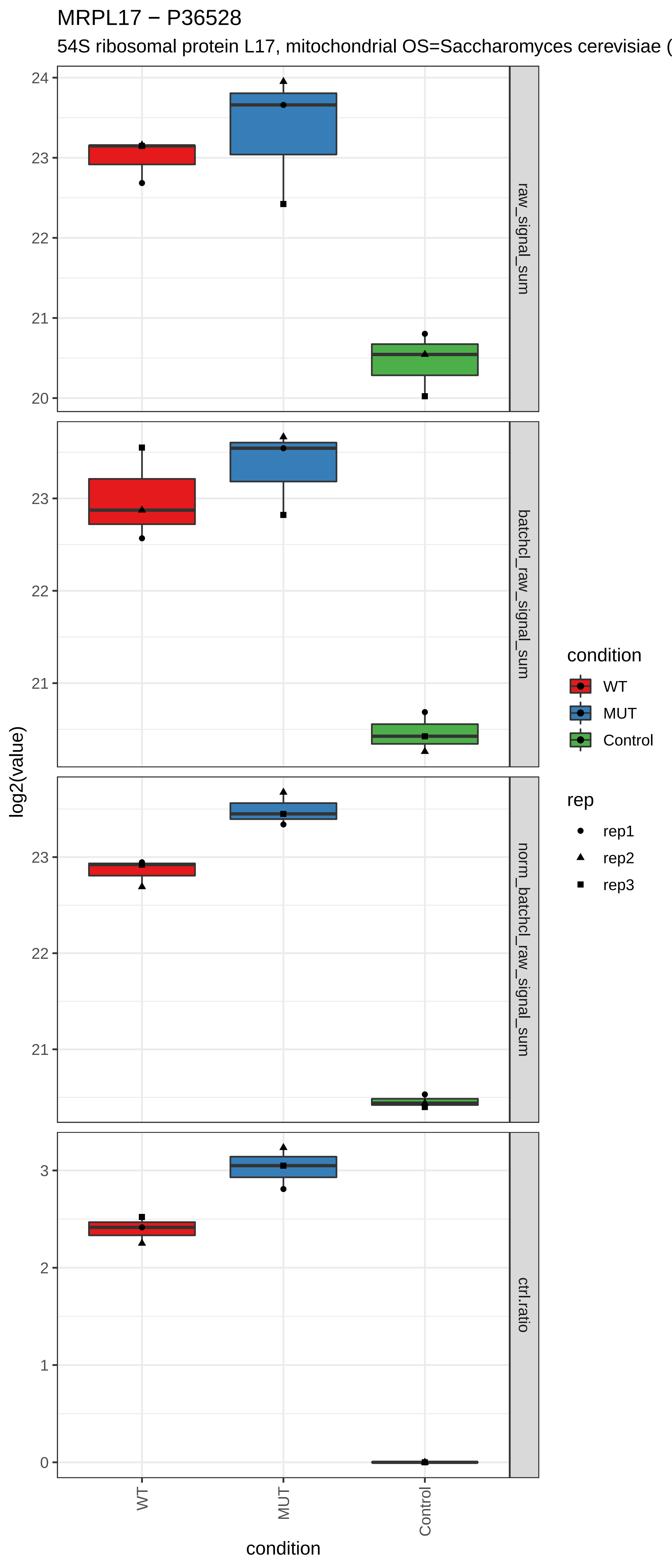

MRPL17 − P36528
54S ribosomal protein L17, mitochondrial OS=Saccharomyces cerevisiae (
log2(value)
condition
raw_signal_sum
batchcl_raw_signal_sum
norm_batchcl_raw_signal_sum
ctrl.ratio
WT
MUT
Control
rep
rep1
rep2
rep3

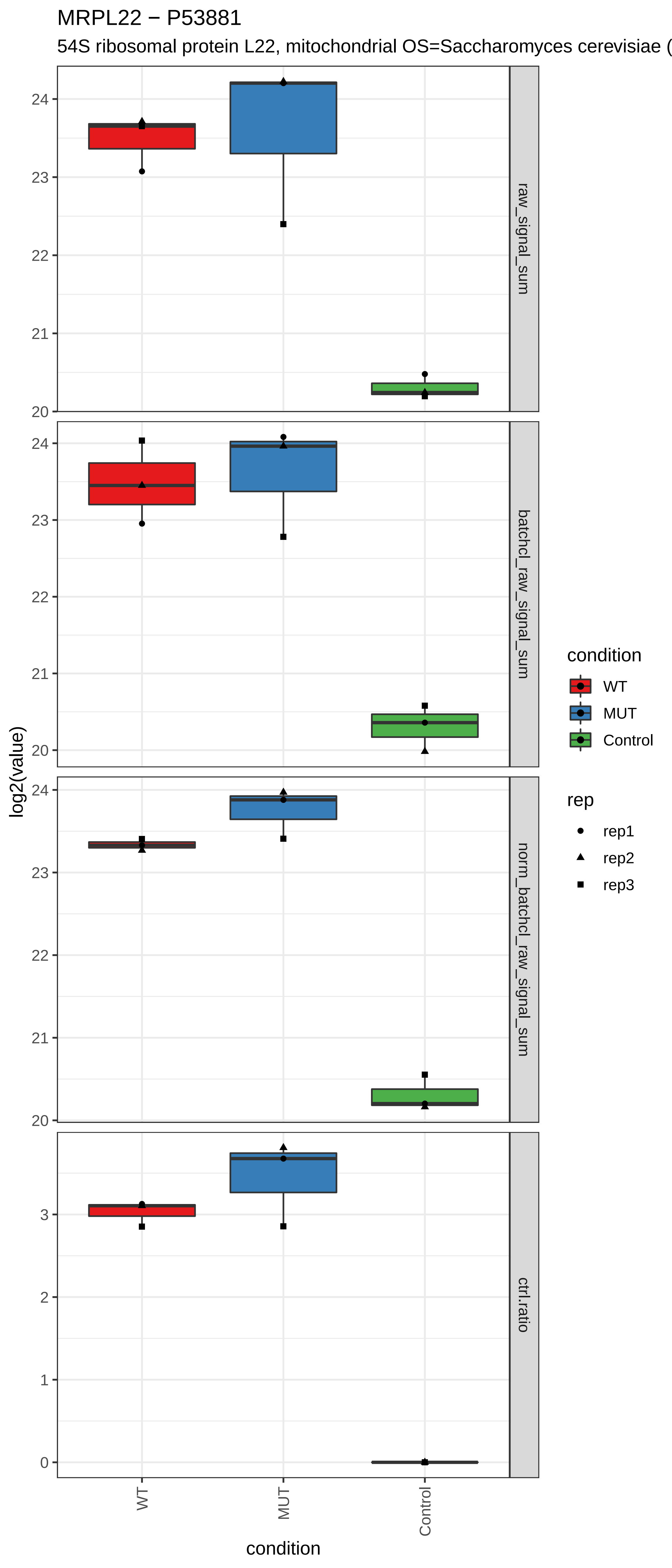
MRPL22 − P53881
54S ribosomal protein L22, mitochondrial OS=Saccharomyces cerevisiae (
raw_signal_sum
batchcl_raw_signal_sum
norm_batchcl_raw_signal_sum
ctrl.ratio
log2(value)
condition
WT
MUT
Control
rep
rep1
rep2
rep3

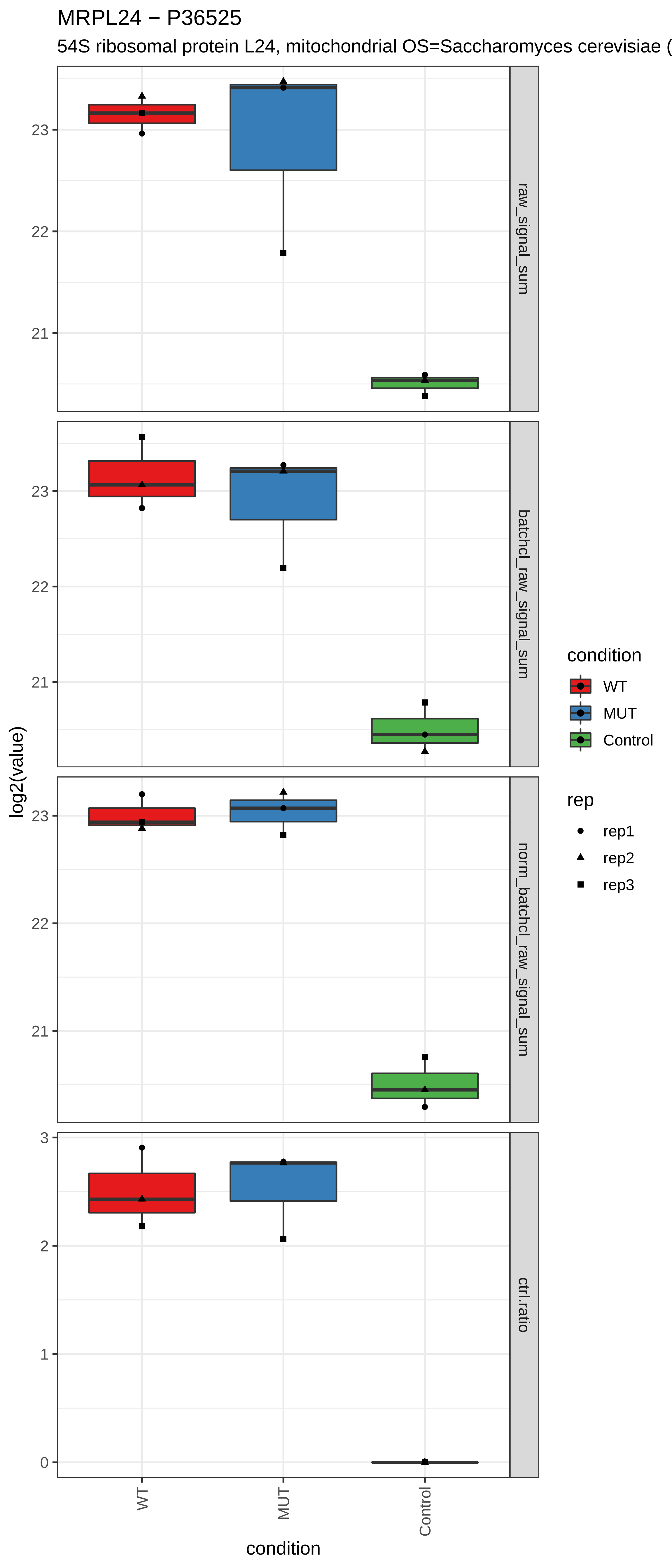

MRPL24 − P36525
54S ribosomal protein L24, mitochondrial OS=Saccharomyces cerevisiae (
raw_signal_sum
batchcl_raw_signal_sum
norm_batchcl_raw_signal_sum
ctrl.ratio
log2(value)
condition
WT
MUT
Control
rep
rep1
rep2
rep3
23
22
21
3
2
1
0

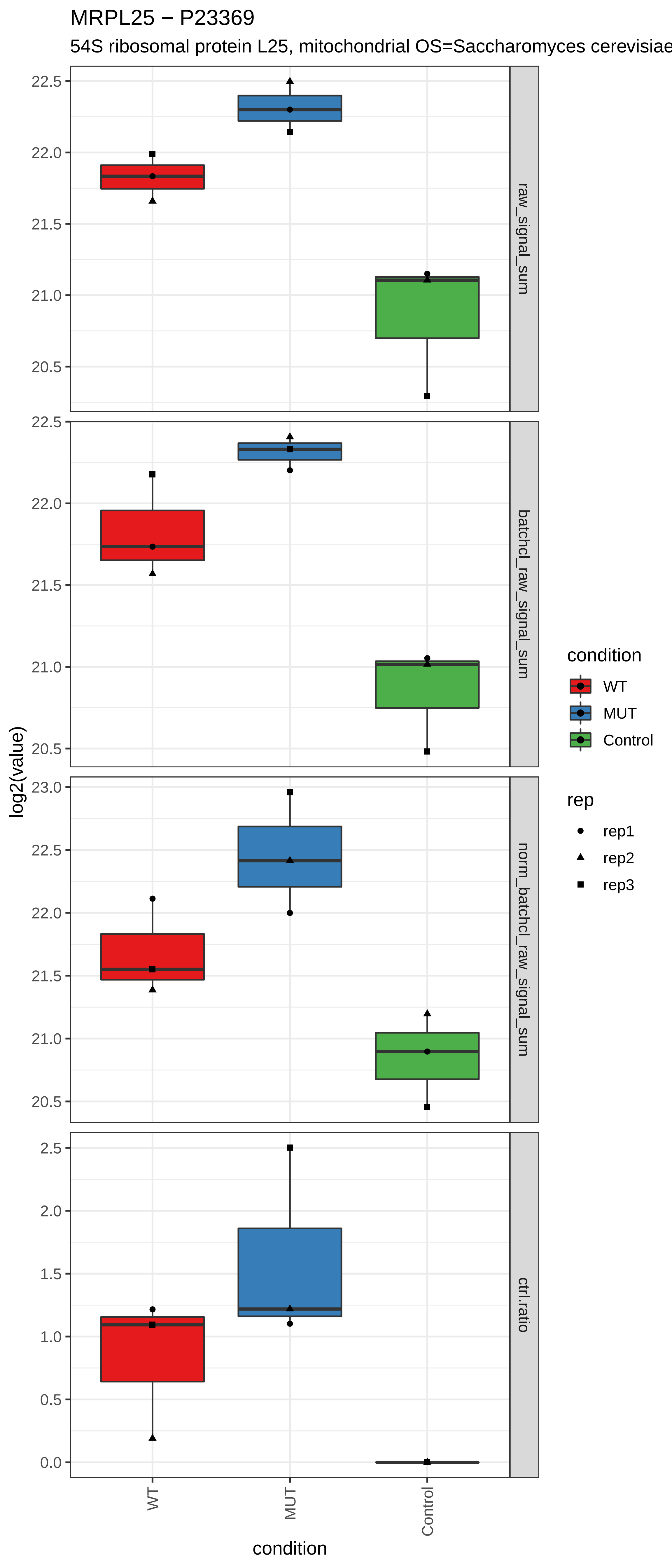

MRPL25 − P23369
54S ribosomal protein L25, mitochondrial OS=Saccharomyces cerevisiae
raw_signal_sum
batchcl_raw_signal_sum
norm_batchcl_raw_signal_sum
ctrl.ratio
log2(value)
condition
WT
MUT
Control
rep
rep1
rep2
rep3

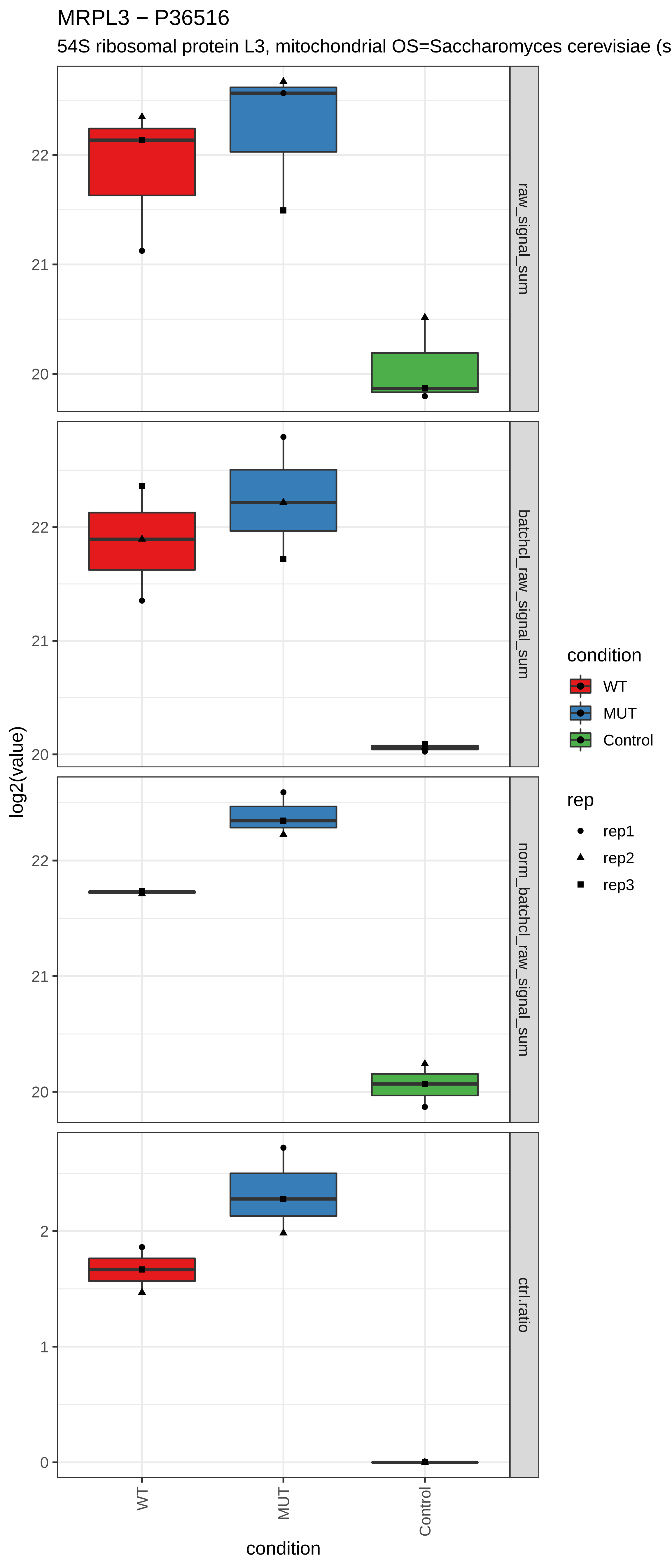

MRPL3 − P36516
54S ribosomal protein L3, mitochondrial OS=Saccharomyces cerevisiae (s
raw_signal_sum
batchcl_raw_signal_sum
norm_batchcl_raw_signal_sum
ctrl.ratio
log2(value)
condition
WT
MUT
Control
rep
rep1
rep2
rep3
22
21
20
2
1
0

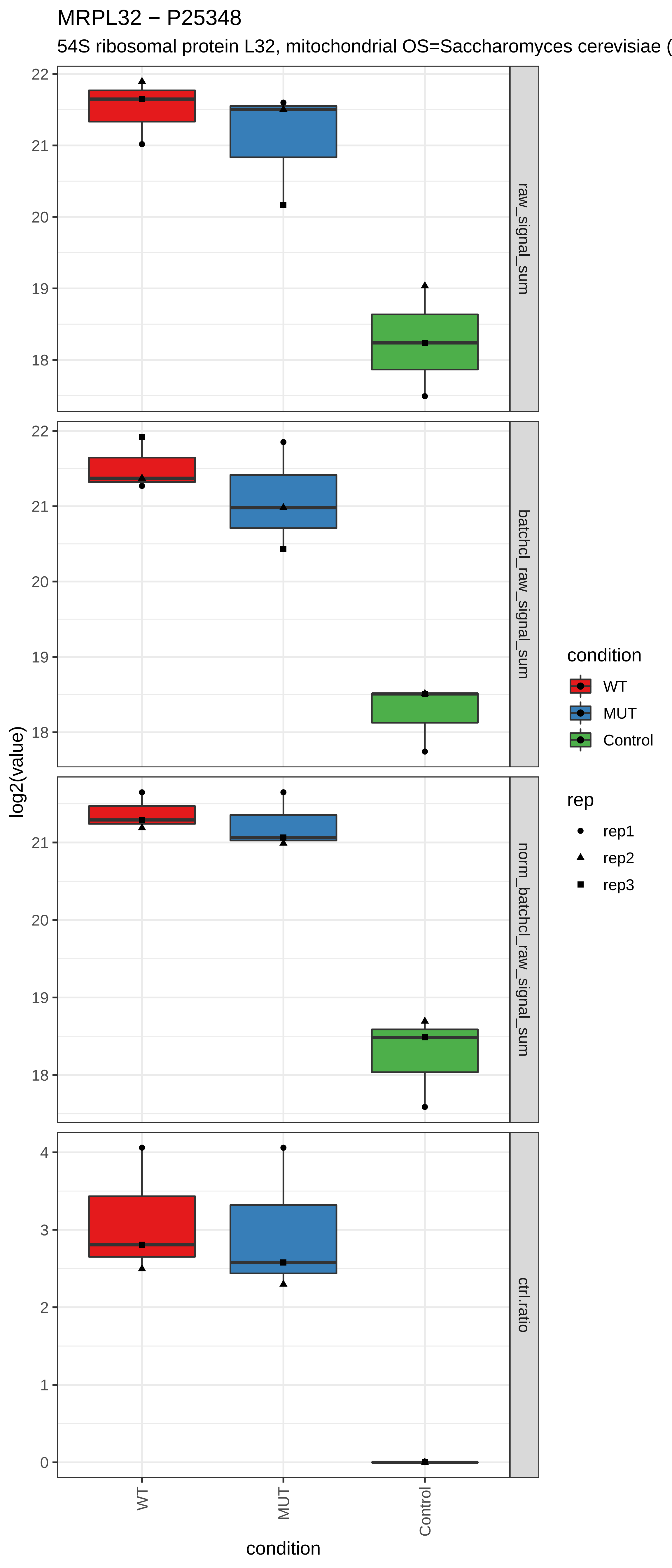
MRPL32 − P25348
54S ribosomal protein L32, mitochondrial OS=Saccharomyces cerevisiae (
raw_signal_sum
batchcl_raw_signal_sum
norm_batchcl_raw_signal_sum
ctrl.ratio
log2(value)
condition
WT
MUT
Control
rep
rep1
rep2
rep3

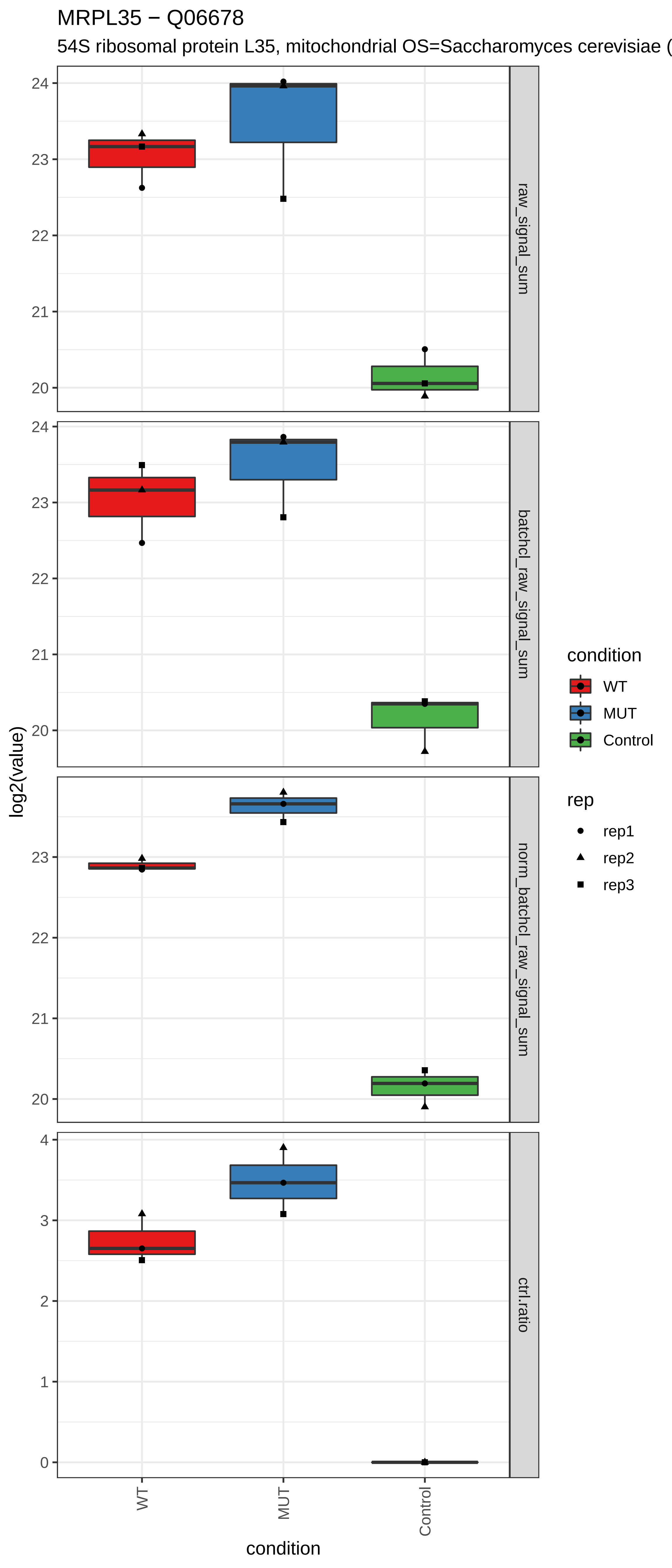

MRPL35 − Q06678
54S ribosomal protein L35, mitochondrial OS=Saccharomyces cerevisiae (
raw_signal_sum
batchcl_raw_signal_sum
norm_batchcl_raw_signal_sum
ctrl.ratio
log2(value)
condition
WT
MUT
Control
rep
rep1
rep2
rep3
24
23
22
21
20
4
3
2
1
0

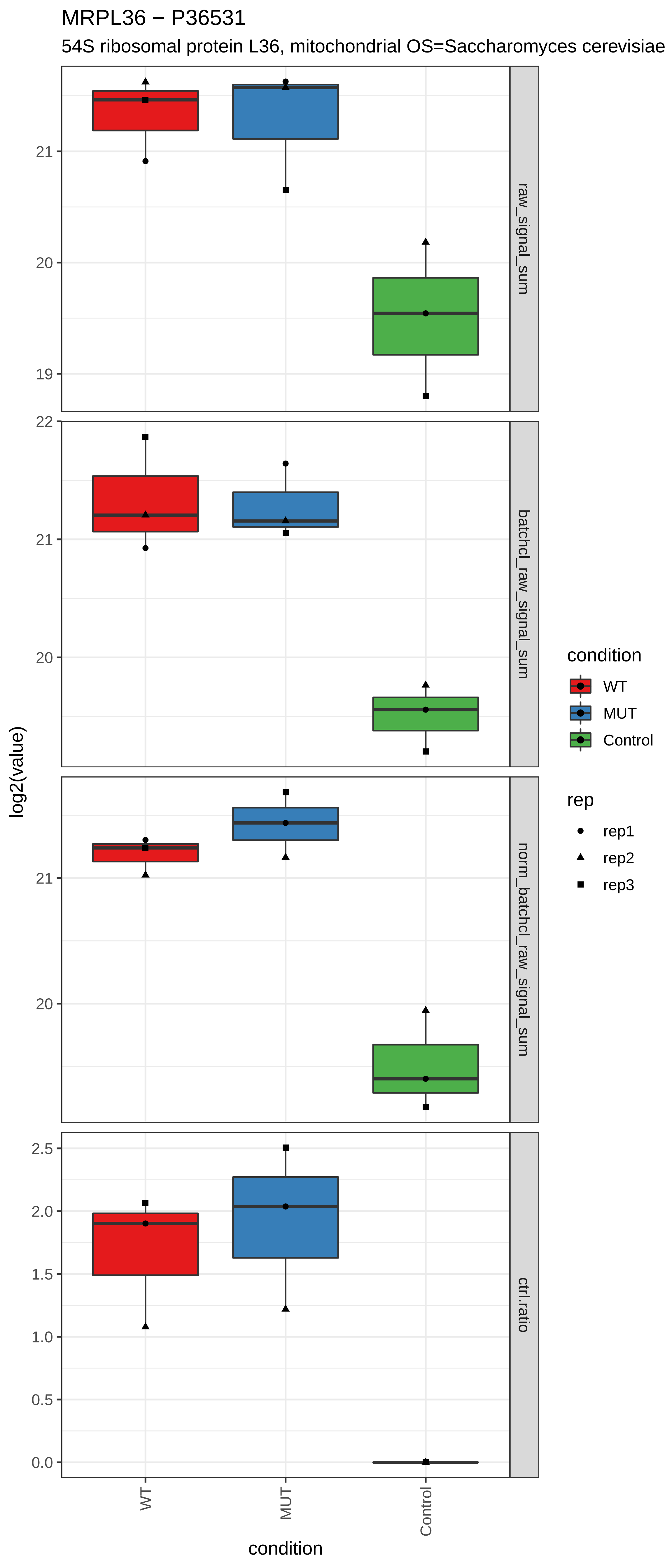

MRPL36 − P36531
54S ribosomal protein L36, mitochondrial OS=Saccharomyces cerevisiae
raw_signal_sum
batchcl_raw_signal_sum
norm_batchcl_raw_signal_sum
ctrl.ratio
log2(value)
condition
WT
MUT
Control
rep
rep1
rep2
rep3

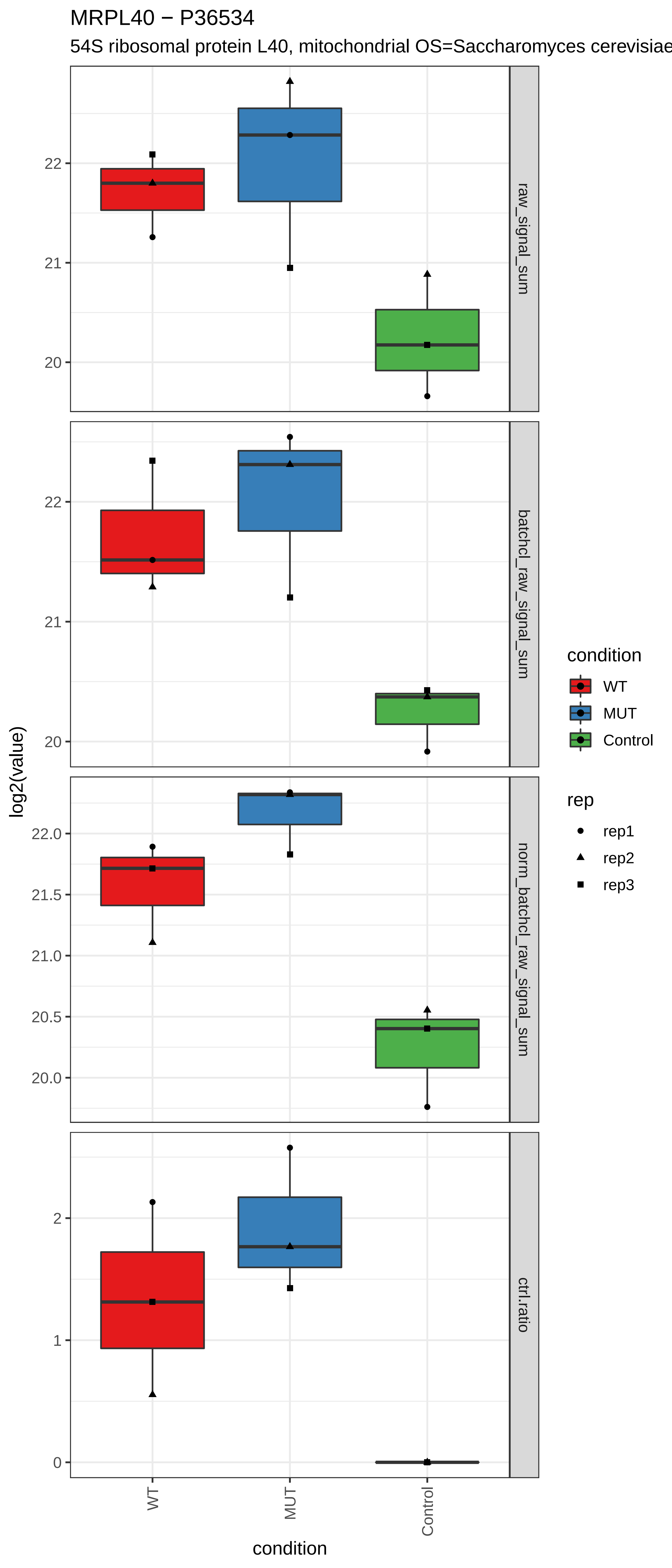

MRPL40 − P36534
54S ribosomal protein L40, mitochondrial OS=Saccharomyces cerevisiae
raw_signal_sum
batchcl_raw_signal_sum
norm_batchcl_raw_signal_sum
ctrl.ratio
log2(value)
22
21
20
22.0
21.5
21.0
20.5
20.0
2
1
0
WT
MUT
Control
condition
condition
WT
MUT
Control
rep
rep1
rep2
rep3

MRPL9 − P31334

54S ribosomal protein L9, mitochondrial OS=Saccharomyces cerevisiae (s

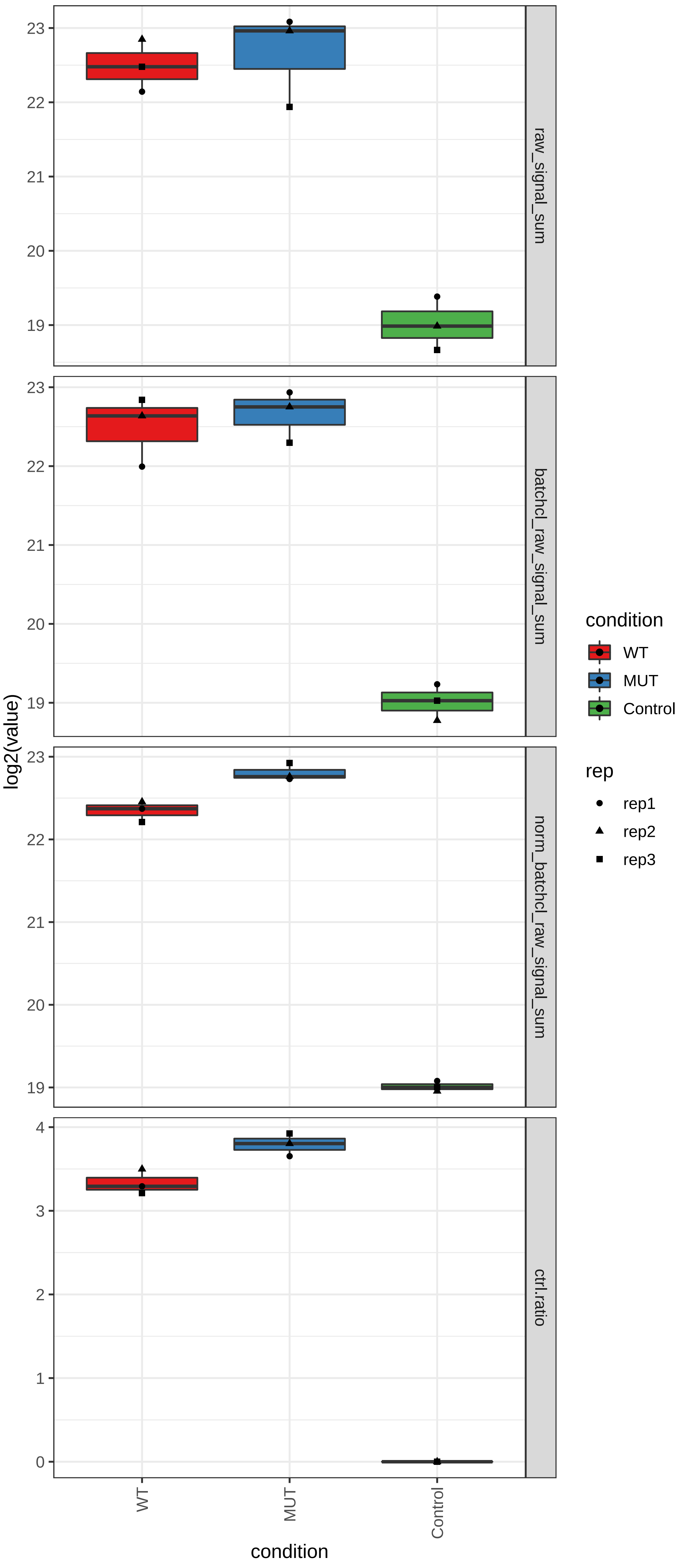

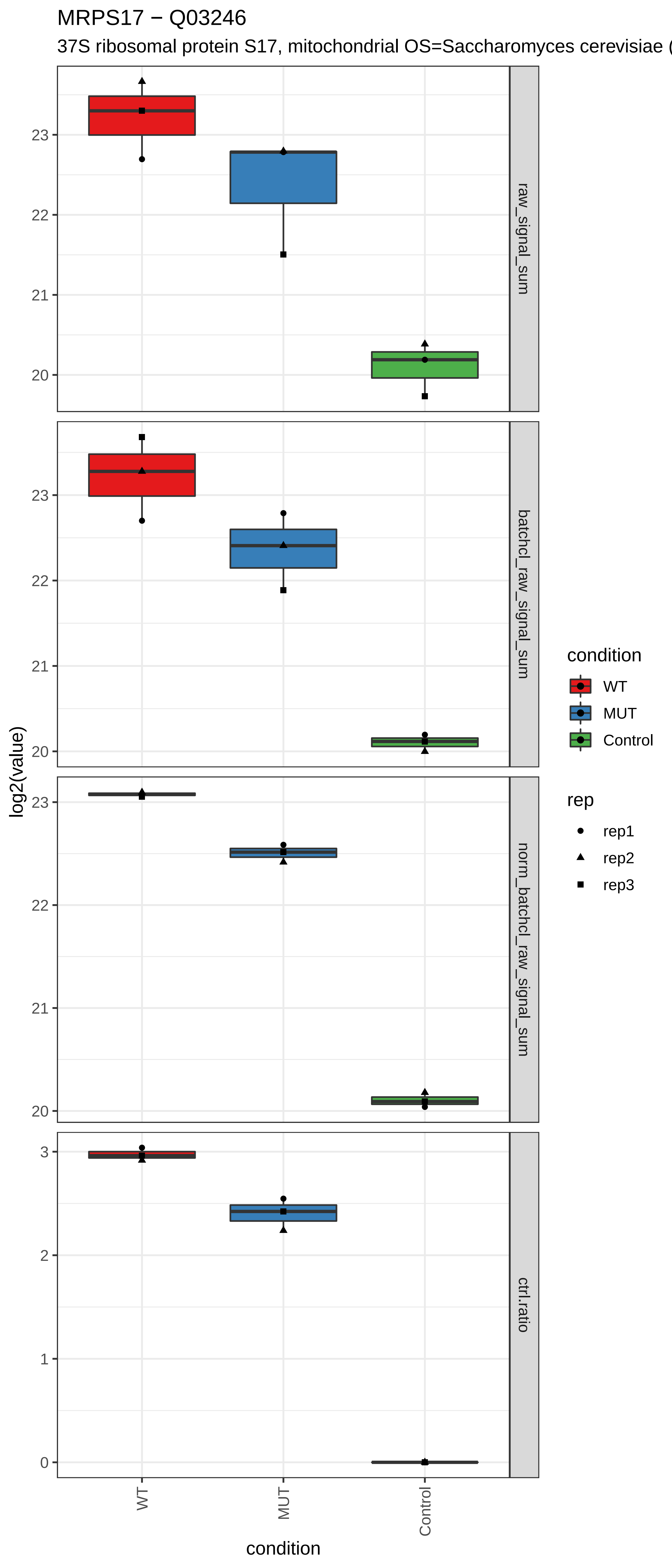
MRPS17 − Q03246
37S ribosomal protein S17, mitochondrial OS=Saccharomyces cerevisiae (
raw_signal_sum
batchcl_raw_signal_sum
norm_batchcl_raw_signal_sum
ctrl.ratio
log2(value)
condition
WT
MUT
Control
rep
rep1
rep2
rep3

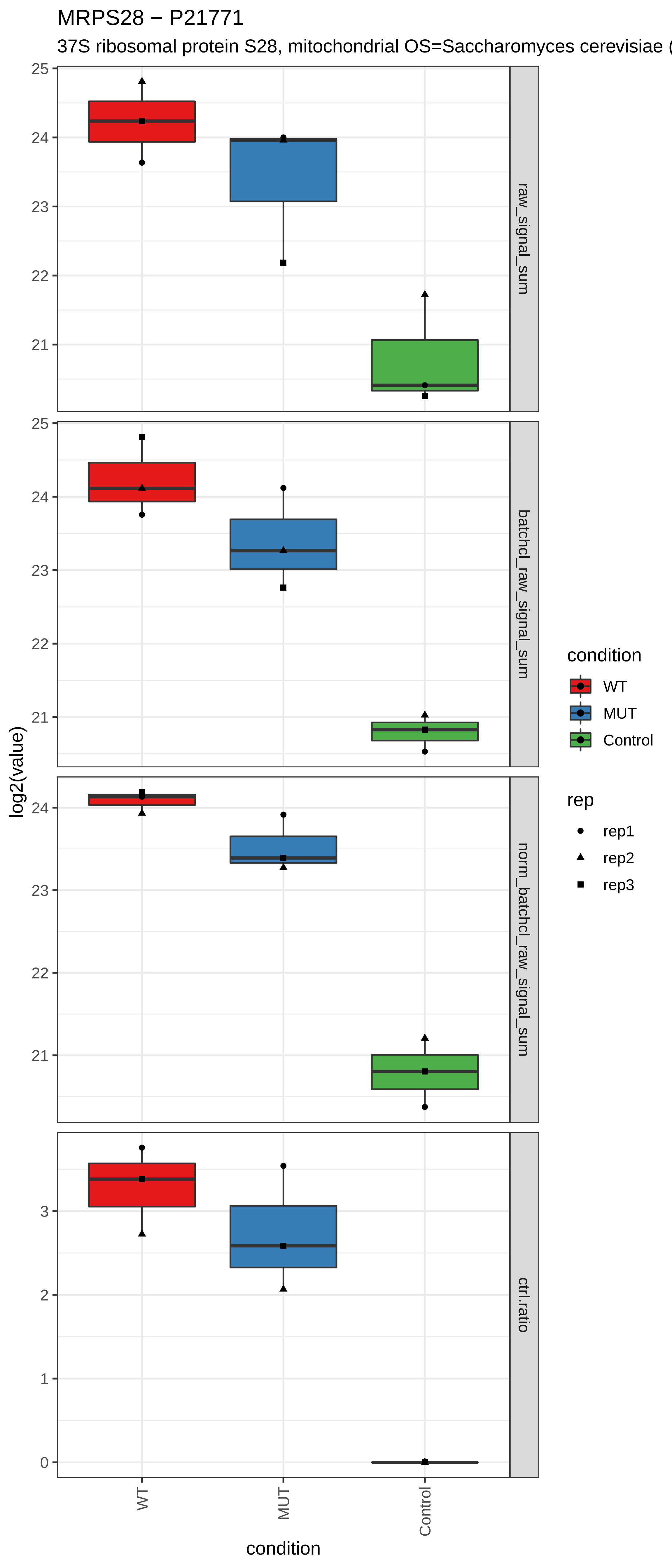

MRPS28 − P21771
37S ribosomal protein S28, mitochondrial OS=Saccharomyces cerevisiae (
log2(value)
raw_signal_sum
batchcl_raw_signal_sum
norm_batchcl_raw_signal_sum
ctrl.ratio
condition
WT
MUT
Control
rep
rep1
rep2
rep3

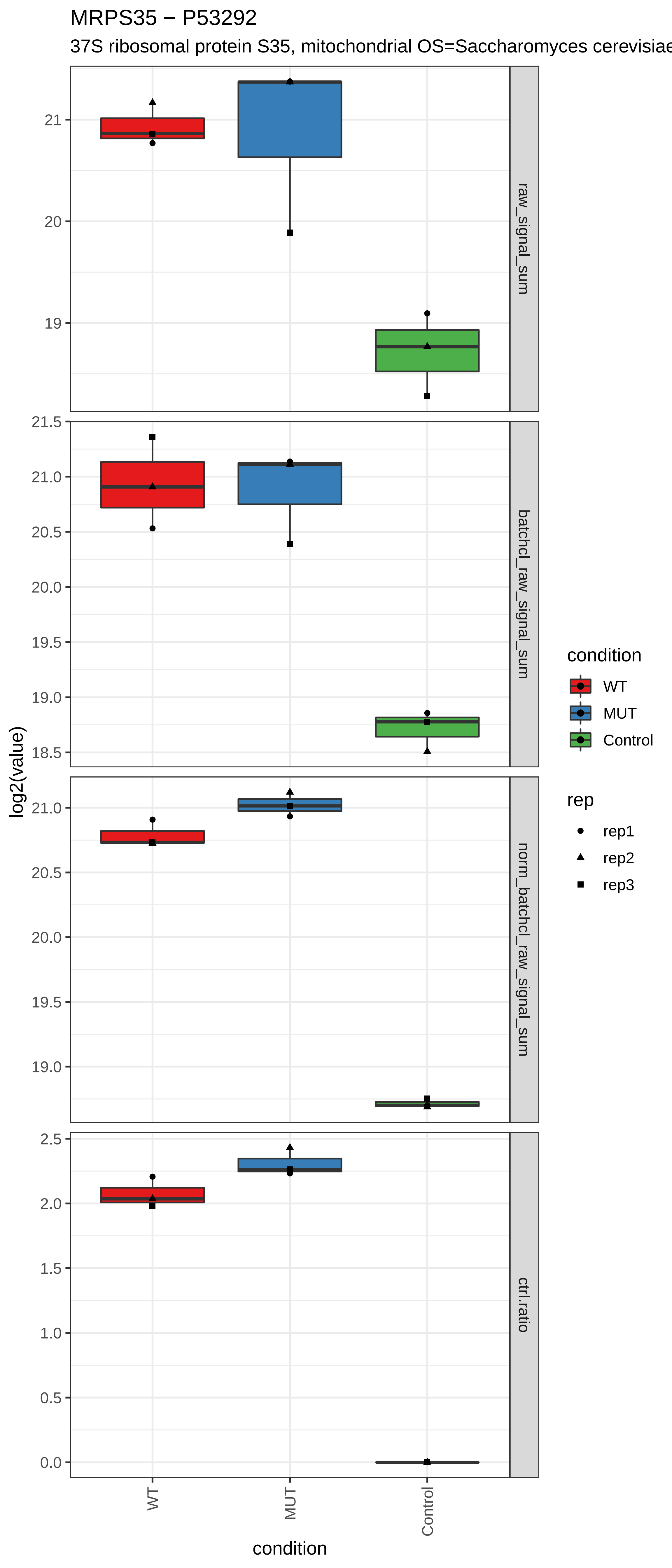

MRPS35 − P53292
37S ribosomal protein S35, mitochondrial OS=Saccharomyces cerevisiae
raw_signal_sum
batchcl_raw_signal_sum
norm_batchcl_raw_signal_sum
ctrl.ratio
log2(value)
condition
WT
MUT
Control
rep
rep1
rep2
rep3
21
20
19
21.5
21.0
20.5
20.0
19.5
19.0
18.5
2.5
2.0
1.5
1.0
0.5
0.0

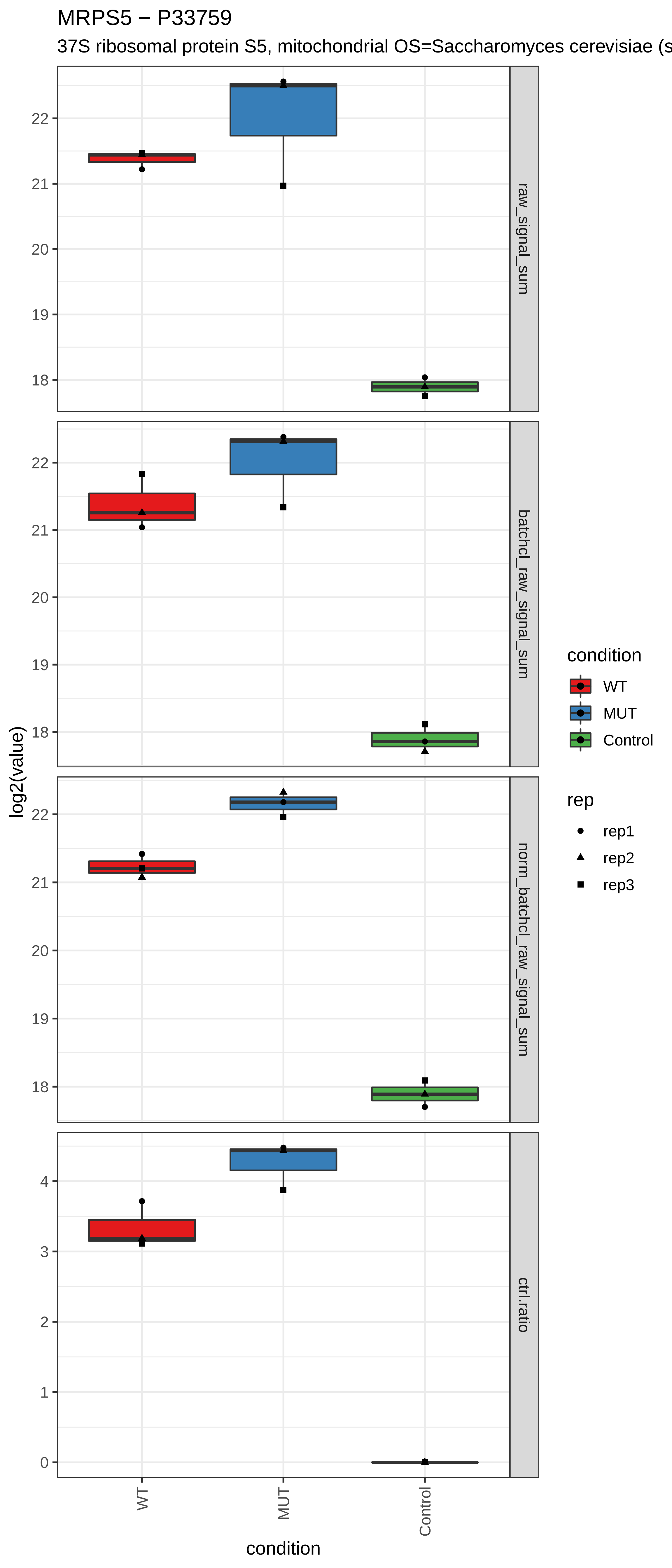

MRPS5 − P33759
37S ribosomal protein S5, mitochondrial OS=Saccharomyces cerevisiae (s
raw_signal_sum
batchcl_raw_signal_sum
norm_batchcl_raw_signal_sum
ctrl.ratio
log2(value)
condition
WT
MUT
Control
rep
rep1
rep2
rep3

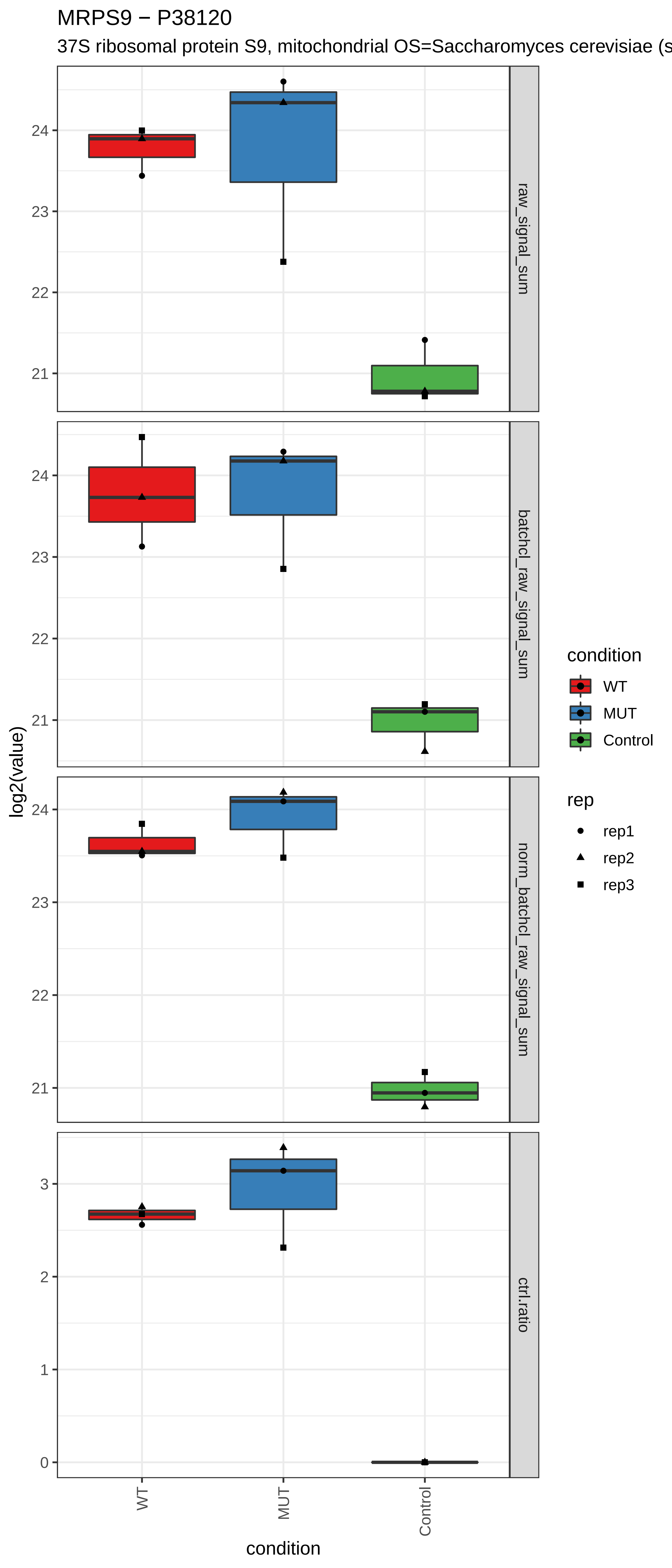
MRPS9 − P38120
37S ribosomal protein S9, mitochondrial OS=Saccharomyces cerevisiae (s
raw_signal_sum
batchcl_raw_signal_sum
norm_batchcl_raw_signal_sum
ctrl.ratio
log2(value)
condition
WT
MUT
Control
rep
rep1
rep2
rep3

MRT4 − P33201

Ribosome assembly factor MRT4 OS=Saccharomyces cerevisiae (strain AT

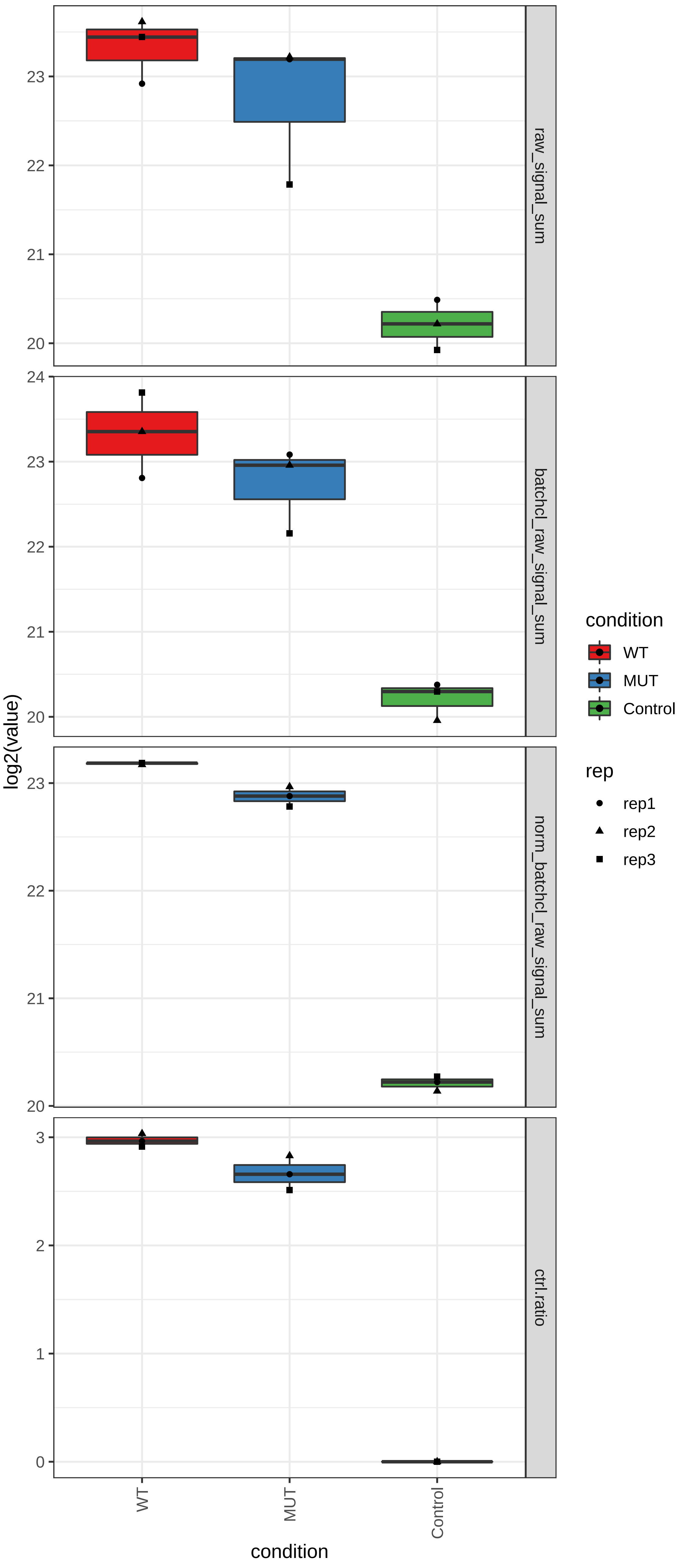

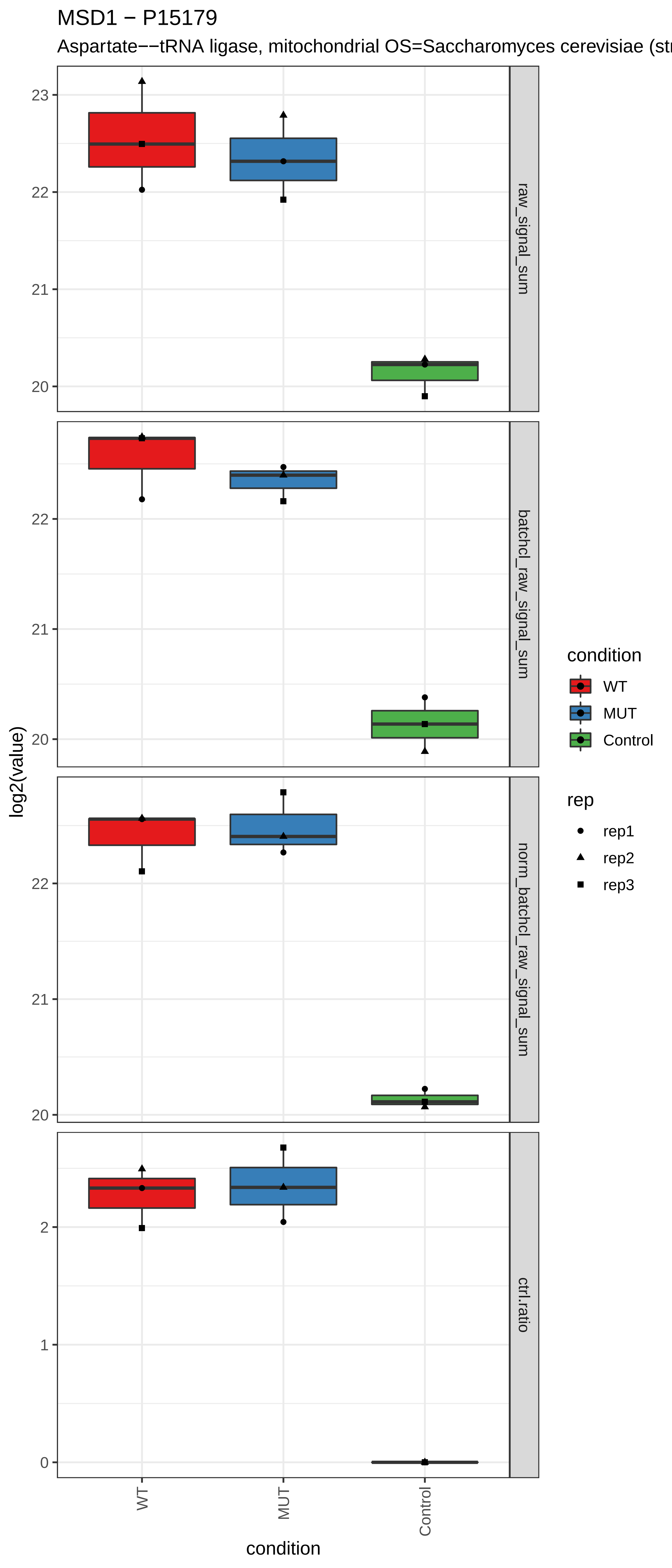

MSD1 − P15179
Aspartate−−tRNA ligase, mitochondrial OS=Saccharomyces cerevisiae (str
raw_signal_sum
batchcl_raw_signal_sum
norm_batchcl_raw_signal_sum
ctrl.ratio
log2(value)
23
22
21
20
0
1
2
WT
MUT
Control
condition
condition
WT
MUT
Control
rep
rep1
rep2
rep3

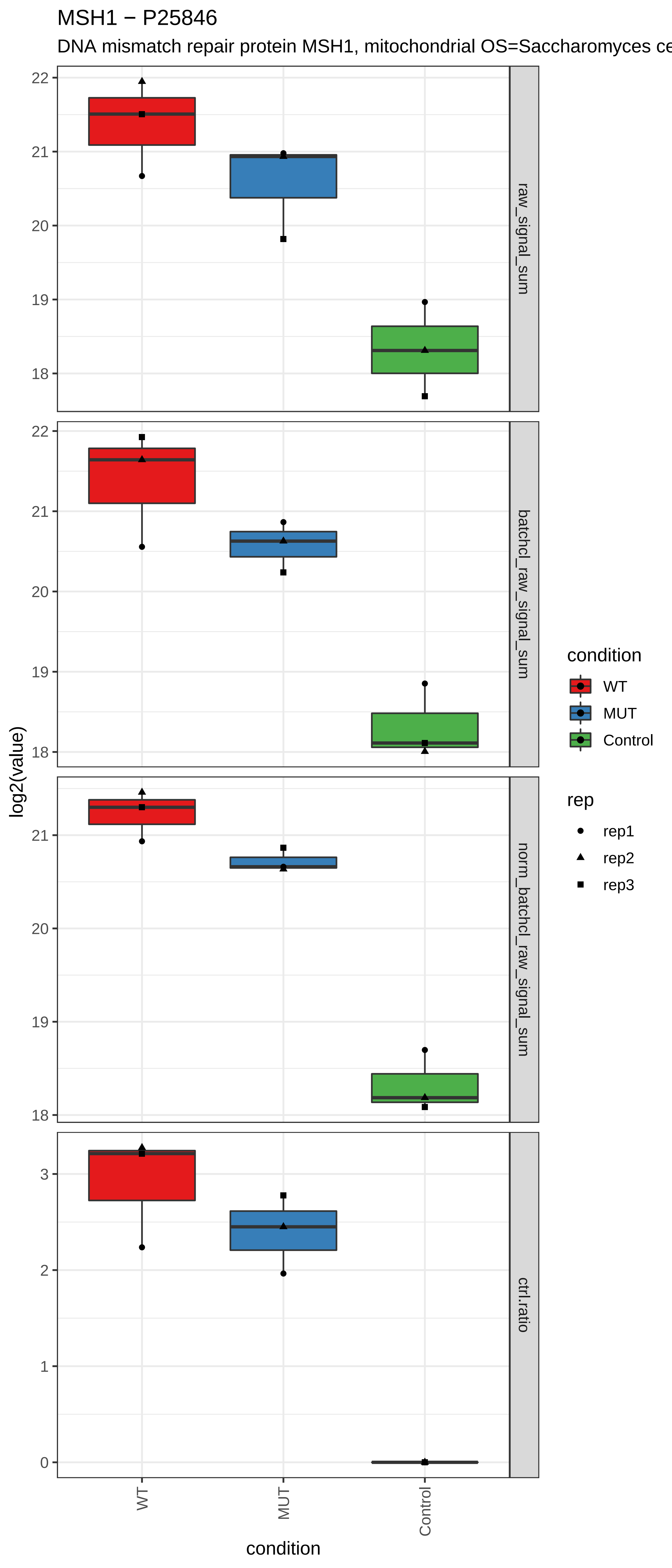

MSH1 − P25846
DNA mismatch repair protein MSH1, mitochondrial OS=Saccharomyces ce
raw_signal_sum
batchcl_raw_signal_sum
norm_batchcl_raw_signal_sum
ctrl.ratio
log2(value)
condition
WT
MUT
Control
rep
rep1
rep2
rep3
22
21
20
19
18
3
2
1
0

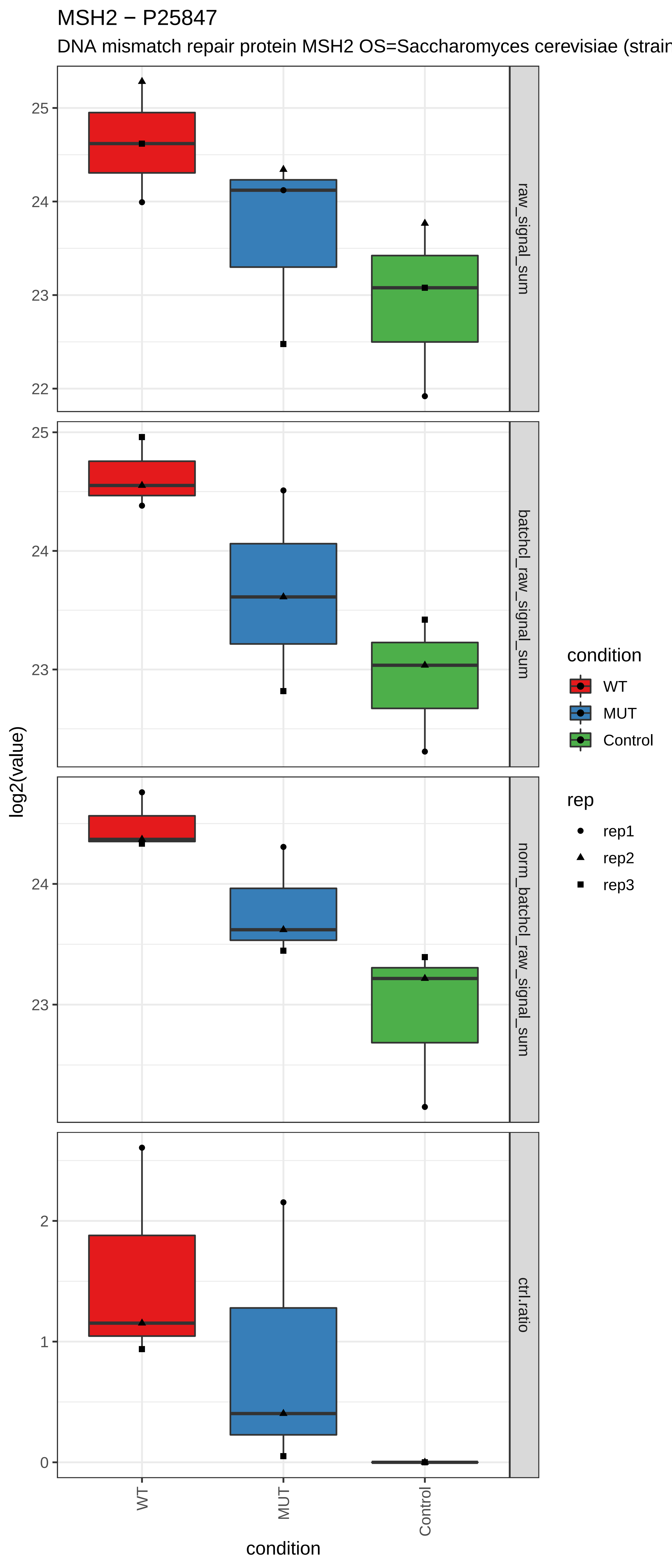

MSH2 − P25847
DNA mismatch repair protein MSH2 OS=Saccharomyces cerevisiae (strain
raw_signal_sum
batchcl_raw_signal_sum
norm_batchcl_raw_signal_sum
ctrl.ratio
log2(value)
condition
WT
MUT
Control
rep
rep1
rep2
rep3
25
24
23
22
2
1
0

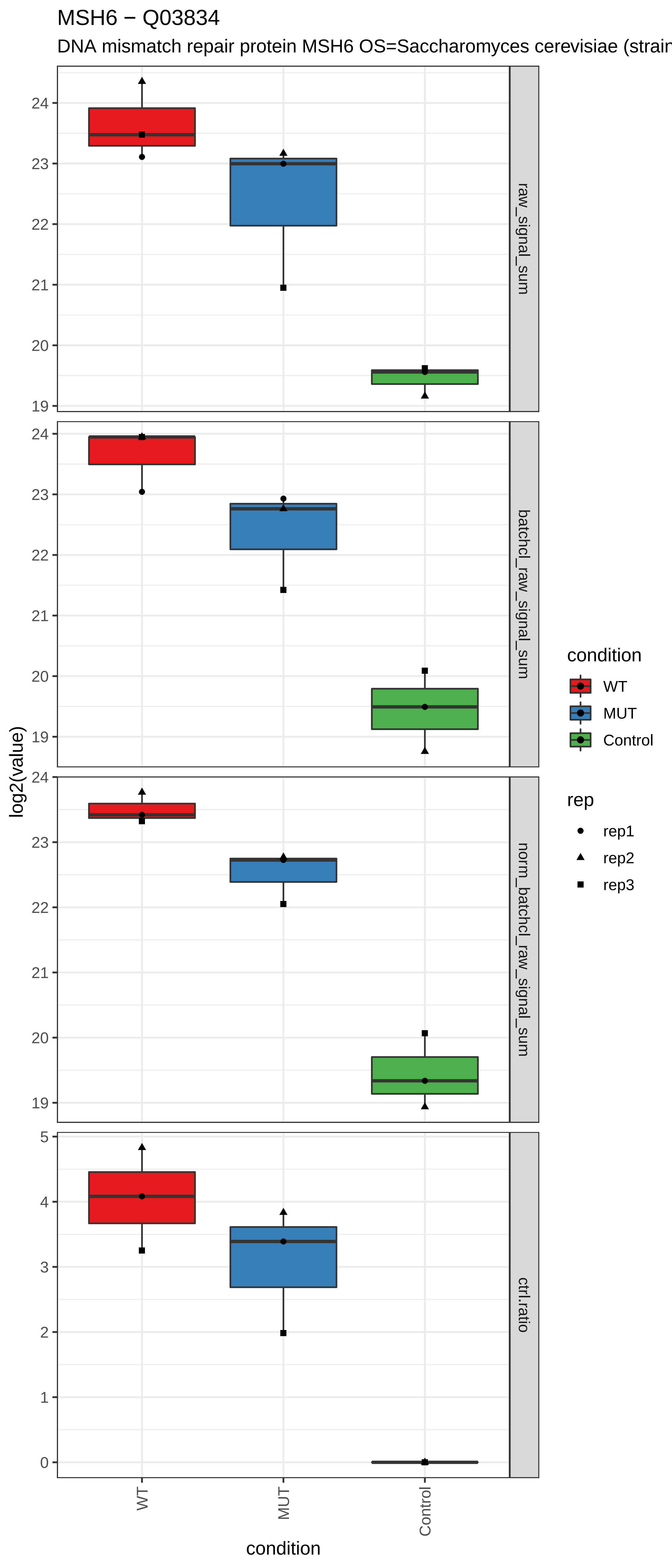

MSH6 − Q03834
DNA mismatch repair protein MSH6 OS=Saccharomyces cerevisiae (strain
raw_signal_sum
batchcl_raw_signal_sum
norm_batchcl_raw_signal_sum
ctrl.ratio
log2(value)
condition
WT
MUT
Control
rep
rep1
rep2
rep3

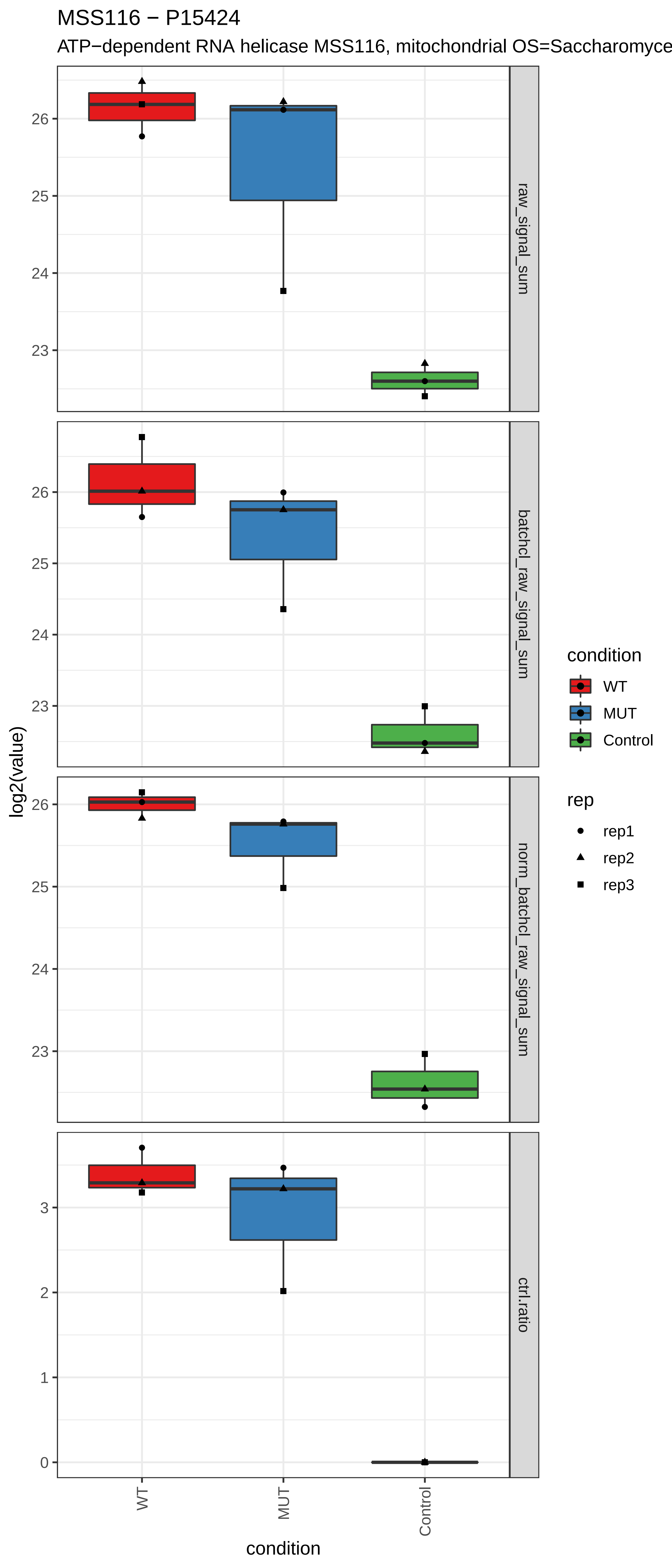

MSS116 − P15424
ATP−dependent RNA helicase MSS116, mitochondrial OS=Saccharomyce
raw_signal_sum
batchcl_raw_signal_sum
norm_batchcl_raw_signal_sum
ctrl.ratio
log2(value)
condition
WT
MUT
Control
rep
rep1
rep2
rep3
26
25
24
23
3
2
1
0

MST1 − P07236

Threonine−−tRNA ligase, mitochondrial OS=Saccharomyces cerevisiae (st

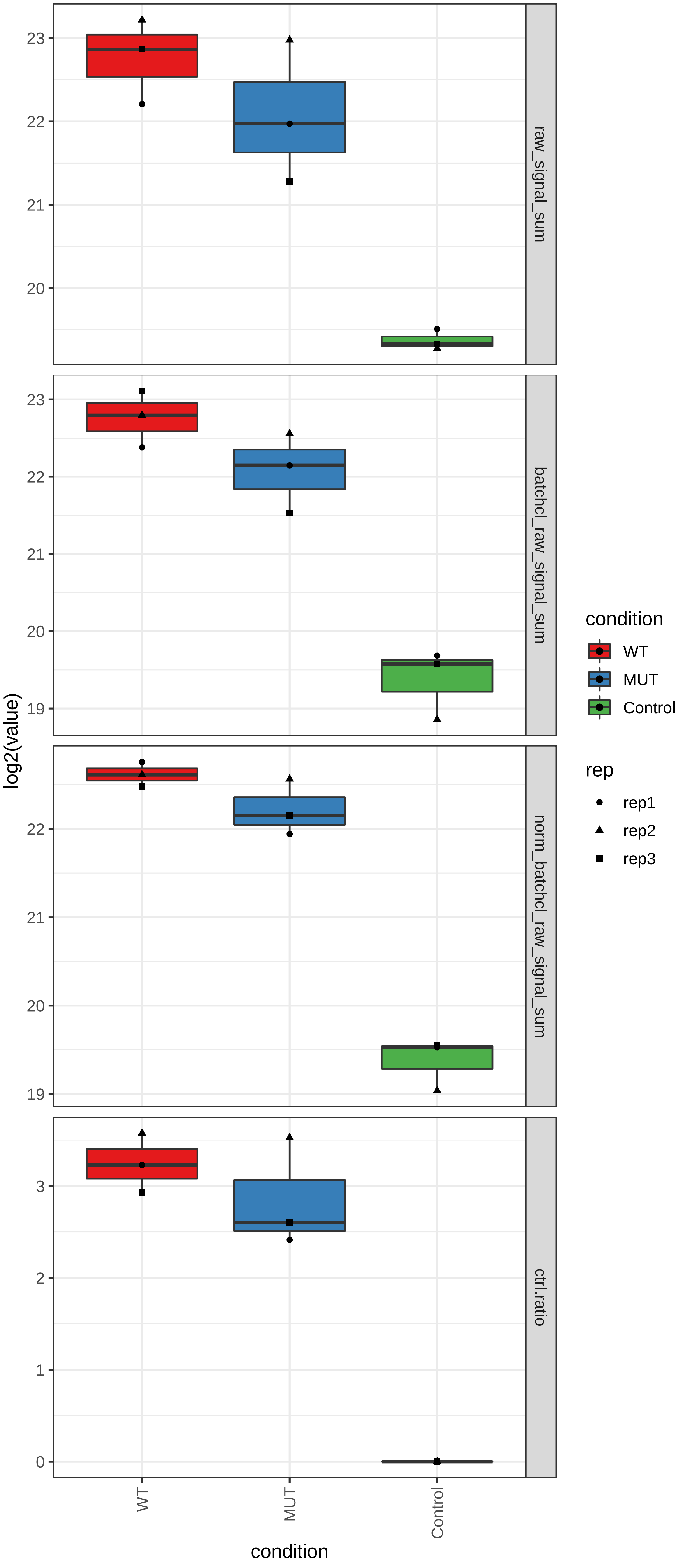

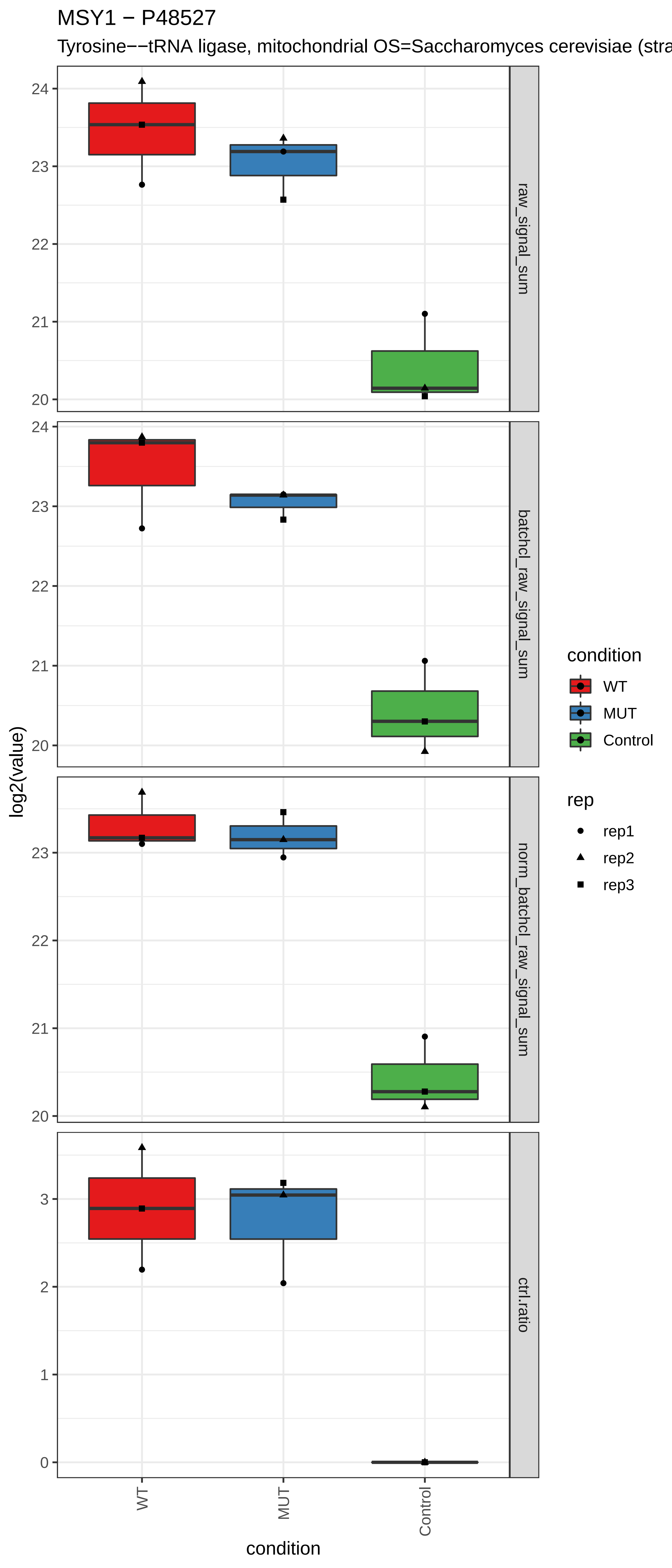
MSY1 − P48527
Tyrosine−−tRNA ligase, mitochondrial OS=Saccharomyces cerevisiae (stra
raw_signal_sum
batchcl_raw_signal_sum
norm_batchcl_raw_signal_sum
ctrl.ratio
log2(value)
condition
WT
MUT
Control
rep
rep1
rep2
rep3

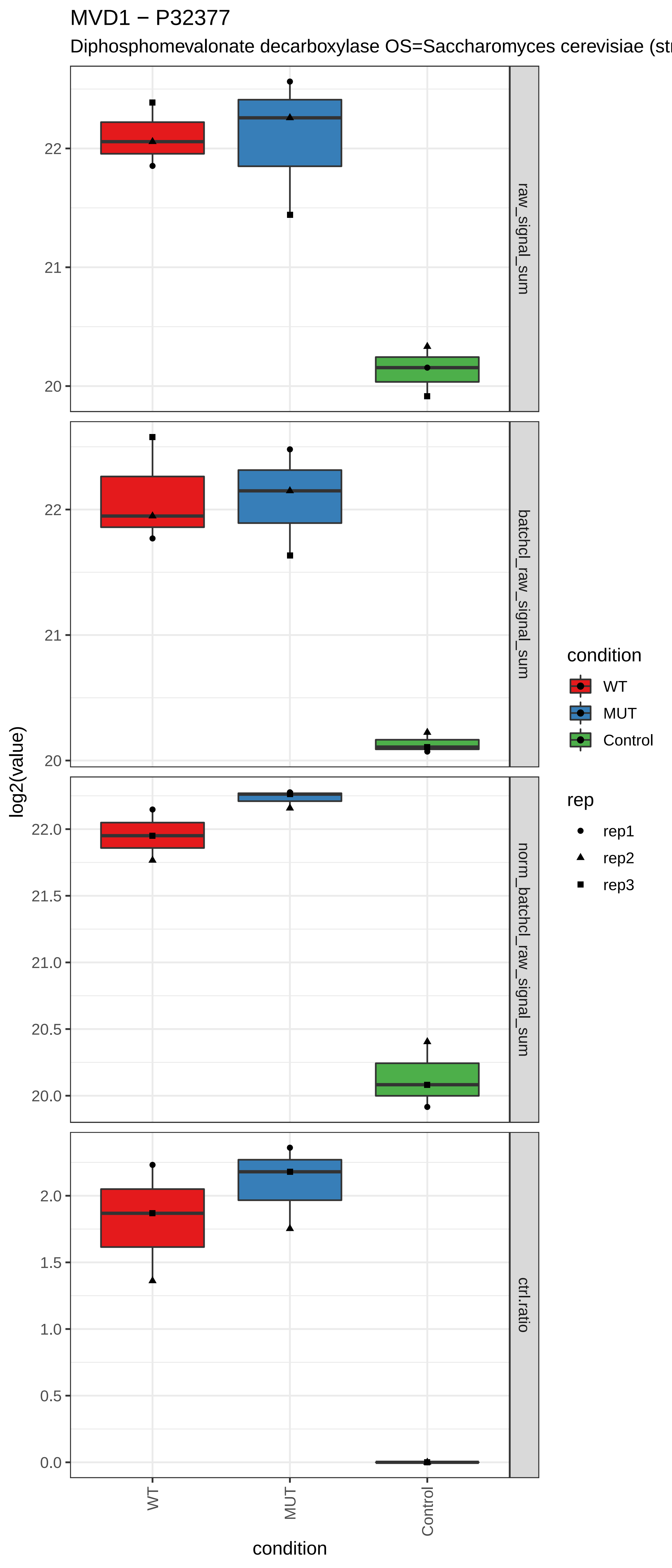

MVD1 − P32377
Diphosphomevalonate decarboxylase OS=Saccharomyces cerevisiae (str
raw_signal_sum
batchcl_raw_signal_sum
norm_batchcl_raw_signal_sum
ctrl.ratio
log2(value)
condition
WT
MUT
Control
rep
rep1
rep2
rep3

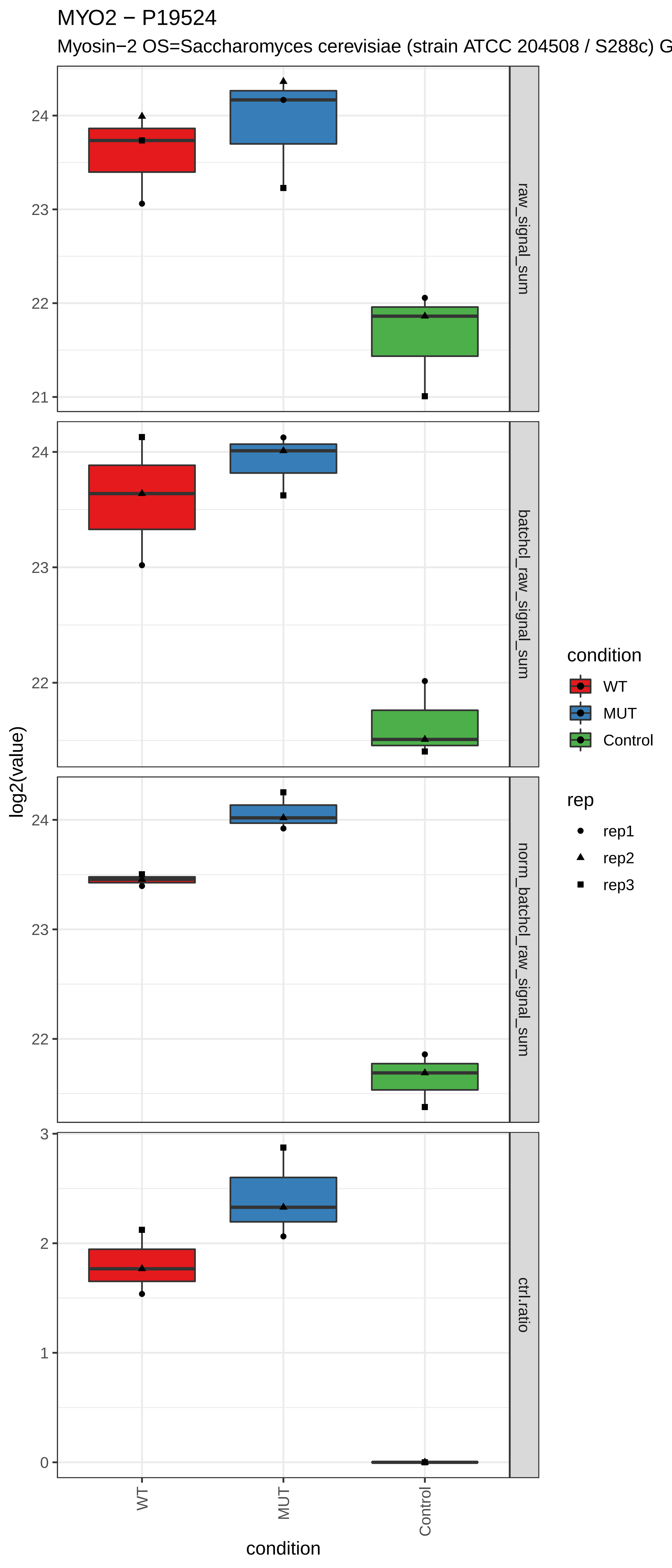

MYO2 − P19524
Myosin−2 OS=Saccharomyces cerevisiae (strain ATCC 204508 / S288c) G
raw_signal_sum
batchcl_raw_signal_sum
norm_batchcl_raw_signal_sum
ctrl.ratio
log2(value)
condition
WT
MUT
Control
rep
rep1
rep2
rep3

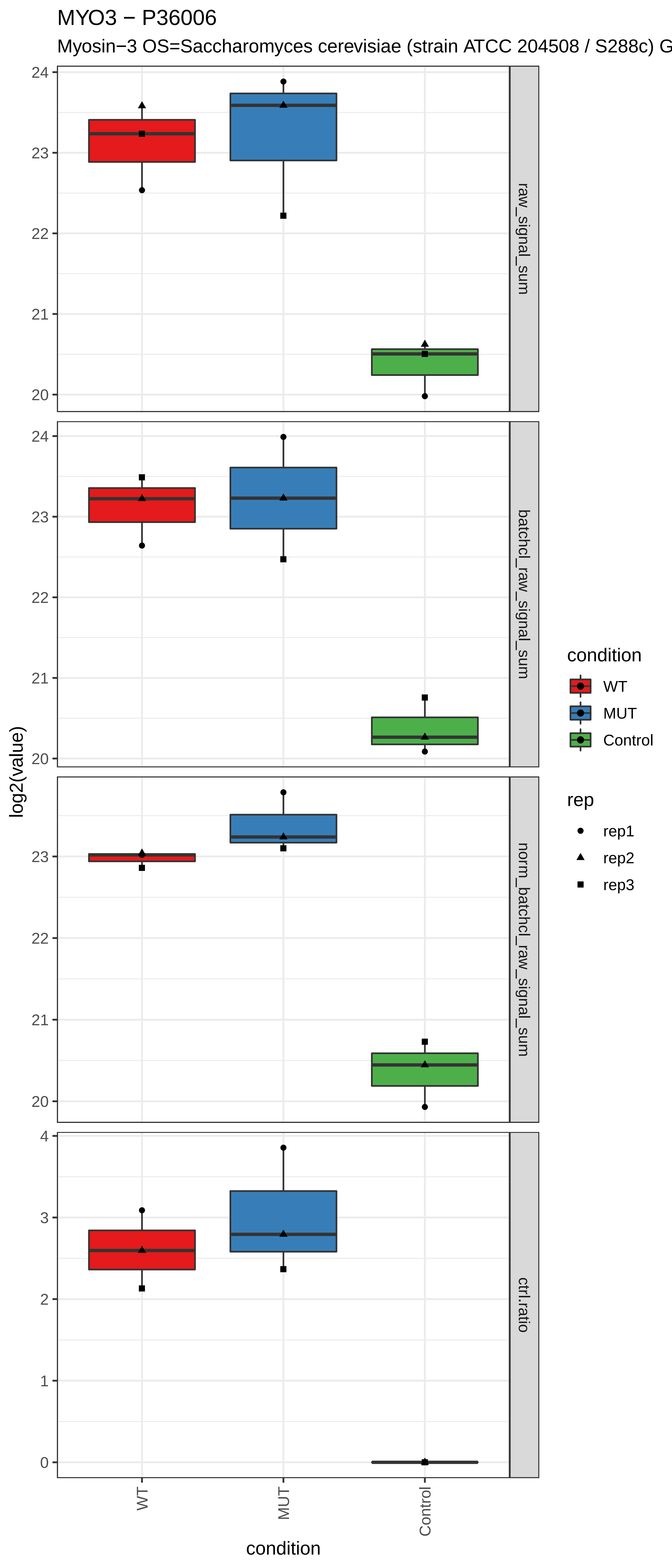

MYO3 − P36006
Myosin−3 OS=Saccharomyces cerevisiae (strain ATCC 204508 / S288c) G
raw_signal_sum
batchcl_raw_signal_sum
norm_batchcl_raw_signal_sum
ctrl.ratio
log2(value)
condition
WT
MUT
Control
rep
rep1
rep2
rep3

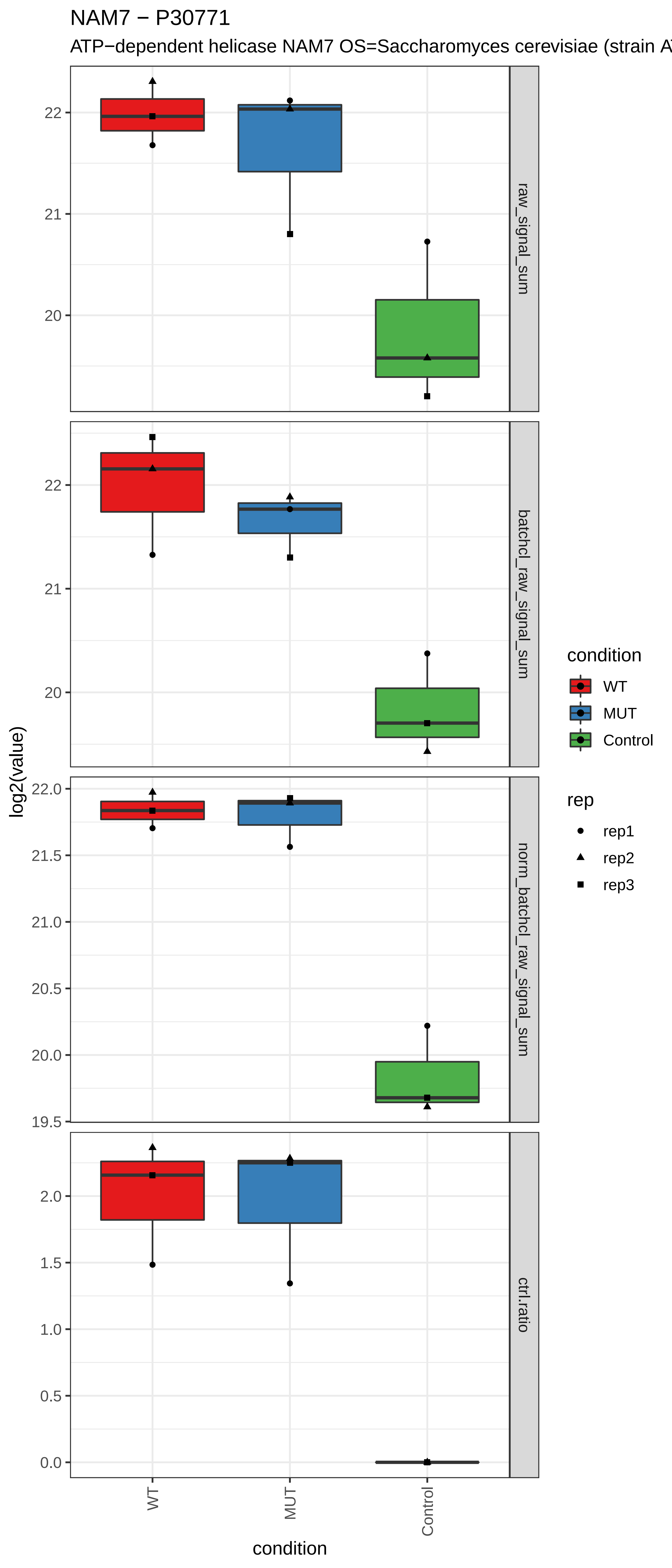

NAM7 − P30771
ATP−dependent helicase NAM7 OS=Saccharomyces cerevisiae (strain A
raw_signal_sum
batchcl_raw_signal_sum
norm_batchcl_raw_signal_sum
ctrl.ratio
log2(value)
condition
WT
MUT
Control
rep
rep1
rep2
rep3
22
21
20
22.0
21.5
21.0
20.5
20.0
19.5
2.0
1.5
1.0
0.5
0.0

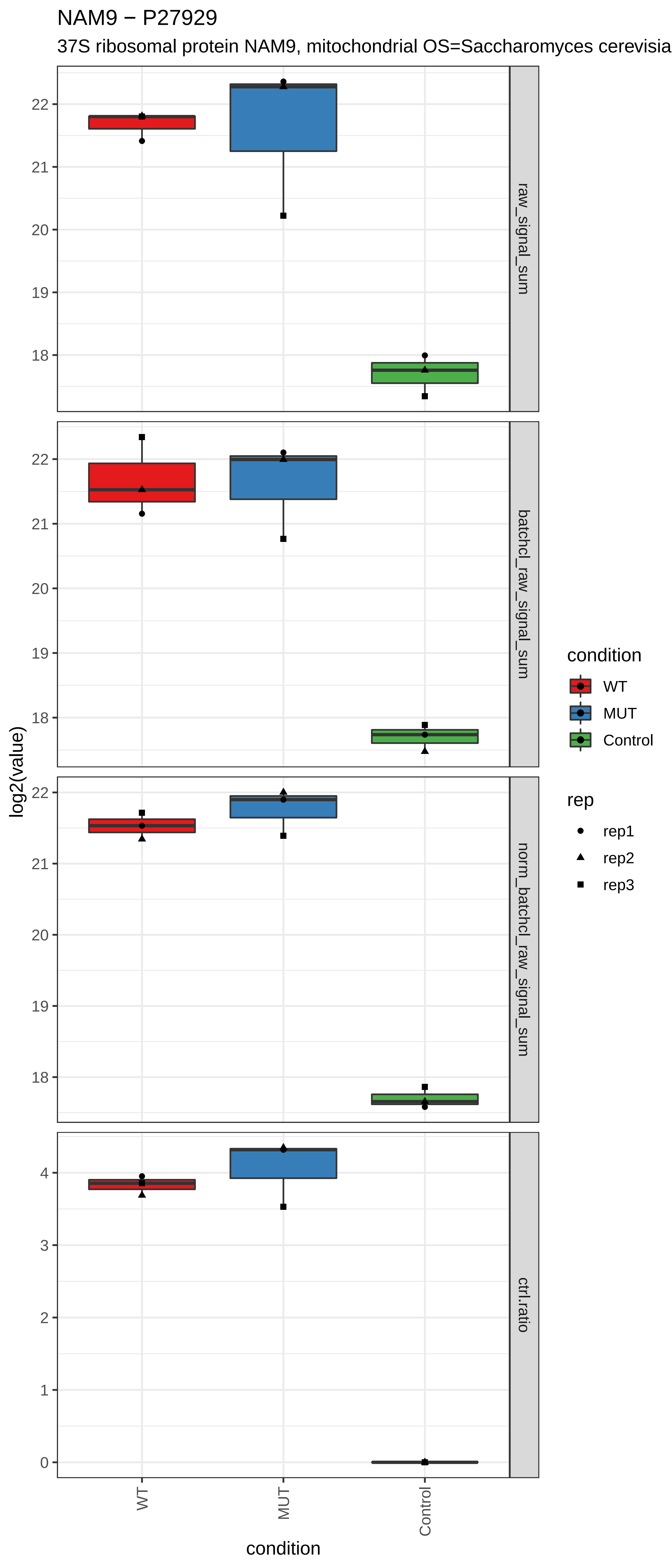
NAM9 − P27929
37S ribosomal protein NAM9, mitochondrial OS=Saccharomyces cerevisia
raw_signal_sum
batchcl_raw_signal_sum
norm_batchcl_raw_signal_sum
ctrl.ratio
log2(value)
condition
WT
MUT
Control
rep
rep1
rep2
rep3
22
21
20
19
18
4
3
2
1
0

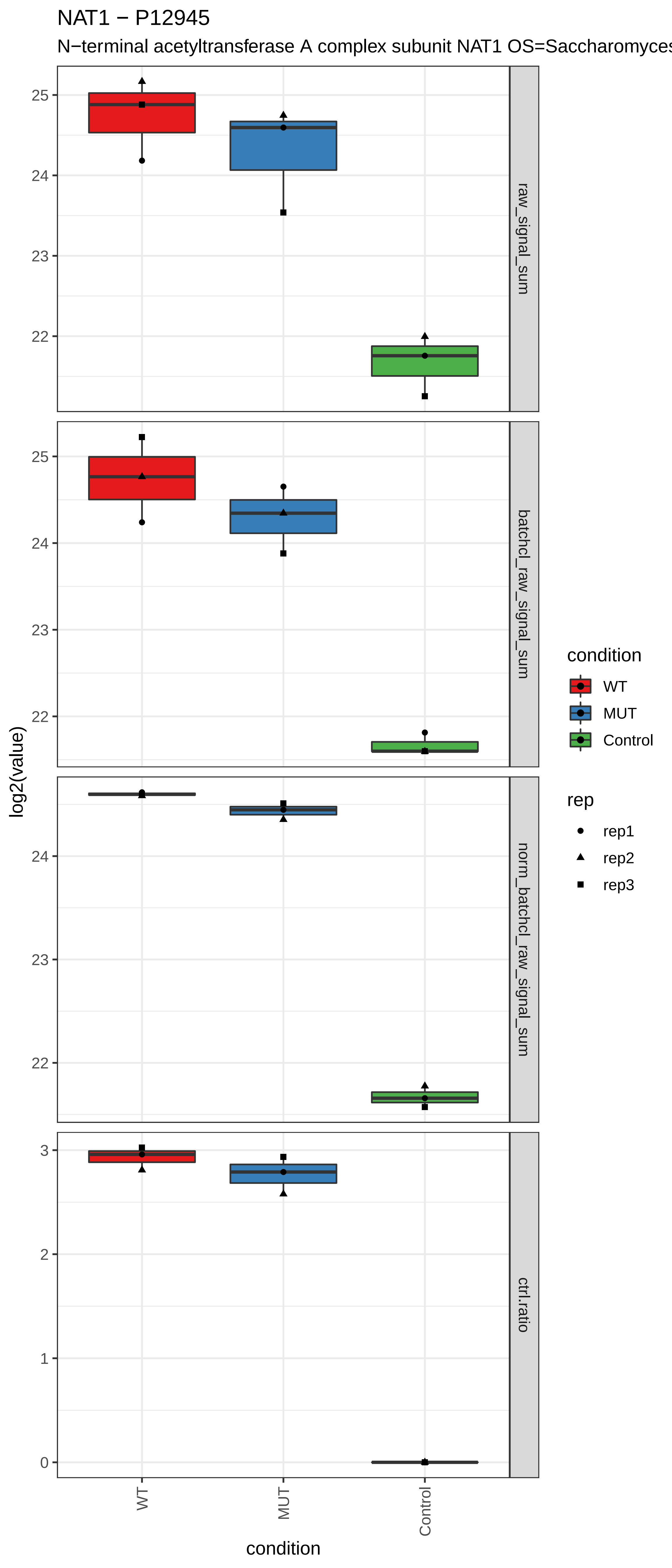
NAT1 − P12945
N−terminal acetyltransferase A complex subunit NAT1 OS=Saccharomyces
raw_signal_sum
batchcl_raw_signal_sum
norm_batchcl_raw_signal_sum
ctrl.ratio
log2(value)
condition
WT
MUT
Control
rep
rep1
rep2
rep3
25
24
23
22
3
2
1
0

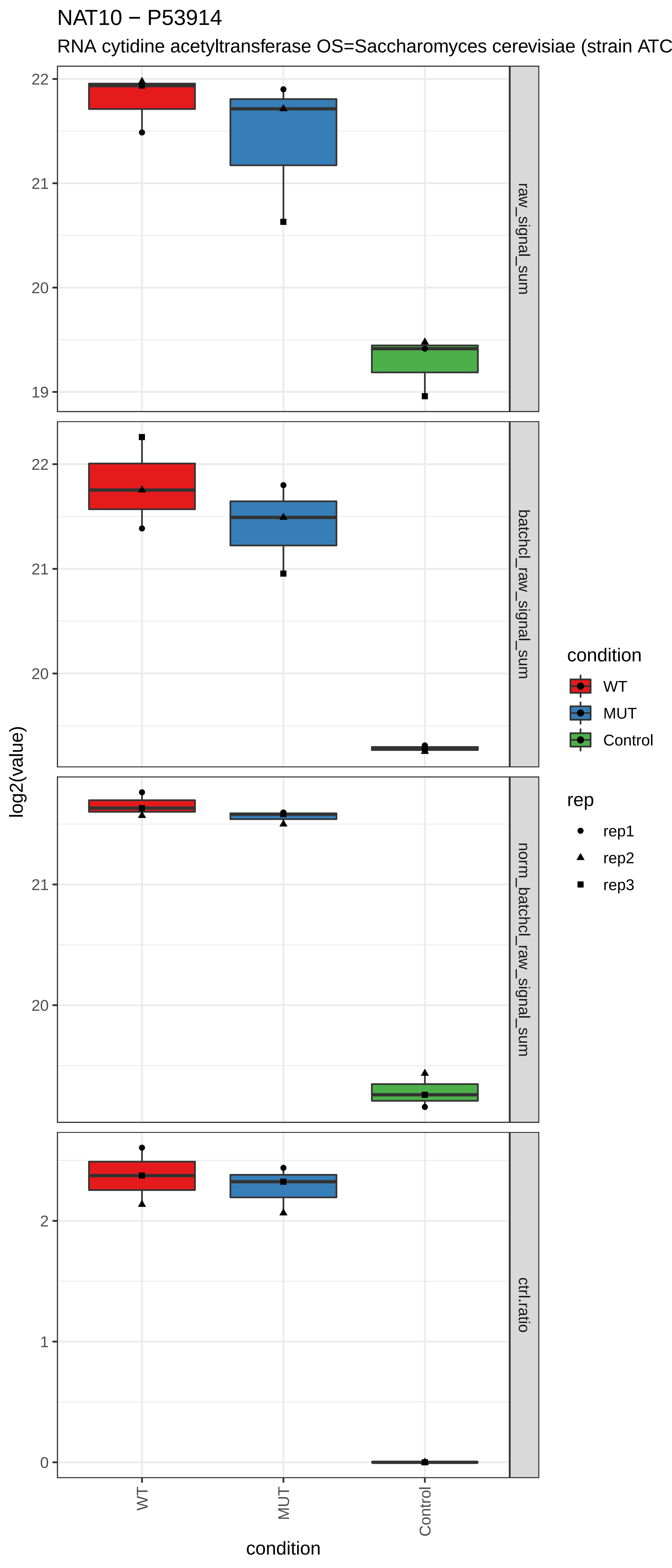
NAT10 − P53914
RNA cytidine acetyltransferase OS=Saccharomyces cerevisiae (strain ATC
raw_signal_sum
batchcl_raw_signal_sum
norm_batchcl_raw_signal_sum
ctrl.ratio
log2(value)
condition
WT
MUT
Control
rep
rep1
rep2
rep3
22
21
20
19
2
1
0

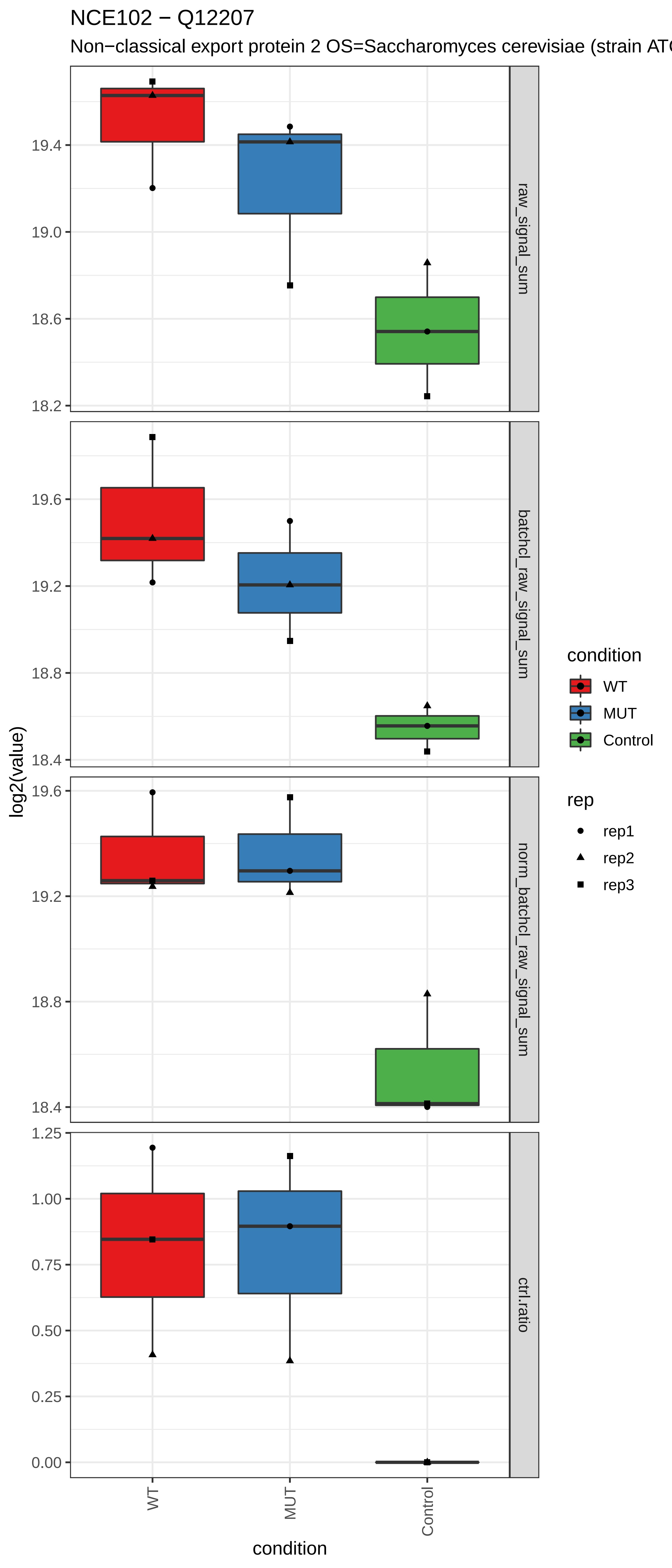

NCE102 − Q12207
Non−classical export protein 2 OS=Saccharomyces cerevisiae (strain ATC
log2(value)
raw_signal_sum
batchcl_raw_signal_sum
norm_batchcl_raw_signal_sum
ctrl.ratio
19.4
19.0
18.6
18.2
19.6
19.2
18.8
18.4
1.25
1.00
0.75
0.50
0.25
0.00
WT
MUT
Control
condition
condition
WT
MUT
Control
rep
rep1
rep2
rep3

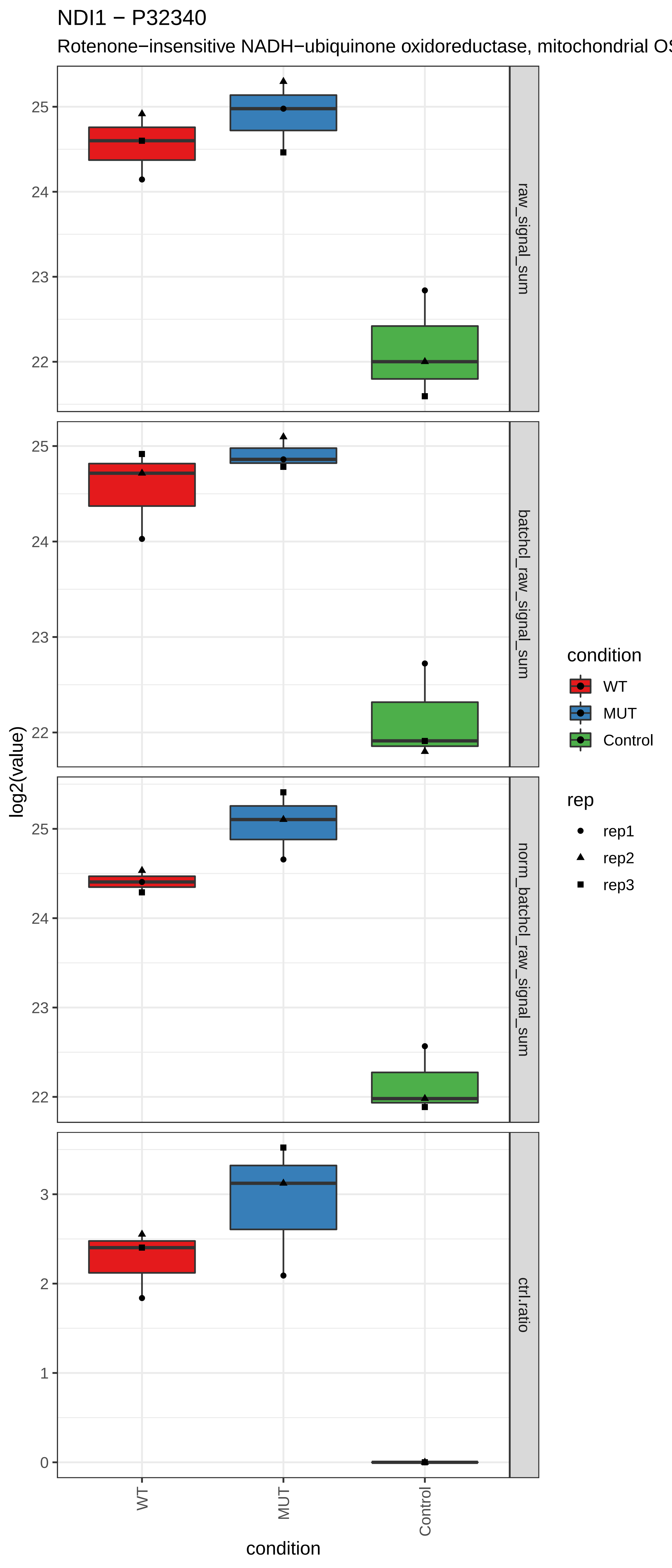

NDI1 − P32340
Rotenone−insensitive NADH−ubiquinone oxidoreductase, mitochondrial OS
raw_signal_sum
batchcl_raw_signal_sum
norm_batchcl_raw_signal_sum
ctrl.ratio
log2(value)
condition
WT
MUT
Control
rep
rep1
rep2
rep3

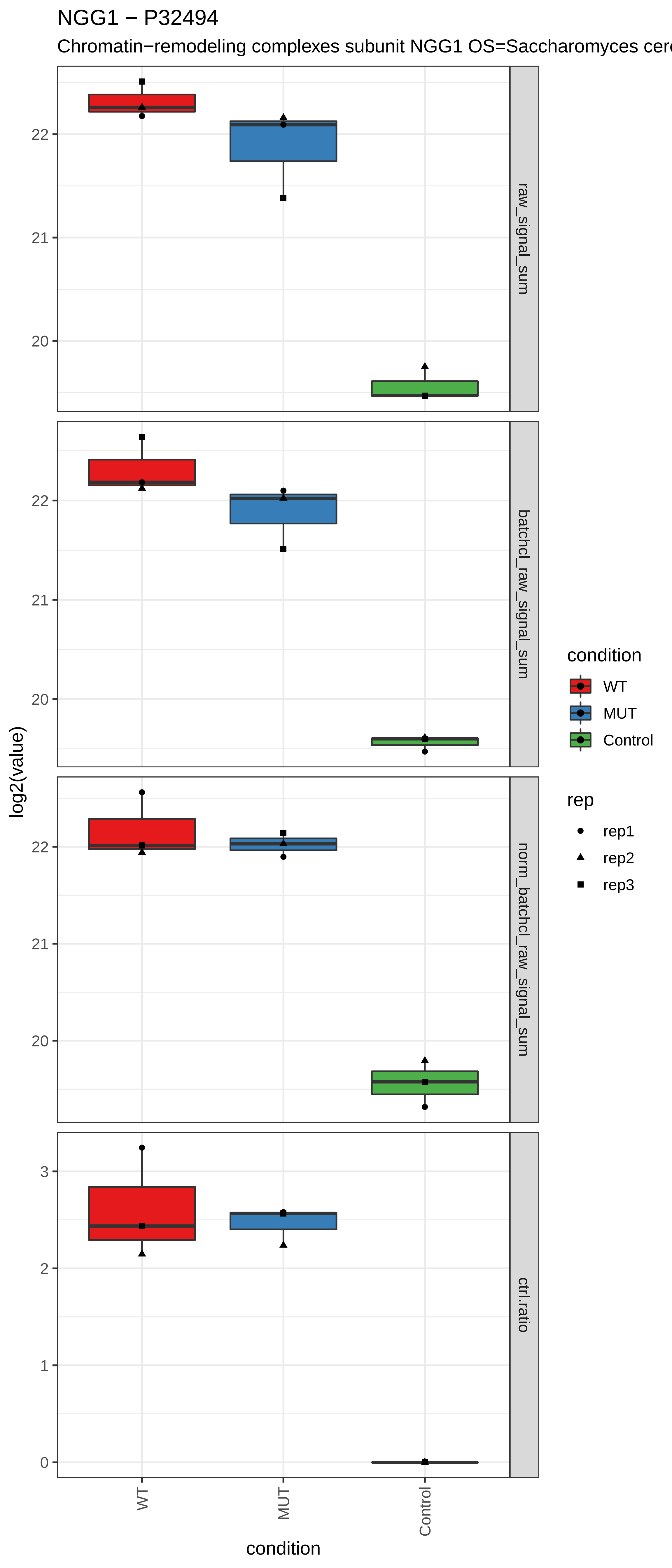

NGG1 − P32494
Chromatin−remodeling complexes subunit NGG1 OS=Saccharomyces cere
raw_signal_sum
batchcl_raw_signal_sum
norm_batchcl_raw_signal_sum
ctrl.ratio
log2(value)
22
21
20
3
2
1
0
WT
MUT
Control
condition
condition
WT
MUT
Control
rep
rep1
rep2
rep3

NHP2 − P32495

H/ACA ribonucleoprotein complex subunit 2 OS=Saccharomyces cerevisiae

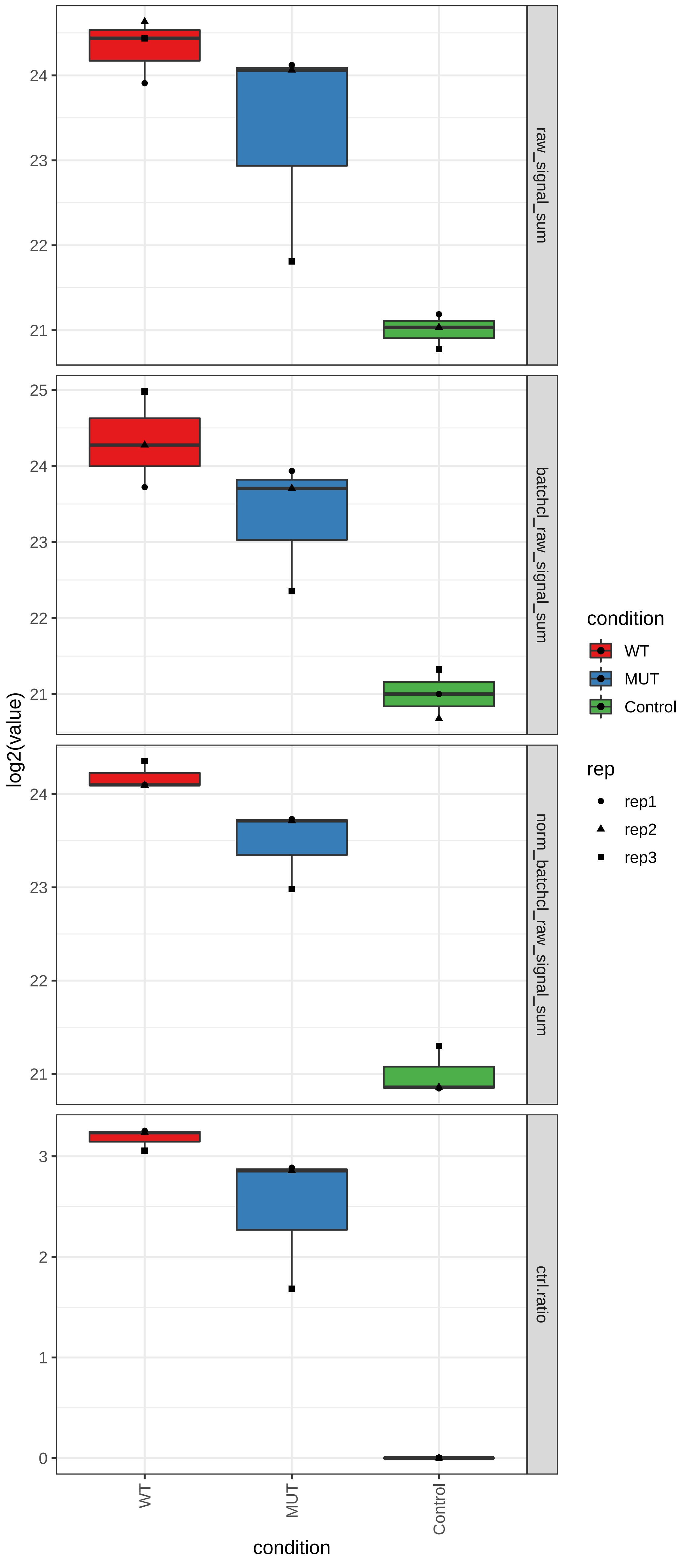

NHP6A − P11632

Non−histone chromosomal protein 6A OS=Saccharomyces cerevisiae (stra

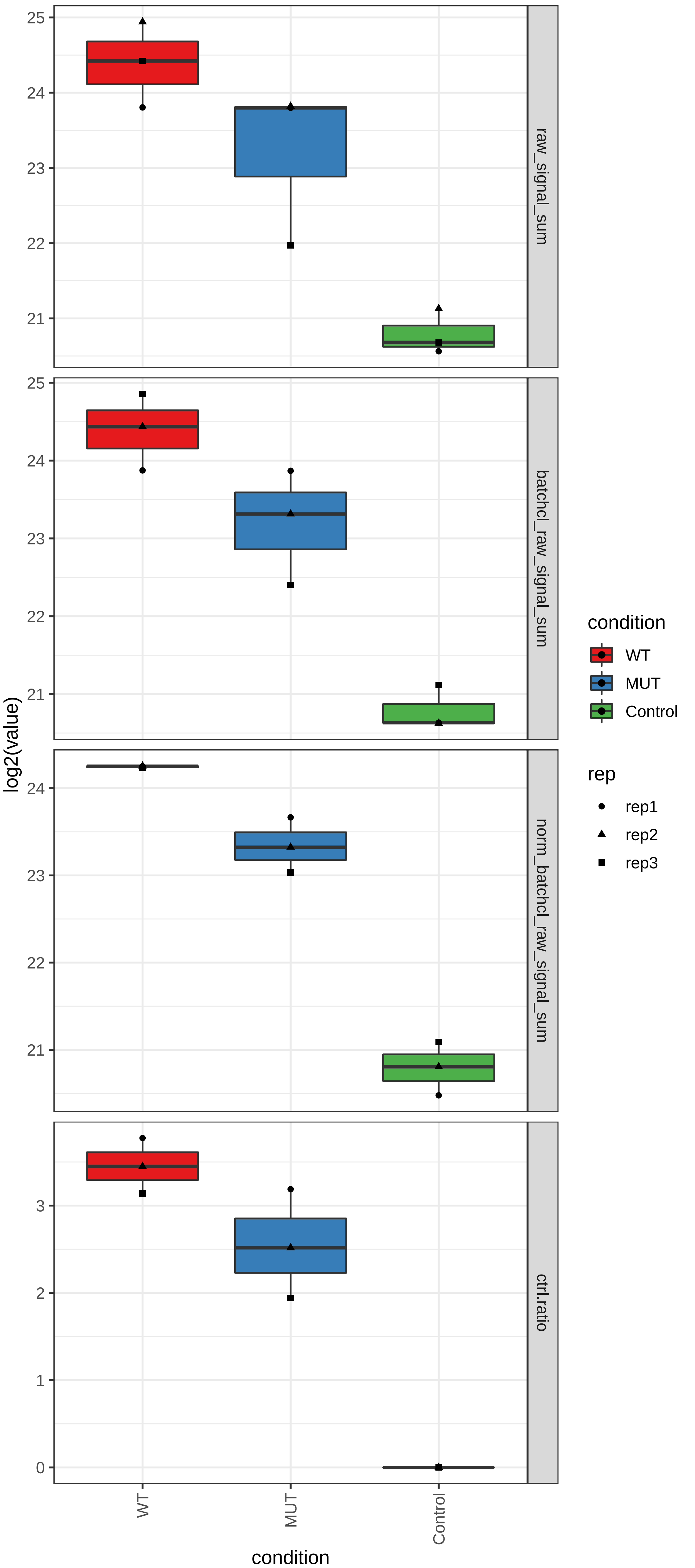

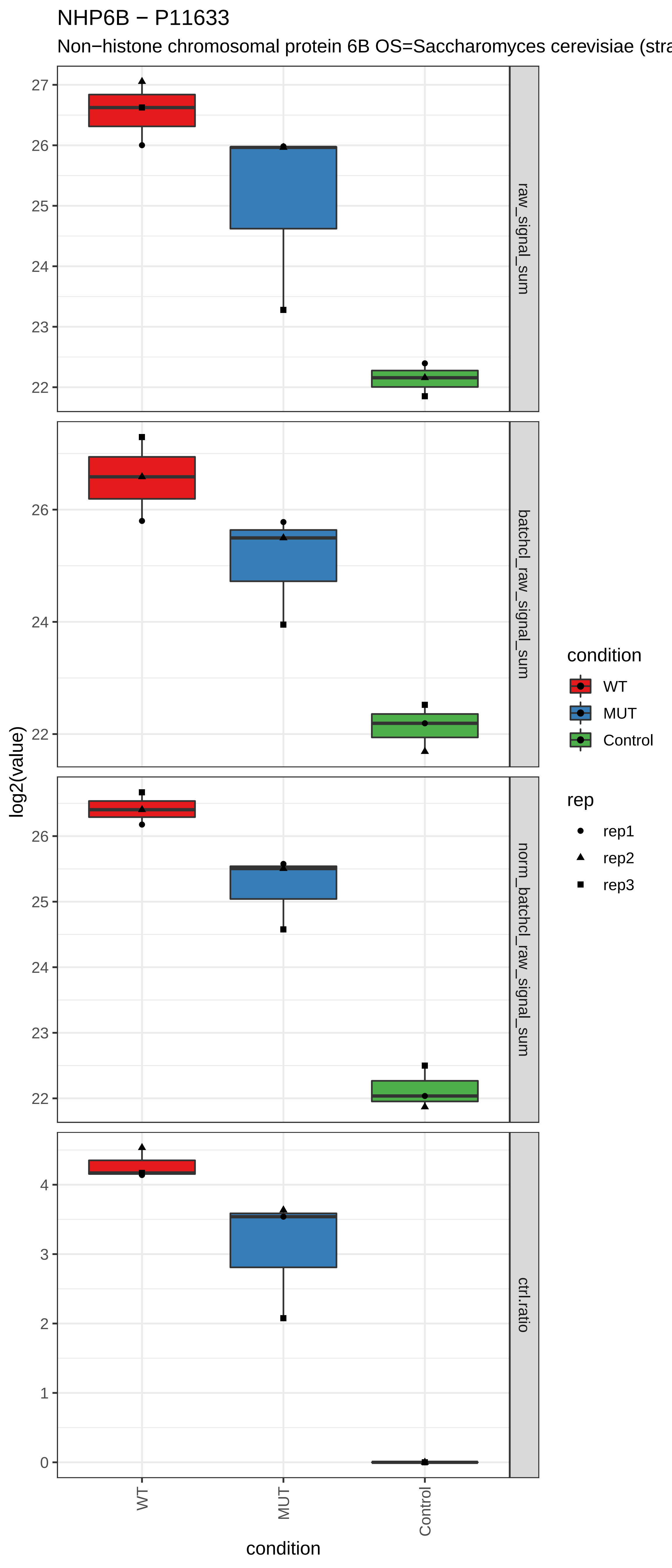
NHP6B − P11633
Non−histone chromosomal protein 6B OS=Saccharomyces cerevisiae (stra
raw_signal_sum
batchcl_raw_signal_sum
norm_batchcl_raw_signal_sum
ctrl.ratio
log2(value)
condition
WT
MUT
Control
rep
rep1
rep2
rep3

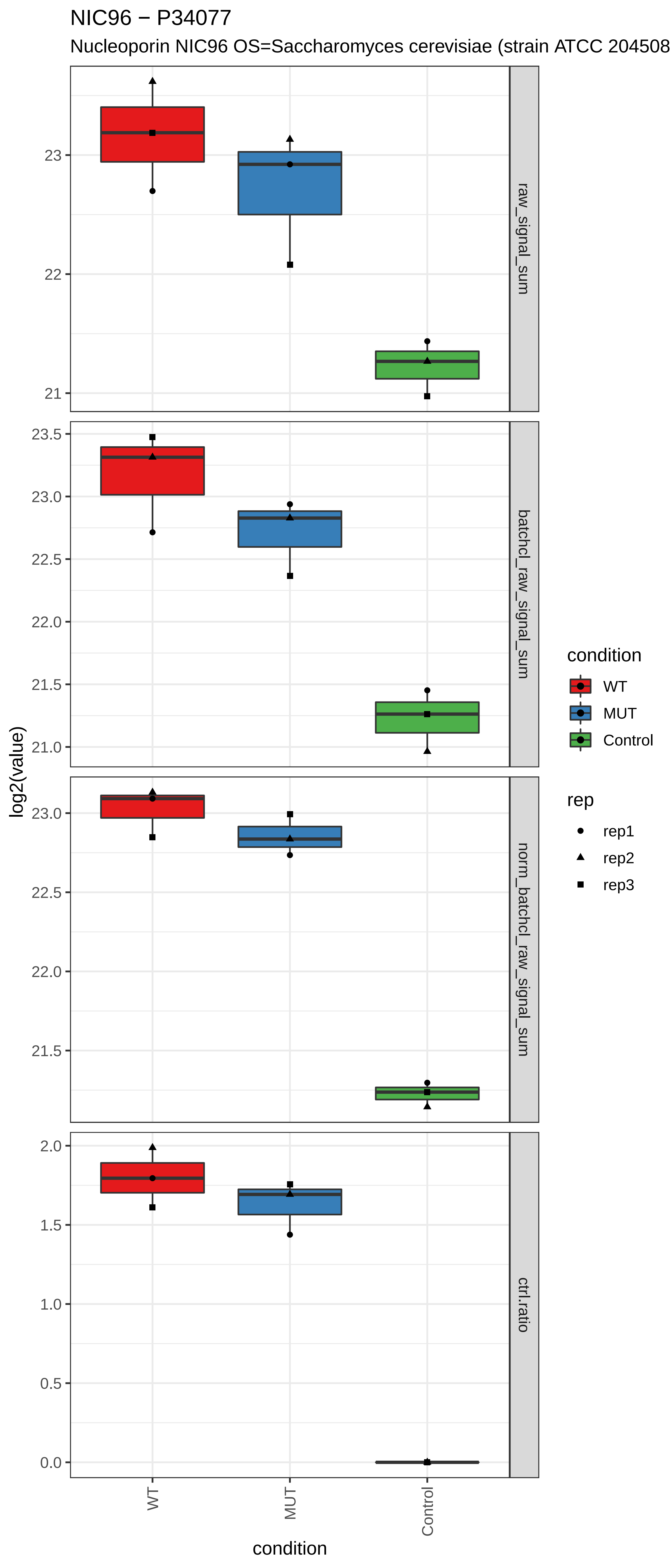
NIC96 − P34077
Nucleoporin NIC96 OS=Saccharomyces cerevisiae (strain ATCC 204508
raw_signal_sum
batchcl_raw_signal_sum
norm_batchcl_raw_signal_sum
ctrl.ratio
log2(value)
condition
WT
MUT
Control
rep
rep1
rep2
rep3
23
22
21
23.5
23.0
22.5
22.0
21.5
21.0
2.0
1.5
1.0
0.5
0.0

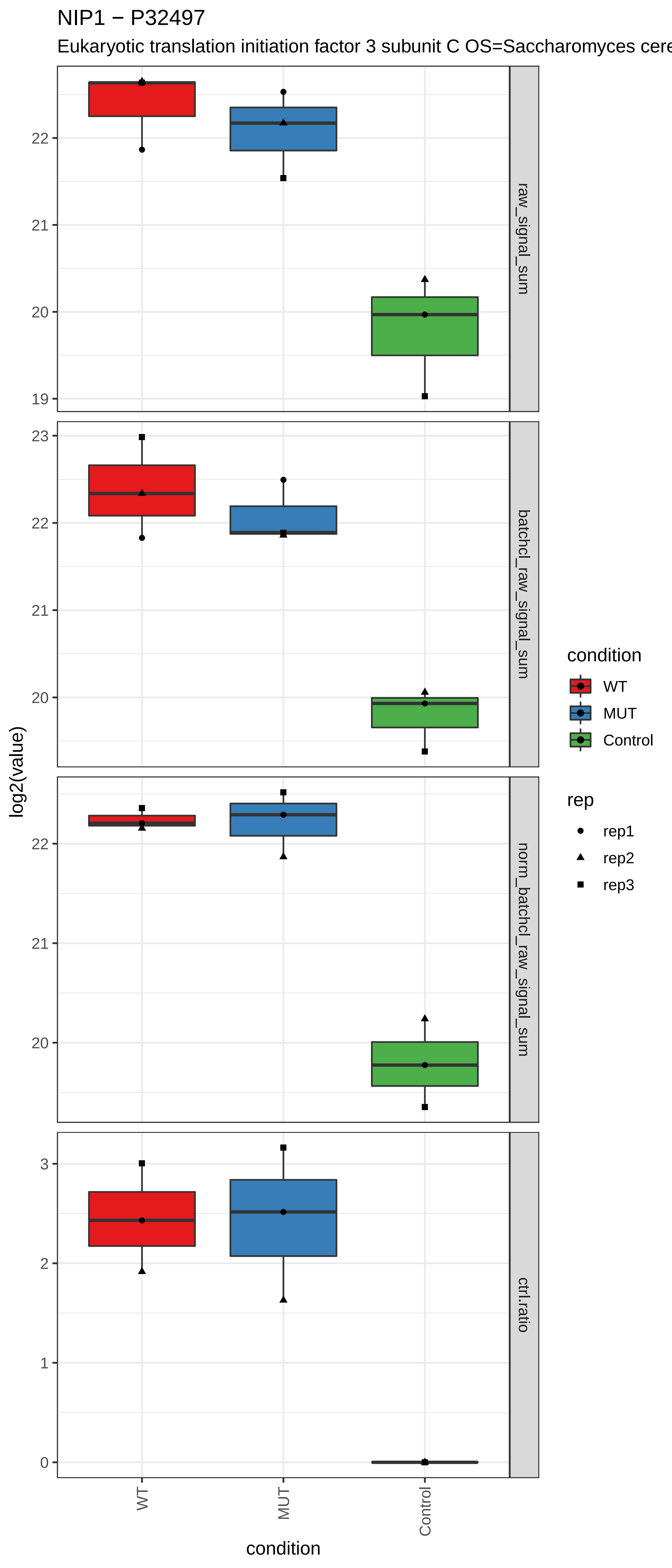

NIP1 − P32497
Eukaryotic translation initiation factor 3 subunit C OS=Saccharomyces cere
raw_signal_sum
batchcl_raw_signal_sum
norm_batchcl_raw_signal_sum
ctrl.ratio
log2(value)
condition
WT
MUT
Control
rep
rep1
rep2
rep3

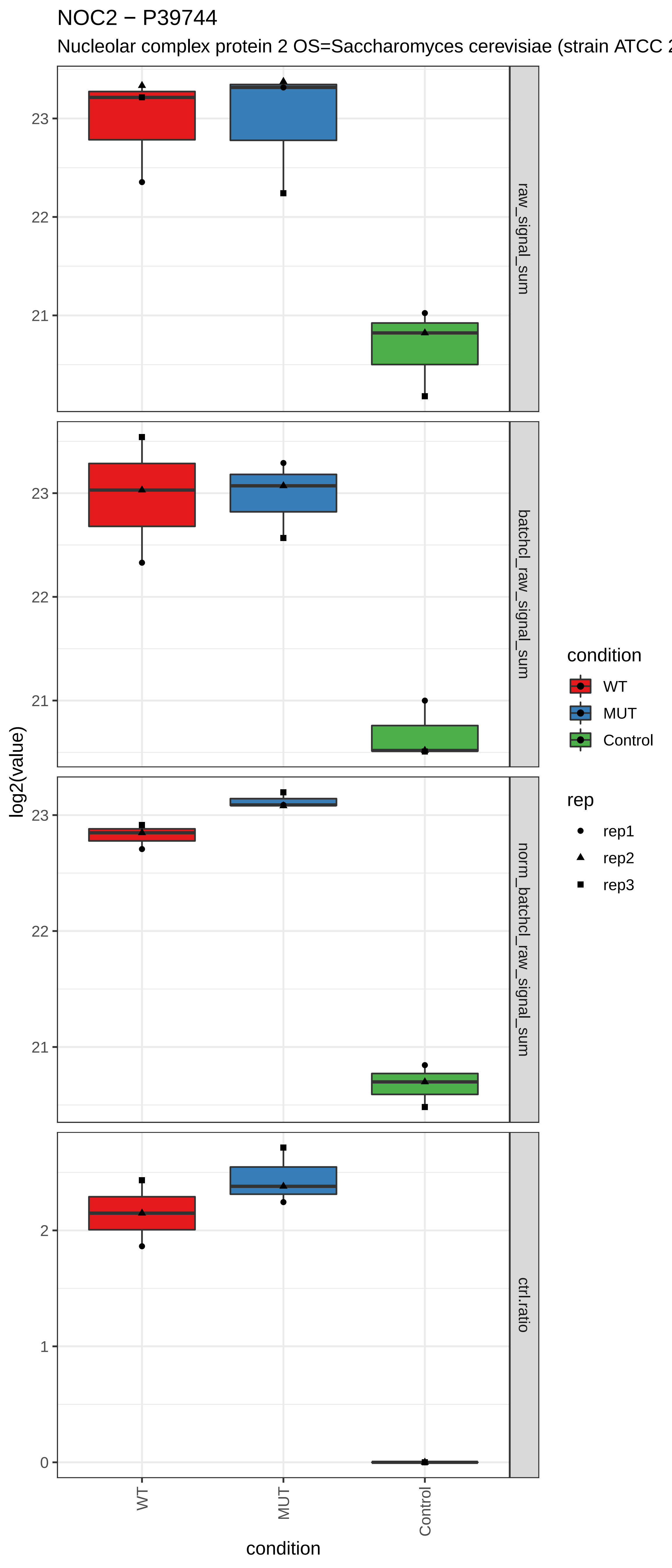

NOC2 − P39744
Nucleolar complex protein 2 OS=Saccharomyces cerevisiae (strain ATCC 2
raw_signal_sum
batchcl_raw_signal_sum
norm_batchcl_raw_signal_sum
ctrl.ratio
log2(value)
condition
WT
MUT
Control
rep
rep1
rep2
rep3
23
22
21
2
1
0

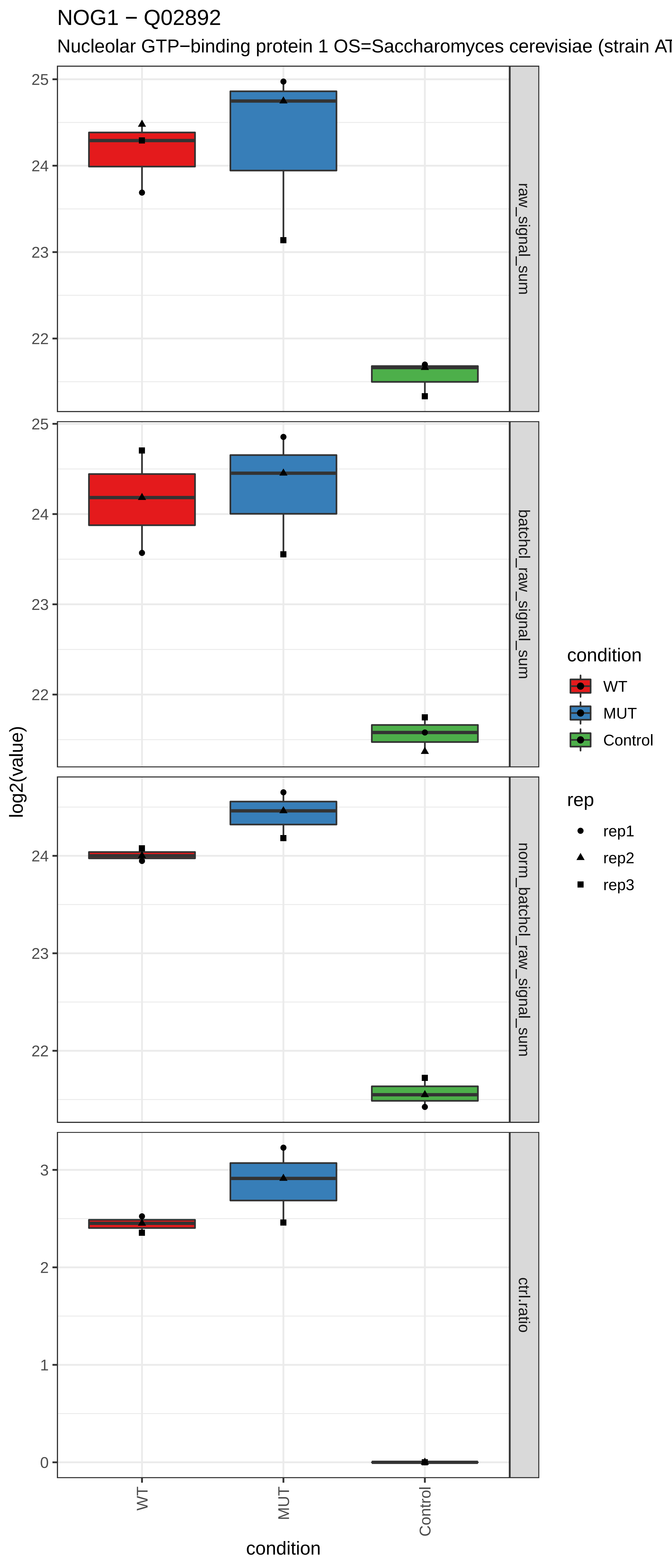

NOG1 − Q02892
Nucleolar GTP−binding protein 1 OS=Saccharomyces cerevisiae (strain AT
raw_signal_sum
batchcl_raw_signal_sum
norm_batchcl_raw_signal_sum
ctrl.ratio
log2(value)
condition
WT
MUT
Control
rep
rep1
rep2
rep3
25
24
23
22
3
2
1
0

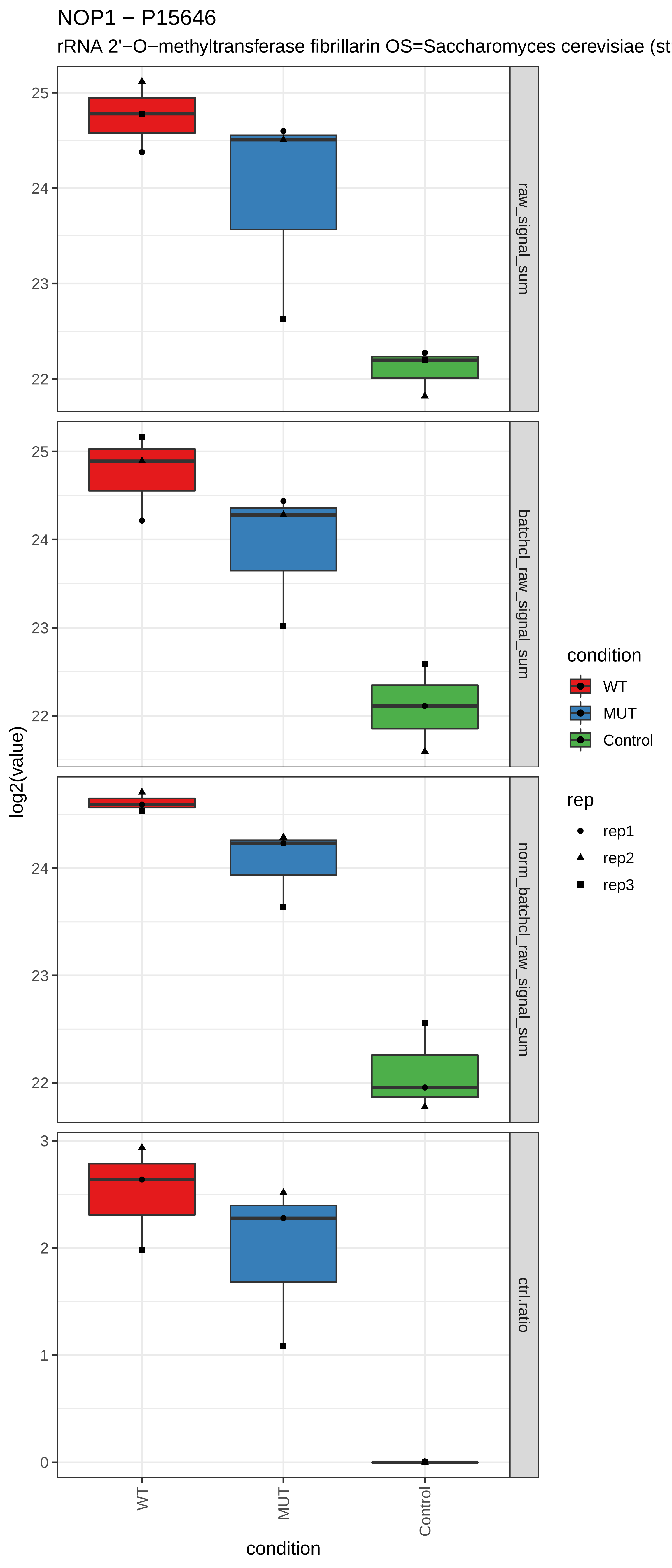
NOP1 − P15646
rRNA 2'−O−methyltransferase fibrillarin OS=Saccharomyces cerevisiae (st
raw_signal_sum
batchcl_raw_signal_sum
norm_batchcl_raw_signal_sum
ctrl.ratio
log2(value)
condition
WT
MUT
Control
rep
rep1
rep2
rep3
25
24
23
22
3
2
1
0

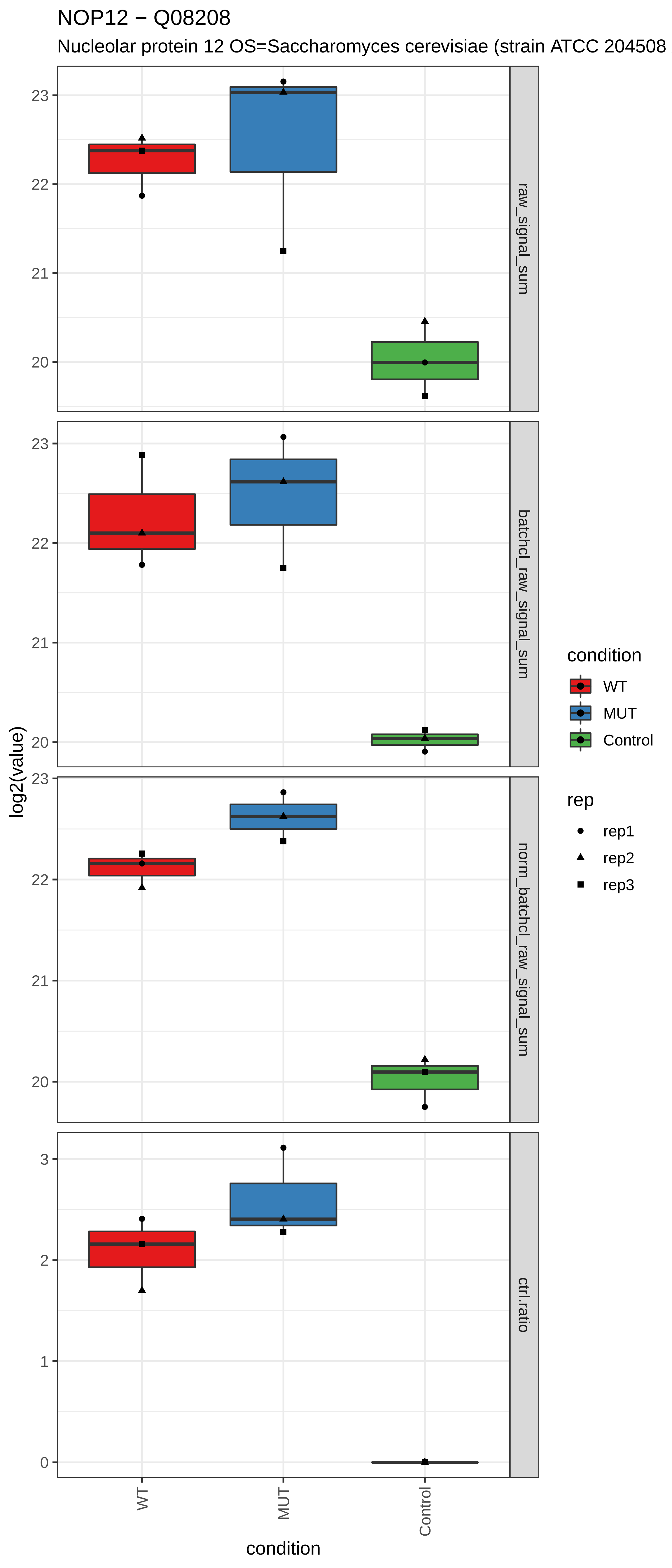
NOP12 − Q08208
Nucleolar protein 12 OS=Saccharomyces cerevisiae (strain ATCC 204508
raw_signal_sum
batchcl_raw_signal_sum
norm_batchcl_raw_signal_sum
ctrl.ratio
log2(value)
condition
WT
MUT
Control
rep
rep1
rep2
rep3
23
22
21
20
3
2
1
0

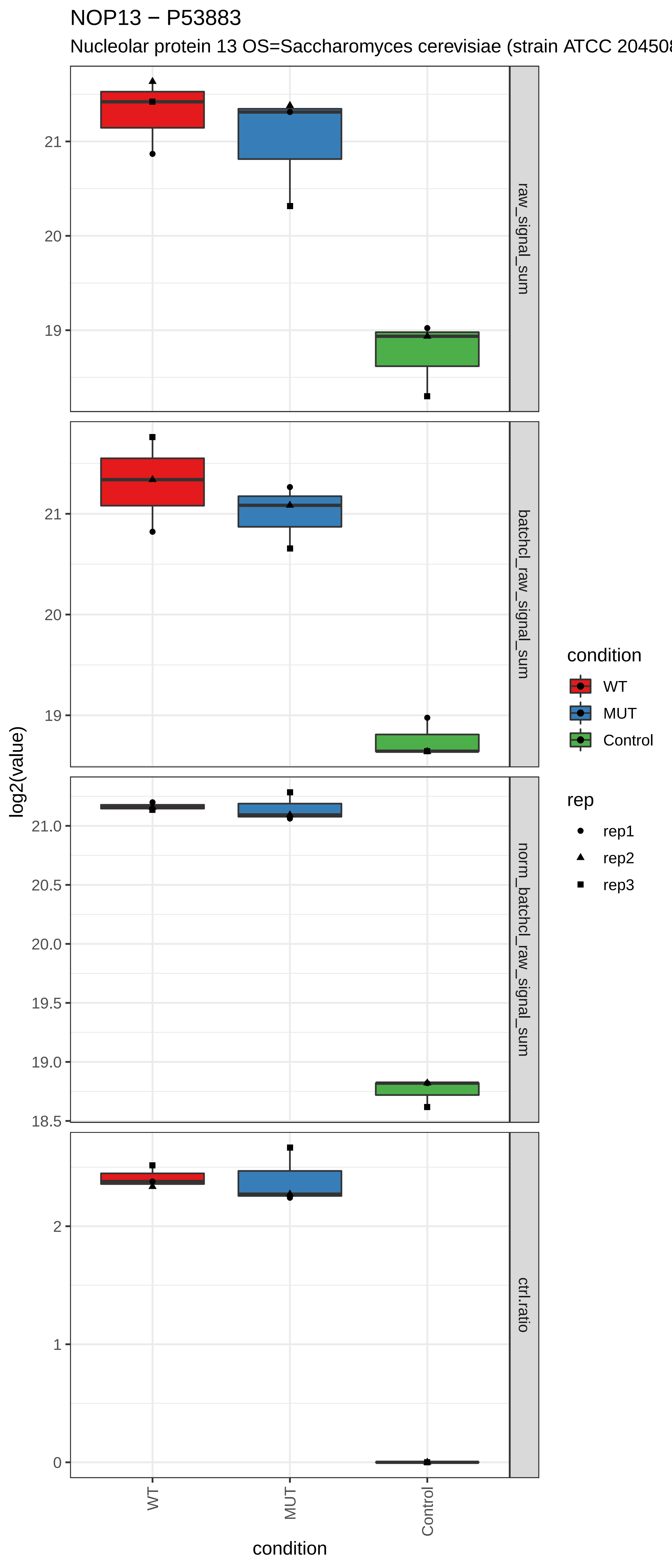

NOP13 − P53883
Nucleolar protein 13 OS=Saccharomyces cerevisiae (strain ATCC 204508
raw_signal_sum
batchcl_raw_signal_sum
norm_batchcl_raw_signal_sum
ctrl.ratio
log2(value)
condition
WT
MUT
Control
rep
rep1
rep2
rep3
21
20
19
21.0
20.5
20.0
19.5
19.0
18.5
2
1
0

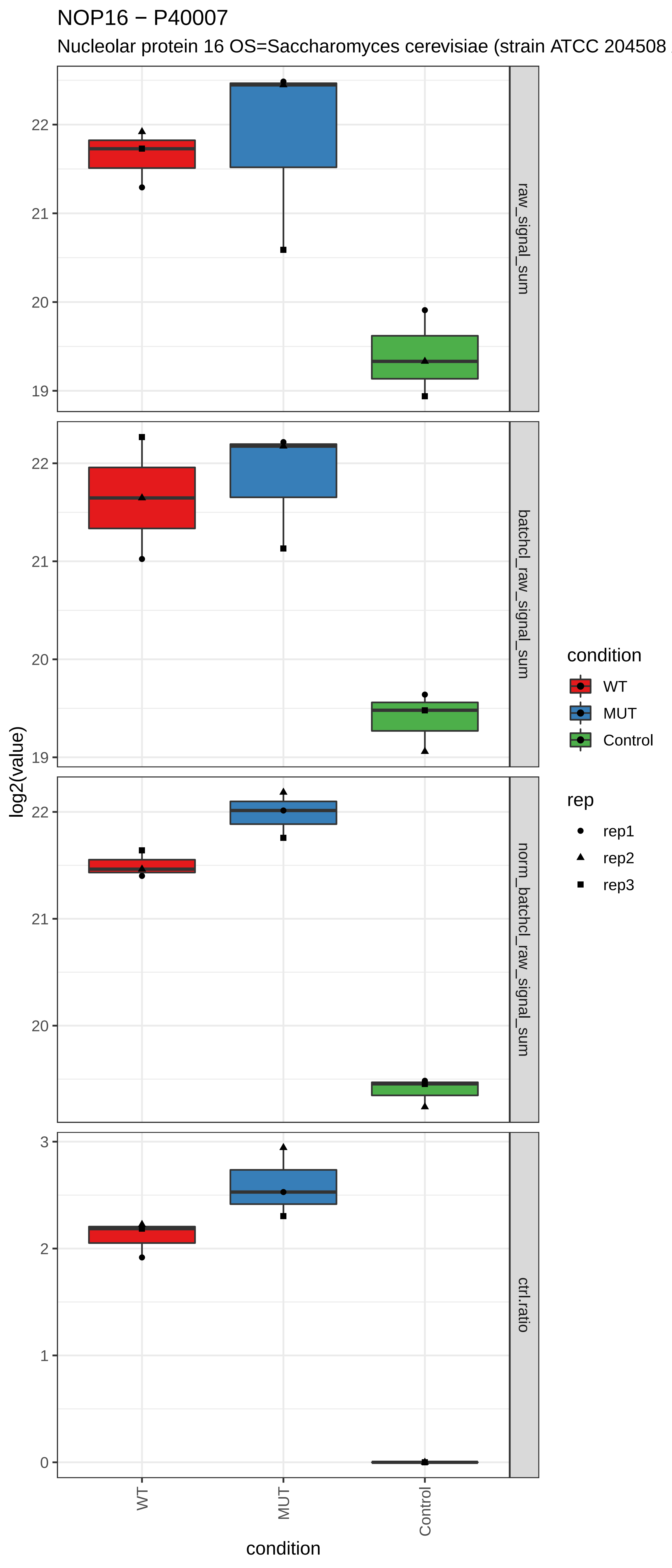

NOP16 − P40007
Nucleolar protein 16 OS=Saccharomyces cerevisiae (strain ATCC 204508
raw_signal_sum
batchcl_raw_signal_sum
norm_batchcl_raw_signal_sum
ctrl.ratio
log2(value)
condition
WT
MUT
Control
rep
rep1
rep2
rep3
22
21
20
19
3
2
1
0

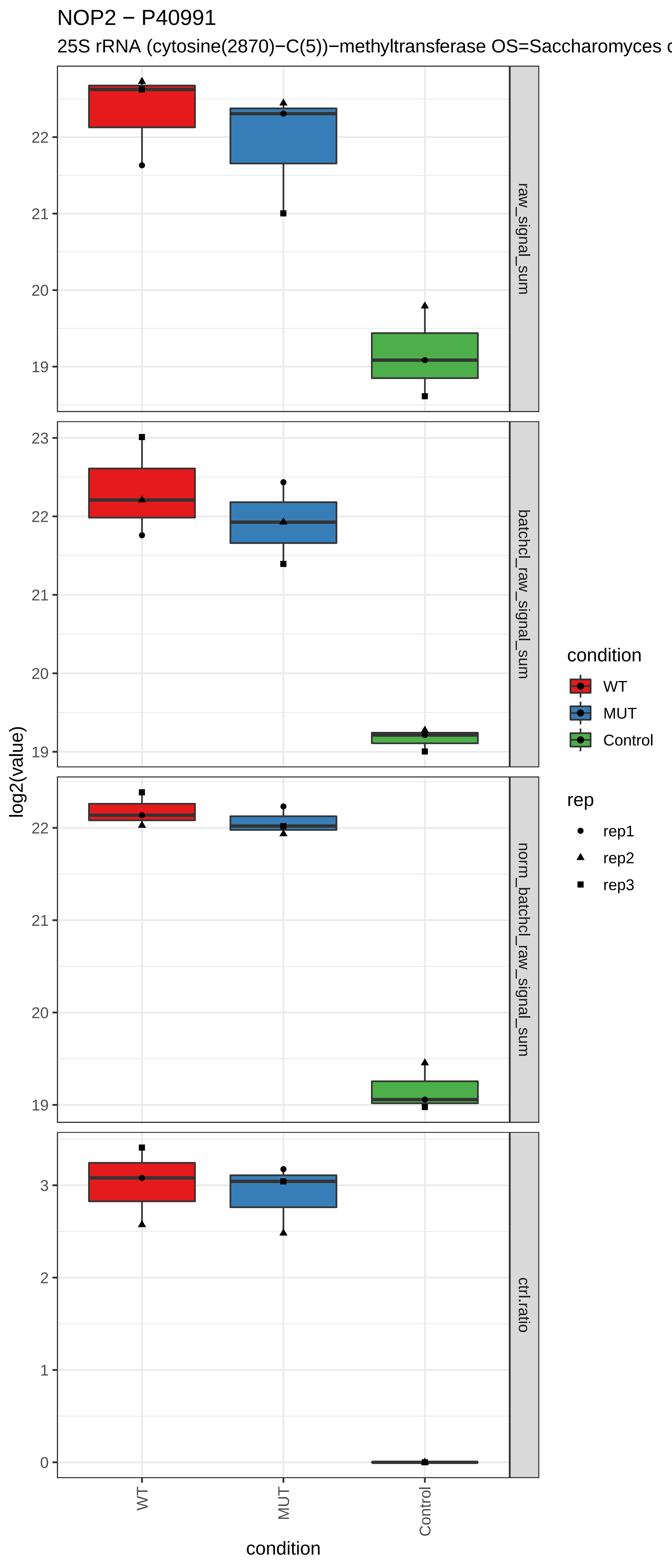
NOP2 − P40991
25S rRNA (cytosine(2870)−C(5))−methyltransferase OS=Saccharomyces c
raw_signal_sum
batchcl_raw_signal_sum
norm_batchcl_raw_signal_sum
ctrl.ratio
log2(value)
condition
WT
MUT
Control
rep
rep1
rep2
rep3
22
21
20
19
23
3
2
1
0

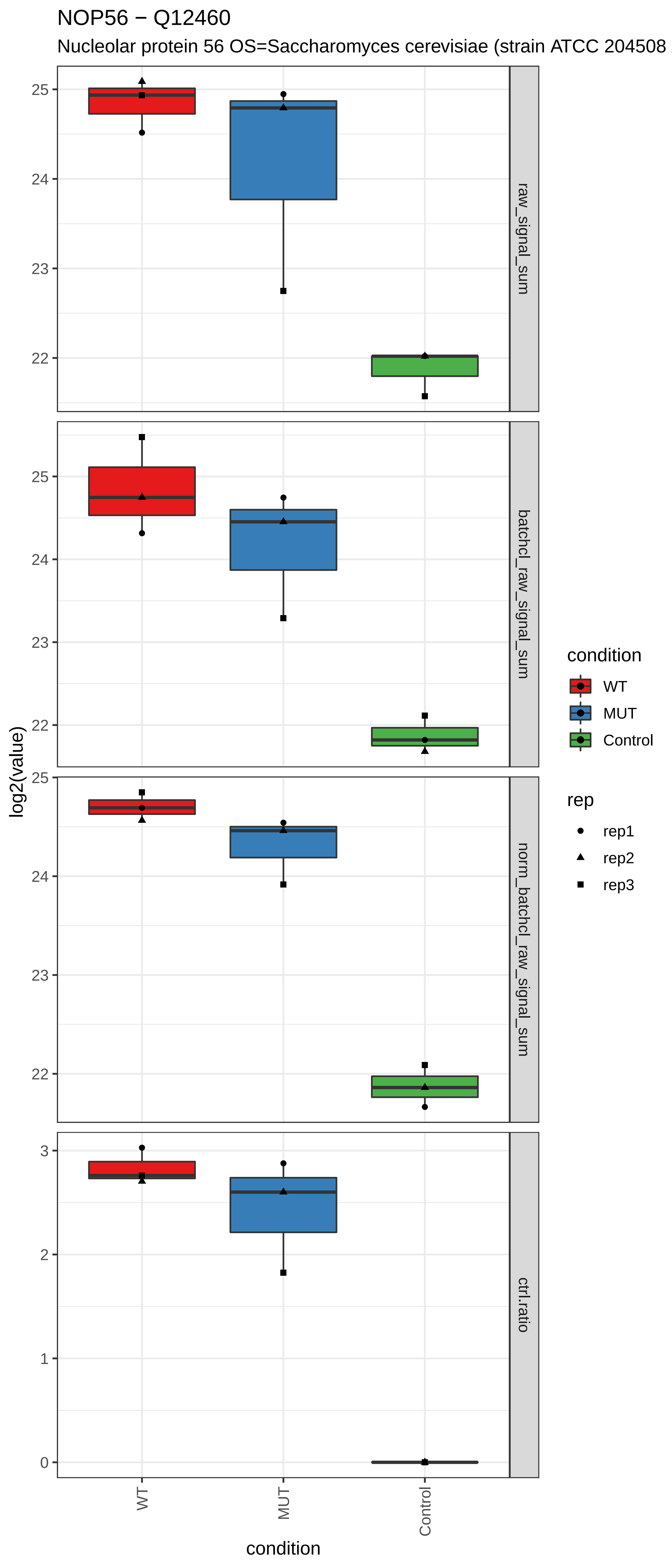

NOP56 − Q12460
Nucleolar protein 56 OS=Saccharomyces cerevisiae (strain ATCC 204508
raw_signal_sum
batchcl_raw_signal_sum
norm_batchcl_raw_signal_sum
ctrl.ratio
log2(value)
condition
WT
MUT
Control
rep
rep1
rep2
rep3
25
24
23
22
3
2
1
0

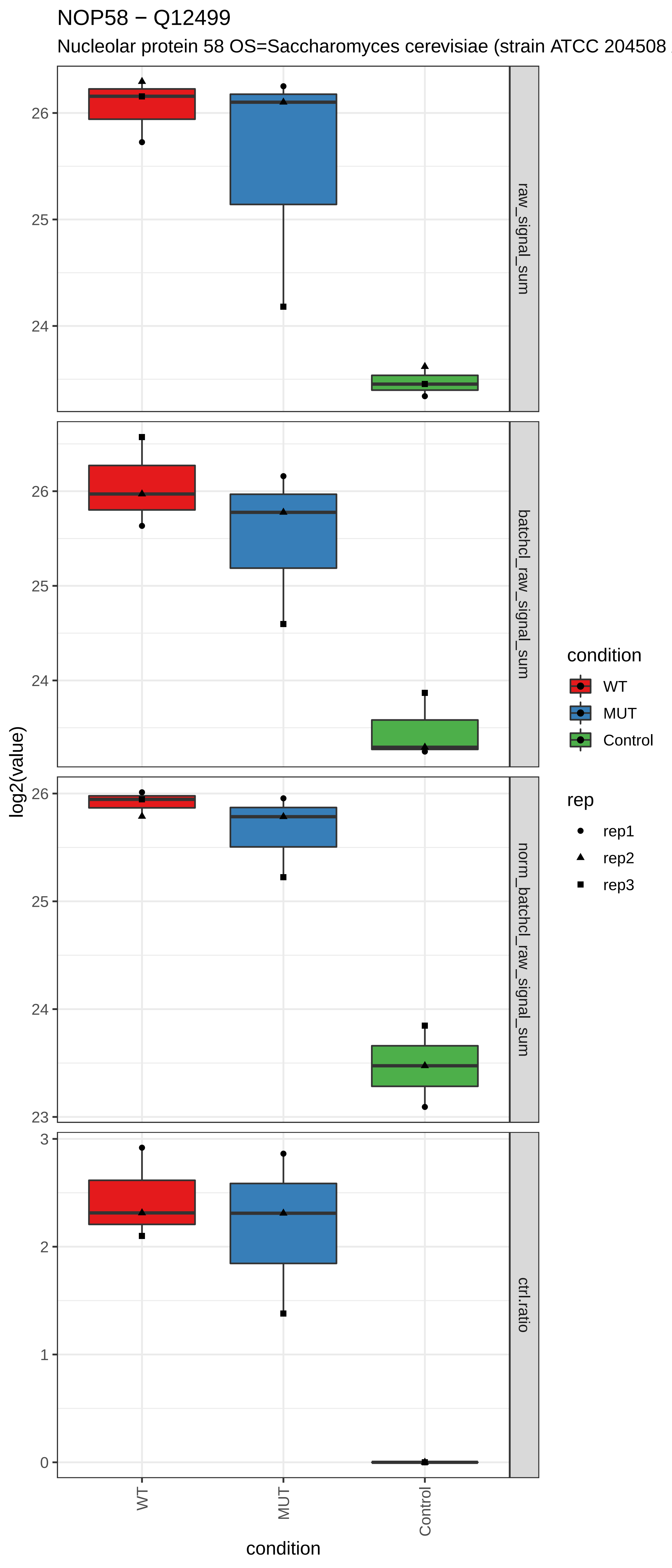

NOP58 − Q12499
Nucleolar protein 58 OS=Saccharomyces cerevisiae (strain ATCC 204508
raw_signal_sum
batchcl_raw_signal_sum
norm_batchcl_raw_signal_sum
ctrl.ratio
log2(value)
26
25
24
23
3
2
1
0
WT
MUT
Control
condition
condition
WT
MUT
Control
rep
rep1
rep2
rep3

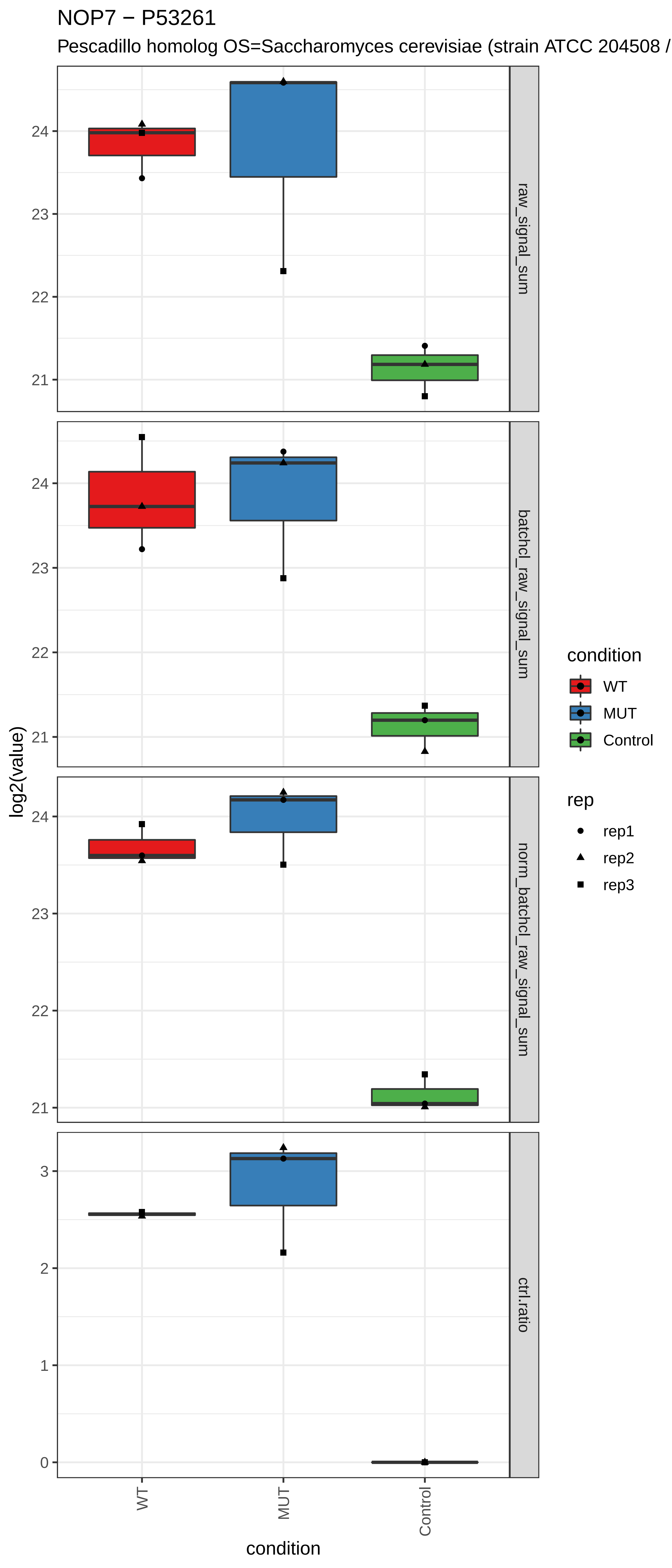
NOP7 − P53261
Pescadillo homolog OS=Saccharomyces cerevisiae (strain ATCC 204508 /
raw_signal_sum
batchcl_raw_signal_sum
norm_batchcl_raw_signal_sum
ctrl.ratio
log2(value)
condition
WT
MUT
Control
rep
rep1
rep2
rep3

NOT5 − Q12514

General negative regulator of transcription subunit 5 OS=Saccharomyces c

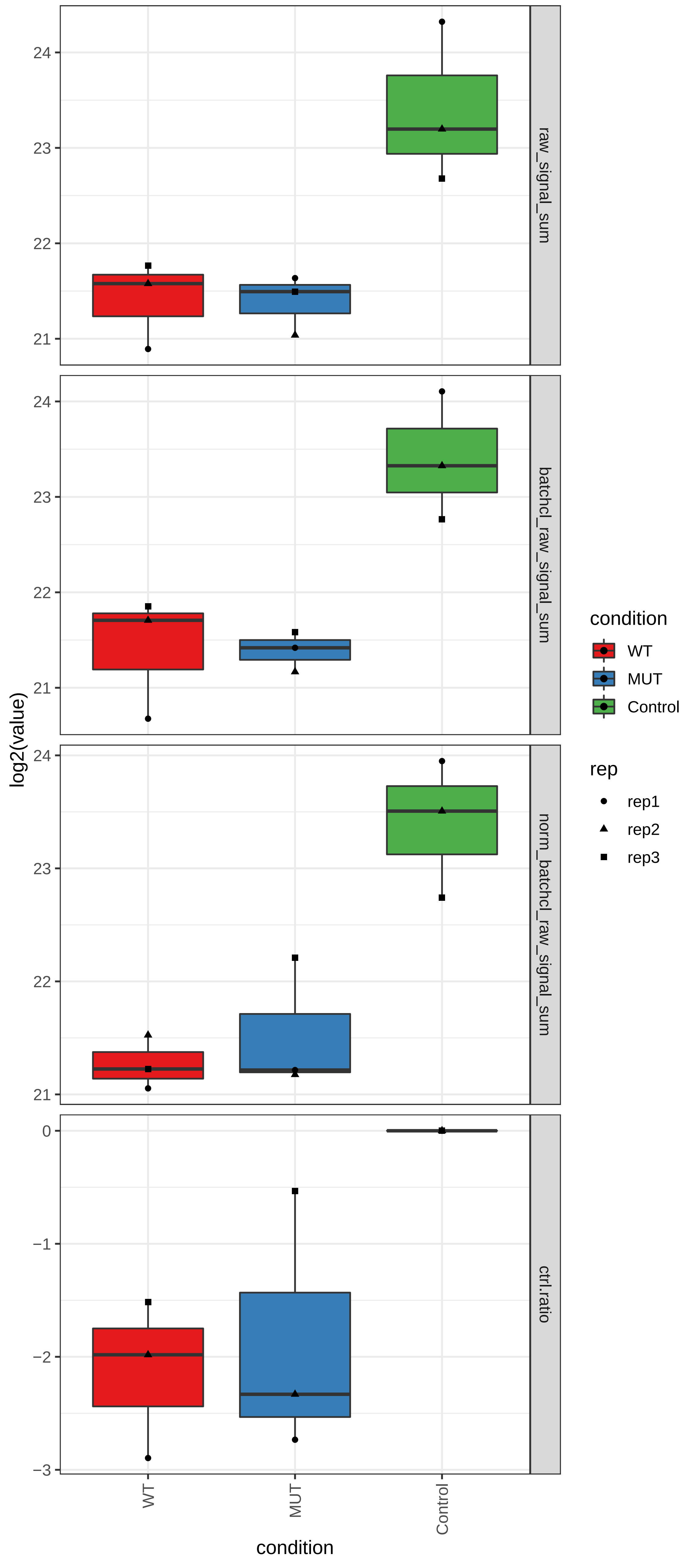

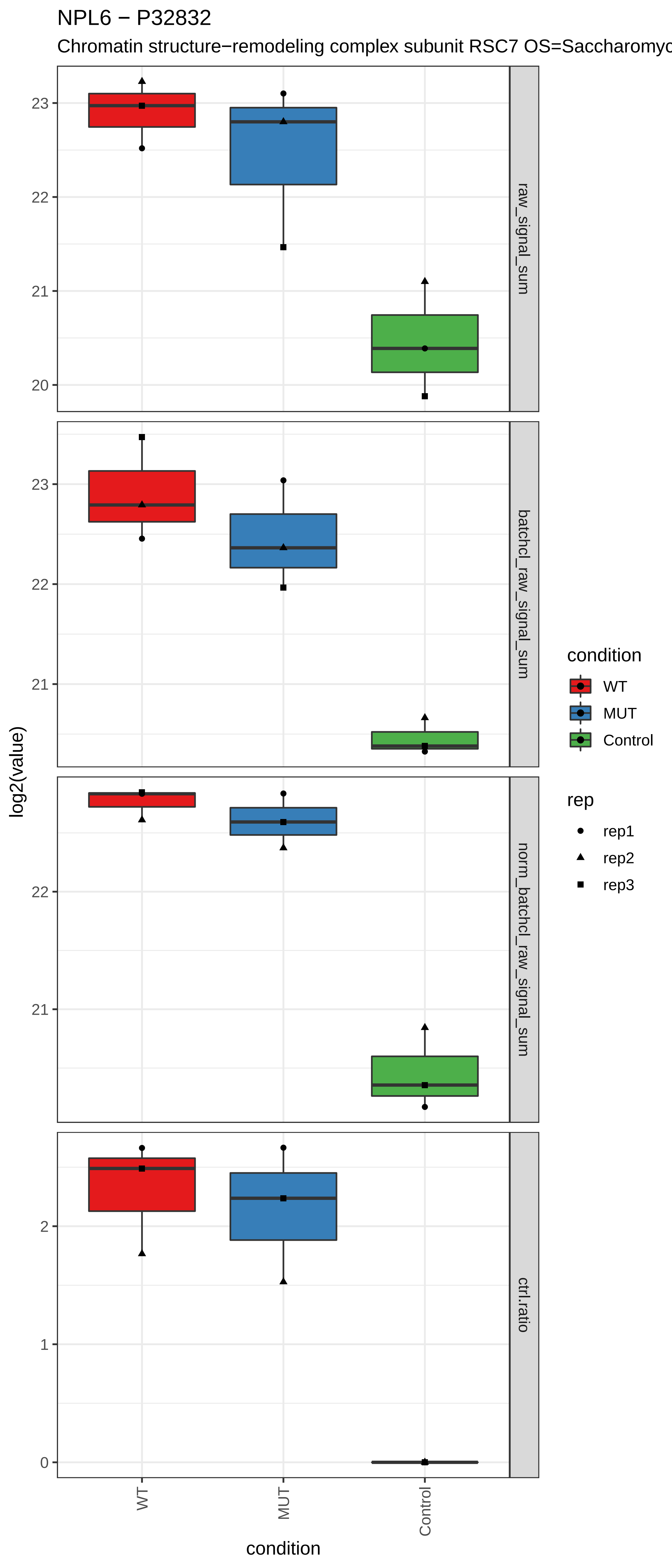
NPL6 − P32832
Chromatin structure−remodeling complex subunit RSC7 OS=Saccharomyc
raw_signal_sum
batchcl_raw_signal_sum
norm_batchcl_raw_signal_sum
ctrl.ratio
log2(value)
condition
WT
MUT
Control
rep
rep1
rep2
rep3
23
22
21
20
2
1
0

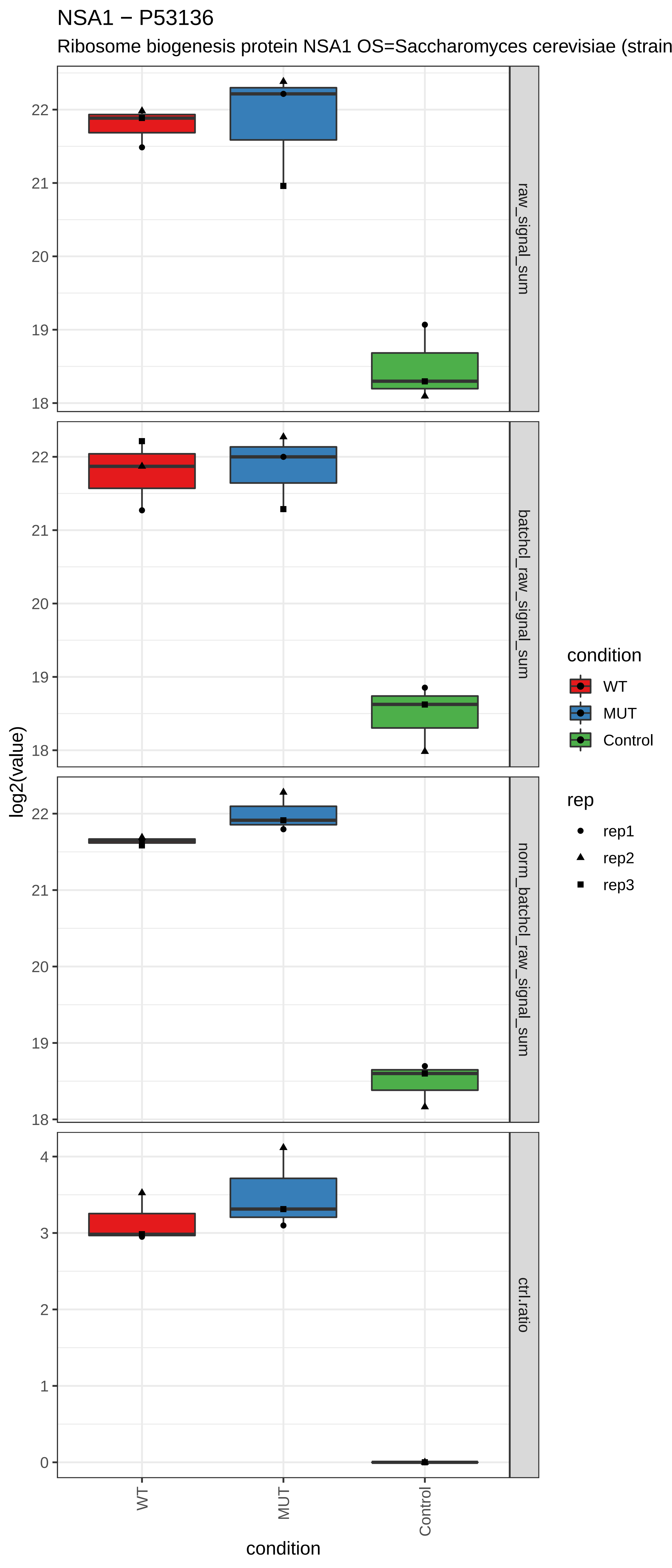

NSA1 − P53136
Ribosome biogenesis protein NSA1 OS=Saccharomyces cerevisiae (strain
raw_signal_sum
batchcl_raw_signal_sum
norm_batchcl_raw_signal_sum
ctrl.ratio
log2(value)
condition
WT
MUT
Control
rep
rep1
rep2
rep3

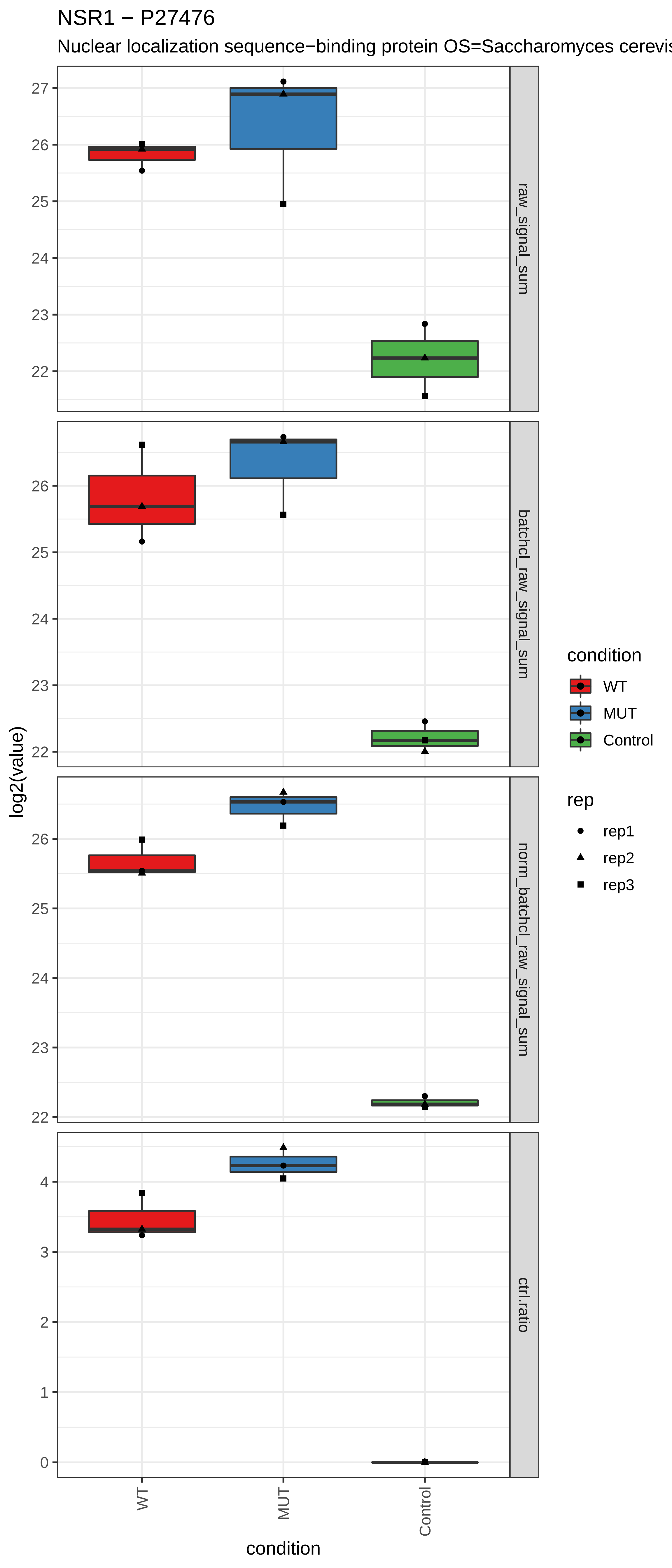
NSR1 − P27476
Nuclear localization sequence−binding protein OS=Saccharomyces cerevis
raw_signal_sum
batchcl_raw_signal_sum
norm_batchcl_raw_signal_sum
ctrl.ratio
log2(value)
condition
WT
MUT
Control
rep
rep1
rep2
rep3
27
26
25
24
23
22
4
3
2
1
0

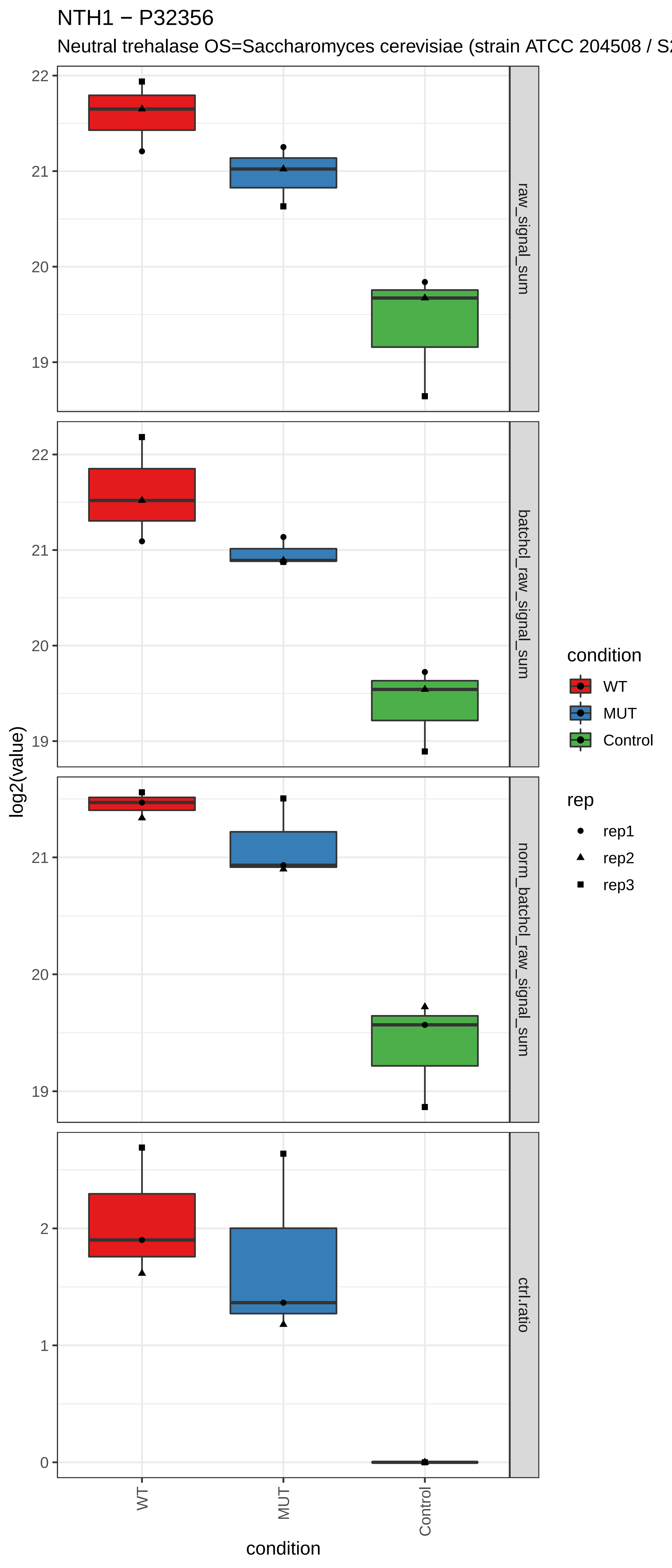

NTH1 − P32356
Neutral trehalase OS=Saccharomyces cerevisiae (strain ATCC 204508 / S2
raw_signal_sum
batchcl_raw_signal_sum
norm_batchcl_raw_signal_sum
ctrl.ratio
log2(value)
condition
WT
MUT
Control
rep
rep1
rep2
rep3

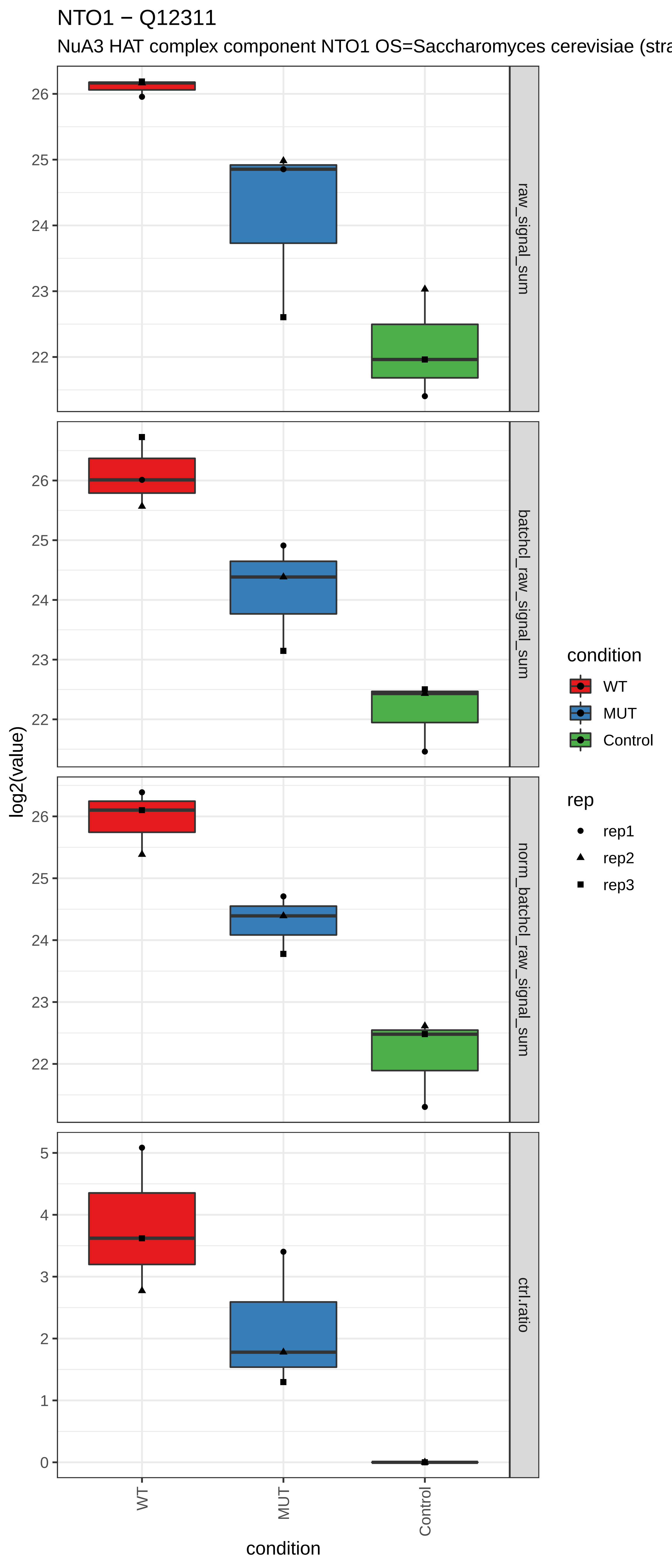

NTO1 − Q12311
NuA3 HAT complex component NTO1 OS=Saccharomyces cerevisiae (stra
raw_signal_sum
batchcl_raw_signal_sum
norm_batchcl_raw_signal_sum
ctrl.ratio
log2(value)
condition
WT
MUT
Control
rep
rep1
rep2
rep3

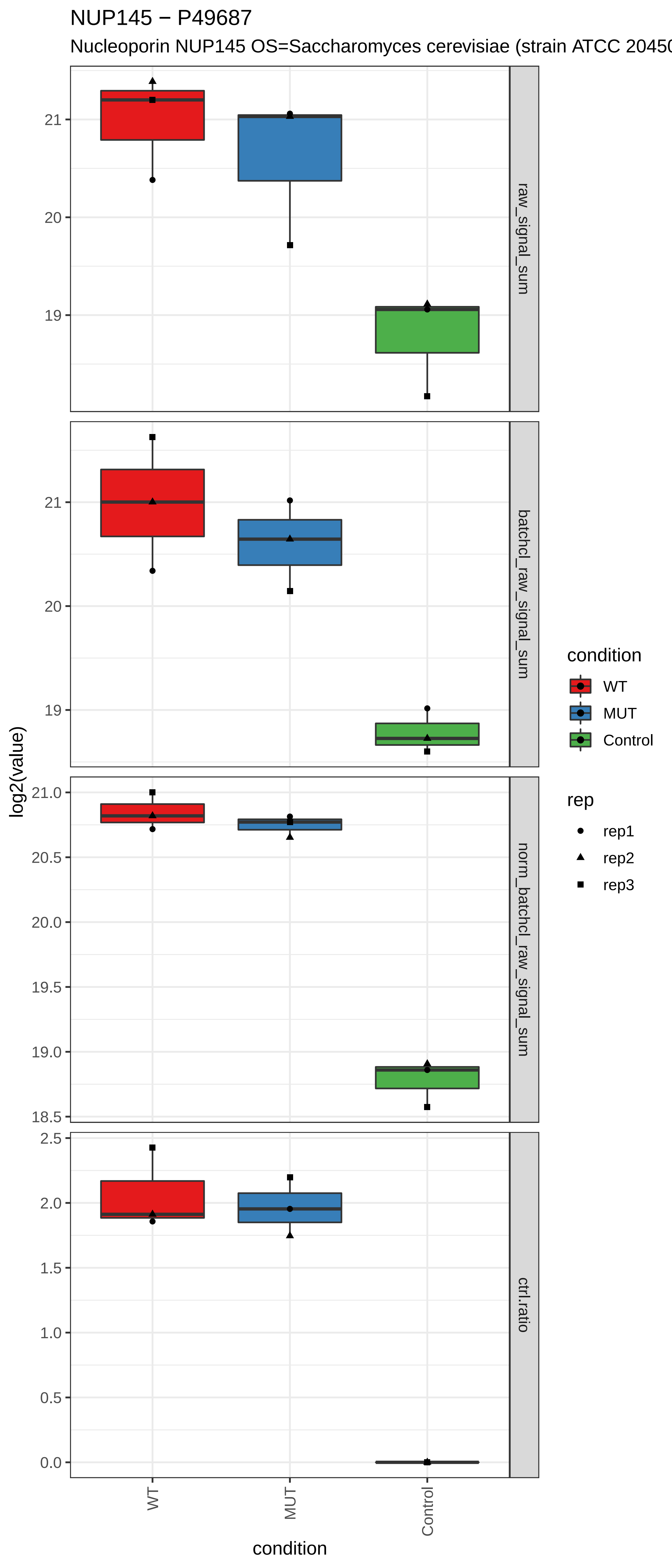

NUP145 − P49687
Nucleoporin NUP145 OS=Saccharomyces cerevisiae (strain ATCC 20450
log2(value)
condition
raw_signal_sum
batchcl_raw_signal_sum
norm_batchcl_raw_signal_sum
ctrl.ratio
WT
MUT
Control
condition
WT
MUT
Control
rep
rep1
rep2
rep3

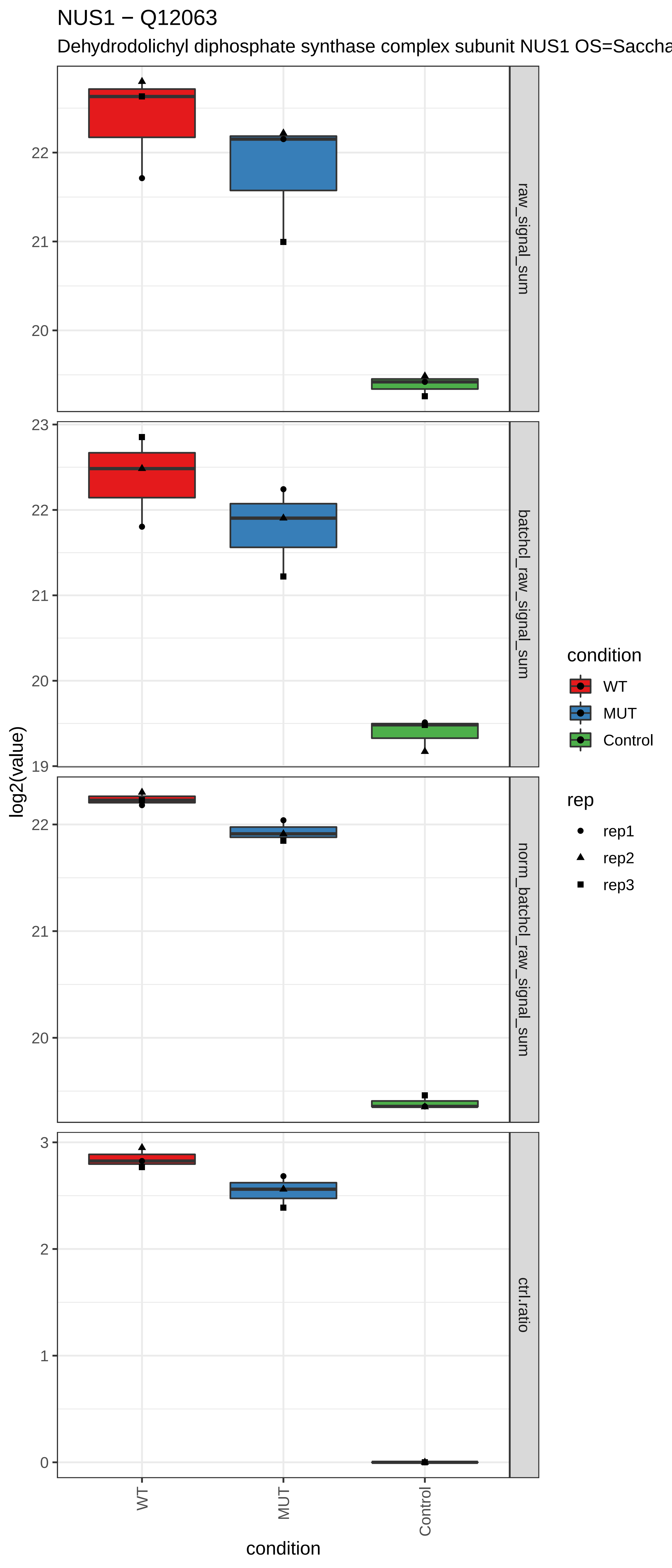
NUS1 − Q12063
Dehydrodolichyl diphosphate synthase complex subunit NUS1 OS=Saccha
raw_signal_sum
batchcl_raw_signal_sum
norm_batchcl_raw_signal_sum
ctrl.ratio
log2(value)
condition
WT
MUT
Control
rep
rep1
rep2
rep3
22
21
20
23
19
3
2
1
0

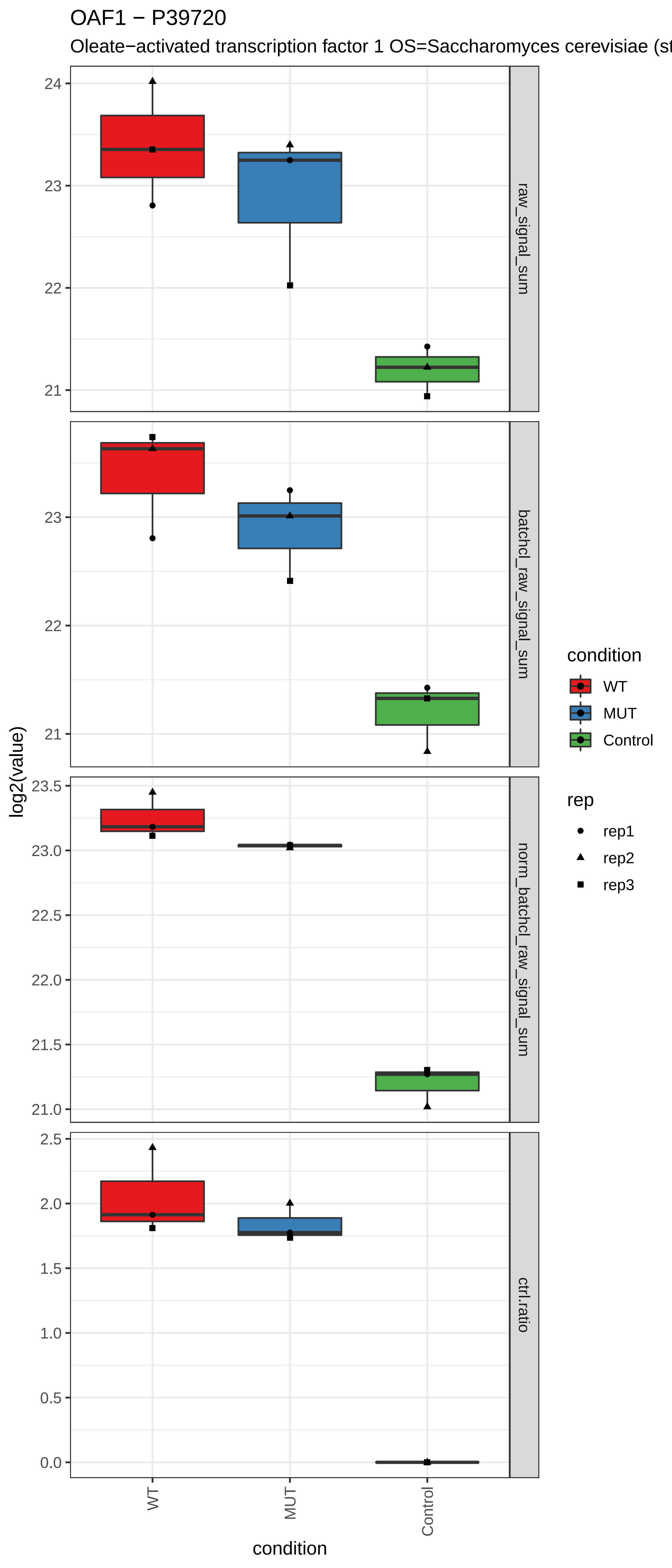

OAF1 − P39720
Oleate−activated transcription factor 1 OS=Saccharomyces cerevisiae (st
raw_signal_sum
batchcl_raw_signal_sum
norm_batchcl_raw_signal_sum
ctrl.ratio
log2(value)
condition
WT
MUT
Control
rep
rep1
rep2
rep3
24
23
22
21
23.5
23.0
22.5
22.0
21.5
21.0
2.5
2.0
1.5
1.0
0.5
0.0

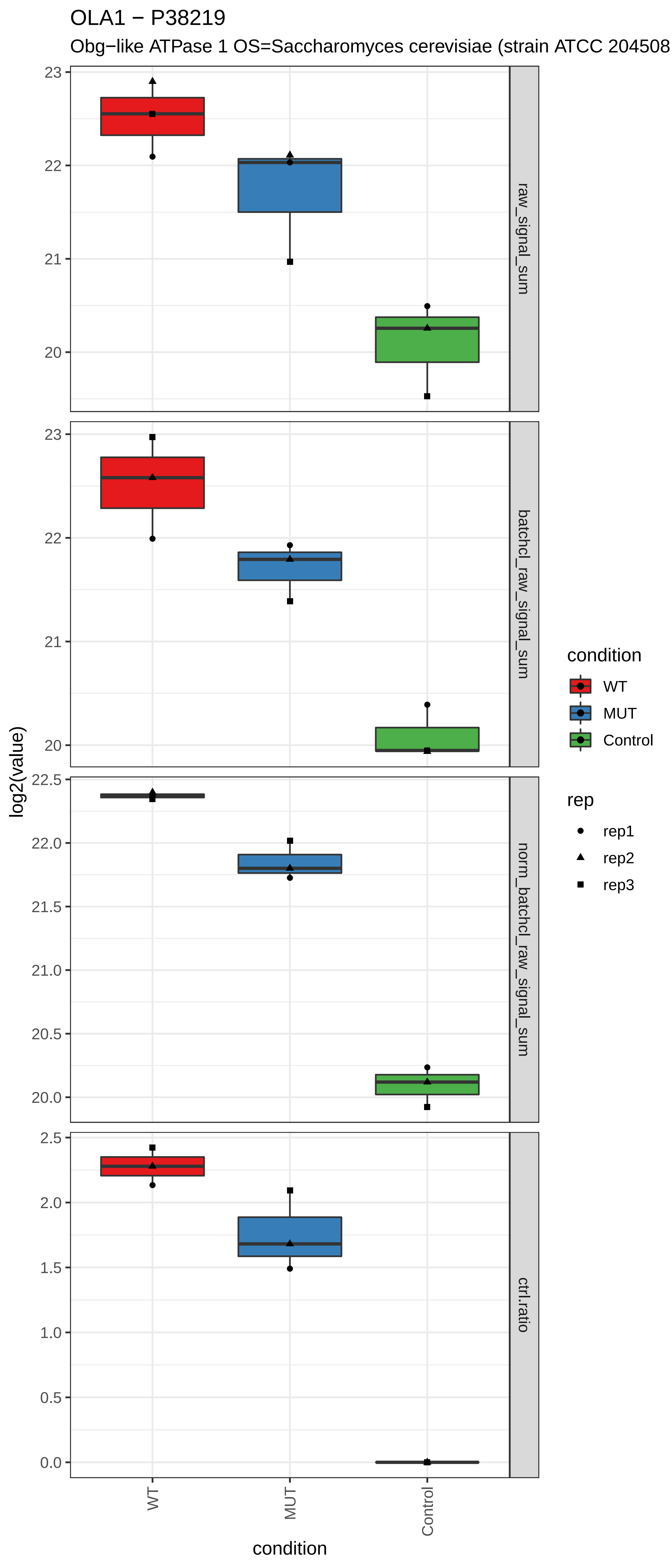

OLA1 − P38219
Obg−like ATPase 1 OS=Saccharomyces cerevisiae (strain ATCC 204508
raw_signal_sum
batchcl_raw_signal_sum
norm_batchcl_raw_signal_sum
ctrl.ratio
log2(value)
condition
WT
MUT
Control
rep
rep1
rep2
rep3

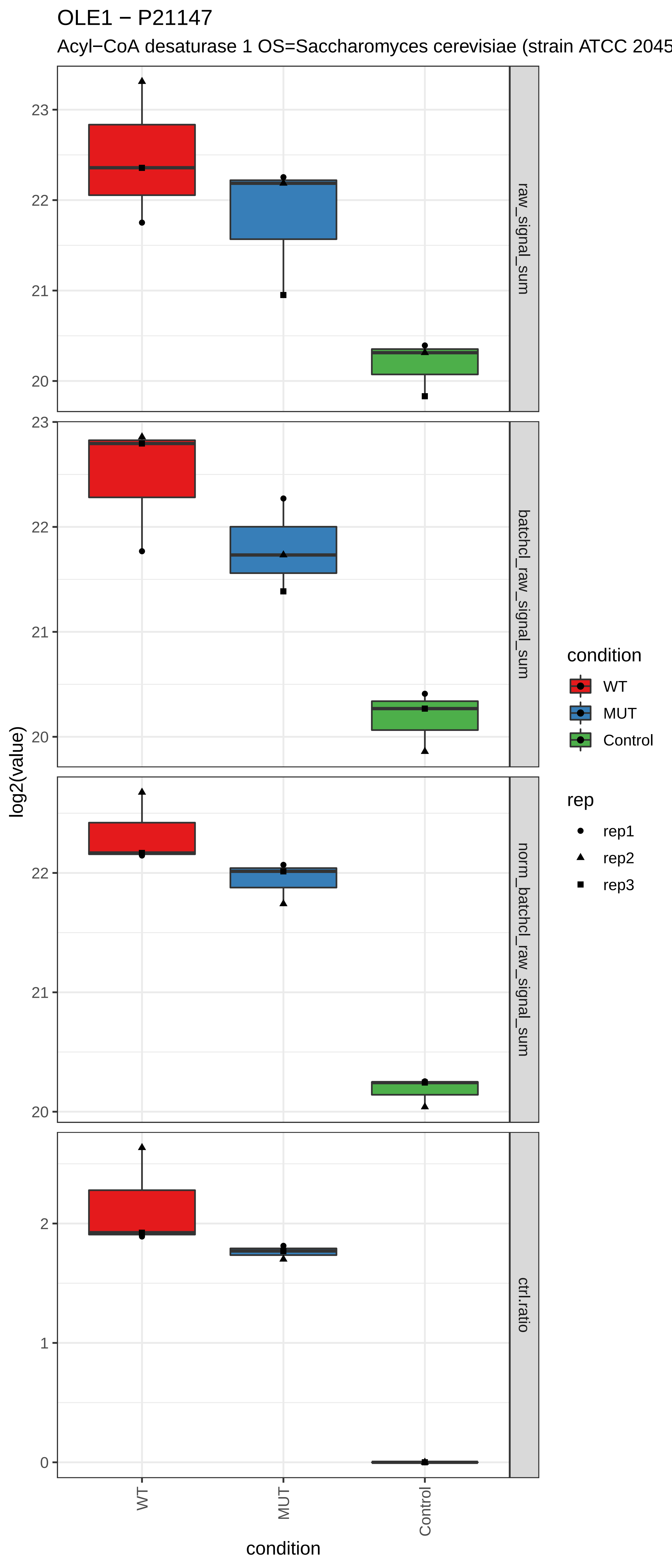

OLE1 − P21147
Acyl−CoA desaturase 1 OS=Saccharomyces cerevisiae (strain ATCC 2045
raw_signal_sum
batchcl_raw_signal_sum
norm_batchcl_raw_signal_sum
ctrl.ratio
log2(value)
condition
WT
MUT
Control
rep
rep1
rep2
rep3

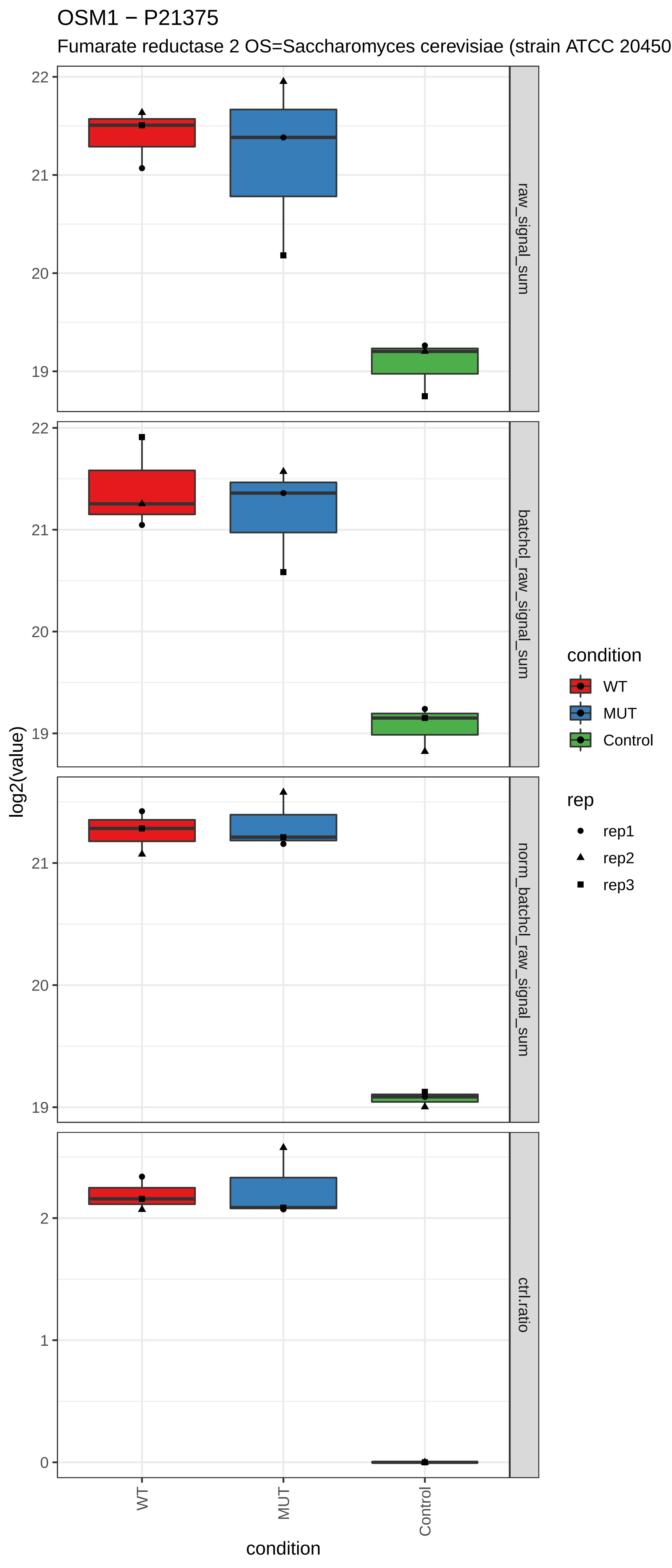
OSM1 − P21375
Fumarate reductase 2 OS=Saccharomyces cerevisiae (strain ATCC 20450
raw_signal_sum
batchcl_raw_signal_sum
norm_batchcl_raw_signal_sum
ctrl.ratio
log2(value)
condition
WT
MUT
Control
rep
rep1
rep2
rep3
22
21
20
19
2
1
0

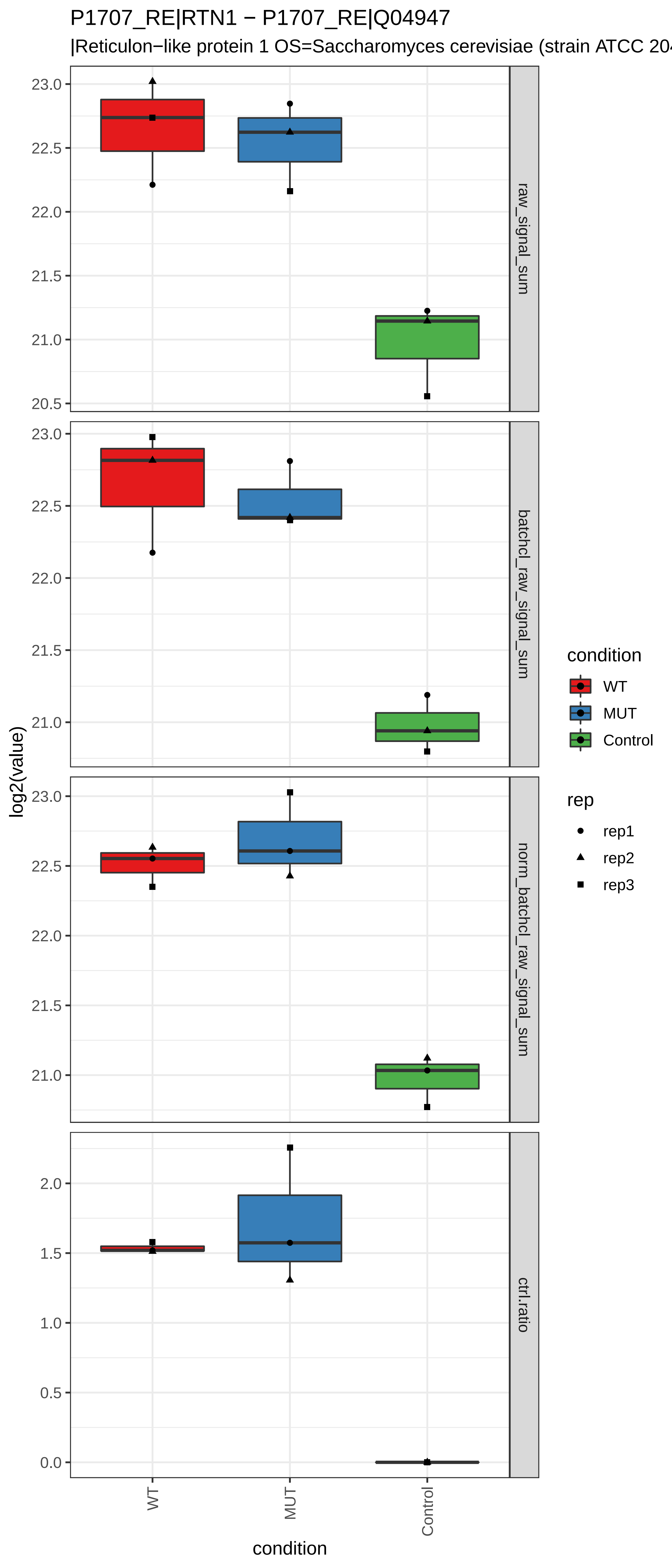
P1707_RE|RTN1 − P1707_RE|Q04947
|Reticulon−like protein 1 OS=Saccharomyces cerevisiae (strain ATCC 204
raw_signal_sum
batchcl_raw_signal_sum
norm_batchcl_raw_signal_sum
ctrl.ratio
log2(value)
condition
WT
MUT
Control
rep
rep1
rep2
rep3
23.0
22.5
22.0
21.5
21.0
20.5
2.0
1.5
1.0
0.5
0.0

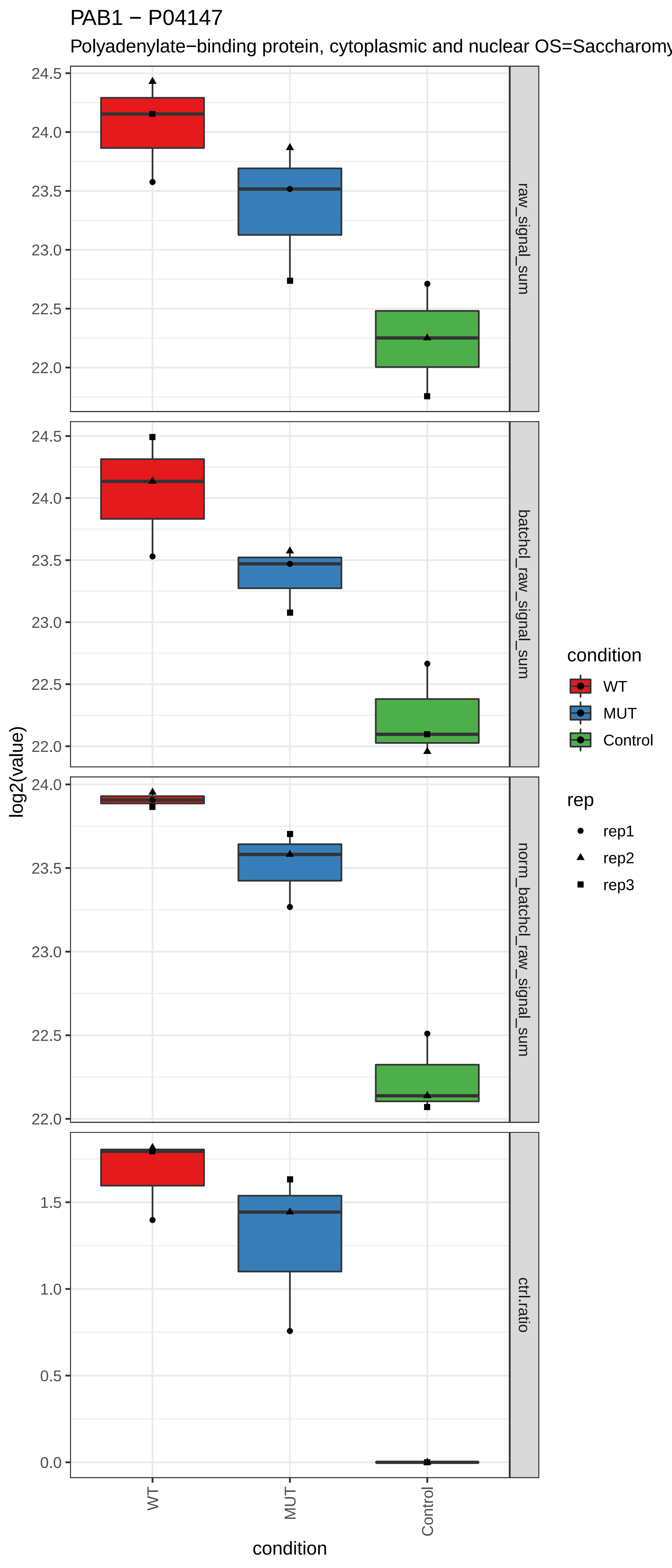

PAB1 − P04147
Polyadenylate−binding protein, cytoplasmic and nuclear OS=Saccharomy
raw_signal_sum
batchcl_raw_signal_sum
norm_batchcl_raw_signal_sum
ctrl.ratio
log2(value)
condition
WT
MUT
Control
rep
rep1
rep2
rep3
24.5
24.0
23.5
23.0
22.5
22.0
1.5
1.0
0.5
0.0

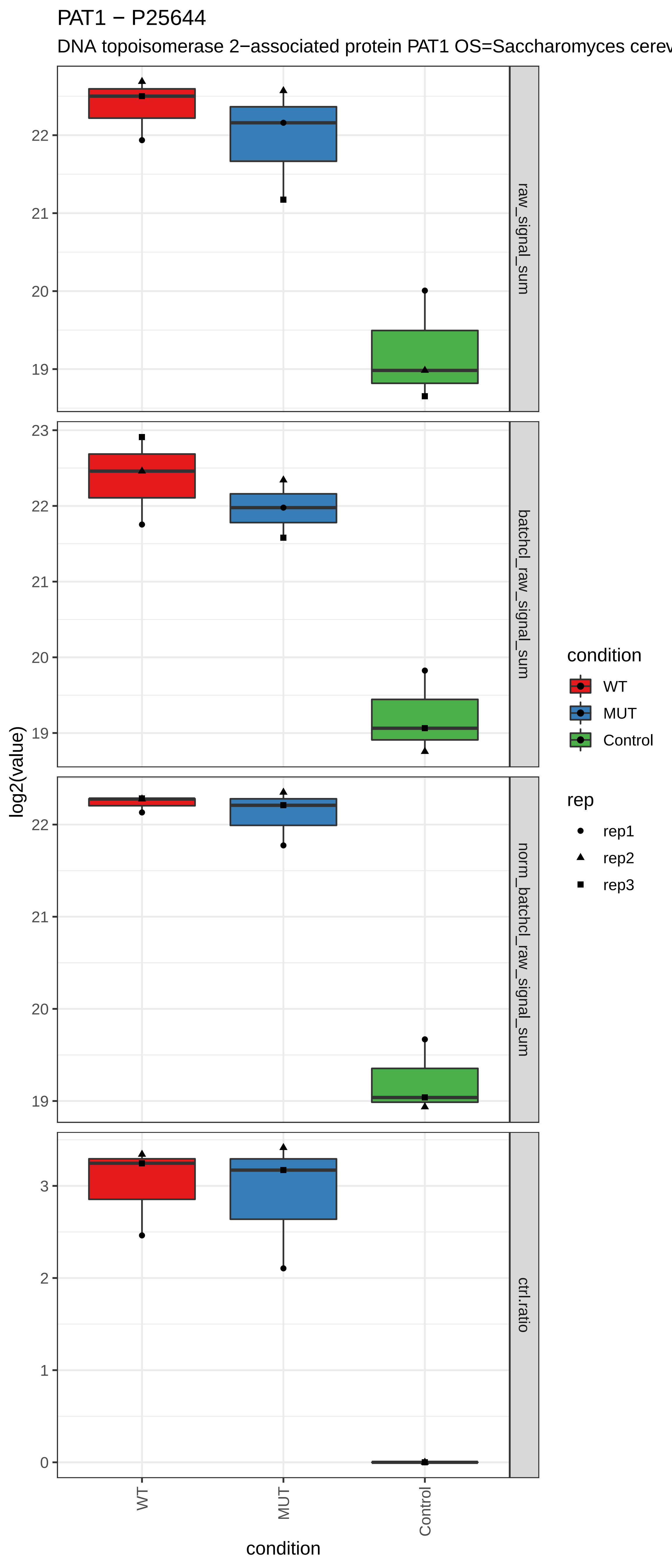

PAT1 − P25644
DNA topoisomerase 2−associated protein PAT1 OS=Saccharomyces cerev
raw_signal_sum
batchcl_raw_signal_sum
norm_batchcl_raw_signal_sum
ctrl.ratio
log2(value)
condition
WT
MUT
Control
rep
rep1
rep2
rep3
22
21
20
19
23
3
2
1
0

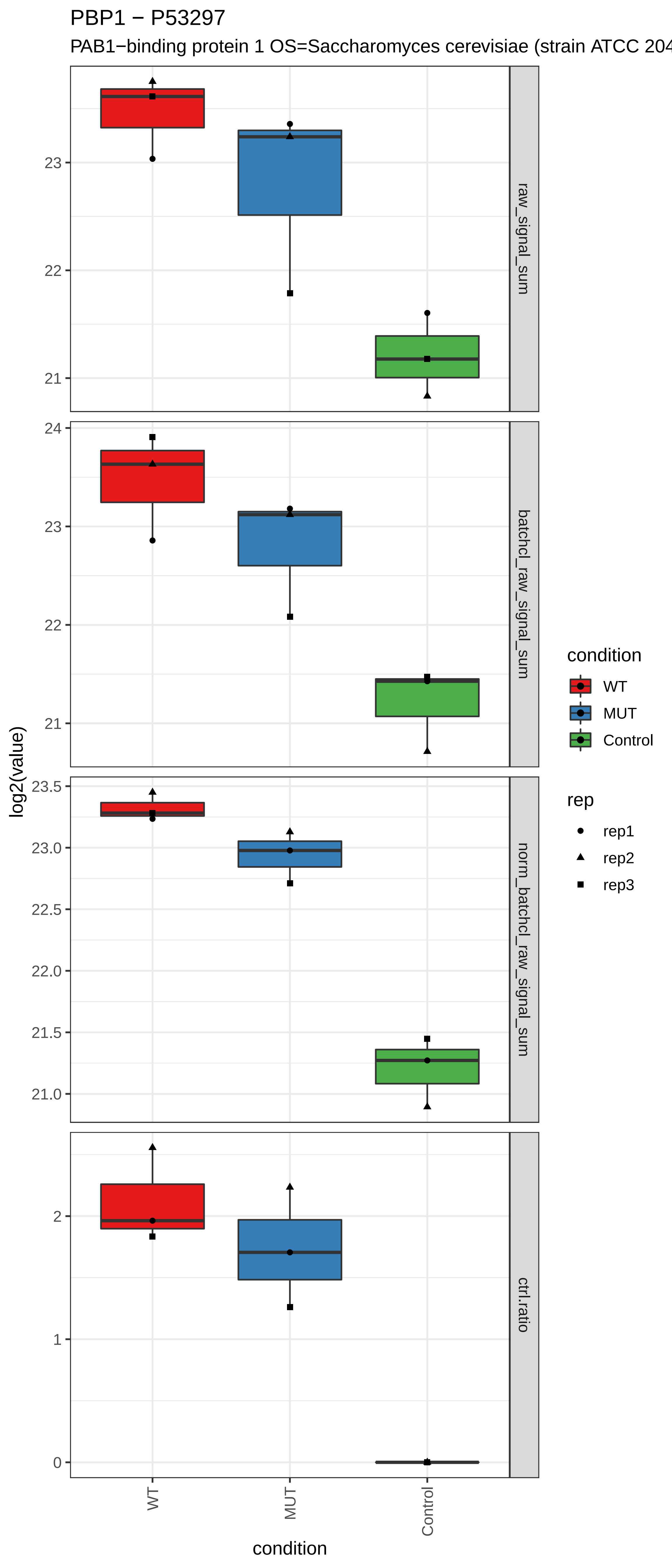
PBP1 − P53297
PAB1−binding protein 1 OS=Saccharomyces cerevisiae (strain ATCC 204
raw_signal_sum
batchcl_raw_signal_sum
norm_batchcl_raw_signal_sum
ctrl.ratio
log2(value)
condition
WT
MUT
Control
rep
rep1
rep2
rep3
23
22
21
24
23.5
23.0
22.5
22.0
21.5
21.0
2
1
0

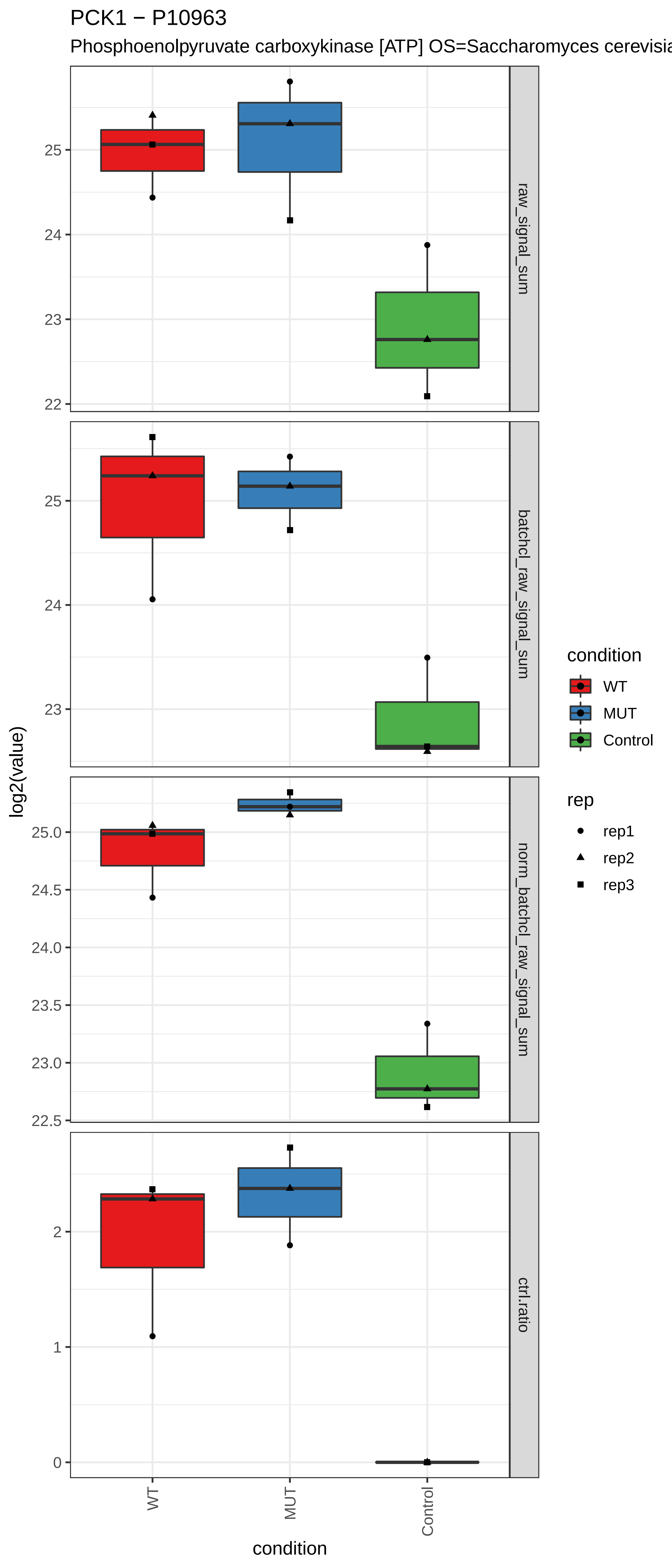

PCK1 − P10963
Phosphoenolpyruvate carboxykinase [ATP] OS=Saccharomyces cerevisia
raw_signal_sum
batchcl_raw_signal_sum
norm_batchcl_raw_signal_sum
ctrl.ratio
log2(value)
condition
WT
MUT
Control
rep
rep1
rep2
rep3
25
24
23
22
25.0
24.5
24.0
23.5
23.0
22.5
2
1
0

PCS60 − P38137

Peroxisomal−coenzyme A synthetase OS=Saccharomyces cerevisiae (stra

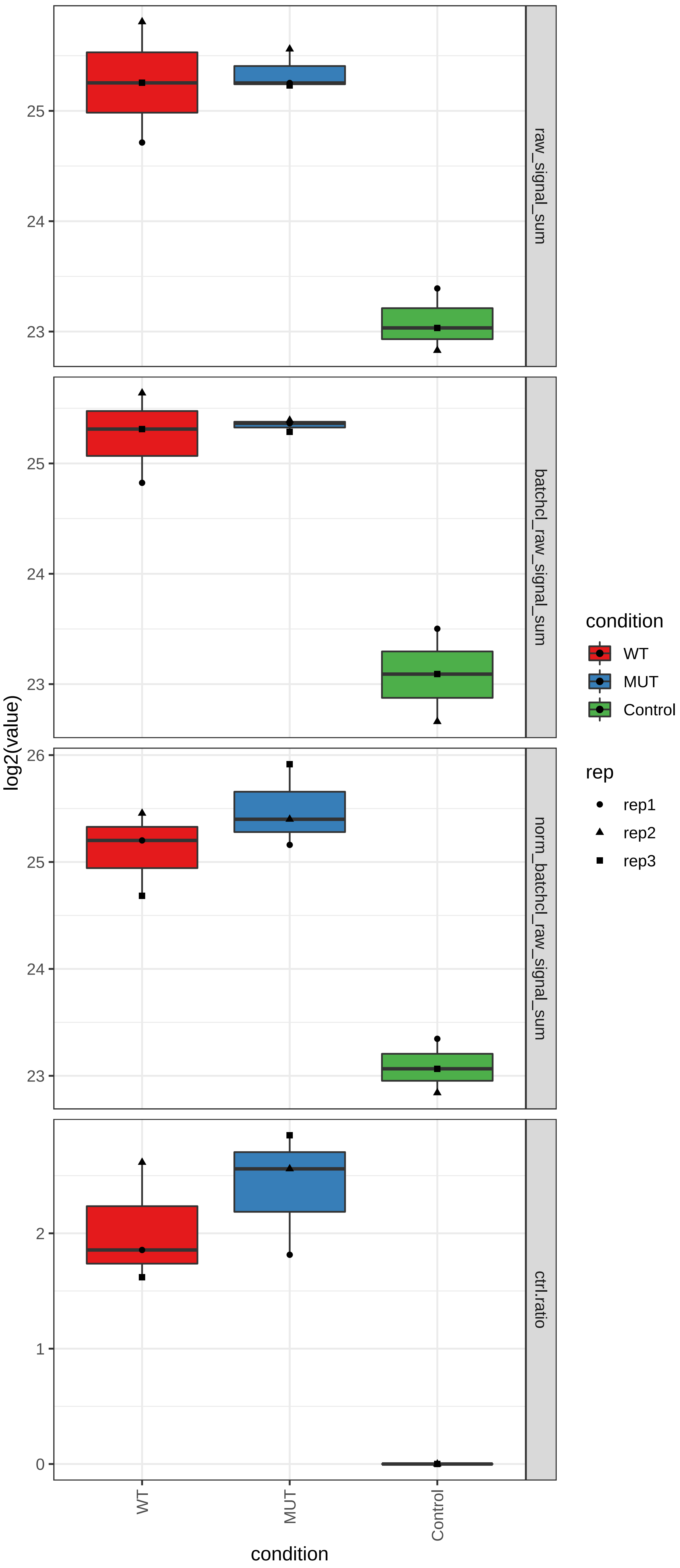

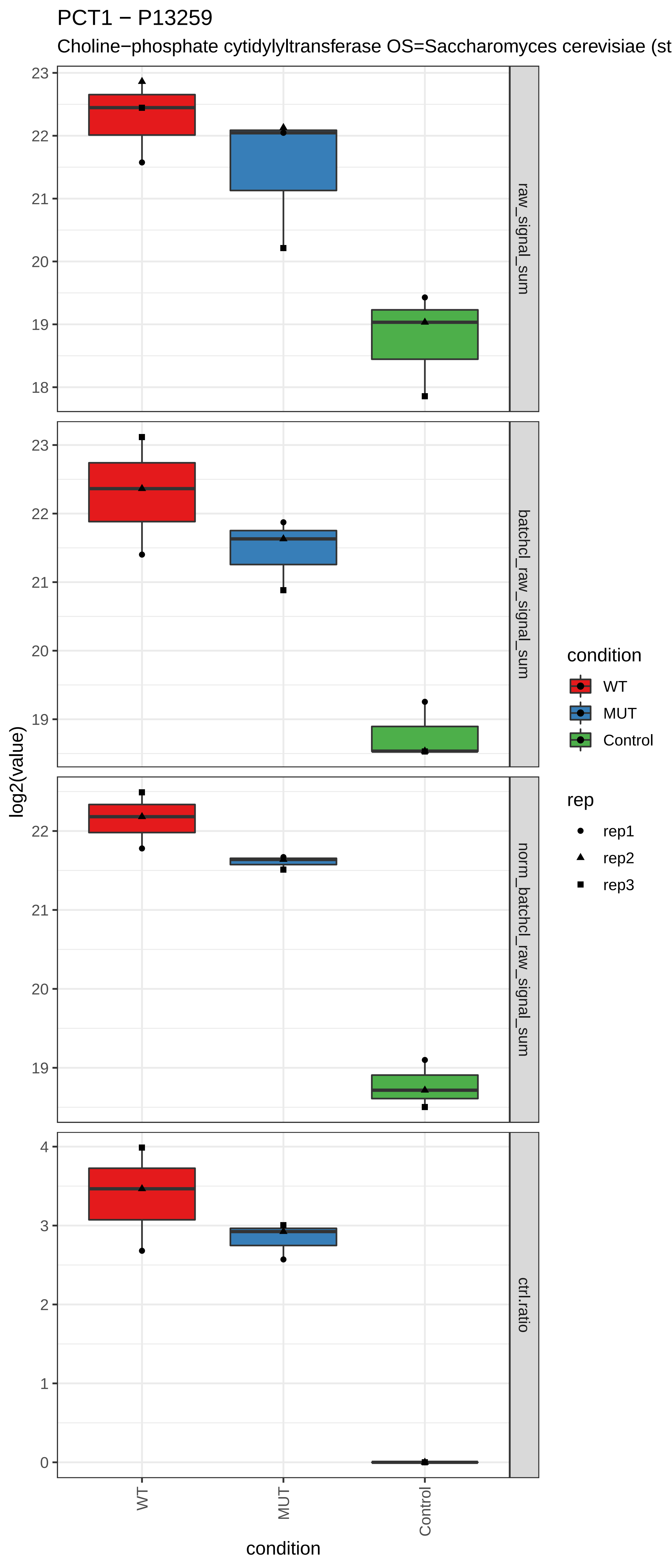

PCT1 − P13259
Choline−phosphate cytidylyltransferase OS=Saccharomyces cerevisiae (st
log2(value)
raw_signal_sum
batchcl_raw_signal_sum
norm_batchcl_raw_signal_sum
ctrl.ratio
WT
MUT
Control
condition
condition
WT
MUT
Control
rep
rep1
rep2
rep3

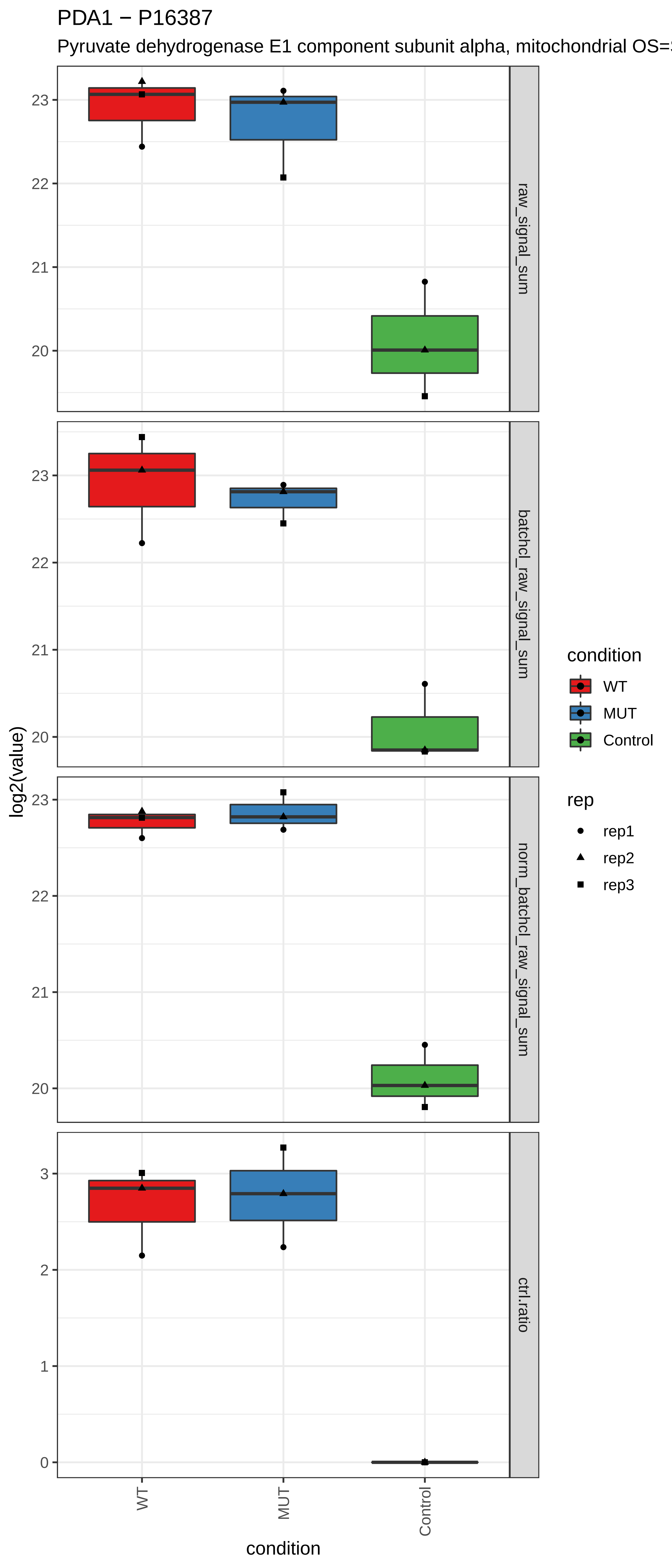
PDA1 − P16387
Pyruvate dehydrogenase E1 component subunit alpha, mitochondrial OS=
raw_signal_sum
batchcl_raw_signal_sum
norm_batchcl_raw_signal_sum
ctrl.ratio
log2(value)
condition
WT
MUT
Control
rep
rep1
rep2
rep3
23
22
21
20
3
2
1
0

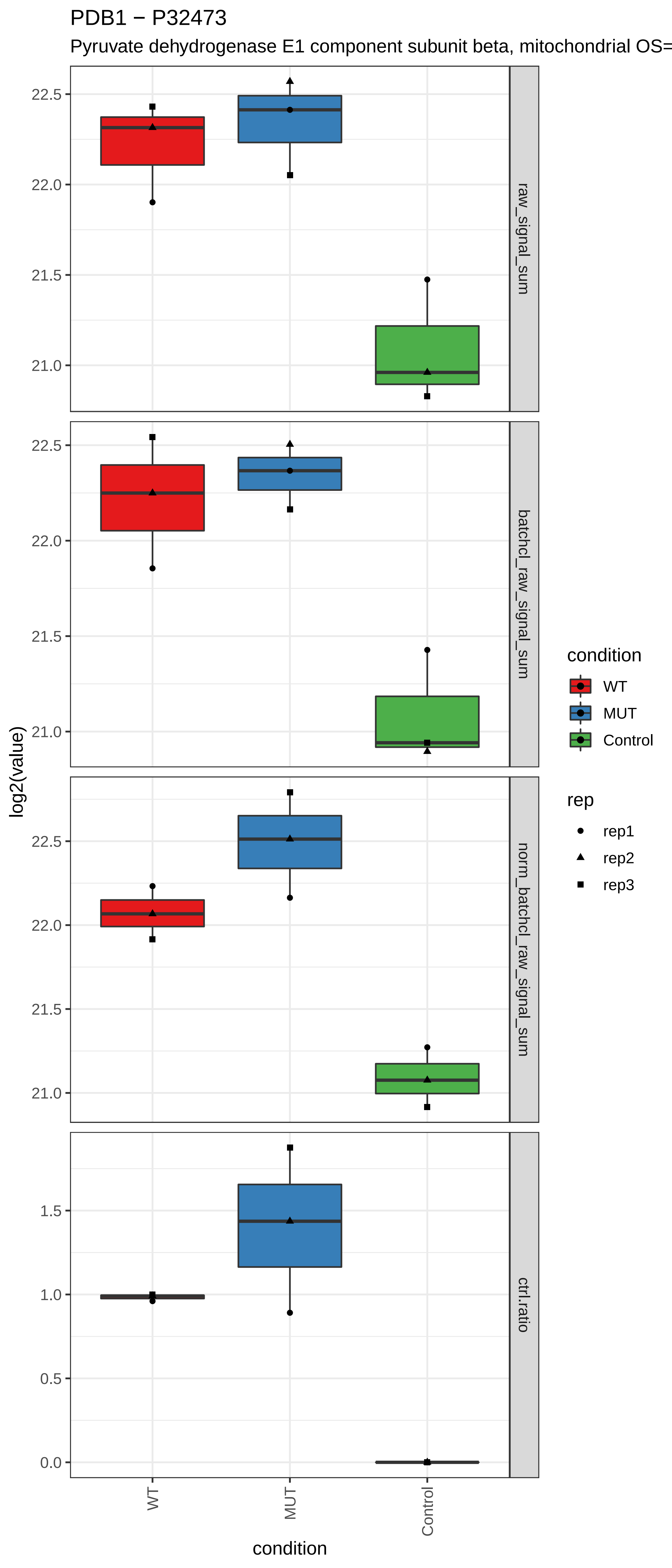

PDB1 − P32473
Pyruvate dehydrogenase E1 component subunit beta, mitochondrial OS=
raw_signal_sum
batchcl_raw_signal_sum
norm_batchcl_raw_signal_sum
ctrl.ratio
22.5
22.0
21.5
21.0
1.5
1.0
0.5
0.0
log2(value)
WT
MUT
Control
condition
rep
rep1
rep2
rep3

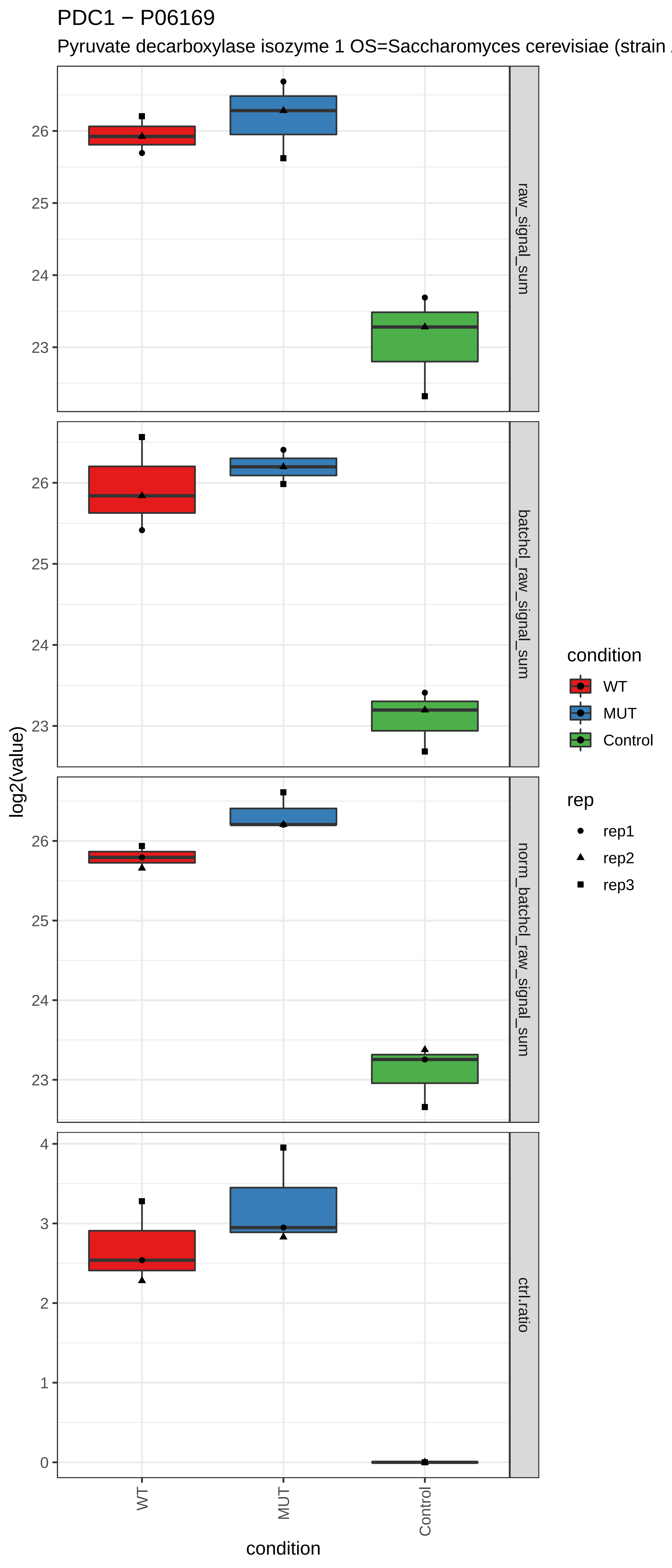
PDC1 − P06169
Pyruvate decarboxylase isozyme 1 OS=Saccharomyces cerevisiae (strain
raw_signal_sum
batchcl_raw_signal_sum
norm_batchcl_raw_signal_sum
ctrl.ratio
log2(value)
condition
WT
MUT
Control
rep
rep1
rep2
rep3

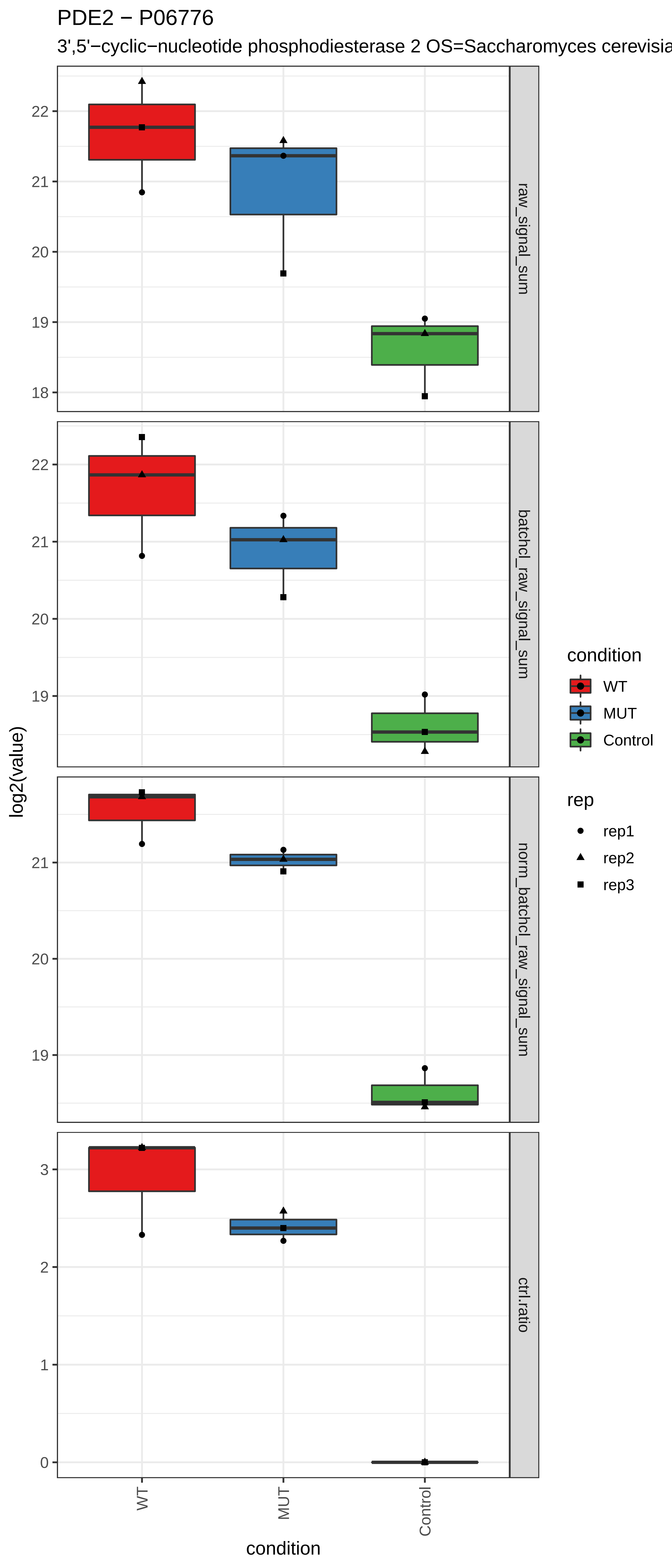

PDE2 − P06776
3',5'−cyclic−nucleotide phosphodiesterase 2 OS=Saccharomyces cerevisia
raw_signal_sum
batchcl_raw_signal_sum
norm_batchcl_raw_signal_sum
ctrl.ratio
log2(value)
condition
WT
MUT
Control
rep
rep1
rep2
rep3
22
21
20
19
18
3
2
1
0

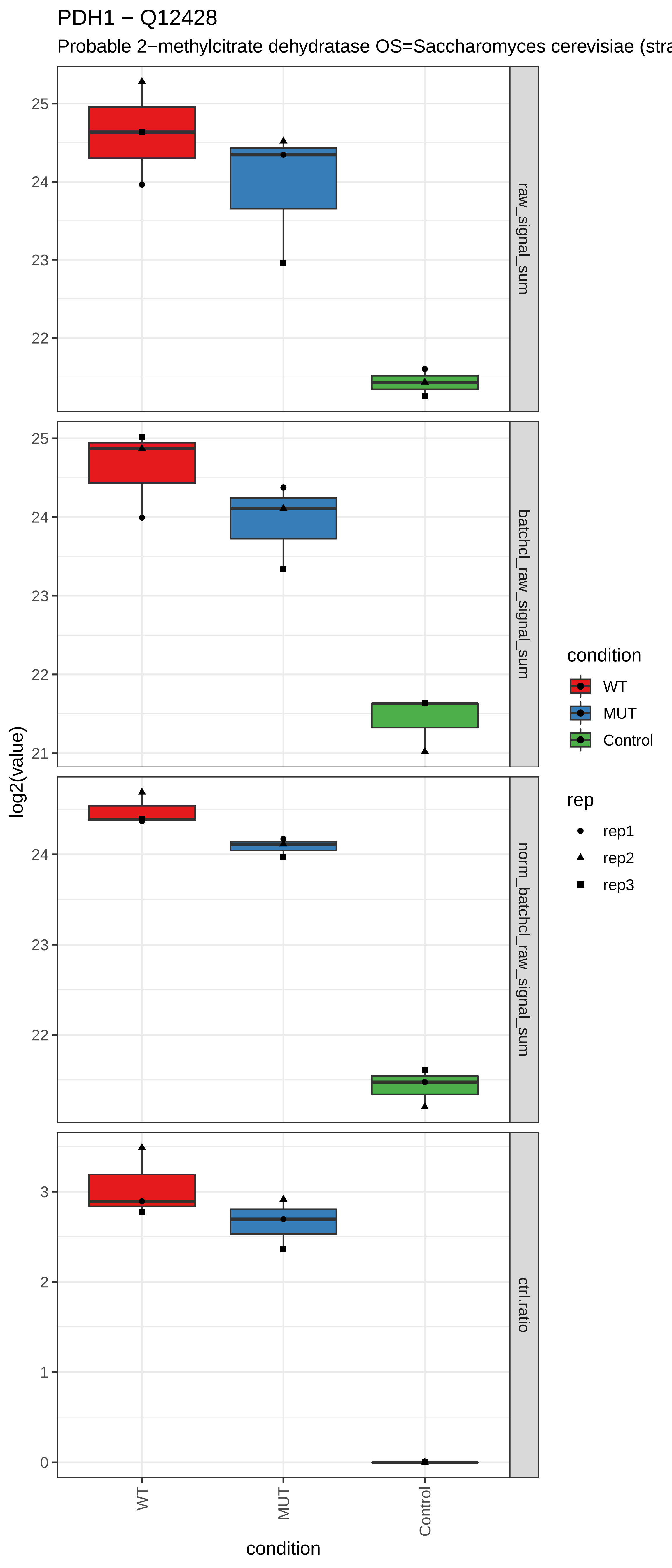
PDH1 − Q12428
Probable 2−methylcitrate dehydratase OS=Saccharomyces cerevisiae (stra
raw_signal_sum
batchcl_raw_signal_sum
norm_batchcl_raw_signal_sum
ctrl.ratio
log2(value)
condition
WT
MUT
Control
condition
WT
MUT
Control
rep
rep1
rep2
rep3
25
24
23
22
21
3
2
1
0

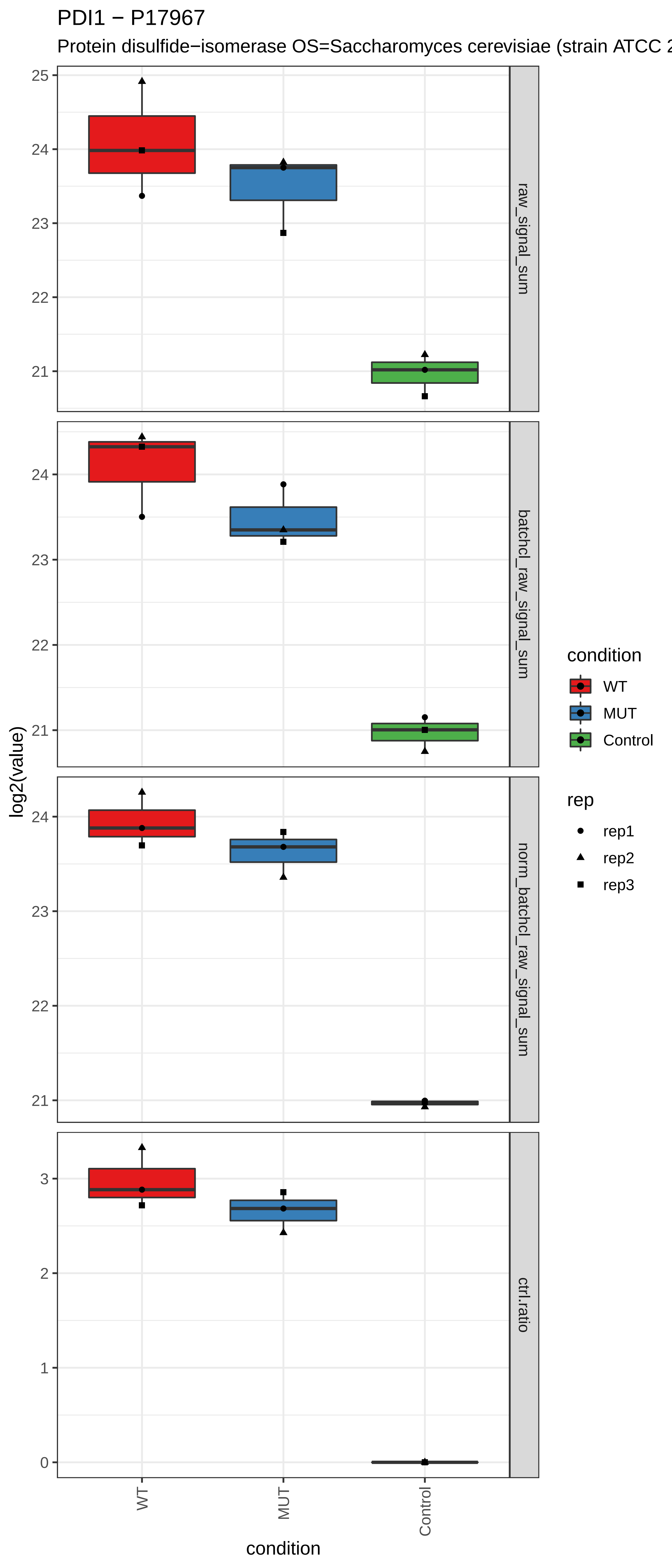
PDI1 − P17967
Protein disulfide−isomerase OS=Saccharomyces cerevisiae (strain ATCC 2
raw_signal_sum
batchcl_raw_signal_sum
norm_batchcl_raw_signal_sum
ctrl.ratio
log2(value)
condition
WT
MUT
Control
rep
rep1
rep2
rep3

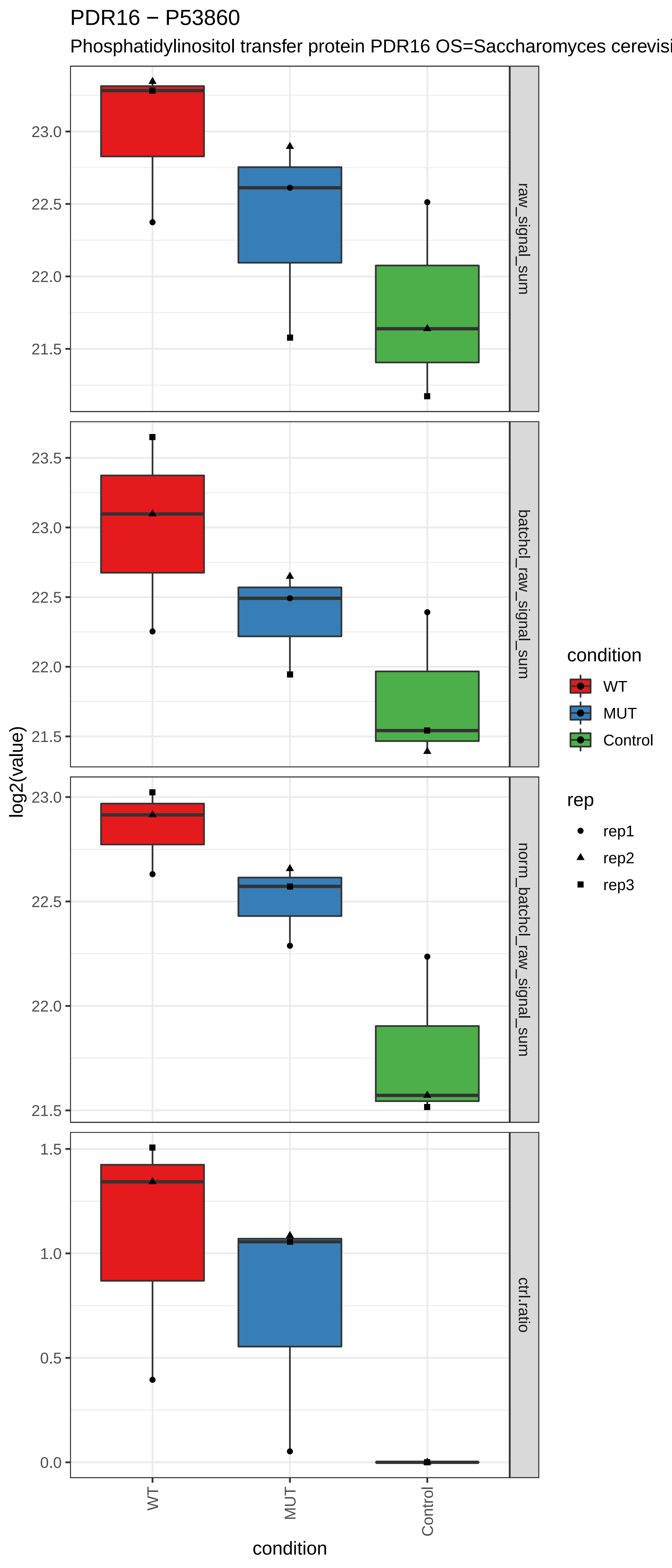

PDR16 − P53860
Phosphatidylinositol transfer protein PDR16 OS=Saccharomyces cerevisi
raw_signal_sum
batchcl_raw_signal_sum
norm_batchcl_raw_signal_sum
ctrl.ratio
log2(value)
condition
WT
MUT
Control
rep
rep1
rep2
rep3
23.0
22.5
22.0
21.5
23.5
1.5
1.0
0.5
0.0

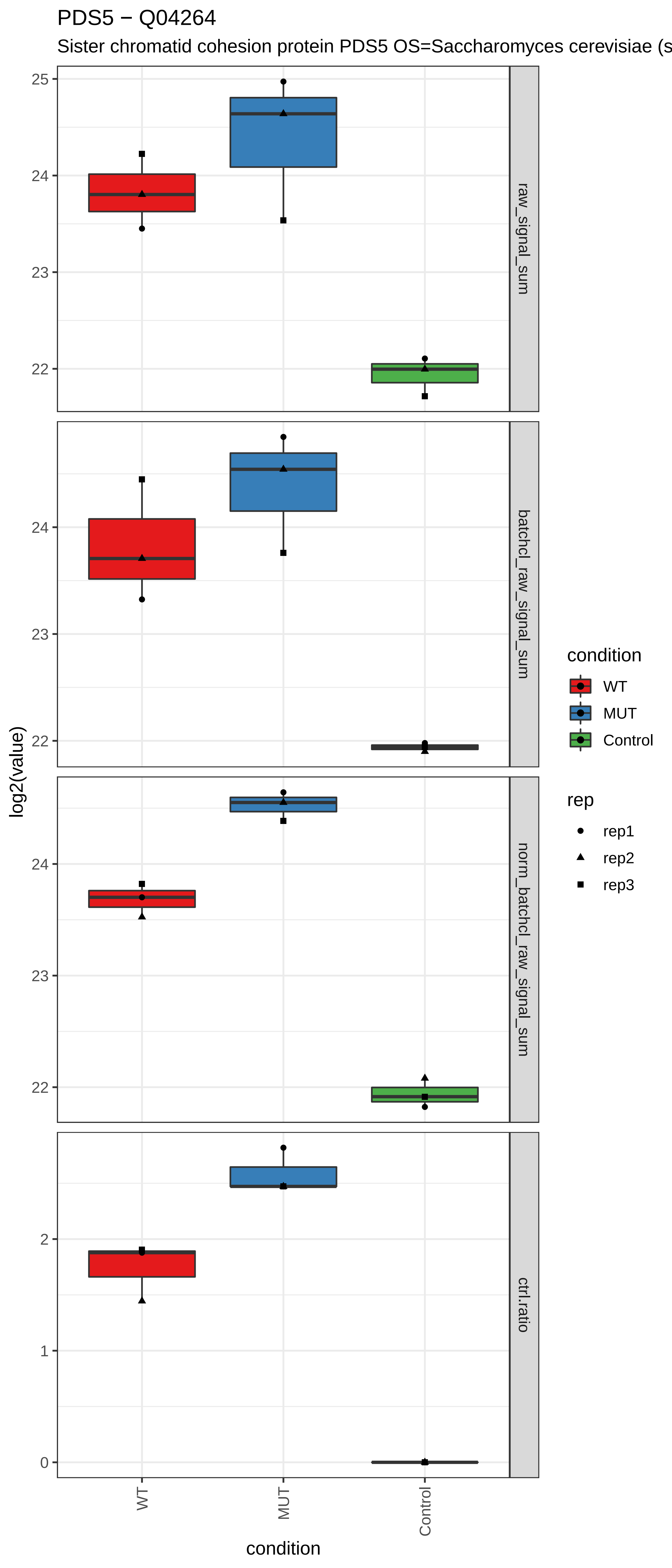

PDS5 − Q04264
Sister chromatid cohesion protein PDS5 OS=Saccharomyces cerevisiae (s
raw_signal_sum
batchcl_raw_signal_sum
norm_batchcl_raw_signal_sum
ctrl.ratio
log2(value)
condition
WT
MUT
Control
rep
rep1
rep2
rep3
25
24
23
22
24
23
22
24
23
22
2
1
0
WT
MUT
Control
condition

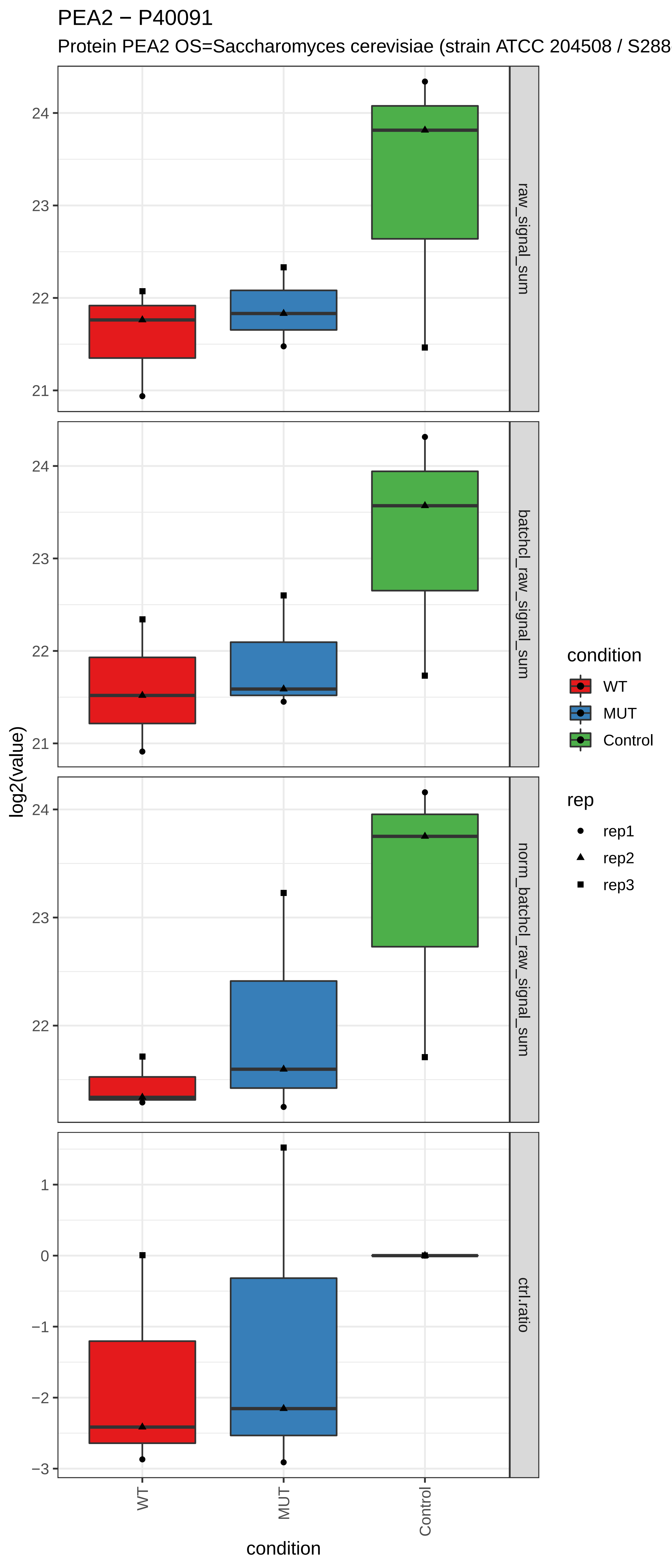

PEA2 − P40091
Protein PEA2 OS=Saccharomyces cerevisiae (strain ATCC 204508 / S288
raw_signal_sum
batchcl_raw_signal_sum
norm_batchcl_raw_signal_sum
ctrl.ratio
log2(value)
condition
WT
MUT
Control
rep
rep1
rep2
rep3
24
23
22
21
1
0
−1
−2
−3

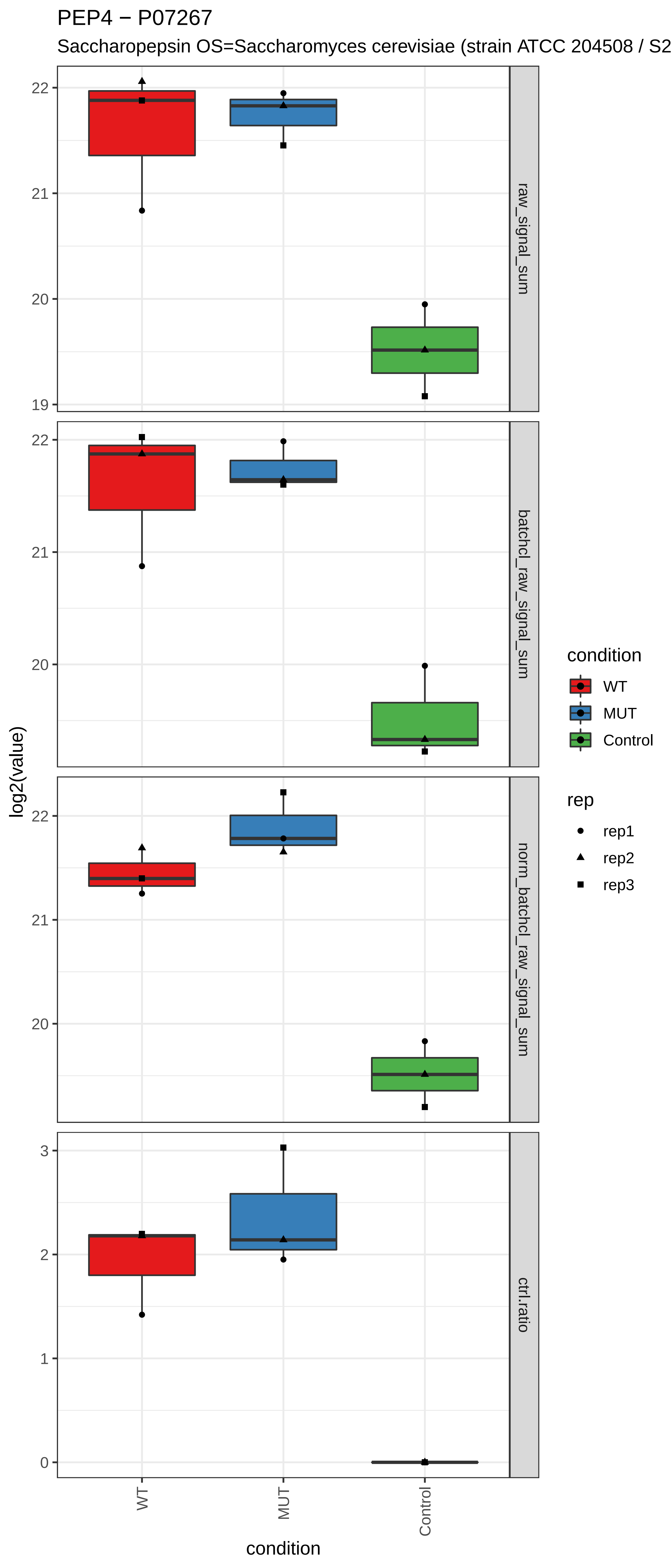
PEP4 − P07267
Saccharopepsin OS=Saccharomyces cerevisiae (strain ATCC 204508 / S2
raw_signal_sum
batchcl_raw_signal_sum
norm_batchcl_raw_signal_sum
ctrl.ratio
log2(value)
condition
WT
MUT
Control
rep
rep1
rep2
rep3
22
21
20
19
3
2
1
0

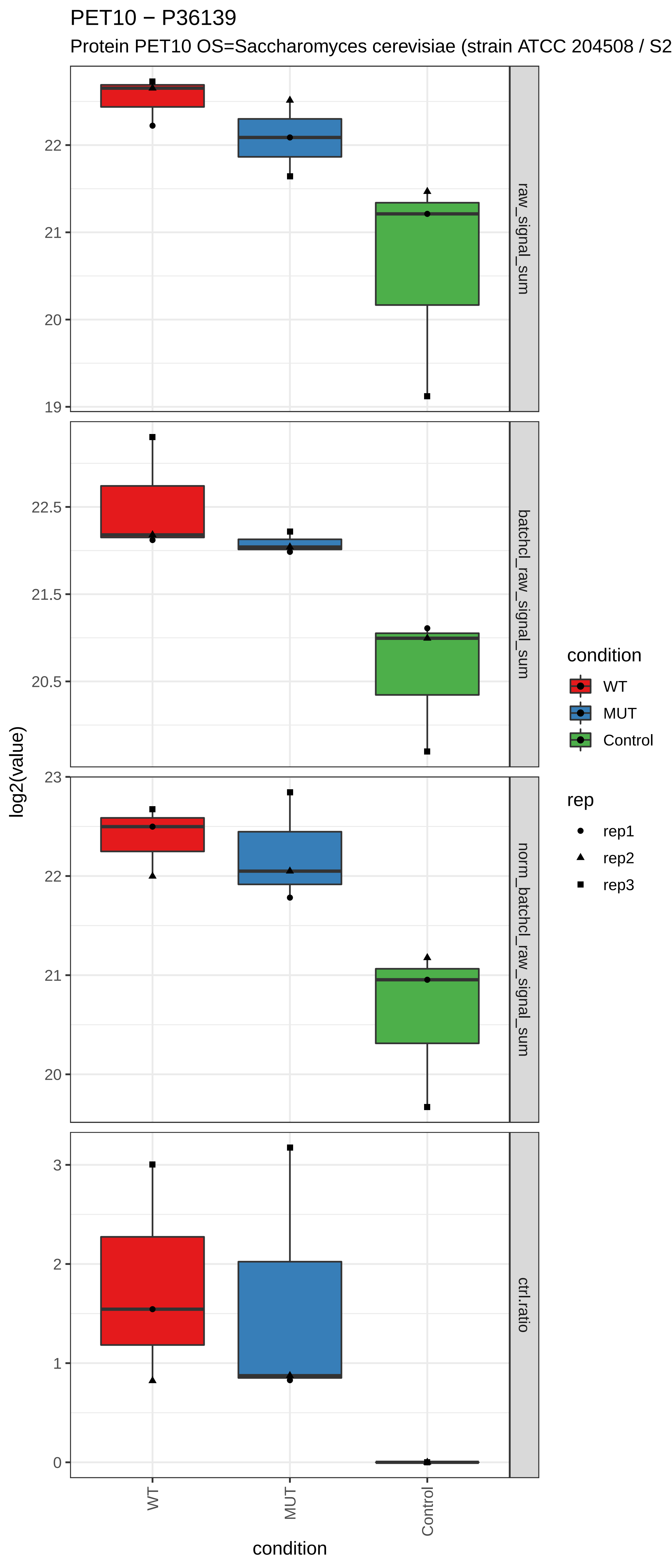

PET10 − P36139
Protein PET10 OS=Saccharomyces cerevisiae (strain ATCC 204508 / S2
log2(value)
raw_signal_sum
batchcl_raw_signal_sum
norm_batchcl_raw_signal_sum
ctrl.ratio
19
20
21
22
20.5
21.5
22.5
20
21
22
23
0
1
2
3
WT
MUT
Control
condition
condition
WT
MUT
Control
rep
rep1
rep2
rep3

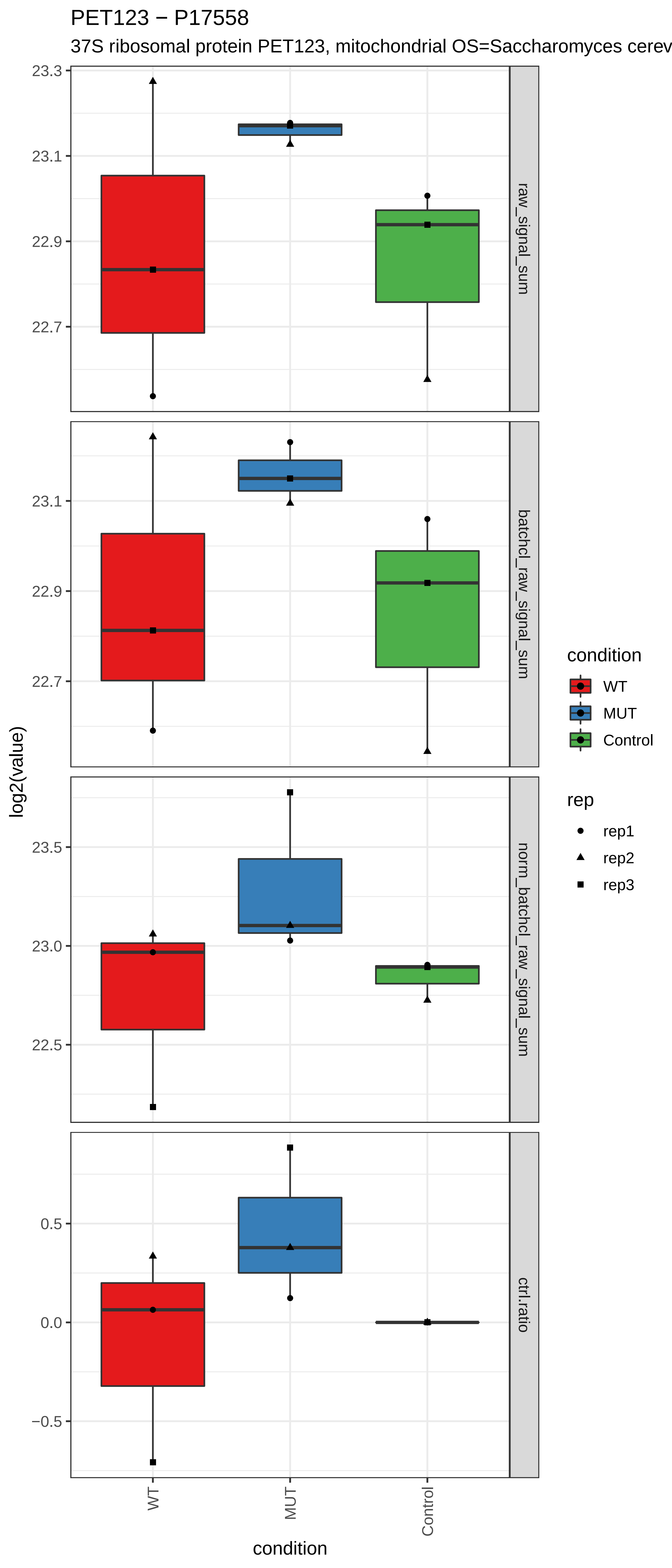

PET123 − P17558
37S ribosomal protein PET123, mitochondrial OS=Saccharomyces cerev
raw_signal_sum
batchcl_raw_signal_sum
norm_batchcl_raw_signal_sum
ctrl.ratio
log2(value)
condition
WT
MUT
Control
rep
rep1
rep2
rep3
23.3
23.1
22.9
22.7
23.5
23.0
22.5
0.5
0.0
−0.5

PET127 − P32606

Putative mitochondrial translation system component PET127 OS=Sacch

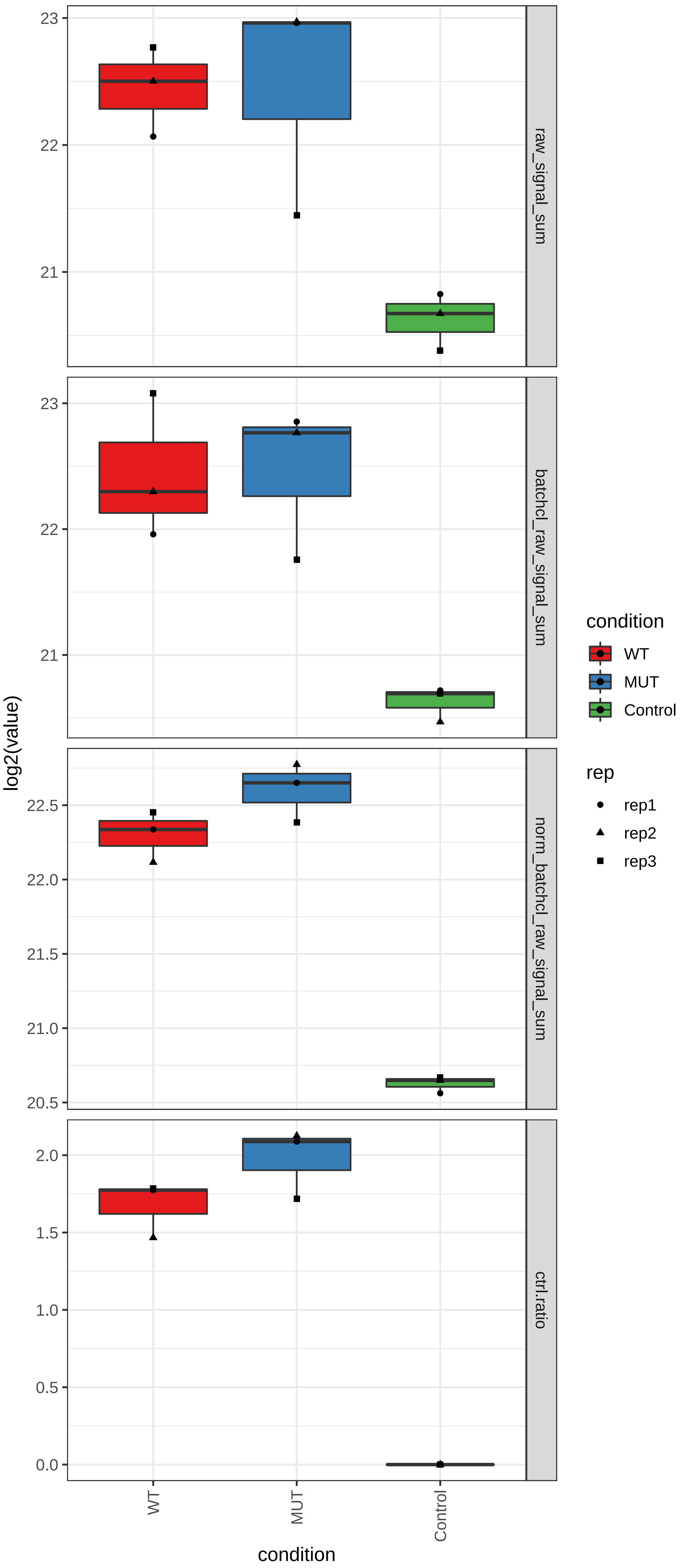

PET9 − P18239

ADP,ATP carrier protein 2 OS=Saccharomyces cerevisiae (strain ATCC 204

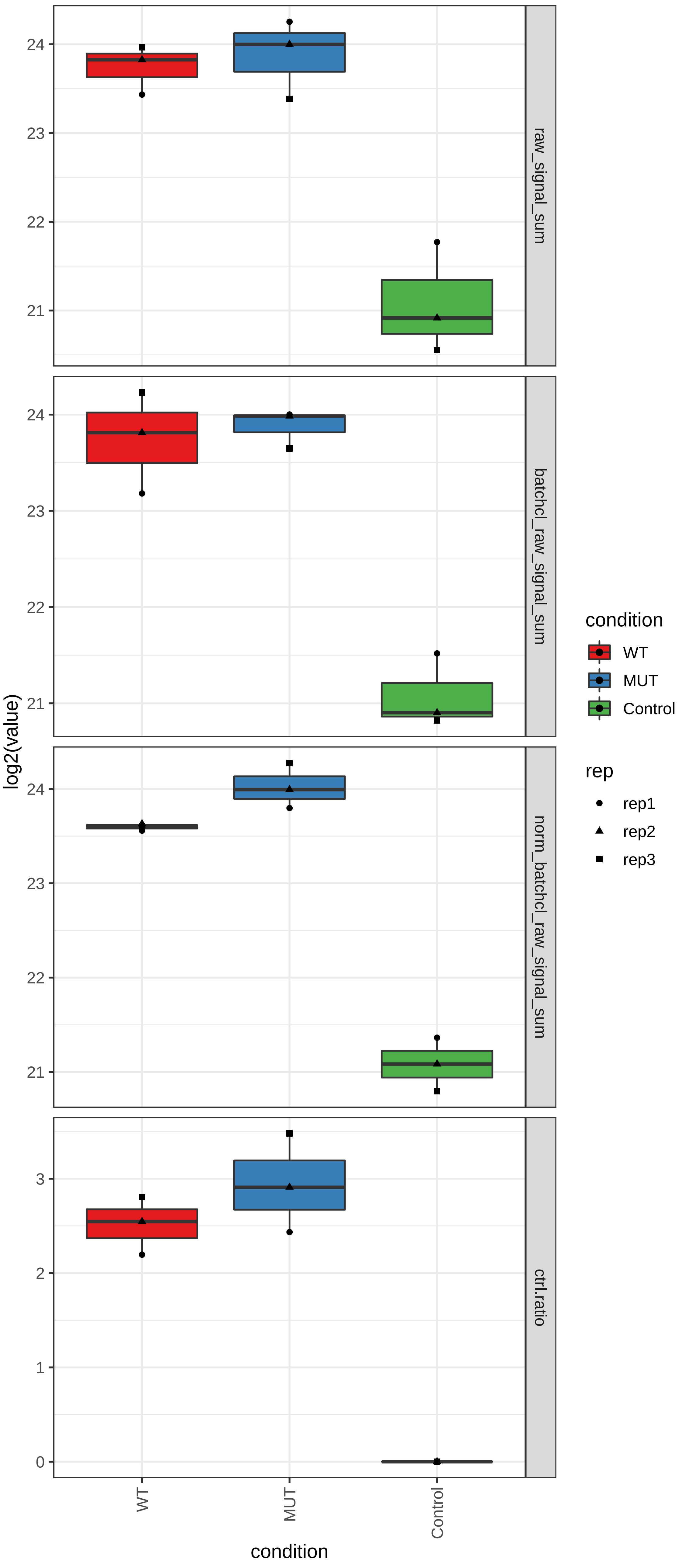

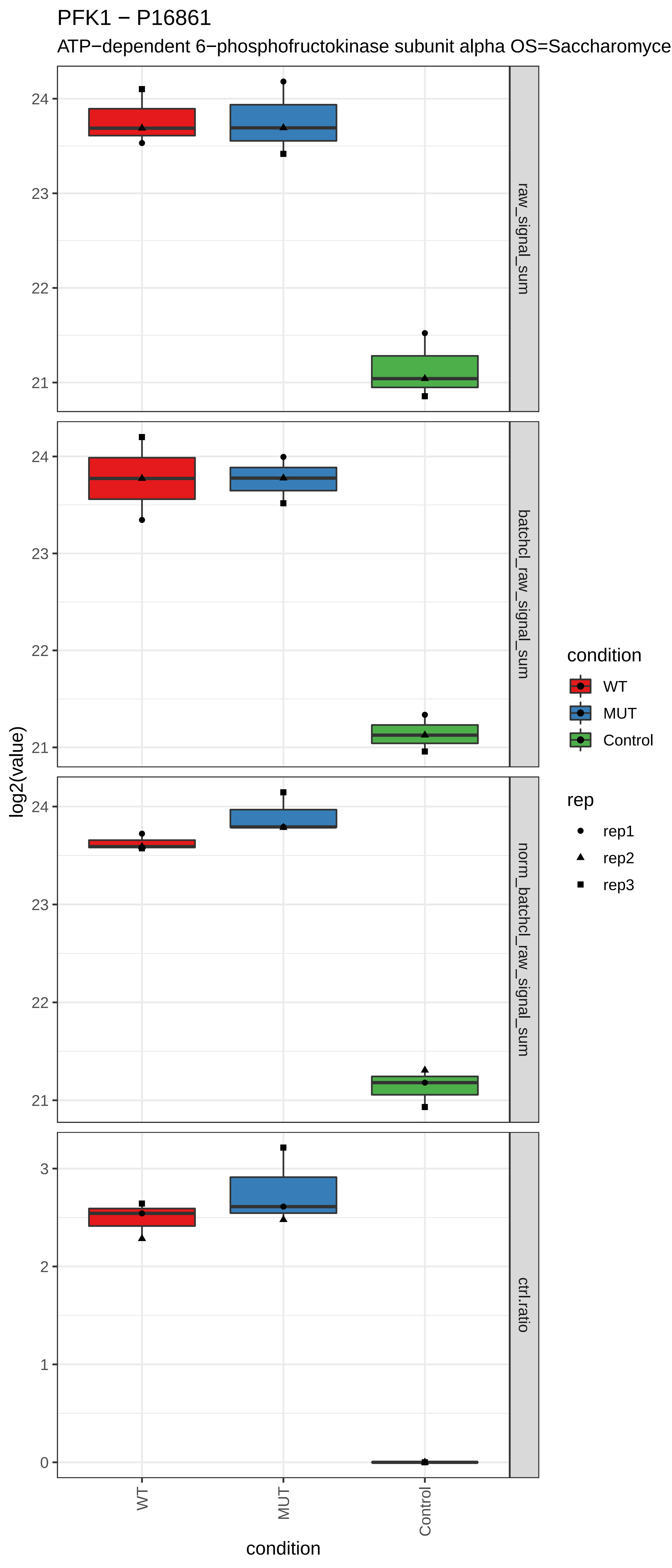
PFK1 − P16861
ATP−dependent 6−phosphofructokinase subunit alpha OS=Saccharomyce
raw_signal_sum
batchcl_raw_signal_sum
norm_batchcl_raw_signal_sum
ctrl.ratio
log2(value)
condition
WT
MUT
Control
rep
rep1
rep2
rep3
24
23
22
21
3
2
1
0

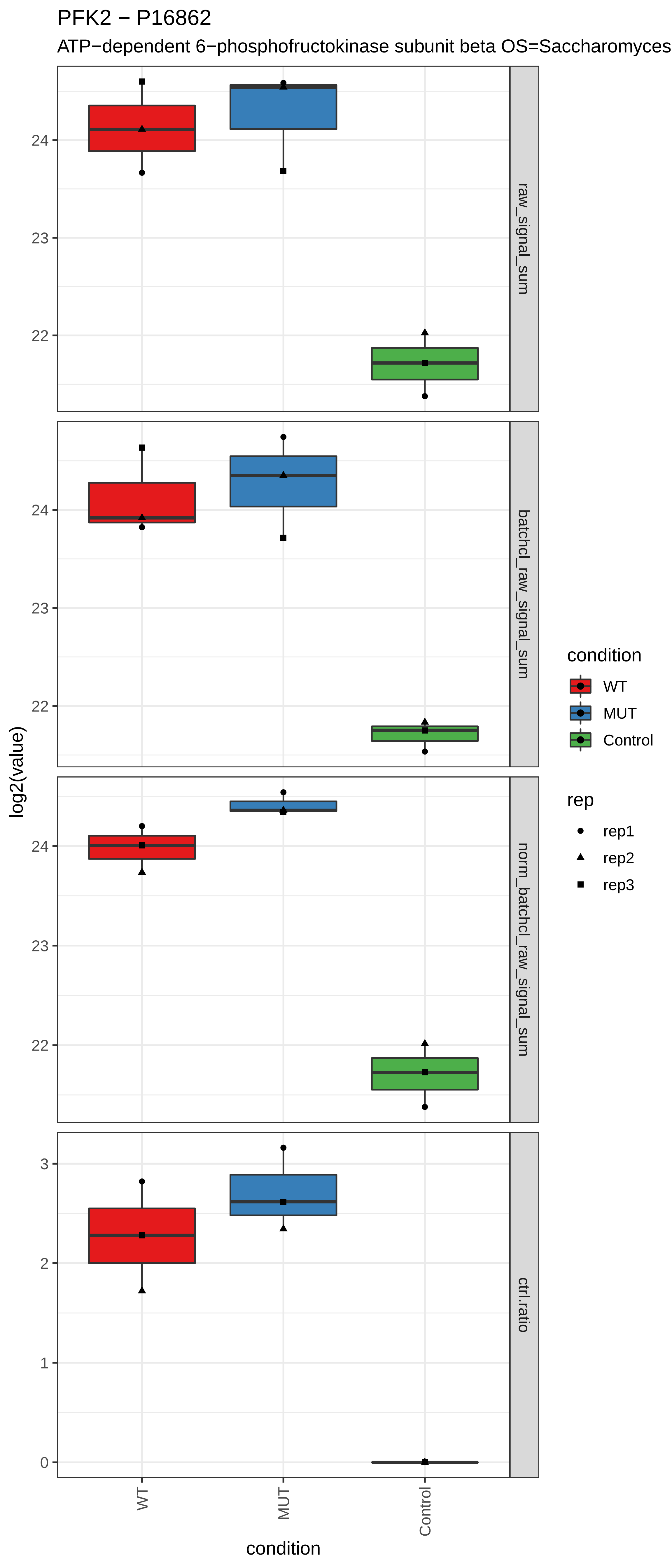
PFK2 − P16862
ATP−dependent 6−phosphofructokinase subunit beta OS=Saccharomyces
raw_signal_sum
batchcl_raw_signal_sum
norm_batchcl_raw_signal_sum
ctrl.ratio
log2(value)
24
23
22
3
2
1
0
WT
MUT
Control
condition
condition
WT
MUT
Control
rep
rep1
rep2
rep3

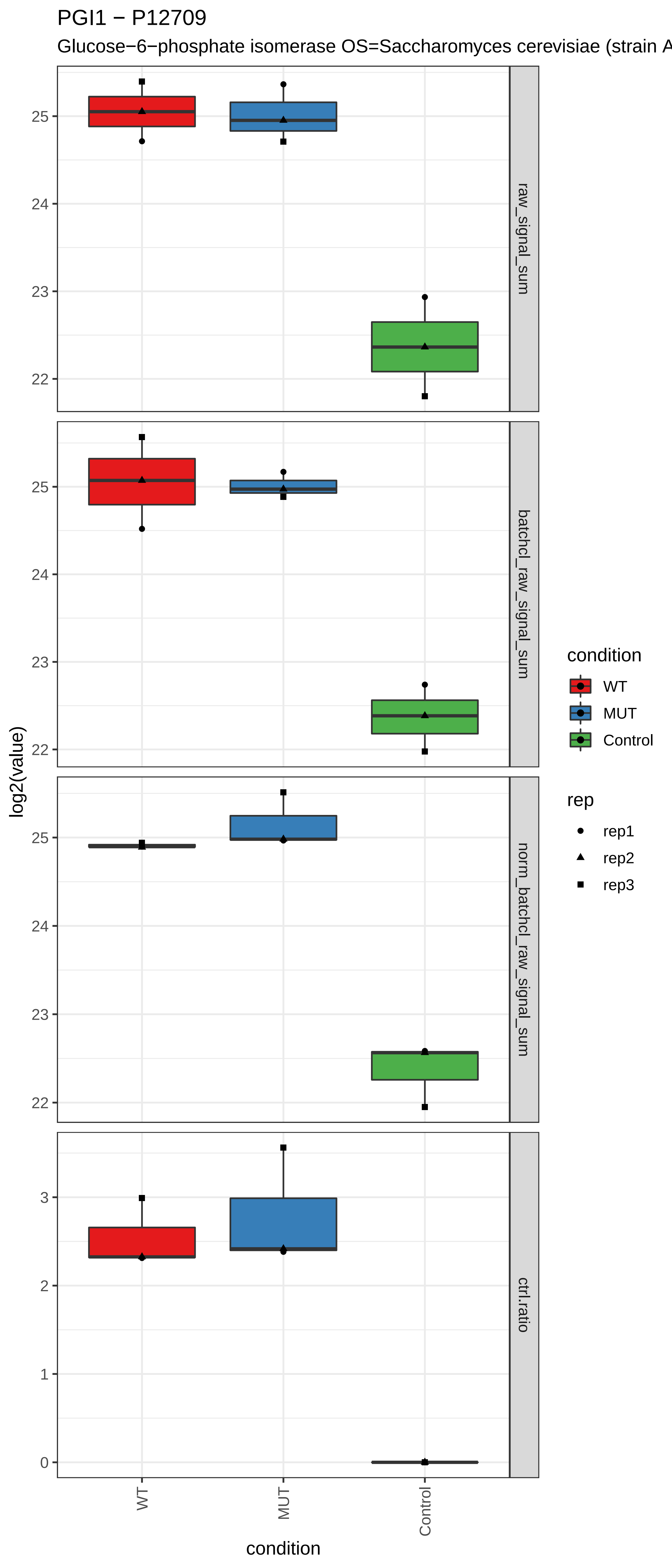

PGI1 − P12709
Glucose−6−phosphate isomerase OS=Saccharomyces cerevisiae (strain A
raw_signal_sum
batchcl_raw_signal_sum
norm_batchcl_raw_signal_sum
ctrl.ratio
log2(value)
condition
WT
MUT
Control
rep
rep1
rep2
rep3
25
24
23
22
3
2
1
0

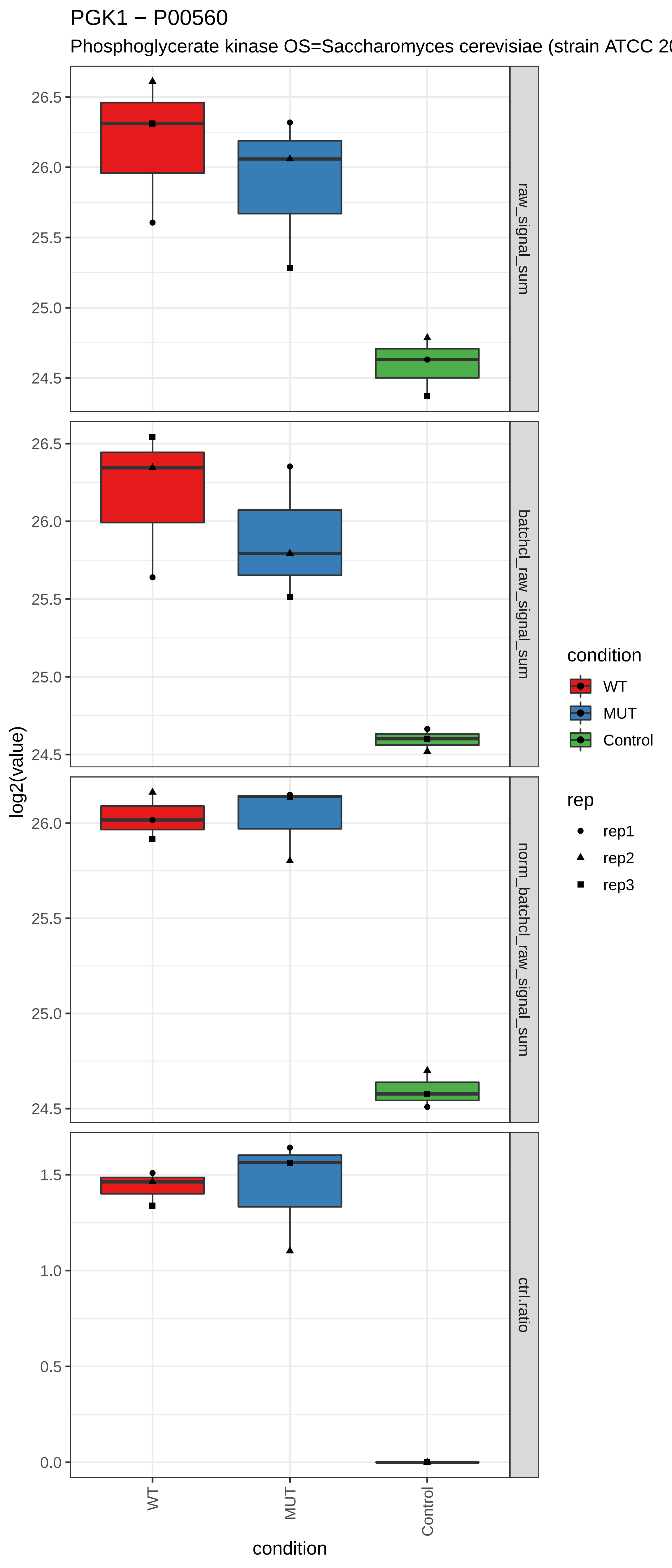

PGK1 − P00560
Phosphoglycerate kinase OS=Saccharomyces cerevisiae (strain ATCC 20
raw_signal_sum
batchcl_raw_signal_sum
norm_batchcl_raw_signal_sum
ctrl.ratio
26.5
26.0
25.5
25.0
24.5
1.5
1.0
0.5
0.0
log2(value)
WT
MUT
Control
condition
condition
WT
MUT
Control
rep
rep1
rep2
rep3

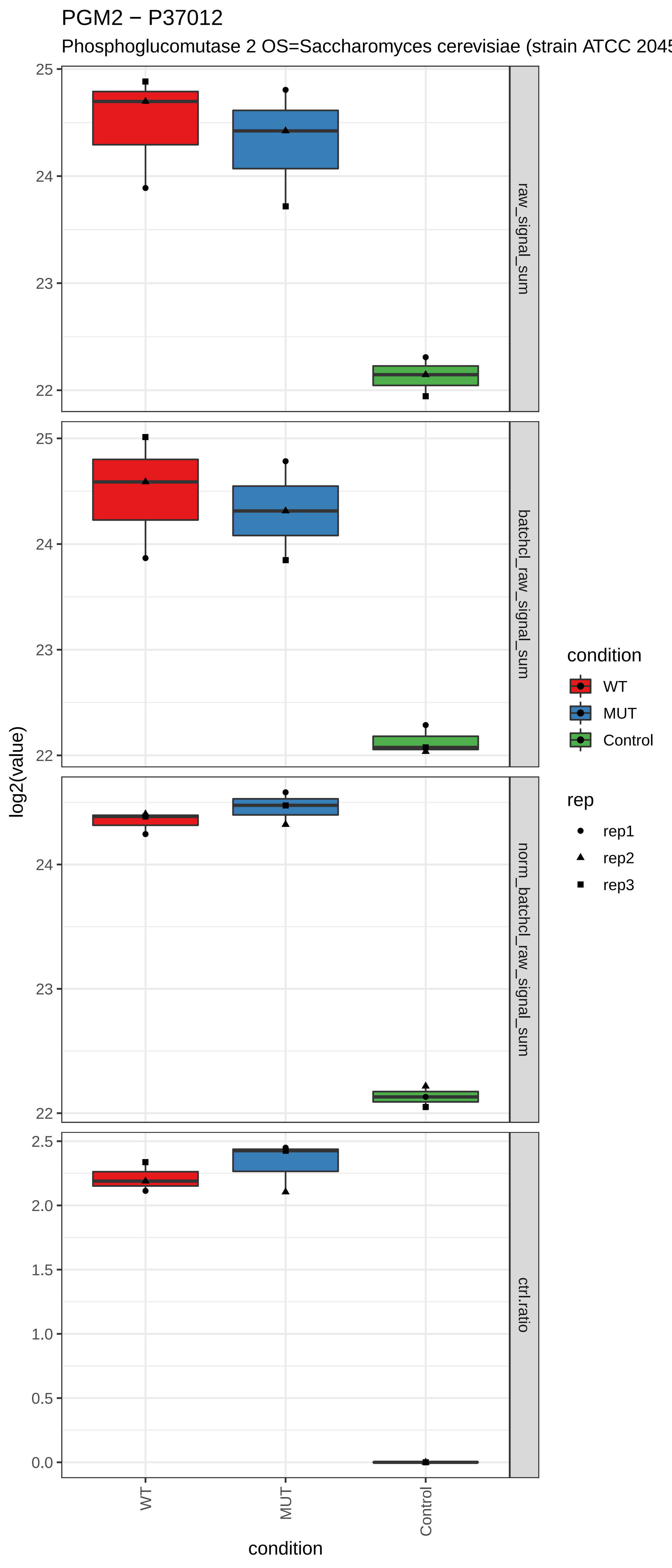

PGM2 − P37012
Phosphoglucomutase 2 OS=Saccharomyces cerevisiae (strain ATCC 2045
log2(value)
condition
raw_signal_sum
batchcl_raw_signal_sum
norm_batchcl_raw_signal_sum
ctrl.ratio
25
24
23
22
2.5
2.0
1.5
1.0
0.5
0.0
WT
MUT
Control
condition
WT
MUT
Control
rep
rep1
rep2
rep3

PHO2 − P07269

Regulatory protein PHO2 OS=Saccharomyces cerevisiae (strain ATCC 204

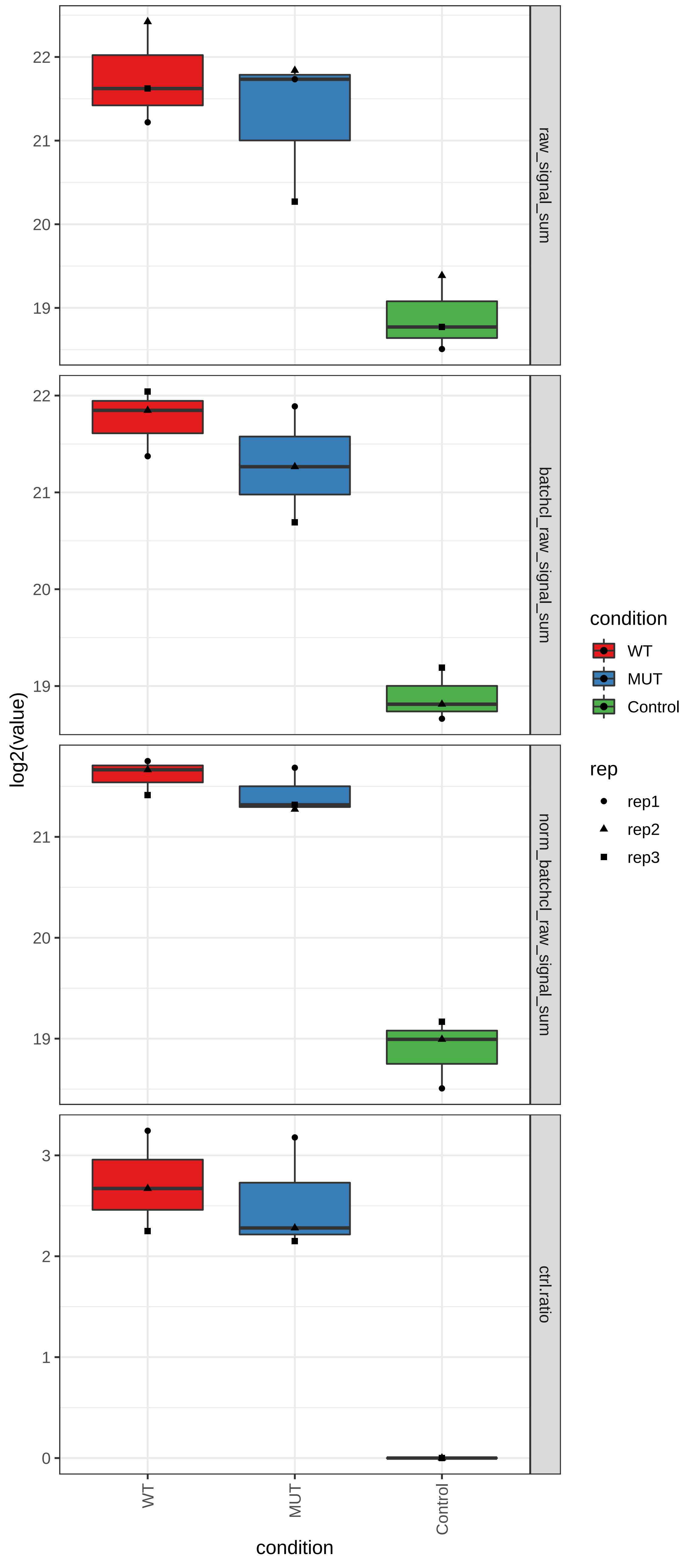

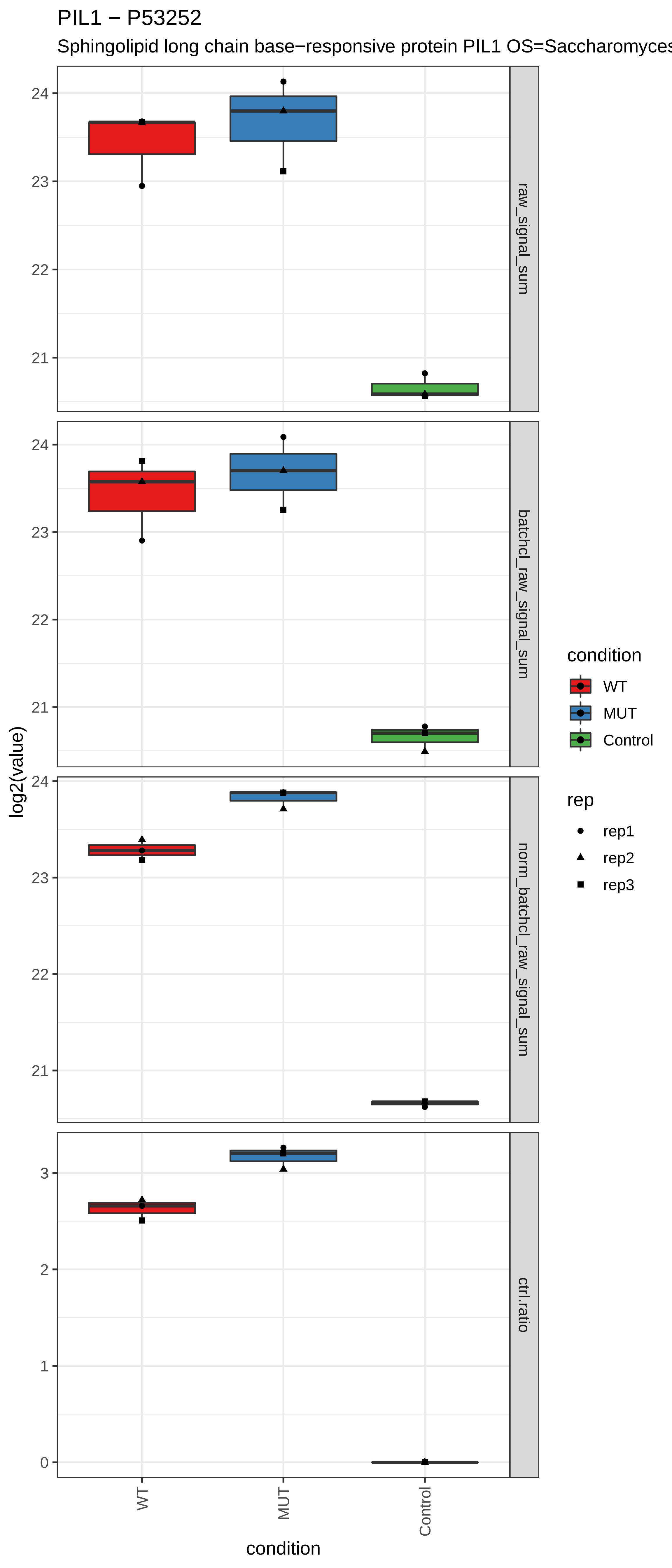
PIL1 − P53252
Sphingolipid long chain base−responsive protein PIL1 OS=Saccharomyces
raw_signal_sum
batchcl_raw_signal_sum
norm_batchcl_raw_signal_sum
ctrl.ratio
log2(value)
condition
WT
MUT
Control
rep
rep1
rep2
rep3
24
23
22
21
3
2
1
0

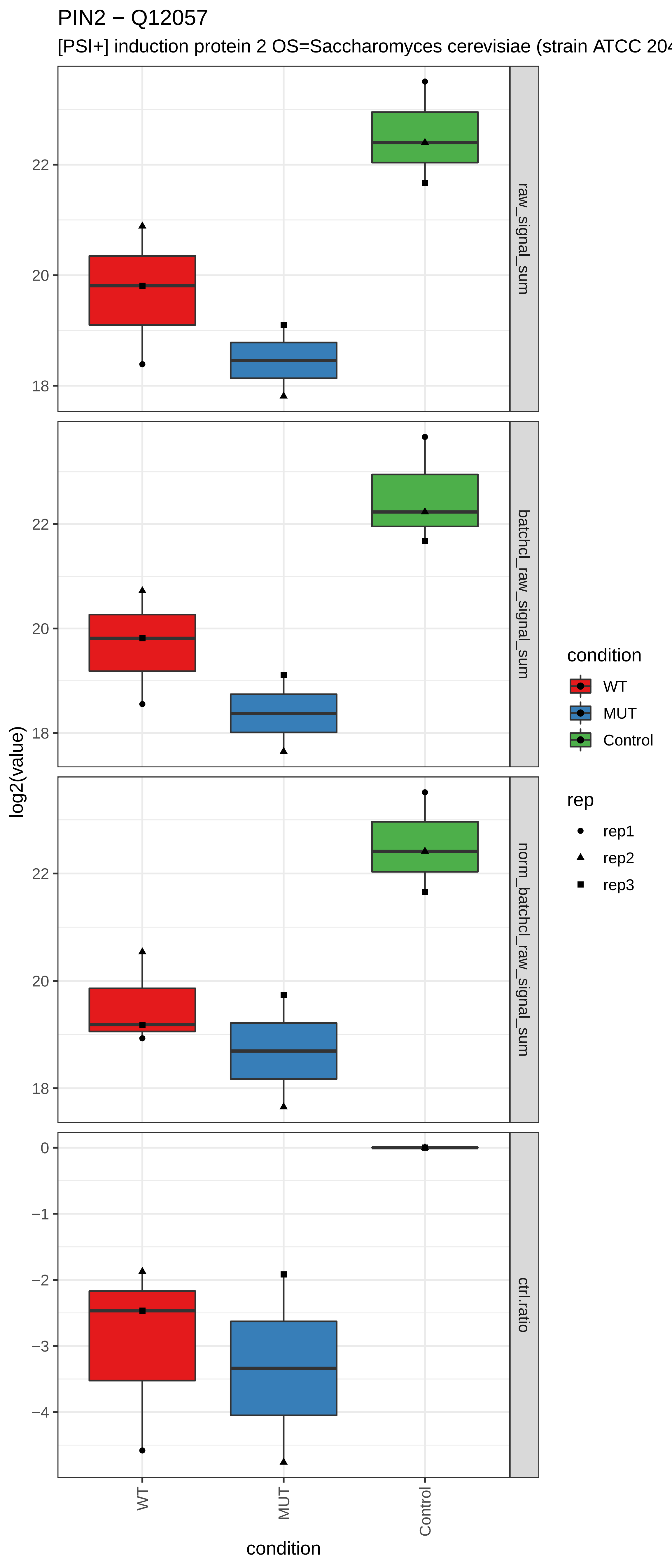

PIN2 − Q12057
[PSI+] induction protein 2 OS=Saccharomyces cerevisiae (strain ATCC 204
raw_signal_sum
batchcl_raw_signal_sum
norm_batchcl_raw_signal_sum
ctrl.ratio
log2(value)
condition
WT
MUT
Control
rep
rep1
rep2
rep3

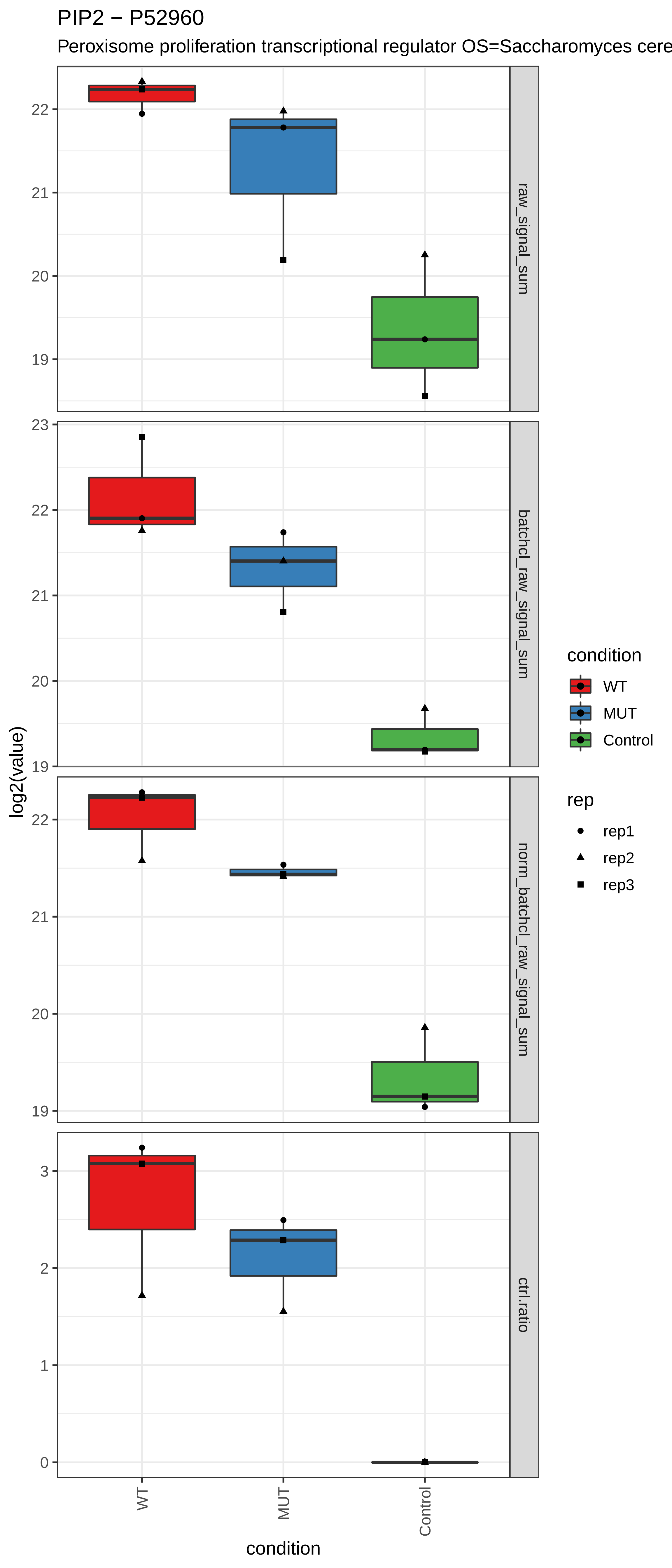
PIP2 − P52960
Peroxisome proliferation transcriptional regulator OS=Saccharomyces cere
raw_signal_sum
batchcl_raw_signal_sum
norm_batchcl_raw_signal_sum
ctrl.ratio
log2(value)
condition
WT
MUT
Control
rep
rep1
rep2
rep3

PMA1 − P05030

Plasma membrane ATPase 1 OS=Saccharomyces cerevisiae (strain ATCC

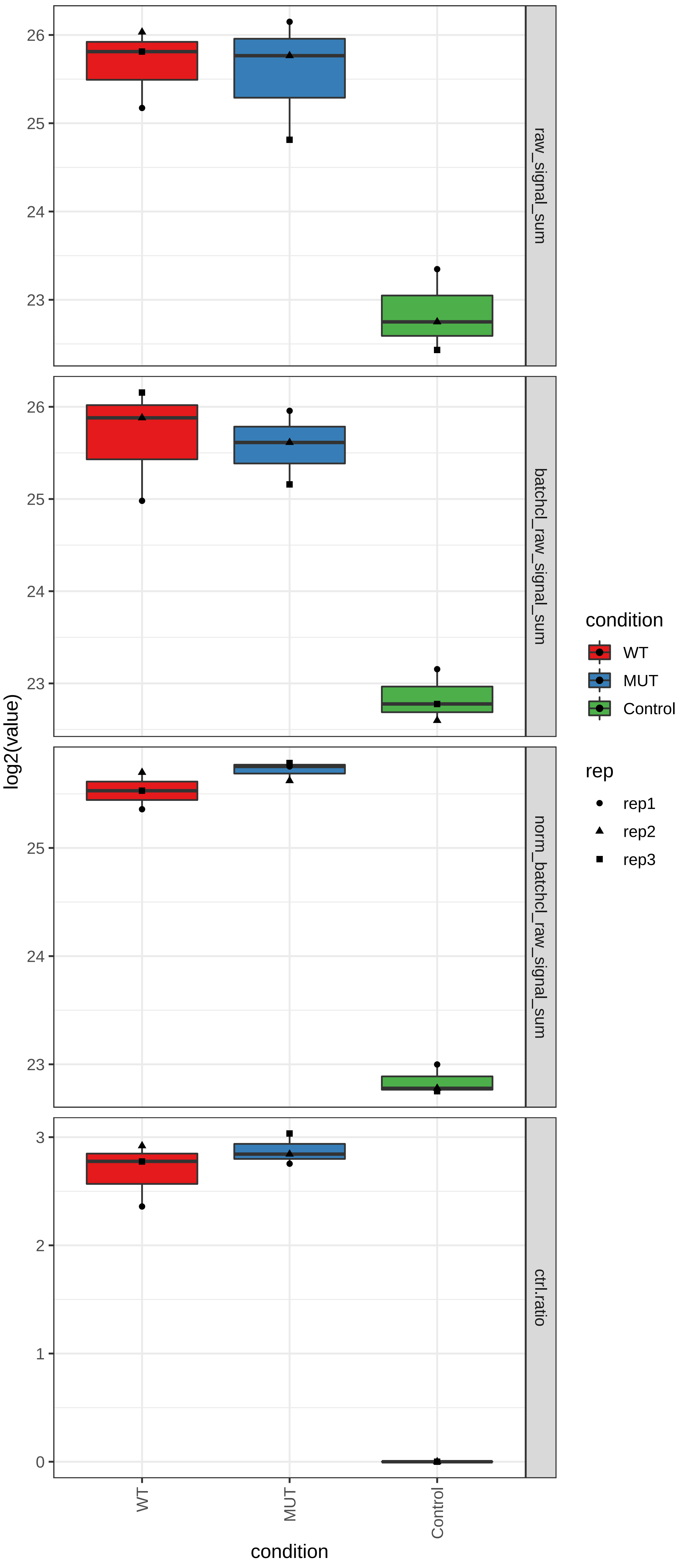

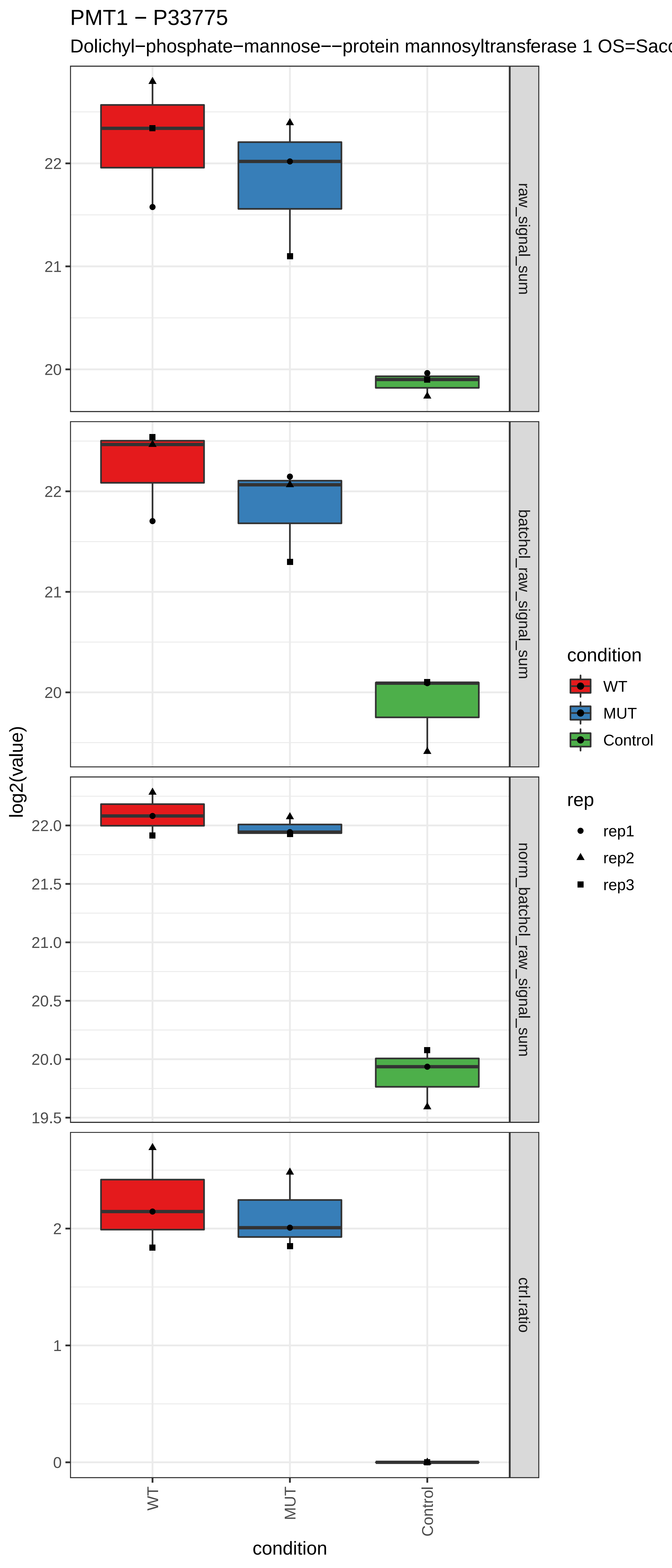

PMT1 − P33775
Dolichyl−phosphate−mannose−−protein mannosyltransferase 1 OS=Sacc
raw_signal_sum
batchcl_raw_signal_sum
norm_batchcl_raw_signal_sum
ctrl.ratio
log2(value)
condition
WT
MUT
Control
rep
rep1
rep2
rep3

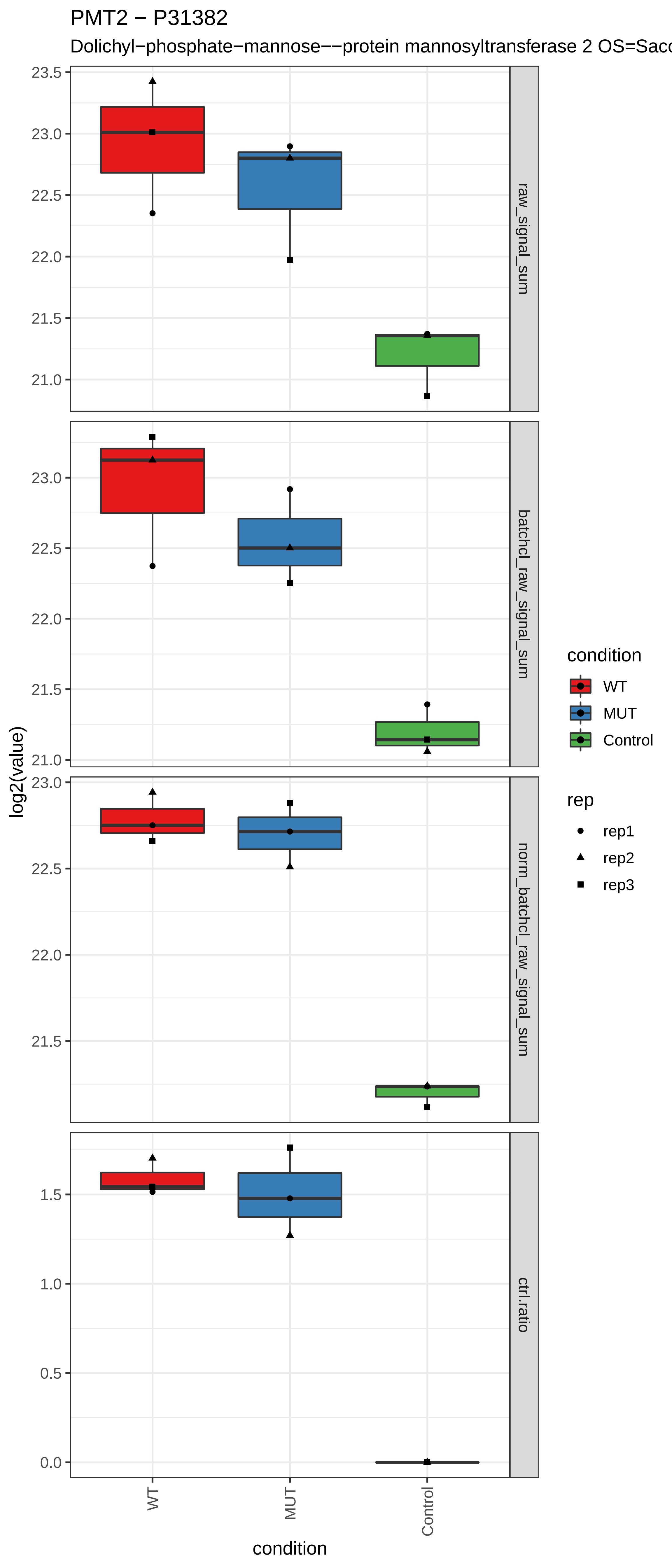

PMT2 − P31382
Dolichyl−phosphate−mannose−−protein mannosyltransferase 2 OS=Sacc
raw_signal_sum
batchcl_raw_signal_sum
norm_batchcl_raw_signal_sum
ctrl.ratio
log2(value)
condition
WT
MUT
Control
rep
rep1
rep2
rep3

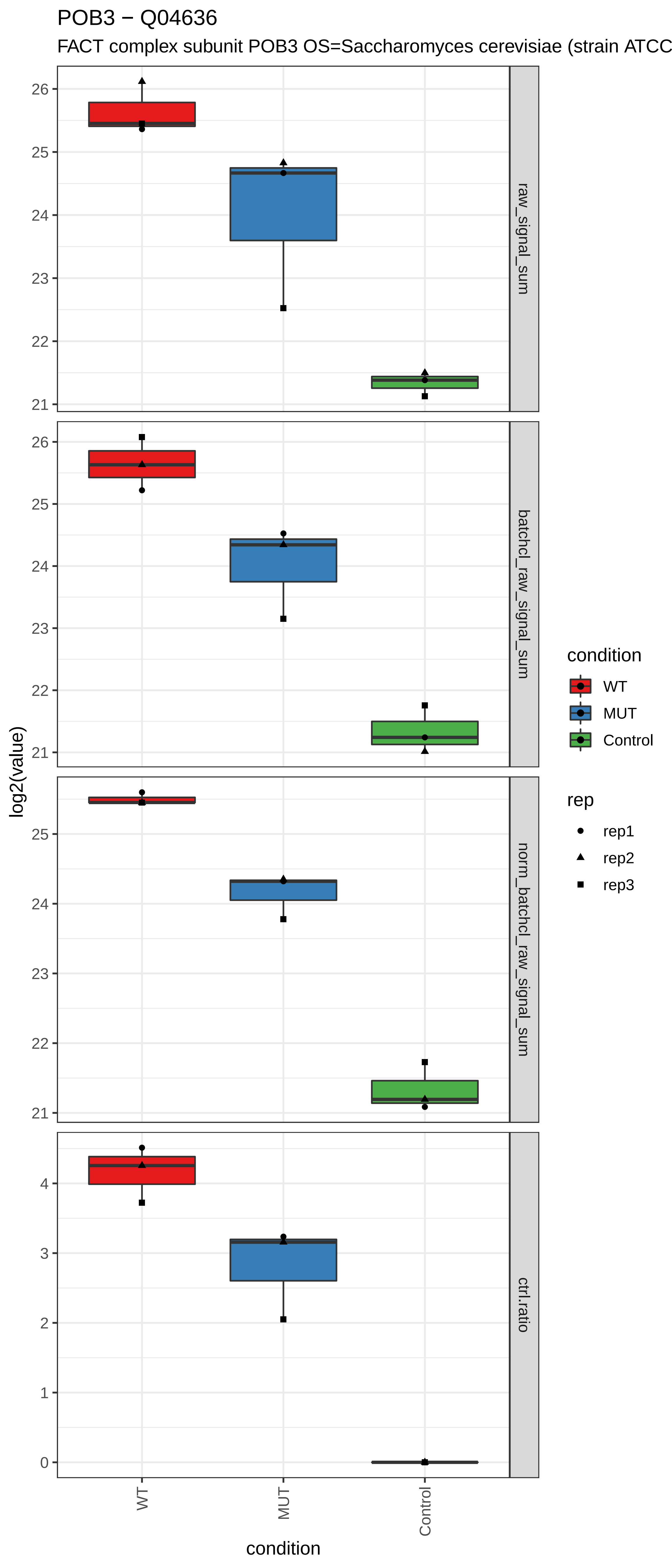

POB3 − Q04636
FACT complex subunit POB3 OS=Saccharomyces cerevisiae (strain ATCC
raw_signal_sum
batchcl_raw_signal_sum
norm_batchcl_raw_signal_sum
ctrl.ratio
log2(value)
condition
WT
MUT
Control
rep
rep1
rep2
rep3

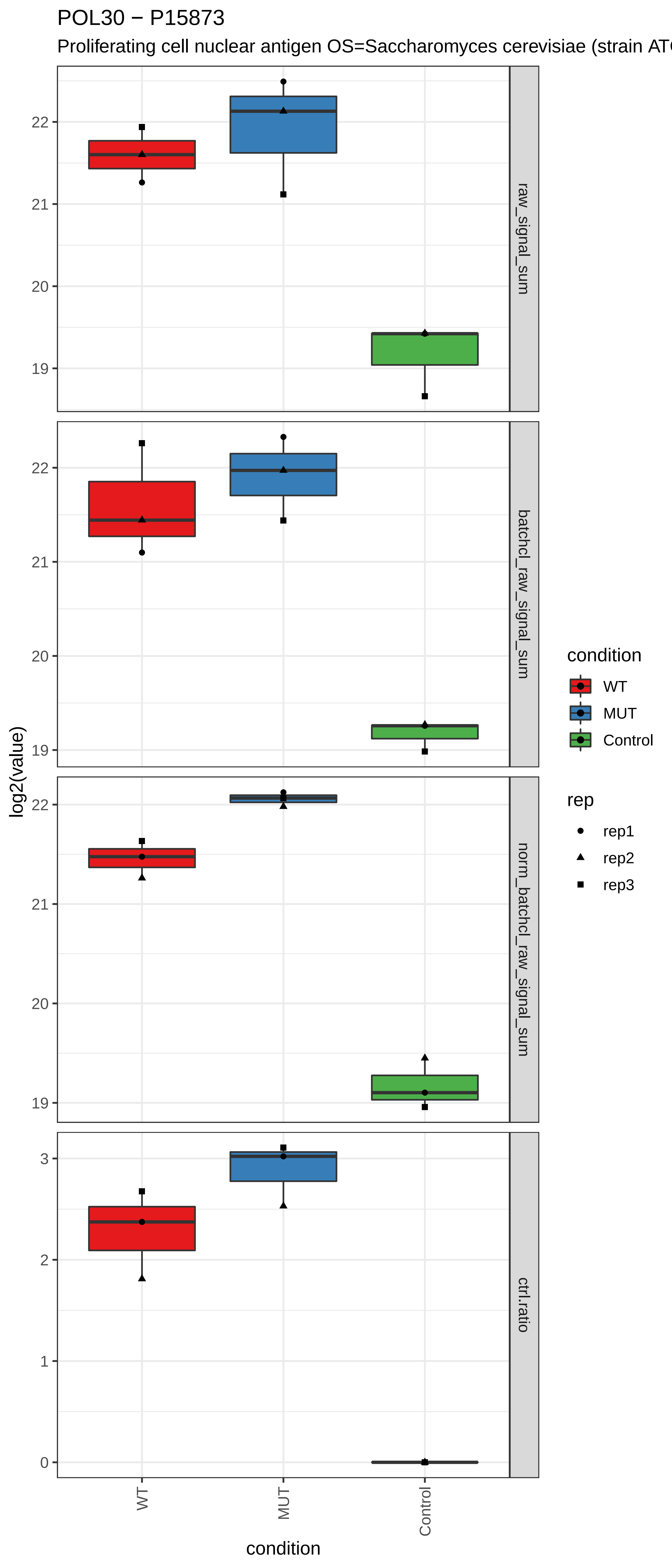

POL30 − P15873
Proliferating cell nuclear antigen OS=Saccharomyces cerevisiae (strain ATC
raw_signal_sum
batchcl_raw_signal_sum
norm_batchcl_raw_signal_sum
ctrl.ratio
log2(value)
condition
WT
MUT
Control
rep
rep1
rep2
rep3
22
21
20
19
3
2
1
0

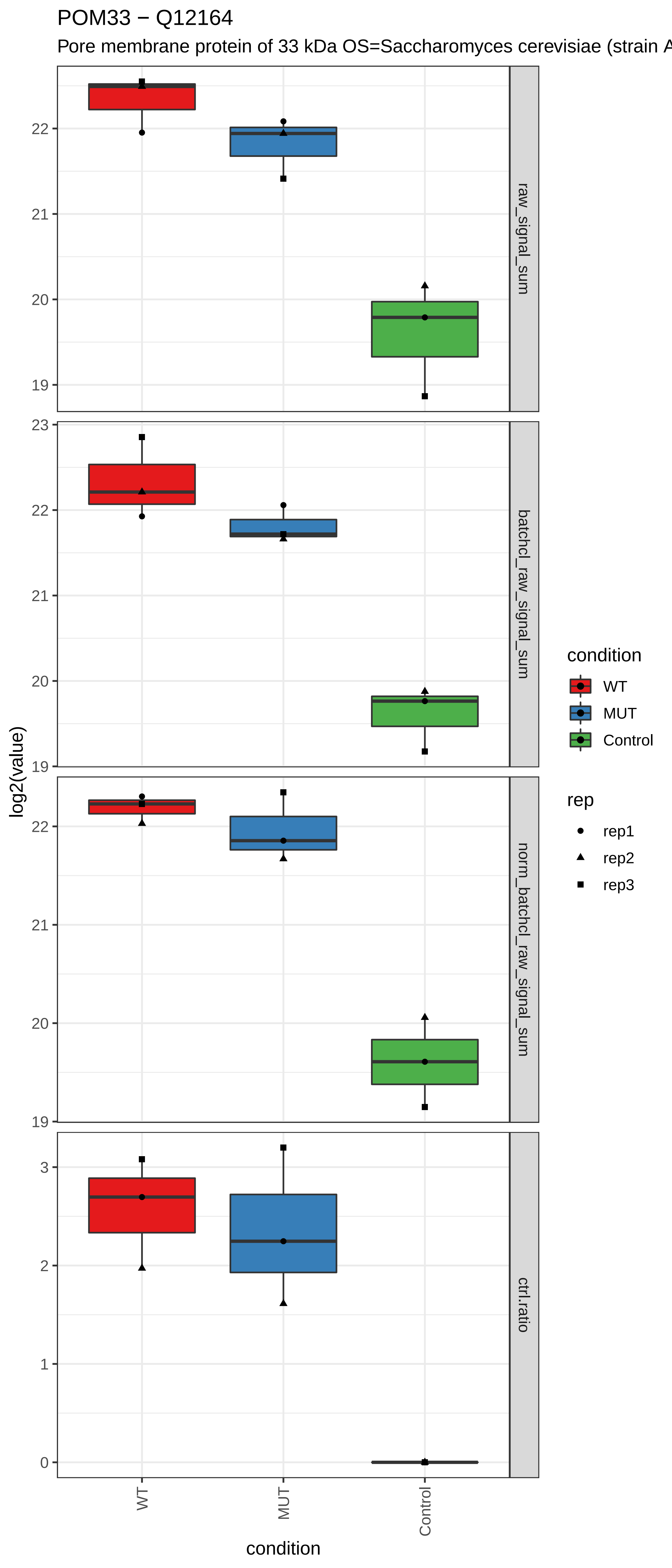
POM33 − Q12164
Pore membrane protein of 33 kDa OS=Saccharomyces cerevisiae (strain A
raw_signal_sum
batchcl_raw_signal_sum
norm_batchcl_raw_signal_sum
ctrl.ratio
log2(value)
condition
WT
MUT
Control
rep
rep1
rep2
rep3

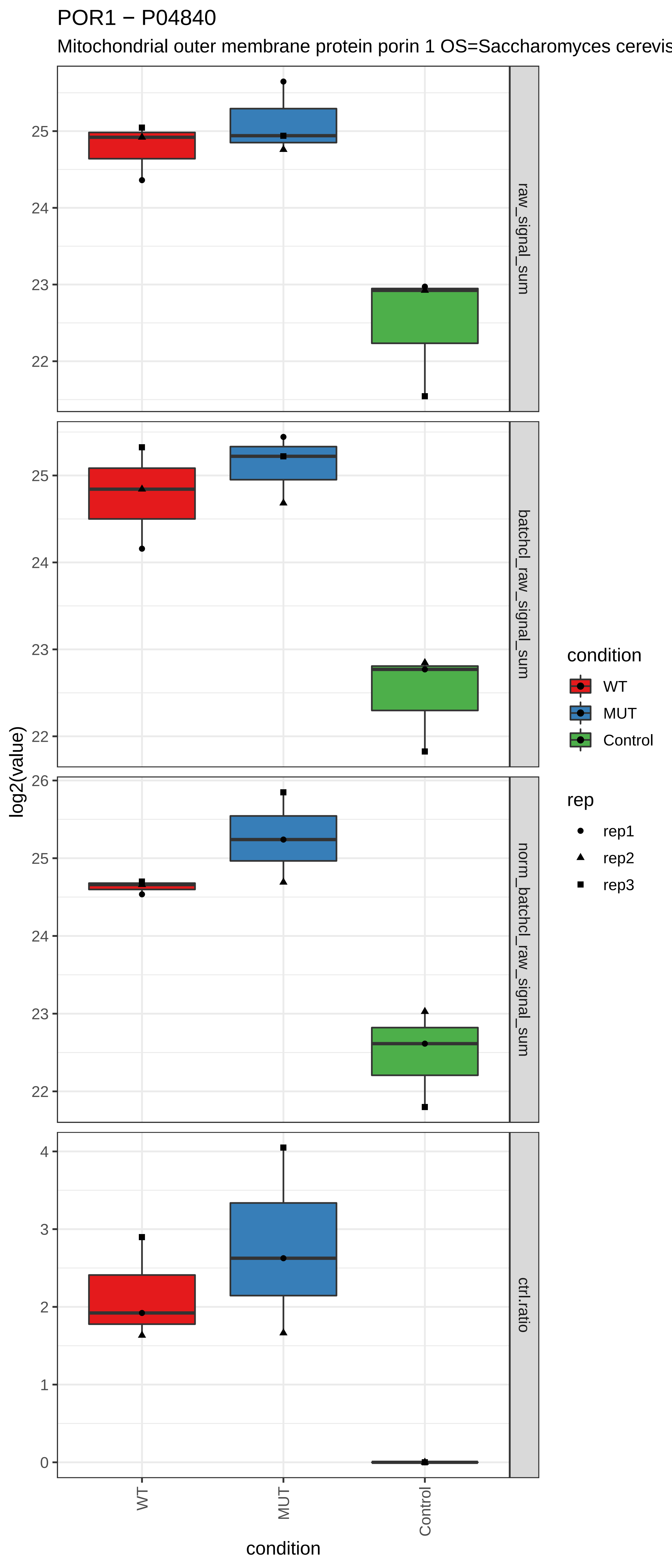
POR1 − P04840
Mitochondrial outer membrane protein porin 1 OS=Saccharomyces cerevis
raw_signal_sum
batchcl_raw_signal_sum
norm_batchcl_raw_signal_sum
ctrl.ratio
log2(value)
condition
WT
MUT
Control
rep
rep1
rep2
rep3
22
23
24
25
26
0
1
2
3
4

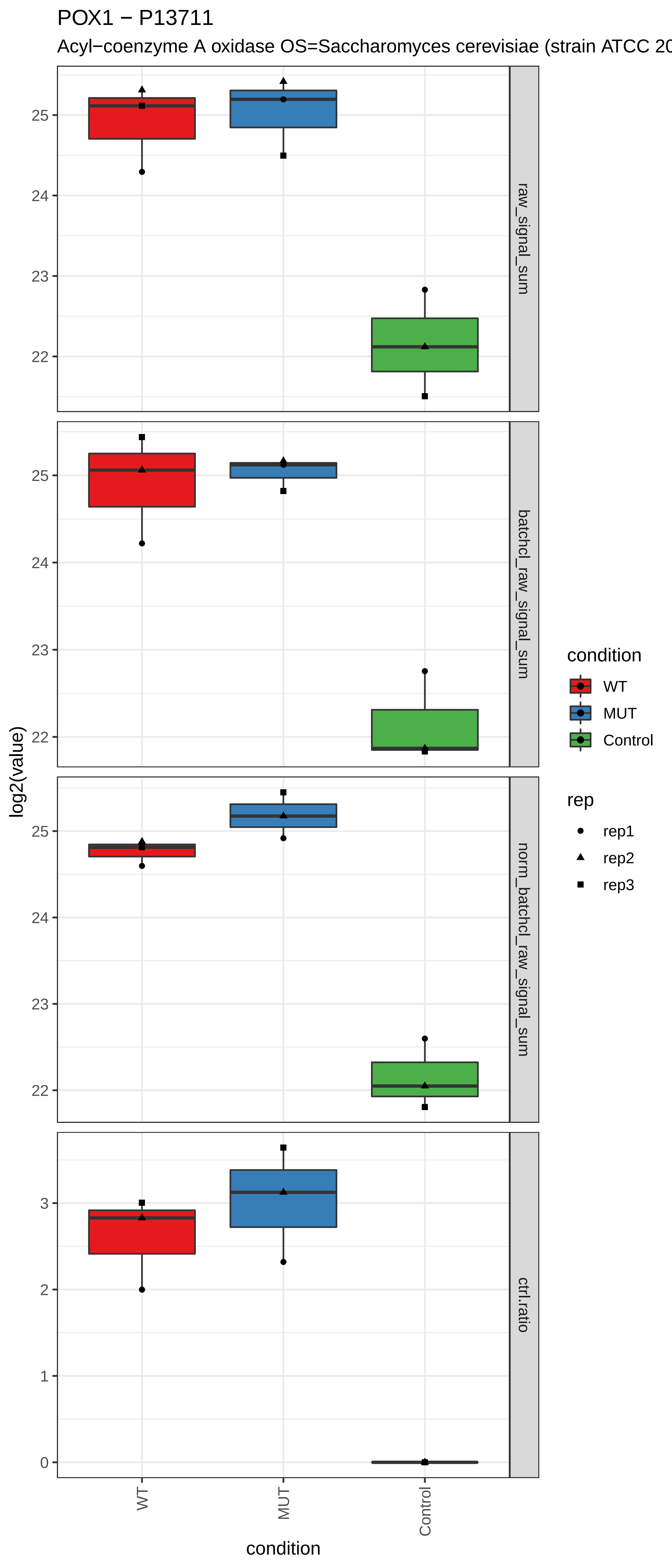
POX1 − P13711
Acyl−coenzyme A oxidase OS=Saccharomyces cerevisiae (strain ATCC 20
raw_signal_sum
batchcl_raw_signal_sum
norm_batchcl_raw_signal_sum
ctrl.ratio
log2(value)
condition
WT
MUT
Control
rep
rep1
rep2
rep3
25
24
23
22
3
2
1
0

PRB1 − P09232

Cerevisin OS=Saccharomyces cerevisiae (strain ATCC 204508 / S288c) (

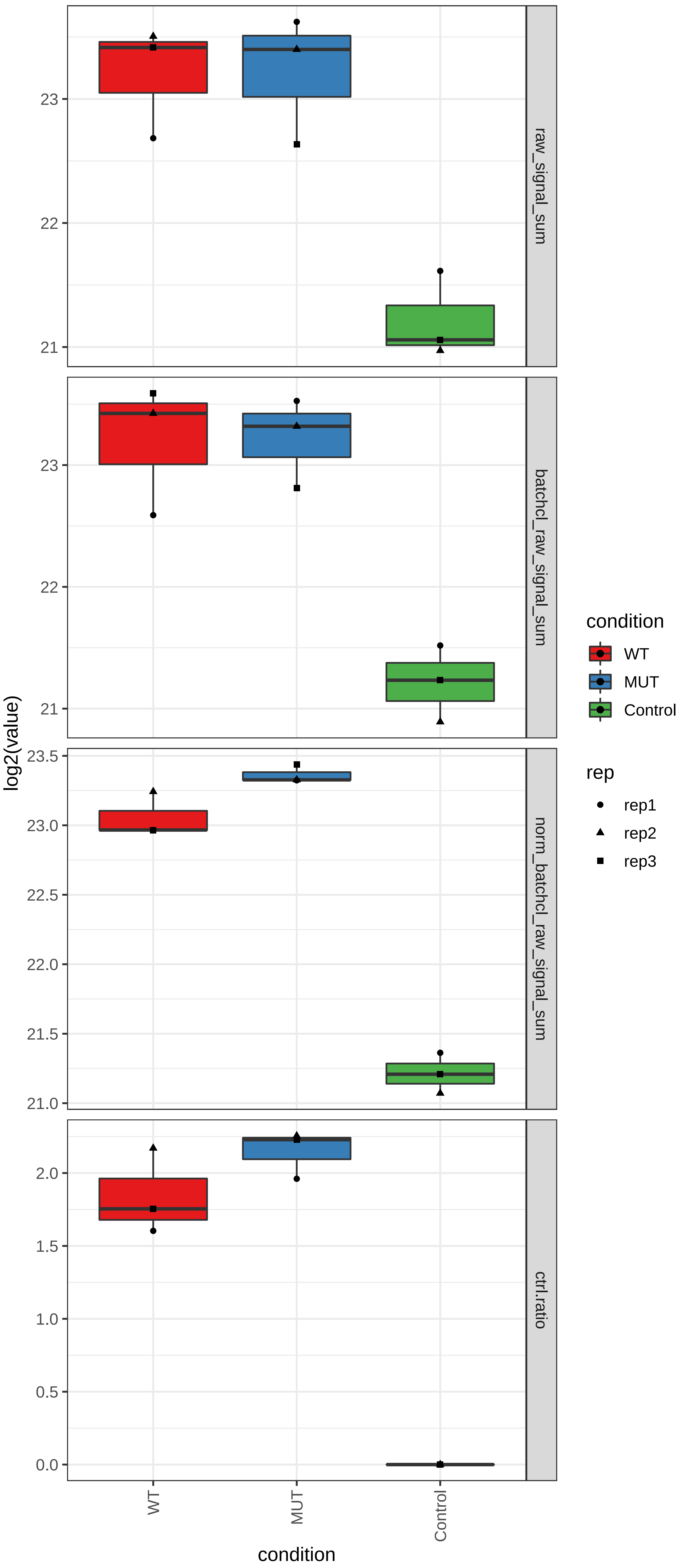

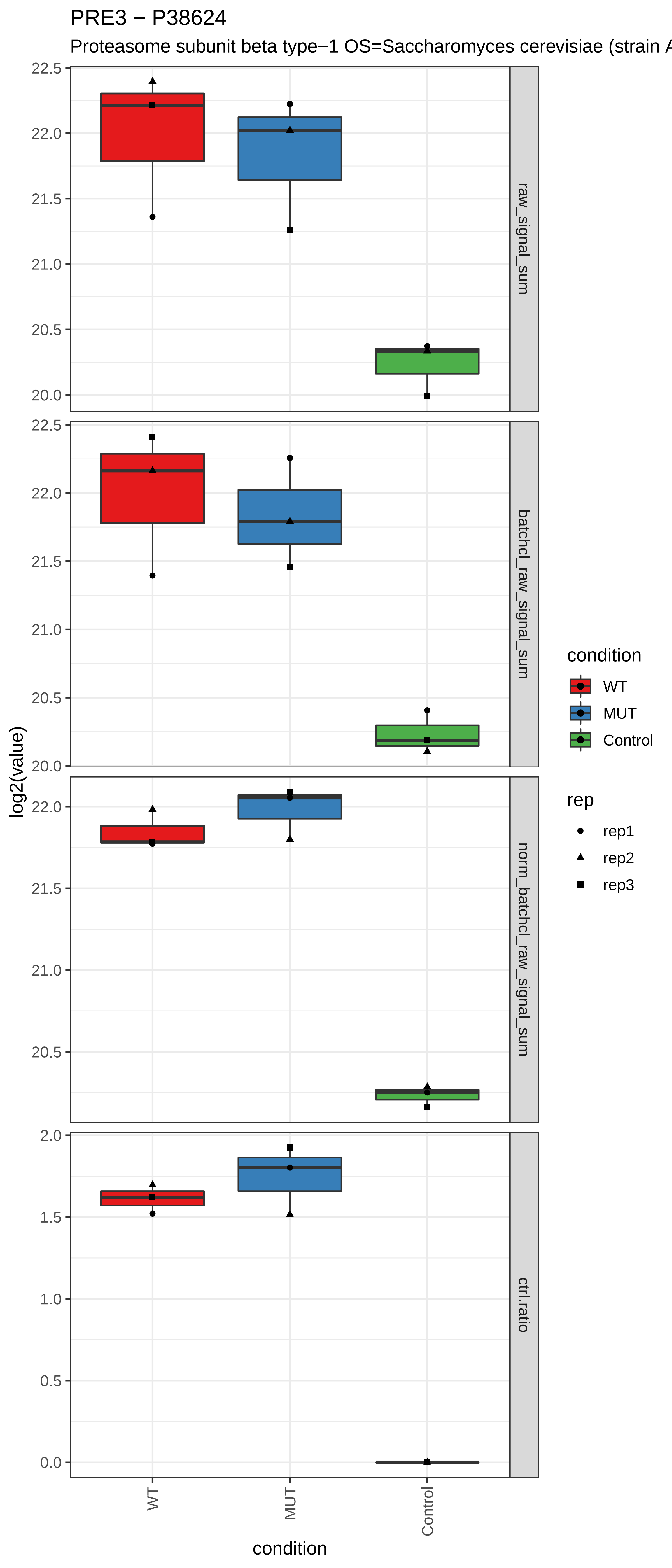

PRE3 − P38624
Proteasome subunit beta type−1 OS=Saccharomyces cerevisiae (strain A
22.5
22.0
21.5
21.0
20.5
20.0
raw_signal_sum
batchcl_raw_signal_sum
norm_batchcl_raw_signal_sum
ctrl.ratio
2.0
1.5
1.0
0.5
0.0
log2(value)
condition
WT
MUT
Control
rep
rep1
rep2
rep3

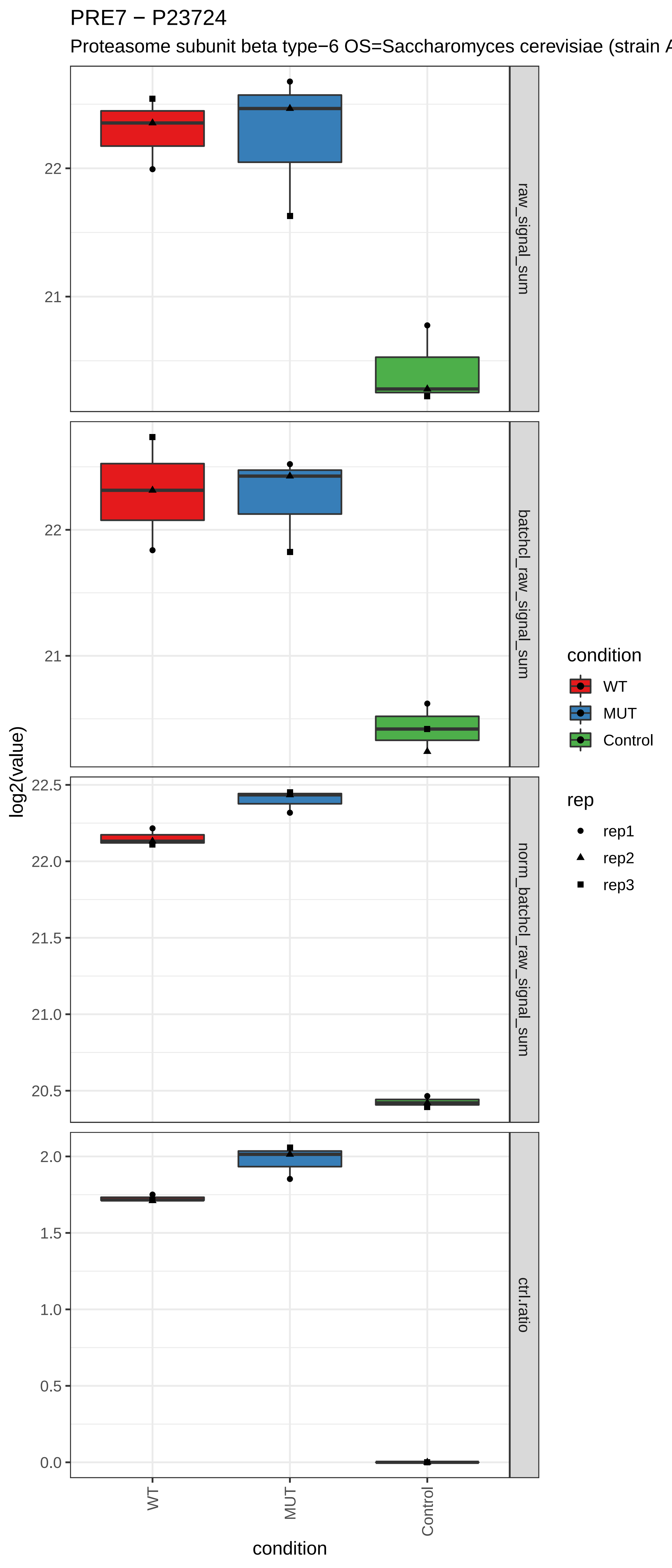
PRE7 − P23724
Proteasome subunit beta type−6 OS=Saccharomyces cerevisiae (strain A
raw_signal_sum
batchcl_raw_signal_sum
norm_batchcl_raw_signal_sum
ctrl.ratio
log2(value)
condition
WT
MUT
Control
rep
rep1
rep2
rep3
22
21
22.5
22.0
21.5
21.0
20.5
2.0
1.5
1.0
0.5
0.0

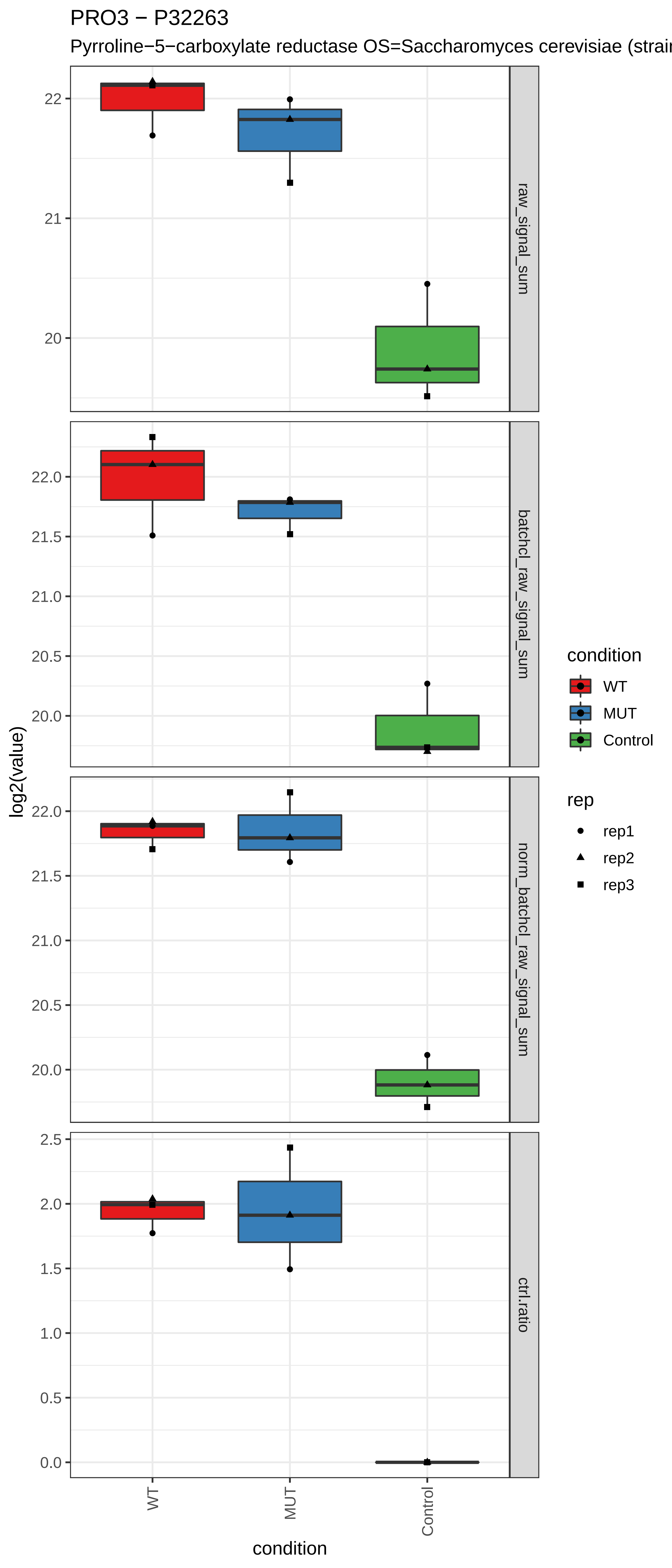

PRO3 − P32263
Pyrroline−5−carboxylate reductase OS=Saccharomyces cerevisiae (strain
raw_signal_sum
batchcl_raw_signal_sum
norm_batchcl_raw_signal_sum
ctrl.ratio
log2(value)
condition
WT
MUT
Control
rep
rep1
rep2
rep3

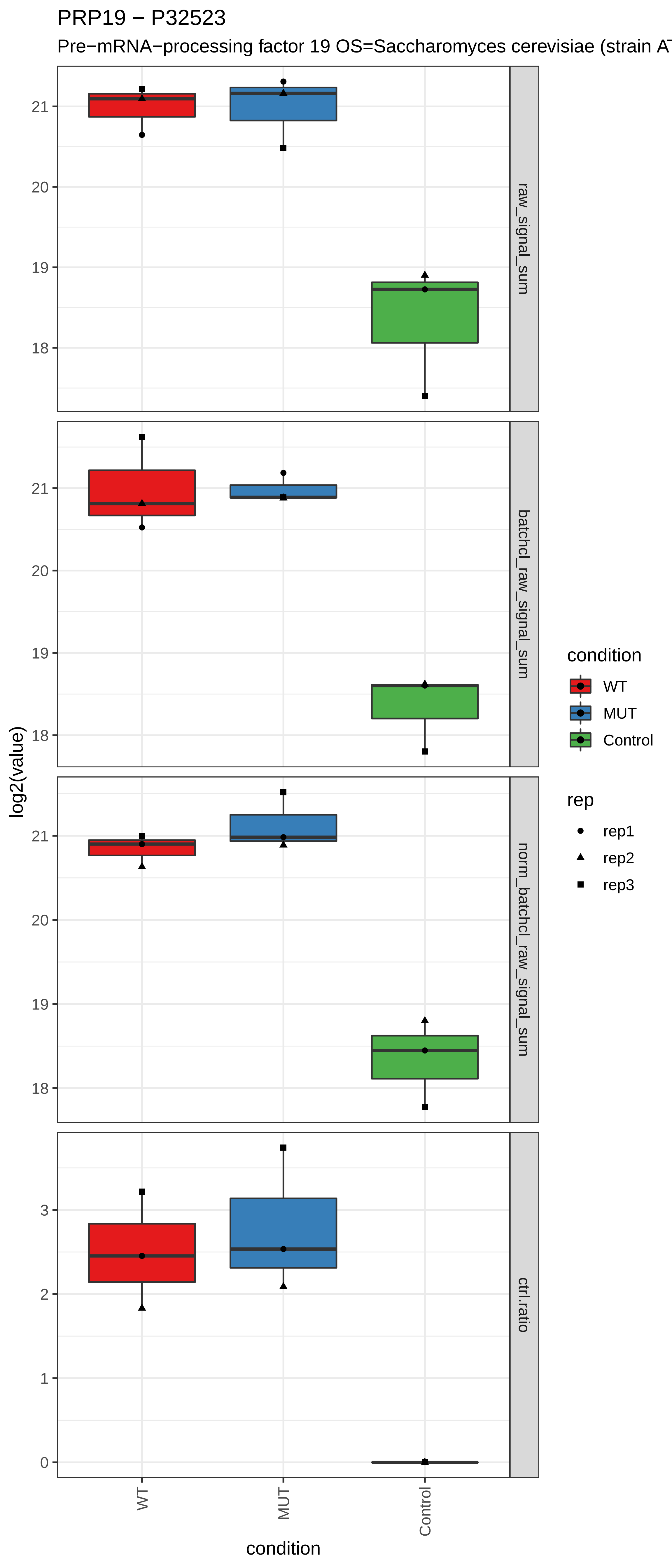
PRP19 − P32523
Pre−mRNA−processing factor 19 OS=Saccharomyces cerevisiae (strain AT
raw_signal_sum
batchcl_raw_signal_sum
norm_batchcl_raw_signal_sum
ctrl.ratio
log2(value)
condition
WT
MUT
Control
rep
rep1
rep2
rep3
21
20
19
18
3
2
1
0

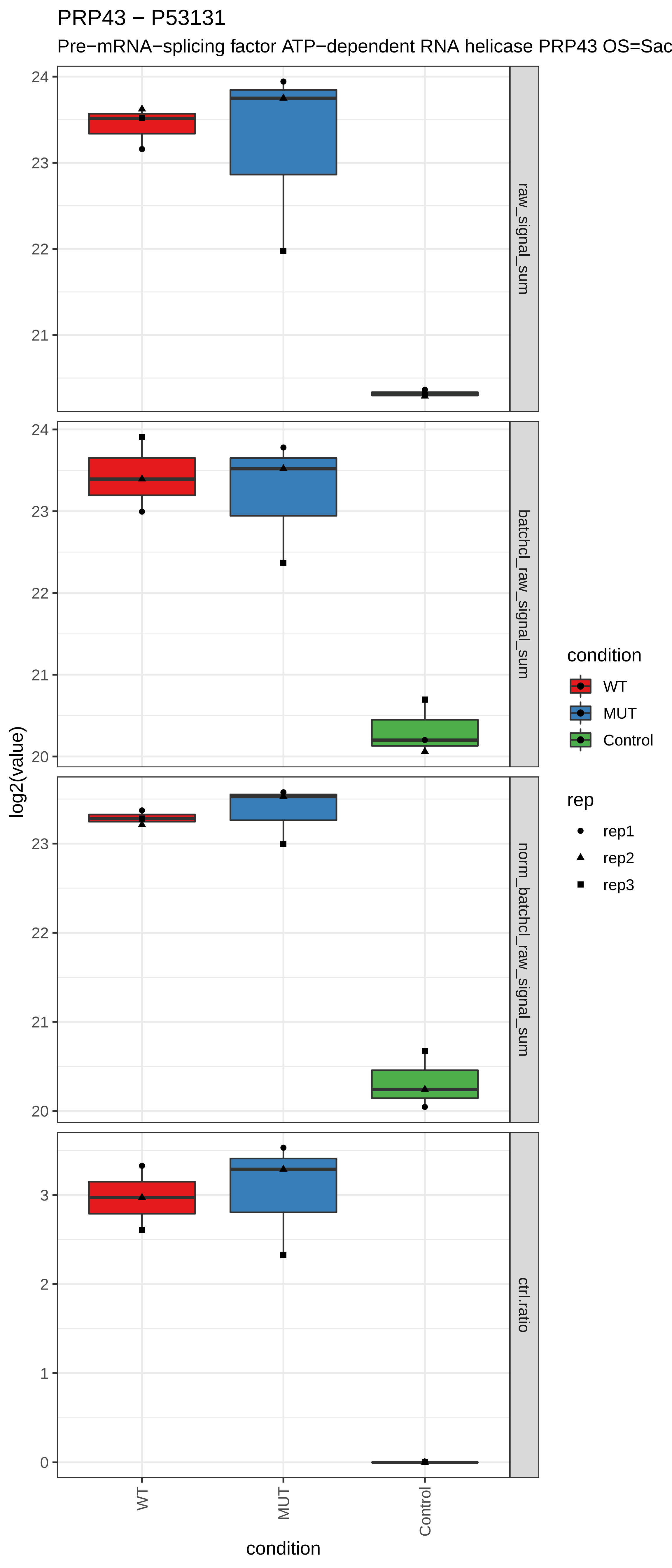
PRP43 − P53131
Pre−mRNA−splicing factor ATP−dependent RNA helicase PRP43 OS=Sac
raw_signal_sum
batchcl_raw_signal_sum
norm_batchcl_raw_signal_sum
ctrl.ratio
log2(value)
condition
WT
MUT
Control
rep
rep1
rep2
rep3
24
23
22
21
20
3
2
1
0

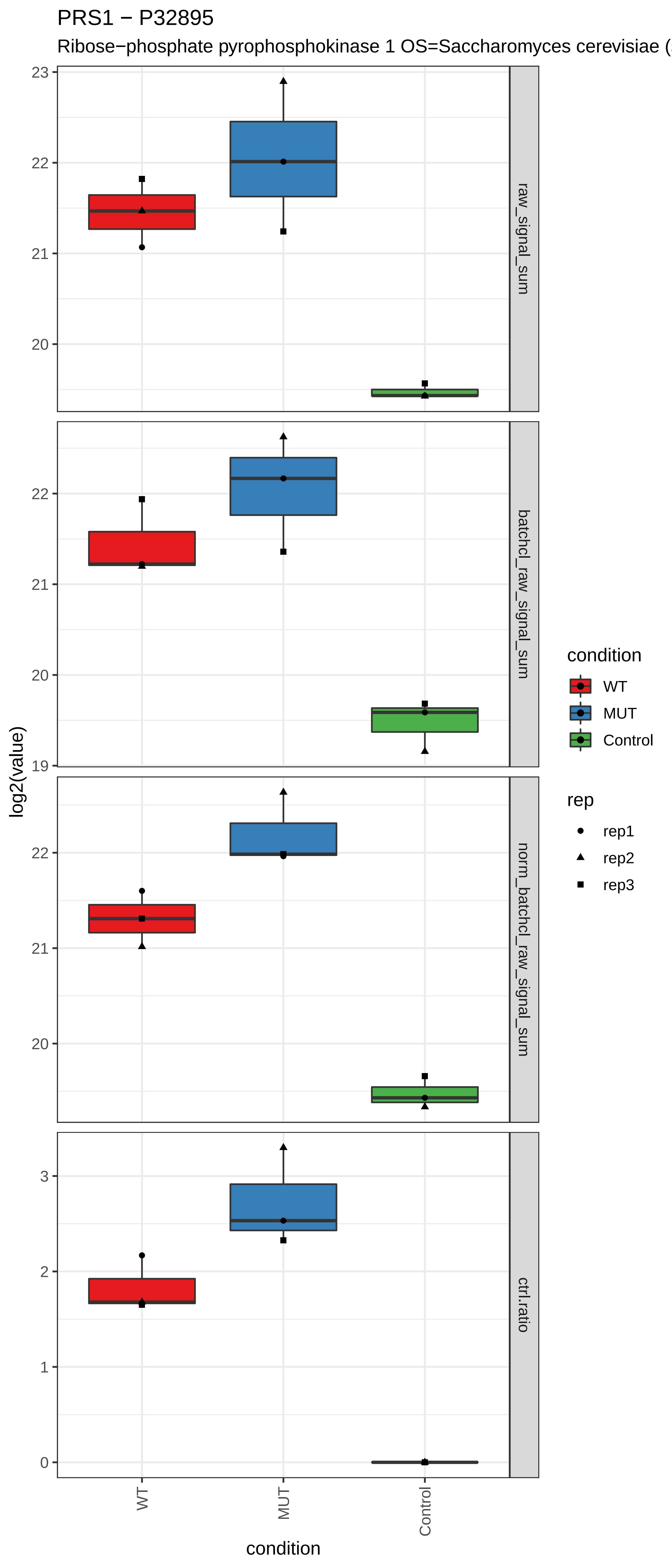
PRS1 − P32895
Ribose−phosphate pyrophosphokinase 1 OS=Saccharomyces cerevisiae (
raw_signal_sum
batchcl_raw_signal_sum
norm_batchcl_raw_signal_sum
ctrl.ratio
log2(value)
condition
WT
MUT
Control
rep
rep1
rep2
rep3
23
22
21
20
19
3
2
1
0

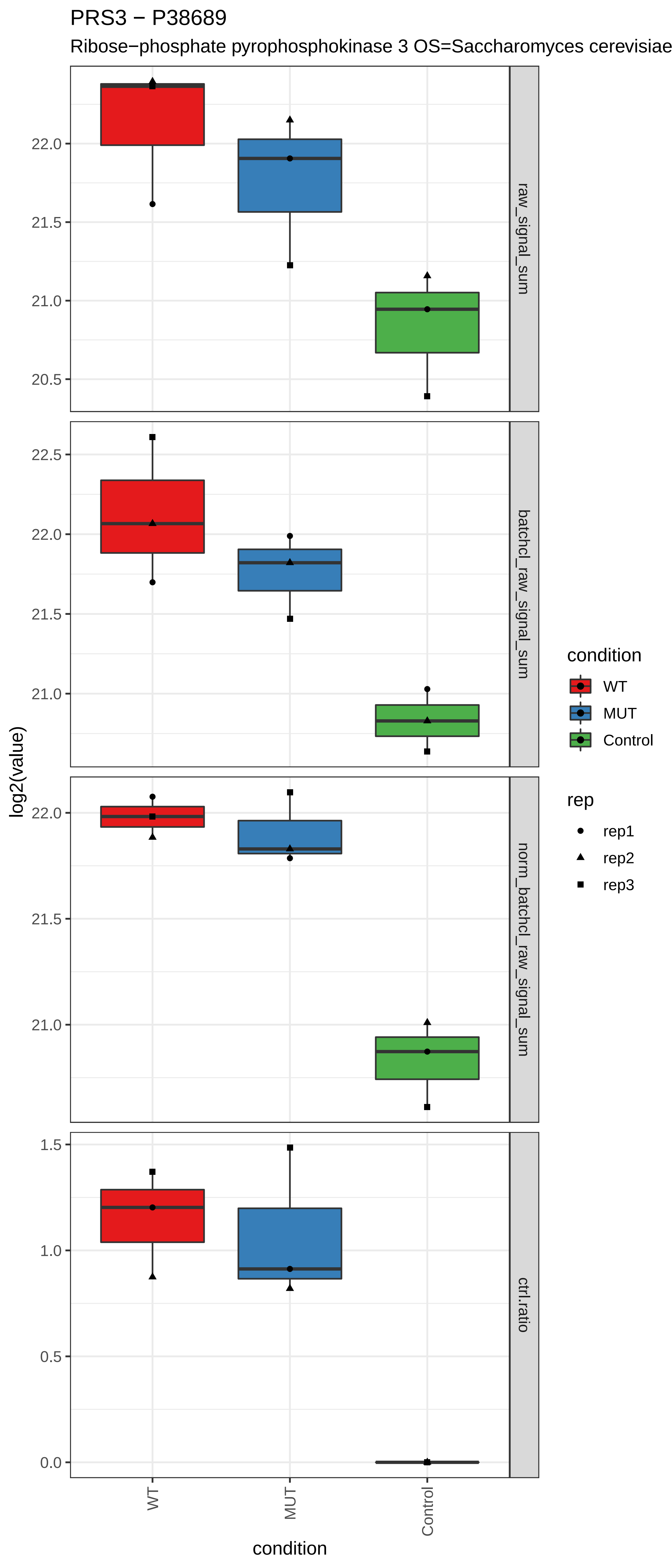
PRS3 − P38689
Ribose−phosphate pyrophosphokinase 3 OS=Saccharomyces cerevisiae
raw_signal_sum
batchcl_raw_signal_sum
norm_batchcl_raw_signal_sum
ctrl.ratio
log2(value)
condition
WT
MUT
Control
rep
rep1
rep2
rep3
22.0
21.5
21.0
20.5
22.5
1.5
1.0
0.5
0.0

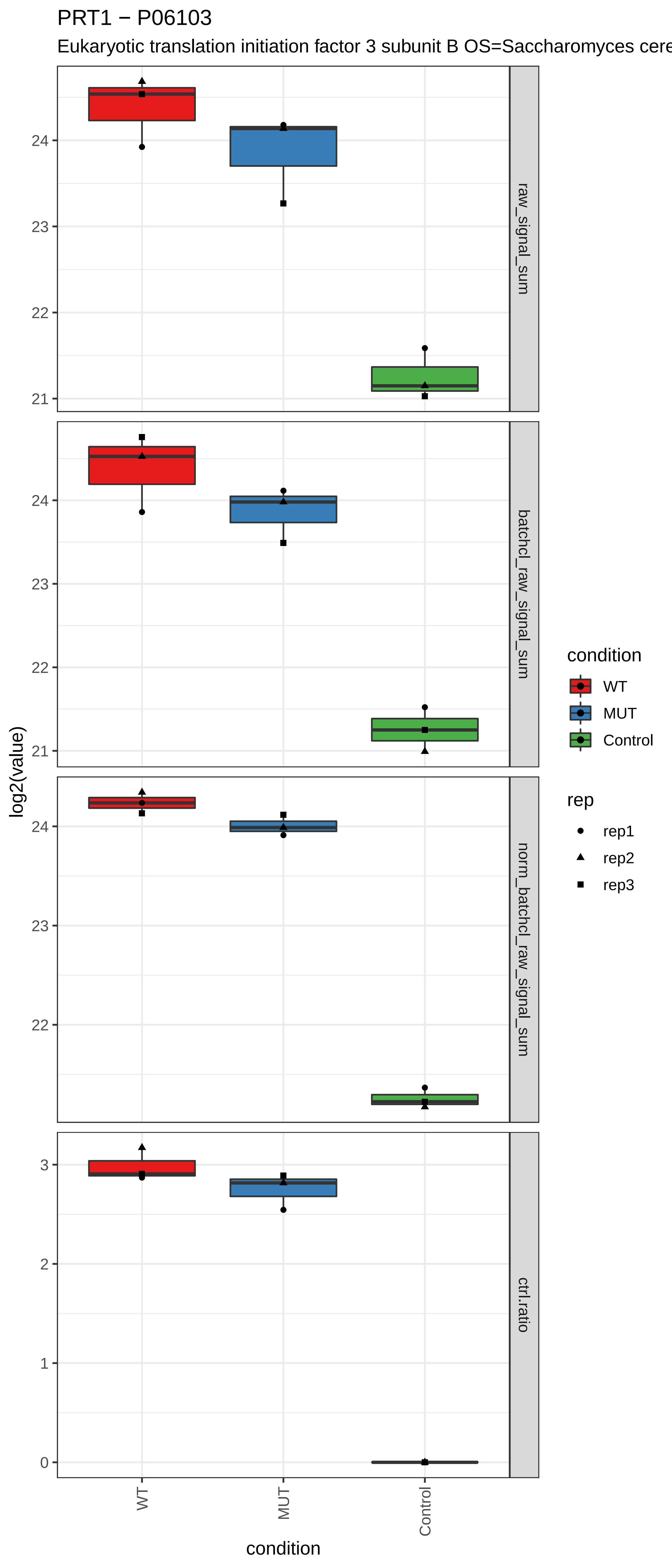

PRT1 − P06103
Eukaryotic translation initiation factor 3 subunit B OS=Saccharomyces cere
raw_signal_sum
batchcl_raw_signal_sum
norm_batchcl_raw_signal_sum
ctrl.ratio
log2(value)
condition
WT
MUT
Control
rep
rep1
rep2
rep3
24
23
22
21
3
2
1
0

PSA1 − P41940

Mannose−1−phosphate guanyltransferase OS=Saccharomyces cerevisiae

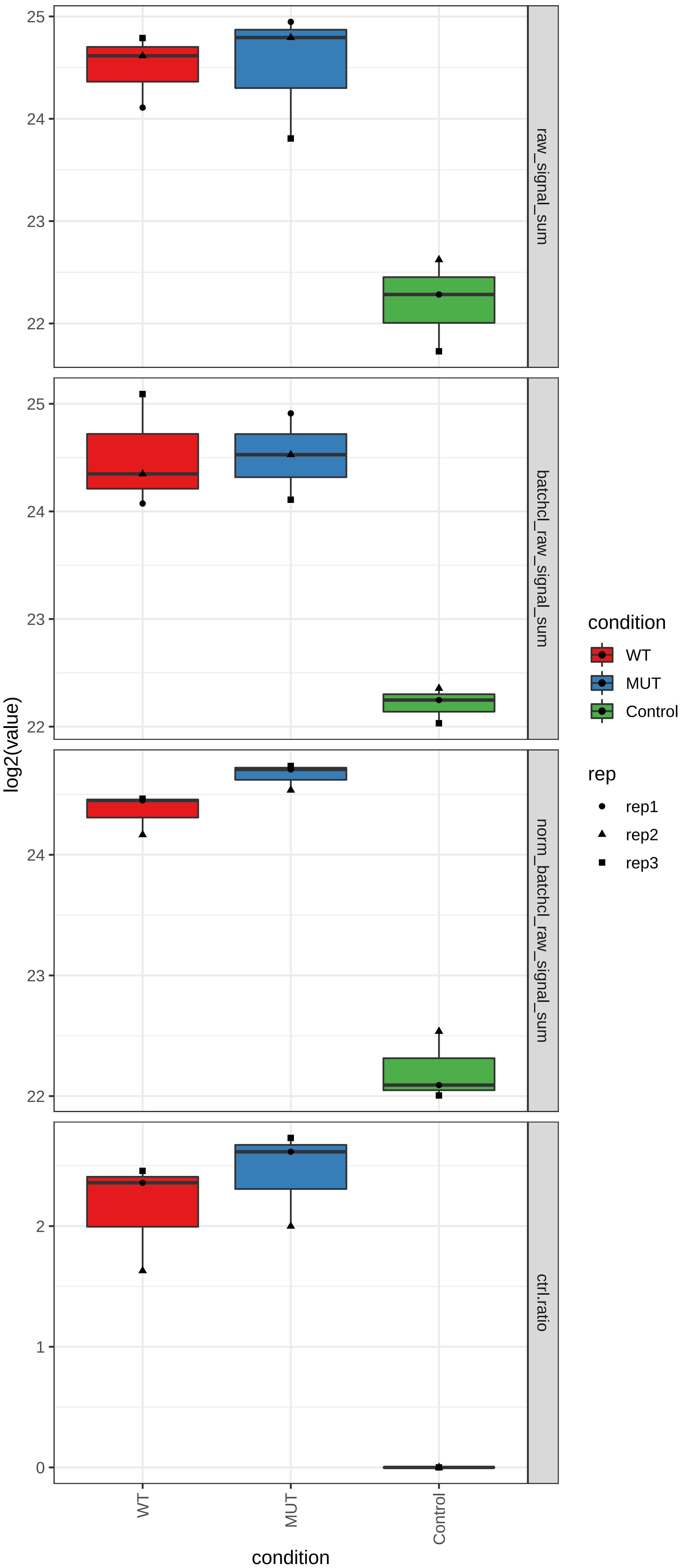

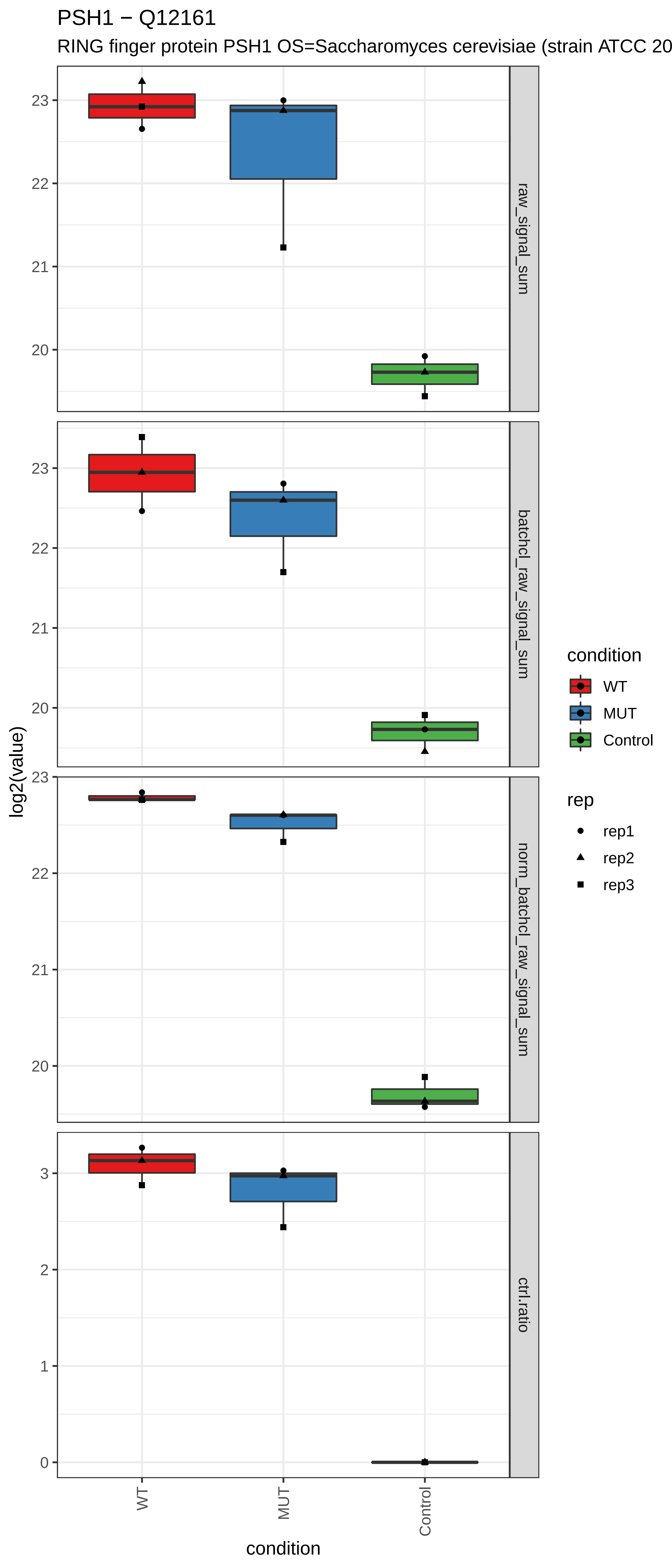

PSH1 − Q12161
RING finger protein PSH1 OS=Saccharomyces cerevisiae (strain ATCC 20
raw_signal_sum
batchcl_raw_signal_sum
norm_batchcl_raw_signal_sum
ctrl.ratio
log2(value)
condition
WT
MUT
Control
rep
rep1
rep2
rep3
23
22
21
20
3
2
1
0

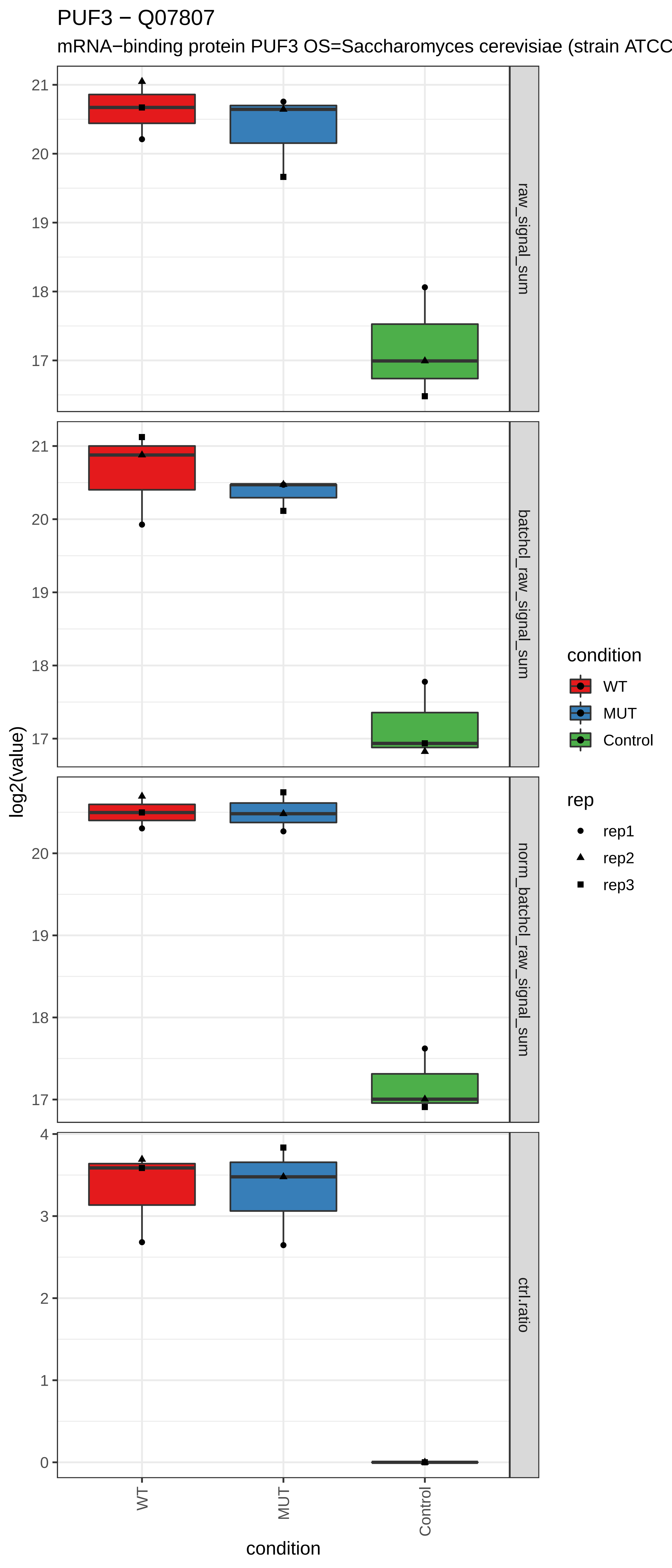
PUF3 − Q07807
mRNA−binding protein PUF3 OS=Saccharomyces cerevisiae (strain ATCC
raw_signal_sum
batchcl_raw_signal_sum
norm_batchcl_raw_signal_sum
ctrl.ratio
log2(value)
condition
WT
MUT
Control
rep
rep1
rep2
rep3
21
20
19
18
17
4
3
2
1
0

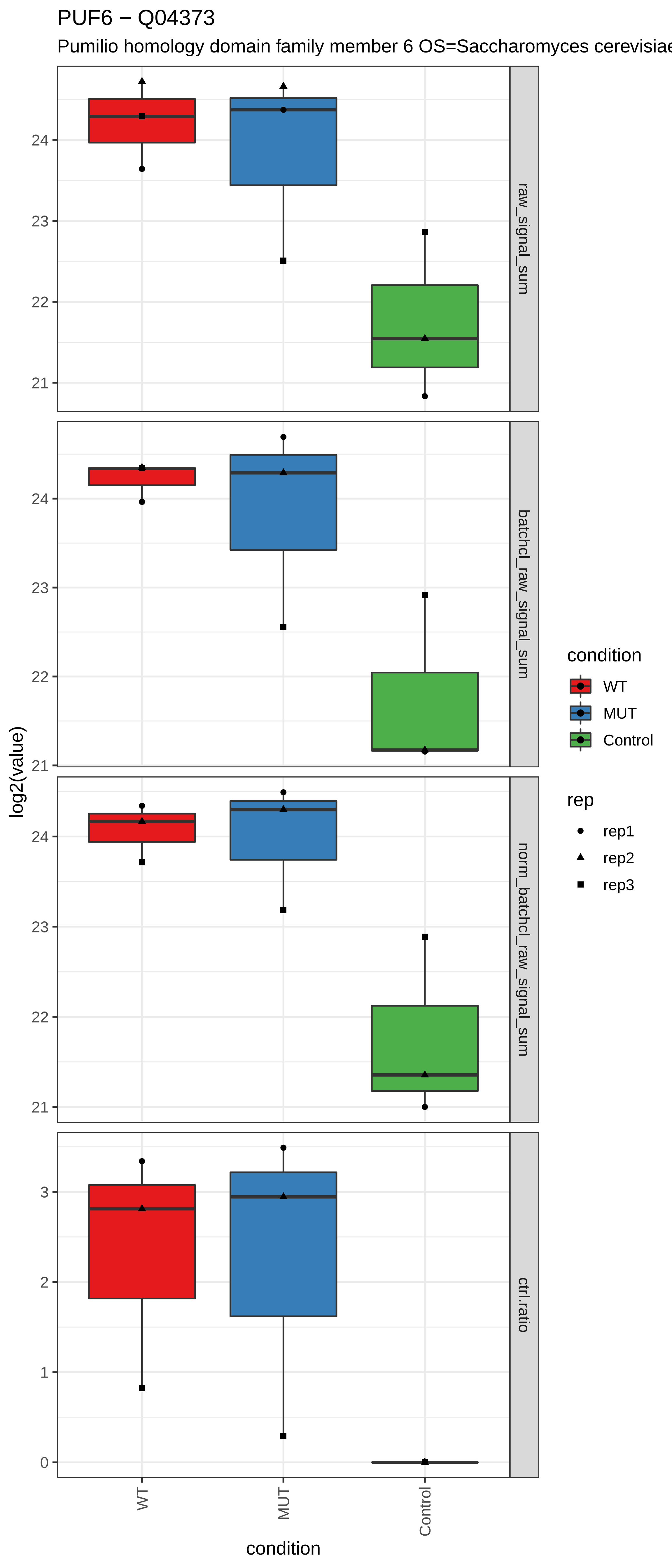

PUF6 − Q04373
Pumilio homology domain family member 6 OS=Saccharomyces cerevisiae
log2(value)
condition
raw_signal_sum
batchcl_raw_signal_sum
norm_batchcl_raw_signal_sum
ctrl.ratio
WT
MUT
Control
condition
WT
MUT
Control
rep
rep1
rep2
rep3

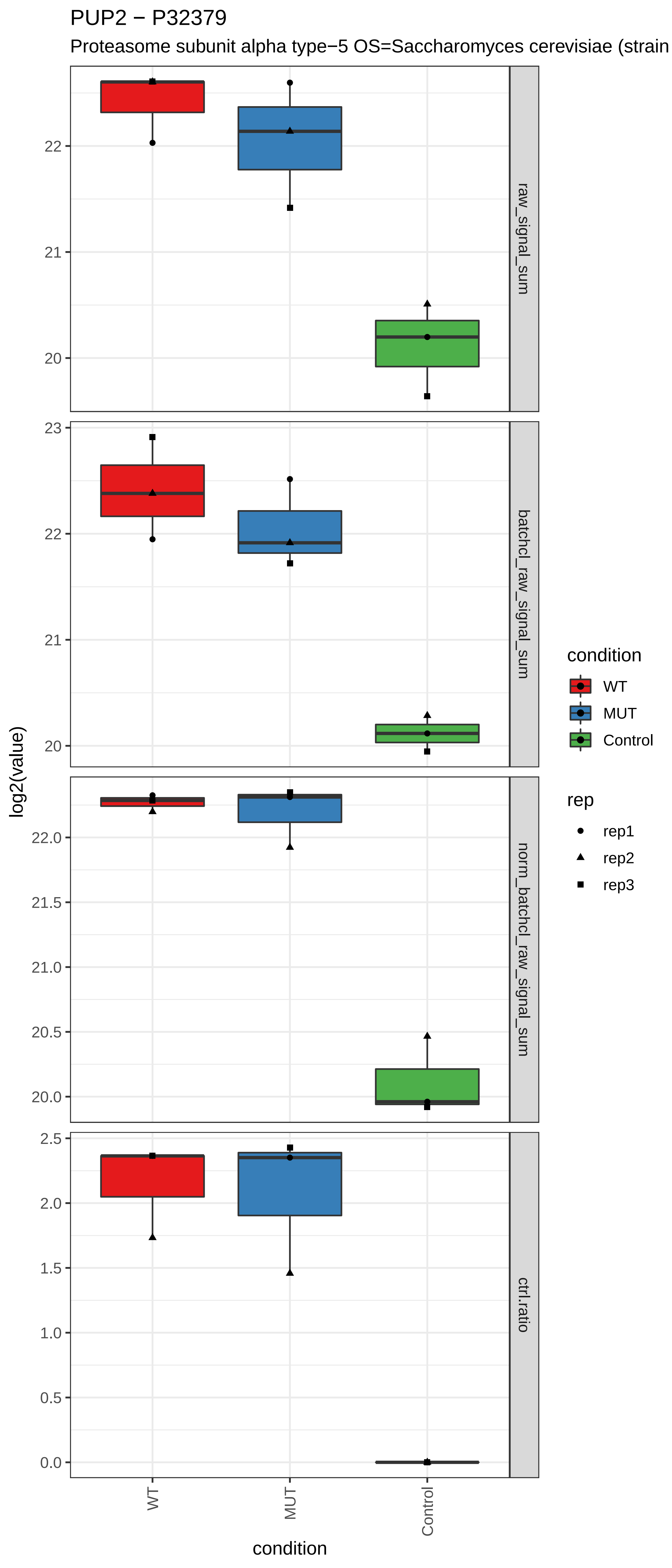
PUP2 − P32379
Proteasome subunit alpha type−5 OS=Saccharomyces cerevisiae (strain
raw_signal_sum
batchcl_raw_signal_sum
norm_batchcl_raw_signal_sum
ctrl.ratio
log2(value)
condition
WT
MUT
Control
rep
rep1
rep2
rep3
22
21
20
23
22.0
21.5
21.0
20.5
20.0
2.5
2.0
1.5
1.0
0.5
0.0

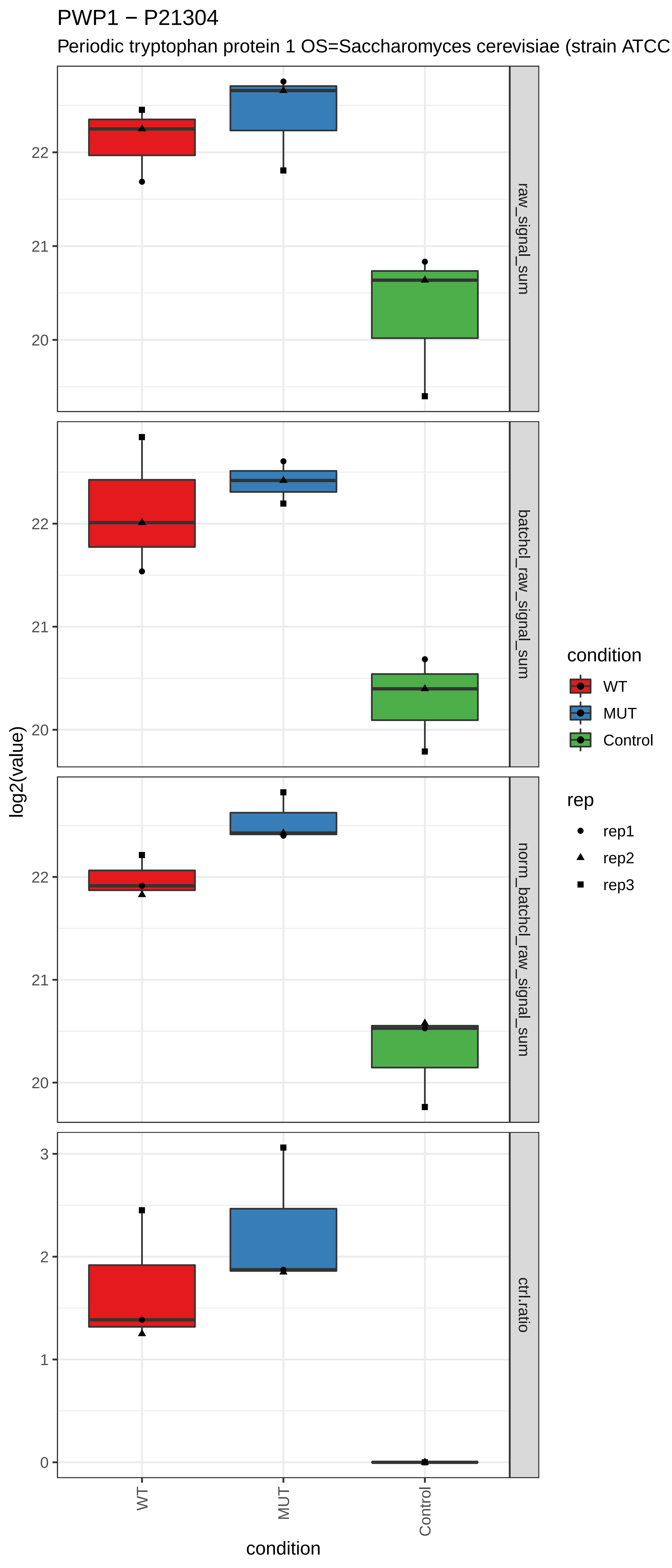

PWP1 − P21304
Periodic tryptophan protein 1 OS=Saccharomyces cerevisiae (strain ATCC
raw_signal_sum
batchcl_raw_signal_sum
norm_batchcl_raw_signal_sum
ctrl.ratio
log2(value)
condition
WT
MUT
Control
rep
rep1
rep2
rep3
22
21
20
3
2
1
0

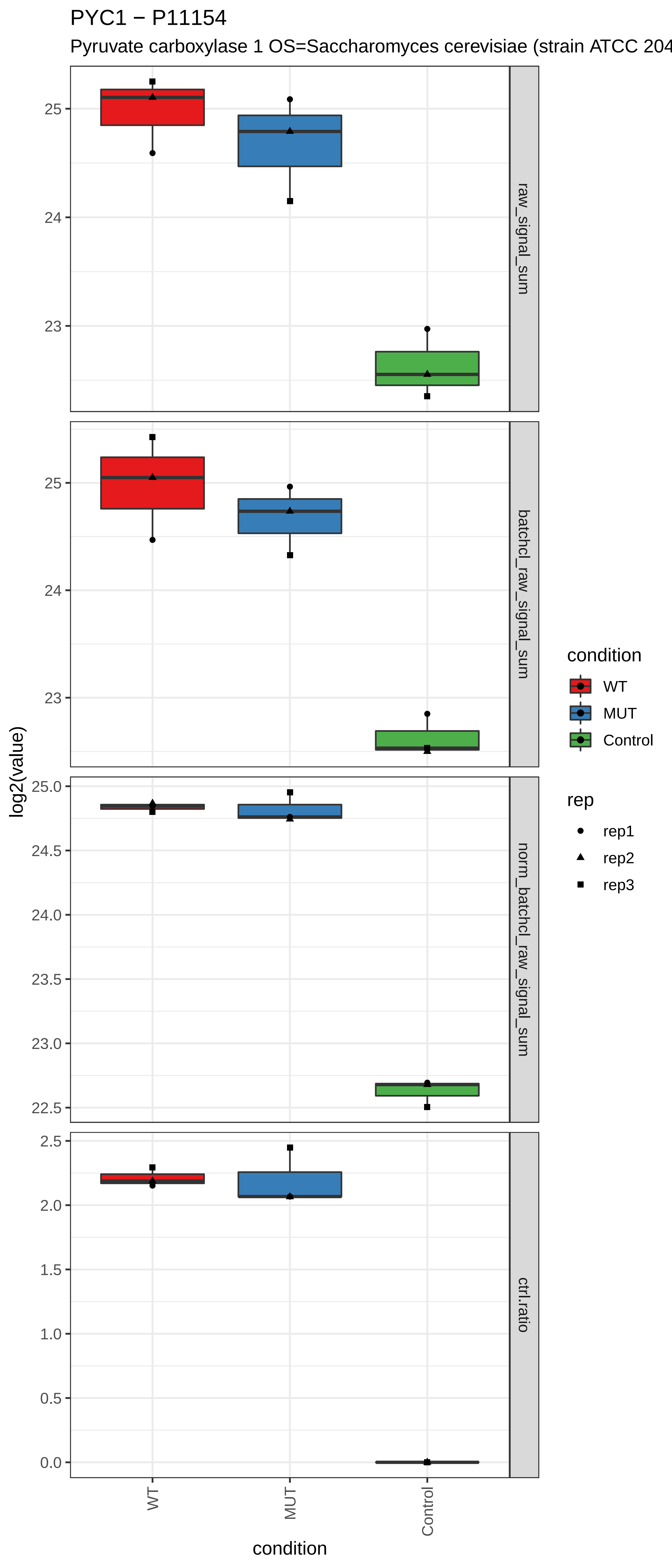

PYC1 − P11154
Pyruvate carboxylase 1 OS=Saccharomyces cerevisiae (strain ATCC 204
raw_signal_sum
batchcl_raw_signal_sum
norm_batchcl_raw_signal_sum
ctrl.ratio
log2(value)
condition
WT
MUT
Control
rep
rep1
rep2
rep3
25
24
23
25.0
24.5
24.0
23.5
23.0
22.5
2.5
2.0
1.5
1.0
0.5
0.0

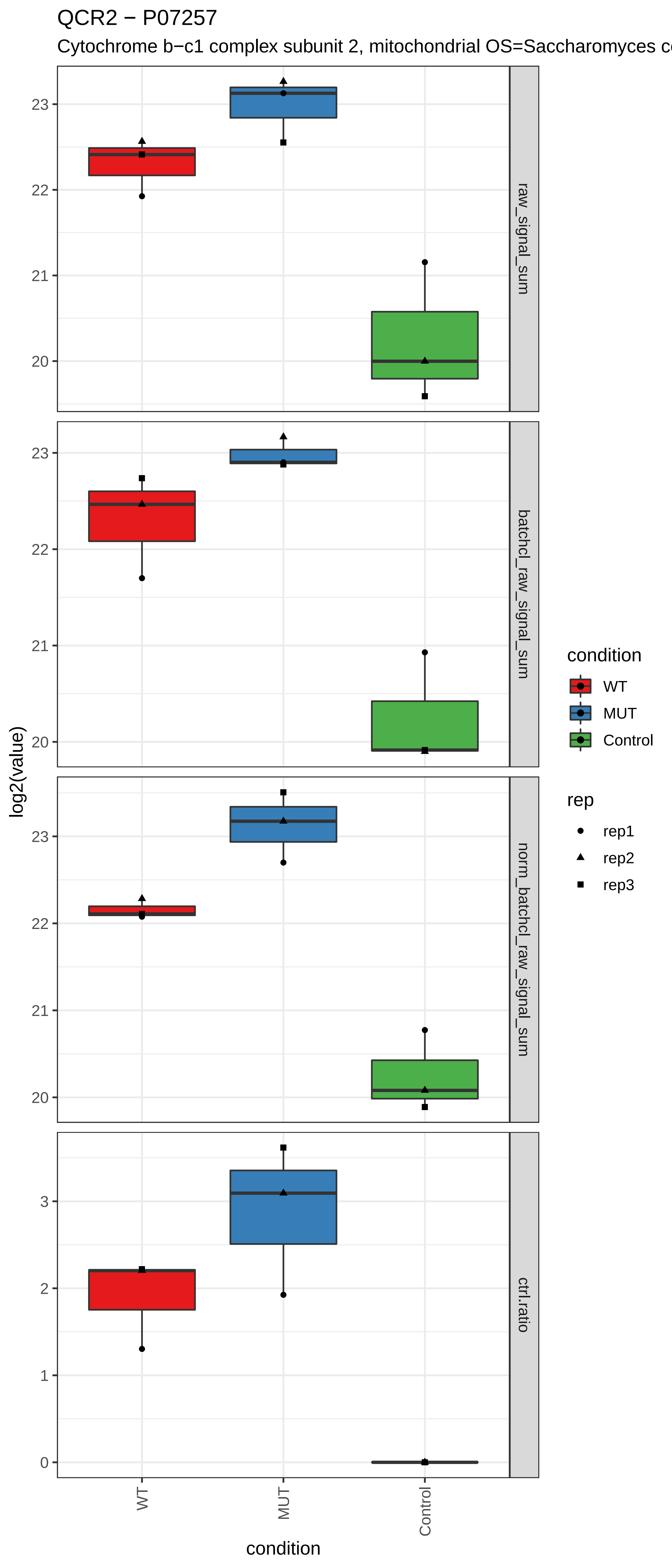

QCR2 − P07257
Cytochrome b−c1 complex subunit 2, mitochondrial OS=Saccharomyces c
log2(value)
raw_signal_sum
batchcl_raw_signal_sum
norm_batchcl_raw_signal_sum
ctrl.ratio
condition
WT
MUT
Control
rep
rep1
rep2
rep3

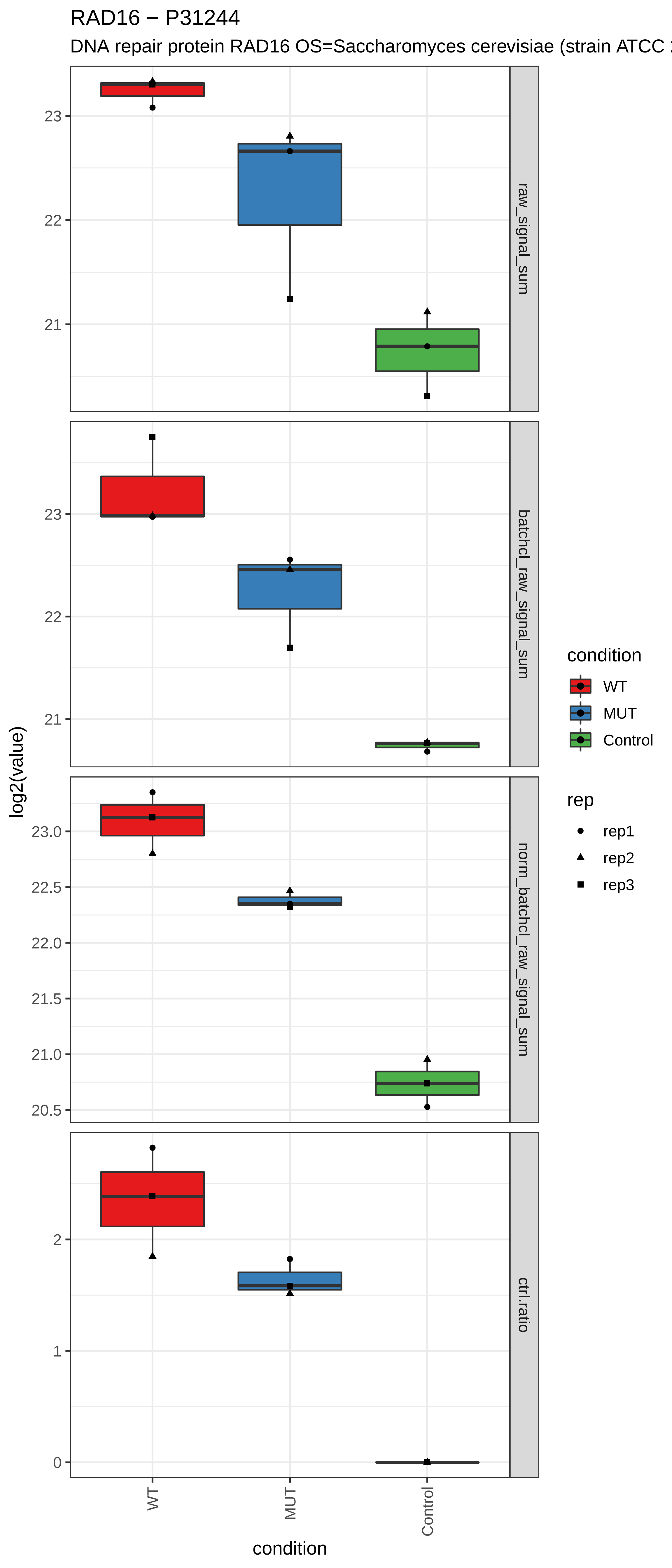

RAD16 − P31244
DNA repair protein RAD16 OS=Saccharomyces cerevisiae (strain ATCC 2
raw_signal_sum
batchcl_raw_signal_sum
norm_batchcl_raw_signal_sum
ctrl.ratio
log2(value)
condition
WT
MUT
Control
rep
rep1
rep2
rep3

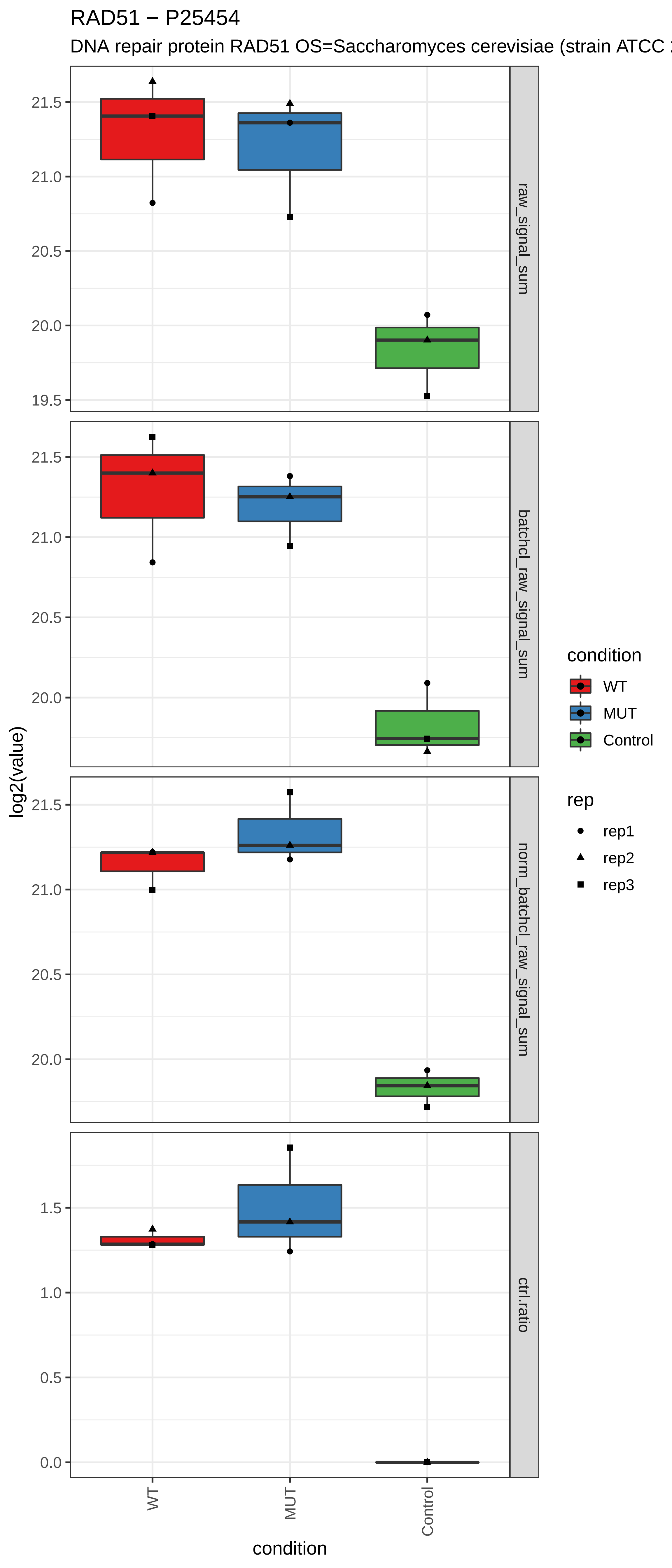

RAD51 − P25454
DNA repair protein RAD51 OS=Saccharomyces cerevisiae (strain ATCC
raw_signal_sum
batchcl_raw_signal_sum
norm_batchcl_raw_signal_sum
ctrl.ratio
21.5
21.0
20.5
20.0
19.5
1.5
1.0
0.5
0.0
log2(value)
WT
MUT
Control
condition
condition
WT
MUT
Control
rep
rep1
rep2
rep3

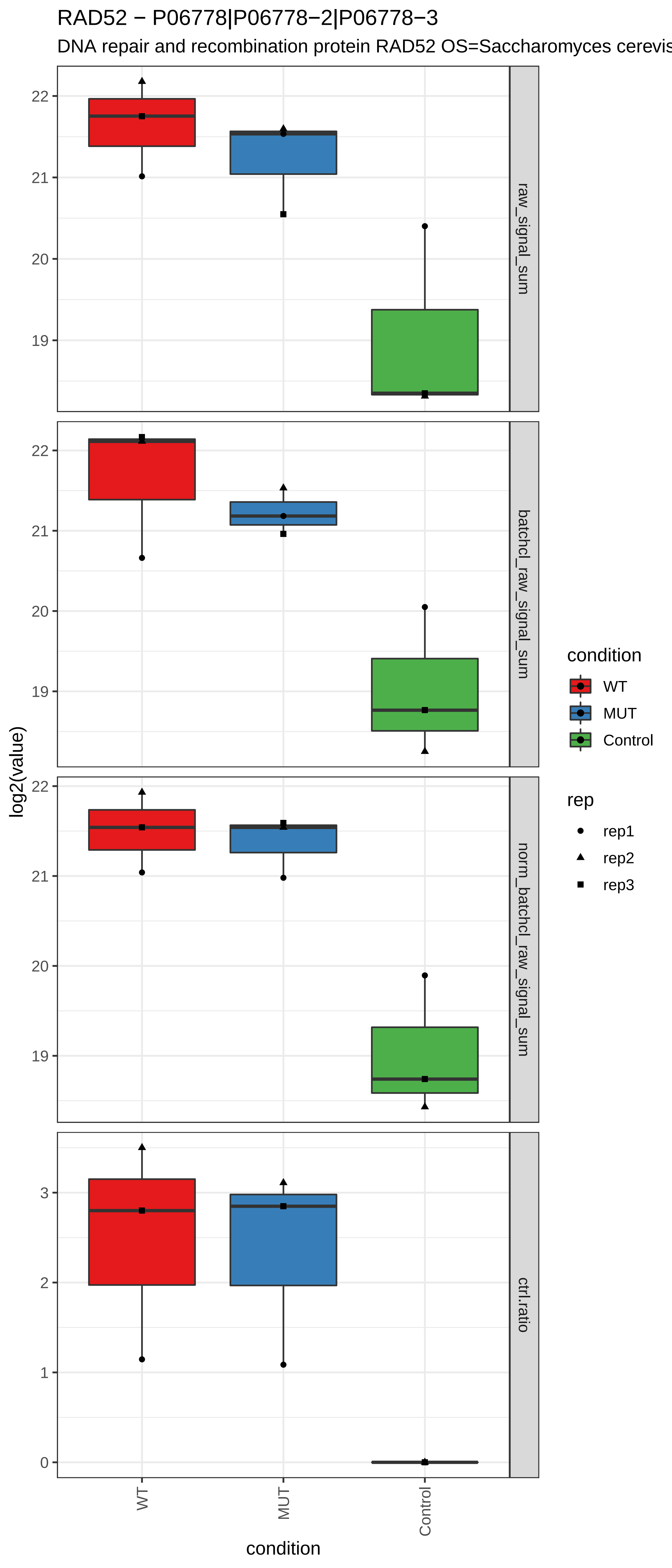

RAD52 − P06778|P06778−2|P06778−3
DNA repair and recombination protein RAD52 OS=Saccharomyces cerevis
raw_signal_sum
batchcl_raw_signal_sum
norm_batchcl_raw_signal_sum
ctrl.ratio
log2(value)
condition
WT
MUT
Control
rep
rep1
rep2
rep3

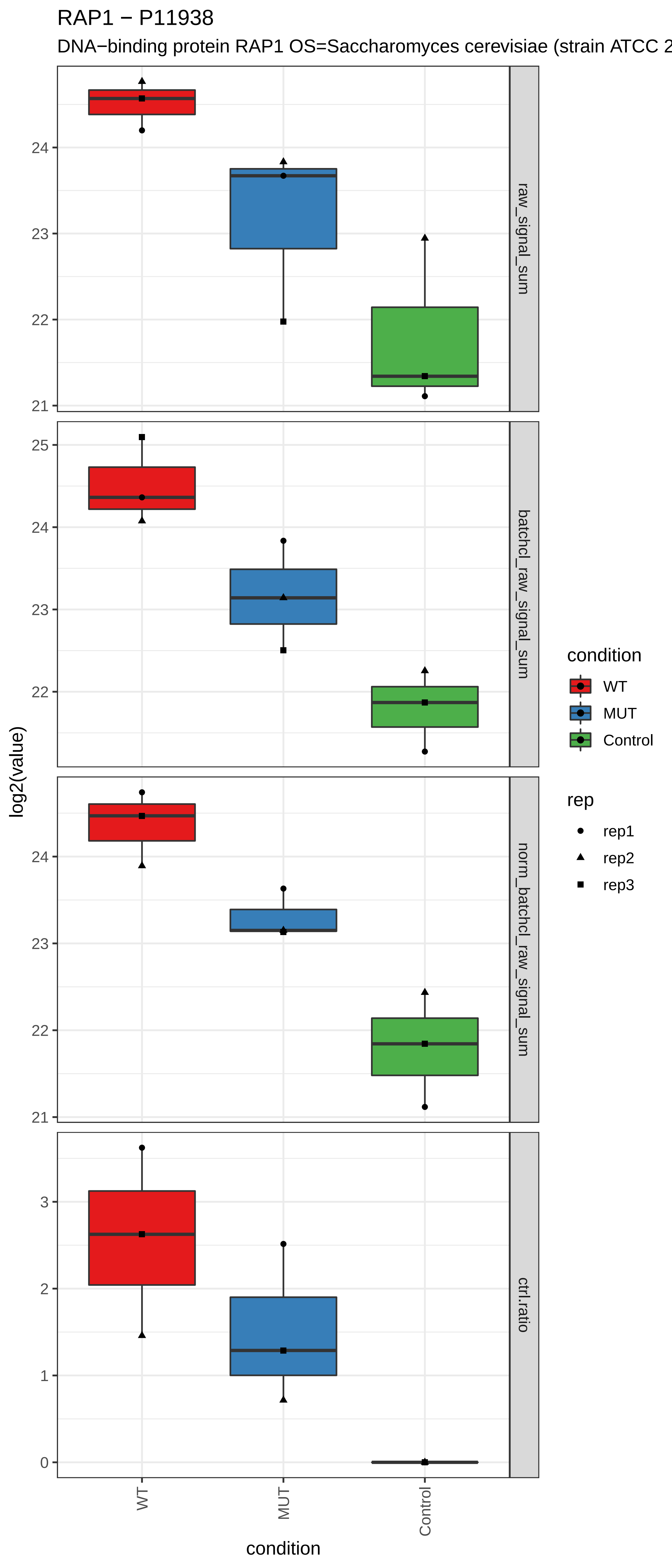
RAP1 − P11938
DNA−binding protein RAP1 OS=Saccharomyces cerevisiae (strain ATCC 2
raw_signal_sum
batchcl_raw_signal_sum
norm_batchcl_raw_signal_sum
ctrl.ratio
log2(value)
condition
WT
MUT
Control
rep
rep1
rep2
rep3

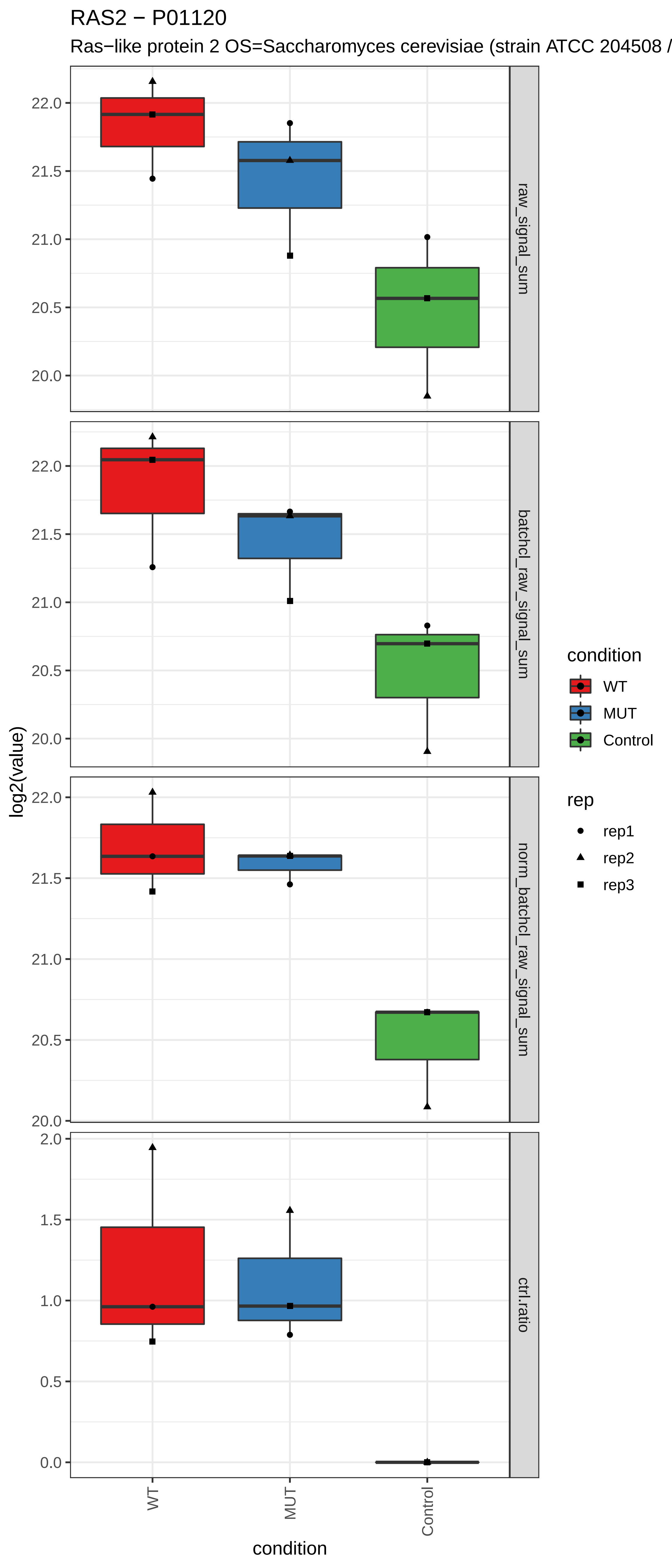

RAS2 − P01120
Ras−like protein 2 OS=Saccharomyces cerevisiae (strain ATCC 204508 /
raw_signal_sum
batchcl_raw_signal_sum
norm_batchcl_raw_signal_sum
ctrl.ratio
22.0
21.5
21.0
20.5
20.0
2.0
1.5
1.0
0.5
0.0
log2(value)
WT
MUT
Control
condition
rep
rep1
rep2
rep3

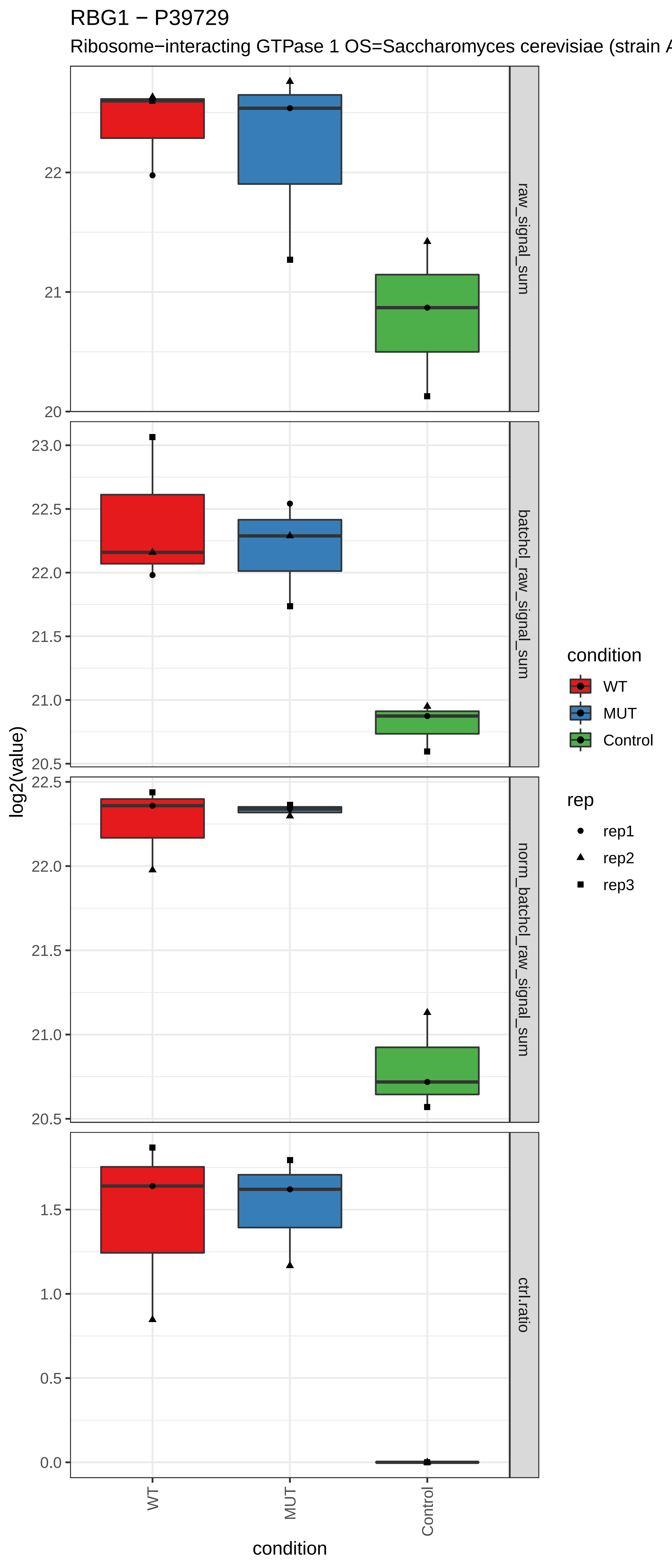

RBG1 − P39729
Ribosome−interacting GTPase 1 OS=Saccharomyces cerevisiae (strain A
raw_signal_sum
batchcl_raw_signal_sum
norm_batchcl_raw_signal_sum
ctrl.ratio
log2(value)
condition
WT
MUT
Control
rep
rep1
rep2
rep3

REB1 − P21538

DNA−binding protein REB1 OS=Saccharomyces cerevisiae (strain ATCC 2

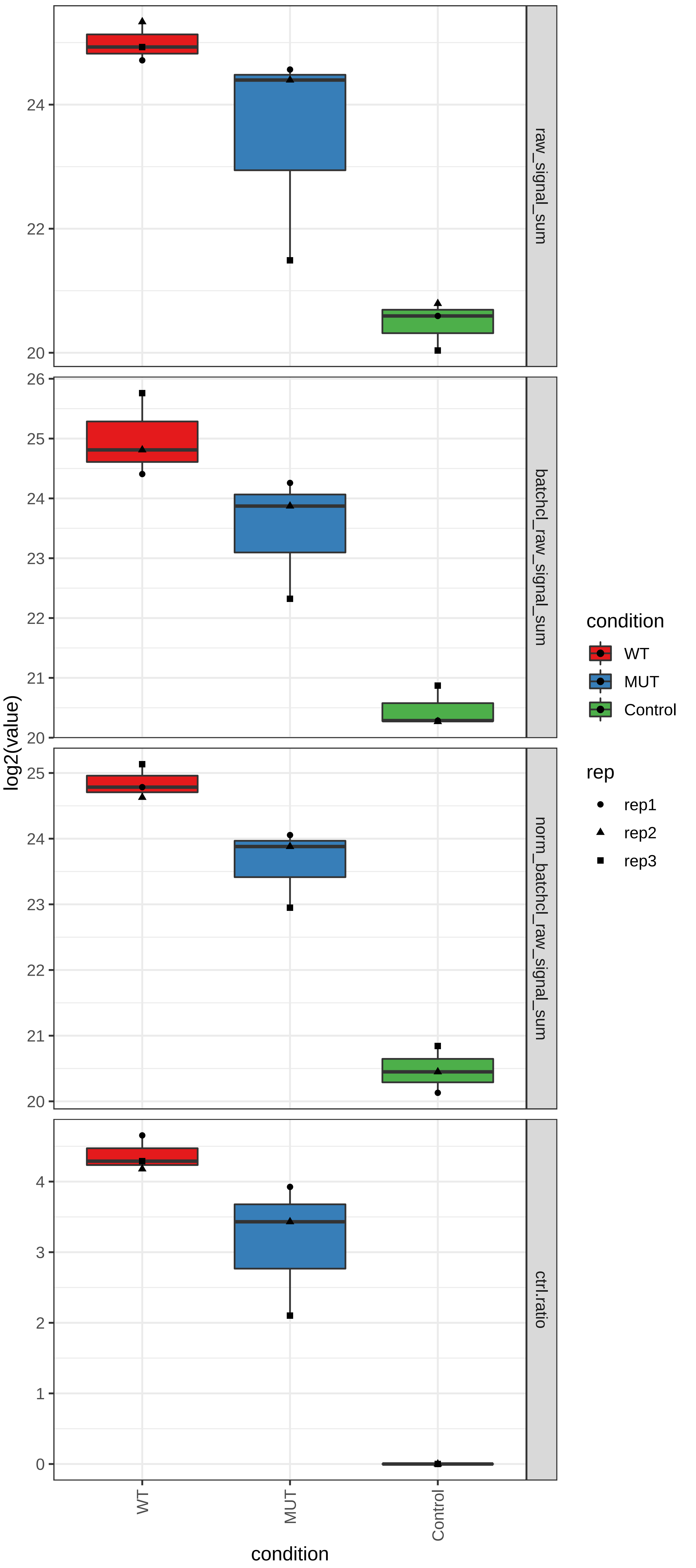

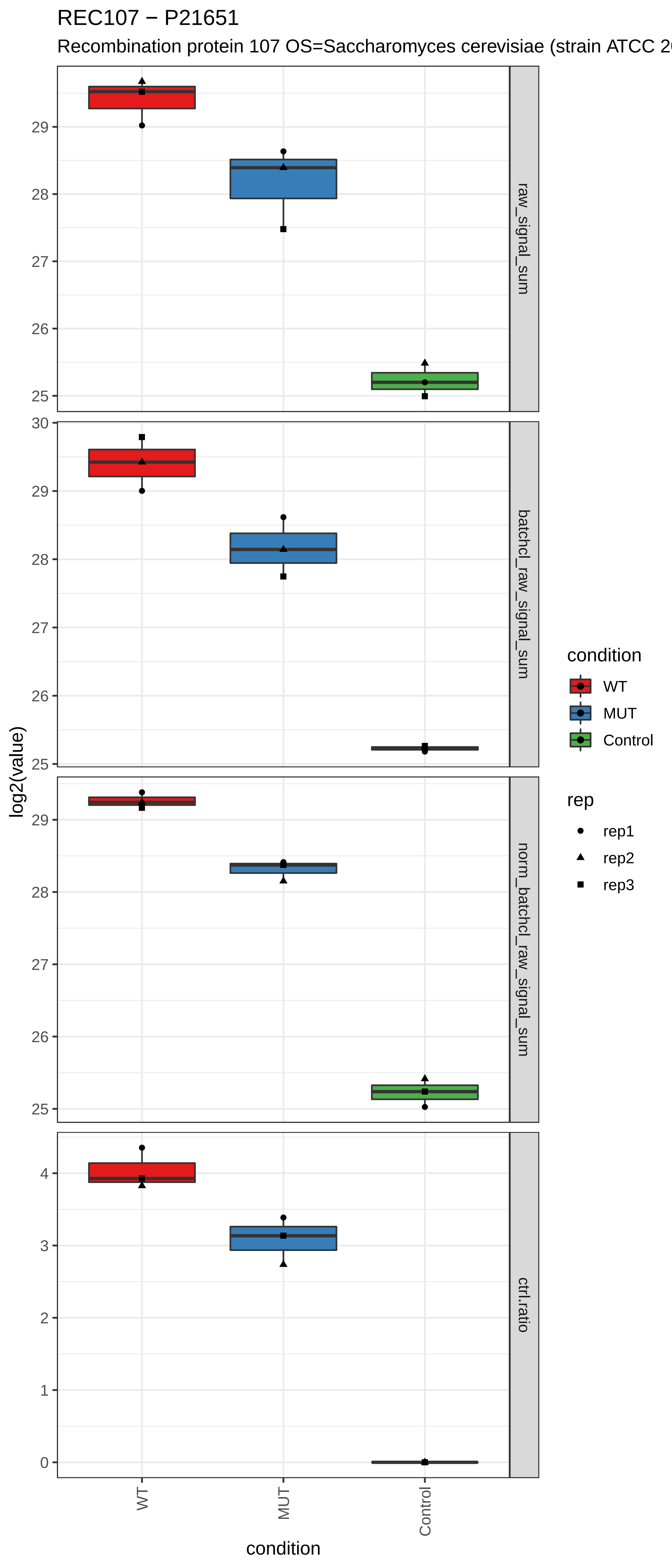
REC107 − P21651
Recombination protein 107 OS=Saccharomyces cerevisiae (strain ATCC 2
raw_signal_sum
batchcl_raw_signal_sum
norm_batchcl_raw_signal_sum
ctrl.ratio
log2(value)
condition
WT
MUT
Control
rep
rep1
rep2
rep3

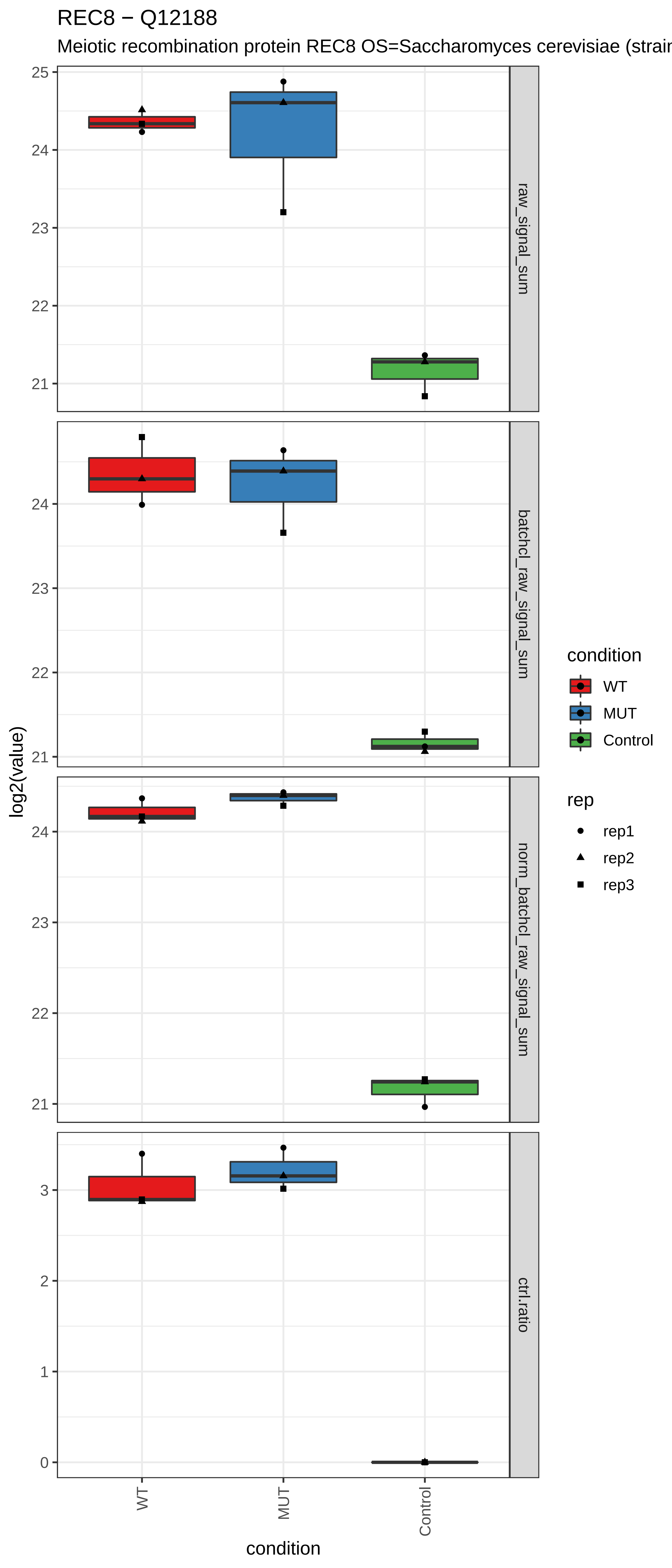
REC8 − Q12188
Meiotic recombination protein REC8 OS=Saccharomyces cerevisiae (strain
raw_signal_sum
batchcl_raw_signal_sum
norm_batchcl_raw_signal_sum
ctrl.ratio
log2(value)
condition
WT
MUT
Control
rep
rep1
rep2
rep3
25
24
23
22
21
3
2
1
0

RED1 − P14291

Protein RED1 OS=Saccharomyces cerevisiae (strain ATCC 204508 / S288

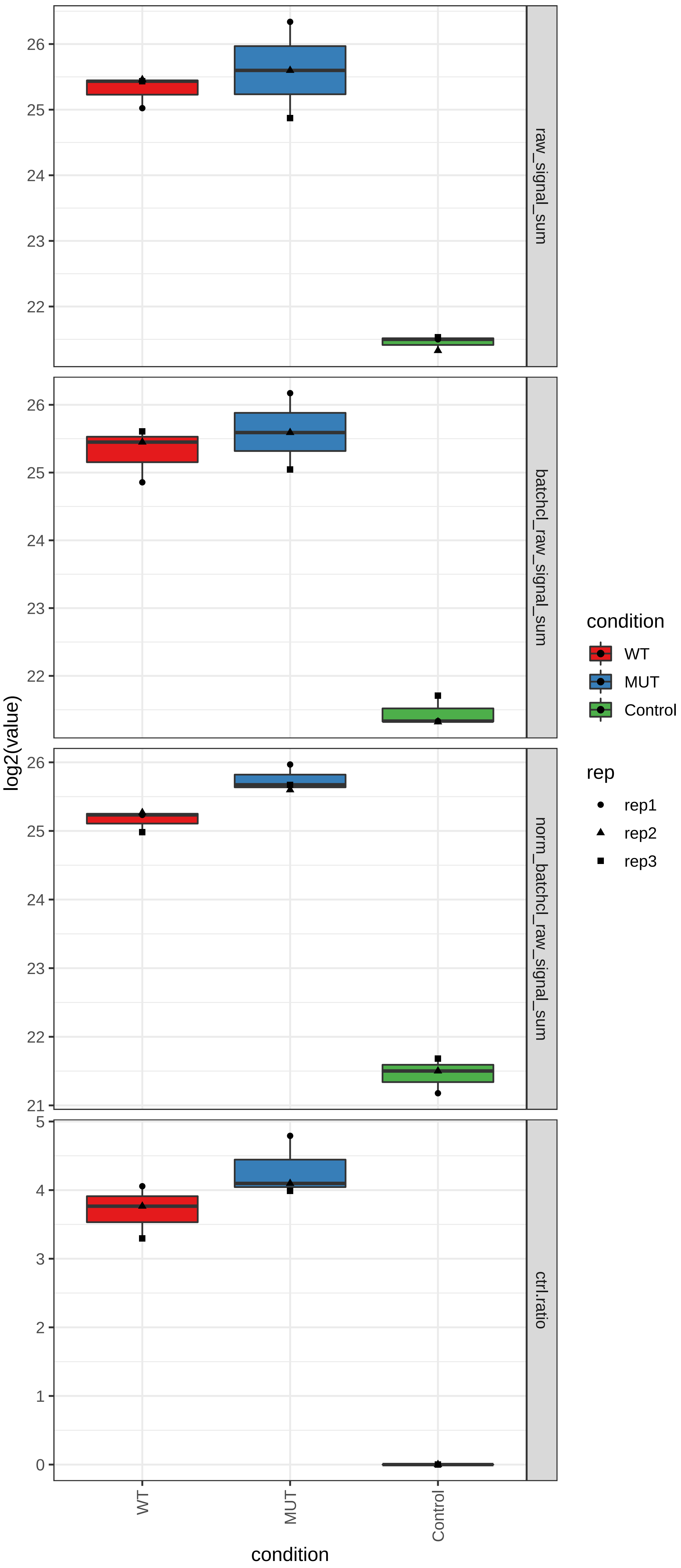

REP1 − P03871

Partitioning protein REP1 OS=Saccharomyces cerevisiae (strain ATCC 204

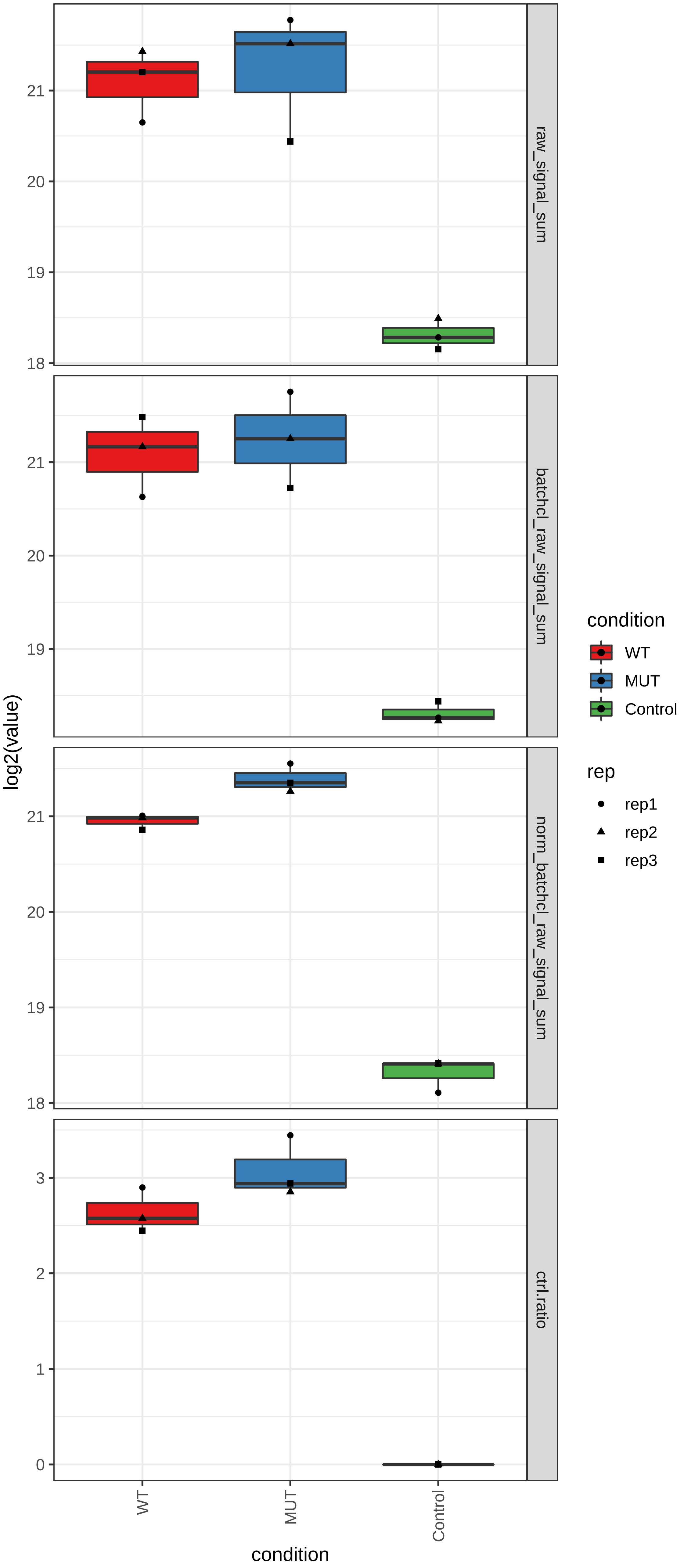

REP2 − P03872

Partitioning protein REP2 OS=Saccharomyces cerevisiae (strain ATCC 204

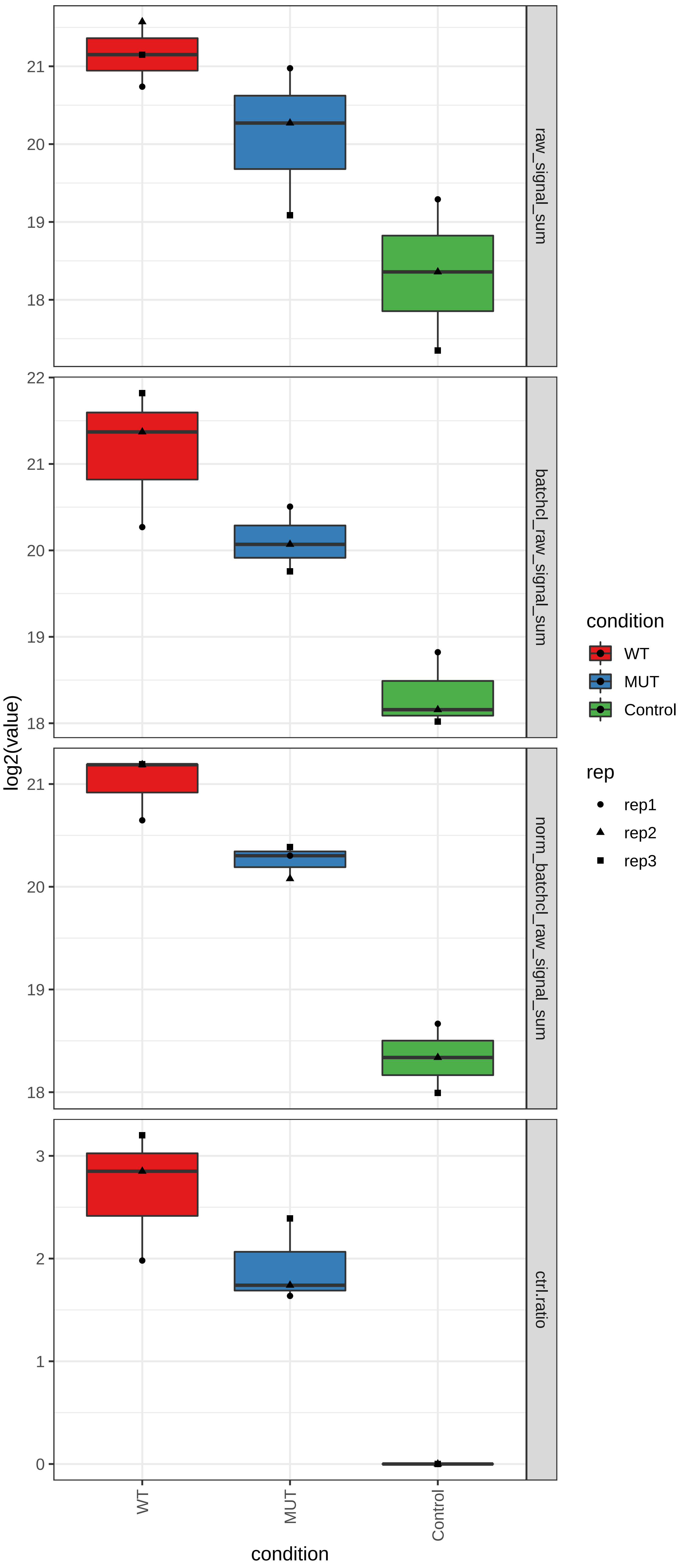

RET2 − P43621

Coatomer subunit delta OS=Saccharomyces cerevisiae (strain ATCC 204

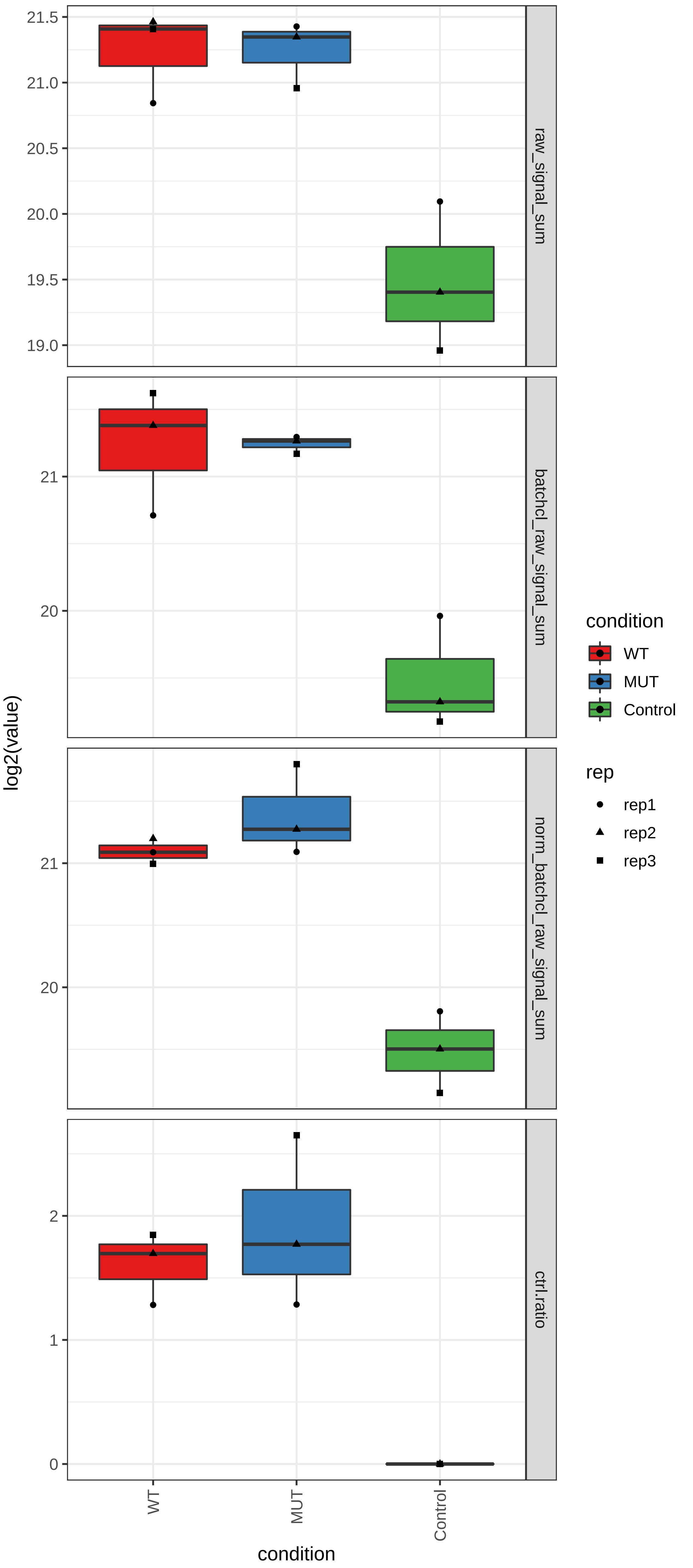

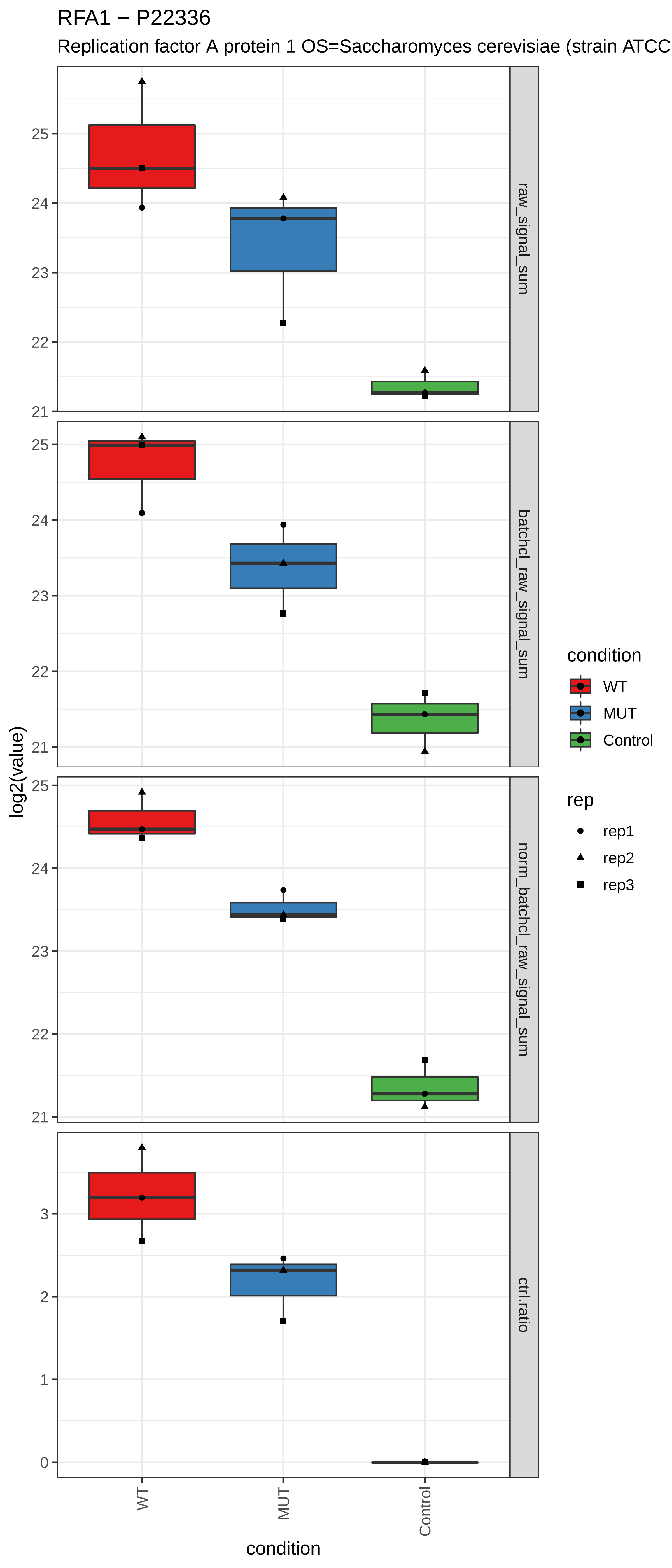

RFA1 − P22336
Replication factor A protein 1 OS=Saccharomyces cerevisiae (strain ATCC
raw_signal_sum
batchcl_raw_signal_sum
norm_batchcl_raw_signal_sum
ctrl.ratio
log2(value)
condition
WT
MUT
Control
rep
rep1
rep2
rep3

RFC1 − P38630

Replication factor C subunit 1 OS=Saccharomyces cerevisiae (strain ATCC

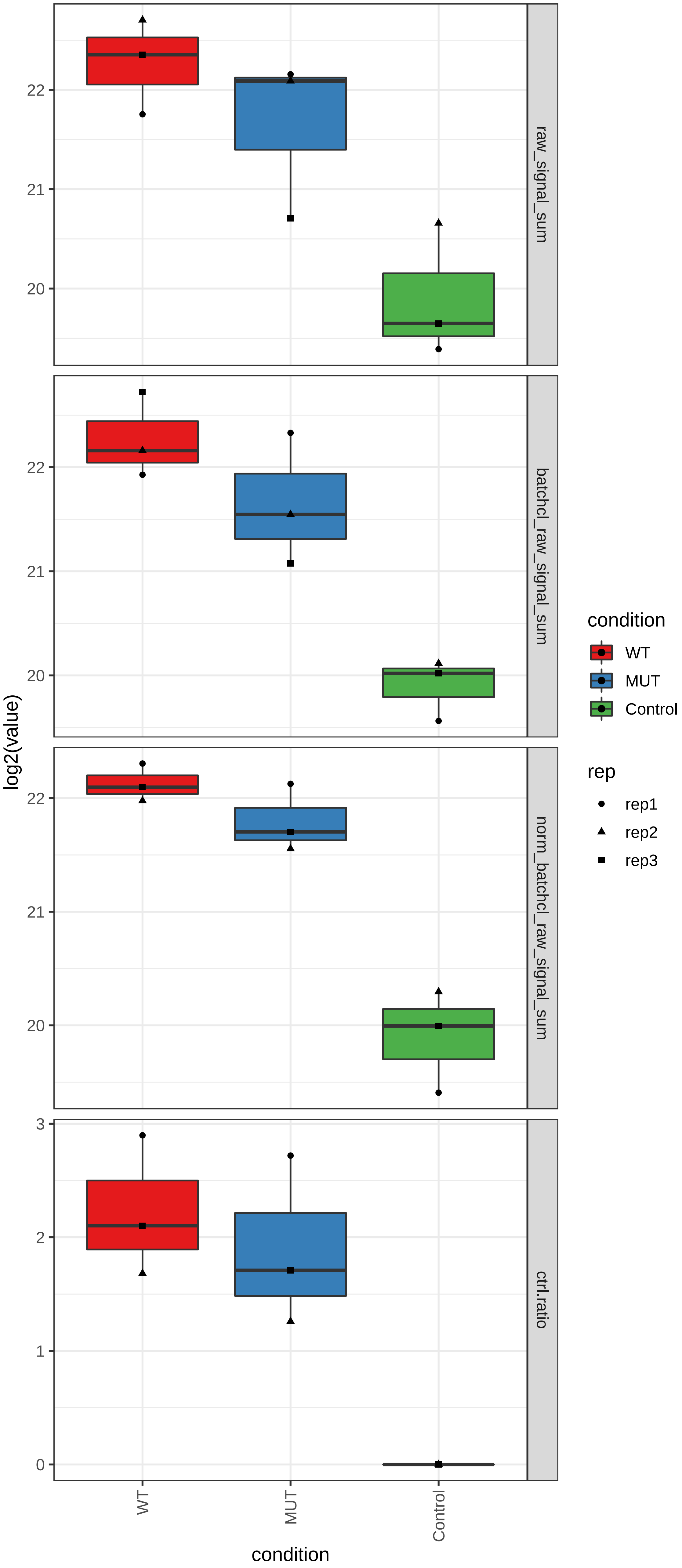

RFC2 − P40348

Replication factor C subunit 2 OS=Saccharomyces cerevisiae (strain ATCC

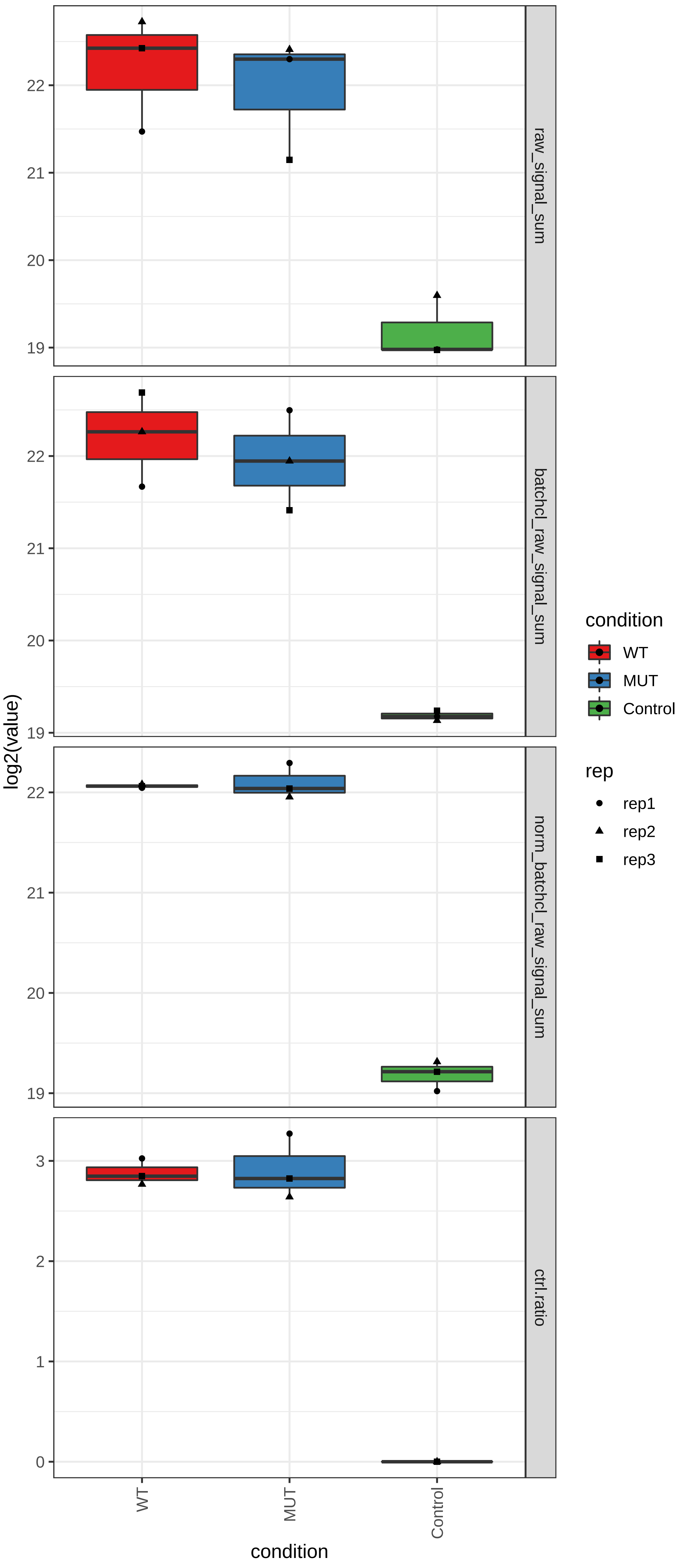

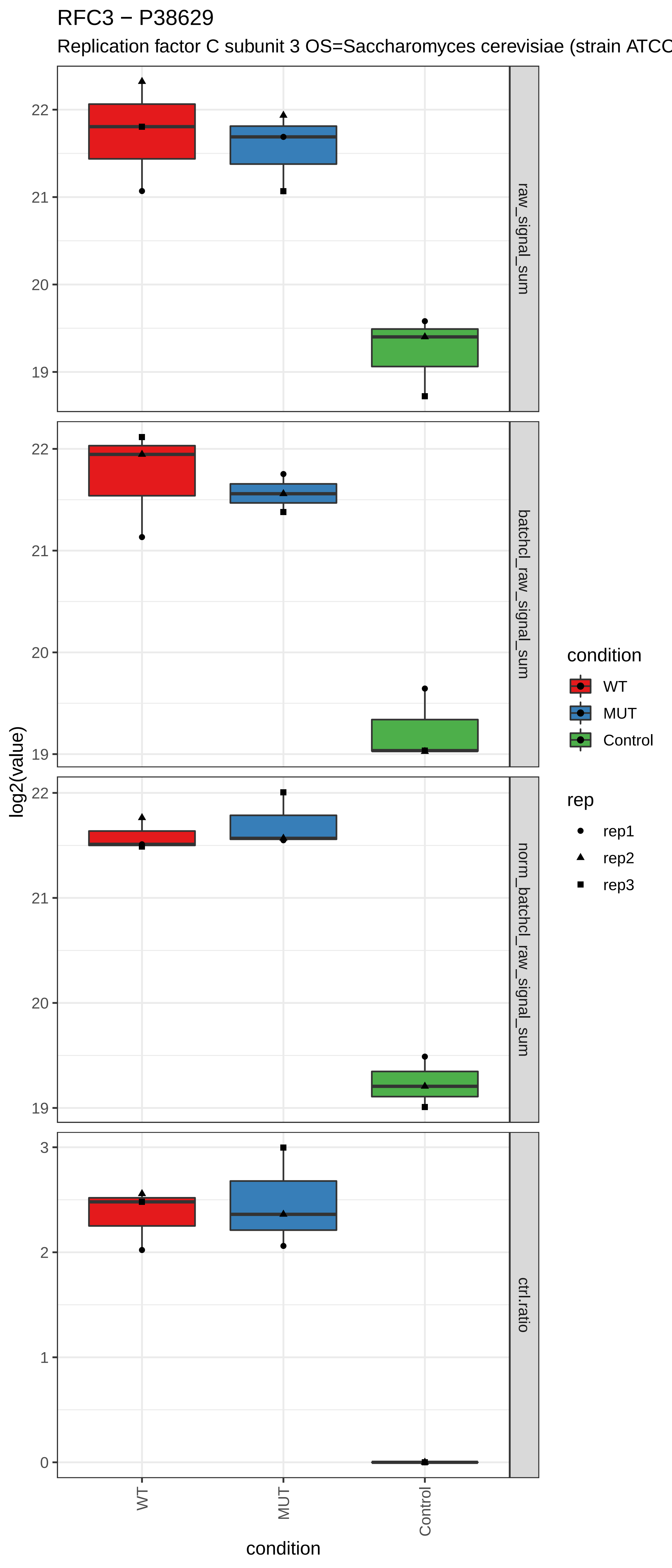

RFC3 − P38629
Replication factor C subunit 3 OS=Saccharomyces cerevisiae (strain ATCC
raw_signal_sum
batchcl_raw_signal_sum
norm_batchcl_raw_signal_sum
ctrl.ratio
log2(value)
condition
WT
MUT
Control
rep
rep1
rep2
rep3

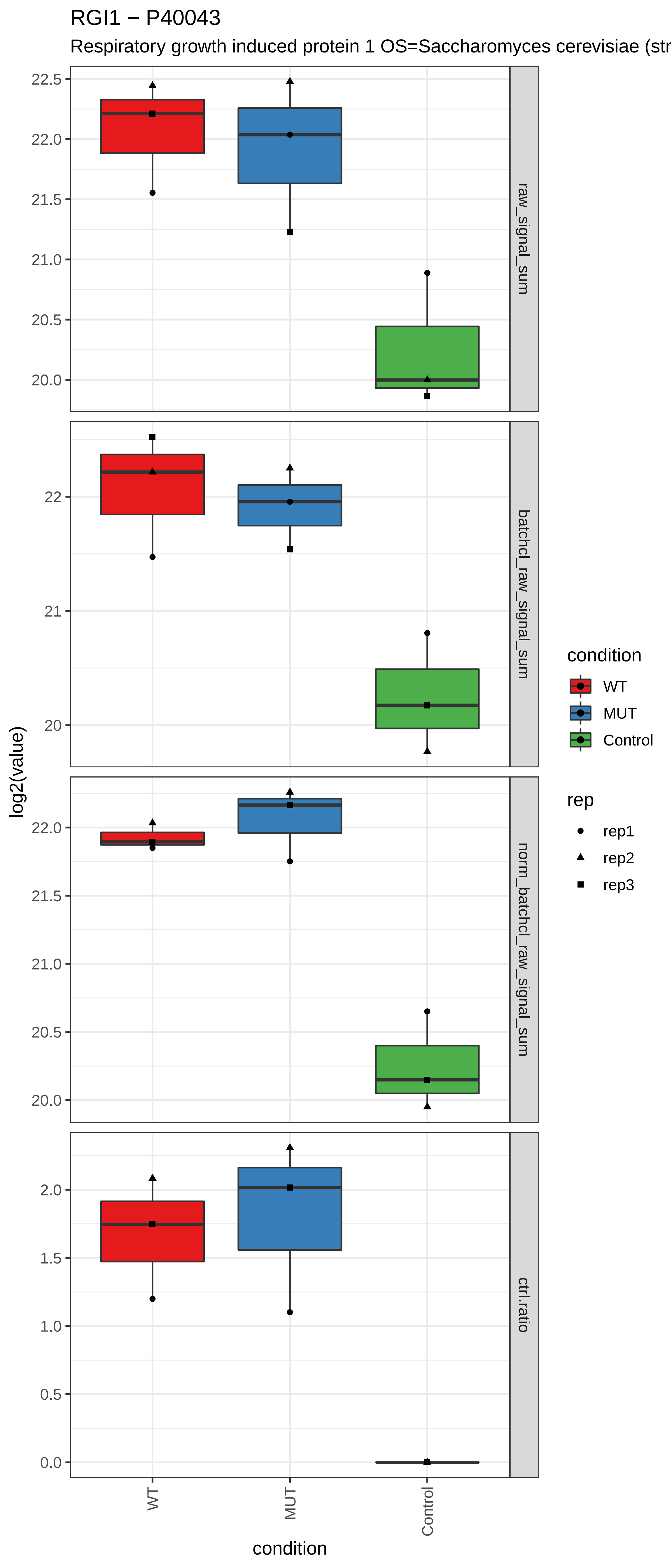

RGI1 − P40043
Respiratory growth induced protein 1 OS=Saccharomyces cerevisiae (str
raw_signal_sum
batchcl_raw_signal_sum
norm_batchcl_raw_signal_sum
ctrl.ratio
log2(value)
condition
WT
MUT
Control
rep
rep1
rep2
rep3
22.5
22.0
21.5
21.0
20.5
20.0
22
21
20
2.0
1.5
1.0
0.5
0.0

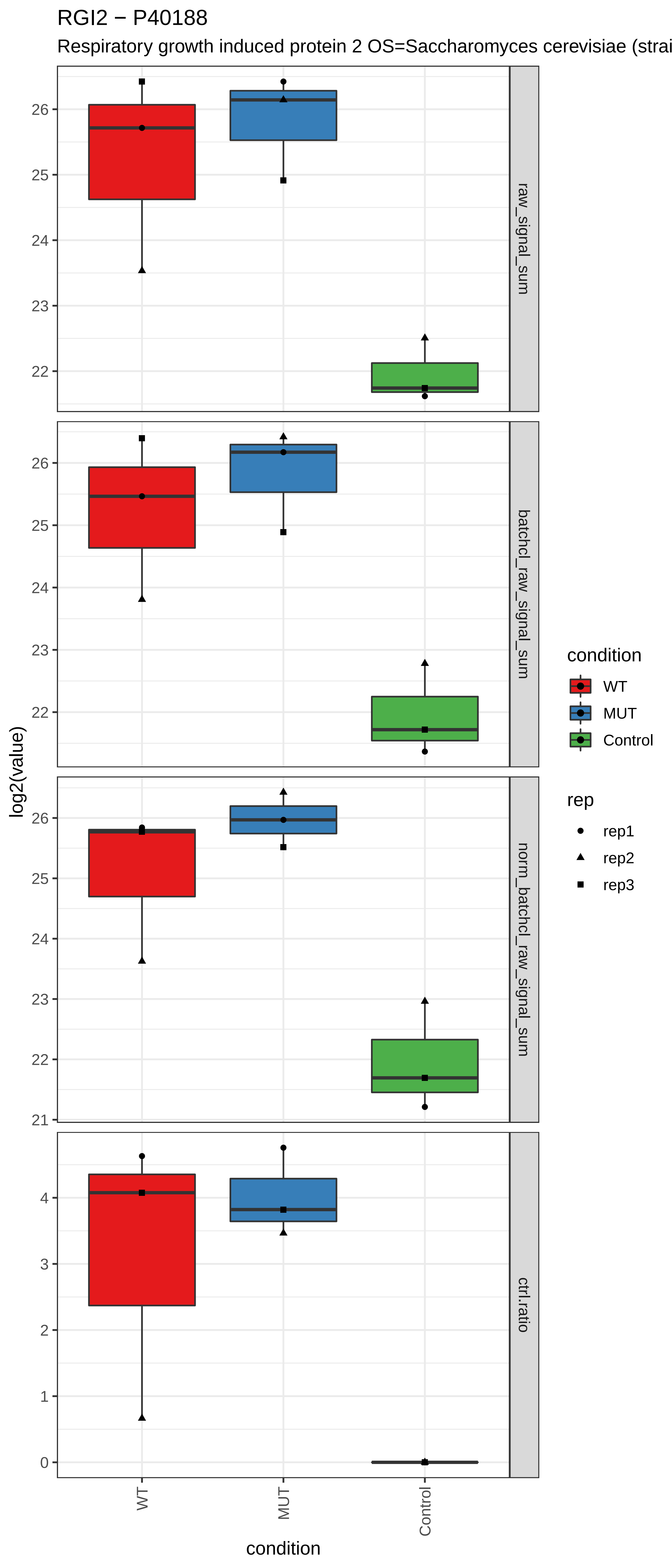

RGI2 − P40188
Respiratory growth induced protein 2 OS=Saccharomyces cerevisiae (strai
raw_signal_sum
batchcl_raw_signal_sum
norm_batchcl_raw_signal_sum
ctrl.ratio
log2(value)
condition
WT
MUT
Control
rep
rep1
rep2
rep3

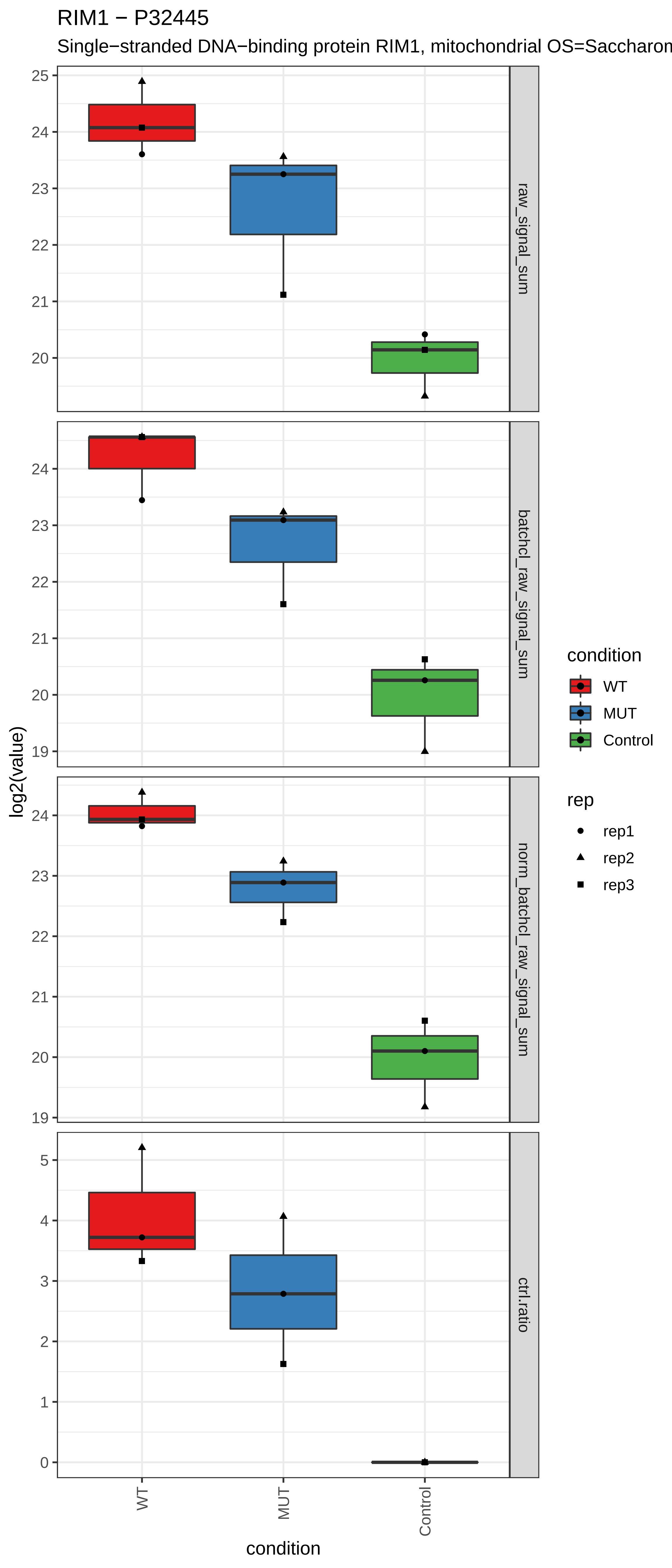
RIM1 − P32445
Single−stranded DNA−binding protein RIM1, mitochondrial OS=Saccharom
raw_signal_sum
batchcl_raw_signal_sum
norm_batchcl_raw_signal_sum
ctrl.ratio
log2(value)
condition
WT
MUT
Control
rep
rep1
rep2
rep3

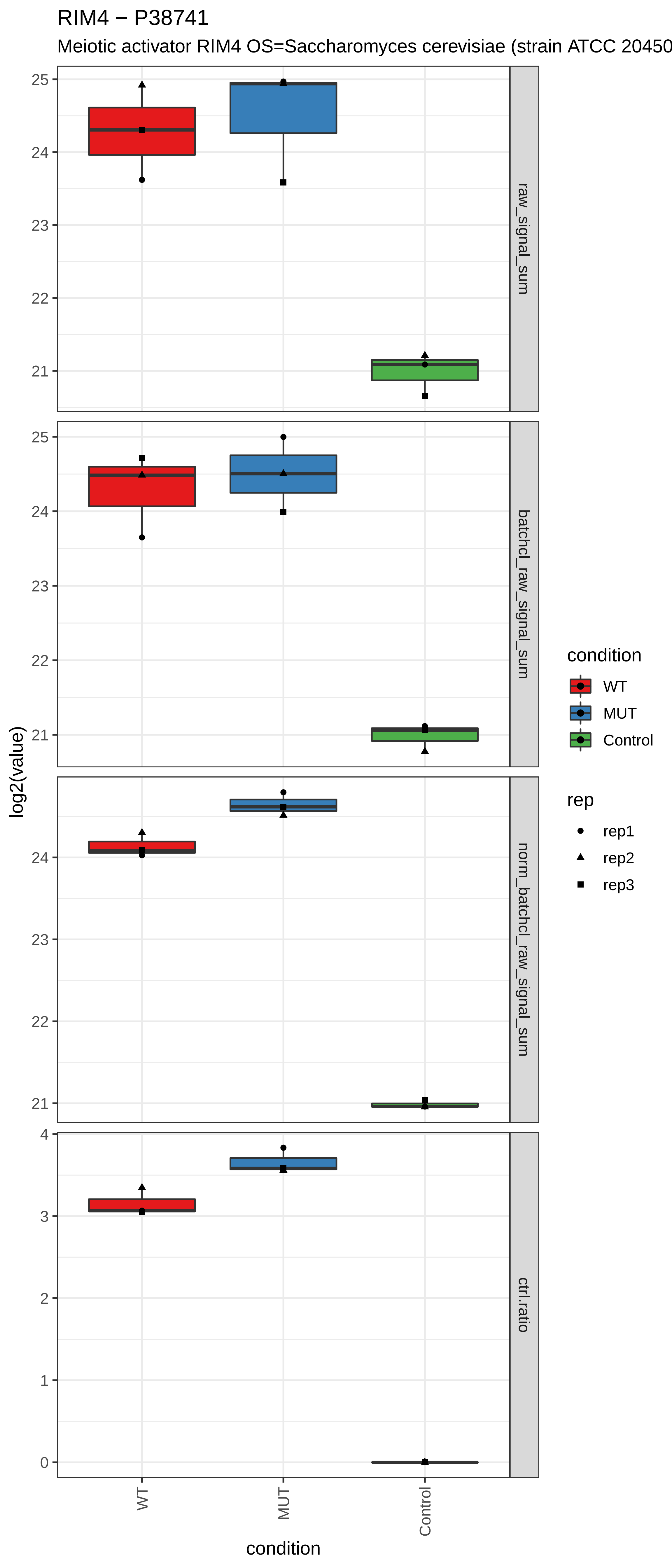
RIM4 − P38741
Meiotic activator RIM4 OS=Saccharomyces cerevisiae (strain ATCC 20450
raw_signal_sum
batchcl_raw_signal_sum
norm_batchcl_raw_signal_sum
ctrl.ratio
log2(value)
condition
WT
MUT
Control
rep
rep1
rep2
rep3
25
24
23
22
21
4
3
2
1
0

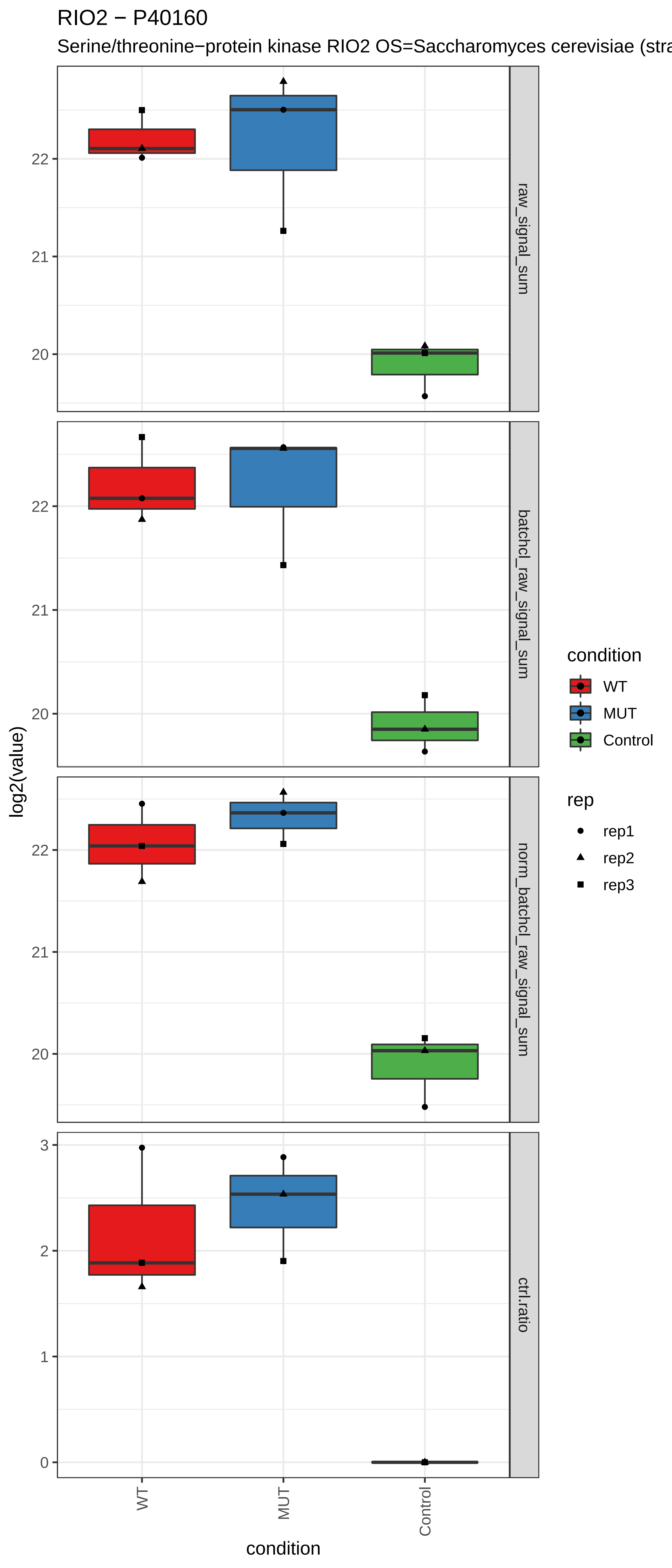

RIO2 − P40160
Serine/threonine−protein kinase RIO2 OS=Saccharomyces cerevisiae (stra
raw_signal_sum
batchcl_raw_signal_sum
norm_batchcl_raw_signal_sum
ctrl.ratio
log2(value)
condition
WT
MUT
Control
condition
WT
MUT
Control
rep
rep1
rep2
rep3

RLI1 − Q03195

Translation initiation factor RLI1 OS=Saccharomyces cerevisiae (strain ATC

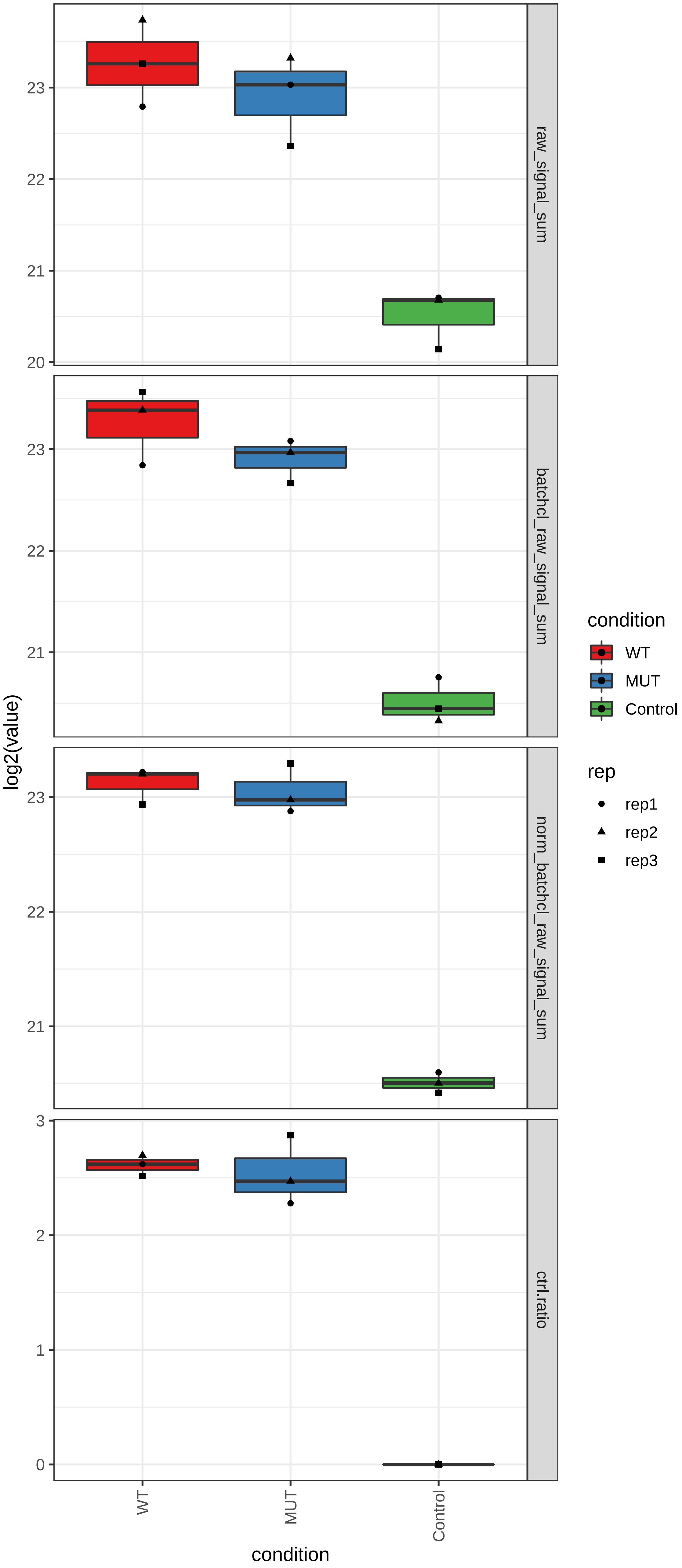

RLP7 − P40693

Ribosome biogenesis protein RLP7 OS=Saccharomyces cerevisiae (strai

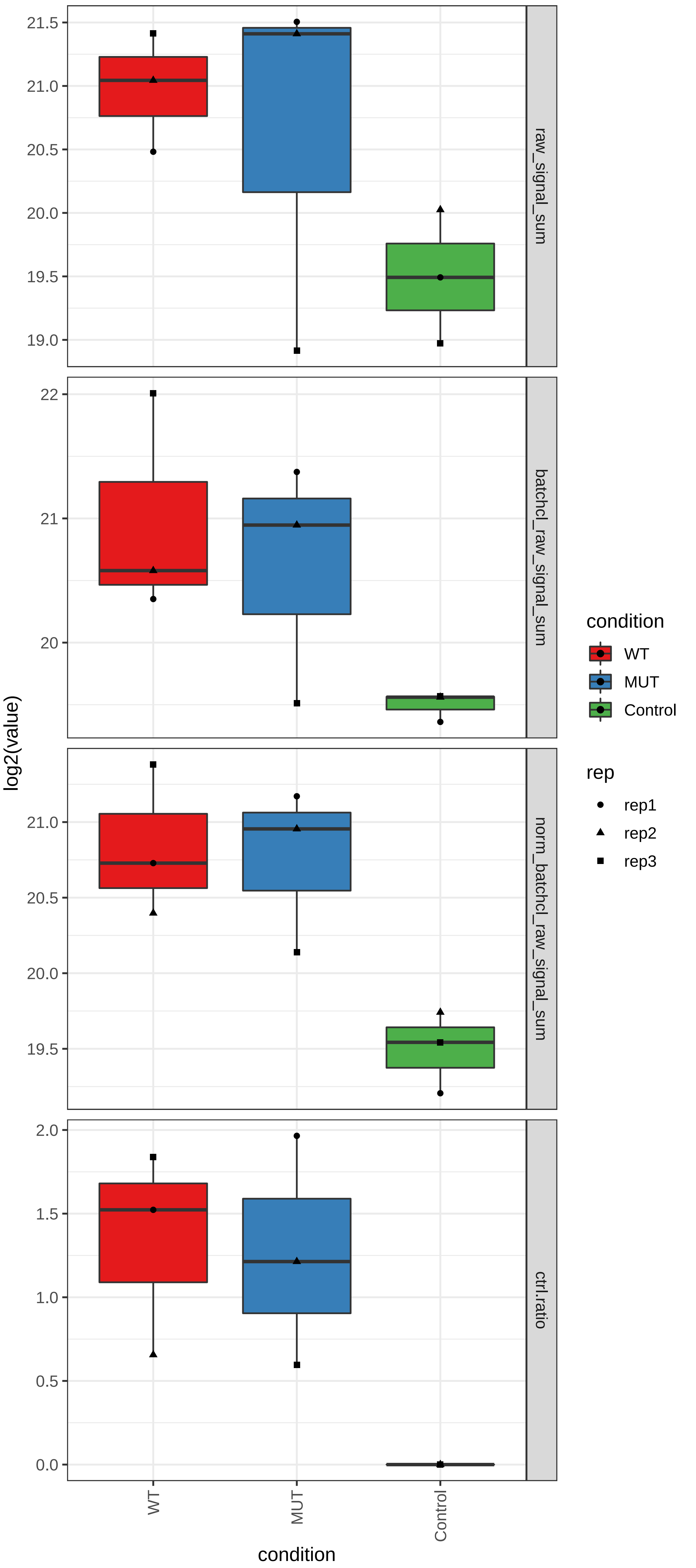

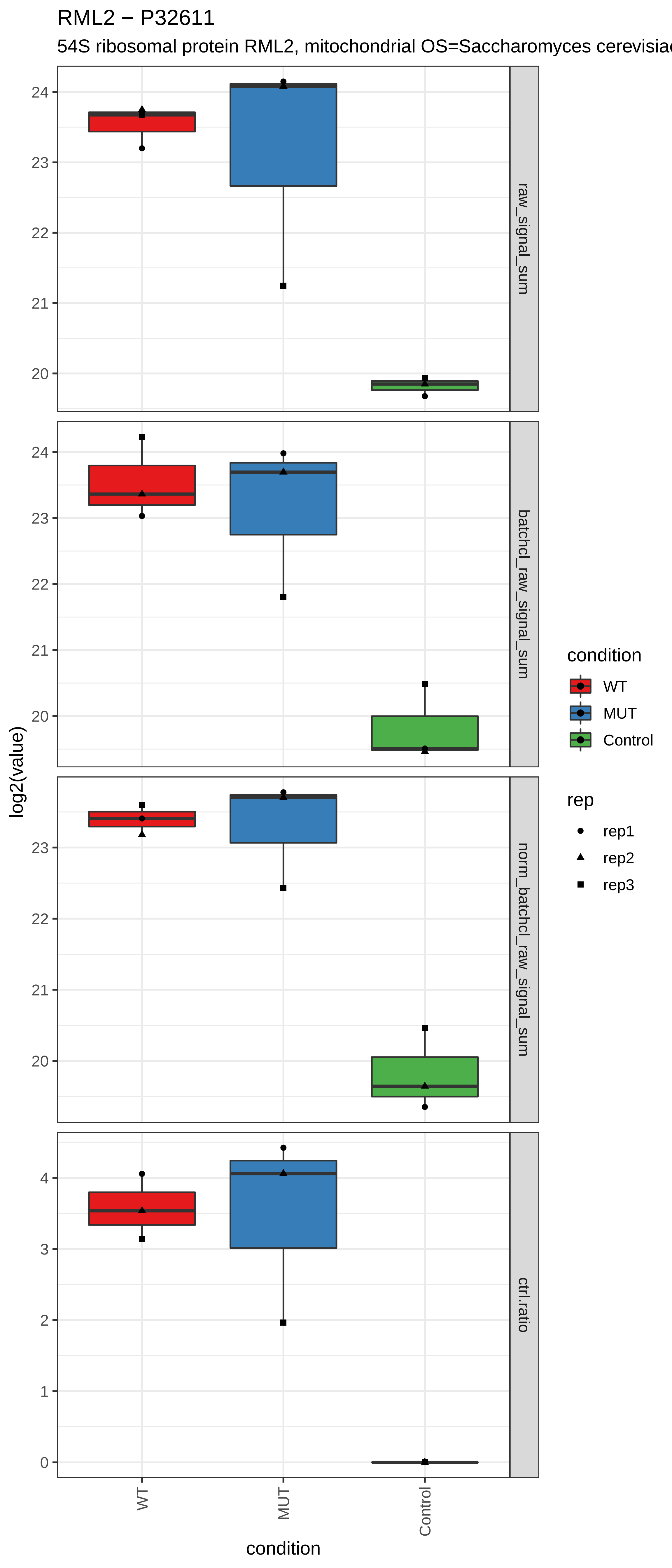
RML2 − P32611
54S ribosomal protein RML2, mitochondrial OS=Saccharomyces cerevisiae
log2(value)
raw_signal_sum
batchcl_raw_signal_sum
norm_batchcl_raw_signal_sum
ctrl.ratio
24
23
22
21
20
4
3
2
1
0
WT
MUT
Control
condition
condition
WT
MUT
Control
rep
rep1
rep2
rep3

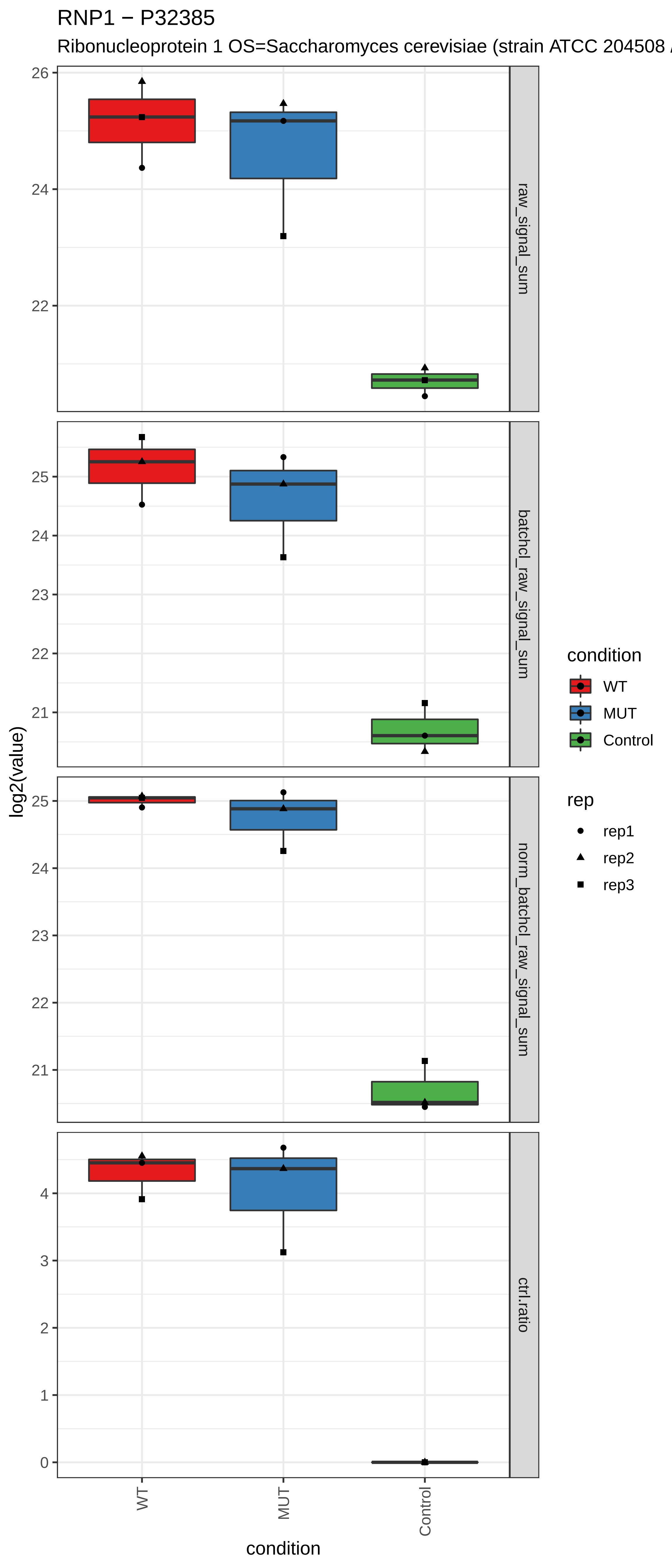
RNP1 − P32385
Ribonucleoprotein 1 OS=Saccharomyces cerevisiae (strain ATCC 204508 /
raw_signal_sum
batchcl_raw_signal_sum
norm_batchcl_raw_signal_sum
ctrl.ratio
log2(value)
condition
WT
MUT
Control
rep
rep1
rep2
rep3
26
24
22
25
24
23
22
21
4
3
2
1
0

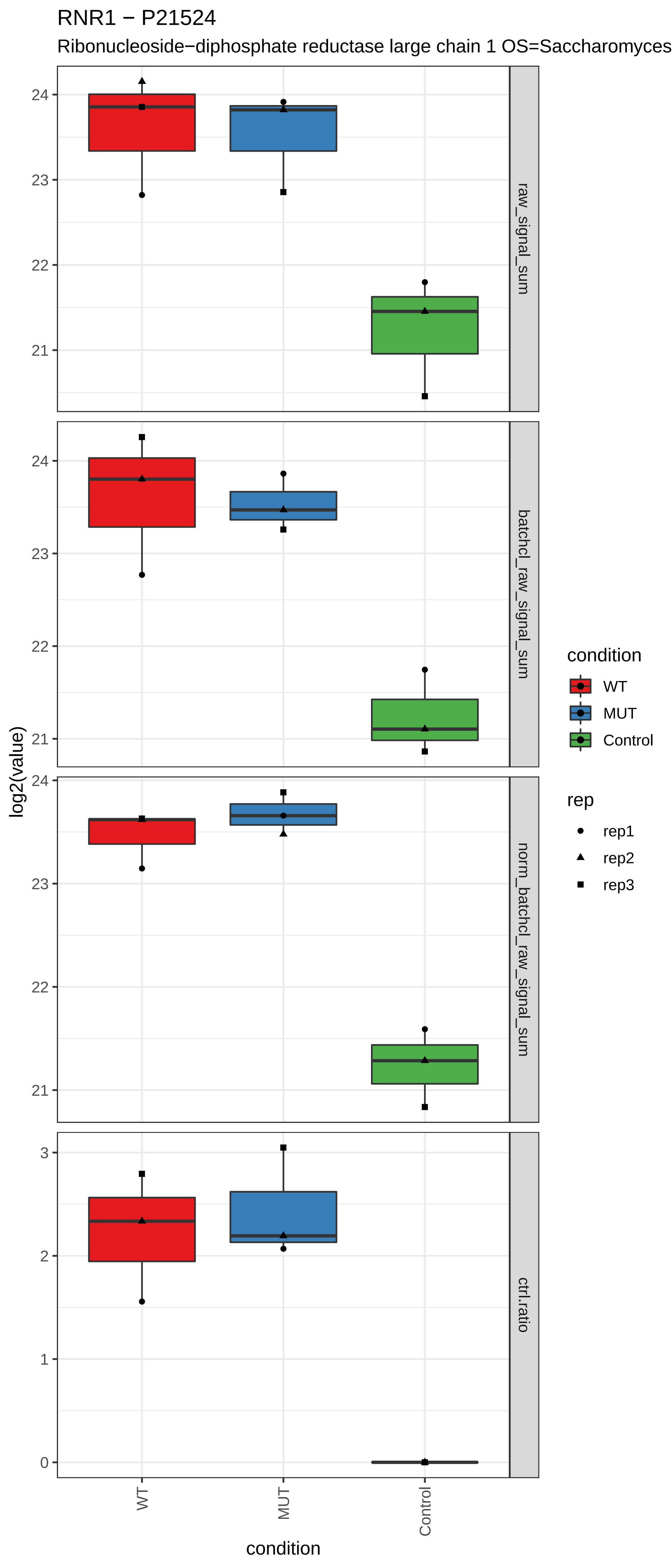

RNR1 − P21524
Ribonucleoside−diphosphate reductase large chain 1 OS=Saccharomyces
raw_signal_sum
batchcl_raw_signal_sum
norm_batchcl_raw_signal_sum
ctrl.ratio
log2(value)
condition
WT
MUT
Control
rep
rep1
rep2
rep3

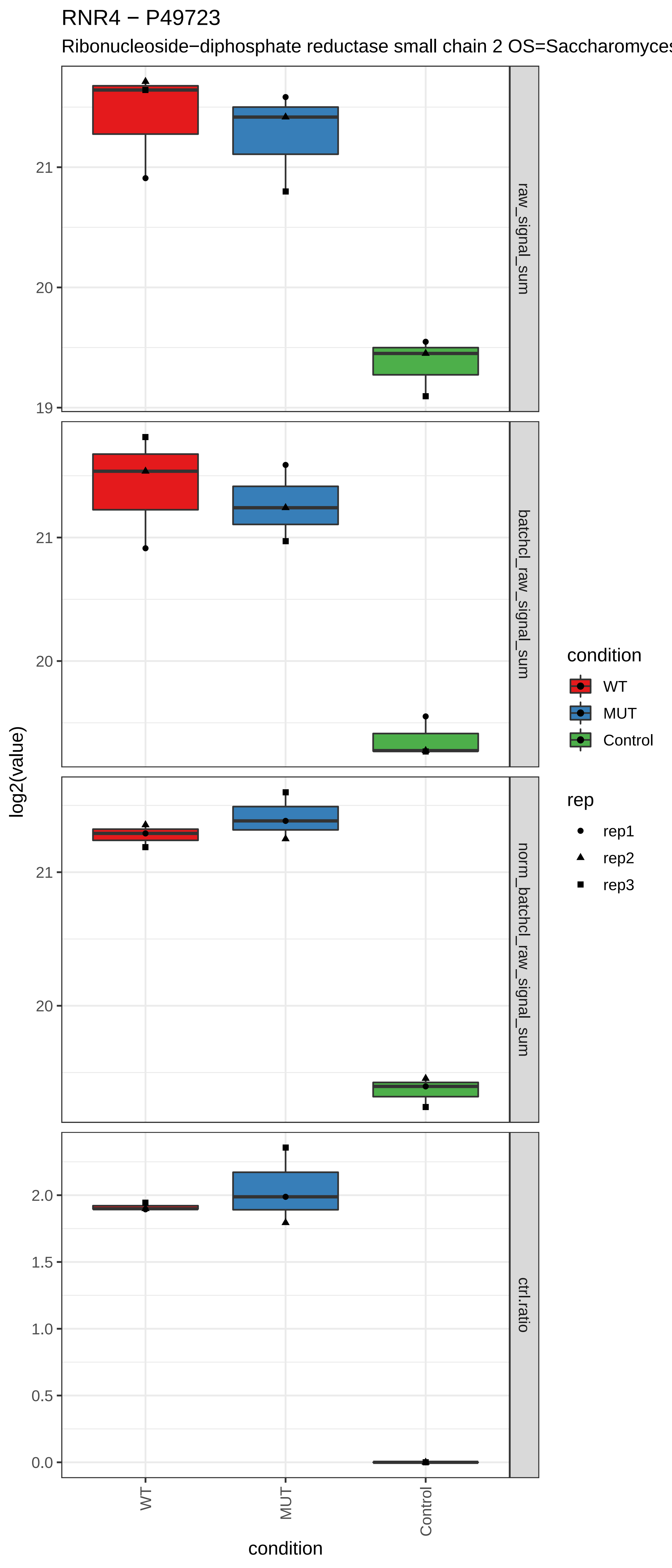

RNR4 − P49723
Ribonucleoside−diphosphate reductase small chain 2 OS=Saccharomyces
raw_signal_sum
batchcl_raw_signal_sum
norm_batchcl_raw_signal_sum
ctrl.ratio
log2(value)
condition
WT
MUT
Control
rep
rep1
rep2
rep3

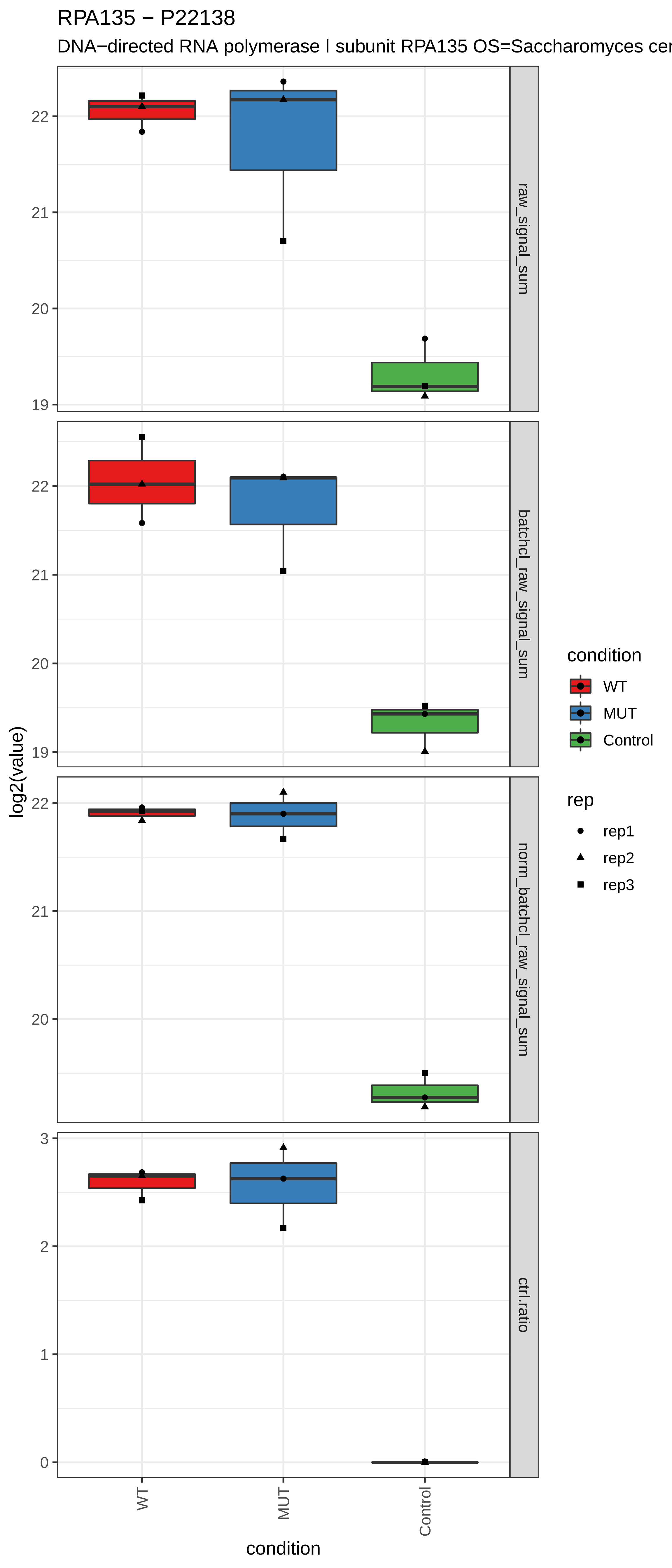

RPA135 − P22138
DNA−directed RNA polymerase I subunit RPA135 OS=Saccharomyces cer
raw_signal_sum
batchcl_raw_signal_sum
norm_batchcl_raw_signal_sum
ctrl.ratio
log2(value)
condition
WT
MUT
Control
rep
rep1
rep2
rep3
22
21
20
19
3
2
1
0

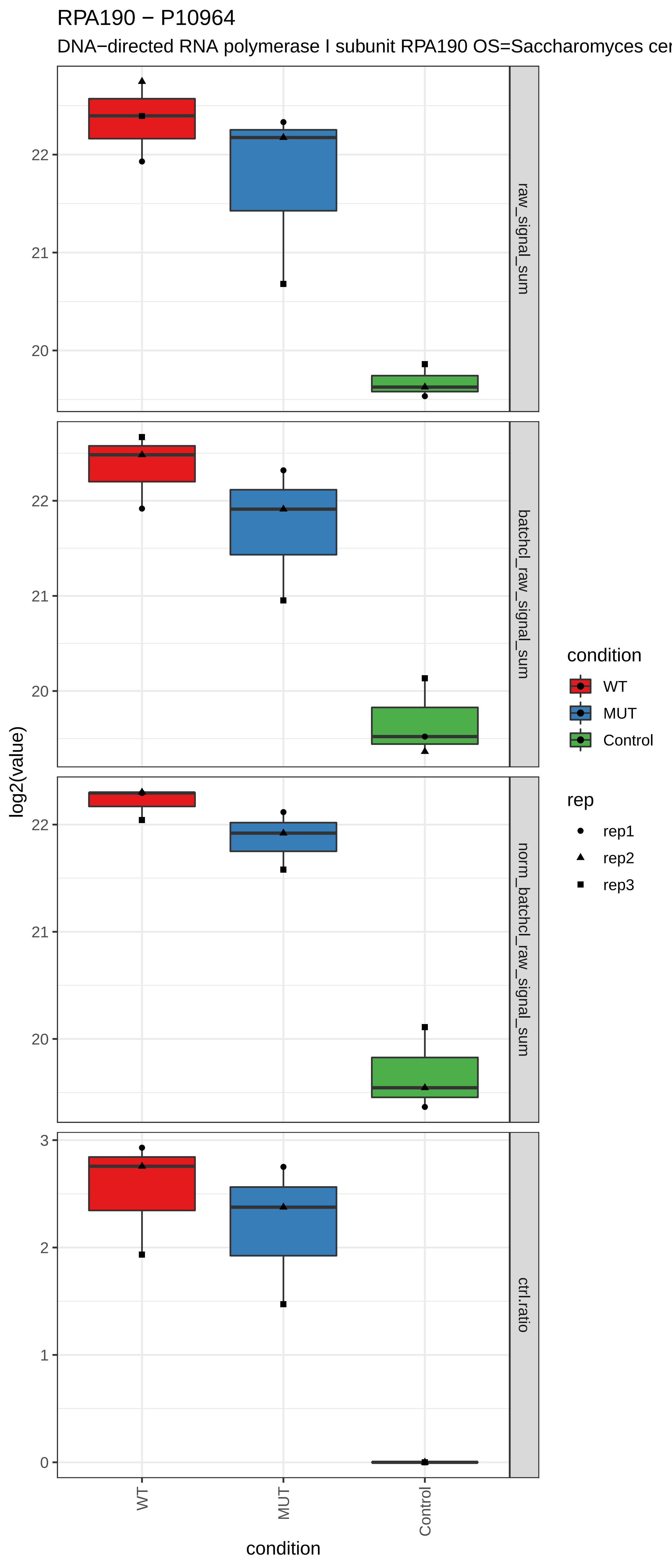
RPA190 − P10964
DNA−directed RNA polymerase I subunit RPA190 OS=Saccharomyces cer
raw_signal_sum
batchcl_raw_signal_sum
norm_batchcl_raw_signal_sum
ctrl.ratio
log2(value)
condition
WT
MUT
Control
rep
rep1
rep2
rep3
22
21
20
3
2
1
0

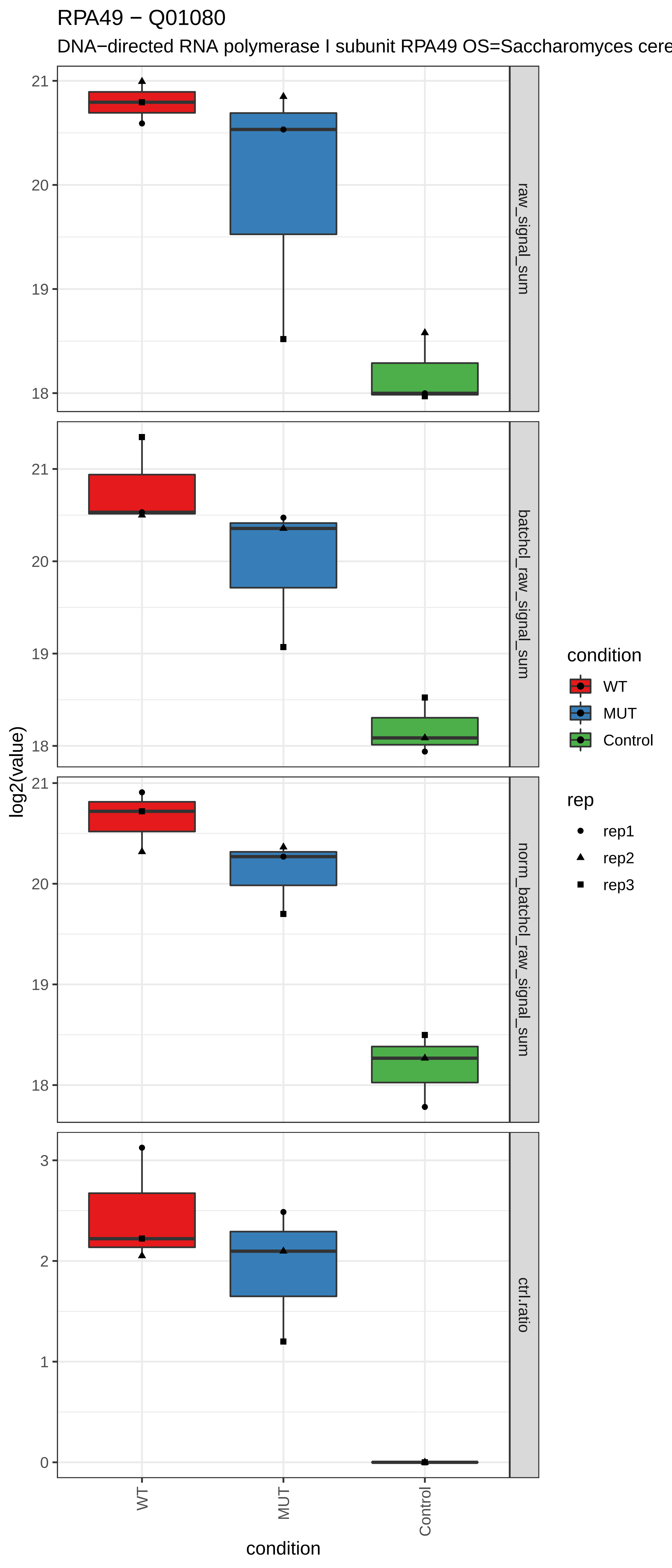
RPA49 − Q01080
DNA−directed RNA polymerase I subunit RPA49 OS=Saccharomyces cere
raw_signal_sum
batchcl_raw_signal_sum
norm_batchcl_raw_signal_sum
ctrl.ratio
log2(value)
condition
WT
MUT
Control
rep
rep1
rep2
rep3
21
20
19
18
3
2
1
0

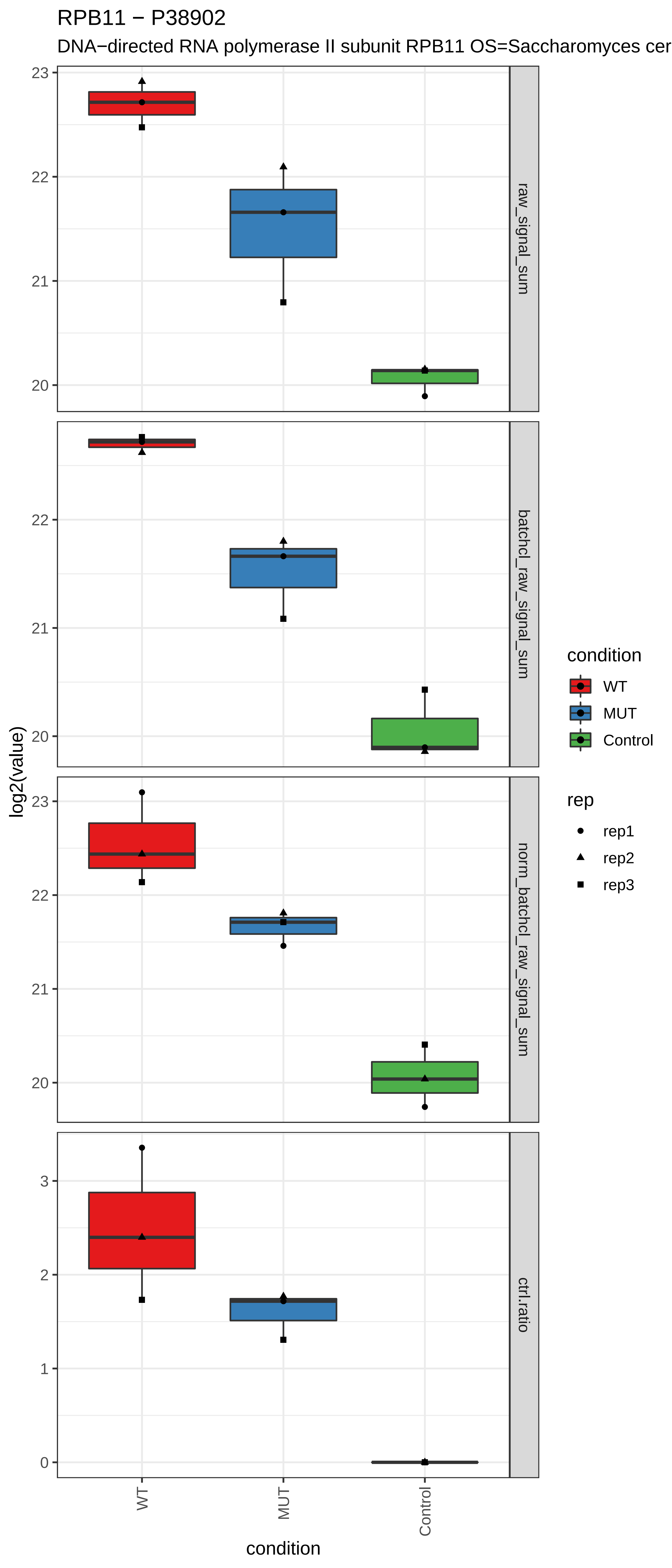
RPB11 − P38902
DNA−directed RNA polymerase II subunit RPB11 OS=Saccharomyces cer
raw_signal_sum
batchcl_raw_signal_sum
norm_batchcl_raw_signal_sum
ctrl.ratio
log2(value)
condition
WT
MUT
Control
rep
rep1
rep2
rep3
23
22
21
20
3
2
1
0

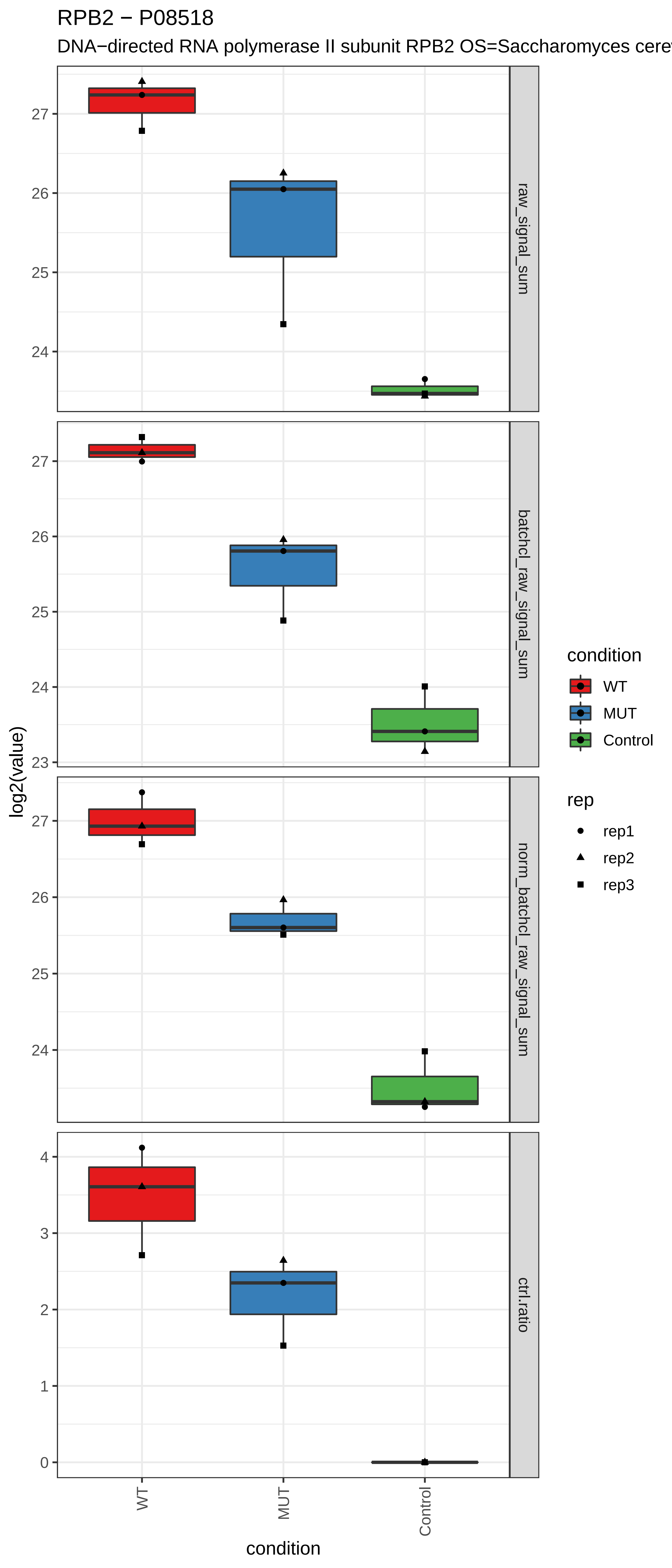
RPB2 − P08518
DNA−directed RNA polymerase II subunit RPB2 OS=Saccharomyces cerev
raw_signal_sum
batchcl_raw_signal_sum
norm_batchcl_raw_signal_sum
ctrl.ratio
log2(value)
condition
WT
MUT
Control
rep
rep1
rep2
rep3

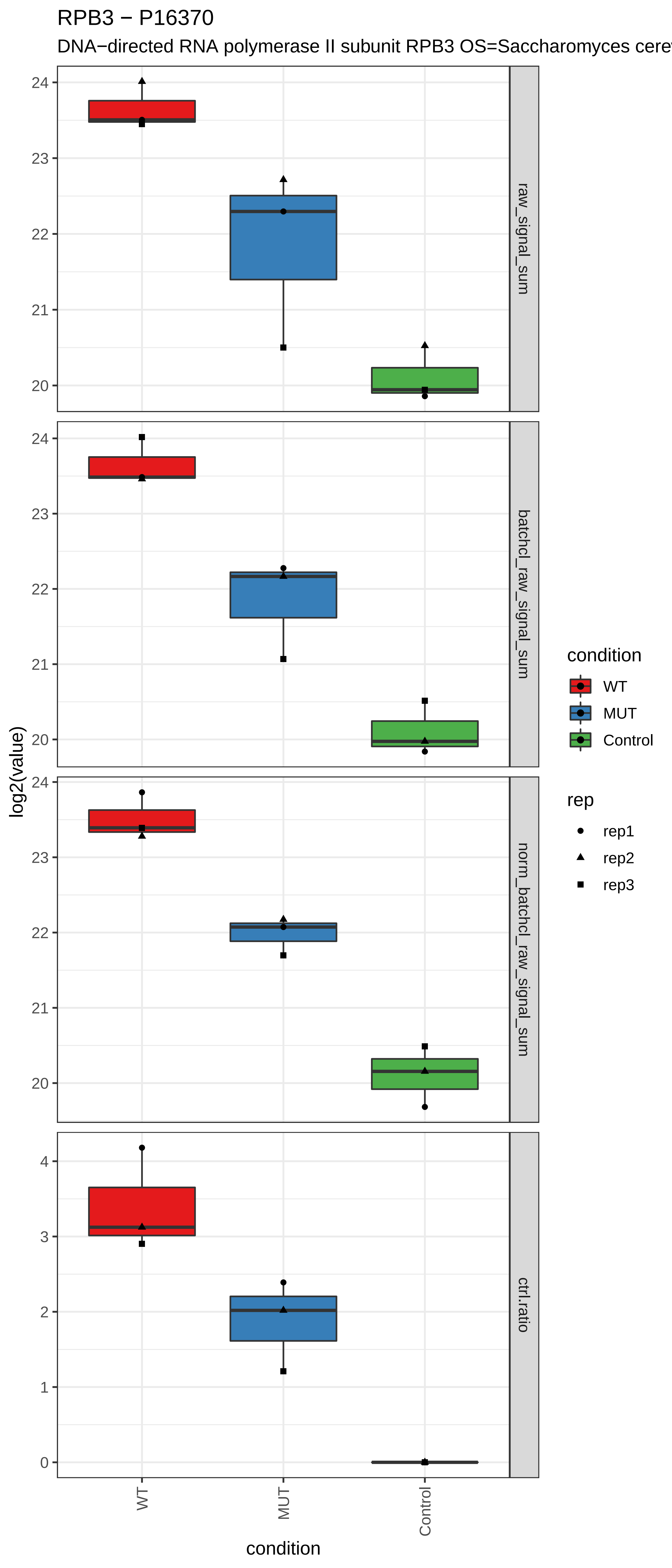
RPB3 − P16370
DNA−directed RNA polymerase II subunit RPB3 OS=Saccharomyces cere
raw_signal_sum
batchcl_raw_signal_sum
norm_batchcl_raw_signal_sum
ctrl.ratio
log2(value)
condition
WT
MUT
Control
rep
rep1
rep2
rep3
24
23
22
21
20
4
3
2
1
0

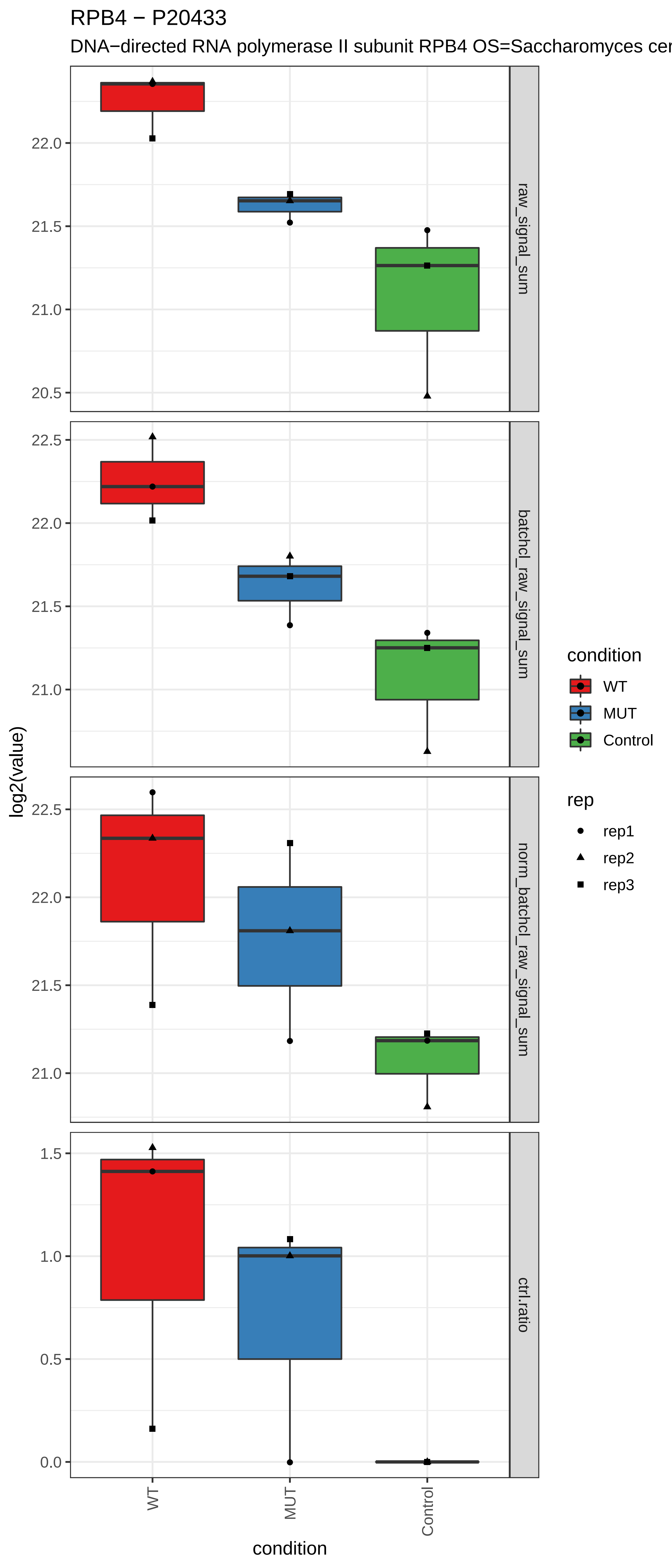

RPB4 − P20433
DNA−directed RNA polymerase II subunit RPB4 OS=Saccharomyces cer
log2(value)
raw_signal_sum
batchcl_raw_signal_sum
norm_batchcl_raw_signal_sum
ctrl.ratio
22.0
21.5
21.0
20.5
22.5
1.5
1.0
0.5
0.0
WT
MUT
Control
condition
rep
rep1
rep2
rep3

RPB5 − P20434

DNA−directed RNA polymerases I, II, and III subunit RPABC1 OS=Saccha

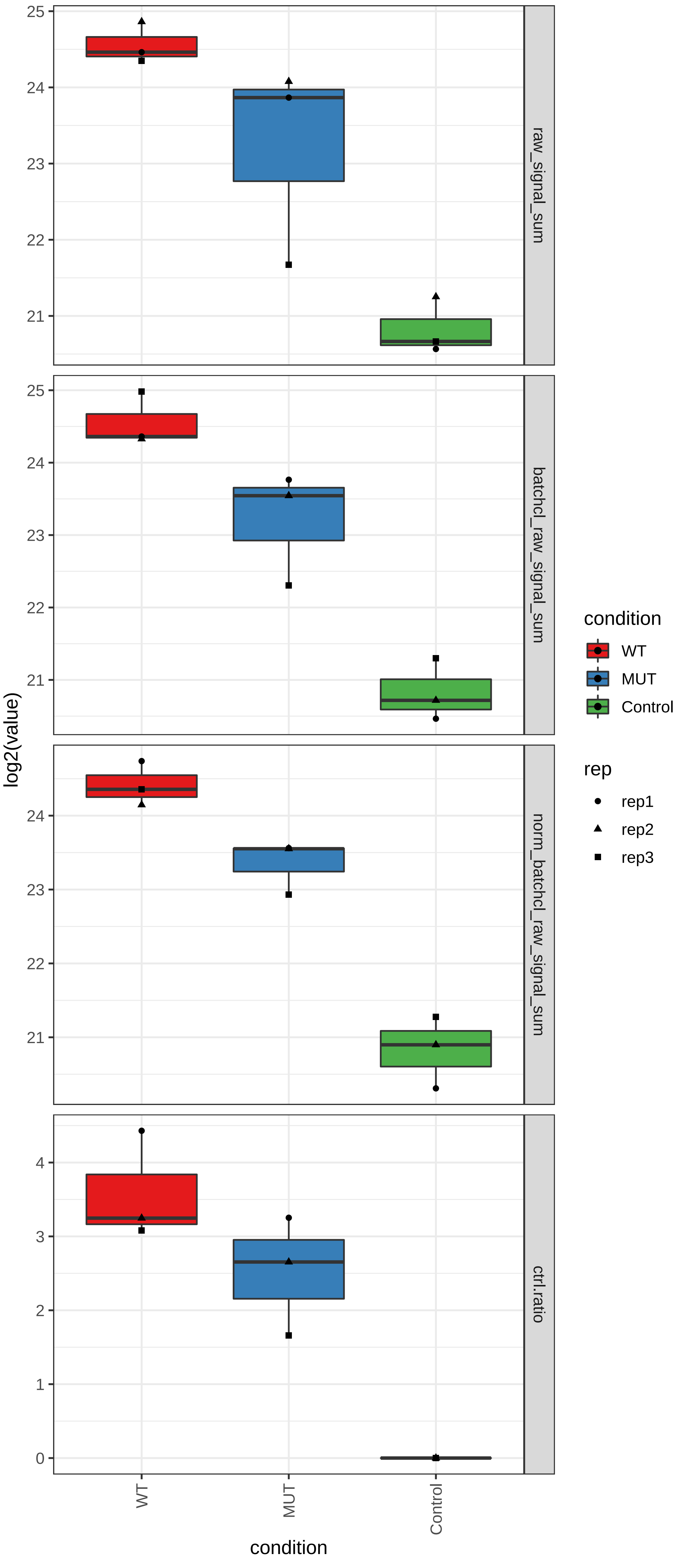

RPB8 − P20436

DNA−directed RNA polymerases I, II, and III subunit RPABC3 OS=Saccha

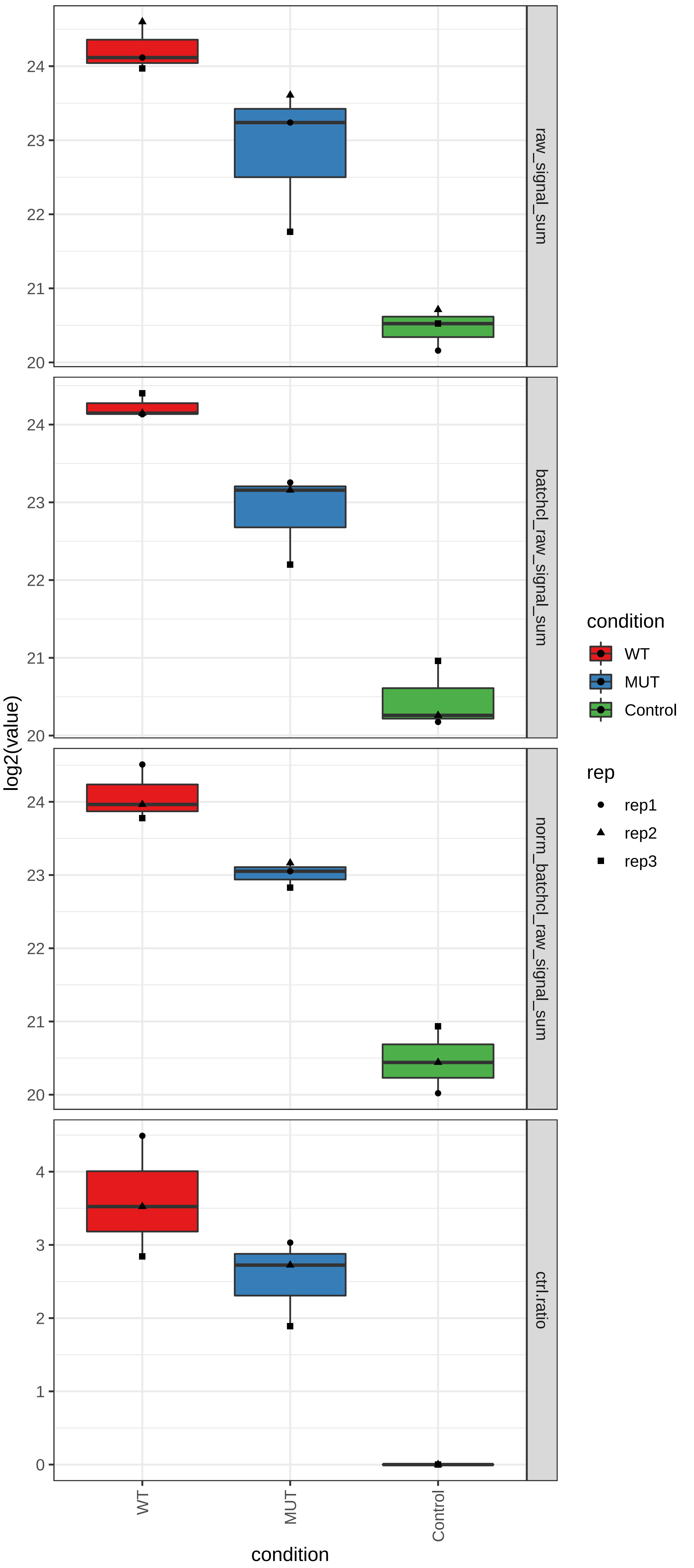

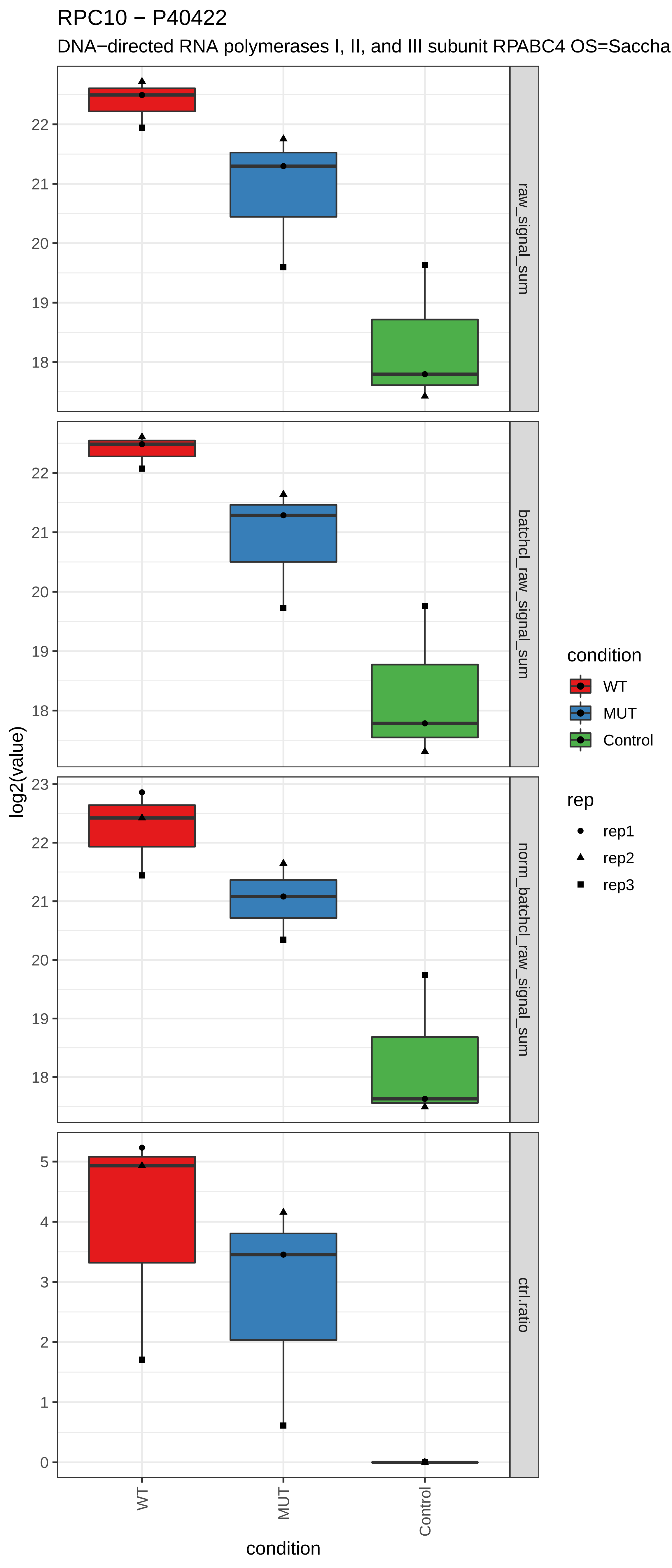

RPC10 − P40422
DNA−directed RNA polymerases I, II, and III subunit RPABC4 OS=Saccha
raw_signal_sum
batchcl_raw_signal_sum
norm_batchcl_raw_signal_sum
ctrl.ratio
log2(value)
condition
WT
MUT
Control
rep
rep1
rep2
rep3

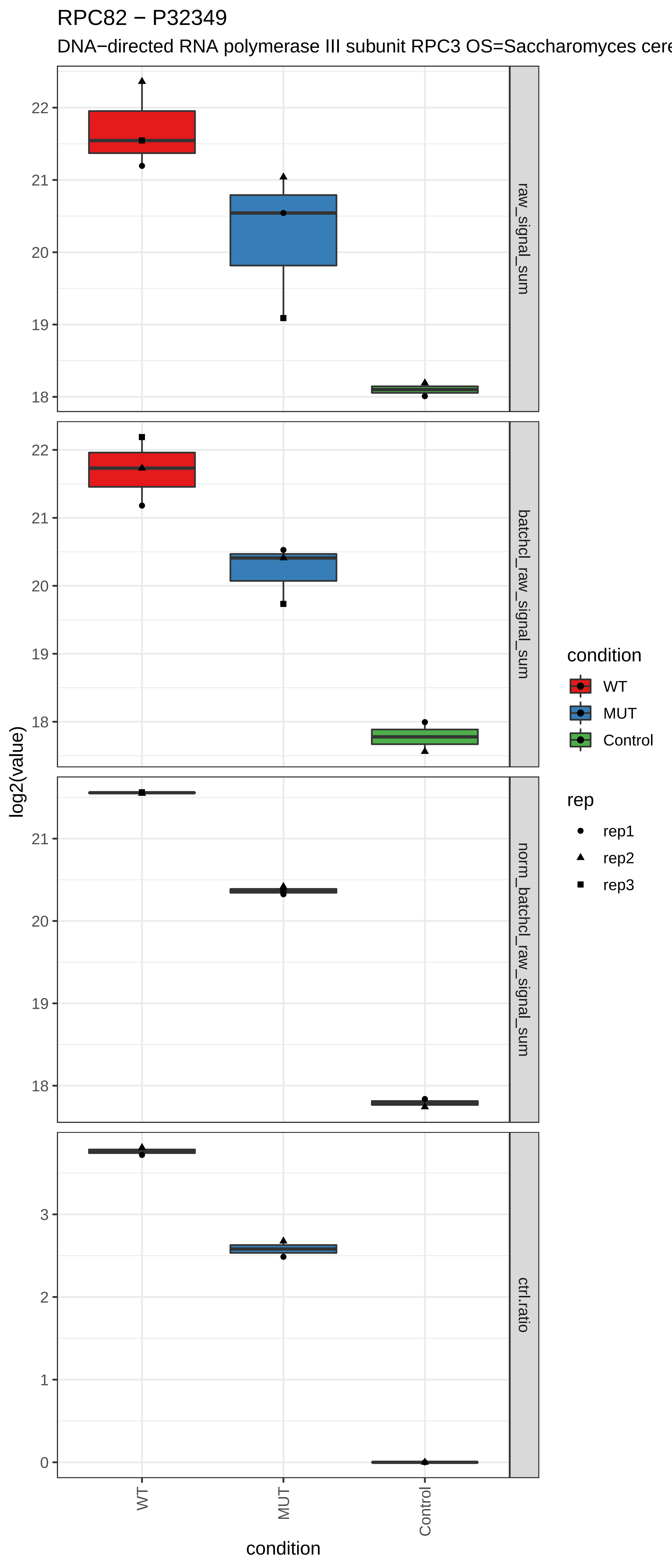
RPC82 − P32349
DNA−directed RNA polymerase III subunit RPC3 OS=Saccharomyces cere
raw_signal_sum
batchcl_raw_signal_sum
norm_batchcl_raw_signal_sum
ctrl.ratio
log2(value)
condition
WT
MUT
Control
rep
rep1
rep2
rep3
22
21
20
19
18
3
2
1
0

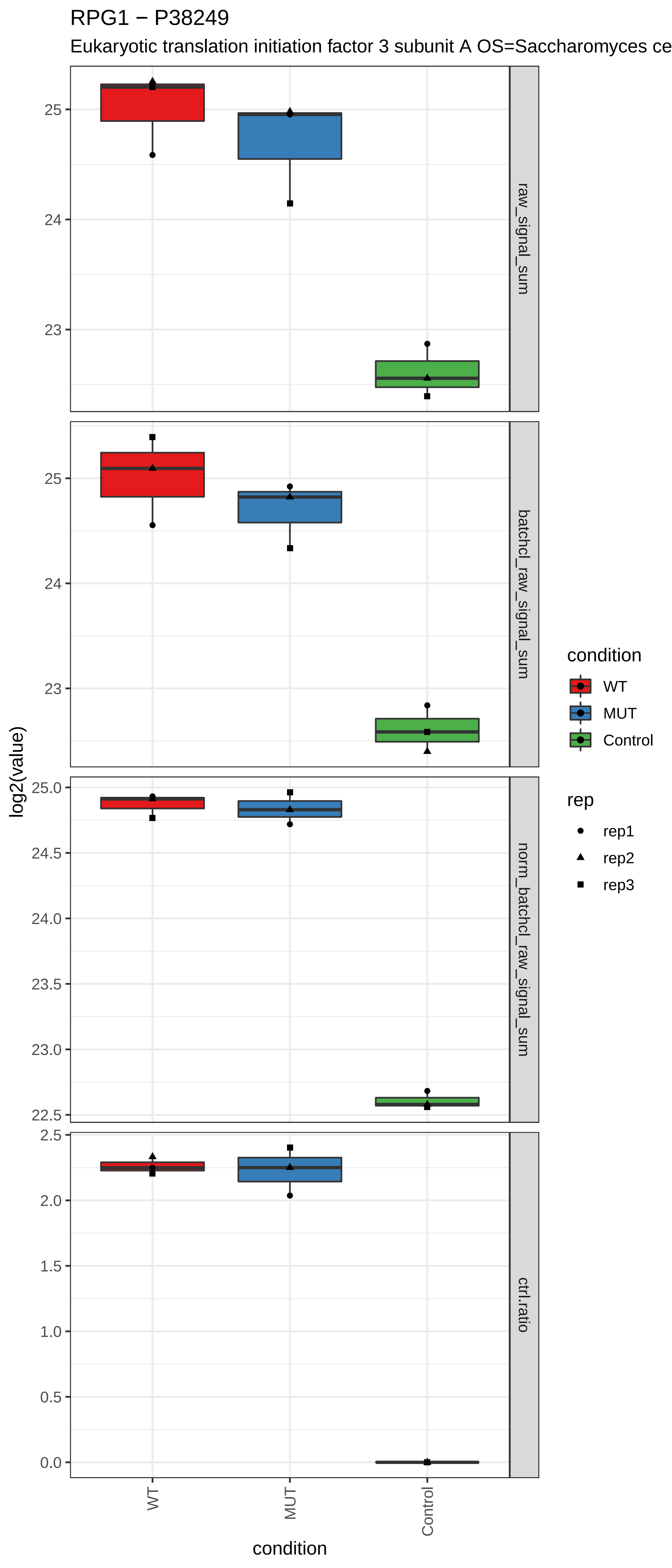
RPG1 − P38249
Eukaryotic translation initiation factor 3 subunit A OS=Saccharomyces ce
raw_signal_sum
batchcl_raw_signal_sum
norm_batchcl_raw_signal_sum
ctrl.ratio
log2(value)
condition
WT
MUT
Control
rep
rep1
rep2
rep3
25
24
23
25.0
24.5
24.0
23.5
23.0
22.5
2.5
2.0
1.5
1.0
0.5
0.0

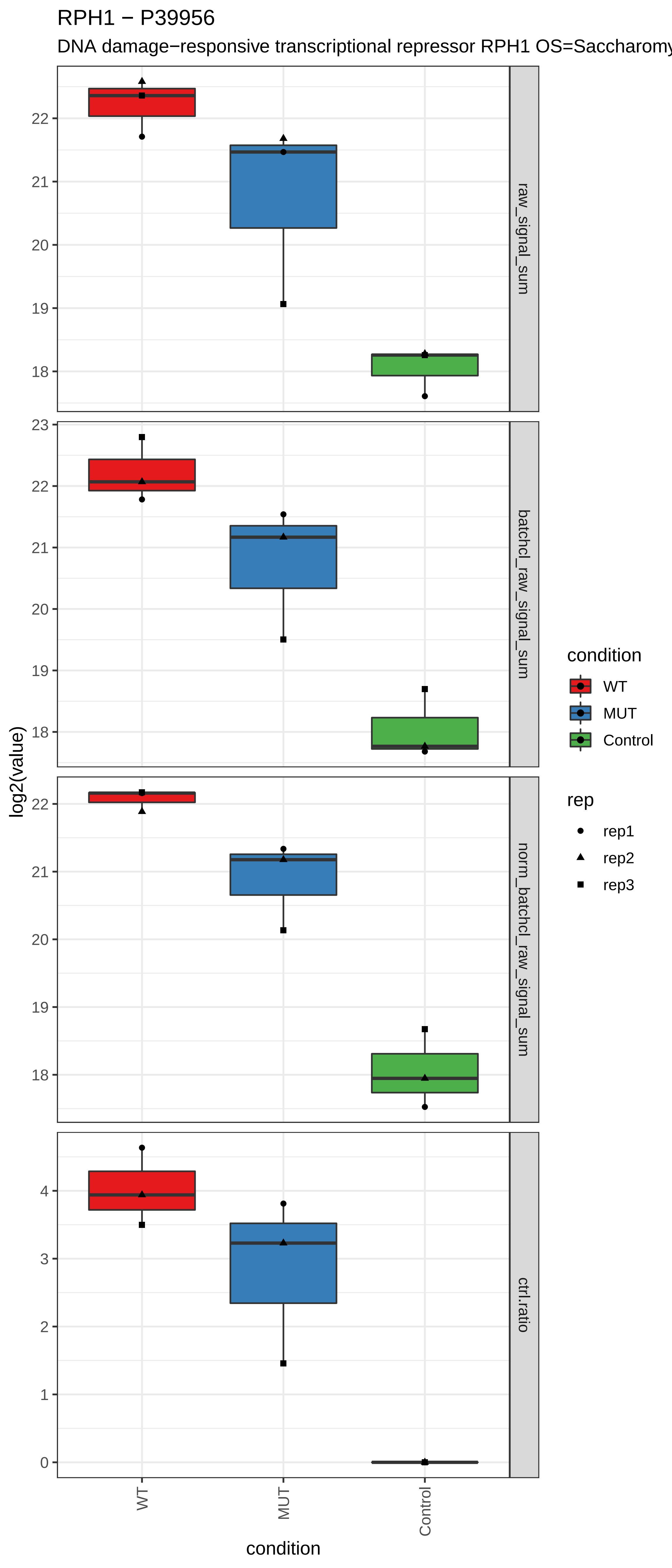
RPH1 − P39956
DNA damage−responsive transcriptional repressor RPH1 OS=Saccharomy
raw_signal_sum
batchcl_raw_signal_sum
norm_batchcl_raw_signal_sum
ctrl.ratio
log2(value)
condition
WT
MUT
Control
rep
rep1
rep2
rep3

RPL10 − P41805

60S ribosomal protein L10 OS=Saccharomyces cerevisiae (strain ATCC 20

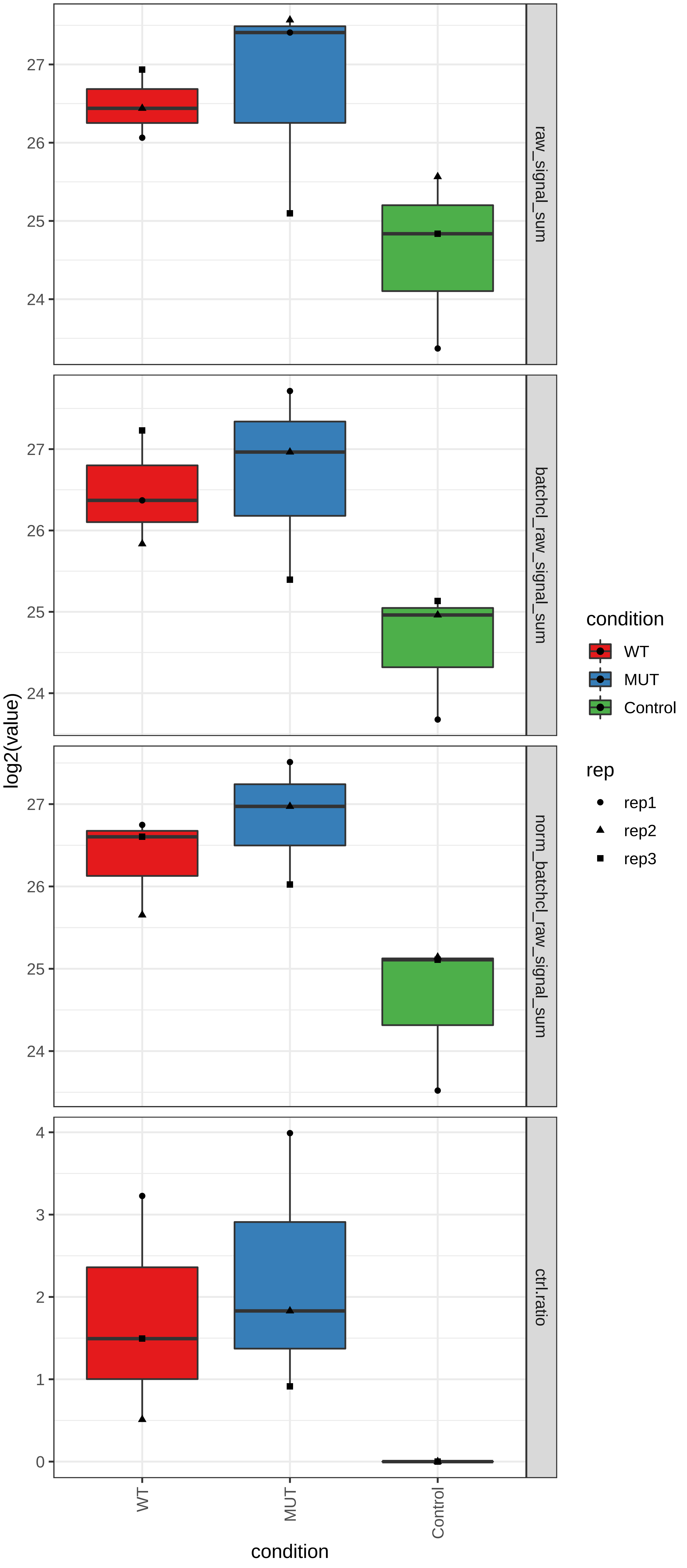

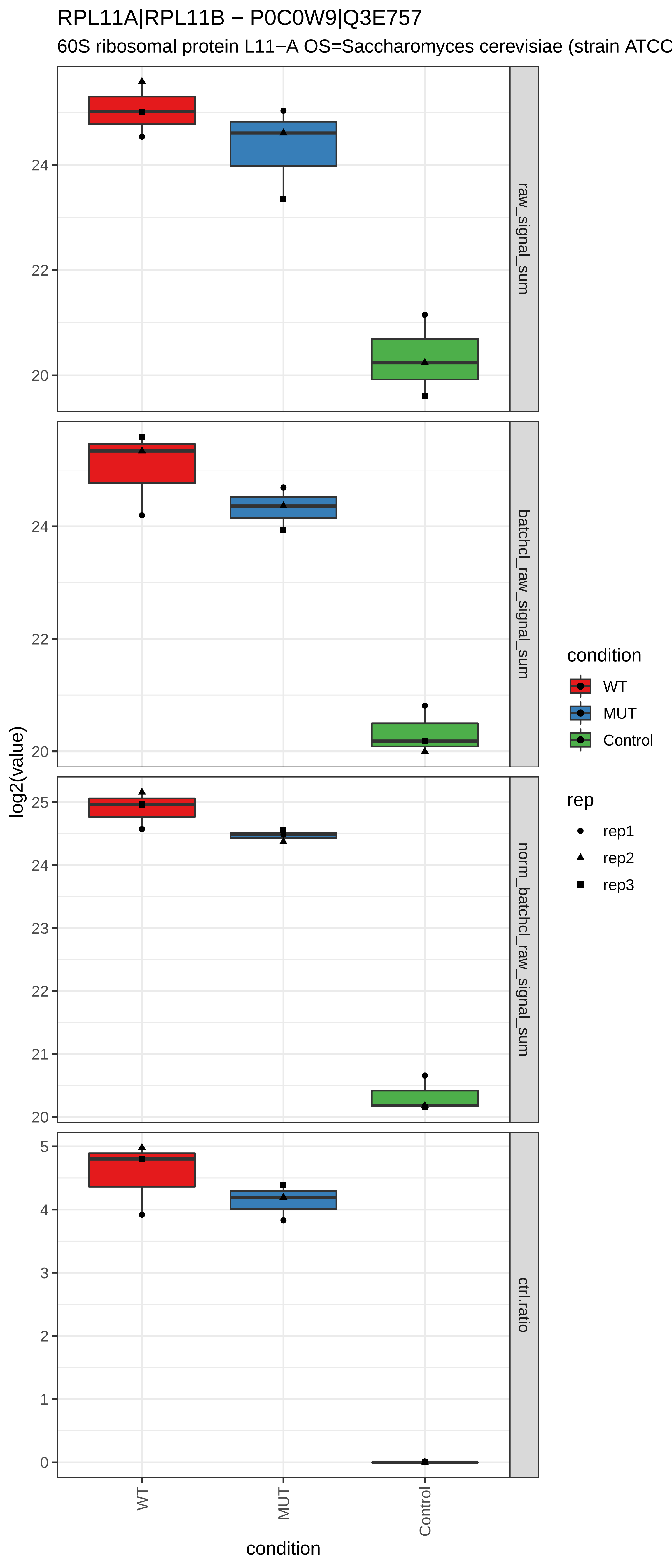

RPL11A|RPL11B − P0C0W9|Q3E757
60S ribosomal protein L11−A OS=Saccharomyces cerevisiae (strain ATCC
raw_signal_sum
batchcl_raw_signal_sum
norm_batchcl_raw_signal_sum
ctrl.ratio
log2(value)
condition
WT
MUT
Control
rep
rep1
rep2
rep3

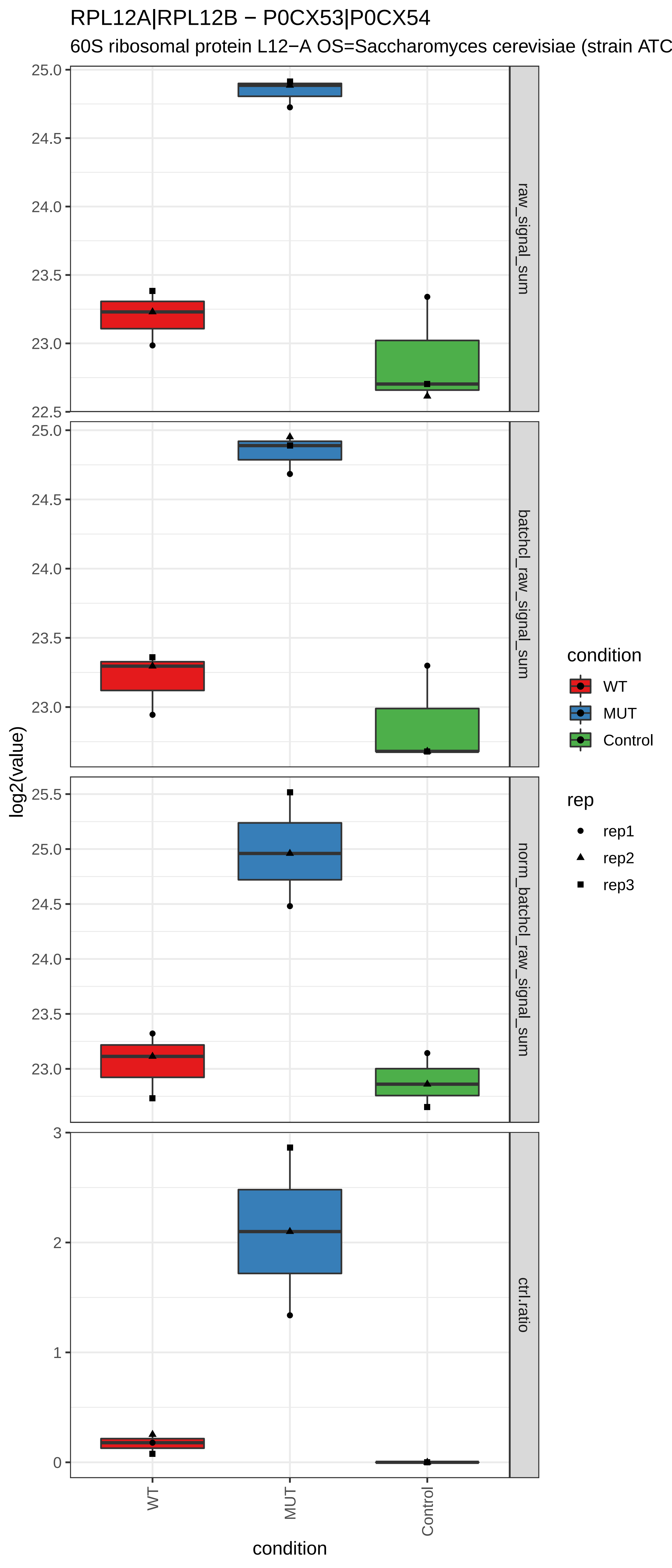
RPL12A|RPL12B − P0CX53|P0CX54
60S ribosomal protein L12−A OS=Saccharomyces cerevisiae (strain ATC
raw_signal_sum
batchcl_raw_signal_sum
norm_batchcl_raw_signal_sum
ctrl.ratio
log2(value)
condition
WT
MUT
Control
rep
rep1
rep2
rep3
25.0
24.5
24.0
23.5
23.0
22.5
25.5
3
2
1
0

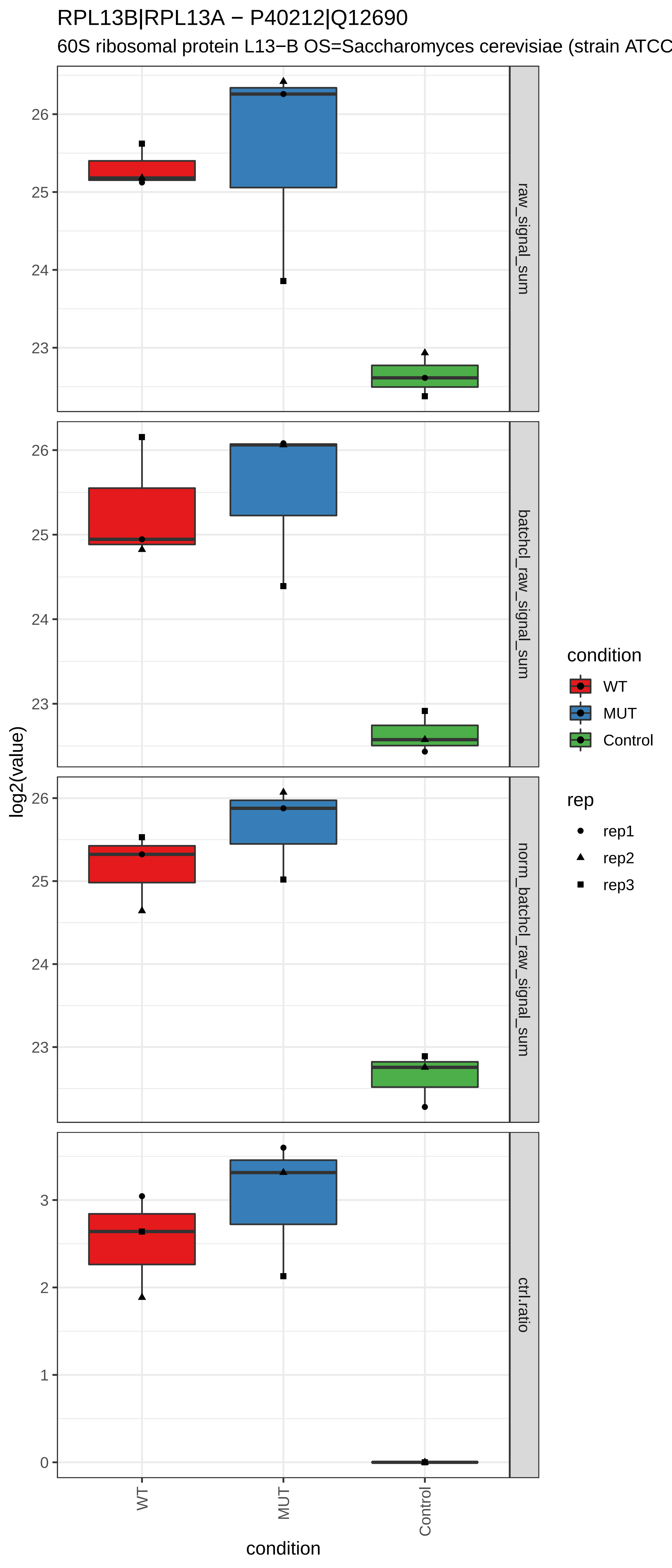

RPL13B|RPL13A − P40212|Q12690
60S ribosomal protein L13−B OS=Saccharomyces cerevisiae (strain ATCC
raw_signal_sum
batchcl_raw_signal_sum
norm_batchcl_raw_signal_sum
ctrl.ratio
log2(value)
condition
WT
MUT
Control
rep
rep1
rep2
rep3

RPL14A|RPL14B − P36105|P38754

60S ribosomal protein L14−A OS=Saccharomyces cerevisiae (strain ATCC

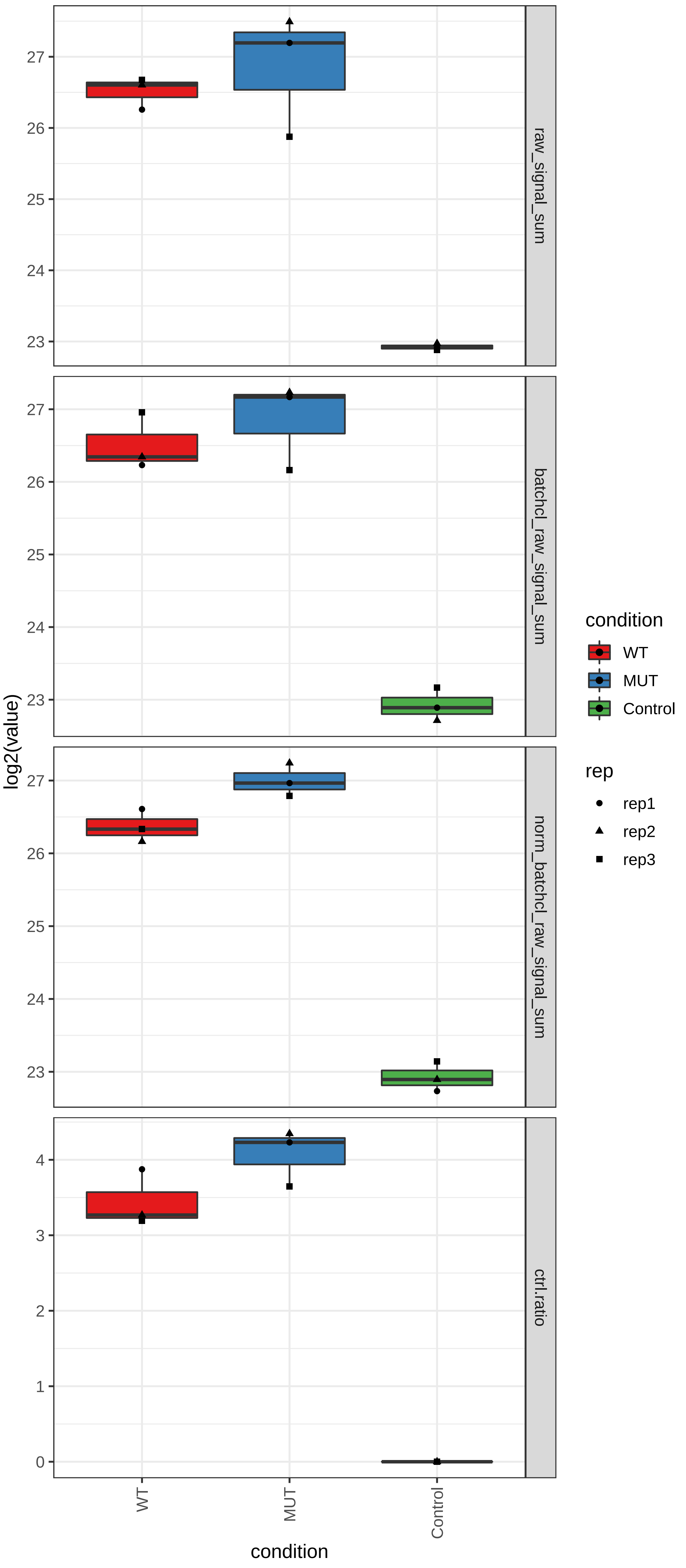

RPL15A − P05748

60S ribosomal protein L15−A OS=Saccharomyces cerevisiae (strain ATCC

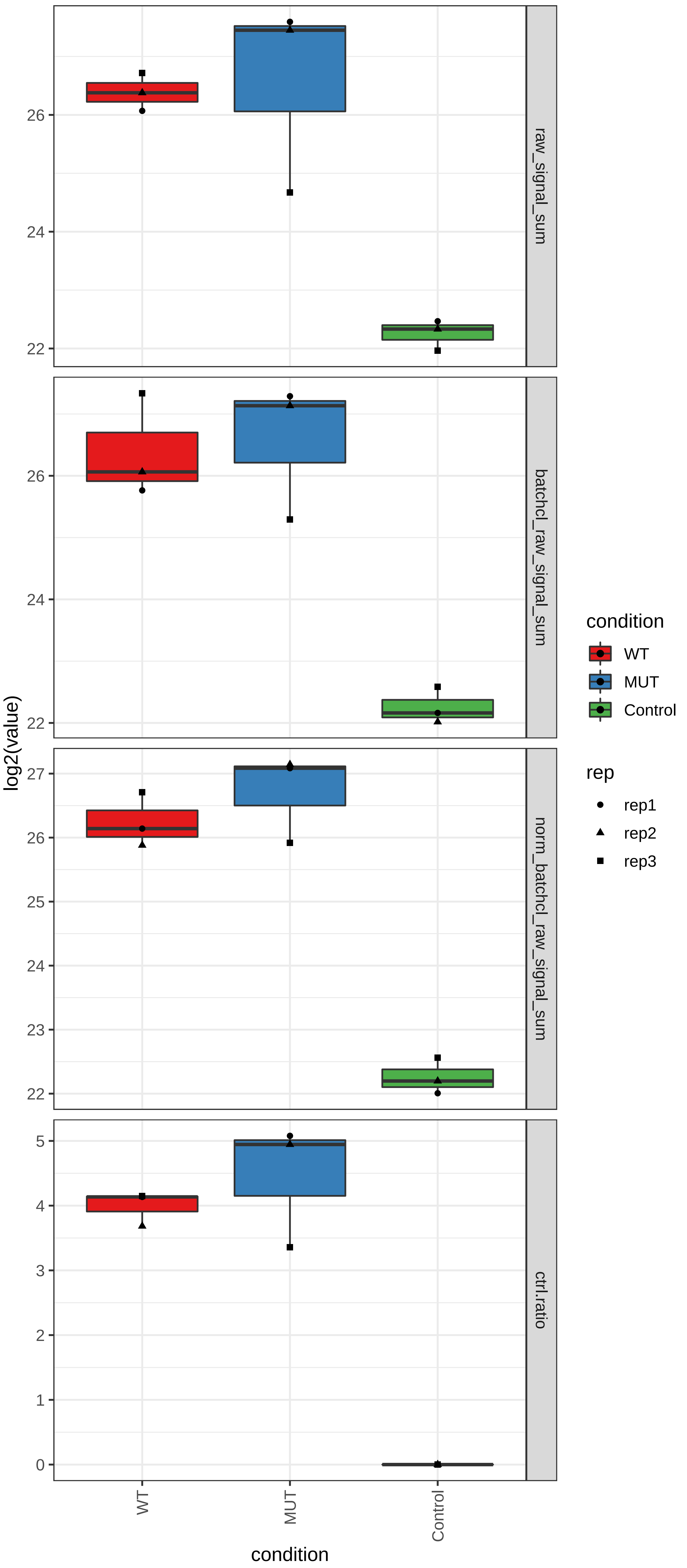

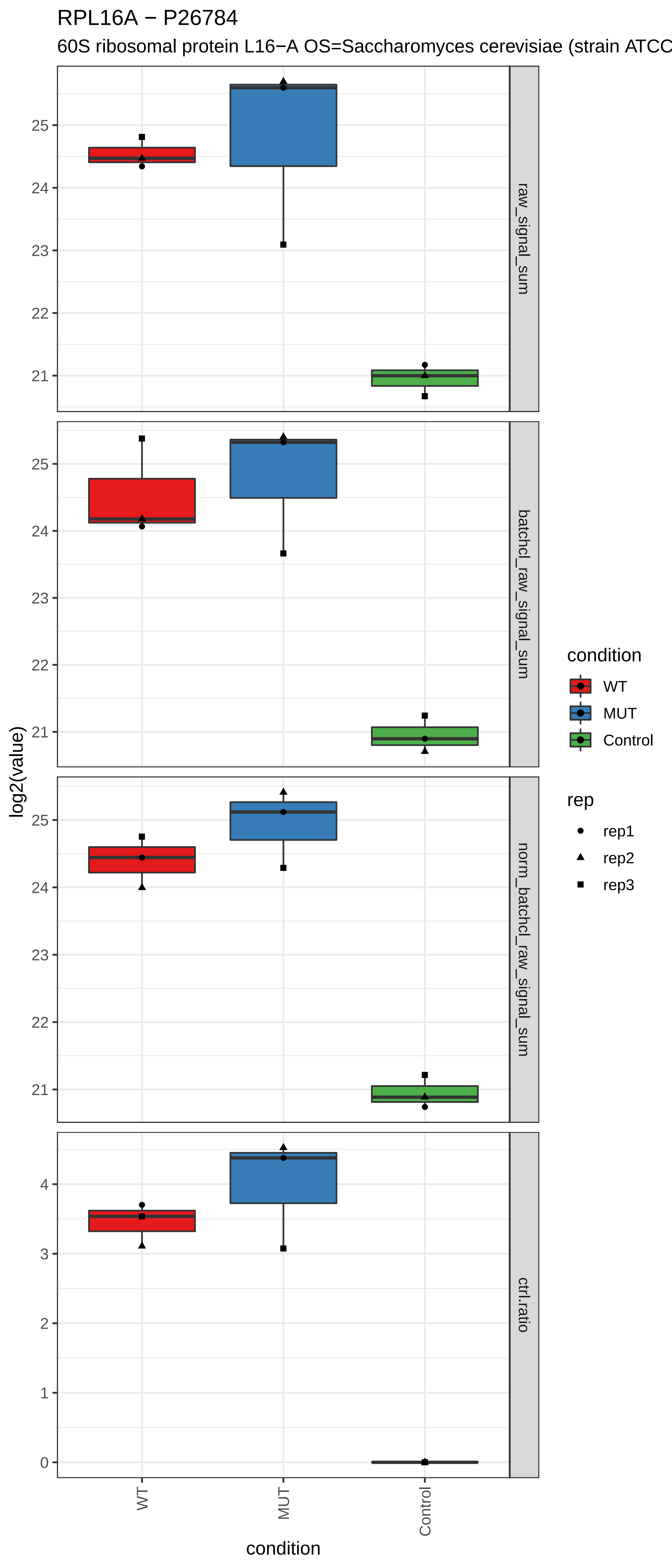

RPL16A − P26784
60S ribosomal protein L16−A OS=Saccharomyces cerevisiae (strain ATCC
raw_signal_sum
batchcl_raw_signal_sum
norm_batchcl_raw_signal_sum
ctrl.ratio
log2(value)
condition
WT
MUT
Control
rep
rep1
rep2
rep3
25
24
23
22
21
4
3
2
1
0

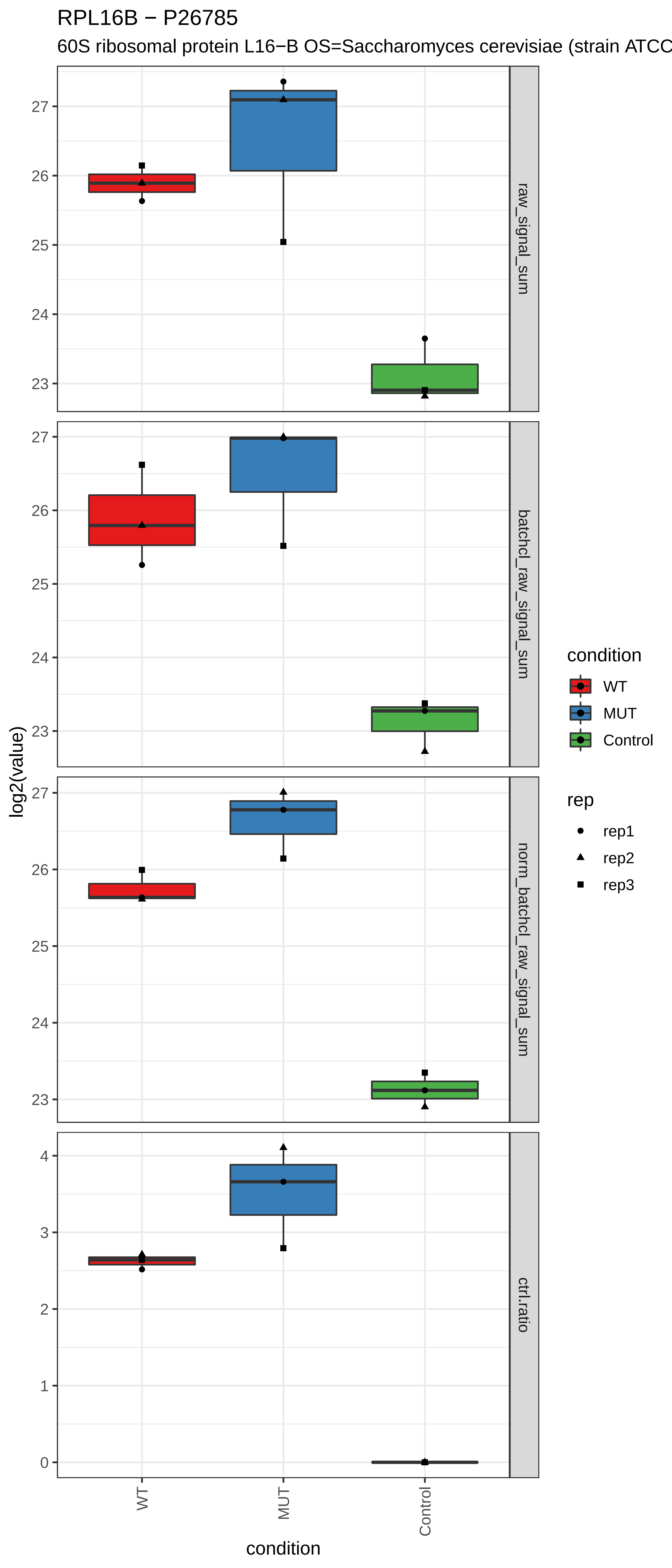

RPL16B − P26785
60S ribosomal protein L16−B OS=Saccharomyces cerevisiae (strain ATCC
raw_signal_sum
batchcl_raw_signal_sum
norm_batchcl_raw_signal_sum
ctrl.ratio
log2(value)
condition
WT
MUT
Control
rep
rep1
rep2
rep3

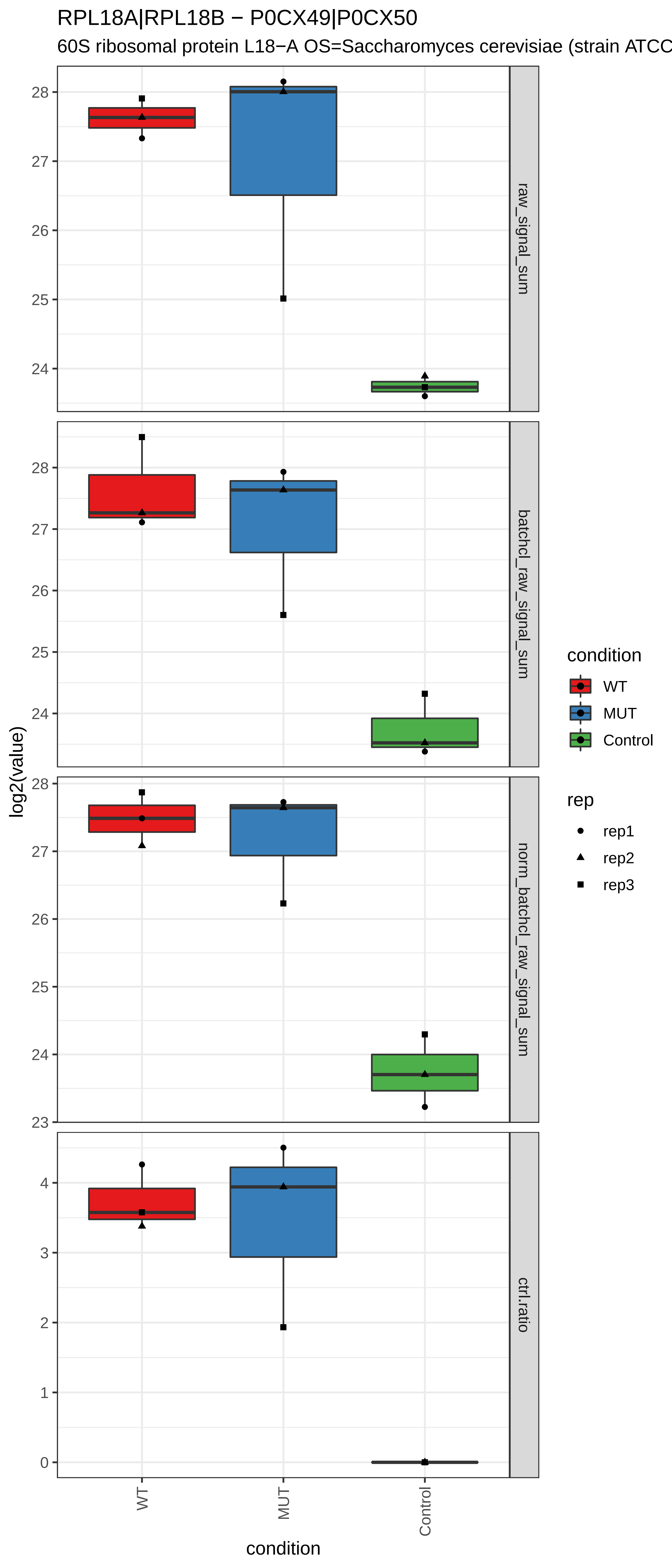
RPL18A|RPL18B − P0CX49|P0CX50
60S ribosomal protein L18−A OS=Saccharomyces cerevisiae (strain ATCC
raw_signal_sum
batchcl_raw_signal_sum
norm_batchcl_raw_signal_sum
ctrl.ratio
log2(value)
condition
WT
MUT
Control
rep
rep1
rep2
rep3

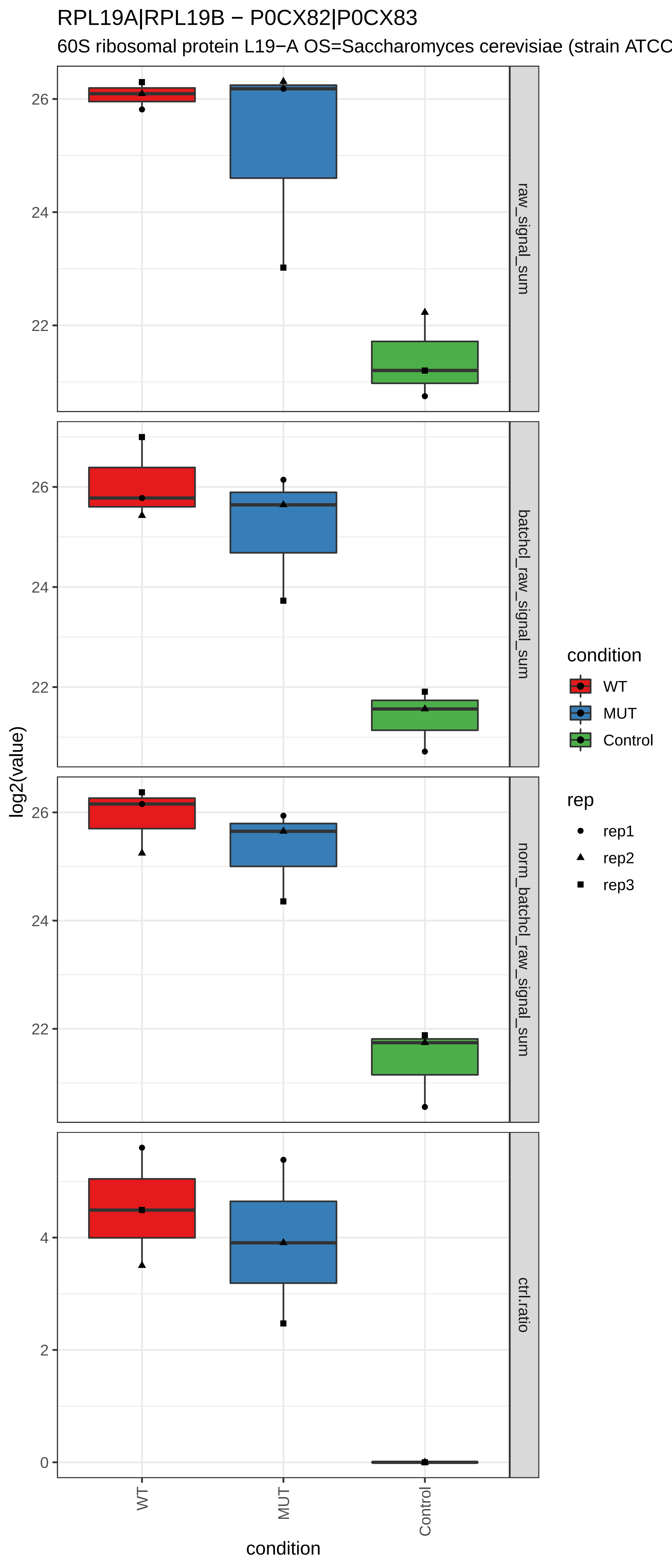
RPL19A|RPL19B − P0CX82|P0CX83
60S ribosomal protein L19−A OS=Saccharomyces cerevisiae (strain ATCC
raw_signal_sum
batchcl_raw_signal_sum
norm_batchcl_raw_signal_sum
ctrl.ratio
log2(value)
condition
WT
MUT
Control
rep
rep1
rep2
rep3
26
24
22
4
2
0

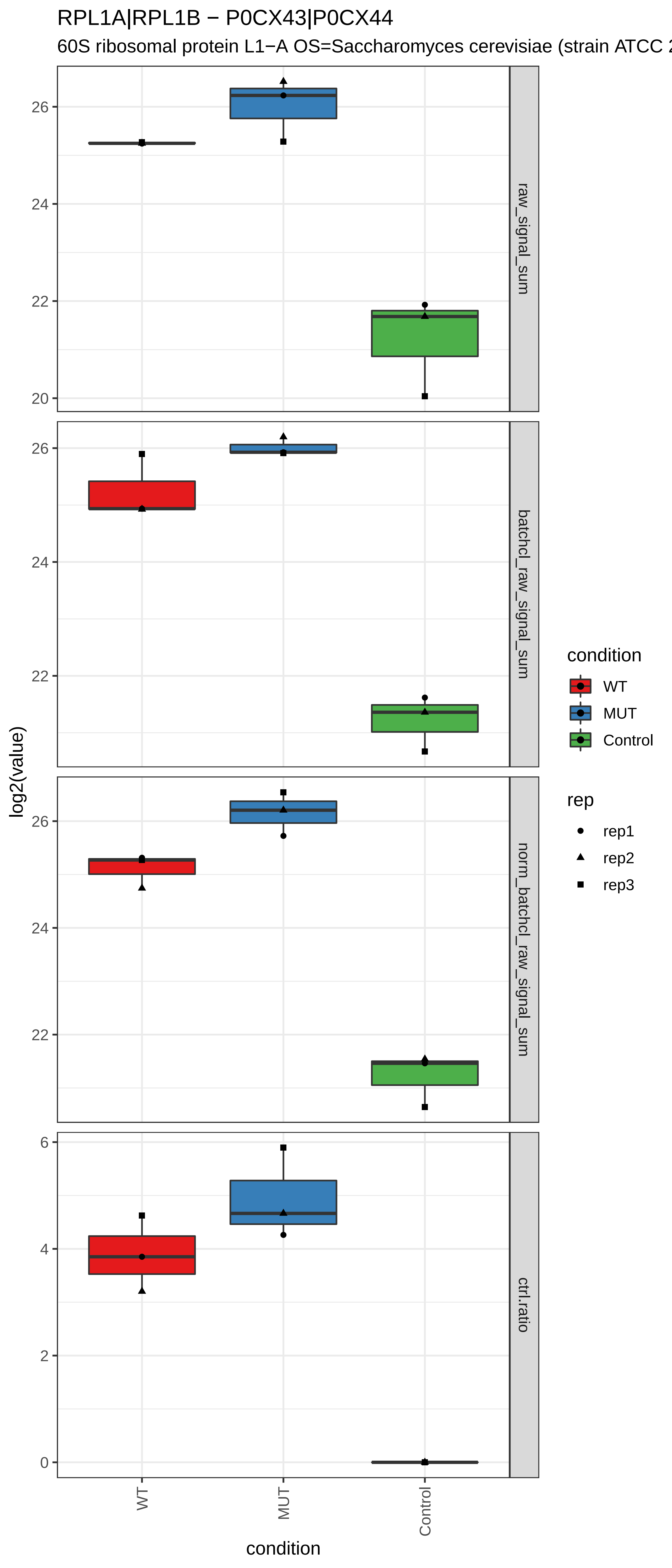

RPL1A|RPL1B − P0CX43|P0CX44
60S ribosomal protein L1−A OS=Saccharomyces cerevisiae (strain ATCC 2
raw_signal_sum
batchcl_raw_signal_sum
norm_batchcl_raw_signal_sum
ctrl.ratio
log2(value)
condition
WT
MUT
Control
rep
rep1
rep2
rep3

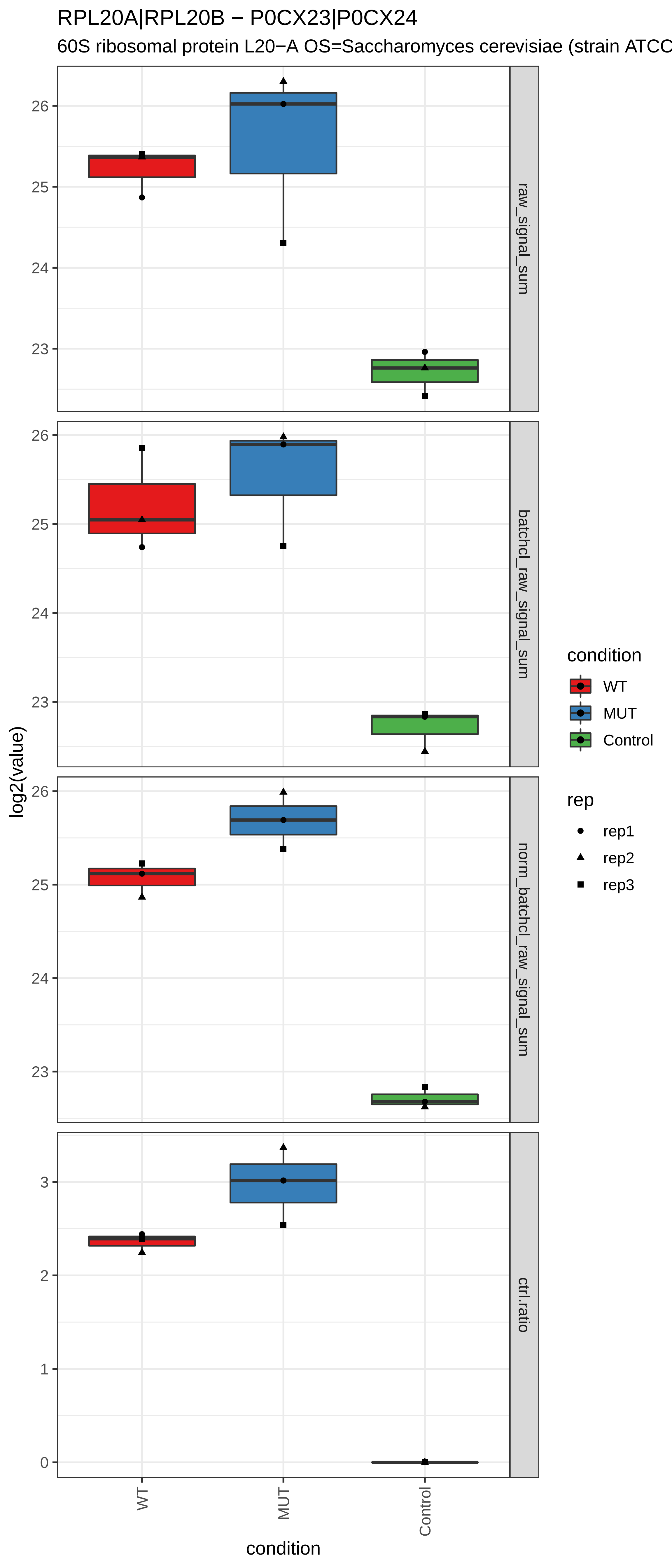
RPL20A|RPL20B − P0CX23|P0CX24
60S ribosomal protein L20−A OS=Saccharomyces cerevisiae (strain ATCC
raw_signal_sum
batchcl_raw_signal_sum
norm_batchcl_raw_signal_sum
ctrl.ratio
log2(value)
condition
WT
MUT
Control
rep
rep1
rep2
rep3

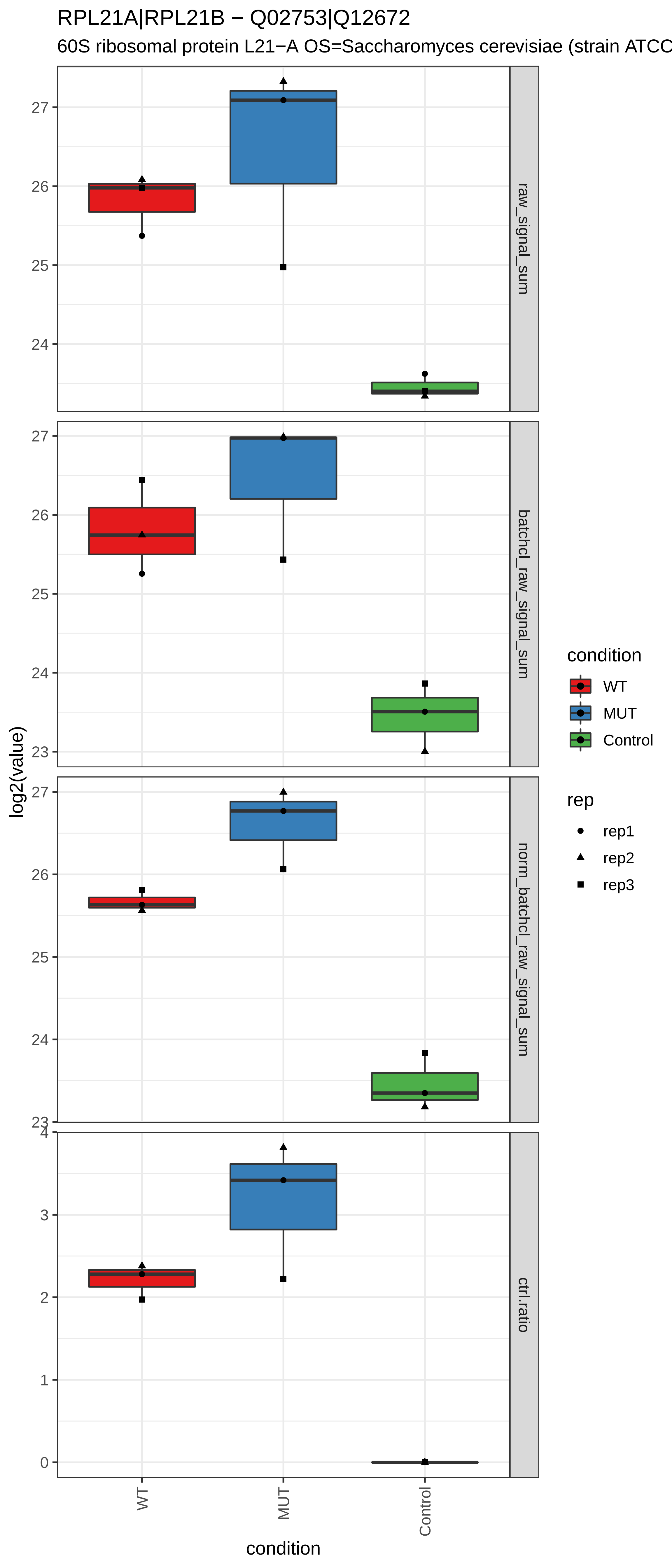

RPL21A|RPL21B − Q02753|Q12672
60S ribosomal protein L21−A OS=Saccharomyces cerevisiae (strain ATCC
raw_signal_sum
batchcl_raw_signal_sum
norm_batchcl_raw_signal_sum
ctrl.ratio
log2(value)
condition
WT
MUT
Control
rep
rep1
rep2
rep3

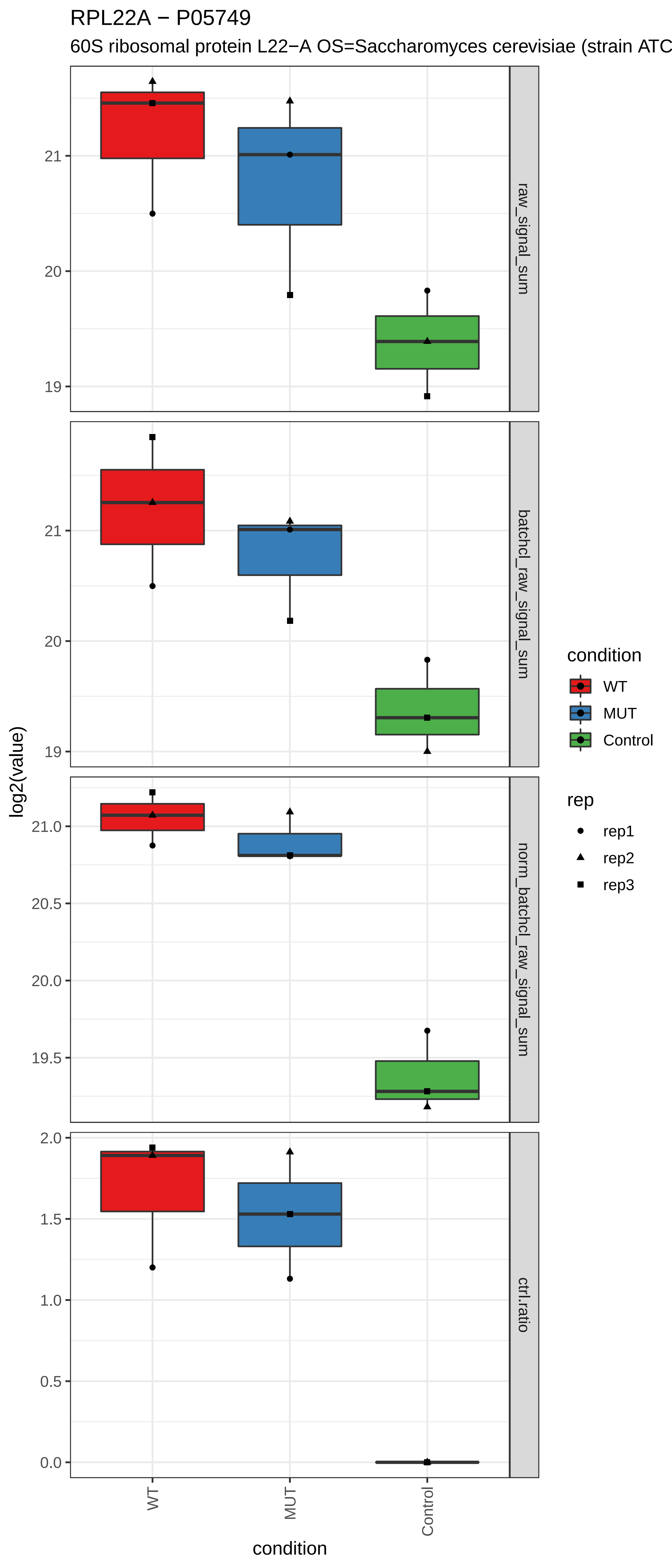

RPL22A − P05749
60S ribosomal protein L22−A OS=Saccharomyces cerevisiae (strain ATC
raw_signal_sum
batchcl_raw_signal_sum
norm_batchcl_raw_signal_sum
ctrl.ratio
log2(value)
condition
WT
MUT
Control
rep
rep1
rep2
rep3

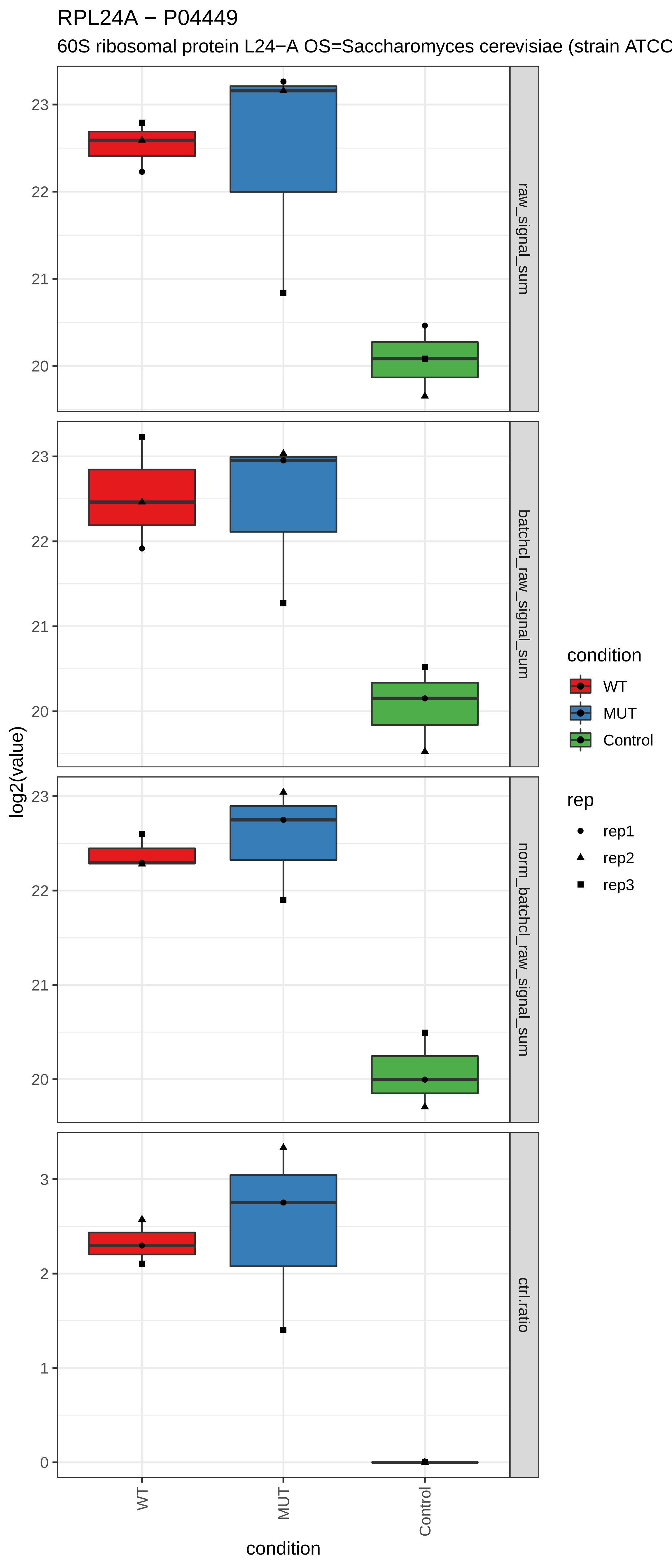

RPL24A − P04449
60S ribosomal protein L24−A OS=Saccharomyces cerevisiae (strain ATCC
raw_signal_sum
batchcl_raw_signal_sum
norm_batchcl_raw_signal_sum
ctrl.ratio
log2(value)
condition
WT
MUT
Control
rep
rep1
rep2
rep3
23
22
21
20
3
2
1
0

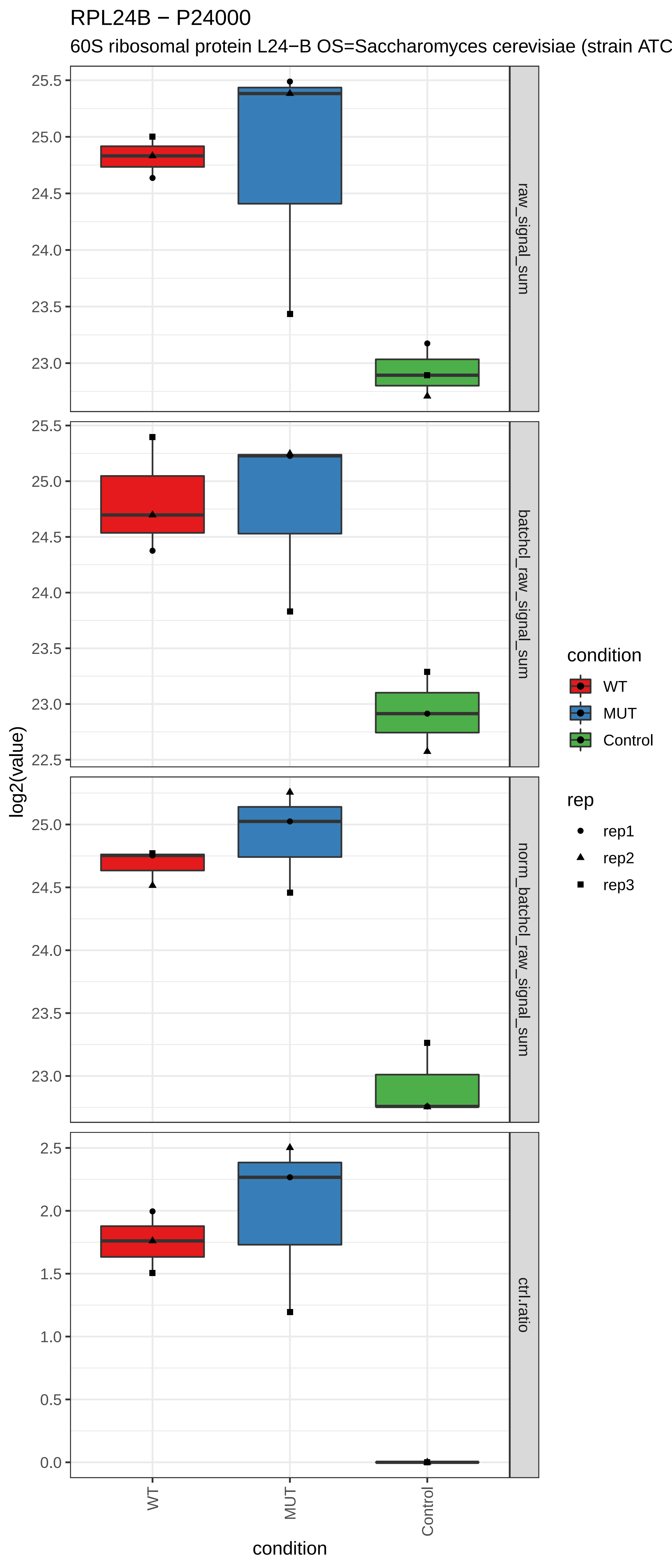

RPL24B − P24000
60S ribosomal protein L24−B OS=Saccharomyces cerevisiae (strain ATC
log2(value)
raw_signal_sum
batchcl_raw_signal_sum
norm_batchcl_raw_signal_sum
ctrl.ratio
25.5
25.0
24.5
24.0
23.5
23.0
22.5
2.5
2.0
1.5
1.0
0.5
0.0
WT
MUT
Control
condition
condition
WT
MUT
Control
rep
rep1
rep2
rep3

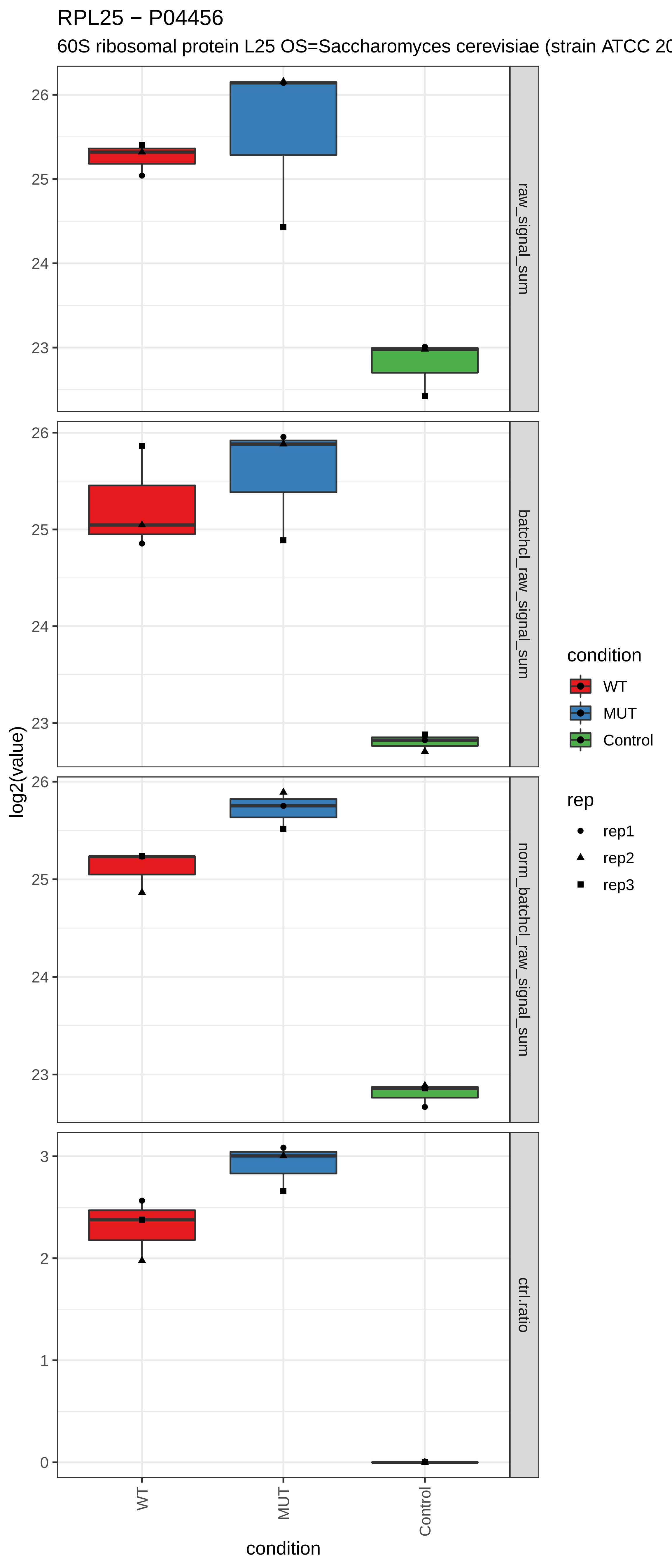

RPL25 − P04456
60S ribosomal protein L25 OS=Saccharomyces cerevisiae (strain ATCC 20
raw_signal_sum
batchcl_raw_signal_sum
norm_batchcl_raw_signal_sum
ctrl.ratio
log2(value)
condition
WT
MUT
Control
rep
rep1
rep2
rep3
26
25
24
23
3
2
1
0

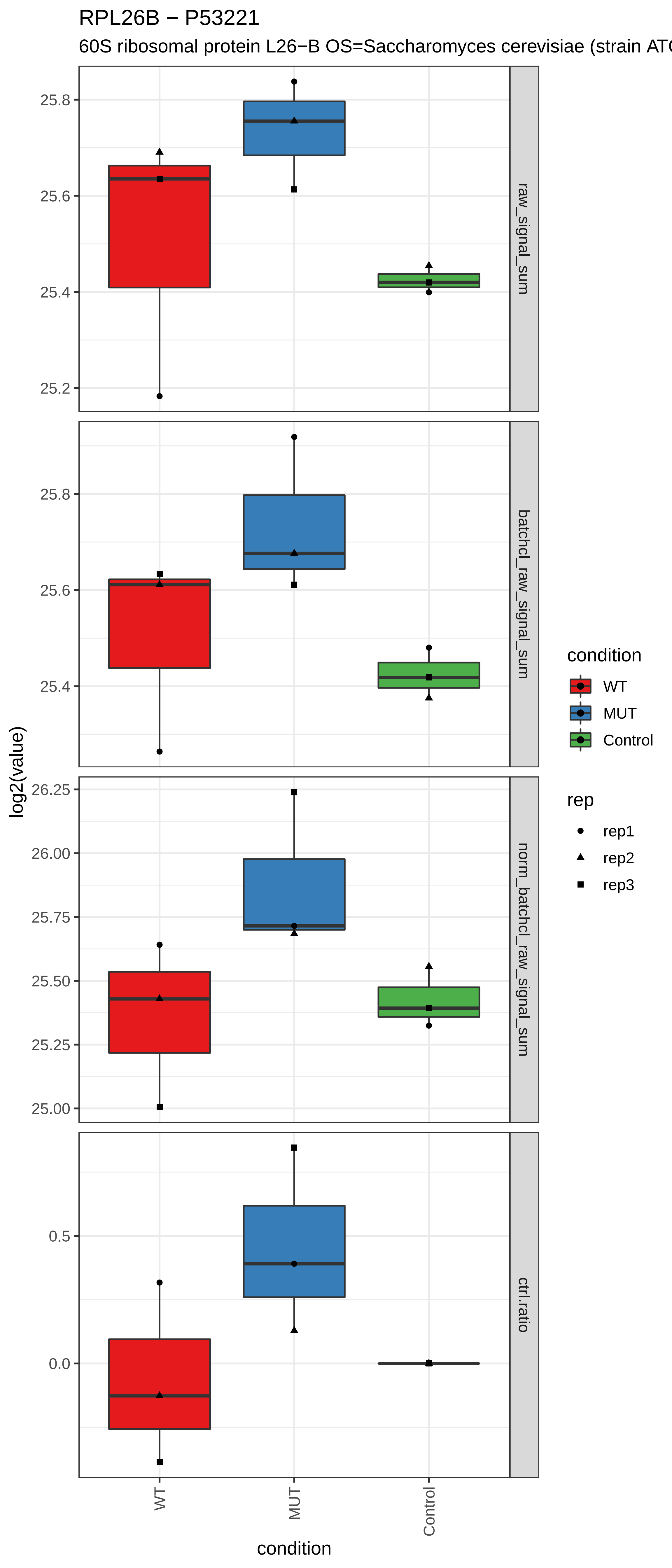

RPL26B − P53221
60S ribosomal protein L26−B OS=Saccharomyces cerevisiae (strain ATC
raw_signal_sum
batchcl_raw_signal_sum
norm_batchcl_raw_signal_sum
ctrl.ratio
log2(value)
25.8
25.6
25.4
25.2
26.25
26.00
25.75
25.50
25.25
25.00
0.5
0.0
WT
MUT
Control
condition
condition
WT
MUT
Control
rep
rep1
rep2
rep3

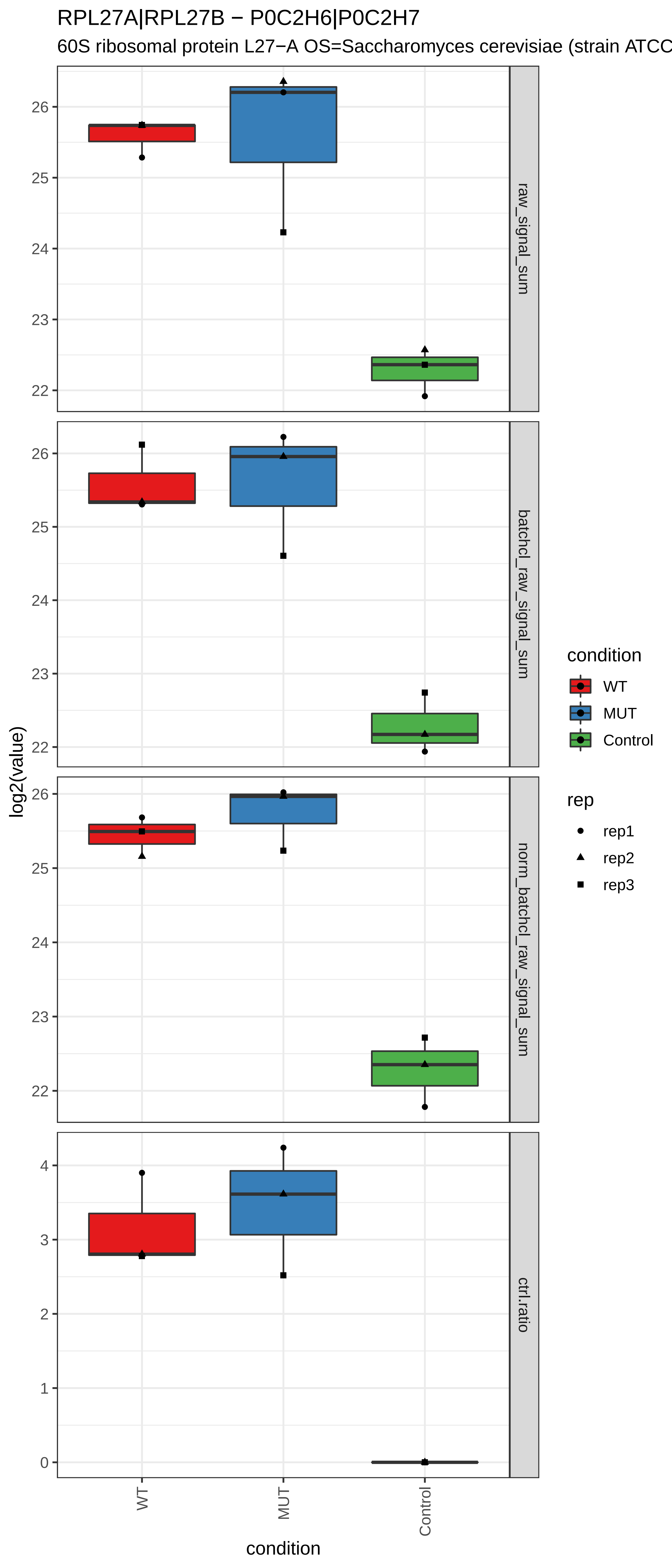
RPL27A|RPL27B − P0C2H6|P0C2H7
60S ribosomal protein L27−A OS=Saccharomyces cerevisiae (strain ATCC
raw_signal_sum
batchcl_raw_signal_sum
norm_batchcl_raw_signal_sum
ctrl.ratio
log2(value)
condition
WT
MUT
Control
rep
rep1
rep2
rep3

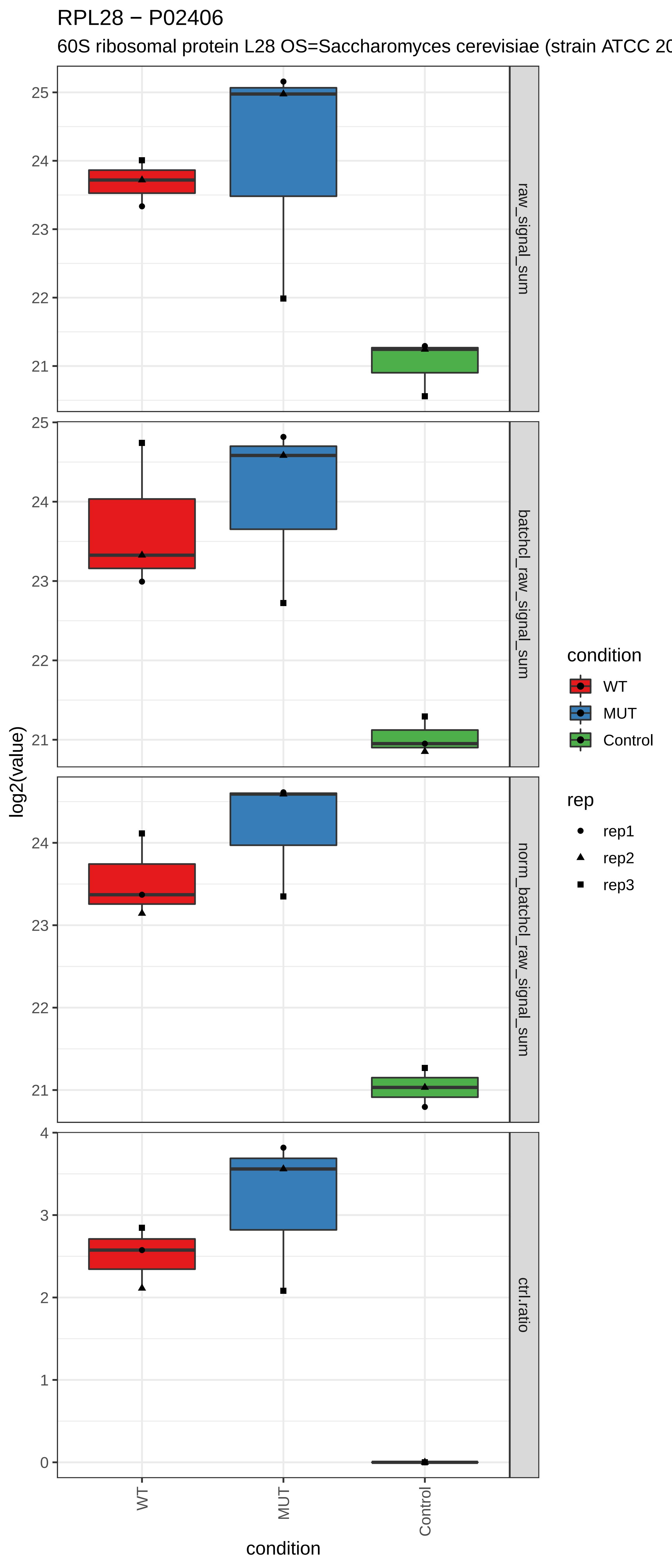
RPL28 − P02406
60S ribosomal protein L28 OS=Saccharomyces cerevisiae (strain ATCC 20
raw_signal_sum
batchcl_raw_signal_sum
norm_batchcl_raw_signal_sum
ctrl.ratio
log2(value)
condition
WT
MUT
Control
rep
rep1
rep2
rep3

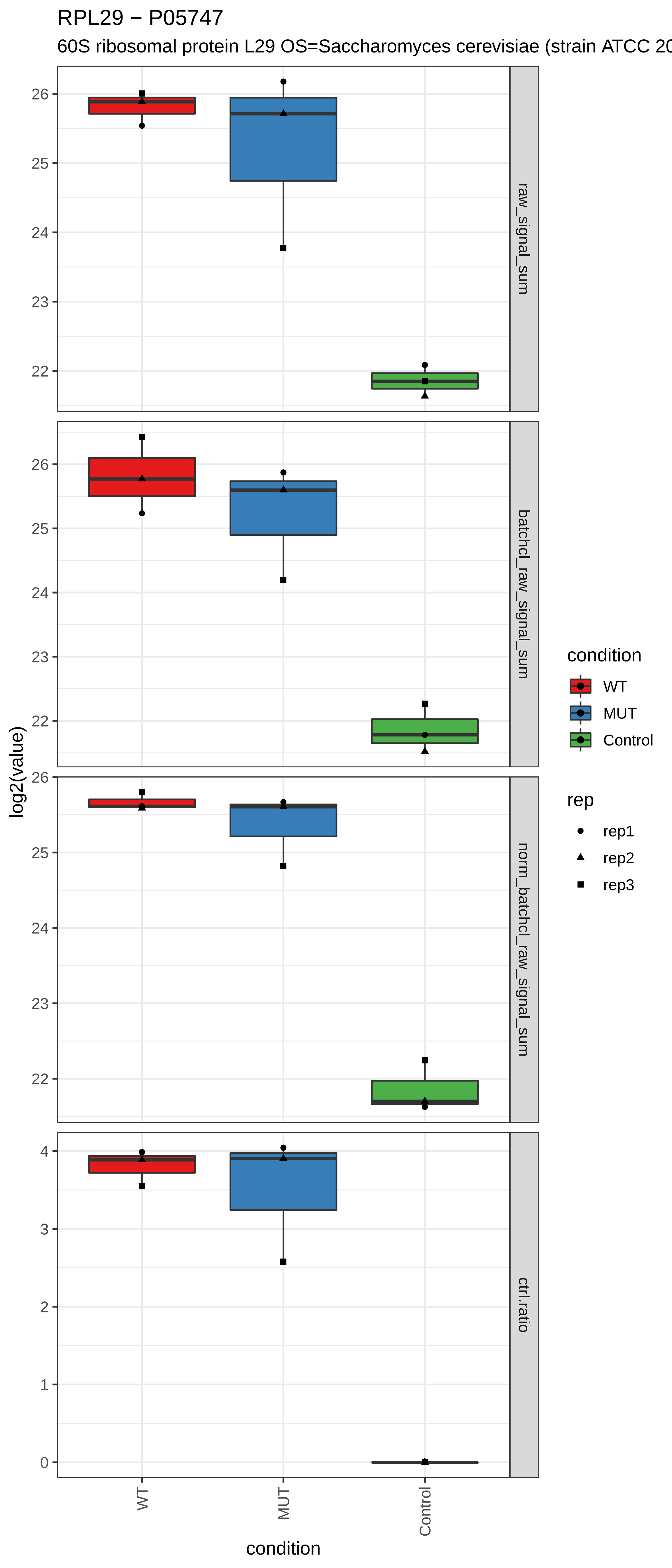

RPL29 − P05747
60S ribosomal protein L29 OS=Saccharomyces cerevisiae (strain ATCC 20
log2(value)
raw_signal_sum
batchcl_raw_signal_sum
norm_batchcl_raw_signal_sum
ctrl.ratio
26
25
24
23
22
4
3
2
1
0
WT
MUT
Control
condition
condition
WT
MUT
Control
rep
rep1
rep2
rep3

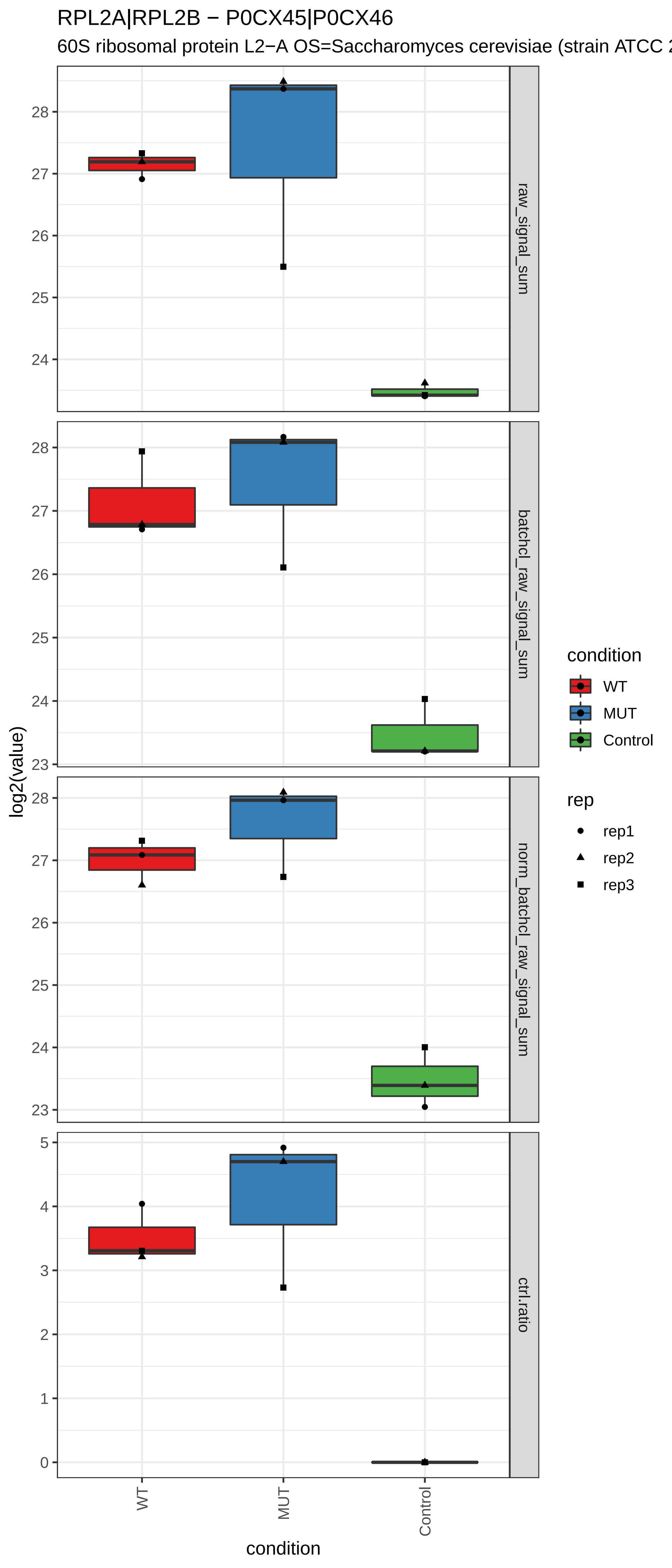
RPL2A|RPL2B − P0CX45|P0CX46
60S ribosomal protein L2−A OS=Saccharomyces cerevisiae (strain ATCC 2
raw_signal_sum
batchcl_raw_signal_sum
norm_batchcl_raw_signal_sum
ctrl.ratio
log2(value)
condition
WT
MUT
Control
condition
WT
MUT
Control
rep
rep1
rep2
rep3

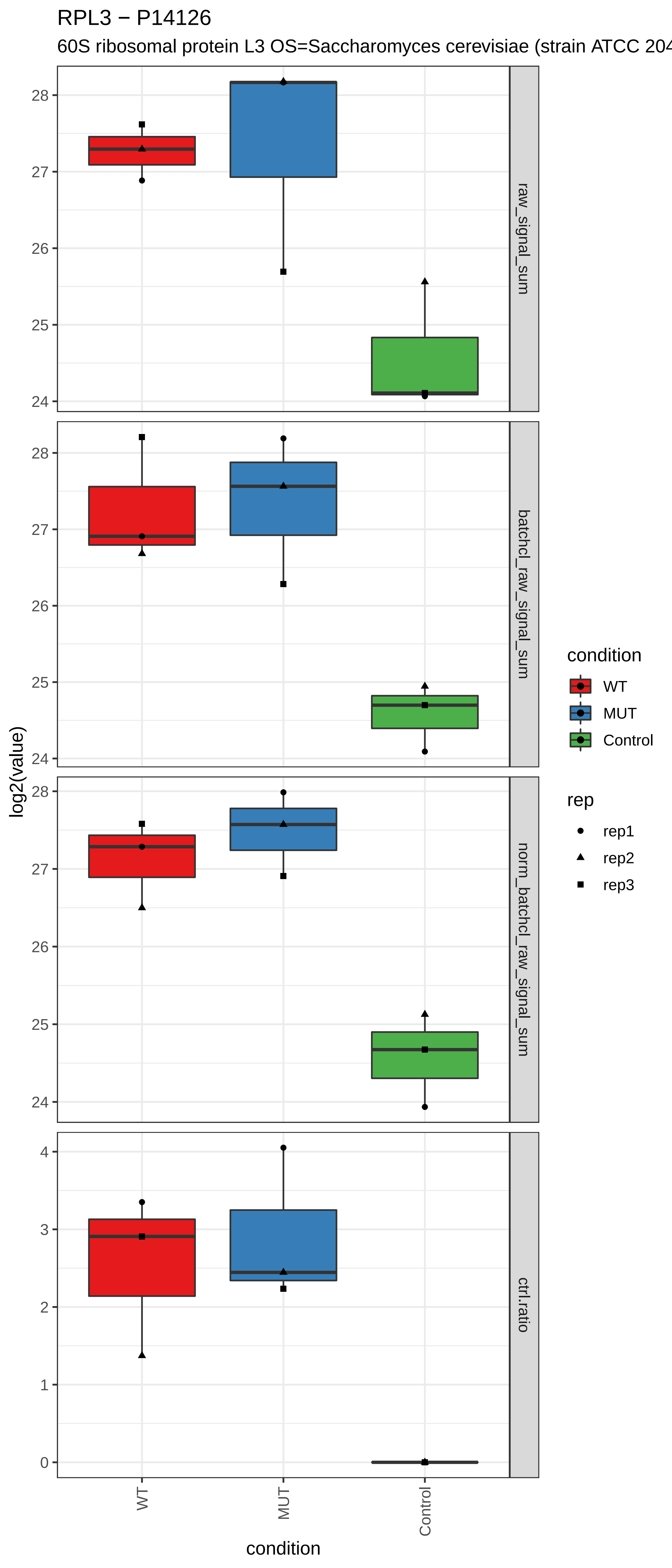
RPL3 − P14126
60S ribosomal protein L3 OS=Saccharomyces cerevisiae (strain ATCC 204
raw_signal_sum
batchcl_raw_signal_sum
norm_batchcl_raw_signal_sum
ctrl.ratio
log2(value)
condition
WT
MUT
Control
rep
rep1
rep2
rep3
28
27
26
25
24
4
3
2
1
0

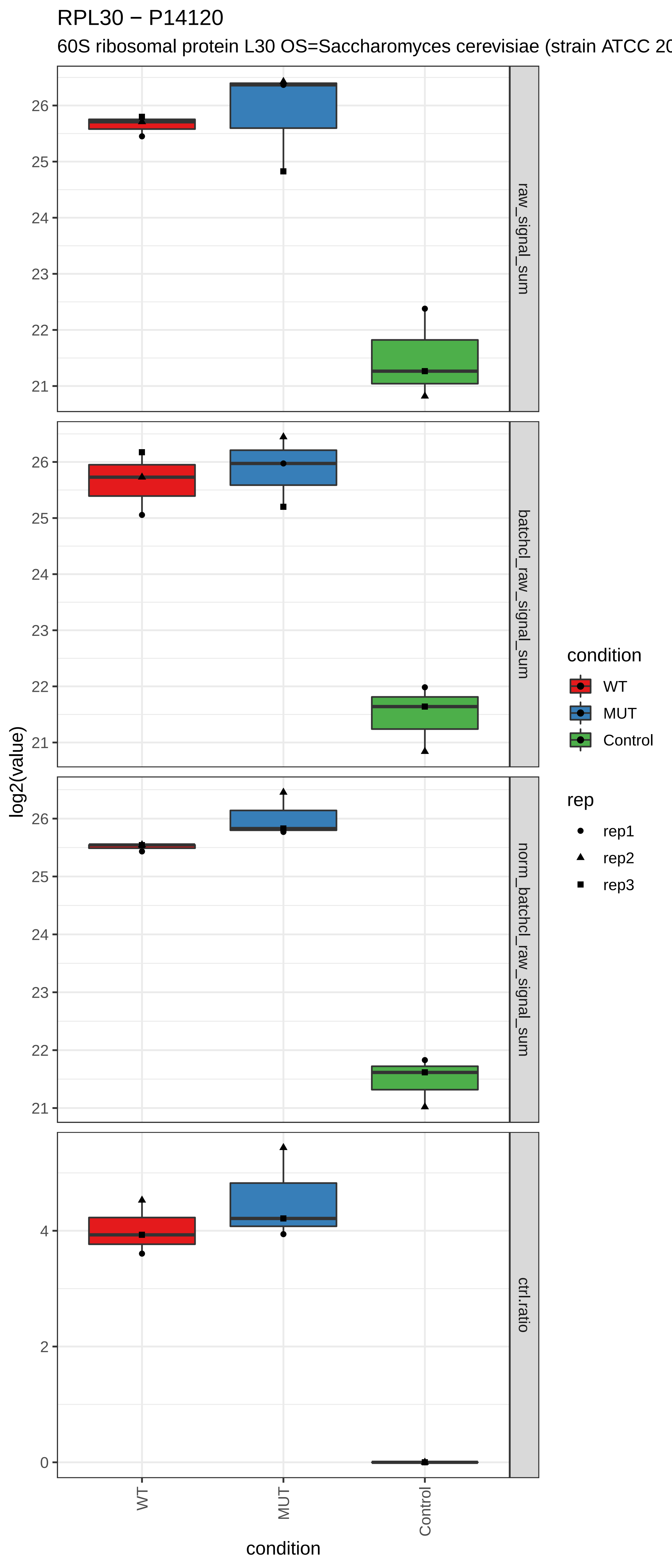

RPL30 − P14120
60S ribosomal protein L30 OS=Saccharomyces cerevisiae (strain ATCC 20
raw_signal_sum
batchcl_raw_signal_sum
norm_batchcl_raw_signal_sum
ctrl.ratio
log2(value)
condition
WT
MUT
Control
rep
rep1
rep2
rep3
26
25
24
23
22
21
4
2
0

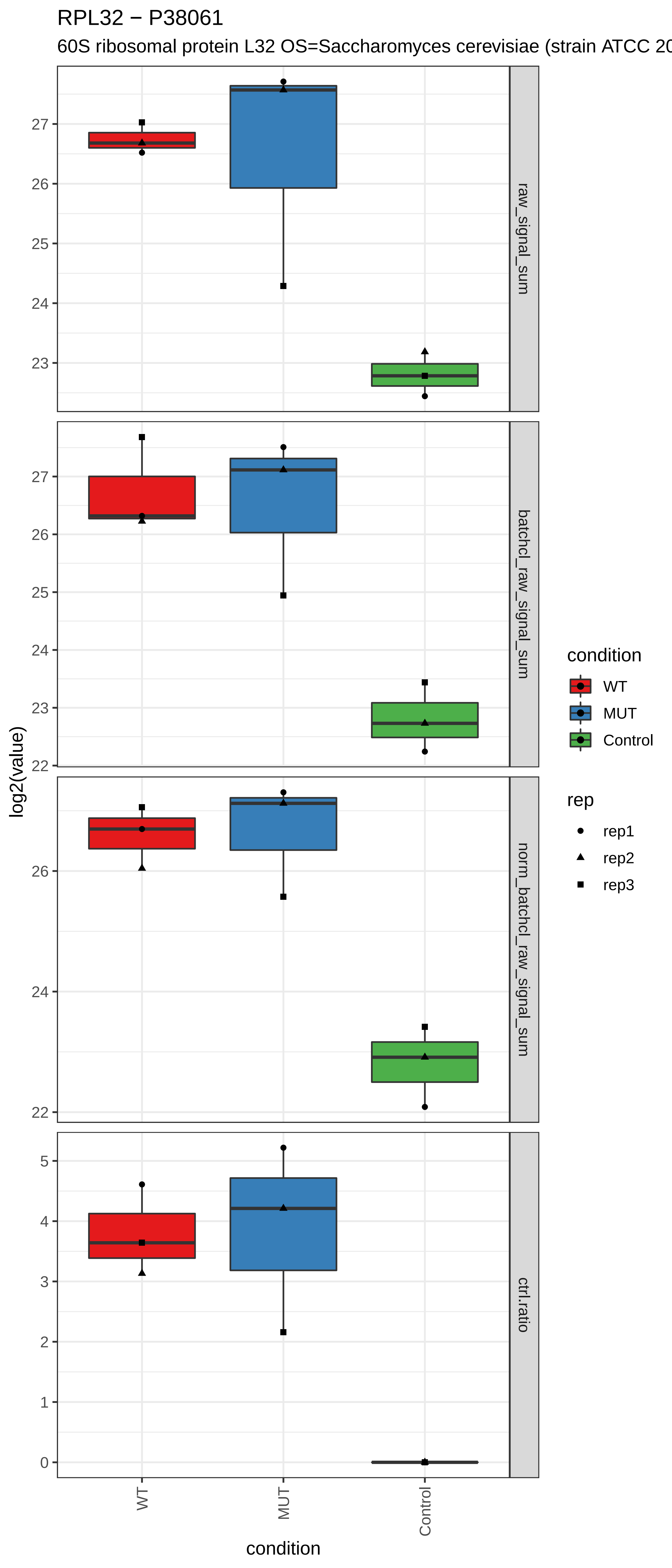

RPL32 − P38061
60S ribosomal protein L32 OS=Saccharomyces cerevisiae (strain ATCC 20
raw_signal_sum
batchcl_raw_signal_sum
norm_batchcl_raw_signal_sum
ctrl.ratio
log2(value)
condition
WT
MUT
Control
condition
WT
MUT
Control
rep
rep1
rep2
rep3
27
26
25
24
23
22
5
4
3
2
1
0

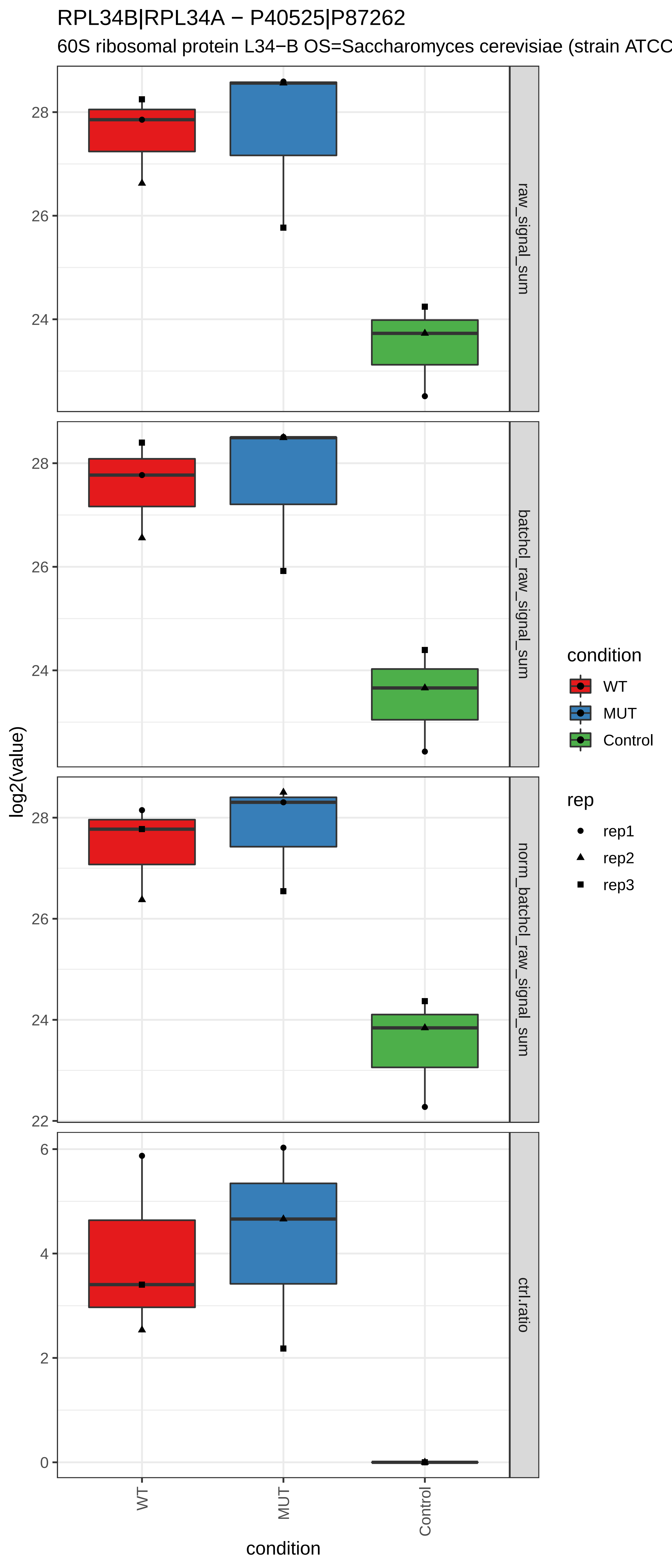

RPL34B|RPL34A − P40525|P87262
60S ribosomal protein L34−B OS=Saccharomyces cerevisiae (strain ATCC
raw_signal_sum
batchcl_raw_signal_sum
norm_batchcl_raw_signal_sum
ctrl.ratio
log2(value)
condition
WT
MUT
Control
rep
rep1
rep2
rep3
28
26
24
22
6
4
2
0

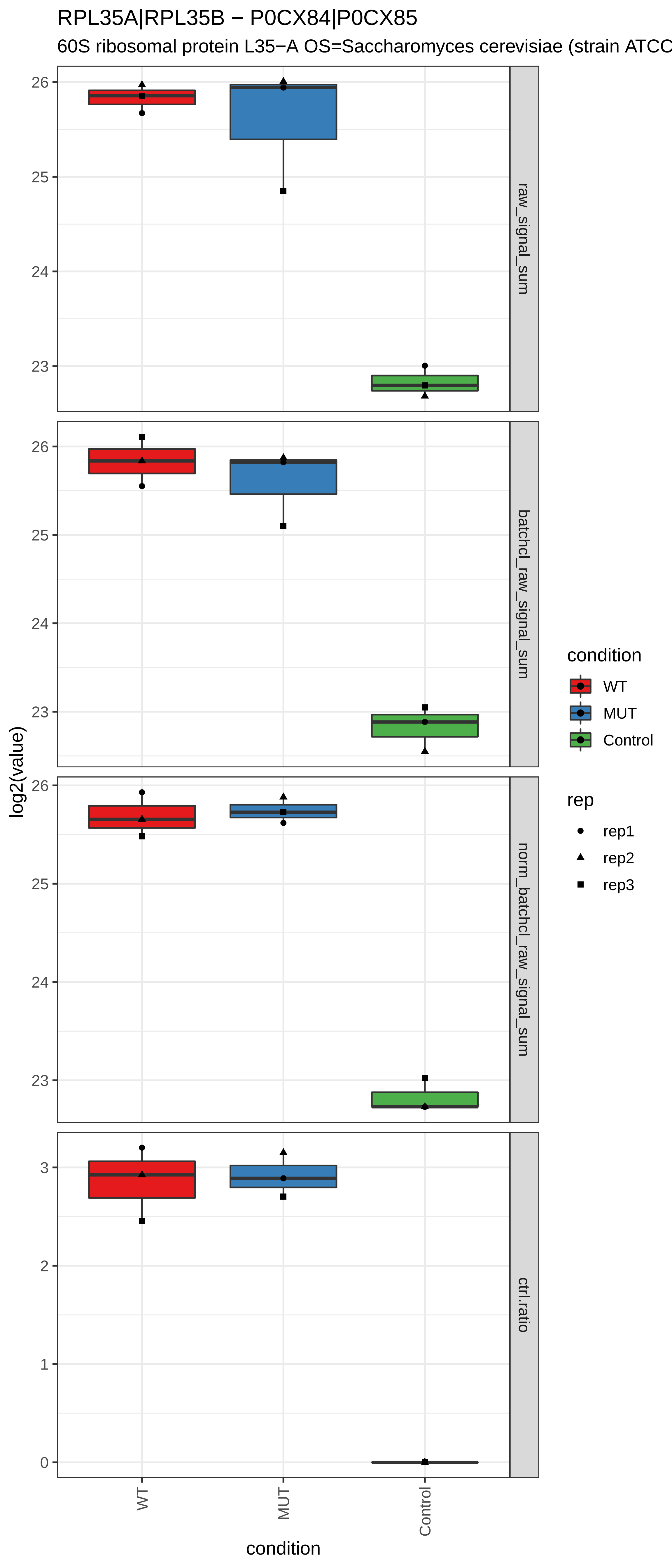
RPL35A|RPL35B − P0CX84|P0CX85
60S ribosomal protein L35−A OS=Saccharomyces cerevisiae (strain ATCC
raw_signal_sum
batchcl_raw_signal_sum
norm_batchcl_raw_signal_sum
ctrl.ratio
log2(value)
condition
WT
MUT
Control
rep
rep1
rep2
rep3
26
25
24
23
3
2
1
0

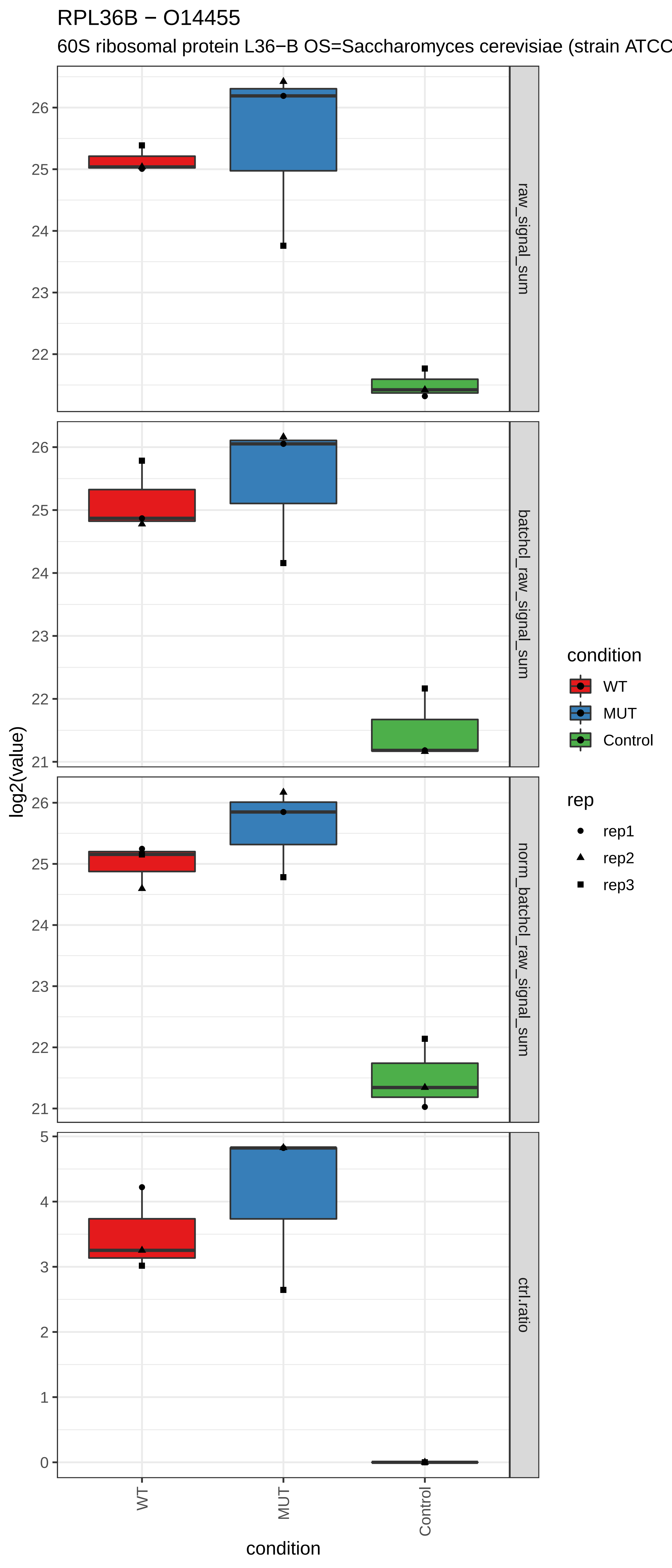

RPL36B − O14455
60S ribosomal protein L36−B OS=Saccharomyces cerevisiae (strain ATCC
raw_signal_sum
batchcl_raw_signal_sum
norm_batchcl_raw_signal_sum
ctrl.ratio
log2(value)
condition
WT
MUT
Control
rep
rep1
rep2
rep3

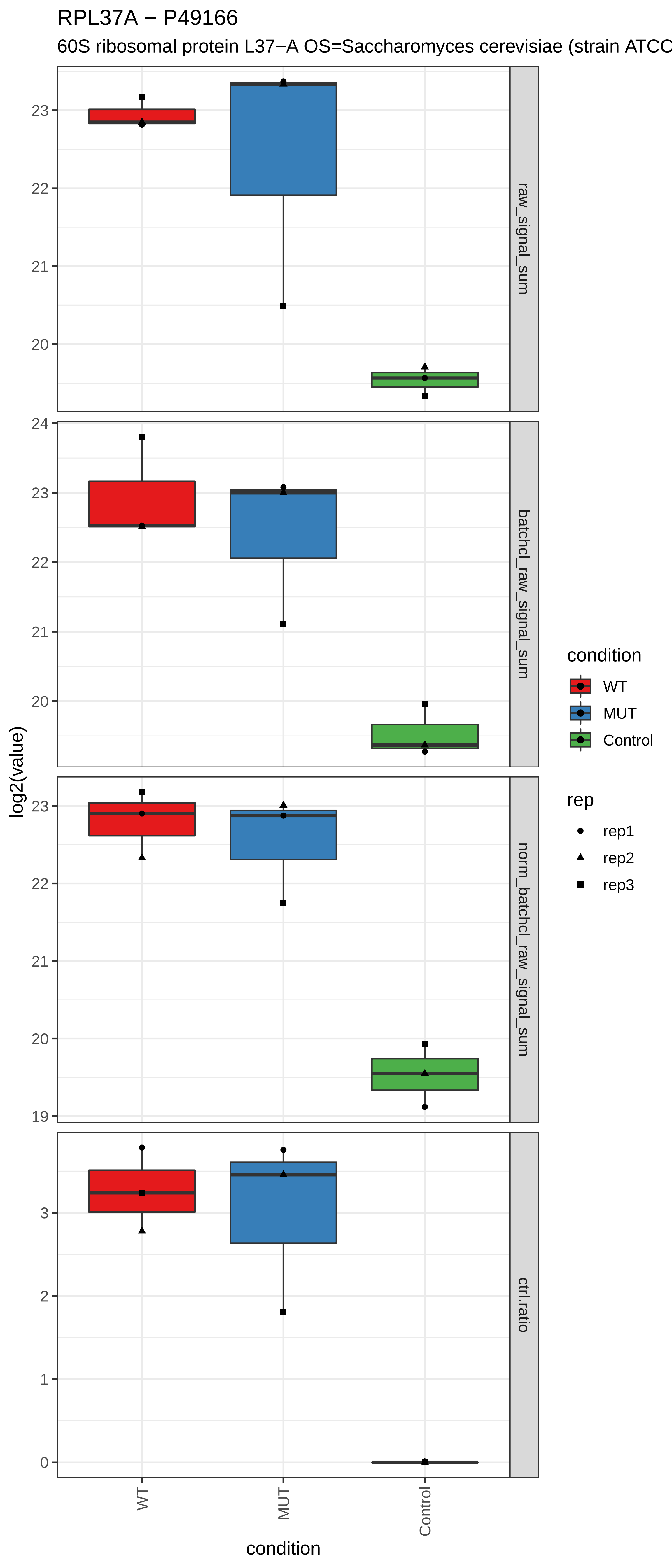
RPL37A − P49166
60S ribosomal protein L37−A OS=Saccharomyces cerevisiae (strain ATCC
raw_signal_sum
batchcl_raw_signal_sum
norm_batchcl_raw_signal_sum
ctrl.ratio
log2(value)
condition
WT
MUT
Control
rep
rep1
rep2
rep3

RPL42A|RPL42B − P0CX27|P0CX28

60S ribosomal protein L42−A OS=Saccharomyces cerevisiae (strain ATCC

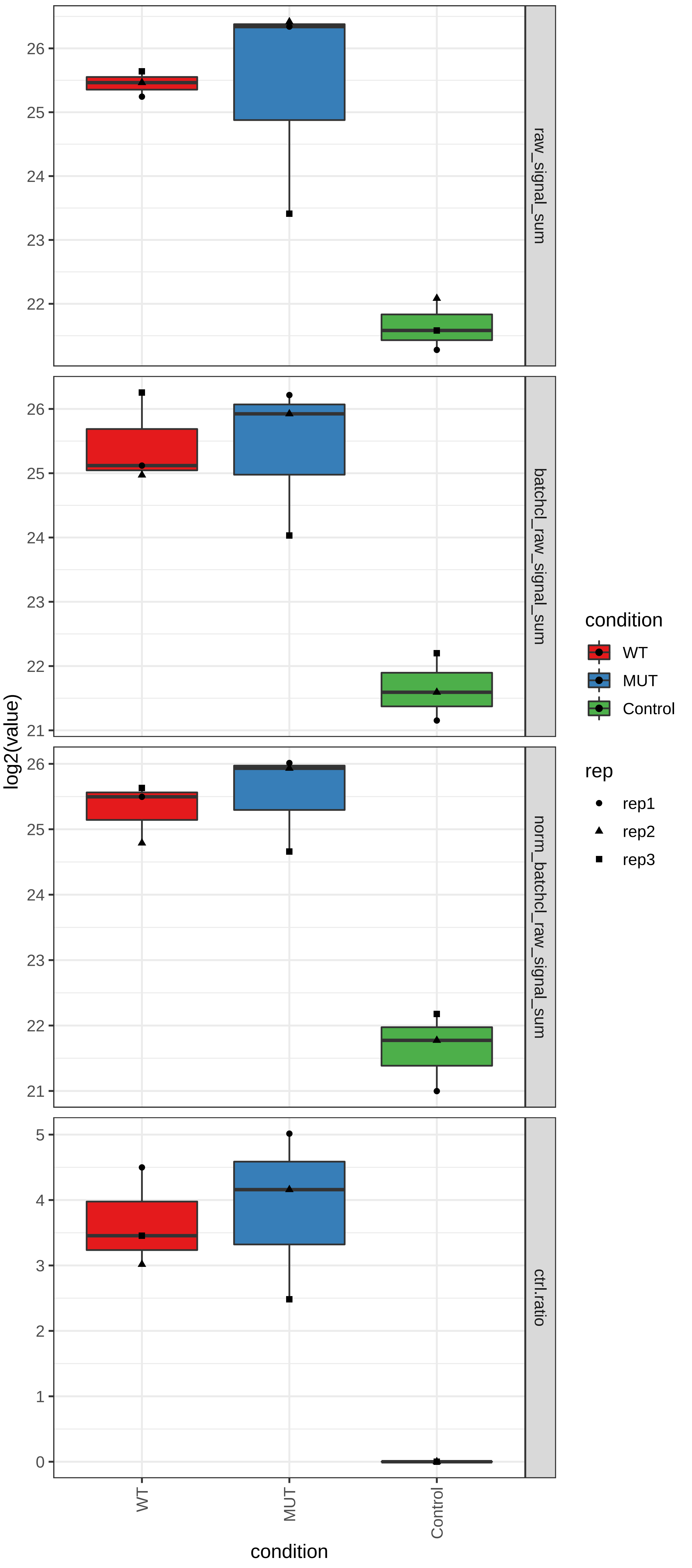

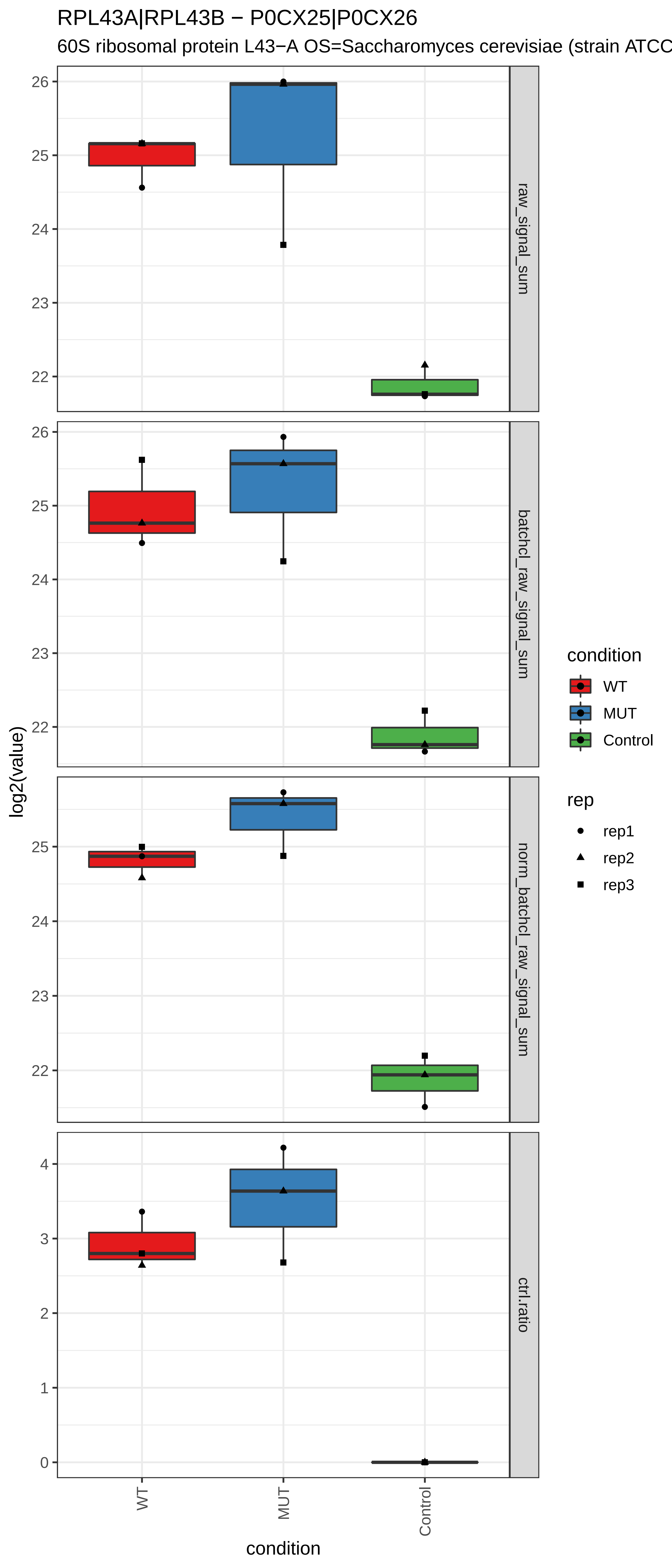

RPL43A|RPL43B − P0CX25|P0CX26
60S ribosomal protein L43−A OS=Saccharomyces cerevisiae (strain ATCC
raw_signal_sum
batchcl_raw_signal_sum
norm_batchcl_raw_signal_sum
ctrl.ratio
log2(value)
condition
WT
MUT
Control
rep
rep1
rep2
rep3

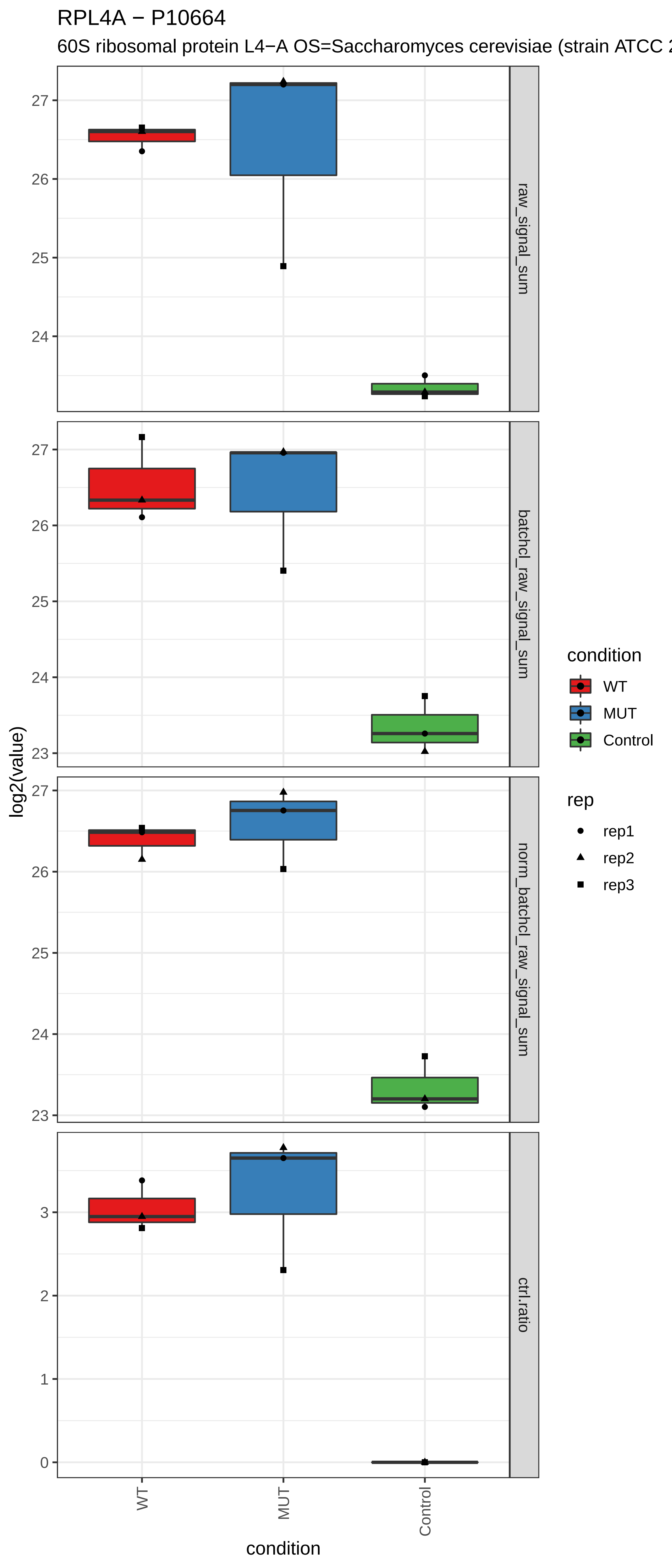

RPL4A − P10664
60S ribosomal protein L4−A OS=Saccharomyces cerevisiae (strain ATCC 2
raw_signal_sum
batchcl_raw_signal_sum
norm_batchcl_raw_signal_sum
ctrl.ratio
log2(value)
condition
WT
MUT
Control
rep
rep1
rep2
rep3

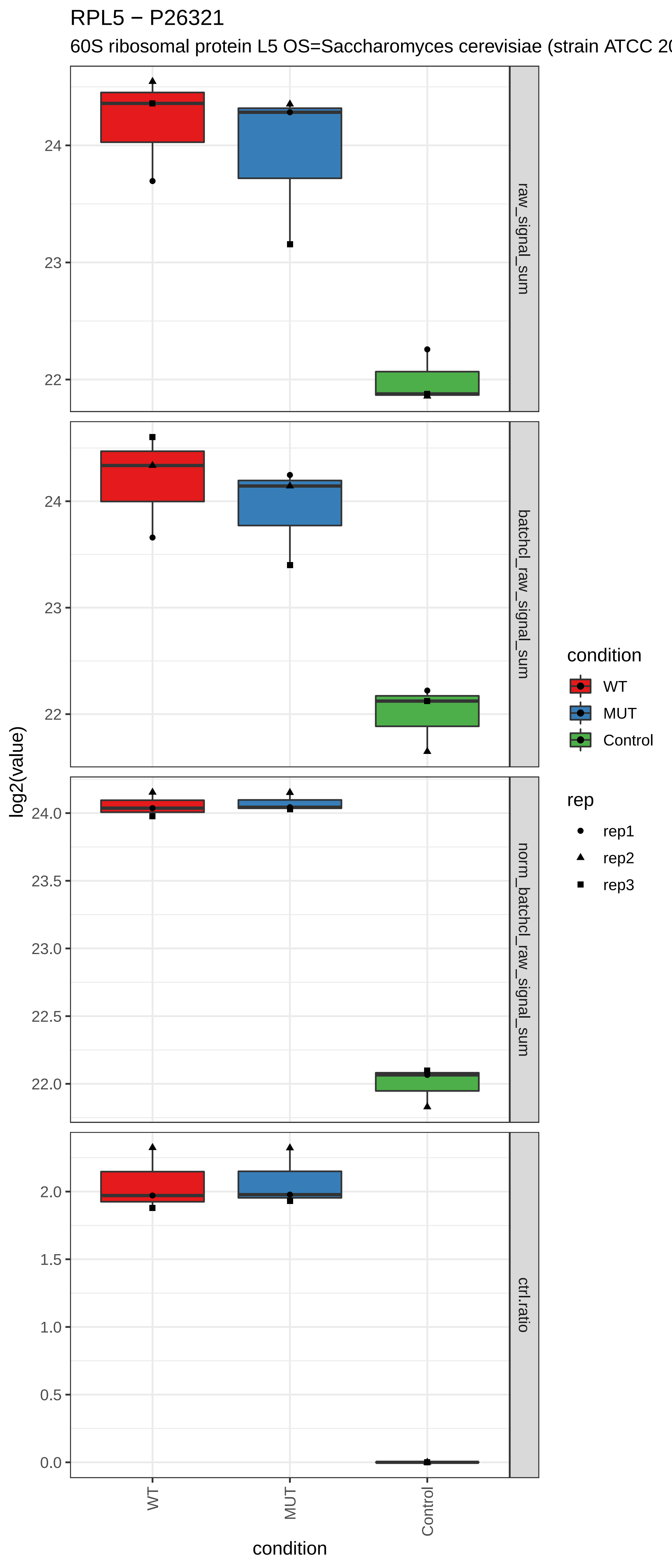
RPL5 − P26321
60S ribosomal protein L5 OS=Saccharomyces cerevisiae (strain ATCC 20
raw_signal_sum
batchcl_raw_signal_sum
norm_batchcl_raw_signal_sum
ctrl.ratio
log2(value)
condition
WT
MUT
Control
rep
rep1
rep2
rep3
24
23
22
24.0
23.5
23.0
22.5
22.0
2.0
1.5
1.0
0.5
0.0

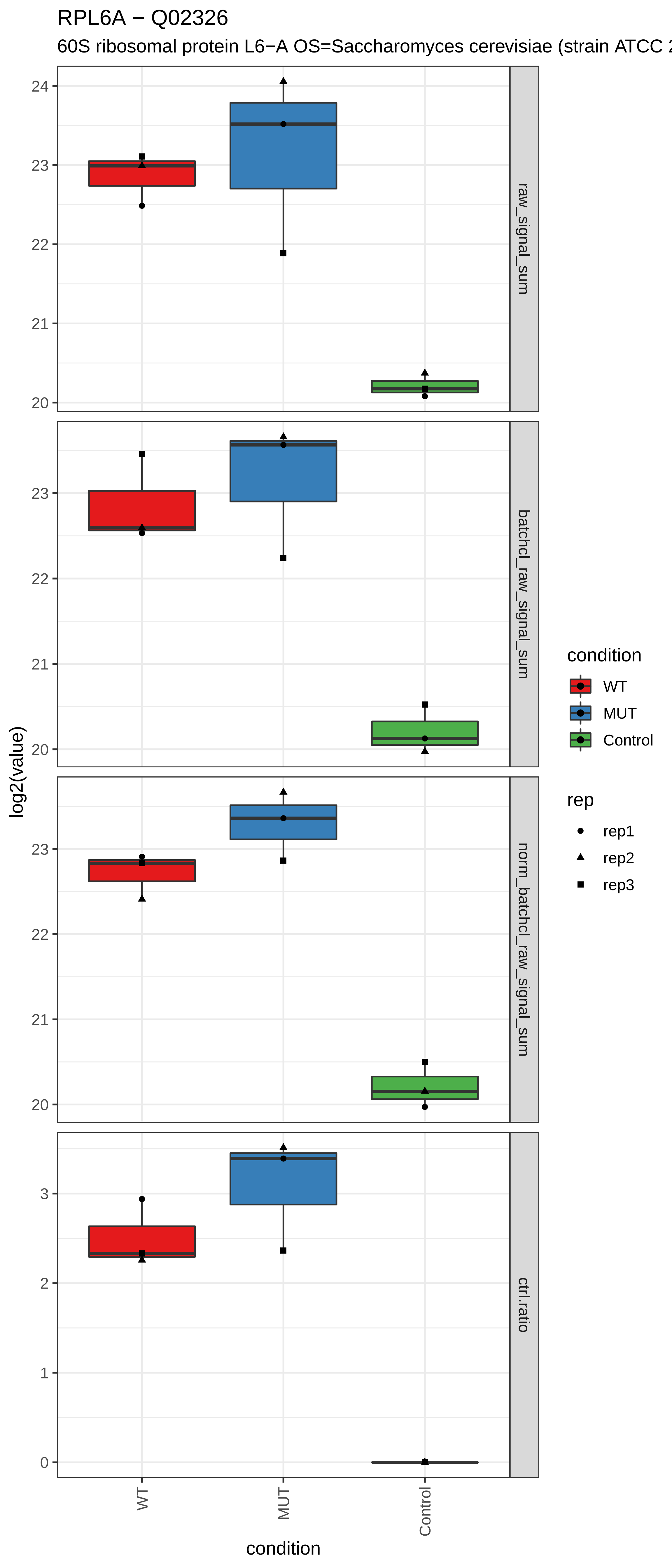
RPL6A − Q02326
60S ribosomal protein L6−A OS=Saccharomyces cerevisiae (strain ATCC 2
raw_signal_sum
batchcl_raw_signal_sum
norm_batchcl_raw_signal_sum
ctrl.ratio
log2(value)
condition
WT
MUT
Control
rep
rep1
rep2
rep3
24
23
22
21
20
3
2
1
0

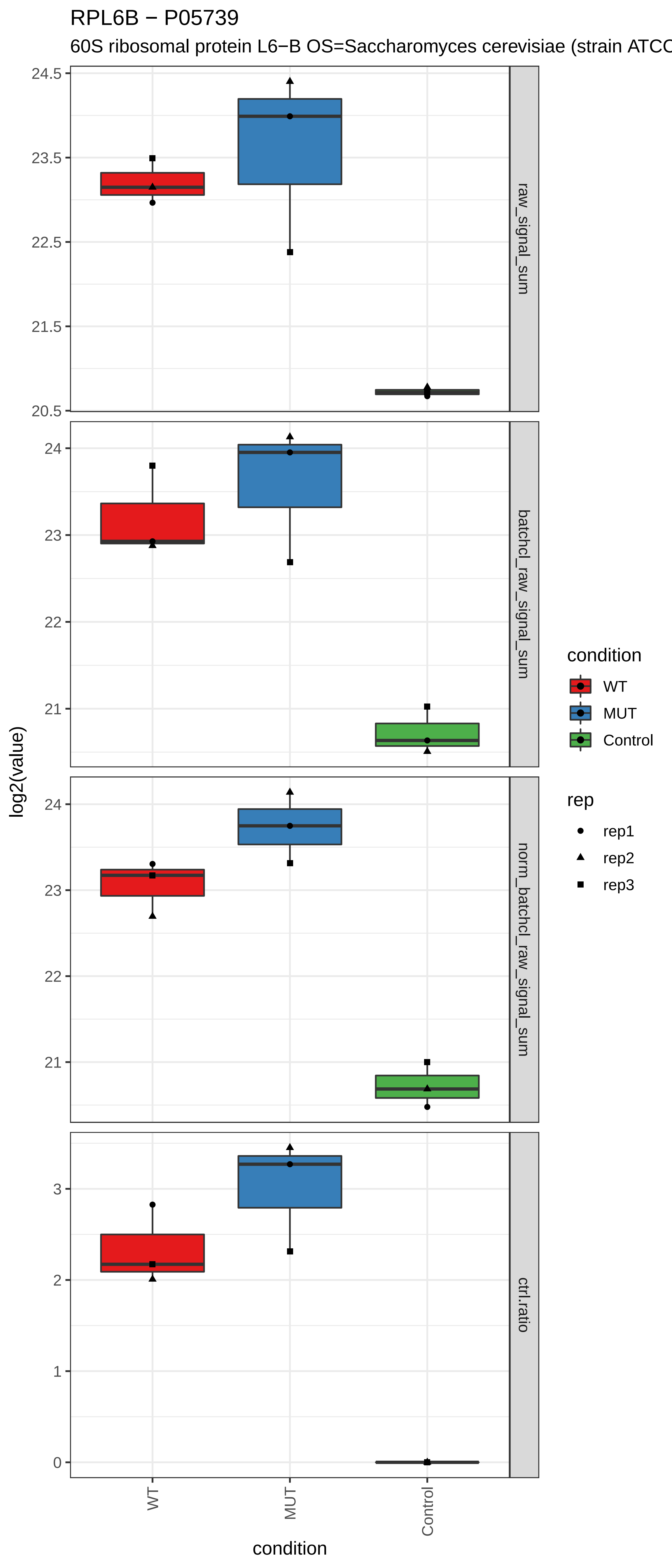

RPL6B − P05739
60S ribosomal protein L6−B OS=Saccharomyces cerevisiae (strain ATCC
raw_signal_sum
batchcl_raw_signal_sum
norm_batchcl_raw_signal_sum
ctrl.ratio
log2(value)
condition
WT
MUT
Control
rep
rep1
rep2
rep3

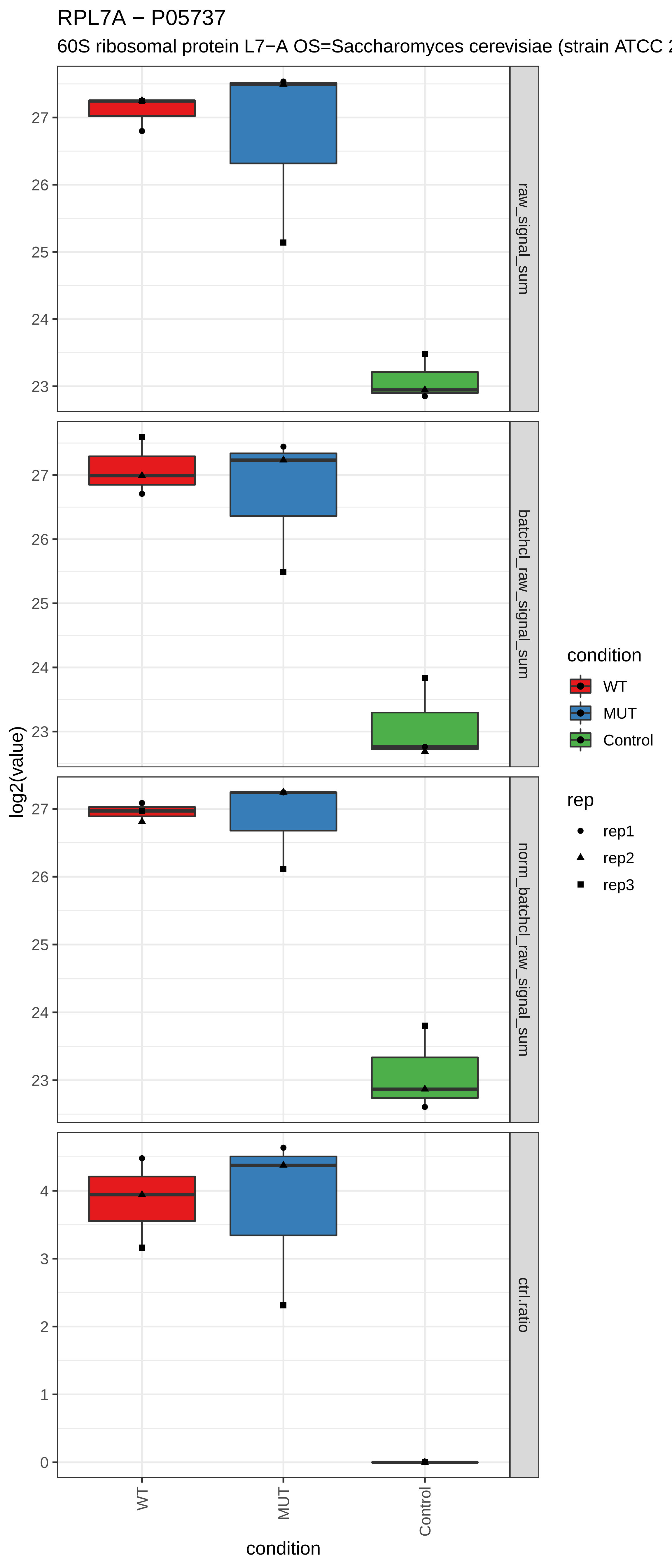

RPL7A − P05737
60S ribosomal protein L7−A OS=Saccharomyces cerevisiae (strain ATCC 2
raw_signal_sum
batchcl_raw_signal_sum
norm_batchcl_raw_signal_sum
ctrl.ratio
log2(value)
condition
WT
MUT
Control
rep
rep1
rep2
rep3

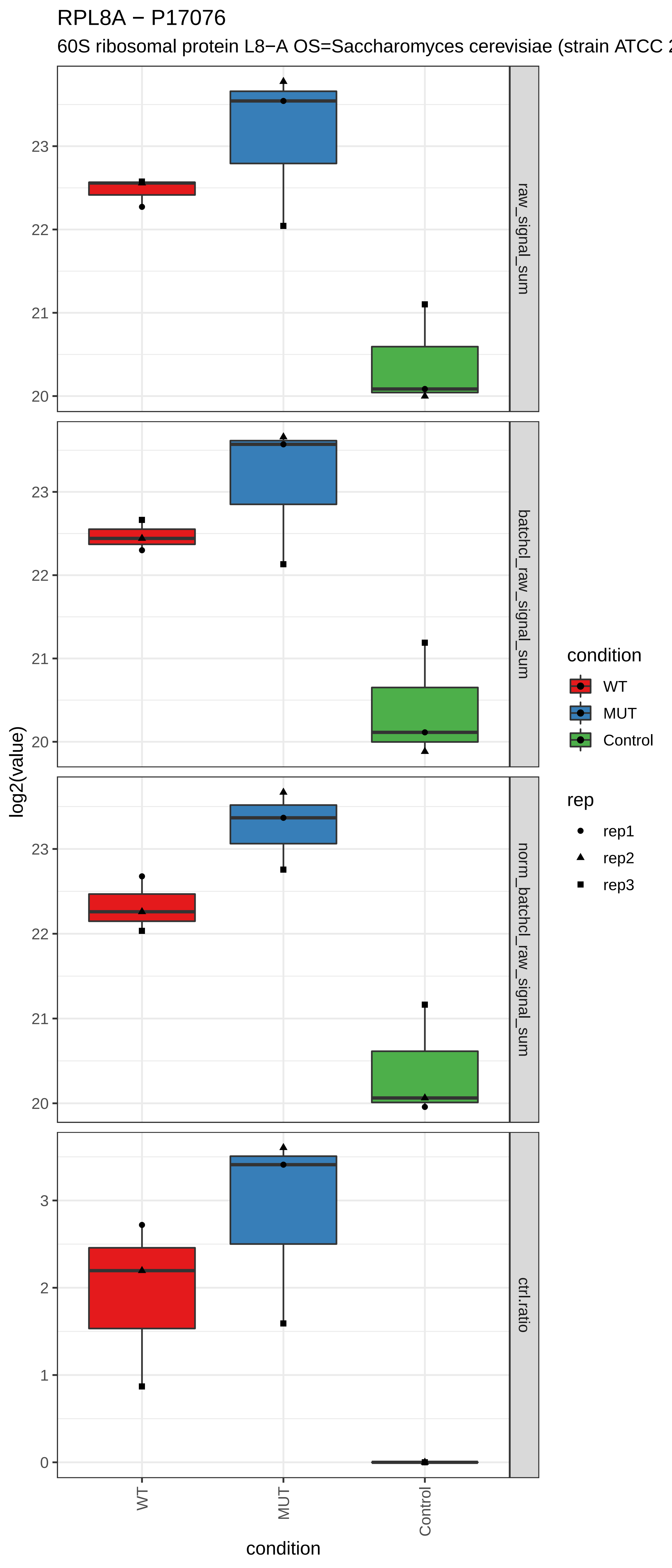

RPL8A − P17076
60S ribosomal protein L8−A OS=Saccharomyces cerevisiae (strain ATCC 2
raw_signal_sum
batchcl_raw_signal_sum
norm_batchcl_raw_signal_sum
ctrl.ratio
log2(value)
condition
WT
MUT
Control
rep
rep1
rep2
rep3

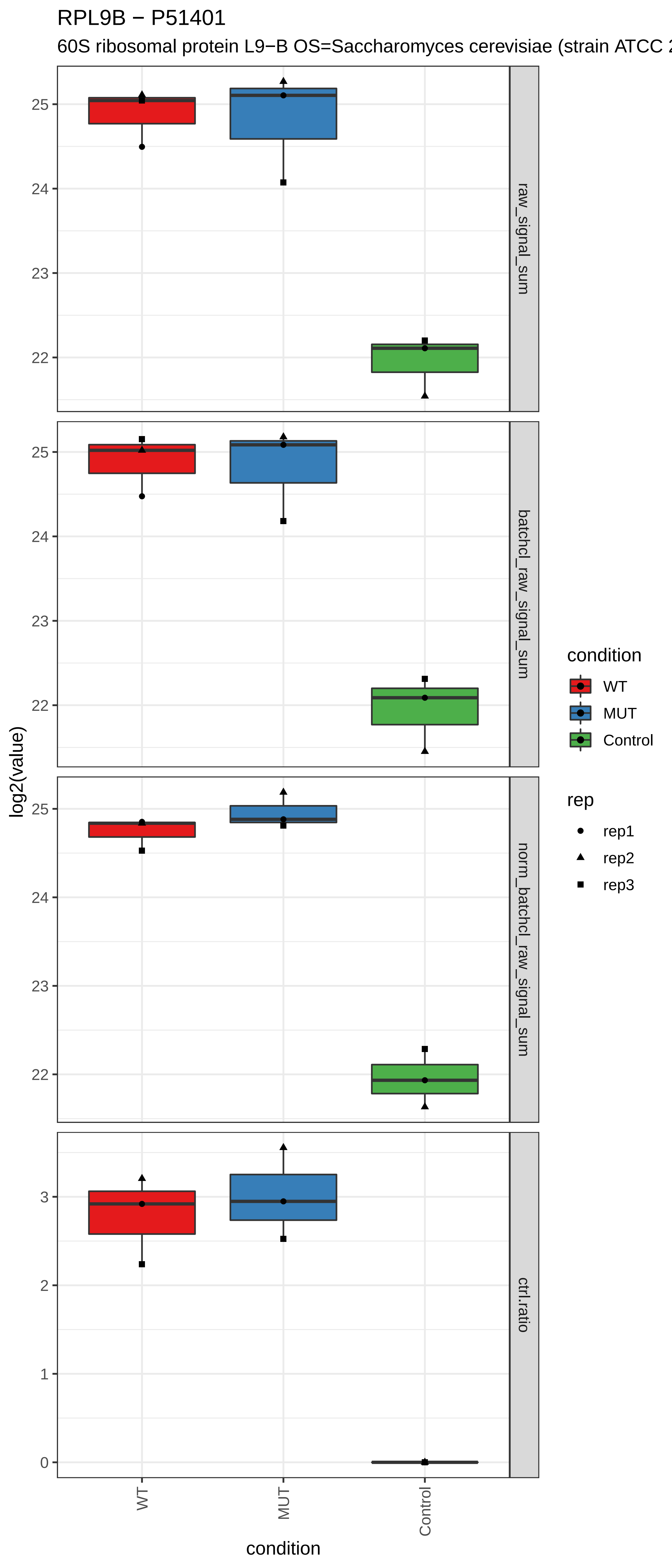
RPL9B − P51401
60S ribosomal protein L9−B OS=Saccharomyces cerevisiae (strain ATCC 2
raw_signal_sum
batchcl_raw_signal_sum
norm_batchcl_raw_signal_sum
ctrl.ratio
log2(value)
condition
WT
MUT
Control
rep
rep1
rep2
rep3

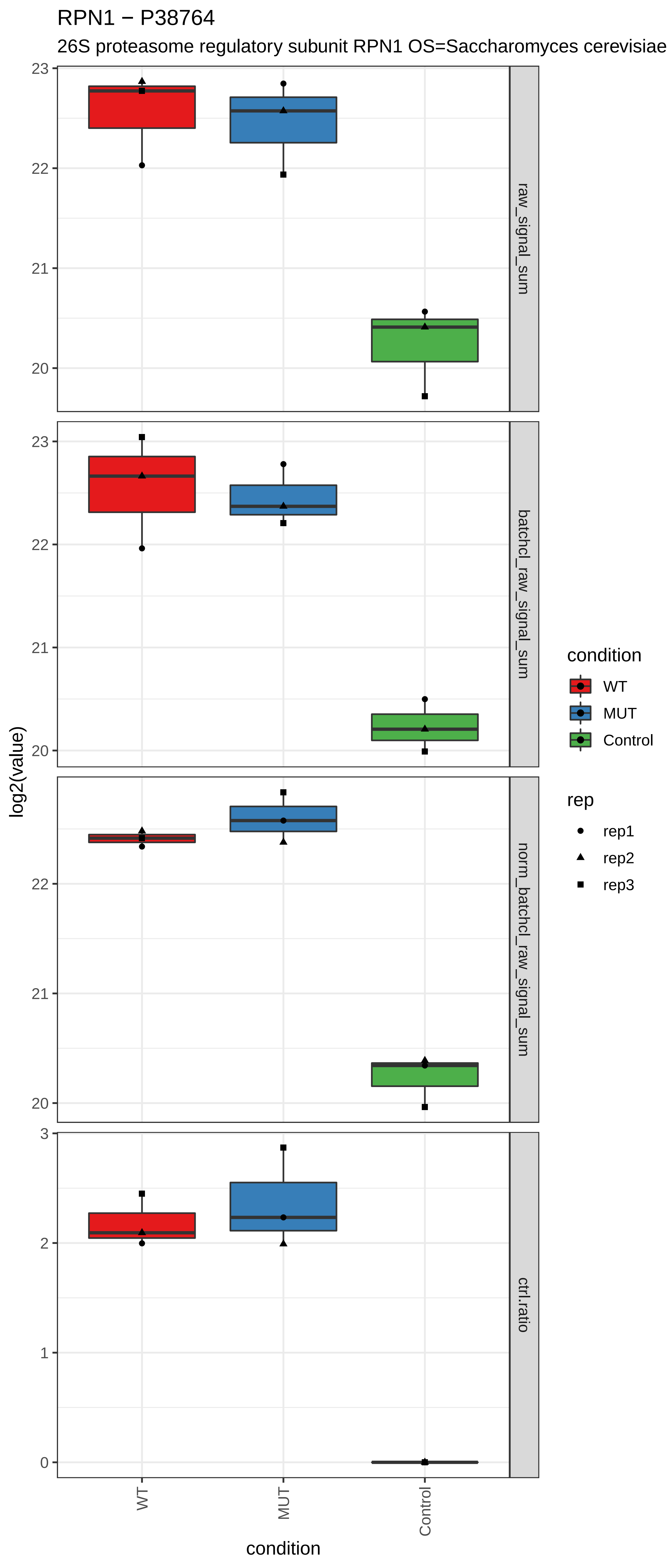

RPN1 − P38764
26S proteasome regulatory subunit RPN1 OS=Saccharomyces cerevisiae
raw_signal_sum
batchcl_raw_signal_sum
norm_batchcl_raw_signal_sum
ctrl.ratio
log2(value)
condition
WT
MUT
Control
rep
rep1
rep2
rep3

RPN2 − P32565

26S proteasome regulatory subunit RPN2 OS=Saccharomyces cerevisiae

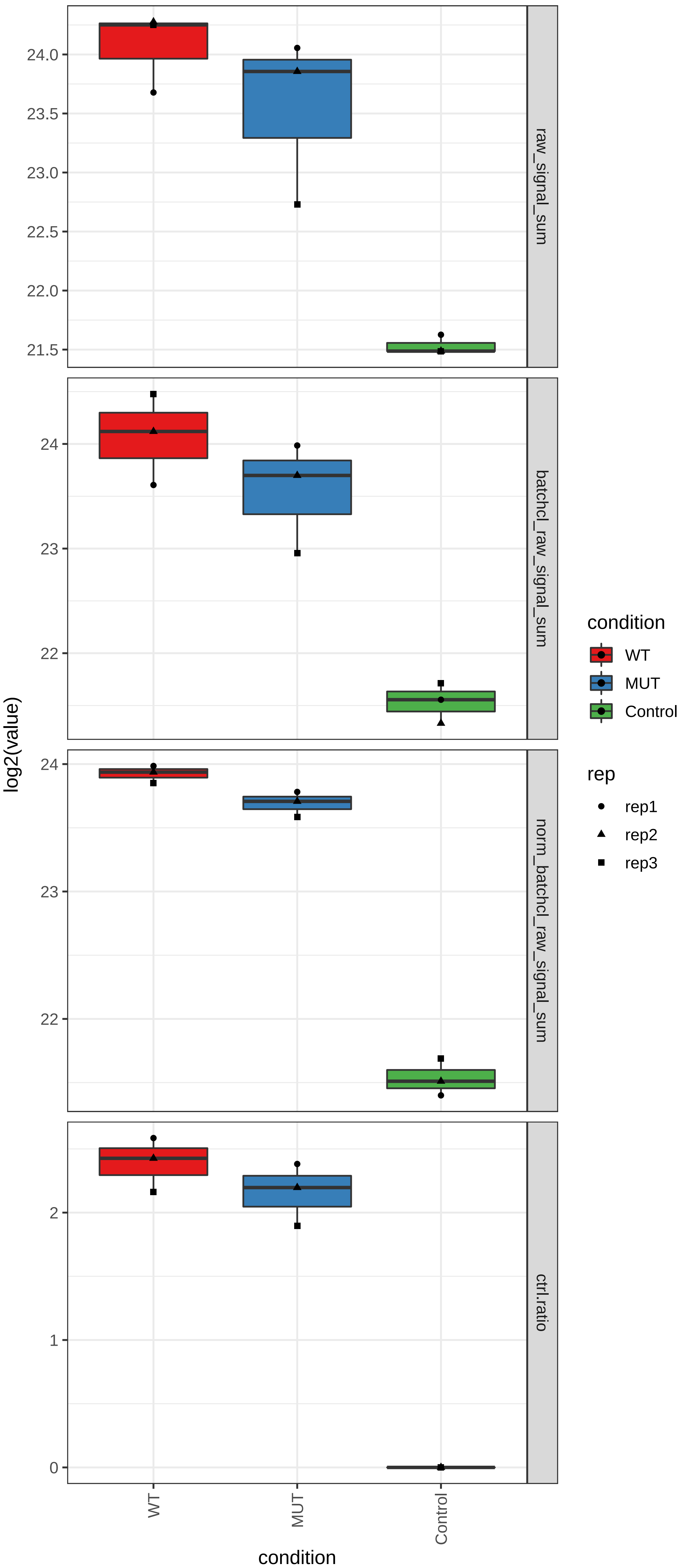

RPN3 − P40016

26S proteasome regulatory subunit RPN3 OS=Saccharomyces cerevisiae

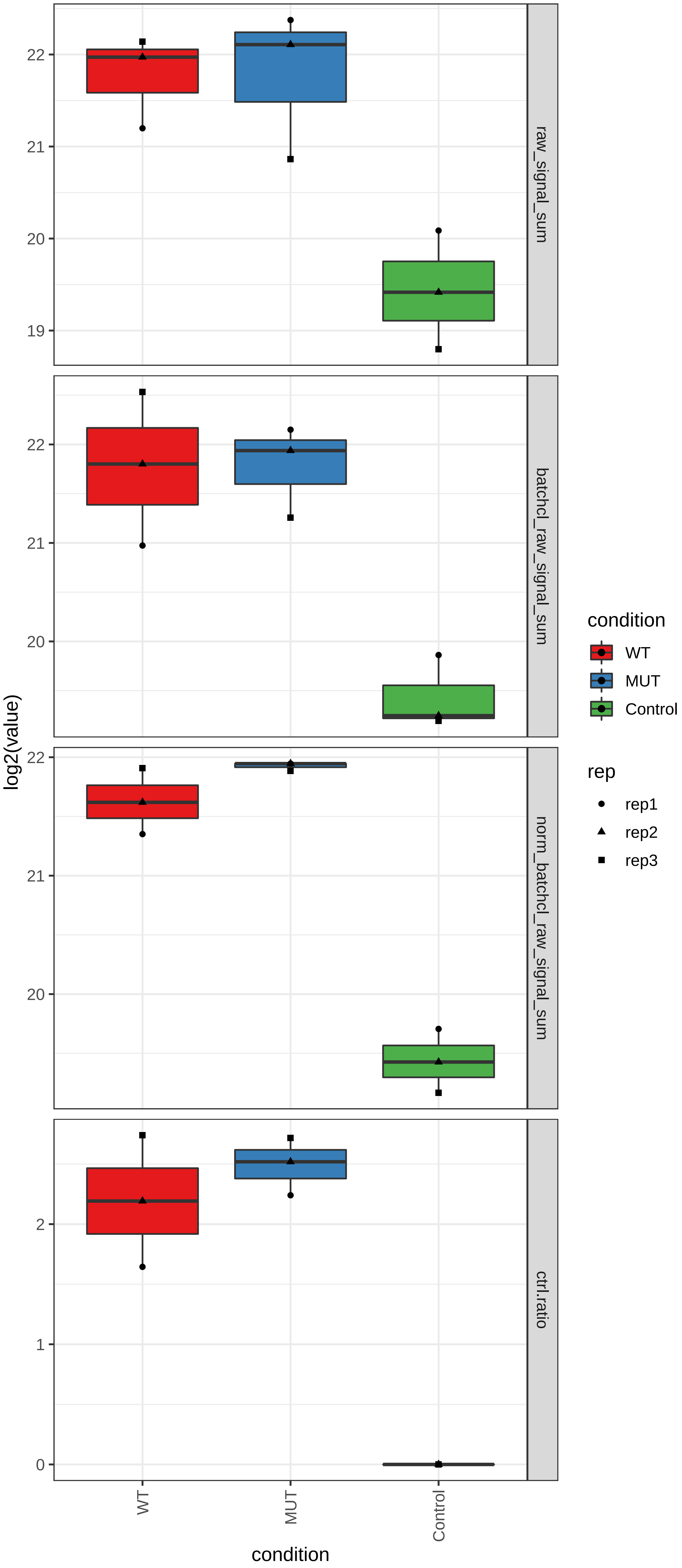

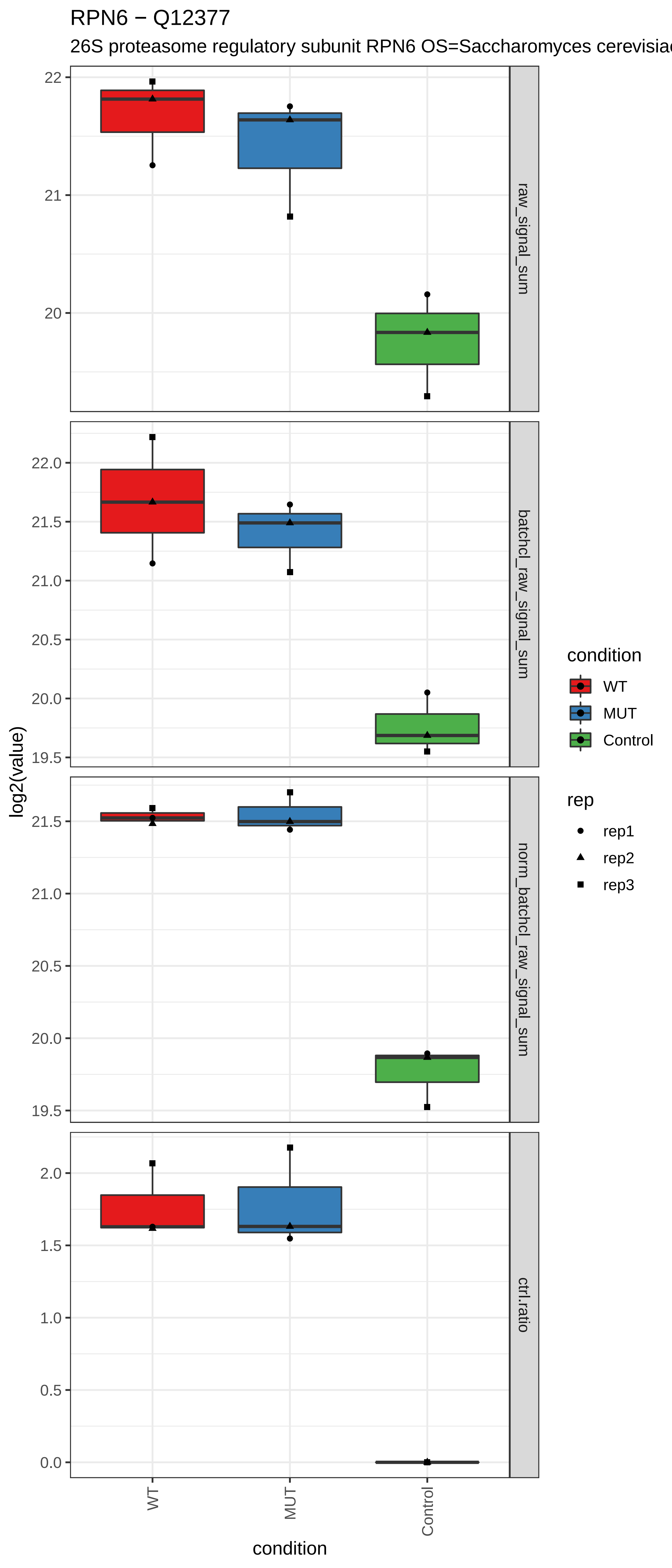
RPN6 − Q12377
26S proteasome regulatory subunit RPN6 OS=Saccharomyces cerevisiae
log2(value)
raw_signal_sum
batchcl_raw_signal_sum
norm_batchcl_raw_signal_sum
ctrl.ratio
condition
WT
MUT
Control
rep
rep1
rep2
rep3
22
21
20
22.0
21.5
21.0
20.5
20.0
19.5
2.0
1.5
1.0
0.5
0.0

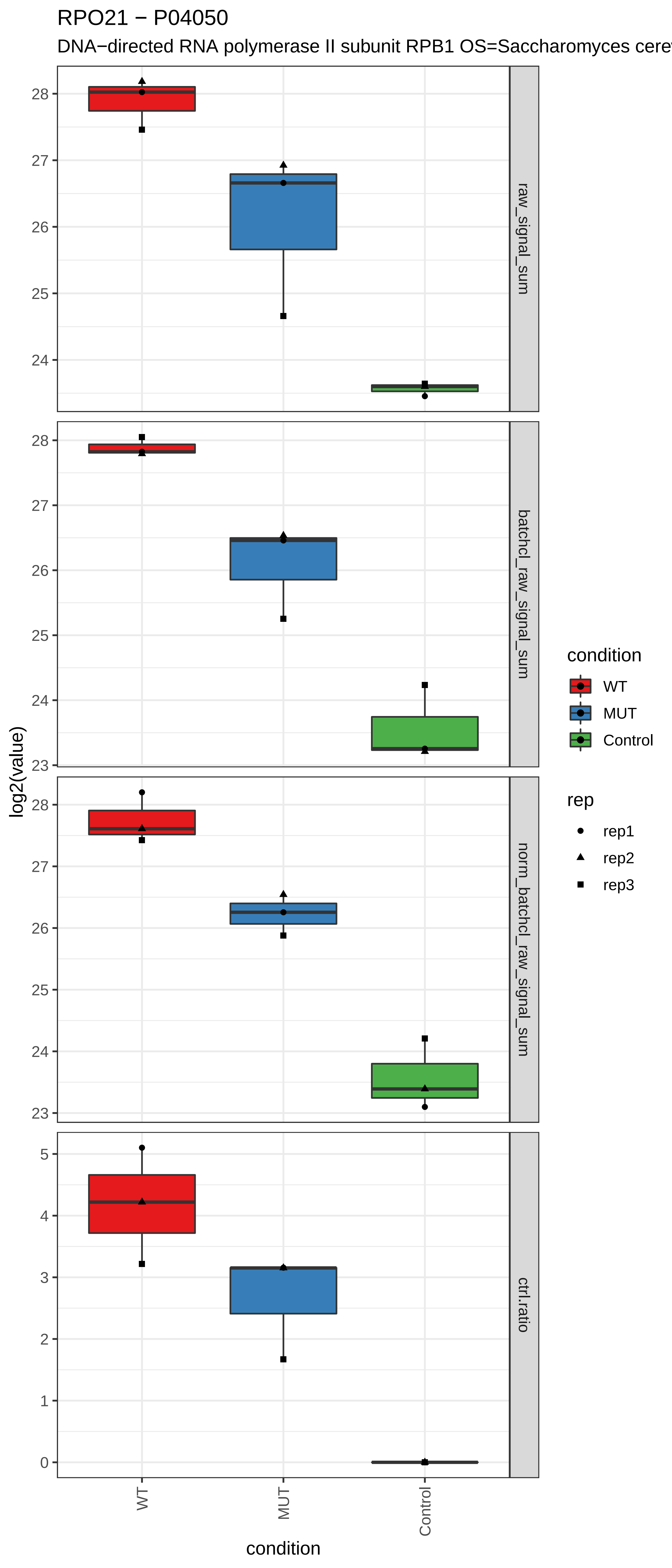

RPO21 − P04050
DNA−directed RNA polymerase II subunit RPB1 OS=Saccharomyces cerev
raw_signal_sum
batchcl_raw_signal_sum
norm_batchcl_raw_signal_sum
ctrl.ratio
log2(value)
condition
WT
MUT
Control
rep
rep1
rep2
rep3

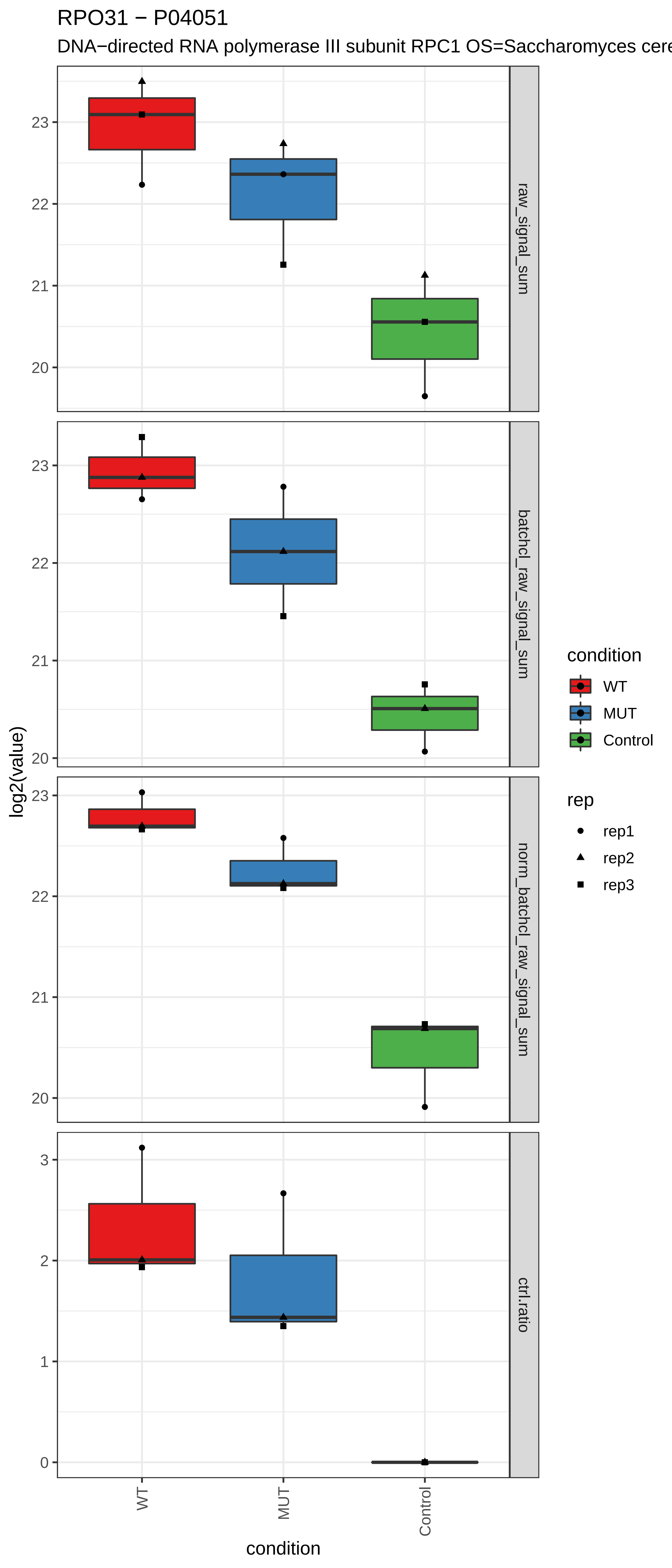

RPO31 − P04051
DNA−directed RNA polymerase III subunit RPC1 OS=Saccharomyces cere
raw_signal_sum
batchcl_raw_signal_sum
norm_batchcl_raw_signal_sum
ctrl.ratio
log2(value)
condition
WT
MUT
Control
rep
rep1
rep2
rep3
23
22
21
20
3
2
1
0

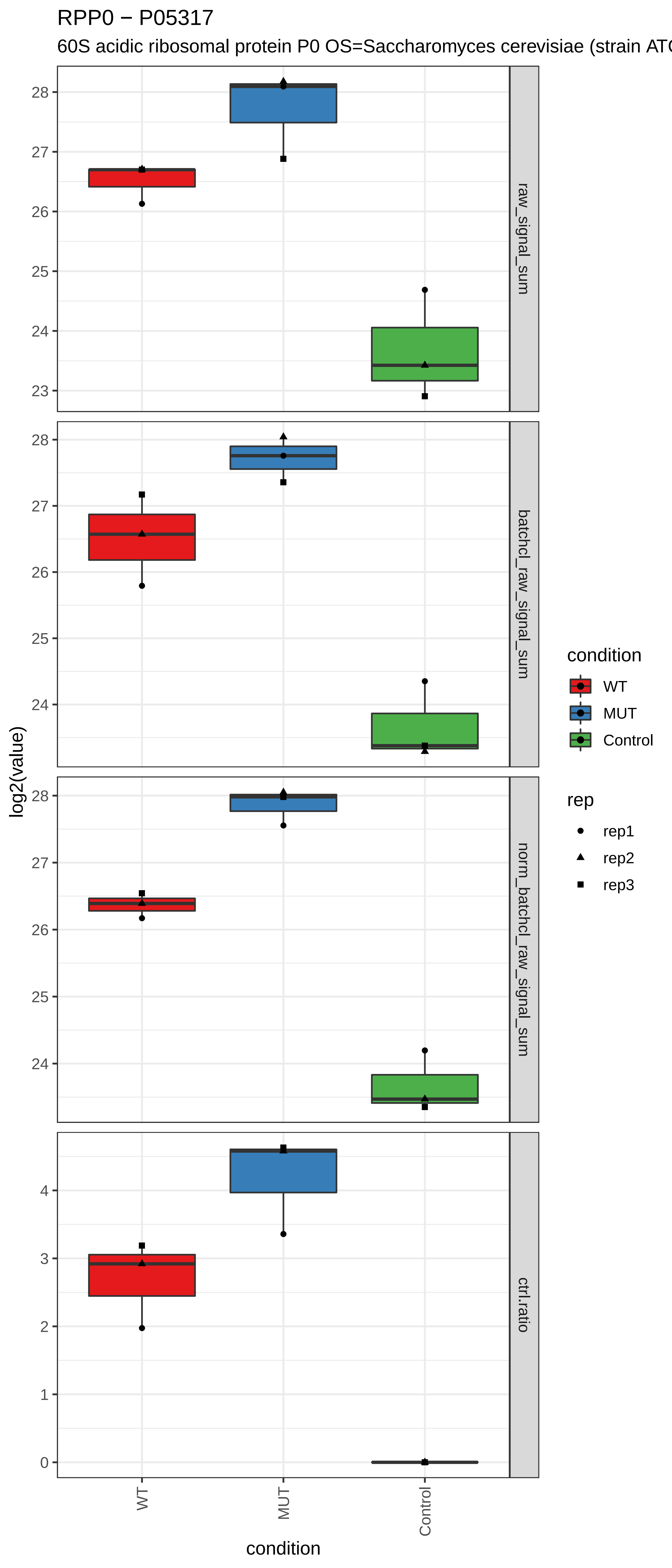

RPP0 − P05317
60S acidic ribosomal protein P0 OS=Saccharomyces cerevisiae (strain ATC
log2(value)
raw_signal_sum
batchcl_raw_signal_sum
norm_batchcl_raw_signal_sum
ctrl.ratio
condition
WT
MUT
Control
rep
rep1
rep2
rep3

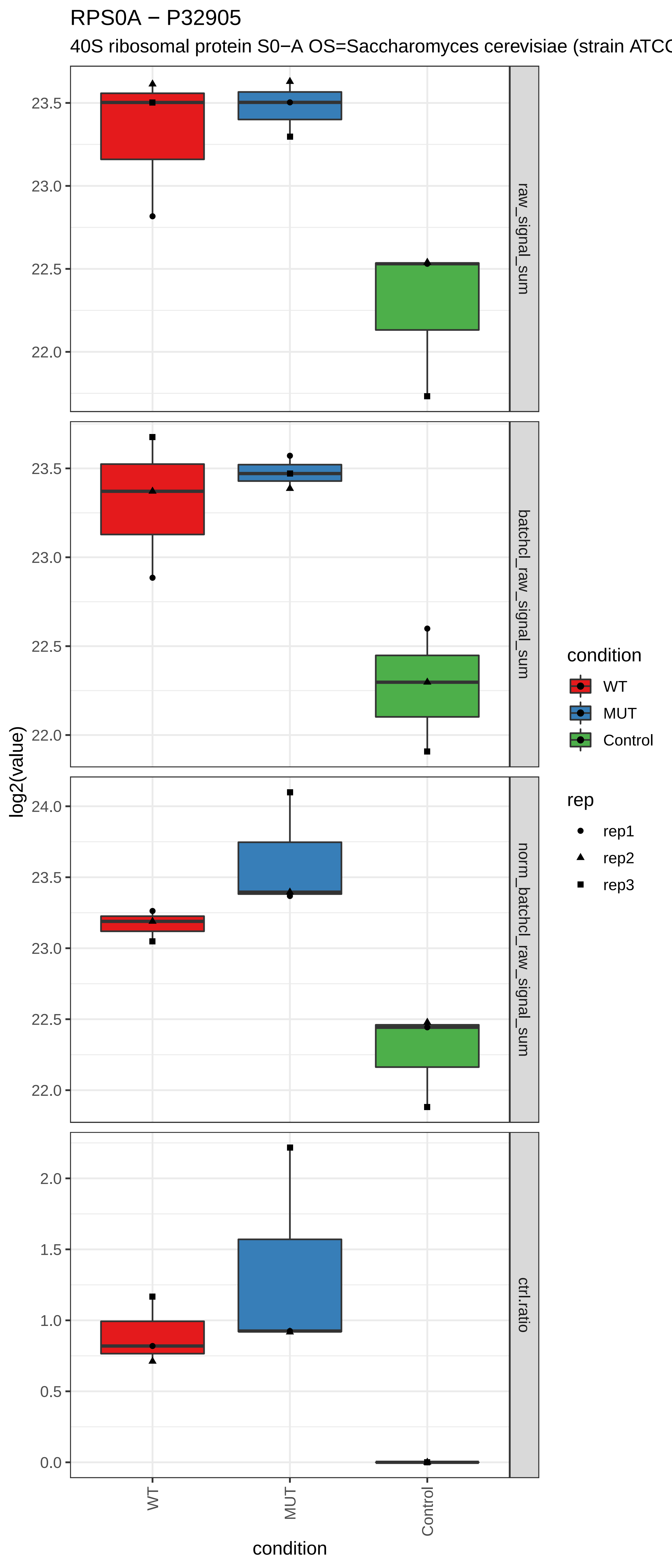

RPS0A − P32905
40S ribosomal protein S0−A OS=Saccharomyces cerevisiae (strain ATCC
raw_signal_sum
batchcl_raw_signal_sum
norm_batchcl_raw_signal_sum
ctrl.ratio
log2(value)
condition
WT
MUT
Control
rep
rep1
rep2
rep3

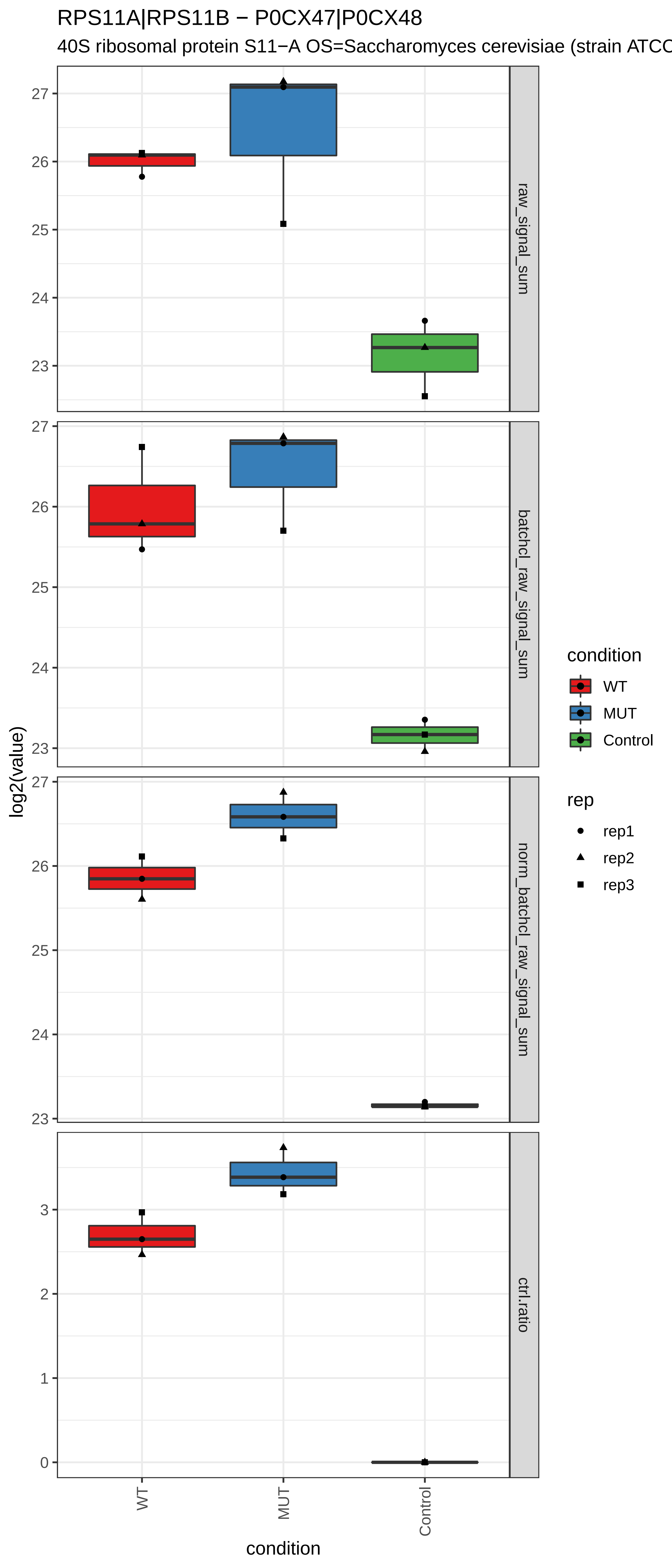
RPS11A|RPS11B − P0CX47|P0CX48
40S ribosomal protein S11−A OS=Saccharomyces cerevisiae (strain ATCC
raw_signal_sum
batchcl_raw_signal_sum
norm_batchcl_raw_signal_sum
ctrl.ratio
log2(value)
condition
WT
MUT
Control
rep
rep1
rep2
rep3
27
26
25
24
23
3
2
1
0

RPS12 − P48589

40S ribosomal protein S12 OS=Saccharomyces cerevisiae (strain ATCC

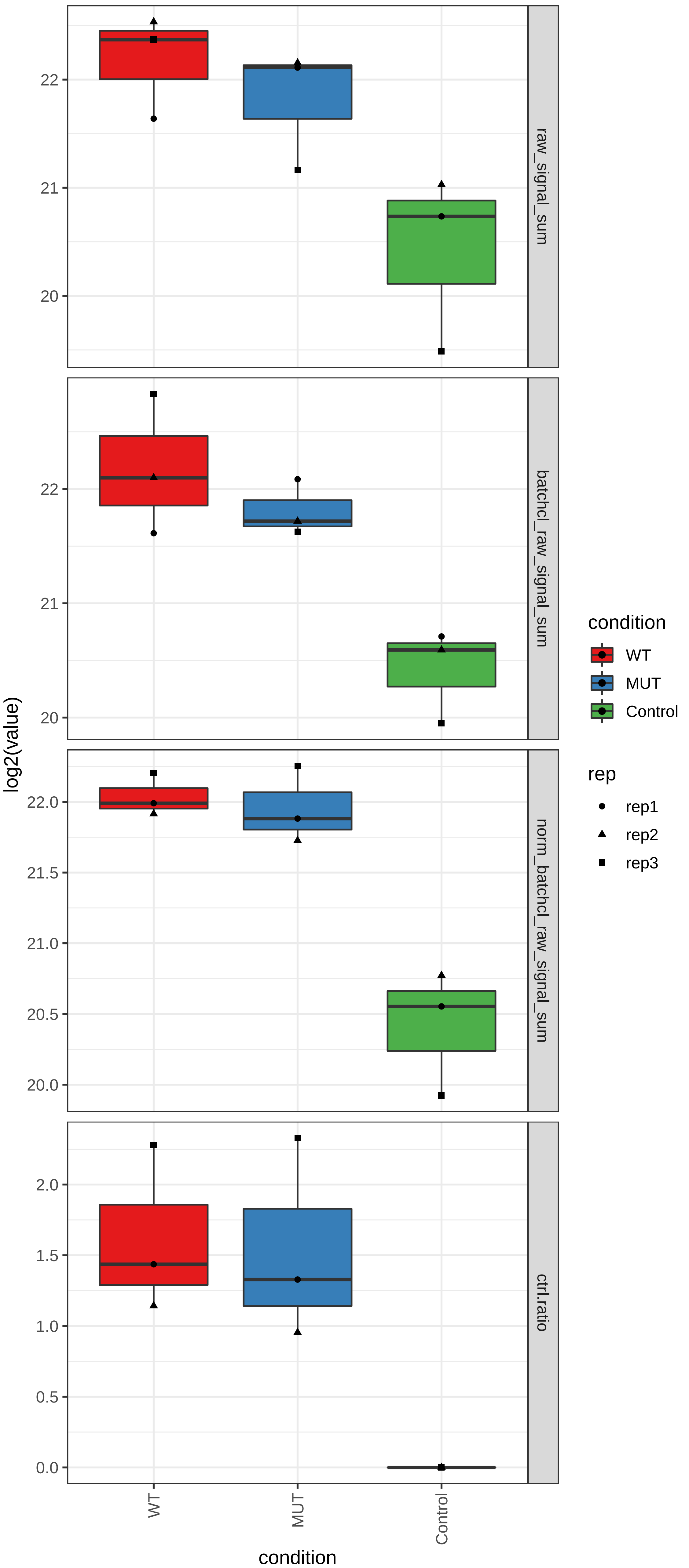

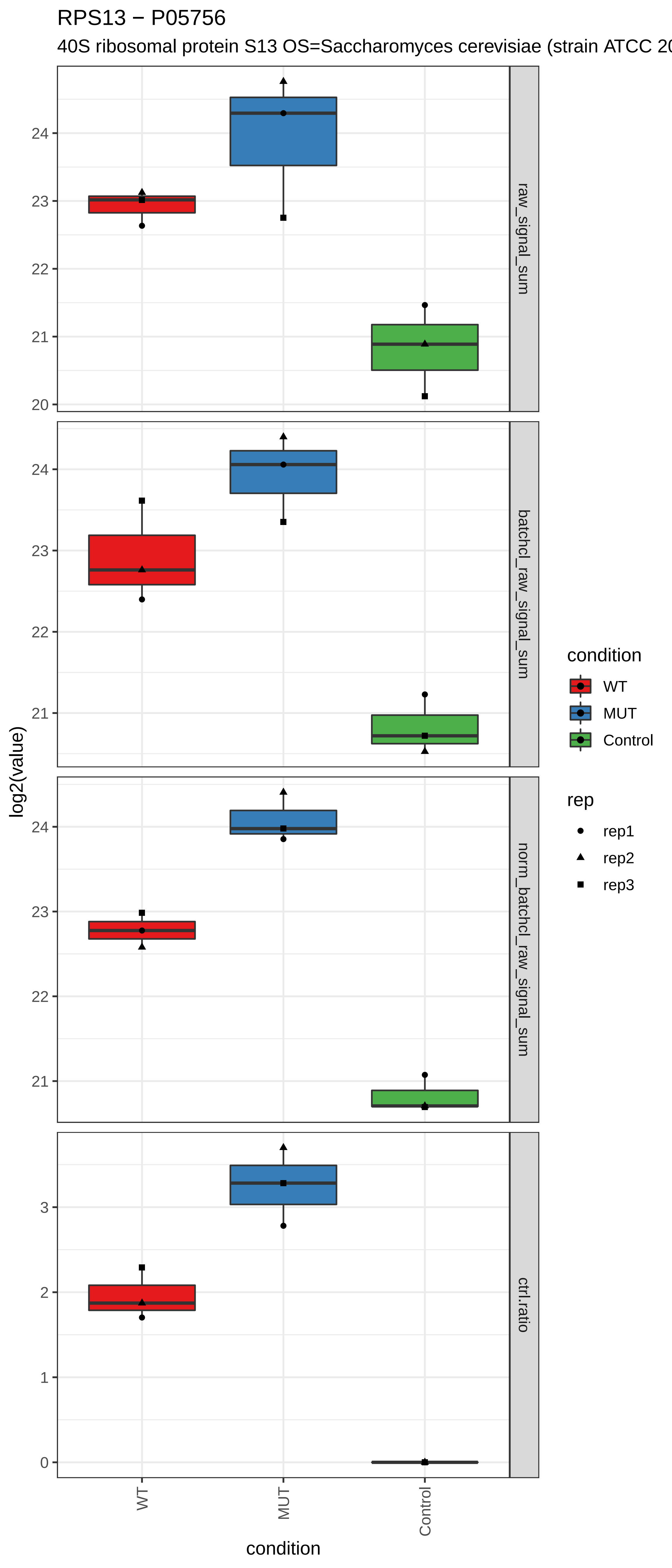
RPS13 − P05756
40S ribosomal protein S13 OS=Saccharomyces cerevisiae (strain ATCC 20
raw_signal_sum
batchcl_raw_signal_sum
norm_batchcl_raw_signal_sum
ctrl.ratio
log2(value)
condition
WT
MUT
Control
rep
rep1
rep2
rep3
20
21
22
23
24
0
1
2
3

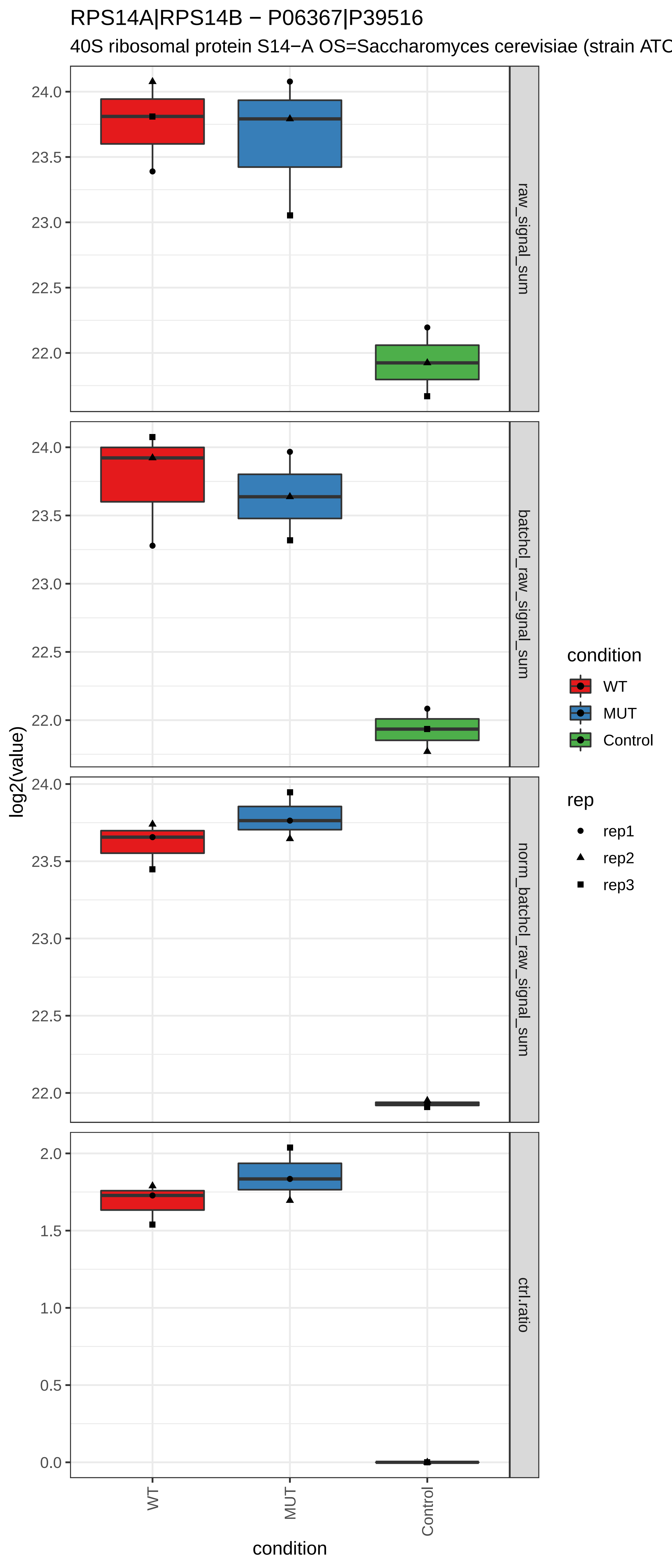
RPS14A|RPS14B − P06367|P39516
40S ribosomal protein S14−A OS=Saccharomyces cerevisiae (strain ATC
raw_signal_sum
batchcl_raw_signal_sum
norm_batchcl_raw_signal_sum
ctrl.ratio
log2(value)
condition
WT
MUT
Control
rep
rep1
rep2
rep3
24.0
23.5
23.0
22.5
22.0
2.0
1.5
1.0
0.5
0.0

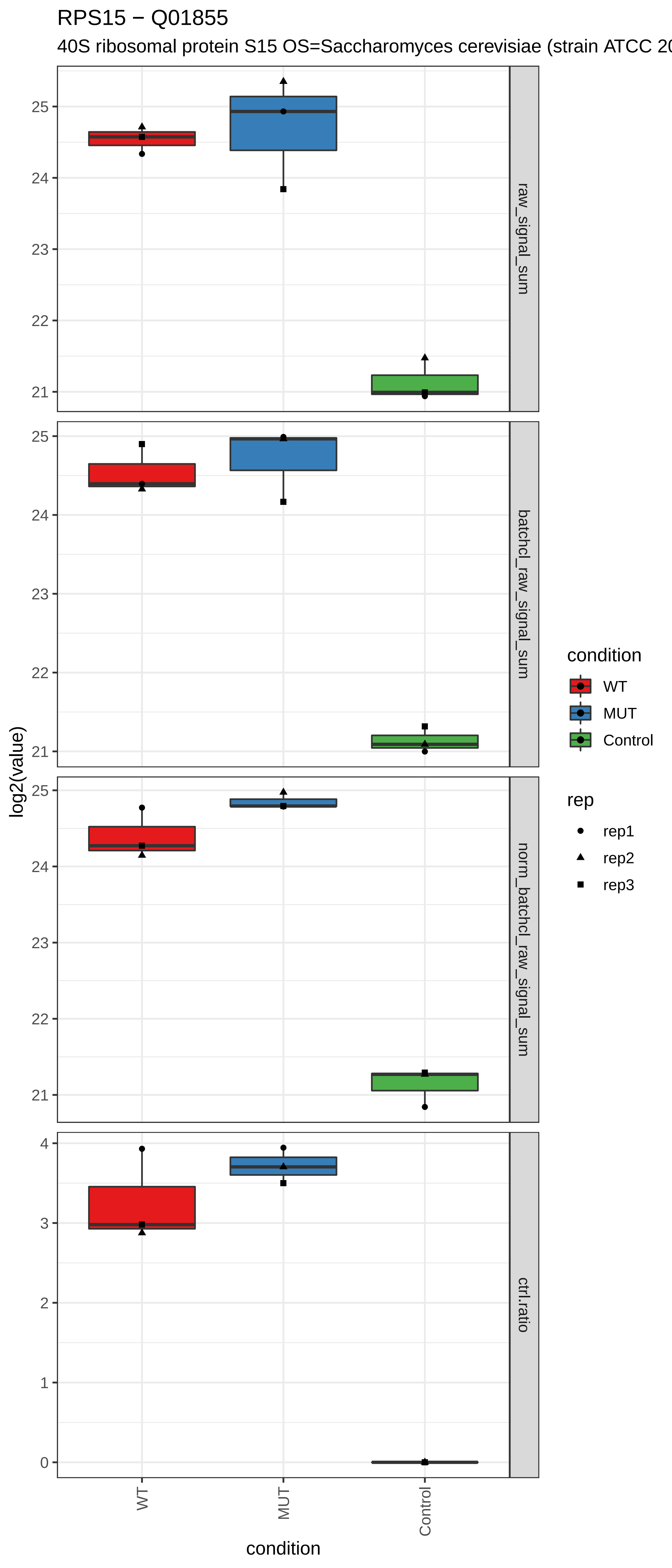

RPS15 − Q01855
40S ribosomal protein S15 OS=Saccharomyces cerevisiae (strain ATCC 20
raw_signal_sum
batchcl_raw_signal_sum
norm_batchcl_raw_signal_sum
ctrl.ratio
log2(value)
condition
WT
MUT
Control
rep
rep1
rep2
rep3

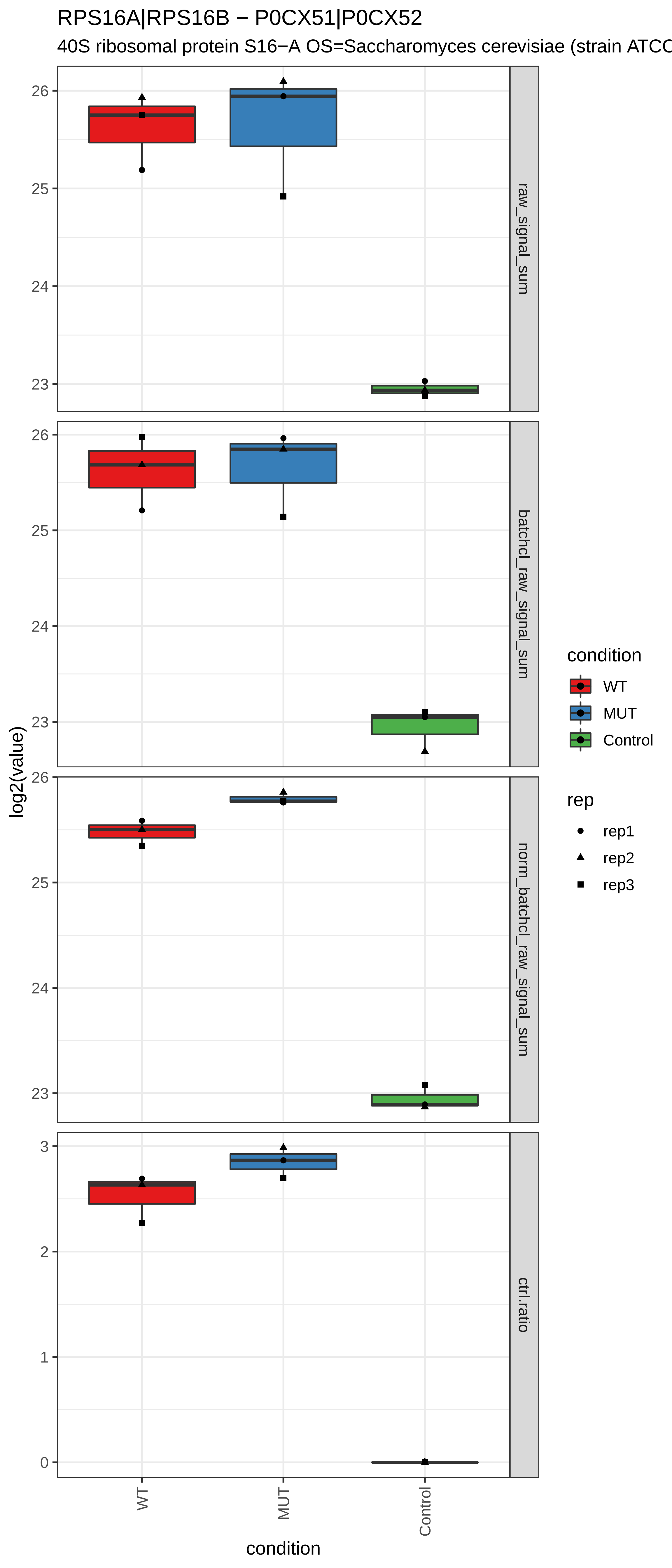

RPS16A|RPS16B − P0CX51|P0CX52
40S ribosomal protein S16−A OS=Saccharomyces cerevisiae (strain ATCC
raw_signal_sum
batchcl_raw_signal_sum
norm_batchcl_raw_signal_sum
ctrl.ratio
log2(value)
condition
WT
MUT
Control
rep
rep1
rep2
rep3
26
25
24
23
3
2
1
0

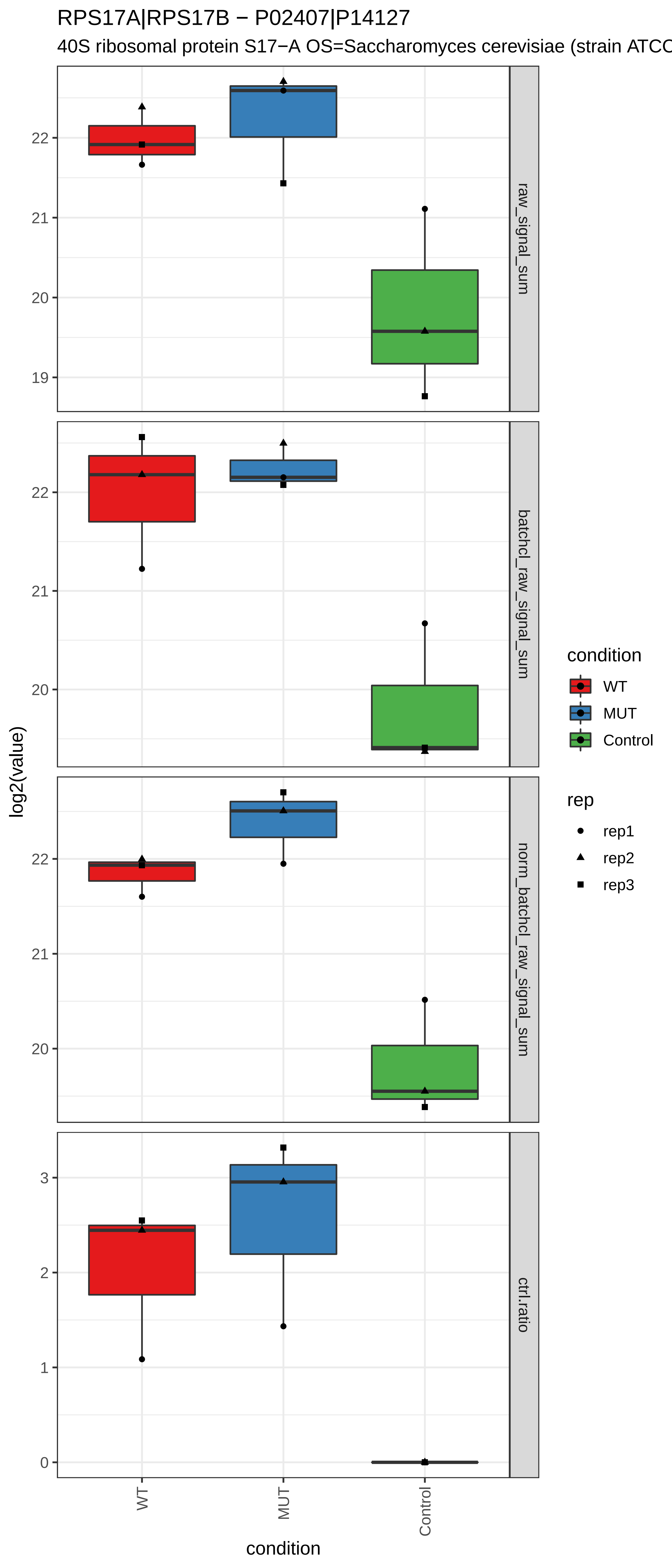

RPS17A|RPS17B − P02407|P14127
40S ribosomal protein S17−A OS=Saccharomyces cerevisiae (strain ATCC
raw_signal_sum
batchcl_raw_signal_sum
norm_batchcl_raw_signal_sum
ctrl.ratio
log2(value)
condition
WT
MUT
Control
rep
rep1
rep2
rep3
22
21
20
19
3
2
1
0

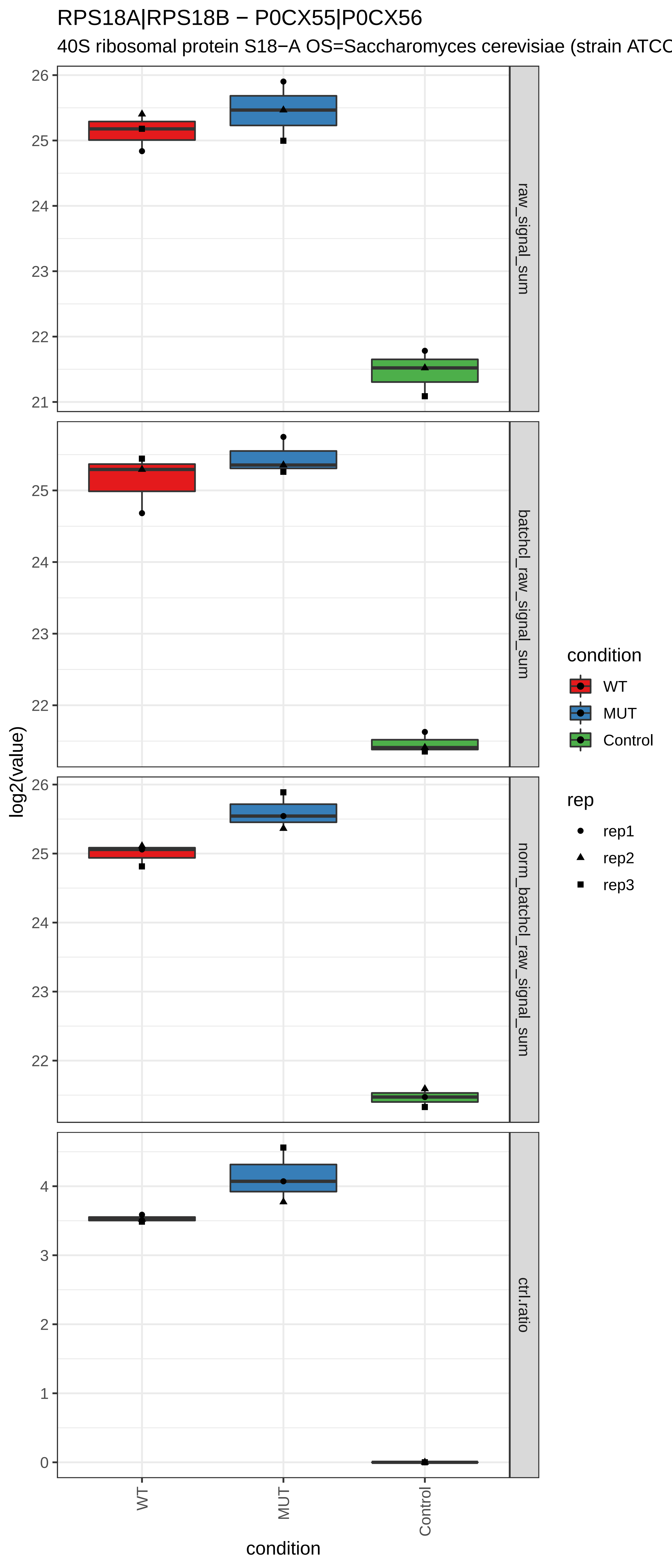

RPS18A|RPS18B − P0CX55|P0CX56
40S ribosomal protein S18−A OS=Saccharomyces cerevisiae (strain ATCC
raw_signal_sum
batchcl_raw_signal_sum
norm_batchcl_raw_signal_sum
ctrl.ratio
log2(value)
condition
WT
MUT
Control
rep
rep1
rep2
rep3

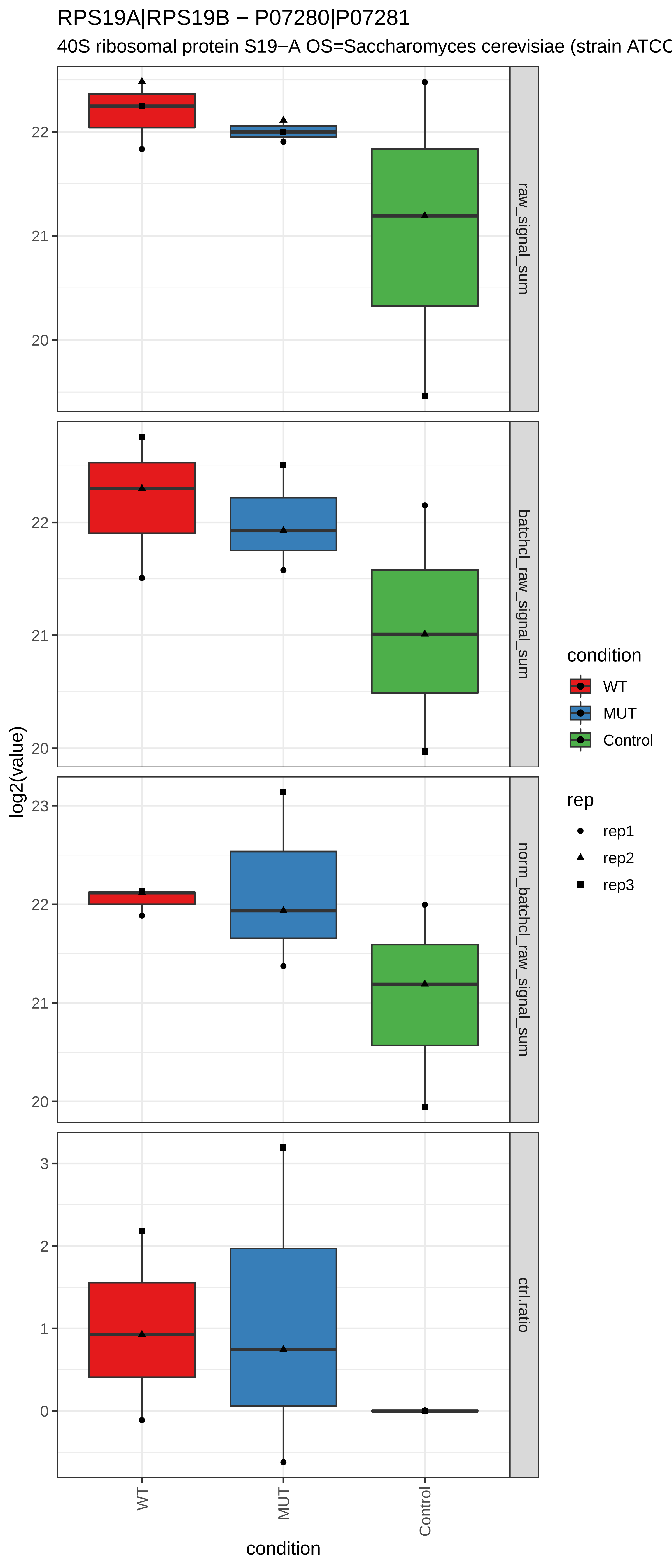

RPS19A|RPS19B − P07280|P07281
40S ribosomal protein S19−A OS=Saccharomyces cerevisiae (strain ATCC
raw_signal_sum
batchcl_raw_signal_sum
norm_batchcl_raw_signal_sum
ctrl.ratio
log2(value)
condition
WT
MUT
Control
rep
rep1
rep2
rep3

RPS1A − P33442

40S ribosomal protein S1−A OS=Saccharomyces cerevisiae (strain ATCC

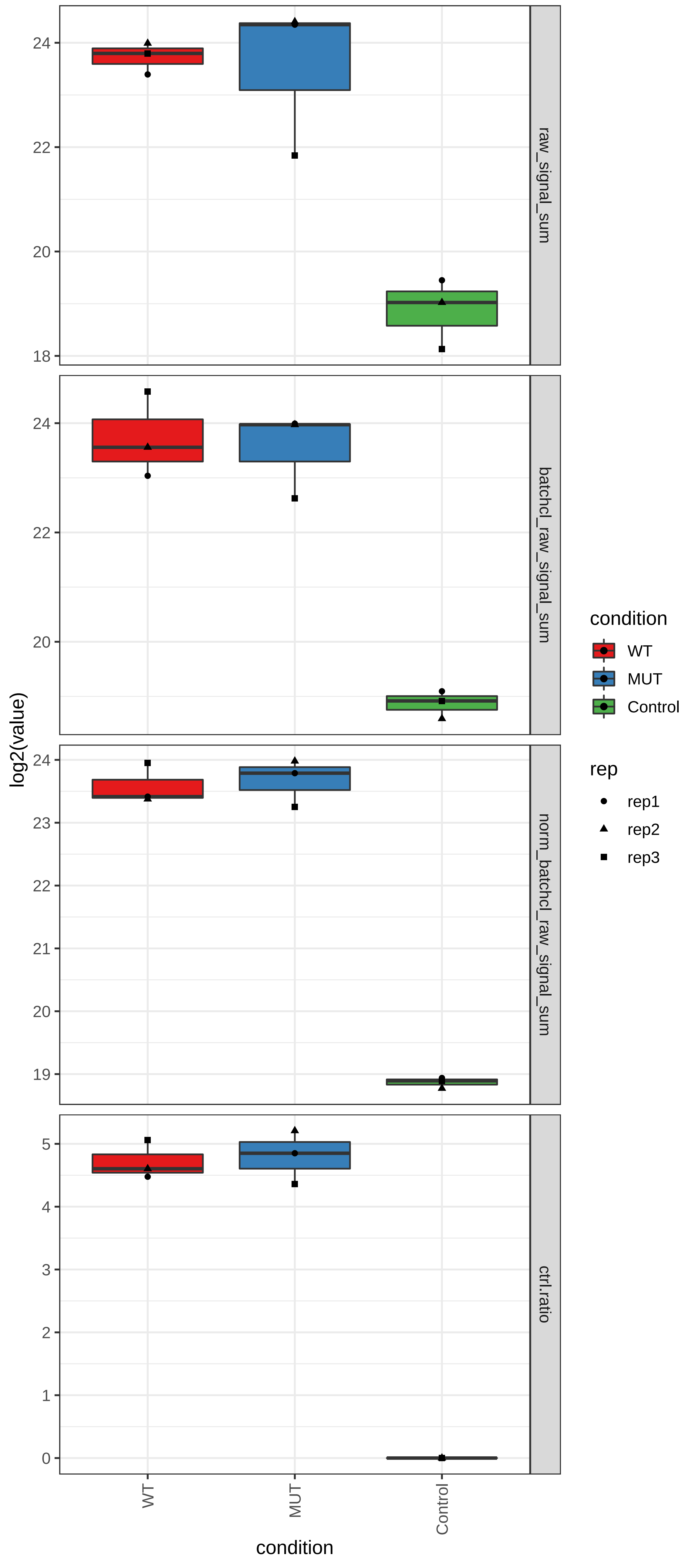

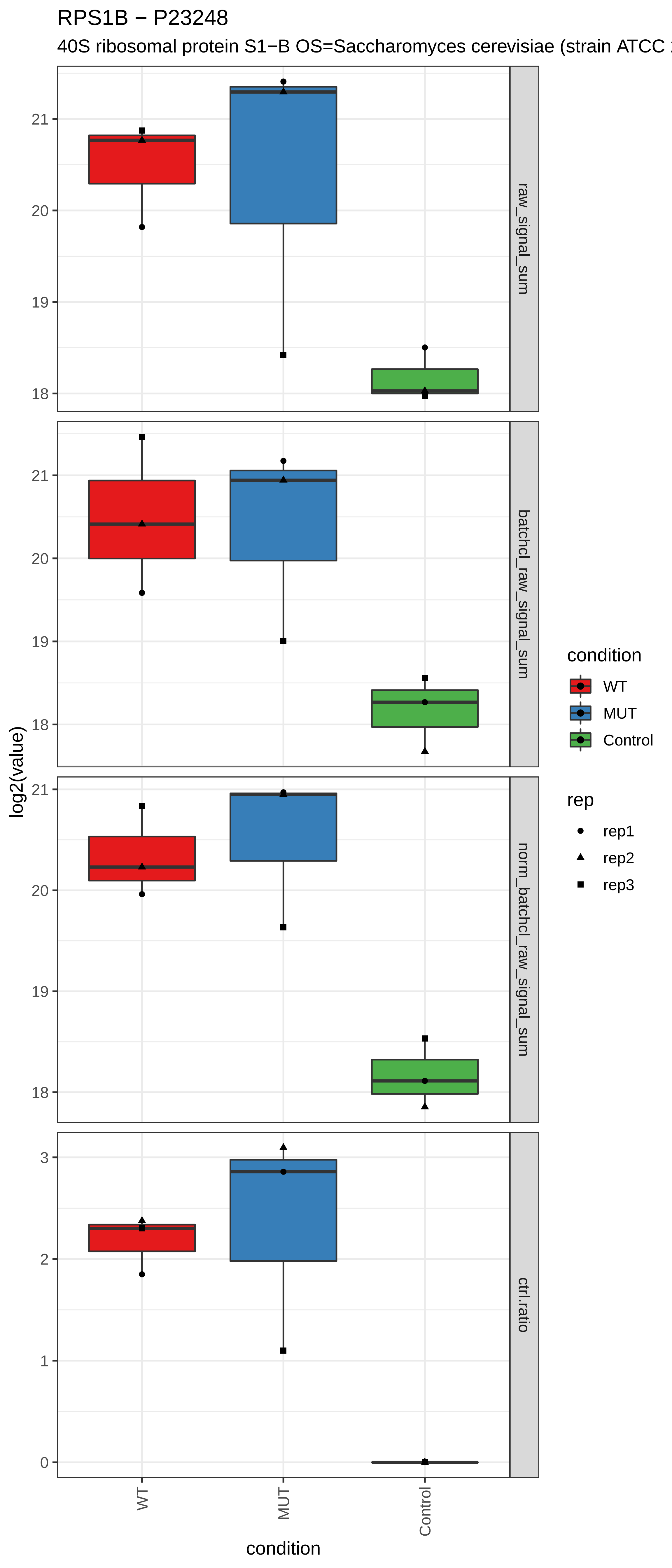
RPS1B − P23248
40S ribosomal protein S1−B OS=Saccharomyces cerevisiae (strain ATCC
raw_signal_sum
batchcl_raw_signal_sum
norm_batchcl_raw_signal_sum
ctrl.ratio
log2(value)
condition
WT
MUT
Control
rep
rep1
rep2
rep3
21
20
19
18
3
2
1
0

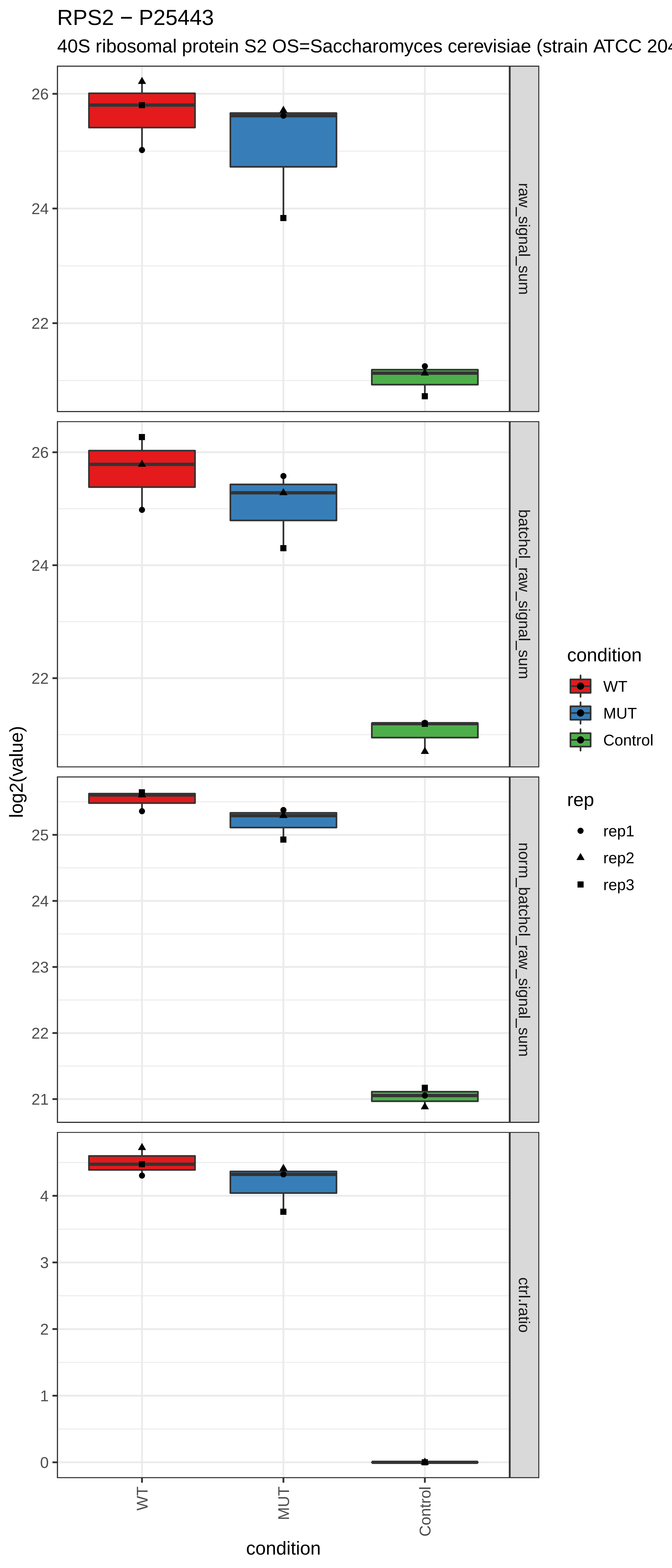
RPS2 − P25443
40S ribosomal protein S2 OS=Saccharomyces cerevisiae (strain ATCC 204
raw_signal_sum
batchcl_raw_signal_sum
norm_batchcl_raw_signal_sum
ctrl.ratio
log2(value)
condition
WT
MUT
Control
rep
rep1
rep2
rep3
26
24
22
25
23
21
4
3
2
1
0

RPS20 − P38701

40S ribosomal protein S20 OS=Saccharomyces cerevisiae (strain ATCC 20

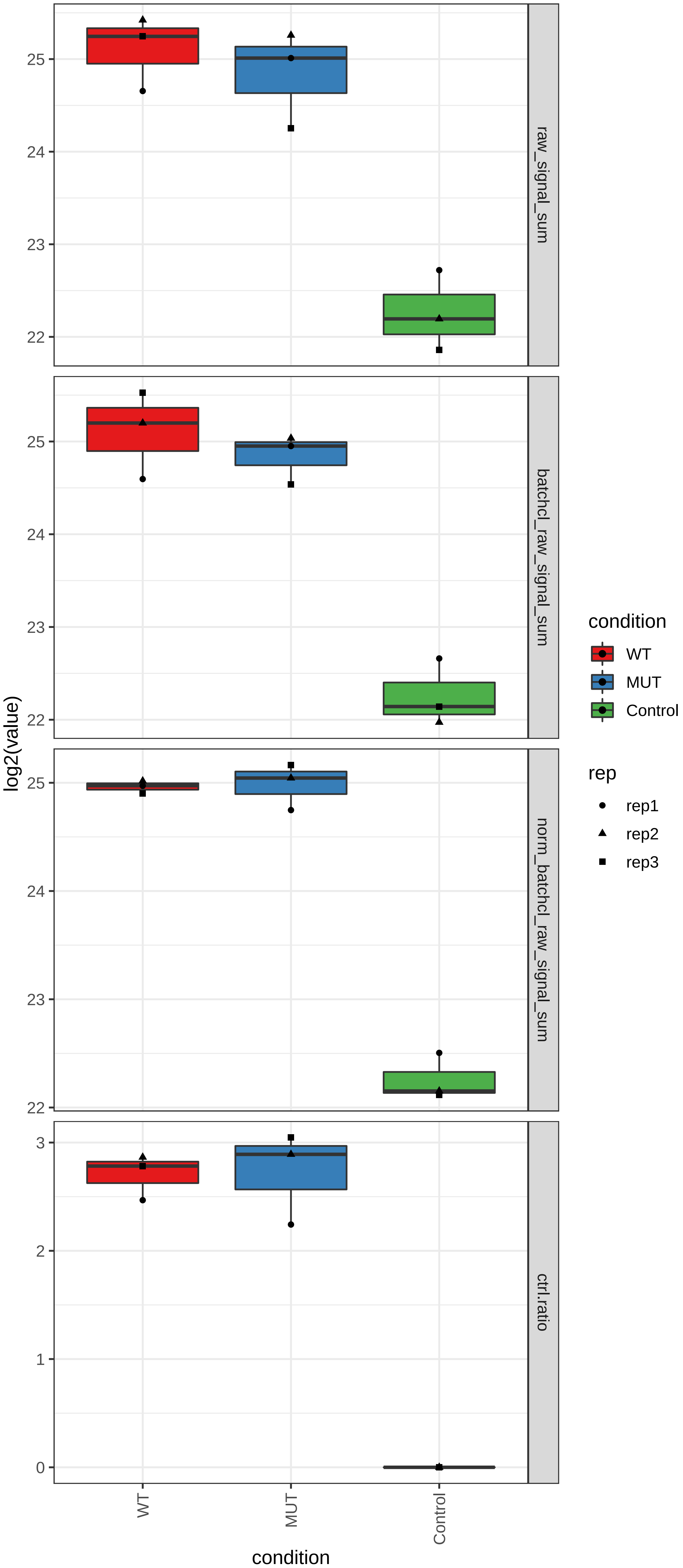

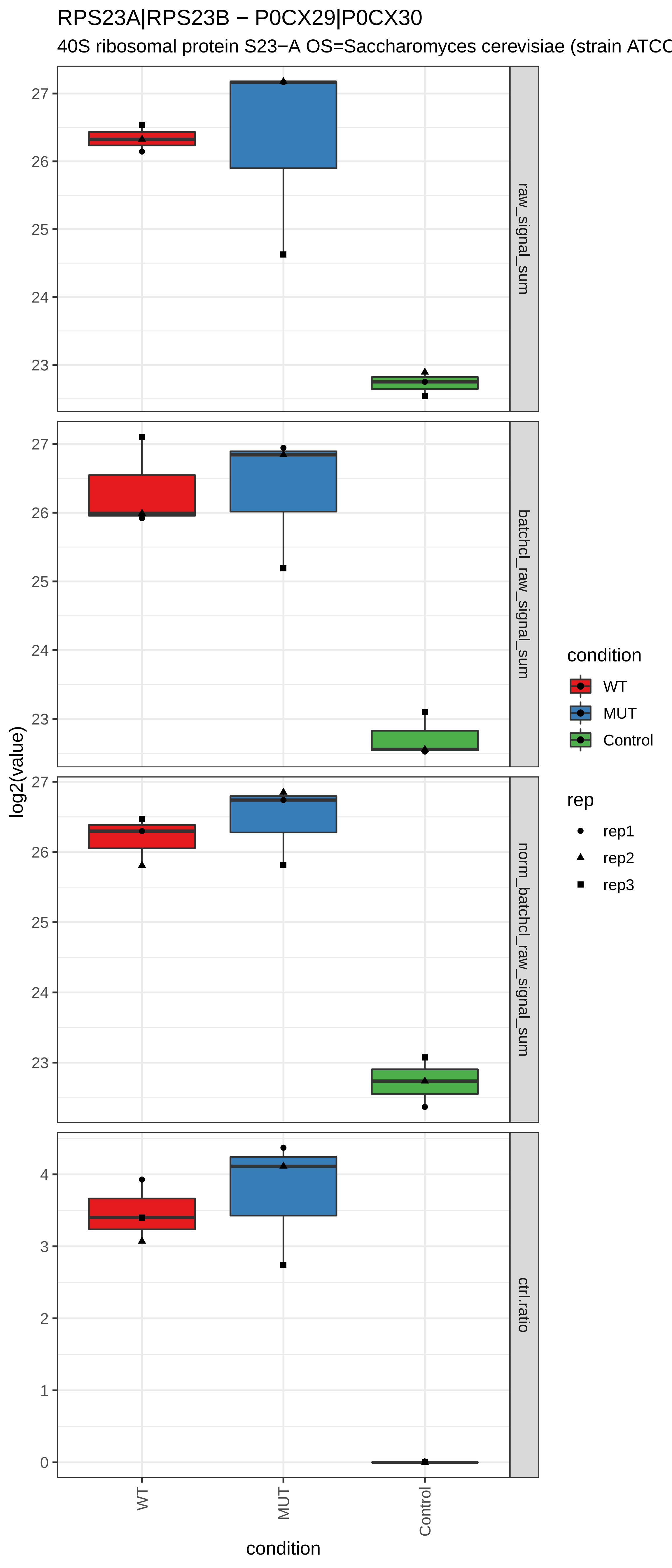

RPS23A|RPS23B − P0CX29|P0CX30
40S ribosomal protein S23−A OS=Saccharomyces cerevisiae (strain ATCC
raw_signal_sum
batchcl_raw_signal_sum
norm_batchcl_raw_signal_sum
ctrl.ratio
log2(value)
condition
WT
MUT
Control
rep
rep1
rep2
rep3

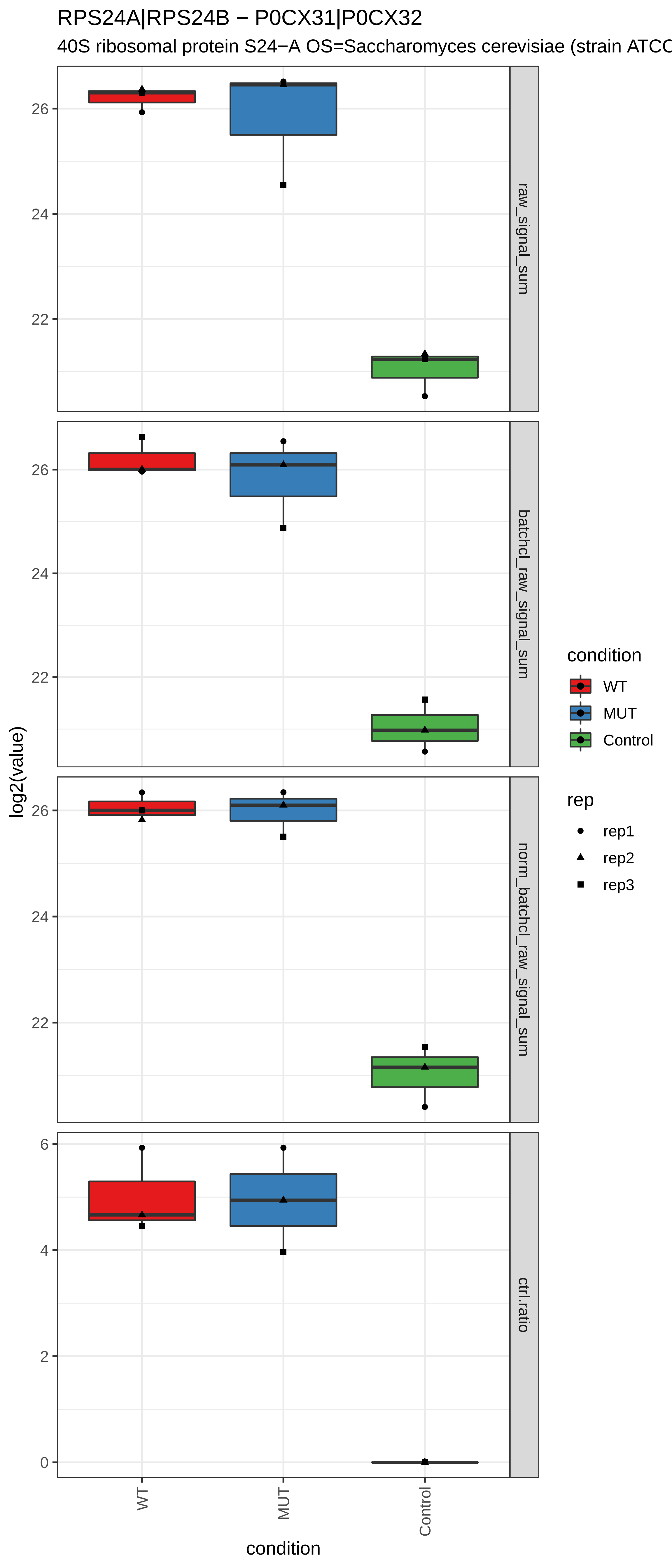

RPS24A|RPS24B − P0CX31|P0CX32
40S ribosomal protein S24−A OS=Saccharomyces cerevisiae (strain ATCC
raw_signal_sum
batchcl_raw_signal_sum
norm_batchcl_raw_signal_sum
ctrl.ratio
log2(value)
condition
WT
MUT
Control
rep
rep1
rep2
rep3

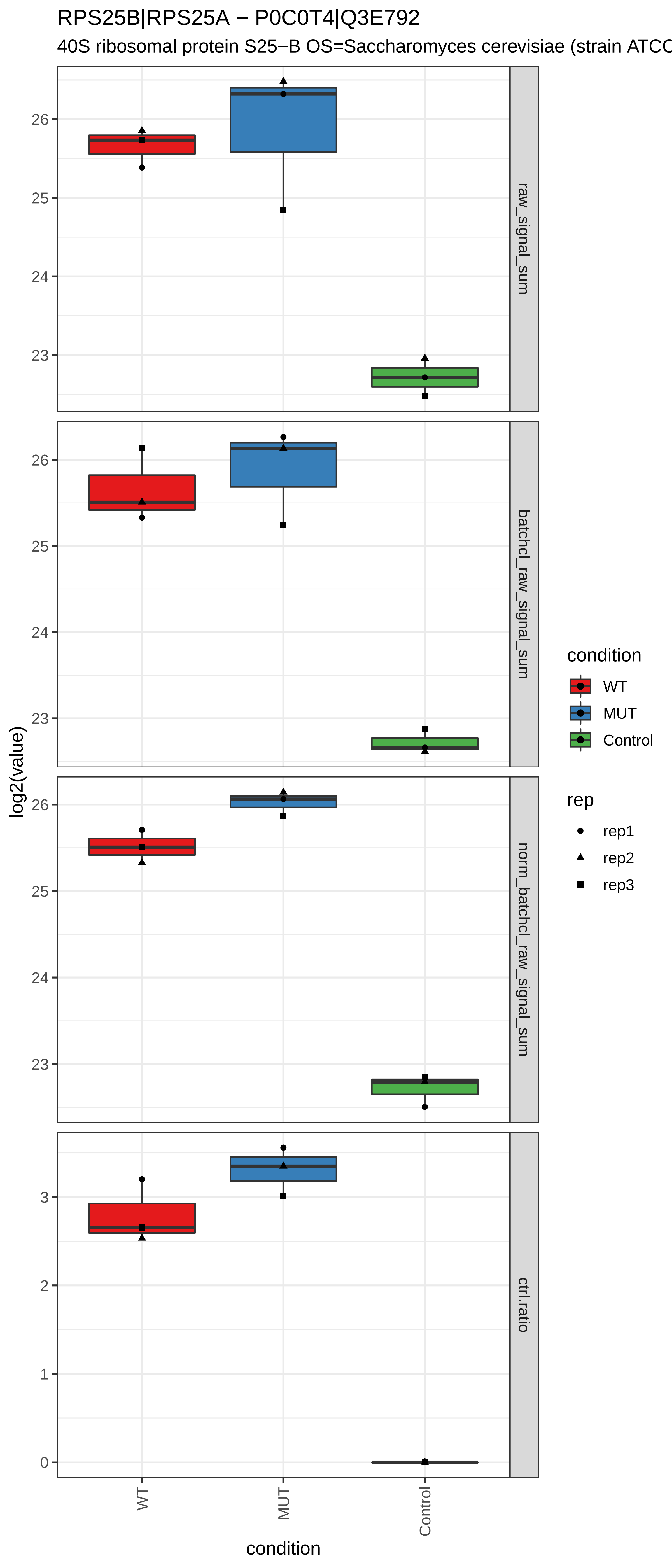

RPS25B|RPS25A − P0C0T4|Q3E792
40S ribosomal protein S25−B OS=Saccharomyces cerevisiae (strain ATCC
raw_signal_sum
batchcl_raw_signal_sum
norm_batchcl_raw_signal_sum
ctrl.ratio
log2(value)
condition
WT
MUT
Control
rep
rep1
rep2
rep3
26
25
24
23
3
2
1
0

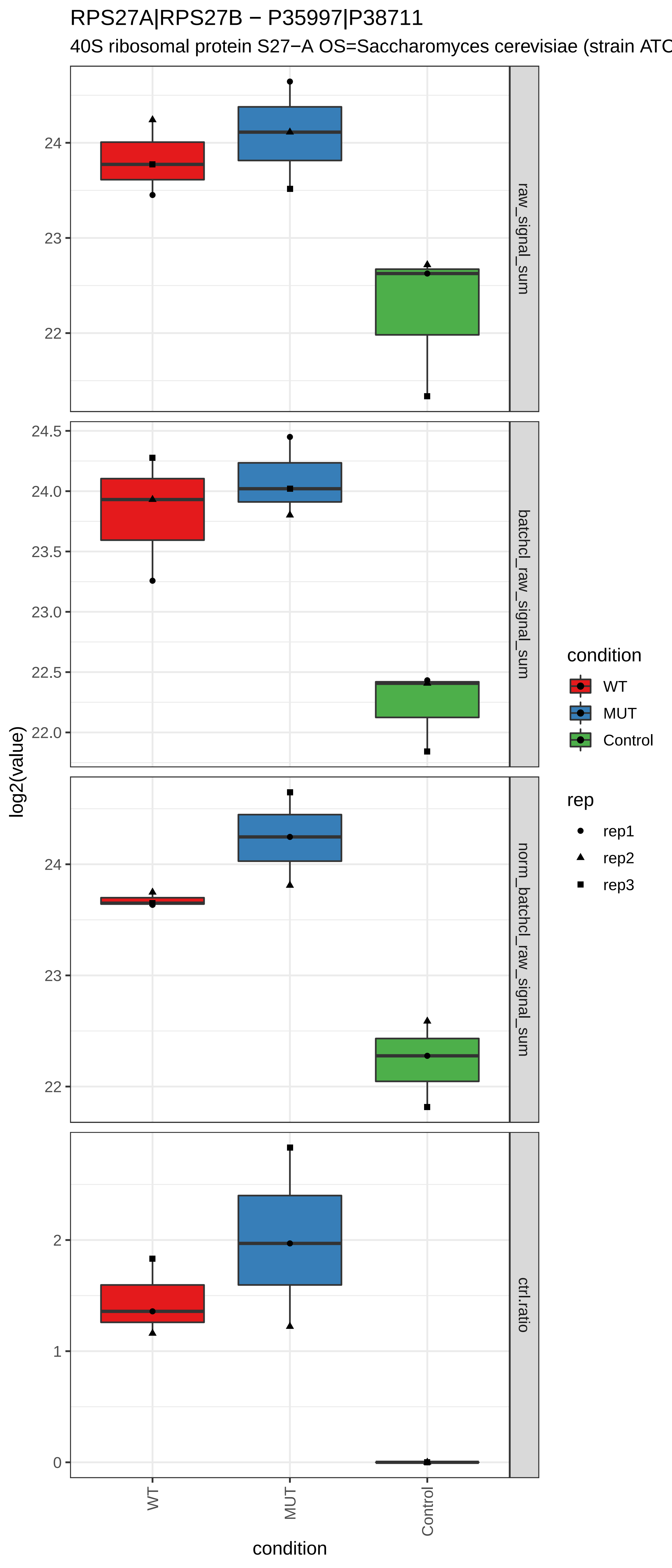

RPS27A|RPS27B − P35997|P38711
40S ribosomal protein S27−A OS=Saccharomyces cerevisiae (strain ATC
raw_signal_sum
batchcl_raw_signal_sum
norm_batchcl_raw_signal_sum
ctrl.ratio
log2(value)
condition
WT
MUT
Control
rep
rep1
rep2
rep3

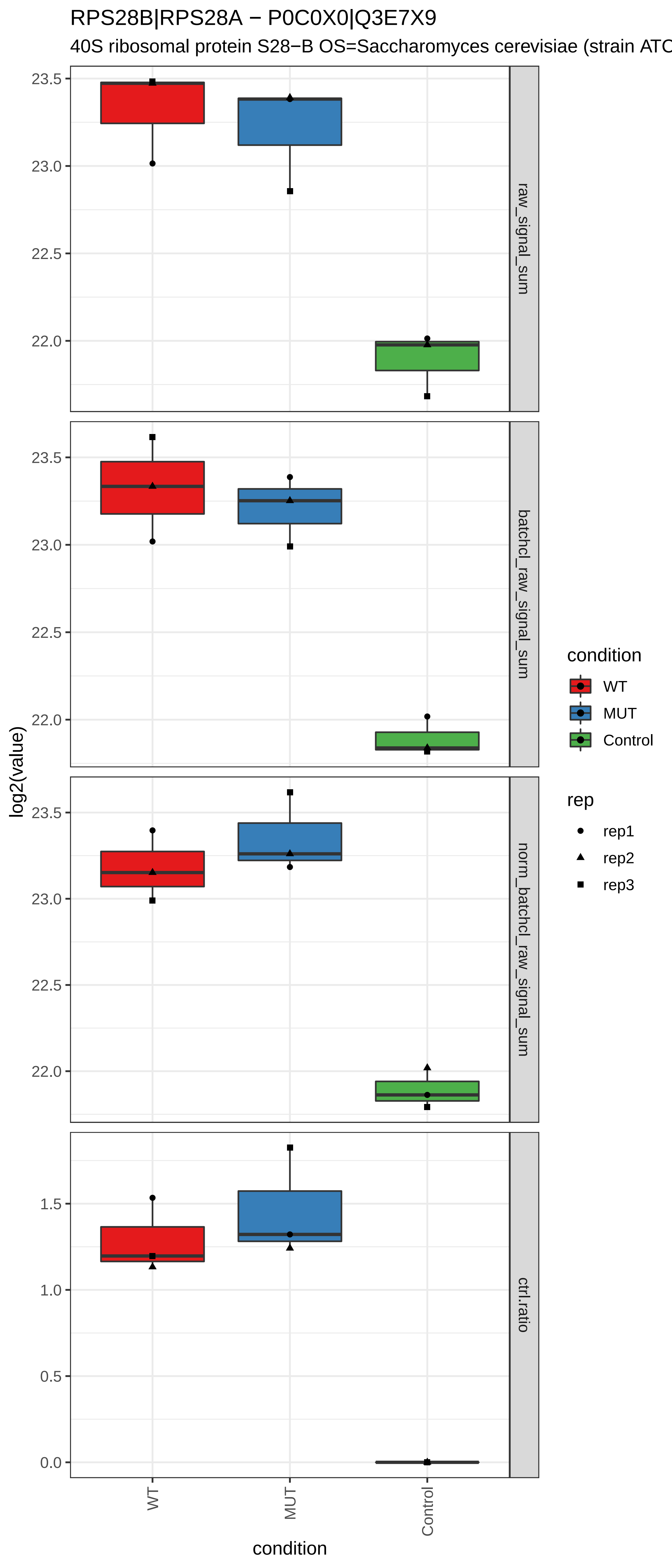

RPS28B|RPS28A − P0C0X0|Q3E7X9
40S ribosomal protein S28−B OS=Saccharomyces cerevisiae (strain ATC
raw_signal_sum
batchcl_raw_signal_sum
norm_batchcl_raw_signal_sum
ctrl.ratio
log2(value)
condition
WT
MUT
Control
rep
rep1
rep2
rep3
23.5
23.0
22.5
22.0
1.5
1.0
0.5
0.0

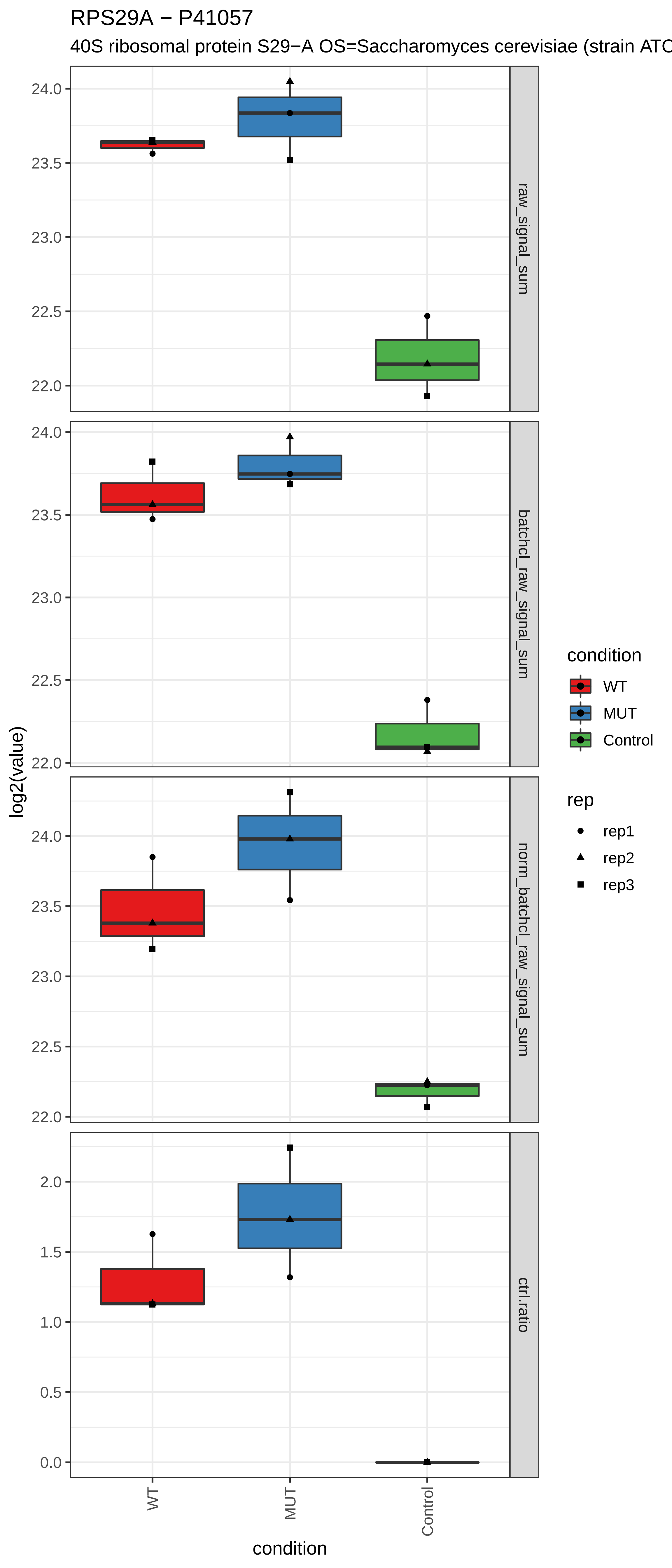
RPS29A − P41057
40S ribosomal protein S29−A OS=Saccharomyces cerevisiae (strain ATC
raw_signal_sum
batchcl_raw_signal_sum
norm_batchcl_raw_signal_sum
ctrl.ratio
log2(value)
condition
WT
MUT
Control
rep
rep1
rep2
rep3
24.0
23.5
23.0
22.5
22.0
2.0
1.5
1.0
0.5
0.0

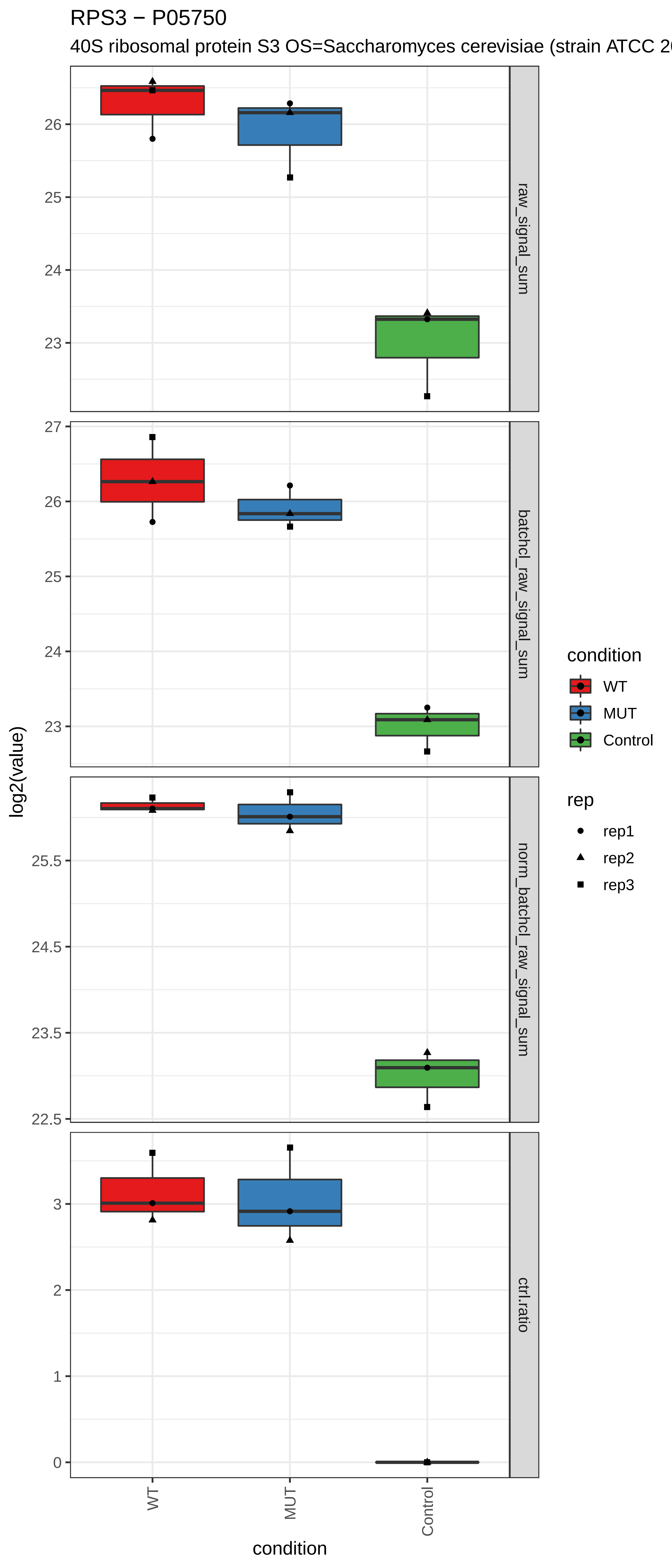
RPS3 − P05750
40S ribosomal protein S3 OS=Saccharomyces cerevisiae (strain ATCC 2
raw_signal_sum
batchcl_raw_signal_sum
norm_batchcl_raw_signal_sum
ctrl.ratio
log2(value)
condition
WT
MUT
Control
rep
rep1
rep2
rep3
26
25
24
23
27
25.5
24.5
23.5
22.5
3
2
1
0

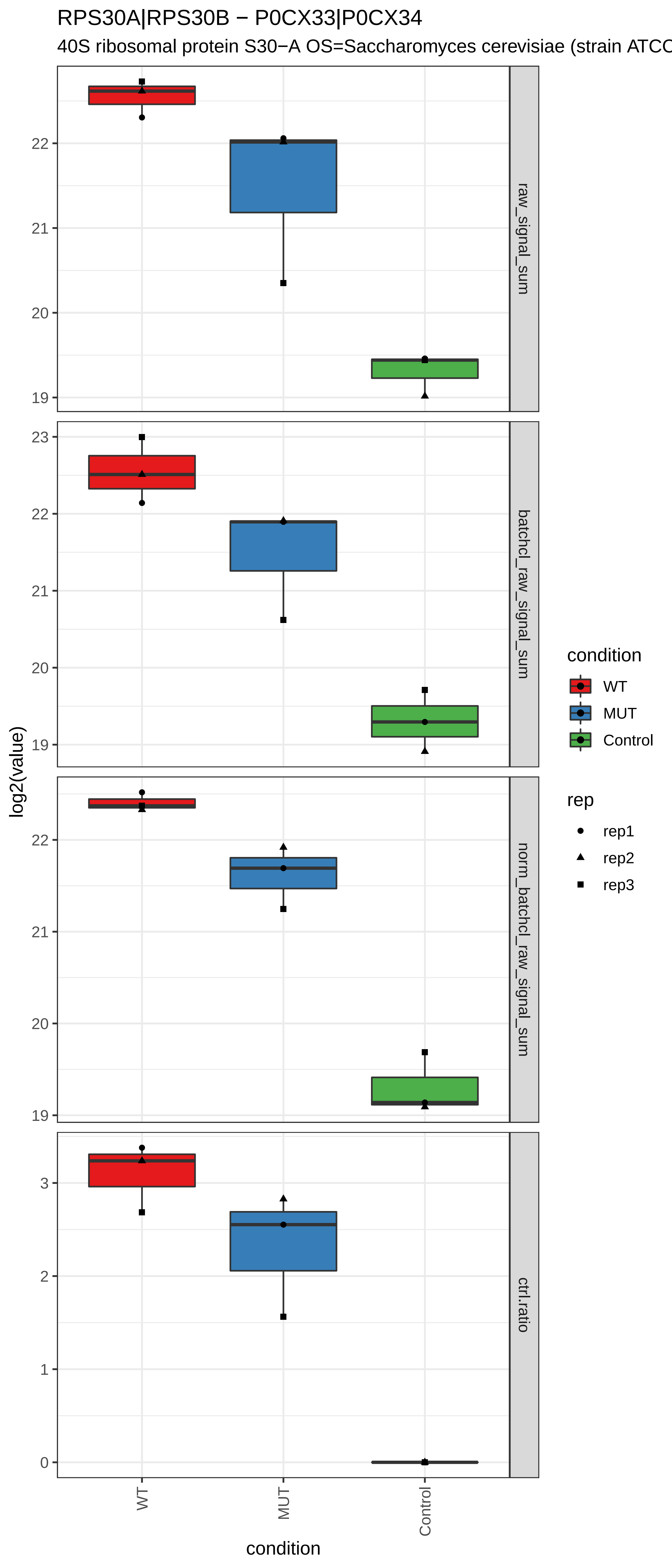
RPS30A|RPS30B − P0CX33|P0CX34
40S ribosomal protein S30−A OS=Saccharomyces cerevisiae (strain ATCC
raw_signal_sum
batchcl_raw_signal_sum
norm_batchcl_raw_signal_sum
ctrl.ratio
log2(value)
condition
WT
MUT
Control
rep
rep1
rep2
rep3
19
20
21
22
23
0
1
2
3

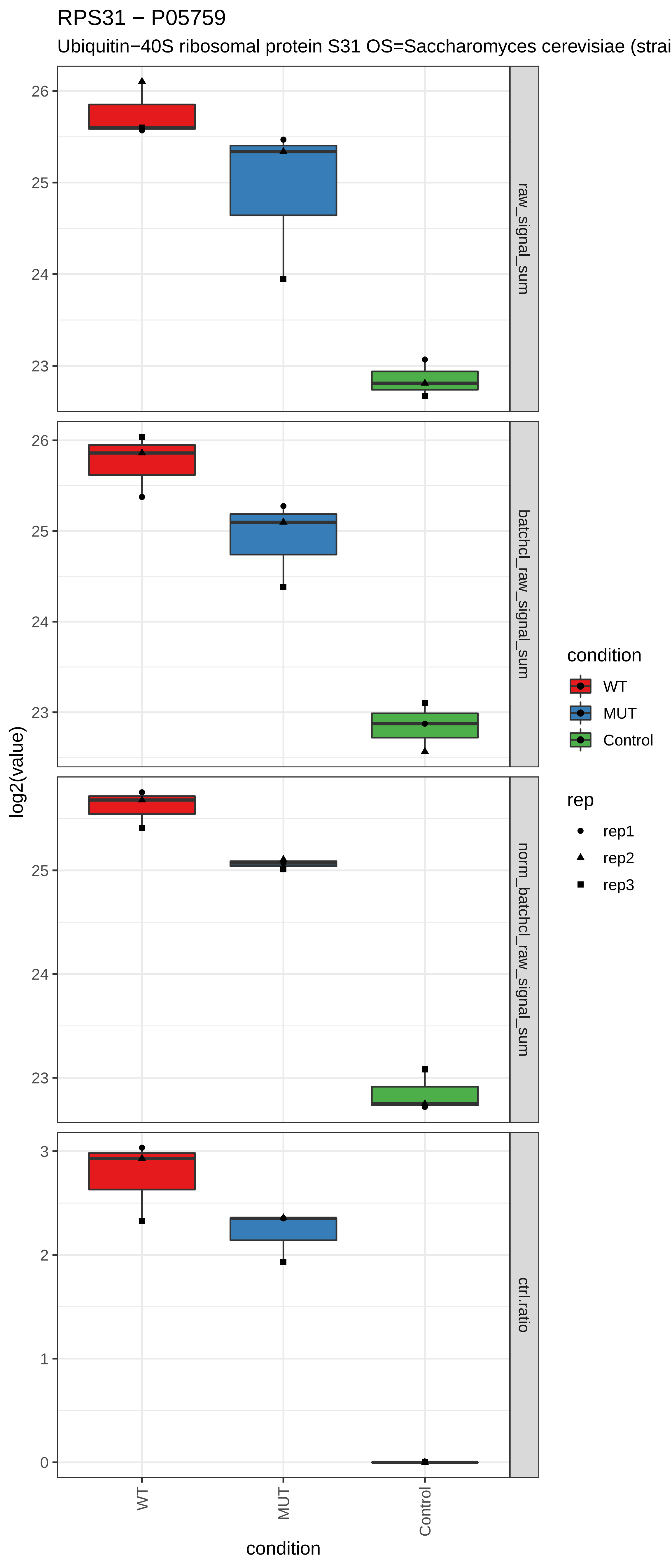

RPS31 − P05759
Ubiquitin−40S ribosomal protein S31 OS=Saccharomyces cerevisiae (strai
raw_signal_sum
batchcl_raw_signal_sum
norm_batchcl_raw_signal_sum
ctrl.ratio
log2(value)
condition
WT
MUT
Control
rep
rep1
rep2
rep3

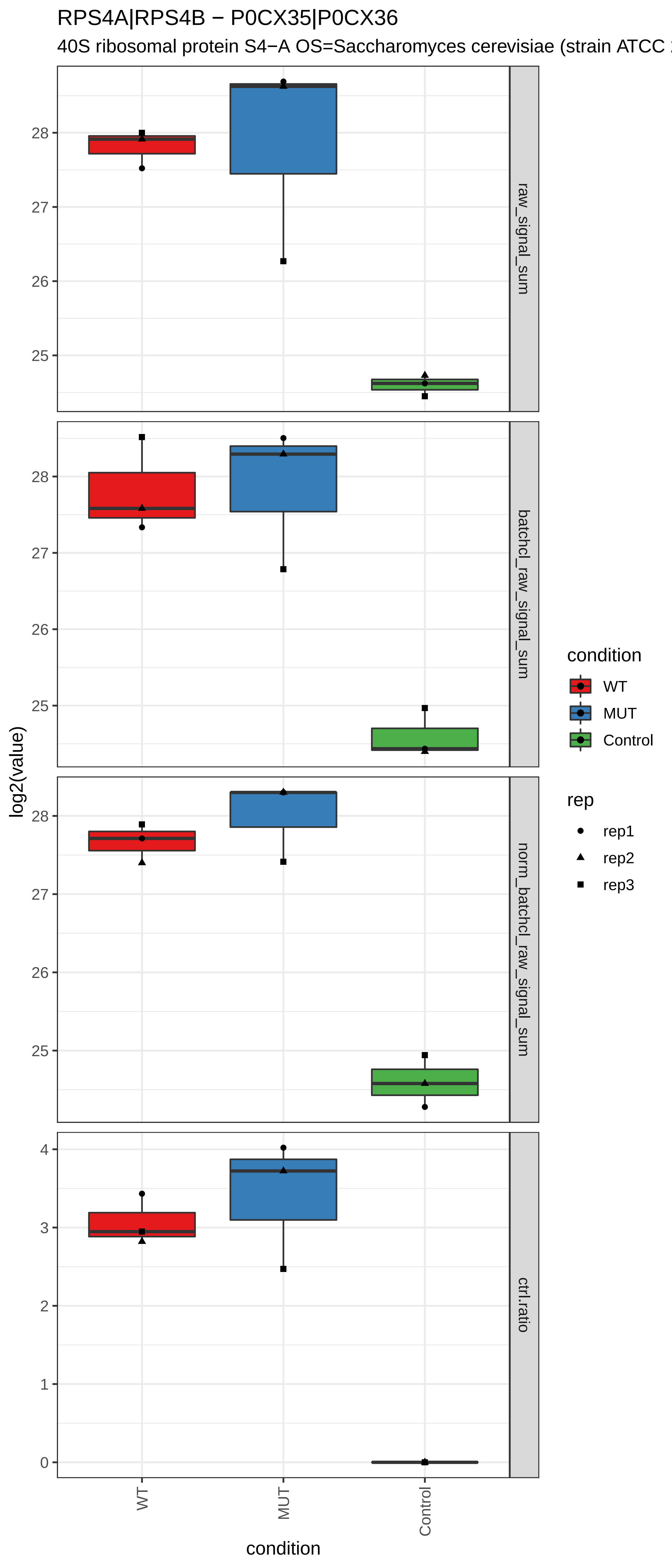

RPS4A|RPS4B − P0CX35|P0CX36
40S ribosomal protein S4−A OS=Saccharomyces cerevisiae (strain ATCC
raw_signal_sum
batchcl_raw_signal_sum
norm_batchcl_raw_signal_sum
ctrl.ratio
log2(value)
condition
WT
MUT
Control
rep
rep1
rep2
rep3
28
27
26
25
4
3
2
1
0

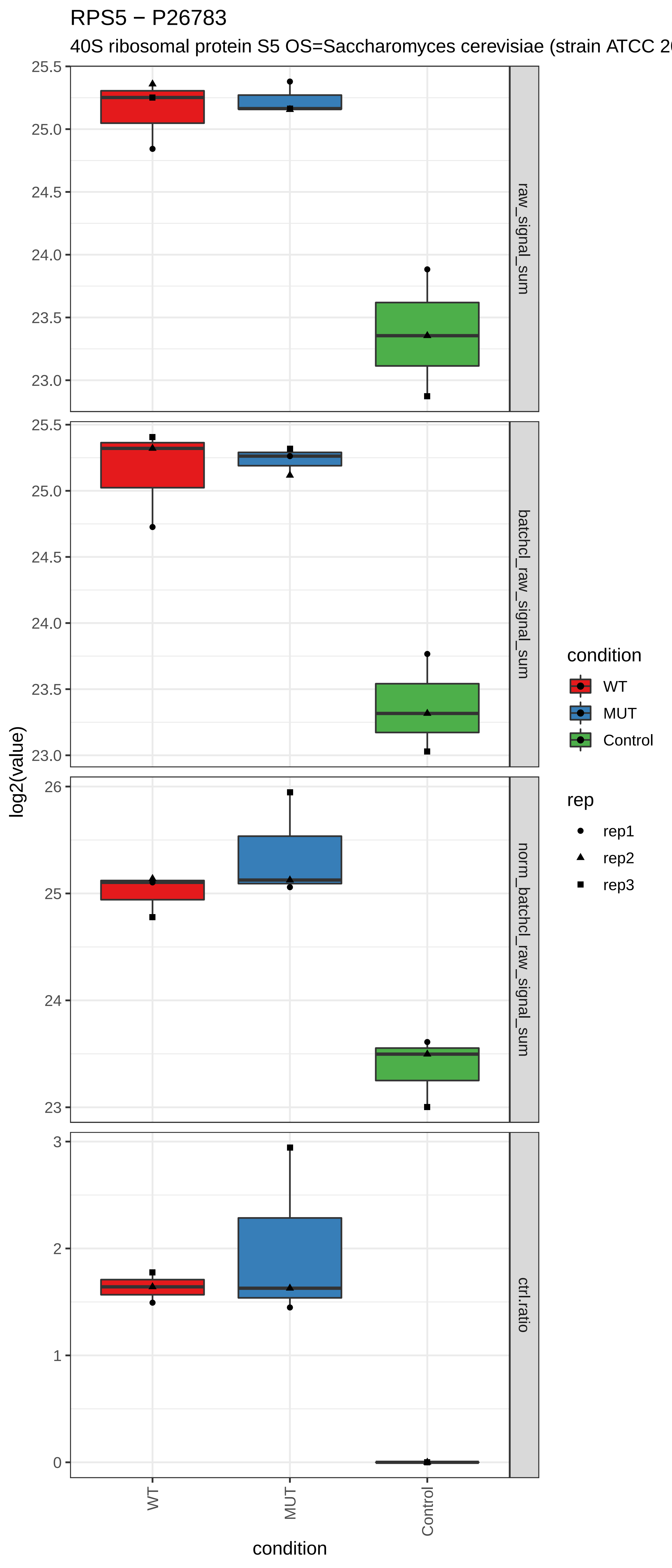

RPS5 − P26783
40S ribosomal protein S5 OS=Saccharomyces cerevisiae (strain ATCC 2
raw_signal_sum
batchcl_raw_signal_sum
norm_batchcl_raw_signal_sum
ctrl.ratio
log2(value)
condition
WT
MUT
Control
rep
rep1
rep2
rep3

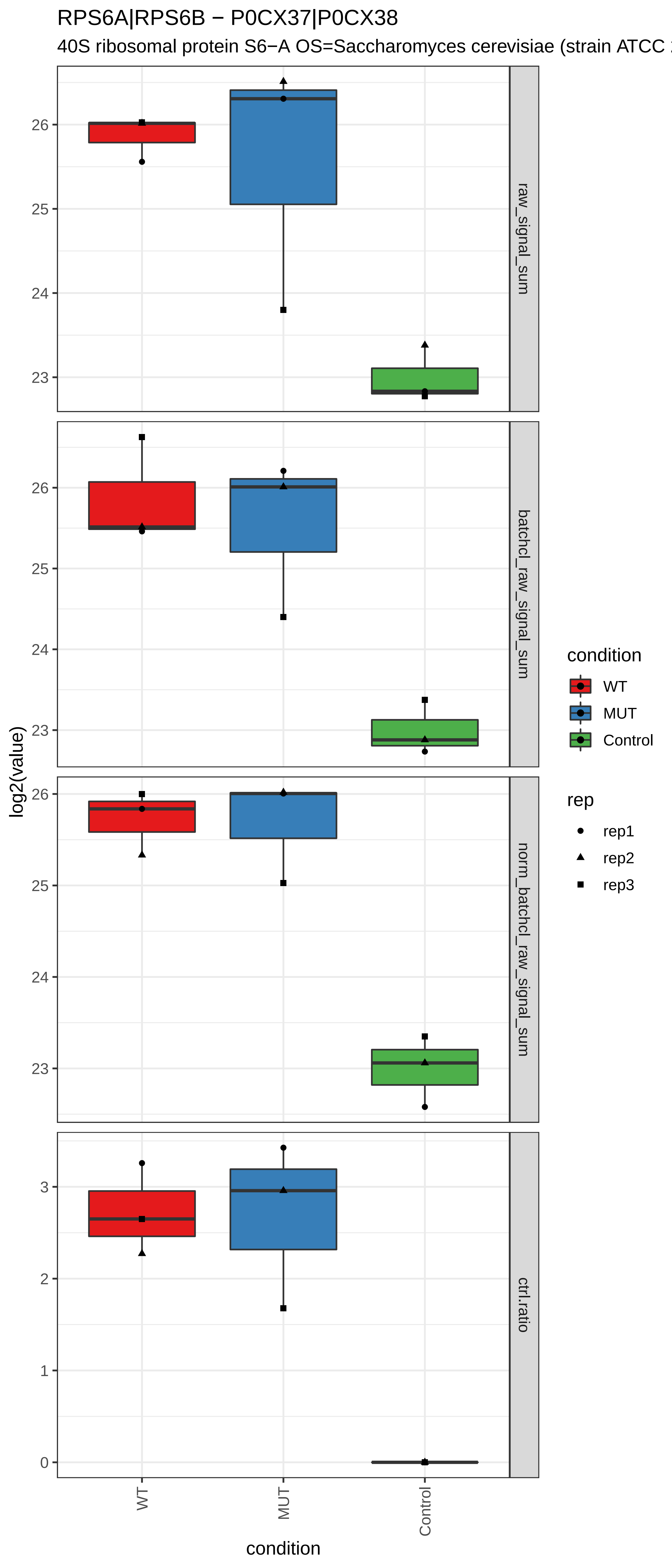
RPS6A|RPS6B − P0CX37|P0CX38
40S ribosomal protein S6−A OS=Saccharomyces cerevisiae (strain ATCC
raw_signal_sum
batchcl_raw_signal_sum
norm_batchcl_raw_signal_sum
ctrl.ratio
log2(value)
condition
WT
MUT
Control
rep
rep1
rep2
rep3
26
25
24
23
3
2
1
0

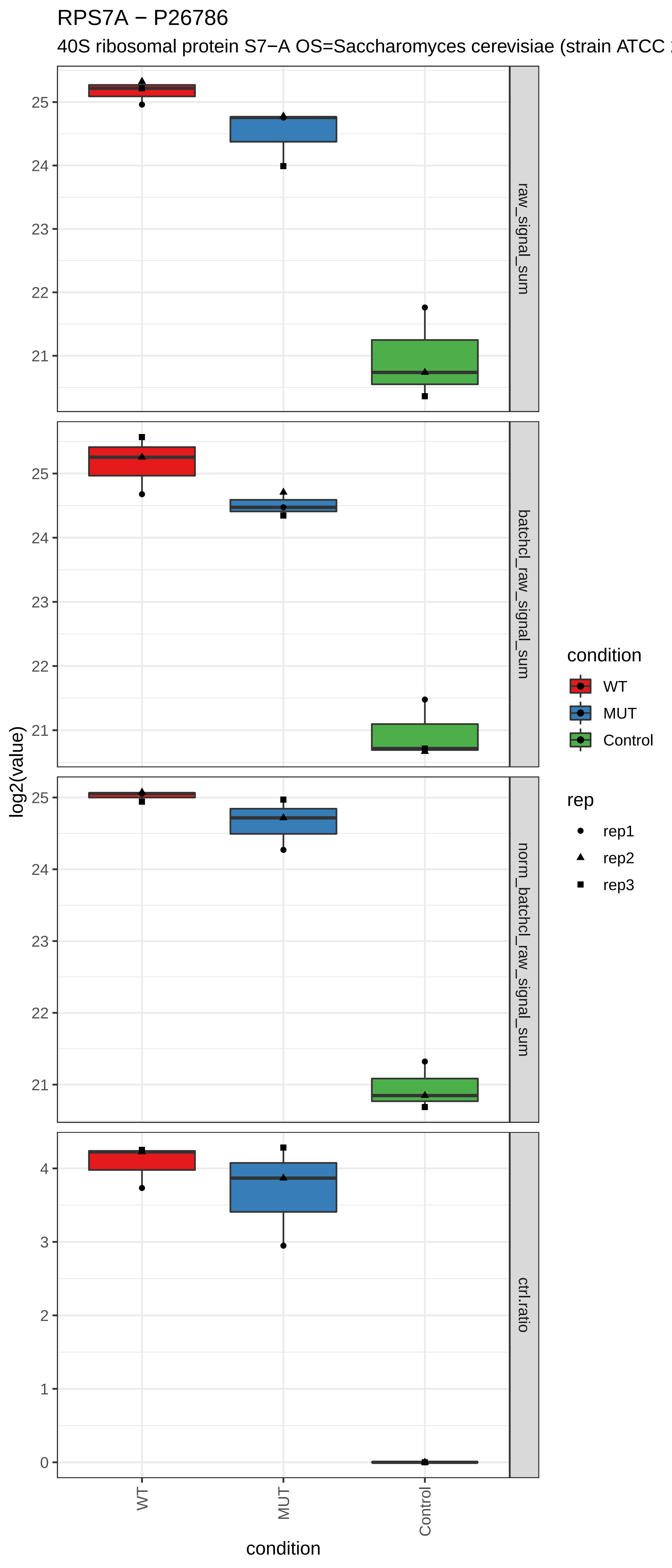

RPS7A − P26786
40S ribosomal protein S7−A OS=Saccharomyces cerevisiae (strain ATCC
raw_signal_sum
batchcl_raw_signal_sum
norm_batchcl_raw_signal_sum
ctrl.ratio
log2(value)
condition
WT
MUT
Control
rep
rep1
rep2
rep3

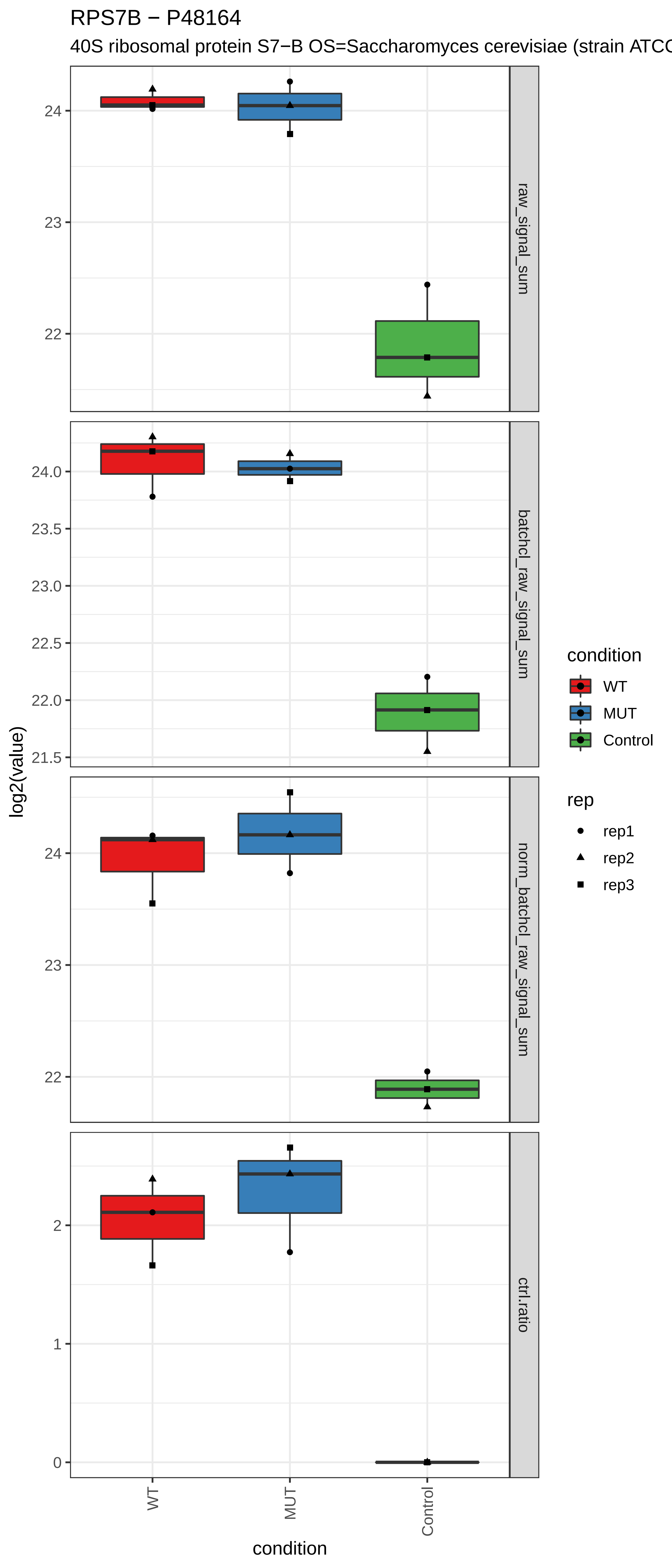
RPS7B − P48164
40S ribosomal protein S7−B OS=Saccharomyces cerevisiae (strain ATCC
raw_signal_sum
batchcl_raw_signal_sum
norm_batchcl_raw_signal_sum
ctrl.ratio
log2(value)
condition
WT
MUT
Control
rep
rep1
rep2
rep3
24
23
22
24.0
23.5
23.0
22.5
22.0
21.5
2
1
0

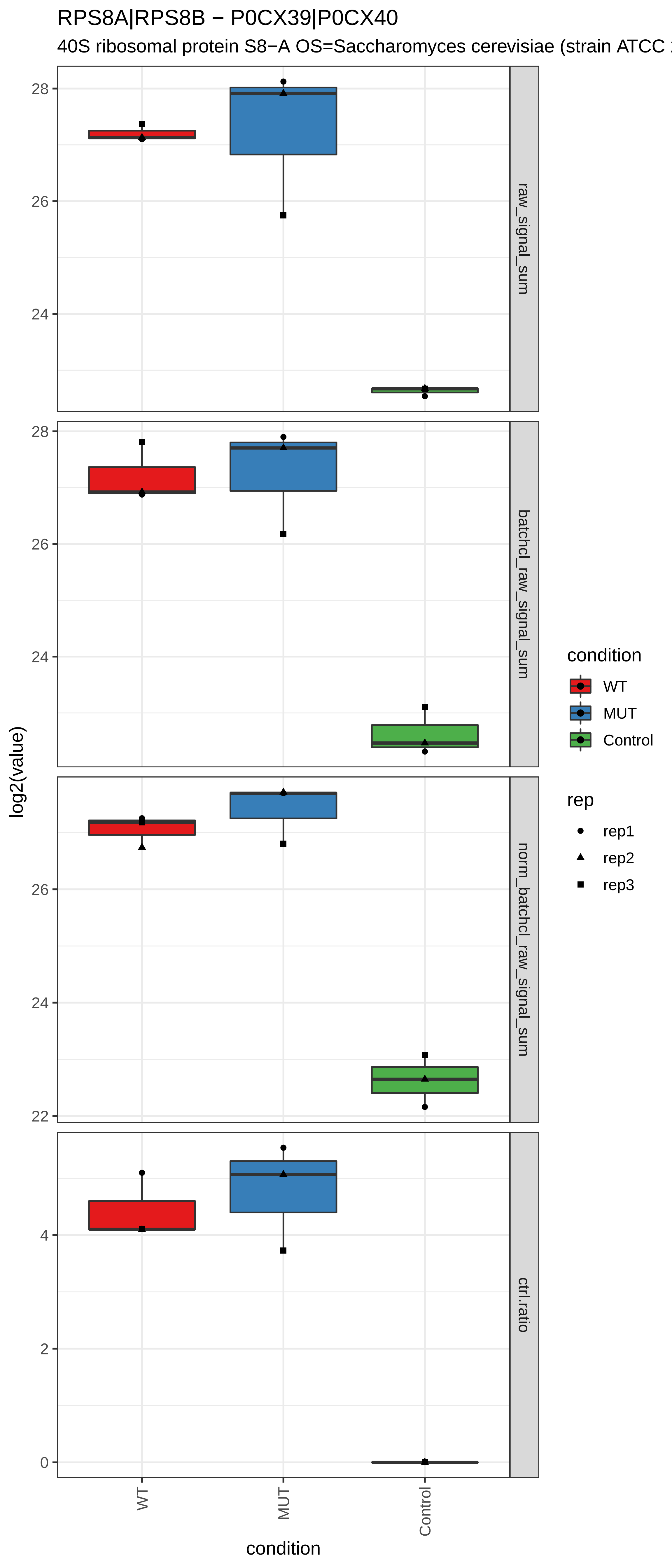

RPS8A|RPS8B − P0CX39|P0CX40
40S ribosomal protein S8−A OS=Saccharomyces cerevisiae (strain ATCC
raw_signal_sum
batchcl_raw_signal_sum
norm_batchcl_raw_signal_sum
ctrl.ratio
log2(value)
condition
WT
MUT
Control
rep
rep1
rep2
rep3
28
26
24
22
4
2
0

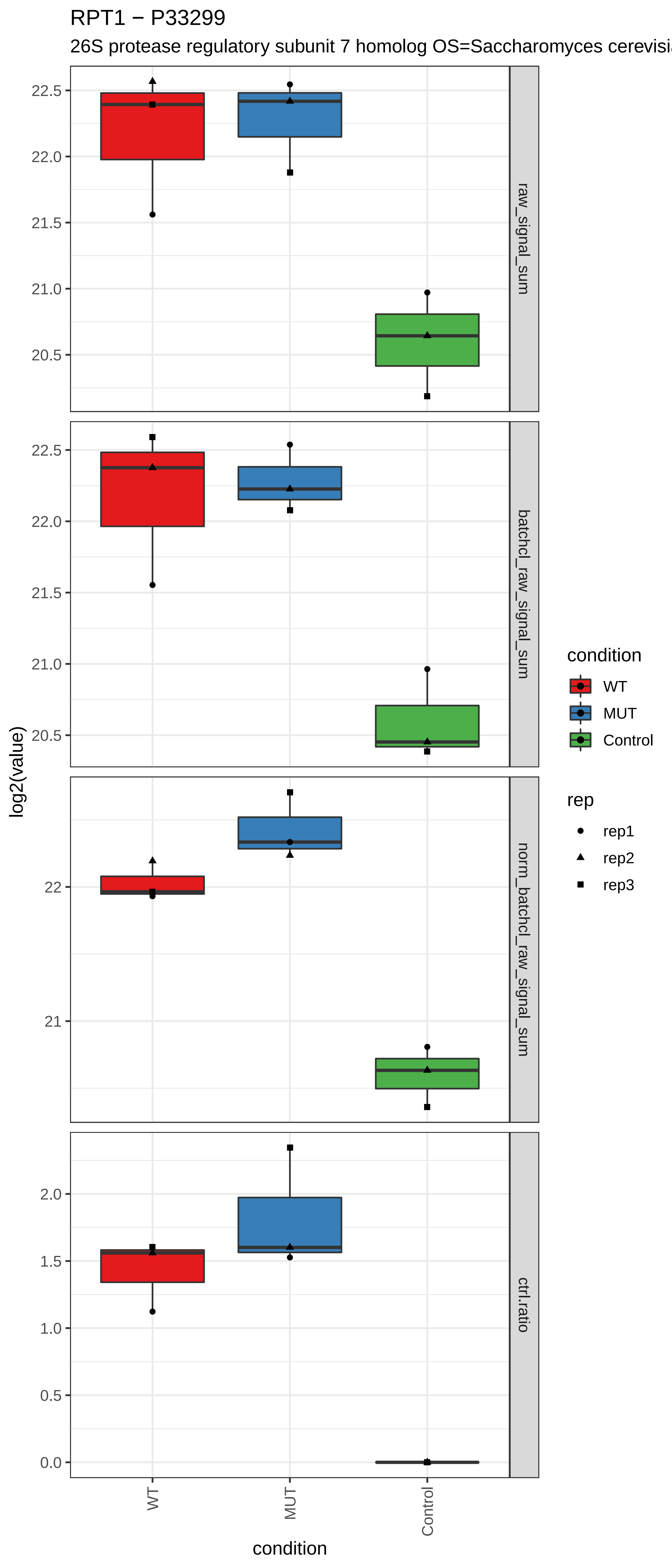
RPT1 − P33299
26S protease regulatory subunit 7 homolog OS=Saccharomyces cerevisi
raw_signal_sum
batchcl_raw_signal_sum
norm_batchcl_raw_signal_sum
ctrl.ratio
log2(value)
condition
WT
MUT
Control
rep
rep1
rep2
rep3

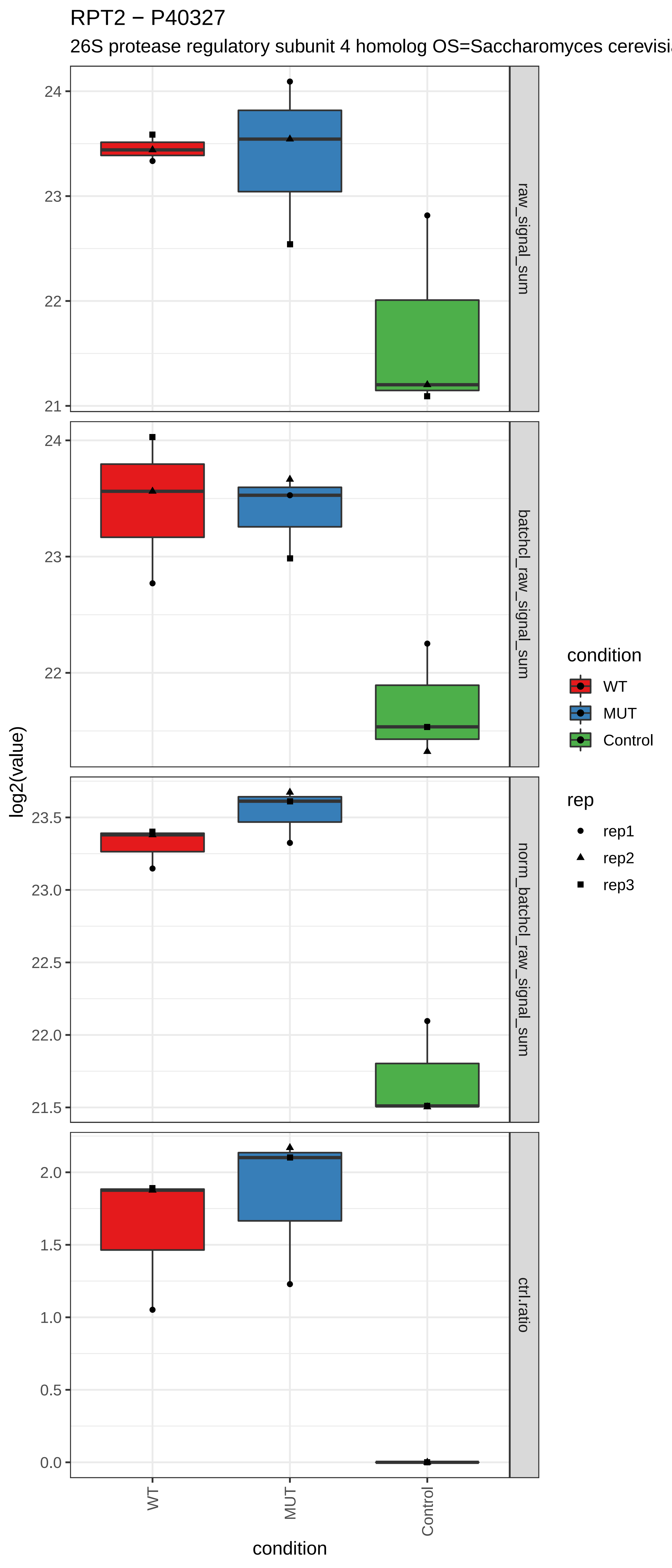

RPT2 − P40327
26S protease regulatory subunit 4 homolog OS=Saccharomyces cerevisi
raw_signal_sum
batchcl_raw_signal_sum
norm_batchcl_raw_signal_sum
ctrl.ratio
log2(value)
condition
WT
MUT
Control
rep
rep1
rep2
rep3
24
23
22
21
23.5
23.0
22.5
22.0
21.5
2.0
1.5
1.0
0.5
0.0

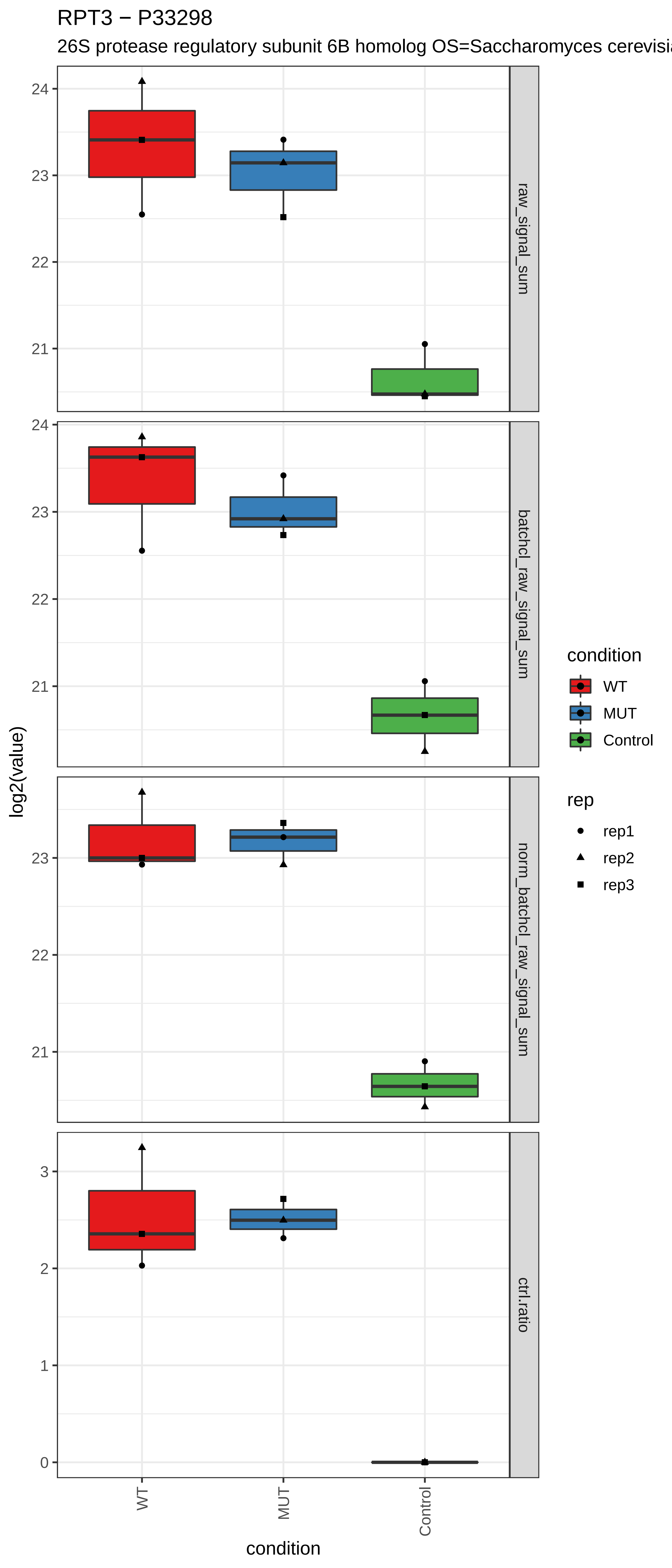
RPT3 − P33298
26S protease regulatory subunit 6B homolog OS=Saccharomyces cerevisia
raw_signal_sum
batchcl_raw_signal_sum
norm_batchcl_raw_signal_sum
ctrl.ratio
log2(value)
condition
WT
MUT
Control
rep
rep1
rep2
rep3
24
23
22
21
3
2
1
0

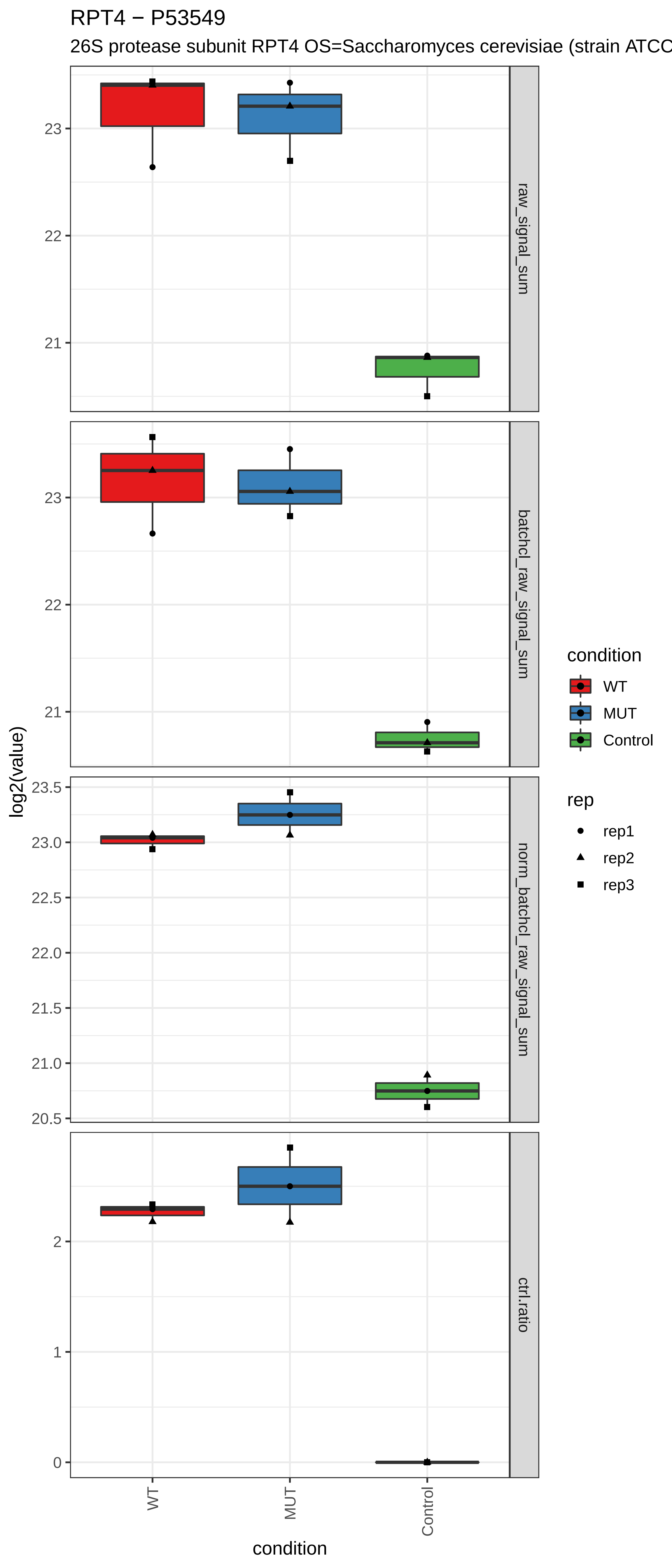

RPT4 − P53549
26S protease subunit RPT4 OS=Saccharomyces cerevisiae (strain ATCC
raw_signal_sum
batchcl_raw_signal_sum
norm_batchcl_raw_signal_sum
ctrl.ratio
log2(value)
condition
WT
MUT
Control
rep
rep1
rep2
rep3

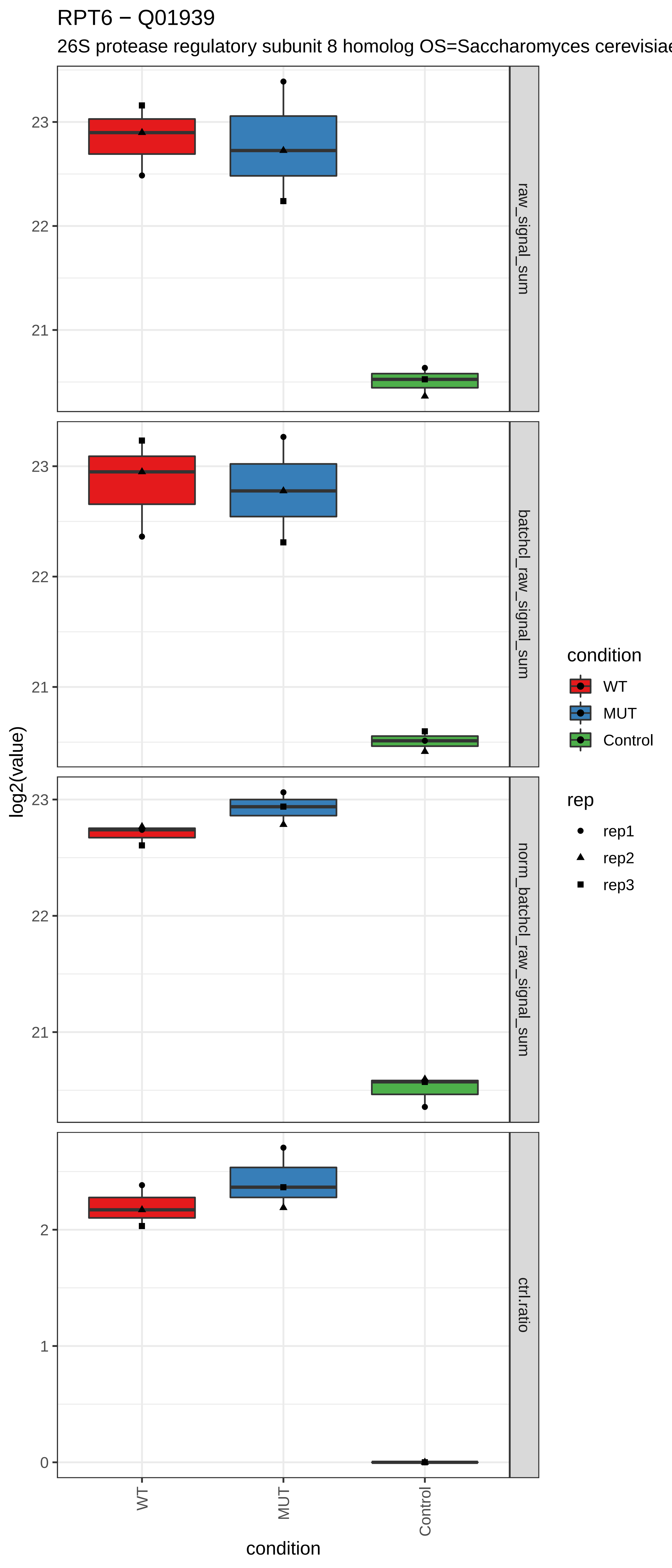
RPT6 − Q01939
26S protease regulatory subunit 8 homolog OS=Saccharomyces cerevisiae
raw_signal_sum
batchcl_raw_signal_sum
norm_batchcl_raw_signal_sum
ctrl.ratio
log2(value)
condition
WT
MUT
Control
rep
rep1
rep2
rep3

RQC2 − Q12532

Ribosome quality control complex subunit 2 OS=Saccharomyces cerevisiae

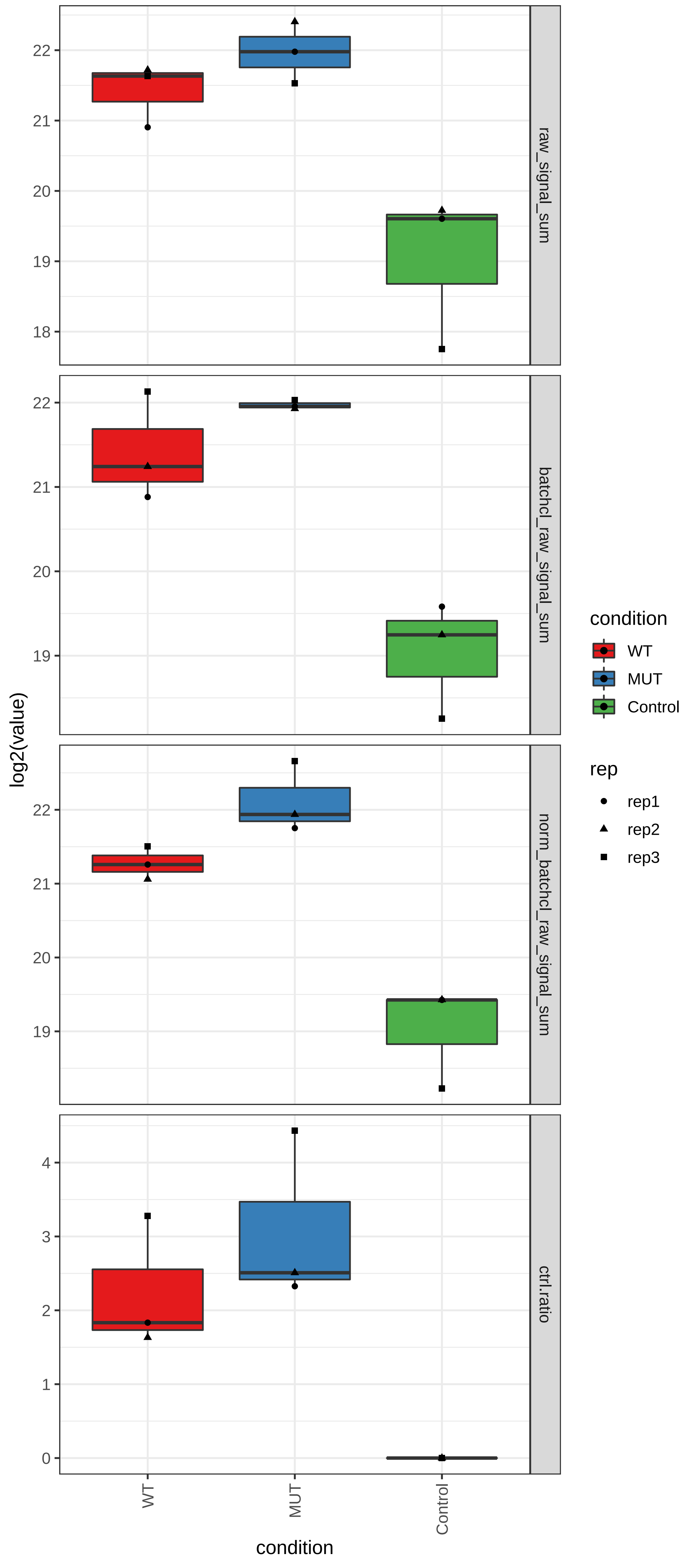

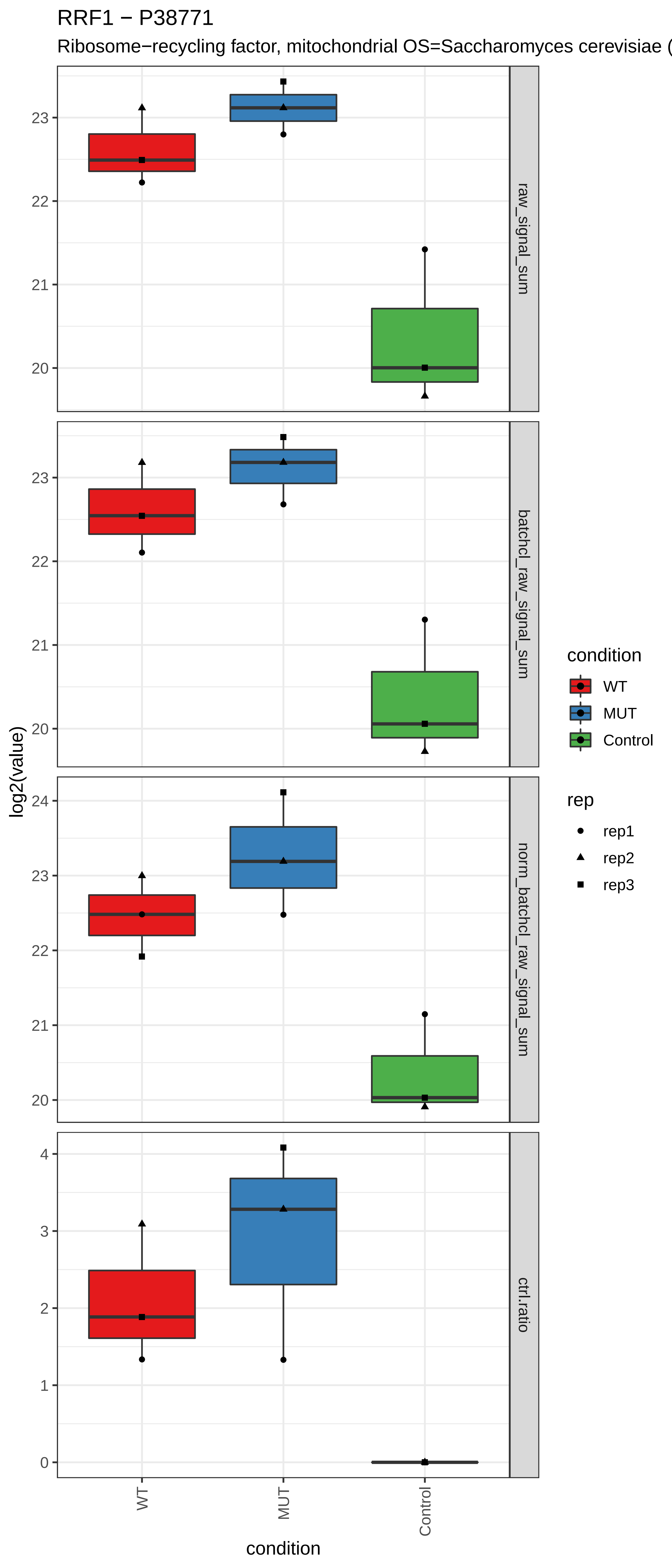
RRF1 − P38771
Ribosome−recycling factor, mitochondrial OS=Saccharomyces cerevisiae (
raw_signal_sum
batchcl_raw_signal_sum
norm_batchcl_raw_signal_sum
ctrl.ratio
log2(value)
condition
WT
MUT
Control
rep
rep1
rep2
rep3
23
22
21
20
24
4
3
2
1
0

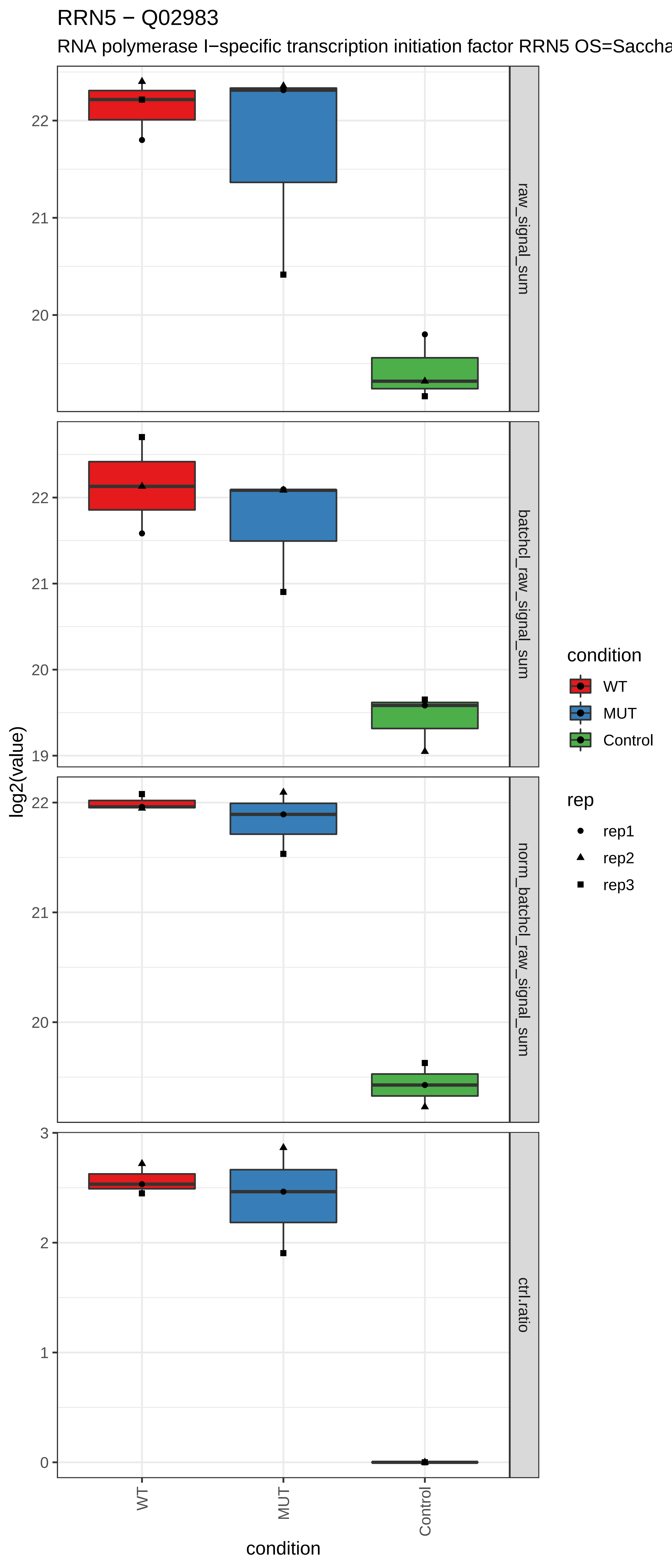
RRN5 − Q02983
RNA polymerase I−specific transcription initiation factor RRN5 OS=Saccha
raw_signal_sum
batchcl_raw_signal_sum
norm_batchcl_raw_signal_sum
ctrl.ratio
log2(value)
condition
WT
MUT
Control
rep
rep1
rep2
rep3
22
21
20
19
3
2
1
0

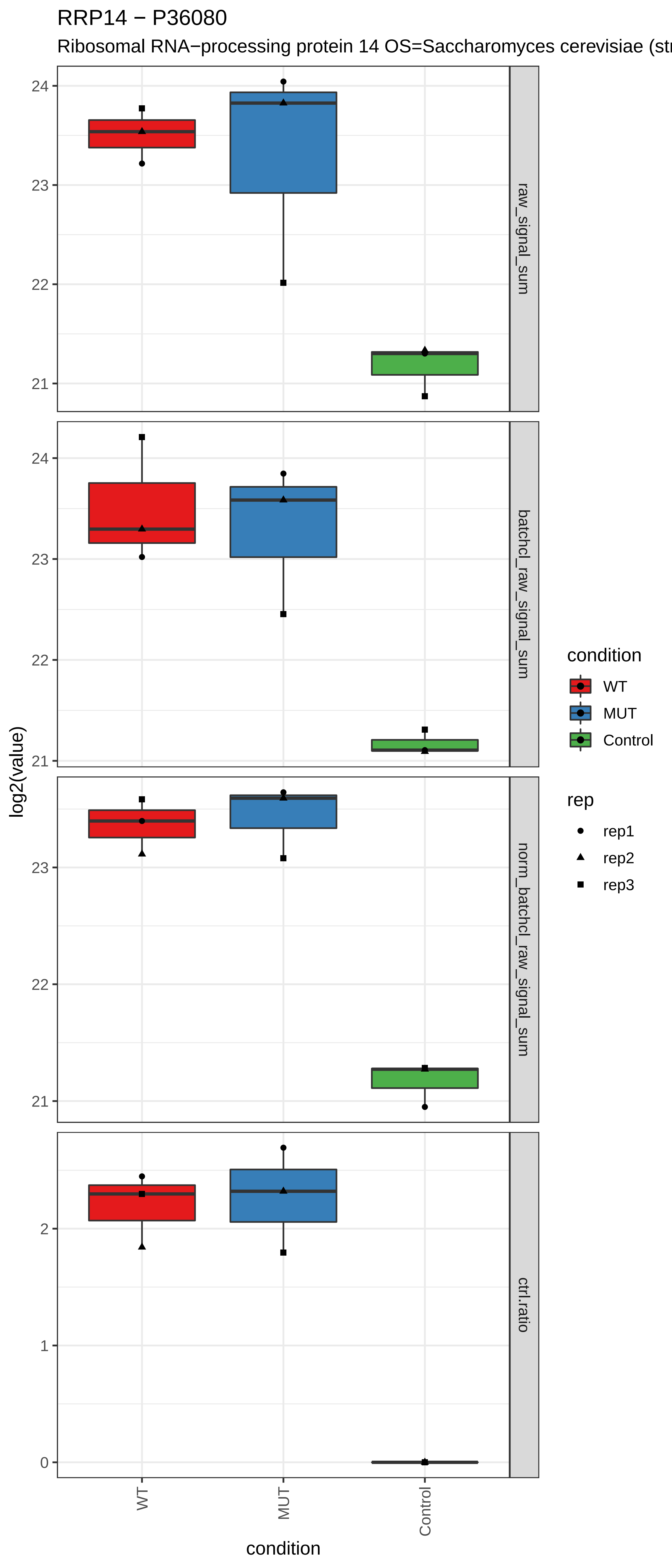
RRP14 − P36080
Ribosomal RNA−processing protein 14 OS=Saccharomyces cerevisiae (str
raw_signal_sum
batchcl_raw_signal_sum
norm_batchcl_raw_signal_sum
ctrl.ratio
log2(value)
condition
WT
MUT
Control
rep
rep1
rep2
rep3

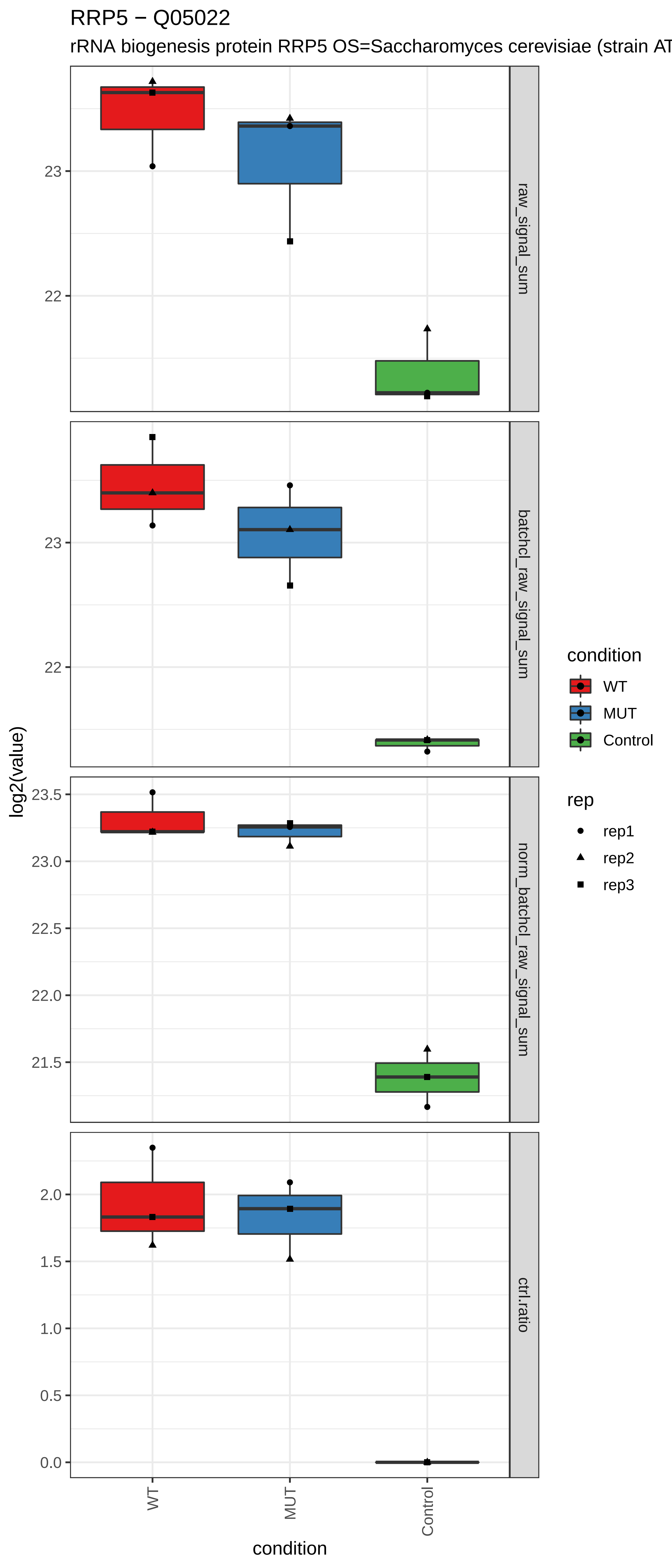
RRP5 − Q05022
rRNA biogenesis protein RRP5 OS=Saccharomyces cerevisiae (strain AT
raw_signal_sum
batchcl_raw_signal_sum
norm_batchcl_raw_signal_sum
ctrl.ratio
23
22
23
22
23.5
23.0
22.5
22.0
21.5
2.0
1.5
1.0
0.5
0.0
log2(value)
WT
MUT
Control
condition
condition
WT
MUT
Control
rep
rep1
rep2
rep3

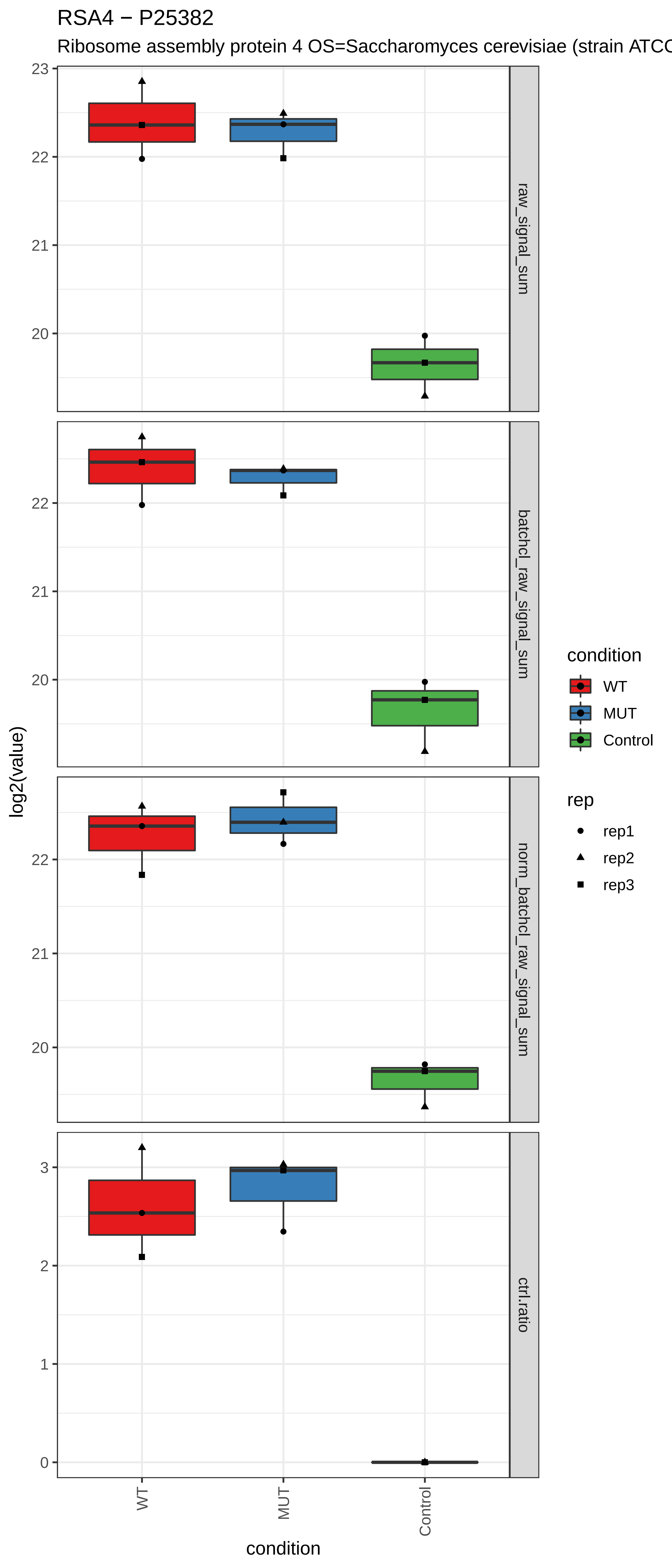

RSA4 − P25382
Ribosome assembly protein 4 OS=Saccharomyces cerevisiae (strain ATCC
raw_signal_sum
batchcl_raw_signal_sum
norm_batchcl_raw_signal_sum
ctrl.ratio
log2(value)
condition
WT
MUT
Control
rep
rep1
rep2
rep3

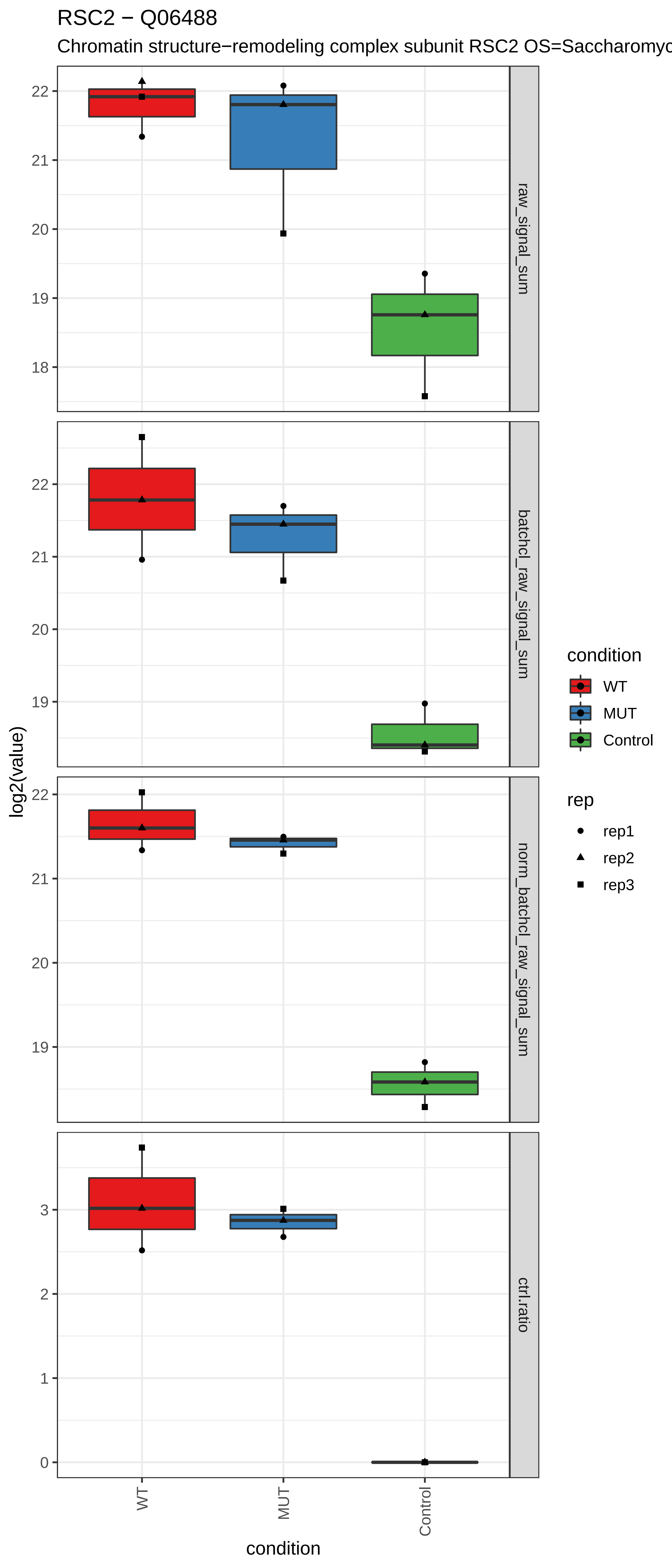

RSC2 − Q06488
Chromatin structure−remodeling complex subunit RSC2 OS=Saccharomyc
raw_signal_sum
batchcl_raw_signal_sum
norm_batchcl_raw_signal_sum
ctrl.ratio
log2(value)
condition
WT
MUT
Control
rep
rep1
rep2
rep3
22
21
20
19
18
3
2
1
0

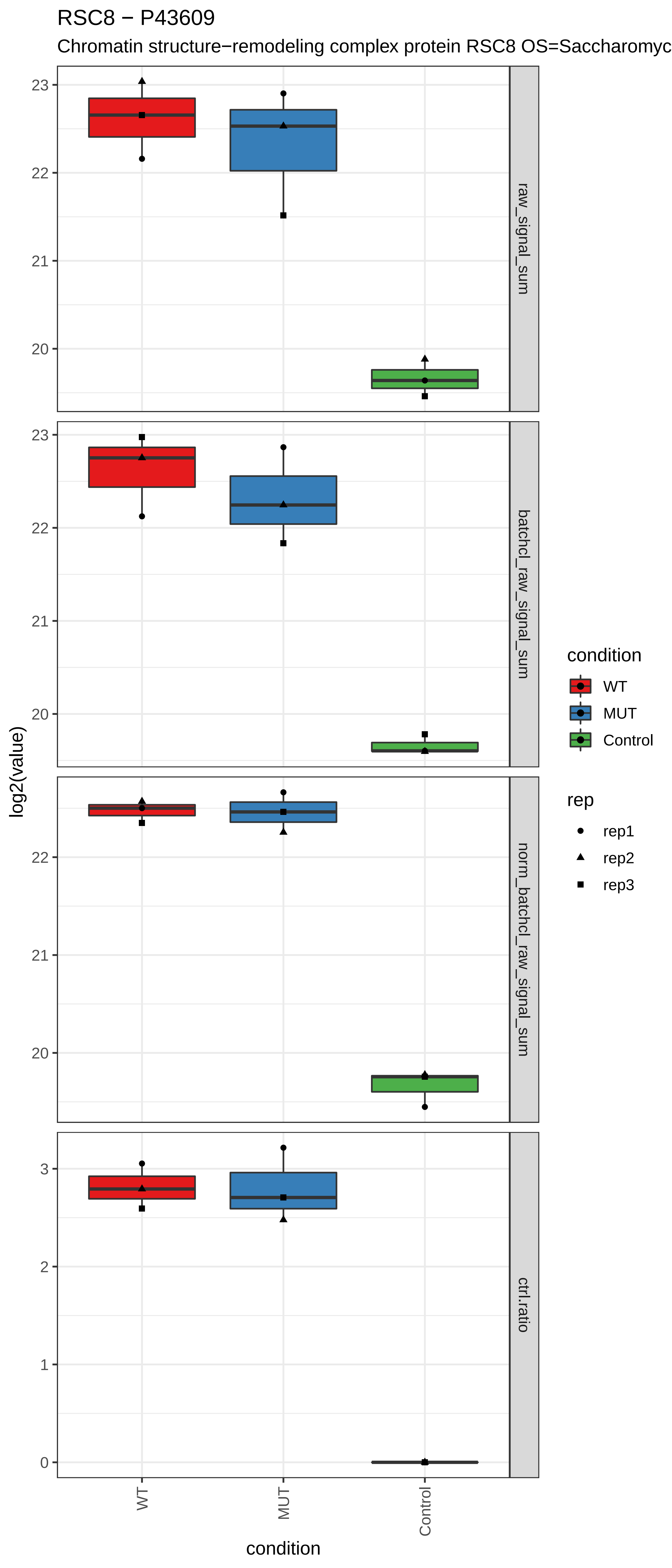

RSC8 − P43609
Chromatin structure−remodeling complex protein RSC8 OS=Saccharomyc
raw_signal_sum
batchcl_raw_signal_sum
norm_batchcl_raw_signal_sum
ctrl.ratio
log2(value)
condition
WT
MUT
Control
rep
rep1
rep2
rep3

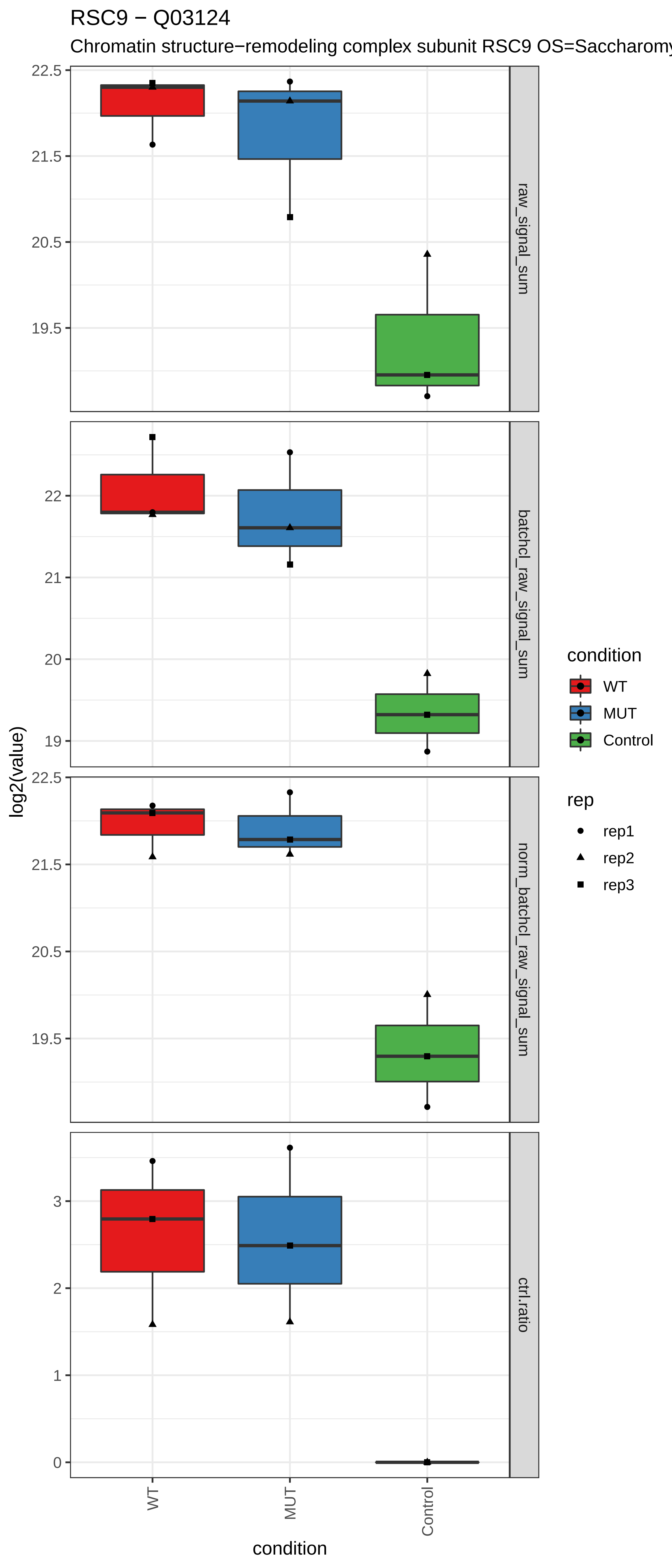

RSC9 − Q03124
Chromatin structure−remodeling complex subunit RSC9 OS=Saccharomy
raw_signal_sum
batchcl_raw_signal_sum
norm_batchcl_raw_signal_sum
ctrl.ratio
log2(value)
condition
WT
MUT
Control
rep
rep1
rep2
rep3
22.5
21.5
20.5
19.5
22
21
20
19
3
2
1
0

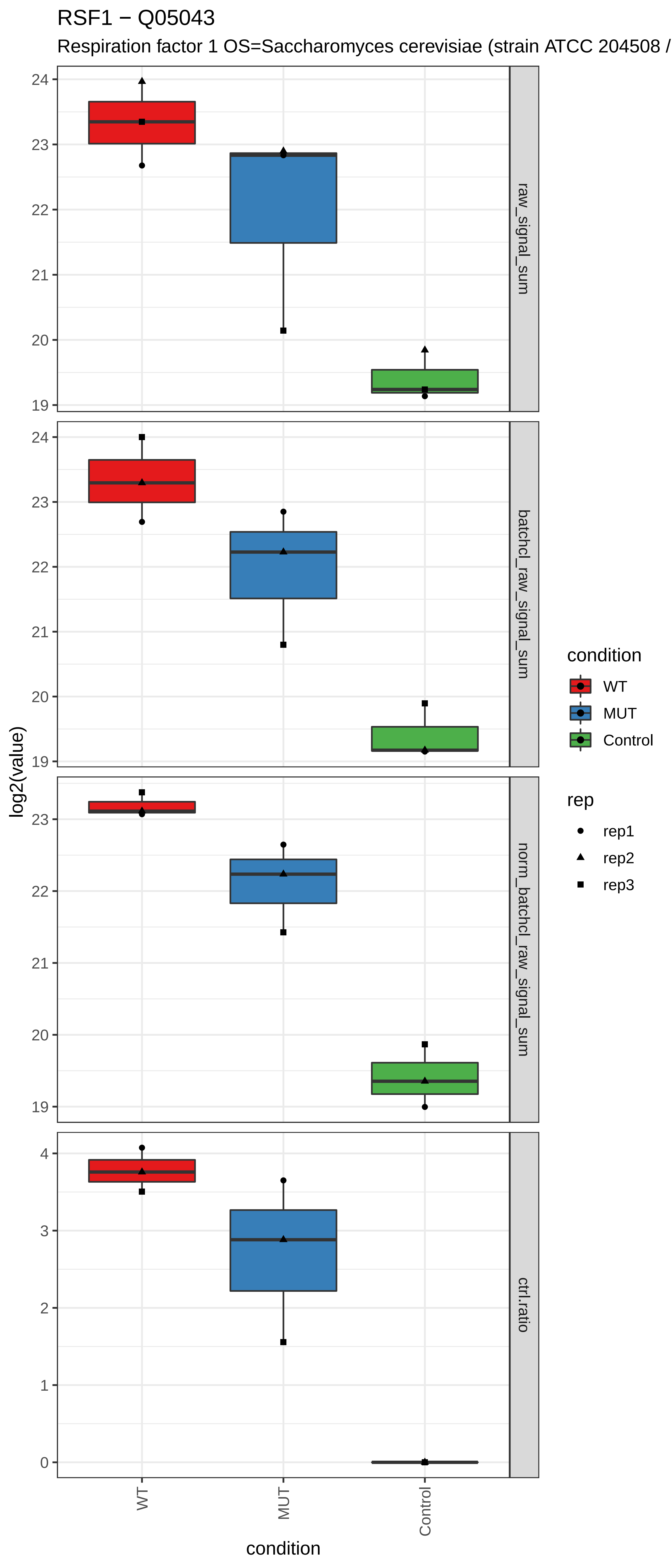
RSF1 − Q05043
Respiration factor 1 OS=Saccharomyces cerevisiae (strain ATCC 204508 /
log2(value)
raw_signal_sum
batchcl_raw_signal_sum
norm_batchcl_raw_signal_sum
ctrl.ratio
WT
MUT
Control
condition
rep
rep1
rep2
rep3
24
23
22
21
20
19
4
3
2
1
0

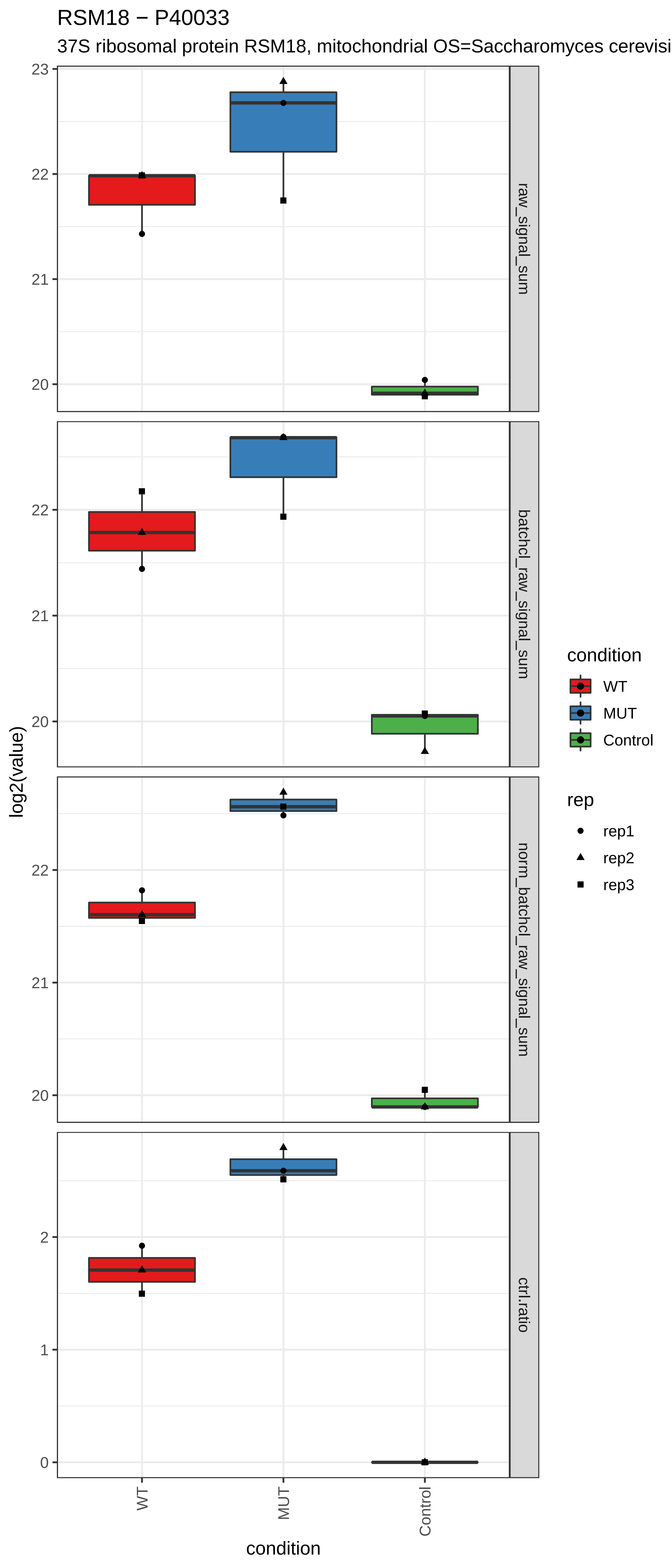

RSM18 − P40033
37S ribosomal protein RSM18, mitochondrial OS=Saccharomyces cerevisi
raw_signal_sum
batchcl_raw_signal_sum
norm_batchcl_raw_signal_sum
ctrl.ratio
log2(value)
condition
WT
MUT
Control
rep
rep1
rep2
rep3
23
22
21
20
2
1
0

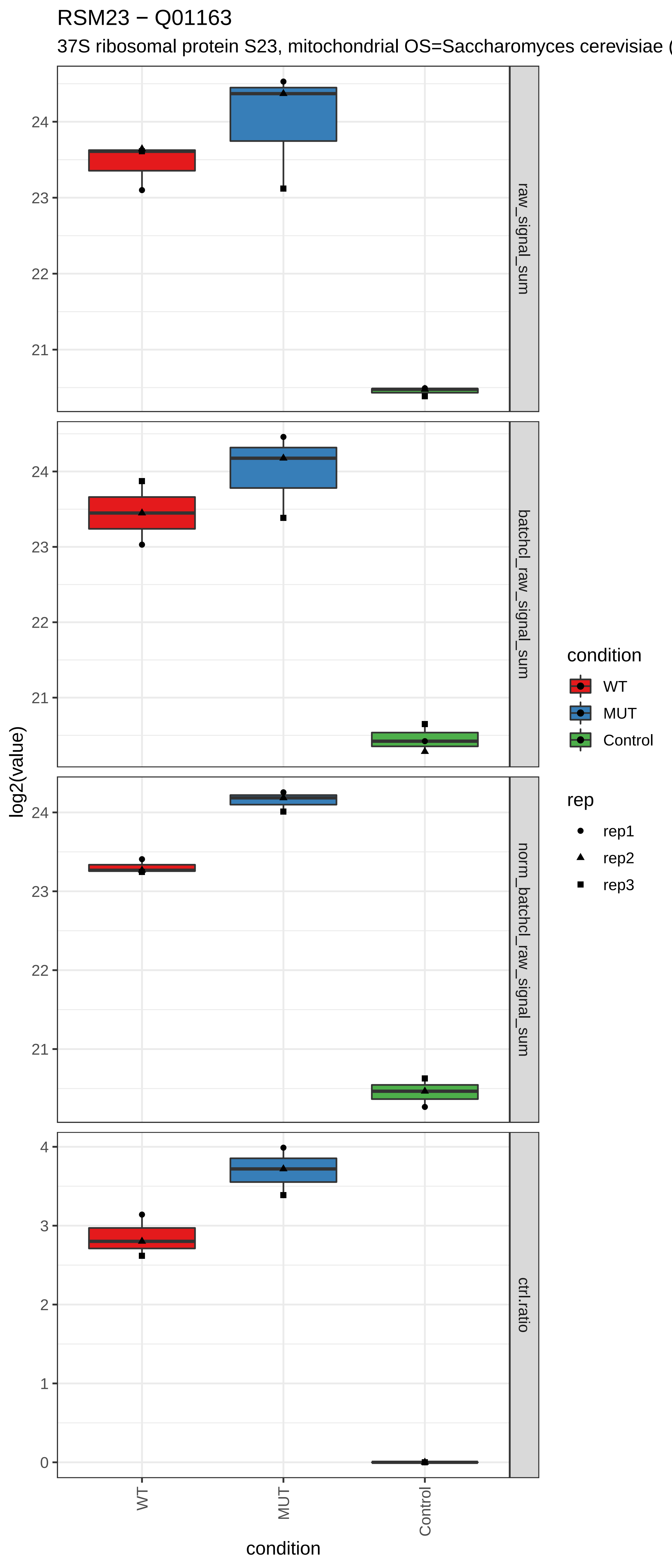
RSM23 − Q01163
37S ribosomal protein S23, mitochondrial OS=Saccharomyces cerevisiae (
raw_signal_sum
batchcl_raw_signal_sum
norm_batchcl_raw_signal_sum
ctrl.ratio
log2(value)
condition
WT
MUT
Control
rep
rep1
rep2
rep3

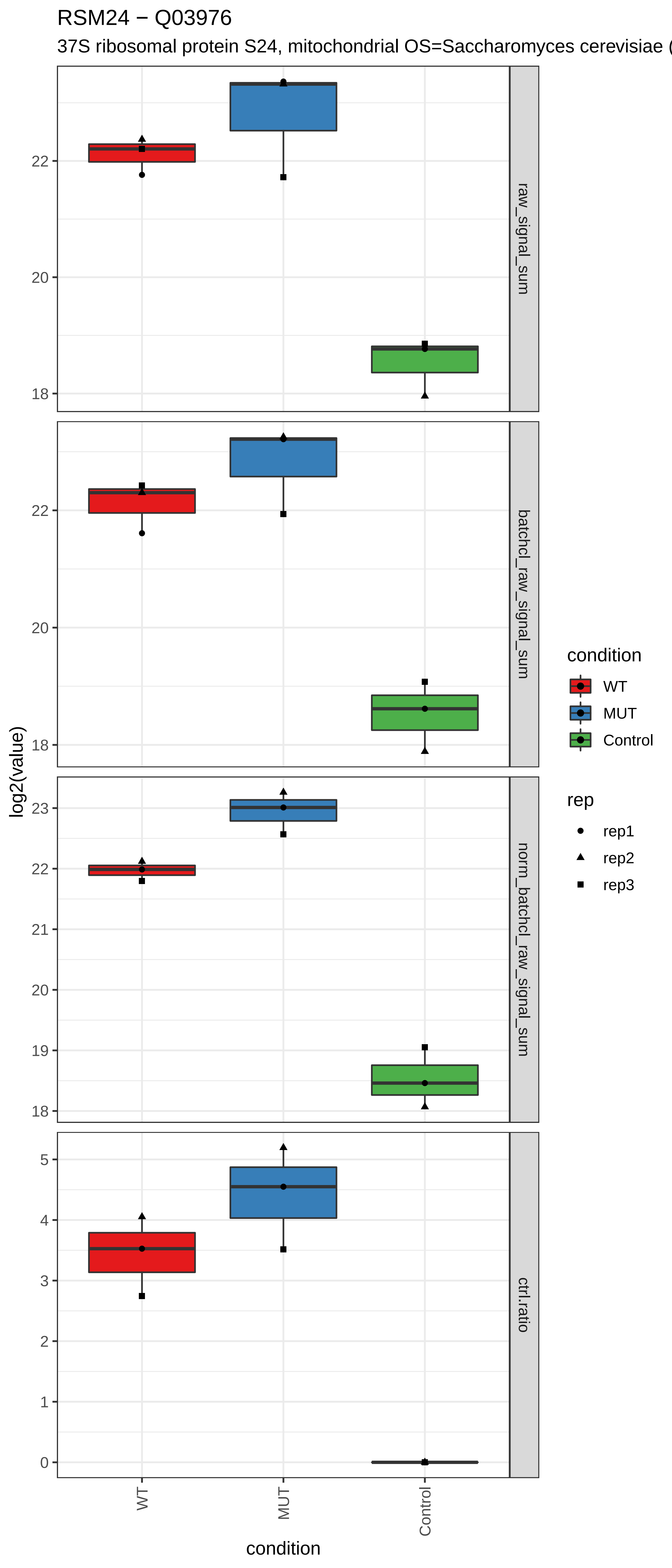
RSM24 − Q03976
37S ribosomal protein S24, mitochondrial OS=Saccharomyces cerevisiae (
raw_signal_sum
batchcl_raw_signal_sum
norm_batchcl_raw_signal_sum
ctrl.ratio
log2(value)
condition
WT
MUT
Control
rep
rep1
rep2
rep3
22
20
18
23
21
19
5
4
3
2
1
0

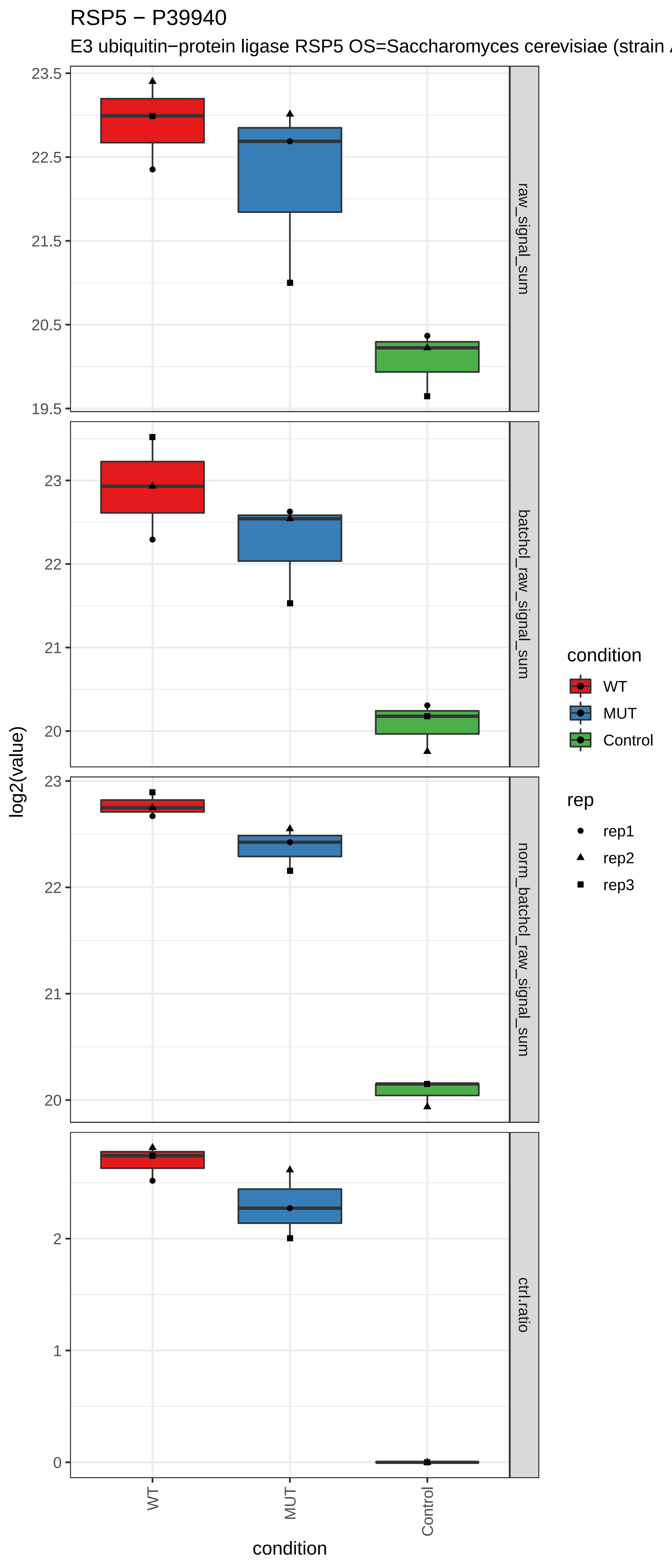
RSP5 − P39940
E3 ubiquitin−protein ligase RSP5 OS=Saccharomyces cerevisiae (strain
raw_signal_sum
batchcl_raw_signal_sum
norm_batchcl_raw_signal_sum
ctrl.ratio
23.5
22.5
21.5
20.5
19.5
23
22
21
20
2
1
0
log2(value)
WT
MUT
Control
condition
rep
rep1
rep2
rep3

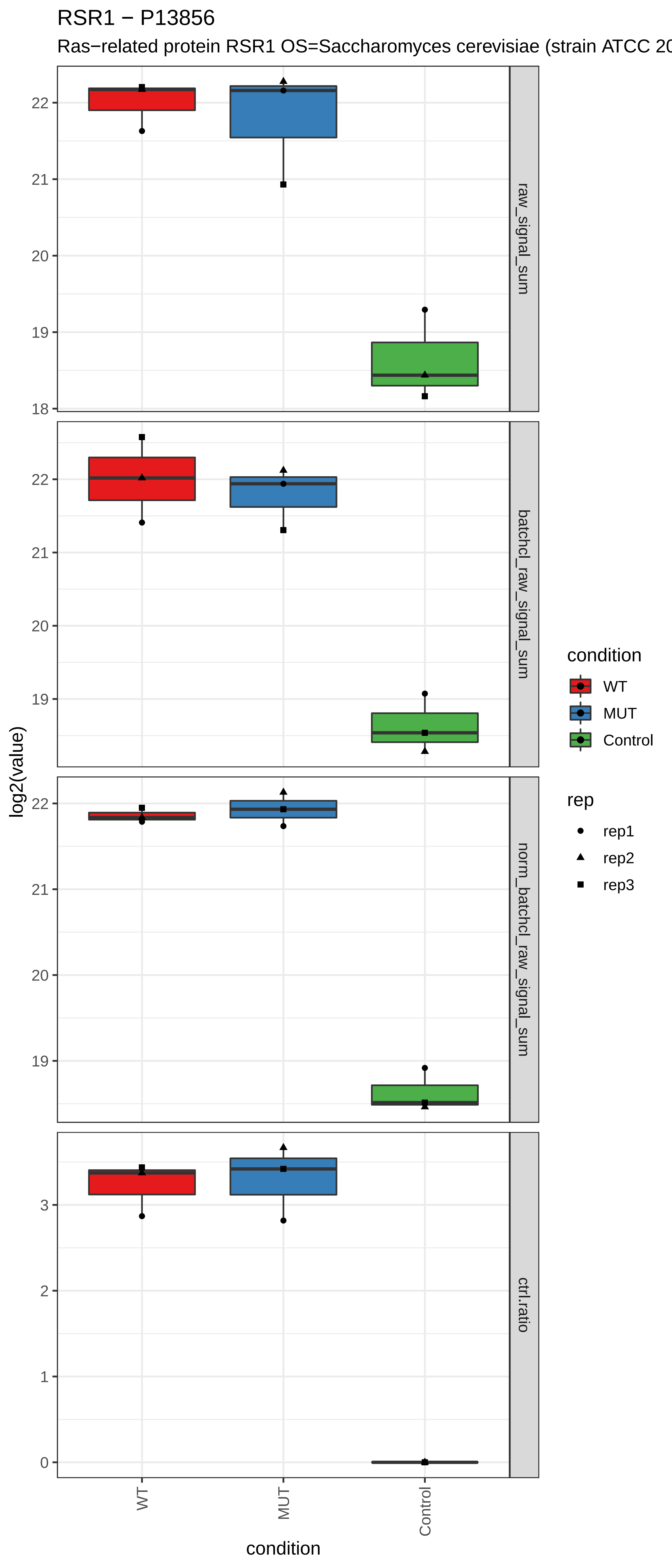

RSR1 − P13856
Ras−related protein RSR1 OS=Saccharomyces cerevisiae (strain ATCC 20
raw_signal_sum
batchcl_raw_signal_sum
norm_batchcl_raw_signal_sum
ctrl.ratio
log2(value)
condition
WT
MUT
Control
rep
rep1
rep2
rep3

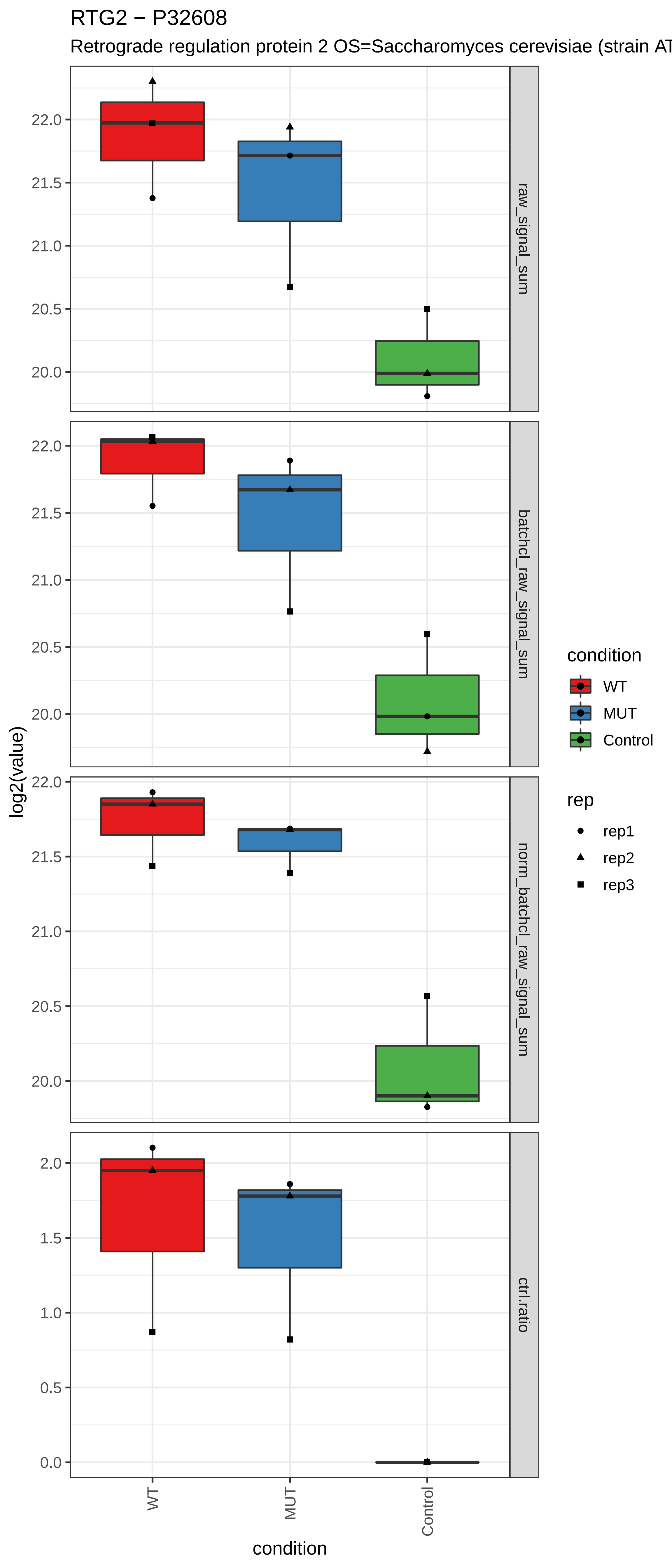
RTG2 − P32608
Retrograde regulation protein 2 OS=Saccharomyces cerevisiae (strain AT
raw_signal_sum
batchcl_raw_signal_sum
norm_batchcl_raw_signal_sum
ctrl.ratio
log2(value)
condition
WT
MUT
Control
rep
rep1
rep2
rep3
22.0
21.5
21.0
20.5
20.0
2.0
1.5
1.0
0.5
0.0

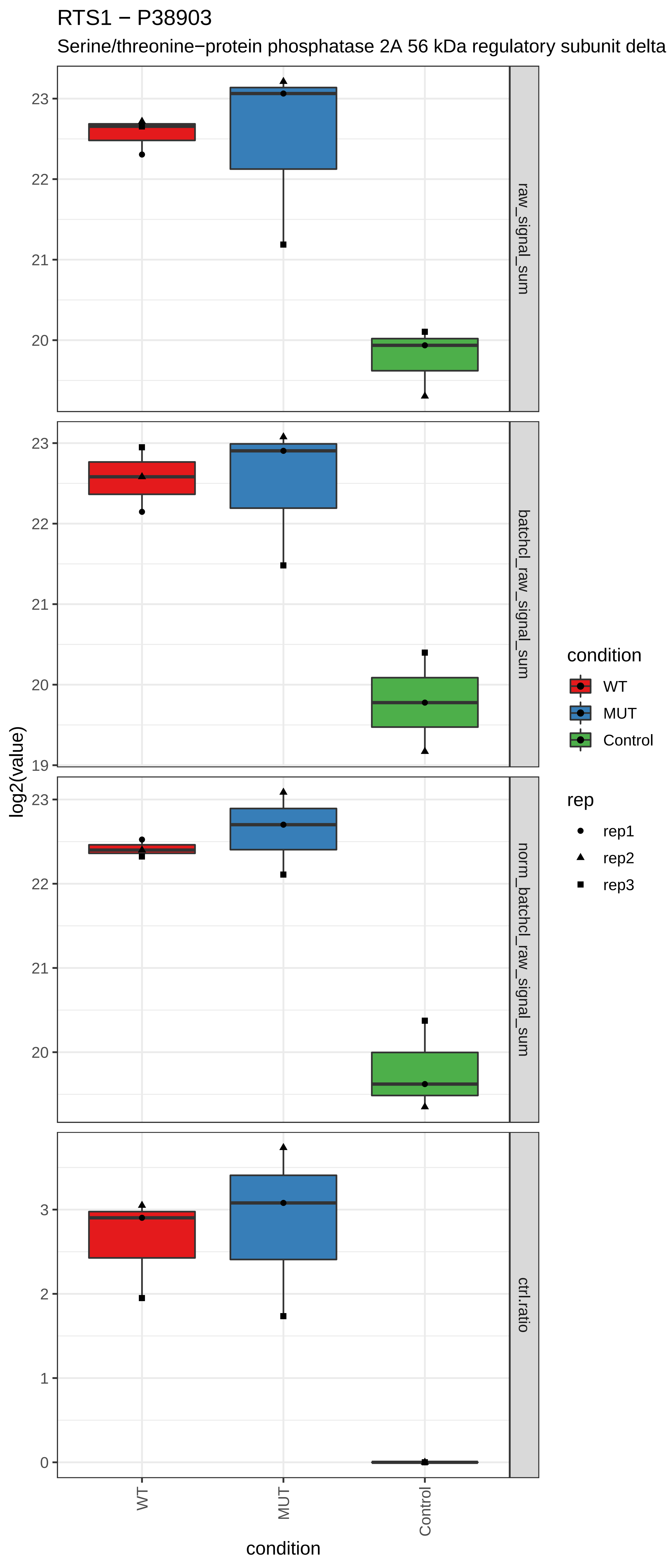

RTS1 − P38903
Serine/threonine−protein phosphatase 2A 56 kDa regulatory subunit delta
raw_signal_sum
batchcl_raw_signal_sum
norm_batchcl_raw_signal_sum
ctrl.ratio
log2(value)
condition
WT
MUT
Control
rep
rep1
rep2
rep3

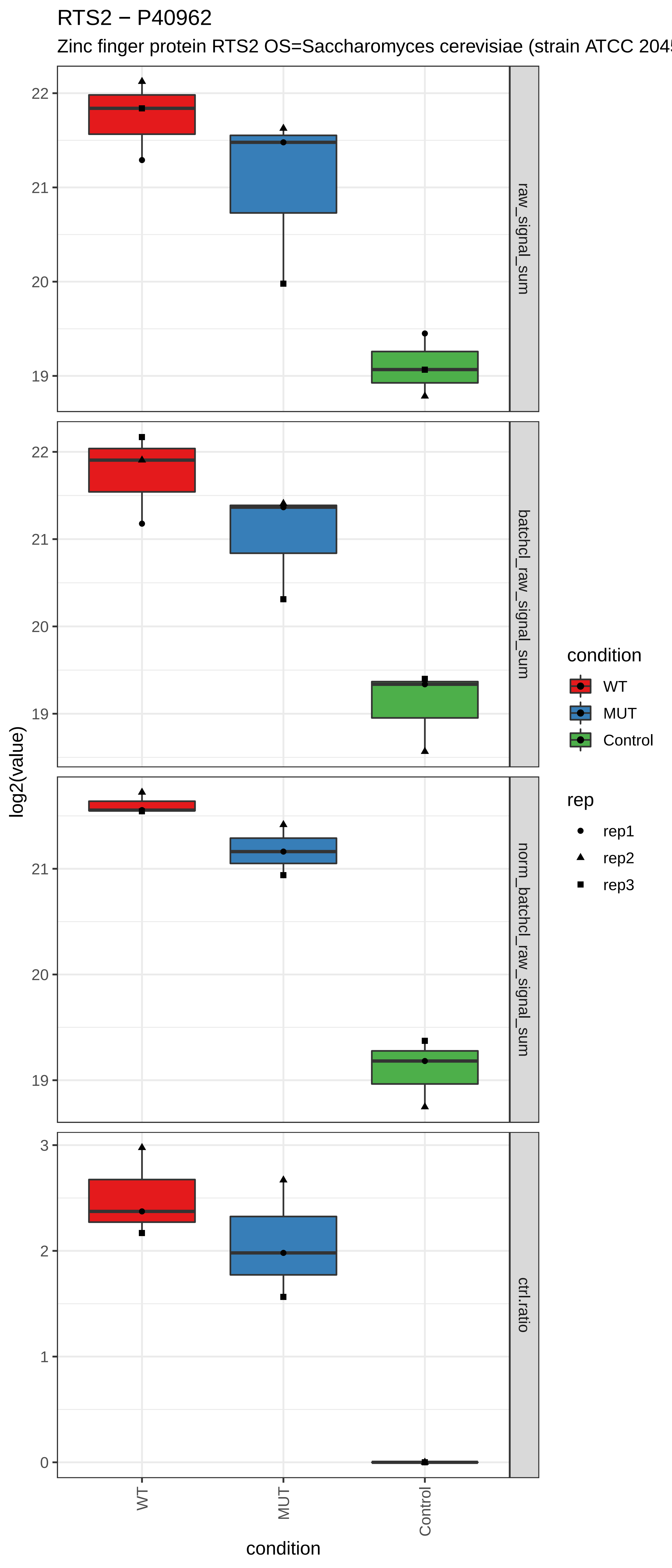

RTS2 − P40962
Zinc finger protein RTS2 OS=Saccharomyces cerevisiae (strain ATCC 2045
raw_signal_sum
batchcl_raw_signal_sum
norm_batchcl_raw_signal_sum
ctrl.ratio
log2(value)
condition
WT
MUT
Control
rep
rep1
rep2
rep3

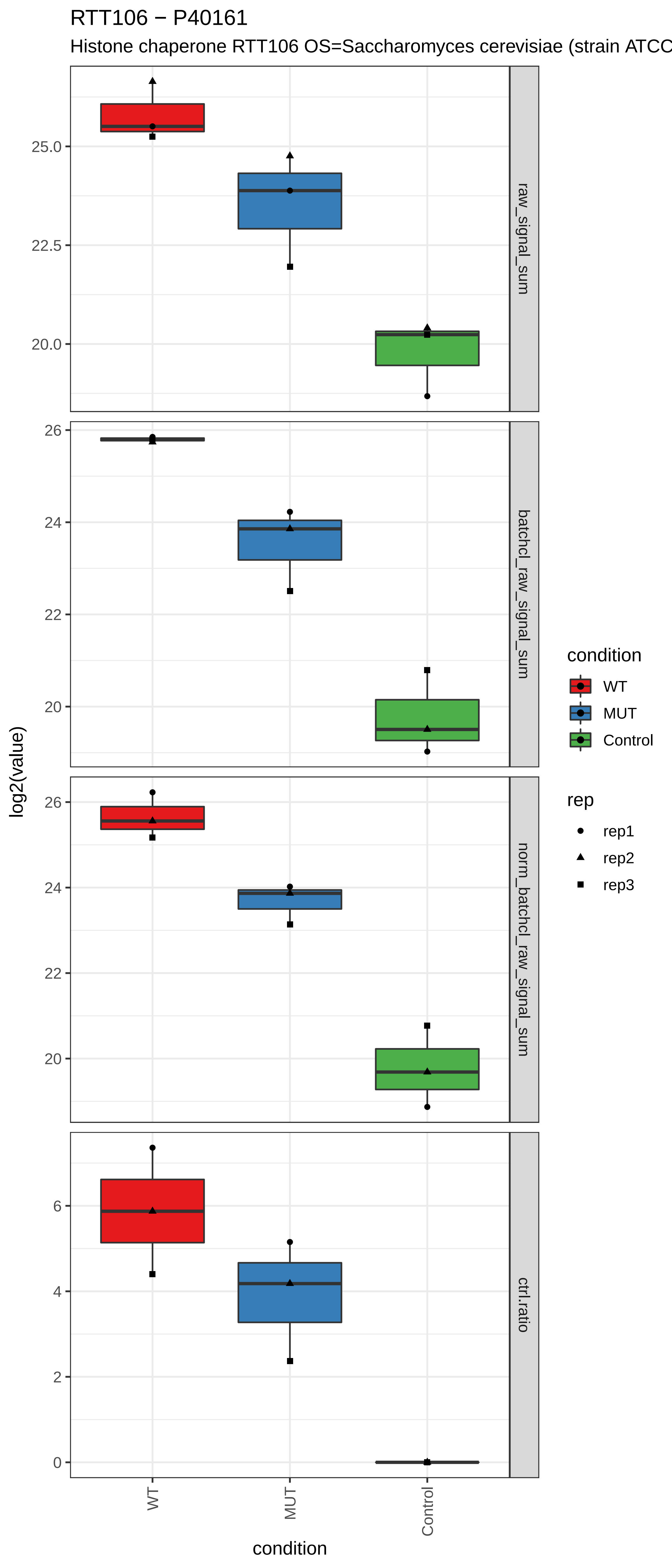
RTT106 − P40161
Histone chaperone RTT106 OS=Saccharomyces cerevisiae (strain ATCC
raw_signal_sum
batchcl_raw_signal_sum
norm_batchcl_raw_signal_sum
ctrl.ratio
log2(value)
condition
WT
MUT
Control
rep
rep1
rep2
rep3
25.0
22.5
20.0
26
24
22
20
6
4
2
0

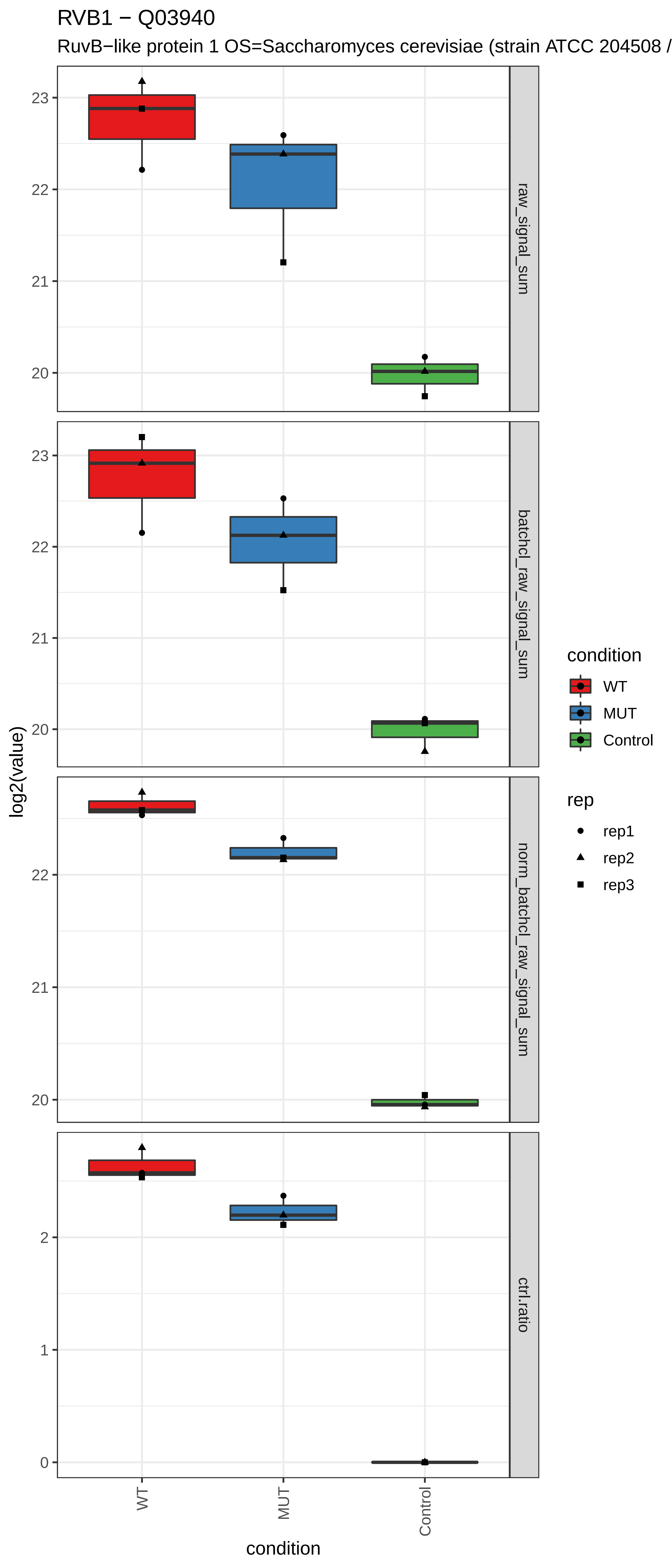

RVB1 − Q03940
RuvB−like protein 1 OS=Saccharomyces cerevisiae (strain ATCC 204508 /
raw_signal_sum
batchcl_raw_signal_sum
norm_batchcl_raw_signal_sum
ctrl.ratio
log2(value)
condition
WT
MUT
Control
rep
rep1
rep2
rep3
23
22
21
20
2
1
0

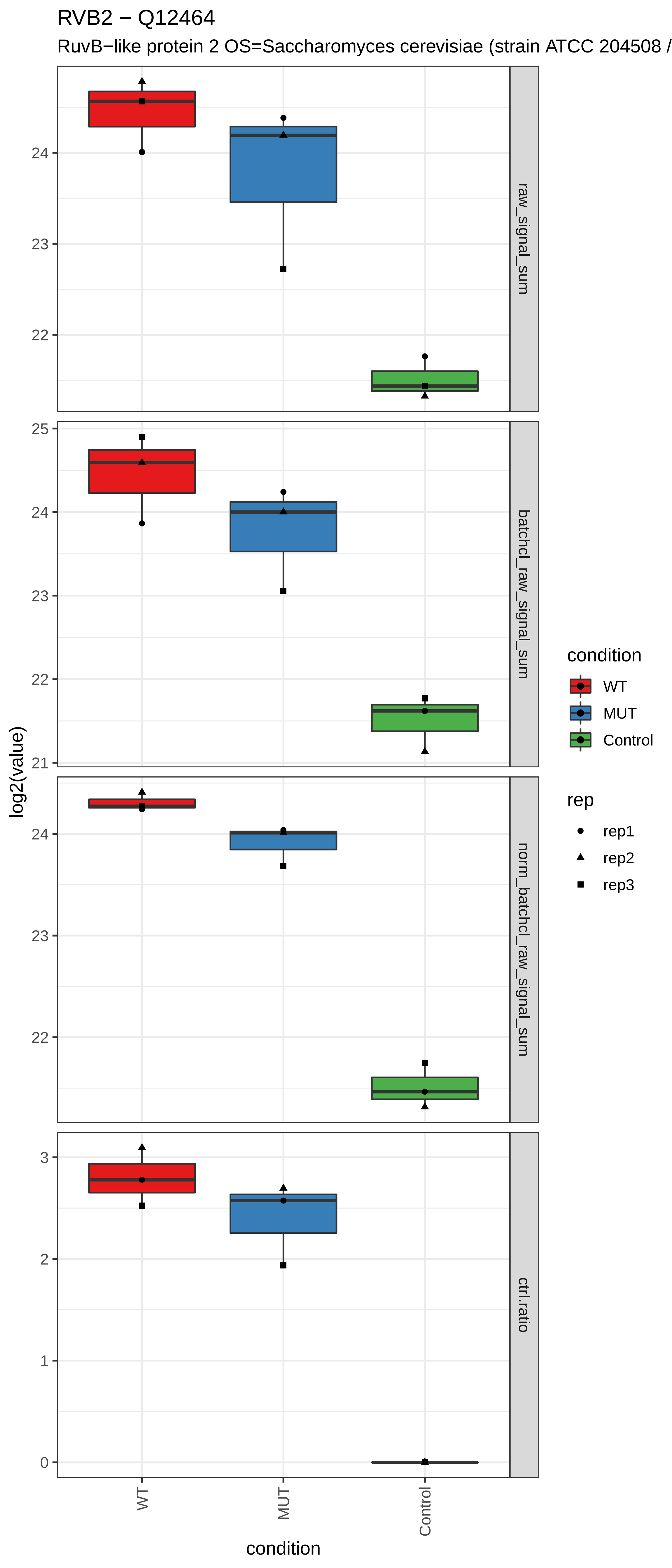

RVB2 − Q12464
RuvB−like protein 2 OS=Saccharomyces cerevisiae (strain ATCC 204508 /
raw_signal_sum
batchcl_raw_signal_sum
norm_batchcl_raw_signal_sum
ctrl.ratio
log2(value)
condition
WT
MUT
Control
rep
rep1
rep2
rep3

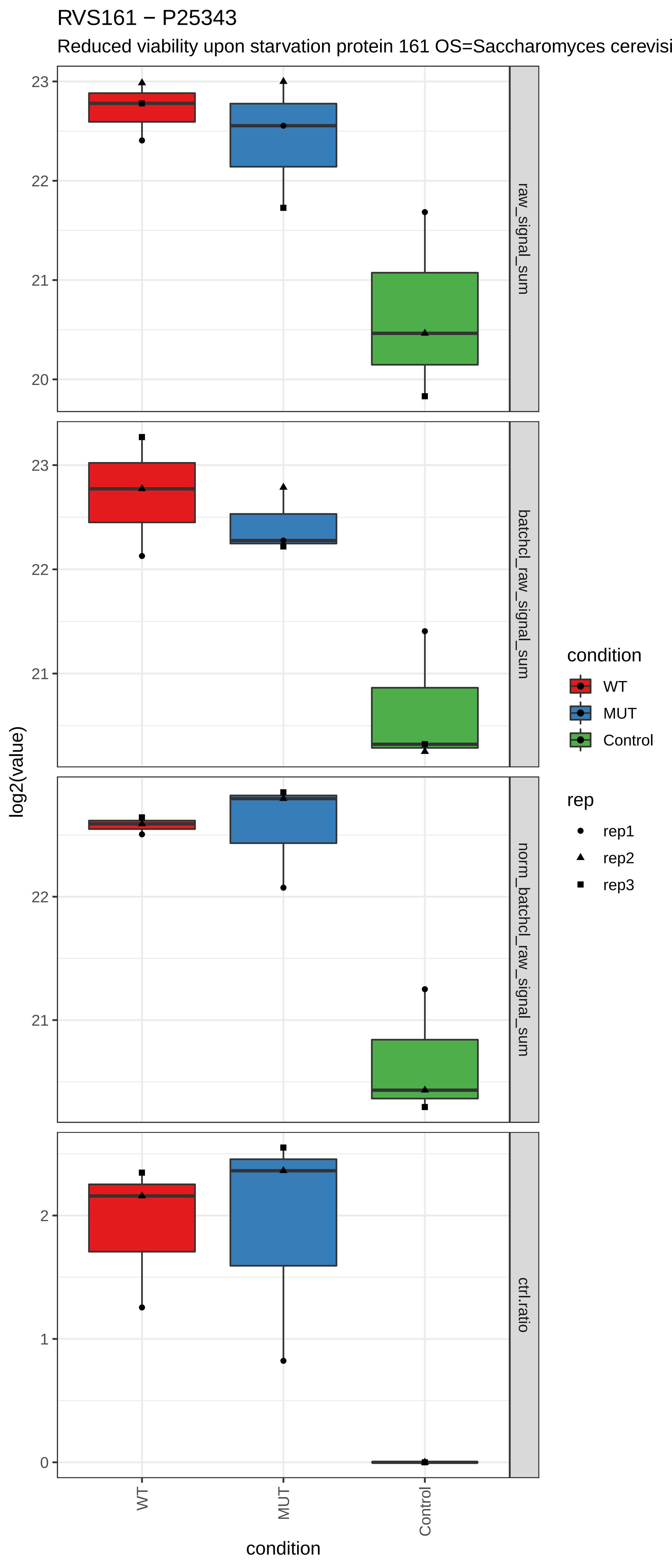

RVS161 − P25343
Reduced viability upon starvation protein 161 OS=Saccharomyces cerevisi
log2(value)
raw_signal_sum
batchcl_raw_signal_sum
norm_batchcl_raw_signal_sum
ctrl.ratio
23
22
21
20
23
22
21
22
21
2
1
0
WT
MUT
Control
condition
condition
WT
MUT
Control
rep
rep1
rep2
rep3

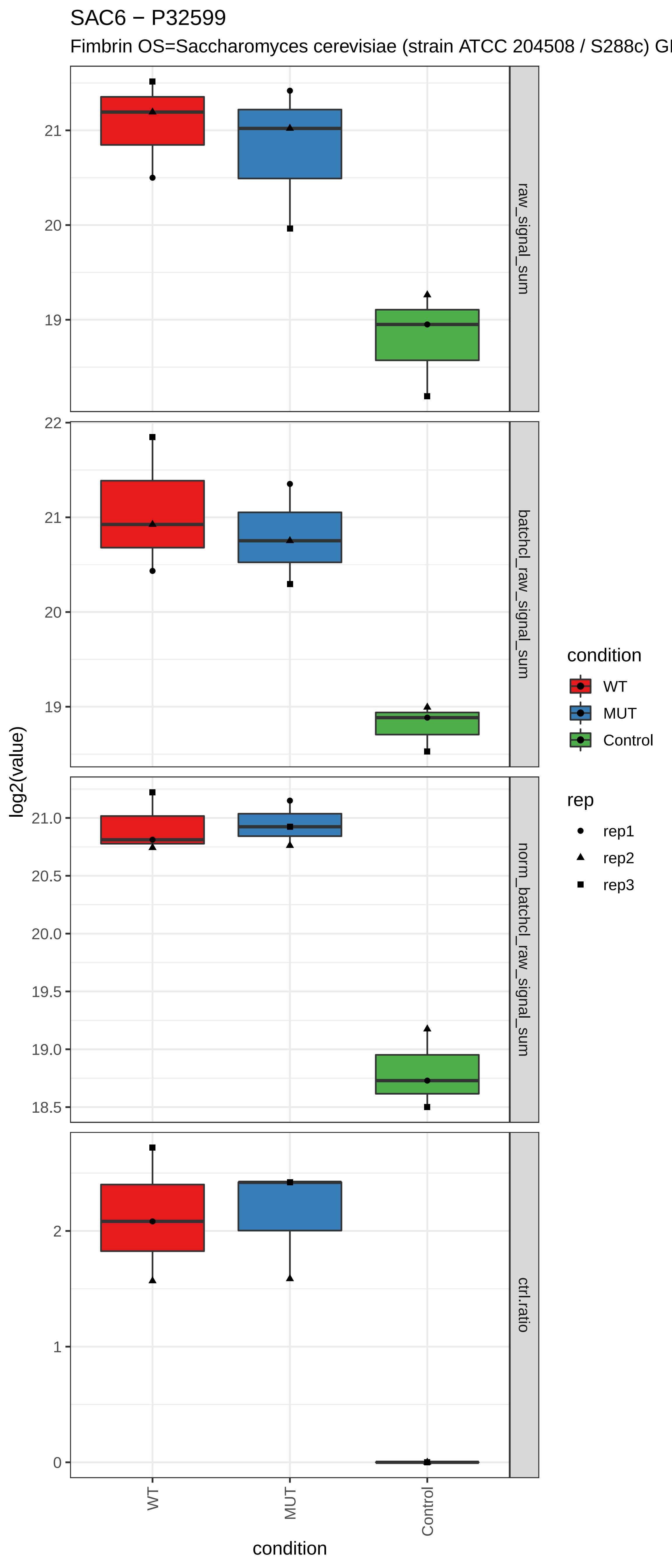

SAC6 − P32599
Fimbrin OS=Saccharomyces cerevisiae (strain ATCC 204508 / S288c) GI
raw_signal_sum
batchcl_raw_signal_sum
norm_batchcl_raw_signal_sum
ctrl.ratio
log2(value)
condition
WT
MUT
Control
rep
rep1
rep2
rep3

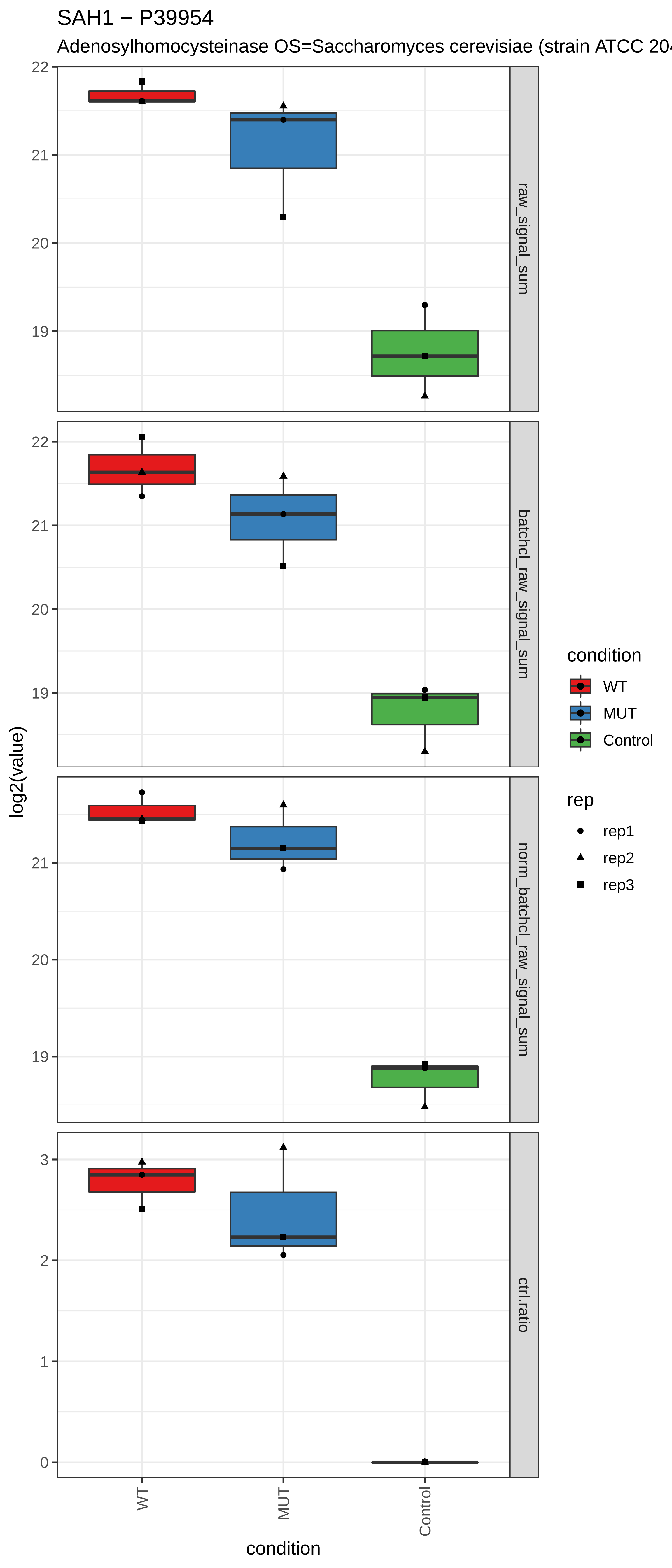
SAH1 − P39954
Adenosylhomocysteinase OS=Saccharomyces cerevisiae (strain ATCC 20
raw_signal_sum
batchcl_raw_signal_sum
norm_batchcl_raw_signal_sum
ctrl.ratio
log2(value)
condition
WT
MUT
Control
rep
rep1
rep2
rep3
22
21
20
19
3
2
1
0

SAM1 − P10659

S−adenosylmethionine synthase 1 OS=Saccharomyces cerevisiae (strain A

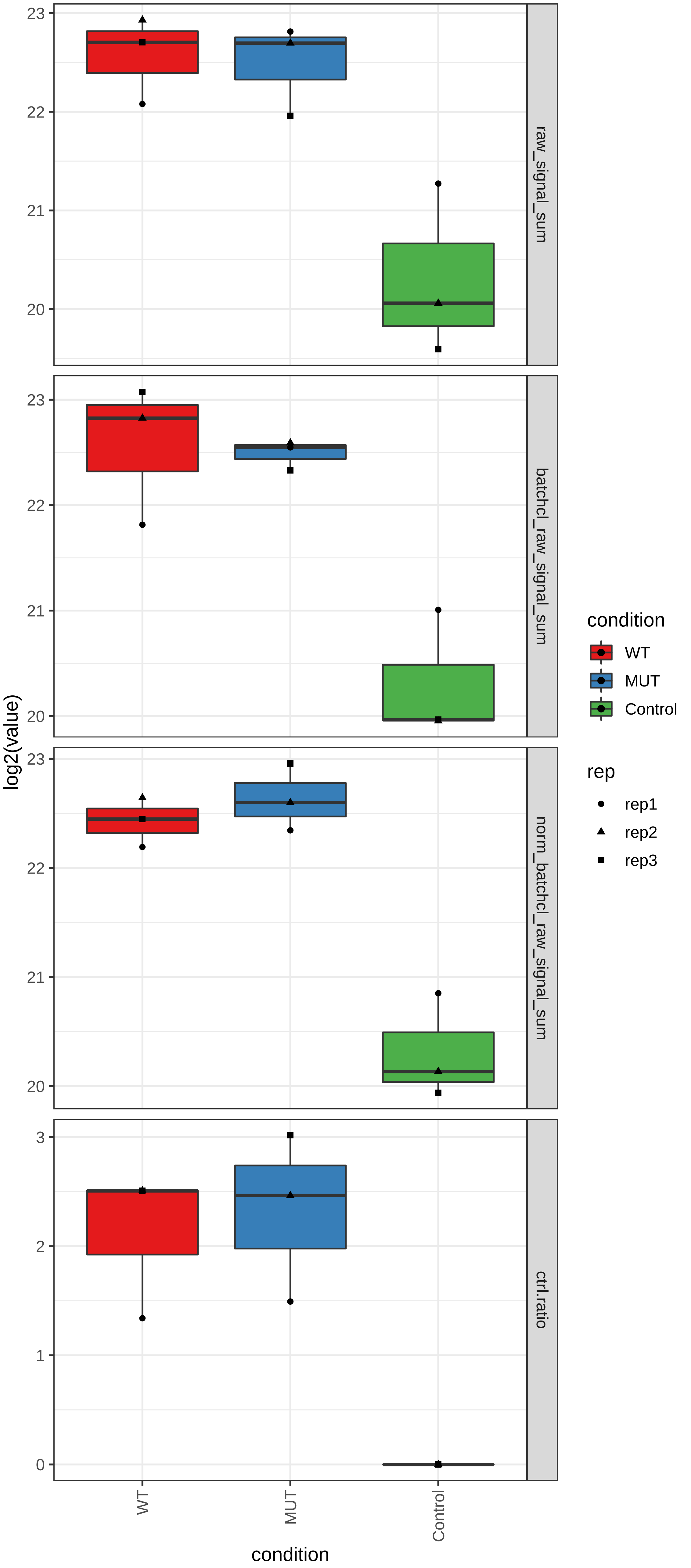

SAM2 − P19358

S−adenosylmethionine synthase 2 OS=Saccharomyces cerevisiae (strain A

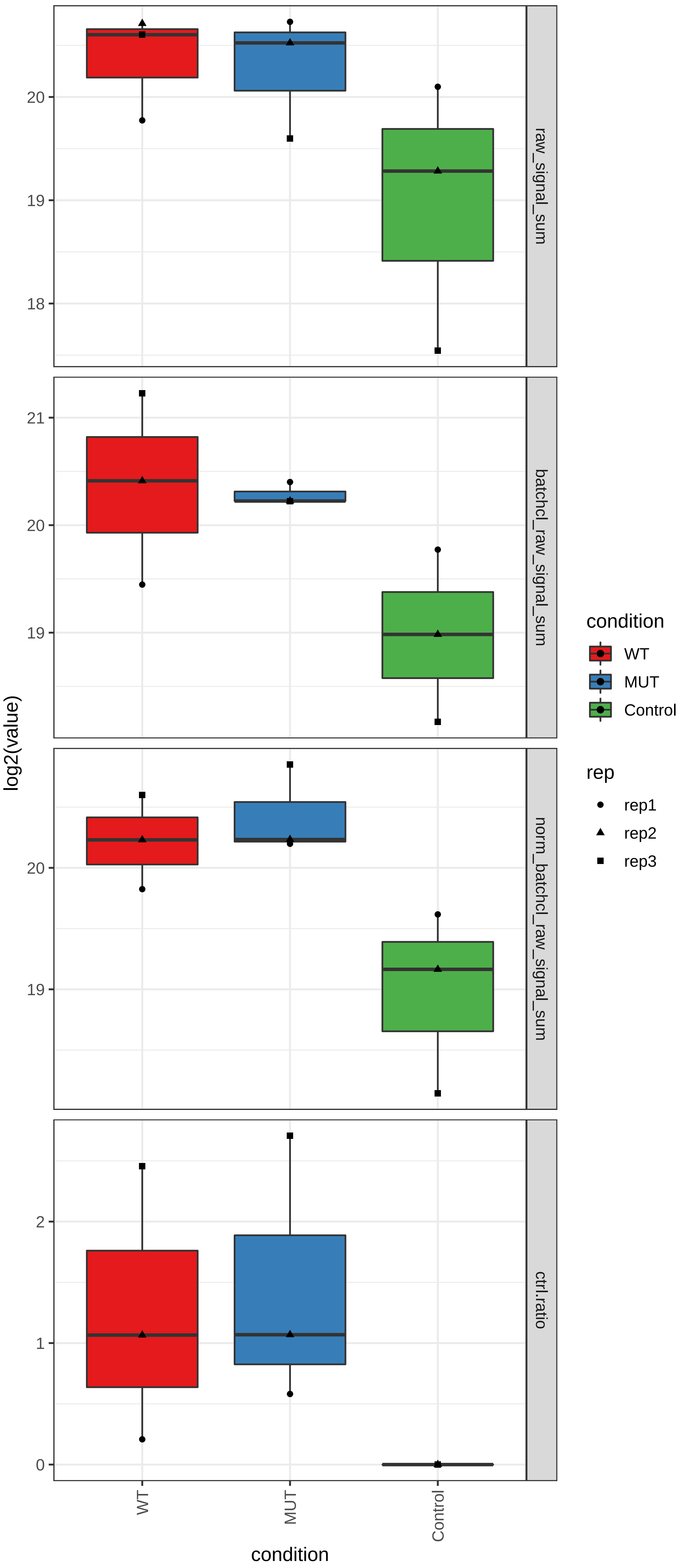

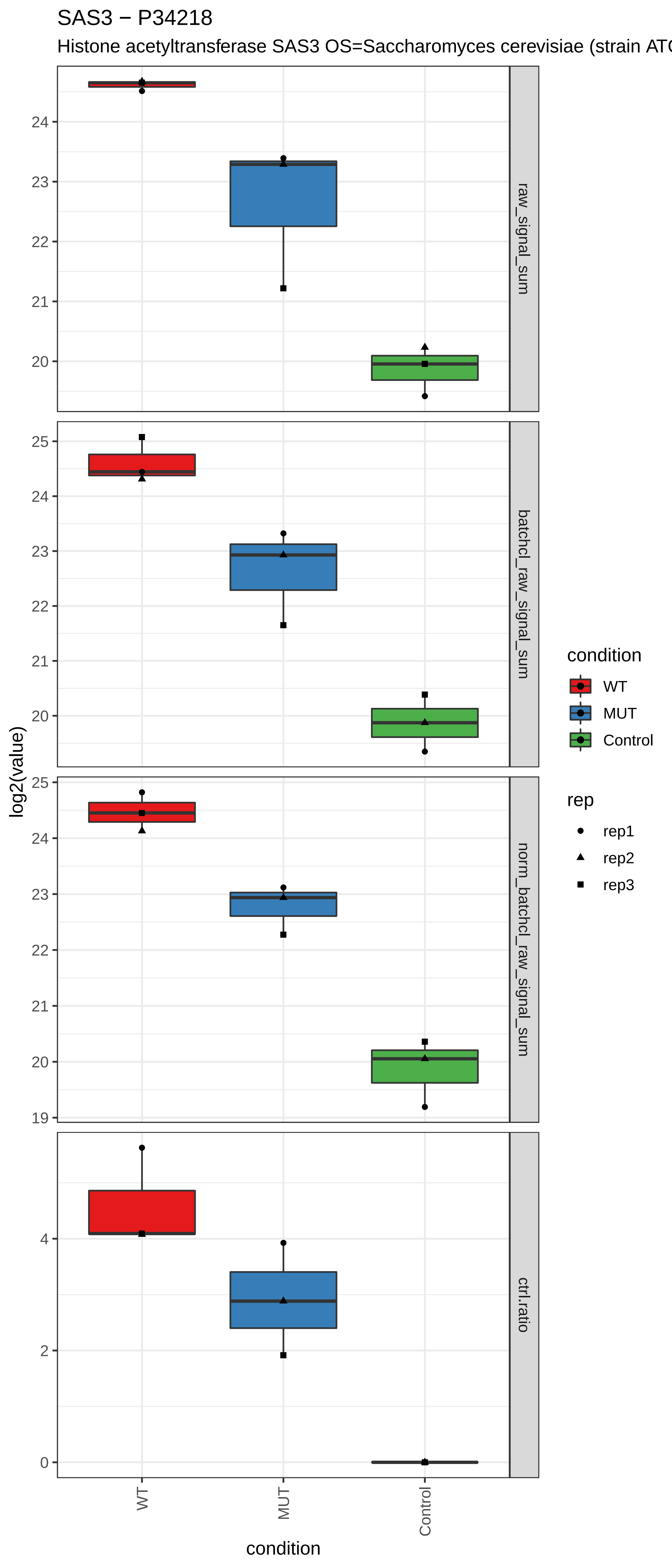
SAS3 − P34218
Histone acetyltransferase SAS3 OS=Saccharomyces cerevisiae (strain ATC
raw_signal_sum
batchcl_raw_signal_sum
norm_batchcl_raw_signal_sum
ctrl.ratio
log2(value)
condition
WT
MUT
Control
rep
rep1
rep2
rep3
24
23
22
21
20
25
19
4
2
0

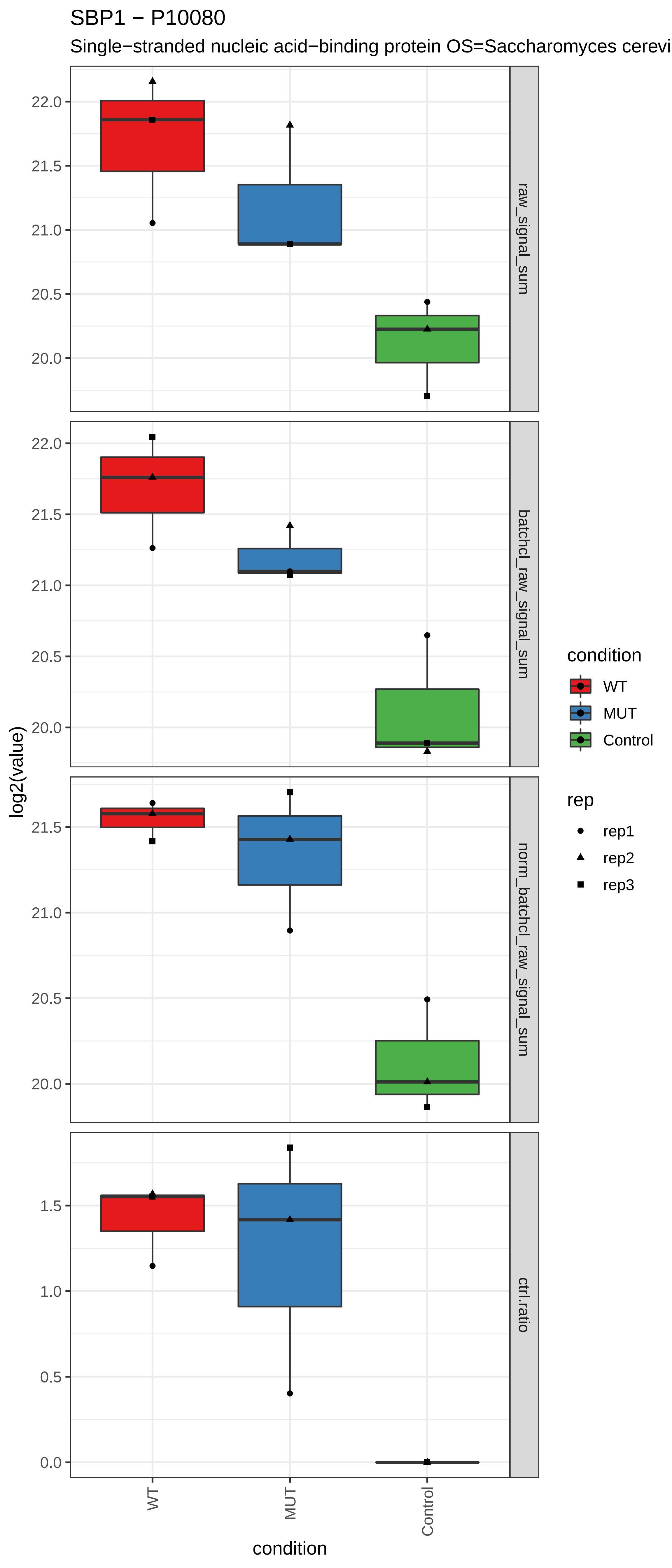
SBP1 − P10080
Single−stranded nucleic acid−binding protein OS=Saccharomyces cerevi
raw_signal_sum
batchcl_raw_signal_sum
norm_batchcl_raw_signal_sum
ctrl.ratio
log2(value)
condition
WT
MUT
Control
rep
rep1
rep2
rep3

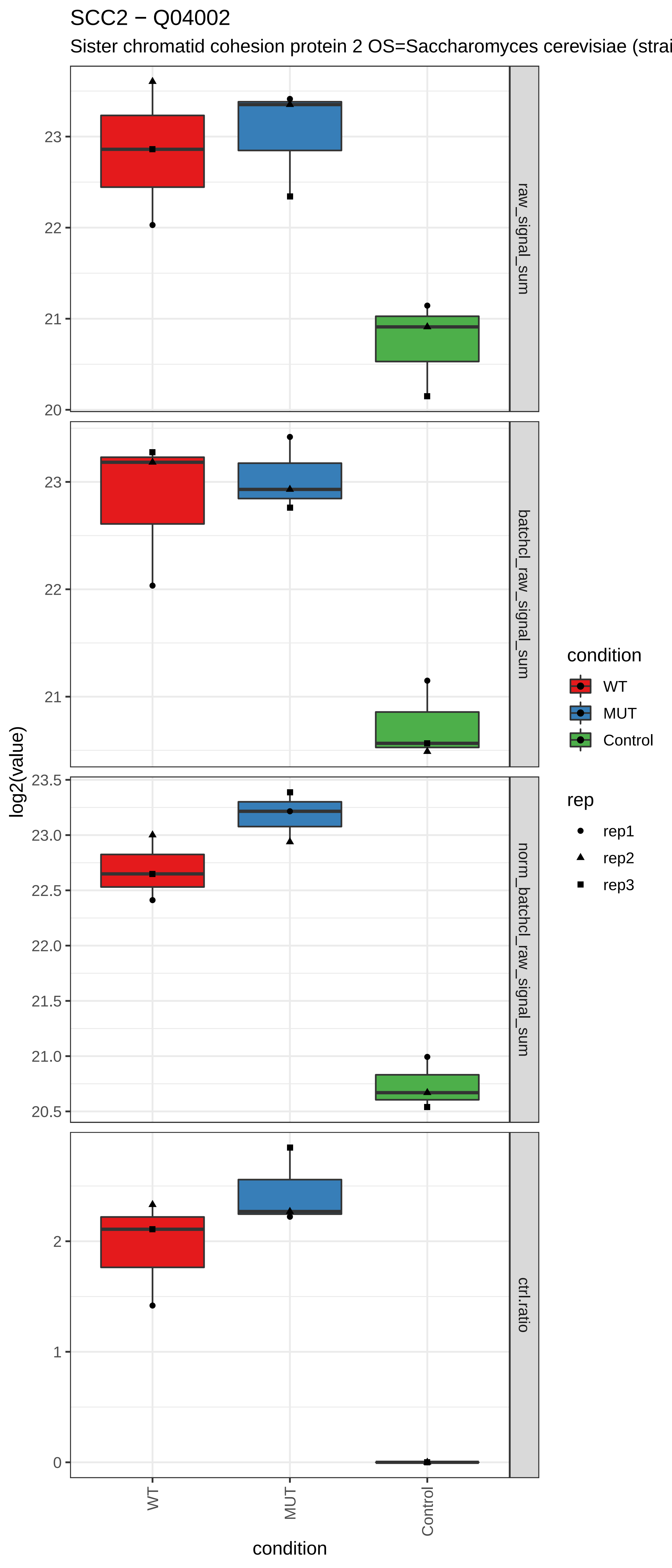

SCC2 − Q04002
Sister chromatid cohesion protein 2 OS=Saccharomyces cerevisiae (strai
raw_signal_sum
batchcl_raw_signal_sum
norm_batchcl_raw_signal_sum
ctrl.ratio
log2(value)
condition
WT
MUT
Control
rep
rep1
rep2
rep3
23
22
21
20
23.5
23.0
22.5
22.0
21.5
21.0
20.5
2
1
0

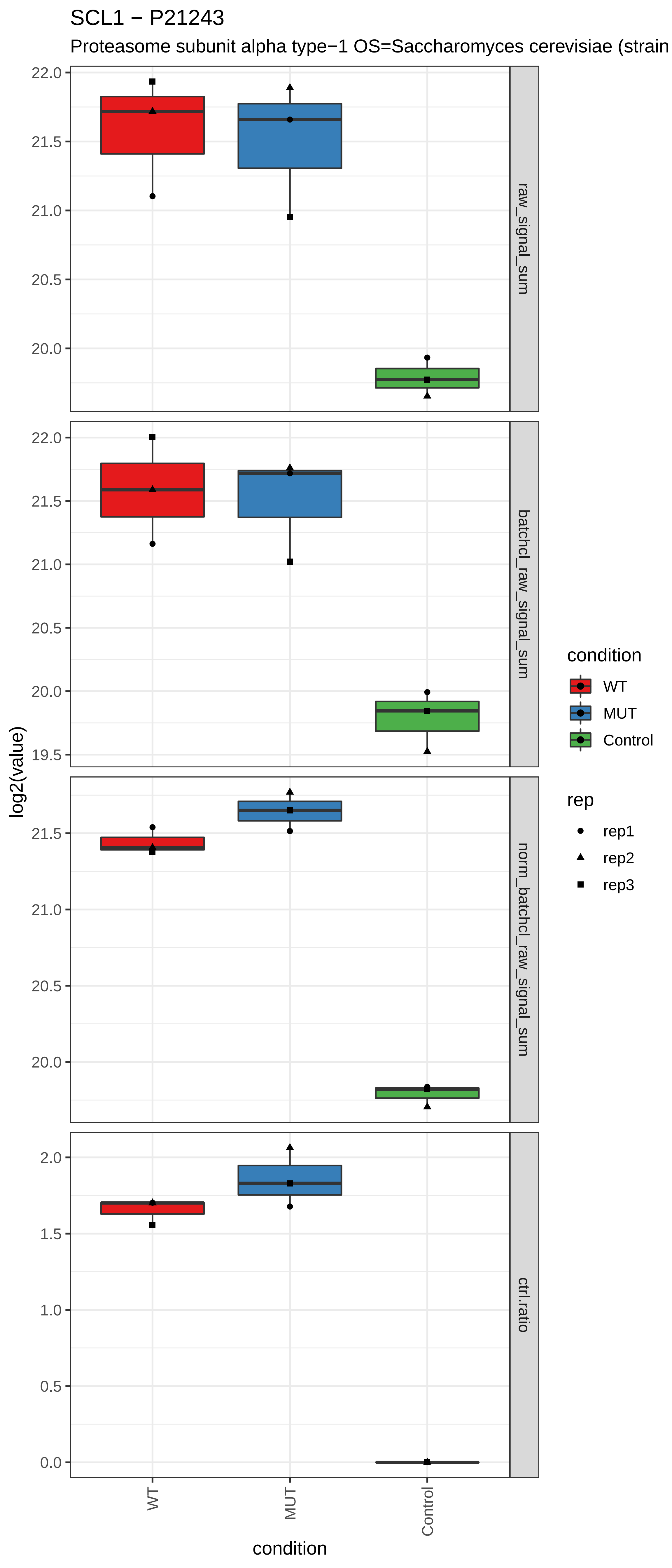

SCL1 − P21243
Proteasome subunit alpha type−1 OS=Saccharomyces cerevisiae (strain
raw_signal_sum
batchcl_raw_signal_sum
norm_batchcl_raw_signal_sum
ctrl.ratio
log2(value)
condition
WT
MUT
Control
rep
rep1
rep2
rep3
22.0
21.5
21.0
20.5
20.0
19.5
2.0
1.5
1.0
0.5
0.0

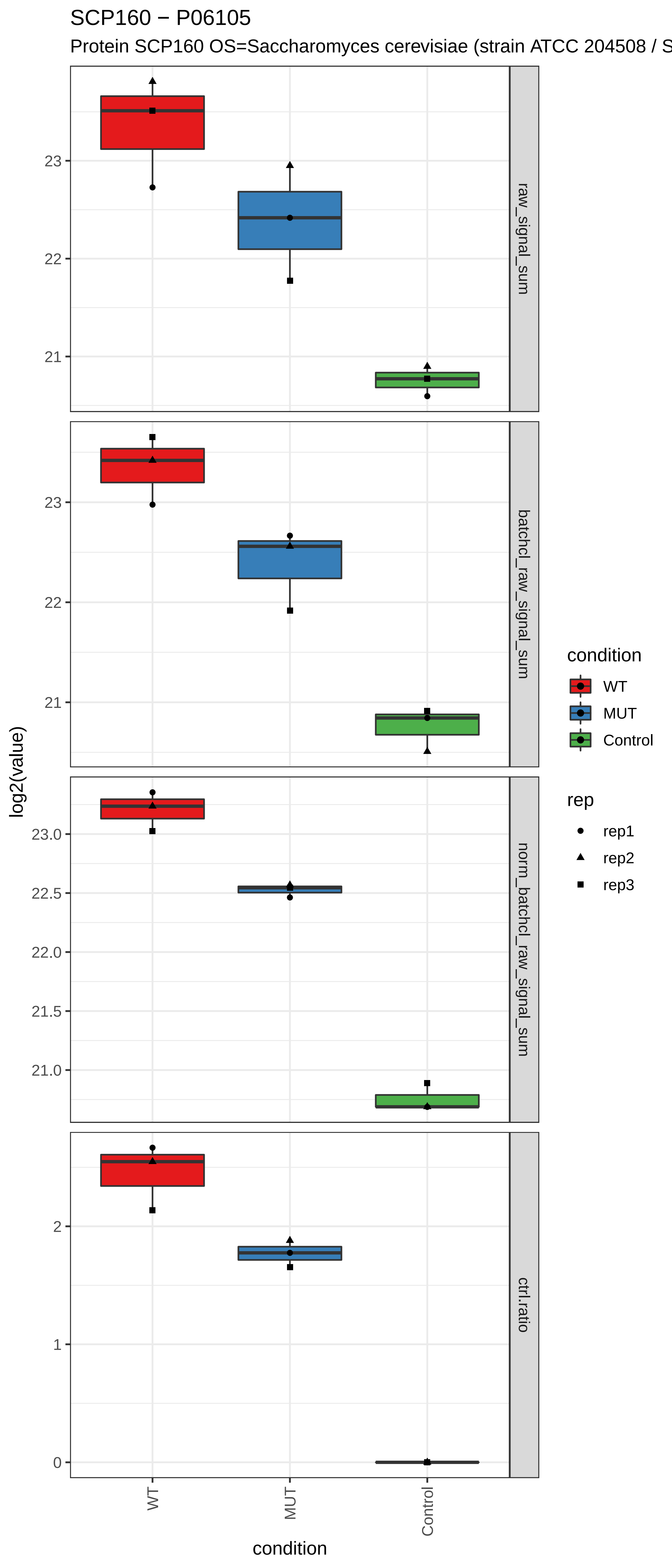
SCP160 − P06105
Protein SCP160 OS=Saccharomyces cerevisiae (strain ATCC 204508 / S
log2(value)
raw_signal_sum
batchcl_raw_signal_sum
norm_batchcl_raw_signal_sum
ctrl.ratio
23
22
21
23.0
22.5
22.0
21.5
21.0
2
1
0
WT
MUT
Control
condition
condition
WT
MUT
Control
rep
rep1
rep2
rep3

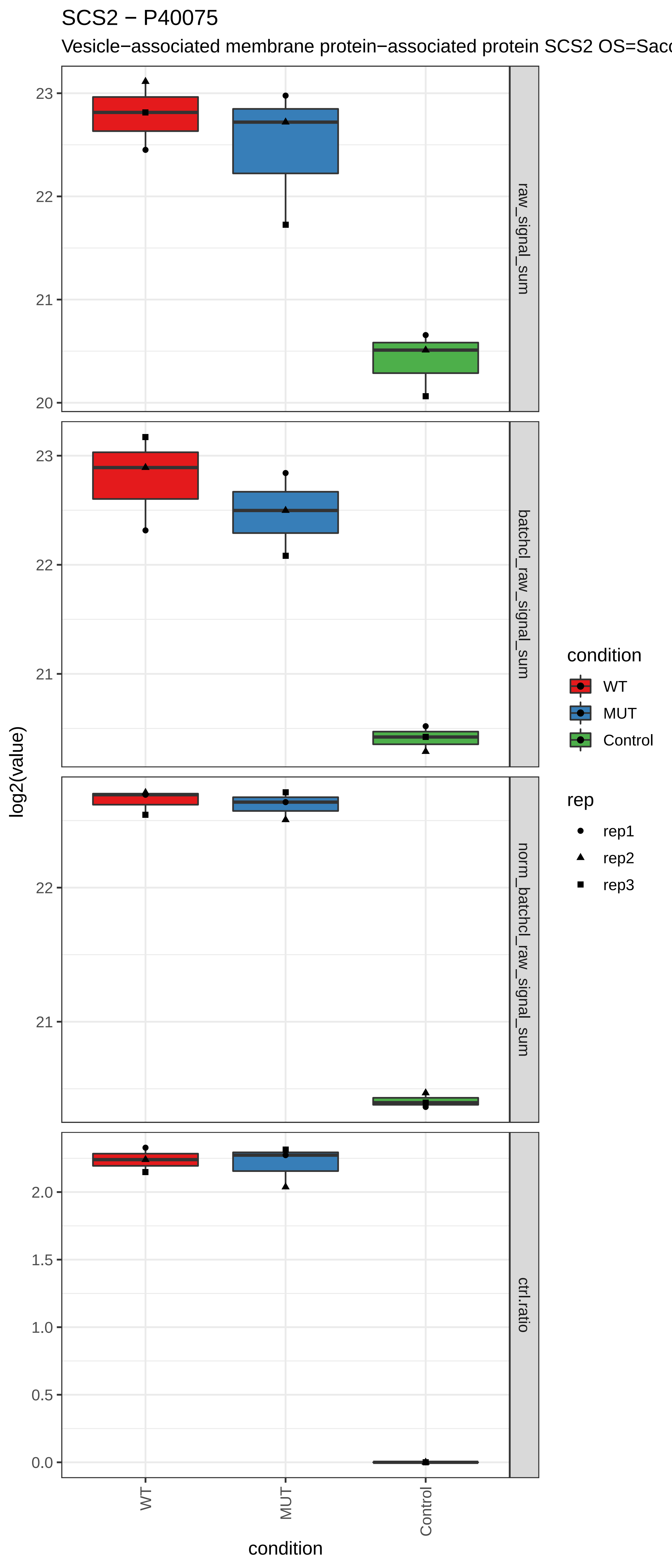

SCS2 − P40075
Vesicle−associated membrane protein−associated protein SCS2 OS=Sacc
raw_signal_sum
batchcl_raw_signal_sum
norm_batchcl_raw_signal_sum
ctrl.ratio
log2(value)
condition
WT
MUT
Control
rep
rep1
rep2
rep3

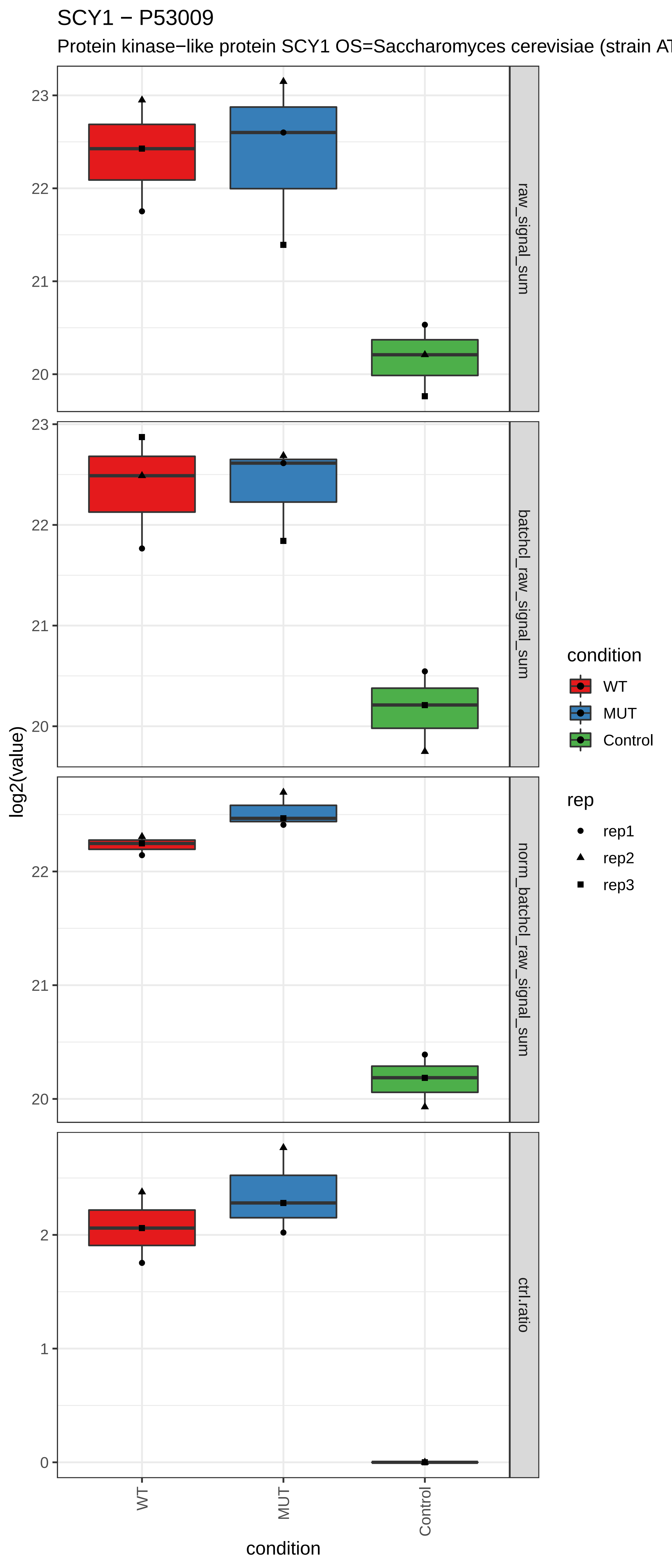

SCY1 − P53009
Protein kinase−like protein SCY1 OS=Saccharomyces cerevisiae (strain AT
raw_signal_sum
batchcl_raw_signal_sum
norm_batchcl_raw_signal_sum
ctrl.ratio
log2(value)
condition
WT
MUT
Control
rep
rep1
rep2
rep3

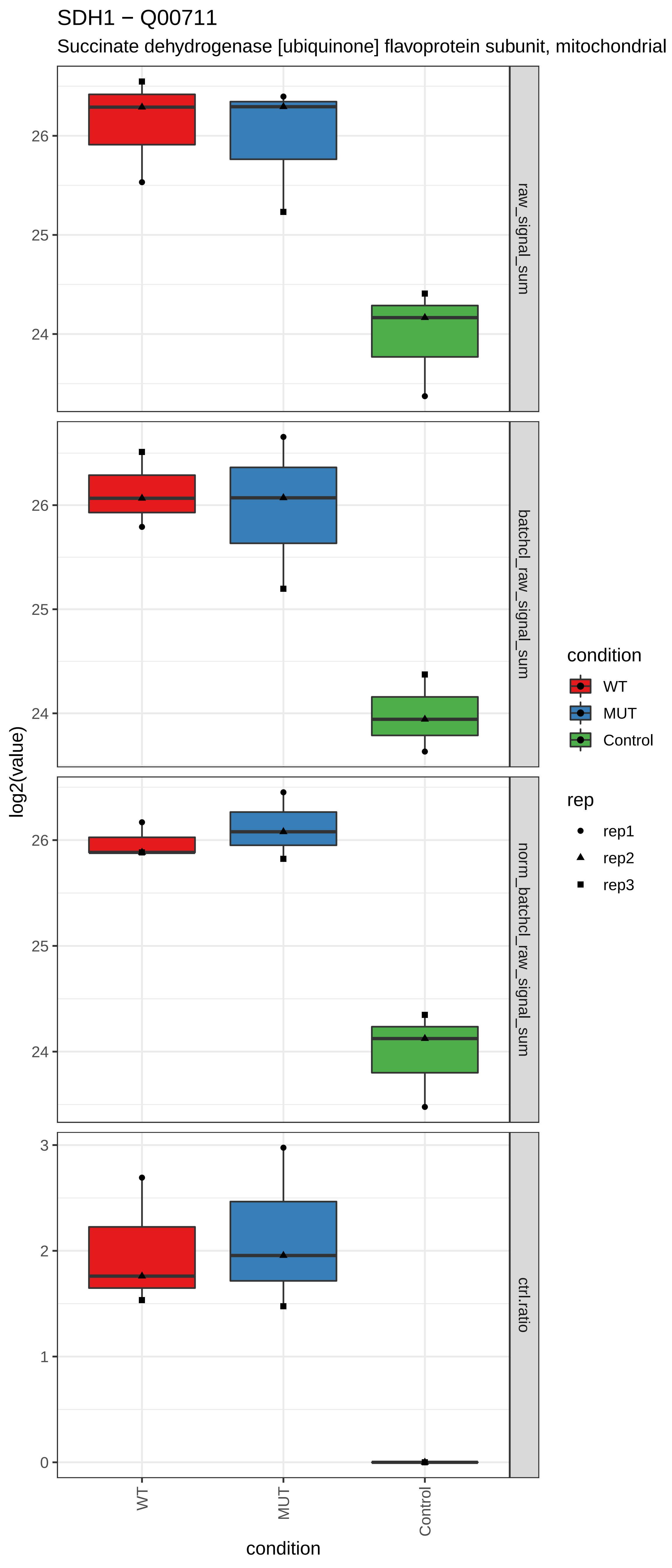
SDH1 − Q00711
Succinate dehydrogenase [ubiquinone] flavoprotein subunit, mitochondrial
raw_signal_sum
batchcl_raw_signal_sum
norm_batchcl_raw_signal_sum
ctrl.ratio
log2(value)
condition
WT
MUT
Control
rep
rep1
rep2
rep3
26
25
24
3
2
1
0

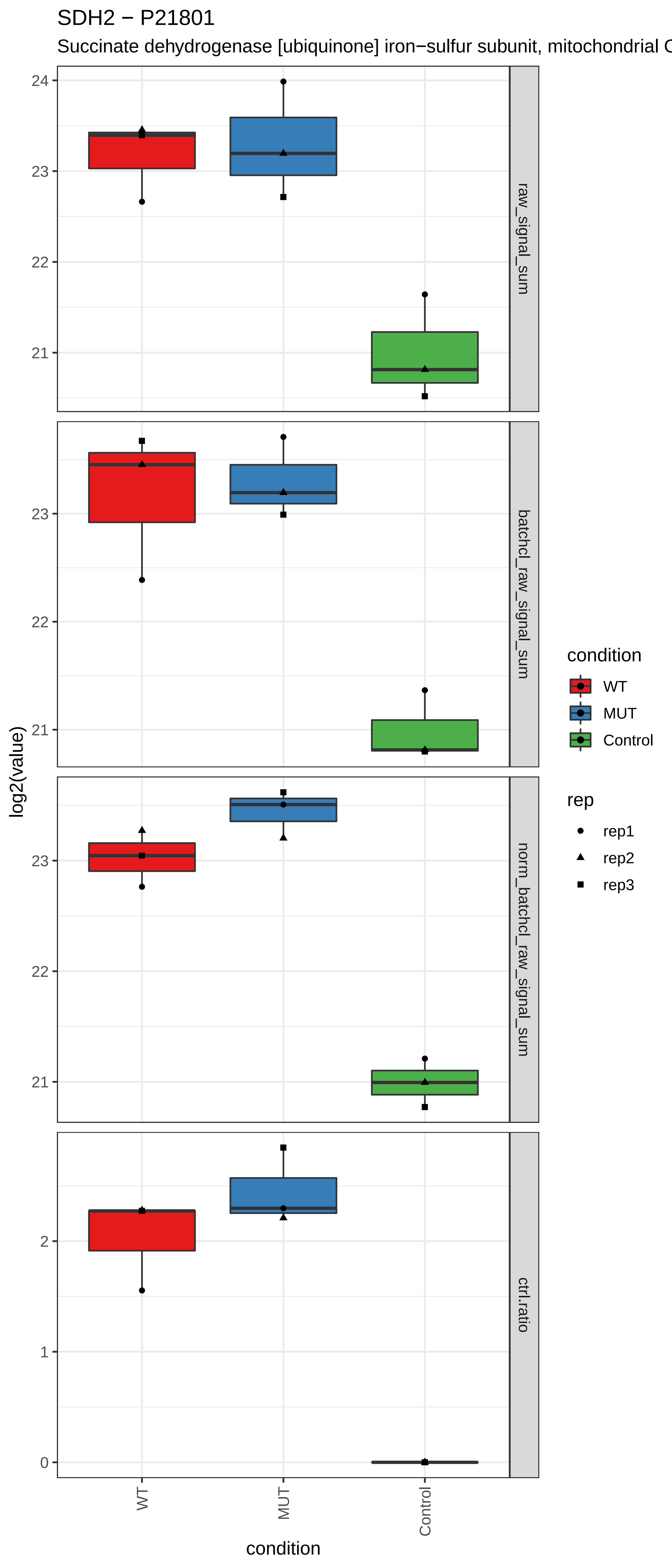

SDH2 − P21801
Succinate dehydrogenase [ubiquinone] iron−sulfur subunit, mitochondrial C
raw_signal_sum
batchcl_raw_signal_sum
norm_batchcl_raw_signal_sum
ctrl.ratio
log2(value)
condition
WT
MUT
Control
rep
rep1
rep2
rep3

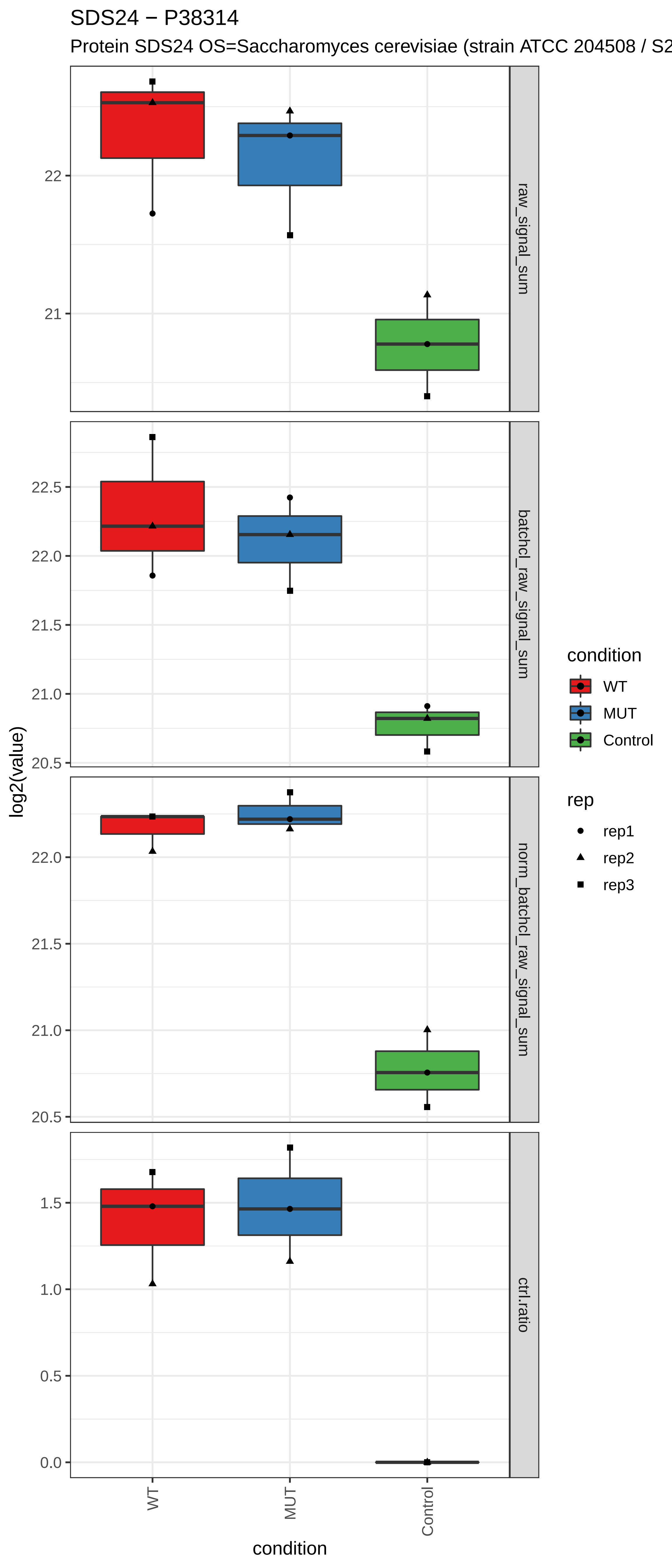

SDS24 − P38314
Protein SDS24 OS=Saccharomyces cerevisiae (strain ATCC 204508 / S2
raw_signal_sum
batchcl_raw_signal_sum
norm_batchcl_raw_signal_sum
ctrl.ratio
log2(value)
condition
WT
MUT
Control
rep
rep1
rep2
rep3
22
21
22.5
22.0
21.5
21.0
20.5
1.5
1.0
0.5
0.0

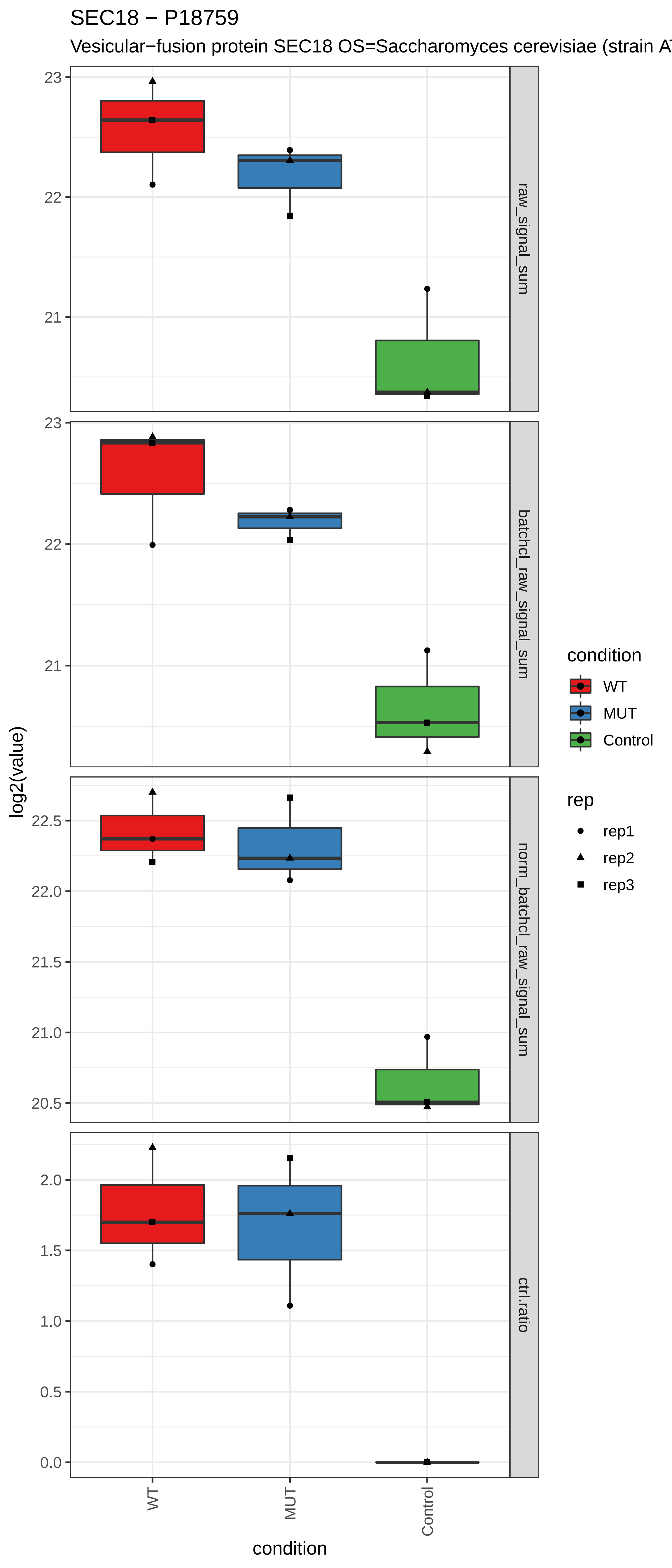

SEC18 − P18759
Vesicular−fusion protein SEC18 OS=Saccharomyces cerevisiae (strain A
raw_signal_sum
batchcl_raw_signal_sum
norm_batchcl_raw_signal_sum
ctrl.ratio
log2(value)
condition
WT
MUT
Control
rep
rep1
rep2
rep3
23
22
21
22.5
22.0
21.5
21.0
20.5
2.0
1.5
1.0
0.5
0.0

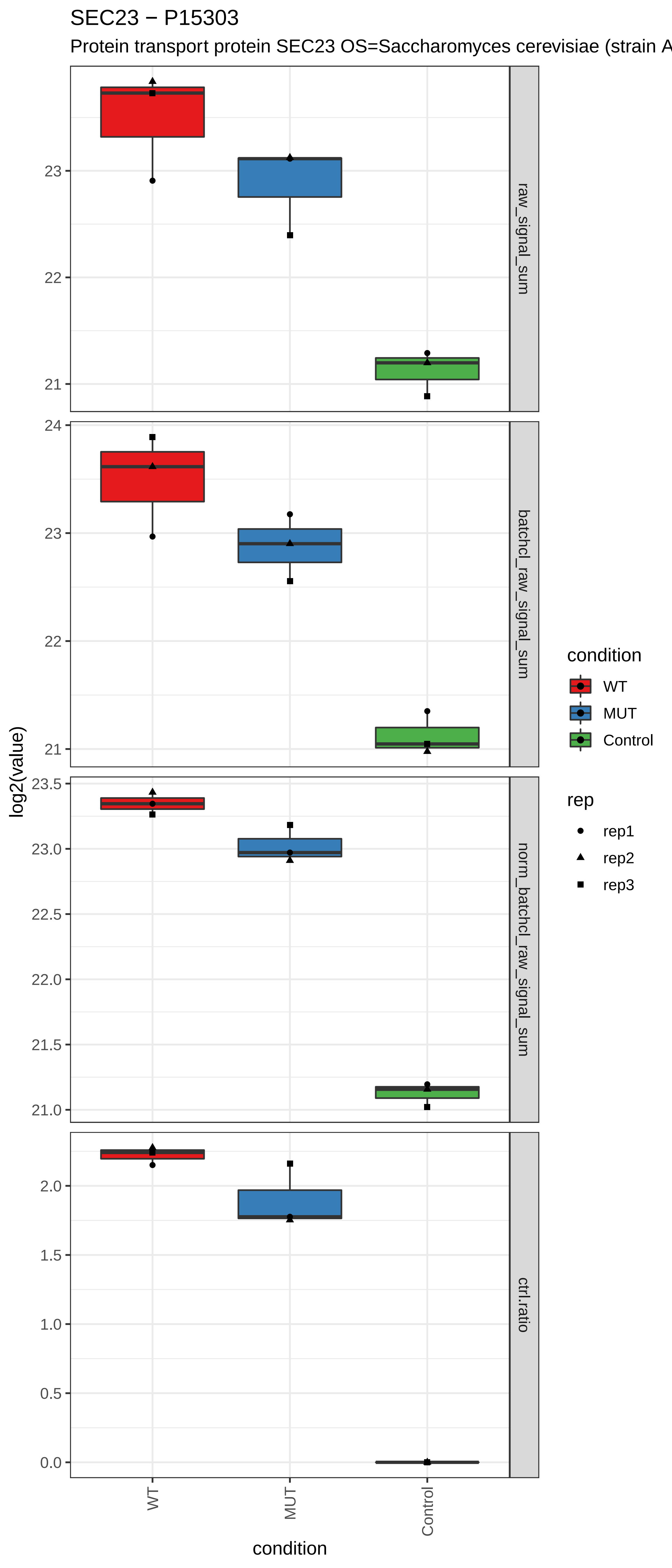

SEC23 − P15303
Protein transport protein SEC23 OS=Saccharomyces cerevisiae (strain A
raw_signal_sum
batchcl_raw_signal_sum
norm_batchcl_raw_signal_sum
ctrl.ratio
log2(value)
condition
WT
MUT
Control
rep
rep1
rep2
rep3

SEC24 − P40482

Protein transport protein SEC24 OS=Saccharomyces cerevisiae (strain AT

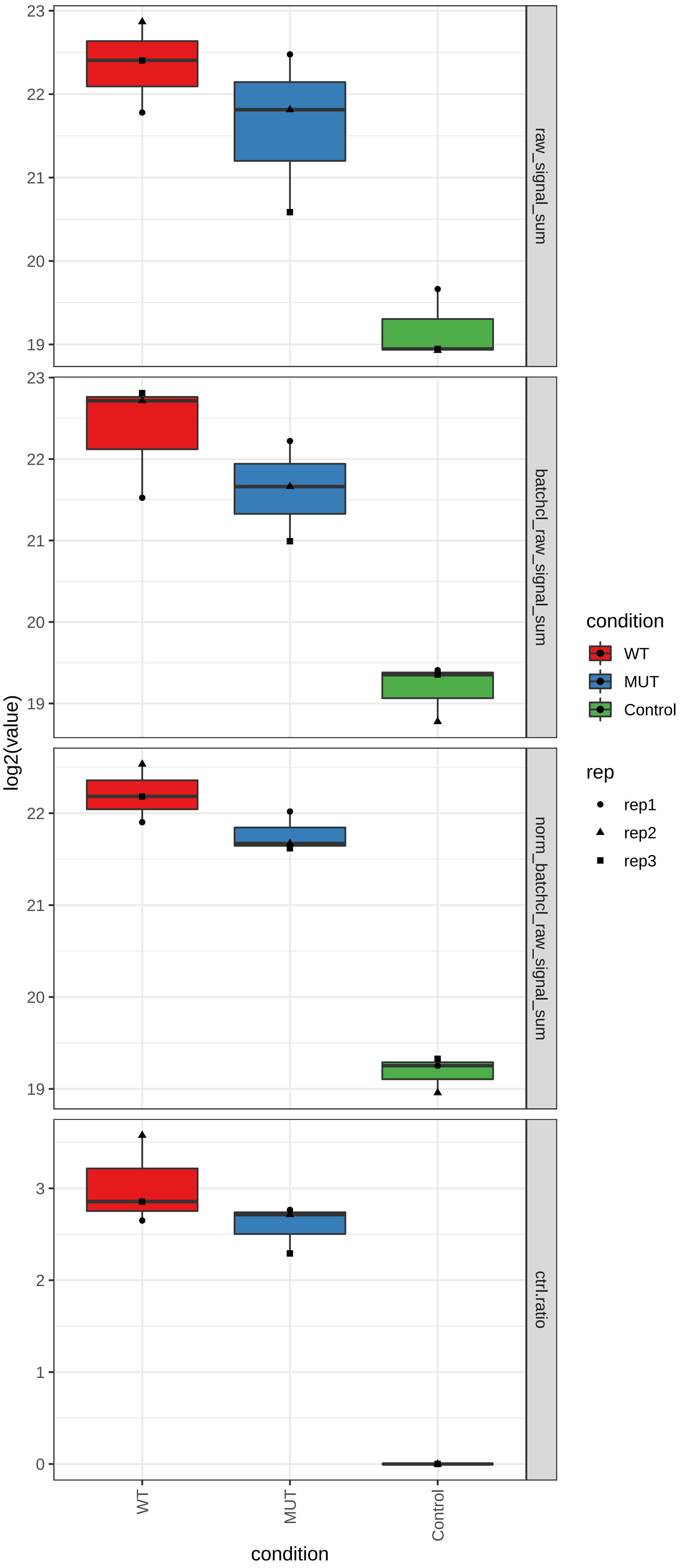

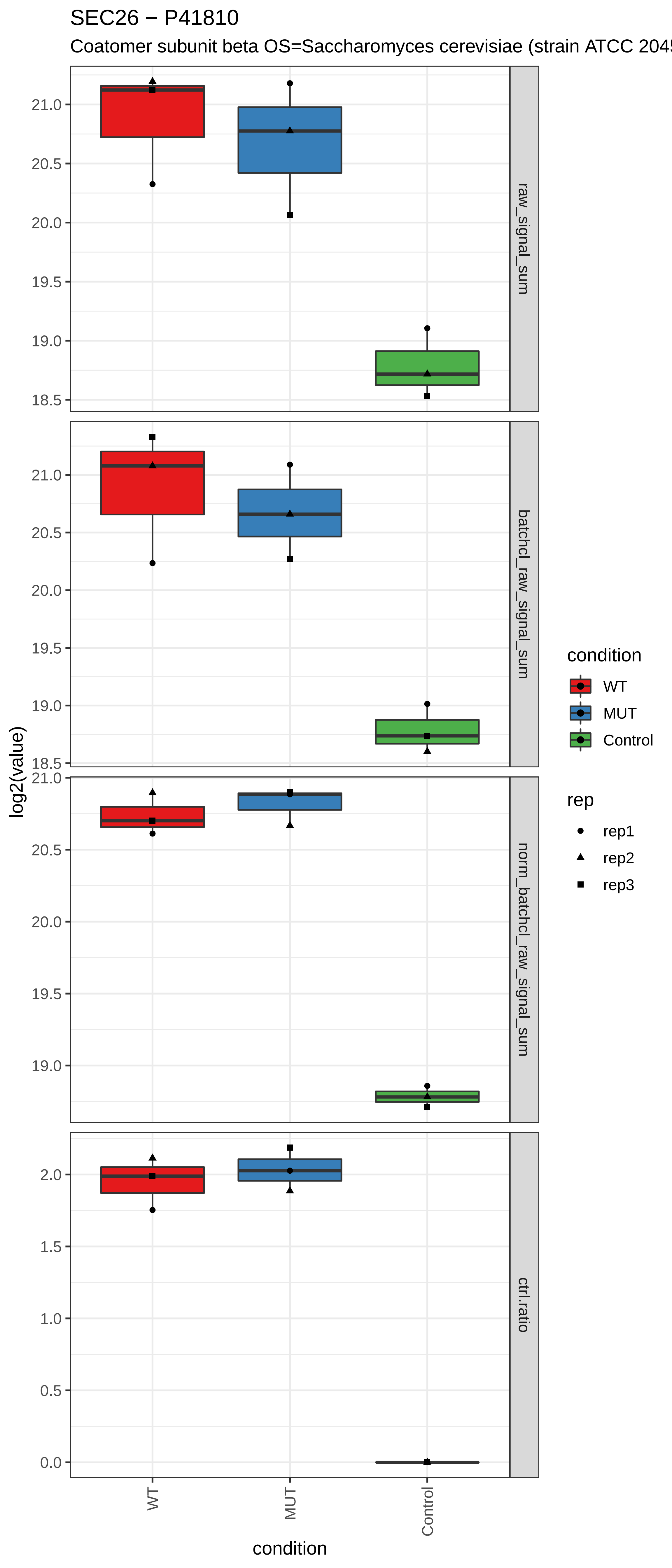

SEC26 − P41810
Coatomer subunit beta OS=Saccharomyces cerevisiae (strain ATCC 204
log2(value)
raw_signal_sum
batchcl_raw_signal_sum
norm_batchcl_raw_signal_sum
ctrl.ratio
21.0
20.5
20.0
19.5
19.0
18.5
2.0
1.5
1.0
0.5
0.0
WT
MUT
Control
condition
condition
WT
MUT
Control
rep
rep1
rep2
rep3

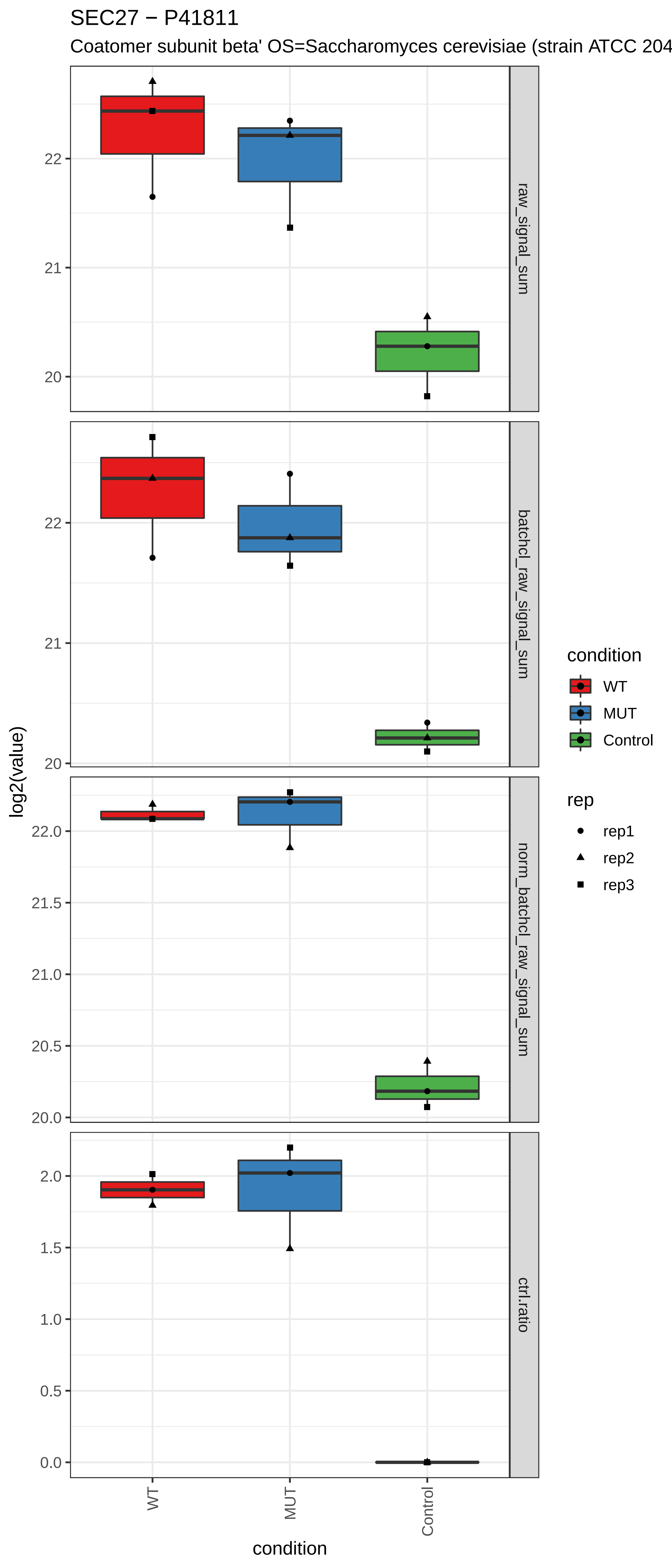

SEC27 − P41811
Coatomer subunit beta' OS=Saccharomyces cerevisiae (strain ATCC 204
raw_signal_sum
batchcl_raw_signal_sum
norm_batchcl_raw_signal_sum
ctrl.ratio
log2(value)
condition
WT
MUT
Control
rep
rep1
rep2
rep3

SEC4 − P07560

Ras−related protein SEC4 OS=Saccharomyces cerevisiae (strain ATCC 20

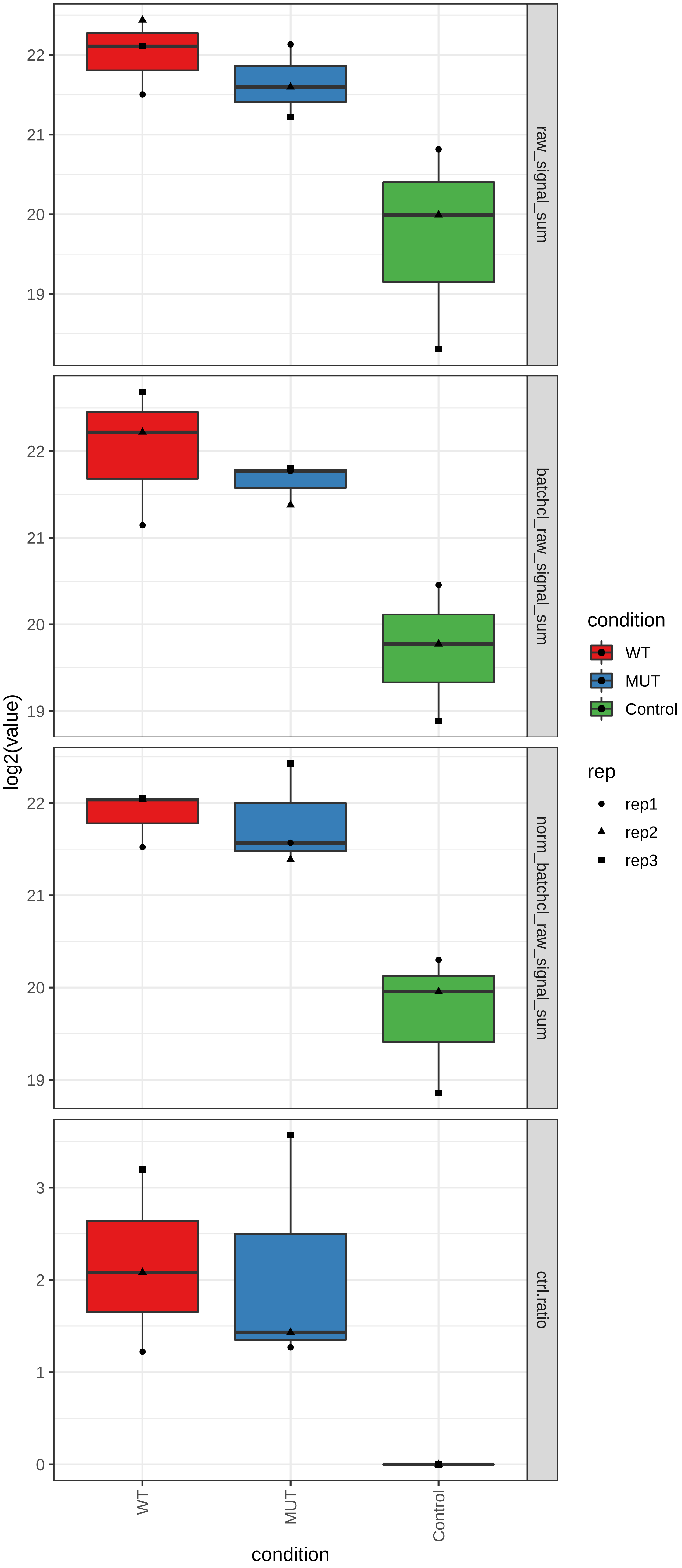

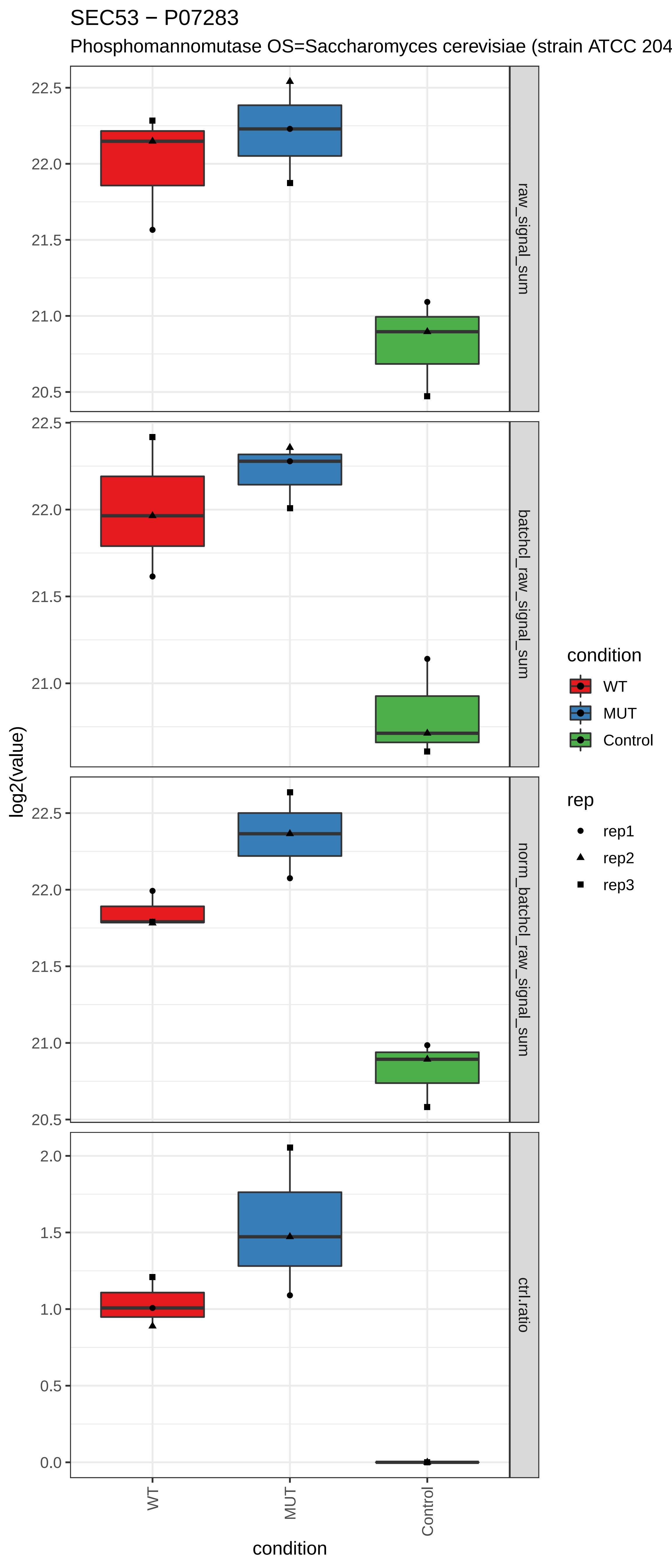

SEC53 − P07283
Phosphomannomutase OS=Saccharomyces cerevisiae (strain ATCC 204
raw_signal_sum
batchcl_raw_signal_sum
norm_batchcl_raw_signal_sum
ctrl.ratio
log2(value)
condition
WT
MUT
Control
rep
rep1
rep2
rep3
22.5
22.0
21.5
21.0
20.5
2.0
1.5
1.0
0.5
0.0

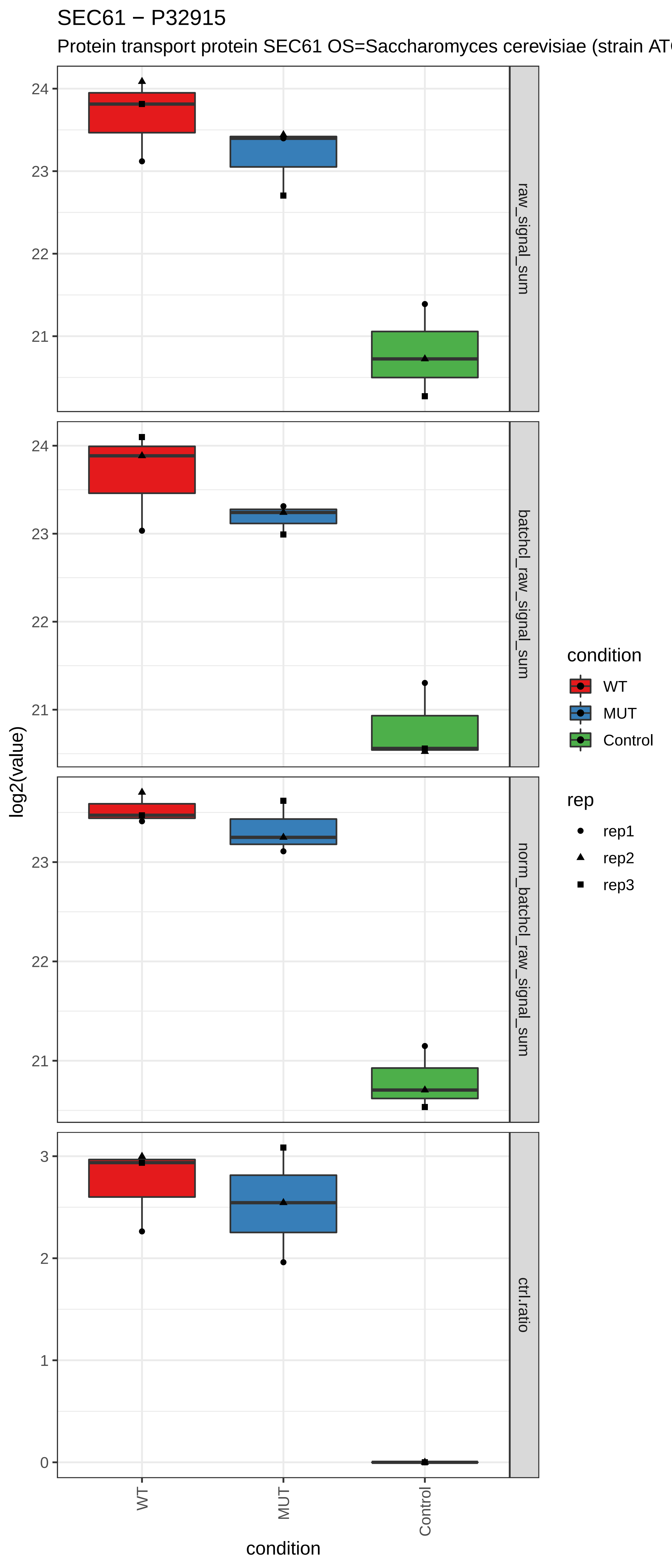

SEC61 − P32915
Protein transport protein SEC61 OS=Saccharomyces cerevisiae (strain AT
log2(value)
raw_signal_sum
batchcl_raw_signal_sum
norm_batchcl_raw_signal_sum
ctrl.ratio
24
23
22
21
3
2
1
0
WT
MUT
Control
condition
condition
WT
MUT
Control
rep
rep1
rep2
rep3

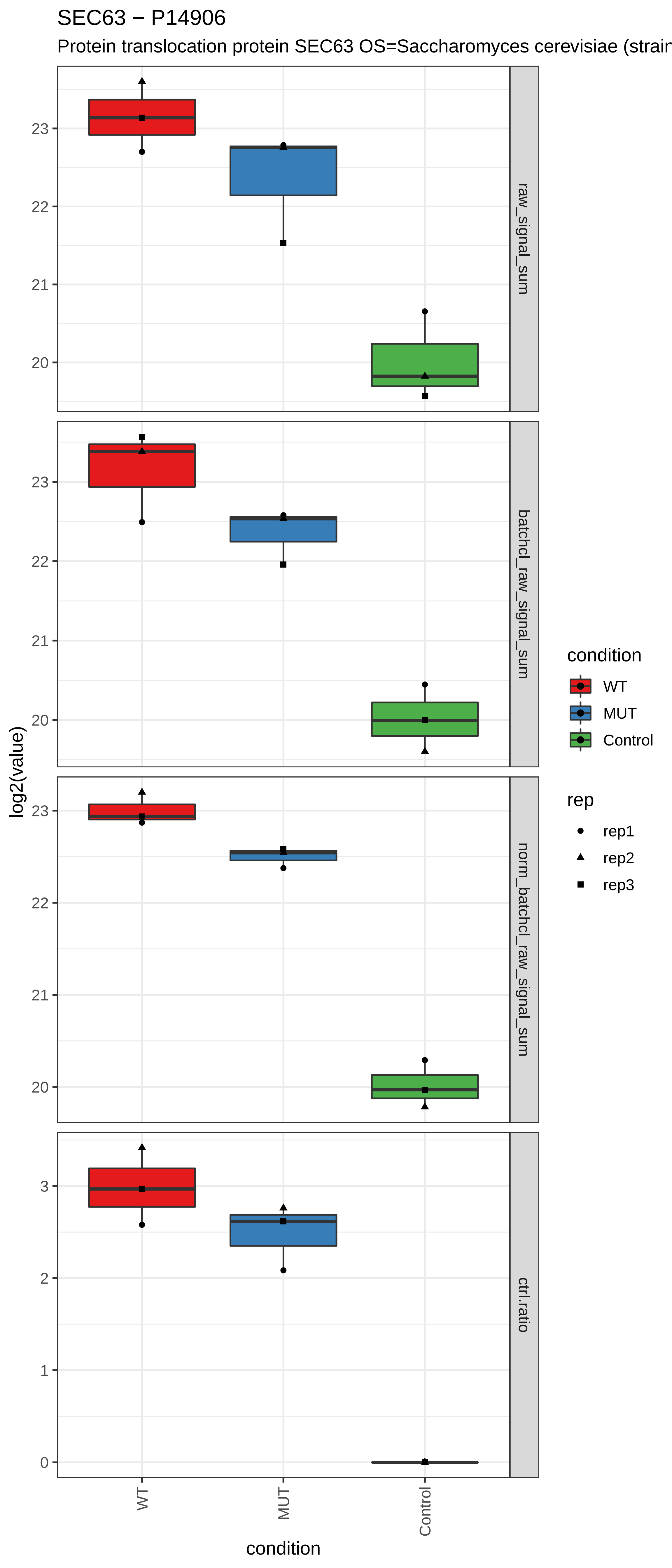

SEC63 − P14906
Protein translocation protein SEC63 OS=Saccharomyces cerevisiae (strain
raw_signal_sum
batchcl_raw_signal_sum
norm_batchcl_raw_signal_sum
ctrl.ratio
log2(value)
condition
WT
MUT
Control
rep
rep1
rep2
rep3

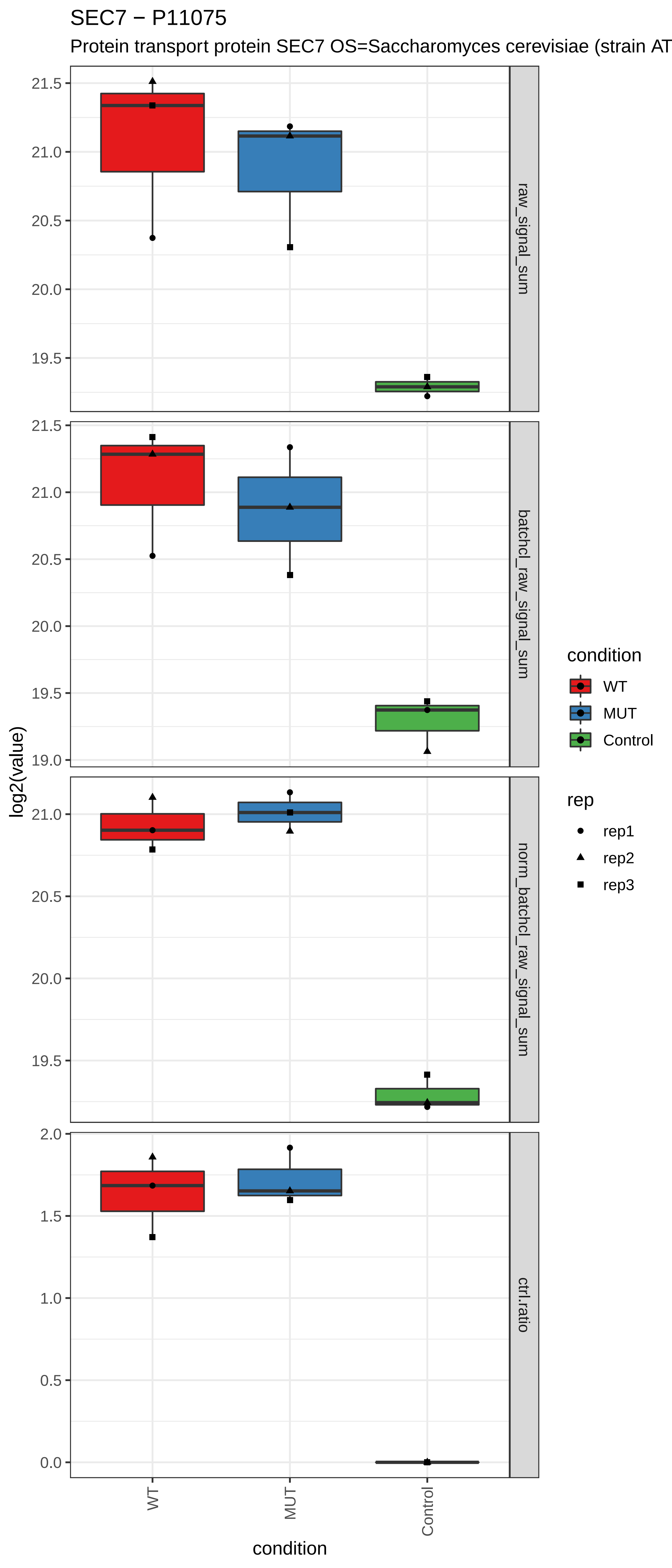

SEC7 − P11075
Protein transport protein SEC7 OS=Saccharomyces cerevisiae (strain AT
raw_signal_sum
batchcl_raw_signal_sum
norm_batchcl_raw_signal_sum
ctrl.ratio
log2(value)
condition
WT
MUT
Control
rep
rep1
rep2
rep3
21.5
21.0
20.5
20.0
19.5
19.0
2.0
1.5
1.0
0.5
0.0

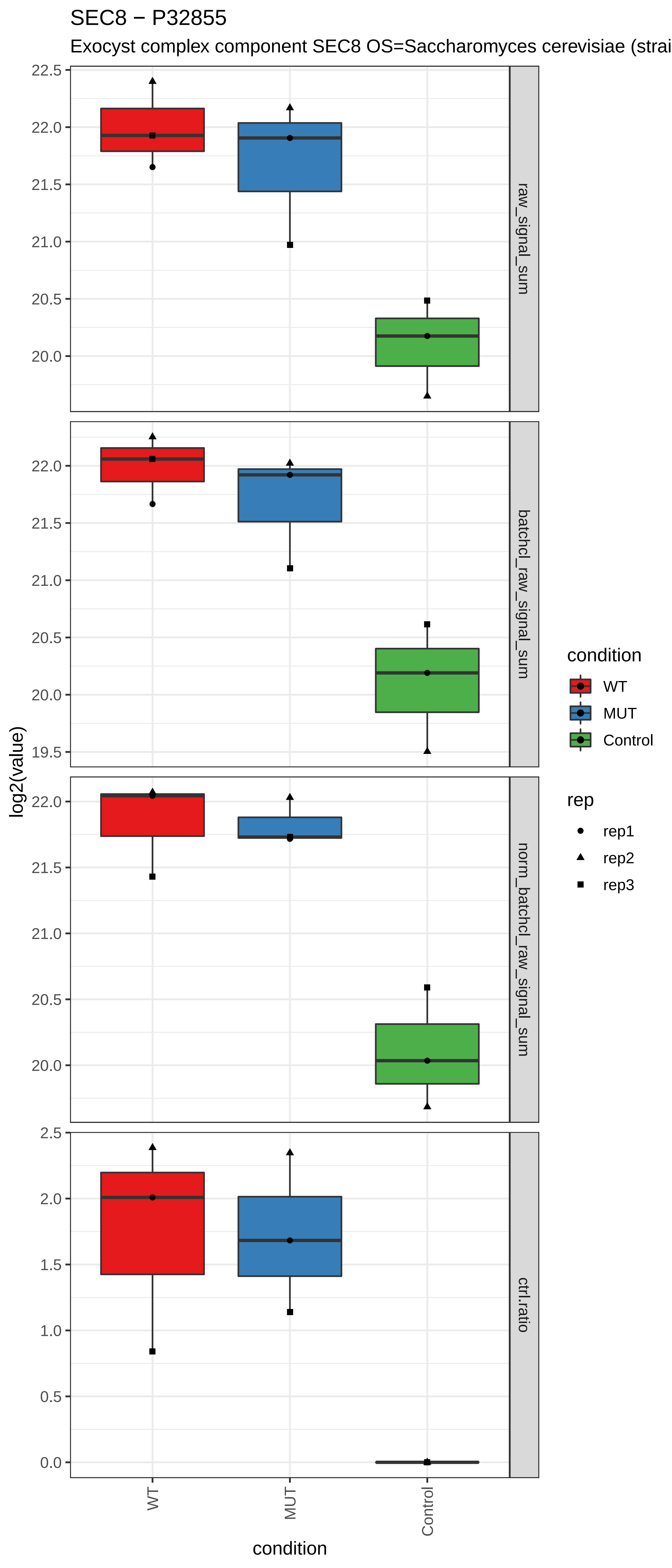
SEC8 − P32855
Exocyst complex component SEC8 OS=Saccharomyces cerevisiae (strai
raw_signal_sum
batchcl_raw_signal_sum
norm_batchcl_raw_signal_sum
ctrl.ratio
log2(value)
condition
WT
MUT
Control
rep
rep1
rep2
rep3
22.5
22.0
21.5
21.0
20.5
20.0
19.5
2.5
2.0
1.5
1.0
0.5
0.0

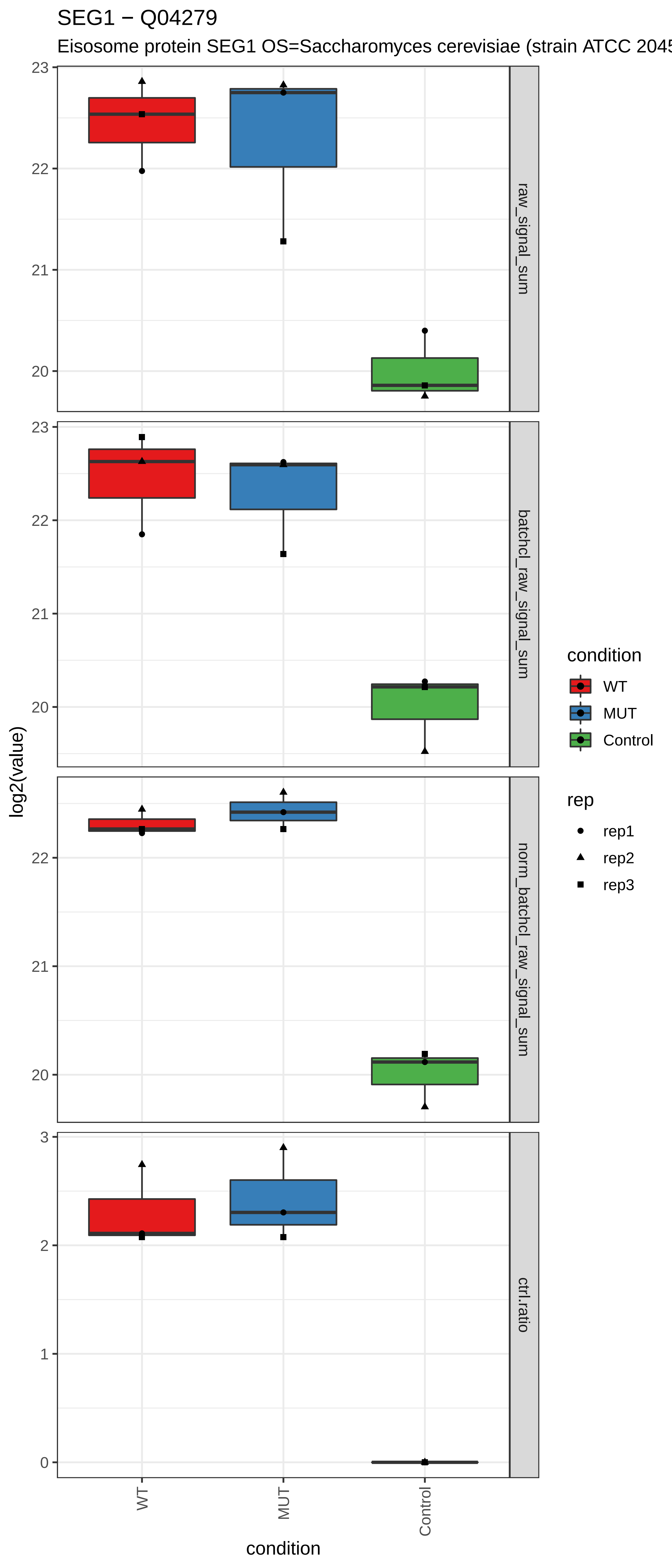
SEG1 − Q04279
Eisosome protein SEG1 OS=Saccharomyces cerevisiae (strain ATCC 2045
raw_signal_sum
batchcl_raw_signal_sum
norm_batchcl_raw_signal_sum
ctrl.ratio
log2(value)
condition
WT
MUT
Control
rep
rep1
rep2
rep3

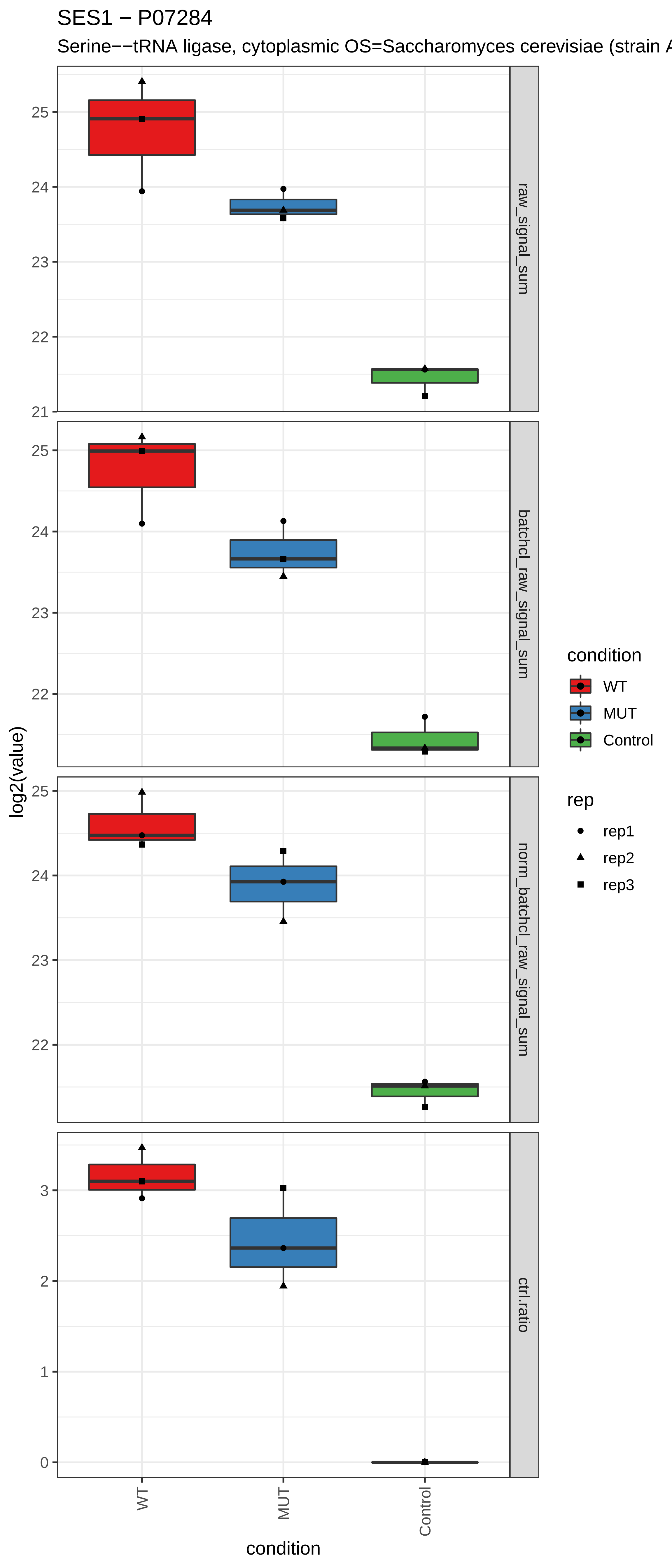
SES1 − P07284
Serine−−tRNA ligase, cytoplasmic OS=Saccharomyces cerevisiae (strain A
raw_signal_sum
batchcl_raw_signal_sum
norm_batchcl_raw_signal_sum
ctrl.ratio
log2(value)
condition
WT
MUT
Control
rep
rep1
rep2
rep3
25
24
23
22
21
3
2
1
0

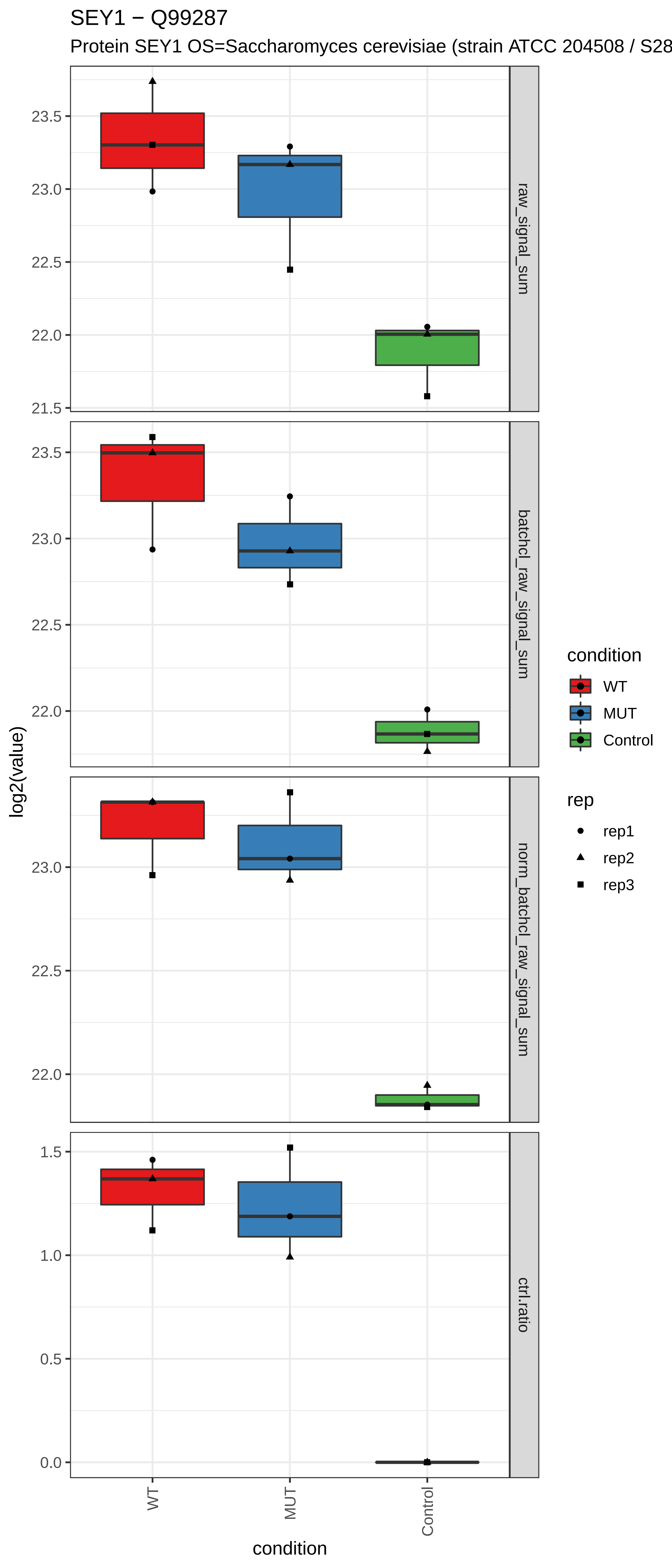

SEY1 − Q99287
Protein SEY1 OS=Saccharomyces cerevisiae (strain ATCC 204508 / S28
raw_signal_sum
batchcl_raw_signal_sum
norm_batchcl_raw_signal_sum
ctrl.ratio
log2(value)
condition
WT
MUT
Control
rep
rep1
rep2
rep3
23.5
23.0
22.5
22.0
21.5
1.5
1.0
0.5
0.0

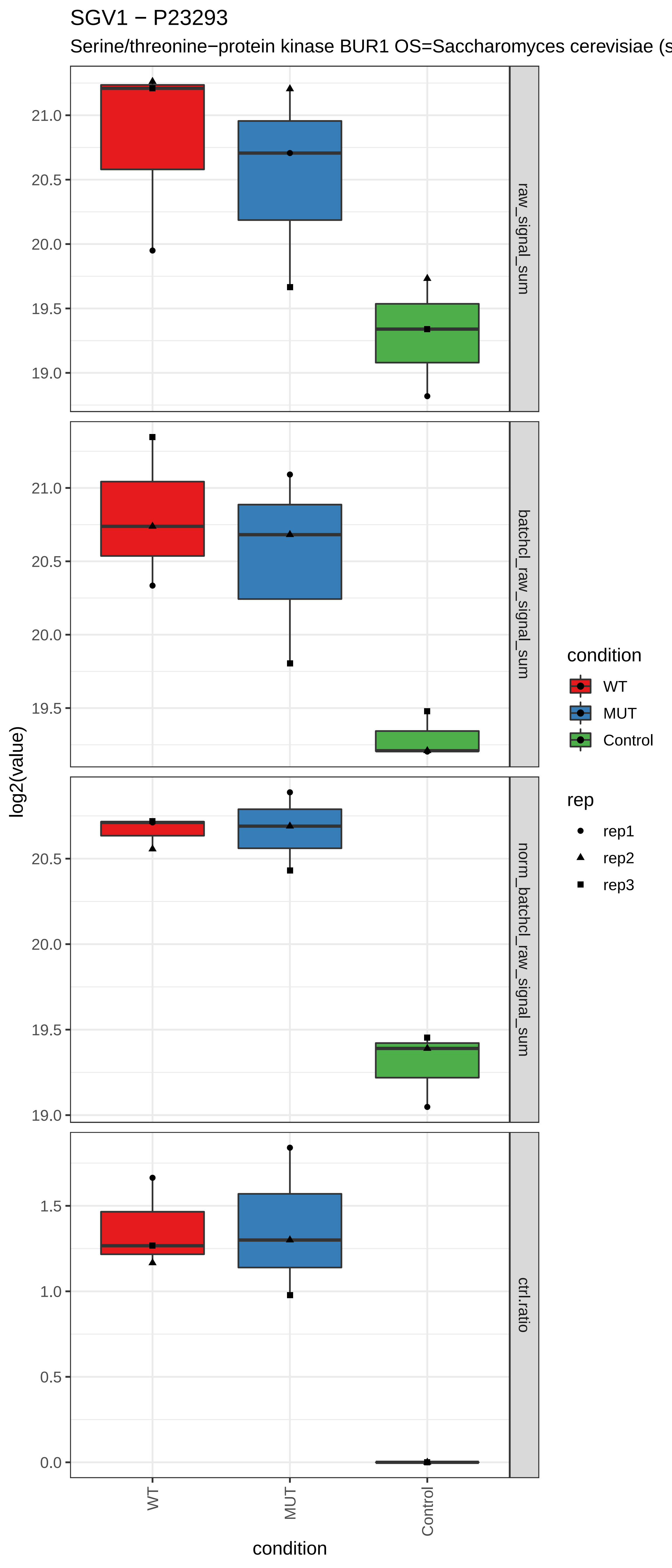
SGV1 − P23293
Serine/threonine−protein kinase BUR1 OS=Saccharomyces cerevisiae (s
raw_signal_sum
batchcl_raw_signal_sum
norm_batchcl_raw_signal_sum
ctrl.ratio
log2(value)
condition
WT
MUT
Control
rep
rep1
rep2
rep3
21.0
20.5
20.0
19.5
19.0
1.5
1.0
0.5
0.0

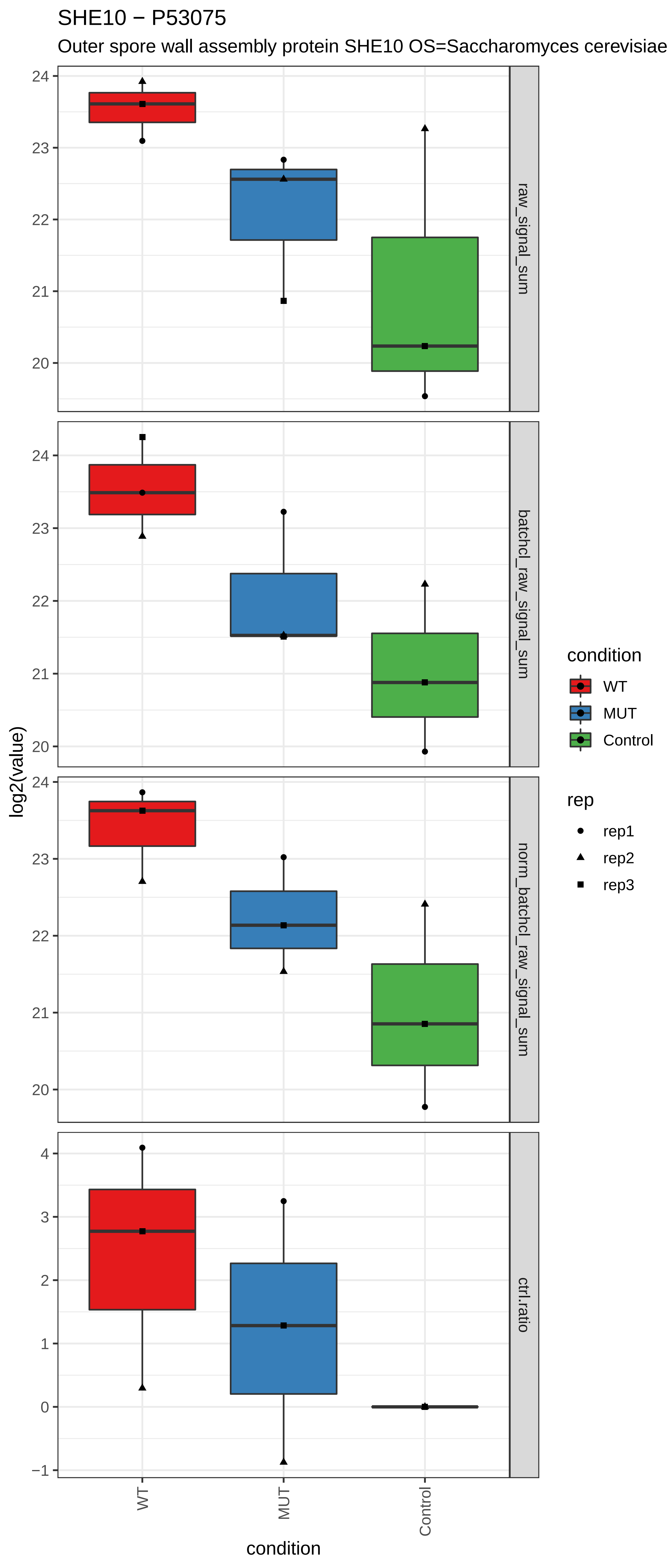

SHE10 − P53075
Outer spore wall assembly protein SHE10 OS=Saccharomyces cerevisiae
log2(value)
raw_signal_sum
batchcl_raw_signal_sum
norm_batchcl_raw_signal_sum
ctrl.ratio
24
23
22
21
20
4
3
2
1
0
−1
WT
MUT
Control
condition
condition
WT
MUT
Control
rep
rep1
rep2
rep3

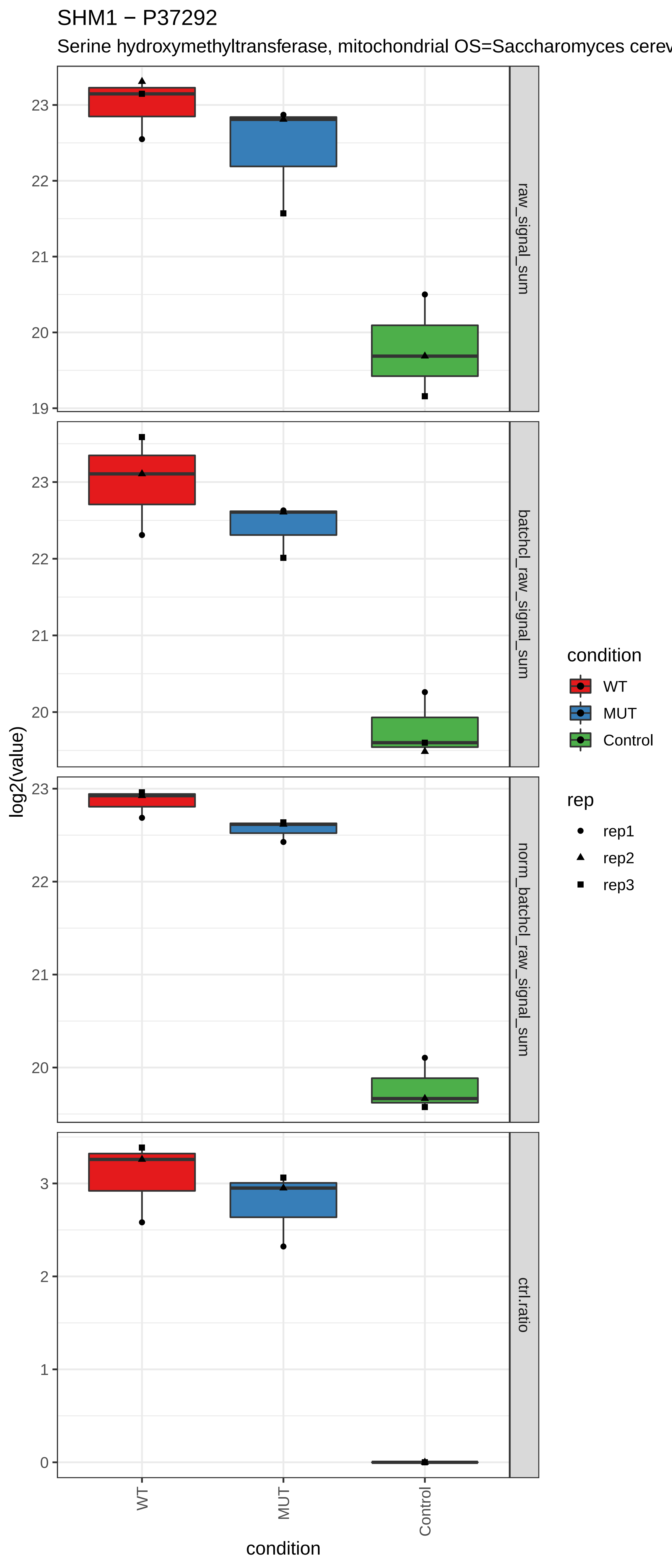

SHM1 − P37292
Serine hydroxymethyltransferase, mitochondrial OS=Saccharomyces cerev
raw_signal_sum
batchcl_raw_signal_sum
norm_batchcl_raw_signal_sum
ctrl.ratio
log2(value)
condition
WT
MUT
Control
rep
rep1
rep2
rep3
23
22
21
20
19
3
2
1
0

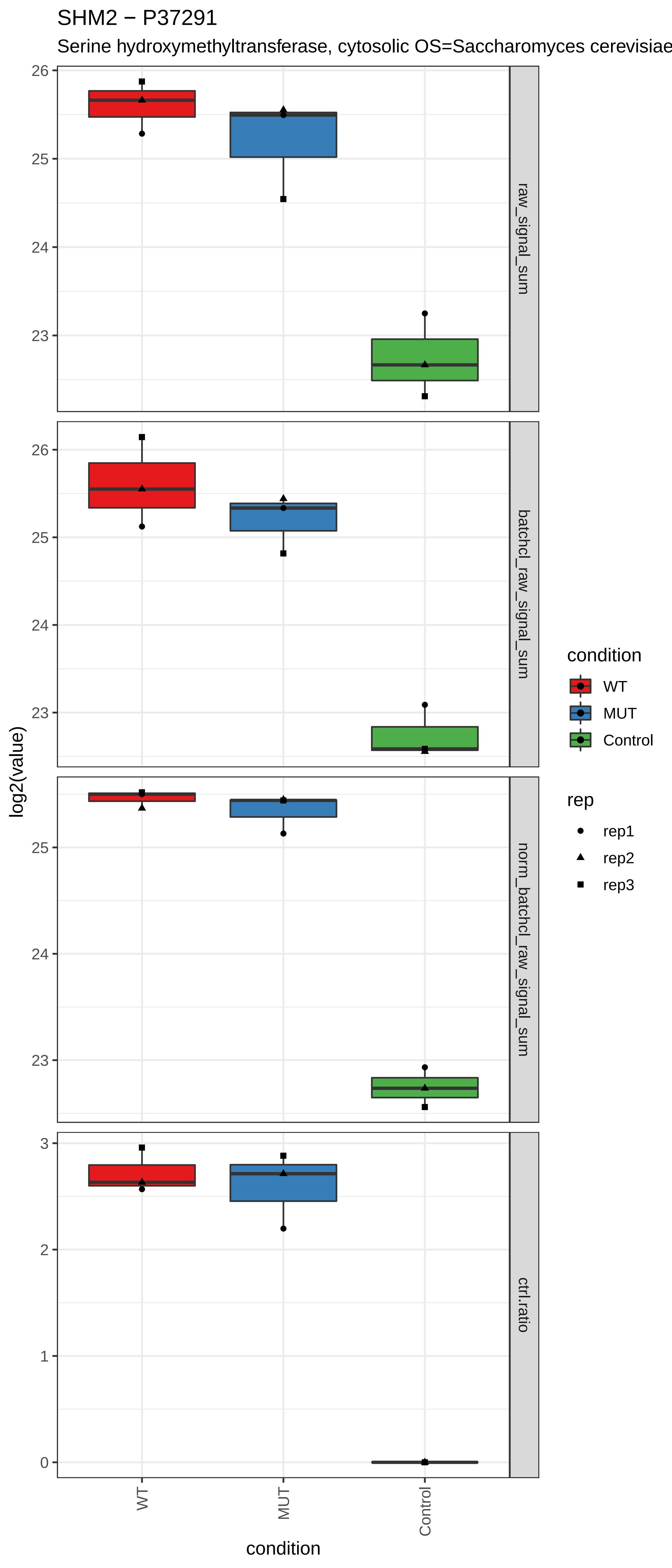

SHM2 − P37291
Serine hydroxymethyltransferase, cytosolic OS=Saccharomyces cerevisiae
raw_signal_sum
batchcl_raw_signal_sum
norm_batchcl_raw_signal_sum
ctrl.ratio
log2(value)
condition
WT
MUT
Control
rep
rep1
rep2
rep3
26
25
24
23
3
2
1
0

SHS1 − Q07657

Seventh homolog of septin 1 OS=Saccharomyces cerevisiae (strain ATCC

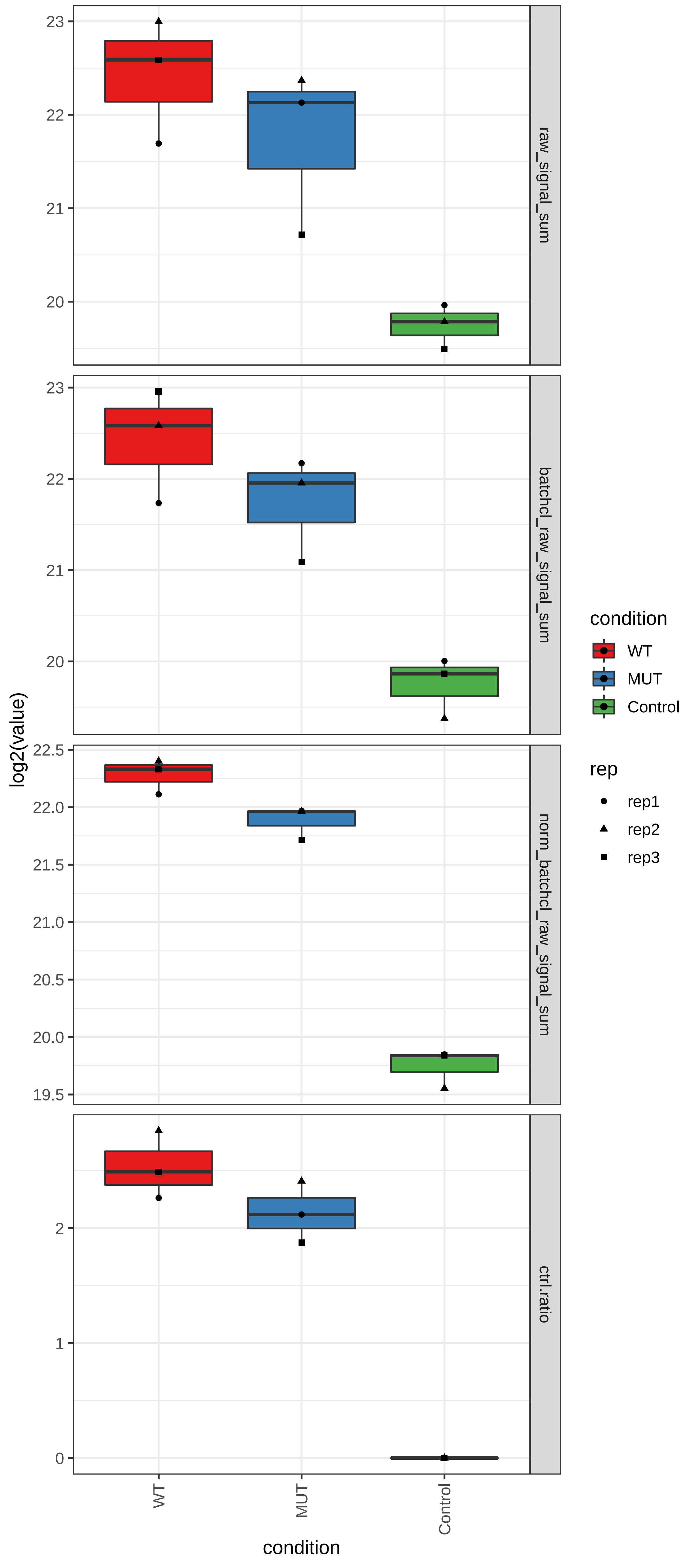

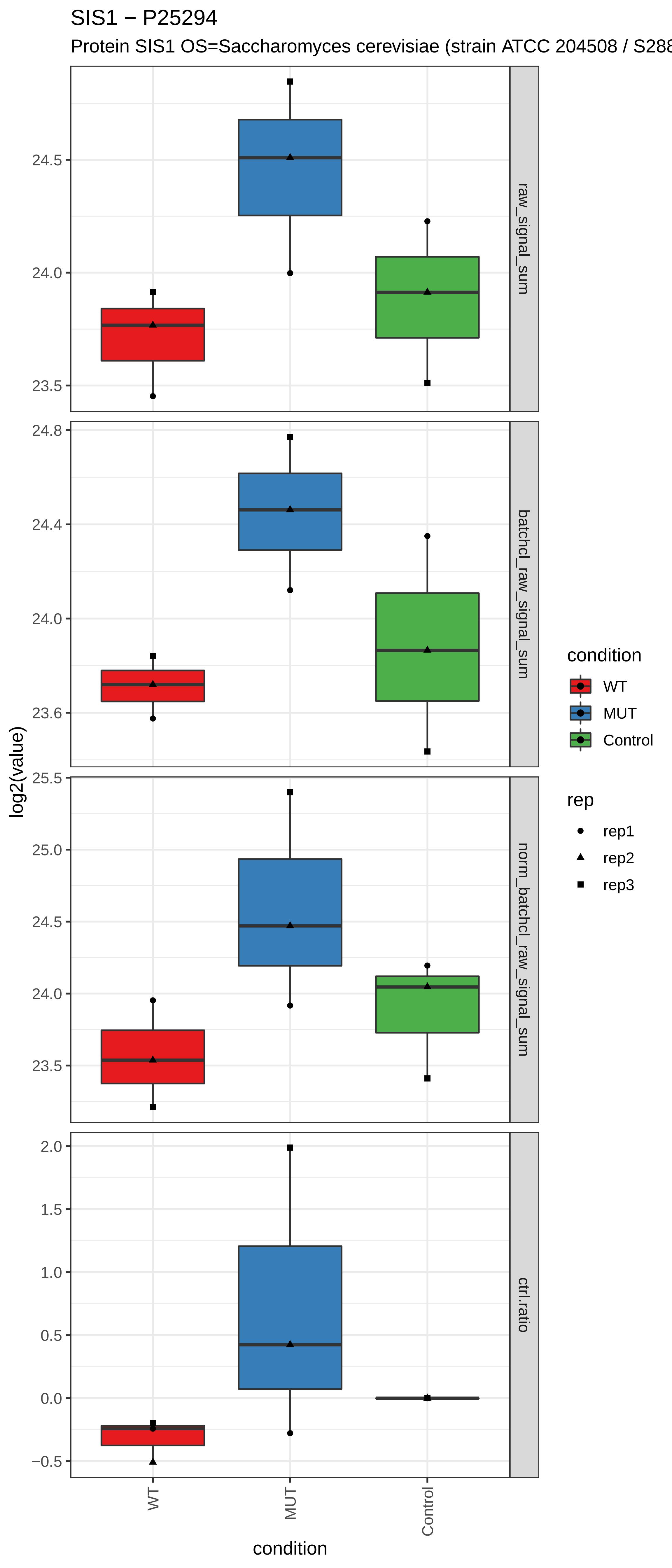

SIS1 − P25294
Protein SIS1 OS=Saccharomyces cerevisiae (strain ATCC 204508 / S288
raw_signal_sum
batchcl_raw_signal_sum
norm_batchcl_raw_signal_sum
ctrl.ratio
log2(value)
condition
WT
MUT
Control
rep
rep1
rep2
rep3

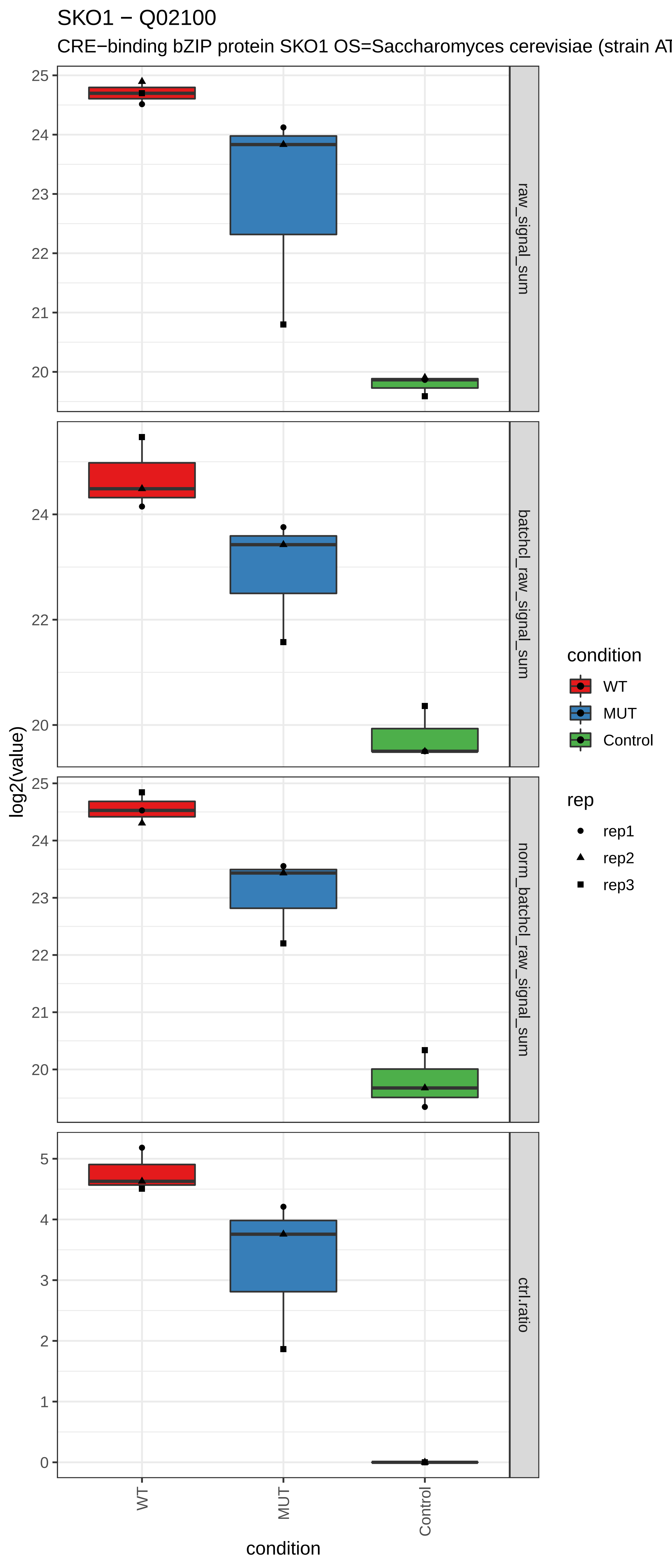

SKO1 − Q02100
CRE−binding bZIP protein SKO1 OS=Saccharomyces cerevisiae (strain AT
log2(value)
raw_signal_sum
batchcl_raw_signal_sum
norm_batchcl_raw_signal_sum
ctrl.ratio
WT
MUT
Control
condition
condition
WT
MUT
Control
rep
rep1
rep2
rep3

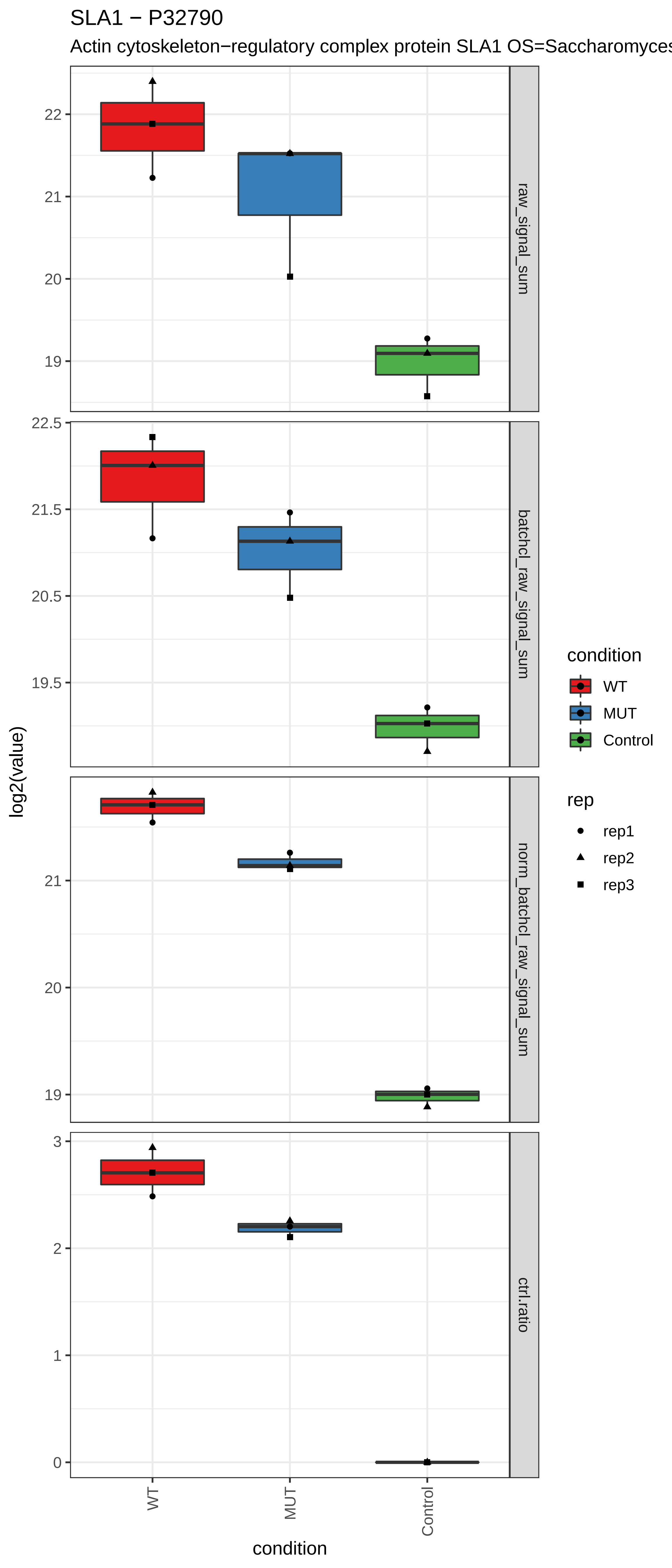
SLA1 − P32790
Actin cytoskeleton−regulatory complex protein SLA1 OS=Saccharomyces
log2(value)
condition
raw_signal_sum
batchcl_raw_signal_sum
norm_batchcl_raw_signal_sum
ctrl.ratio
WT
MUT
Control
condition
WT
MUT
Control
rep
rep1
rep2
rep3
22
21
20
19
22.5
21.5
20.5
19.5
3
2
1
0

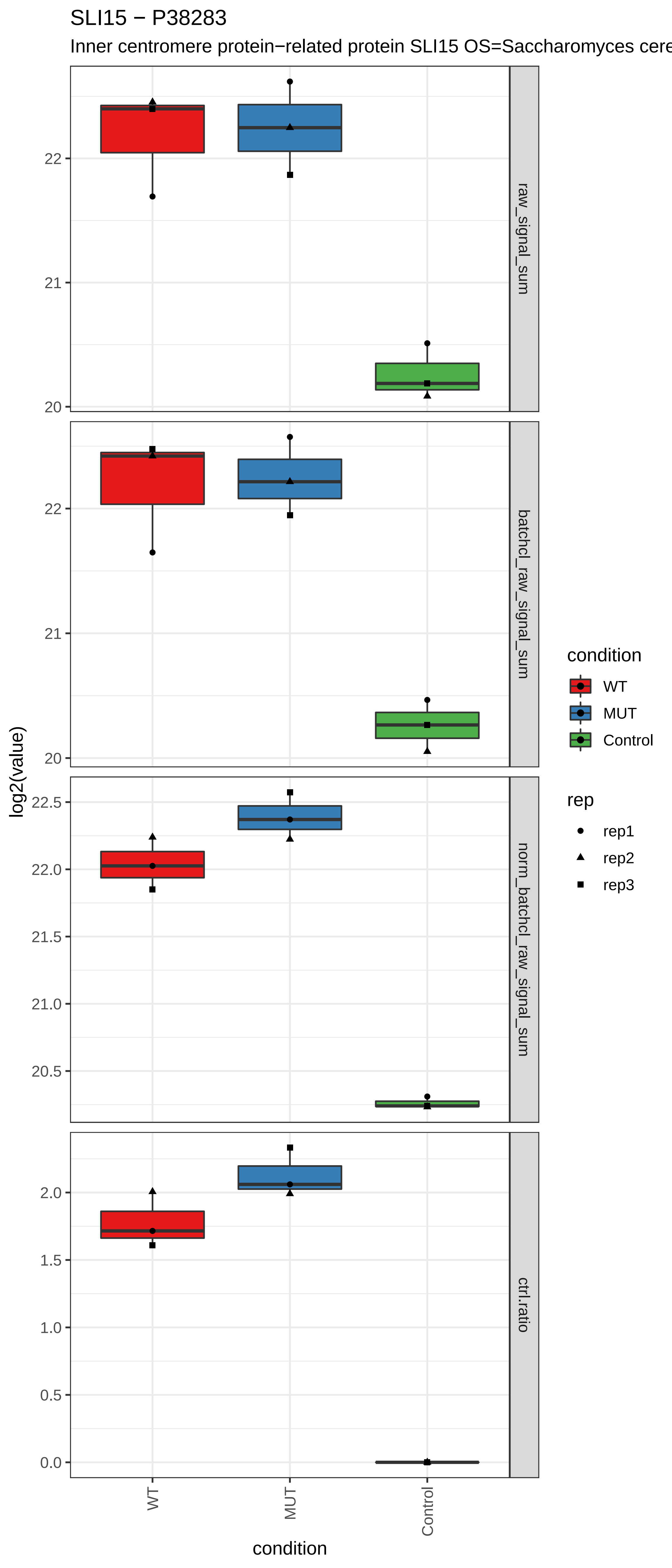

SLI15 − P38283
Inner centromere protein−related protein SLI15 OS=Saccharomyces cere
raw_signal_sum
batchcl_raw_signal_sum
norm_batchcl_raw_signal_sum
ctrl.ratio
log2(value)
condition
WT
MUT
Control
rep
rep1
rep2
rep3

SLM1 − P40485

Phosphatidylinositol 4,5−bisphosphate−binding protein SLM1 OS=Sacchar

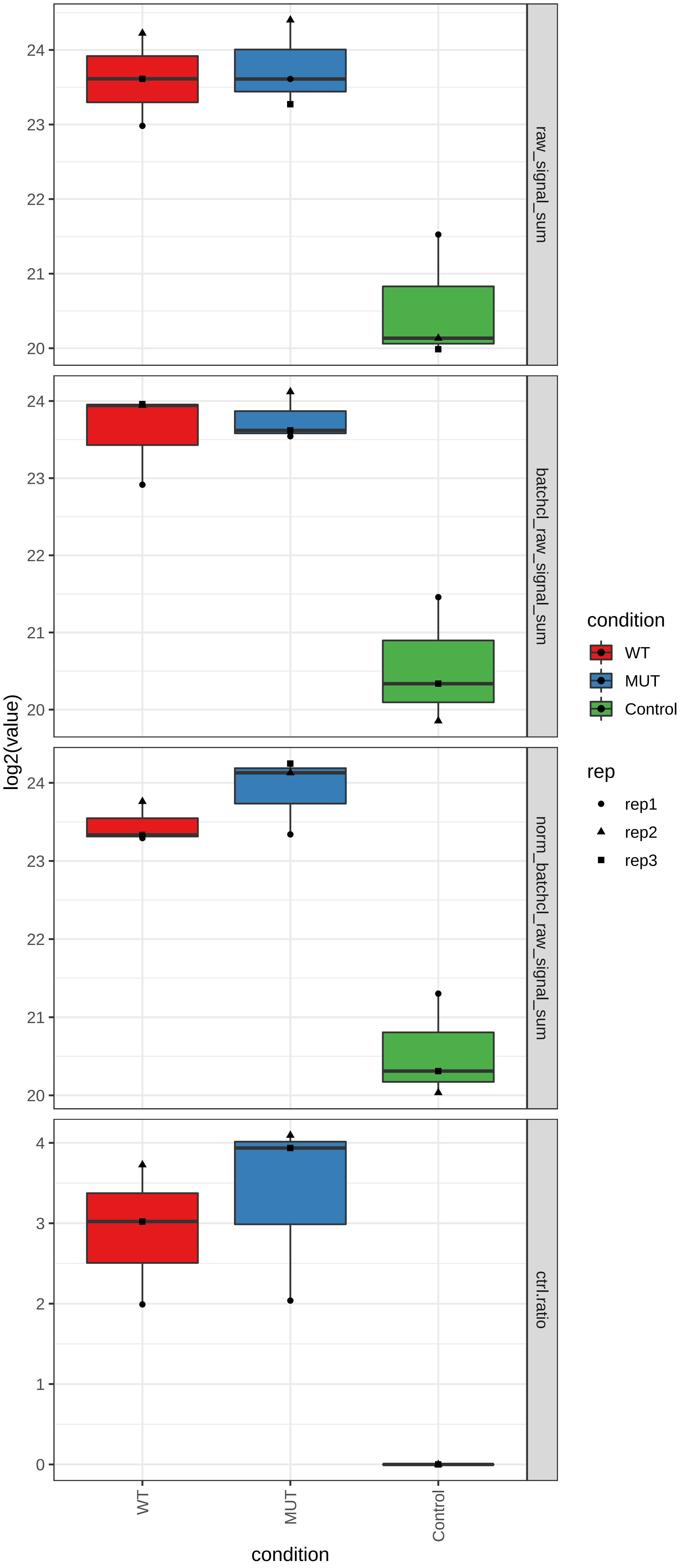

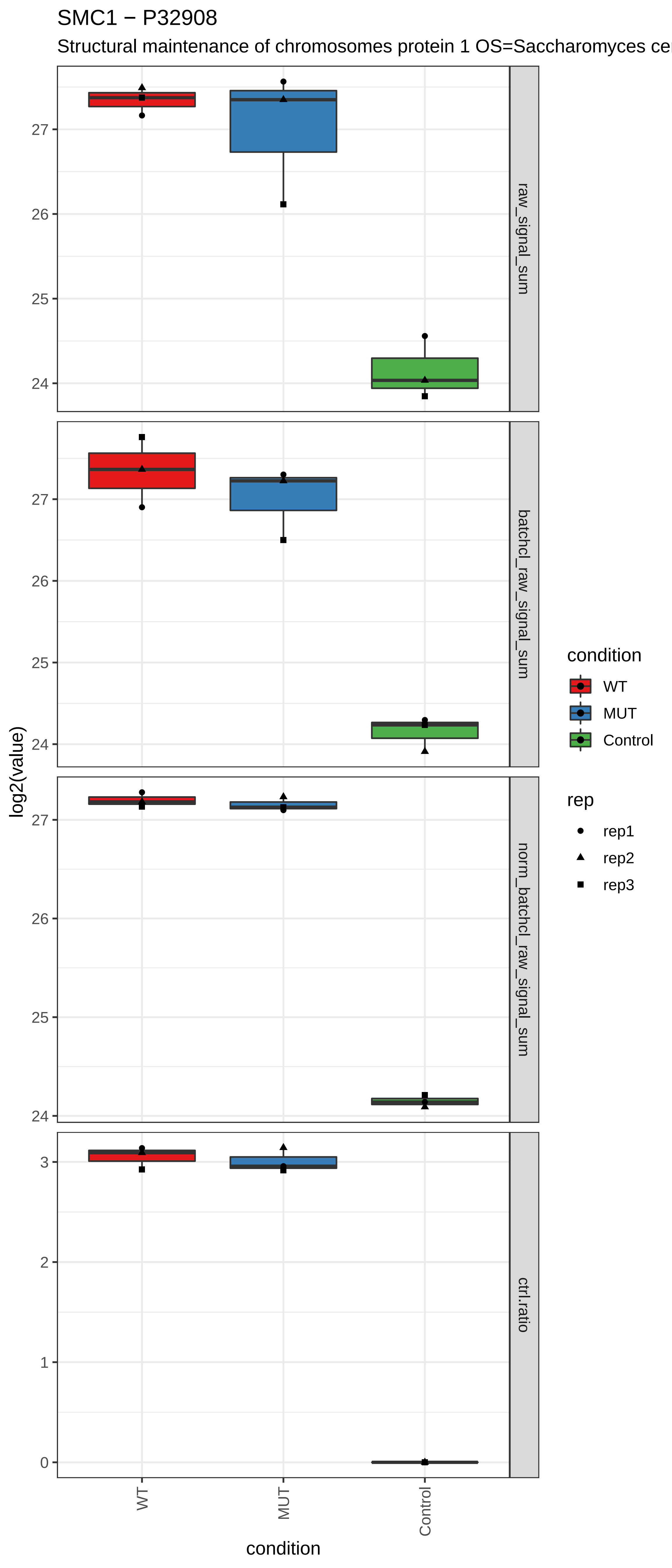
SMC1 − P32908
Structural maintenance of chromosomes protein 1 OS=Saccharomyces ce
raw_signal_sum
batchcl_raw_signal_sum
norm_batchcl_raw_signal_sum
ctrl.ratio
log2(value)
condition
WT
MUT
Control
rep
rep1
rep2
rep3
27
26
25
24
3
2
1
0

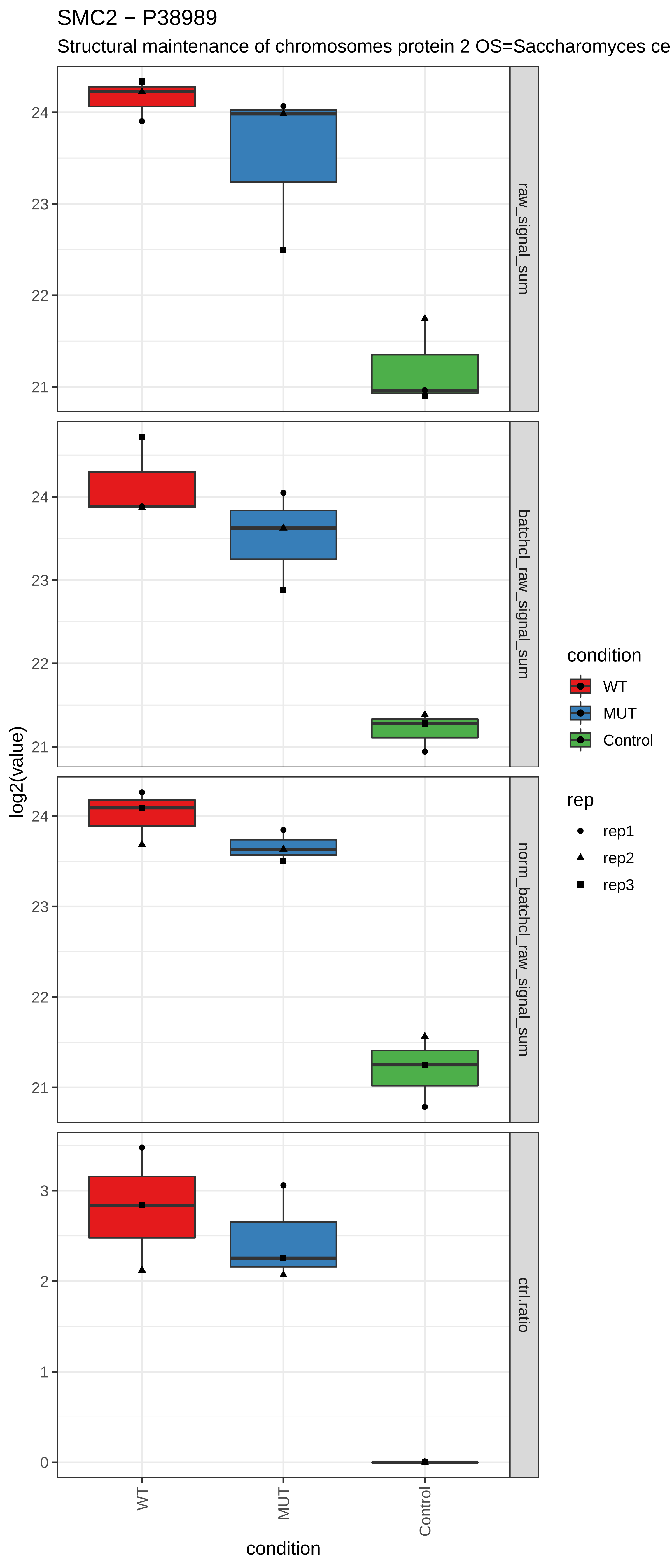
SMC2 − P38989
Structural maintenance of chromosomes protein 2 OS=Saccharomyces ce
raw_signal_sum
batchcl_raw_signal_sum
norm_batchcl_raw_signal_sum
ctrl.ratio
log2(value)
condition
WT
MUT
Control
rep
rep1
rep2
rep3

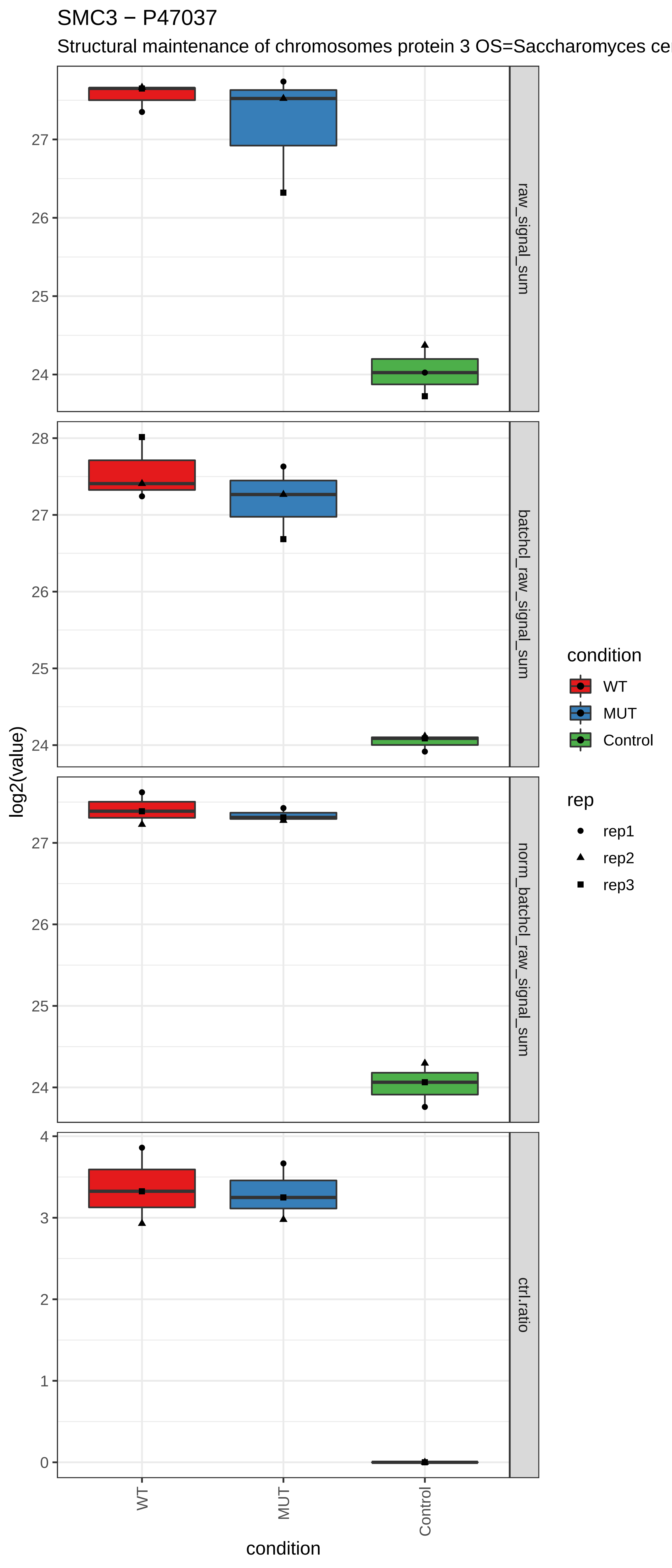

SMC3 − P47037
Structural maintenance of chromosomes protein 3 OS=Saccharomyces ce
raw_signal_sum
batchcl_raw_signal_sum
norm_batchcl_raw_signal_sum
ctrl.ratio
log2(value)
condition
WT
MUT
Control
rep
rep1
rep2
rep3

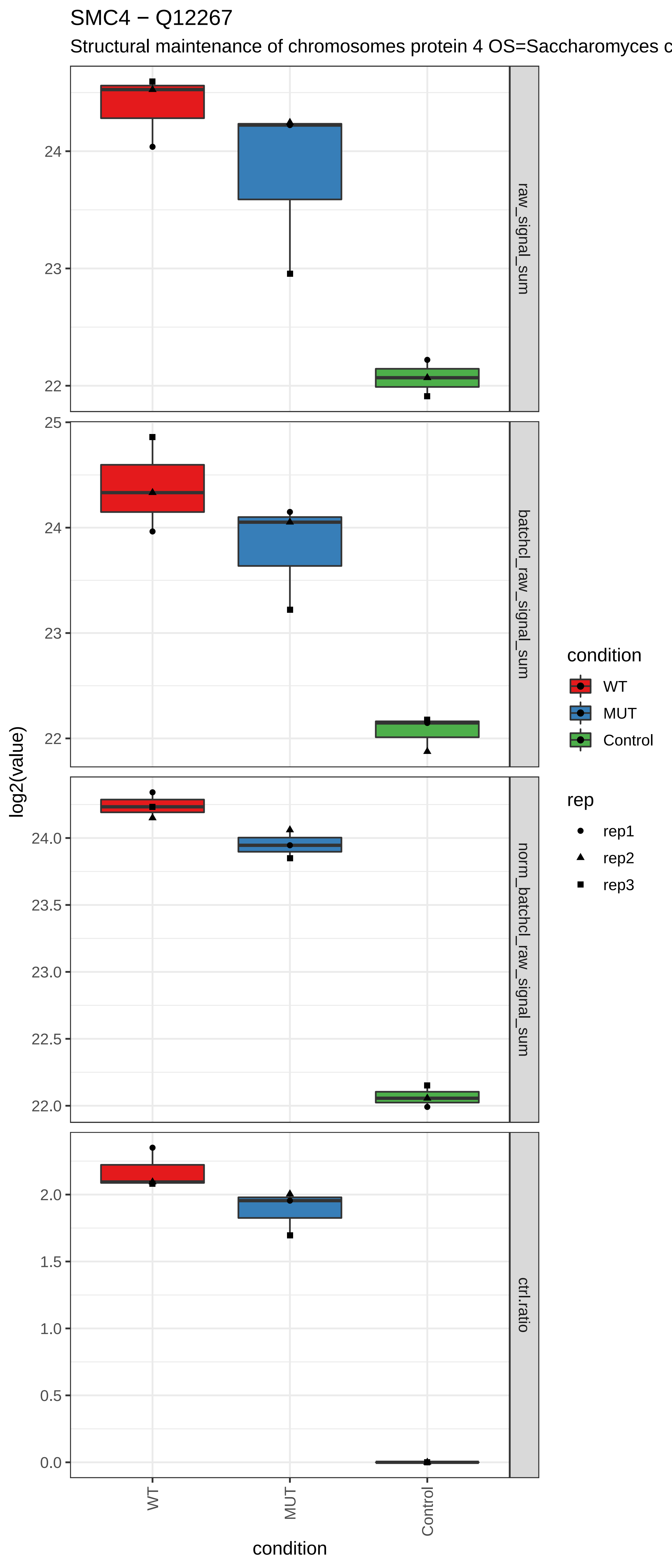

SMC4 − Q12267
Structural maintenance of chromosomes protein 4 OS=Saccharomyces c
raw_signal_sum
batchcl_raw_signal_sum
norm_batchcl_raw_signal_sum
ctrl.ratio
log2(value)
condition
WT
MUT
Control
rep
rep1
rep2
rep3

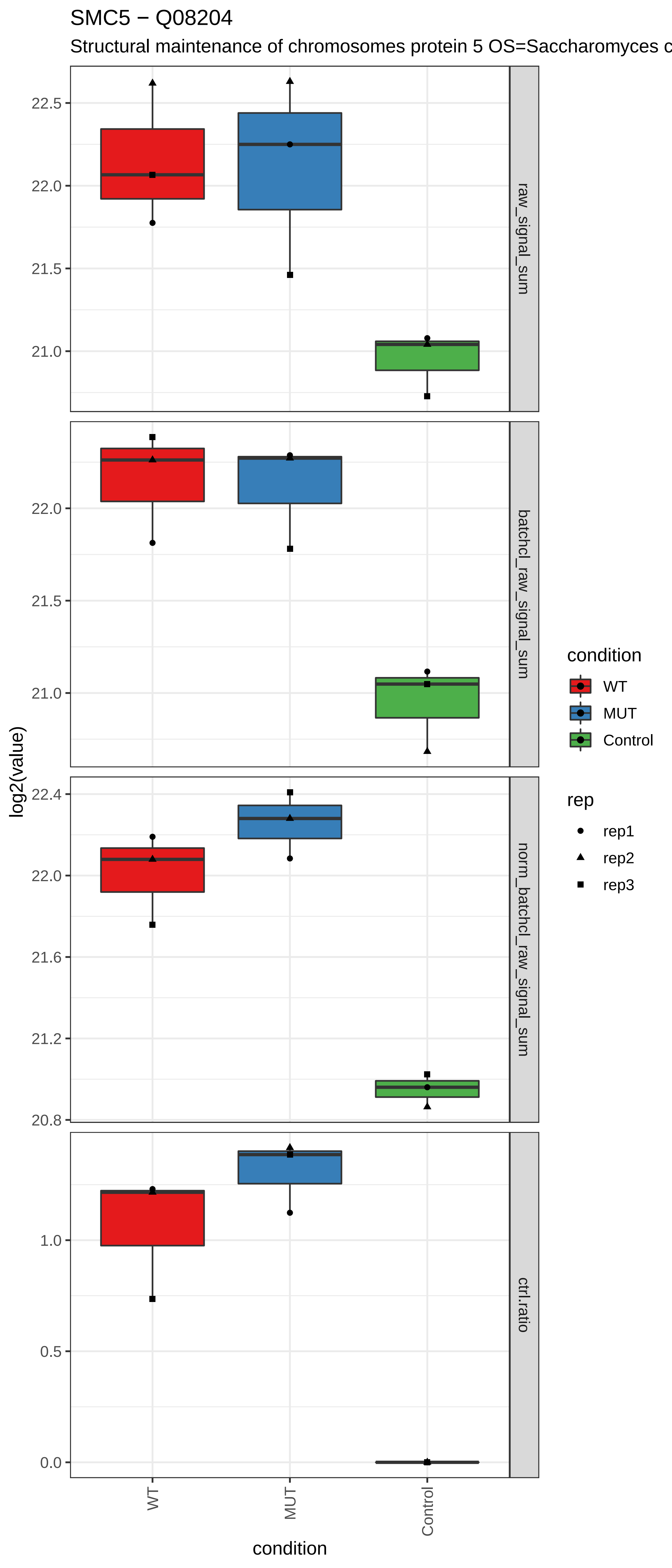

SMC5 − Q08204
Structural maintenance of chromosomes protein 5 OS=Saccharomyces c
raw_signal_sum
batchcl_raw_signal_sum
norm_batchcl_raw_signal_sum
ctrl.ratio
22.5
22.0
21.5
21.0
22.0
21.5
21.0
22.4
22.0
21.6
21.2
20.8
1.0
0.5
0.0
log2(value)
WT
MUT
Control
condition
condition
WT
MUT
Control
rep
rep1
rep2
rep3

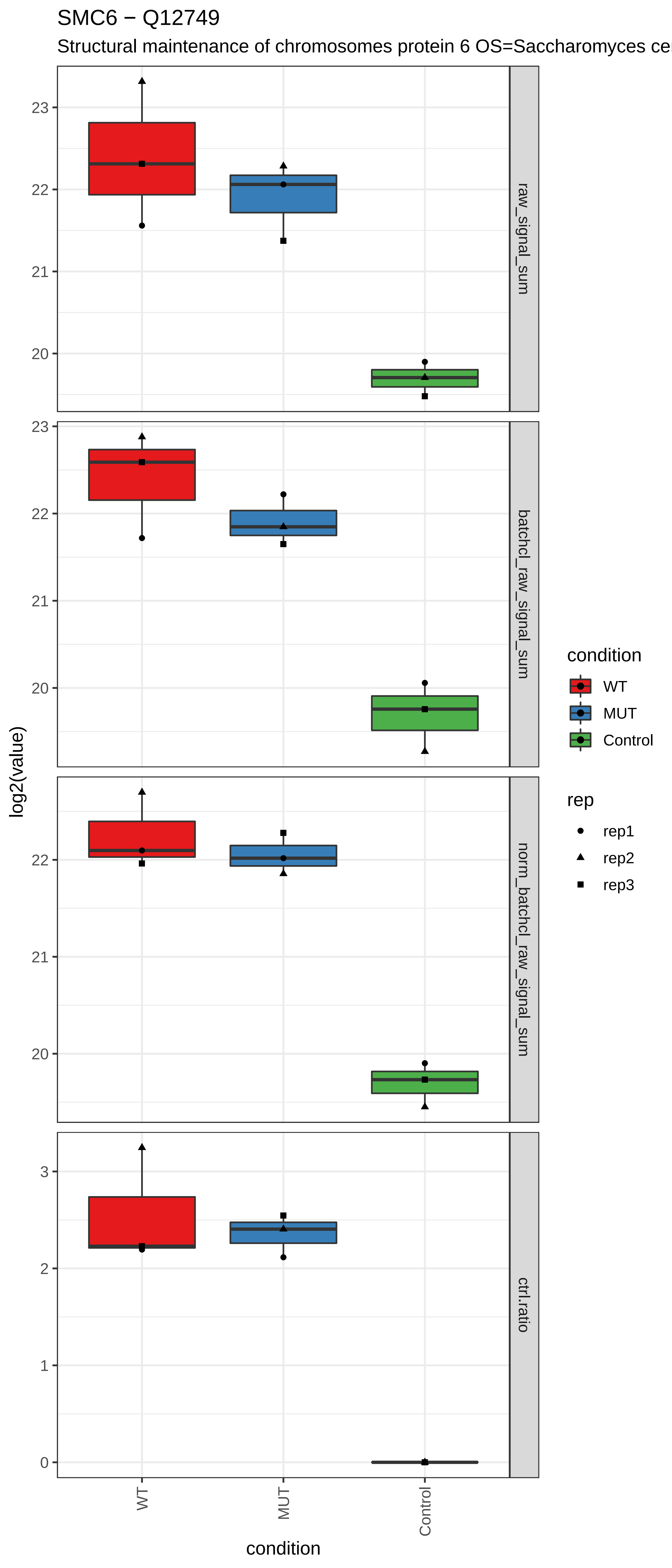
SMC6 − Q12749
Structural maintenance of chromosomes protein 6 OS=Saccharomyces ce
raw_signal_sum
batchcl_raw_signal_sum
norm_batchcl_raw_signal_sum
ctrl.ratio
log2(value)
condition
WT
MUT
Control
rep
rep1
rep2
rep3

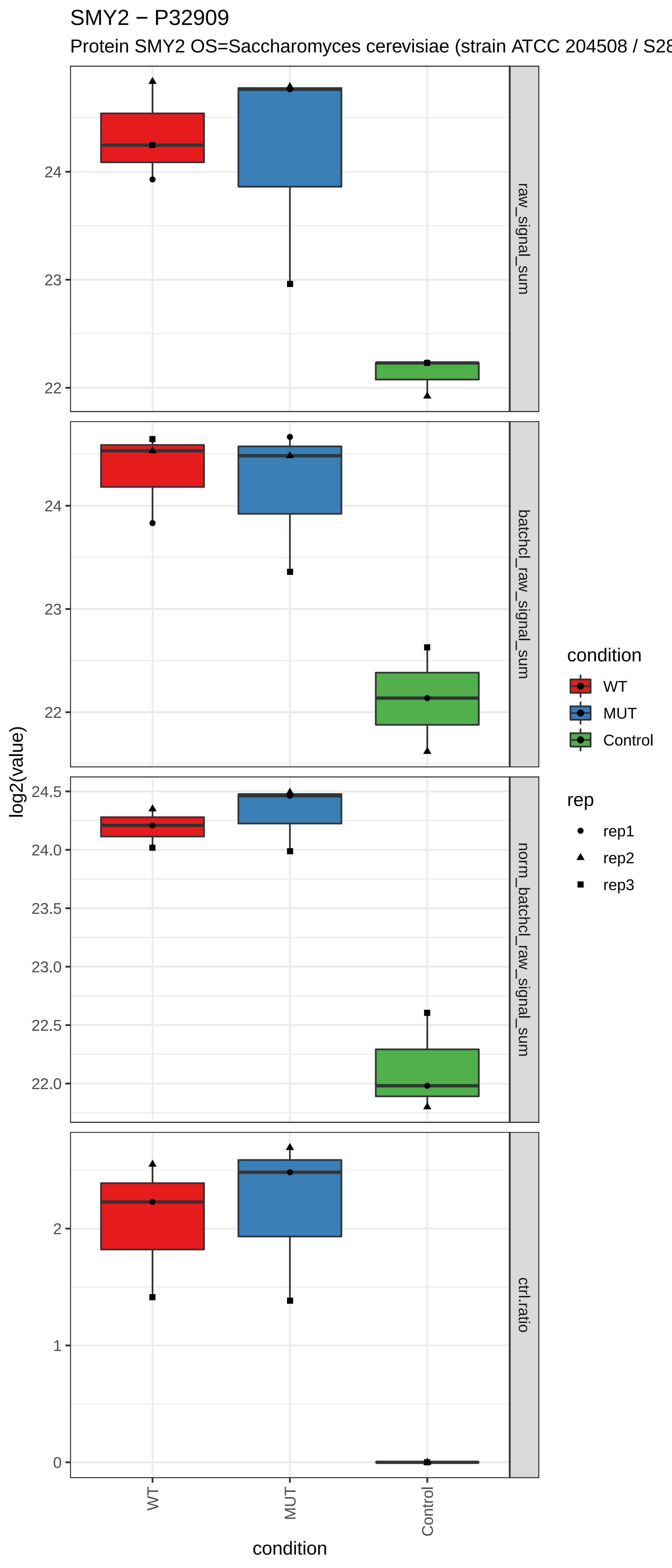
SMY2 − P32909
Protein SMY2 OS=Saccharomyces cerevisiae (strain ATCC 204508 / S28
raw_signal_sum
batchcl_raw_signal_sum
norm_batchcl_raw_signal_sum
ctrl.ratio
log2(value)
condition
WT
MUT
Control
rep
rep1
rep2
rep3

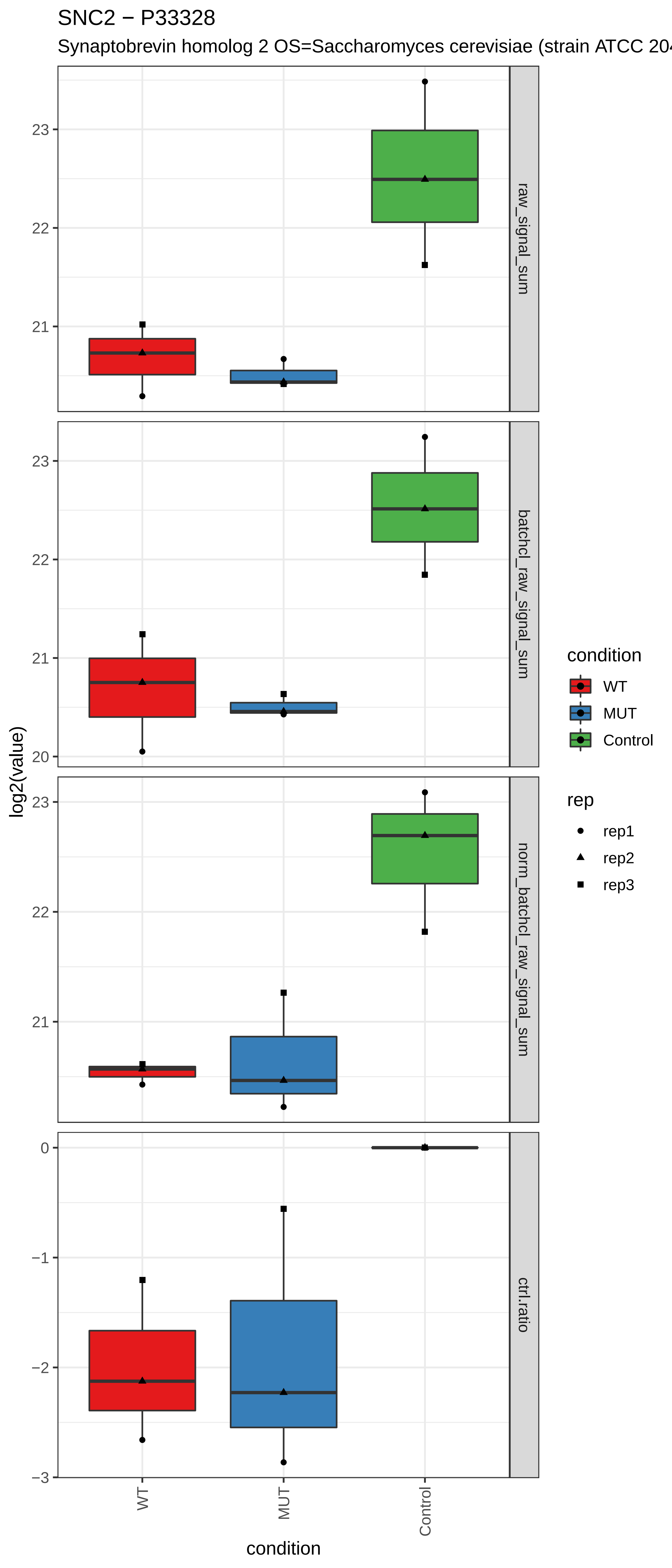

SNC2 − P33328
Synaptobrevin homolog 2 OS=Saccharomyces cerevisiae (strain ATCC 204
raw_signal_sum
batchcl_raw_signal_sum
norm_batchcl_raw_signal_sum
ctrl.ratio
log2(value)
condition
WT
MUT
Control
rep
rep1
rep2
rep3
23
22
21
20
0
−1
−2
−3

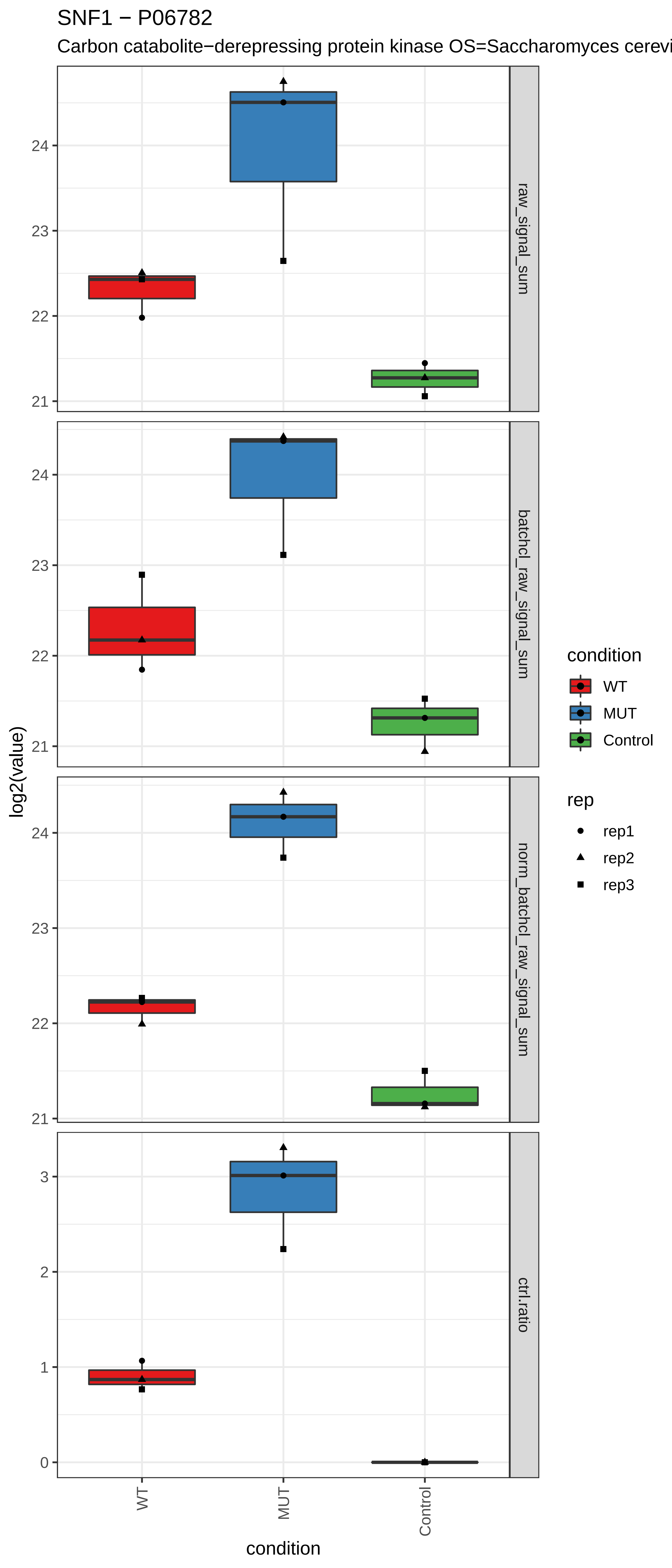

SNF1 − P06782
Carbon catabolite−derepressing protein kinase OS=Saccharomyces cerevi
raw_signal_sum
batchcl_raw_signal_sum
norm_batchcl_raw_signal_sum
ctrl.ratio
log2(value)
condition
WT
MUT
Control
rep
rep1
rep2
rep3

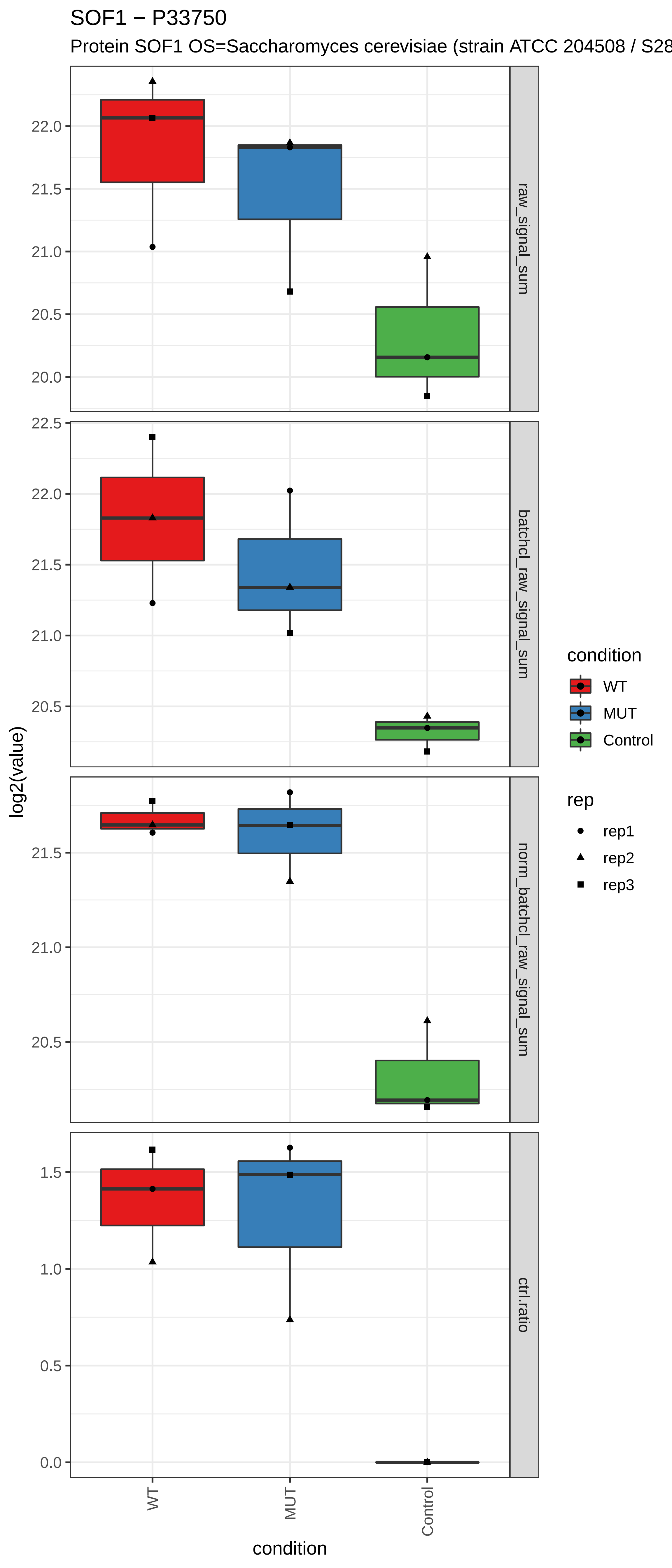
SOF1 − P33750
Protein SOF1 OS=Saccharomyces cerevisiae (strain ATCC 204508 / S28
raw_signal_sum
batchcl_raw_signal_sum
norm_batchcl_raw_signal_sum
ctrl.ratio
log2(value)
condition
WT
MUT
Control
rep
rep1
rep2
rep3
22.0
21.5
21.0
20.5
20.0
22.5
1.5
1.0
0.5
0.0

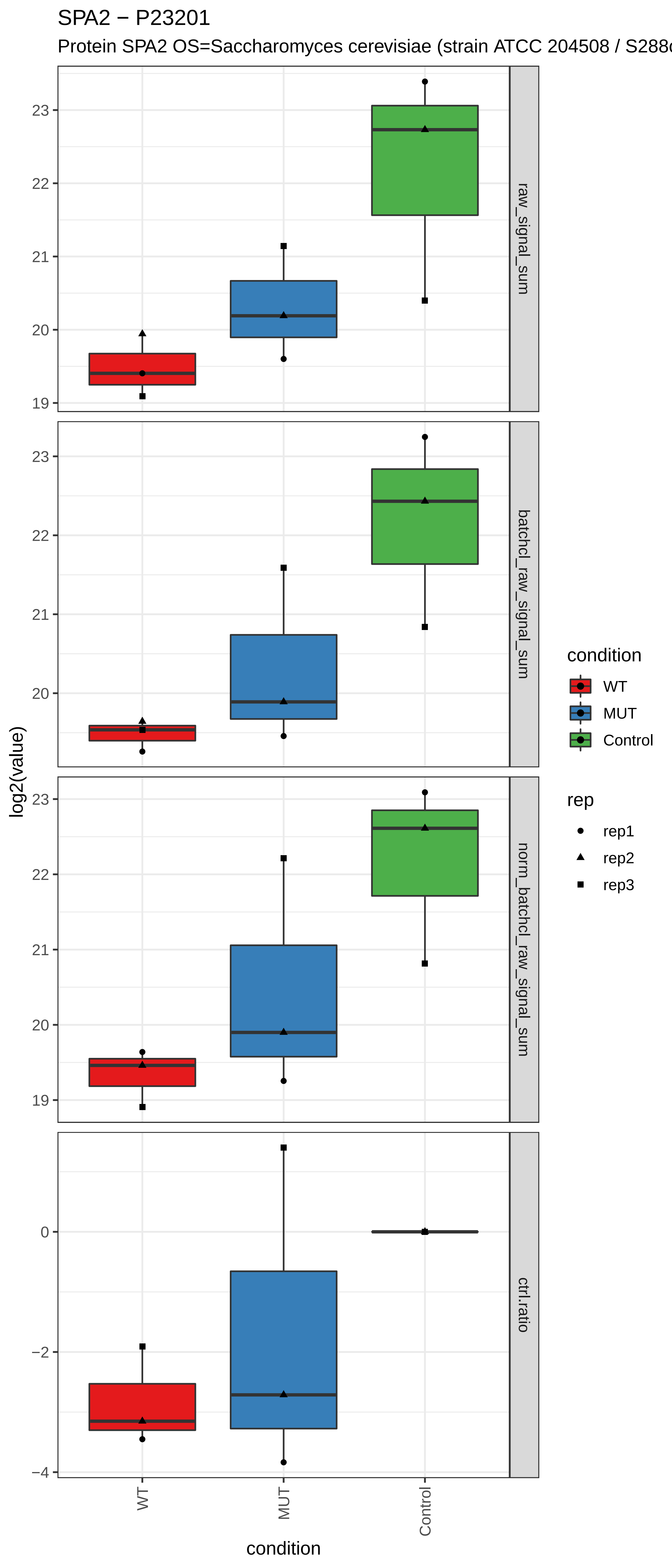

SPA2 − P23201
Protein SPA2 OS=Saccharomyces cerevisiae (strain ATCC 204508 / S288c
raw_signal_sum
batchcl_raw_signal_sum
norm_batchcl_raw_signal_sum
ctrl.ratio
log2(value)
condition
WT
MUT
Control
rep
rep1
rep2
rep3

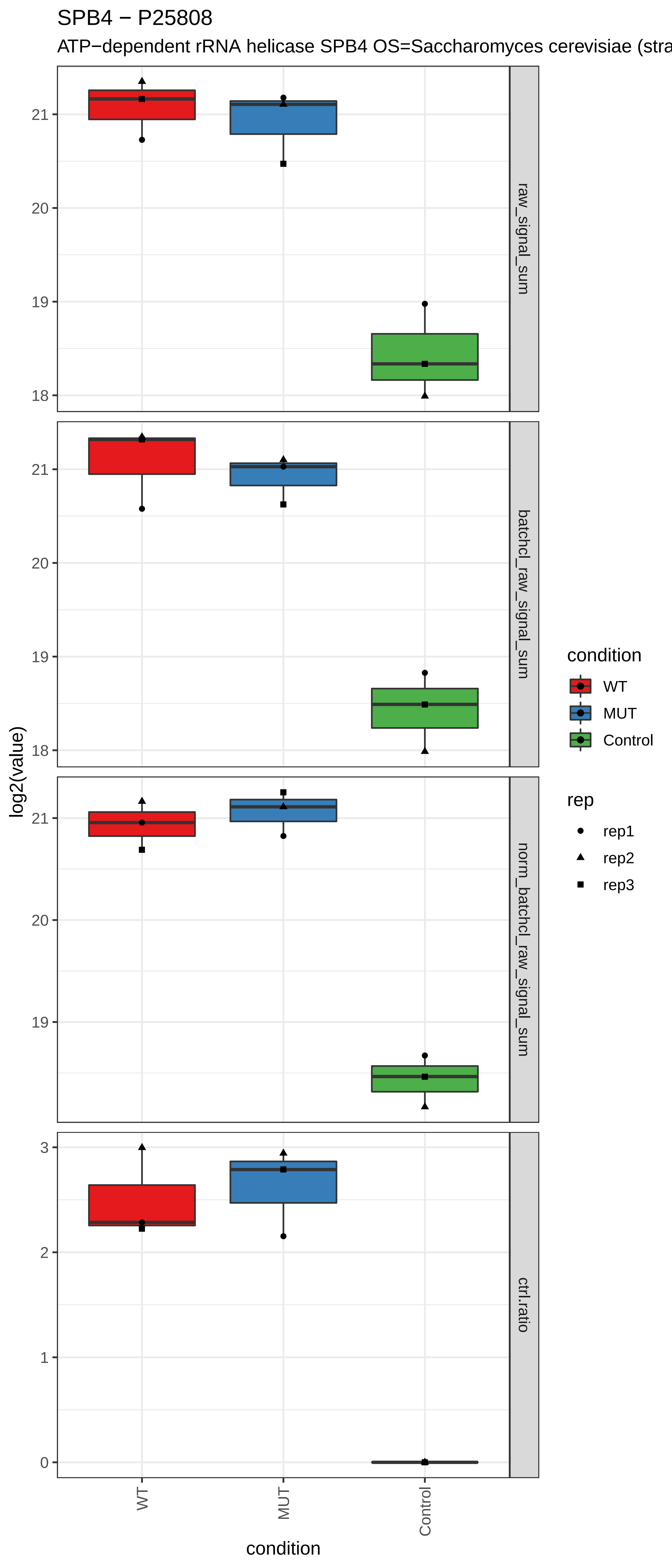

SPB4 − P25808
ATP−dependent rRNA helicase SPB4 OS=Saccharomyces cerevisiae (stra
raw_signal_sum
batchcl_raw_signal_sum
norm_batchcl_raw_signal_sum
ctrl.ratio
log2(value)
condition
WT
MUT
Control
rep
rep1
rep2
rep3

SPF1 − P39986

Manganese−transporting ATPase 1 OS=Saccharomyces cerevisiae (strain

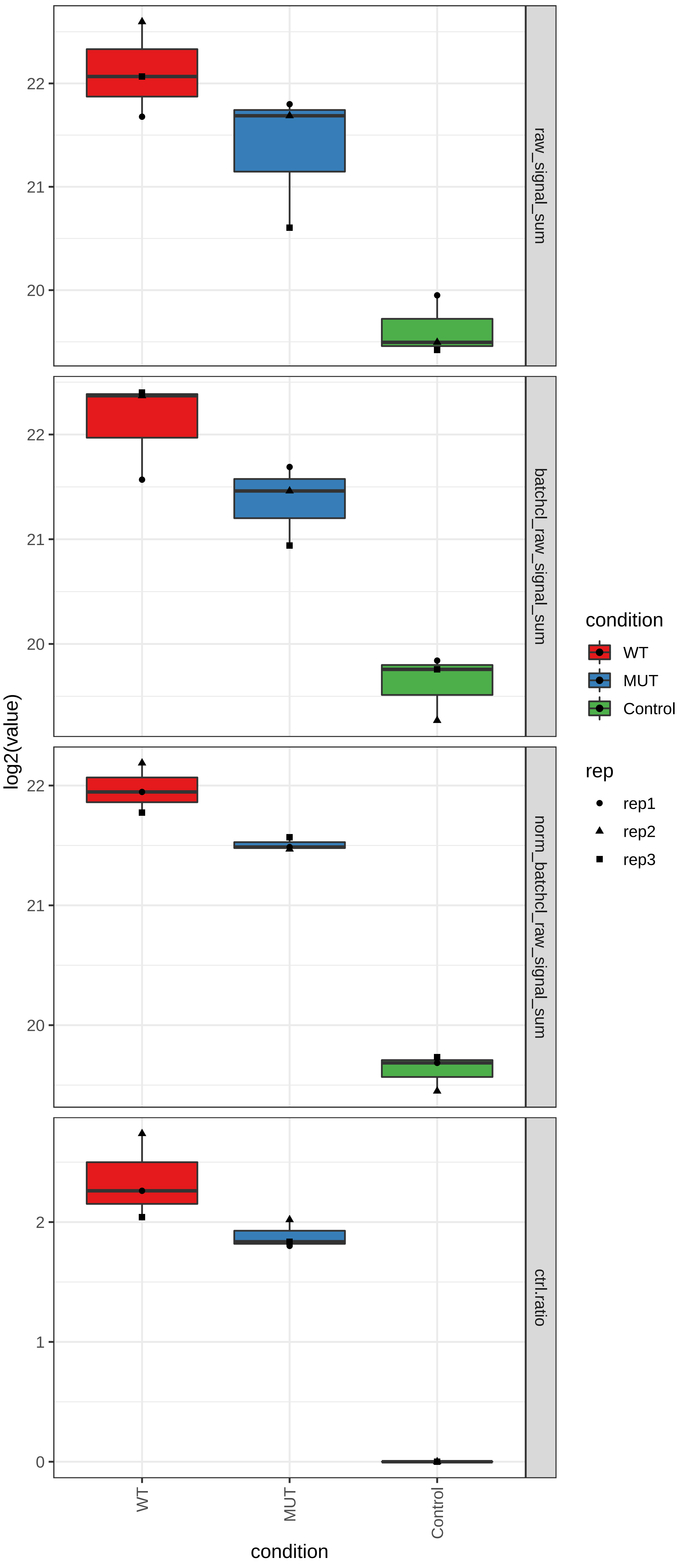

SPO14 − P36126

Phospholipase D1 OS=Saccharomyces cerevisiae (strain ATCC 204508 / S

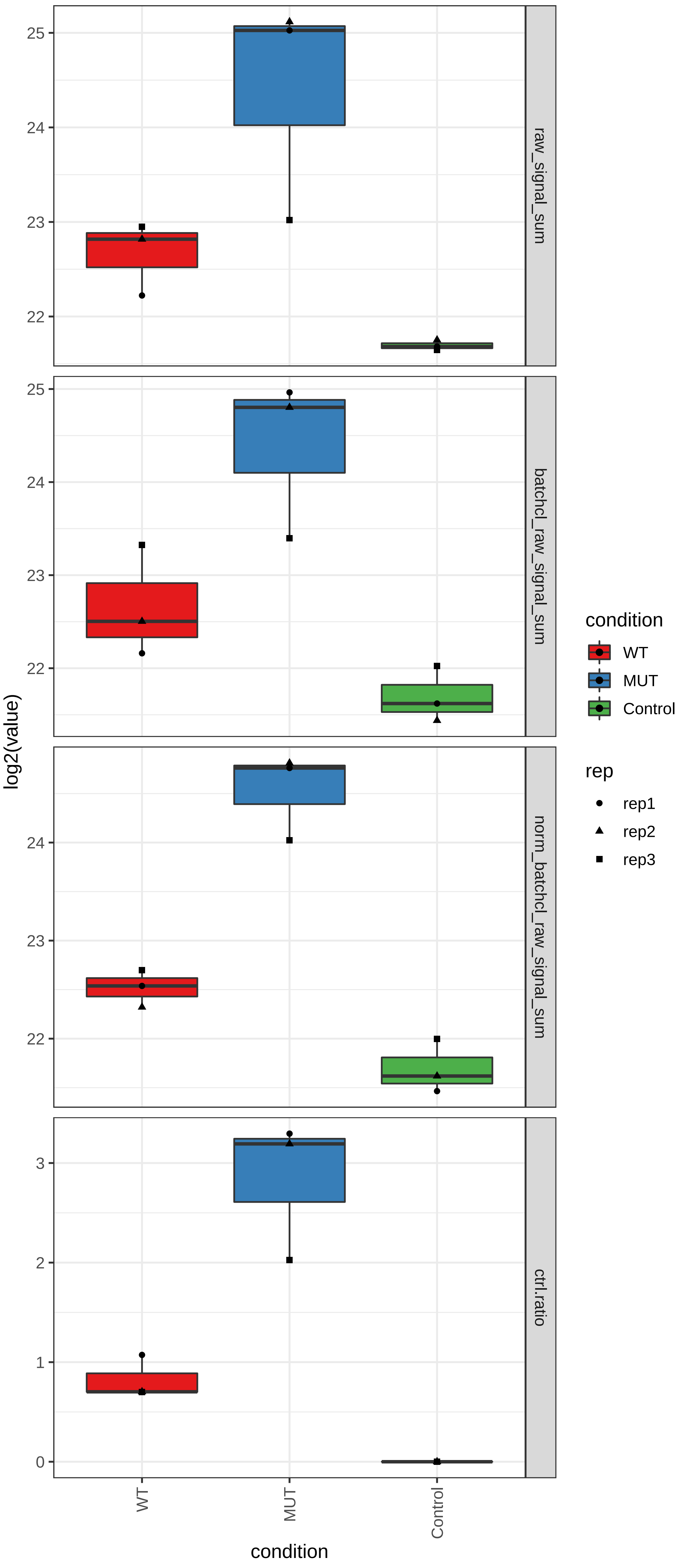

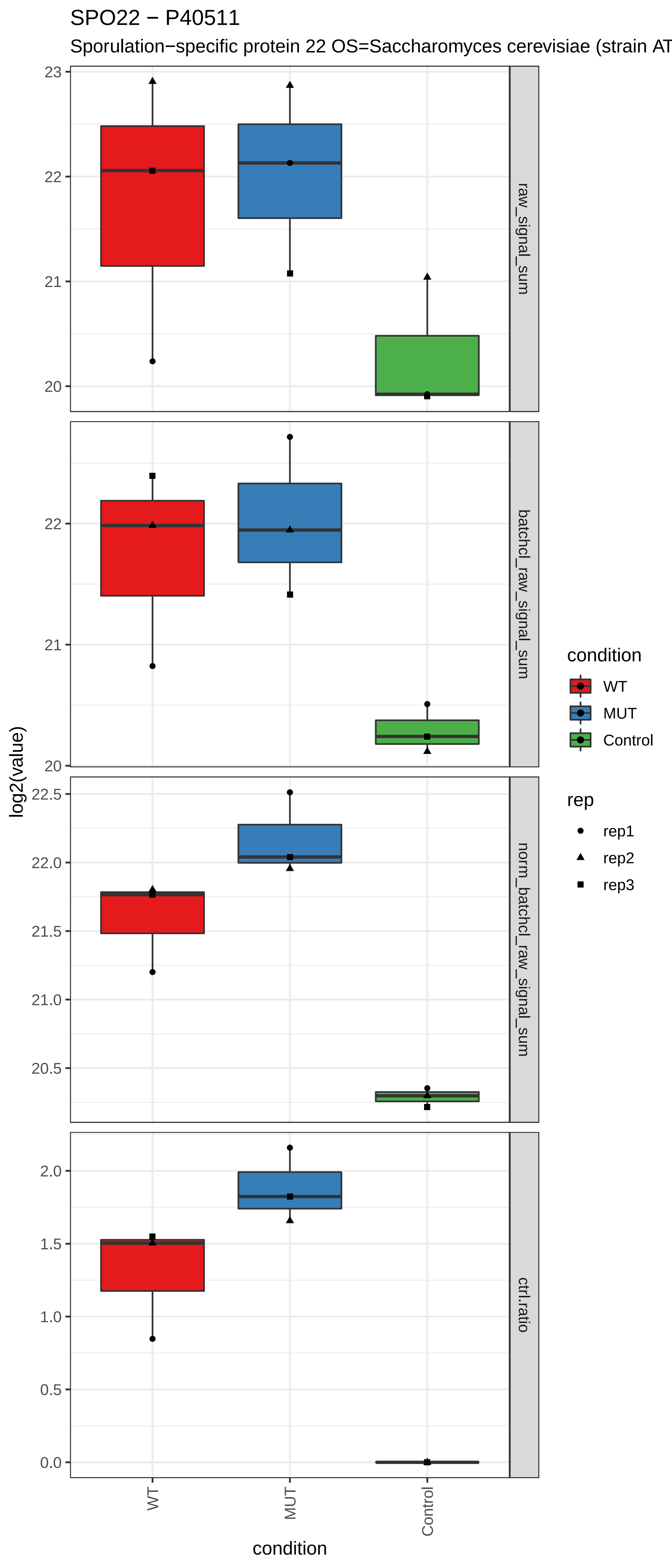
SPO22 − P40511
Sporulation−specific protein 22 OS=Saccharomyces cerevisiae (strain AT
raw_signal_sum
batchcl_raw_signal_sum
norm_batchcl_raw_signal_sum
ctrl.ratio
log2(value)
condition
WT
MUT
Control
rep
rep1
rep2
rep3
23
22
21
20
22.5
22.0
21.5
21.0
20.5
2.0
1.5
1.0
0.5
0.0

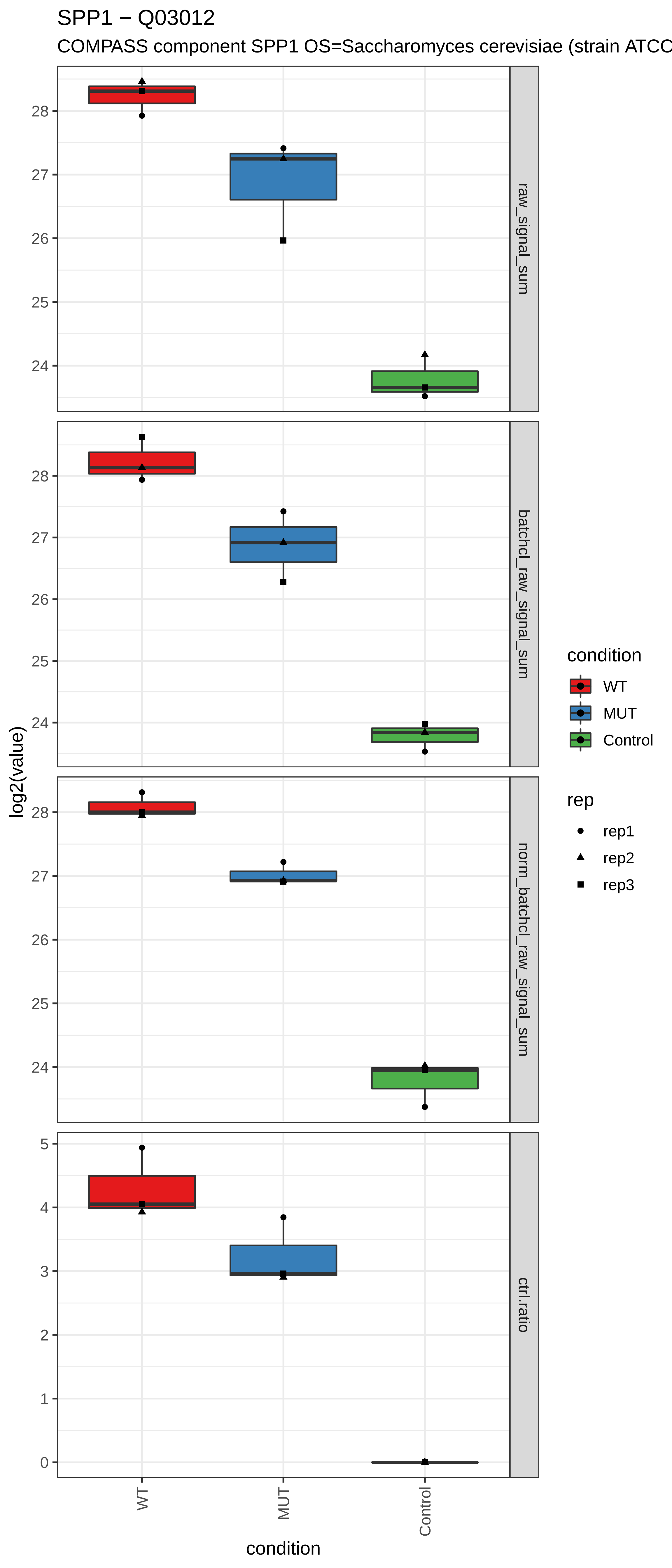

SPP1 − Q03012
COMPASS component SPP1 OS=Saccharomyces cerevisiae (strain ATCC
raw_signal_sum
batchcl_raw_signal_sum
norm_batchcl_raw_signal_sum
ctrl.ratio
log2(value)
condition
WT
MUT
Control
rep
rep1
rep2
rep3

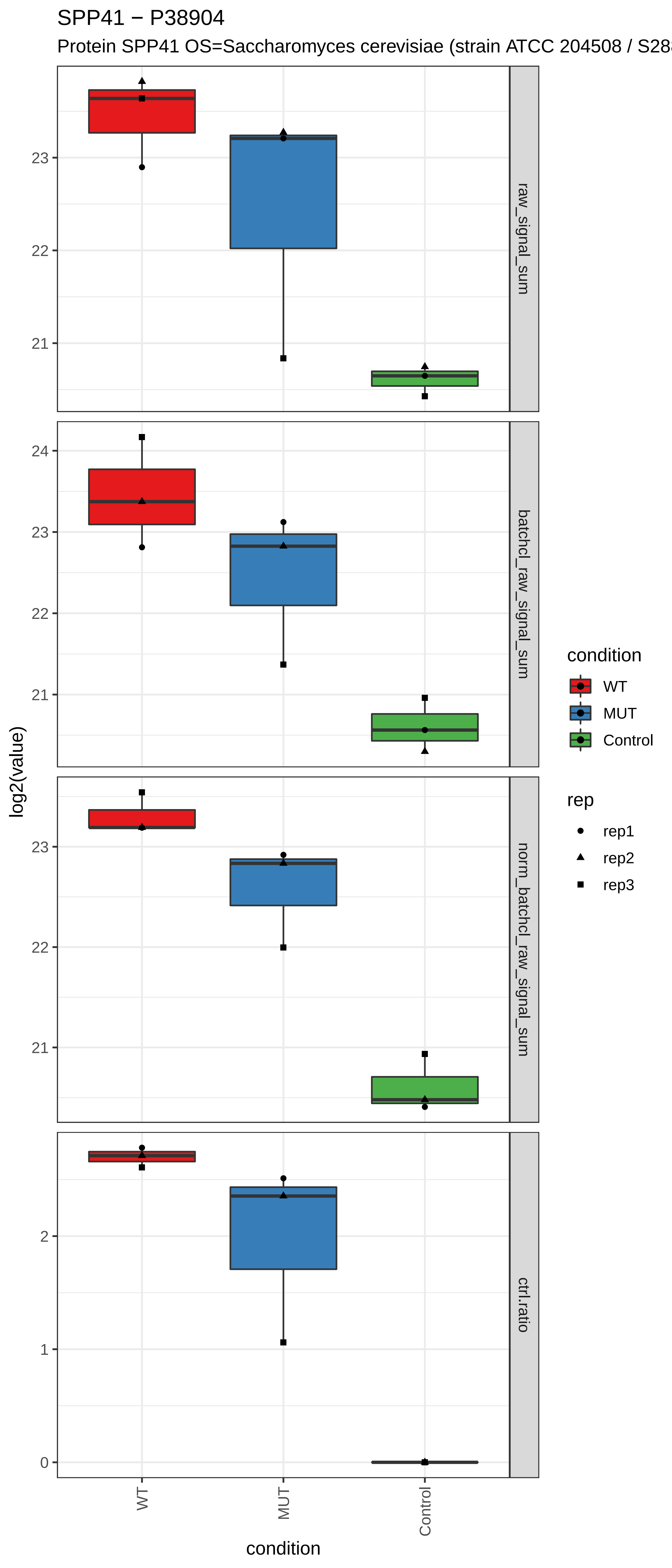

SPP41 − P38904
Protein SPP41 OS=Saccharomyces cerevisiae (strain ATCC 204508 / S288
raw_signal_sum
batchcl_raw_signal_sum
norm_batchcl_raw_signal_sum
ctrl.ratio
log2(value)
condition
WT
MUT
Control
rep
rep1
rep2
rep3
23
22
21
24
2
1
0

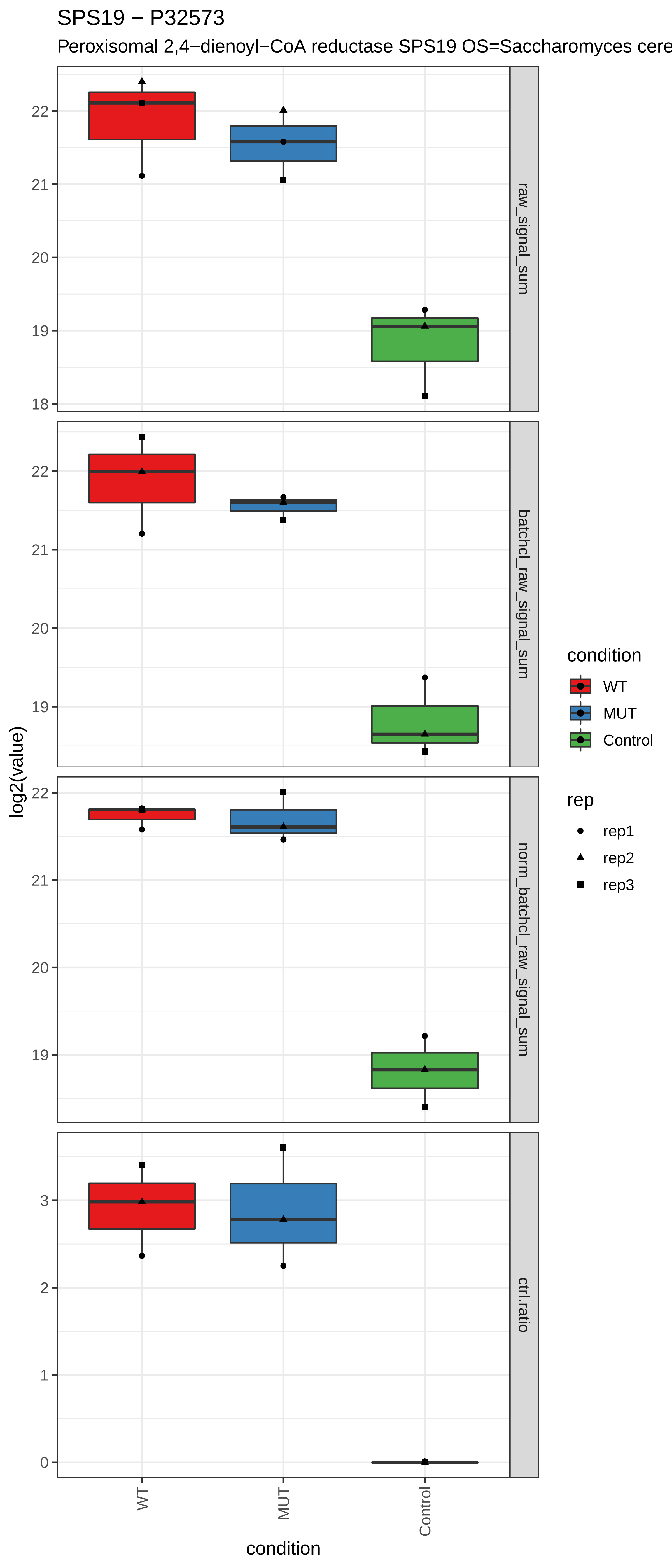

SPS19 − P32573
Peroxisomal 2,4−dienoyl−CoA reductase SPS19 OS=Saccharomyces cere
log2(value)
raw_signal_sum
batchcl_raw_signal_sum
norm_batchcl_raw_signal_sum
ctrl.ratio
22
21
20
19
18
3
2
1
0
WT
MUT
Control
condition
condition
WT
MUT
Control
rep
rep1
rep2
rep3

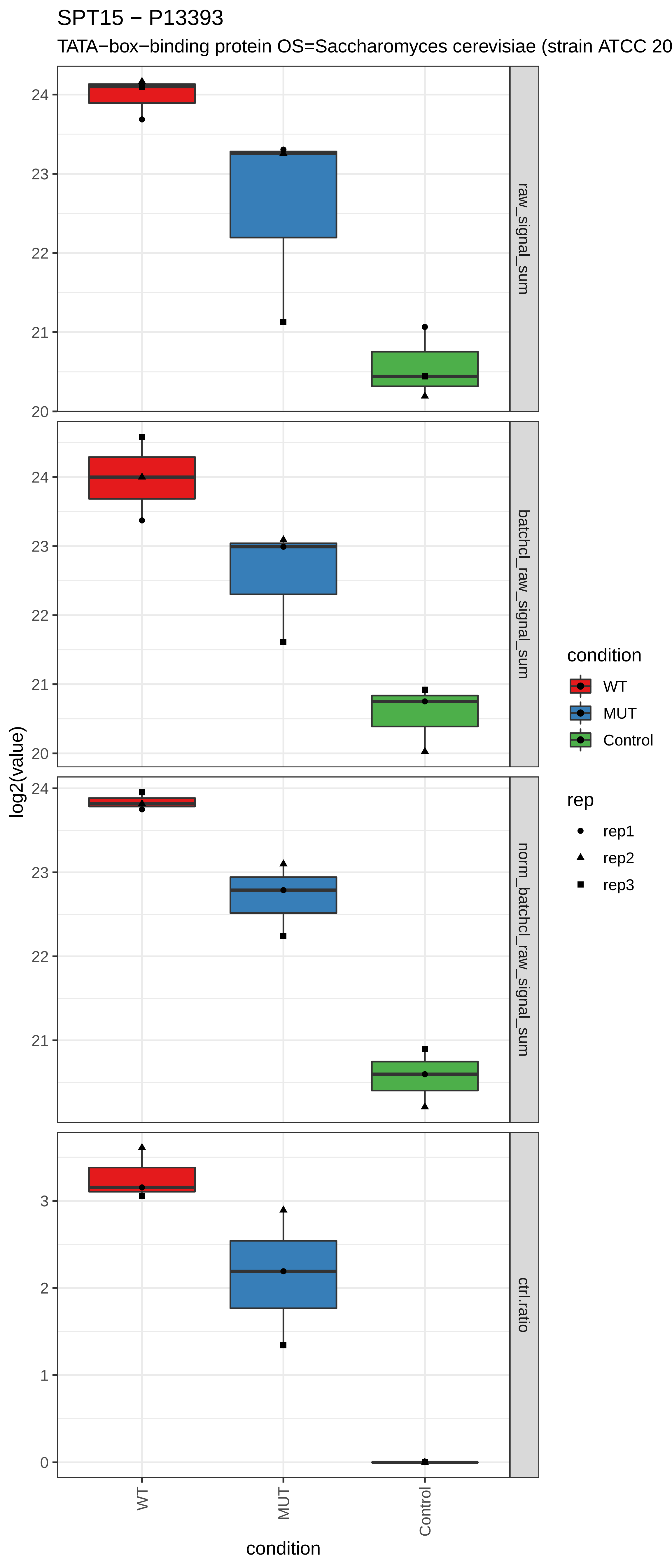
SPT15 − P13393
TATA−box−binding protein OS=Saccharomyces cerevisiae (strain ATCC 20
raw_signal_sum
batchcl_raw_signal_sum
norm_batchcl_raw_signal_sum
ctrl.ratio
log2(value)
condition
WT
MUT
Control
rep
rep1
rep2
rep3

SPT16 − P32558

FACT complex subunit SPT16 OS=Saccharomyces cerevisiae (strain ATCC

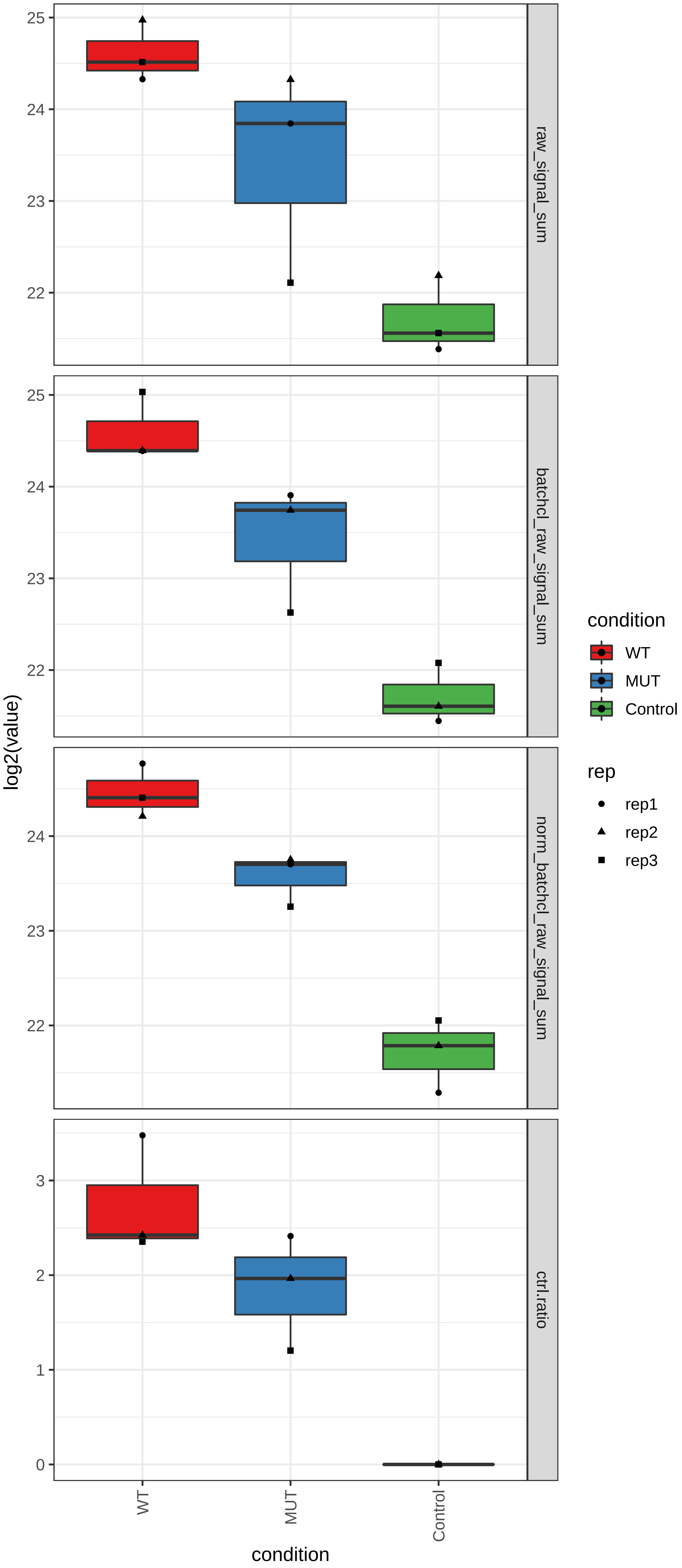

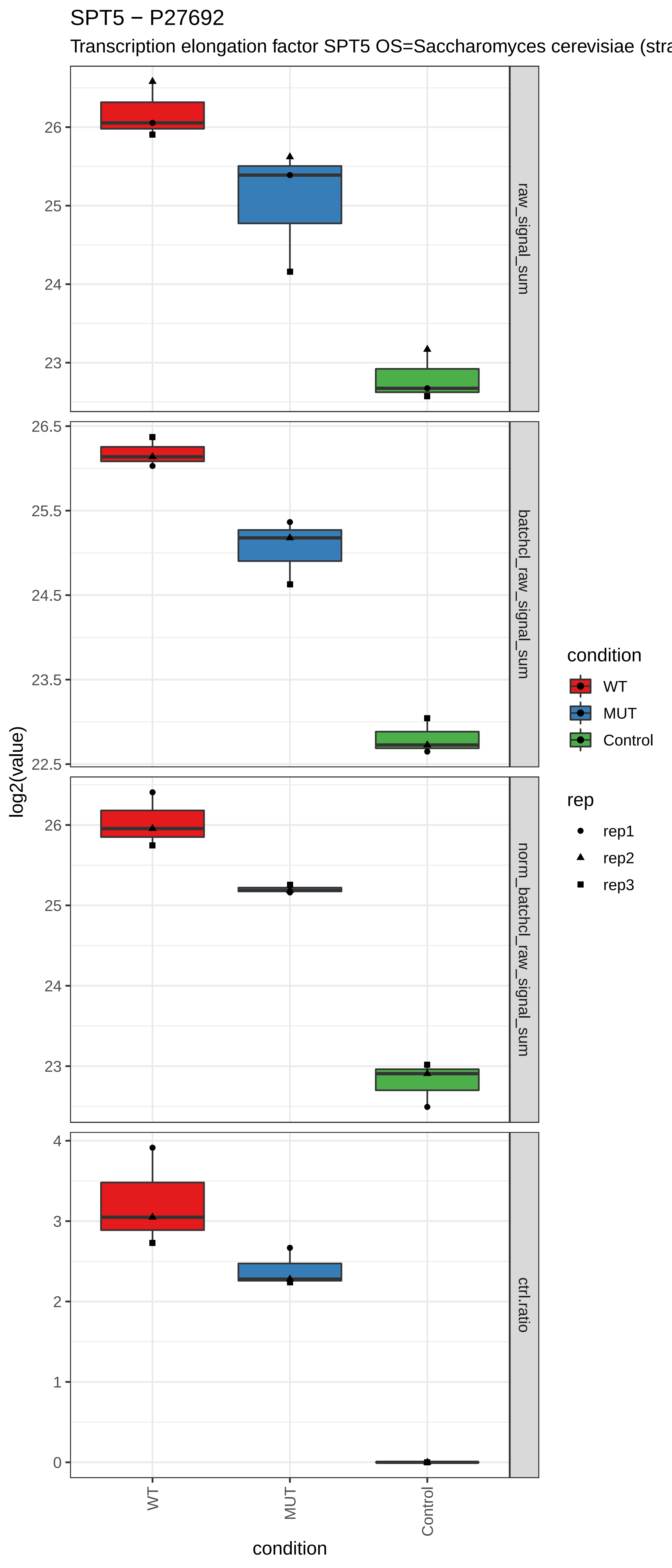

SPT5 − P27692
Transcription elongation factor SPT5 OS=Saccharomyces cerevisiae (stra
raw_signal_sum
batchcl_raw_signal_sum
norm_batchcl_raw_signal_sum
ctrl.ratio
log2(value)
condition
WT
MUT
Control
rep
rep1
rep2
rep3
26
25
24
23
26.5
25.5
24.5
23.5
22.5
4
3
2
1
0

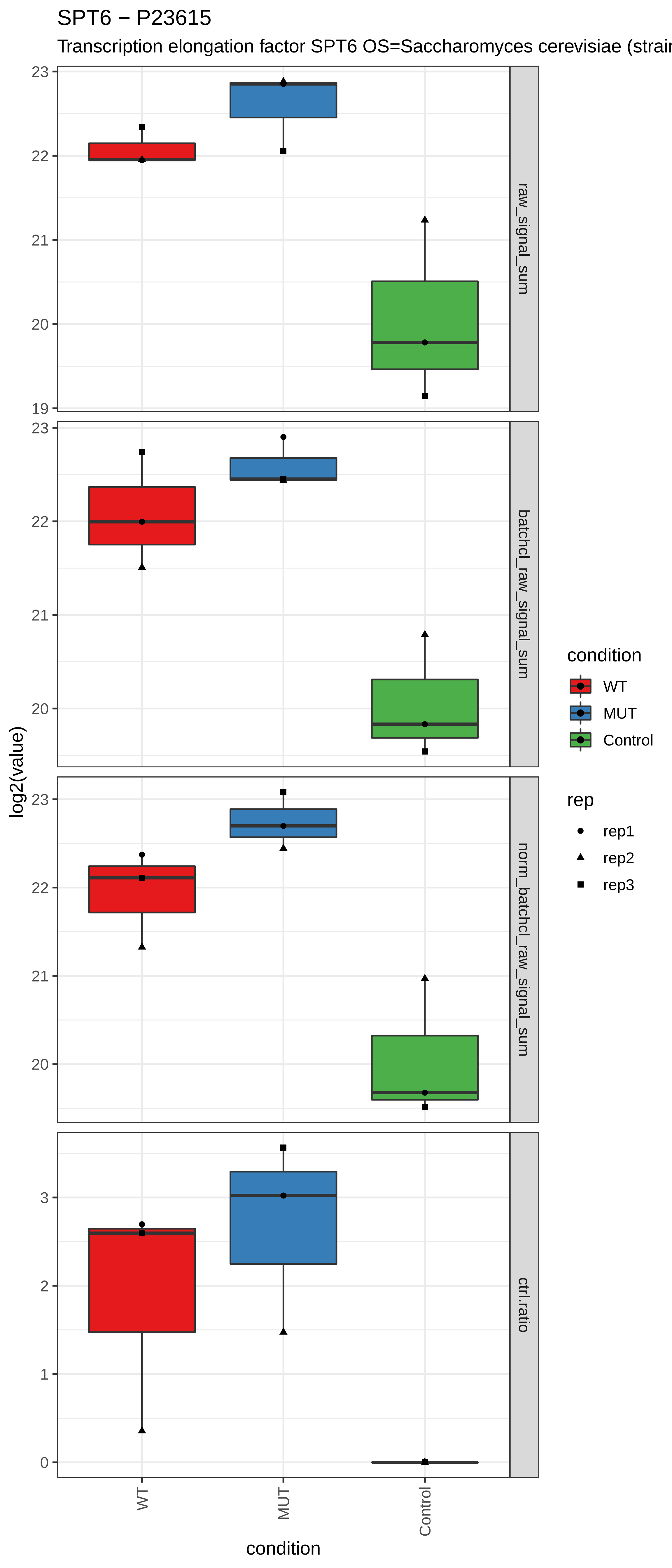

SPT6 − P23615
Transcription elongation factor SPT6 OS=Saccharomyces cerevisiae (strain
raw_signal_sum
batchcl_raw_signal_sum
norm_batchcl_raw_signal_sum
ctrl.ratio
log2(value)
condition
WT
MUT
Control
rep
rep1
rep2
rep3

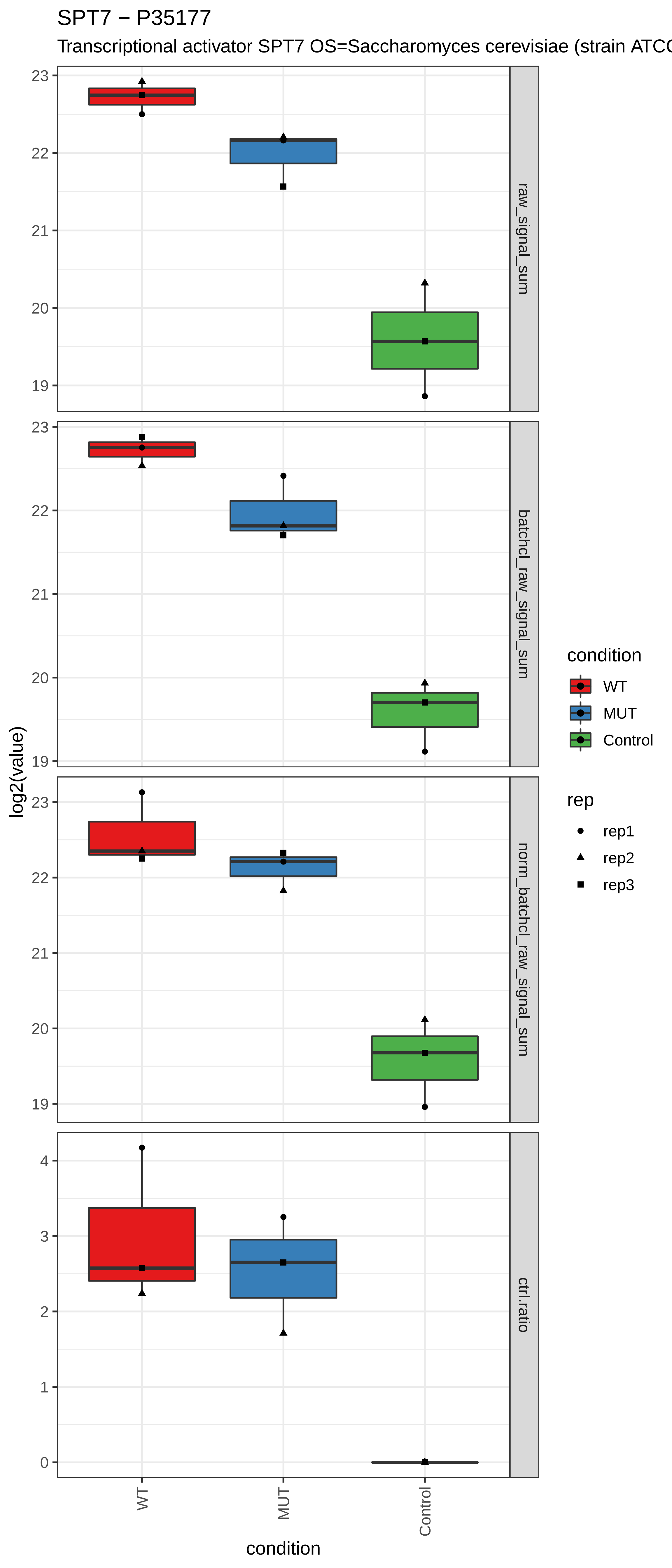
SPT7 − P35177
Transcriptional activator SPT7 OS=Saccharomyces cerevisiae (strain ATCC
raw_signal_sum
batchcl_raw_signal_sum
norm_batchcl_raw_signal_sum
ctrl.ratio
log2(value)
condition
WT
MUT
Control
rep
rep1
rep2
rep3
23
22
21
20
19
4
3
2
1
0

SRL2 − Q12020

Protein SRL2 OS=Saccharomyces cerevisiae (strain ATCC 204508 / S28

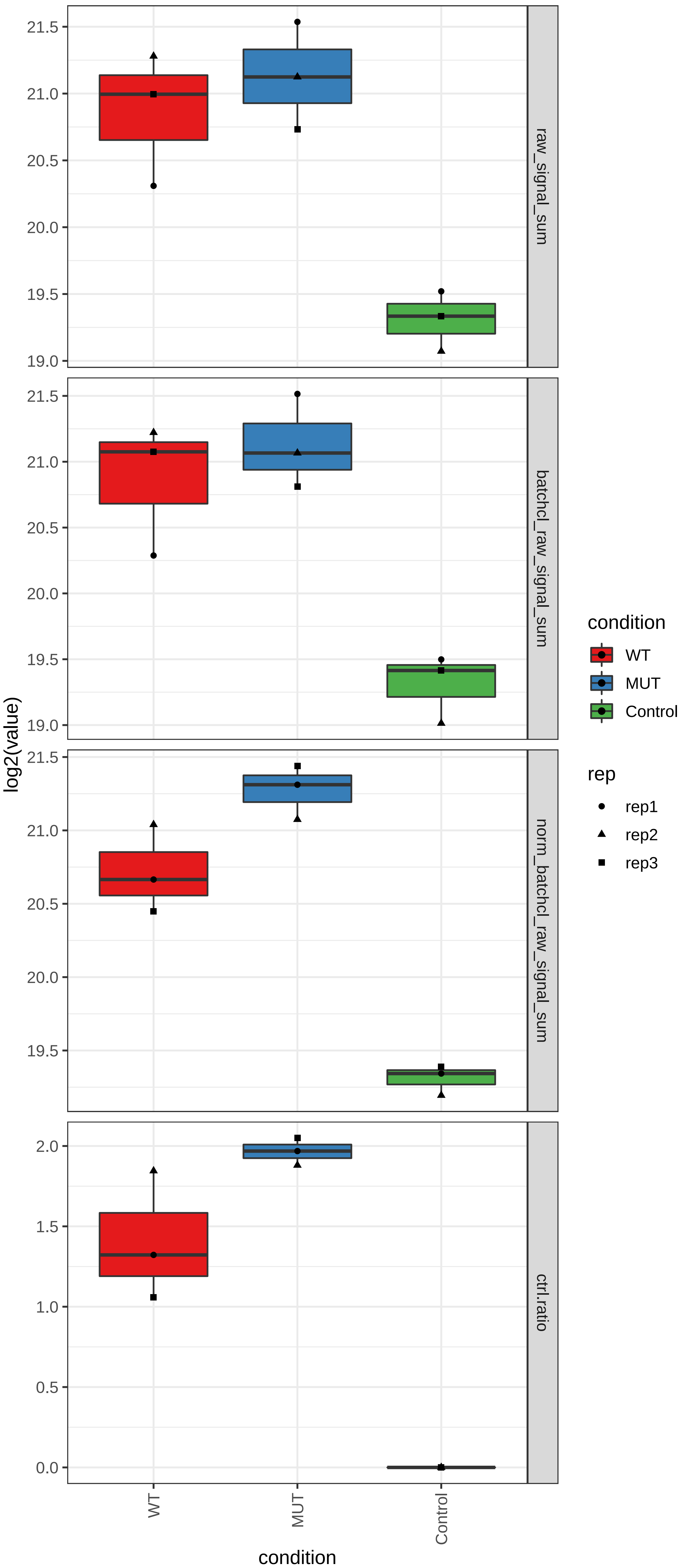

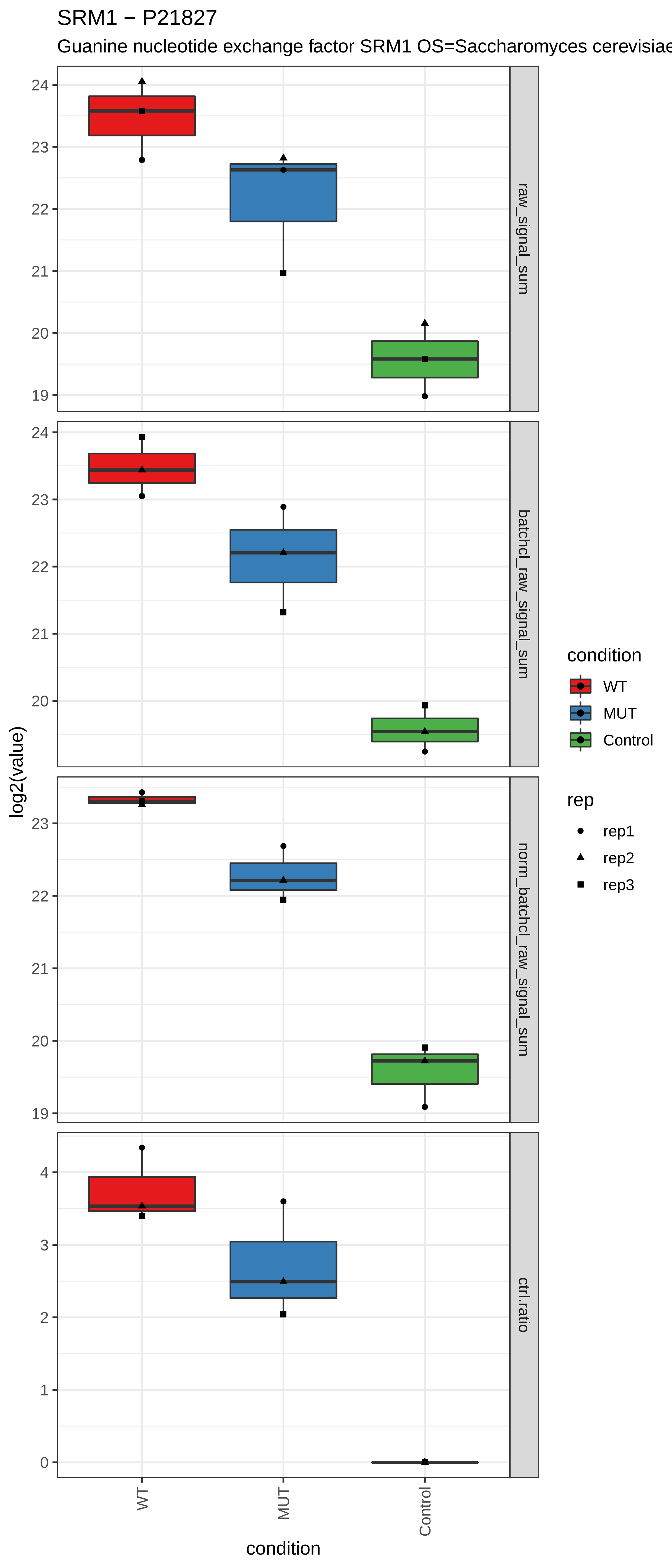

SRM1 − P21827
Guanine nucleotide exchange factor SRM1 OS=Saccharomyces cerevisiae
raw_signal_sum
batchcl_raw_signal_sum
norm_batchcl_raw_signal_sum
ctrl.ratio
log2(value)
condition
WT
MUT
Control
rep
rep1
rep2
rep3

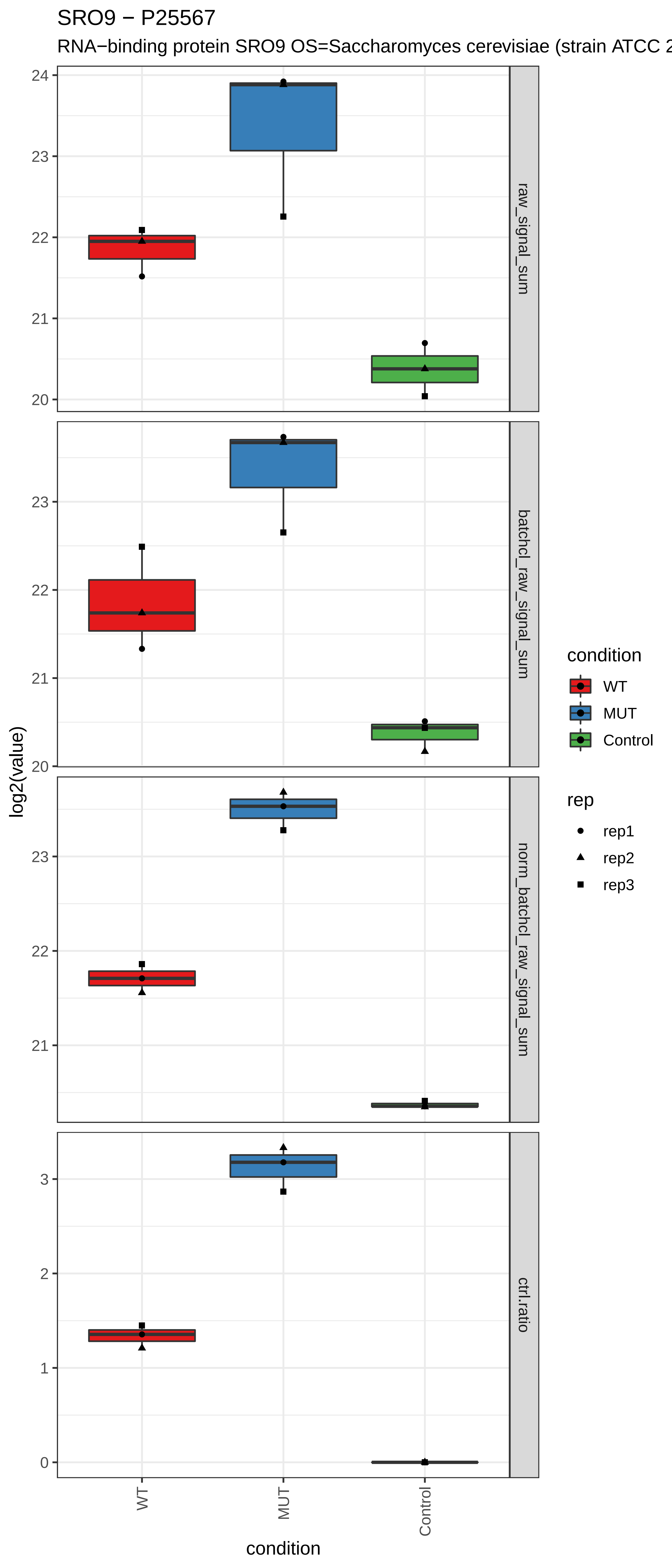

SRO9 − P25567
RNA−binding protein SRO9 OS=Saccharomyces cerevisiae (strain ATCC 2
raw_signal_sum
batchcl_raw_signal_sum
norm_batchcl_raw_signal_sum
ctrl.ratio
log2(value)
condition
WT
MUT
Control
rep
rep1
rep2
rep3

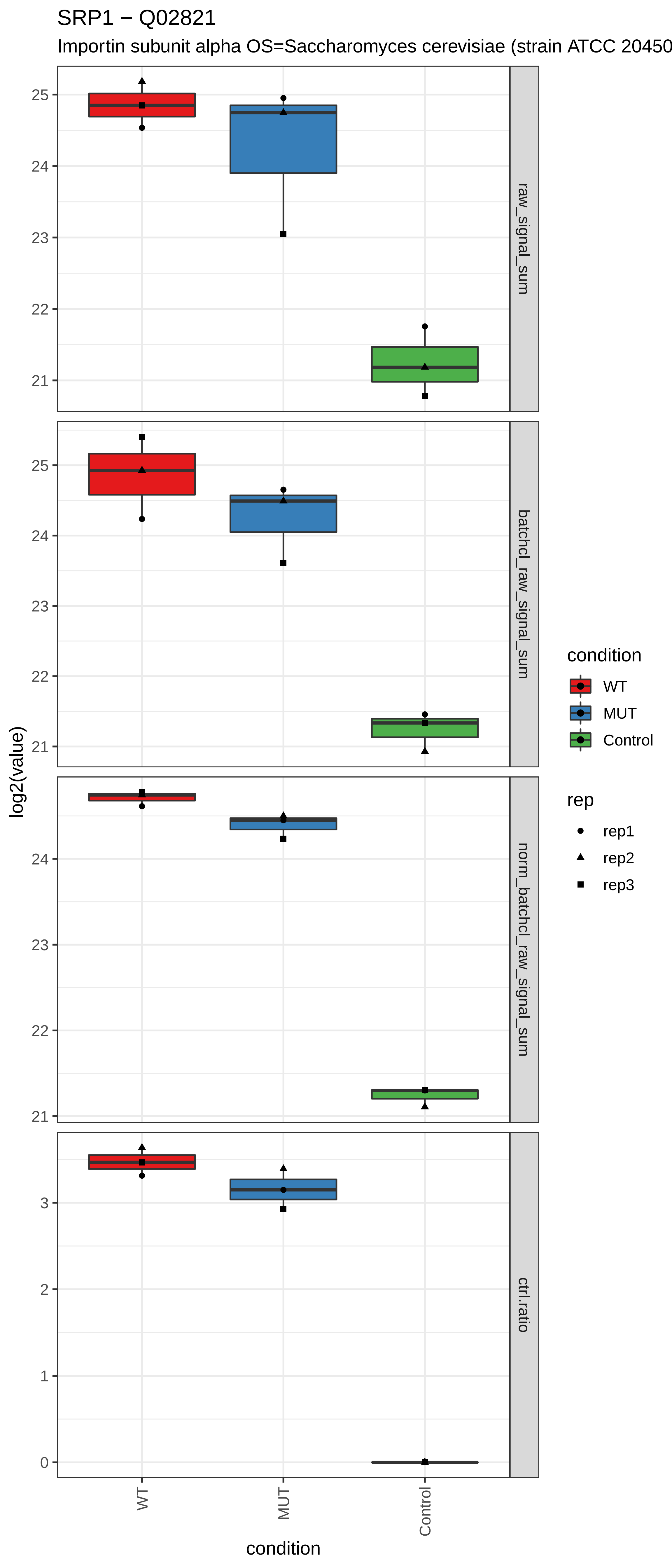

SRP1 − Q02821
Importin subunit alpha OS=Saccharomyces cerevisiae (strain ATCC 20450
raw_signal_sum
batchcl_raw_signal_sum
norm_batchcl_raw_signal_sum
ctrl.ratio
log2(value)
condition
WT
MUT
Control
rep
rep1
rep2
rep3

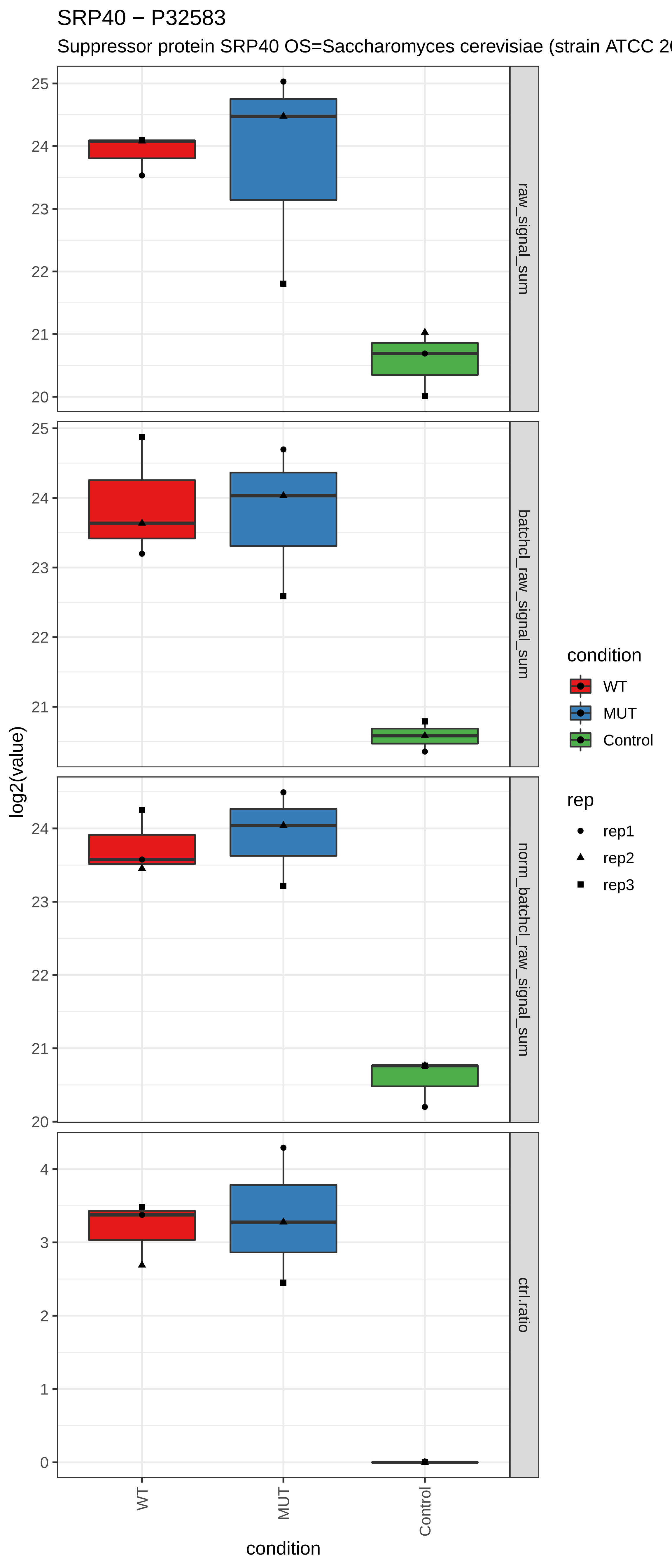
SRP40 − P32583
Suppressor protein SRP40 OS=Saccharomyces cerevisiae (strain ATCC 2
raw_signal_sum
batchcl_raw_signal_sum
norm_batchcl_raw_signal_sum
ctrl.ratio
log2(value)
condition
WT
MUT
Control
rep
rep1
rep2
rep3

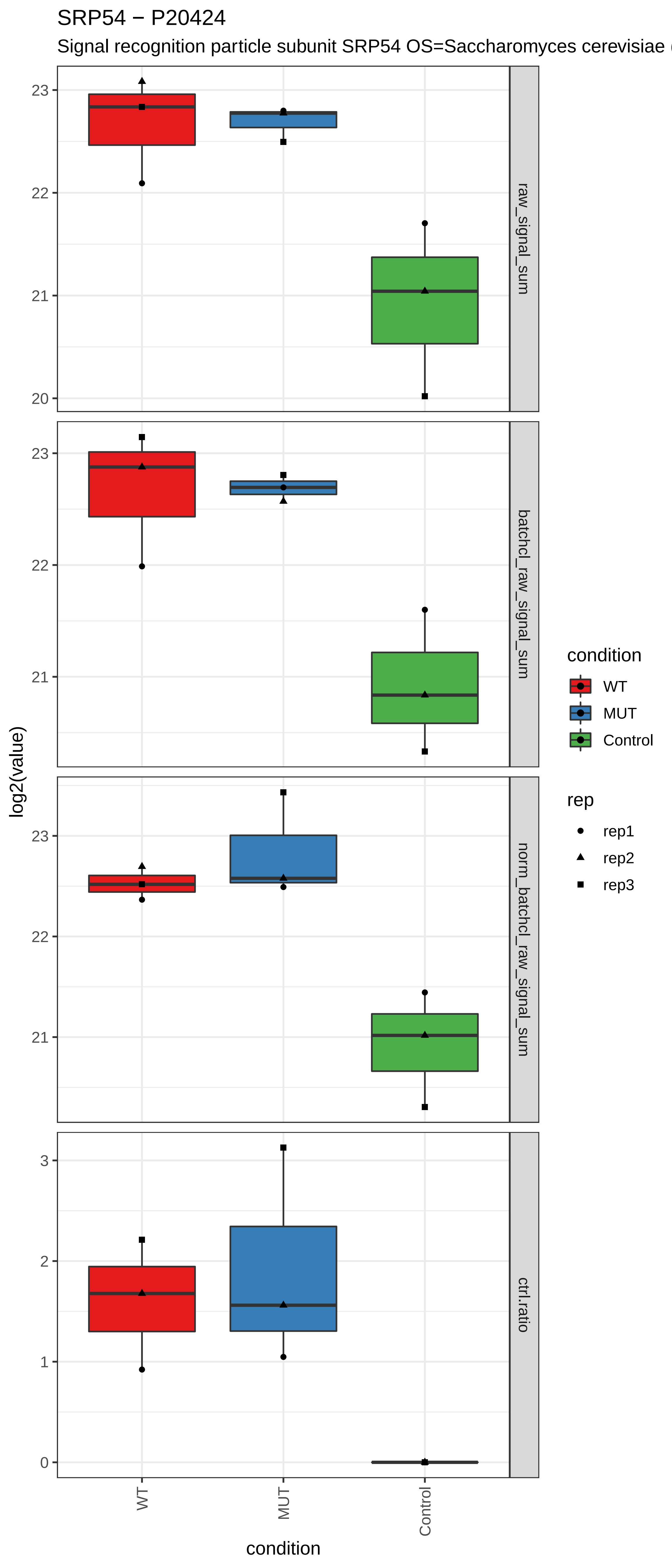

SRP54 − P20424
Signal recognition particle subunit SRP54 OS=Saccharomyces cerevisiae
raw_signal_sum
batchcl_raw_signal_sum
norm_batchcl_raw_signal_sum
ctrl.ratio
log2(value)
condition
WT
MUT
Control
rep
rep1
rep2
rep3

SRP72 − P38688

Signal recognition particle subunit SRP72 OS=Saccharomyces cerevisiae (

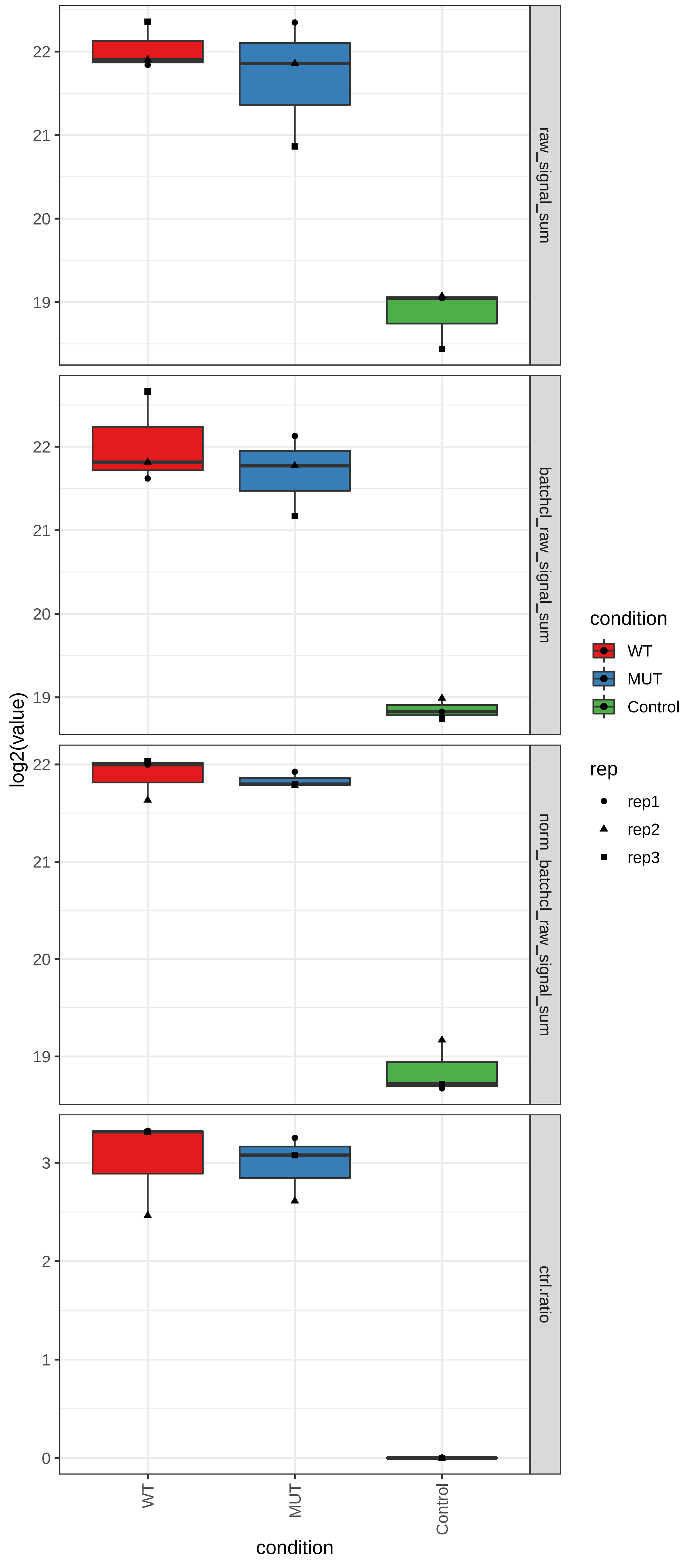

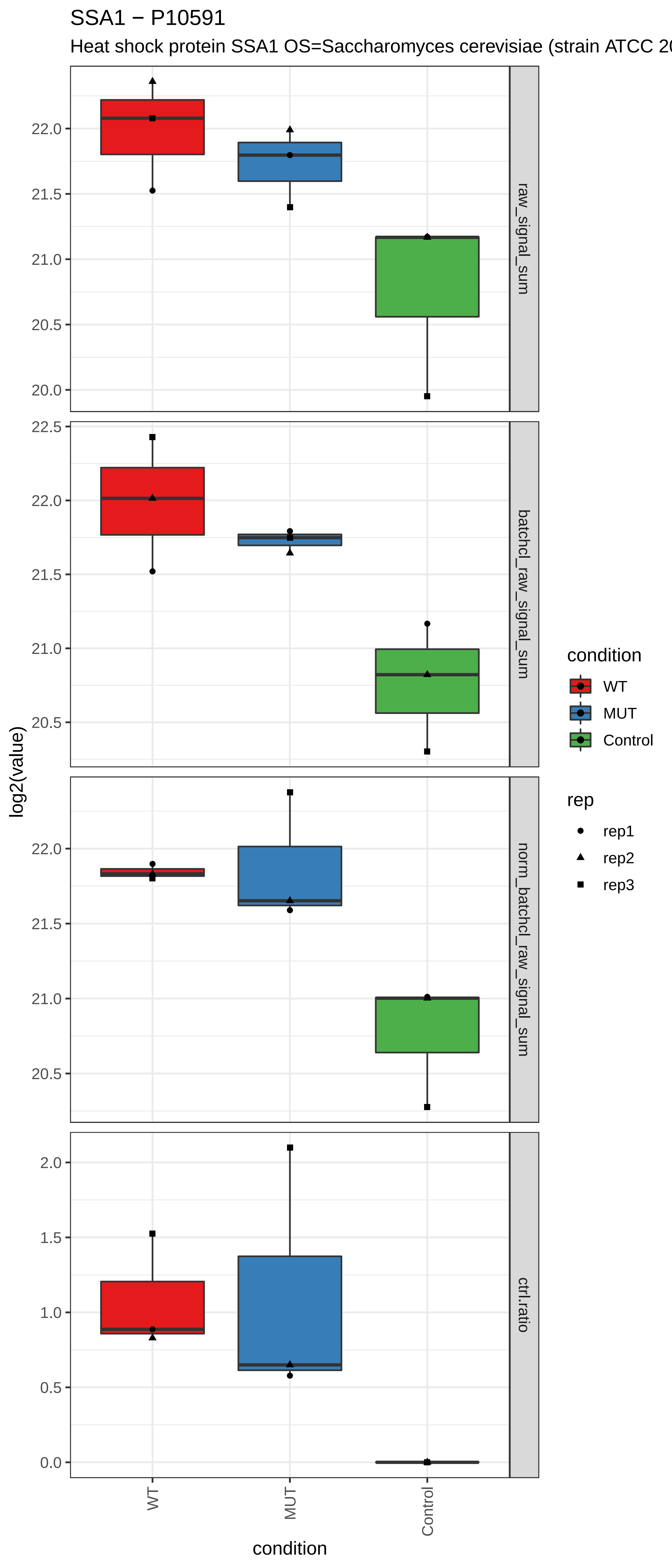
SSA1 − P10591
Heat shock protein SSA1 OS=Saccharomyces cerevisiae (strain ATCC 20
raw_signal_sum
batchcl_raw_signal_sum
norm_batchcl_raw_signal_sum
ctrl.ratio
log2(value)
condition
WT
MUT
Control
rep
rep1
rep2
rep3
22.0
21.5
21.0
20.5
20.0
22.5
2.0
1.5
1.0
0.5
0.0

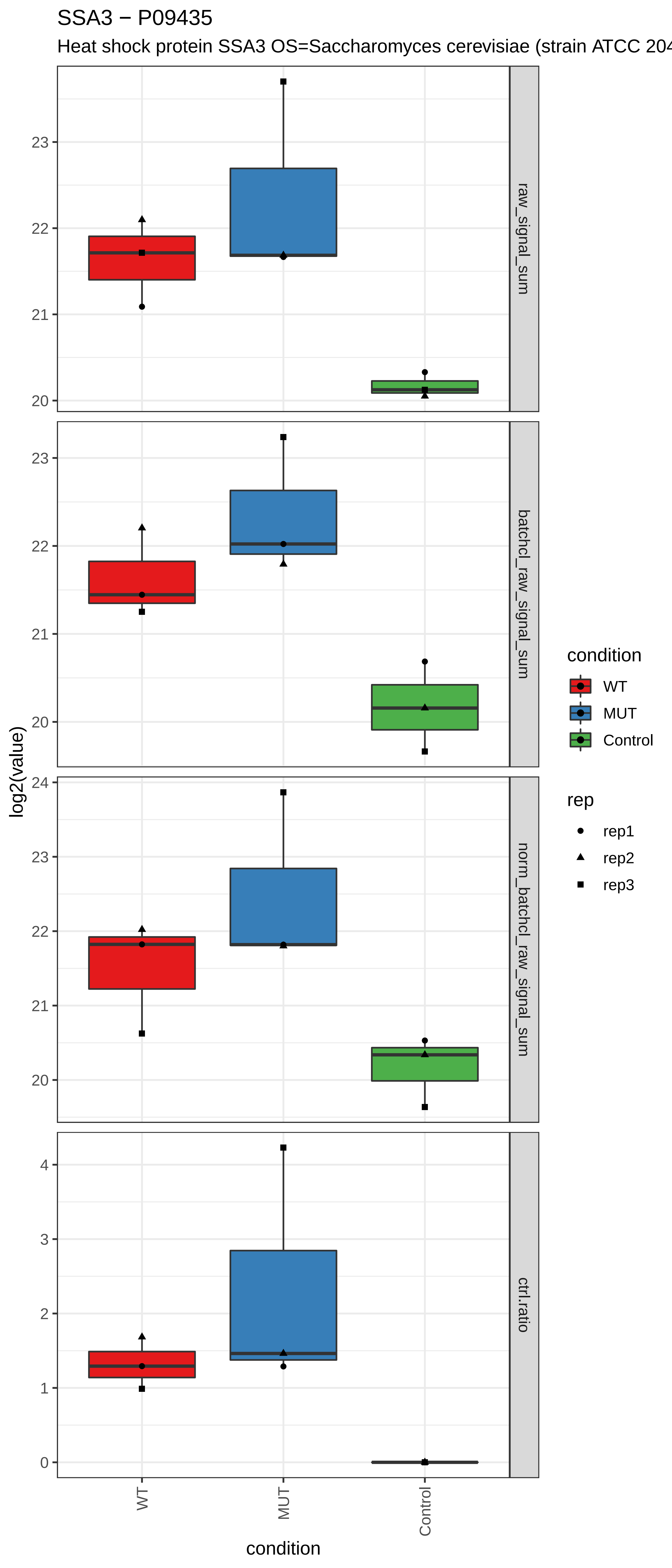

SSA3 − P09435
Heat shock protein SSA3 OS=Saccharomyces cerevisiae (strain ATCC 204
raw_signal_sum
batchcl_raw_signal_sum
norm_batchcl_raw_signal_sum
ctrl.ratio
log2(value)
condition
WT
MUT
Control
rep
rep1
rep2
rep3

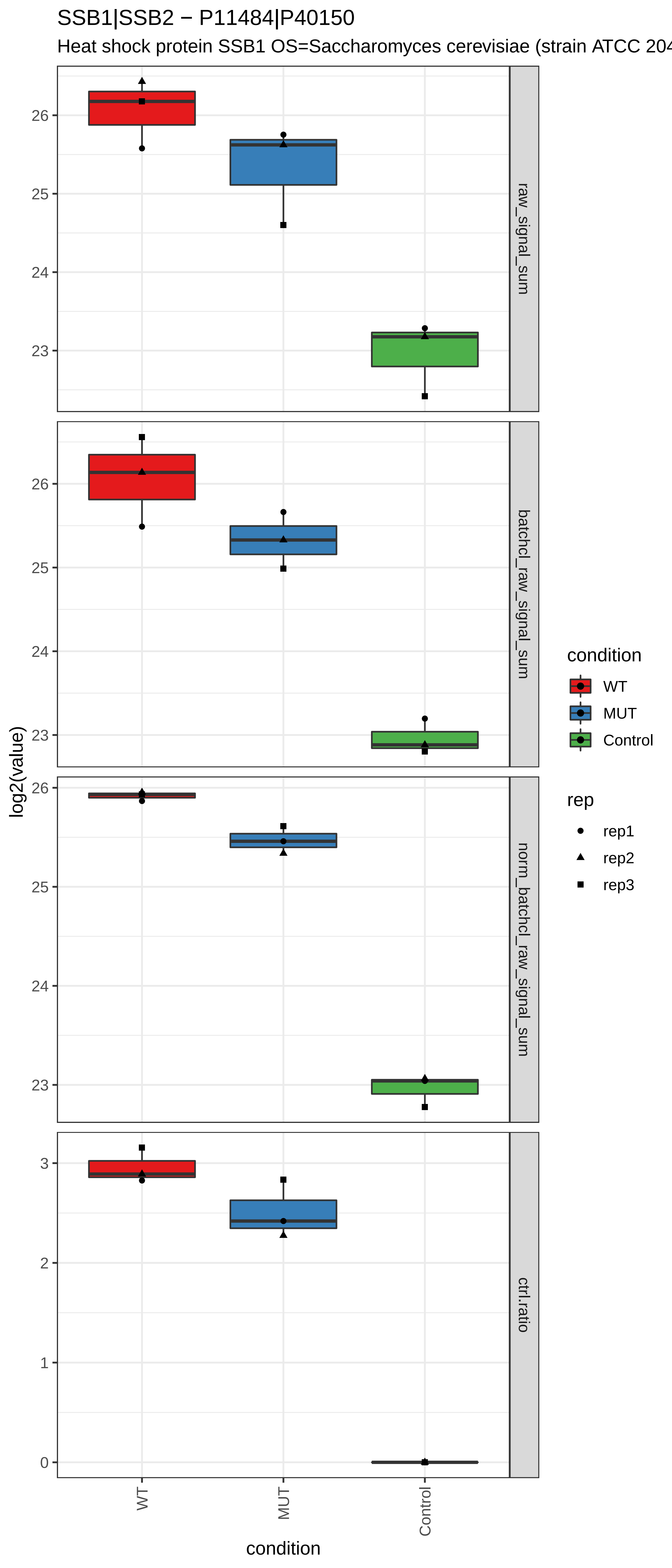

SSB1|SSB2 − P11484|P40150
Heat shock protein SSB1 OS=Saccharomyces cerevisiae (strain ATCC 204
raw_signal_sum
batchcl_raw_signal_sum
norm_batchcl_raw_signal_sum
ctrl.ratio
log2(value)
condition
WT
MUT
Control
rep
rep1
rep2
rep3

SSC1 − P0CS90

Heat shock protein SSC1, mitochondrial OS=Saccharomyces cerevisiae (s

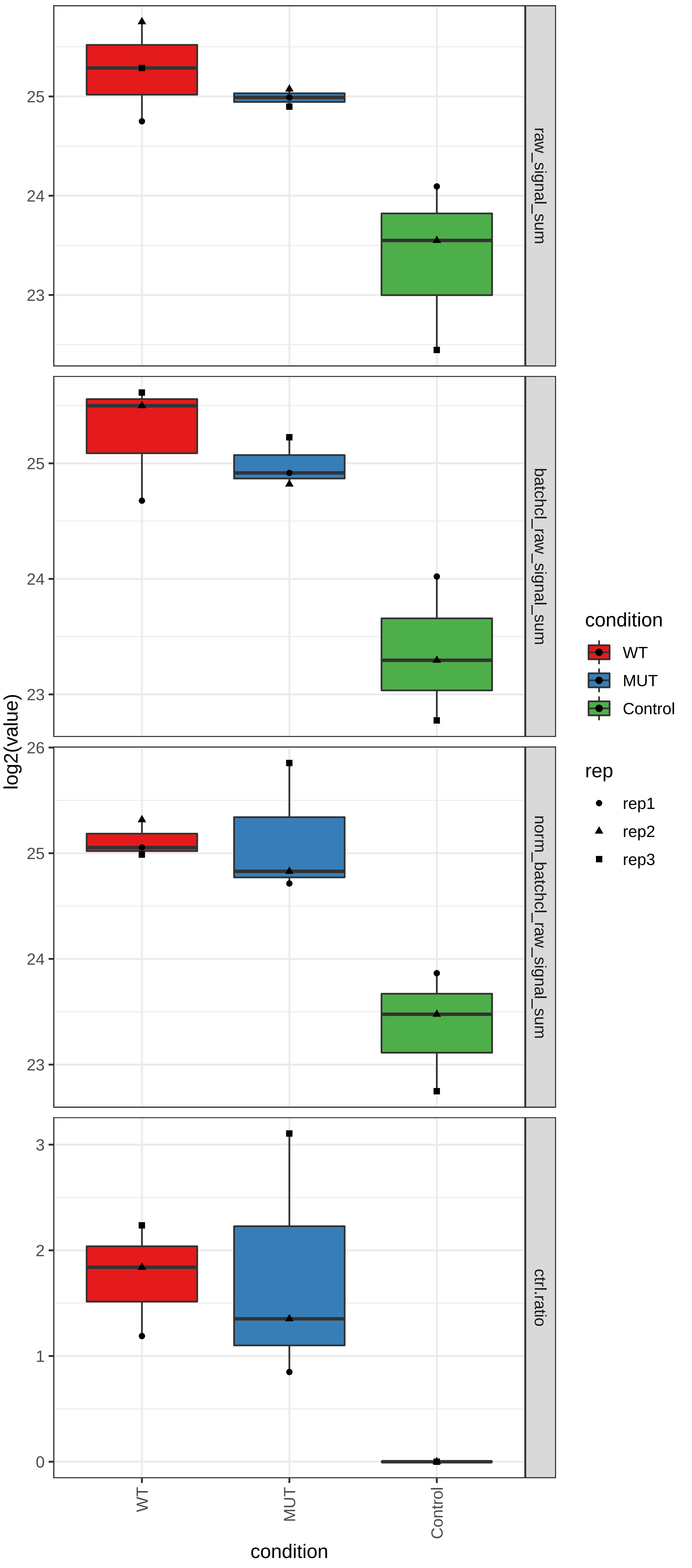

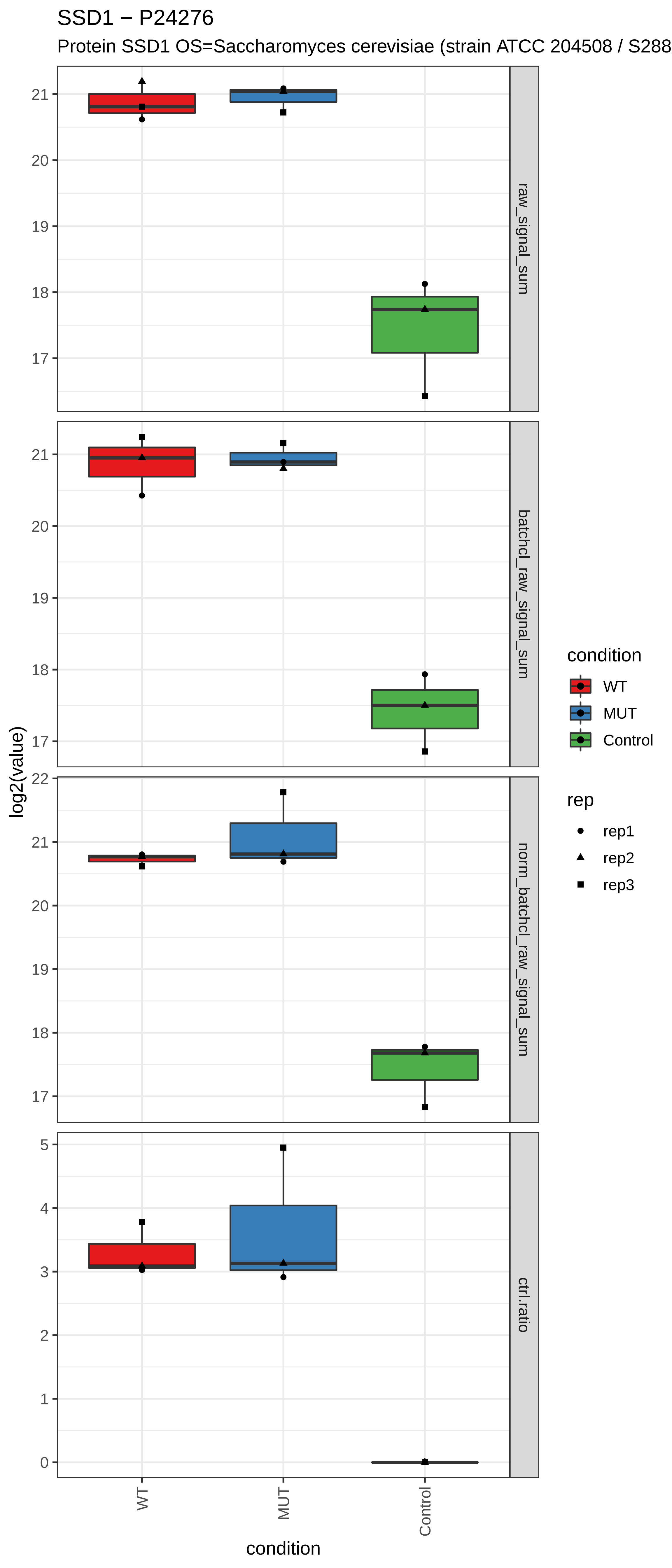

SSD1 − P24276
Protein SSD1 OS=Saccharomyces cerevisiae (strain ATCC 204508 / S288
raw_signal_sum
batchcl_raw_signal_sum
norm_batchcl_raw_signal_sum
ctrl.ratio
log2(value)
condition
WT
MUT
Control
rep
rep1
rep2
rep3

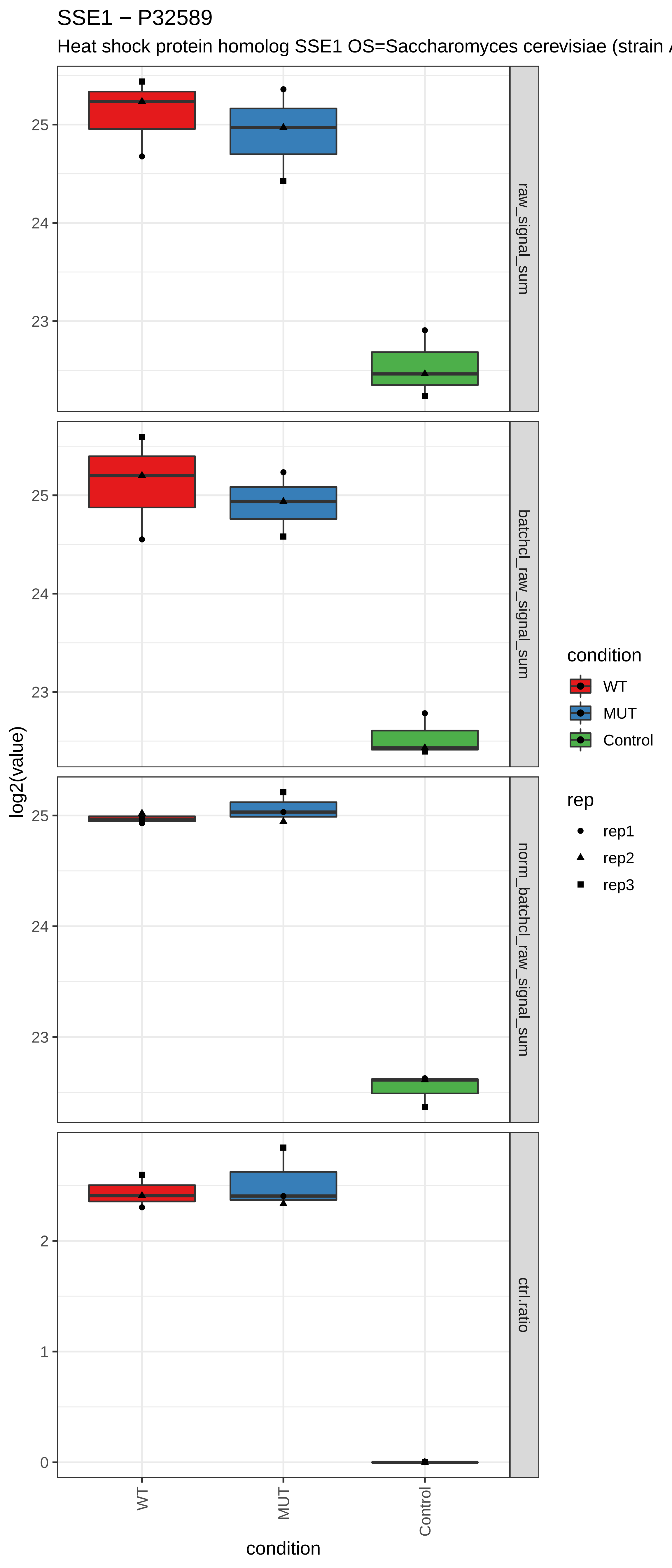

SSE1 − P32589
Heat shock protein homolog SSE1 OS=Saccharomyces cerevisiae (strain
raw_signal_sum
batchcl_raw_signal_sum
norm_batchcl_raw_signal_sum
ctrl.ratio
log2(value)
25
24
23
2
1
0
WT
MUT
Control
condition
rep
rep1
rep2
rep3

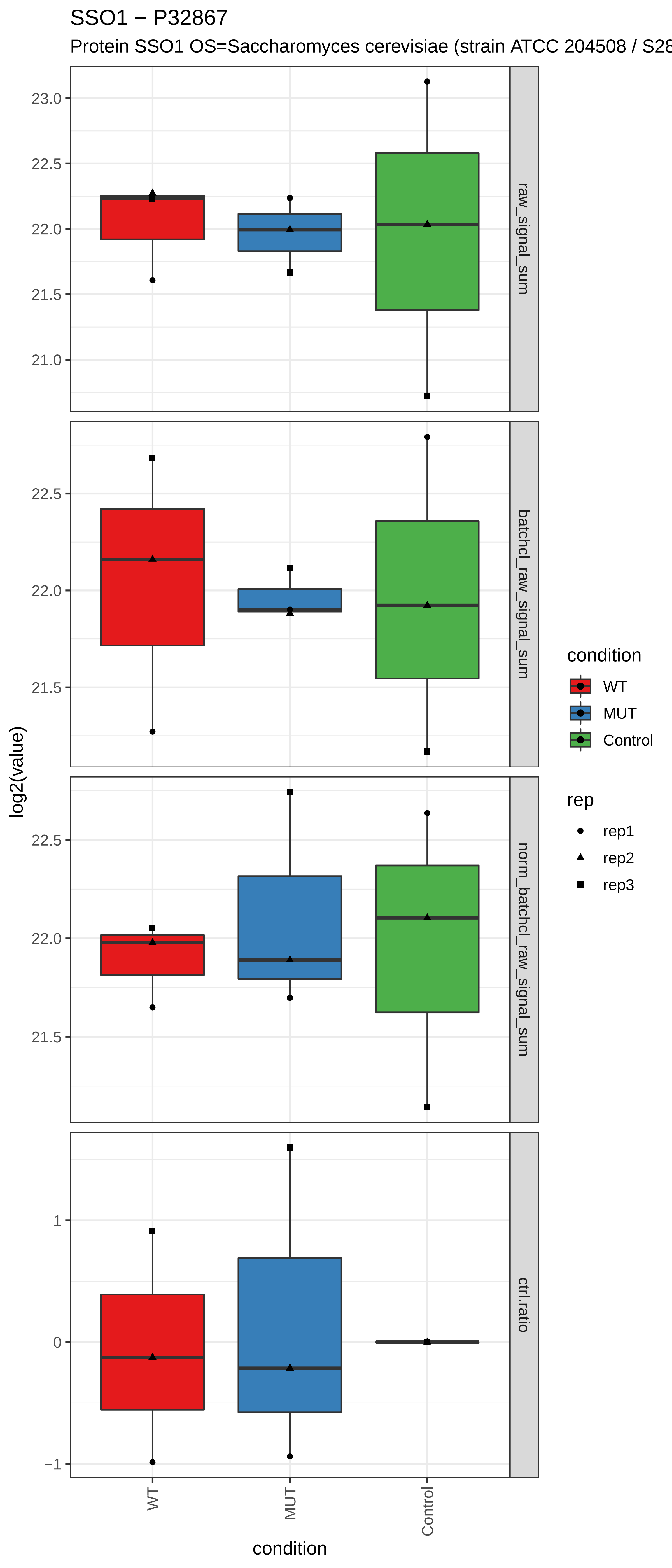

SSO1 − P32867
Protein SSO1 OS=Saccharomyces cerevisiae (strain ATCC 204508 / S28
raw_signal_sum
batchcl_raw_signal_sum
norm_batchcl_raw_signal_sum
ctrl.ratio
log2(value)
condition
WT
MUT
Control
rep
rep1
rep2
rep3

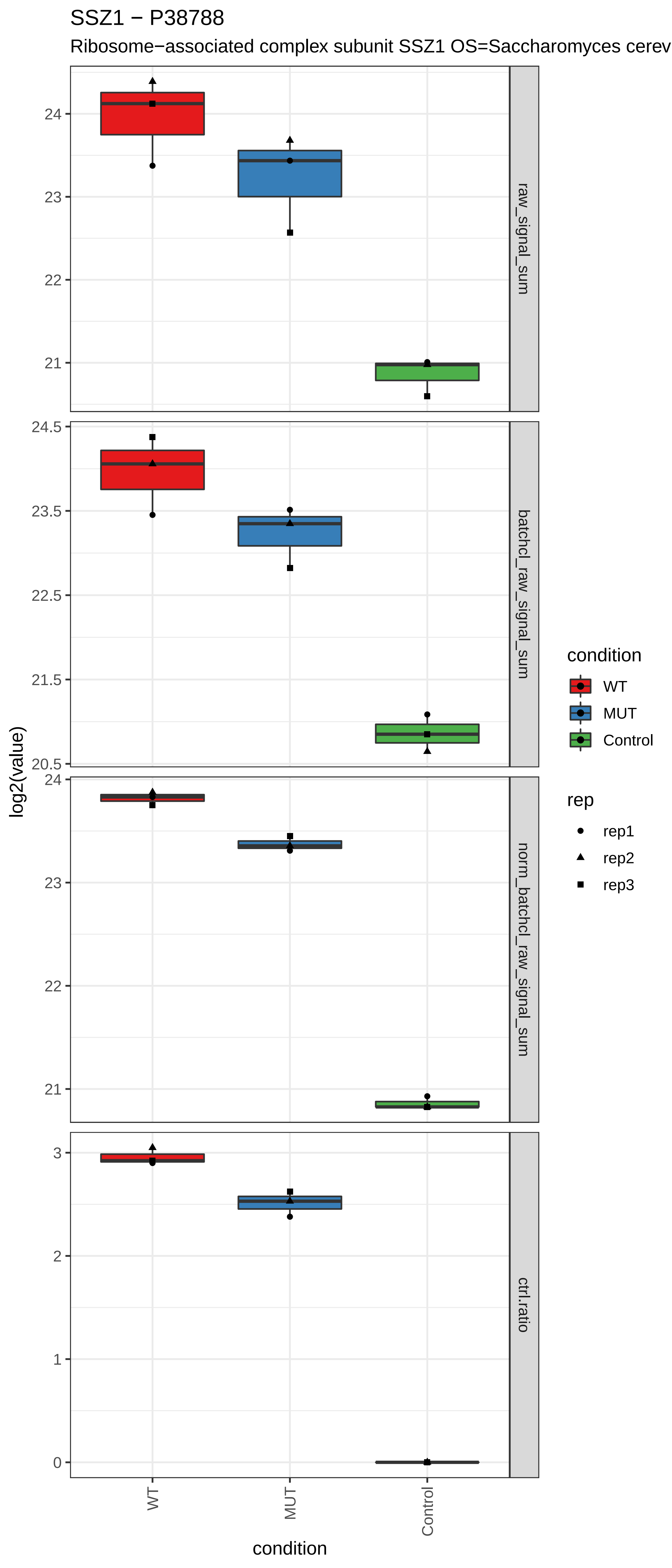
SSZ1 − P38788
Ribosome−associated complex subunit SSZ1 OS=Saccharomyces cerev
raw_signal_sum
batchcl_raw_signal_sum
norm_batchcl_raw_signal_sum
ctrl.ratio
log2(value)
condition
WT
MUT
Control
rep
rep1
rep2
rep3
24
23
22
21
24.5
23.5
22.5
21.5
20.5
3
2
1
0

STE24 − P47154

CAAX prenyl protease 1 OS=Saccharomyces cerevisiae (strain ATCC 2045

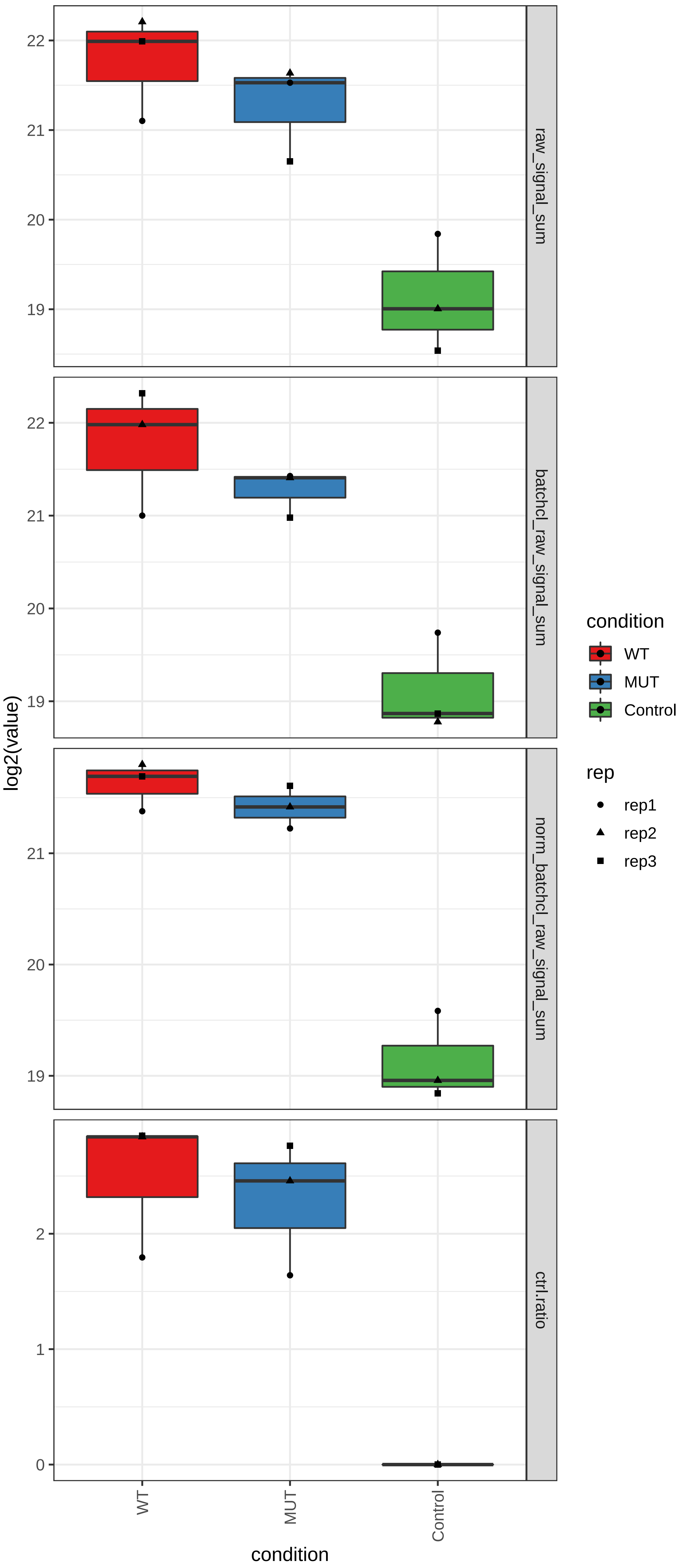

STH1 − P32597

Nuclear protein STH1/NPS1 OS=Saccharomyces cerevisiae (strain ATCC

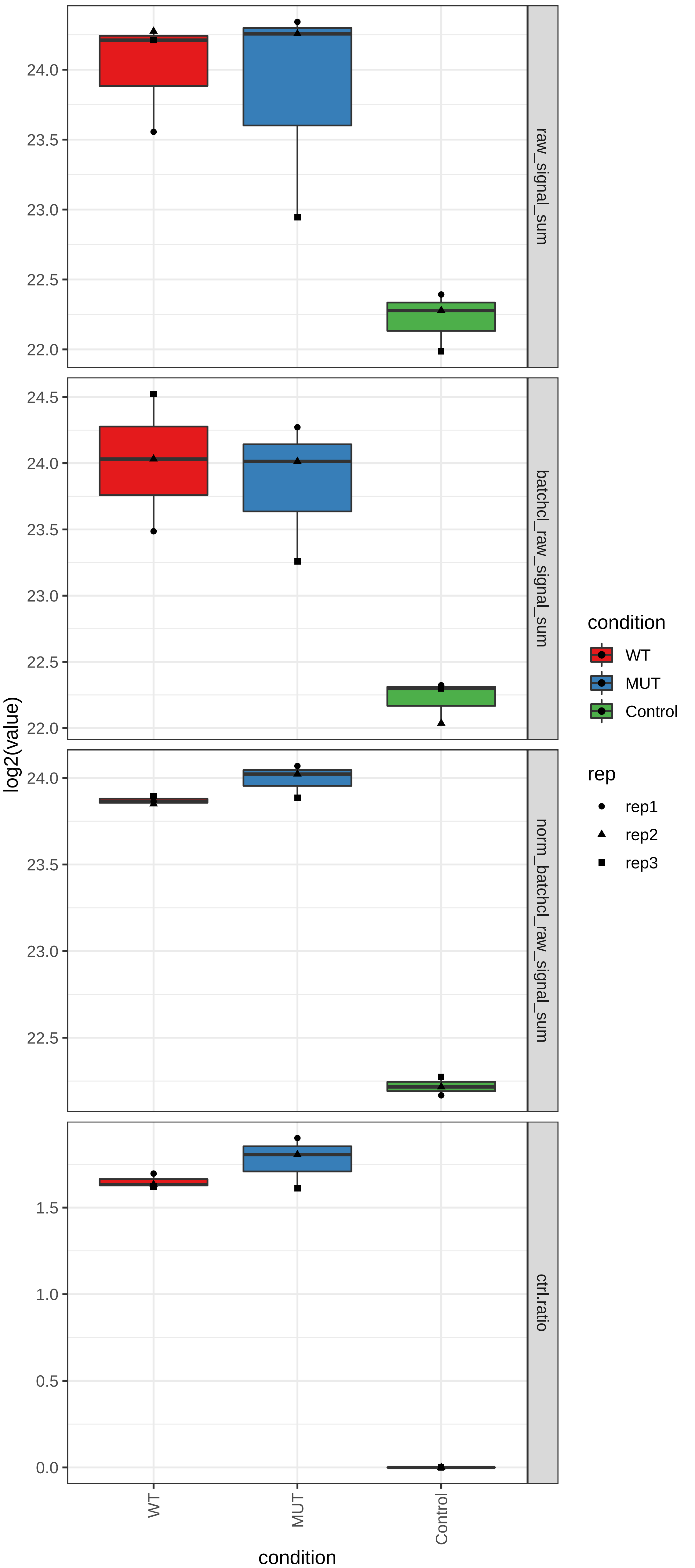

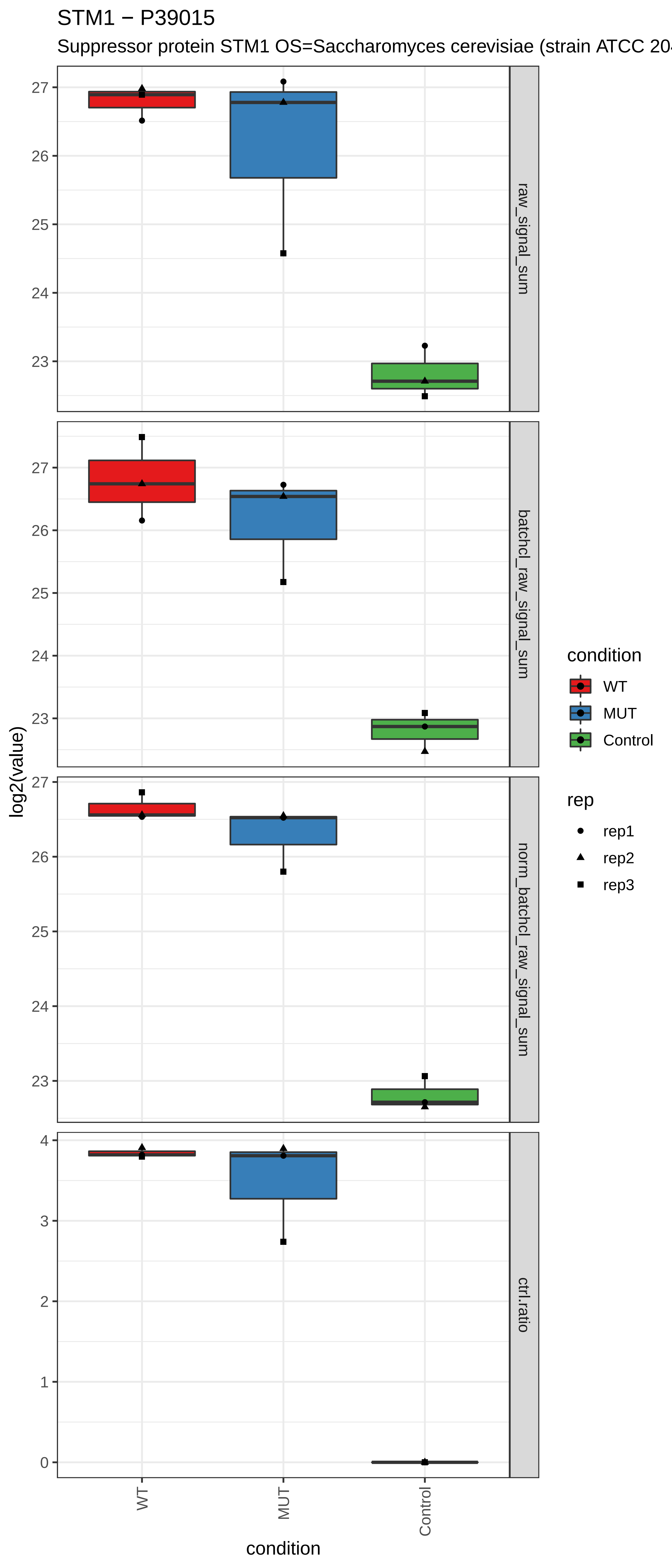
STM1 − P39015
Suppressor protein STM1 OS=Saccharomyces cerevisiae (strain ATCC 20
raw_signal_sum
batchcl_raw_signal_sum
norm_batchcl_raw_signal_sum
ctrl.ratio
log2(value)
condition
WT
MUT
Control
rep
rep1
rep2
rep3
27
26
25
24
23
4
3
2
1
0

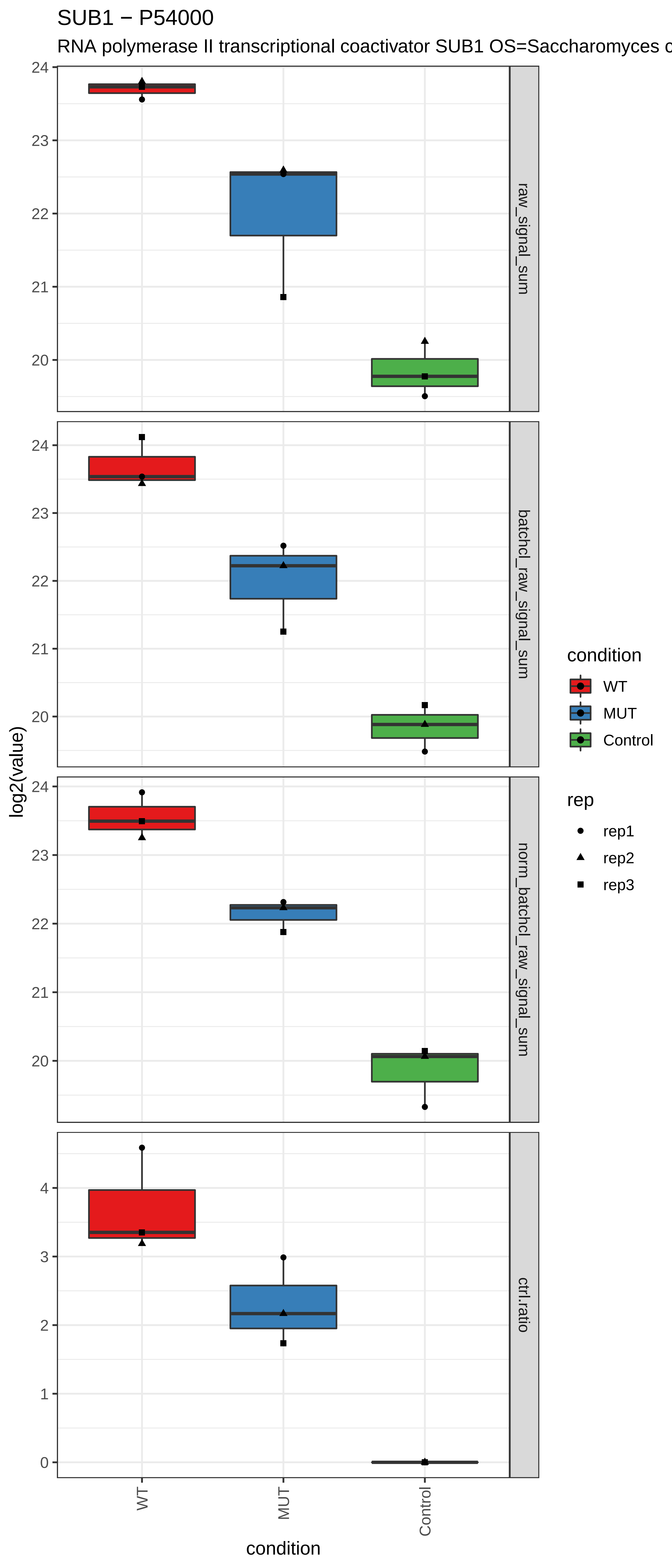

SUB1 − P54000
RNA polymerase II transcriptional coactivator SUB1 OS=Saccharomyces c
raw_signal_sum
batchcl_raw_signal_sum
norm_batchcl_raw_signal_sum
ctrl.ratio
log2(value)
condition
WT
MUT
Control
rep
rep1
rep2
rep3

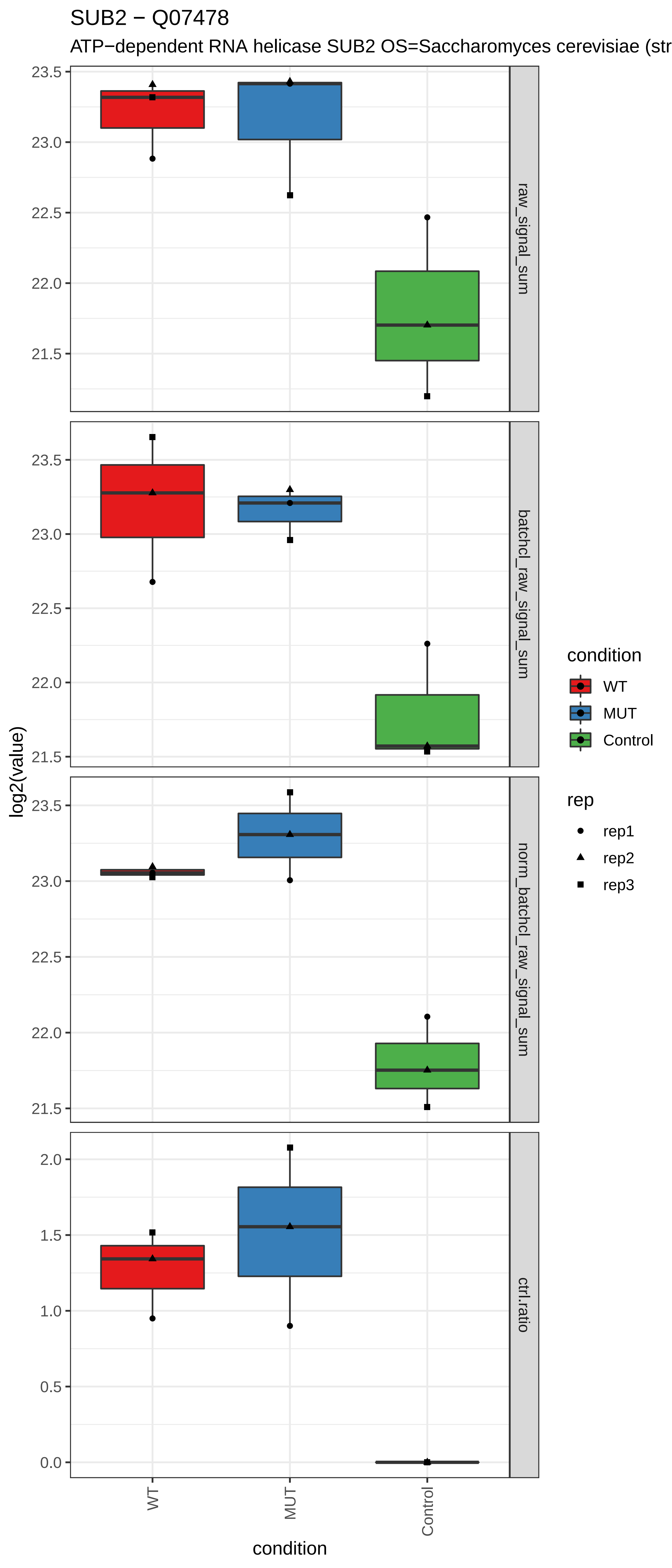

SUB2 − Q07478
ATP−dependent RNA helicase SUB2 OS=Saccharomyces cerevisiae (str
raw_signal_sum
batchcl_raw_signal_sum
norm_batchcl_raw_signal_sum
ctrl.ratio
log2(value)
condition
WT
MUT
Control
rep
rep1
rep2
rep3
23.5
23.0
22.5
22.0
21.5
2.0
1.5
1.0
0.5
0.0

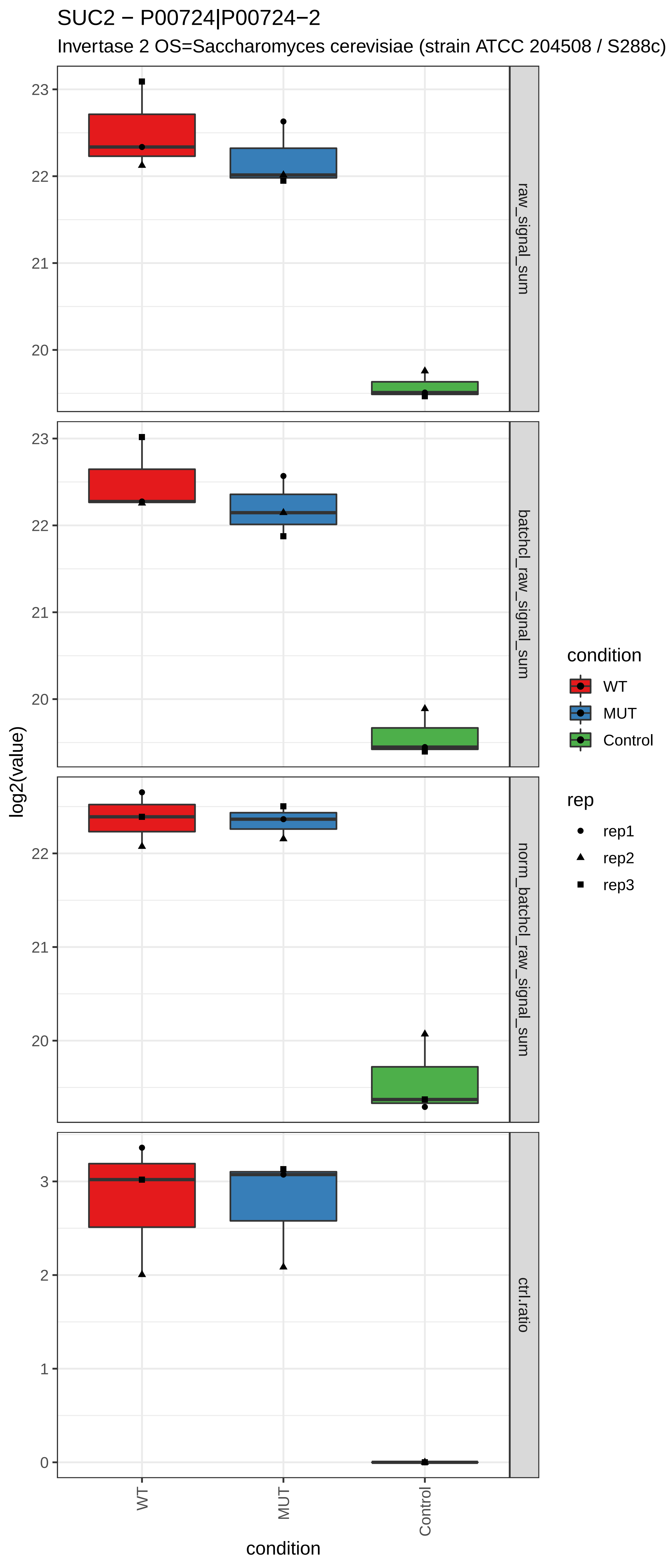

SUC2 − P00724|P00724−2
Invertase 2 OS=Saccharomyces cerevisiae (strain ATCC 204508 / S288c)
raw_signal_sum
batchcl_raw_signal_sum
norm_batchcl_raw_signal_sum
ctrl.ratio
log2(value)
condition
WT
MUT
Control
rep
rep1
rep2
rep3

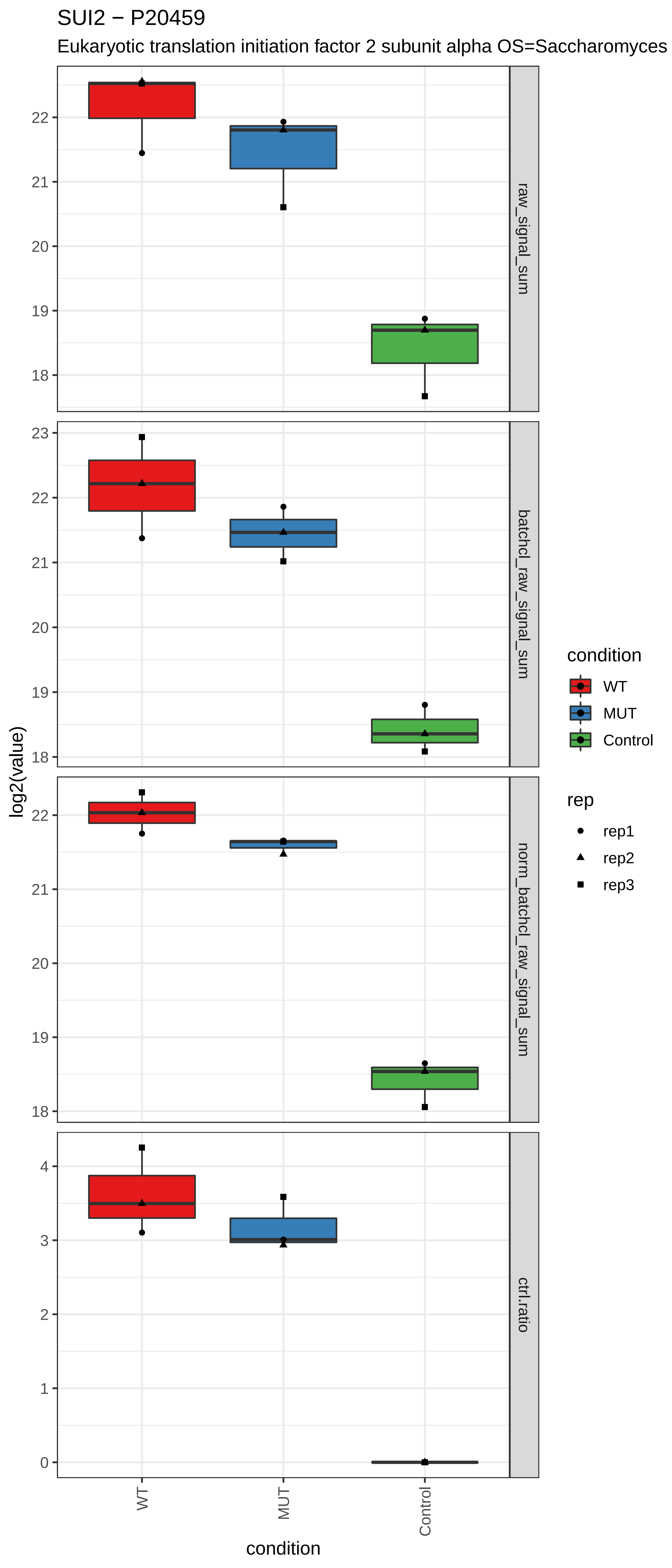
SUI2 − P20459
Eukaryotic translation initiation factor 2 subunit alpha OS=Saccharomyces
raw_signal_sum
batchcl_raw_signal_sum
norm_batchcl_raw_signal_sum
ctrl.ratio
log2(value)
condition
WT
MUT
Control
rep
rep1
rep2
rep3
22
21
20
19
18
23
4
3
2
1
0

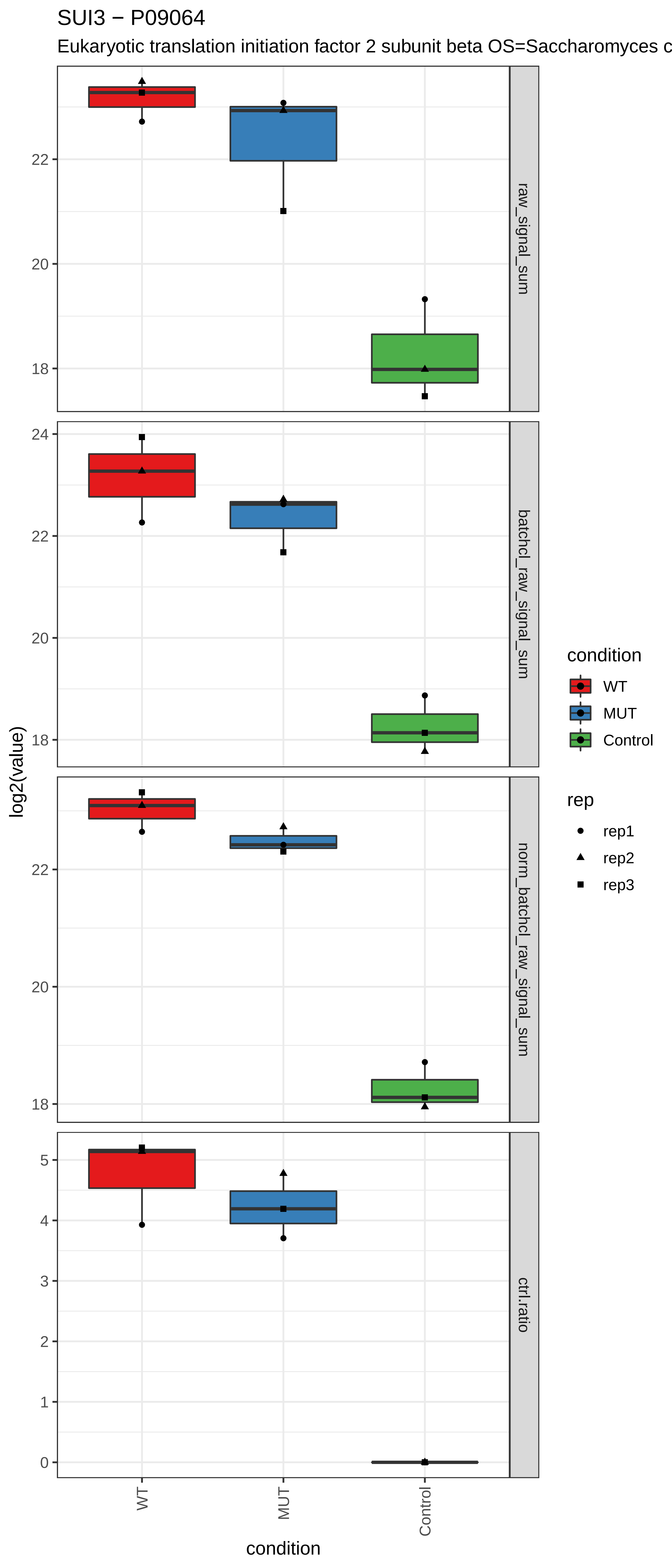

SUI3 − P09064
Eukaryotic translation initiation factor 2 subunit beta OS=Saccharomyces c
raw_signal_sum
batchcl_raw_signal_sum
norm_batchcl_raw_signal_sum
ctrl.ratio
log2(value)
condition
WT
MUT
Control
rep
rep1
rep2
rep3
22
20
18
24
5
4
3
2
1
0

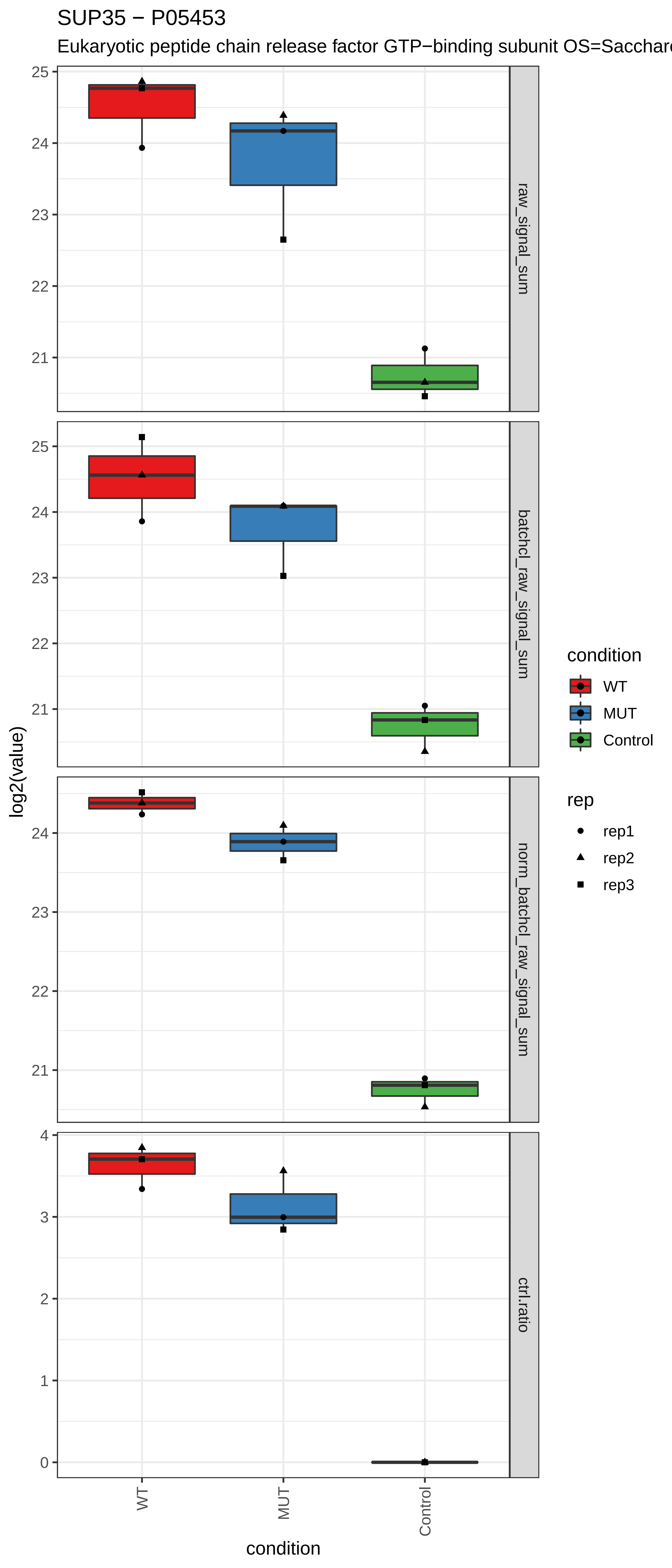

SUP35 − P05453
Eukaryotic peptide chain release factor GTP−binding subunit OS=Sacchar
raw_signal_sum
batchcl_raw_signal_sum
norm_batchcl_raw_signal_sum
ctrl.ratio
log2(value)
condition
WT
MUT
Control
rep
rep1
rep2
rep3

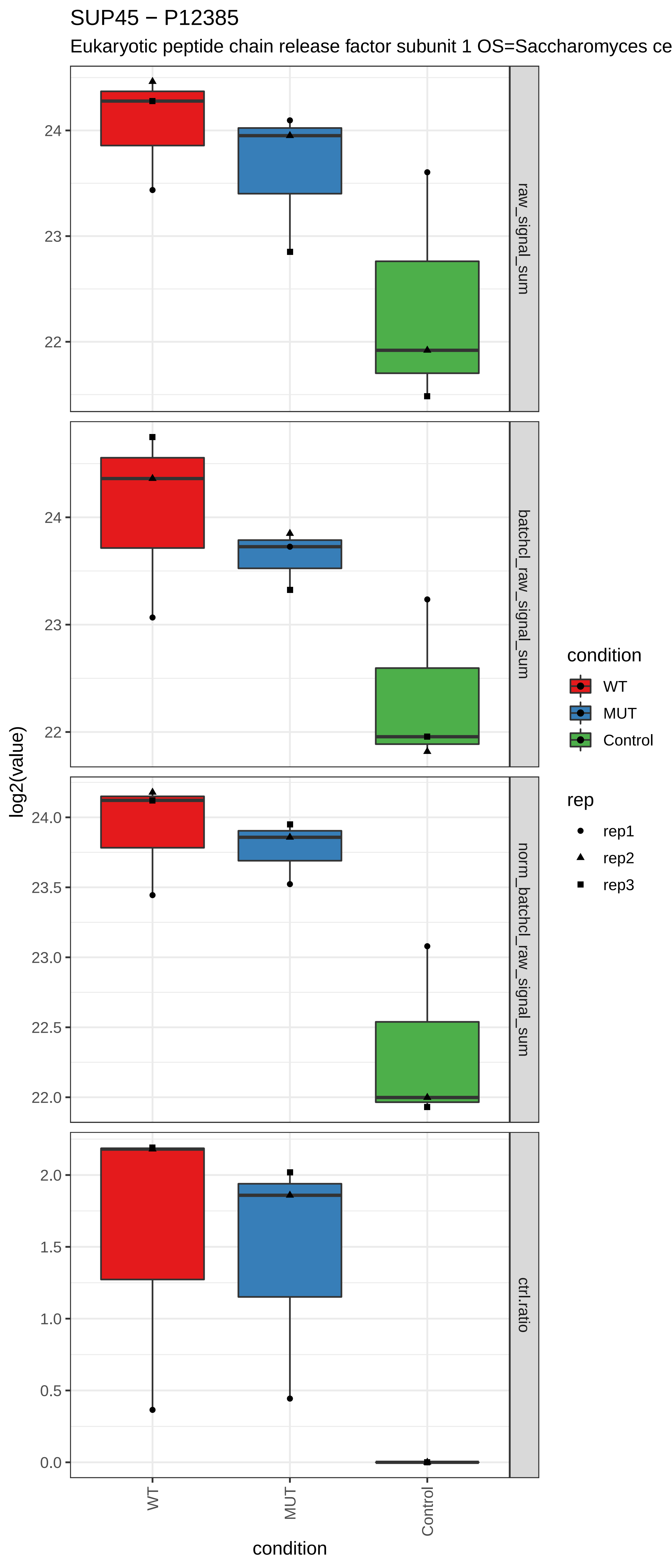
SUP45 − P12385
Eukaryotic peptide chain release factor subunit 1 OS=Saccharomyces ce
raw_signal_sum
batchcl_raw_signal_sum
norm_batchcl_raw_signal_sum
ctrl.ratio
log2(value)
condition
WT
MUT
Control
rep
rep1
rep2
rep3
24
23
22
24.0
23.5
23.0
22.5
22.0
2.0
1.5
1.0
0.5
0.0

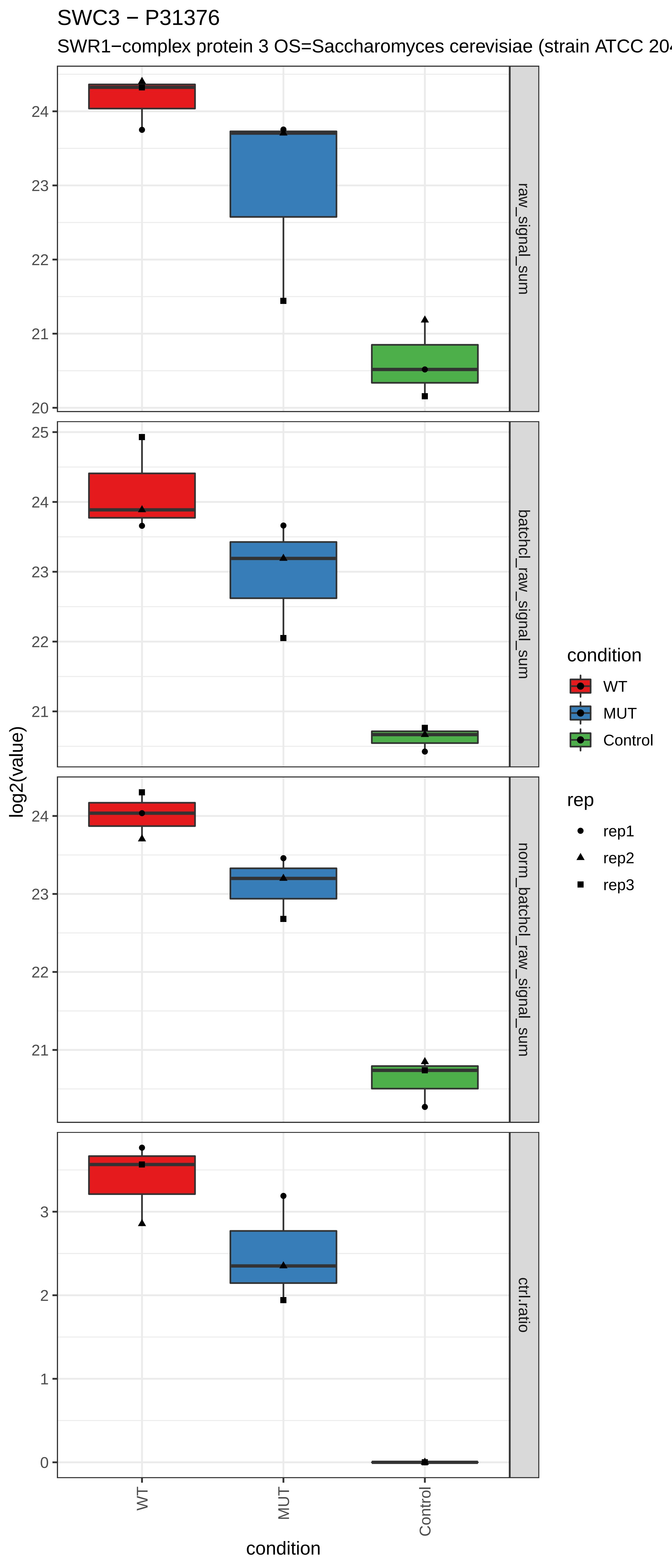

SWC3 − P31376
SWR1−complex protein 3 OS=Saccharomyces cerevisiae (strain ATCC 20
raw_signal_sum
batchcl_raw_signal_sum
norm_batchcl_raw_signal_sum
ctrl.ratio
log2(value)
condition
WT
MUT
Control
rep
rep1
rep2
rep3

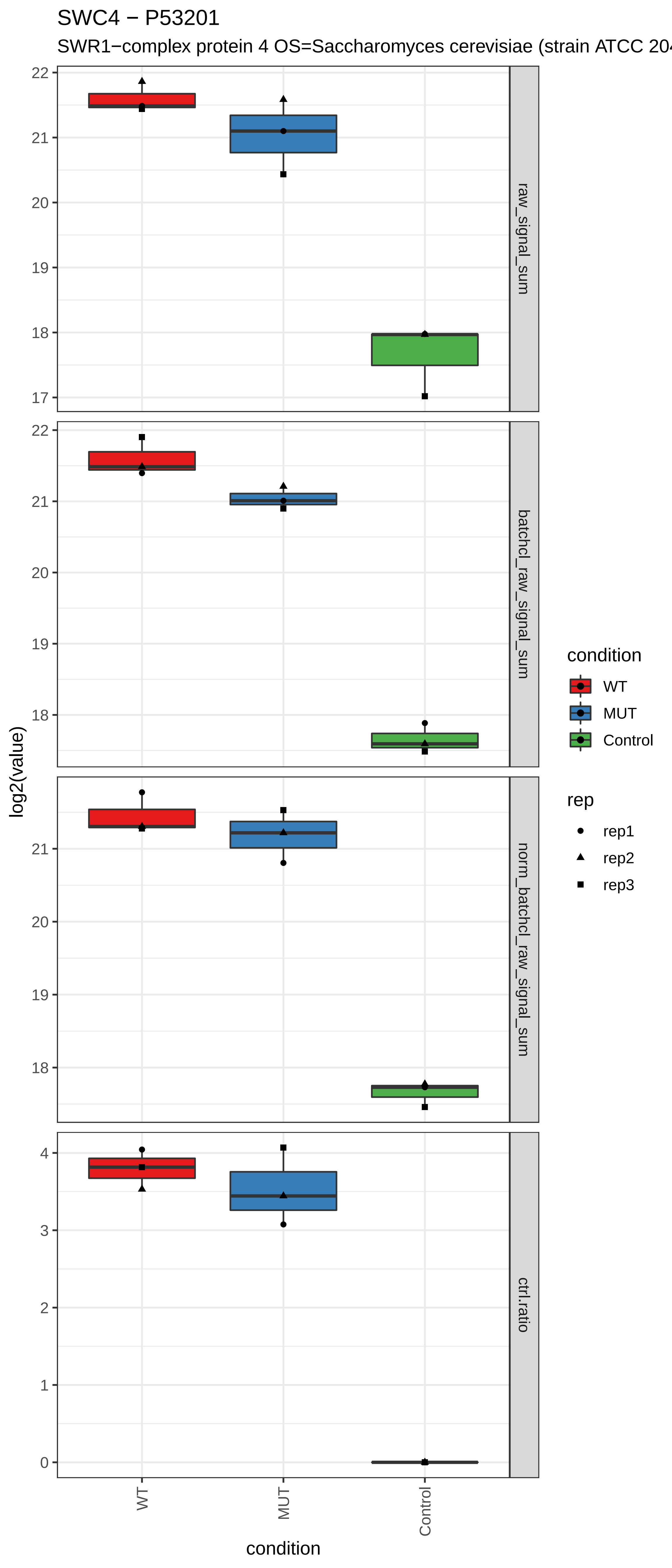

SWC4 − P53201
SWR1−complex protein 4 OS=Saccharomyces cerevisiae (strain ATCC 204
log2(value)
raw_signal_sum
batchcl_raw_signal_sum
norm_batchcl_raw_signal_sum
ctrl.ratio
condition
WT
MUT
Control
rep
rep1
rep2
rep3

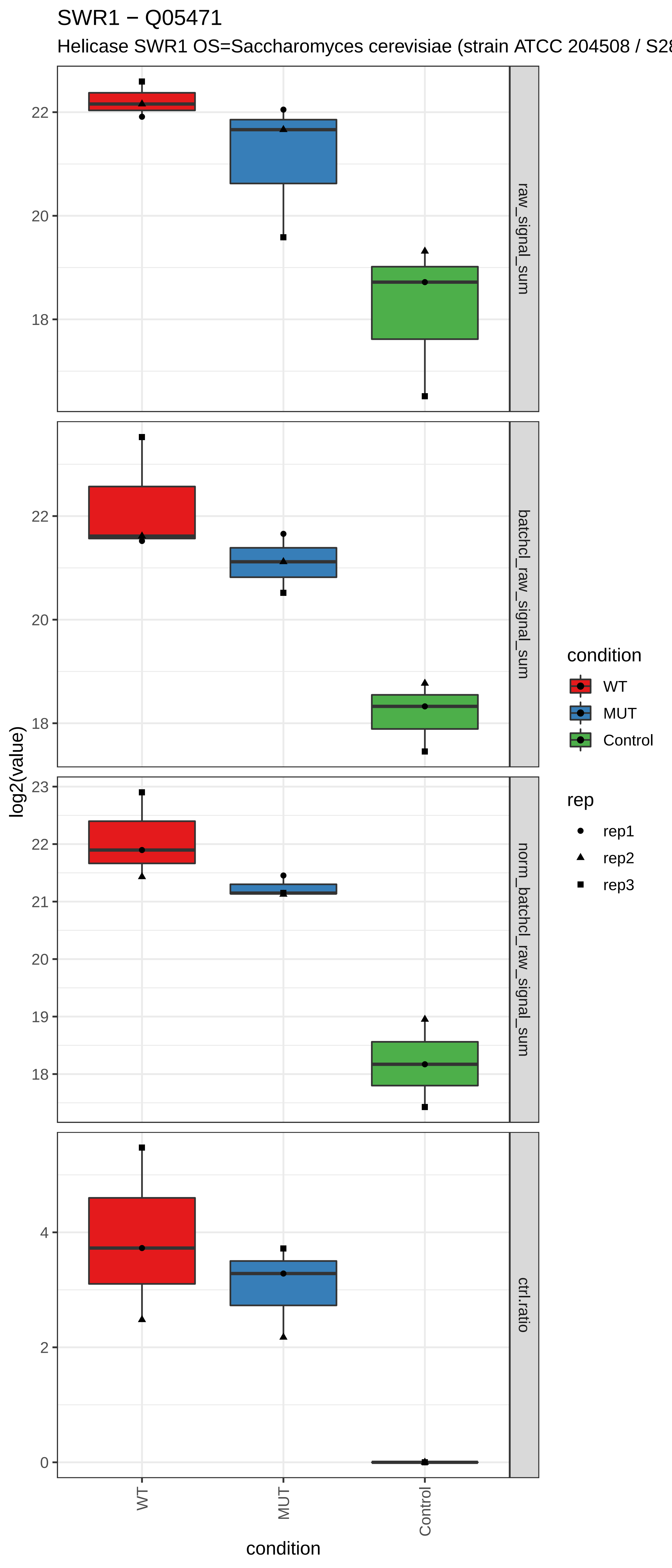

SWR1 − Q05471
Helicase SWR1 OS=Saccharomyces cerevisiae (strain ATCC 204508 / S28
log2(value)
condition
WT
MUT
Control
raw_signal_sum
batchcl_raw_signal_sum
norm_batchcl_raw_signal_sum
ctrl.ratio
22
20
18
23
21
19
4
2
0
condition
WT
MUT
Control
rep
rep1
rep2
rep3

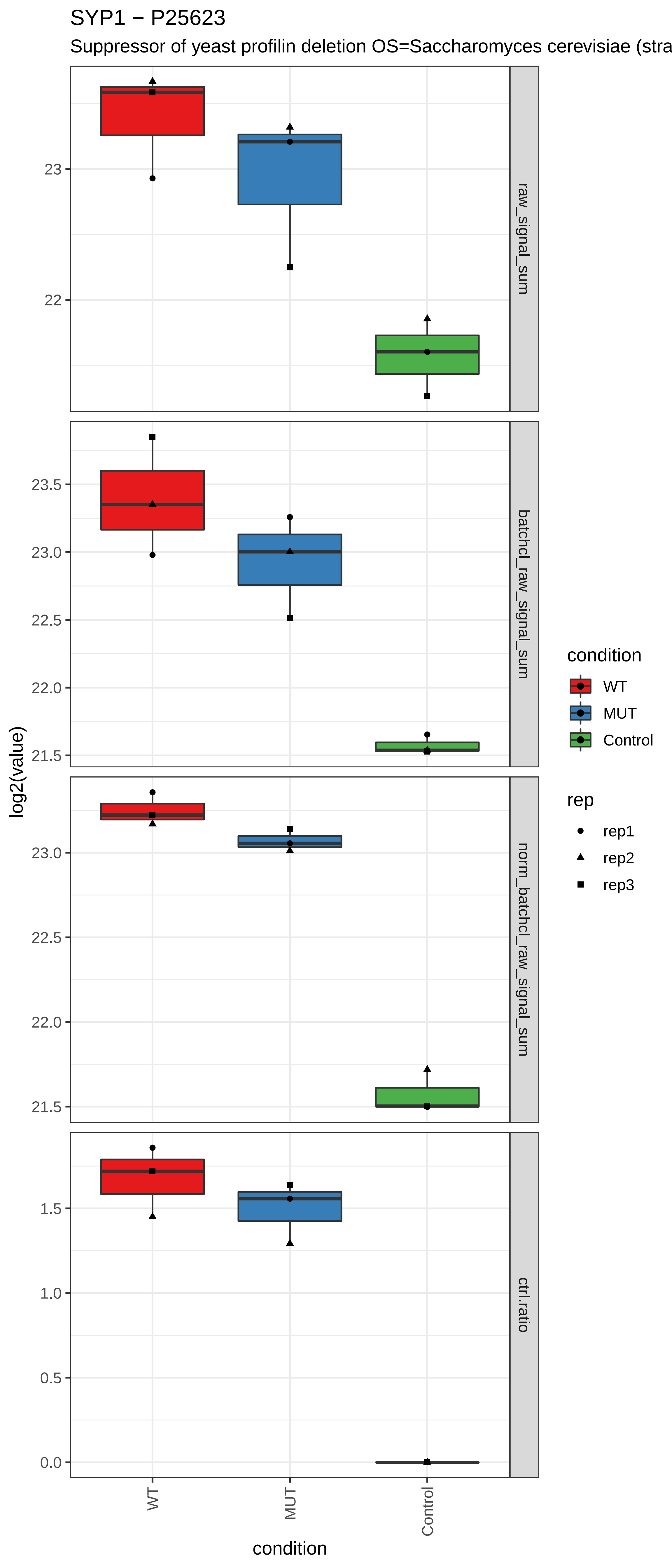

SYP1 − P25623
Suppressor of yeast profilin deletion OS=Saccharomyces cerevisiae (stra
raw_signal_sum
batchcl_raw_signal_sum
norm_batchcl_raw_signal_sum
ctrl.ratio
log2(value)
condition
WT
MUT
Control
rep
rep1
rep2
rep3
23
22
23.5
23.0
22.5
22.0
21.5
1.5
1.0
0.5
0.0

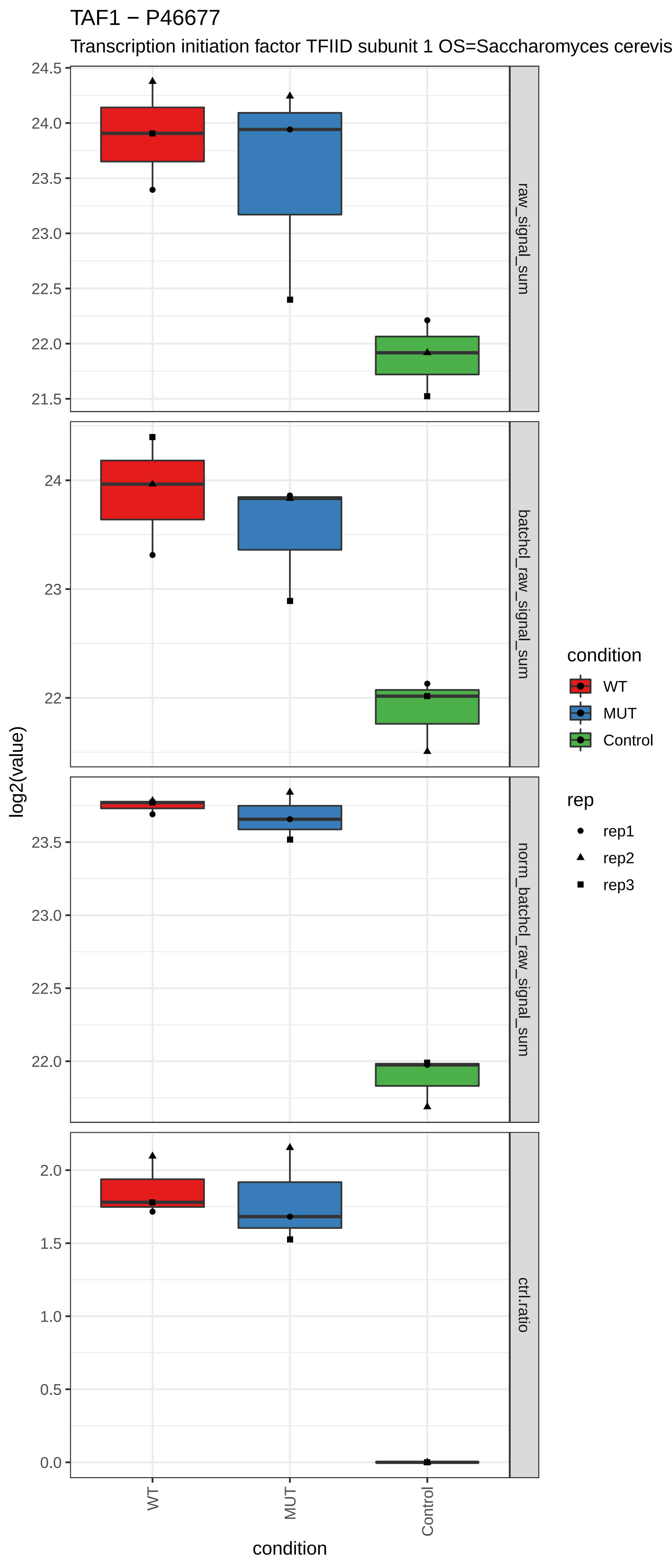

TAF1 − P46677
Transcription initiation factor TFIID subunit 1 OS=Saccharomyces cerevis
raw_signal_sum
batchcl_raw_signal_sum
norm_batchcl_raw_signal_sum
ctrl.ratio
log2(value)
condition
WT
MUT
Control
rep
rep1
rep2
rep3

TAF14 − P35189

Transcription initiation factor TFIID subunit 14 OS=Saccharomyces cerevisi

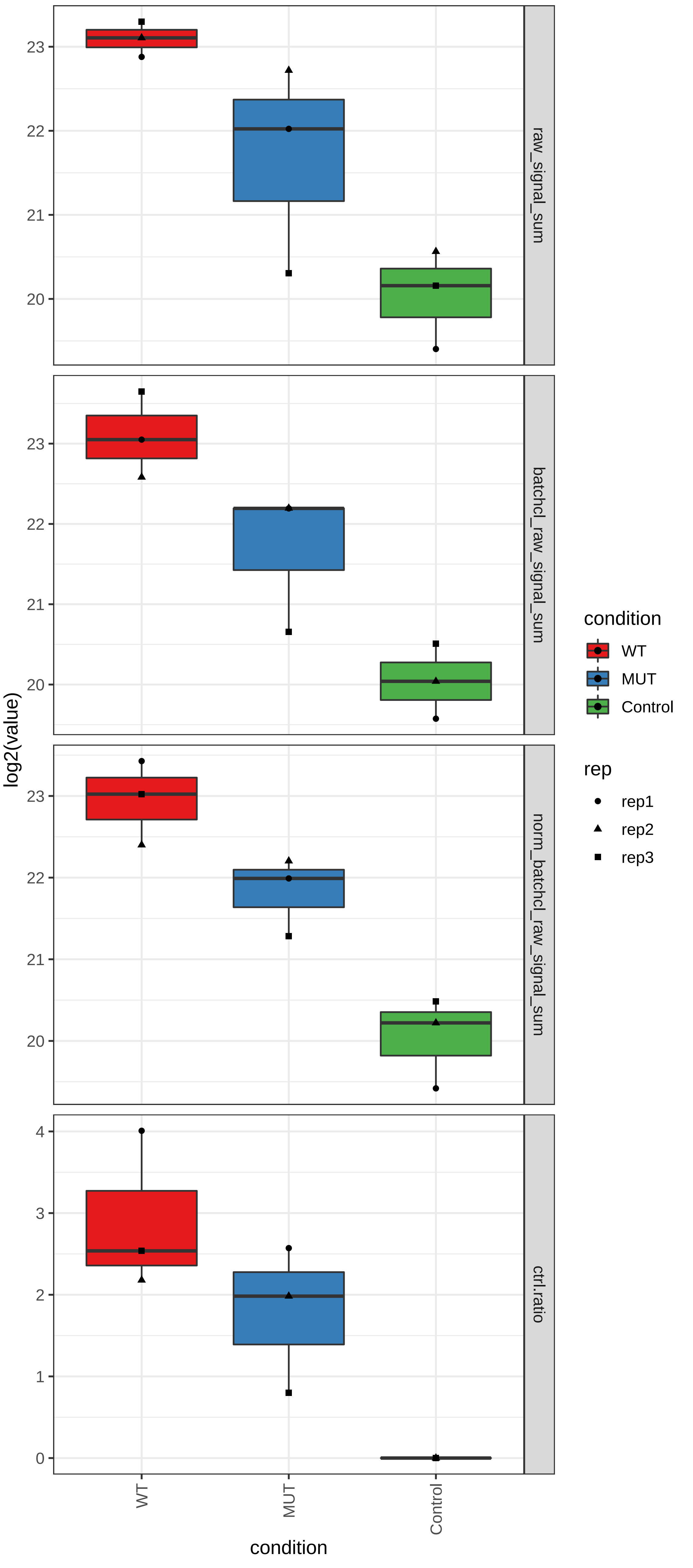

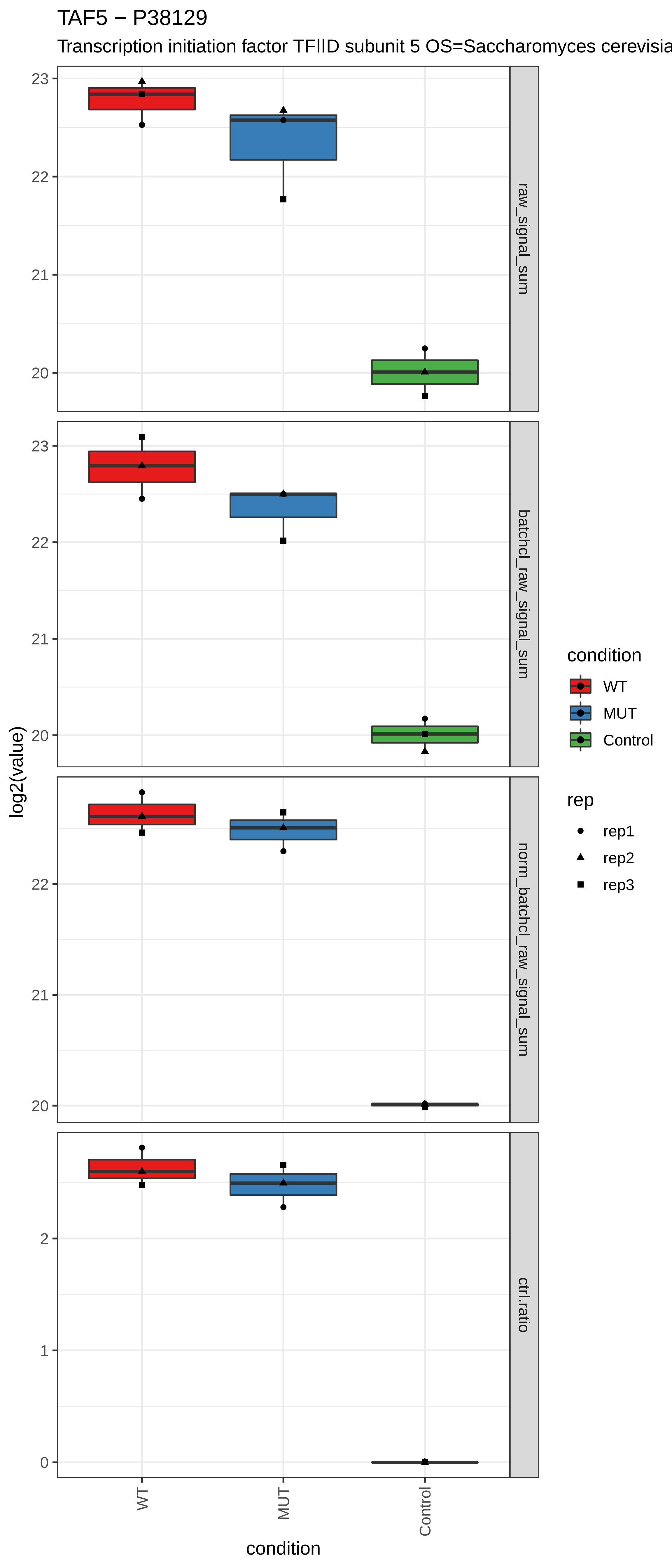
TAF5 − P38129
Transcription initiation factor TFIID subunit 5 OS=Saccharomyces cerevisia
raw_signal_sum
batchcl_raw_signal_sum
norm_batchcl_raw_signal_sum
ctrl.ratio
log2(value)
23
22
21
20
2
1
0
WT
MUT
Control
condition
condition
WT
MUT
Control
rep
rep1
rep2
rep3

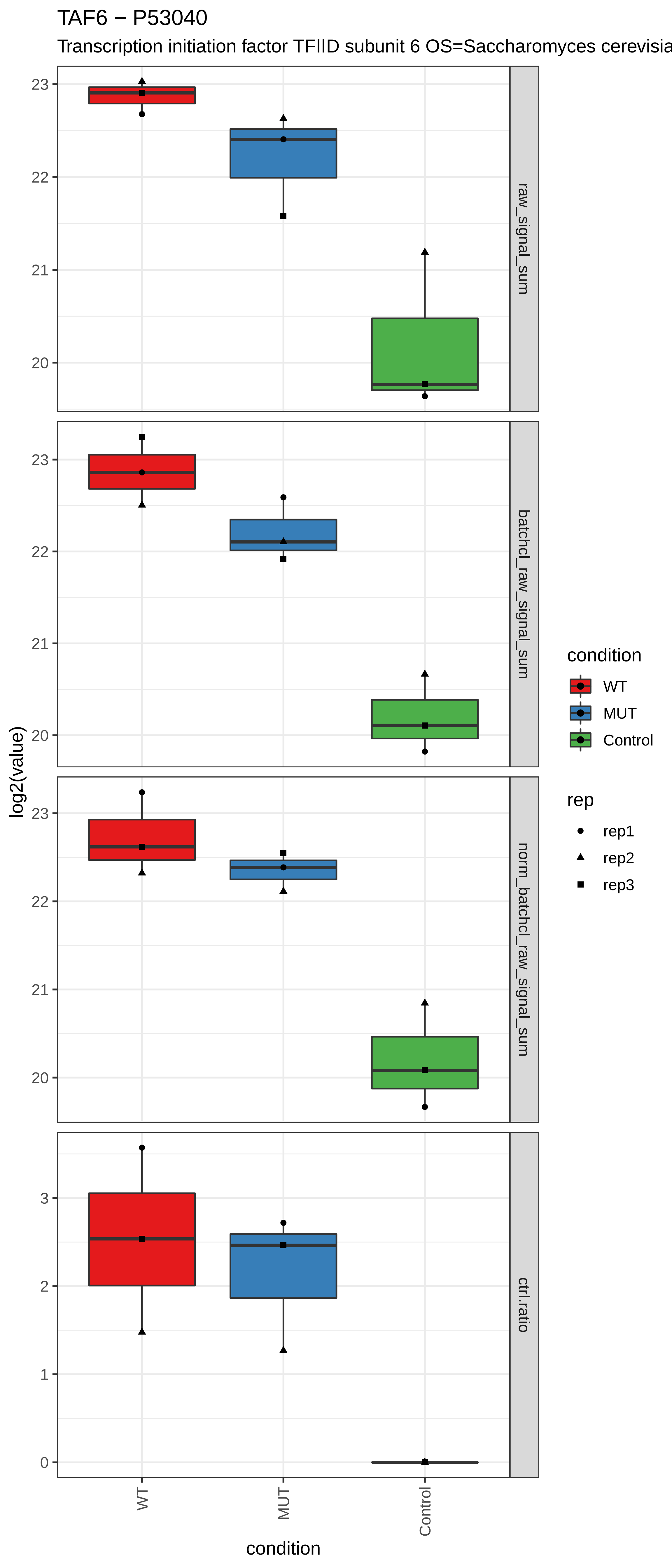

TAF6 − P53040
Transcription initiation factor TFIID subunit 6 OS=Saccharomyces cerevisia
raw_signal_sum
batchcl_raw_signal_sum
norm_batchcl_raw_signal_sum
ctrl.ratio
log2(value)
condition
WT
MUT
Control
rep
rep1
rep2
rep3
23
22
21
20
3
2
1
0

TAL1 − P15019

Transaldolase OS=Saccharomyces cerevisiae (strain ATCC 204508 / S288

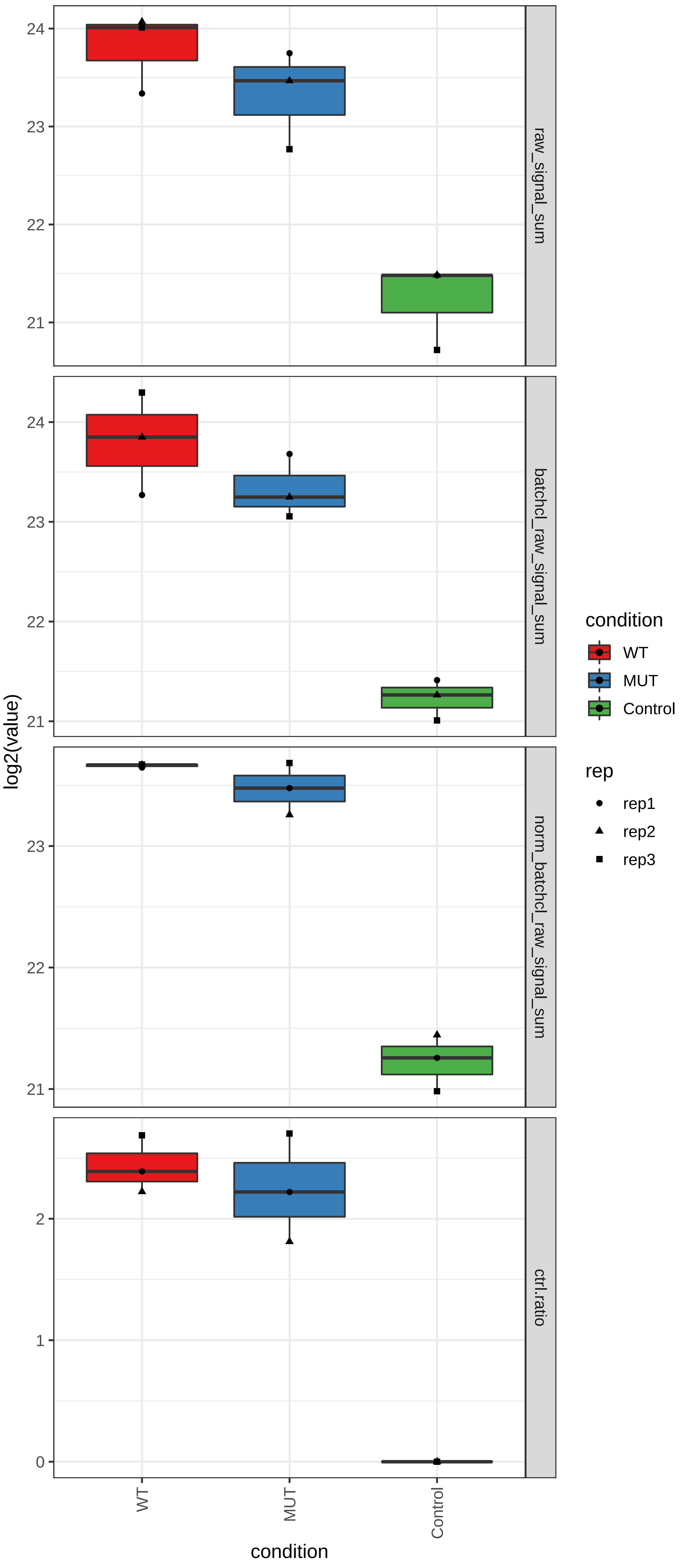

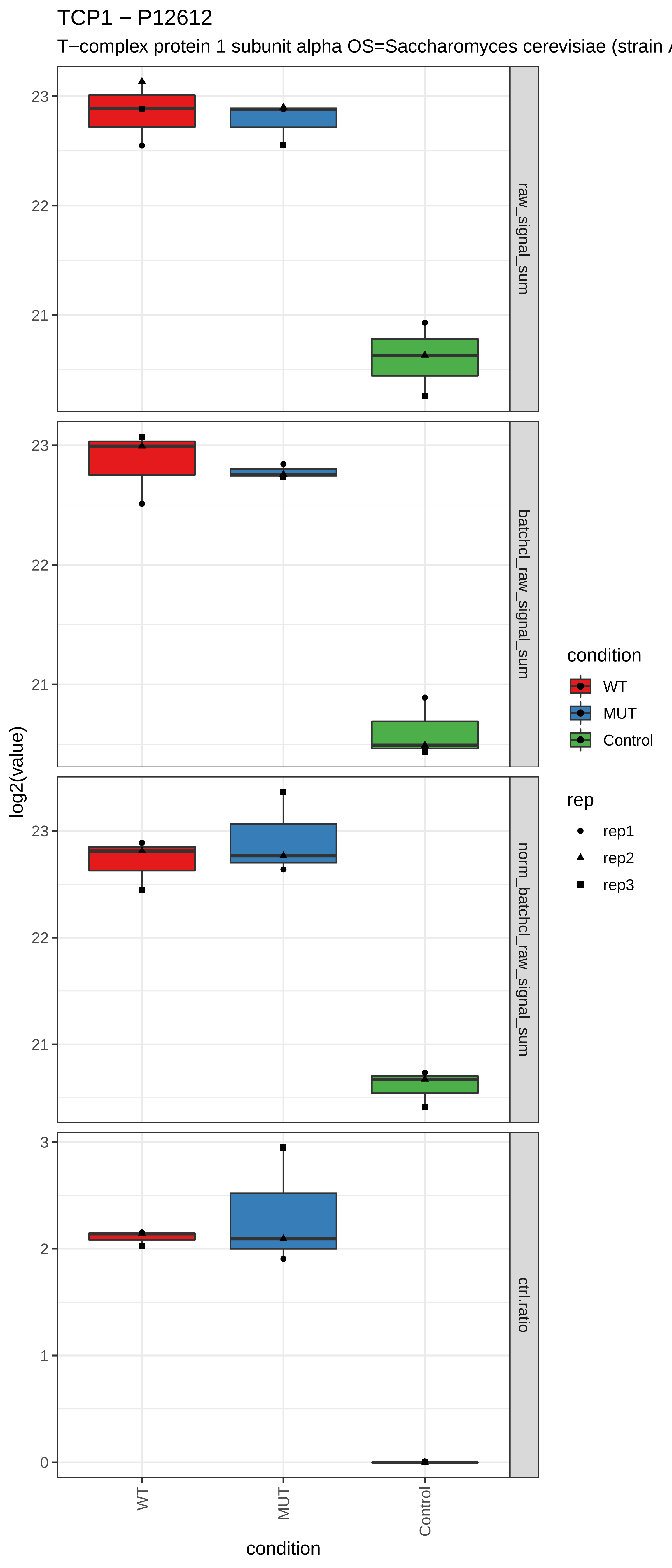

TCP1 − P12612
T−complex protein 1 subunit alpha OS=Saccharomyces cerevisiae (strain
raw_signal_sum
batchcl_raw_signal_sum
norm_batchcl_raw_signal_sum
ctrl.ratio
log2(value)
condition
WT
MUT
Control
rep
rep1
rep2
rep3

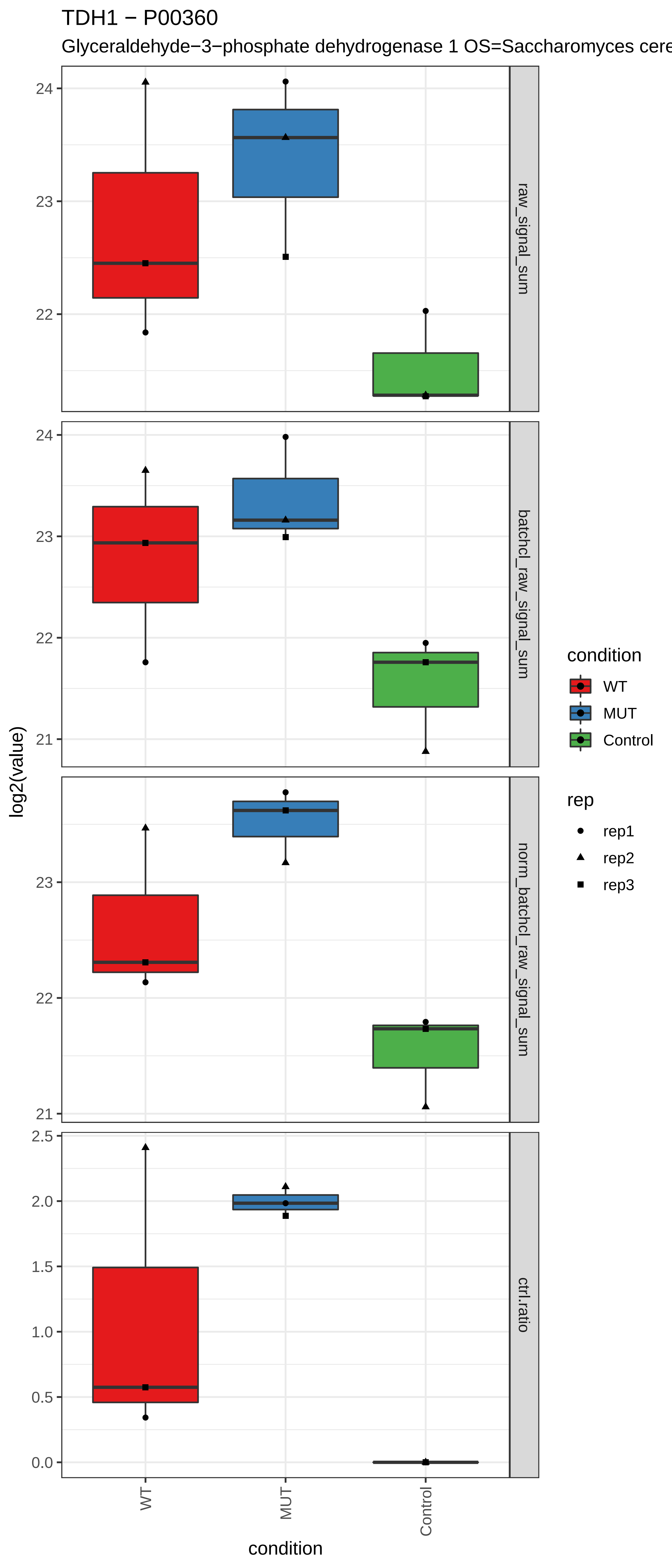
TDH1 − P00360
Glyceraldehyde−3−phosphate dehydrogenase 1 OS=Saccharomyces cere
raw_signal_sum
batchcl_raw_signal_sum
norm_batchcl_raw_signal_sum
ctrl.ratio
log2(value)
condition
WT
MUT
Control
rep
rep1
rep2
rep3
24
23
22
21
2.5
2.0
1.5
1.0
0.5
0.0

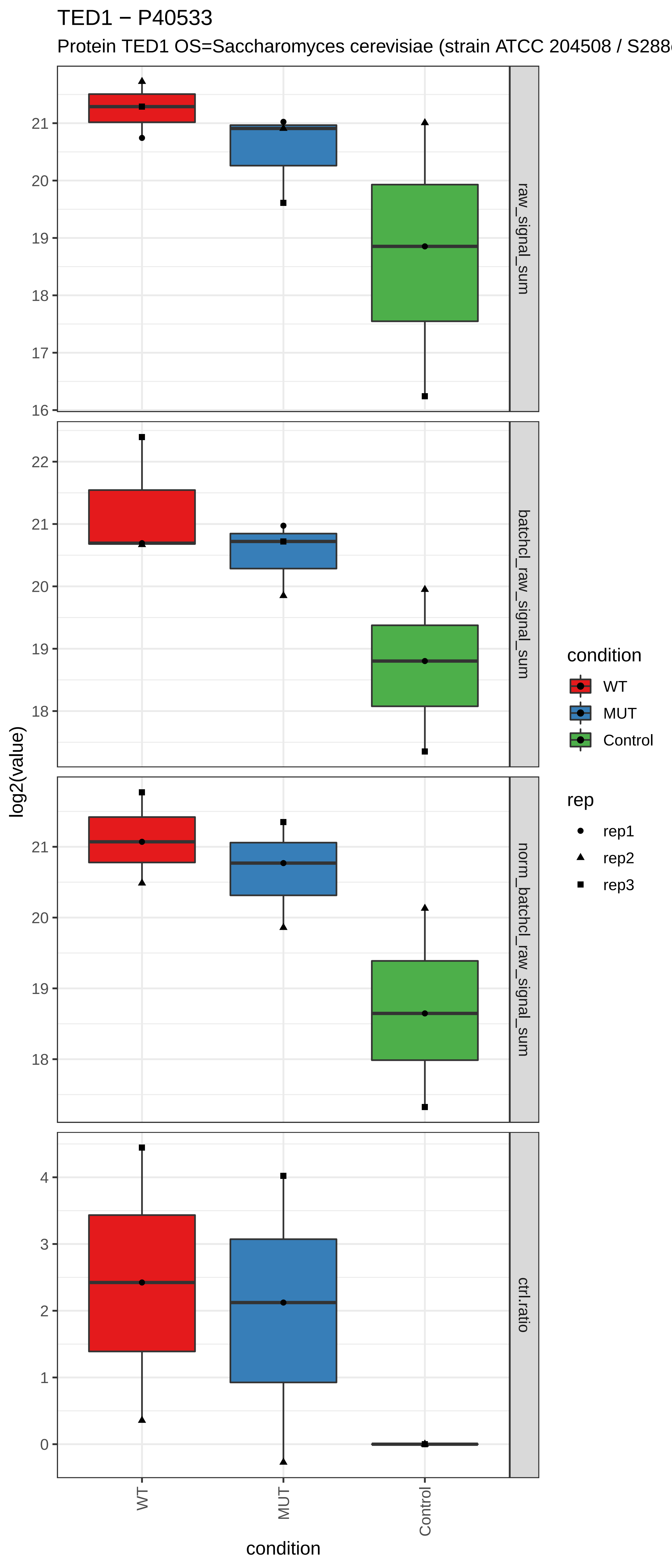

TED1 − P40533
Protein TED1 OS=Saccharomyces cerevisiae (strain ATCC 204508 / S288c
raw_signal_sum
batchcl_raw_signal_sum
norm_batchcl_raw_signal_sum
ctrl.ratio
log2(value)
condition
WT
MUT
Control
rep
rep1
rep2
rep3

TEF1 − P02994

Elongation factor 1−alpha OS=Saccharomyces cerevisiae (strain ATCC 2

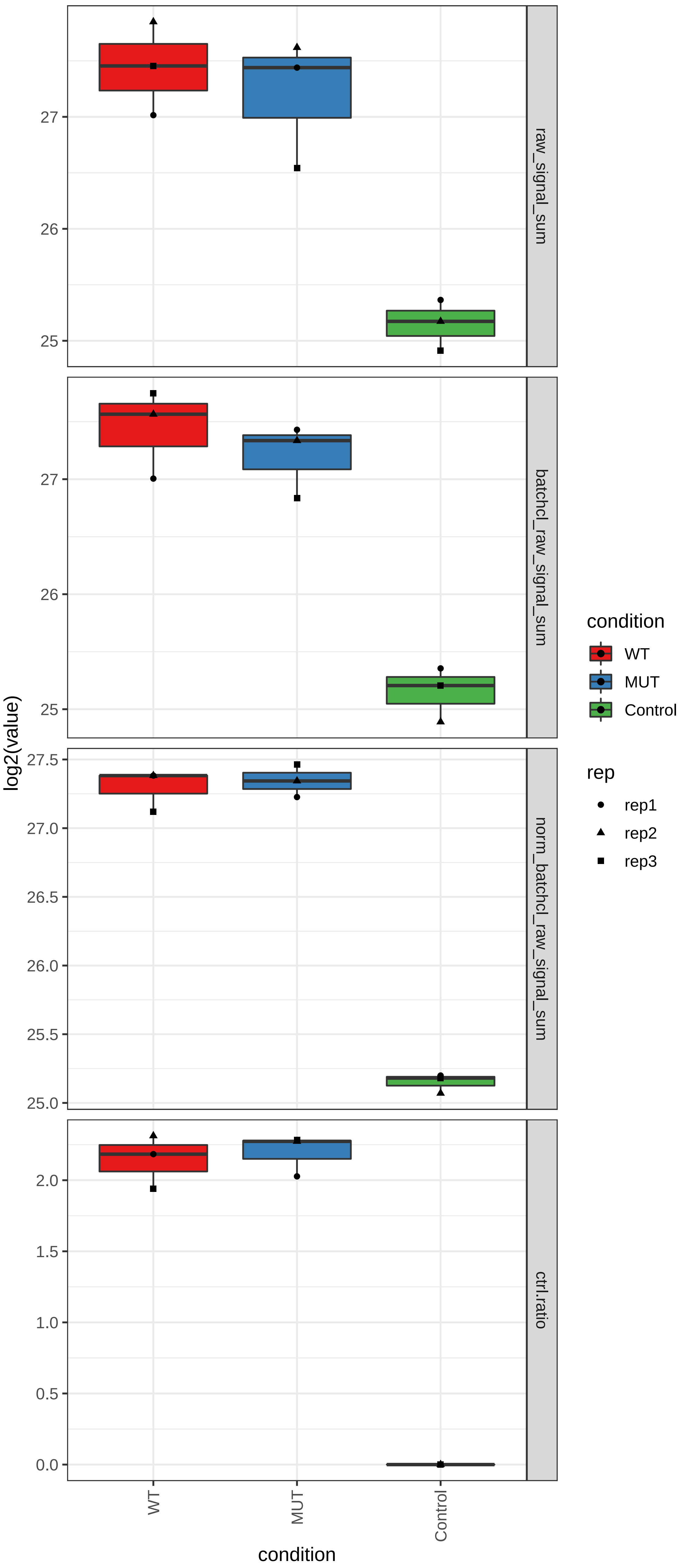

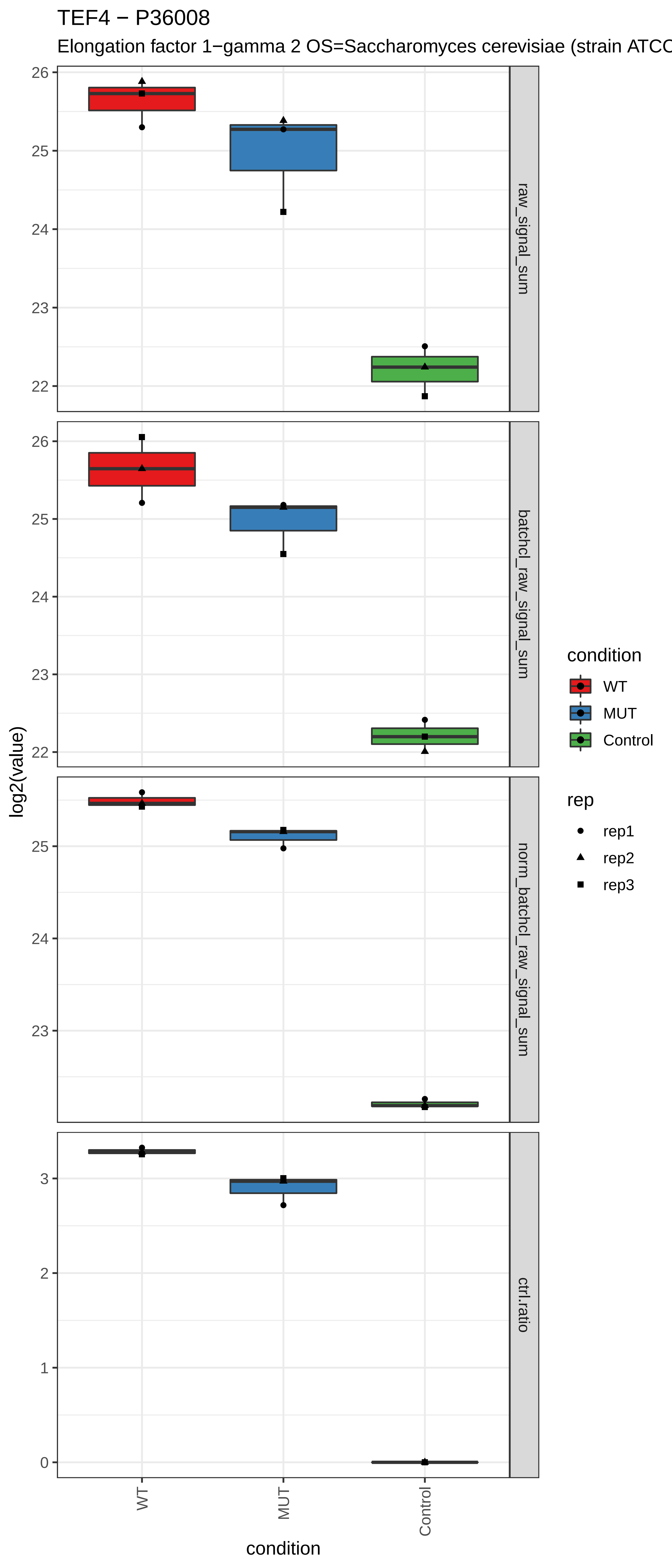

TEF4 − P36008
Elongation factor 1−gamma 2 OS=Saccharomyces cerevisiae (strain ATCC
raw_signal_sum
batchcl_raw_signal_sum
norm_batchcl_raw_signal_sum
ctrl.ratio
log2(value)
condition
WT
MUT
Control
rep
rep1
rep2
rep3

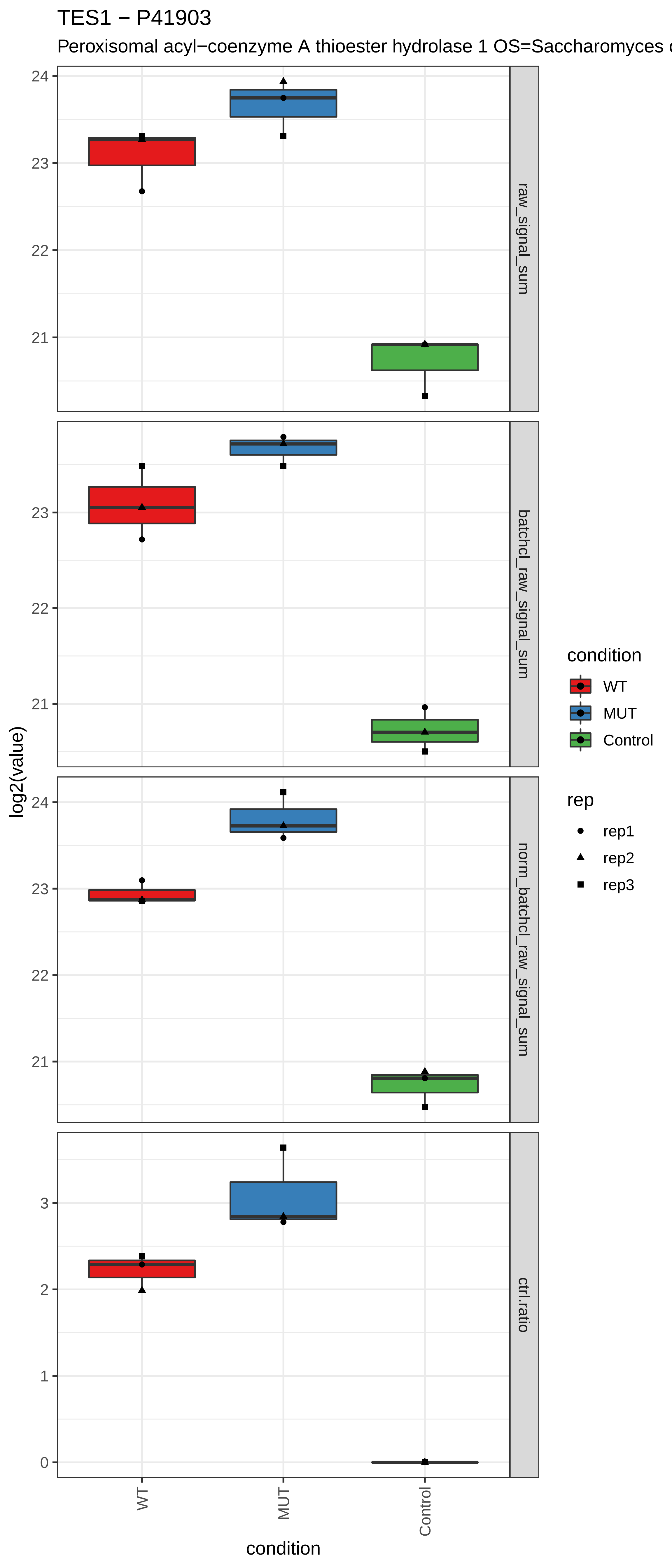

TES1 − P41903
Peroxisomal acyl−coenzyme A thioester hydrolase 1 OS=Saccharomyces c
raw_signal_sum
batchcl_raw_signal_sum
norm_batchcl_raw_signal_sum
ctrl.ratio
log2(value)
condition
WT
MUT
Control
rep
rep1
rep2
rep3

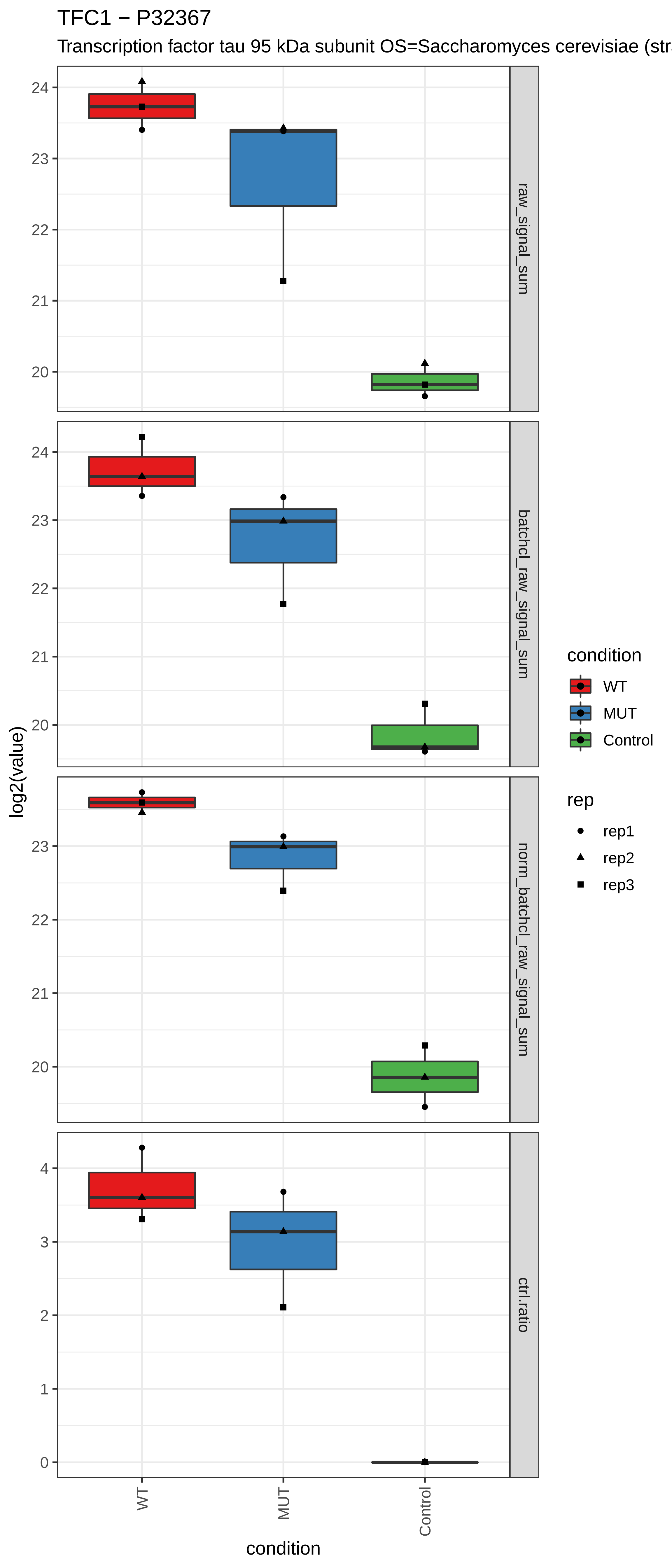

TFC1 − P32367
Transcription factor tau 95 kDa subunit OS=Saccharomyces cerevisiae (str
raw_signal_sum
batchcl_raw_signal_sum
norm_batchcl_raw_signal_sum
ctrl.ratio
log2(value)
condition
WT
MUT
Control
rep
rep1
rep2
rep3

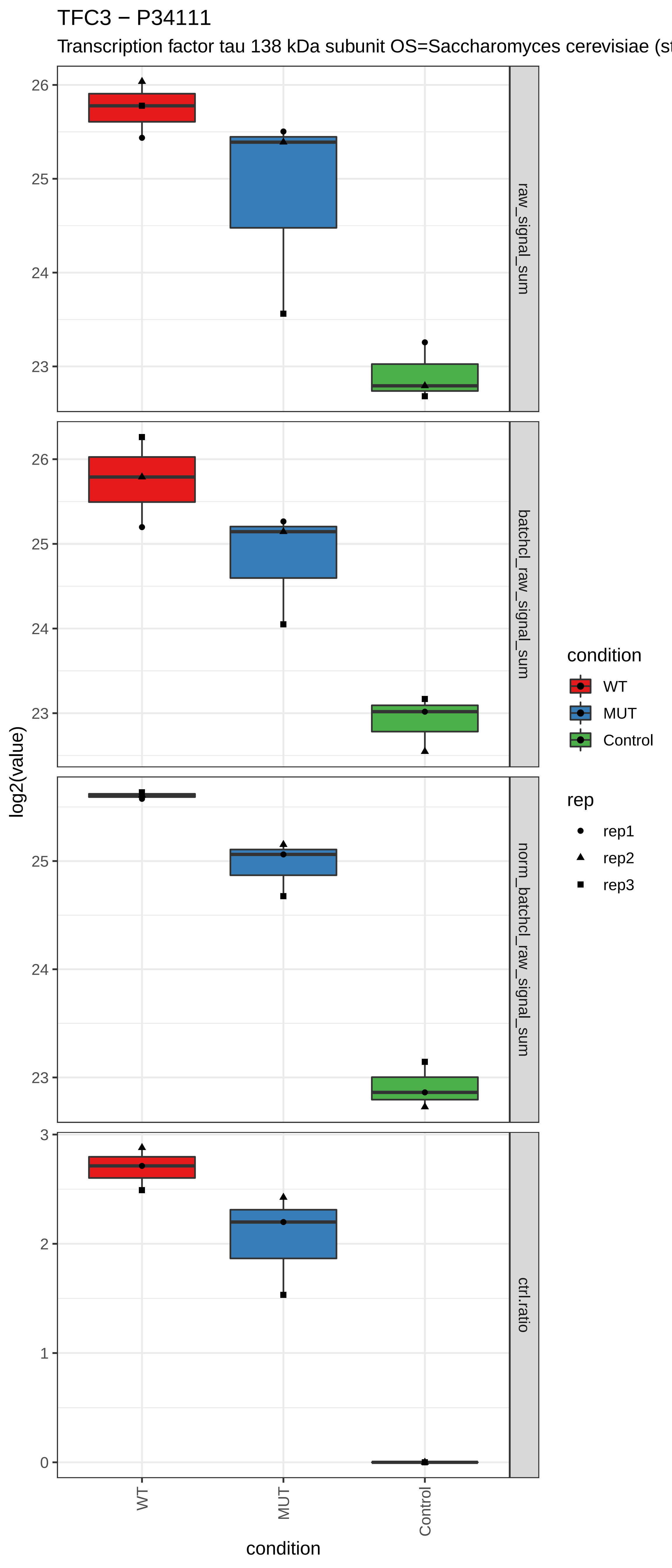

TFC3 − P34111
Transcription factor tau 138 kDa subunit OS=Saccharomyces cerevisiae (st
raw_signal_sum
batchcl_raw_signal_sum
norm_batchcl_raw_signal_sum
ctrl.ratio
log2(value)
condition
WT
MUT
Control
rep
rep1
rep2
rep3
26
25
24
23
3
2
1
0

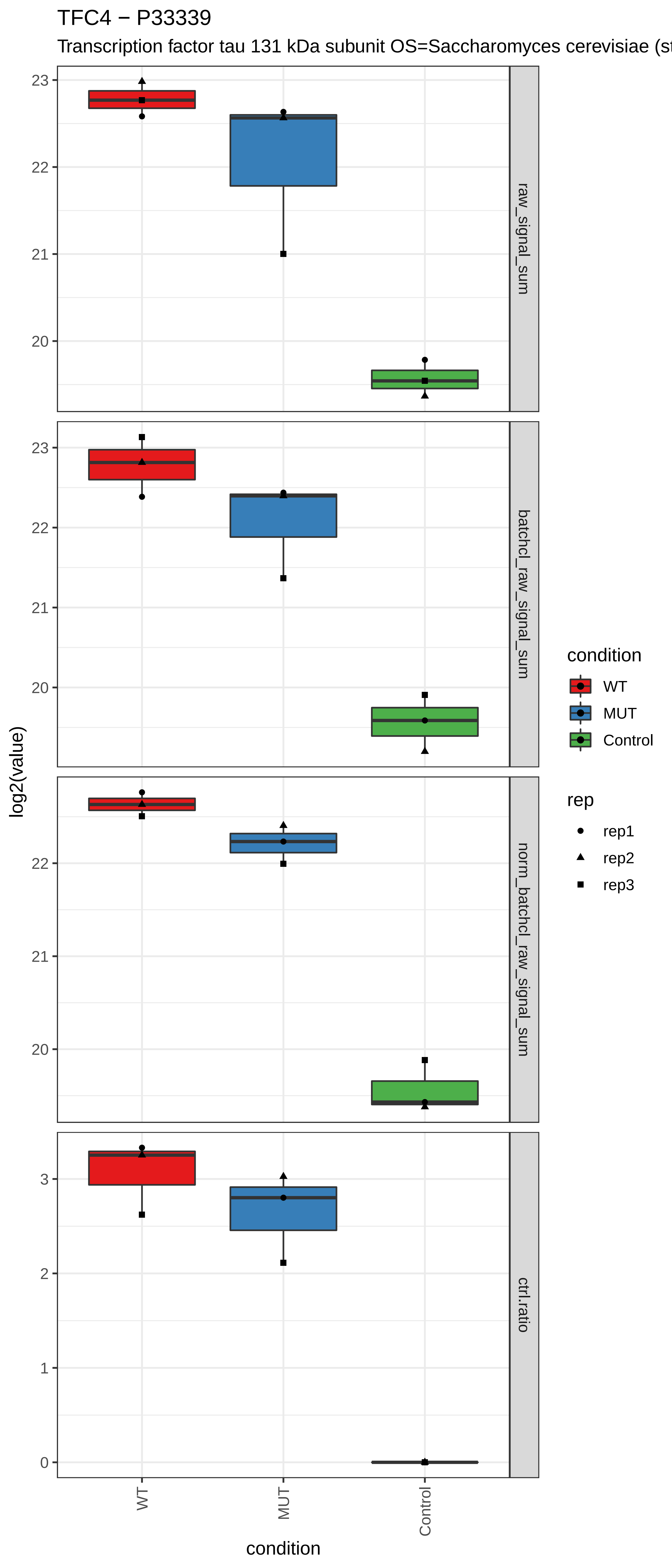

TFC4 − P33339
Transcription factor tau 131 kDa subunit OS=Saccharomyces cerevisiae (st
raw_signal_sum
batchcl_raw_signal_sum
norm_batchcl_raw_signal_sum
ctrl.ratio
log2(value)
condition
WT
MUT
Control
rep
rep1
rep2
rep3

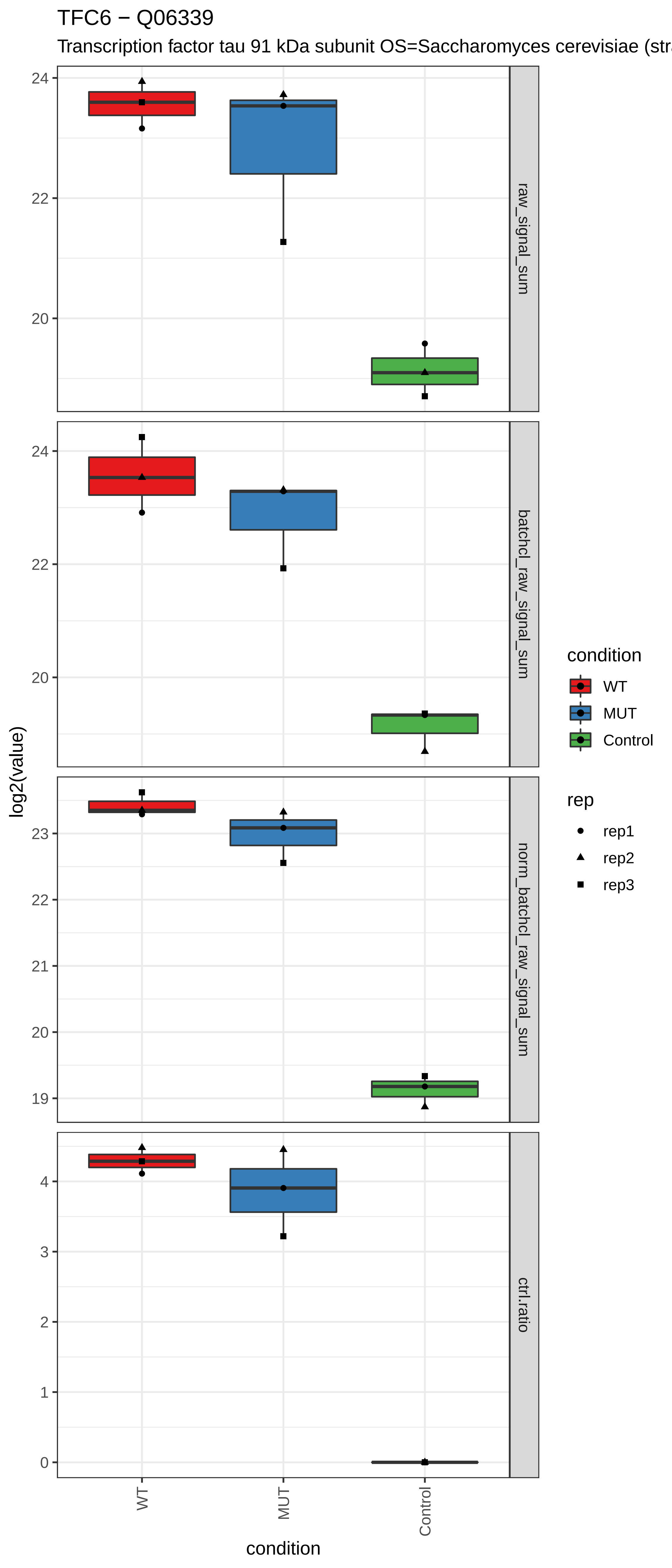

TFC6 − Q06339
Transcription factor tau 91 kDa subunit OS=Saccharomyces cerevisiae (str
raw_signal_sum
batchcl_raw_signal_sum
norm_batchcl_raw_signal_sum
ctrl.ratio
log2(value)
condition
WT
MUT
Control
rep
rep1
rep2
rep3
24
22
20
19
23
21
4
3
2
1
0

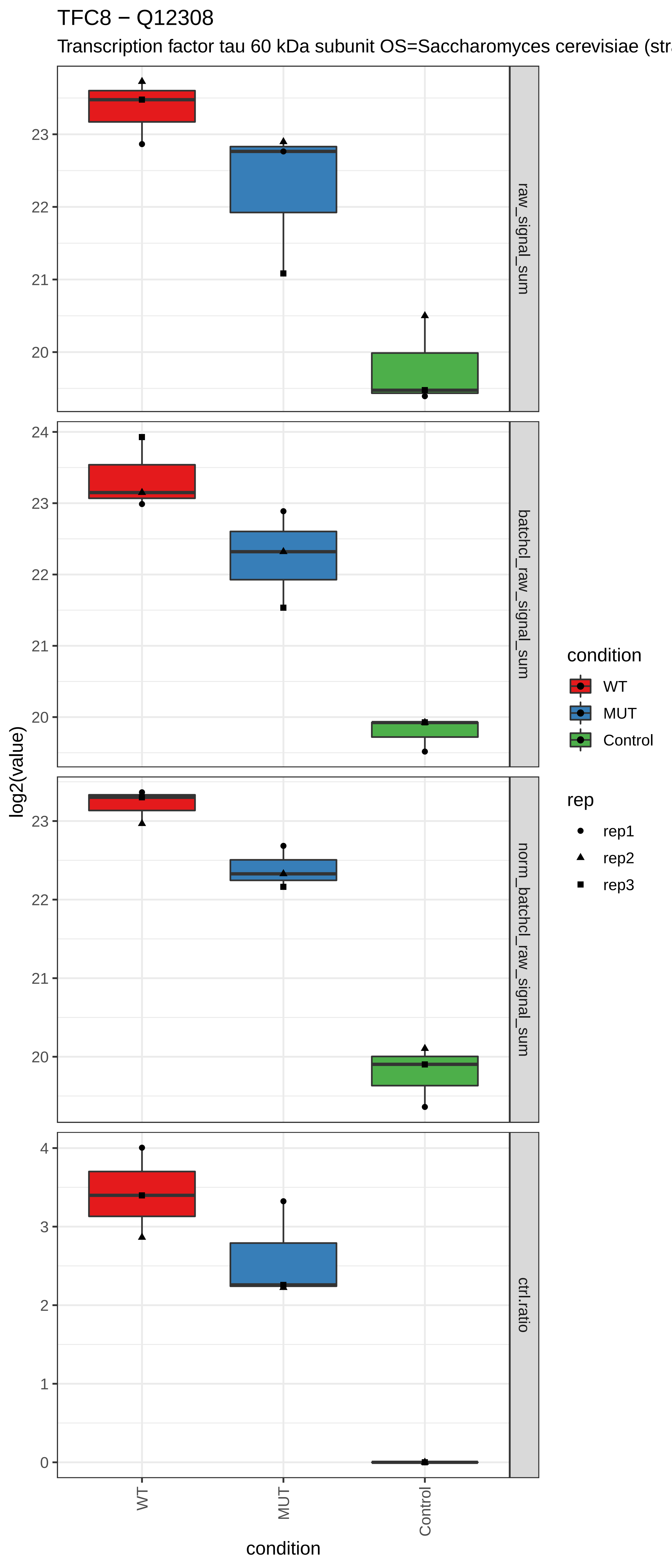
TFC8 − Q12308
Transcription factor tau 60 kDa subunit OS=Saccharomyces cerevisiae (str
raw_signal_sum
batchcl_raw_signal_sum
norm_batchcl_raw_signal_sum
ctrl.ratio
log2(value)
condition
WT
MUT
Control
rep
rep1
rep2
rep3

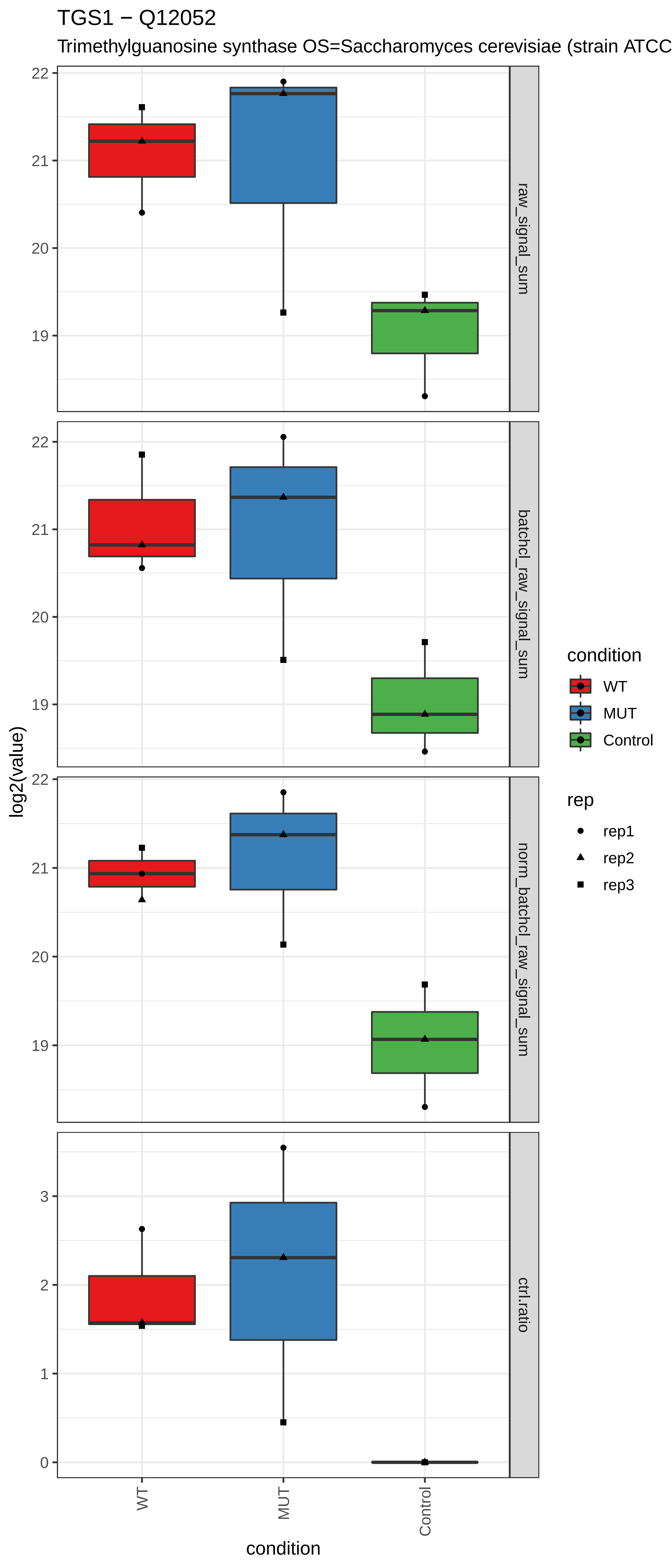

TGS1 − Q12052
Trimethylguanosine synthase OS=Saccharomyces cerevisiae (strain ATCC
raw_signal_sum
batchcl_raw_signal_sum
norm_batchcl_raw_signal_sum
ctrl.ratio
log2(value)
condition
WT
MUT
Control
rep
rep1
rep2
rep3
22
21
20
19
3
2
1
0

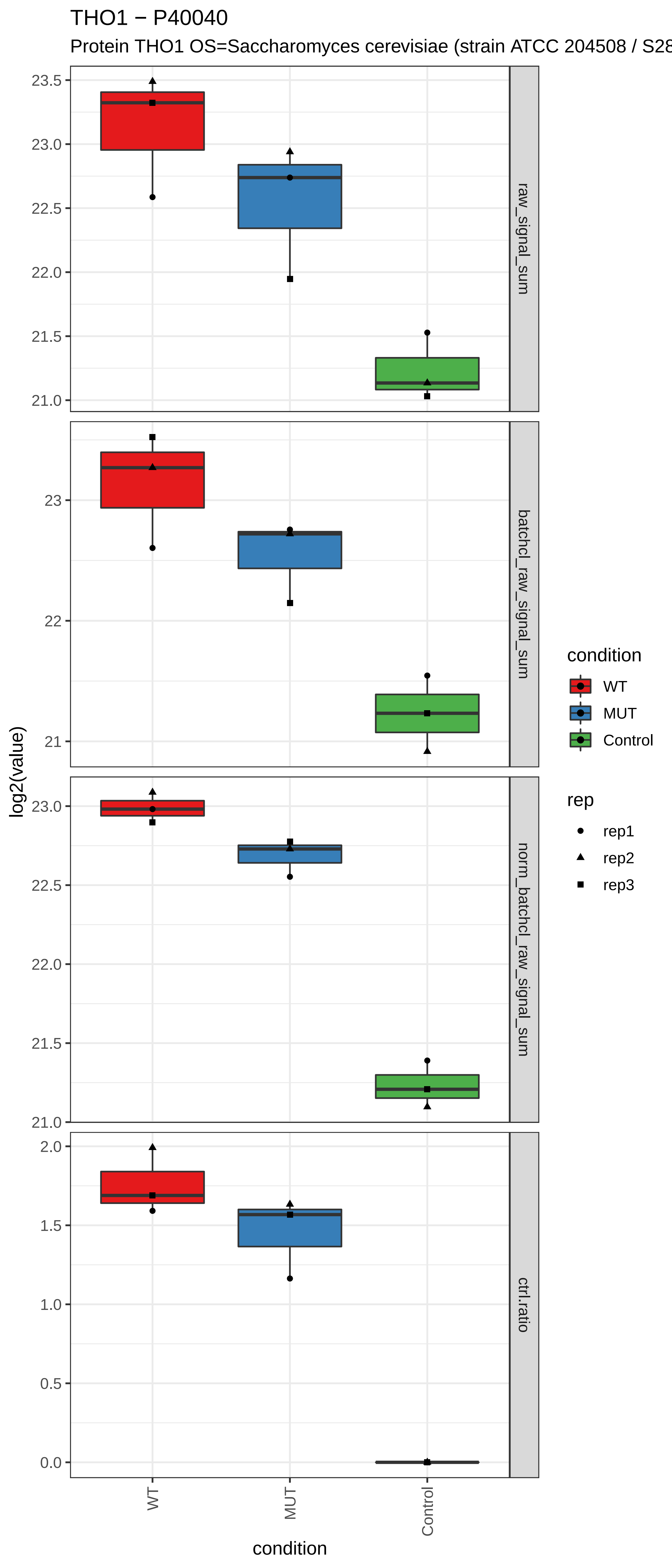
THO1 − P40040
Protein THO1 OS=Saccharomyces cerevisiae (strain ATCC 204508 / S28
raw_signal_sum
batchcl_raw_signal_sum
norm_batchcl_raw_signal_sum
ctrl.ratio
log2(value)
condition
WT
MUT
Control
rep
rep1
rep2
rep3
23.5
23.0
22.5
22.0
21.5
21.0
23
22
21
2.0
1.5
1.0
0.5
0.0

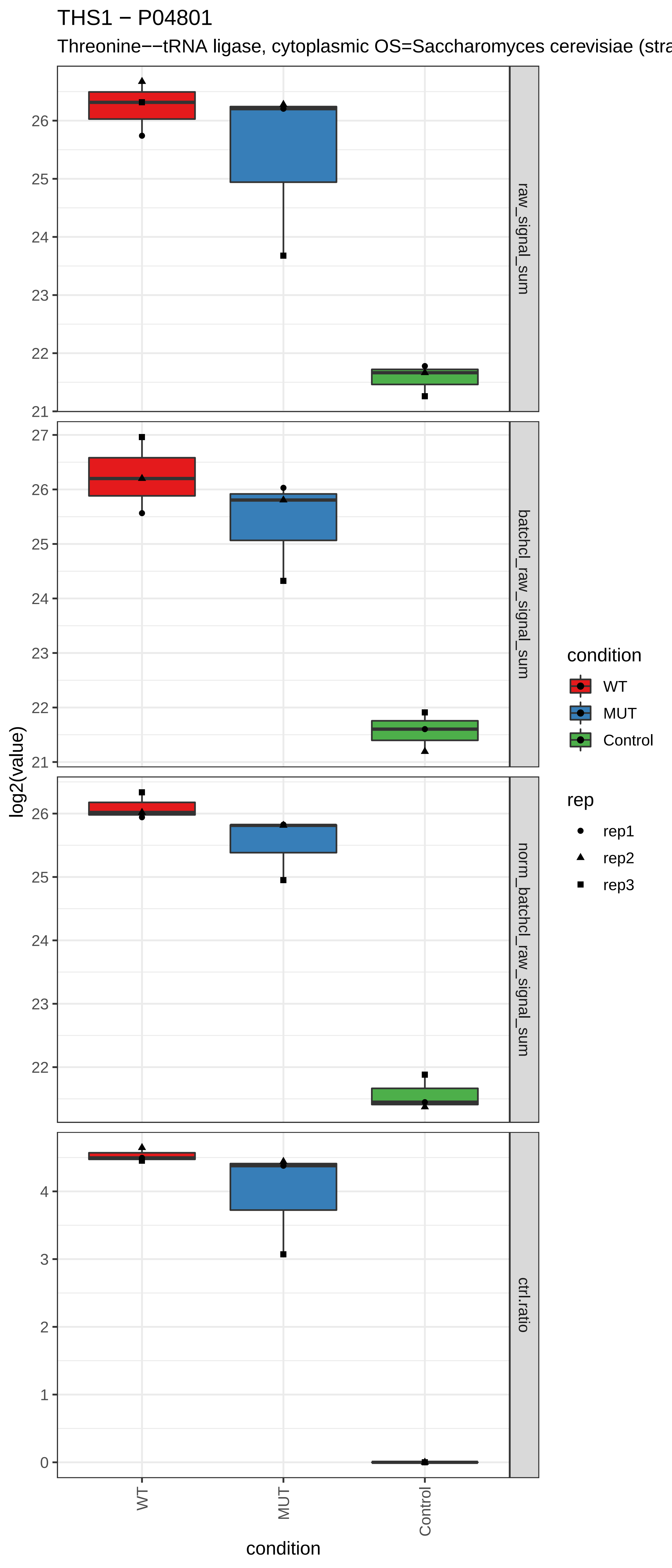
THS1 − P04801
Threonine−−tRNA ligase, cytoplasmic OS=Saccharomyces cerevisiae (stra
raw_signal_sum
batchcl_raw_signal_sum
norm_batchcl_raw_signal_sum
ctrl.ratio
log2(value)
condition
WT
MUT
Control
rep
rep1
rep2
rep3

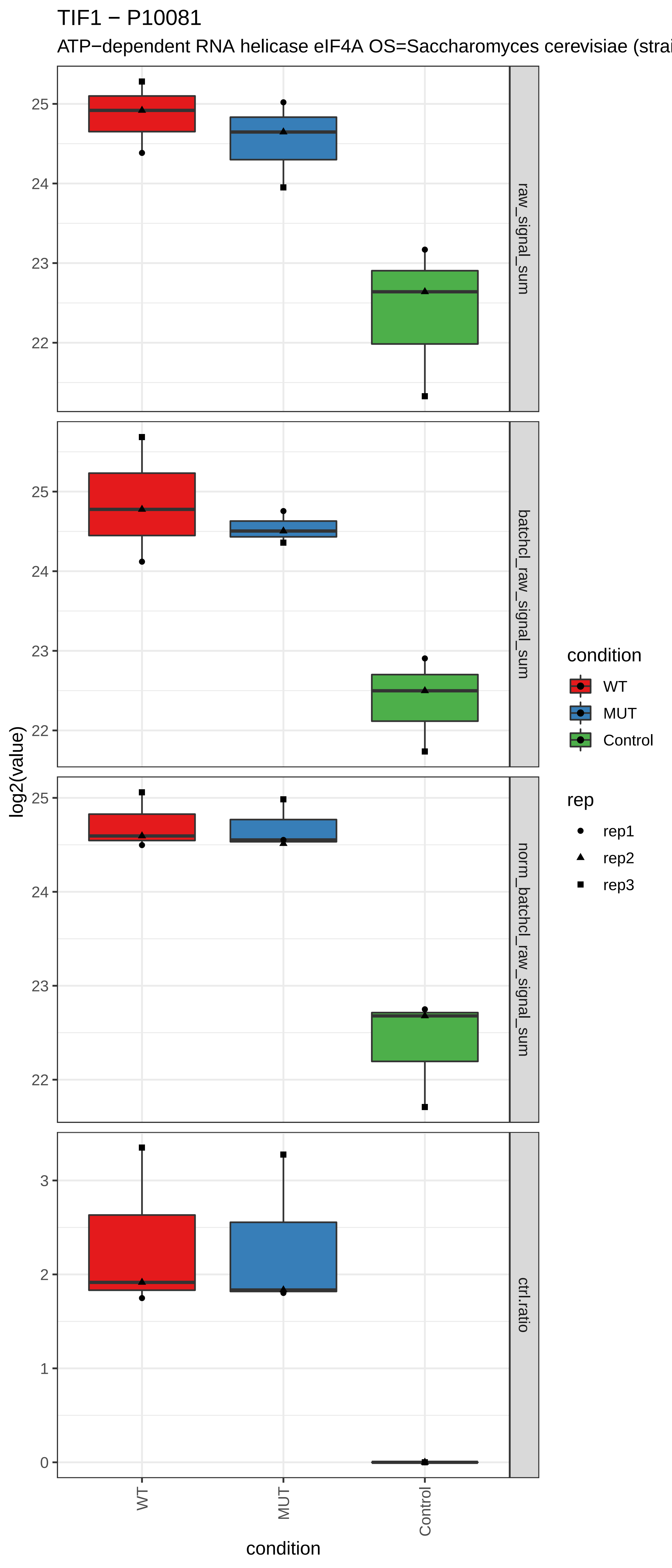
TIF1 − P10081
ATP−dependent RNA helicase eIF4A OS=Saccharomyces cerevisiae (strai
raw_signal_sum
batchcl_raw_signal_sum
norm_batchcl_raw_signal_sum
ctrl.ratio
log2(value)
condition
WT
MUT
Control
rep
rep1
rep2
rep3
25
24
23
22
3
2
1
0

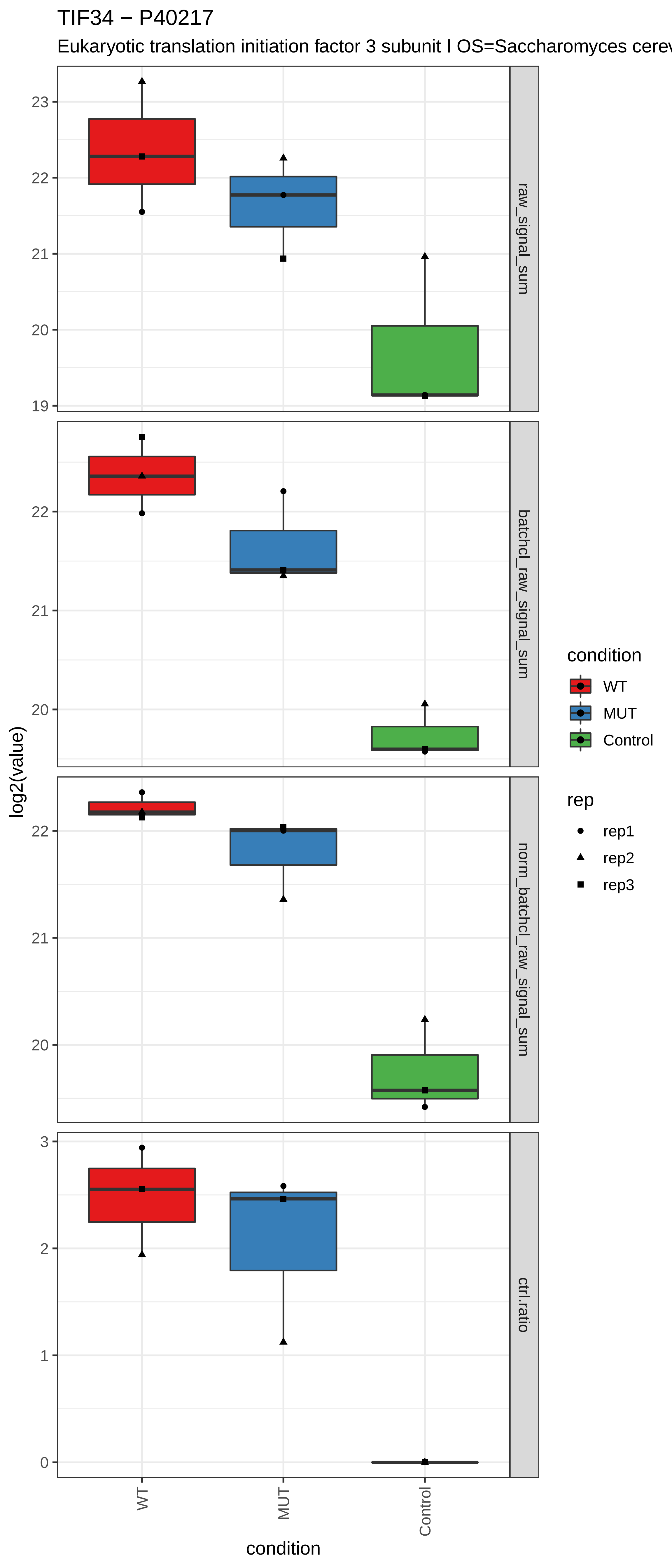
TIF34 − P40217
Eukaryotic translation initiation factor 3 subunit I OS=Saccharomyces cerev
raw_signal_sum
batchcl_raw_signal_sum
norm_batchcl_raw_signal_sum
ctrl.ratio
log2(value)
condition
WT
MUT
Control
rep
rep1
rep2
rep3
23
22
21
20
19
3
2
1
0

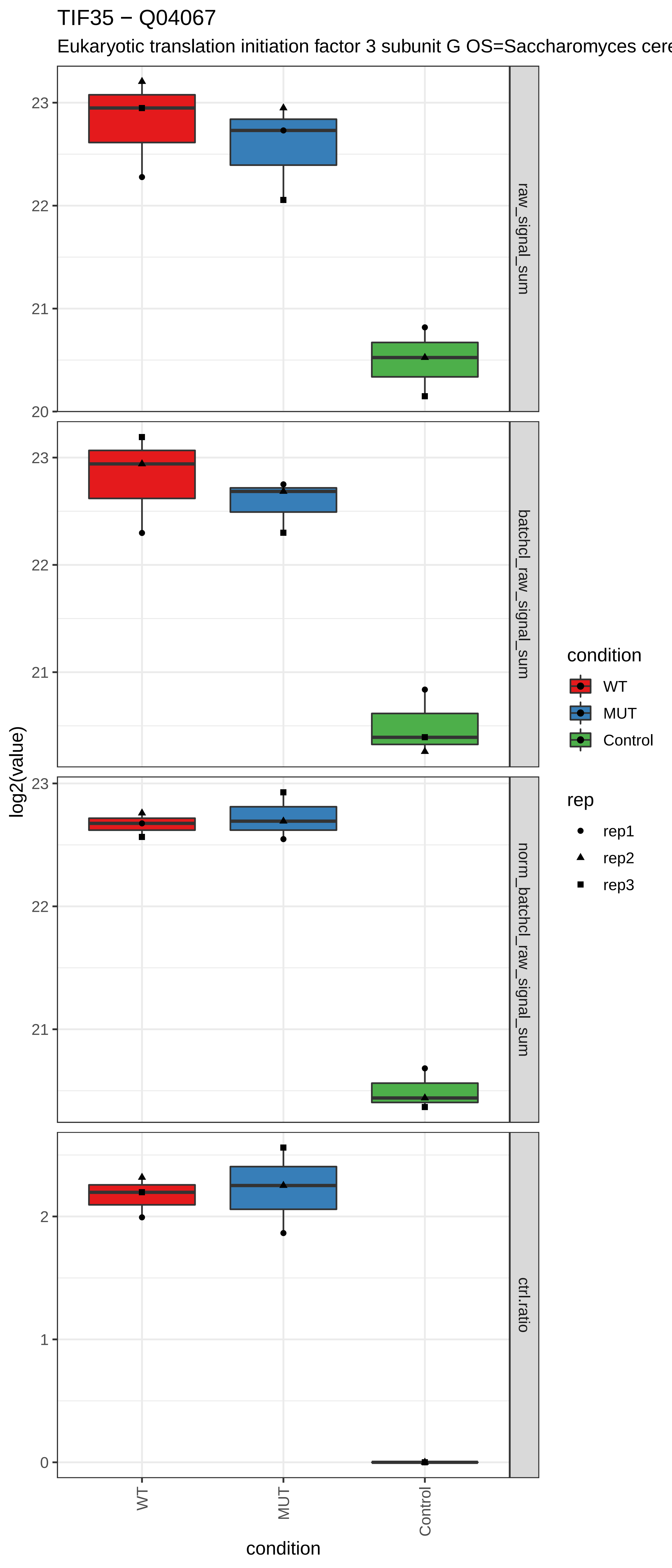
TIF35 − Q04067
Eukaryotic translation initiation factor 3 subunit G OS=Saccharomyces cere
raw_signal_sum
batchcl_raw_signal_sum
norm_batchcl_raw_signal_sum
ctrl.ratio
log2(value)
condition
WT
MUT
Control
rep
rep1
rep2
rep3
23
22
21
20
2
1
0

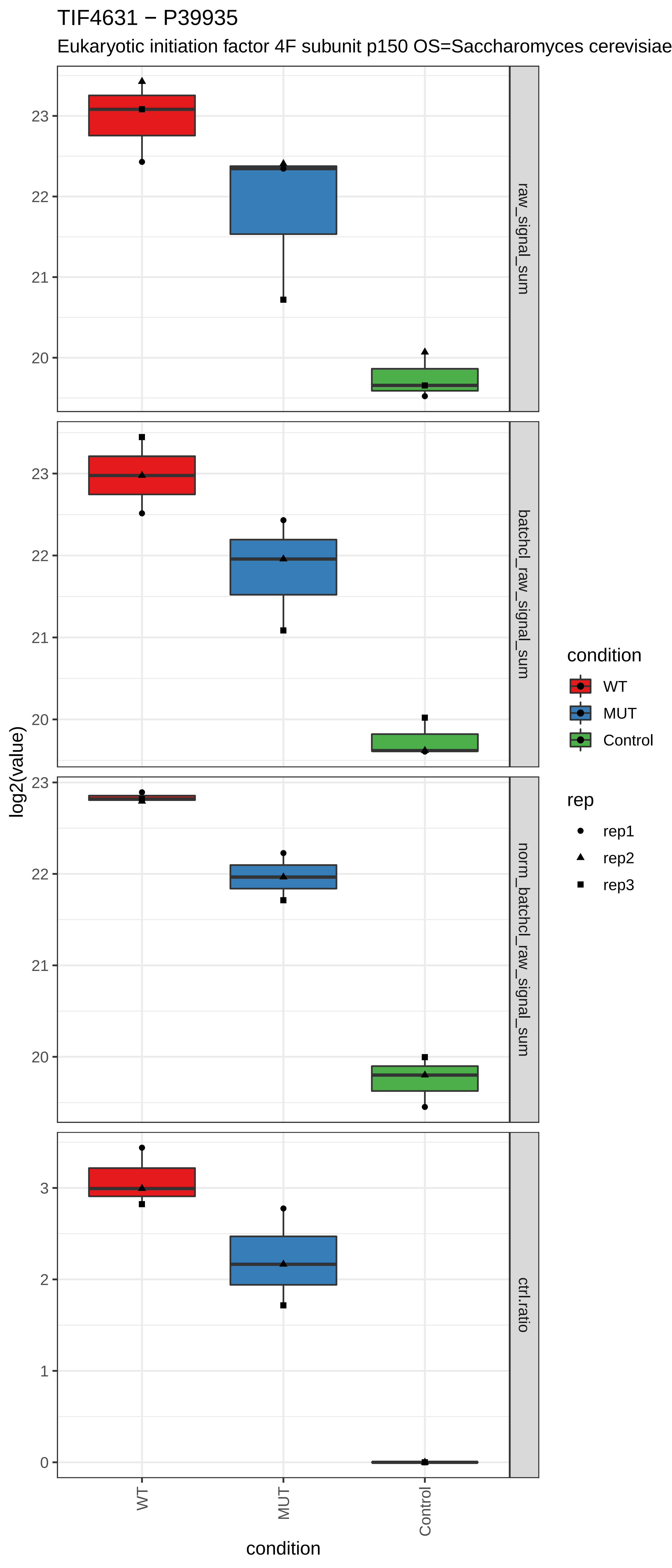

TIF4631 − P39935
Eukaryotic initiation factor 4F subunit p150 OS=Saccharomyces cerevisiae
log2(value)
raw_signal_sum
batchcl_raw_signal_sum
norm_batchcl_raw_signal_sum
ctrl.ratio
23
22
21
20
3
2
1
0
WT
MUT
Control
condition
condition
WT
MUT
Control
rep
rep1
rep2
rep3

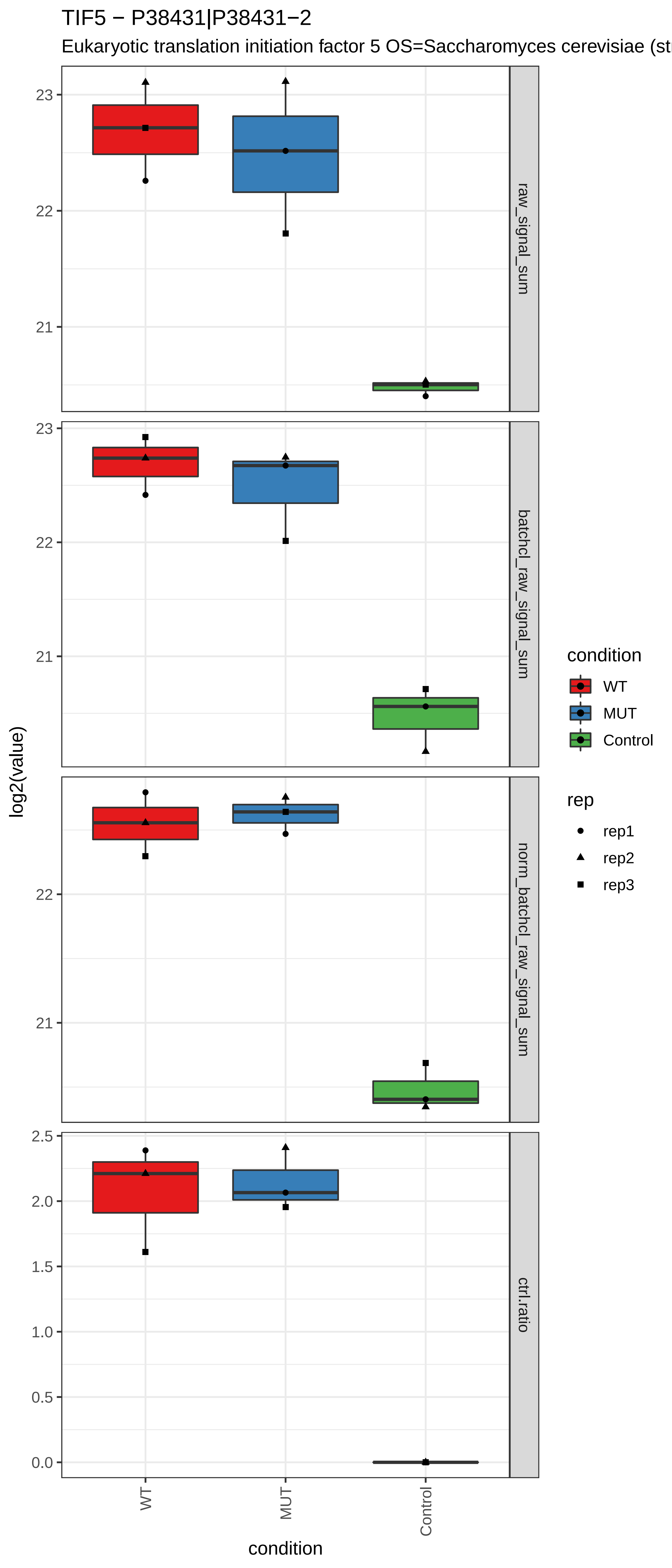

TIF5 − P38431|P38431−2
Eukaryotic translation initiation factor 5 OS=Saccharomyces cerevisiae (st
log2(value)
raw_signal_sum
batchcl_raw_signal_sum
norm_batchcl_raw_signal_sum
ctrl.ratio
23
22
21
2.5
2.0
1.5
1.0
0.5
0.0
WT
MUT
Control
condition
condition
WT
MUT
Control
rep
rep1
rep2
rep3

TIM44 − Q01852

Mitochondrial import inner membrane translocase subunit TIM44 OS=Sacc

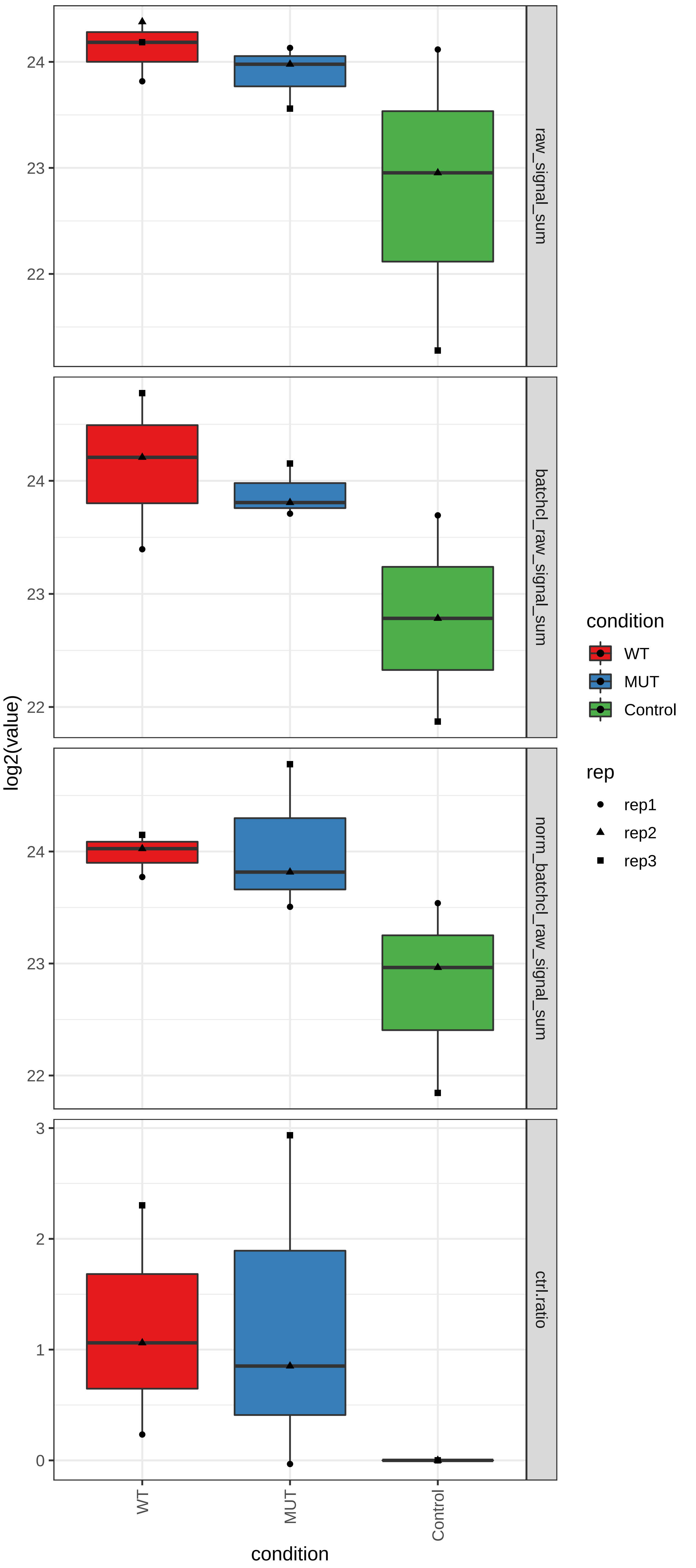

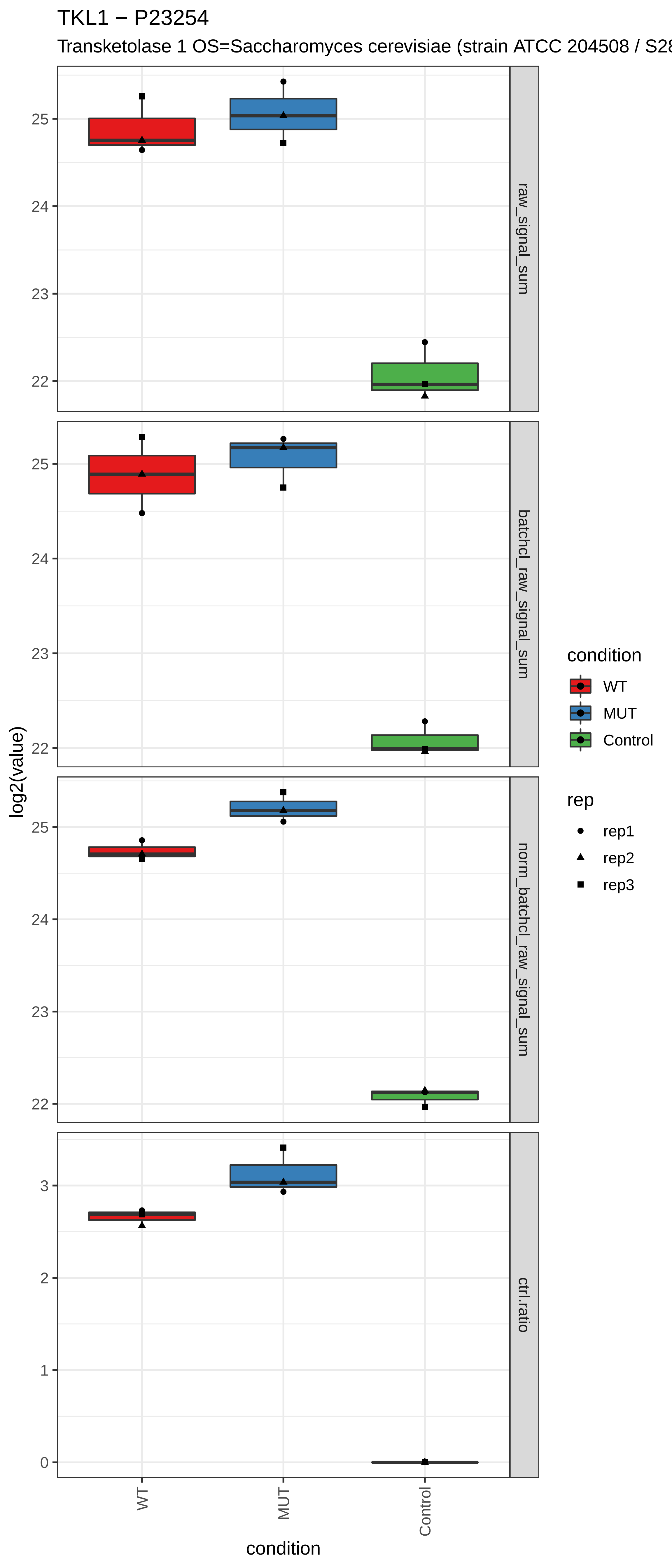
TKL1 − P23254
Transketolase 1 OS=Saccharomyces cerevisiae (strain ATCC 204508 / S28
raw_signal_sum
batchcl_raw_signal_sum
norm_batchcl_raw_signal_sum
ctrl.ratio
log2(value)
condition
WT
MUT
Control
rep
rep1
rep2
rep3
25
24
23
22
3
2
1
0

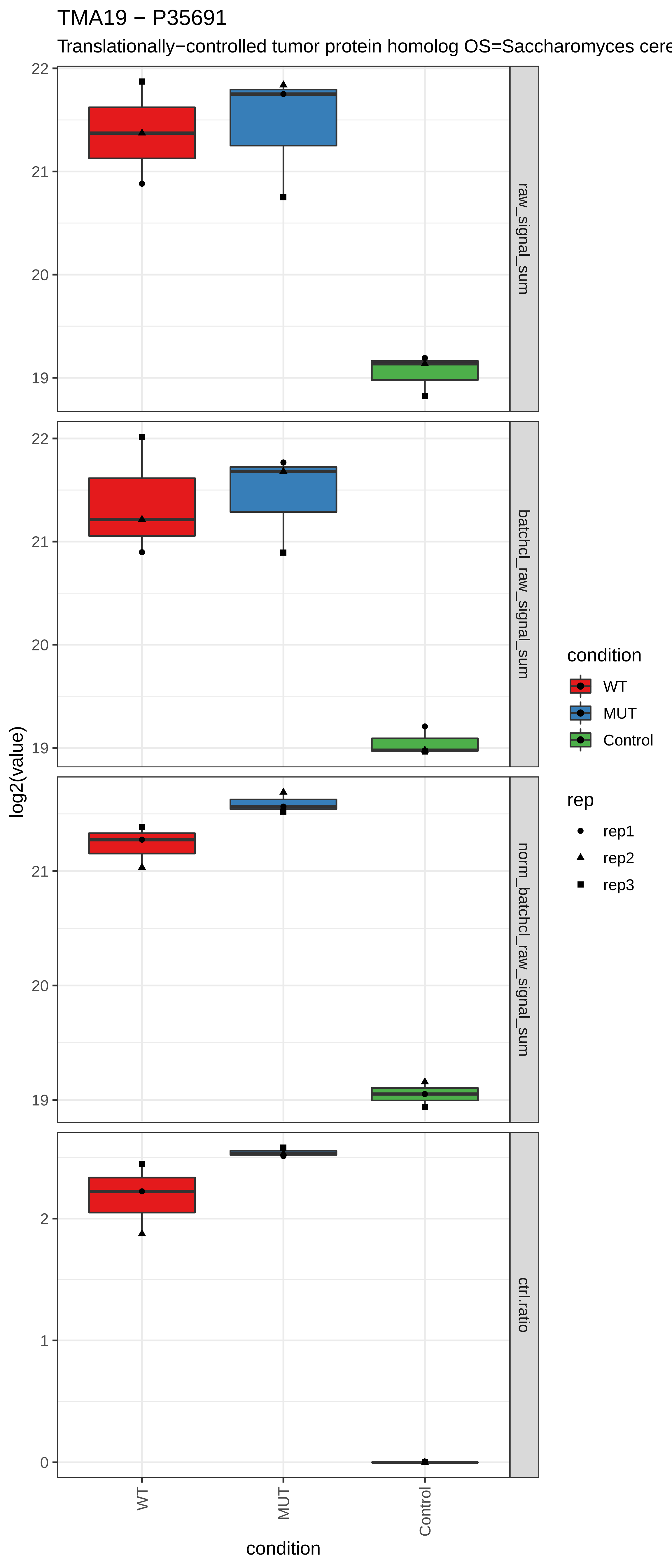

TMA19 − P35691
Translationally−controlled tumor protein homolog OS=Saccharomyces cere
raw_signal_sum
batchcl_raw_signal_sum
norm_batchcl_raw_signal_sum
ctrl.ratio
log2(value)
condition
WT
MUT
Control
rep
rep1
rep2
rep3
22
21
20
19
2
1
0

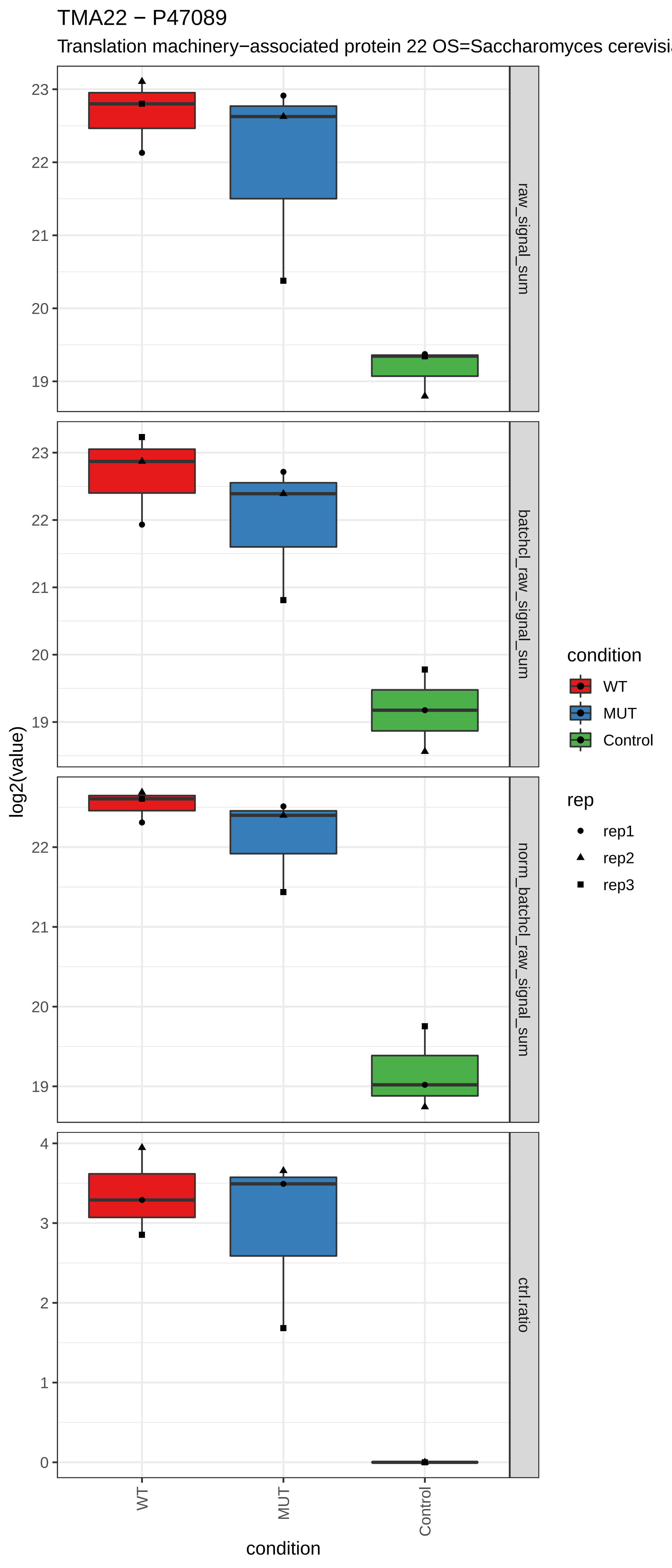

TMA22 − P47089
Translation machinery−associated protein 22 OS=Saccharomyces cerevisi
raw_signal_sum
batchcl_raw_signal_sum
norm_batchcl_raw_signal_sum
ctrl.ratio
log2(value)
condition
WT
MUT
Control
rep
rep1
rep2
rep3

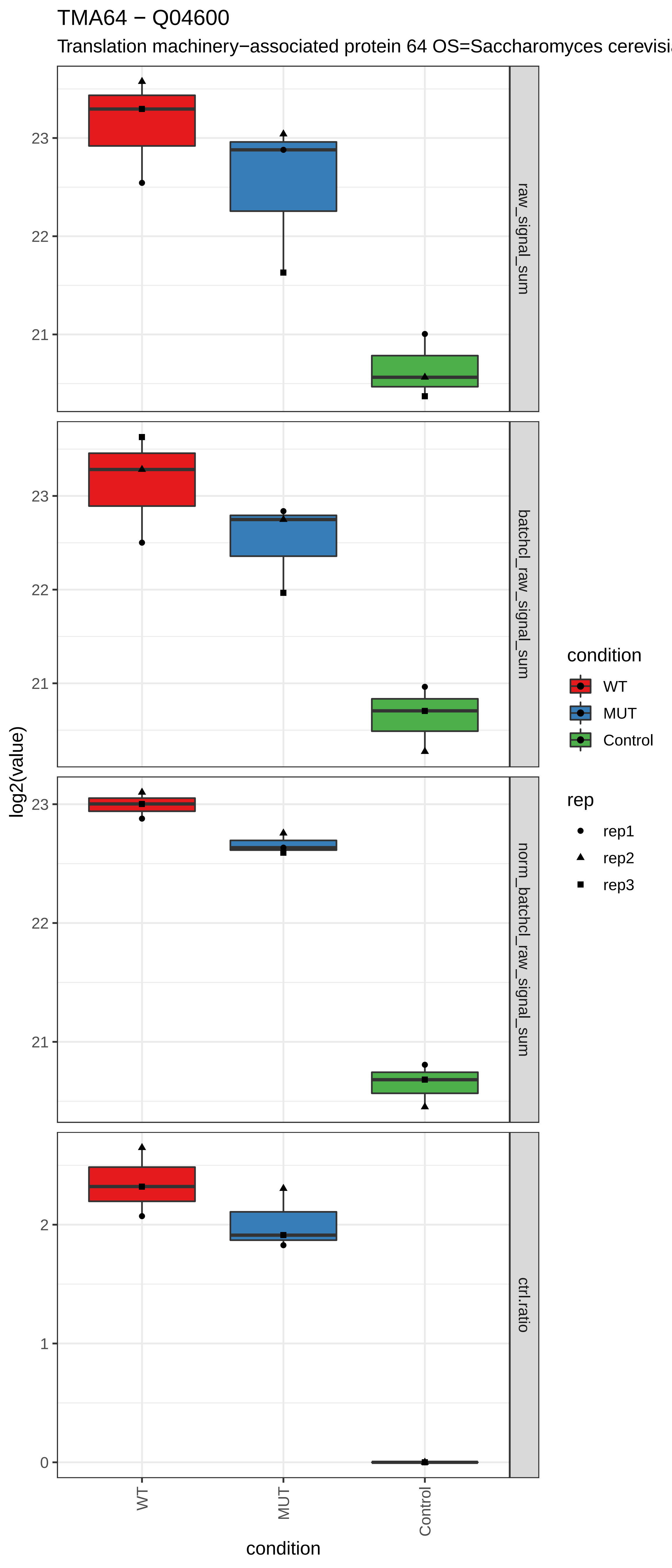

TMA64 − Q04600
Translation machinery−associated protein 64 OS=Saccharomyces cerevisi
raw_signal_sum
batchcl_raw_signal_sum
norm_batchcl_raw_signal_sum
ctrl.ratio
log2(value)
condition
WT
MUT
Control
rep
rep1
rep2
rep3
23
22
21
2
1
0

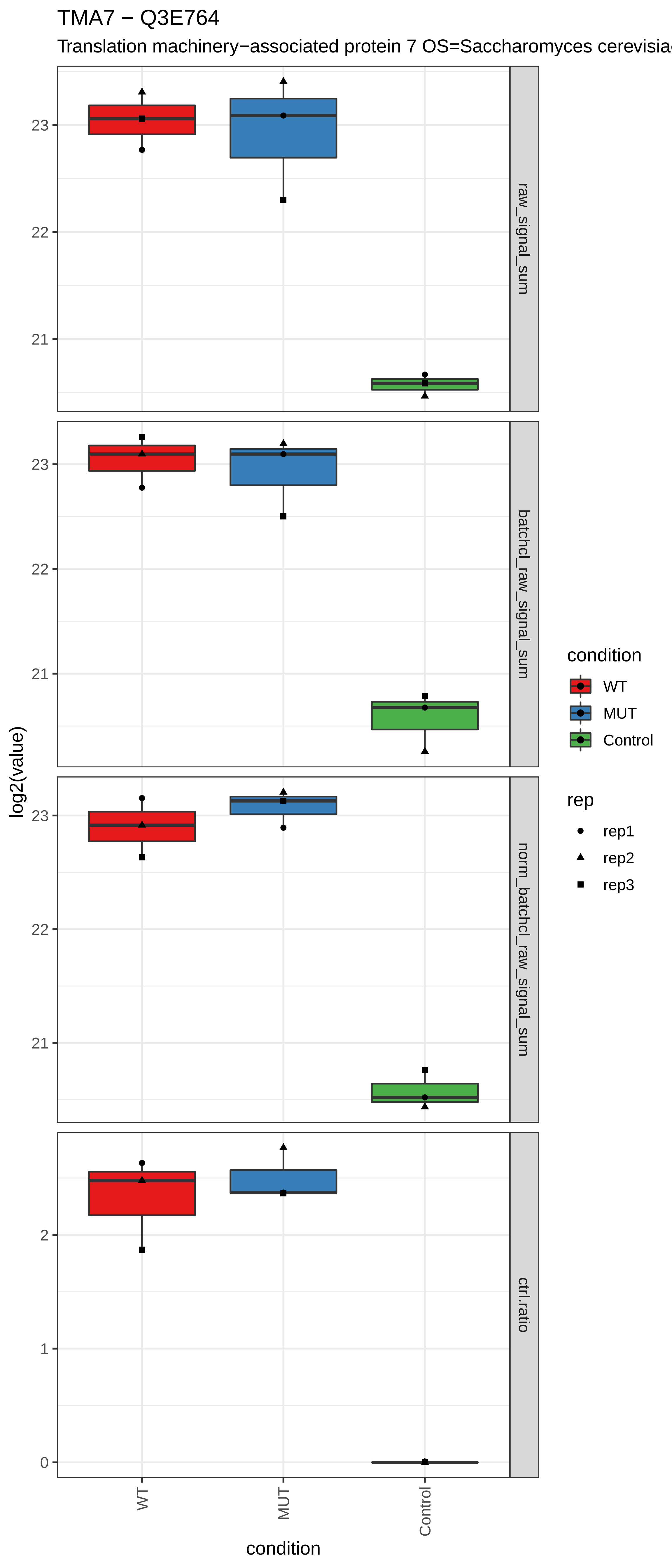
TMA7 − Q3E764
Translation machinery−associated protein 7 OS=Saccharomyces cerevisiae
raw_signal_sum
batchcl_raw_signal_sum
norm_batchcl_raw_signal_sum
ctrl.ratio
log2(value)
23
22
21
2
1
0
WT
MUT
Control
condition
condition
WT
MUT
Control
rep
rep1
rep2
rep3

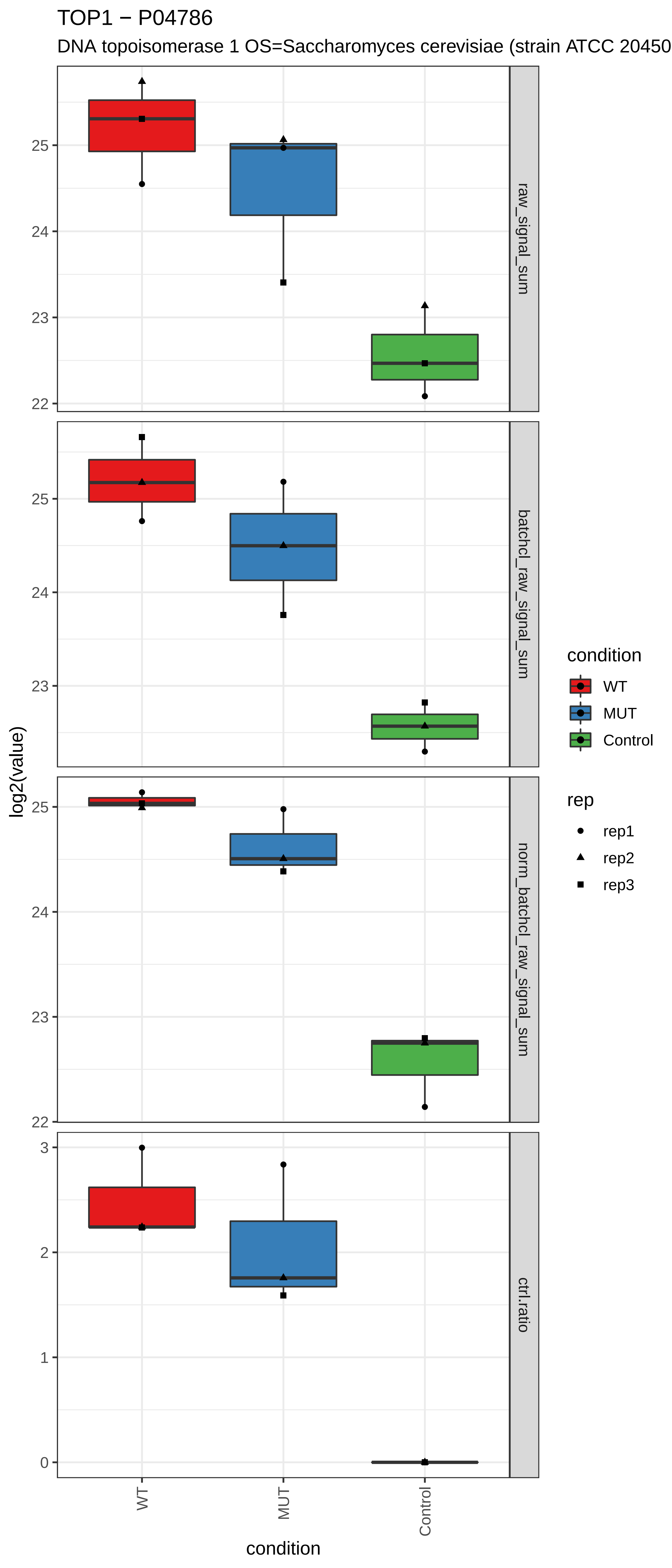
TOP1 − P04786
DNA topoisomerase 1 OS=Saccharomyces cerevisiae (strain ATCC 20450
raw_signal_sum
batchcl_raw_signal_sum
norm_batchcl_raw_signal_sum
ctrl.ratio
log2(value)
condition
WT
MUT
Control
rep
rep1
rep2
rep3
25
24
23
22
3
2
1
0

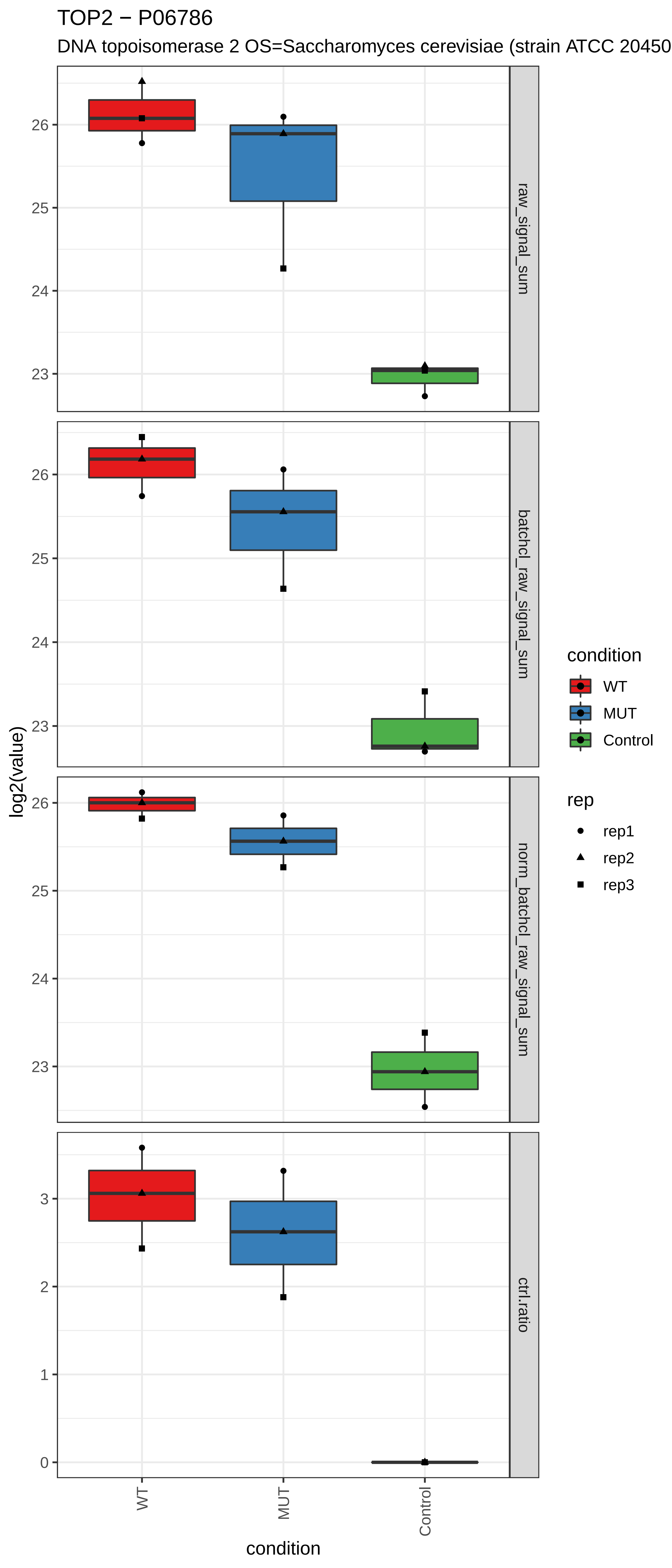

TOP2 − P06786
DNA topoisomerase 2 OS=Saccharomyces cerevisiae (strain ATCC 20450
raw_signal_sum
batchcl_raw_signal_sum
norm_batchcl_raw_signal_sum
ctrl.ratio
log2(value)
condition
WT
MUT
Control
rep
rep1
rep2
rep3
26
25
24
23
3
2
1
0

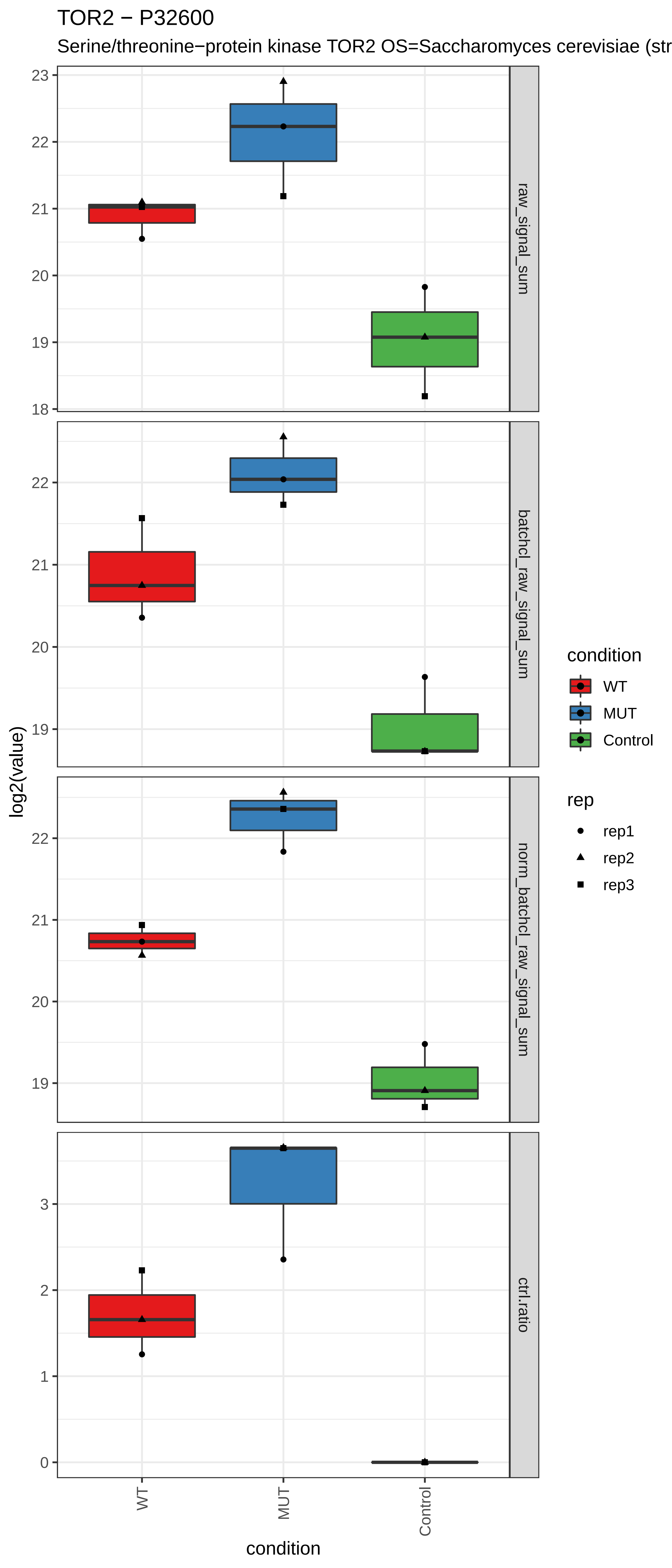

TOR2 − P32600
Serine/threonine−protein kinase TOR2 OS=Saccharomyces cerevisiae (str
raw_signal_sum
batchcl_raw_signal_sum
norm_batchcl_raw_signal_sum
ctrl.ratio
log2(value)
condition
WT
MUT
Control
rep
rep1
rep2
rep3

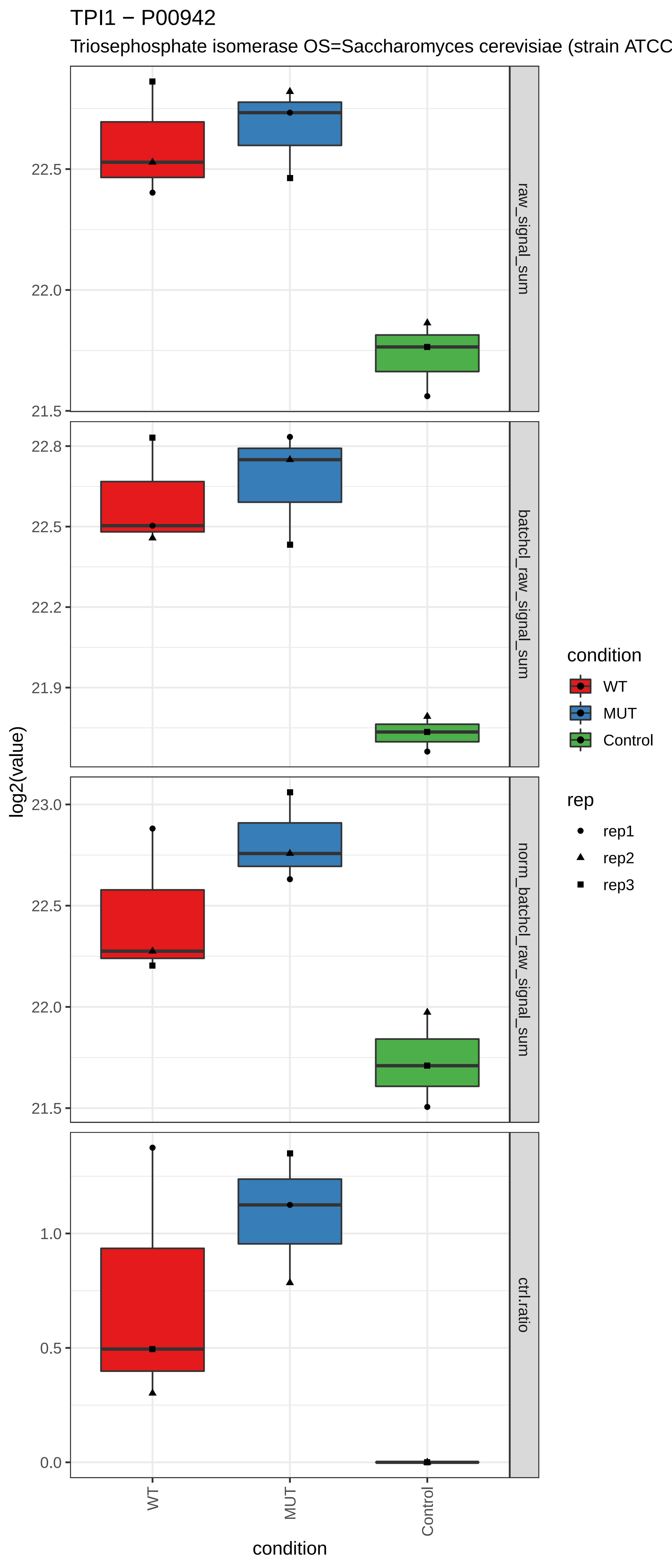

TPI1 − P00942
Triosephosphate isomerase OS=Saccharomyces cerevisiae (strain ATCC
raw_signal_sum
batchcl_raw_signal_sum
norm_batchcl_raw_signal_sum
ctrl.ratio
22.5
22.0
21.5
22.8
22.5
22.2
21.9
23.0
22.5
22.0
21.5
1.0
0.5
0.0
log2(value)
WT
MUT
Control
condition
condition
WT
MUT
Control
rep
rep1
rep2
rep3

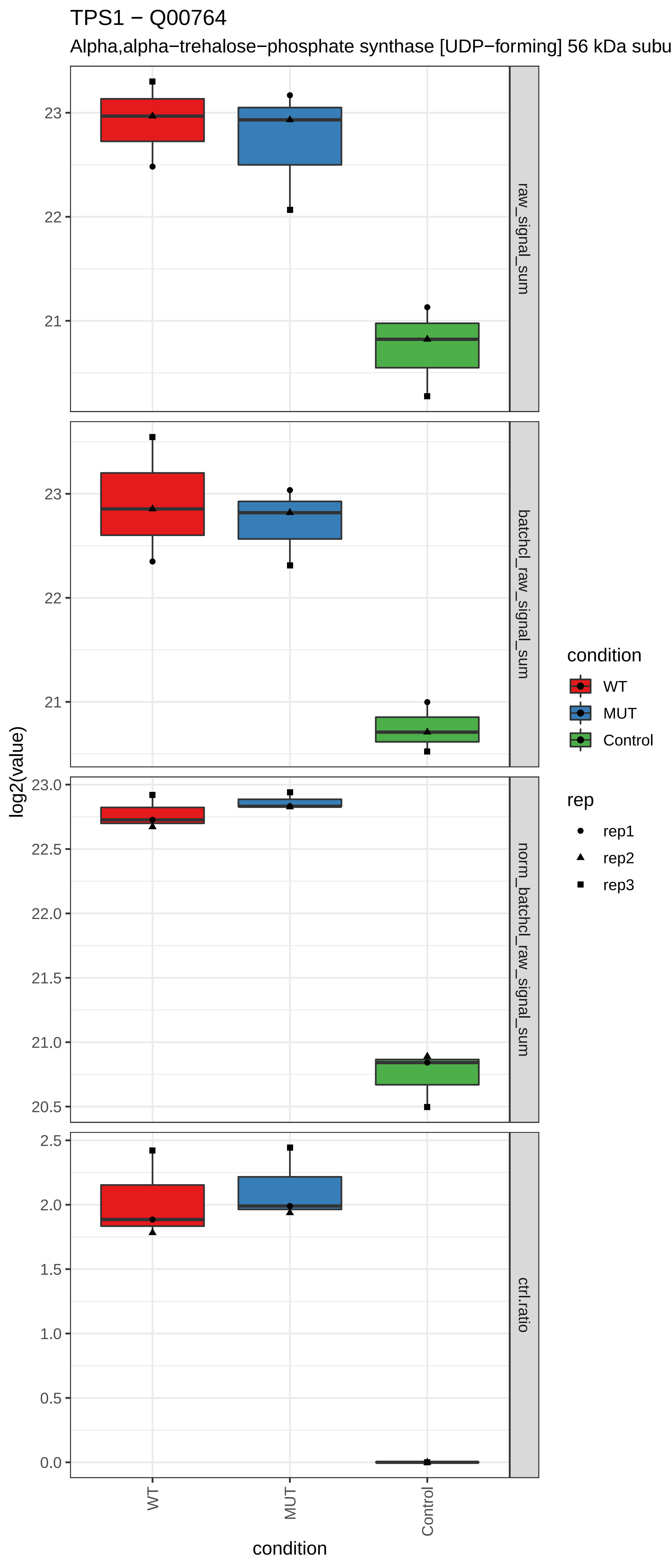

TPS1 − Q00764
Alpha,alpha−trehalose−phosphate synthase [UDP−forming] 56 kDa subu
raw_signal_sum
batchcl_raw_signal_sum
norm_batchcl_raw_signal_sum
ctrl.ratio
log2(value)
condition
WT
MUT
Control
rep
rep1
rep2
rep3
23
22
21
23.0
22.5
22.0
21.5
21.0
20.5
2.5
2.0
1.5
1.0
0.5
0.0

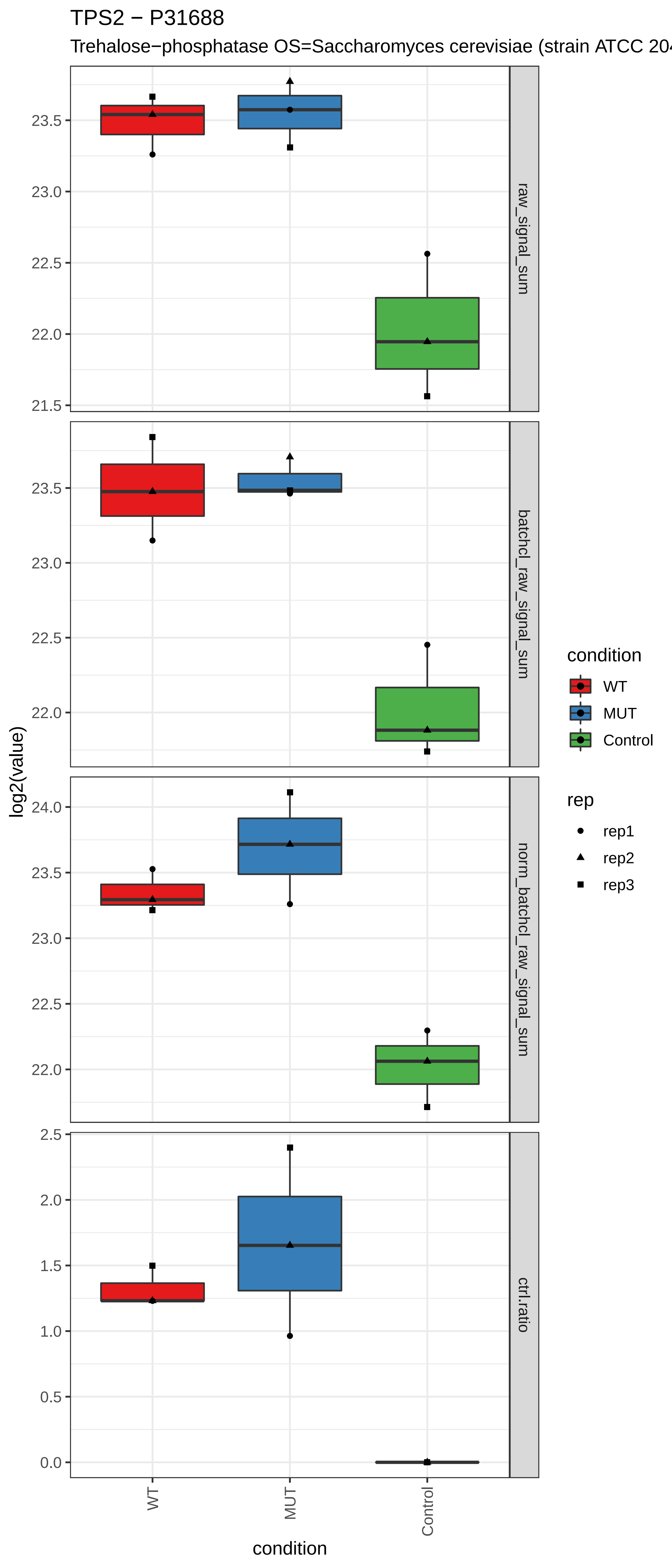

TPS2 − P31688
Trehalose−phosphatase OS=Saccharomyces cerevisiae (strain ATCC 20
raw_signal_sum
batchcl_raw_signal_sum
norm_batchcl_raw_signal_sum
ctrl.ratio
log2(value)
condition
WT
MUT
Control
rep
rep1
rep2
rep3

TRA1 − P38811

Transcription−associated protein 1 OS=Saccharomyces cerevisiae (strain A

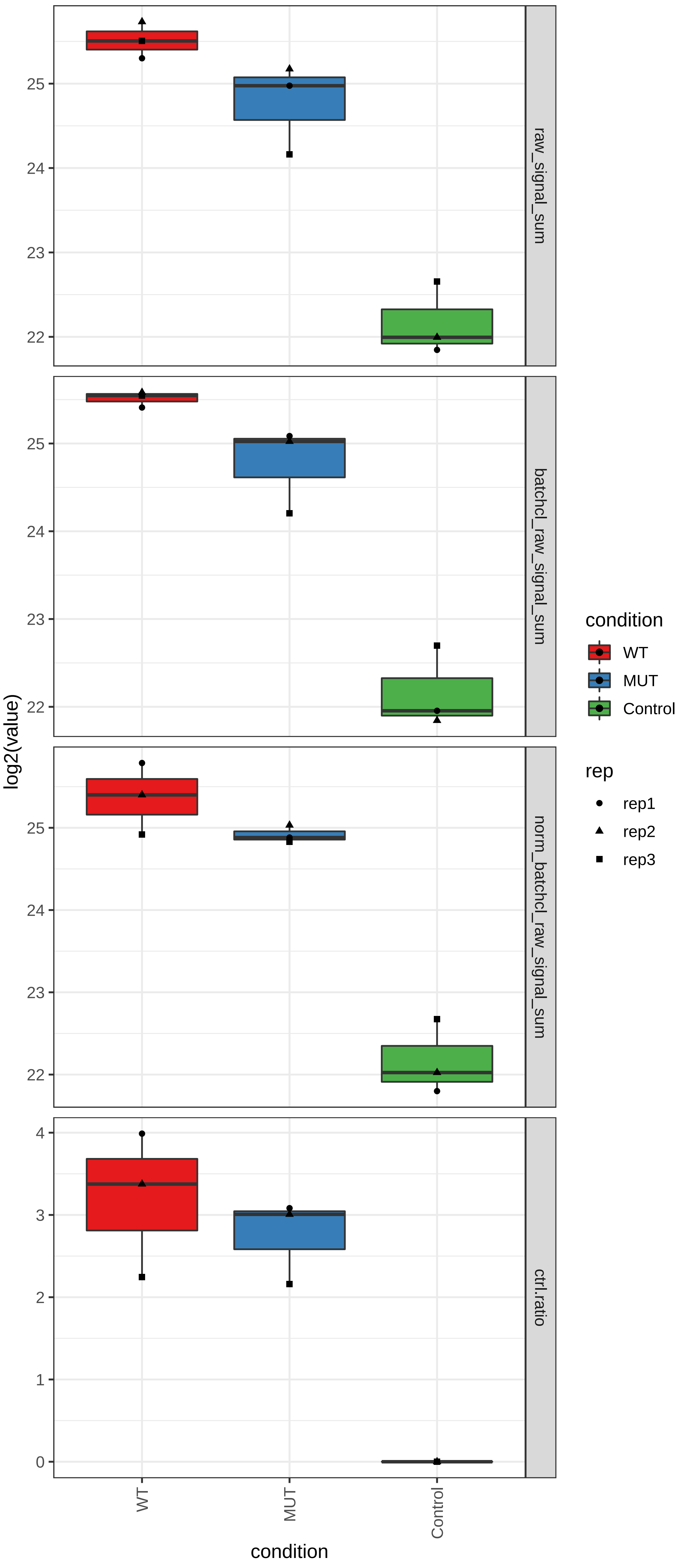

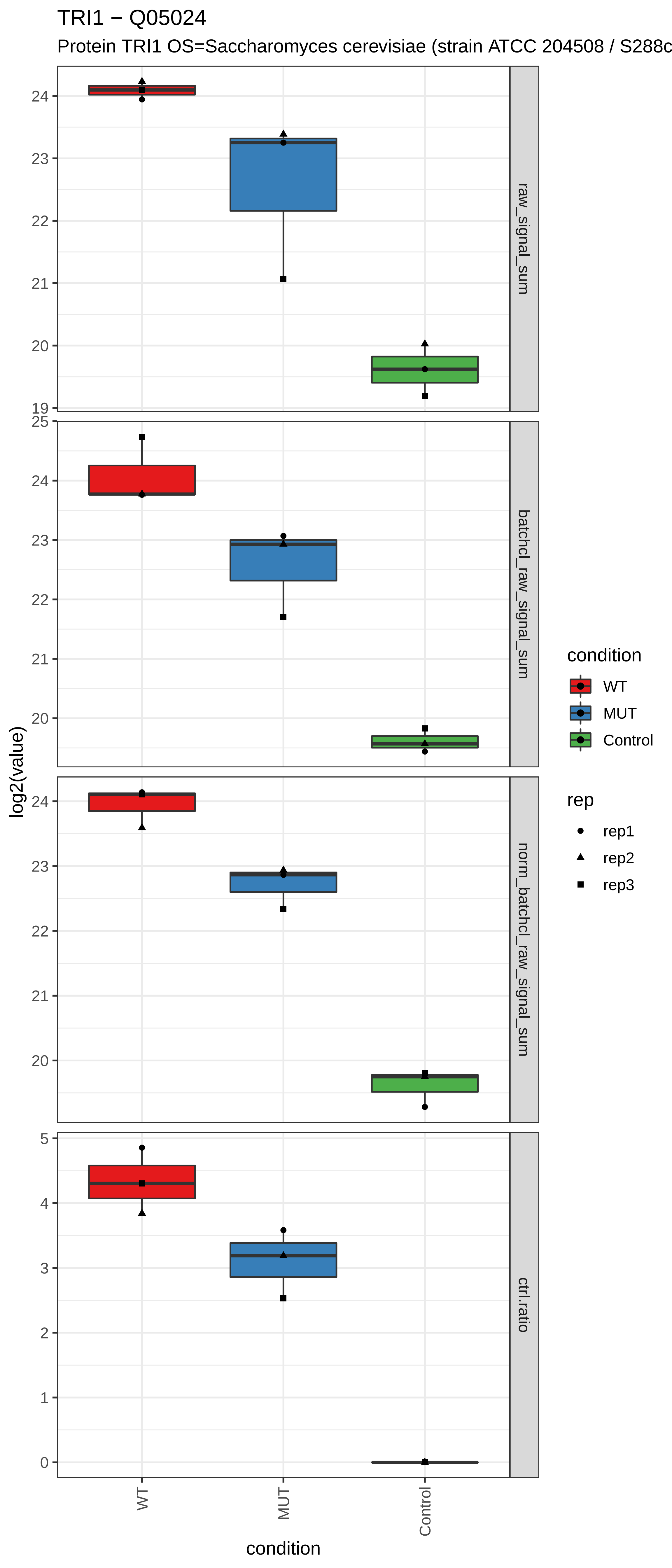
TRI1 − Q05024
Protein TRI1 OS=Saccharomyces cerevisiae (strain ATCC 204508 / S288c
raw_signal_sum
batchcl_raw_signal_sum
norm_batchcl_raw_signal_sum
ctrl.ratio
log2(value)
condition
WT
MUT
Control
rep
rep1
rep2
rep3

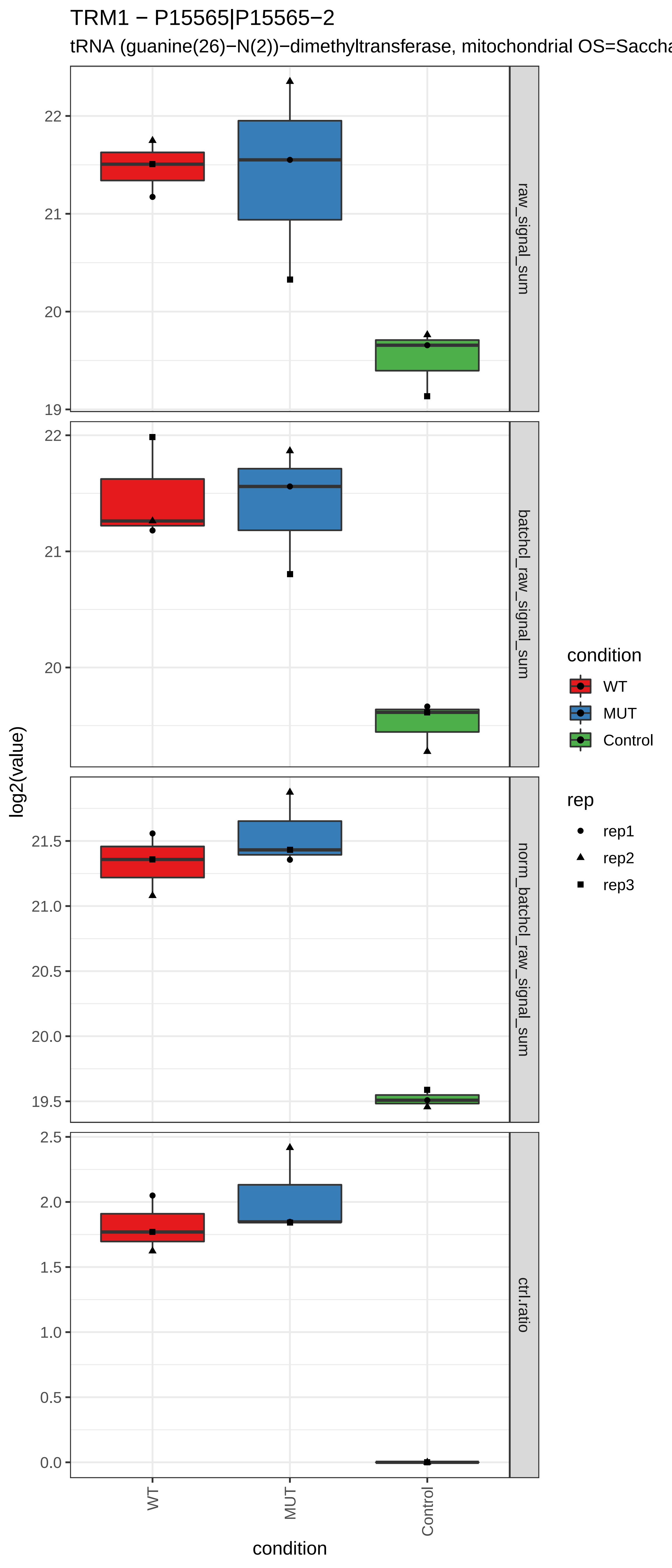

TRM1 − P15565|P15565−2
tRNA (guanine(26)−N(2))−dimethyltransferase, mitochondrial OS=Saccha
raw_signal_sum
batchcl_raw_signal_sum
norm_batchcl_raw_signal_sum
ctrl.ratio
log2(value)
condition
WT
MUT
Control
rep
rep1
rep2
rep3
22
21
20
19
21.5
21.0
20.5
20.0
19.5
2.5
2.0
1.5
1.0
0.5
0.0

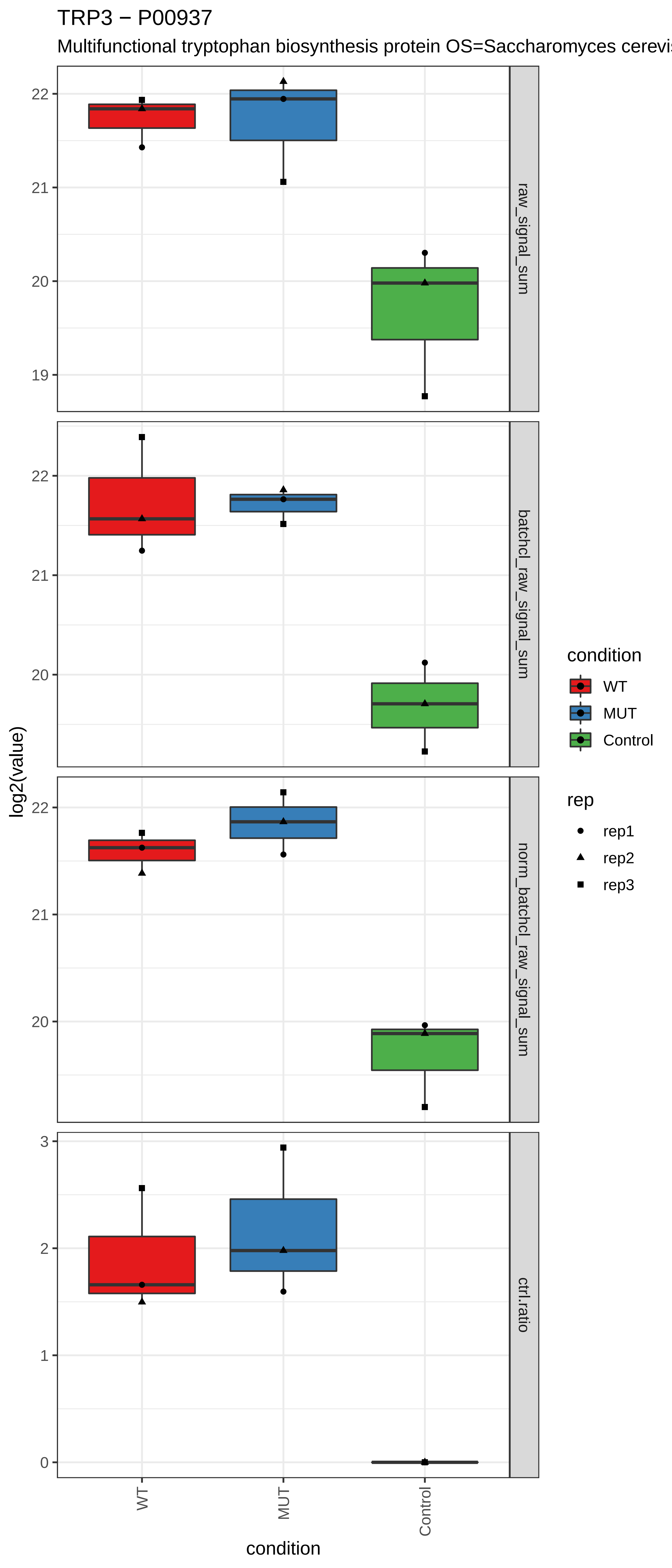

TRP3 − P00937
Multifunctional tryptophan biosynthesis protein OS=Saccharomyces cerevis
raw_signal_sum
batchcl_raw_signal_sum
norm_batchcl_raw_signal_sum
ctrl.ratio
log2(value)
condition
WT
MUT
Control
rep
rep1
rep2
rep3

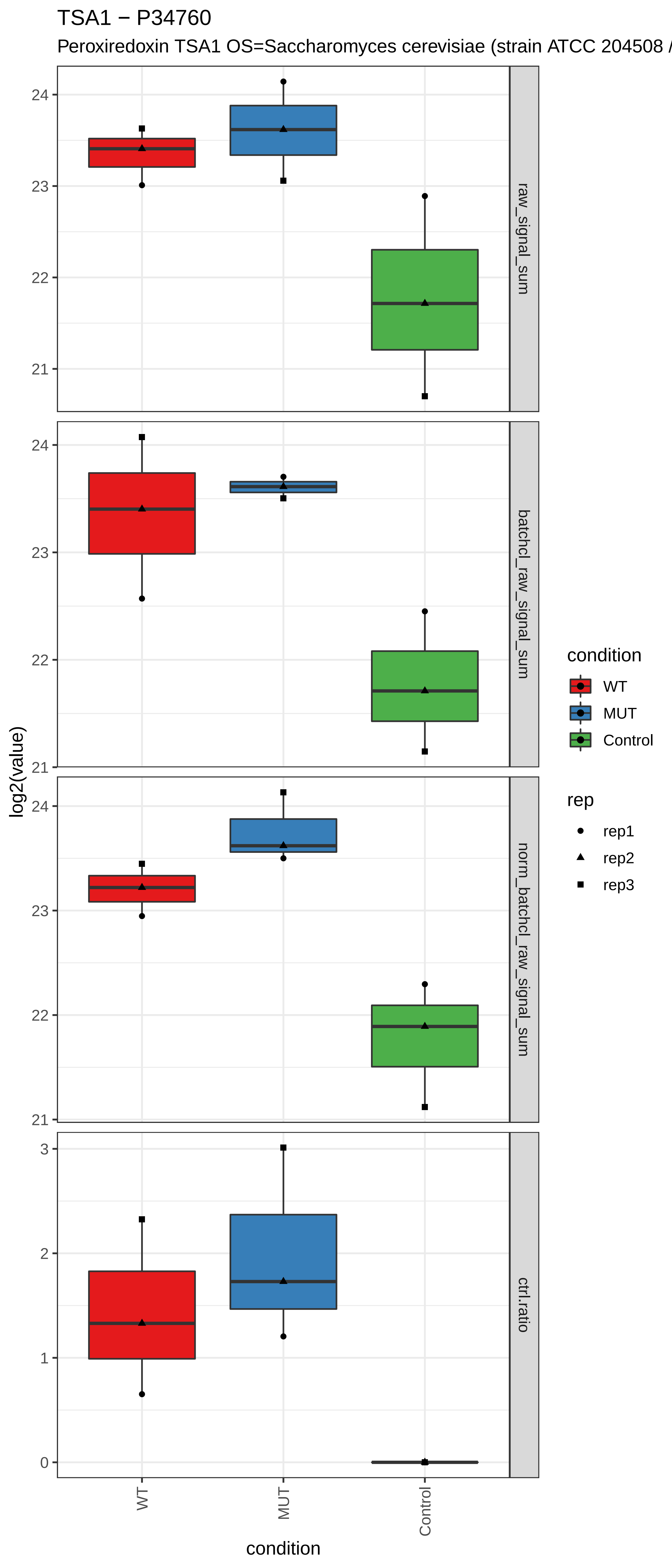

TSA1 − P34760
Peroxiredoxin TSA1 OS=Saccharomyces cerevisiae (strain ATCC 204508 /
raw_signal_sum
batchcl_raw_signal_sum
norm_batchcl_raw_signal_sum
ctrl.ratio
log2(value)
condition
WT
MUT
Control
rep
rep1
rep2
rep3
24
23
22
21
3
2
1
0

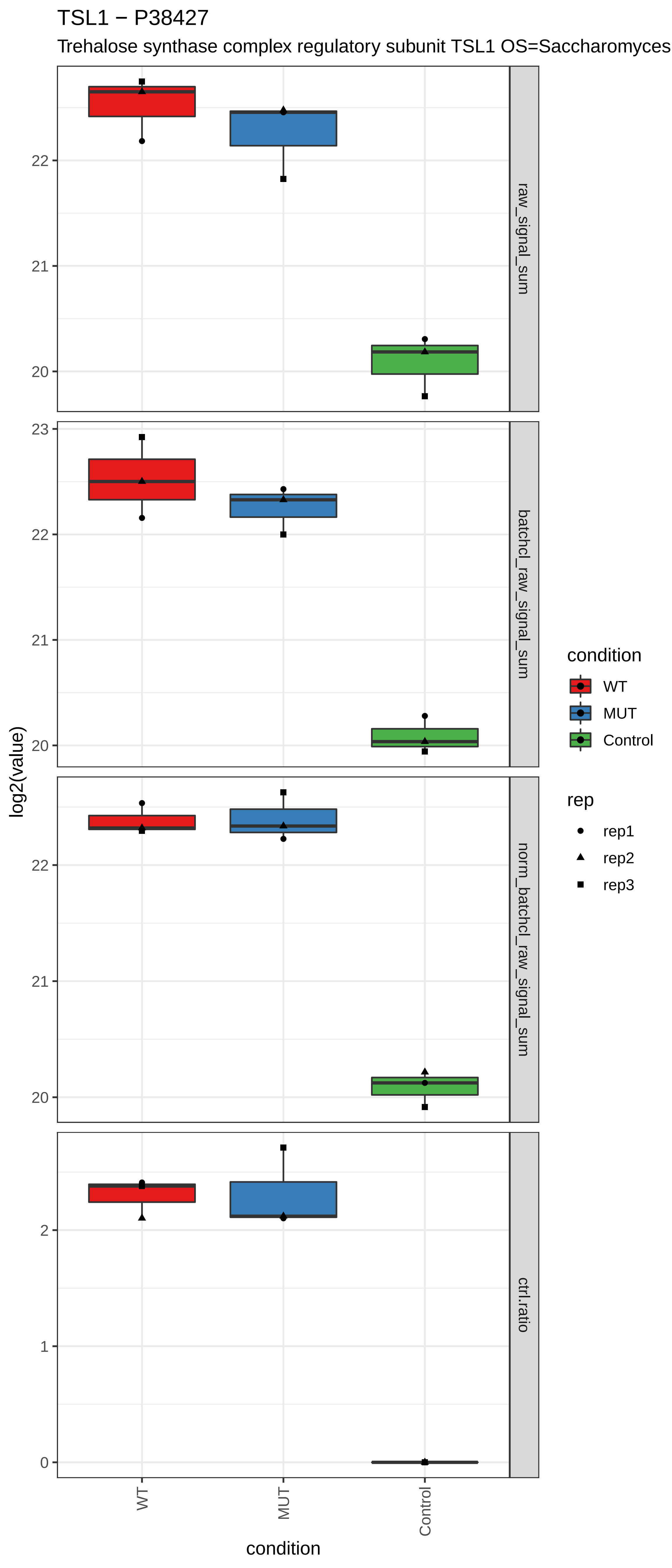
TSL1 − P38427
Trehalose synthase complex regulatory subunit TSL1 OS=Saccharomyces
raw_signal_sum
batchcl_raw_signal_sum
norm_batchcl_raw_signal_sum
ctrl.ratio
log2(value)
condition
WT
MUT
Control
rep
rep1
rep2
rep3
22
21
20
23
2
1
0

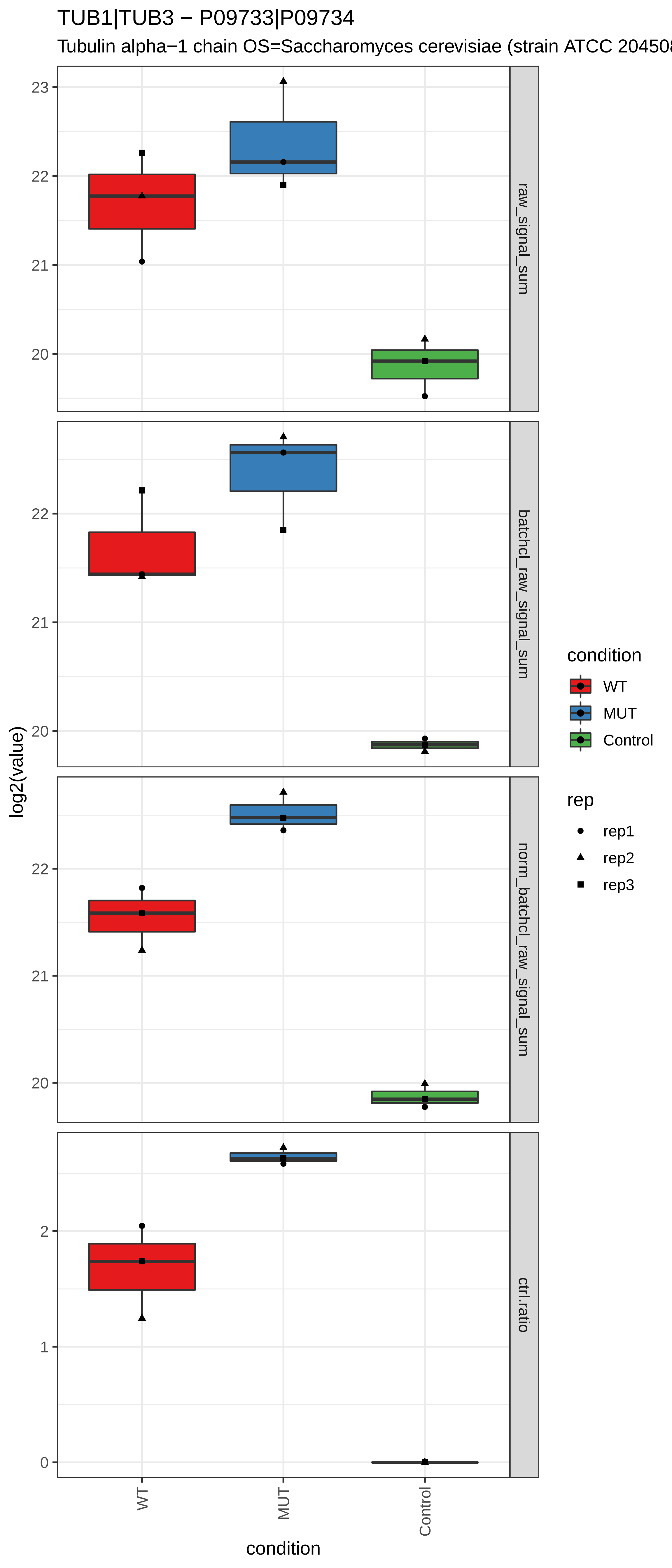
TUB1|TUB3 − P09733|P09734
Tubulin alpha−1 chain OS=Saccharomyces cerevisiae (strain ATCC 204508
raw_signal_sum
batchcl_raw_signal_sum
norm_batchcl_raw_signal_sum
ctrl.ratio
log2(value)
condition
WT
MUT
Control
rep
rep1
rep2
rep3
23
22
21
20
2
1
0

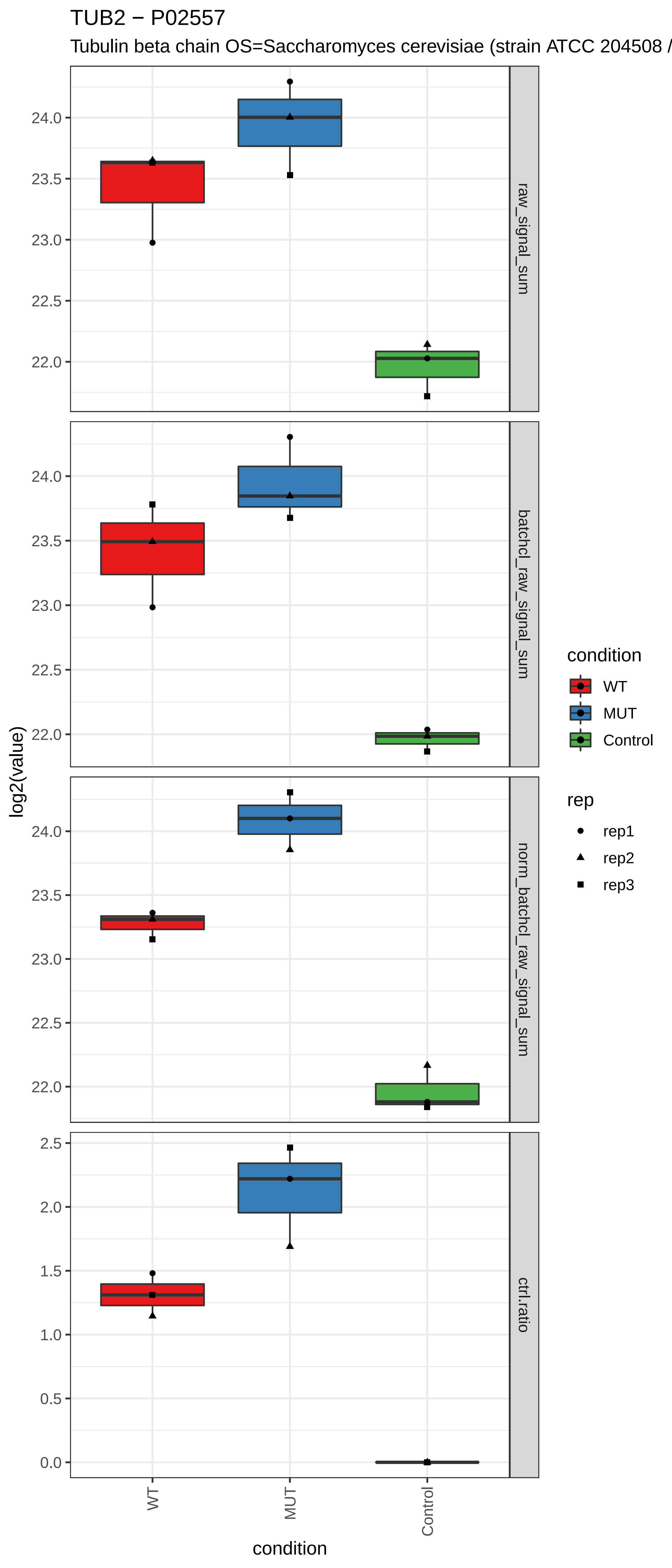

TUB2 − P02557
Tubulin beta chain OS=Saccharomyces cerevisiae (strain ATCC 204508 /
raw_signal_sum
batchcl_raw_signal_sum
norm_batchcl_raw_signal_sum
ctrl.ratio
log2(value)
condition
WT
MUT
Control
rep
rep1
rep2
rep3
24.0
23.5
23.0
22.5
22.0
2.5
2.0
1.5
1.0
0.5
0.0

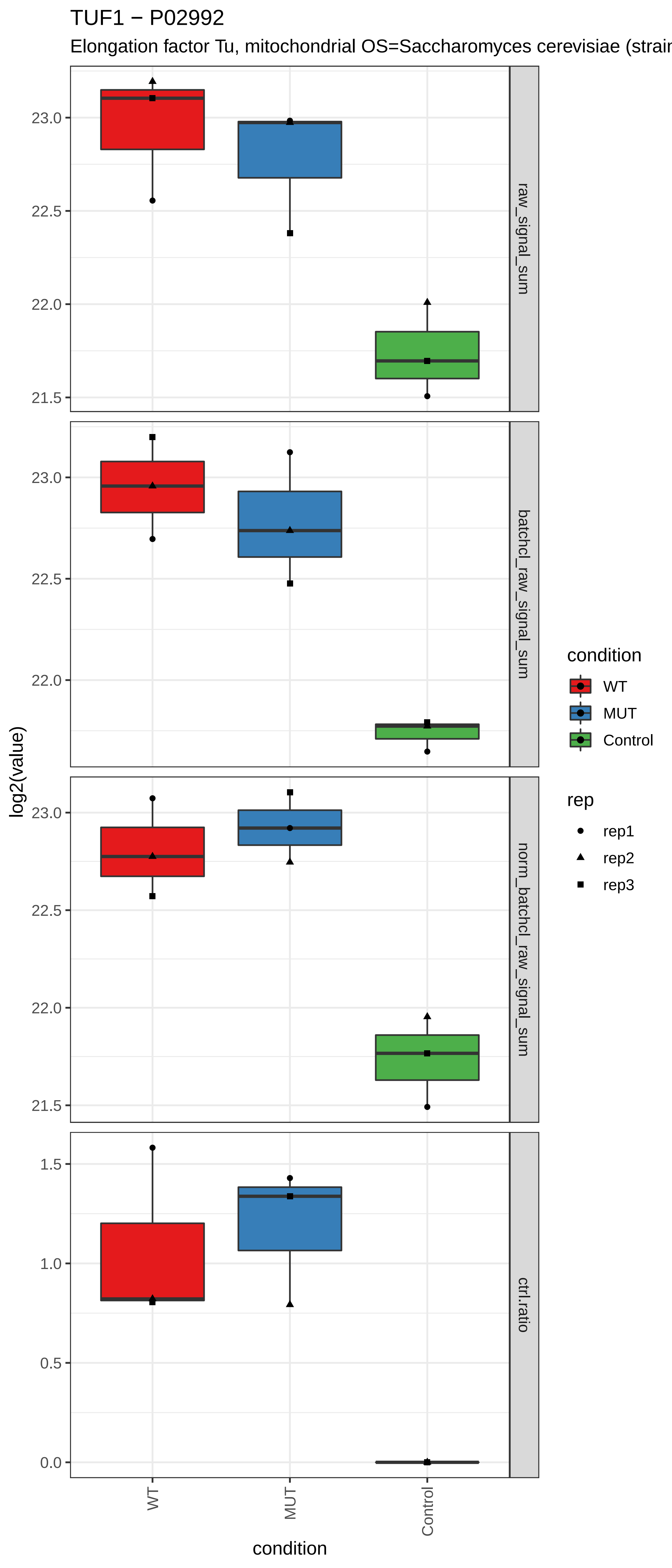

TUF1 − P02992
Elongation factor Tu, mitochondrial OS=Saccharomyces cerevisiae (strain
raw_signal_sum
batchcl_raw_signal_sum
norm_batchcl_raw_signal_sum
ctrl.ratio
23.0
22.5
22.0
21.5
1.5
1.0
0.5
0.0
log2(value)
WT
MUT
Control
condition
condition
WT
MUT
Control
rep
rep1
rep2
rep3

TYS1 − P36421

Tyrosine−−tRNA ligase, cytoplasmic OS=Saccharomyces cerevisiae (strain

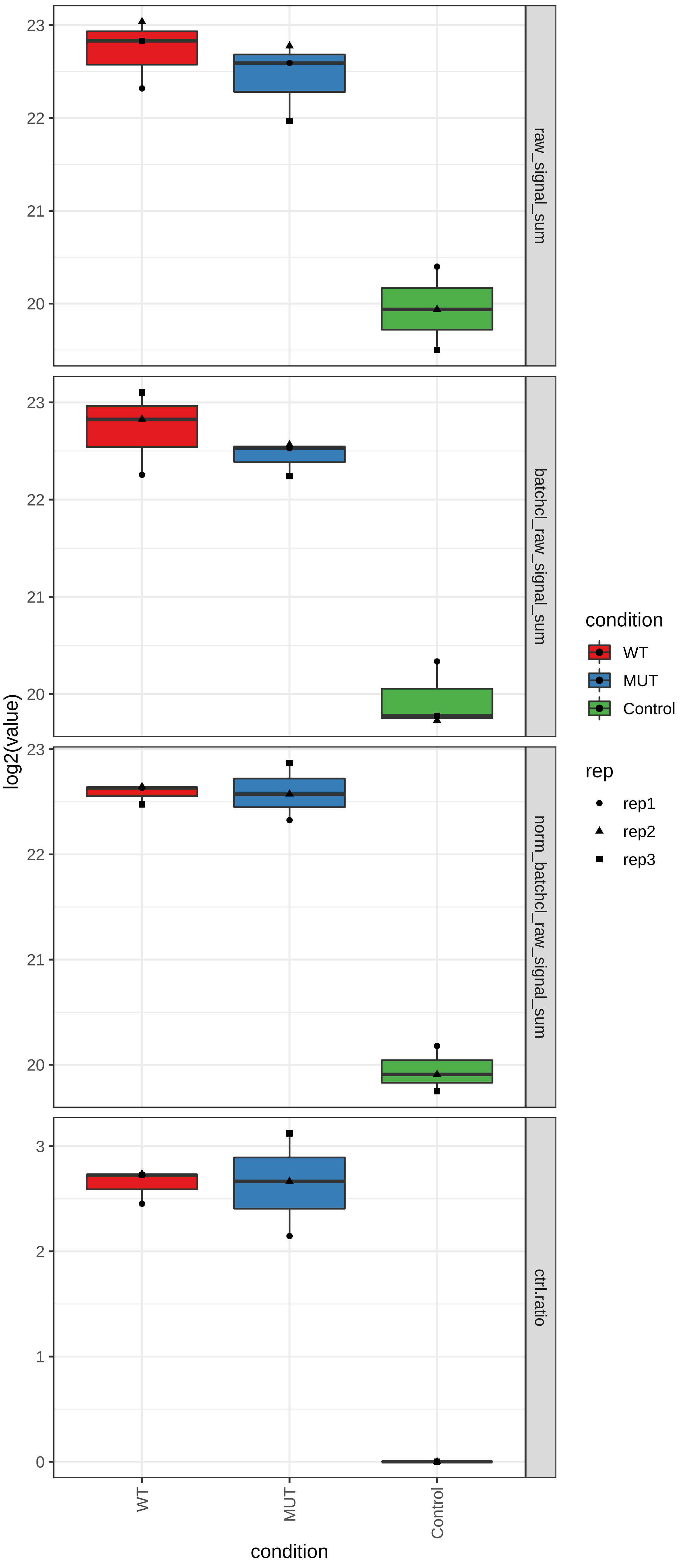

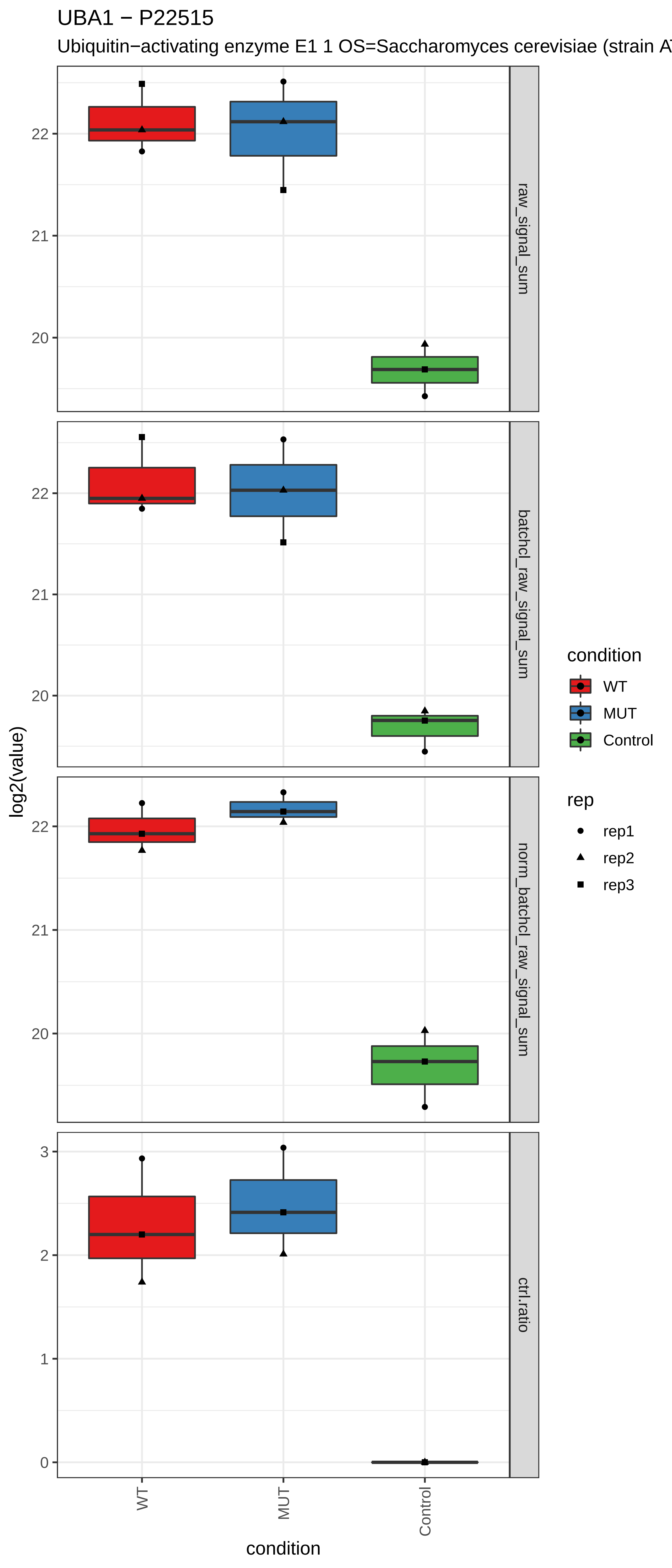
UBA1 − P22515
Ubiquitin−activating enzyme E1 1 OS=Saccharomyces cerevisiae (strain A
raw_signal_sum
batchcl_raw_signal_sum
norm_batchcl_raw_signal_sum
ctrl.ratio
log2(value)
condition
WT
MUT
Control
rep
rep1
rep2
rep3
22
21
20
3
2
1
0

UBP3 − Q01477

Ubiquitin carboxyl−terminal hydrolase 3 OS=Saccharomyces cerevisiae (st

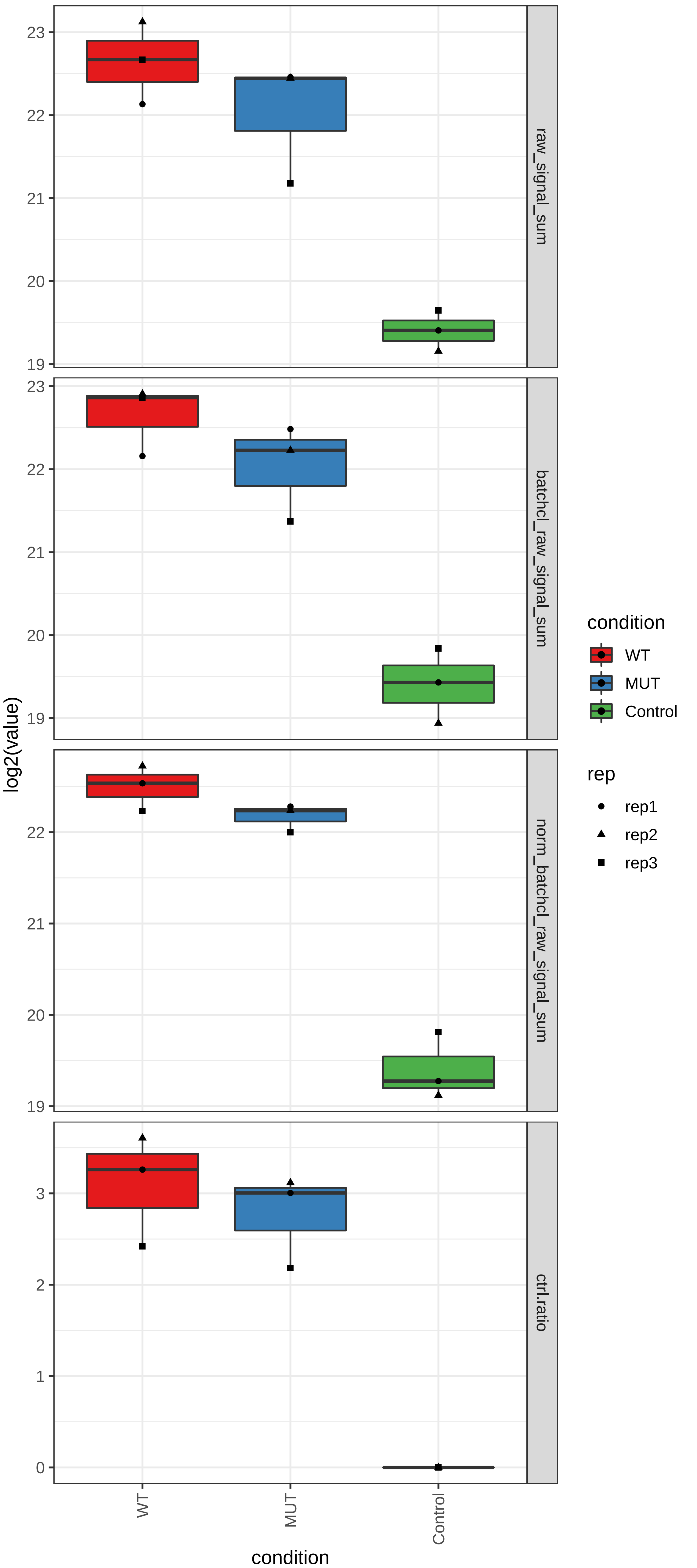

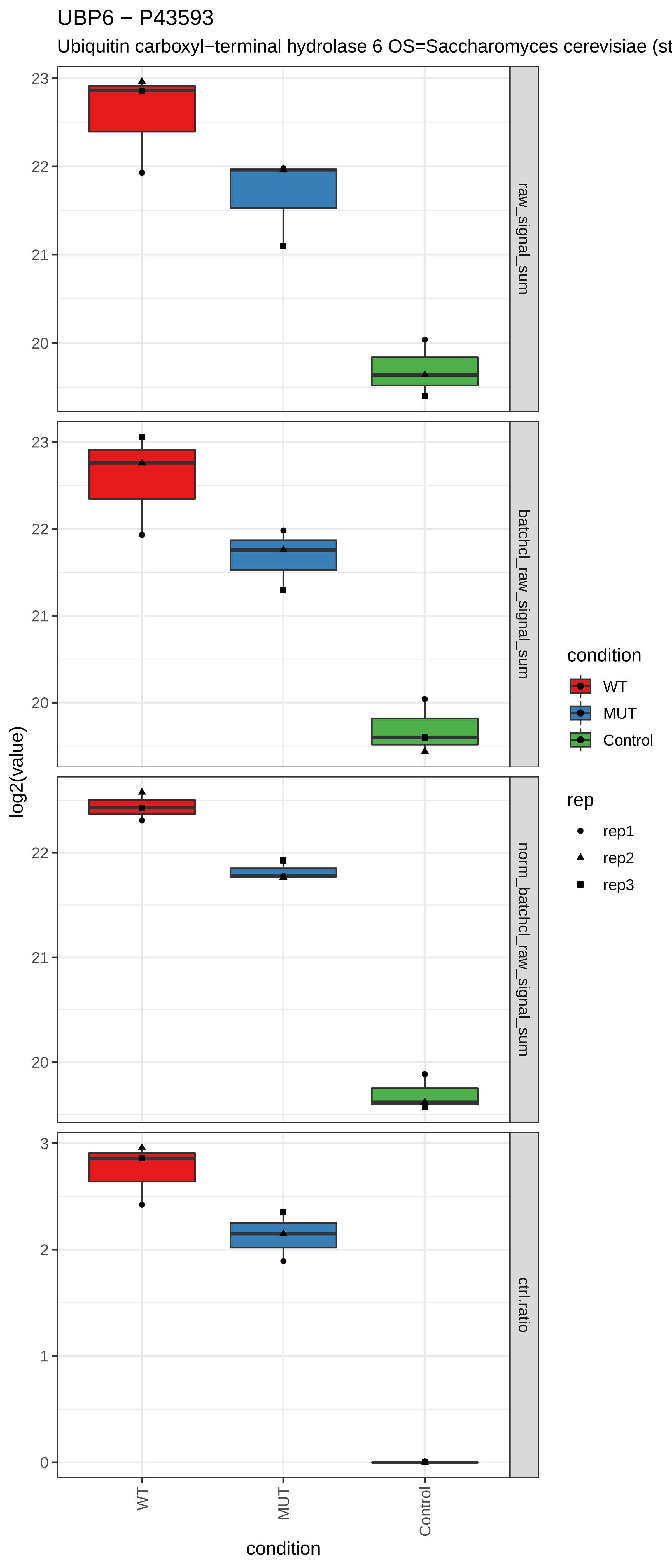
UBP6 − P43593
Ubiquitin carboxyl−terminal hydrolase 6 OS=Saccharomyces cerevisiae (st
raw_signal_sum
batchcl_raw_signal_sum
norm_batchcl_raw_signal_sum
ctrl.ratio
log2(value)
condition
WT
MUT
Control
rep
rep1
rep2
rep3
23
22
21
20
3
2
1
0

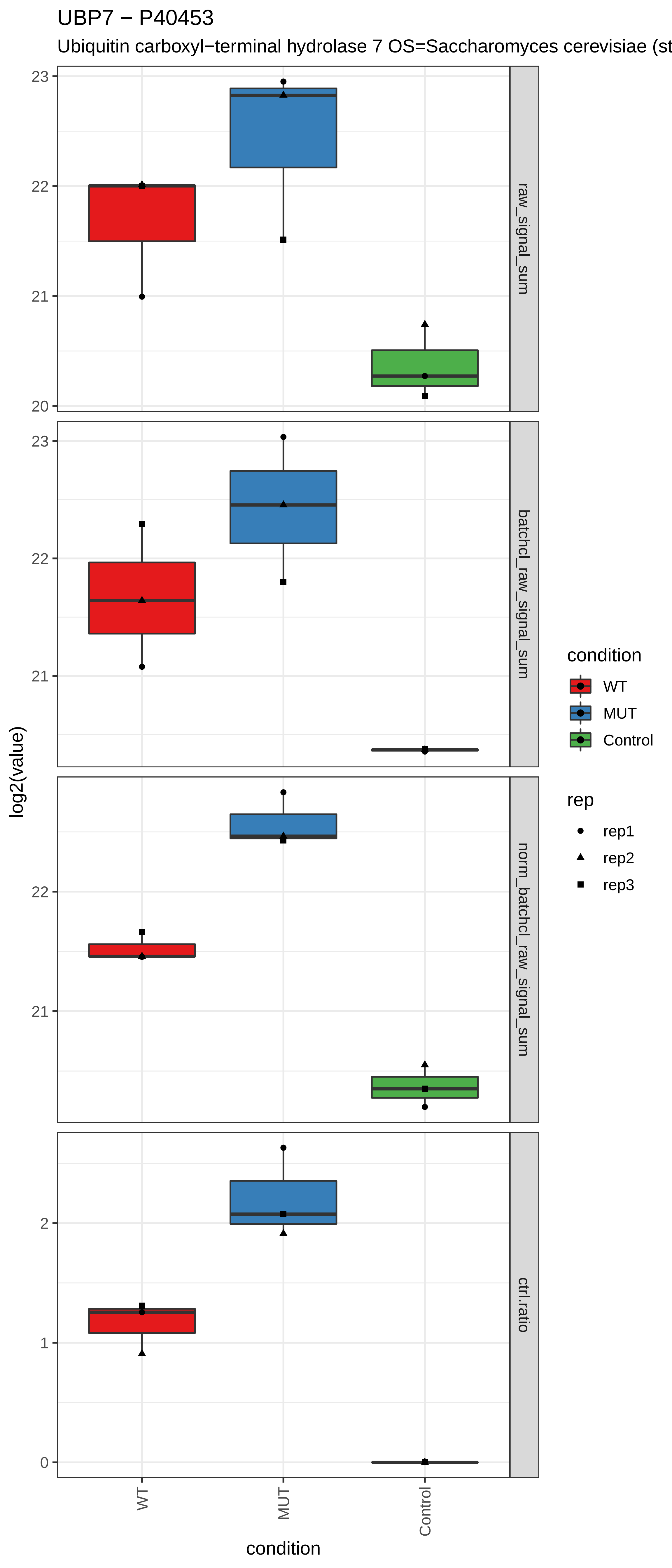

UBP7 − P40453
Ubiquitin carboxyl−terminal hydrolase 7 OS=Saccharomyces cerevisiae (st
raw_signal_sum
batchcl_raw_signal_sum
norm_batchcl_raw_signal_sum
ctrl.ratio
log2(value)
condition
WT
MUT
Control
rep
rep1
rep2
rep3

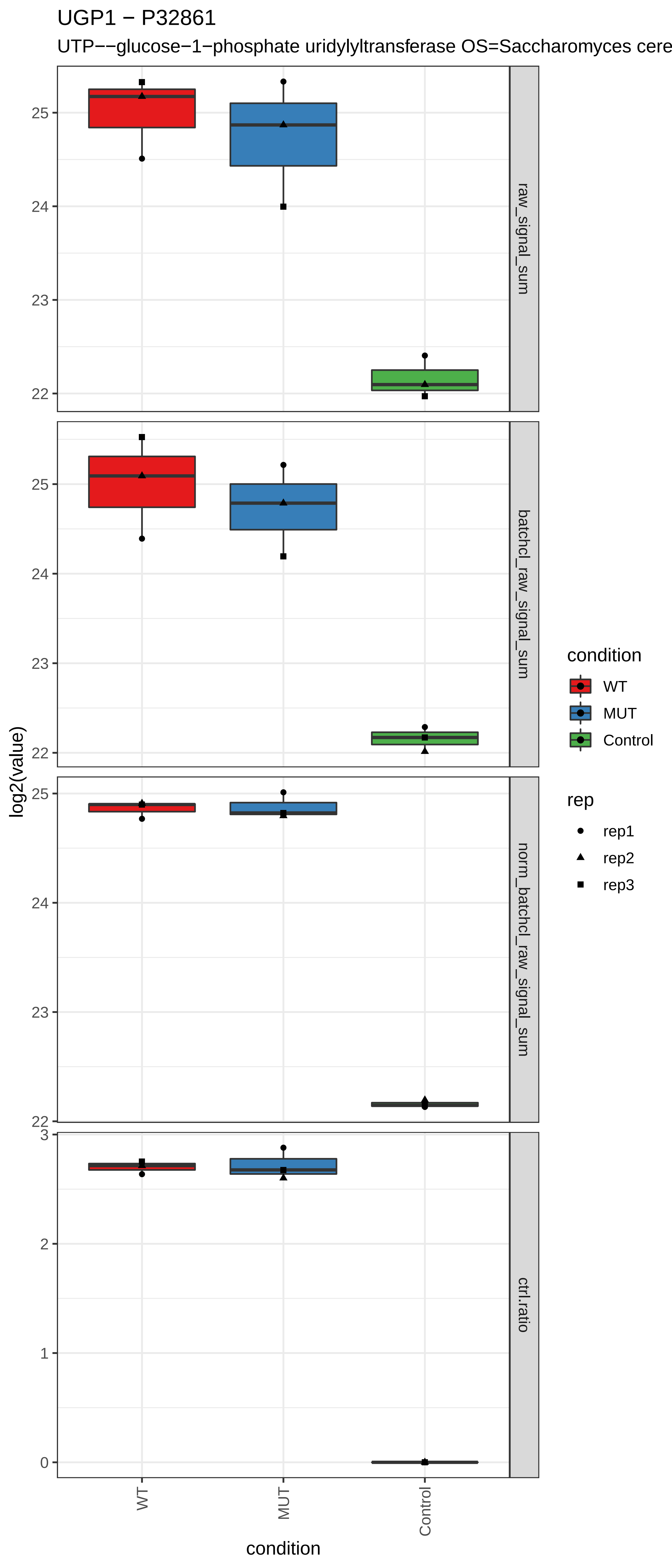

UGP1 − P32861
UTP−−glucose−1−phosphate uridylyltransferase OS=Saccharomyces cere
raw_signal_sum
batchcl_raw_signal_sum
norm_batchcl_raw_signal_sum
ctrl.ratio
log2(value)
condition
WT
MUT
Control
rep
rep1
rep2
rep3

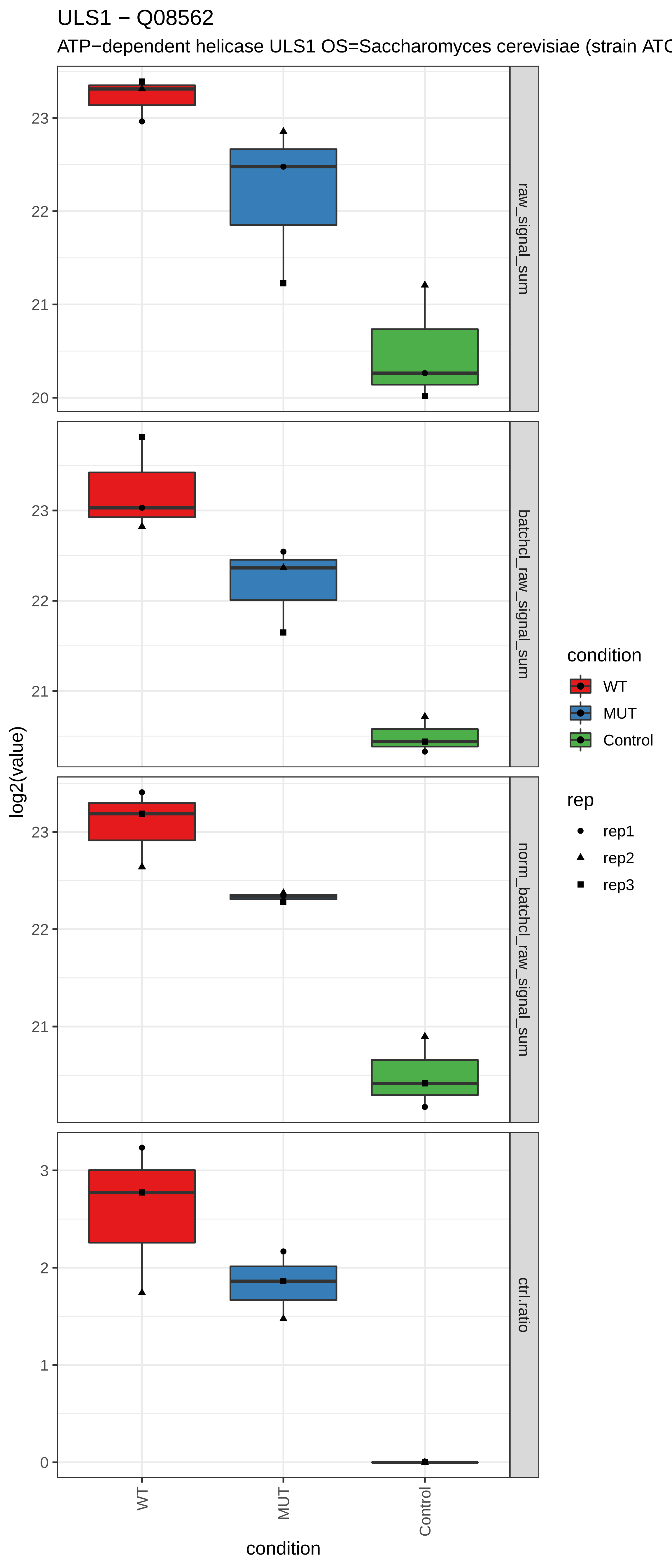

ULS1 − Q08562
ATP−dependent helicase ULS1 OS=Saccharomyces cerevisiae (strain ATC
raw_signal_sum
batchcl_raw_signal_sum
norm_batchcl_raw_signal_sum
ctrl.ratio
log2(value)
condition
WT
MUT
Control
rep
rep1
rep2
rep3
23
22
21
20
3
2
1
0

UME6 − P39001

Transcriptional regulatory protein UME6 OS=Saccharomyces cerevisiae (

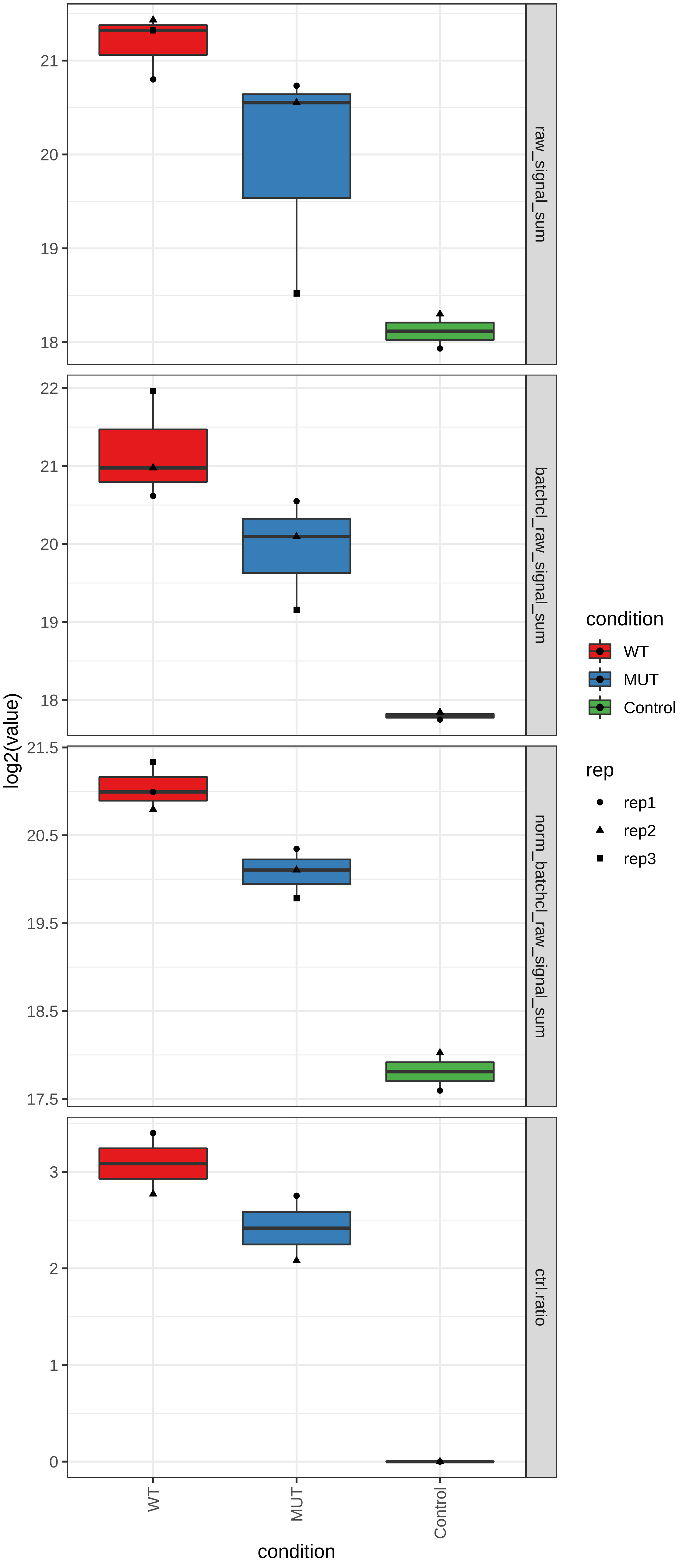

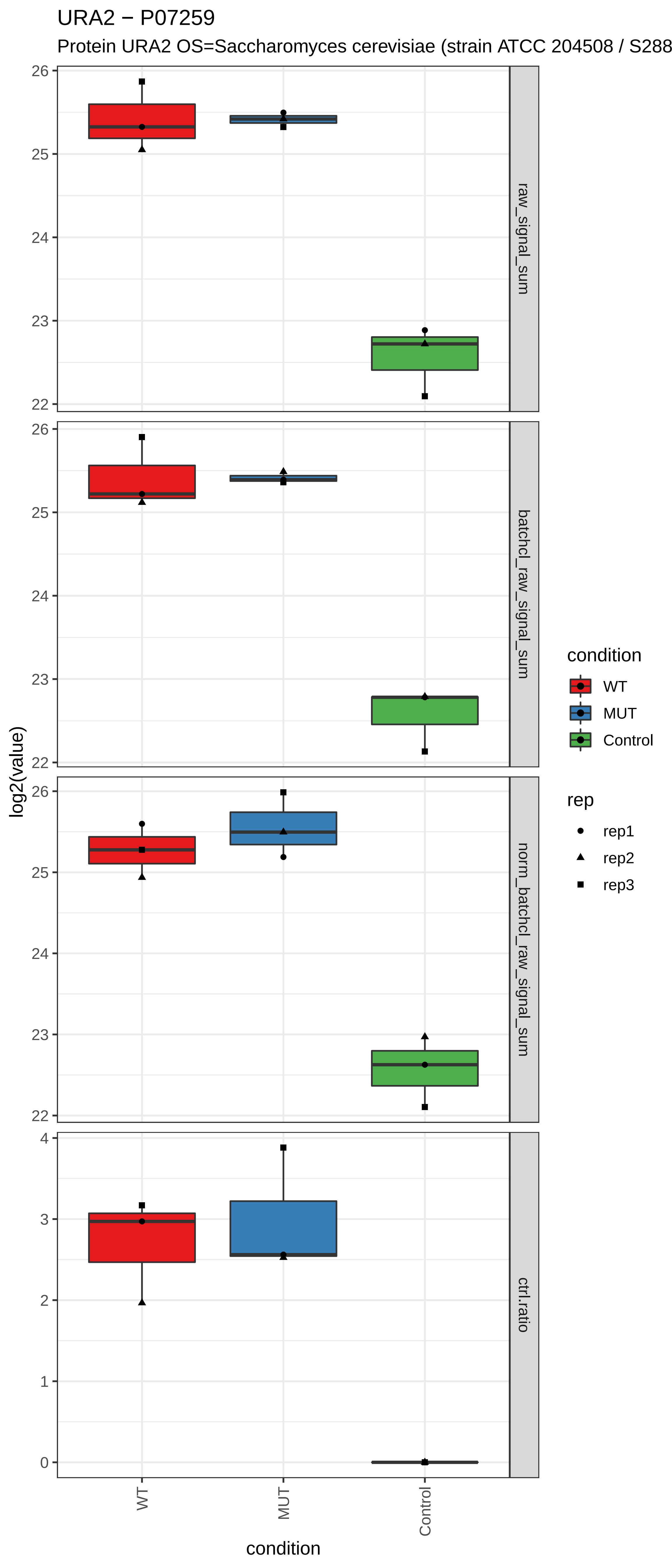

URA2 − P07259
Protein URA2 OS=Saccharomyces cerevisiae (strain ATCC 204508 / S288
raw_signal_sum
batchcl_raw_signal_sum
norm_batchcl_raw_signal_sum
ctrl.ratio
log2(value)
condition
WT
MUT
Control
rep
rep1
rep2
rep3
26
25
24
23
22
4
3
2
1
0

UTP23 − Q12339

rRNA−processing protein UTP23 OS=Saccharomyces cerevisiae (strain AT

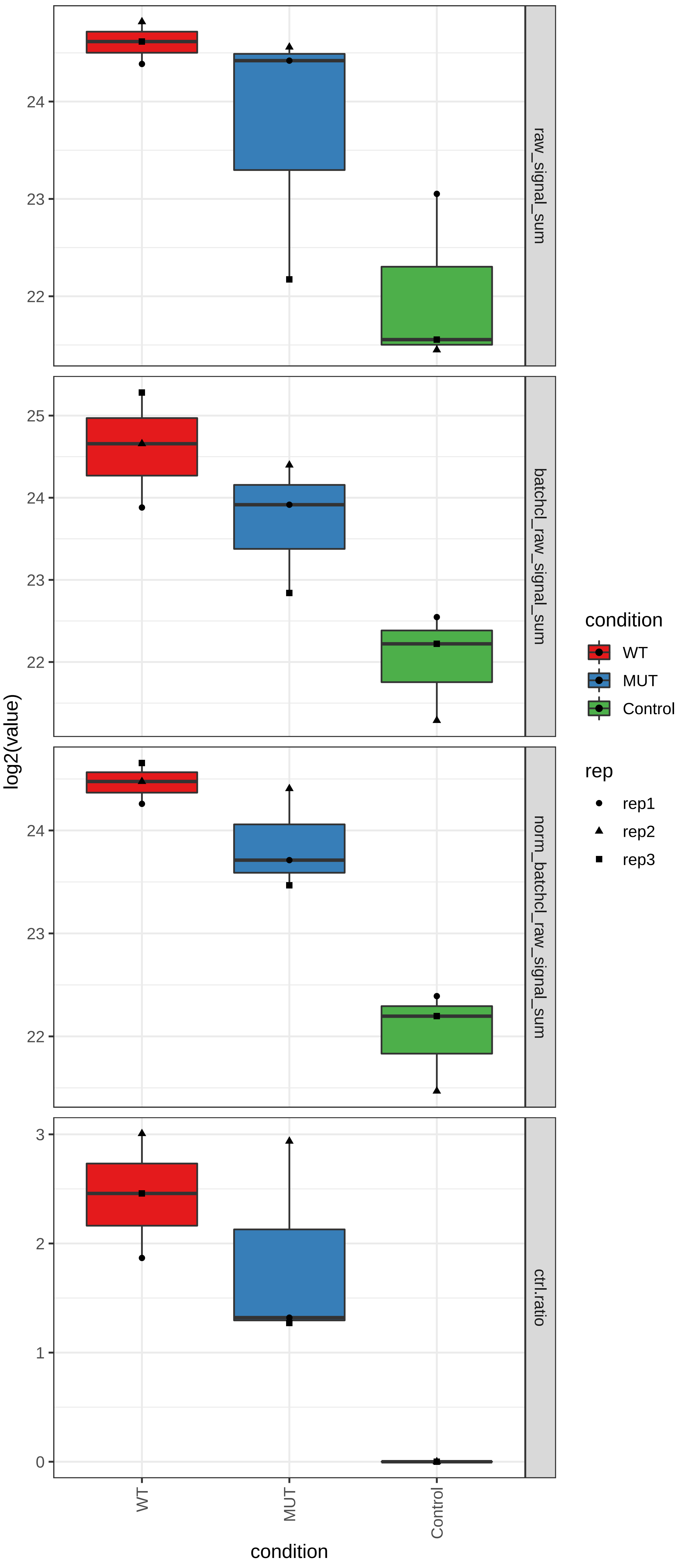

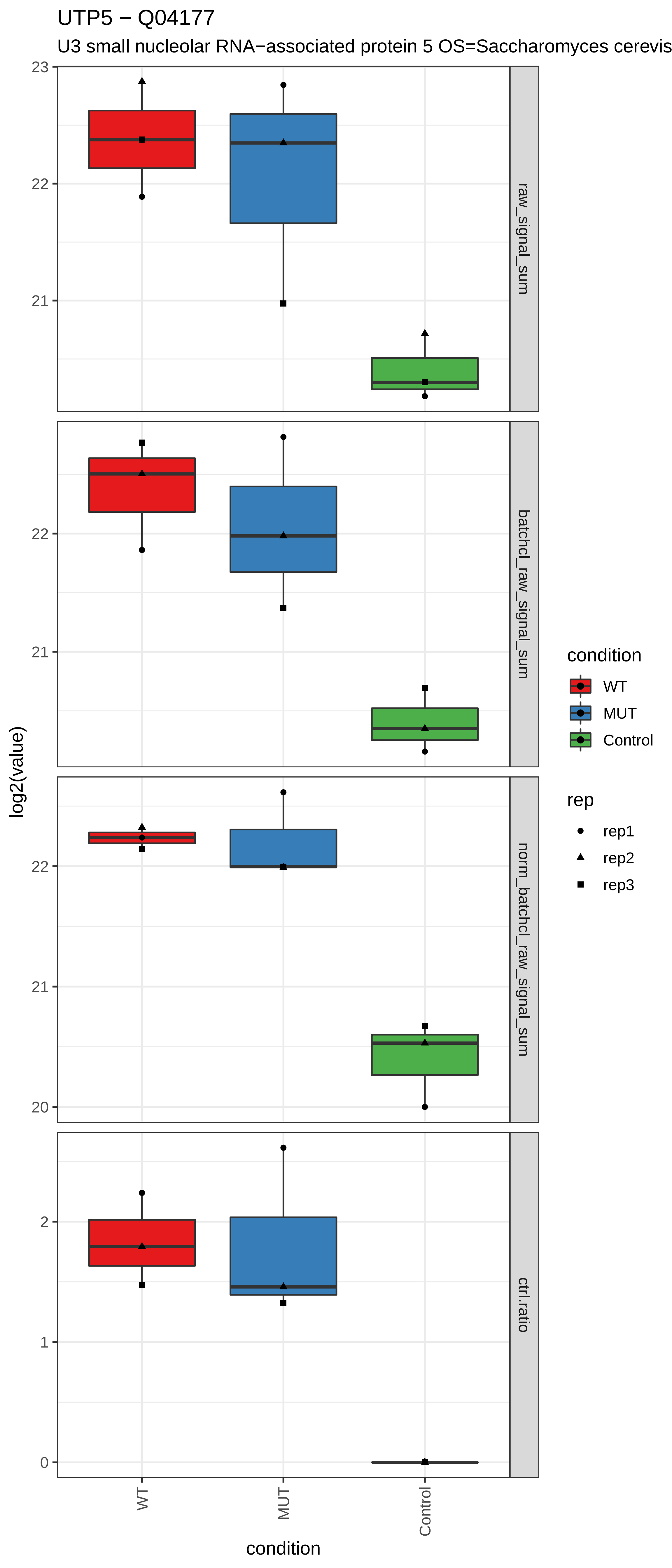

UTP5 − Q04177
U3 small nucleolar RNA−associated protein 5 OS=Saccharomyces cerevis
raw_signal_sum
batchcl_raw_signal_sum
norm_batchcl_raw_signal_sum
ctrl.ratio
log2(value)
condition
WT
MUT
Control
rep
rep1
rep2
rep3
23
22
21
20
2
1
0

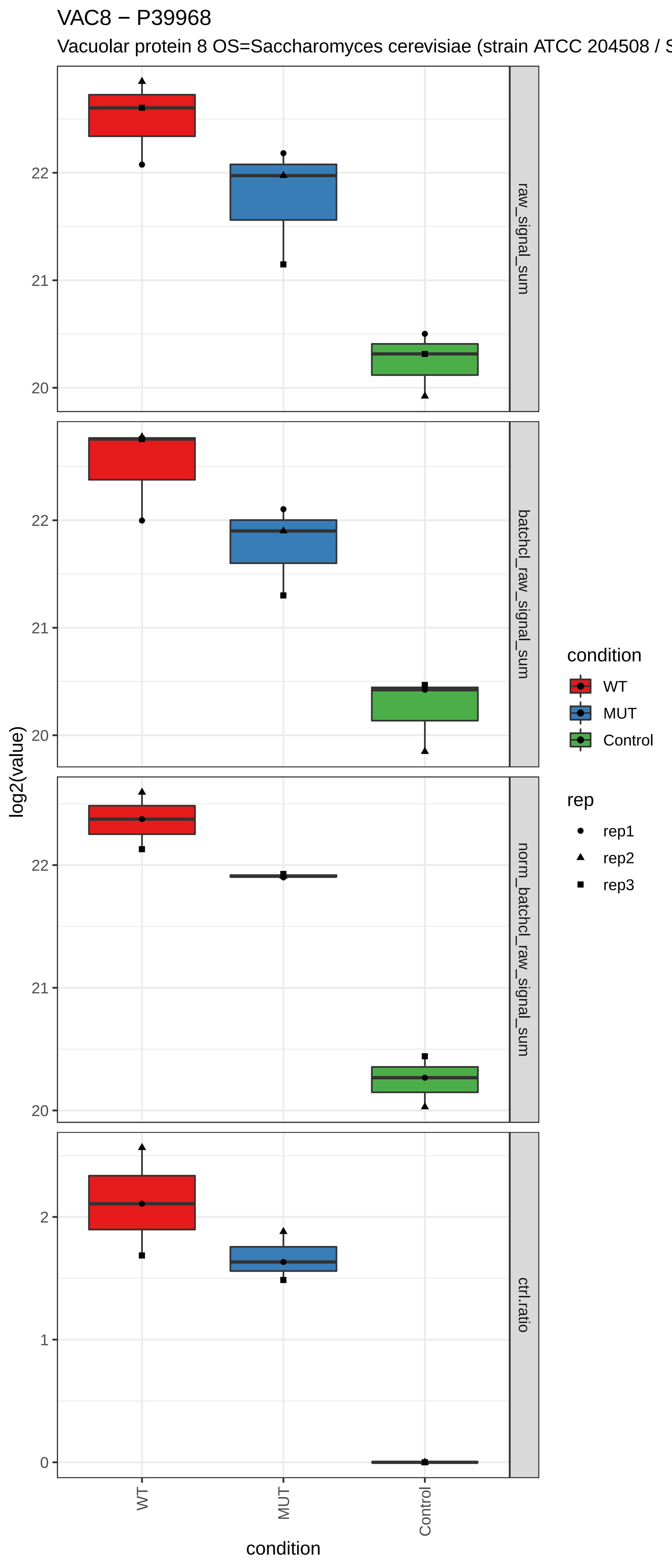
VAC8 − P39968
Vacuolar protein 8 OS=Saccharomyces cerevisiae (strain ATCC 204508 / S
raw_signal_sum
batchcl_raw_signal_sum
norm_batchcl_raw_signal_sum
ctrl.ratio
log2(value)
condition
WT
MUT
Control
rep
rep1
rep2
rep3
22
21
20
2
1
0

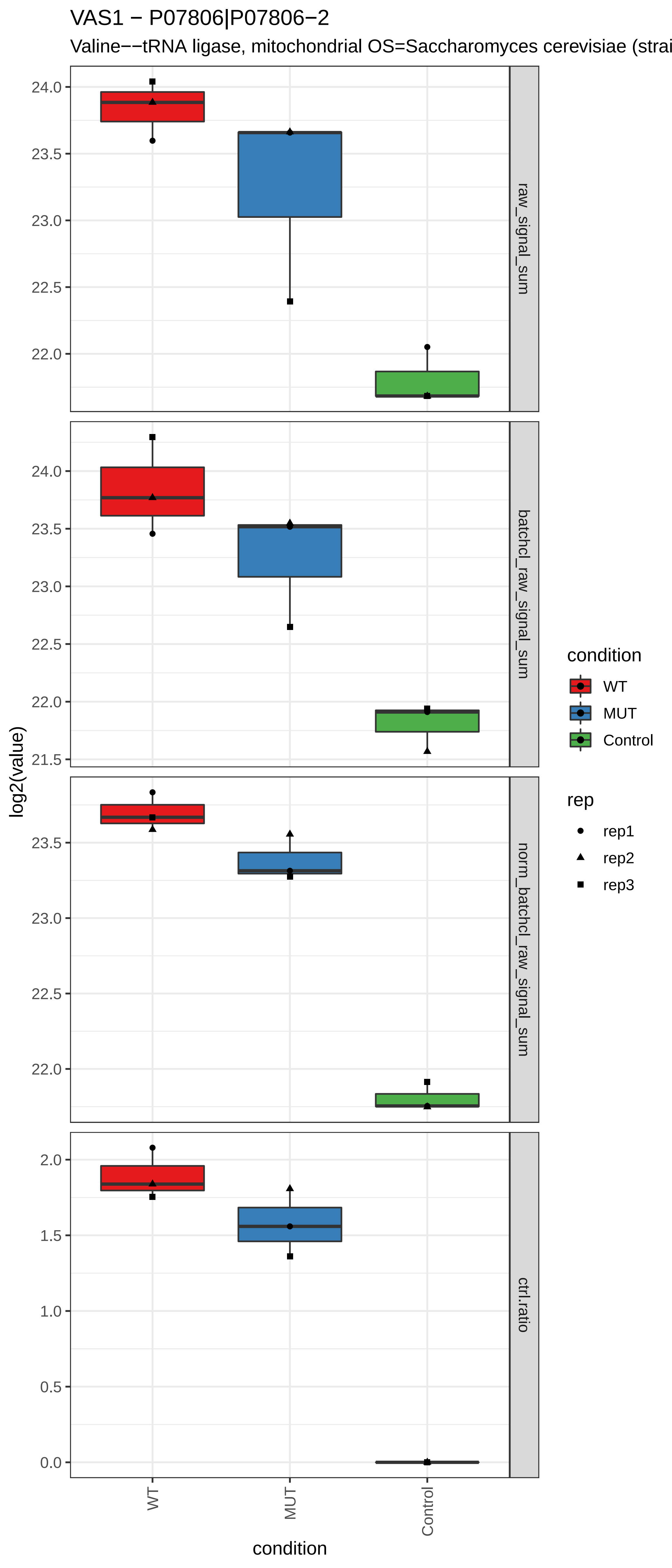
VAS1 − P07806|P07806−2
Valine−−tRNA ligase, mitochondrial OS=Saccharomyces cerevisiae (strai
raw_signal_sum
batchcl_raw_signal_sum
norm_batchcl_raw_signal_sum
ctrl.ratio
log2(value)
condition
WT
MUT
Control
rep
rep1
rep2
rep3

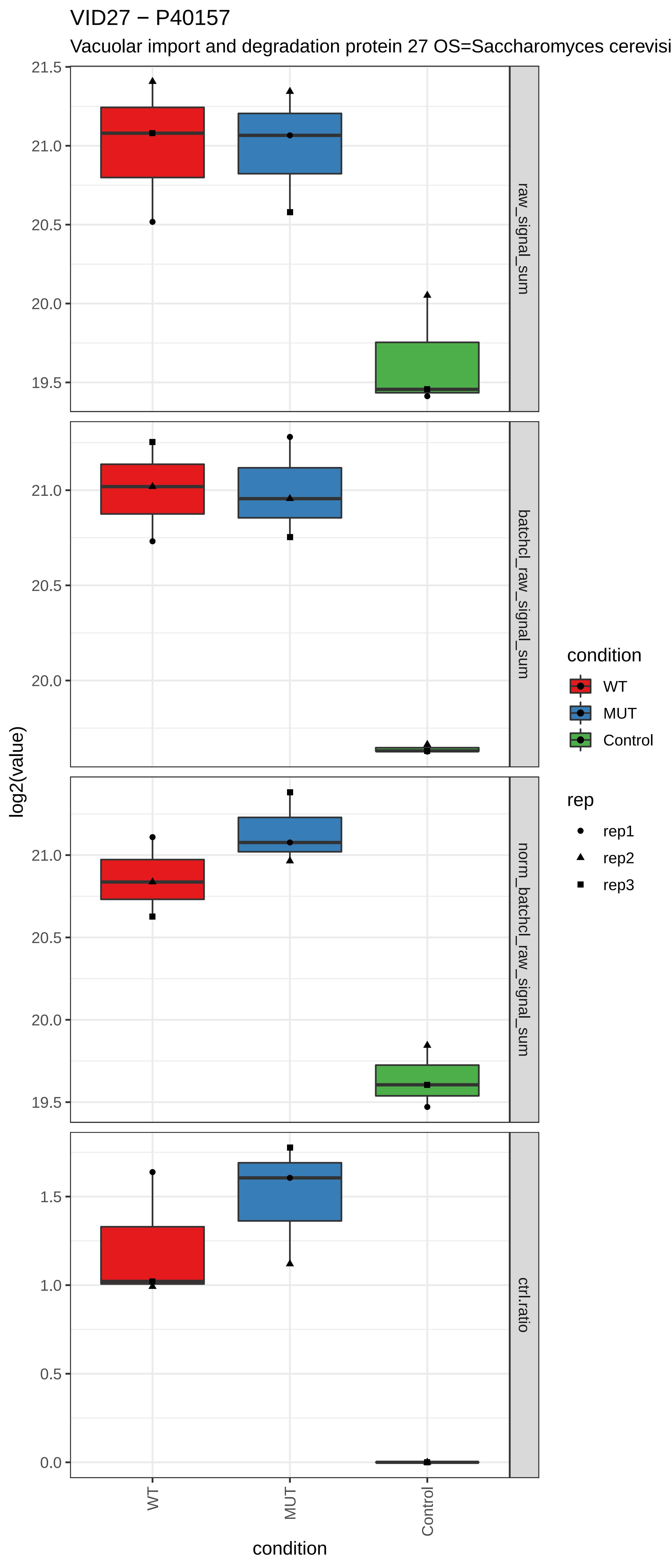

VID27 − P40157
Vacuolar import and degradation protein 27 OS=Saccharomyces cerevisi
log2(value)
raw_signal_sum
batchcl_raw_signal_sum
norm_batchcl_raw_signal_sum
ctrl.ratio
21.5
21.0
20.5
20.0
19.5
1.5
1.0
0.5
0.0
WT
MUT
Control
condition
condition
WT
MUT
Control
rep
rep1
rep2
rep3

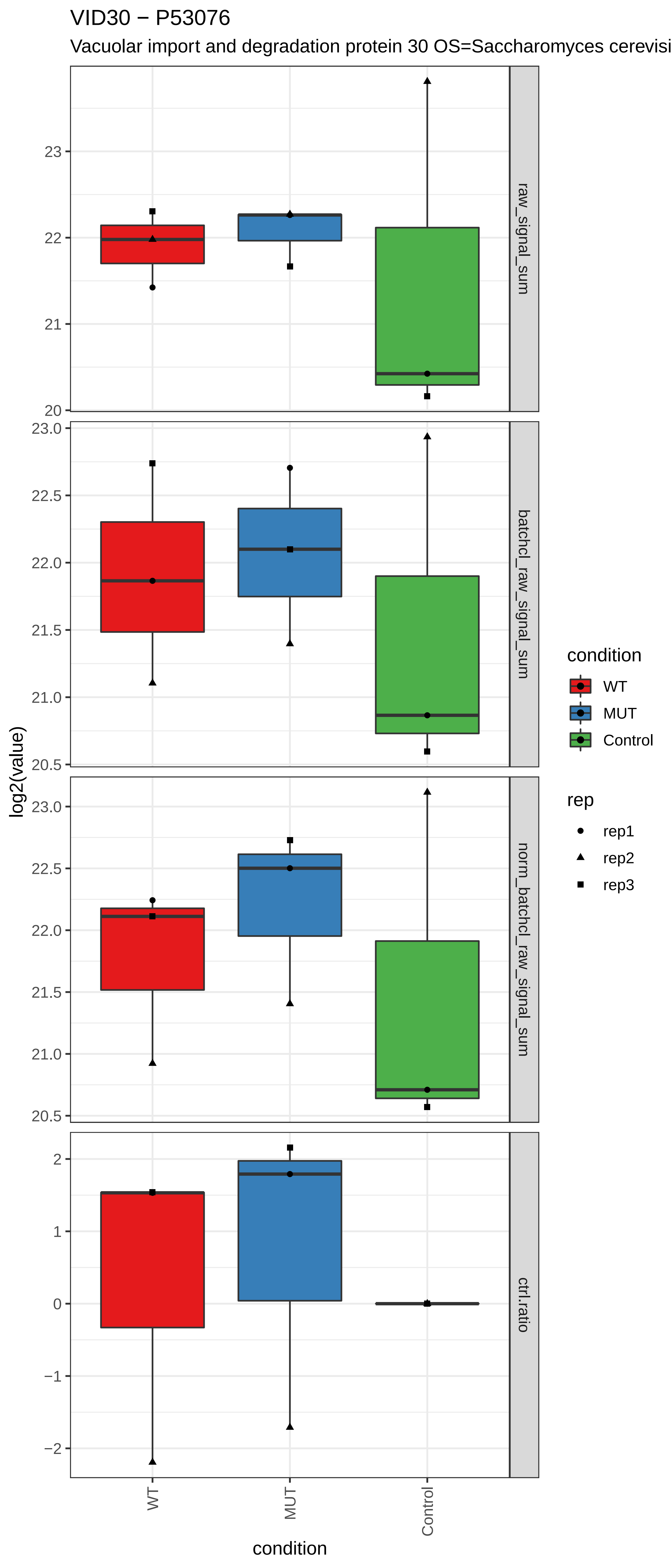
VID30 − P53076
Vacuolar import and degradation protein 30 OS=Saccharomyces cerevisi
raw_signal_sum
batchcl_raw_signal_sum
norm_batchcl_raw_signal_sum
ctrl.ratio
log2(value)
condition
WT
MUT
Control
rep
rep1
rep2
rep3
23
22
21
20
23.0
22.5
22.0
21.5
21.0
20.5
2
1
0
−1
−2

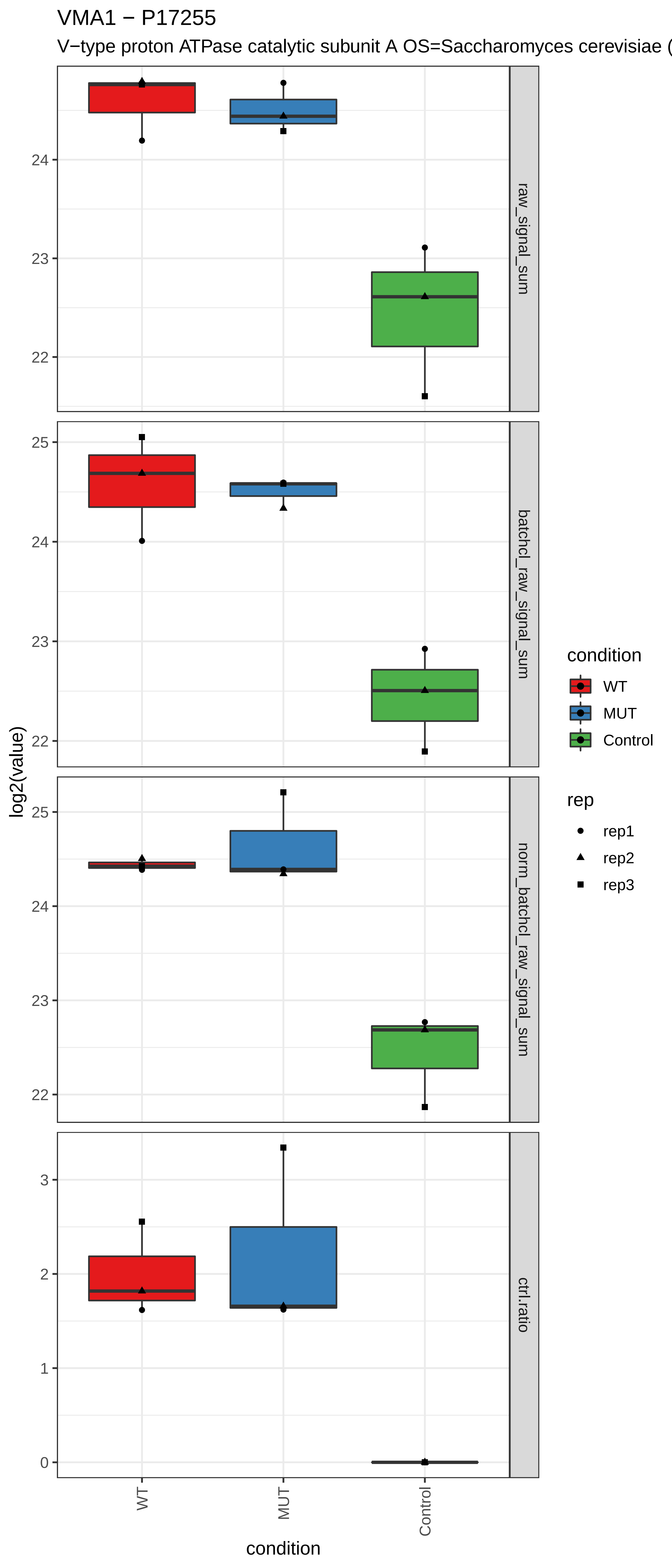

VMA1 − P17255
V−type proton ATPase catalytic subunit A OS=Saccharomyces cerevisiae (
raw_signal_sum
batchcl_raw_signal_sum
norm_batchcl_raw_signal_sum
ctrl.ratio
log2(value)
condition
WT
MUT
Control
rep
rep1
rep2
rep3

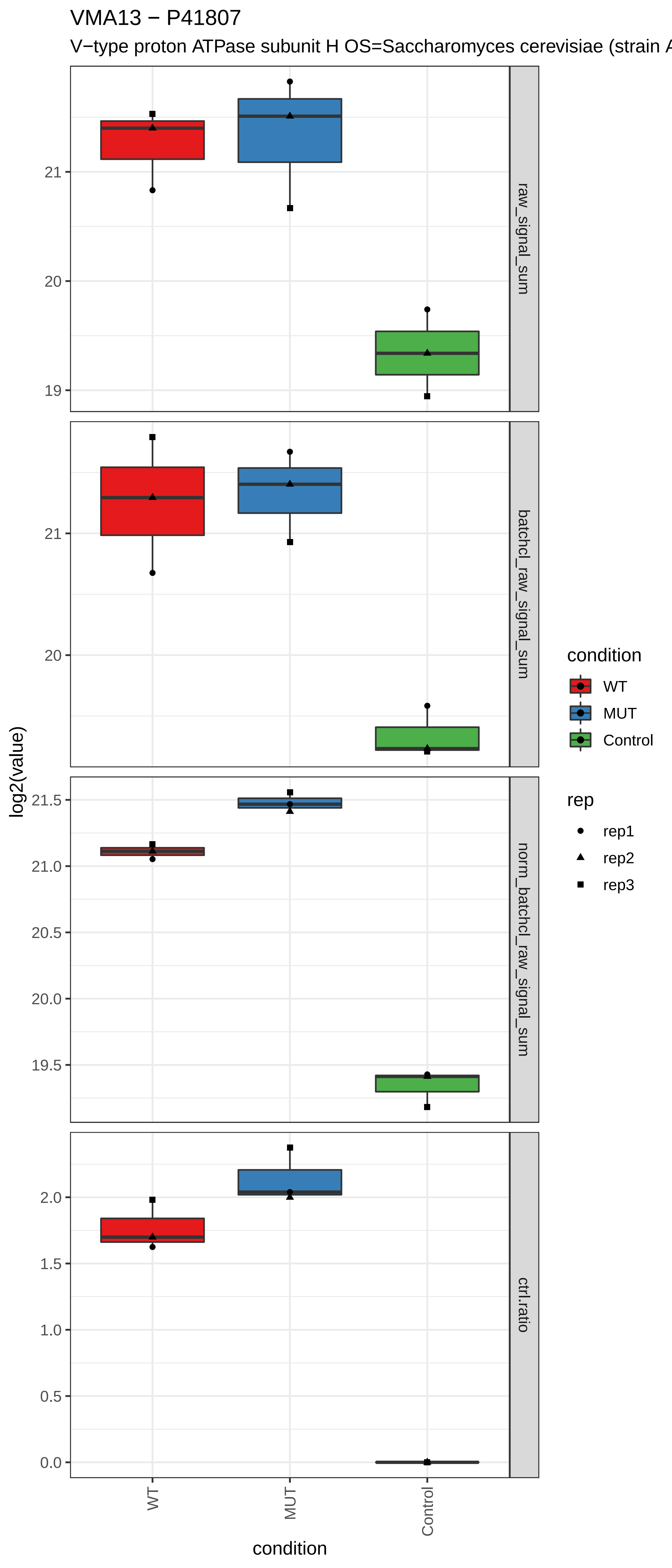
VMA13 − P41807
V−type proton ATPase subunit H OS=Saccharomyces cerevisiae (strain A
raw_signal_sum
batchcl_raw_signal_sum
norm_batchcl_raw_signal_sum
ctrl.ratio
log2(value)
21
20
19
21.5
21.0
20.5
20.0
19.5
2.0
1.5
1.0
0.5
0.0
WT
MUT
Control
condition
condition
WT
MUT
Control
rep
rep1
rep2
rep3

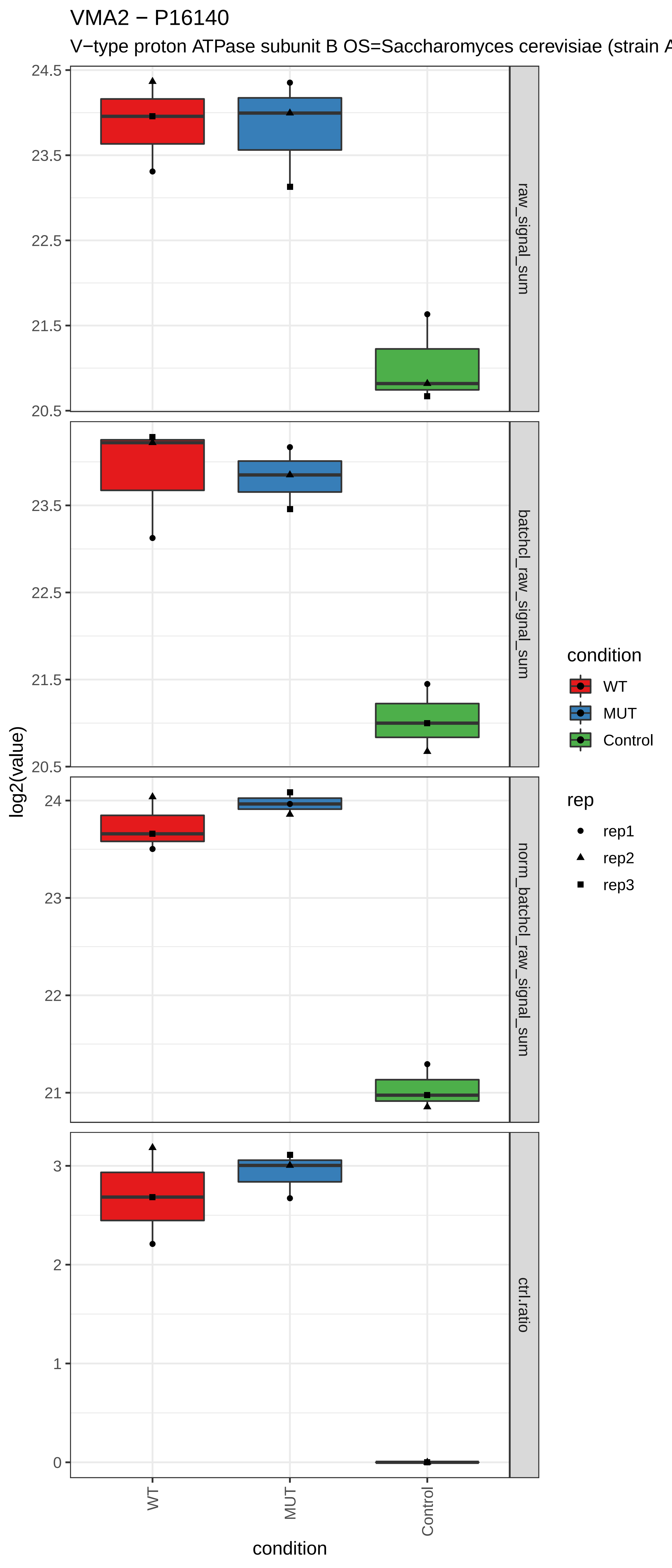
VMA2 − P16140
V−type proton ATPase subunit B OS=Saccharomyces cerevisiae (strain A
log2(value)
raw_signal_sum
batchcl_raw_signal_sum
norm_batchcl_raw_signal_sum
ctrl.ratio
24.5
23.5
22.5
21.5
20.5
24
23
22
21
3
2
1
0
WT
MUT
Control
condition
condition
WT
MUT
Control
rep
rep1
rep2
rep3

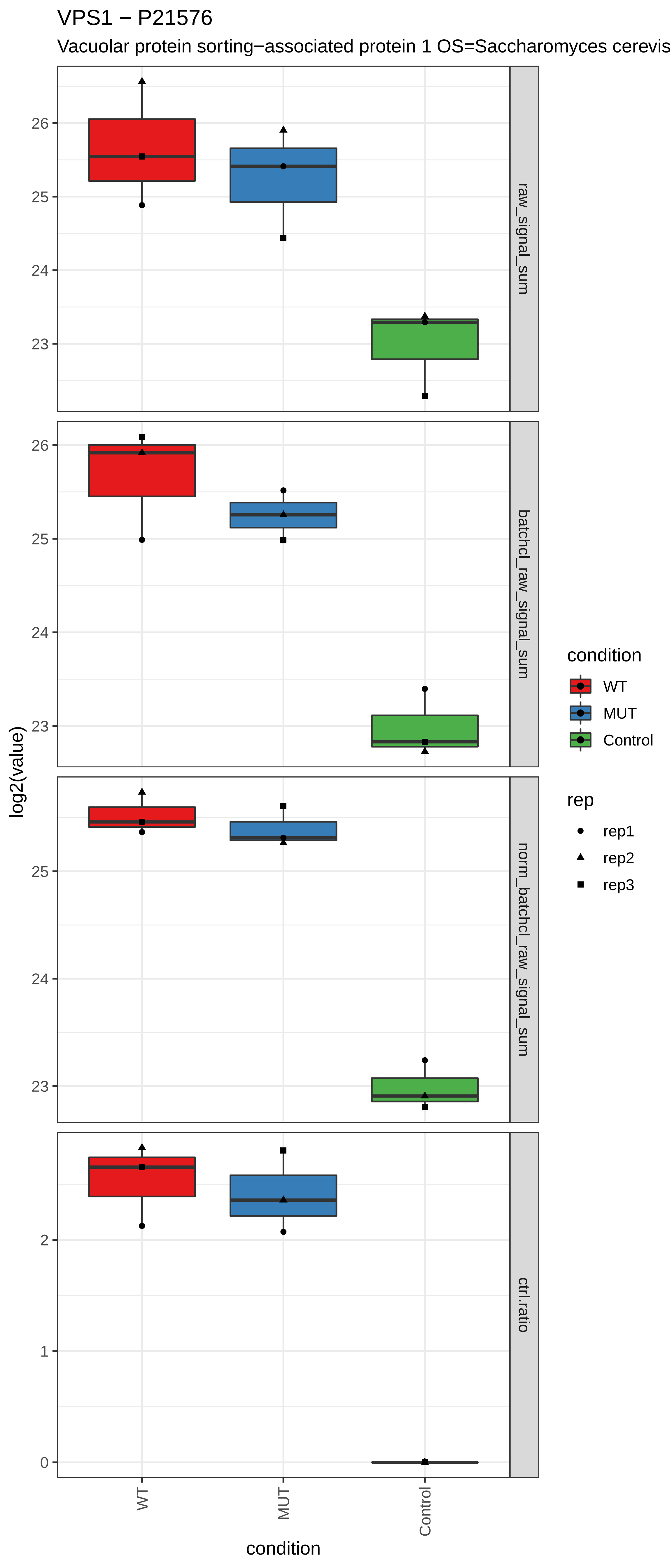

VPS1 − P21576
Vacuolar protein sorting−associated protein 1 OS=Saccharomyces cerevis
raw_signal_sum
batchcl_raw_signal_sum
norm_batchcl_raw_signal_sum
ctrl.ratio
log2(value)
condition
WT
MUT
Control
rep
rep1
rep2
rep3
26
25
24
23
2
1
0

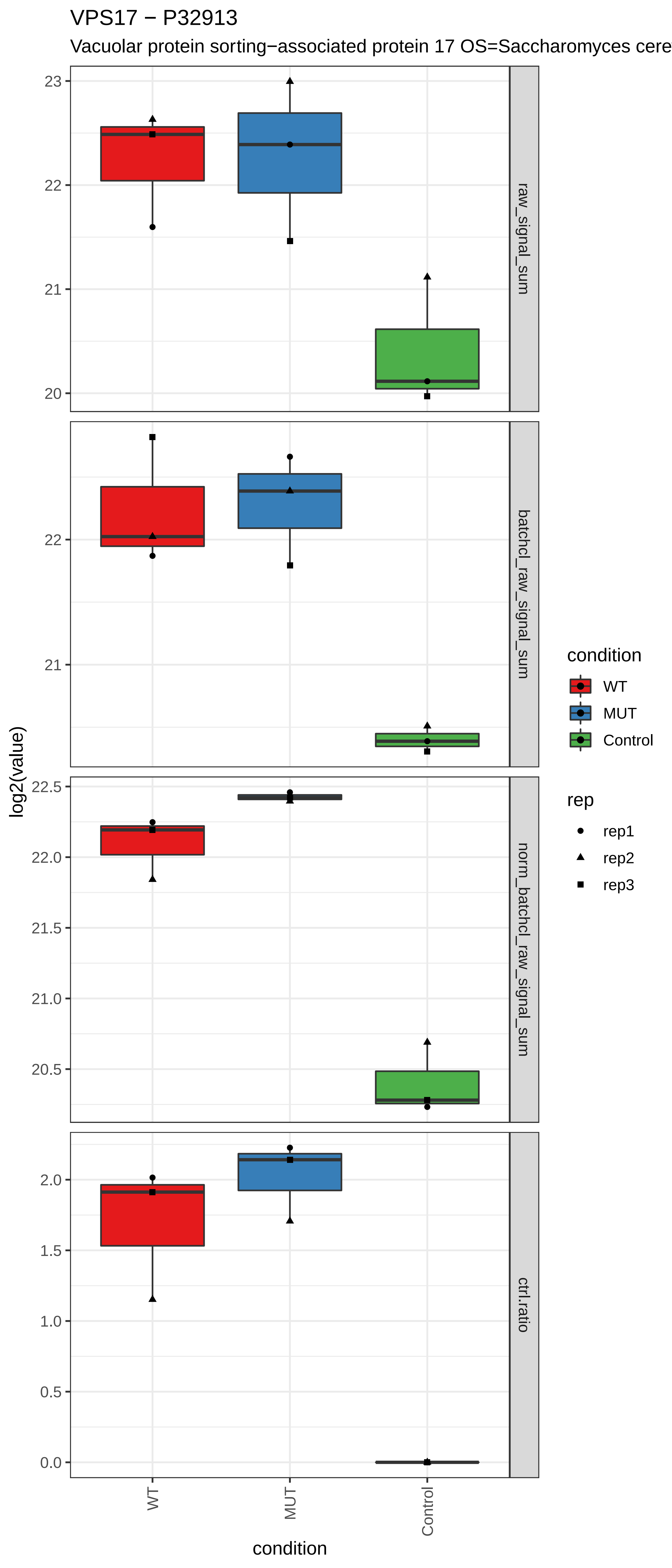

VPS17 − P32913
Vacuolar protein sorting−associated protein 17 OS=Saccharomyces cere
raw_signal_sum
batchcl_raw_signal_sum
norm_batchcl_raw_signal_sum
ctrl.ratio
log2(value)
condition
WT
MUT
Control
rep
rep1
rep2
rep3

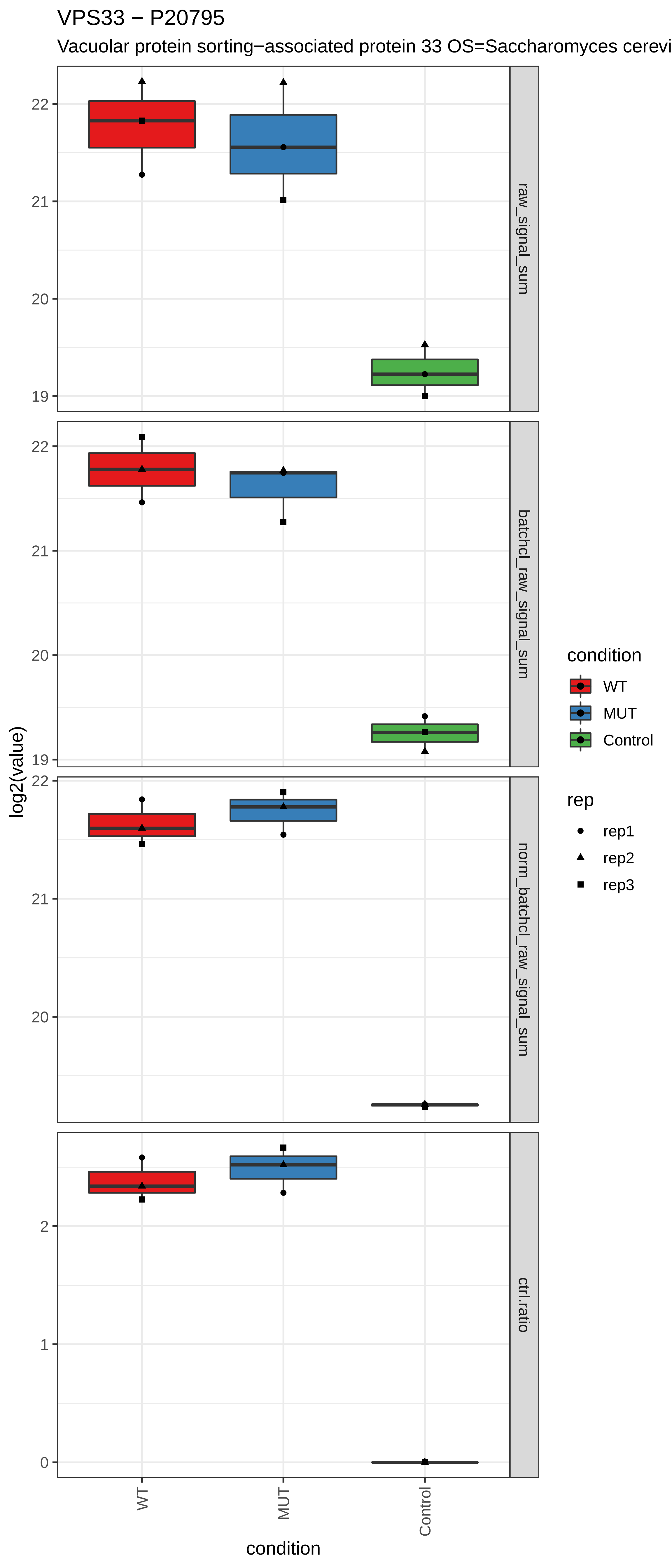

VPS33 − P20795
Vacuolar protein sorting−associated protein 33 OS=Saccharomyces cerevi
raw_signal_sum
batchcl_raw_signal_sum
norm_batchcl_raw_signal_sum
ctrl.ratio
log2(value)
condition
WT
MUT
Control
rep
rep1
rep2
rep3
22
21
20
19
2
1
0

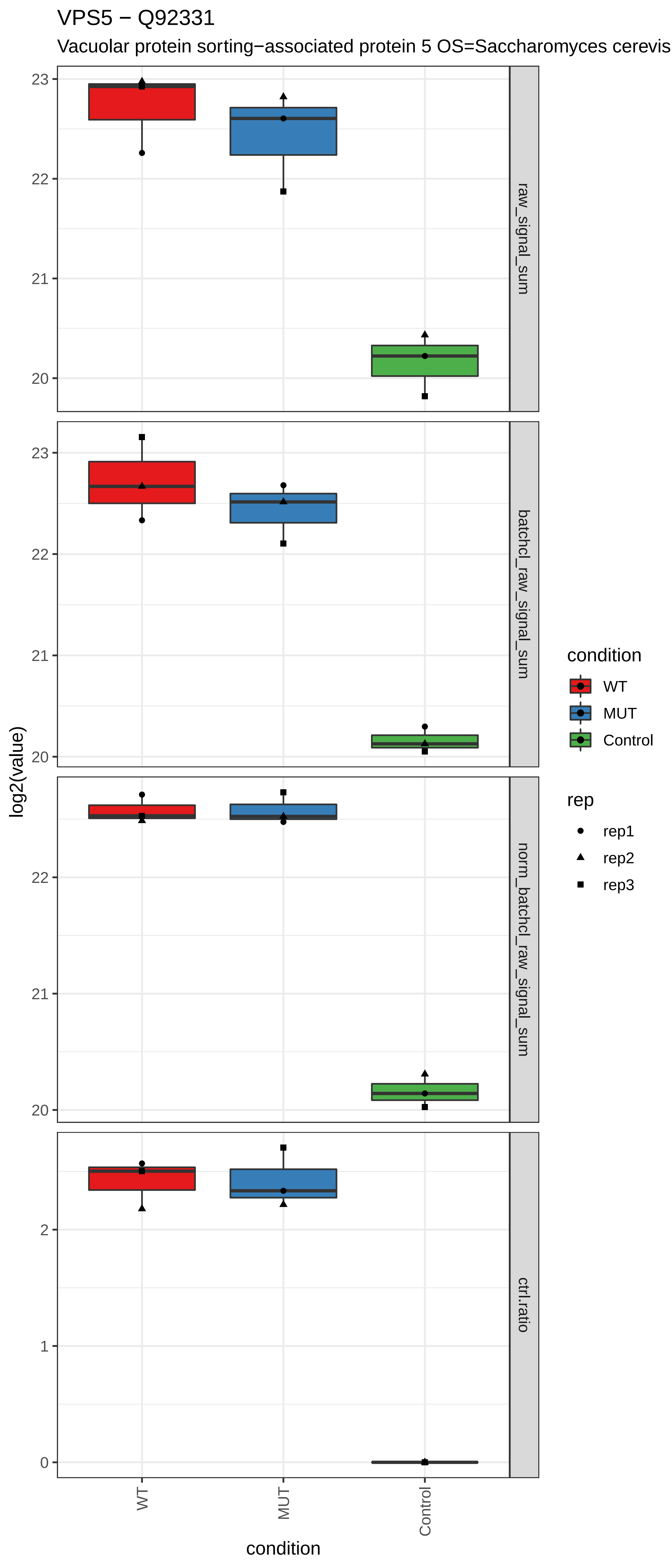
VPS5 − Q92331
Vacuolar protein sorting−associated protein 5 OS=Saccharomyces cerevis
log2(value)
raw_signal_sum
batchcl_raw_signal_sum
norm_batchcl_raw_signal_sum
ctrl.ratio
condition
WT
MUT
Control
rep
rep1
rep2
rep3
23
22
21
20
2
1
0

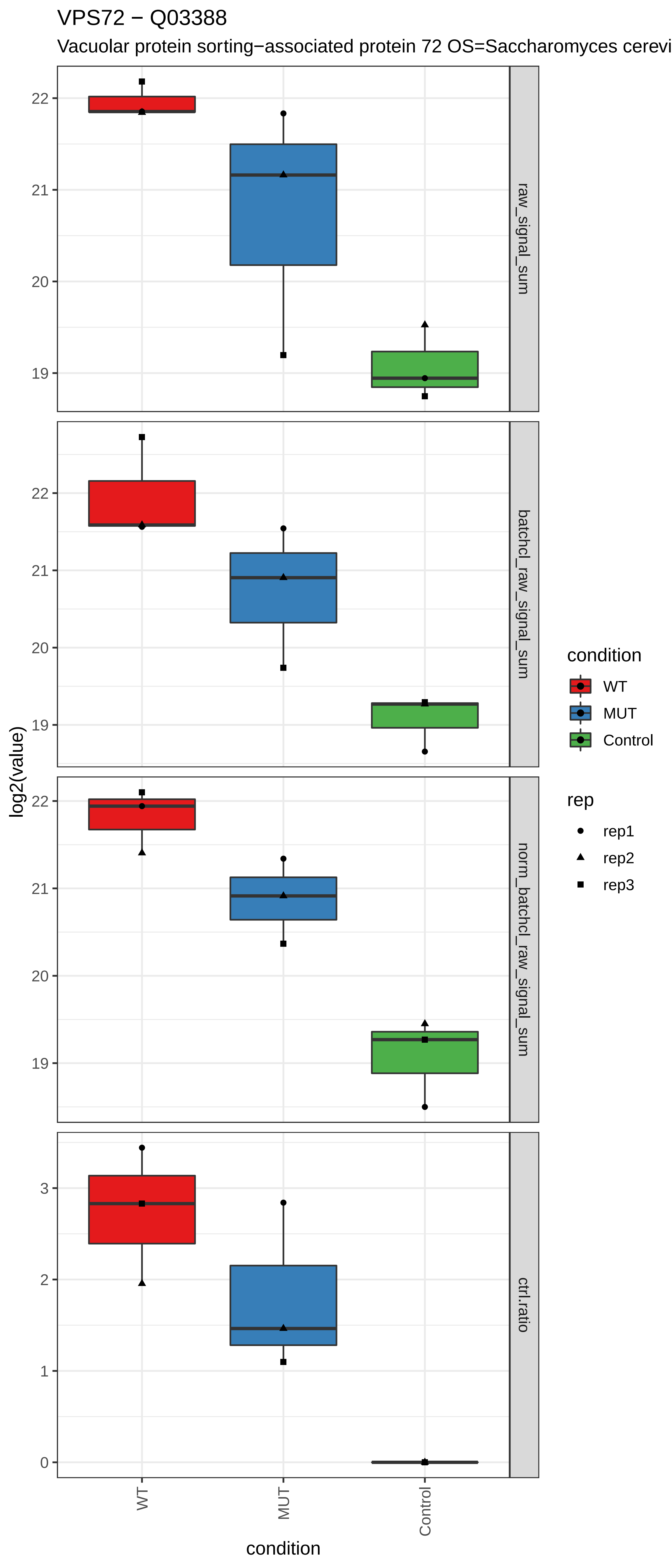

VPS72 − Q03388
Vacuolar protein sorting−associated protein 72 OS=Saccharomyces cerevi
raw_signal_sum
batchcl_raw_signal_sum
norm_batchcl_raw_signal_sum
ctrl.ratio
log2(value)
condition
WT
MUT
Control
rep
rep1
rep2
rep3

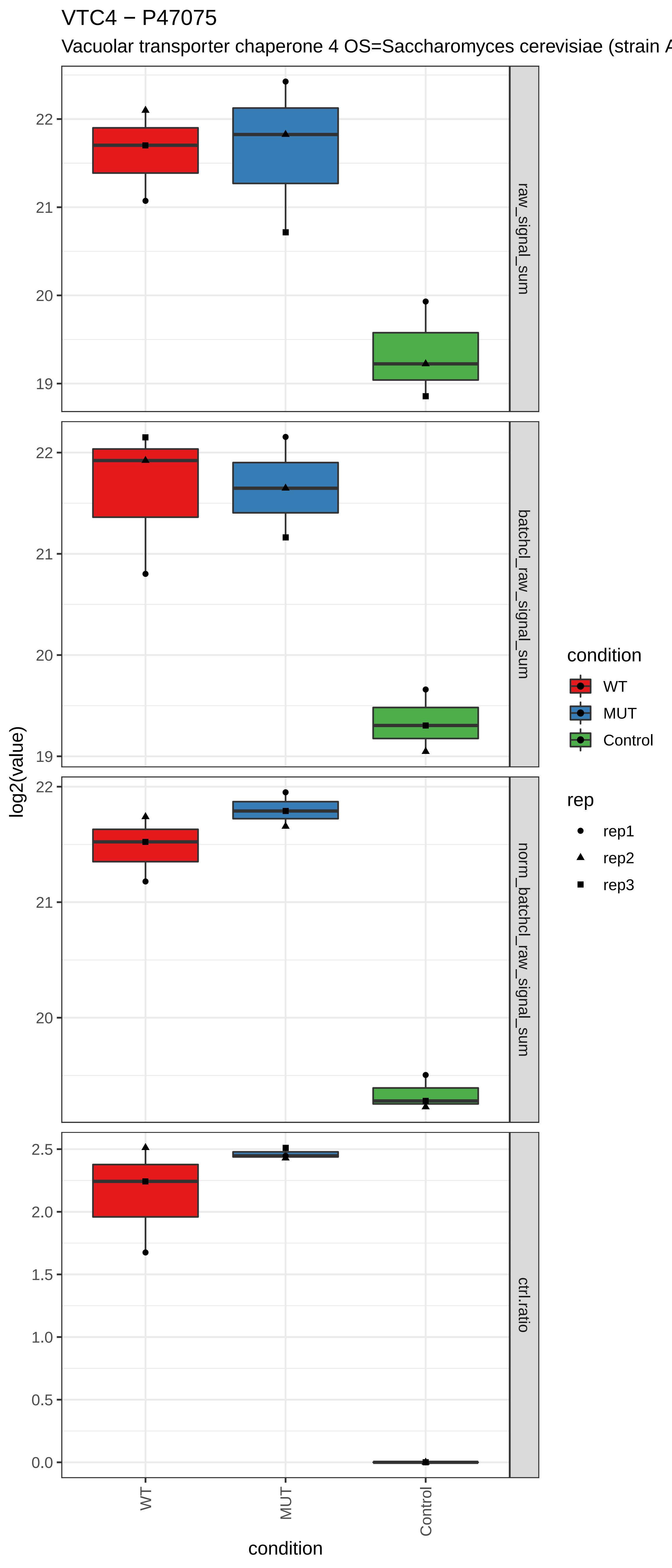

VTC4 − P47075
Vacuolar transporter chaperone 4 OS=Saccharomyces cerevisiae (strain A
raw_signal_sum
batchcl_raw_signal_sum
norm_batchcl_raw_signal_sum
ctrl.ratio
log2(value)
condition
WT
MUT
Control
rep
rep1
rep2
rep3

WRS1 − Q12109

Tryptophan−−tRNA ligase, cytoplasmic OS=Saccharomyces cerevisiae (str

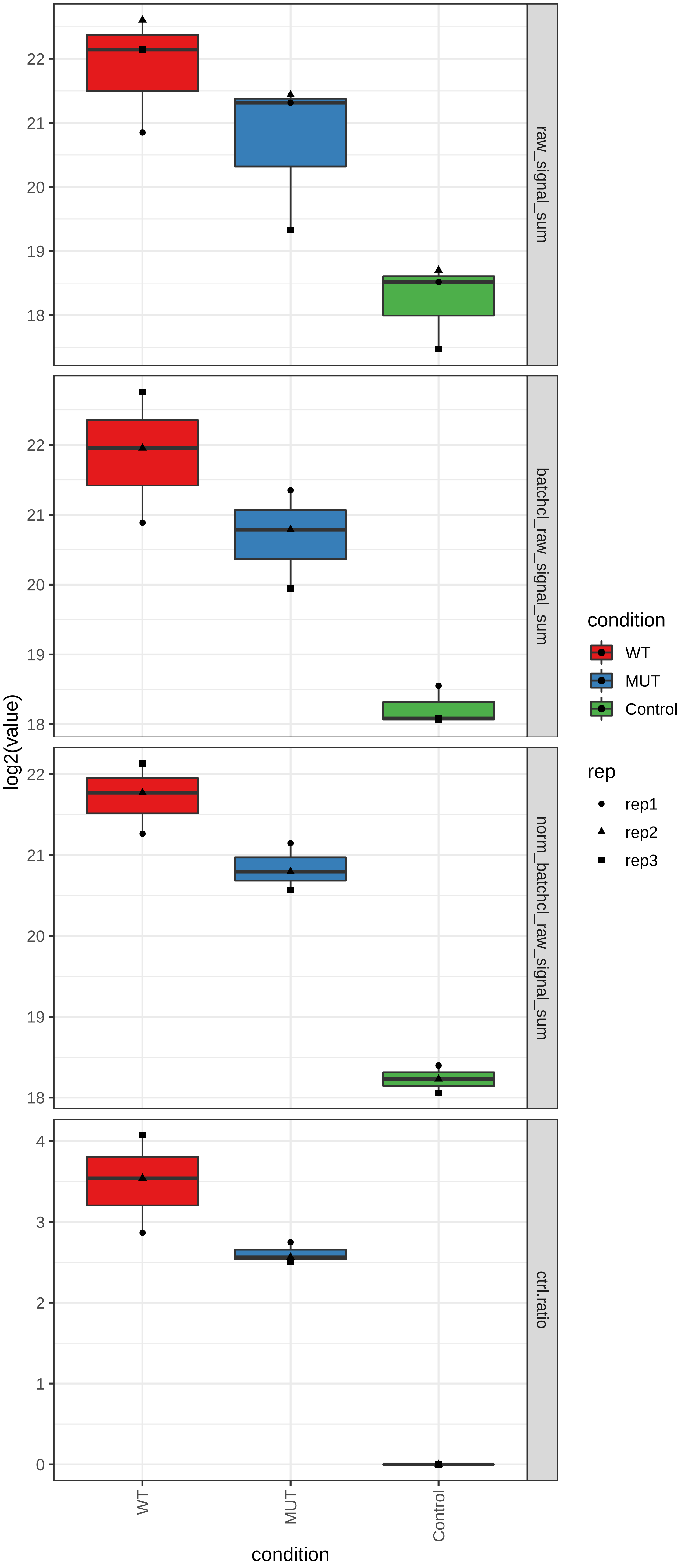

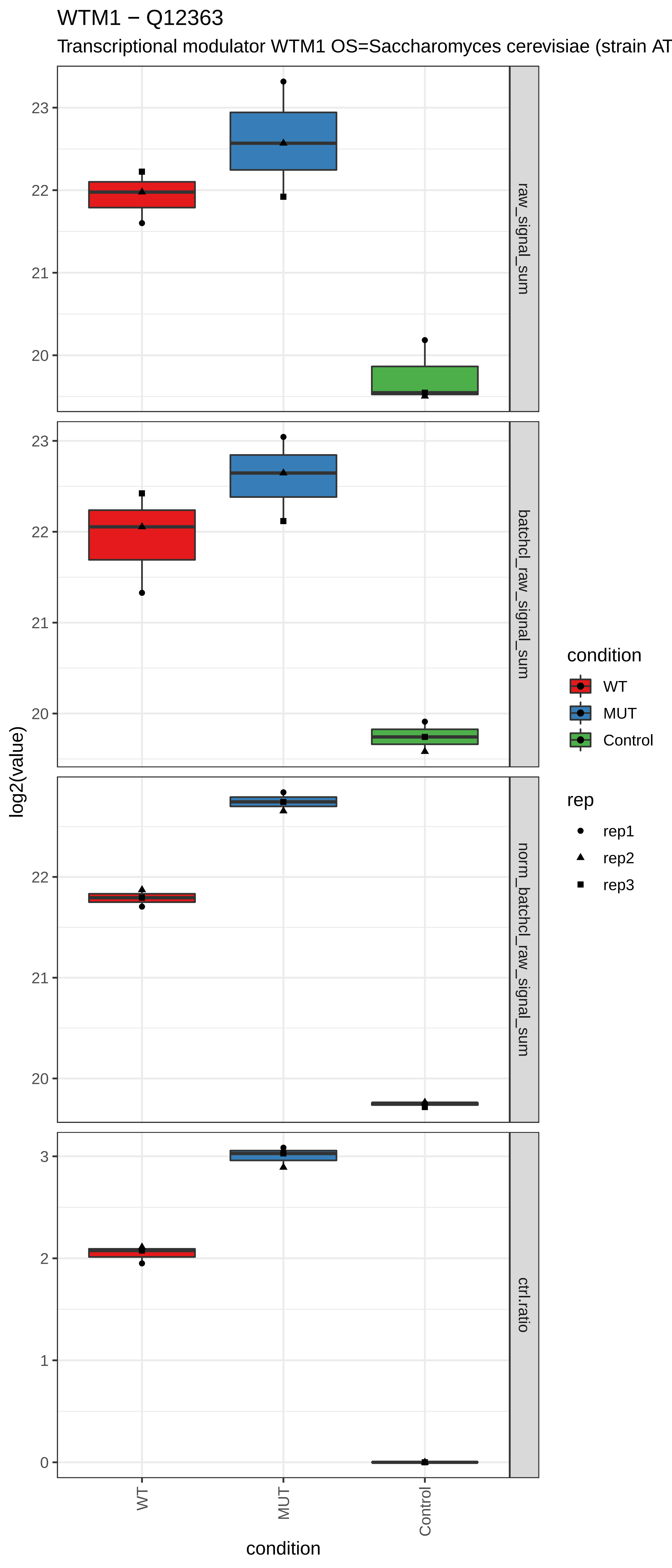

WTM1 − Q12363
Transcriptional modulator WTM1 OS=Saccharomyces cerevisiae (strain AT
raw_signal_sum
batchcl_raw_signal_sum
norm_batchcl_raw_signal_sum
ctrl.ratio
log2(value)
condition
WT
MUT
Control
rep
rep1
rep2
rep3

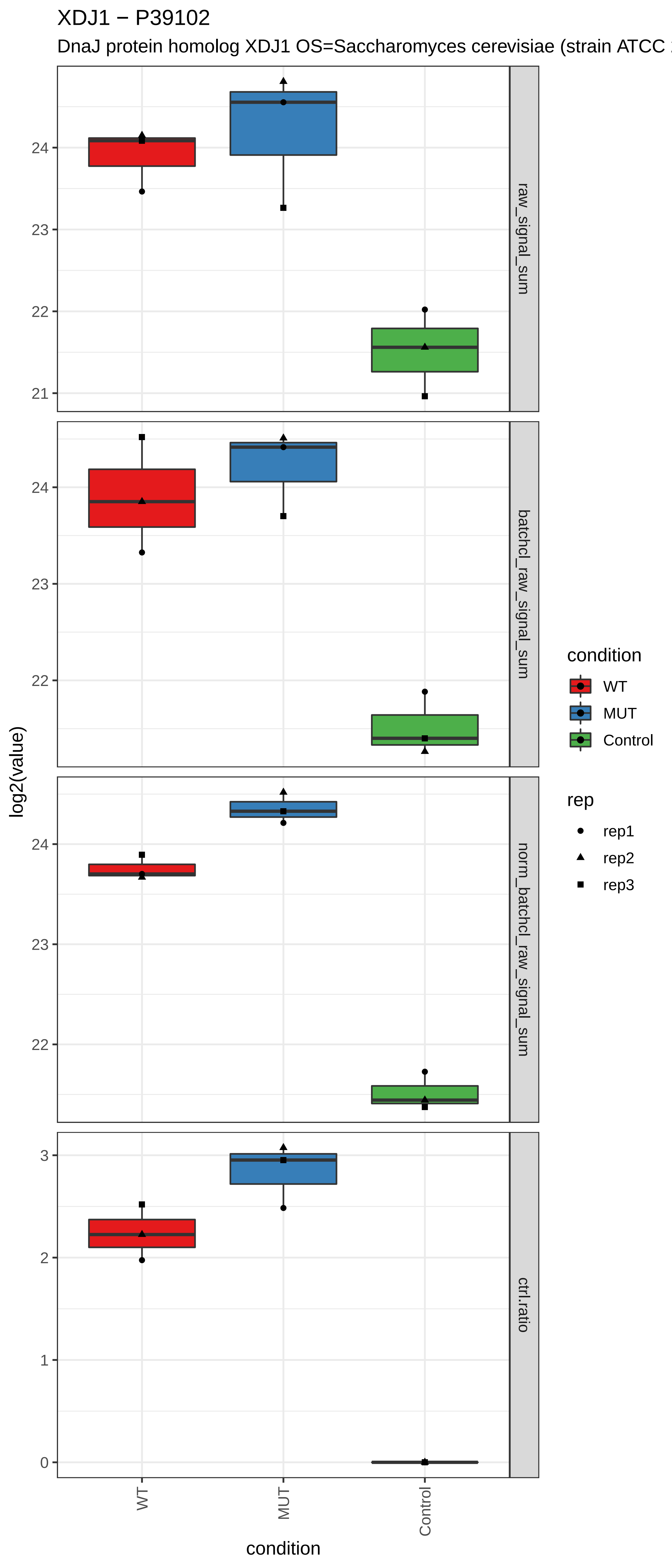

XDJ1 − P39102
DnaJ protein homolog XDJ1 OS=Saccharomyces cerevisiae (strain ATCC
log2(value)
raw_signal_sum
batchcl_raw_signal_sum
norm_batchcl_raw_signal_sum
ctrl.ratio
condition
WT
MUT
Control
rep
rep1
rep2
rep3

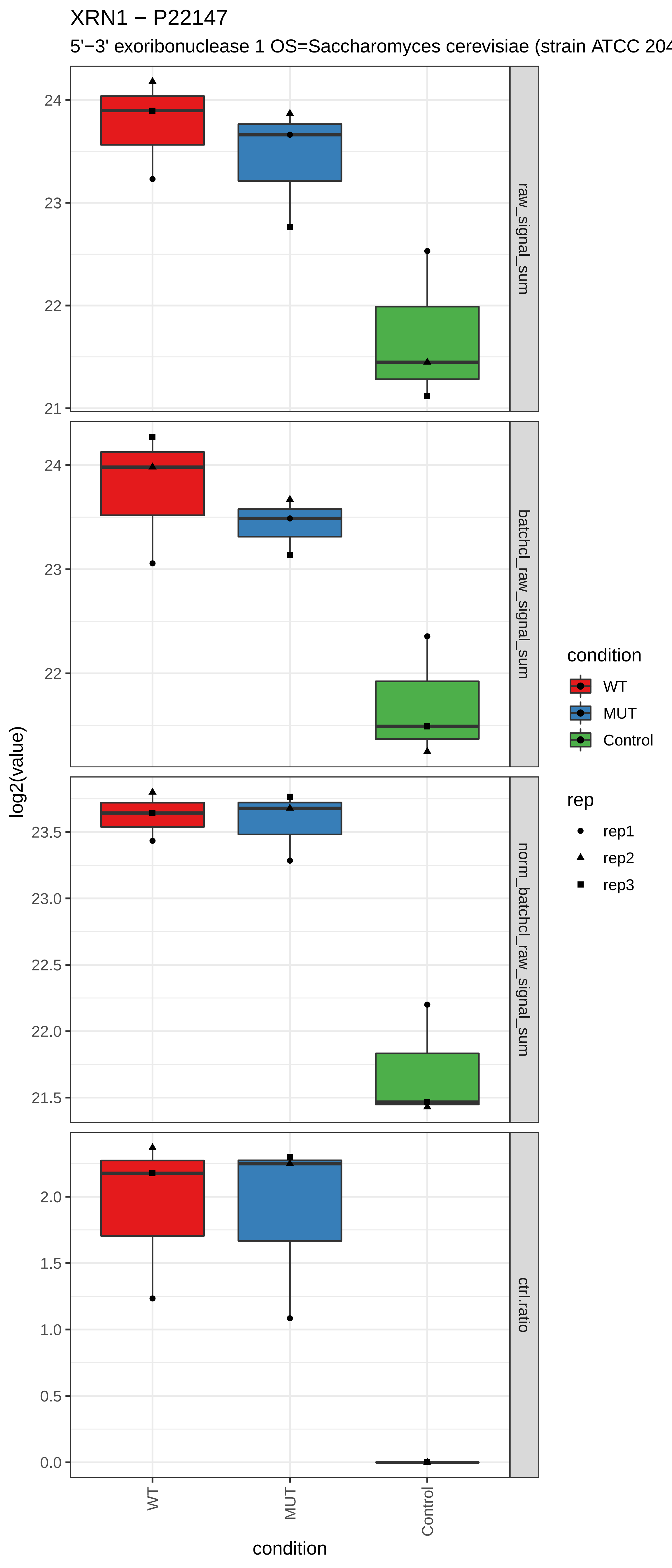
XRN1 − P22147
5'−3' exoribonuclease 1 OS=Saccharomyces cerevisiae (strain ATCC 204
raw_signal_sum
batchcl_raw_signal_sum
norm_batchcl_raw_signal_sum
ctrl.ratio
log2(value)
condition
WT
MUT
Control
rep
rep1
rep2
rep3
24
23
22
21
23.5
23.0
22.5
22.0
21.5
2.0
1.5
1.0
0.5
0.0

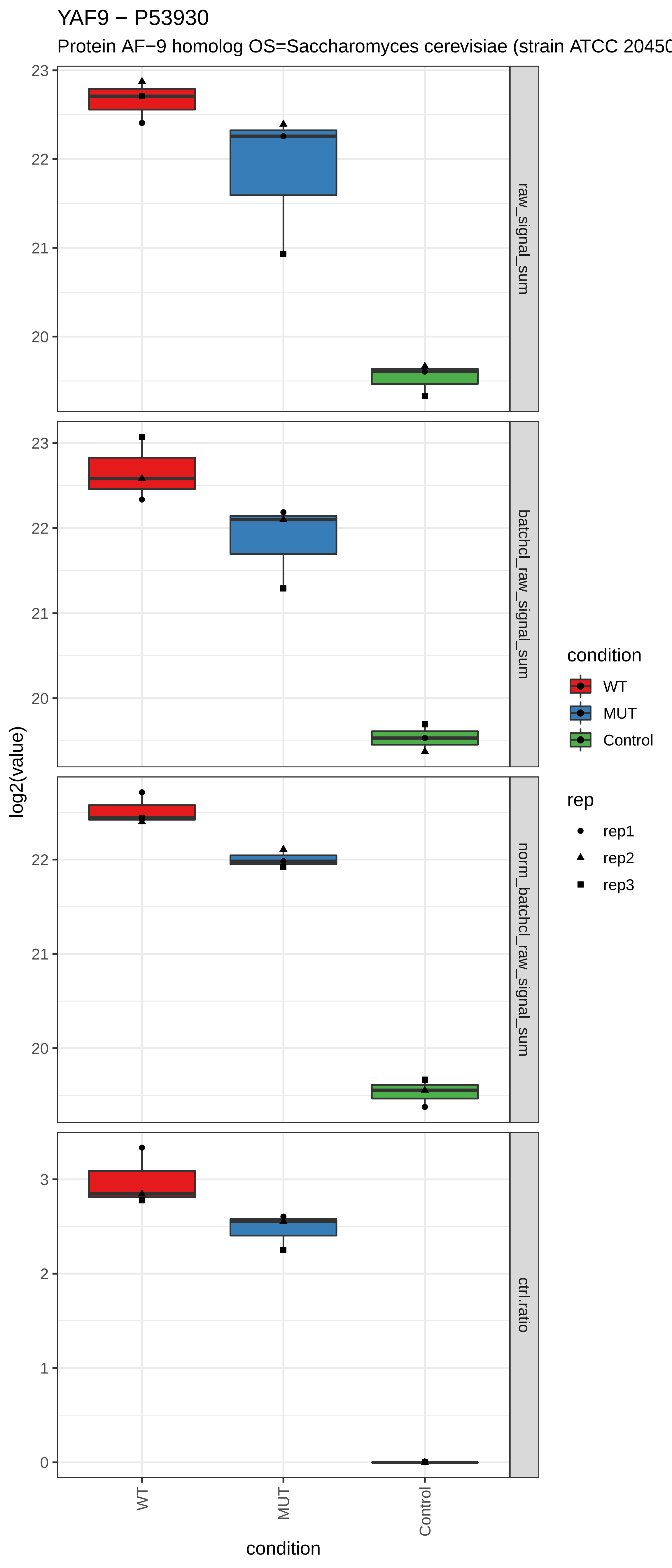

YAF9 − P53930
Protein AF−9 homolog OS=Saccharomyces cerevisiae (strain ATCC 20450
raw_signal_sum
batchcl_raw_signal_sum
norm_batchcl_raw_signal_sum
ctrl.ratio
log2(value)
condition
WT
MUT
Control
rep
rep1
rep2
rep3
23
22
21
20
3
2
1
0

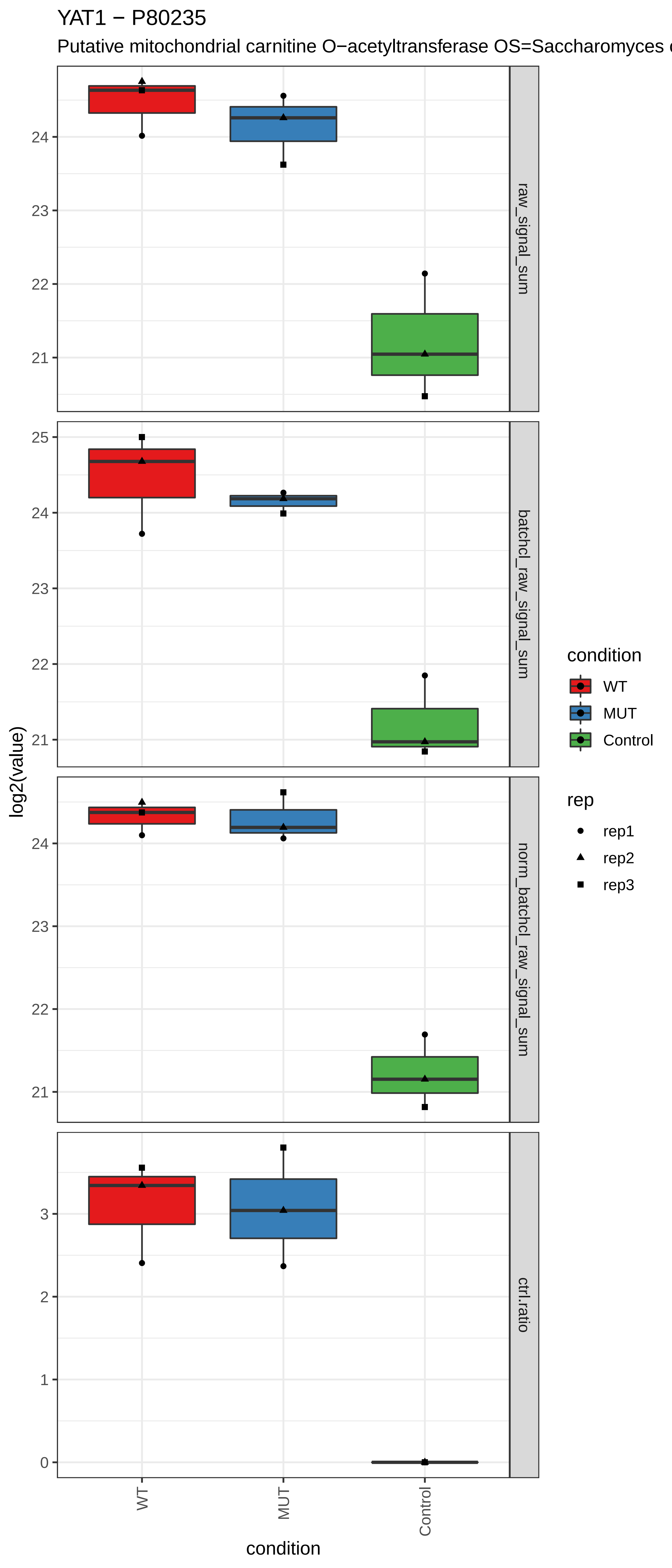

YAT1 − P80235
Putative mitochondrial carnitine O−acetyltransferase OS=Saccharomyces c
raw_signal_sum
batchcl_raw_signal_sum
norm_batchcl_raw_signal_sum
ctrl.ratio
log2(value)
condition
WT
MUT
Control
rep
rep1
rep2
rep3

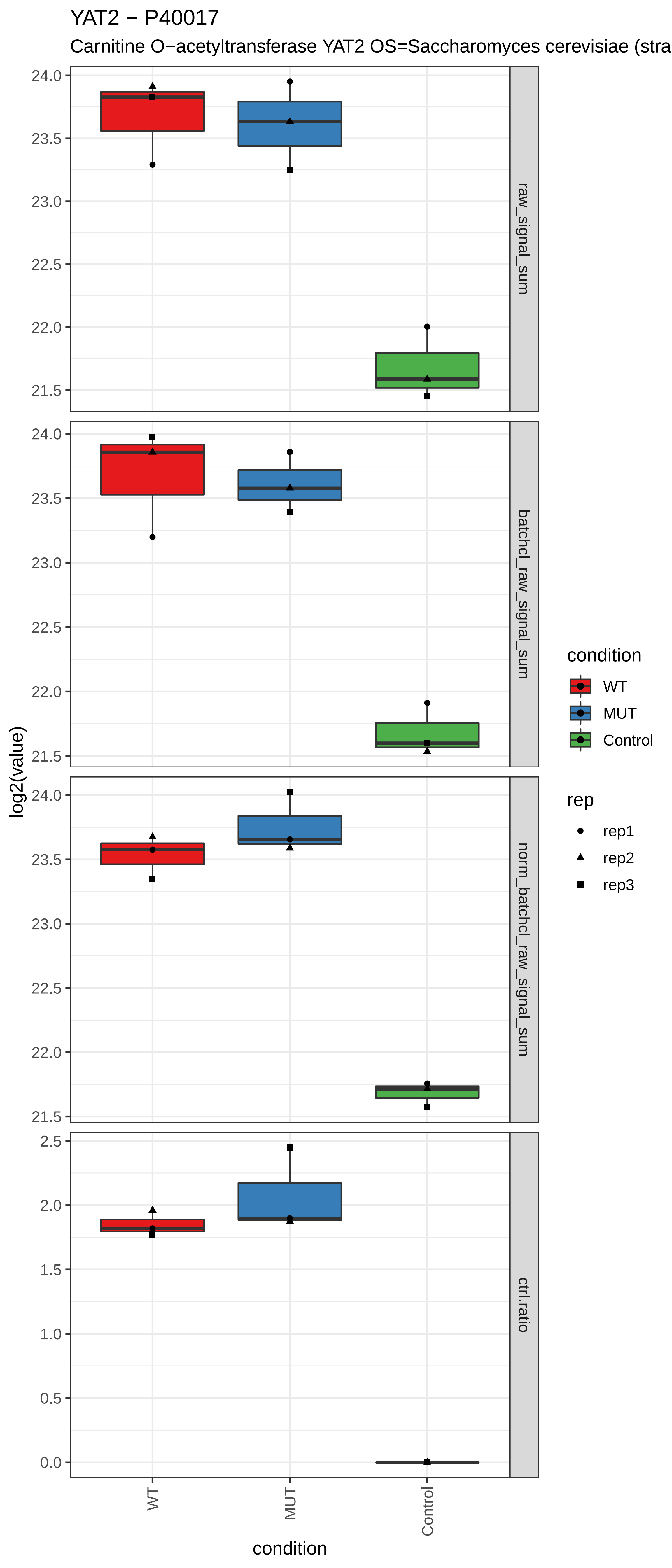

YAT2 − P40017
Carnitine O−acetyltransferase YAT2 OS=Saccharomyces cerevisiae (stra
log2(value)
condition
raw_signal_sum
batchcl_raw_signal_sum
norm_batchcl_raw_signal_sum
ctrl.ratio
24.0
23.5
23.0
22.5
22.0
21.5
2.5
2.0
1.5
1.0
0.5
0.0
WT
MUT
Control
condition
WT
MUT
Control
rep
rep1
rep2
rep3

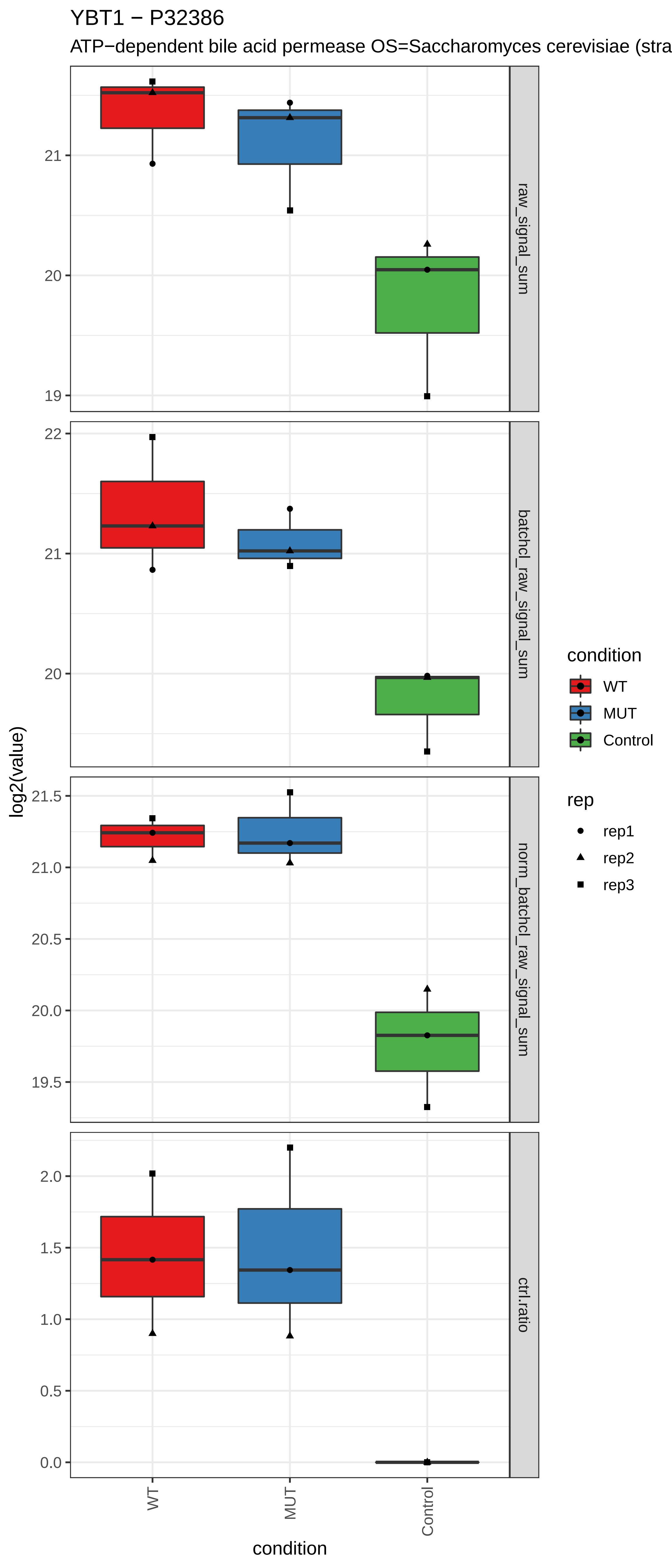

YBT1 − P32386
ATP−dependent bile acid permease OS=Saccharomyces cerevisiae (stra
raw_signal_sum
batchcl_raw_signal_sum
norm_batchcl_raw_signal_sum
ctrl.ratio
log2(value)
condition
WT
MUT
Control
rep
rep1
rep2
rep3

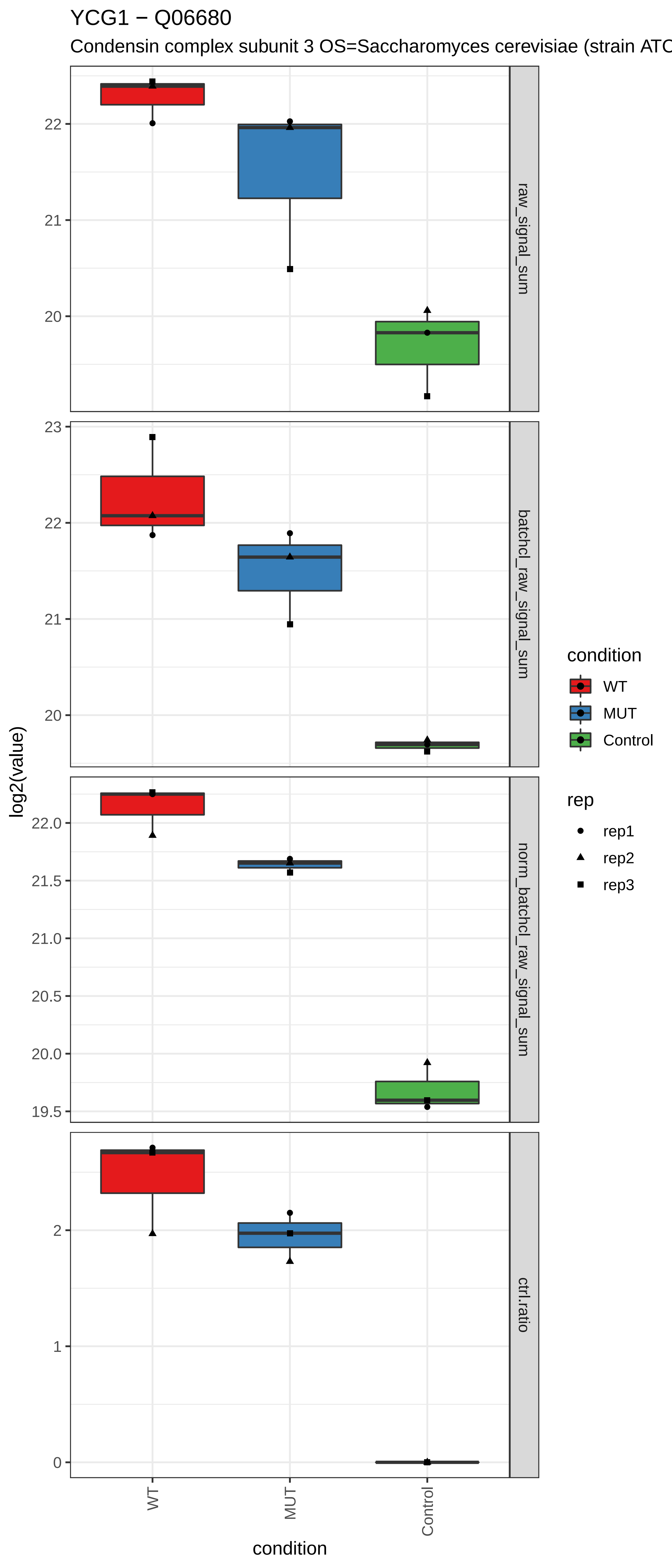

YCG1 − Q06680
Condensin complex subunit 3 OS=Saccharomyces cerevisiae (strain ATC
raw_signal_sum
batchcl_raw_signal_sum
norm_batchcl_raw_signal_sum
ctrl.ratio
log2(value)
condition
WT
MUT
Control
rep
rep1
rep2
rep3

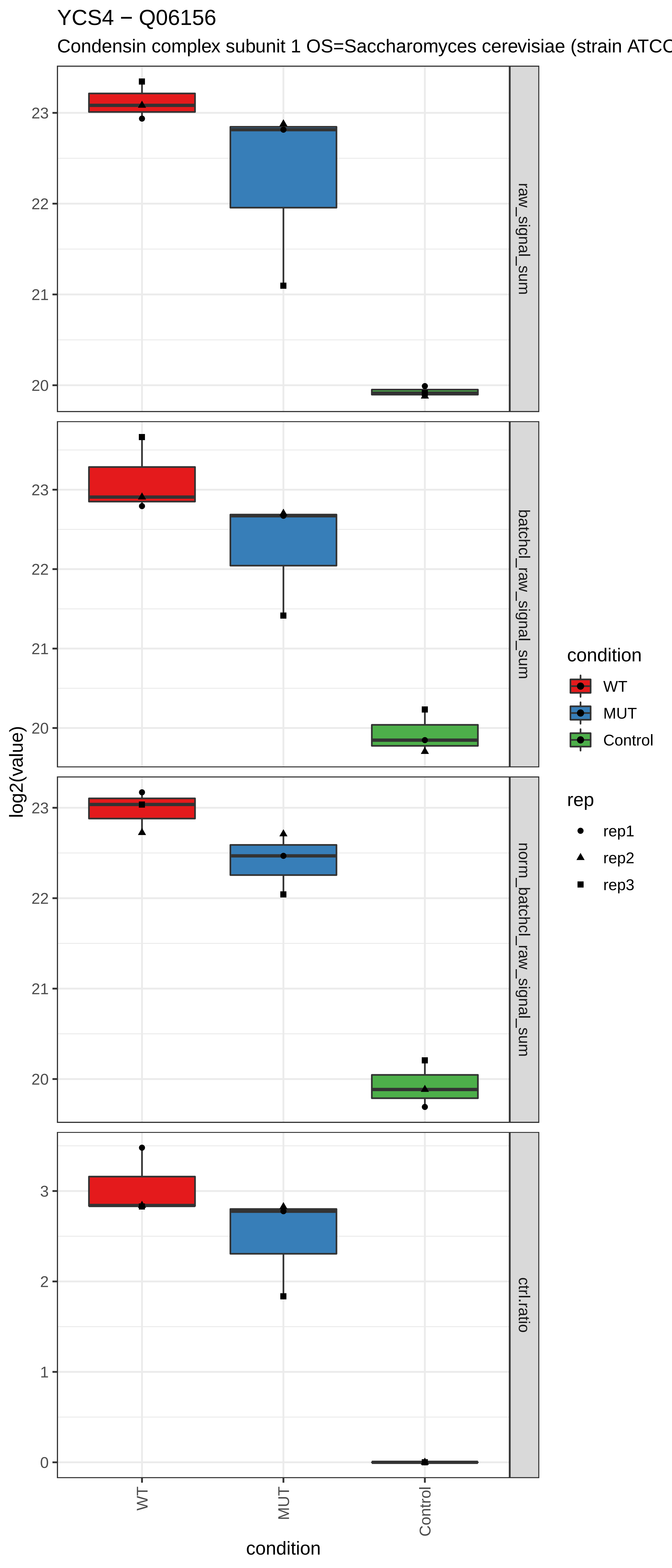

YCS4 − Q06156
Condensin complex subunit 1 OS=Saccharomyces cerevisiae (strain ATCC
raw_signal_sum
batchcl_raw_signal_sum
norm_batchcl_raw_signal_sum
ctrl.ratio
log2(value)
condition
WT
MUT
Control
rep
rep1
rep2
rep3
23
22
21
20
3
2
1
0

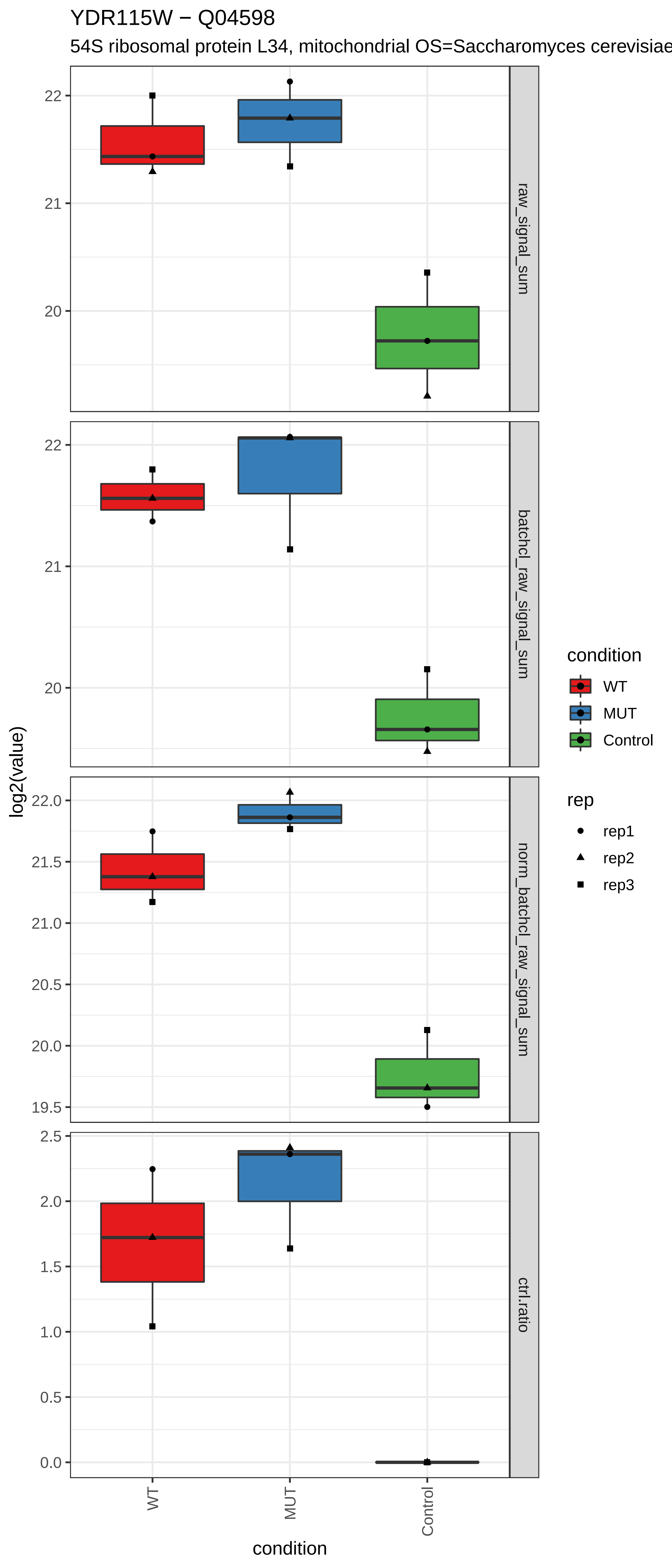

YDR115W − Q04598
54S ribosomal protein L34, mitochondrial OS=Saccharomyces cerevisiae
raw_signal_sum
batchcl_raw_signal_sum
norm_batchcl_raw_signal_sum
ctrl.ratio
log2(value)
condition
WT
MUT
Control
rep
rep1
rep2
rep3

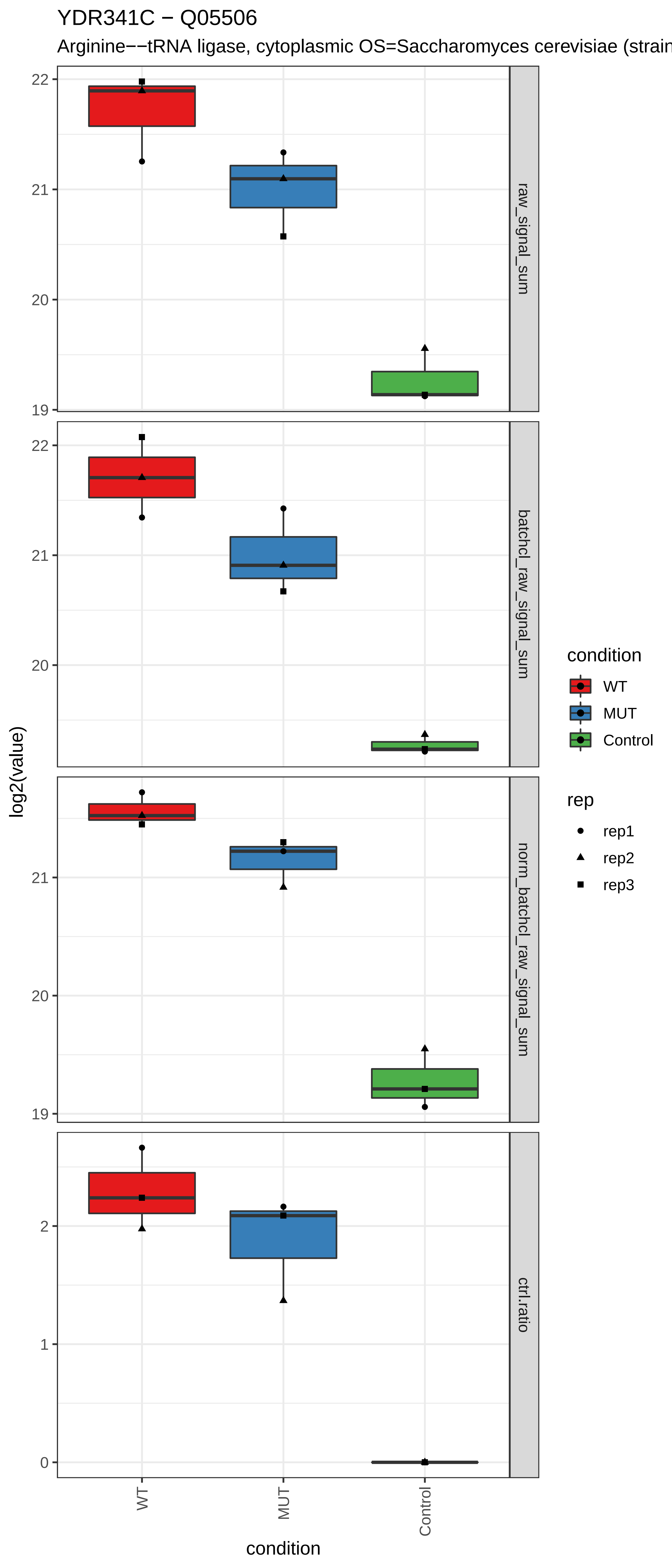
YDR341C − Q05506
Arginine−−tRNA ligase, cytoplasmic OS=Saccharomyces cerevisiae (strain
raw_signal_sum
batchcl_raw_signal_sum
norm_batchcl_raw_signal_sum
ctrl.ratio
log2(value)
condition
WT
MUT
Control
rep
rep1
rep2
rep3
22
21
20
19
2
1
0
WT
MUT
Control
condition

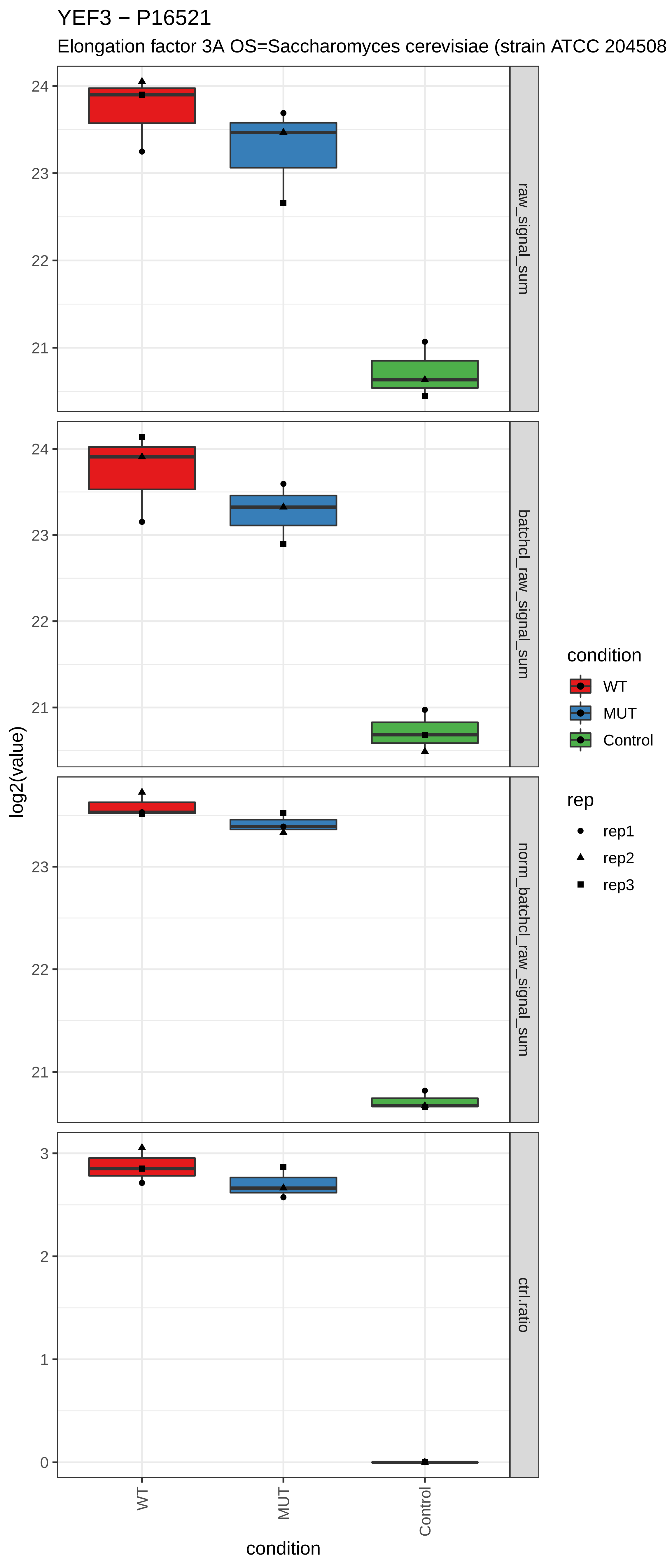
YEF3 − P16521
Elongation factor 3A OS=Saccharomyces cerevisiae (strain ATCC 204508
raw_signal_sum
batchcl_raw_signal_sum
norm_batchcl_raw_signal_sum
ctrl.ratio
log2(value)
condition
WT
MUT
Control
rep
rep1
rep2
rep3

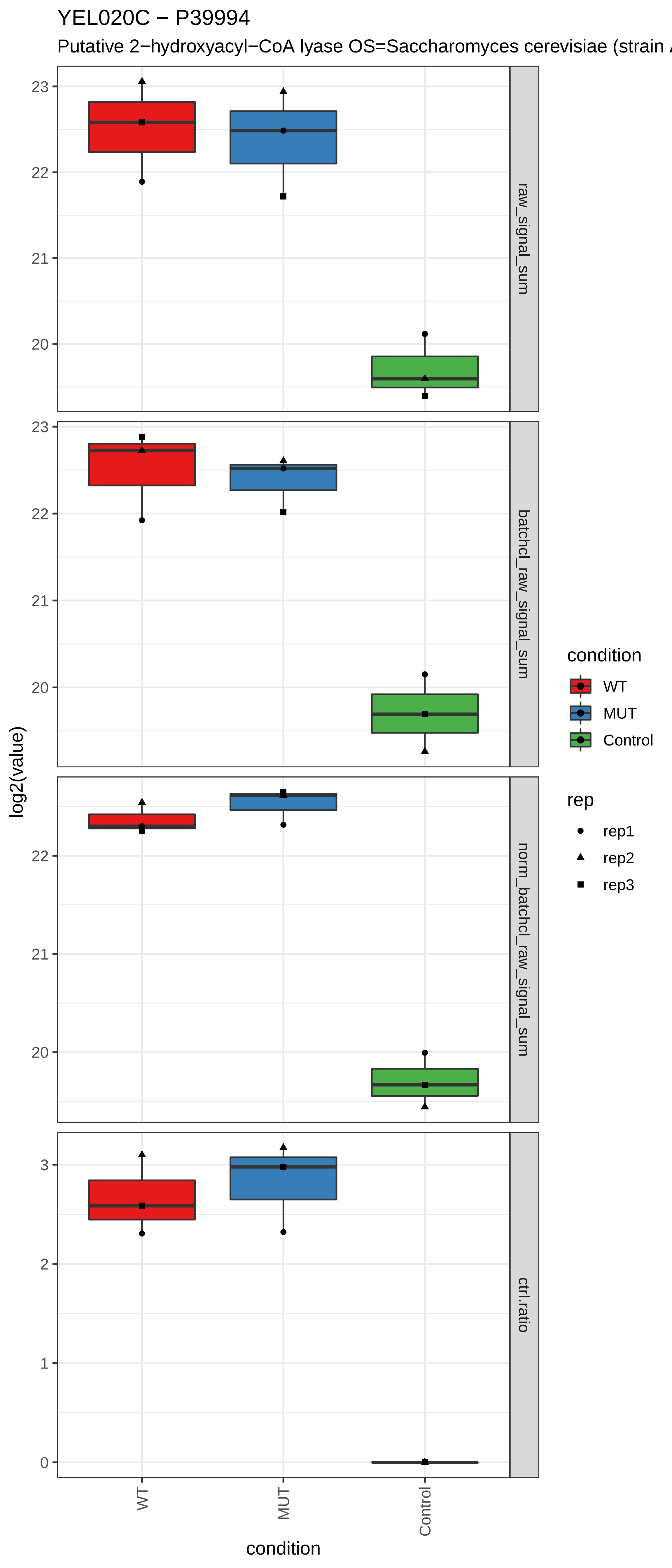
YEL020C − P39994
Putative 2−hydroxyacyl−CoA lyase OS=Saccharomyces cerevisiae (strain
raw_signal_sum
batchcl_raw_signal_sum
norm_batchcl_raw_signal_sum
ctrl.ratio
log2(value)
condition
WT
MUT
Control
rep
rep1
rep2
rep3
23
22
21
20
3
2
1
0

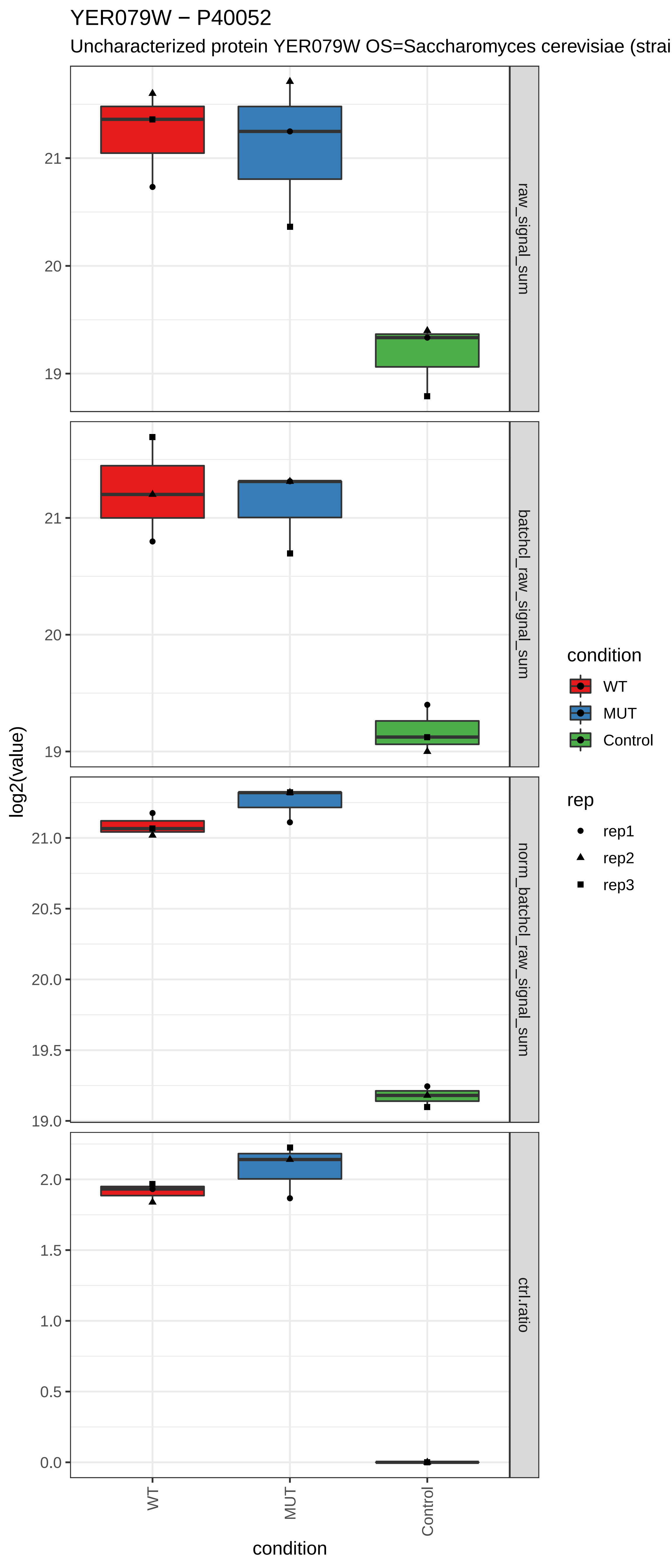
YER079W − P40052
Uncharacterized protein YER079W OS=Saccharomyces cerevisiae (strai
raw_signal_sum
batchcl_raw_signal_sum
norm_batchcl_raw_signal_sum
ctrl.ratio
log2(value)
condition
WT
MUT
Control
rep
rep1
rep2
rep3
21
20
19
21.0
20.5
20.0
19.5
19.0
2.0
1.5
1.0
0.5
0.0

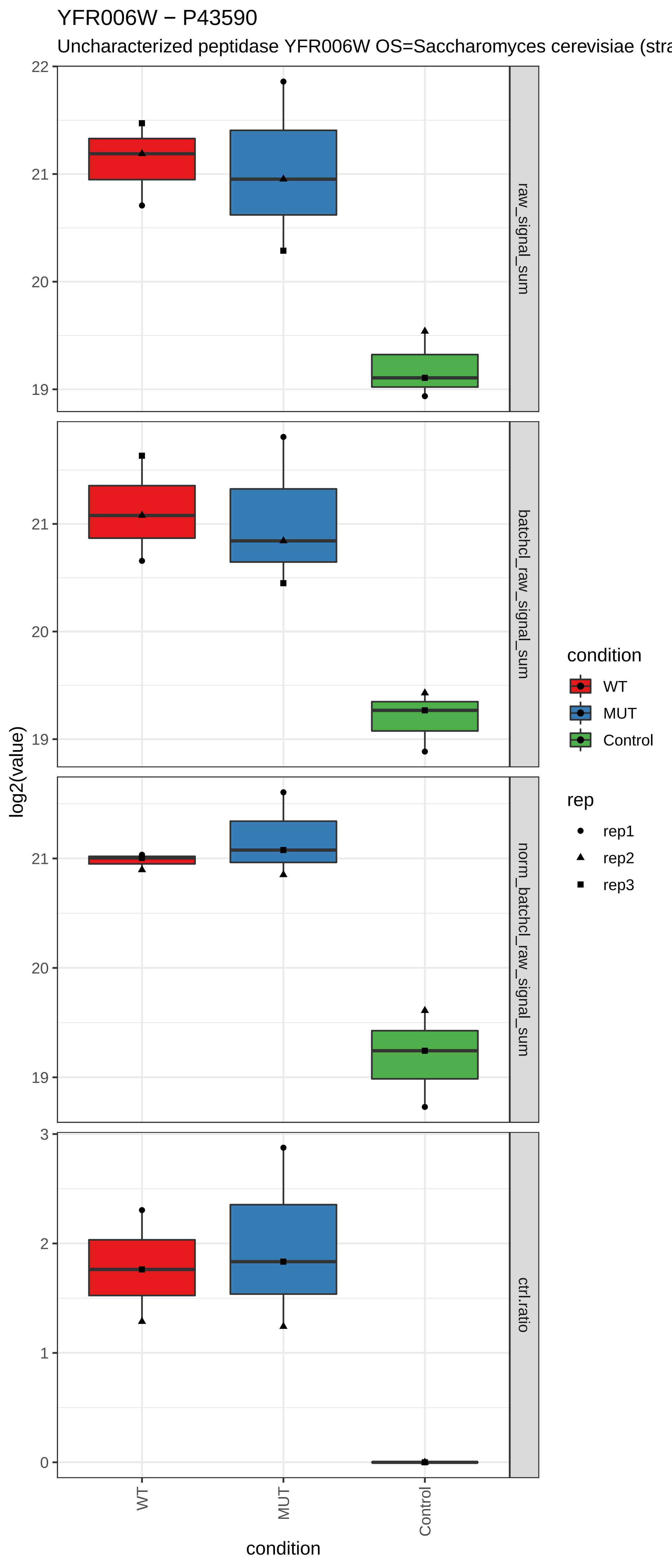

YFR006W − P43590
Uncharacterized peptidase YFR006W OS=Saccharomyces cerevisiae (stra
log2(value)
raw_signal_sum
batchcl_raw_signal_sum
norm_batchcl_raw_signal_sum
ctrl.ratio
22
21
20
19
3
2
1
0
WT
MUT
Control
condition
condition
WT
MUT
Control
rep
rep1
rep2
rep3

YGL081W − P53156

Uncharacterized protein YGL081W OS=Saccharomyces cerevisiae (strai

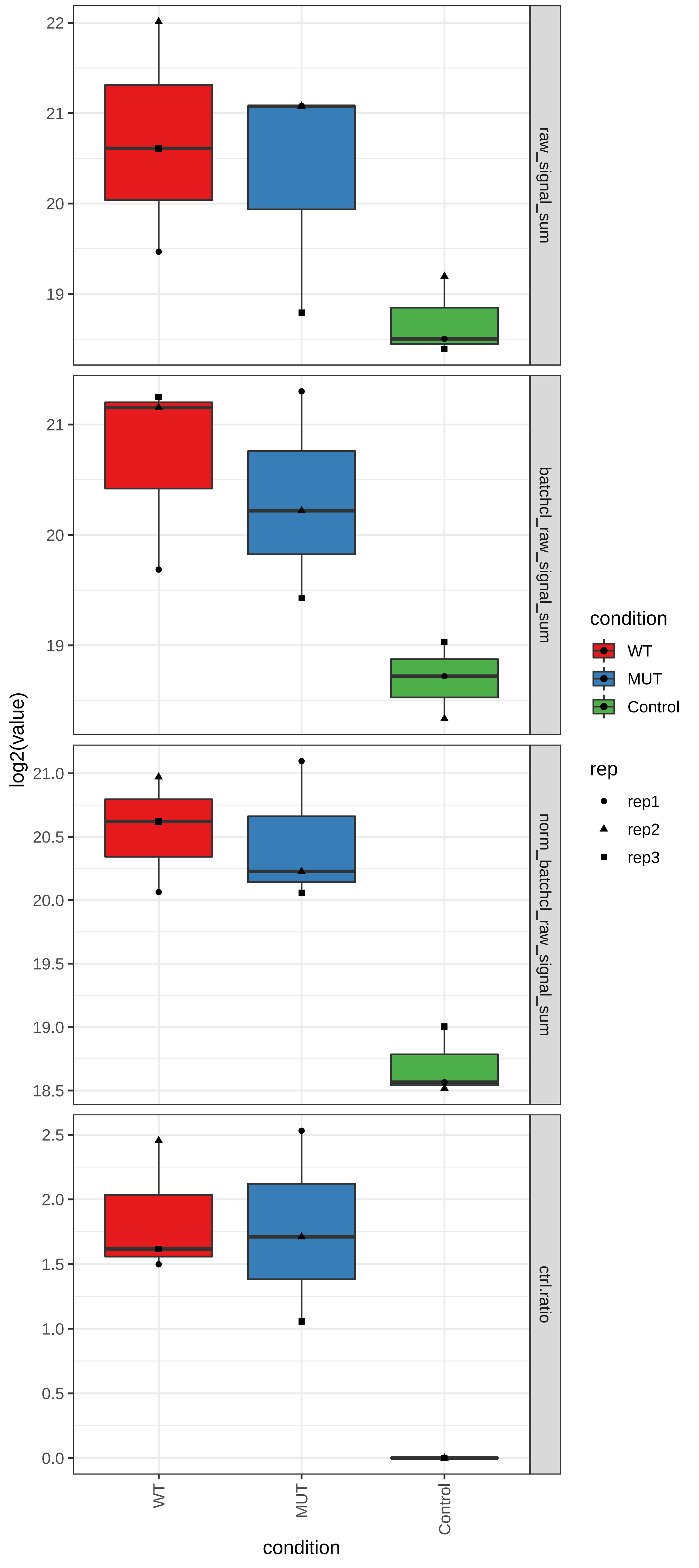

YGL108C − P53139

Uncharacterized protein YGL108C OS=Saccharomyces cerevisiae (strain A

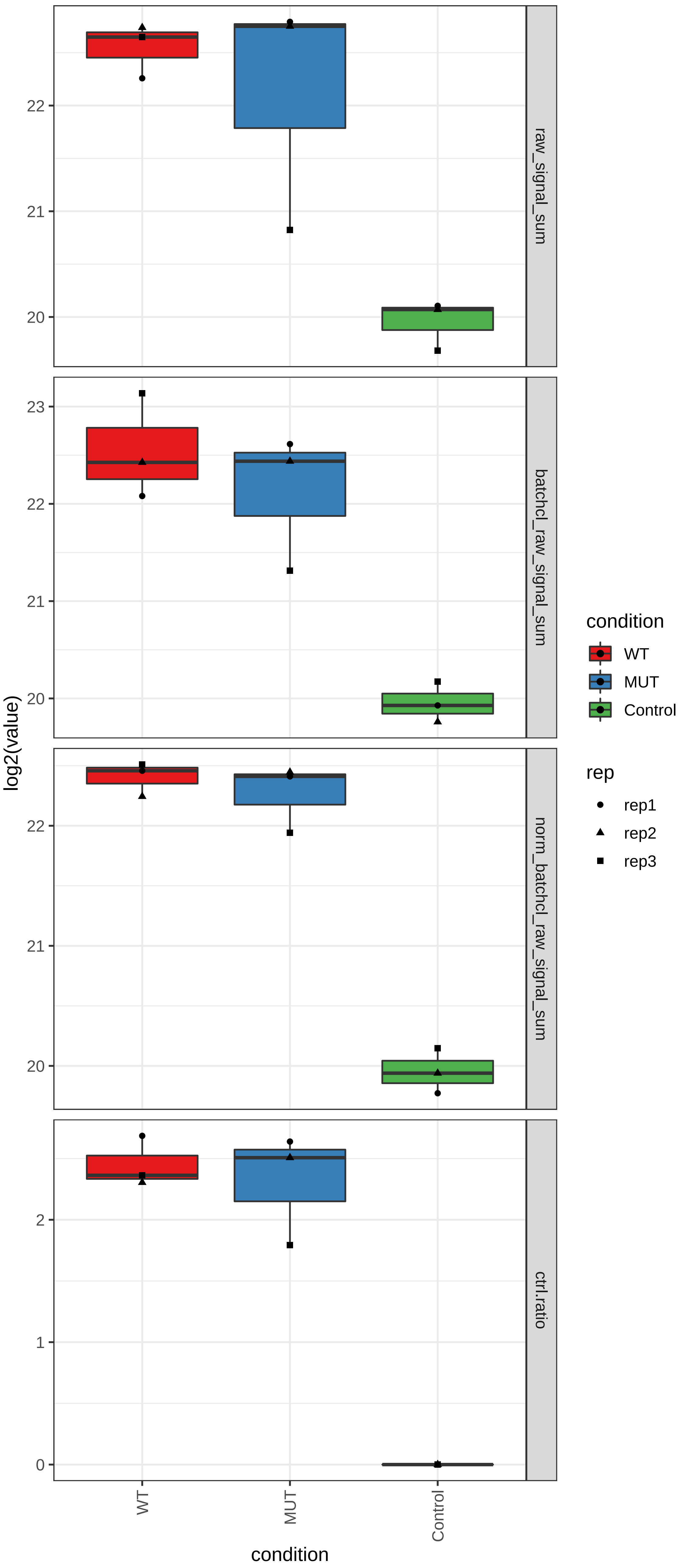

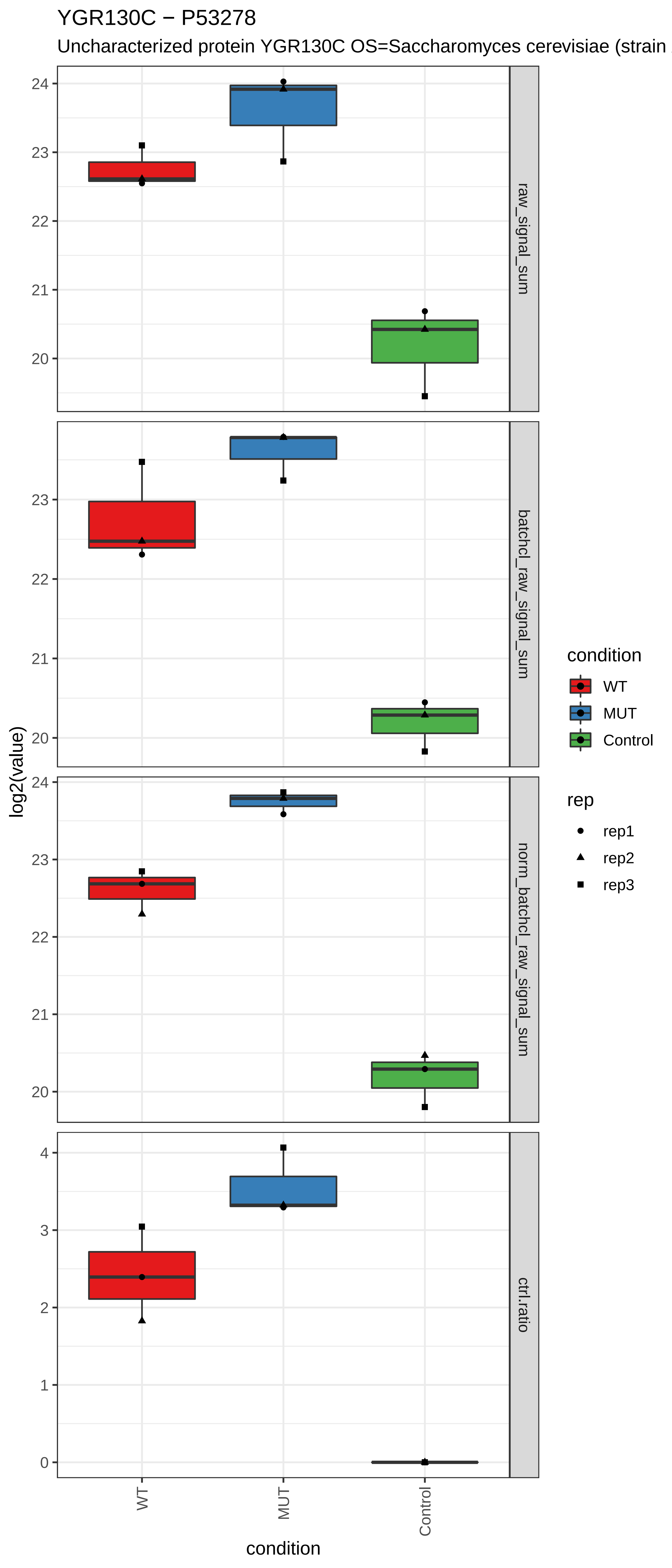

YGR130C − P53278
Uncharacterized protein YGR130C OS=Saccharomyces cerevisiae (strain
raw_signal_sum
batchcl_raw_signal_sum
norm_batchcl_raw_signal_sum
ctrl.ratio
log2(value)
condition
WT
MUT
Control
rep
rep1
rep2
rep3

YGR169C−A − Q3E772

Uncharacterized protein YGR169C−A OS=Saccharomyces cerevisiae (stra

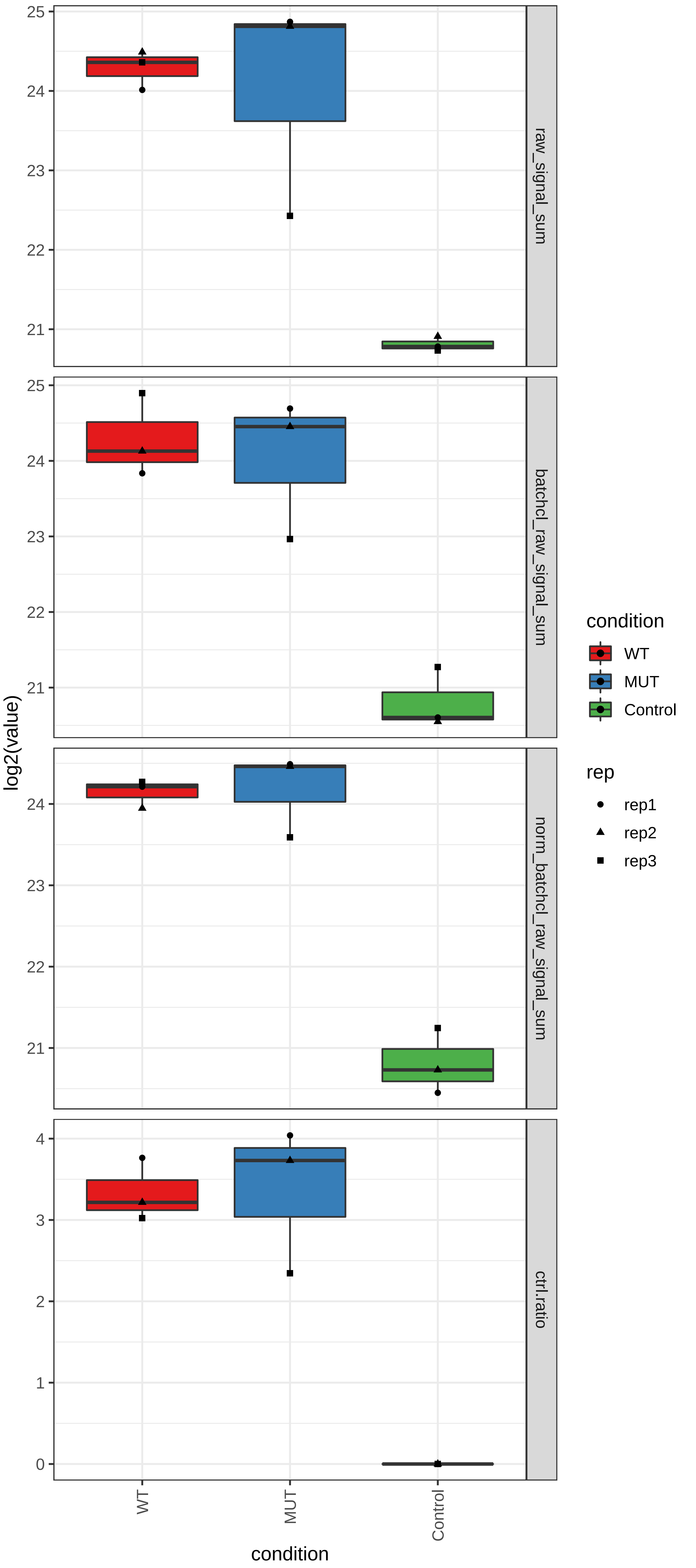

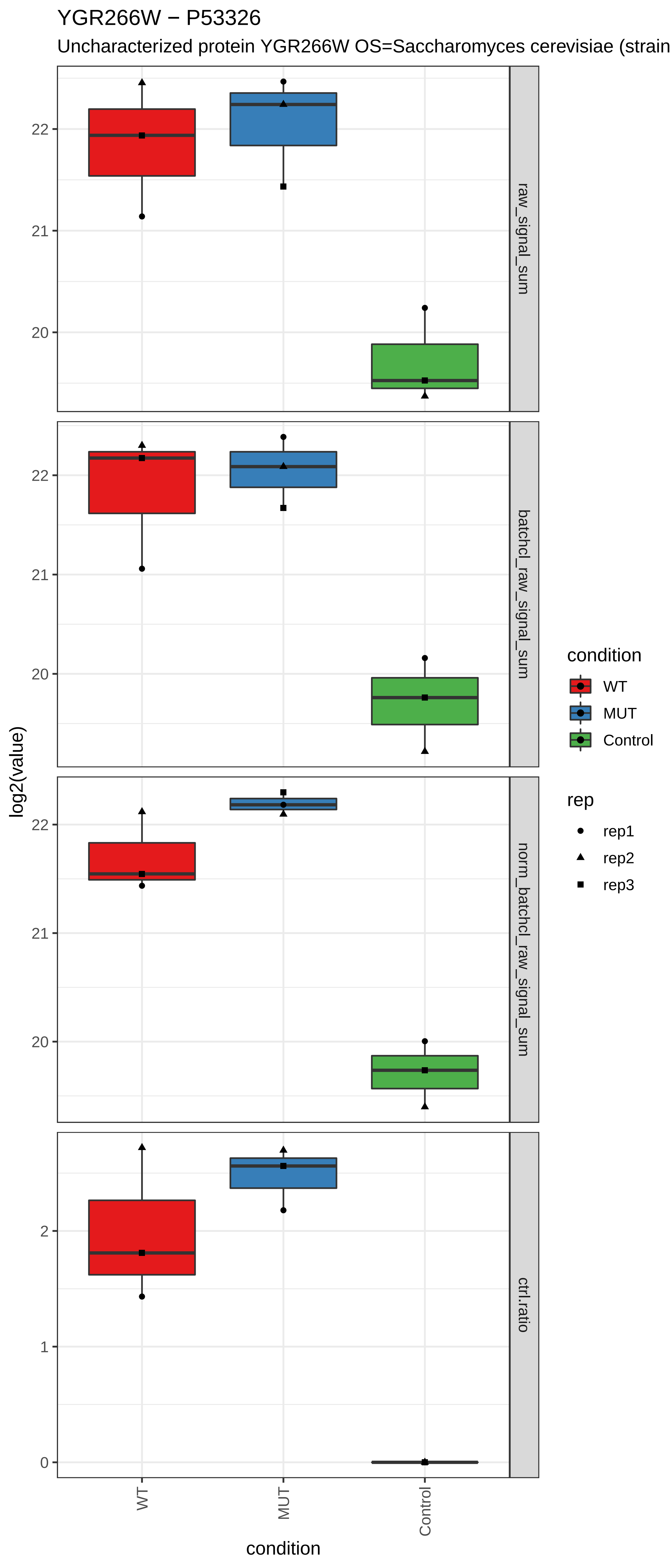

YGR266W − P53326
Uncharacterized protein YGR266W OS=Saccharomyces cerevisiae (strain
raw_signal_sum
batchcl_raw_signal_sum
norm_batchcl_raw_signal_sum
ctrl.ratio
log2(value)
22
21
20
2
1
0
WT
MUT
Control
condition
condition
WT
MUT
Control
rep
rep1
rep2
rep3

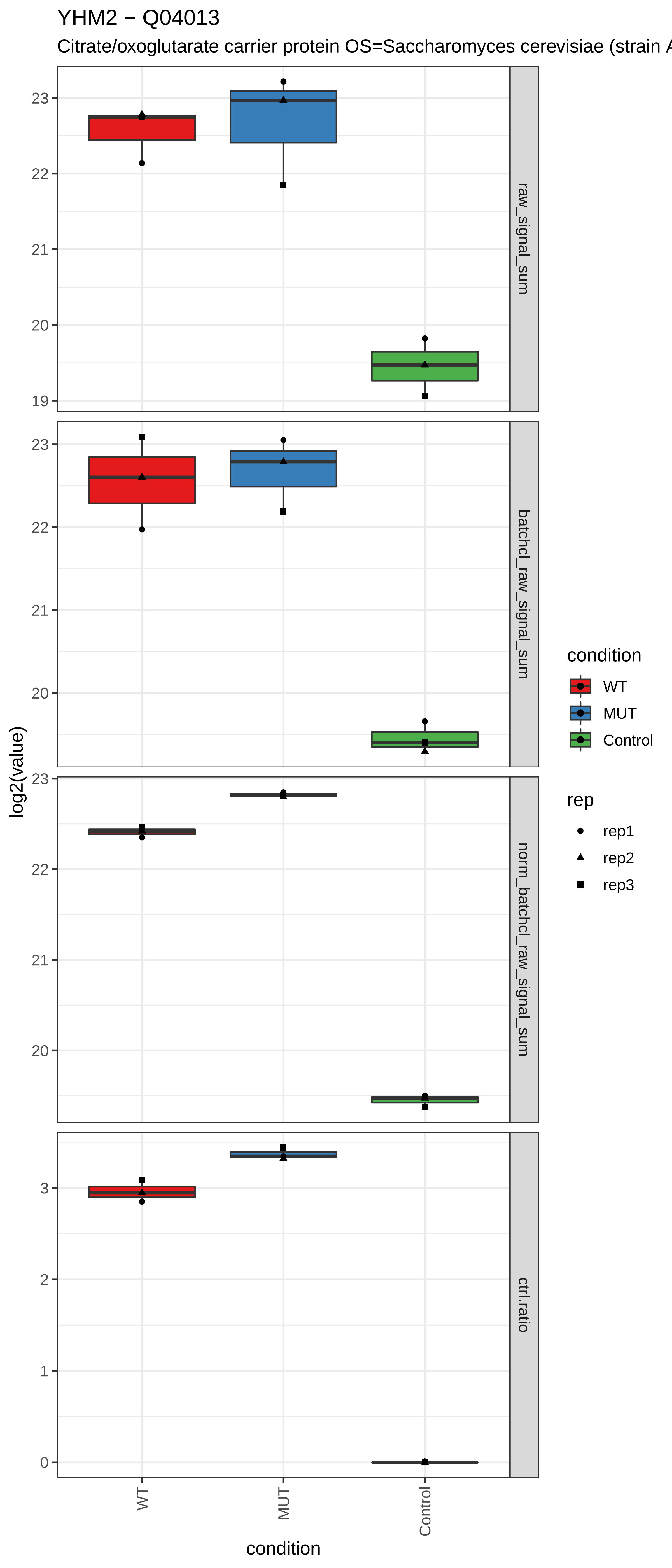
YHM2 − Q04013
Citrate/oxoglutarate carrier protein OS=Saccharomyces cerevisiae (strain A
raw_signal_sum
batchcl_raw_signal_sum
norm_batchcl_raw_signal_sum
ctrl.ratio
log2(value)
condition
WT
MUT
Control
rep
rep1
rep2
rep3

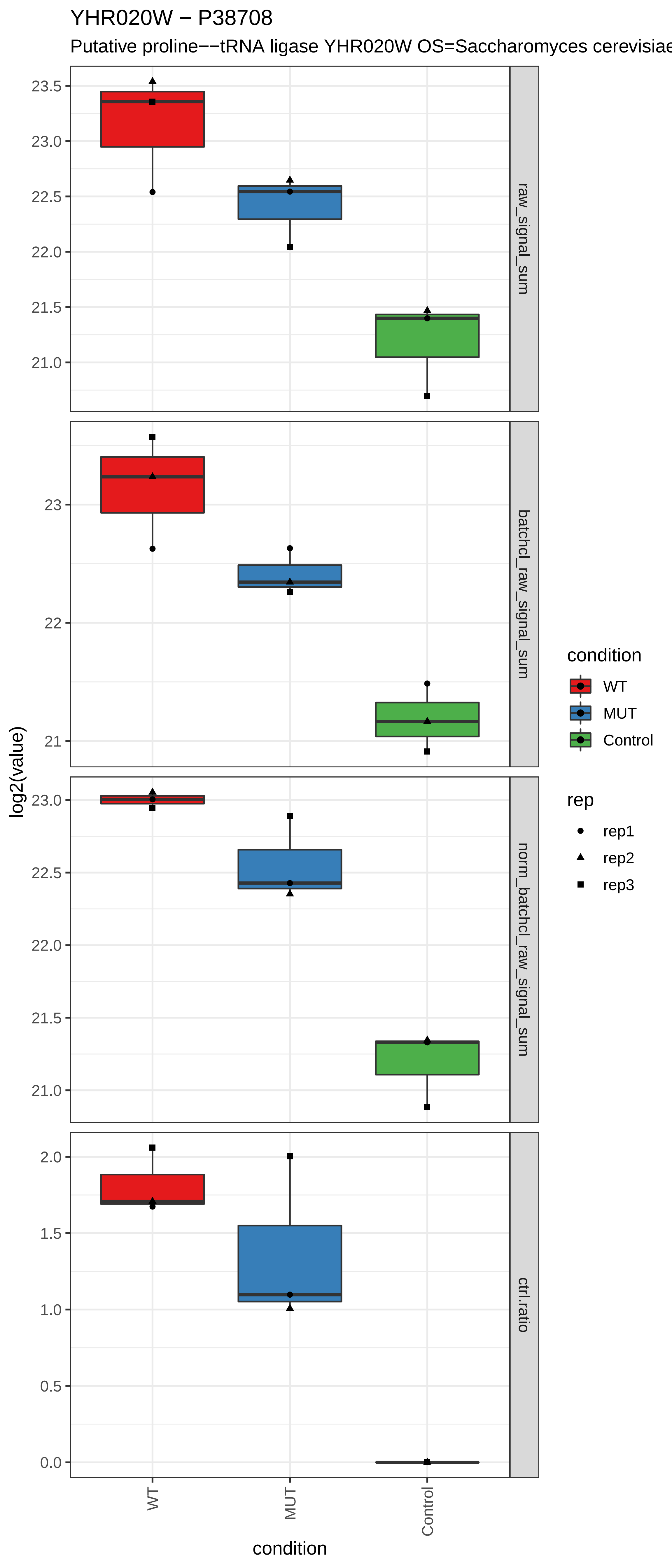

YHR020W − P38708
Putative proline−−tRNA ligase YHR020W OS=Saccharomyces cerevisiae
raw_signal_sum
batchcl_raw_signal_sum
norm_batchcl_raw_signal_sum
ctrl.ratio
log2(value)
condition
WT
MUT
Control
rep
rep1
rep2
rep3

YHR097C − P38809

Uncharacterized protein YHR097C OS=Saccharomyces cerevisiae (strain

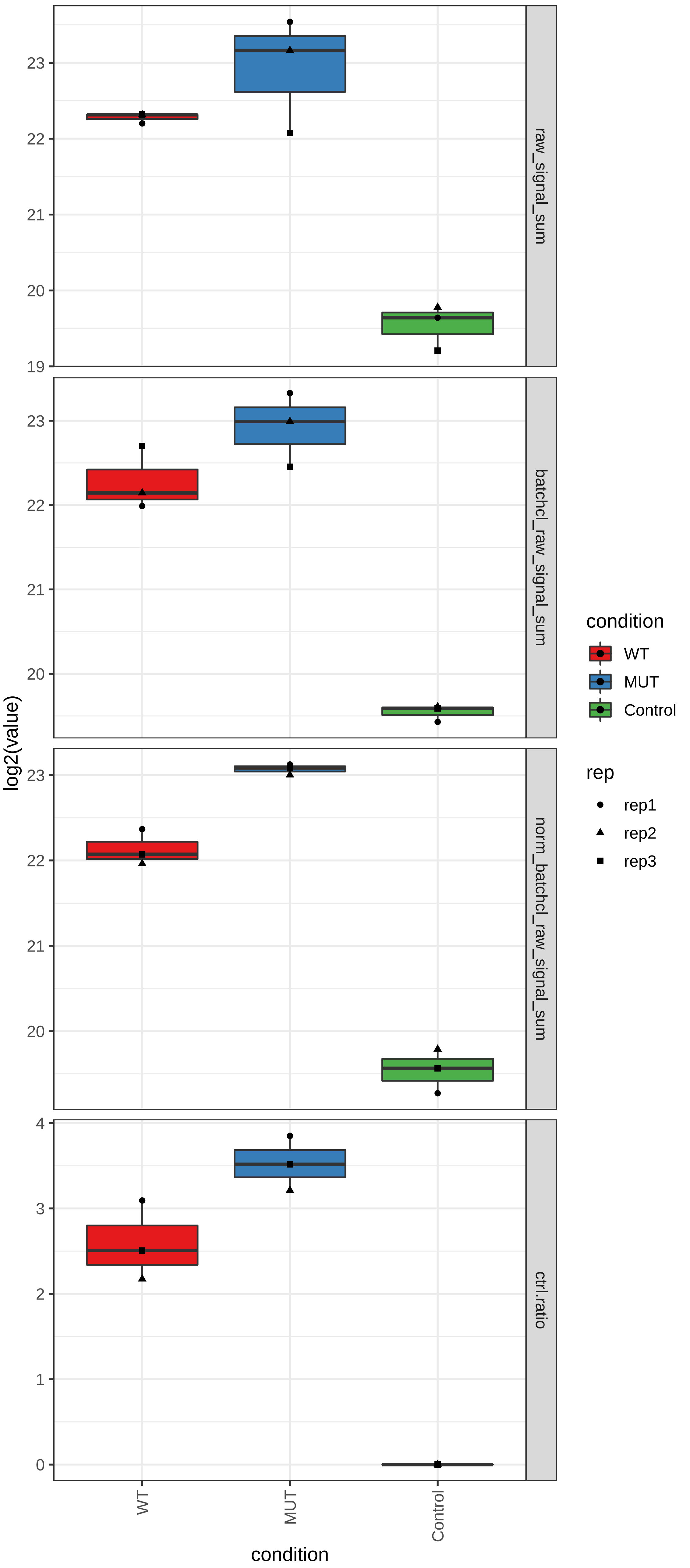

YJR005C−A − Q3E827

Uncharacterized protein YJR005C−A OS=Saccharomyces cerevisiae (strai

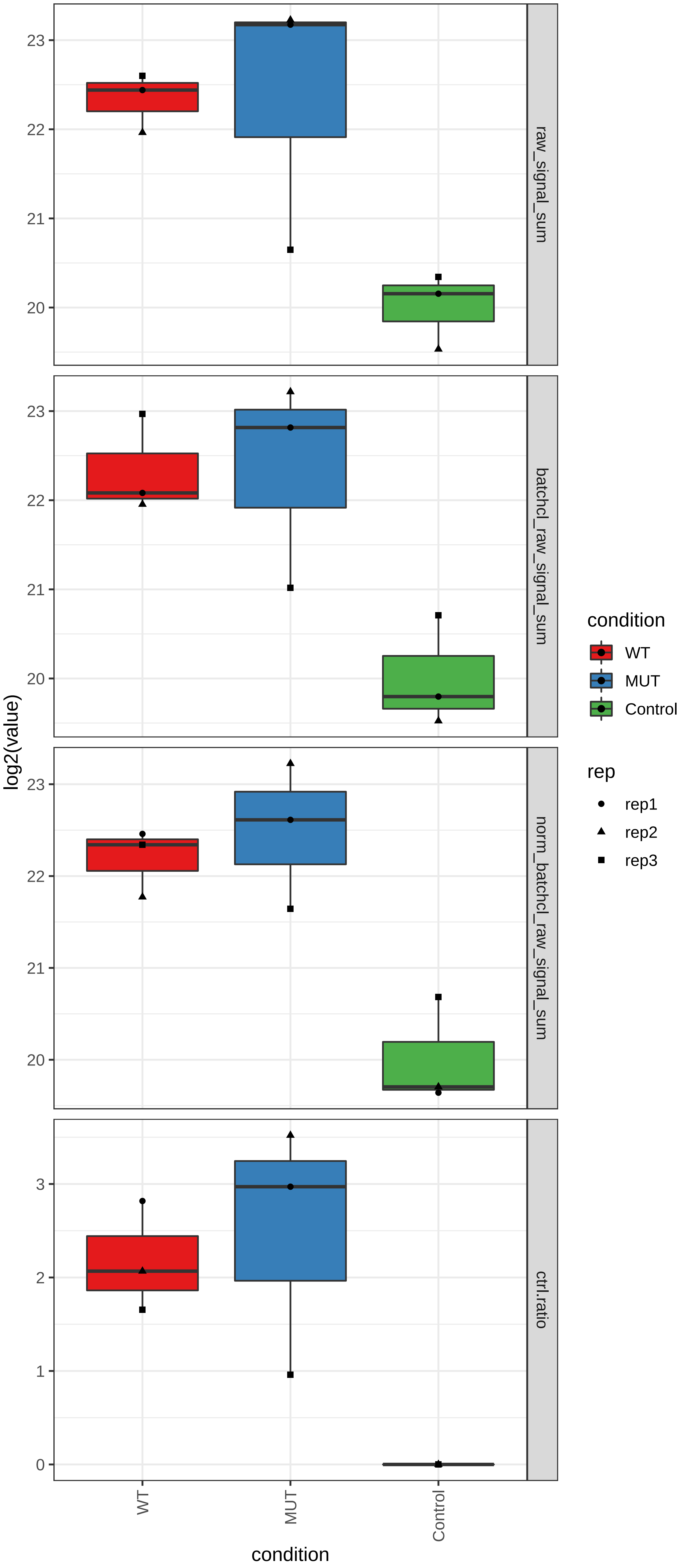

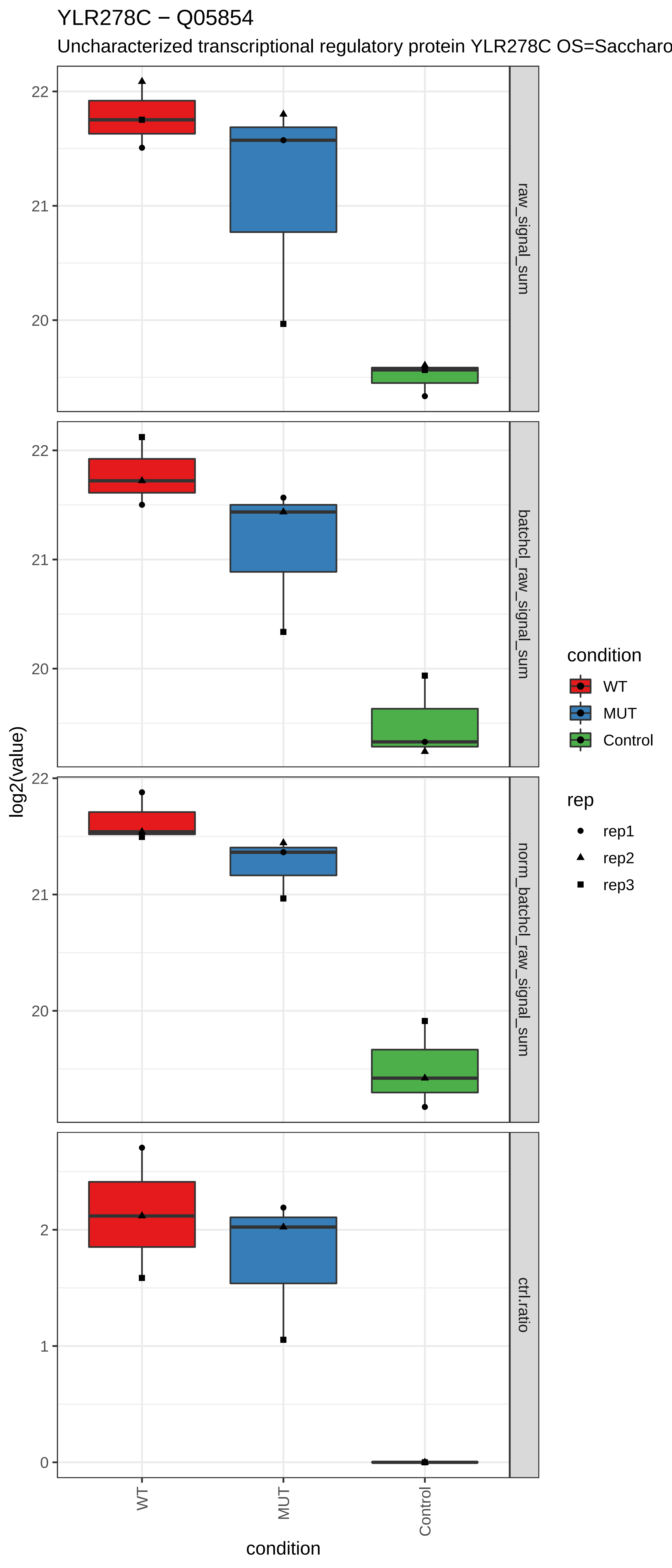
YLR278C − Q05854
Uncharacterized transcriptional regulatory protein YLR278C OS=Saccharo
raw_signal_sum
batchcl_raw_signal_sum
norm_batchcl_raw_signal_sum
ctrl.ratio
log2(value)
condition
WT
MUT
Control
rep
rep1
rep2
rep3
22
21
20
2
1
0

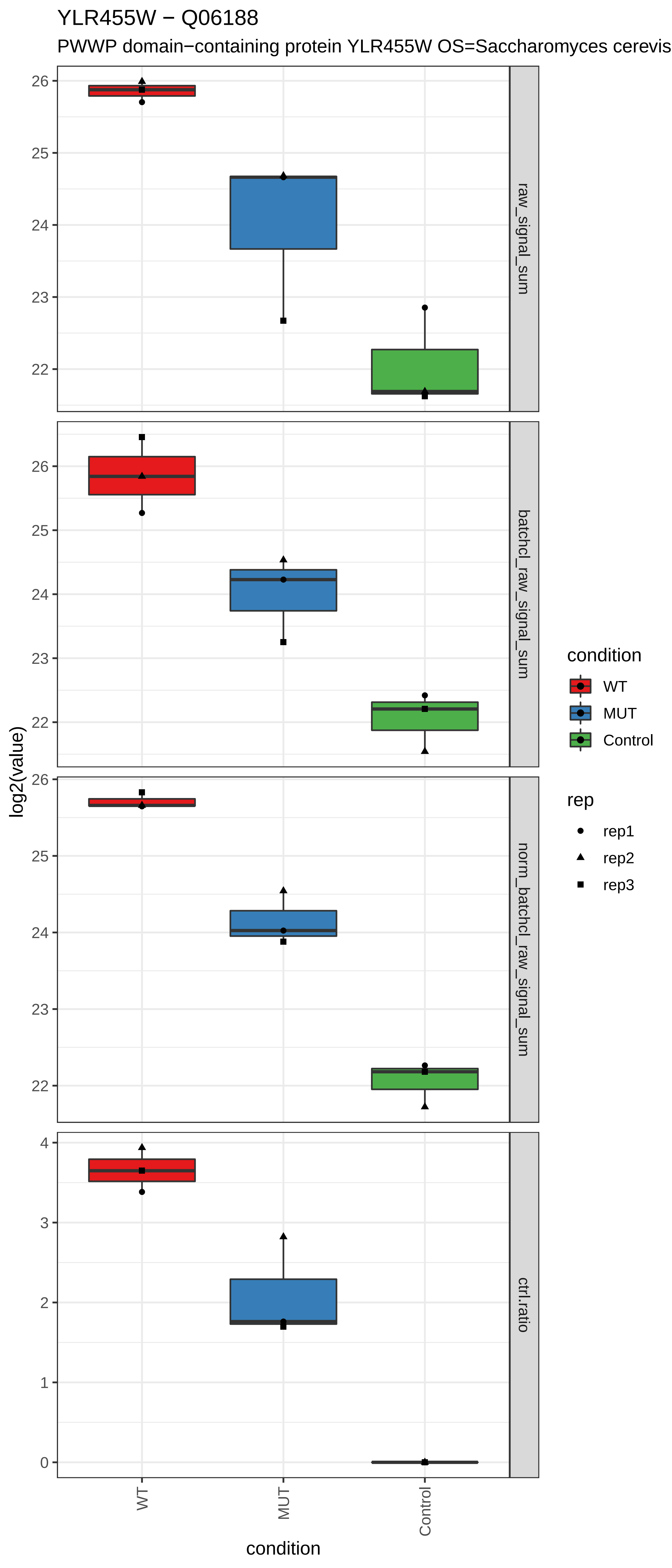

YLR455W − Q06188
PWWP domain−containing protein YLR455W OS=Saccharomyces cerevis
log2(value)
raw_signal_sum
batchcl_raw_signal_sum
norm_batchcl_raw_signal_sum
ctrl.ratio
condition
WT
MUT
Control
rep
rep1
rep2
rep3

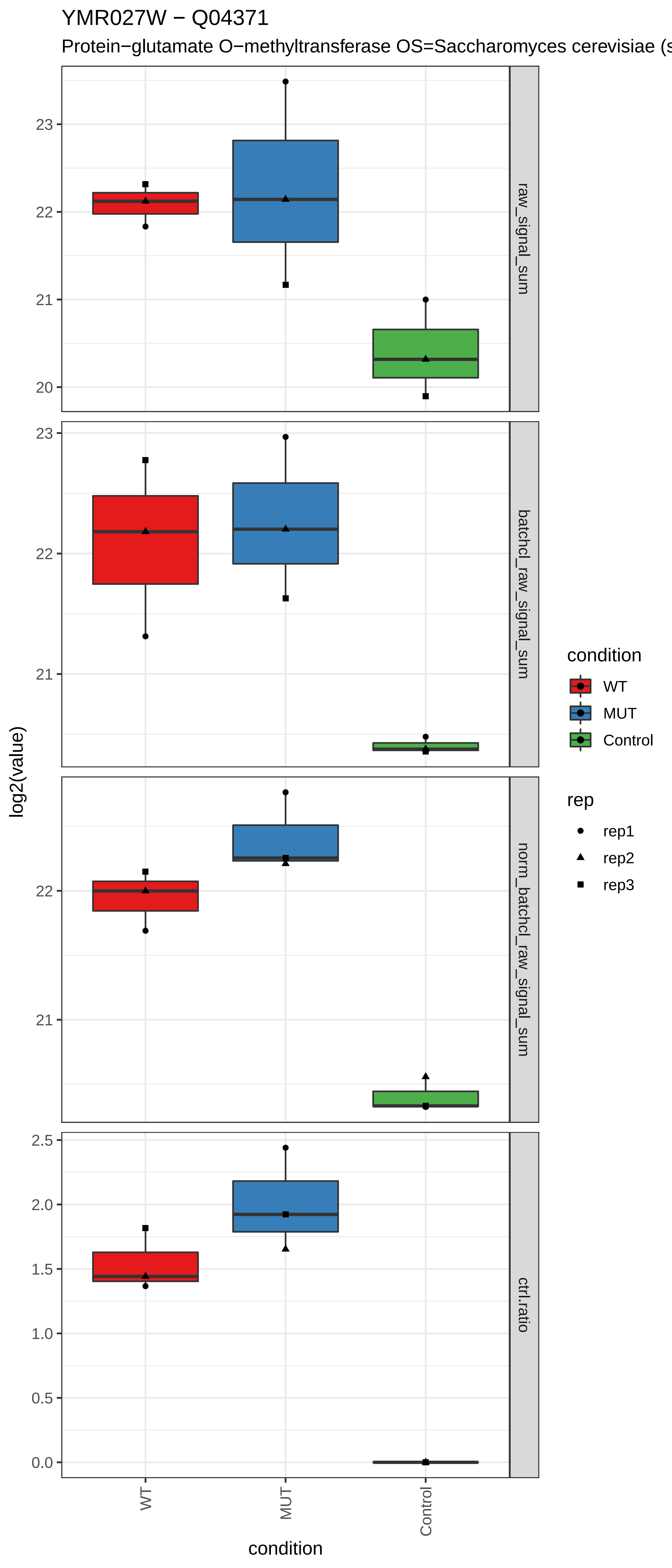

YMR027W − Q04371
Protein−glutamate O−methyltransferase OS=Saccharomyces cerevisiae (s
raw_signal_sum
batchcl_raw_signal_sum
norm_batchcl_raw_signal_sum
ctrl.ratio
log2(value)
condition
WT
MUT
Control
rep
rep1
rep2
rep3
23
22
21
20
2.5
2.0
1.5
1.0
0.5
0.0

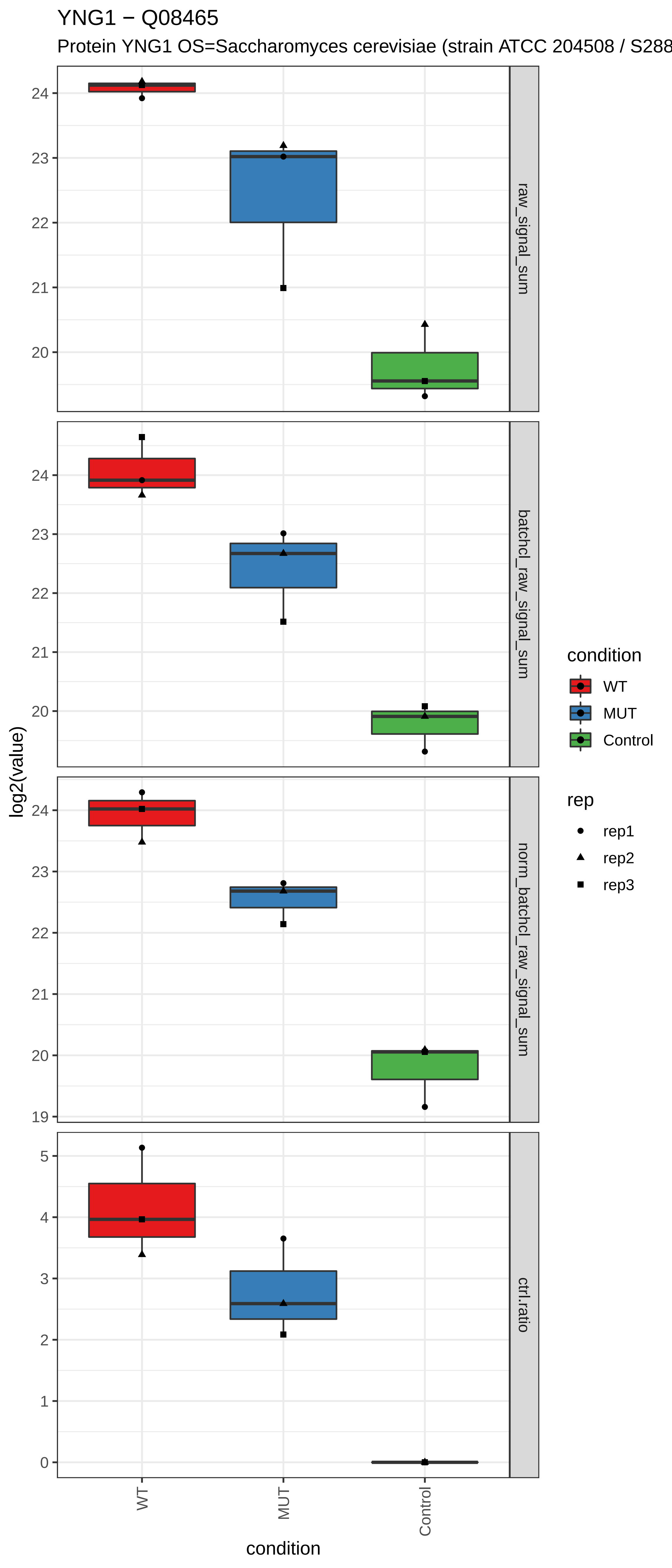

YNG1 − Q08465
Protein YNG1 OS=Saccharomyces cerevisiae (strain ATCC 204508 / S288
raw_signal_sum
batchcl_raw_signal_sum
norm_batchcl_raw_signal_sum
ctrl.ratio
log2(value)
condition
WT
MUT
Control
rep
rep1
rep2
rep3

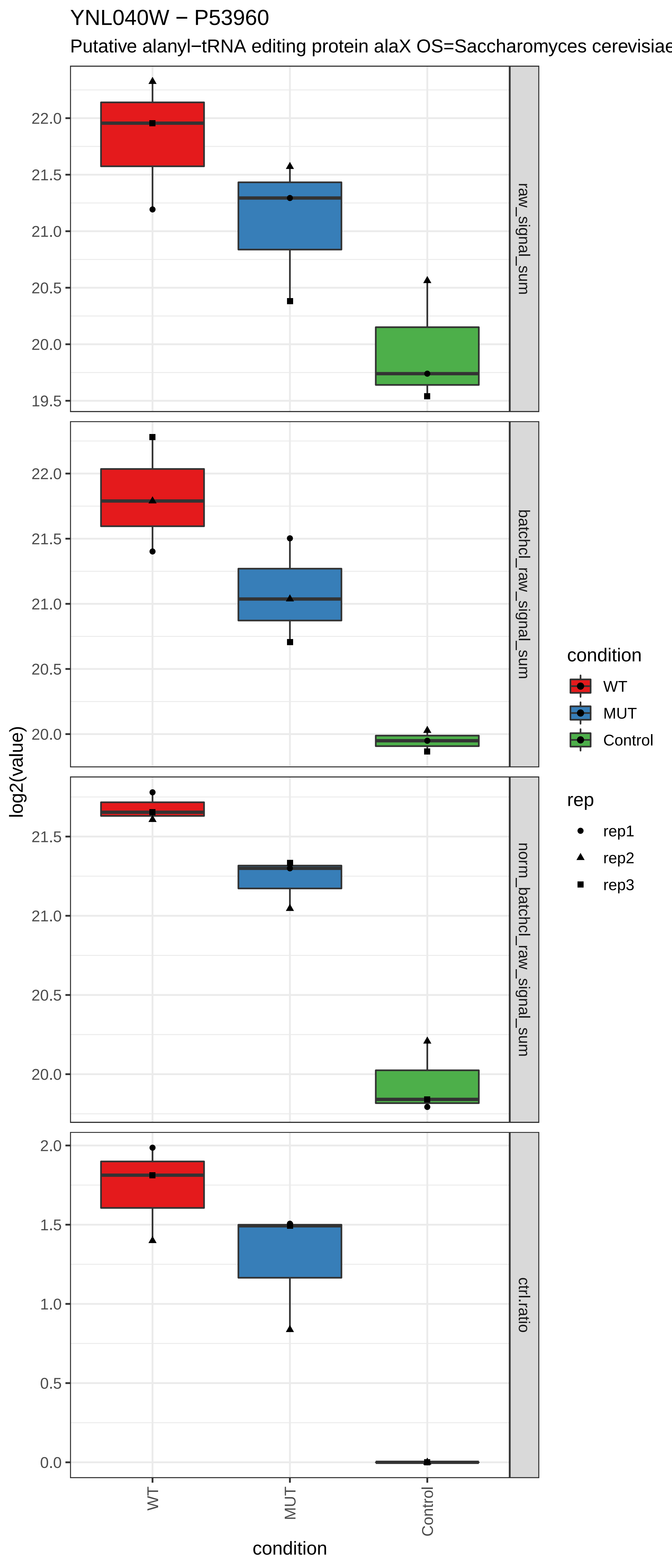
YNL040W − P53960
Putative alanyl−tRNA editing protein alaX OS=Saccharomyces cerevisiae
raw_signal_sum
batchcl_raw_signal_sum
norm_batchcl_raw_signal_sum
ctrl.ratio
log2(value)
condition
WT
MUT
Control
rep
rep1
rep2
rep3
22.0
21.5
21.0
20.5
20.0
19.5
2.0
1.5
1.0
0.5
0.0

YNL115C − P53925

Uncharacterized vacuolar membrane protein YNL115C OS=Saccharomy

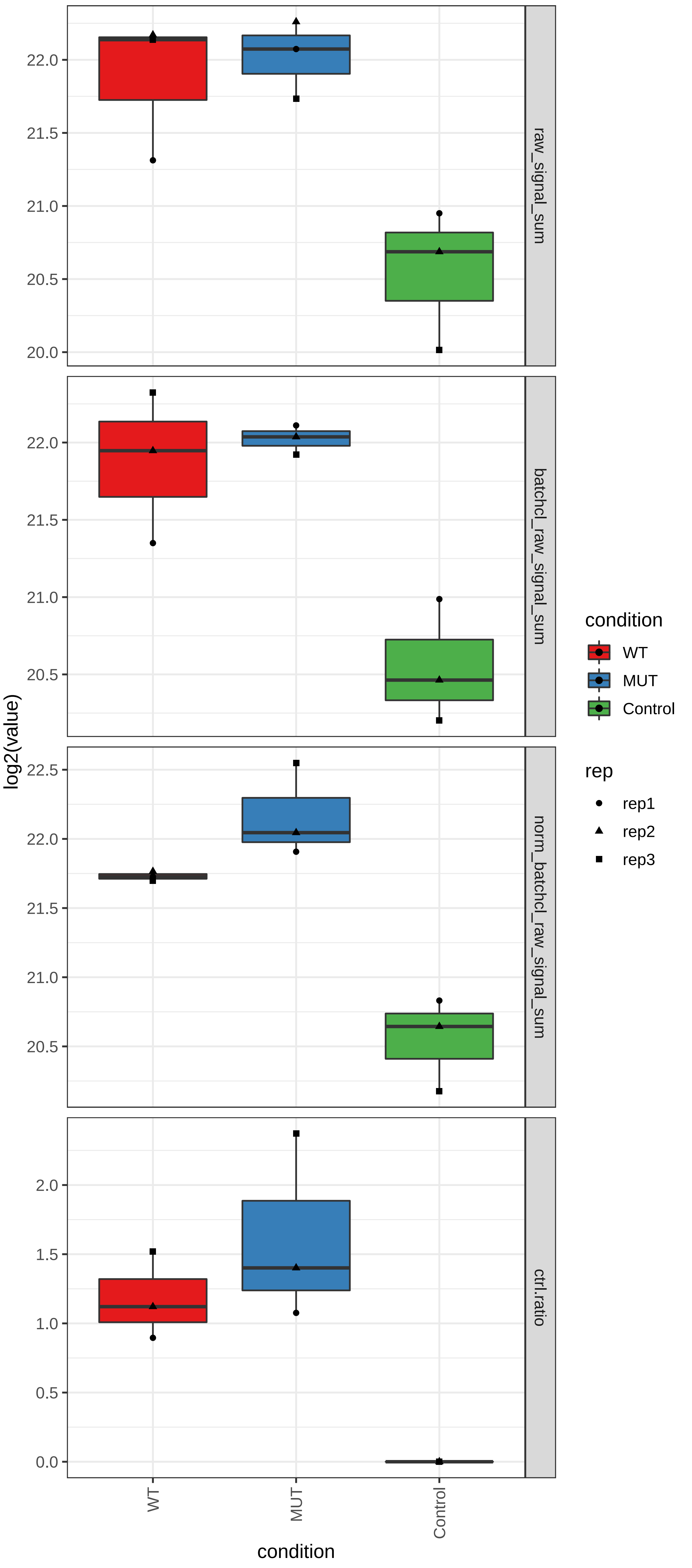

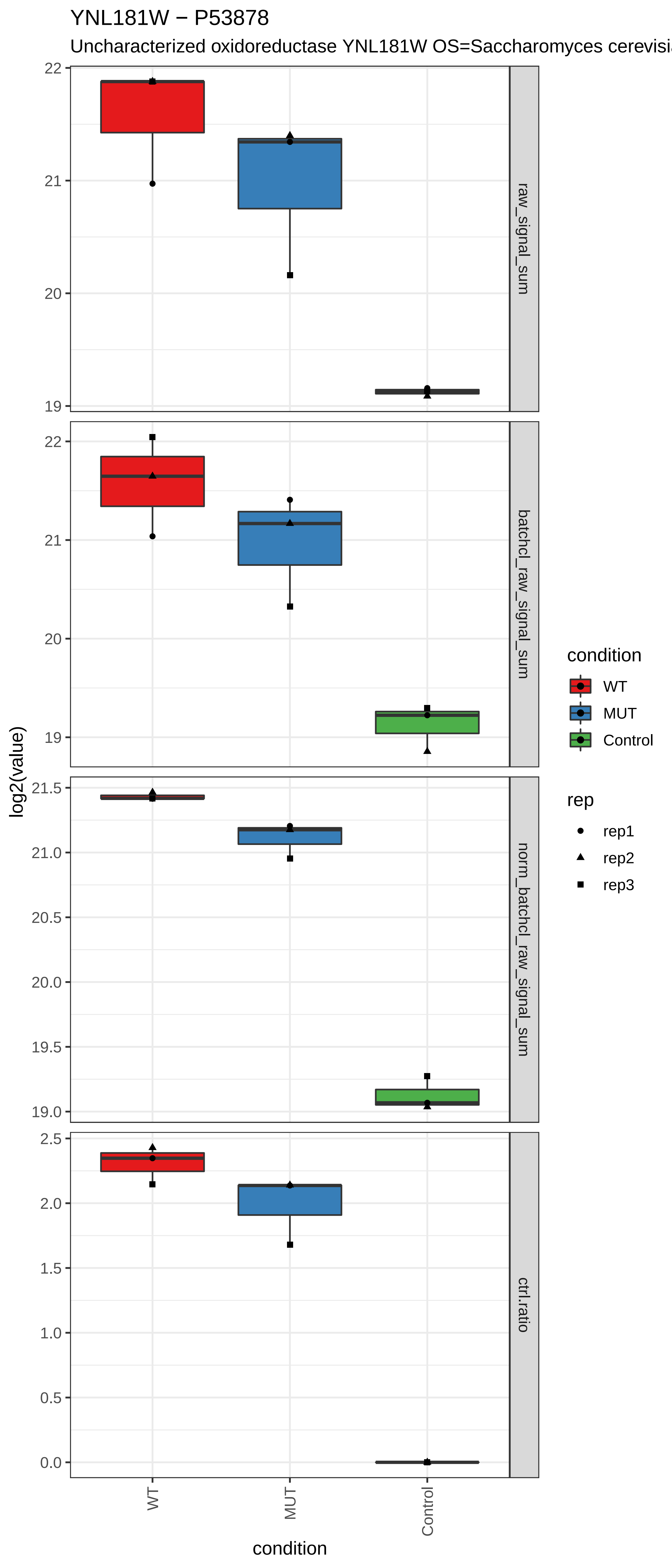

YNL181W − P53878
Uncharacterized oxidoreductase YNL181W OS=Saccharomyces cerevisi
raw_signal_sum
batchcl_raw_signal_sum
norm_batchcl_raw_signal_sum
ctrl.ratio
log2(value)
condition
WT
MUT
Control
rep
rep1
rep2
rep3

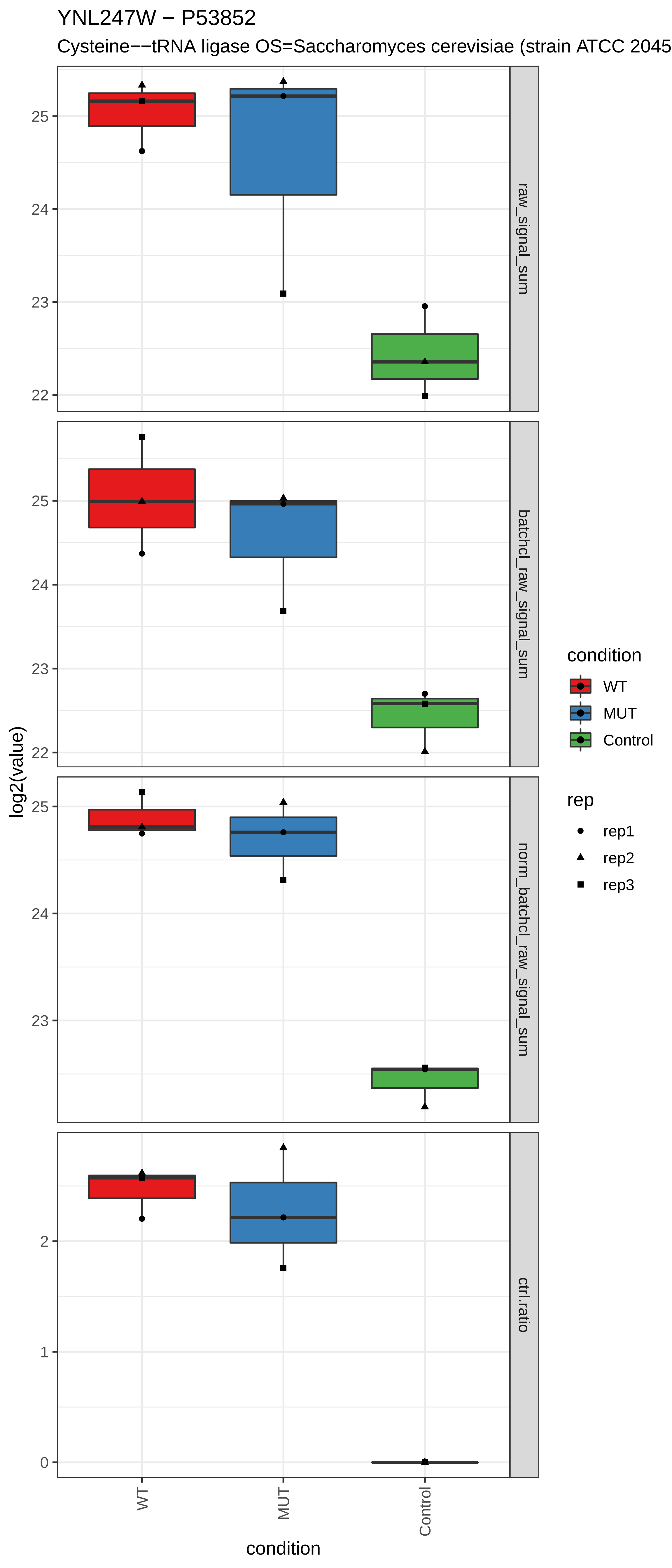
YNL247W − P53852
Cysteine−−tRNA ligase OS=Saccharomyces cerevisiae (strain ATCC 2045
raw_signal_sum
batchcl_raw_signal_sum
norm_batchcl_raw_signal_sum
ctrl.ratio
log2(value)
condition
WT
MUT
Control
rep
rep1
rep2
rep3
25
24
23
22
2
1
0

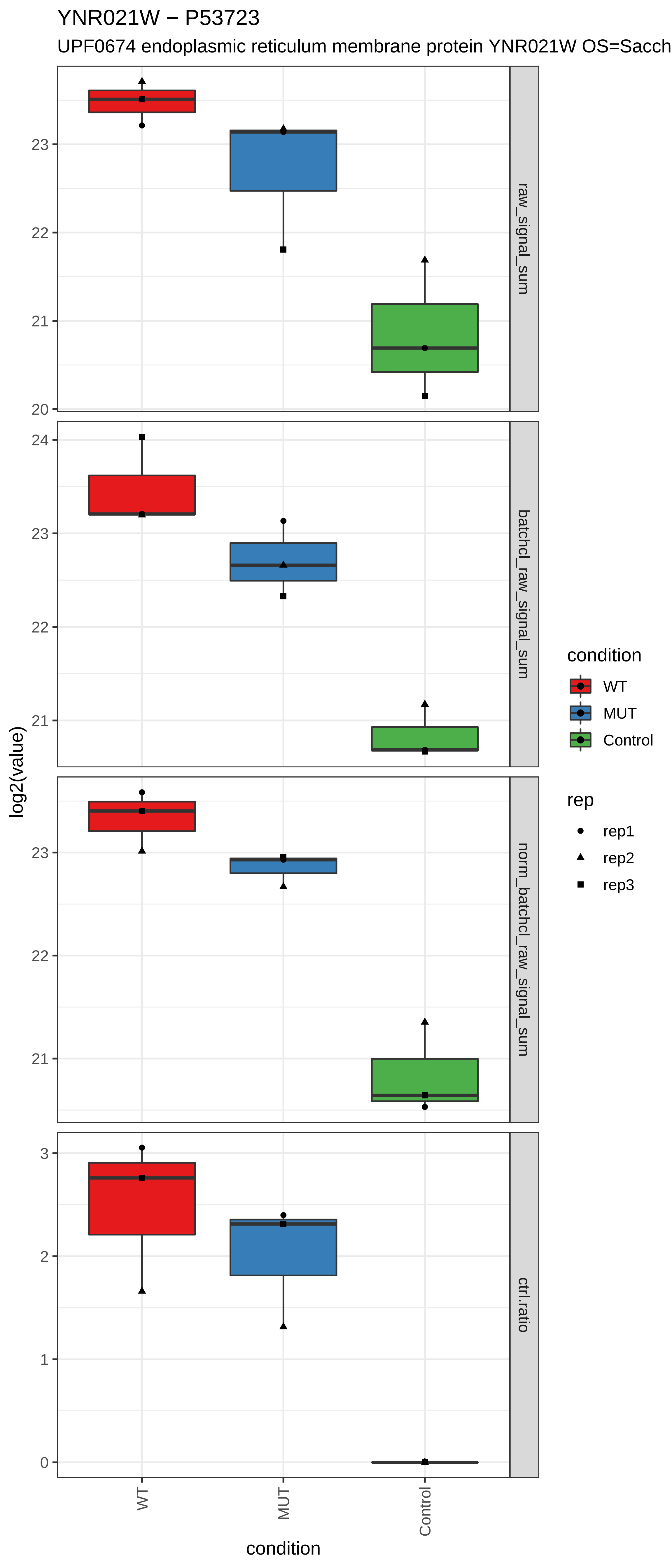
YNR021W − P53723
UPF0674 endoplasmic reticulum membrane protein YNR021W OS=Sacch
raw_signal_sum
batchcl_raw_signal_sum
norm_batchcl_raw_signal_sum
ctrl.ratio
log2(value)
condition
WT
MUT
Control
rep
rep1
rep2
rep3

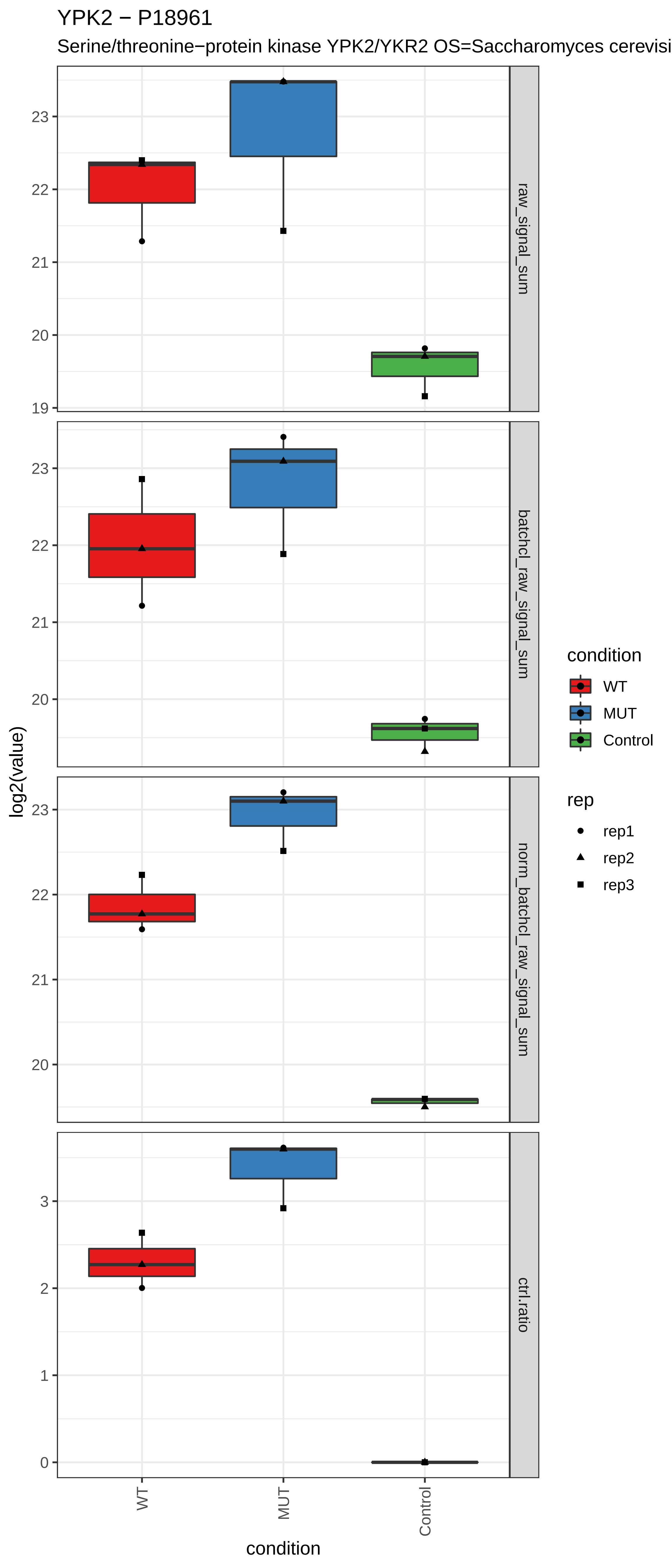
YPK2 − P18961
Serine/threonine−protein kinase YPK2/YKR2 OS=Saccharomyces cerevisi
raw_signal_sum
batchcl_raw_signal_sum
norm_batchcl_raw_signal_sum
ctrl.ratio
log2(value)
condition
WT
MUT
Control
condition
WT
MUT
Control
rep
rep1
rep2
rep3
19
20
21
22
23
0
1
2
3

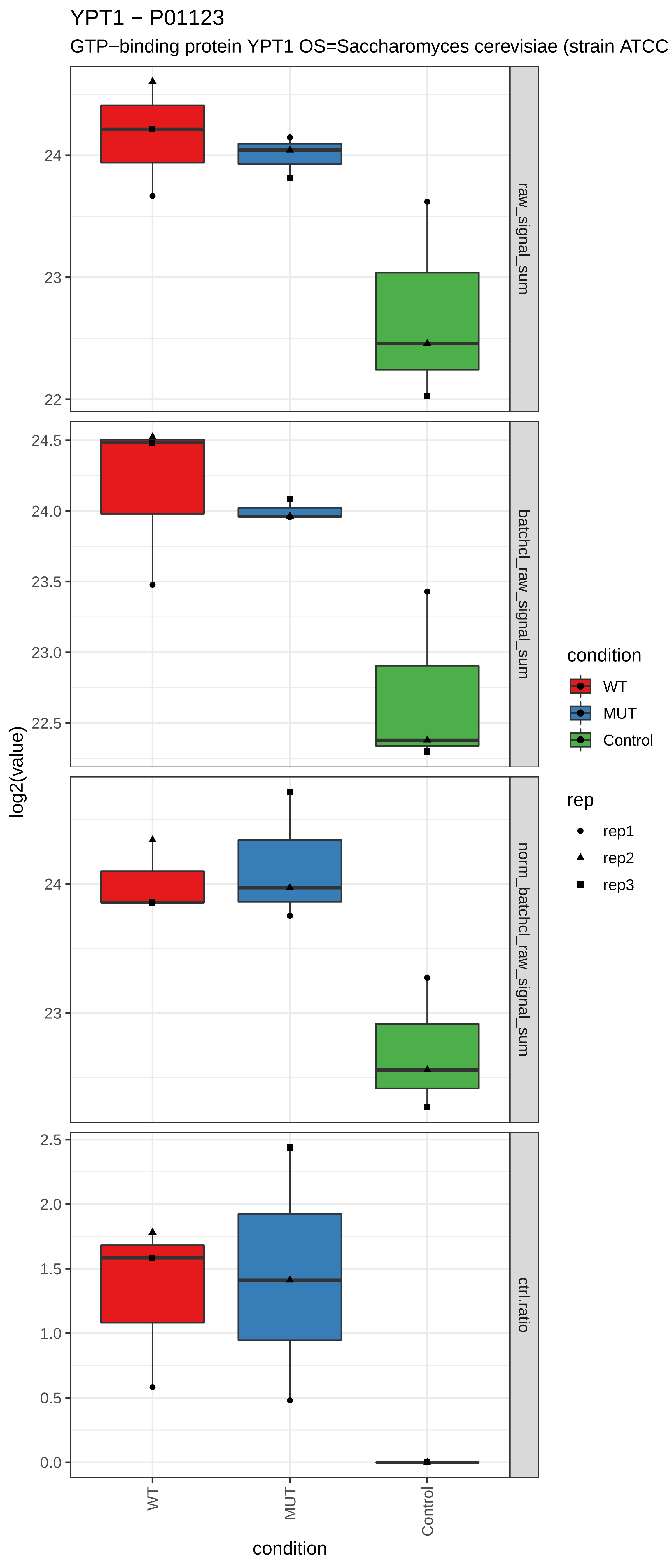
YPT1 − P01123
GTP−binding protein YPT1 OS=Saccharomyces cerevisiae (strain ATCC
raw_signal_sum
batchcl_raw_signal_sum
norm_batchcl_raw_signal_sum
ctrl.ratio
log2(value)
condition
WT
MUT
Control
rep
rep1
rep2
rep3
24
23
22
24.5
24.0
23.5
23.0
22.5
2.5
2.0
1.5
1.0
0.5
0.0

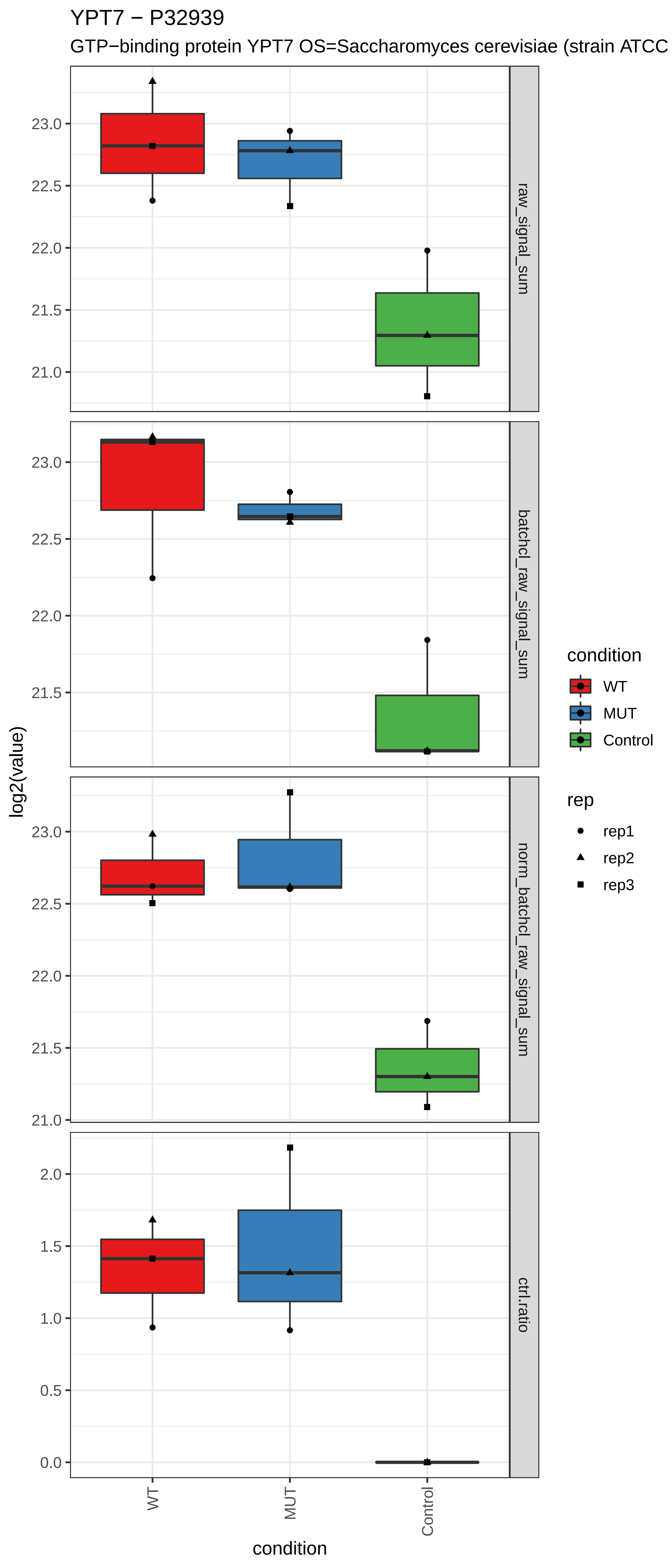
YPT7 − P32939
GTP−binding protein YPT7 OS=Saccharomyces cerevisiae (strain ATCC
raw_signal_sum
batchcl_raw_signal_sum
norm_batchcl_raw_signal_sum
ctrl.ratio
log2(value)
condition
WT
MUT
Control
rep
rep1
rep2
rep3
23.0
22.5
22.0
21.5
21.0
2.0
1.5
1.0
0.5
0.0

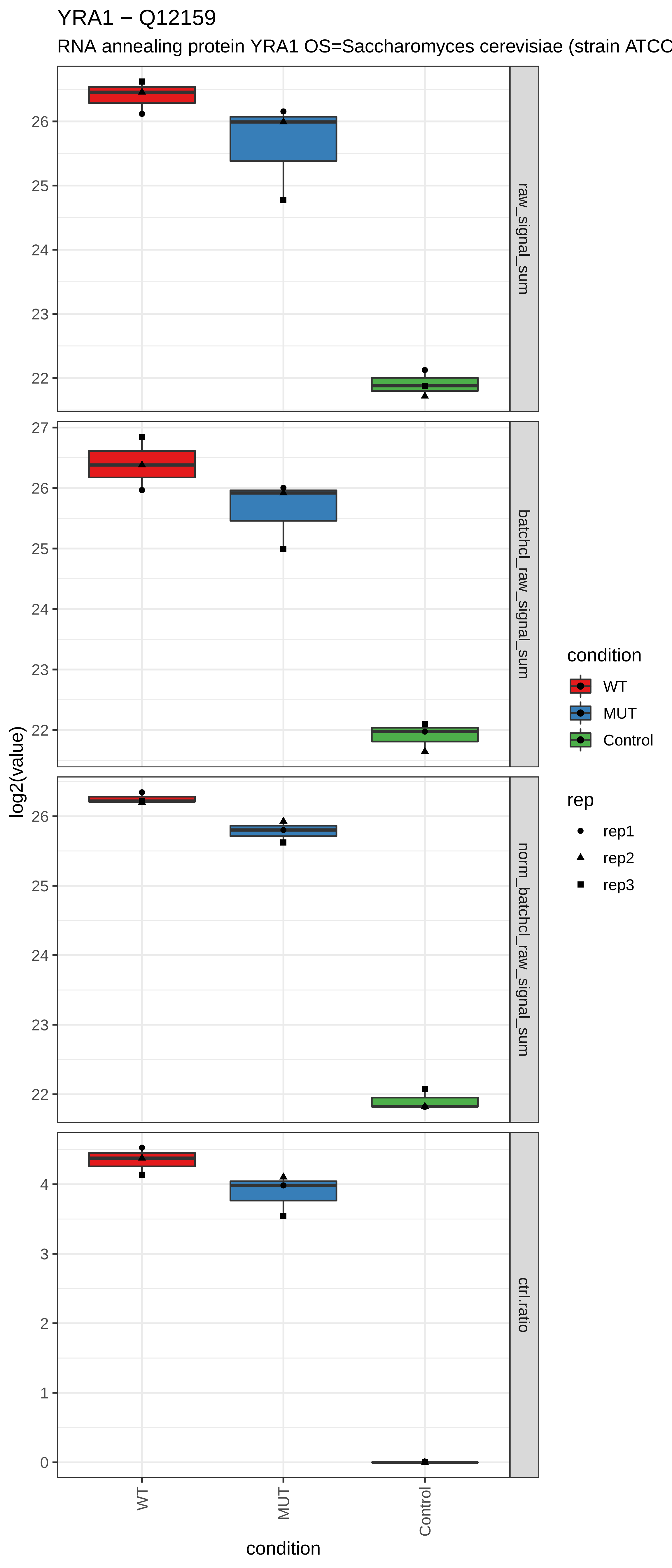
YRA1 − Q12159
RNA annealing protein YRA1 OS=Saccharomyces cerevisiae (strain ATCC
raw_signal_sum
batchcl_raw_signal_sum
norm_batchcl_raw_signal_sum
ctrl.ratio
log2(value)
condition
WT
MUT
Control
rep
rep1
rep2
rep3
26
25
24
23
22
27
4
3
2
1
0

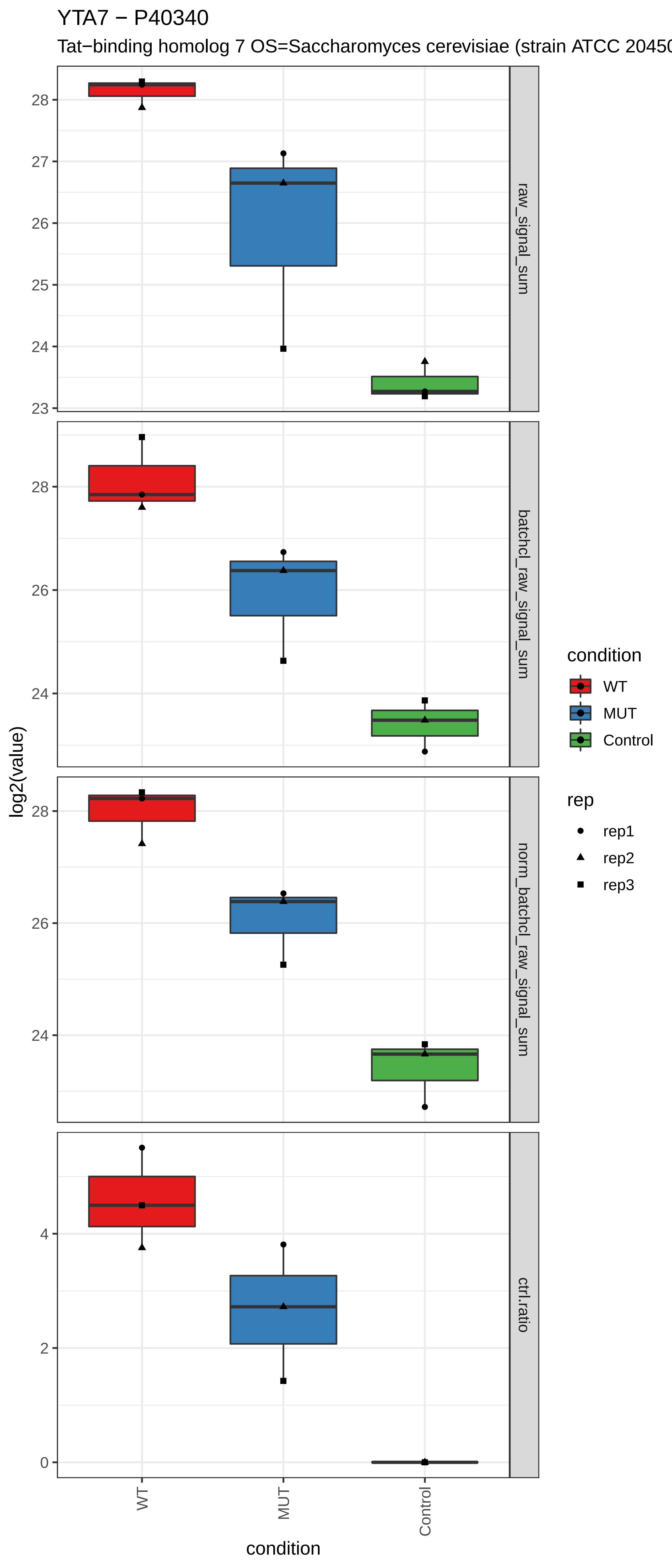

YTA7 − P40340
Tat−binding homolog 7 OS=Saccharomyces cerevisiae (strain ATCC 20450
log2(value)
raw_signal_sum
batchcl_raw_signal_sum
norm_batchcl_raw_signal_sum
ctrl.ratio
28
27
26
25
24
23
28
26
24
28
26
24
4
2
0
WT
MUT
Control
condition
condition
WT
MUT
Control
rep
rep1
rep2
rep3

YTM1 − Q12024

Ribosome biogenesis protein YTM1 OS=Saccharomyces cerevisiae (strain

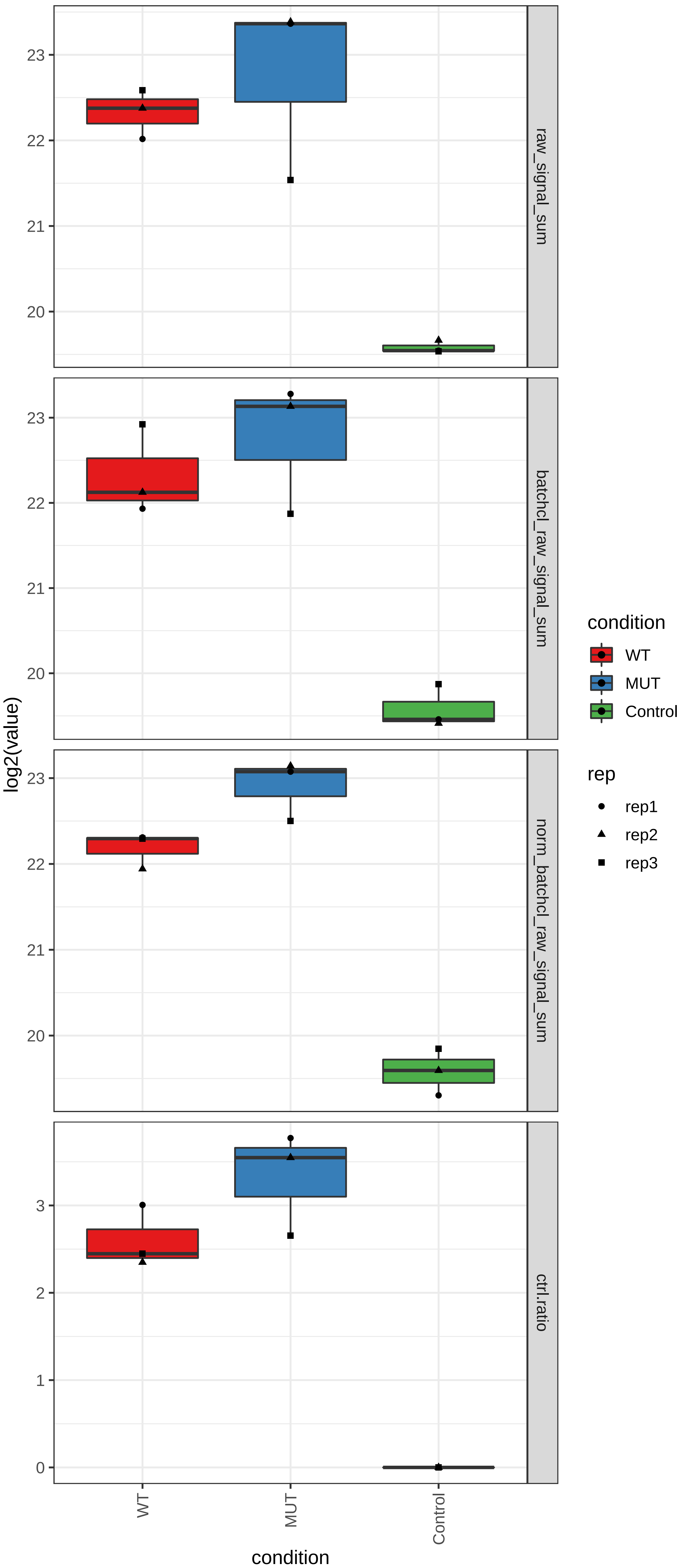

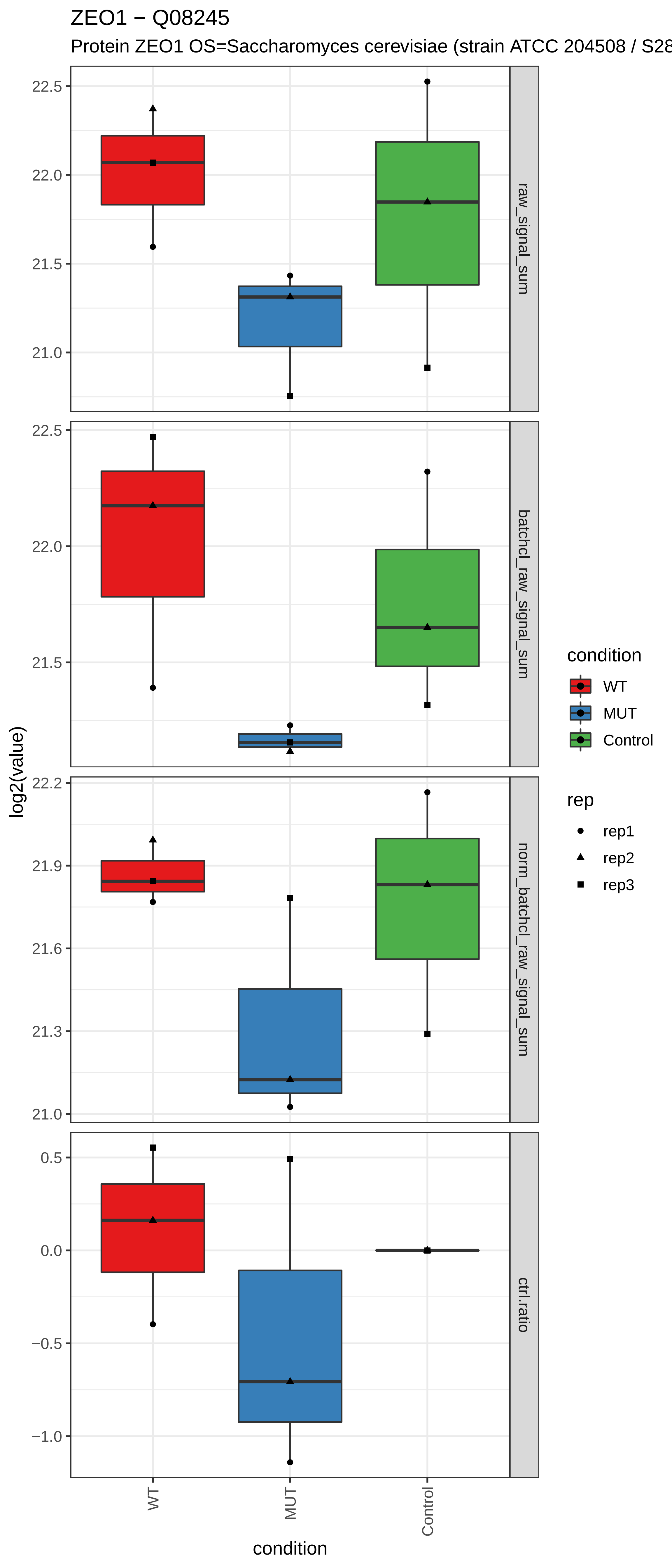
ZEO1 − Q08245
Protein ZEO1 OS=Saccharomyces cerevisiae (strain ATCC 204508 / S28
raw_signal_sum
batchcl_raw_signal_sum
norm_batchcl_raw_signal_sum
ctrl.ratio
log2(value)
condition
WT
MUT
Control
rep
rep1
rep2
rep3
22.5
22.0
21.5
21.0
22.2
21.9
21.6
21.3
0.5
0.0
−0.5
−1.0

ZIP1 − P31111

Synaptonemal complex protein ZIP1 OS=Saccharomyces cerevisiae (strain

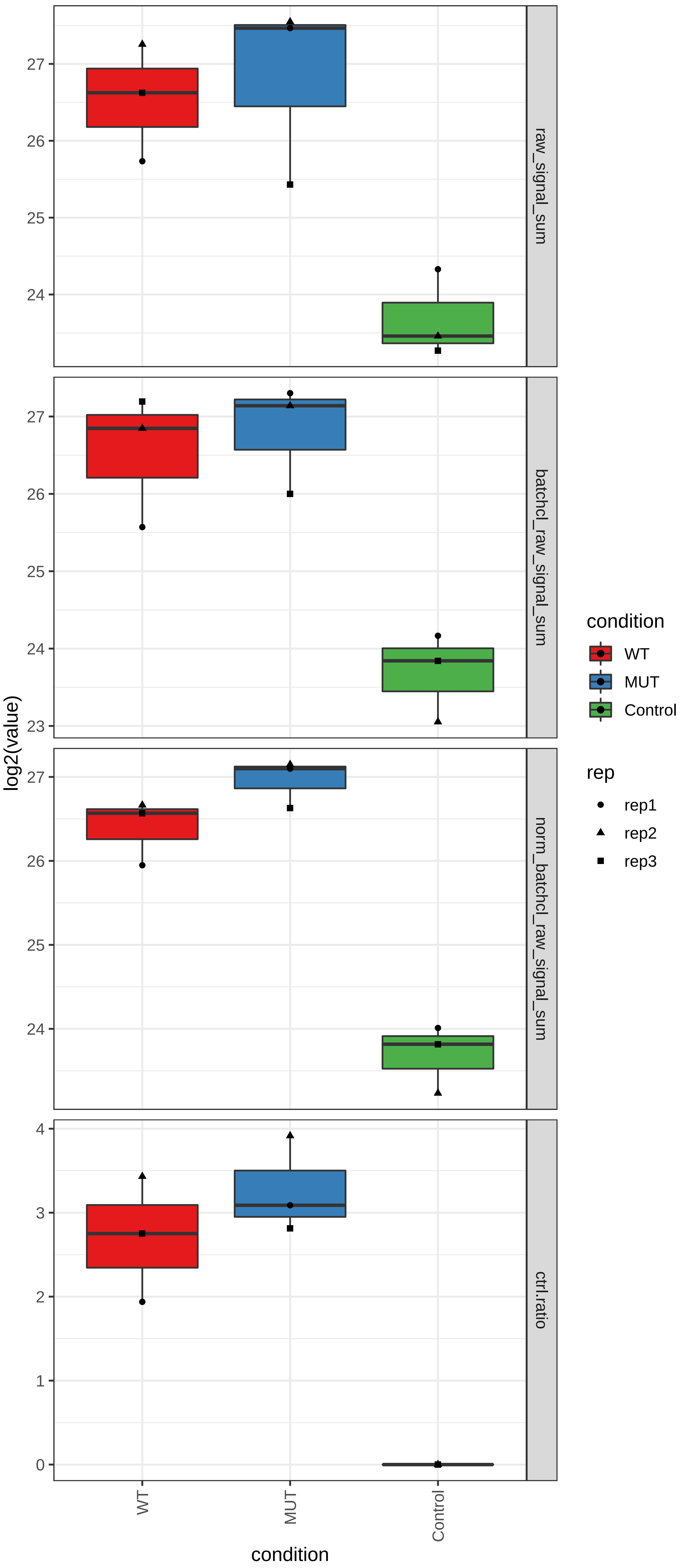

ZTA1 − P38230

Probable quinone oxidoreductase OS=Saccharomyces cerevisiae (strain

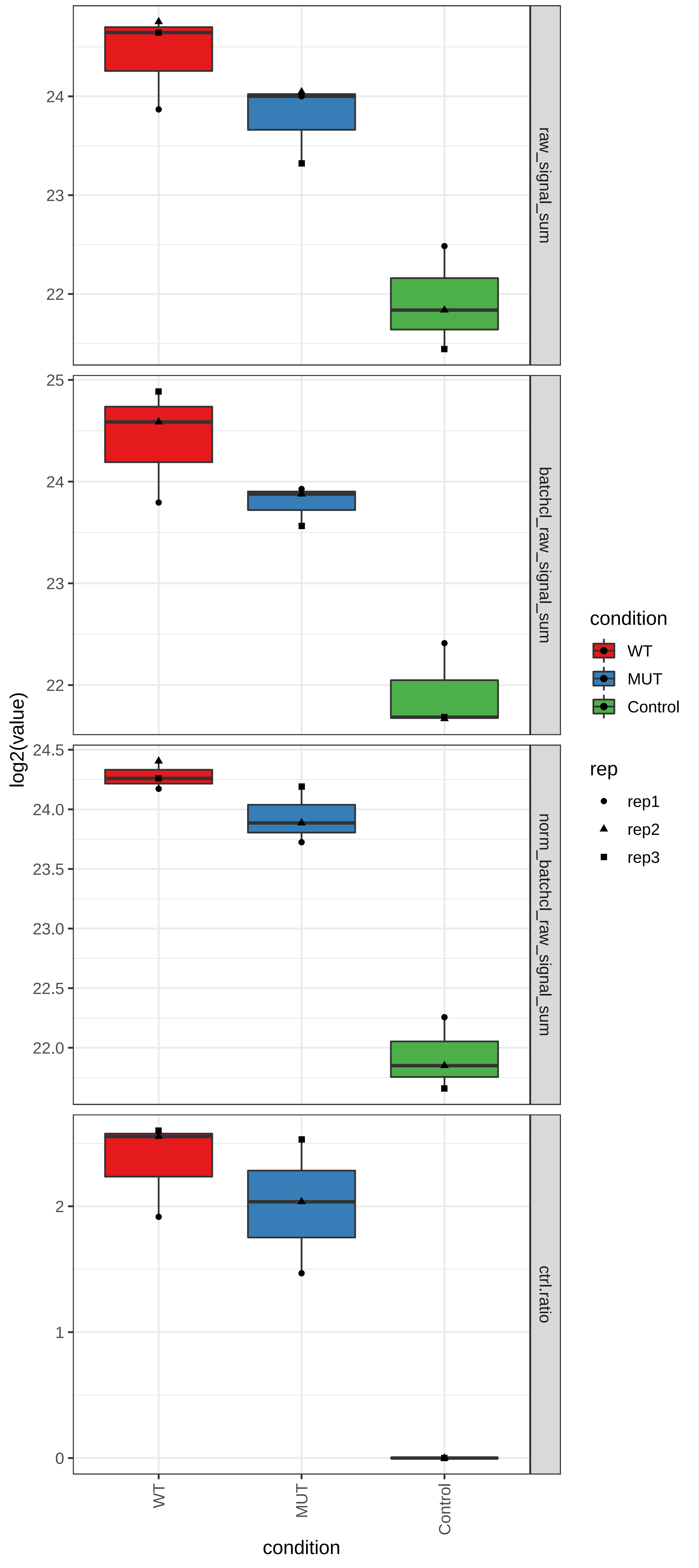

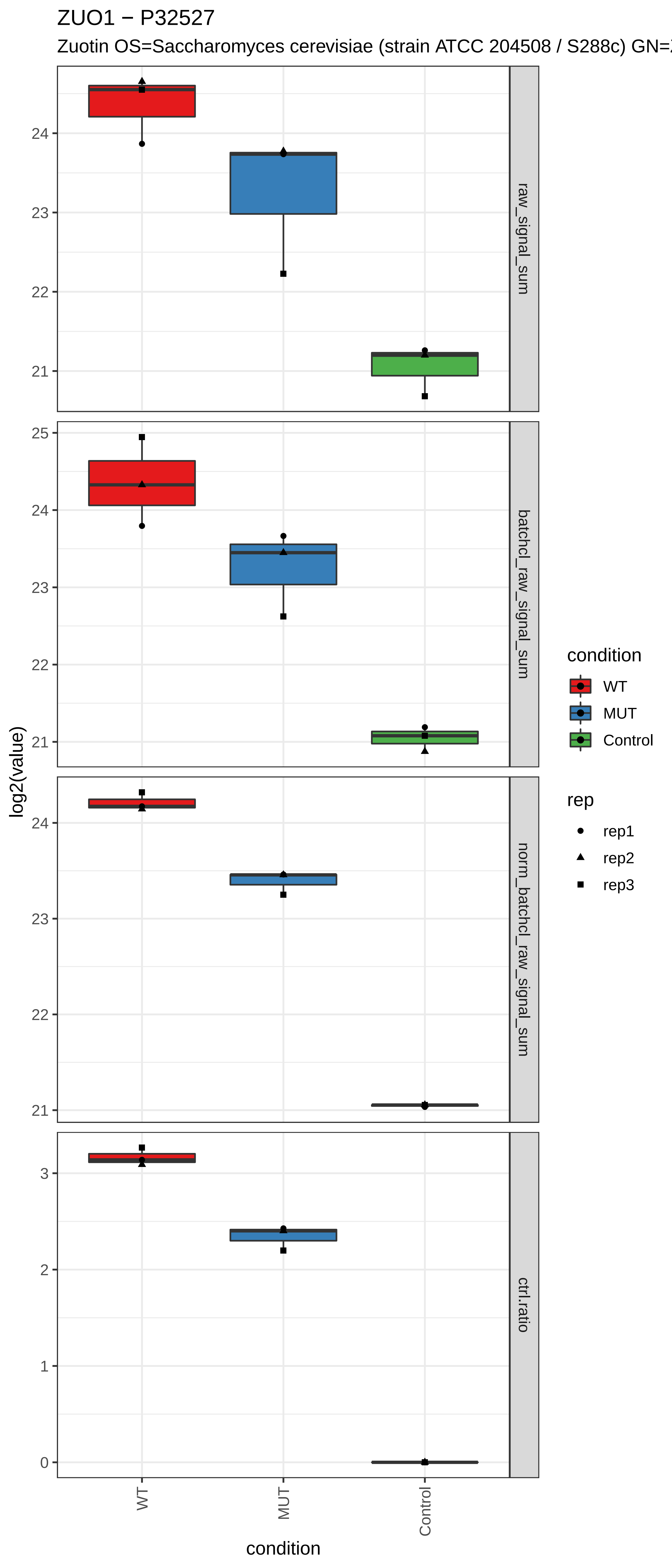

ZUO1 − P32527
Zuotin OS=Saccharomyces cerevisiae (strain ATCC 204508 / S288c) GN=
raw_signal_sum
batchcl_raw_signal_sum
norm_batchcl_raw_signal_sum
ctrl.ratio
log2(value)
condition
WT
MUT
Control
rep
rep1
rep2
rep3
