# Supplementary material for: Novel mechanistic insights into the role of Mer2 as the keystone of meiotic DNA break formation
Source: eLife. 2021 Dec 24;10:e72330. doi: 10.7554/eLife.72330 (PMC8848140; doi:10.7554/eLife.72330)
Supplement: Source data 2. [file Clustering_heatmap_hits_hclust_10_cluster_741_proteins_V1.pdf › data_analysis_results_V1/Clustering_heatmap_hits_hclust_10_cluster_741_proteins_V1.pdf]

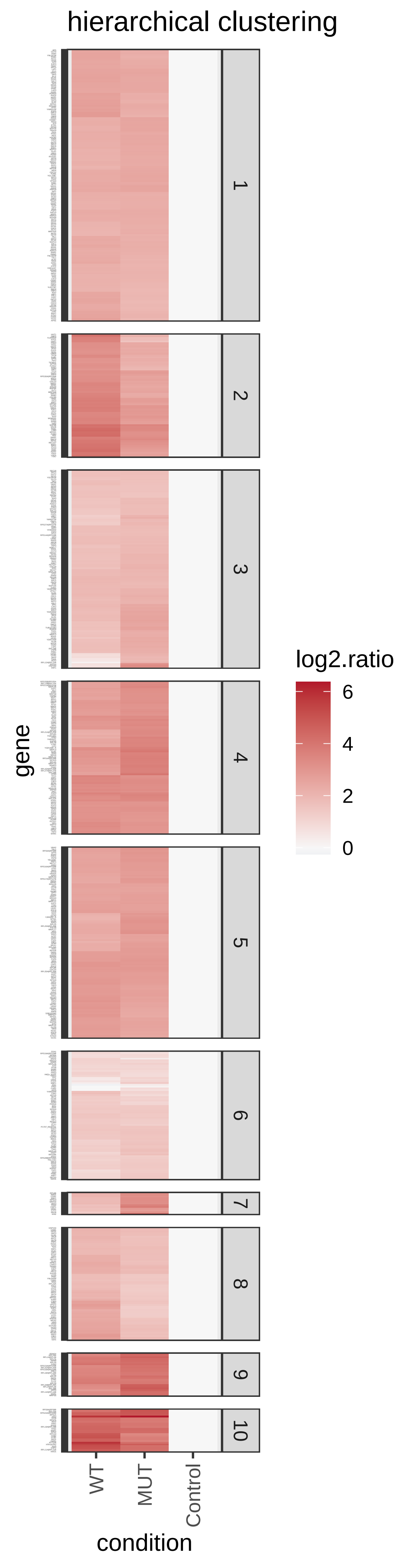
hierarchical clustering
gene
1
2
3
4
5
6
7
8
9
10
WT
MUT
Control
condition
log2.ratio
6
4
2
0
