# Supplementary material for: Novel mechanistic insights into the role of Mer2 as the keystone of meiotic DNA break formation
Source: eLife. 2021 Dec 24;10:e72330. doi: 10.7554/eLife.72330 (PMC8848140; doi:10.7554/eLife.72330)
Supplement: Source data 2. [file hit_proteins_V1.pdf › data_analysis_results_V1/hit_proteins_V1.pdf]

ABF1 − P14164

ARS−binding factor 1 OS=Saccharomyces cerevisiae (strain ATCC 204508

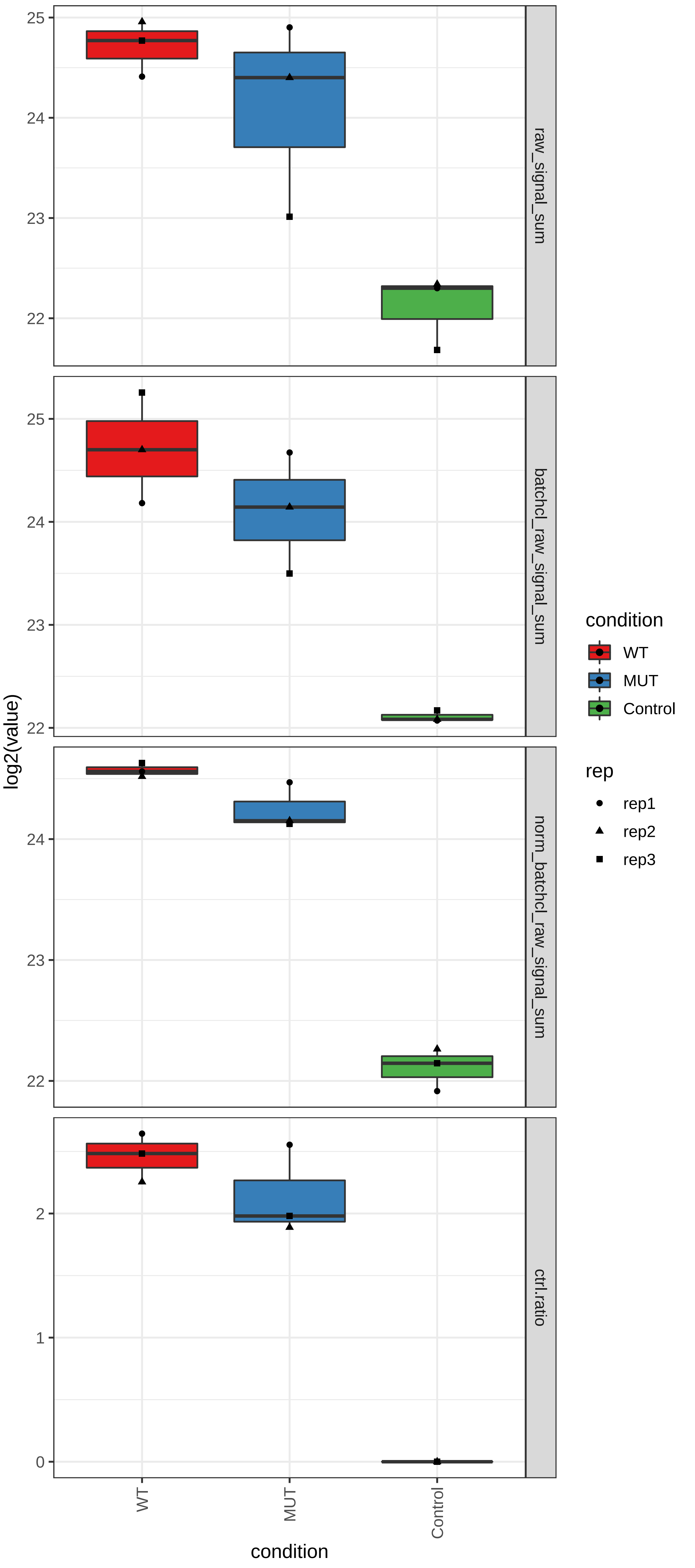

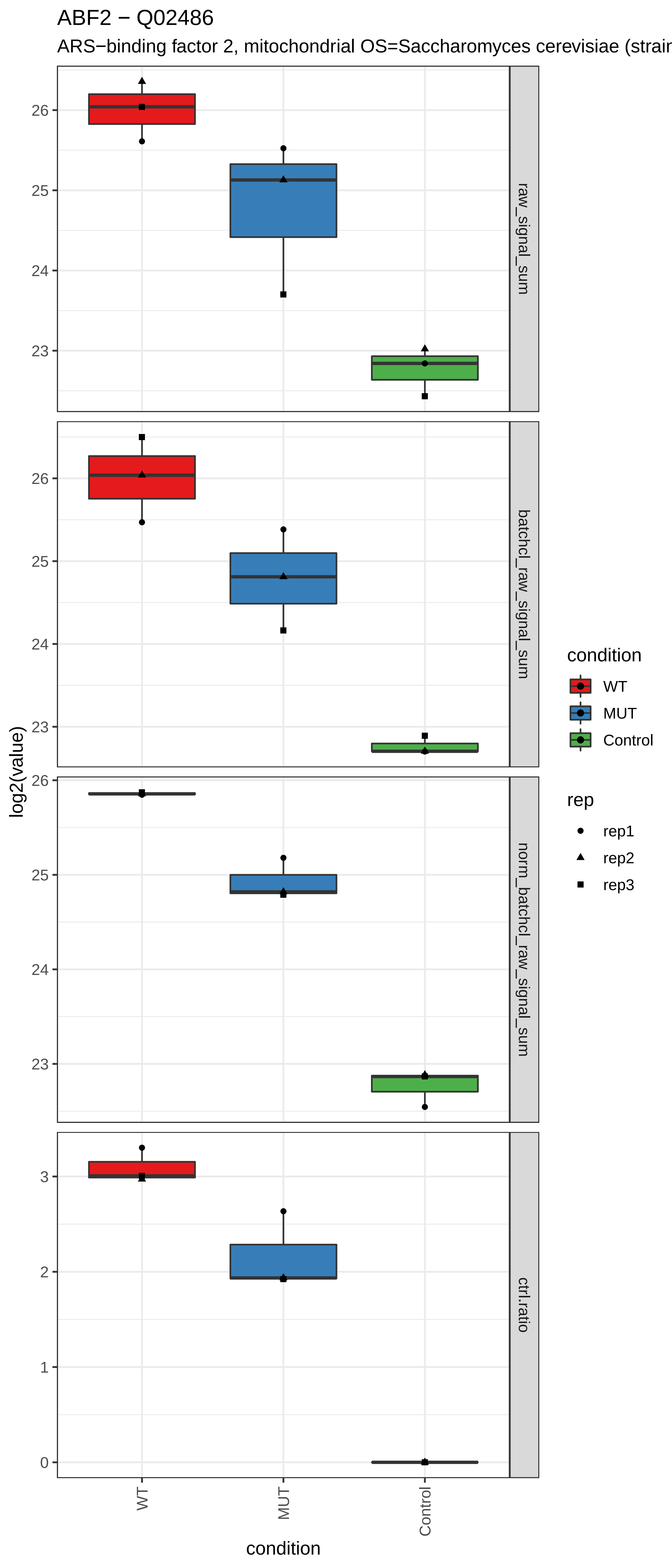
ABF2 − Q02486
ARS−binding factor 2, mitochondrial OS=Saccharomyces cerevisiae (strain
raw_signal_sum
batchcl_raw_signal_sum
norm_batchcl_raw_signal_sum
ctrl.ratio
log2(value)
condition
WT
MUT
Control
rep
rep1
rep2
rep3
26
25
24
23
3
2
1
0

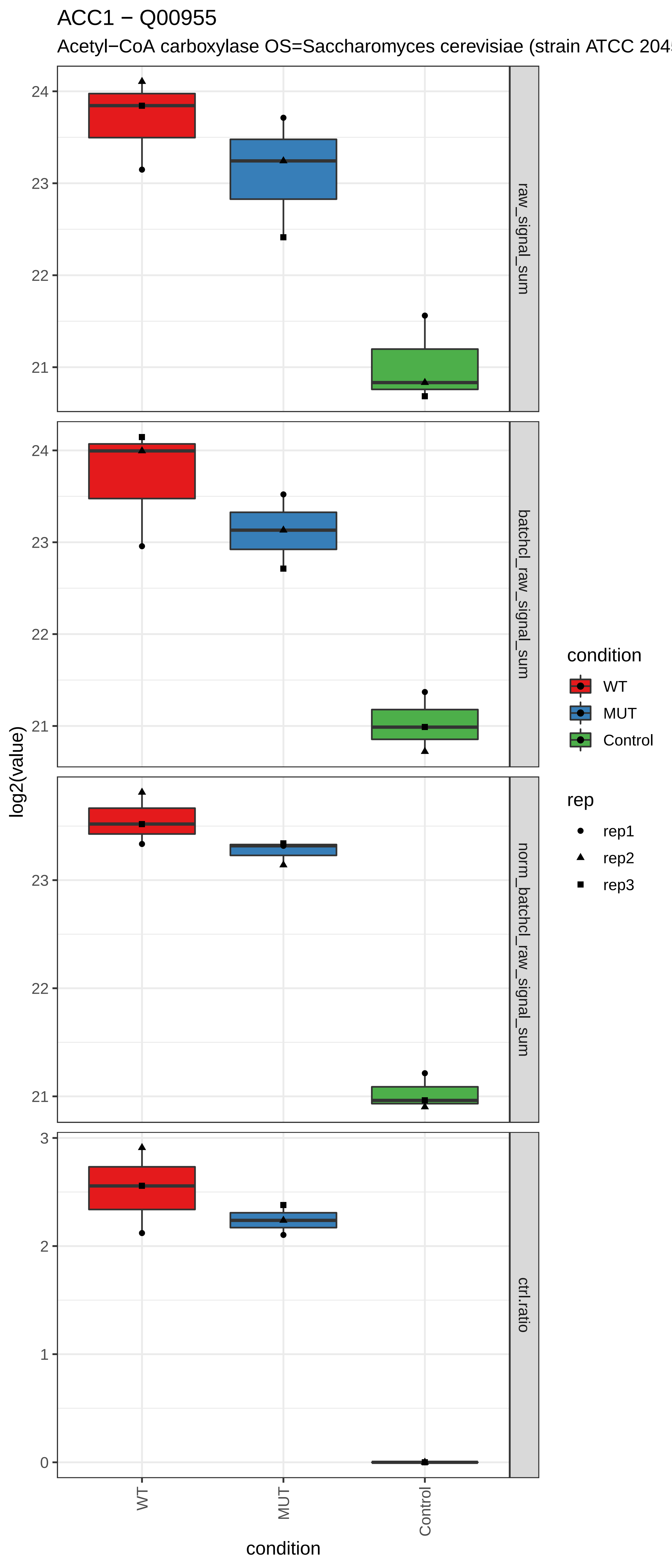

ACC1 − Q00955
Acetyl−CoA carboxylase OS=Saccharomyces cerevisiae (strain ATCC 204
raw_signal_sum
batchcl_raw_signal_sum
norm_batchcl_raw_signal_sum
ctrl.ratio
log2(value)
condition
WT
MUT
Control
rep
rep1
rep2
rep3
24
23
22
21
3
2
1
0

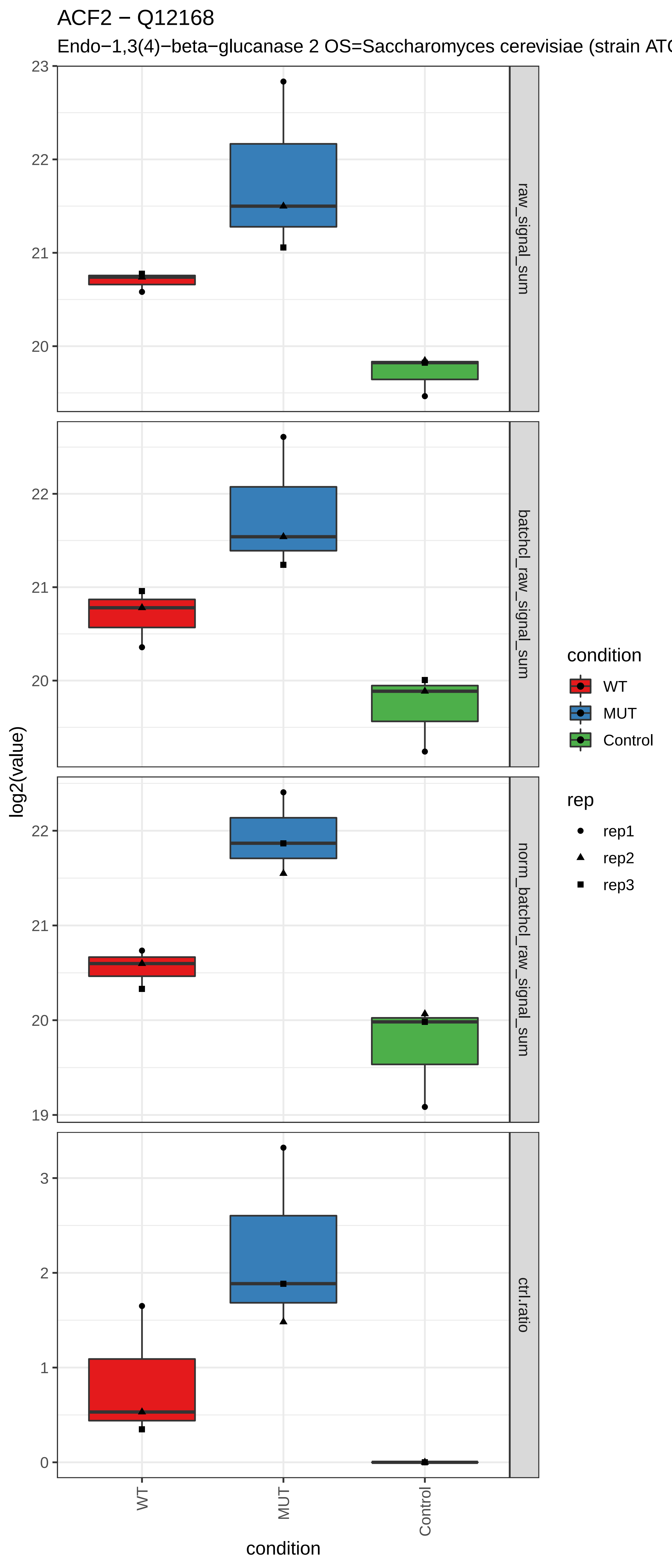

ACF2 − Q12168
Endo−1,3(4)−beta−glucanase 2 OS=Saccharomyces cerevisiae (strain ATC
raw_signal_sum
batchcl_raw_signal_sum
norm_batchcl_raw_signal_sum
ctrl.ratio
log2(value)
condition
WT
MUT
Control
rep
rep1
rep2
rep3

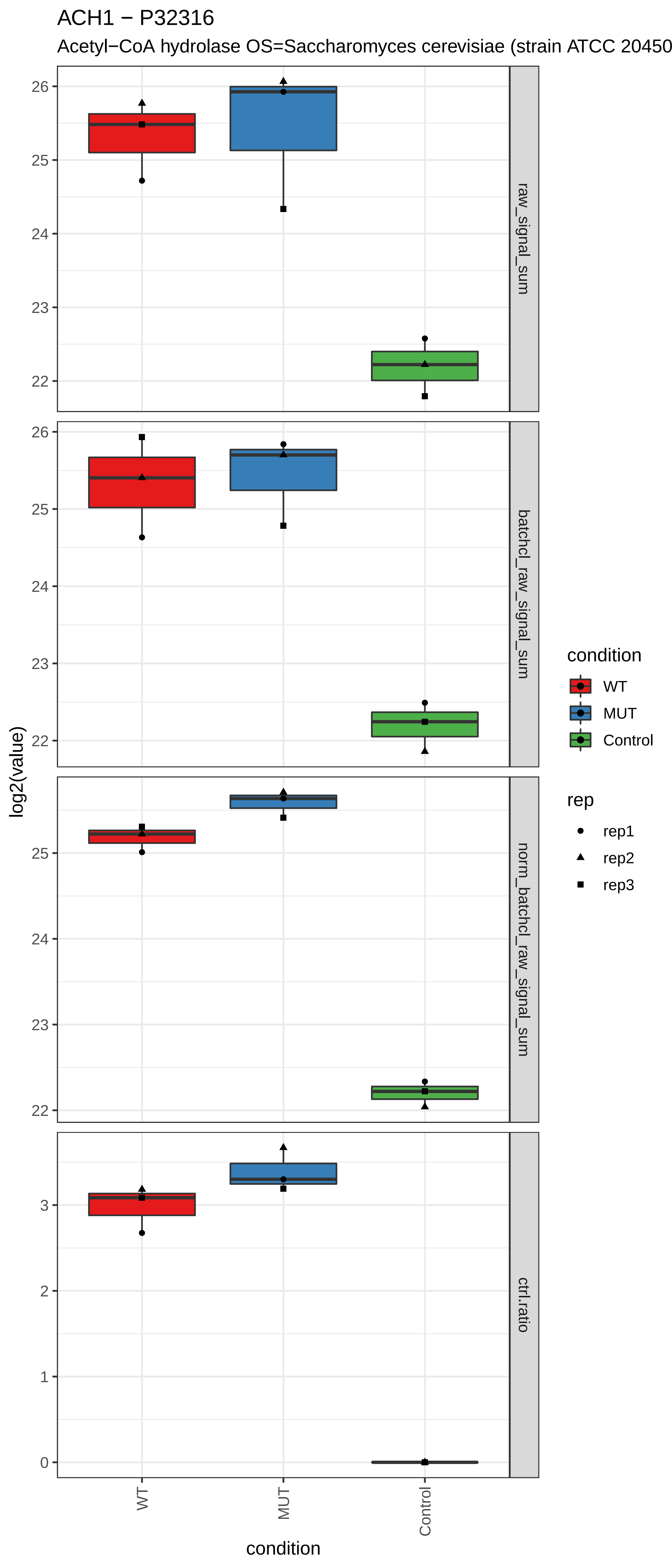

ACH1 − P32316
Acetyl−CoA hydrolase OS=Saccharomyces cerevisiae (strain ATCC 20450
raw_signal_sum
batchcl_raw_signal_sum
norm_batchcl_raw_signal_sum
ctrl.ratio
log2(value)
condition
WT
MUT
Control
rep
rep1
rep2
rep3

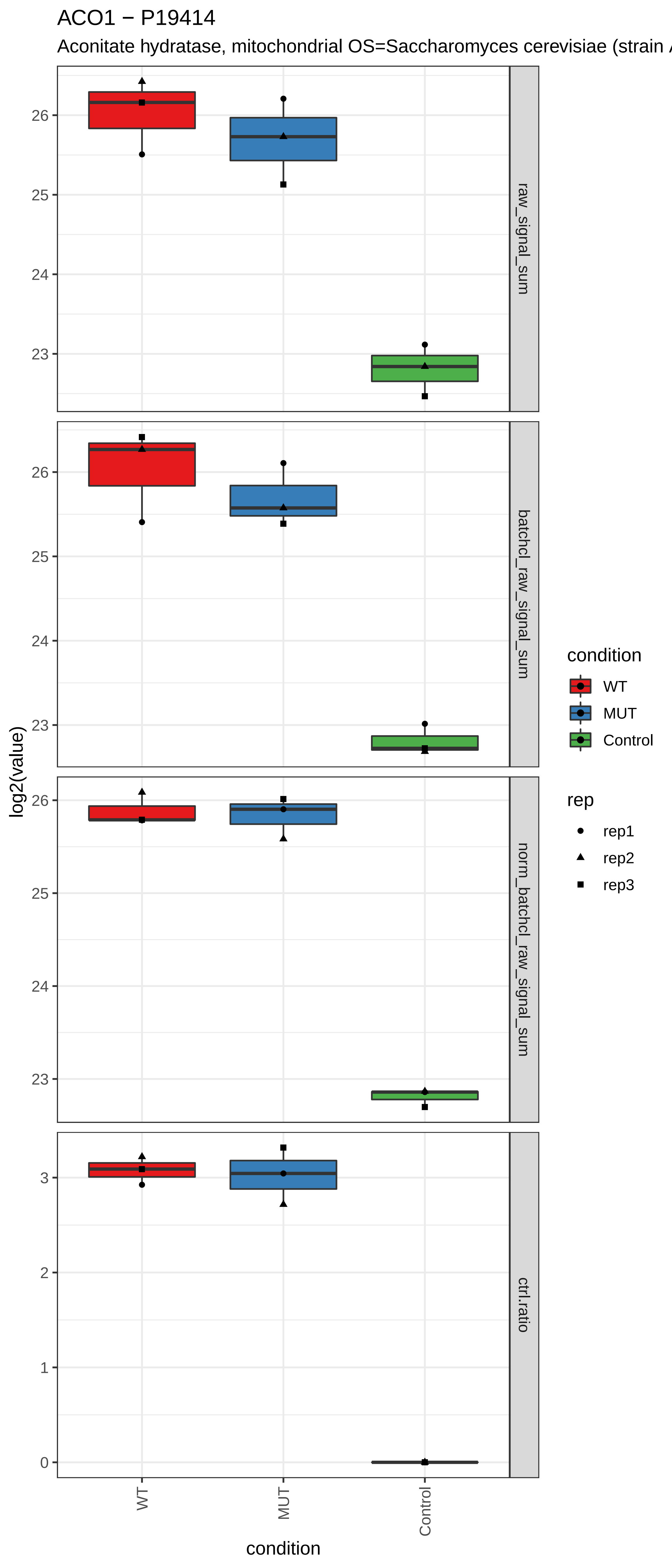

ACO1 − P19414
Aconitate hydratase, mitochondrial OS=Saccharomyces cerevisiae (strain
raw_signal_sum
batchcl_raw_signal_sum
norm_batchcl_raw_signal_sum
ctrl.ratio
log2(value)
condition
WT
MUT
Control
rep
rep1
rep2
rep3
26
25
24
23
3
2
1
0

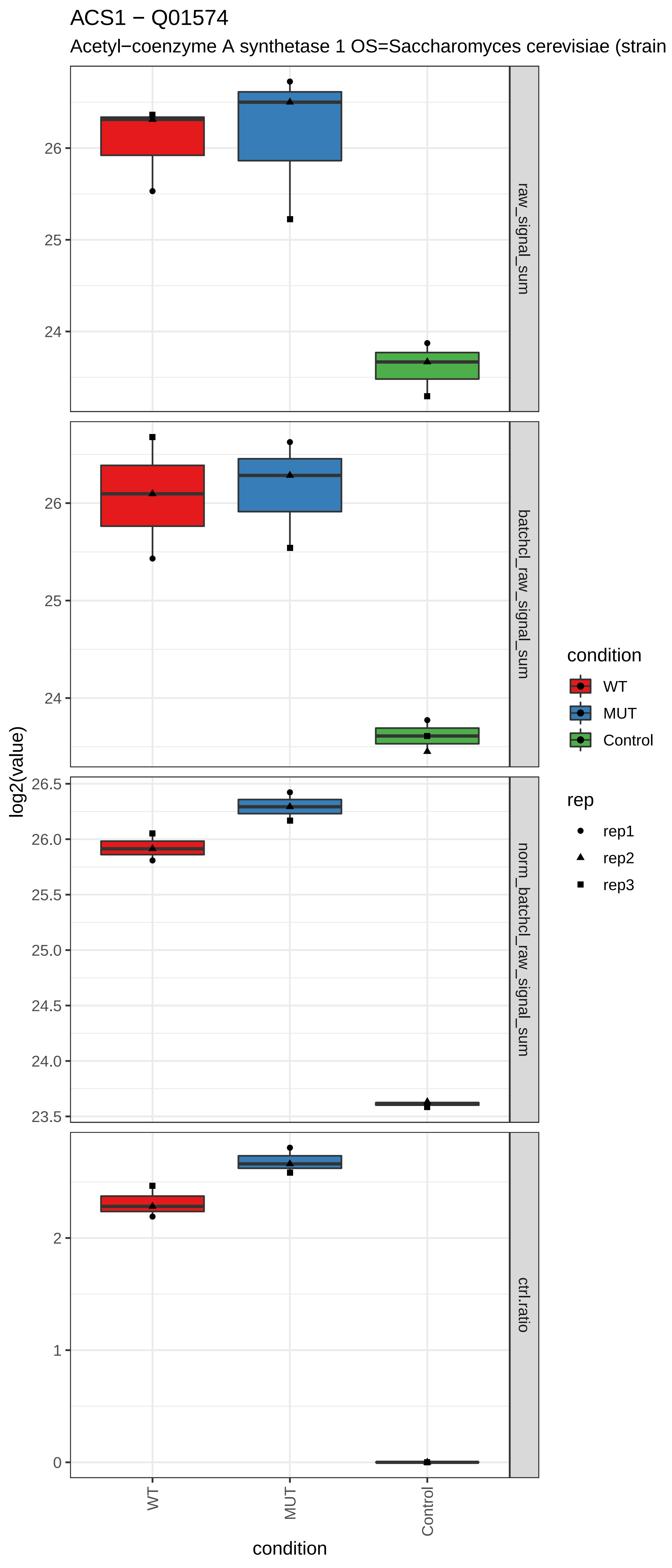

ACS1 − Q01574
Acetyl−coenzyme A synthetase 1 OS=Saccharomyces cerevisiae (strain
raw_signal_sum
batchcl_raw_signal_sum
norm_batchcl_raw_signal_sum
ctrl.ratio
log2(value)
condition
WT
MUT
Control
rep
rep1
rep2
rep3

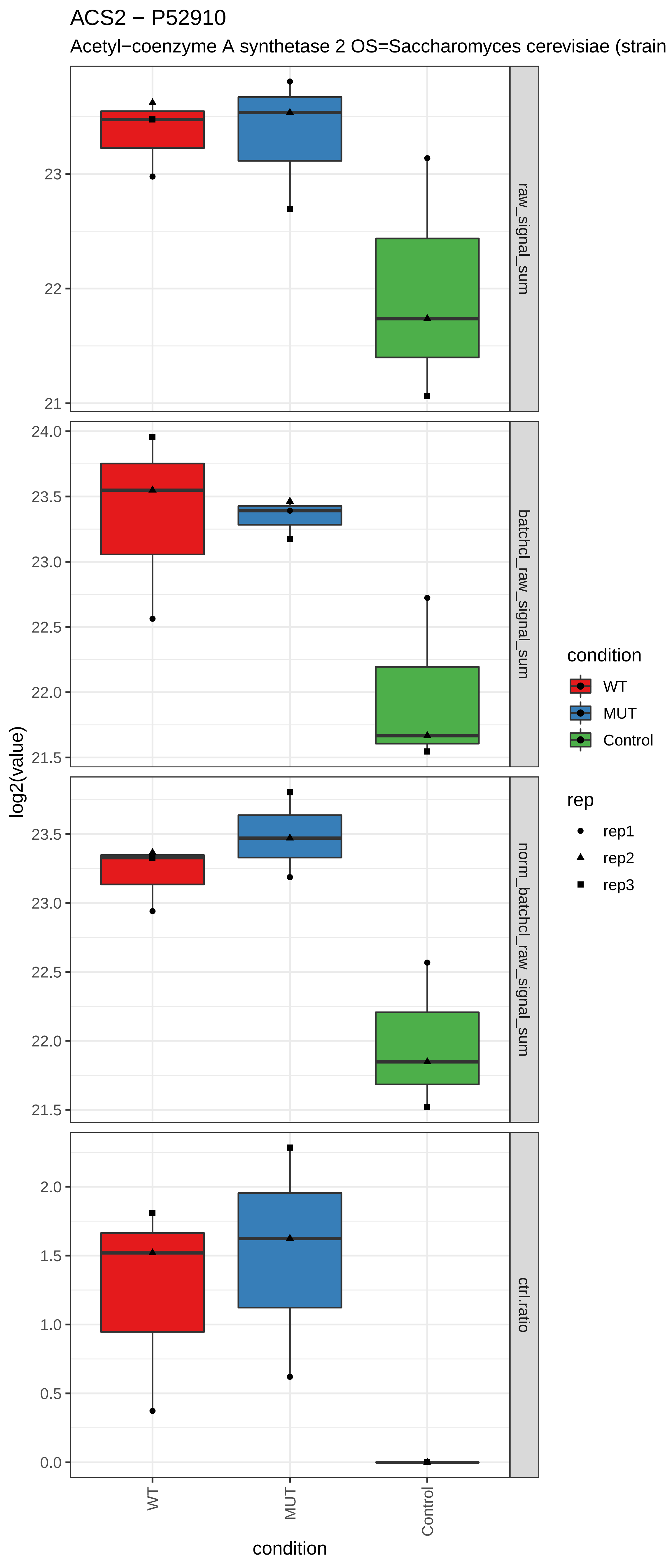
ACS2 − P52910
Acetyl−coenzyme A synthetase 2 OS=Saccharomyces cerevisiae (strain
raw_signal_sum
batchcl_raw_signal_sum
norm_batchcl_raw_signal_sum
ctrl.ratio
log2(value)
condition
WT
MUT
Control
rep
rep1
rep2
rep3

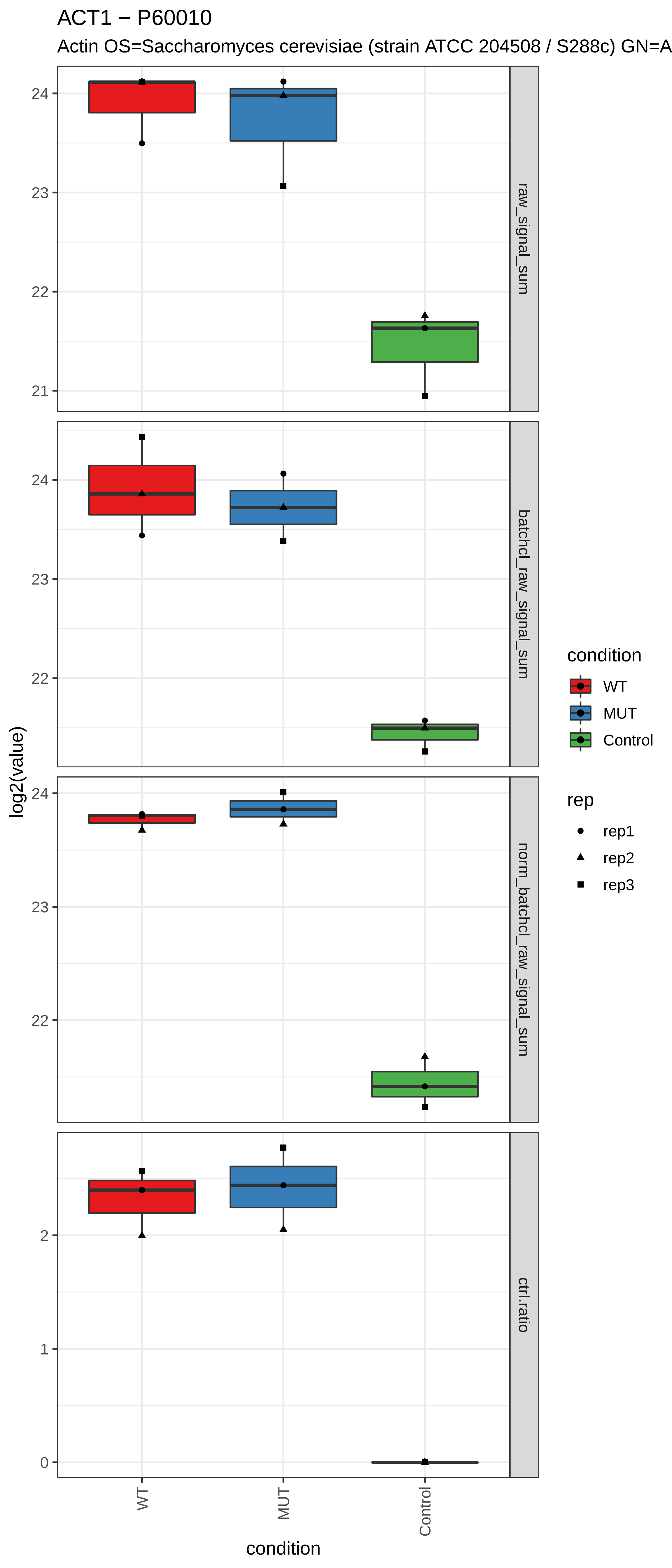
ACT1 − P60010
Actin OS=Saccharomyces cerevisiae (strain ATCC 204508 / S288c) GN=A
raw_signal_sum
batchcl_raw_signal_sum
norm_batchcl_raw_signal_sum
ctrl.ratio
log2(value)
condition
WT
MUT
Control
rep
rep1
rep2
rep3
24
23
22
21
2
1
0

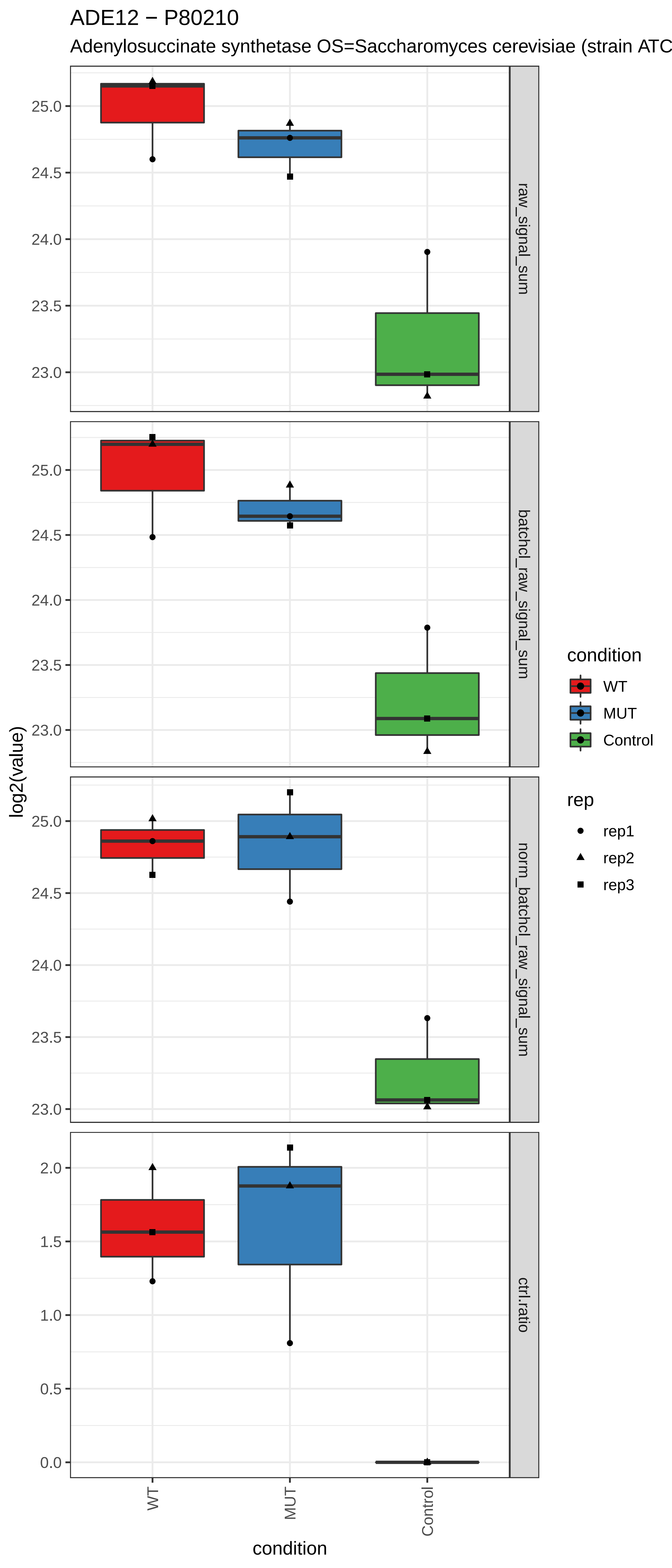

ADE12 − P80210
Adenylosuccinate synthetase OS=Saccharomyces cerevisiae (strain ATC
raw_signal_sum
batchcl_raw_signal_sum
norm_batchcl_raw_signal_sum
ctrl.ratio
log2(value)
condition
WT
MUT
Control
rep
rep1
rep2
rep3

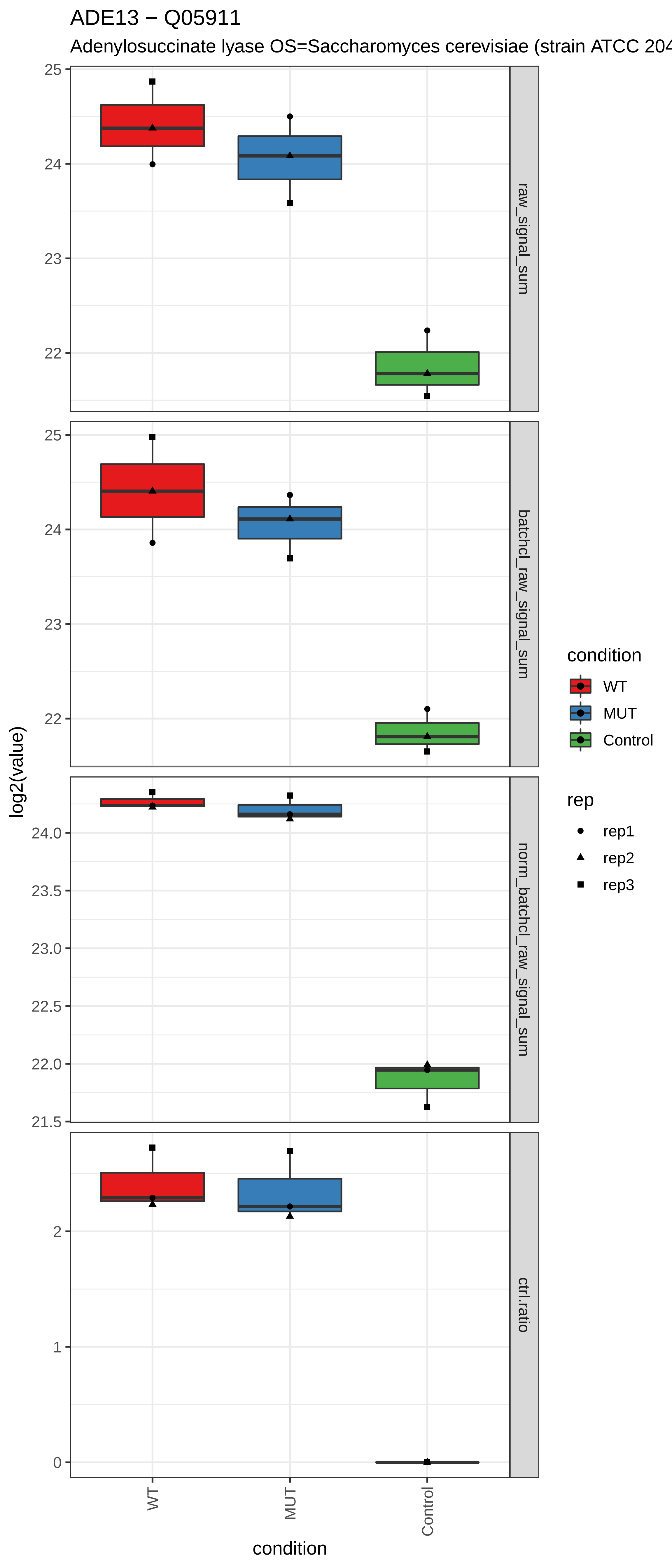
ADE13 − Q05911
Adenylosuccinate lyase OS=Saccharomyces cerevisiae (strain ATCC 204
raw_signal_sum
batchcl_raw_signal_sum
norm_batchcl_raw_signal_sum
ctrl.ratio
log2(value)
condition
WT
MUT
Control
rep
rep1
rep2
rep3

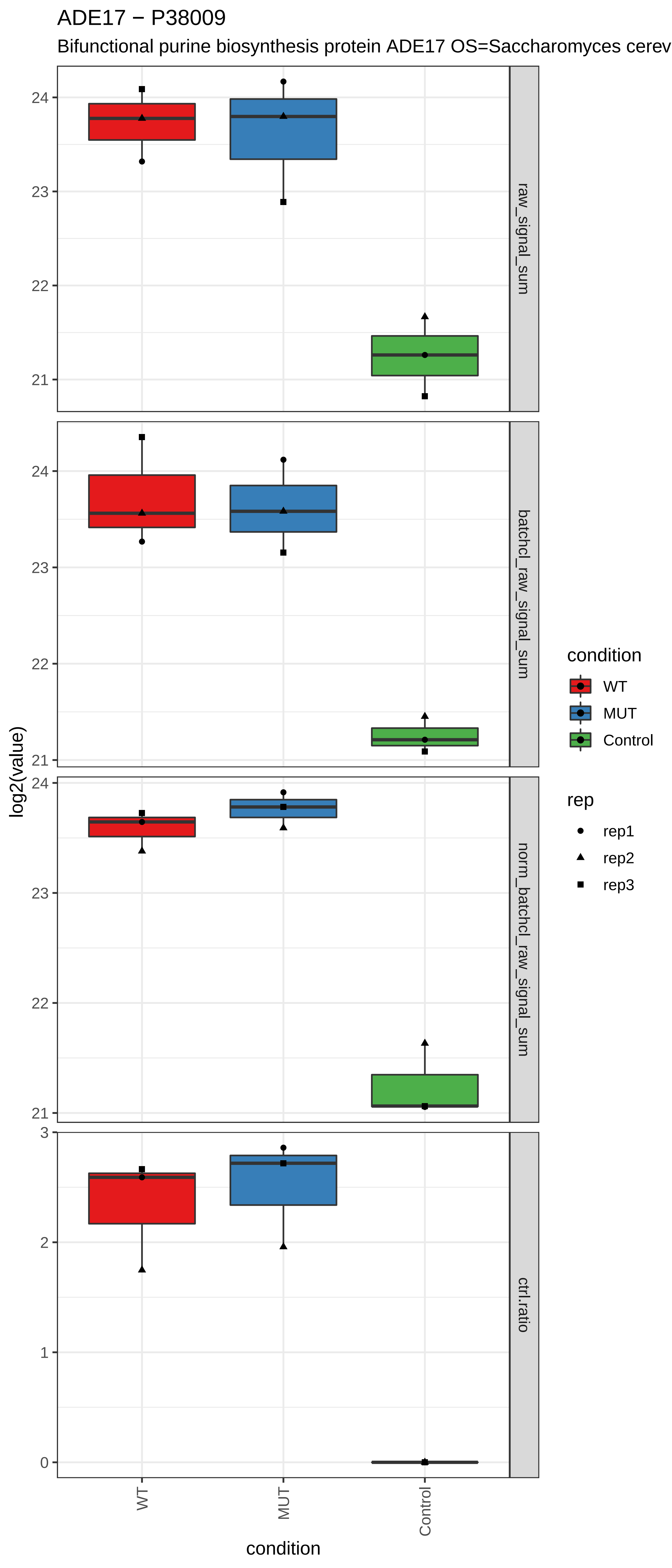

ADE17 − P38009
Bifunctional purine biosynthesis protein ADE17 OS=Saccharomyces cerev
raw_signal_sum
batchcl_raw_signal_sum
norm_batchcl_raw_signal_sum
ctrl.ratio
log2(value)
condition
WT
MUT
Control
rep
rep1
rep2
rep3
24
23
22
21
3
2
1
0

ADE2 − P21264

Phosphoribosylaminoimidazole carboxylase OS=Saccharomyces cerevisia

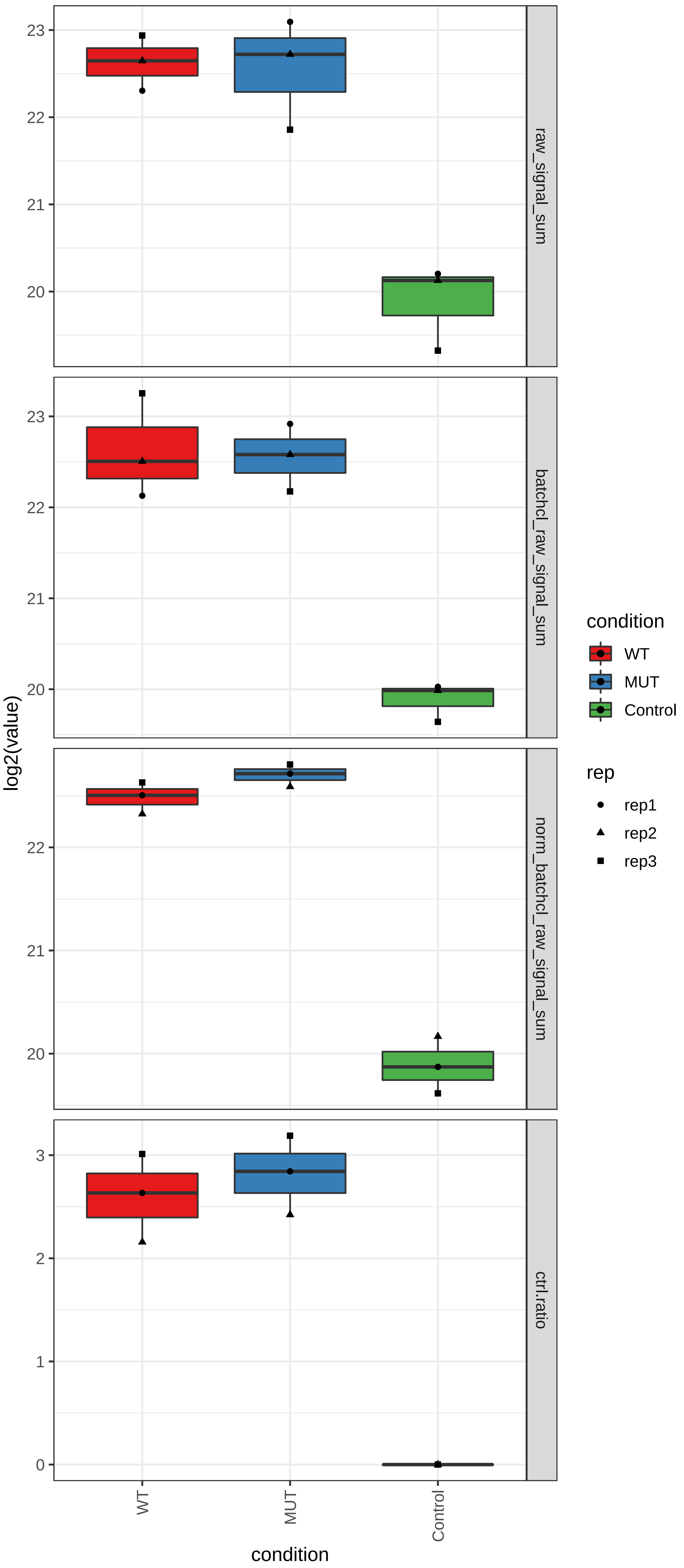

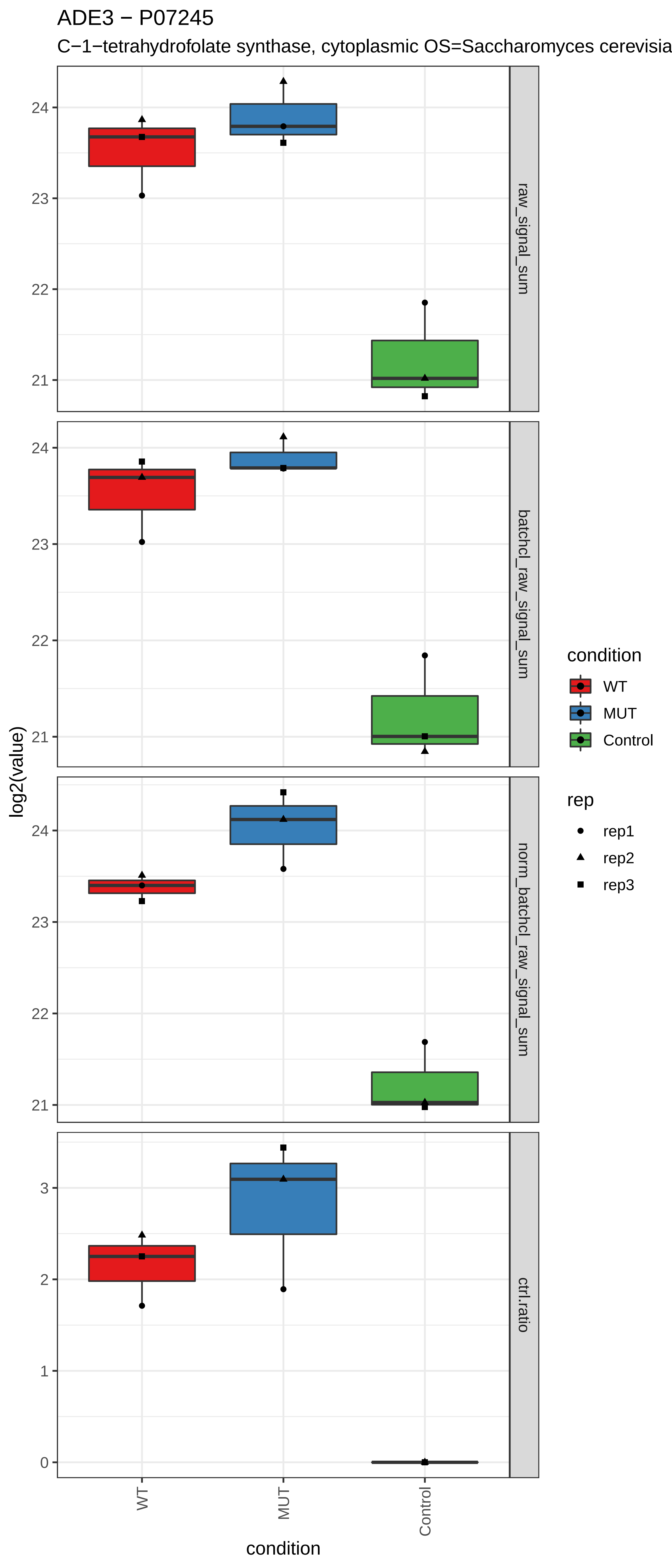
ADE3 − P07245
C−1−tetrahydrofolate synthase, cytoplasmic OS=Saccharomyces cerevisia
raw_signal_sum
batchcl_raw_signal_sum
norm_batchcl_raw_signal_sum
ctrl.ratio
log2(value)
condition
WT
MUT
Control
rep
rep1
rep2
rep3

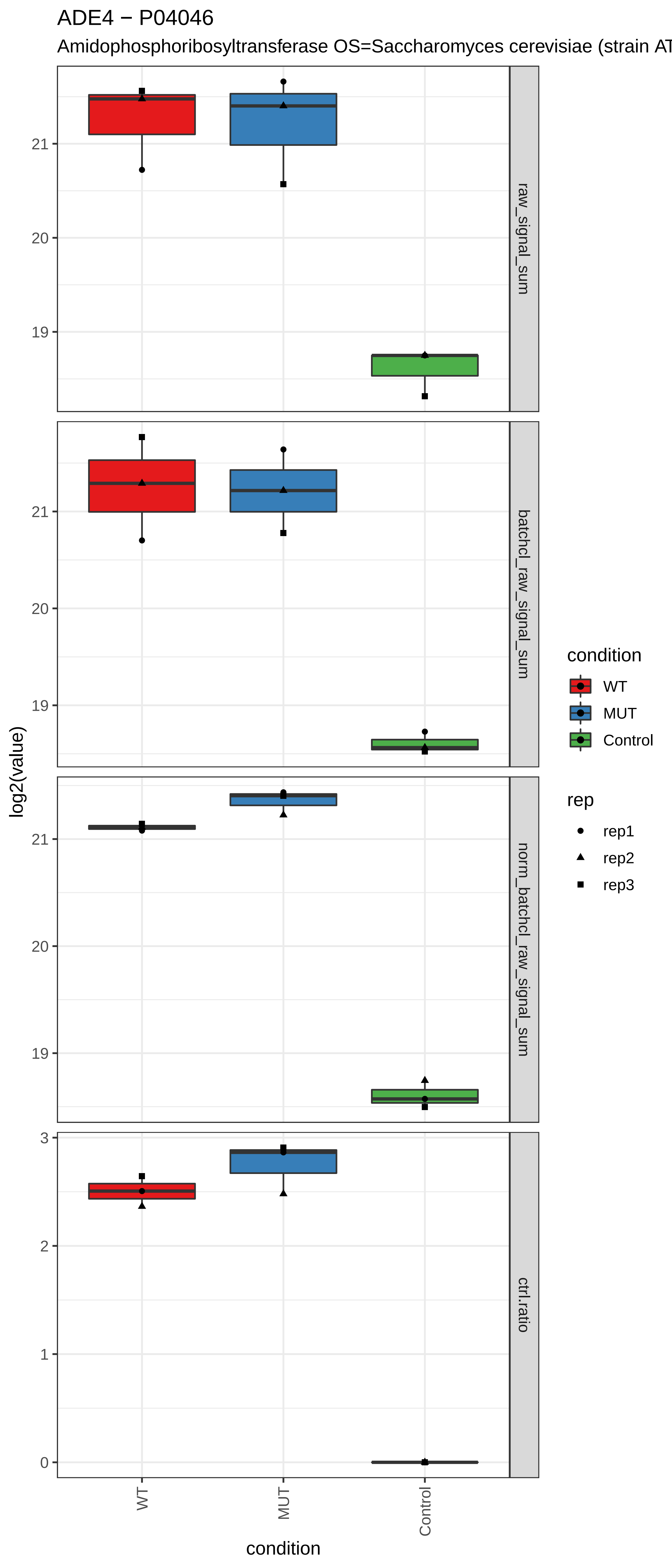

ADE4 − P04046
Amidophosphoribosyltransferase OS=Saccharomyces cerevisiae (strain AT
raw_signal_sum
batchcl_raw_signal_sum
norm_batchcl_raw_signal_sum
ctrl.ratio
log2(value)
condition
WT
MUT
Control
rep
rep1
rep2
rep3
21
20
19
3
2
1
0

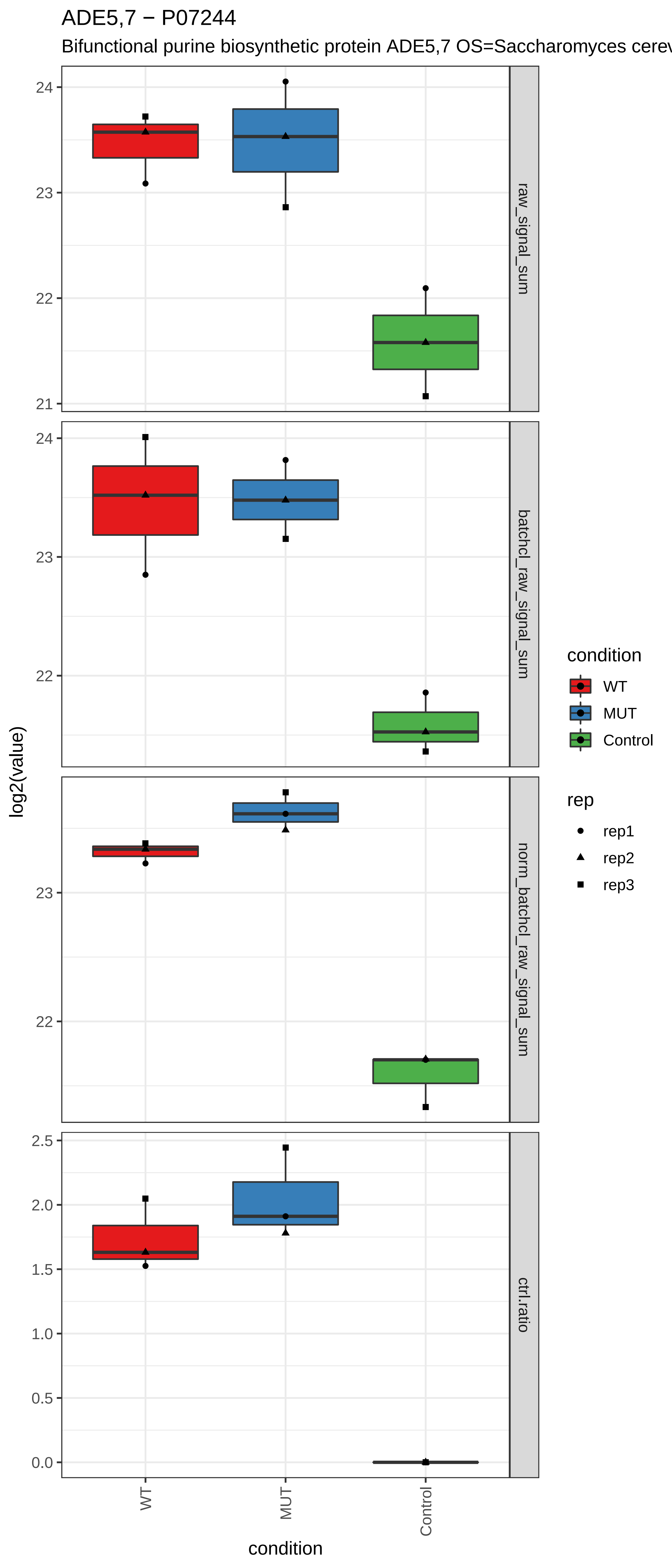

ADE5,7 − P07244
Bifunctional purine biosynthetic protein ADE5,7 OS=Saccharomyces cerev
raw_signal_sum
batchcl_raw_signal_sum
norm_batchcl_raw_signal_sum
ctrl.ratio
log2(value)
condition
WT
MUT
Control
rep
rep1
rep2
rep3

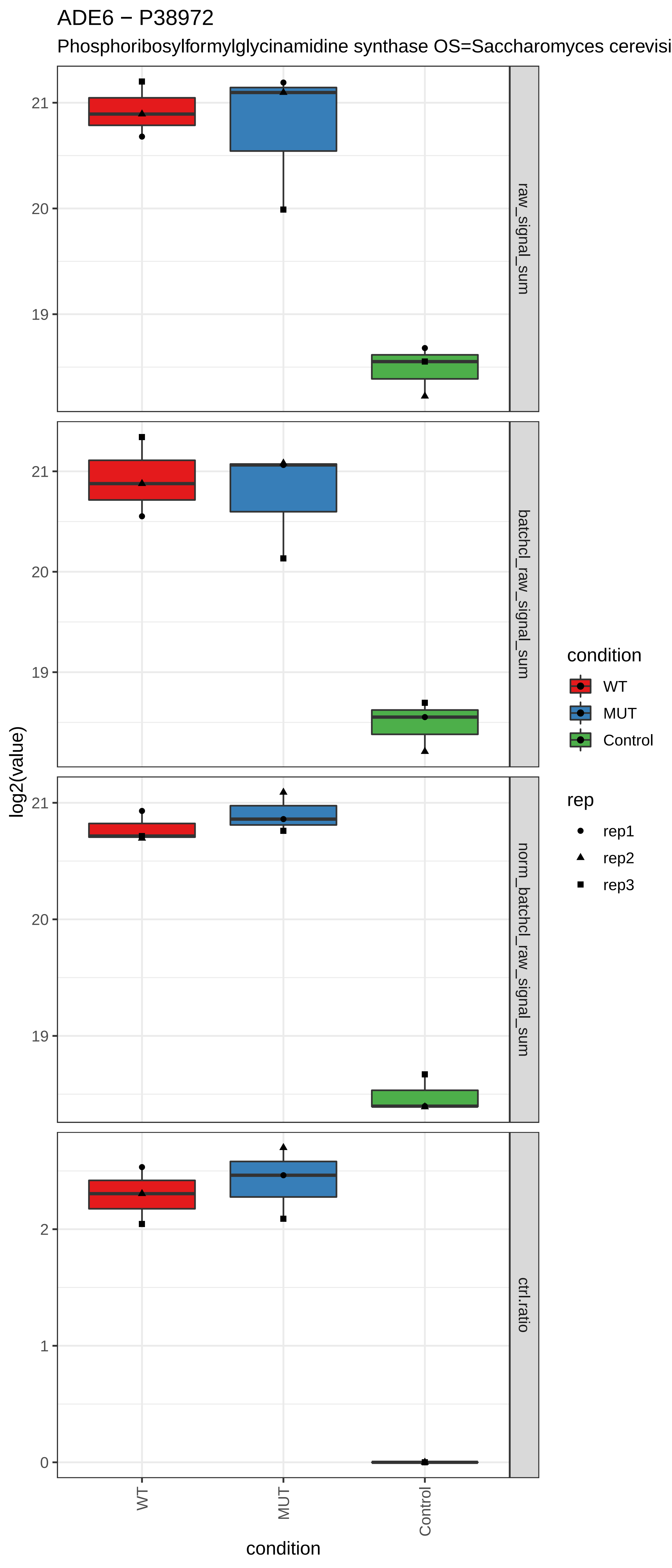

ADE6 − P38972
Phosphoribosylformylglycinamidine synthase OS=Saccharomyces cerevisi
raw_signal_sum
batchcl_raw_signal_sum
norm_batchcl_raw_signal_sum
ctrl.ratio
log2(value)
condition
WT
MUT
Control
rep
rep1
rep2
rep3
21
20
19
2
1
0

ADH1 − P00330

Alcohol dehydrogenase 1 OS=Saccharomyces cerevisiae (strain ATCC 2

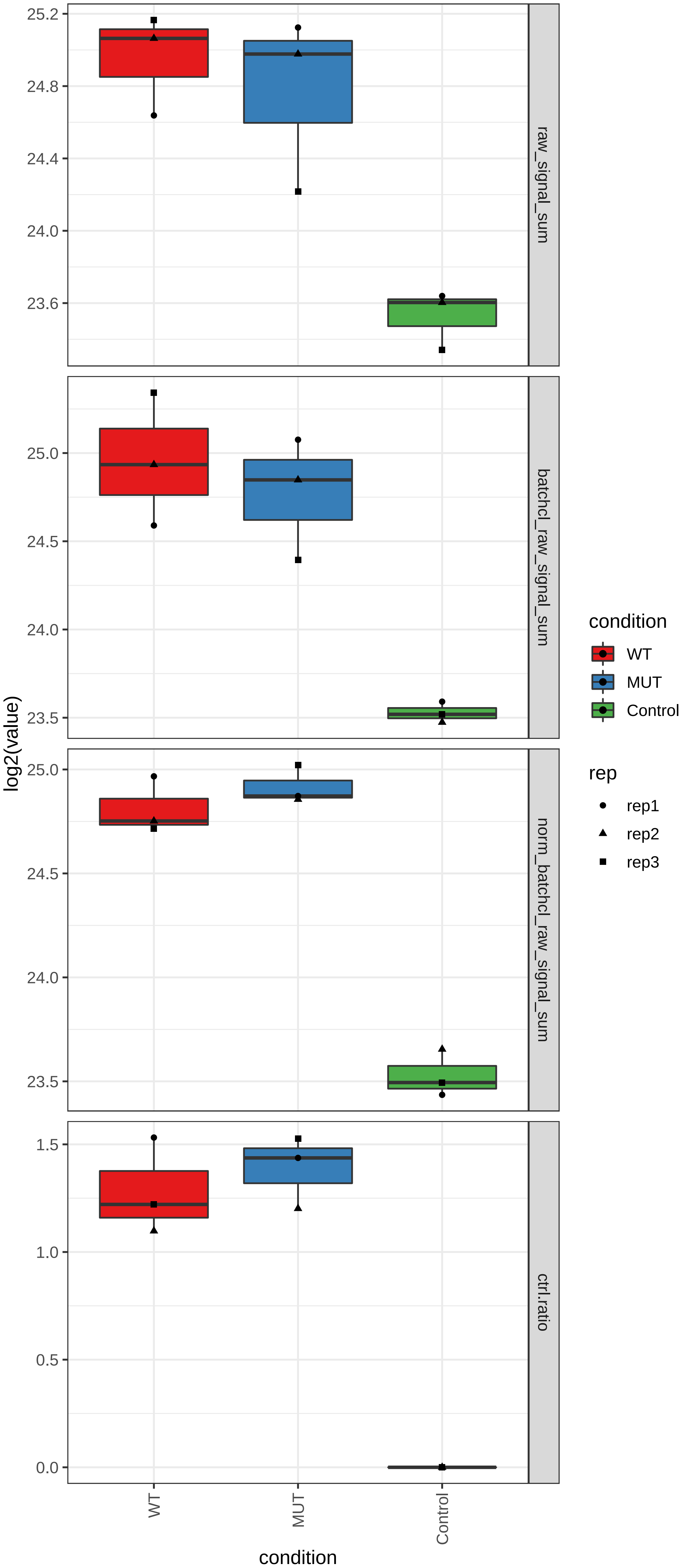

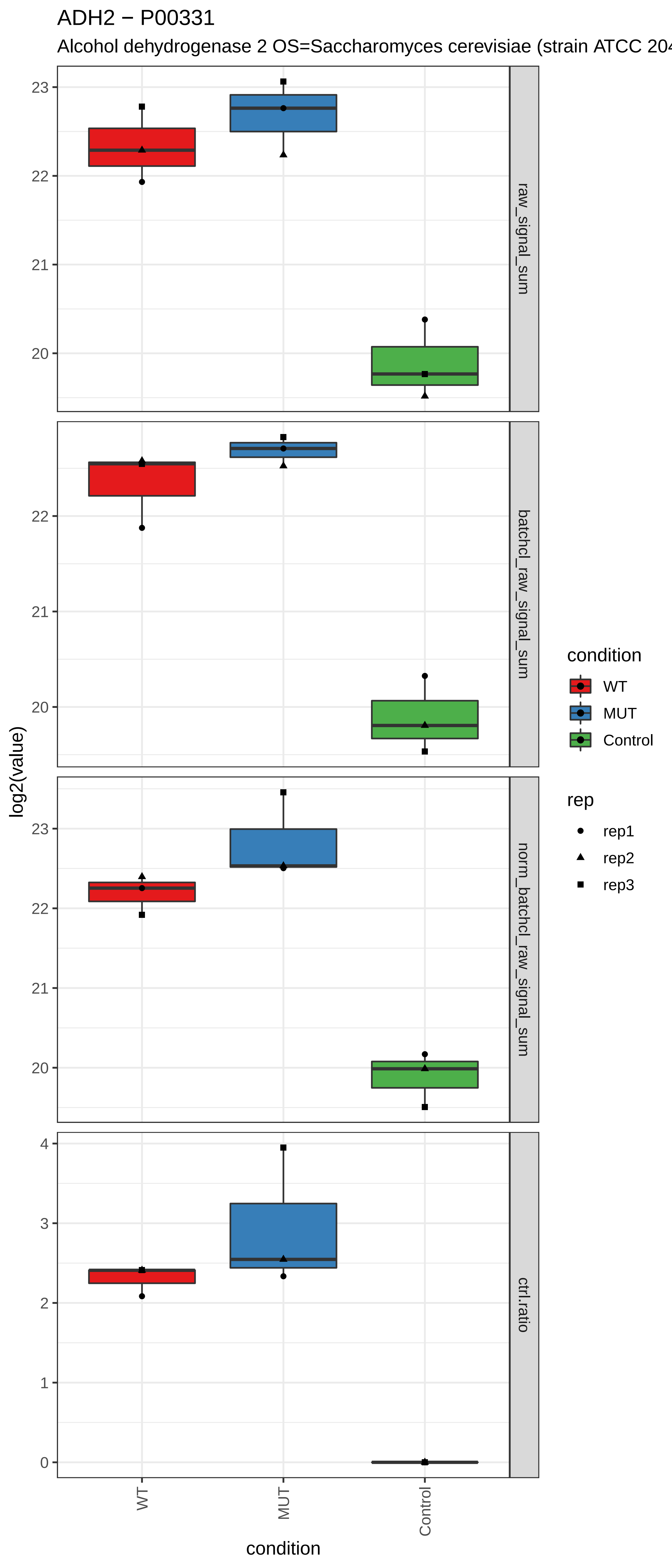

ADH2 − P00331
Alcohol dehydrogenase 2 OS=Saccharomyces cerevisiae (strain ATCC 204
raw_signal_sum
batchcl_raw_signal_sum
norm_batchcl_raw_signal_sum
ctrl.ratio
log2(value)
condition
WT
MUT
Control
rep
rep1
rep2
rep3

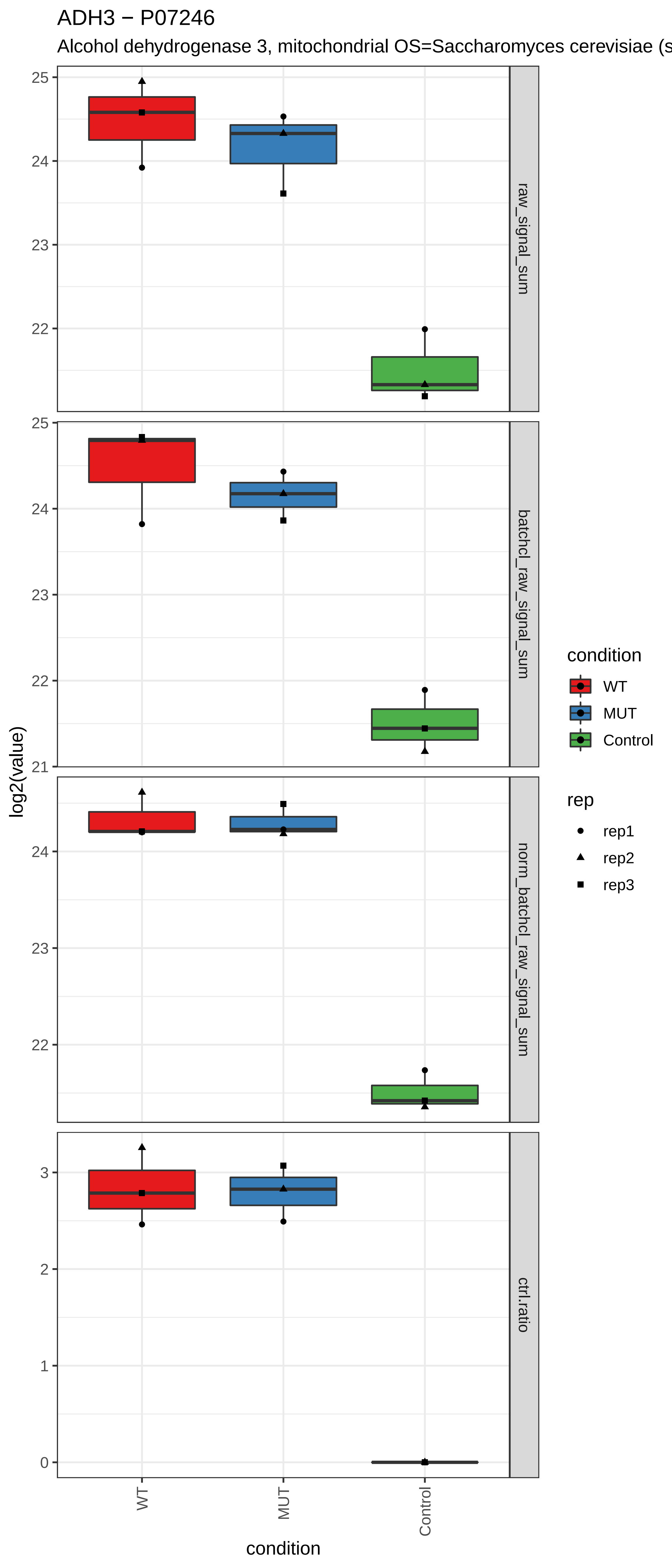

ADH3 − P07246
Alcohol dehydrogenase 3, mitochondrial OS=Saccharomyces cerevisiae (s
raw_signal_sum
batchcl_raw_signal_sum
norm_batchcl_raw_signal_sum
ctrl.ratio
log2(value)
condition
WT
MUT
Control
rep
rep1
rep2
rep3
25
24
23
22
21
3
2
1
0

ADR1 − P07248

Regulatory protein ADR1 OS=Saccharomyces cerevisiae (strain ATCC 204

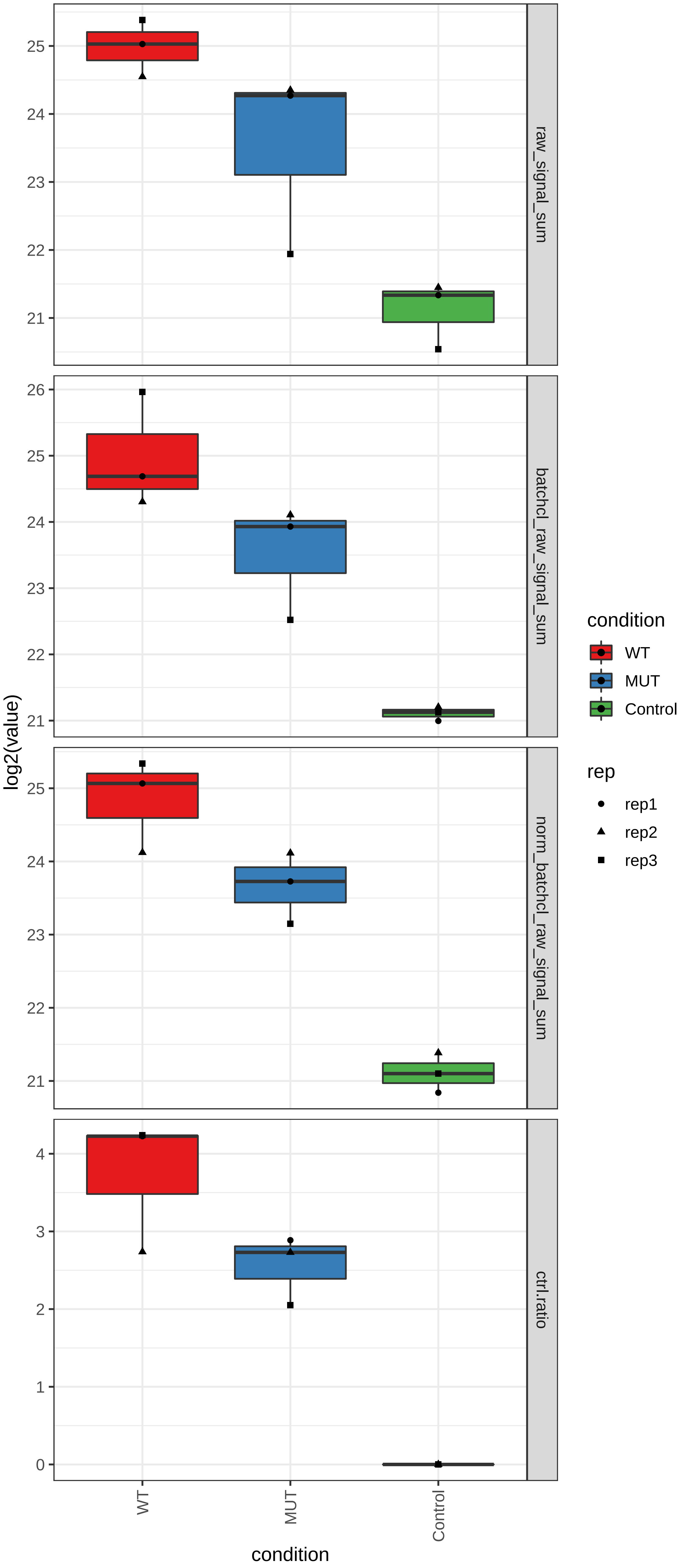

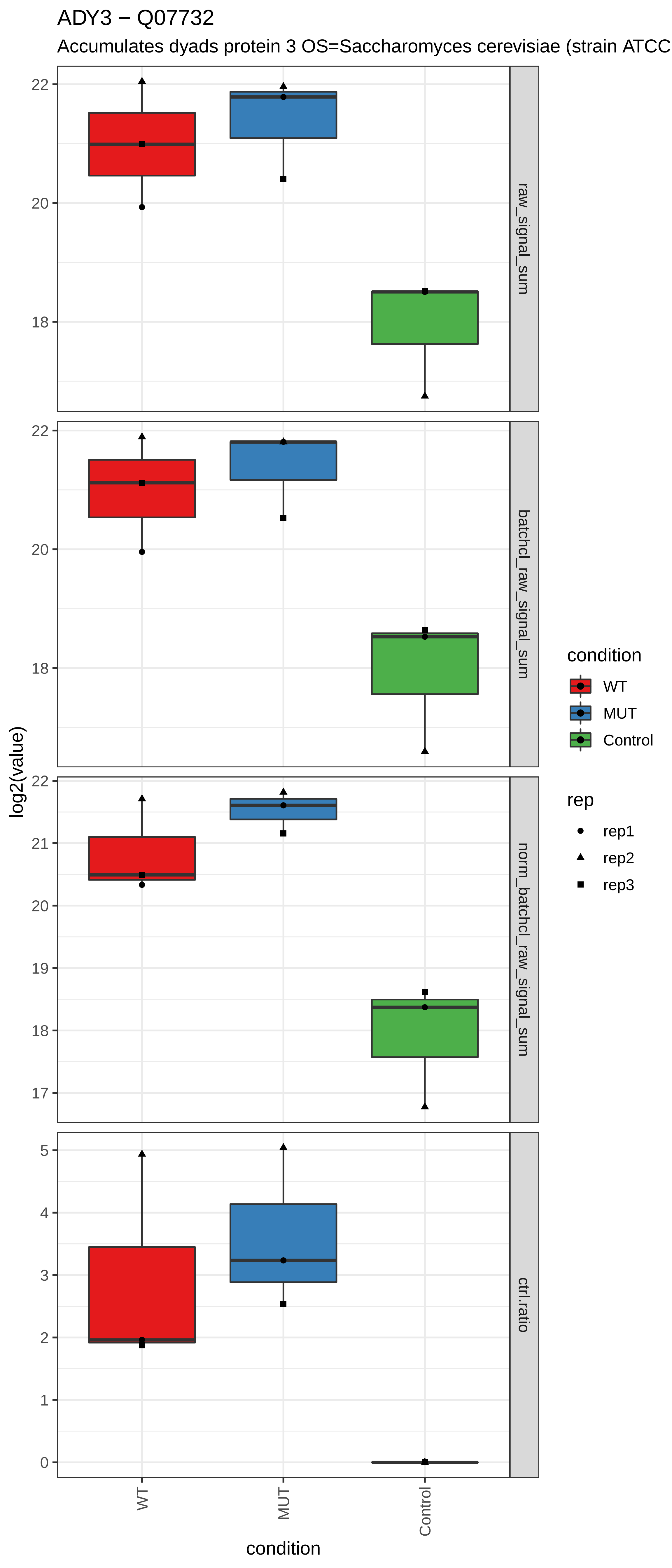

ADY3 − Q07732
Accumulates dyads protein 3 OS=Saccharomyces cerevisiae (strain ATCC
raw_signal_sum
batchcl_raw_signal_sum
norm_batchcl_raw_signal_sum
ctrl.ratio
log2(value)
condition
WT
MUT
Control
rep
rep1
rep2
rep3

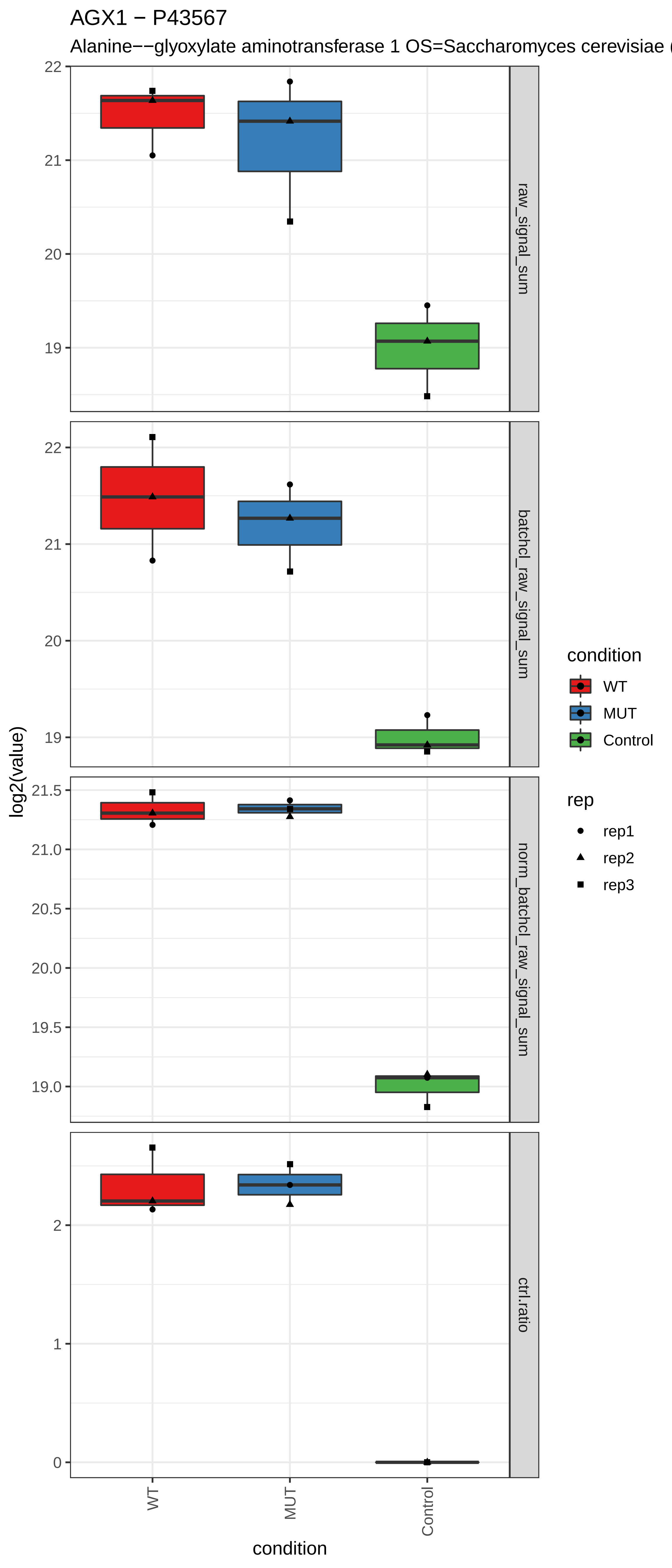

AGX1 − P43567
Alanine−−glyoxylate aminotransferase 1 OS=Saccharomyces cerevisiae (
raw_signal_sum
batchcl_raw_signal_sum
norm_batchcl_raw_signal_sum
ctrl.ratio
log2(value)
condition
WT
MUT
Control
rep
rep1
rep2
rep3

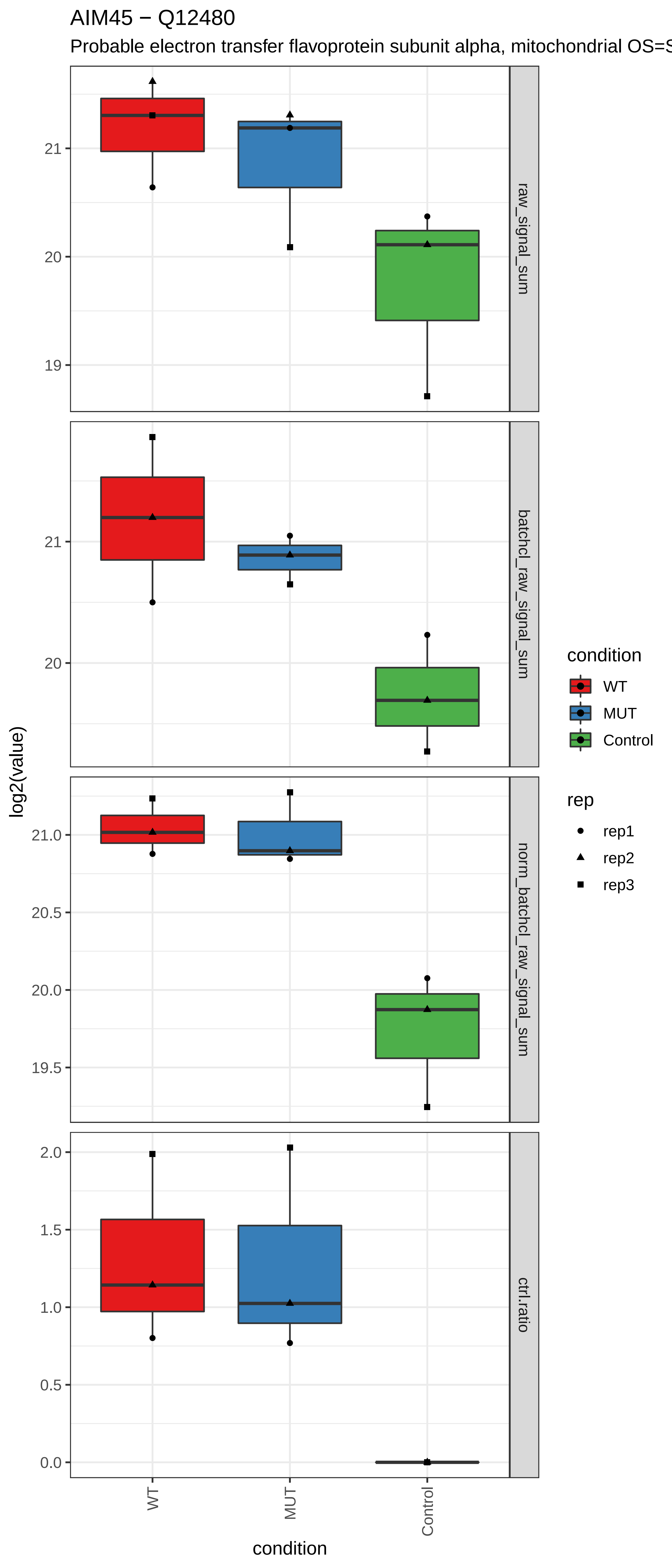
AIM45 − Q12480
Probable electron transfer flavoprotein subunit alpha, mitochondrial OS=S
raw_signal_sum
batchcl_raw_signal_sum
norm_batchcl_raw_signal_sum
ctrl.ratio
log2(value)
condition
WT
MUT
Control
rep
rep1
rep2
rep3
21
20
19
21.0
20.5
20.0
19.5
2.0
1.5
1.0
0.5
0.0

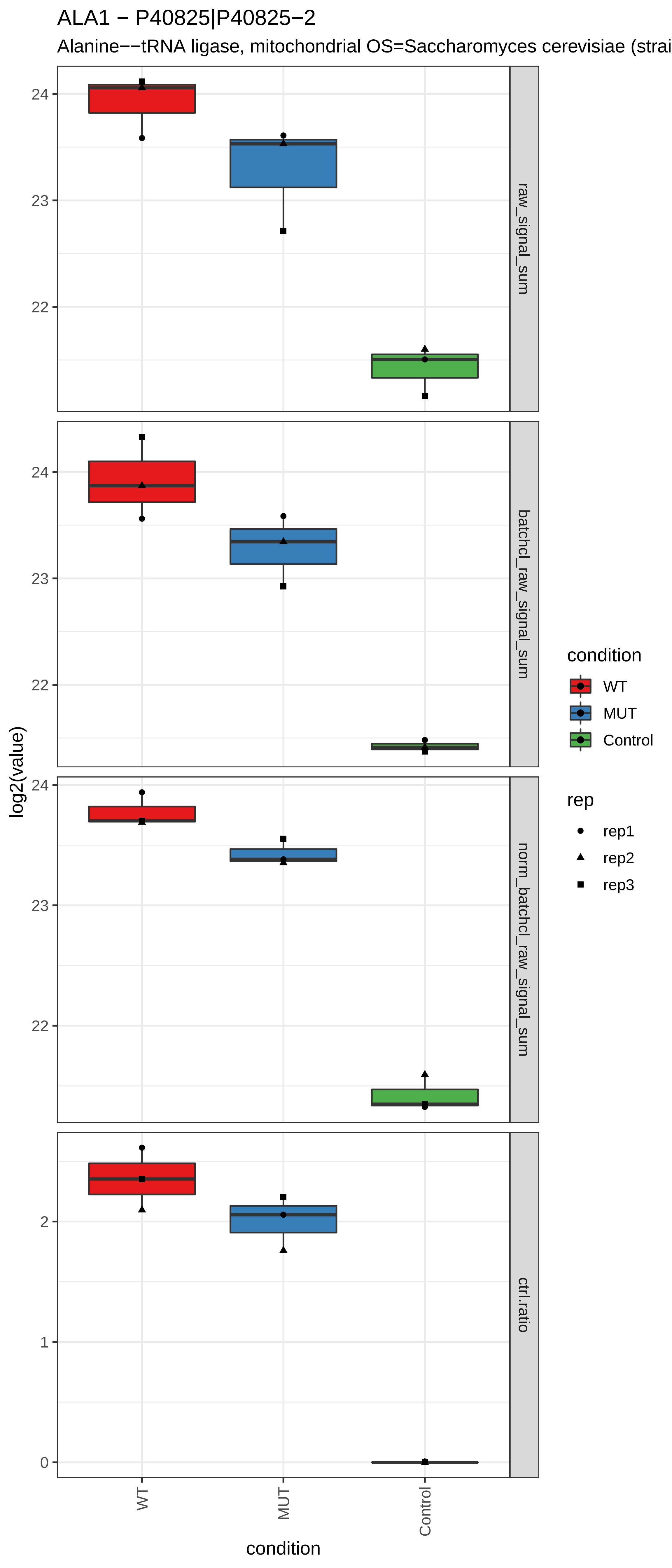

ALA1 − P40825|P40825−2
Alanine−−tRNA ligase, mitochondrial OS=Saccharomyces cerevisiae (strai
raw_signal_sum
batchcl_raw_signal_sum
norm_batchcl_raw_signal_sum
ctrl.ratio
log2(value)
24
23
22
2
1
0
WT
MUT
Control
condition
condition
WT
MUT
Control
rep
rep1
rep2
rep3

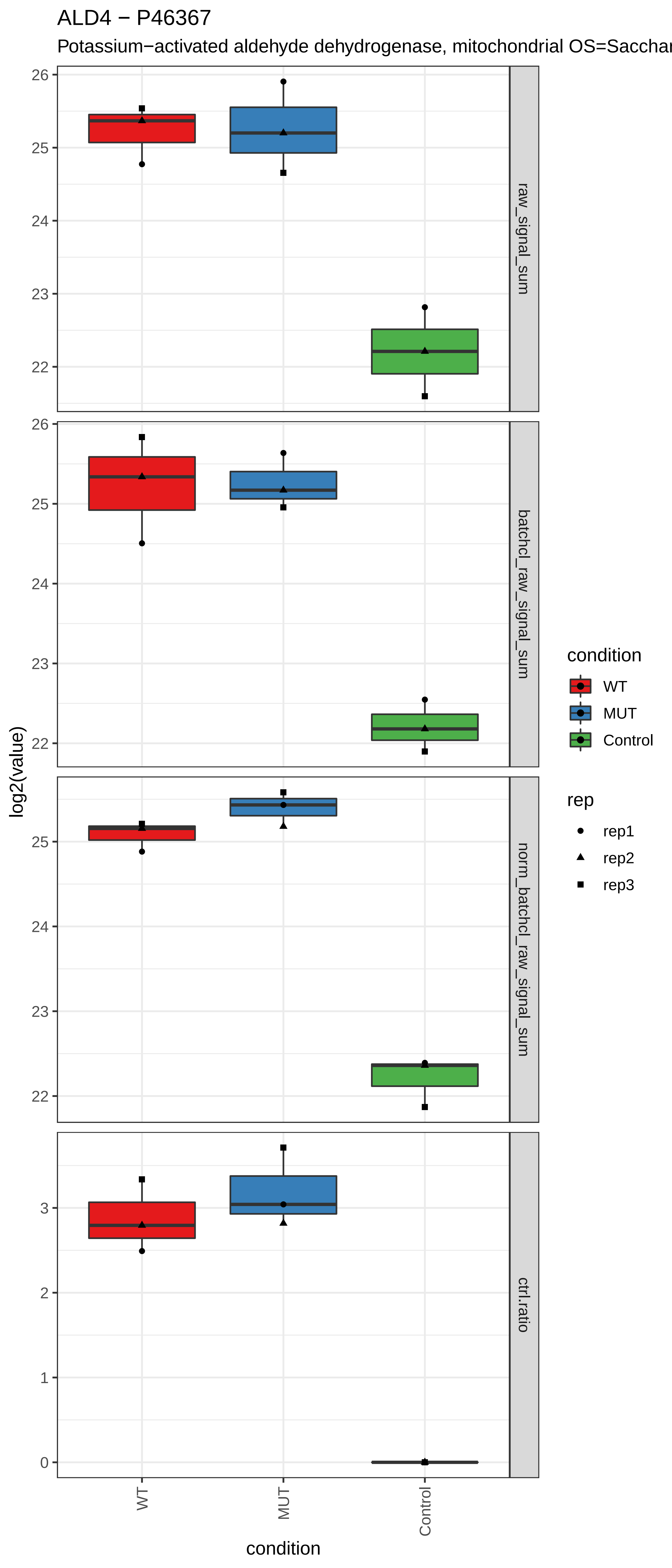

ALD4 − P46367
Potassium−activated aldehyde dehydrogenase, mitochondrial OS=Sacchar
raw_signal_sum
batchcl_raw_signal_sum
norm_batchcl_raw_signal_sum
ctrl.ratio
log2(value)
condition
WT
MUT
Control
rep
rep1
rep2
rep3

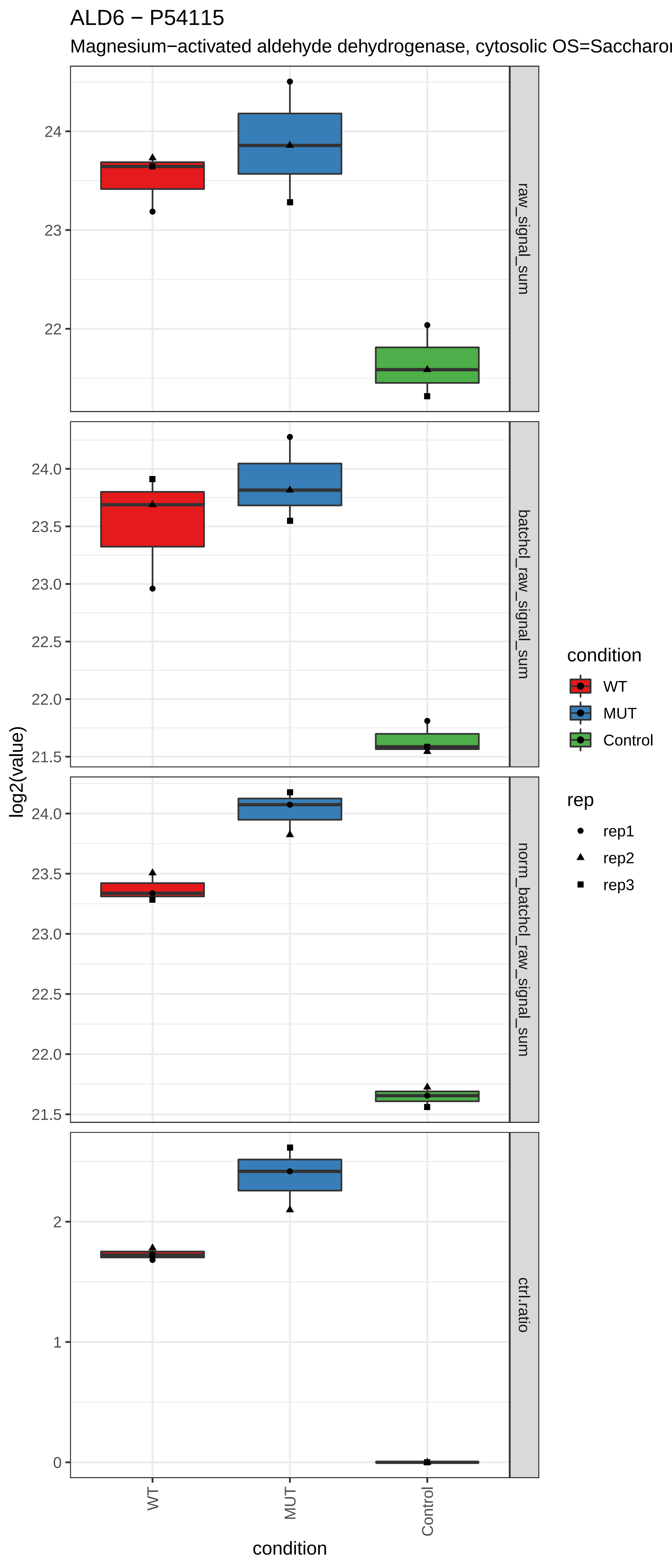

ALD6 − P54115
Magnesium−activated aldehyde dehydrogenase, cytosolic OS=Saccharo
raw_signal_sum
batchcl_raw_signal_sum
norm_batchcl_raw_signal_sum
ctrl.ratio
log2(value)
condition
WT
MUT
Control
rep
rep1
rep2
rep3

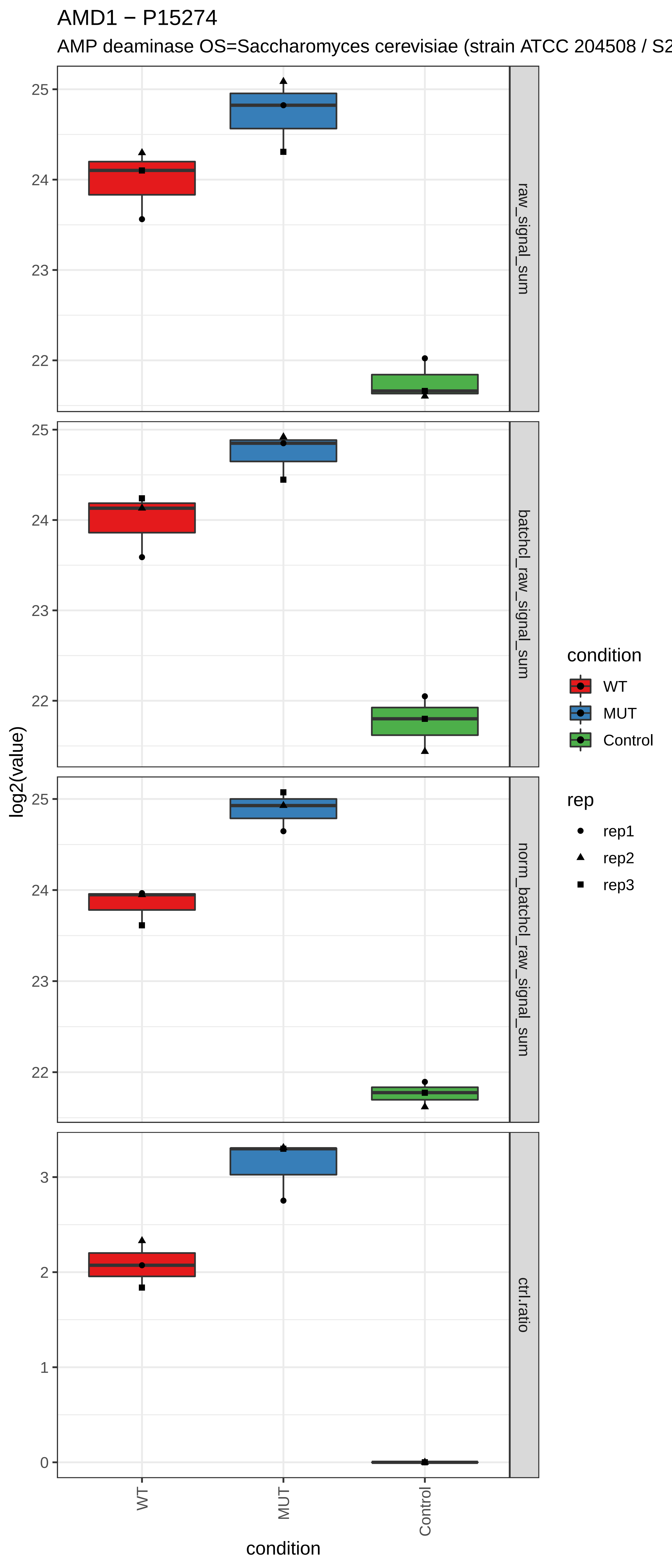
AMD1 − P15274
AMP deaminase OS=Saccharomyces cerevisiae (strain ATCC 204508 / S2
raw_signal_sum
batchcl_raw_signal_sum
norm_batchcl_raw_signal_sum
ctrl.ratio
log2(value)
condition
WT
MUT
Control
rep
rep1
rep2
rep3
25
24
23
22
3
2
1
0

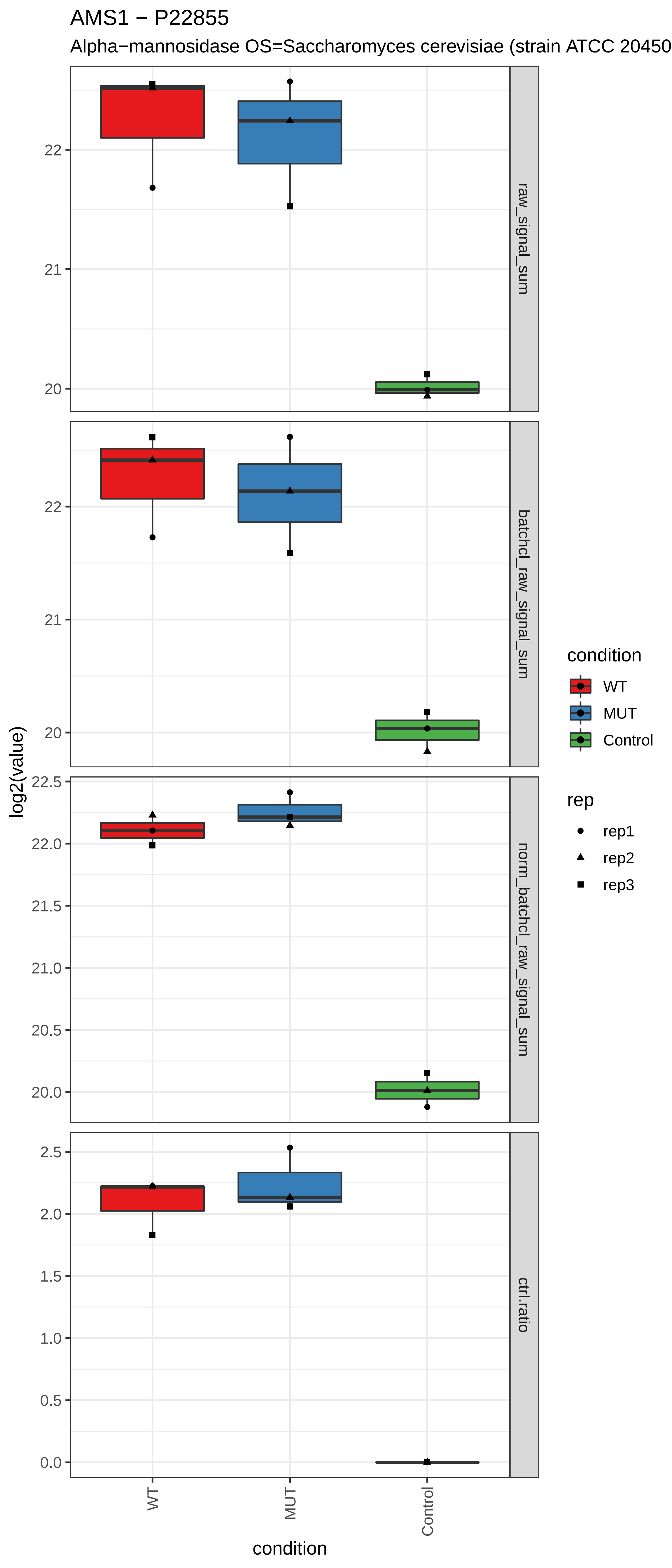
AMS1 − P22855
Alpha−mannosidase OS=Saccharomyces cerevisiae (strain ATCC 20450
raw_signal_sum
batchcl_raw_signal_sum
norm_batchcl_raw_signal_sum
ctrl.ratio
log2(value)
condition
WT
MUT
Control
rep
rep1
rep2
rep3
22
21
20
22.5
22.0
21.5
21.0
20.5
20.0
2.5
2.0
1.5
1.0
0.5
0.0

APE2 − P32454

Aminopeptidase 2, mitochondrial OS=Saccharomyces cerevisiae (strain AT

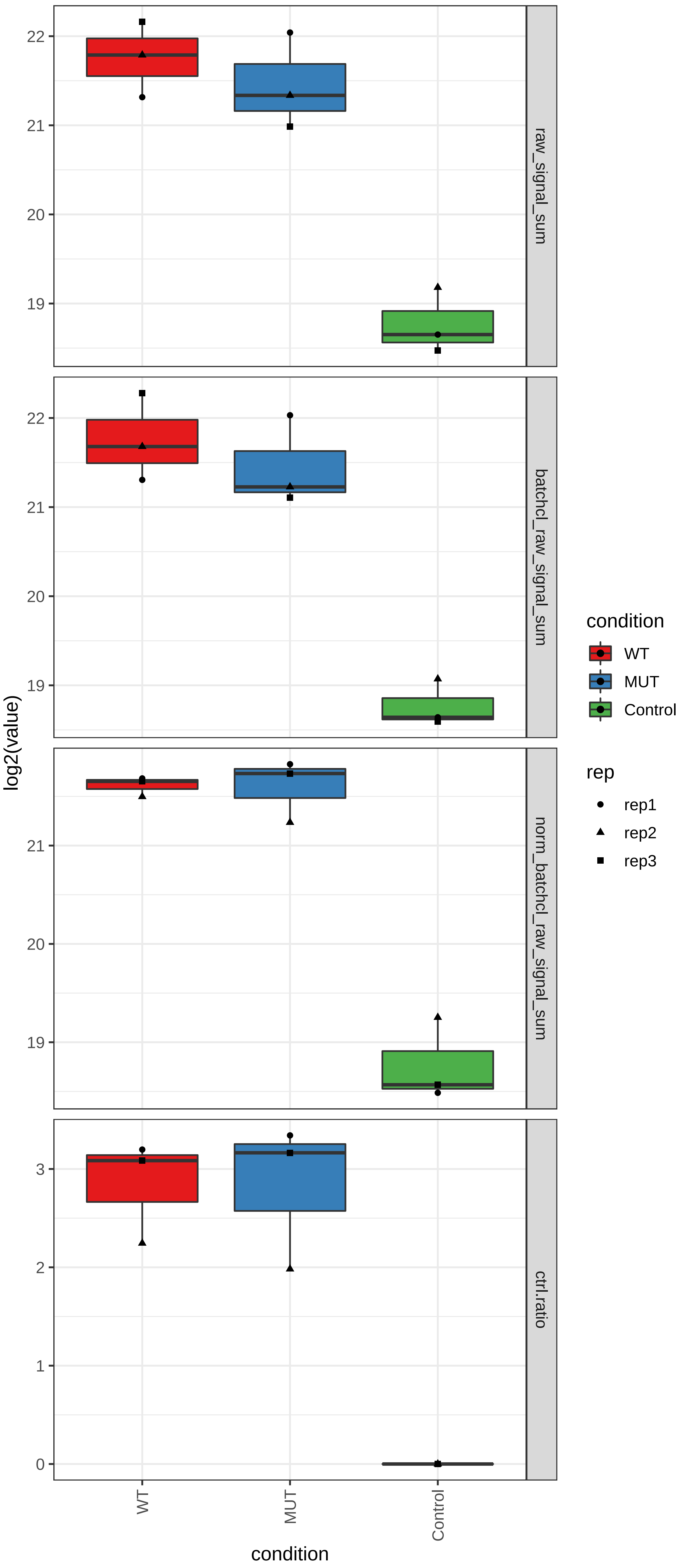

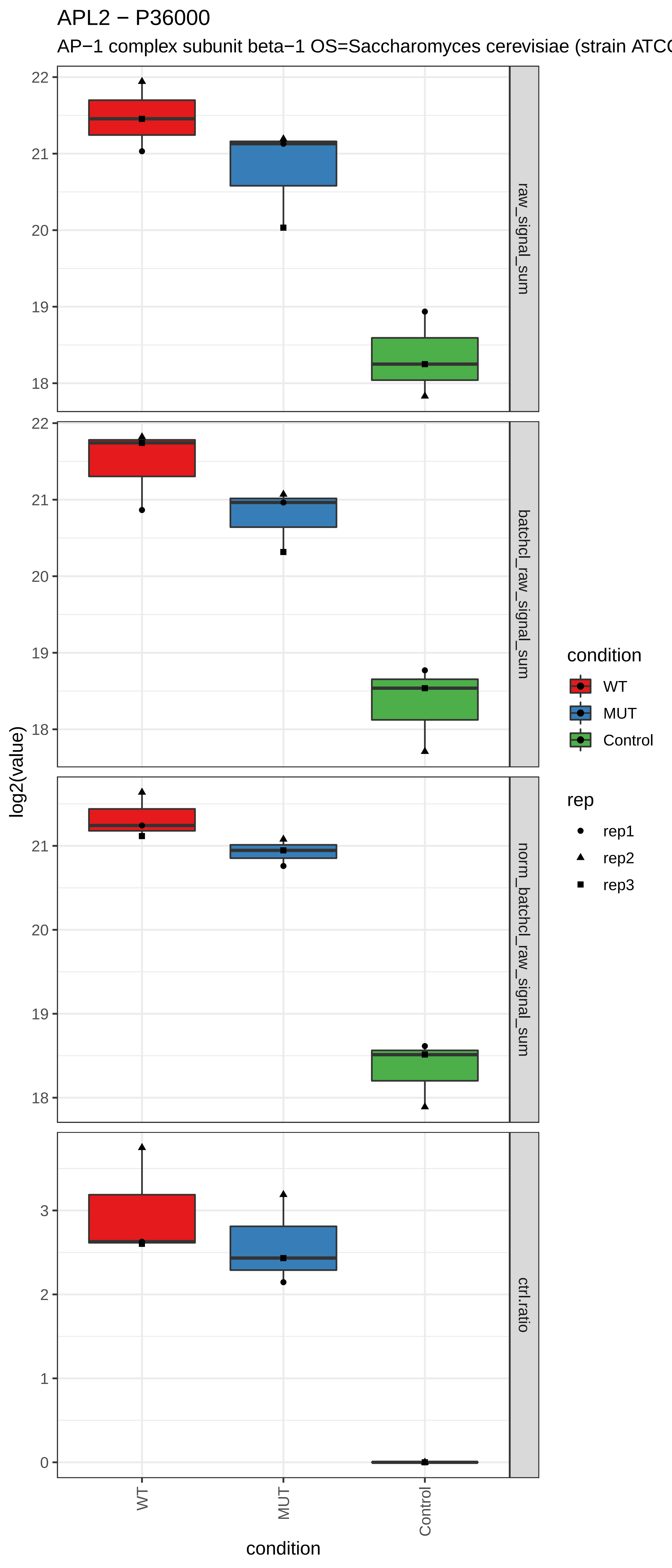
APL2 − P36000
AP−1 complex subunit beta−1 OS=Saccharomyces cerevisiae (strain ATCC
raw_signal_sum
batchcl_raw_signal_sum
norm_batchcl_raw_signal_sum
ctrl.ratio
log2(value)
condition
WT
MUT
Control
rep
rep1
rep2
rep3

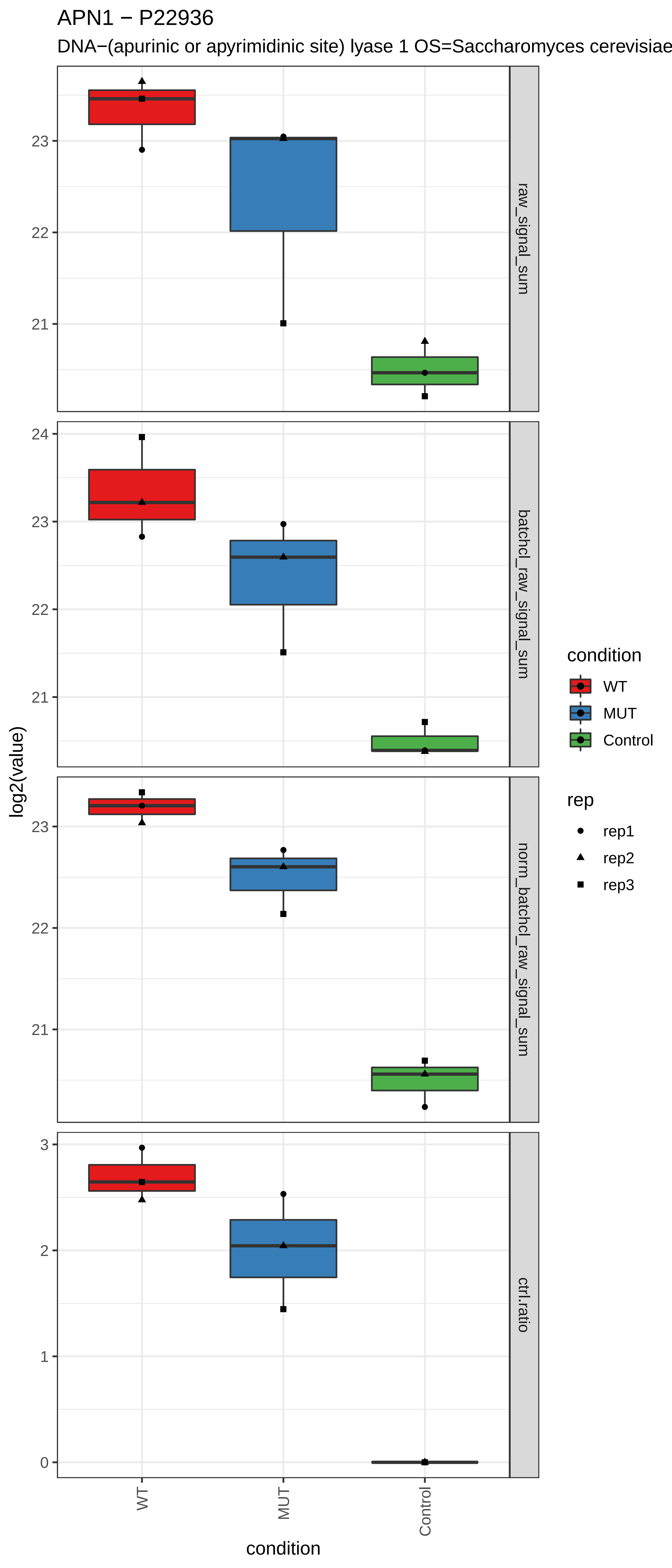
APN1 − P22936
DNA−(apurinic or apyrimidinic site) lyase 1 OS=Saccharomyces cerevisiae
raw_signal_sum
batchcl_raw_signal_sum
norm_batchcl_raw_signal_sum
ctrl.ratio
log2(value)
condition
WT
MUT
Control
rep
rep1
rep2
rep3

ARB1 − P40024

ABC transporter ATP−binding protein ARB1 OS=Saccharomyces cerevisia

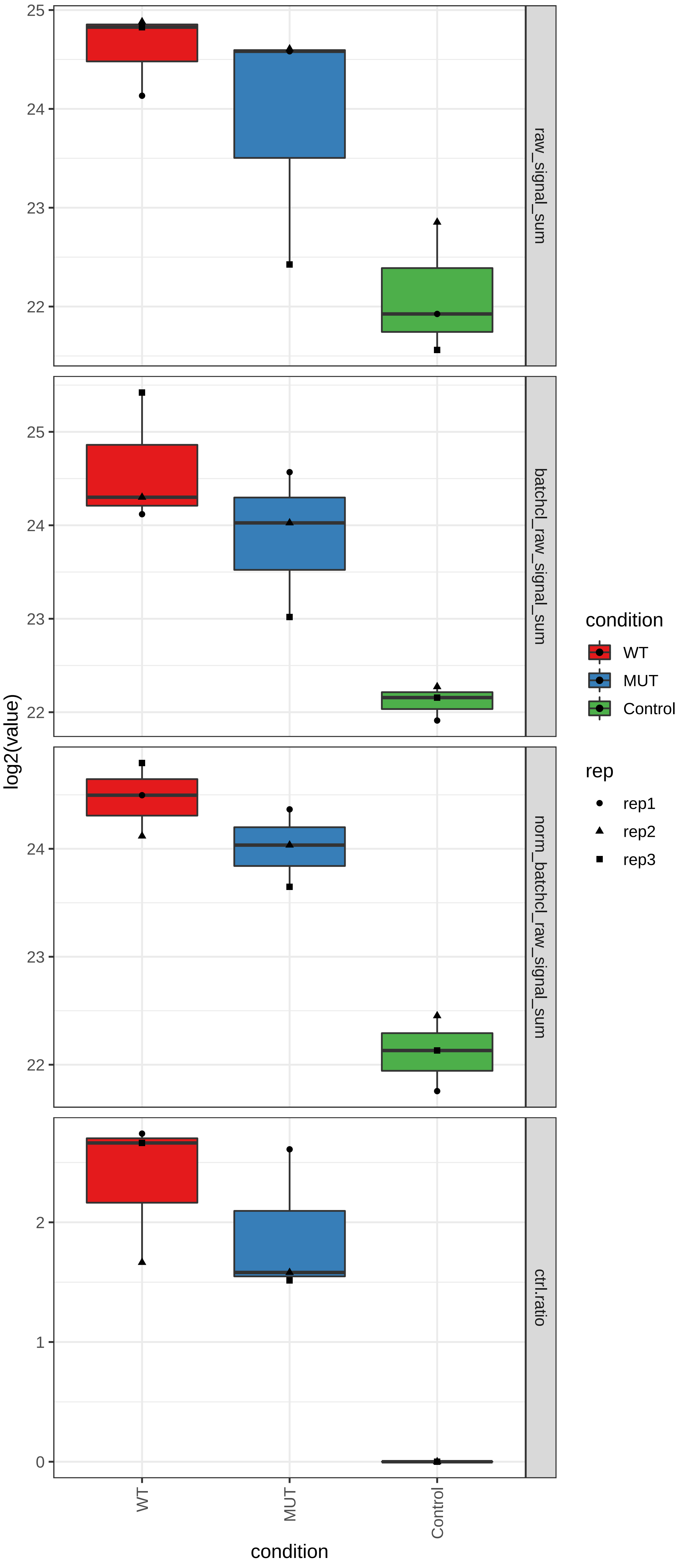

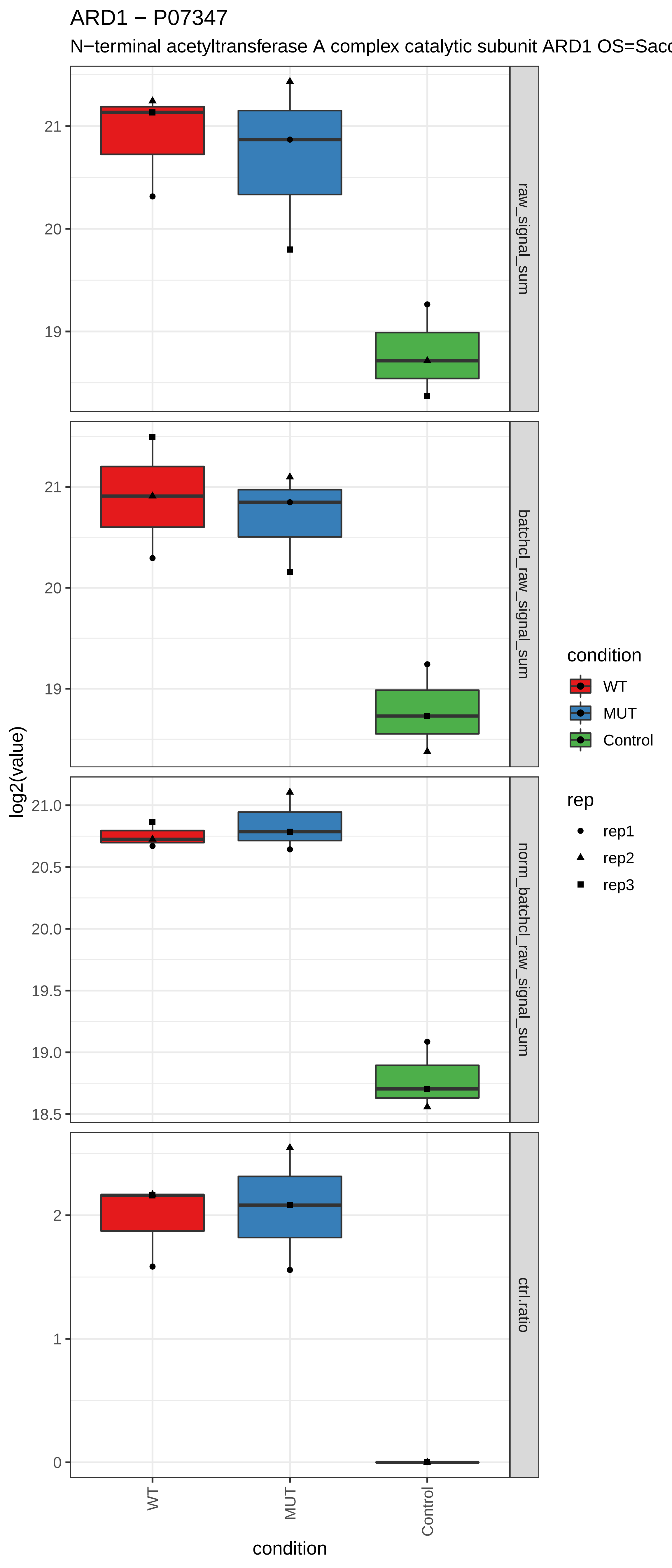

ARD1 − P07347
N−terminal acetyltransferase A complex catalytic subunit ARD1 OS=Sacc
raw_signal_sum
batchcl_raw_signal_sum
norm_batchcl_raw_signal_sum
ctrl.ratio
log2(value)
condition
WT
MUT
Control
rep
rep1
rep2
rep3

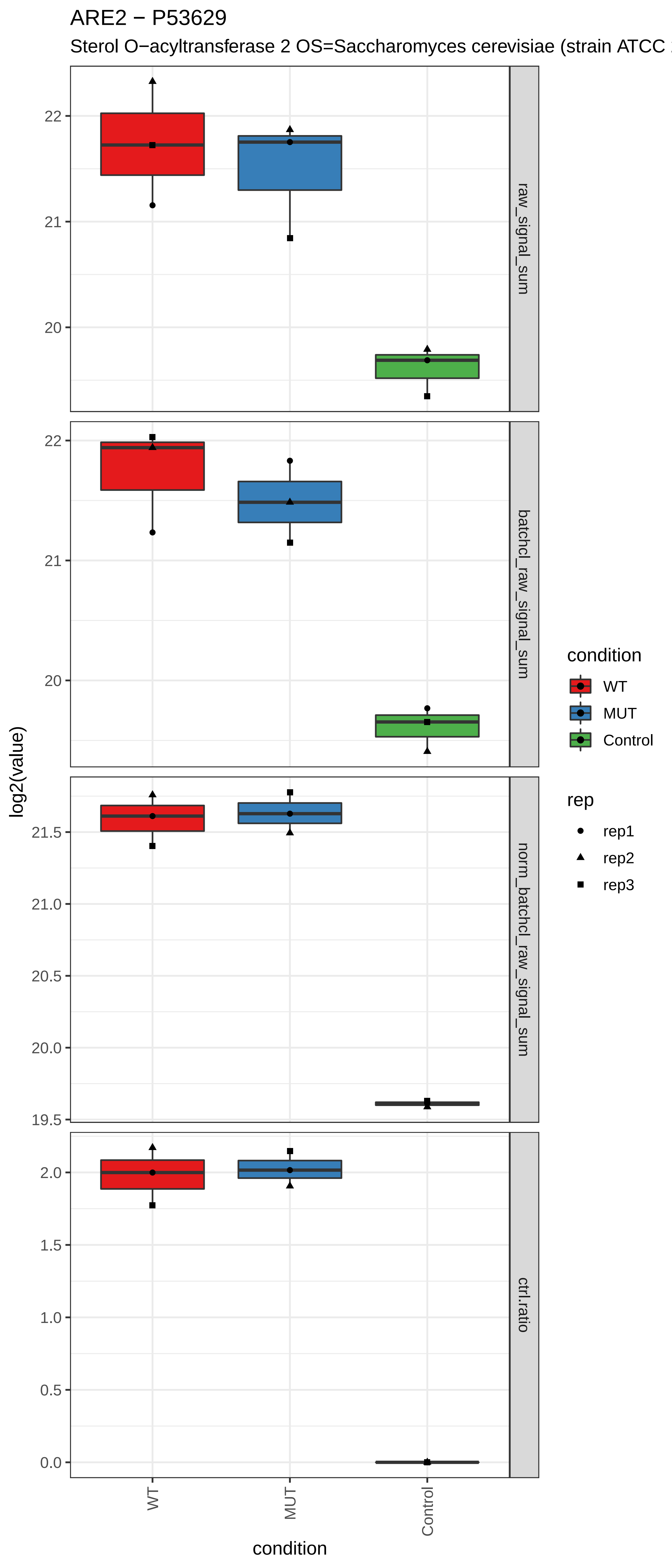

ARE2 − P53629
Sterol O−acyltransferase 2 OS=Saccharomyces cerevisiae (strain ATCC
log2(value)
raw_signal_sum
batchcl_raw_signal_sum
norm_batchcl_raw_signal_sum
ctrl.ratio
22
21
20
21.5
21.0
20.5
20.0
19.5
2.0
1.5
1.0
0.5
0.0
WT
MUT
Control
condition
condition
WT
MUT
Control
rep
rep1
rep2
rep3

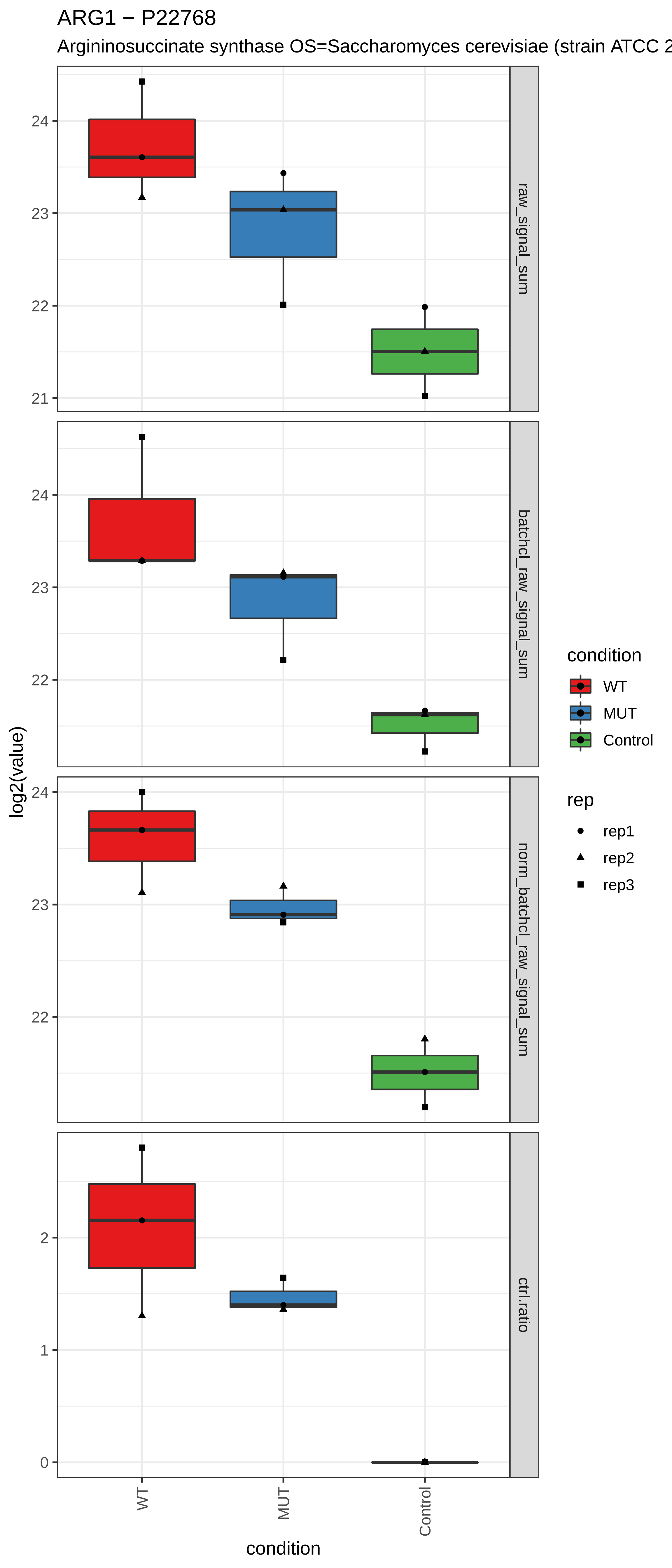
ARG1 − P22768
Argininosuccinate synthase OS=Saccharomyces cerevisiae (strain ATCC 2
raw_signal_sum
batchcl_raw_signal_sum
norm_batchcl_raw_signal_sum
ctrl.ratio
log2(value)
condition
WT
MUT
Control
rep
rep1
rep2
rep3
24
23
22
21
2
1
0

ARL1 − P38116

ADP−ribosylation factor−like protein 1 OS=Saccharomyces cerevisiae (stra

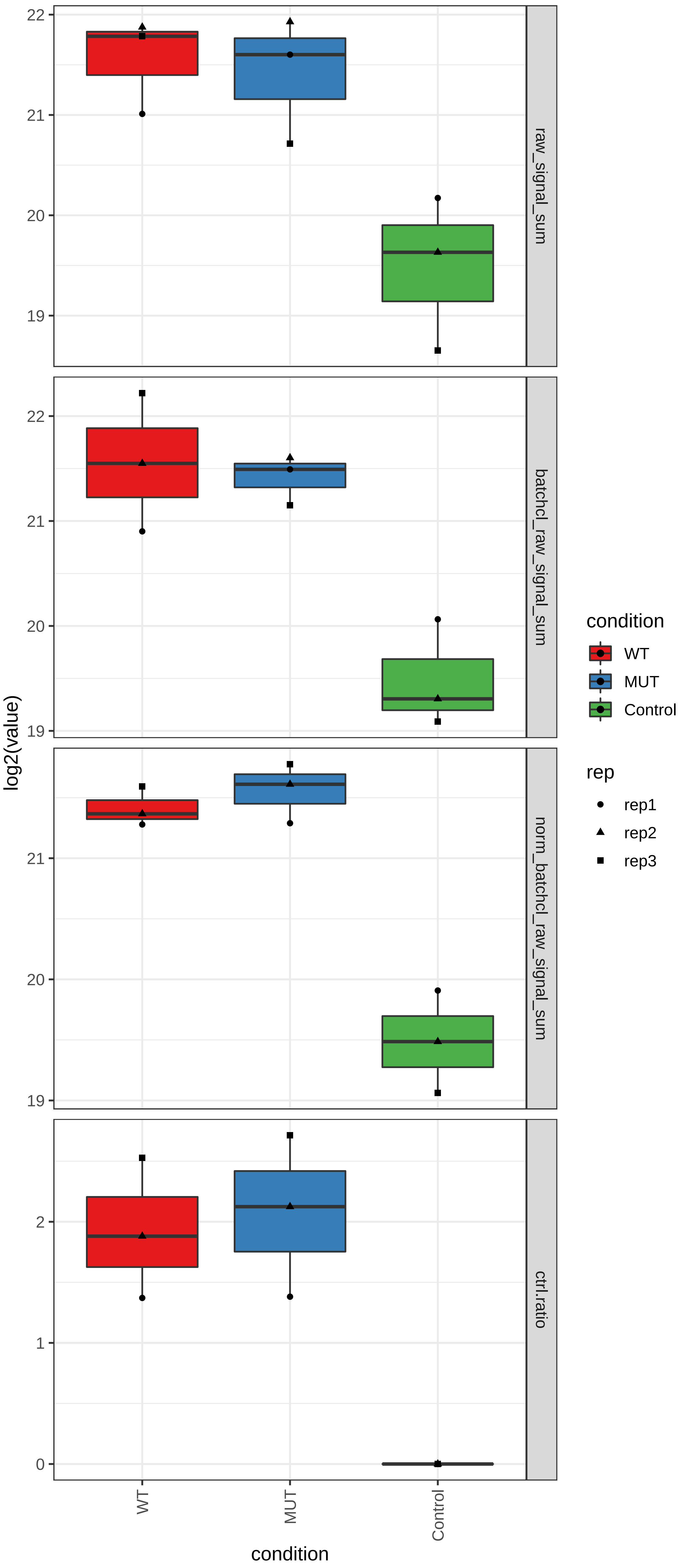

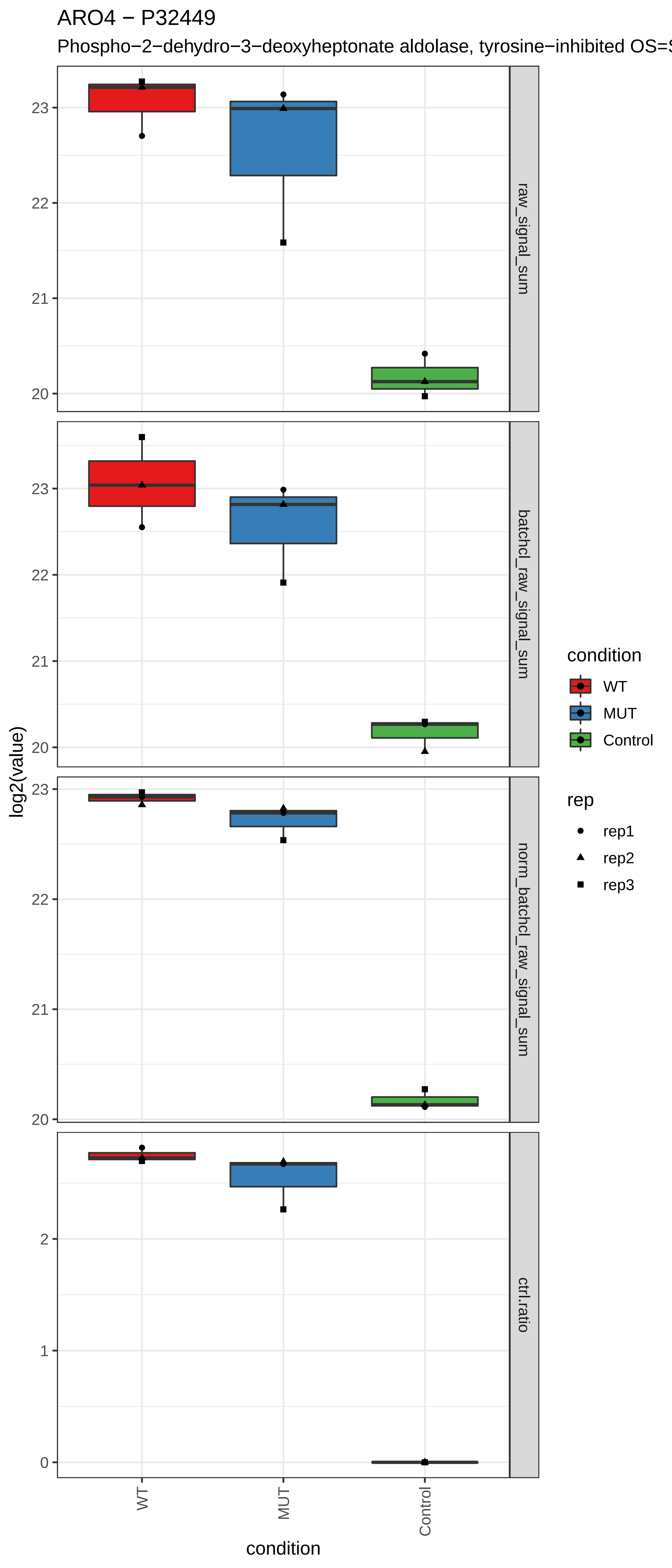

ARO4 − P32449
Phospho−2−dehydro−3−deoxyheptonate aldolase, tyrosine−inhibited OS=S
raw_signal_sum
batchcl_raw_signal_sum
norm_batchcl_raw_signal_sum
ctrl.ratio
log2(value)
condition
WT
MUT
Control
rep
rep1
rep2
rep3

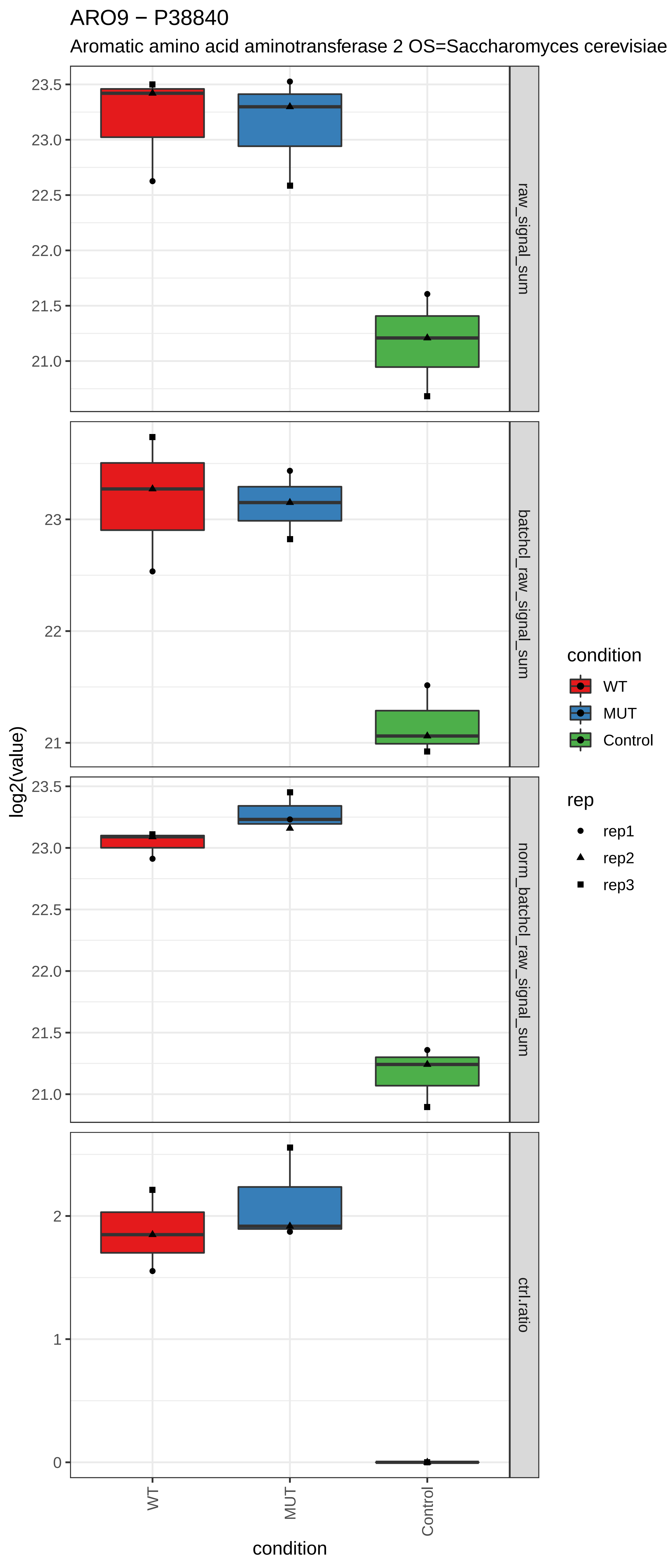

ARO9 − P38840
Aromatic amino acid aminotransferase 2 OS=Saccharomyces cerevisiae
raw_signal_sum
batchcl_raw_signal_sum
norm_batchcl_raw_signal_sum
ctrl.ratio
log2(value)
condition
WT
MUT
Control
rep
rep1
rep2
rep3
23.5
23.0
22.5
22.0
21.5
21.0
23
22
21
2
1
0

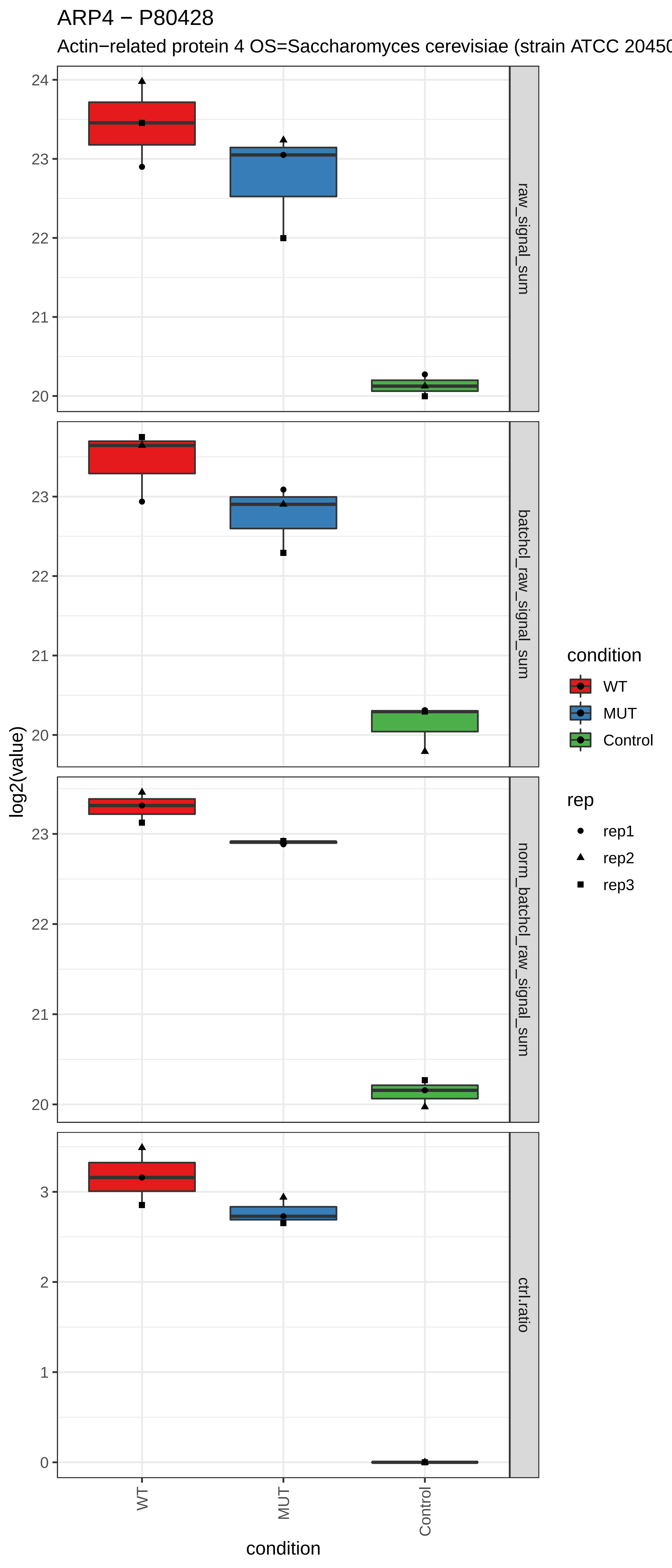

ARP4 − P80428
Actin−related protein 4 OS=Saccharomyces cerevisiae (strain ATCC 20450
raw_signal_sum
batchcl_raw_signal_sum
norm_batchcl_raw_signal_sum
ctrl.ratio
log2(value)
condition
WT
MUT
Control
rep
rep1
rep2
rep3
24
23
22
21
20
3
2
1
0

ARP7 − Q12406

Actin−related protein 7 OS=Saccharomyces cerevisiae (strain ATCC 2045

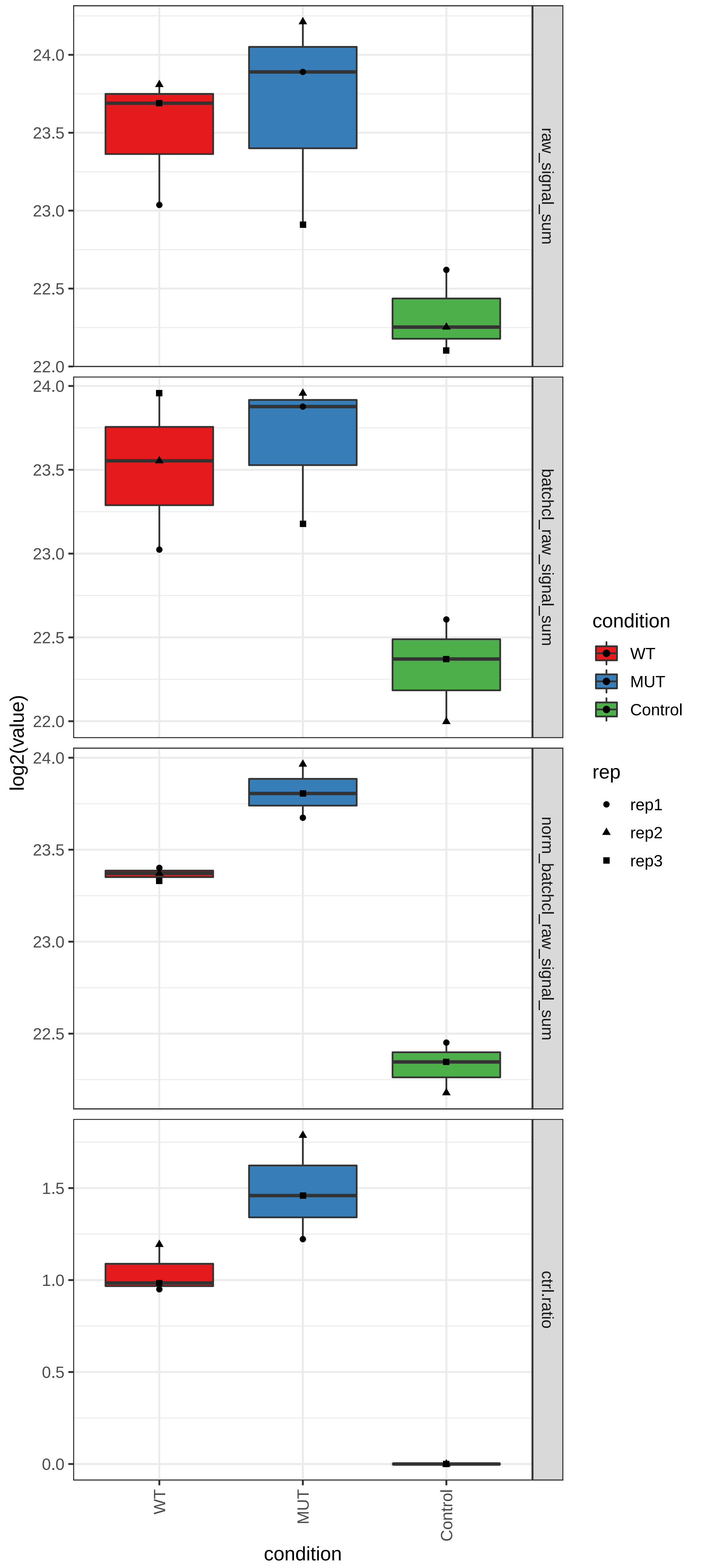

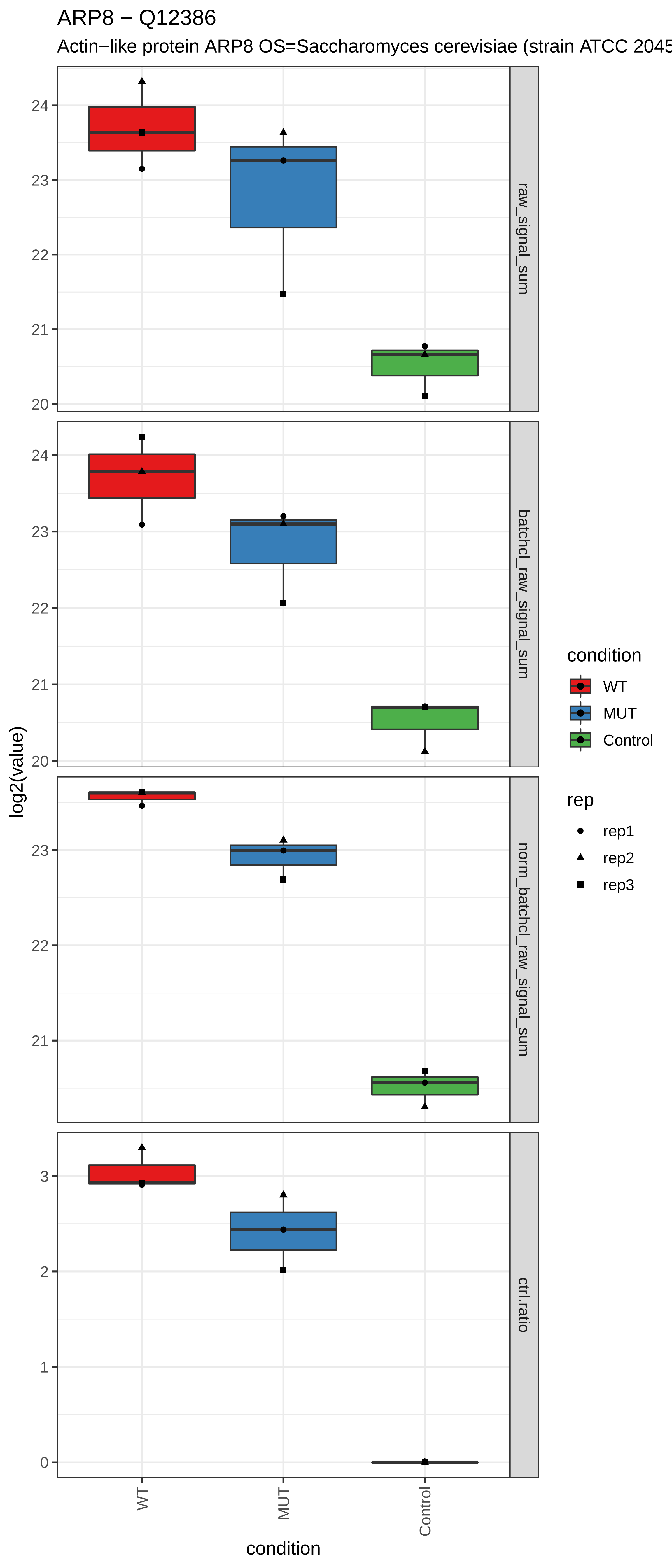

ARP8 − Q12386
Actin−like protein ARP8 OS=Saccharomyces cerevisiae (strain ATCC 2045
raw_signal_sum
batchcl_raw_signal_sum
norm_batchcl_raw_signal_sum
ctrl.ratio
log2(value)
condition
WT
MUT
Control
rep
rep1
rep2
rep3

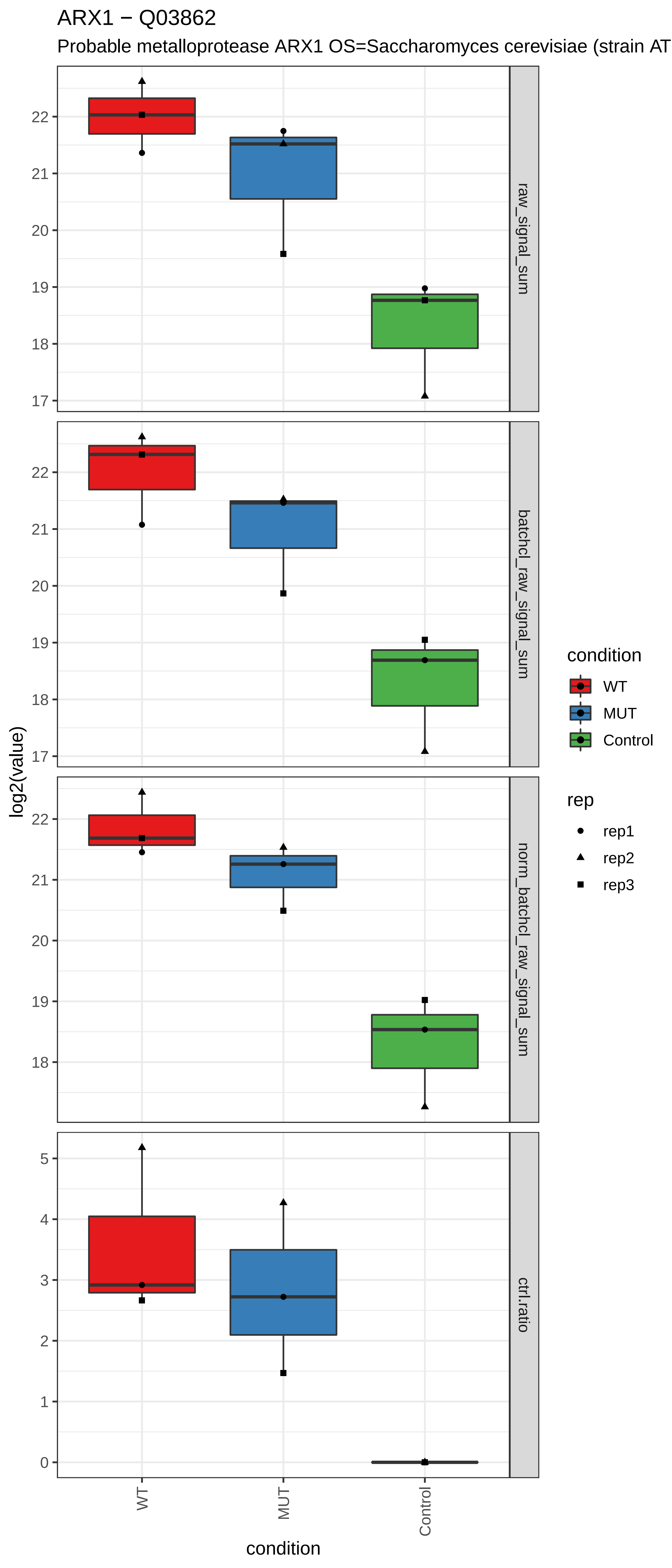

ARX1 − Q03862
Probable metalloprotease ARX1 OS=Saccharomyces cerevisiae (strain AT
raw_signal_sum
batchcl_raw_signal_sum
norm_batchcl_raw_signal_sum
ctrl.ratio
log2(value)
condition
WT
MUT
Control
rep
rep1
rep2
rep3

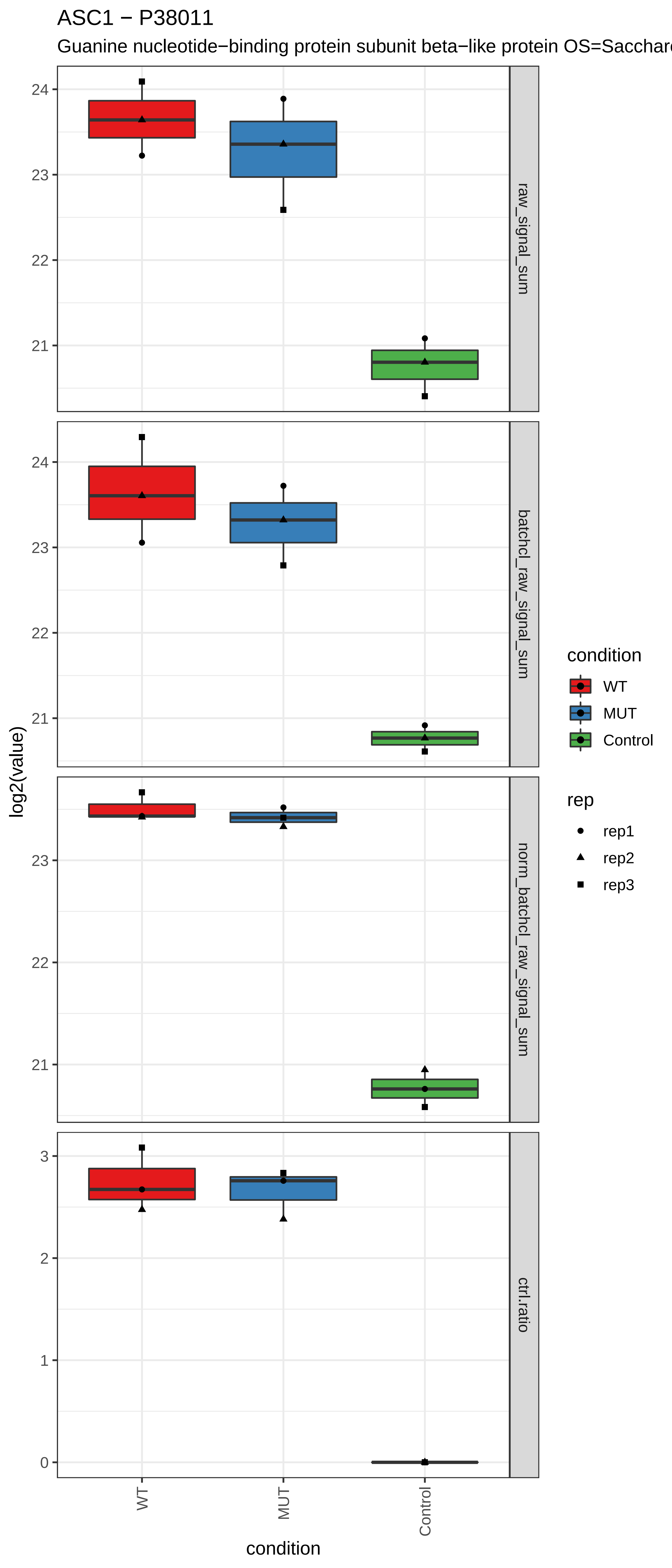
ASC1 − P38011
Guanine nucleotide−binding protein subunit beta−like protein OS=Sacchar
raw_signal_sum
batchcl_raw_signal_sum
norm_batchcl_raw_signal_sum
ctrl.ratio
log2(value)
condition
WT
MUT
Control
rep
rep1
rep2
rep3
24
23
22
21
3
2
1
0

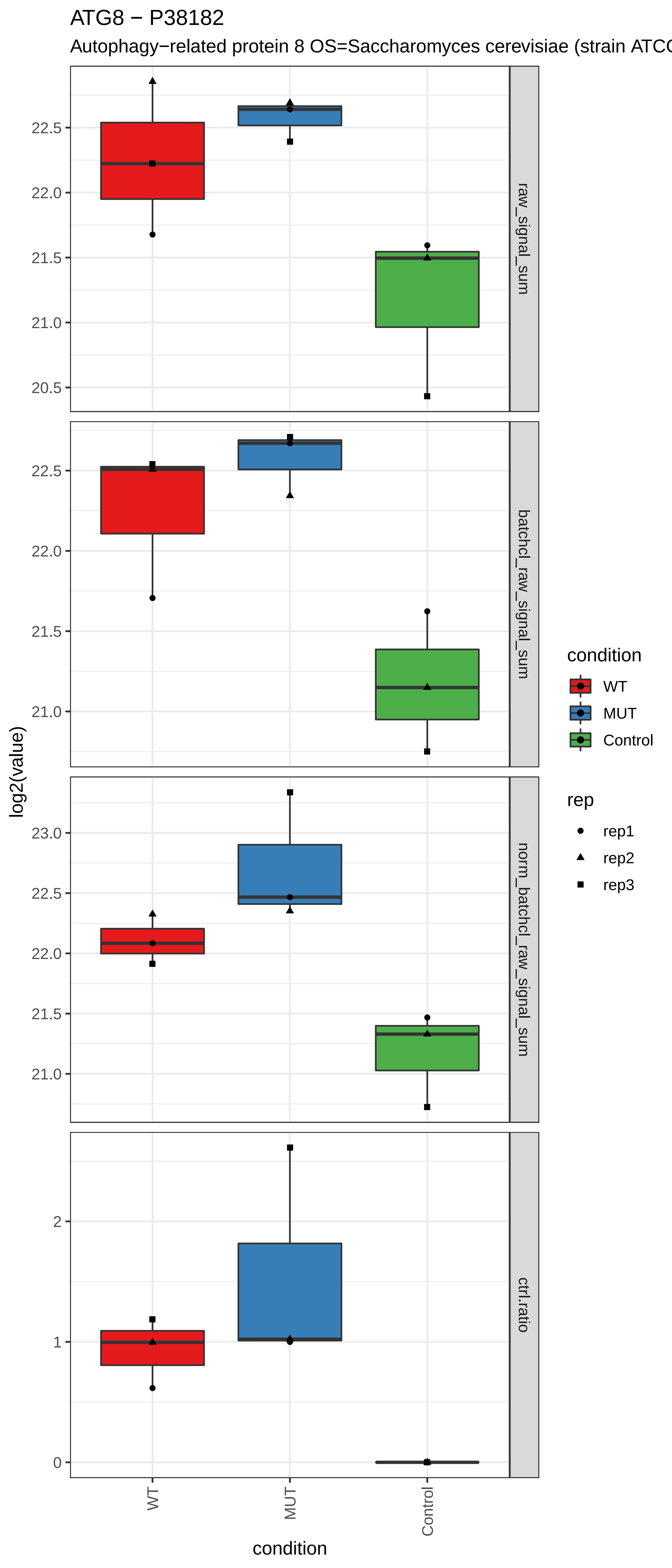
ATG8 − P38182
Autophagy−related protein 8 OS=Saccharomyces cerevisiae (strain ATCC
raw_signal_sum
batchcl_raw_signal_sum
norm_batchcl_raw_signal_sum
ctrl.ratio
log2(value)
condition
WT
MUT
Control
rep
rep1
rep2
rep3
22.5
22.0
21.5
21.0
20.5
23.0
2
1
0

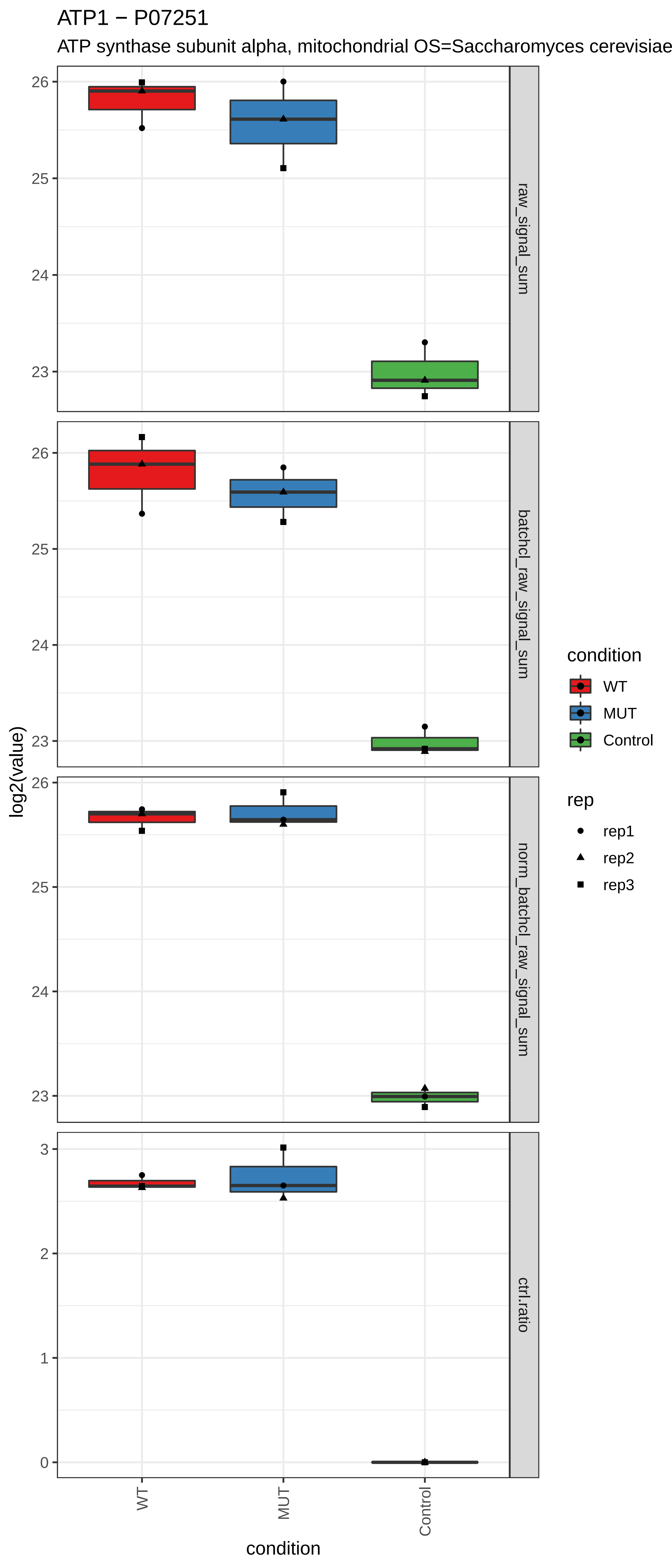

ATP1 − P07251
ATP synthase subunit alpha, mitochondrial OS=Saccharomyces cerevisiae
raw_signal_sum
batchcl_raw_signal_sum
norm_batchcl_raw_signal_sum
ctrl.ratio
log2(value)
condition
WT
MUT
Control
rep
rep1
rep2
rep3

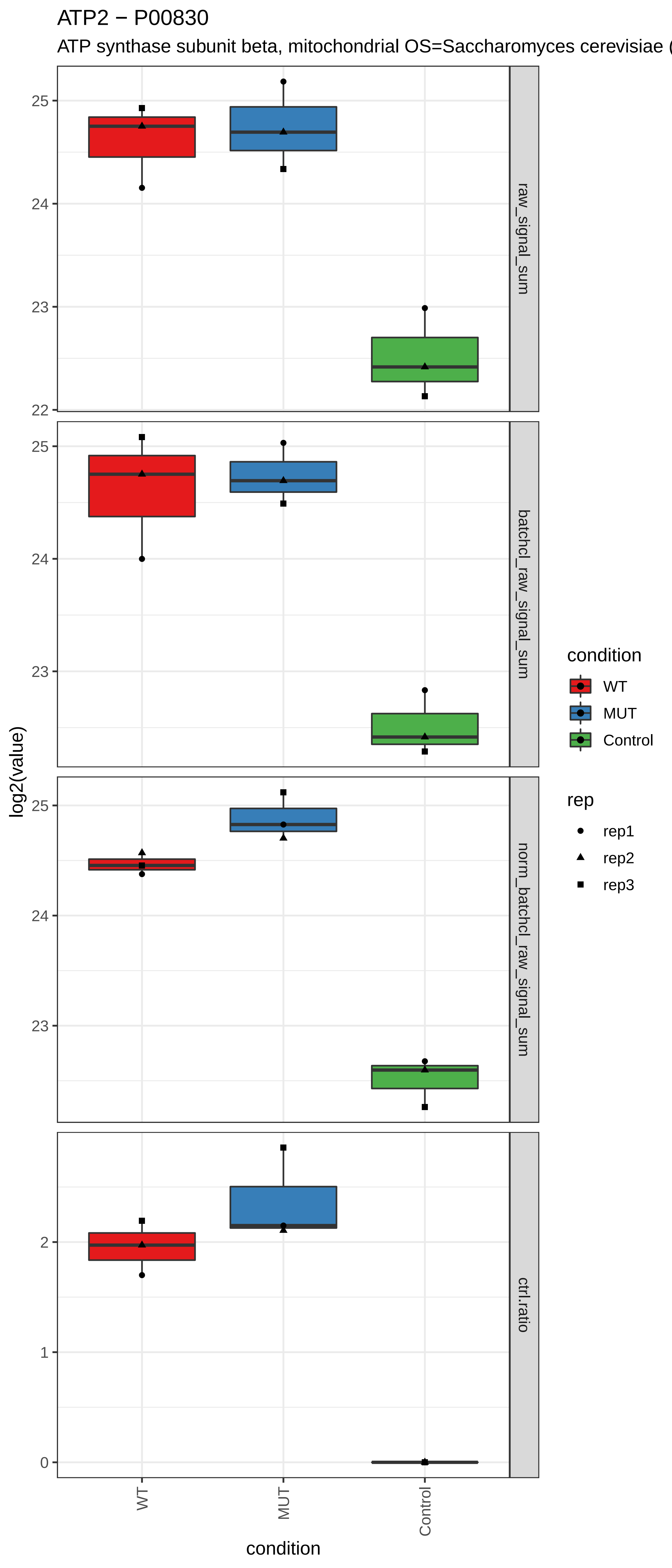

ATP2 − P00830
ATP synthase subunit beta, mitochondrial OS=Saccharomyces cerevisiae (
raw_signal_sum
batchcl_raw_signal_sum
norm_batchcl_raw_signal_sum
ctrl.ratio
log2(value)
condition
WT
MUT
Control
rep
rep1
rep2
rep3
25
24
23
22
2
1
0

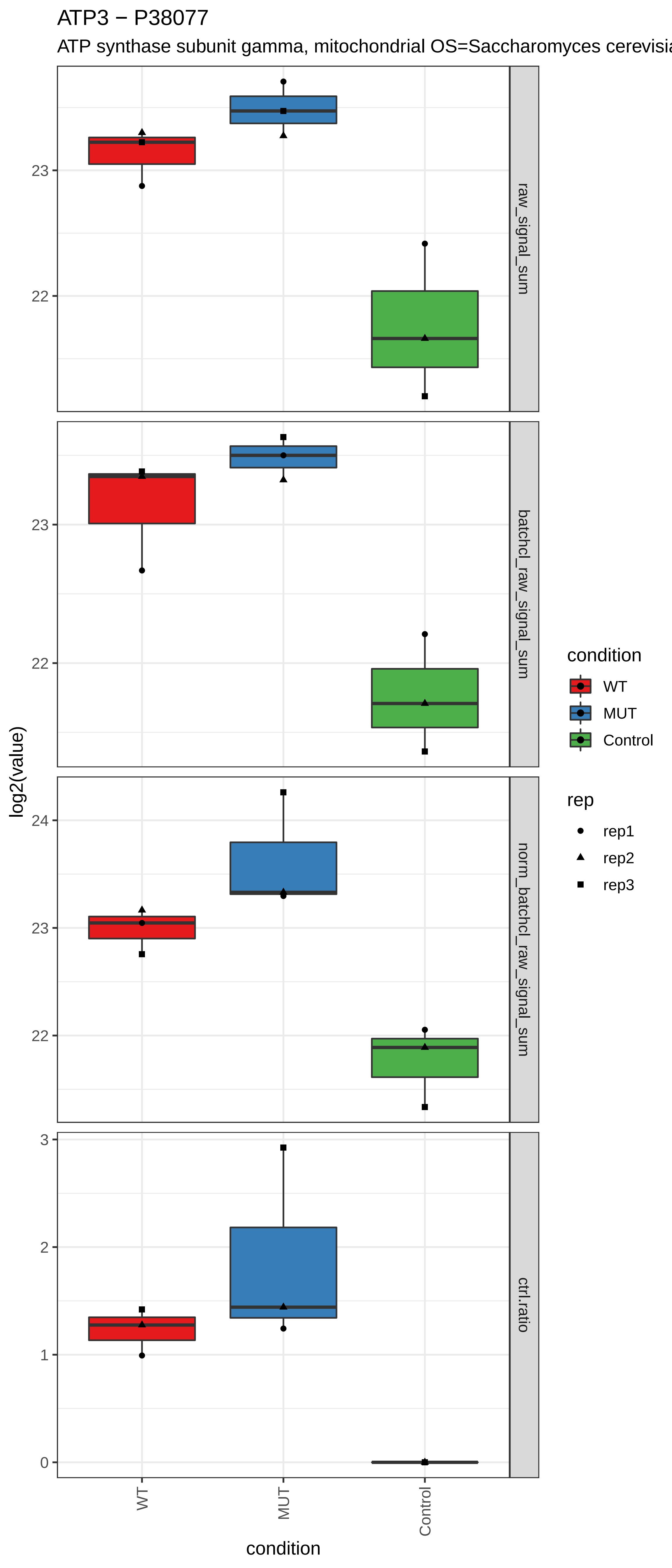

ATP3 − P38077
ATP synthase subunit gamma, mitochondrial OS=Saccharomyces cerevisia
raw_signal_sum
batchcl_raw_signal_sum
norm_batchcl_raw_signal_sum
ctrl.ratio
log2(value)
condition
WT
MUT
Control
rep
rep1
rep2
rep3

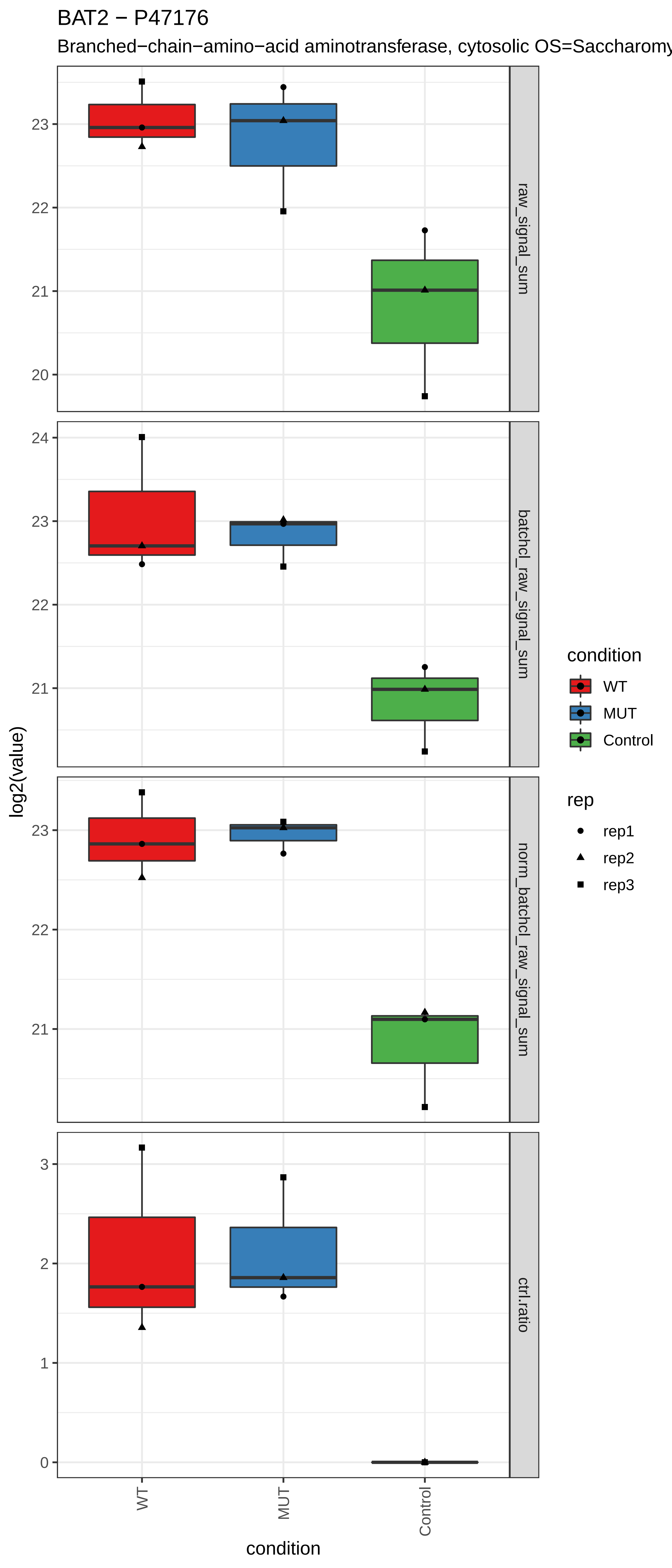

BAT2 − P47176
Branched−chain−amino−acid aminotransferase, cytosolic OS=Saccharomy
raw_signal_sum
batchcl_raw_signal_sum
norm_batchcl_raw_signal_sum
ctrl.ratio
log2(value)
condition
WT
MUT
Control
rep
rep1
rep2
rep3

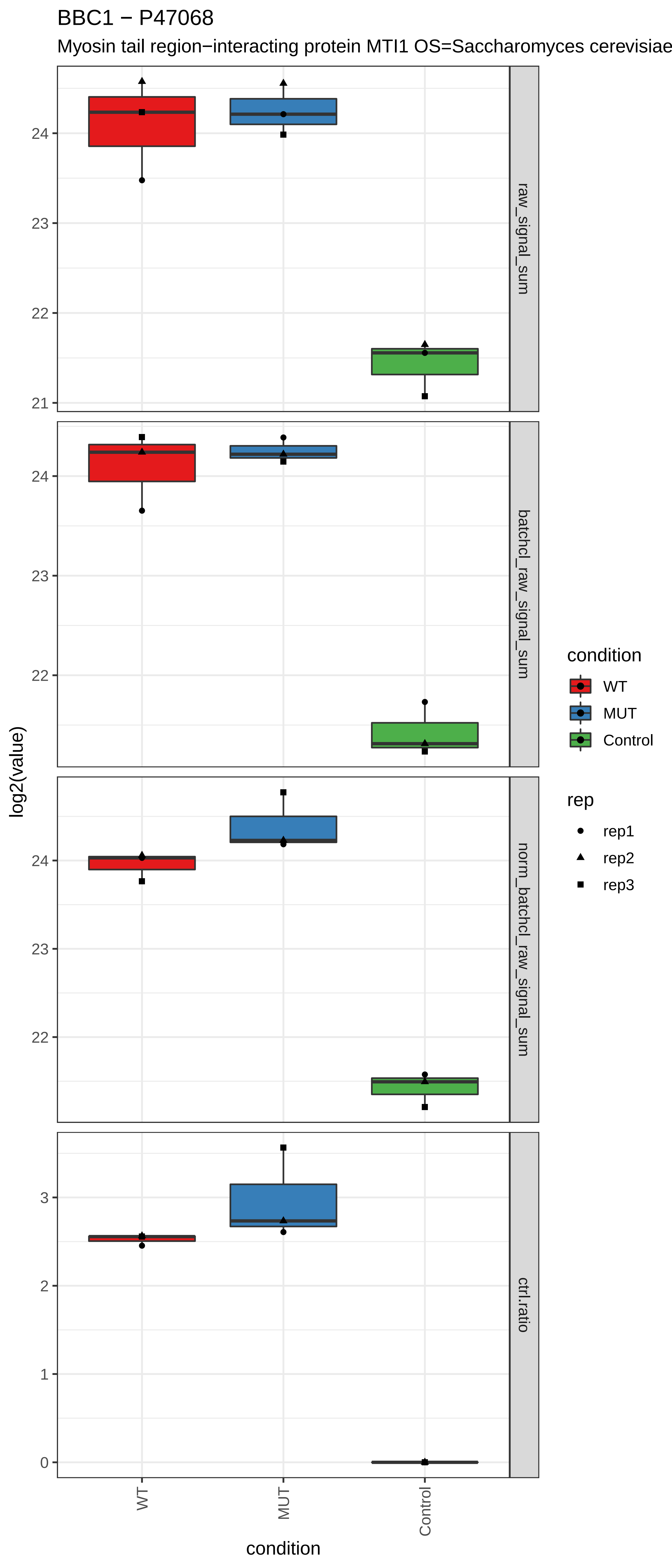
BBC1 − P47068
Myosin tail region−interacting protein MTI1 OS=Saccharomyces cerevisiae
raw_signal_sum
batchcl_raw_signal_sum
norm_batchcl_raw_signal_sum
ctrl.ratio
log2(value)
condition
WT
MUT
Control
rep
rep1
rep2
rep3
24
23
22
21
3
2
1
0

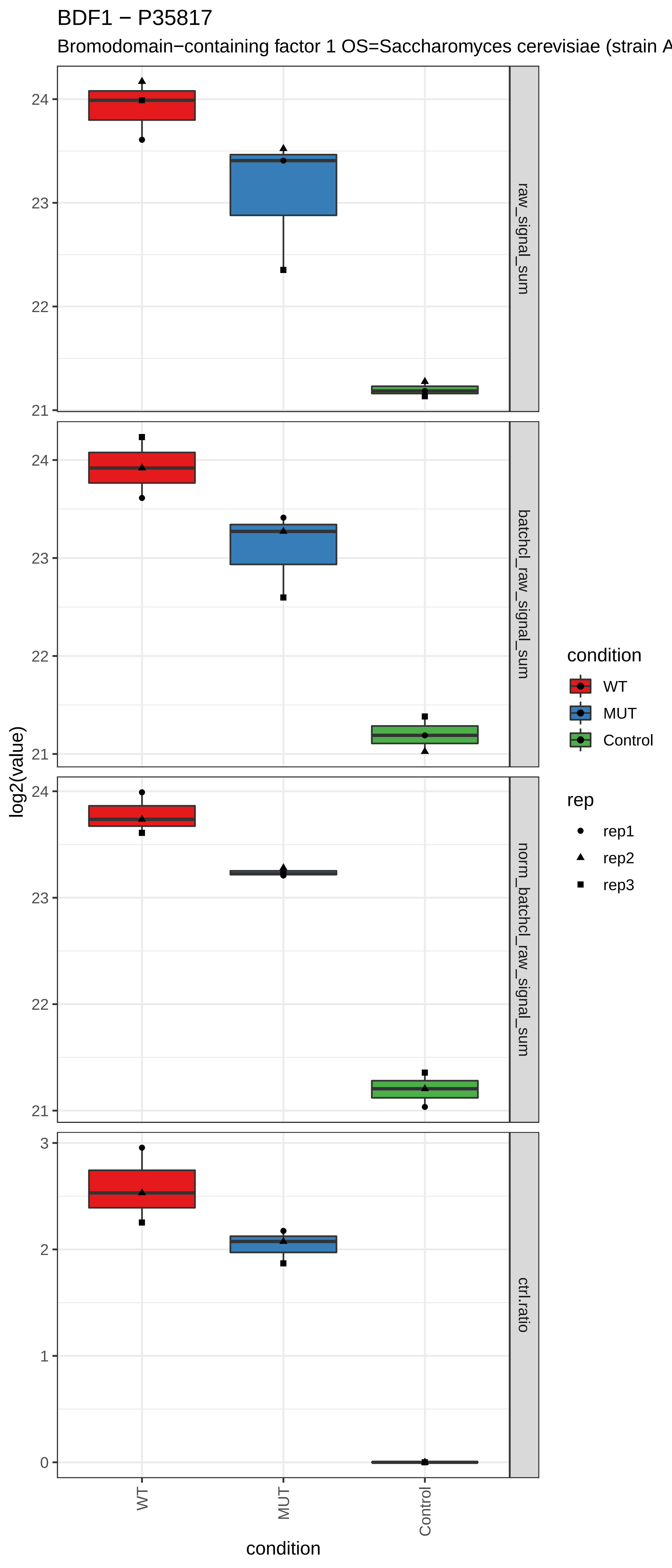

BDF1 − P35817
Bromodomain−containing factor 1 OS=Saccharomyces cerevisiae (strain A
raw_signal_sum
batchcl_raw_signal_sum
norm_batchcl_raw_signal_sum
ctrl.ratio
log2(value)
condition
WT
MUT
Control
rep
rep1
rep2
rep3
24
23
22
21
3
2
1
0

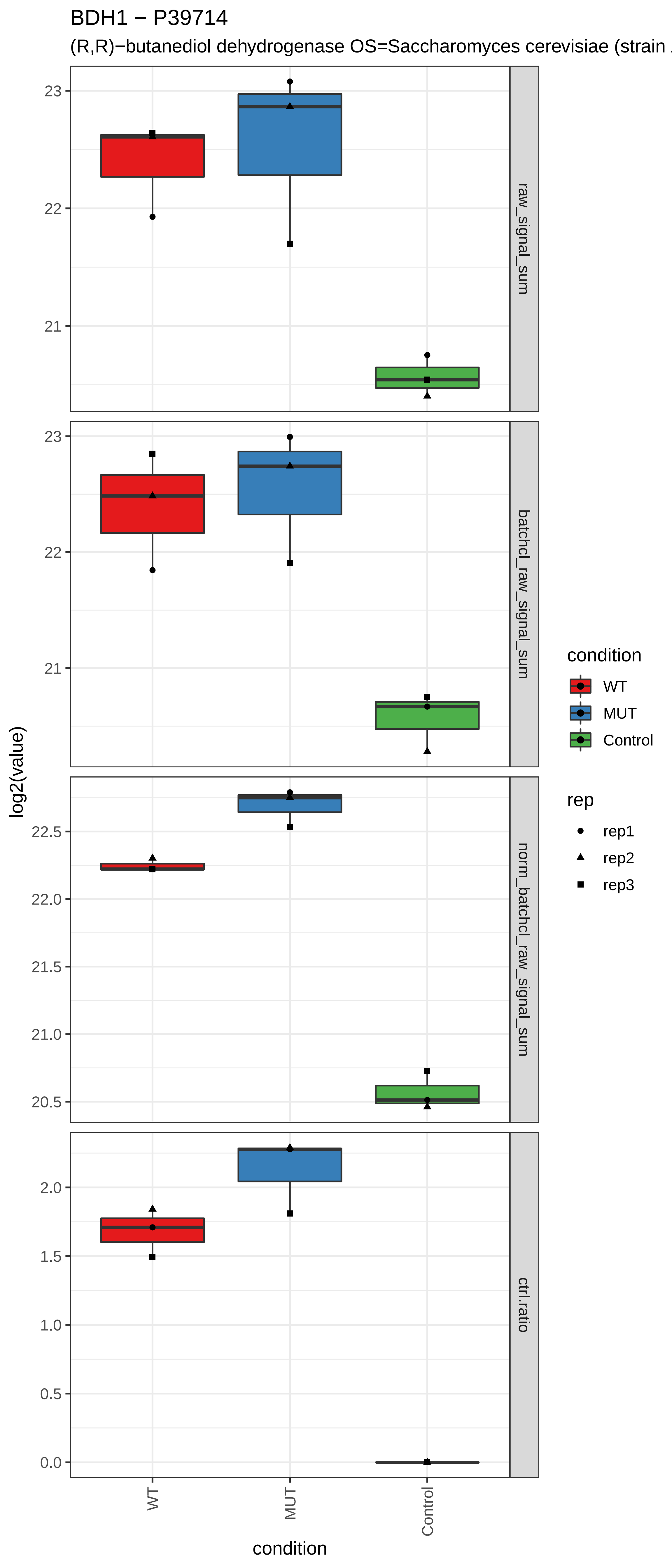

BDH1 − P39714
(R,R)−butanediol dehydrogenase OS=Saccharomyces cerevisiae (strain
raw_signal_sum
batchcl_raw_signal_sum
norm_batchcl_raw_signal_sum
ctrl.ratio
log2(value)
condition
WT
MUT
Control
rep
rep1
rep2
rep3

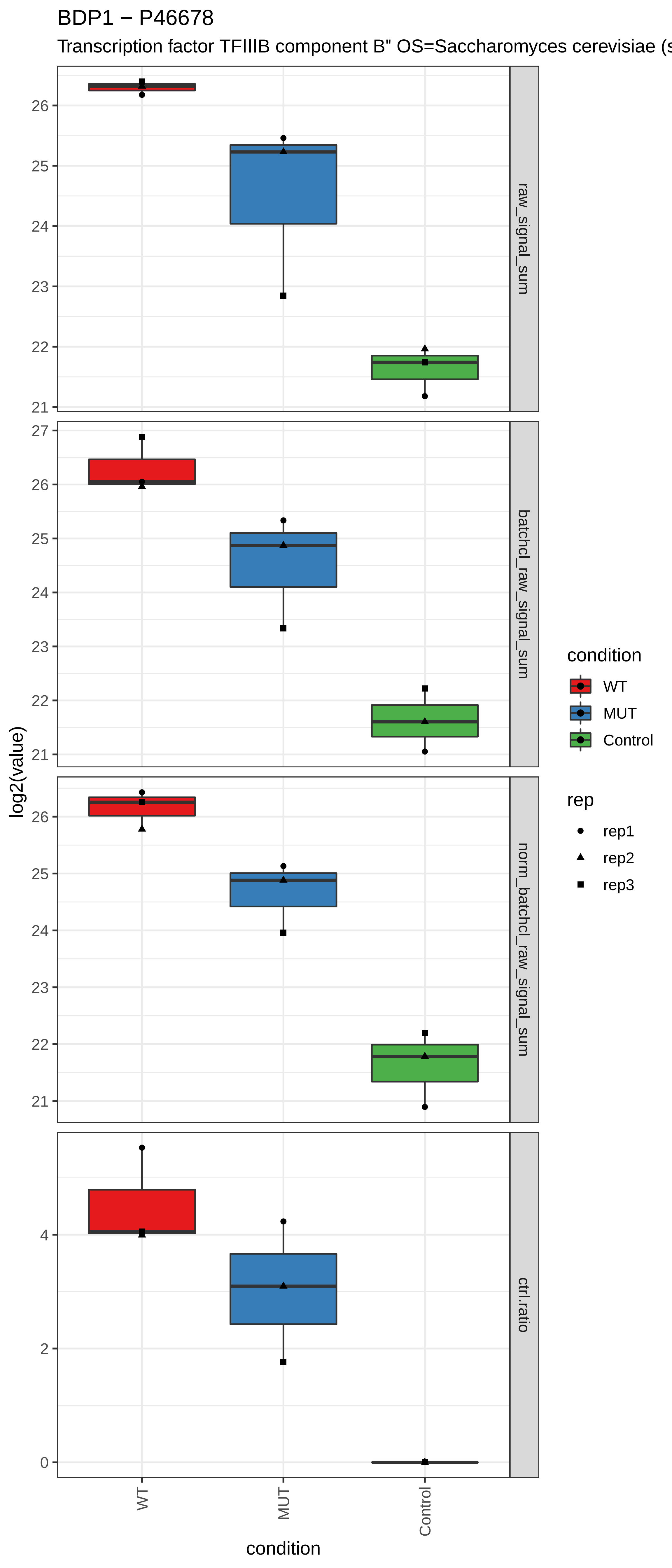
BDP1 − P46678
Transcription factor TFIIIB component B" OS=Saccharomyces cerevisiae (s
raw_signal_sum
batchcl_raw_signal_sum
norm_batchcl_raw_signal_sum
ctrl.ratio
log2(value)
condition
WT
MUT
Control
rep
rep1
rep2
rep3

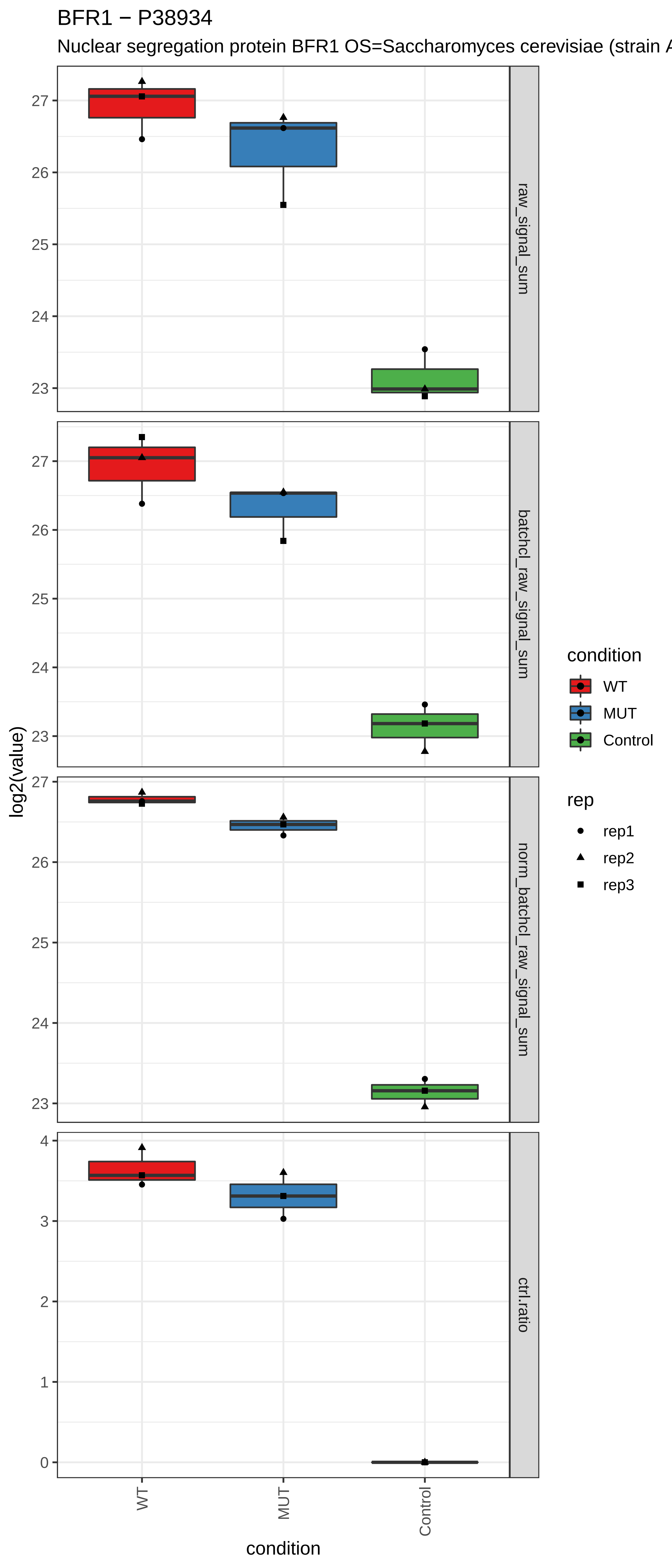

BFR1 − P38934
Nuclear segregation protein BFR1 OS=Saccharomyces cerevisiae (strain A
raw_signal_sum
batchcl_raw_signal_sum
norm_batchcl_raw_signal_sum
ctrl.ratio
log2(value)
condition
WT
MUT
Control
rep
rep1
rep2
rep3

BGL2 − P15703

Glucan 1,3−beta−glucosidase OS=Saccharomyces cerevisiae (strain ATCC

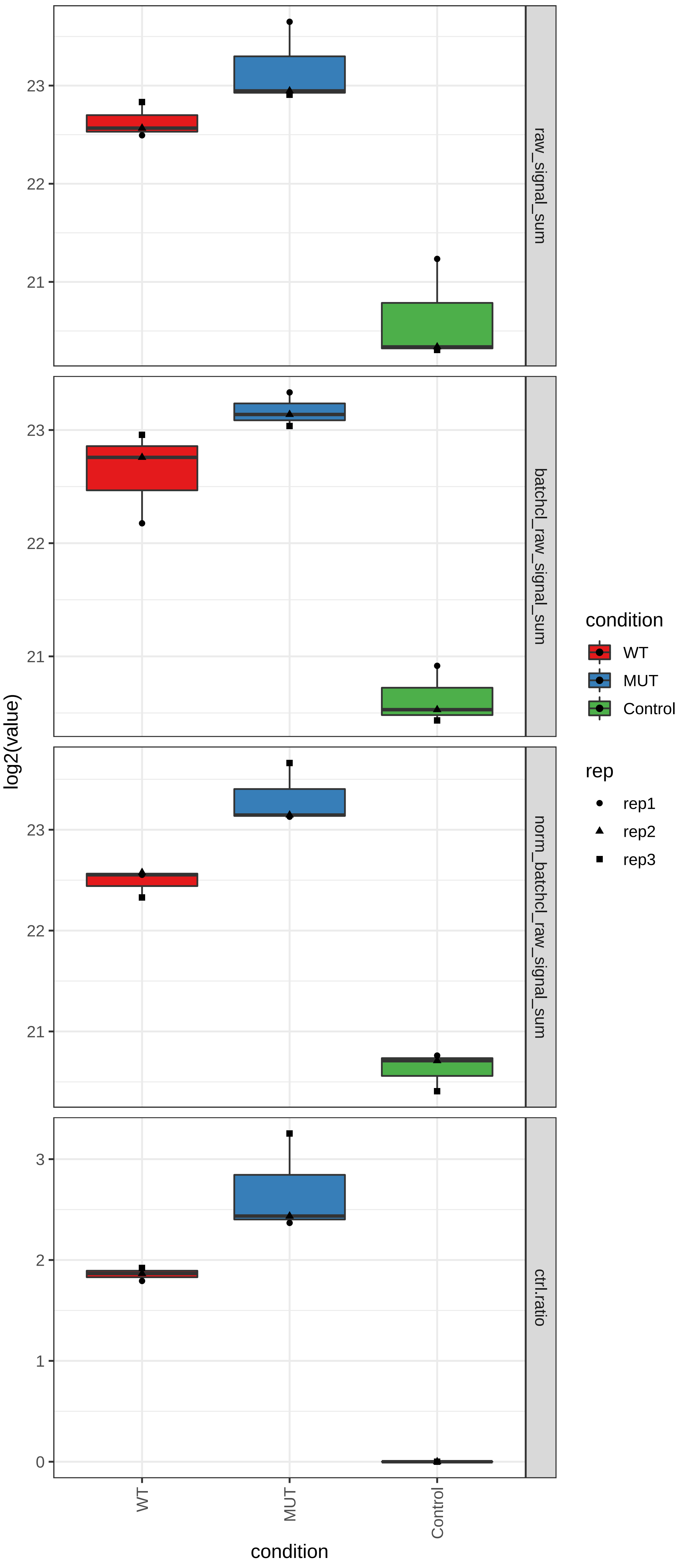

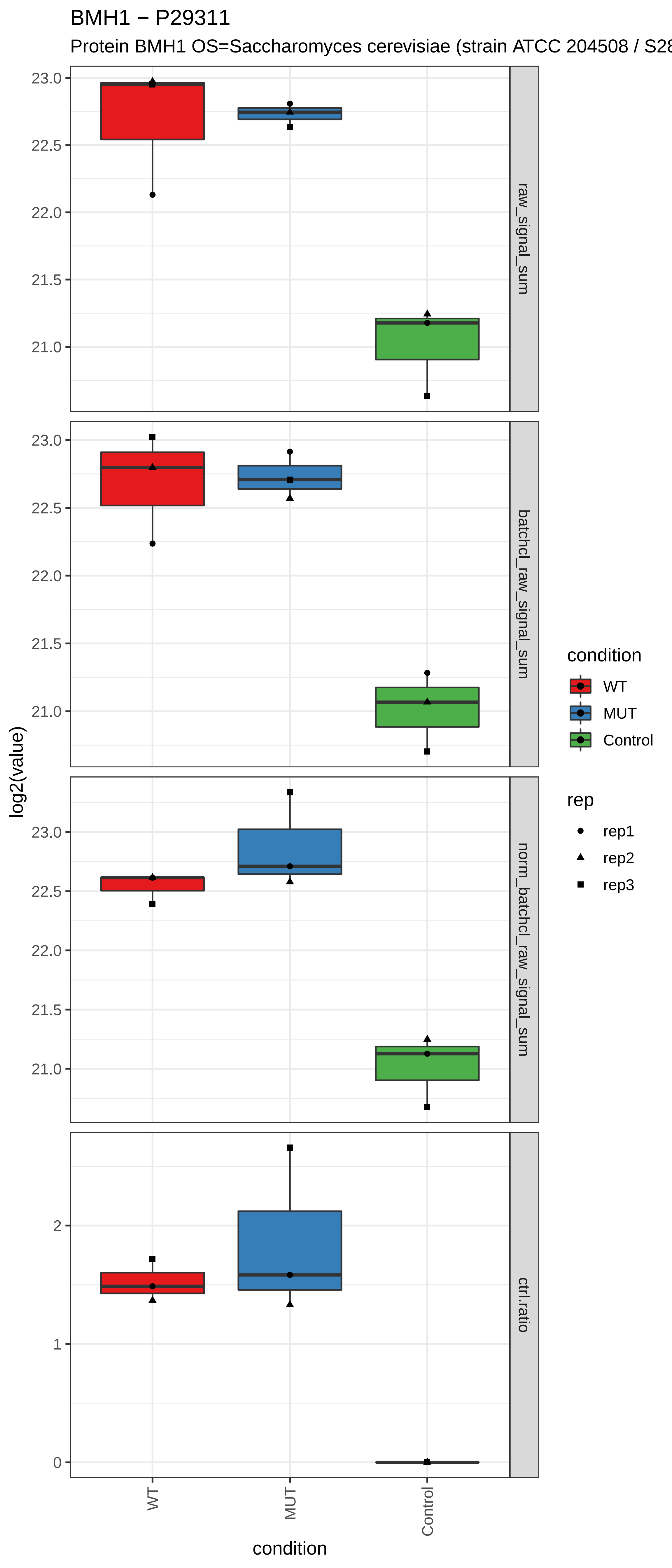

BMH1 − P29311
Protein BMH1 OS=Saccharomyces cerevisiae (strain ATCC 204508 / S28
raw_signal_sum
batchcl_raw_signal_sum
norm_batchcl_raw_signal_sum
ctrl.ratio
log2(value)
condition
WT
MUT
Control
rep
rep1
rep2
rep3
23.0
22.5
22.0
21.5
21.0
2
1
0

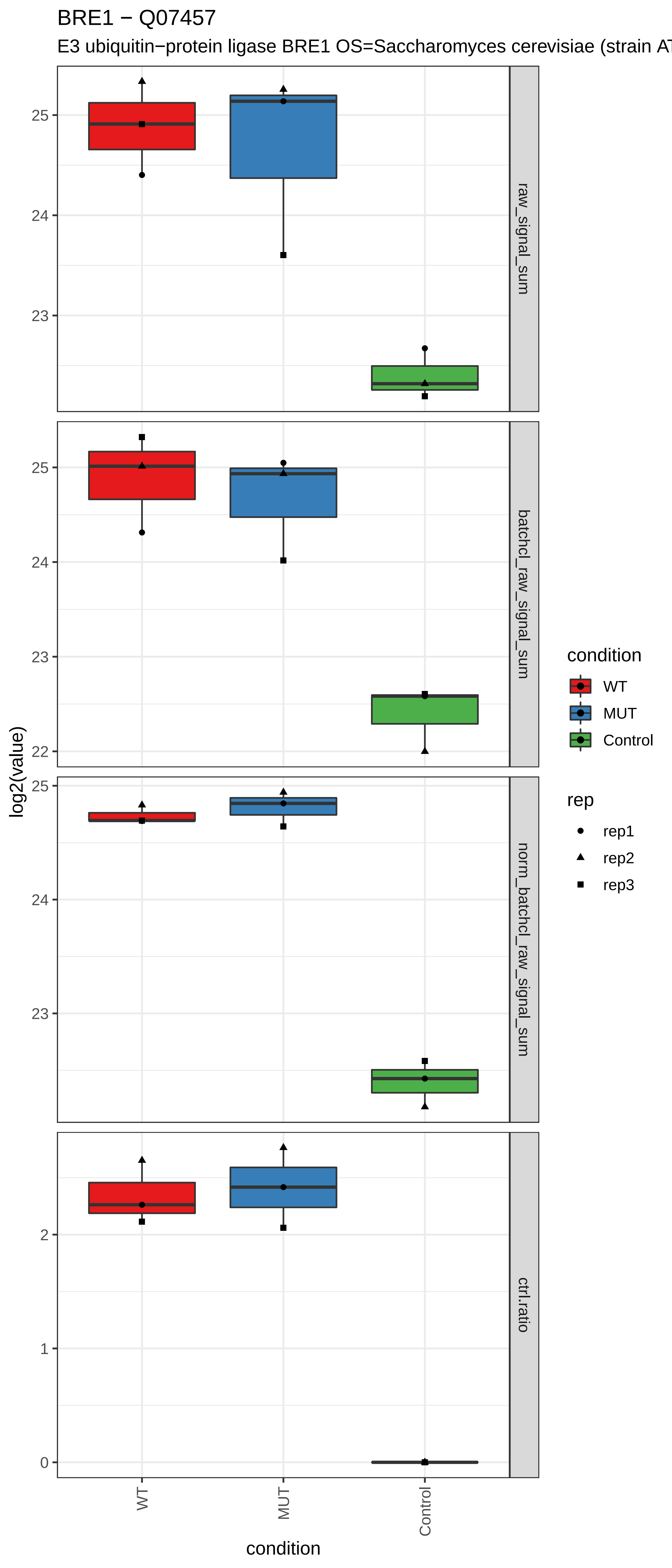
BRE1 − Q07457
E3 ubiquitin−protein ligase BRE1 OS=Saccharomyces cerevisiae (strain AT
raw_signal_sum
batchcl_raw_signal_sum
norm_batchcl_raw_signal_sum
ctrl.ratio
log2(value)
condition
WT
MUT
Control
rep
rep1
rep2
rep3

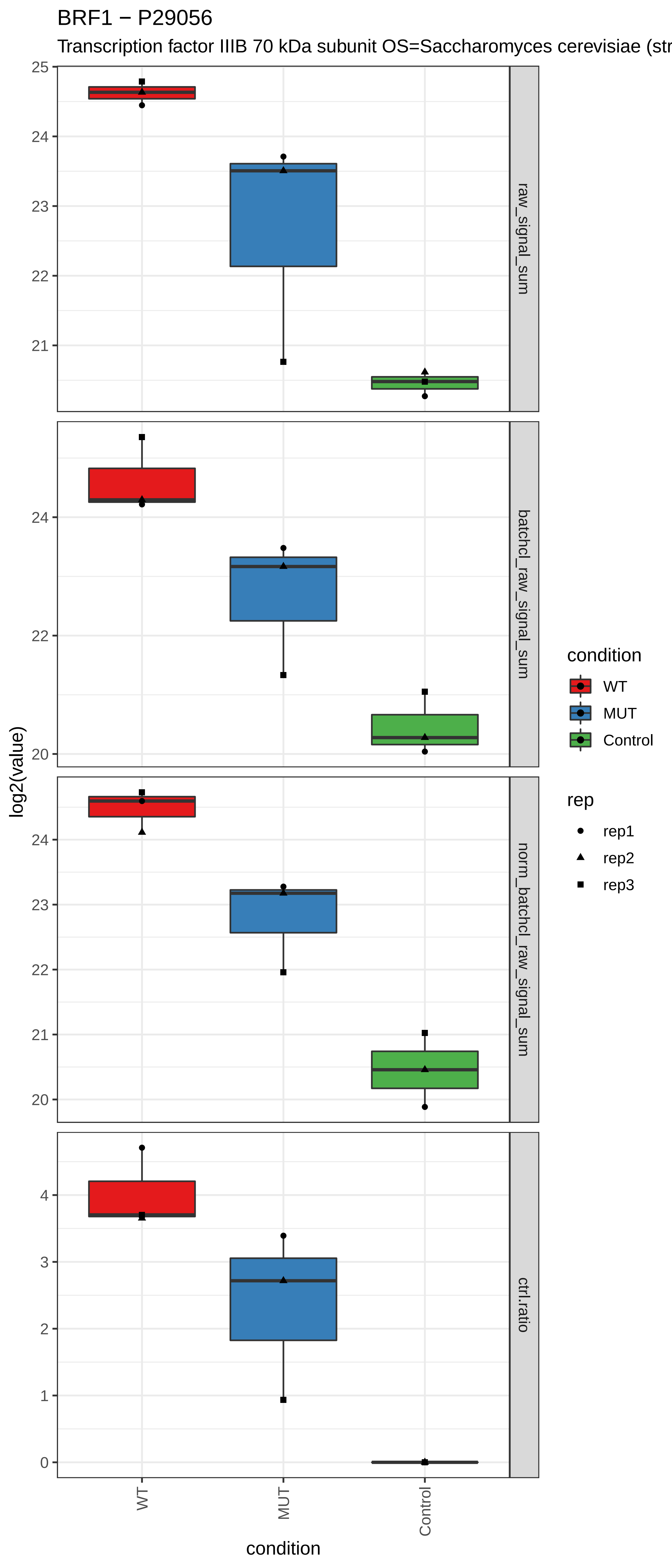

BRF1 − P29056
Transcription factor IIIB 70 kDa subunit OS=Saccharomyces cerevisiae (str
raw_signal_sum
batchcl_raw_signal_sum
norm_batchcl_raw_signal_sum
ctrl.ratio
log2(value)
condition
WT
MUT
Control
rep
rep1
rep2
rep3

BRN1 − P38170

Condensin complex subunit 2 OS=Saccharomyces cerevisiae (strain ATCC

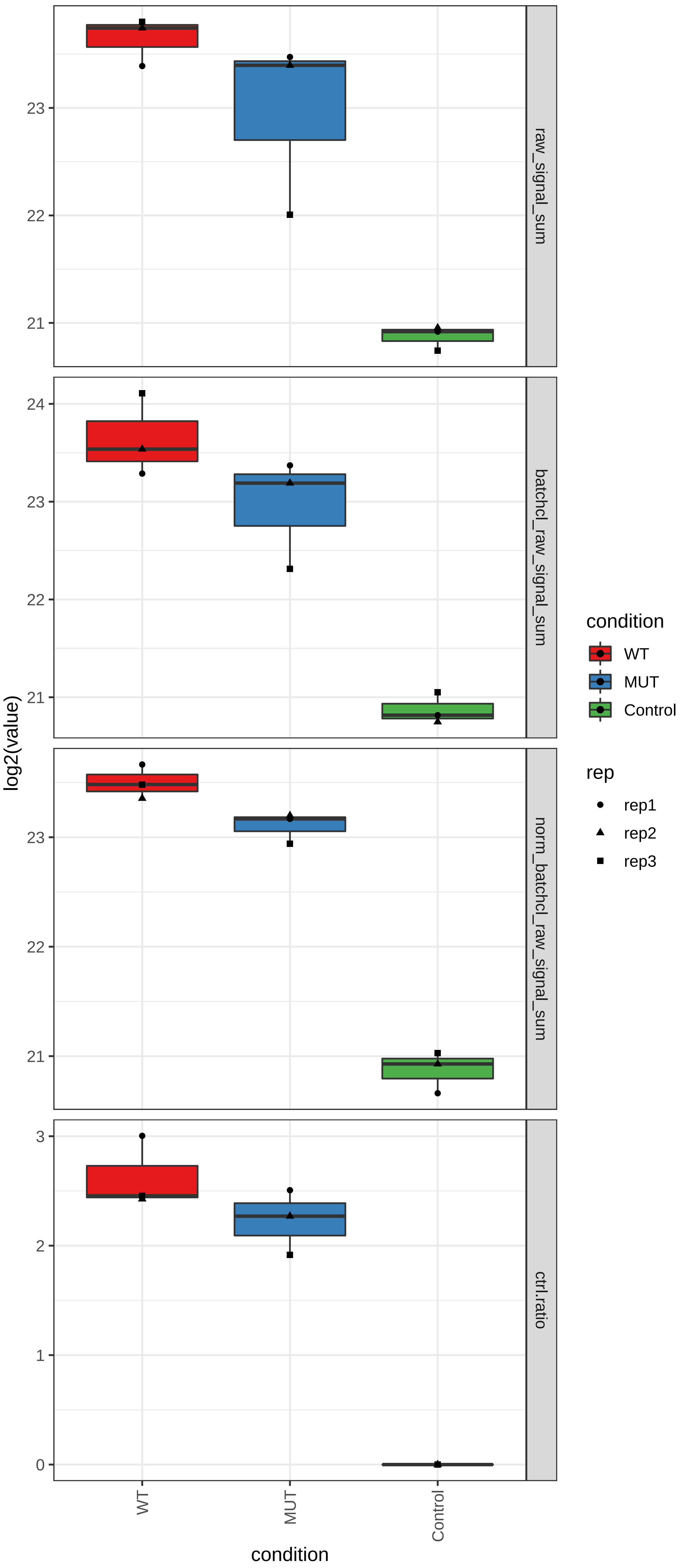

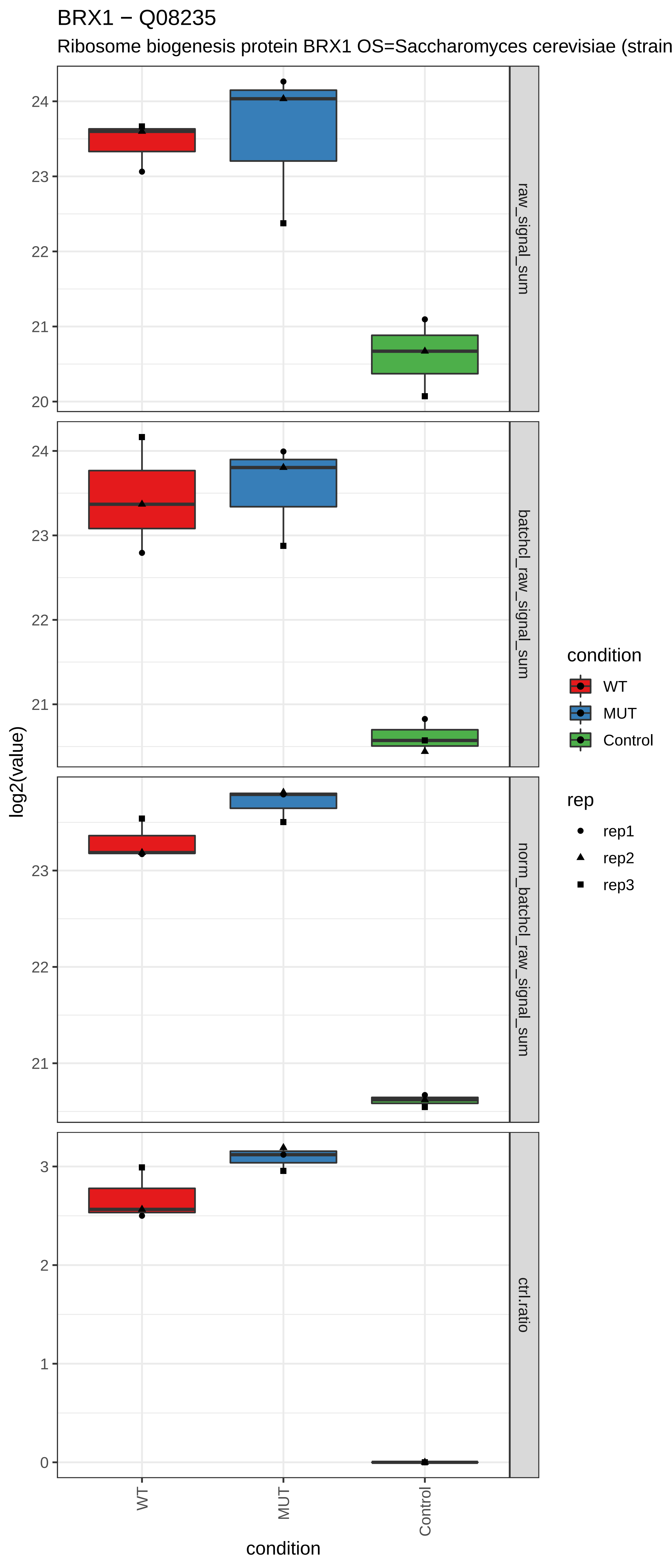

BRX1 − Q08235
Ribosome biogenesis protein BRX1 OS=Saccharomyces cerevisiae (strain
raw_signal_sum
batchcl_raw_signal_sum
norm_batchcl_raw_signal_sum
ctrl.ratio
log2(value)
condition
WT
MUT
Control
rep
rep1
rep2
rep3

CAD1 − P24813

AP−1−like transcription activator YAP2 OS=Saccharomyces cerevisiae (str

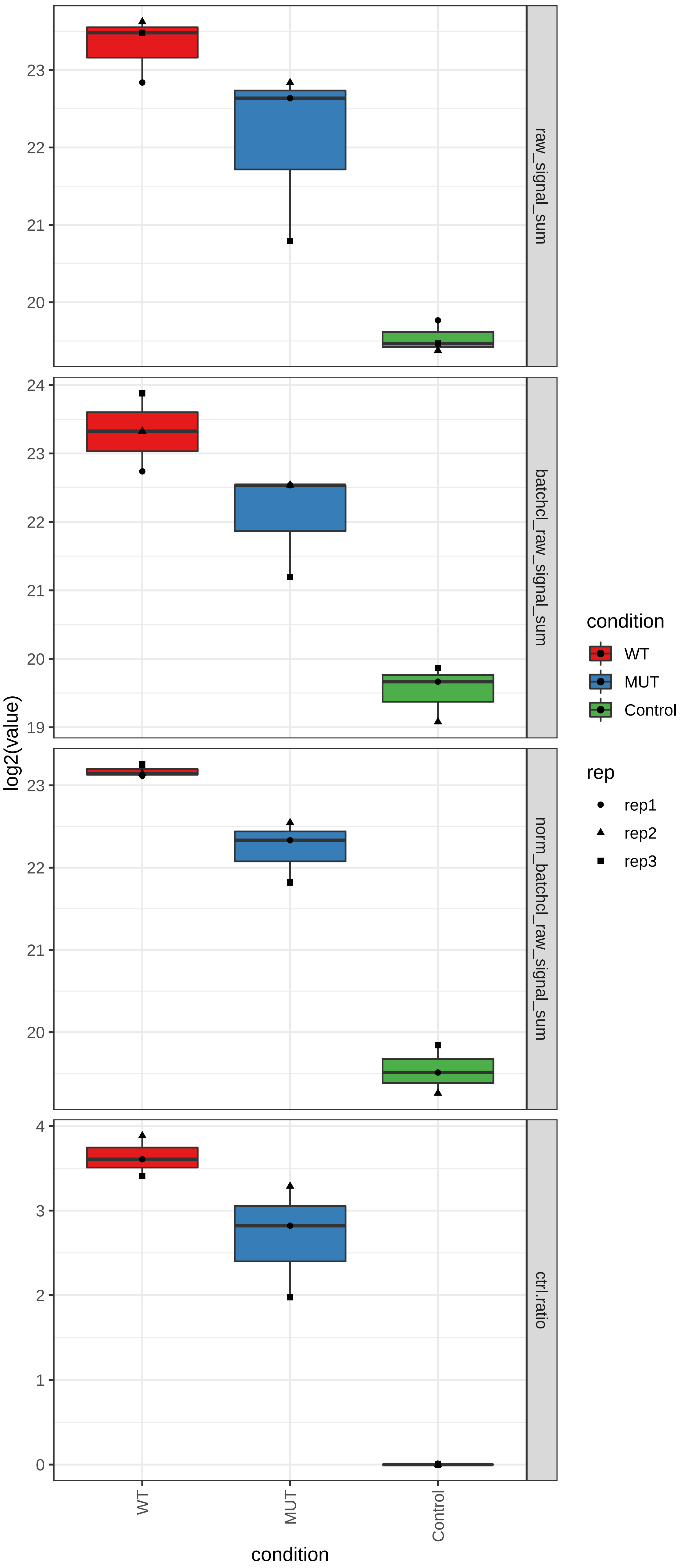

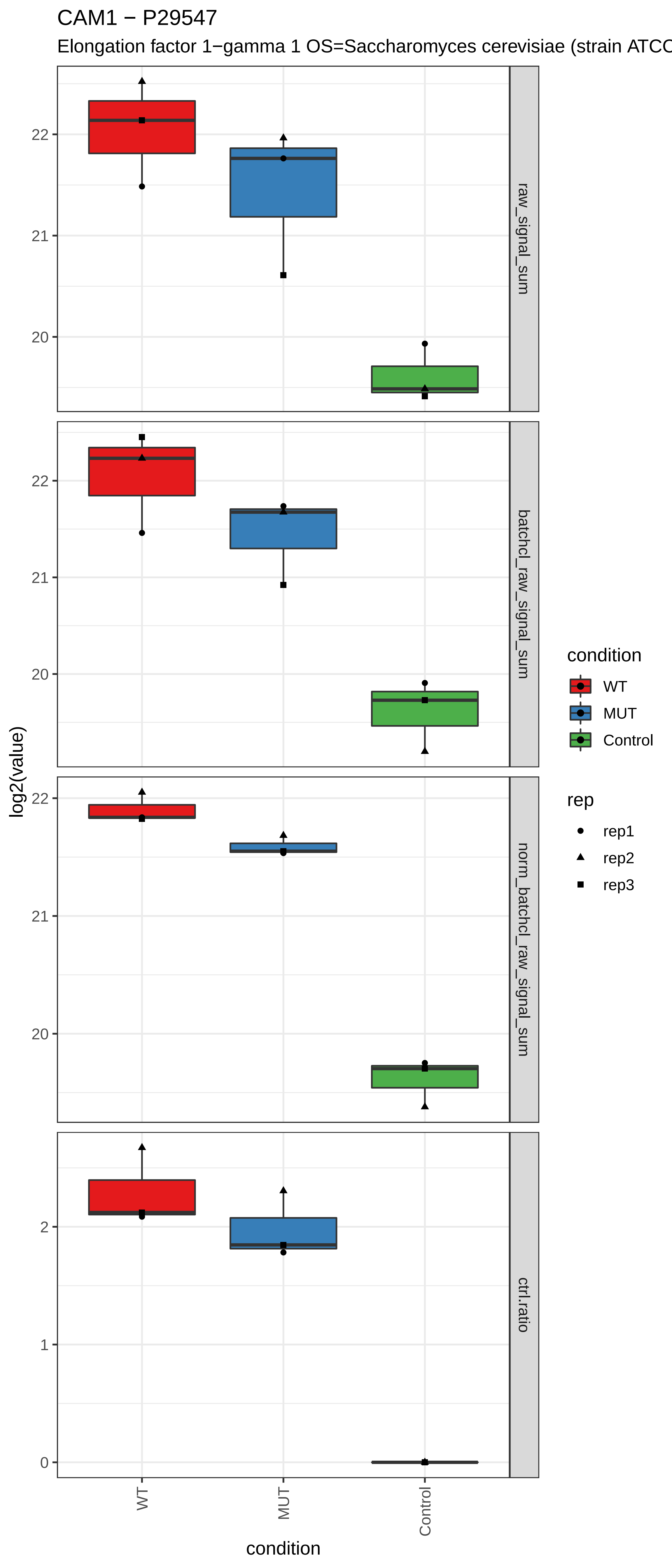

CAM1 − P29547
Elongation factor 1−gamma 1 OS=Saccharomyces cerevisiae (strain ATCC
log2(value)
raw_signal_sum
batchcl_raw_signal_sum
norm_batchcl_raw_signal_sum
ctrl.ratio
22
21
20
2
1
0
WT
MUT
Control
condition
condition
WT
MUT
Control
rep
rep1
rep2
rep3

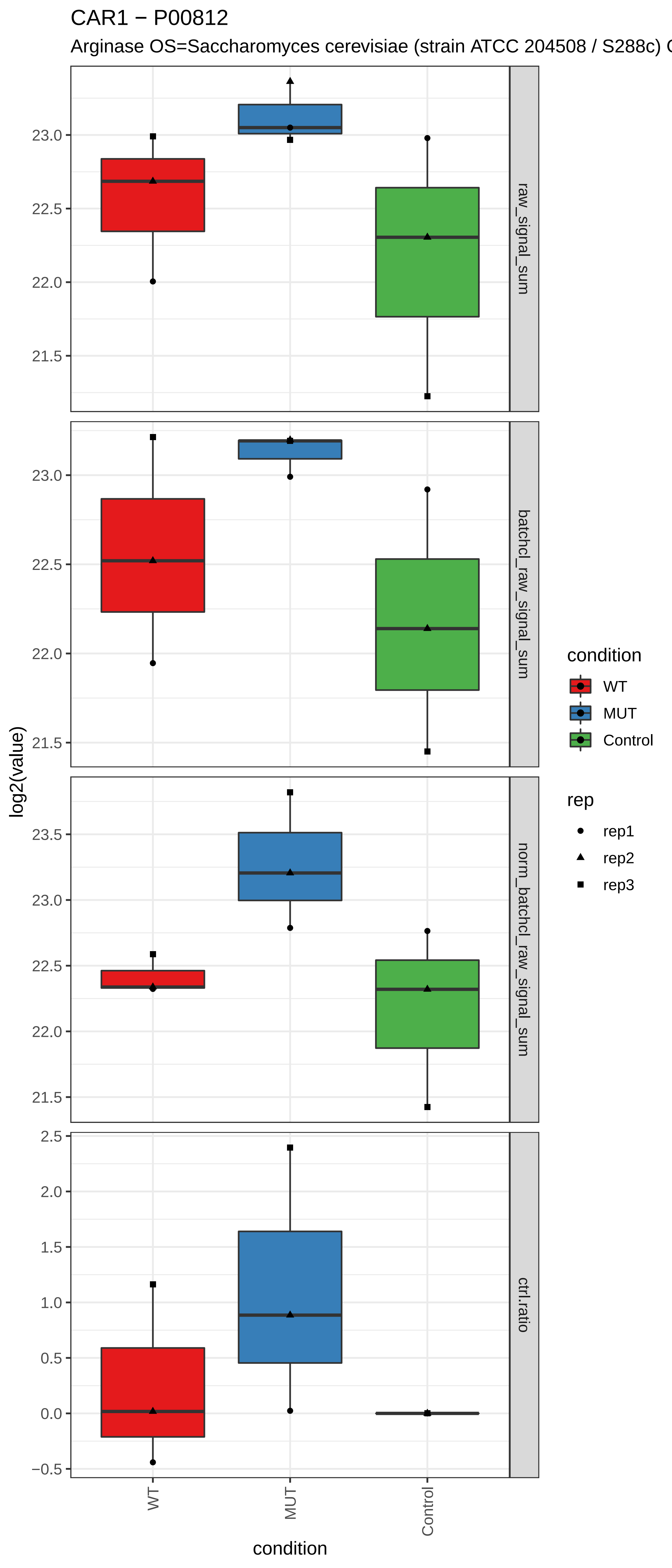

CAR1 − P00812
Arginase OS=Saccharomyces cerevisiae (strain ATCC 204508 / S288c) G
raw_signal_sum
batchcl_raw_signal_sum
norm_batchcl_raw_signal_sum
ctrl.ratio
log2(value)
condition
WT
MUT
Control
rep
rep1
rep2
rep3

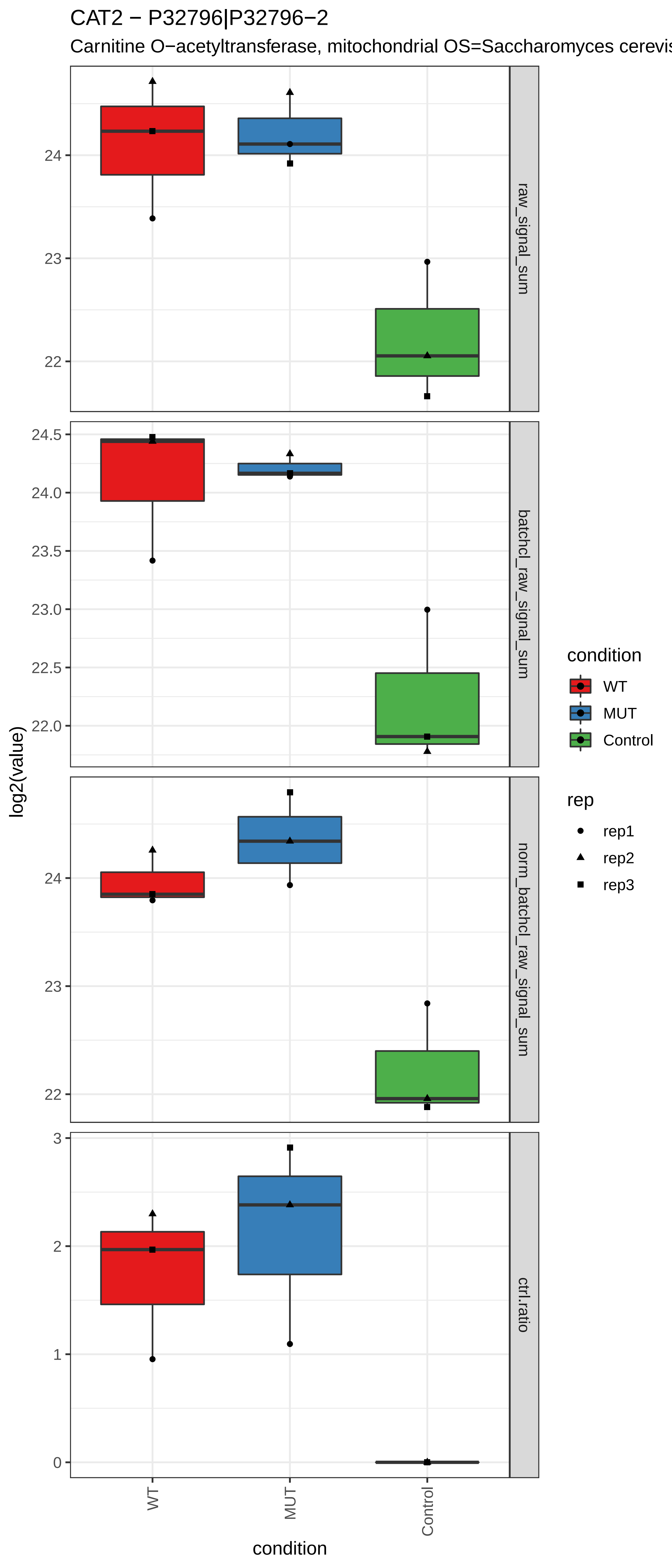

CAT2 − P32796|P32796−2
Carnitine O−acetyltransferase, mitochondrial OS=Saccharomyces cerevis
raw_signal_sum
batchcl_raw_signal_sum
norm_batchcl_raw_signal_sum
ctrl.ratio
log2(value)
condition
WT
MUT
Control
rep
rep1
rep2
rep3

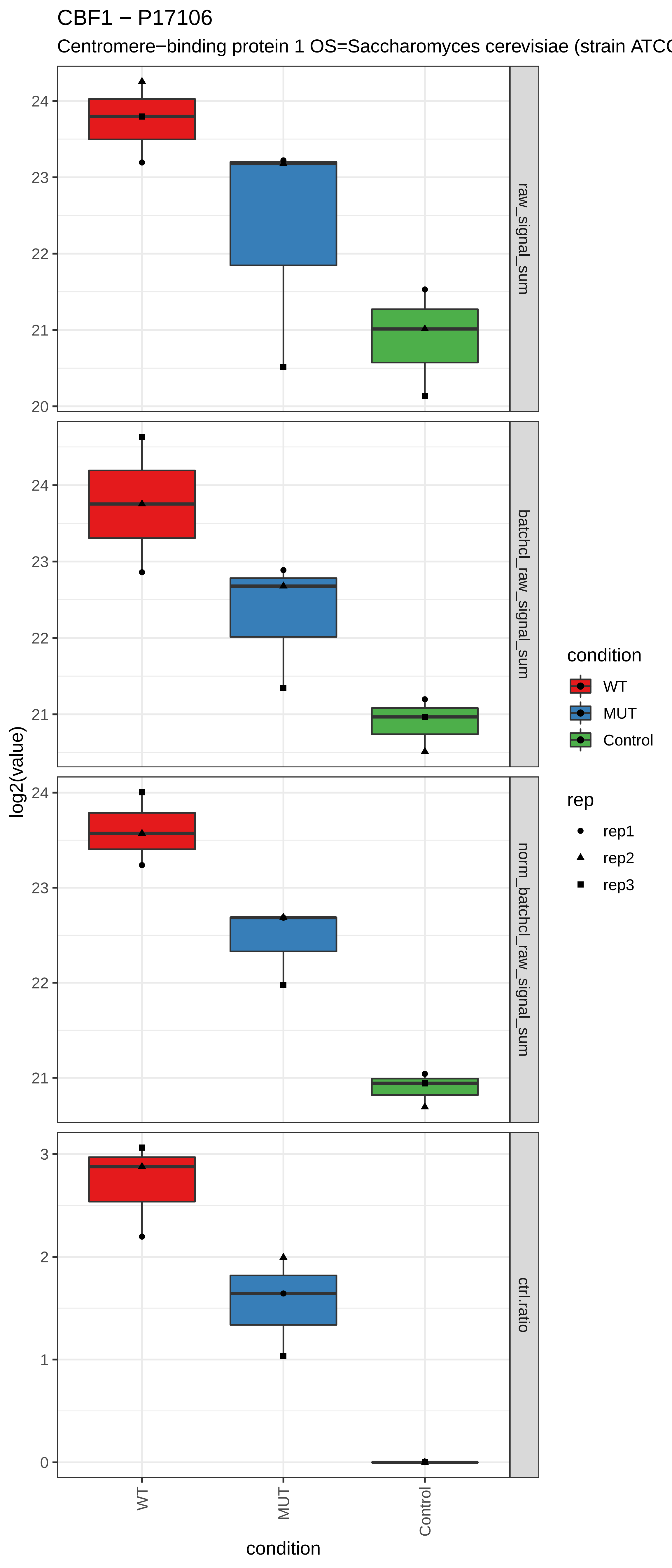

CBF1 − P17106
Centromere−binding protein 1 OS=Saccharomyces cerevisiae (strain ATCC
raw_signal_sum
batchcl_raw_signal_sum
norm_batchcl_raw_signal_sum
ctrl.ratio
log2(value)
condition
WT
MUT
Control
rep
rep1
rep2
rep3

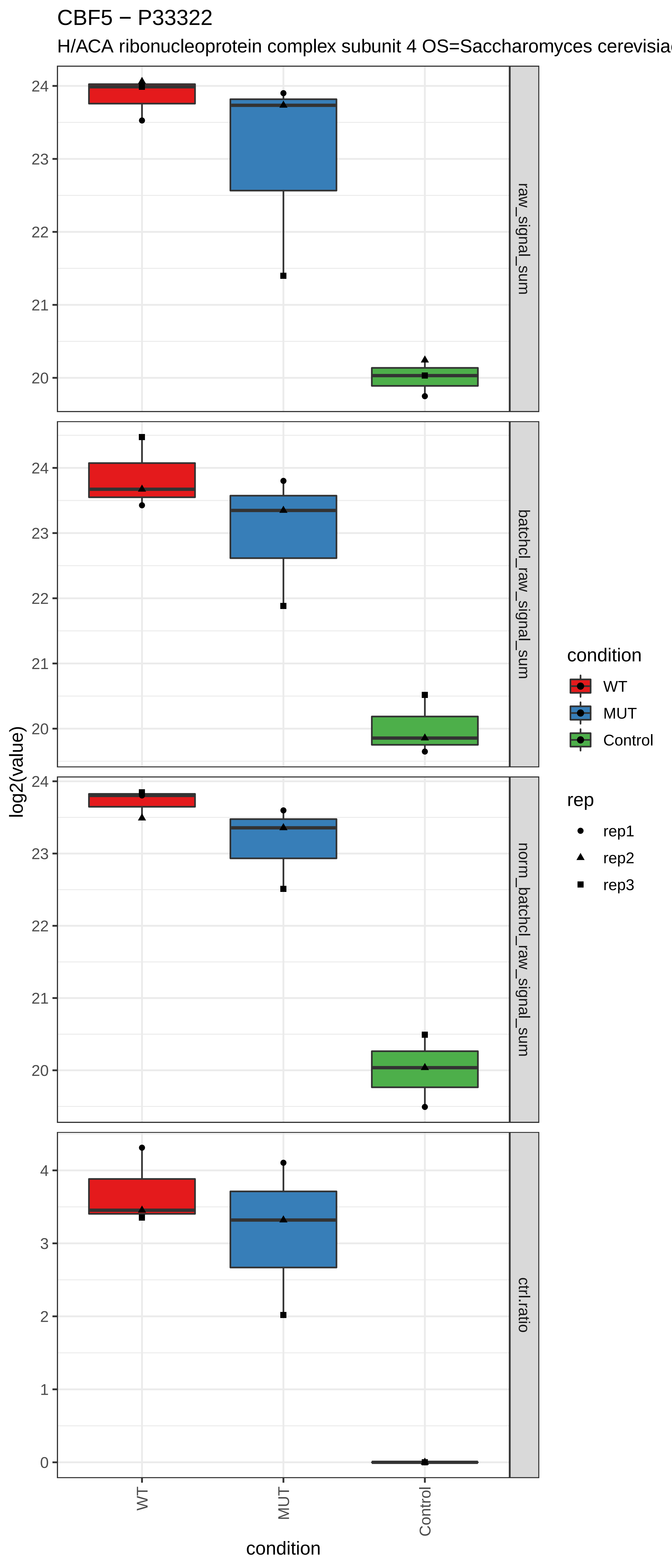

CBF5 − P33322
H/ACA ribonucleoprotein complex subunit 4 OS=Saccharomyces cerevisiae
log2(value)
raw_signal_sum
batchcl_raw_signal_sum
norm_batchcl_raw_signal_sum
ctrl.ratio
WT
MUT
Control
condition
condition
WT
MUT
Control
rep
rep1
rep2
rep3

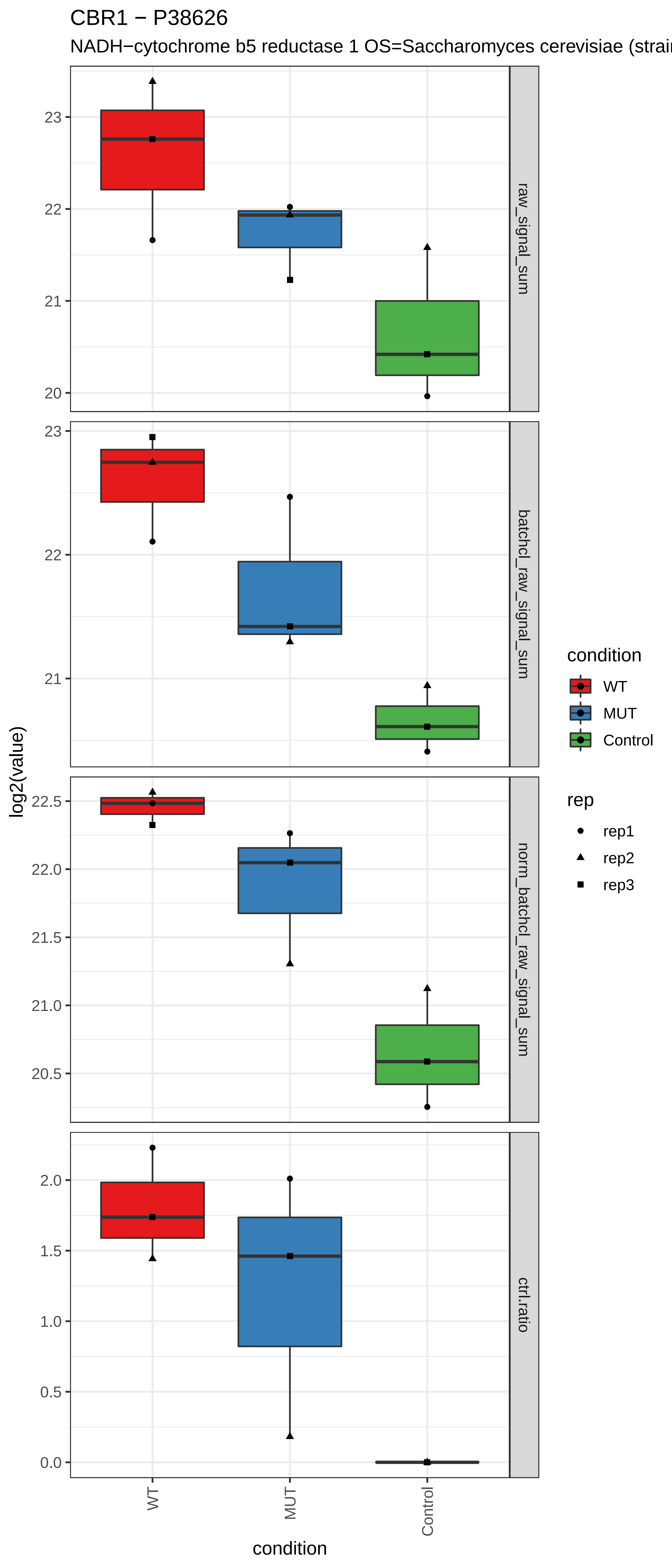
CBR1 − P38626
NADH−cytochrome b5 reductase 1 OS=Saccharomyces cerevisiae (strai
raw_signal_sum
batchcl_raw_signal_sum
norm_batchcl_raw_signal_sum
ctrl.ratio
log2(value)
condition
WT
MUT
Control
rep
rep1
rep2
rep3
23
22
21
20
22.5
22.0
21.5
21.0
20.5
2.0
1.5
1.0
0.5
0.0

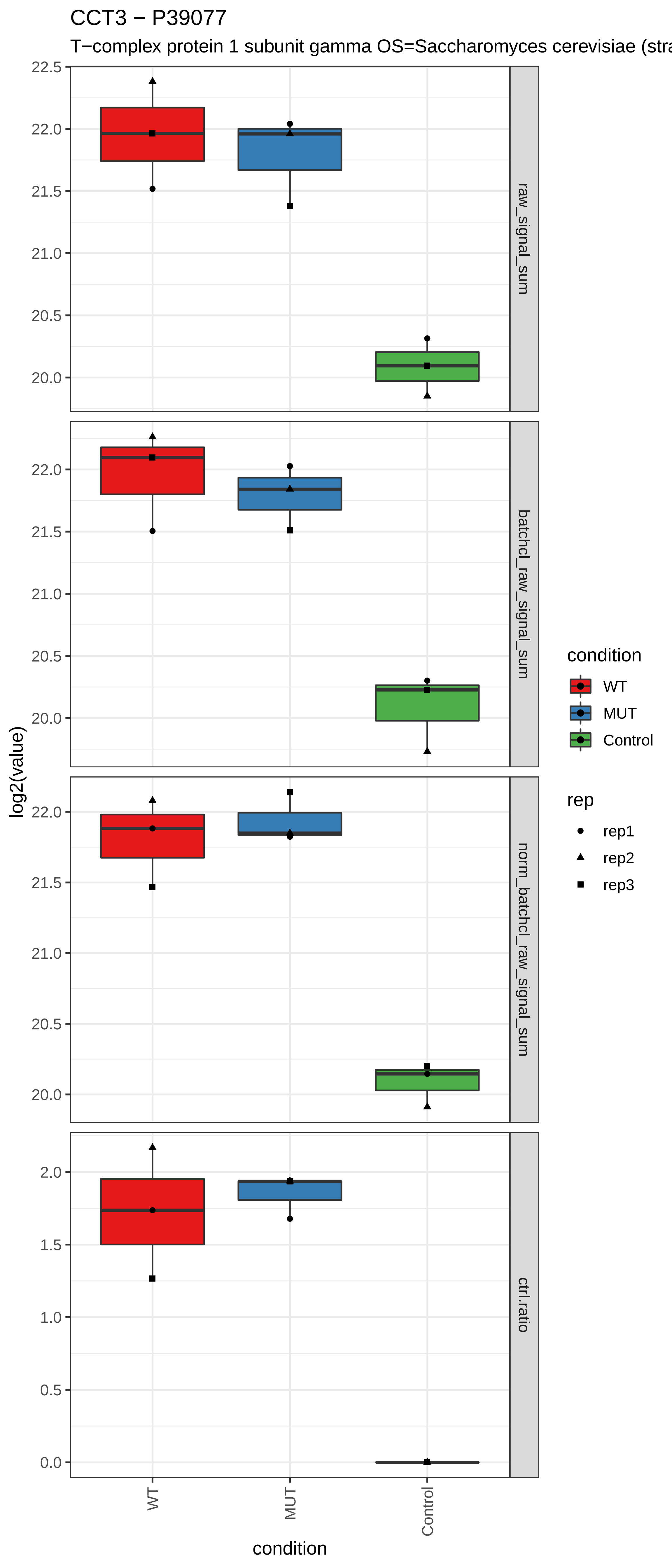

CCT3 − P39077
T−complex protein 1 subunit gamma OS=Saccharomyces cerevisiae (stra
raw_signal_sum
batchcl_raw_signal_sum
norm_batchcl_raw_signal_sum
ctrl.ratio
log2(value)
condition
WT
MUT
Control
rep
rep1
rep2
rep3

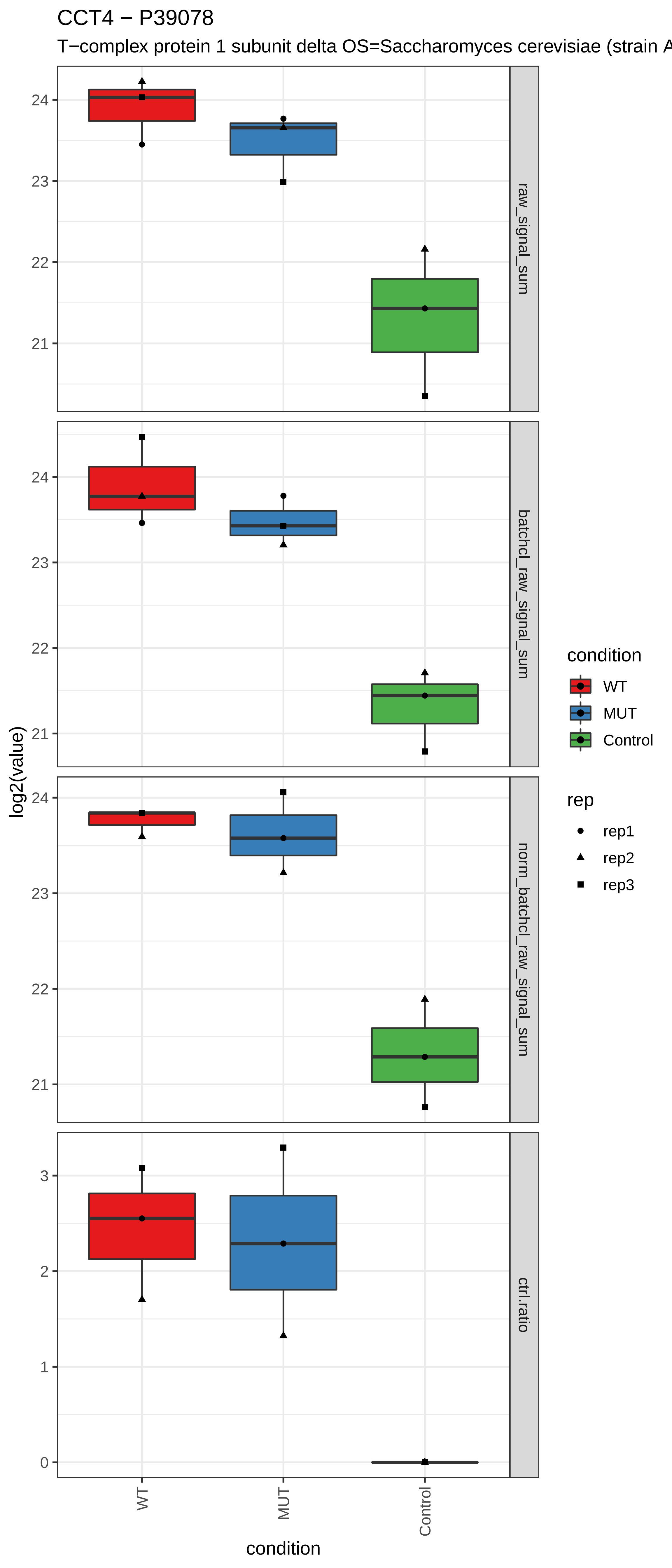
CCT4 − P39078
T−complex protein 1 subunit delta OS=Saccharomyces cerevisiae (strain A
raw_signal_sum
batchcl_raw_signal_sum
norm_batchcl_raw_signal_sum
ctrl.ratio
log2(value)
condition
WT
MUT
Control
rep
rep1
rep2
rep3

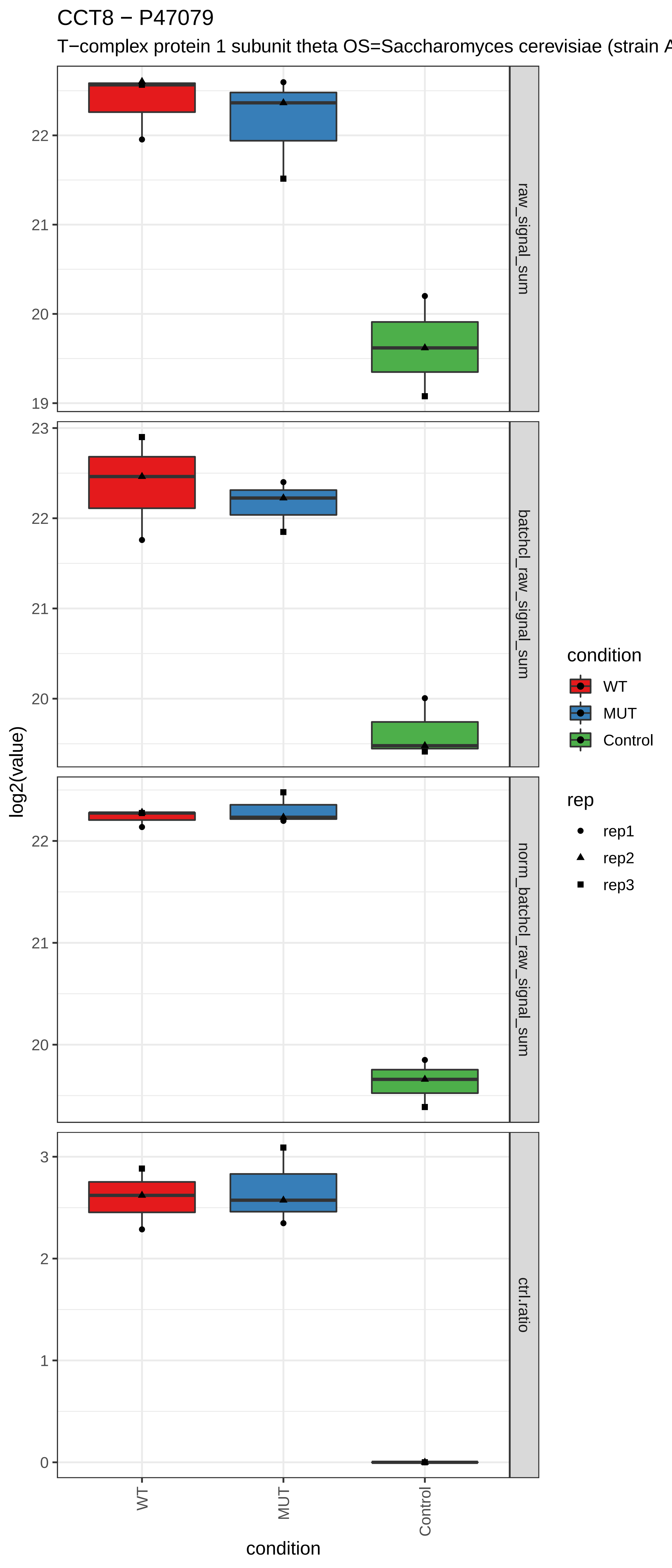
CCT8 − P47079
T−complex protein 1 subunit theta OS=Saccharomyces cerevisiae (strain A
raw_signal_sum
batchcl_raw_signal_sum
norm_batchcl_raw_signal_sum
ctrl.ratio
log2(value)
condition
WT
MUT
Control
rep
rep1
rep2
rep3

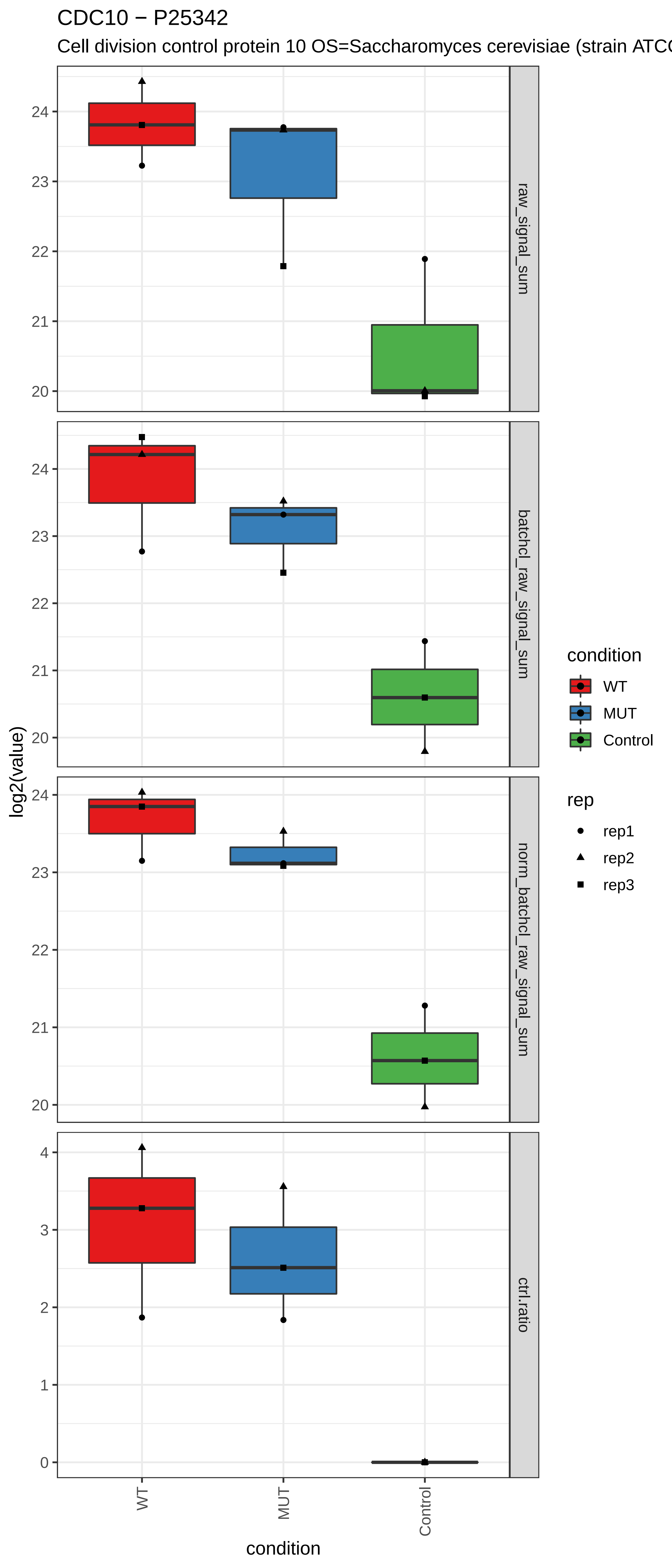

CDC10 − P25342
Cell division control protein 10 OS=Saccharomyces cerevisiae (strain ATCC
raw_signal_sum
batchcl_raw_signal_sum
norm_batchcl_raw_signal_sum
ctrl.ratio
log2(value)
condition
WT
MUT
Control
rep
rep1
rep2
rep3
24
23
22
21
20
4
3
2
1
0

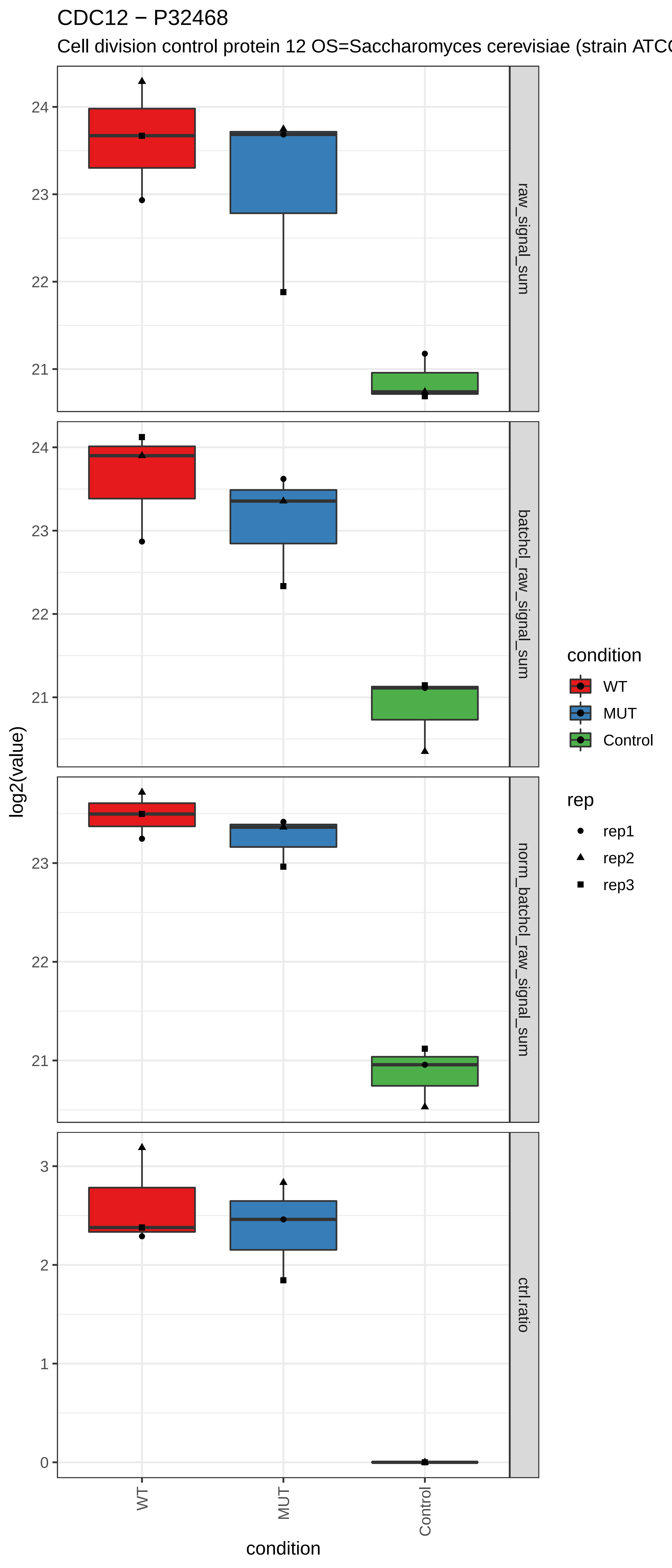
CDC12 − P32468
Cell division control protein 12 OS=Saccharomyces cerevisiae (strain ATCC
raw_signal_sum
batchcl_raw_signal_sum
norm_batchcl_raw_signal_sum
ctrl.ratio
log2(value)
condition
WT
MUT
Control
rep
rep1
rep2
rep3
24
23
22
21
3
2
1
0

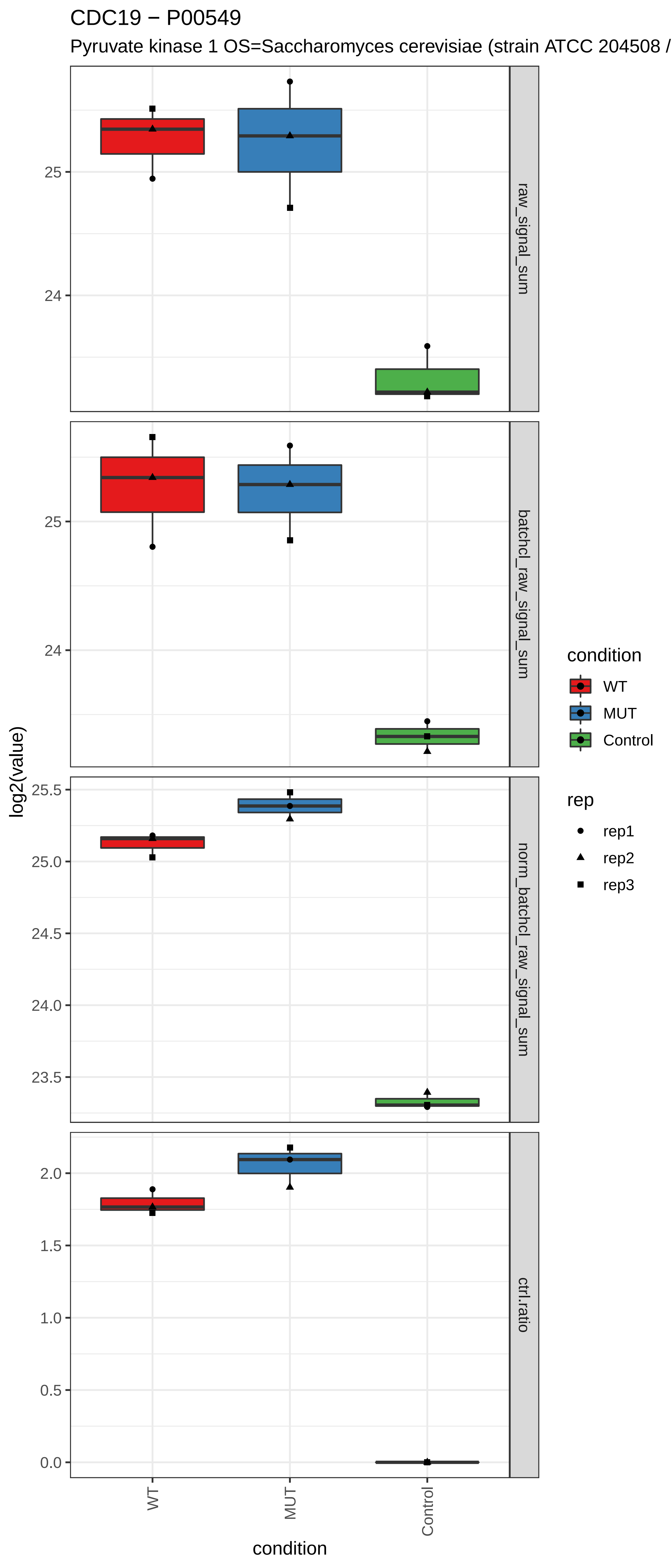

CDC19 − P00549
Pyruvate kinase 1 OS=Saccharomyces cerevisiae (strain ATCC 204508 /
raw_signal_sum
batchcl_raw_signal_sum
norm_batchcl_raw_signal_sum
ctrl.ratio
25
24
25.5
25.0
24.5
24.0
23.5
2.0
1.5
1.0
0.5
0.0
log2(value)
WT
MUT
Control
condition
condition
WT
MUT
Control
rep
rep1
rep2
rep3

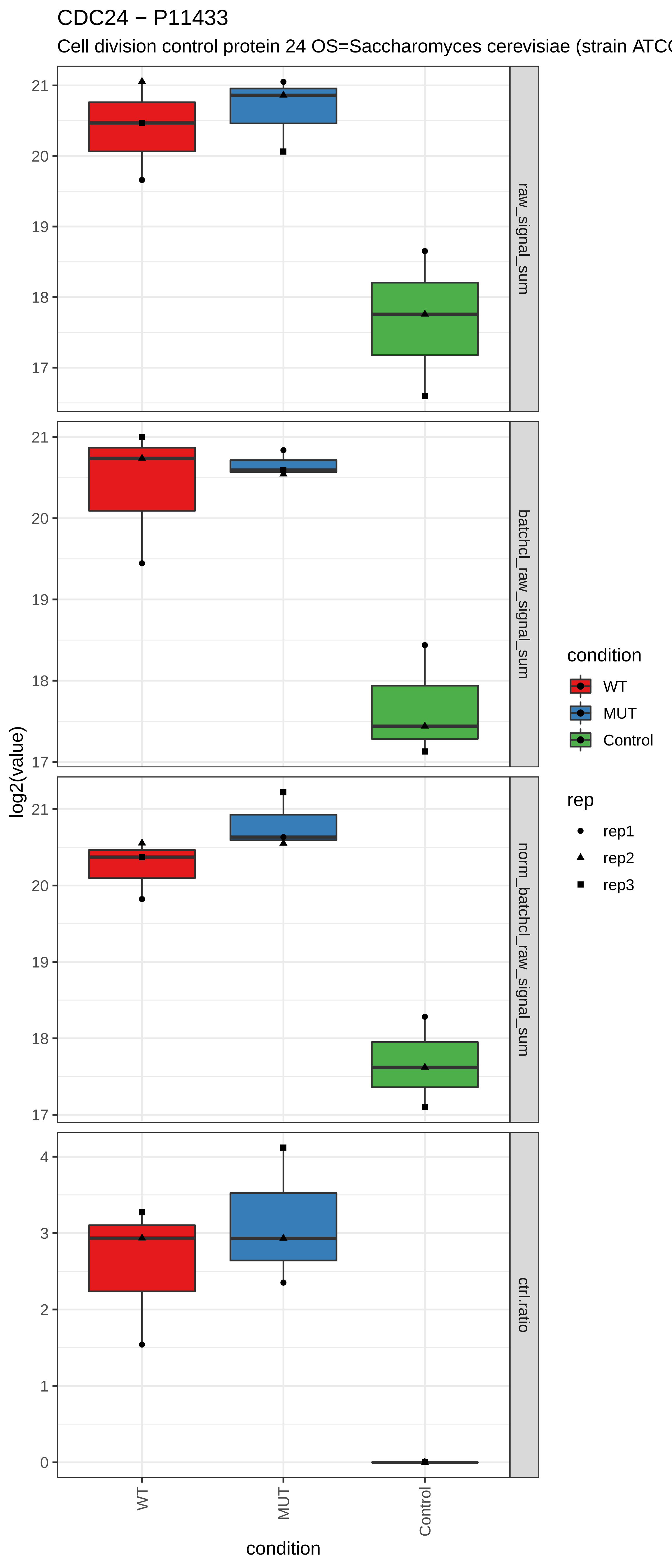
CDC24 − P11433
Cell division control protein 24 OS=Saccharomyces cerevisiae (strain ATCC
raw_signal_sum
batchcl_raw_signal_sum
norm_batchcl_raw_signal_sum
ctrl.ratio
log2(value)
condition
WT
MUT
Control
rep
rep1
rep2
rep3
21
20
19
18
17
4
3
2
1
0

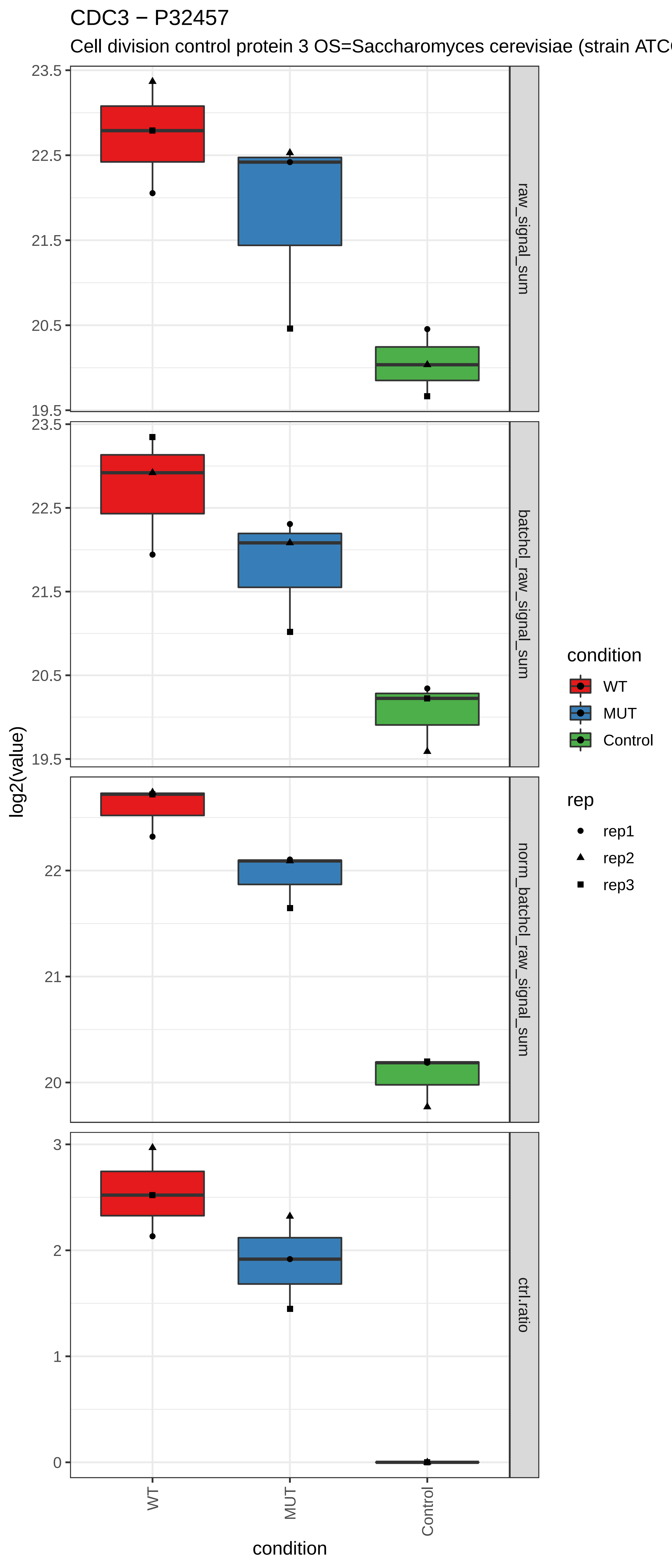

CDC3 − P32457
Cell division control protein 3 OS=Saccharomyces cerevisiae (strain ATC
raw_signal_sum
batchcl_raw_signal_sum
norm_batchcl_raw_signal_sum
ctrl.ratio
log2(value)
condition
WT
MUT
Control
rep
rep1
rep2
rep3

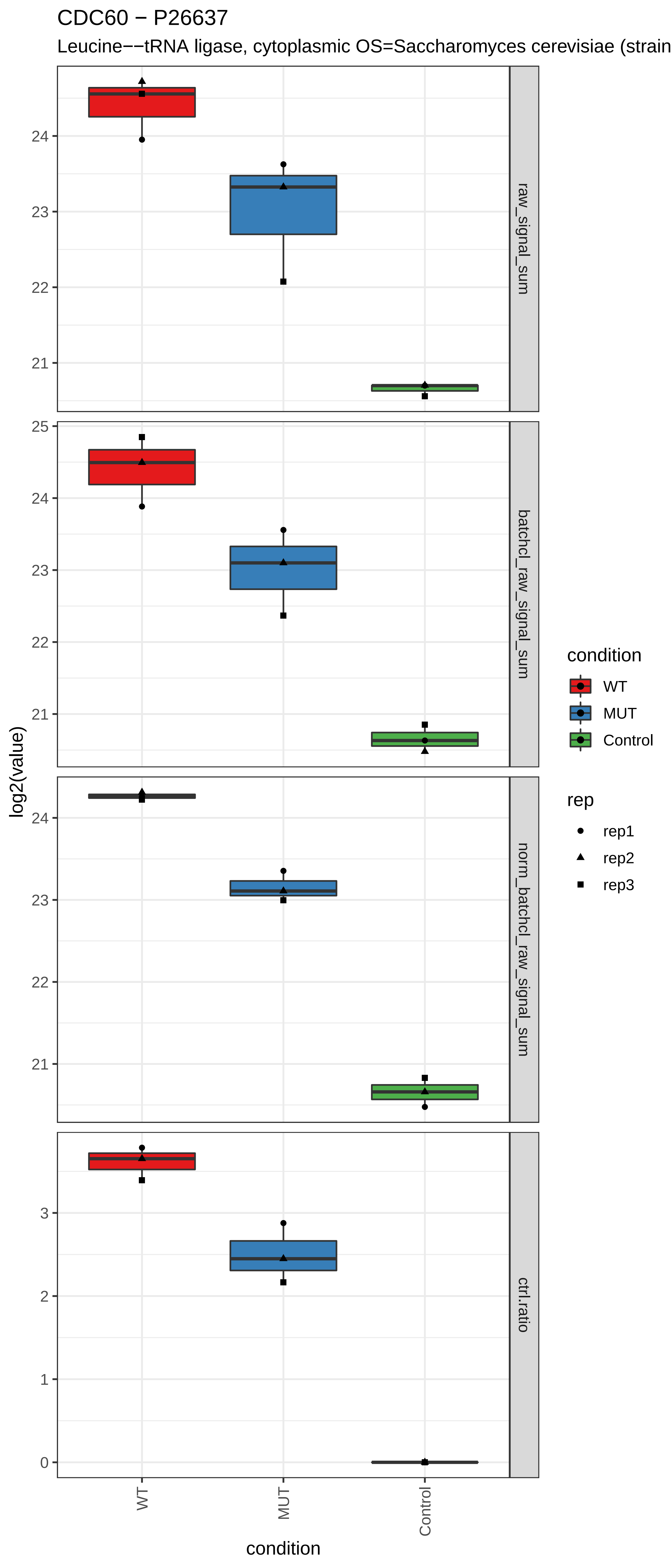
CDC60 − P26637
Leucine−−tRNA ligase, cytoplasmic OS=Saccharomyces cerevisiae (strain
raw_signal_sum
batchcl_raw_signal_sum
norm_batchcl_raw_signal_sum
ctrl.ratio
log2(value)
condition
WT
MUT
Control
condition
WT
MUT
Control
rep
rep1
rep2
rep3

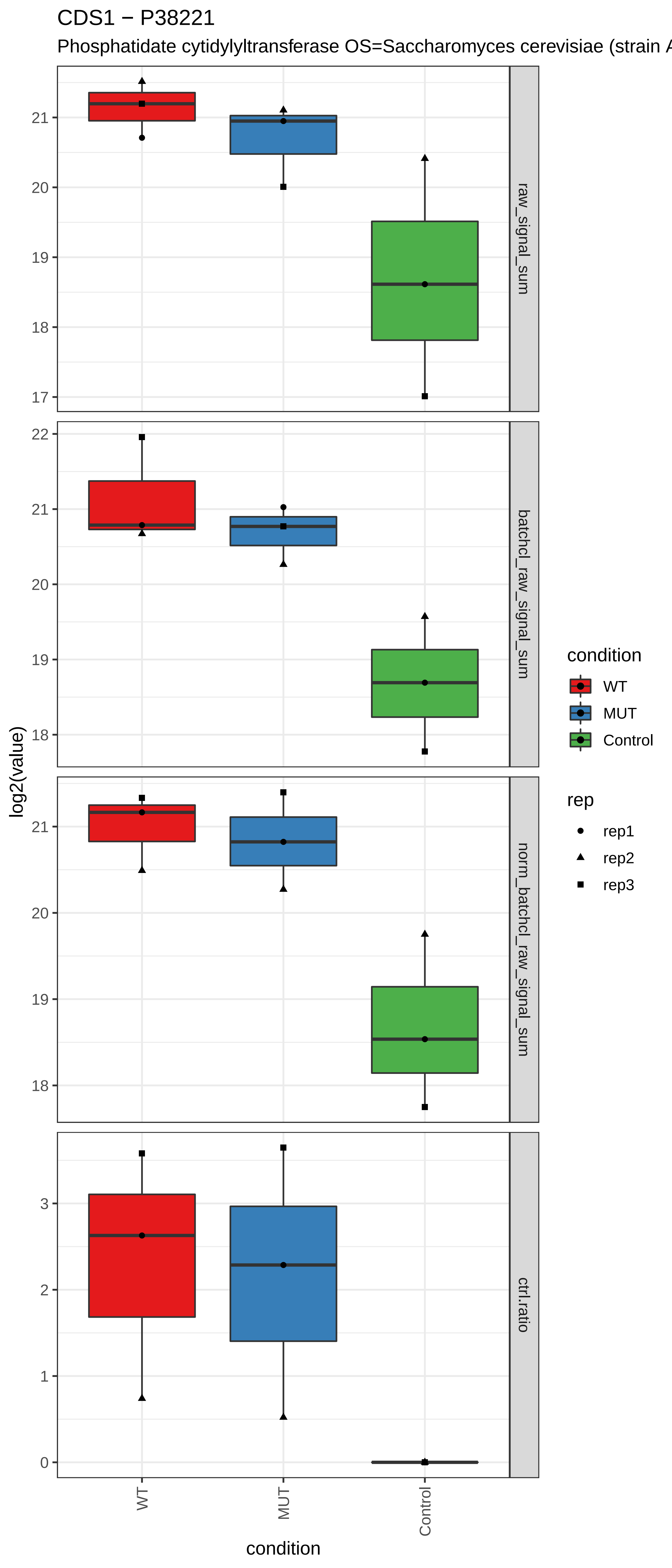

CDS1 − P38221
Phosphatidate cytidylyltransferase OS=Saccharomyces cerevisiae (strain A
raw_signal_sum
batchcl_raw_signal_sum
norm_batchcl_raw_signal_sum
ctrl.ratio
log2(value)
condition
WT
MUT
Control
rep
rep1
rep2
rep3

CFT2 − Q12102

Cleavage factor two protein 2 OS=Saccharomyces cerevisiae (strain ATC

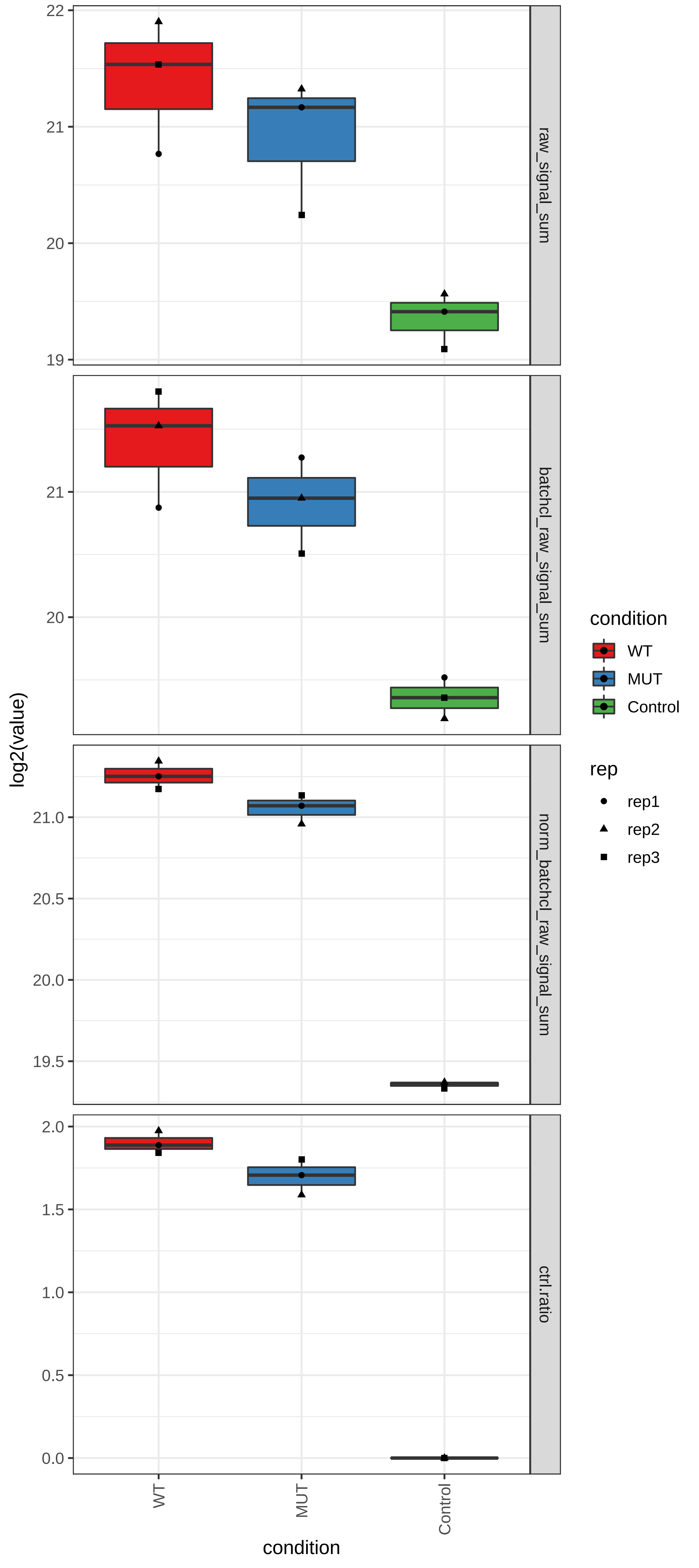

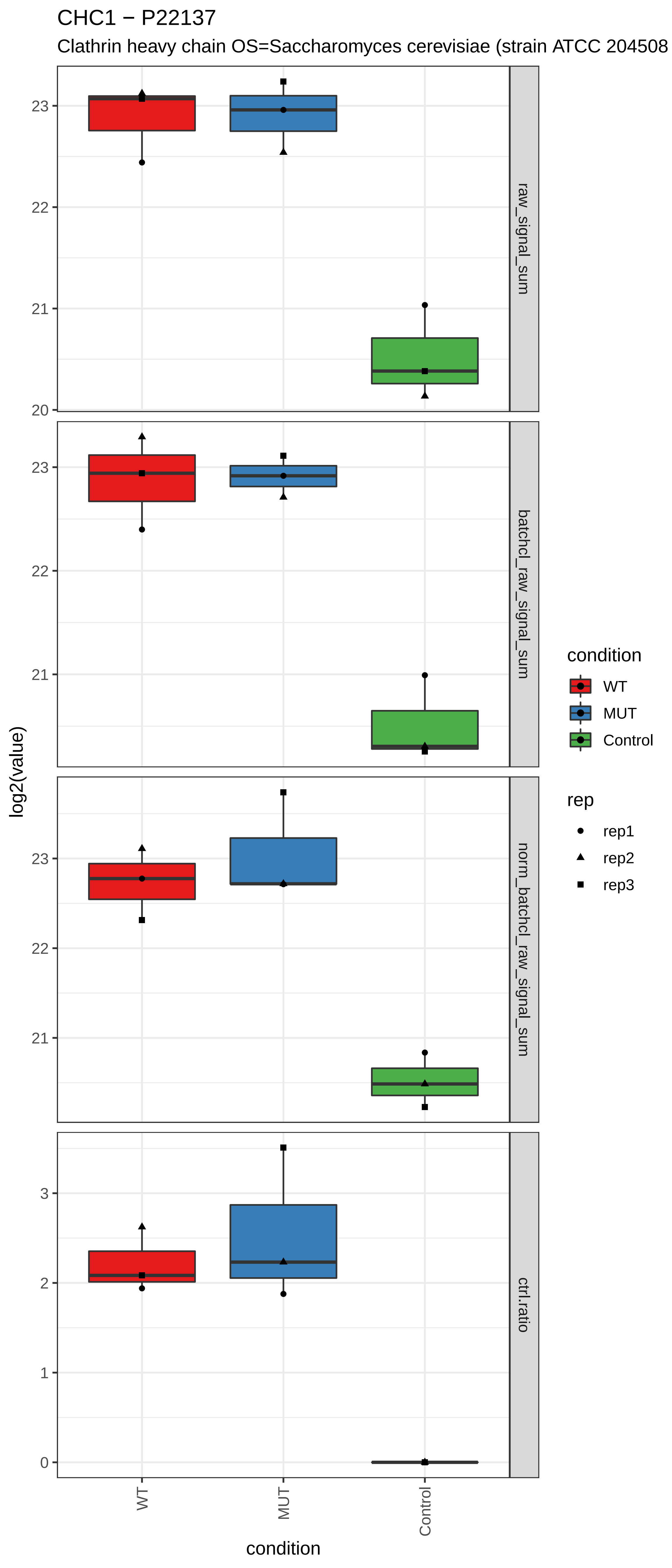
CHC1 − P22137
Clathrin heavy chain OS=Saccharomyces cerevisiae (strain ATCC 204508
raw_signal_sum
batchcl_raw_signal_sum
norm_batchcl_raw_signal_sum
ctrl.ratio
log2(value)
condition
WT
MUT
Control
rep
rep1
rep2
rep3

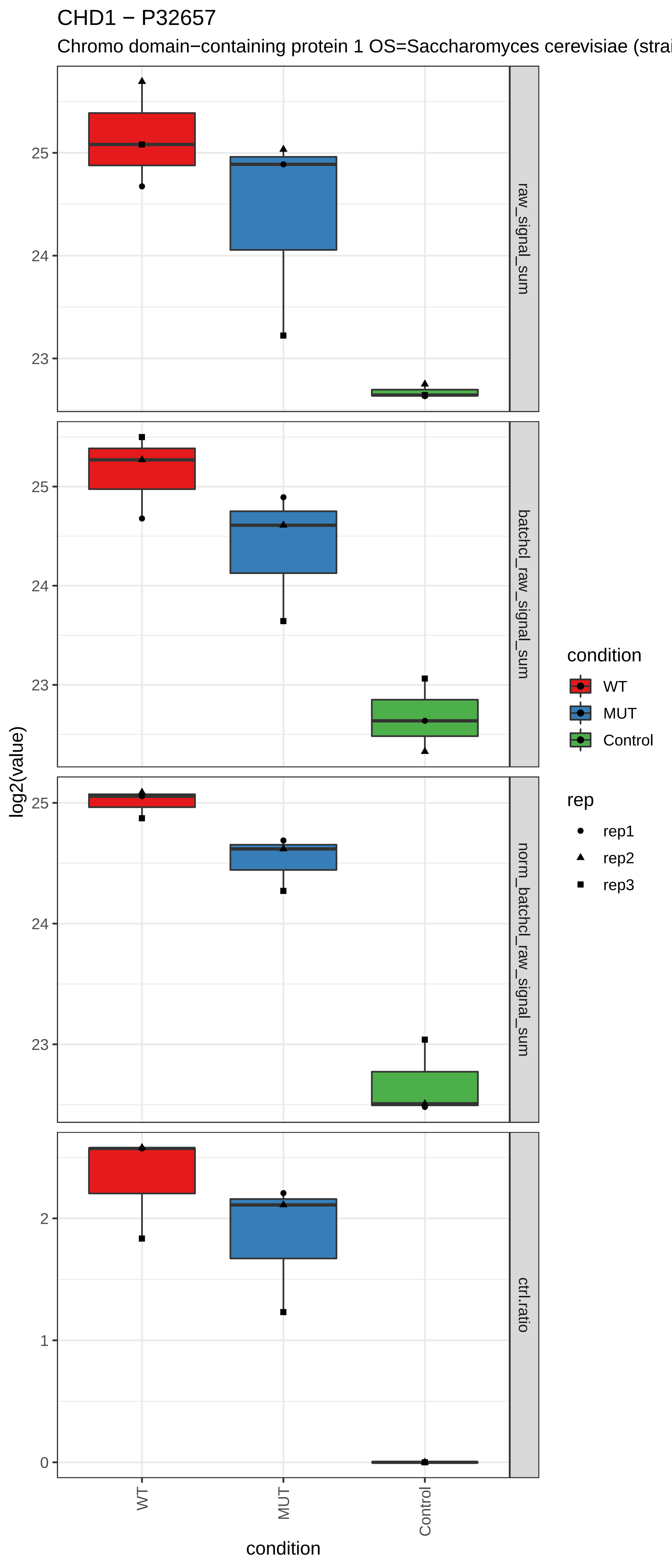
CHD1 − P32657
Chromo domain−containing protein 1 OS=Saccharomyces cerevisiae (strai
raw_signal_sum
batchcl_raw_signal_sum
norm_batchcl_raw_signal_sum
ctrl.ratio
log2(value)
25
24
23
25
24
23
25
24
23
2
1
0
WT
MUT
Control
condition
condition
WT
MUT
Control
rep
rep1
rep2
rep3

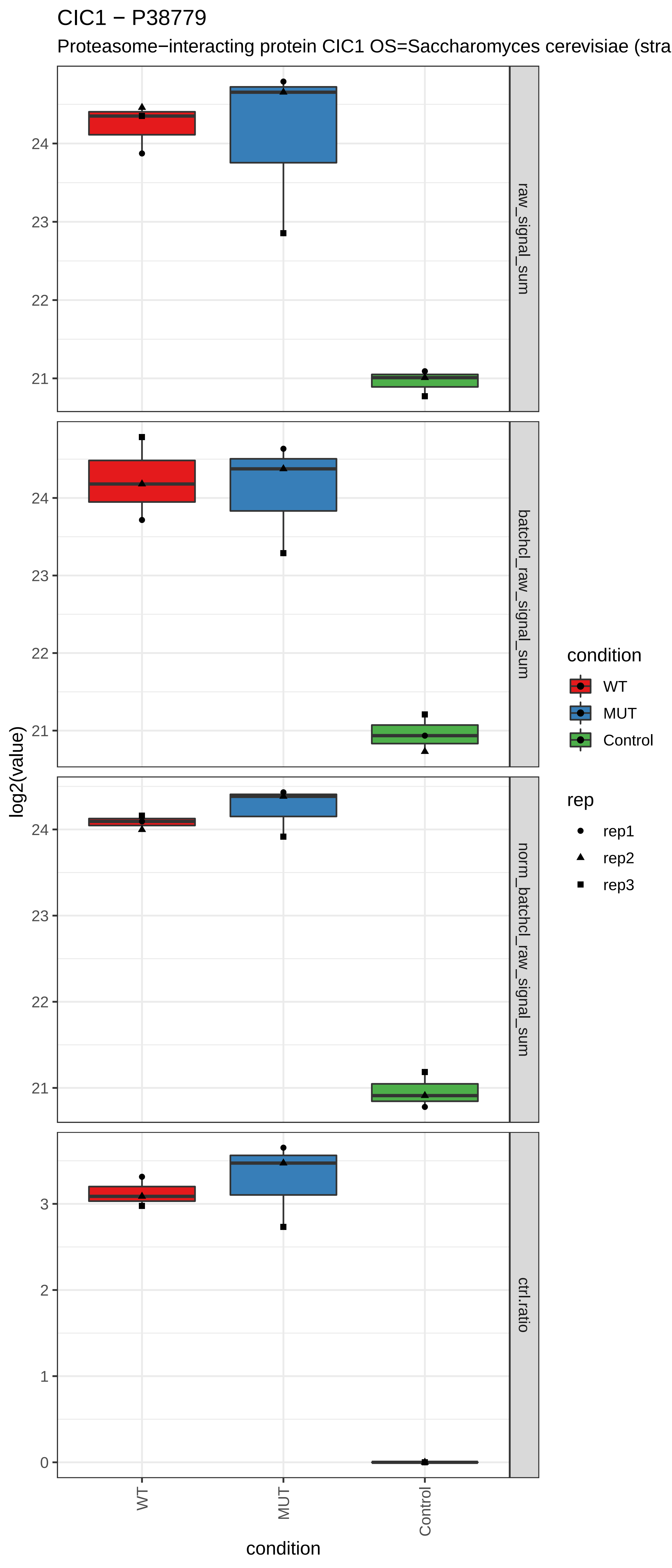

CIC1 − P38779
Proteasome−interacting protein CIC1 OS=Saccharomyces cerevisiae (stra
raw_signal_sum
batchcl_raw_signal_sum
norm_batchcl_raw_signal_sum
ctrl.ratio
log2(value)
condition
WT
MUT
Control
rep
rep1
rep2
rep3

CIT1 − P00890

Citrate synthase, mitochondrial OS=Saccharomyces cerevisiae (strain ATC

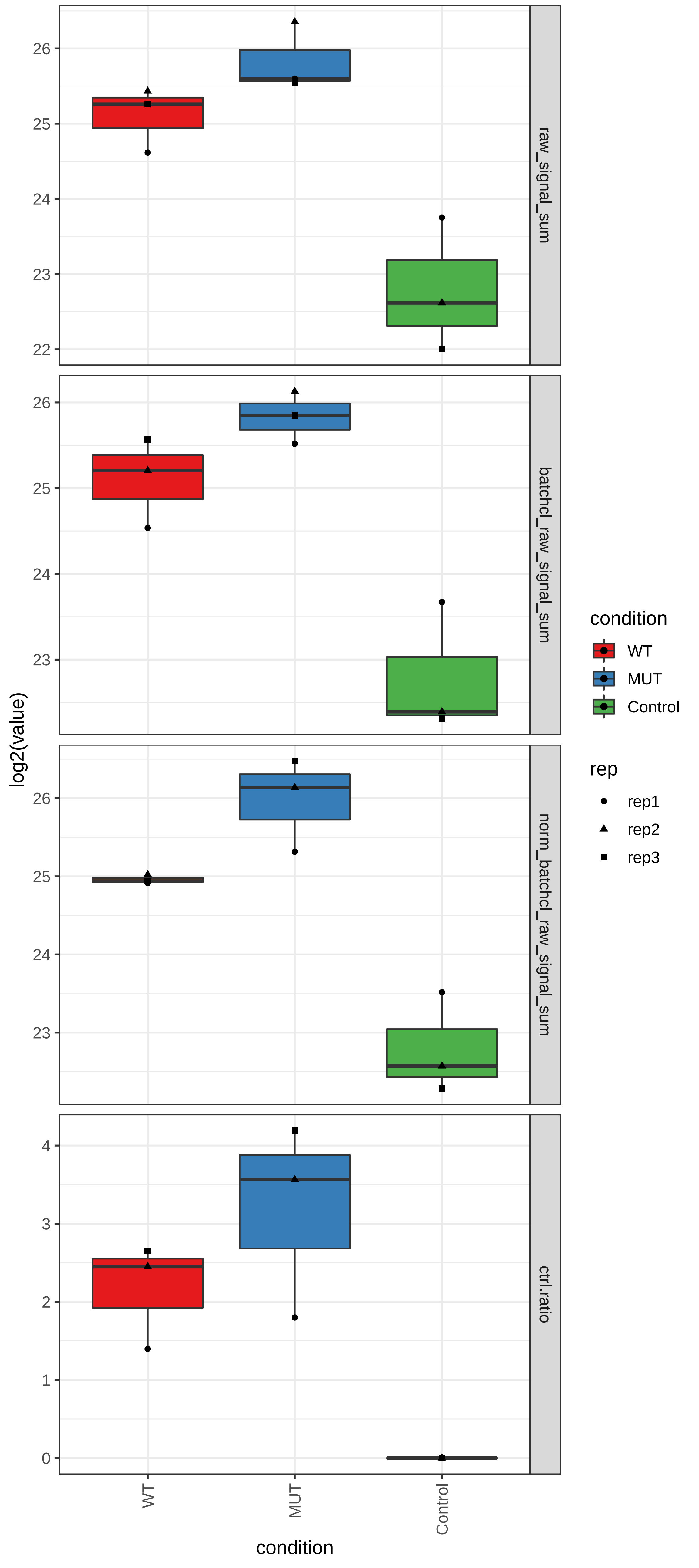

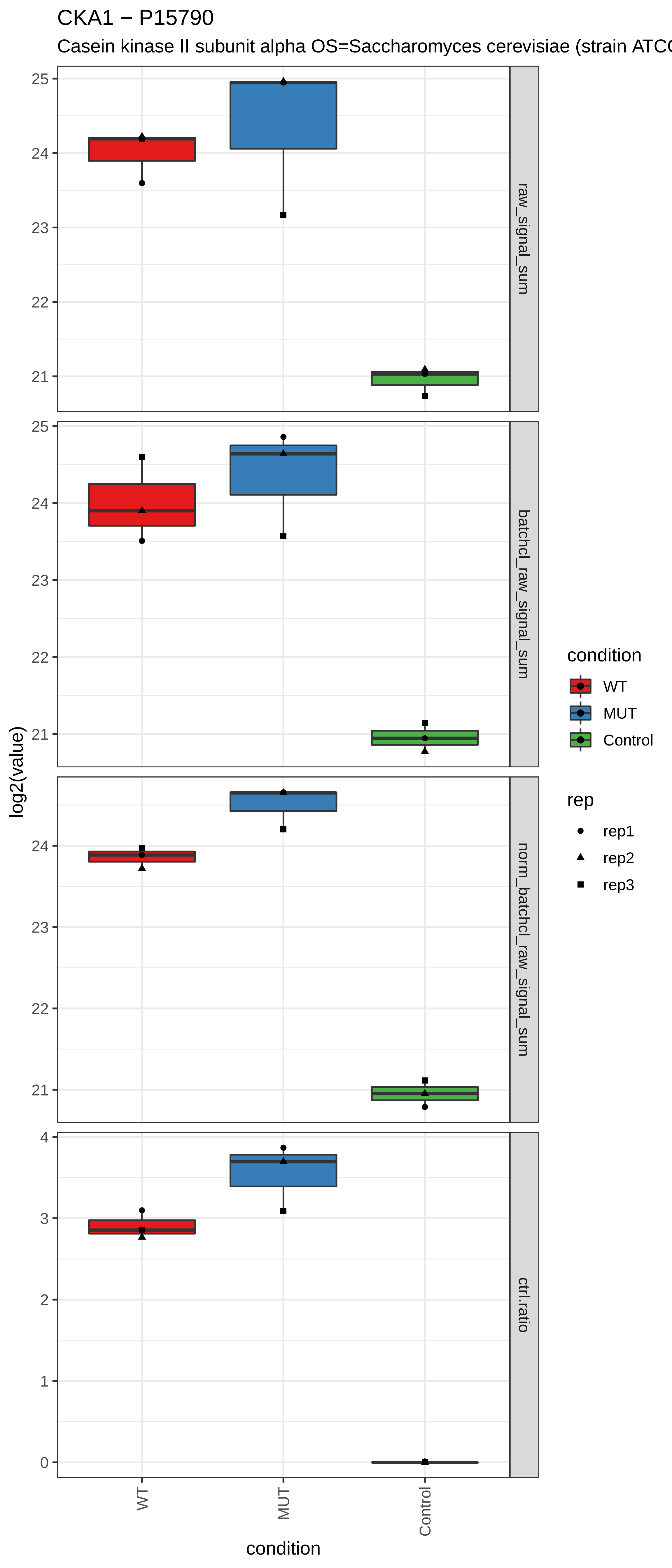

CKA1 − P15790
Casein kinase II subunit alpha OS=Saccharomyces cerevisiae (strain ATCC
raw_signal_sum
batchcl_raw_signal_sum
norm_batchcl_raw_signal_sum
ctrl.ratio
log2(value)
condition
WT
MUT
Control
rep
rep1
rep2
rep3

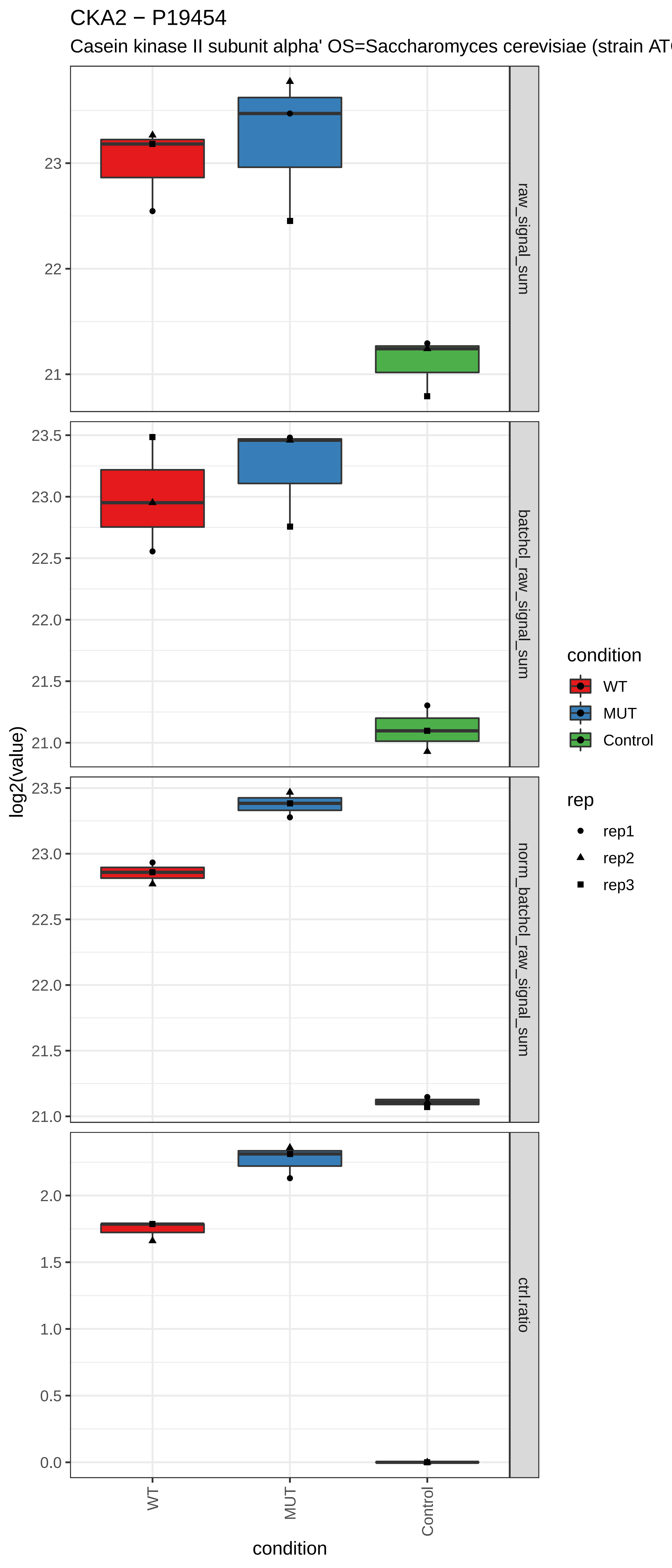
CKA2 − P19454
Casein kinase II subunit alpha' OS=Saccharomyces cerevisiae (strain ATC
raw_signal_sum
batchcl_raw_signal_sum
norm_batchcl_raw_signal_sum
ctrl.ratio
log2(value)
condition
WT
MUT
Control
rep
rep1
rep2
rep3
23
22
21
23.5
23.0
22.5
22.0
21.5
21.0
2.0
1.5
1.0
0.5
0.0

CLU1 − Q03690

Clustered mitochondria protein 1 OS=Saccharomyces cerevisiae (strain AT

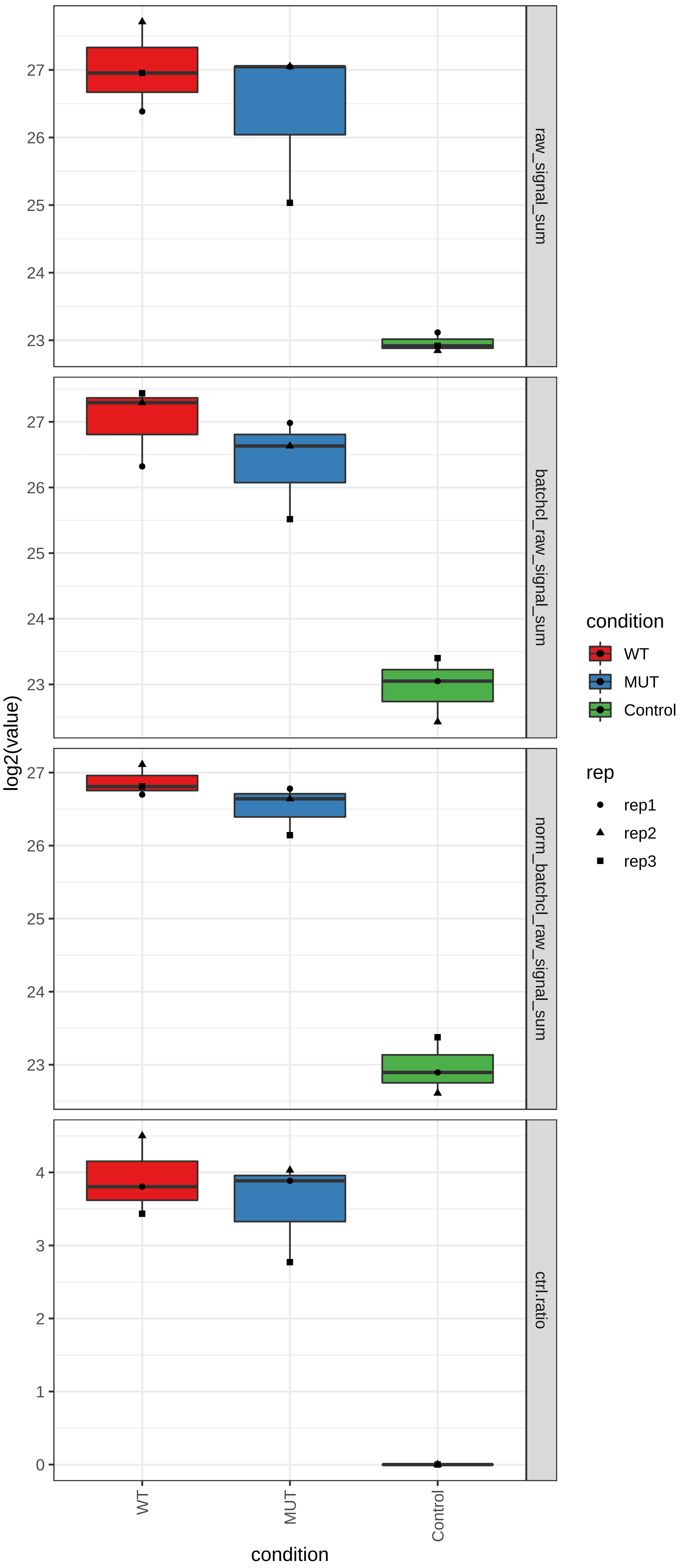

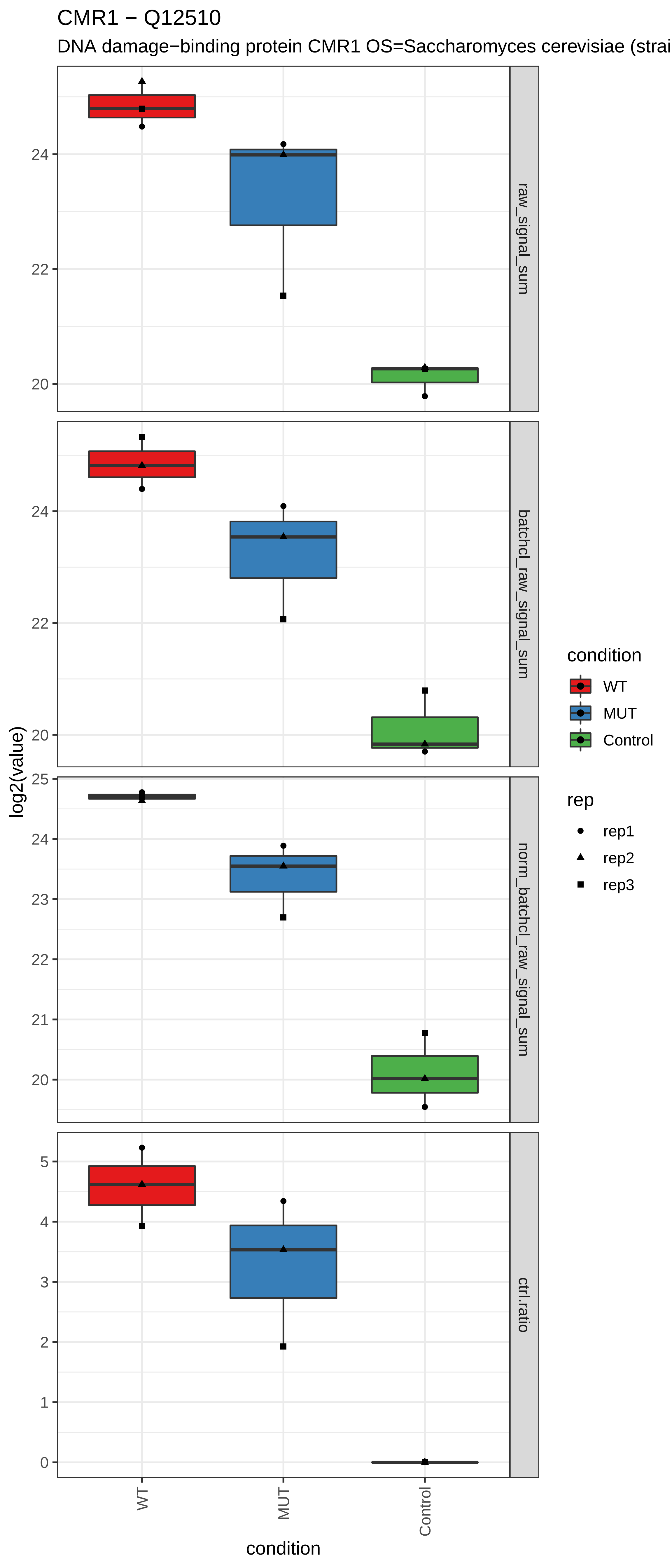

CMR1 − Q12510
DNA damage−binding protein CMR1 OS=Saccharomyces cerevisiae (strai
raw_signal_sum
batchcl_raw_signal_sum
norm_batchcl_raw_signal_sum
ctrl.ratio
log2(value)
condition
WT
MUT
Control
rep
rep1
rep2
rep3

CNM67 − P53865

Chaotic nuclear migration protein 67 OS=Saccharomyces cerevisiae (strain

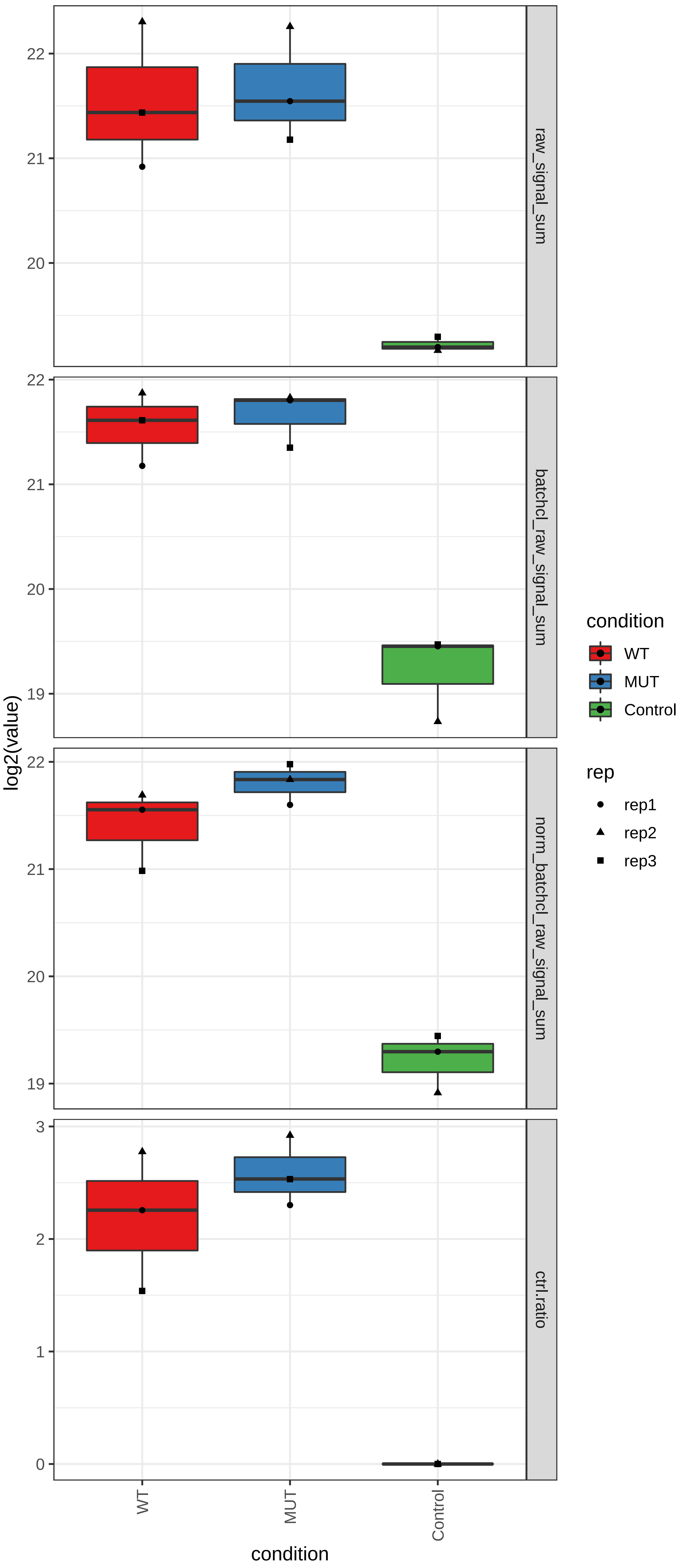

COP1 − P53622

Coatomer subunit alpha OS=Saccharomyces cerevisiae (strain ATCC 204

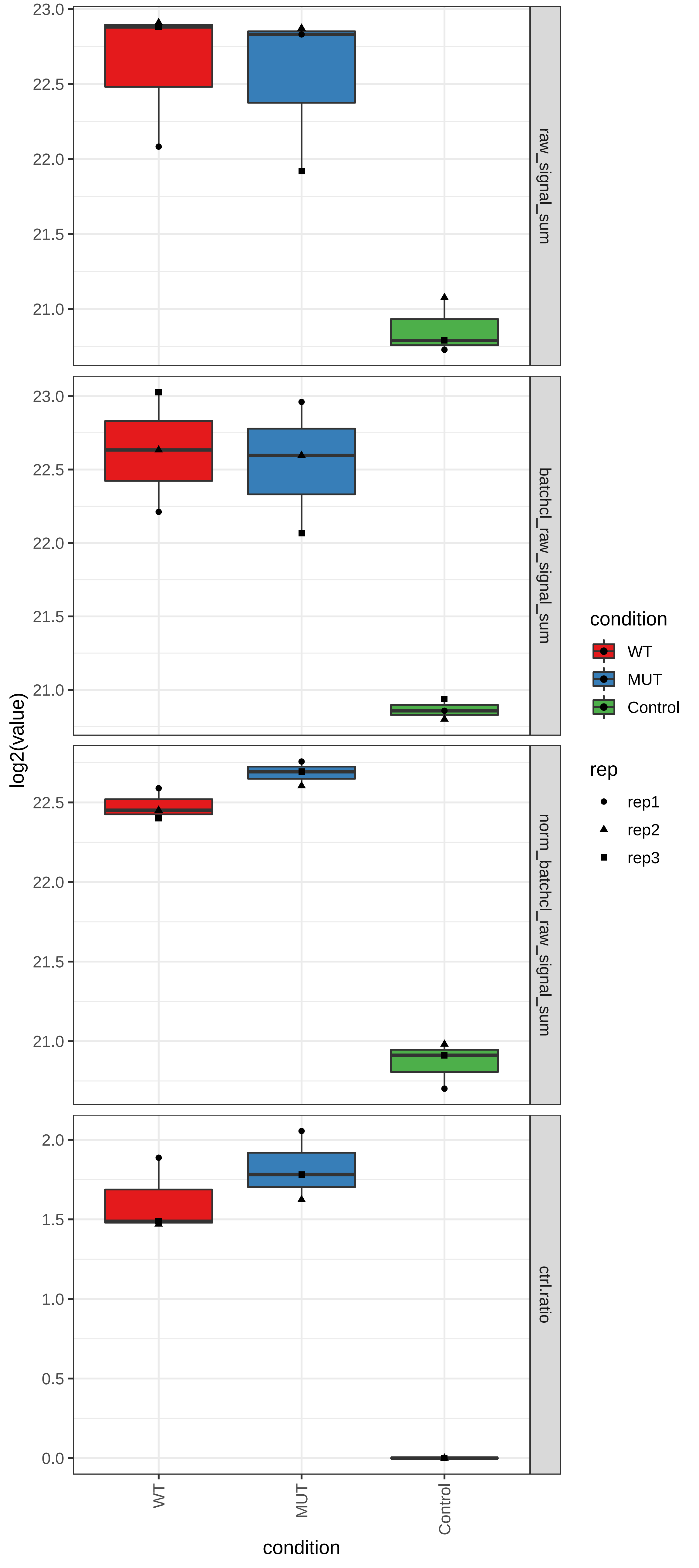

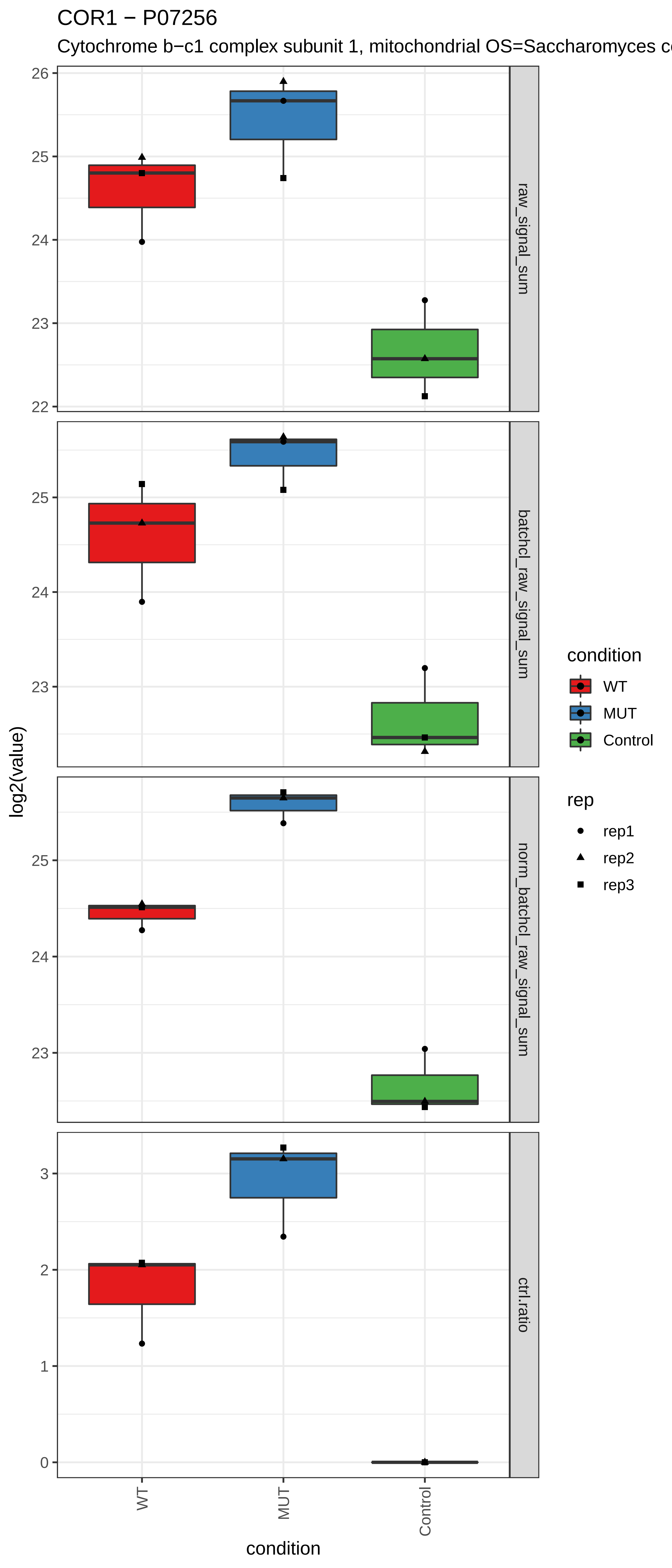
COR1 − P07256
Cytochrome b−c1 complex subunit 1, mitochondrial OS=Saccharomyces c
raw_signal_sum
batchcl_raw_signal_sum
norm_batchcl_raw_signal_sum
ctrl.ratio
log2(value)
condition
WT
MUT
Control
rep
rep1
rep2
rep3

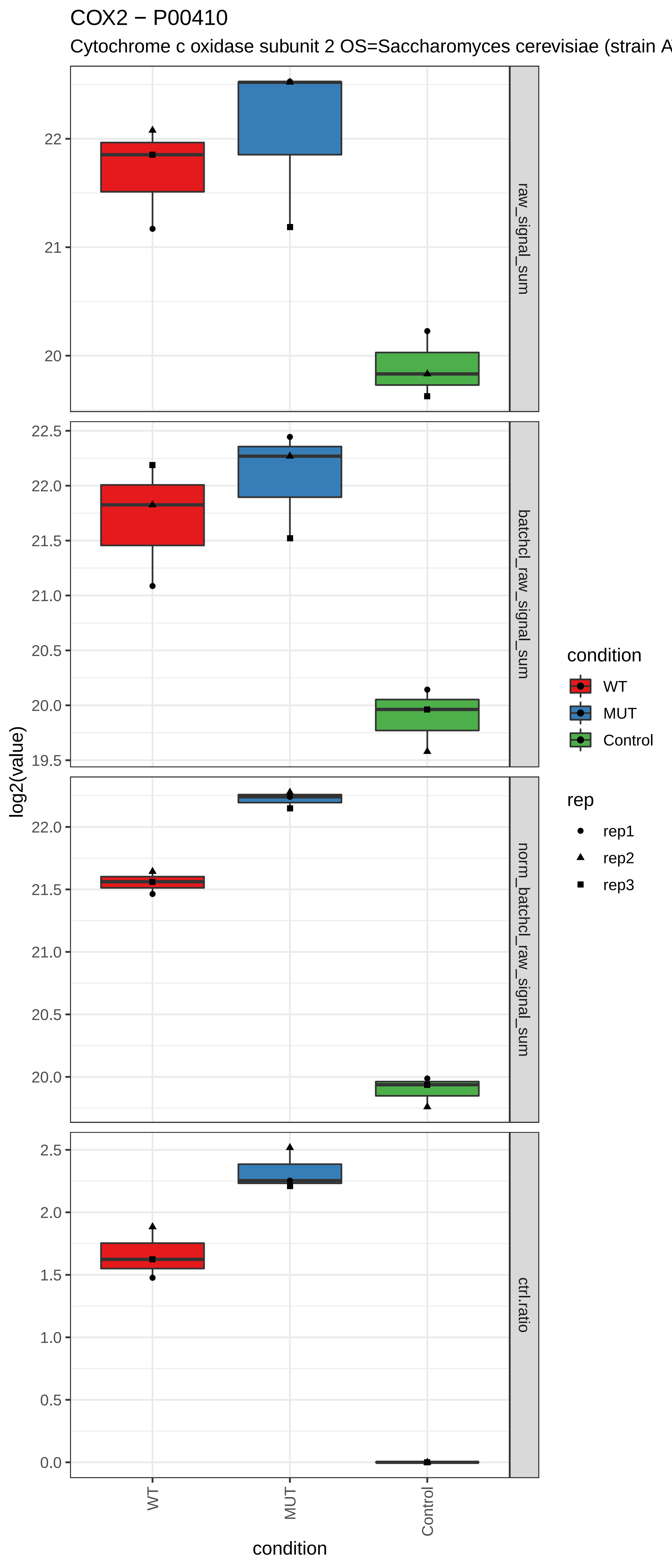

COX2 − P00410
Cytochrome c oxidase subunit 2 OS=Saccharomyces cerevisiae (strain A
raw_signal_sum
batchcl_raw_signal_sum
norm_batchcl_raw_signal_sum
ctrl.ratio
log2(value)
condition
WT
MUT
Control
rep
rep1
rep2
rep3

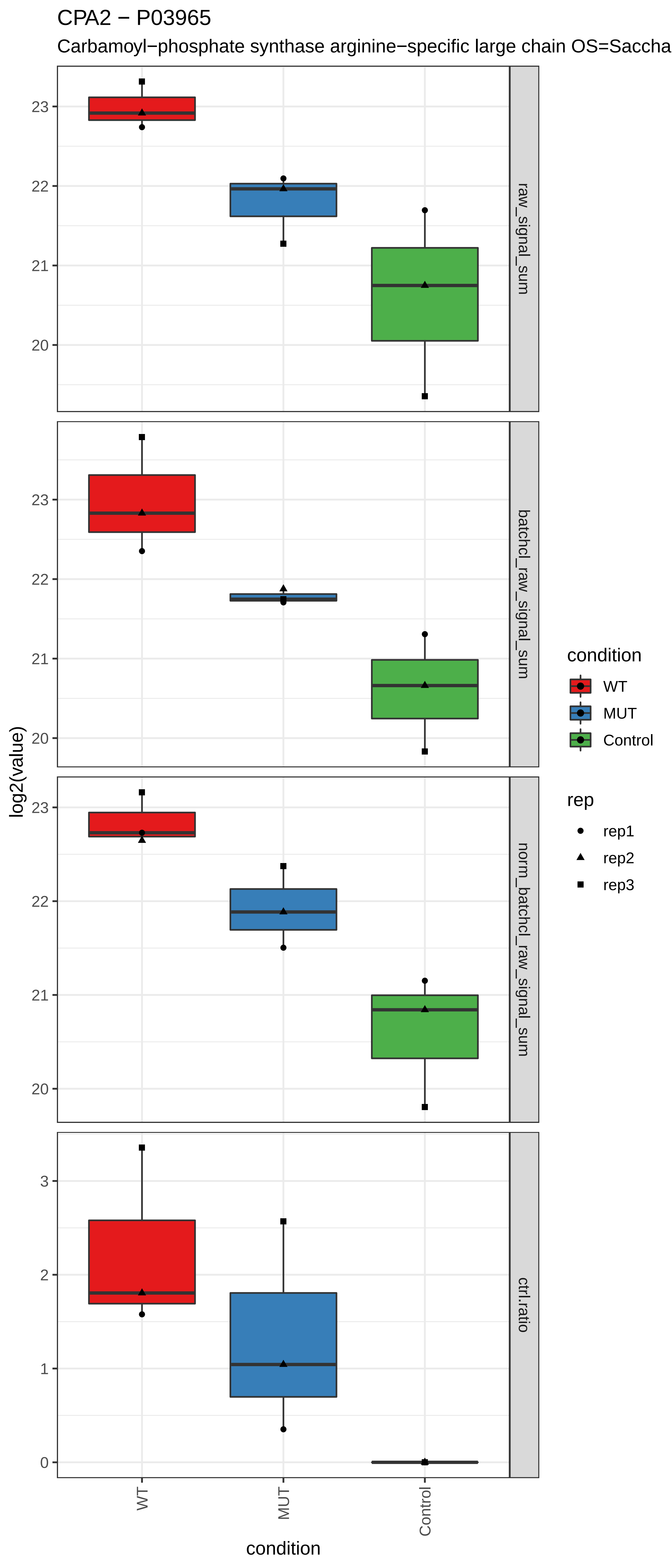
CPA2 − P03965
Carbamoyl−phosphate synthase arginine−specific large chain OS=Saccha
raw_signal_sum
batchcl_raw_signal_sum
norm_batchcl_raw_signal_sum
ctrl.ratio
log2(value)
condition
WT
MUT
Control
rep
rep1
rep2
rep3

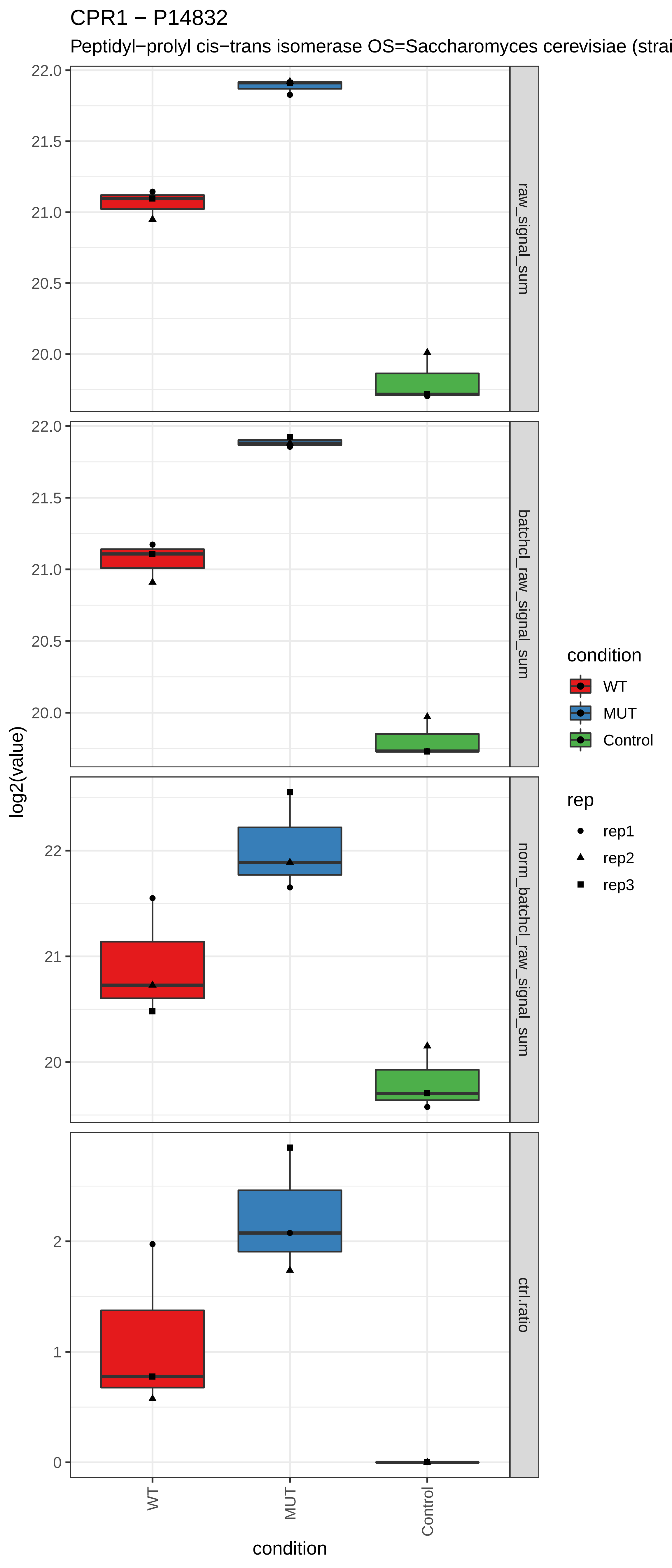

CPR1 − P14832
Peptidyl−prolyl cis−trans isomerase OS=Saccharomyces cerevisiae (strai
raw_signal_sum
batchcl_raw_signal_sum
norm_batchcl_raw_signal_sum
ctrl.ratio
log2(value)
condition
WT
MUT
Control
rep
rep1
rep2
rep3

CPS1 − P27614

Carboxypeptidase S OS=Saccharomyces cerevisiae (strain ATCC 204508

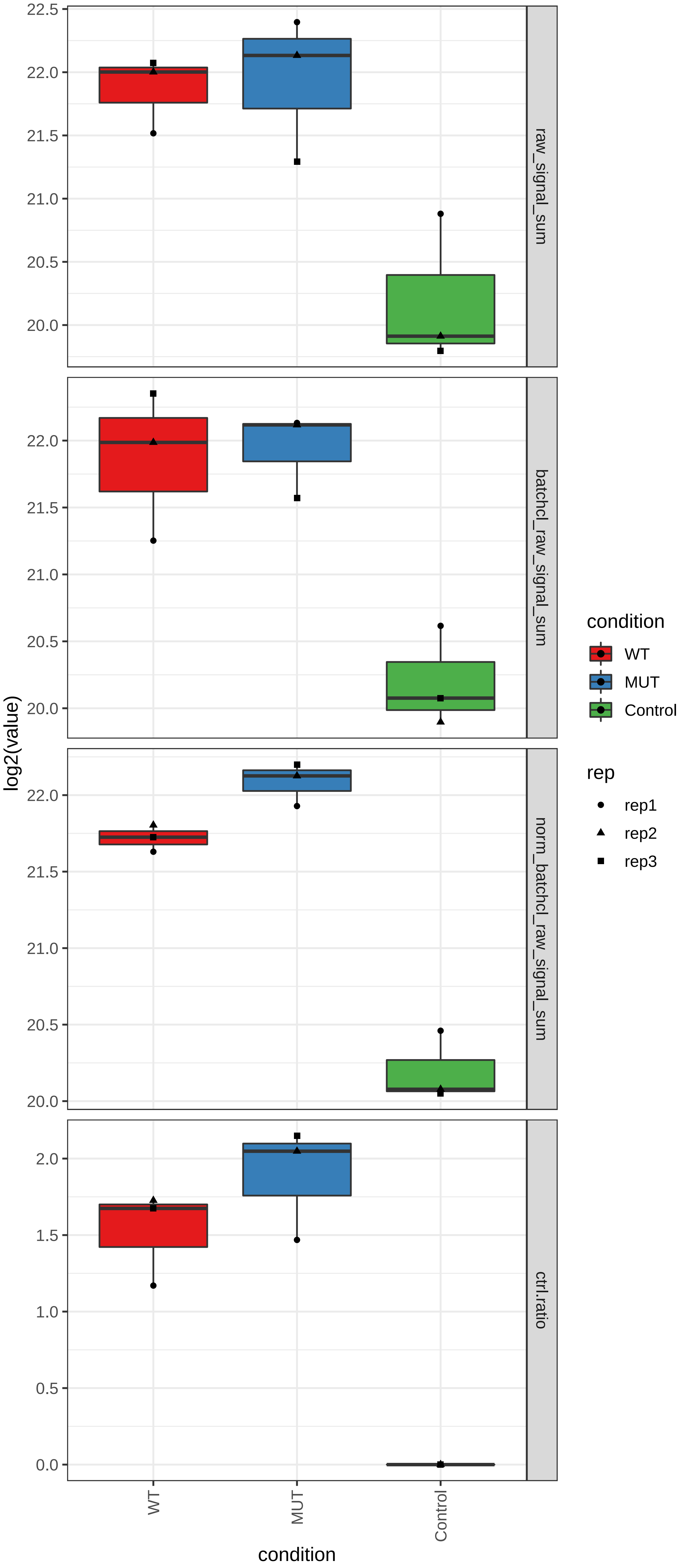

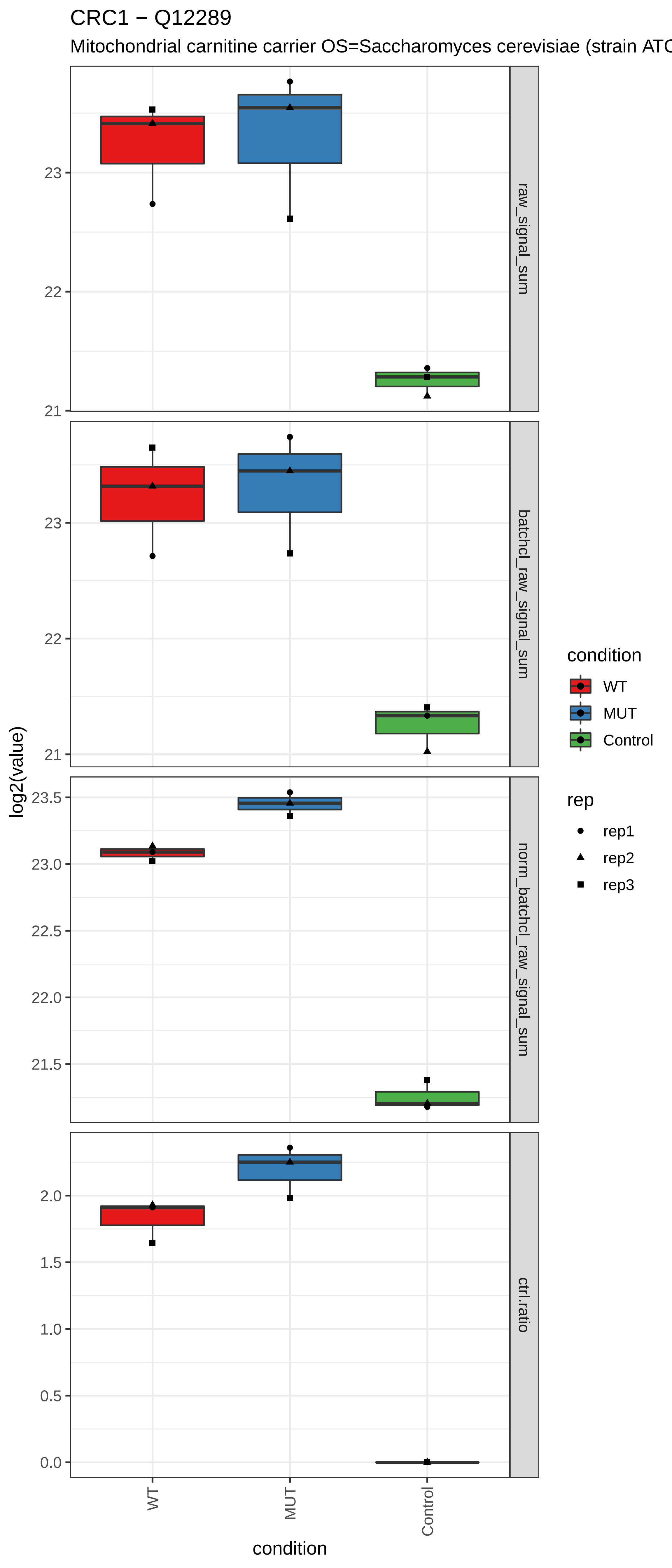

CRC1 − Q12289
Mitochondrial carnitine carrier OS=Saccharomyces cerevisiae (strain ATC
raw_signal_sum
batchcl_raw_signal_sum
norm_batchcl_raw_signal_sum
ctrl.ratio
log2(value)
condition
WT
MUT
Control
rep
rep1
rep2
rep3
23
22
21
23.5
23.0
22.5
22.0
21.5
2.0
1.5
1.0
0.5
0.0

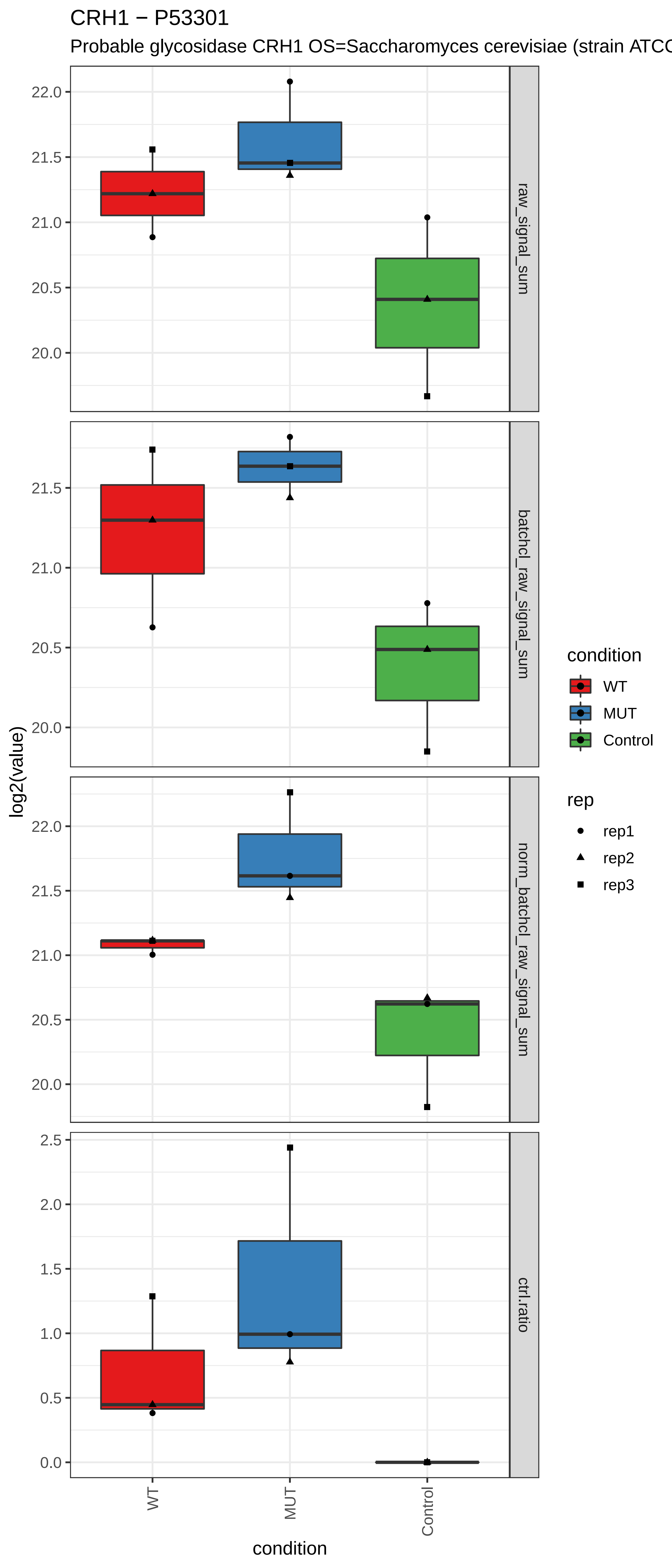
CRH1 − P53301
Probable glycosidase CRH1 OS=Saccharomyces cerevisiae (strain ATCC
raw_signal_sum
batchcl_raw_signal_sum
norm_batchcl_raw_signal_sum
ctrl.ratio
log2(value)
condition
WT
MUT
Control
rep
rep1
rep2
rep3
22.0
21.5
21.0
20.5
20.0
2.5
2.0
1.5
1.0
0.5
0.0

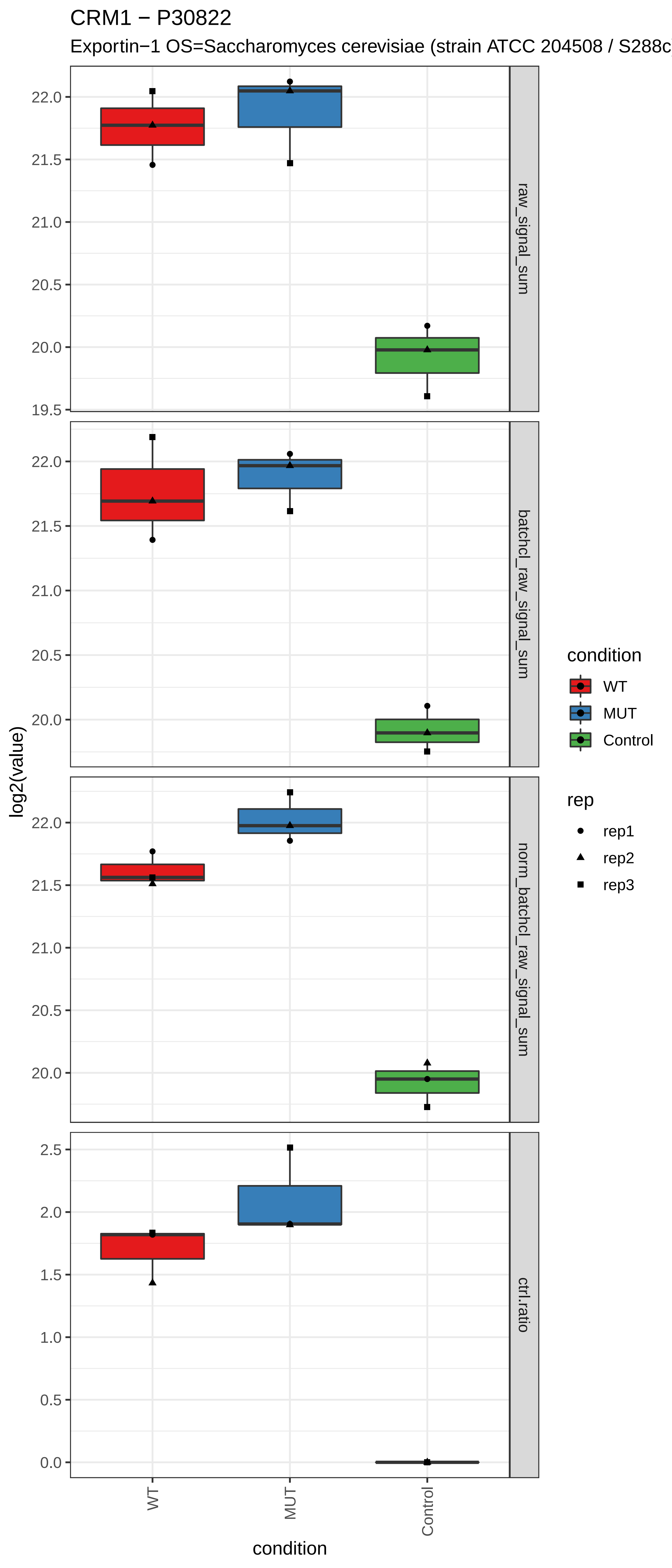

CRM1 − P30822
Exportin−1 OS=Saccharomyces cerevisiae (strain ATCC 204508 / S288c)
raw_signal_sum
batchcl_raw_signal_sum
norm_batchcl_raw_signal_sum
ctrl.ratio
log2(value)
condition
WT
MUT
Control
rep
rep1
rep2
rep3

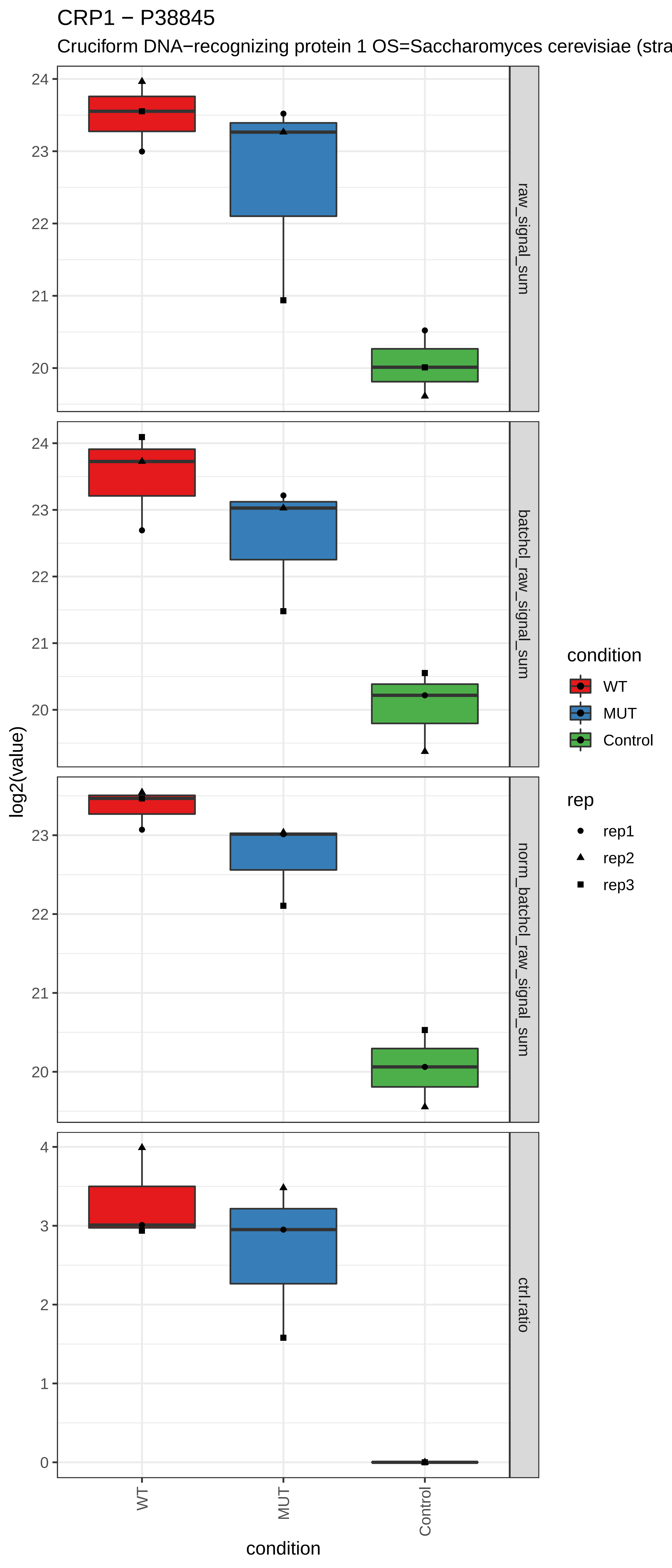
CRP1 − P38845
Cruciform DNA−recognizing protein 1 OS=Saccharomyces cerevisiae (stra
raw_signal_sum
batchcl_raw_signal_sum
norm_batchcl_raw_signal_sum
ctrl.ratio
log2(value)
condition
WT
MUT
Control
rep
rep1
rep2
rep3
24
23
22
21
20
4
3
2
1
0

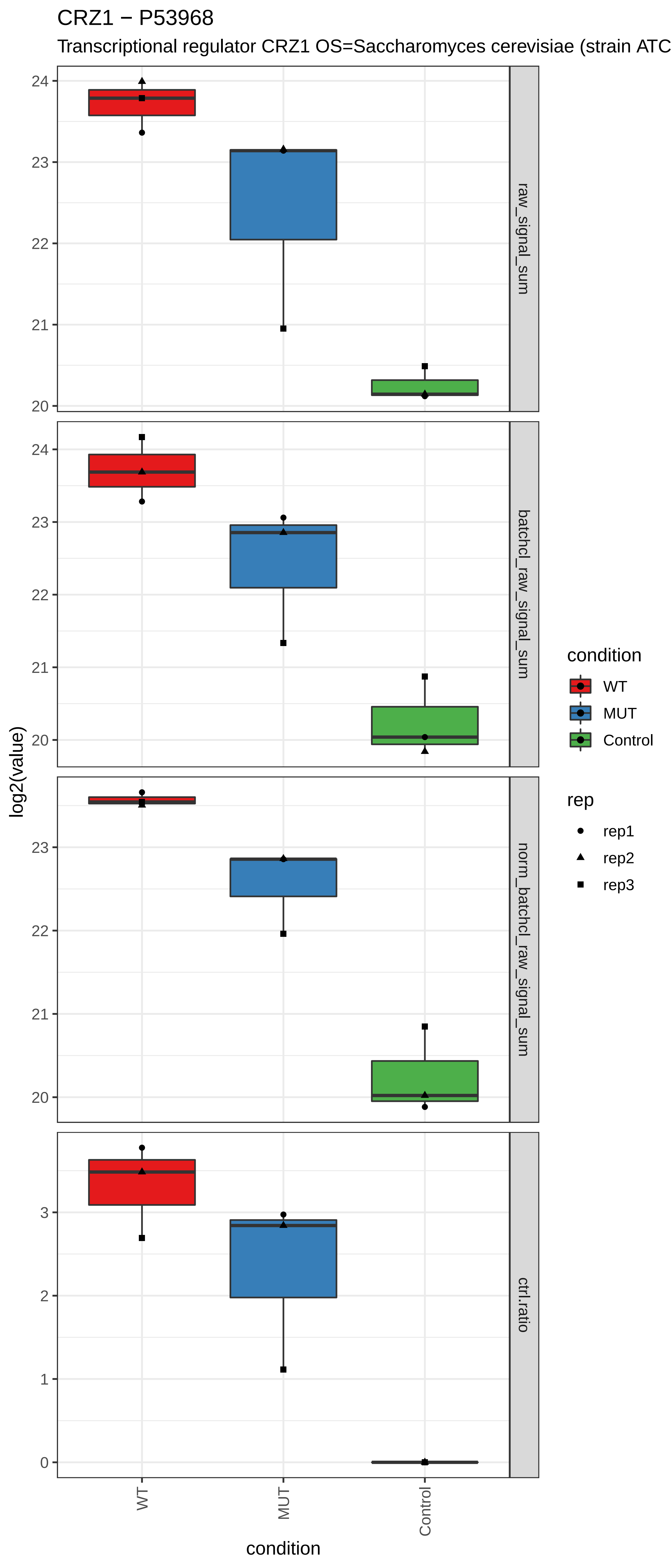
CRZ1 − P53968
Transcriptional regulator CRZ1 OS=Saccharomyces cerevisiae (strain ATC
raw_signal_sum
batchcl_raw_signal_sum
norm_batchcl_raw_signal_sum
ctrl.ratio
log2(value)
condition
WT
MUT
Control
rep
rep1
rep2
rep3

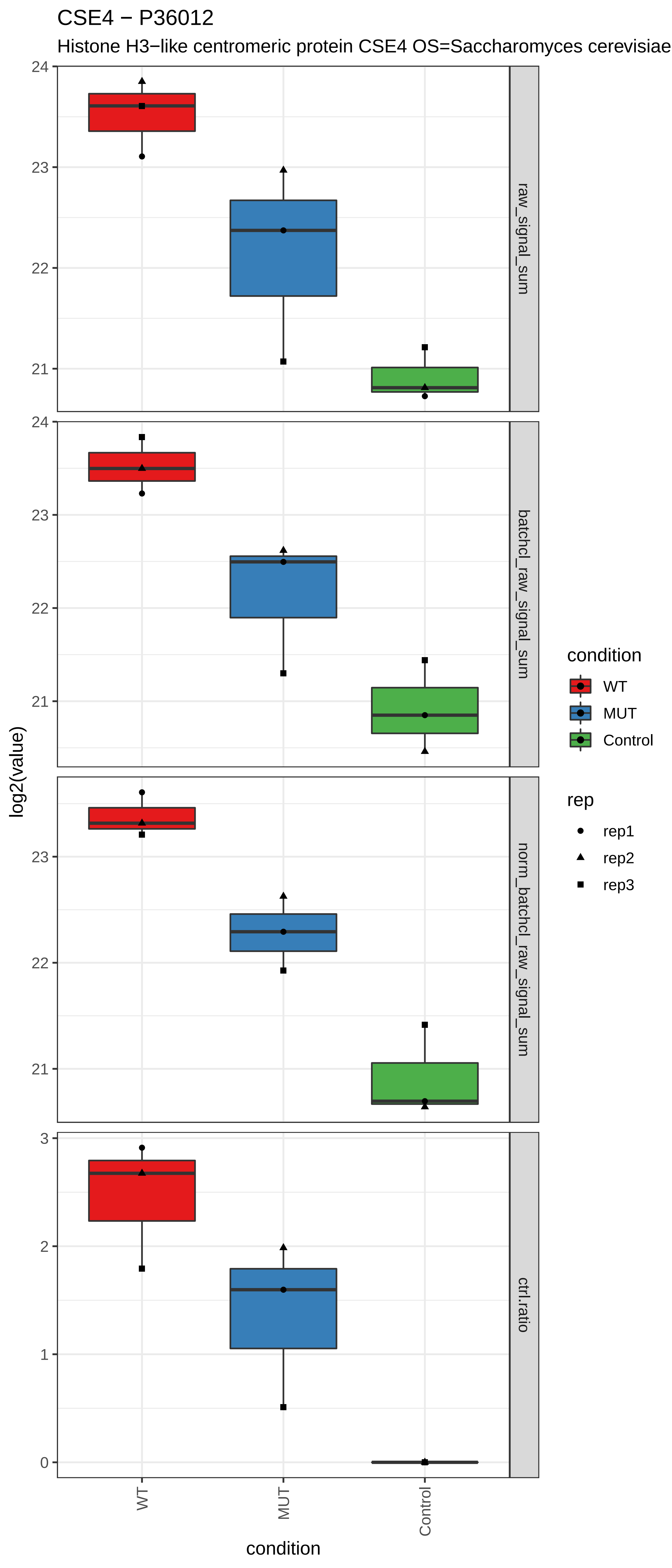

CSE4 − P36012
Histone H3−like centromeric protein CSE4 OS=Saccharomyces cerevisiae
raw_signal_sum
batchcl_raw_signal_sum
norm_batchcl_raw_signal_sum
ctrl.ratio
log2(value)
condition
WT
MUT
Control
rep
rep1
rep2
rep3

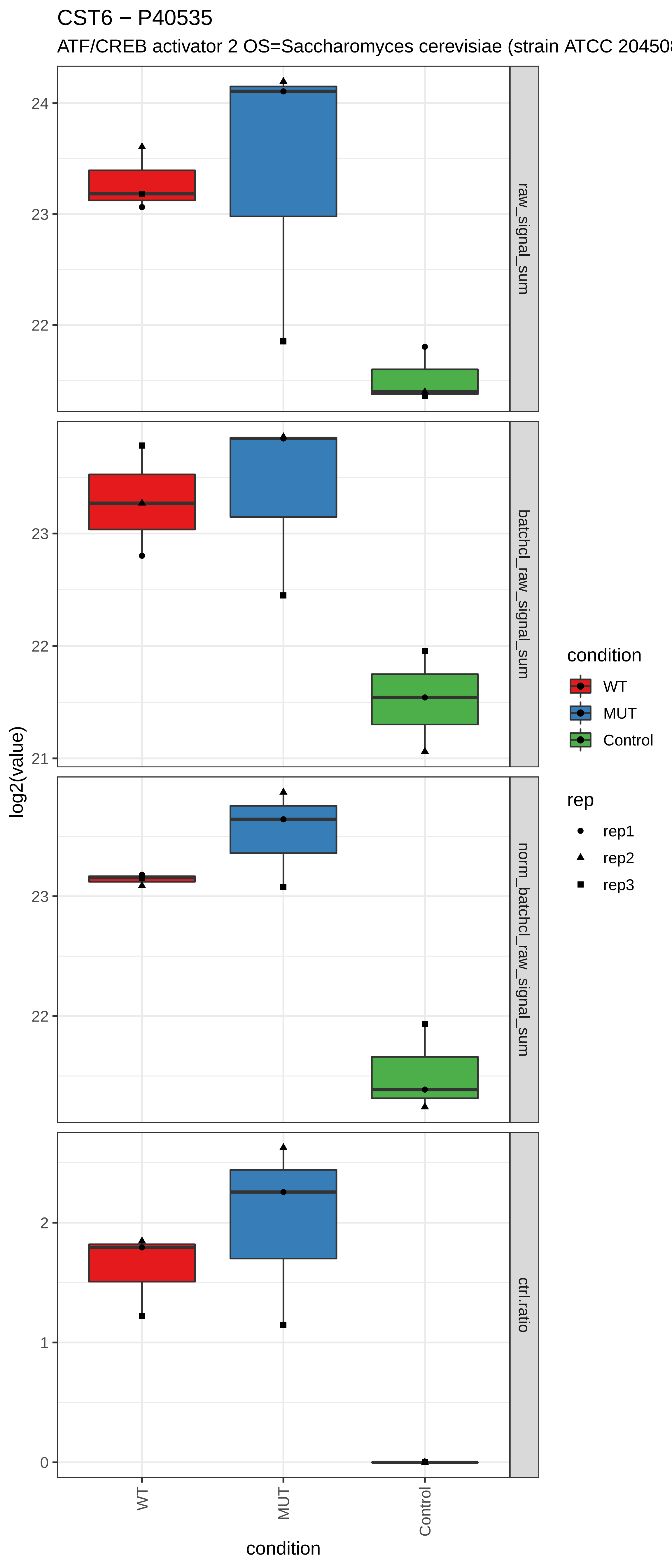
CST6 − P40535
ATF/CREB activator 2 OS=Saccharomyces cerevisiae (strain ATCC 20450
raw_signal_sum
batchcl_raw_signal_sum
norm_batchcl_raw_signal_sum
ctrl.ratio
log2(value)
condition
WT
MUT
Control
rep
rep1
rep2
rep3
24
23
22
21
2
1
0

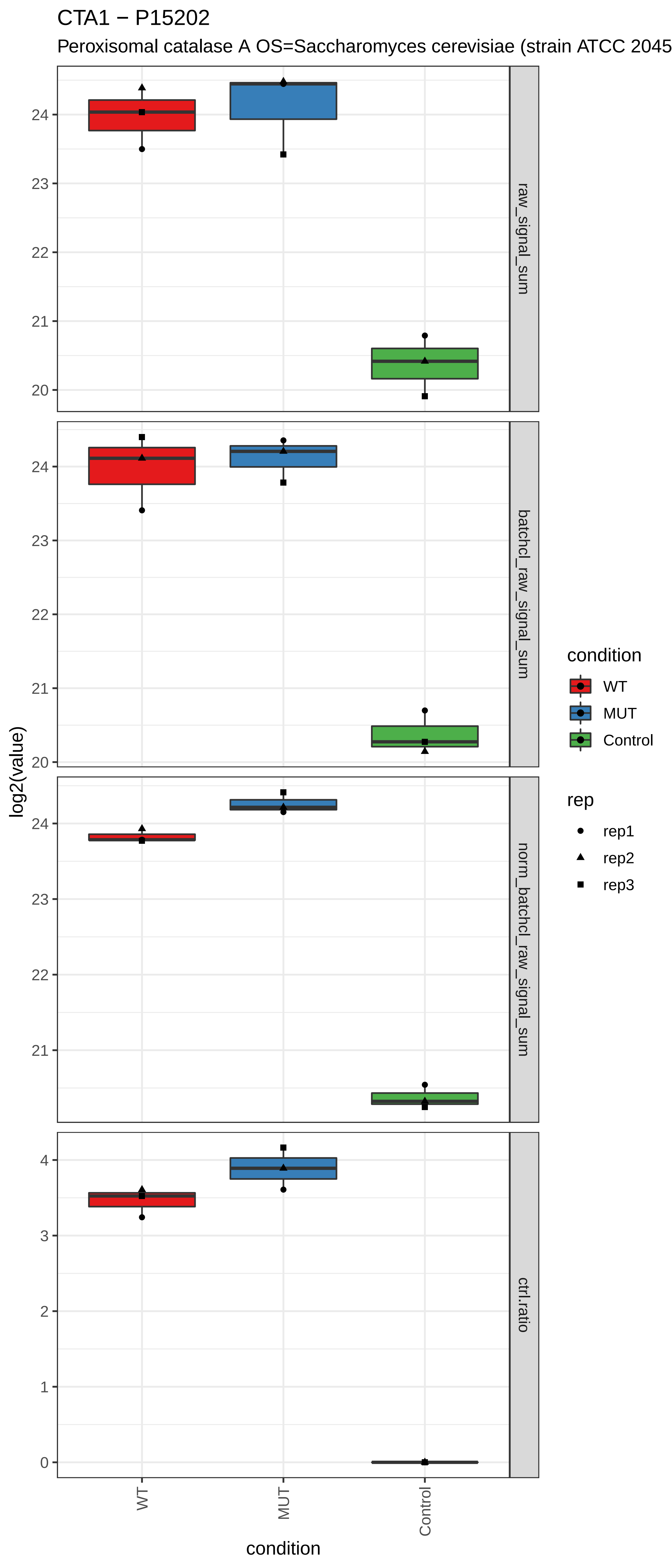

CTA1 − P15202
Peroxisomal catalase A OS=Saccharomyces cerevisiae (strain ATCC 2045
raw_signal_sum
batchcl_raw_signal_sum
norm_batchcl_raw_signal_sum
ctrl.ratio
log2(value)
condition
WT
MUT
Control
rep
rep1
rep2
rep3

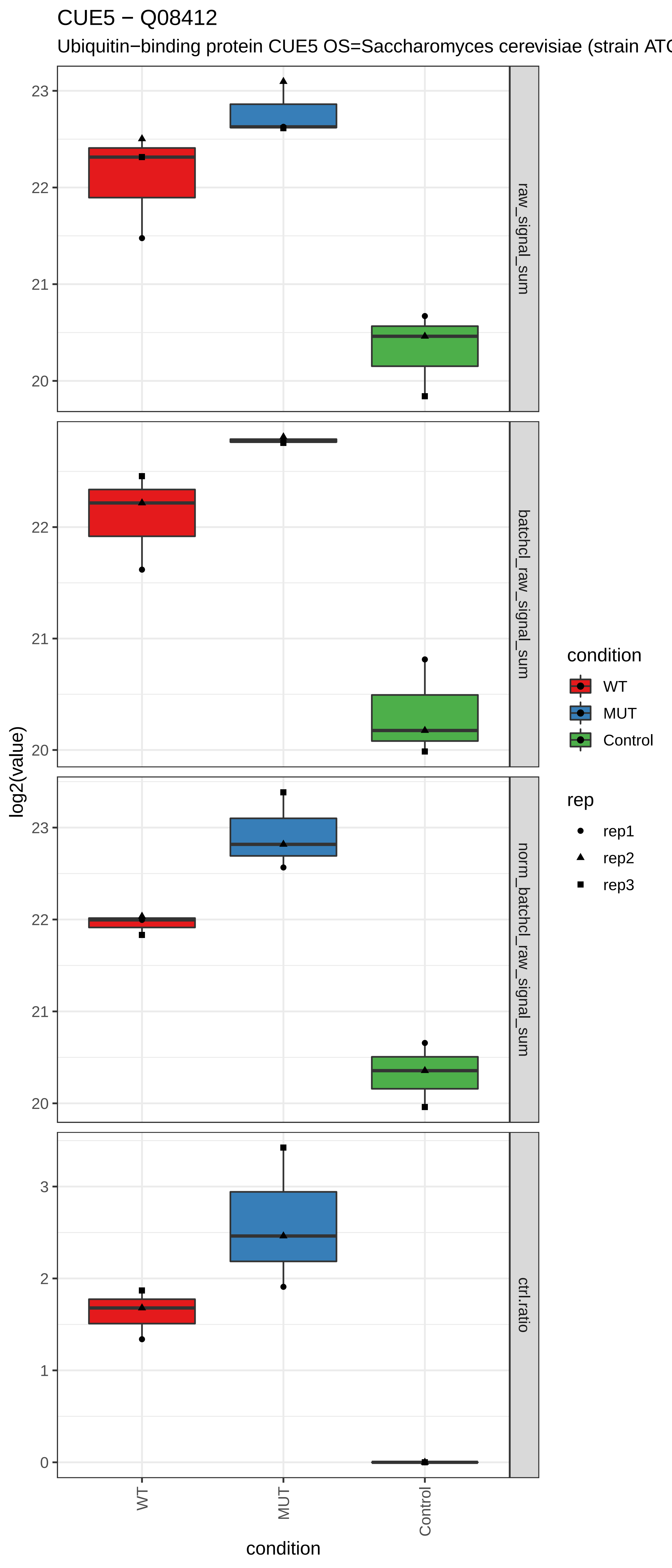

CUE5 − Q08412
Ubiquitin−binding protein CUE5 OS=Saccharomyces cerevisiae (strain ATC
raw_signal_sum
batchcl_raw_signal_sum
norm_batchcl_raw_signal_sum
ctrl.ratio
log2(value)
condition
WT
MUT
Control
rep
rep1
rep2
rep3

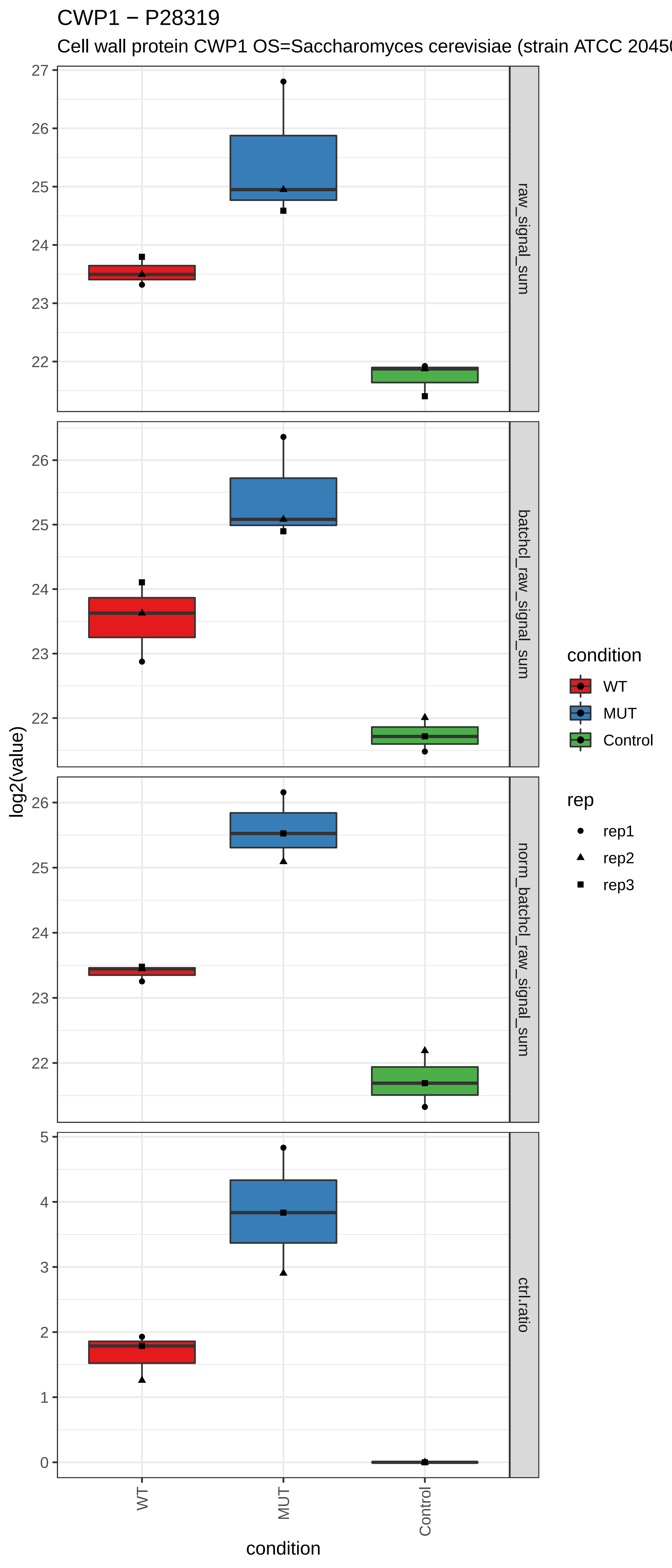
CWP1 − P28319
Cell wall protein CWP1 OS=Saccharomyces cerevisiae (strain ATCC 2045
raw_signal_sum
batchcl_raw_signal_sum
norm_batchcl_raw_signal_sum
ctrl.ratio
log2(value)
condition
WT
MUT
Control
rep
rep1
rep2
rep3

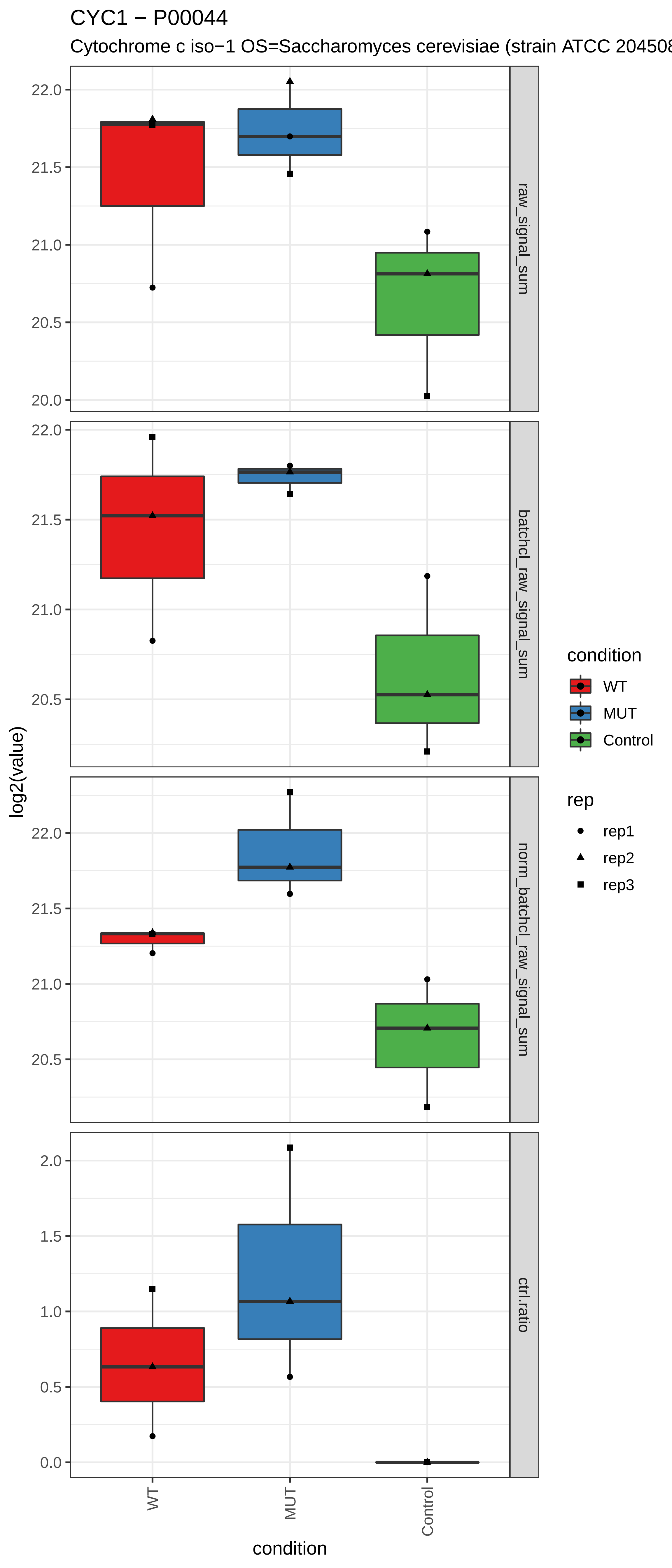

CYC1 − P00044
Cytochrome c iso−1 OS=Saccharomyces cerevisiae (strain ATCC 204508
raw_signal_sum
batchcl_raw_signal_sum
norm_batchcl_raw_signal_sum
ctrl.ratio
log2(value)
condition
WT
MUT
Control
rep
rep1
rep2
rep3

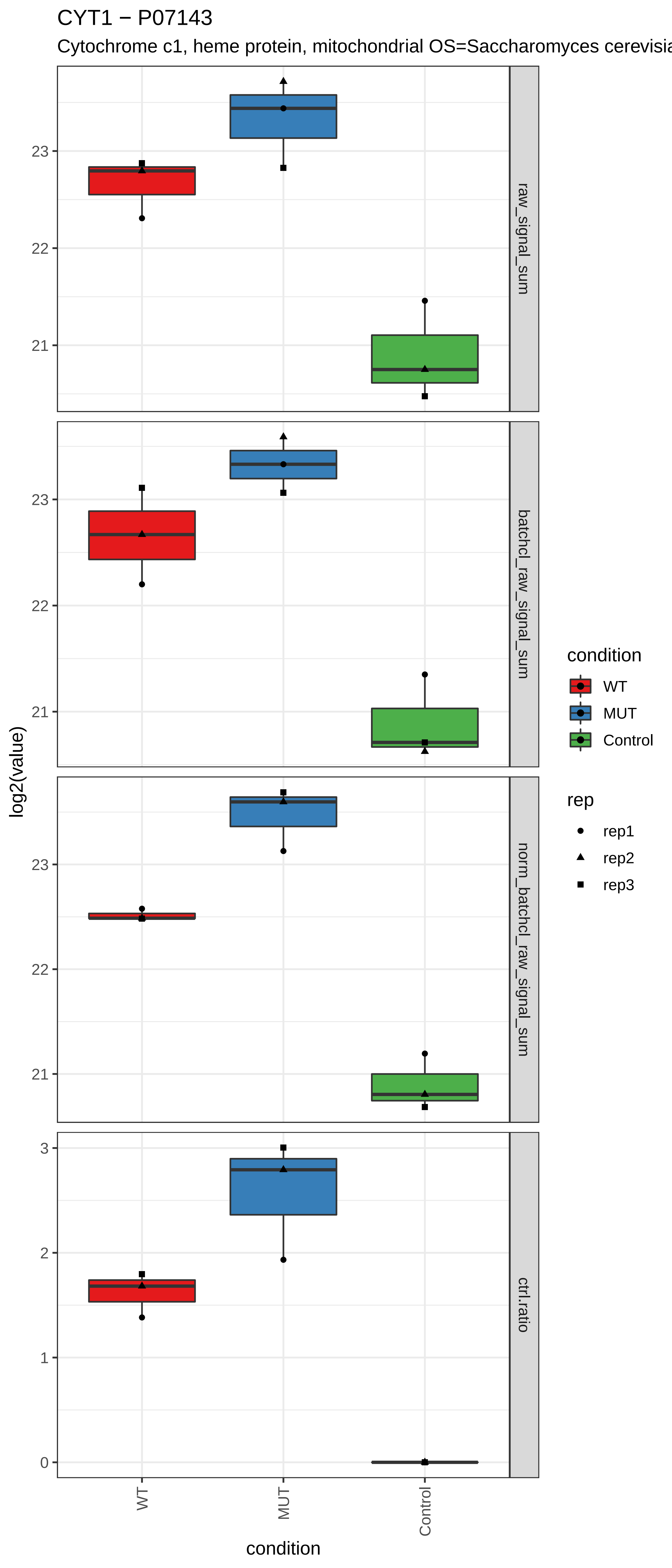

CYT1 − P07143
Cytochrome c1, heme protein, mitochondrial OS=Saccharomyces cerevisia
raw_signal_sum
batchcl_raw_signal_sum
norm_batchcl_raw_signal_sum
ctrl.ratio
log2(value)
23
22
21
3
2
1
0
WT
MUT
Control
condition
condition
WT
MUT
Control
rep
rep1
rep2
rep3

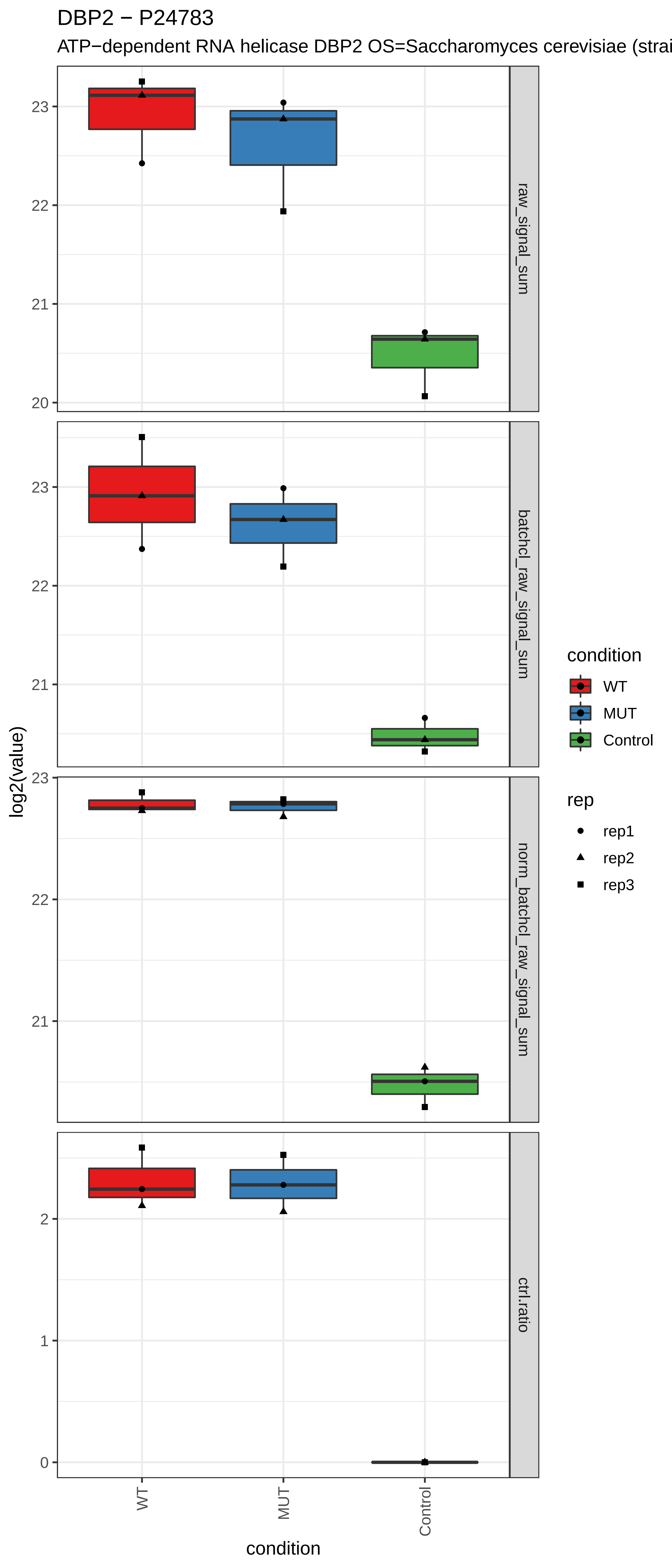

DBP2 − P24783
ATP−dependent RNA helicase DBP2 OS=Saccharomyces cerevisiae (strai
raw_signal_sum
batchcl_raw_signal_sum
norm_batchcl_raw_signal_sum
ctrl.ratio
log2(value)
condition
WT
MUT
Control
rep
rep1
rep2
rep3

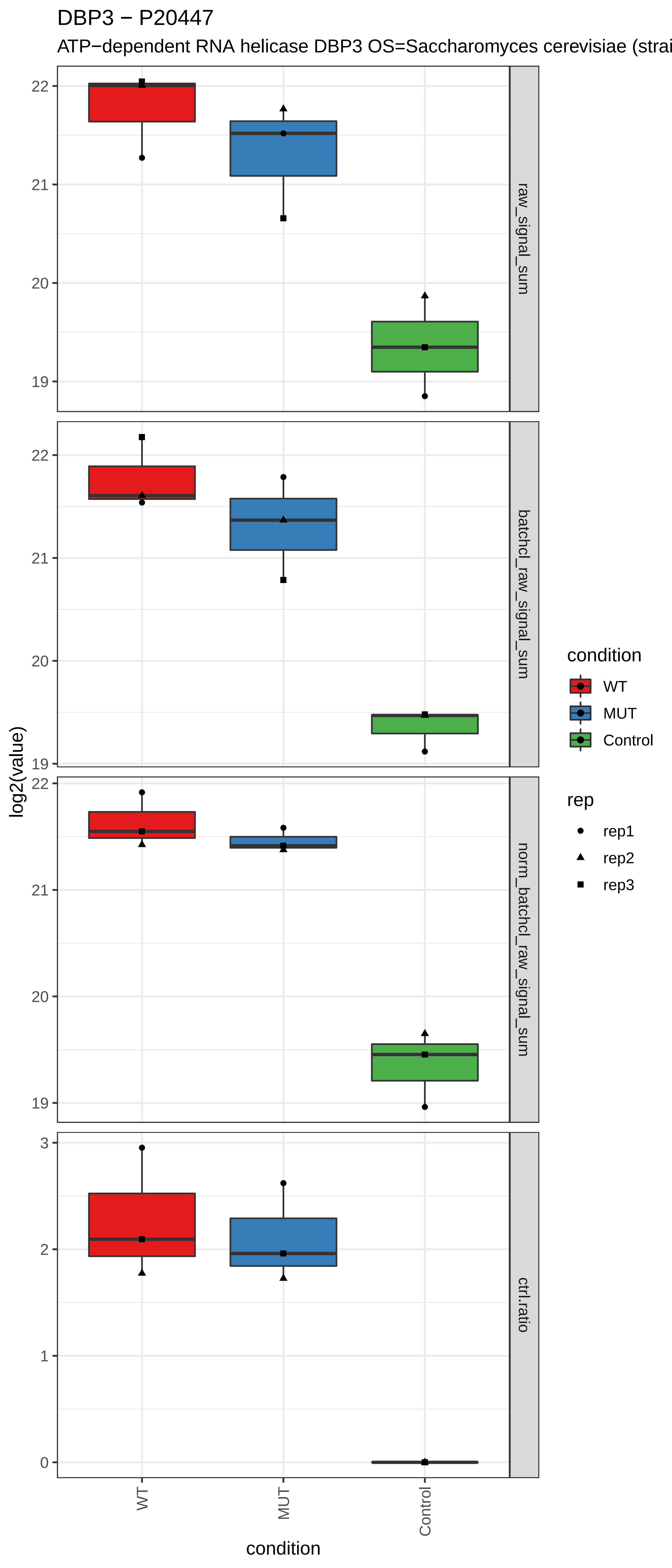
DBP3 − P20447
ATP−dependent RNA helicase DBP3 OS=Saccharomyces cerevisiae (strai
raw_signal_sum
batchcl_raw_signal_sum
norm_batchcl_raw_signal_sum
ctrl.ratio
log2(value)
condition
WT
MUT
Control
rep
rep1
rep2
rep3
22
21
20
19
3
2
1
0

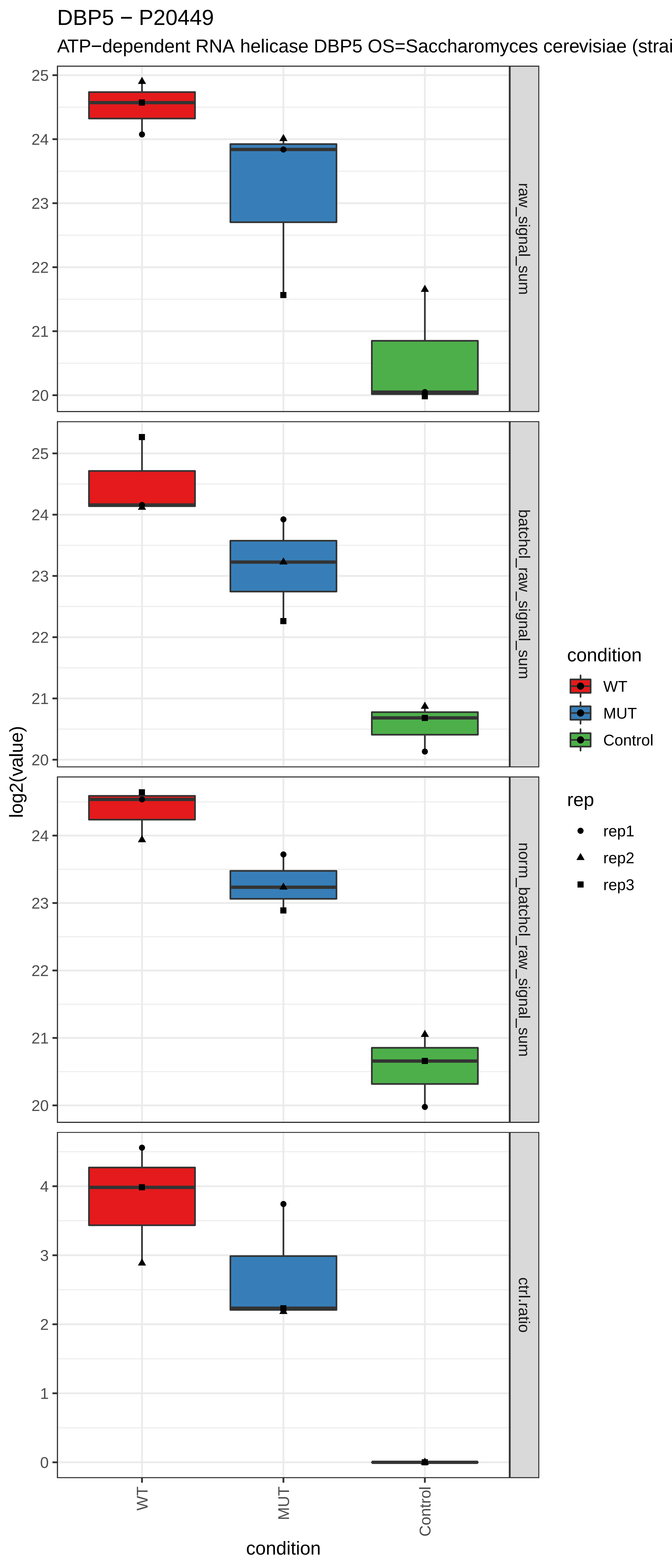

DBP5 − P20449
ATP−dependent RNA helicase DBP5 OS=Saccharomyces cerevisiae (strai
raw_signal_sum
batchcl_raw_signal_sum
norm_batchcl_raw_signal_sum
ctrl.ratio
log2(value)
condition
WT
MUT
Control
rep
rep1
rep2
rep3

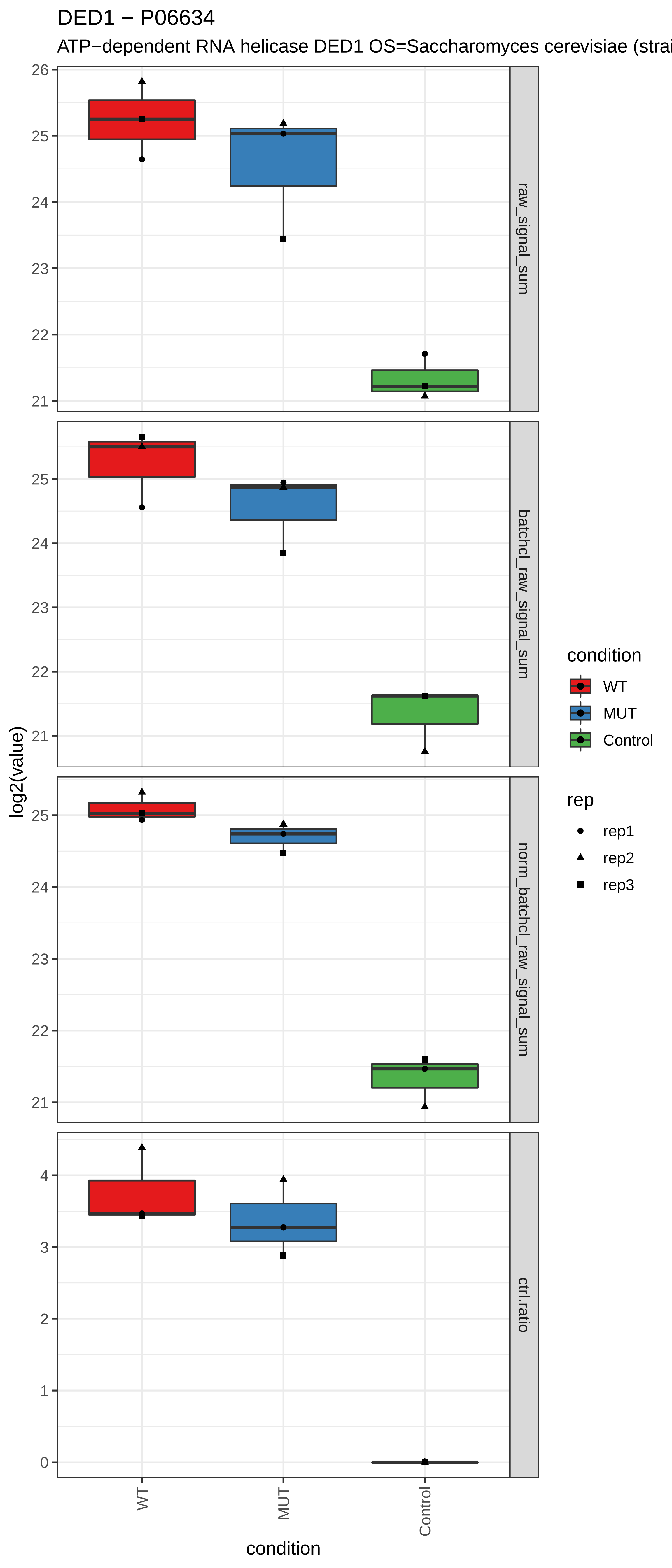

DED1 − P06634
ATP−dependent RNA helicase DED1 OS=Saccharomyces cerevisiae (strai
raw_signal_sum
batchcl_raw_signal_sum
norm_batchcl_raw_signal_sum
ctrl.ratio
log2(value)
condition
WT
MUT
Control
rep
rep1
rep2
rep3

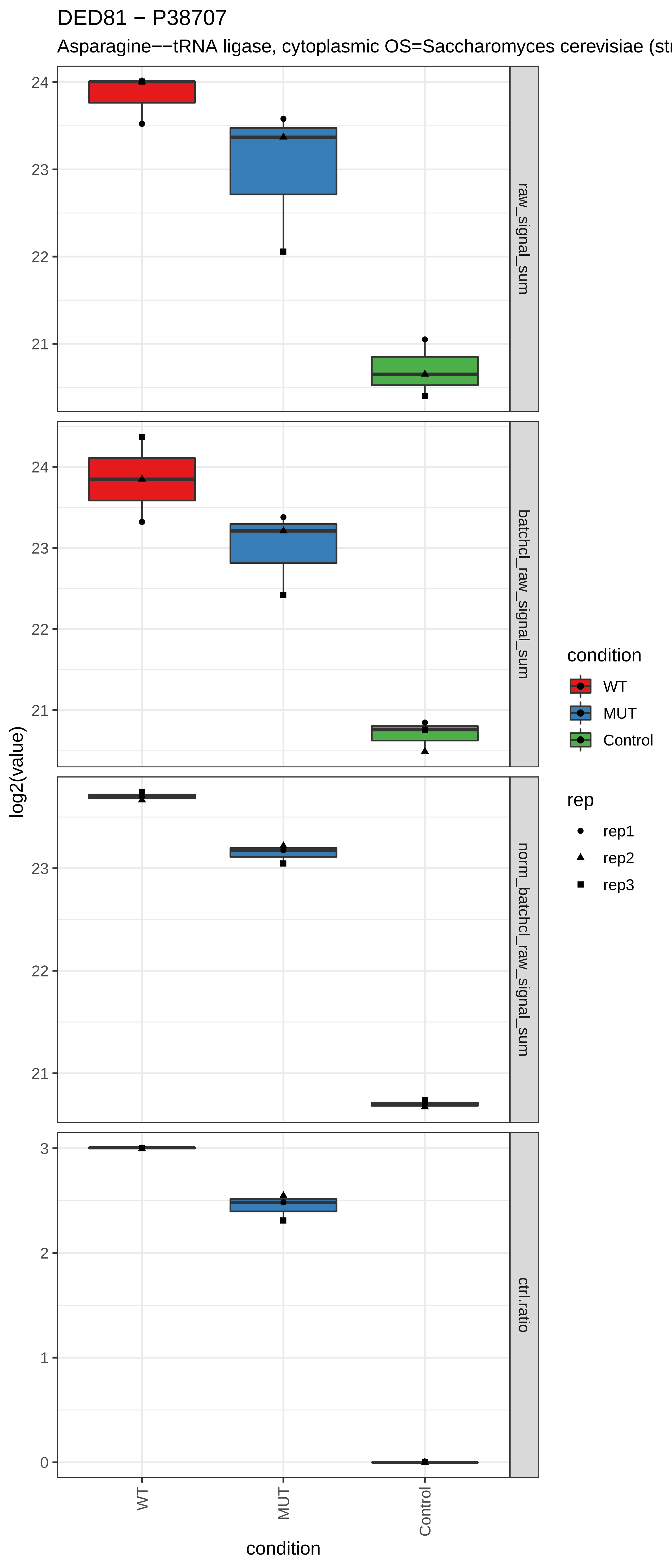

DED81 − P38707
Asparagine−−tRNA ligase, cytoplasmic OS=Saccharomyces cerevisiae (str
raw_signal_sum
batchcl_raw_signal_sum
norm_batchcl_raw_signal_sum
ctrl.ratio
log2(value)
condition
WT
MUT
Control
rep
rep1
rep2
rep3

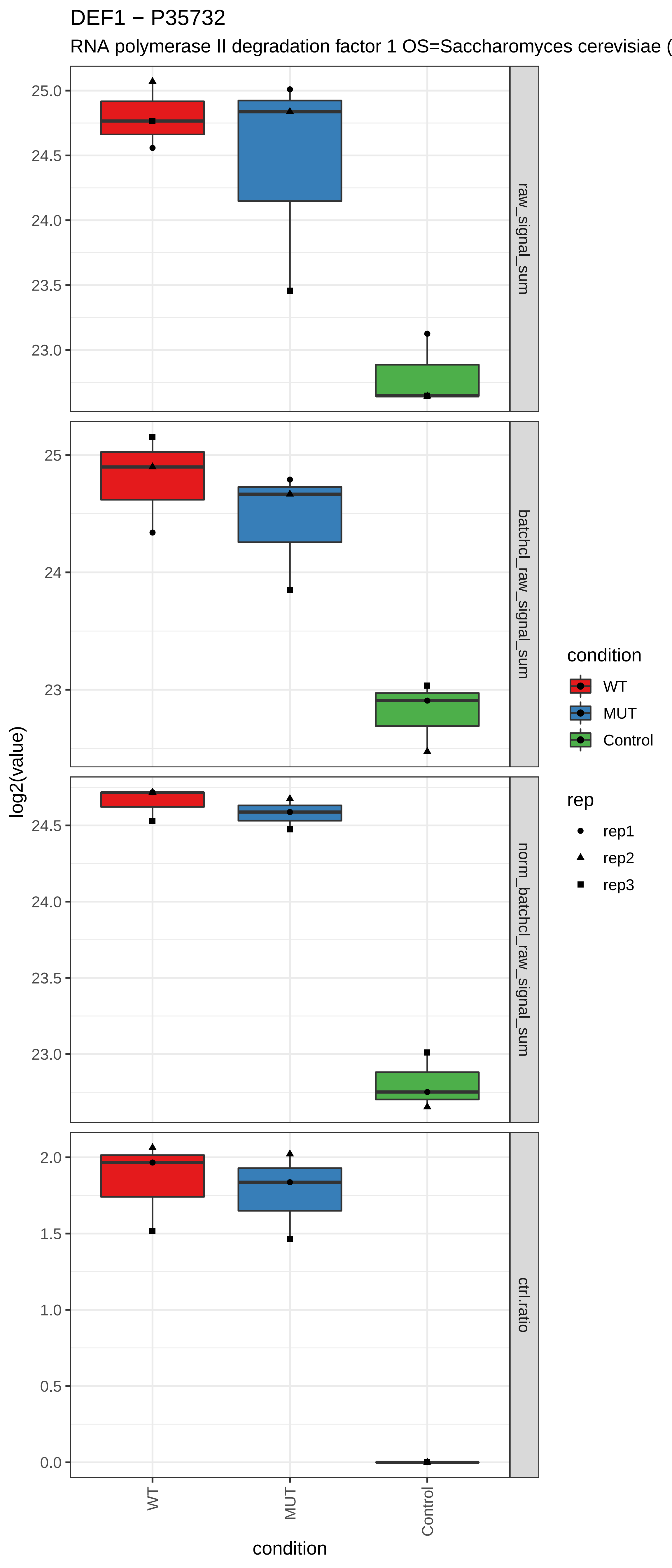
DEF1 − P35732
RNA polymerase II degradation factor 1 OS=Saccharomyces cerevisiae (
raw_signal_sum
batchcl_raw_signal_sum
norm_batchcl_raw_signal_sum
ctrl.ratio
log2(value)
condition
WT
MUT
Control
rep
rep1
rep2
rep3
25.0
24.5
24.0
23.5
23.0
25
24
23
2.0
1.5
1.0
0.5
0.0

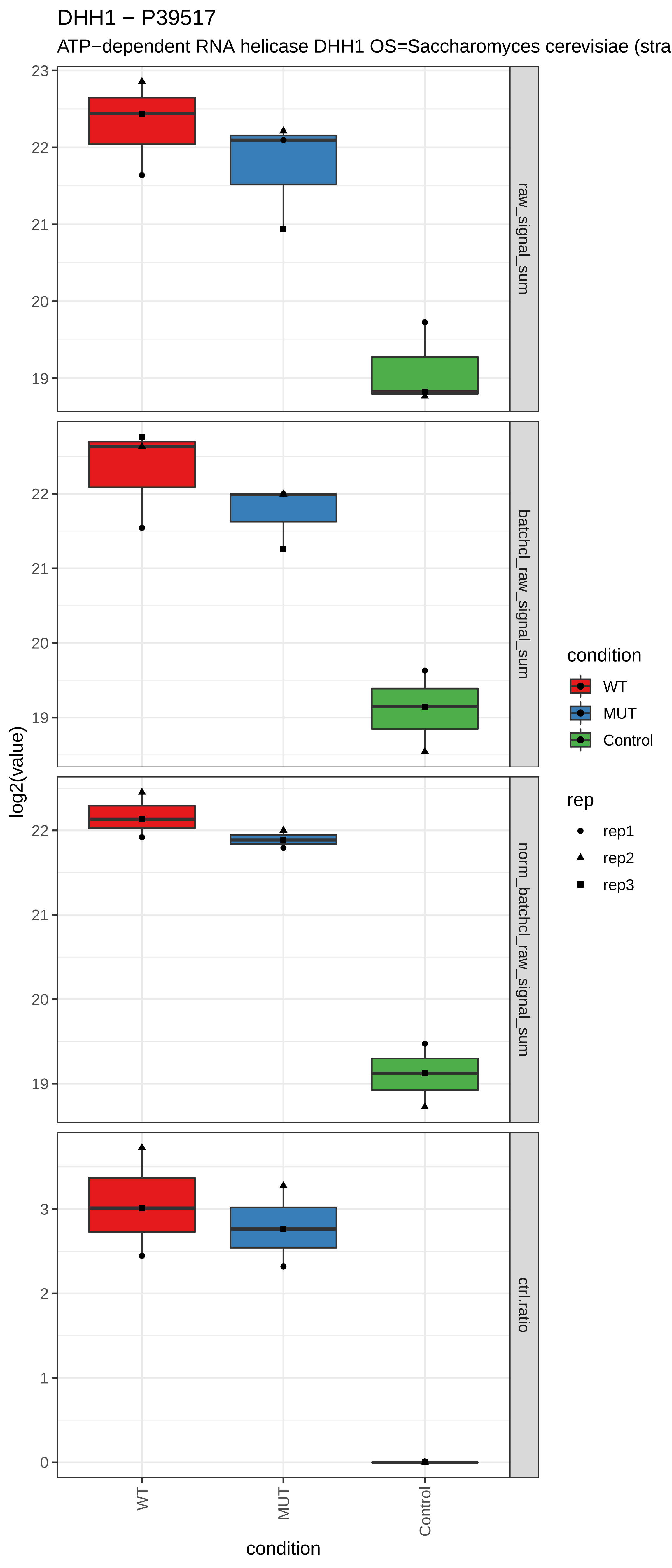

DHH1 − P39517
ATP−dependent RNA helicase DHH1 OS=Saccharomyces cerevisiae (stra
raw_signal_sum
batchcl_raw_signal_sum
norm_batchcl_raw_signal_sum
ctrl.ratio
log2(value)
condition
WT
MUT
Control
rep
rep1
rep2
rep3

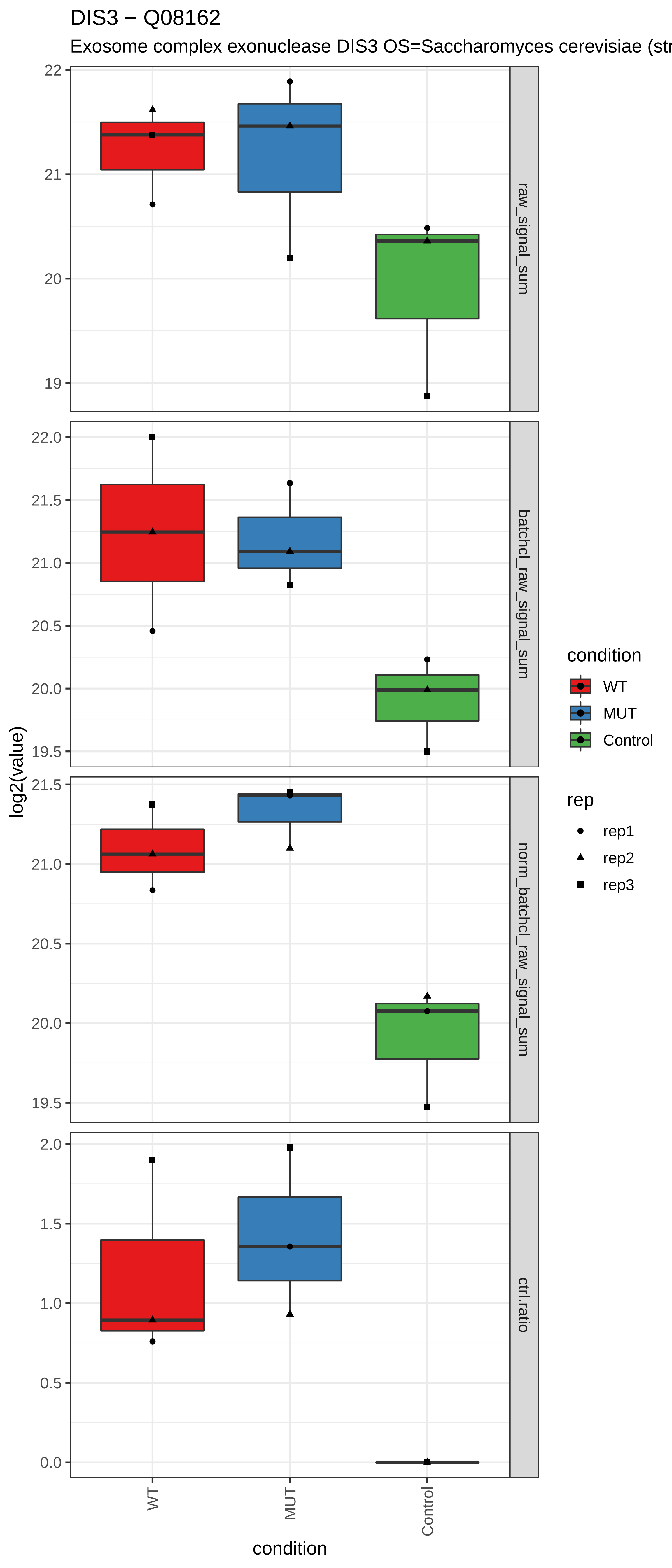

DIS3 − Q08162
Exosome complex exonuclease DIS3 OS=Saccharomyces cerevisiae (str
raw_signal_sum
batchcl_raw_signal_sum
norm_batchcl_raw_signal_sum
ctrl.ratio
log2(value)
condition
WT
MUT
Control
rep
rep1
rep2
rep3

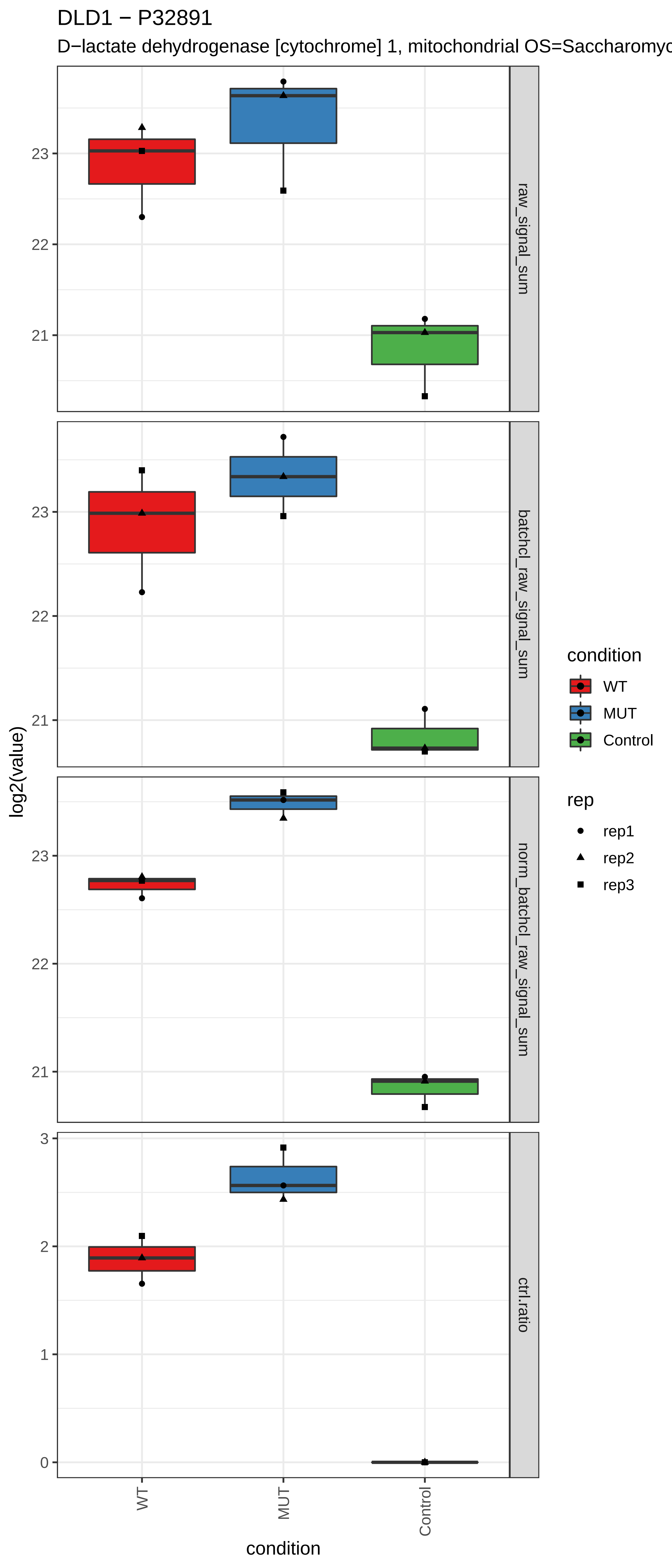

DLD1 − P32891
D−lactate dehydrogenase [cytochrome] 1, mitochondrial OS=Saccharomyc
raw_signal_sum
batchcl_raw_signal_sum
norm_batchcl_raw_signal_sum
ctrl.ratio
log2(value)
condition
WT
MUT
Control
rep
rep1
rep2
rep3

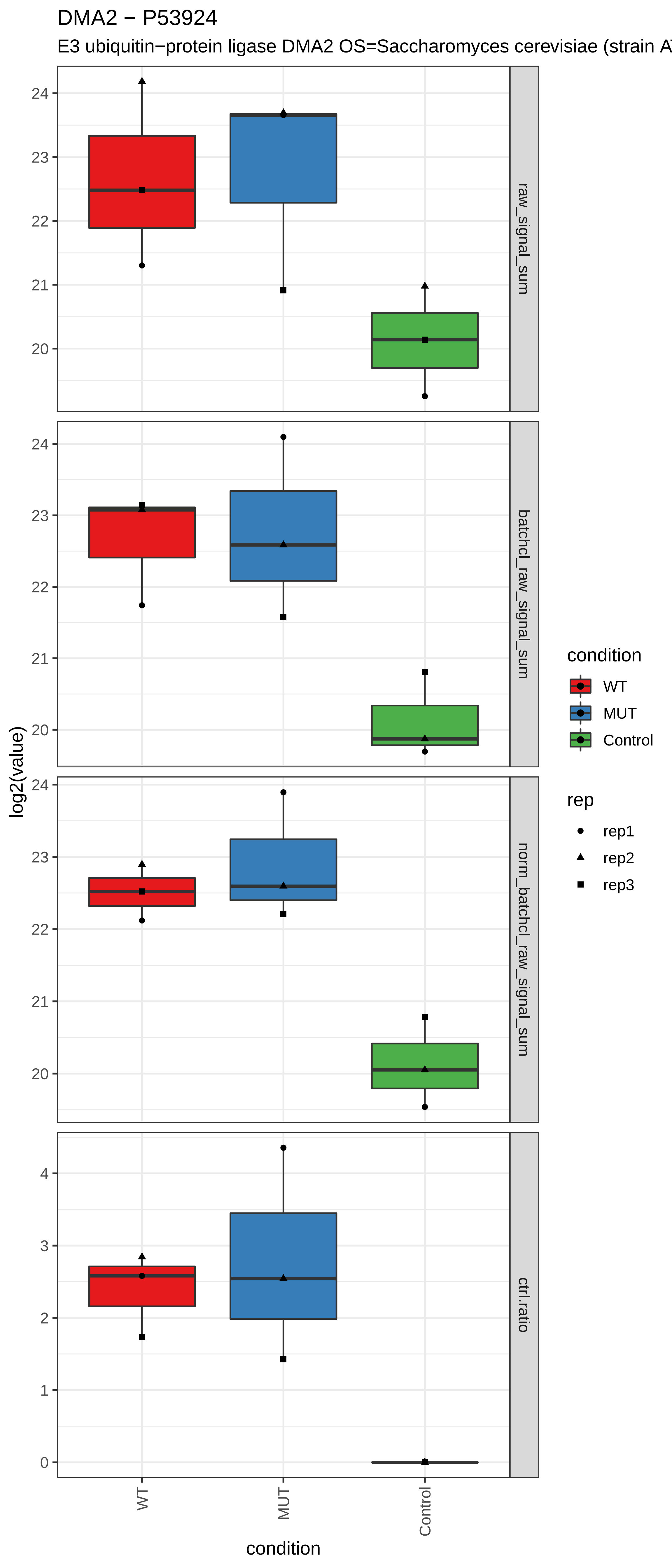

DMA2 − P53924
E3 ubiquitin−protein ligase DMA2 OS=Saccharomyces cerevisiae (strain A
raw_signal_sum
batchcl_raw_signal_sum
norm_batchcl_raw_signal_sum
ctrl.ratio
log2(value)
condition
WT
MUT
Control
rep
rep1
rep2
rep3

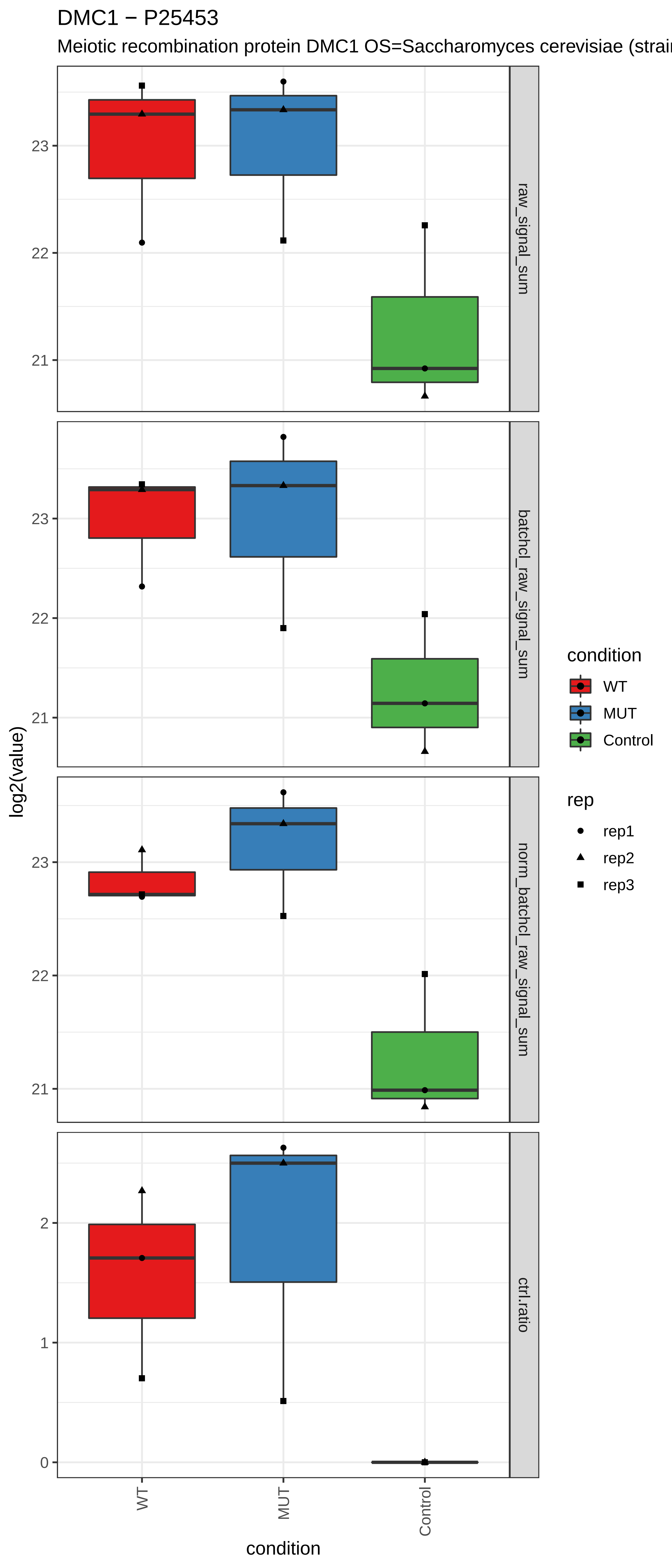

DMC1 − P25453
Meiotic recombination protein DMC1 OS=Saccharomyces cerevisiae (strai
raw_signal_sum
batchcl_raw_signal_sum
norm_batchcl_raw_signal_sum
ctrl.ratio
log2(value)
23
22
21
2
1
0
WT
MUT
Control
condition
condition
WT
MUT
Control
rep
rep1
rep2
rep3

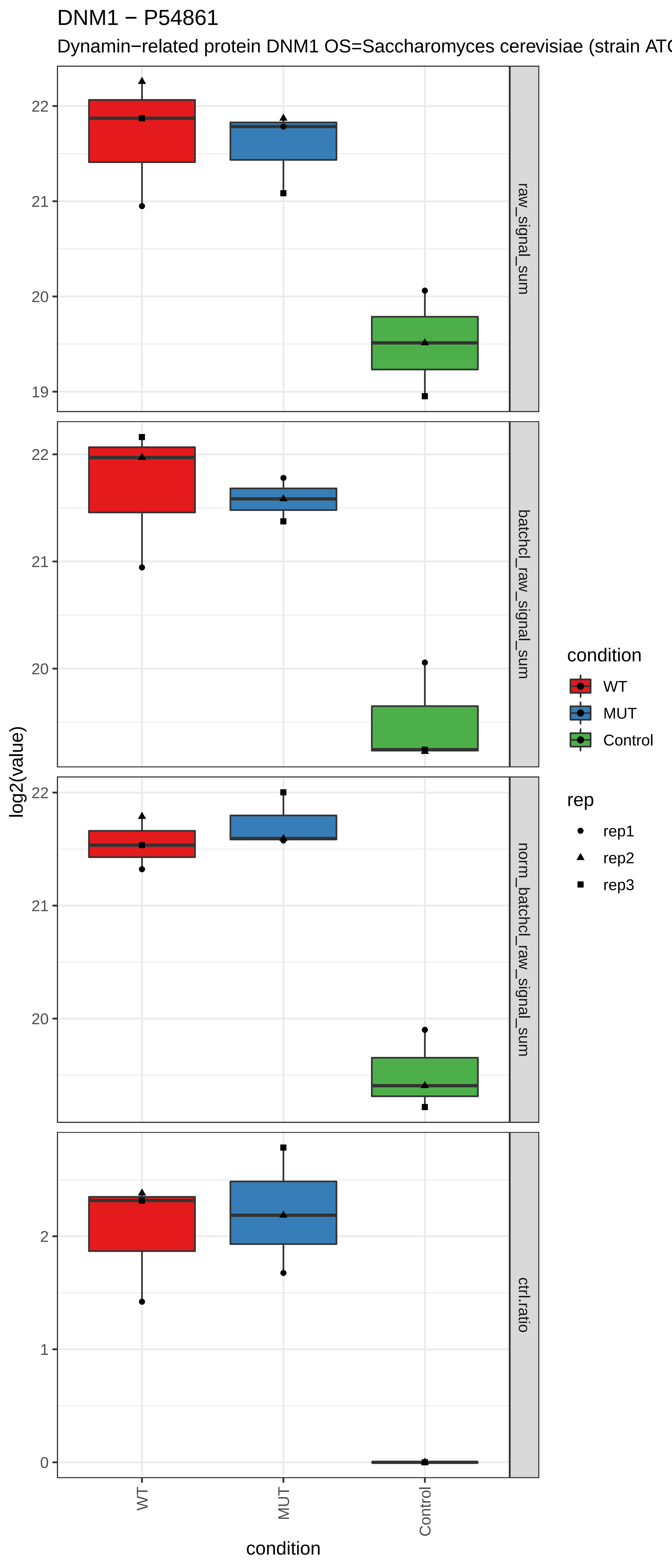
DNM1 − P54861
Dynamin−related protein DNM1 OS=Saccharomyces cerevisiae (strain ATC
raw_signal_sum
batchcl_raw_signal_sum
norm_batchcl_raw_signal_sum
ctrl.ratio
log2(value)
condition
WT
MUT
Control
rep
rep1
rep2
rep3
22
21
20
19
2
1
0

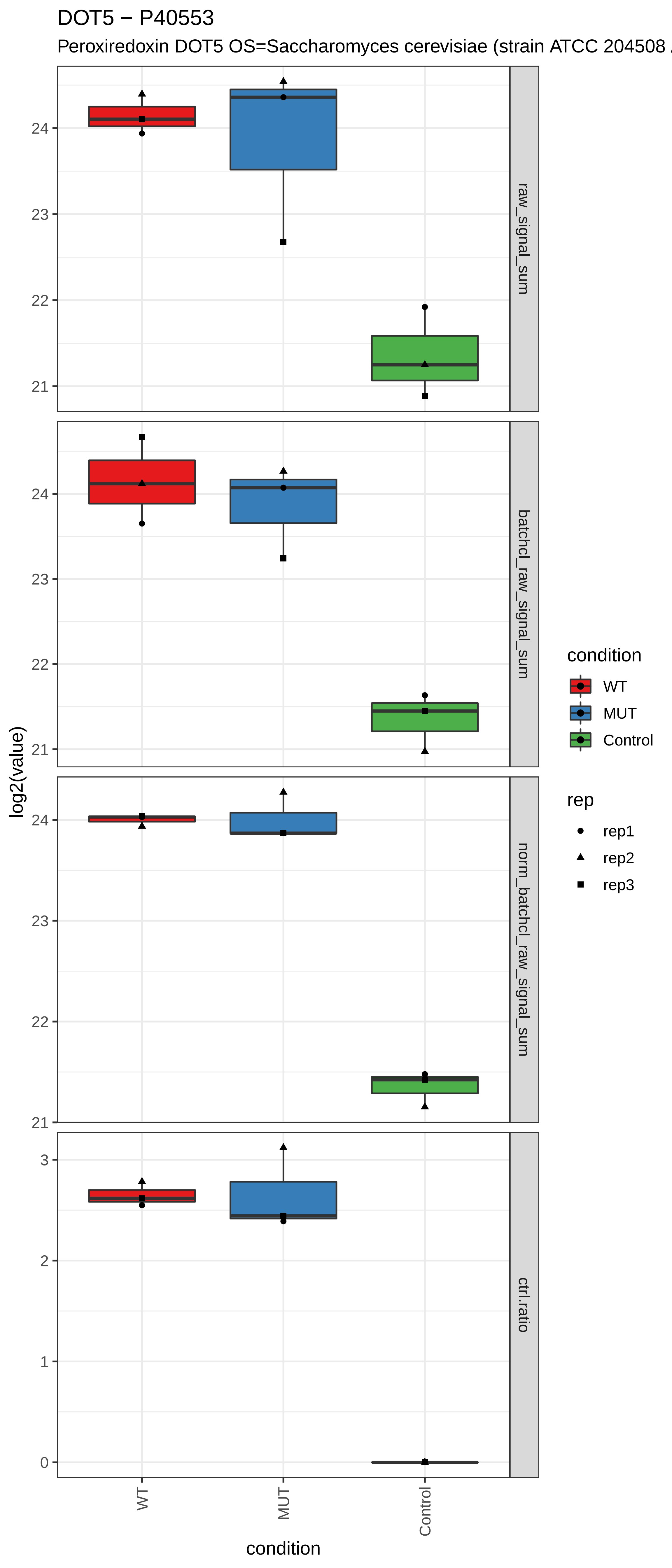

DOT5 − P40553
Peroxiredoxin DOT5 OS=Saccharomyces cerevisiae (strain ATCC 204508 /
raw_signal_sum
batchcl_raw_signal_sum
norm_batchcl_raw_signal_sum
ctrl.ratio
log2(value)
condition
WT
MUT
Control
rep
rep1
rep2
rep3

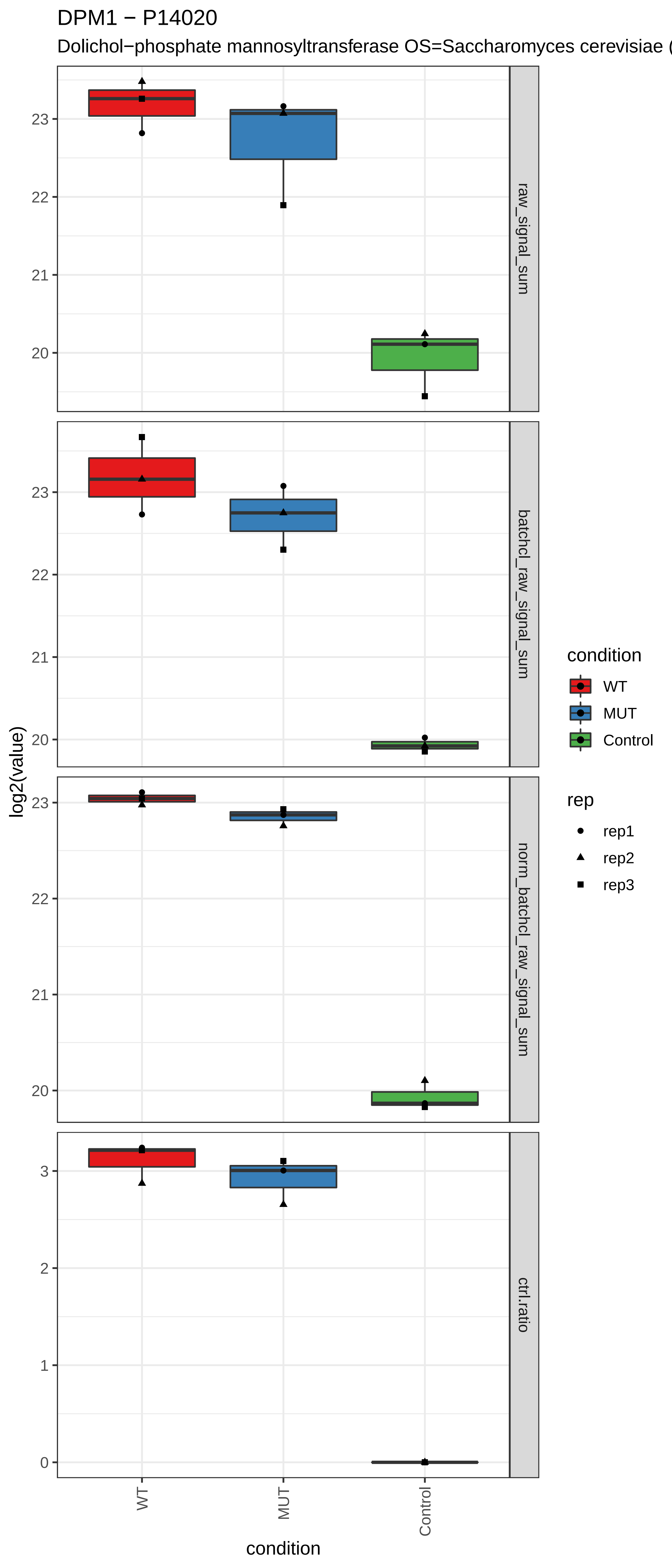

DPM1 − P14020
Dolichol−phosphate mannosyltransferase OS=Saccharomyces cerevisiae (
raw_signal_sum
batchcl_raw_signal_sum
norm_batchcl_raw_signal_sum
ctrl.ratio
log2(value)
condition
WT
MUT
Control
rep
rep1
rep2
rep3

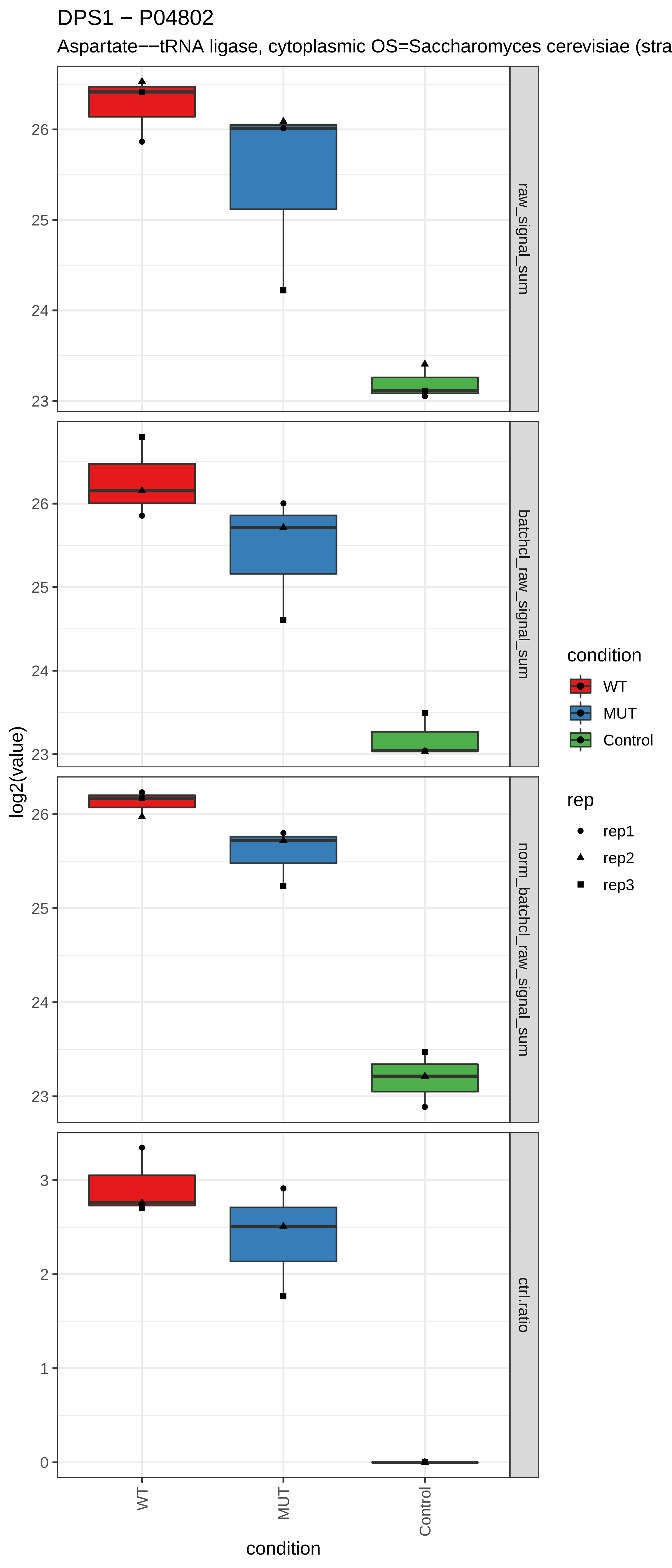

DPS1 − P04802
Aspartate−−tRNA ligase, cytoplasmic OS=Saccharomyces cerevisiae (stra
raw_signal_sum
batchcl_raw_signal_sum
norm_batchcl_raw_signal_sum
ctrl.ratio
log2(value)
condition
WT
MUT
Control
rep
rep1
rep2
rep3

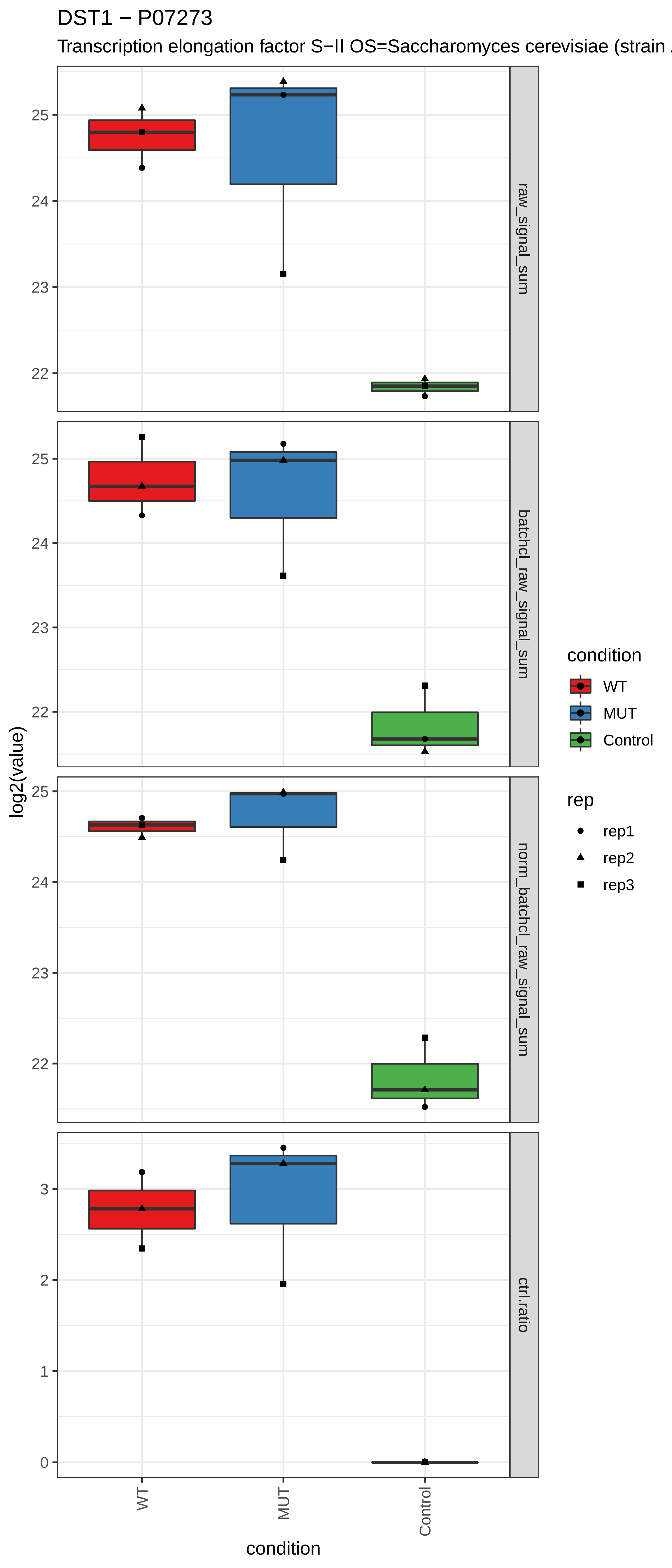
DST1 − P07273
Transcription elongation factor S−II OS=Saccharomyces cerevisiae (strain /
raw_signal_sum
batchcl_raw_signal_sum
norm_batchcl_raw_signal_sum
ctrl.ratio
log2(value)
condition
WT
MUT
Control
rep
rep1
rep2
rep3

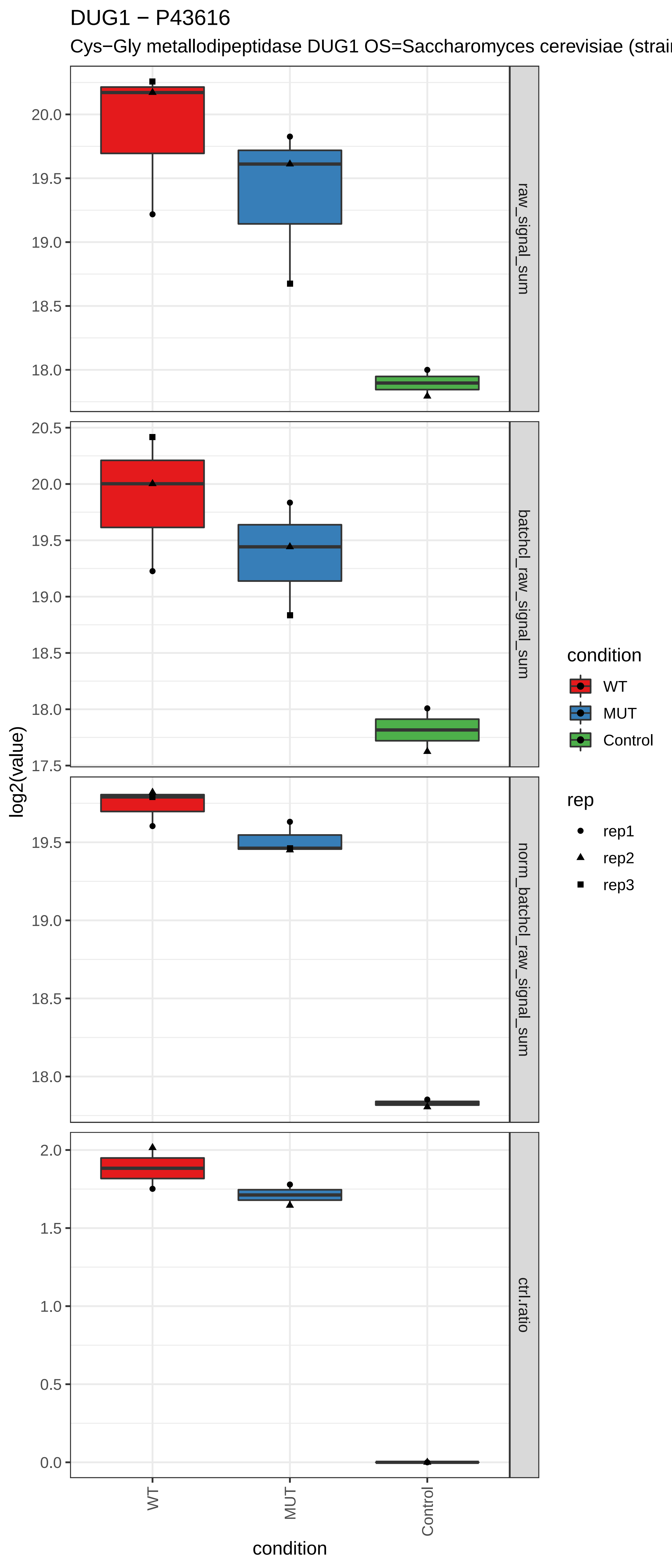

DUG1 − P43616
Cys−Gly metallodipeptidase DUG1 OS=Saccharomyces cerevisiae (strai
raw_signal_sum
batchcl_raw_signal_sum
norm_batchcl_raw_signal_sum
ctrl.ratio
log2(value)
condition
WT
MUT
Control
rep
rep1
rep2
rep3

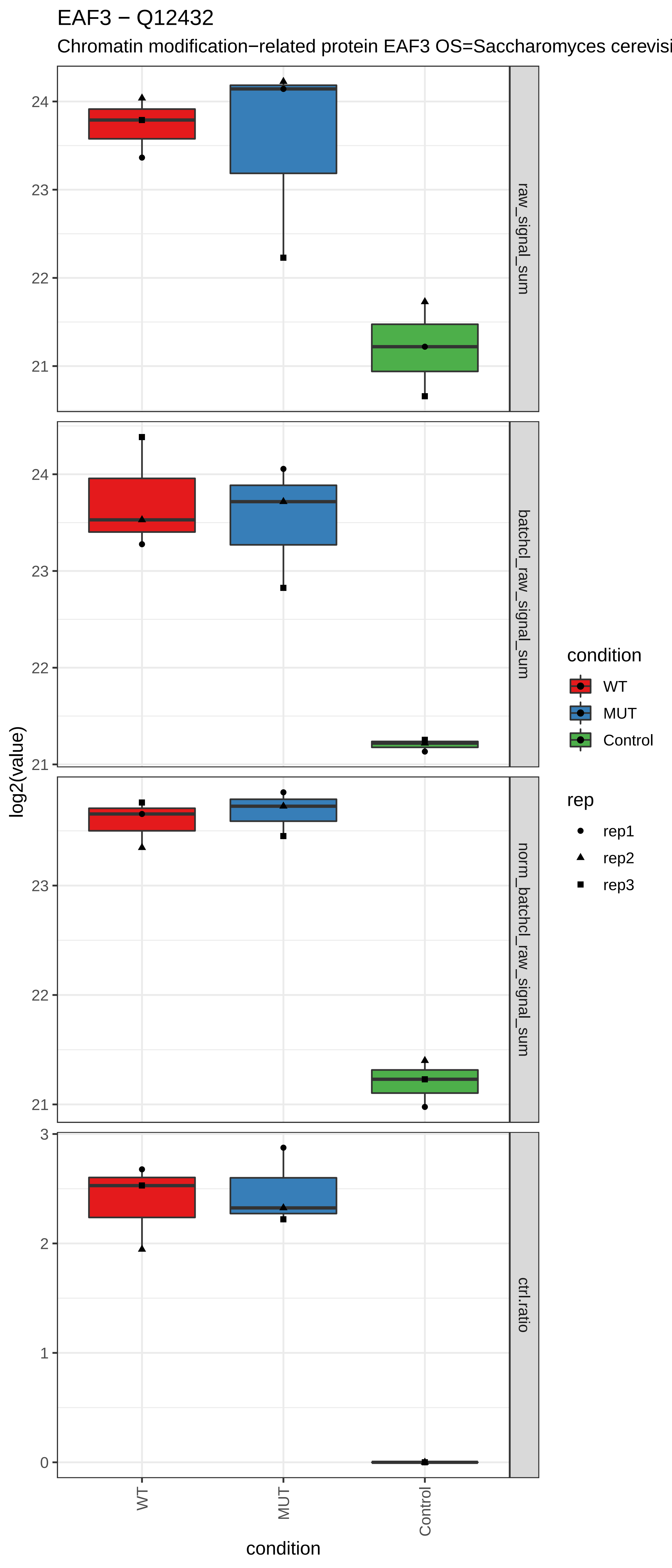

EAF3 − Q12432
Chromatin modification−related protein EAF3 OS=Saccharomyces cerevisi
raw_signal_sum
batchcl_raw_signal_sum
norm_batchcl_raw_signal_sum
ctrl.ratio
log2(value)
condition
WT
MUT
Control
rep
rep1
rep2
rep3

EBP2 − P36049

rRNA−processing protein EBP2 OS=Saccharomyces cerevisiae (strain ATC

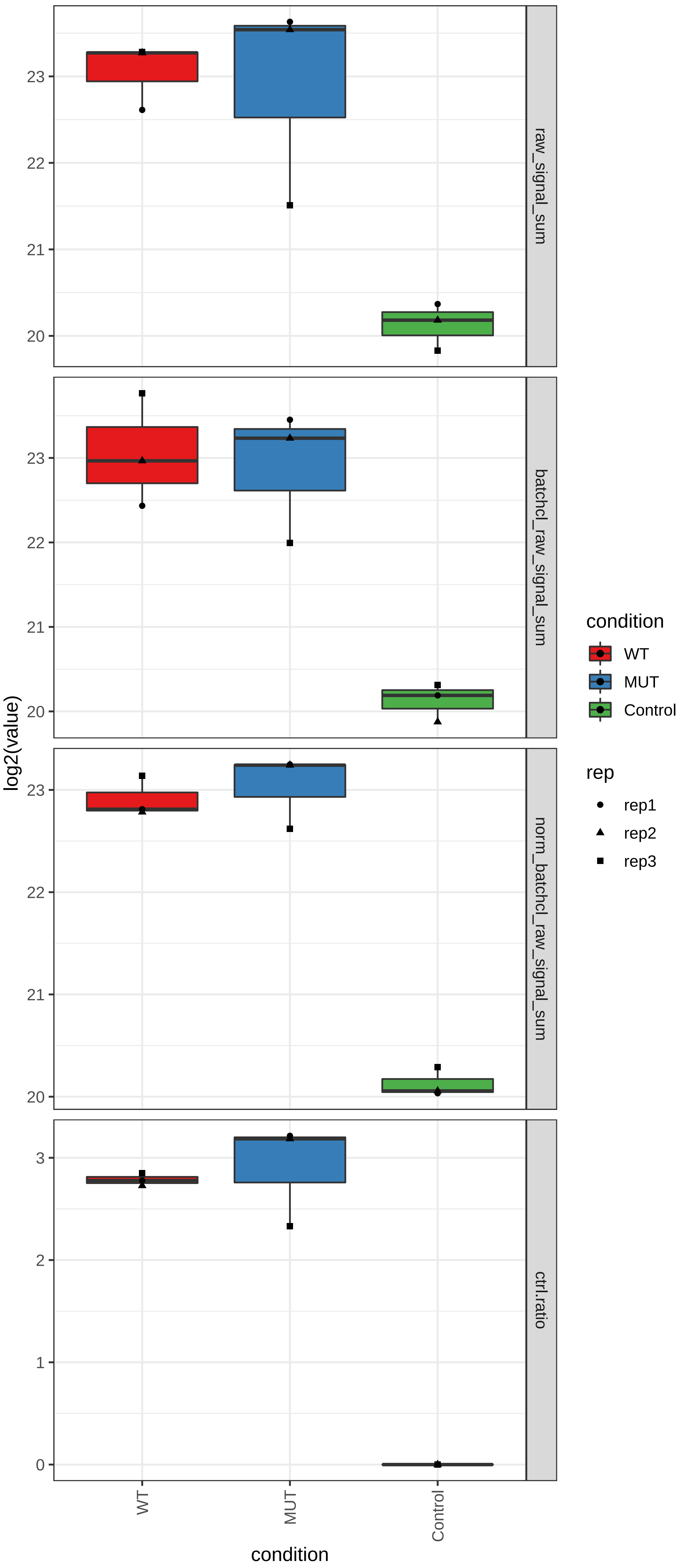

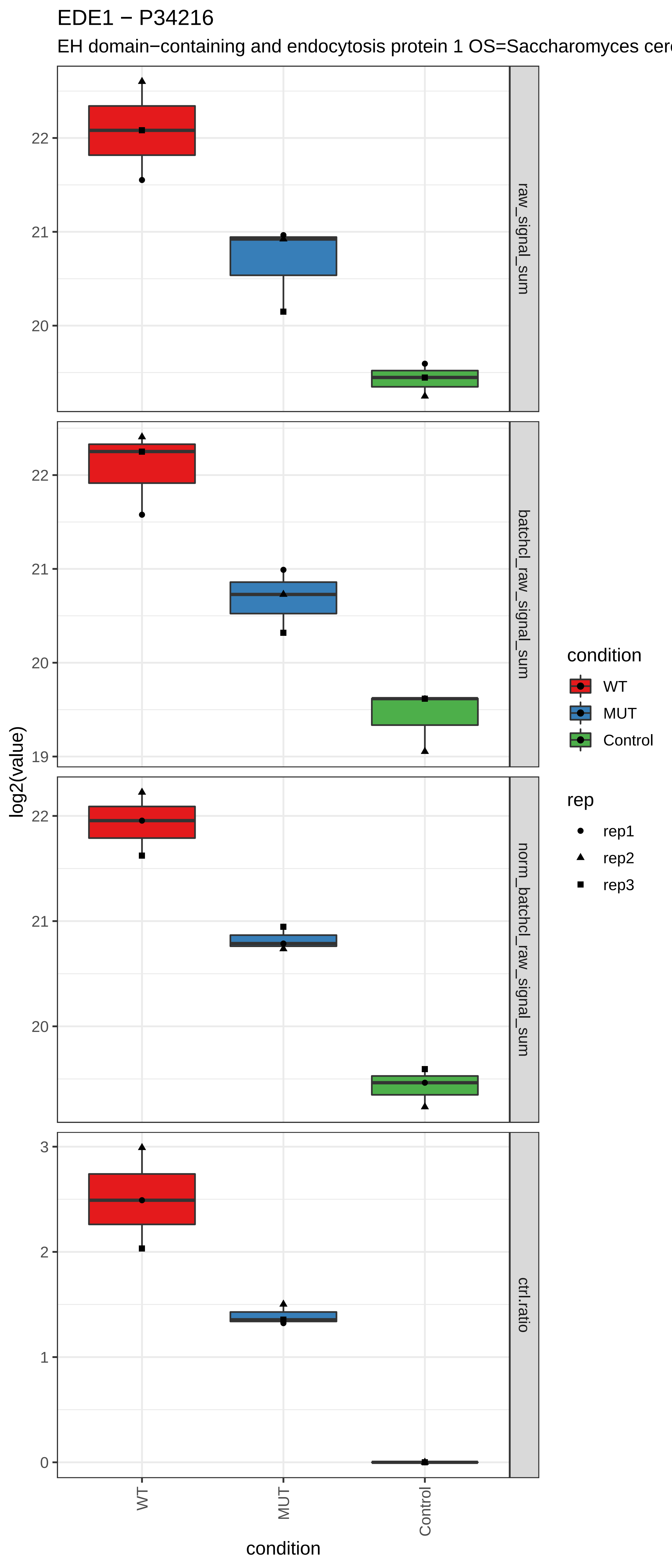

EDE1 − P34216
EH domain−containing and endocytosis protein 1 OS=Saccharomyces cere
raw_signal_sum
batchcl_raw_signal_sum
norm_batchcl_raw_signal_sum
ctrl.ratio
log2(value)
condition
WT
MUT
Control
rep
rep1
rep2
rep3
22
21
20
19
3
2
1
0

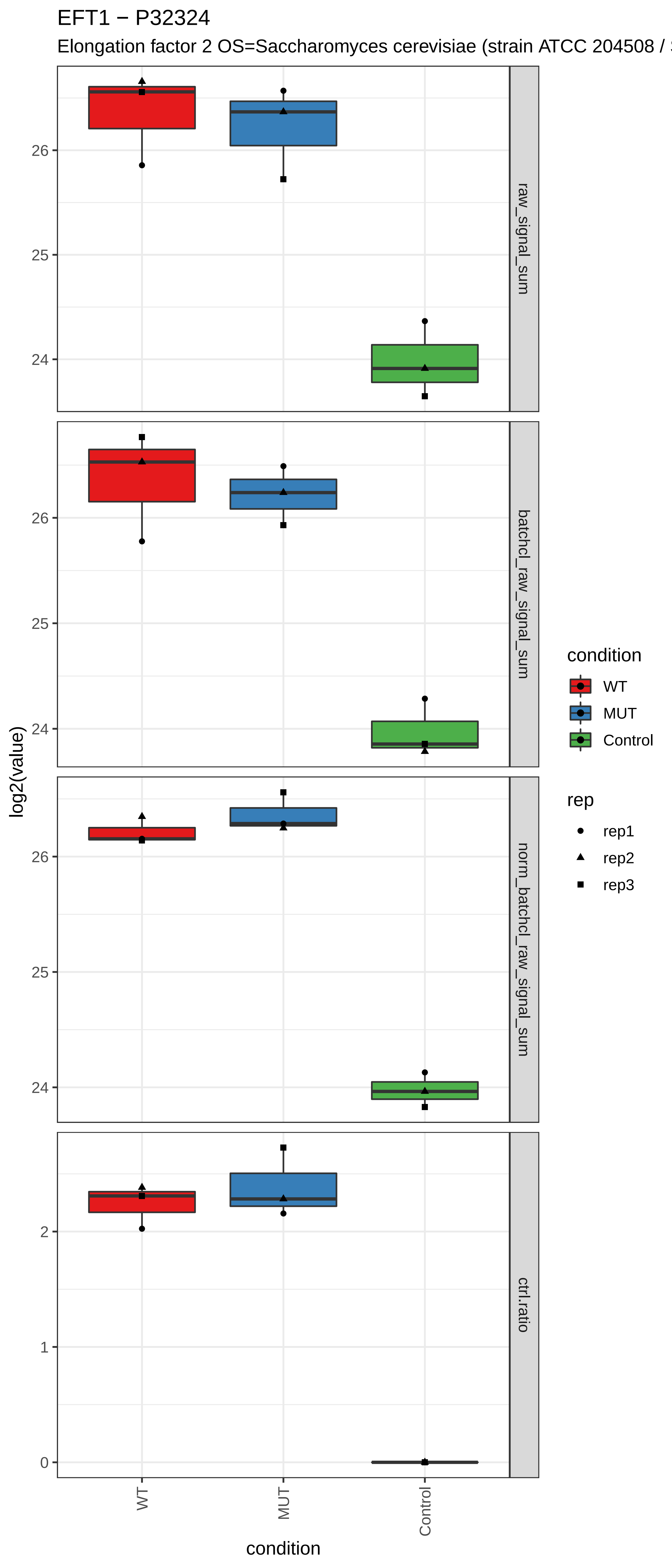
EFT1 − P32324
Elongation factor 2 OS=Saccharomyces cerevisiae (strain ATCC 204508 /
raw_signal_sum
batchcl_raw_signal_sum
norm_batchcl_raw_signal_sum
ctrl.ratio
log2(value)
26
25
24
2
1
0
WT
MUT
Control
condition
condition
WT
MUT
Control
rep
rep1
rep2
rep3

EGD1 − Q02642

Nascent polypeptide−associated complex subunit beta−1 OS=Saccharomy

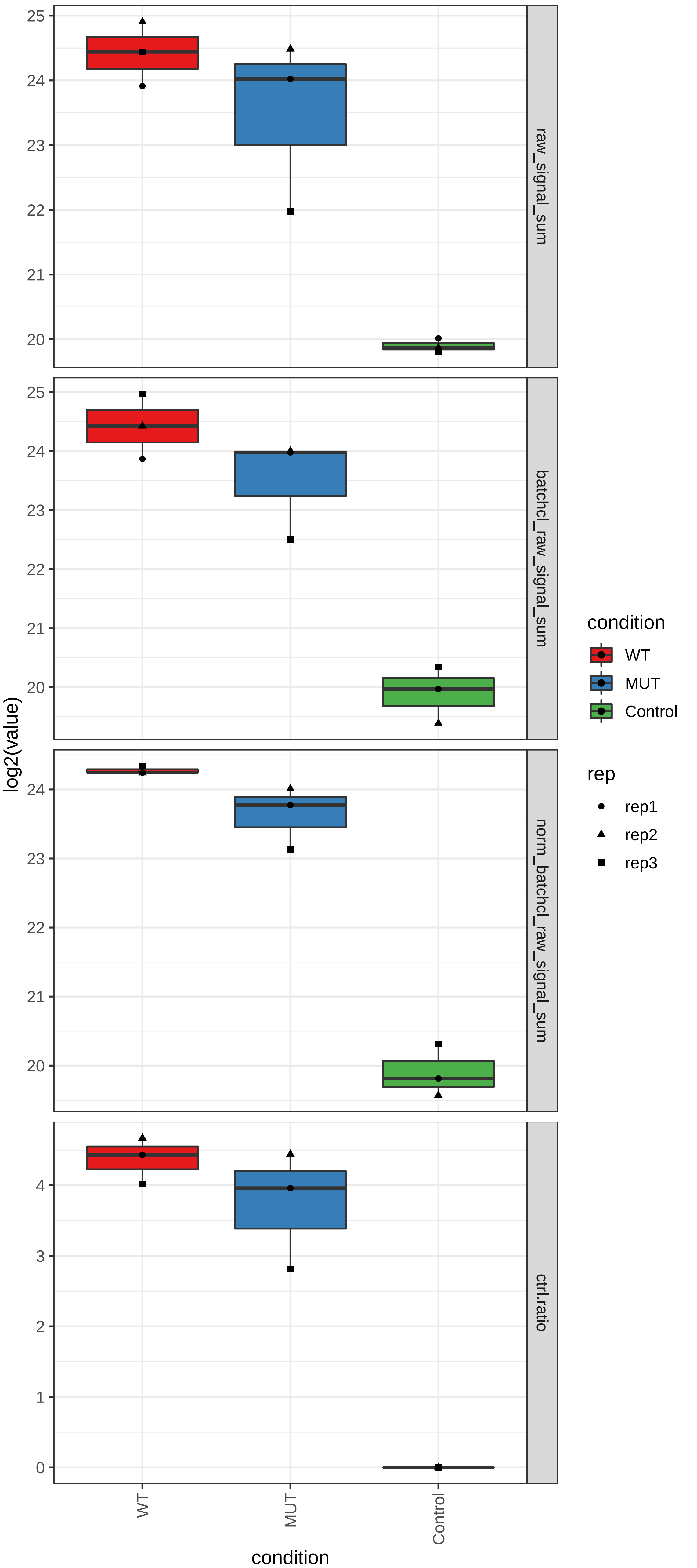

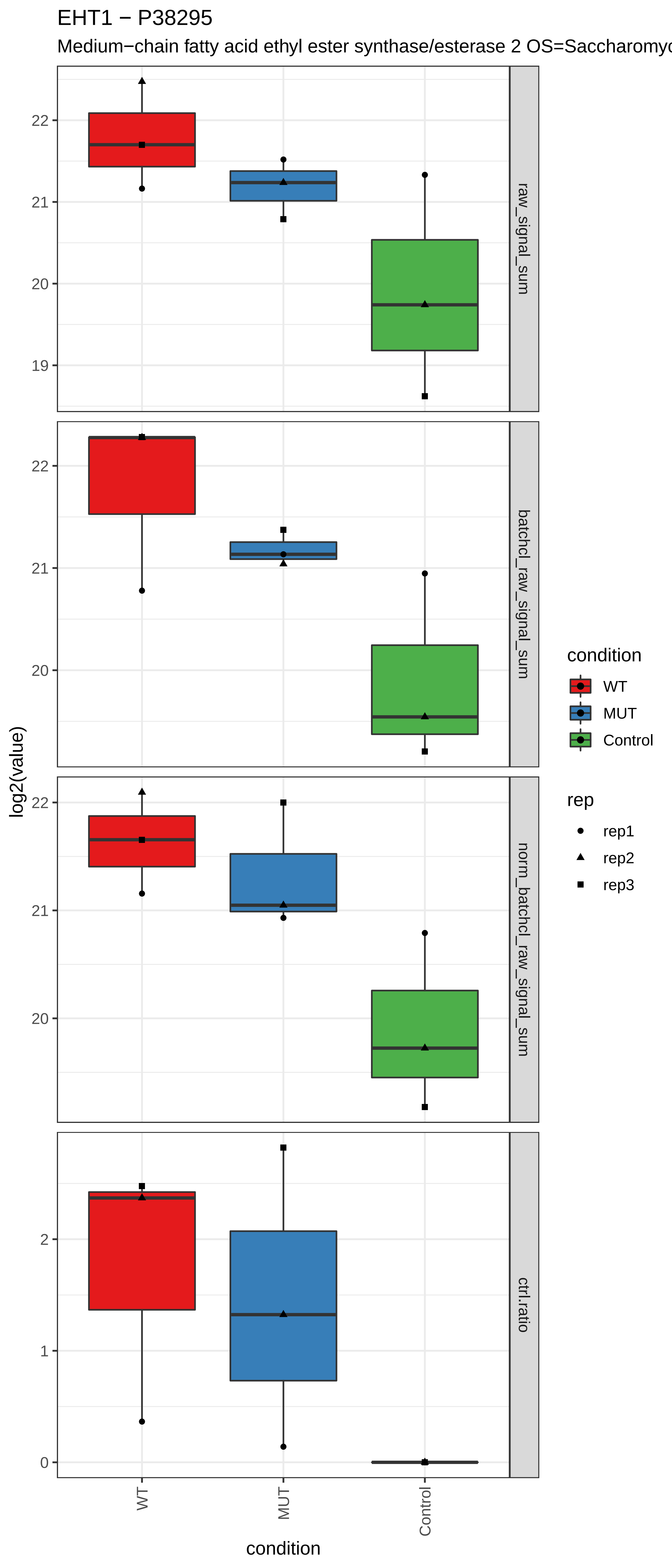
EHT1 − P38295
Medium−chain fatty acid ethyl ester synthase/esterase 2 OS=Saccharomyc
raw_signal_sum
batchcl_raw_signal_sum
norm_batchcl_raw_signal_sum
ctrl.ratio
log2(value)
condition
WT
MUT
Control
rep
rep1
rep2
rep3
22
21
20
19
2
1
0

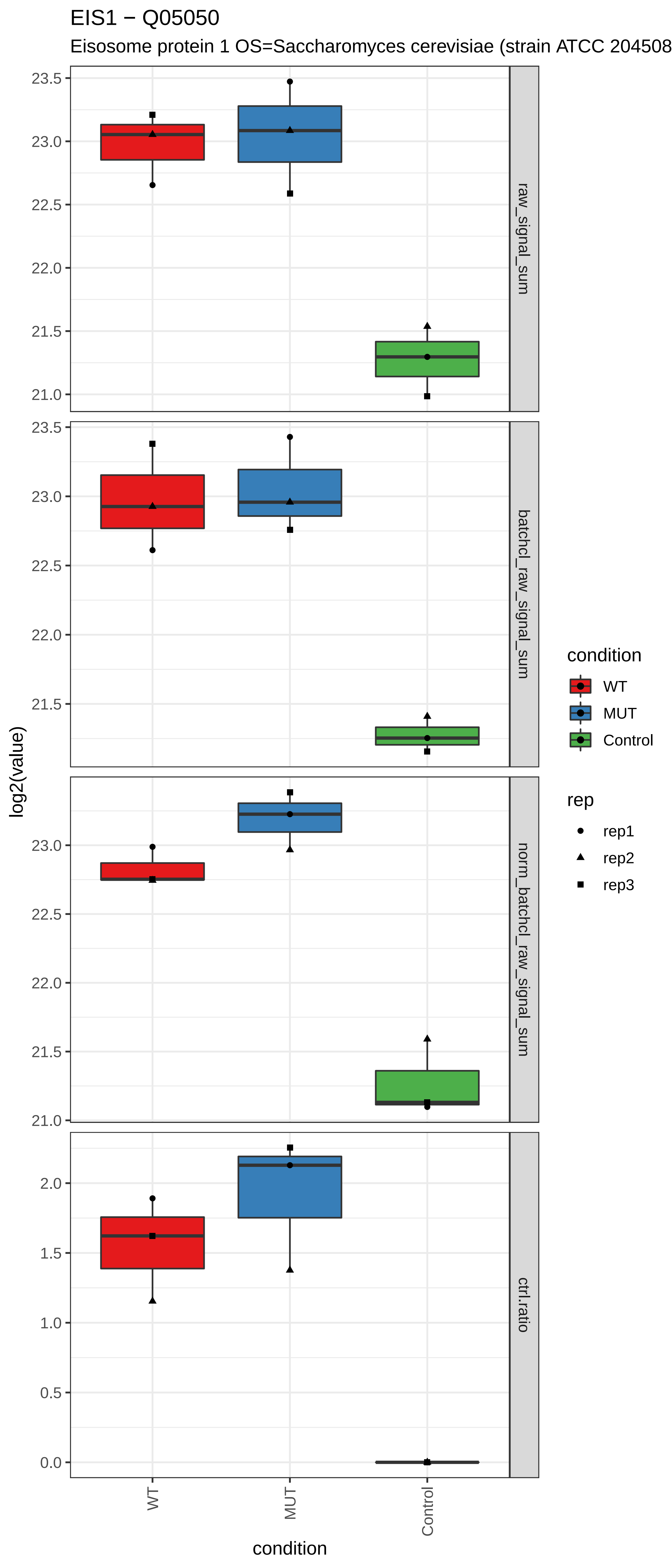

EIS1 − Q05050
Eisosome protein 1 OS=Saccharomyces cerevisiae (strain ATCC 204508
log2(value)
raw_signal_sum
batchcl_raw_signal_sum
norm_batchcl_raw_signal_sum
ctrl.ratio
23.5
23.0
22.5
22.0
21.5
21.0
2.0
1.5
1.0
0.5
0.0
WT
MUT
Control
condition
condition
WT
MUT
Control
rep
rep1
rep2
rep3

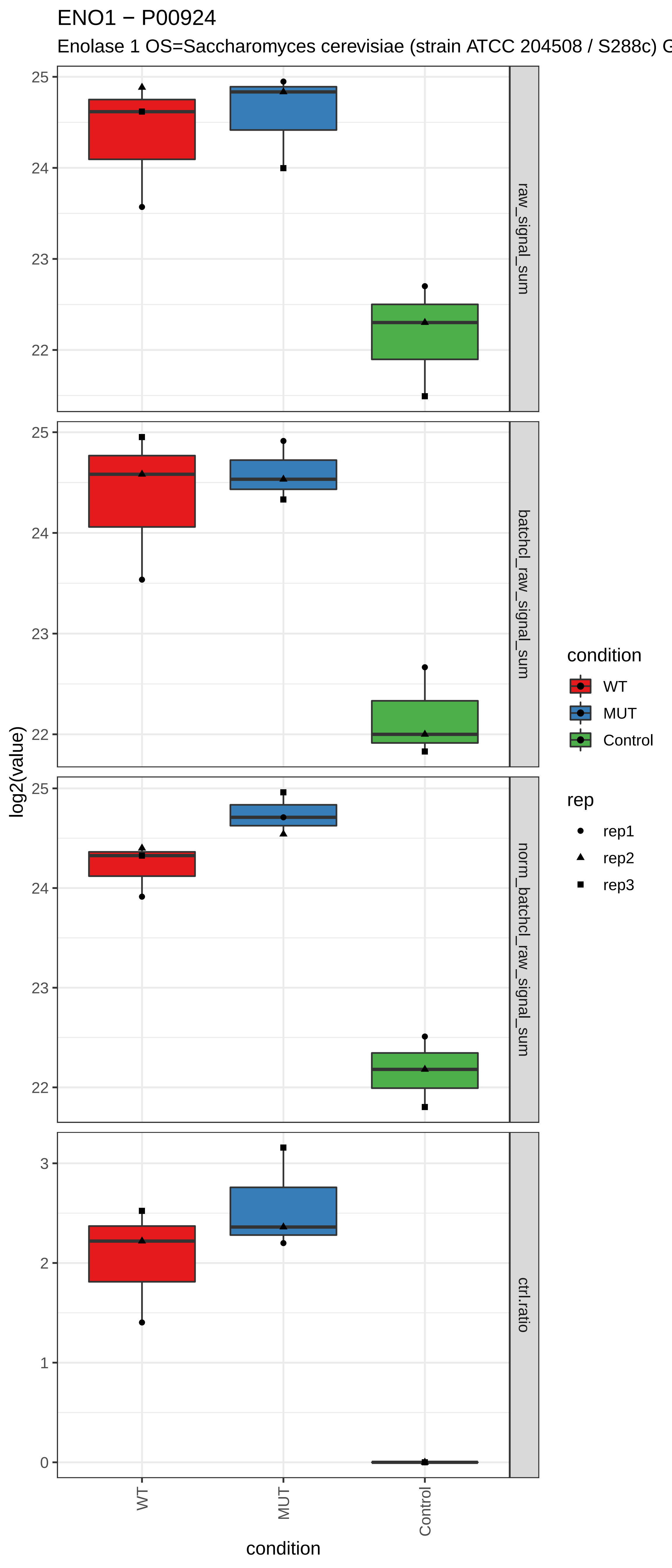

ENO1 − P00924
Enolase 1 OS=Saccharomyces cerevisiae (strain ATCC 204508 / S288c) G
raw_signal_sum
batchcl_raw_signal_sum
norm_batchcl_raw_signal_sum
ctrl.ratio
log2(value)
condition
WT
MUT
Control
rep
rep1
rep2
rep3

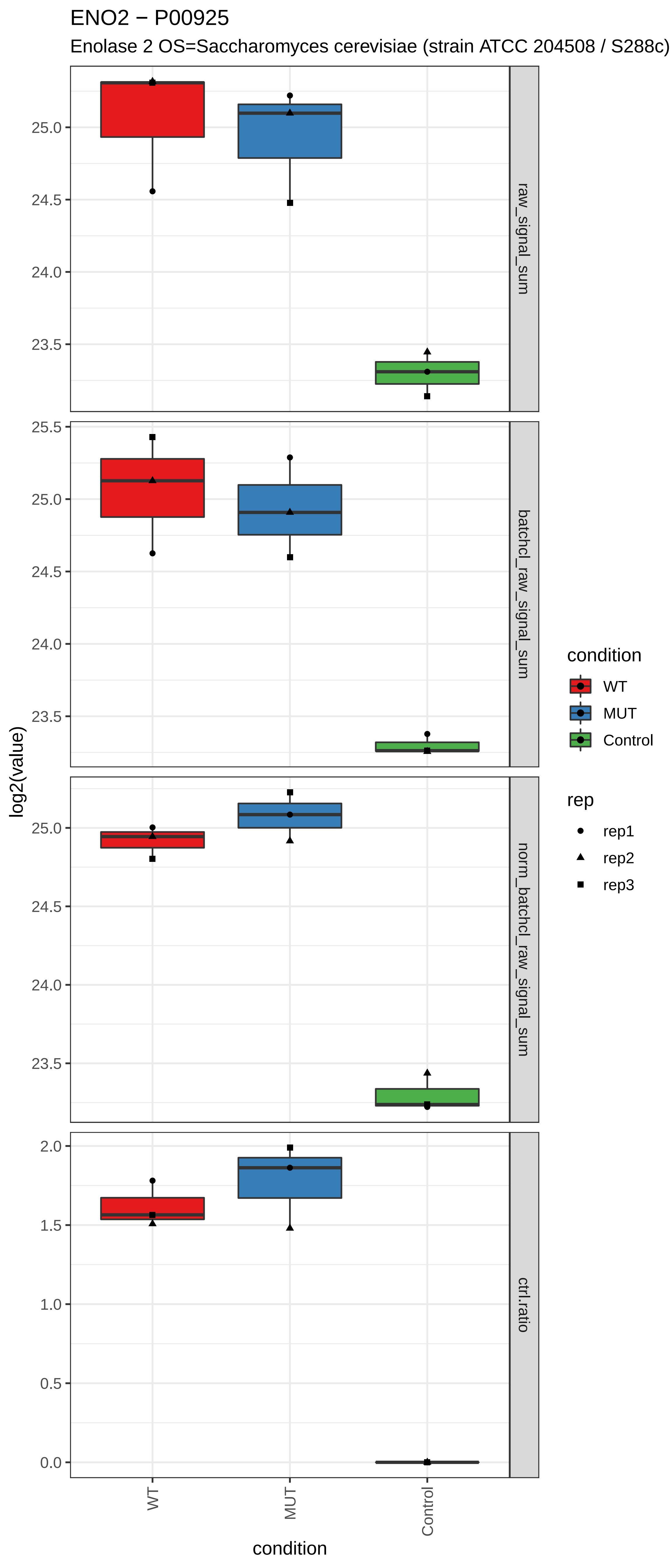
ENO2 − P00925
Enolase 2 OS=Saccharomyces cerevisiae (strain ATCC 204508 / S288c)
raw_signal_sum
batchcl_raw_signal_sum
norm_batchcl_raw_signal_sum
ctrl.ratio
log2(value)
condition
WT
MUT
Control
rep
rep1
rep2
rep3
25.0
24.5
24.0
23.5
25.5
2.0
1.5
1.0
0.5
0.0

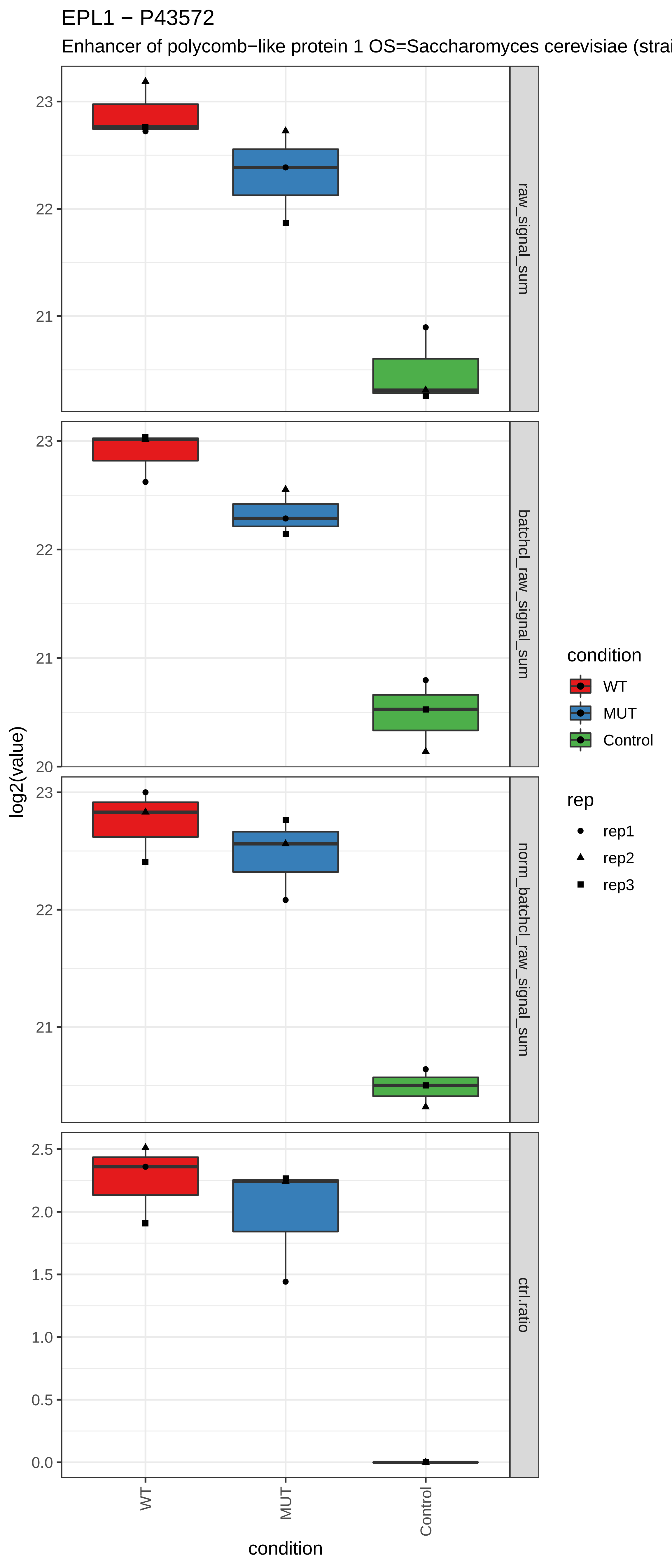
EPL1 − P43572
Enhancer of polycomb−like protein 1 OS=Saccharomyces cerevisiae (strai
raw_signal_sum
batchcl_raw_signal_sum
norm_batchcl_raw_signal_sum
ctrl.ratio
log2(value)
condition
WT
MUT
Control
rep
rep1
rep2
rep3
23
22
21
20
2.5
2.0
1.5
1.0
0.5
0.0

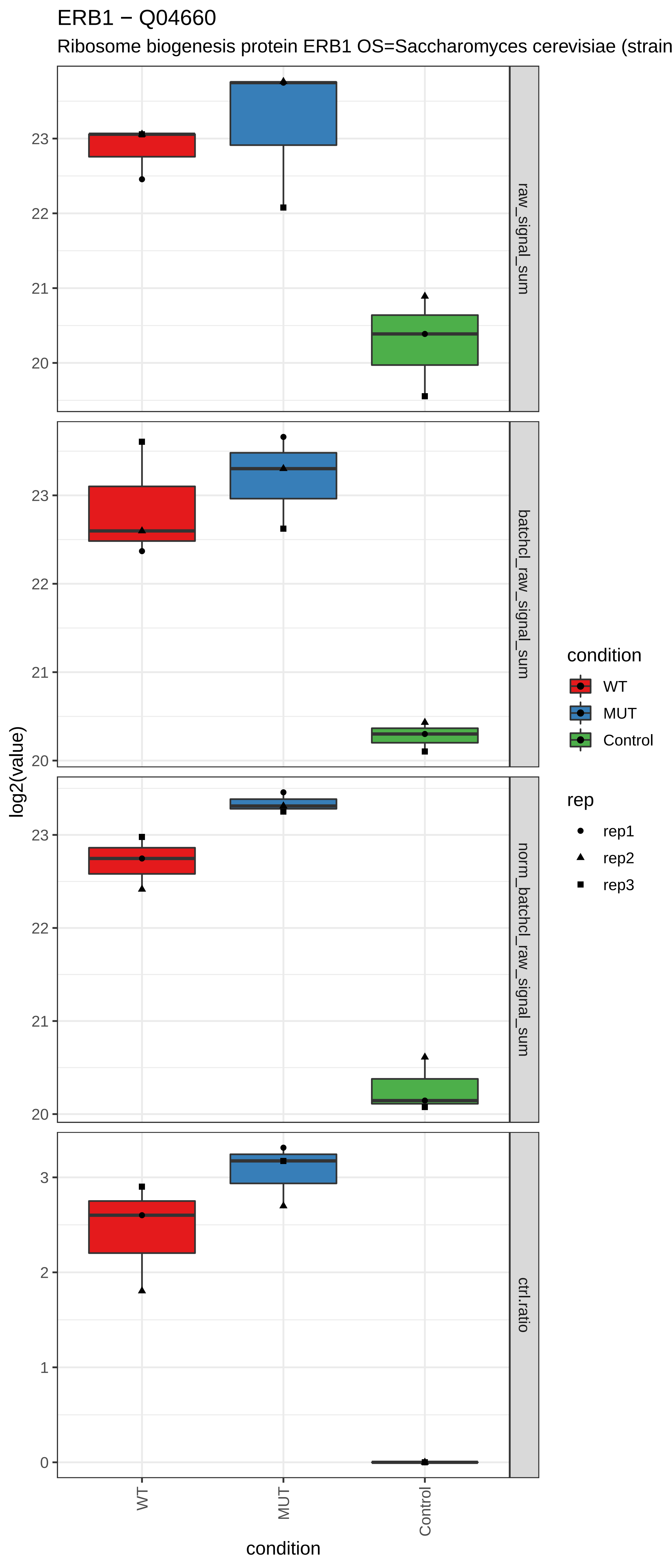

ERB1 − Q04660
Ribosome biogenesis protein ERB1 OS=Saccharomyces cerevisiae (strain
raw_signal_sum
batchcl_raw_signal_sum
norm_batchcl_raw_signal_sum
ctrl.ratio
log2(value)
condition
WT
MUT
Control
rep
rep1
rep2
rep3

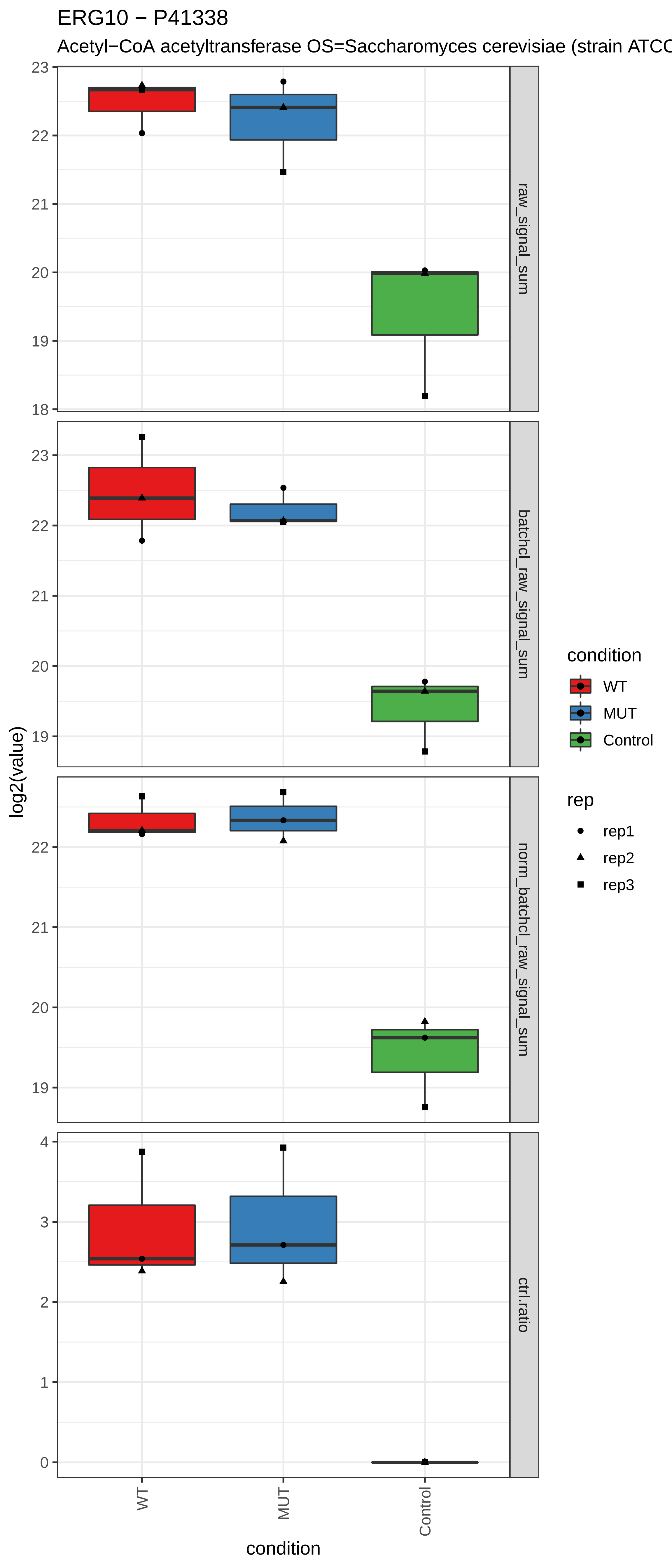

ERG10 − P41338
Acetyl−CoA acetyltransferase OS=Saccharomyces cerevisiae (strain ATCC
raw_signal_sum
batchcl_raw_signal_sum
norm_batchcl_raw_signal_sum
ctrl.ratio
log2(value)
condition
WT
MUT
Control
rep
rep1
rep2
rep3

ERG13 − P54839

Hydroxymethylglutaryl−CoA synthase OS=Saccharomyces cerevisiae (st

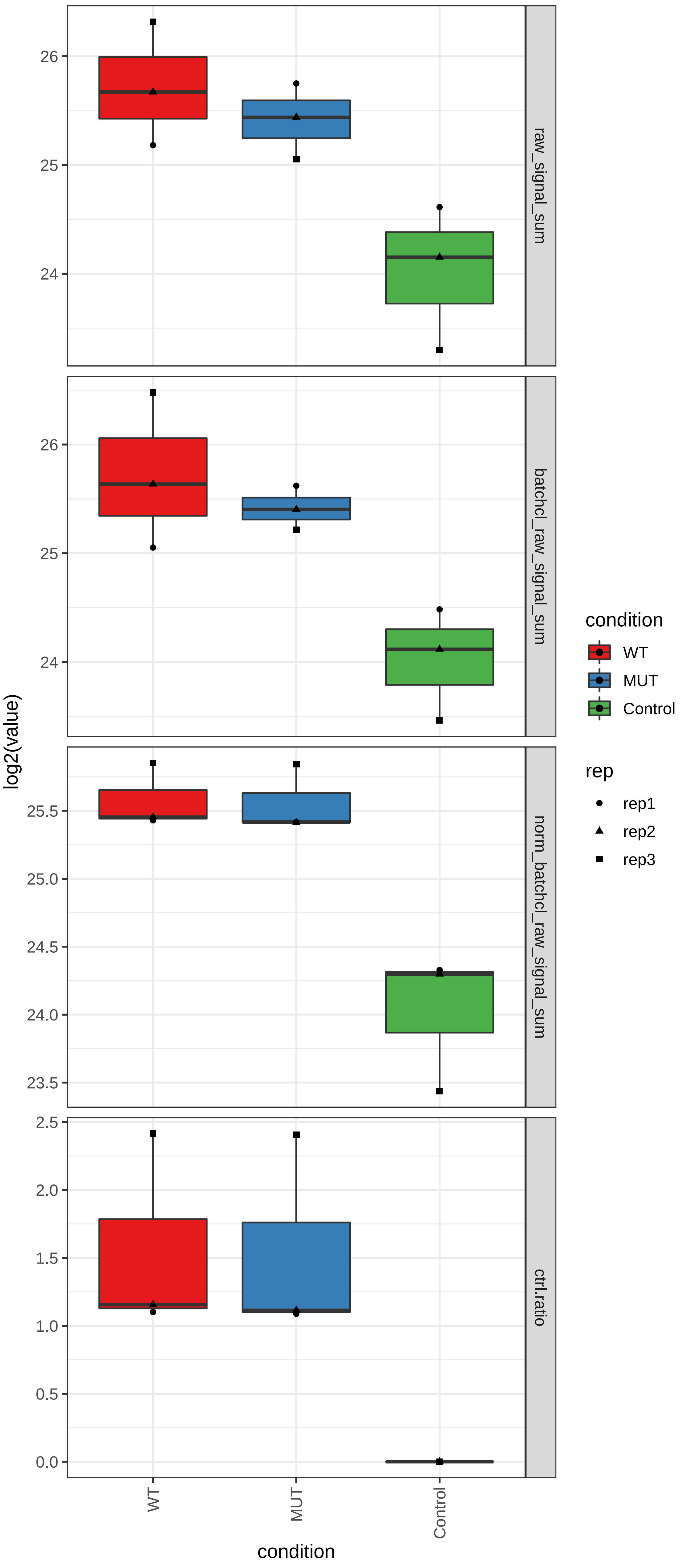

ERG20 − P08524

Farnesyl pyrophosphate synthase OS=Saccharomyces cerevisiae (strain

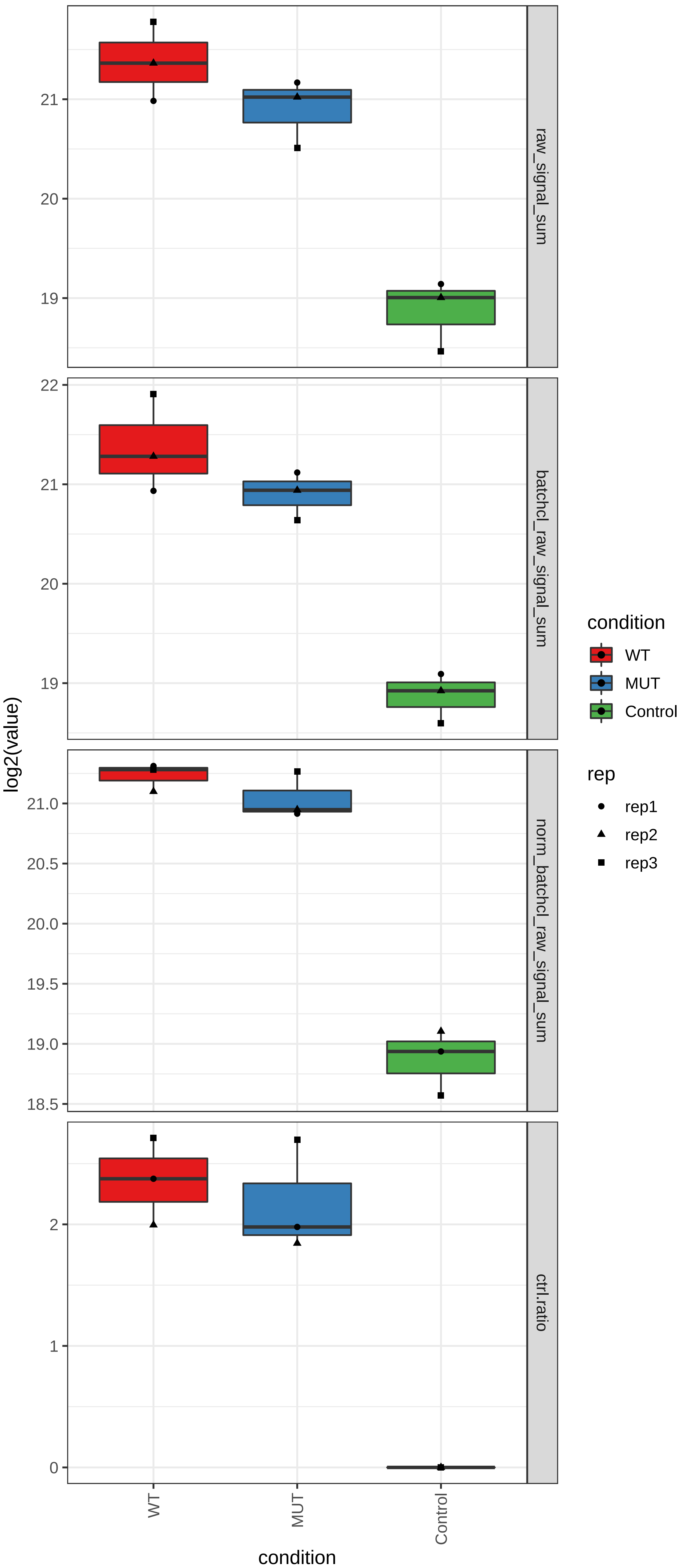

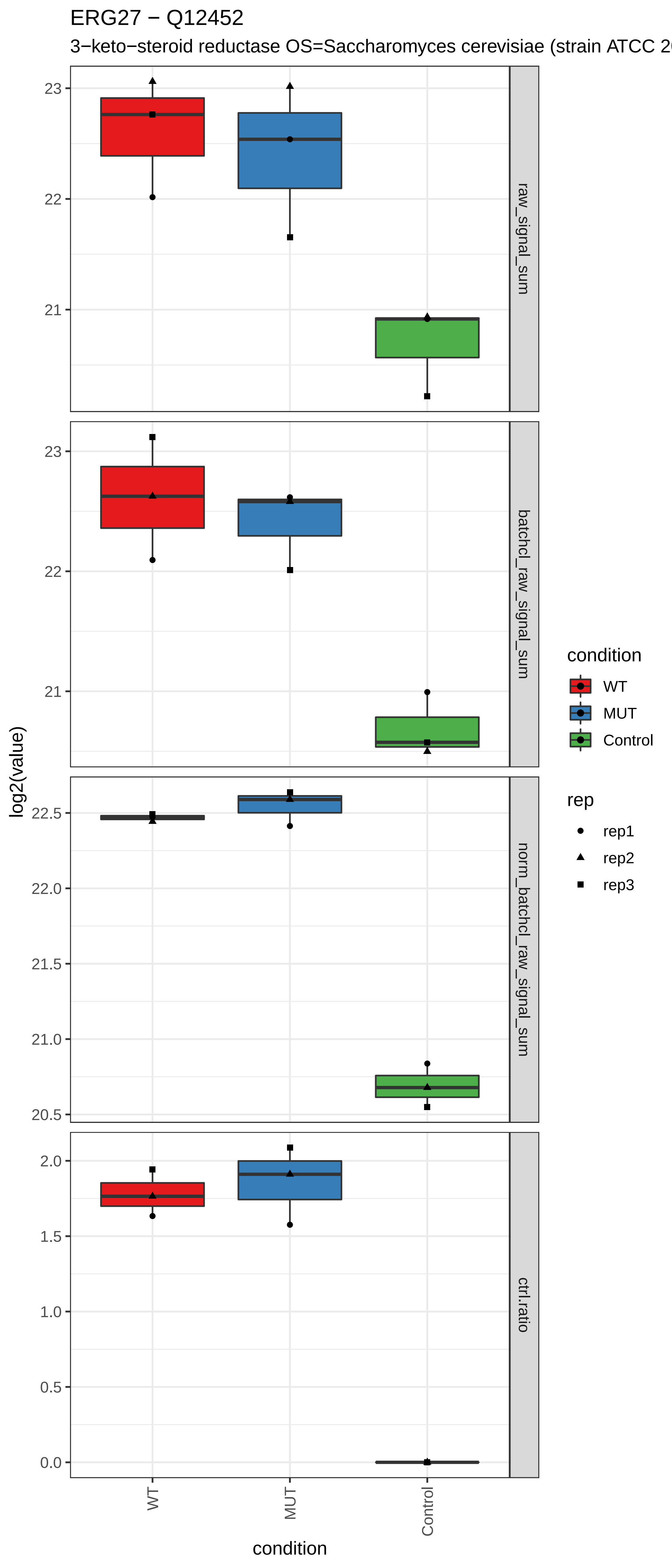

ERG27 − Q12452
3−keto−steroid reductase OS=Saccharomyces cerevisiae (strain ATCC 2
raw_signal_sum
batchcl_raw_signal_sum
norm_batchcl_raw_signal_sum
ctrl.ratio
log2(value)
condition
WT
MUT
Control
rep
rep1
rep2
rep3
23
22
21
22.5
22.0
21.5
21.0
20.5
2.0
1.5
1.0
0.5
0.0

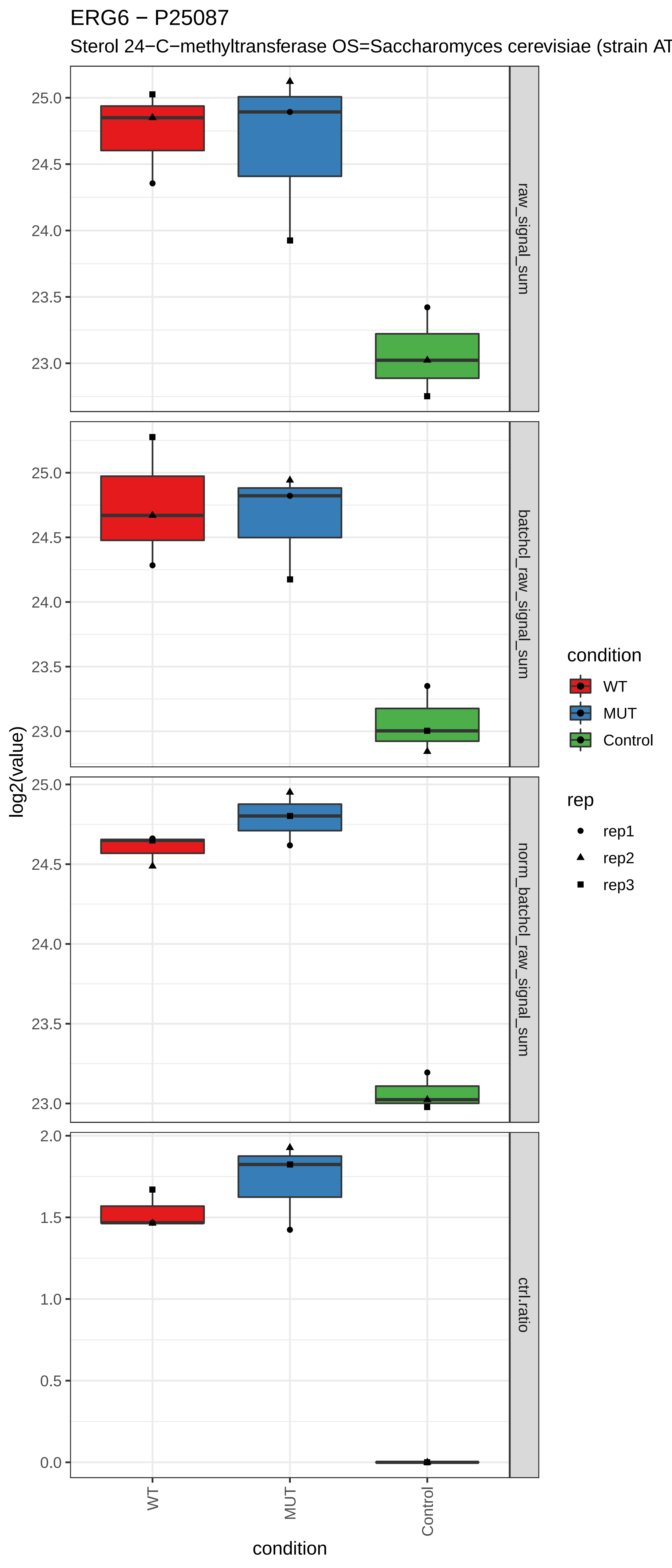

ERG6 − P25087
Sterol 24−C−methyltransferase OS=Saccharomyces cerevisiae (strain AT
raw_signal_sum
batchcl_raw_signal_sum
norm_batchcl_raw_signal_sum
ctrl.ratio
log2(value)
condition
WT
MUT
Control
rep
rep1
rep2
rep3
25.0
24.5
24.0
23.5
23.0
2.0
1.5
1.0
0.5
0.0

ERV29 − P53337

ER−derived vesicles protein ERV29 OS=Saccharomyces cerevisiae (strain

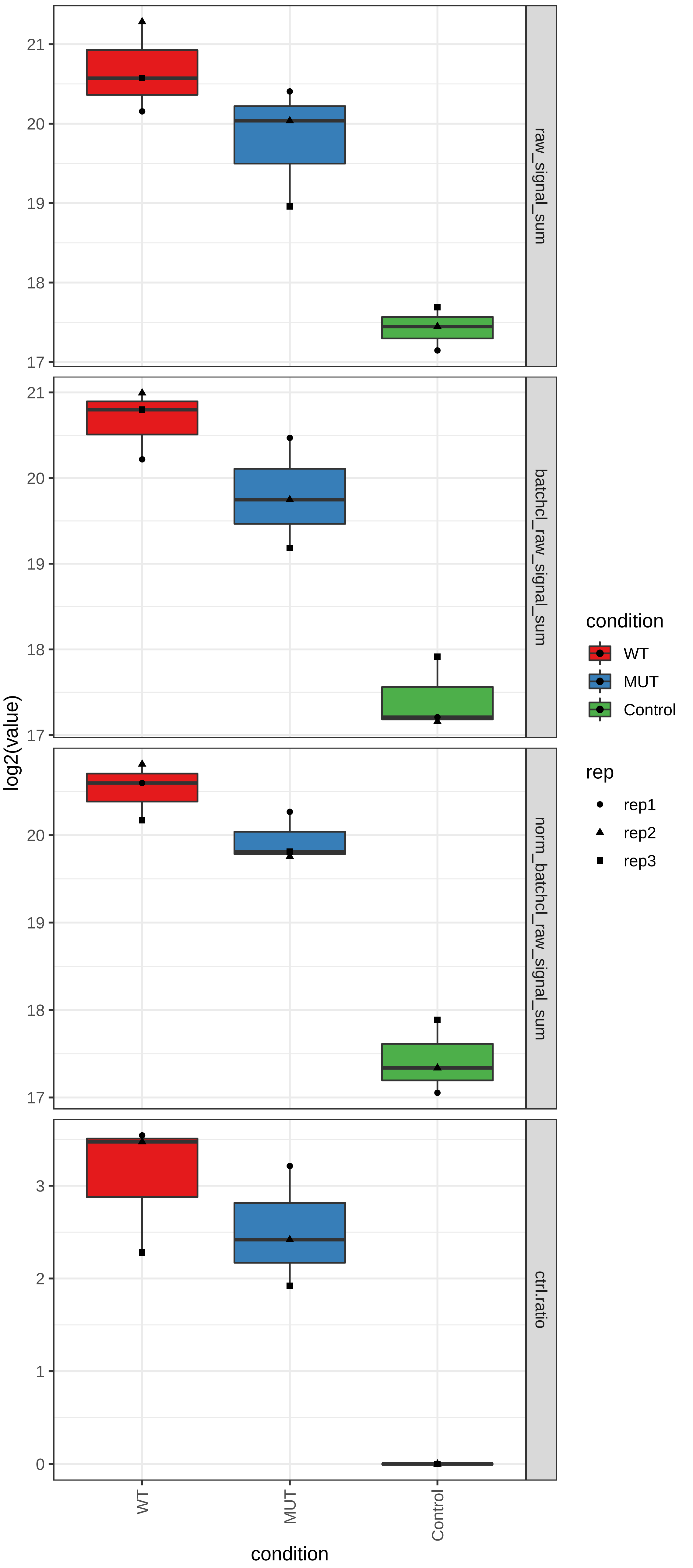

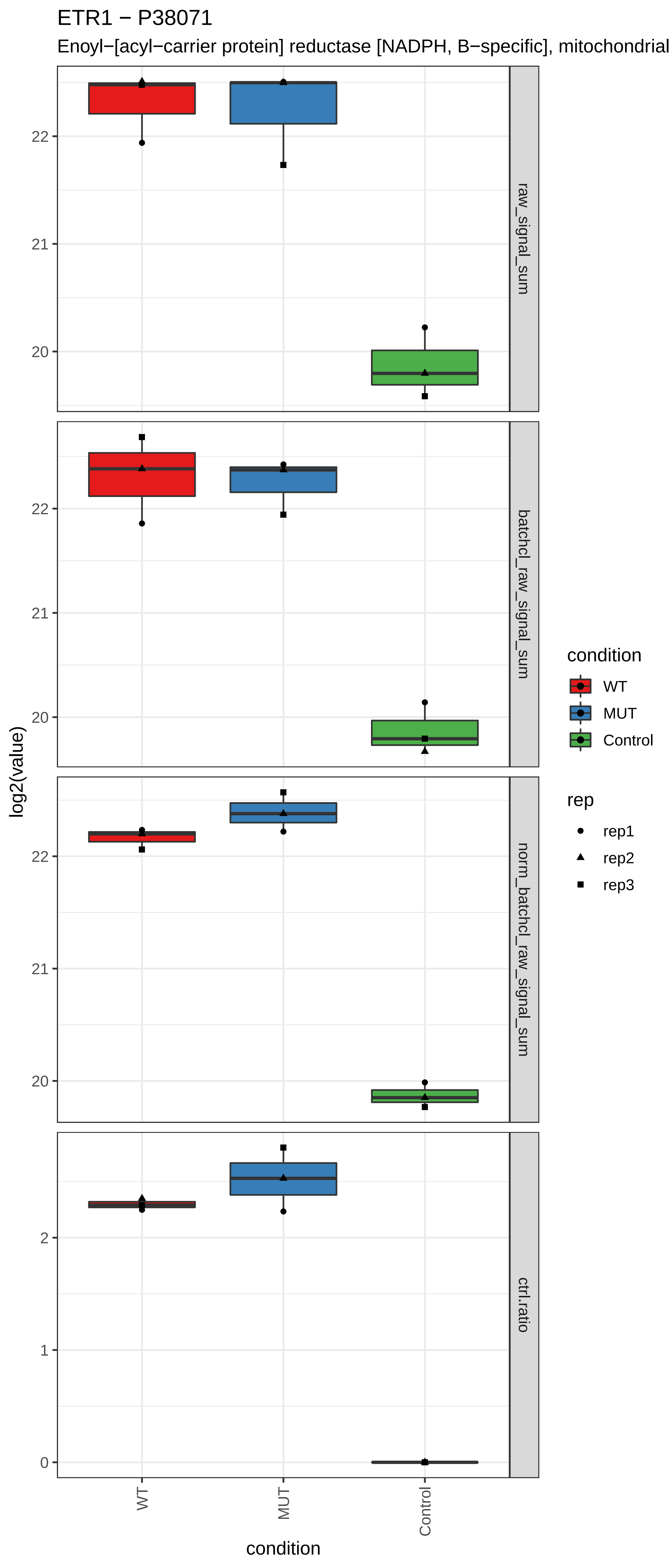
ETR1 − P38071
Enoyl−[acyl−carrier protein] reductase [NADPH, B−specific], mitochondrial
raw_signal_sum
batchcl_raw_signal_sum
norm_batchcl_raw_signal_sum
ctrl.ratio
log2(value)
condition
WT
MUT
Control
rep
rep1
rep2
rep3
22
21
20
2
1
0

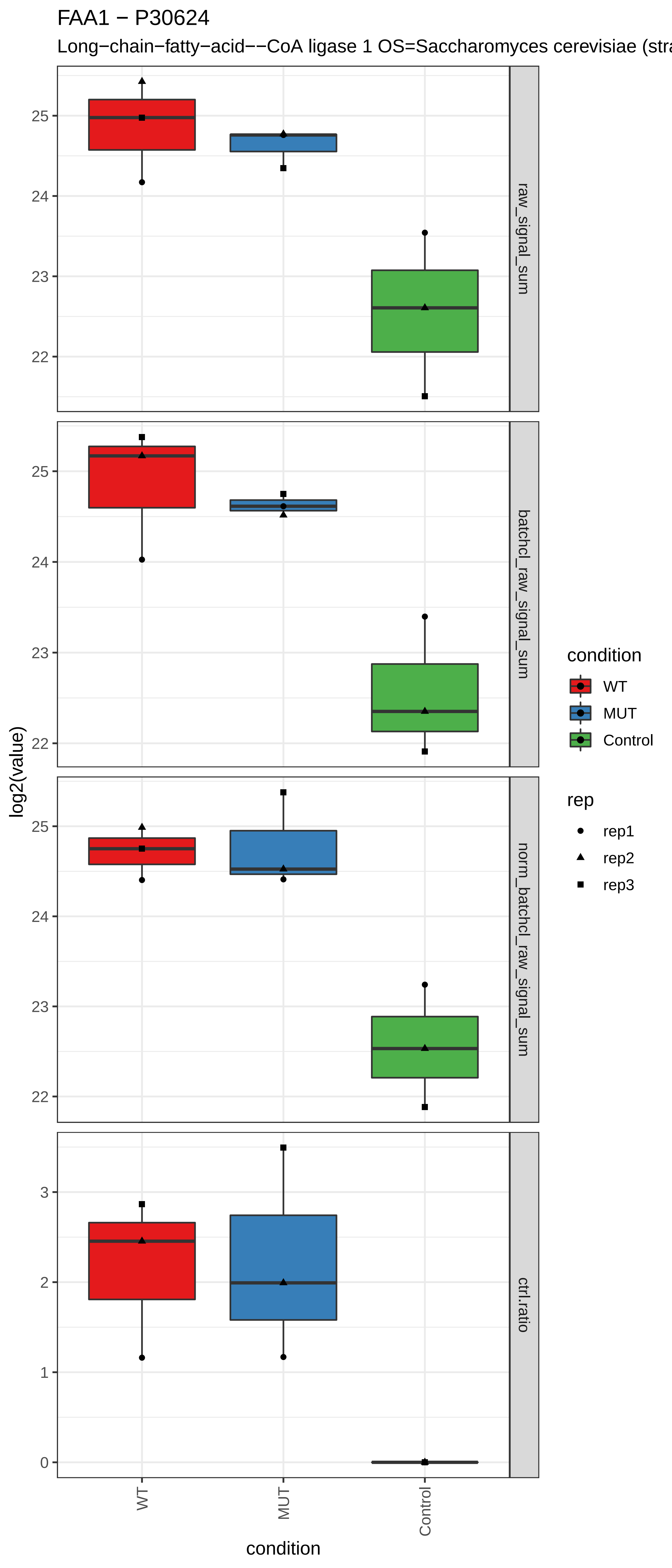

FAA1 − P30624
Long−chain−fatty−acid−−CoA ligase 1 OS=Saccharomyces cerevisiae (stra
raw_signal_sum
batchcl_raw_signal_sum
norm_batchcl_raw_signal_sum
ctrl.ratio
log2(value)
condition
WT
MUT
Control
rep
rep1
rep2
rep3

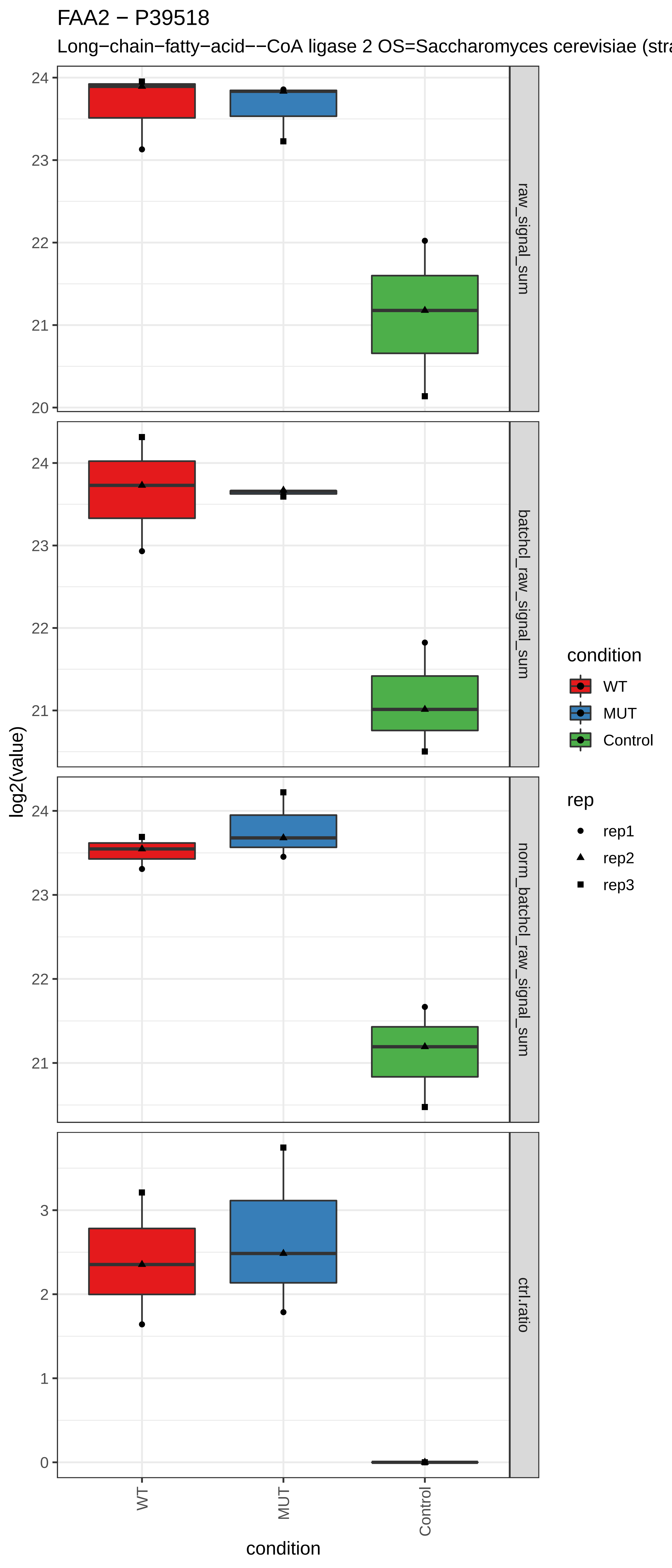

FAA2 − P39518
Long−chain−fatty−acid−−CoA ligase 2 OS=Saccharomyces cerevisiae (stra
raw_signal_sum
batchcl_raw_signal_sum
norm_batchcl_raw_signal_sum
ctrl.ratio
log2(value)
condition
WT
MUT
Control
rep
rep1
rep2
rep3
24
23
22
21
20
3
2
1
0

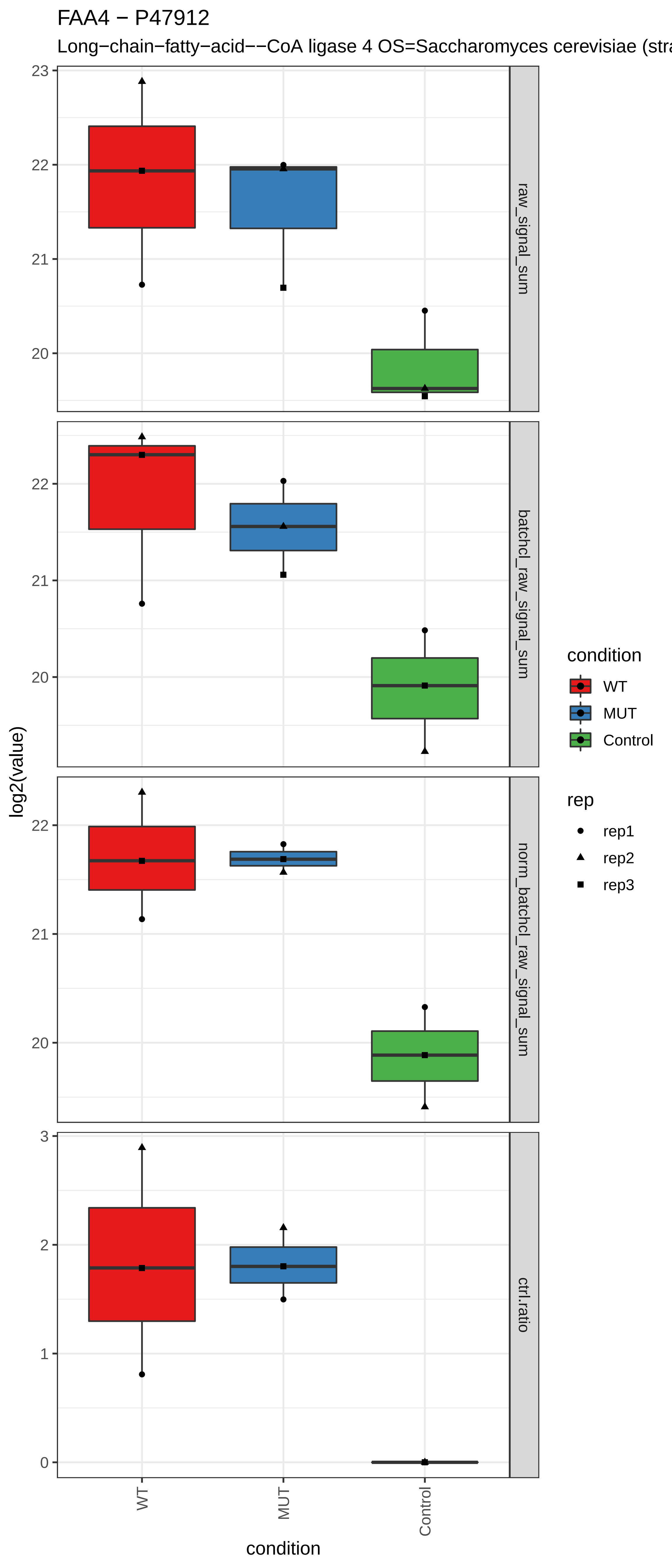

FAA4 − P47912
Long−chain−fatty−acid−−CoA ligase 4 OS=Saccharomyces cerevisiae (stra
raw_signal_sum
batchcl_raw_signal_sum
norm_batchcl_raw_signal_sum
ctrl.ratio
log2(value)
condition
WT
MUT
Control
rep
rep1
rep2
rep3

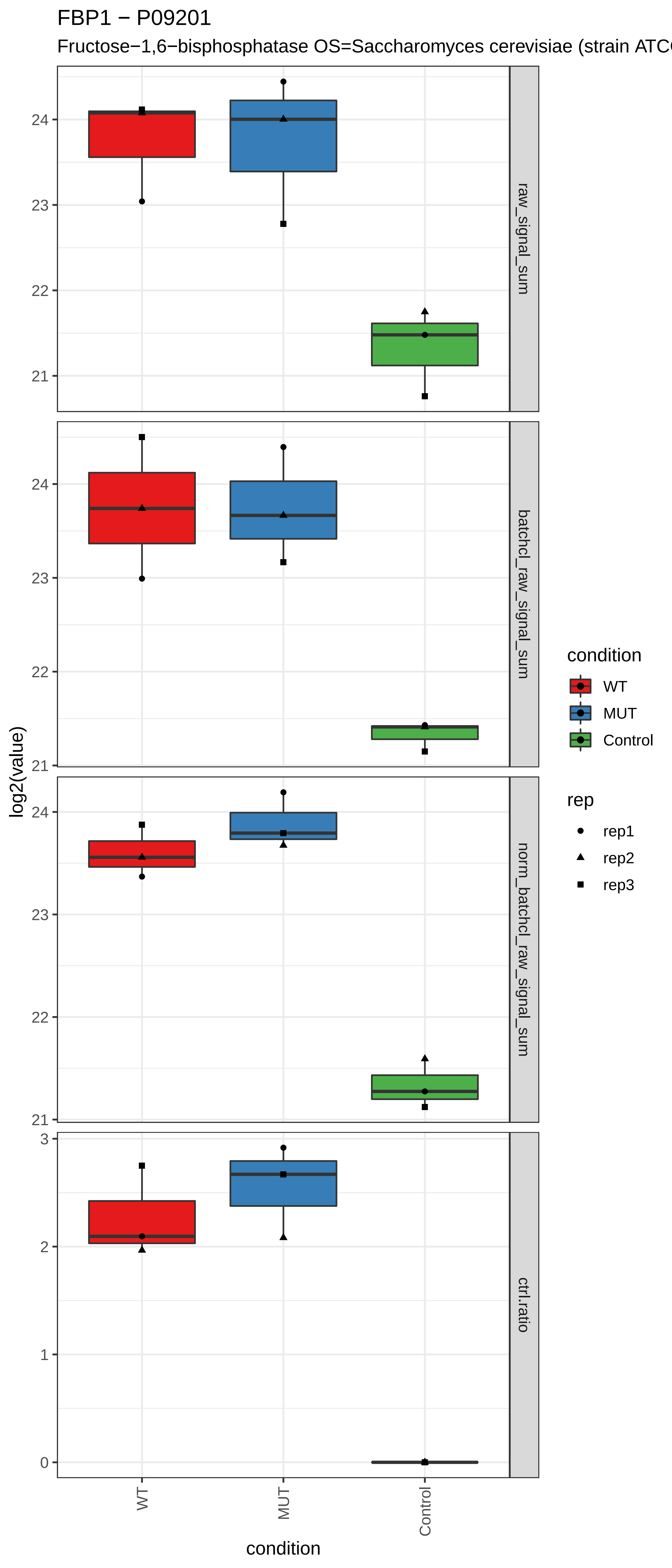
FBP1 − P09201
Fructose−1,6−bisphosphatase OS=Saccharomyces cerevisiae (strain ATC
raw_signal_sum
batchcl_raw_signal_sum
norm_batchcl_raw_signal_sum
ctrl.ratio
log2(value)
condition
WT
MUT
Control
rep
rep1
rep2
rep3
24
23
22
21
3
2
1
0

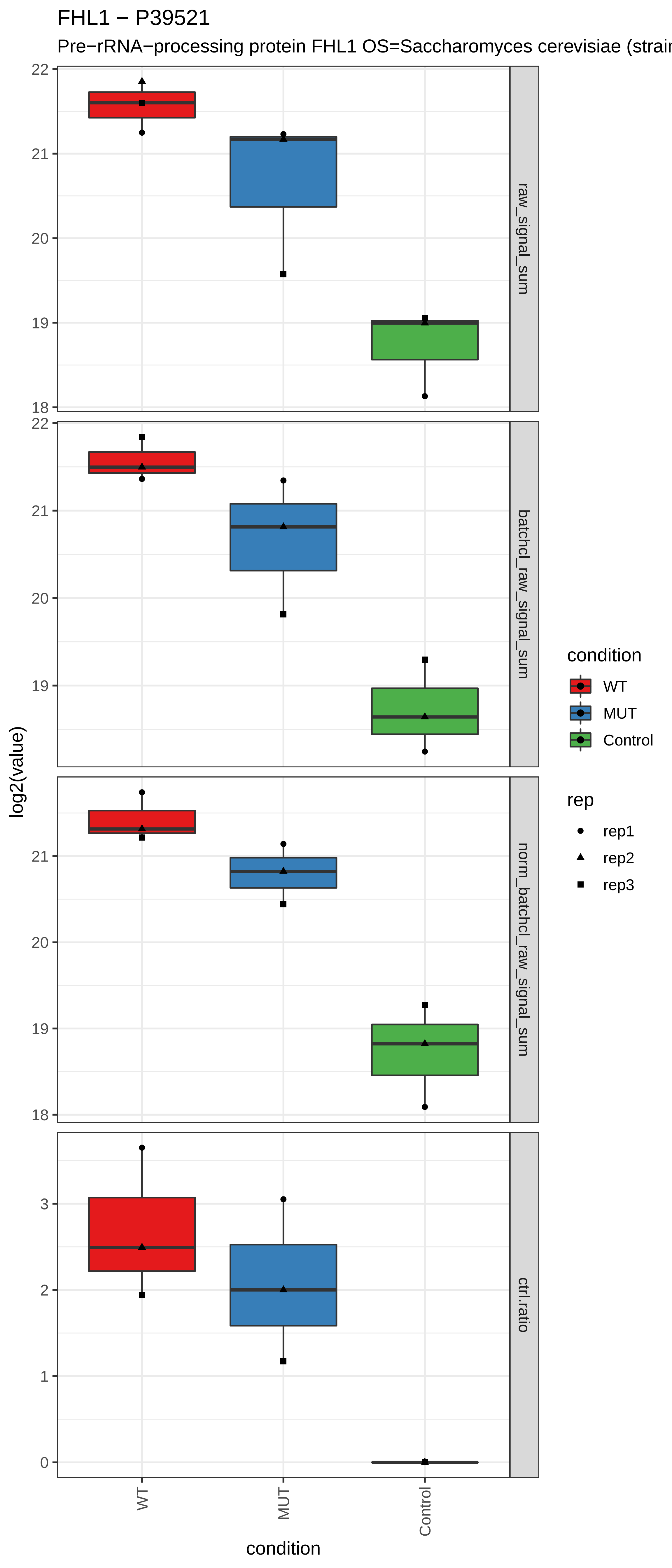

FHL1 − P39521
Pre−rRNA−processing protein FHL1 OS=Saccharomyces cerevisiae (strain
raw_signal_sum
batchcl_raw_signal_sum
norm_batchcl_raw_signal_sum
ctrl.ratio
log2(value)
condition
WT
MUT
Control
rep
rep1
rep2
rep3

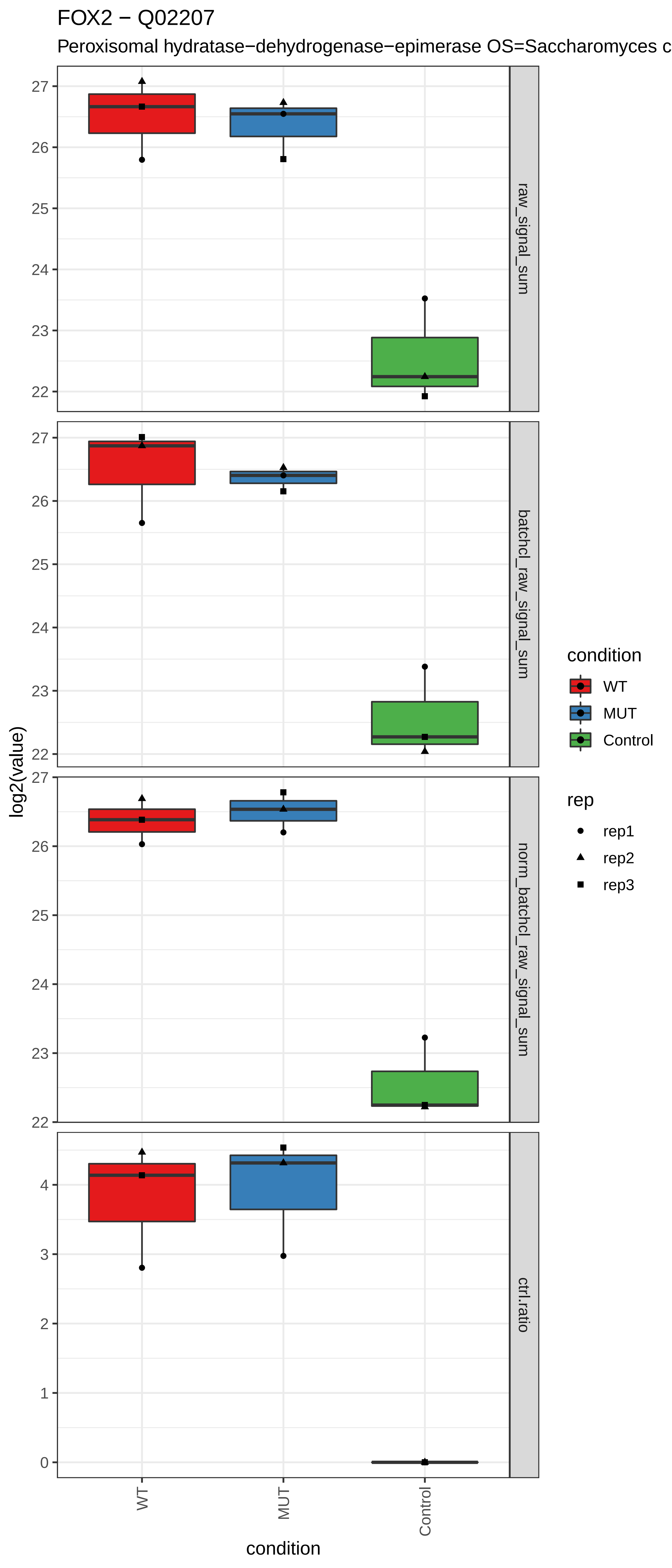

FOX2 − Q02207
Peroxisomal hydratase−dehydrogenase−epimerase OS=Saccharomyces c
raw_signal_sum
batchcl_raw_signal_sum
norm_batchcl_raw_signal_sum
ctrl.ratio
log2(value)
condition
WT
MUT
Control
rep
rep1
rep2
rep3

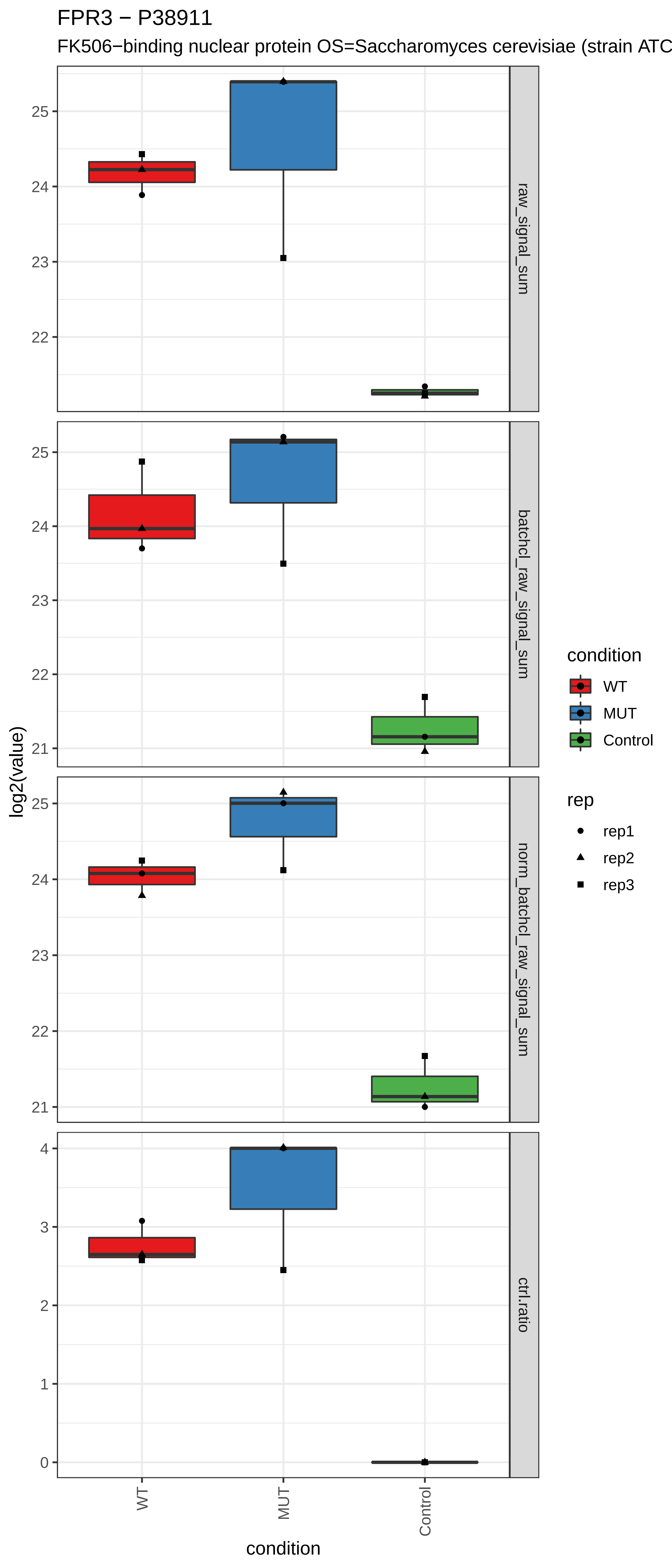

FPR3 − P38911
FK506−binding nuclear protein OS=Saccharomyces cerevisiae (strain ATC
raw_signal_sum
batchcl_raw_signal_sum
norm_batchcl_raw_signal_sum
ctrl.ratio
log2(value)
condition
WT
MUT
Control
rep
rep1
rep2
rep3

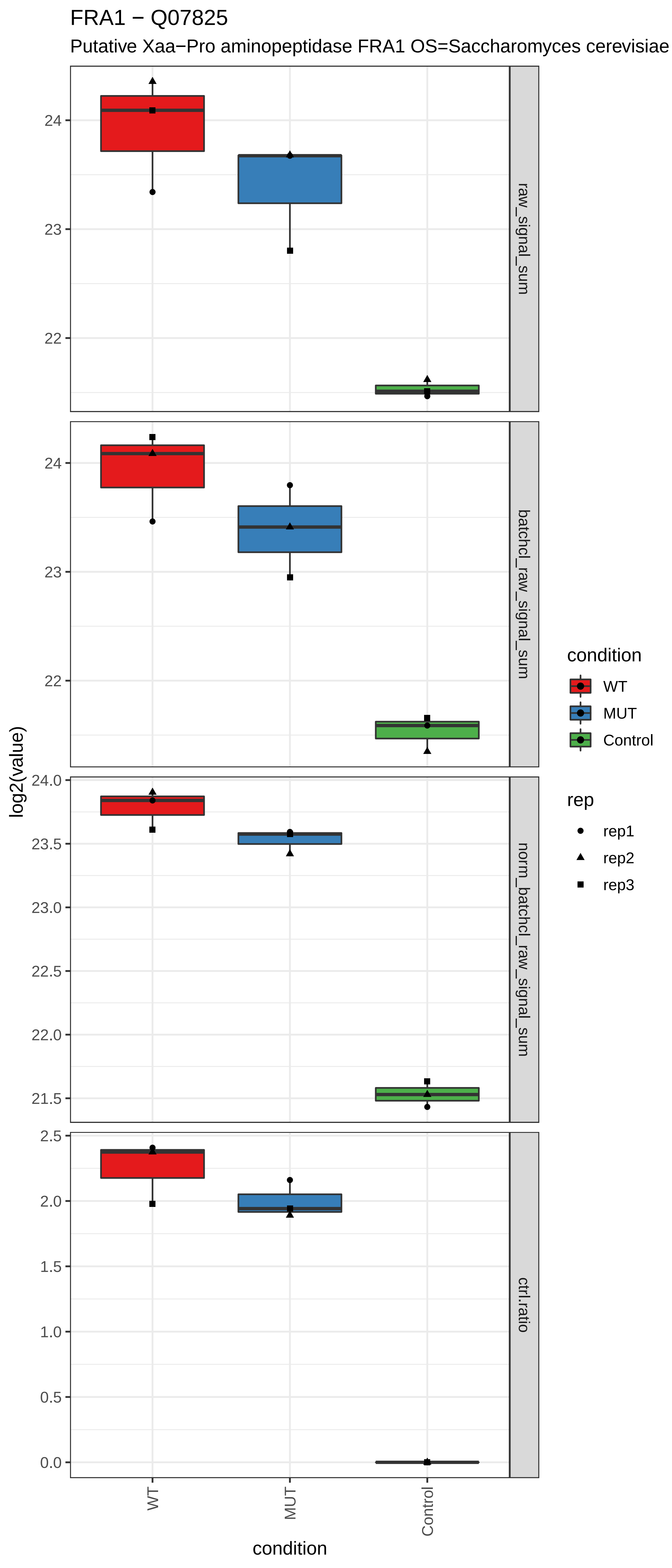
FRA1 − Q07825
Putative Xaa−Pro aminopeptidase FRA1 OS=Saccharomyces cerevisiae
raw_signal_sum
batchcl_raw_signal_sum
norm_batchcl_raw_signal_sum
ctrl.ratio
log2(value)
condition
WT
MUT
Control
rep
rep1
rep2
rep3

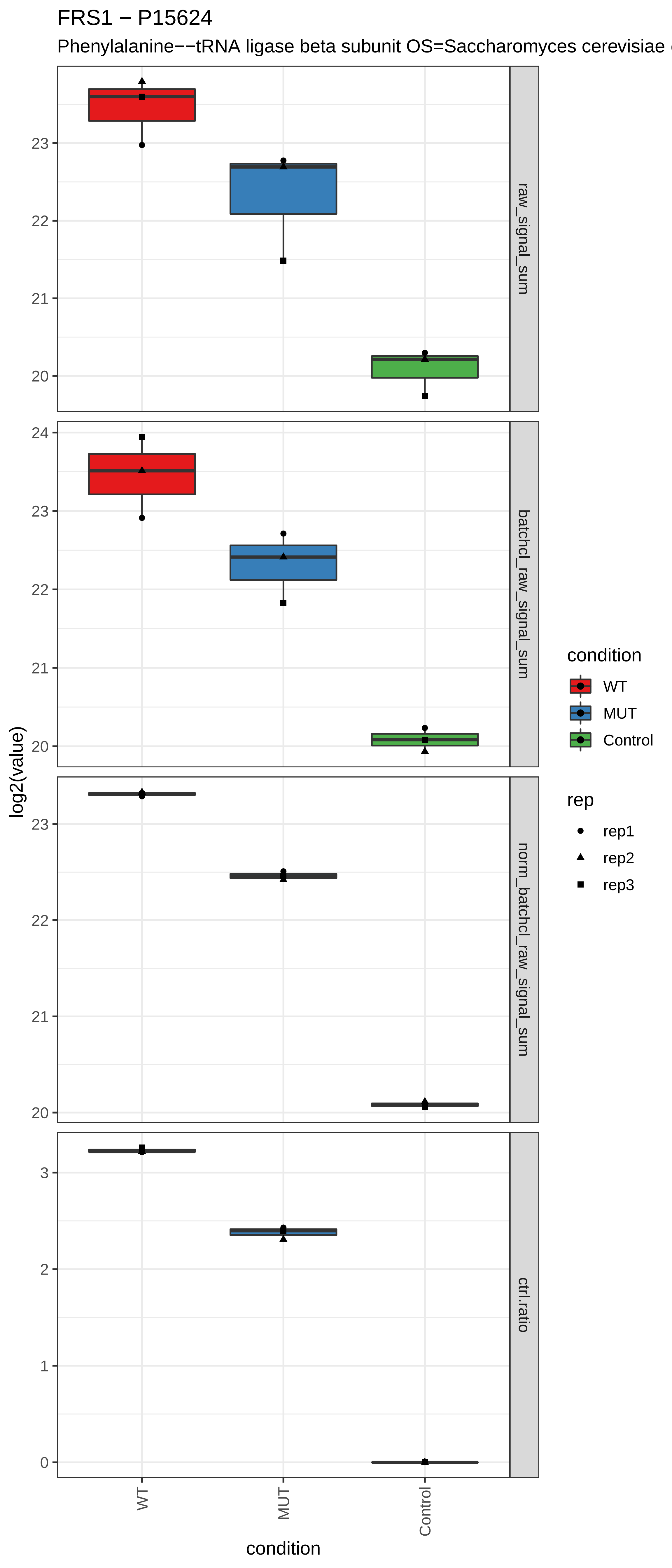

FRS1 − P15624
Phenylalanine−−tRNA ligase beta subunit OS=Saccharomyces cerevisiae
raw_signal_sum
batchcl_raw_signal_sum
norm_batchcl_raw_signal_sum
ctrl.ratio
log2(value)
condition
WT
MUT
Control
rep
rep1
rep2
rep3

FRS2 − P15625

Phenylalanine−−tRNA ligase alpha subunit OS=Saccharomyces cerevisiae

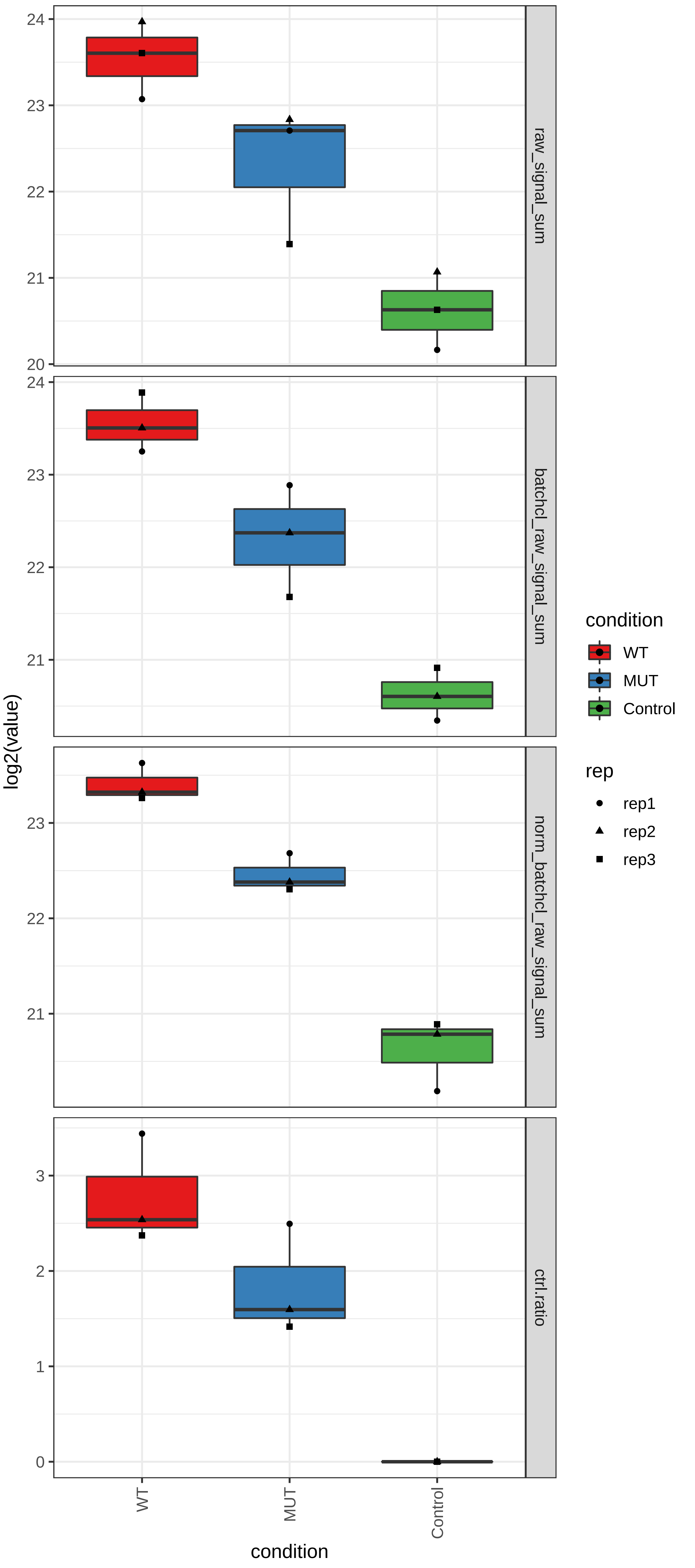

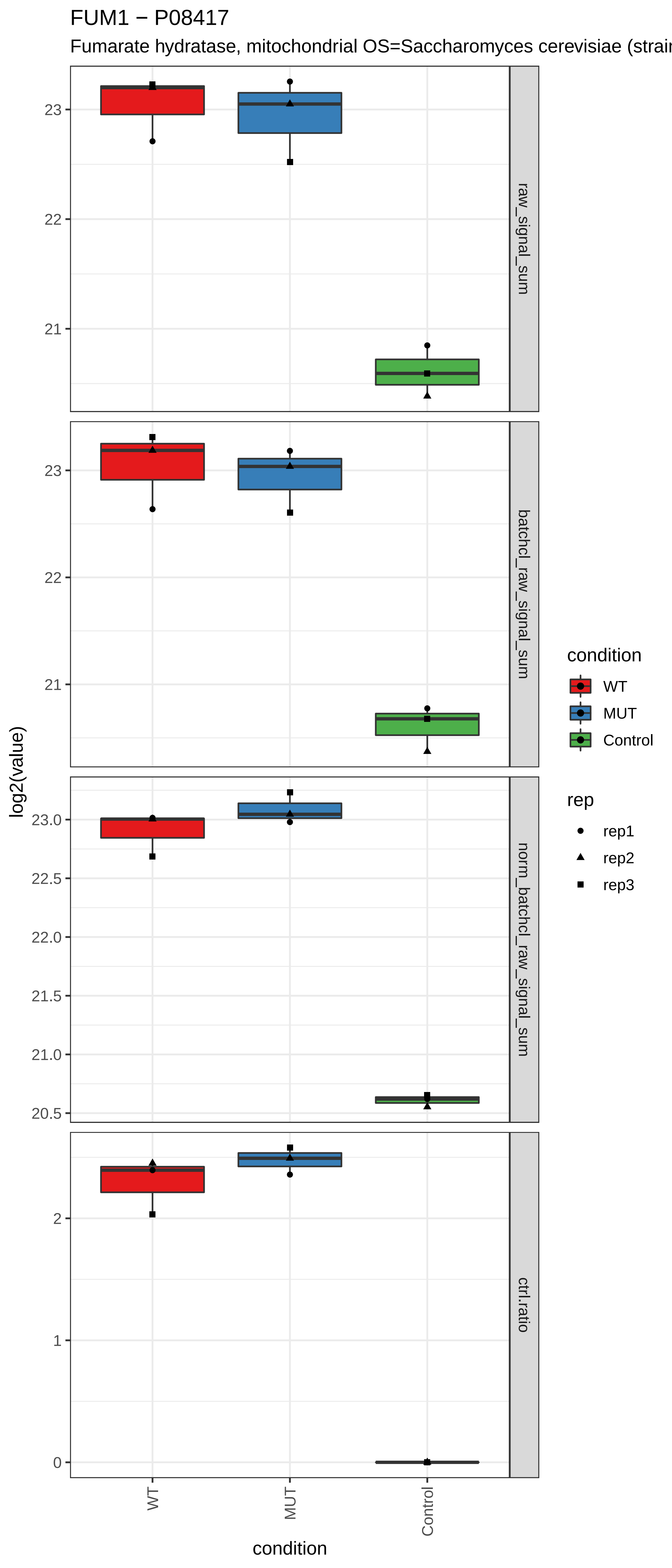

FUM1 − P08417
Fumarate hydratase, mitochondrial OS=Saccharomyces cerevisiae (strain
raw_signal_sum
batchcl_raw_signal_sum
norm_batchcl_raw_signal_sum
ctrl.ratio
log2(value)
condition
WT
MUT
Control
rep
rep1
rep2
rep3
23
22
21
23.0
22.5
22.0
21.5
21.0
20.5
2
1
0

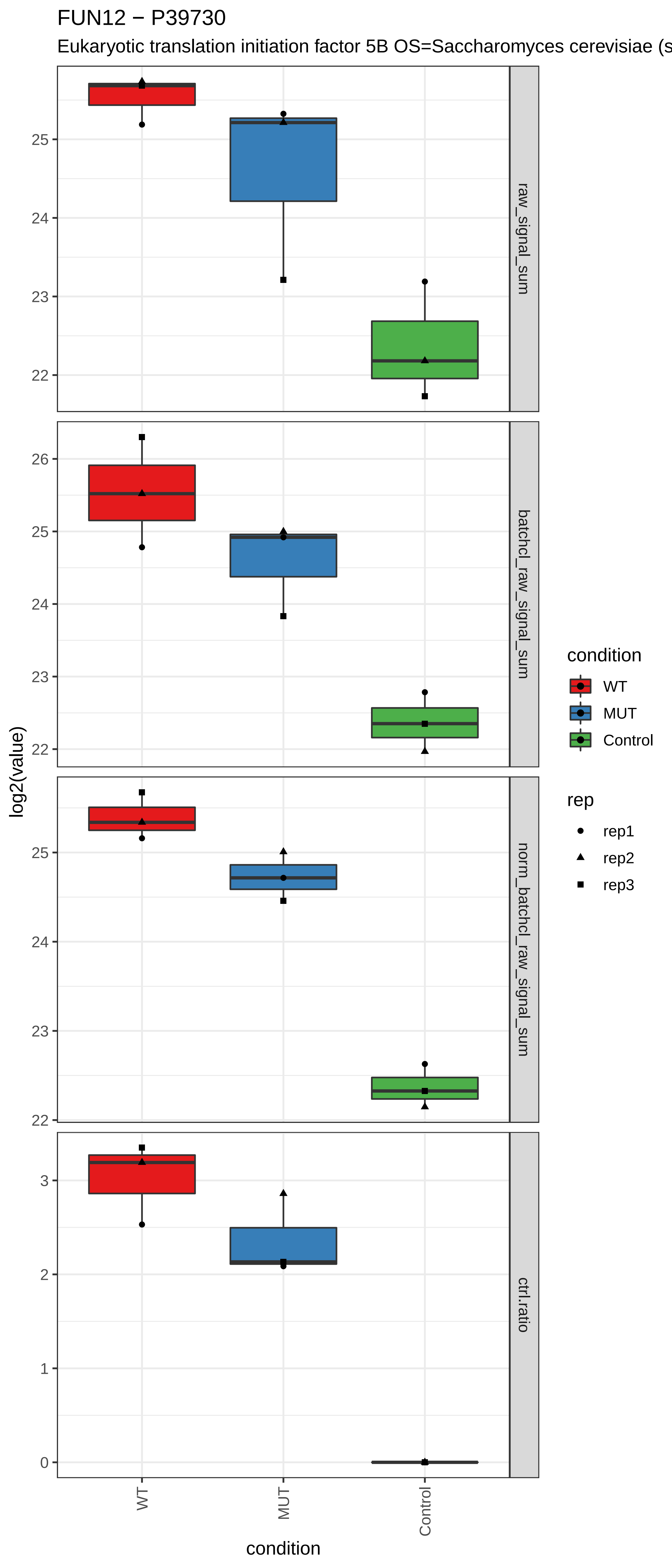
FUN12 − P39730
Eukaryotic translation initiation factor 5B OS=Saccharomyces cerevisiae (s
raw_signal_sum
batchcl_raw_signal_sum
norm_batchcl_raw_signal_sum
ctrl.ratio
log2(value)
condition
WT
MUT
Control
rep
rep1
rep2
rep3
25
24
23
22
26
3
2
1
0

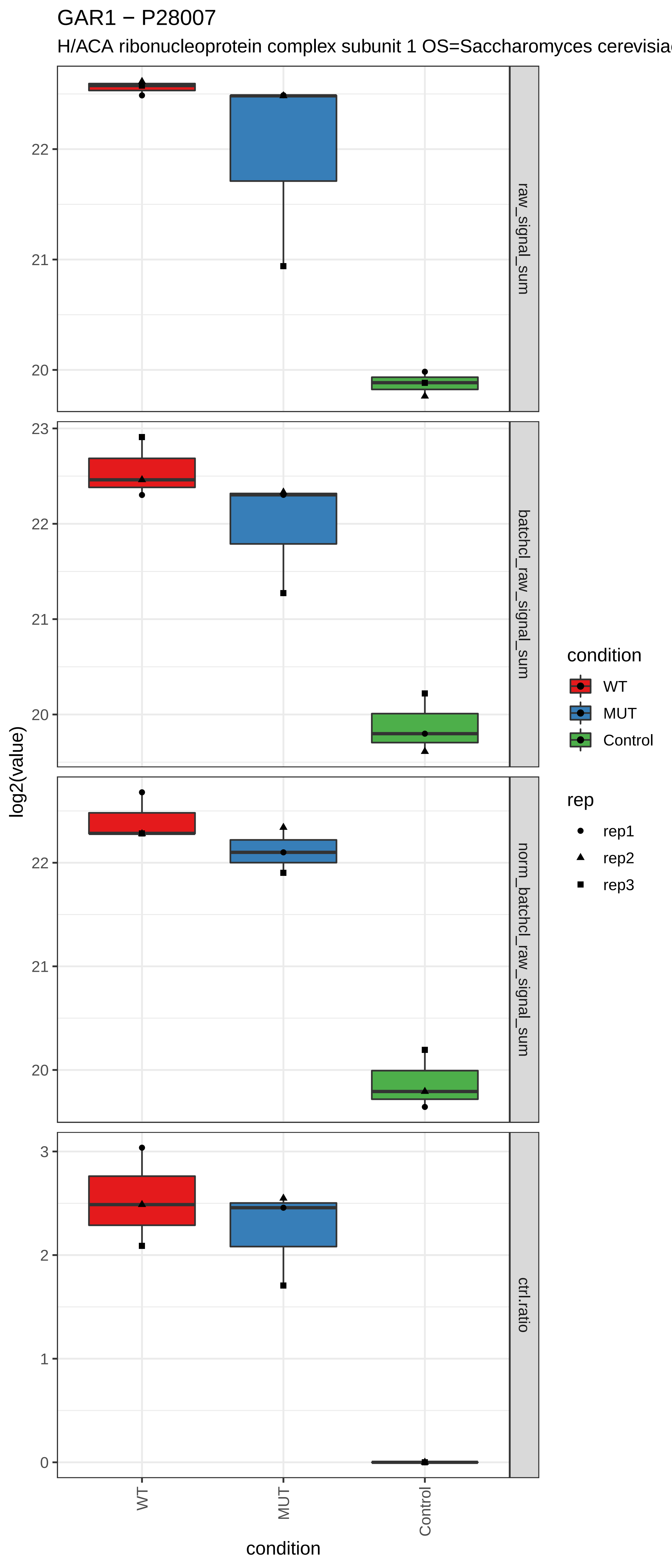

GAR1 − P28007
H/ACA ribonucleoprotein complex subunit 1 OS=Saccharomyces cerevisiae
raw_signal_sum
batchcl_raw_signal_sum
norm_batchcl_raw_signal_sum
ctrl.ratio
log2(value)
condition
WT
MUT
Control
rep
rep1
rep2
rep3

GAS1 − P22146

1,3−beta−glucanosyltransferase GAS1 OS=Saccharomyces cerevisiae (str

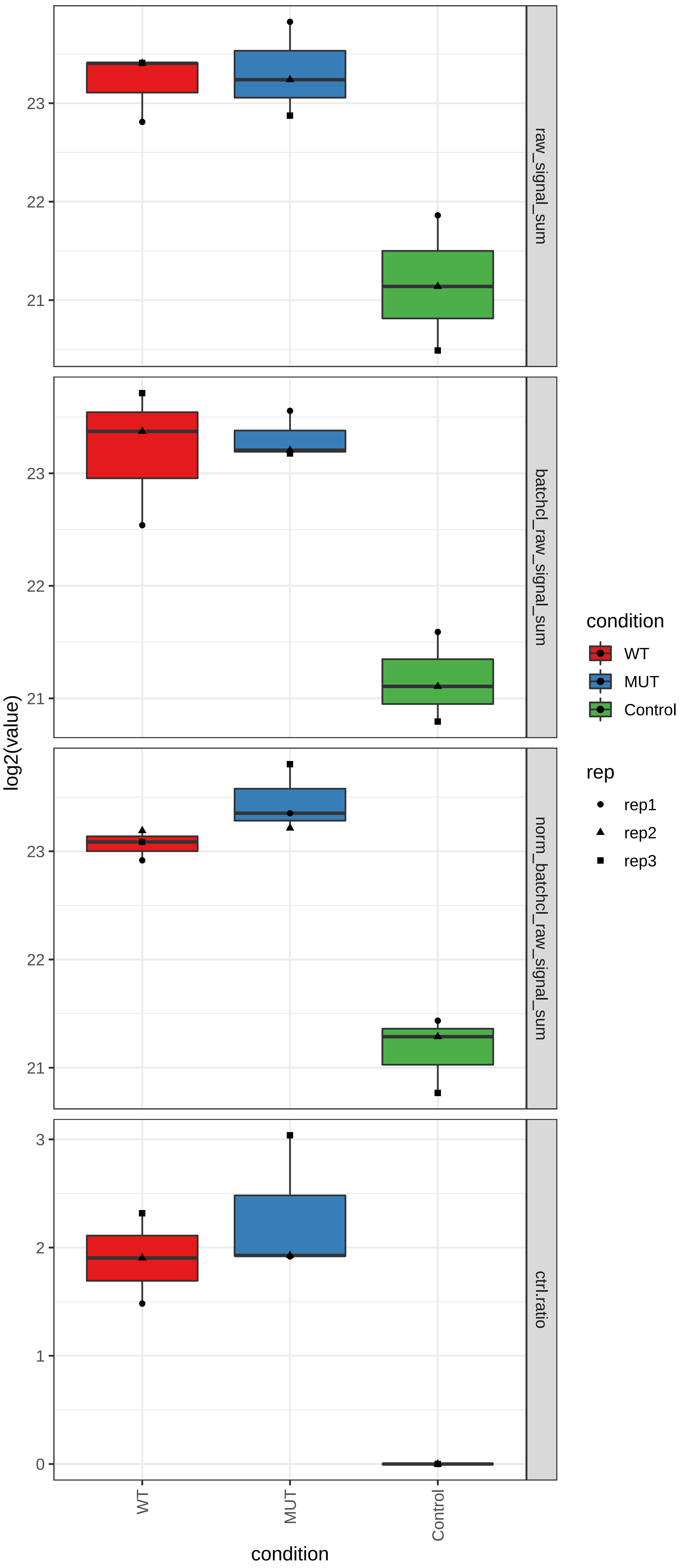

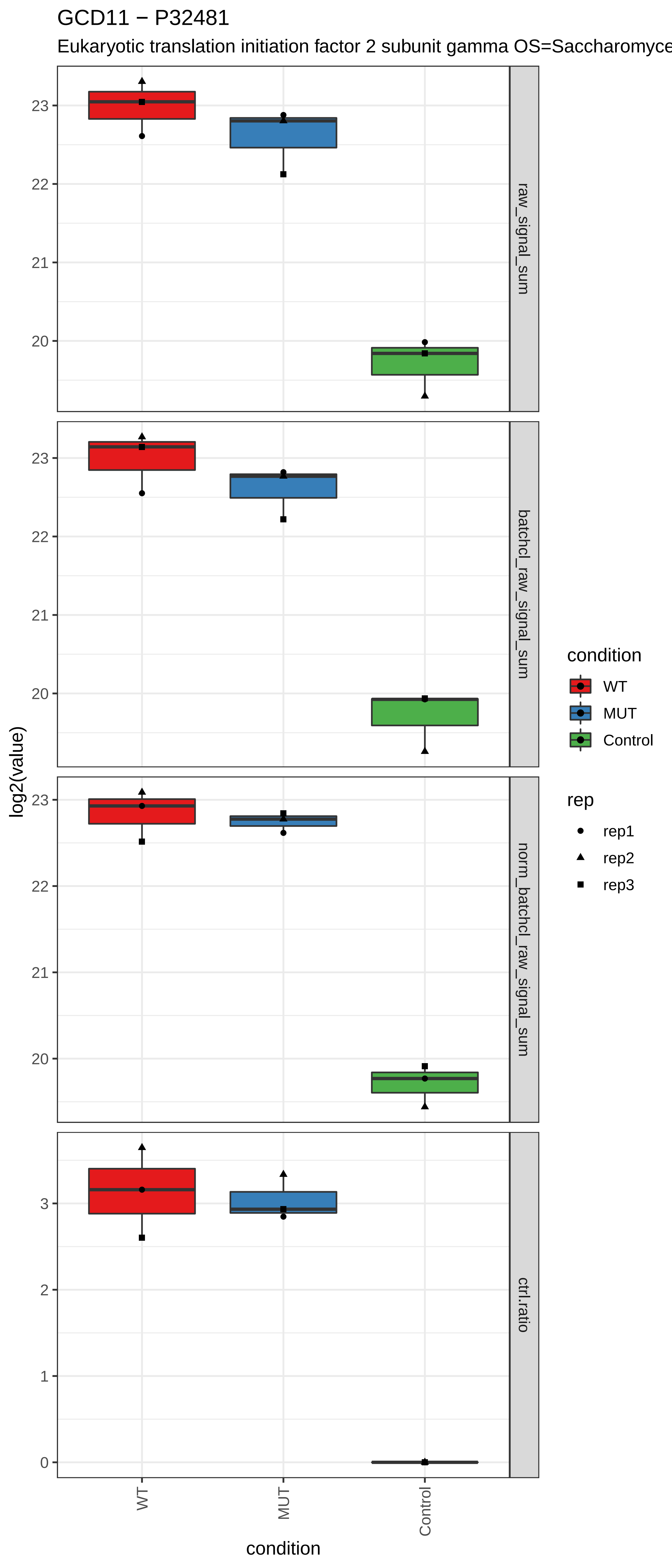

GCD11 − P32481
Eukaryotic translation initiation factor 2 subunit gamma OS=Saccharomyce
raw_signal_sum
batchcl_raw_signal_sum
norm_batchcl_raw_signal_sum
ctrl.ratio
log2(value)
condition
WT
MUT
Control
rep
rep1
rep2
rep3

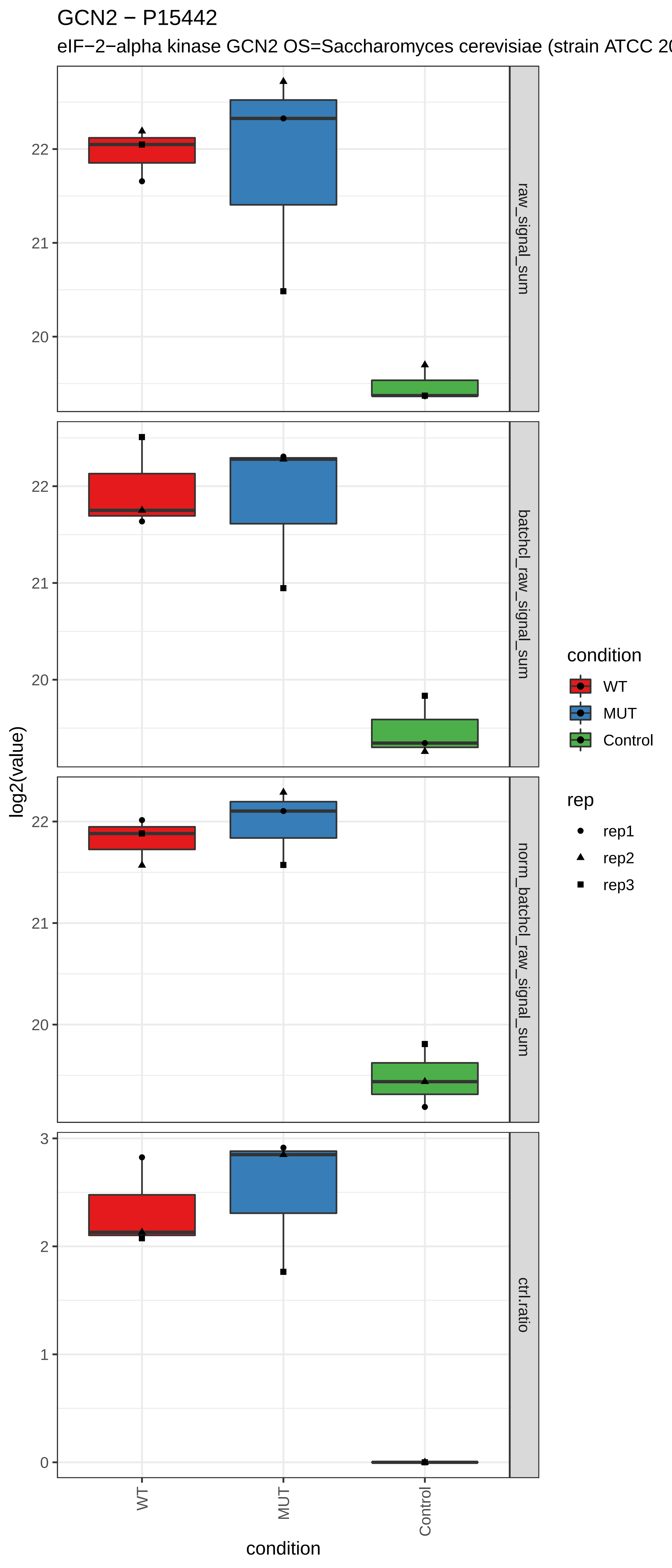

GCN2 − P15442
eIF−2−alpha kinase GCN2 OS=Saccharomyces cerevisiae (strain ATCC 20
raw_signal_sum
batchcl_raw_signal_sum
norm_batchcl_raw_signal_sum
ctrl.ratio
log2(value)
condition
WT
MUT
Control
rep
rep1
rep2
rep3
22
21
20
3
2
1
0

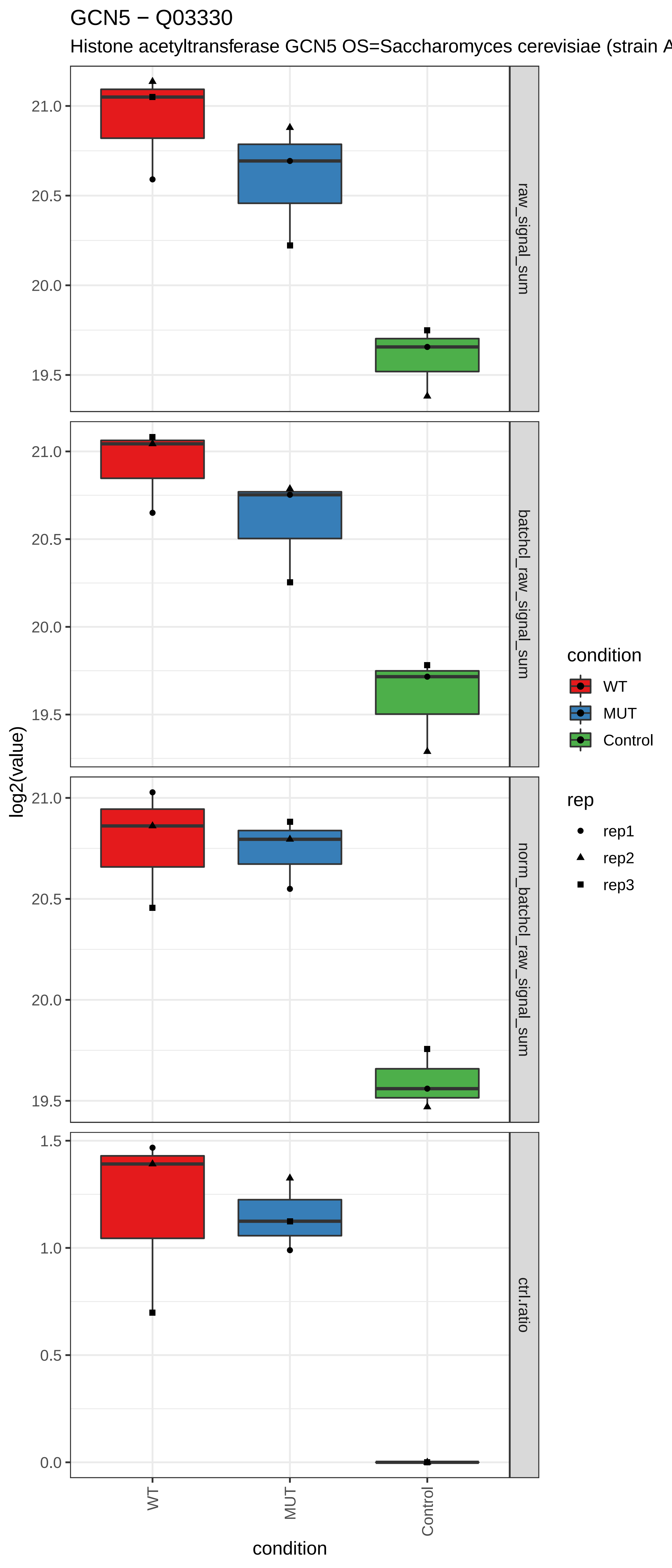

GCN5 − Q03330
Histone acetyltransferase GCN5 OS=Saccharomyces cerevisiae (strain A
log2(value)
raw_signal_sum
batchcl_raw_signal_sum
norm_batchcl_raw_signal_sum
ctrl.ratio
21.0
20.5
20.0
19.5
1.5
1.0
0.5
0.0
WT
MUT
Control
condition
rep
rep1
rep2
rep3

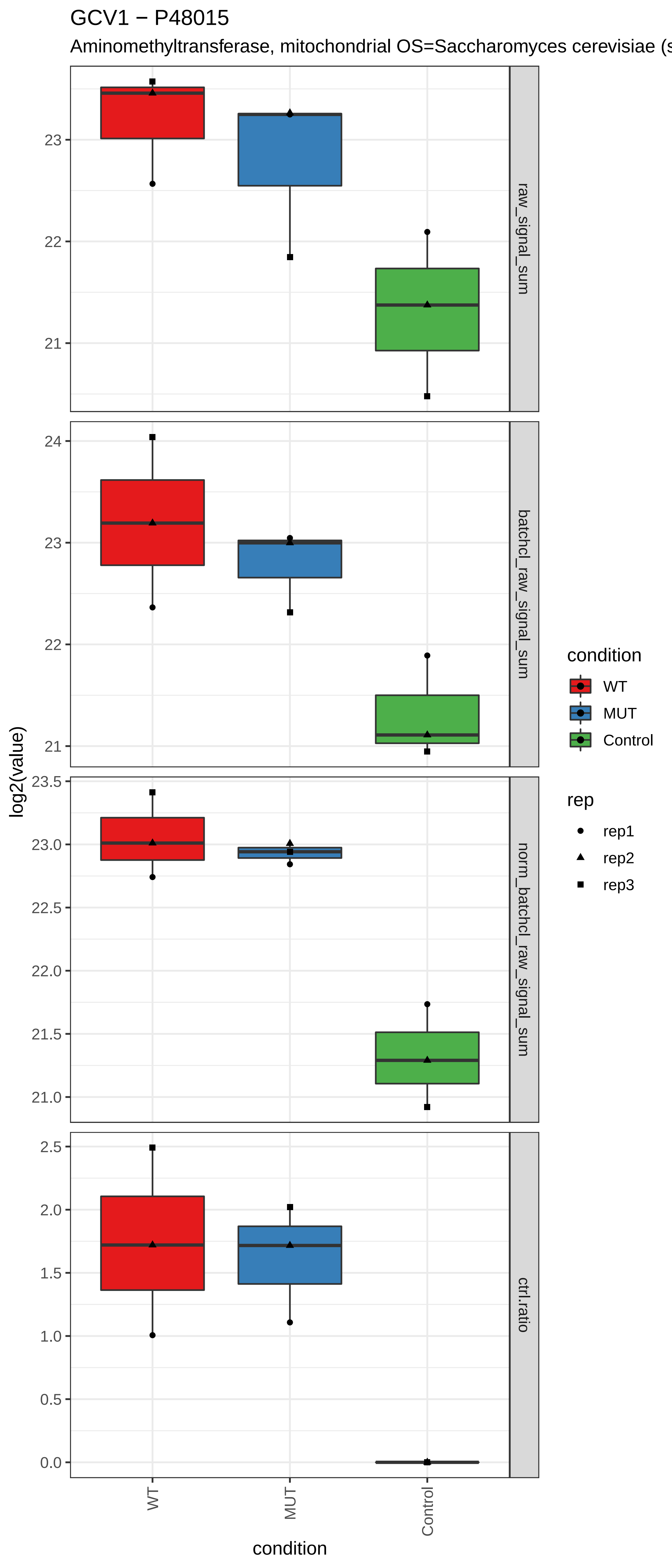
GCV1 − P48015
Aminomethyltransferase, mitochondrial OS=Saccharomyces cerevisiae (s
raw_signal_sum
batchcl_raw_signal_sum
norm_batchcl_raw_signal_sum
ctrl.ratio
log2(value)
condition
WT
MUT
Control
condition
rep
rep1
rep2
rep3

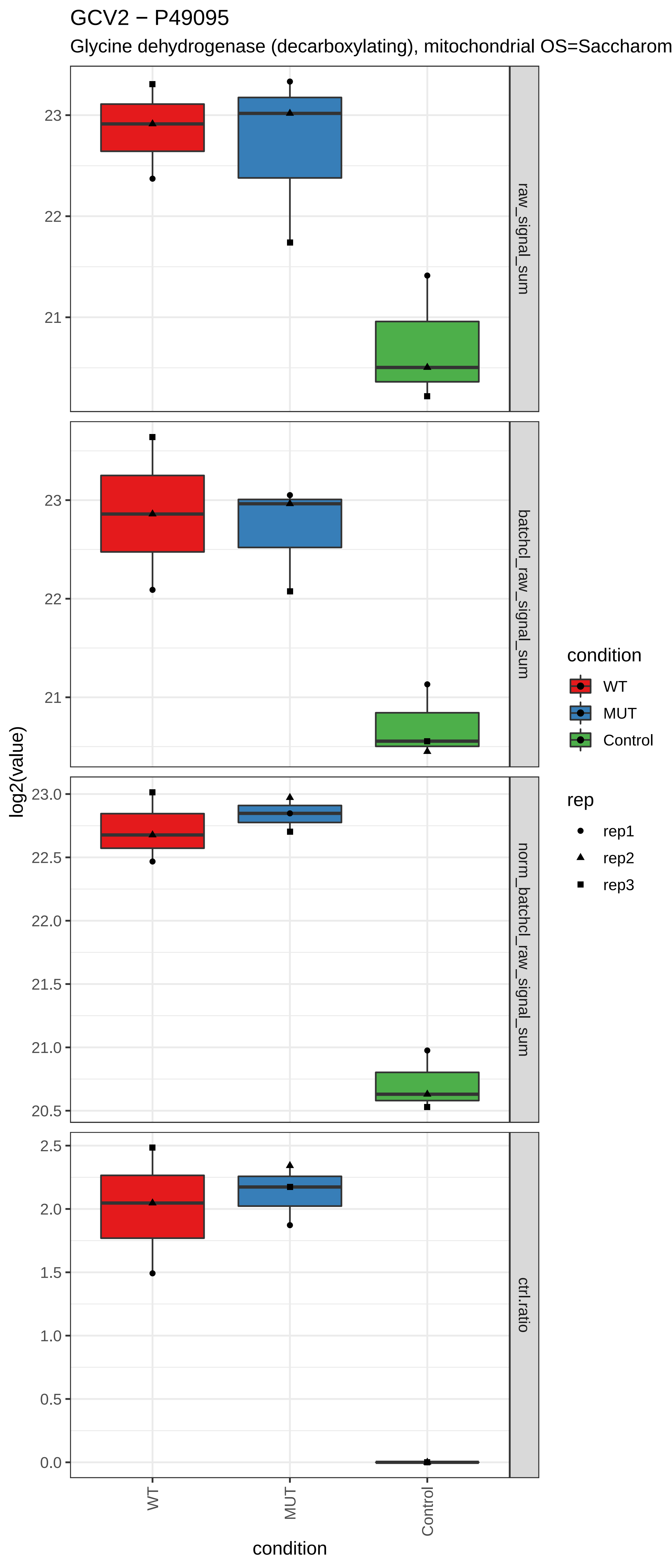

GCV2 − P49095
Glycine dehydrogenase (decarboxylating), mitochondrial OS=Saccharom
raw_signal_sum
batchcl_raw_signal_sum
norm_batchcl_raw_signal_sum
ctrl.ratio
log2(value)
condition
WT
MUT
Control
rep
rep1
rep2
rep3

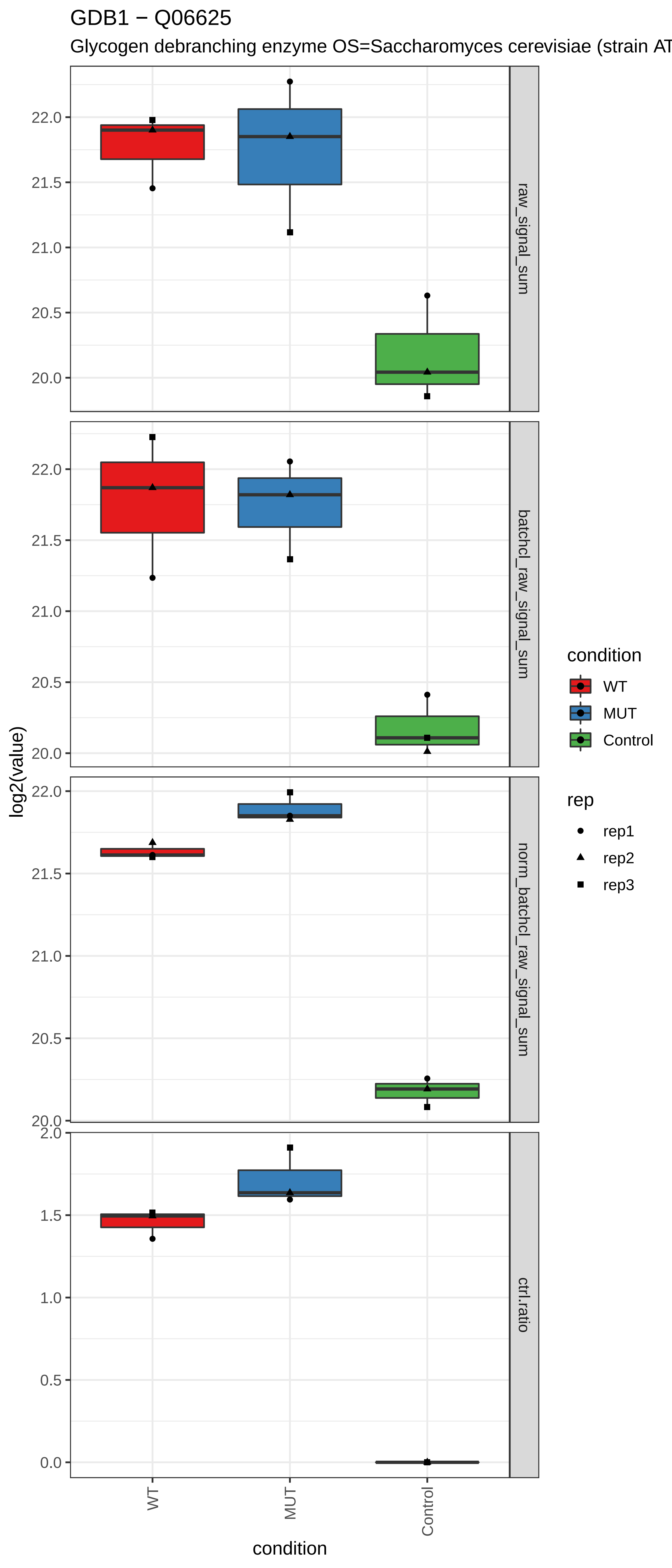

GDB1 − Q06625
Glycogen debranching enzyme OS=Saccharomyces cerevisiae (strain AT
raw_signal_sum
batchcl_raw_signal_sum
norm_batchcl_raw_signal_sum
ctrl.ratio
log2(value)
condition
WT
MUT
Control
rep
rep1
rep2
rep3
22.0
21.5
21.0
20.5
20.0
2.0
1.5
1.0
0.5
0.0

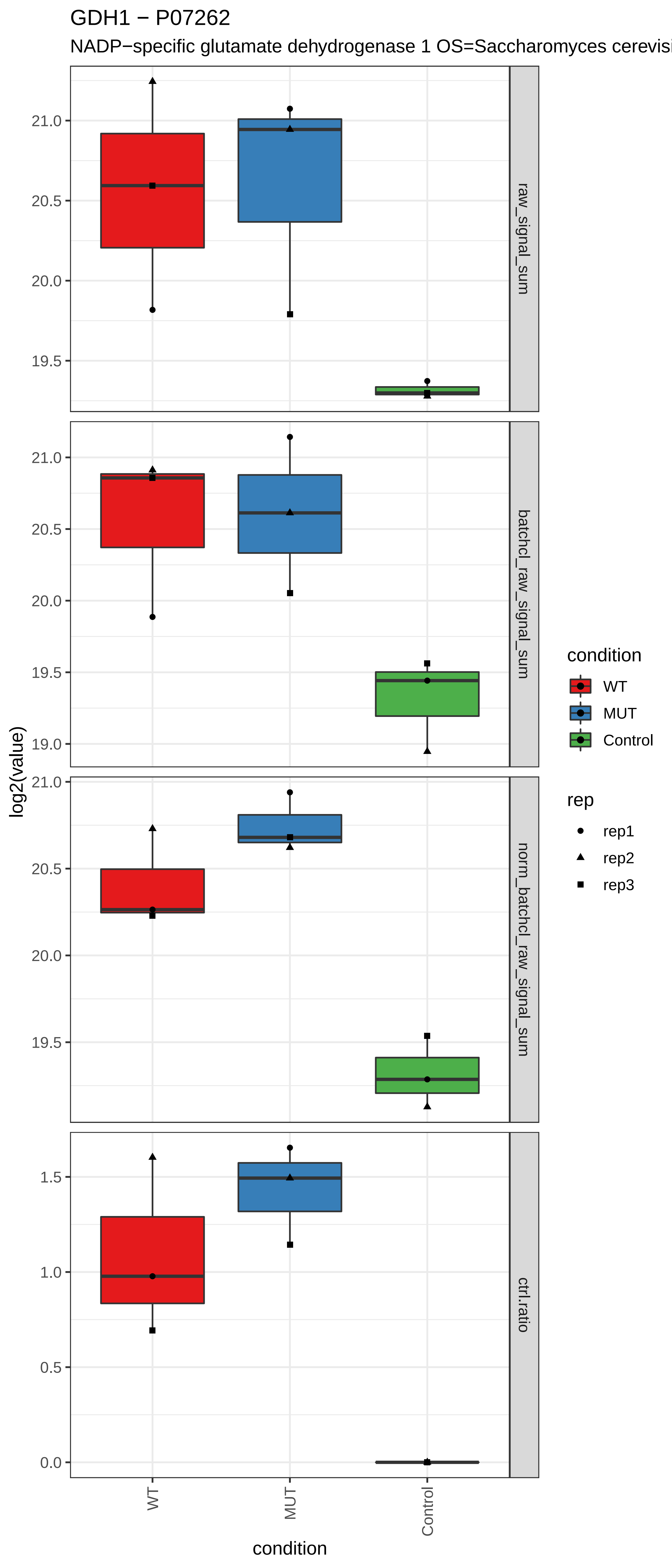
GDH1 − P07262
NADP−specific glutamate dehydrogenase 1 OS=Saccharomyces cerevisi
raw_signal_sum
batchcl_raw_signal_sum
norm_batchcl_raw_signal_sum
ctrl.ratio
log2(value)
condition
WT
MUT
Control
rep
rep1
rep2
rep3
21.0
20.5
20.0
19.5
19.0
1.5
1.0
0.5
0.0

GDH2 − P33327

NAD−specific glutamate dehydrogenase OS=Saccharomyces cerevisiae (s

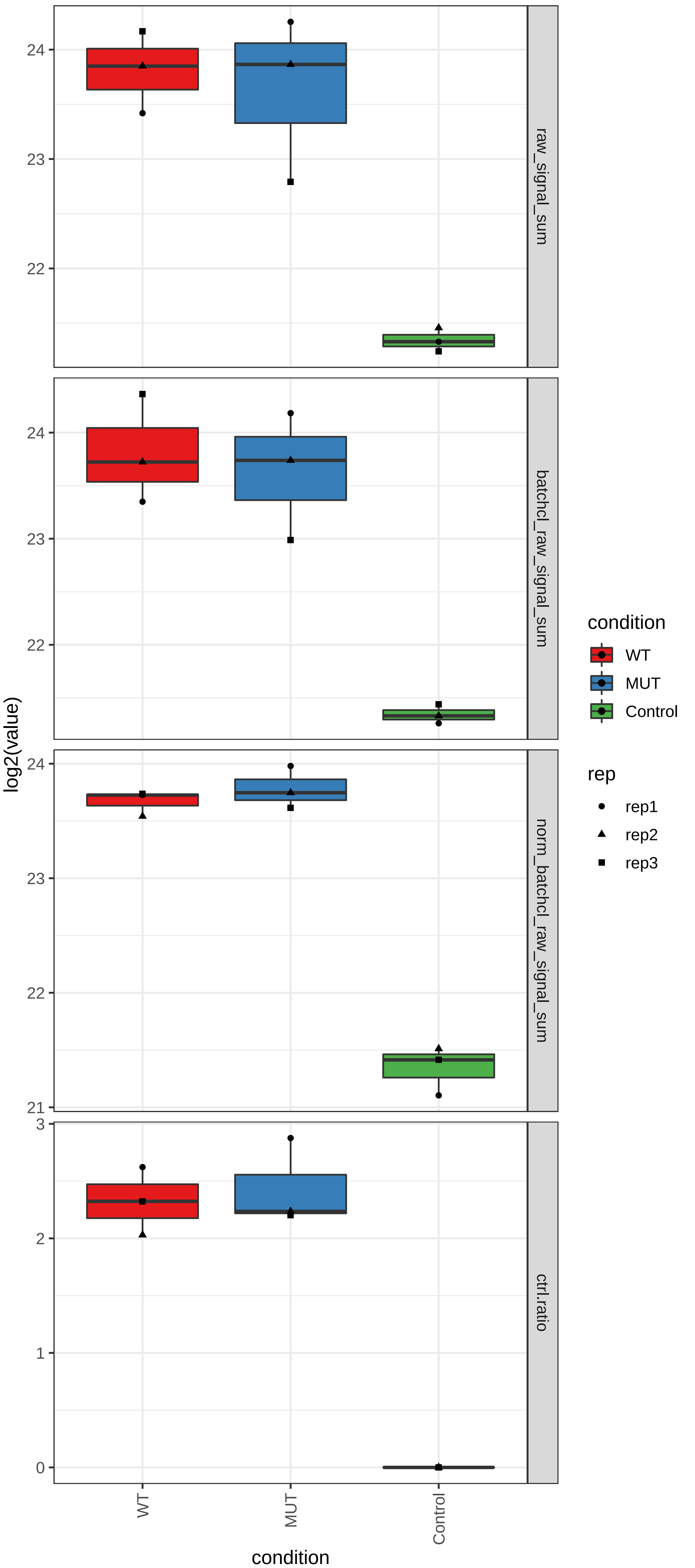

GDI1 − P39958

Rab GDP−dissociation inhibitor OS=Saccharomyces cerevisiae (strain AT

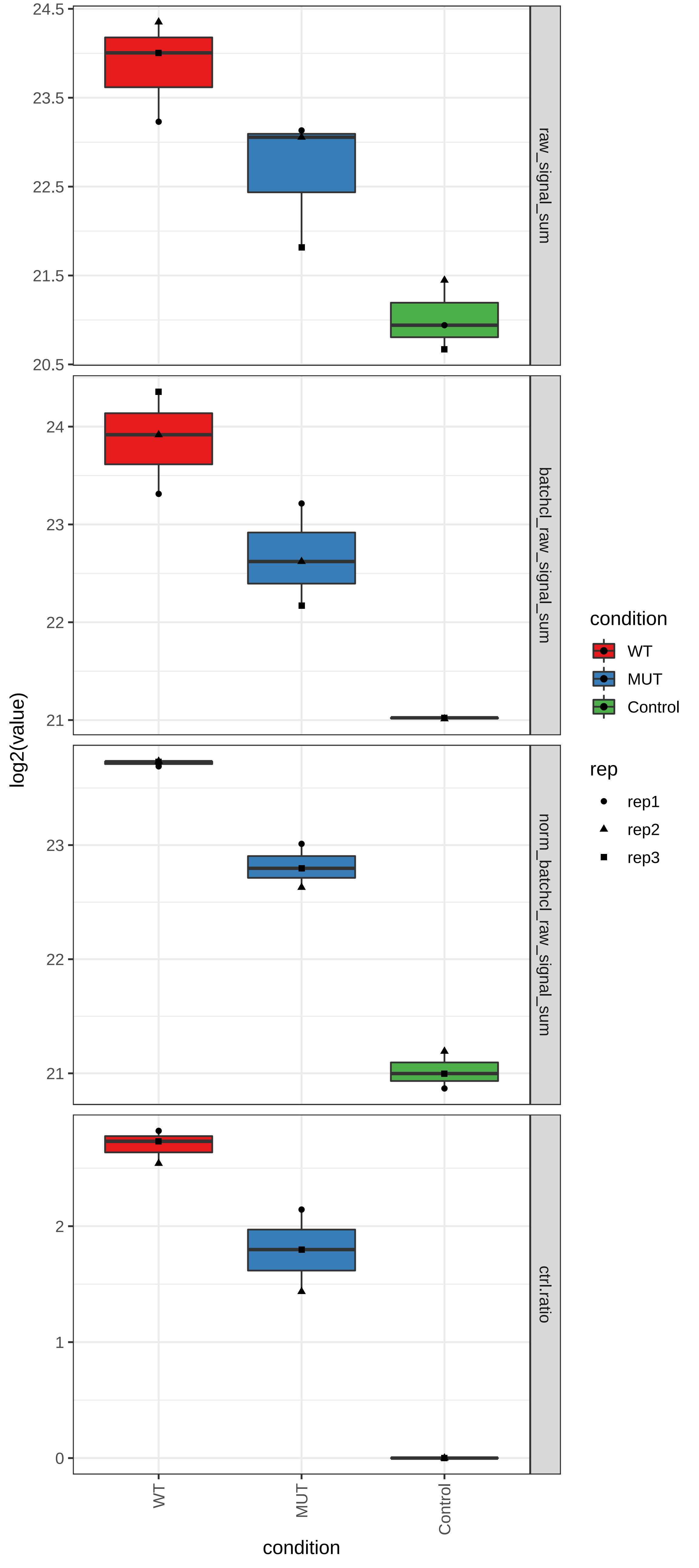

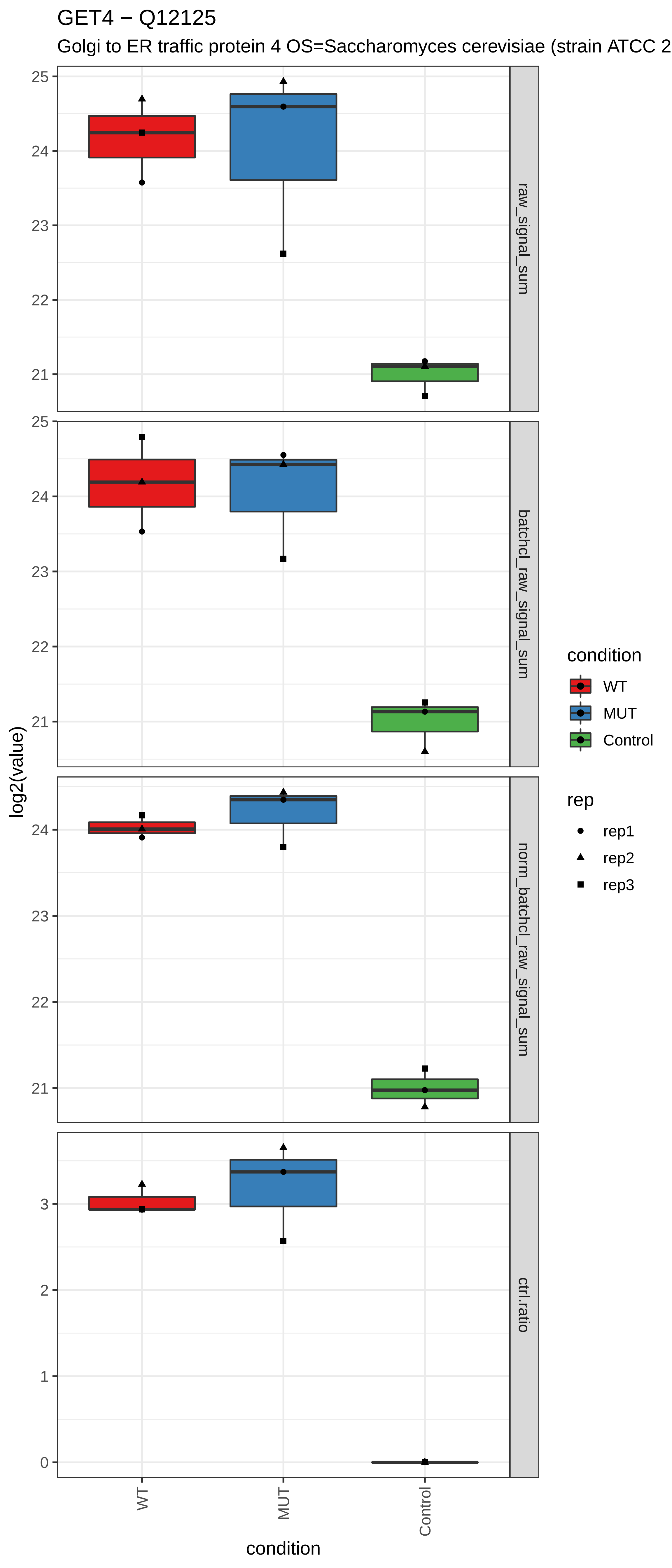
GET4 − Q12125
Golgi to ER traffic protein 4 OS=Saccharomyces cerevisiae (strain ATCC 2
raw_signal_sum
batchcl_raw_signal_sum
norm_batchcl_raw_signal_sum
ctrl.ratio
log2(value)
condition
WT
MUT
Control
rep
rep1
rep2
rep3

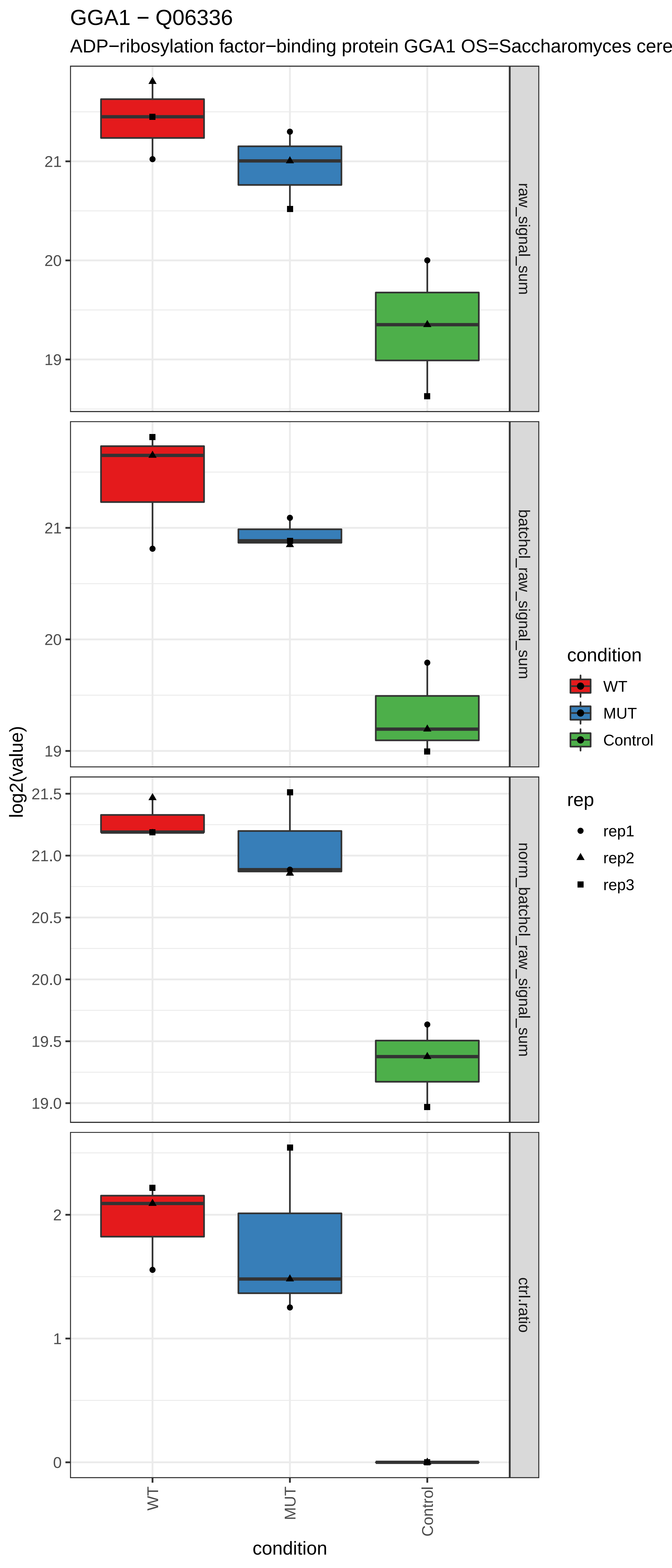
GGA1 − Q06336
ADP−ribosylation factor−binding protein GGA1 OS=Saccharomyces cere
raw_signal_sum
batchcl_raw_signal_sum
norm_batchcl_raw_signal_sum
ctrl.ratio
log2(value)
condition
WT
MUT
Control
rep
rep1
rep2
rep3
21
20
19
21.5
21.0
20.5
20.0
19.5
19.0
2
1
0

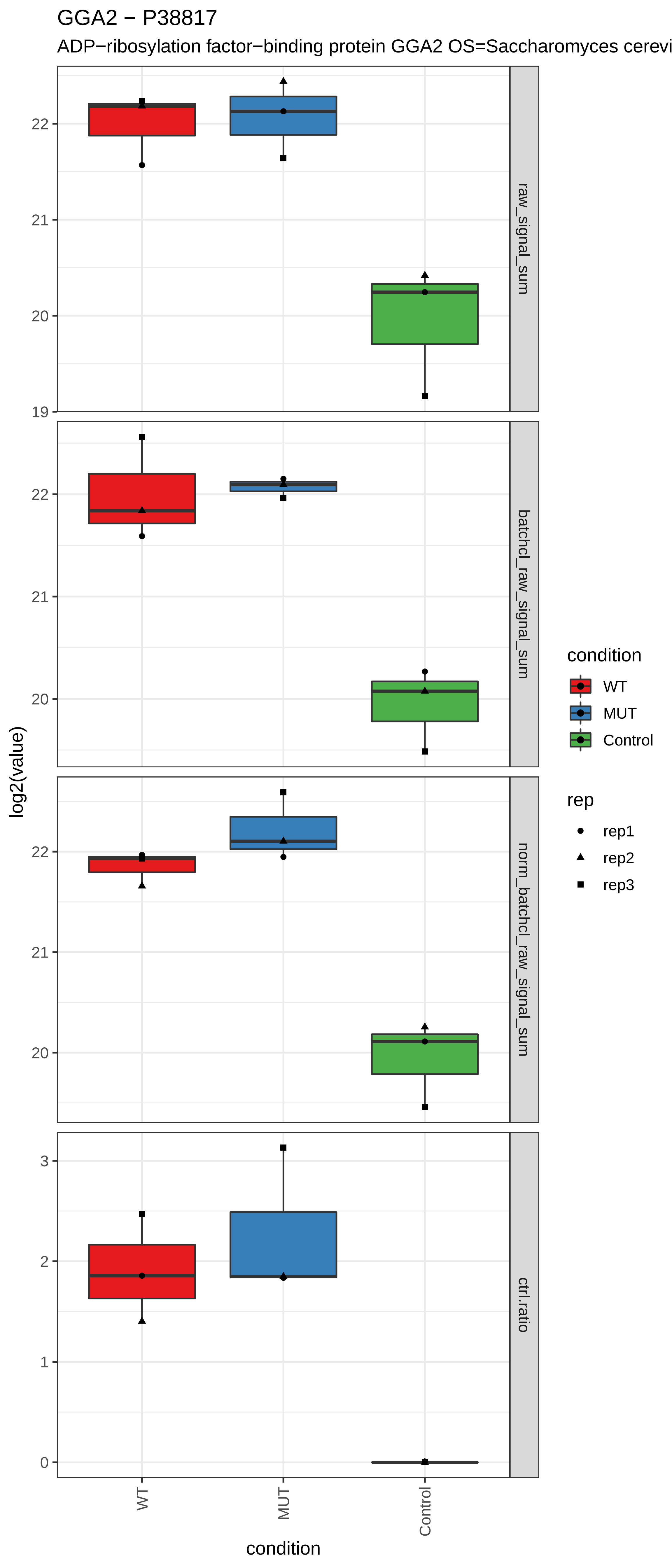
GGA2 − P38817
ADP−ribosylation factor−binding protein GGA2 OS=Saccharomyces cerevi
raw_signal_sum
batchcl_raw_signal_sum
norm_batchcl_raw_signal_sum
ctrl.ratio
log2(value)
condition
WT
MUT
Control
rep
rep1
rep2
rep3

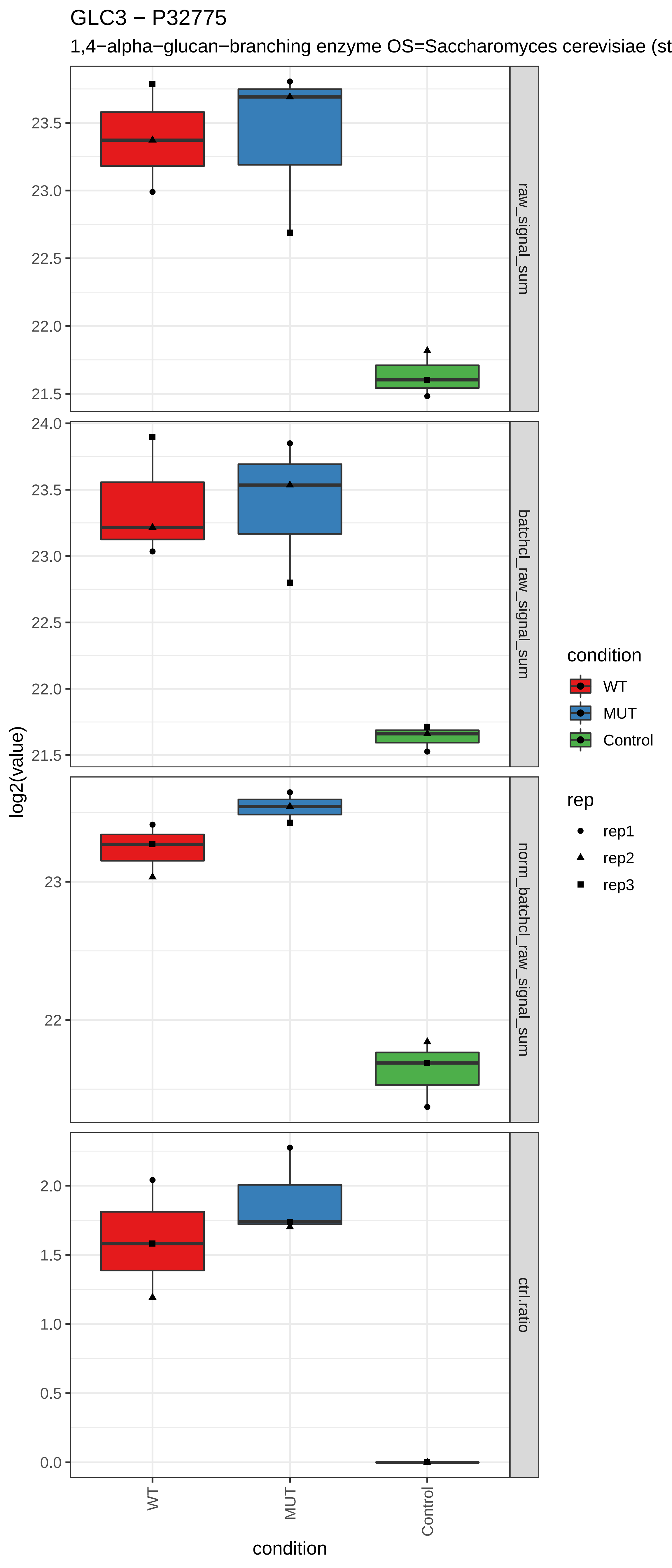

GLC3 − P32775
1,4−alpha−glucan−branching enzyme OS=Saccharomyces cerevisiae (st
raw_signal_sum
batchcl_raw_signal_sum
norm_batchcl_raw_signal_sum
ctrl.ratio
log2(value)
condition
WT
MUT
Control
rep
rep1
rep2
rep3
23.5
23.0
22.5
22.0
21.5
24.0
23
22
2.0
1.5
1.0
0.5
0.0

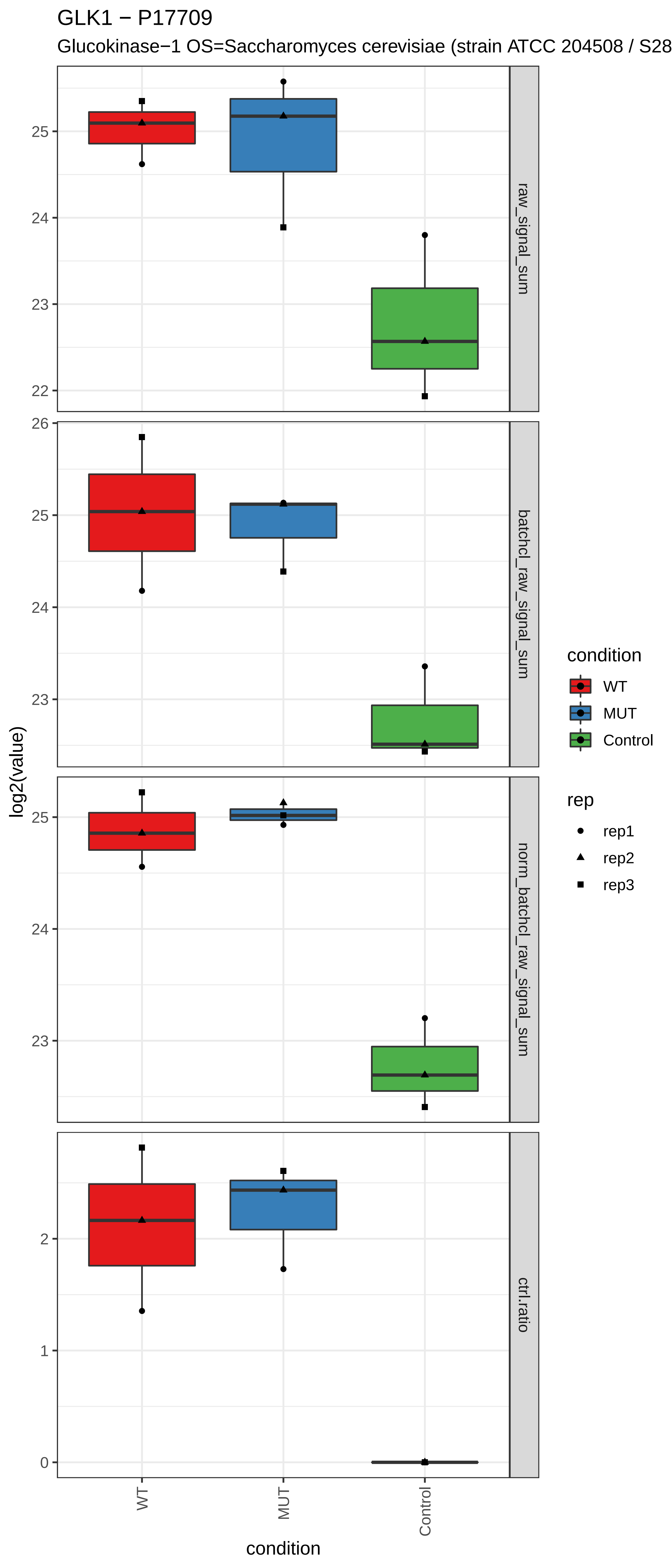

GLK1 − P17709
Glucokinase−1 OS=Saccharomyces cerevisiae (strain ATCC 204508 / S28
raw_signal_sum
batchcl_raw_signal_sum
norm_batchcl_raw_signal_sum
ctrl.ratio
log2(value)
condition
WT
MUT
Control
rep
rep1
rep2
rep3

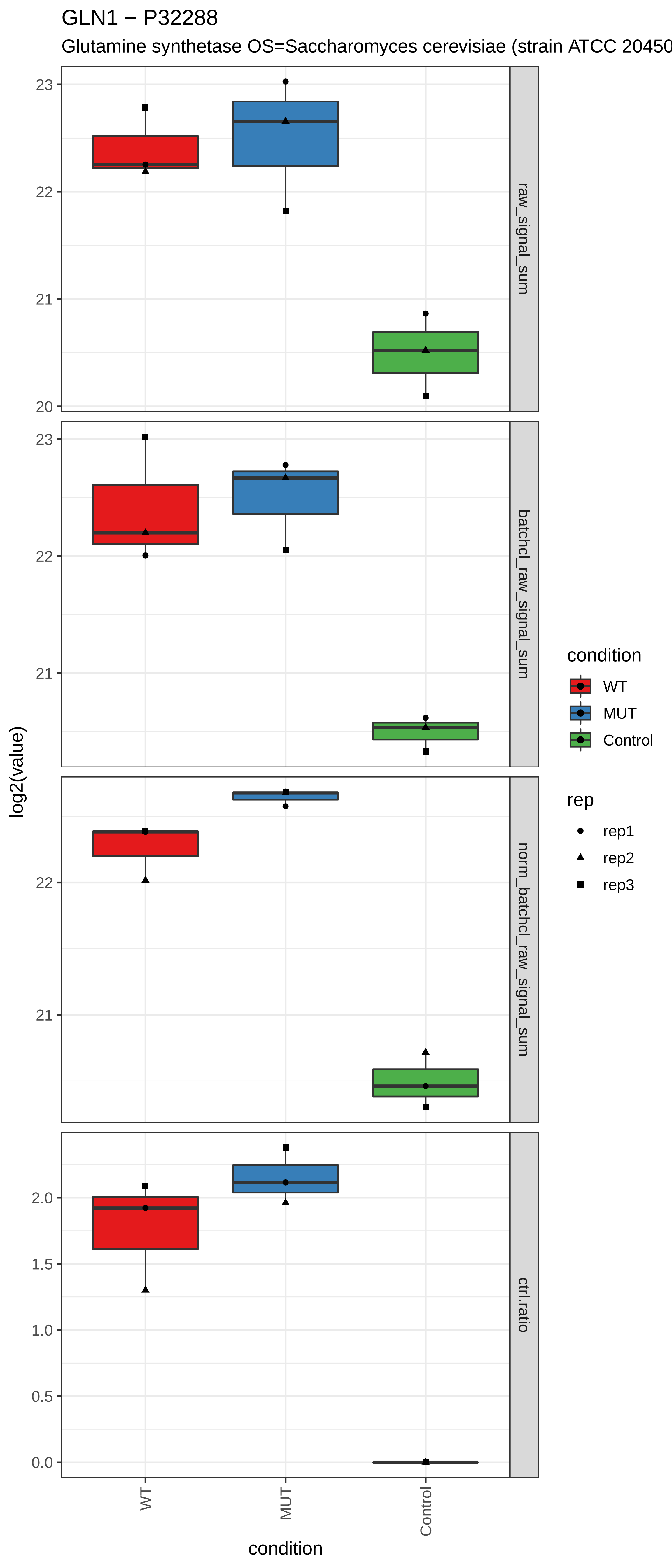
GLN1 − P32288
Glutamine synthetase OS=Saccharomyces cerevisiae (strain ATCC 20450
raw_signal_sum
batchcl_raw_signal_sum
norm_batchcl_raw_signal_sum
ctrl.ratio
log2(value)
condition
WT
MUT
Control
rep
rep1
rep2
rep3

GLO3 − P38682

ADP−ribosylation factor GTPase−activating protein GLO3 OS=Saccharomy

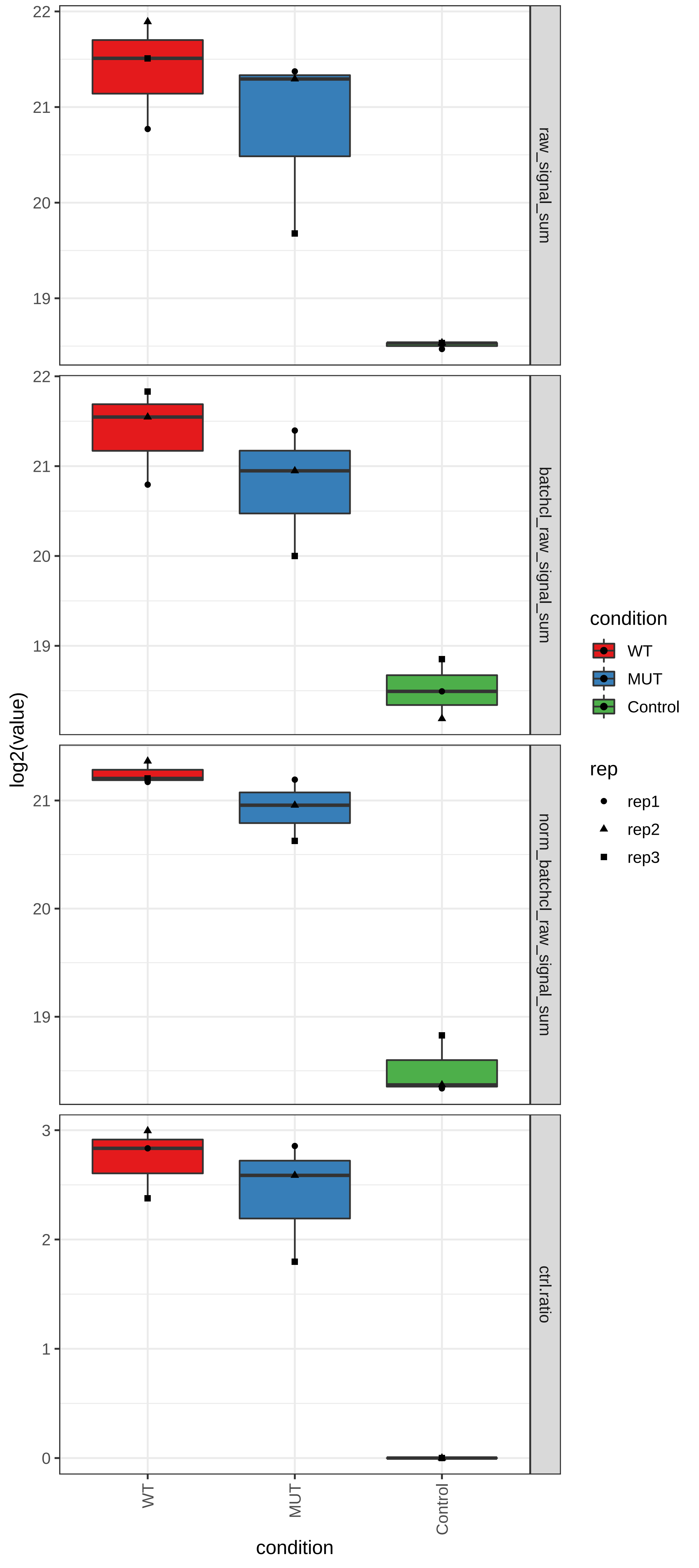

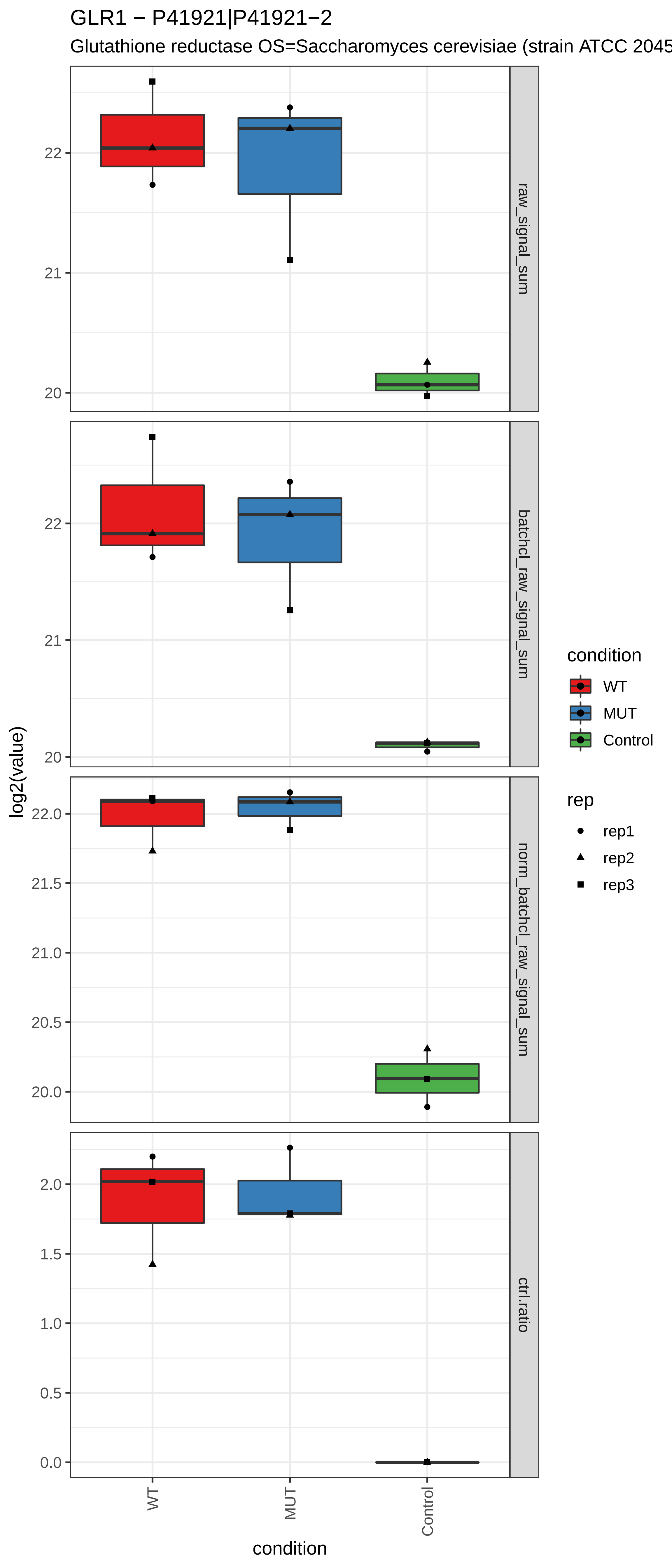
GLR1 − P41921|P41921−2
Glutathione reductase OS=Saccharomyces cerevisiae (strain ATCC 2045
raw_signal_sum
batchcl_raw_signal_sum
norm_batchcl_raw_signal_sum
ctrl.ratio
log2(value)
condition
WT
MUT
Control
rep
rep1
rep2
rep3
22
21
20
22.0
21.5
21.0
20.5
20.0
2.0
1.5
1.0
0.5
0.0

GLT1 − Q12680

Glutamate synthase [NADH] OS=Saccharomyces cerevisiae (strain ATCC

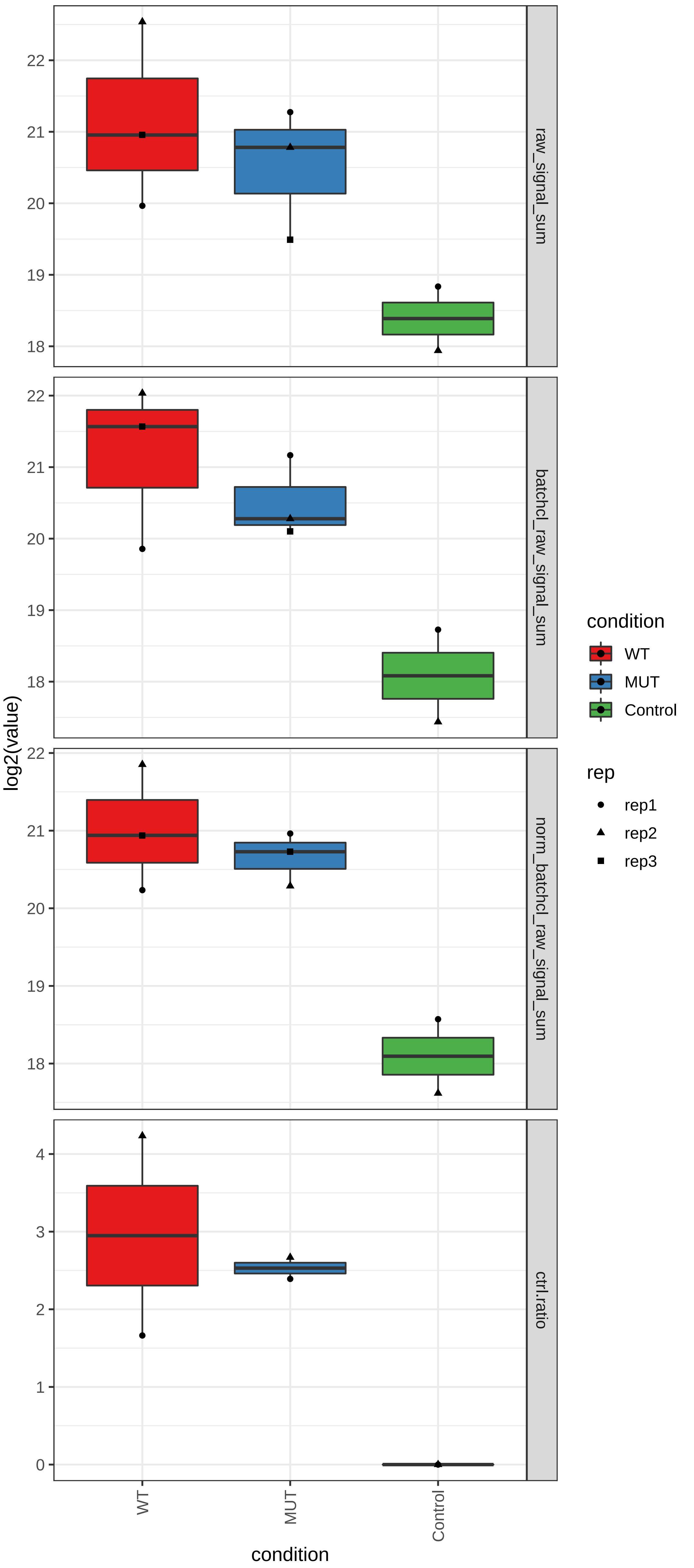

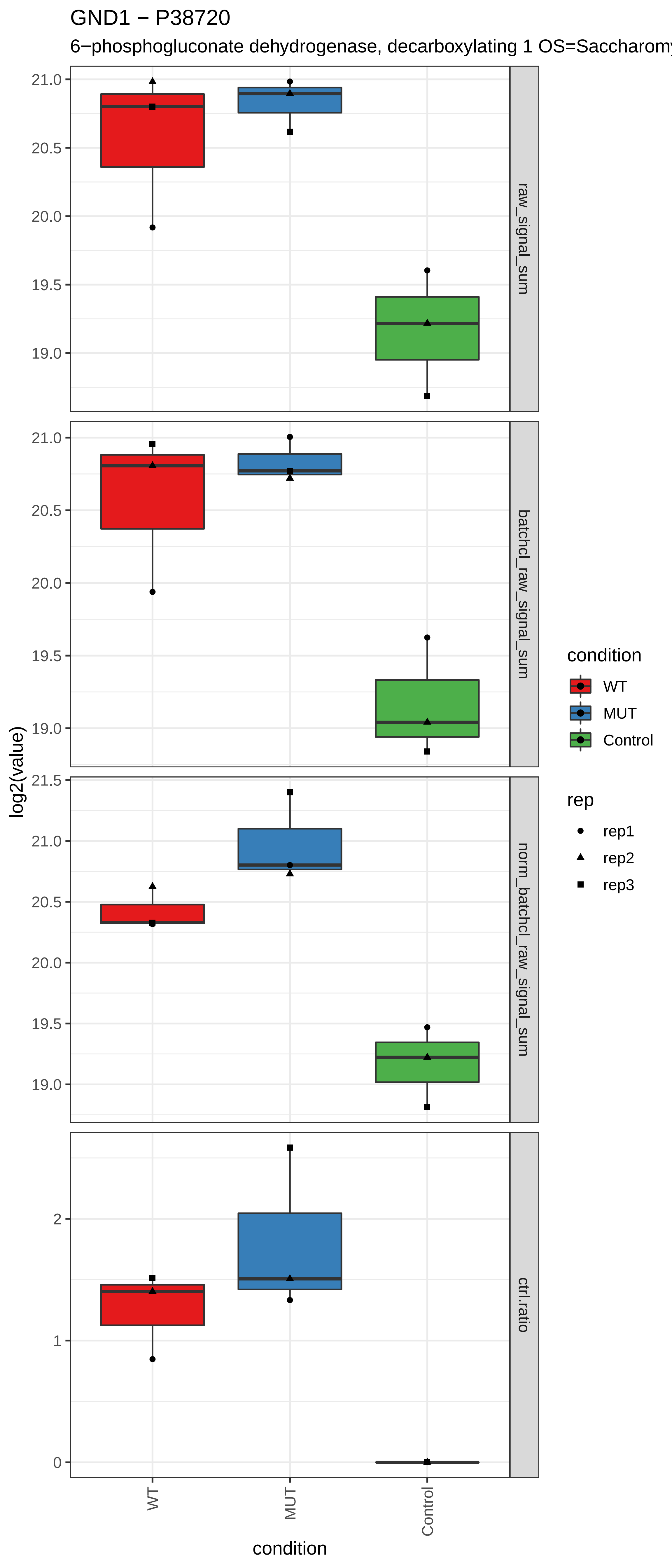

GND1 − P38720
6−phosphogluconate dehydrogenase, decarboxylating 1 OS=Saccharomy
raw_signal_sum
batchcl_raw_signal_sum
norm_batchcl_raw_signal_sum
ctrl.ratio
log2(value)
condition
WT
MUT
Control
rep
rep1
rep2
rep3
21.0
20.5
20.0
19.5
19.0
21.5
2
1
0

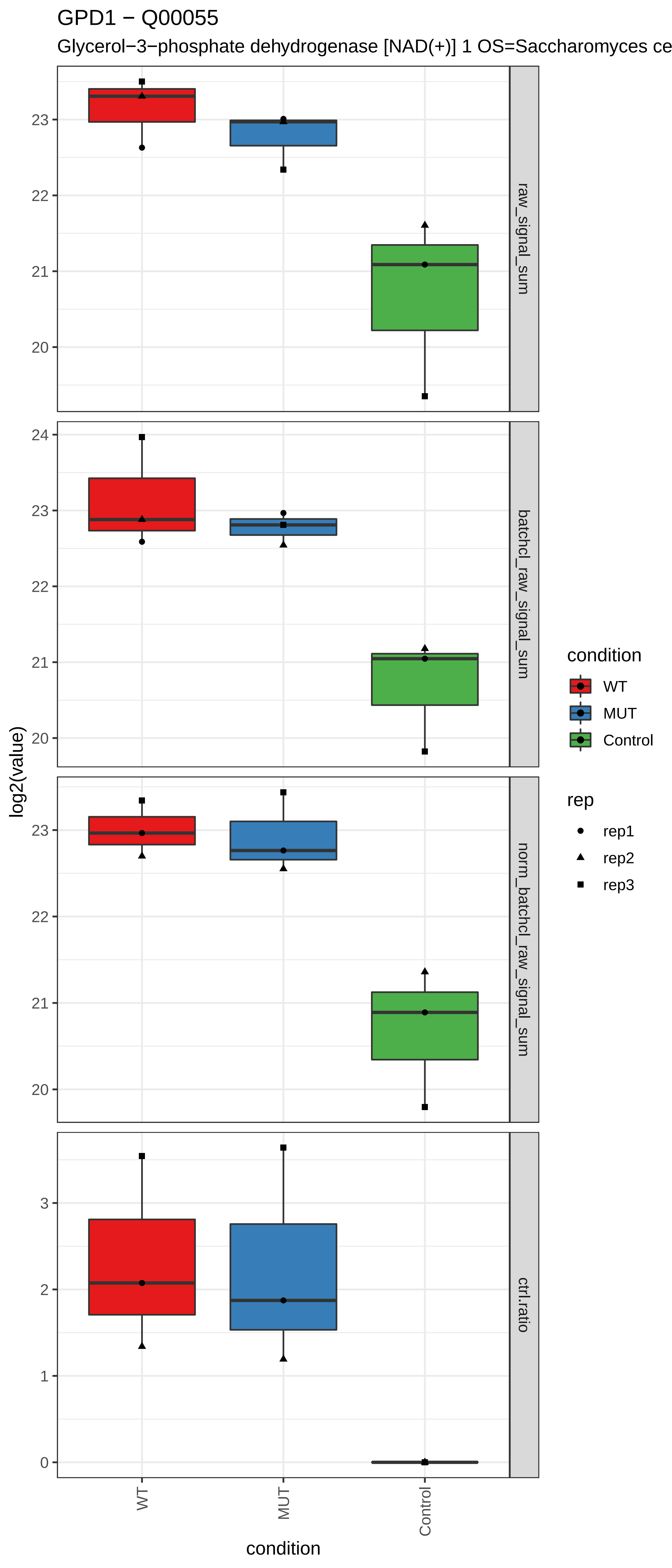

GPD1 − Q00055
Glycerol−3−phosphate dehydrogenase [NAD(+)] 1 OS=Saccharomyces ce
raw_signal_sum
batchcl_raw_signal_sum
norm_batchcl_raw_signal_sum
ctrl.ratio
log2(value)
condition
WT
MUT
Control
rep
rep1
rep2
rep3

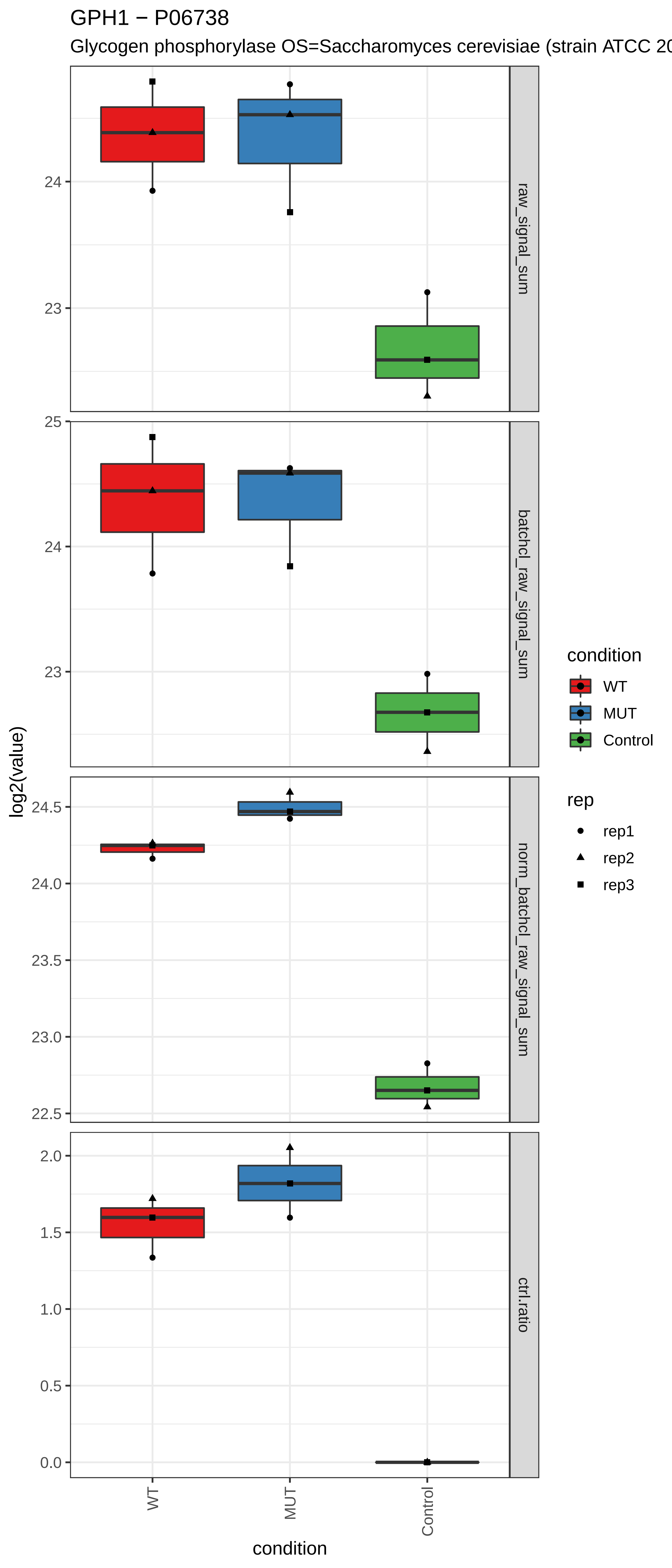
GPH1 − P06738
Glycogen phosphorylase OS=Saccharomyces cerevisiae (strain ATCC 20
raw_signal_sum
batchcl_raw_signal_sum
norm_batchcl_raw_signal_sum
ctrl.ratio
log2(value)
condition
WT
MUT
Control
rep
rep1
rep2
rep3

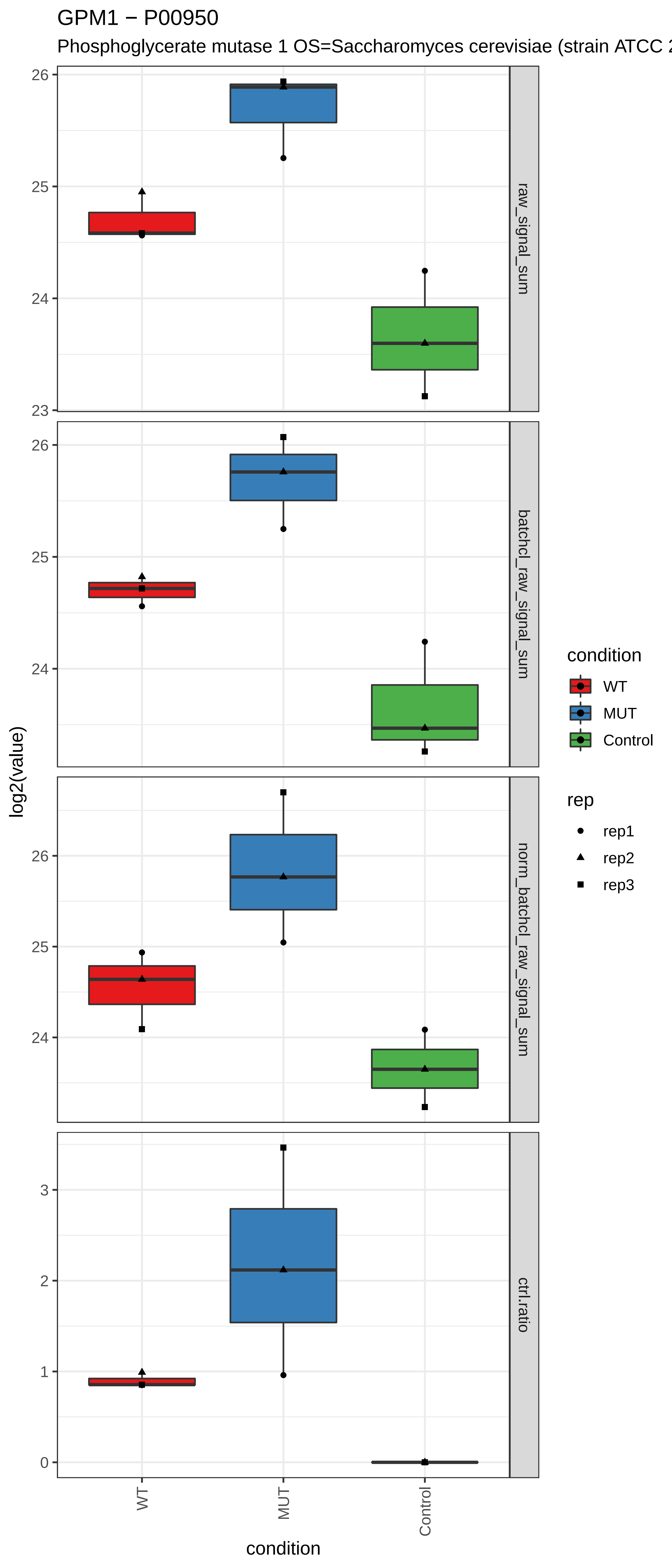
GPM1 − P00950
Phosphoglycerate mutase 1 OS=Saccharomyces cerevisiae (strain ATCC 2
raw_signal_sum
batchcl_raw_signal_sum
norm_batchcl_raw_signal_sum
ctrl.ratio
log2(value)
condition
WT
MUT
Control
rep
rep1
rep2
rep3

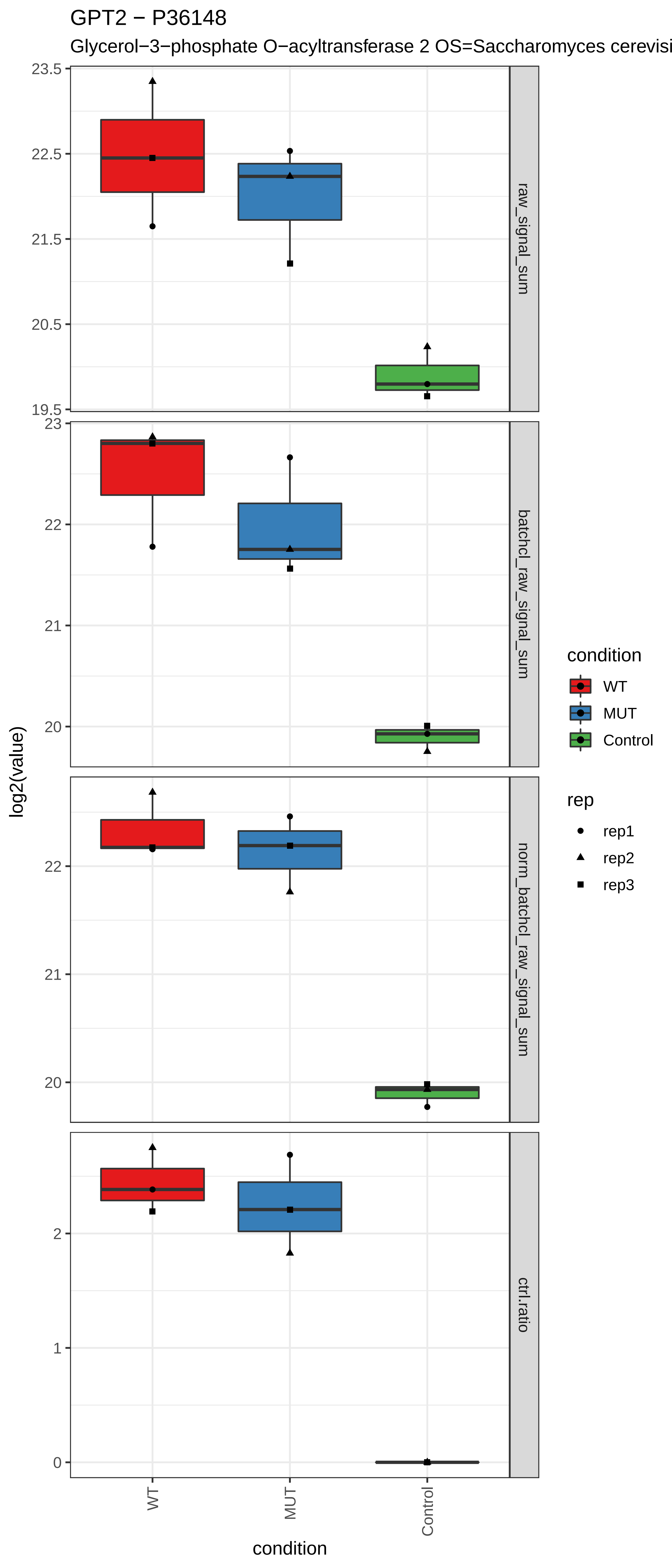

GPT2 − P36148
Glycerol−3−phosphate O−acyltransferase 2 OS=Saccharomyces cerevisi
raw_signal_sum
batchcl_raw_signal_sum
norm_batchcl_raw_signal_sum
ctrl.ratio
log2(value)
condition
WT
MUT
Control
rep
rep1
rep2
rep3

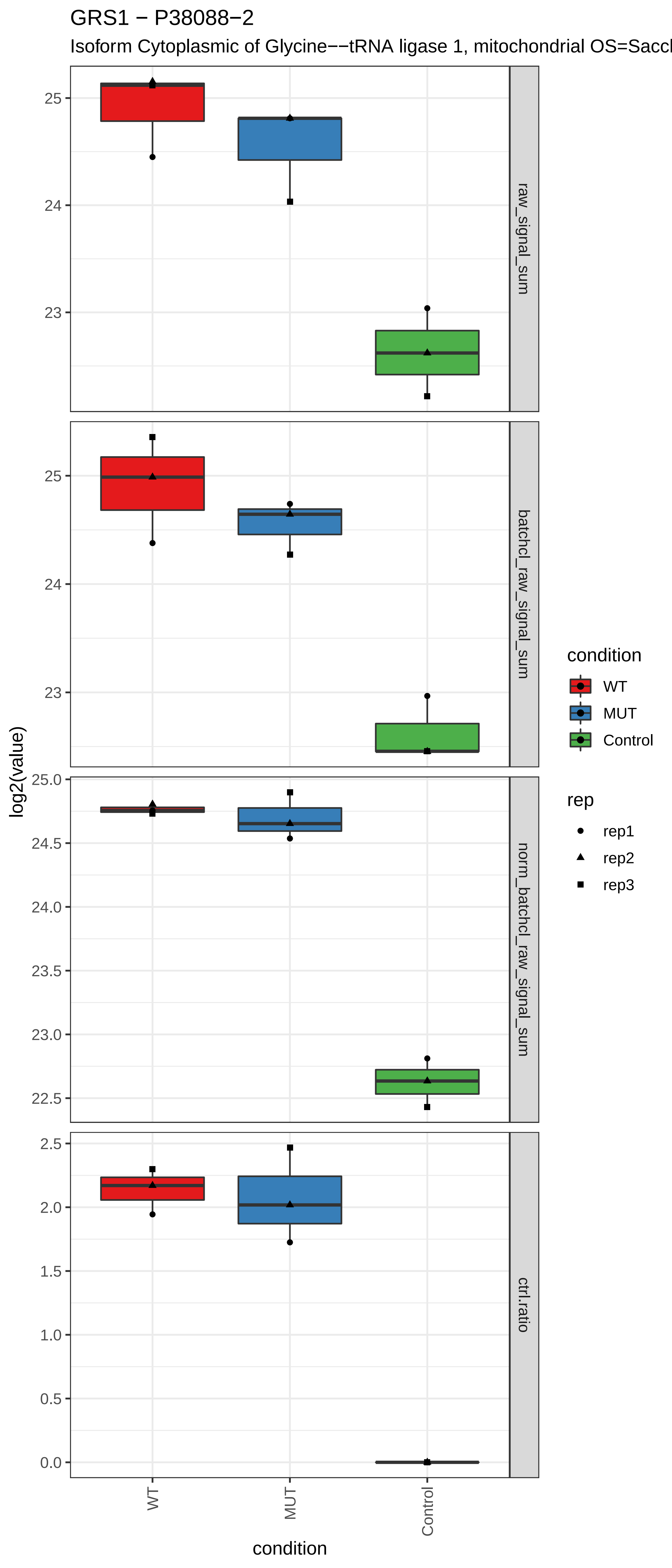

GRS1 − P38088−2
Isoform Cytoplasmic of Glycine−−tRNA ligase 1, mitochondrial OS=Saccl
raw_signal_sum
batchcl_raw_signal_sum
norm_batchcl_raw_signal_sum
ctrl.ratio
log2(value)
condition
WT
MUT
Control
rep
rep1
rep2
rep3
25
24
23
25.0
24.5
24.0
23.5
23.0
22.5
2.5
2.0
1.5
1.0
0.5
0.0

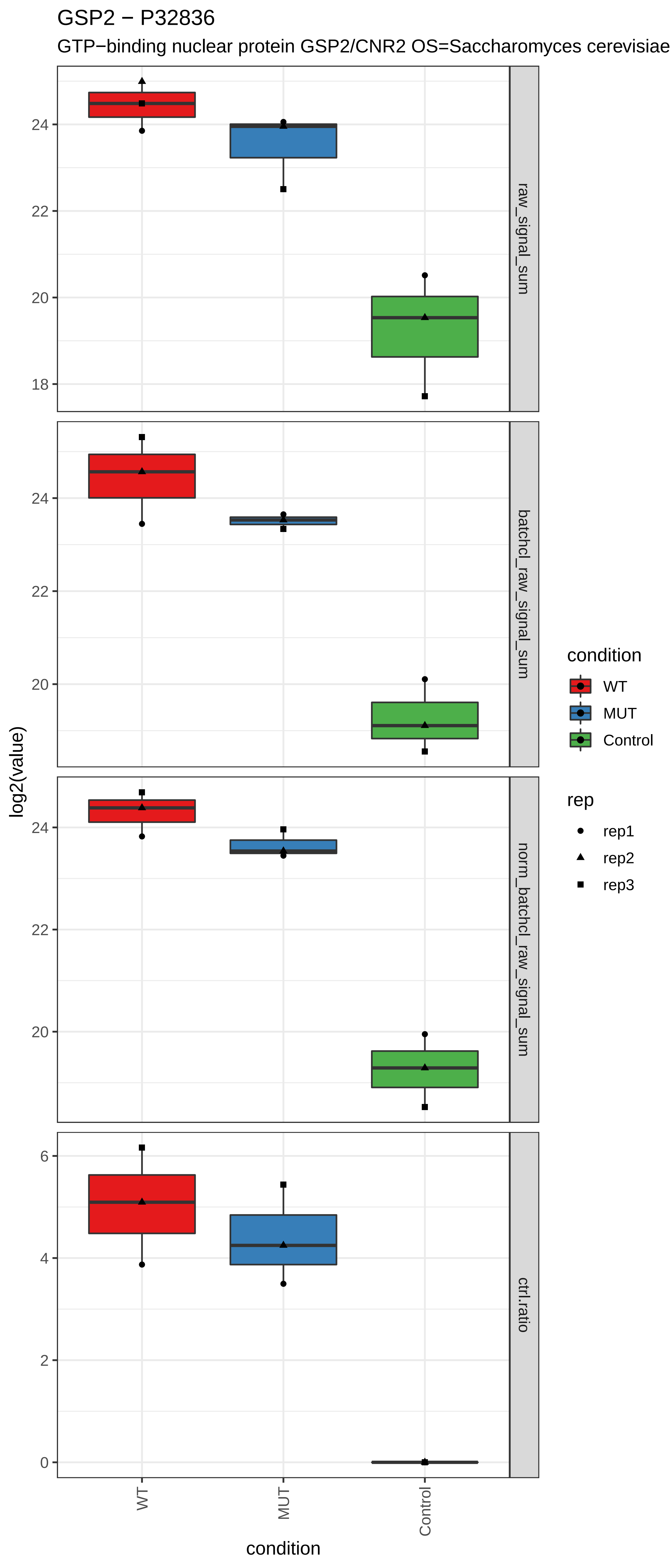

GSP2 − P32836
GTP−binding nuclear protein GSP2/CNR2 OS=Saccharomyces cerevisiae
log2(value)
raw_signal_sum
batchcl_raw_signal_sum
norm_batchcl_raw_signal_sum
ctrl.ratio
24
22
20
18
6
4
2
0
WT
MUT
Control
condition
condition
WT
MUT
Control
rep
rep1
rep2
rep3

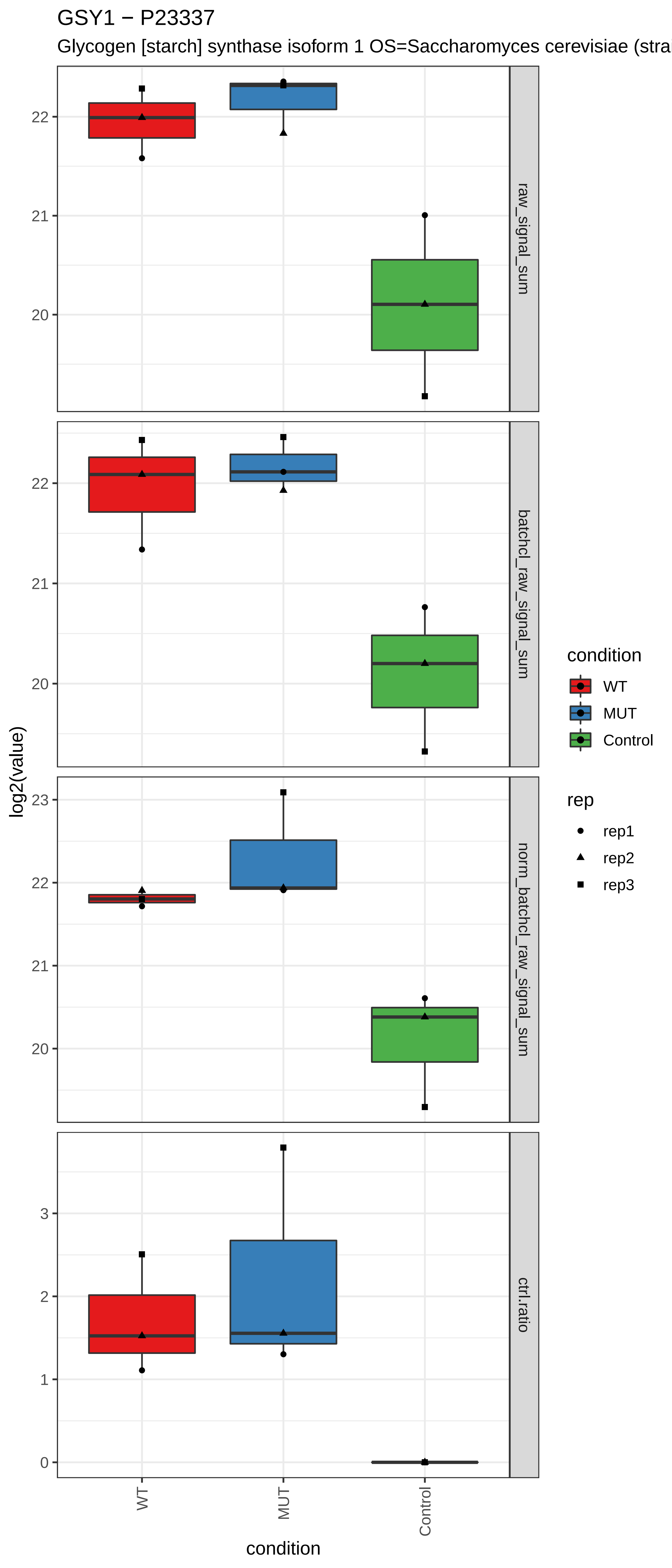

GSY1 − P23337
Glycogen [starch] synthase isoform 1 OS=Saccharomyces cerevisiae (stra
raw_signal_sum
batchcl_raw_signal_sum
norm_batchcl_raw_signal_sum
ctrl.ratio
log2(value)
condition
WT
MUT
Control
rep
rep1
rep2
rep3

GTS1 − P40956

Protein GTS1 OS=Saccharomyces cerevisiae (strain ATCC 204508 / S288

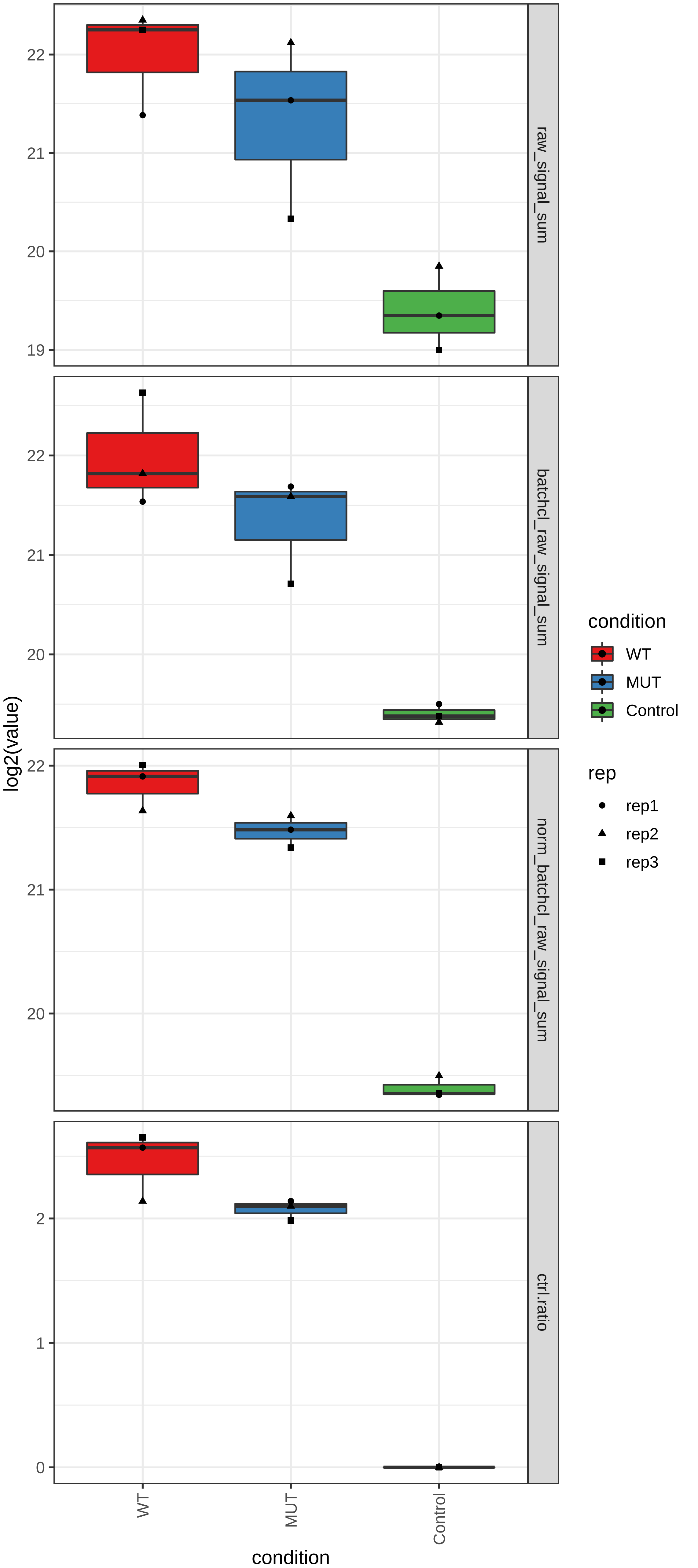

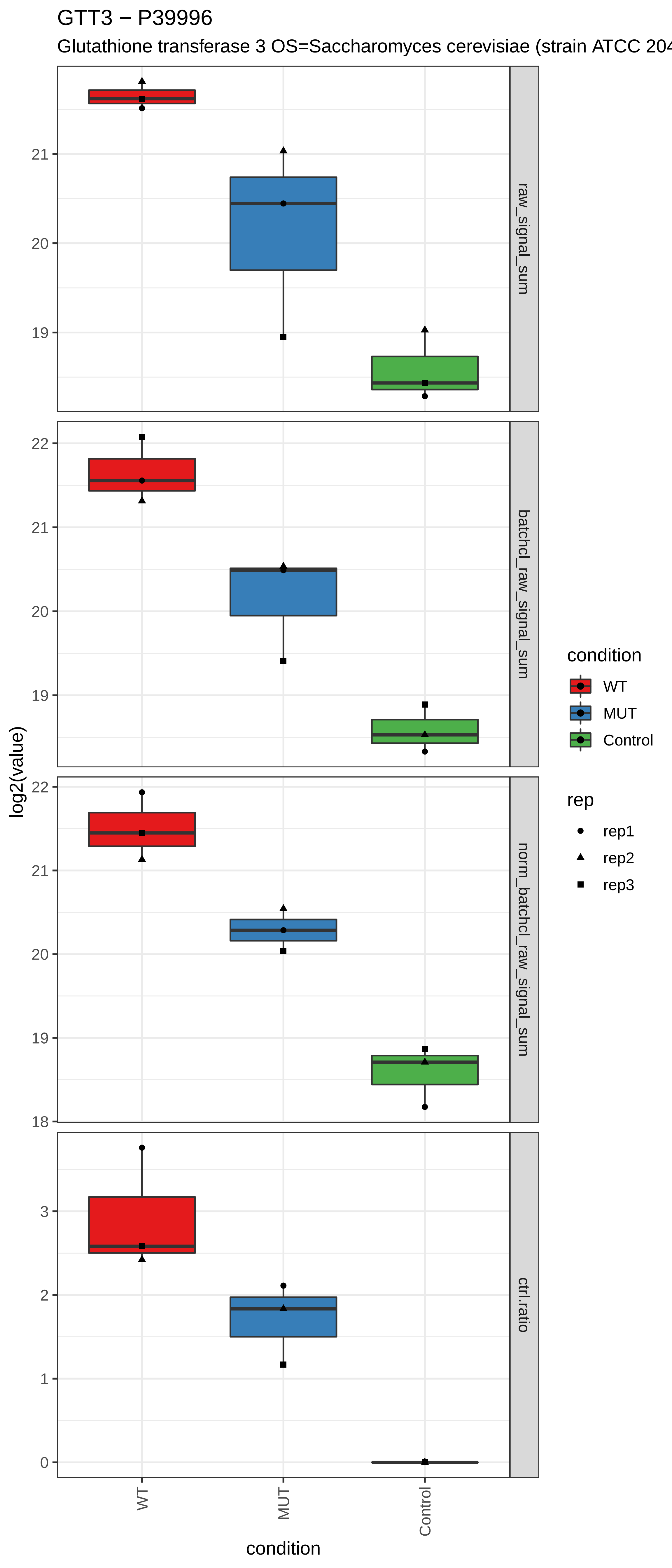

GTT3 − P39996
Glutathione transferase 3 OS=Saccharomyces cerevisiae (strain ATCC 204
raw_signal_sum
batchcl_raw_signal_sum
norm_batchcl_raw_signal_sum
ctrl.ratio
log2(value)
condition
WT
MUT
Control
rep
rep1
rep2
rep3

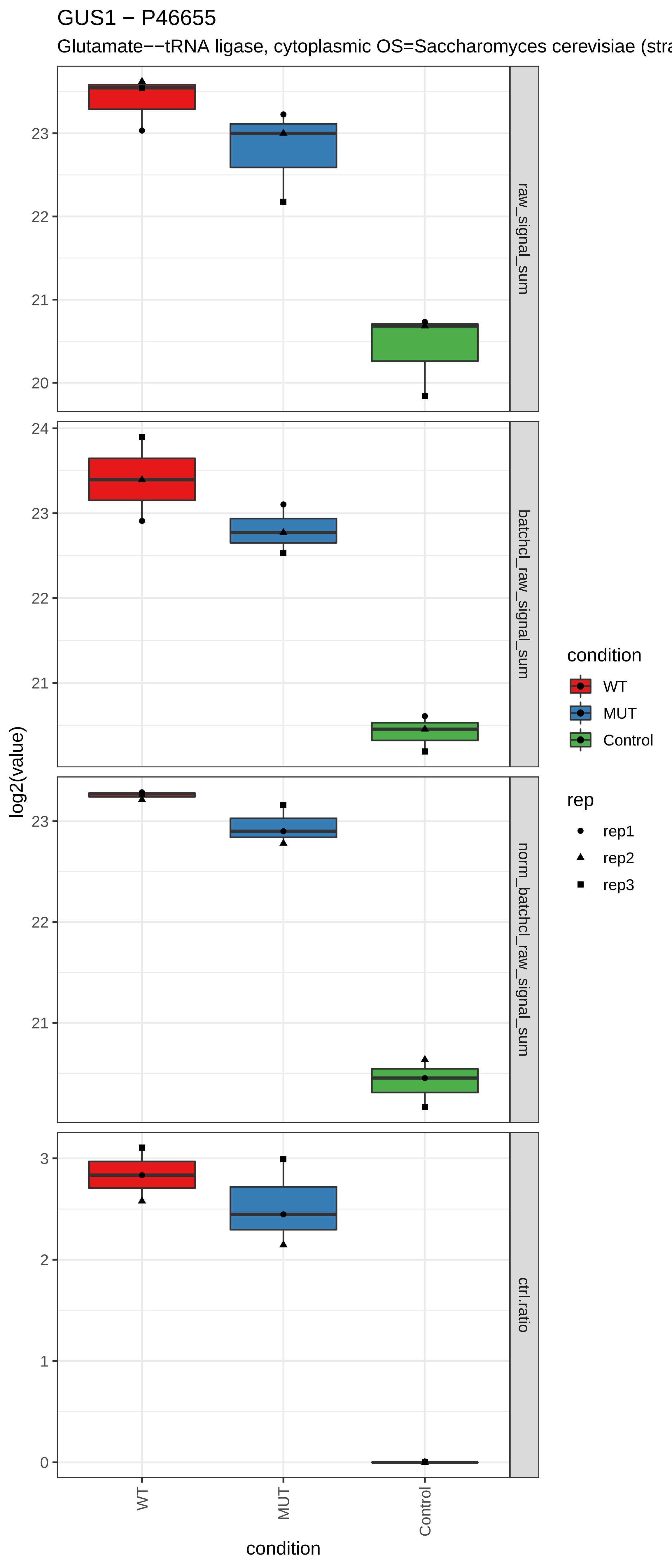

GUS1 − P46655
Glutamate−−tRNA ligase, cytoplasmic OS=Saccharomyces cerevisiae (stra
raw_signal_sum
batchcl_raw_signal_sum
norm_batchcl_raw_signal_sum
ctrl.ratio
log2(value)
condition
WT
MUT
Control
rep
rep1
rep2
rep3

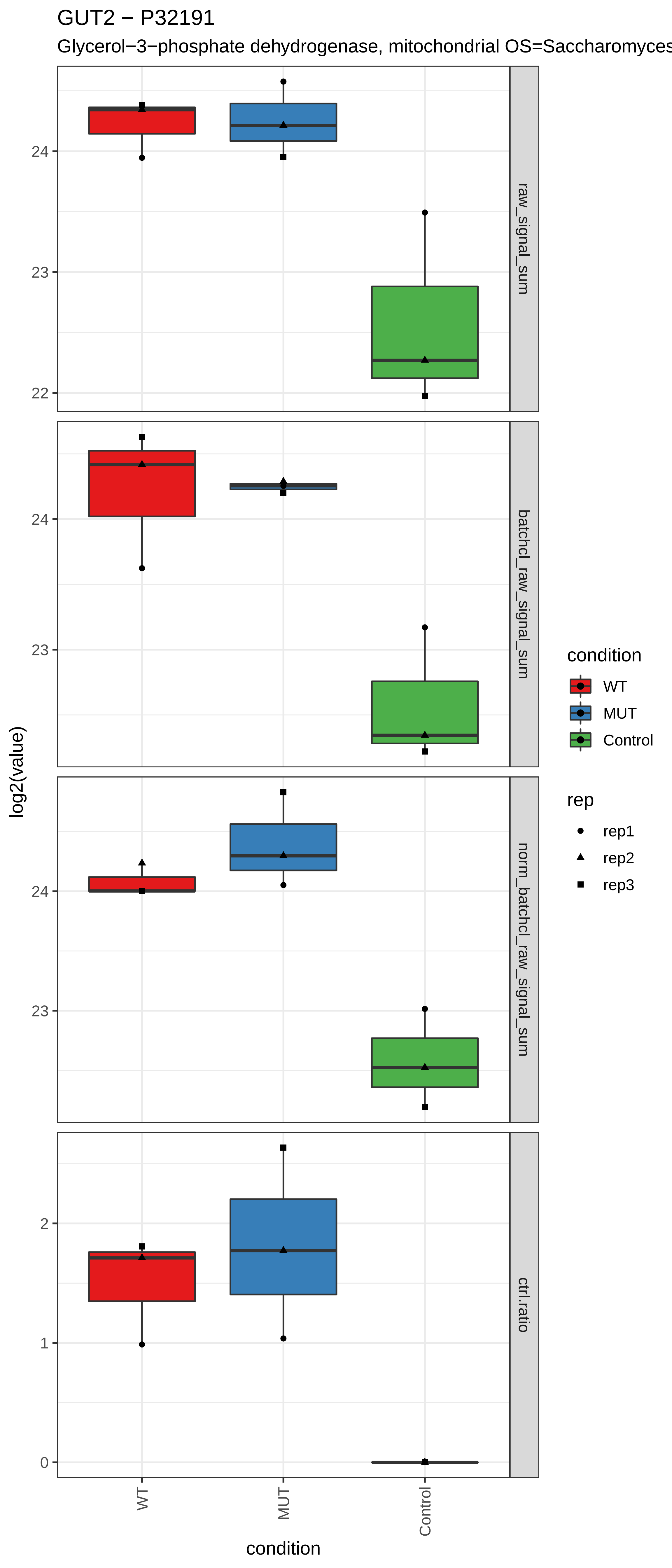

GUT2 − P32191
Glycerol−3−phosphate dehydrogenase, mitochondrial OS=Saccharomyces
raw_signal_sum
batchcl_raw_signal_sum
norm_batchcl_raw_signal_sum
ctrl.ratio
log2(value)
condition
WT
MUT
Control
rep
rep1
rep2
rep3
24
23
22
2
1
0

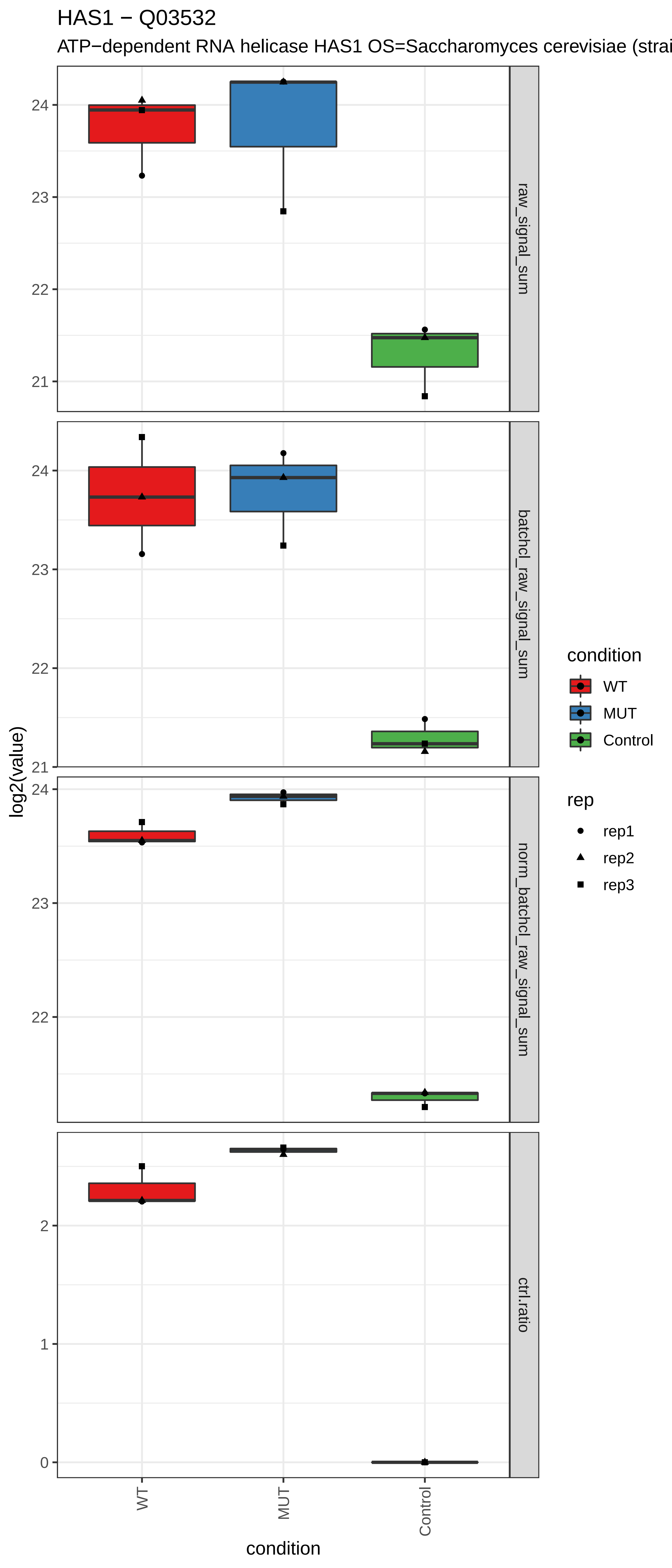
HAS1 − Q03532
ATP−dependent RNA helicase HAS1 OS=Saccharomyces cerevisiae (strai
raw_signal_sum
batchcl_raw_signal_sum
norm_batchcl_raw_signal_sum
ctrl.ratio
log2(value)
condition
WT
MUT
Control
rep
rep1
rep2
rep3

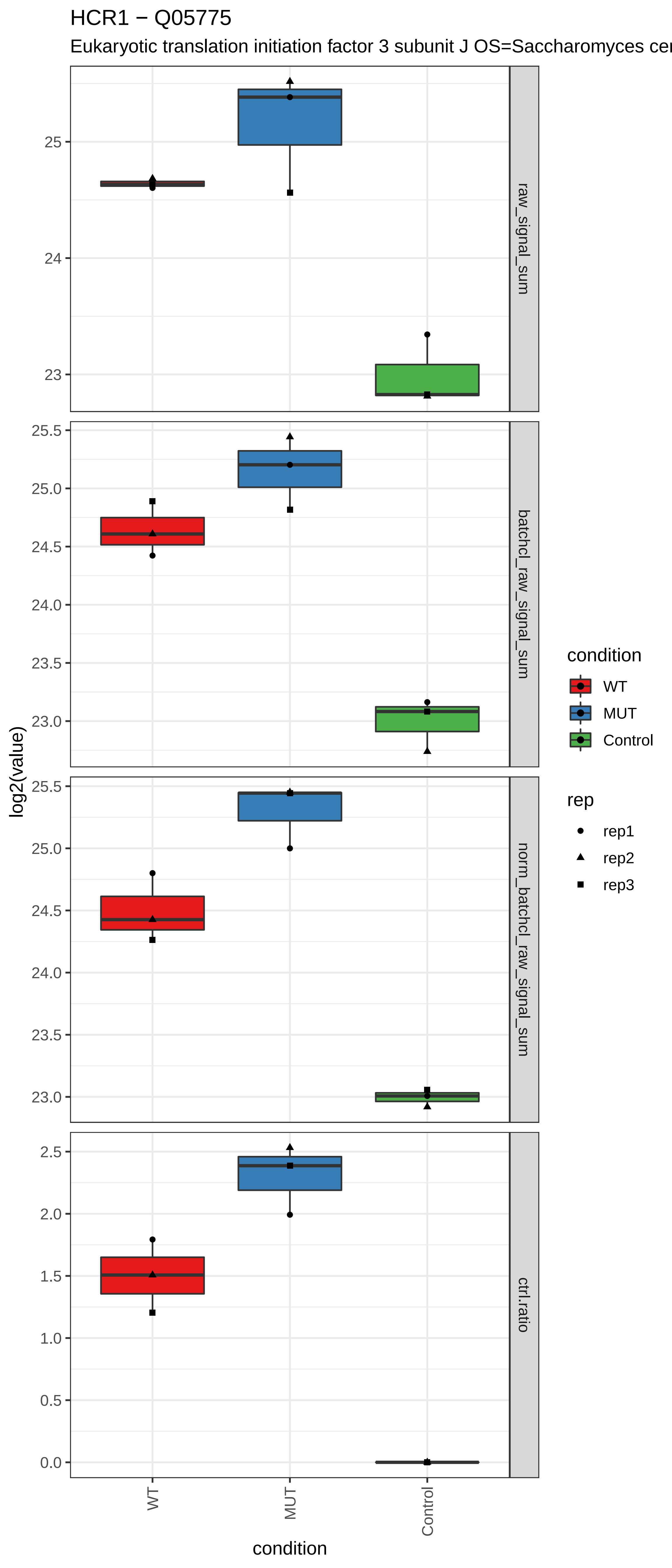

HCR1 − Q05775
Eukaryotic translation initiation factor 3 subunit J OS=Saccharomyces cer
raw_signal_sum
batchcl_raw_signal_sum
norm_batchcl_raw_signal_sum
ctrl.ratio
log2(value)
condition
WT
MUT
Control
rep
rep1
rep2
rep3
25
24
23
25.5
25.0
24.5
24.0
23.5
23.0
2.5
2.0
1.5
1.0
0.5
0.0

HEF3 − P53978

Elongation factor 3B OS=Saccharomyces cerevisiae (strain ATCC 204508

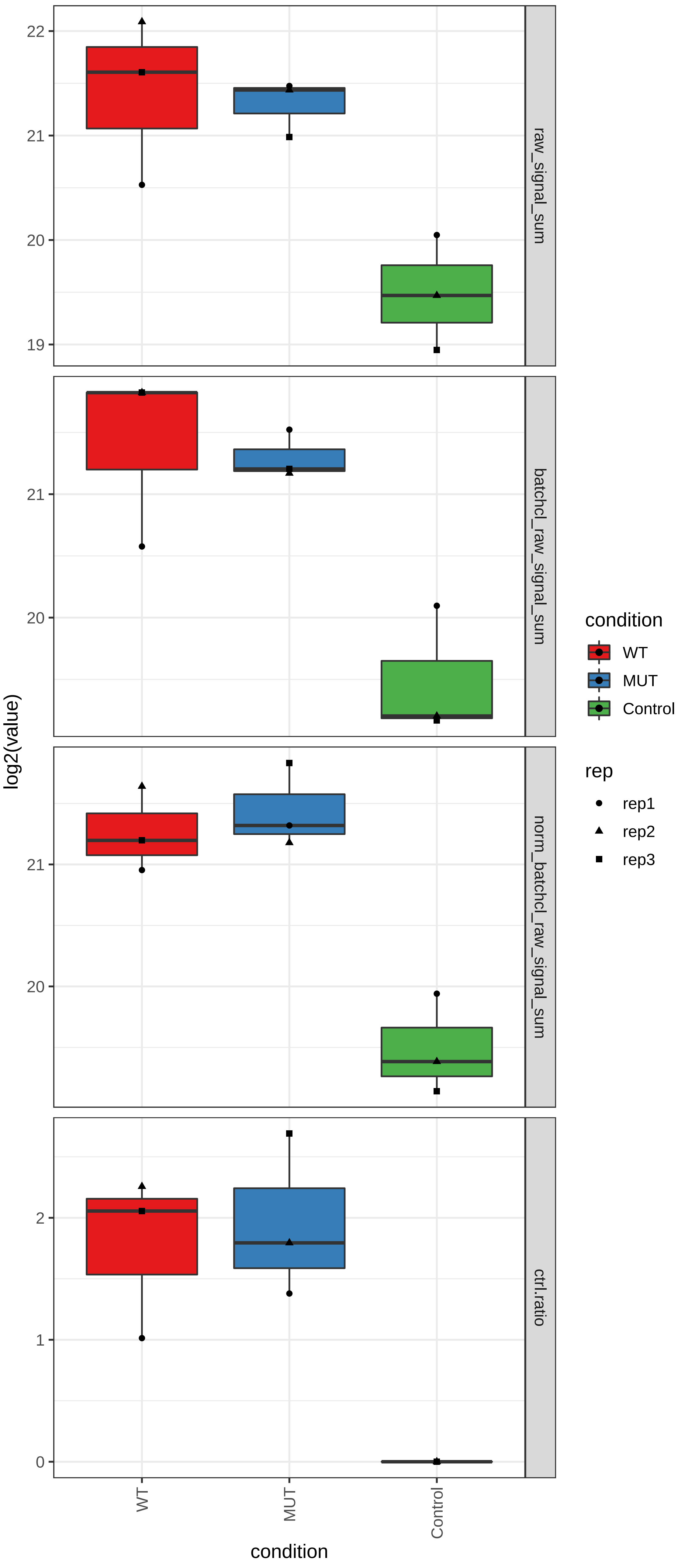

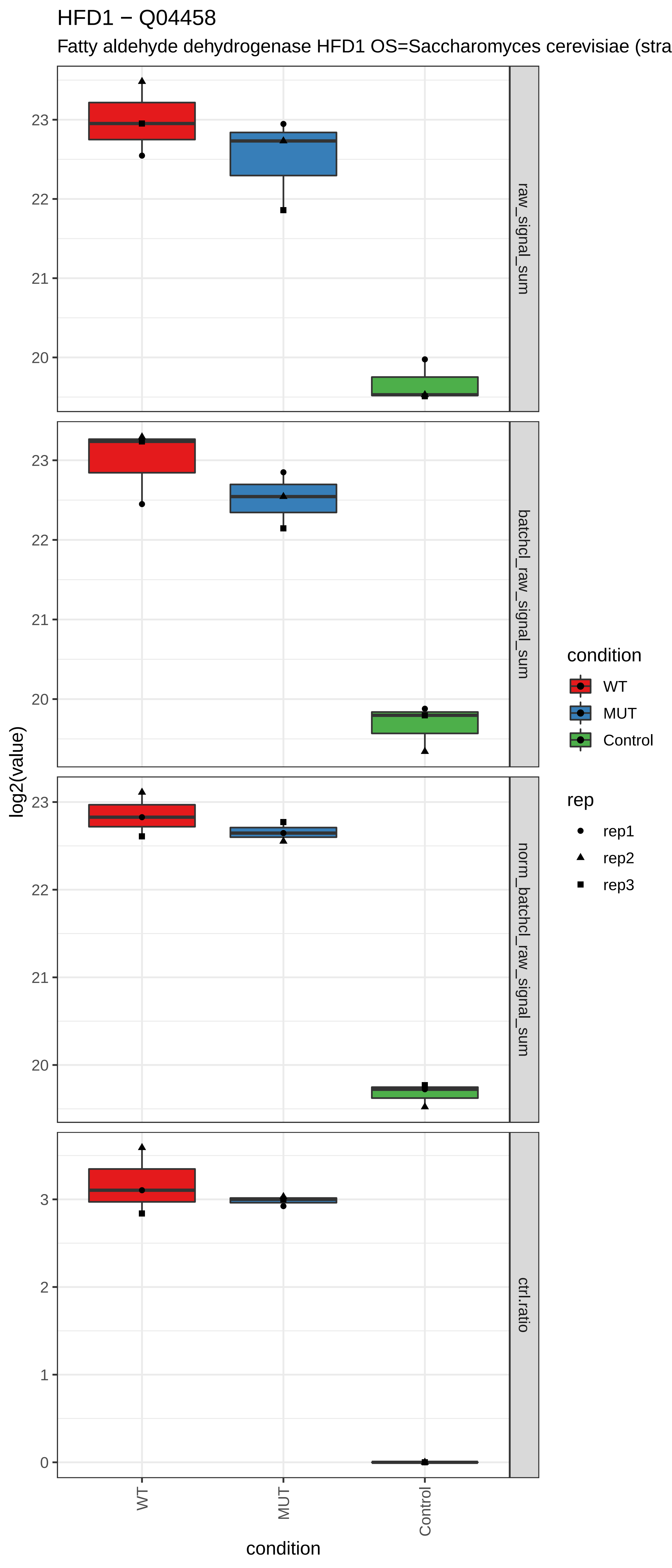

HFD1 − Q04458
Fatty aldehyde dehydrogenase HFD1 OS=Saccharomyces cerevisiae (stra
raw_signal_sum
batchcl_raw_signal_sum
norm_batchcl_raw_signal_sum
ctrl.ratio
log2(value)
condition
WT
MUT
Control
rep
rep1
rep2
rep3

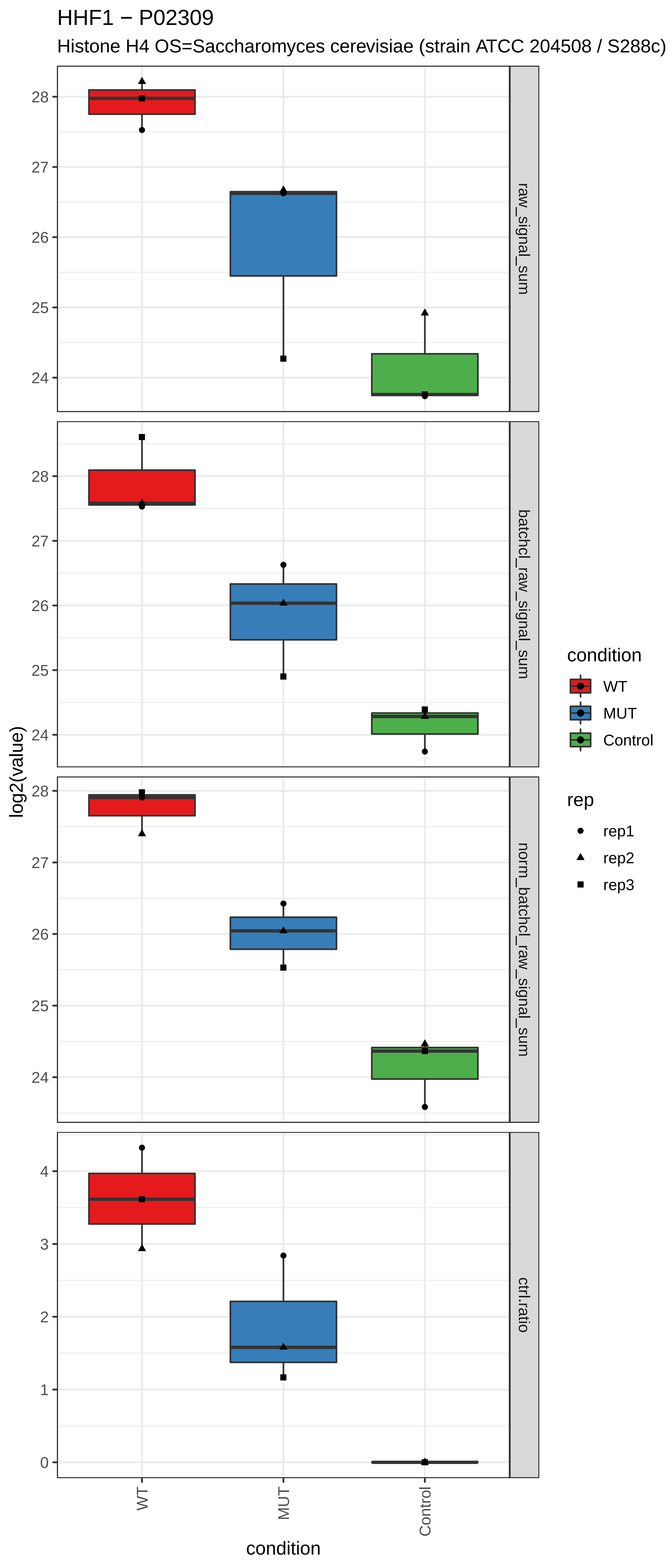

HHF1 − P02309
Histone H4 OS=Saccharomyces cerevisiae (strain ATCC 204508 / S288c)
raw_signal_sum
batchcl_raw_signal_sum
norm_batchcl_raw_signal_sum
ctrl.ratio
log2(value)
condition
WT
MUT
Control
rep
rep1
rep2
rep3

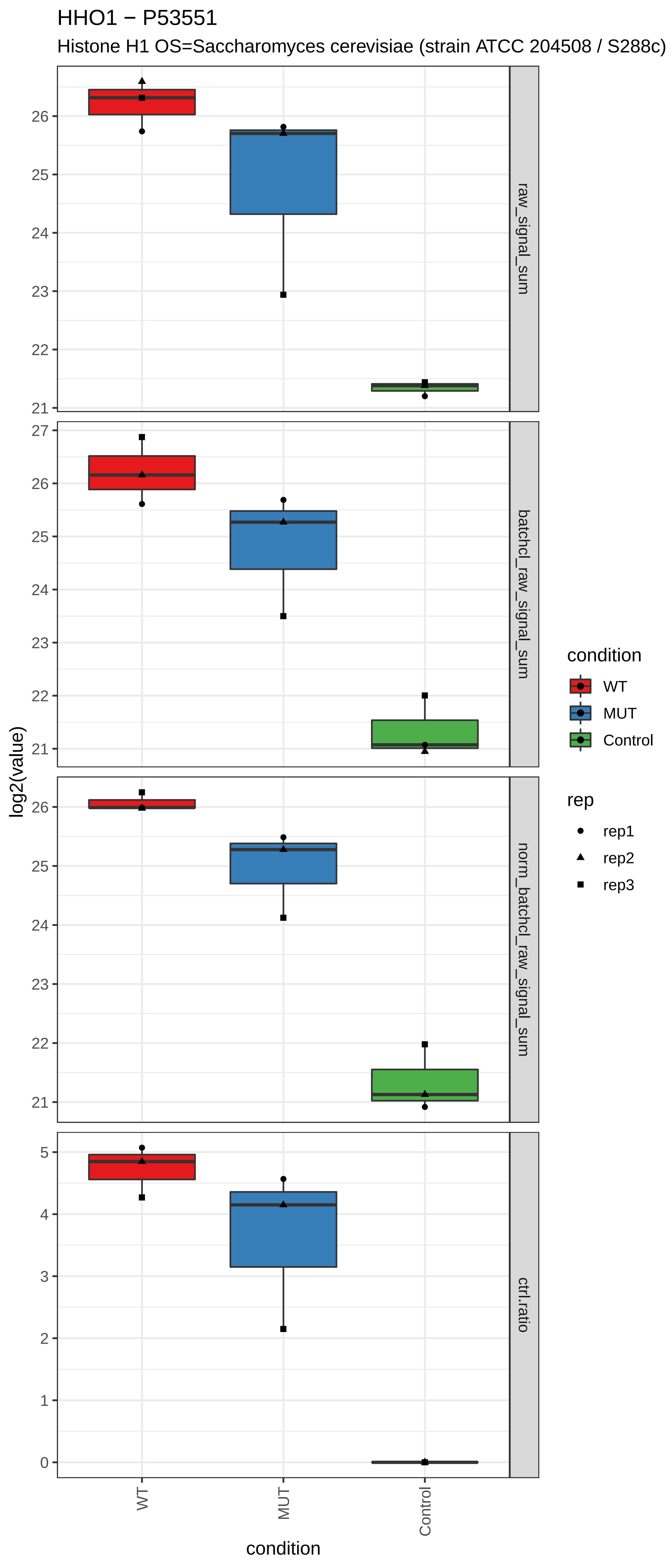

HHO1 − P53551
Histone H1 OS=Saccharomyces cerevisiae (strain ATCC 204508 / S288c)
raw_signal_sum
batchcl_raw_signal_sum
norm_batchcl_raw_signal_sum
ctrl.ratio
log2(value)
condition
WT
MUT
Control
rep
rep1
rep2
rep3

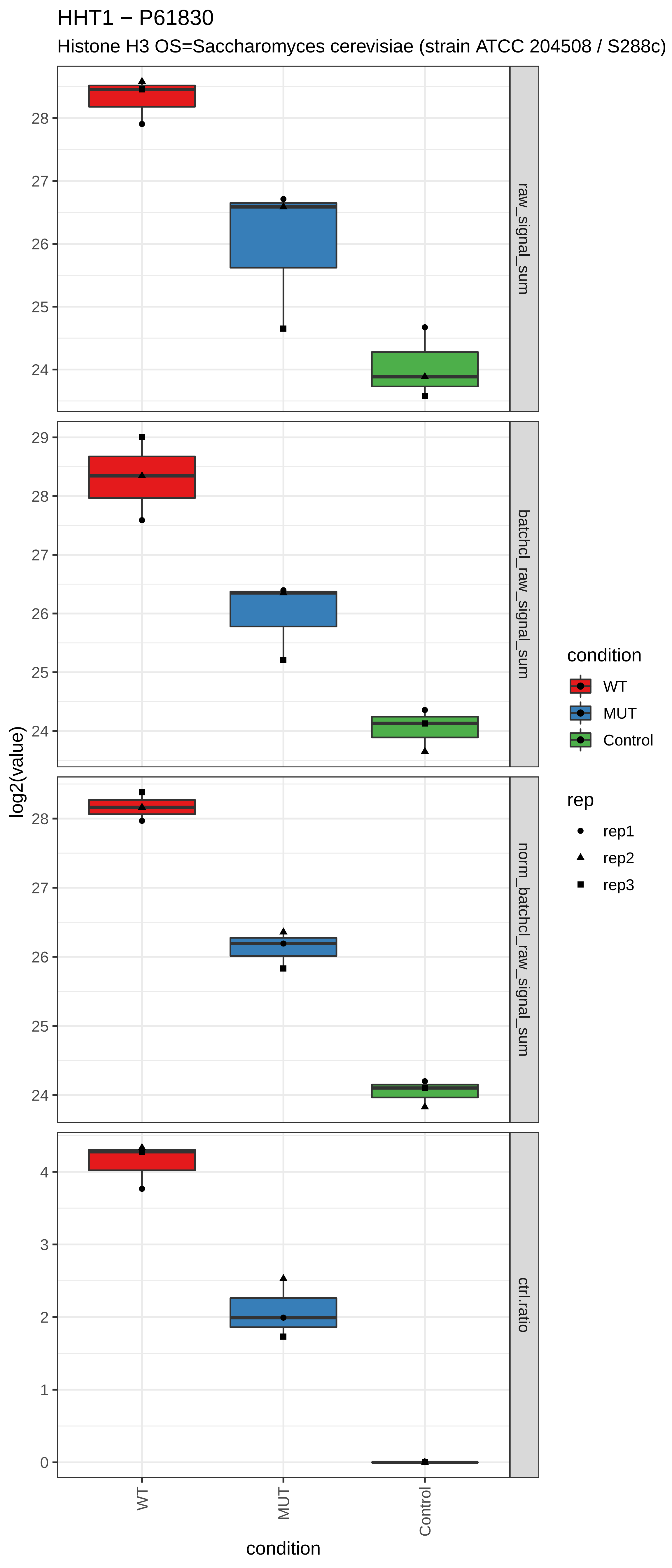
HHT1 − P61830
Histone H3 OS=Saccharomyces cerevisiae (strain ATCC 204508 / S288c)
raw_signal_sum
batchcl_raw_signal_sum
norm_batchcl_raw_signal_sum
ctrl.ratio
log2(value)
condition
WT
MUT
Control
rep
rep1
rep2
rep3

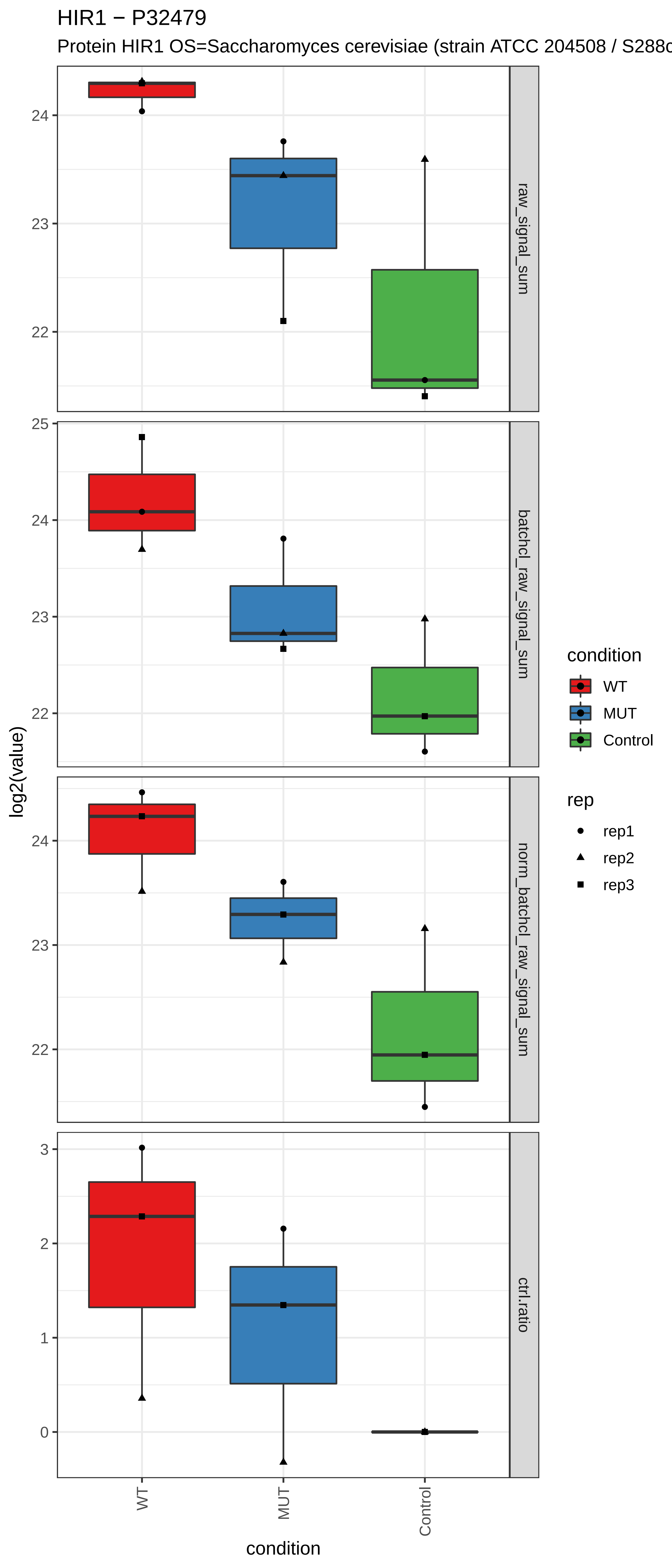

HIR1 − P32479
Protein HIR1 OS=Saccharomyces cerevisiae (strain ATCC 204508 / S288c
raw_signal_sum
batchcl_raw_signal_sum
norm_batchcl_raw_signal_sum
ctrl.ratio
log2(value)
condition
WT
MUT
Control
rep
rep1
rep2
rep3

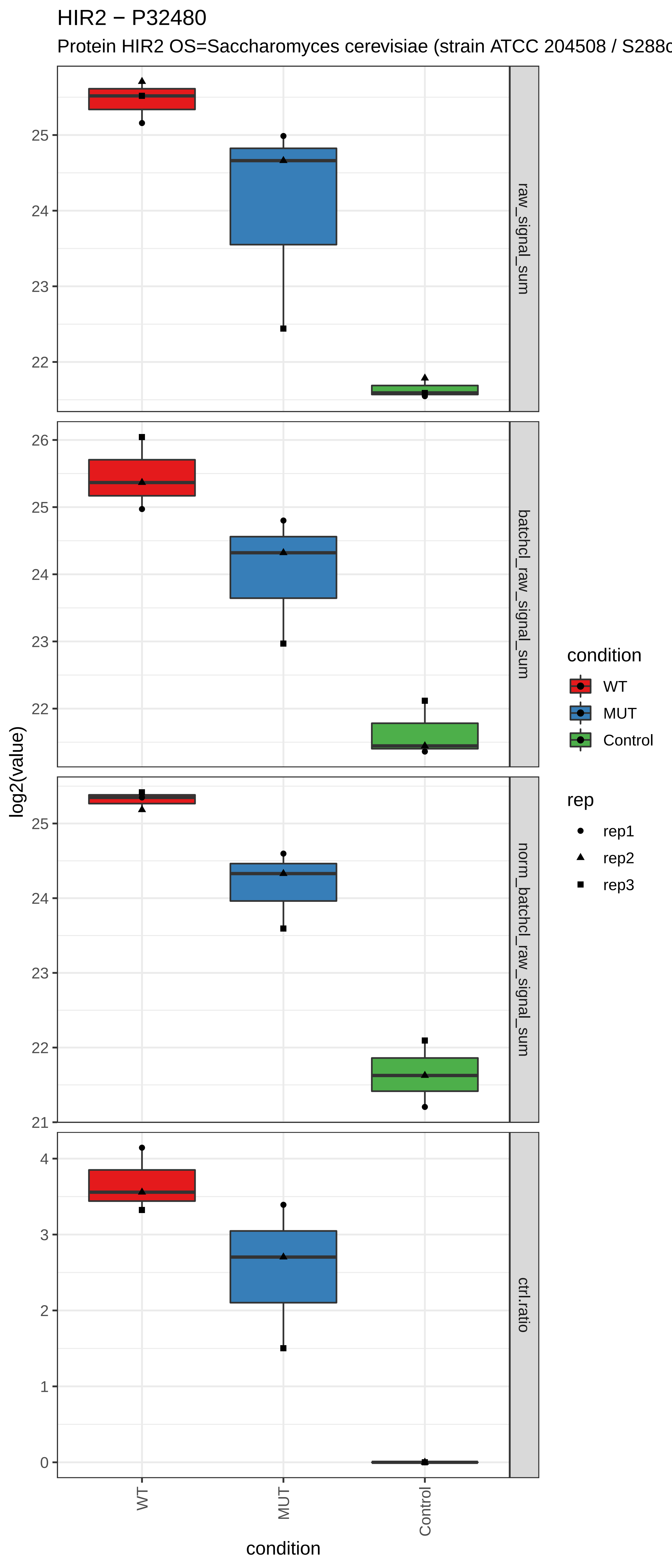
HIR2 − P32480
Protein HIR2 OS=Saccharomyces cerevisiae (strain ATCC 204508 / S288c
raw_signal_sum
batchcl_raw_signal_sum
norm_batchcl_raw_signal_sum
ctrl.ratio
log2(value)
condition
WT
MUT
Control
rep
rep1
rep2
rep3

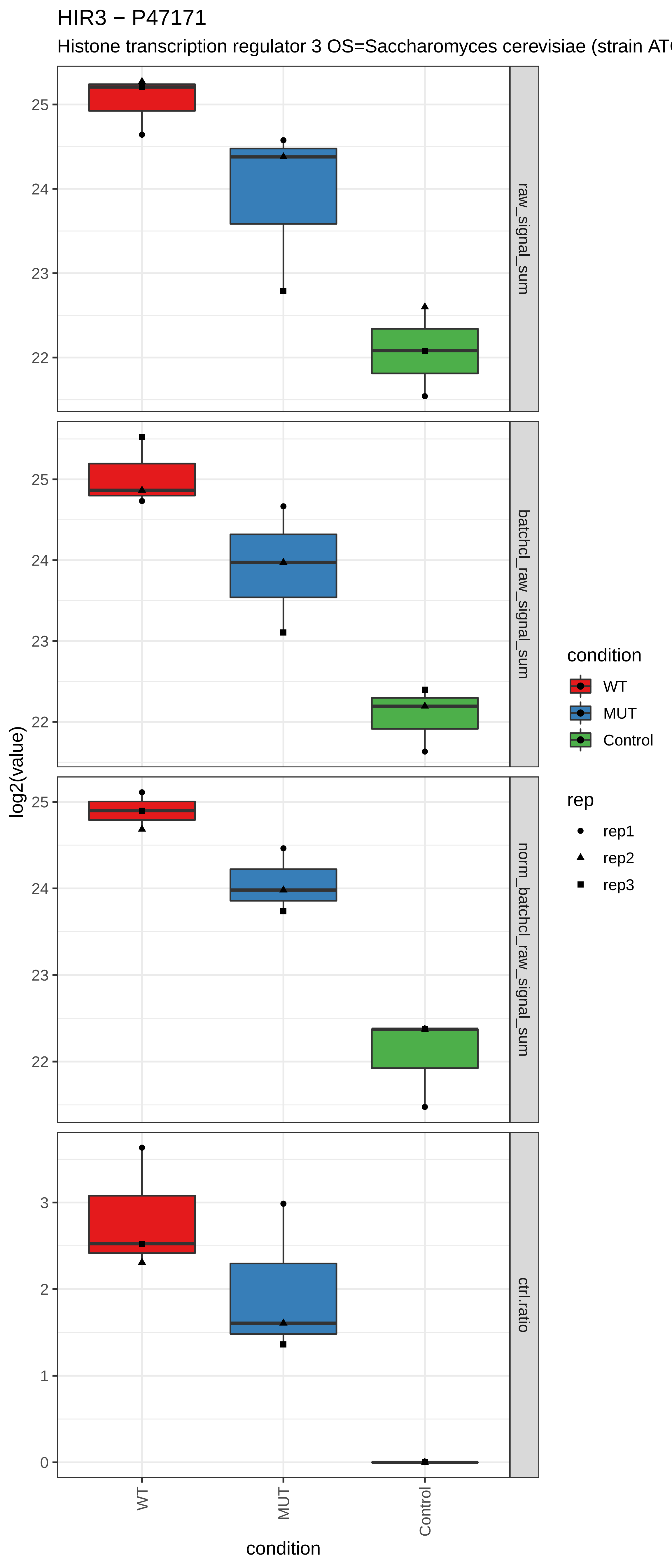

HIR3 − P47171
Histone transcription regulator 3 OS=Saccharomyces cerevisiae (strain ATC
raw_signal_sum
batchcl_raw_signal_sum
norm_batchcl_raw_signal_sum
ctrl.ratio
log2(value)
condition
WT
MUT
Control
rep
rep1
rep2
rep3

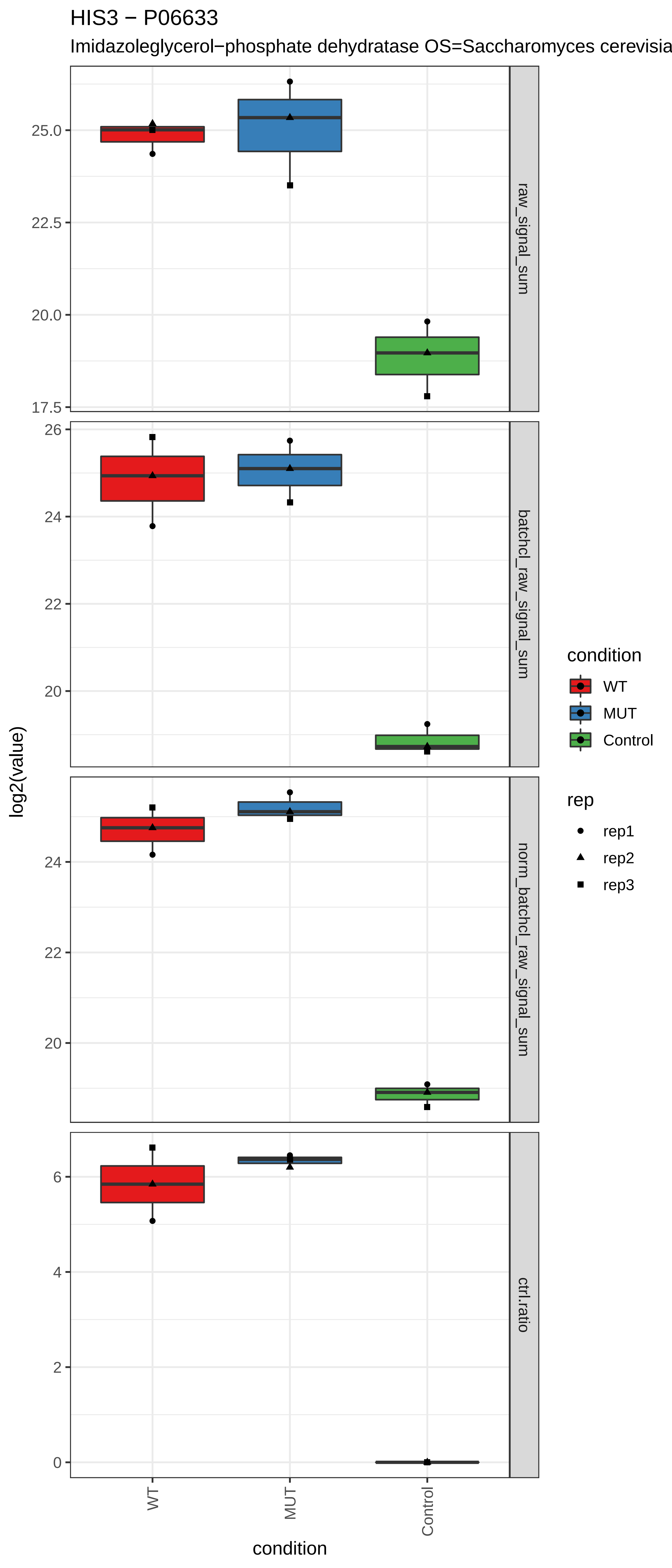

HIS3 − P06633
Imidazoleglycerol−phosphate dehydratase OS=Saccharomyces cerevisia
raw_signal_sum
batchcl_raw_signal_sum
norm_batchcl_raw_signal_sum
ctrl.ratio
log2(value)
condition
WT
MUT
Control
rep
rep1
rep2
rep3
25.0
22.5
20.0
17.5
26
24
22
20
6
4
2
0

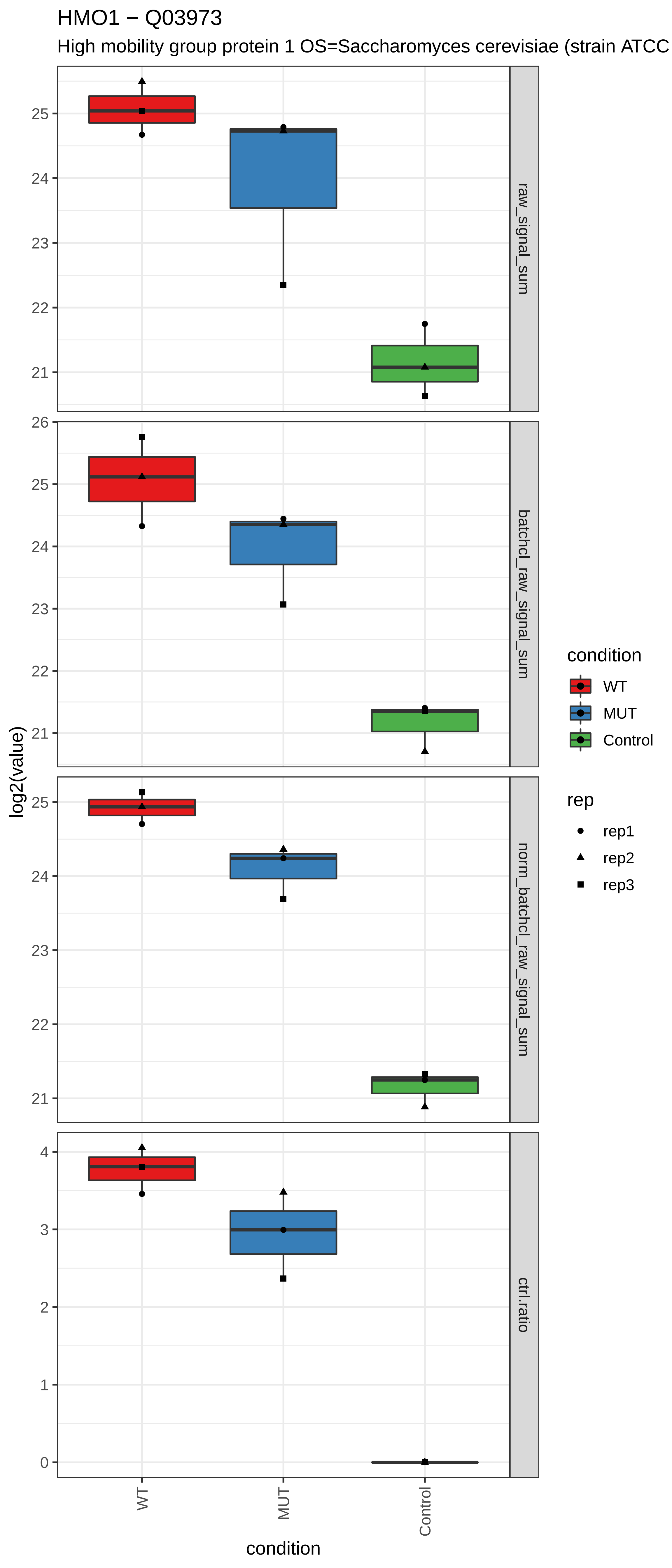

HMO1 − Q03973
High mobility group protein 1 OS=Saccharomyces cerevisiae (strain ATCC
raw_signal_sum
batchcl_raw_signal_sum
norm_batchcl_raw_signal_sum
ctrl.ratio
log2(value)
condition
WT
MUT
Control
rep
rep1
rep2
rep3

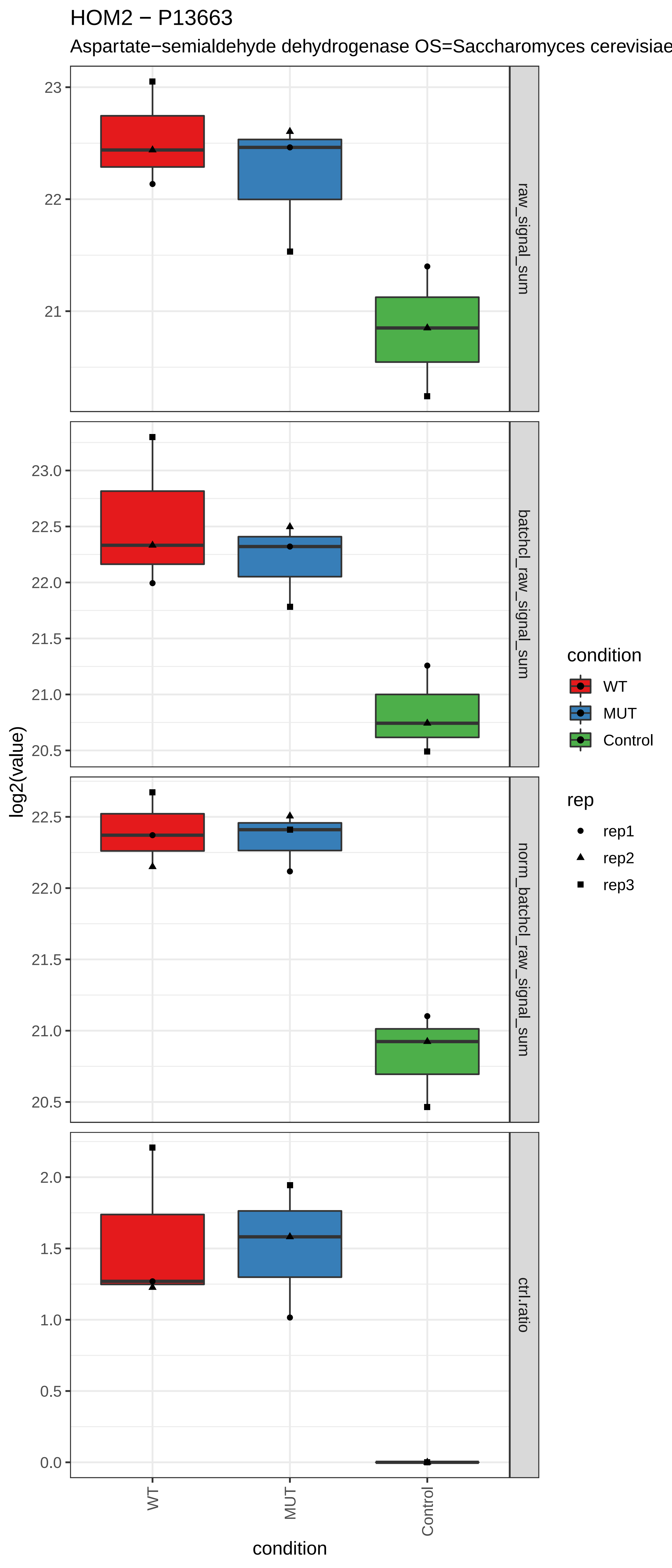
HOM2 − P13663
Aspartate−semialdehyde dehydrogenase OS=Saccharomyces cerevisiae
raw_signal_sum
batchcl_raw_signal_sum
norm_batchcl_raw_signal_sum
ctrl.ratio
log2(value)
condition
WT
MUT
Control
rep
rep1
rep2
rep3

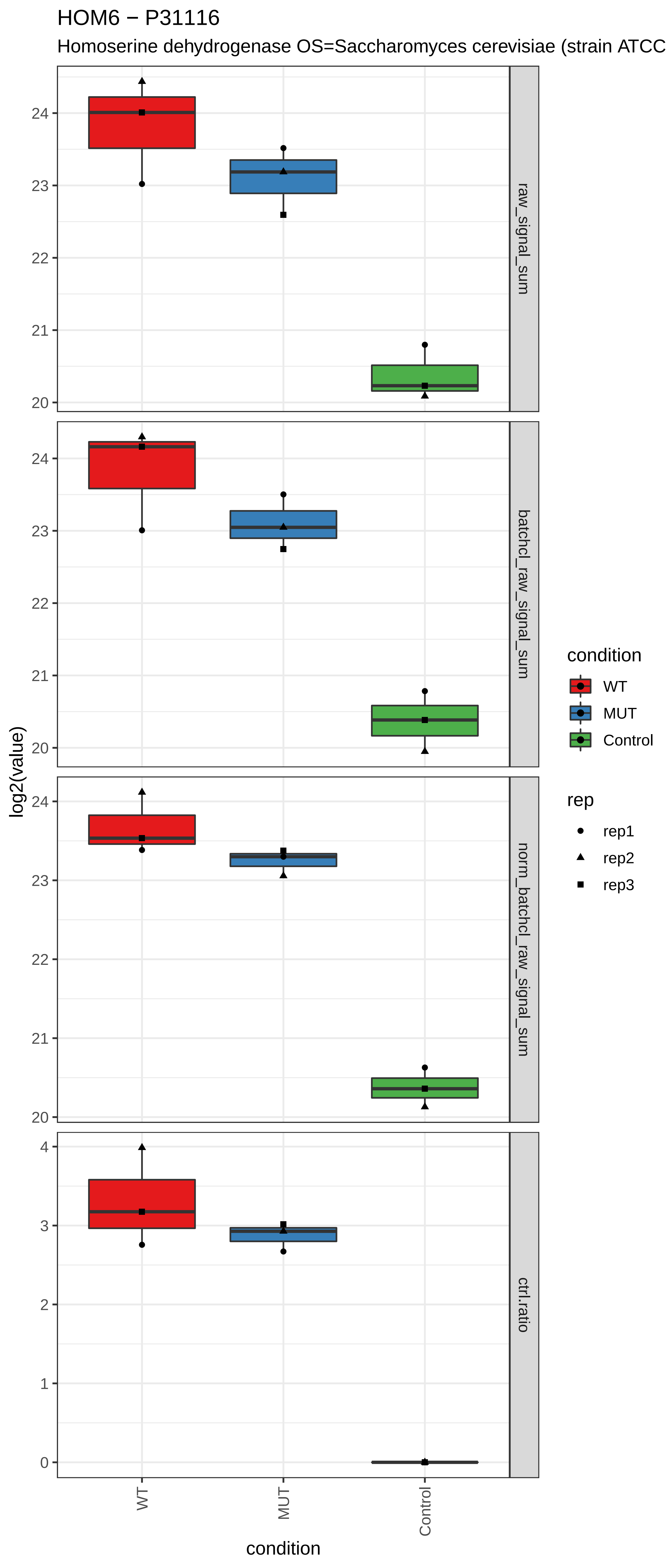

HOM6 − P31116
Homoserine dehydrogenase OS=Saccharomyces cerevisiae (strain ATCC
raw_signal_sum
batchcl_raw_signal_sum
norm_batchcl_raw_signal_sum
ctrl.ratio
log2(value)
condition
WT
MUT
Control
rep
rep1
rep2
rep3

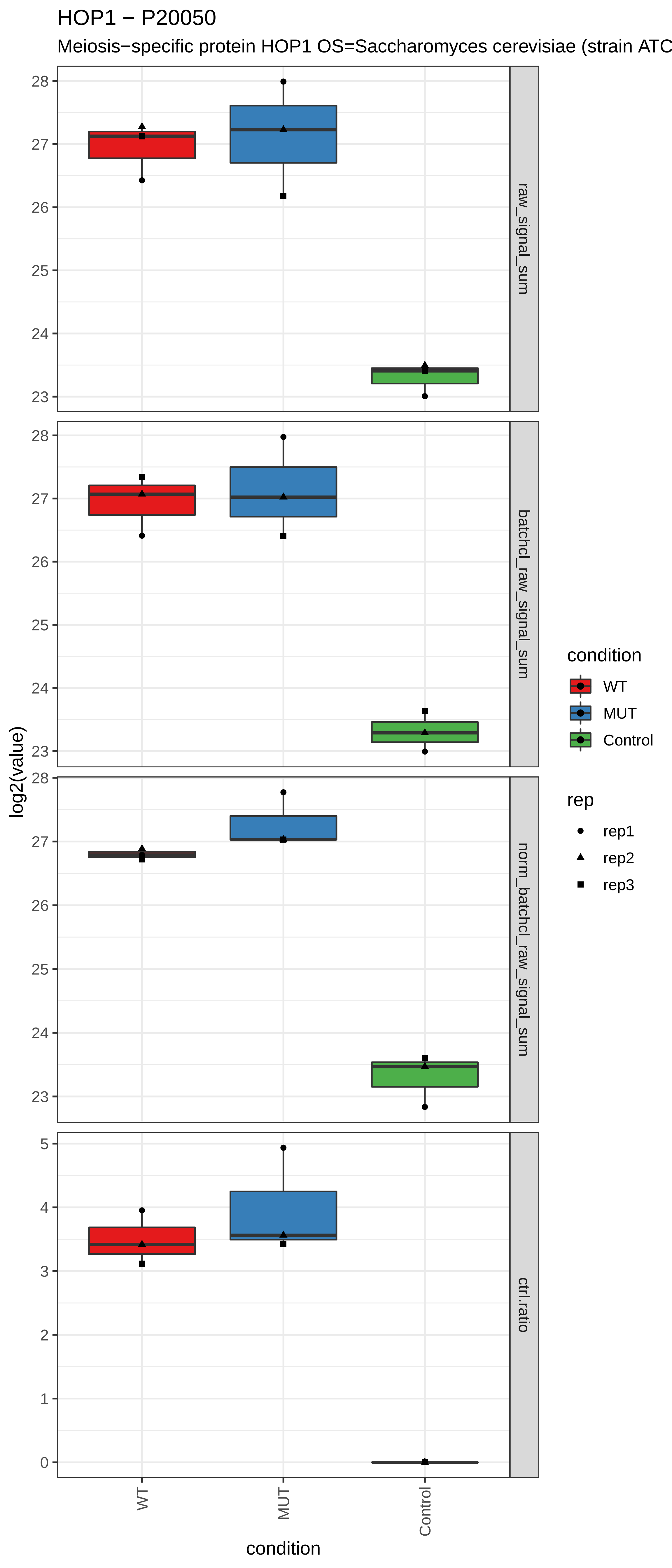

HOP1 − P20050
Meiosis−specific protein HOP1 OS=Saccharomyces cerevisiae (strain ATC
raw_signal_sum
batchcl_raw_signal_sum
norm_batchcl_raw_signal_sum
ctrl.ratio
log2(value)
condition
WT
MUT
Control
rep
rep1
rep2
rep3

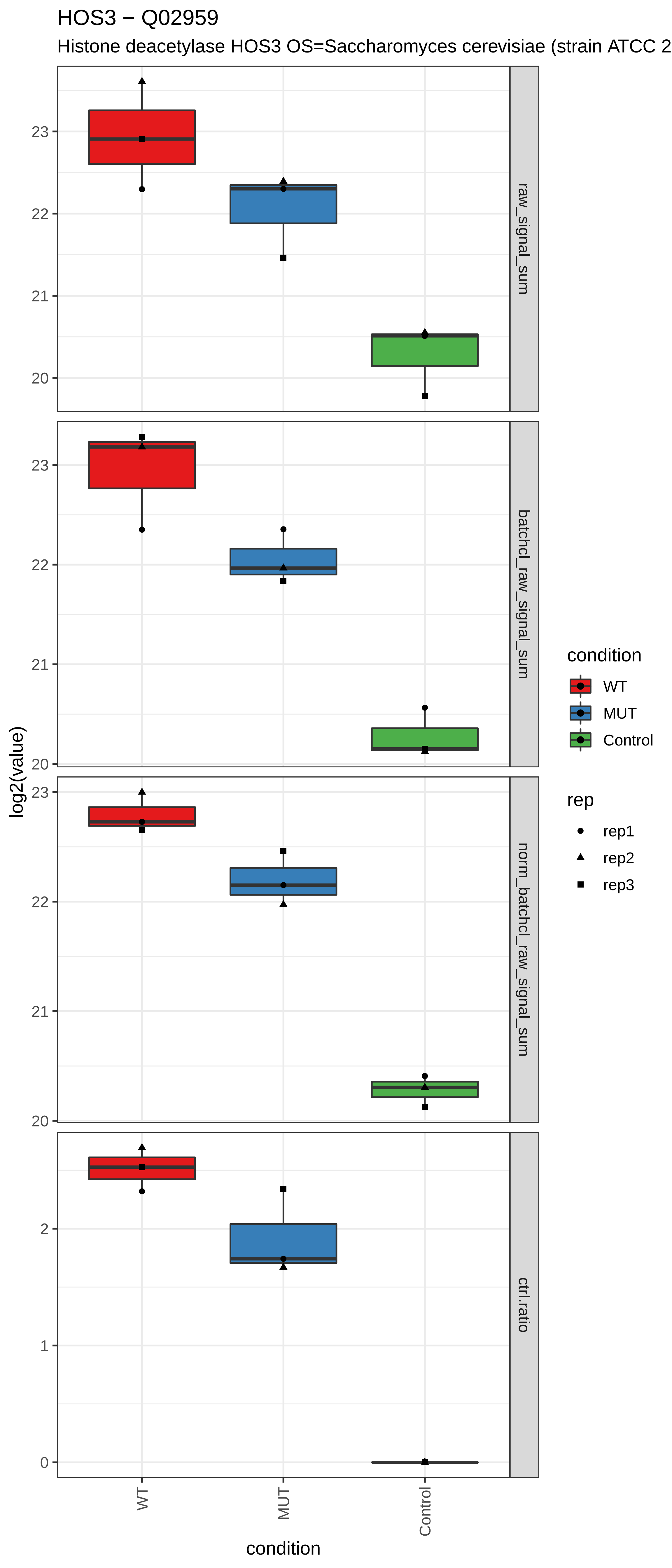

HOS3 − Q02959
Histone deacetylase HOS3 OS=Saccharomyces cerevisiae (strain ATCC 2
raw_signal_sum
batchcl_raw_signal_sum
norm_batchcl_raw_signal_sum
ctrl.ratio
log2(value)
condition
WT
MUT
Control
rep
rep1
rep2
rep3

HPC2 − Q01448

Histone promoter control protein 2 OS=Saccharomyces cerevisiae (strain A

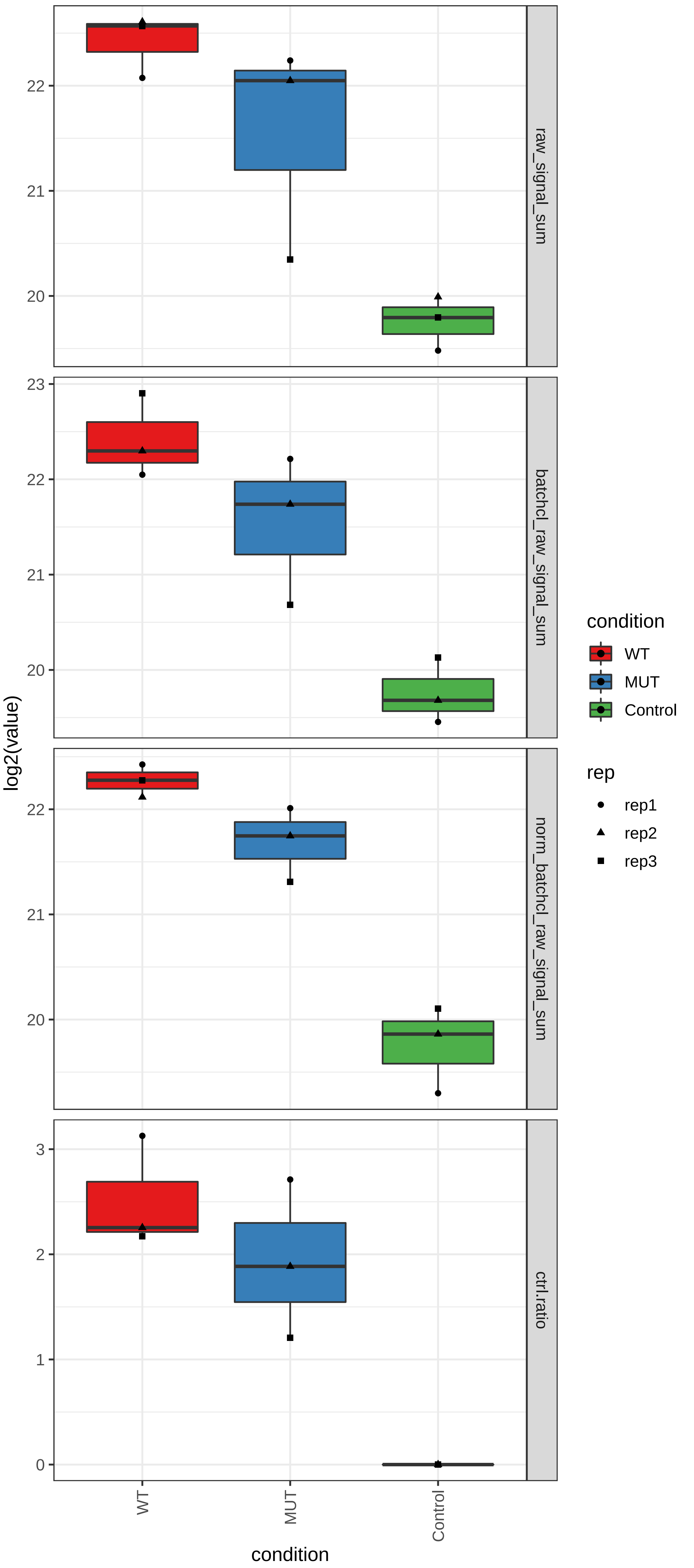

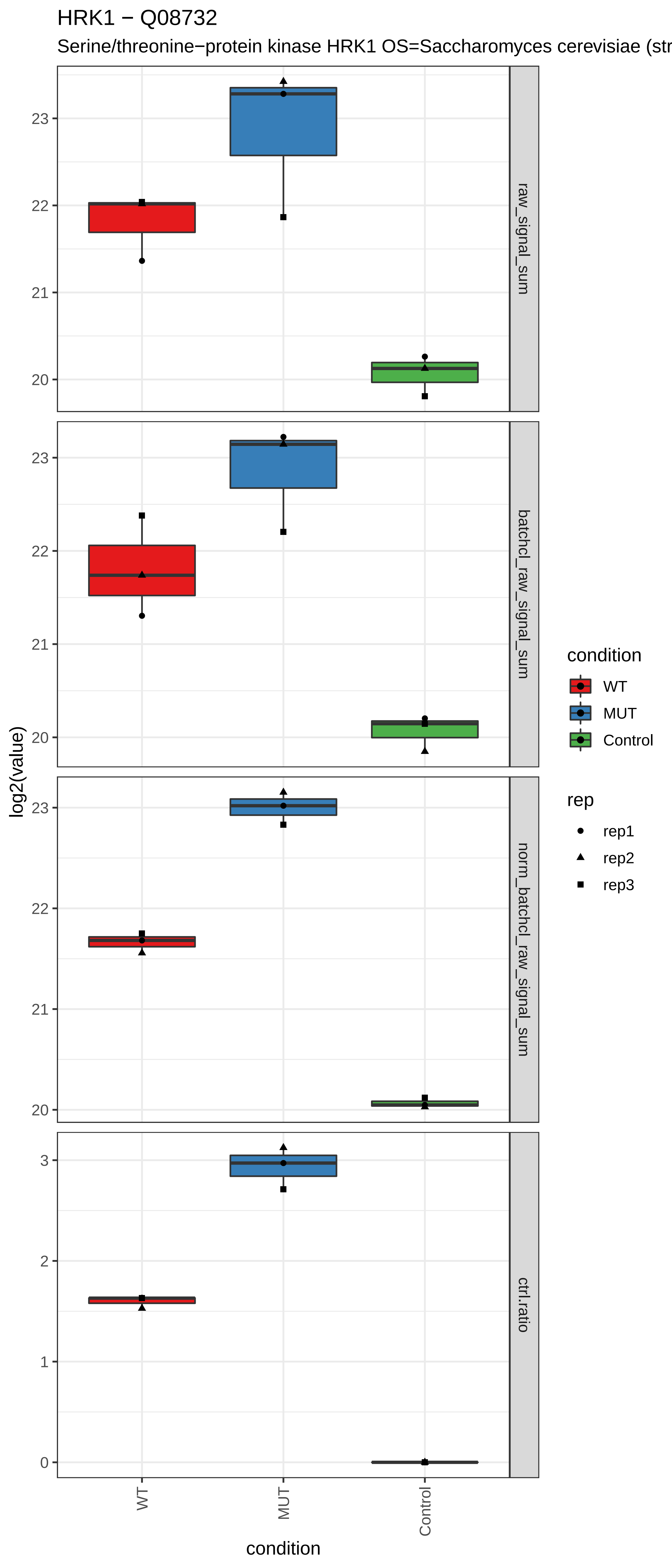

HRK1 − Q08732
Serine/threonine−protein kinase HRK1 OS=Saccharomyces cerevisiae (str
raw_signal_sum
batchcl_raw_signal_sum
norm_batchcl_raw_signal_sum
ctrl.ratio
log2(value)
condition
WT
MUT
Control
rep
rep1
rep2
rep3

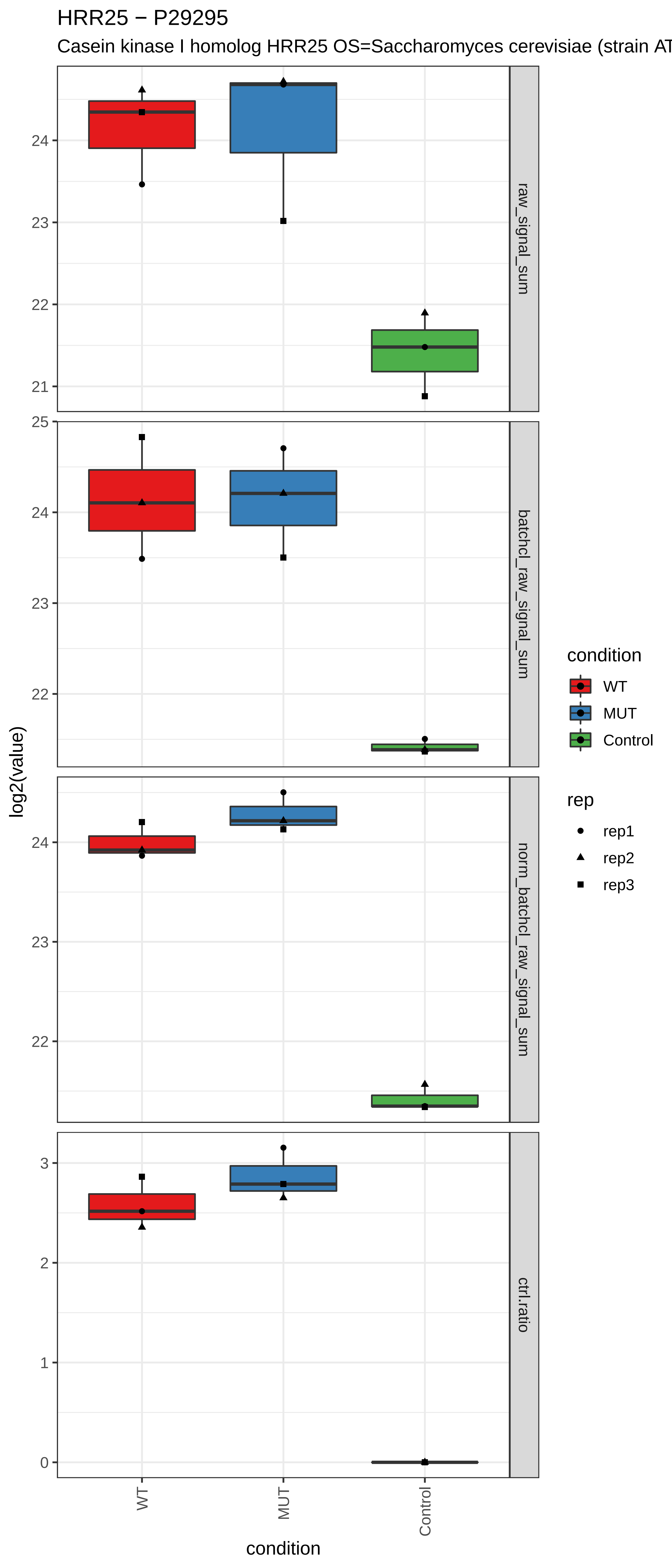
HRR25 − P29295
Casein kinase I homolog HRR25 OS=Saccharomyces cerevisiae (strain AT
raw_signal_sum
batchcl_raw_signal_sum
norm_batchcl_raw_signal_sum
ctrl.ratio
log2(value)
condition
WT
MUT
Control
rep
rep1
rep2
rep3

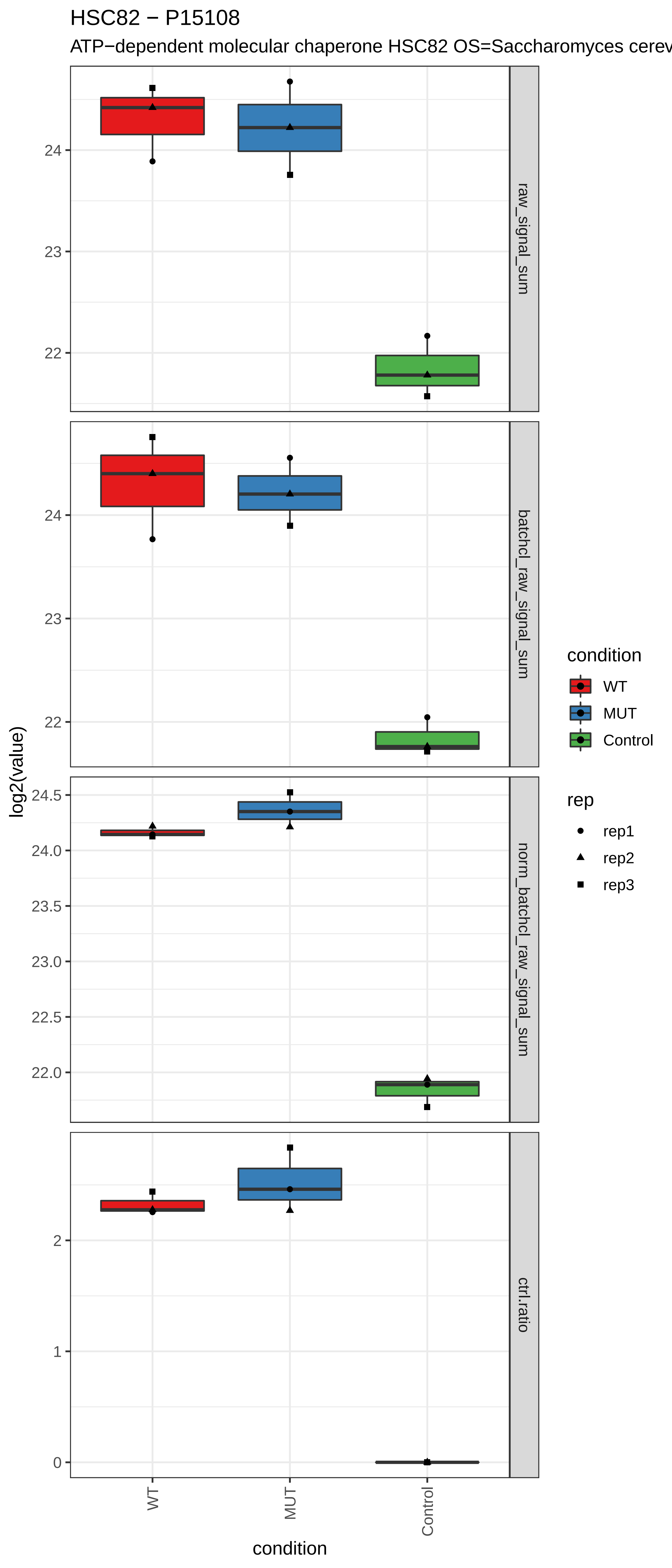
HSC82 − P15108
ATP−dependent molecular chaperone HSC82 OS=Saccharomyces cerev
raw_signal_sum
batchcl_raw_signal_sum
norm_batchcl_raw_signal_sum
ctrl.ratio
log2(value)
condition
WT
MUT
Control
rep
rep1
rep2
rep3
24
23
22
24.5
24.0
23.5
23.0
22.5
22.0
2
1
0

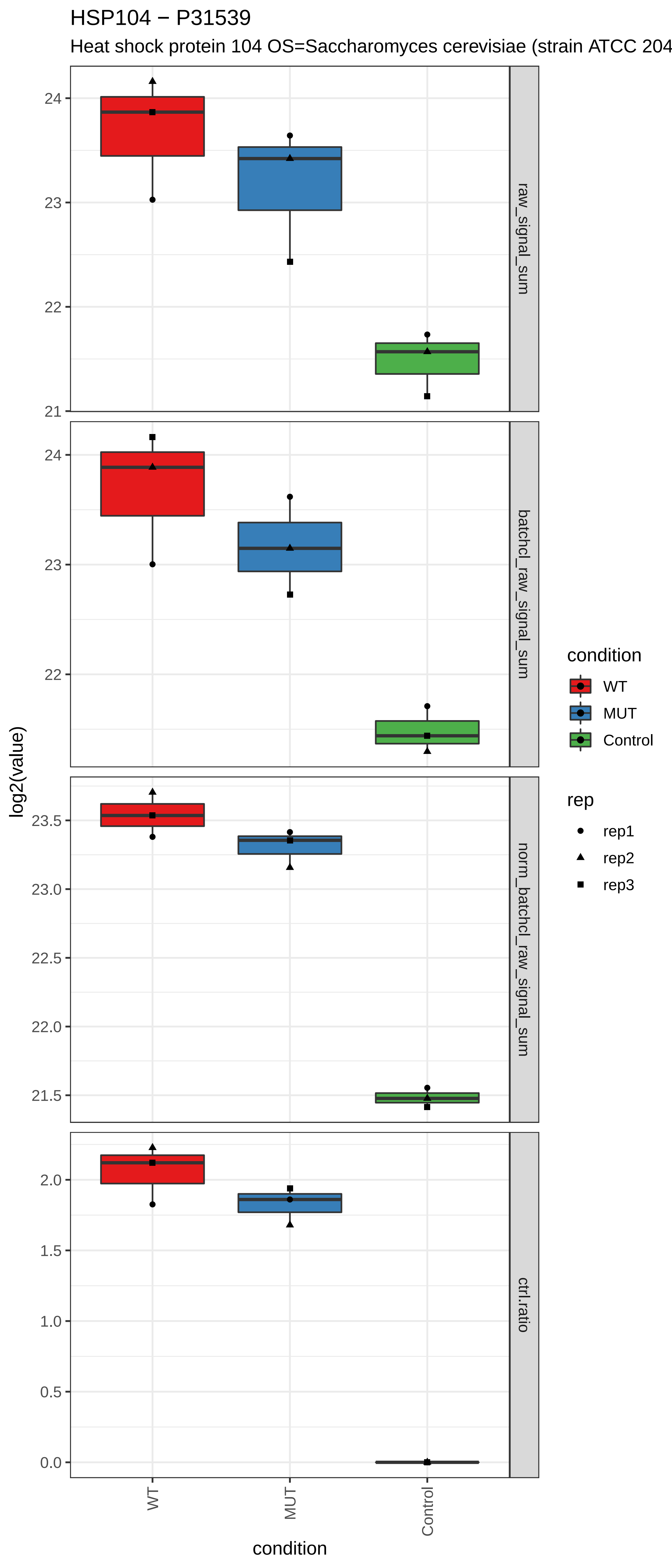

HSP104 − P31539
Heat shock protein 104 OS=Saccharomyces cerevisiae (strain ATCC 204
raw_signal_sum
batchcl_raw_signal_sum
norm_batchcl_raw_signal_sum
ctrl.ratio
log2(value)
condition
WT
MUT
Control
rep
rep1
rep2
rep3

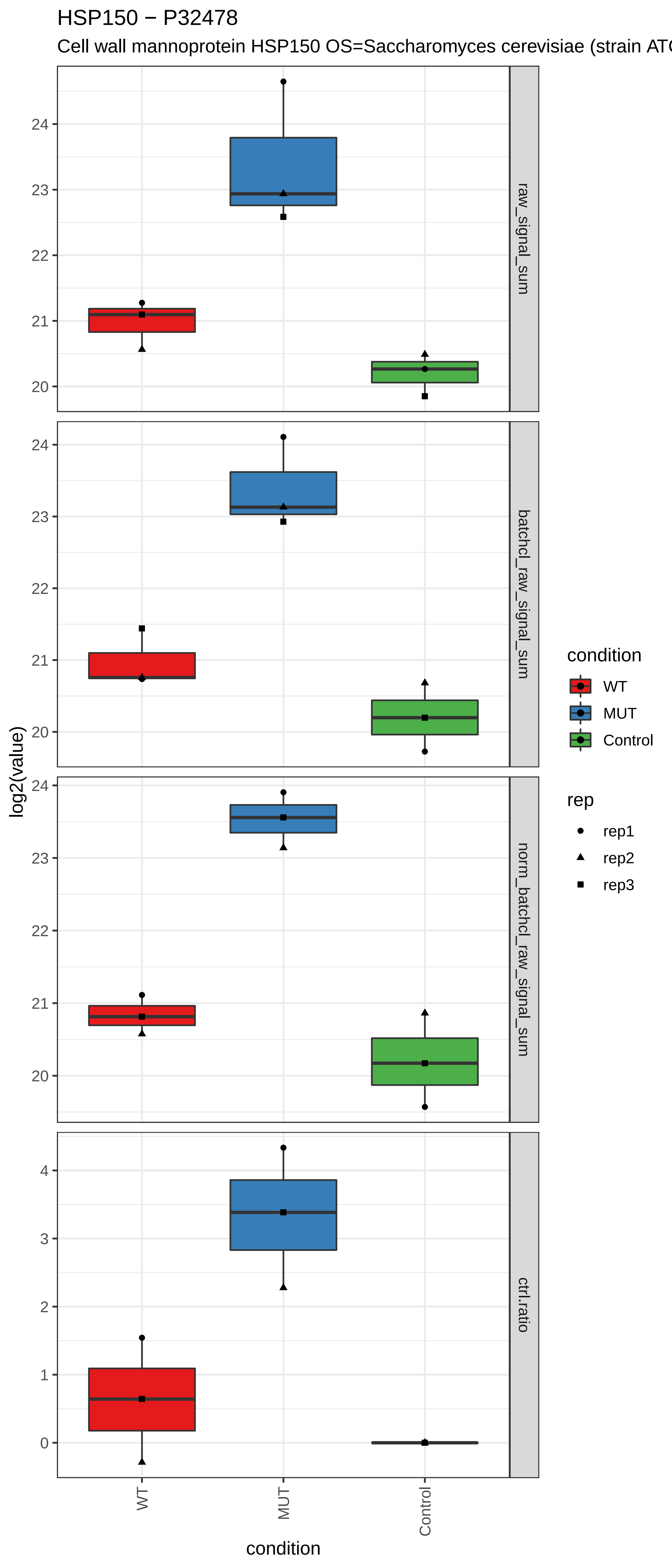
HSP150 − P32478
Cell wall mannoprotein HSP150 OS=Saccharomyces cerevisiae (strain ATC
raw_signal_sum
batchcl_raw_signal_sum
norm_batchcl_raw_signal_sum
ctrl.ratio
log2(value)
condition
WT
MUT
Control
rep
rep1
rep2
rep3
24
23
22
21
20
4
3
2
1
0

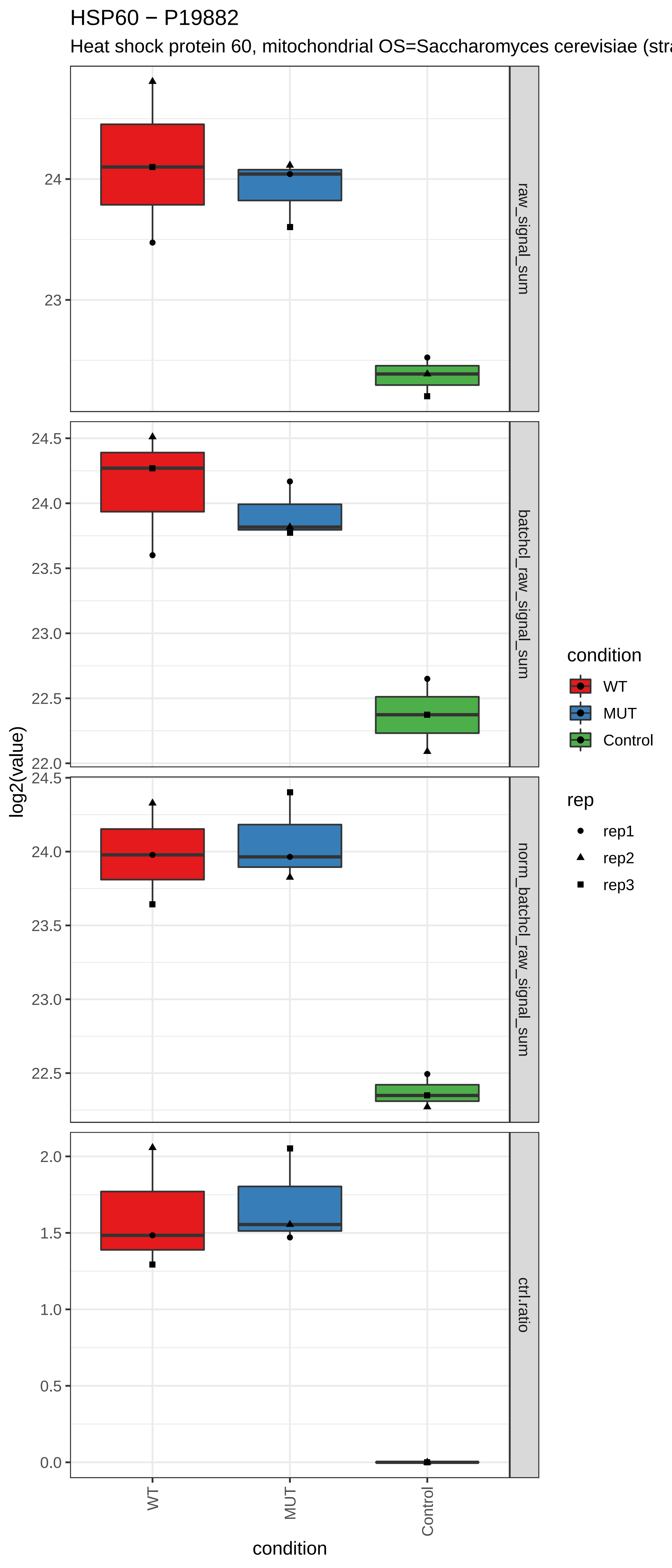

HSP60 − P19882
Heat shock protein 60, mitochondrial OS=Saccharomyces cerevisiae (stra
raw_signal_sum
batchcl_raw_signal_sum
norm_batchcl_raw_signal_sum
ctrl.ratio
log2(value)
condition
WT
MUT
Control
rep
rep1
rep2
rep3
24
23
24.5
24.0
23.5
23.0
22.5
22.0
2.0
1.5
1.0
0.5
0.0

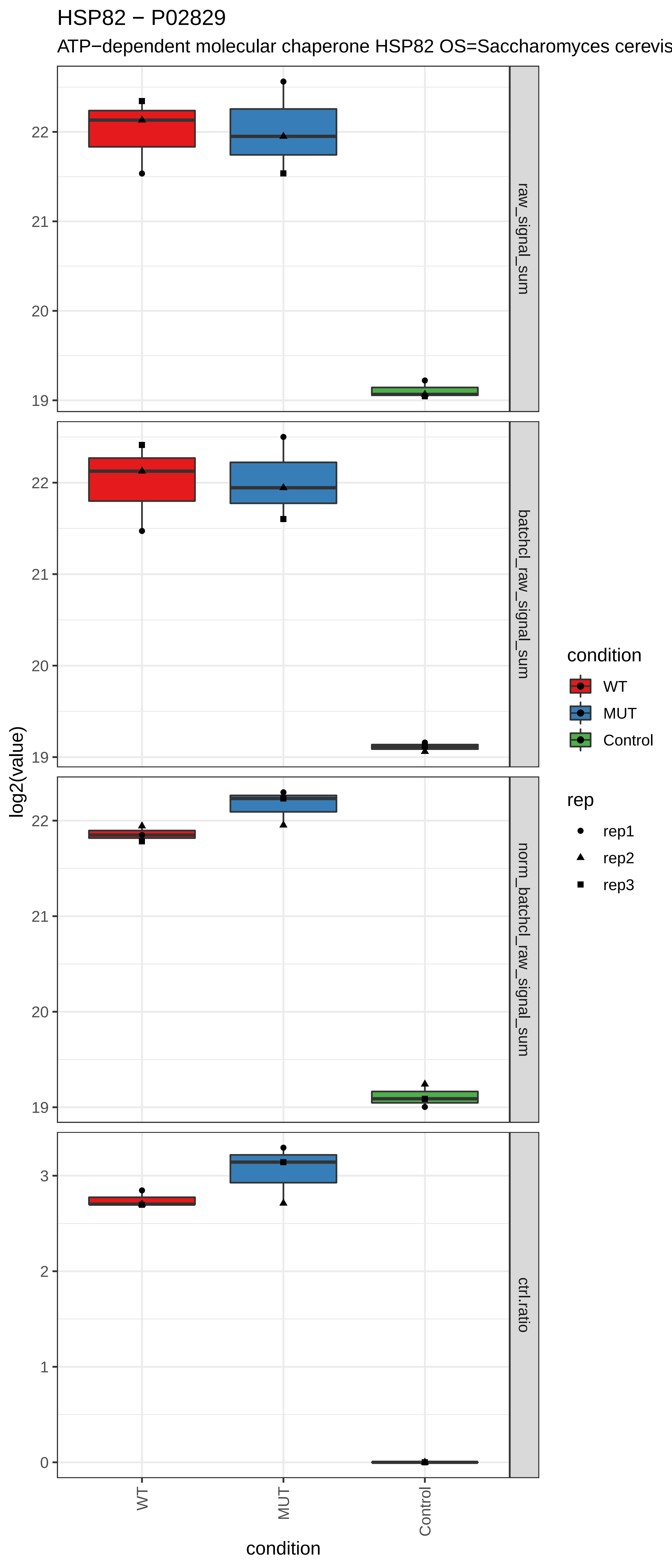

HSP82 − P02829
ATP−dependent molecular chaperone HSP82 OS=Saccharomyces cerevis
raw_signal_sum
batchcl_raw_signal_sum
norm_batchcl_raw_signal_sum
ctrl.ratio
log2(value)
condition
WT
MUT
Control
rep
rep1
rep2
rep3

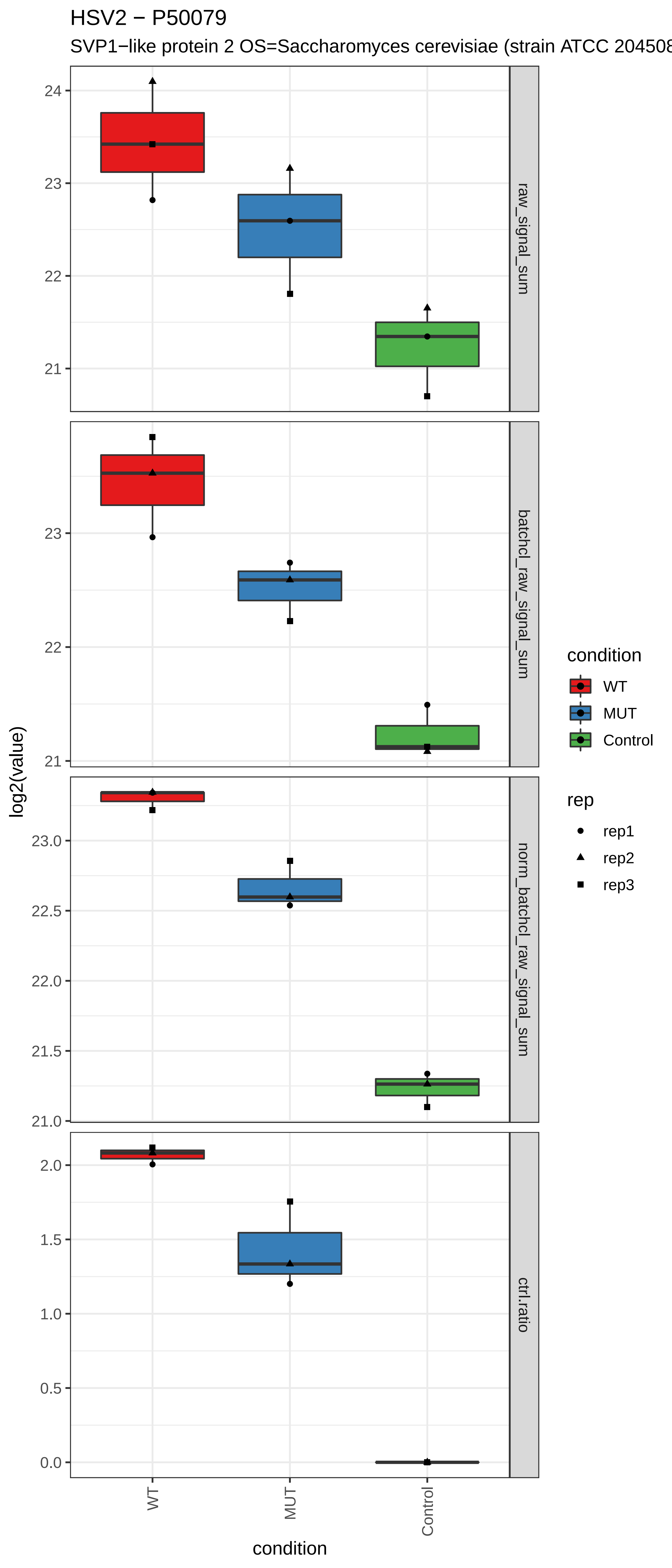

HSV2 − P50079
SVP1−like protein 2 OS=Saccharomyces cerevisiae (strain ATCC 204508
raw_signal_sum
batchcl_raw_signal_sum
norm_batchcl_raw_signal_sum
ctrl.ratio
log2(value)
condition
WT
MUT
Control
rep
rep1
rep2
rep3

HTA1|HTA2 − P04911|P04912

Histone H2A.1 OS=Saccharomyces cerevisiae (strain ATCC 204508 / S288

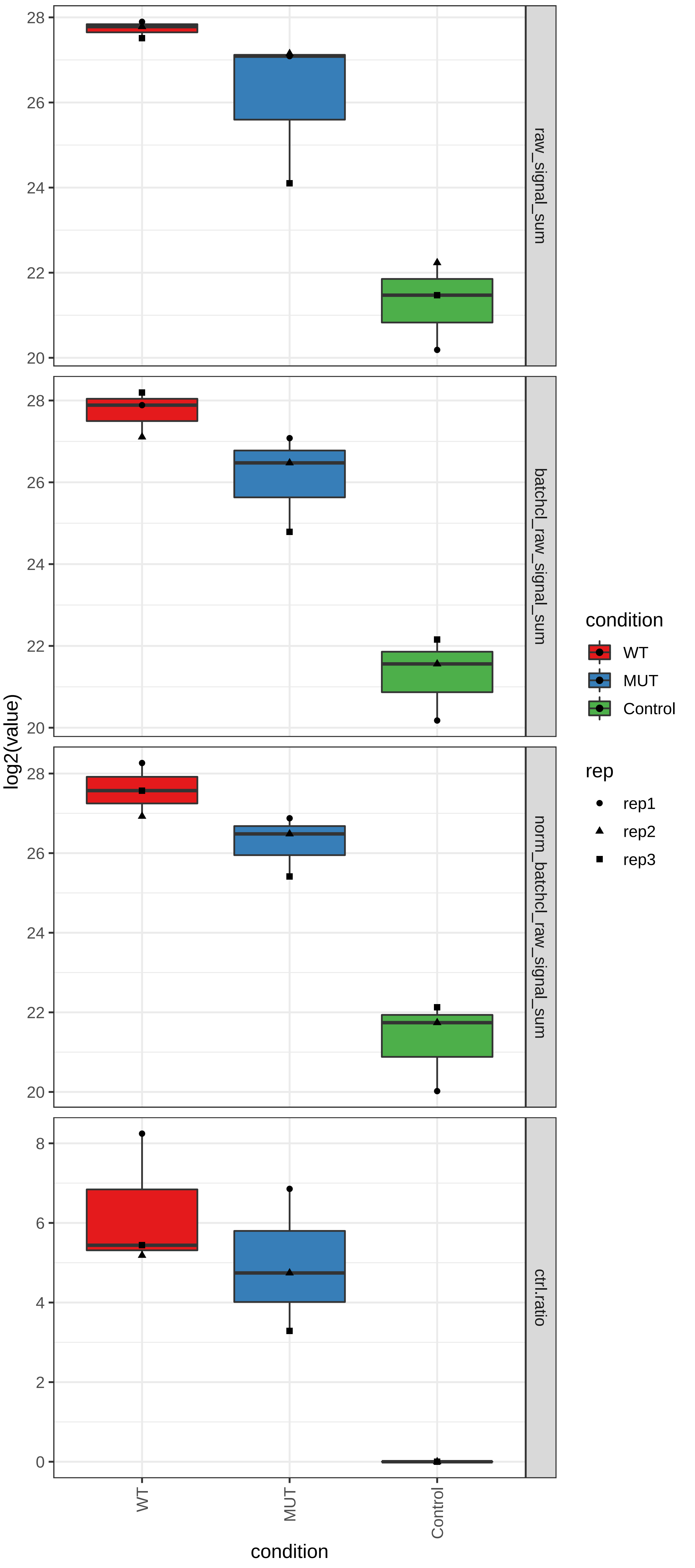

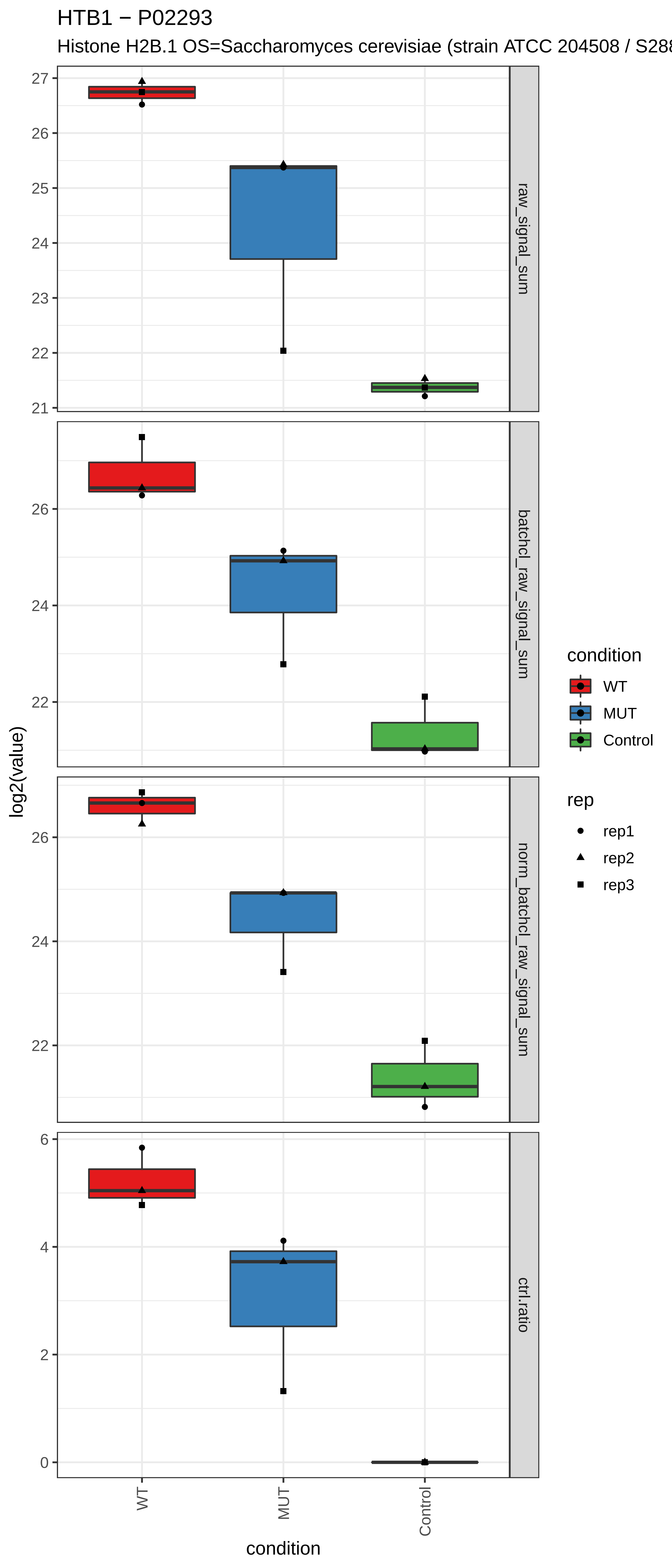

HTB1 − P02293
Histone H2B.1 OS=Saccharomyces cerevisiae (strain ATCC 204508 / S288
raw_signal_sum
batchcl_raw_signal_sum
norm_batchcl_raw_signal_sum
ctrl.ratio
log2(value)
condition
WT
MUT
Control
rep
rep1
rep2
rep3
27
26
25
24
23
22
21
6
4
2
0

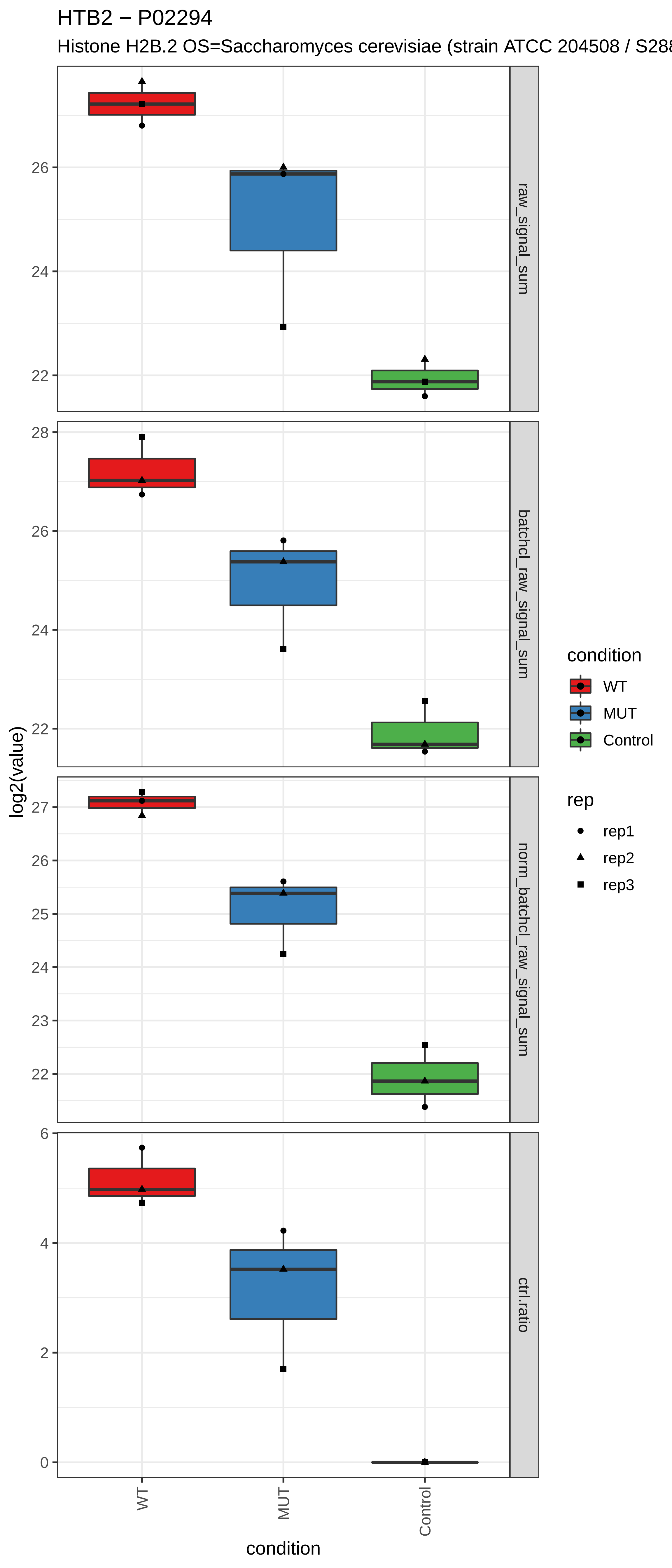

HTB2 − P02294
Histone H2B.2 OS=Saccharomyces cerevisiae (strain ATCC 204508 / S288
raw_signal_sum
batchcl_raw_signal_sum
norm_batchcl_raw_signal_sum
ctrl.ratio
log2(value)
condition
WT
MUT
Control
rep
rep1
rep2
rep3

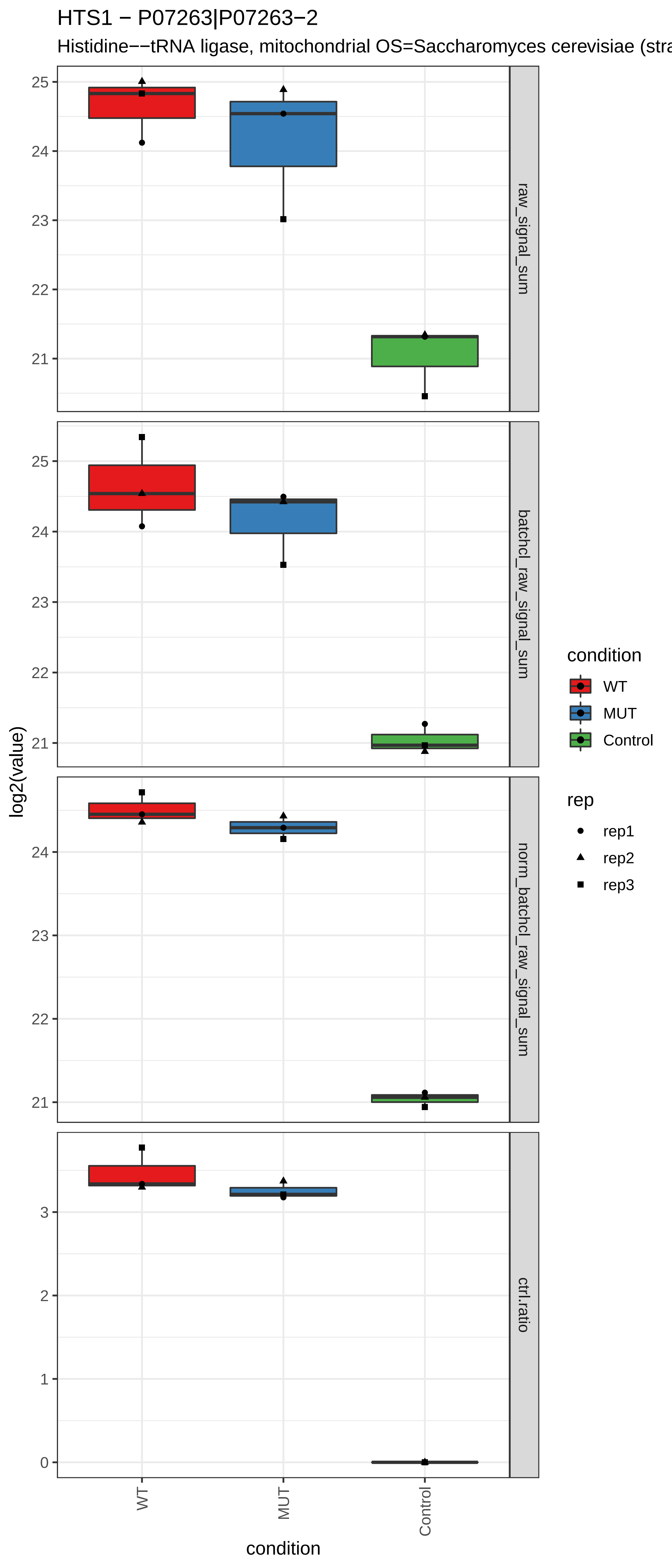

HTS1 − P07263|P07263−2
Histidine−−tRNA ligase, mitochondrial OS=Saccharomyces cerevisiae (stra
raw_signal_sum
batchcl_raw_signal_sum
norm_batchcl_raw_signal_sum
ctrl.ratio
log2(value)
condition
WT
MUT
Control
rep
rep1
rep2
rep3
25
24
23
22
21
3
2
1
0

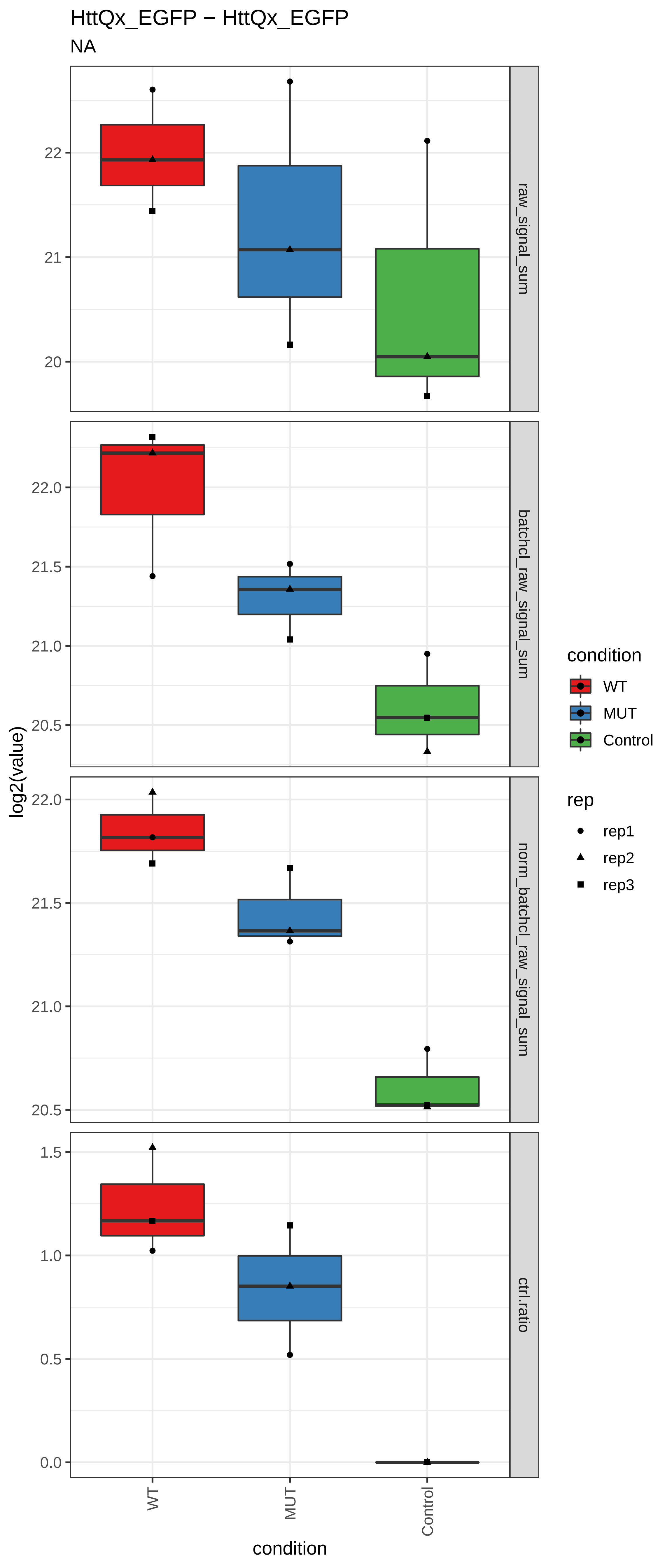

HttQx_EGFP − HttQx_EGFP
NA
raw_signal_sum
batchcl_raw_signal_sum
norm_batchcl_raw_signal_sum
ctrl.ratio
log2(value)
condition
WT
MUT
Control
rep
rep1
rep2
rep3
22
21
20
22.0
21.5
21.0
20.5
1.5
1.0
0.5
0.0

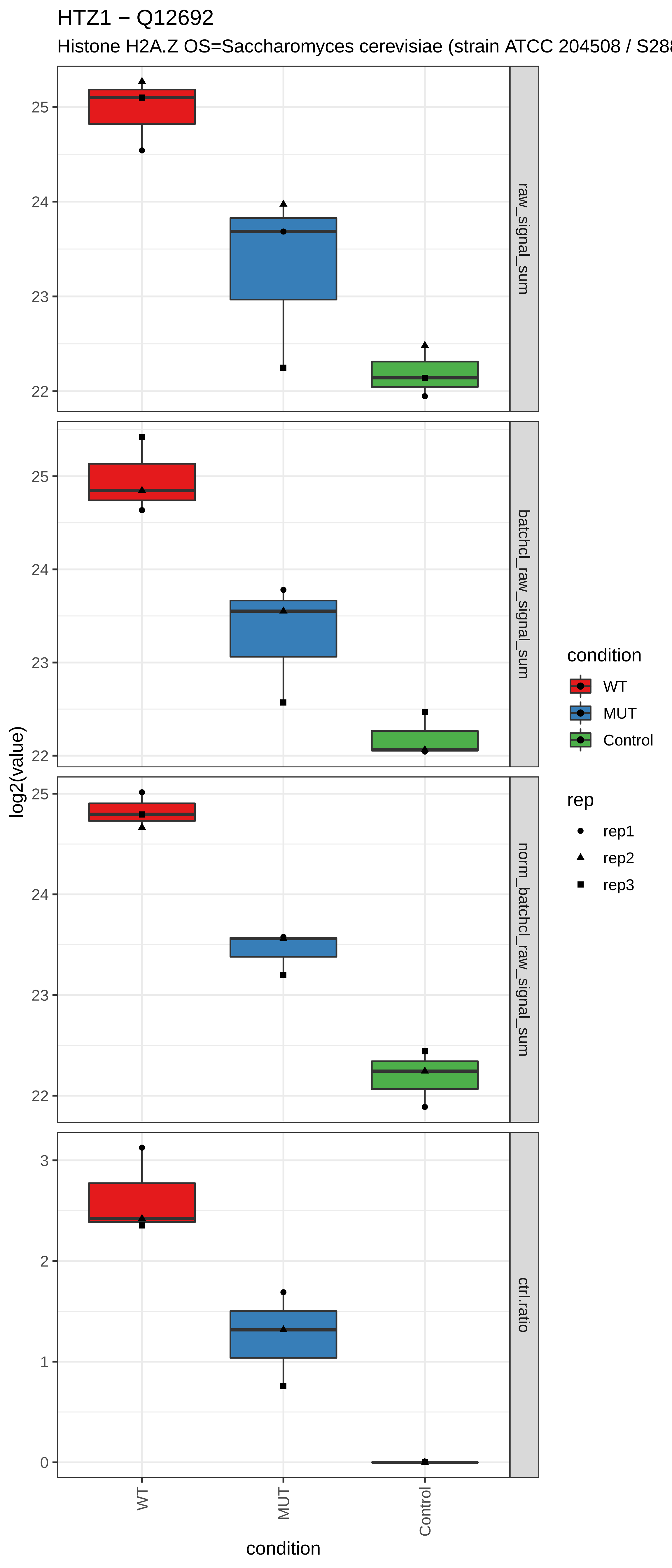

HTZ1 − Q12692
Histone H2A.Z OS=Saccharomyces cerevisiae (strain ATCC 204508 / S288
log2(value)
condition
WT
MUT
Control
raw_signal_sum
batchcl_raw_signal_sum
norm_batchcl_raw_signal_sum
ctrl.ratio
condition
WT
MUT
Control
rep
rep1
rep2
rep3

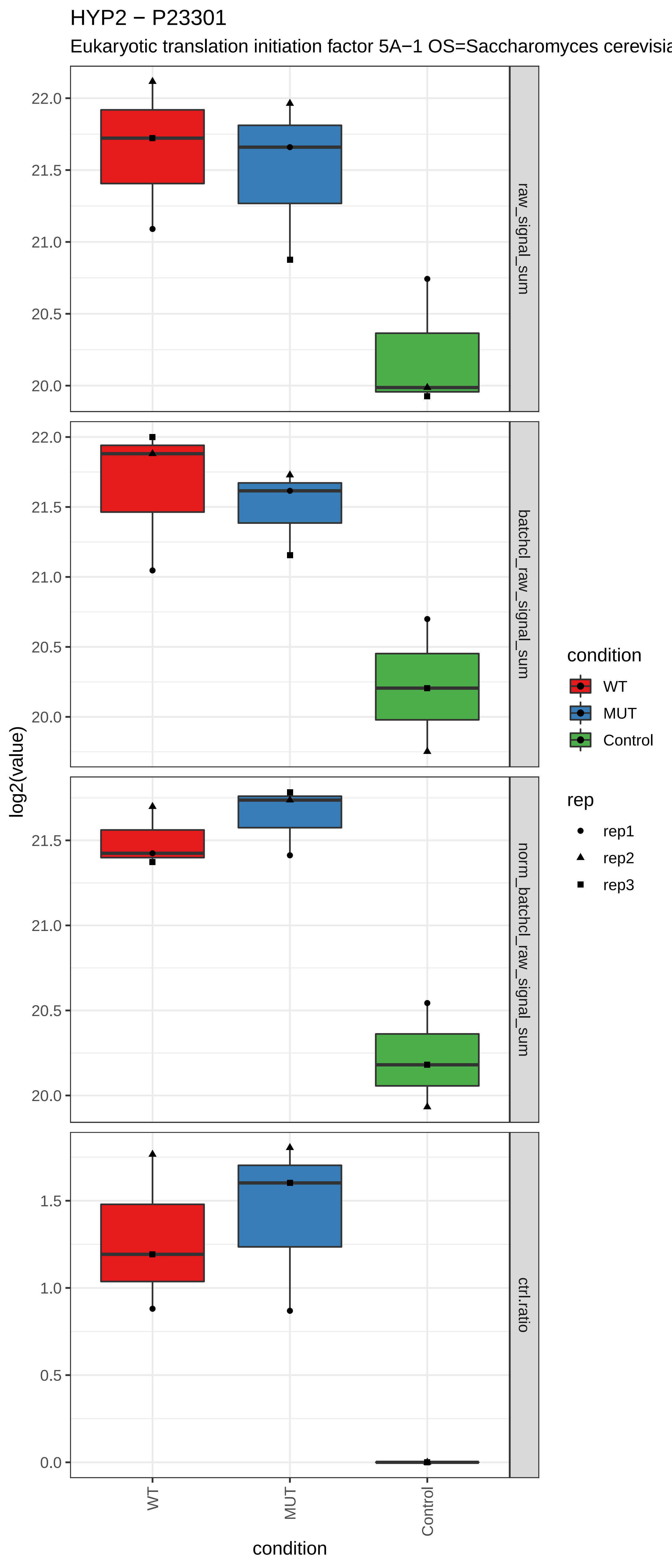
HYP2 − P23301
Eukaryotic translation initiation factor 5A−1 OS=Saccharomyces cerevisia
raw_signal_sum
batchcl_raw_signal_sum
norm_batchcl_raw_signal_sum
ctrl.ratio
log2(value)
condition
WT
MUT
Control
rep
rep1
rep2
rep3
22.0
21.5
21.0
20.5
20.0
1.5
1.0
0.5
0.0

ICL1 − P28240

Isocitrate lyase OS=Saccharomyces cerevisiae (strain ATCC 204508 / S28

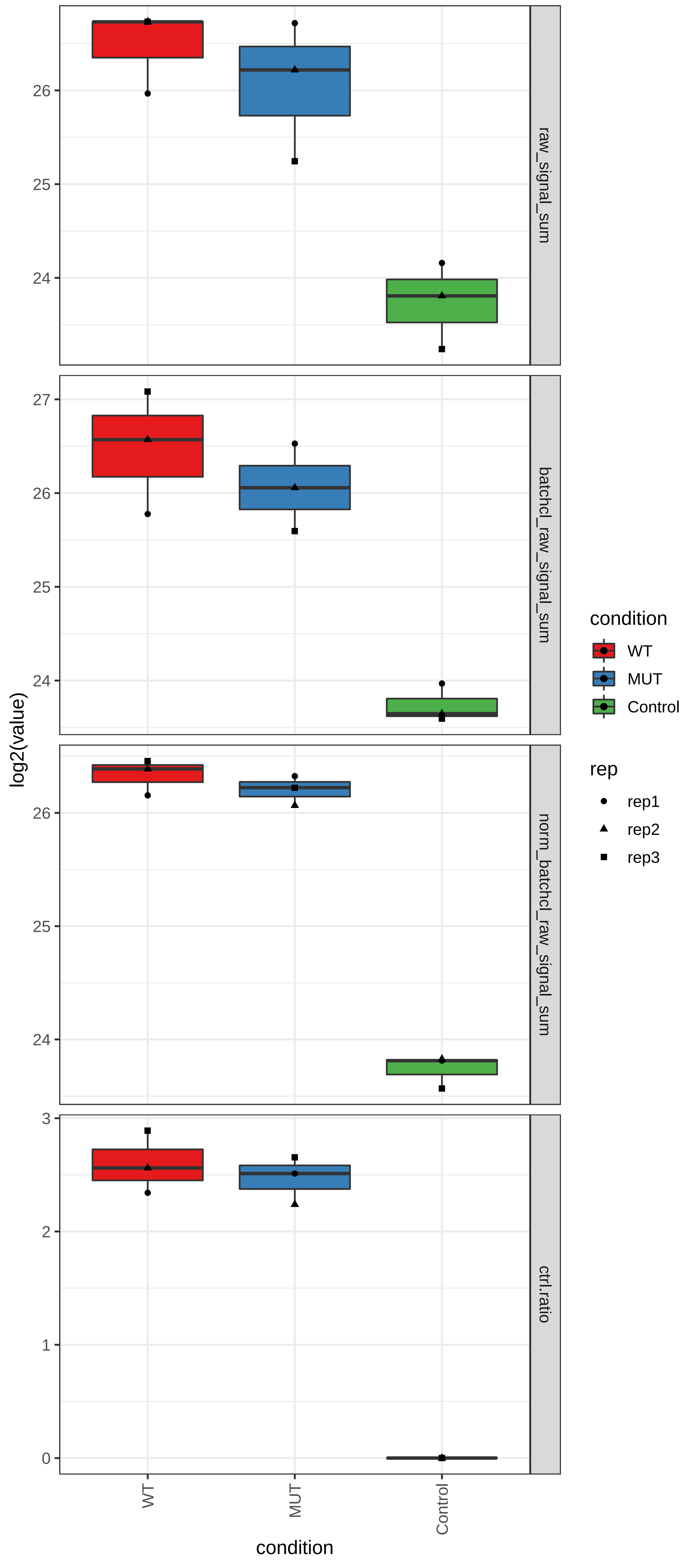

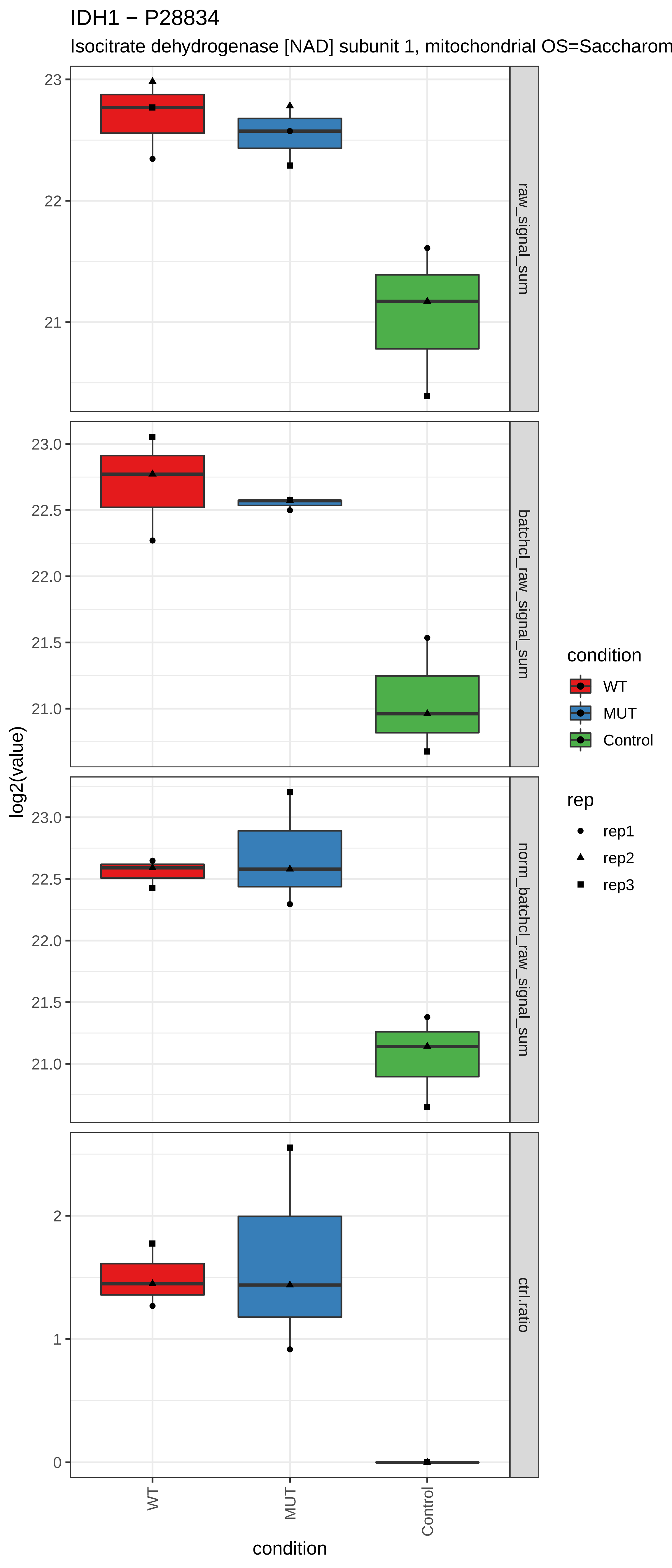

IDH1 − P28834
Isocitrate dehydrogenase [NAD] subunit 1, mitochondrial OS=Saccharom
raw_signal_sum
batchcl_raw_signal_sum
norm_batchcl_raw_signal_sum
ctrl.ratio
log2(value)
condition
WT
MUT
Control
rep
rep1
rep2
rep3

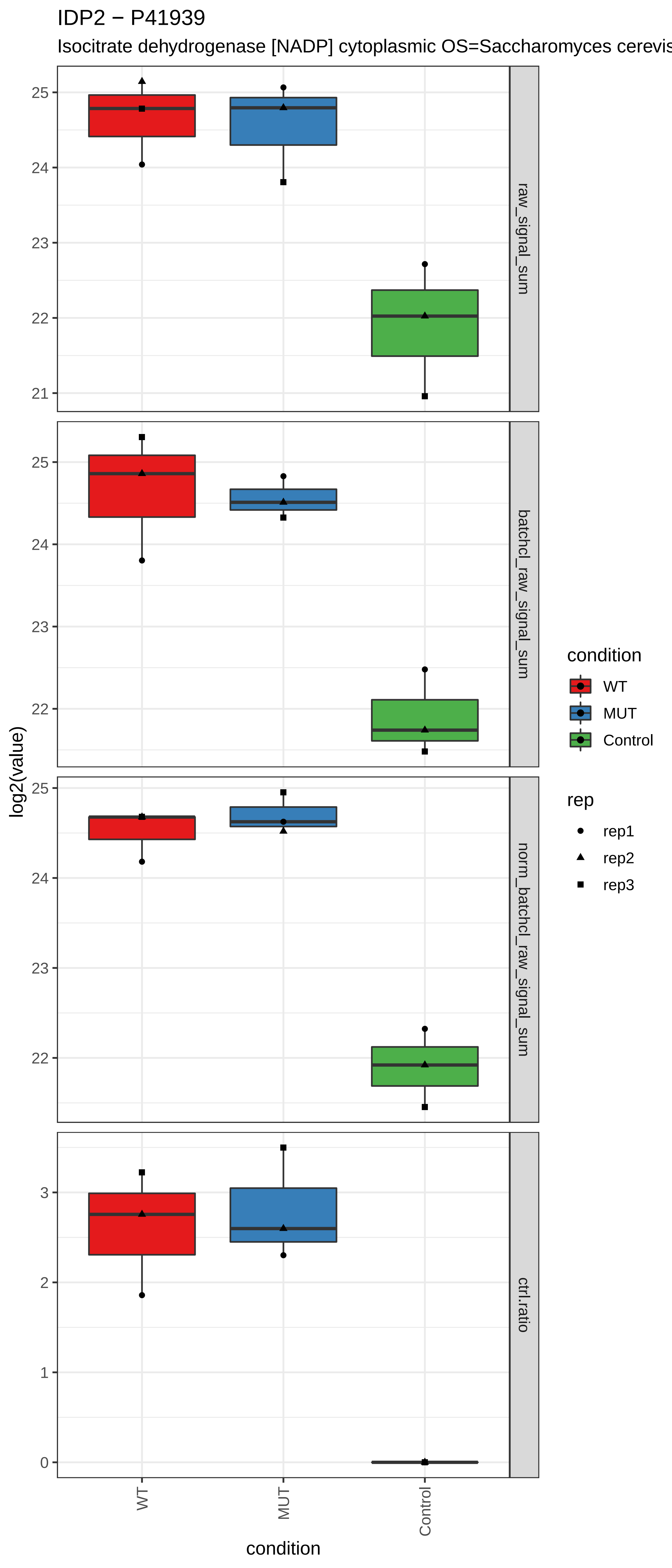

IDP2 − P41939
Isocitrate dehydrogenase [NADP] cytoplasmic OS=Saccharomyces cerevis
raw_signal_sum
batchcl_raw_signal_sum
norm_batchcl_raw_signal_sum
ctrl.ratio
log2(value)
condition
WT
MUT
Control
rep
rep1
rep2
rep3
25
24
23
22
21
3
2
1
0

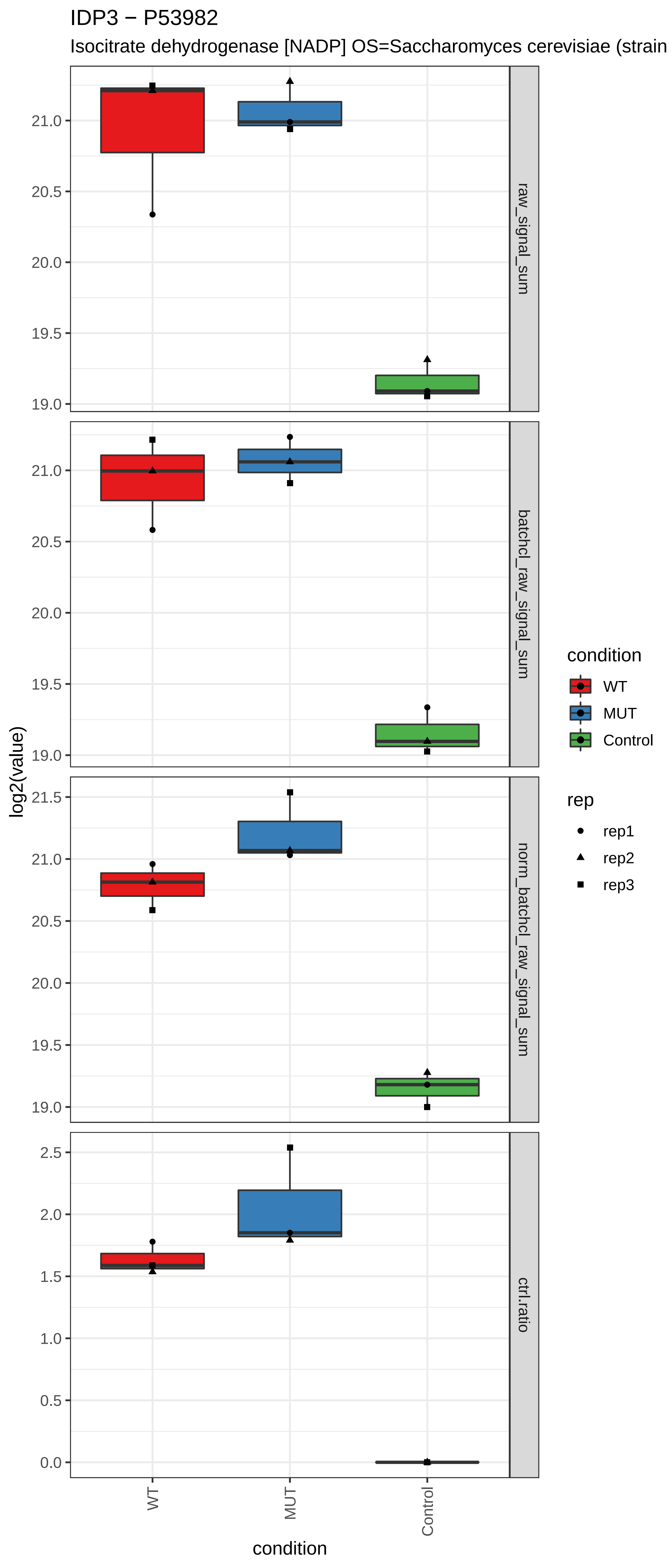

IDP3 − P53982
Isocitrate dehydrogenase [NADP] OS=Saccharomyces cerevisiae (strain
raw_signal_sum
batchcl_raw_signal_sum
norm_batchcl_raw_signal_sum
ctrl.ratio
log2(value)
condition
WT
MUT
Control
rep
rep1
rep2
rep3
21.5
21.0
20.5
20.0
19.5
19.0
2.5
2.0
1.5
1.0
0.5
0.0

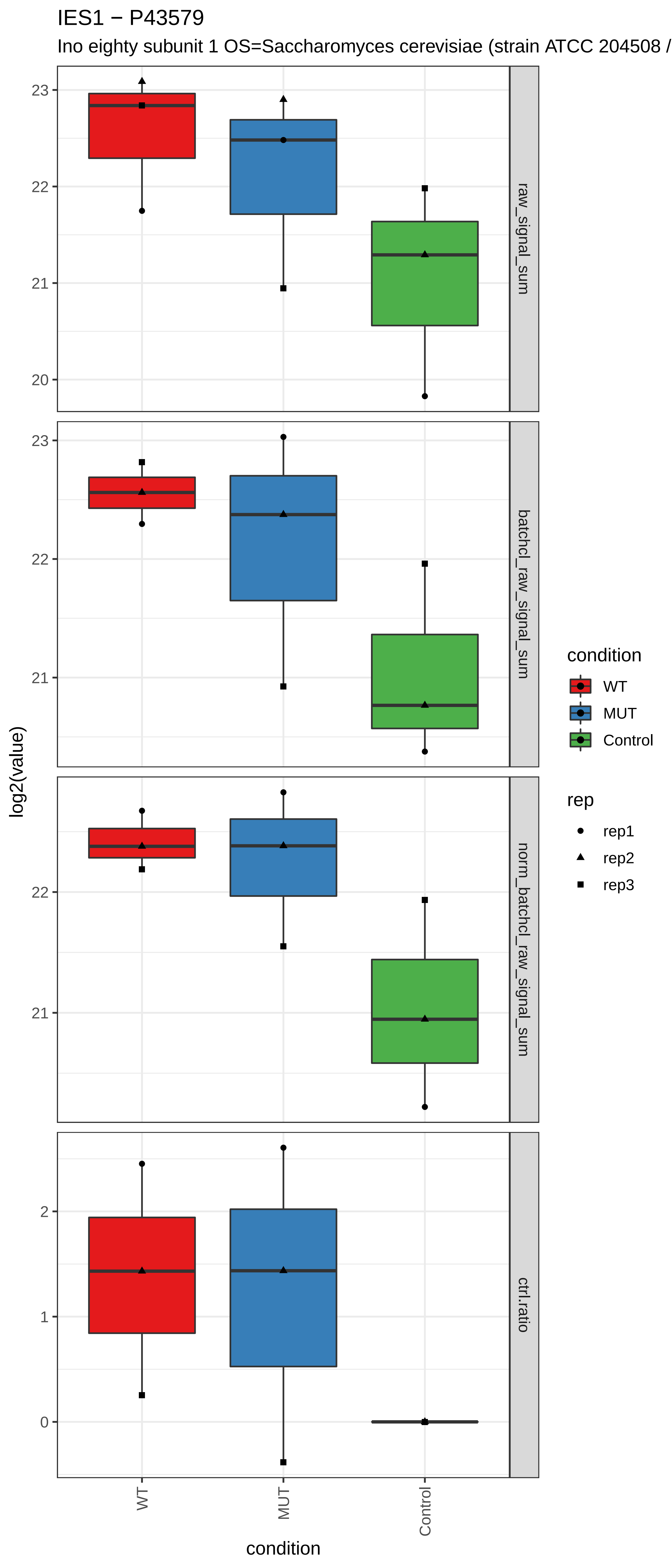

IES1 − P43579
Ino eighty subunit 1 OS=Saccharomyces cerevisiae (strain ATCC 204508 /
raw_signal_sum
batchcl_raw_signal_sum
norm_batchcl_raw_signal_sum
ctrl.ratio
log2(value)
condition
WT
MUT
Control
rep
rep1
rep2
rep3

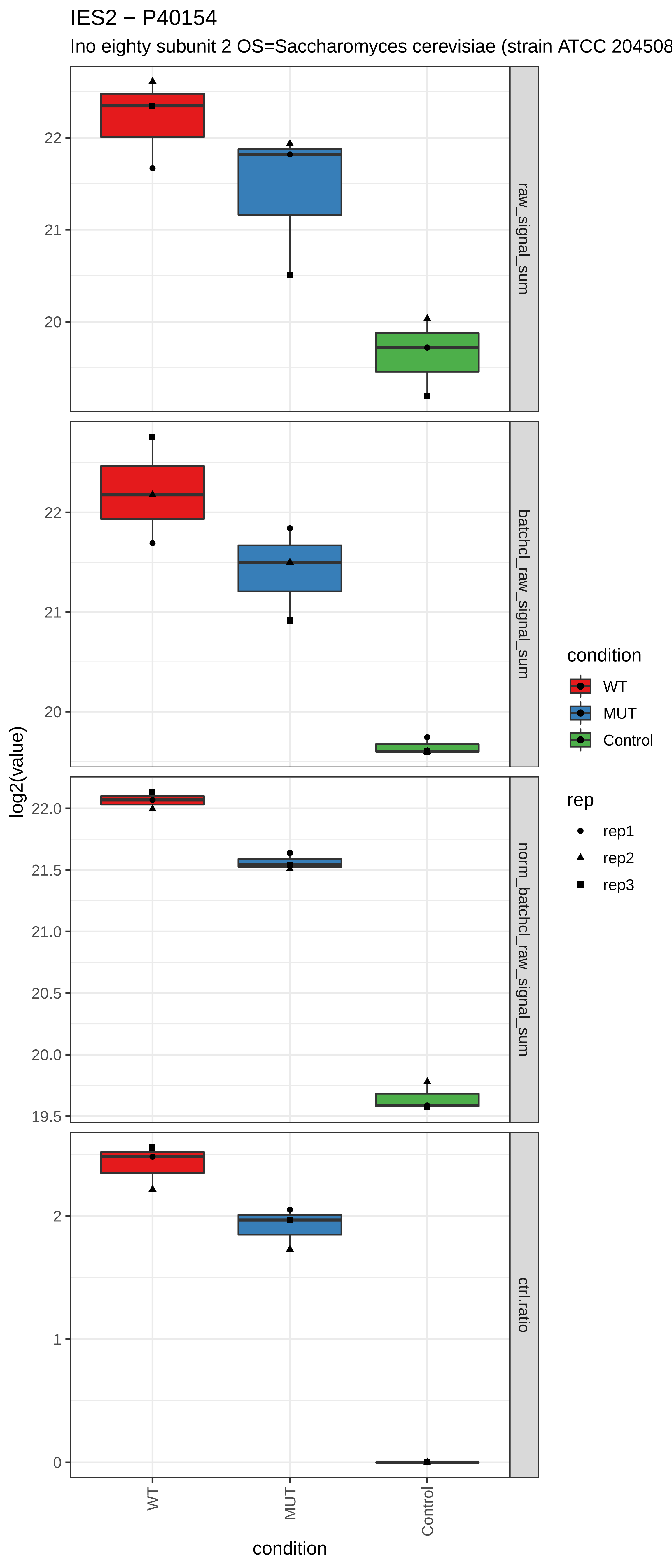

IES2 − P40154
Ino eighty subunit 2 OS=Saccharomyces cerevisiae (strain ATCC 204508
raw_signal_sum
batchcl_raw_signal_sum
norm_batchcl_raw_signal_sum
ctrl.ratio
log2(value)
condition
WT
MUT
Control
rep
rep1
rep2
rep3
22
21
20
22.0
21.5
21.0
20.5
20.0
19.5
2
1
0

IFM1 − P25038

Translation initiation factor IF−2, mitochondrial OS=Saccharomyces cerev

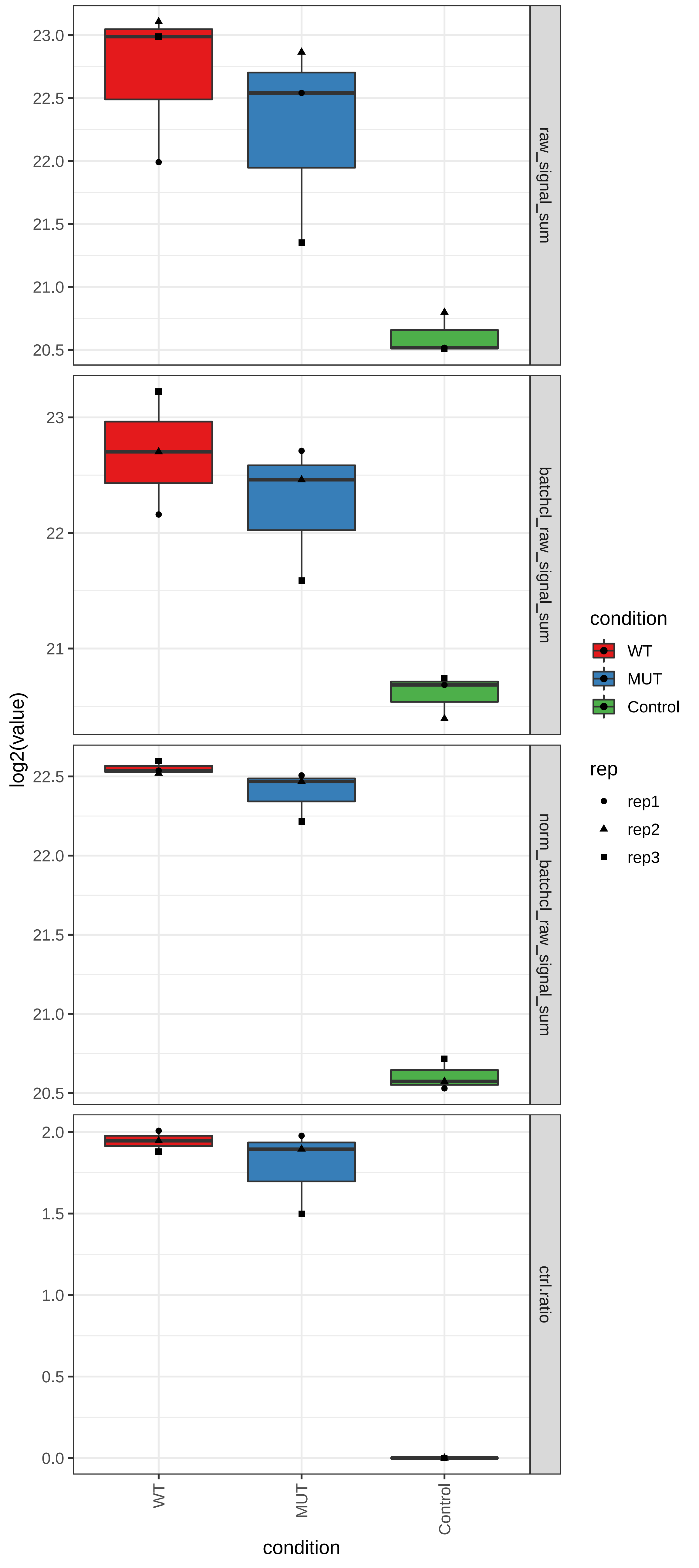

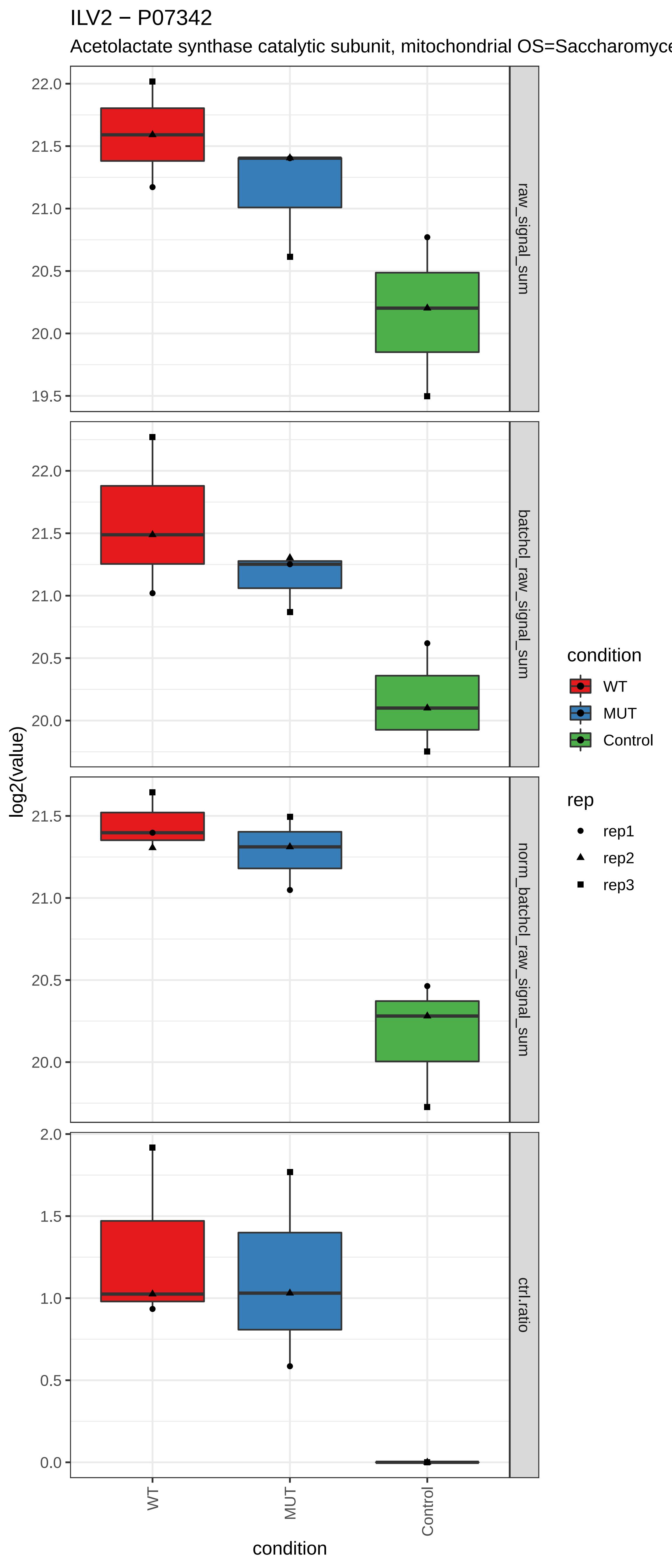

ILV2 − P07342
Acetolactate synthase catalytic subunit, mitochondrial OS=Saccharomyce
raw_signal_sum
batchcl_raw_signal_sum
norm_batchcl_raw_signal_sum
ctrl.ratio
log2(value)
condition
WT
MUT
Control
rep
rep1
rep2
rep3

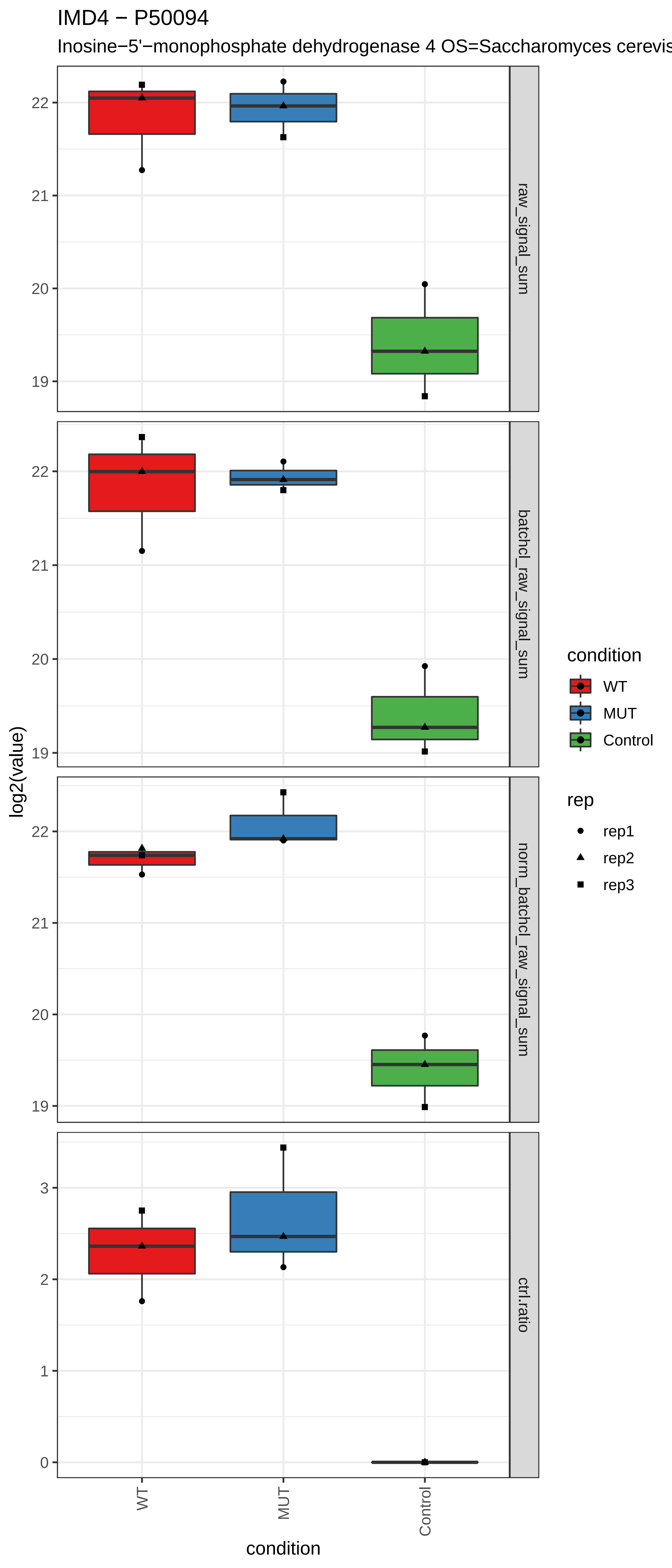

IMD4 − P50094
Inosine−5'−monophosphate dehydrogenase 4 OS=Saccharomyces cerevis
raw_signal_sum
batchcl_raw_signal_sum
norm_batchcl_raw_signal_sum
ctrl.ratio
log2(value)
22
21
20
19
3
2
1
0
WT
MUT
Control
condition
condition
WT
MUT
Control
rep
rep1
rep2
rep3

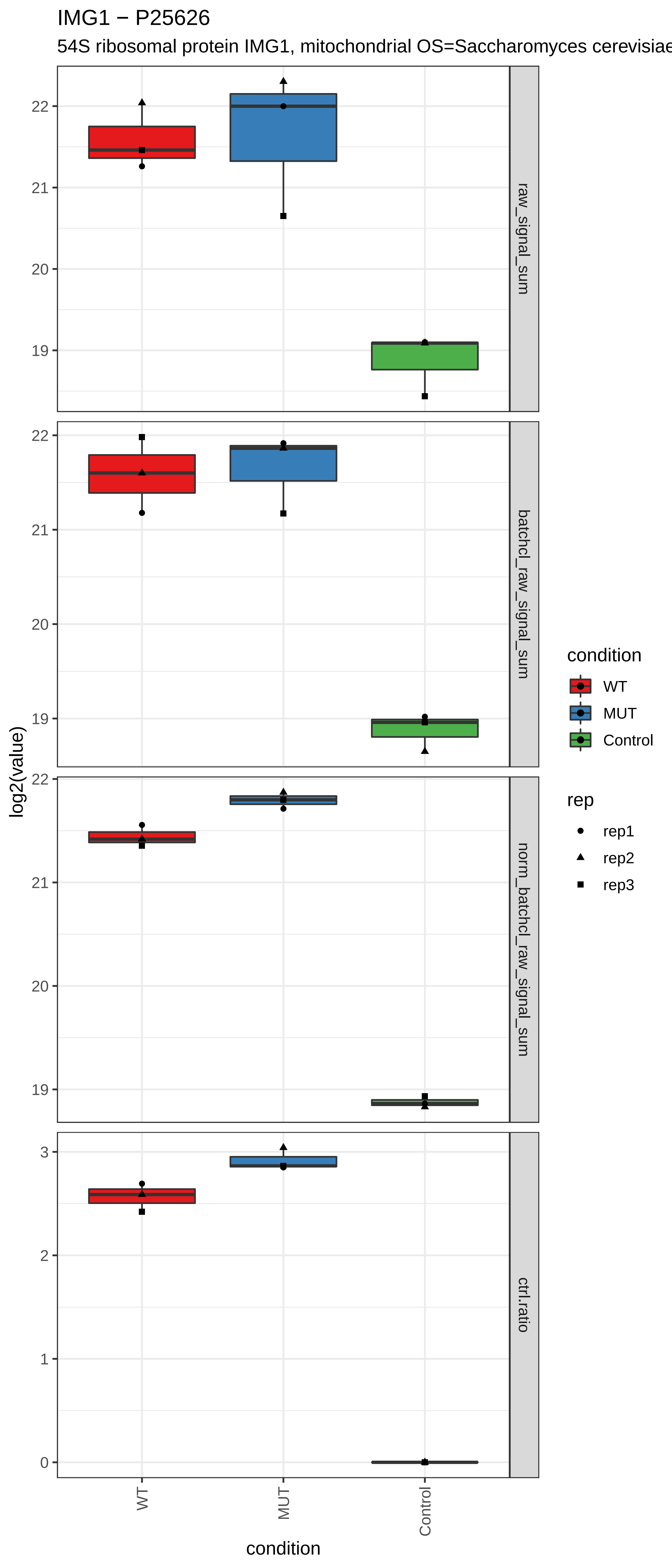

IMG1 − P25626
54S ribosomal protein IMG1, mitochondrial OS=Saccharomyces cerevisiae
raw_signal_sum
batchcl_raw_signal_sum
norm_batchcl_raw_signal_sum
ctrl.ratio
log2(value)
condition
WT
MUT
Control
rep
rep1
rep2
rep3
22
21
20
19
3
2
1
0

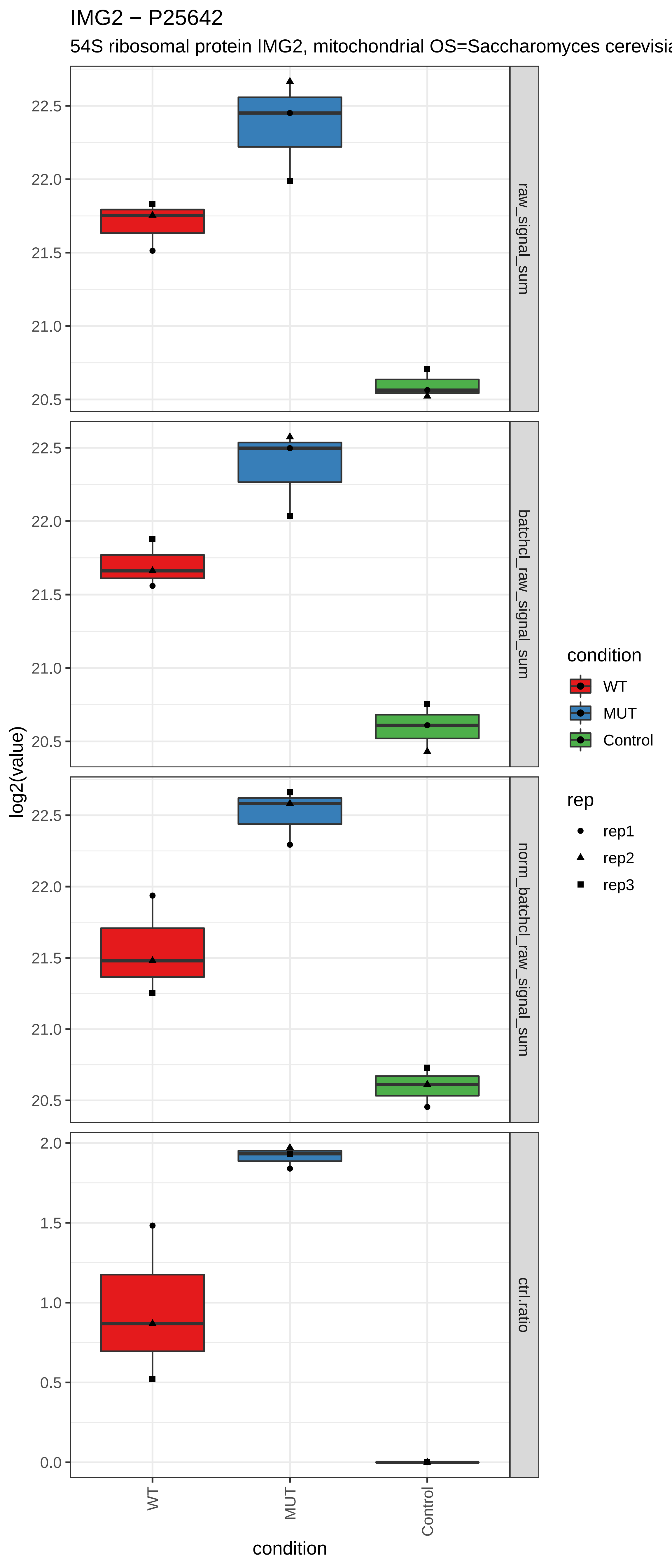

IMG2 − P25642
54S ribosomal protein IMG2, mitochondrial OS=Saccharomyces cerevisia
raw_signal_sum
batchcl_raw_signal_sum
norm_batchcl_raw_signal_sum
ctrl.ratio
log2(value)
condition
WT
MUT
Control
rep
rep1
rep2
rep3

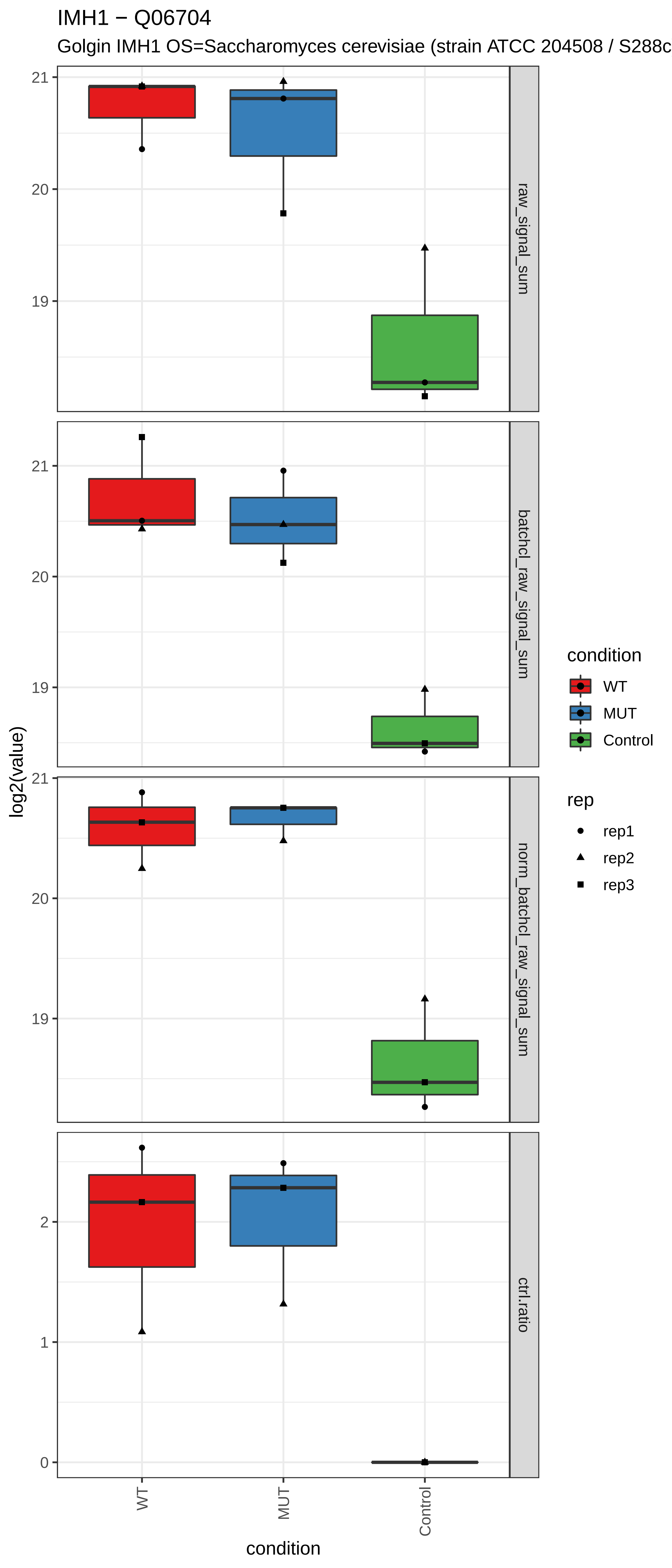

IMH1 − Q06704
Golgin IMH1 OS=Saccharomyces cerevisiae (strain ATCC 204508 / S288c
raw_signal_sum
batchcl_raw_signal_sum
norm_batchcl_raw_signal_sum
ctrl.ratio
log2(value)
condition
WT
MUT
Control
rep
rep1
rep2
rep3
21
20
19
2
1
0

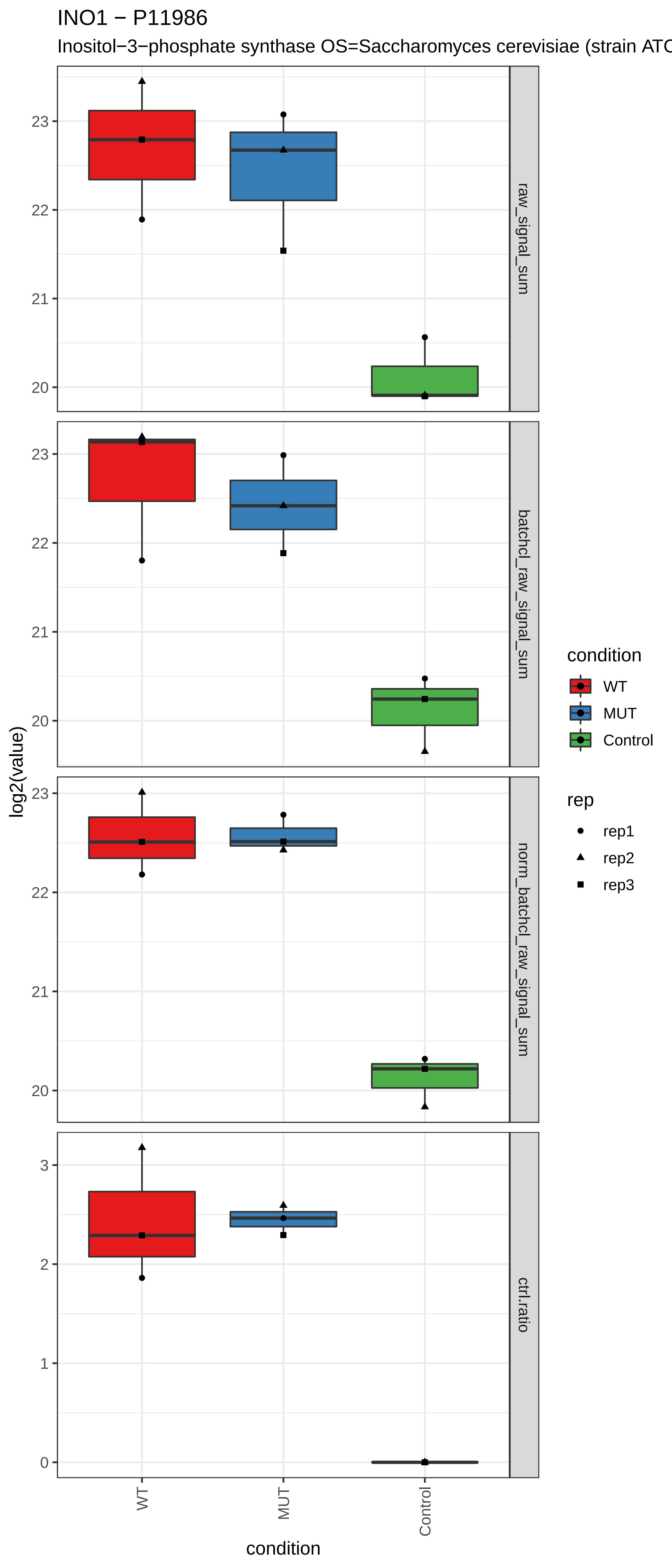

INO1 − P11986
Inositol−3−phosphate synthase OS=Saccharomyces cerevisiae (strain ATC
log2(value)
condition
raw_signal_sum
batchcl_raw_signal_sum
norm_batchcl_raw_signal_sum
ctrl.ratio
WT
MUT
Control
rep
rep1
rep2
rep3

INO80 − P53115

Putative DNA helicase INO80 OS=Saccharomyces cerevisiae (strain ATCC

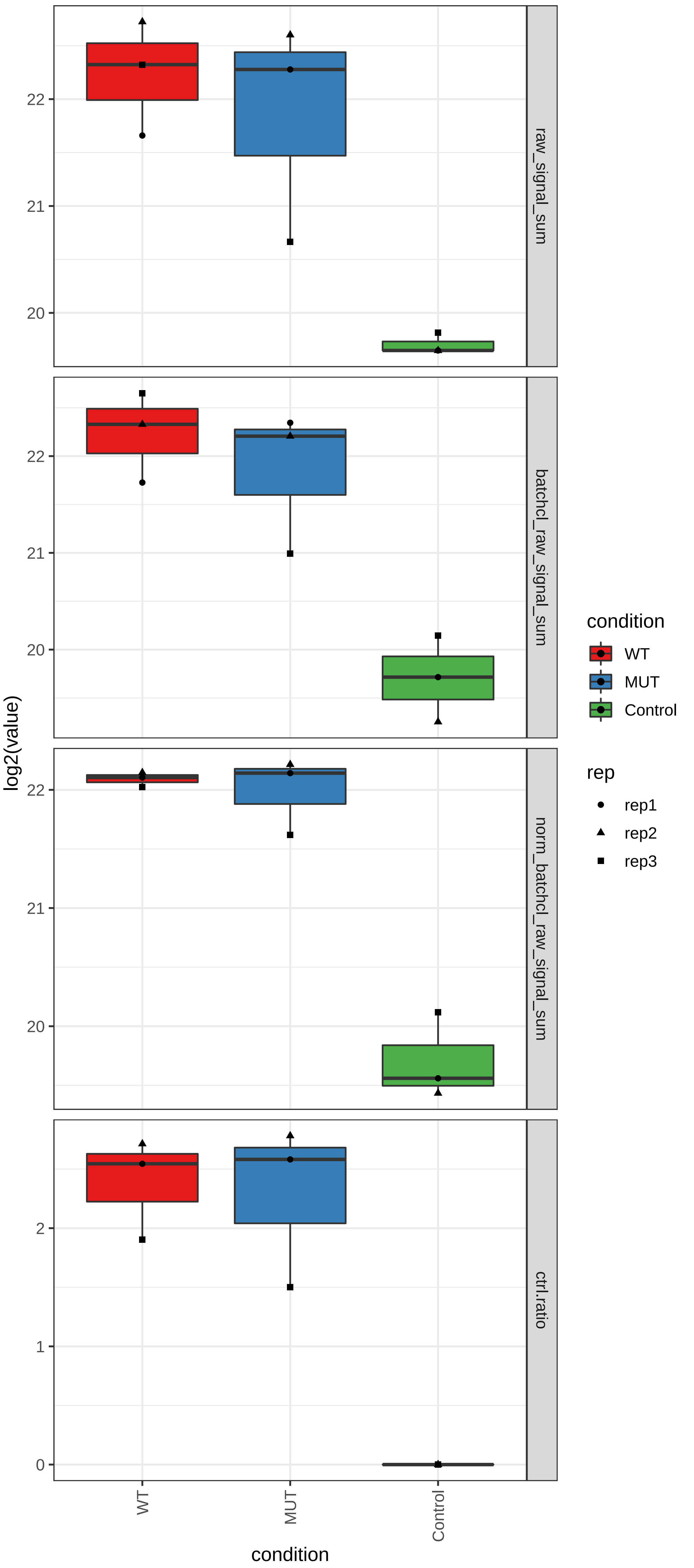

IOC2 − Q12072

ISWI one complex protein 2 OS=Saccharomyces cerevisiae (strain ATCC 2

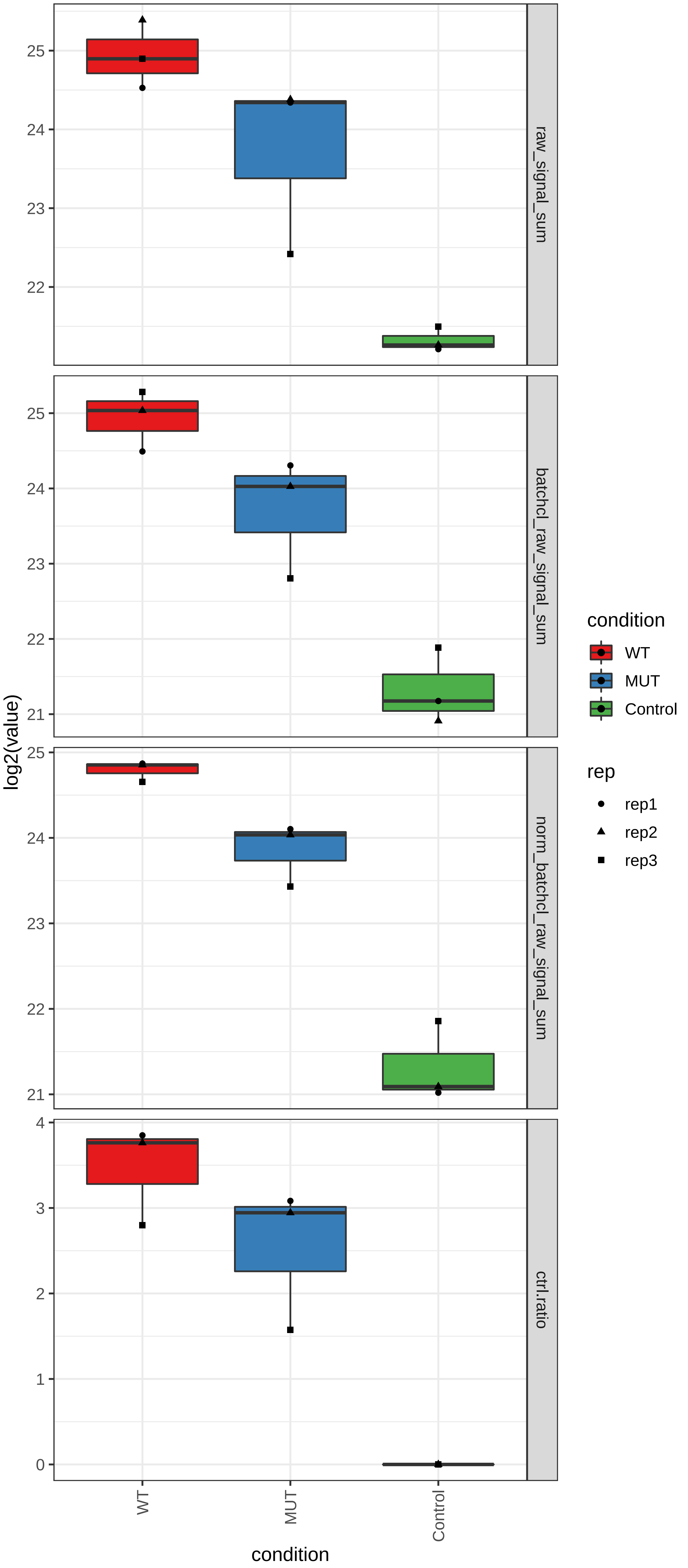

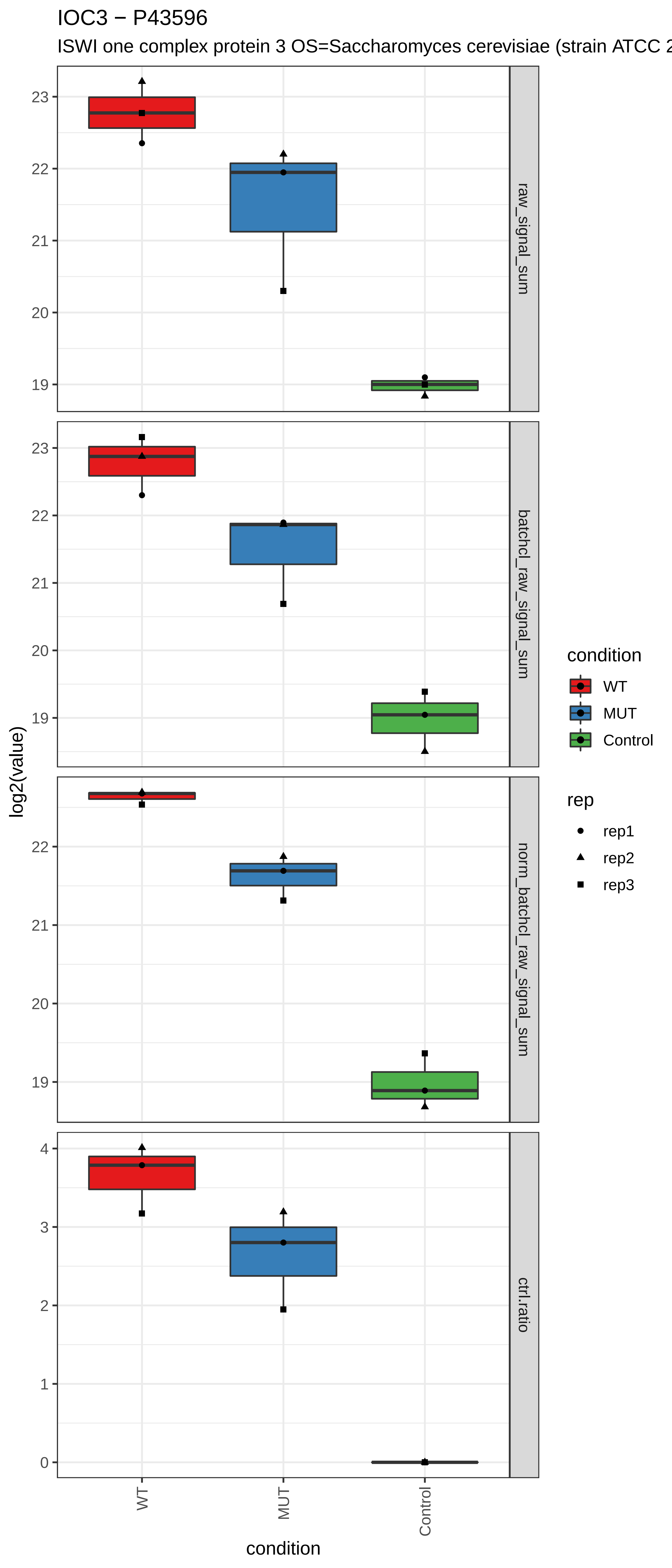

IOC3 − P43596
ISWI one complex protein 3 OS=Saccharomyces cerevisiae (strain ATCC 2
raw_signal_sum
batchcl_raw_signal_sum
norm_batchcl_raw_signal_sum
ctrl.ratio
log2(value)
condition
WT
MUT
Control
rep
rep1
rep2
rep3

IPI3 − P53877

Pre−rRNA−processing protein IPI3 OS=Saccharomyces cerevisiae (strain

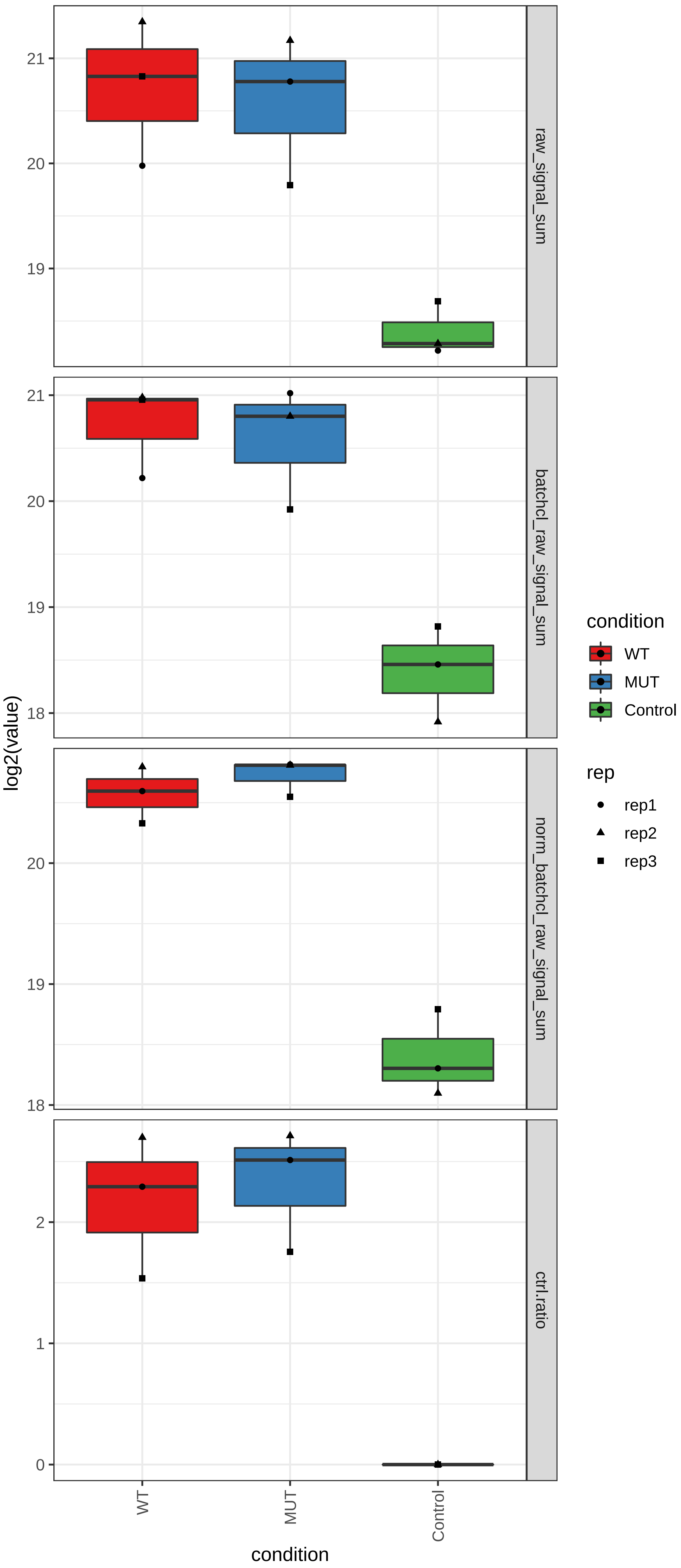

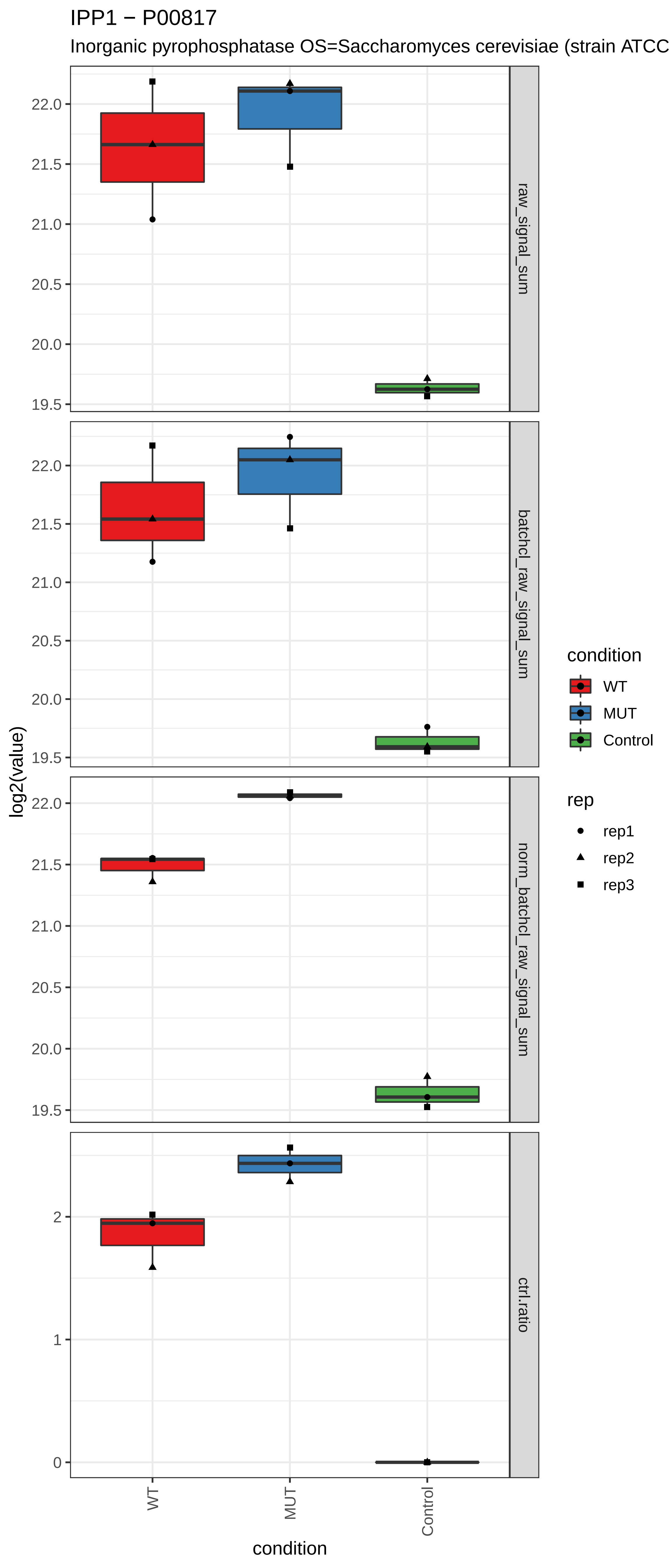

IPP1 − P00817
Inorganic pyrophosphatase OS=Saccharomyces cerevisiae (strain ATCC
raw_signal_sum
batchcl_raw_signal_sum
norm_batchcl_raw_signal_sum
ctrl.ratio
log2(value)
condition
WT
MUT
Control
rep
rep1
rep2
rep3
22.0
21.5
21.0
20.5
20.0
19.5
2
1
0

IRR1 − P40541

Cohesin subunit SCC3 OS=Saccharomyces cerevisiae (strain ATCC 20450

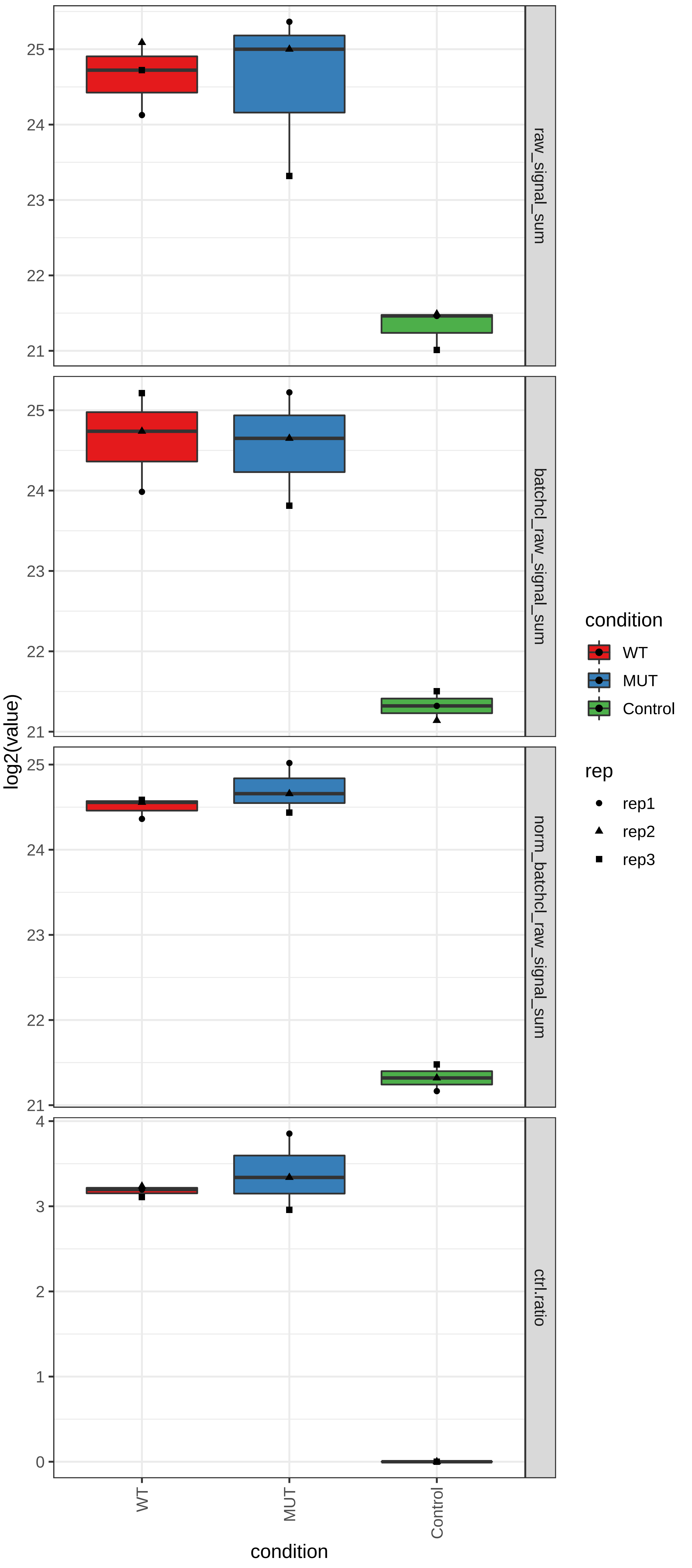

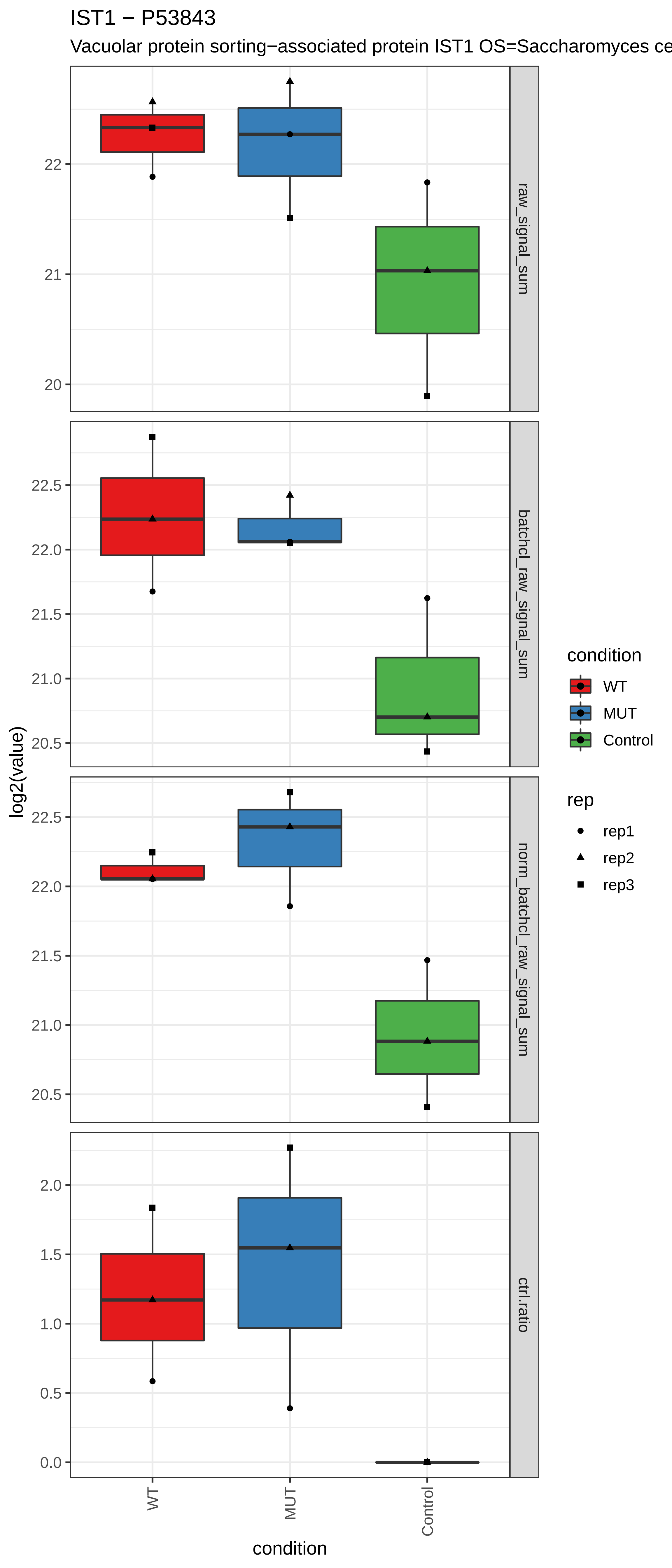

IST1 − P53843
Vacuolar protein sorting−associated protein IST1 OS=Saccharomyces ce
raw_signal_sum
batchcl_raw_signal_sum
norm_batchcl_raw_signal_sum
ctrl.ratio
log2(value)
condition
WT
MUT
Control
rep
rep1
rep2
rep3

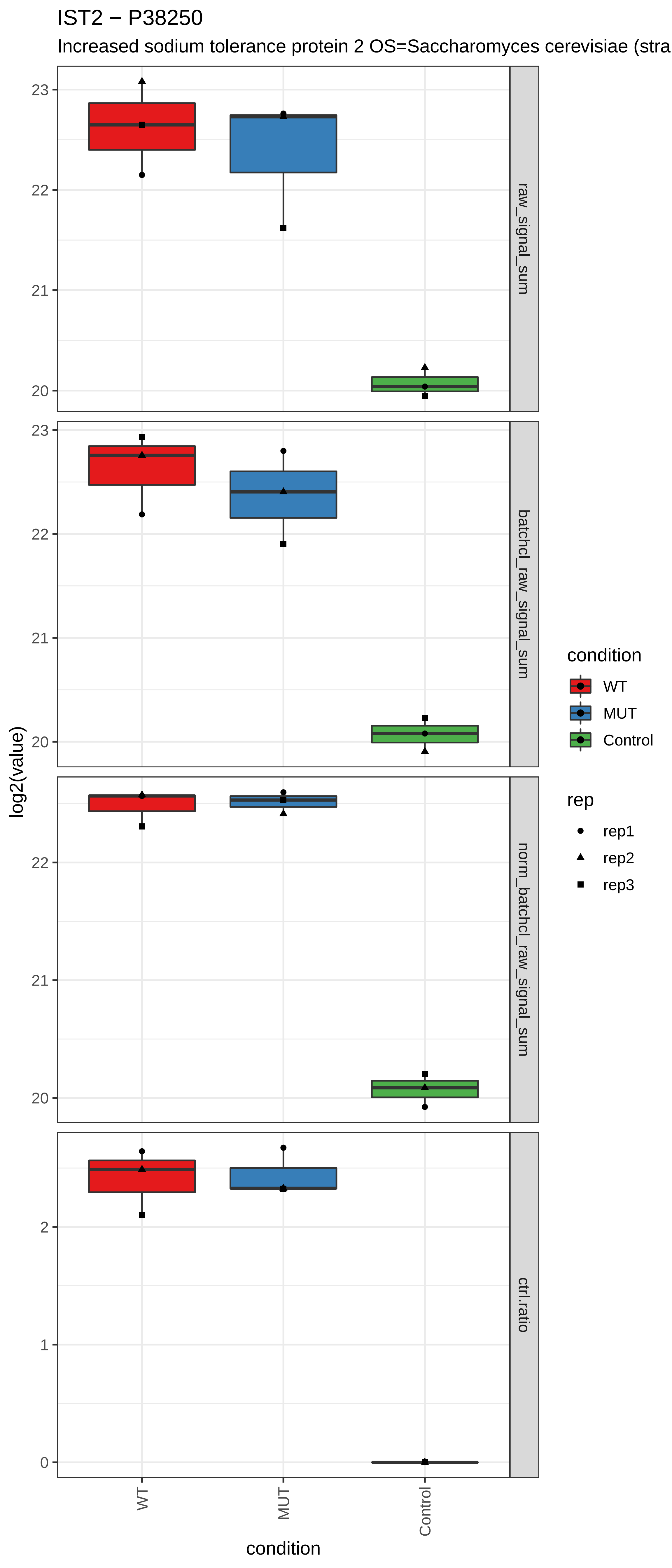

IST2 − P38250
Increased sodium tolerance protein 2 OS=Saccharomyces cerevisiae (strai
raw_signal_sum
batchcl_raw_signal_sum
norm_batchcl_raw_signal_sum
ctrl.ratio
log2(value)
condition
WT
MUT
Control
rep
rep1
rep2
rep3
23
22
21
20
2
1
0

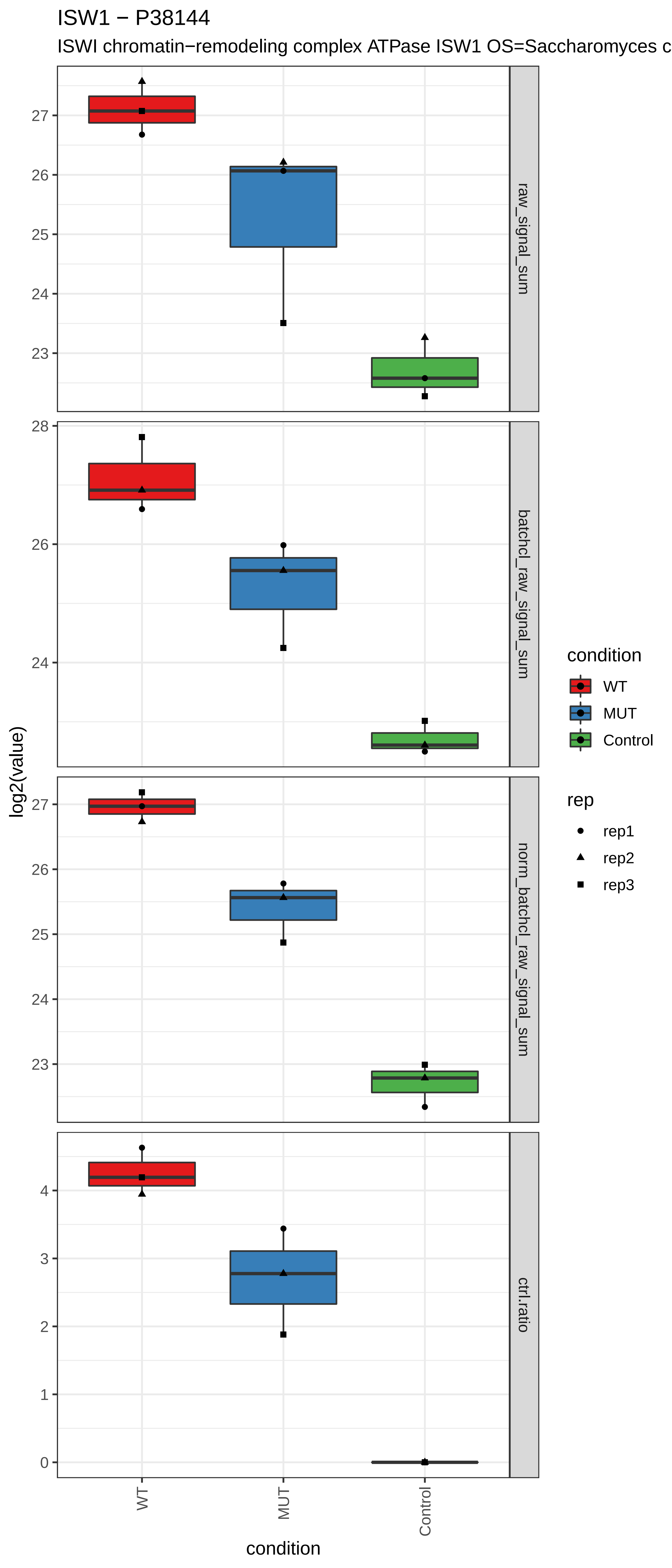

ISW1 − P38144
ISWI chromatin−remodeling complex ATPase ISW1 OS=Saccharomyces c
raw_signal_sum
batchcl_raw_signal_sum
norm_batchcl_raw_signal_sum
ctrl.ratio
log2(value)
condition
WT
MUT
Control
rep
rep1
rep2
rep3

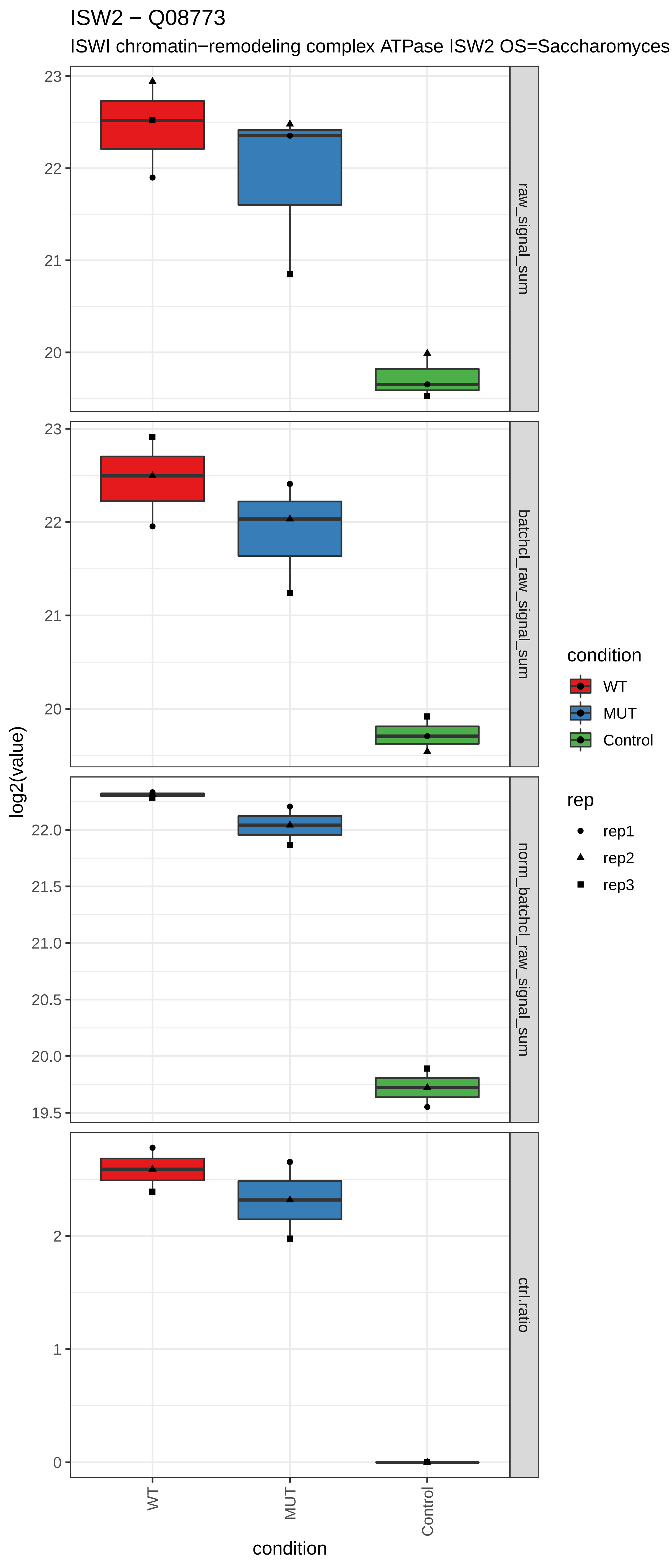

ISW2 − Q08773
ISWI chromatin−remodeling complex ATPase ISW2 OS=Saccharomyces
raw_signal_sum
batchcl_raw_signal_sum
norm_batchcl_raw_signal_sum
ctrl.ratio
23
22
21
20
22.0
21.5
21.0
20.5
20.0
19.5
2
1
0
log2(value)
WT
MUT
Control
condition
rep
rep1
rep2
rep3

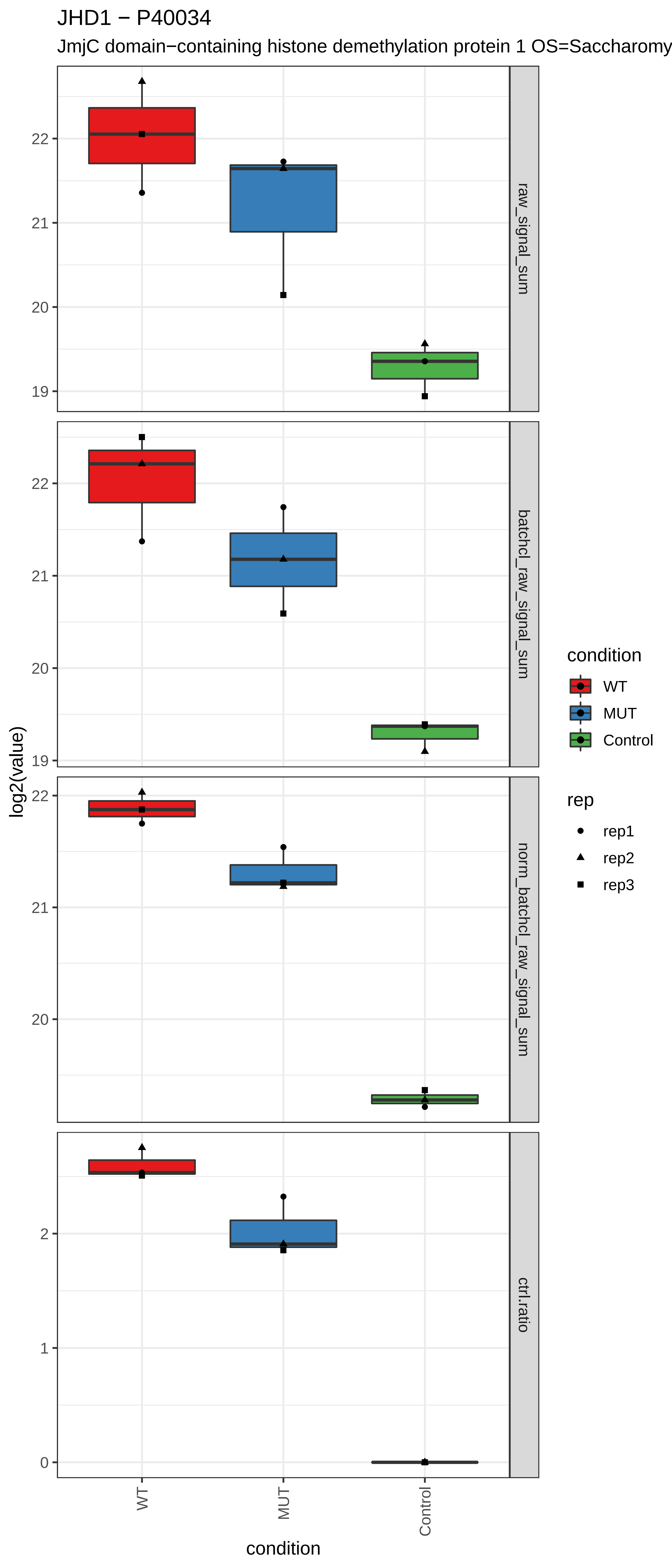

JHD1 − P40034
JmjC domain−containing histone demethylation protein 1 OS=Saccharomy
raw_signal_sum
batchcl_raw_signal_sum
norm_batchcl_raw_signal_sum
ctrl.ratio
log2(value)
condition
WT
MUT
Control
rep
rep1
rep2
rep3
22
21
20
19
2
1
0

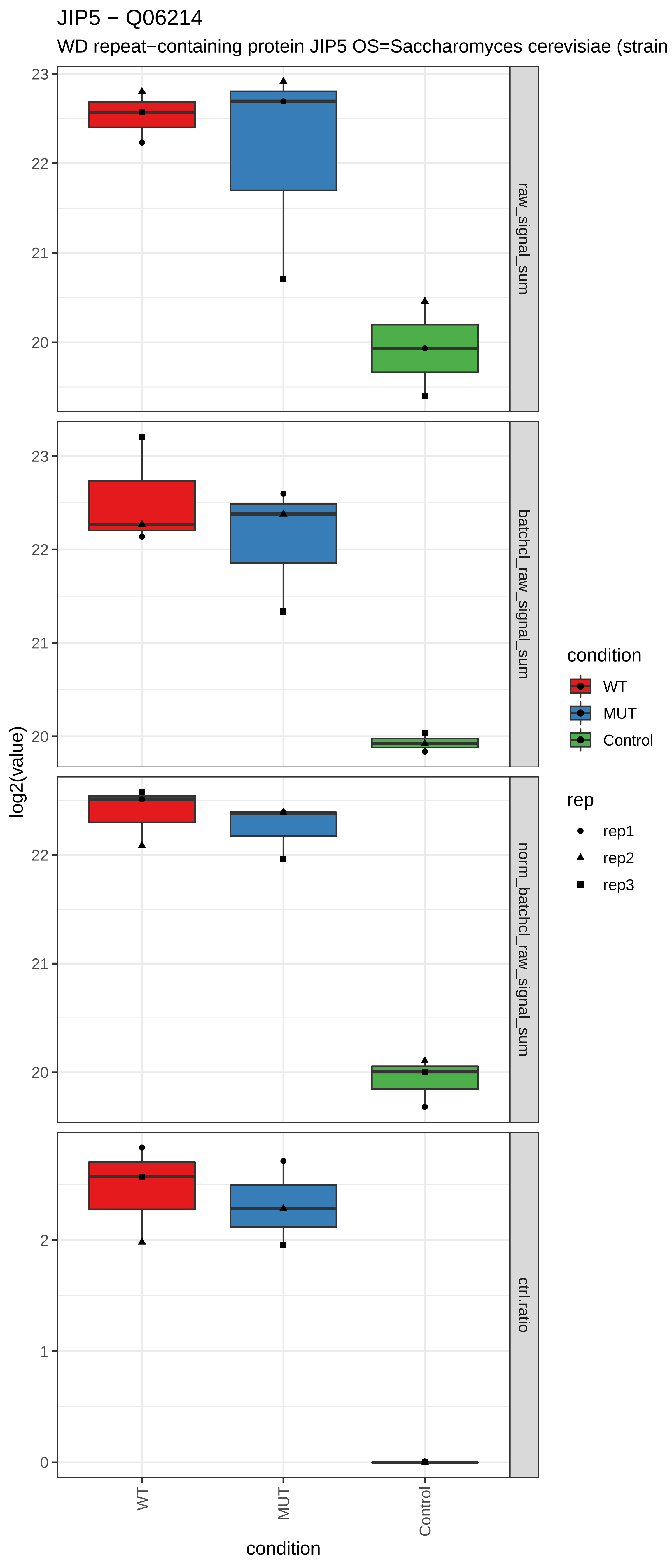

JIP5 − Q06214
WD repeat−containing protein JIP5 OS=Saccharomyces cerevisiae (strain
raw_signal_sum
batchcl_raw_signal_sum
norm_batchcl_raw_signal_sum
ctrl.ratio
log2(value)
condition
WT
MUT
Control
rep
rep1
rep2
rep3

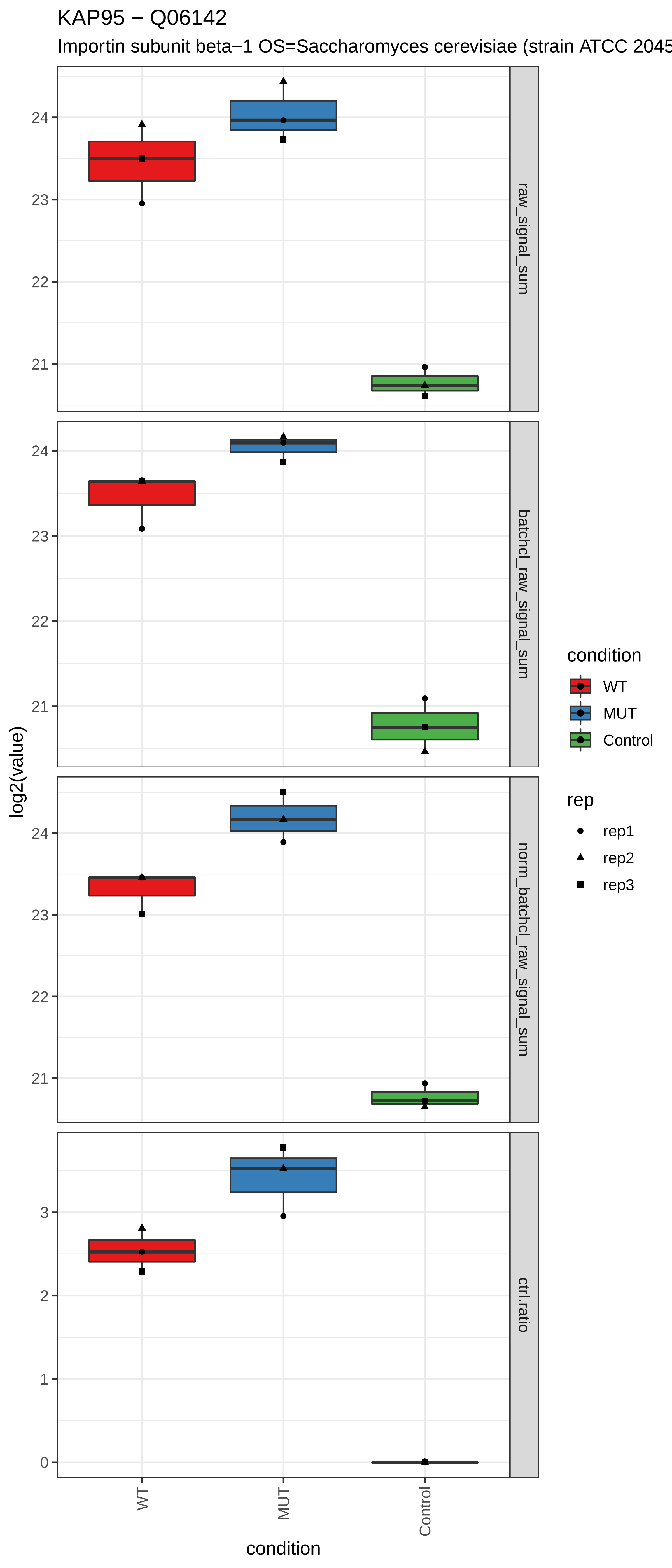

KAP95 − Q06142
Importin subunit beta−1 OS=Saccharomyces cerevisiae (strain ATCC 2045
raw_signal_sum
batchcl_raw_signal_sum
norm_batchcl_raw_signal_sum
ctrl.ratio
24
23
22
21
3
2
1
0
log2(value)
WT
MUT
Control
condition
condition
WT
MUT
Control
rep
rep1
rep2
rep3

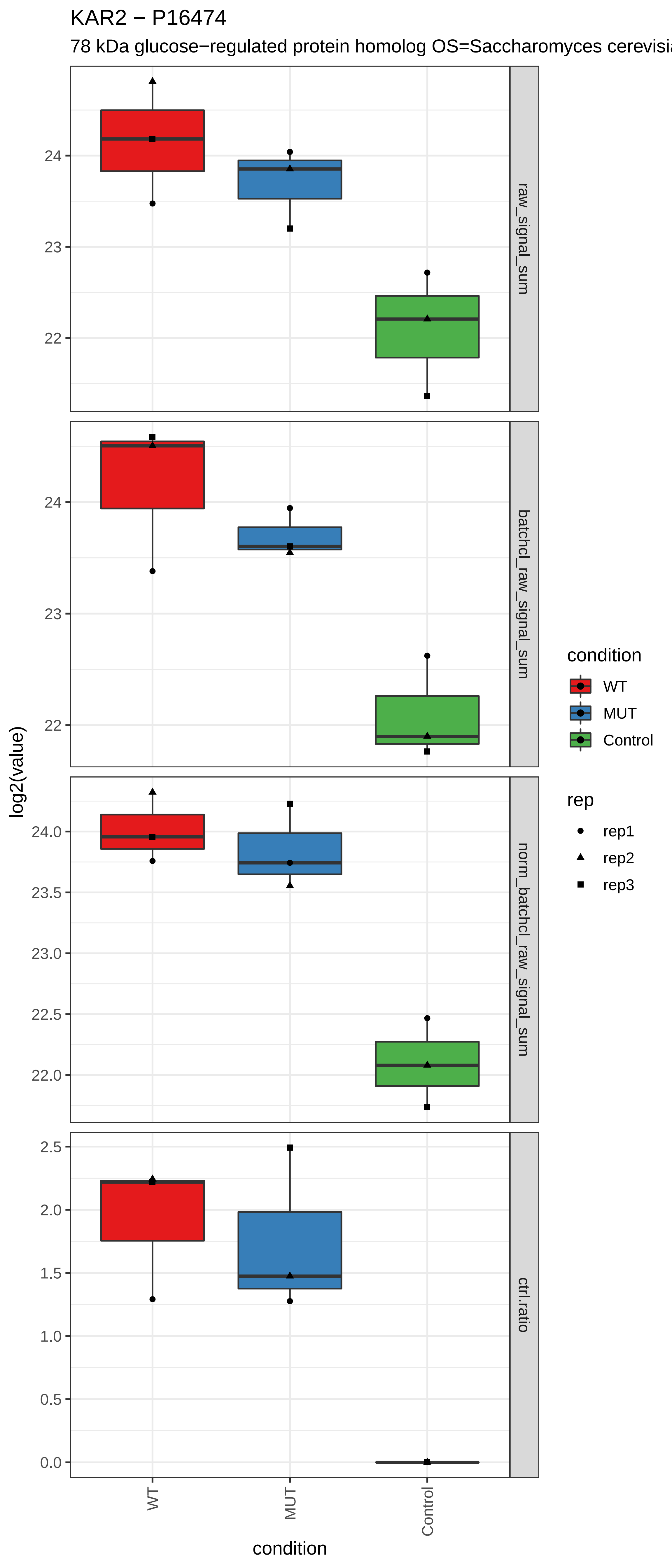
KAR2 − P16474
78 kDa glucose−regulated protein homolog OS=Saccharomyces cerevisia
raw_signal_sum
batchcl_raw_signal_sum
norm_batchcl_raw_signal_sum
ctrl.ratio
log2(value)
condition
WT
MUT
Control
rep
rep1
rep2
rep3

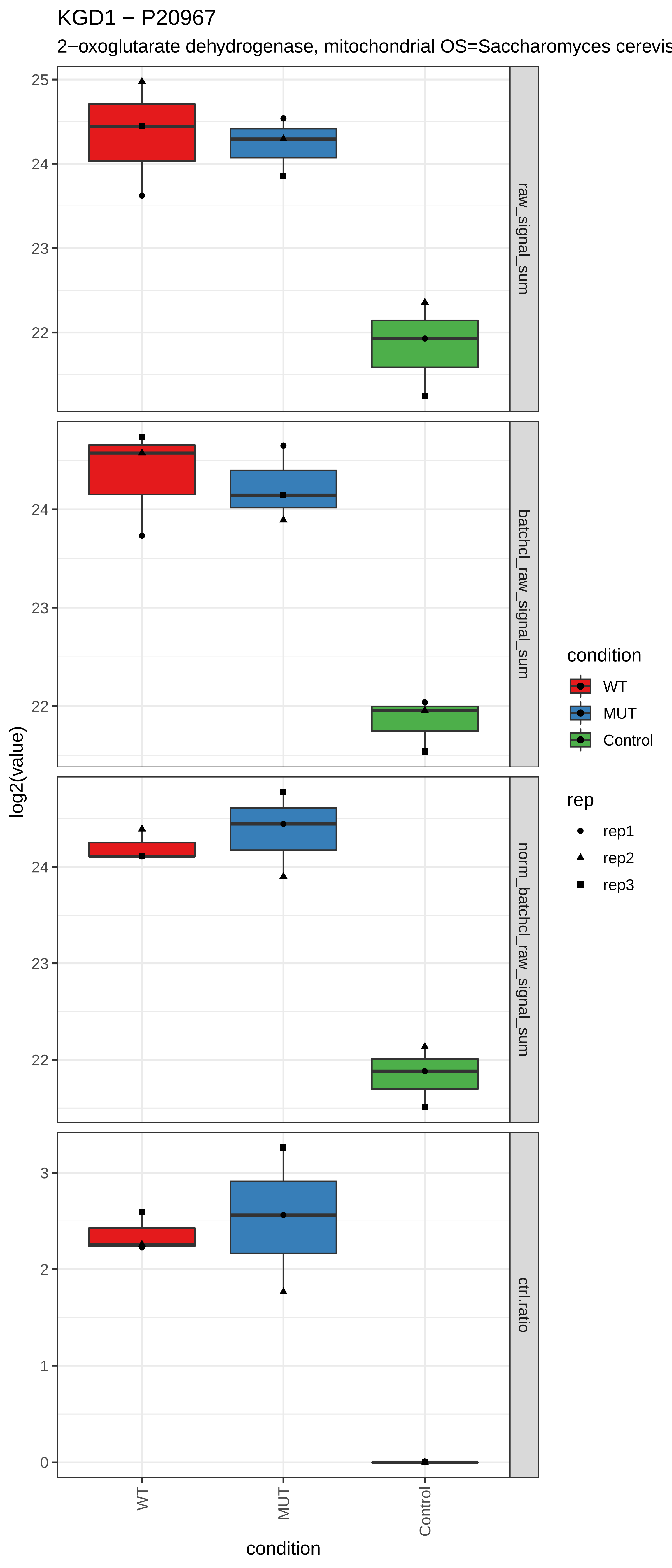

KGD1 − P20967
2−oxoglutarate dehydrogenase, mitochondrial OS=Saccharomyces cerevis
raw_signal_sum
batchcl_raw_signal_sum
norm_batchcl_raw_signal_sum
ctrl.ratio
log2(value)
condition
WT
MUT
Control
rep
rep1
rep2
rep3

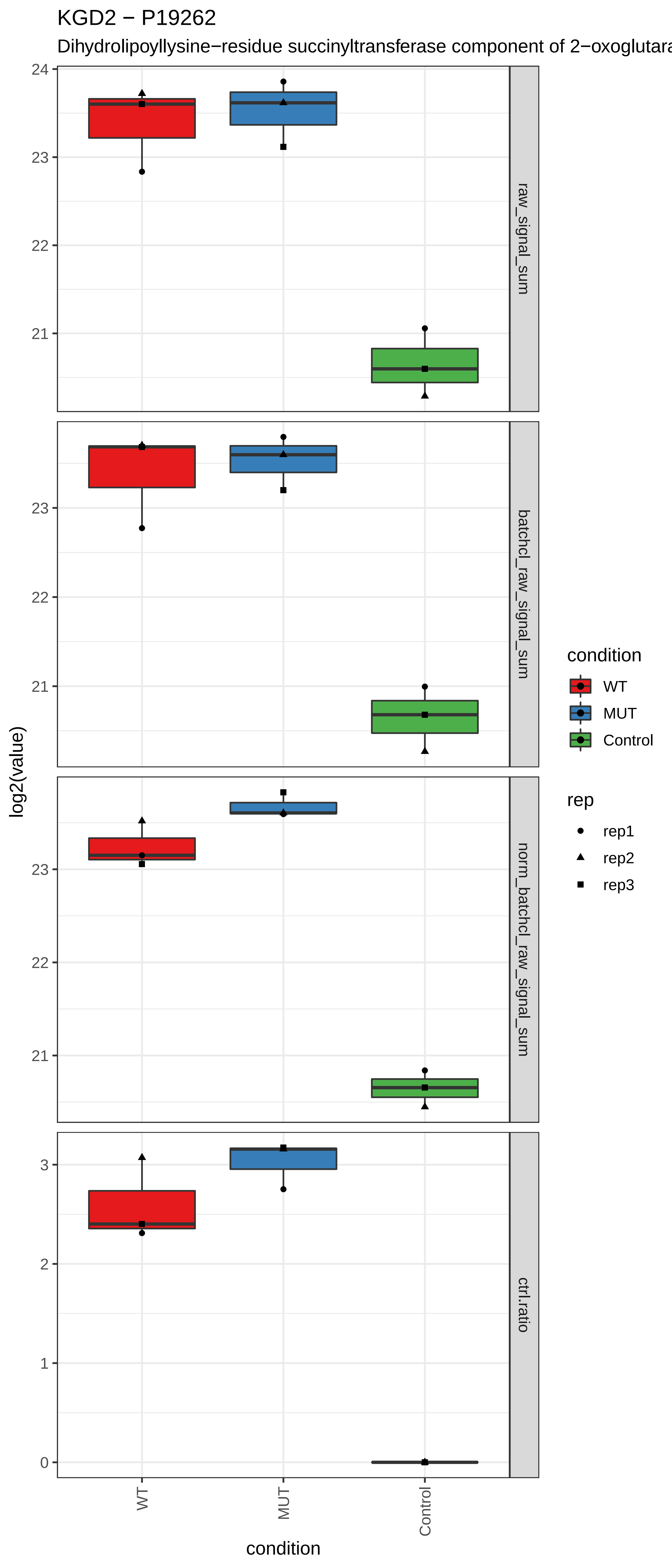
KGD2 − P19262
Dihydrolipoyllysine−residue succinyltransferase component of 2−oxoglutara
raw_signal_sum
batchcl_raw_signal_sum
norm_batchcl_raw_signal_sum
ctrl.ratio
log2(value)
condition
WT
MUT
Control
condition
WT
MUT
Control
rep
rep1
rep2
rep3

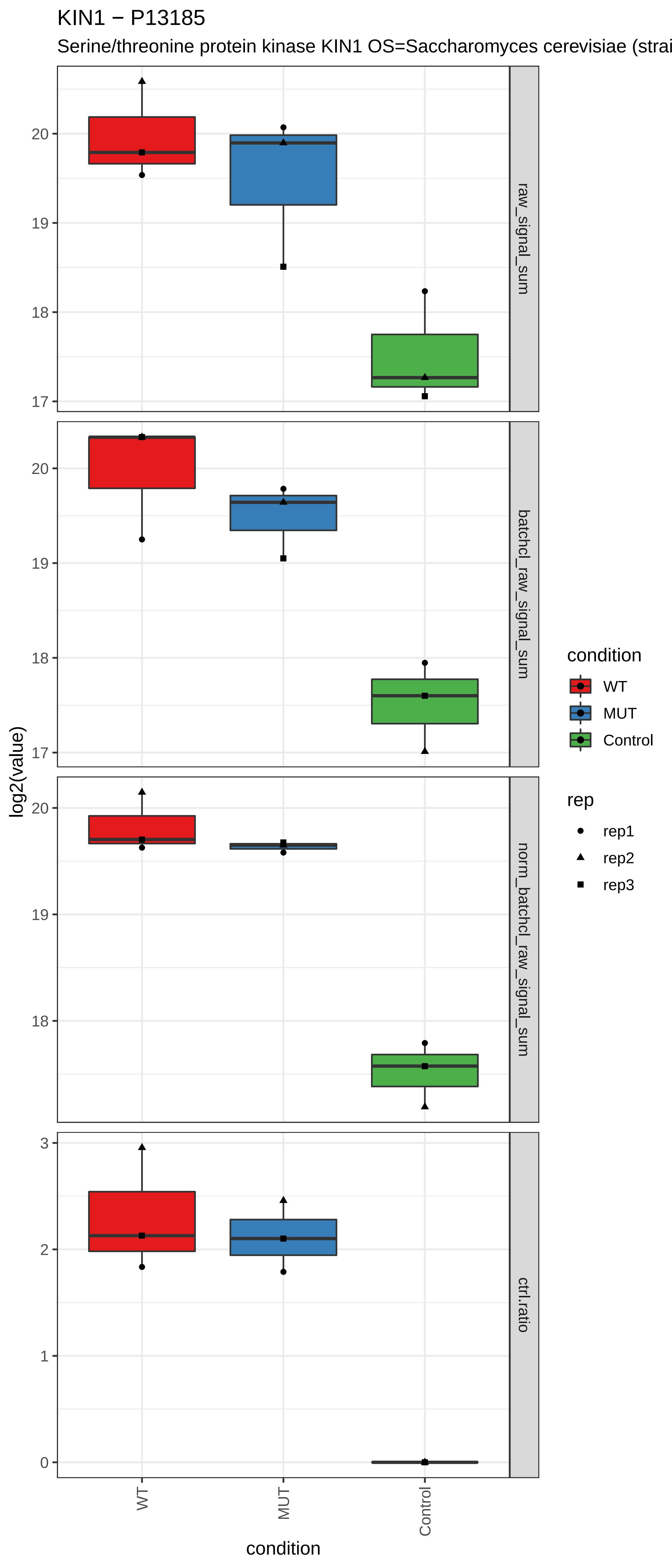
KIN1 − P13185
Serine/threonine protein kinase KIN1 OS=Saccharomyces cerevisiae (strai
raw_signal_sum
batchcl_raw_signal_sum
norm_batchcl_raw_signal_sum
ctrl.ratio
log2(value)
condition
WT
MUT
Control
rep
rep1
rep2
rep3
17
18
19
20
0
1
2
3

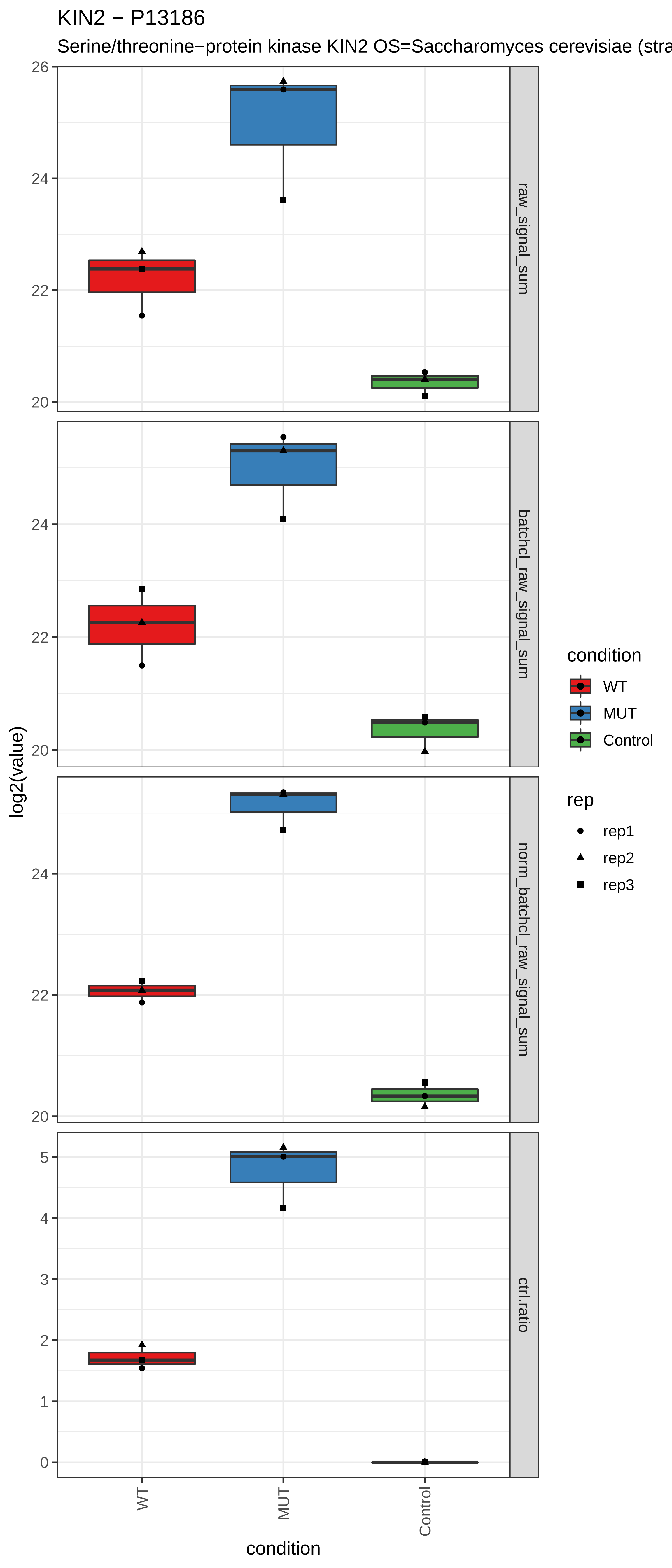

KIN2 − P13186
Serine/threonine−protein kinase KIN2 OS=Saccharomyces cerevisiae (stra
raw_signal_sum
batchcl_raw_signal_sum
norm_batchcl_raw_signal_sum
ctrl.ratio
log2(value)
condition
WT
MUT
Control
rep
rep1
rep2
rep3
26
24
22
20
5
4
3
2
1
0

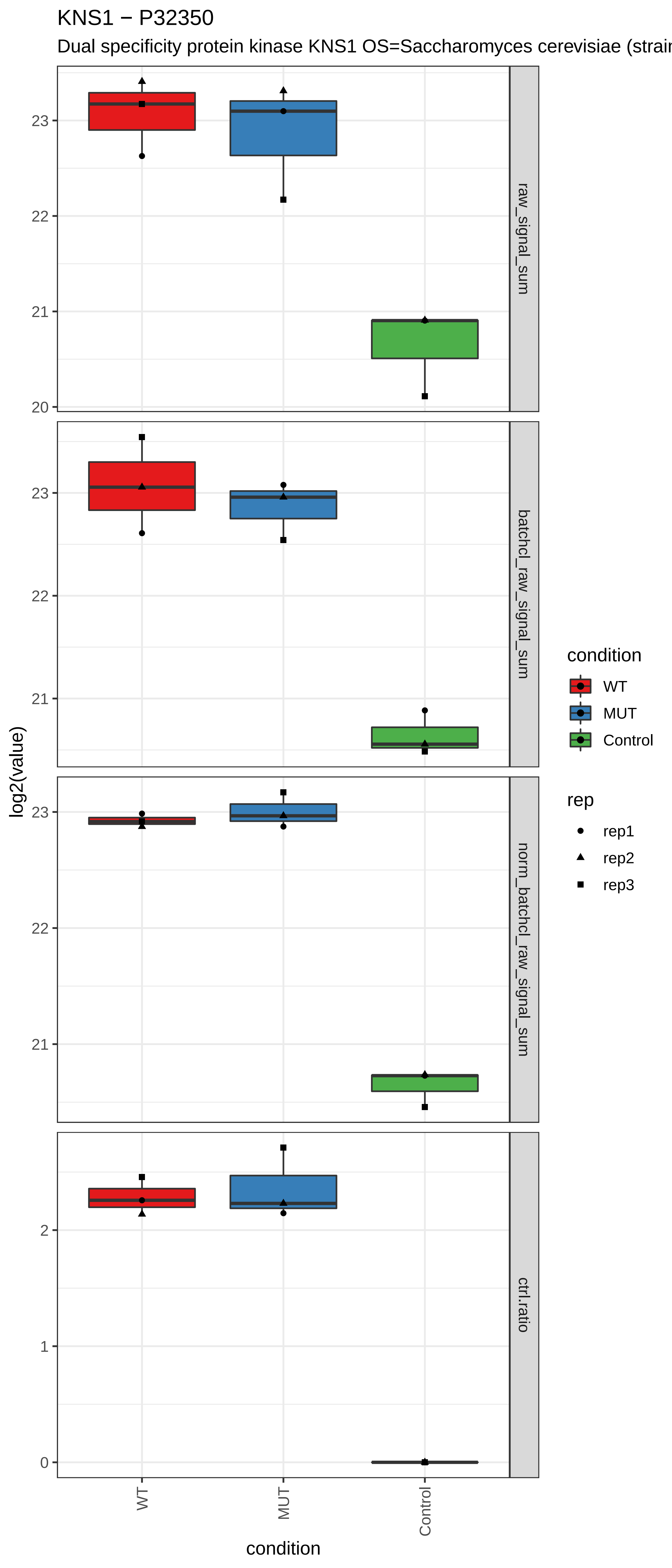

KNS1 − P32350
Dual specificity protein kinase KNS1 OS=Saccharomyces cerevisiae (strain
raw_signal_sum
batchcl_raw_signal_sum
norm_batchcl_raw_signal_sum
ctrl.ratio
log2(value)
condition
WT
MUT
Control
rep
rep1
rep2
rep3

KRS1 − P15180

Lysine−−tRNA ligase, cytoplasmic OS=Saccharomyces cerevisiae (strain A

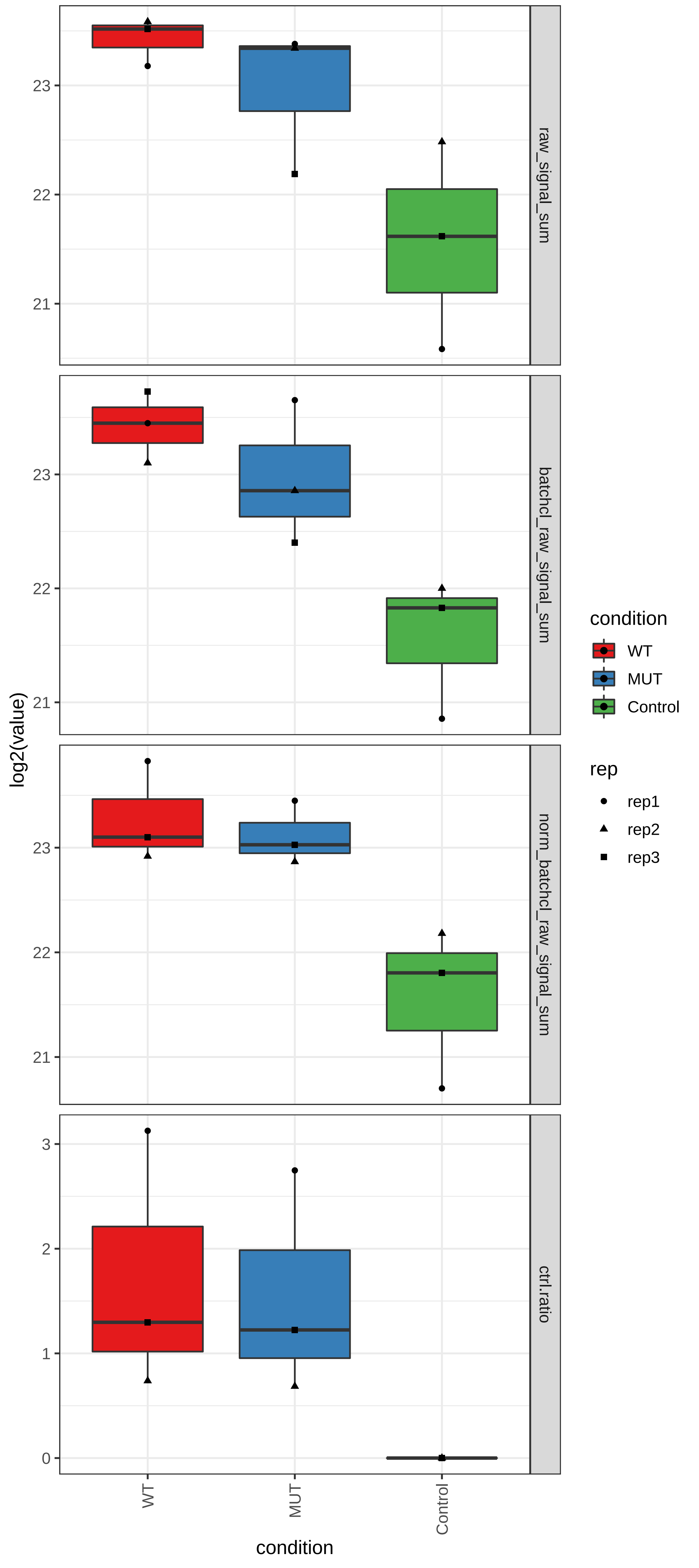

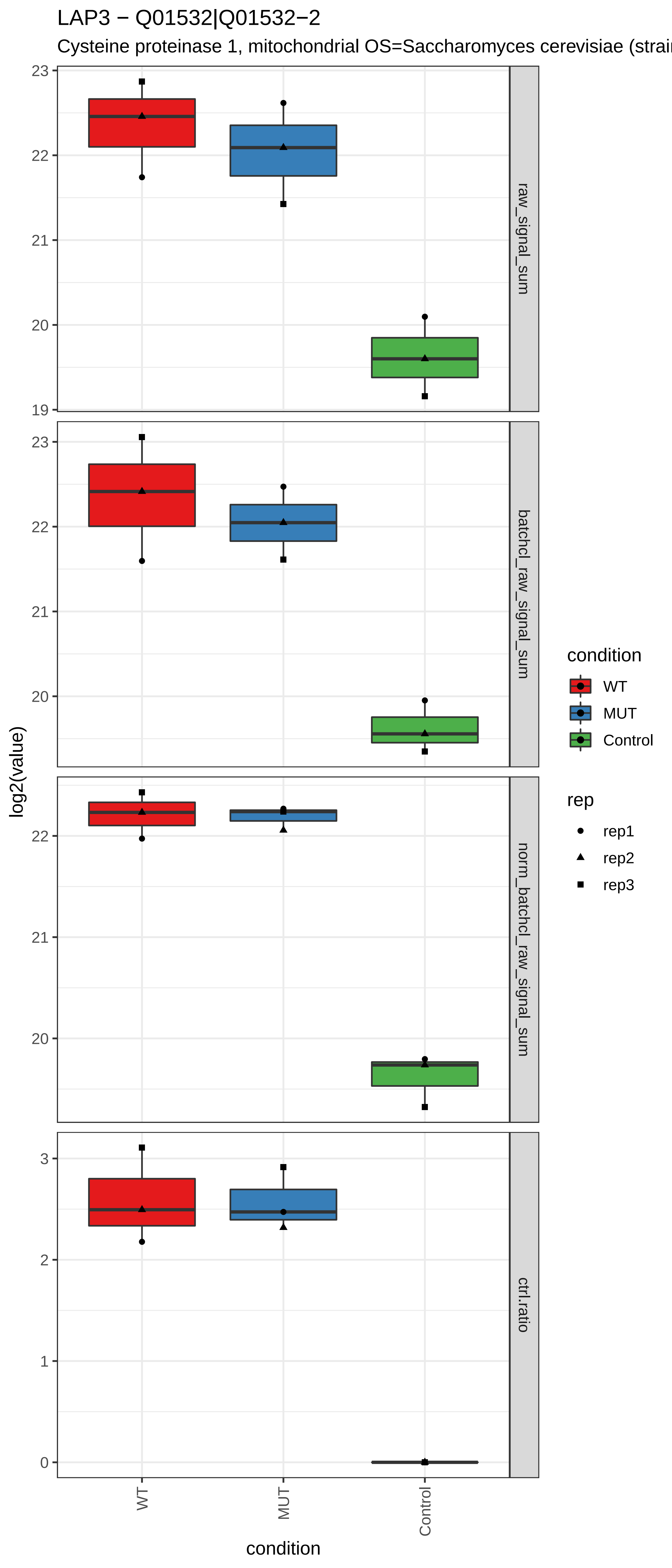
LAP3 − Q01532|Q01532−2
Cysteine proteinase 1, mitochondrial OS=Saccharomyces cerevisiae (strai
raw_signal_sum
batchcl_raw_signal_sum
norm_batchcl_raw_signal_sum
ctrl.ratio
log2(value)
condition
WT
MUT
Control
rep
rep1
rep2
rep3
23
22
21
20
19
3
2
1
0

LAT1 − P12695

Dihydrolipoyllysine−residue acetyltransferase component of pyruvate dehyd

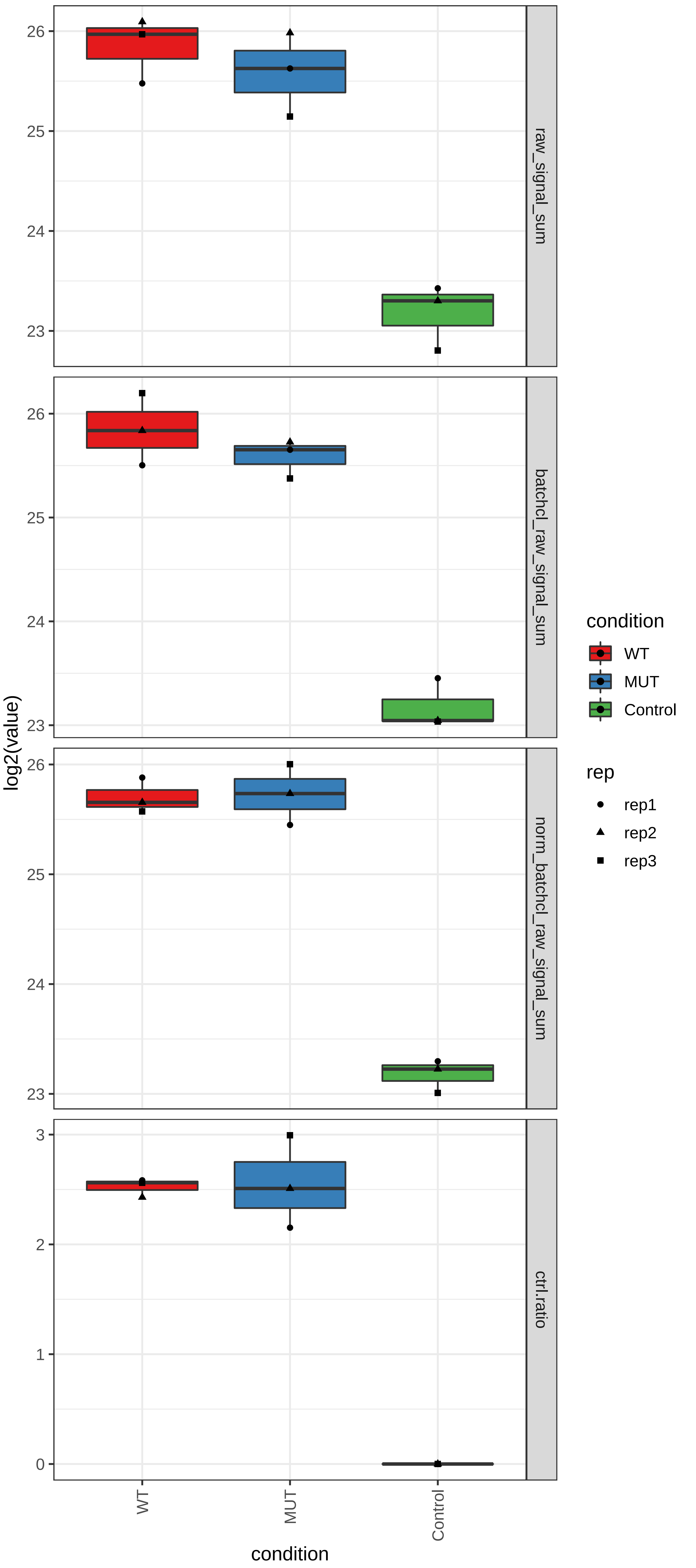

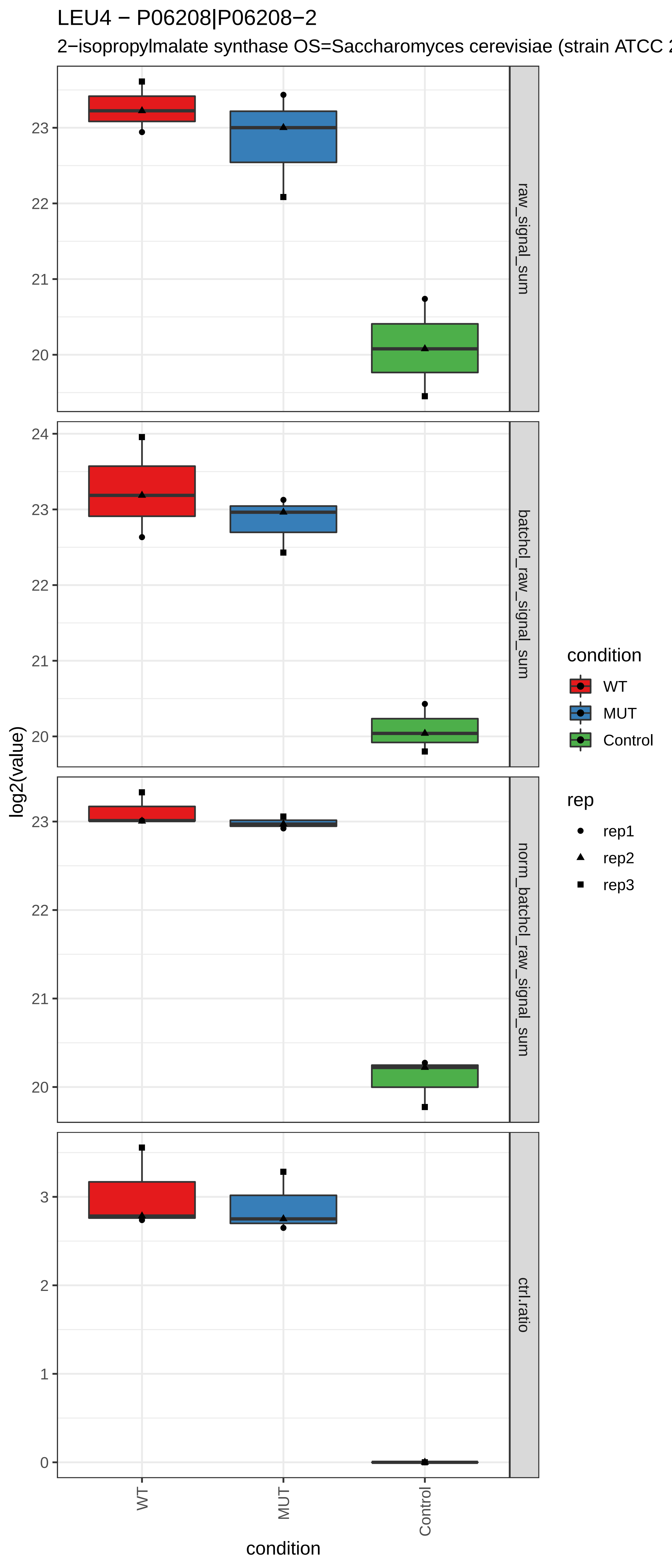

LEU4 − P06208|P06208−2
2−isopropylmalate synthase OS=Saccharomyces cerevisiae (strain ATCC 2
raw_signal_sum
batchcl_raw_signal_sum
norm_batchcl_raw_signal_sum
ctrl.ratio
log2(value)
condition
WT
MUT
Control
rep
rep1
rep2
rep3

LOC1 − P43586

60S ribosomal subunit assembly/export protein LOC1 OS=Saccharomyces

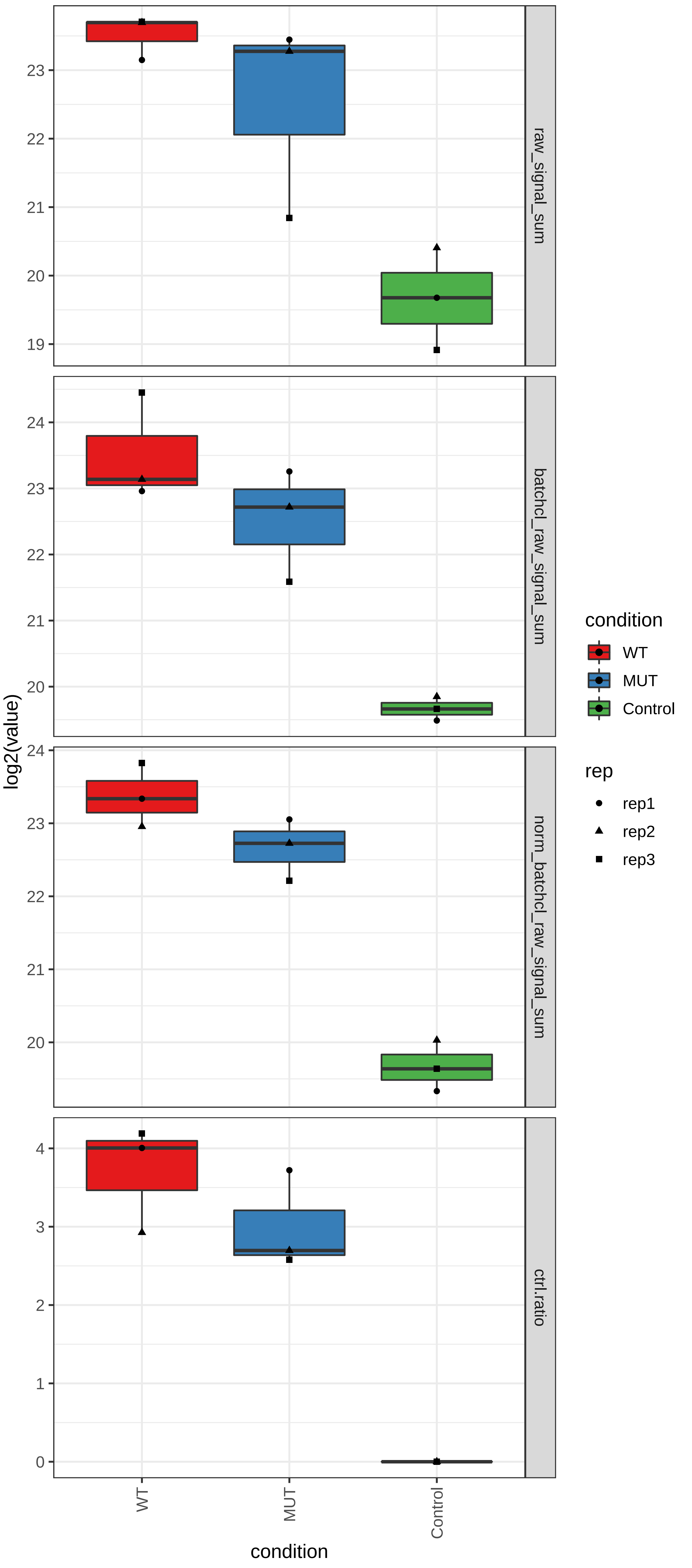

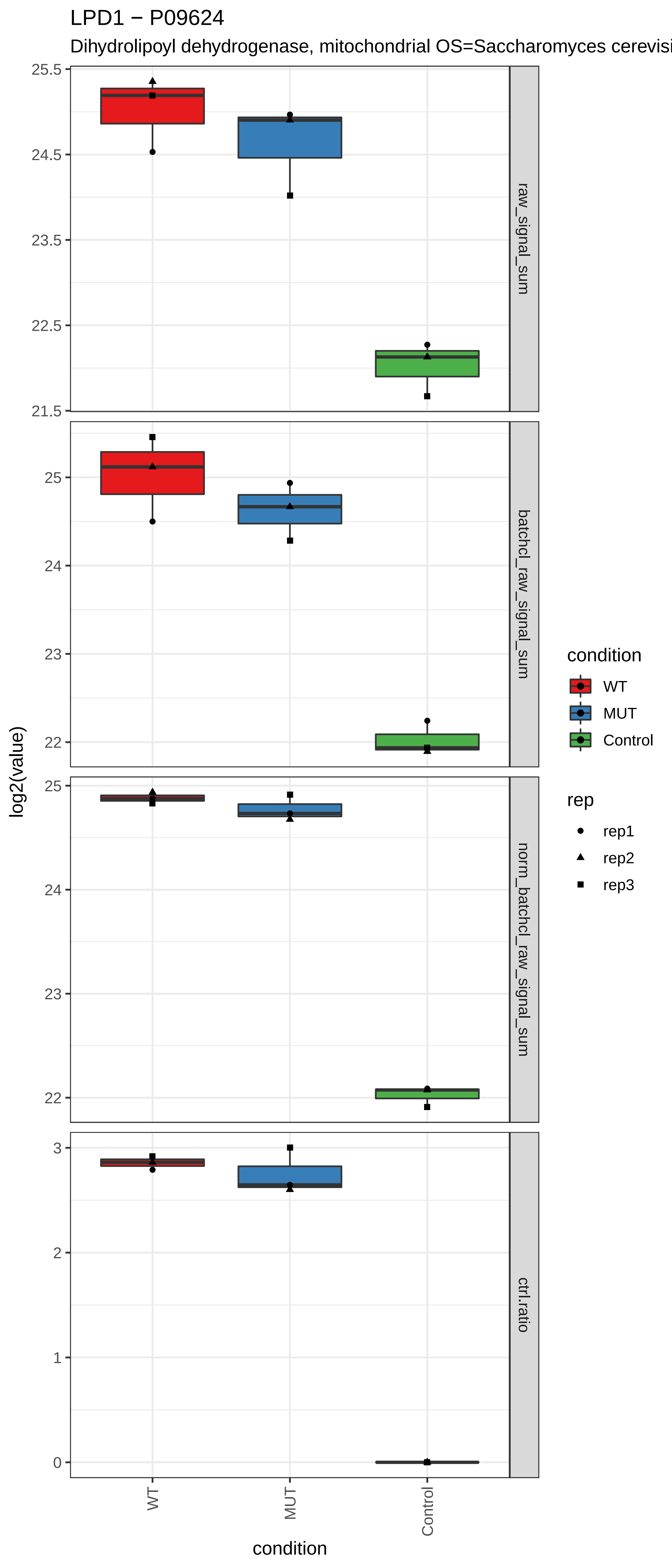
LPD1 − P09624
Dihydrolipoyl dehydrogenase, mitochondrial OS=Saccharomyces cerevisi
raw_signal_sum
batchcl_raw_signal_sum
norm_batchcl_raw_signal_sum
ctrl.ratio
log2(value)
condition
WT
MUT
Control
rep
rep1
rep2
rep3
25.5
24.5
23.5
22.5
21.5
25
24
23
22
3
2
1
0

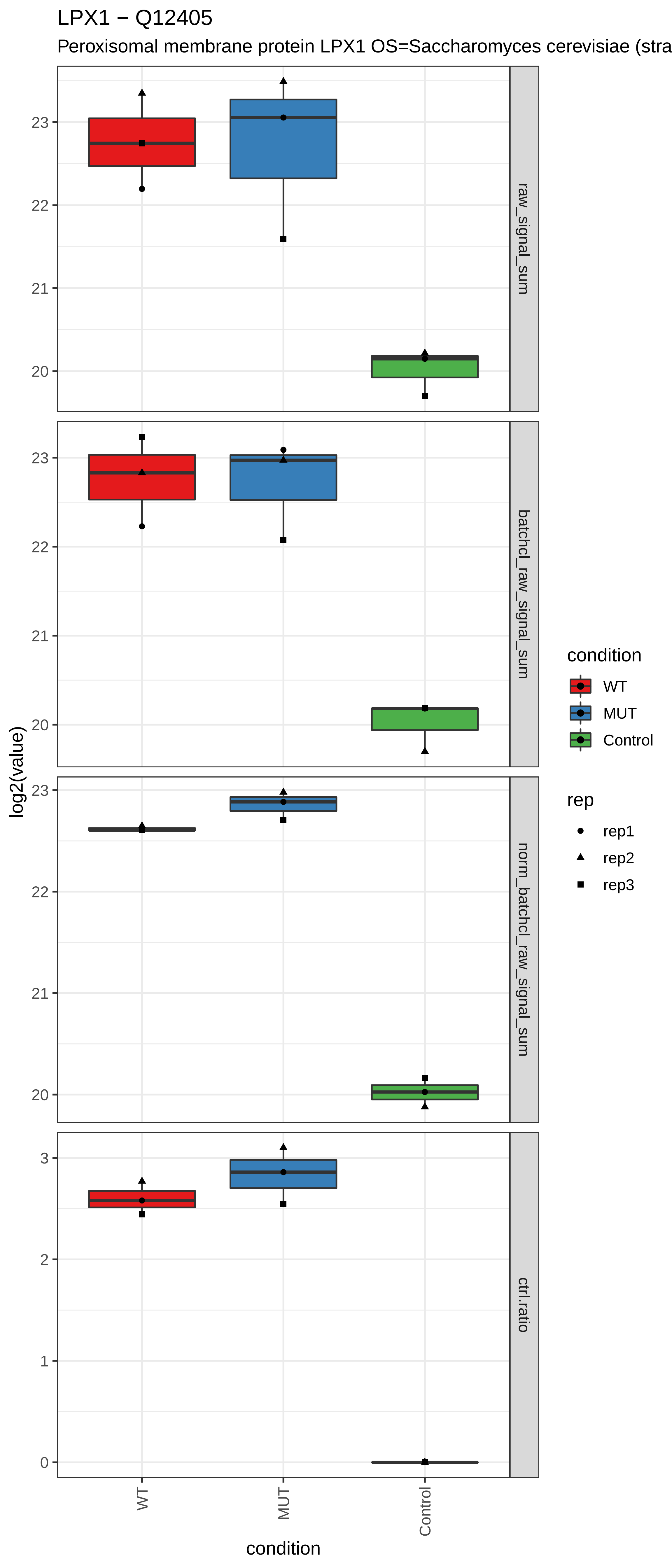

LPX1 − Q12405
Peroxisomal membrane protein LPX1 OS=Saccharomyces cerevisiae (stra
raw_signal_sum
batchcl_raw_signal_sum
norm_batchcl_raw_signal_sum
ctrl.ratio
log2(value)
condition
WT
MUT
Control
rep
rep1
rep2
rep3

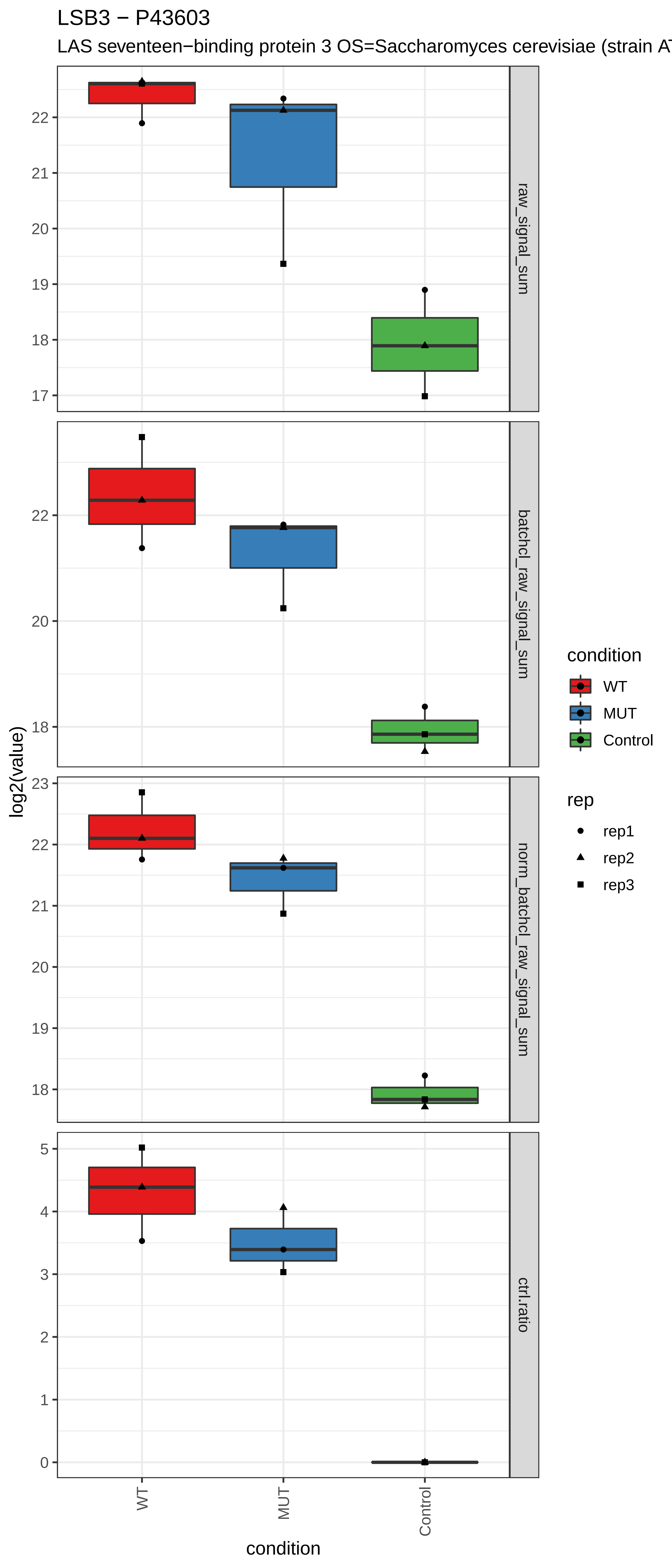

LSB3 − P43603
LAS seventeen−binding protein 3 OS=Saccharomyces cerevisiae (strain AT
raw_signal_sum
batchcl_raw_signal_sum
norm_batchcl_raw_signal_sum
ctrl.ratio
log2(value)
condition
WT
MUT
Control
rep
rep1
rep2
rep3

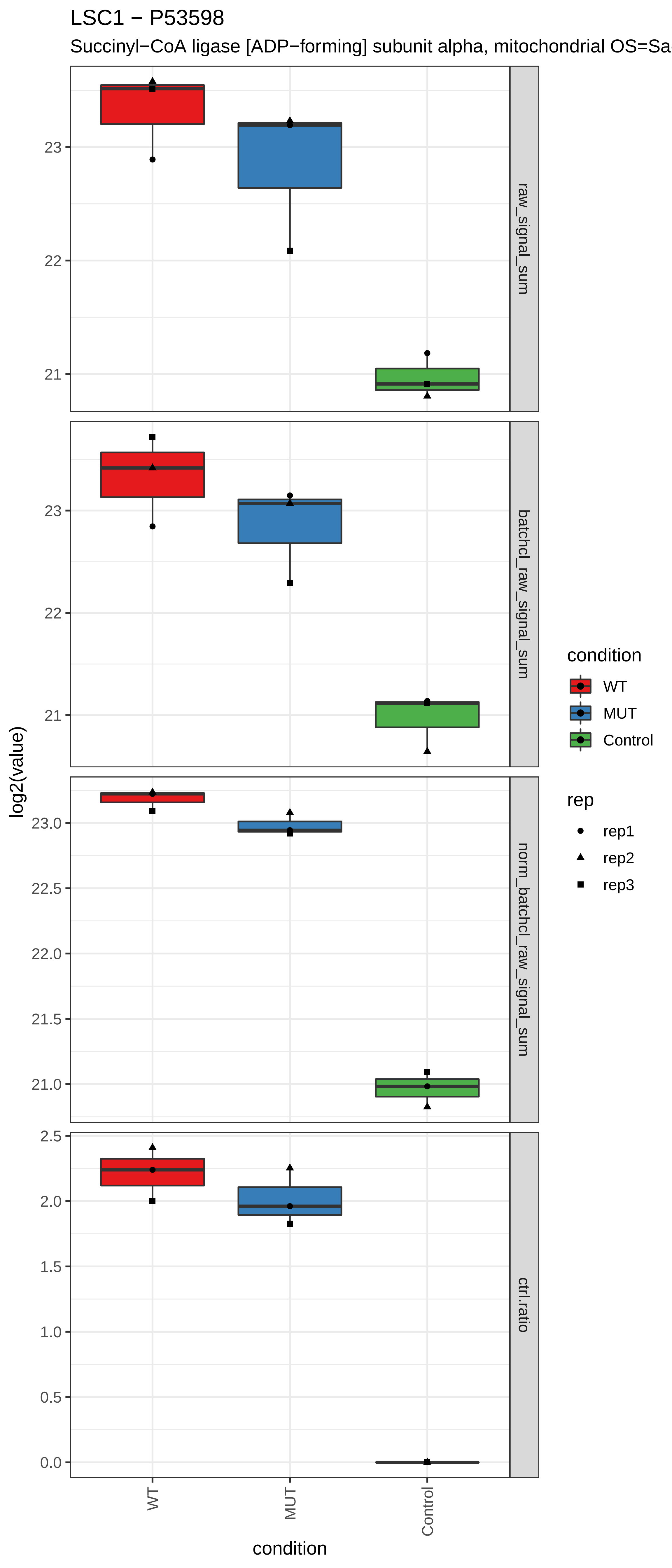

LSC1 − P53598
Succinyl−CoA ligase [ADP−forming] subunit alpha, mitochondrial OS=Sa
raw_signal_sum
batchcl_raw_signal_sum
norm_batchcl_raw_signal_sum
ctrl.ratio
log2(value)
condition
WT
MUT
Control
rep
rep1
rep2
rep3
23
22
21
23.0
22.5
22.0
21.5
21.0
2.5
2.0
1.5
1.0
0.5
0.0

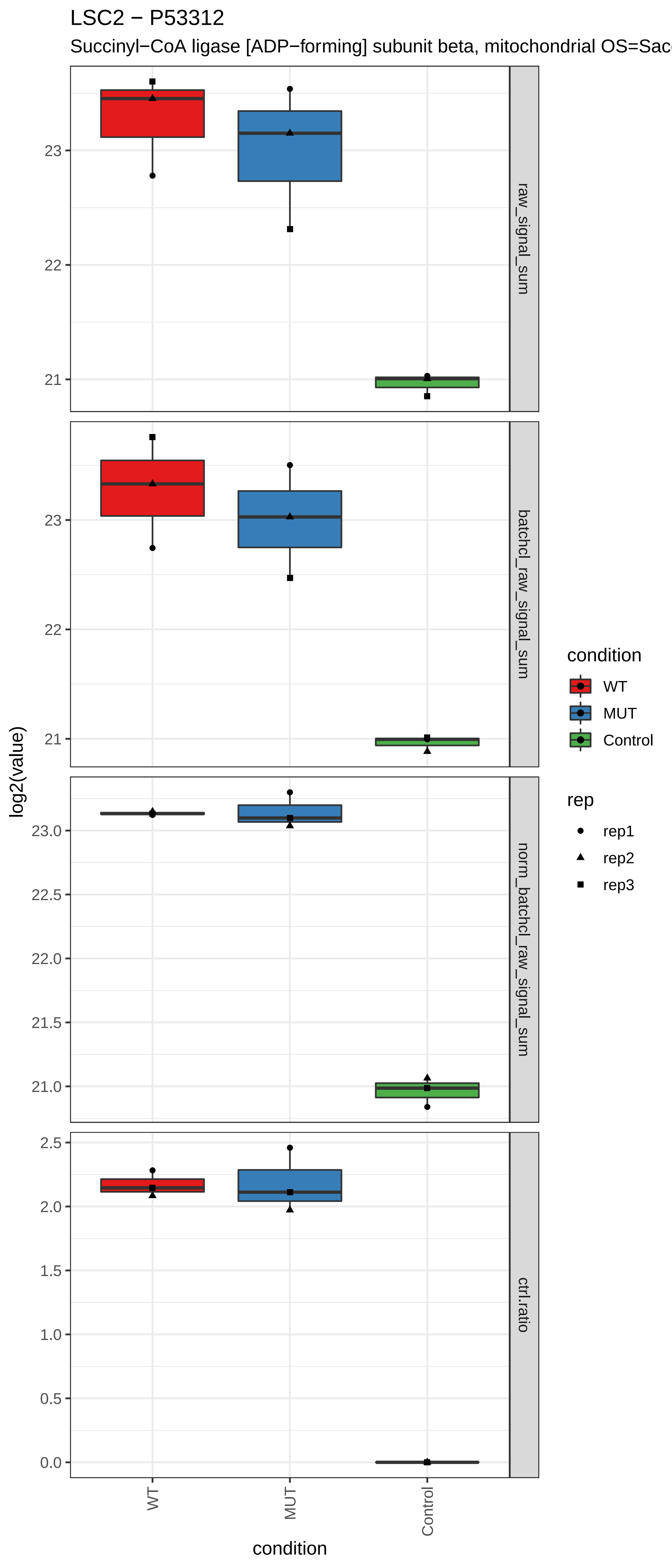

LSC2 − P53312
Succinyl−CoA ligase [ADP−forming] subunit beta, mitochondrial OS=Sac
raw_signal_sum
batchcl_raw_signal_sum
norm_batchcl_raw_signal_sum
ctrl.ratio
log2(value)
condition
WT
MUT
Control
rep
rep1
rep2
rep3

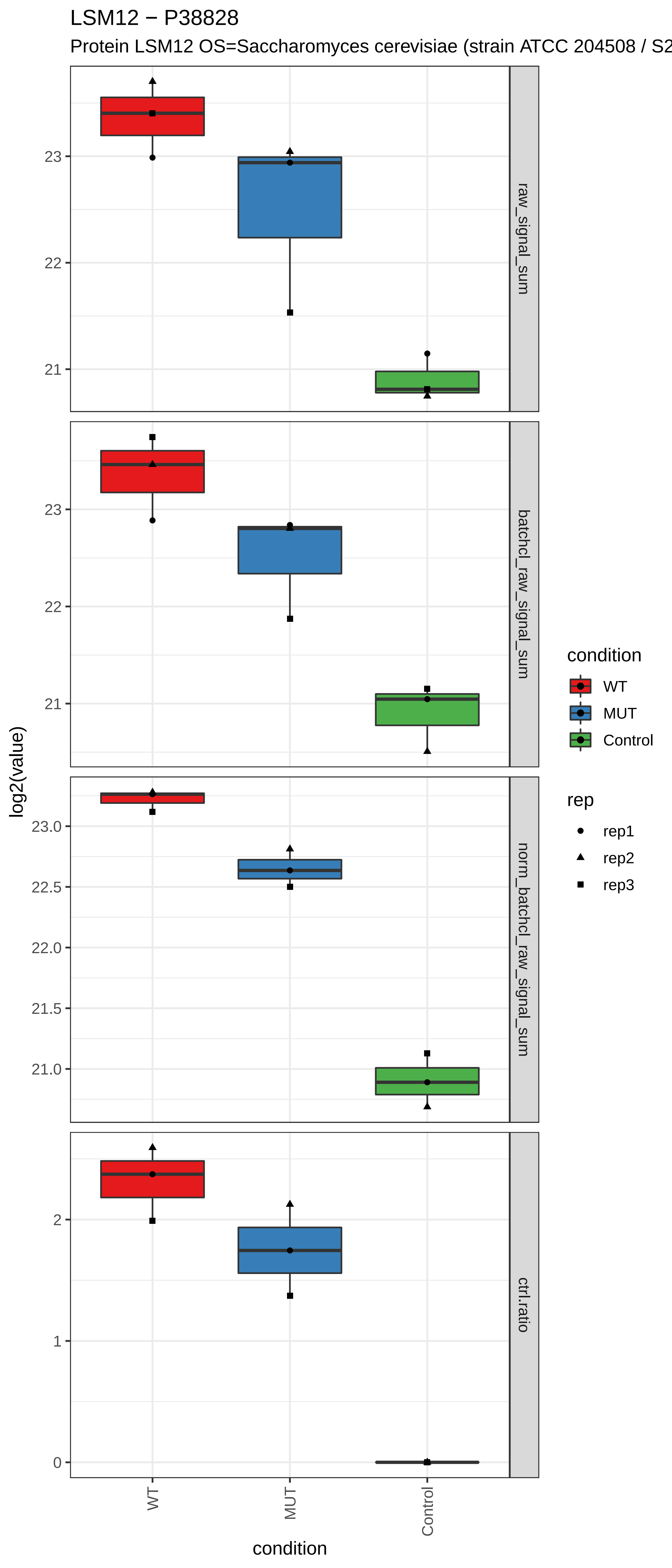

LSM12 − P38828
Protein LSM12 OS=Saccharomyces cerevisiae (strain ATCC 204508 / S2
raw_signal_sum
batchcl_raw_signal_sum
norm_batchcl_raw_signal_sum
ctrl.ratio
log2(value)
condition
WT
MUT
Control
rep
rep1
rep2
rep3
23
22
21
23.0
22.5
22.0
21.5
21.0
2
1
0

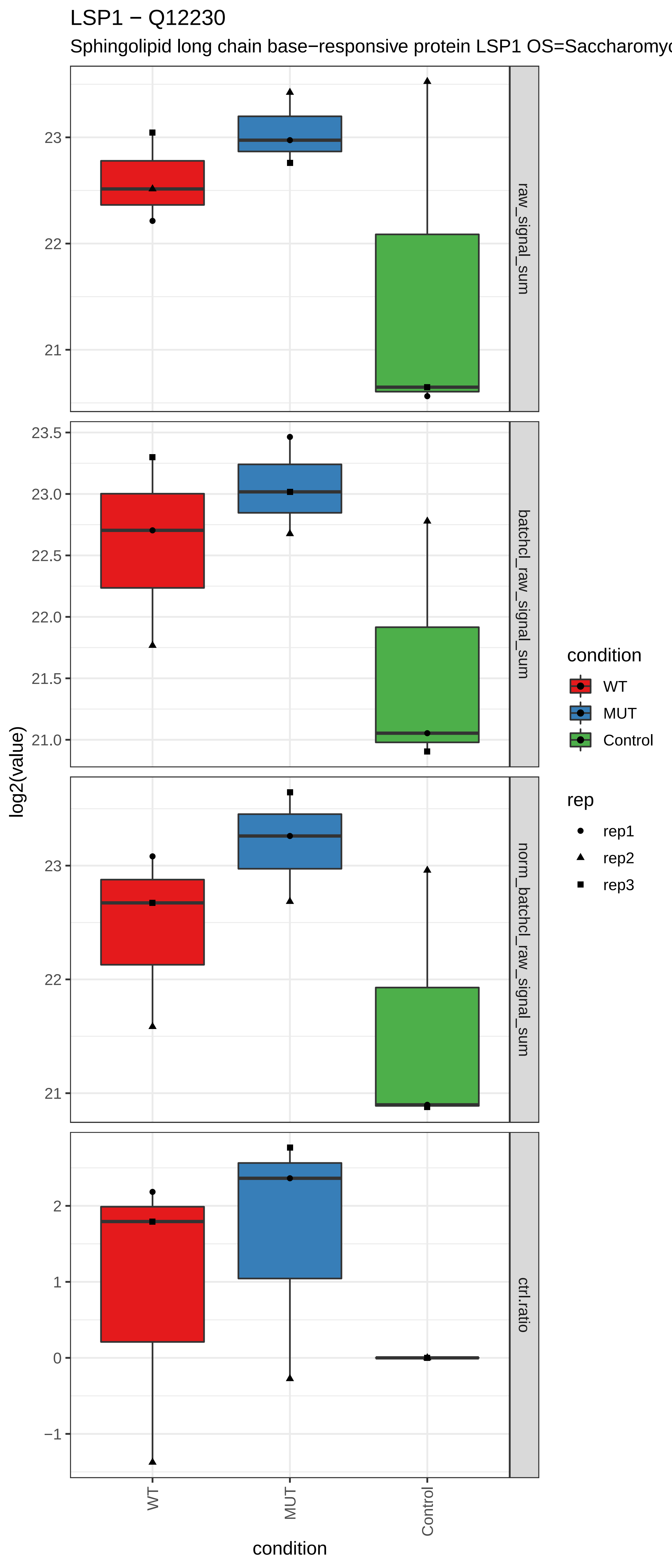

LSP1 − Q12230
Sphingolipid long chain base−responsive protein LSP1 OS=Saccharomyc
raw_signal_sum
batchcl_raw_signal_sum
norm_batchcl_raw_signal_sum
ctrl.ratio
log2(value)
condition
WT
MUT
Control
rep
rep1
rep2
rep3

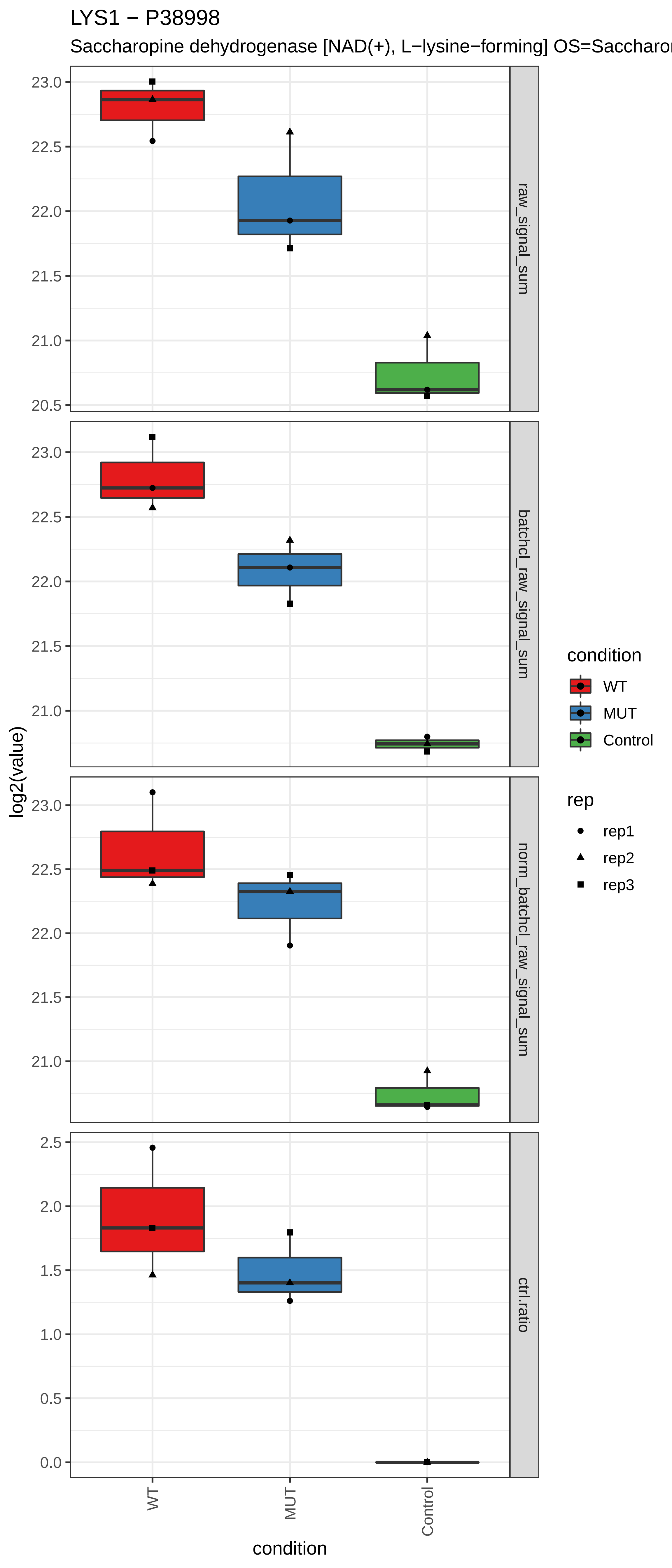
LYS1 − P38998
Saccharopine dehydrogenase [NAD(+), L−lysine−forming] OS=Saccharon
raw_signal_sum
batchcl_raw_signal_sum
norm_batchcl_raw_signal_sum
ctrl.ratio
log2(value)
condition
WT
MUT
Control
rep
rep1
rep2
rep3
23.0
22.5
22.0
21.5
21.0
20.5
2.5
2.0
1.5
1.0
0.5
0.0

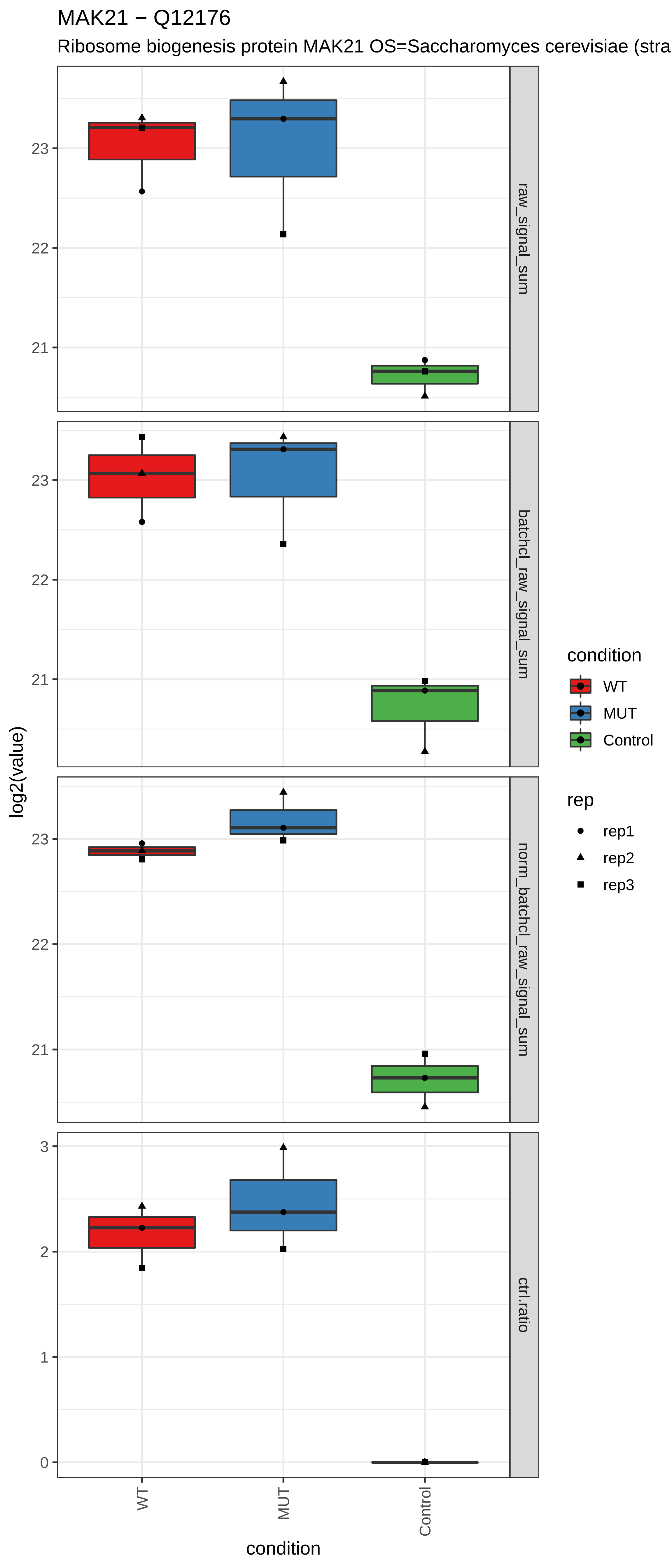
MAK21 − Q12176
Ribosome biogenesis protein MAK21 OS=Saccharomyces cerevisiae (stra
raw_signal_sum
batchcl_raw_signal_sum
norm_batchcl_raw_signal_sum
ctrl.ratio
log2(value)
condition
WT
MUT
Control
rep
rep1
rep2
rep3
23
22
21
3
2
1
0

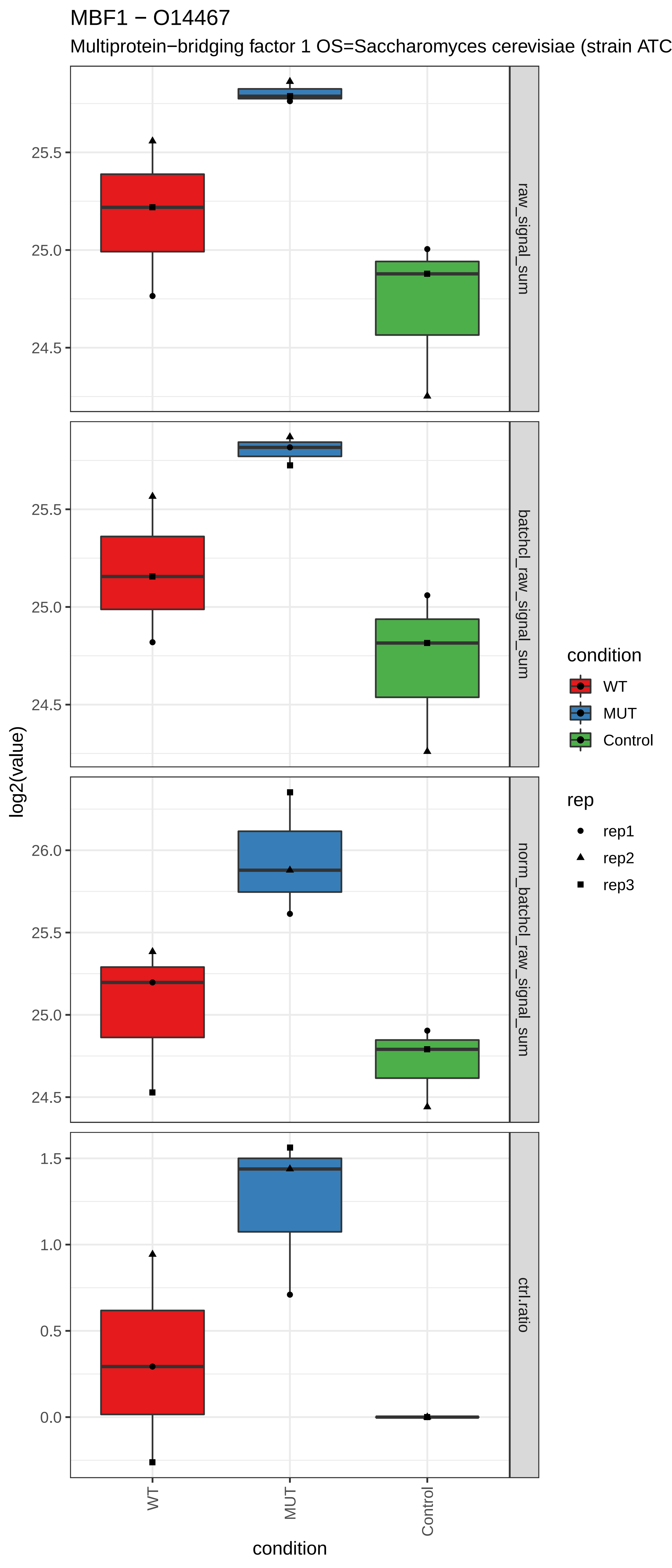

MBF1 − O14467
Multiprotein−bridging factor 1 OS=Saccharomyces cerevisiae (strain ATC
raw_signal_sum
batchcl_raw_signal_sum
norm_batchcl_raw_signal_sum
ctrl.ratio
log2(value)
condition
WT
MUT
Control
rep
rep1
rep2
rep3
25.5
25.0
24.5
26.0
1.5
1.0
0.5
0.0

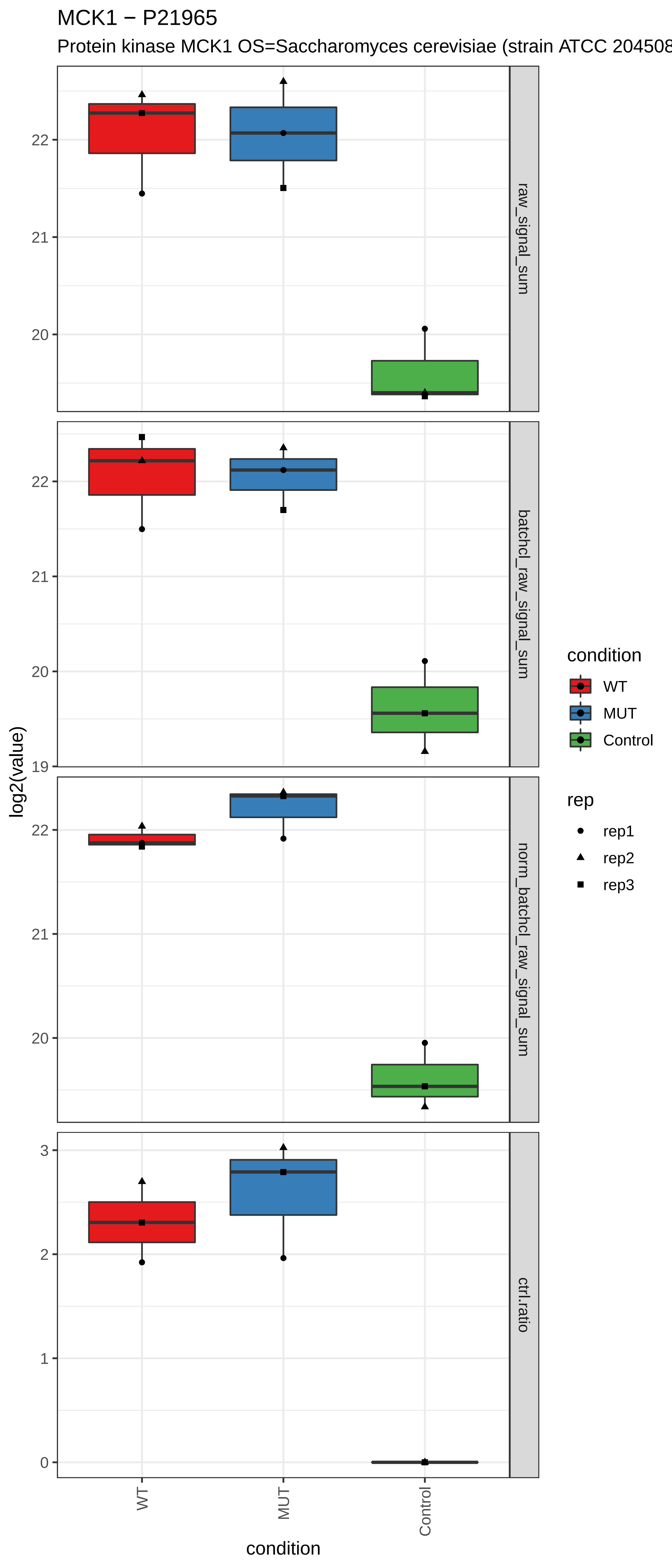

MCK1 − P21965
Protein kinase MCK1 OS=Saccharomyces cerevisiae (strain ATCC 204508
raw_signal_sum
batchcl_raw_signal_sum
norm_batchcl_raw_signal_sum
ctrl.ratio
log2(value)
condition
WT
MUT
Control
rep
rep1
rep2
rep3
22
21
20
19
3
2
1
0

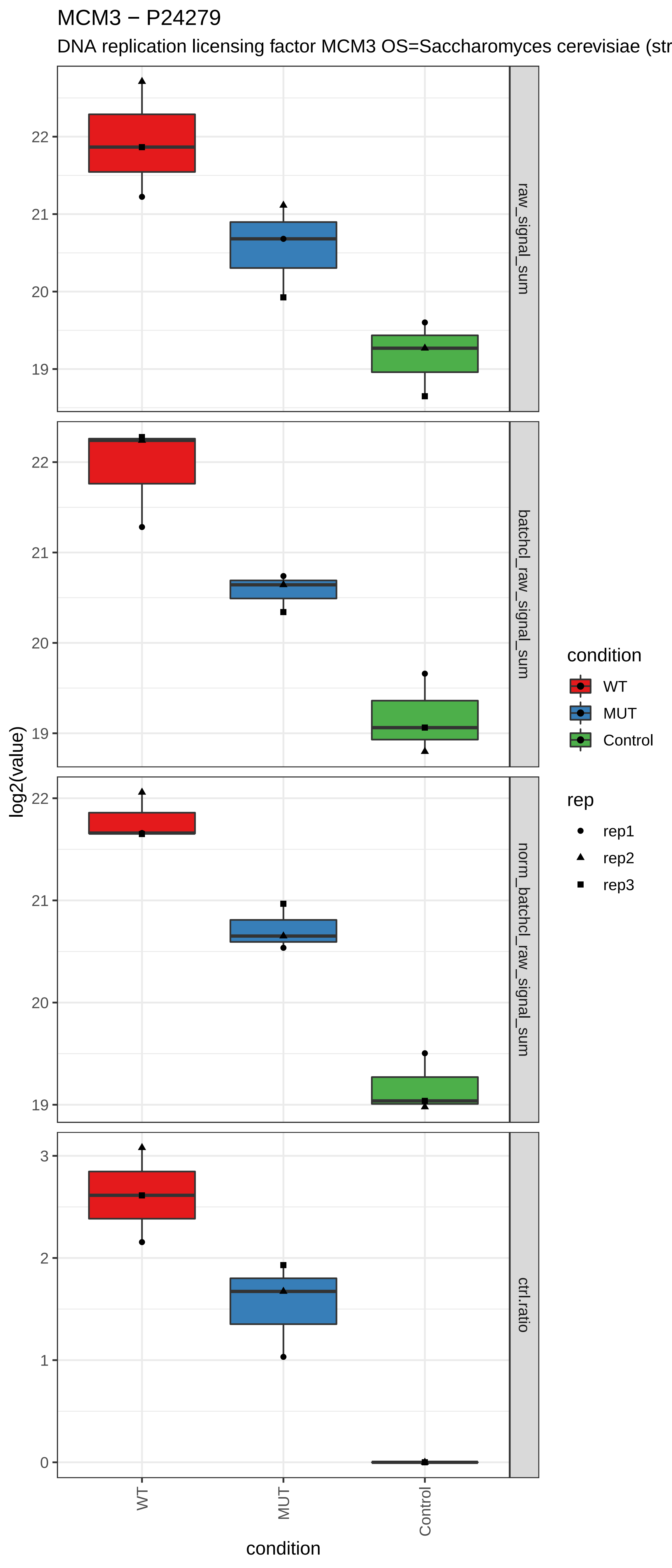
MCM3 − P24279
DNA replication licensing factor MCM3 OS=Saccharomyces cerevisiae (str
raw_signal_sum
batchcl_raw_signal_sum
norm_batchcl_raw_signal_sum
ctrl.ratio
log2(value)
condition
WT
MUT
Control
rep
rep1
rep2
rep3

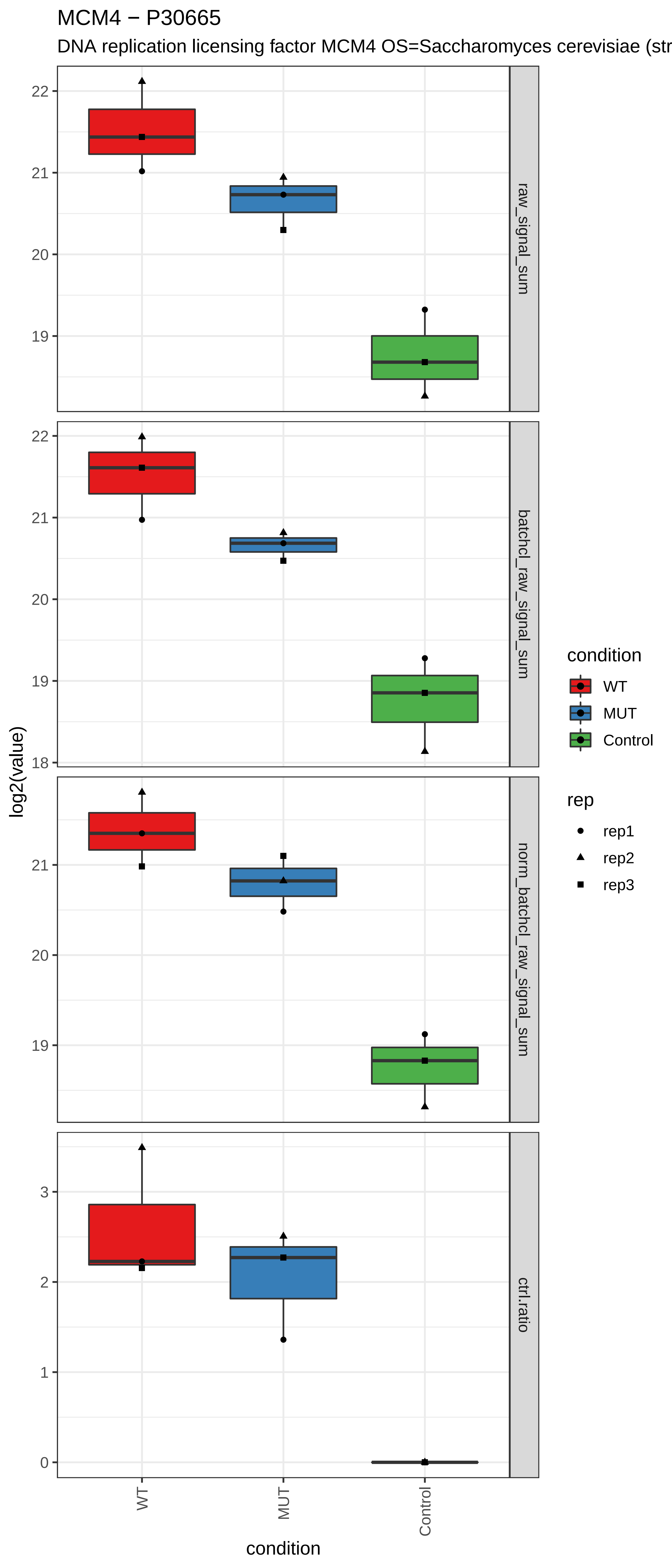
MCM4 − P30665
DNA replication licensing factor MCM4 OS=Saccharomyces cerevisiae (str
raw_signal_sum
batchcl_raw_signal_sum
norm_batchcl_raw_signal_sum
ctrl.ratio
log2(value)
condition
WT
MUT
Control
rep
rep1
rep2
rep3

MDG1 − P53885

Signal transduction protein MDG1 OS=Saccharomyces cerevisiae (strain A

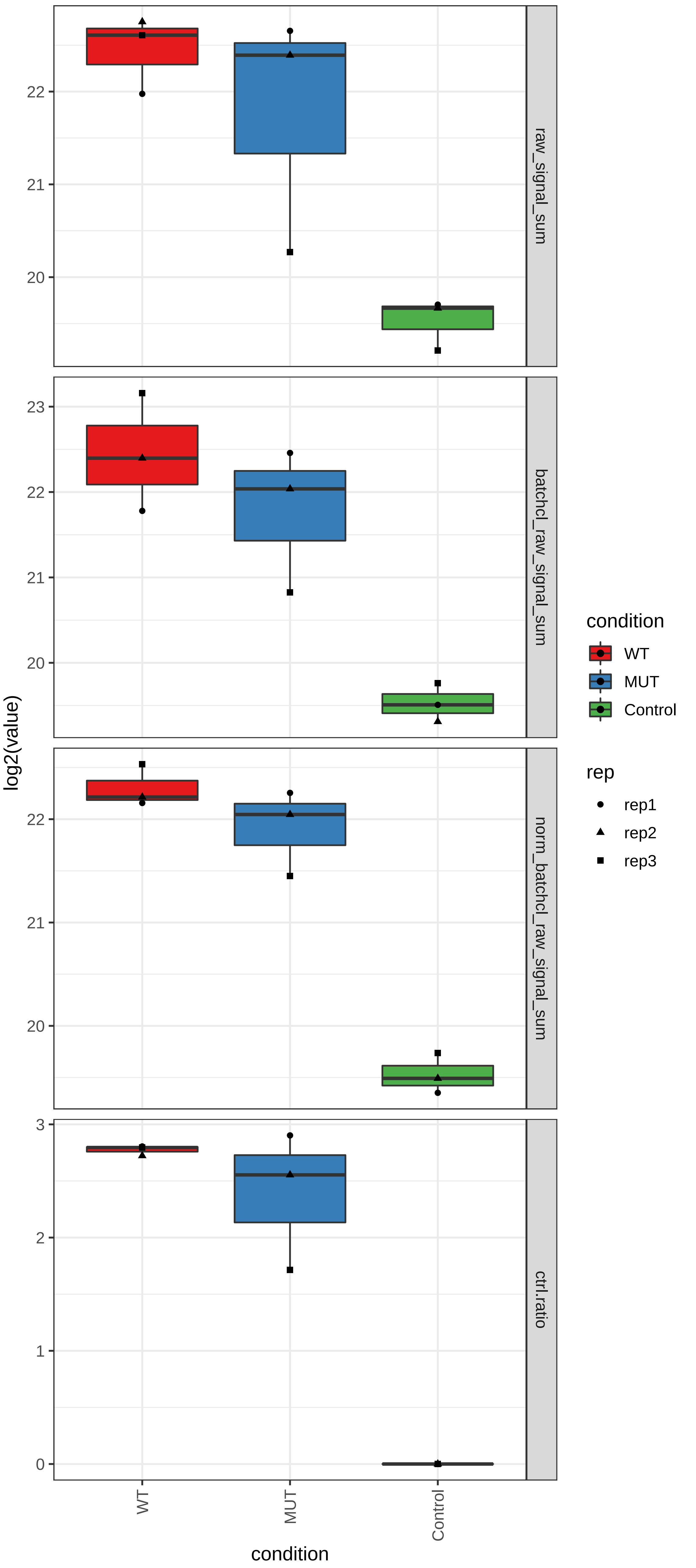

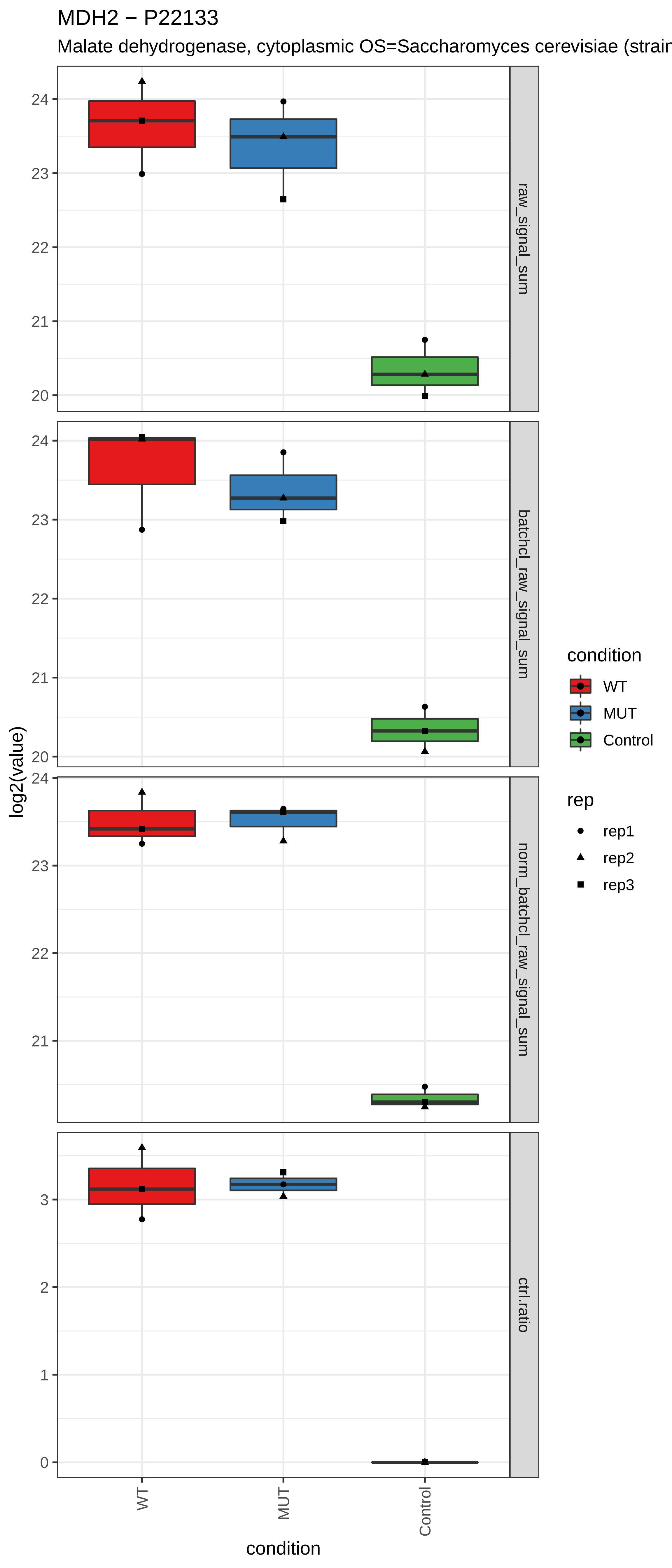

MDH2 − P22133
Malate dehydrogenase, cytoplasmic OS=Saccharomyces cerevisiae (strain
raw_signal_sum
batchcl_raw_signal_sum
norm_batchcl_raw_signal_sum
ctrl.ratio
log2(value)
condition
WT
MUT
Control
rep
rep1
rep2
rep3

MDH3 − P32419

Malate dehydrogenase, peroxisomal OS=Saccharomyces cerevisiae (strain

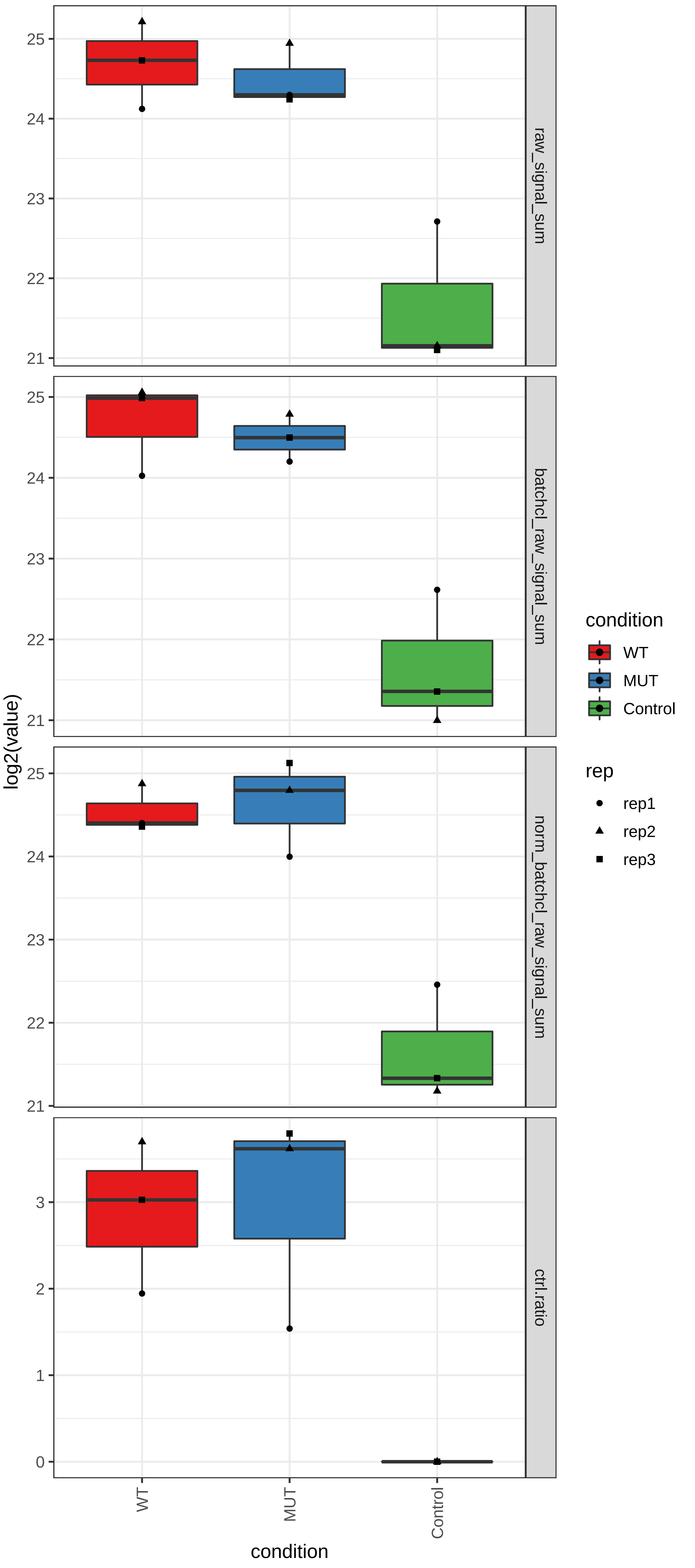

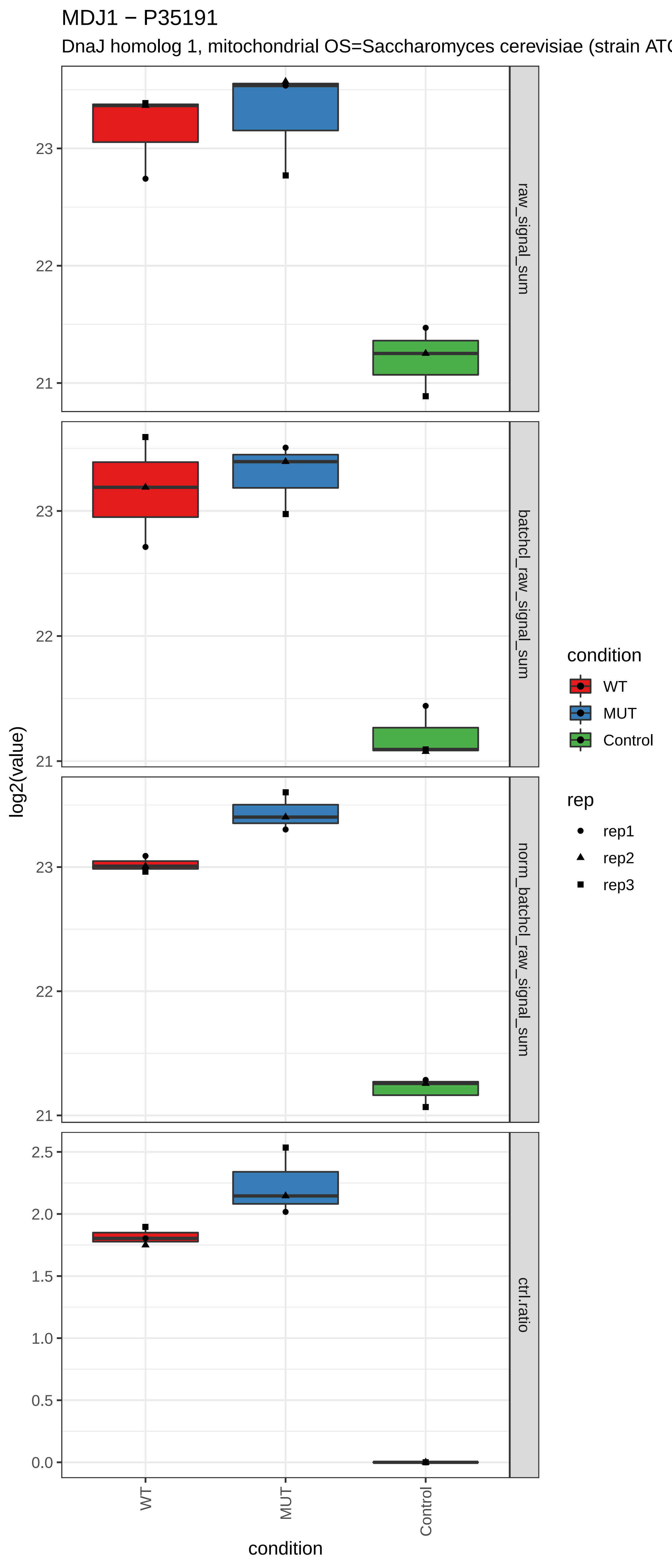
MDJ1 − P35191
DnaJ homolog 1, mitochondrial OS=Saccharomyces cerevisiae (strain ATC
raw_signal_sum
batchcl_raw_signal_sum
norm_batchcl_raw_signal_sum
ctrl.ratio
log2(value)
condition
WT
MUT
Control
rep
rep1
rep2
rep3
23
22
21
2.5
2.0
1.5
1.0
0.5
0.0

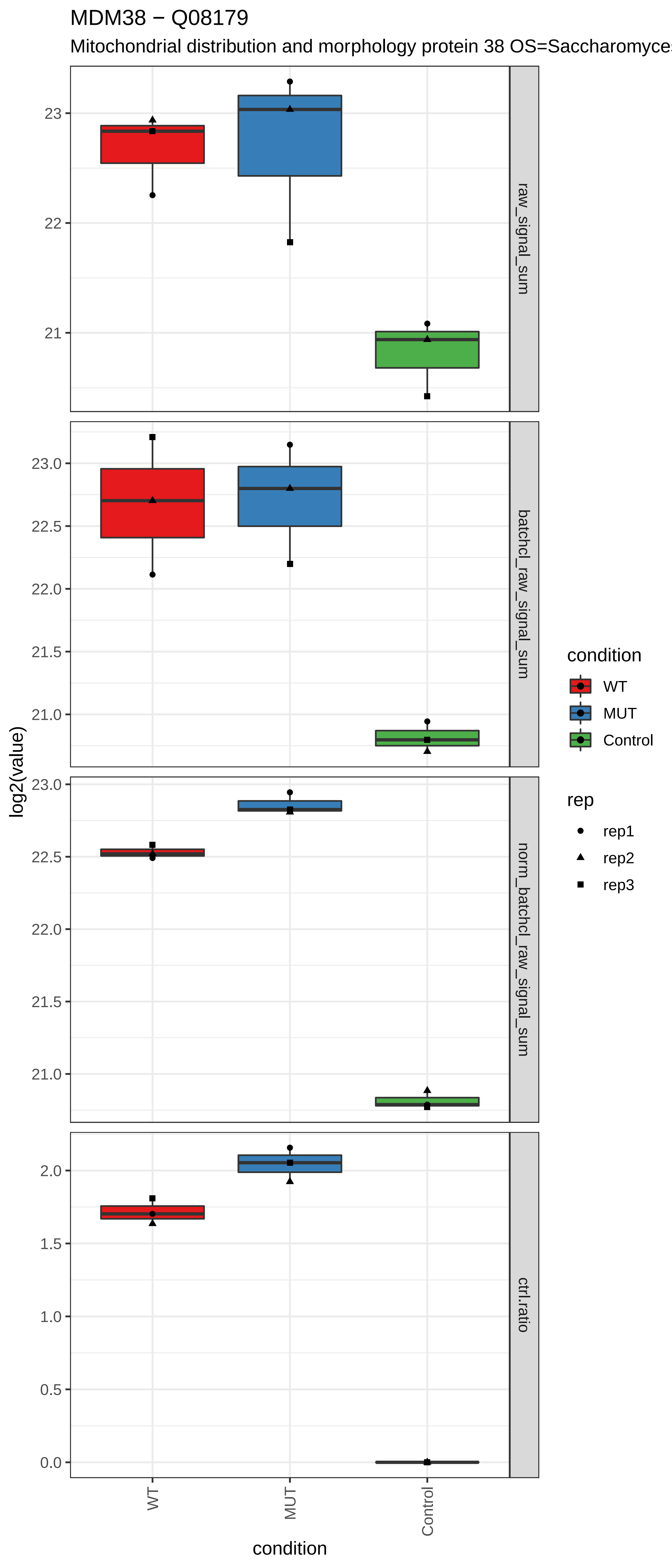
MDM38 − Q08179
Mitochondrial distribution and morphology protein 38 OS=Saccharomyces
raw_signal_sum
batchcl_raw_signal_sum
norm_batchcl_raw_signal_sum
ctrl.ratio
log2(value)
condition
WT
MUT
Control
rep
rep1
rep2
rep3
23
22
21
23.0
22.5
22.0
21.5
21.0
2.0
1.5
1.0
0.5
0.0

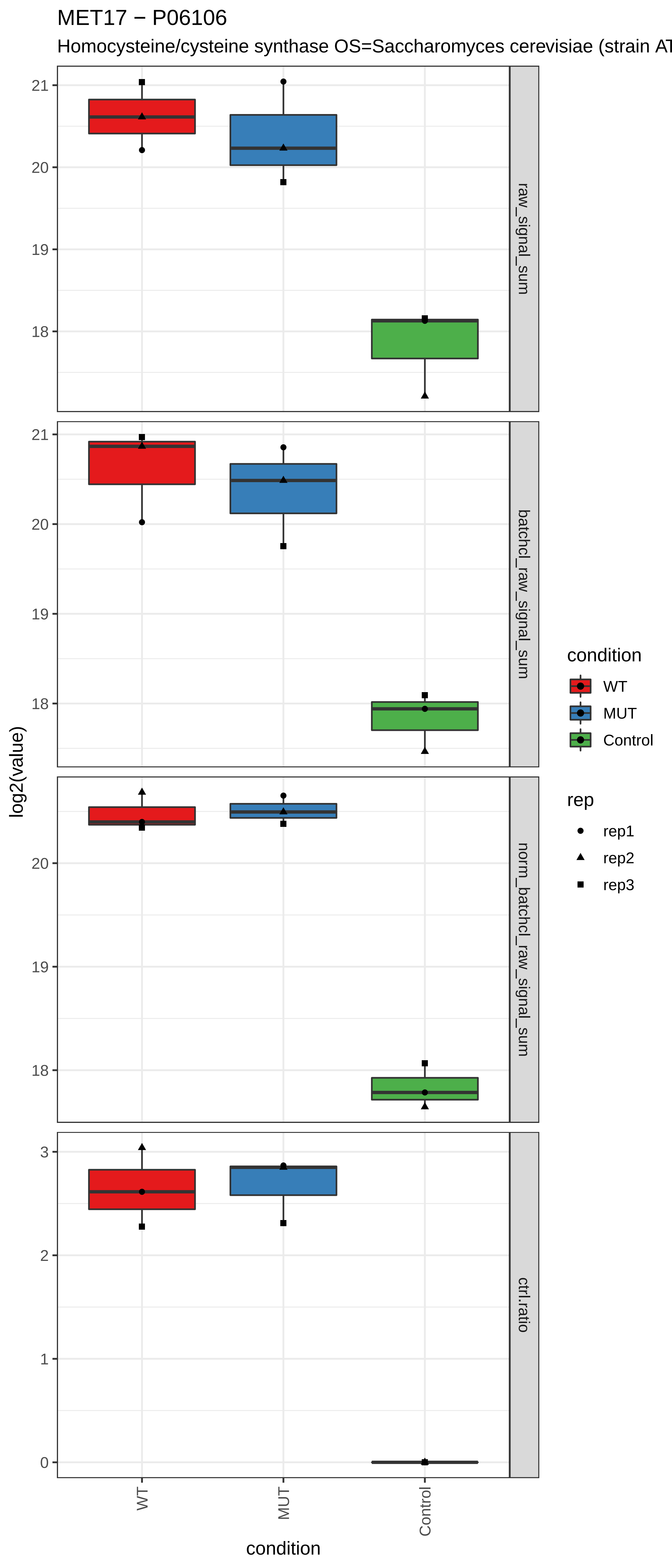
MET17 − P06106
Homocysteine/cysteine synthase OS=Saccharomyces cerevisiae (strain AT
raw_signal_sum
batchcl_raw_signal_sum
norm_batchcl_raw_signal_sum
ctrl.ratio
log2(value)
condition
WT
MUT
Control
rep
rep1
rep2
rep3
21
20
19
18
3
2
1
0

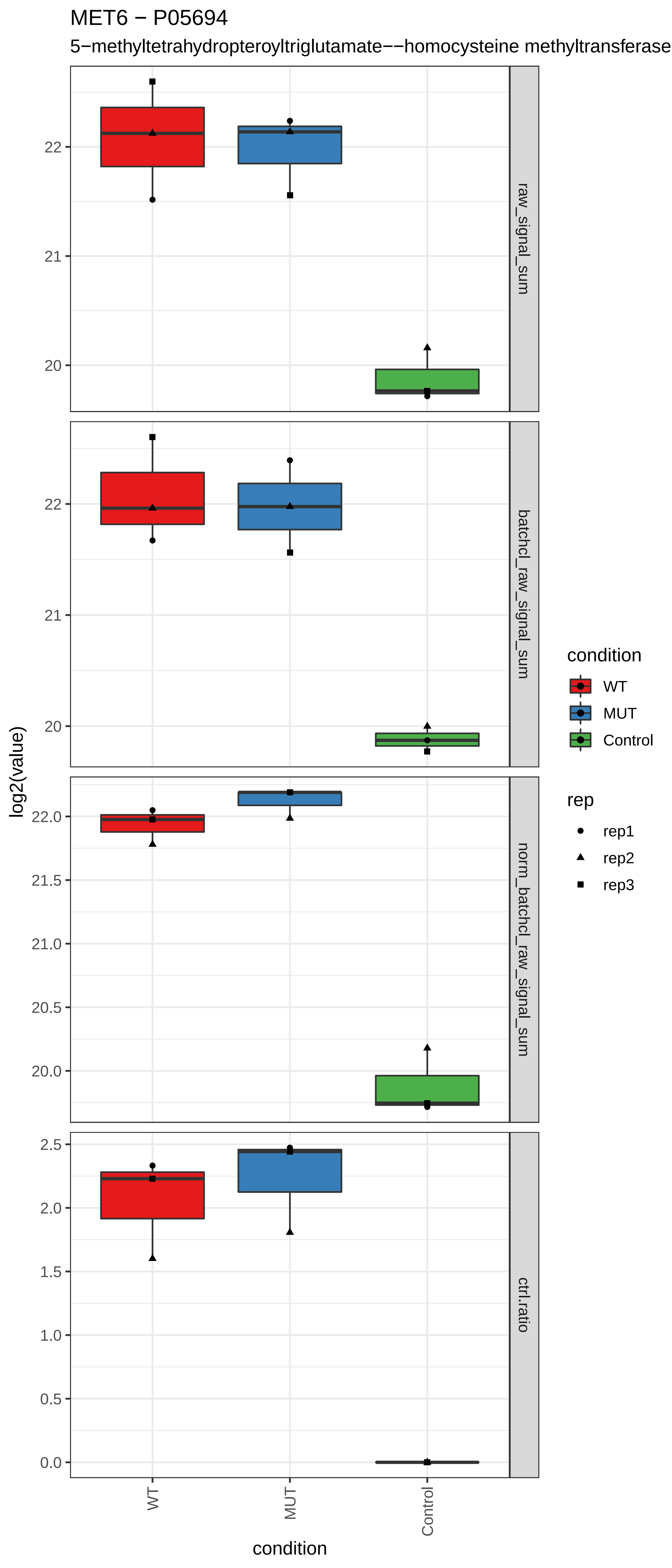

MET6 − P05694
5−methyltetrahydropteroyltriglutamate−−homocysteine methyltransferase
raw_signal_sum
batchcl_raw_signal_sum
norm_batchcl_raw_signal_sum
ctrl.ratio
log2(value)
condition
WT
MUT
Control
rep
rep1
rep2
rep3

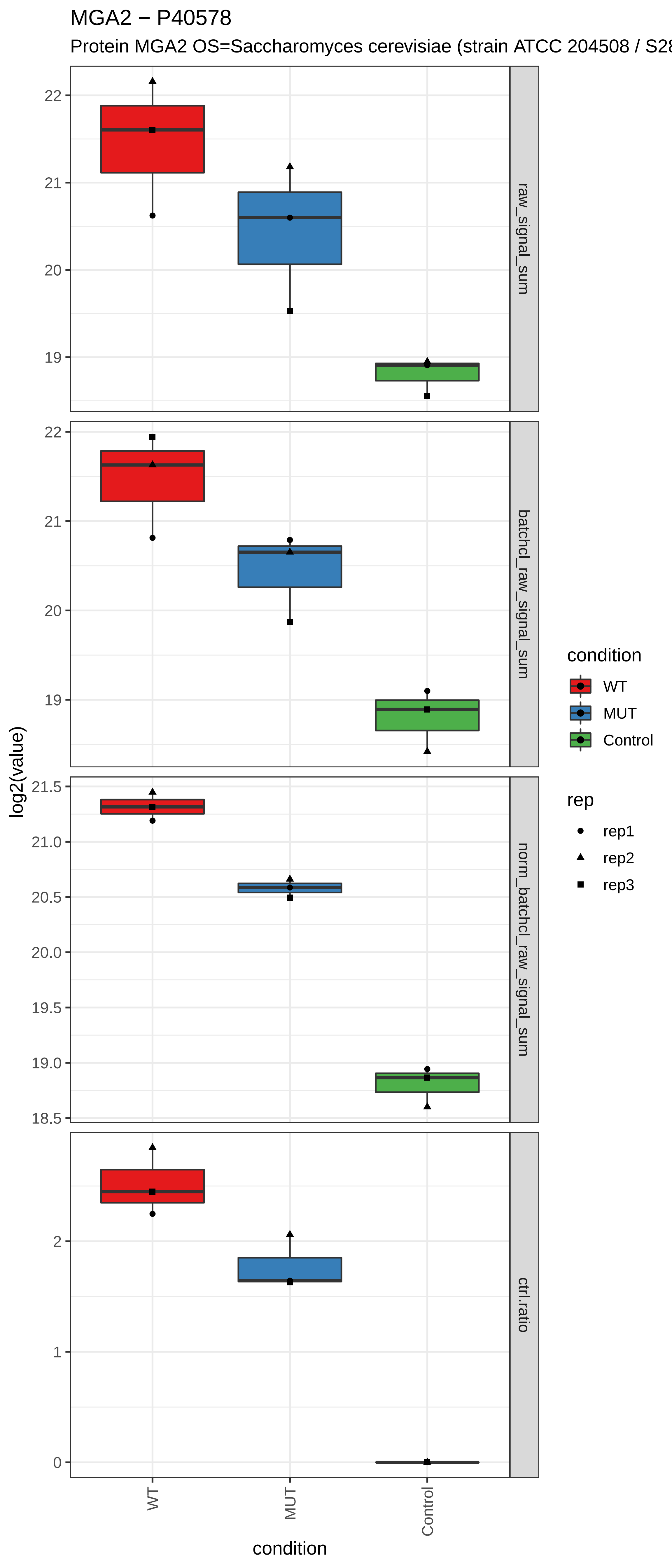

MGA2 − P40578
Protein MGA2 OS=Saccharomyces cerevisiae (strain ATCC 204508 / S28
raw_signal_sum
batchcl_raw_signal_sum
norm_batchcl_raw_signal_sum
ctrl.ratio
log2(value)
condition
WT
MUT
Control
rep
rep1
rep2
rep3

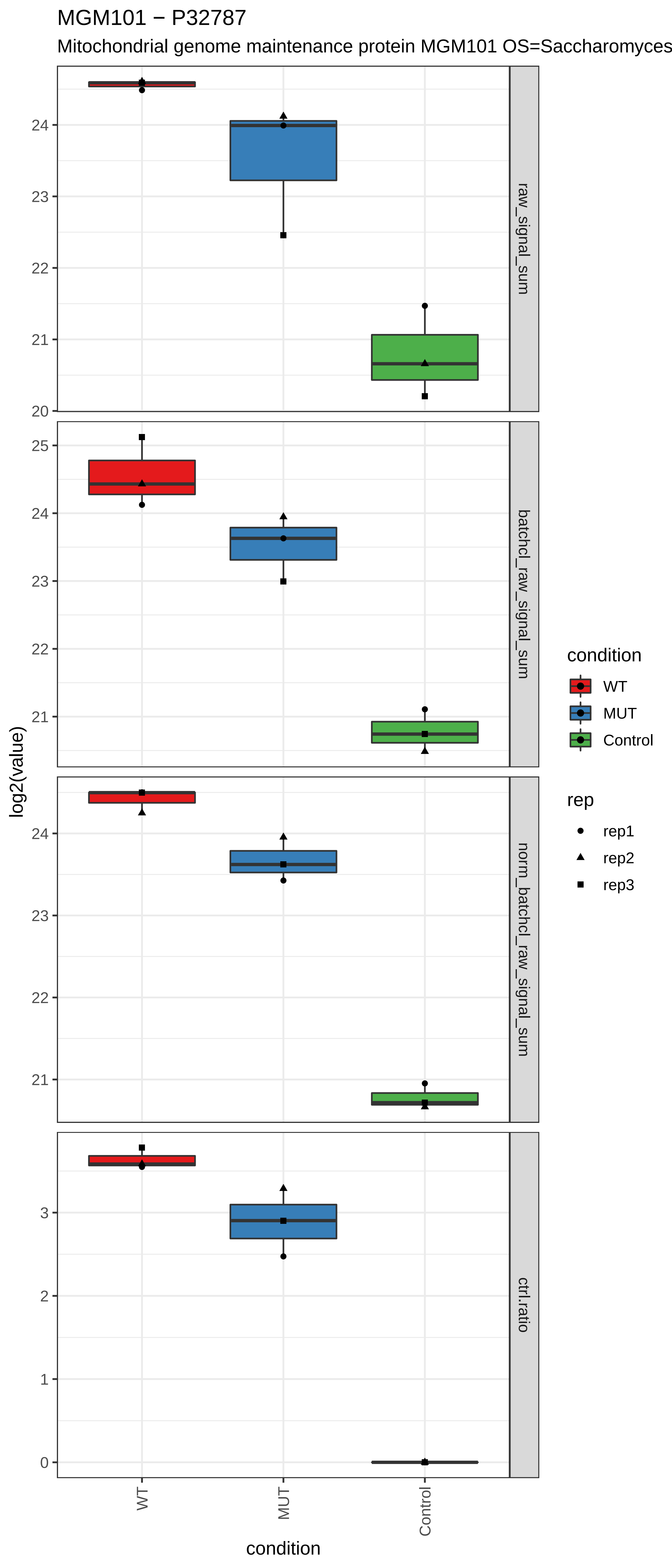

MGM101 − P32787
Mitochondrial genome maintenance protein MGM101 OS=Saccharomyces
raw_signal_sum
batchcl_raw_signal_sum
norm_batchcl_raw_signal_sum
ctrl.ratio
log2(value)
condition
WT
MUT
Control
rep
rep1
rep2
rep3

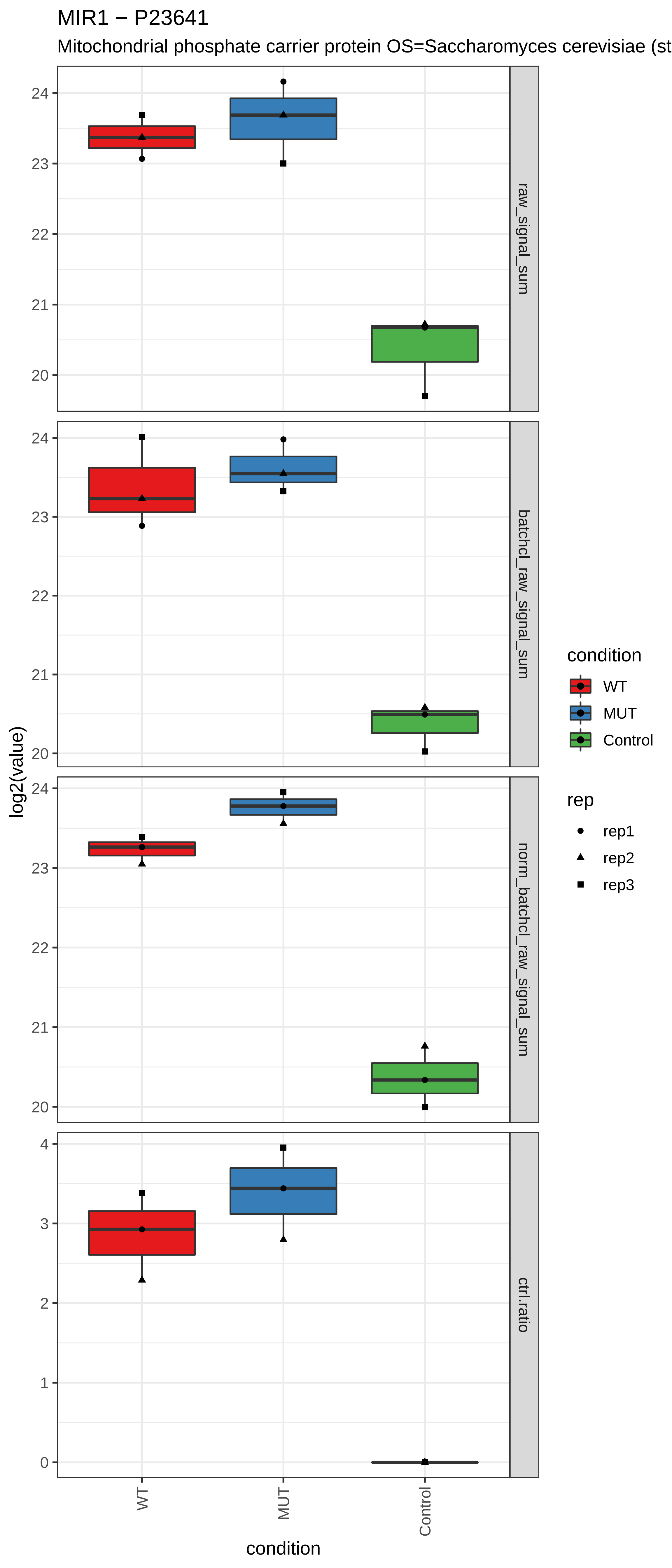
MIR1 − P23641
Mitochondrial phosphate carrier protein OS=Saccharomyces cerevisiae (st
raw_signal_sum
batchcl_raw_signal_sum
norm_batchcl_raw_signal_sum
ctrl.ratio
log2(value)
condition
WT
MUT
Control
rep
rep1
rep2
rep3
24
23
22
21
20
4
3
2
1
0

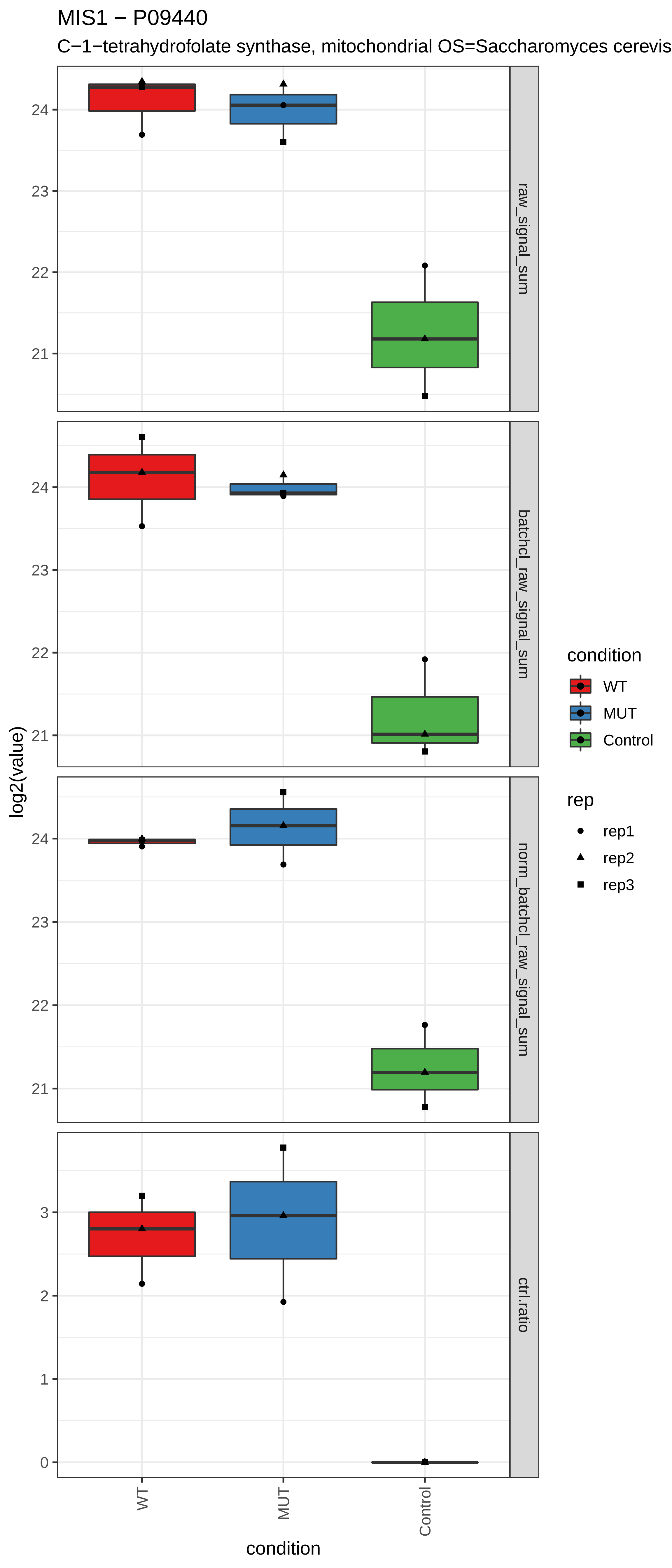
MIS1 − P09440
C−1−tetrahydrofolate synthase, mitochondrial OS=Saccharomyces cerevis
raw_signal_sum
batchcl_raw_signal_sum
norm_batchcl_raw_signal_sum
ctrl.ratio
log2(value)
condition
WT
MUT
Control
rep
rep1
rep2
rep3
24
23
22
21
3
2
1
0

MKT1 − P40850

Protein MKT1 OS=Saccharomyces cerevisiae (strain ATCC 204508 / S288

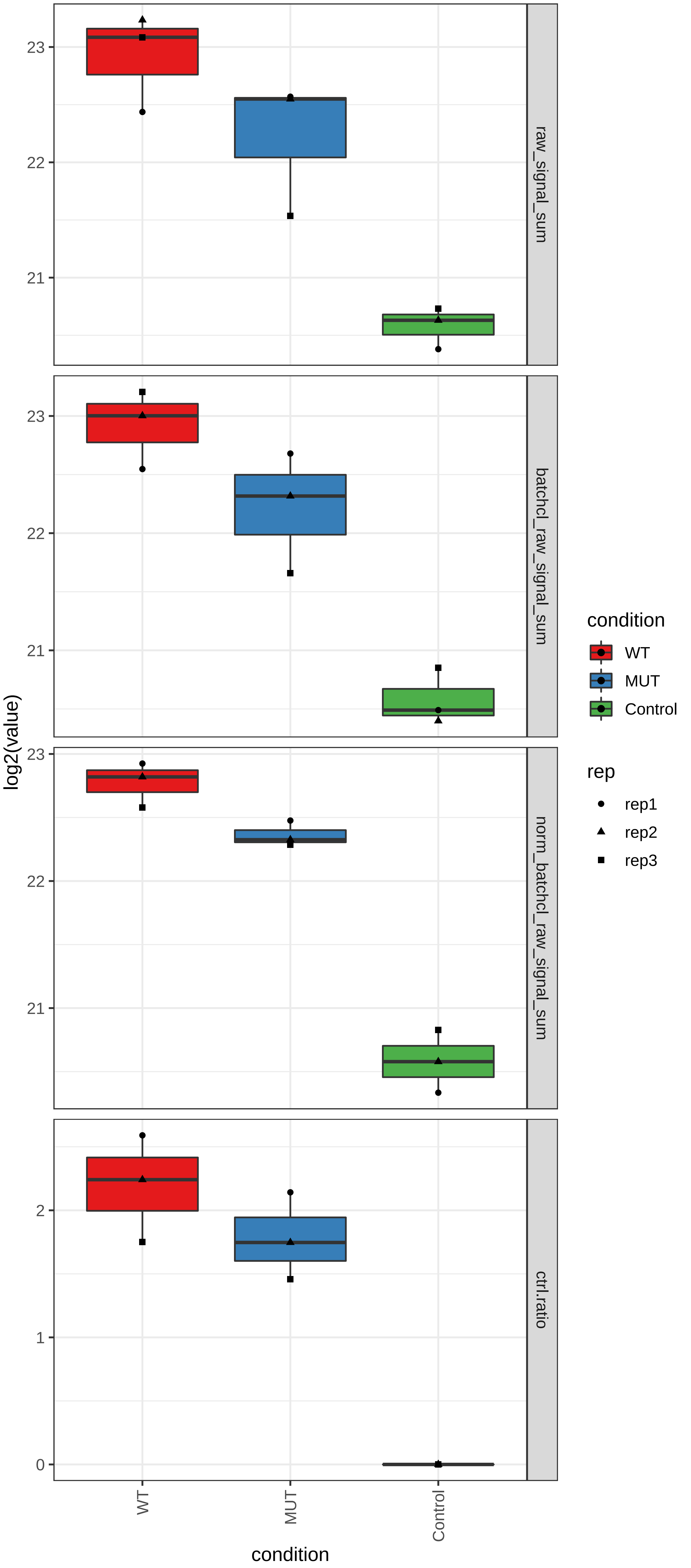

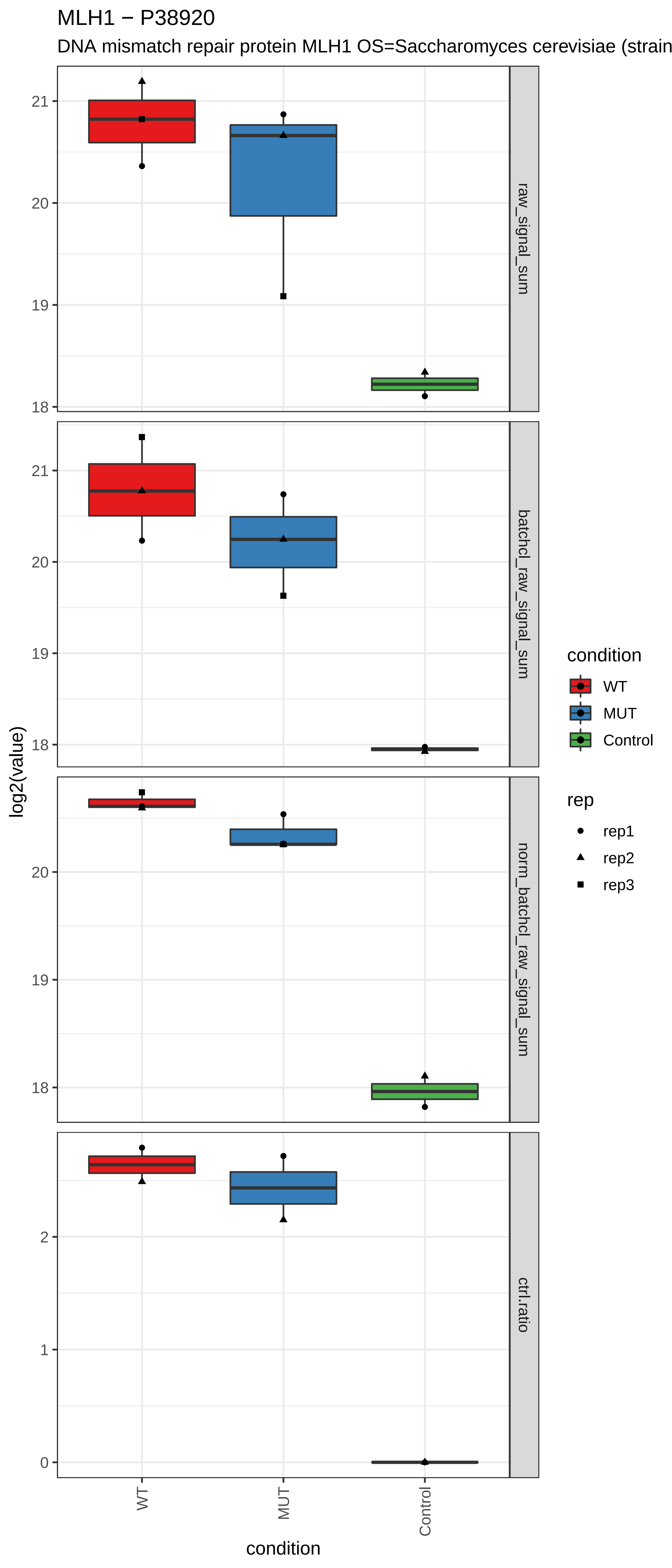
MLH1 − P38920
DNA mismatch repair protein MLH1 OS=Saccharomyces cerevisiae (strain
raw_signal_sum
batchcl_raw_signal_sum
norm_batchcl_raw_signal_sum
ctrl.ratio
log2(value)
condition
WT
MUT
Control
rep
rep1
rep2
rep3
21
20
19
18
2
1
0

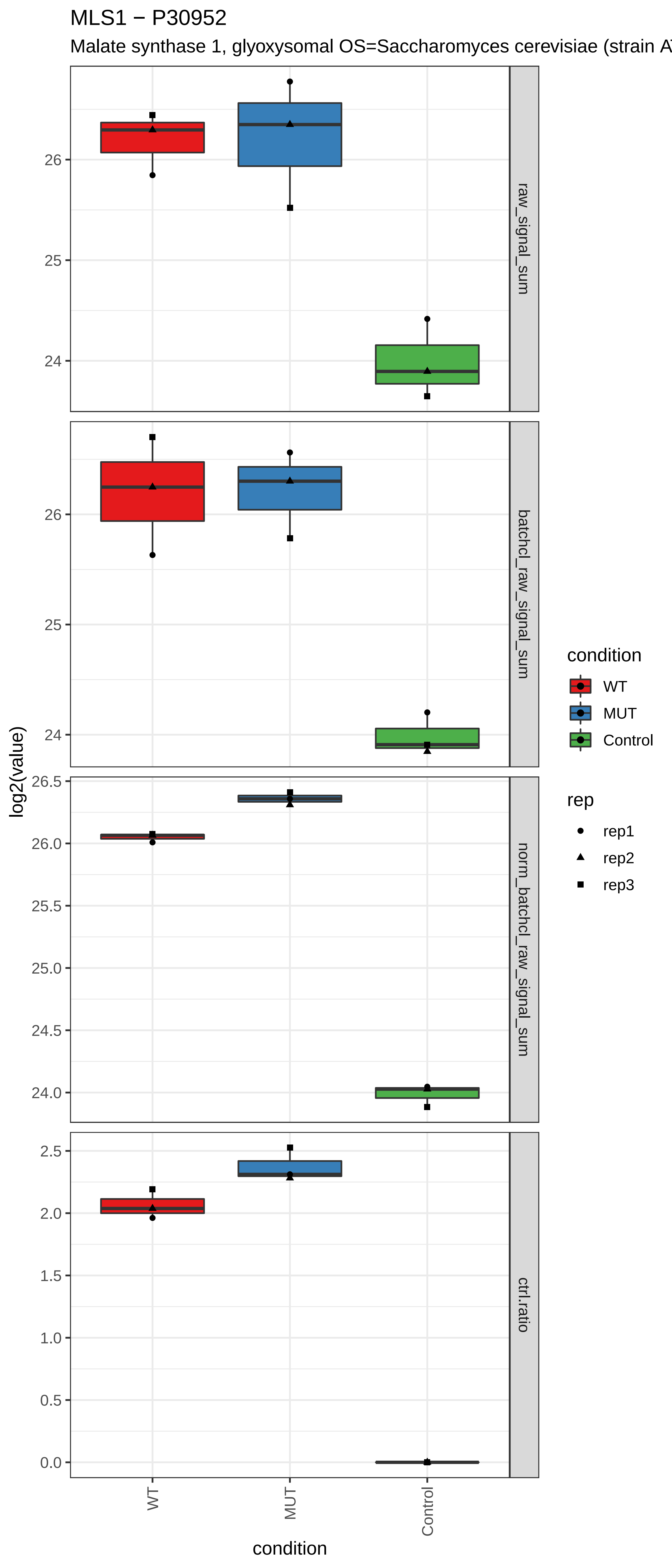
MLS1 − P30952
Malate synthase 1, glyoxysomal OS=Saccharomyces cerevisiae (strain A
raw_signal_sum
batchcl_raw_signal_sum
norm_batchcl_raw_signal_sum
ctrl.ratio
log2(value)
condition
WT
MUT
Control
rep
rep1
rep2
rep3
26
25
24
26.5
26.0
25.5
25.0
24.5
24.0
2.5
2.0
1.5
1.0
0.5
0.0

MND1 − P53102

Meiotic nuclear division protein 1 OS=Saccharomyces cerevisiae (strain AT

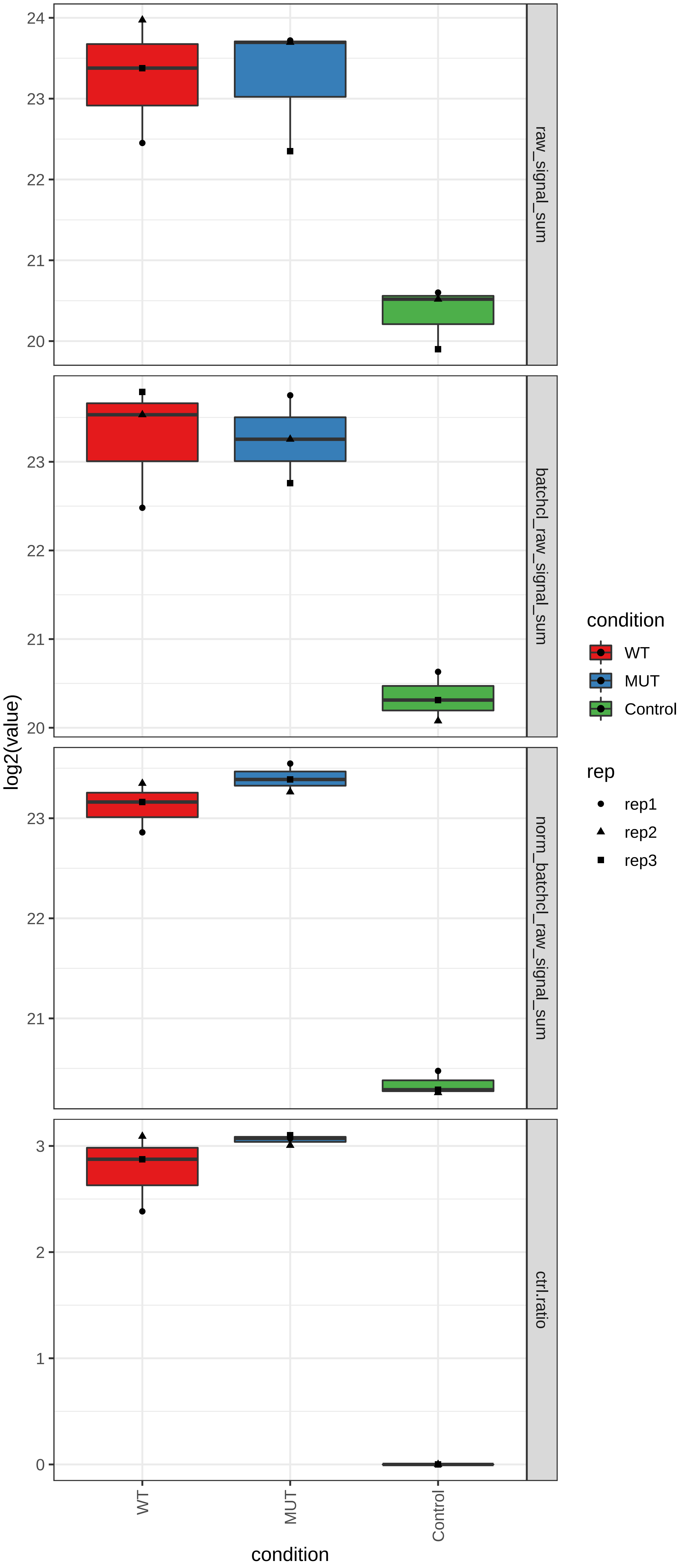

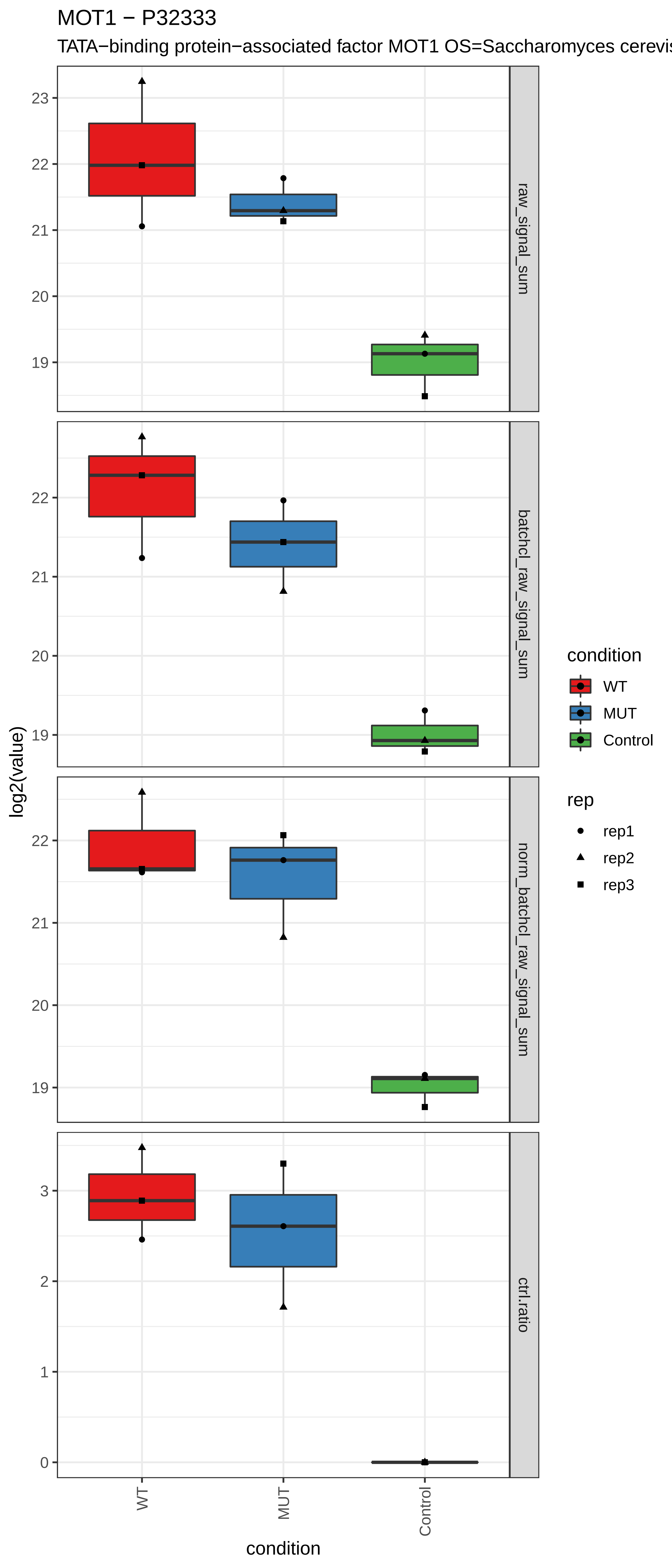

MOT1 − P32333
TATA−binding protein−associated factor MOT1 OS=Saccharomyces cerevis
raw_signal_sum
batchcl_raw_signal_sum
norm_batchcl_raw_signal_sum
ctrl.ratio
log2(value)
condition
WT
MUT
Control
rep
rep1
rep2
rep3
23
22
21
20
19
3
2
1
0

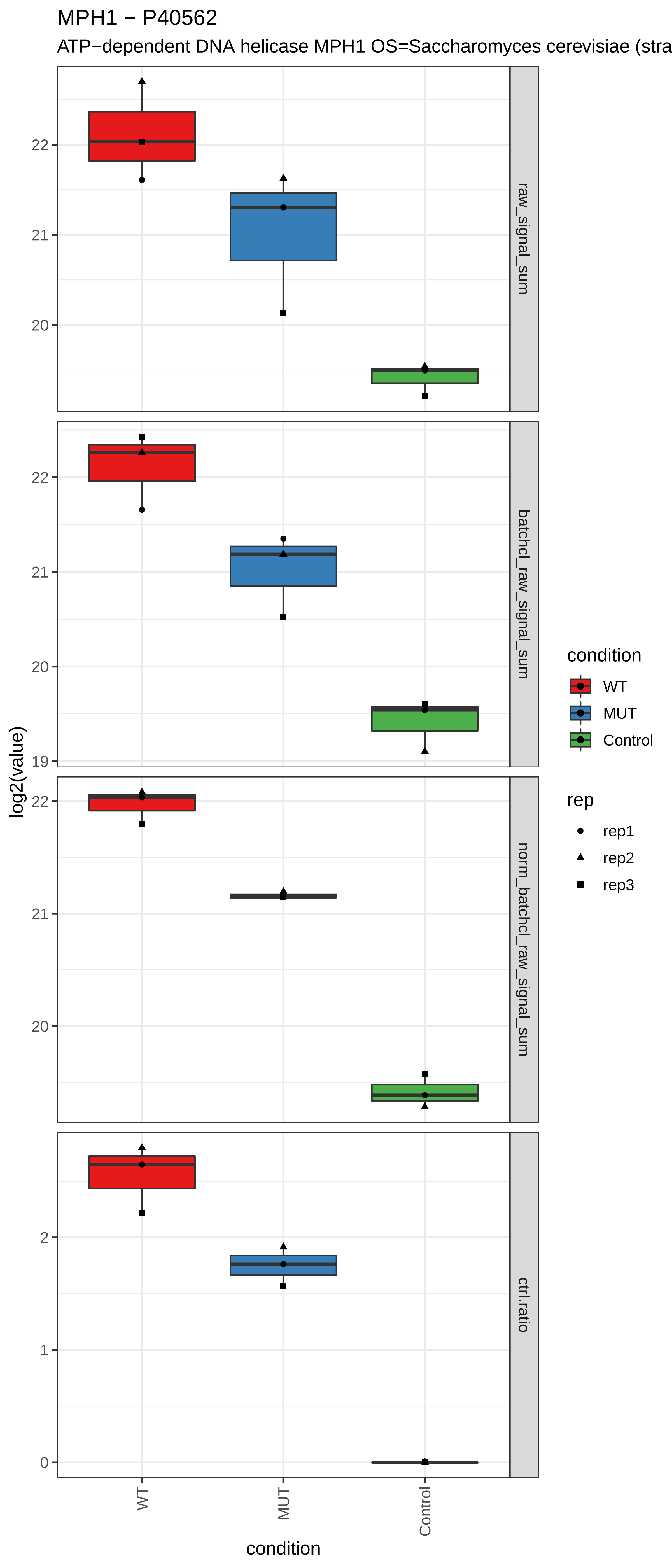

MPH1 − P40562
ATP−dependent DNA helicase MPH1 OS=Saccharomyces cerevisiae (stra
raw_signal_sum
batchcl_raw_signal_sum
norm_batchcl_raw_signal_sum
ctrl.ratio
log2(value)
condition
WT
MUT
Control
rep
rep1
rep2
rep3

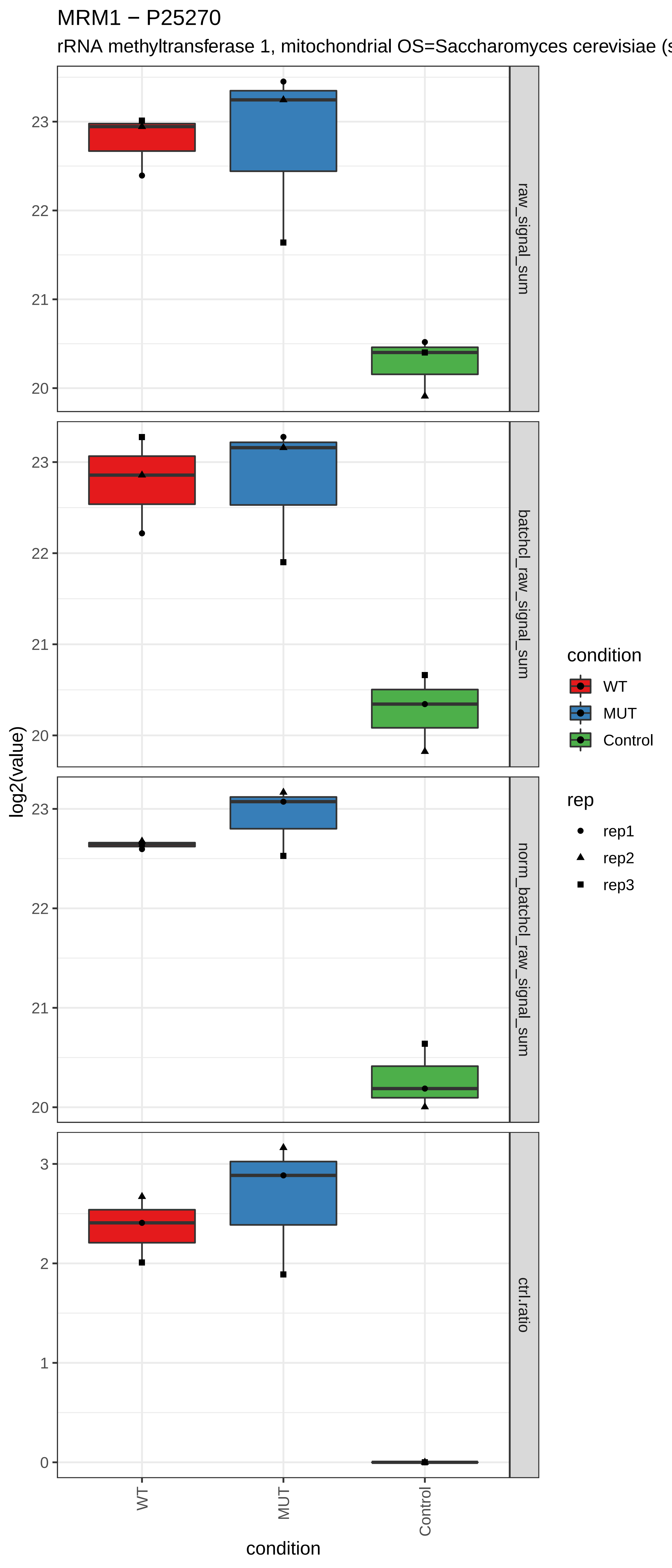
MRM1 − P25270
rRNA methyltransferase 1, mitochondrial OS=Saccharomyces cerevisiae (s
raw_signal_sum
batchcl_raw_signal_sum
norm_batchcl_raw_signal_sum
ctrl.ratio
log2(value)
condition
WT
MUT
Control
rep
rep1
rep2
rep3

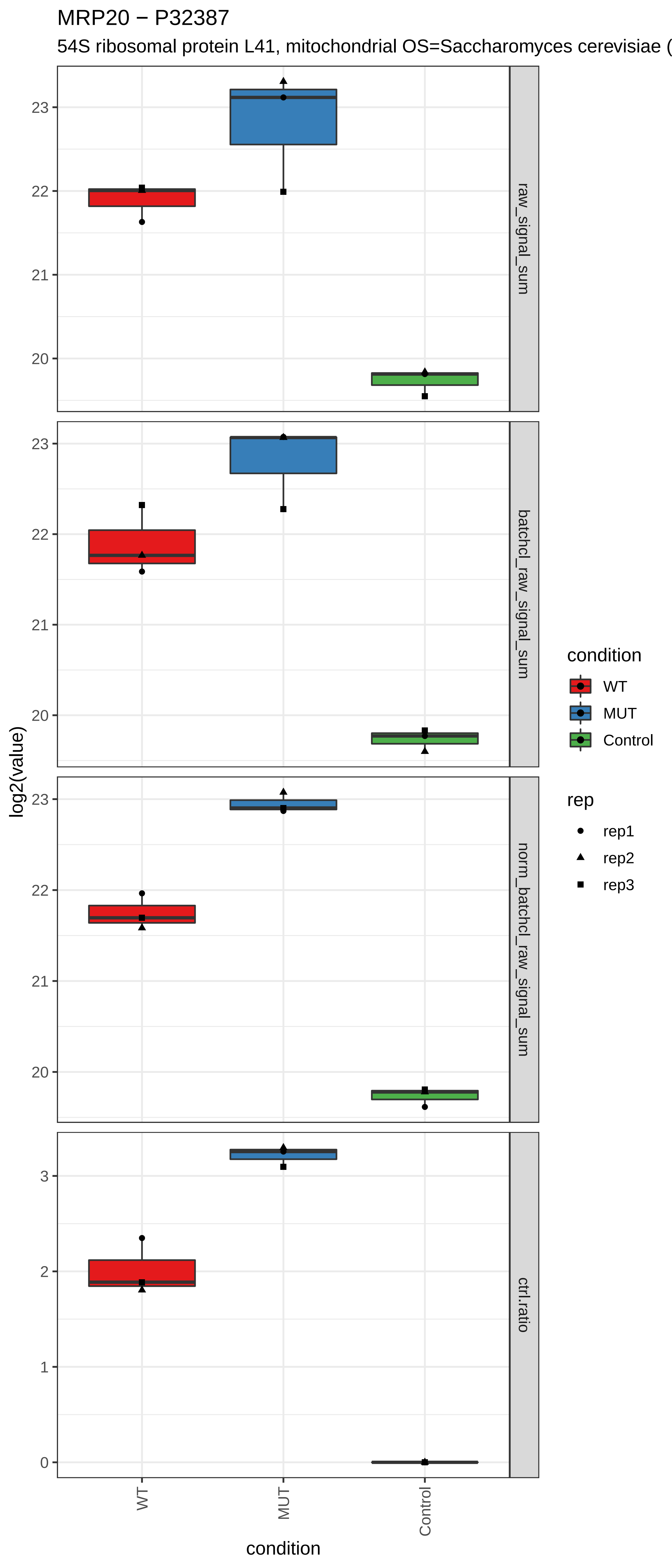

MRP20 − P32387
54S ribosomal protein L41, mitochondrial OS=Saccharomyces cerevisiae (
raw_signal_sum
batchcl_raw_signal_sum
norm_batchcl_raw_signal_sum
ctrl.ratio
log2(value)
condition
WT
MUT
Control
rep
rep1
rep2
rep3

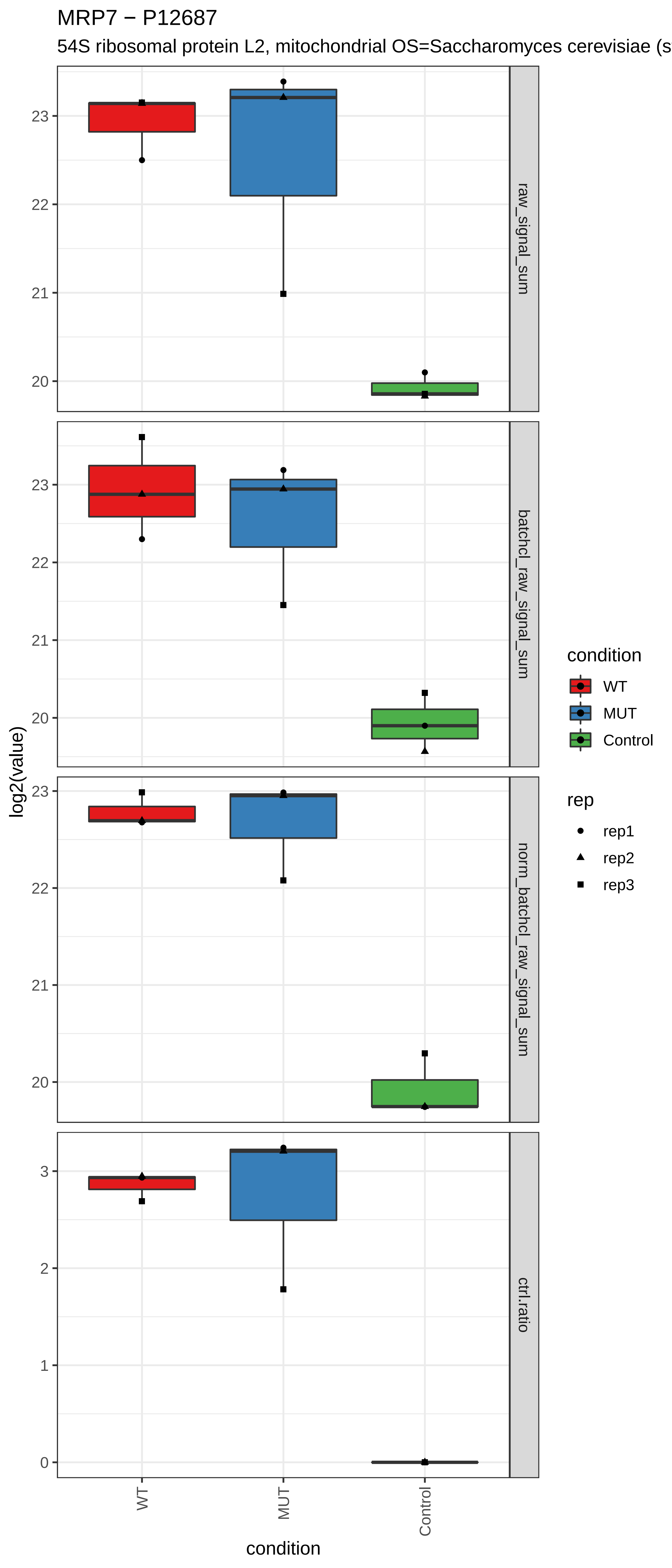

MRP7 − P12687
54S ribosomal protein L2, mitochondrial OS=Saccharomyces cerevisiae (s
raw_signal_sum
batchcl_raw_signal_sum
norm_batchcl_raw_signal_sum
ctrl.ratio
log2(value)
condition
WT
MUT
Control
rep
rep1
rep2
rep3

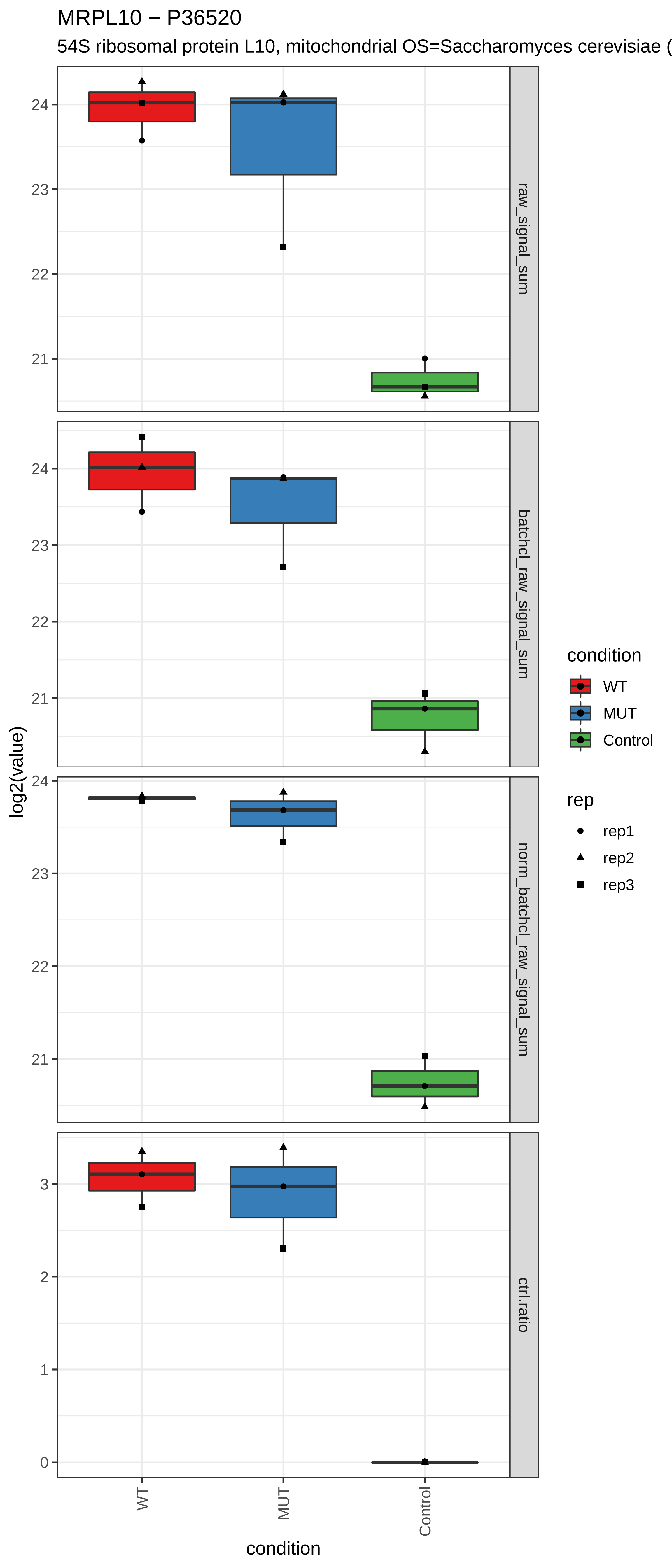

MRPL10 − P36520
54S ribosomal protein L10, mitochondrial OS=Saccharomyces cerevisiae (
raw_signal_sum
batchcl_raw_signal_sum
norm_batchcl_raw_signal_sum
ctrl.ratio
log2(value)
condition
WT
MUT
Control
rep
rep1
rep2
rep3
24
23
22
21
3
2
1
0

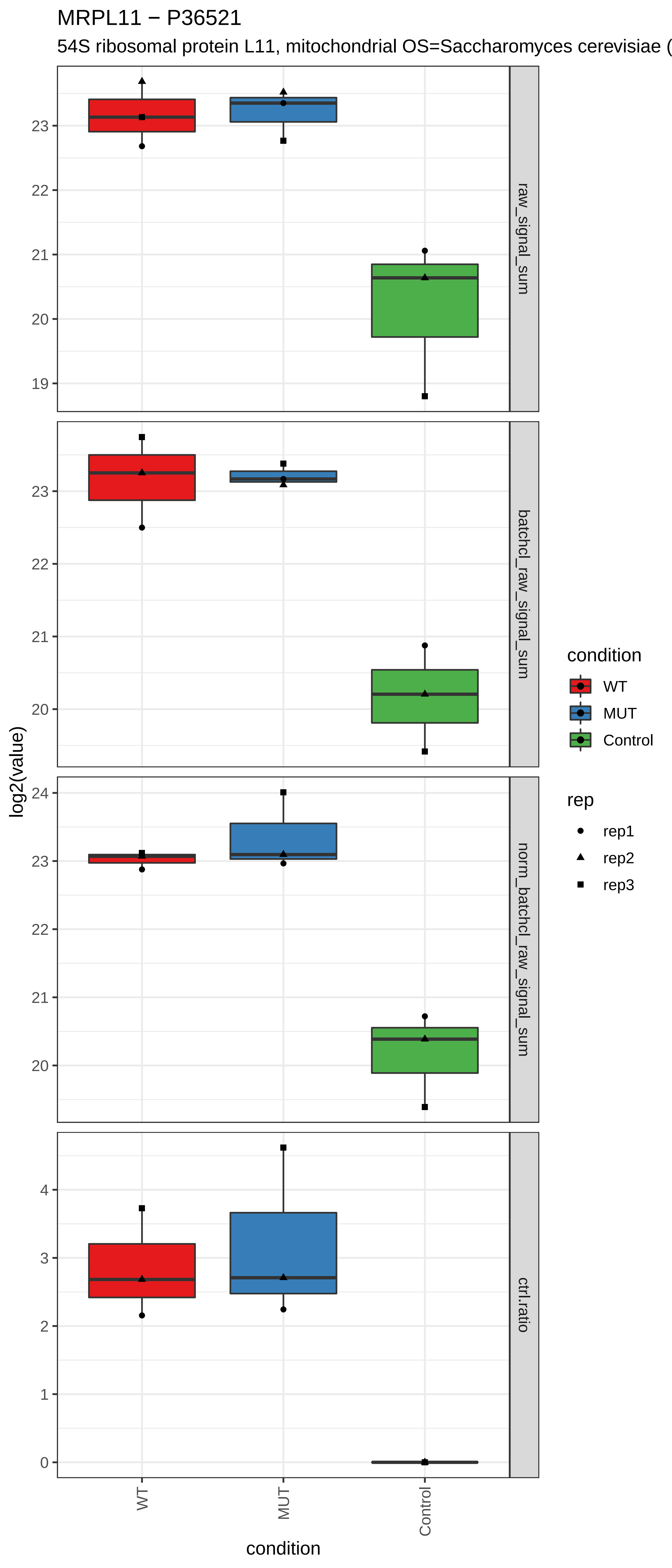

MRPL11 − P36521
54S ribosomal protein L11, mitochondrial OS=Saccharomyces cerevisiae (
raw_signal_sum
batchcl_raw_signal_sum
norm_batchcl_raw_signal_sum
ctrl.ratio
log2(value)
condition
WT
MUT
Control
rep
rep1
rep2
rep3

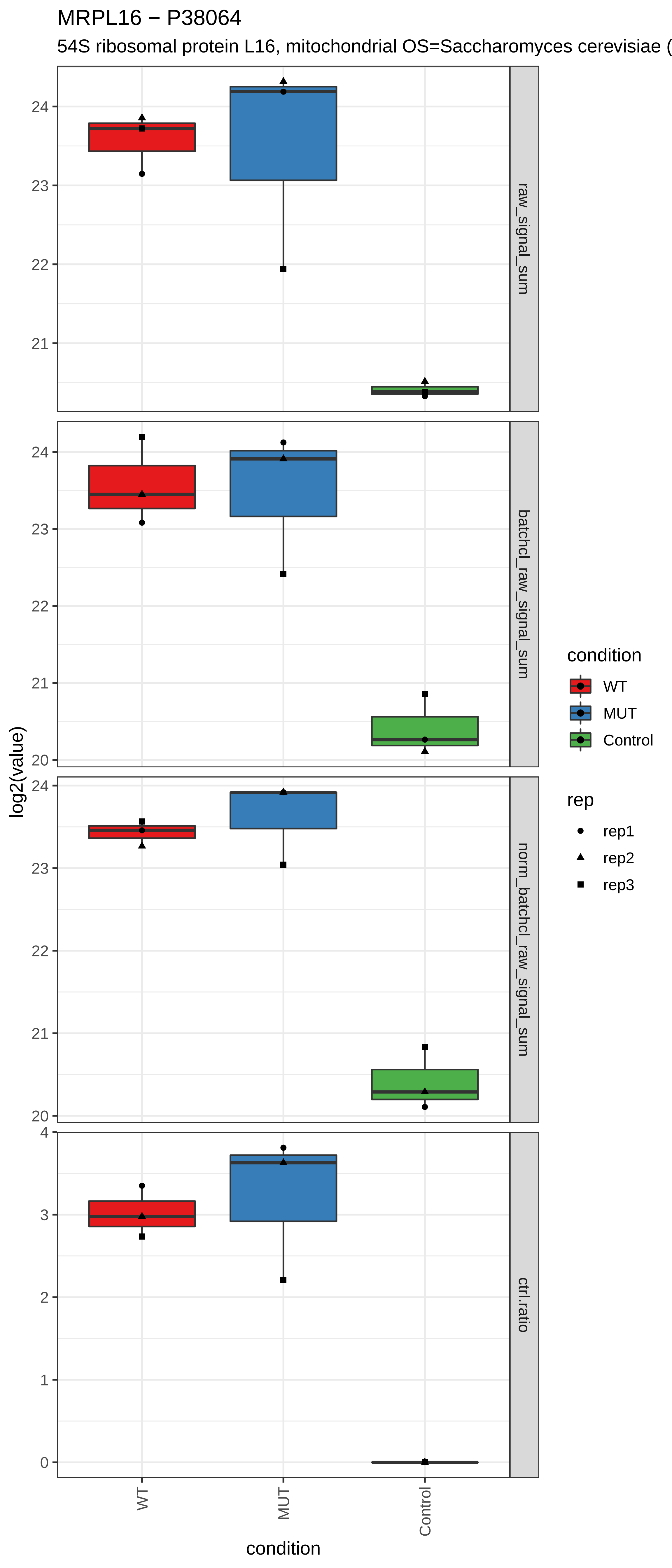
MRPL16 − P38064
54S ribosomal protein L16, mitochondrial OS=Saccharomyces cerevisiae (
raw_signal_sum
batchcl_raw_signal_sum
norm_batchcl_raw_signal_sum
ctrl.ratio
log2(value)
condition
WT
MUT
Control
rep
rep1
rep2
rep3

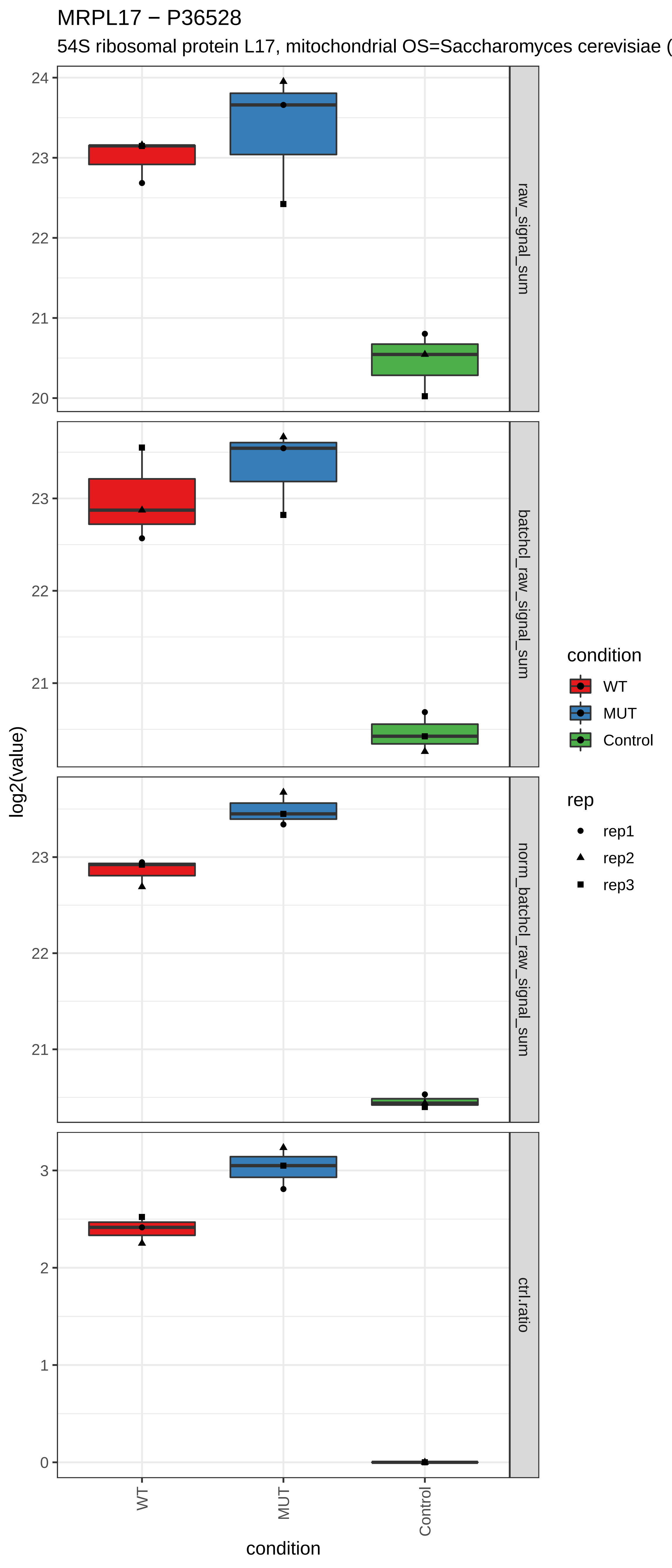

MRPL17 − P36528
54S ribosomal protein L17, mitochondrial OS=Saccharomyces cerevisiae (
raw_signal_sum
batchcl_raw_signal_sum
norm_batchcl_raw_signal_sum
ctrl.ratio
log2(value)
condition
WT
MUT
Control
rep
rep1
rep2
rep3
24
23
22
21
20
3
2
1
0

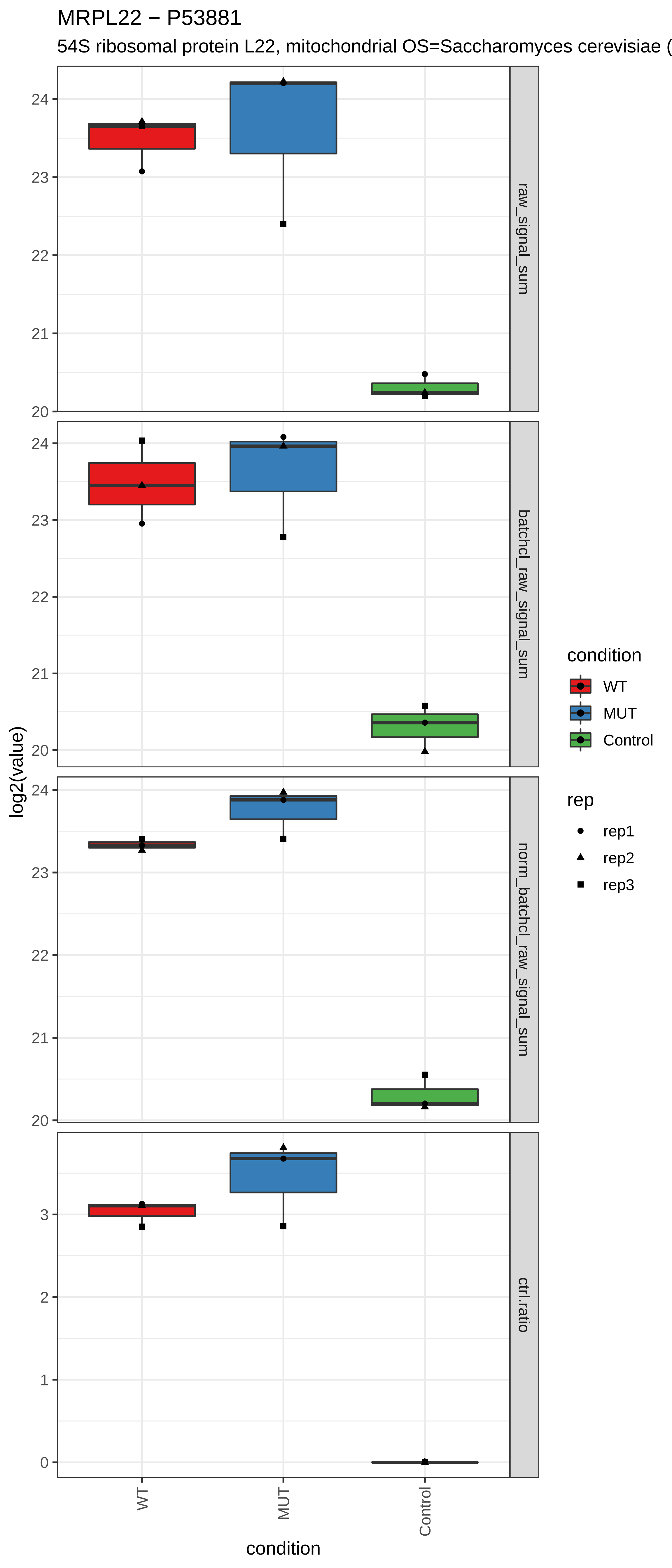
MRPL22 − P53881
54S ribosomal protein L22, mitochondrial OS=Saccharomyces cerevisiae (
raw_signal_sum
batchcl_raw_signal_sum
norm_batchcl_raw_signal_sum
ctrl.ratio
log2(value)
condition
WT
MUT
Control
rep
rep1
rep2
rep3
24
23
22
21
20
3
2
1
0

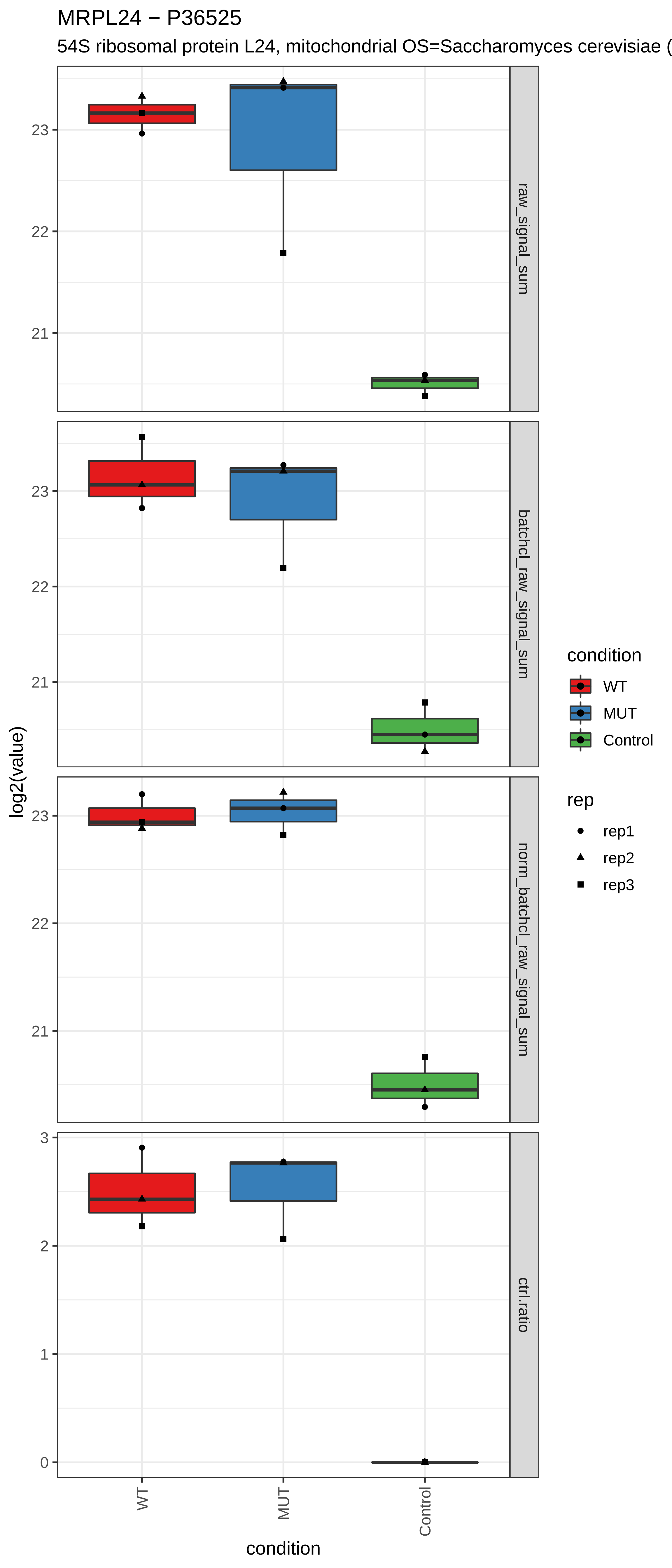
MRPL24 − P36525
54S ribosomal protein L24, mitochondrial OS=Saccharomyces cerevisiae (
raw_signal_sum
batchcl_raw_signal_sum
norm_batchcl_raw_signal_sum
ctrl.ratio
log2(value)
condition
WT
MUT
Control
rep
rep1
rep2
rep3
23
22
21
3
2
1
0

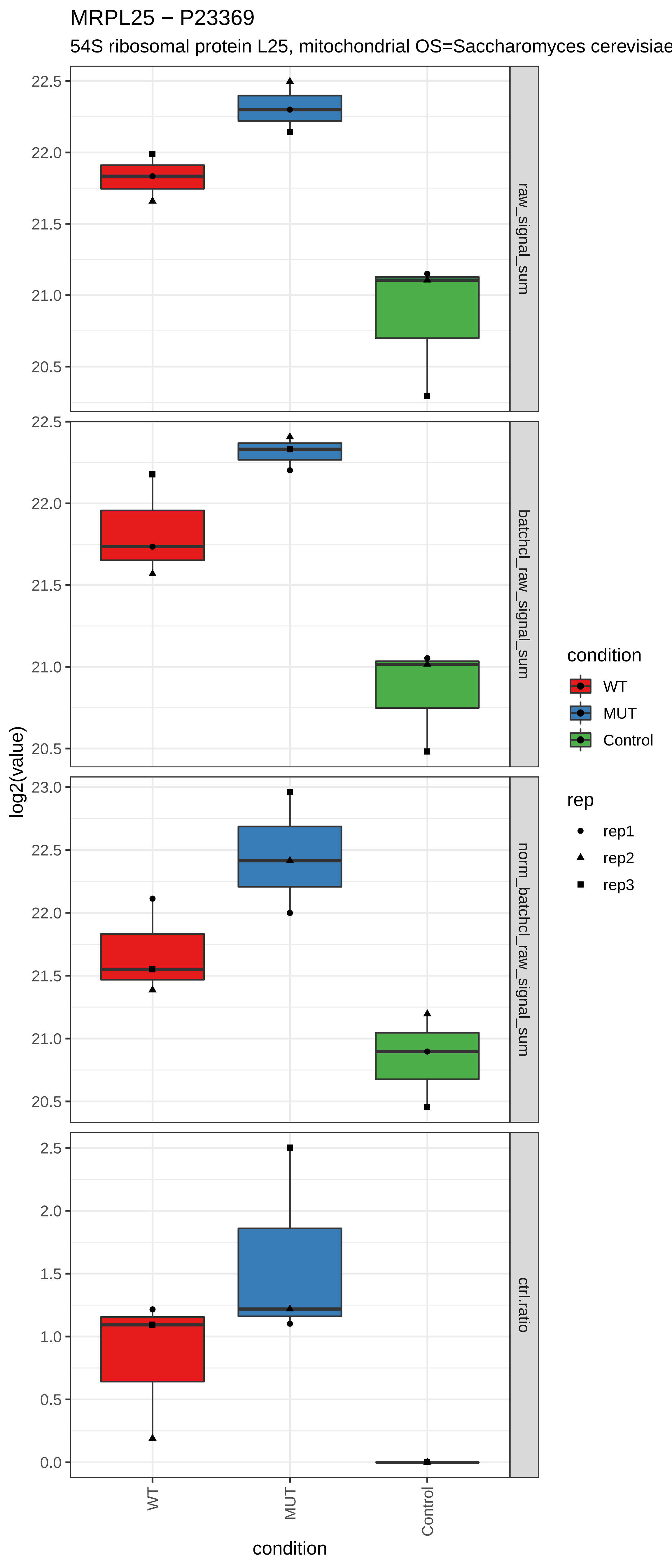

MRPL25 − P23369
54S ribosomal protein L25, mitochondrial OS=Saccharomyces cerevisiae
raw_signal_sum
batchcl_raw_signal_sum
norm_batchcl_raw_signal_sum
ctrl.ratio
log2(value)
condition
WT
MUT
Control
rep
rep1
rep2
rep3

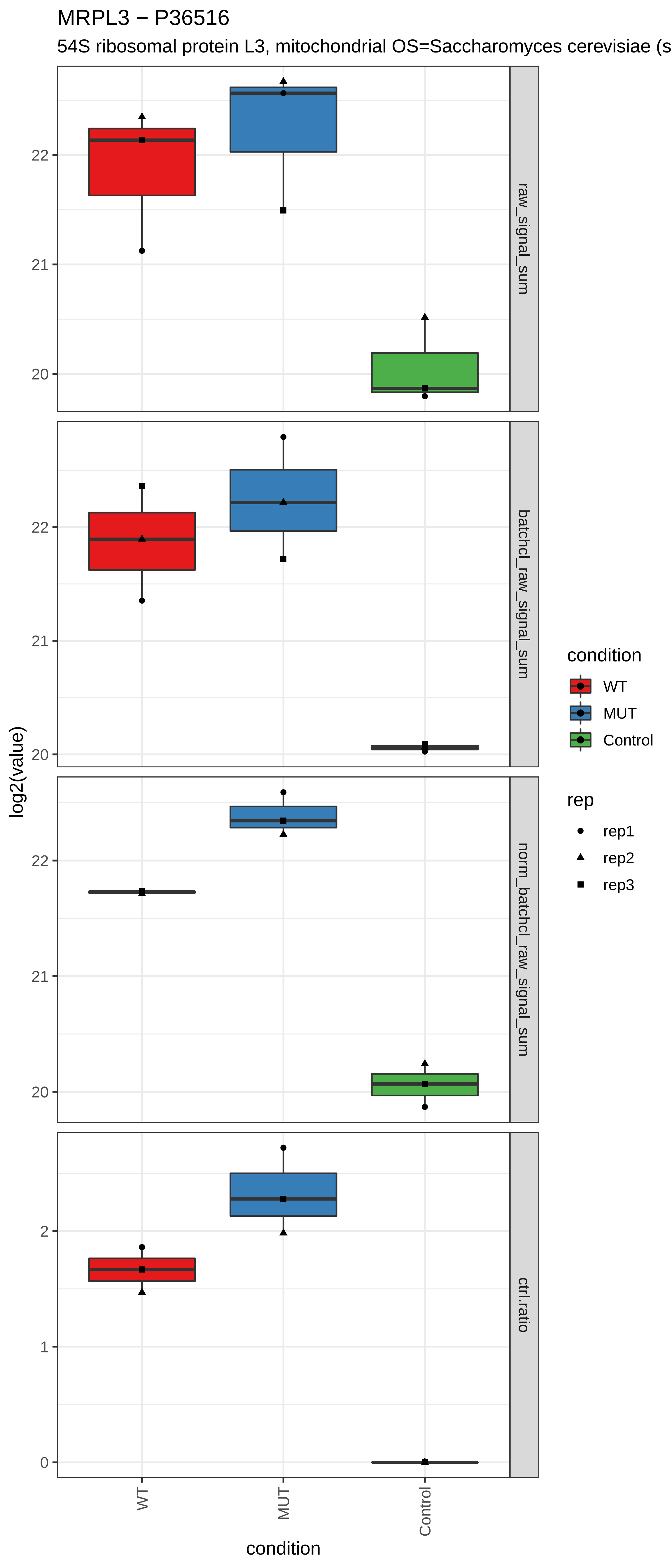

MRPL3 − P36516
54S ribosomal protein L3, mitochondrial OS=Saccharomyces cerevisiae (s
raw_signal_sum
batchcl_raw_signal_sum
norm_batchcl_raw_signal_sum
ctrl.ratio
log2(value)
condition
WT
MUT
Control
rep
rep1
rep2
rep3

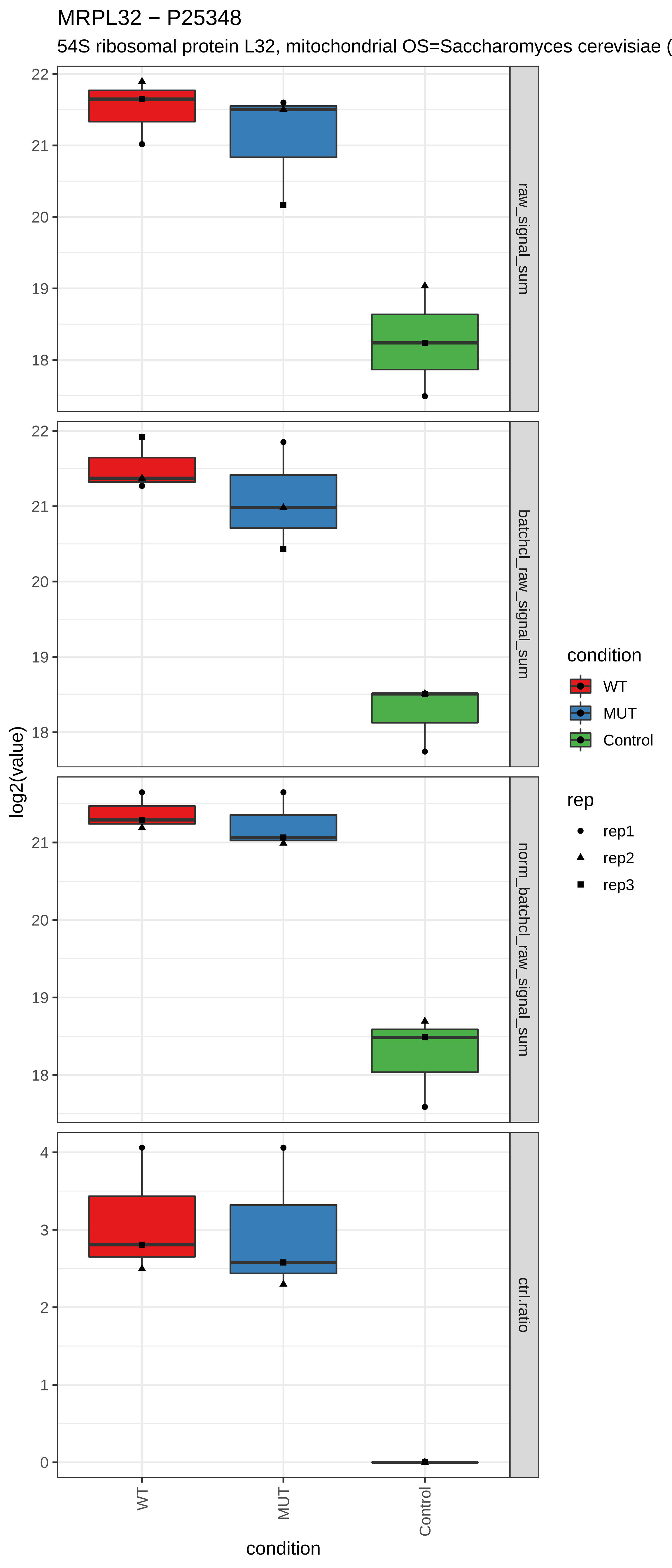

MRPL32 − P25348
54S ribosomal protein L32, mitochondrial OS=Saccharomyces cerevisiae (
raw_signal_sum
batchcl_raw_signal_sum
norm_batchcl_raw_signal_sum
ctrl.ratio
log2(value)
condition
WT
MUT
Control
rep
rep1
rep2
rep3
22
21
20
19
18
4
3
2
1
0

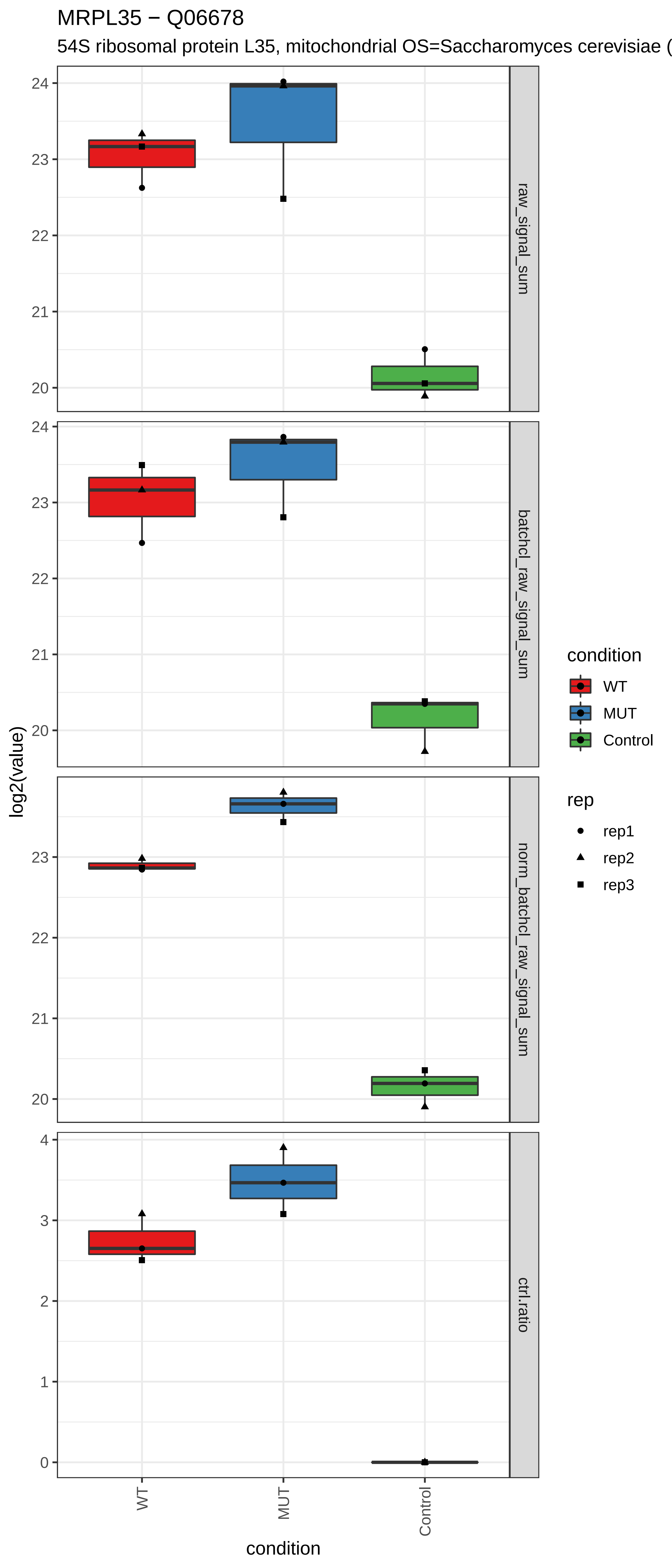
MRPL35 − Q06678
54S ribosomal protein L35, mitochondrial OS=Saccharomyces cerevisiae (
raw_signal_sum
batchcl_raw_signal_sum
norm_batchcl_raw_signal_sum
ctrl.ratio
log2(value)
condition
WT
MUT
Control
rep
rep1
rep2
rep3

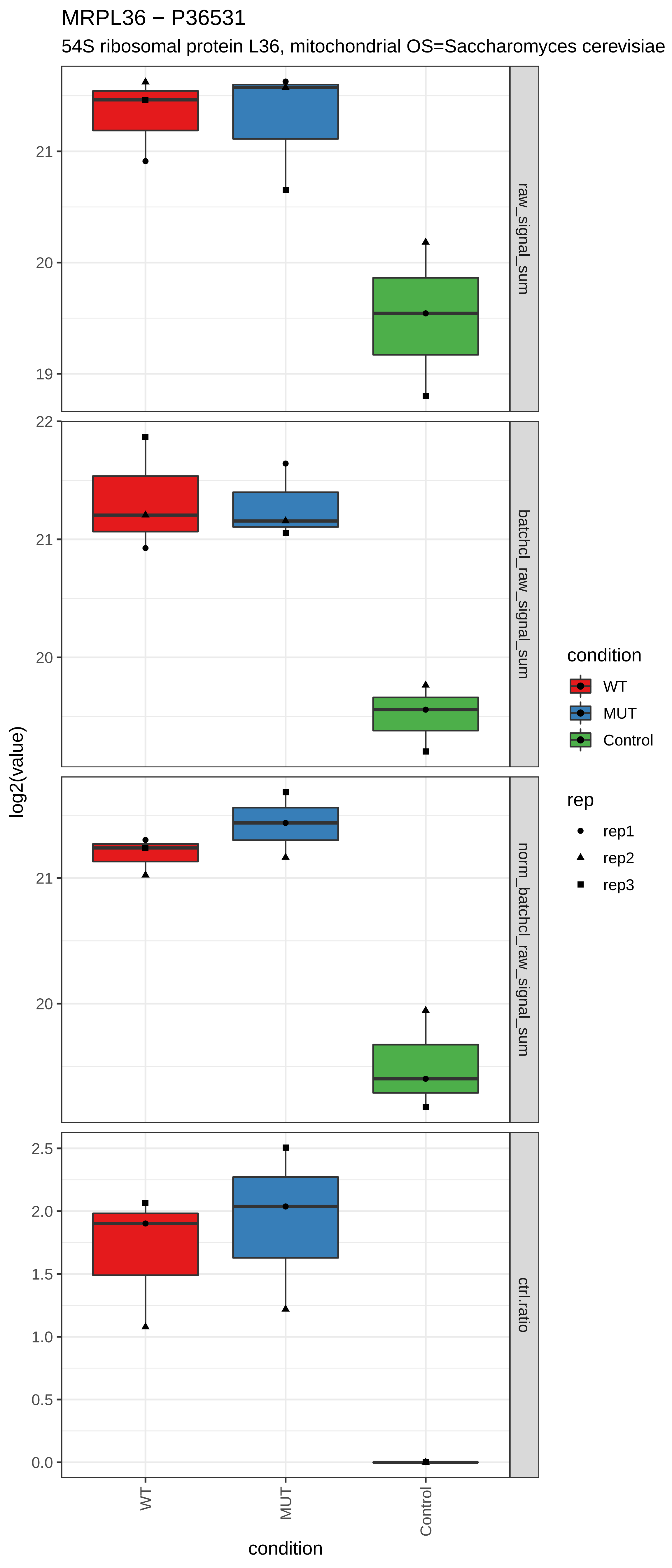
MRPL36 − P36531
54S ribosomal protein L36, mitochondrial OS=Saccharomyces cerevisiae
raw_signal_sum
batchcl_raw_signal_sum
norm_batchcl_raw_signal_sum
ctrl.ratio
log2(value)
condition
WT
MUT
Control
rep
rep1
rep2
rep3
21
20
19
22
2.5
2.0
1.5
1.0
0.5
0.0

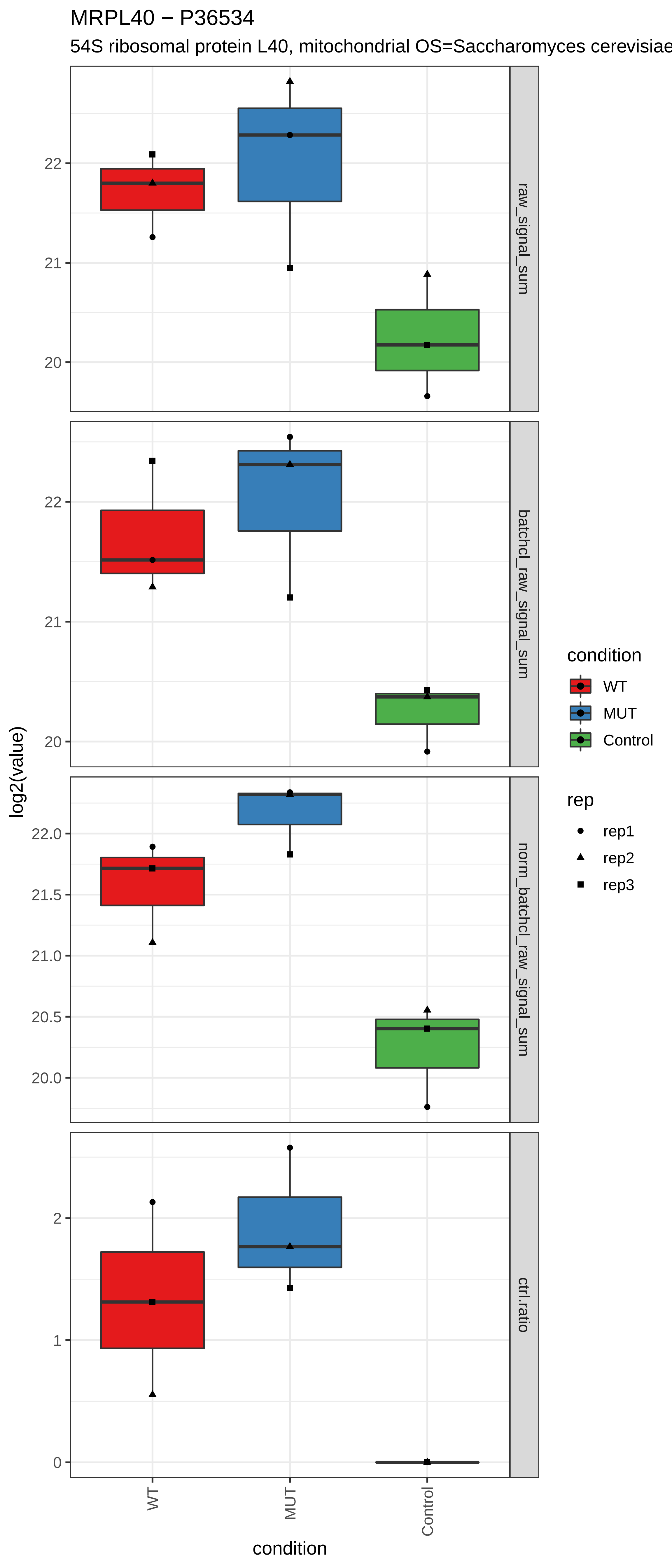

MRPL40 − P36534
54S ribosomal protein L40, mitochondrial OS=Saccharomyces cerevisiae
raw_signal_sum
batchcl_raw_signal_sum
norm_batchcl_raw_signal_sum
ctrl.ratio
log2(value)
22
21
20
22.0
21.5
21.0
20.5
20.0
2
1
0
WT
MUT
Control
condition
condition
WT
MUT
Control
rep
rep1
rep2
rep3

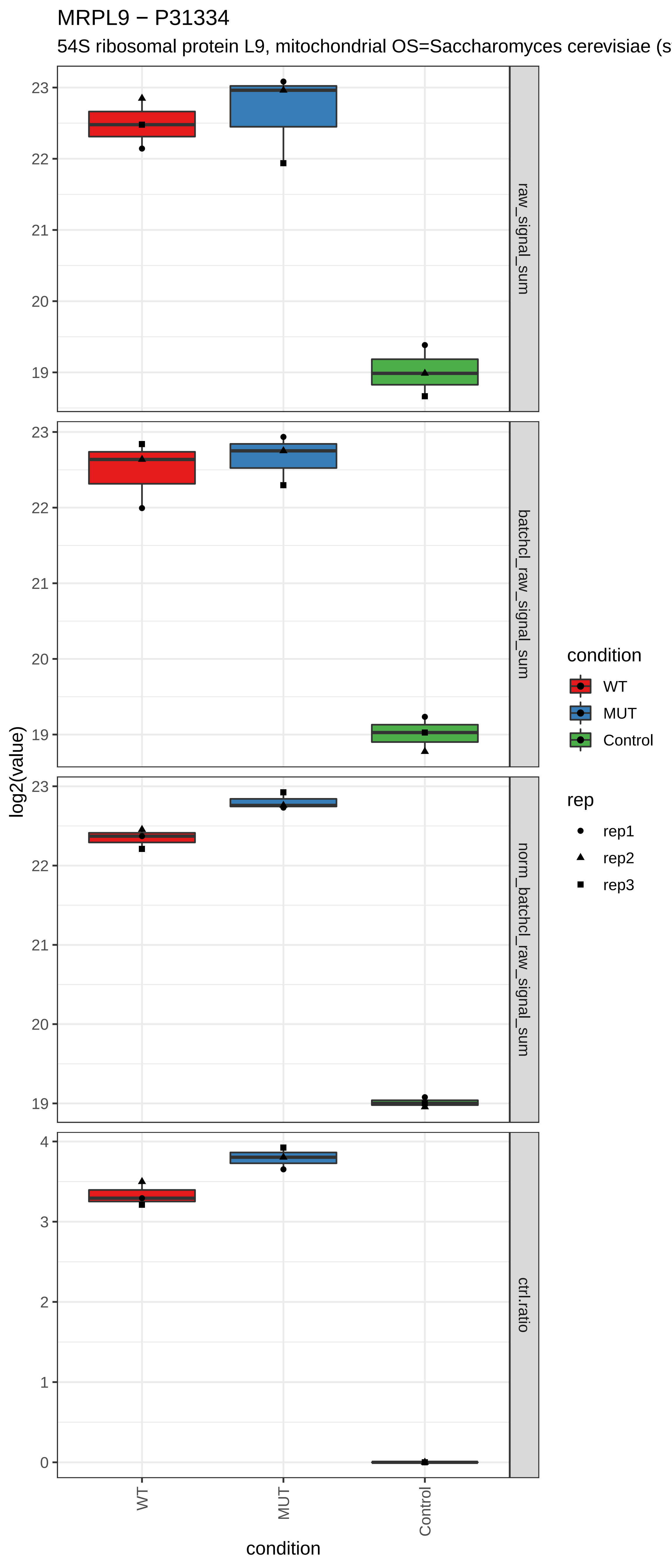

MRPL9 − P31334
54S ribosomal protein L9, mitochondrial OS=Saccharomyces cerevisiae (s
raw_signal_sum
batchcl_raw_signal_sum
norm_batchcl_raw_signal_sum
ctrl.ratio
log2(value)
condition
WT
MUT
Control
rep
rep1
rep2
rep3

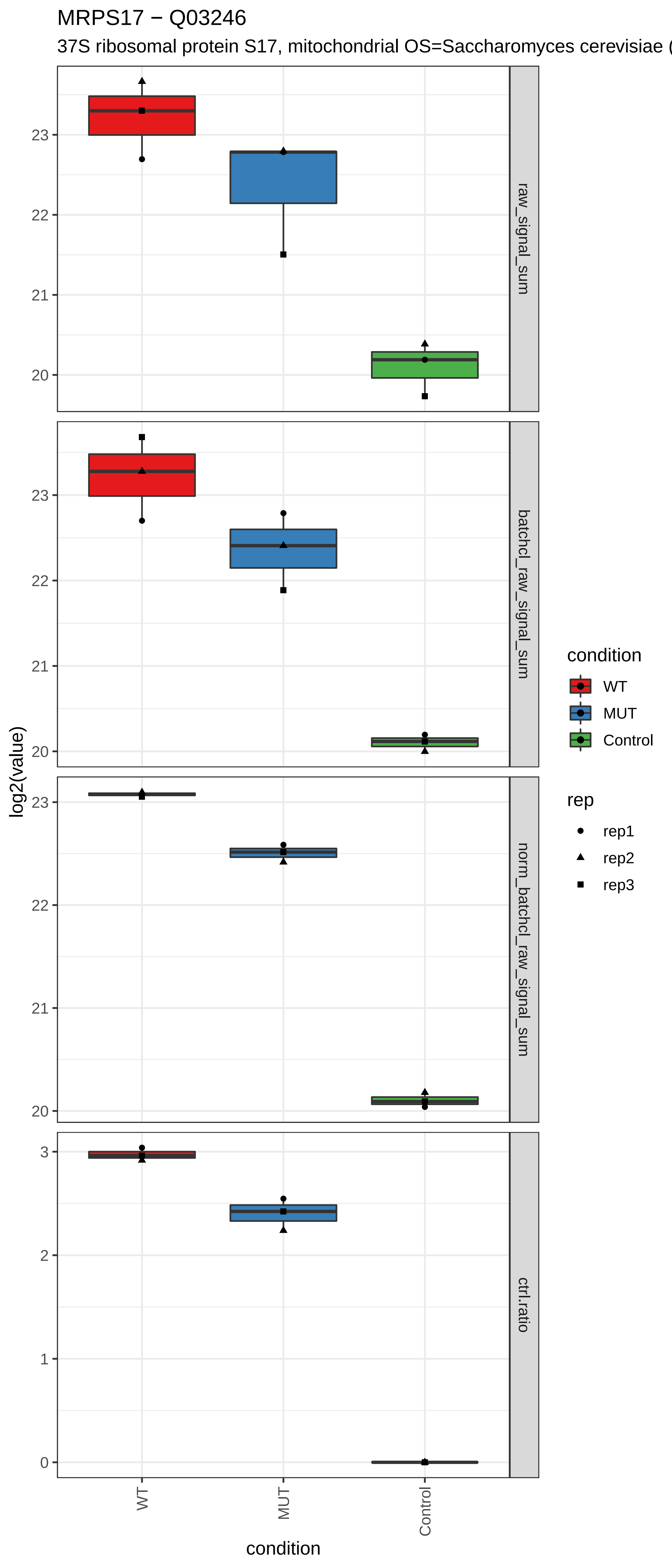

MRPS17 − Q03246
37S ribosomal protein S17, mitochondrial OS=Saccharomyces cerevisiae (
raw_signal_sum
batchcl_raw_signal_sum
norm_batchcl_raw_signal_sum
ctrl.ratio
log2(value)
condition
WT
MUT
Control
rep
rep1
rep2
rep3

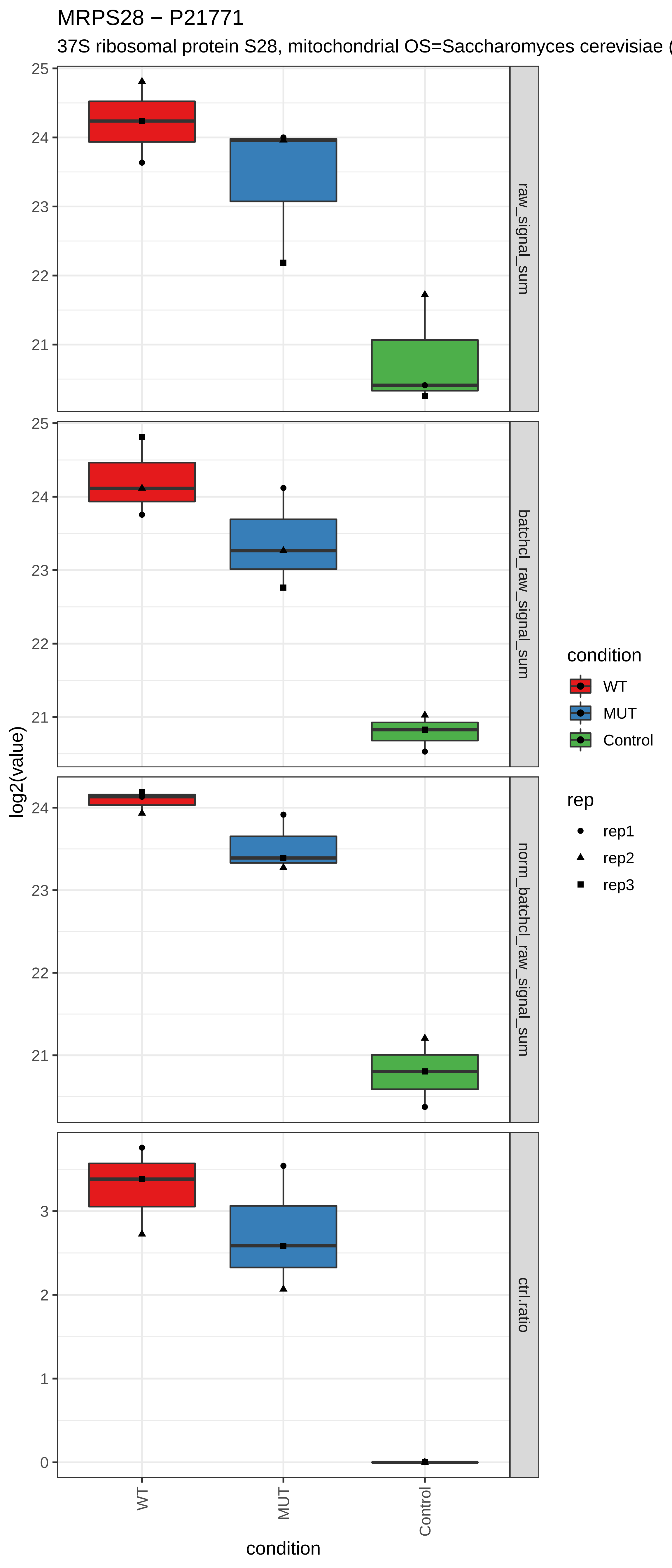

MRPS28 − P21771
37S ribosomal protein S28, mitochondrial OS=Saccharomyces cerevisiae (
raw_signal_sum
batchcl_raw_signal_sum
norm_batchcl_raw_signal_sum
ctrl.ratio
log2(value)
condition
WT
MUT
Control
rep
rep1
rep2
rep3

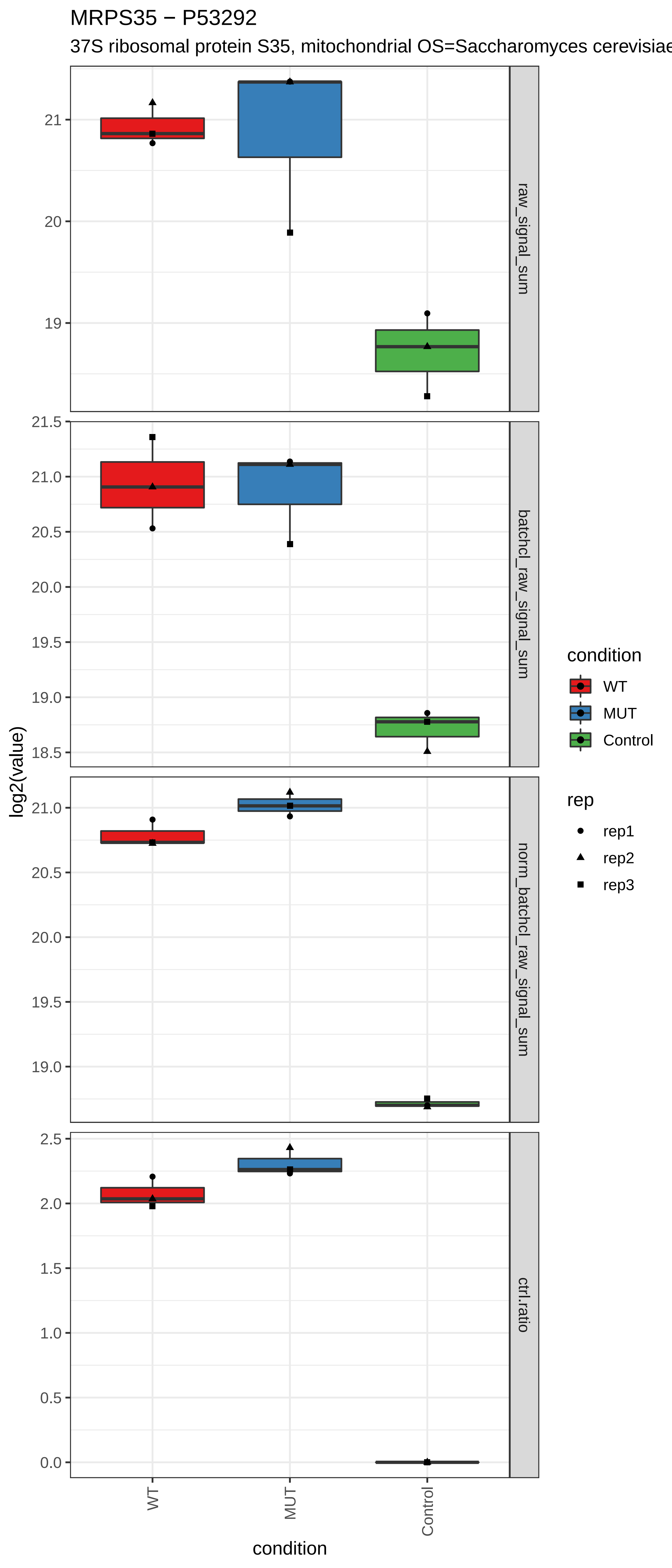
MRPS35 − P53292
37S ribosomal protein S35, mitochondrial OS=Saccharomyces cerevisiae
raw_signal_sum
batchcl_raw_signal_sum
norm_batchcl_raw_signal_sum
ctrl.ratio
log2(value)
condition
WT
MUT
Control
rep
rep1
rep2
rep3
21
20
19
21.5
21.0
20.5
20.0
19.5
19.0
18.5
2.5
2.0
1.5
1.0
0.5
0.0

MRPS5 − P33759

37S ribosomal protein S5, mitochondrial OS=Saccharomyces cerevisiae (s

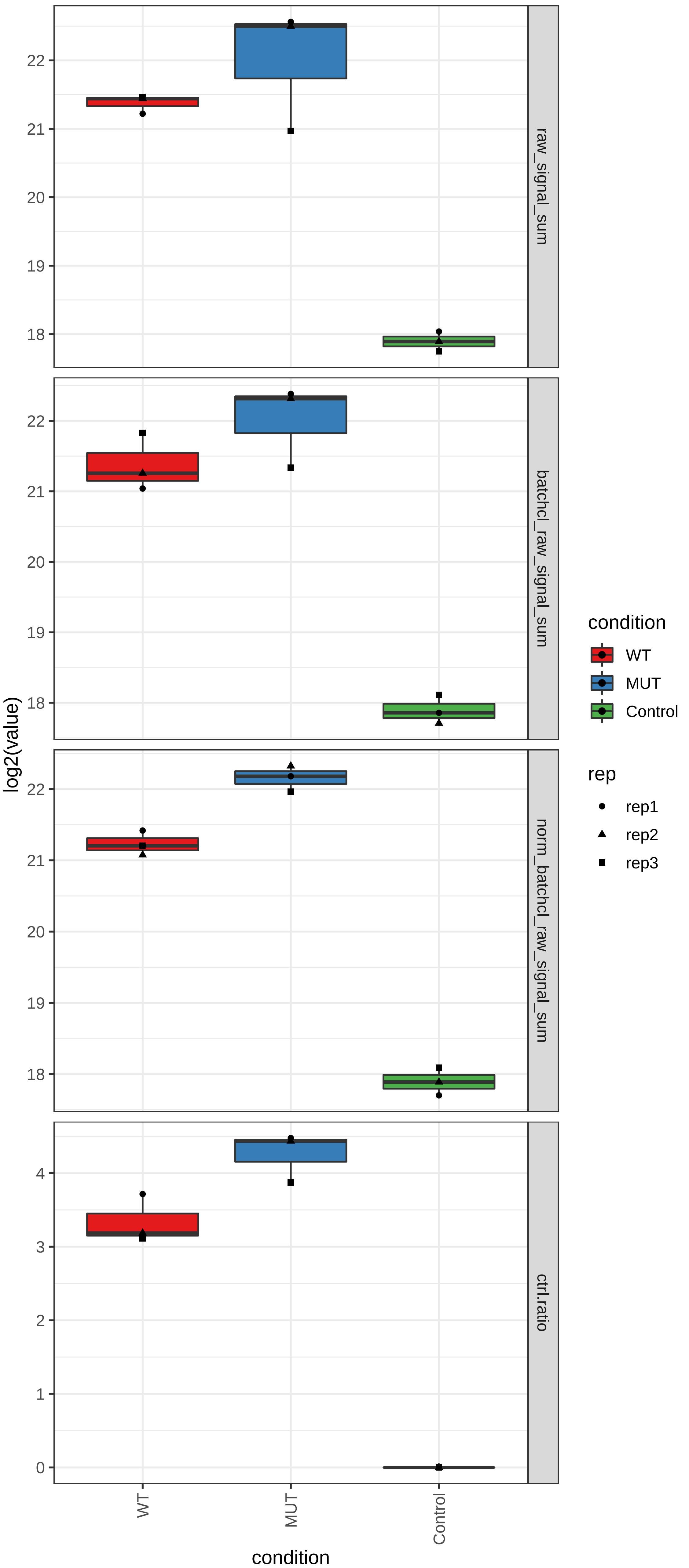

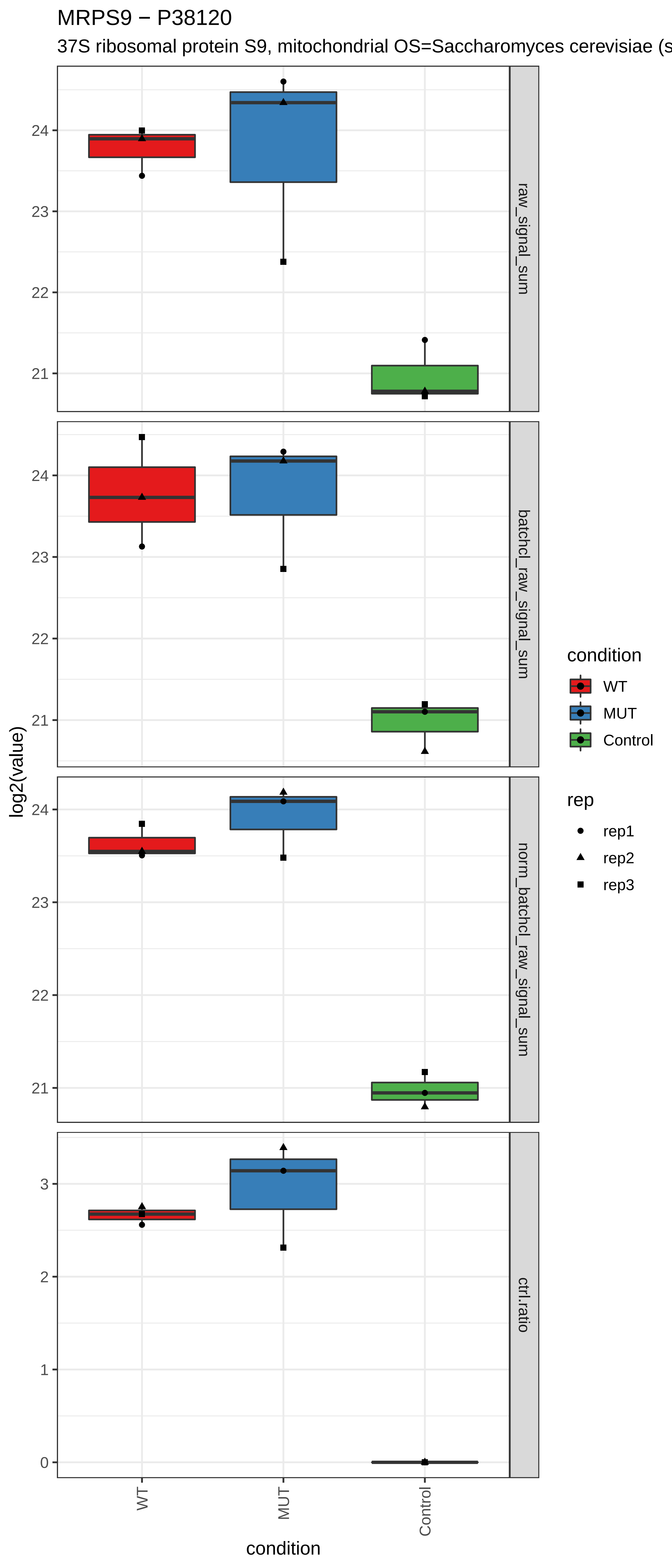

MRPS9 − P38120
37S ribosomal protein S9, mitochondrial OS=Saccharomyces cerevisiae (s
raw_signal_sum
batchcl_raw_signal_sum
norm_batchcl_raw_signal_sum
ctrl.ratio
log2(value)
condition
WT
MUT
Control
rep
rep1
rep2
rep3

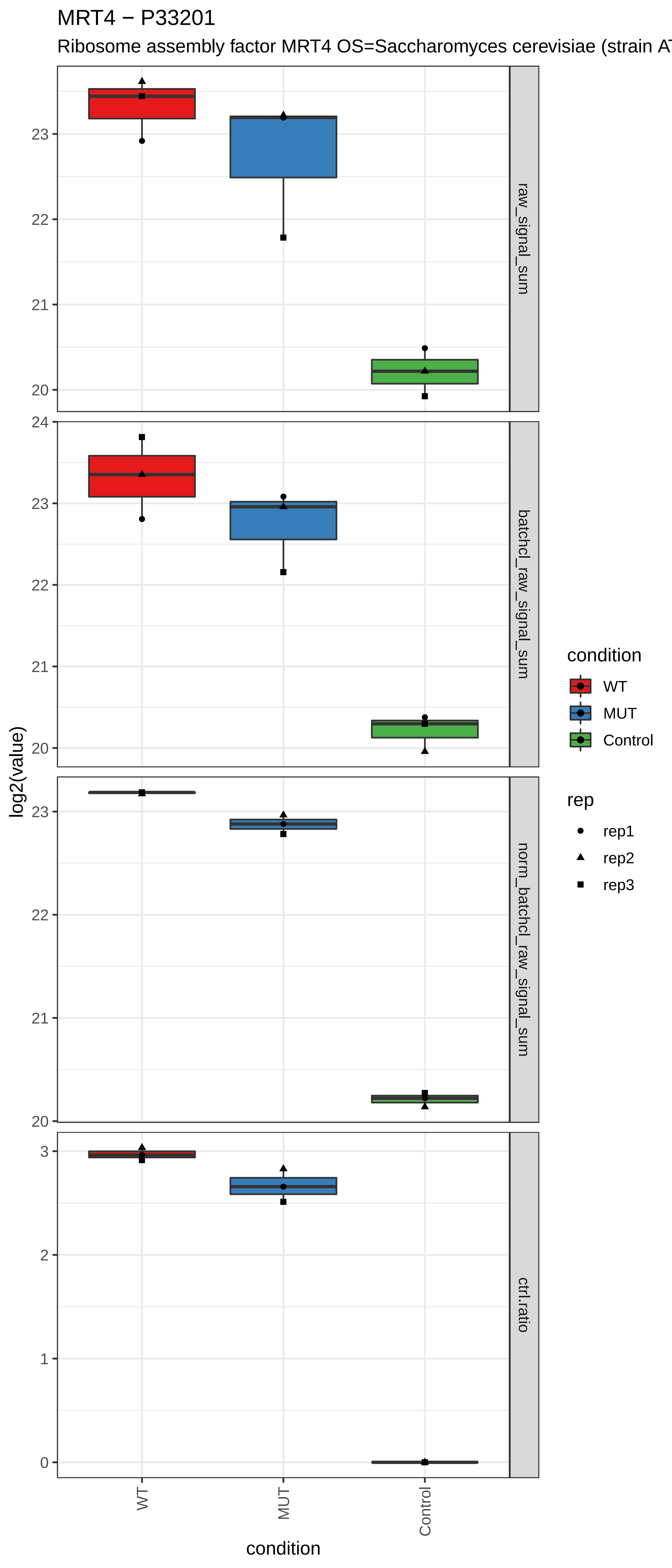
MRT4 − P33201
Ribosome assembly factor MRT4 OS=Saccharomyces cerevisiae (strain AT
raw_signal_sum
batchcl_raw_signal_sum
norm_batchcl_raw_signal_sum
ctrl.ratio
log2(value)
condition
WT
MUT
Control
rep
rep1
rep2
rep3
24
23
22
21
20
3
2
1
0

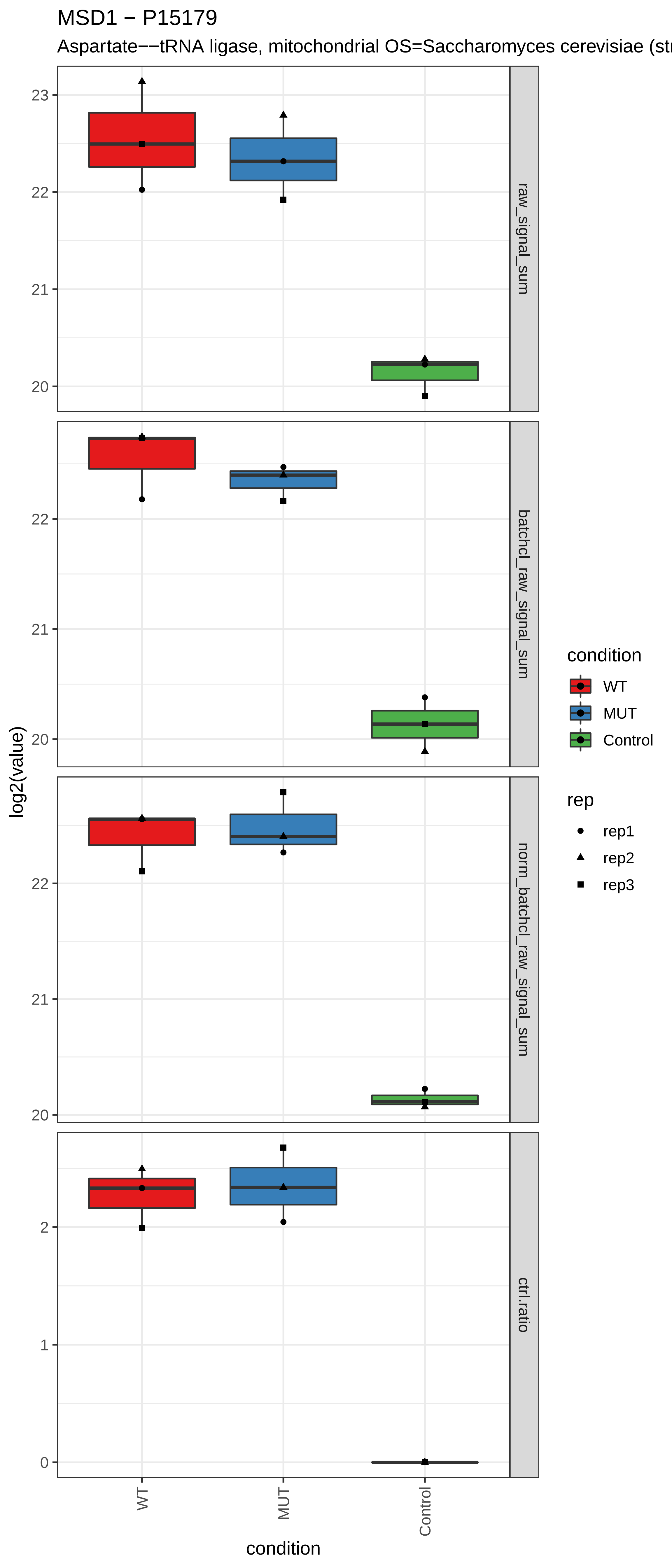

MSD1 − P15179
Aspartate−−tRNA ligase, mitochondrial OS=Saccharomyces cerevisiae (str
raw_signal_sum
batchcl_raw_signal_sum
norm_batchcl_raw_signal_sum
ctrl.ratio
log2(value)
condition
WT
MUT
Control
rep
rep1
rep2
rep3

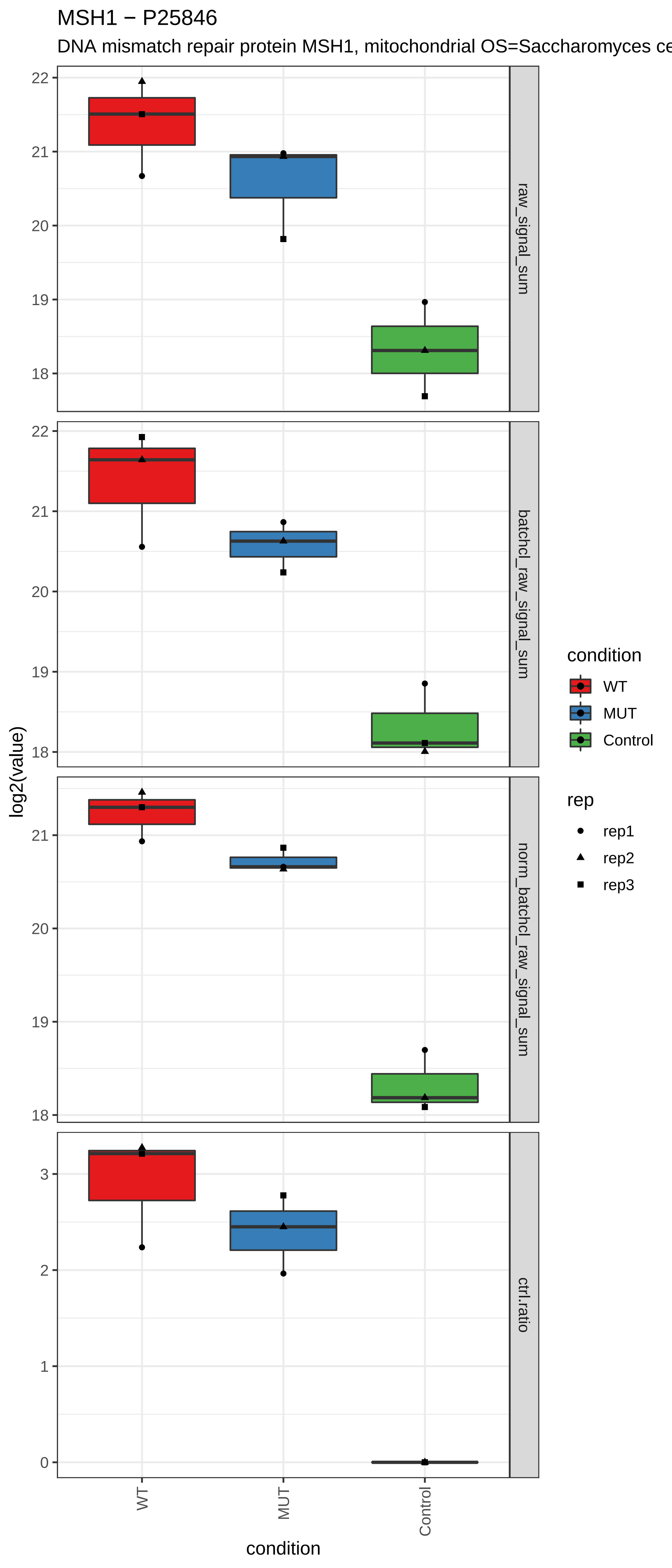

MSH1 − P25846
DNA mismatch repair protein MSH1, mitochondrial OS=Saccharomyces ce
raw_signal_sum
batchcl_raw_signal_sum
norm_batchcl_raw_signal_sum
ctrl.ratio
log2(value)
condition
WT
MUT
Control
rep
rep1
rep2
rep3

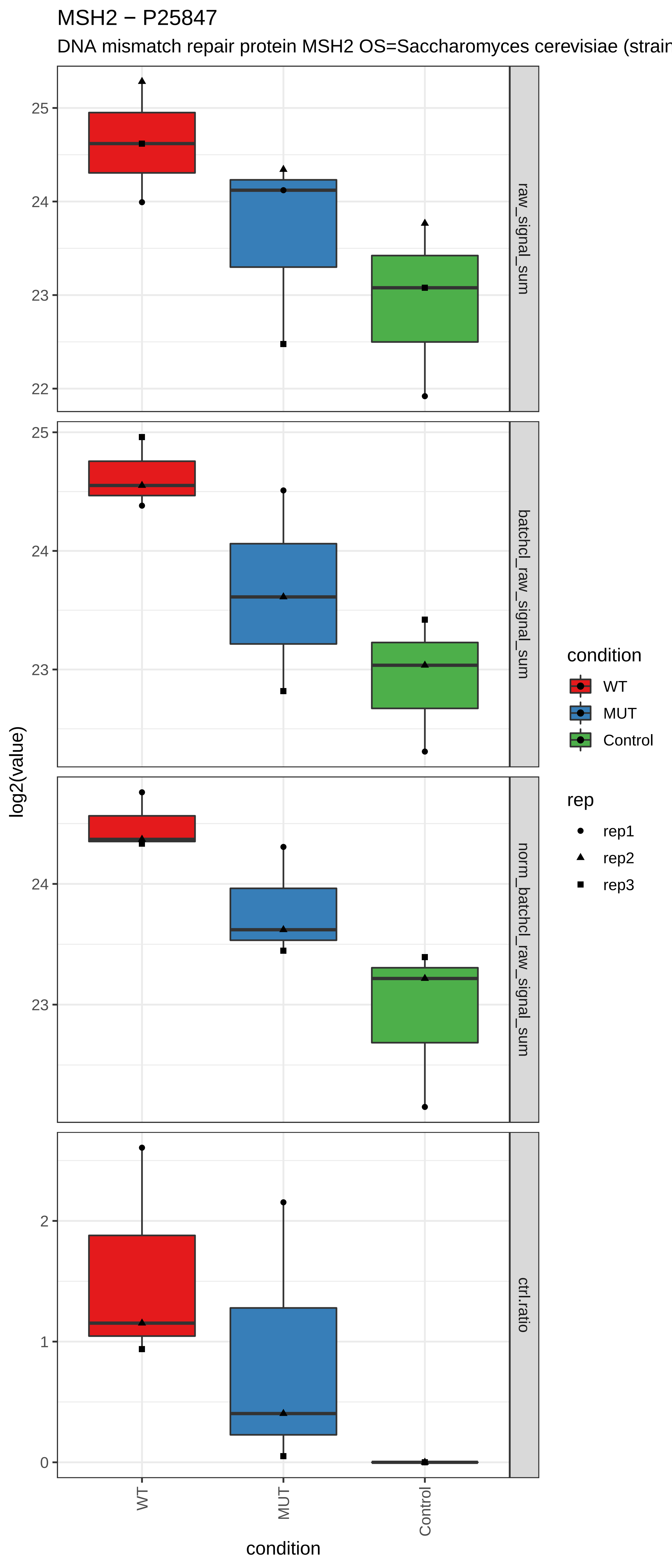
MSH2 − P25847
DNA mismatch repair protein MSH2 OS=Saccharomyces cerevisiae (strain
raw_signal_sum
batchcl_raw_signal_sum
norm_batchcl_raw_signal_sum
ctrl.ratio
log2(value)
condition
WT
MUT
Control
rep
rep1
rep2
rep3
25
24
23
22
2
1
0

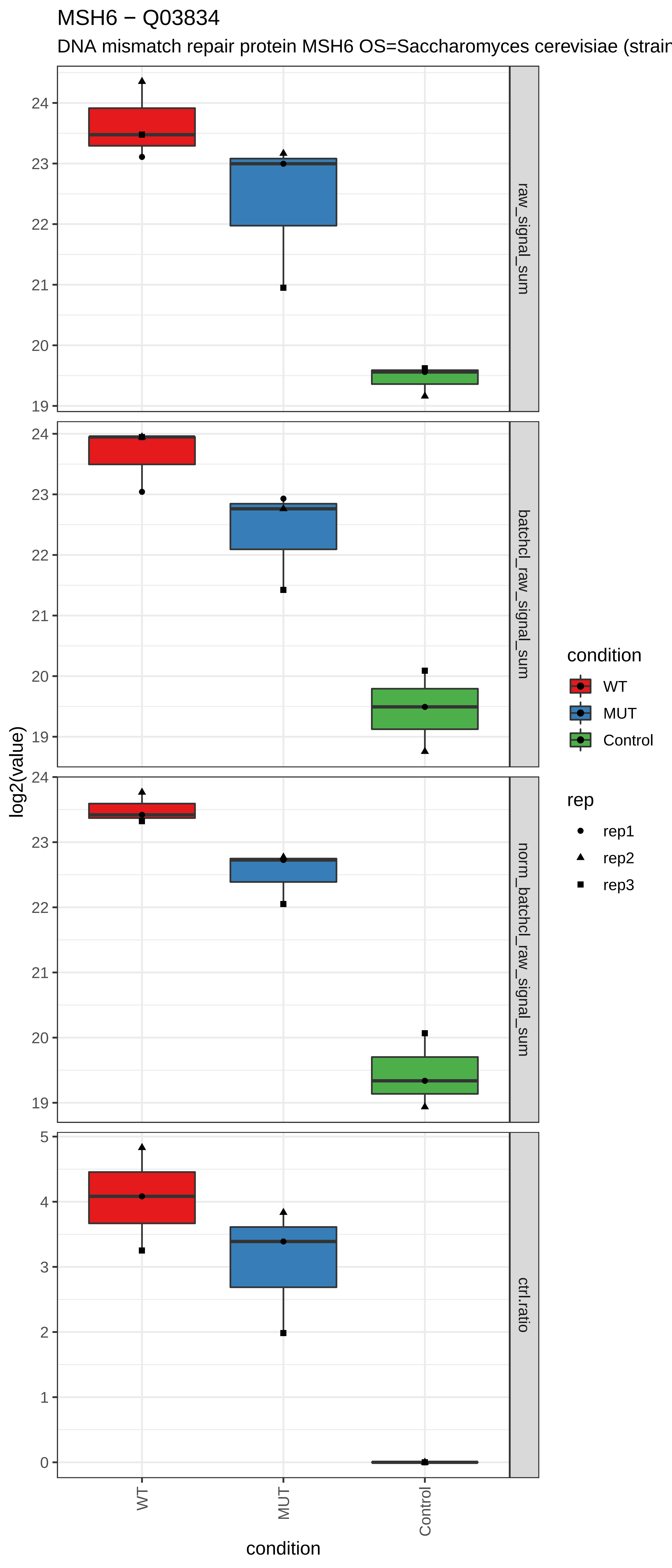
MSH6 − Q03834
DNA mismatch repair protein MSH6 OS=Saccharomyces cerevisiae (strain
raw_signal_sum
batchcl_raw_signal_sum
norm_batchcl_raw_signal_sum
ctrl.ratio
log2(value)
condition
WT
MUT
Control
rep
rep1
rep2
rep3

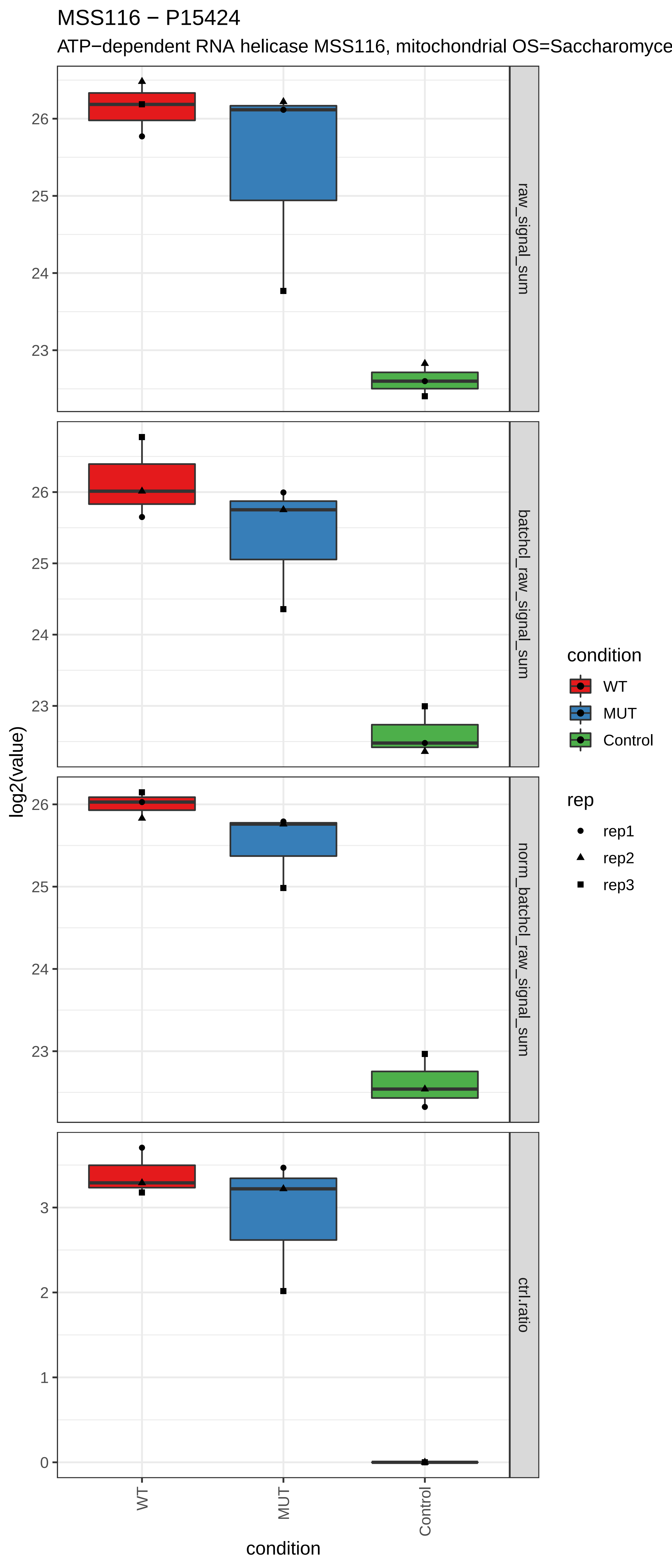

MSS116 − P15424
ATP−dependent RNA helicase MSS116, mitochondrial OS=Saccharomyce
log2(value)
raw_signal_sum
batchcl_raw_signal_sum
norm_batchcl_raw_signal_sum
ctrl.ratio
26
25
24
23
3
2
1
0
WT
MUT
Control
condition
condition
WT
MUT
Control
rep
rep1
rep2
rep3

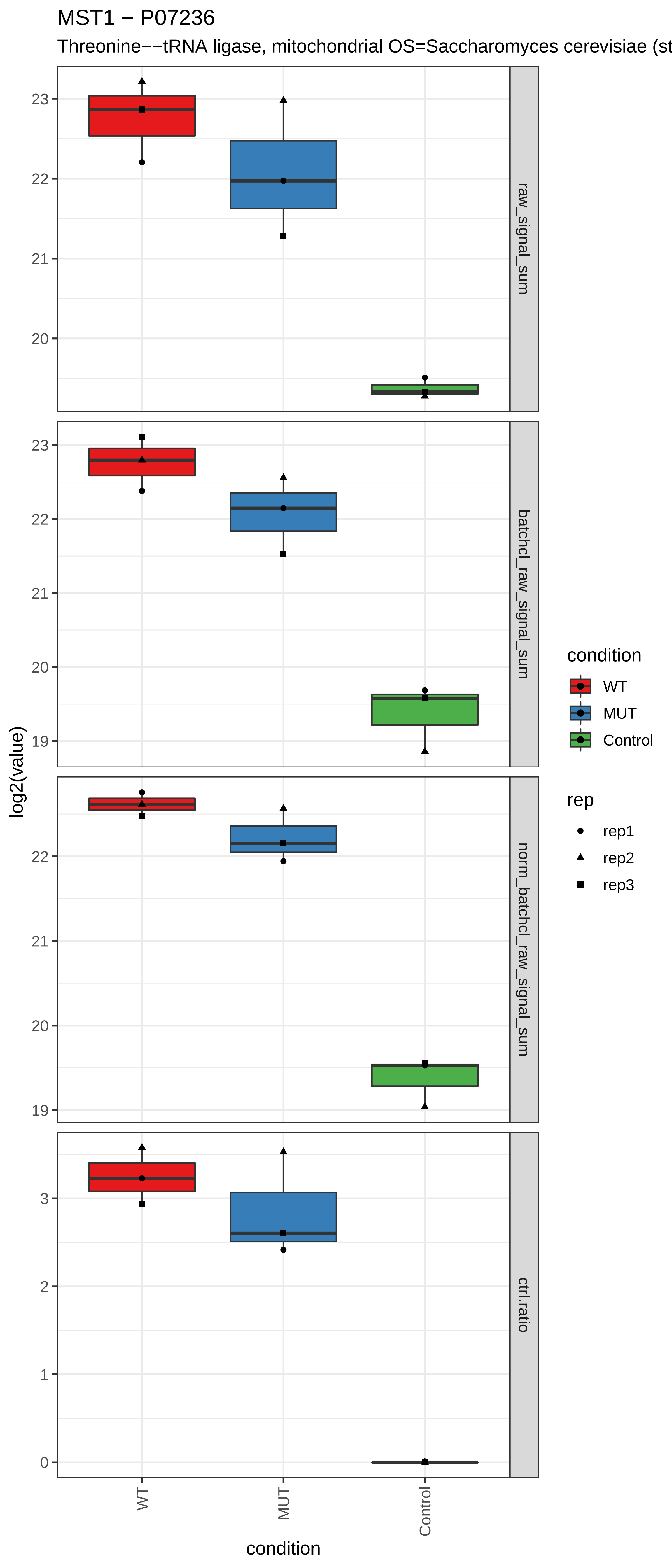

MST1 − P07236
Threonine−−tRNA ligase, mitochondrial OS=Saccharomyces cerevisiae (st
raw_signal_sum
batchcl_raw_signal_sum
norm_batchcl_raw_signal_sum
ctrl.ratio
log2(value)
condition
WT
MUT
Control
rep
rep1
rep2
rep3
23
22
21
20
19
3
2
1
0

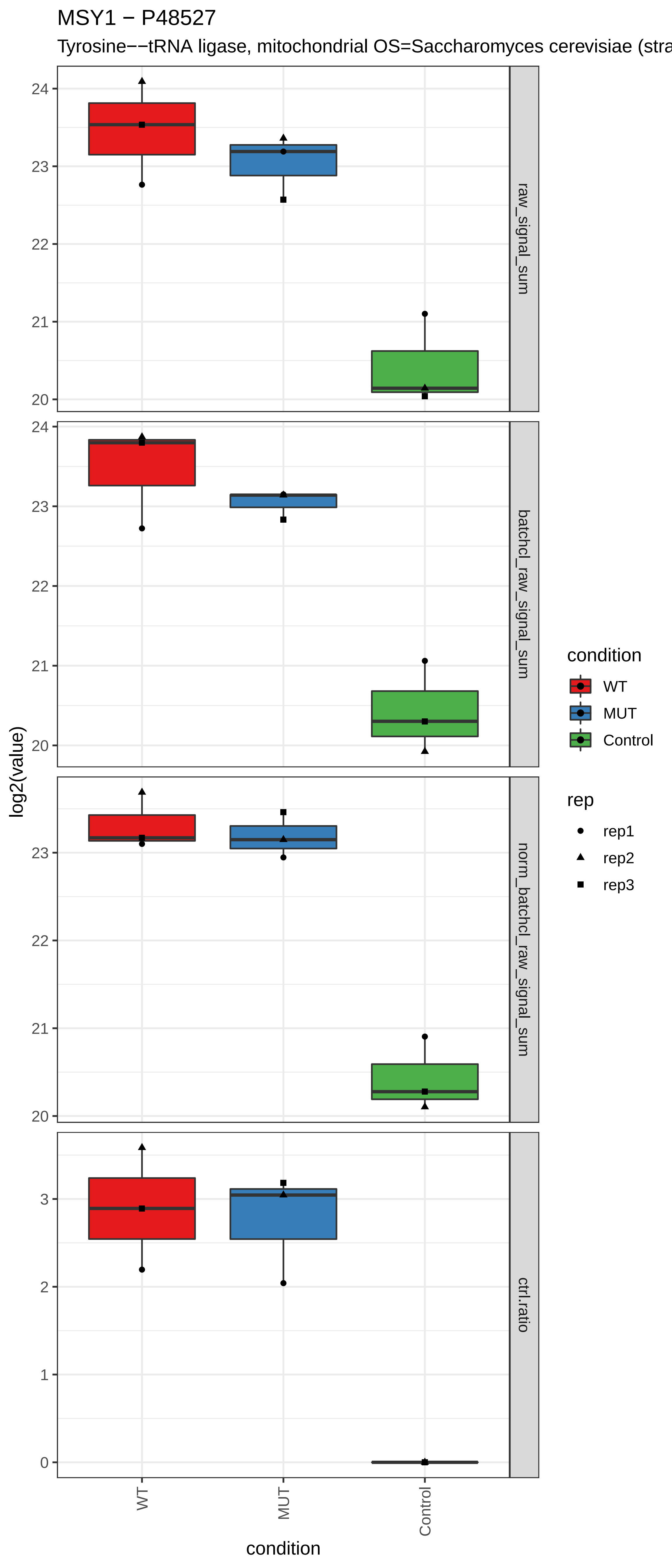

MSY1 − P48527
Tyrosine−−tRNA ligase, mitochondrial OS=Saccharomyces cerevisiae (stra
raw_signal_sum
batchcl_raw_signal_sum
norm_batchcl_raw_signal_sum
ctrl.ratio
log2(value)
condition
WT
MUT
Control
rep
rep1
rep2
rep3

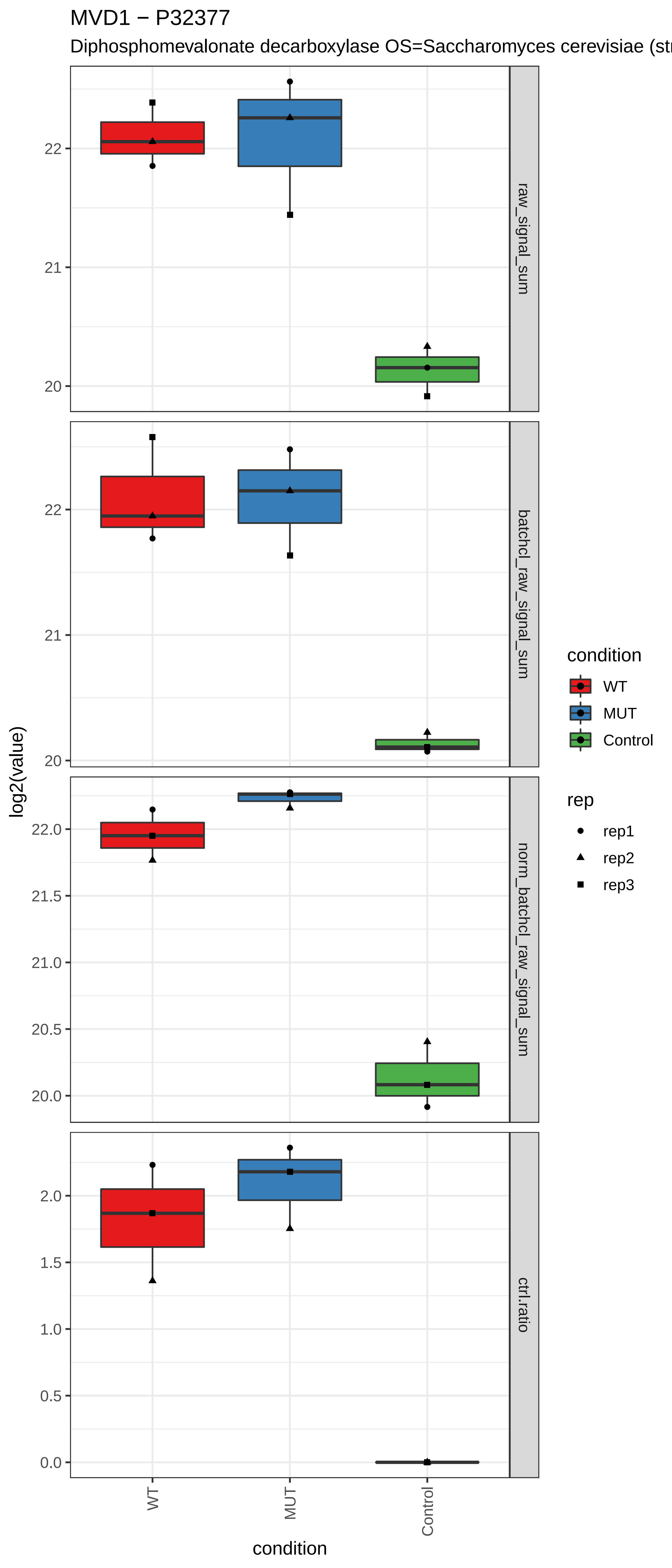
MVD1 − P32377
Diphosphomevalonate decarboxylase OS=Saccharomyces cerevisiae (str
raw_signal_sum
batchcl_raw_signal_sum
norm_batchcl_raw_signal_sum
ctrl.ratio
log2(value)
condition
WT
MUT
Control
rep
rep1
rep2
rep3
22
21
20
22.0
21.5
21.0
20.5
20.0
2.0
1.5
1.0
0.5
0.0

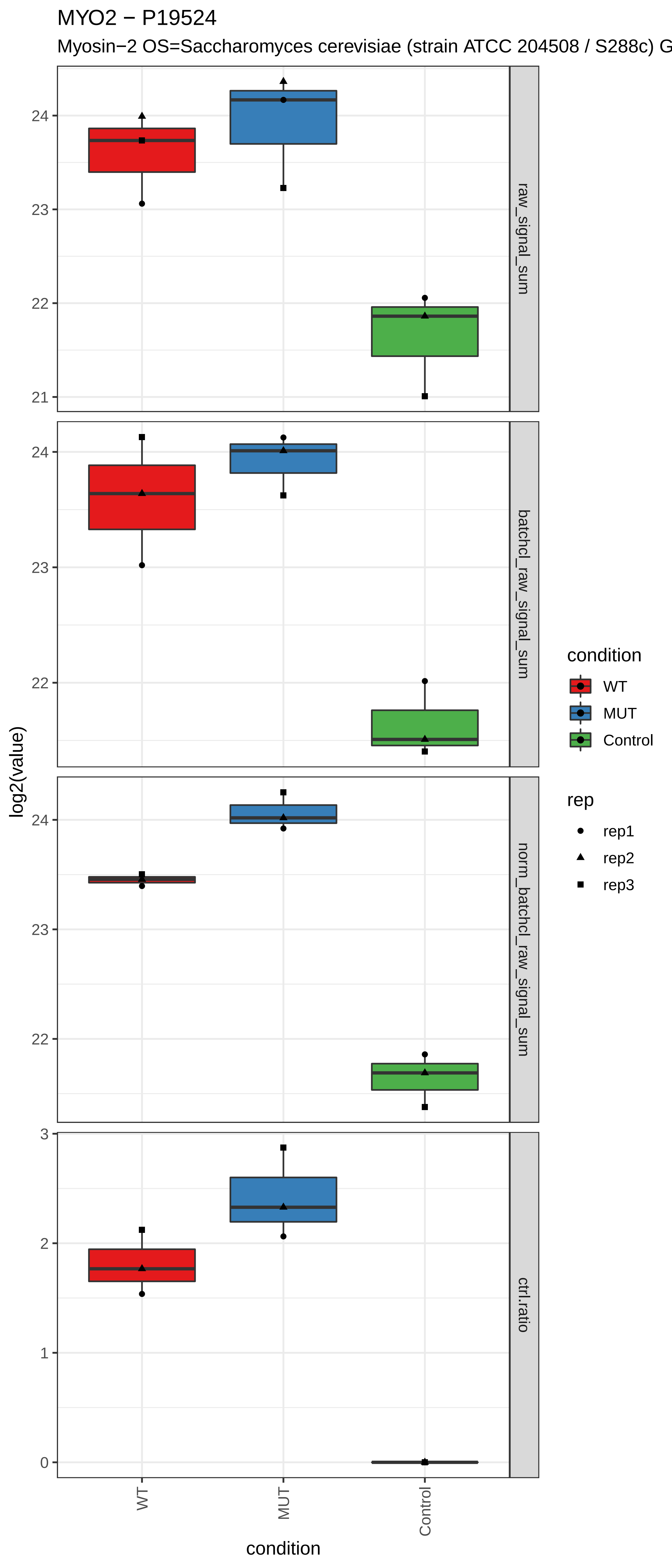

MYO2 − P19524
Myosin−2 OS=Saccharomyces cerevisiae (strain ATCC 204508 / S288c) G
raw_signal_sum
batchcl_raw_signal_sum
norm_batchcl_raw_signal_sum
ctrl.ratio
log2(value)
condition
WT
MUT
Control
rep
rep1
rep2
rep3
24
23
22
21
3
2
1
0

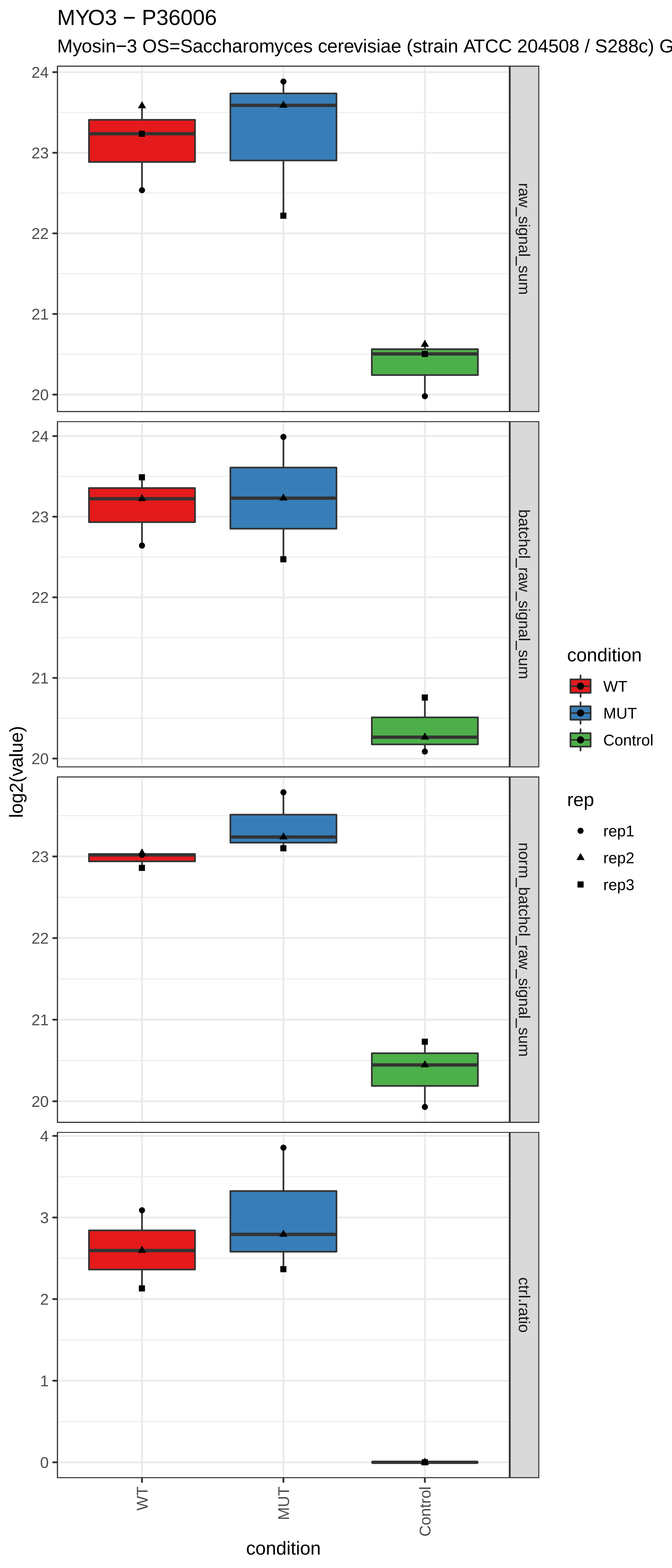

MYO3 − P36006
Myosin−3 OS=Saccharomyces cerevisiae (strain ATCC 204508 / S288c) G
raw_signal_sum
batchcl_raw_signal_sum
norm_batchcl_raw_signal_sum
ctrl.ratio
log2(value)
condition
WT
MUT
Control
rep
rep1
rep2
rep3

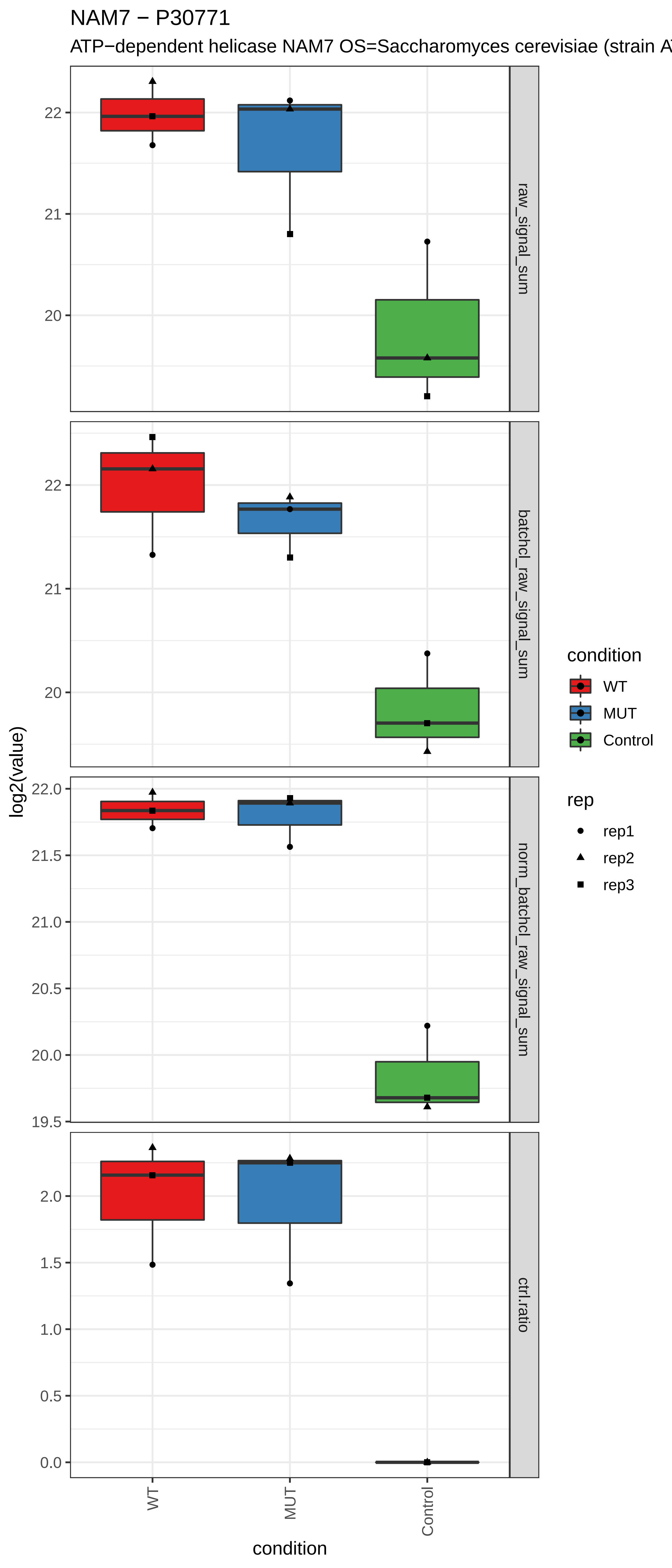

NAM7 − P30771
ATP−dependent helicase NAM7 OS=Saccharomyces cerevisiae (strain A
raw_signal_sum
batchcl_raw_signal_sum
norm_batchcl_raw_signal_sum
ctrl.ratio
log2(value)
condition
WT
MUT
Control
rep
rep1
rep2
rep3

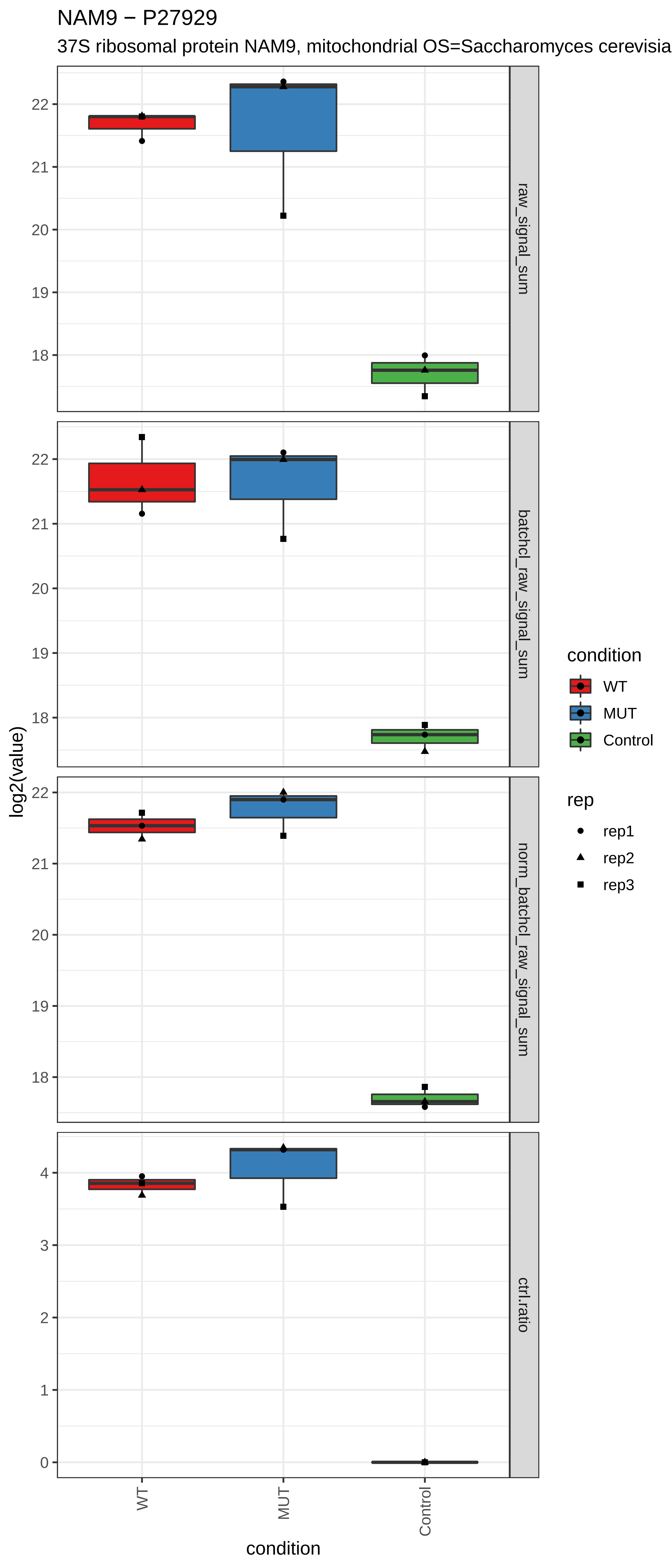

NAM9 − P27929
37S ribosomal protein NAM9, mitochondrial OS=Saccharomyces cerevisia
raw_signal_sum
batchcl_raw_signal_sum
norm_batchcl_raw_signal_sum
ctrl.ratio
log2(value)
condition
WT
MUT
Control
rep
rep1
rep2
rep3
22
21
20
19
18
4
3
2
1
0

NAT1 − P12945

N−terminal acetyltransferase A complex subunit NAT1 OS=Saccharomyces

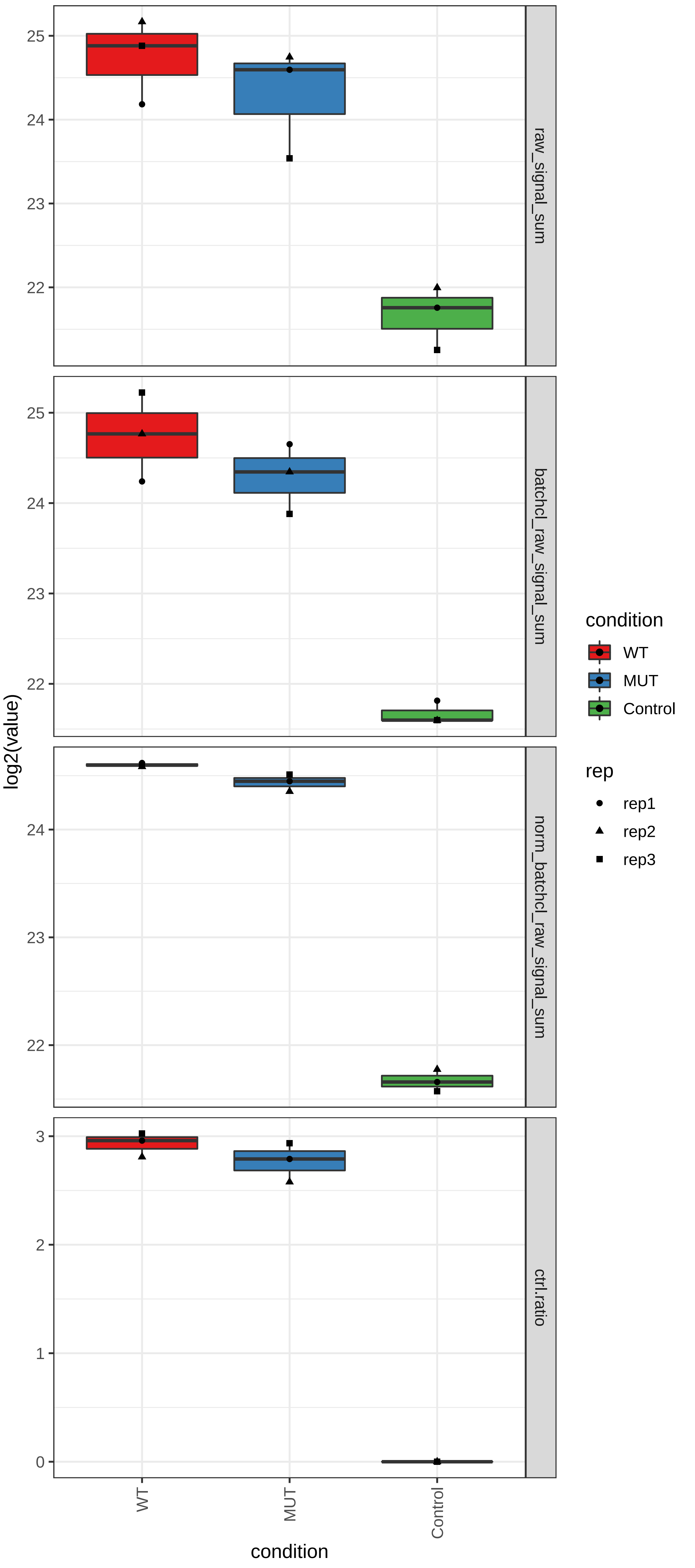

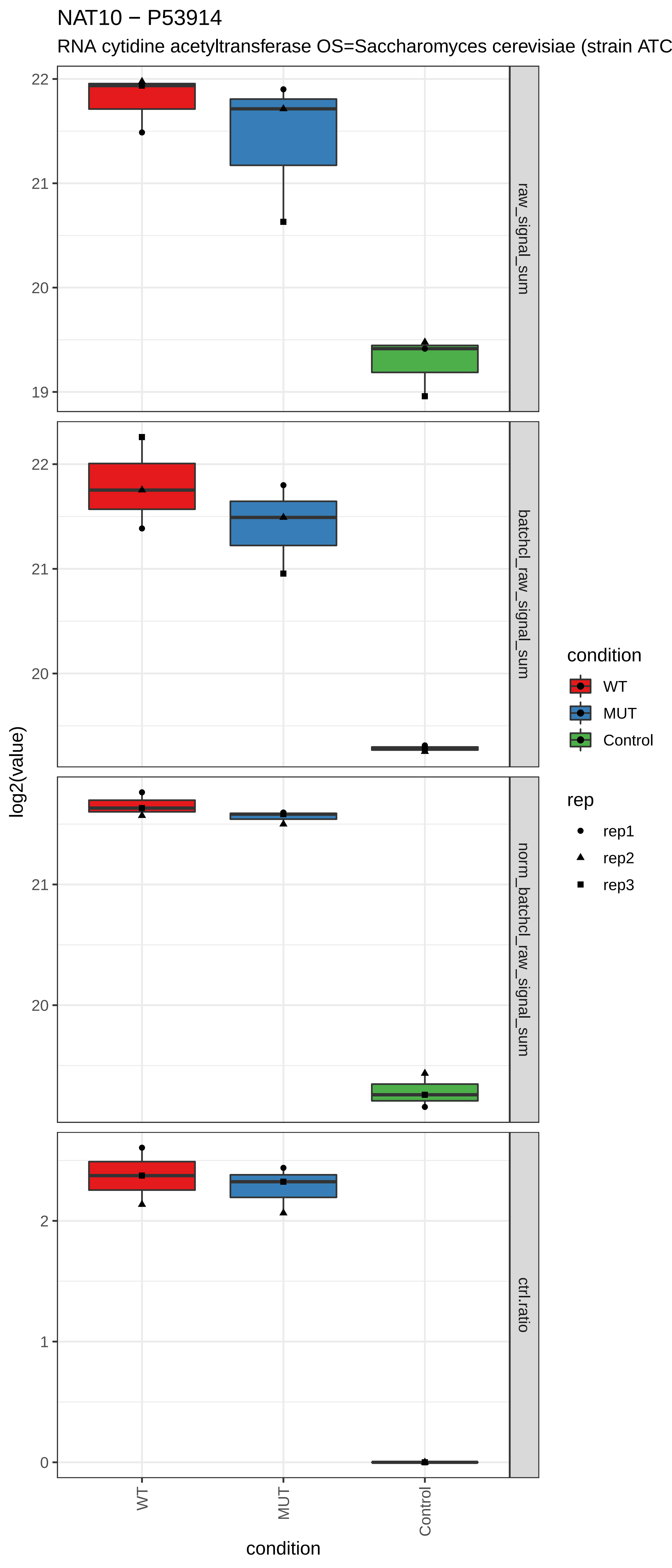
NAT10 − P53914
RNA cytidine acetyltransferase OS=Saccharomyces cerevisiae (strain ATC
raw_signal_sum
batchcl_raw_signal_sum
norm_batchcl_raw_signal_sum
ctrl.ratio
log2(value)
condition
WT
MUT
Control
rep
rep1
rep2
rep3
22
21
20
19
2
1
0

NDI1 − P32340

Rotenone−insensitive NADH−ubiquinone oxidoreductase, mitochondrial OS

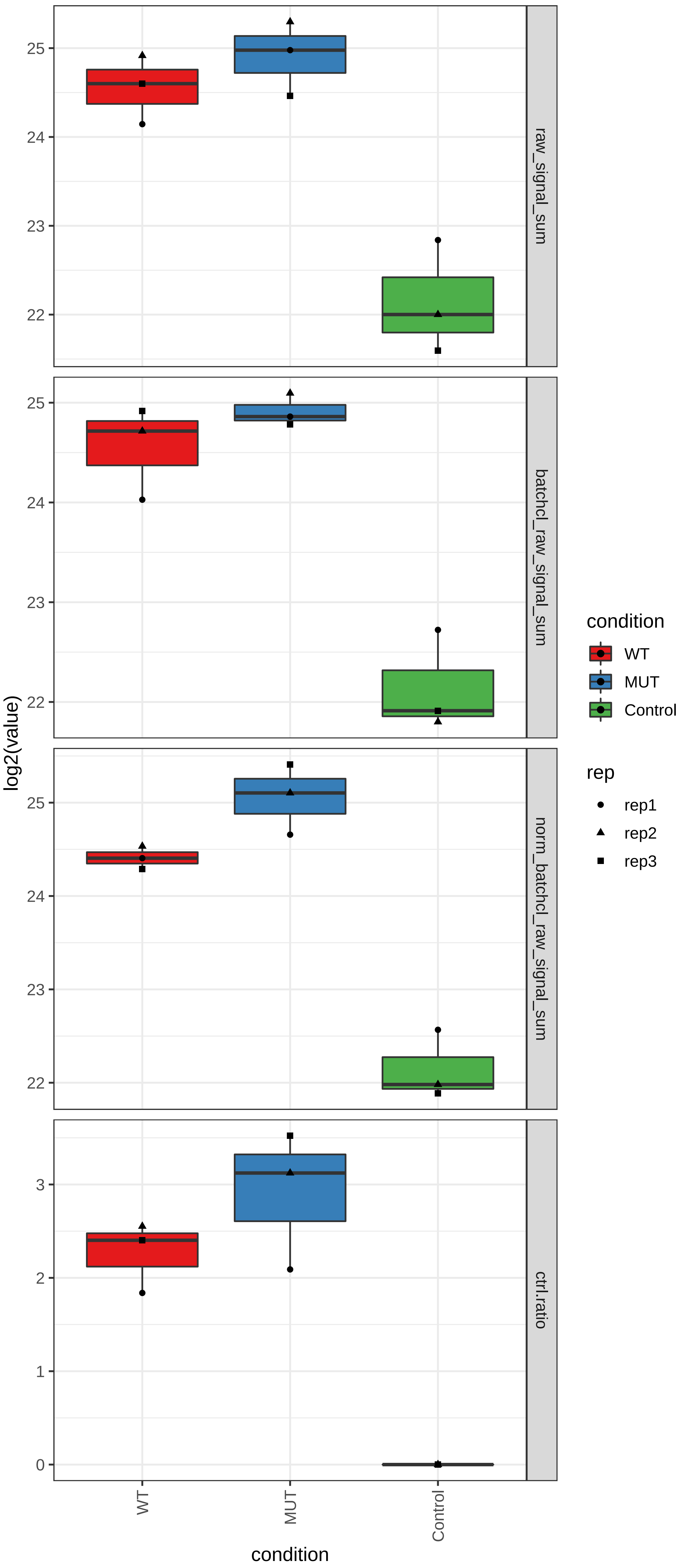

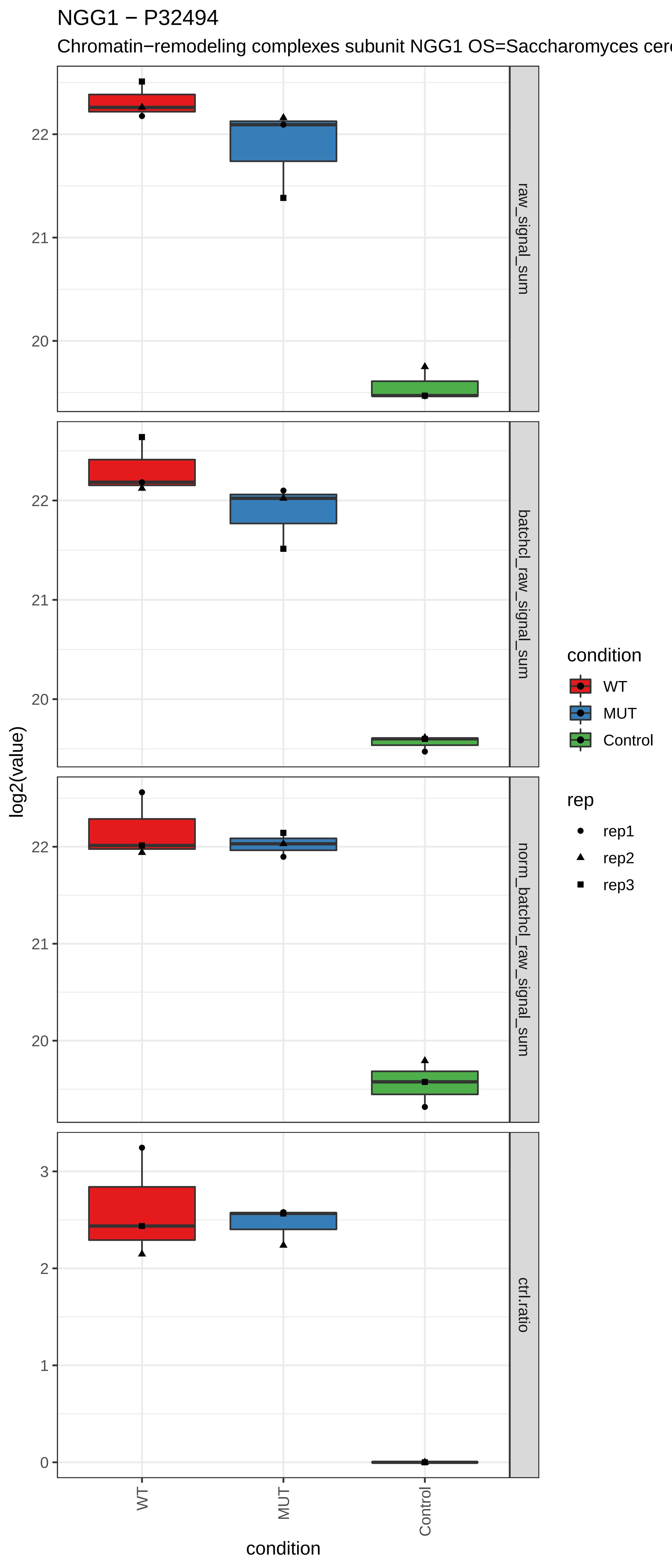

NGG1 − P32494
Chromatin−remodeling complexes subunit NGG1 OS=Saccharomyces cere
raw_signal_sum
batchcl_raw_signal_sum
norm_batchcl_raw_signal_sum
ctrl.ratio
log2(value)
condition
WT
MUT
Control
rep
rep1
rep2
rep3
22
21
20
3
2
1
0

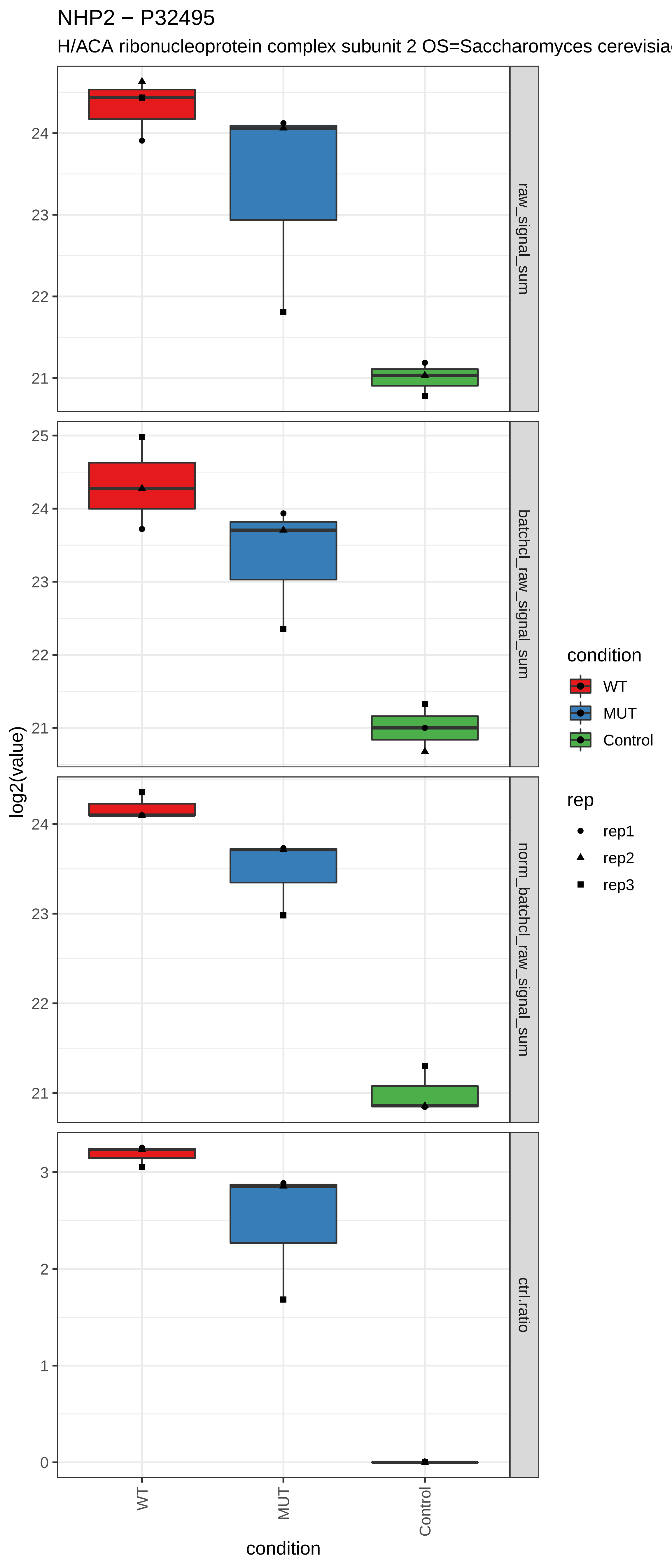
NHP2 − P32495
H/ACA ribonucleoprotein complex subunit 2 OS=Saccharomyces cerevisiae
raw_signal_sum
batchcl_raw_signal_sum
norm_batchcl_raw_signal_sum
ctrl.ratio
log2(value)
condition
WT
MUT
Control
rep
rep1
rep2
rep3

NHP6A − P11632

Non−histone chromosomal protein 6A OS=Saccharomyces cerevisiae (stra

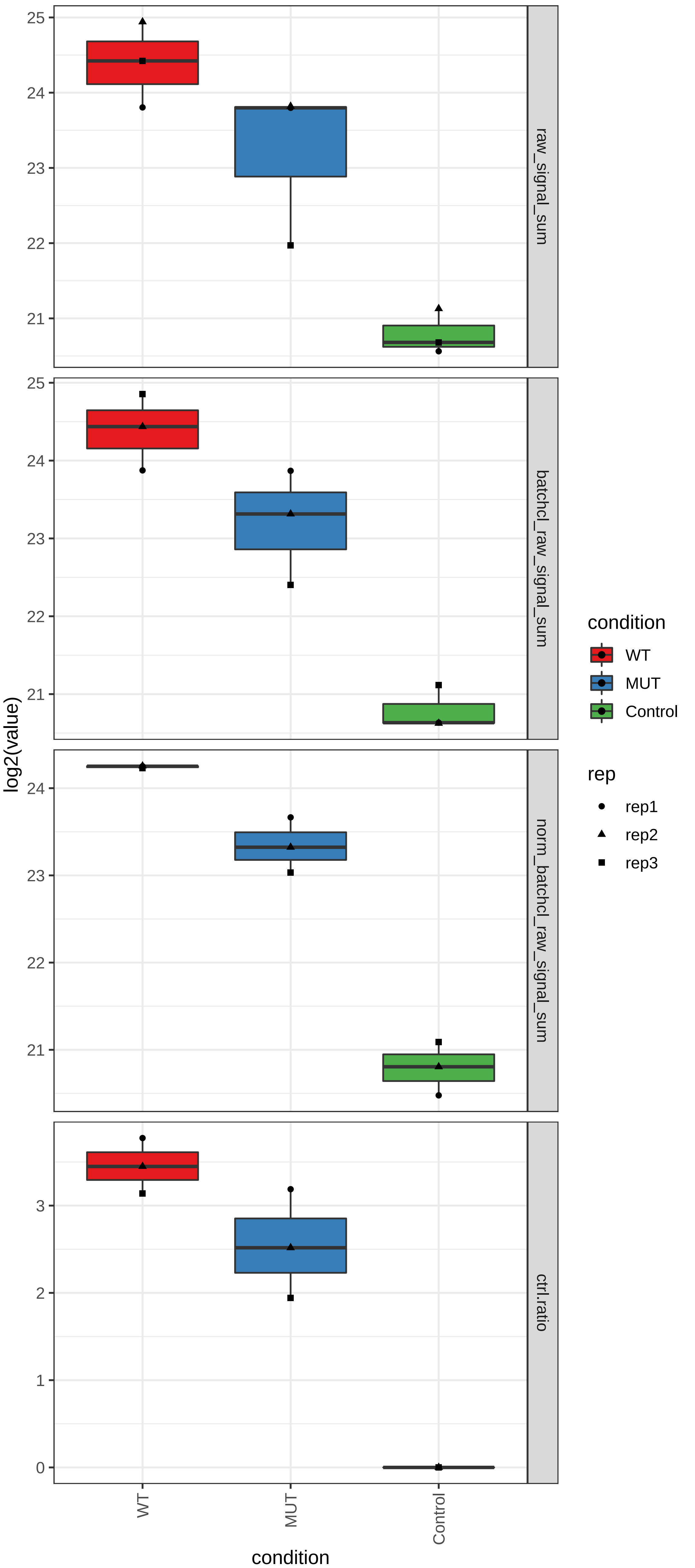

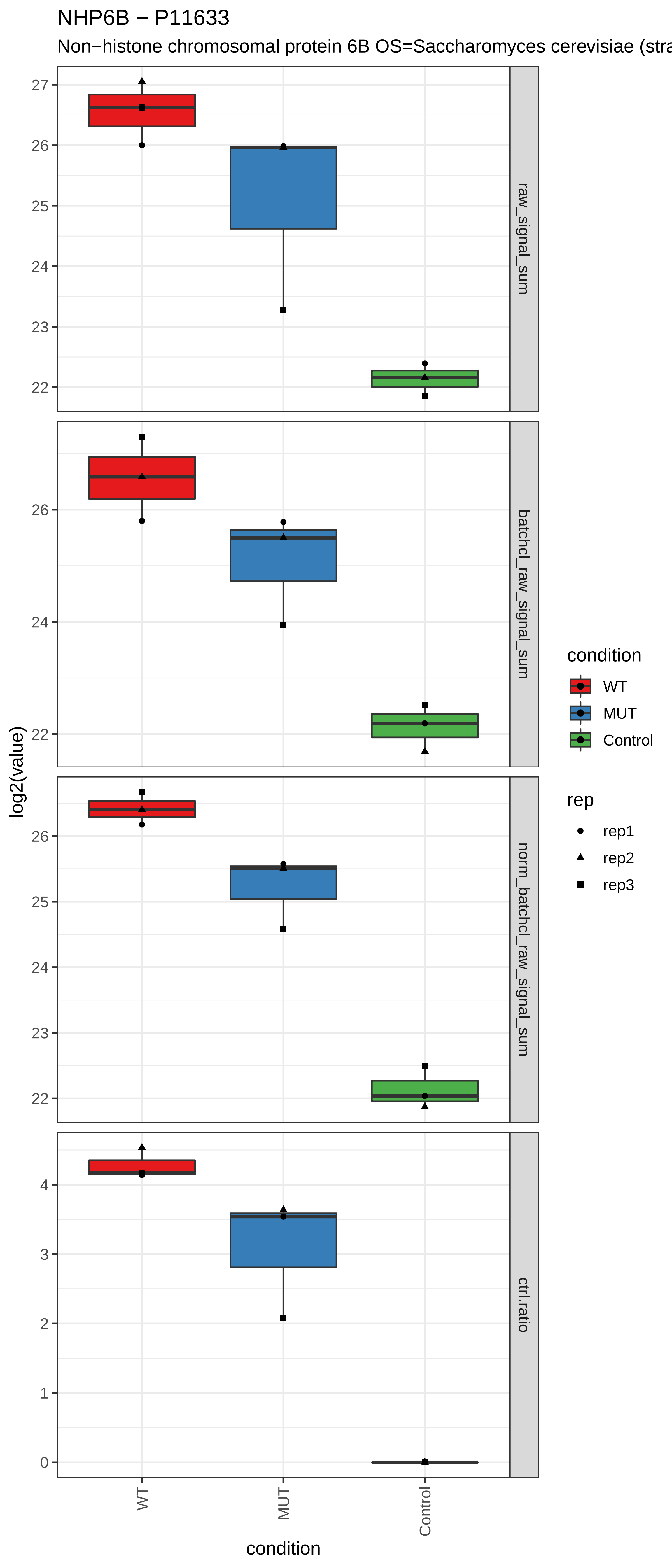

NHP6B − P11633
Non−histone chromosomal protein 6B OS=Saccharomyces cerevisiae (stra
raw_signal_sum
batchcl_raw_signal_sum
norm_batchcl_raw_signal_sum
ctrl.ratio
log2(value)
condition
WT
MUT
Control
rep
rep1
rep2
rep3

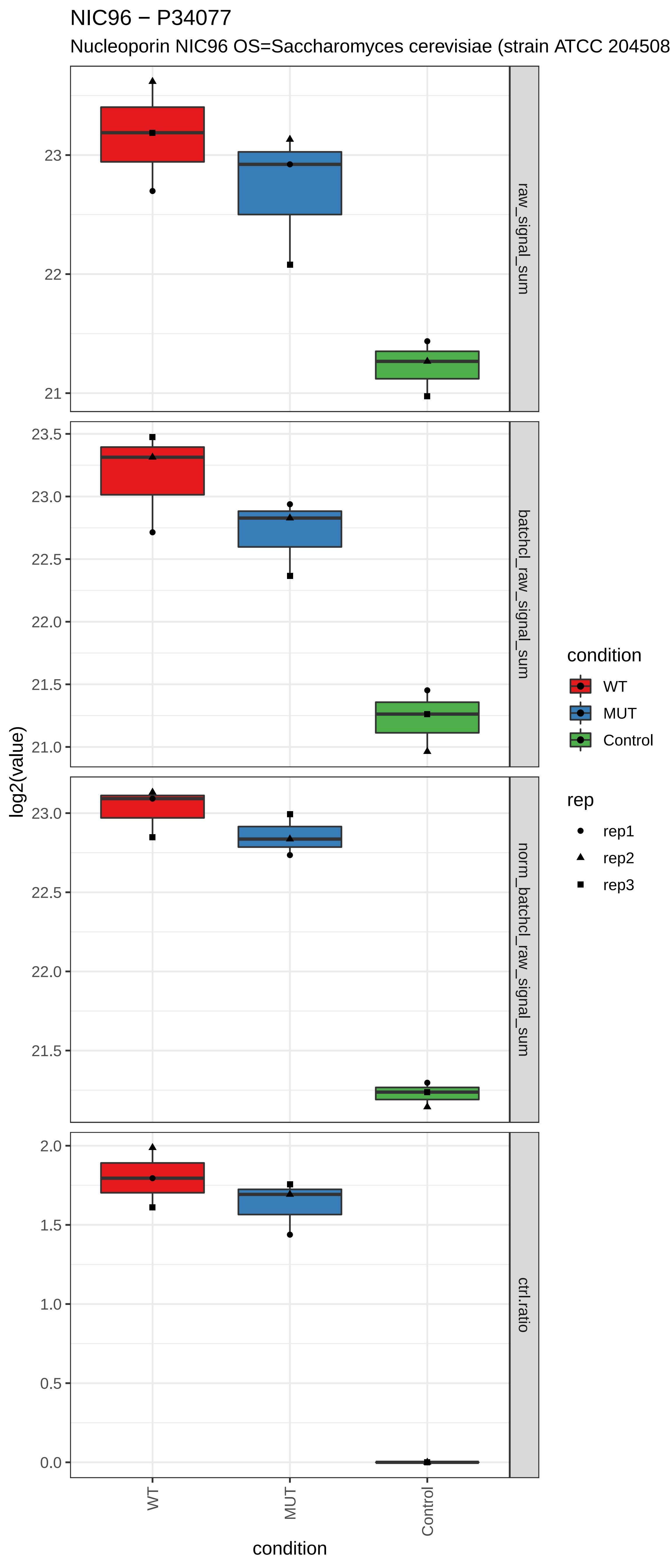

NIC96 − P34077
Nucleoporin NIC96 OS=Saccharomyces cerevisiae (strain ATCC 204508
raw_signal_sum
batchcl_raw_signal_sum
norm_batchcl_raw_signal_sum
ctrl.ratio
log2(value)
condition
WT
MUT
Control
rep
rep1
rep2
rep3

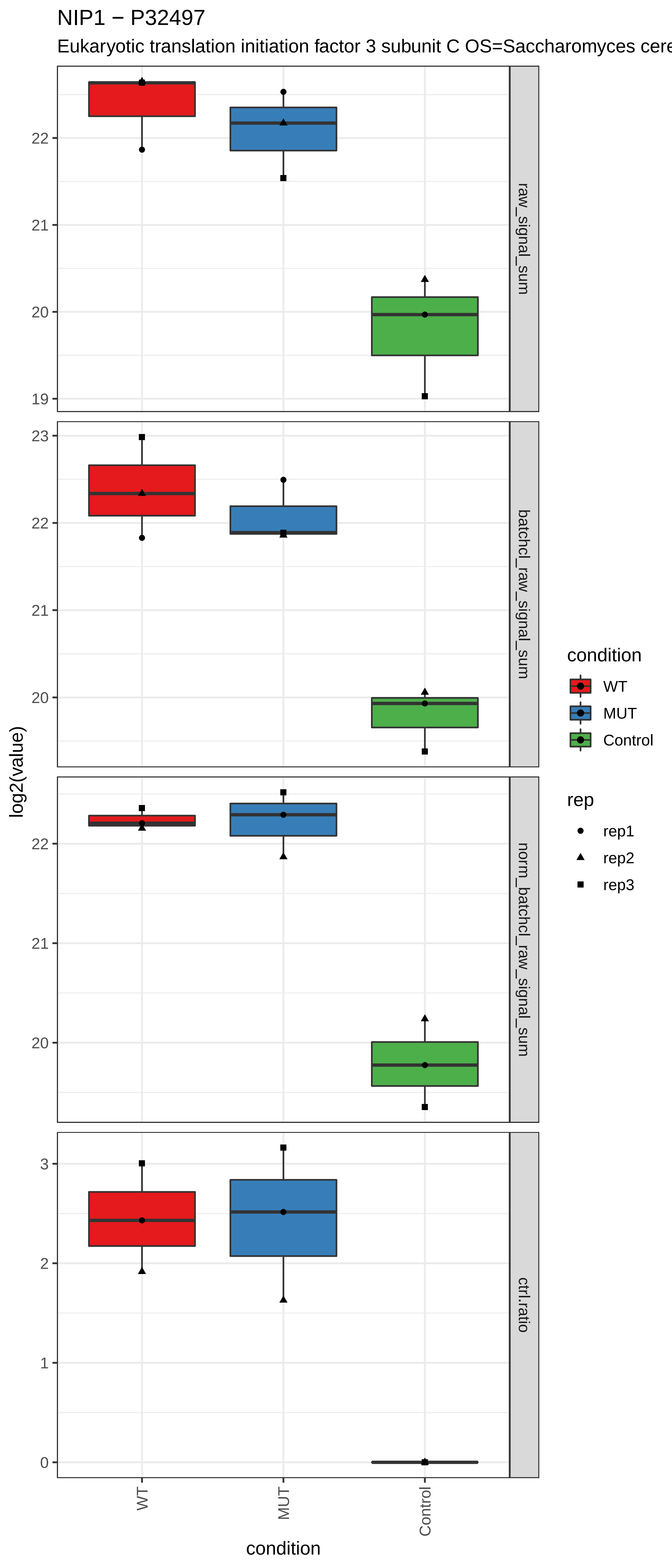
NIP1 − P32497
Eukaryotic translation initiation factor 3 subunit C OS=Saccharomyces cere
raw_signal_sum
batchcl_raw_signal_sum
norm_batchcl_raw_signal_sum
ctrl.ratio
log2(value)
condition
WT
MUT
Control
rep
rep1
rep2
rep3

NOC2 − P39744

Nucleolar complex protein 2 OS=Saccharomyces cerevisiae (strain ATCC 2

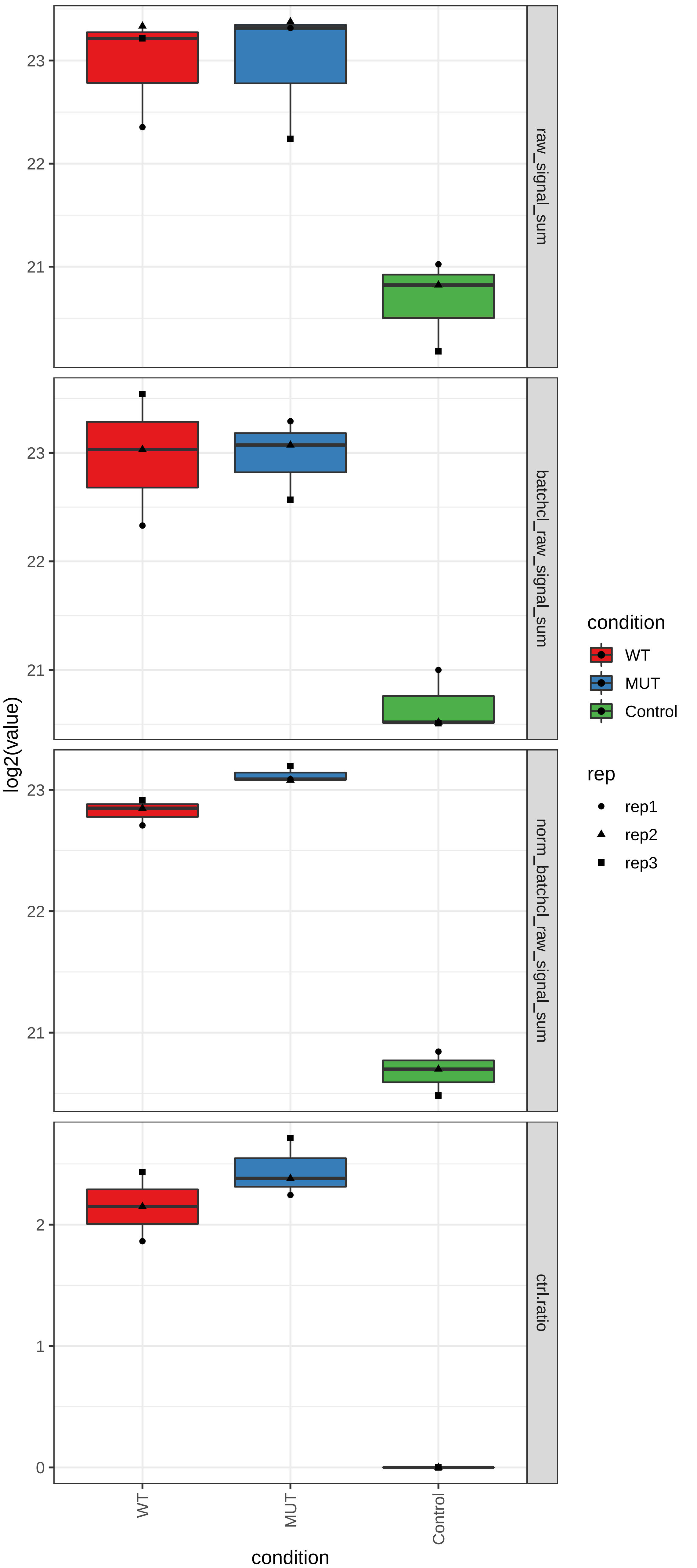

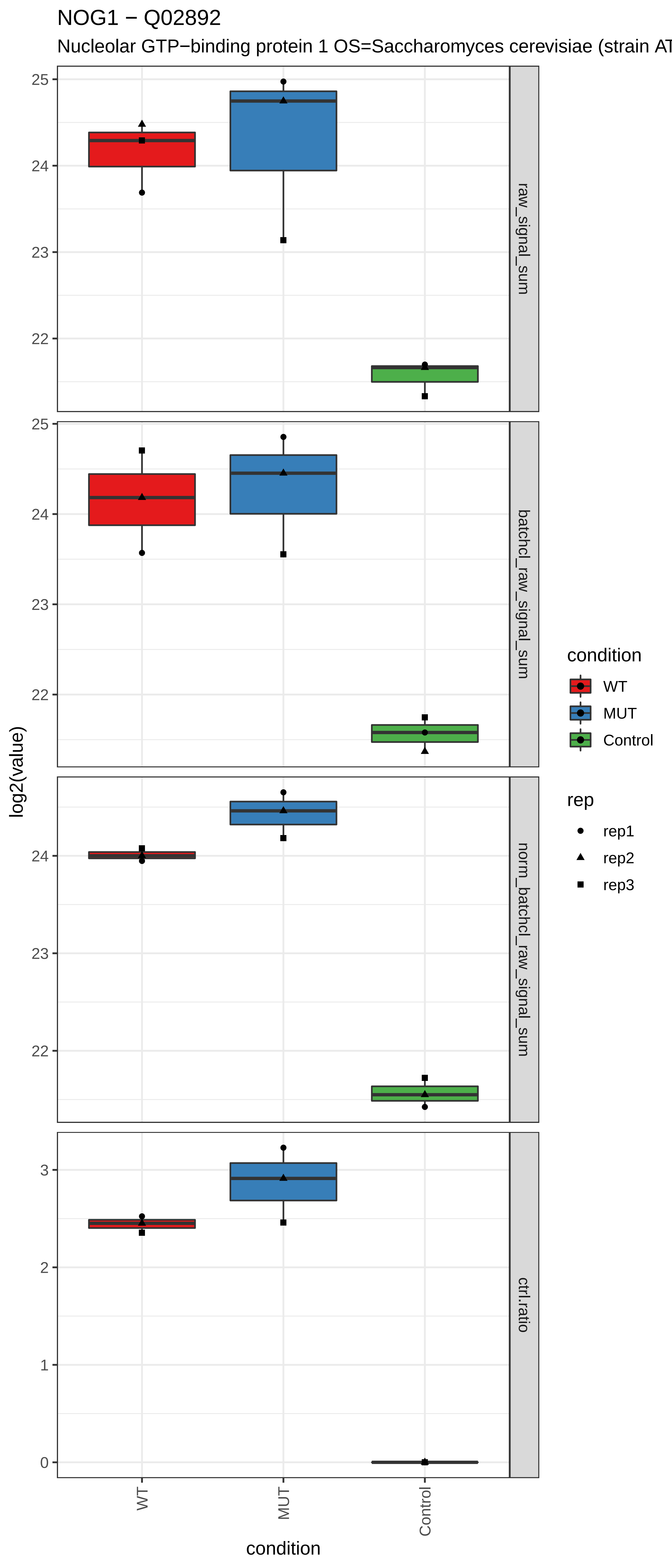

NOG1 − Q02892
Nucleolar GTP−binding protein 1 OS=Saccharomyces cerevisiae (strain AT
raw_signal_sum
batchcl_raw_signal_sum
norm_batchcl_raw_signal_sum
ctrl.ratio
log2(value)
condition
WT
MUT
Control
rep
rep1
rep2
rep3
25
24
23
22
3
2
1
0

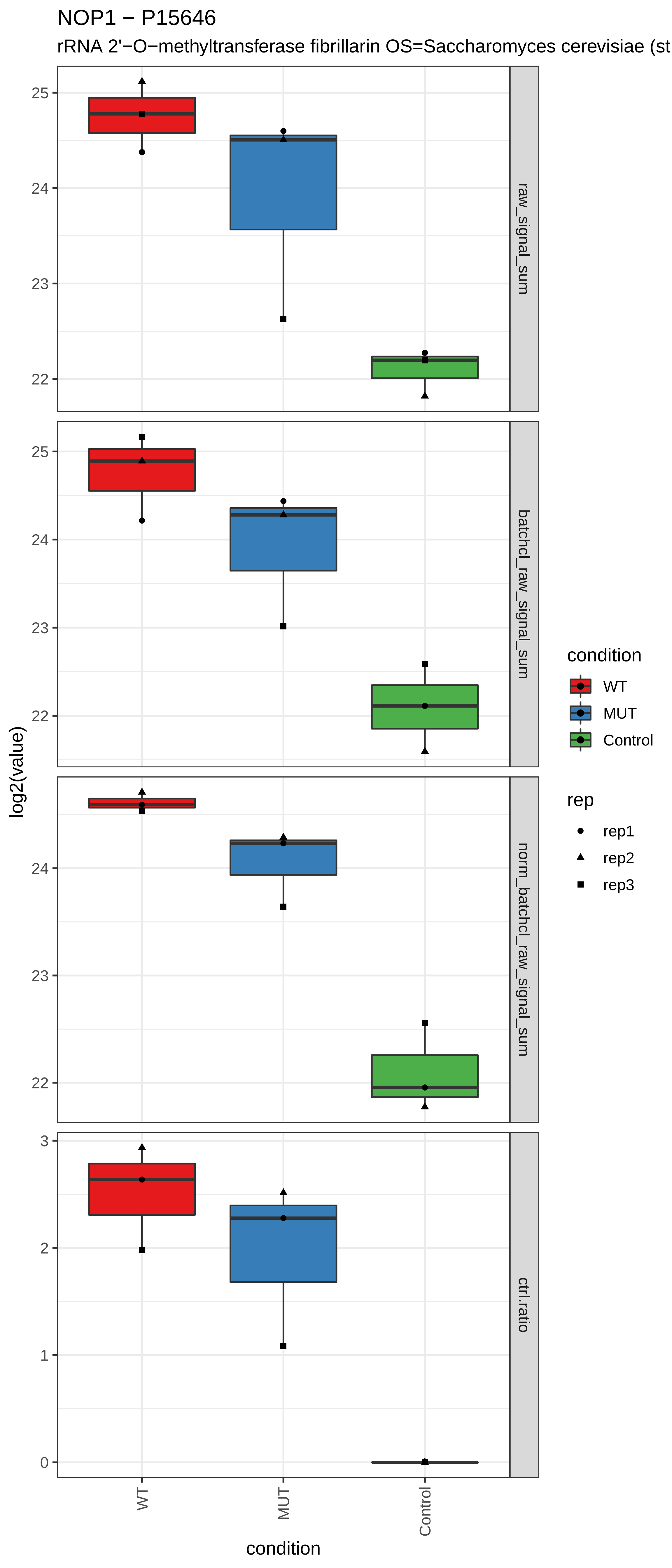

NOP1 − P15646
rRNA 2'−O−methyltransferase fibrillarin OS=Saccharomyces cerevisiae (st
raw_signal_sum
batchcl_raw_signal_sum
norm_batchcl_raw_signal_sum
ctrl.ratio
log2(value)
condition
WT
MUT
Control
rep
rep1
rep2
rep3

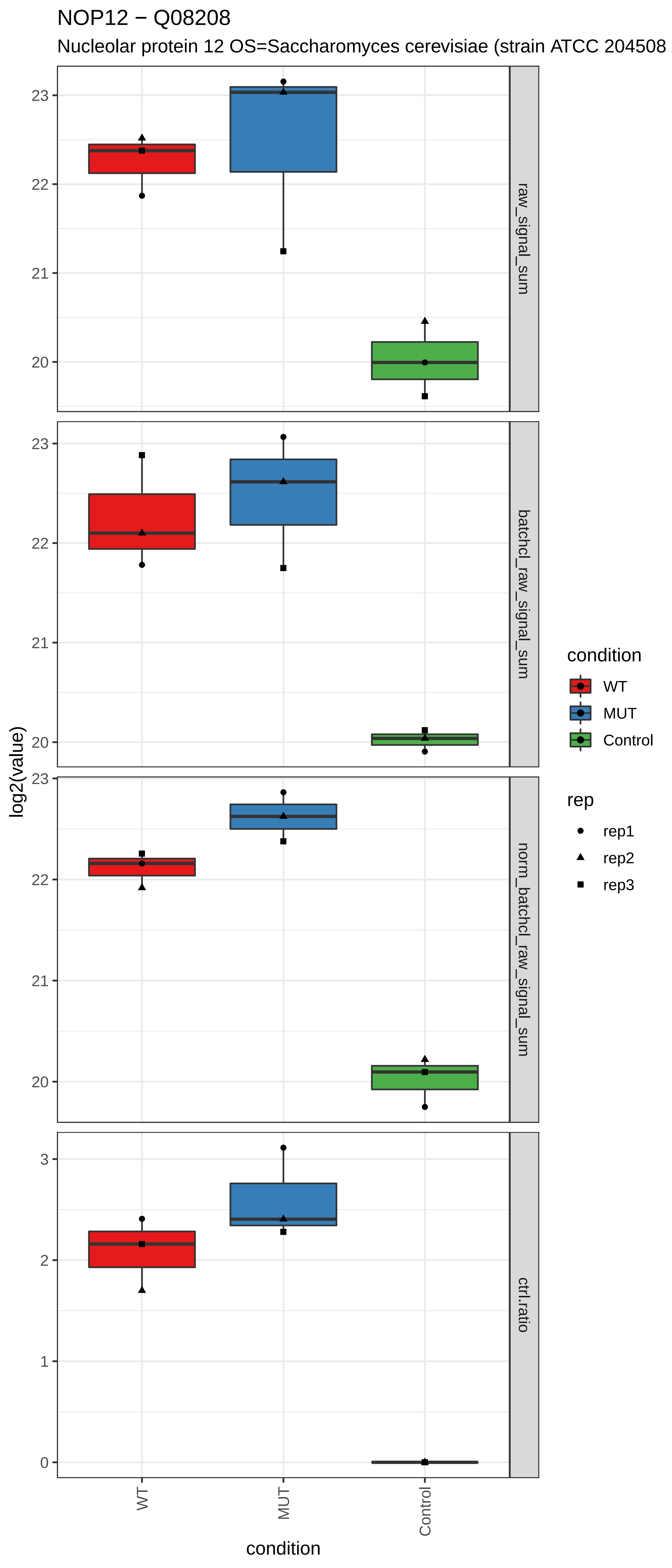

NOP12 − Q08208
Nucleolar protein 12 OS=Saccharomyces cerevisiae (strain ATCC 204508
raw_signal_sum
batchcl_raw_signal_sum
norm_batchcl_raw_signal_sum
ctrl.ratio
log2(value)
condition
WT
MUT
Control
rep
rep1
rep2
rep3

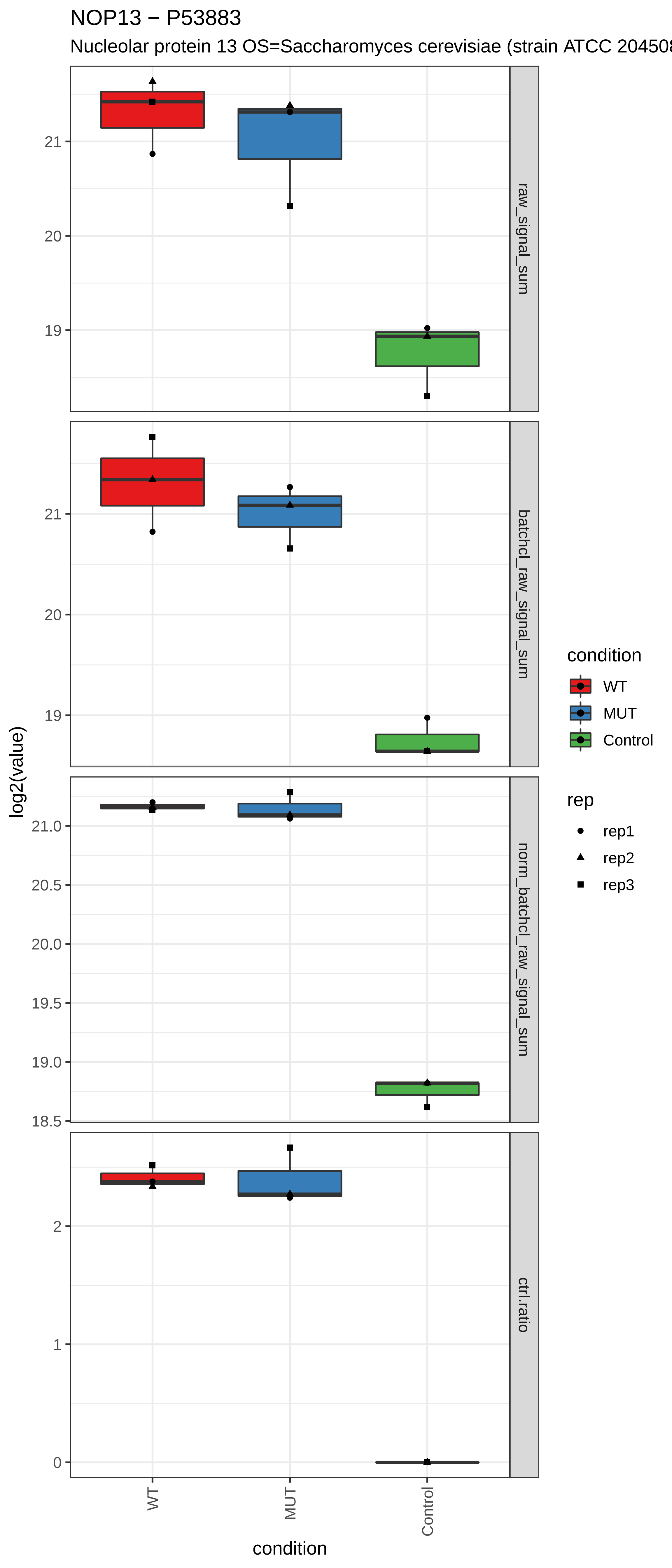

NOP13 − P53883
Nucleolar protein 13 OS=Saccharomyces cerevisiae (strain ATCC 204508
raw_signal_sum
batchcl_raw_signal_sum
norm_batchcl_raw_signal_sum
ctrl.ratio
log2(value)
condition
WT
MUT
Control
rep
rep1
rep2
rep3
21
20
19
21.0
20.5
20.0
19.5
19.0
18.5
2
1
0

NOP16 − P40007

Nucleolar protein 16 OS=Saccharomyces cerevisiae (strain ATCC 204508

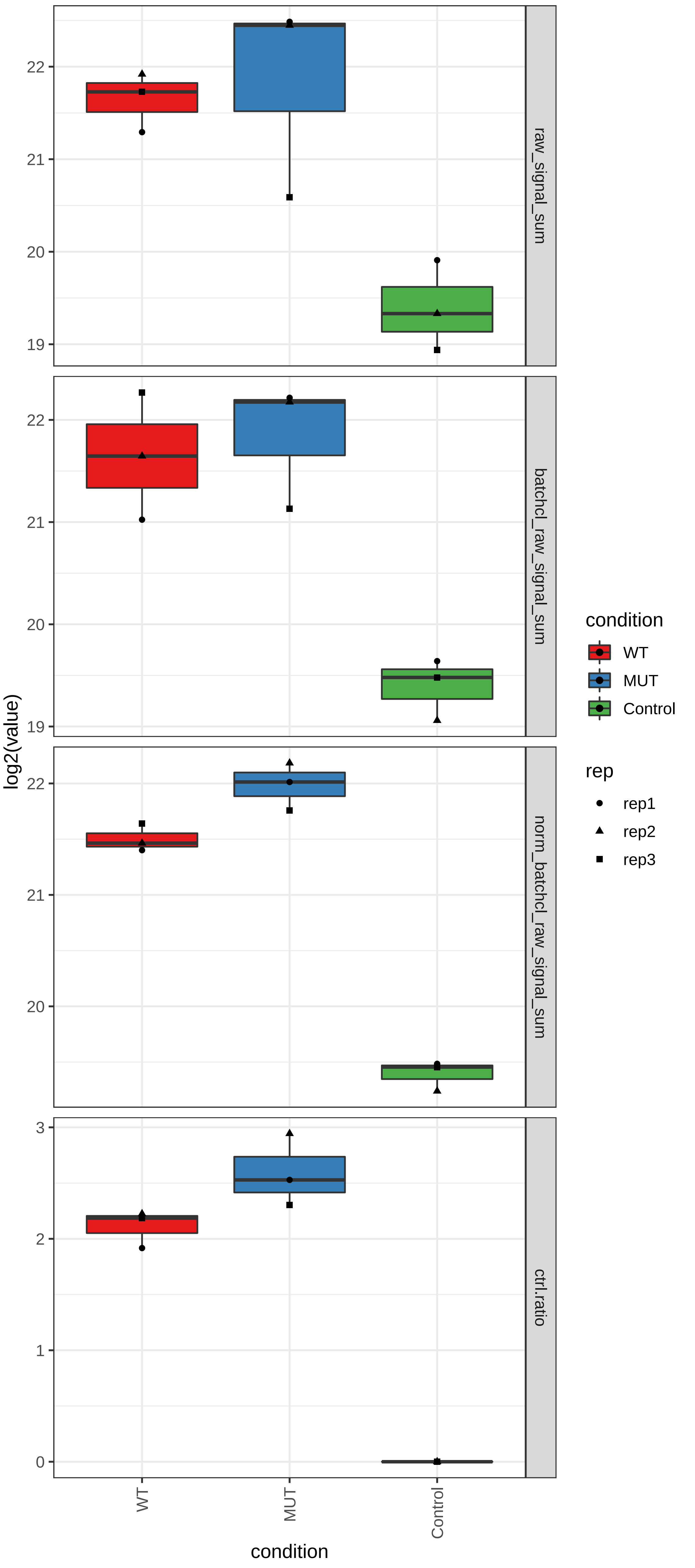

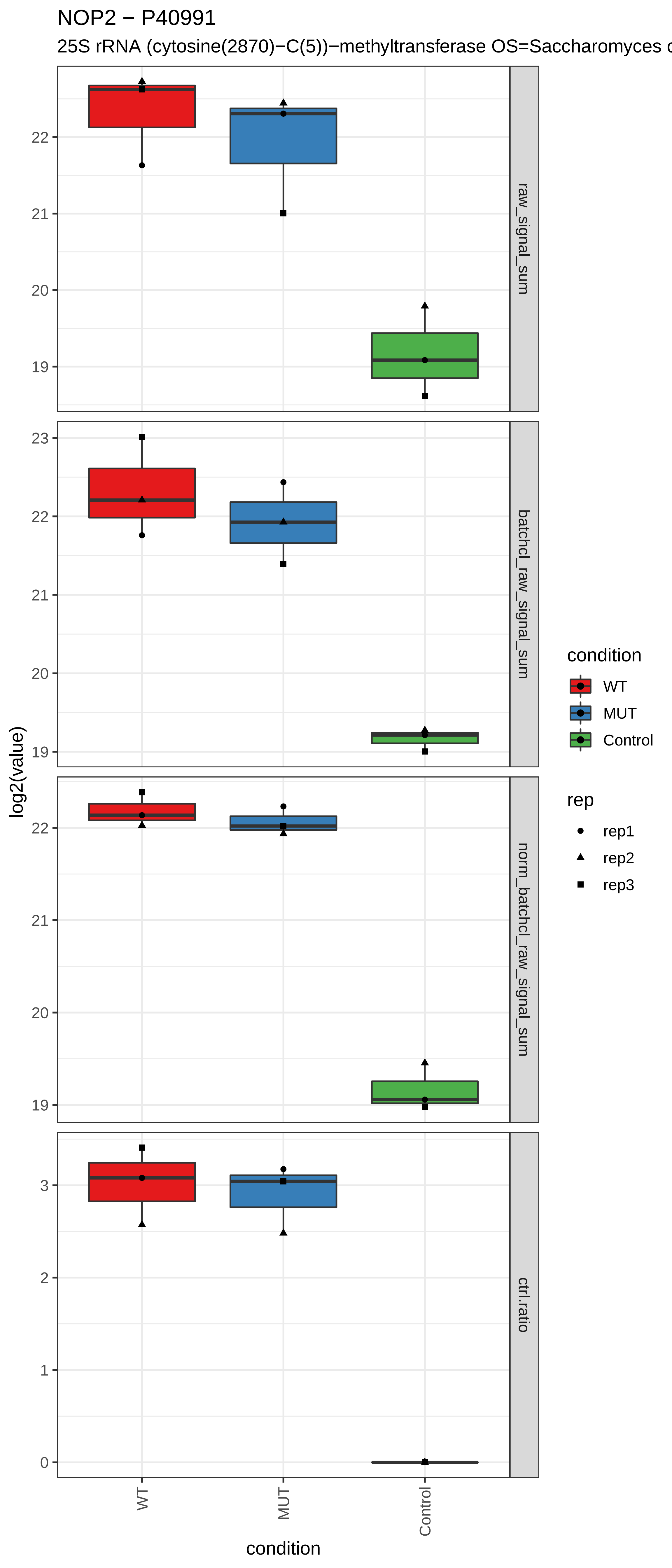
NOP2 − P40991
25S rRNA (cytosine(2870)−C(5))−methyltransferase OS=Saccharomyces c
raw_signal_sum
batchcl_raw_signal_sum
norm_batchcl_raw_signal_sum
ctrl.ratio
log2(value)
condition
WT
MUT
Control
rep
rep1
rep2
rep3

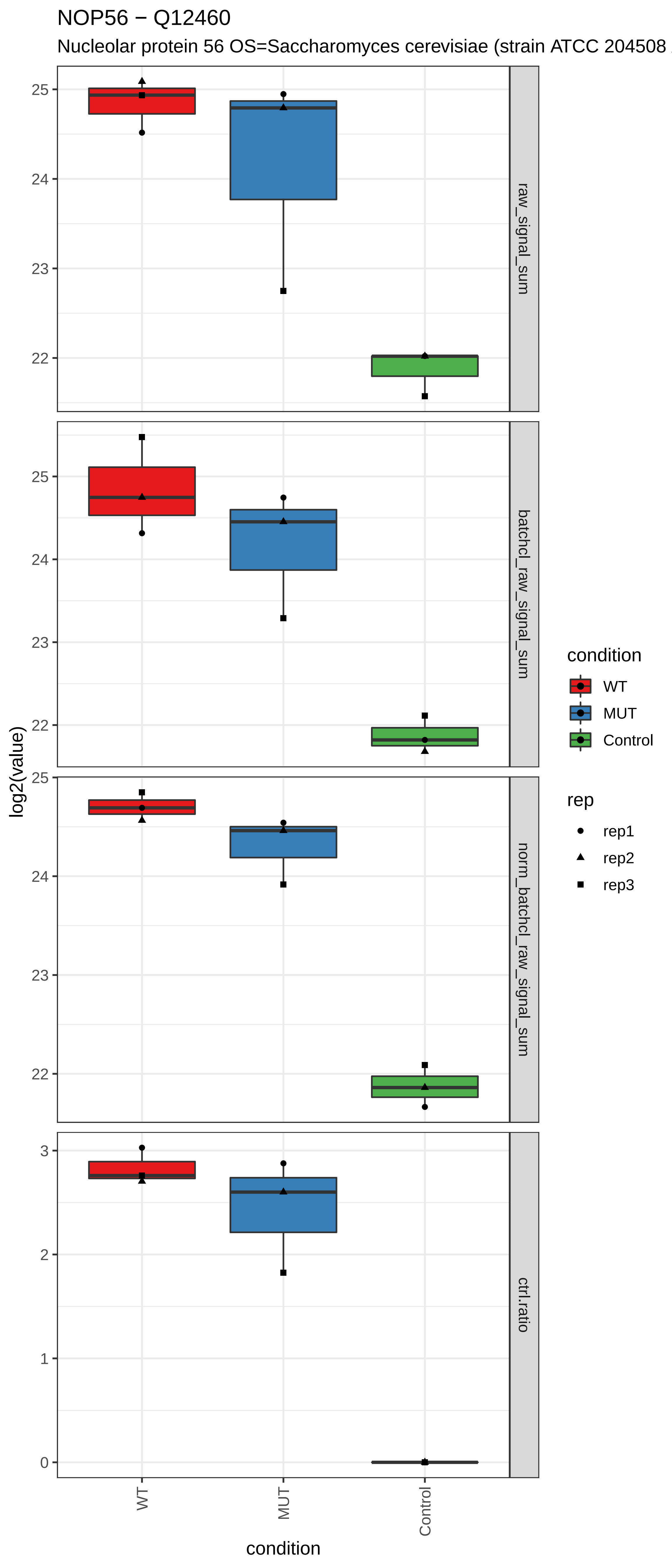

NOP56 − Q12460
Nucleolar protein 56 OS=Saccharomyces cerevisiae (strain ATCC 204508
raw_signal_sum
batchcl_raw_signal_sum
norm_batchcl_raw_signal_sum
ctrl.ratio
log2(value)
condition
WT
MUT
Control
rep
rep1
rep2
rep3

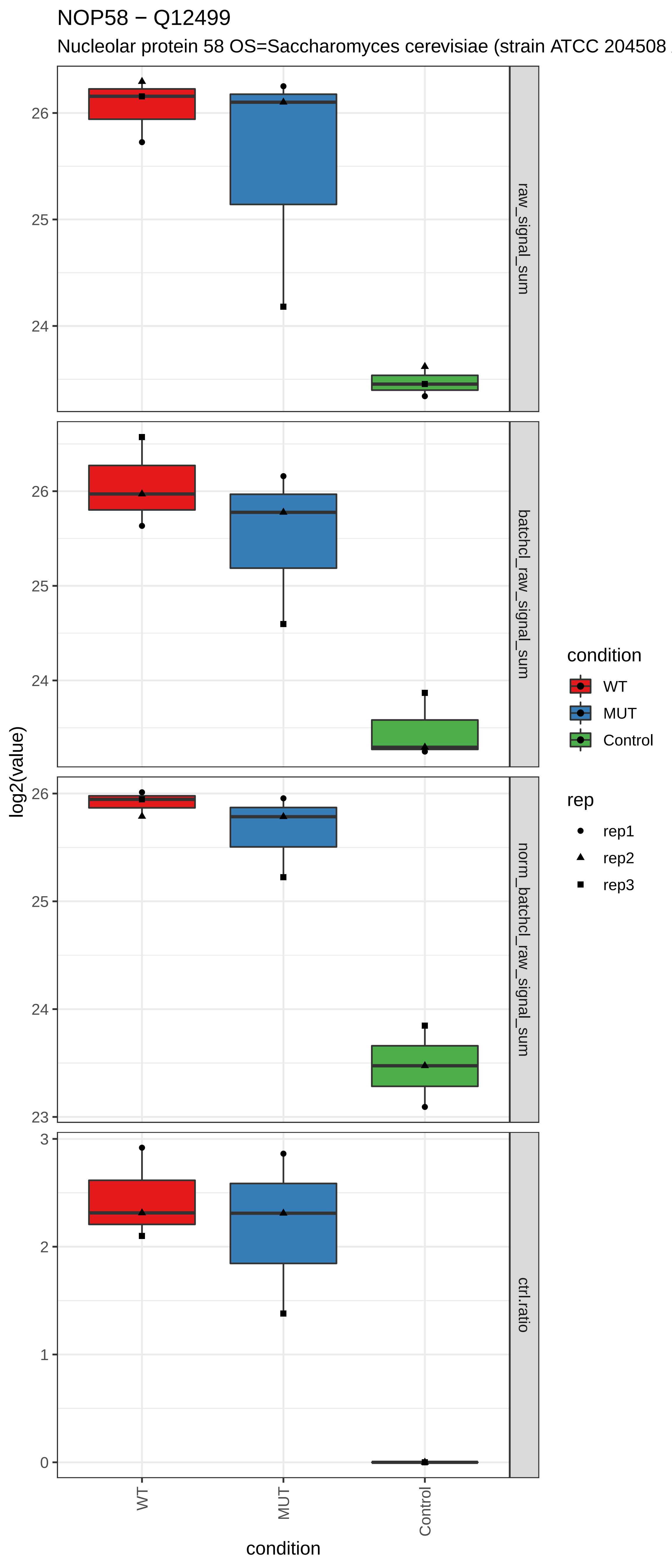

NOP58 − Q12499
Nucleolar protein 58 OS=Saccharomyces cerevisiae (strain ATCC 204508
raw_signal_sum
batchcl_raw_signal_sum
norm_batchcl_raw_signal_sum
ctrl.ratio
log2(value)
condition
WT
MUT
Control
rep
rep1
rep2
rep3

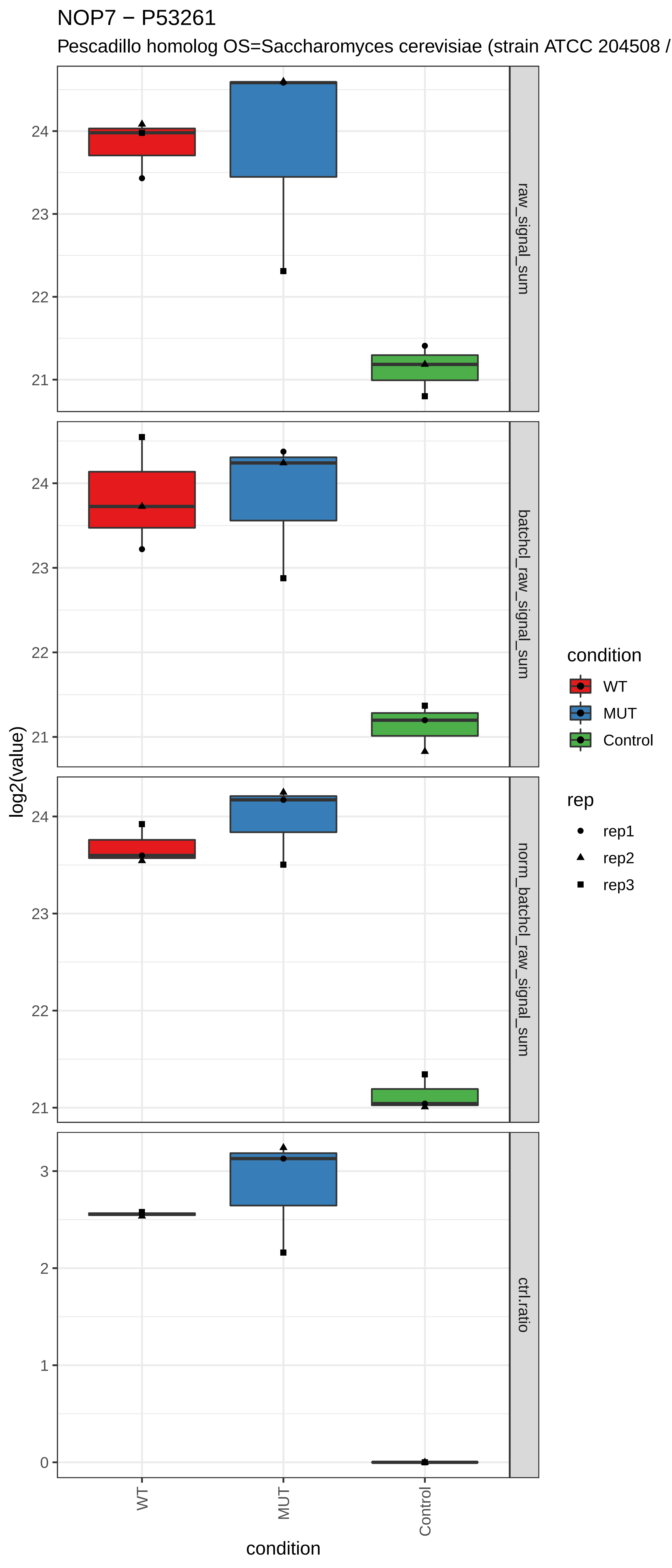

NOP7 − P53261
Pescadillo homolog OS=Saccharomyces cerevisiae (strain ATCC 204508 /
raw_signal_sum
batchcl_raw_signal_sum
norm_batchcl_raw_signal_sum
ctrl.ratio
log2(value)
condition
WT
MUT
Control
rep
rep1
rep2
rep3
24
23
22
21
3
2
1
0

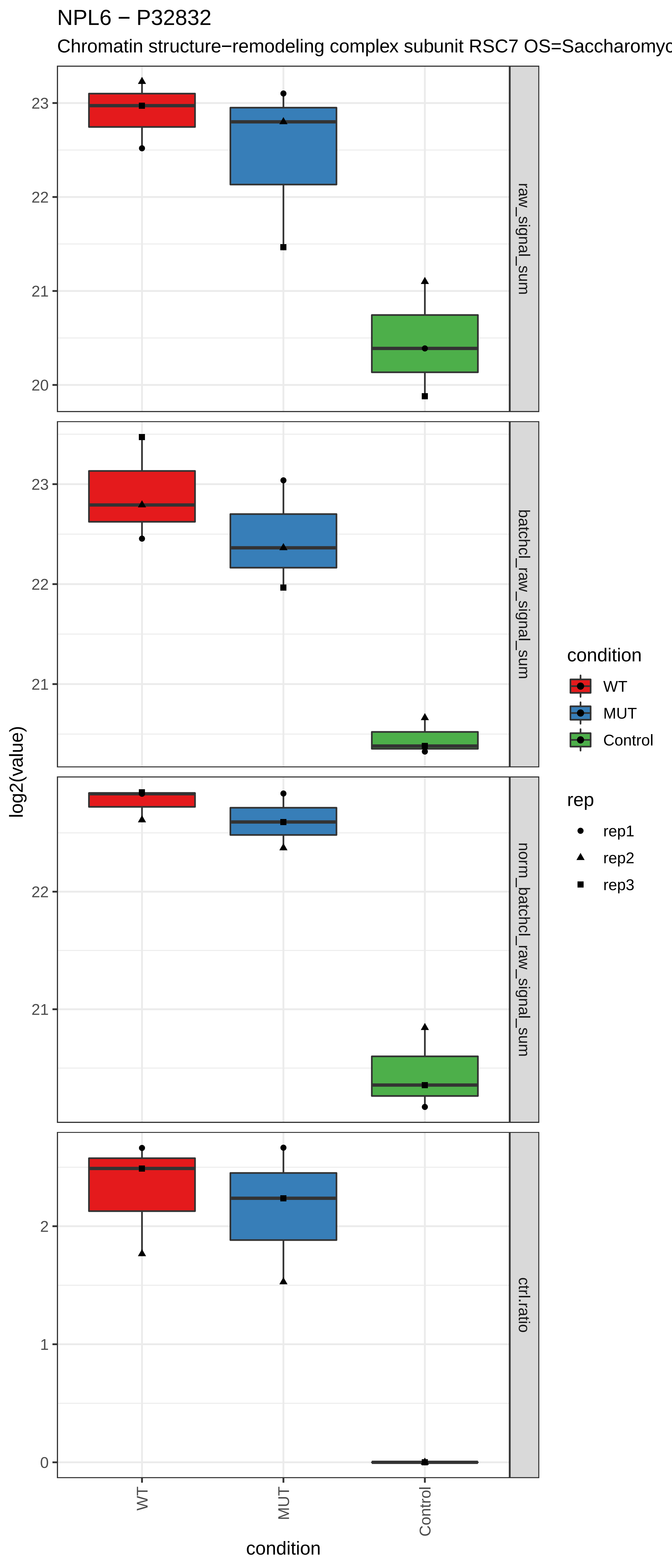

NPL6 − P32832
Chromatin structure−remodeling complex subunit RSC7 OS=Saccharomyc
raw_signal_sum
batchcl_raw_signal_sum
norm_batchcl_raw_signal_sum
ctrl.ratio
log2(value)
condition
WT
MUT
Control
rep
rep1
rep2
rep3

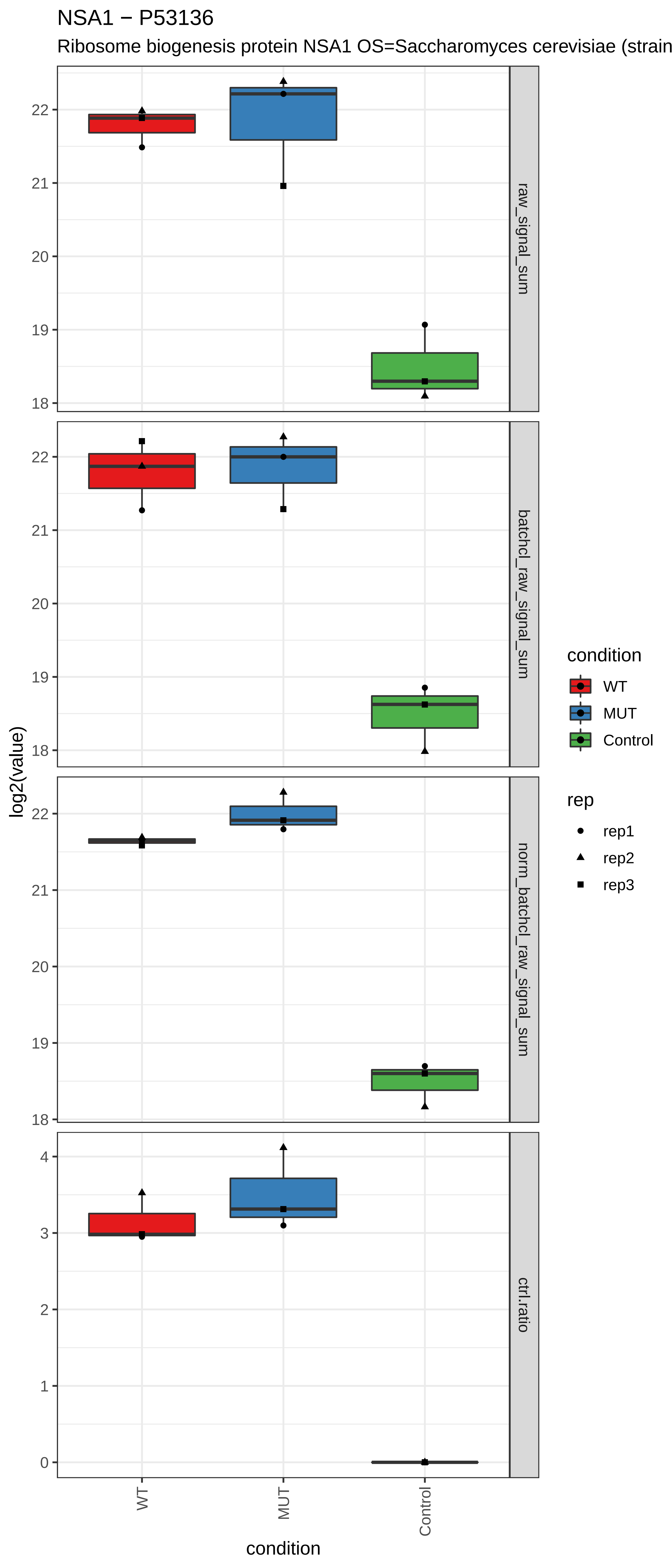

NSA1 − P53136
Ribosome biogenesis protein NSA1 OS=Saccharomyces cerevisiae (strain
raw_signal_sum
batchcl_raw_signal_sum
norm_batchcl_raw_signal_sum
ctrl.ratio
log2(value)
condition
WT
MUT
Control
rep
rep1
rep2
rep3

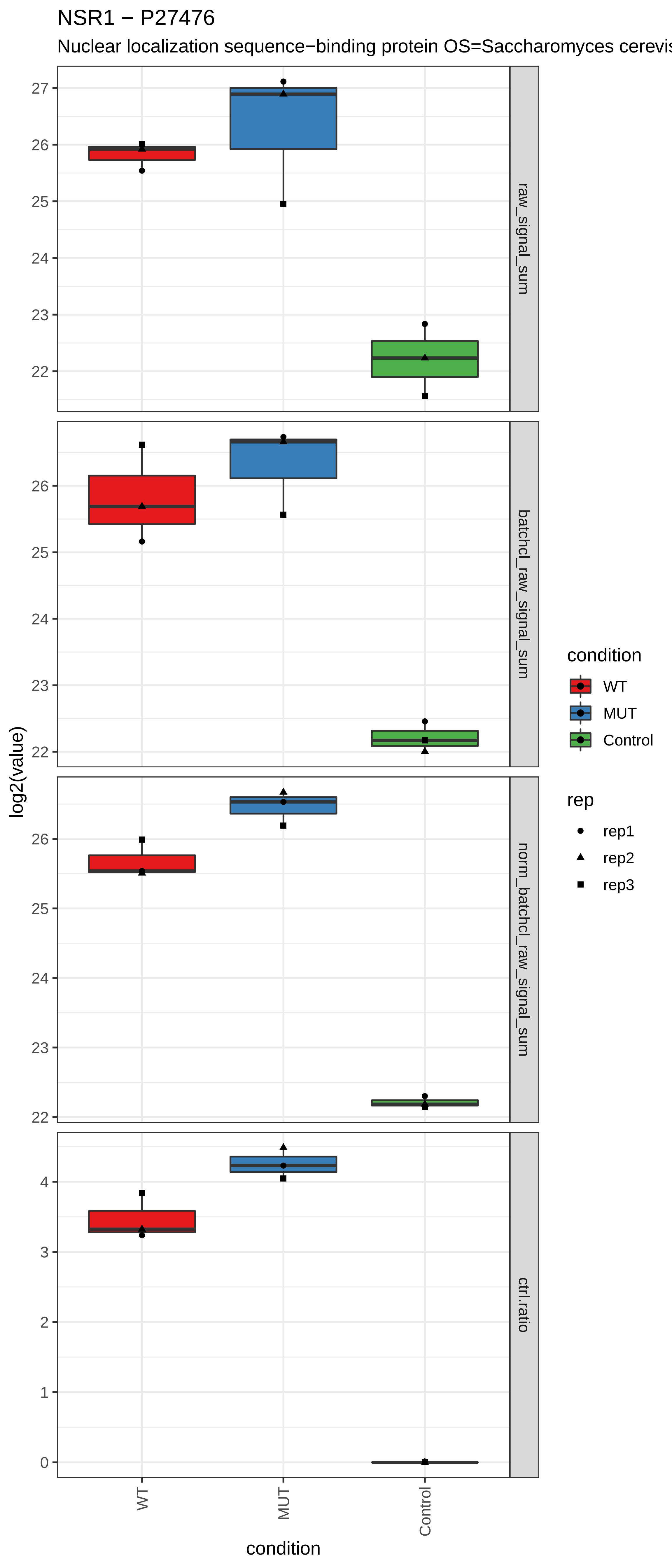

NSR1 − P27476
Nuclear localization sequence−binding protein OS=Saccharomyces cerevis
raw_signal_sum
batchcl_raw_signal_sum
norm_batchcl_raw_signal_sum
ctrl.ratio
log2(value)
condition
WT
MUT
Control
rep
rep1
rep2
rep3

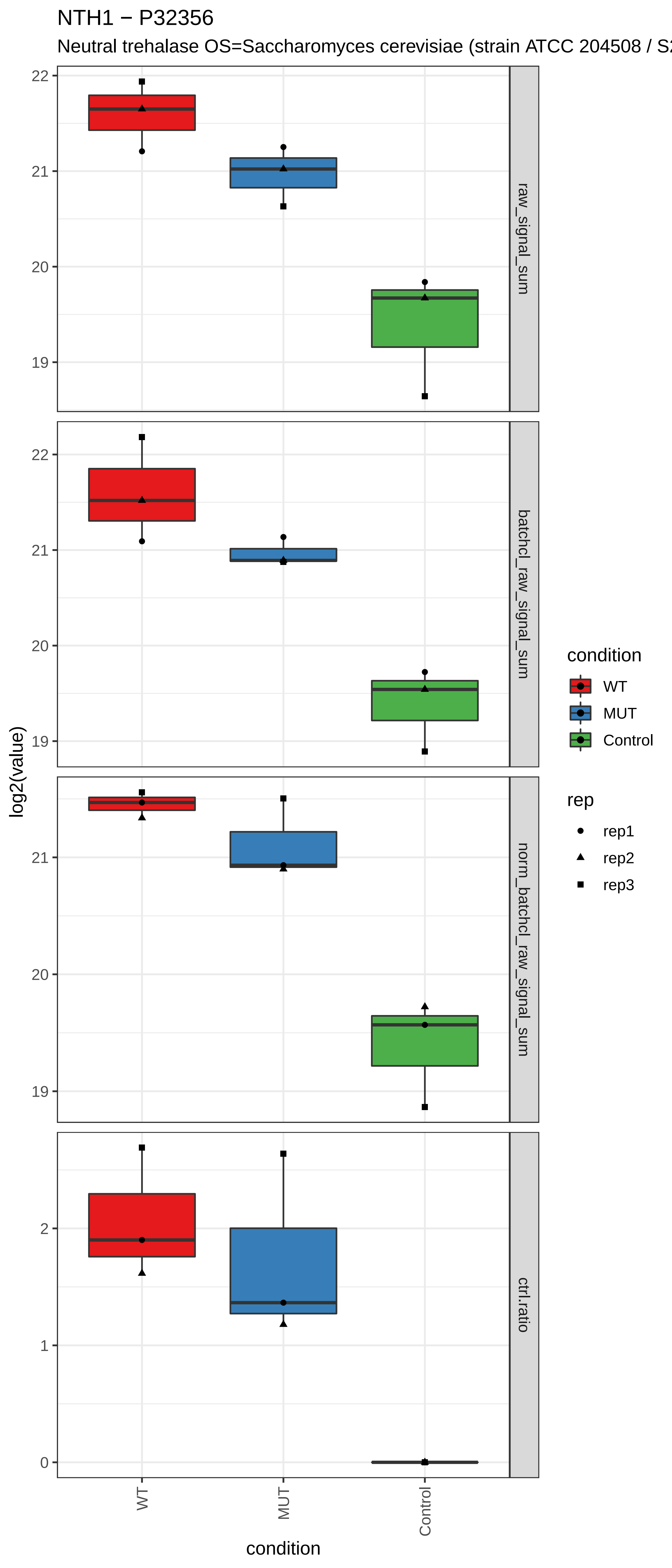

NTH1 − P32356
Neutral trehalase OS=Saccharomyces cerevisiae (strain ATCC 204508 / S2
raw_signal_sum
batchcl_raw_signal_sum
norm_batchcl_raw_signal_sum
ctrl.ratio
log2(value)
condition
WT
MUT
Control
rep
rep1
rep2
rep3

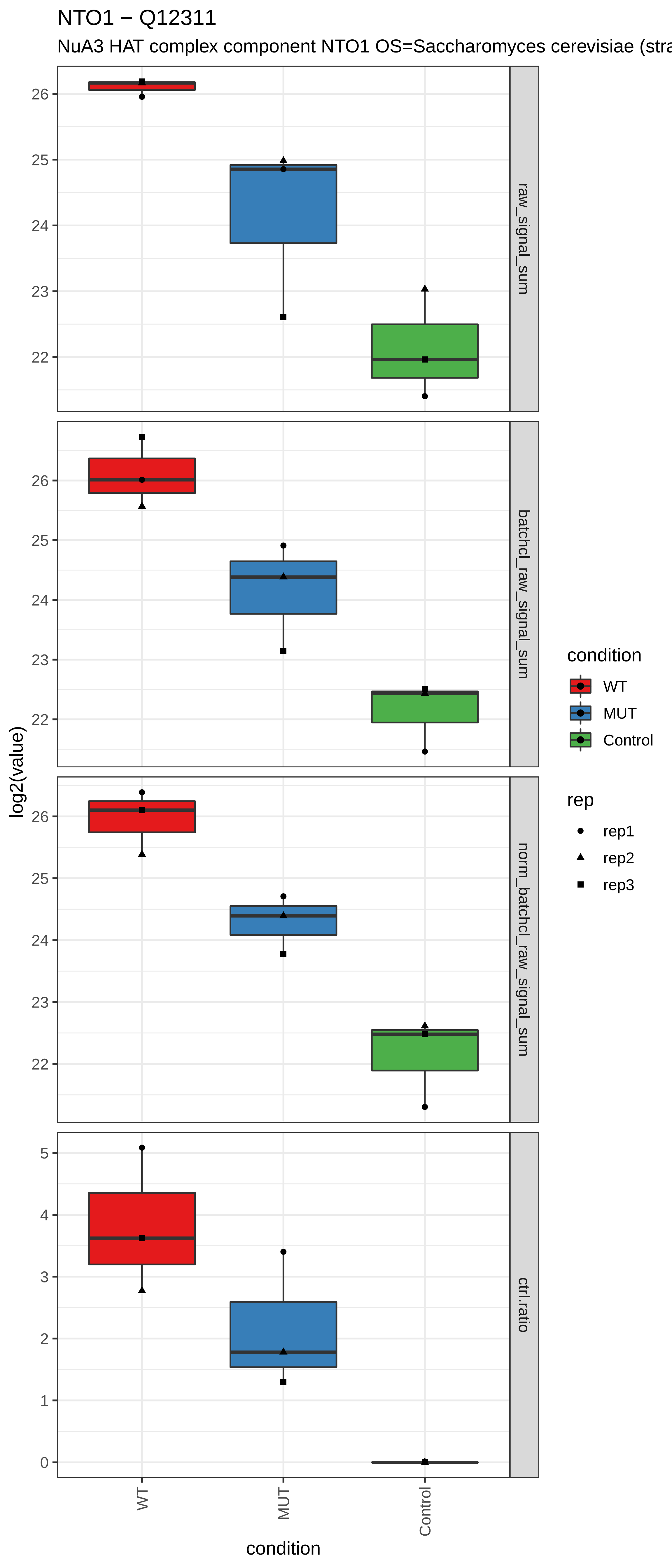

NTO1 − Q12311
NuA3 HAT complex component NTO1 OS=Saccharomyces cerevisiae (stra
raw_signal_sum
batchcl_raw_signal_sum
norm_batchcl_raw_signal_sum
ctrl.ratio
log2(value)
condition
WT
MUT
Control
rep
rep1
rep2
rep3

NUP145 − P49687

Nucleoporin NUP145 OS=Saccharomyces cerevisiae (strain ATCC 20450

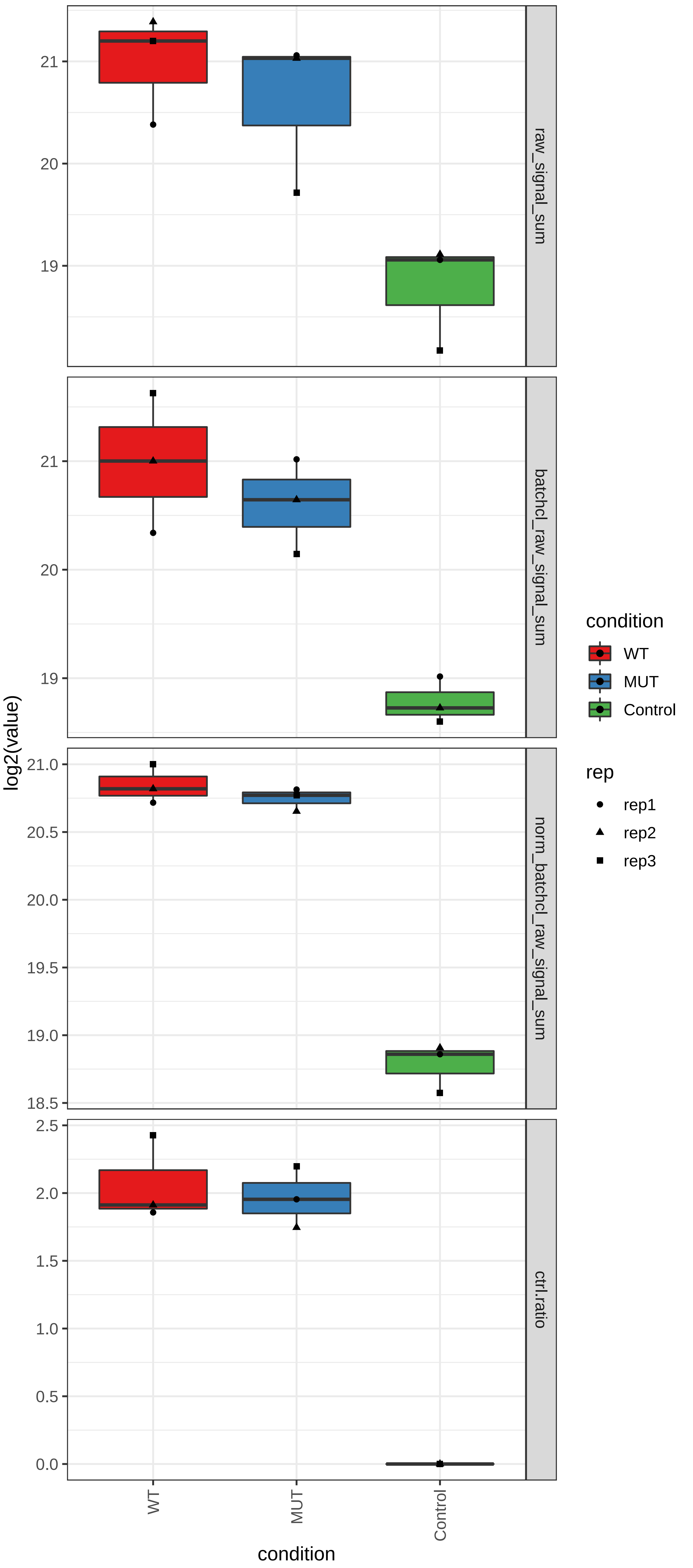

NUS1 − Q12063

Dehydrodolichyl diphosphate synthase complex subunit NUS1 OS=Saccha

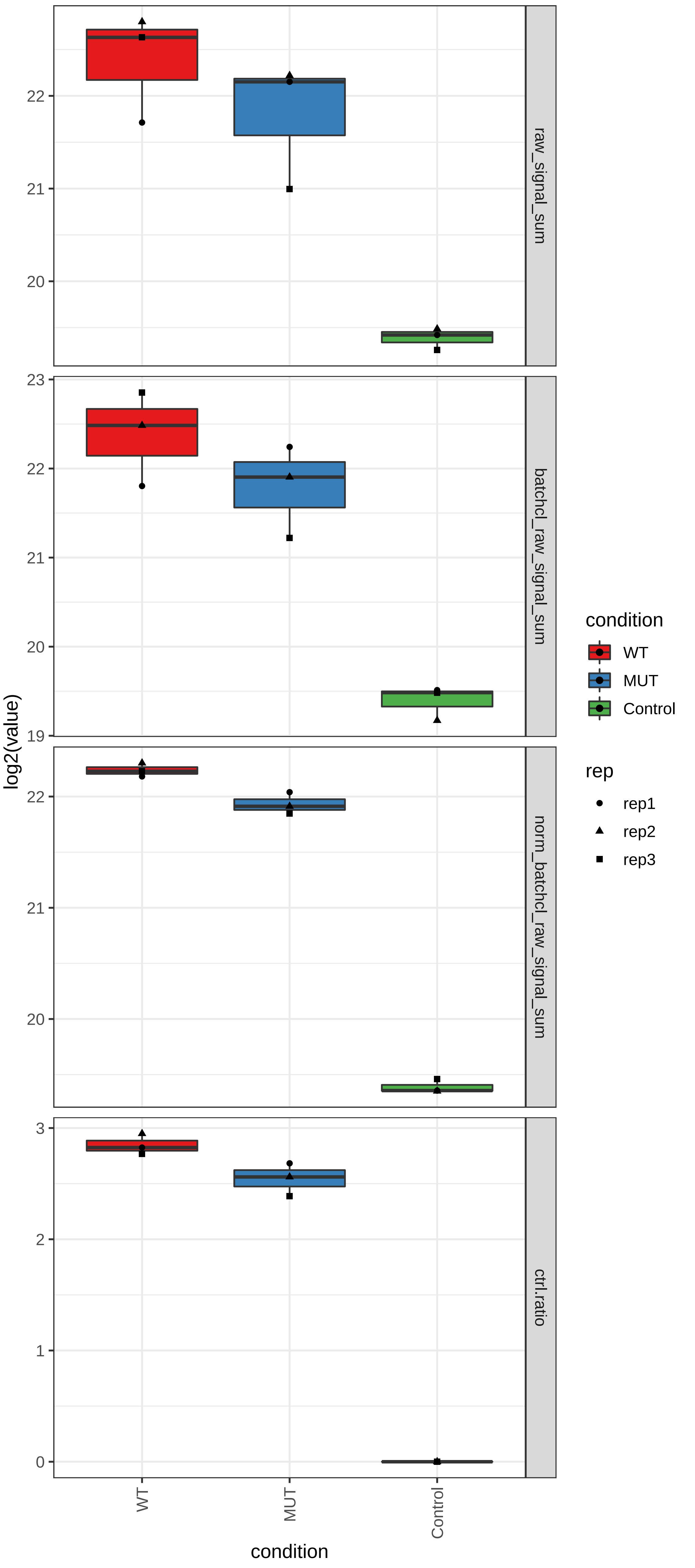

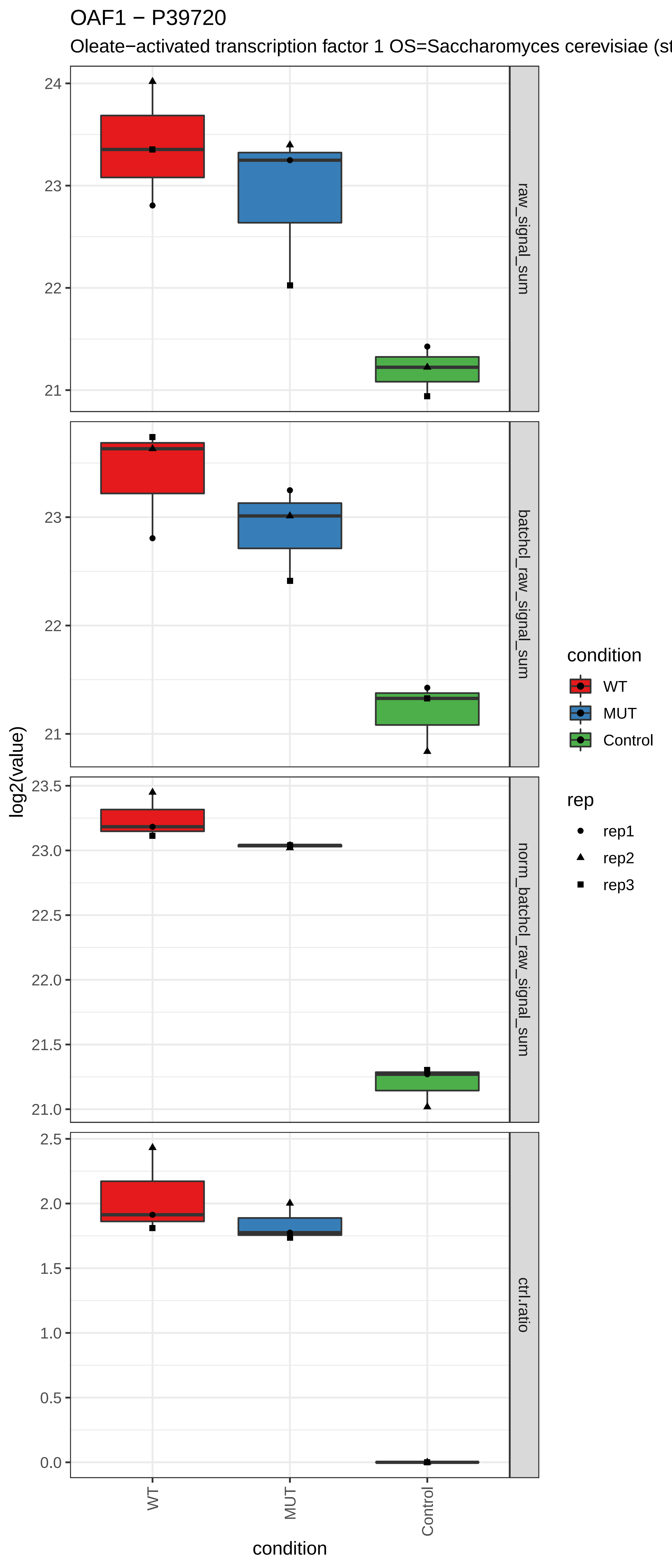
OAF1 − P39720
Oleate−activated transcription factor 1 OS=Saccharomyces cerevisiae (st
raw_signal_sum
batchcl_raw_signal_sum
norm_batchcl_raw_signal_sum
ctrl.ratio
log2(value)
condition
WT
MUT
Control
rep
rep1
rep2
rep3
24
23
22
21
23.5
23.0
22.5
22.0
21.5
21.0
2.5
2.0
1.5
1.0
0.5
0.0

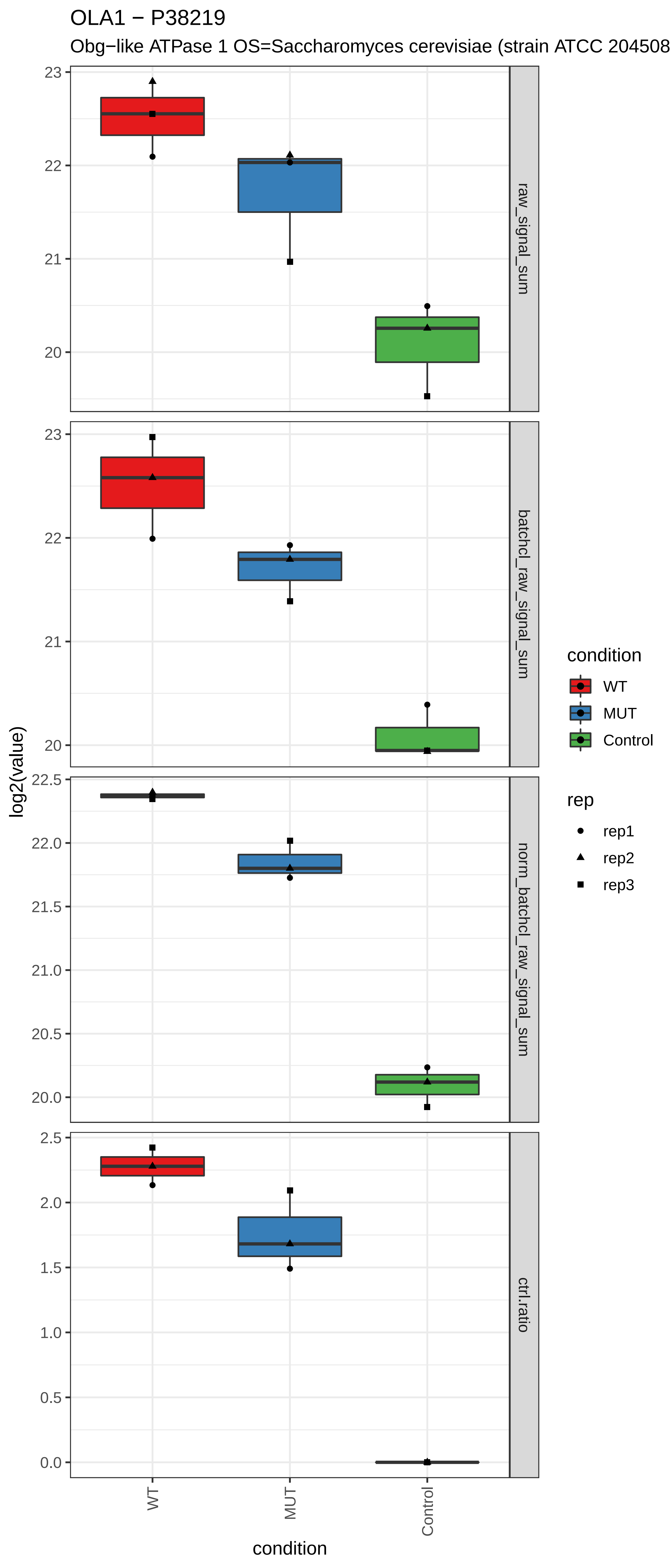

OLA1 − P38219
Obg−like ATPase 1 OS=Saccharomyces cerevisiae (strain ATCC 204508
raw_signal_sum
batchcl_raw_signal_sum
norm_batchcl_raw_signal_sum
ctrl.ratio
log2(value)
condition
WT
MUT
Control
rep
rep1
rep2
rep3

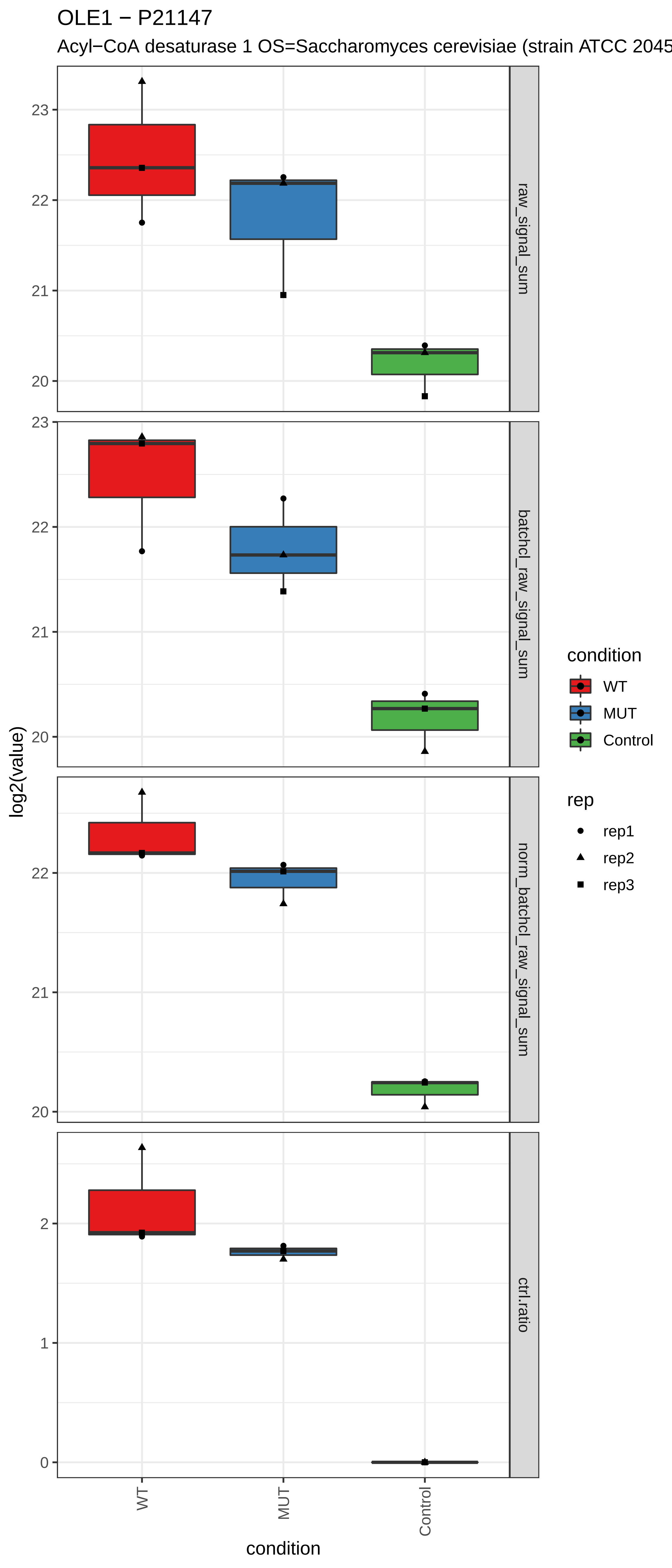

OLE1 − P21147
Acyl−CoA desaturase 1 OS=Saccharomyces cerevisiae (strain ATCC 2045
raw_signal_sum
batchcl_raw_signal_sum
norm_batchcl_raw_signal_sum
ctrl.ratio
log2(value)
condition
WT
MUT
Control
rep
rep1
rep2
rep3

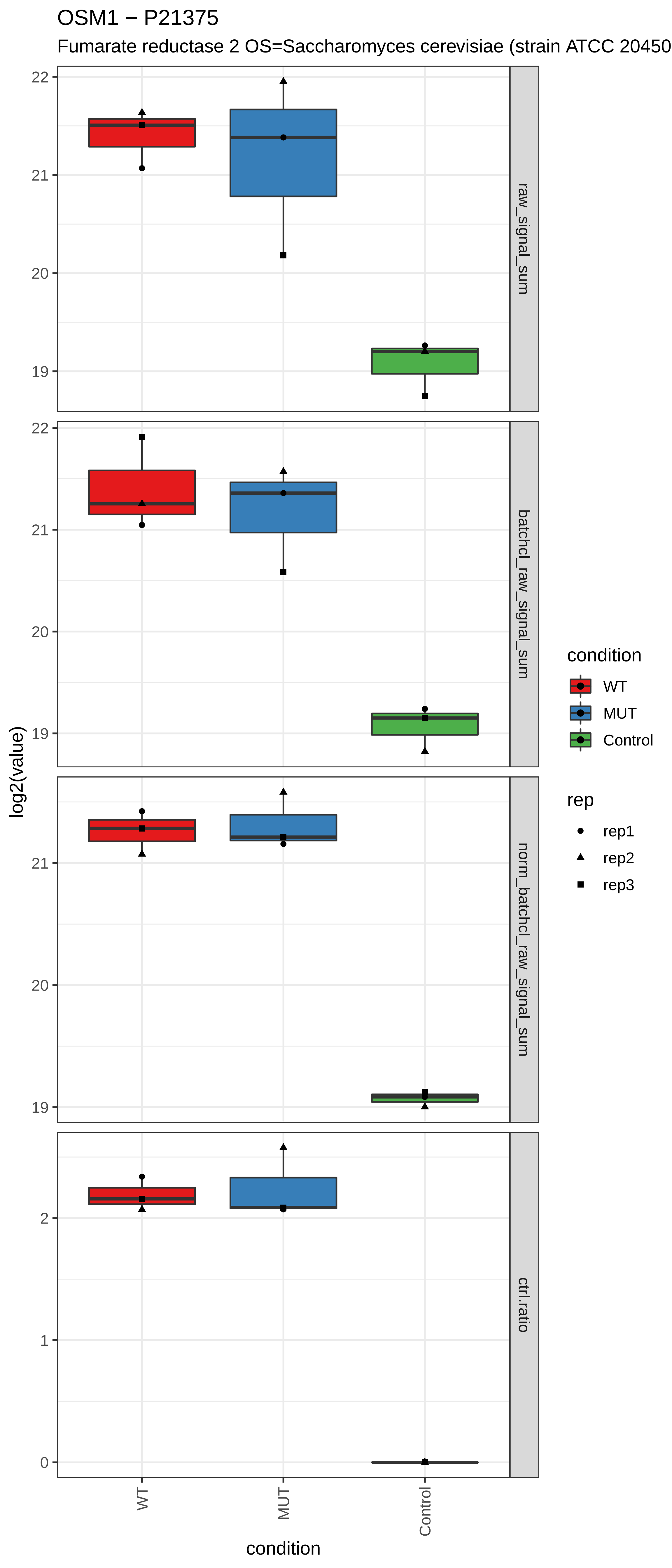
OSM1 − P21375
Fumarate reductase 2 OS=Saccharomyces cerevisiae (strain ATCC 20450
log2(value)
raw_signal_sum
batchcl_raw_signal_sum
norm_batchcl_raw_signal_sum
ctrl.ratio
condition
WT
MUT
Control
rep
rep1
rep2
rep3

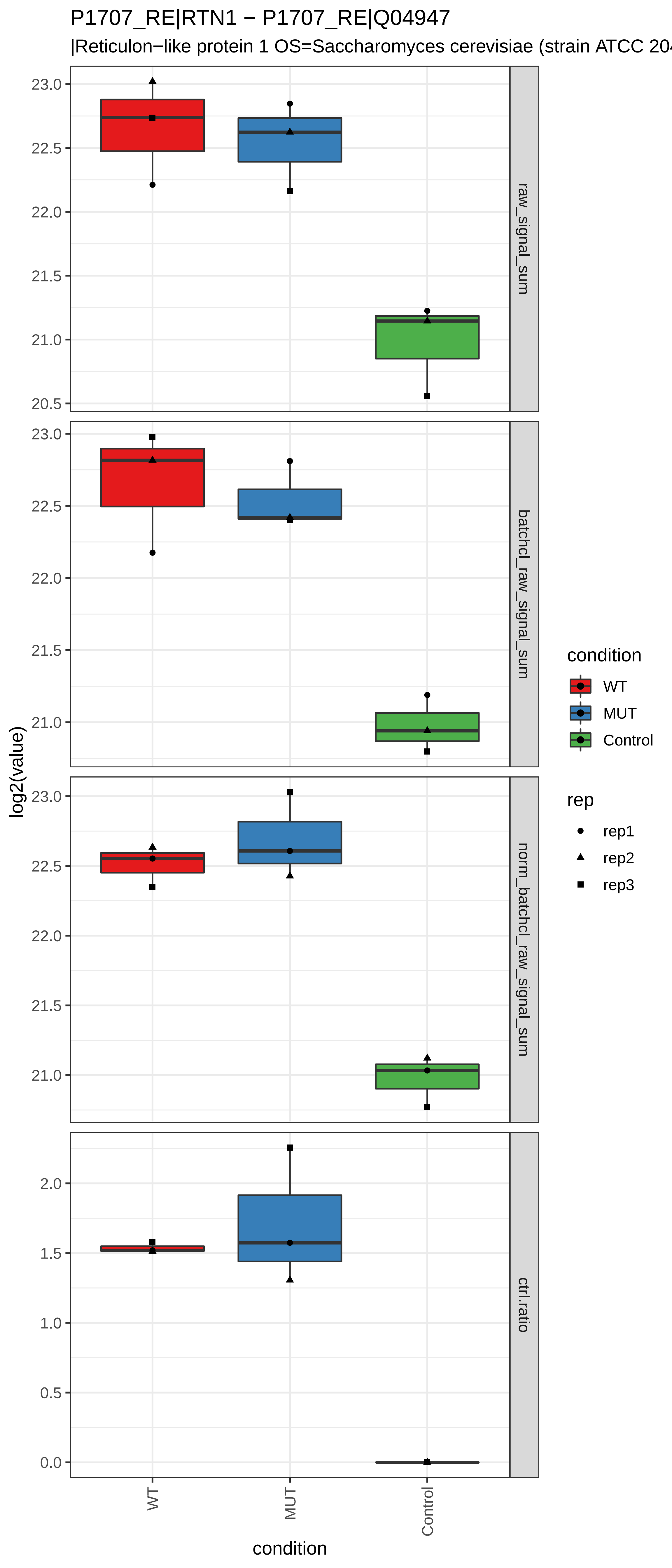
P1707_RE|RTN1 − P1707_RE|Q04947
|Reticulon−like protein 1 OS=Saccharomyces cerevisiae (strain ATCC 20
raw_signal_sum
batchcl_raw_signal_sum
norm_batchcl_raw_signal_sum
ctrl.ratio
log2(value)
condition
WT
MUT
Control
rep
rep1
rep2
rep3
23.0
22.5
22.0
21.5
21.0
20.5
2.0
1.5
1.0
0.5
0.0

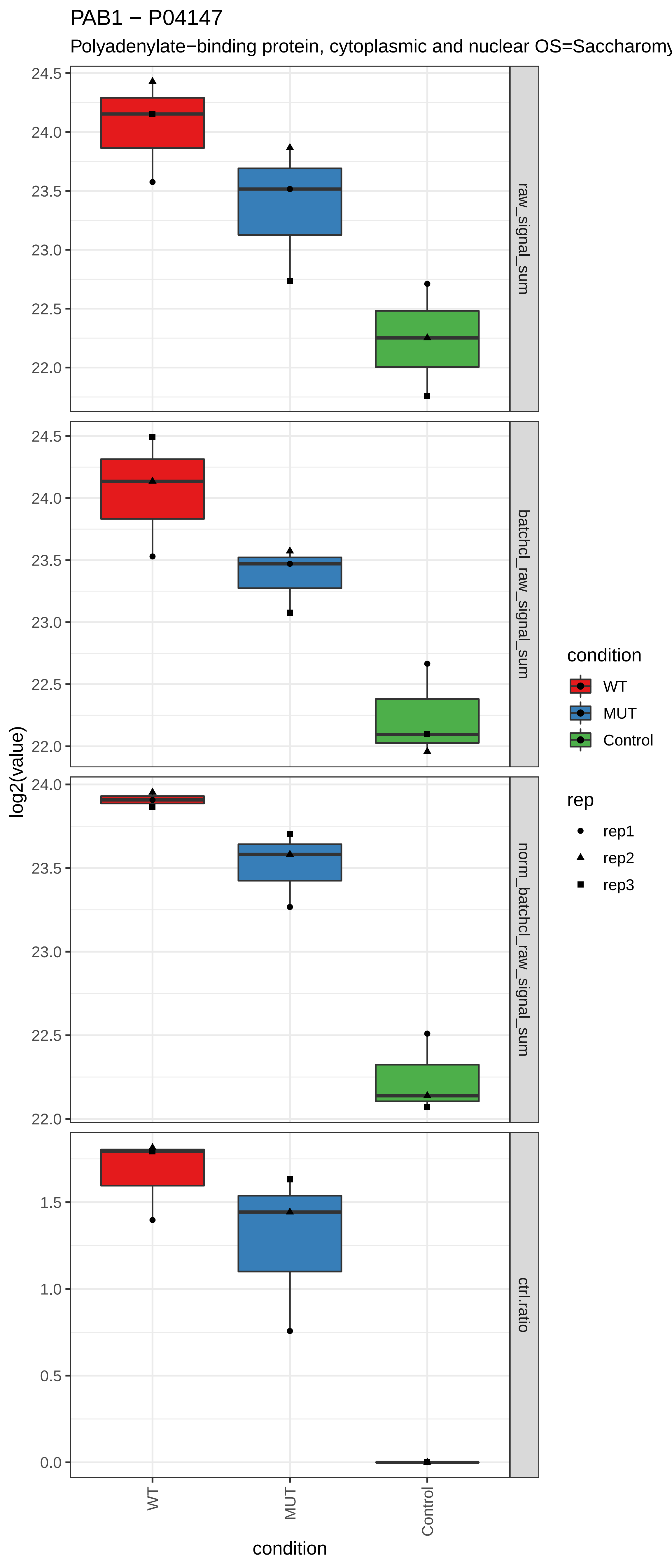
PAB1 − P04147
Polyadenylate−binding protein, cytoplasmic and nuclear OS=Saccharomy
log2(value)
raw_signal_sum
batchcl_raw_signal_sum
norm_batchcl_raw_signal_sum
ctrl.ratio
24.5
24.0
23.5
23.0
22.5
22.0
1.5
1.0
0.5
0.0
WT
MUT
Control
condition
condition
WT
MUT
Control
rep
rep1
rep2
rep3

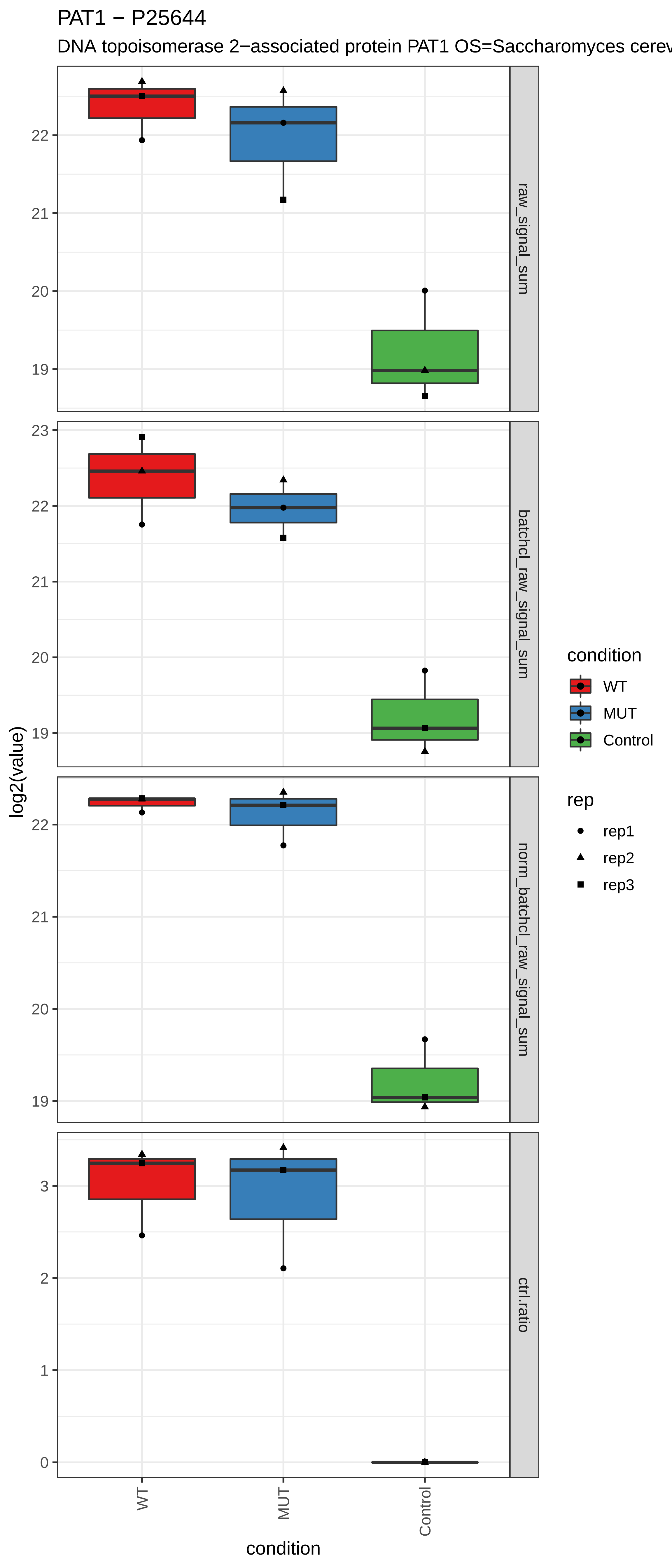

PAT1 − P25644
DNA topoisomerase 2−associated protein PAT1 OS=Saccharomyces cerev
raw_signal_sum
batchcl_raw_signal_sum
norm_batchcl_raw_signal_sum
ctrl.ratio
log2(value)
condition
WT
MUT
Control
rep
rep1
rep2
rep3

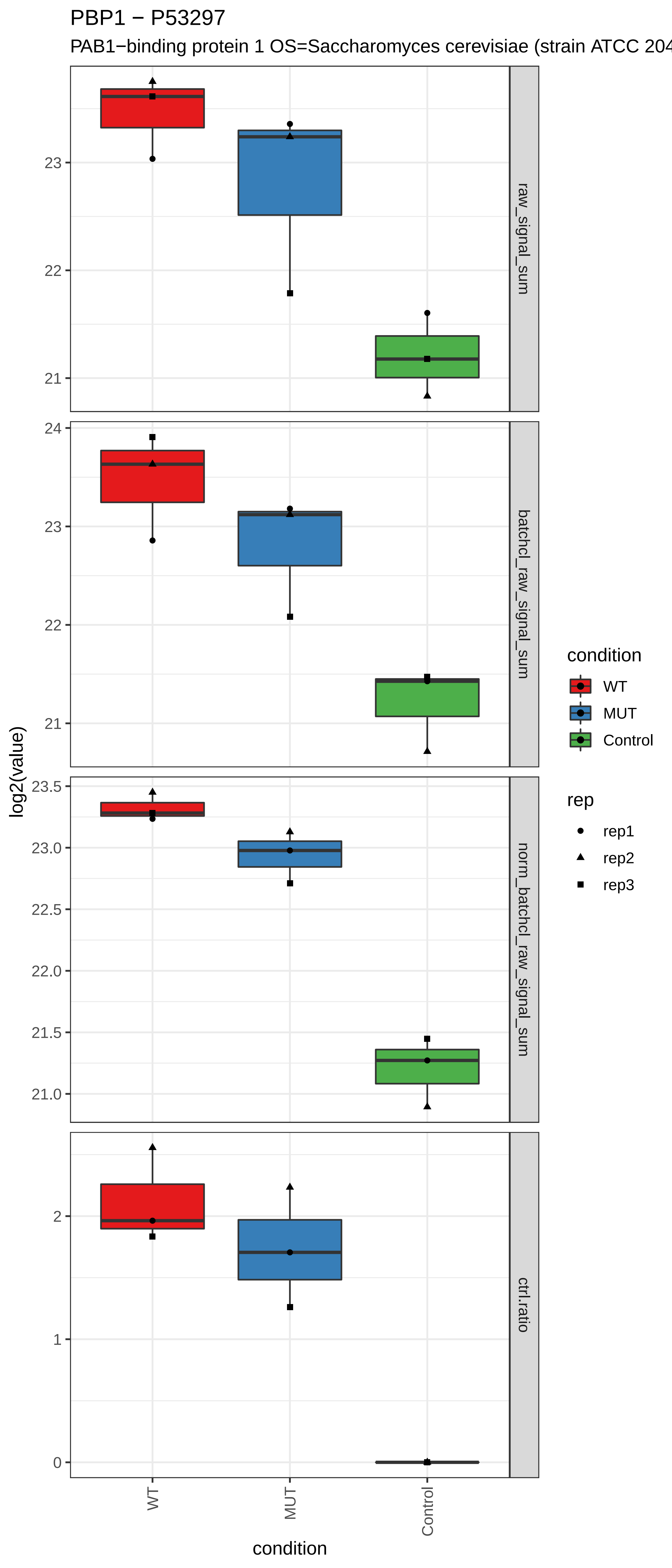
PBP1 − P53297
PAB1−binding protein 1 OS=Saccharomyces cerevisiae (strain ATCC 204
raw_signal_sum
batchcl_raw_signal_sum
norm_batchcl_raw_signal_sum
ctrl.ratio
log2(value)
condition
WT
MUT
Control
rep
rep1
rep2
rep3
23
22
21
24
23.5
23.0
22.5
22.0
21.5
21.0
2
1
0

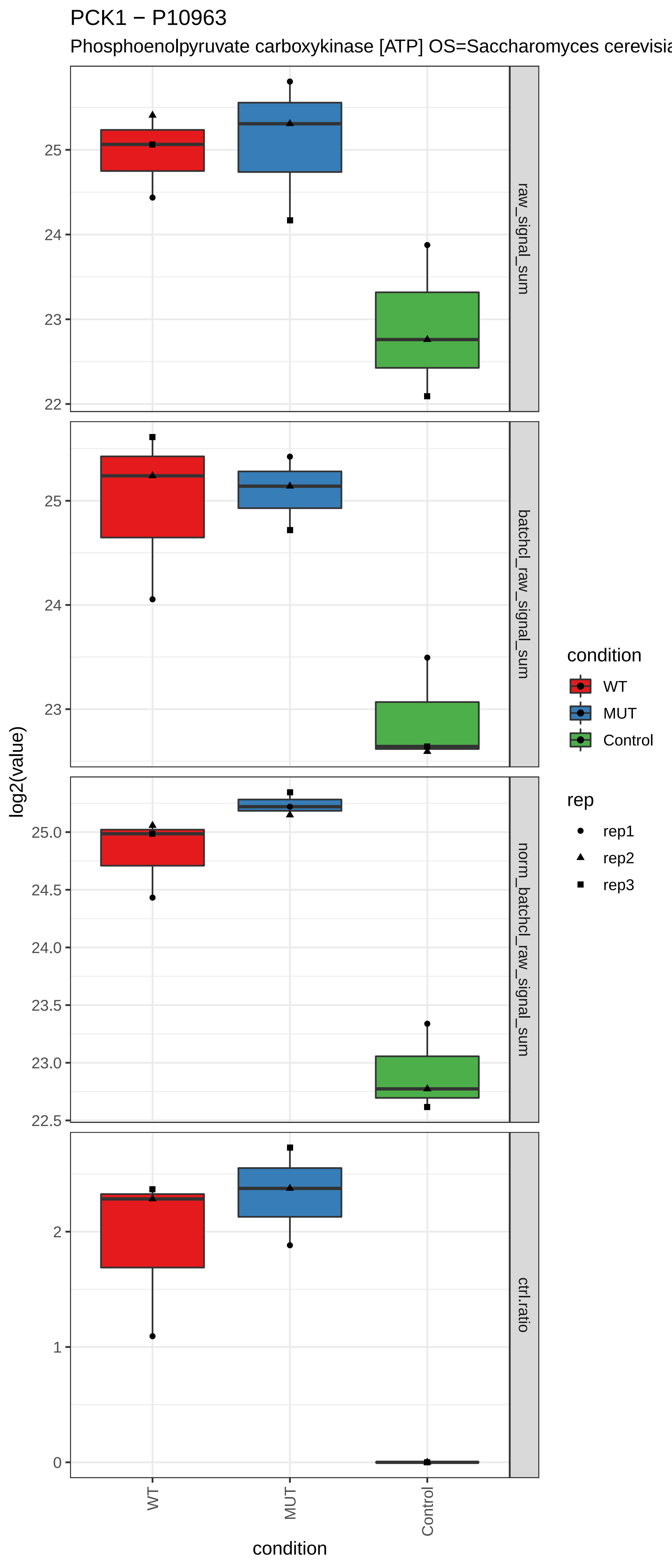

PCK1 − P10963
Phosphoenolpyruvate carboxykinase [ATP] OS=Saccharomyces cerevisia
raw_signal_sum
batchcl_raw_signal_sum
norm_batchcl_raw_signal_sum
ctrl.ratio
log2(value)
condition
WT
MUT
Control
rep
rep1
rep2
rep3

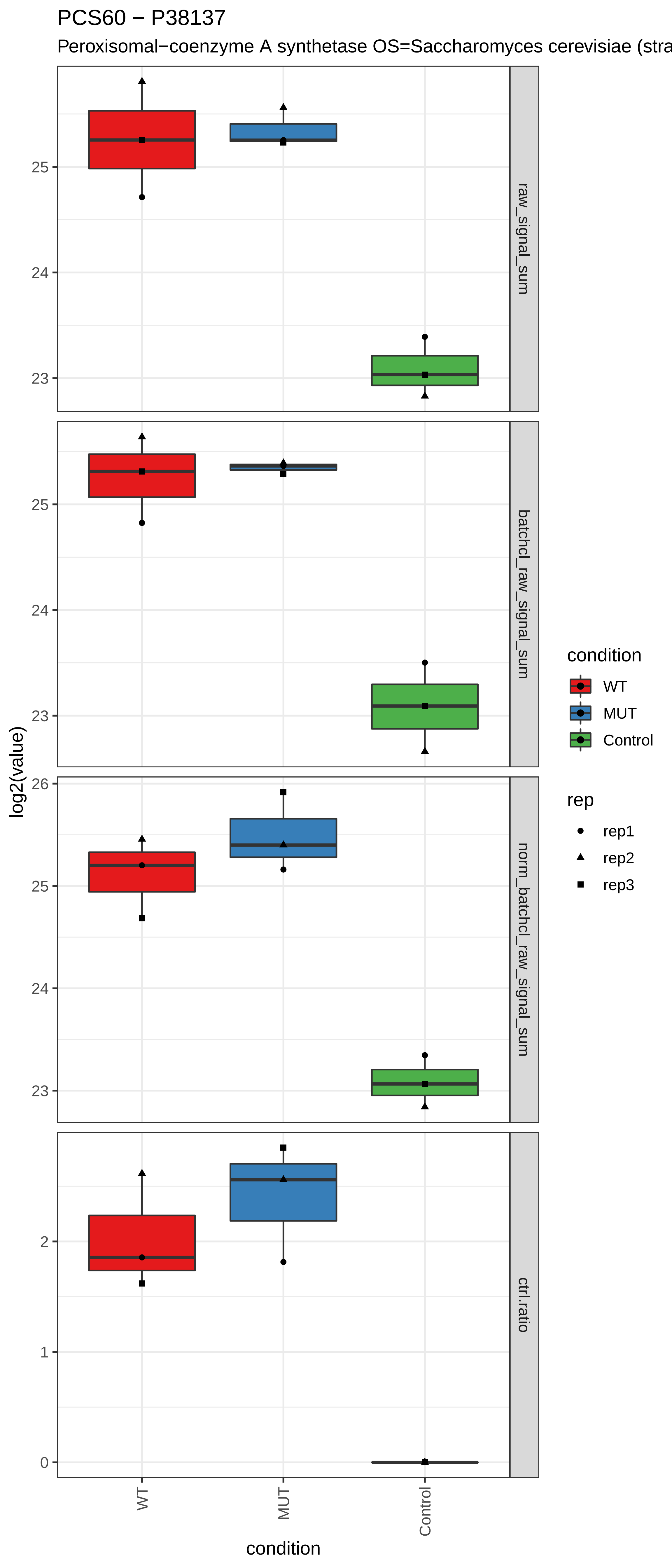
PCS60 − P38137
Peroxisomal−coenzyme A synthetase OS=Saccharomyces cerevisiae (stra
raw_signal_sum
batchcl_raw_signal_sum
norm_batchcl_raw_signal_sum
ctrl.ratio
log2(value)
condition
WT
MUT
Control
rep
rep1
rep2
rep3
25
24
23
26
2
1
0

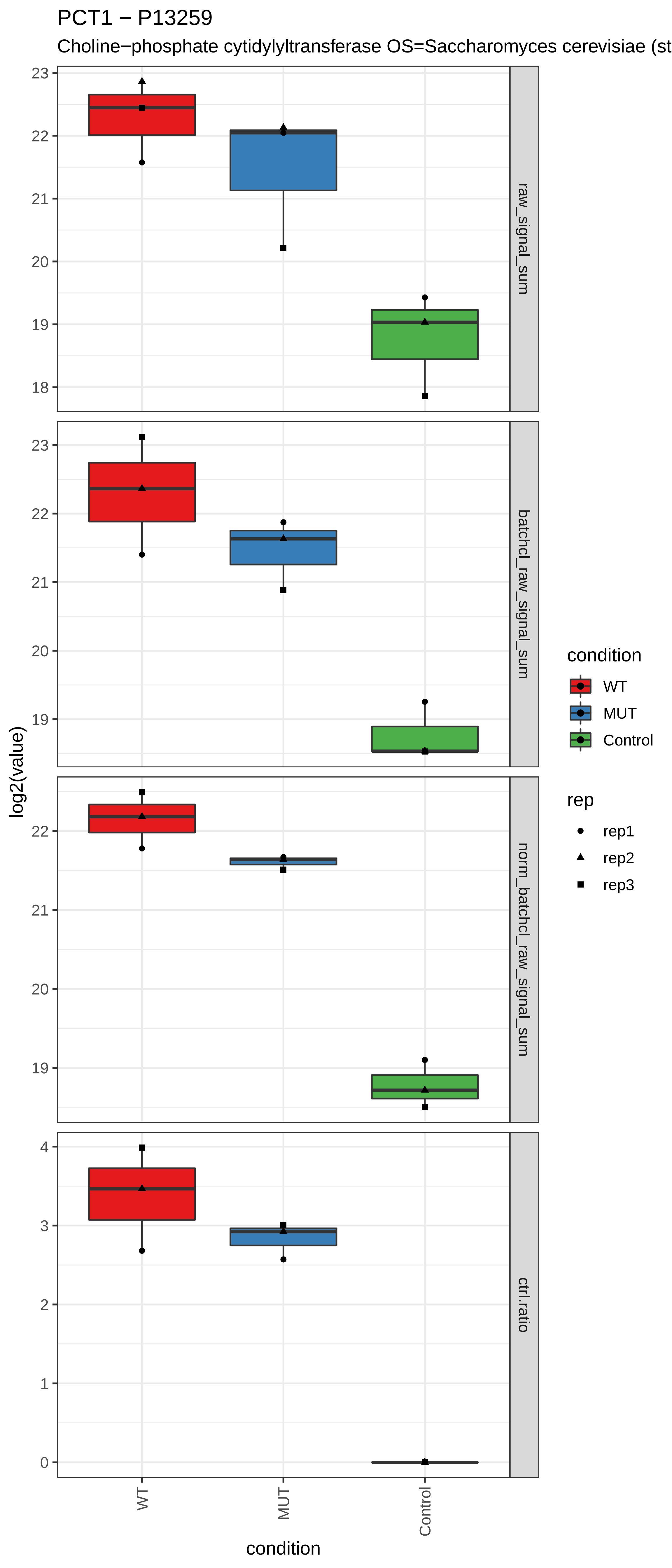

PCT1 − P13259
Choline−phosphate cytidylyltransferase OS=Saccharomyces cerevisiae (st
log2(value)
raw_signal_sum
batchcl_raw_signal_sum
norm_batchcl_raw_signal_sum
ctrl.ratio
23
22
21
20
19
18
0
1
2
3
4
WT
MUT
Control
condition
condition
WT
MUT
Control
rep
rep1
rep2
rep3

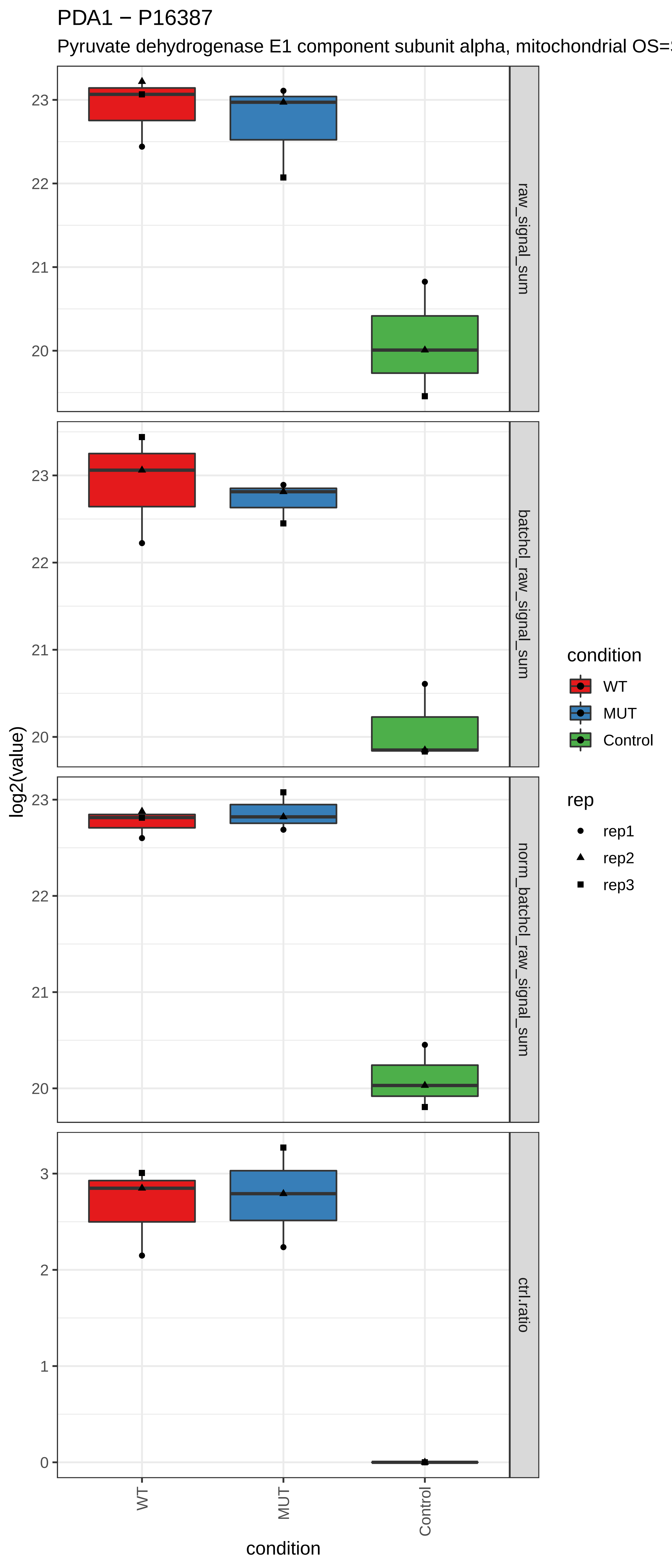

PDA1 − P16387
Pyruvate dehydrogenase E1 component subunit alpha, mitochondrial OS=
raw_signal_sum
batchcl_raw_signal_sum
norm_batchcl_raw_signal_sum
ctrl.ratio
log2(value)
condition
WT
MUT
Control
rep
rep1
rep2
rep3

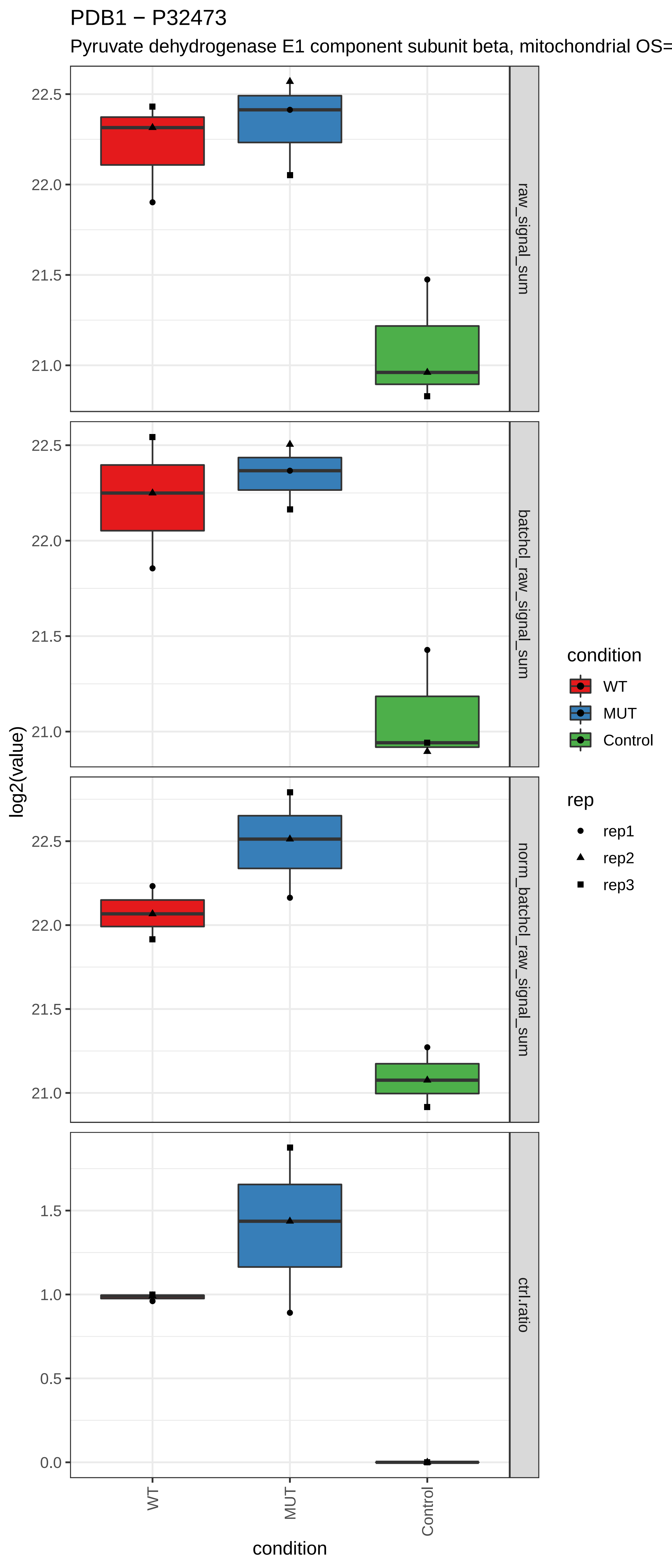

PDB1 − P32473
Pyruvate dehydrogenase E1 component subunit beta, mitochondrial OS=
raw_signal_sum
batchcl_raw_signal_sum
norm_batchcl_raw_signal_sum
ctrl.ratio
log2(value)
condition
WT
MUT
Control
rep
rep1
rep2
rep3
22.5
22.0
21.5
21.0
1.5
1.0
0.5
0.0

PDC1 − P06169

Pyruvate decarboxylase isozyme 1 OS=Saccharomyces cerevisiae (strain

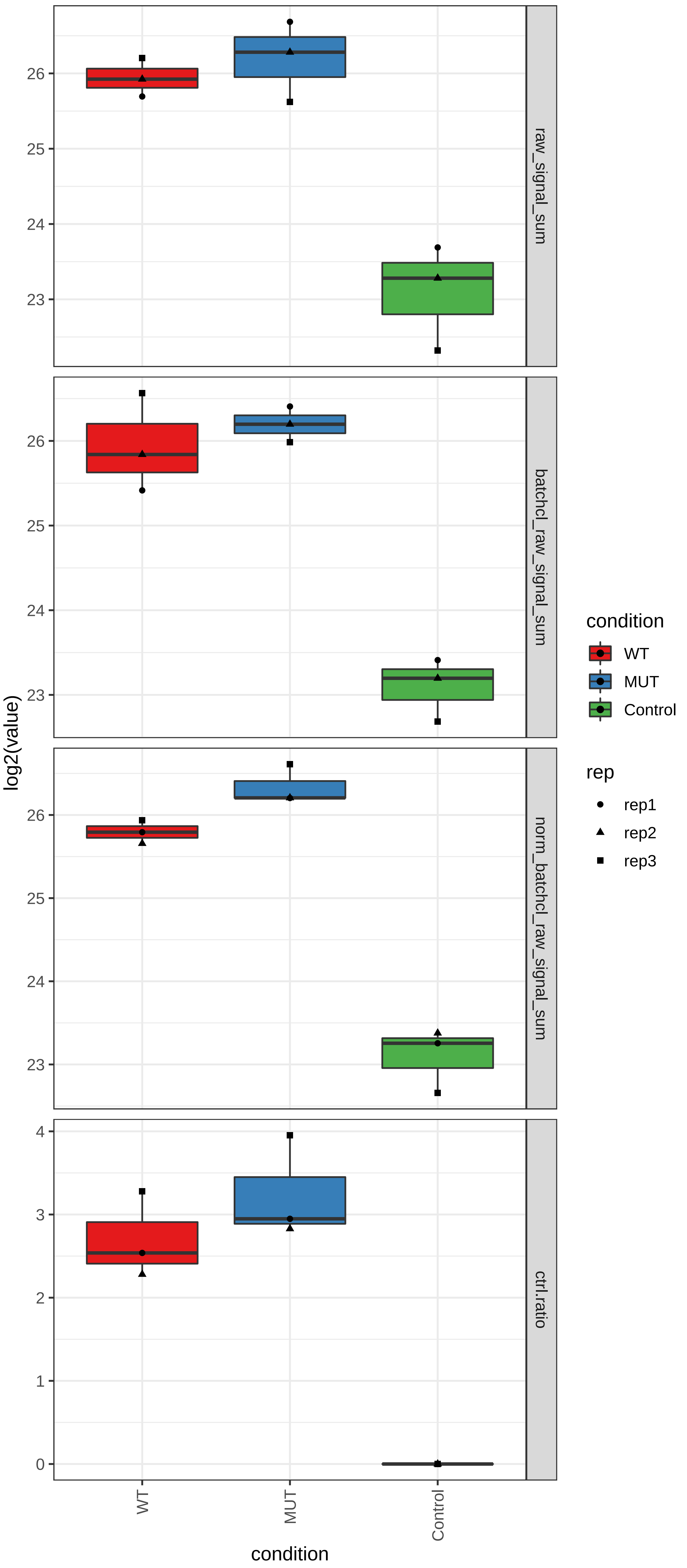

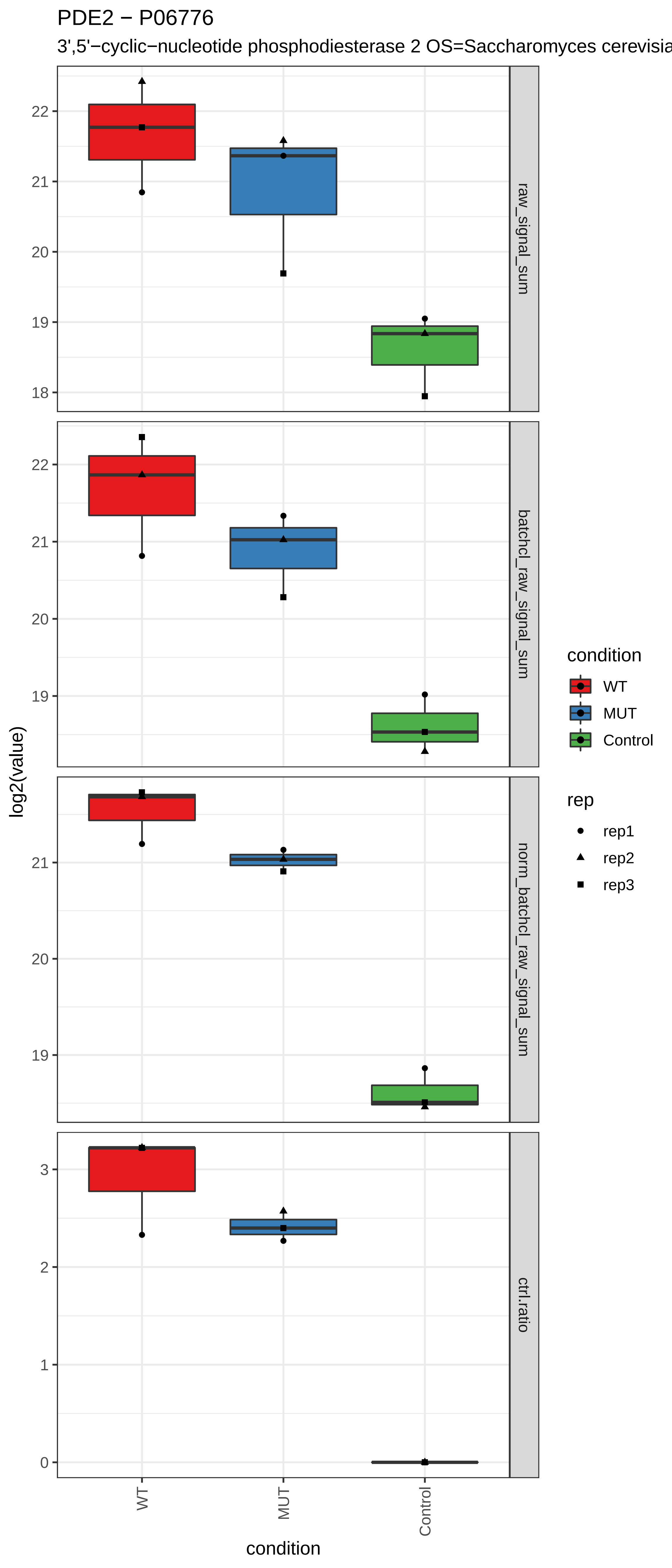
PDE2 − P06776
3',5'−cyclic−nucleotide phosphodiesterase 2 OS=Saccharomyces cerevisia
raw_signal_sum
batchcl_raw_signal_sum
norm_batchcl_raw_signal_sum
ctrl.ratio
log2(value)
condition
WT
MUT
Control
rep
rep1
rep2
rep3

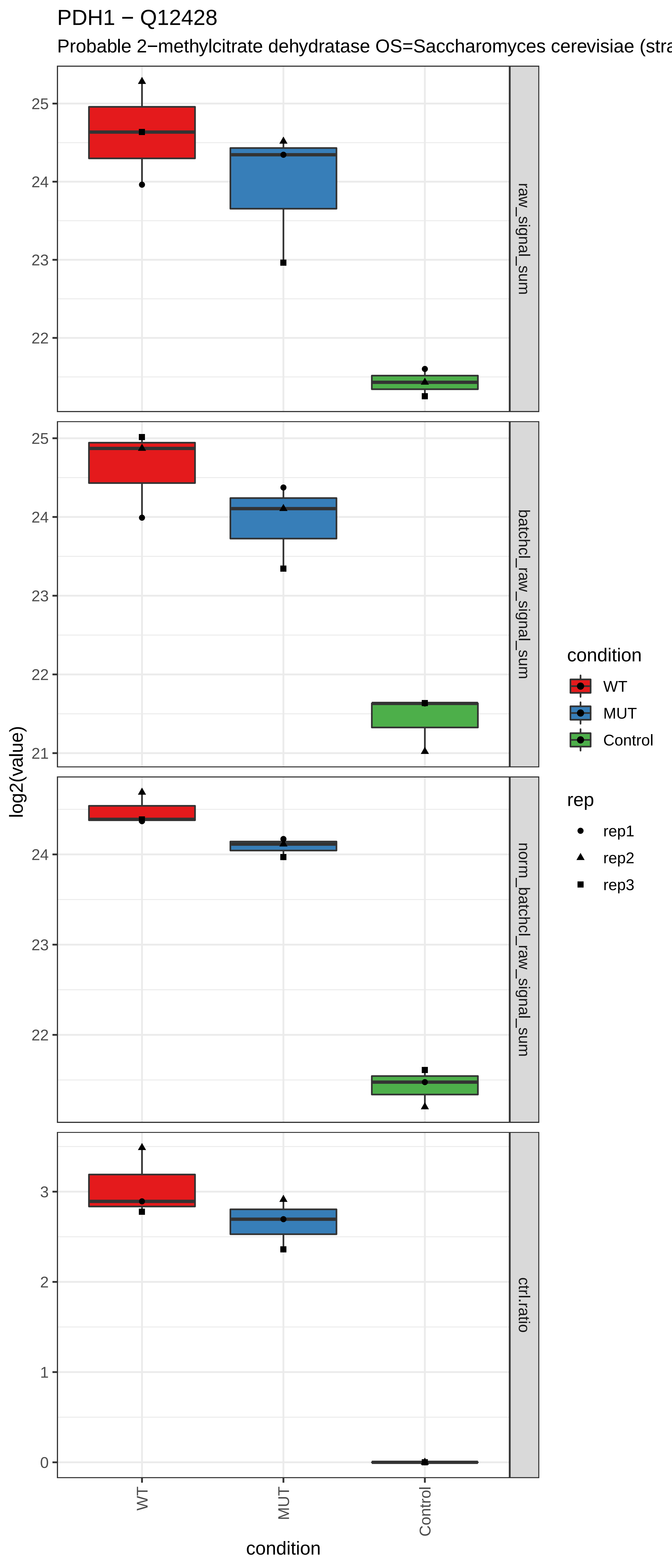

PDH1 − Q12428
Probable 2−methylcitrate dehydratase OS=Saccharomyces cerevisiae (stra
raw_signal_sum
batchcl_raw_signal_sum
norm_batchcl_raw_signal_sum
ctrl.ratio
log2(value)
condition
WT
MUT
Control
rep
rep1
rep2
rep3

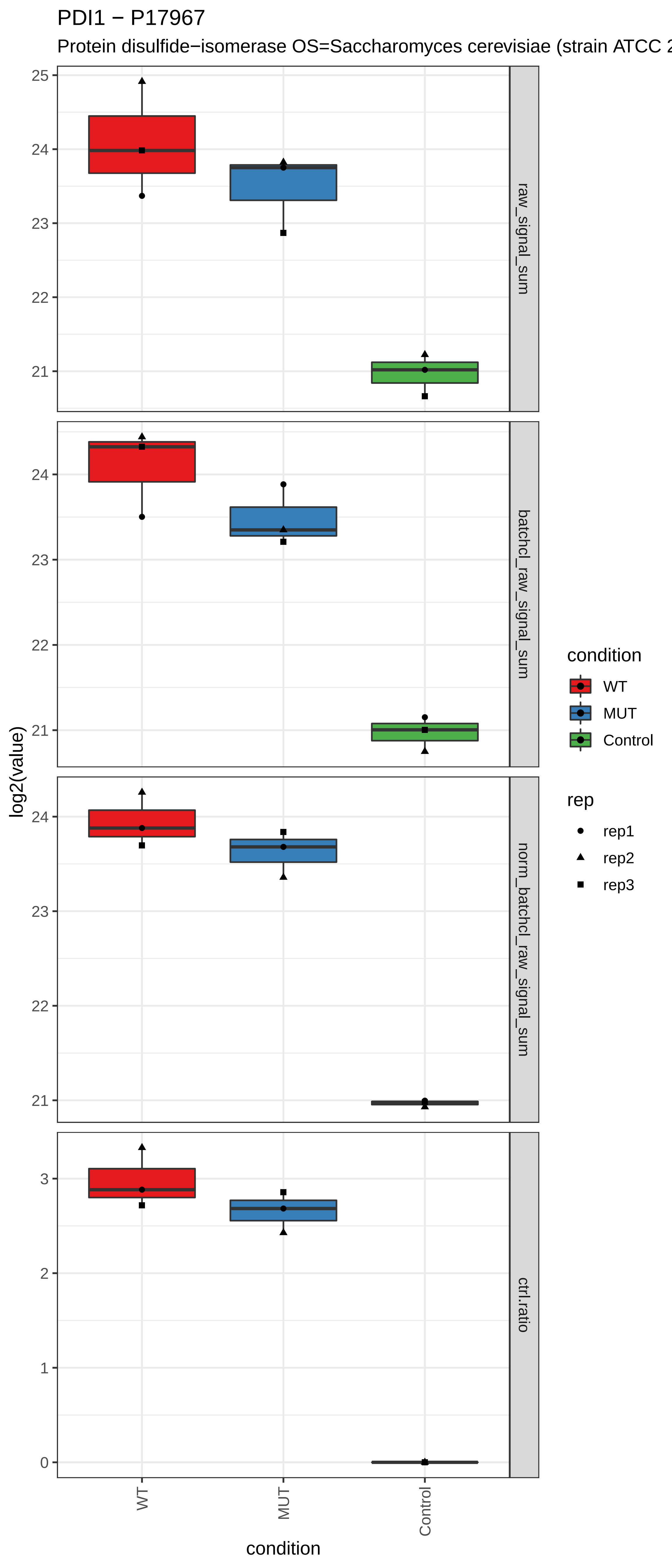

PDI1 − P17967
Protein disulfide−isomerase OS=Saccharomyces cerevisiae (strain ATCC 2
raw_signal_sum
batchcl_raw_signal_sum
norm_batchcl_raw_signal_sum
ctrl.ratio
log2(value)
condition
WT
MUT
Control
rep
rep1
rep2
rep3

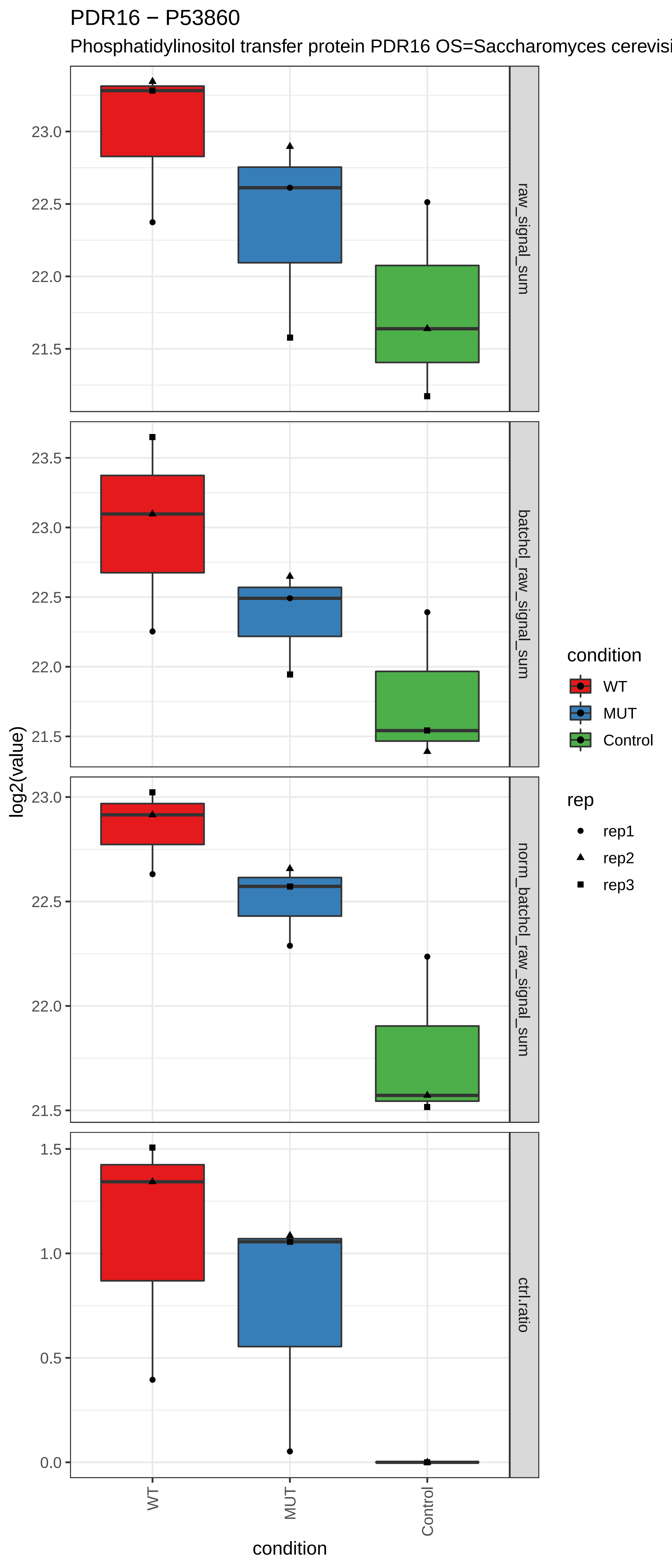

PDR16 − P53860
Phosphatidylinositol transfer protein PDR16 OS=Saccharomyces cerevisi
raw_signal_sum
batchcl_raw_signal_sum
norm_batchcl_raw_signal_sum
ctrl.ratio
log2(value)
condition
WT
MUT
Control
rep
rep1
rep2
rep3

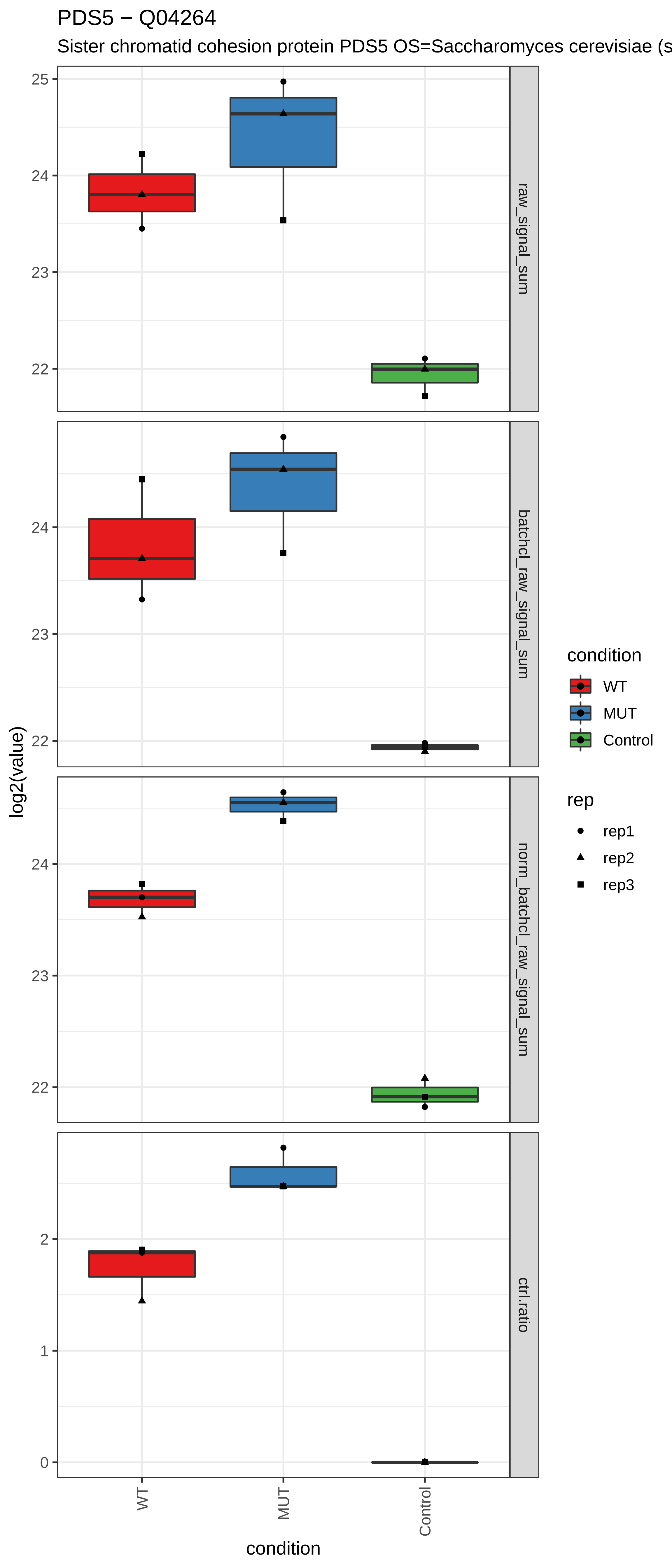

PDS5 − Q04264
Sister chromatid cohesion protein PDS5 OS=Saccharomyces cerevisiae (s
raw_signal_sum
batchcl_raw_signal_sum
norm_batchcl_raw_signal_sum
ctrl.ratio
log2(value)
condition
WT
MUT
Control
rep
rep1
rep2
rep3
25
24
23
22
2
1
0

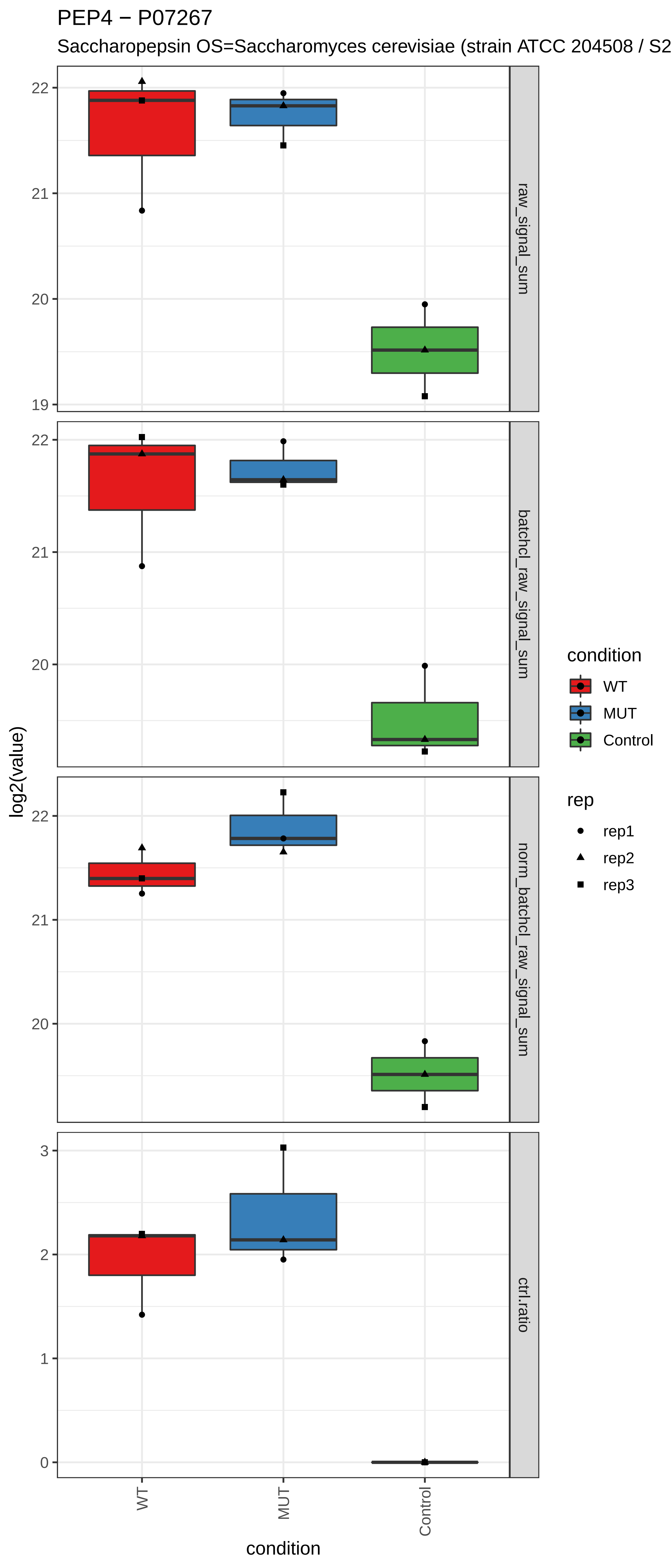

PEP4 − P07267
Saccharopepsin OS=Saccharomyces cerevisiae (strain ATCC 204508 / S2
raw_signal_sum
batchcl_raw_signal_sum
norm_batchcl_raw_signal_sum
ctrl.ratio
log2(value)
condition
WT
MUT
Control
rep
rep1
rep2
rep3
22
21
20
19
3
2
1
0

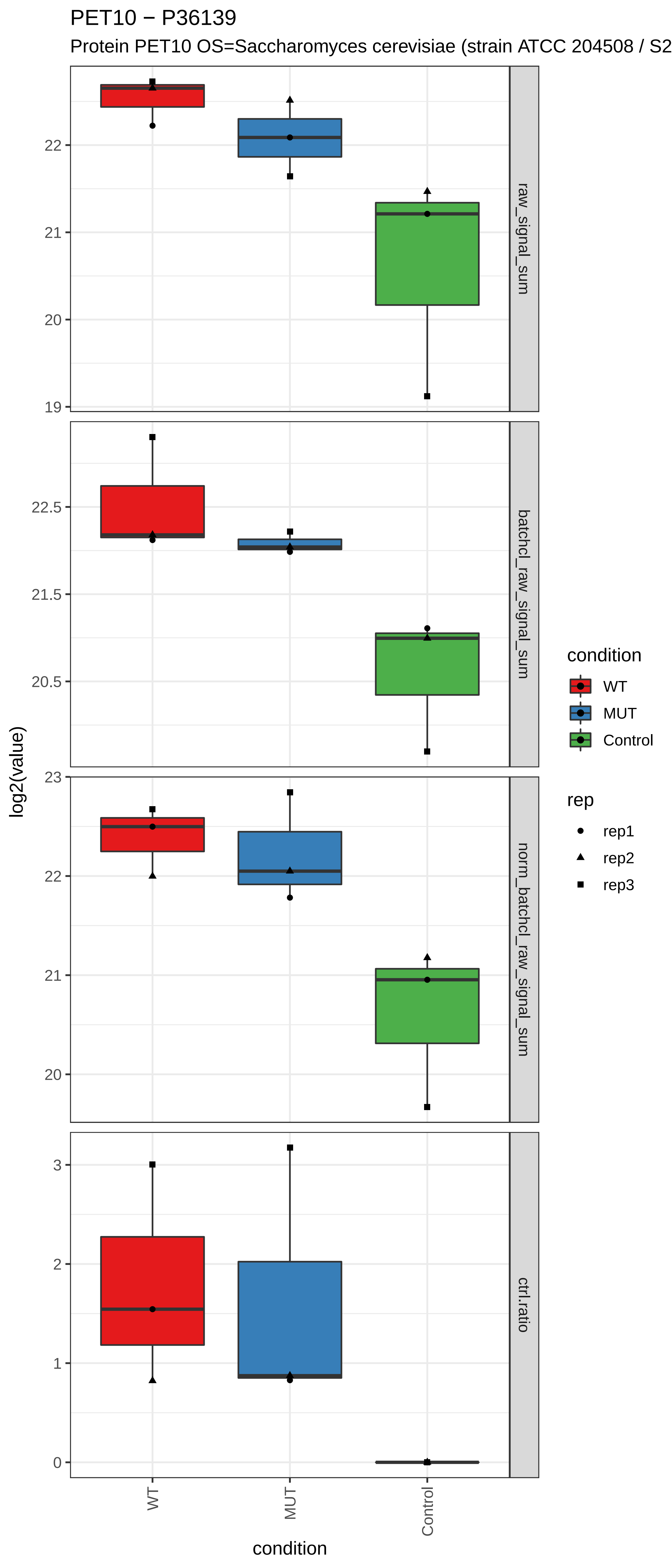

PET10 − P36139
Protein PET10 OS=Saccharomyces cerevisiae (strain ATCC 204508 / S2
raw_signal_sum
batchcl_raw_signal_sum
norm_batchcl_raw_signal_sum
ctrl.ratio
log2(value)
condition
WT
MUT
Control
rep
rep1
rep2
rep3

PET127 − P32606

Putative mitochondrial translation system component PET127 OS=Sacch

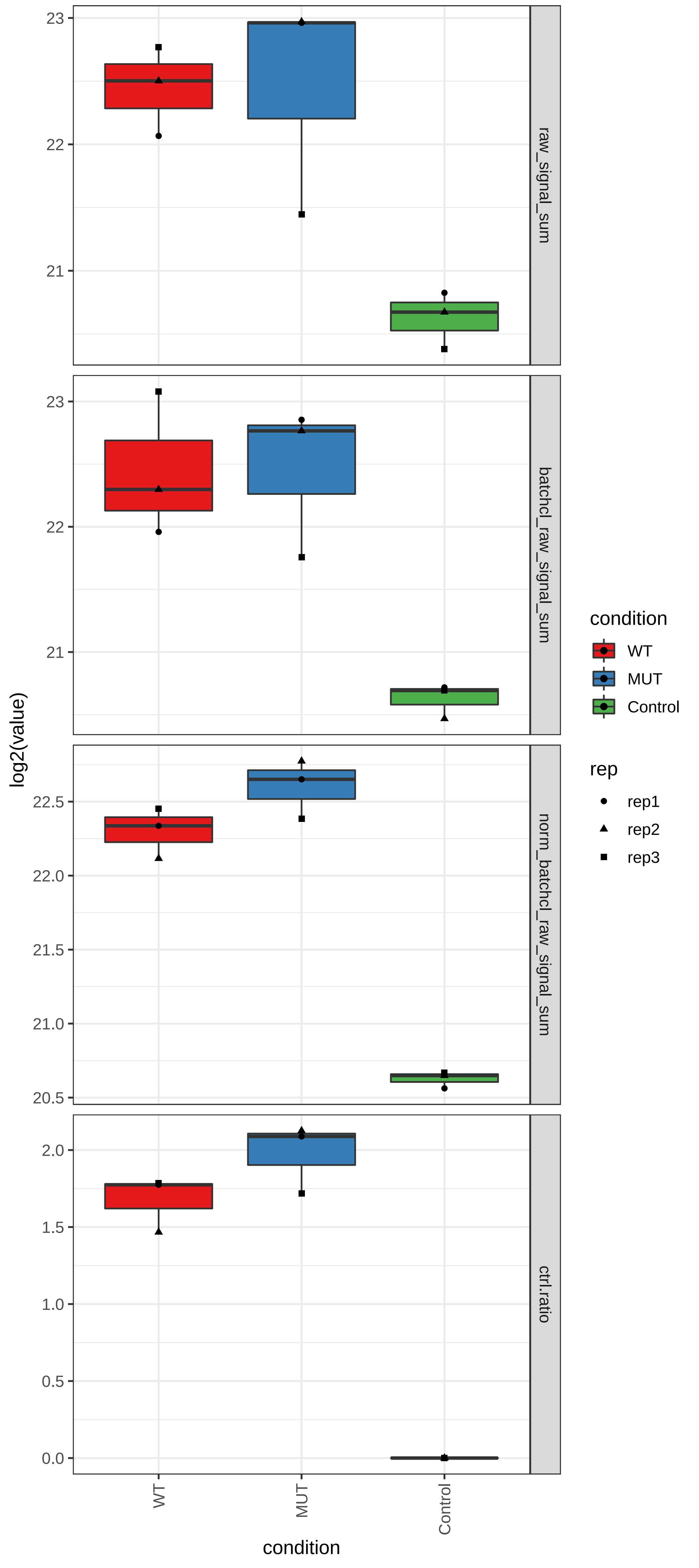

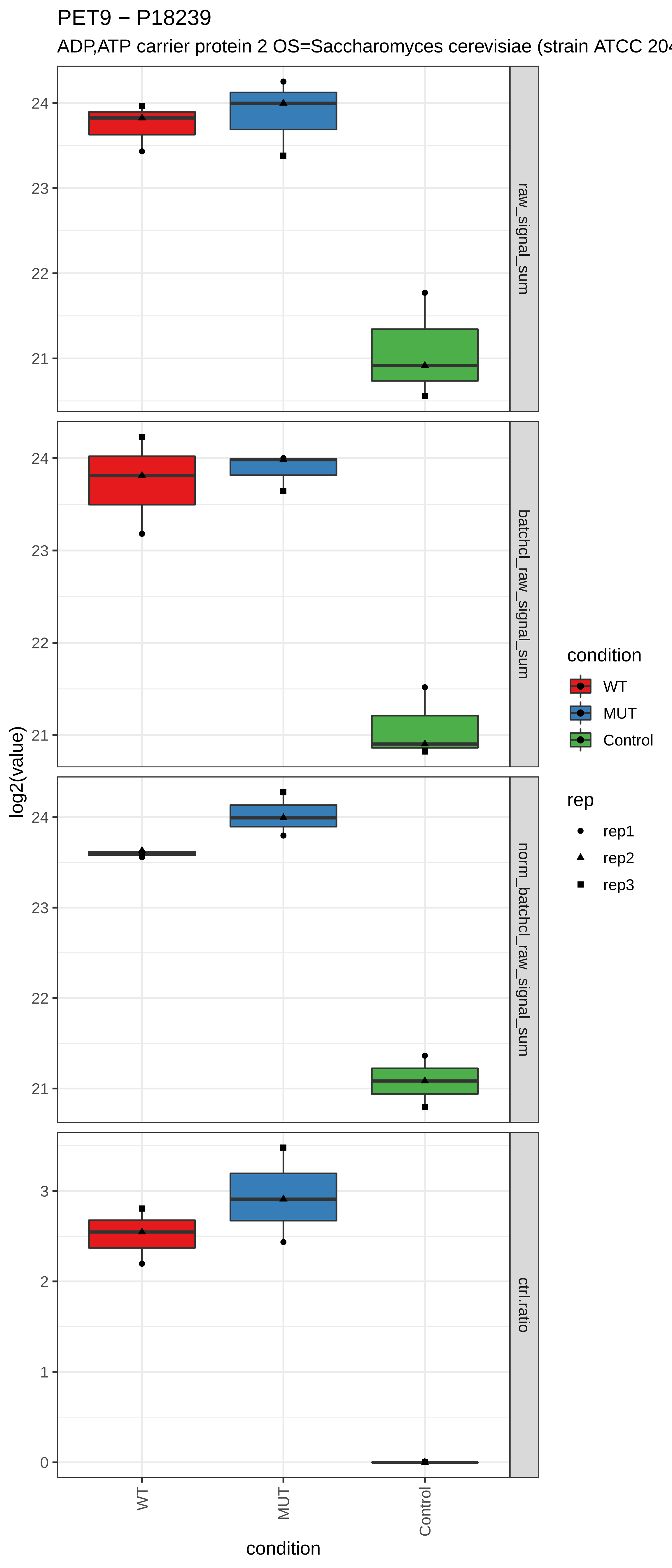
PET9 − P18239
ADP,ATP carrier protein 2 OS=Saccharomyces cerevisiae (strain ATCC 204
raw_signal_sum
batchcl_raw_signal_sum
norm_batchcl_raw_signal_sum
ctrl.ratio
log2(value)
condition
WT
MUT
Control
rep
rep1
rep2
rep3
24
23
22
21
3
2
1
0

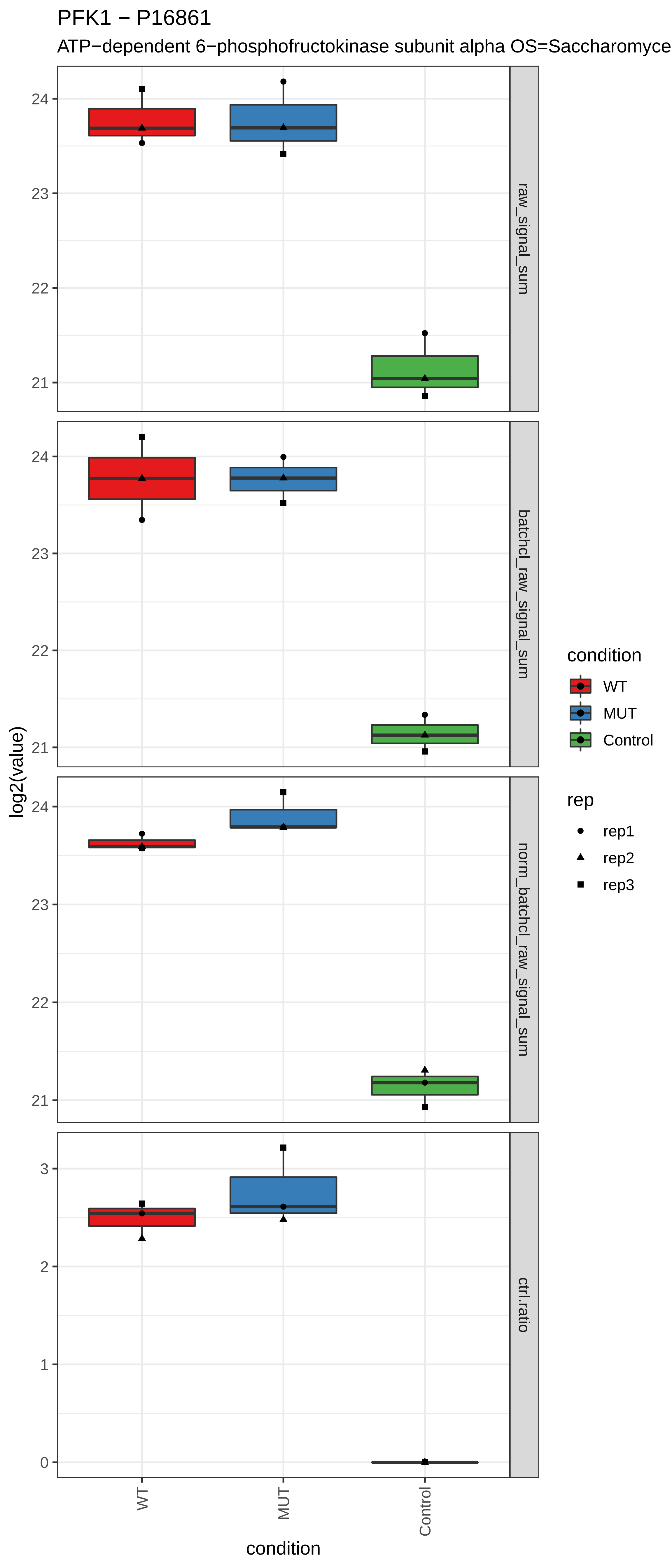
PFK1 − P16861
ATP−dependent 6−phosphofructokinase subunit alpha OS=Saccharomyce
raw_signal_sum
batchcl_raw_signal_sum
norm_batchcl_raw_signal_sum
ctrl.ratio
log2(value)
condition
WT
MUT
Control
rep
rep1
rep2
rep3
24
23
22
21
3
2
1
0

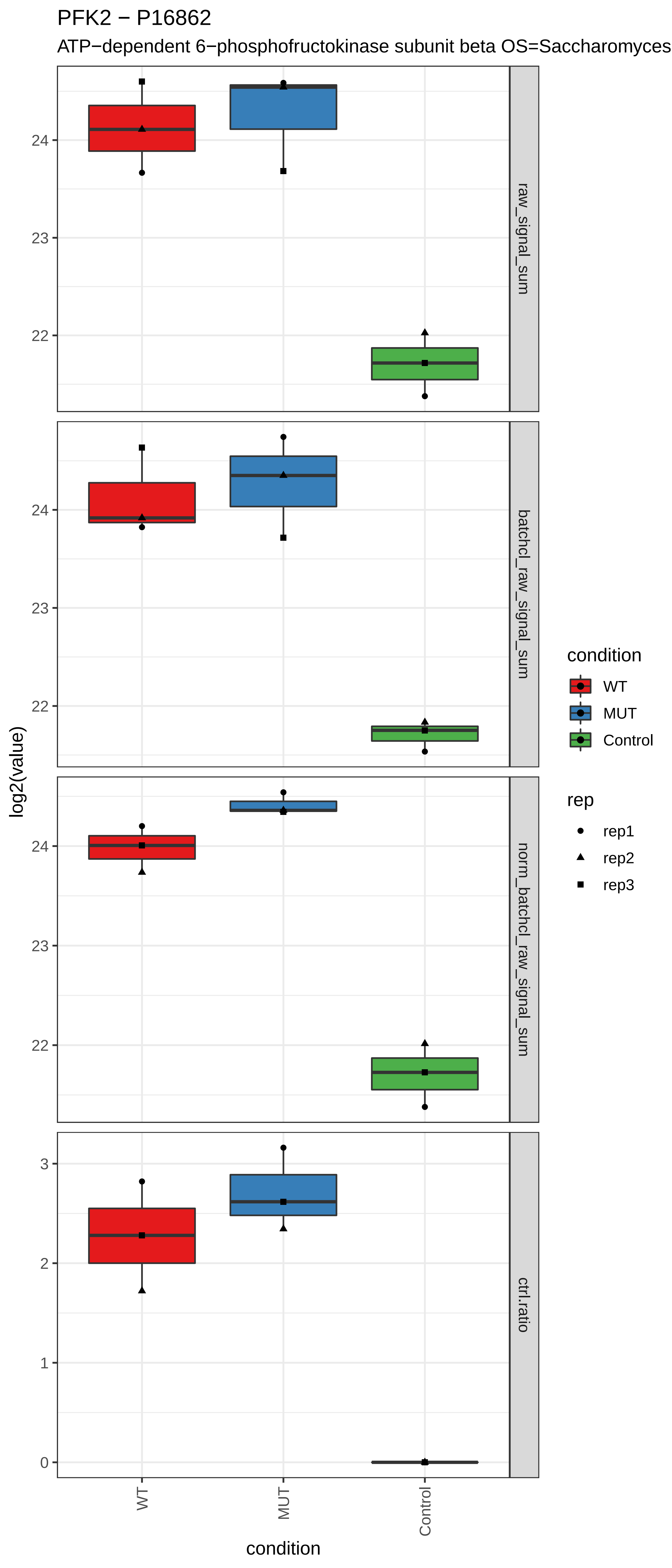
PFK2 − P16862
ATP−dependent 6−phosphofructokinase subunit beta OS=Saccharomyces
raw_signal_sum
batchcl_raw_signal_sum
norm_batchcl_raw_signal_sum
ctrl.ratio
log2(value)
condition
WT
MUT
Control
rep
rep1
rep2
rep3
24
23
22
3
2
1
0

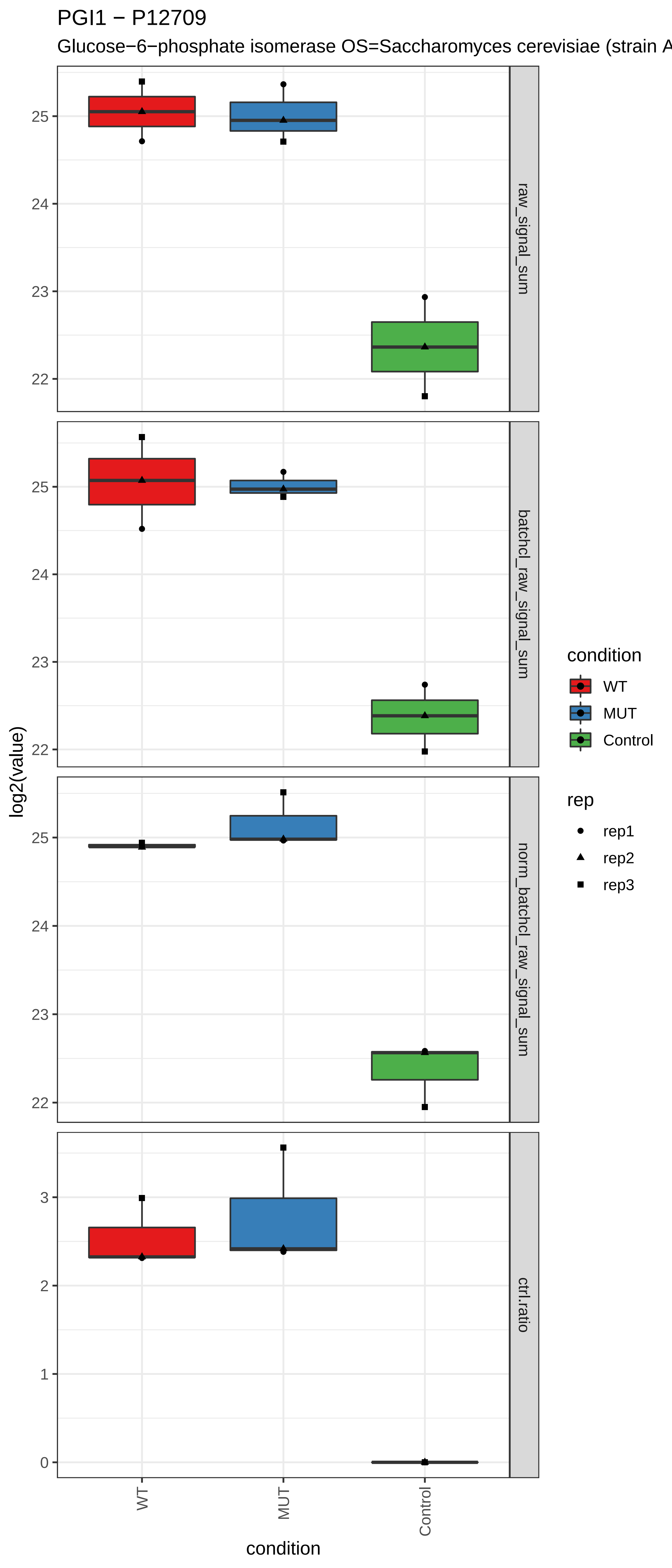

PGI1 − P12709
Glucose−6−phosphate isomerase OS=Saccharomyces cerevisiae (strain A
raw_signal_sum
batchcl_raw_signal_sum
norm_batchcl_raw_signal_sum
ctrl.ratio
log2(value)
condition
WT
MUT
Control
rep
rep1
rep2
rep3

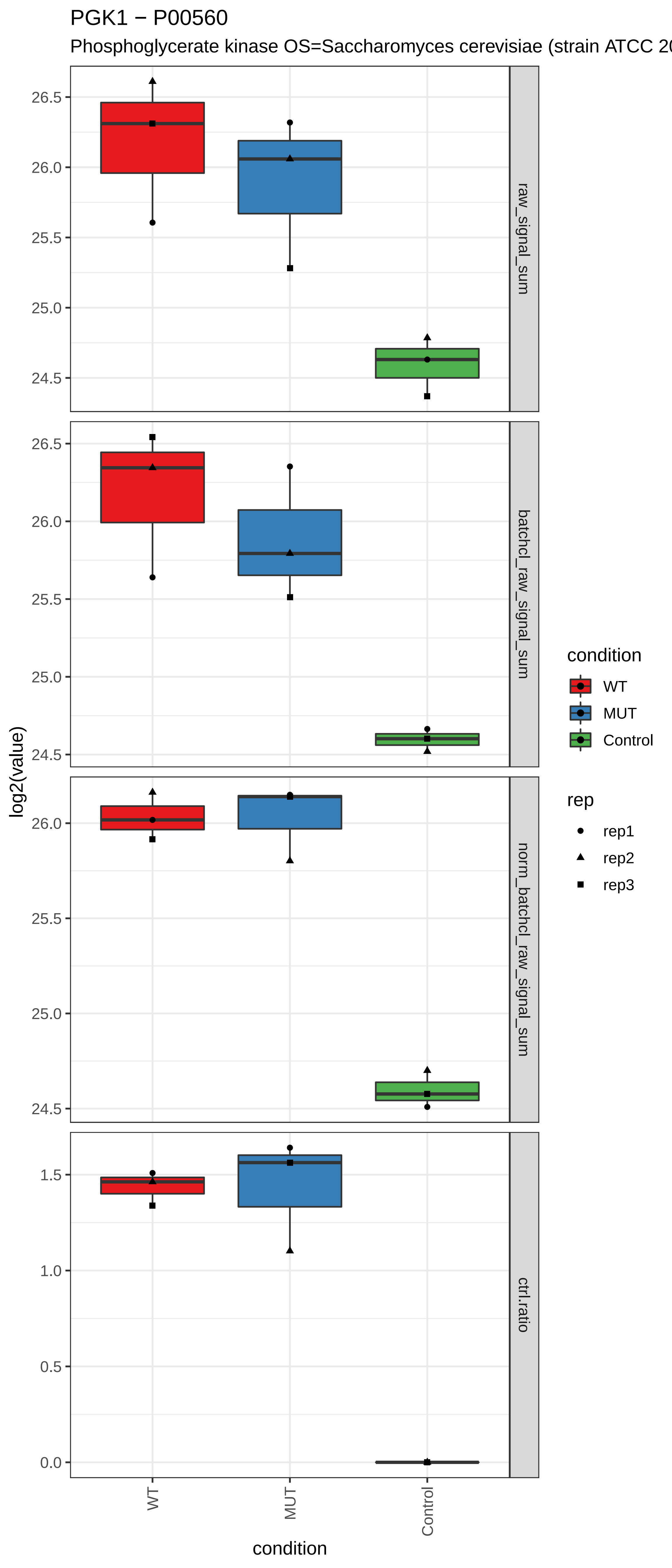
PGK1 − P00560
Phosphoglycerate kinase OS=Saccharomyces cerevisiae (strain ATCC 20
raw_signal_sum
batchcl_raw_signal_sum
norm_batchcl_raw_signal_sum
ctrl.ratio
log2(value)
condition
WT
MUT
Control
rep
rep1
rep2
rep3
26.5
26.0
25.5
25.0
24.5
1.5
1.0
0.5
0.0

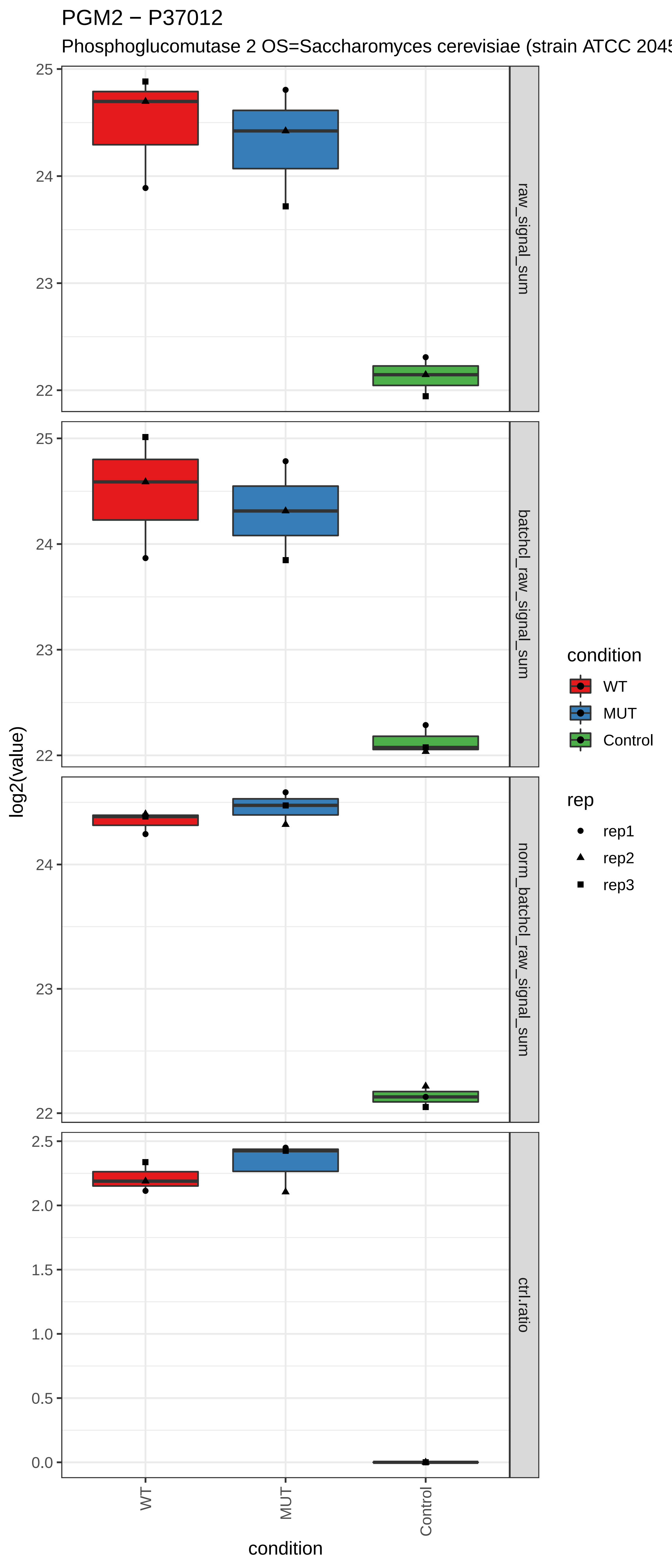
PGM2 − P37012
Phosphoglucomutase 2 OS=Saccharomyces cerevisiae (strain ATCC 2045
log2(value)
condition
raw_signal_sum
batchcl_raw_signal_sum
norm_batchcl_raw_signal_sum
ctrl.ratio
WT
MUT
Control
rep
rep1
rep2
rep3
25
24
23
22
2.5
2.0
1.5
1.0
0.5
0.0

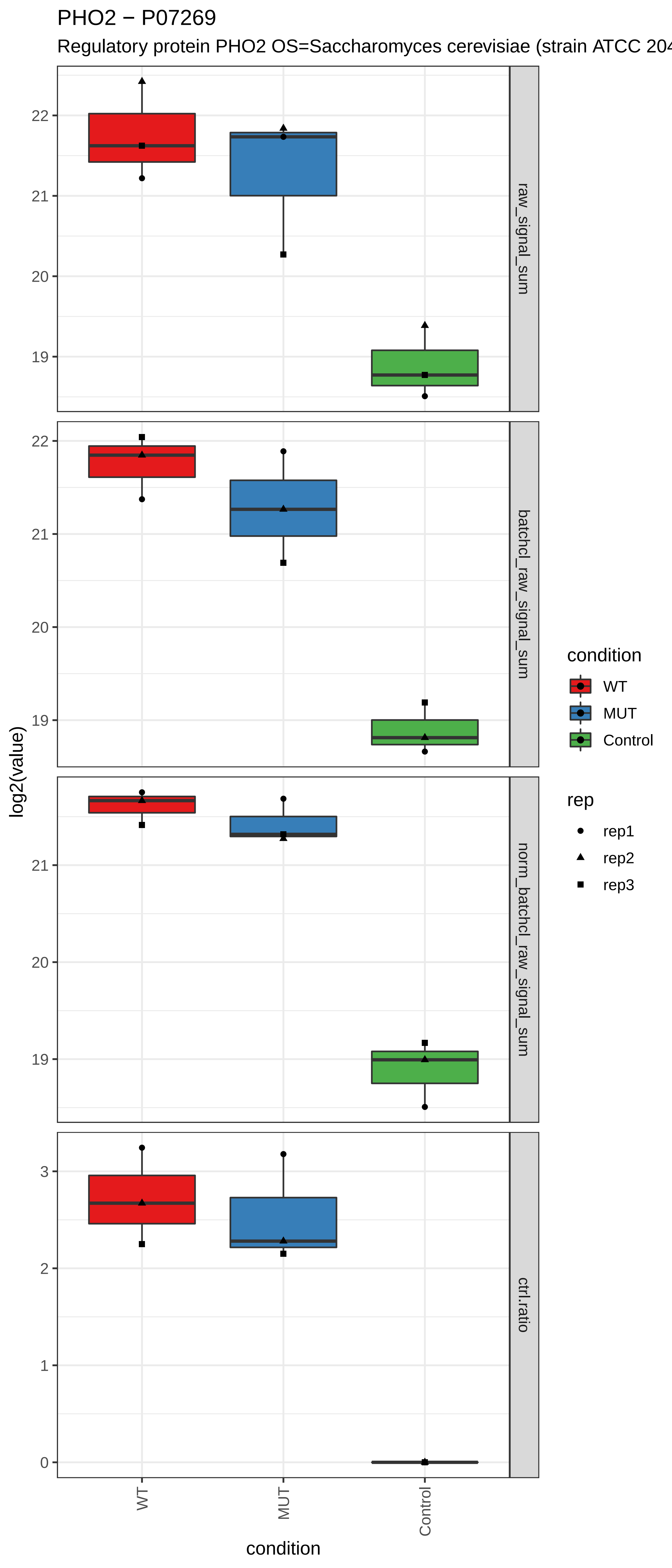

PHO2 − P07269
Regulatory protein PHO2 OS=Saccharomyces cerevisiae (strain ATCC 204
raw_signal_sum
batchcl_raw_signal_sum
norm_batchcl_raw_signal_sum
ctrl.ratio
log2(value)
condition
WT
MUT
Control
rep
rep1
rep2
rep3
22
21
20
19
3
2
1
0

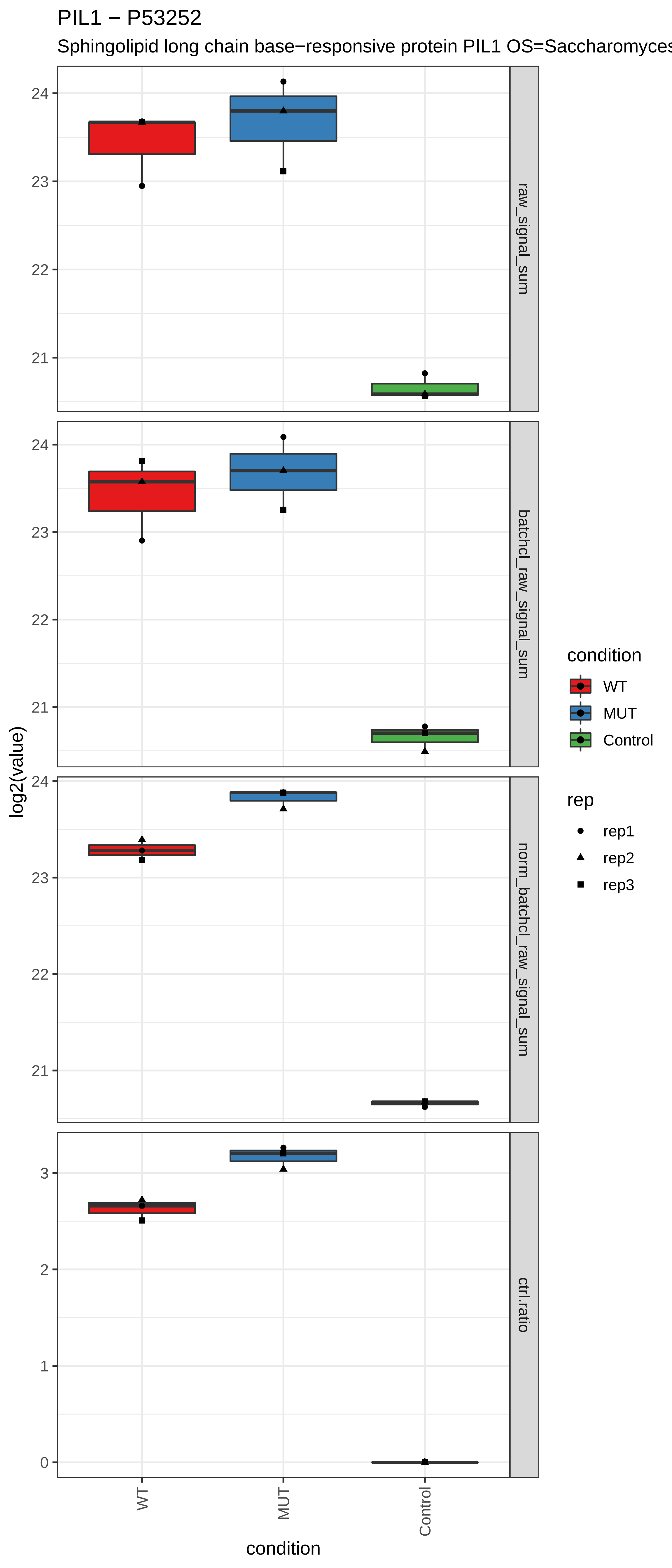
PIL1 − P53252
Sphingolipid long chain base−responsive protein PIL1 OS=Saccharomyces
log2(value)
raw_signal_sum
batchcl_raw_signal_sum
norm_batchcl_raw_signal_sum
ctrl.ratio
24
23
22
21
3
2
1
0
WT
MUT
Control
condition
condition
WT
MUT
Control
rep
rep1
rep2
rep3

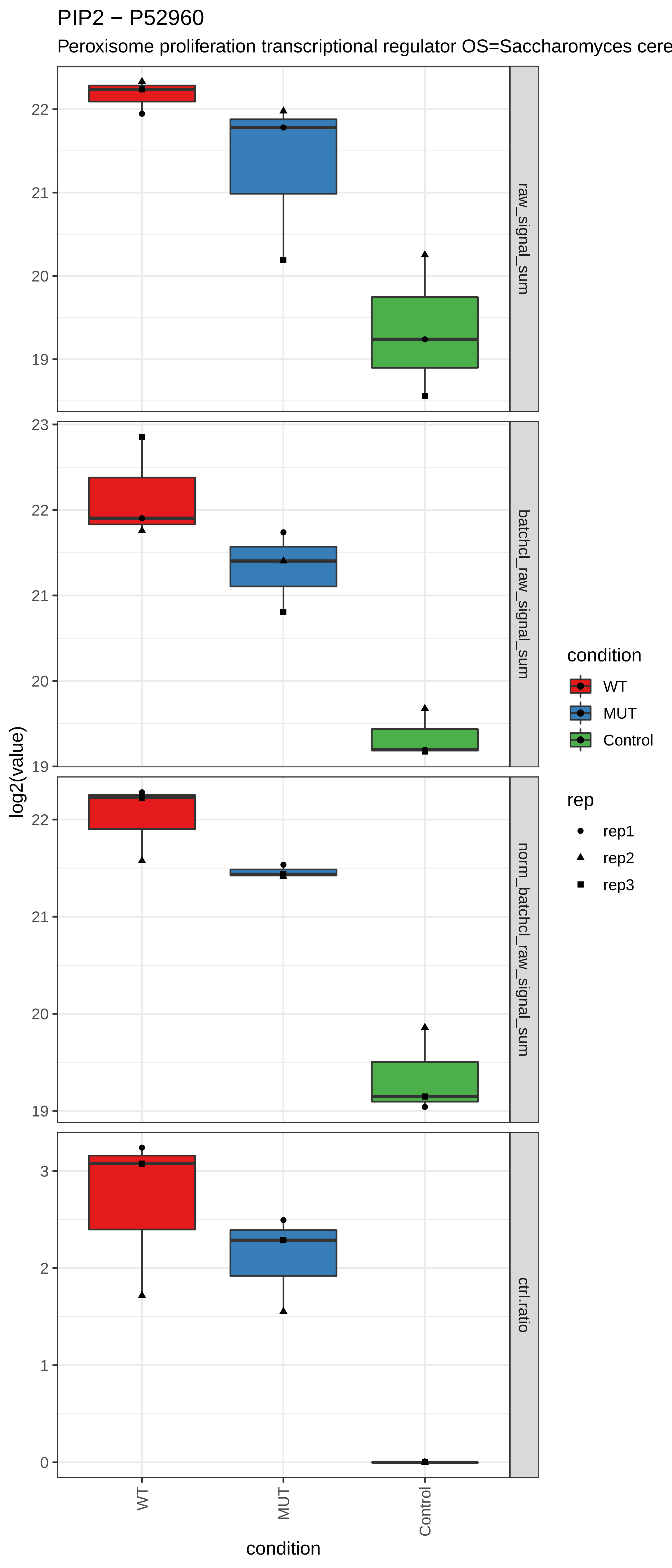

PIP2 − P52960
Peroxisome proliferation transcriptional regulator OS=Saccharomyces cere
raw_signal_sum
batchcl_raw_signal_sum
norm_batchcl_raw_signal_sum
ctrl.ratio
log2(value)
condition
WT
MUT
Control
rep
rep1
rep2
rep3

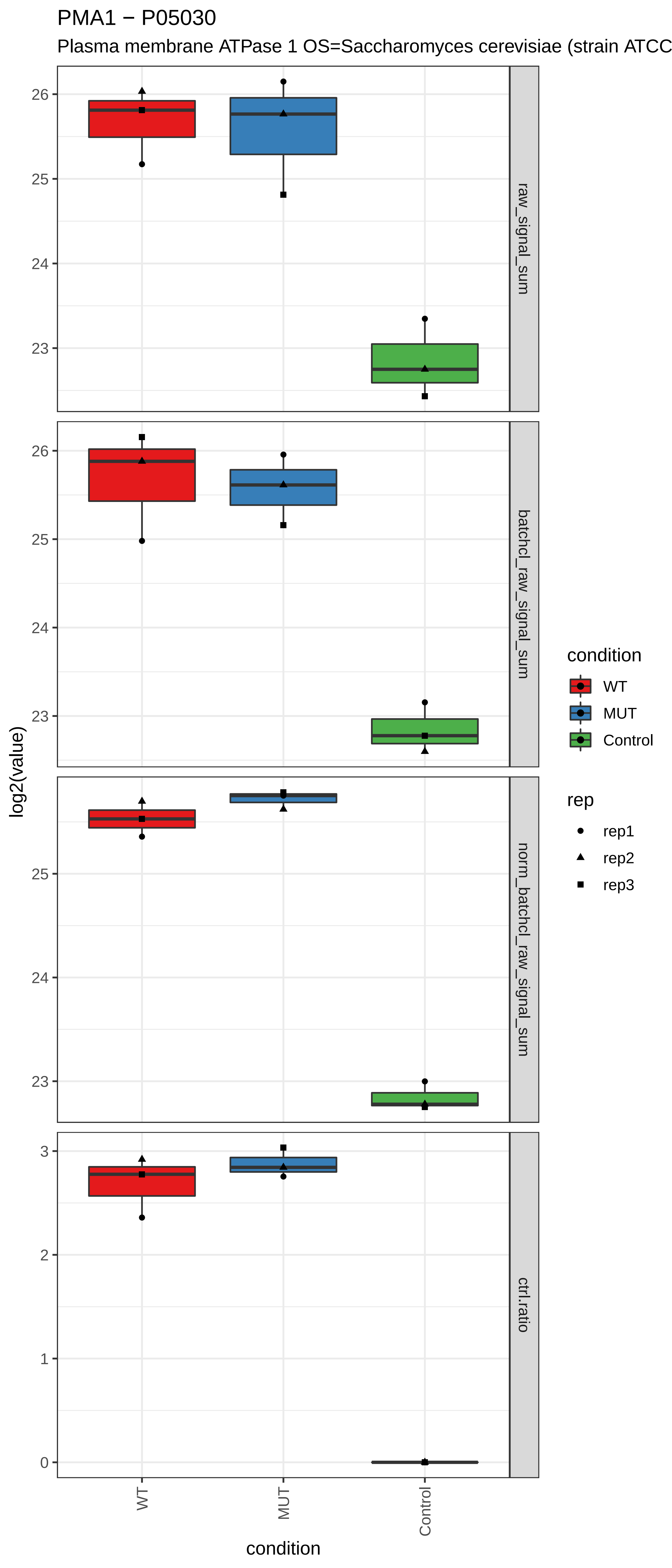
PMA1 − P05030
Plasma membrane ATPase 1 OS=Saccharomyces cerevisiae (strain ATCC
raw_signal_sum
batchcl_raw_signal_sum
norm_batchcl_raw_signal_sum
ctrl.ratio
log2(value)
condition
WT
MUT
Control
rep
rep1
rep2
rep3

PMT1 − P33775

Dolichyl−phosphate−mannose−−protein mannosyltransferase 1 OS=Sacc

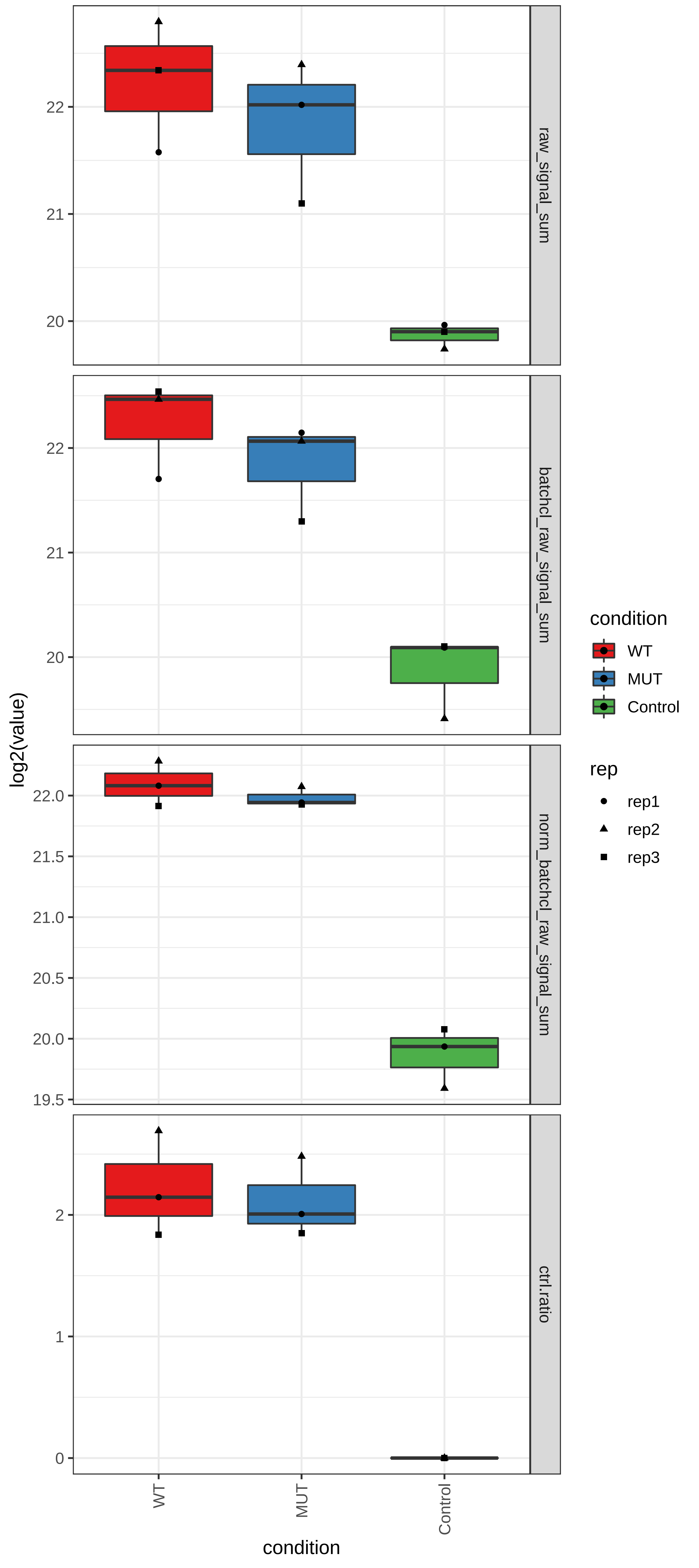

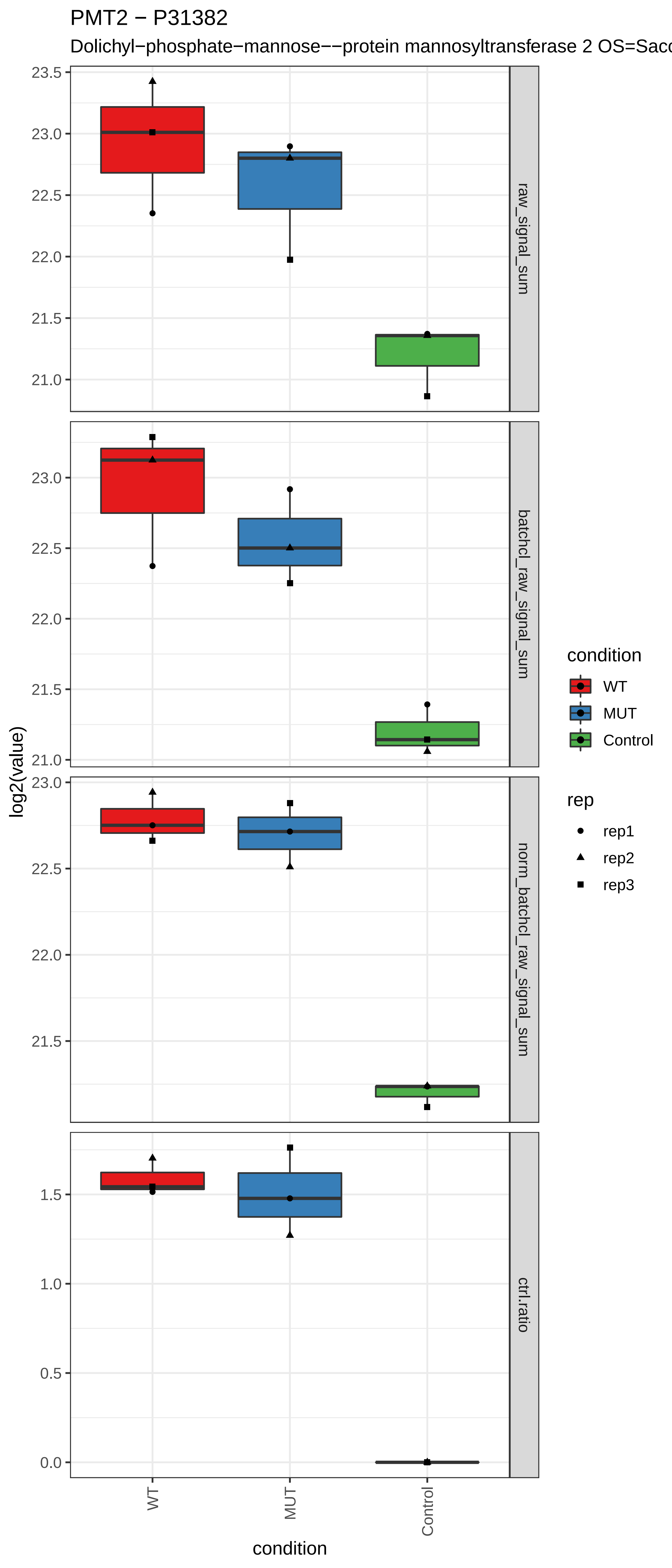

PMT2 − P31382
Dolichyl−phosphate−mannose−−protein mannosyltransferase 2 OS=Sacc
raw_signal_sum
batchcl_raw_signal_sum
norm_batchcl_raw_signal_sum
ctrl.ratio
log2(value)
condition
WT
MUT
Control
condition
WT
MUT
Control
rep
rep1
rep2
rep3

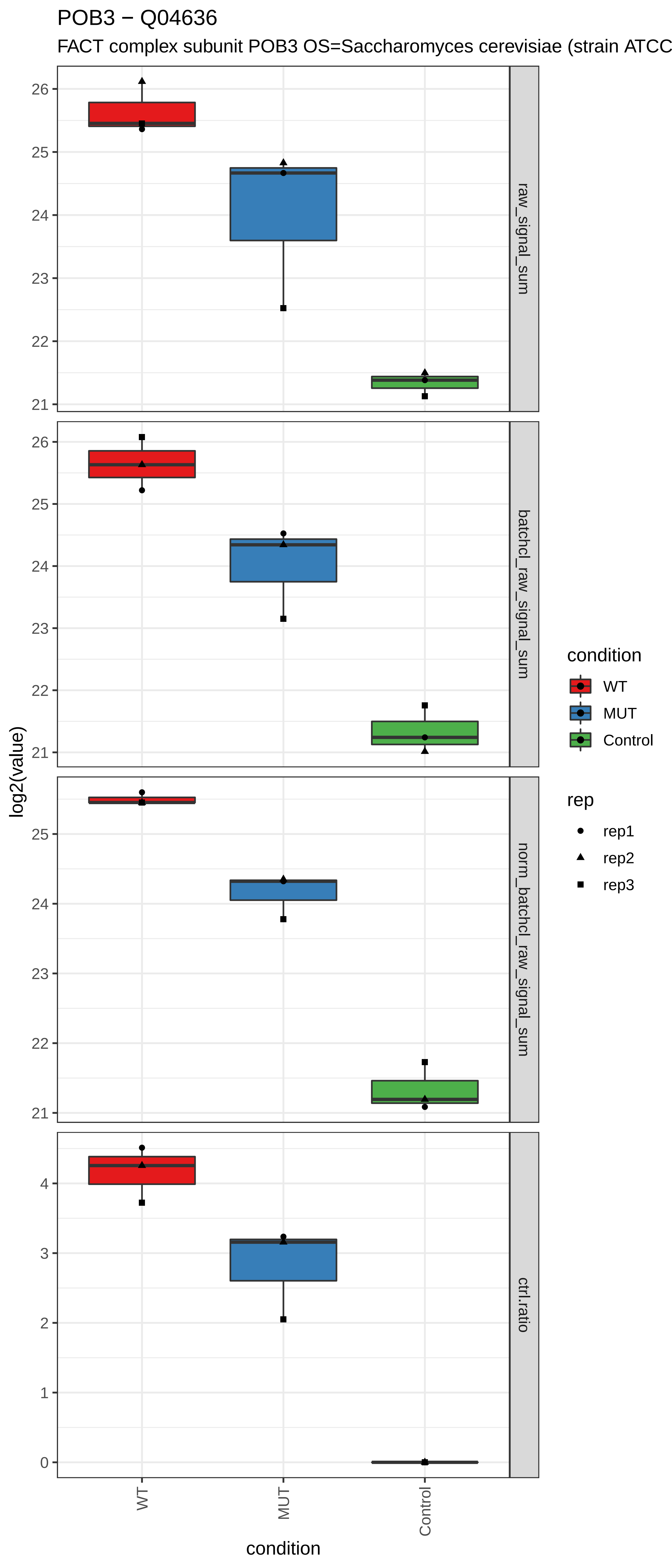

POB3 − Q04636
FACT complex subunit POB3 OS=Saccharomyces cerevisiae (strain ATCC
raw_signal_sum
batchcl_raw_signal_sum
norm_batchcl_raw_signal_sum
ctrl.ratio
log2(value)
condition
WT
MUT
Control
rep
rep1
rep2
rep3

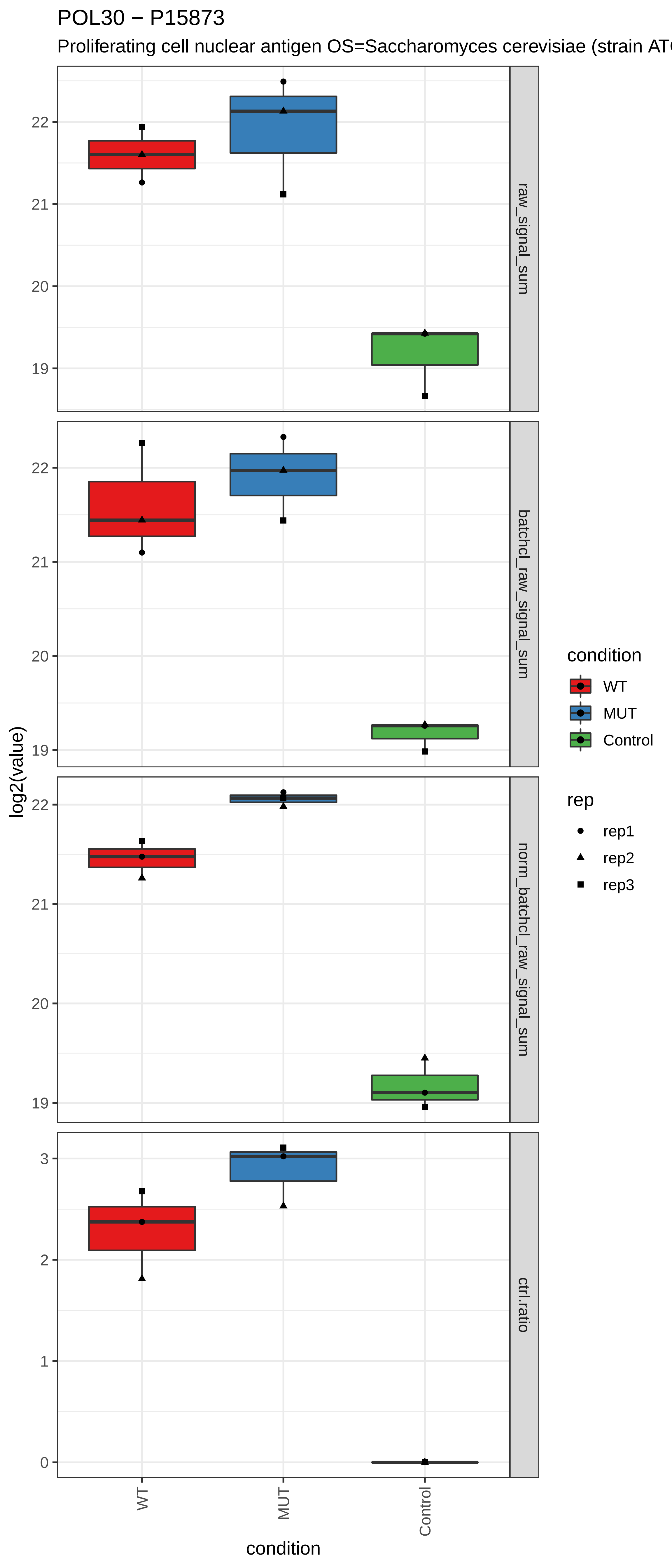

POL30 − P15873
Proliferating cell nuclear antigen OS=Saccharomyces cerevisiae (strain ATC
raw_signal_sum
batchcl_raw_signal_sum
norm_batchcl_raw_signal_sum
ctrl.ratio
log2(value)
condition
WT
MUT
Control
rep
rep1
rep2
rep3

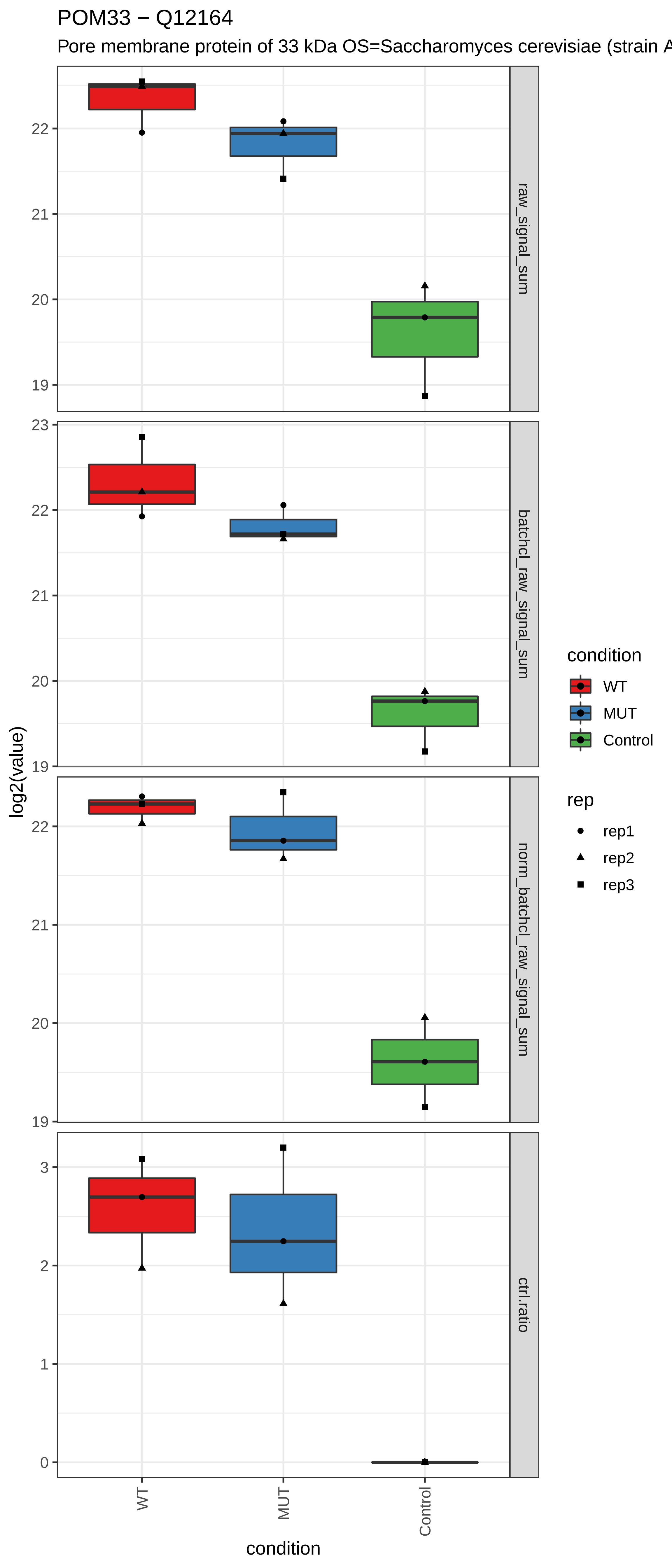
POM33 − Q12164
Pore membrane protein of 33 kDa OS=Saccharomyces cerevisiae (strain A
raw_signal_sum
batchcl_raw_signal_sum
norm_batchcl_raw_signal_sum
ctrl.ratio
log2(value)
condition
WT
MUT
Control
rep
rep1
rep2
rep3
23
22
21
20
19
3
2
1
0

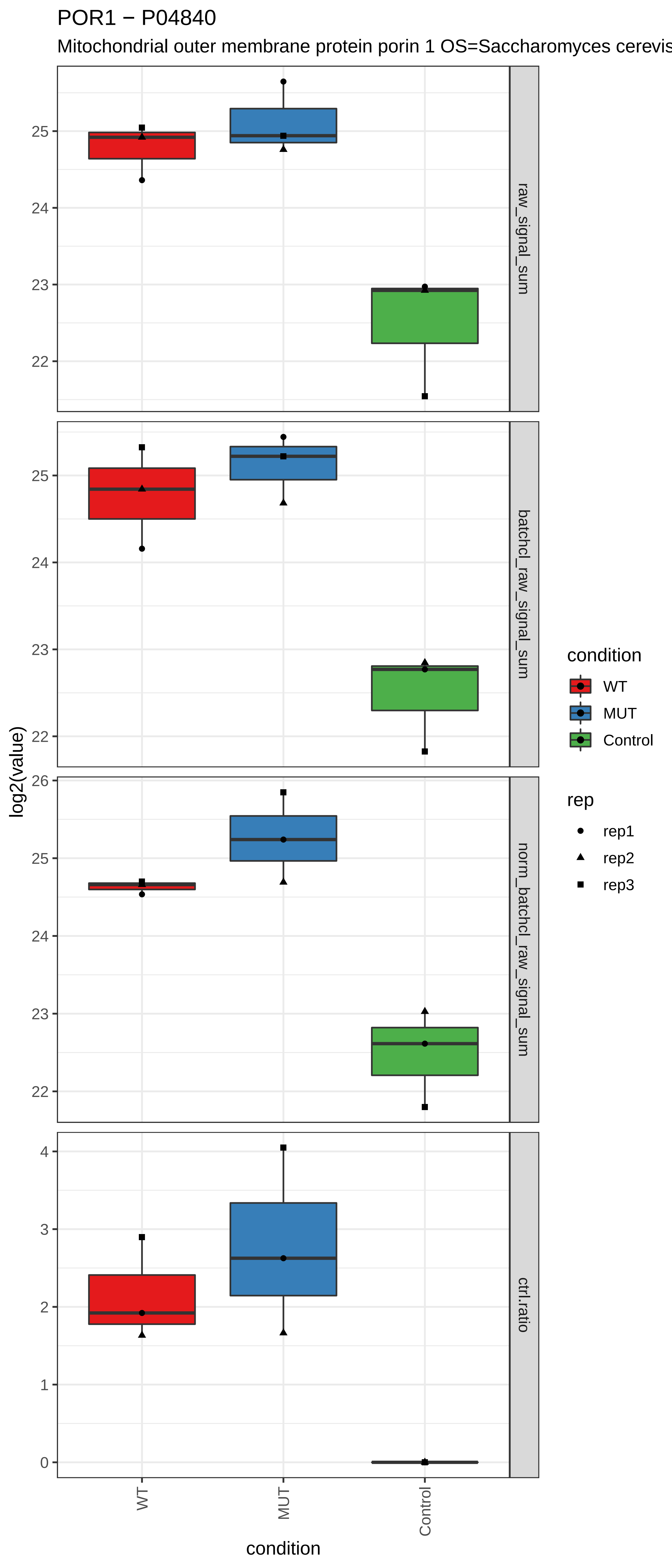
POR1 − P04840
Mitochondrial outer membrane protein porin 1 OS=Saccharomyces cerevis
raw_signal_sum
batchcl_raw_signal_sum
norm_batchcl_raw_signal_sum
ctrl.ratio
log2(value)
condition
WT
MUT
Control
rep
rep1
rep2
rep3
26
25
24
23
22
4
3
2
1
0

POX1 − P13711

Acyl−coenzyme A oxidase OS=Saccharomyces cerevisiae (strain ATCC 20

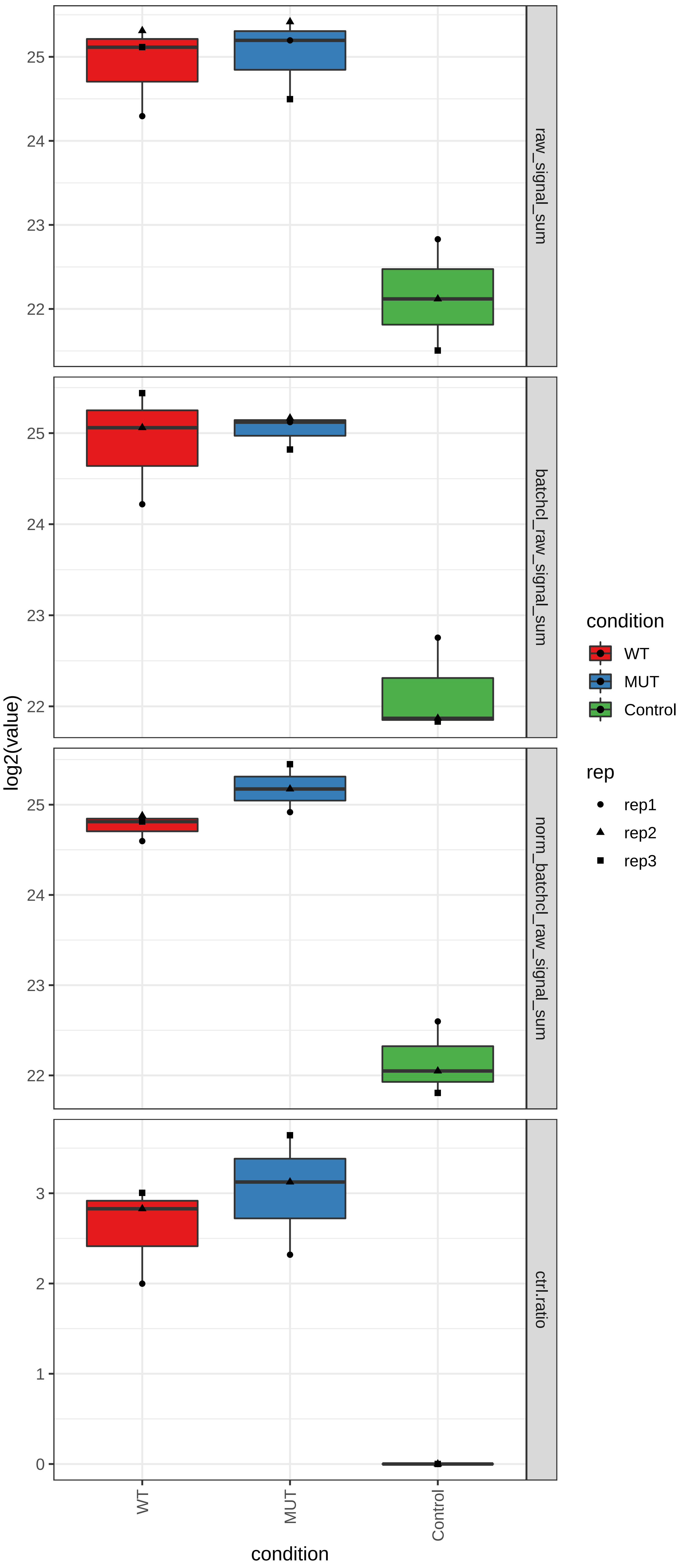

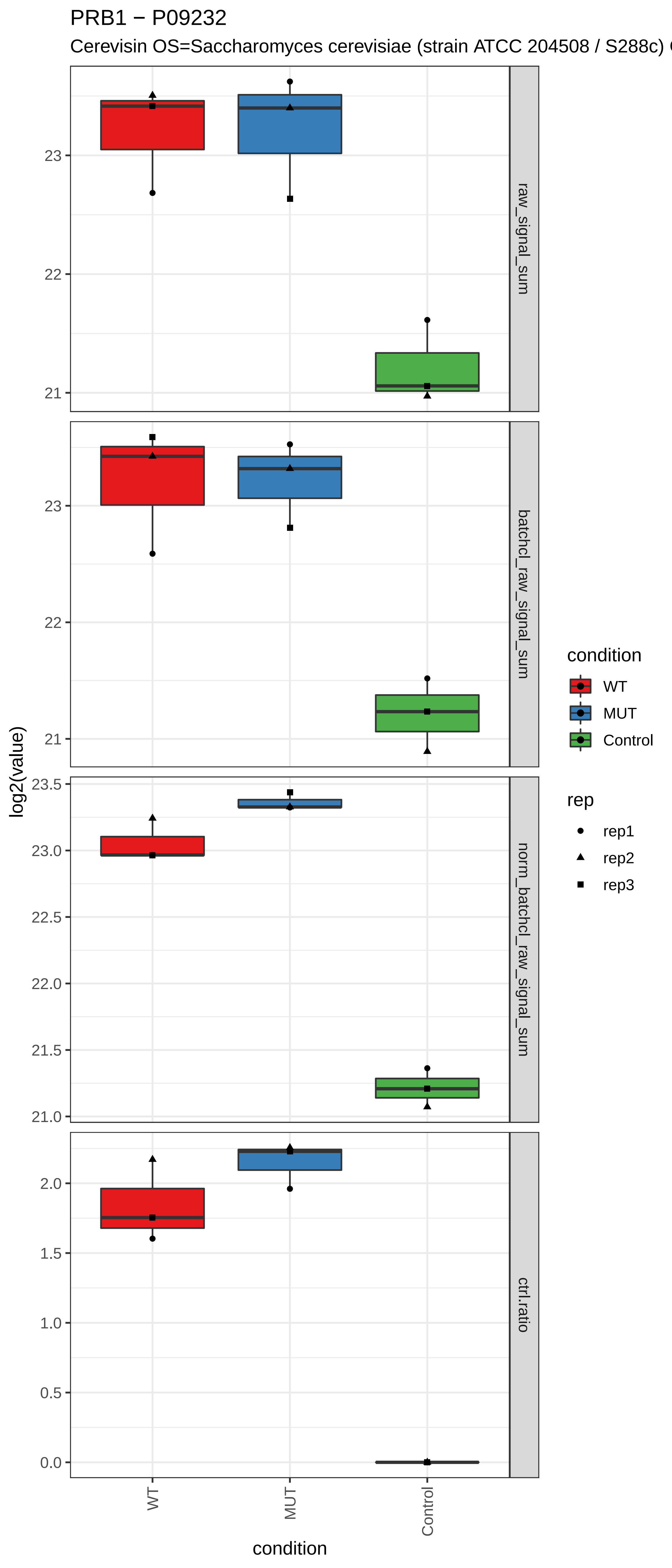

PRB1 − P09232
Cerevisin OS=Saccharomyces cerevisiae (strain ATCC 204508 / S288c)
raw_signal_sum
batchcl_raw_signal_sum
norm_batchcl_raw_signal_sum
ctrl.ratio
log2(value)
condition
WT
MUT
Control
rep
rep1
rep2
rep3

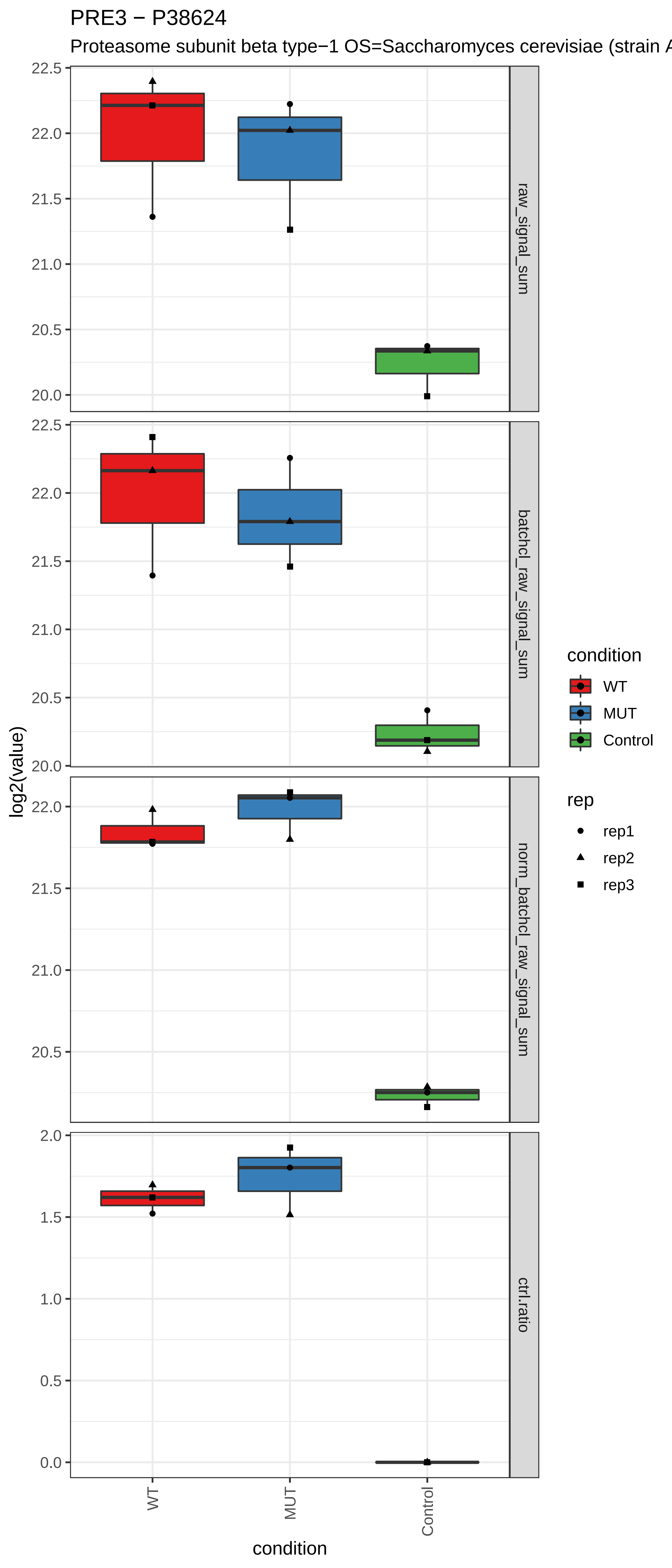

PRE3 − P38624
Proteasome subunit beta type−1 OS=Saccharomyces cerevisiae (strain A
raw_signal_sum
batchcl_raw_signal_sum
norm_batchcl_raw_signal_sum
ctrl.ratio
log2(value)
condition
WT
MUT
Control
rep
rep1
rep2
rep3
22.5
22.0
21.5
21.0
20.5
20.0
2.0
1.5
1.0
0.5
0.0

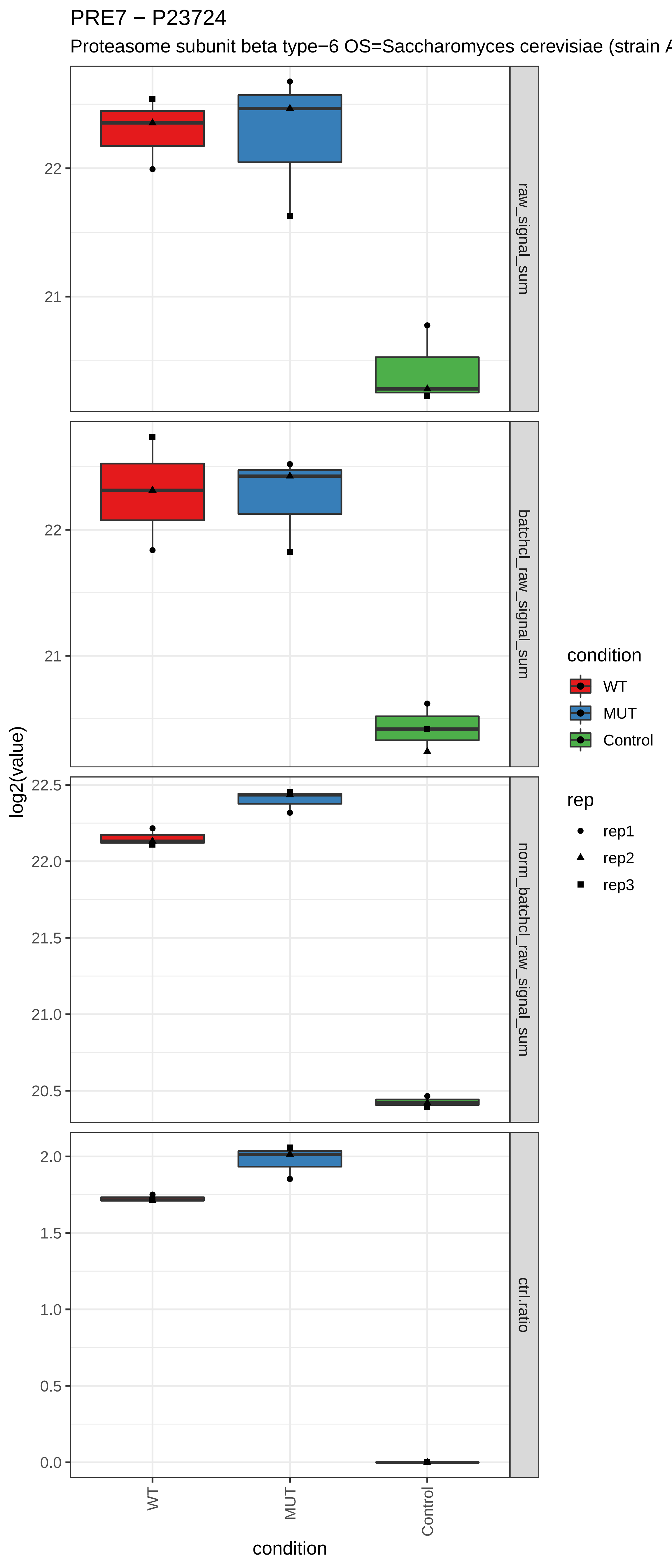
PRE7 − P23724
Proteasome subunit beta type−6 OS=Saccharomyces cerevisiae (strain A
raw_signal_sum
batchcl_raw_signal_sum
norm_batchcl_raw_signal_sum
ctrl.ratio
log2(value)
condition
WT
MUT
Control
rep
rep1
rep2
rep3
22
21
22.5
22.0
21.5
21.0
20.5
2.0
1.5
1.0
0.5
0.0

PRO3 − P32263

Pyrroline−5−carboxylate reductase OS=Saccharomyces cerevisiae (strain

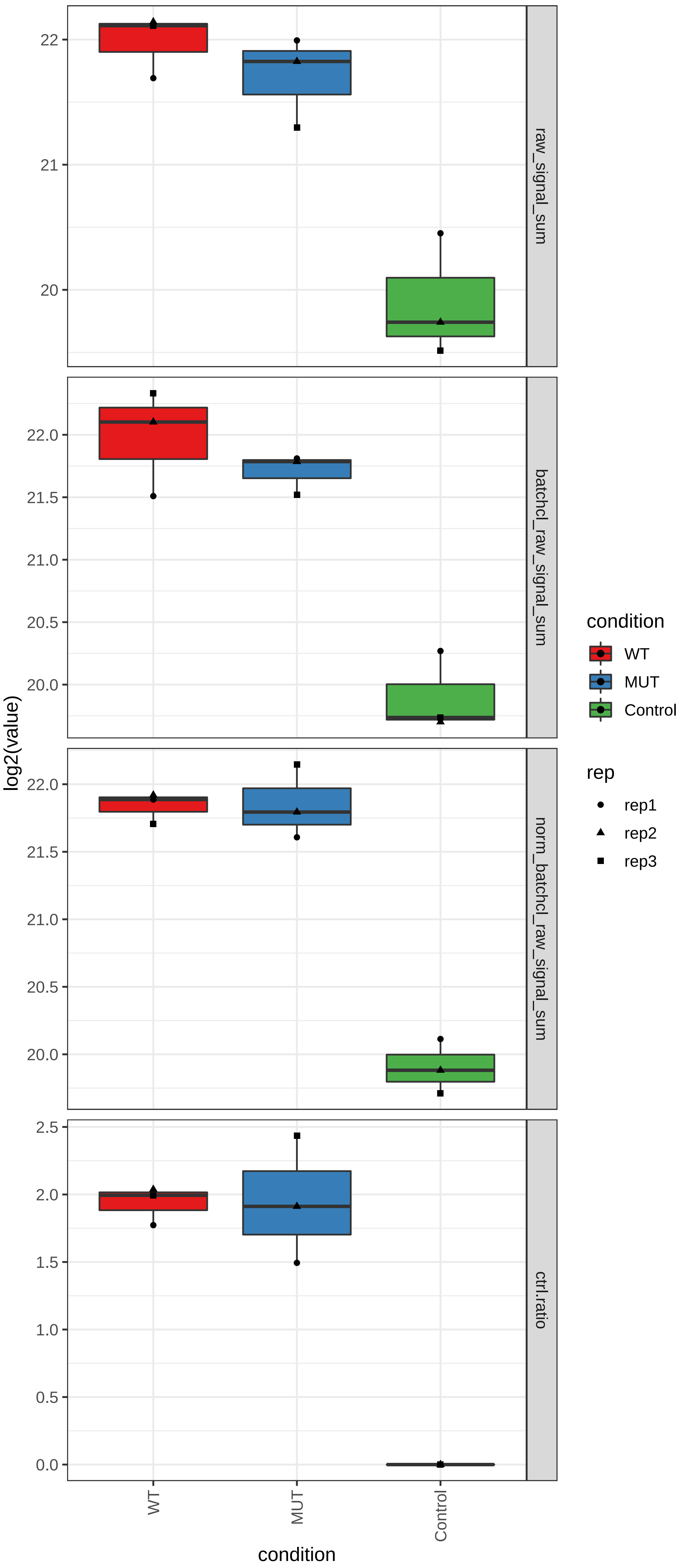

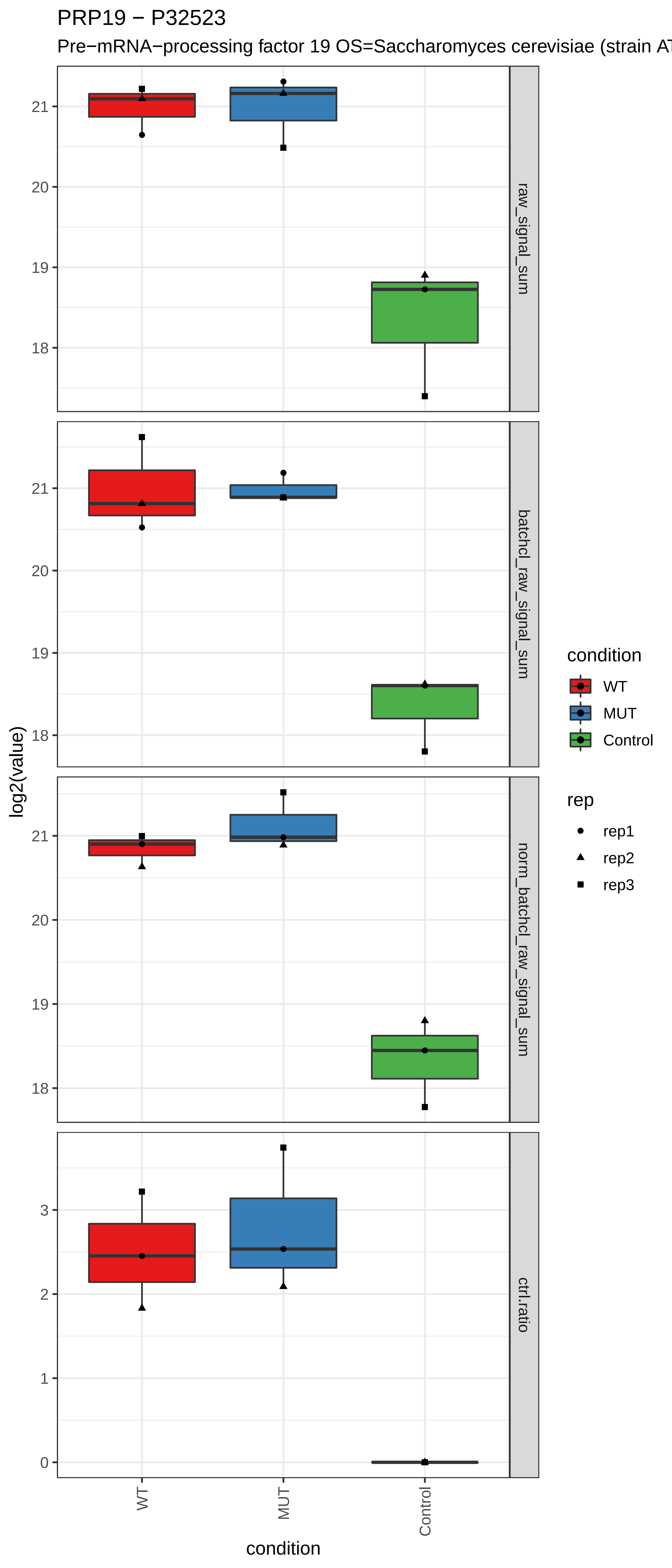

PRP19 − P32523
Pre−mRNA−processing factor 19 OS=Saccharomyces cerevisiae (strain AT
raw_signal_sum
batchcl_raw_signal_sum
norm_batchcl_raw_signal_sum
ctrl.ratio
log2(value)
condition
WT
MUT
Control
rep
rep1
rep2
rep3

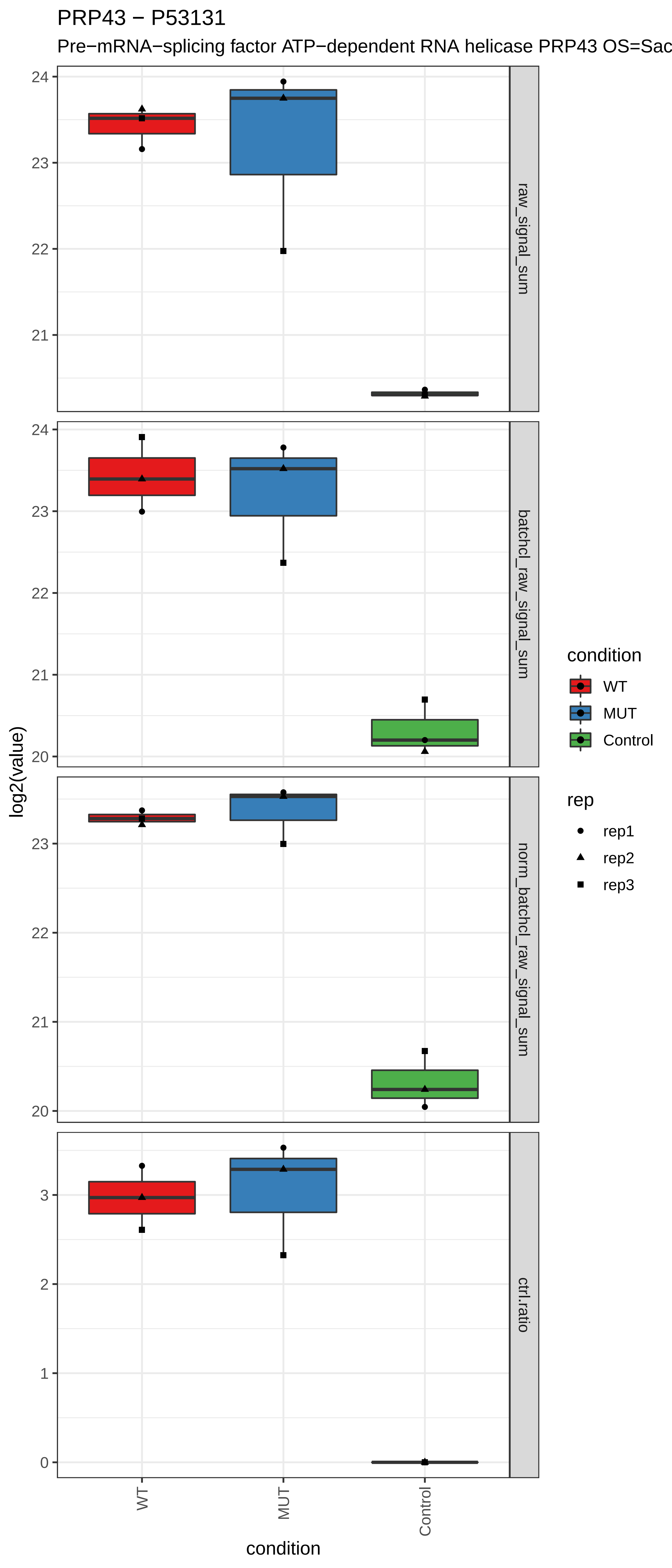
PRP43 − P53131
Pre−mRNA−splicing factor ATP−dependent RNA helicase PRP43 OS=Sac
raw_signal_sum
batchcl_raw_signal_sum
norm_batchcl_raw_signal_sum
ctrl.ratio
log2(value)
condition
WT
MUT
Control
rep
rep1
rep2
rep3

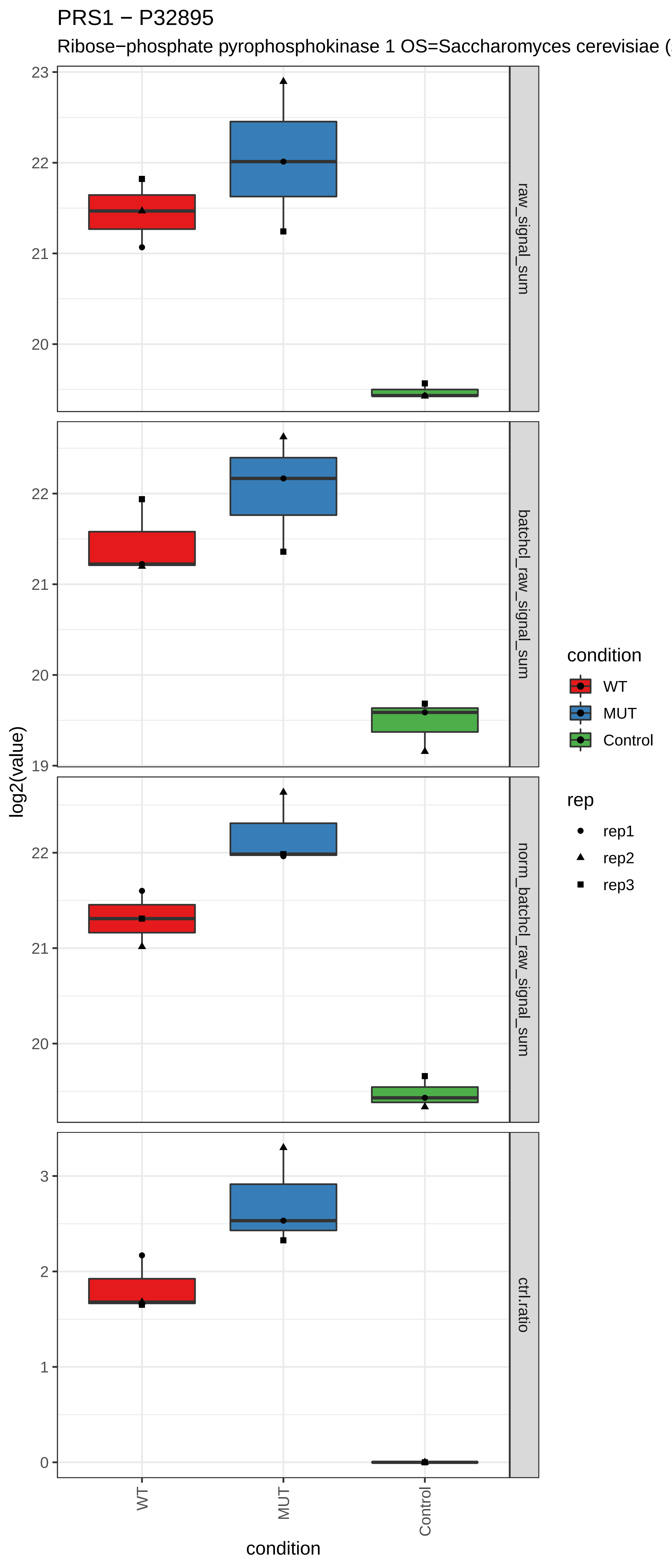
PRS1 − P32895
Ribose−phosphate pyrophosphokinase 1 OS=Saccharomyces cerevisiae (
raw_signal_sum
batchcl_raw_signal_sum
norm_batchcl_raw_signal_sum
ctrl.ratio
log2(value)
condition
WT
MUT
Control
rep
rep1
rep2
rep3

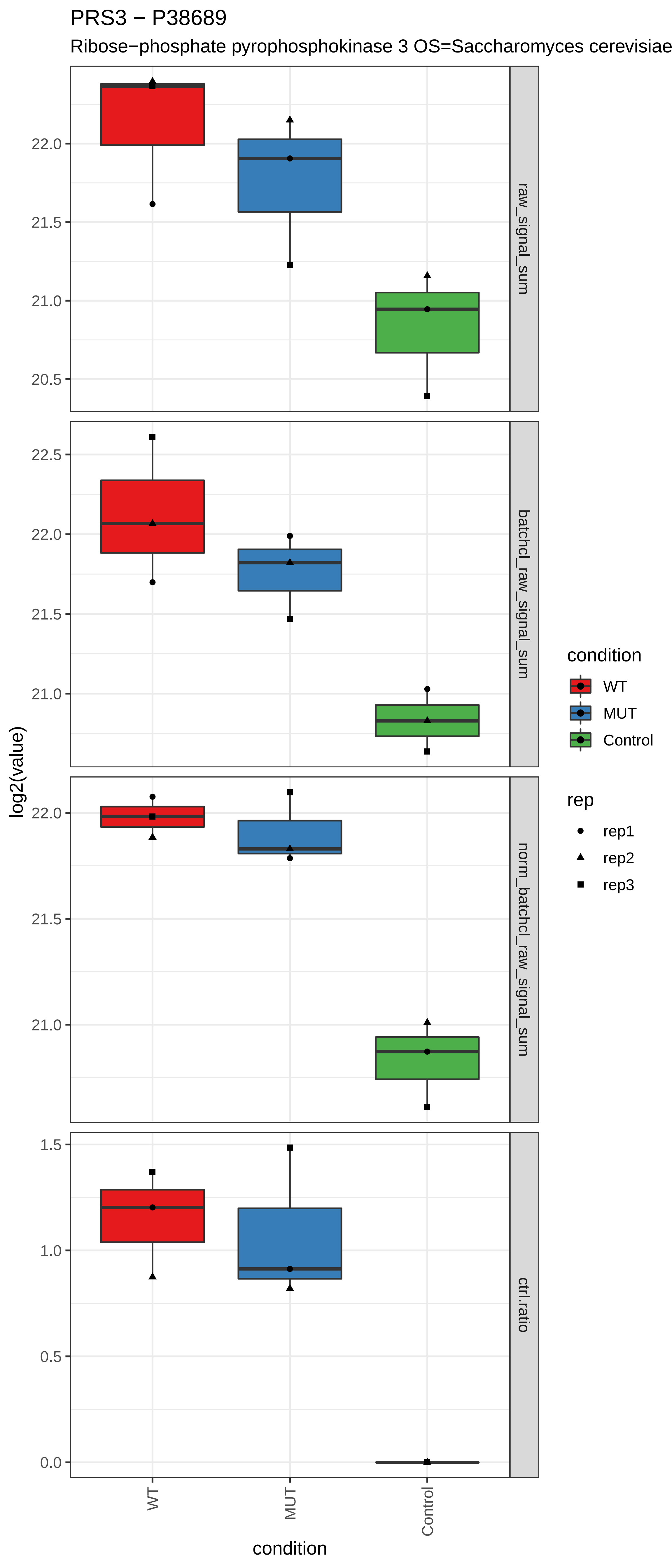

PRS3 − P38689
Ribose−phosphate pyrophosphokinase 3 OS=Saccharomyces cerevisiae
raw_signal_sum
batchcl_raw_signal_sum
norm_batchcl_raw_signal_sum
ctrl.ratio
log2(value)
condition
WT
MUT
Control
rep
rep1
rep2
rep3
22.0
21.5
21.0
20.5
22.5
1.5
1.0
0.5
0.0

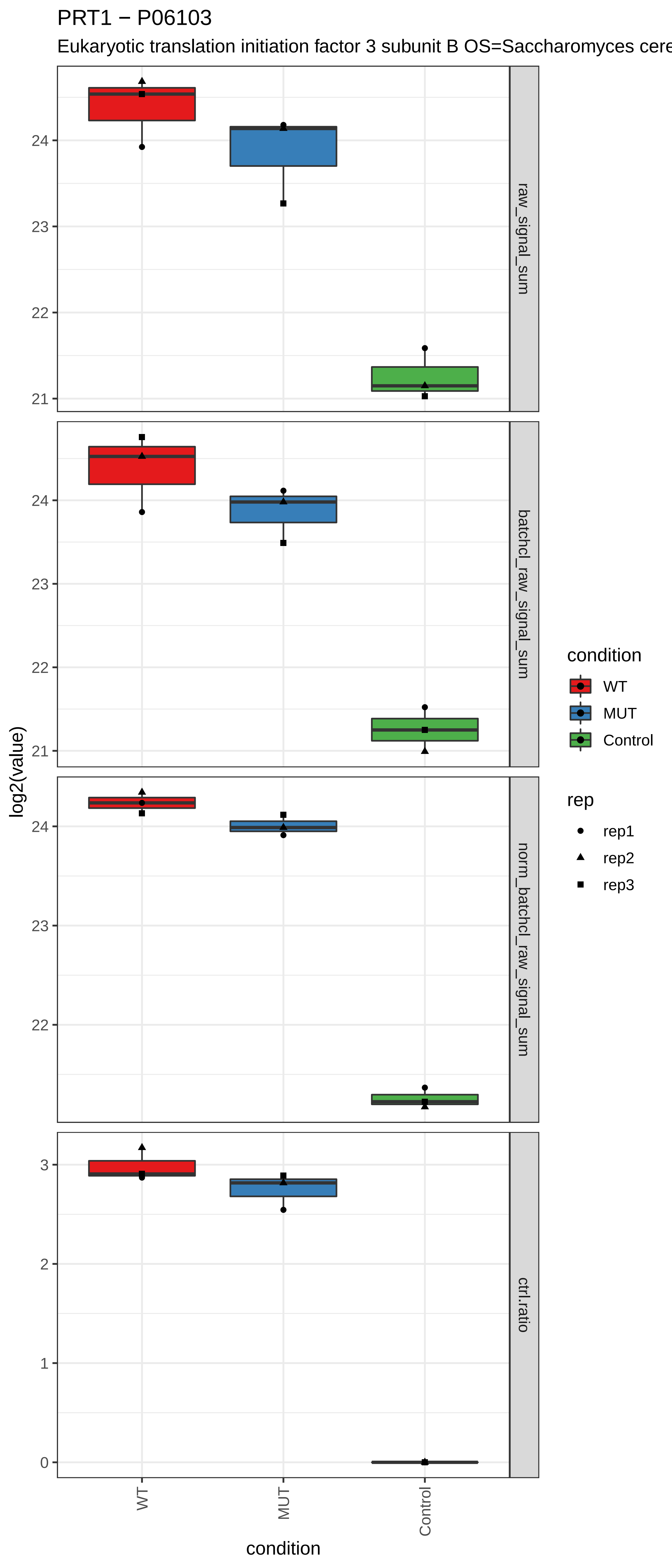

PRT1 − P06103
Eukaryotic translation initiation factor 3 subunit B OS=Saccharomyces cere
raw_signal_sum
batchcl_raw_signal_sum
norm_batchcl_raw_signal_sum
ctrl.ratio
log2(value)
condition
WT
MUT
Control
rep
rep1
rep2
rep3
24
23
22
21
3
2
1
0

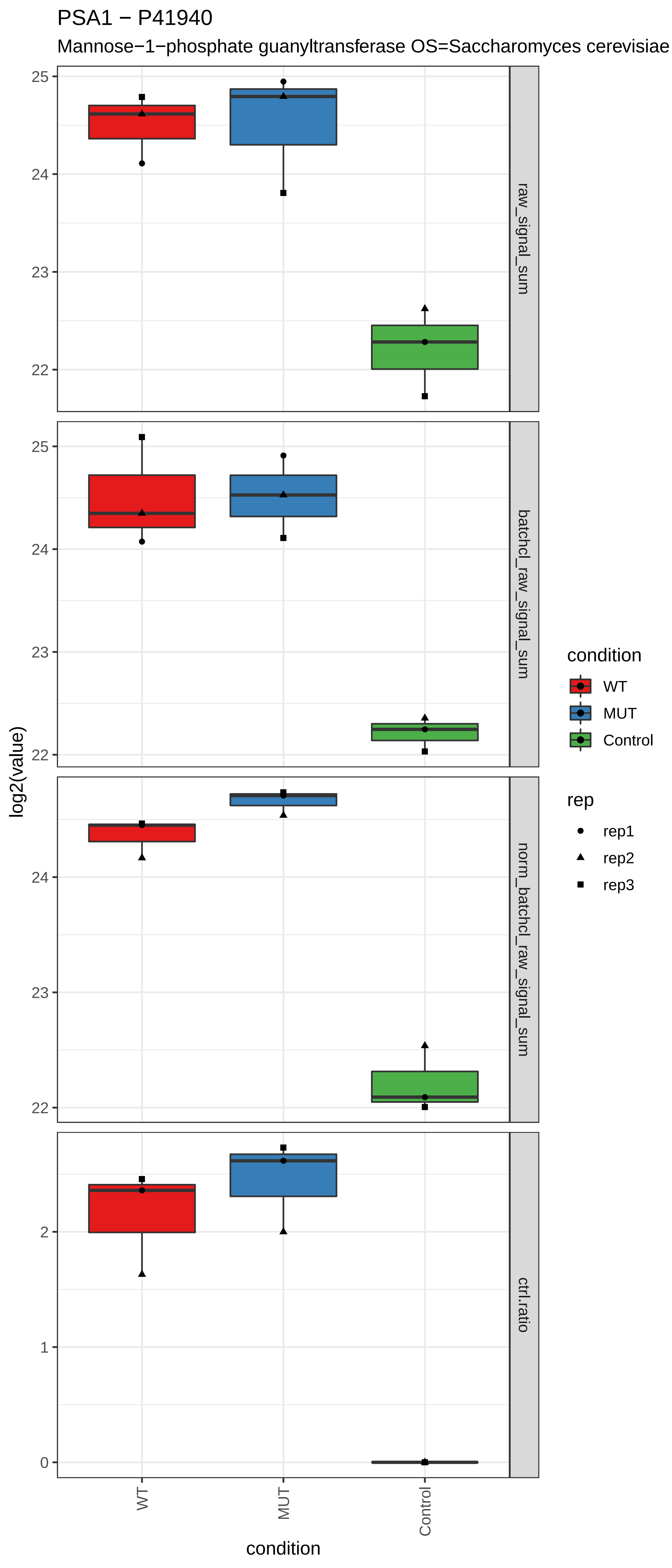

PSA1 − P41940
Mannose−1−phosphate guanyltransferase OS=Saccharomyces cerevisiae
raw_signal_sum
batchcl_raw_signal_sum
norm_batchcl_raw_signal_sum
ctrl.ratio
log2(value)
condition
WT
MUT
Control
rep
rep1
rep2
rep3
25
24
23
22
2
1
0

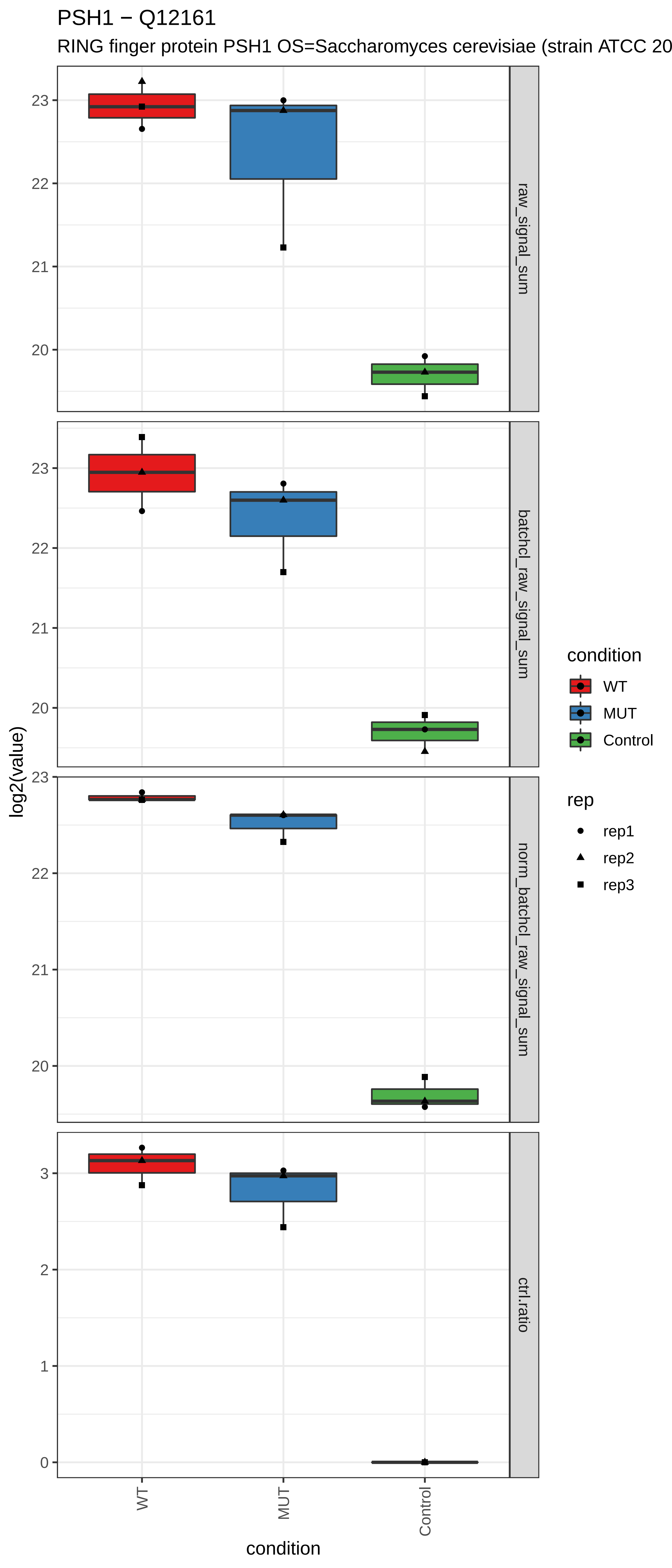

PSH1 − Q12161
RING finger protein PSH1 OS=Saccharomyces cerevisiae (strain ATCC 20
raw_signal_sum
batchcl_raw_signal_sum
norm_batchcl_raw_signal_sum
ctrl.ratio
log2(value)
condition
WT
MUT
Control
rep
rep1
rep2
rep3

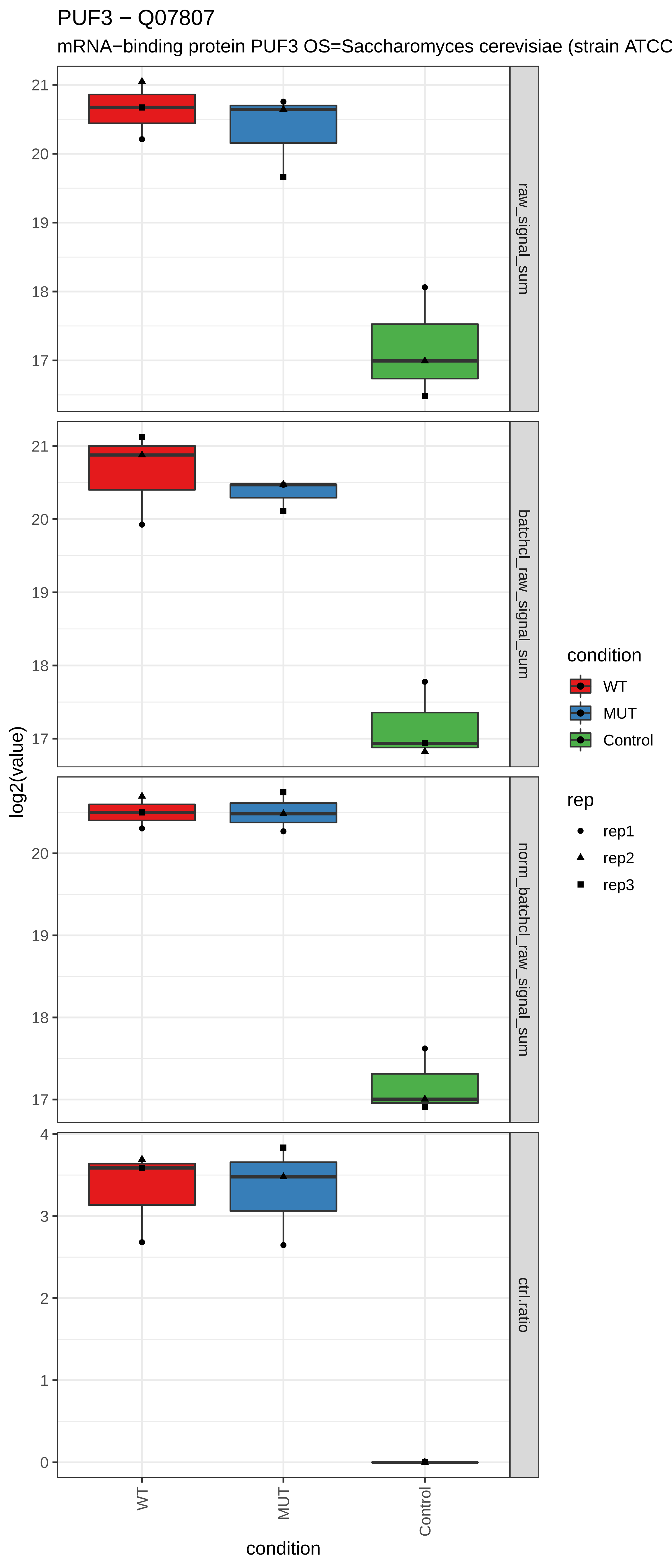
PUF3 − Q07807
mRNA−binding protein PUF3 OS=Saccharomyces cerevisiae (strain ATCC
raw_signal_sum
batchcl_raw_signal_sum
norm_batchcl_raw_signal_sum
ctrl.ratio
log2(value)
condition
WT
MUT
Control
rep
rep1
rep2
rep3
21
20
19
18
17
4
3
2
1
0

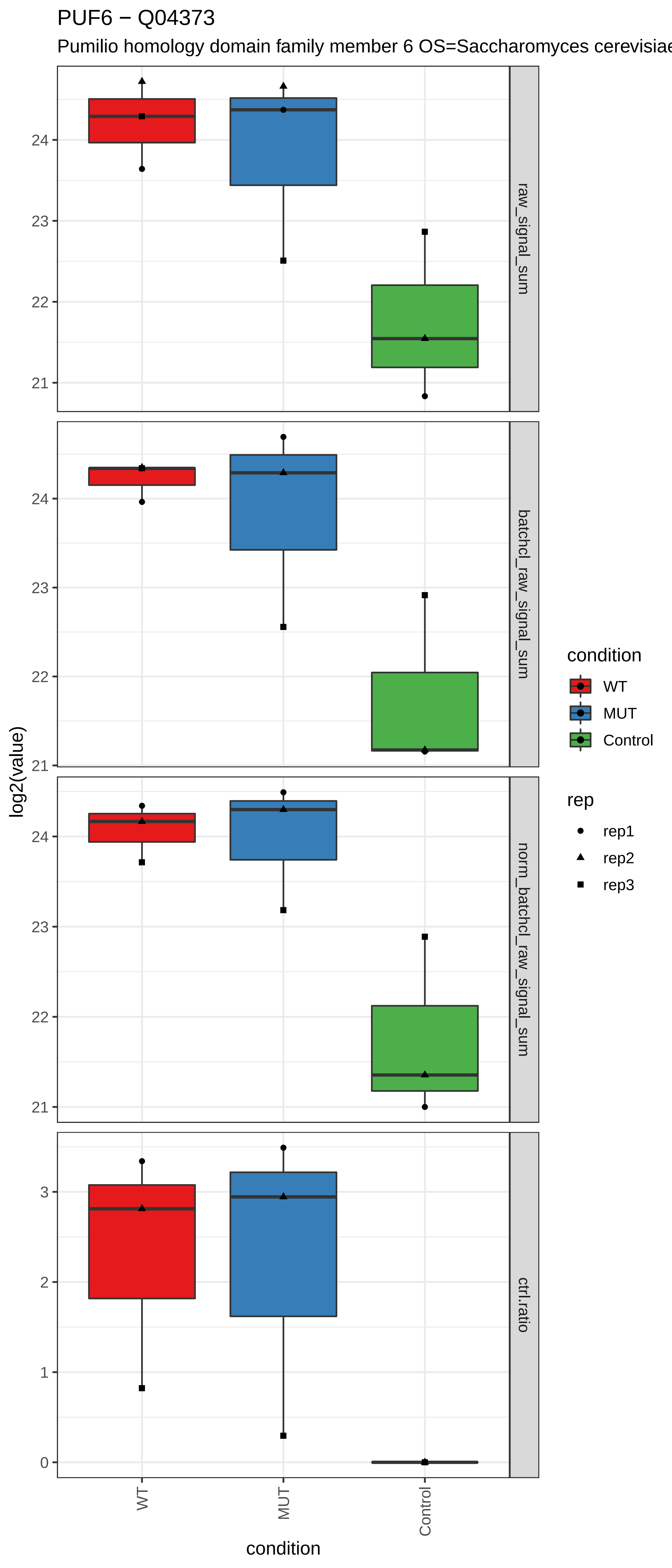

PUF6 − Q04373
Pumilio homology domain family member 6 OS=Saccharomyces cerevisiae
raw_signal_sum
batchcl_raw_signal_sum
norm_batchcl_raw_signal_sum
ctrl.ratio
log2(value)
condition
WT
MUT
Control
rep
rep1
rep2
rep3

PUP2 − P32379

Proteasome subunit alpha type−5 OS=Saccharomyces cerevisiae (strain

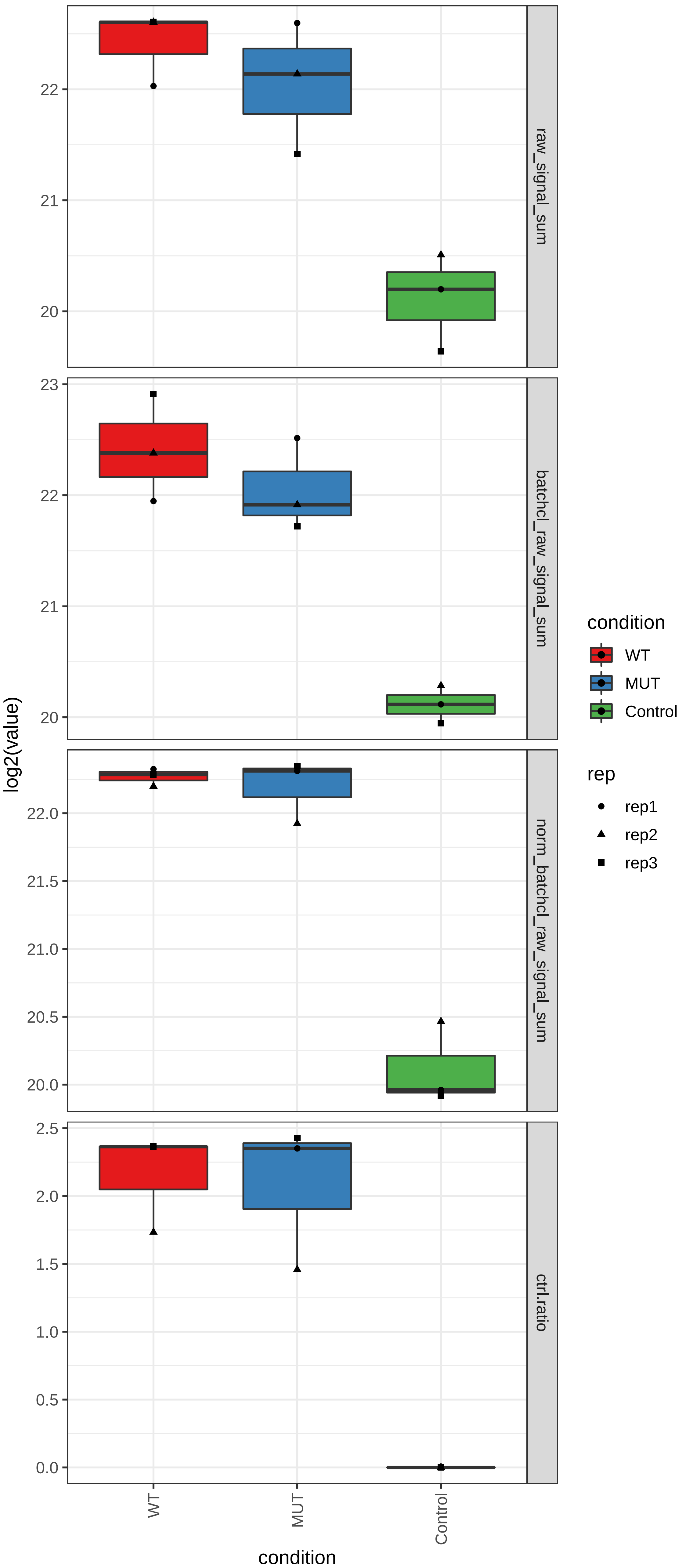

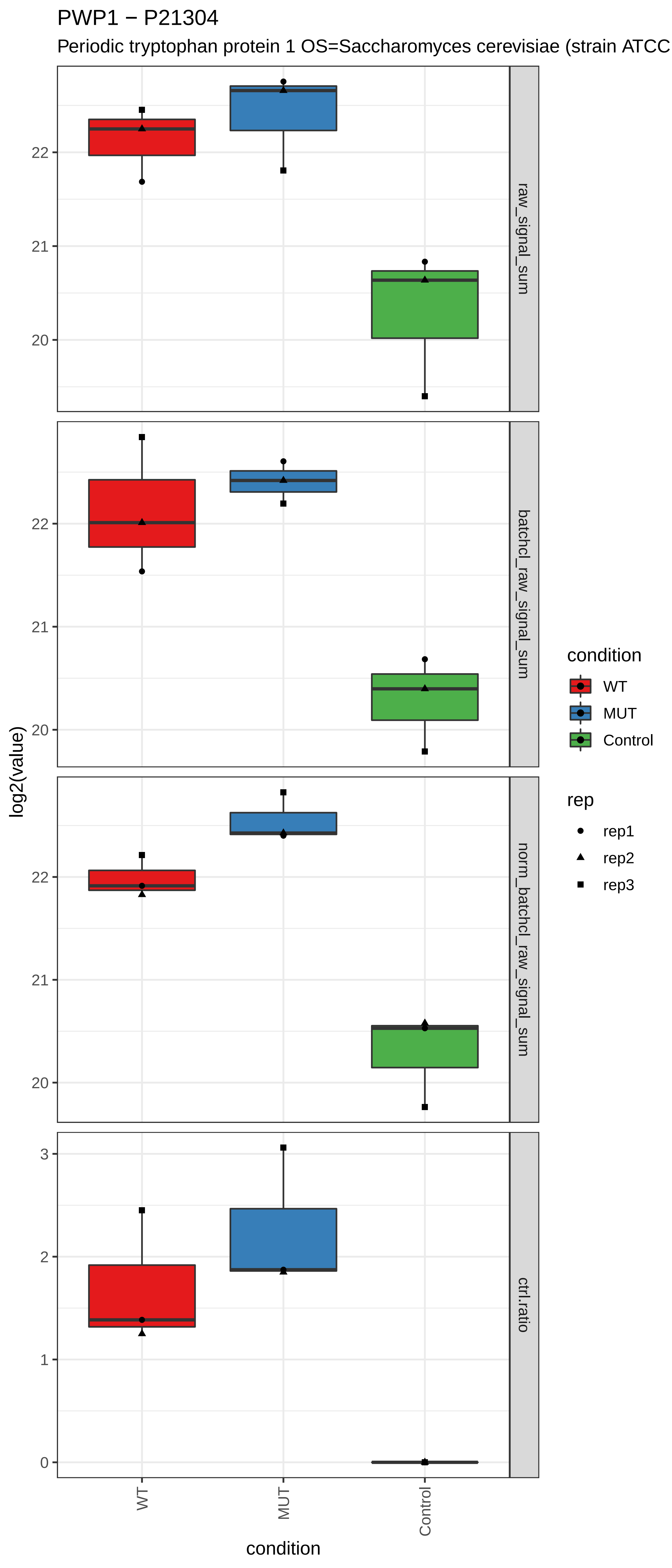
PWP1 − P21304
Periodic tryptophan protein 1 OS=Saccharomyces cerevisiae (strain ATCC
raw_signal_sum
batchcl_raw_signal_sum
norm_batchcl_raw_signal_sum
ctrl.ratio
log2(value)
condition
WT
MUT
Control
rep
rep1
rep2
rep3
22
21
20
3
2
1
0

PYC1 − P11154

Pyruvate carboxylase 1 OS=Saccharomyces cerevisiae (strain ATCC 204

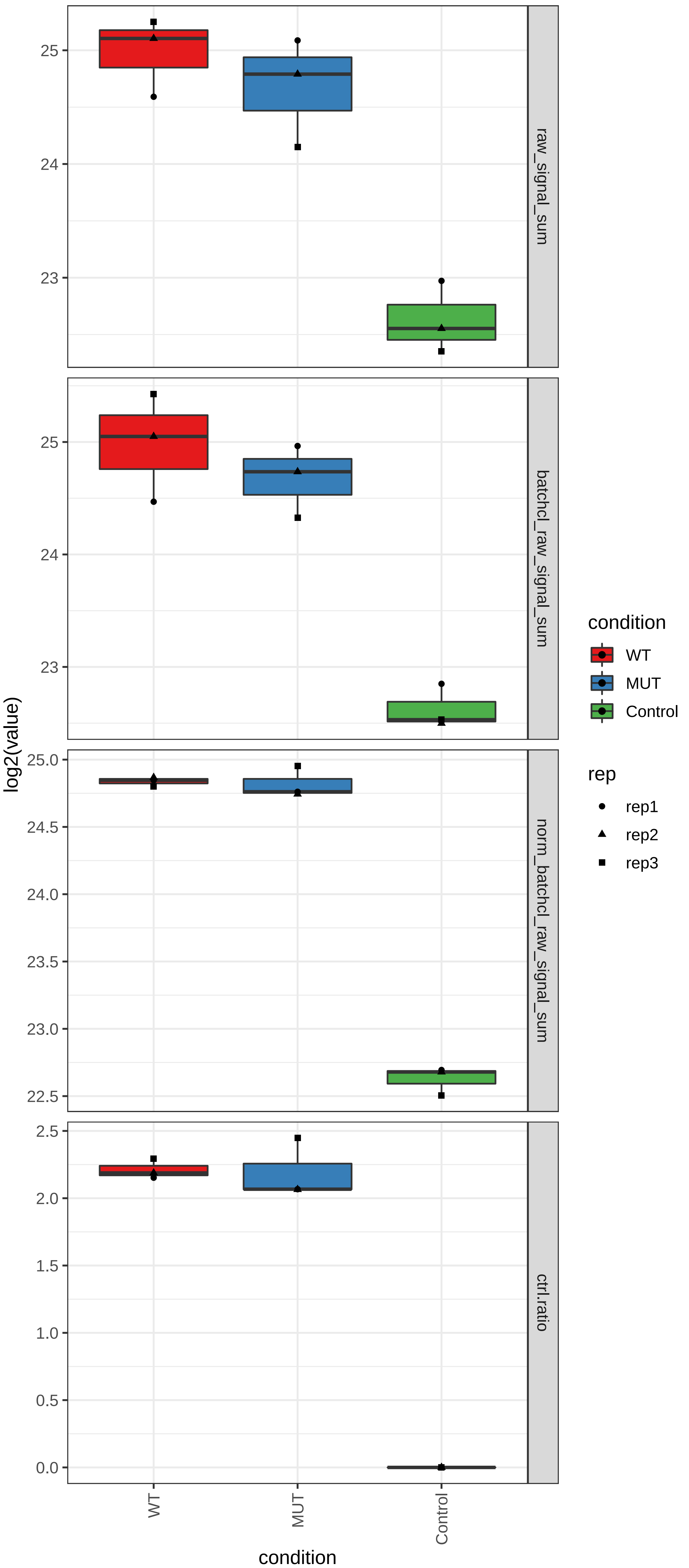

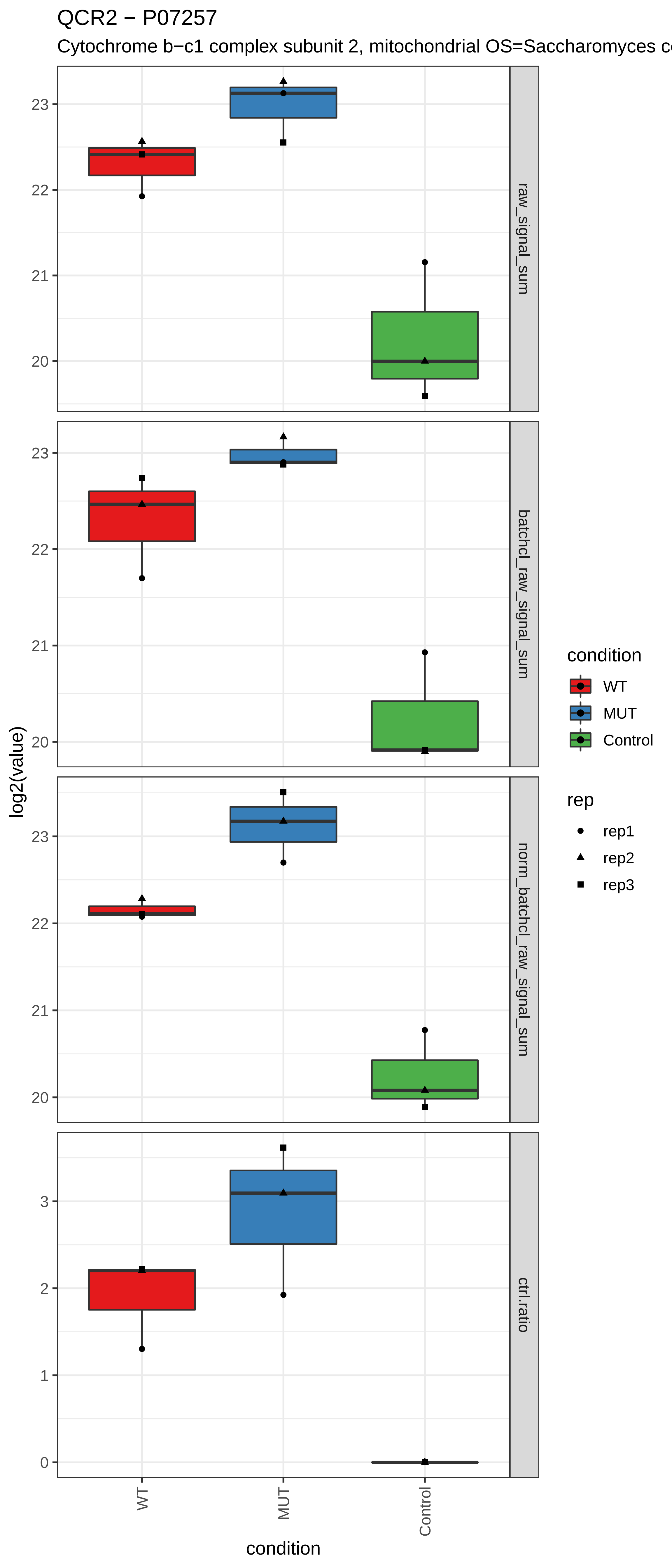

QCR2 − P07257
Cytochrome b−c1 complex subunit 2, mitochondrial OS=Saccharomyces c
log2(value)
raw_signal_sum
batchcl_raw_signal_sum
norm_batchcl_raw_signal_sum
ctrl.ratio
23
22
21
20
3
2
1
0
WT
MUT
Control
condition
condition
WT
MUT
Control
rep
rep1
rep2
rep3

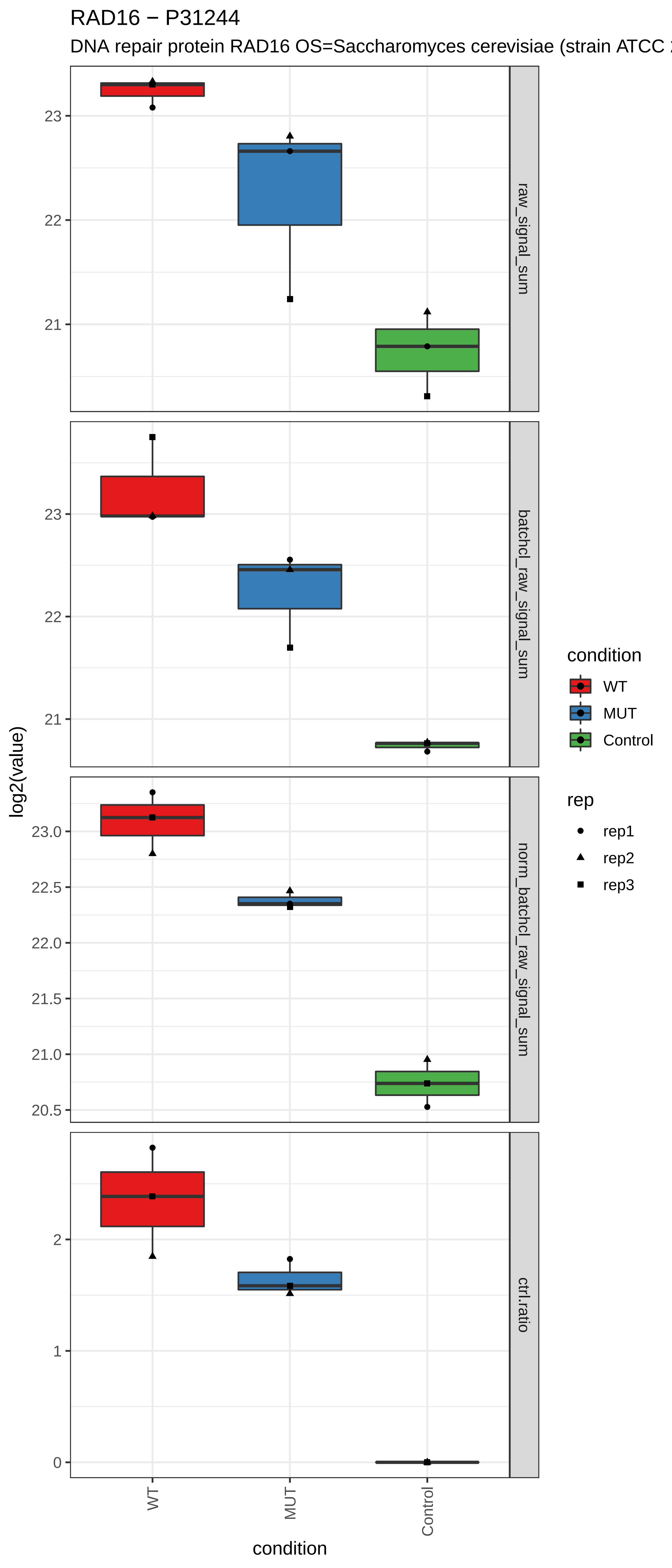
RAD16 − P31244
DNA repair protein RAD16 OS=Saccharomyces cerevisiae (strain ATCC 2
raw_signal_sum
batchcl_raw_signal_sum
norm_batchcl_raw_signal_sum
ctrl.ratio
log2(value)
condition
WT
MUT
Control
rep
rep1
rep2
rep3
23
22
21
23.0
22.5
22.0
21.5
21.0
20.5
2
1
0

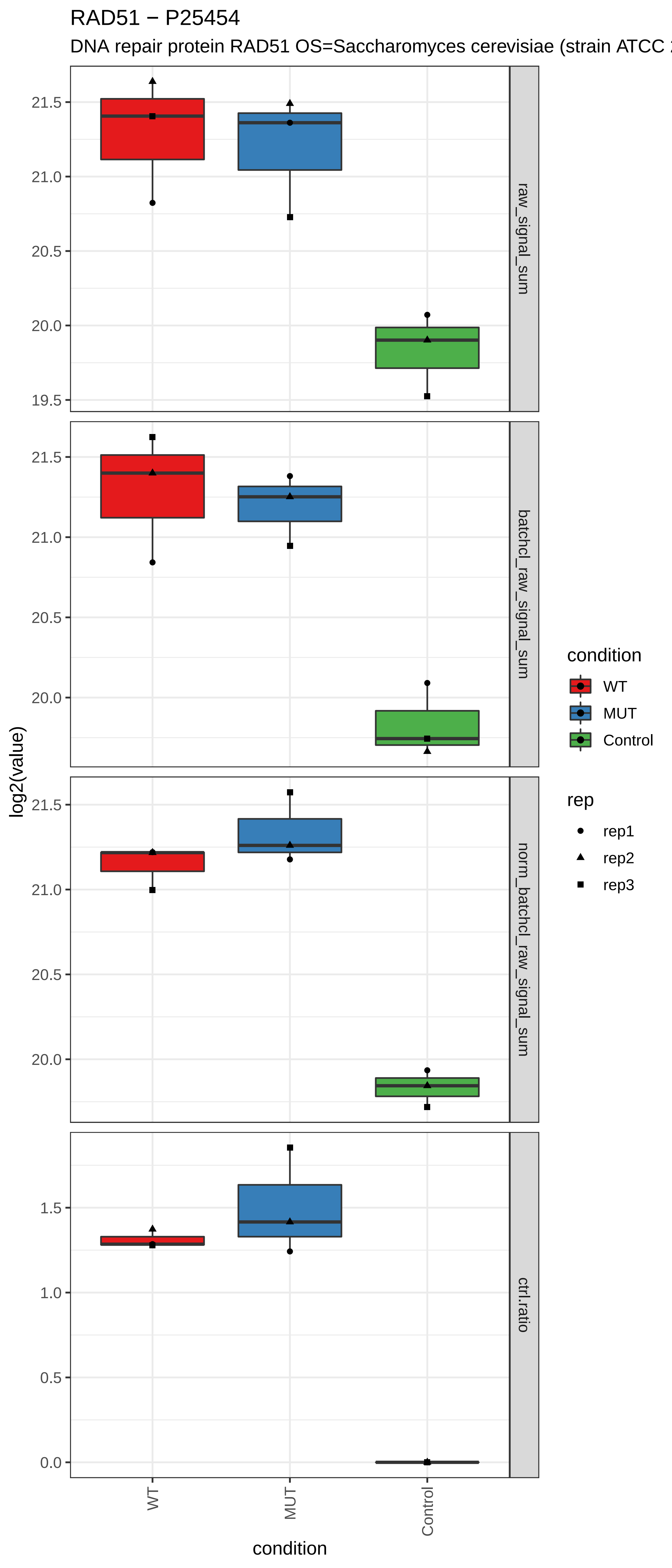

RAD51 − P25454
DNA repair protein RAD51 OS=Saccharomyces cerevisiae (strain ATCC
raw_signal_sum
batchcl_raw_signal_sum
norm_batchcl_raw_signal_sum
ctrl.ratio
21.5
21.0
20.5
20.0
19.5
1.5
1.0
0.5
0.0
log2(value)
WT
MUT
Control
condition
rep
rep1
rep2
rep3

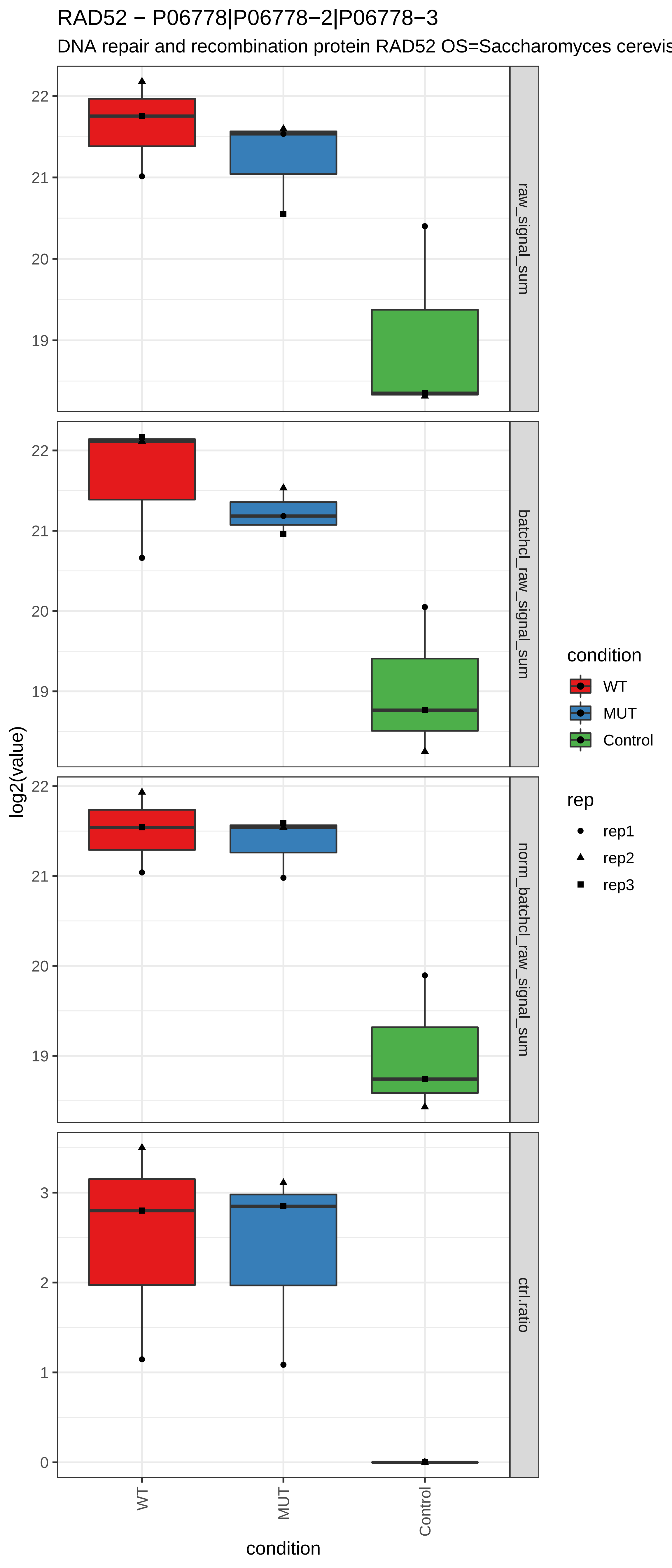

RAD52 − P06778|P06778−2|P06778−3
DNA repair and recombination protein RAD52 OS=Saccharomyces cerevis
raw_signal_sum
batchcl_raw_signal_sum
norm_batchcl_raw_signal_sum
ctrl.ratio
log2(value)
condition
WT
MUT
Control
rep
rep1
rep2
rep3

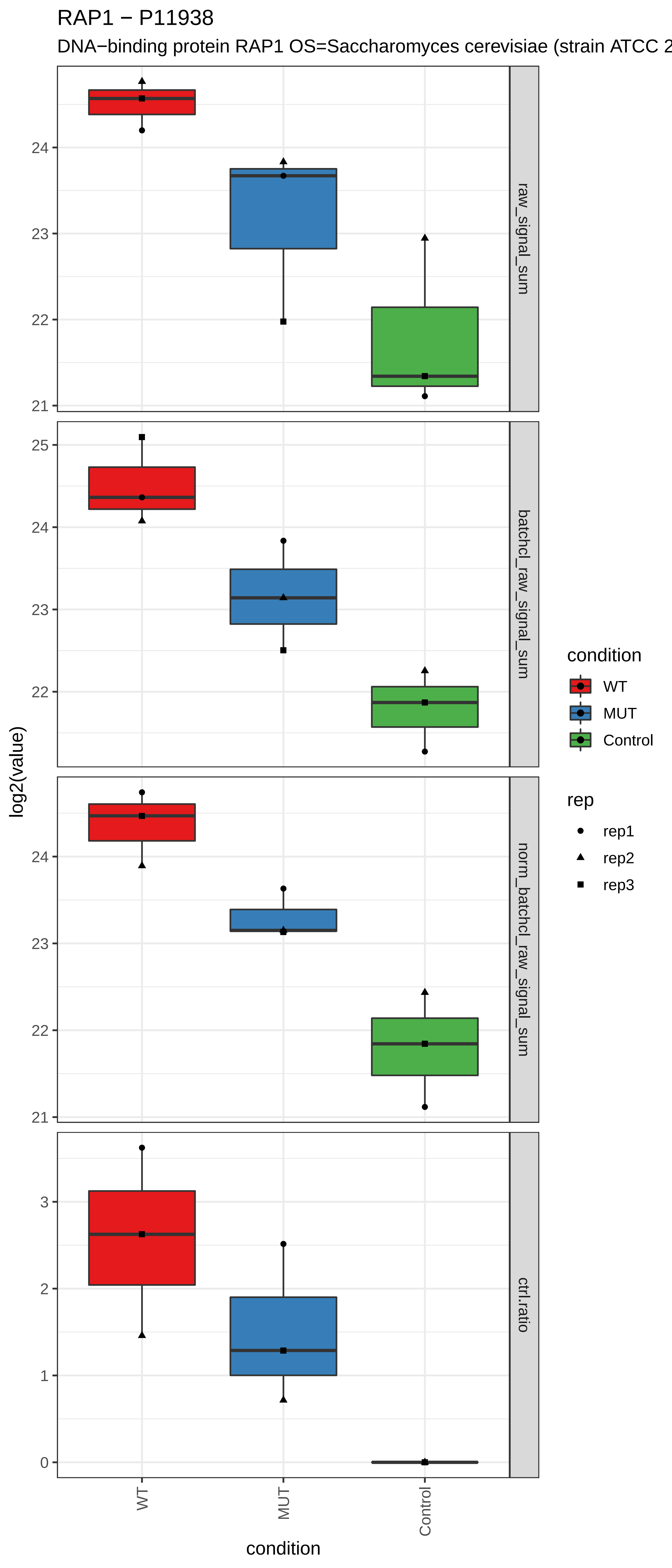
RAP1 − P11938
DNA−binding protein RAP1 OS=Saccharomyces cerevisiae (strain ATCC 2
raw_signal_sum
batchcl_raw_signal_sum
norm_batchcl_raw_signal_sum
ctrl.ratio
log2(value)
condition
WT
MUT
Control
rep
rep1
rep2
rep3

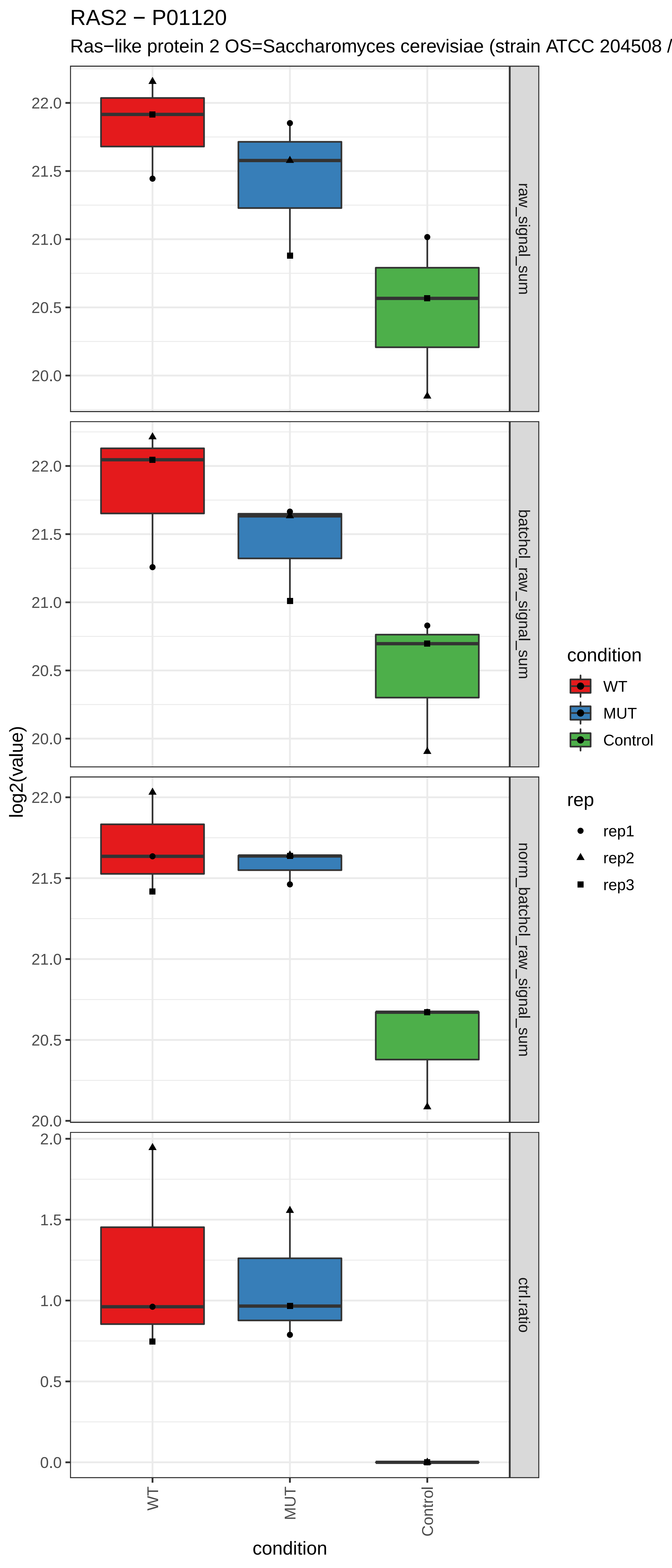

RAS2 − P01120
Ras−like protein 2 OS=Saccharomyces cerevisiae (strain ATCC 204508 /
log2(value)
raw_signal_sum
batchcl_raw_signal_sum
norm_batchcl_raw_signal_sum
ctrl.ratio
22.0
21.5
21.0
20.5
20.0
2.0
1.5
1.0
0.5
0.0
WT
MUT
Control
condition
condition
WT
MUT
Control
rep
rep1
rep2
rep3

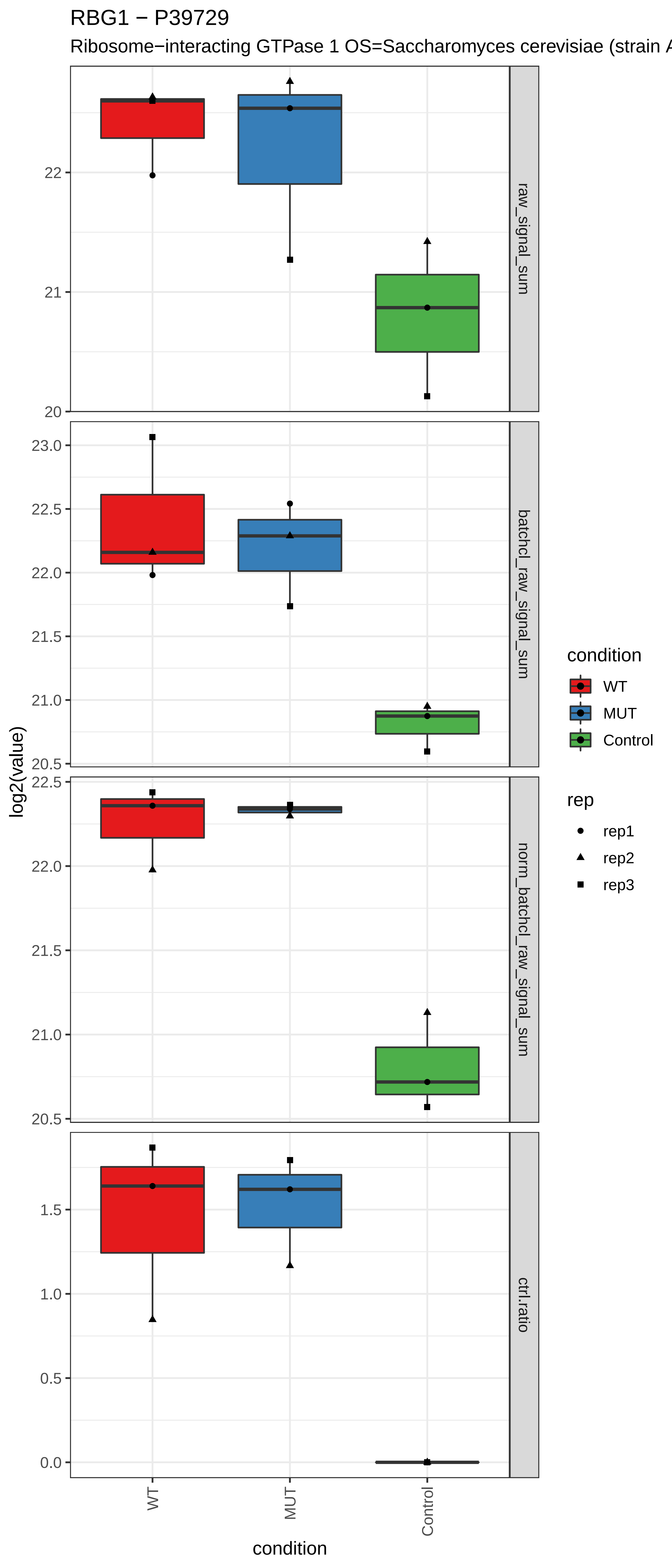
RBG1 − P39729
Ribosome−interacting GTPase 1 OS=Saccharomyces cerevisiae (strain A
raw_signal_sum
batchcl_raw_signal_sum
norm_batchcl_raw_signal_sum
ctrl.ratio
log2(value)
condition
WT
MUT
Control
rep
rep1
rep2
rep3
22
21
20
23.0
22.5
22.0
21.5
21.0
20.5
1.5
1.0
0.5
0.0

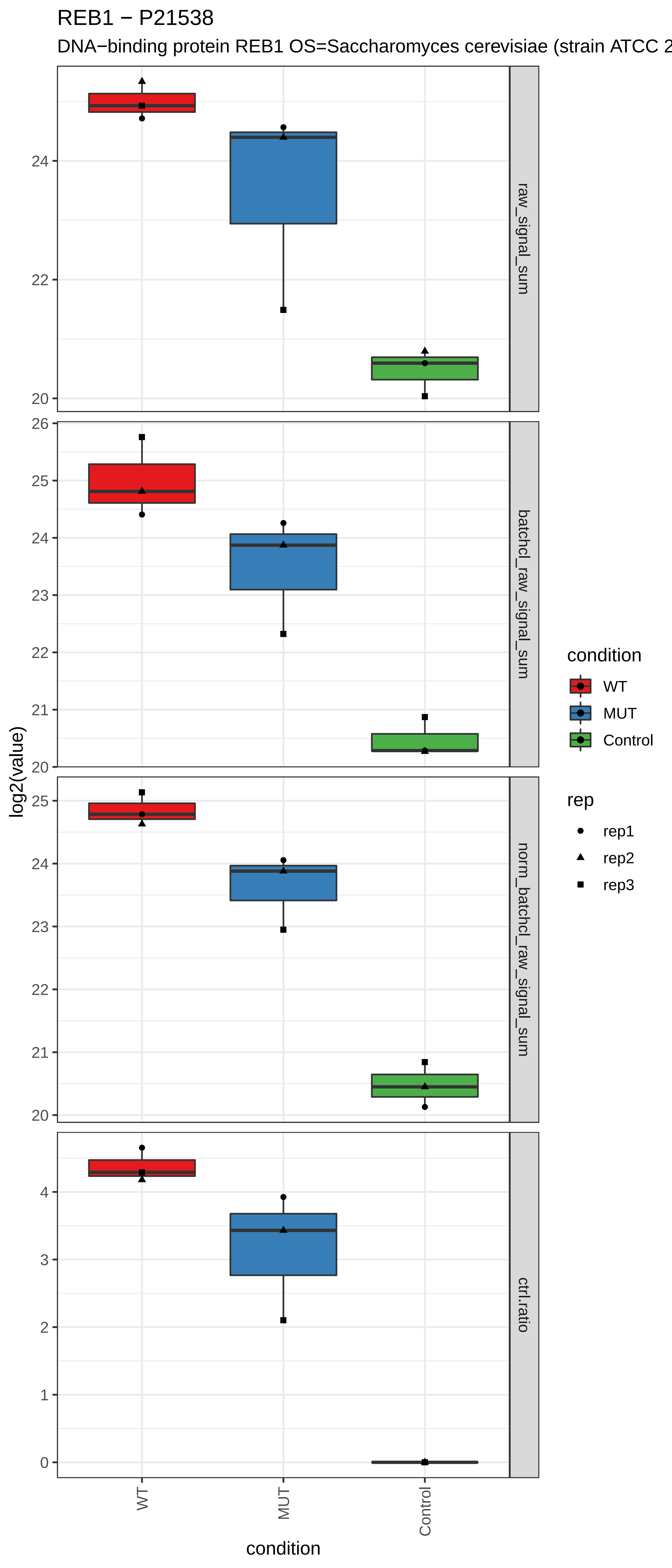

REB1 − P21538
DNA−binding protein REB1 OS=Saccharomyces cerevisiae (strain ATCC 2
raw_signal_sum
batchcl_raw_signal_sum
norm_batchcl_raw_signal_sum
ctrl.ratio
log2(value)
condition
WT
MUT
Control
rep
rep1
rep2
rep3

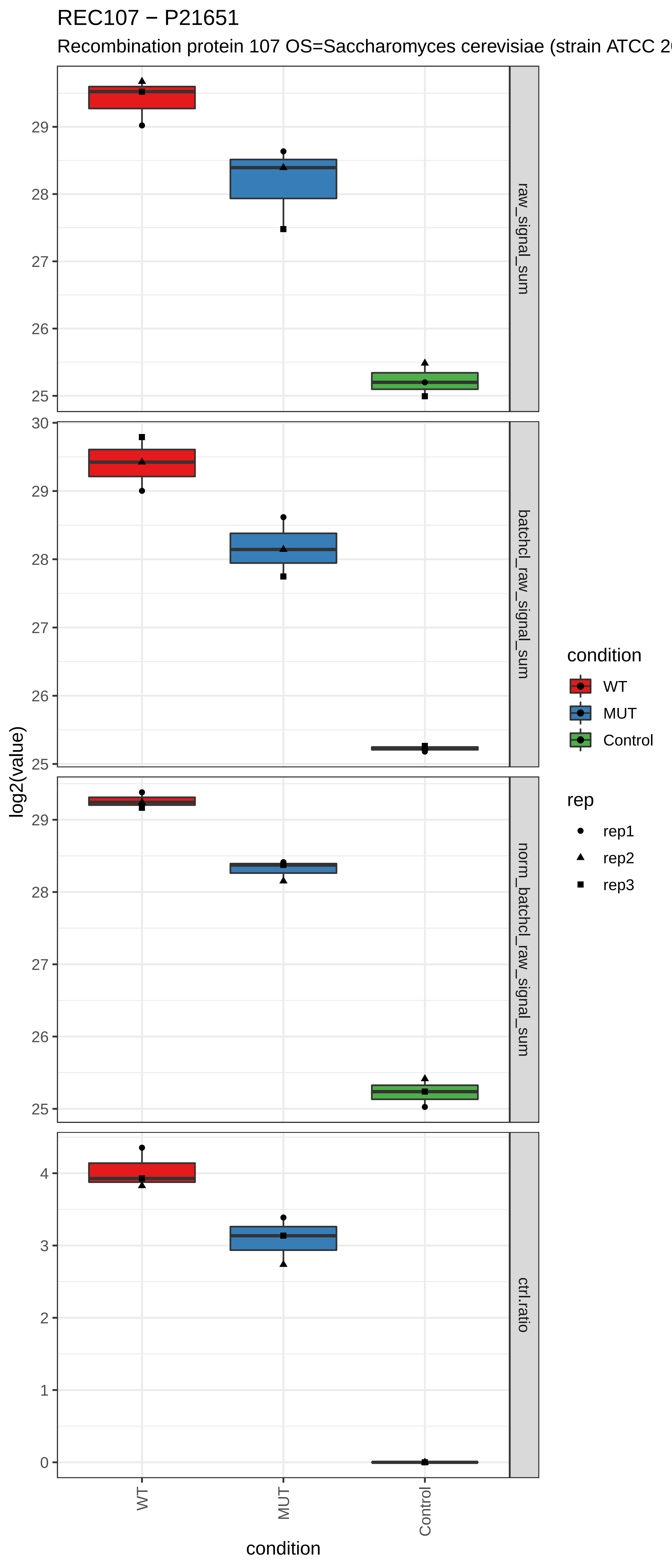

REC107 − P21651
Recombination protein 107 OS=Saccharomyces cerevisiae (strain ATCC 2
raw_signal_sum
batchcl_raw_signal_sum
norm_batchcl_raw_signal_sum
ctrl.ratio
log2(value)
condition
WT
MUT
Control
condition
WT
MUT
Control
rep
rep1
rep2
rep3

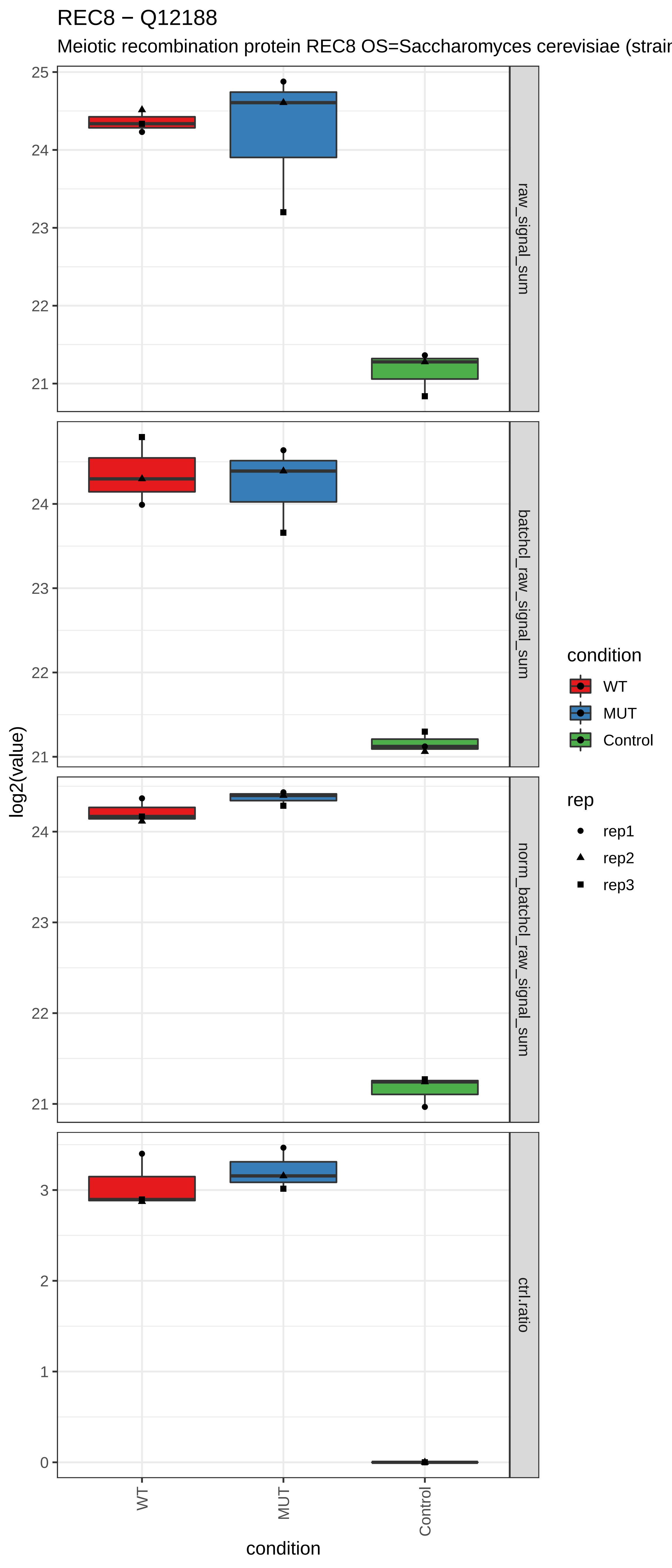

REC8 − Q12188
Meiotic recombination protein REC8 OS=Saccharomyces cerevisiae (strain
log2(value)
raw_signal_sum
batchcl_raw_signal_sum
norm_batchcl_raw_signal_sum
ctrl.ratio
condition
WT
MUT
Control
rep
rep1
rep2
rep3

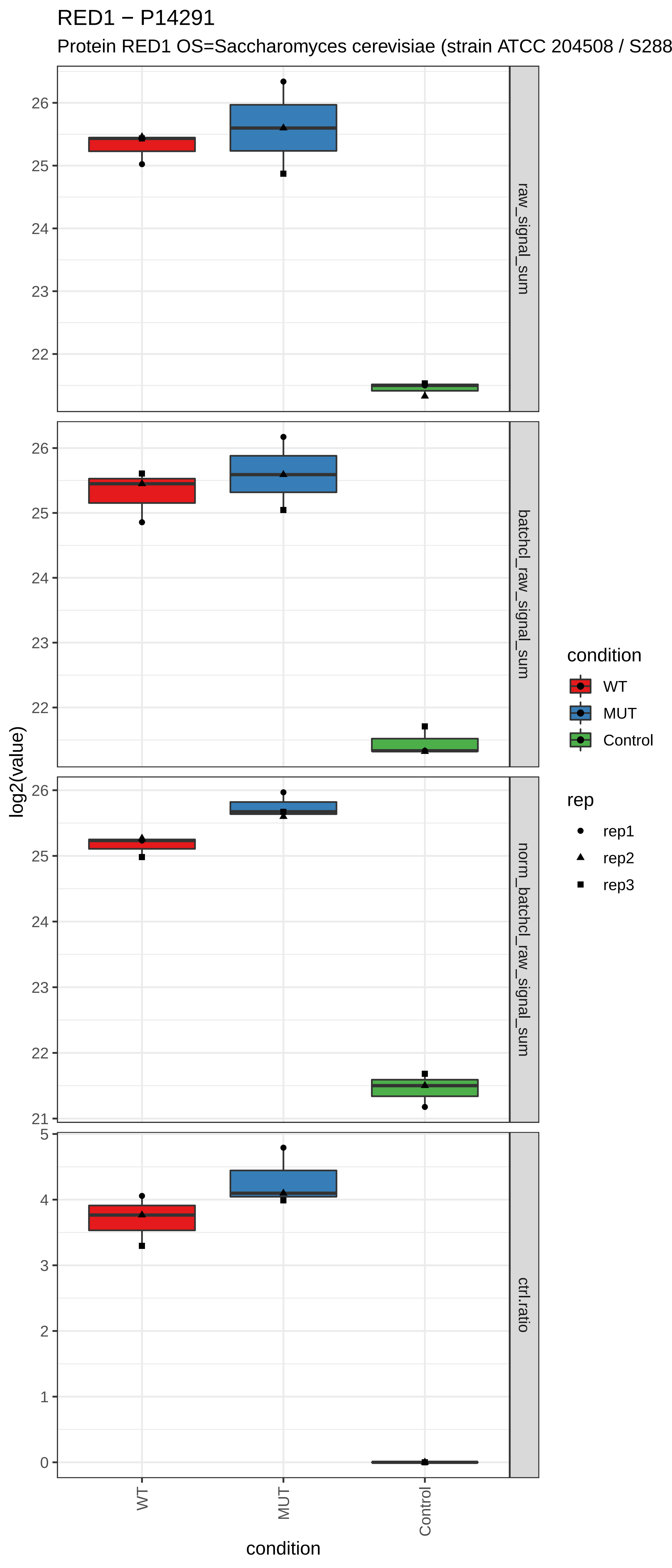

RED1 − P14291
Protein RED1 OS=Saccharomyces cerevisiae (strain ATCC 204508 / S288
raw_signal_sum
batchcl_raw_signal_sum
norm_batchcl_raw_signal_sum
ctrl.ratio
log2(value)
condition
WT
MUT
Control
rep
rep1
rep2
rep3

REP1 − P03871

Partitioning protein REP1 OS=Saccharomyces cerevisiae (strain ATCC 204

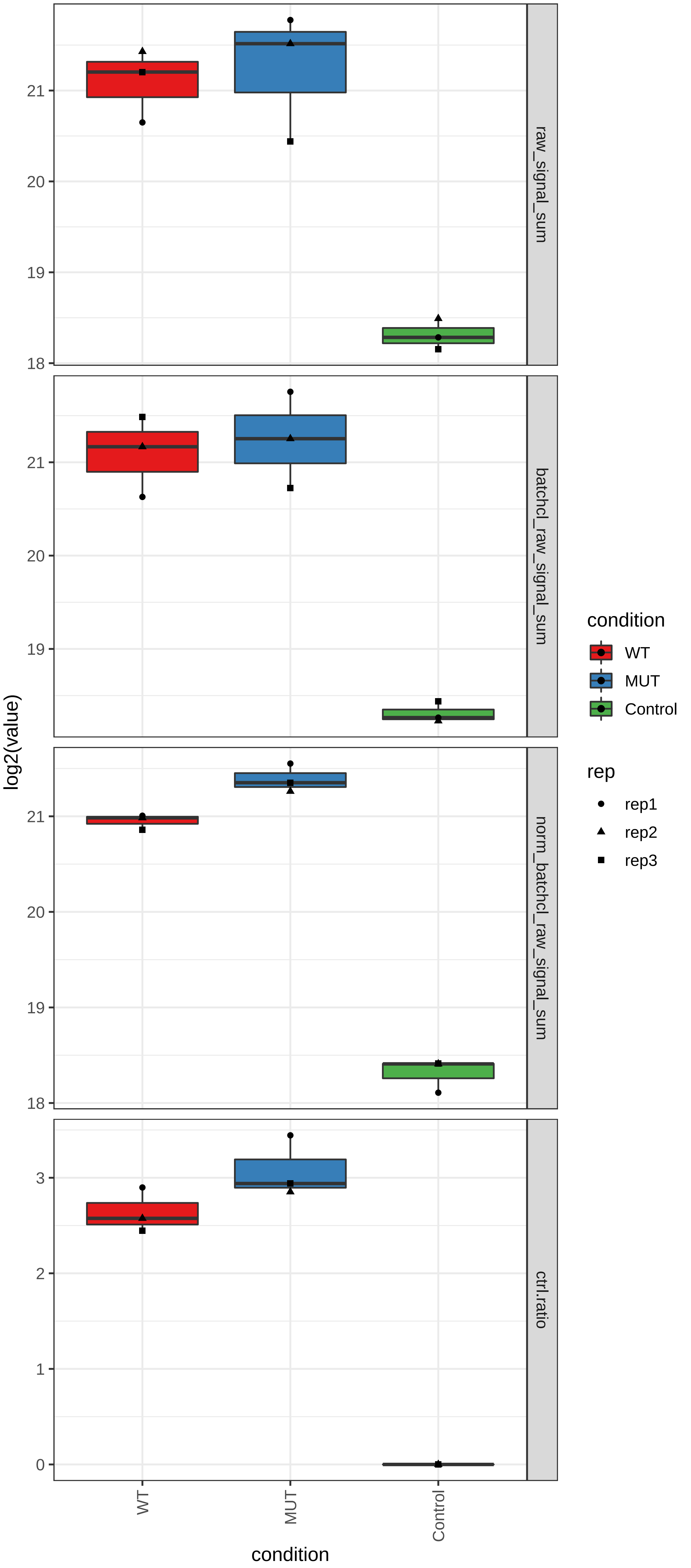

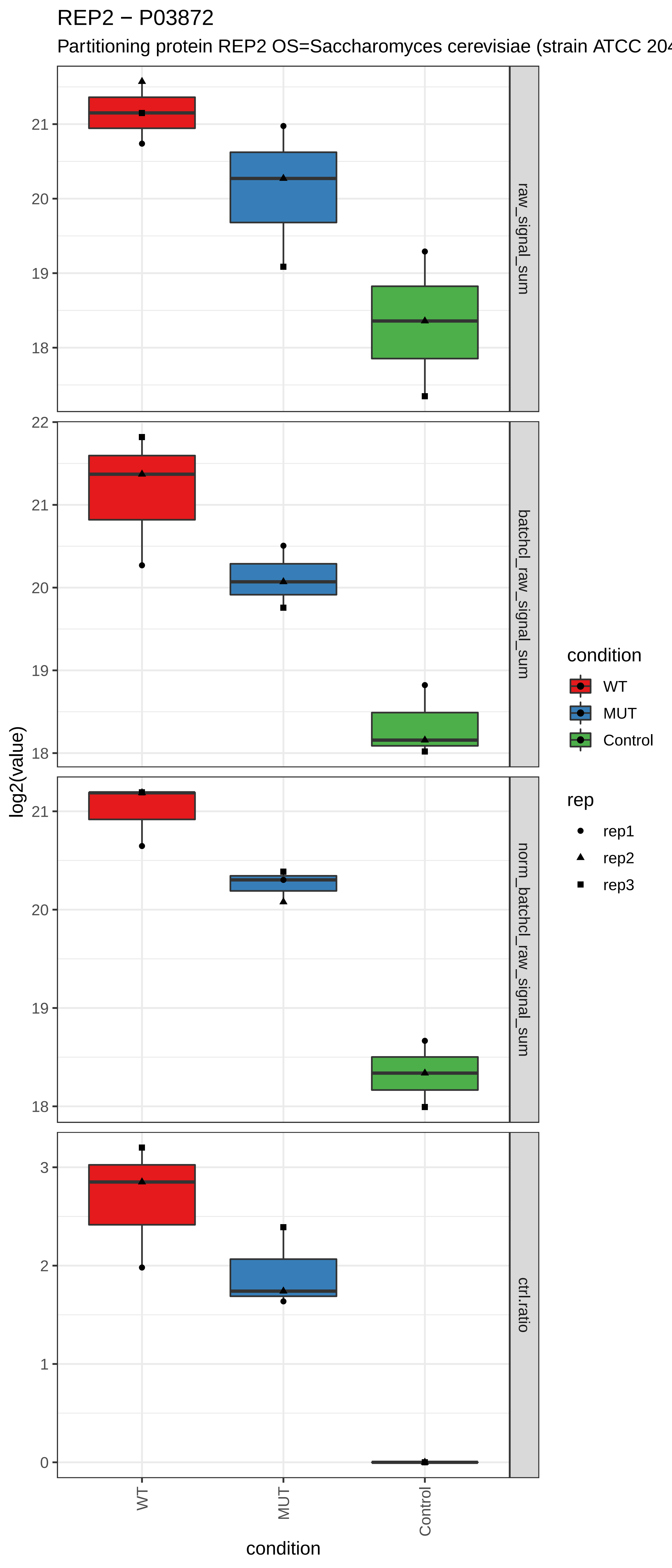

REP2 − P03872
Partitioning protein REP2 OS=Saccharomyces cerevisiae (strain ATCC 204
raw_signal_sum
batchcl_raw_signal_sum
norm_batchcl_raw_signal_sum
ctrl.ratio
log2(value)
condition
WT
MUT
Control
rep
rep1
rep2
rep3

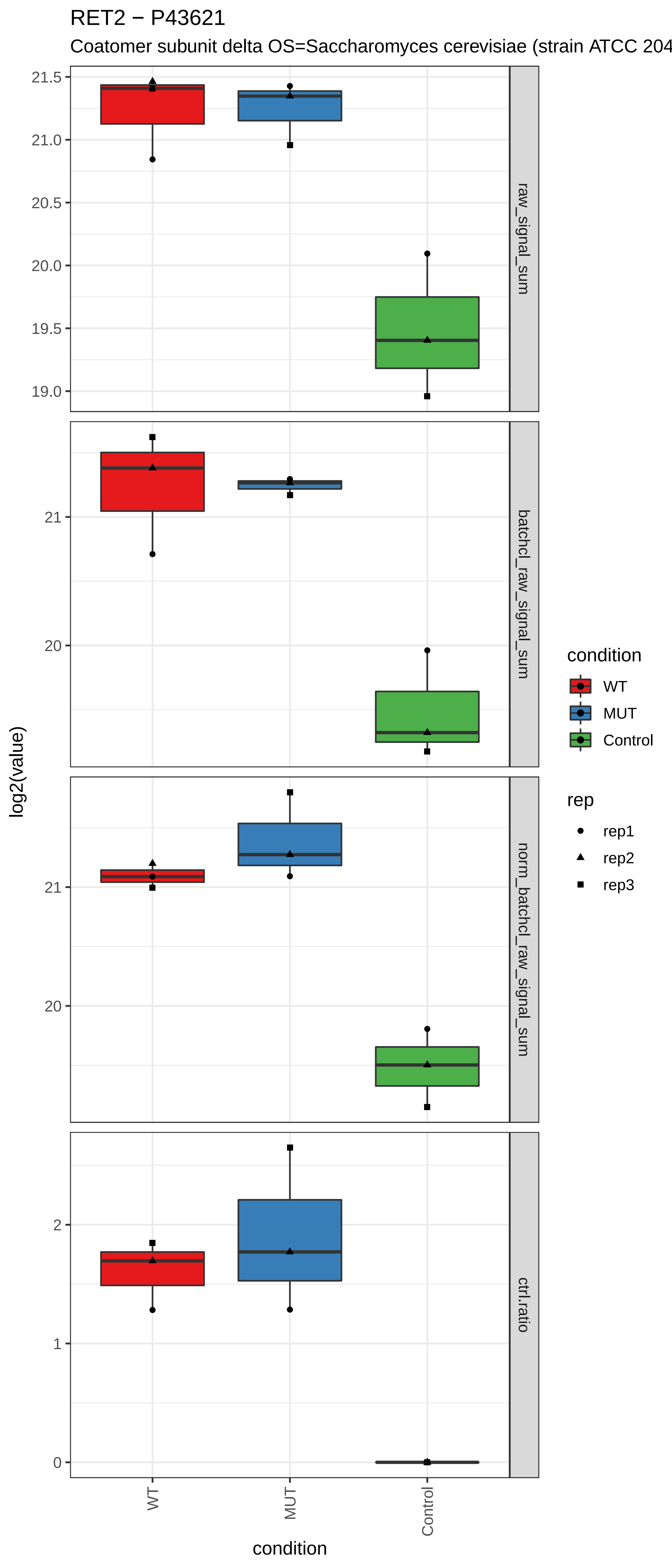
RET2 − P43621
Coatomer subunit delta OS=Saccharomyces cerevisiae (strain ATCC 204
raw_signal_sum
batchcl_raw_signal_sum
norm_batchcl_raw_signal_sum
ctrl.ratio
log2(value)
condition
WT
MUT
Control
rep
rep1
rep2
rep3
21.5
21.0
20.5
20.0
19.5
19.0
21
20
2
1
0

RFA1 − P22336

Replication factor A protein 1 OS=Saccharomyces cerevisiae (strain ATCC

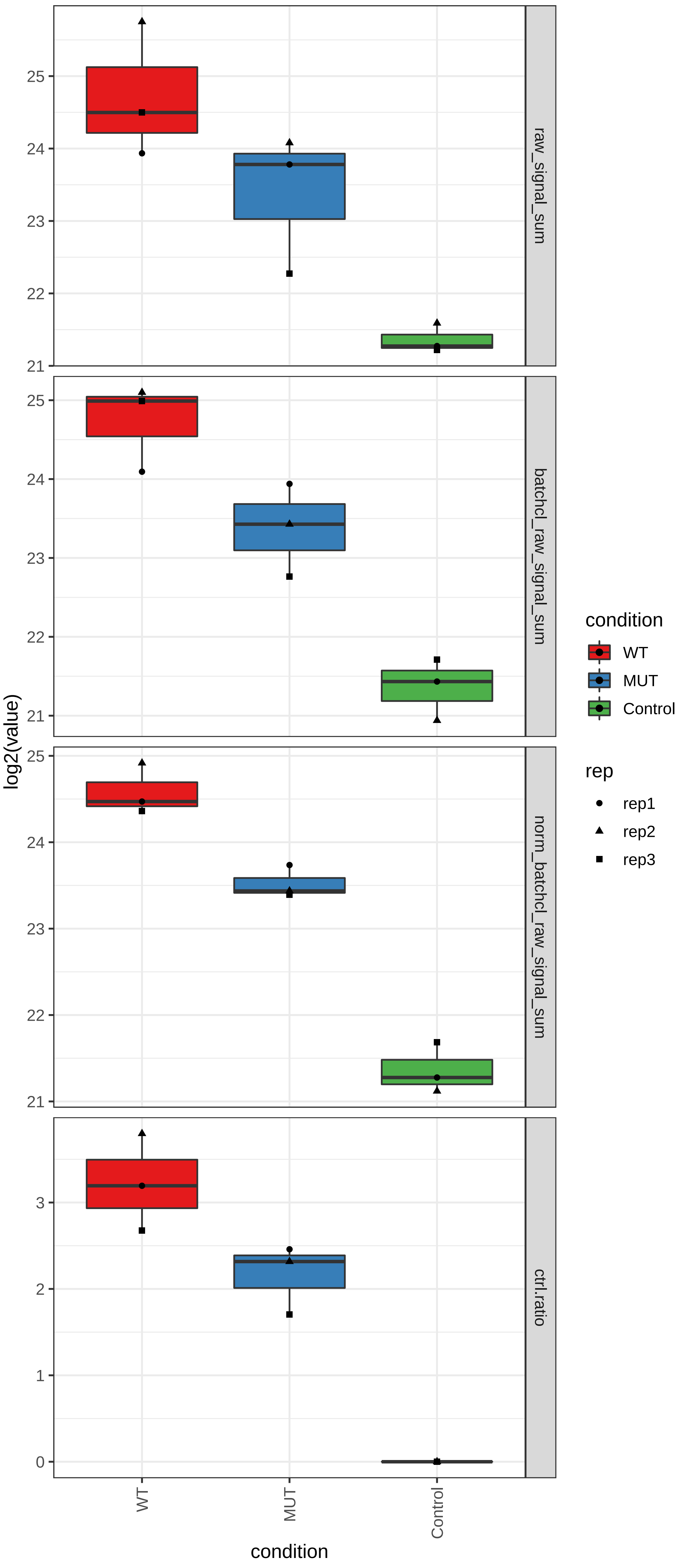

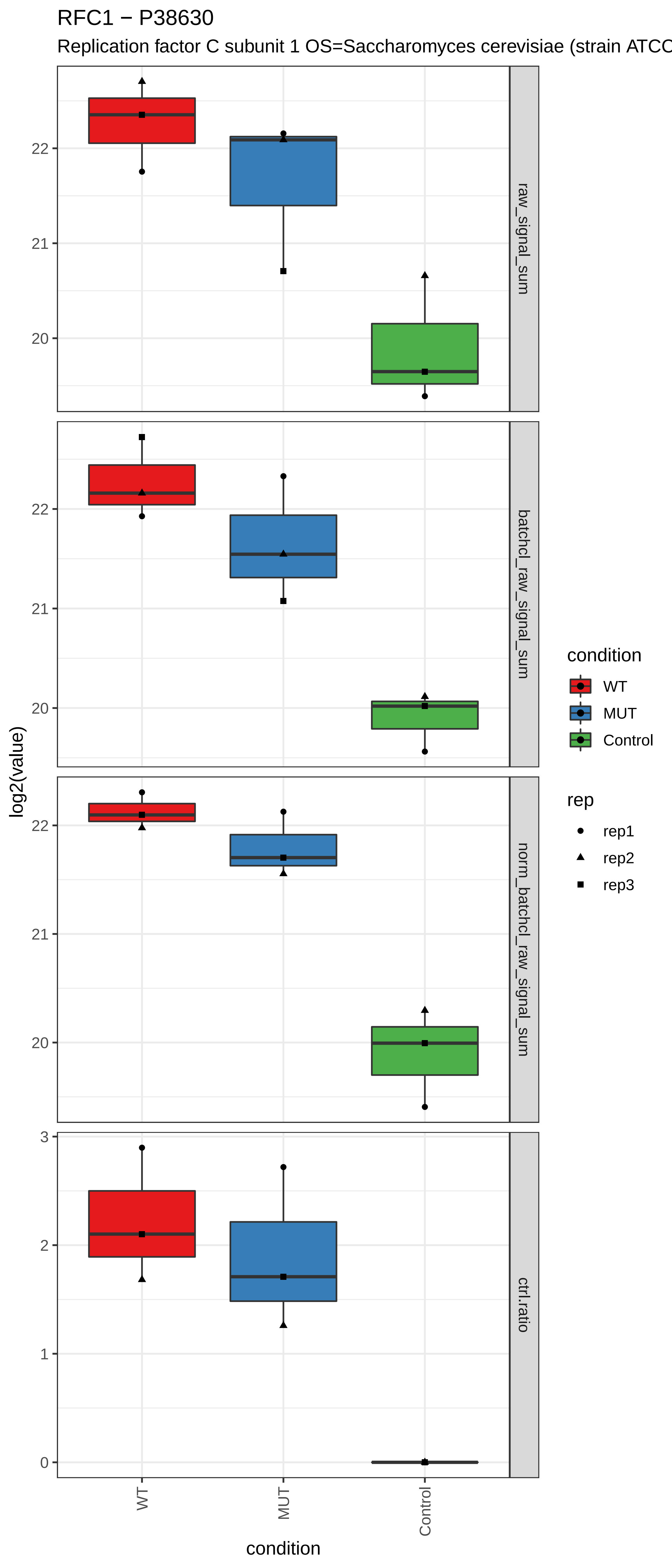

RFC1 − P38630
Replication factor C subunit 1 OS=Saccharomyces cerevisiae (strain ATCC
raw_signal_sum
batchcl_raw_signal_sum
norm_batchcl_raw_signal_sum
ctrl.ratio
log2(value)
condition
WT
MUT
Control
rep
rep1
rep2
rep3
22
21
20
3
2
1
0

RFC2 − P40348

Replication factor C subunit 2 OS=Saccharomyces cerevisiae (strain ATCC

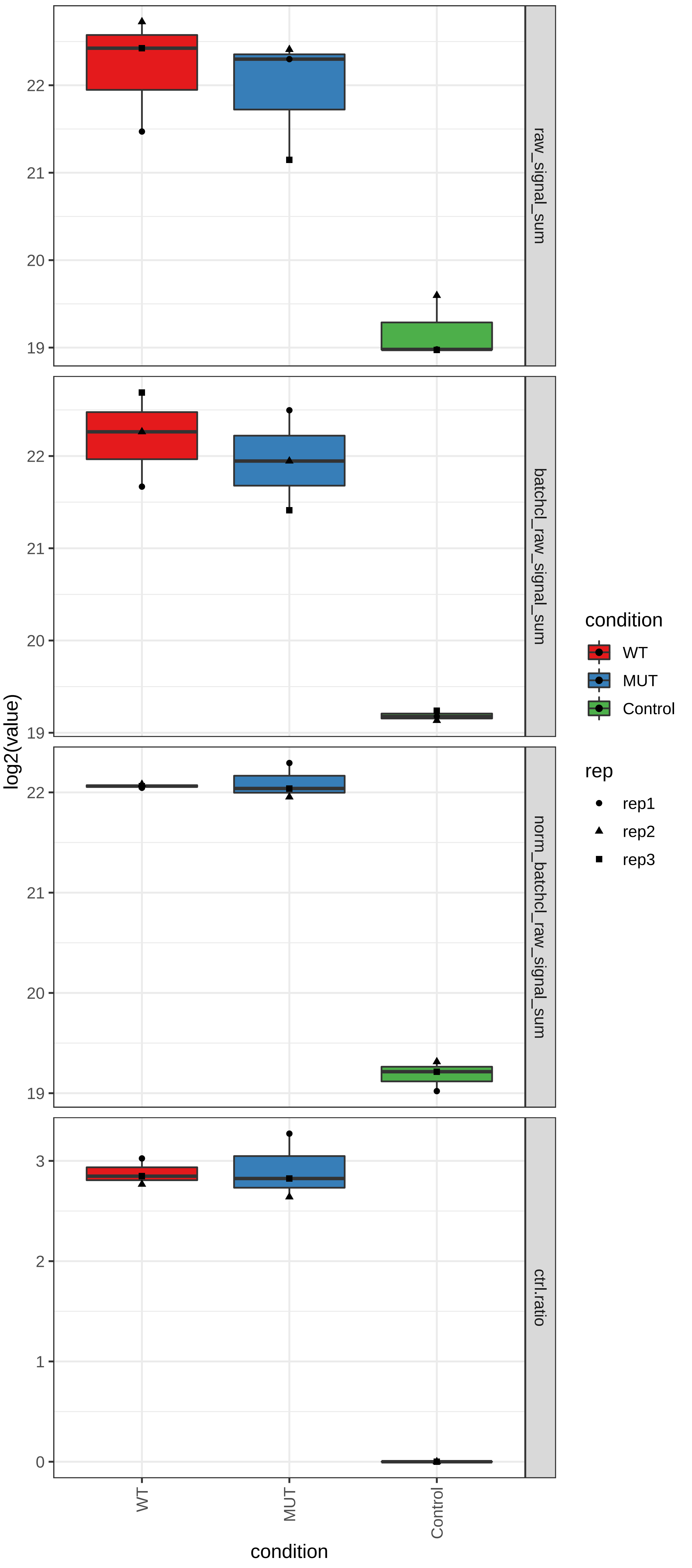

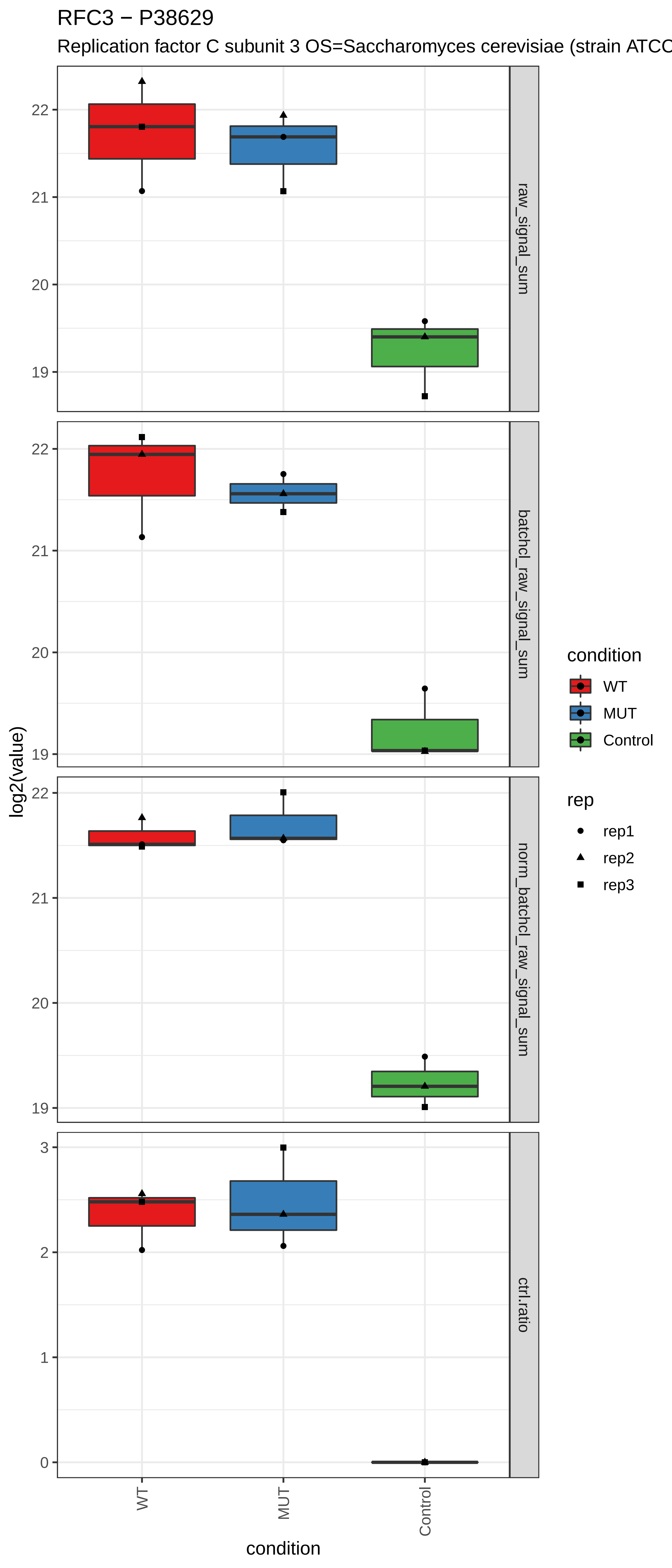
RFC3 − P38629
Replication factor C subunit 3 OS=Saccharomyces cerevisiae (strain ATCC
raw_signal_sum
batchcl_raw_signal_sum
norm_batchcl_raw_signal_sum
ctrl.ratio
log2(value)
condition
WT
MUT
Control
rep
rep1
rep2
rep3
22
21
20
19
3
2
1
0

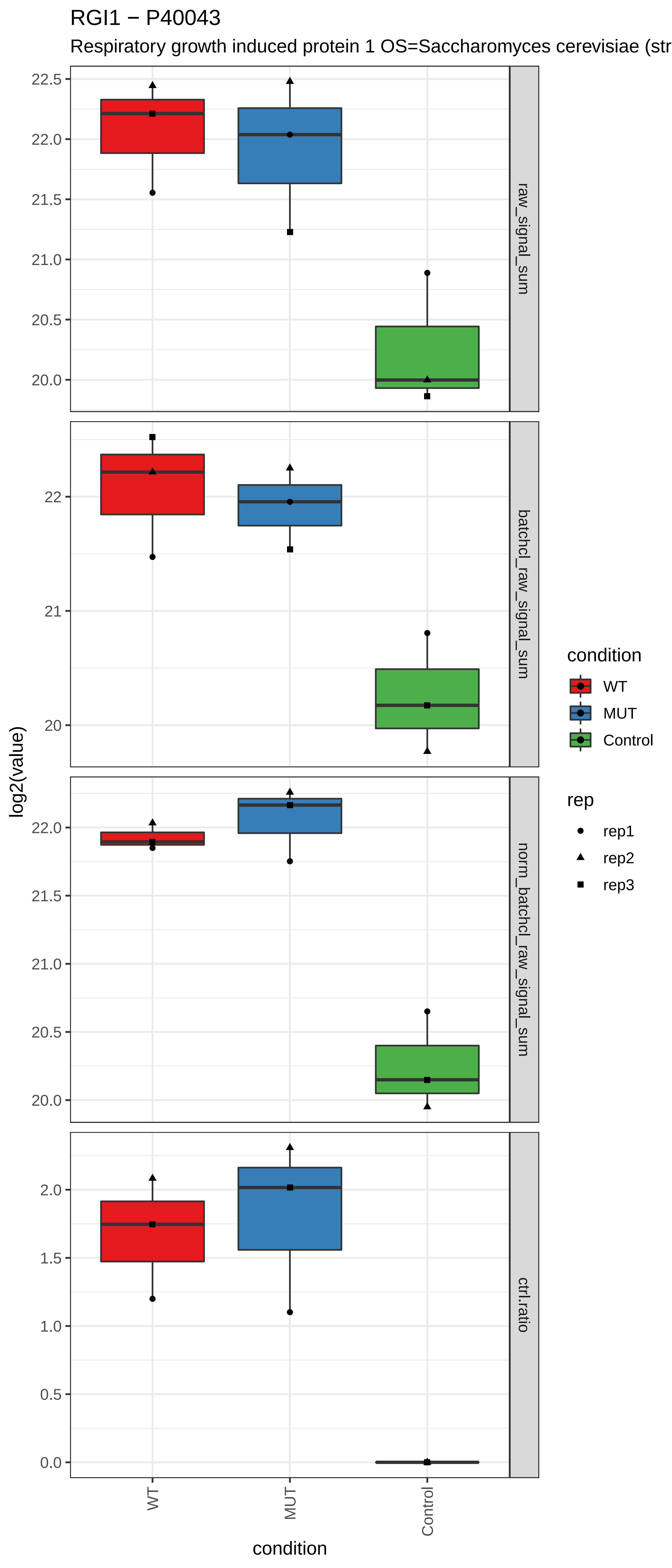

RGI1 − P40043
Respiratory growth induced protein 1 OS=Saccharomyces cerevisiae (str
log2(value)
condition
raw_signal_sum
batchcl_raw_signal_sum
norm_batchcl_raw_signal_sum
ctrl.ratio
WT
MUT
Control
rep
rep1
rep2
rep3
22.5
22.0
21.5
21.0
20.5
20.0
22
21
20
2.0
1.5
1.0
0.5
0.0

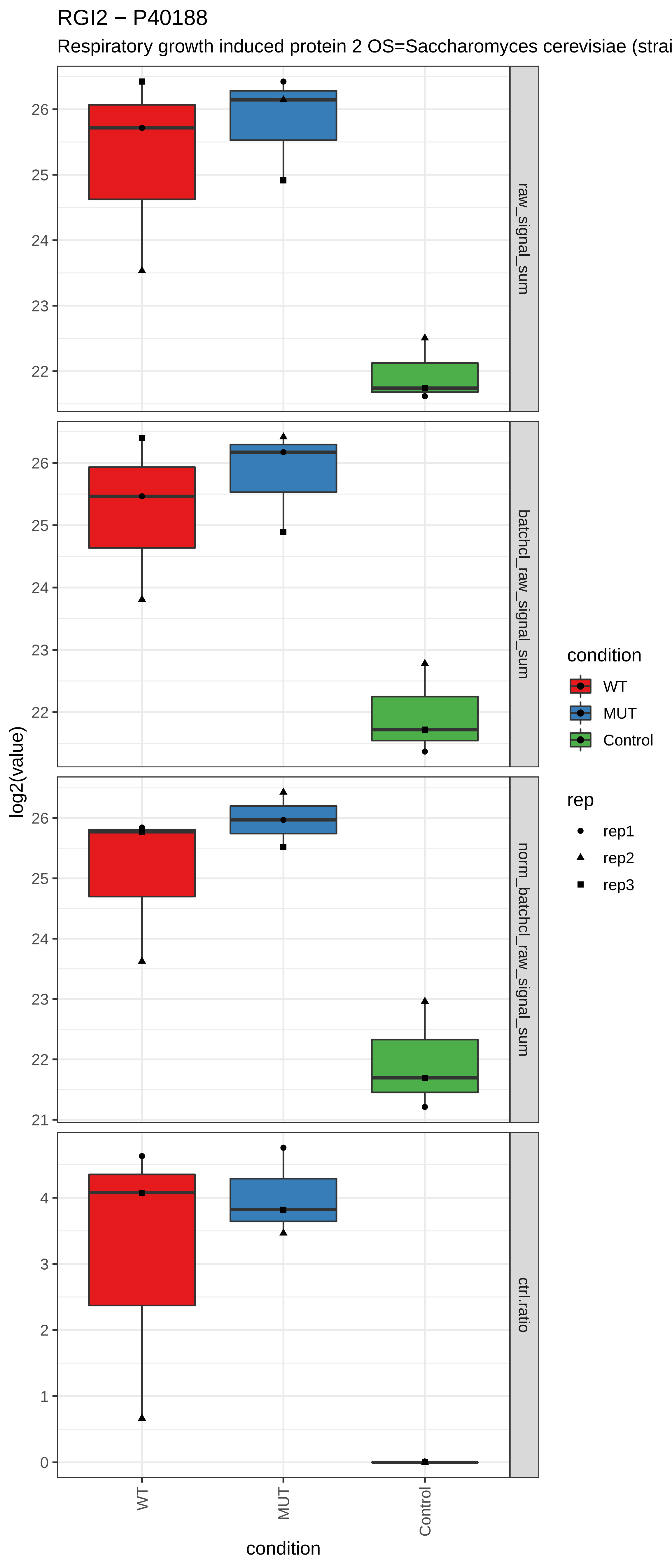

RGI2 − P40188
Respiratory growth induced protein 2 OS=Saccharomyces cerevisiae (strai
raw_signal_sum
batchcl_raw_signal_sum
norm_batchcl_raw_signal_sum
ctrl.ratio
log2(value)
condition
WT
MUT
Control
rep
rep1
rep2
rep3
26
25
24
23
22
21
4
3
2
1
0

RIM1 − P32445

Single−stranded DNA−binding protein RIM1, mitochondrial OS=Saccharon

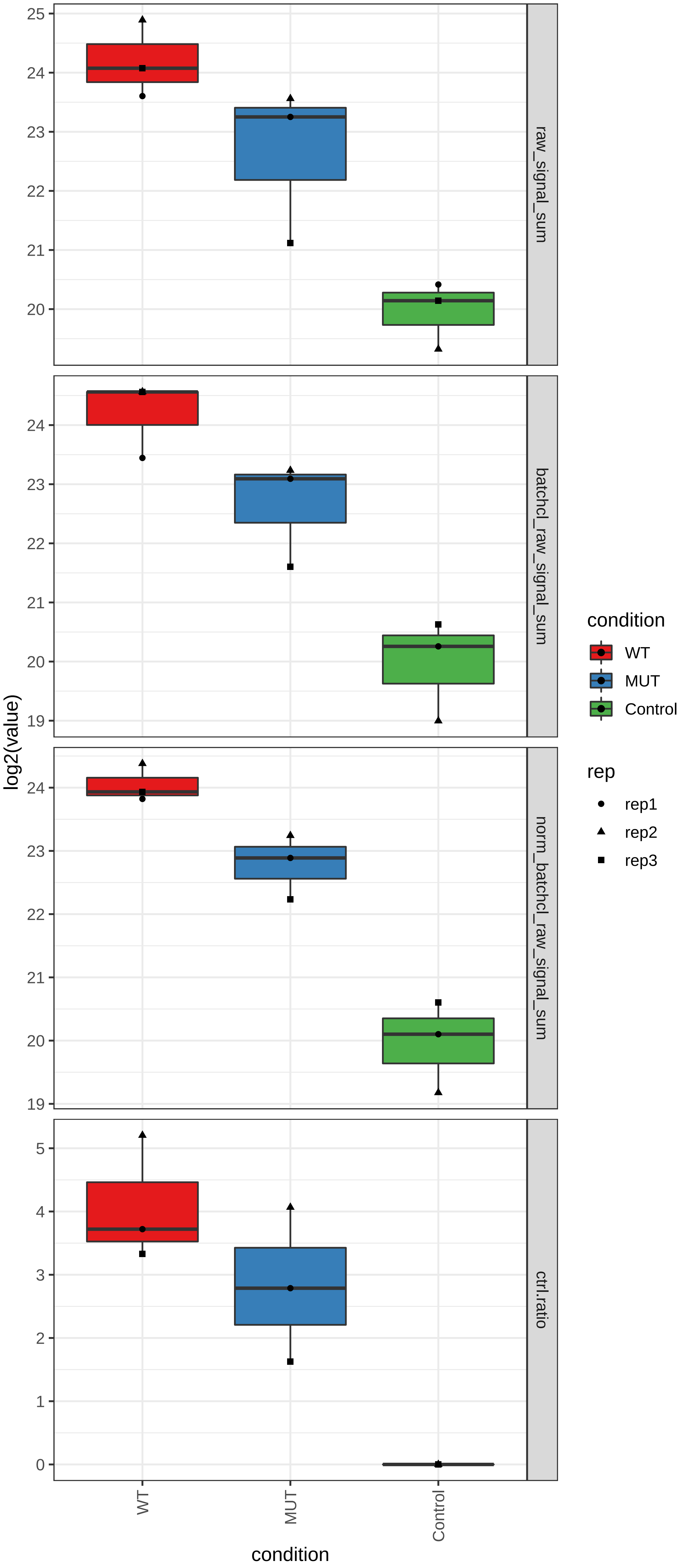

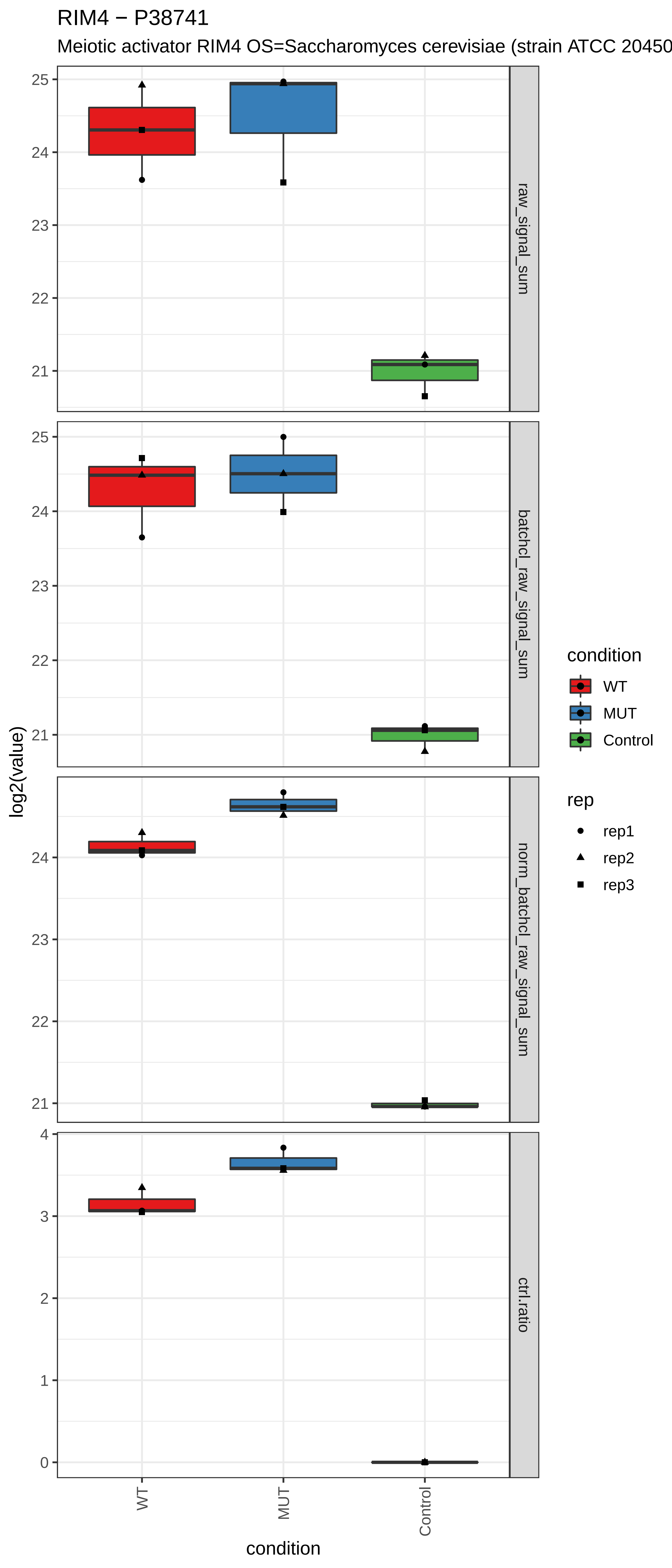

RIM4 − P38741
Meiotic activator RIM4 OS=Saccharomyces cerevisiae (strain ATCC 20450
raw_signal_sum
batchcl_raw_signal_sum
norm_batchcl_raw_signal_sum
ctrl.ratio
log2(value)
condition
WT
MUT
Control
rep
rep1
rep2
rep3

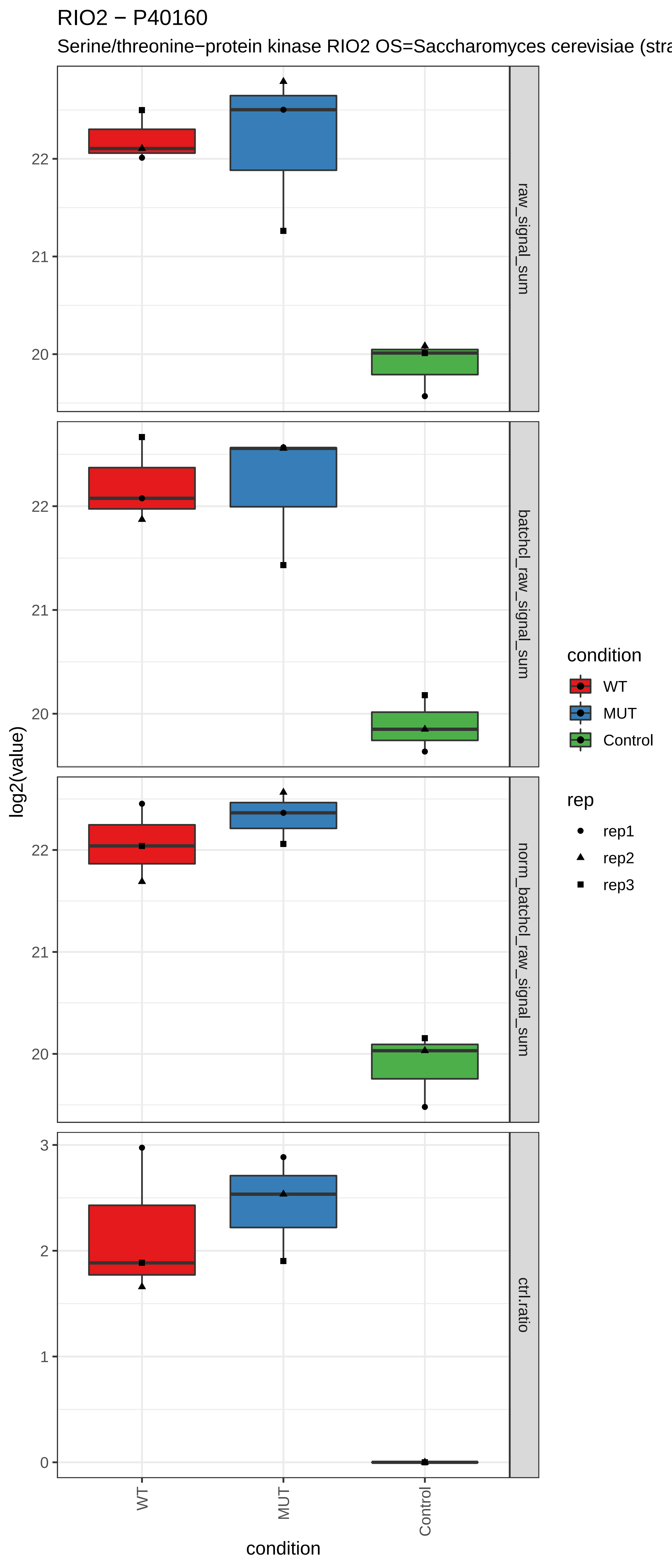

RIO2 − P40160
Serine/threonine−protein kinase RIO2 OS=Saccharomyces cerevisiae (stra
raw_signal_sum
batchcl_raw_signal_sum
norm_batchcl_raw_signal_sum
ctrl.ratio
log2(value)
condition
WT
MUT
Control
rep
rep1
rep2
rep3

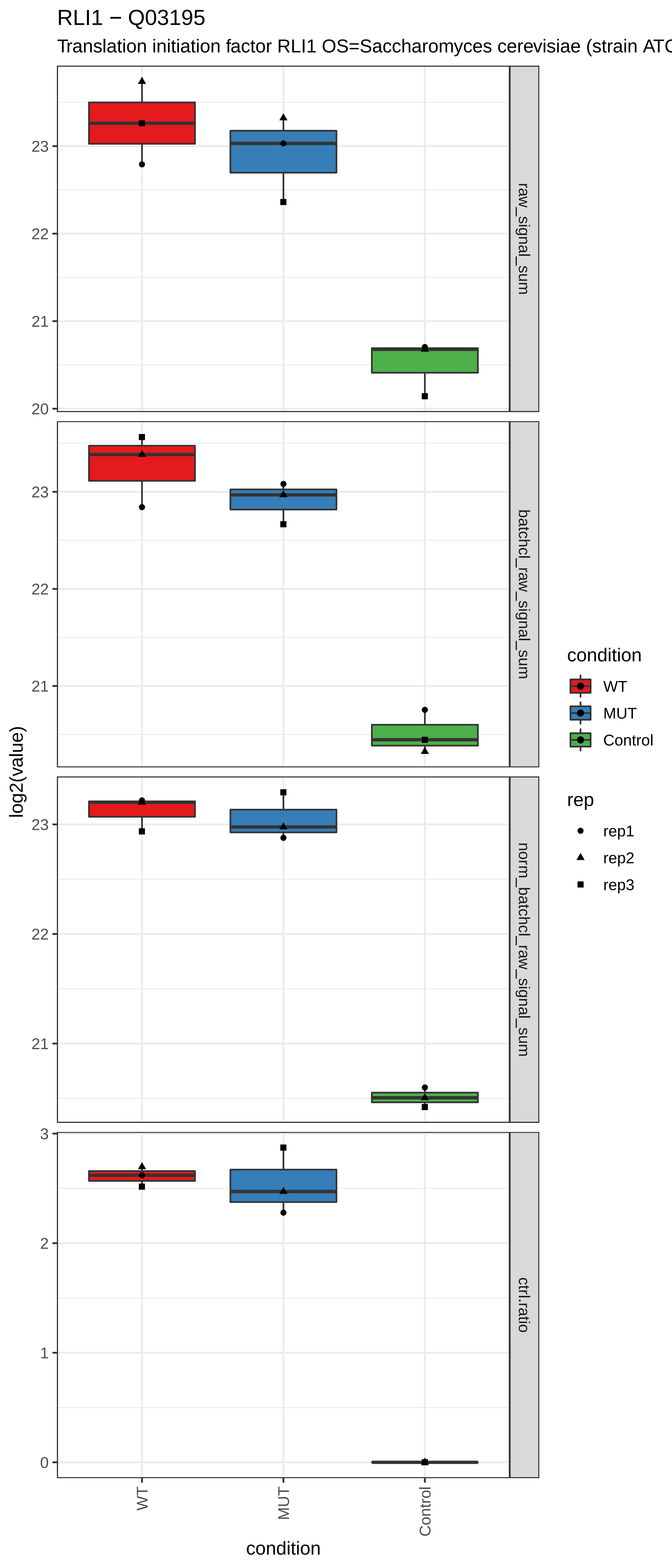

RLI1 − Q03195
Translation initiation factor RLI1 OS=Saccharomyces cerevisiae (strain ATC
raw_signal_sum
batchcl_raw_signal_sum
norm_batchcl_raw_signal_sum
ctrl.ratio
log2(value)
condition
WT
MUT
Control
rep
rep1
rep2
rep3

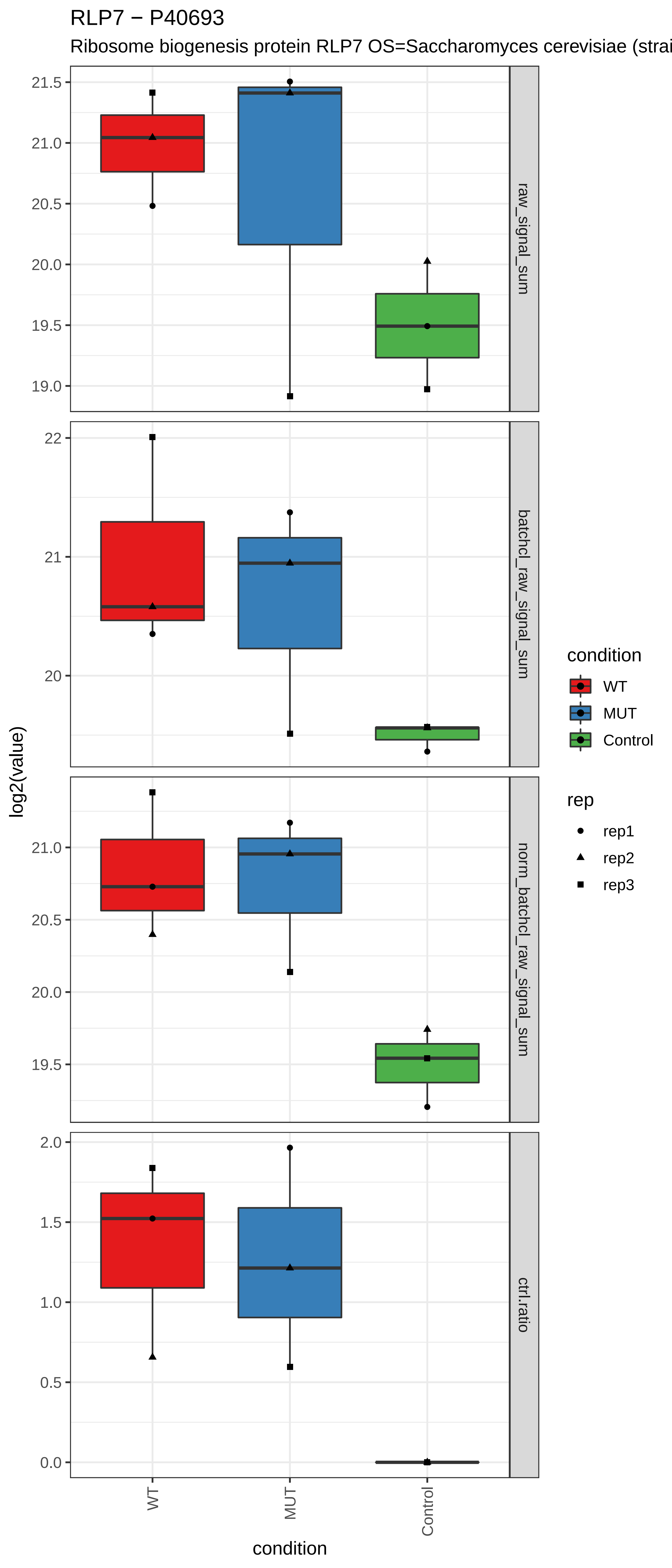

RLP7 − P40693
Ribosome biogenesis protein RLP7 OS=Saccharomyces cerevisiae (strai
raw_signal_sum
batchcl_raw_signal_sum
norm_batchcl_raw_signal_sum
ctrl.ratio
log2(value)
condition
WT
MUT
Control
rep
rep1
rep2
rep3

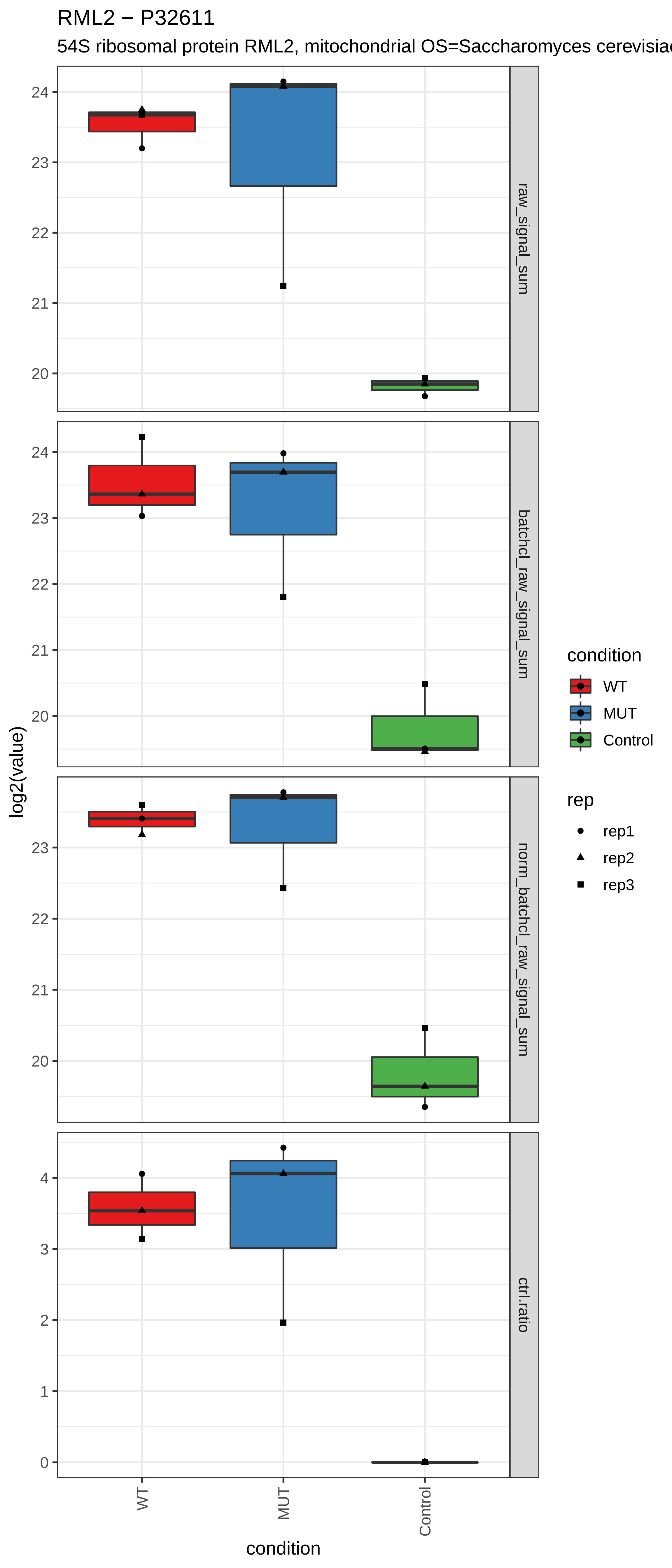

RML2 − P32611
54S ribosomal protein RML2, mitochondrial OS=Saccharomyces cerevisiae
raw_signal_sum
batchcl_raw_signal_sum
norm_batchcl_raw_signal_sum
ctrl.ratio
log2(value)
condition
WT
MUT
Control
rep
rep1
rep2
rep3

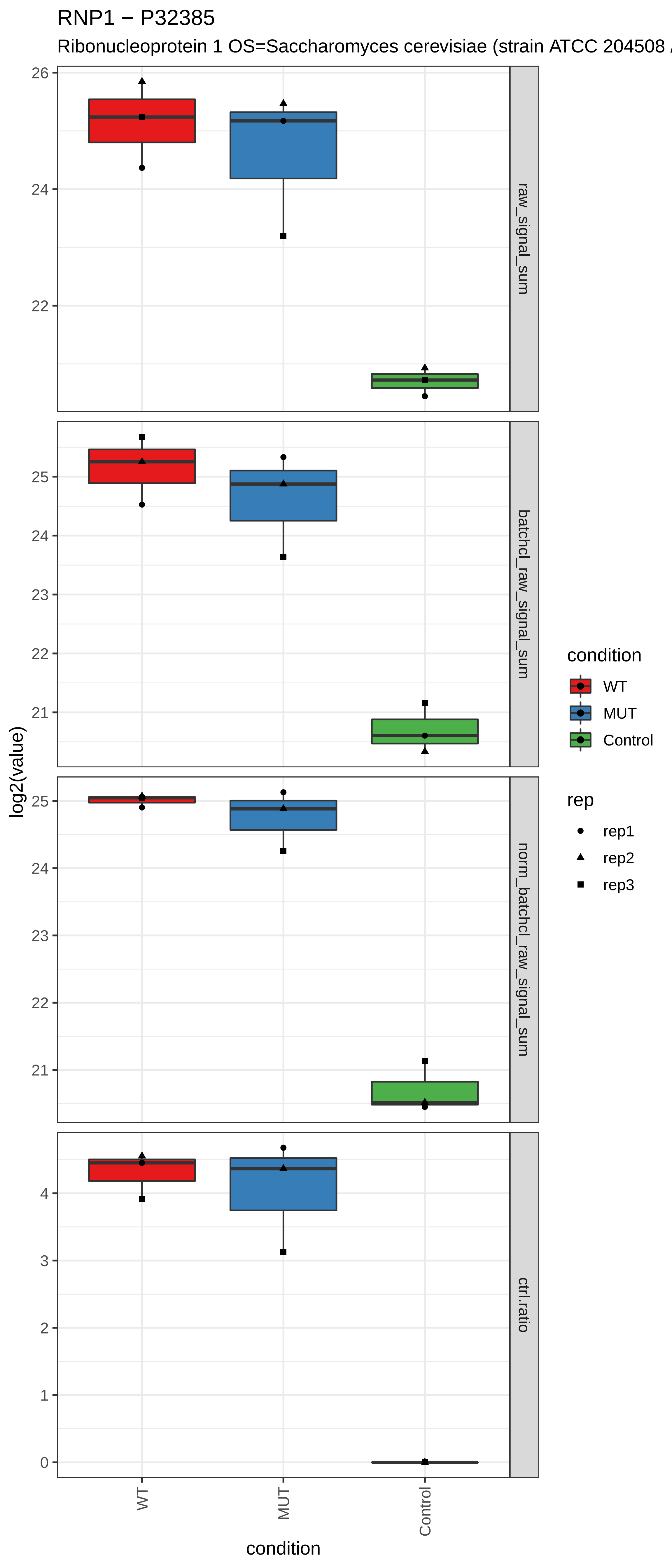
RNP1 − P32385
Ribonucleoprotein 1 OS=Saccharomyces cerevisiae (strain ATCC 204508 /
raw_signal_sum
batchcl_raw_signal_sum
norm_batchcl_raw_signal_sum
ctrl.ratio
log2(value)
condition
WT
MUT
Control
rep
rep1
rep2
rep3

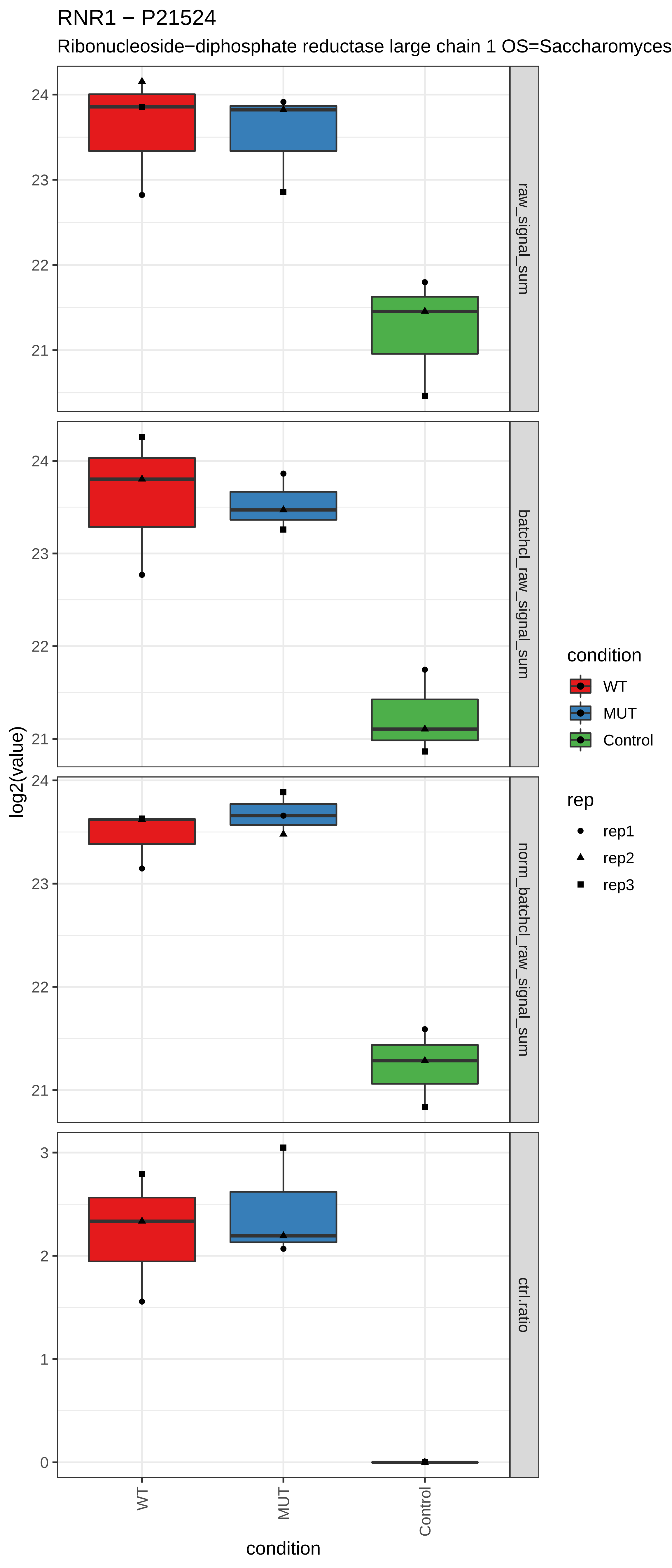

RNR1 − P21524
Ribonucleoside−diphosphate reductase large chain 1 OS=Saccharomyces
raw_signal_sum
batchcl_raw_signal_sum
norm_batchcl_raw_signal_sum
ctrl.ratio
log2(value)
condition
WT
MUT
Control
rep
rep1
rep2
rep3

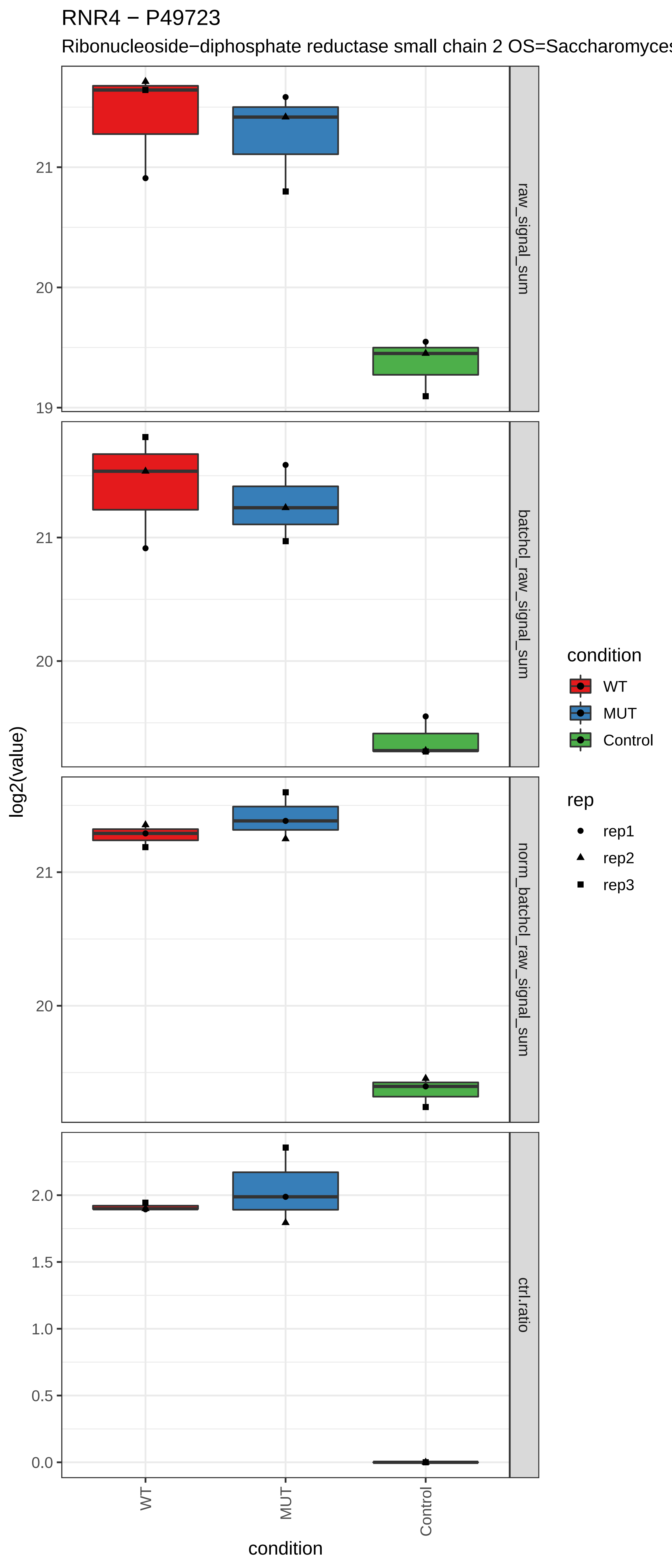

RNR4 − P49723
Ribonucleoside−diphosphate reductase small chain 2 OS=Saccharomyces
raw_signal_sum
batchcl_raw_signal_sum
norm_batchcl_raw_signal_sum
ctrl.ratio
log2(value)
condition
WT
MUT
Control
rep
rep1
rep2
rep3

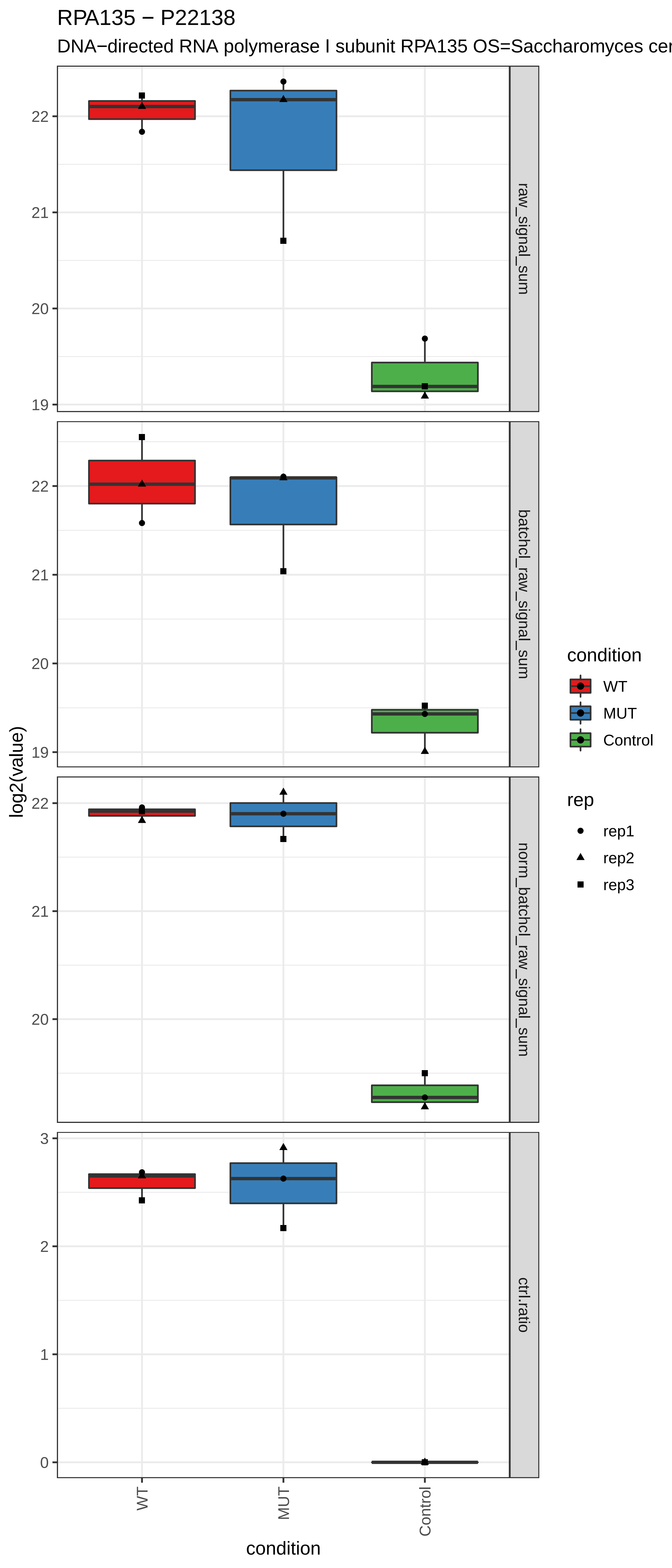

RPA135 − P22138
DNA−directed RNA polymerase I subunit RPA135 OS=Saccharomyces cer
raw_signal_sum
batchcl_raw_signal_sum
norm_batchcl_raw_signal_sum
ctrl.ratio
log2(value)
condition
WT
MUT
Control
rep
rep1
rep2
rep3
22
21
20
19
3
2
1
0

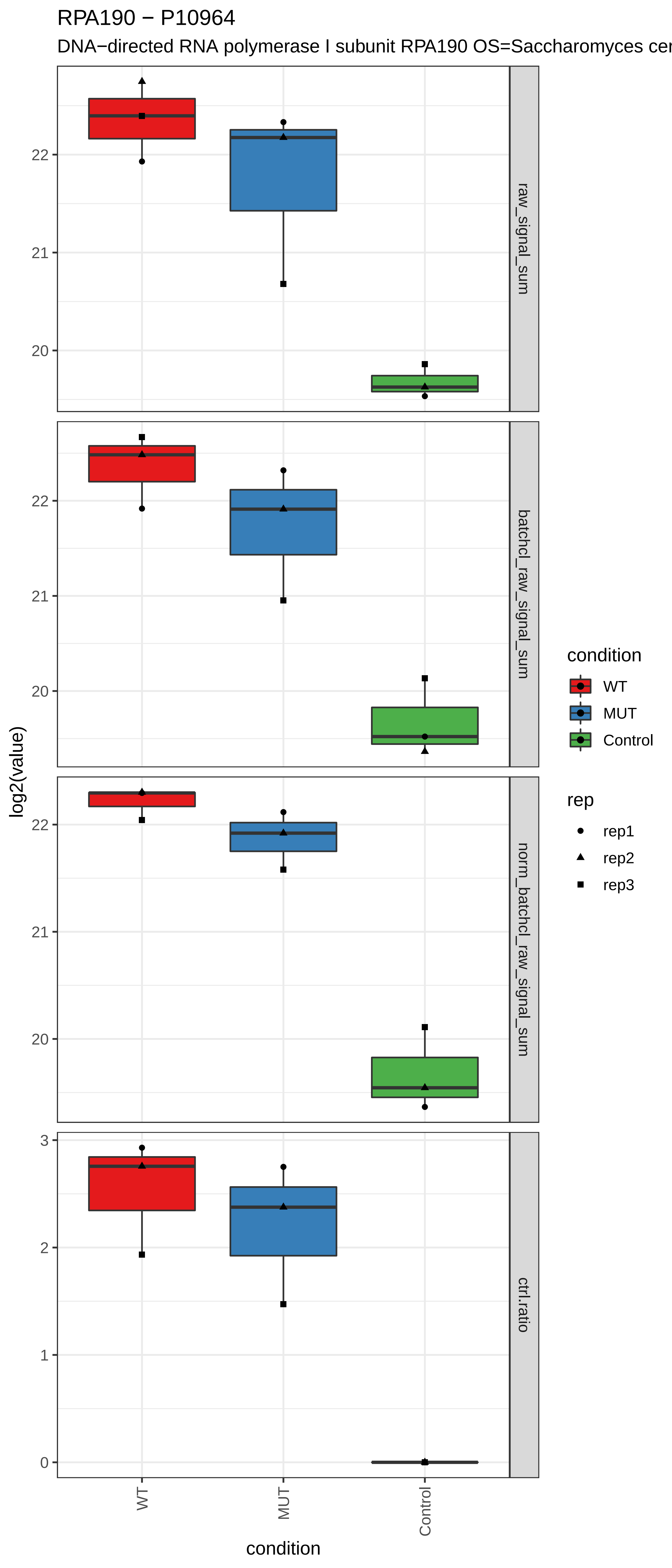

RPA190 − P10964
DNA−directed RNA polymerase I subunit RPA190 OS=Saccharomyces cer
raw_signal_sum
batchcl_raw_signal_sum
norm_batchcl_raw_signal_sum
ctrl.ratio
log2(value)
condition
WT
MUT
Control
rep
rep1
rep2
rep3

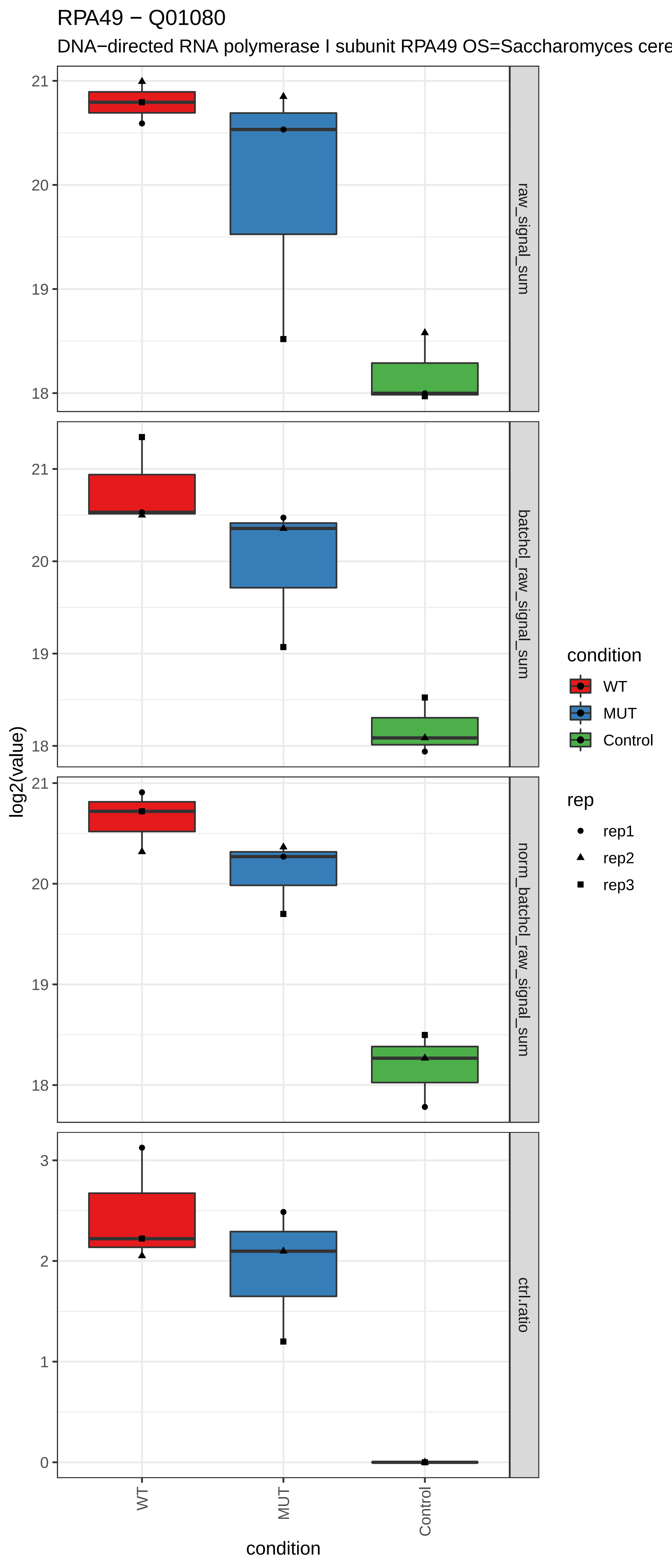

RPA49 − Q01080
DNA−directed RNA polymerase I subunit RPA49 OS=Saccharomyces cere
raw_signal_sum
batchcl_raw_signal_sum
norm_batchcl_raw_signal_sum
ctrl.ratio
log2(value)
condition
WT
MUT
Control
rep
rep1
rep2
rep3

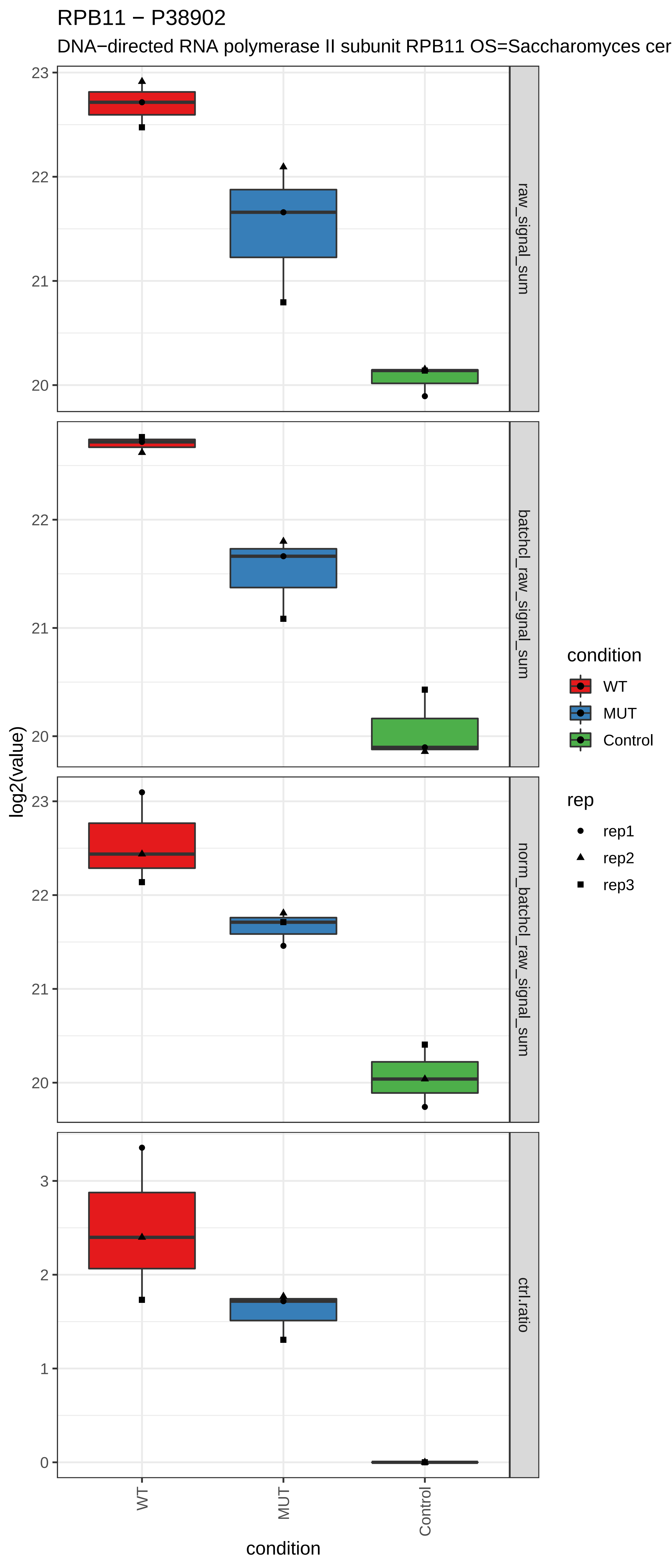
RPB11 − P38902
DNA−directed RNA polymerase II subunit RPB11 OS=Saccharomyces cer
raw_signal_sum
batchcl_raw_signal_sum
norm_batchcl_raw_signal_sum
ctrl.ratio
log2(value)
condition
WT
MUT
Control
rep
rep1
rep2
rep3

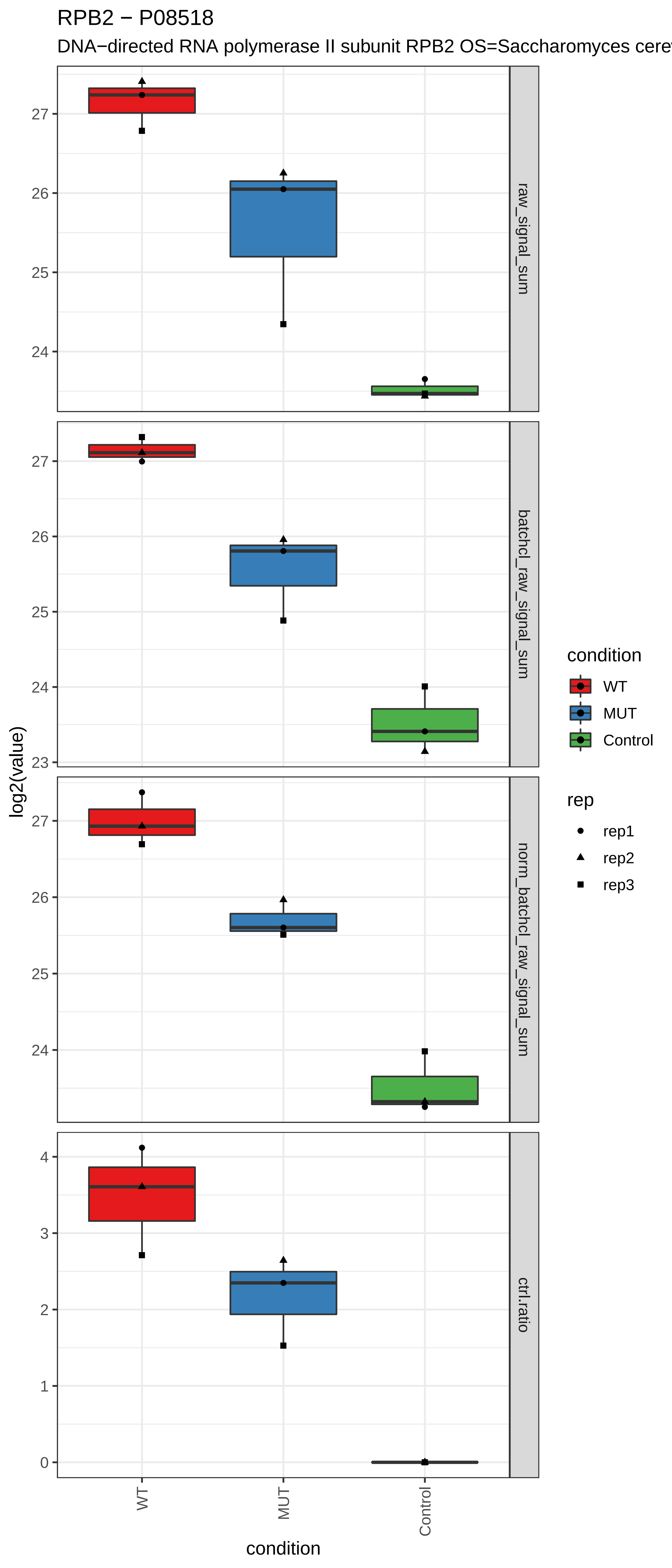

RPB2 − P08518
DNA−directed RNA polymerase II subunit RPB2 OS=Saccharomyces cere
raw_signal_sum
batchcl_raw_signal_sum
norm_batchcl_raw_signal_sum
ctrl.ratio
log2(value)
condition
WT
MUT
Control
rep
rep1
rep2
rep3

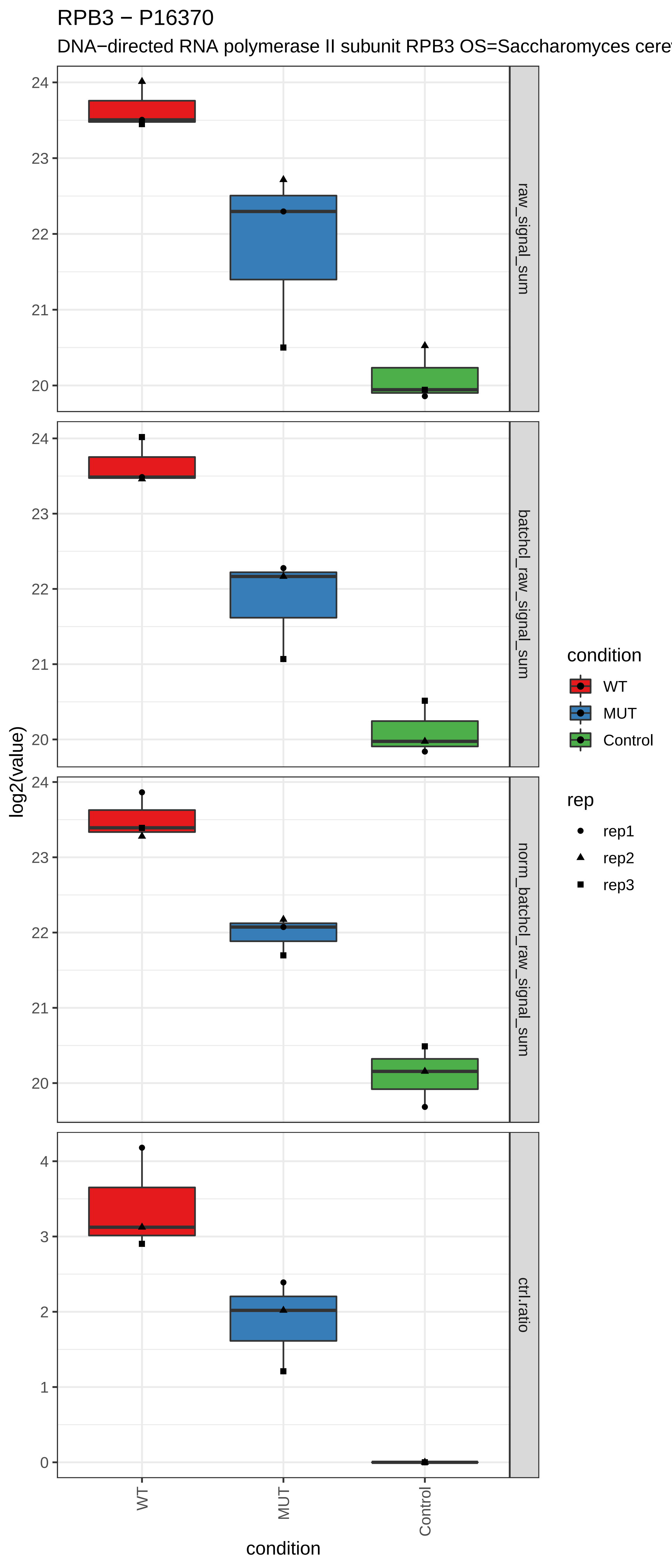

RPB3 − P16370
DNA−directed RNA polymerase II subunit RPB3 OS=Saccharomyces cere
raw_signal_sum
batchcl_raw_signal_sum
norm_batchcl_raw_signal_sum
ctrl.ratio
log2(value)
condition
WT
MUT
Control
rep
rep1
rep2
rep3

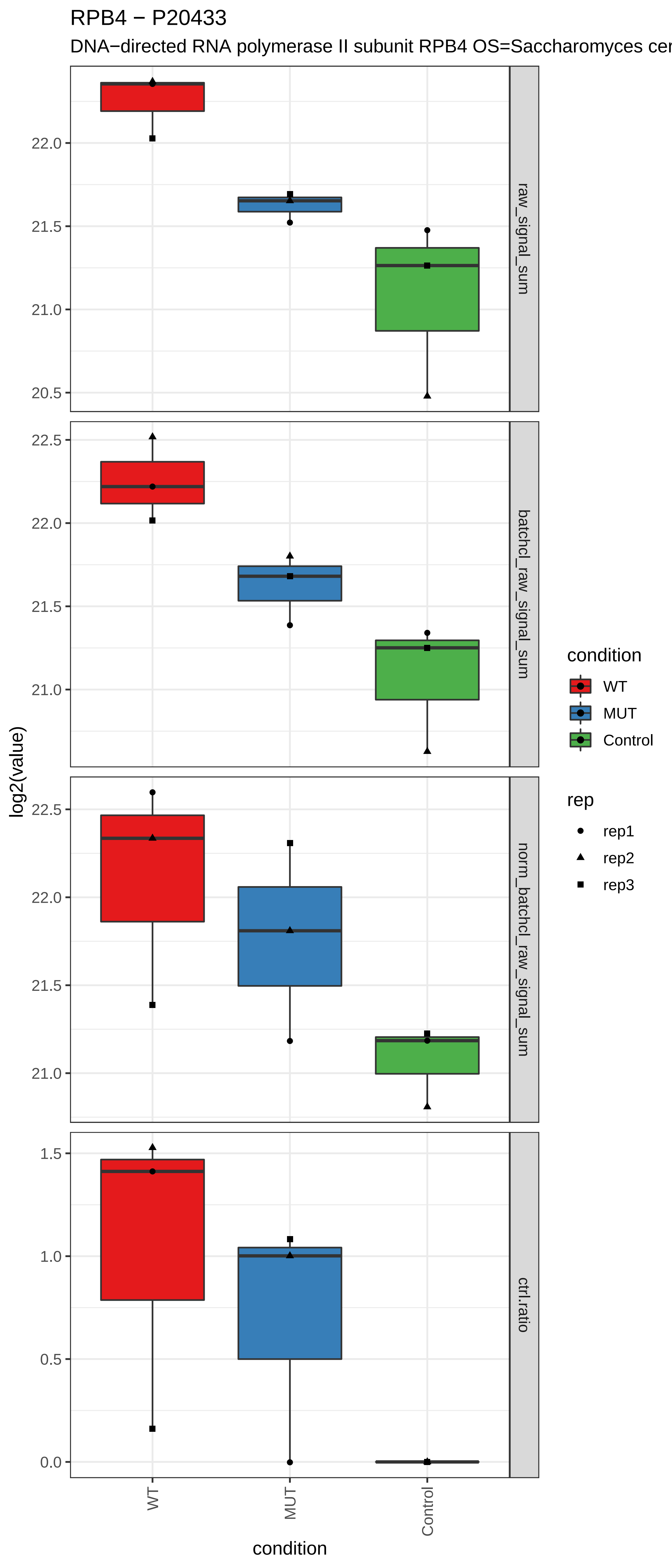

RPB4 − P20433
DNA−directed RNA polymerase II subunit RPB4 OS=Saccharomyces cer
raw_signal_sum
batchcl_raw_signal_sum
norm_batchcl_raw_signal_sum
ctrl.ratio
log2(value)
condition
WT
MUT
Control
rep
rep1
rep2
rep3

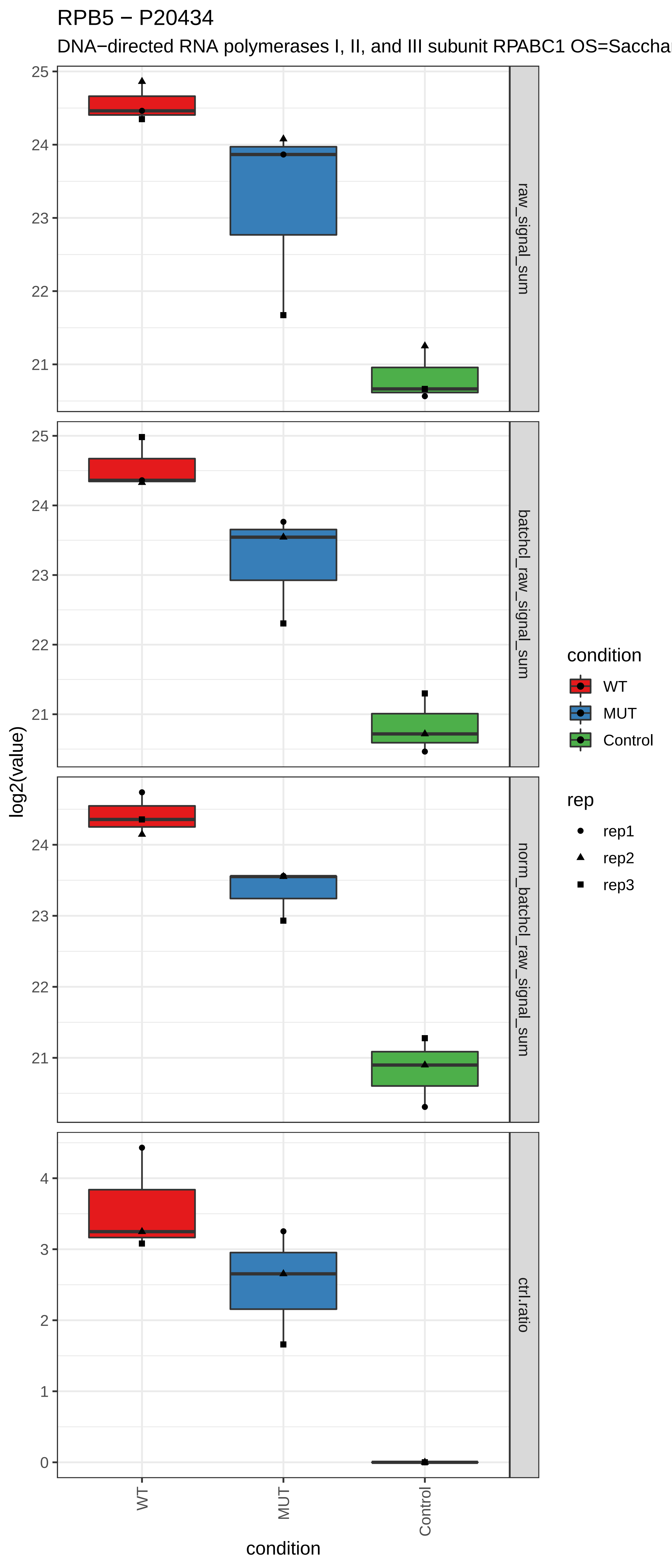

RPB5 − P20434
DNA−directed RNA polymerases I, II, and III subunit RPABC1 OS=Saccha
log2(value)
raw_signal_sum
batchcl_raw_signal_sum
norm_batchcl_raw_signal_sum
ctrl.ratio
condition
WT
MUT
Control
rep
rep1
rep2
rep3

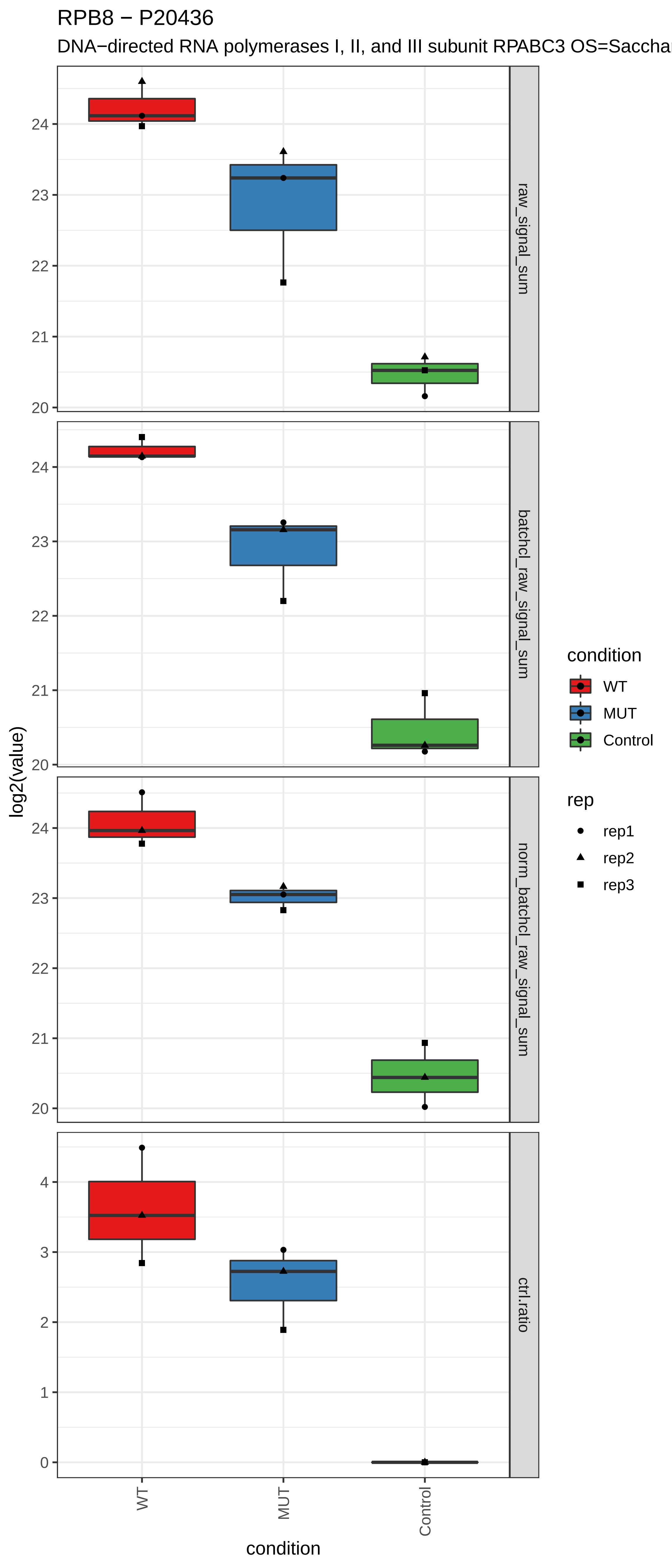

RPB8 − P20436
DNA−directed RNA polymerases I, II, and III subunit RPABC3 OS=Saccha
raw_signal_sum
batchcl_raw_signal_sum
norm_batchcl_raw_signal_sum
ctrl.ratio
log2(value)
condition
WT
MUT
Control
rep
rep1
rep2
rep3

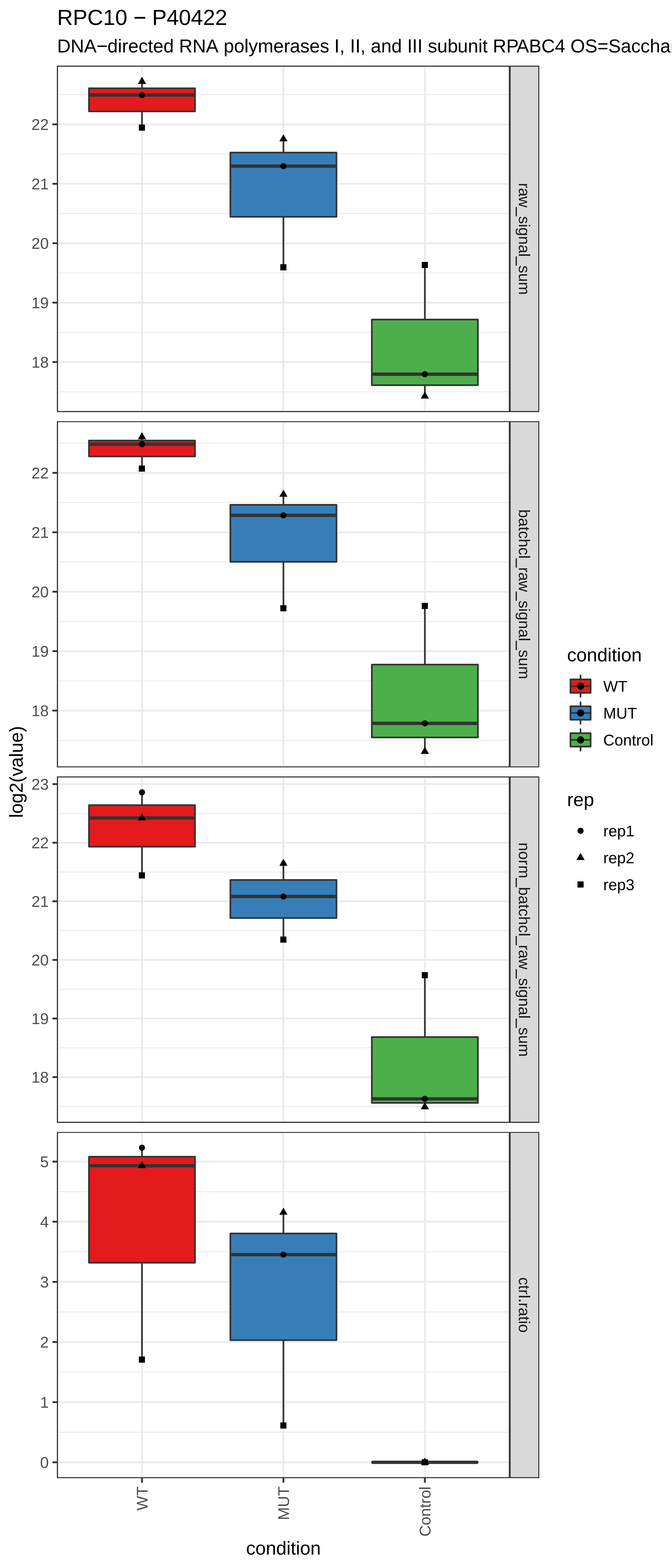

RPC10 − P40422
DNA−directed RNA polymerases I, II, and III subunit RPABC4 OS=Saccha
raw_signal_sum
batchcl_raw_signal_sum
norm_batchcl_raw_signal_sum
ctrl.ratio
log2(value)
condition
WT
MUT
Control
rep
rep1
rep2
rep3

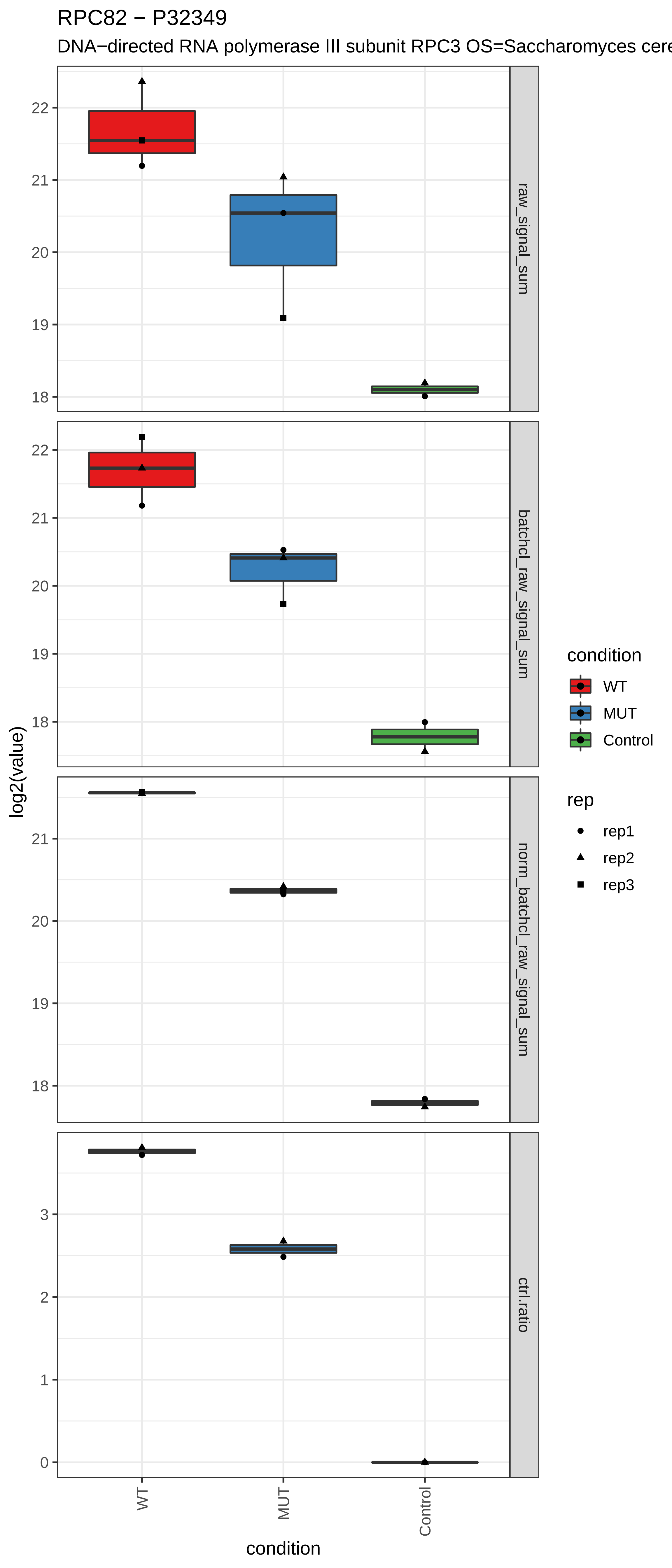
RPC82 − P32349
DNA−directed RNA polymerase III subunit RPC3 OS=Saccharomyces cere
raw_signal_sum
batchcl_raw_signal_sum
norm_batchcl_raw_signal_sum
ctrl.ratio
log2(value)
condition
WT
MUT
Control
rep
rep1
rep2
rep3

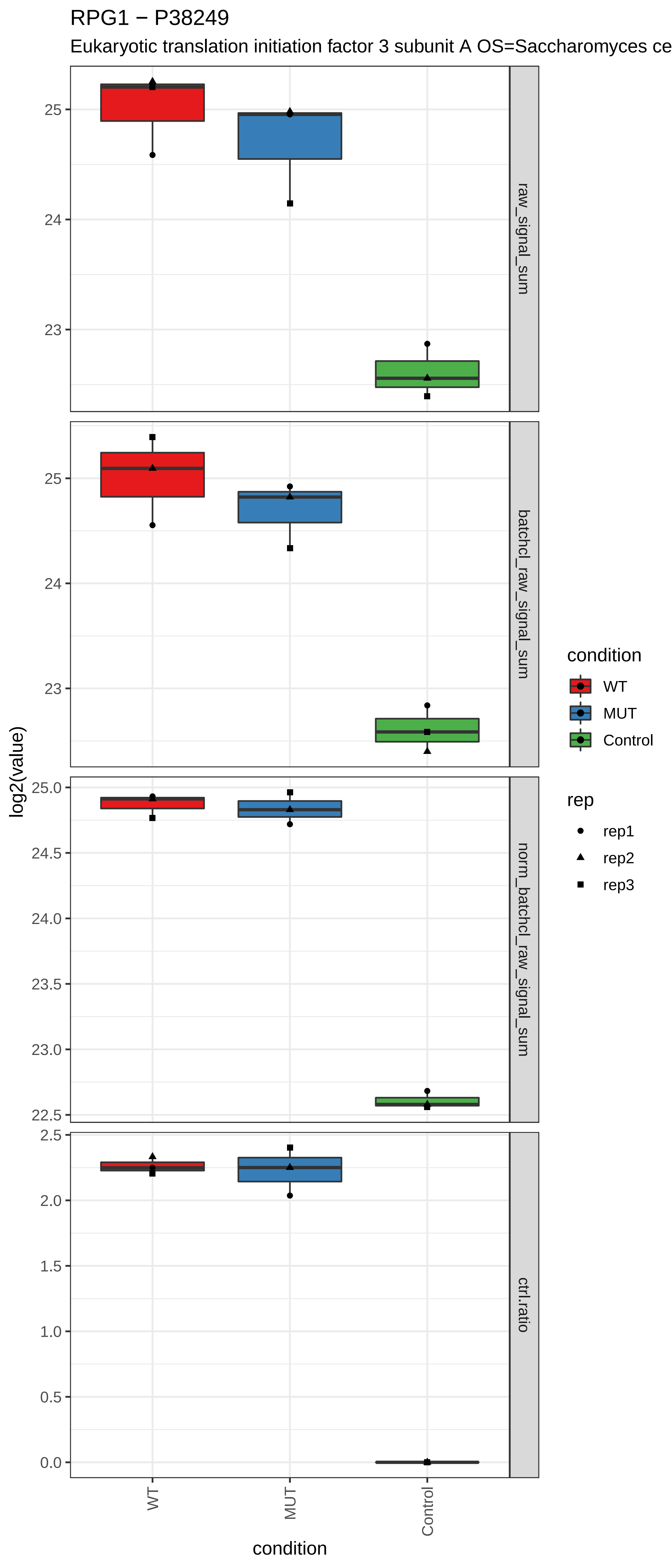

RPG1 − P38249
Eukaryotic translation initiation factor 3 subunit A OS=Saccharomyces ce
raw_signal_sum
batchcl_raw_signal_sum
norm_batchcl_raw_signal_sum
ctrl.ratio
log2(value)
condition
WT
MUT
Control
rep
rep1
rep2
rep3
25
24
23
25.0
24.5
24.0
23.5
23.0
22.5
2.5
2.0
1.5
1.0
0.5
0.0

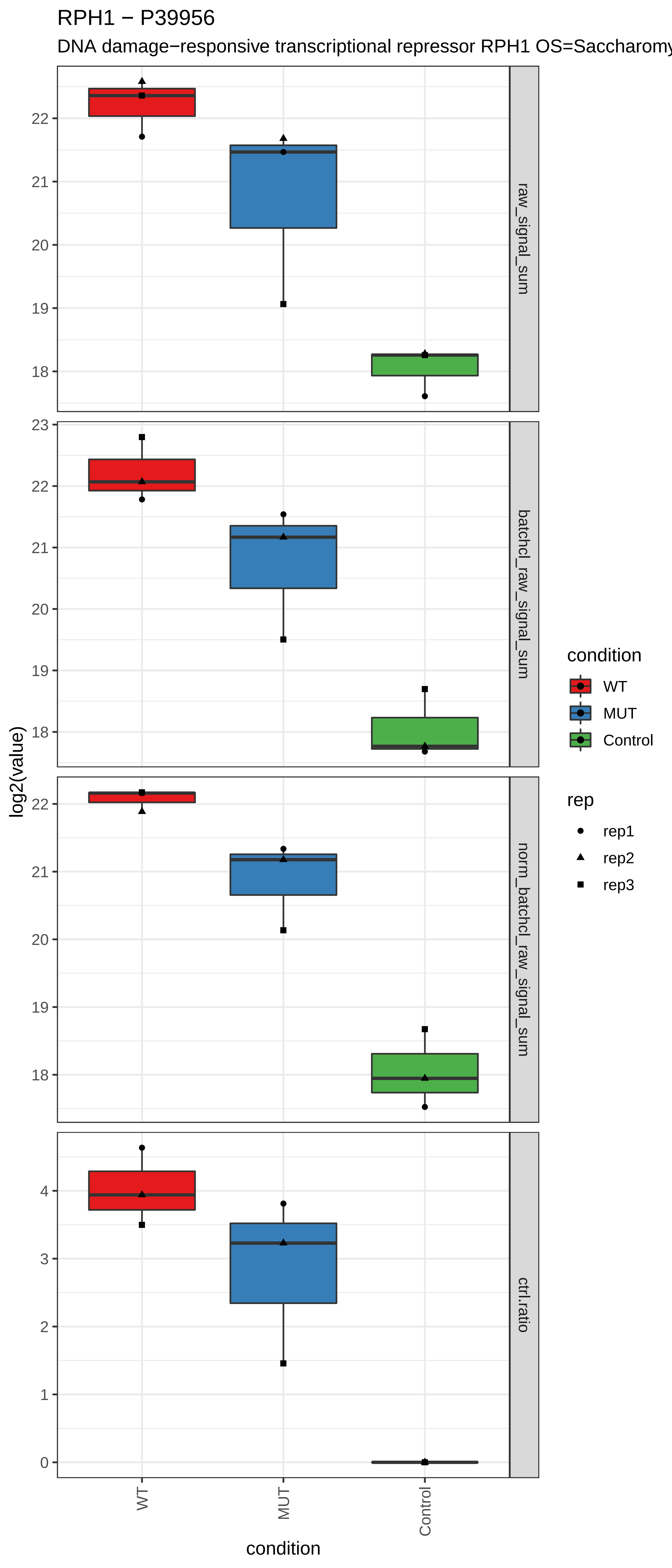

RPH1 − P39956
DNA damage−responsive transcriptional repressor RPH1 OS=Saccharomy
raw_signal_sum
batchcl_raw_signal_sum
norm_batchcl_raw_signal_sum
ctrl.ratio
log2(value)
condition
WT
MUT
Control
rep
rep1
rep2
rep3

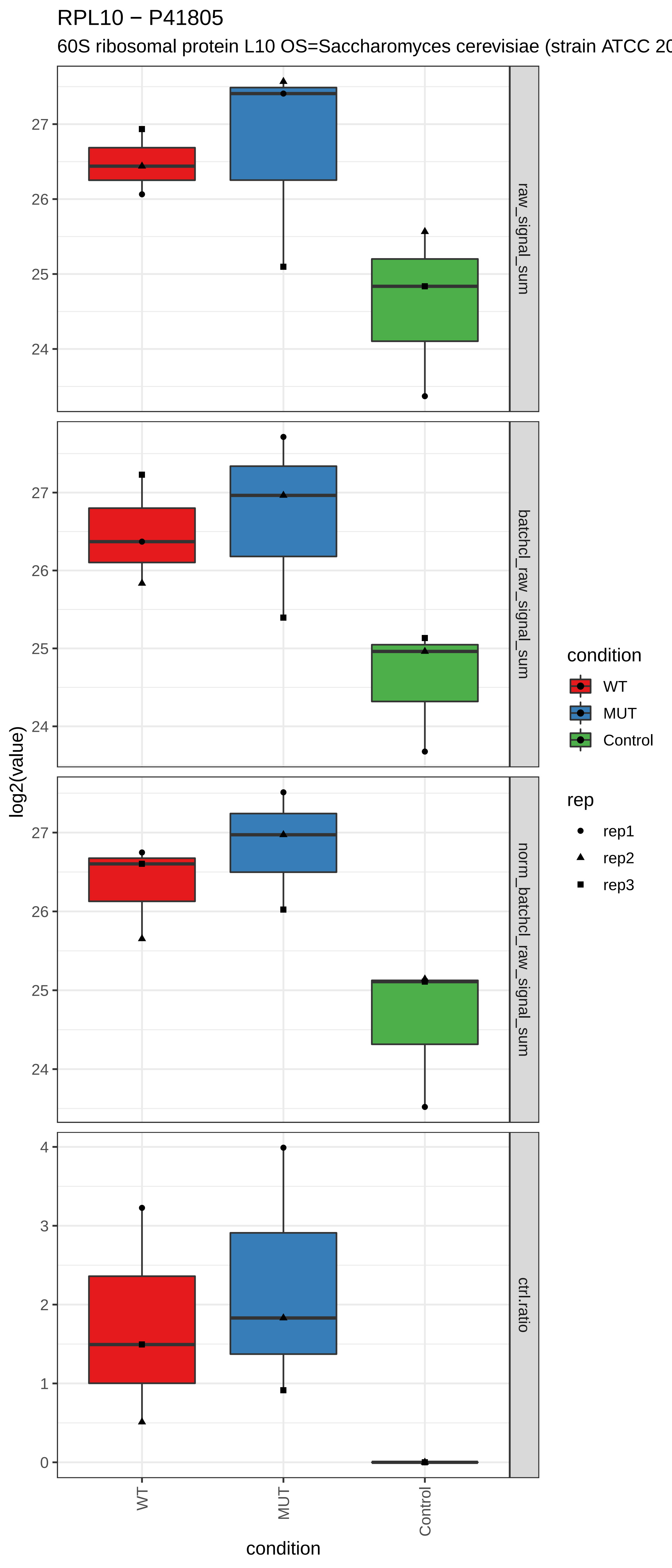
RPL10 − P41805
60S ribosomal protein L10 OS=Saccharomyces cerevisiae (strain ATCC 20
raw_signal_sum
batchcl_raw_signal_sum
norm_batchcl_raw_signal_sum
ctrl.ratio
log2(value)
condition
WT
MUT
Control
rep
rep1
rep2
rep3

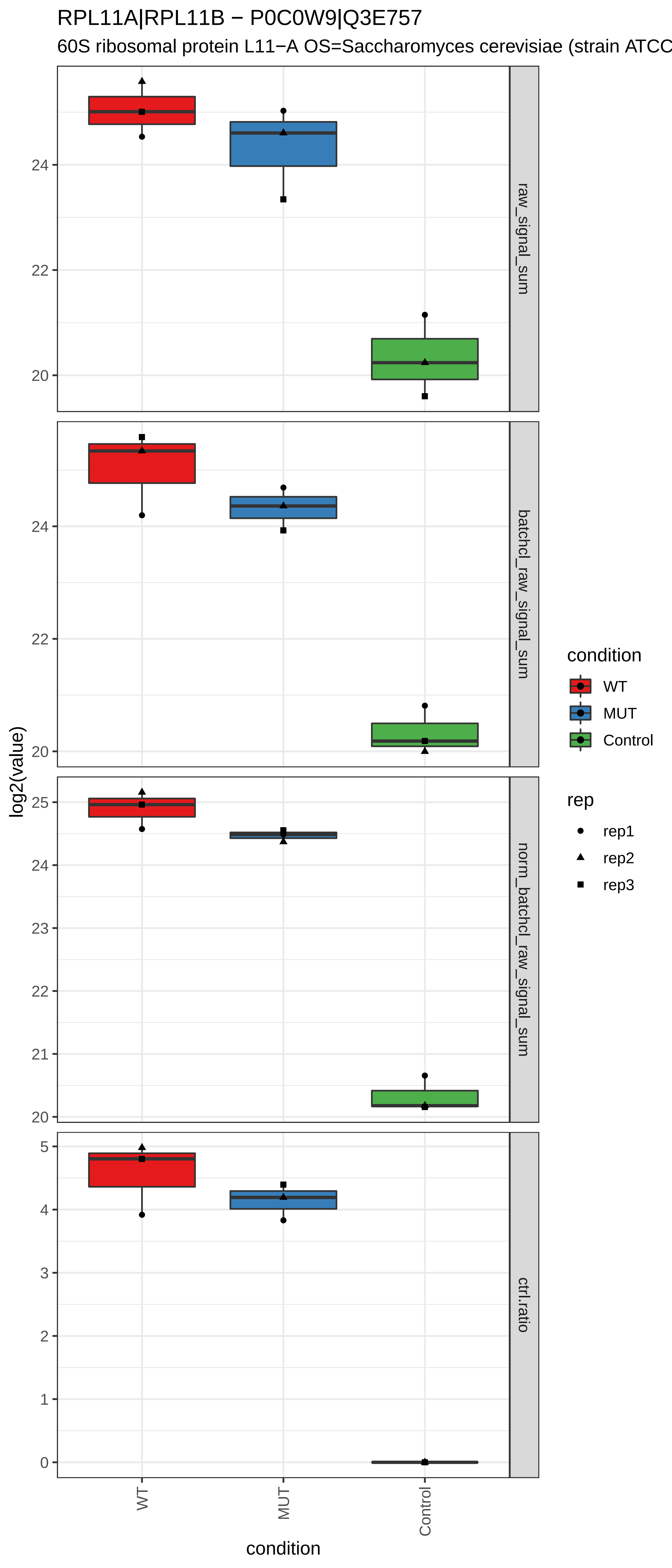

RPL11A|RPL11B − P0C0W9|Q3E757
60S ribosomal protein L11−A OS=Saccharomyces cerevisiae (strain ATCC
raw_signal_sum
batchcl_raw_signal_sum
norm_batchcl_raw_signal_sum
ctrl.ratio
log2(value)
condition
WT
MUT
Control
rep
rep1
rep2
rep3

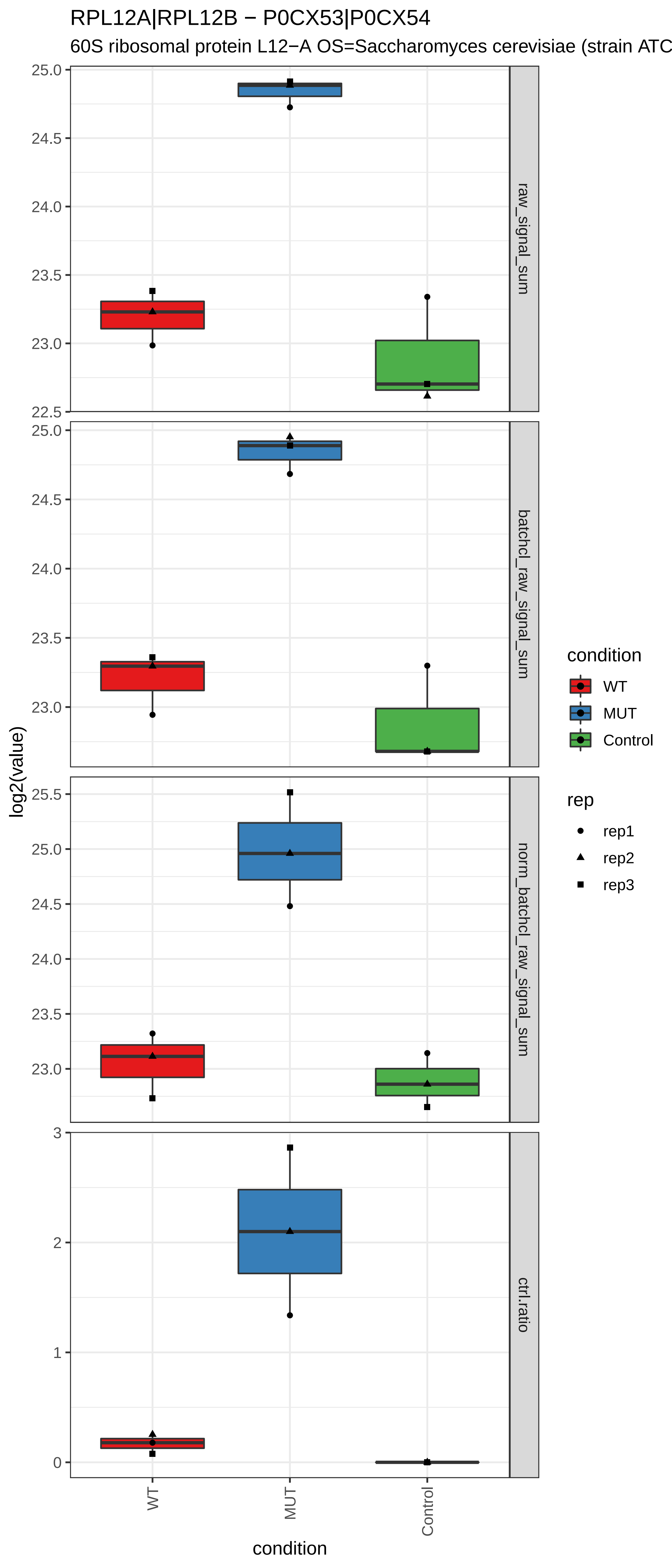

RPL12A|RPL12B − P0CX53|P0CX54
60S ribosomal protein L12−A OS=Saccharomyces cerevisiae (strain ATC
raw_signal_sum
batchcl_raw_signal_sum
norm_batchcl_raw_signal_sum
ctrl.ratio
log2(value)
condition
WT
MUT
Control
rep
rep1
rep2
rep3
25.0
24.5
24.0
23.5
23.0
22.5
25.5
3
2
1
0

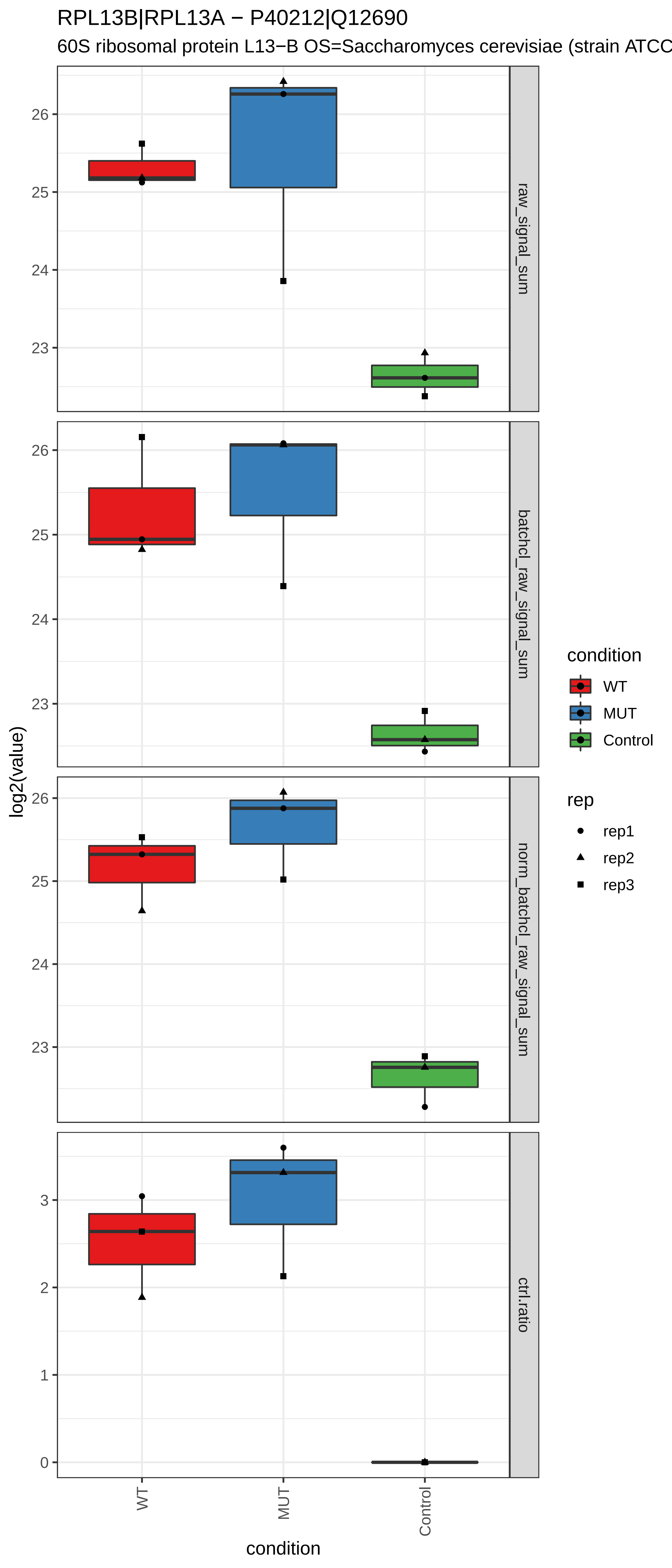
RPL13B|RPL13A − P40212|Q12690
60S ribosomal protein L13−B OS=Saccharomyces cerevisiae (strain ATCC
raw_signal_sum
batchcl_raw_signal_sum
norm_batchcl_raw_signal_sum
ctrl.ratio
log2(value)
condition
WT
MUT
Control
rep
rep1
rep2
rep3
condition

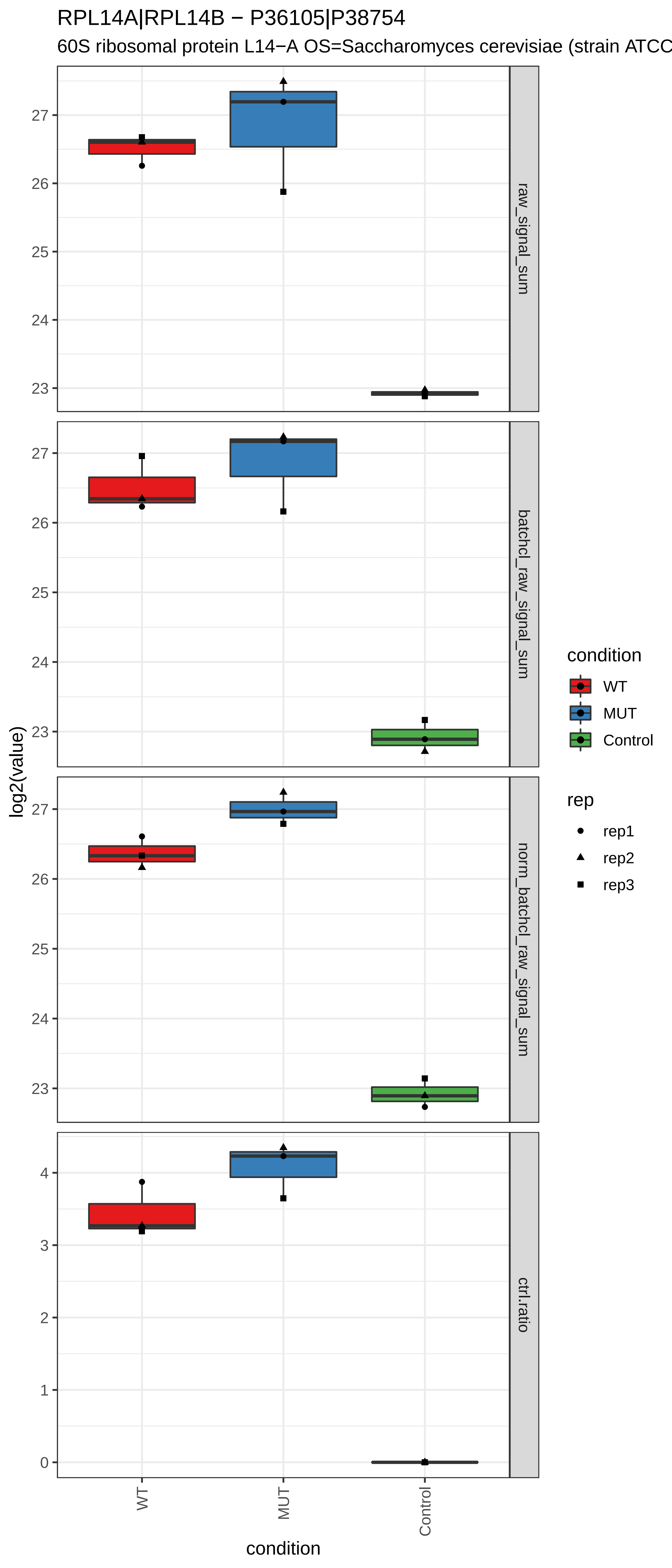
RPL14A|RPL14B − P36105|P38754
60S ribosomal protein L14−A OS=Saccharomyces cerevisiae (strain ATCC
raw_signal_sum
batchcl_raw_signal_sum
norm_batchcl_raw_signal_sum
ctrl.ratio
log2(value)
condition
WT
MUT
Control
rep
rep1
rep2
rep3
27
26
25
24
23
4
3
2
1
0

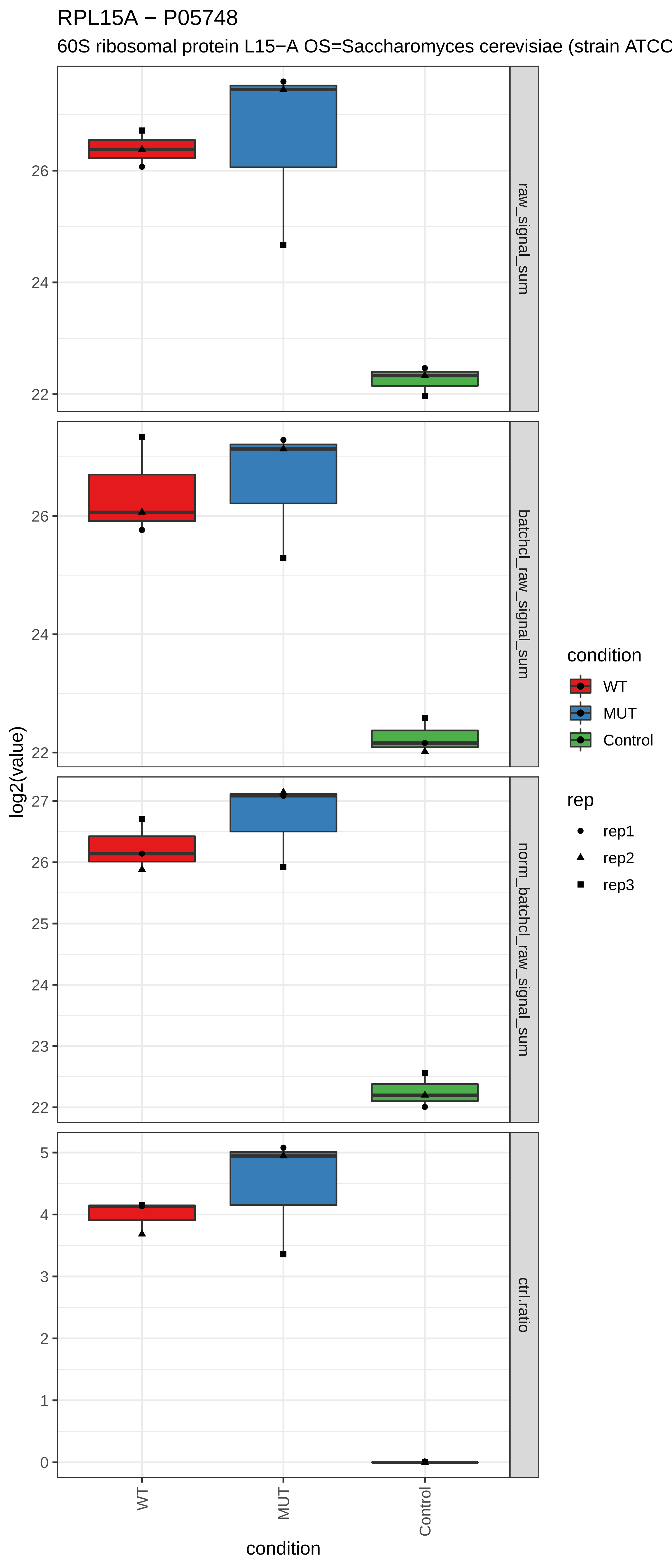

RPL15A − P05748
60S ribosomal protein L15−A OS=Saccharomyces cerevisiae (strain ATCC
raw_signal_sum
batchcl_raw_signal_sum
norm_batchcl_raw_signal_sum
ctrl.ratio
log2(value)
condition
WT
MUT
Control
rep
rep1
rep2
rep3

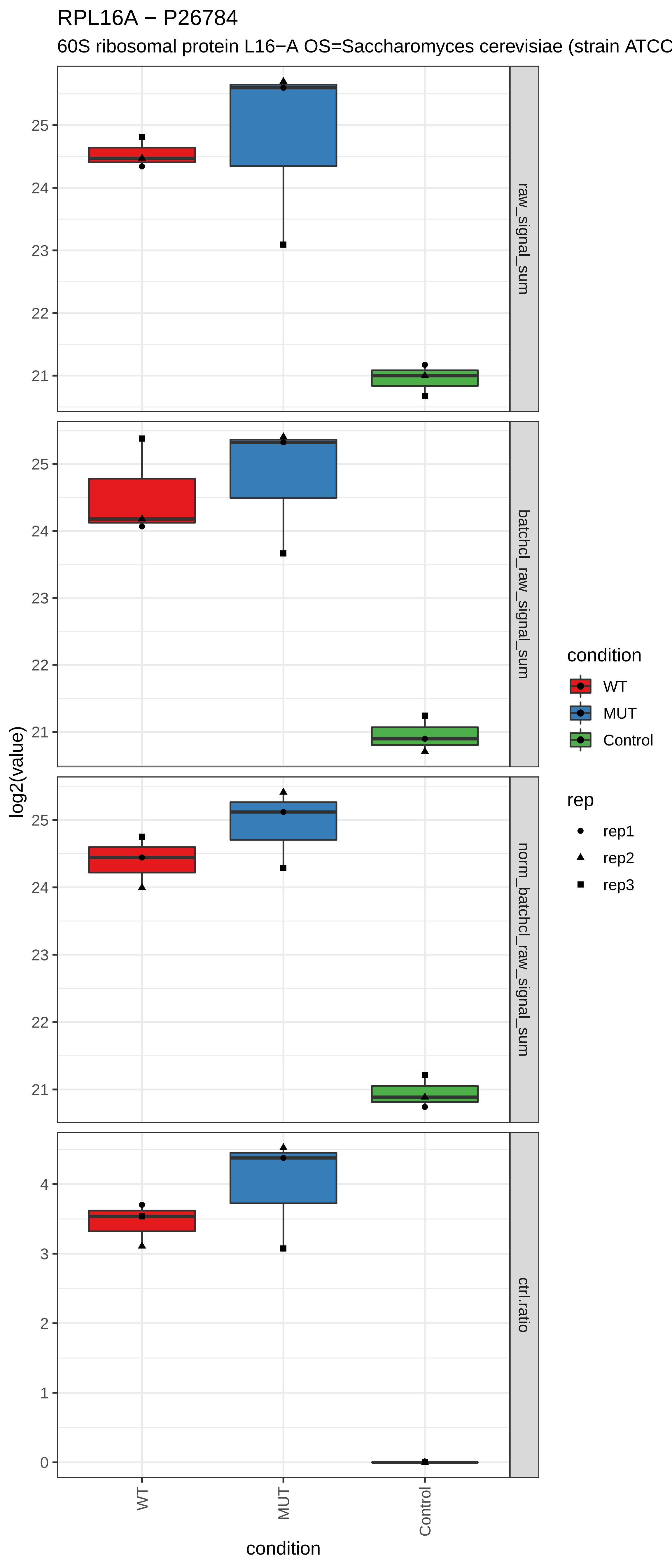

RPL16A − P26784
60S ribosomal protein L16−A OS=Saccharomyces cerevisiae (strain ATCC
raw_signal_sum
batchcl_raw_signal_sum
norm_batchcl_raw_signal_sum
ctrl.ratio
log2(value)
condition
WT
MUT
Control
rep
rep1
rep2
rep3

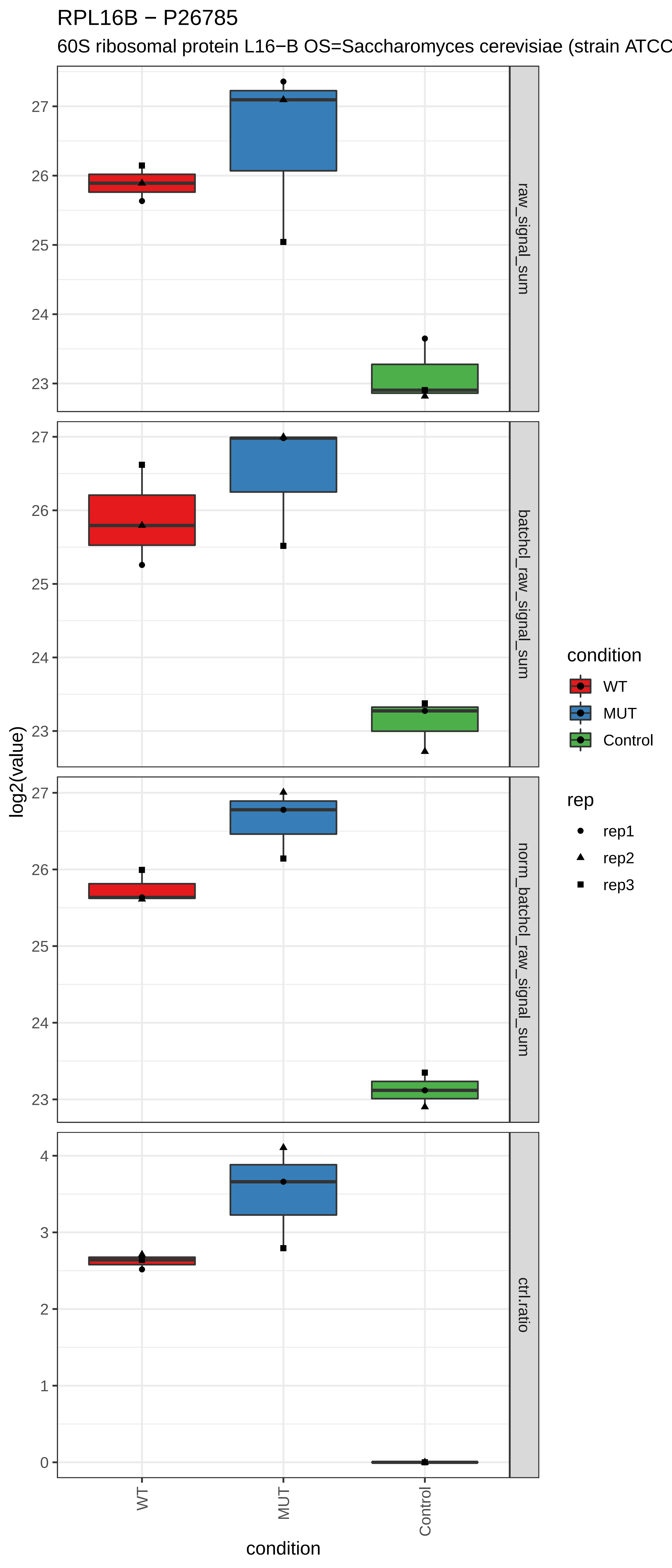
RPL16B − P26785
60S ribosomal protein L16−B OS=Saccharomyces cerevisiae (strain ATCC
raw_signal_sum
batchcl_raw_signal_sum
norm_batchcl_raw_signal_sum
ctrl.ratio
log2(value)
condition
WT
MUT
Control
rep
rep1
rep2
rep3

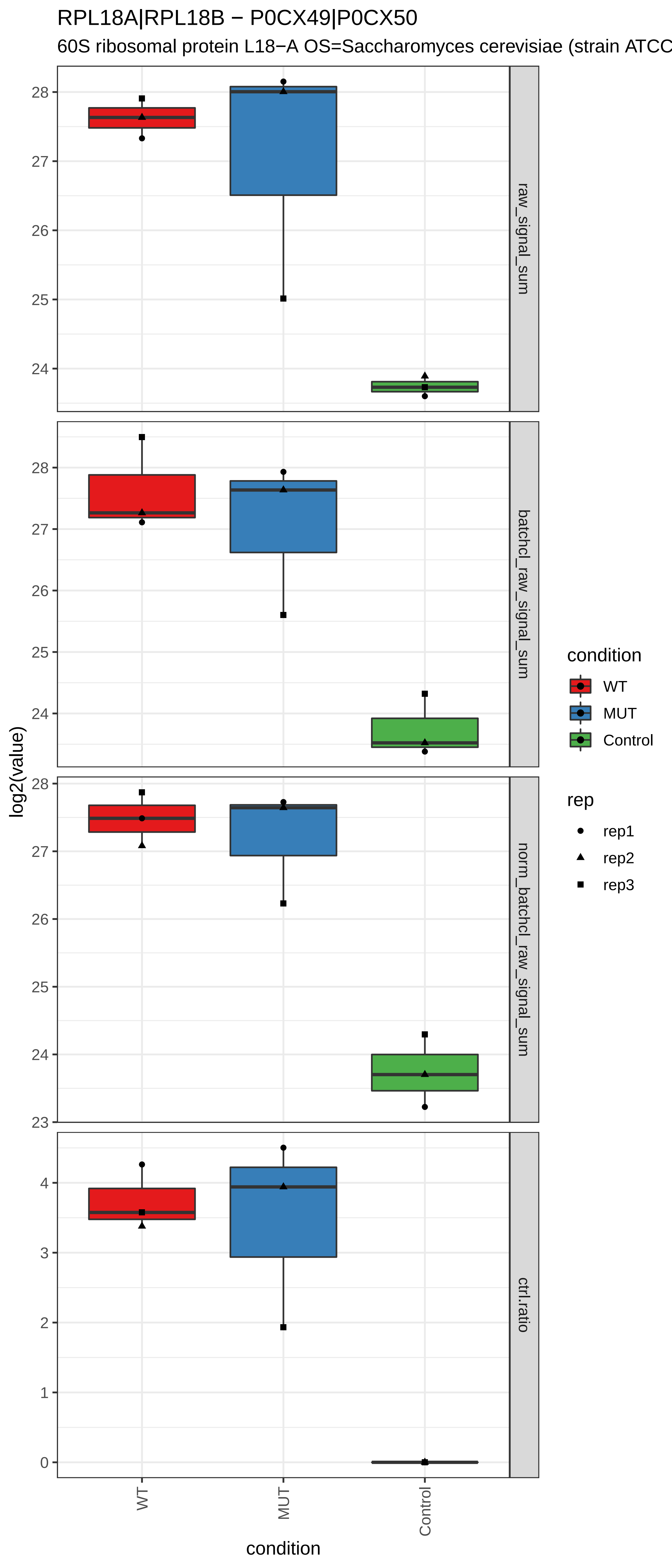
RPL18A|RPL18B − P0CX49|P0CX50
60S ribosomal protein L18−A OS=Saccharomyces cerevisiae (strain ATCC
raw_signal_sum
batchcl_raw_signal_sum
norm_batchcl_raw_signal_sum
ctrl.ratio
log2(value)
condition
WT
MUT
Control
rep
rep1
rep2
rep3

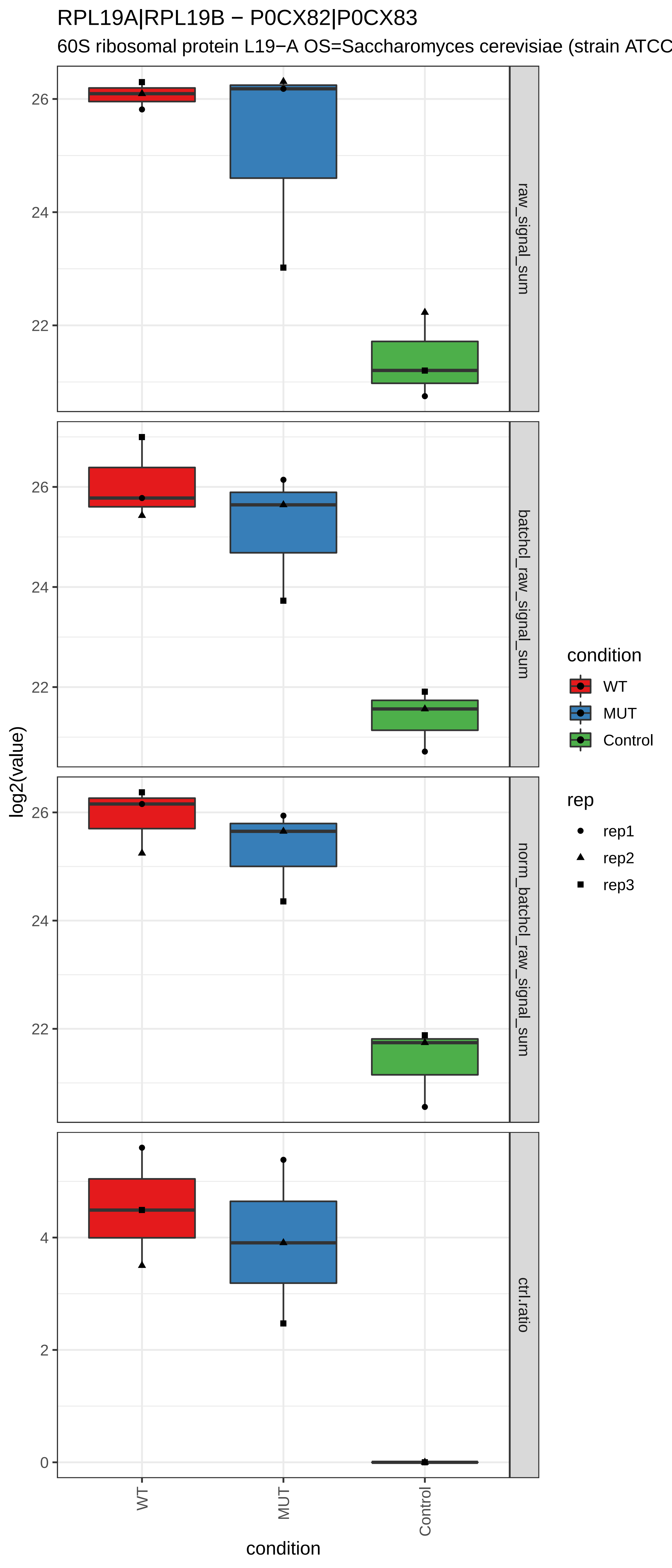

RPL19A|RPL19B − P0CX82|P0CX83
60S ribosomal protein L19−A OS=Saccharomyces cerevisiae (strain ATCC
raw_signal_sum
batchcl_raw_signal_sum
norm_batchcl_raw_signal_sum
ctrl.ratio
log2(value)
condition
WT
MUT
Control
rep
rep1
rep2
rep3
26
24
22
4
2
0

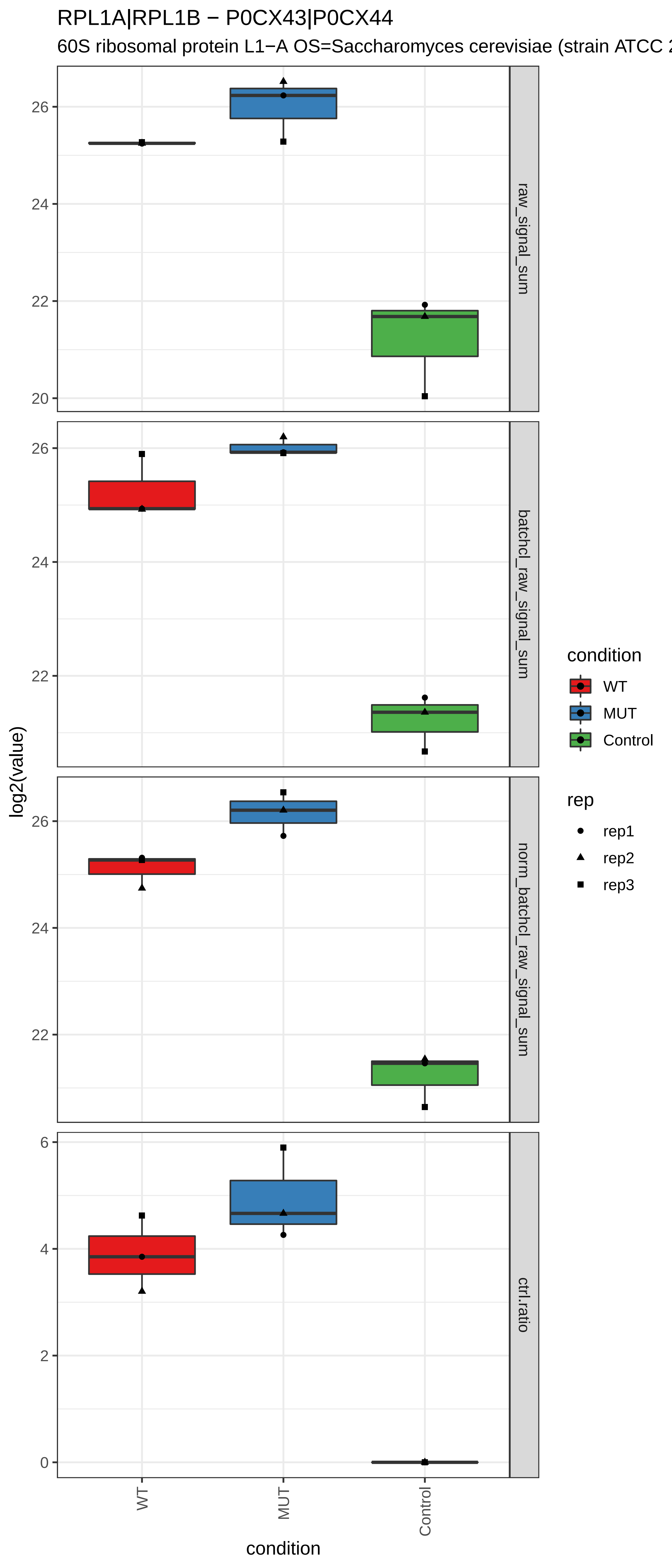

RPL1A|RPL1B − P0CX43|P0CX44
60S ribosomal protein L1−A OS=Saccharomyces cerevisiae (strain ATCC 2
raw_signal_sum
batchcl_raw_signal_sum
norm_batchcl_raw_signal_sum
ctrl.ratio
log2(value)
condition
WT
MUT
Control
rep
rep1
rep2
rep3

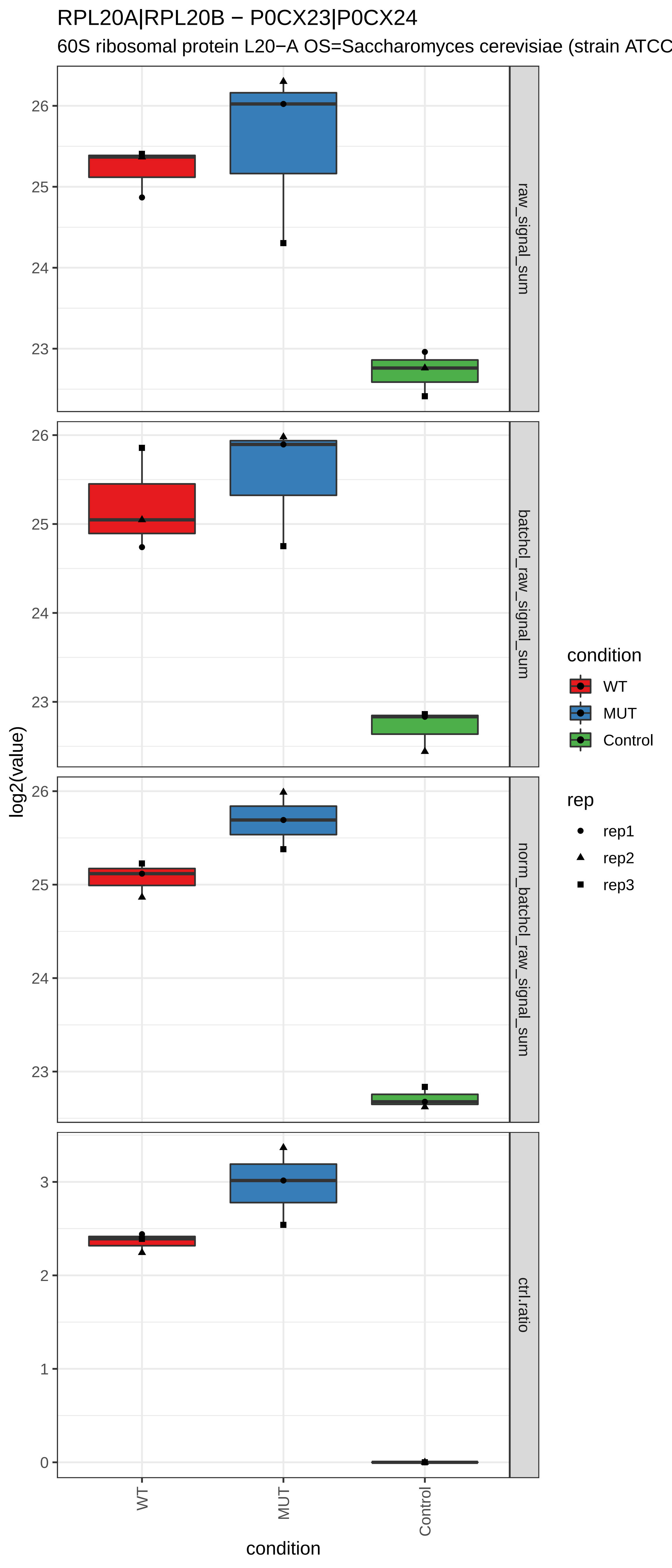

RPL20A|RPL20B − P0CX23|P0CX24
60S ribosomal protein L20−A OS=Saccharomyces cerevisiae (strain ATCC
raw_signal_sum
batchcl_raw_signal_sum
norm_batchcl_raw_signal_sum
ctrl.ratio
log2(value)
condition
WT
MUT
Control
rep
rep1
rep2
rep3
26
25
24
23
3
2
1
0

RPL21A|RPL21B − Q02753|Q12672

60S ribosomal protein L21−A OS=Saccharomyces cerevisiae (strain ATCC

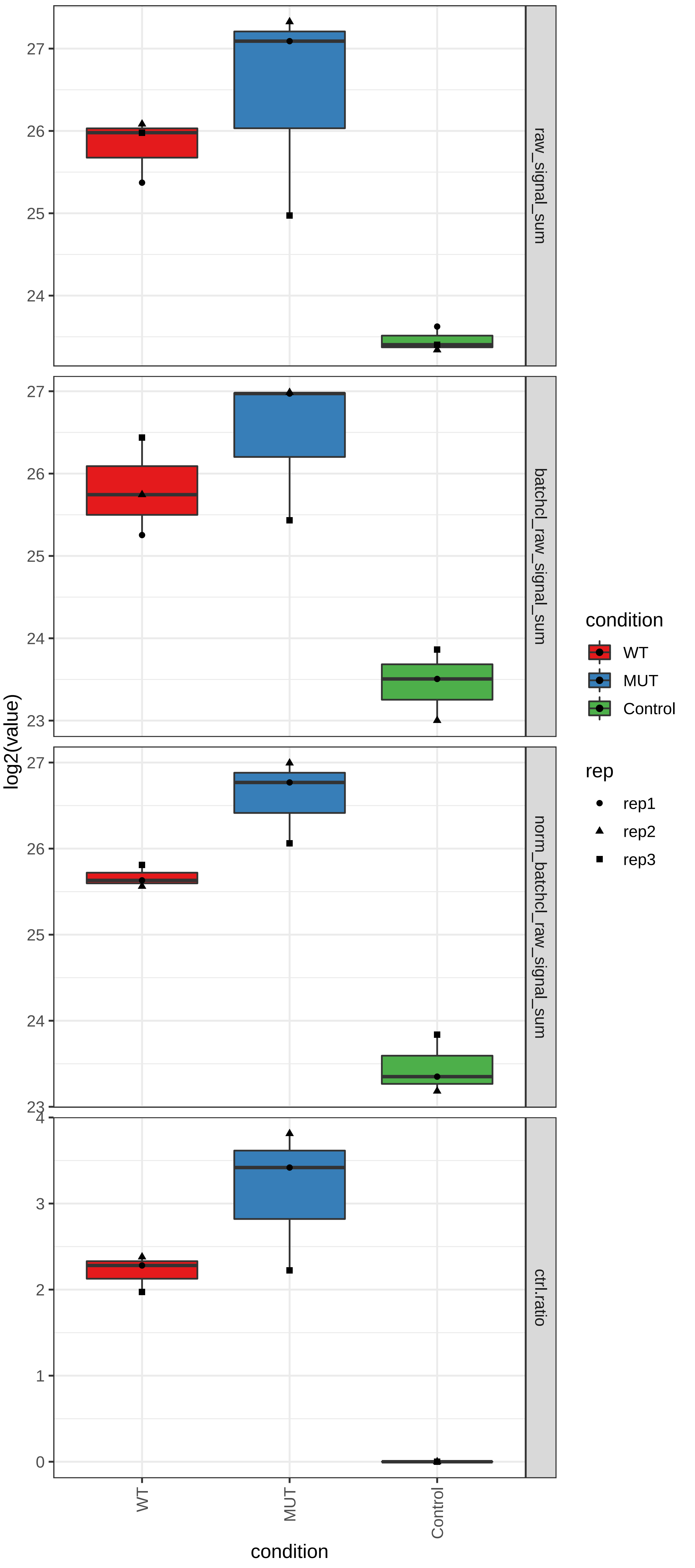

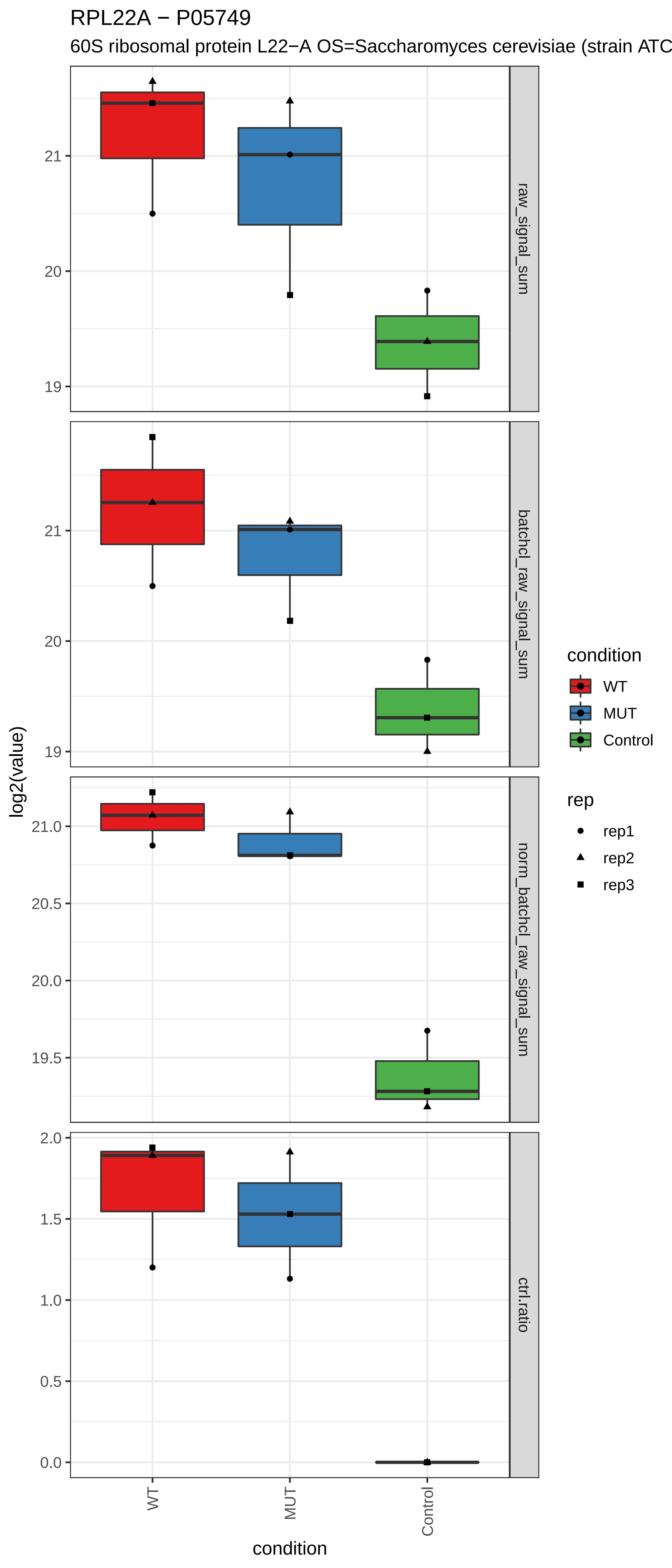

RPL22A − P05749
60S ribosomal protein L22−A OS=Saccharomyces cerevisiae (strain ATC
raw_signal_sum
batchcl_raw_signal_sum
norm_batchcl_raw_signal_sum
ctrl.ratio
log2(value)
condition
WT
MUT
Control
rep
rep1
rep2
rep3
21
20
19
21.0
20.5
20.0
19.5
2.0
1.5
1.0
0.5
0.0

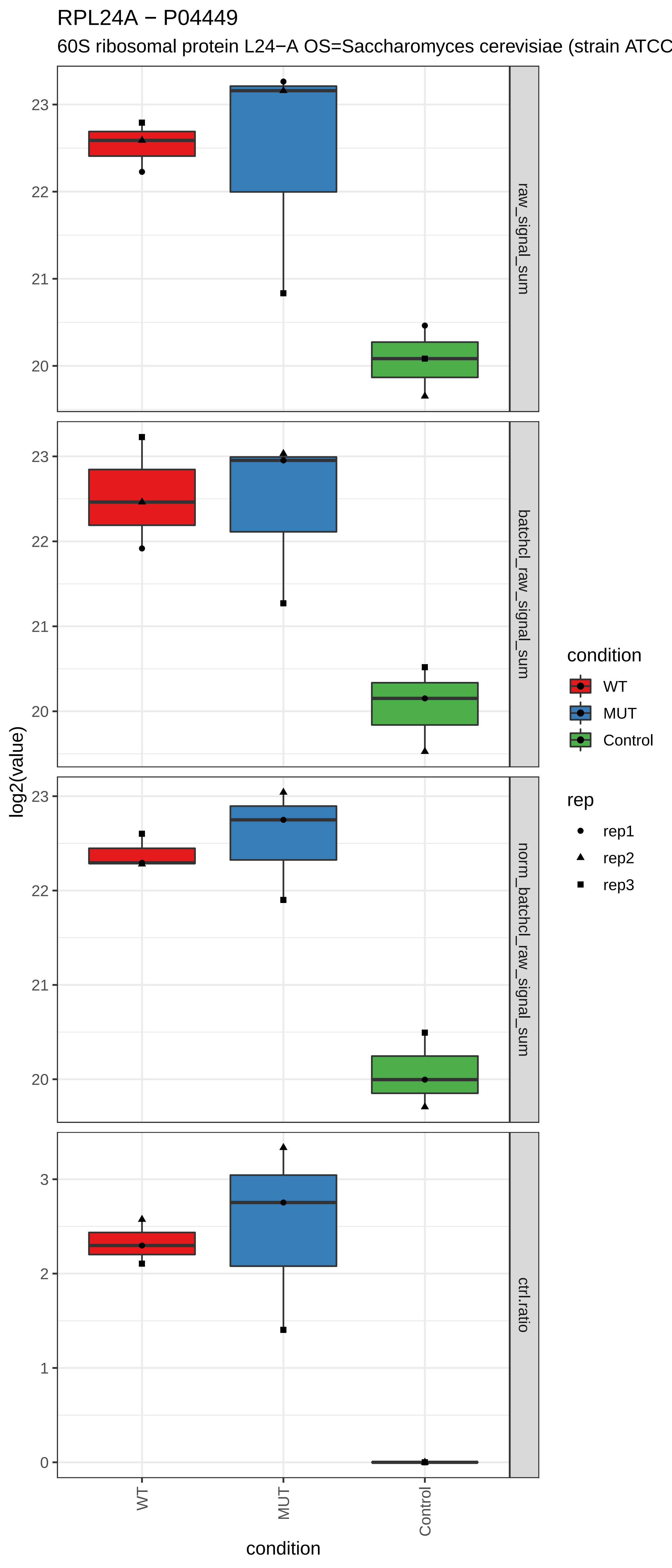

RPL24A − P04449
60S ribosomal protein L24−A OS=Saccharomyces cerevisiae (strain ATCC
raw_signal_sum
batchcl_raw_signal_sum
norm_batchcl_raw_signal_sum
ctrl.ratio
log2(value)
condition
WT
MUT
Control
rep
rep1
rep2
rep3

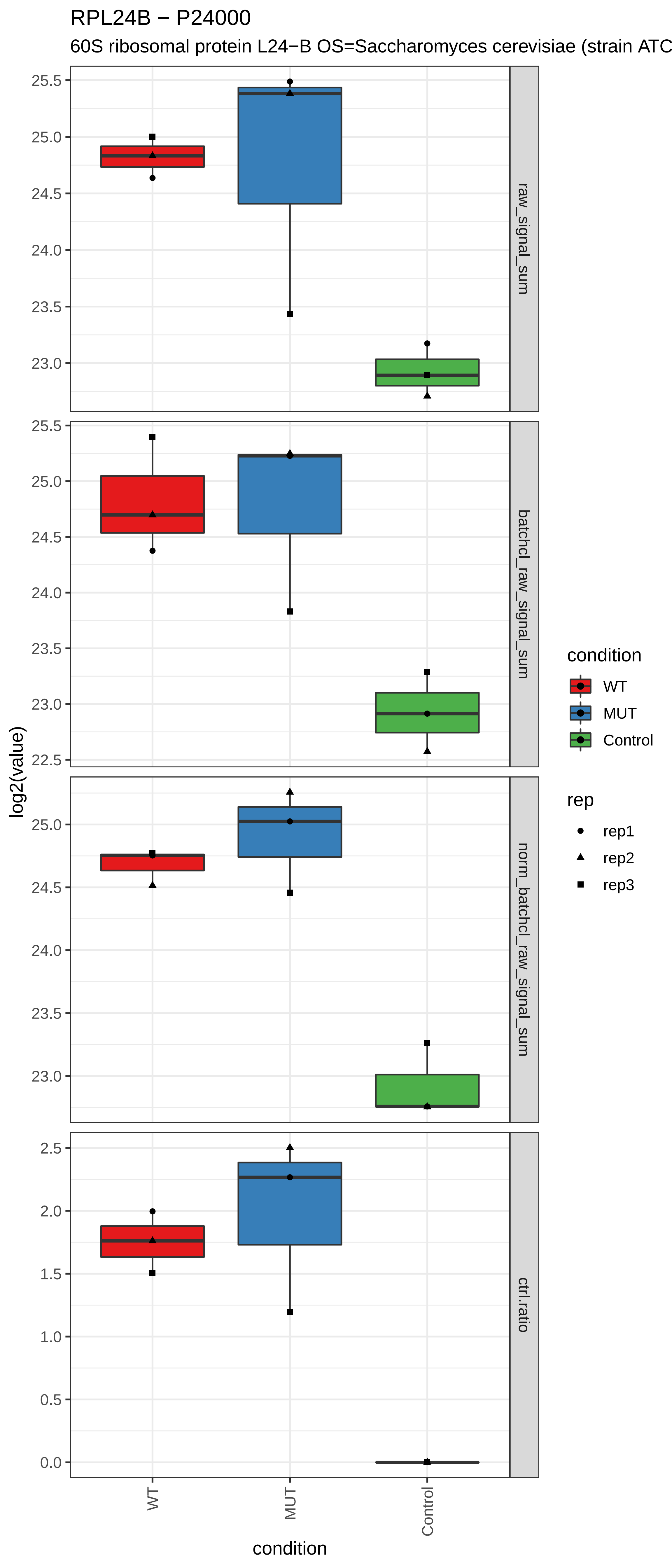

RPL24B − P24000
60S ribosomal protein L24−B OS=Saccharomyces cerevisiae (strain ATC
raw_signal_sum
batchcl_raw_signal_sum
norm_batchcl_raw_signal_sum
ctrl.ratio
log2(value)
condition
WT
MUT
Control
rep
rep1
rep2
rep3
25.5
25.0
24.5
24.0
23.5
23.0
22.5
2.5
2.0
1.5
1.0
0.5
0.0

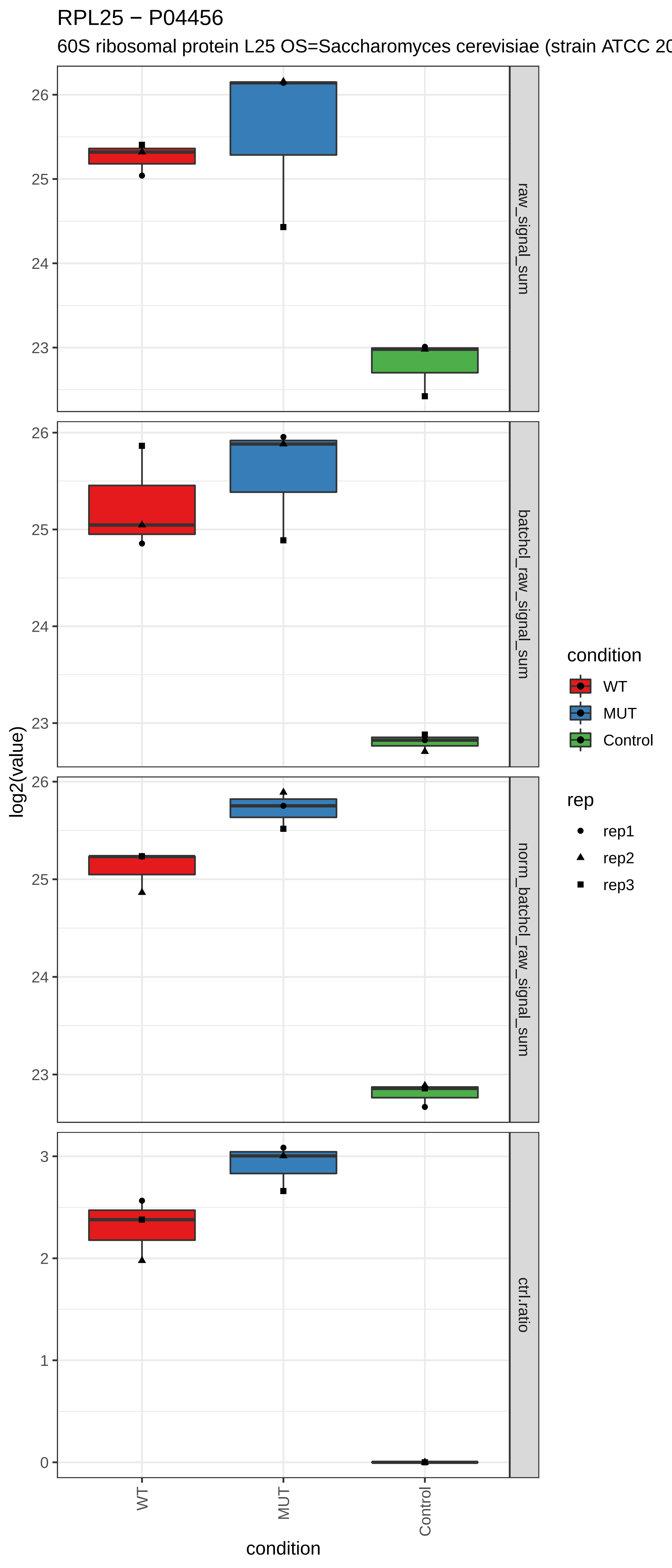
RPL25 − P04456
60S ribosomal protein L25 OS=Saccharomyces cerevisiae (strain ATCC 20
raw_signal_sum
batchcl_raw_signal_sum
norm_batchcl_raw_signal_sum
ctrl.ratio
log2(value)
condition
WT
MUT
Control
rep
rep1
rep2
rep3
26
25
24
23
3
2
1
0

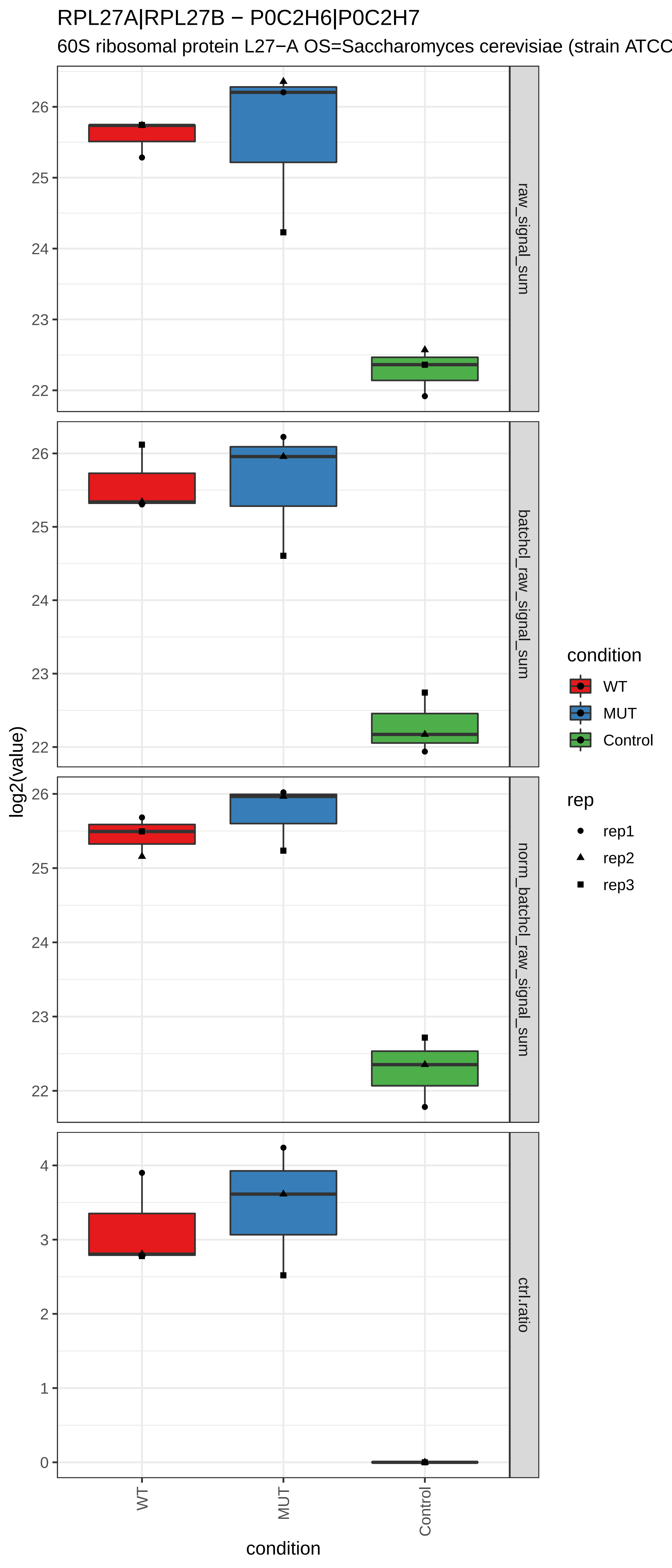

RPL27A|RPL27B − P0C2H6|P0C2H7
60S ribosomal protein L27−A OS=Saccharomyces cerevisiae (strain ATCC
raw_signal_sum
batchcl_raw_signal_sum
norm_batchcl_raw_signal_sum
ctrl.ratio
log2(value)
condition
WT
MUT
Control
rep
rep1
rep2
rep3

RPL28 − P02406

60S ribosomal protein L28 OS=Saccharomyces cerevisiae (strain ATCC 20

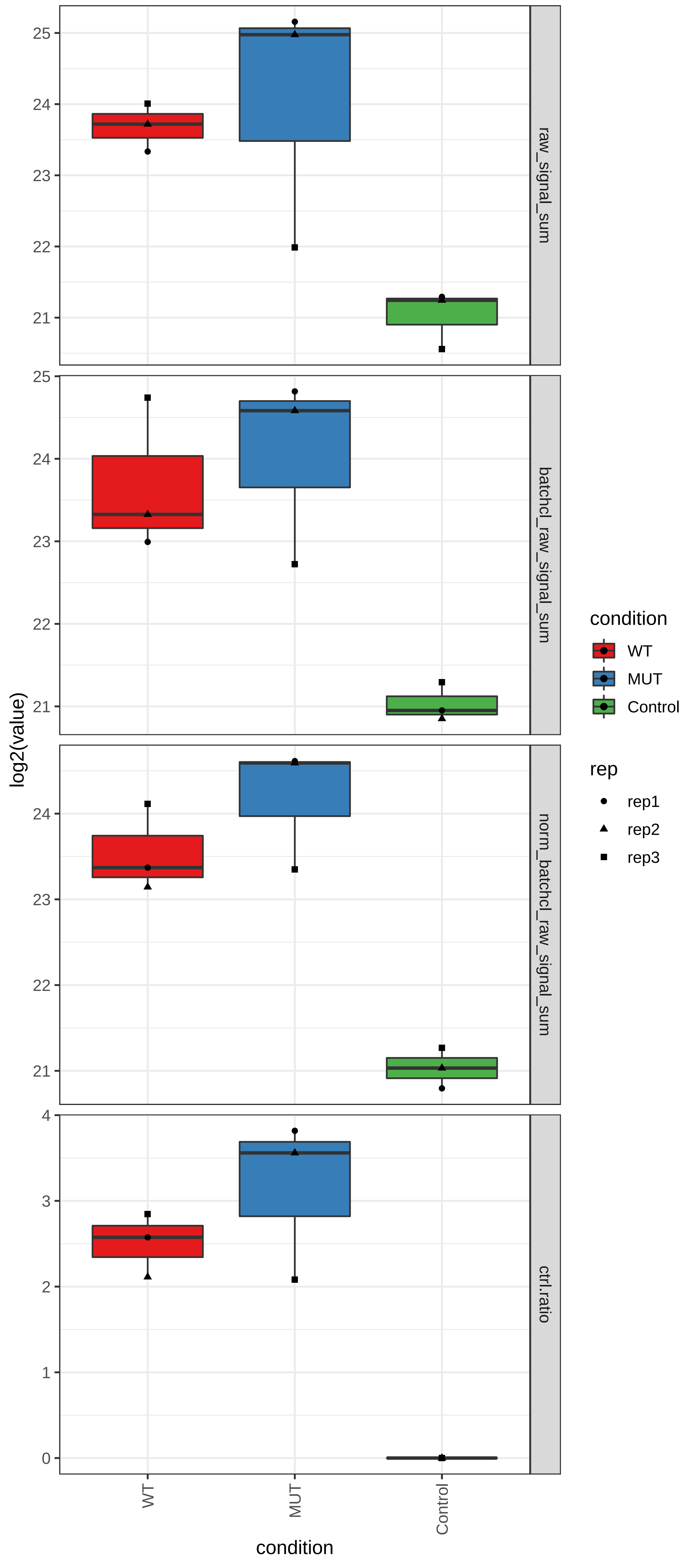

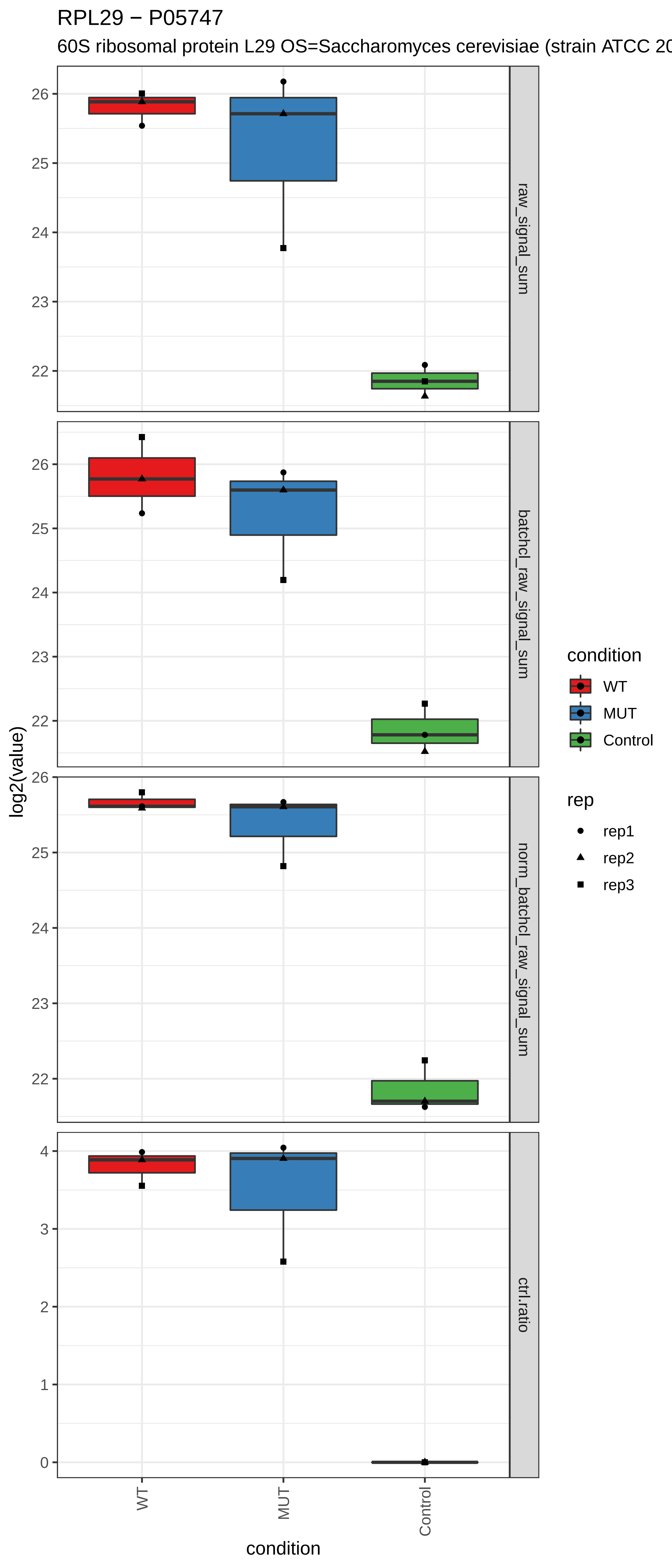

RPL29 − P05747
60S ribosomal protein L29 OS=Saccharomyces cerevisiae (strain ATCC 20
raw_signal_sum
batchcl_raw_signal_sum
norm_batchcl_raw_signal_sum
ctrl.ratio
log2(value)
condition
WT
MUT
Control
rep
rep1
rep2
rep3

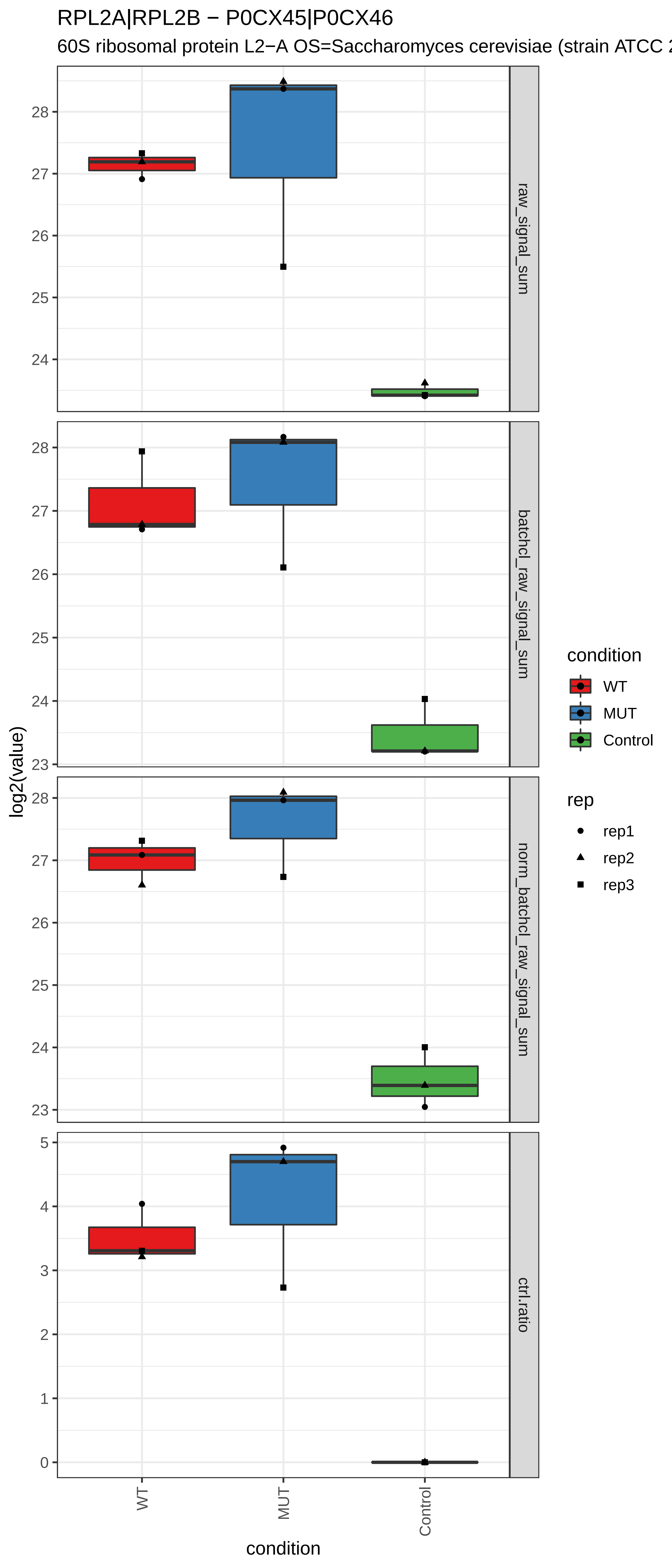
RPL2A|RPL2B − P0CX45|P0CX46
60S ribosomal protein L2−A OS=Saccharomyces cerevisiae (strain ATCC 2
raw_signal_sum
batchcl_raw_signal_sum
norm_batchcl_raw_signal_sum
ctrl.ratio
log2(value)
condition
WT
MUT
Control
rep
rep1
rep2
rep3

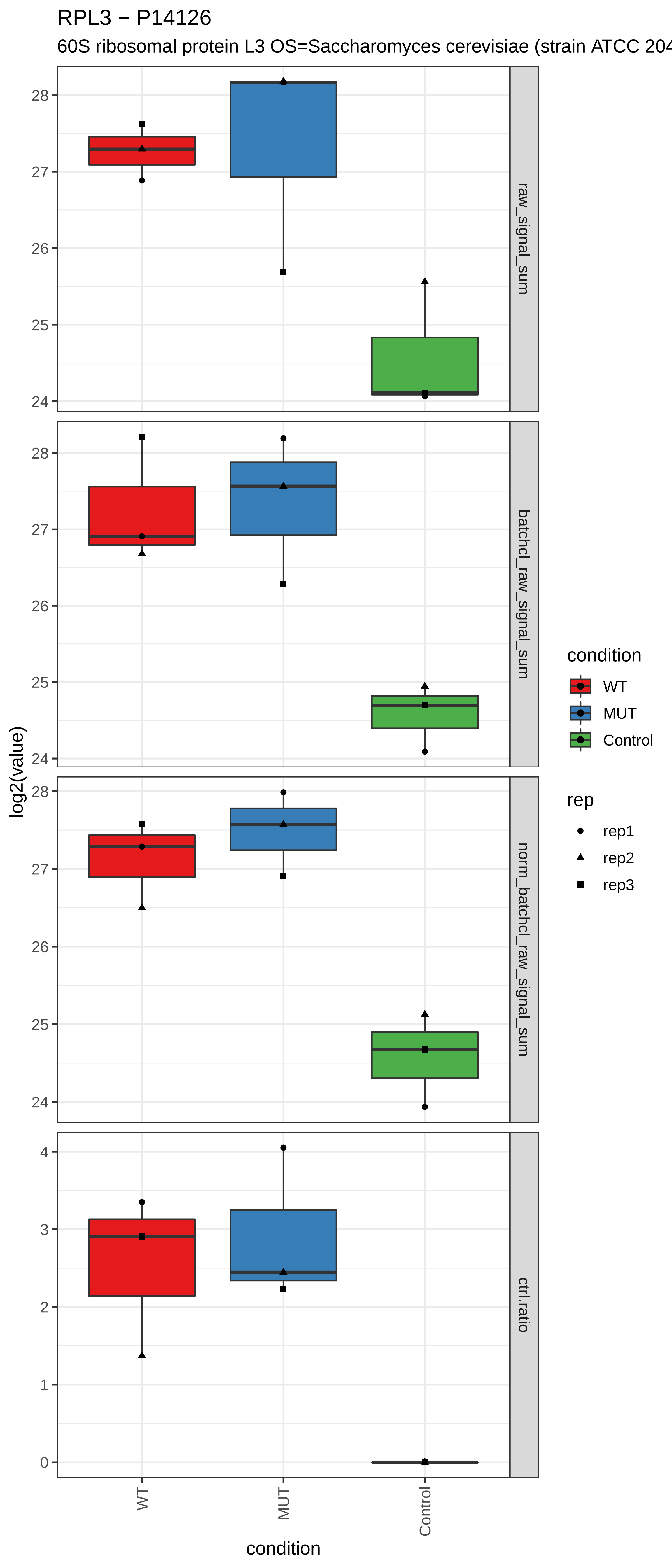

RPL3 − P14126
60S ribosomal protein L3 OS=Saccharomyces cerevisiae (strain ATCC 204
raw_signal_sum
batchcl_raw_signal_sum
norm_batchcl_raw_signal_sum
ctrl.ratio
log2(value)
condition
WT
MUT
Control
rep
rep1
rep2
rep3

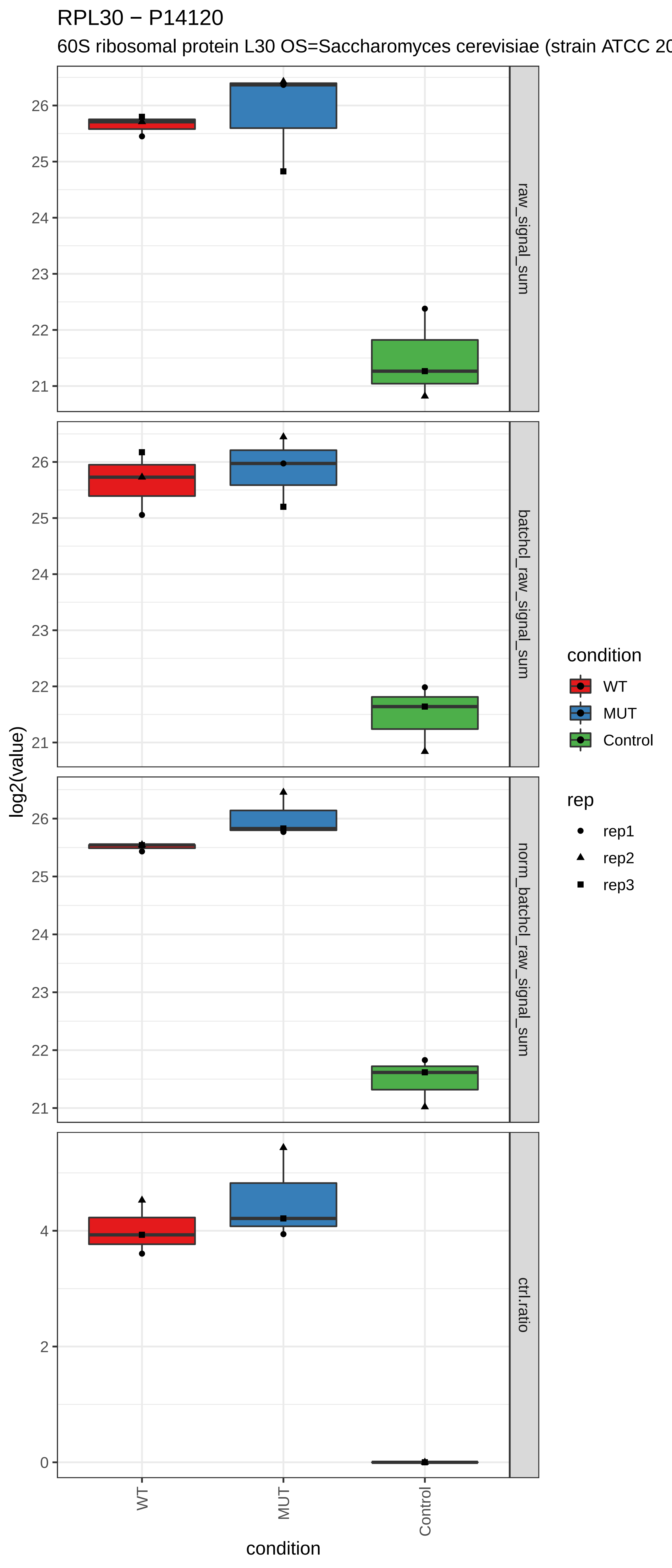

RPL30 − P14120
60S ribosomal protein L30 OS=Saccharomyces cerevisiae (strain ATCC 20
raw_signal_sum
batchcl_raw_signal_sum
norm_batchcl_raw_signal_sum
ctrl.ratio
log2(value)
condition
WT
MUT
Control
rep
rep1
rep2
rep3

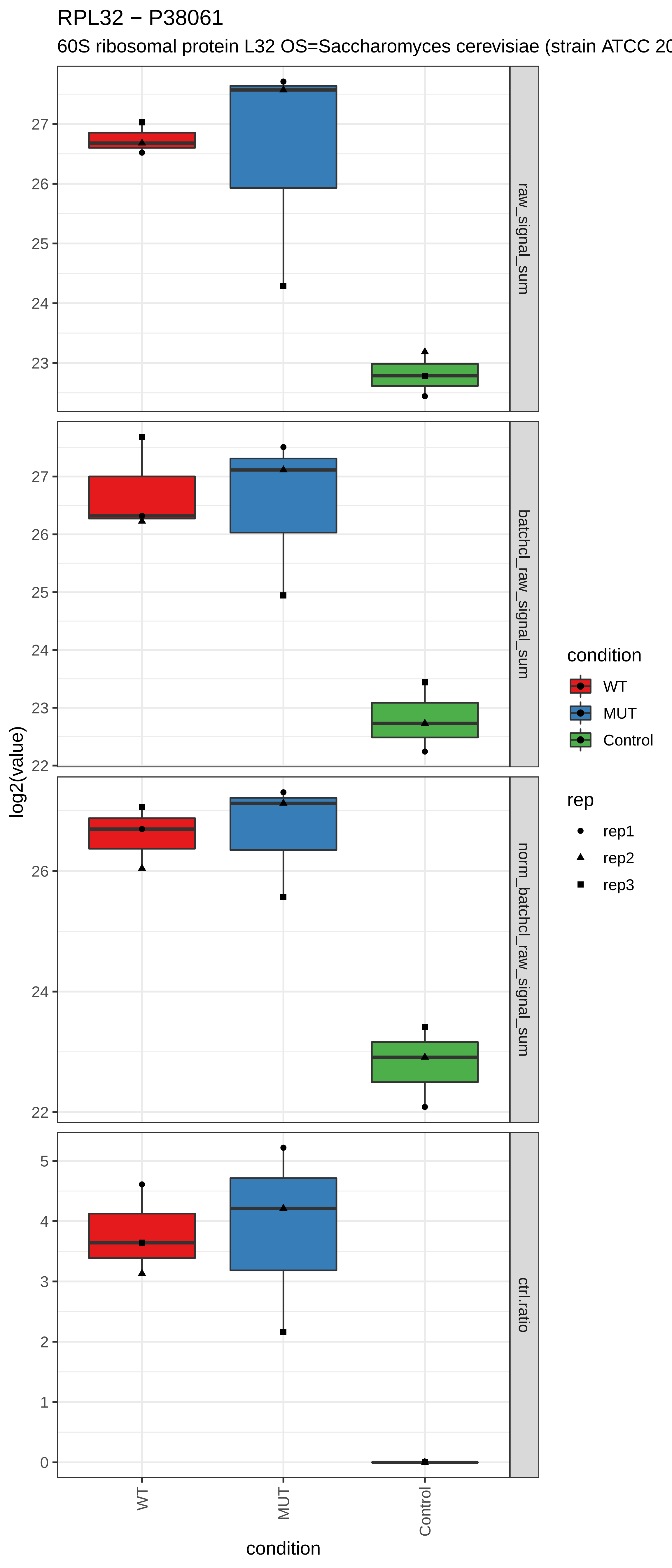

RPL32 − P38061
60S ribosomal protein L32 OS=Saccharomyces cerevisiae (strain ATCC 20
raw_signal_sum
batchcl_raw_signal_sum
norm_batchcl_raw_signal_sum
ctrl.ratio
log2(value)
condition
WT
MUT
Control
rep
rep1
rep2
rep3
27
26
25
24
23
22
5
4
3
2
1
0

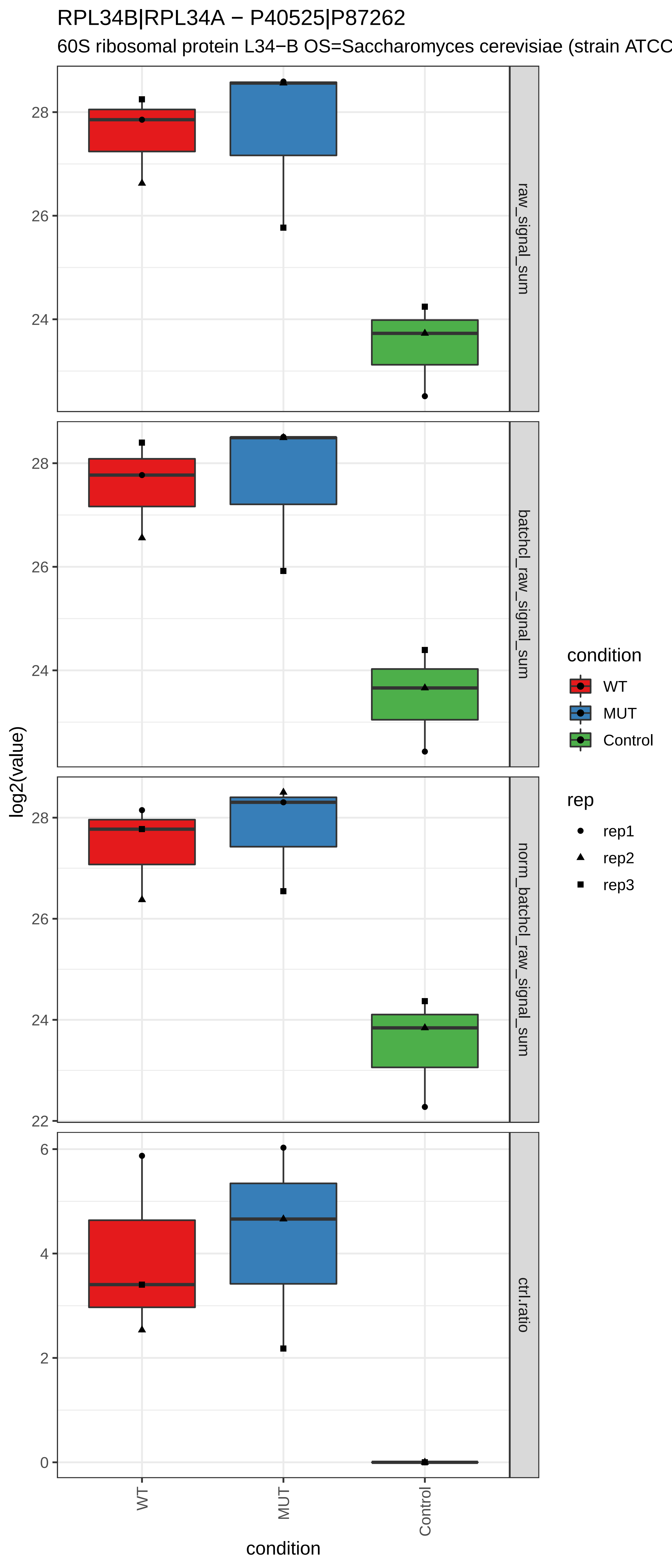
RPL34B|RPL34A − P40525|P87262
60S ribosomal protein L34−B OS=Saccharomyces cerevisiae (strain ATCC
raw_signal_sum
batchcl_raw_signal_sum
norm_batchcl_raw_signal_sum
ctrl.ratio
log2(value)
condition
WT
MUT
Control
rep
rep1
rep2
rep3

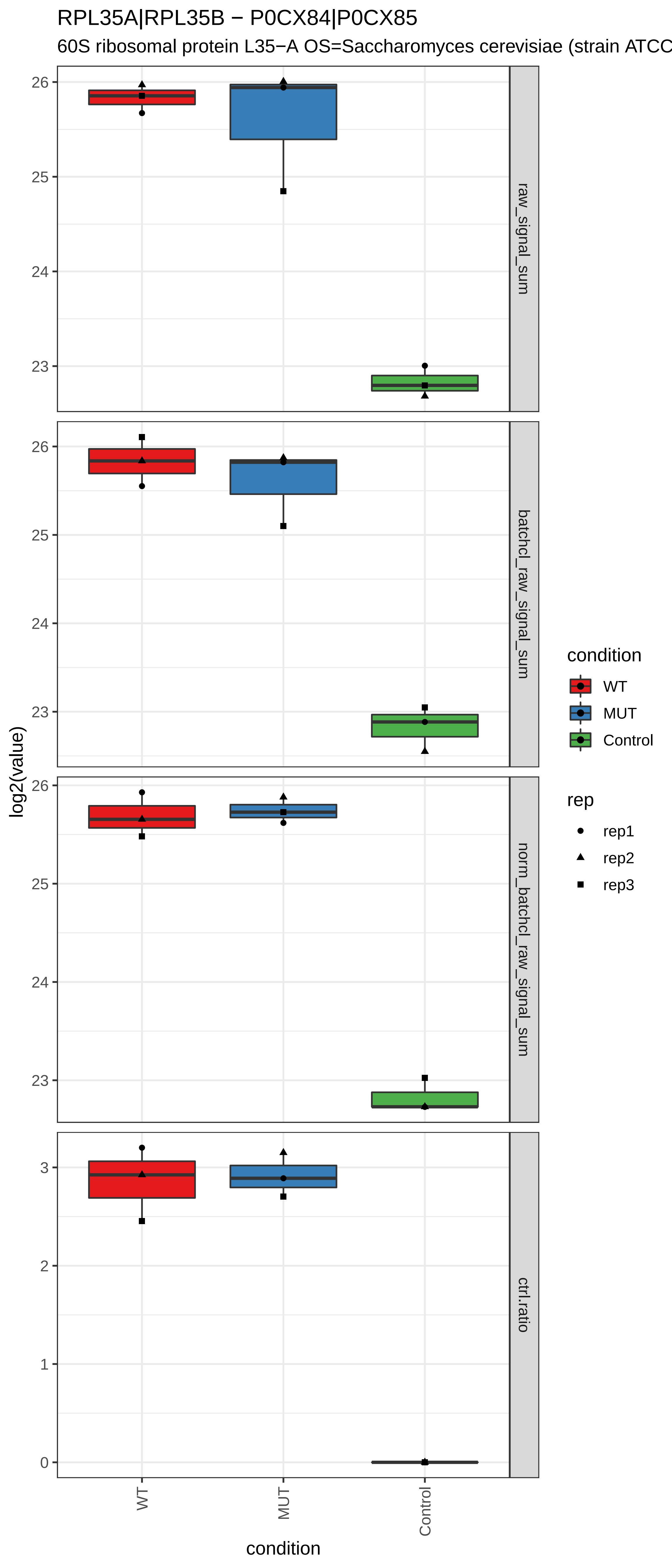

RPL35A|RPL35B − P0CX84|P0CX85
60S ribosomal protein L35−A OS=Saccharomyces cerevisiae (strain ATCC
raw_signal_sum
batchcl_raw_signal_sum
norm_batchcl_raw_signal_sum
ctrl.ratio
log2(value)
condition
WT
MUT
Control
rep
rep1
rep2
rep3

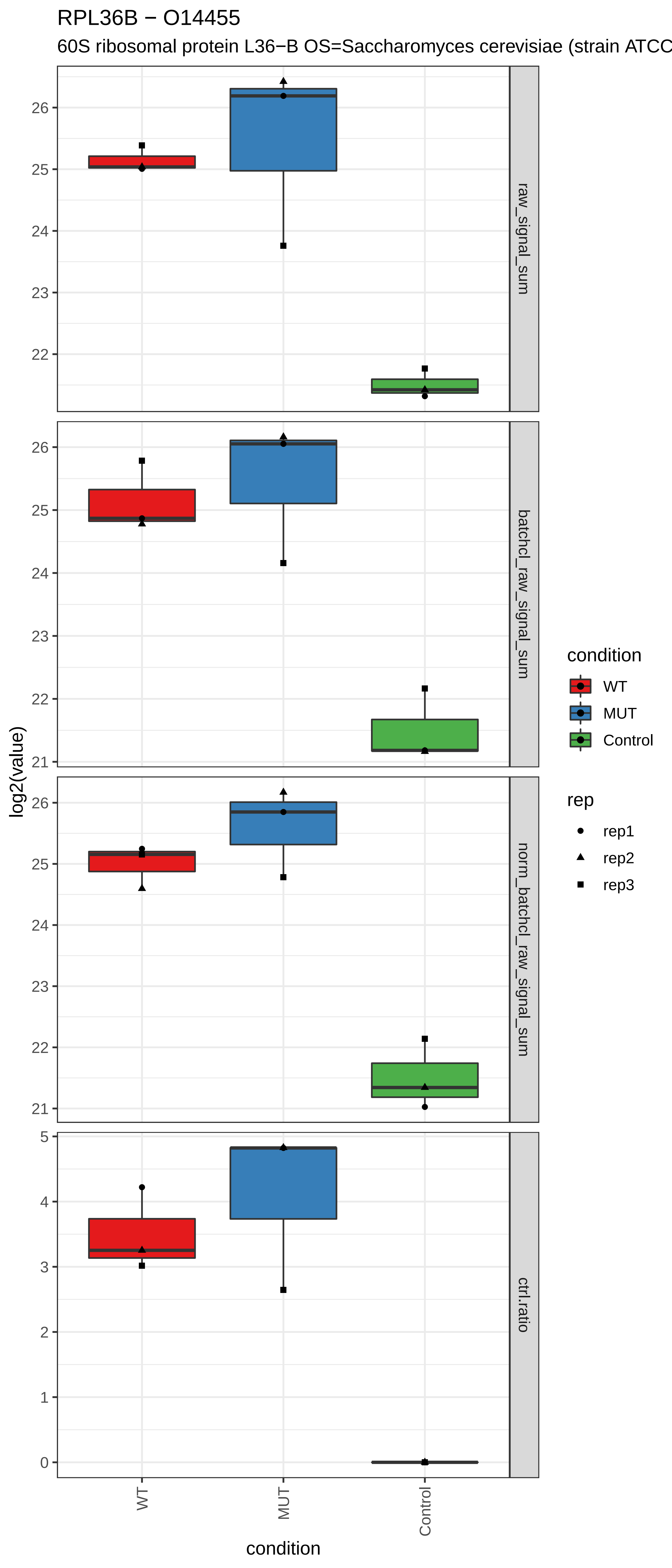

RPL36B − O14455
60S ribosomal protein L36−B OS=Saccharomyces cerevisiae (strain ATCC
raw_signal_sum
batchcl_raw_signal_sum
norm_batchcl_raw_signal_sum
ctrl.ratio
log2(value)
condition
WT
MUT
Control
rep
rep1
rep2
rep3
26
25
24
23
22
21
5
4
3
2
1
0

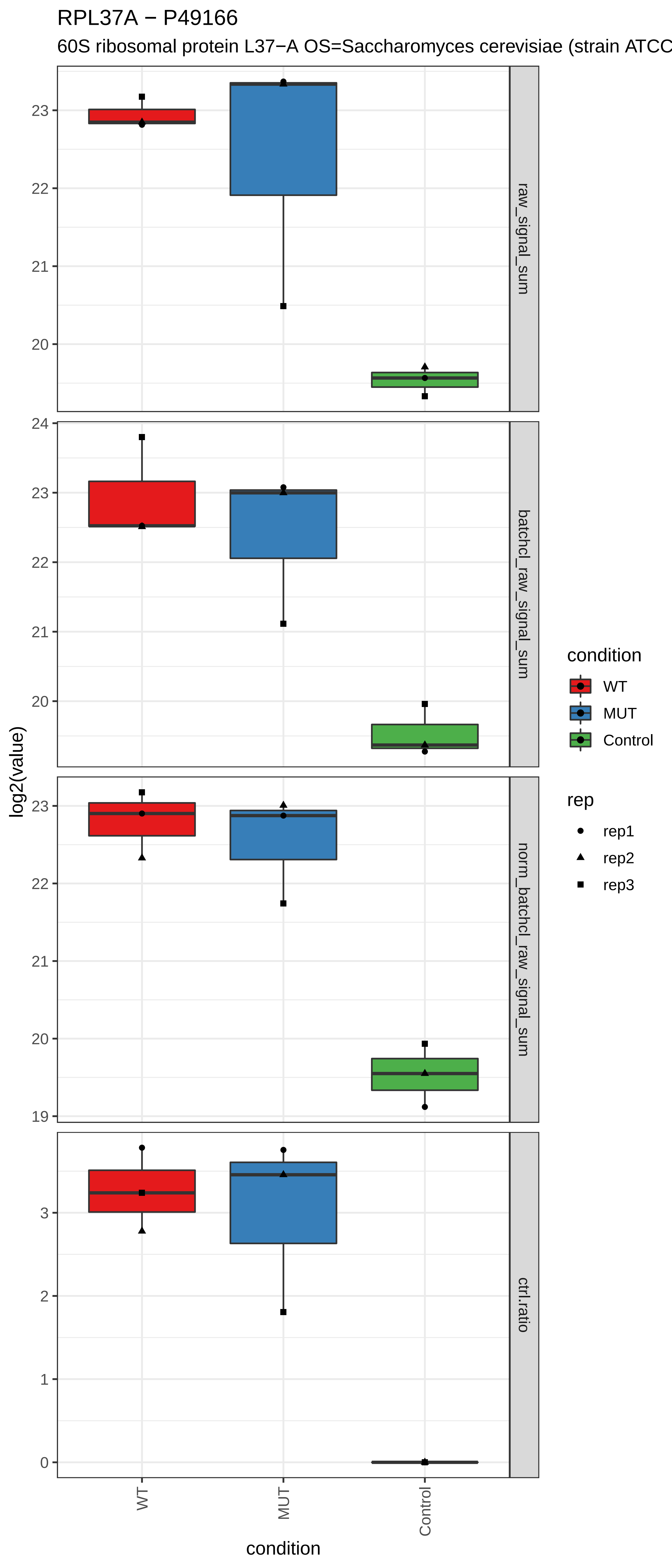

RPL37A − P49166
60S ribosomal protein L37−A OS=Saccharomyces cerevisiae (strain ATCC
raw_signal_sum
batchcl_raw_signal_sum
norm_batchcl_raw_signal_sum
ctrl.ratio
log2(value)
condition
WT
MUT
Control
rep
rep1
rep2
rep3

RPL42A|RPL42B − P0CX27|P0CX28

60S ribosomal protein L42−A OS=Saccharomyces cerevisiae (strain ATCC

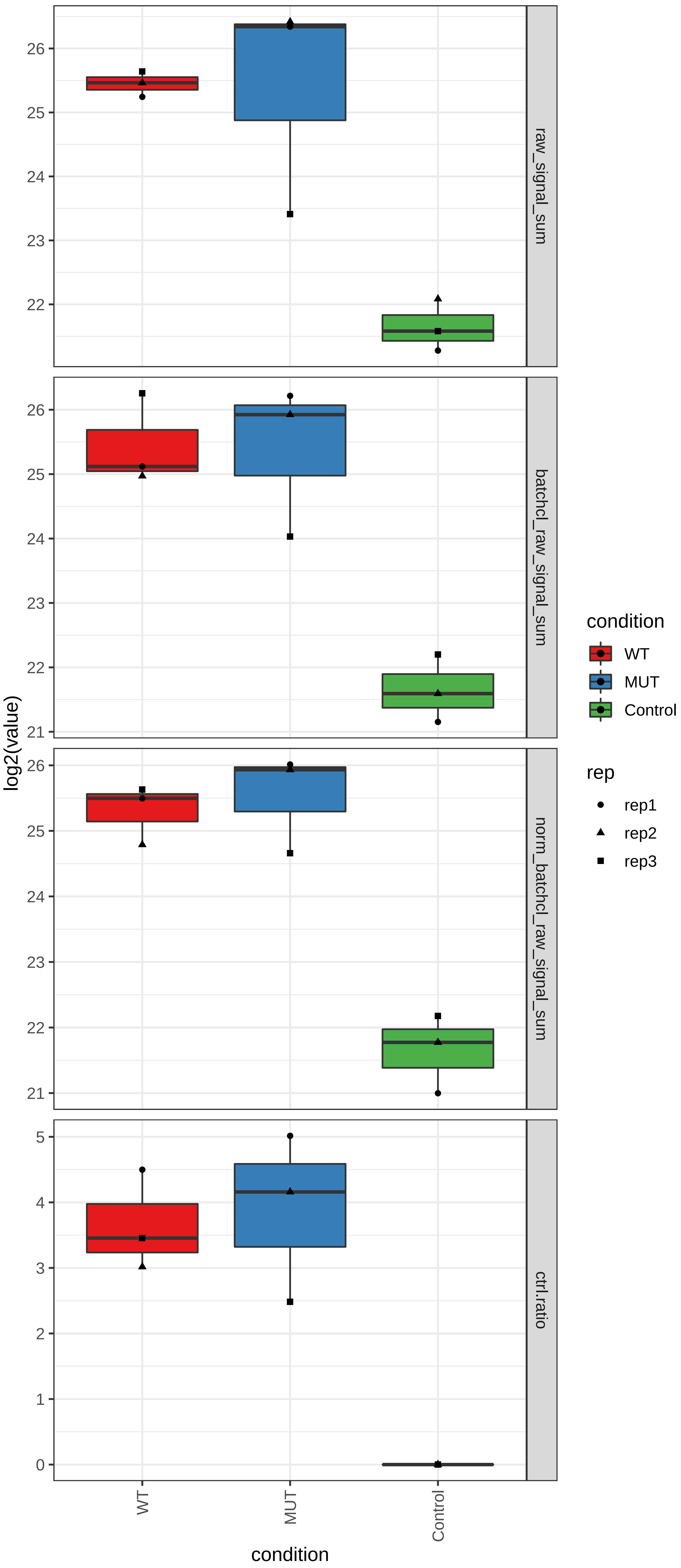

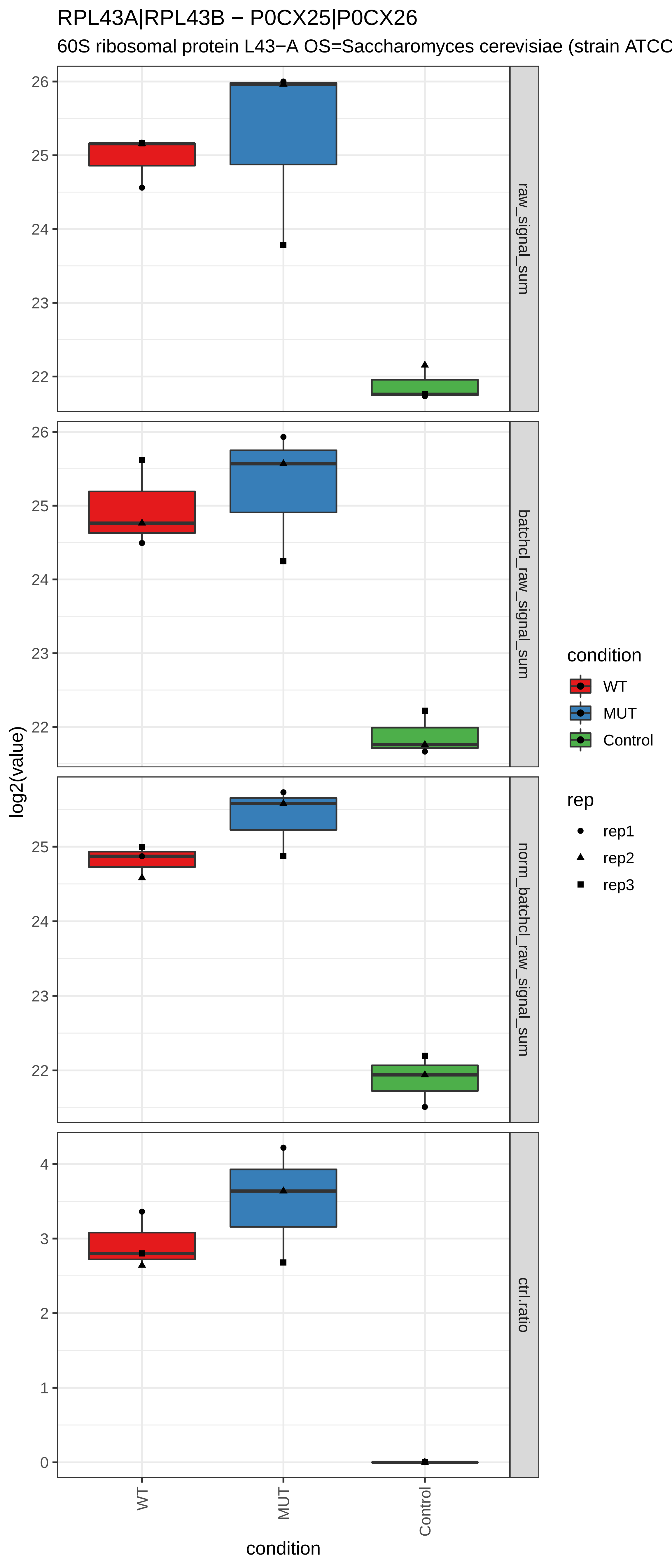

RPL43A|RPL43B − P0CX25|P0CX26
60S ribosomal protein L43−A OS=Saccharomyces cerevisiae (strain ATCC
raw_signal_sum
batchcl_raw_signal_sum
norm_batchcl_raw_signal_sum
ctrl.ratio
log2(value)
condition
WT
MUT
Control
rep
rep1
rep2
rep3

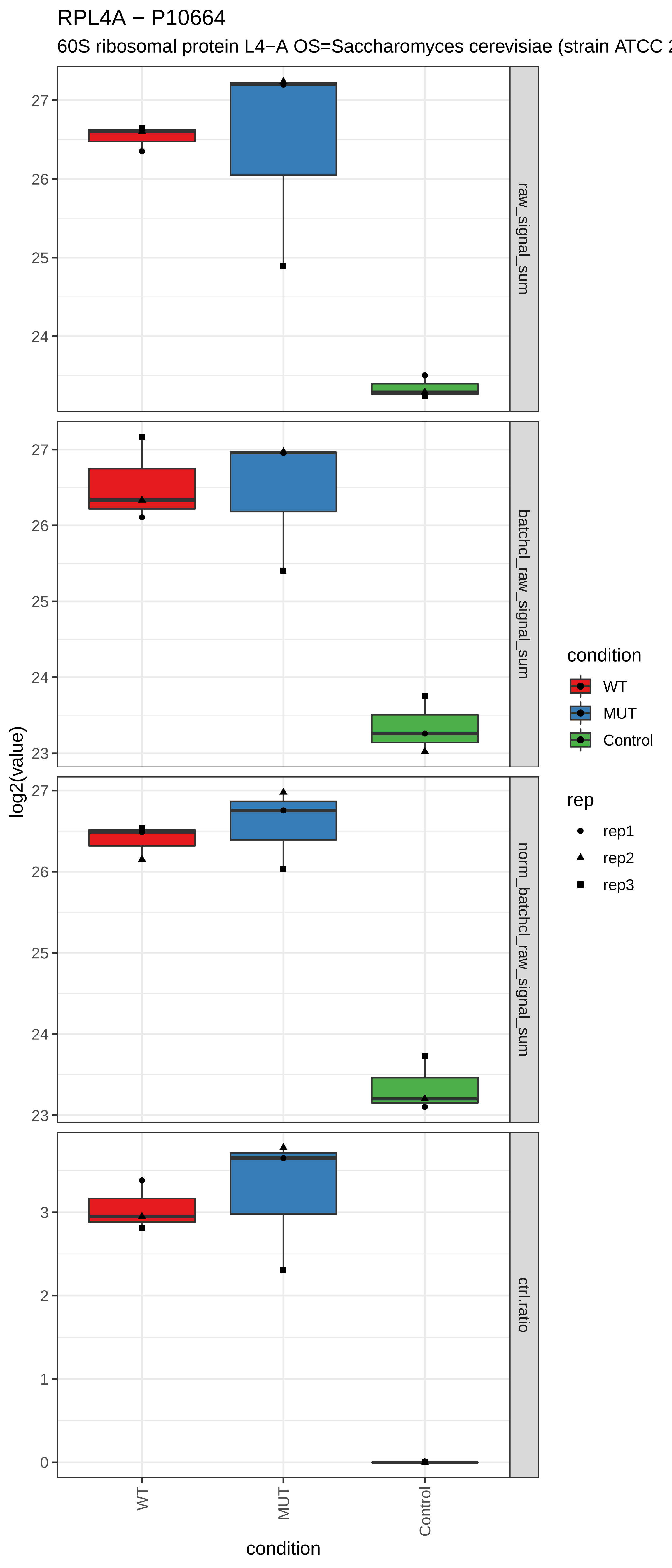

RPL4A − P10664
60S ribosomal protein L4−A OS=Saccharomyces cerevisiae (strain ATCC 2
raw_signal_sum
batchcl_raw_signal_sum
norm_batchcl_raw_signal_sum
ctrl.ratio
log2(value)
condition
WT
MUT
Control
rep
rep1
rep2
rep3

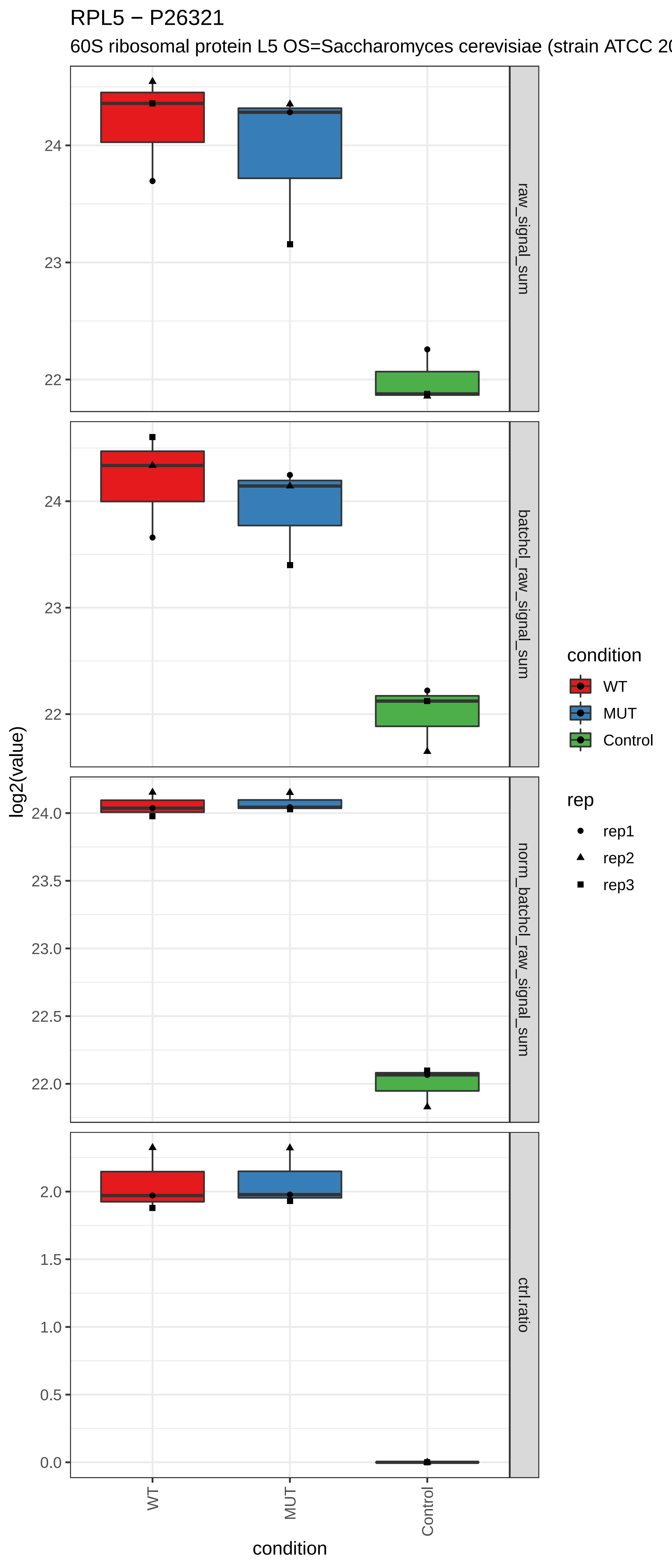

RPL5 − P26321
60S ribosomal protein L5 OS=Saccharomyces cerevisiae (strain ATCC 20
raw_signal_sum
batchcl_raw_signal_sum
norm_batchcl_raw_signal_sum
ctrl.ratio
log2(value)
condition
WT
MUT
Control
rep
rep1
rep2
rep3
24
23
22
24.0
23.5
23.0
22.5
22.0
2.0
1.5
1.0
0.5
0.0

RPL6A − Q02326

60S ribosomal protein L6−A OS=Saccharomyces cerevisiae (strain ATCC 2

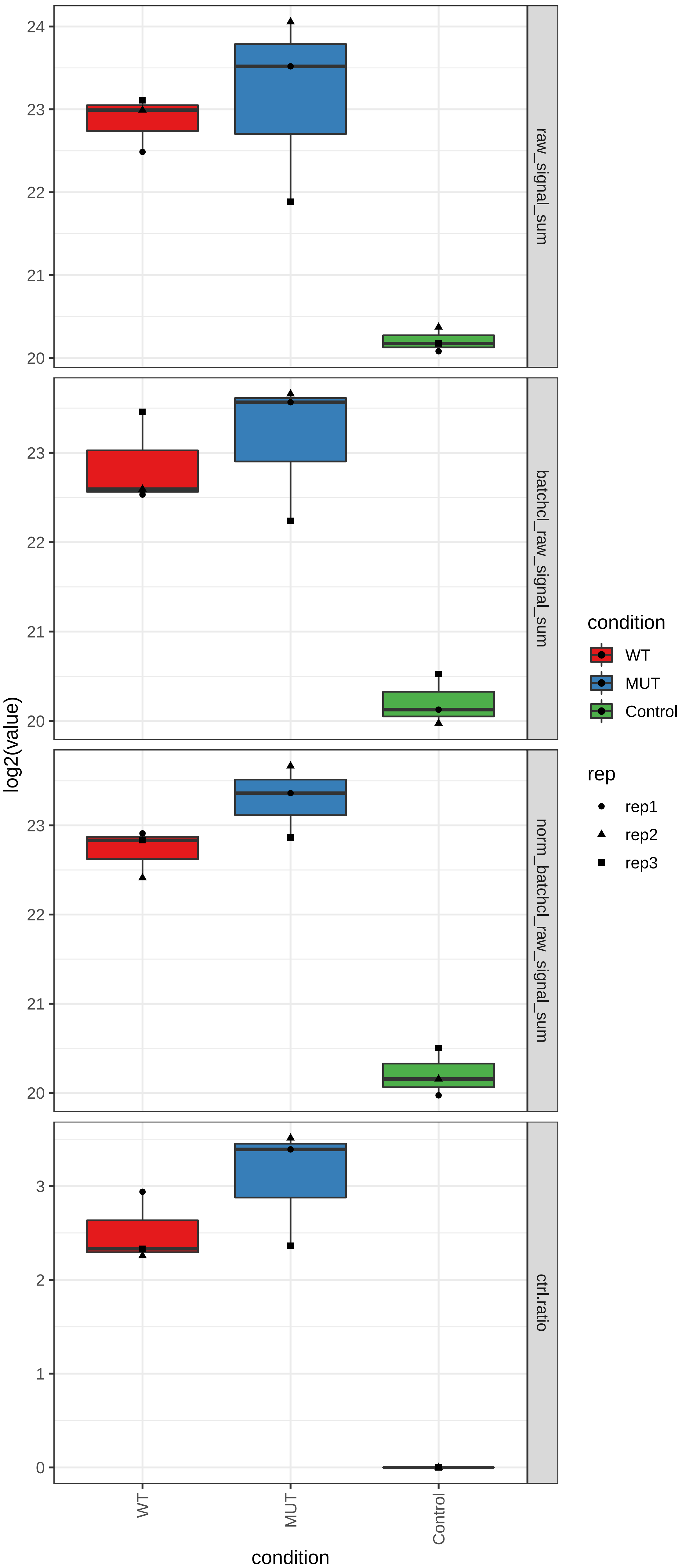

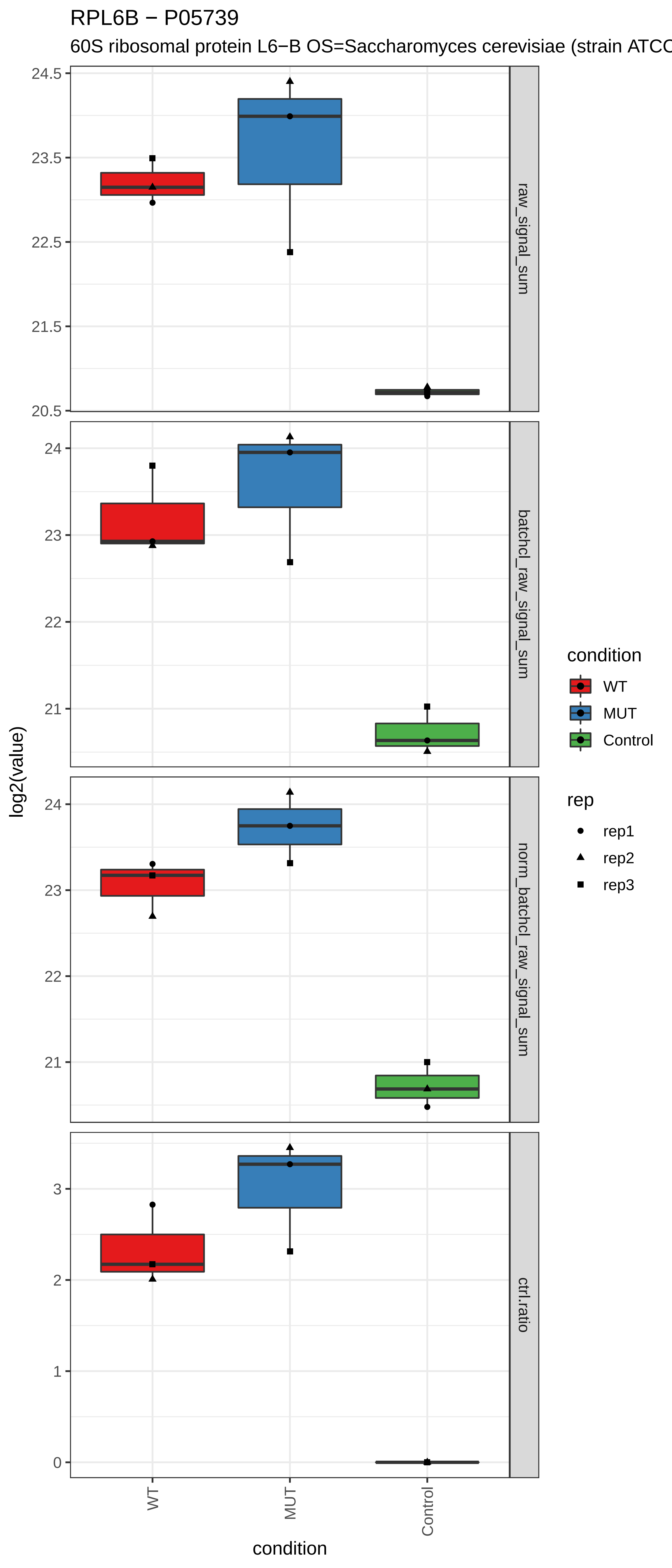
RPL6B − P05739
60S ribosomal protein L6−B OS=Saccharomyces cerevisiae (strain ATCC
raw_signal_sum
batchcl_raw_signal_sum
norm_batchcl_raw_signal_sum
ctrl.ratio
log2(value)
condition
WT
MUT
Control
rep
rep1
rep2
rep3
24.5
23.5
22.5
21.5
20.5
24
23
22
21
3
2
1
0

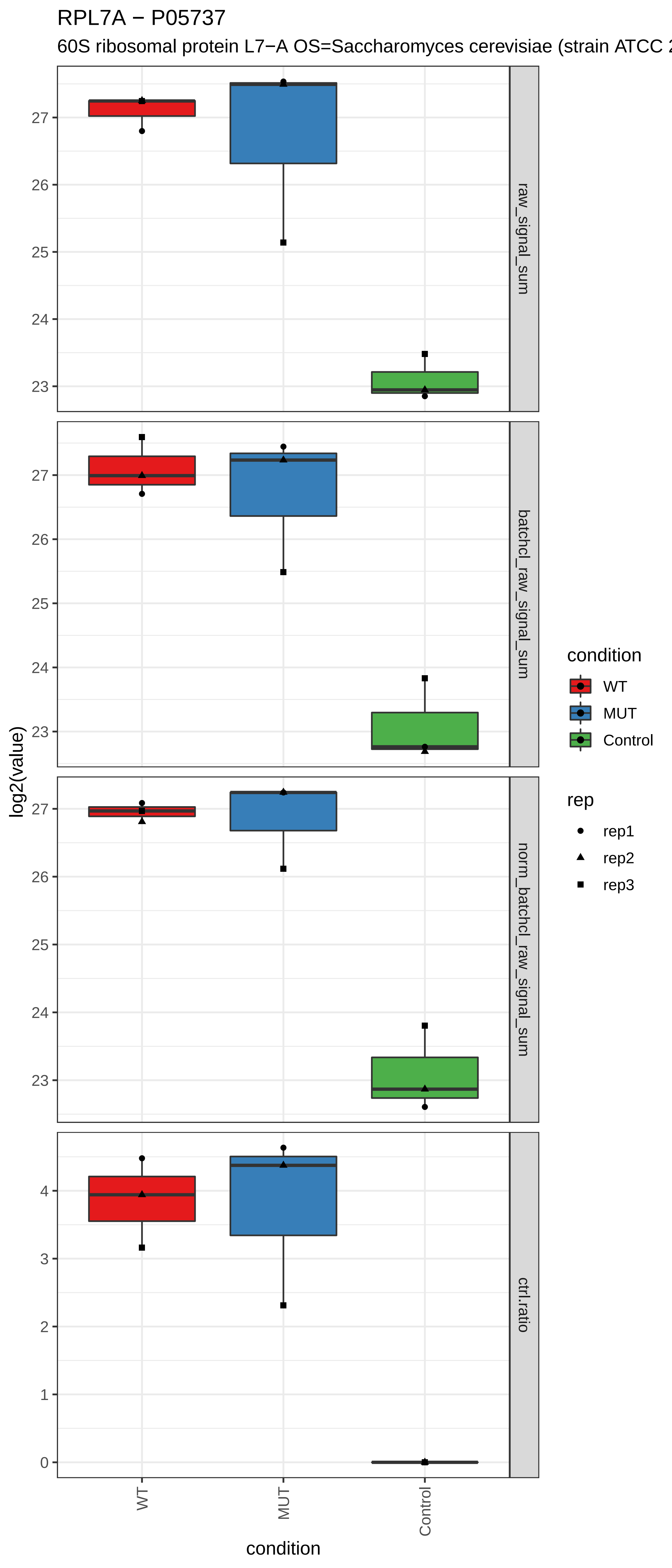

RPL7A − P05737
60S ribosomal protein L7−A OS=Saccharomyces cerevisiae (strain ATCC 2
raw_signal_sum
batchcl_raw_signal_sum
norm_batchcl_raw_signal_sum
ctrl.ratio
log2(value)
condition
WT
MUT
Control
rep
rep1
rep2
rep3

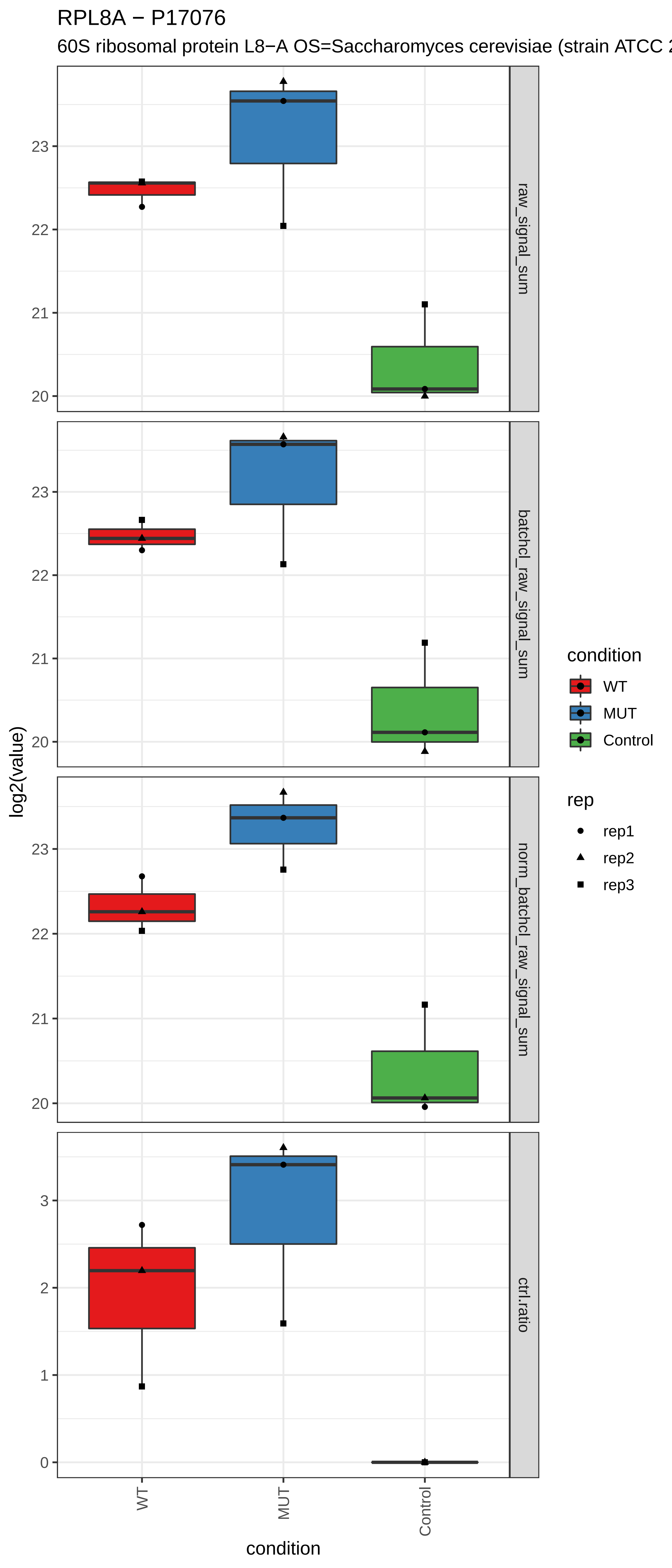

RPL8A − P17076
60S ribosomal protein L8−A OS=Saccharomyces cerevisiae (strain ATCC 2
raw_signal_sum
batchcl_raw_signal_sum
norm_batchcl_raw_signal_sum
ctrl.ratio
log2(value)
condition
WT
MUT
Control
rep
rep1
rep2
rep3

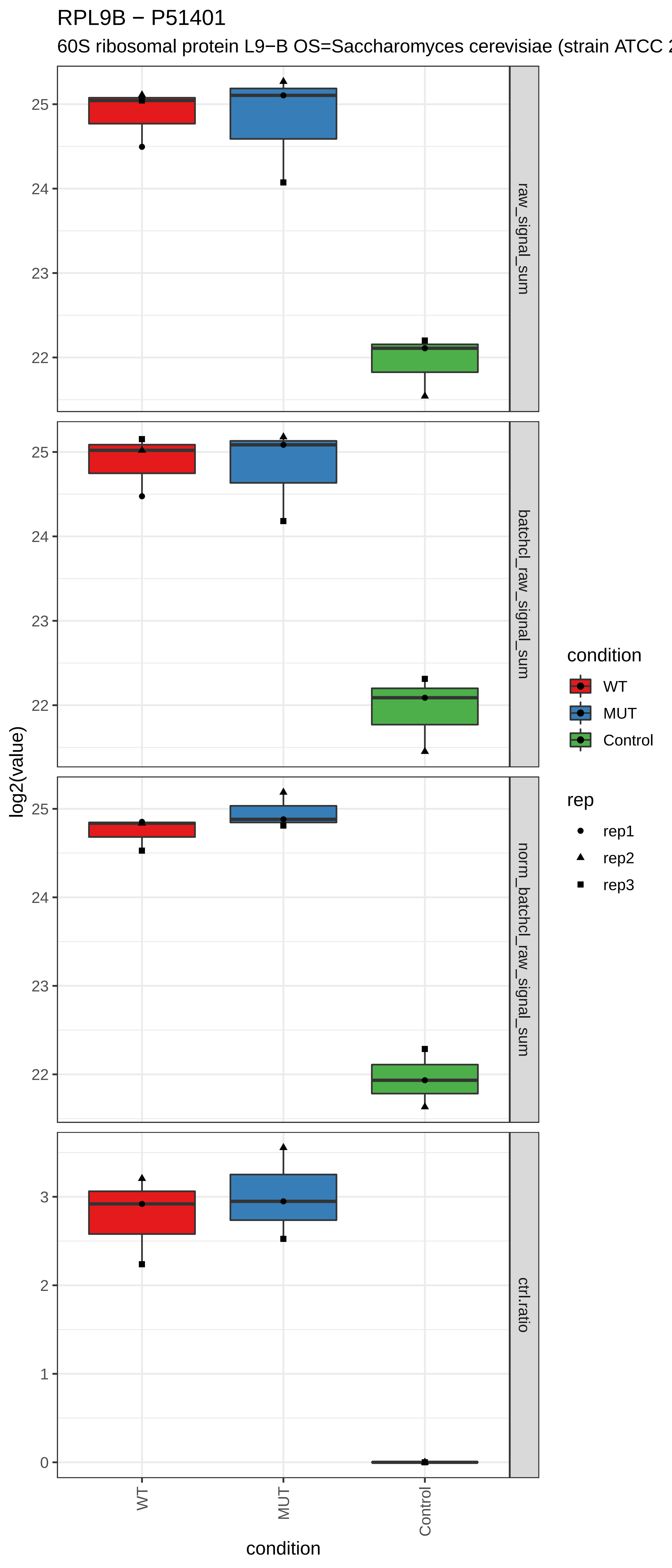
RPL9B − P51401
60S ribosomal protein L9−B OS=Saccharomyces cerevisiae (strain ATCC 2
raw_signal_sum
batchcl_raw_signal_sum
norm_batchcl_raw_signal_sum
ctrl.ratio
log2(value)
condition
WT
MUT
Control
rep
rep1
rep2
rep3
25
24
23
22
3
2
1
0

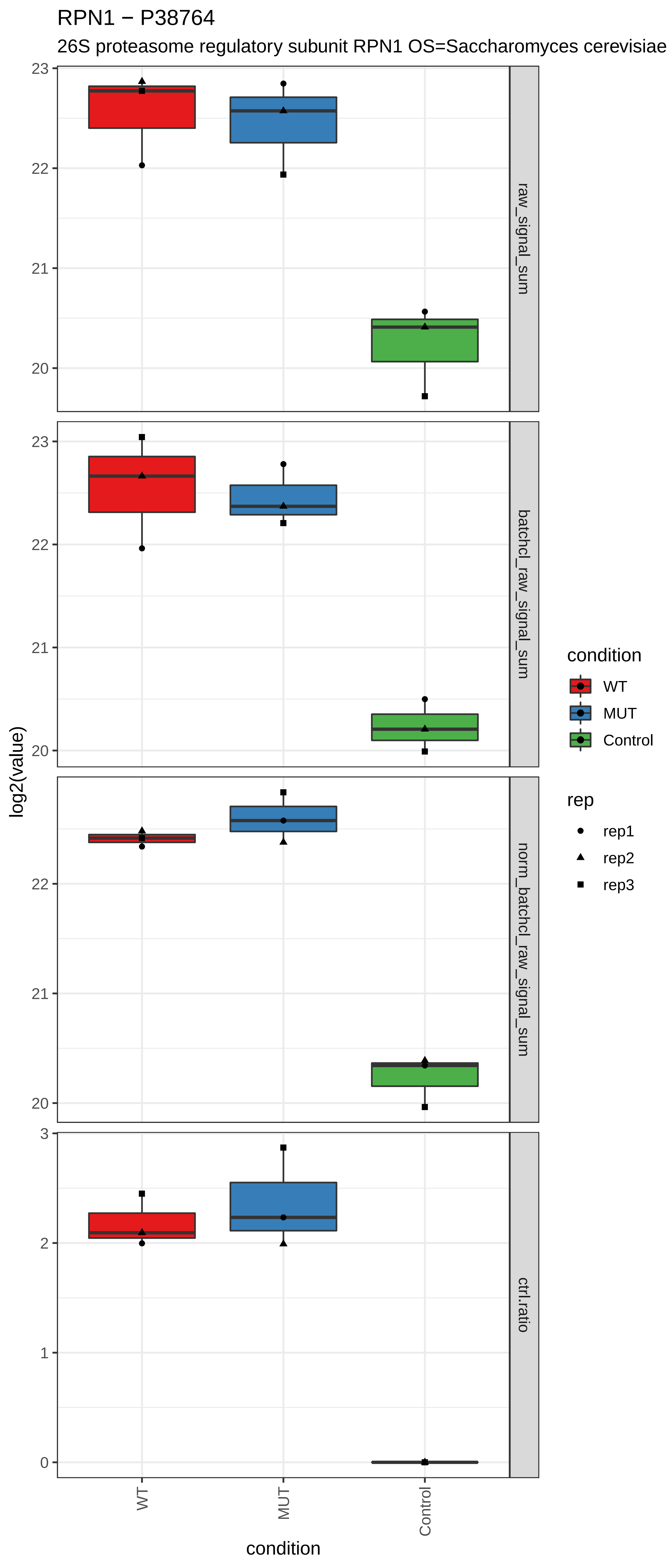
RPN1 − P38764
26S proteasome regulatory subunit RPN1 OS=Saccharomyces cerevisiae
raw_signal_sum
batchcl_raw_signal_sum
norm_batchcl_raw_signal_sum
ctrl.ratio
log2(value)
condition
WT
MUT
Control
rep
rep1
rep2
rep3
23
22
21
20
3
2
1
0

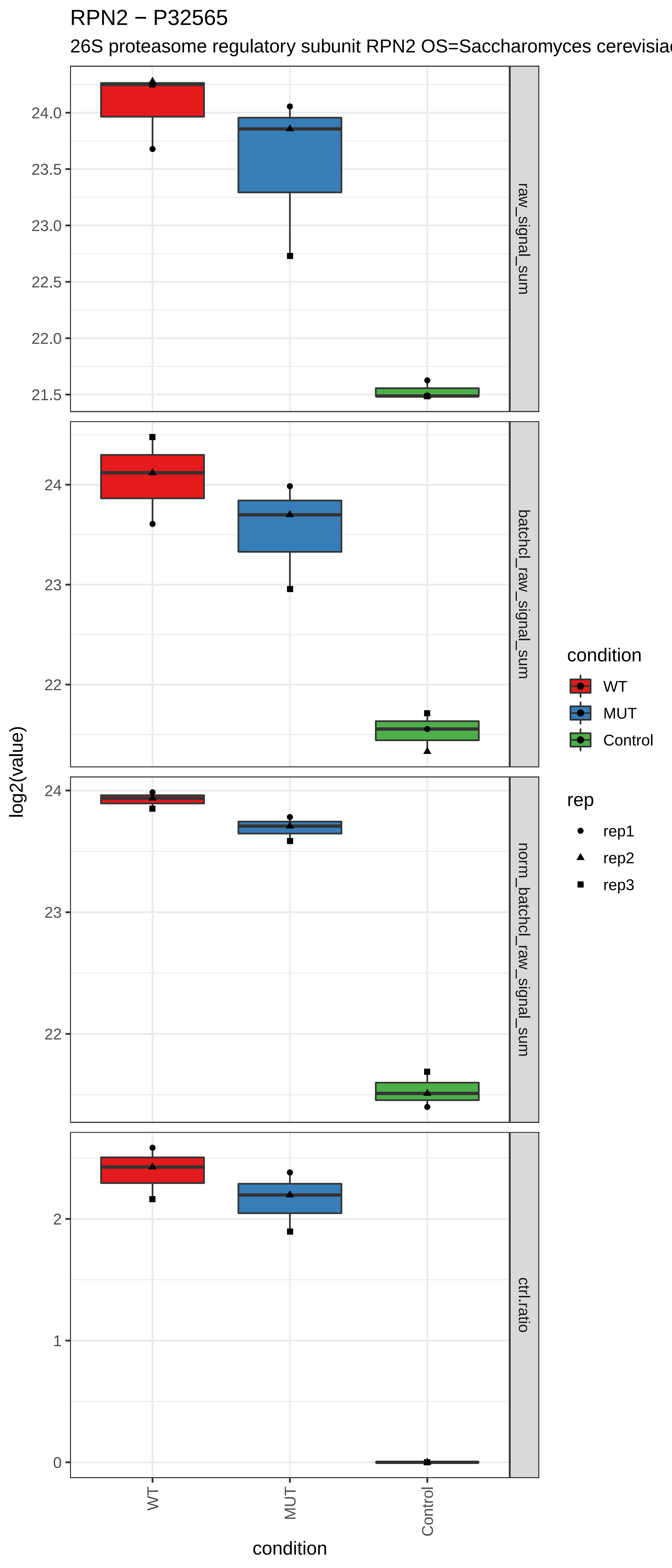
RPN2 − P32565
26S proteasome regulatory subunit RPN2 OS=Saccharomyces cerevisiae
raw_signal_sum
batchcl_raw_signal_sum
norm_batchcl_raw_signal_sum
ctrl.ratio
24.0
23.5
23.0
22.5
22.0
21.5
24
23
22
2
1
0
log2(value)
WT
MUT
Control
condition
rep
rep1
rep2
rep3

RPN3 − P40016

26S proteasome regulatory subunit RPN3 OS=Saccharomyces cerevisiae

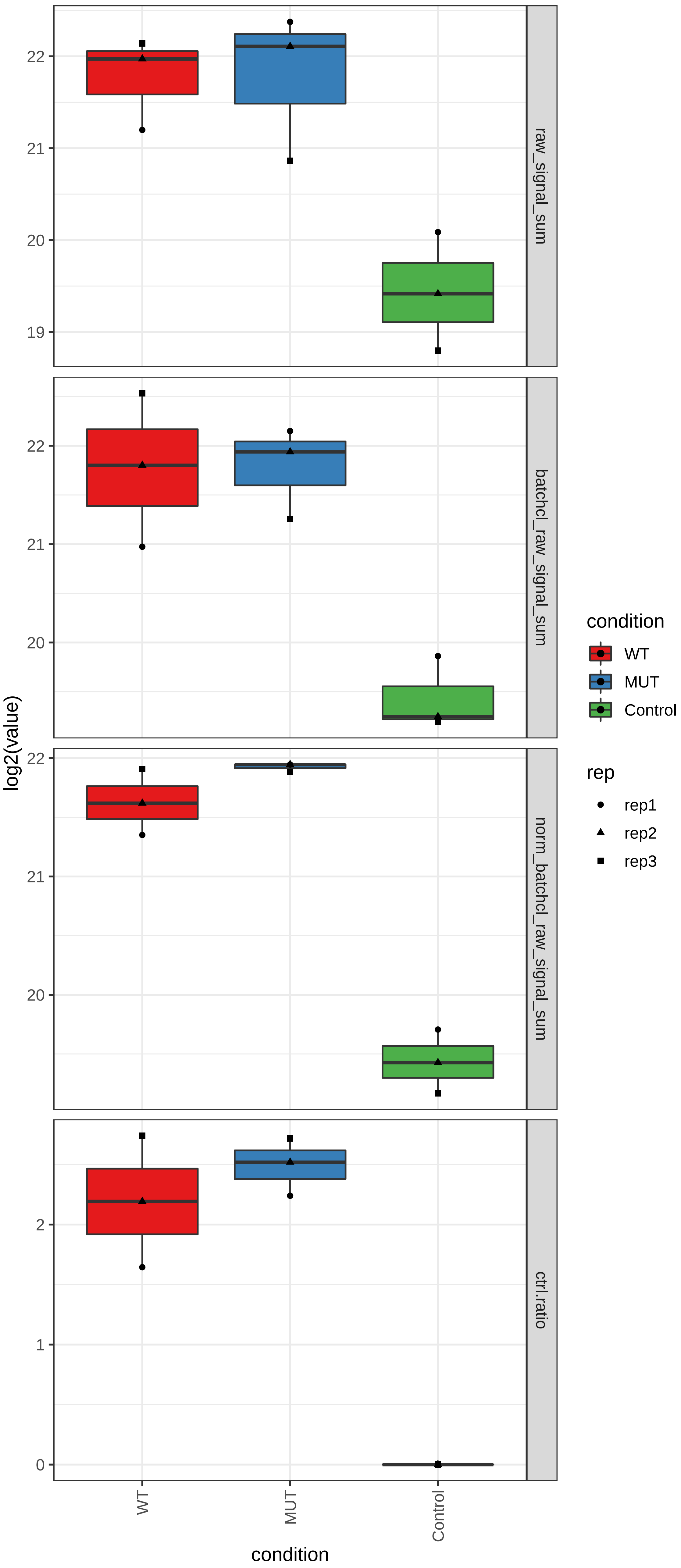

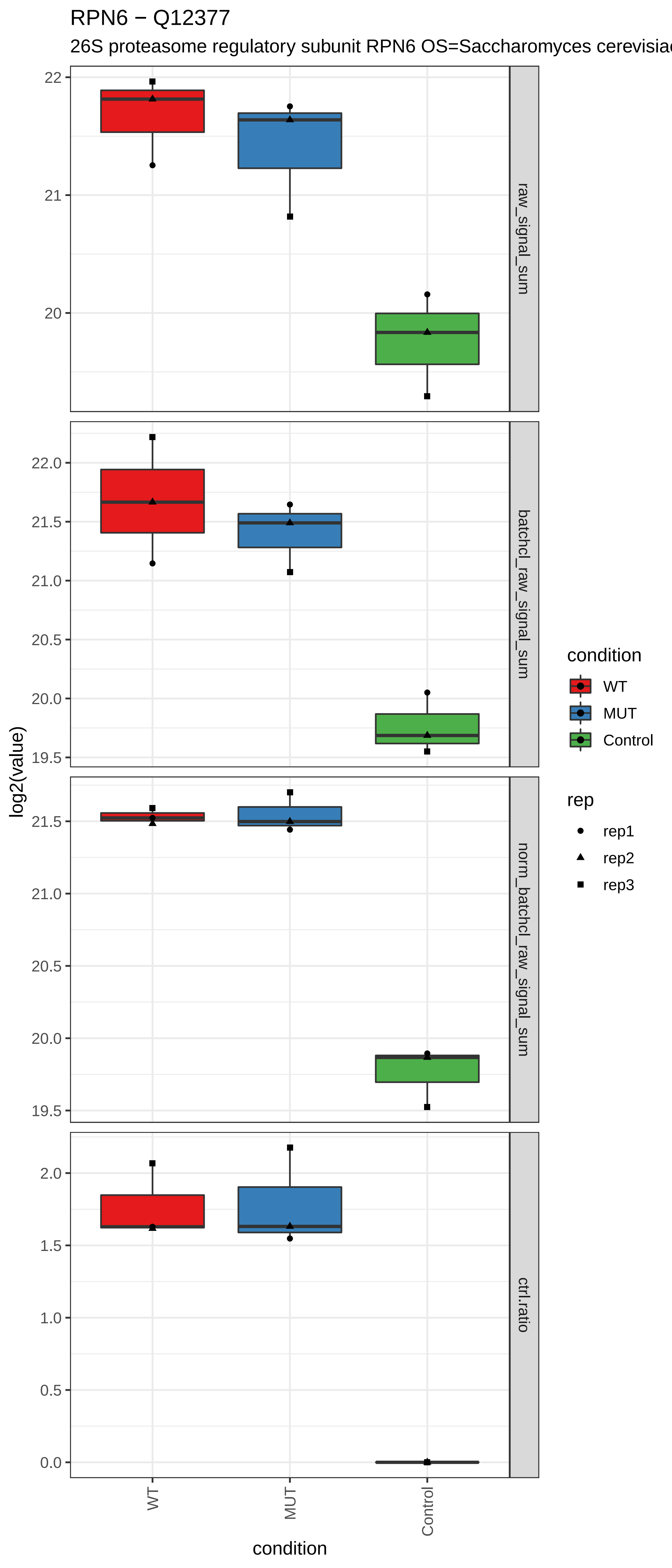
RPN6 − Q12377
26S proteasome regulatory subunit RPN6 OS=Saccharomyces cerevisiae
raw_signal_sum
batchcl_raw_signal_sum
norm_batchcl_raw_signal_sum
ctrl.ratio
log2(value)
condition
WT
MUT
Control
rep
rep1
rep2
rep3
22
21
20
22.0
21.5
21.0
20.5
20.0
19.5
2.0
1.5
1.0
0.5
0.0

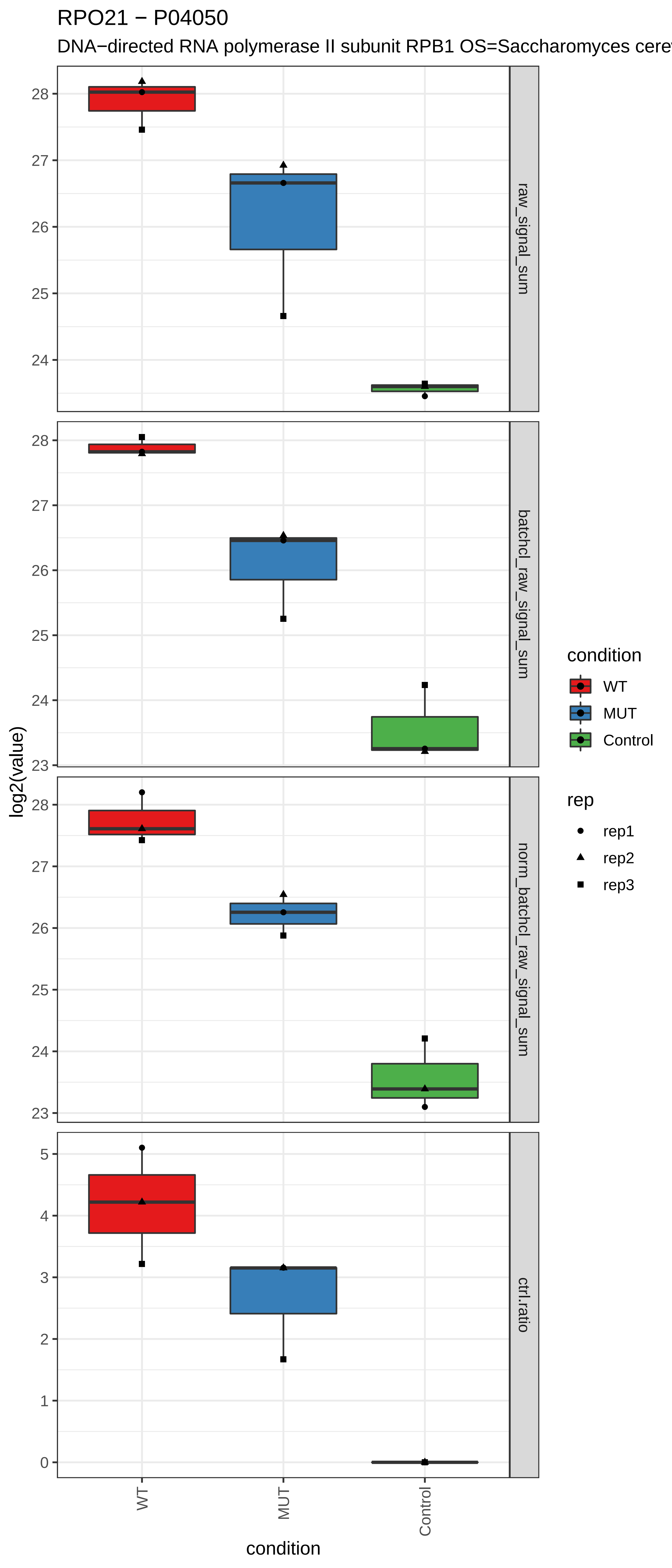

RPO21 − P04050
DNA−directed RNA polymerase II subunit RPB1 OS=Saccharomyces cere
raw_signal_sum
batchcl_raw_signal_sum
norm_batchcl_raw_signal_sum
ctrl.ratio
log2(value)
condition
WT
MUT
Control
rep
rep1
rep2
rep3

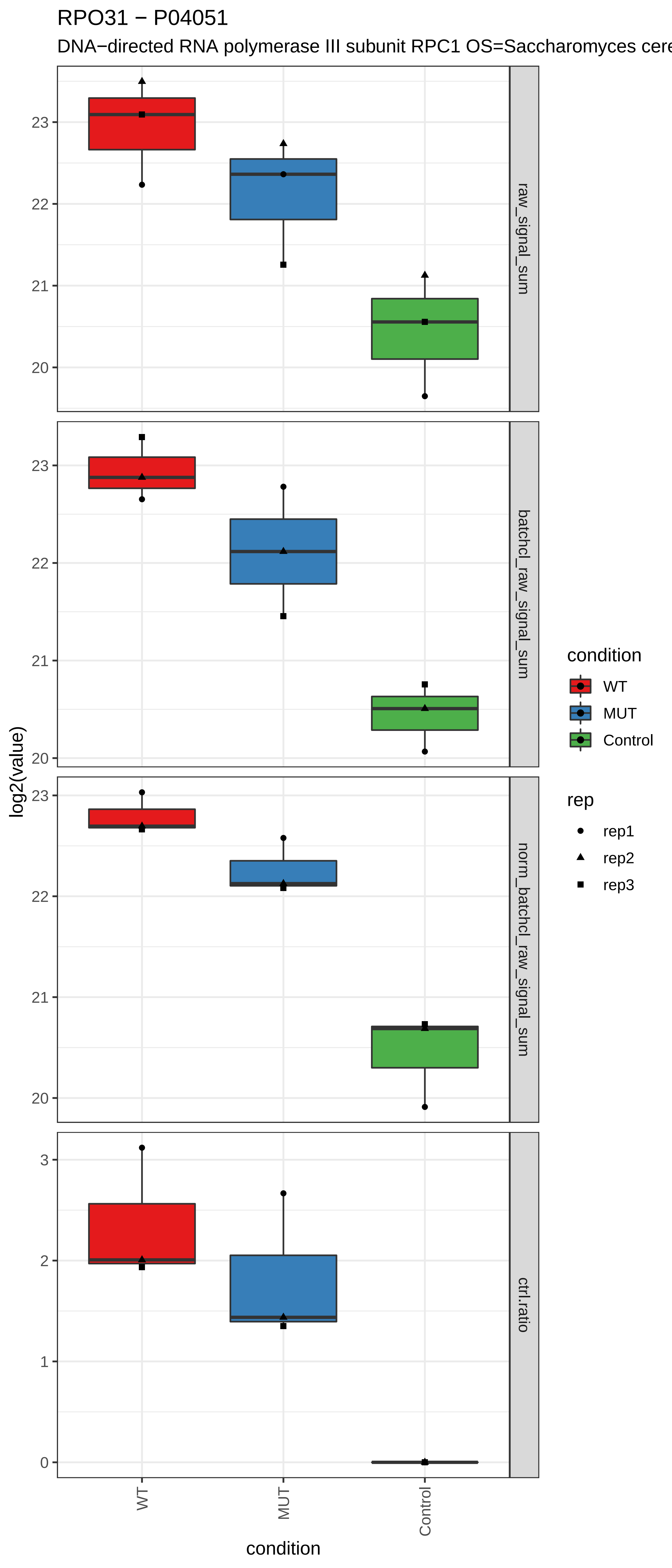

RPO31 − P04051
DNA−directed RNA polymerase III subunit RPC1 OS=Saccharomyces cere
raw_signal_sum
batchcl_raw_signal_sum
norm_batchcl_raw_signal_sum
ctrl.ratio
log2(value)
condition
WT
MUT
Control
rep
rep1
rep2
rep3

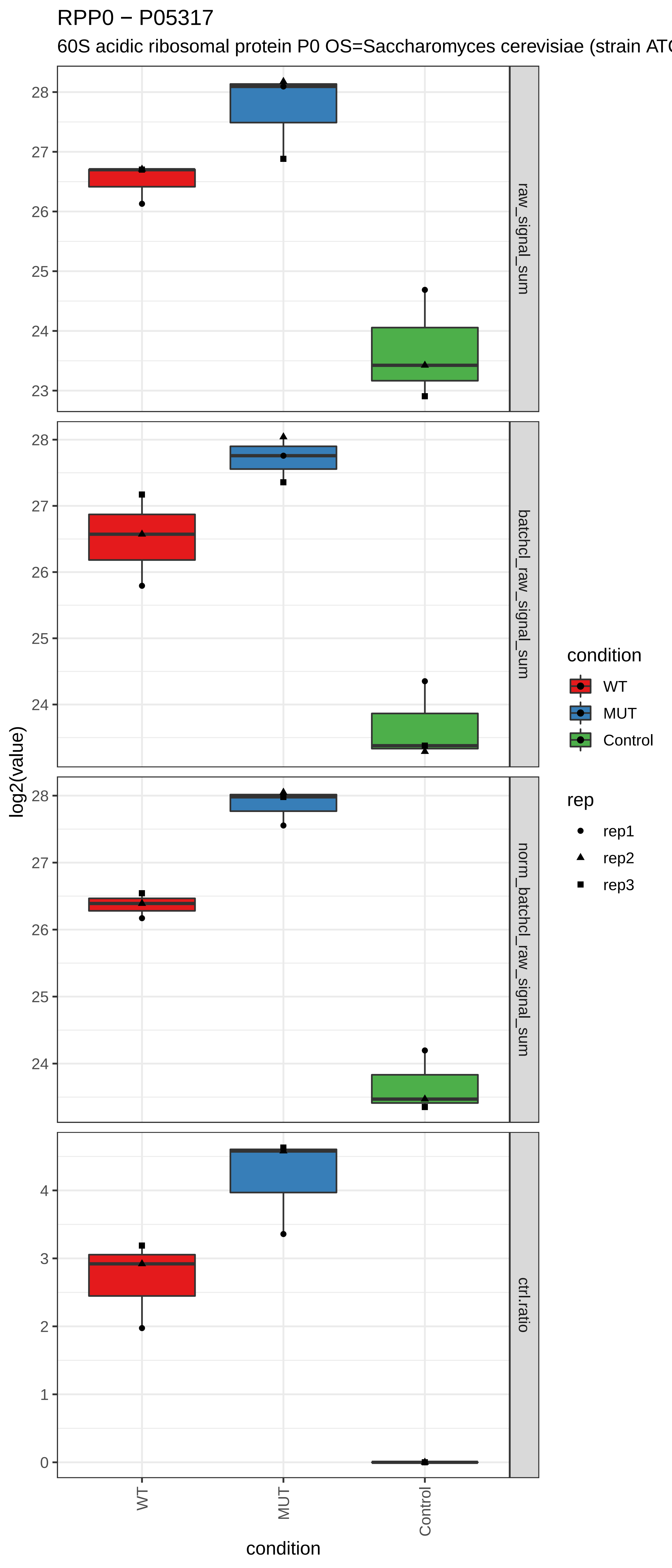

RPP0 − P05317
60S acidic ribosomal protein P0 OS=Saccharomyces cerevisiae (strain ATC
raw_signal_sum
batchcl_raw_signal_sum
norm_batchcl_raw_signal_sum
ctrl.ratio
log2(value)
condition
WT
MUT
Control
rep
rep1
rep2
rep3

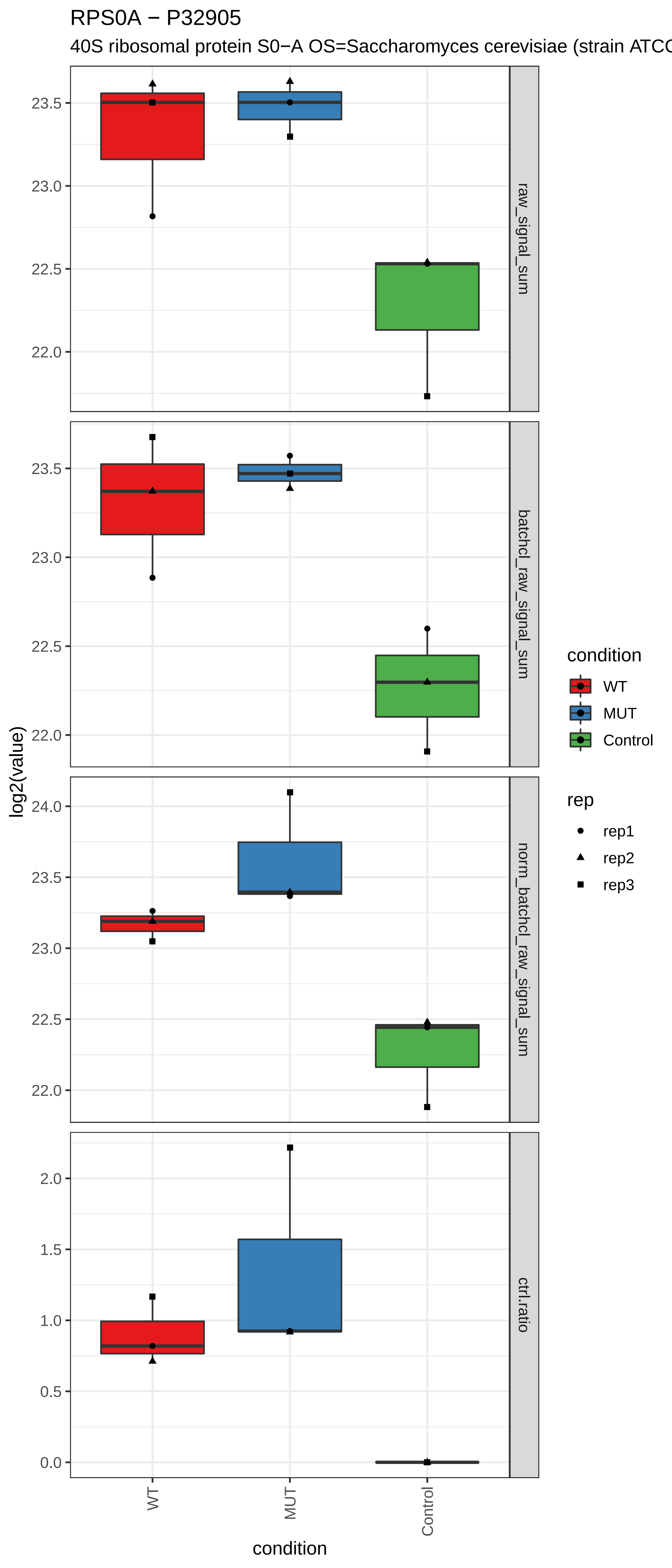

RPS0A − P32905
40S ribosomal protein S0−A OS=Saccharomyces cerevisiae (strain ATCC
raw_signal_sum
batchcl_raw_signal_sum
norm_batchcl_raw_signal_sum
ctrl.ratio
log2(value)
condition
WT
MUT
Control
rep
rep1
rep2
rep3
23.5
23.0
22.5
22.0
24.0
2.0
1.5
1.0
0.5
0.0

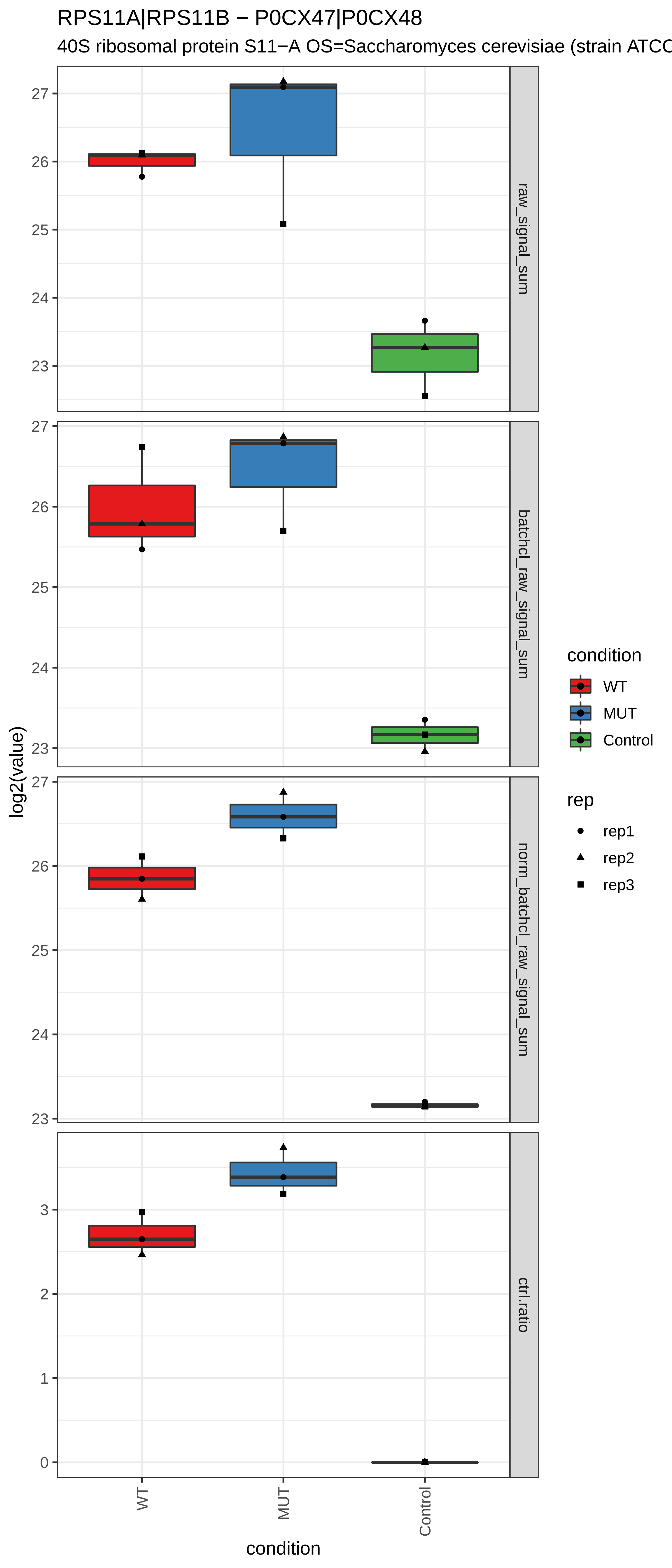
RPS11A|RPS11B − P0CX47|P0CX48
40S ribosomal protein S11−A OS=Saccharomyces cerevisiae (strain ATCC
raw_signal_sum
batchcl_raw_signal_sum
norm_batchcl_raw_signal_sum
ctrl.ratio
log2(value)
condition
WT
MUT
Control
rep
rep1
rep2
rep3

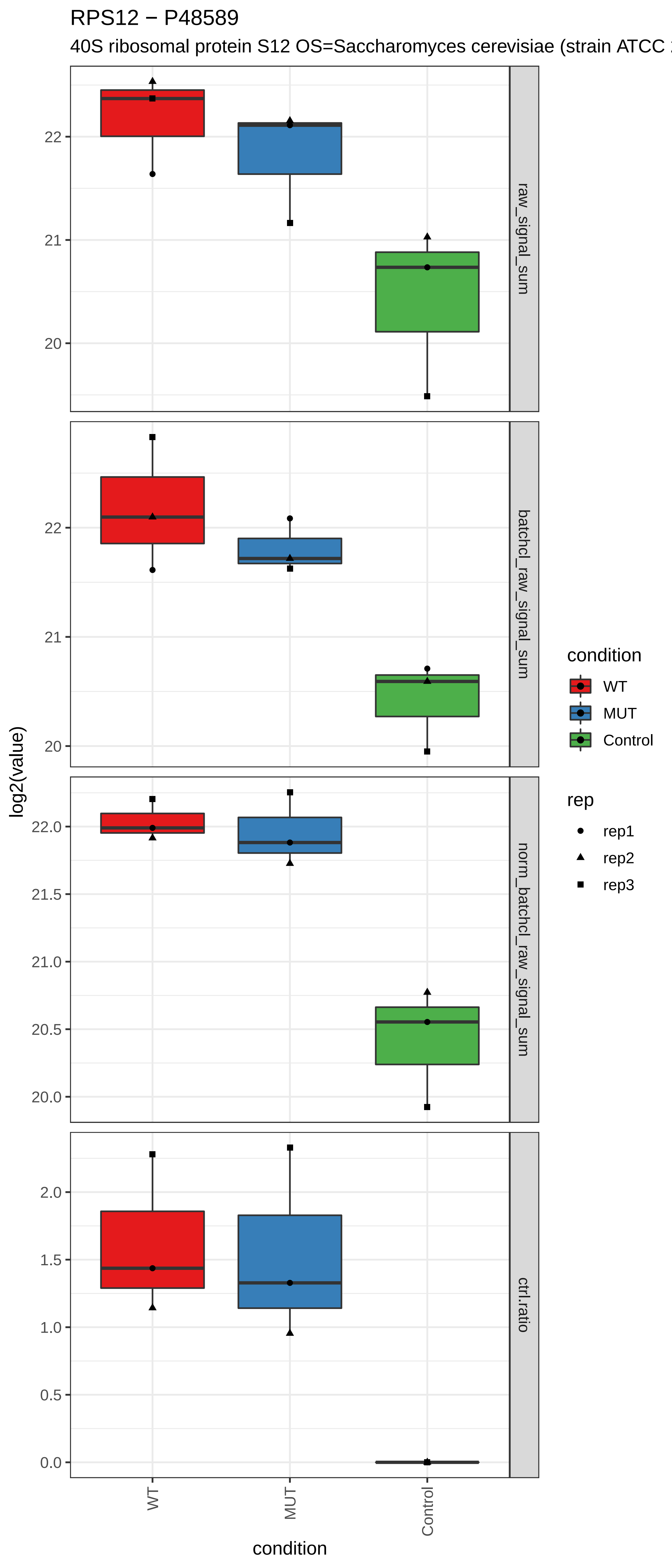

RPS12 − P48589
40S ribosomal protein S12 OS=Saccharomyces cerevisiae (strain ATCC
raw_signal_sum
batchcl_raw_signal_sum
norm_batchcl_raw_signal_sum
ctrl.ratio
log2(value)
condition
WT
MUT
Control
rep
rep1
rep2
rep3
22
21
20
22.0
21.5
21.0
20.5
20.0
2.0
1.5
1.0
0.5
0.0

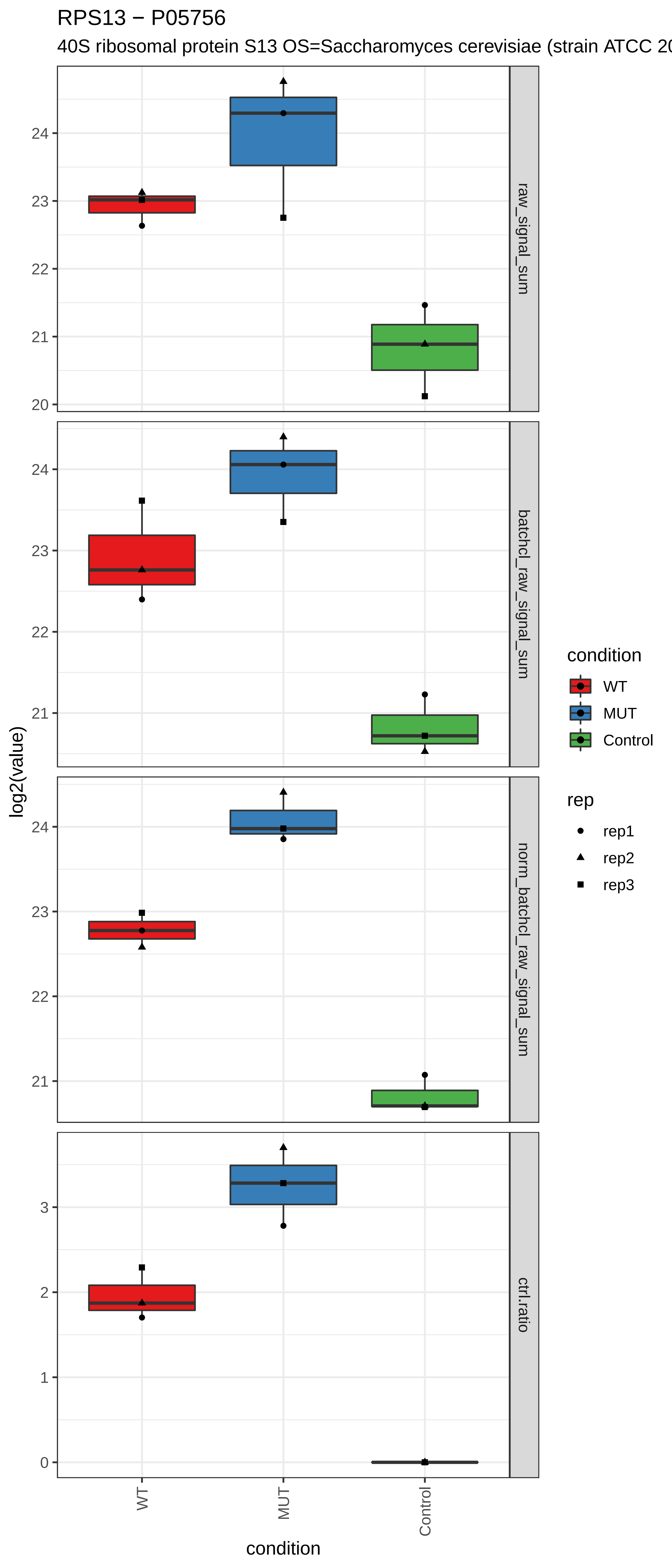

RPS13 − P05756
40S ribosomal protein S13 OS=Saccharomyces cerevisiae (strain ATCC 20
raw_signal_sum
batchcl_raw_signal_sum
norm_batchcl_raw_signal_sum
ctrl.ratio
log2(value)
condition
WT
MUT
Control
rep
rep1
rep2
rep3

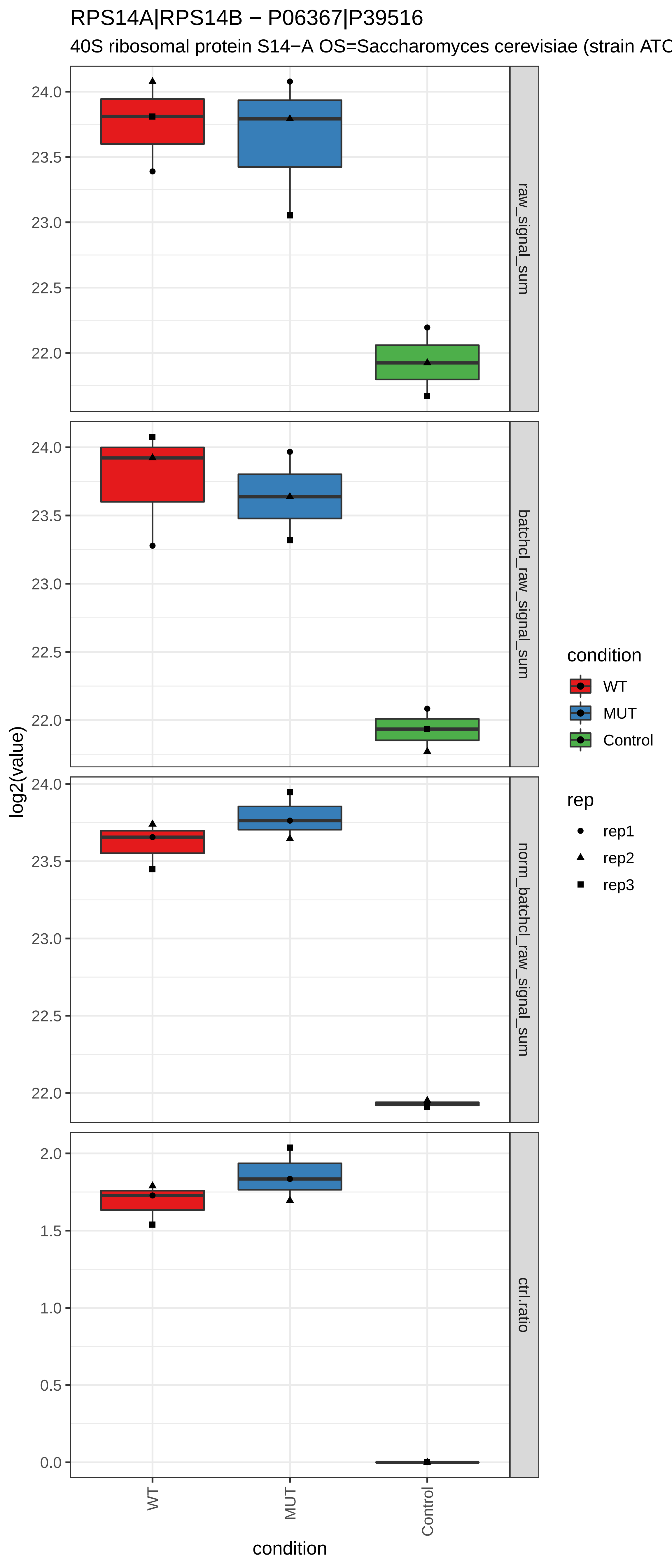

RPS14A|RPS14B − P06367|P39516
40S ribosomal protein S14−A OS=Saccharomyces cerevisiae (strain ATC
raw_signal_sum
batchcl_raw_signal_sum
norm_batchcl_raw_signal_sum
ctrl.ratio
log2(value)
condition
WT
MUT
Control
rep
rep1
rep2
rep3

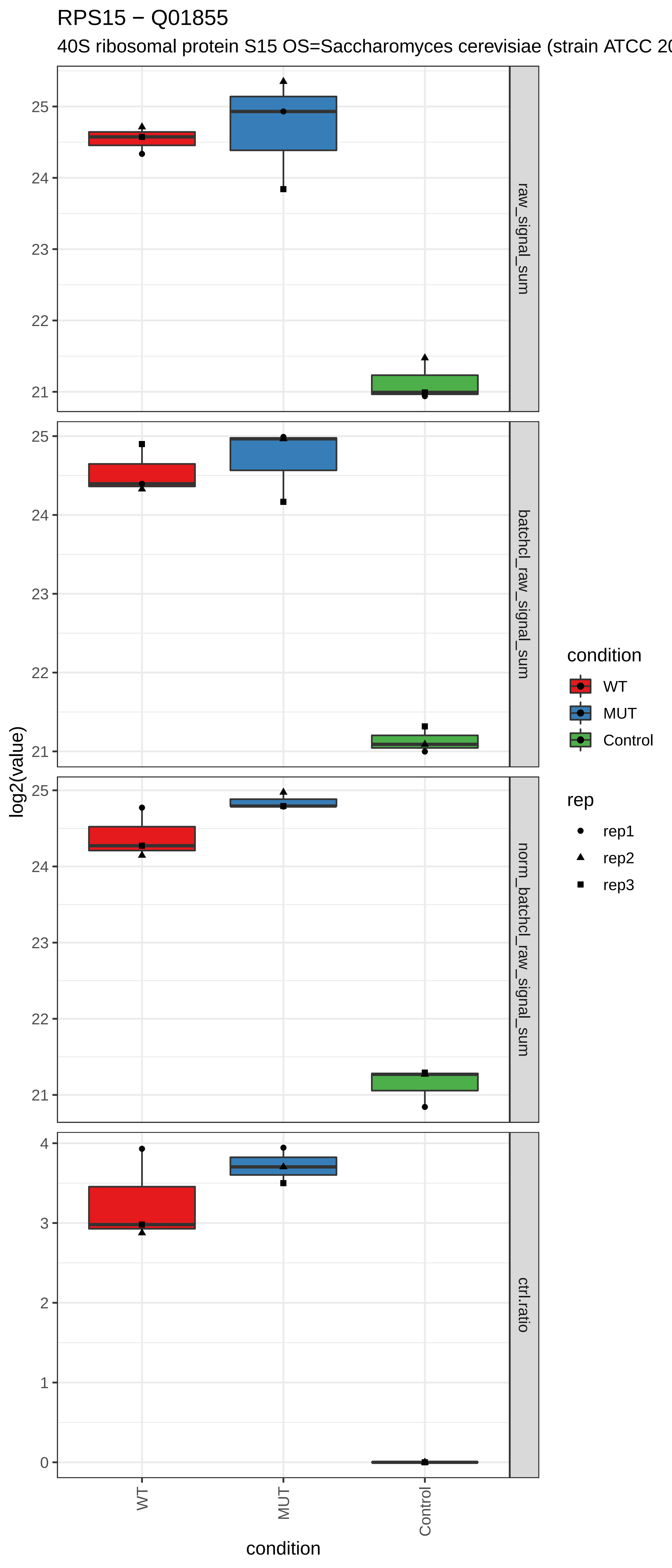

RPS15 − Q01855
40S ribosomal protein S15 OS=Saccharomyces cerevisiae (strain ATCC 20
raw_signal_sum
batchcl_raw_signal_sum
norm_batchcl_raw_signal_sum
ctrl.ratio
log2(value)
condition
WT
MUT
Control
rep
rep1
rep2
rep3

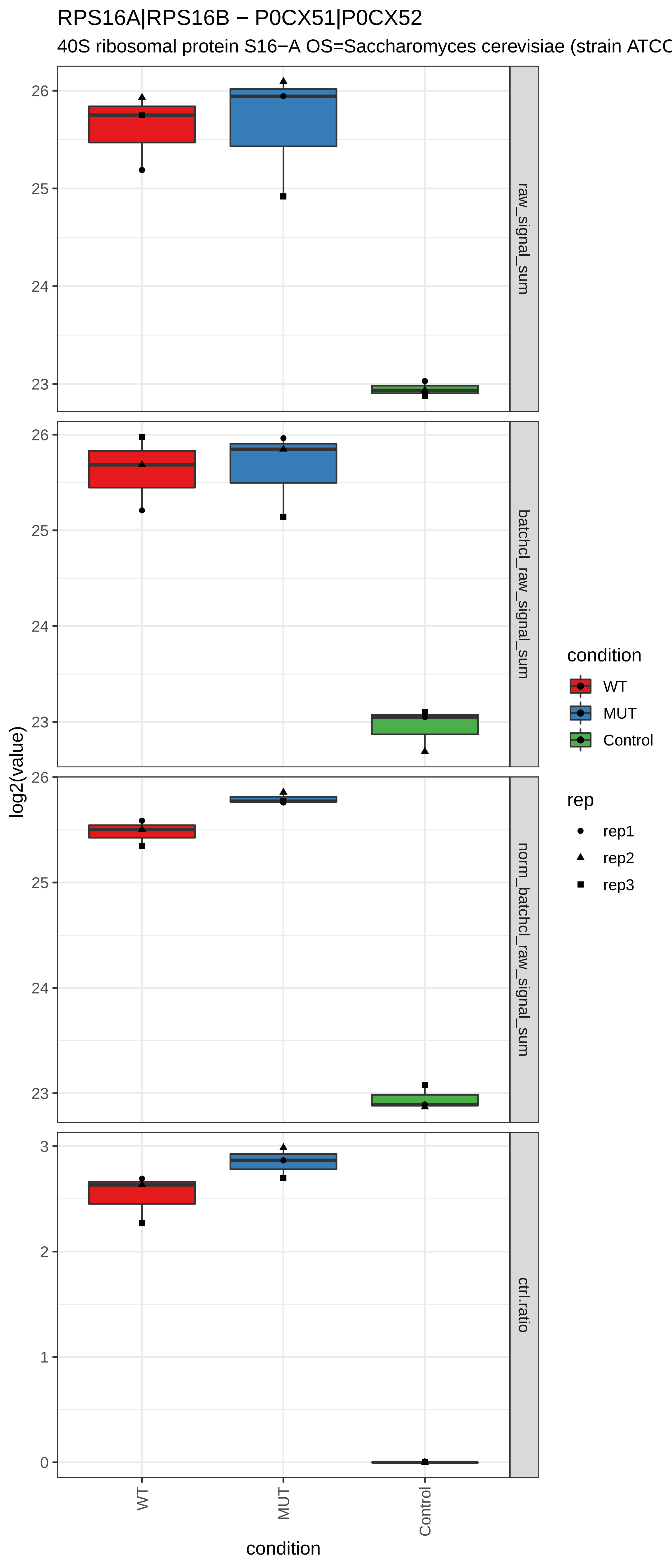

RPS16A|RPS16B − P0CX51|P0CX52
40S ribosomal protein S16−A OS=Saccharomyces cerevisiae (strain ATCC
raw_signal_sum
batchcl_raw_signal_sum
norm_batchcl_raw_signal_sum
ctrl.ratio
log2(value)
condition
WT
MUT
Control
rep
rep1
rep2
rep3
26
25
24
23
3
2
1
0

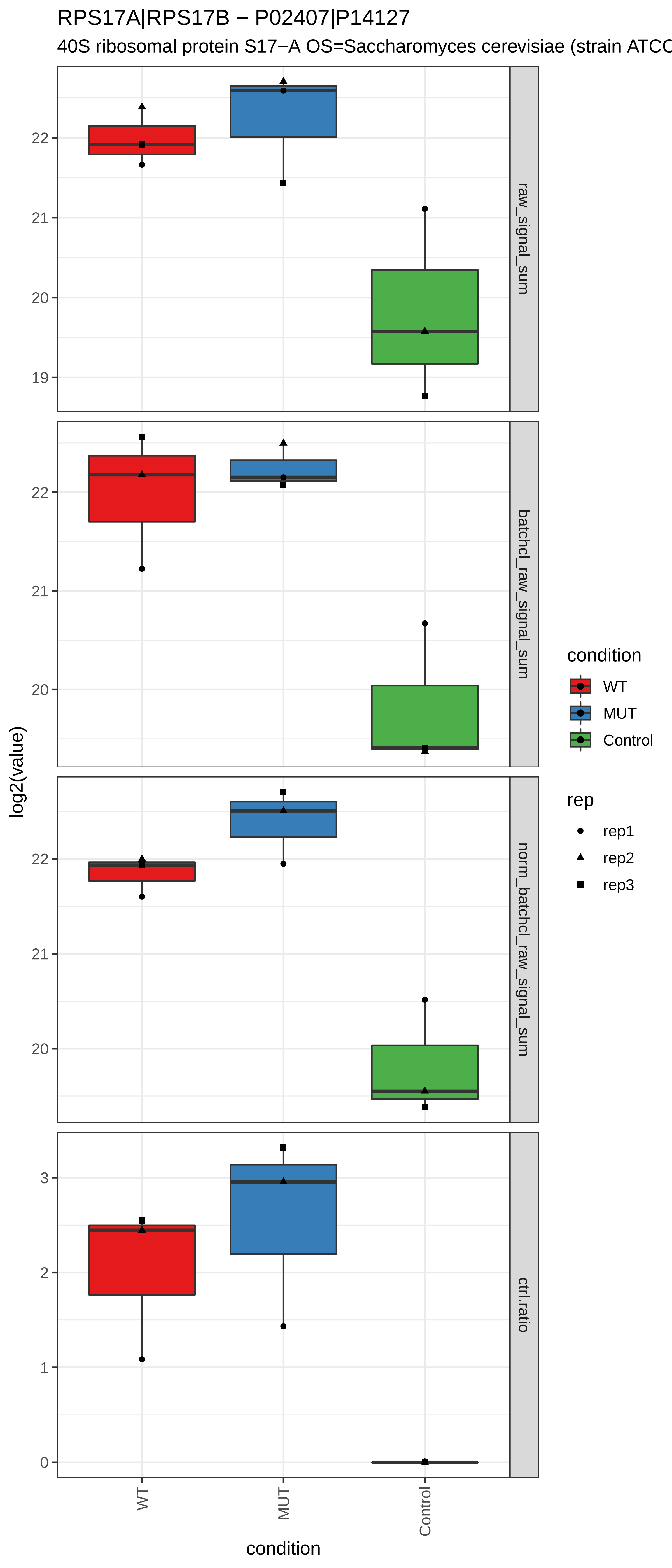

RPS17A|RPS17B − P02407|P14127
40S ribosomal protein S17−A OS=Saccharomyces cerevisiae (strain ATCC
raw_signal_sum
batchcl_raw_signal_sum
norm_batchcl_raw_signal_sum
ctrl.ratio
log2(value)
condition
WT
MUT
Control
rep
rep1
rep2
rep3

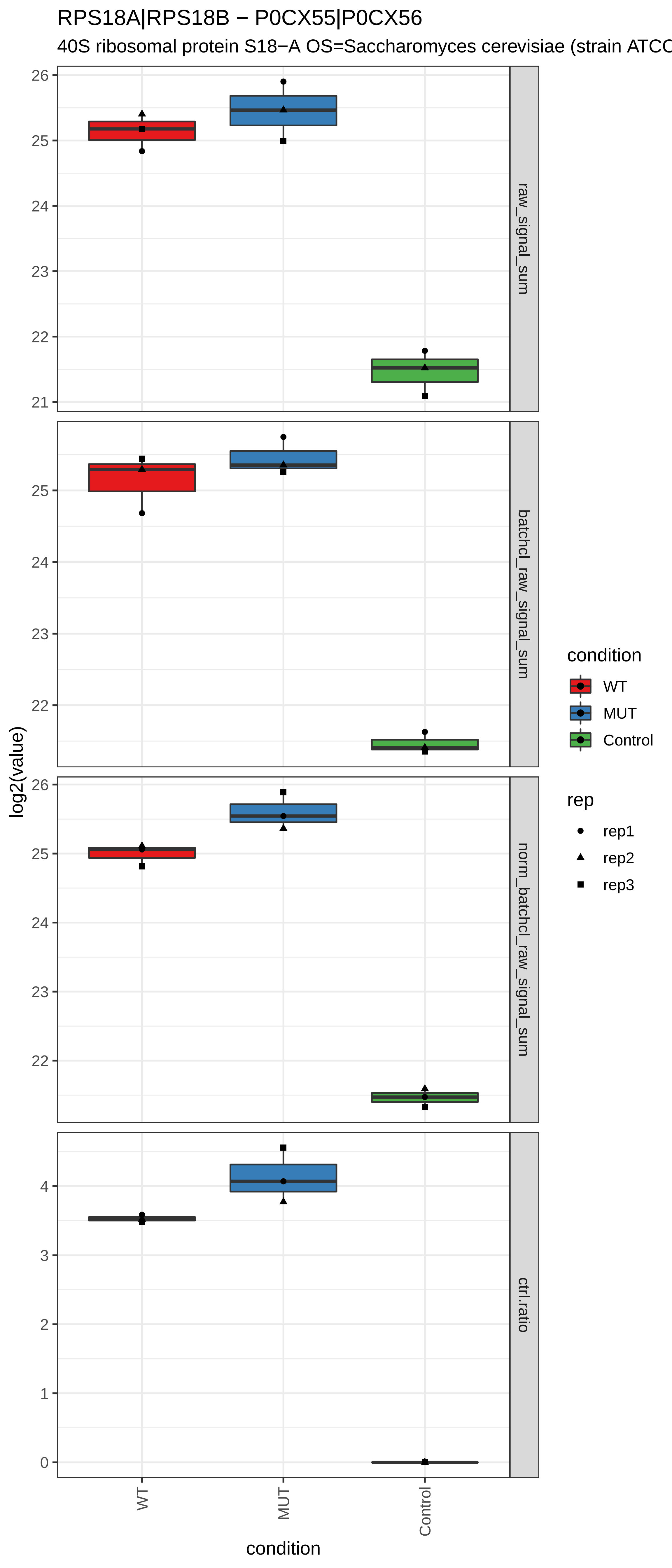
RPS18A|RPS18B − P0CX55|P0CX56
40S ribosomal protein S18−A OS=Saccharomyces cerevisiae (strain ATCC
raw_signal_sum
batchcl_raw_signal_sum
norm_batchcl_raw_signal_sum
ctrl.ratio
log2(value)
condition
WT
MUT
Control
rep
rep1
rep2
rep3
26
25
24
23
22
21
4
3
2
1
0

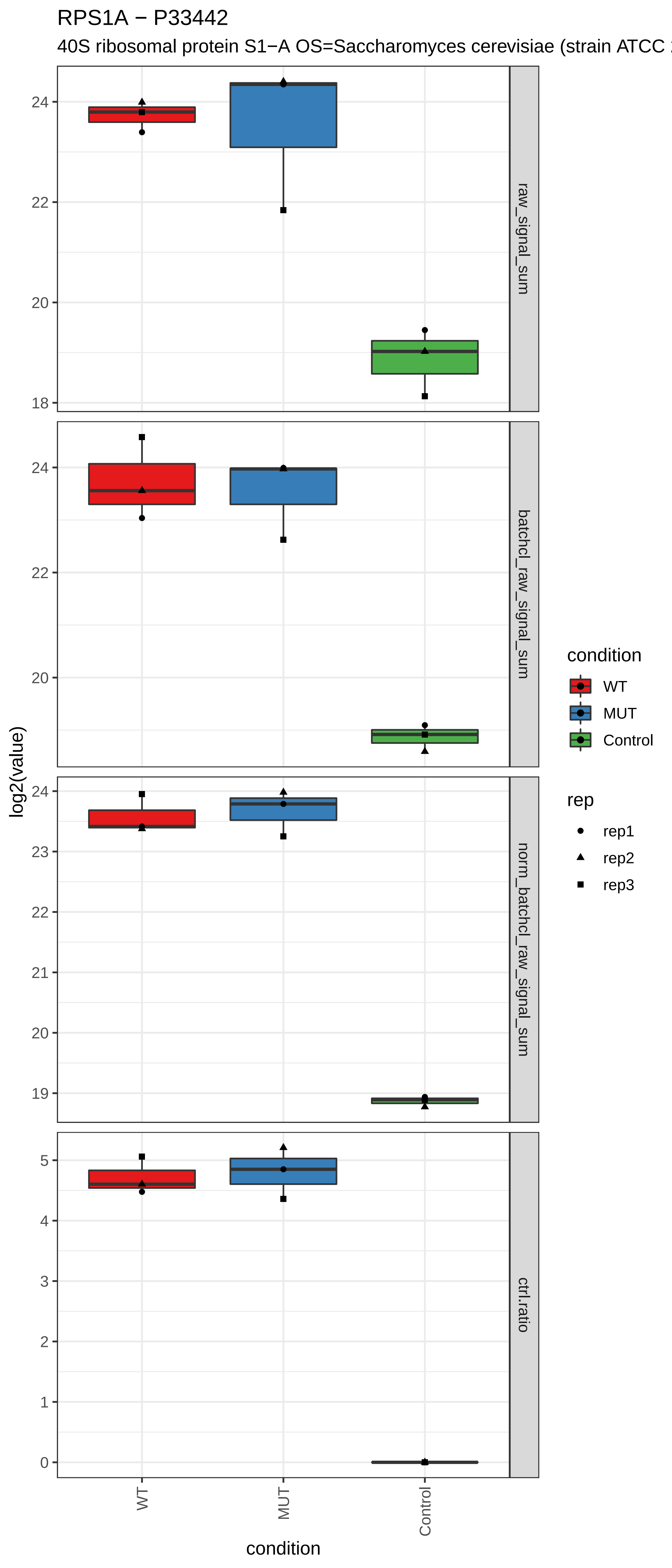
RPS1A − P33442
40S ribosomal protein S1−A OS=Saccharomyces cerevisiae (strain ATCC
raw_signal_sum
batchcl_raw_signal_sum
norm_batchcl_raw_signal_sum
ctrl.ratio
log2(value)
condition
WT
MUT
Control
rep
rep1
rep2
rep3

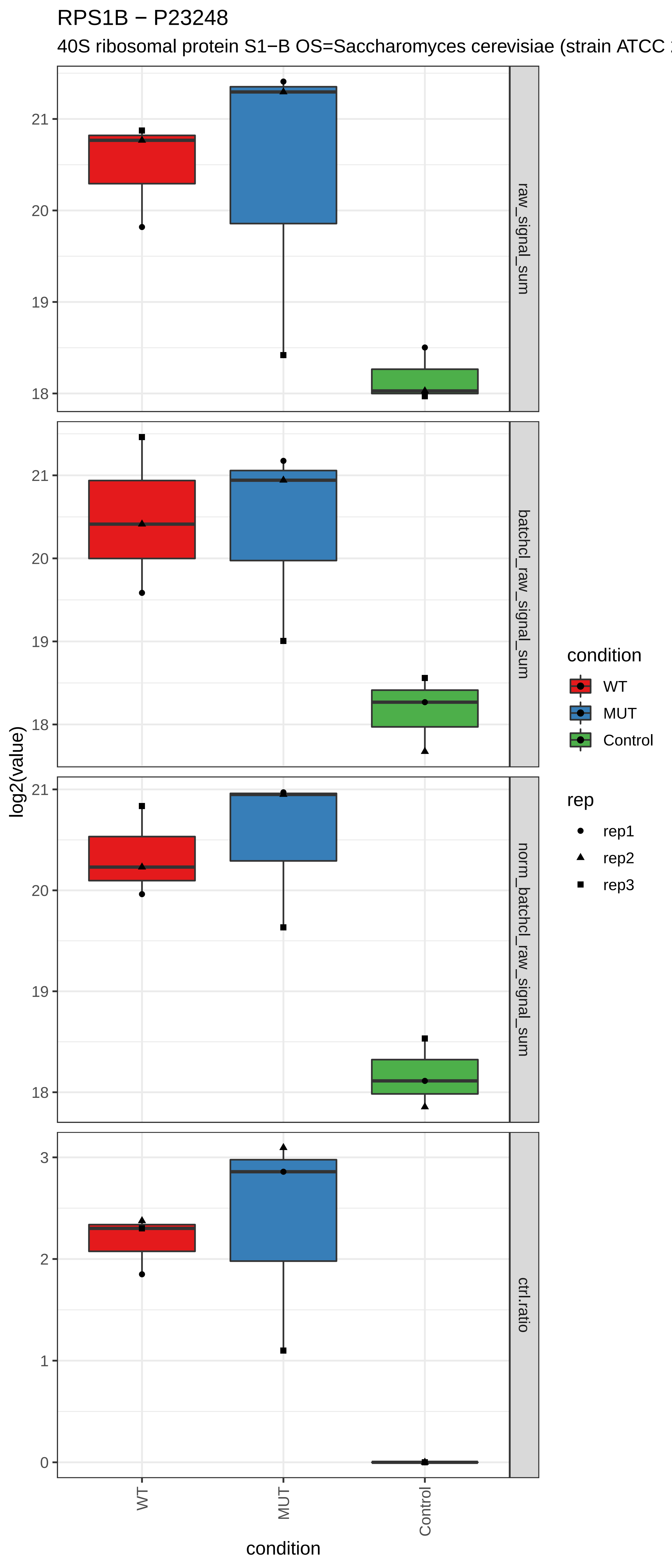

RPS1B − P23248
40S ribosomal protein S1−B OS=Saccharomyces cerevisiae (strain ATCC
raw_signal_sum
batchcl_raw_signal_sum
norm_batchcl_raw_signal_sum
ctrl.ratio
log2(value)
condition
WT
MUT
Control
rep
rep1
rep2
rep3
21
20
19
18
3
2
1
0

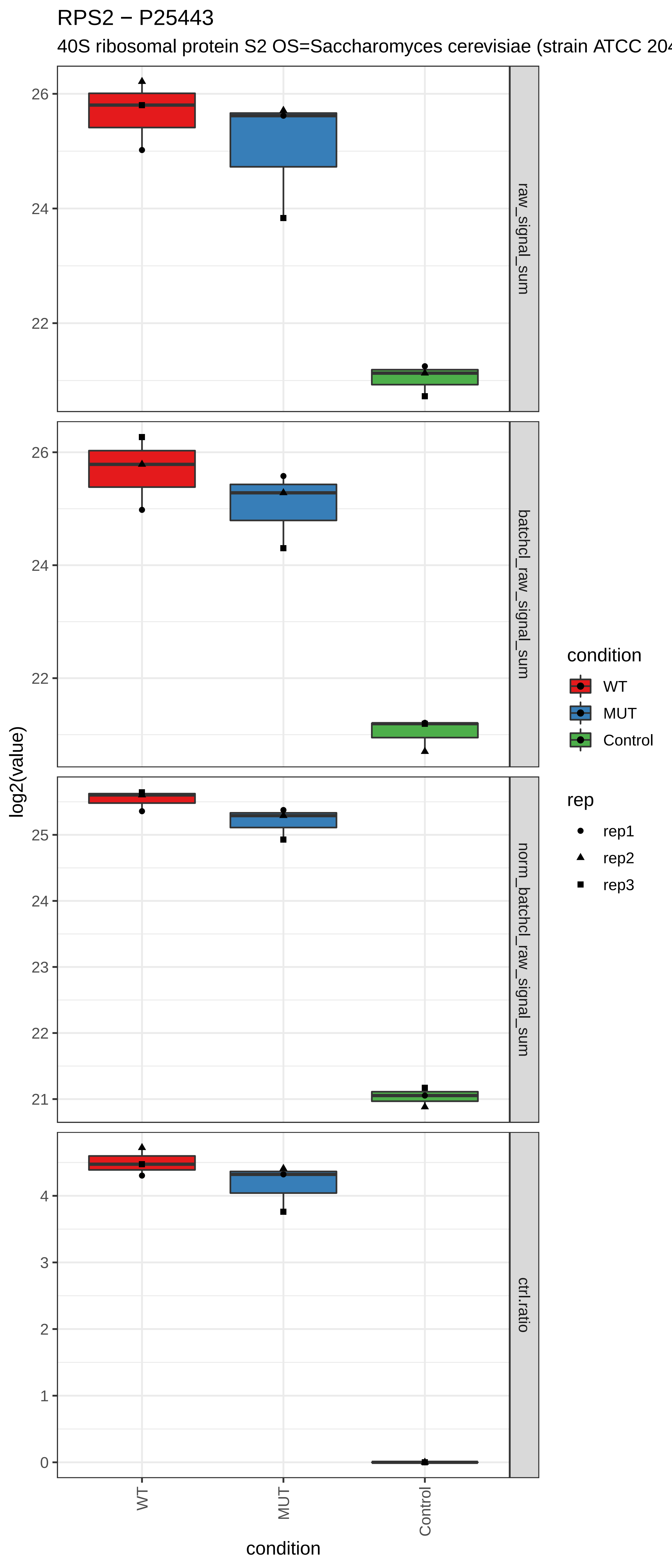

RPS2 − P25443
40S ribosomal protein S2 OS=Saccharomyces cerevisiae (strain ATCC 204
raw_signal_sum
batchcl_raw_signal_sum
norm_batchcl_raw_signal_sum
ctrl.ratio
log2(value)
condition
WT
MUT
Control
rep
rep1
rep2
rep3

RPS20 − P38701

40S ribosomal protein S20 OS=Saccharomyces cerevisiae (strain ATCC 20

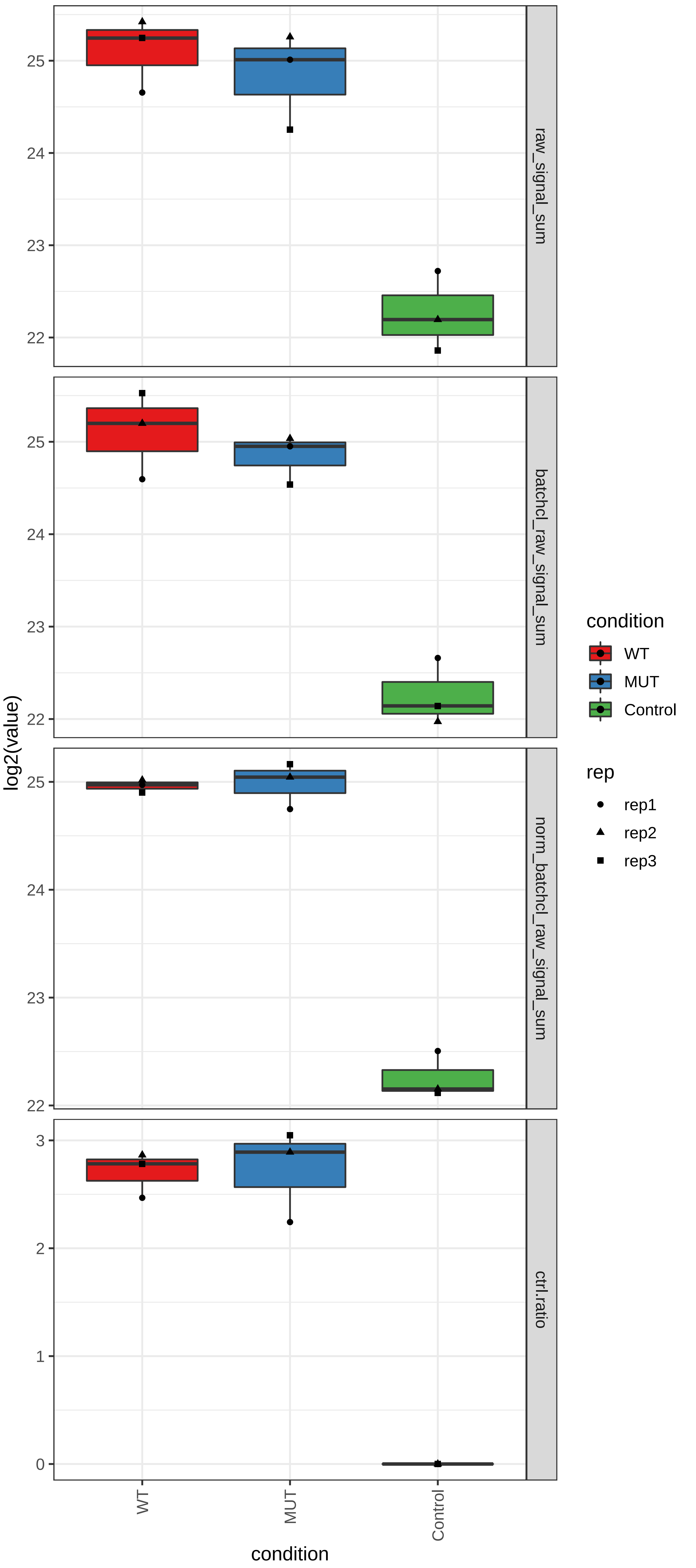

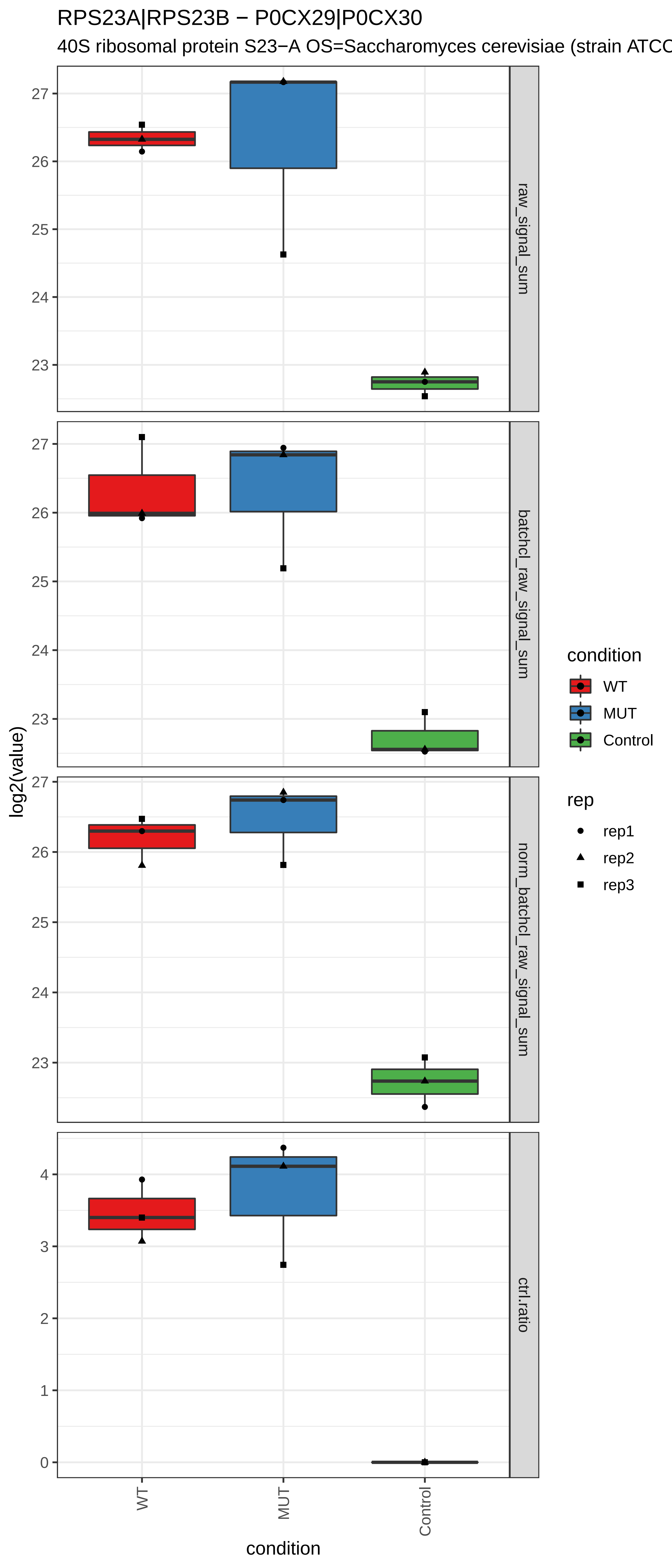
RPS23A|RPS23B − P0CX29|P0CX30
40S ribosomal protein S23−A OS=Saccharomyces cerevisiae (strain ATCC
raw_signal_sum
batchcl_raw_signal_sum
norm_batchcl_raw_signal_sum
ctrl.ratio
log2(value)
condition
WT
MUT
Control
rep
rep1
rep2
rep3

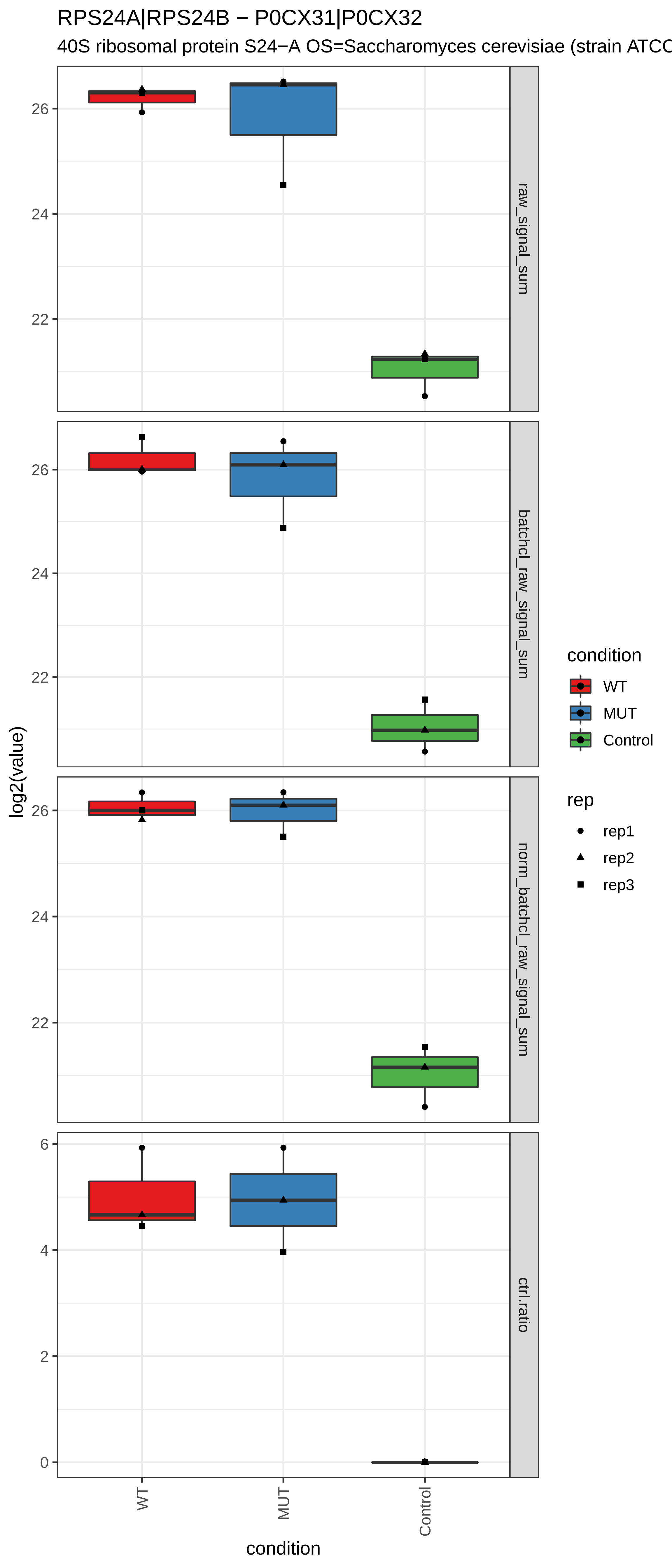

RPS24A|RPS24B − P0CX31|P0CX32
40S ribosomal protein S24−A OS=Saccharomyces cerevisiae (strain ATCC
raw_signal_sum
batchcl_raw_signal_sum
norm_batchcl_raw_signal_sum
ctrl.ratio
log2(value)
condition
WT
MUT
Control
rep
rep1
rep2
rep3
26
24
22
6
4
2
0

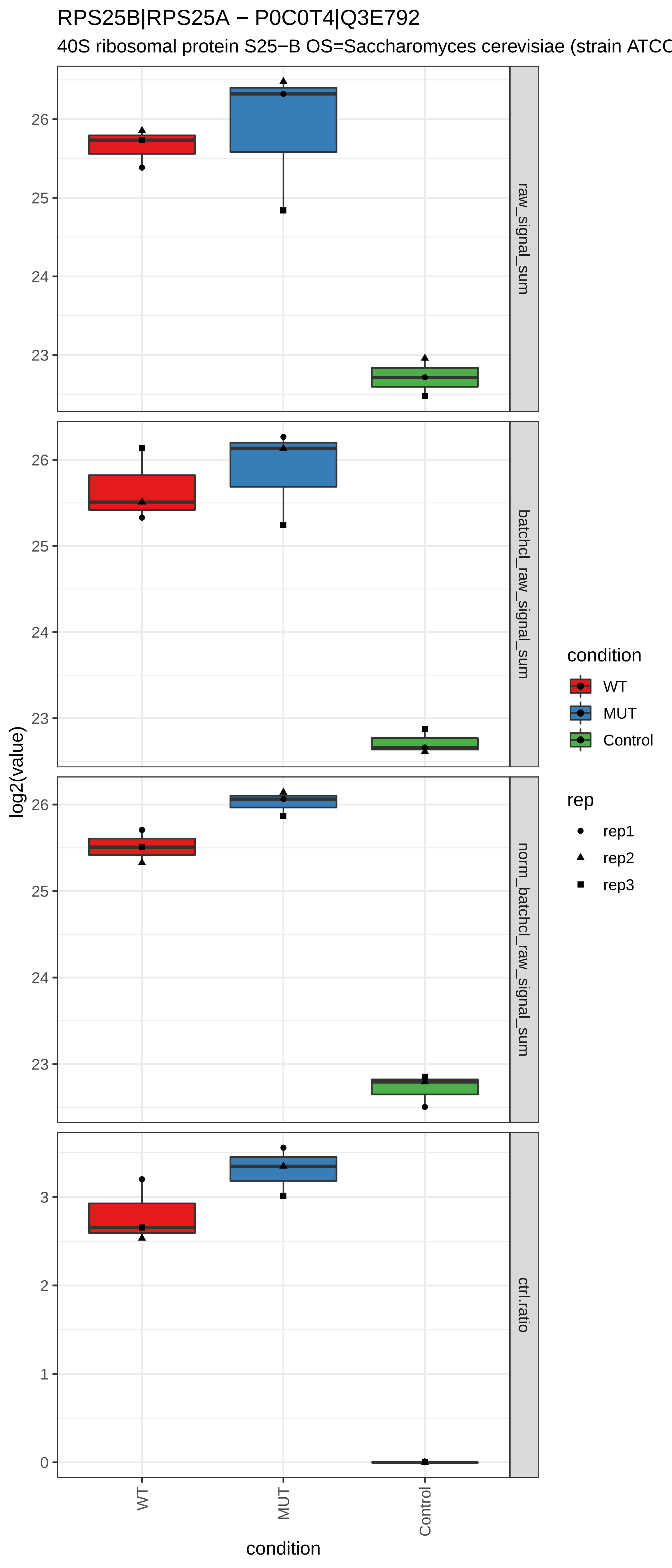

RPS25B|RPS25A − P0C0T4|Q3E792
40S ribosomal protein S25−B OS=Saccharomyces cerevisiae (strain ATCC
raw_signal_sum
batchcl_raw_signal_sum
norm_batchcl_raw_signal_sum
ctrl.ratio
log2(value)
condition
WT
MUT
Control
rep
rep1
rep2
rep3
26
25
24
23
3
2
1
0

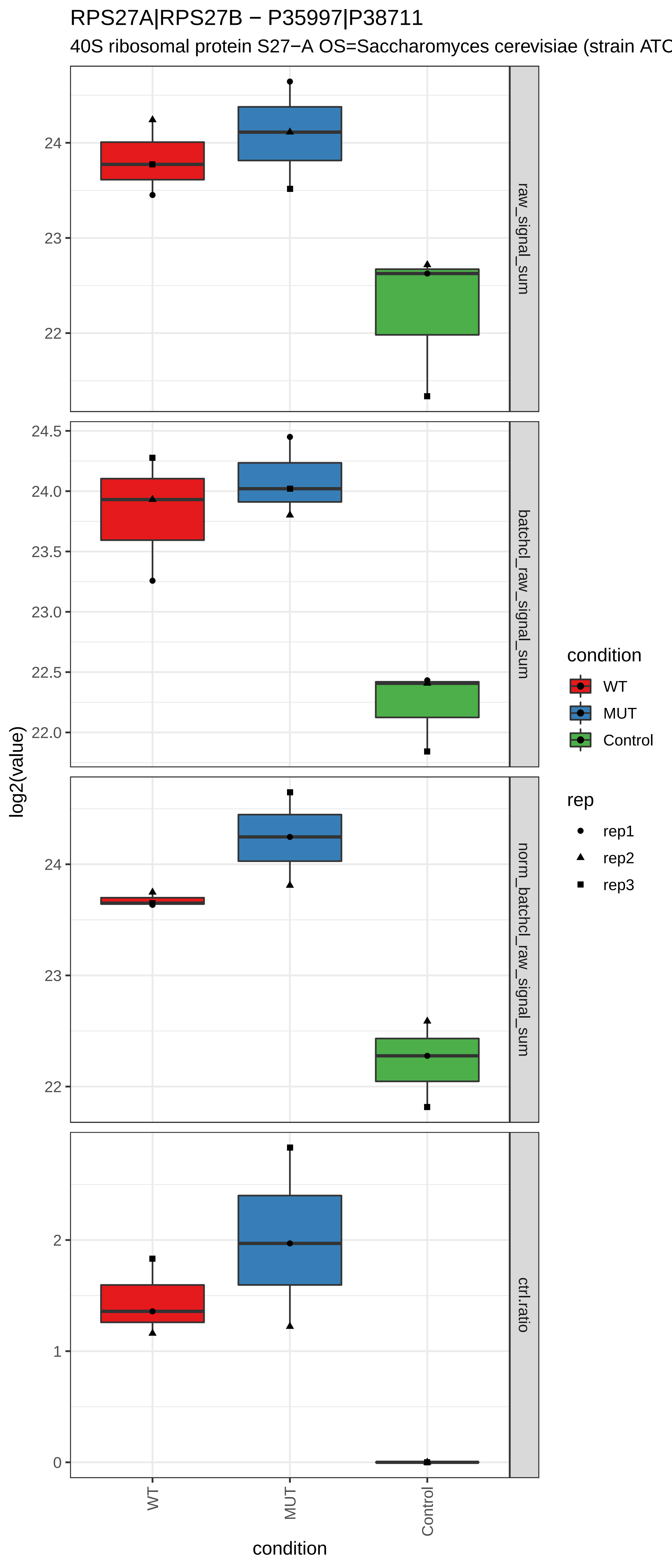
RPS27A|RPS27B − P35997|P38711
40S ribosomal protein S27−A OS=Saccharomyces cerevisiae (strain ATC
raw_signal_sum
batchcl_raw_signal_sum
norm_batchcl_raw_signal_sum
ctrl.ratio
log2(value)
condition
WT
MUT
Control
rep
rep1
rep2
rep3
24
23
22
24.5
24.0
23.5
23.0
22.5
22.0
2
1
0

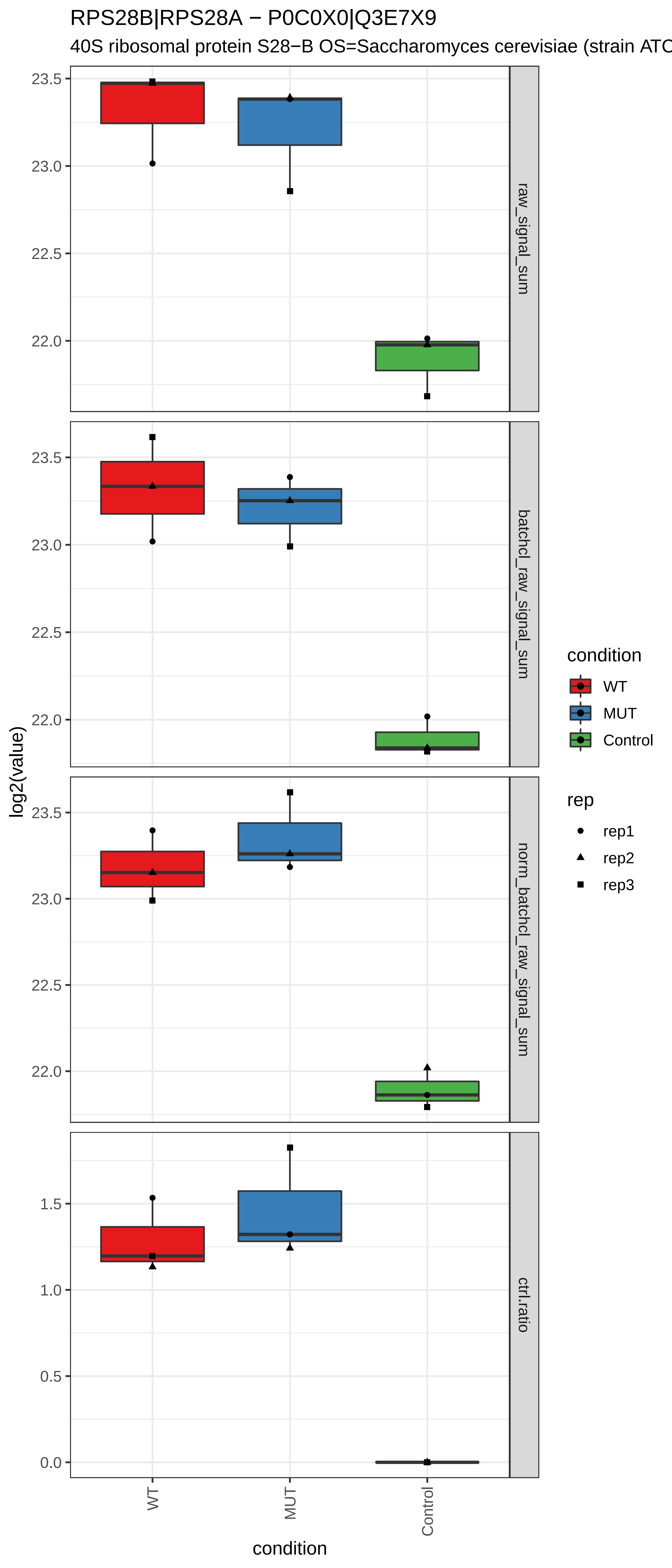

RPS28B|RPS28A − P0C0X0|Q3E7X9
40S ribosomal protein S28−B OS=Saccharomyces cerevisiae (strain ATC
raw_signal_sum
batchcl_raw_signal_sum
norm_batchcl_raw_signal_sum
ctrl.ratio
log2(value)
condition
WT
MUT
Control
rep
rep1
rep2
rep3
23.5
23.0
22.5
22.0
1.5
1.0
0.5
0.0

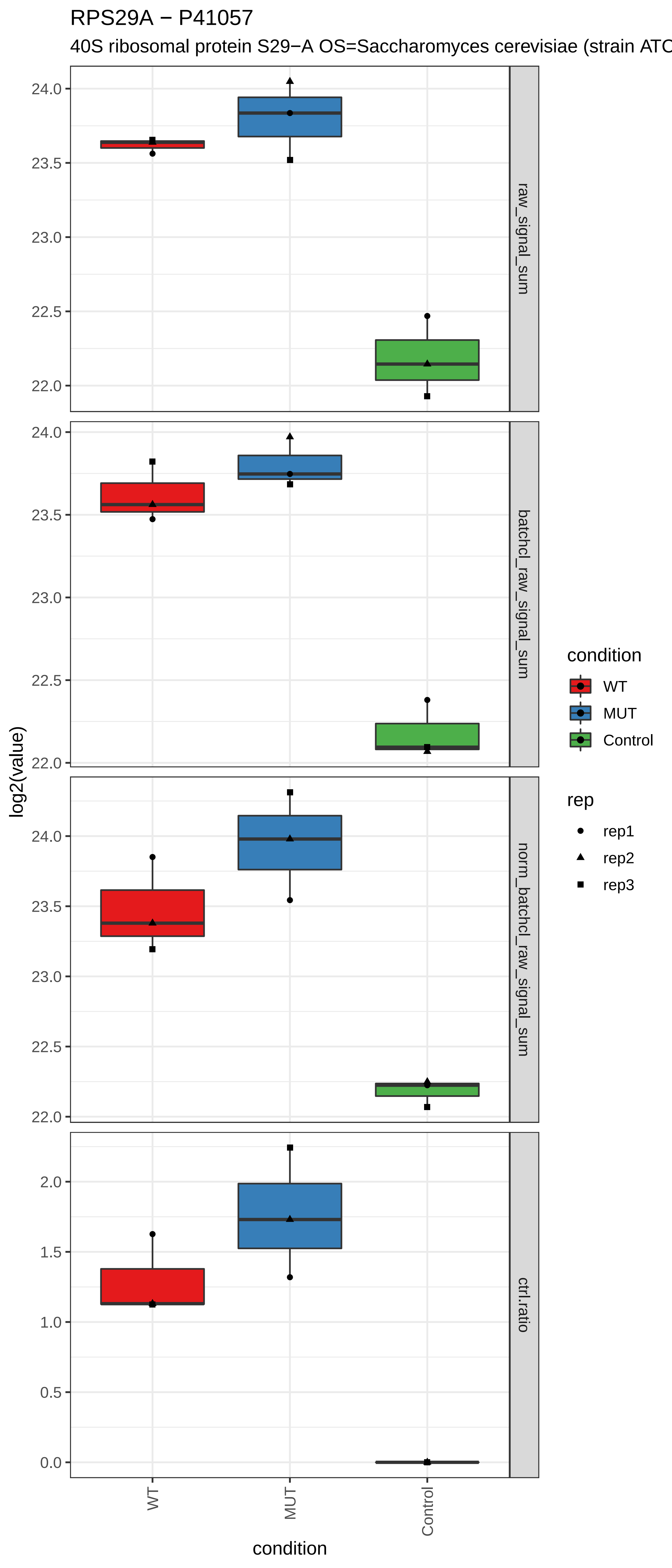
RPS29A − P41057
40S ribosomal protein S29−A OS=Saccharomyces cerevisiae (strain ATC
raw_signal_sum
batchcl_raw_signal_sum
norm_batchcl_raw_signal_sum
ctrl.ratio
log2(value)
condition
WT
MUT
Control
rep
rep1
rep2
rep3
24.0
23.5
23.0
22.5
22.0
2.0
1.5
1.0
0.5
0.0

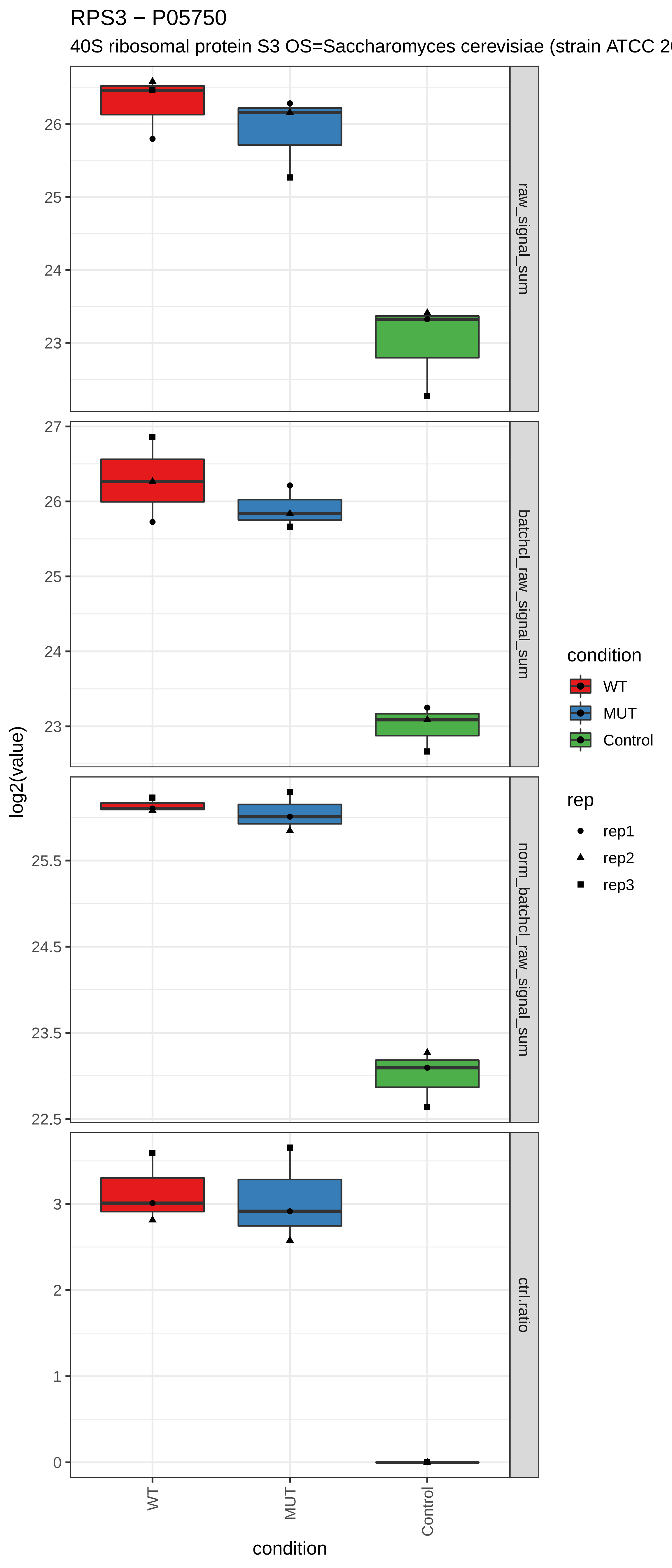

RPS3 − P05750
40S ribosomal protein S3 OS=Saccharomyces cerevisiae (strain ATCC 2
raw_signal_sum
batchcl_raw_signal_sum
norm_batchcl_raw_signal_sum
ctrl.ratio
log2(value)
condition
WT
MUT
Control
rep
rep1
rep2
rep3

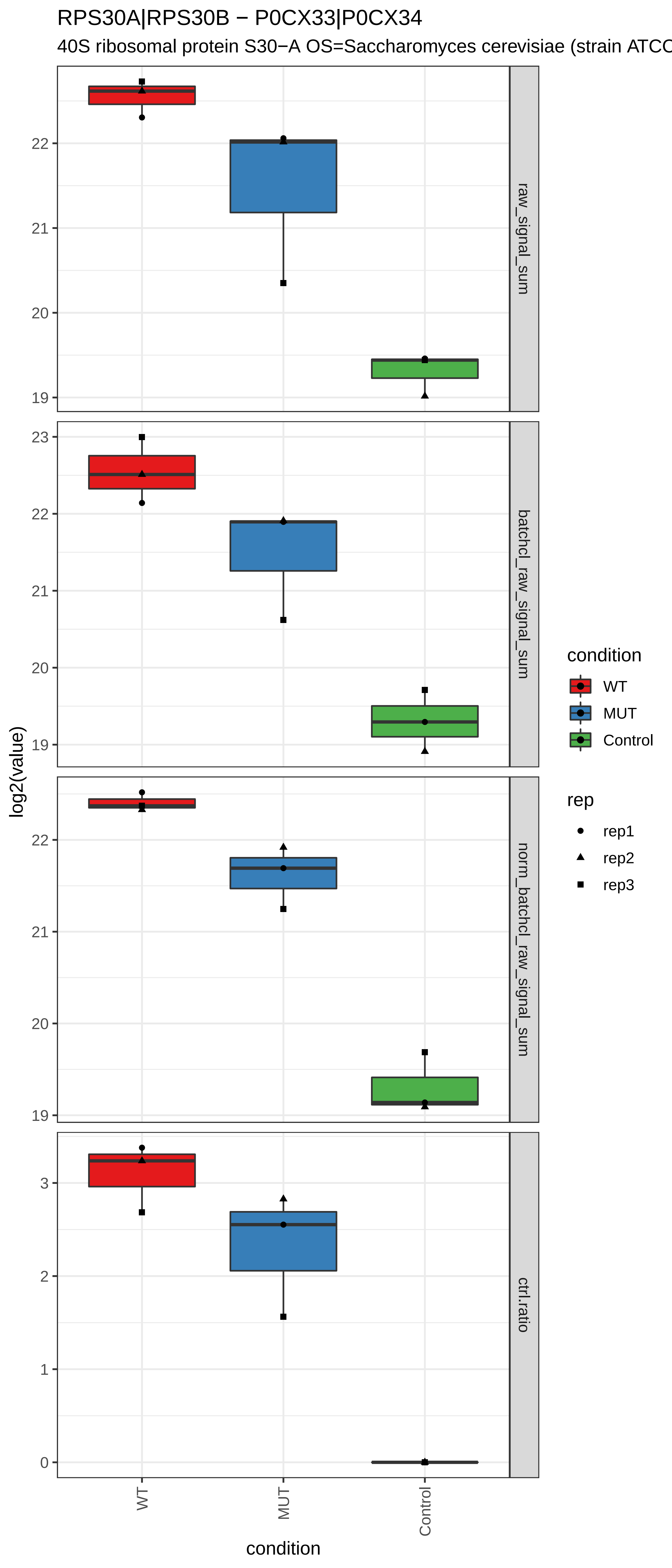

RPS30A|RPS30B − P0CX33|P0CX34
40S ribosomal protein S30−A OS=Saccharomyces cerevisiae (strain ATCC
raw_signal_sum
batchcl_raw_signal_sum
norm_batchcl_raw_signal_sum
ctrl.ratio
log2(value)
condition
WT
MUT
Control
rep
rep1
rep2
rep3

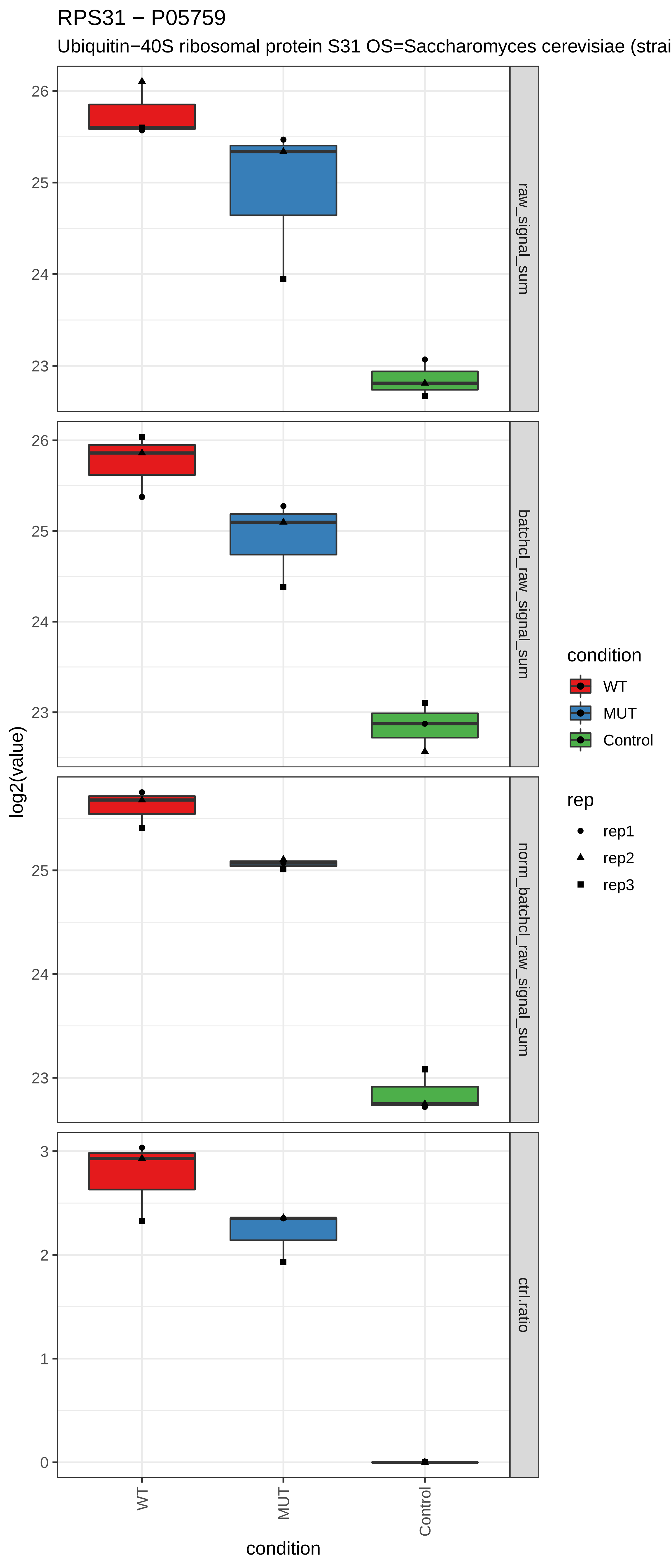
RPS31 − P05759
Ubiquitin−40S ribosomal protein S31 OS=Saccharomyces cerevisiae (strai
raw_signal_sum
batchcl_raw_signal_sum
norm_batchcl_raw_signal_sum
ctrl.ratio
log2(value)
condition
WT
MUT
Control
rep
rep1
rep2
rep3
26
25
24
23
3
2
1
0

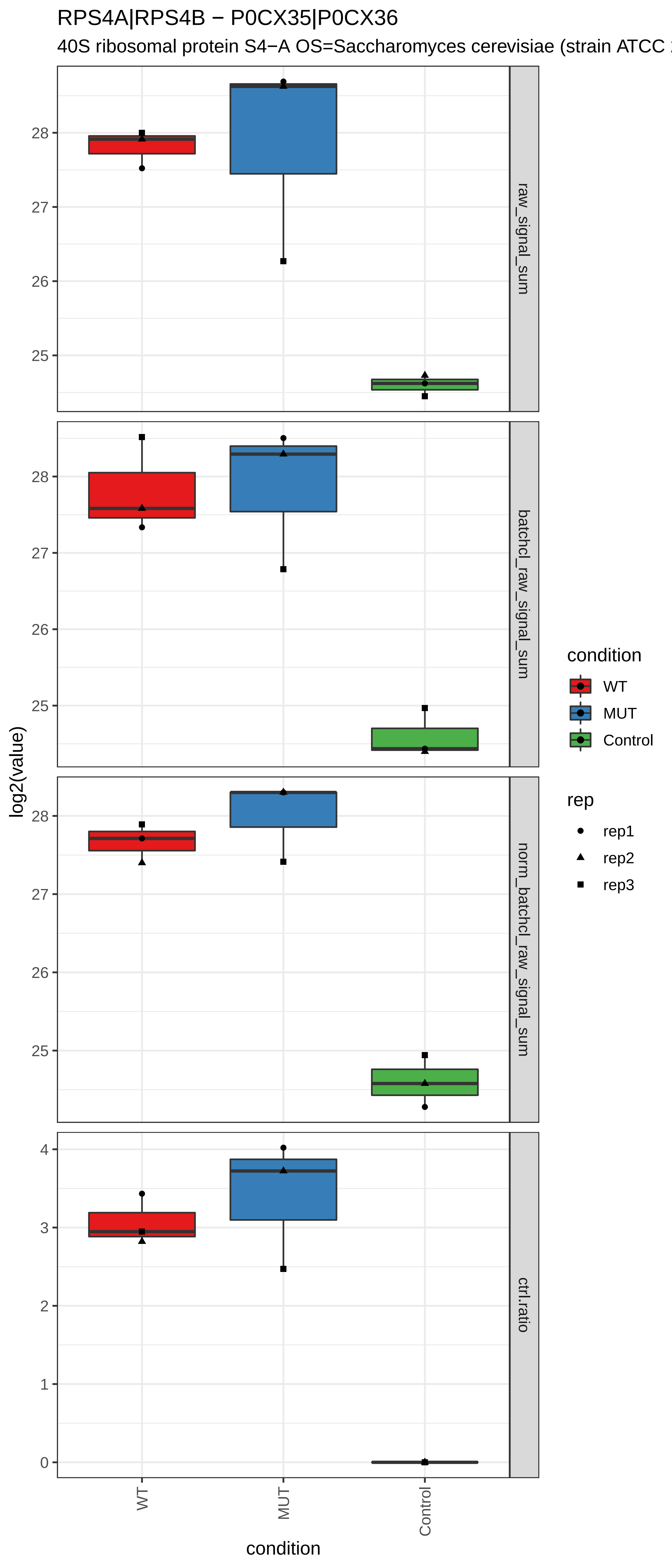

RPS4A|RPS4B − P0CX35|P0CX36
40S ribosomal protein S4−A OS=Saccharomyces cerevisiae (strain ATCC
raw_signal_sum
batchcl_raw_signal_sum
norm_batchcl_raw_signal_sum
ctrl.ratio
log2(value)
condition
WT
MUT
Control
rep
rep1
rep2
rep3
28
27
26
25
4
3
2
1
0

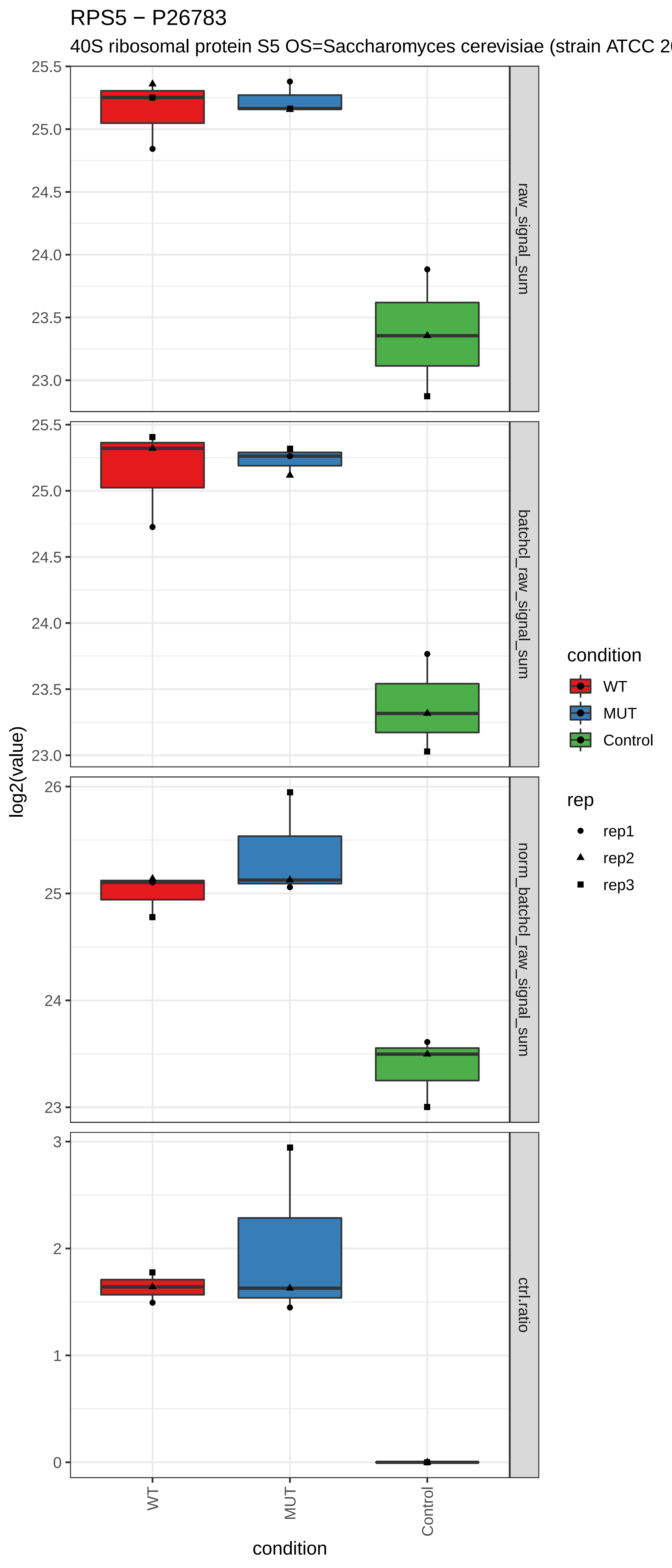

RPS5 − P26783
40S ribosomal protein S5 OS=Saccharomyces cerevisiae (strain ATCC 2
raw_signal_sum
batchcl_raw_signal_sum
norm_batchcl_raw_signal_sum
ctrl.ratio
log2(value)
condition
WT
MUT
Control
rep
rep1
rep2
rep3

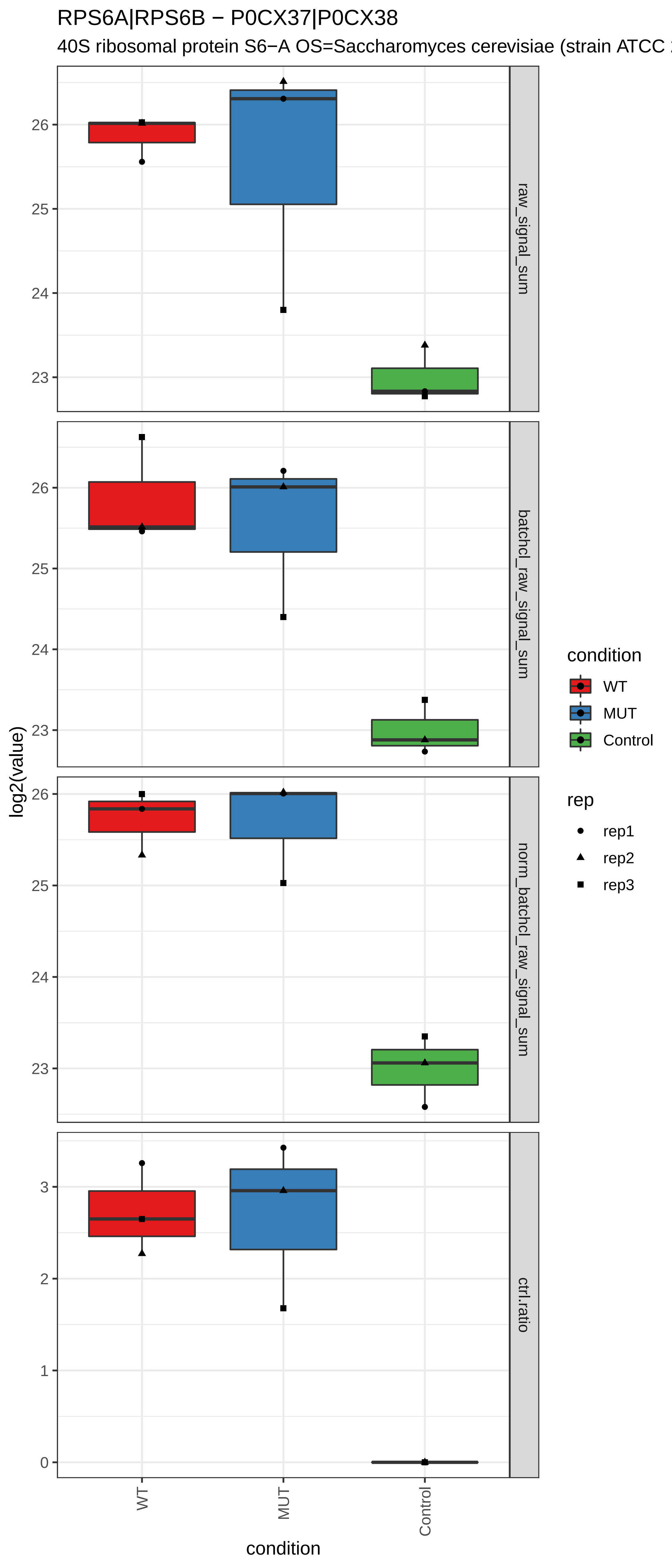
RPS6A|RPS6B − P0CX37|P0CX38
40S ribosomal protein S6−A OS=Saccharomyces cerevisiae (strain ATCC
raw_signal_sum
batchcl_raw_signal_sum
norm_batchcl_raw_signal_sum
ctrl.ratio
log2(value)
condition
WT
MUT
Control
rep
rep1
rep2
rep3
26
25
24
23
3
2
1
0
WT
MUT
Control
condition

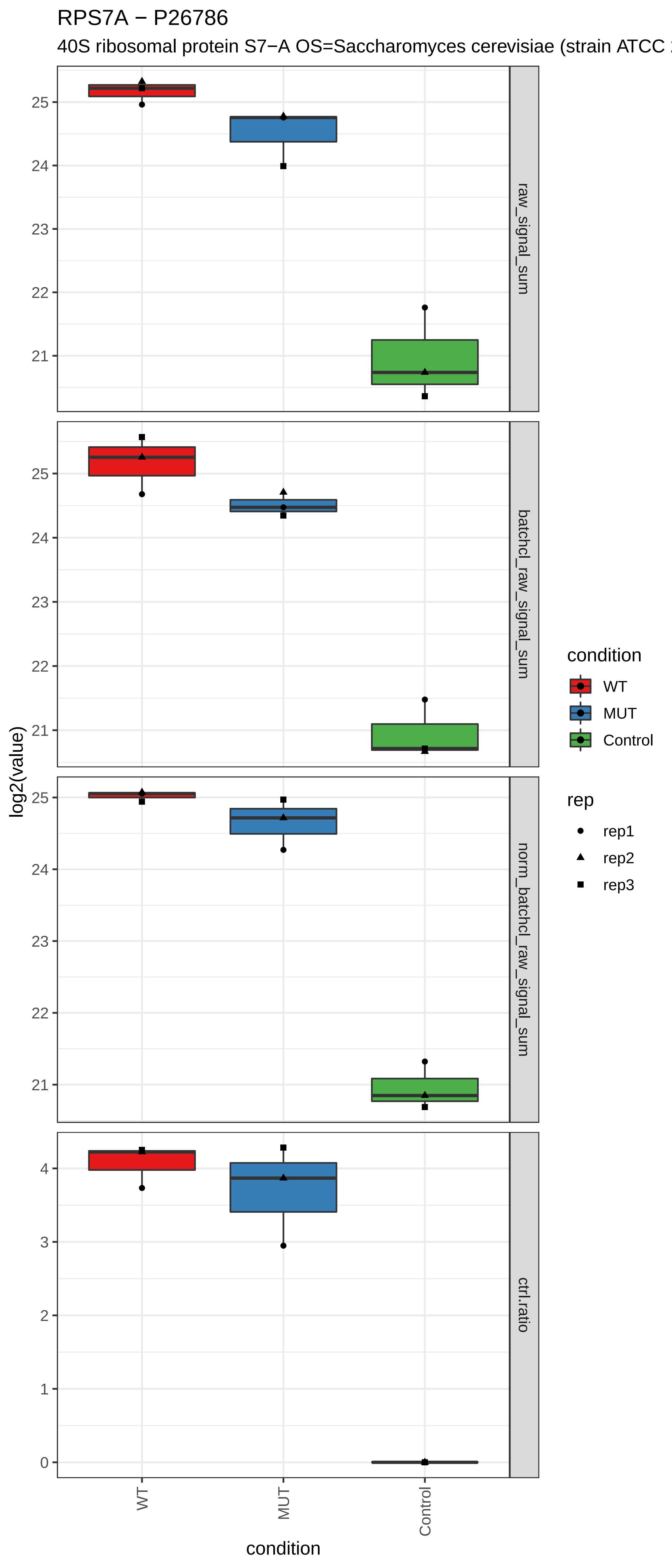

RPS7A − P26786
40S ribosomal protein S7−A OS=Saccharomyces cerevisiae (strain ATCC
raw_signal_sum
batchcl_raw_signal_sum
norm_batchcl_raw_signal_sum
ctrl.ratio
log2(value)
condition
WT
MUT
Control
rep
rep1
rep2
rep3

RPS7B − P48164

40S ribosomal protein S7−B OS=Saccharomyces cerevisiae (strain ATCC

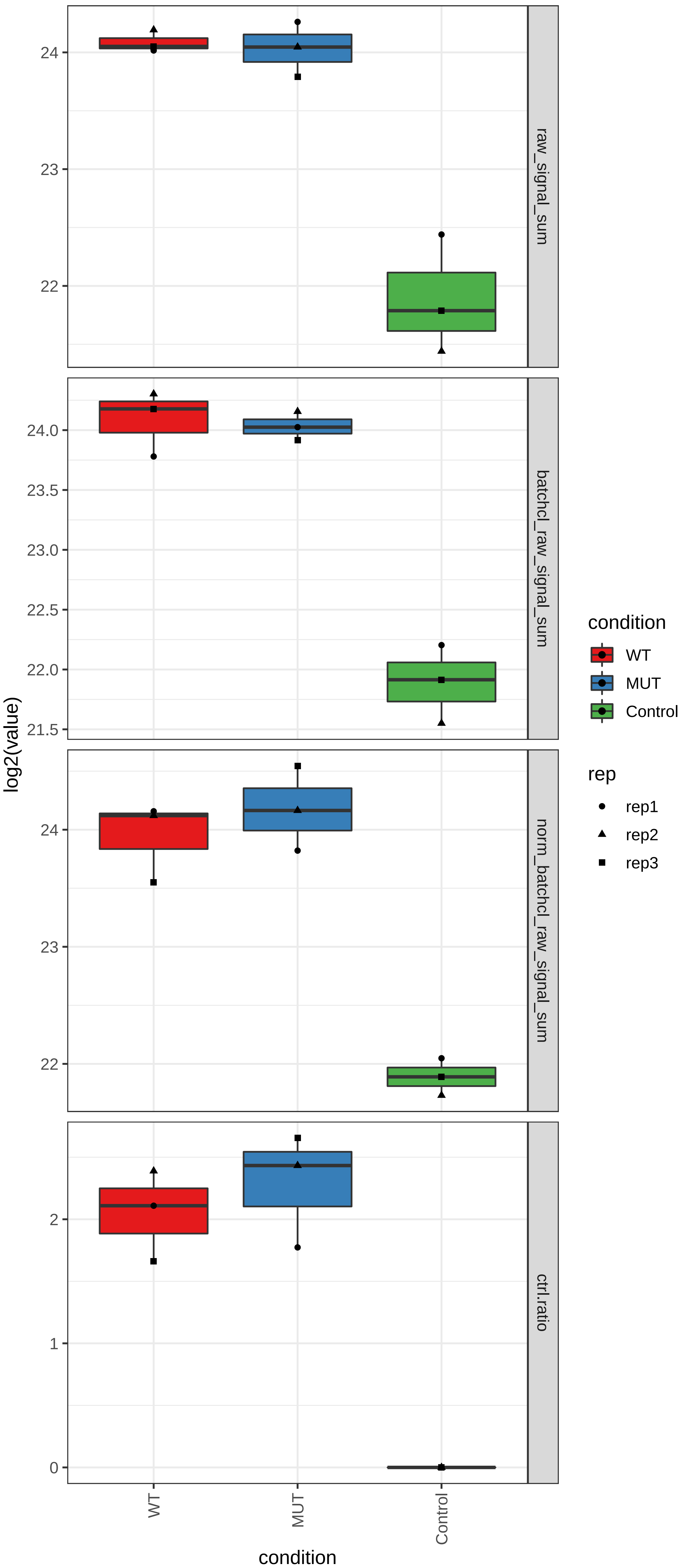

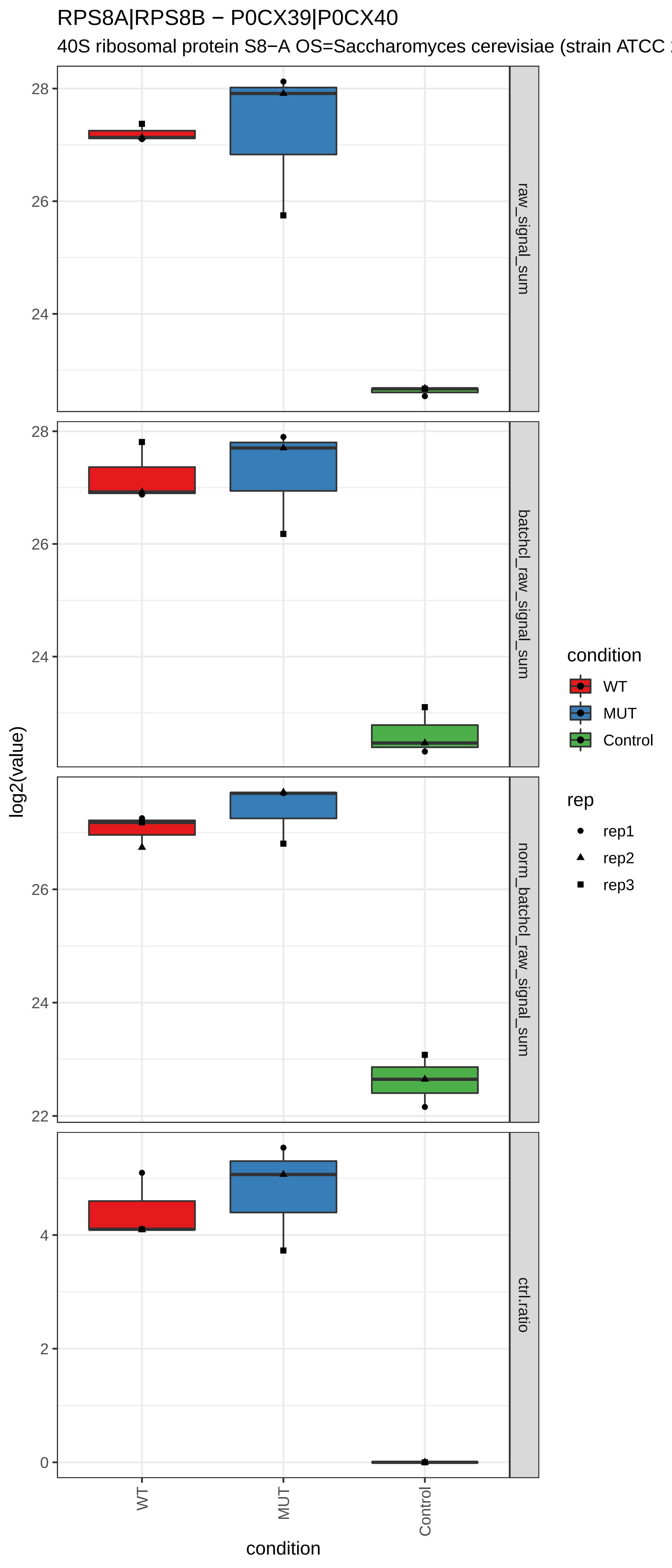
RPS8A|RPS8B − P0CX39|P0CX40
40S ribosomal protein S8−A OS=Saccharomyces cerevisiae (strain ATCC
raw_signal_sum
batchcl_raw_signal_sum
norm_batchcl_raw_signal_sum
ctrl.ratio
log2(value)
condition
WT
MUT
Control
rep
rep1
rep2
rep3
28
26
24
22
4
2
0

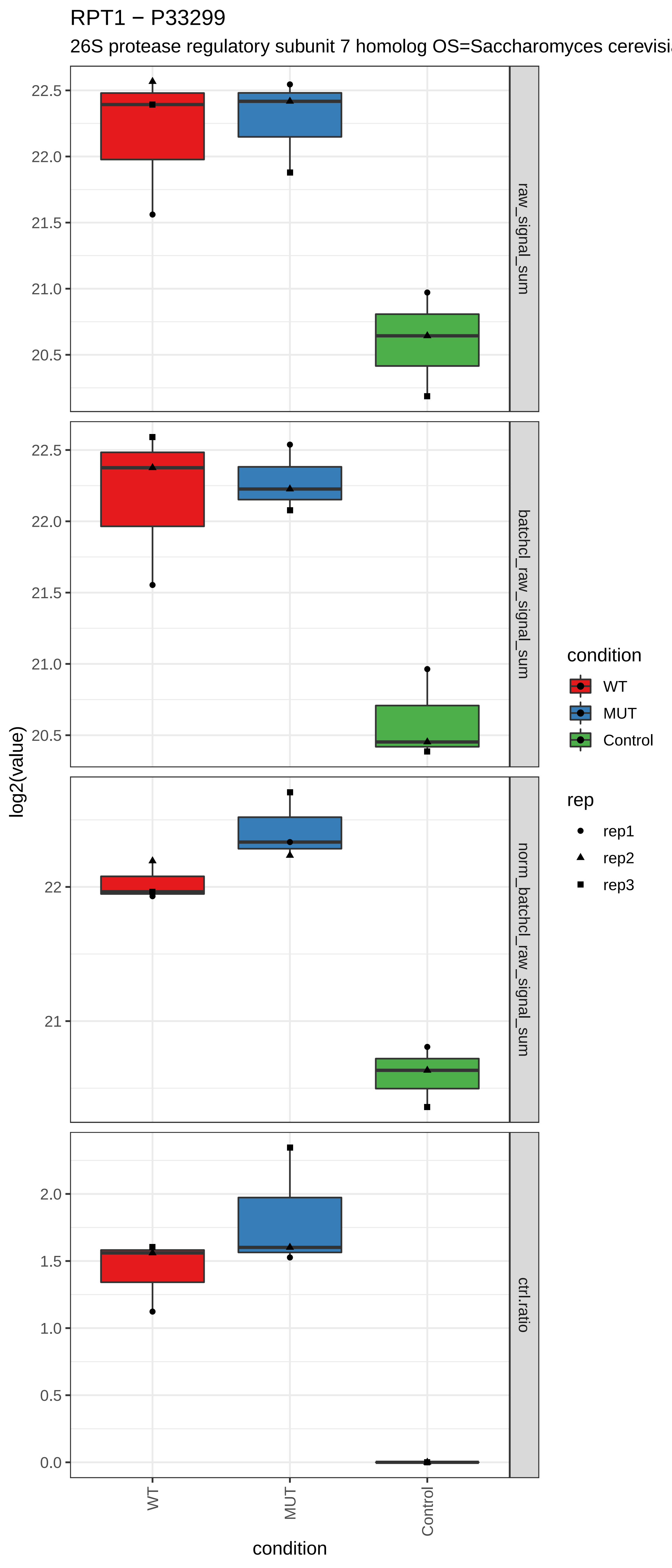
RPT1 − P33299
26S protease regulatory subunit 7 homolog OS=Saccharomyces cerevisi
raw_signal_sum
batchcl_raw_signal_sum
norm_batchcl_raw_signal_sum
ctrl.ratio
log2(value)
condition
WT
MUT
Control
rep
rep1
rep2
rep3

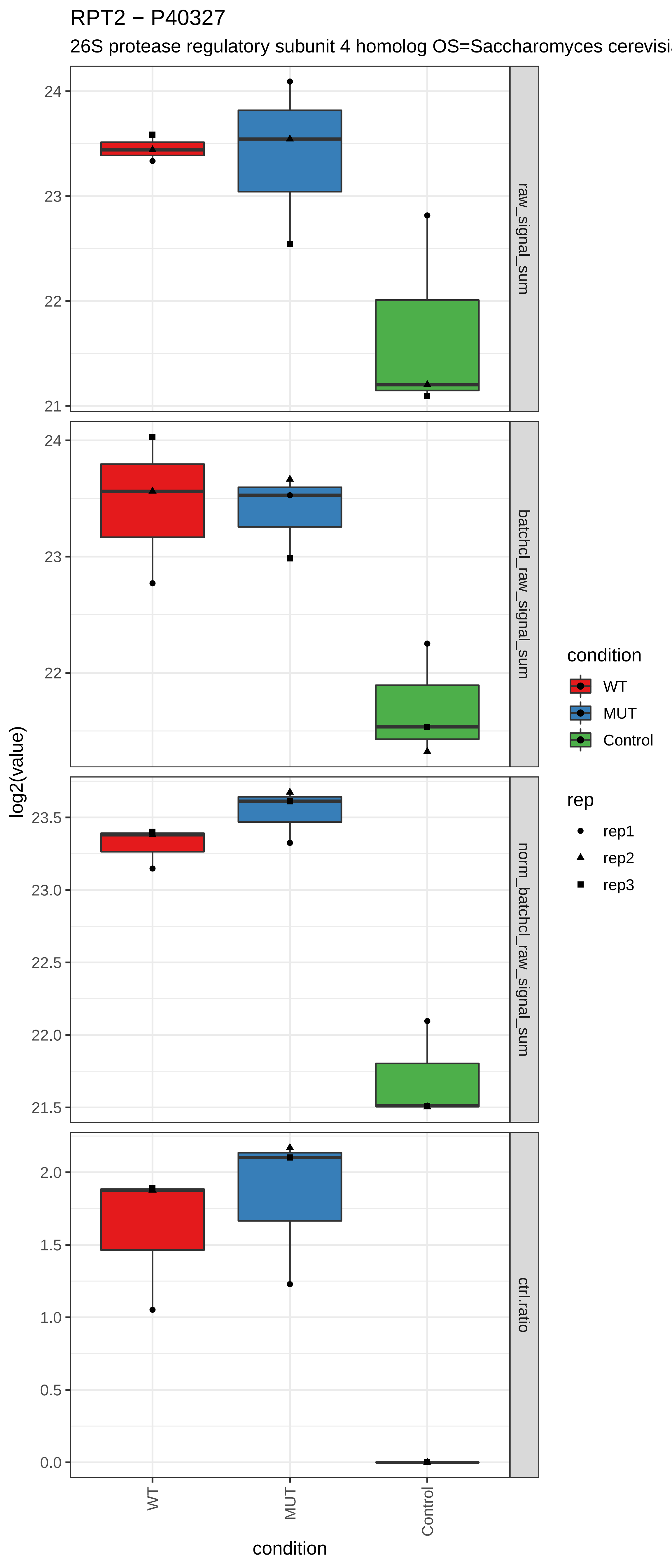
RPT2 − P40327
26S protease regulatory subunit 4 homolog OS=Saccharomyces cerevisi
log2(value)
raw_signal_sum
batchcl_raw_signal_sum
norm_batchcl_raw_signal_sum
ctrl.ratio
24
23
22
21
23.5
23.0
22.5
22.0
21.5
2.0
1.5
1.0
0.5
0.0
WT
MUT
Control
condition
condition
WT
MUT
Control
rep
rep1
rep2
rep3

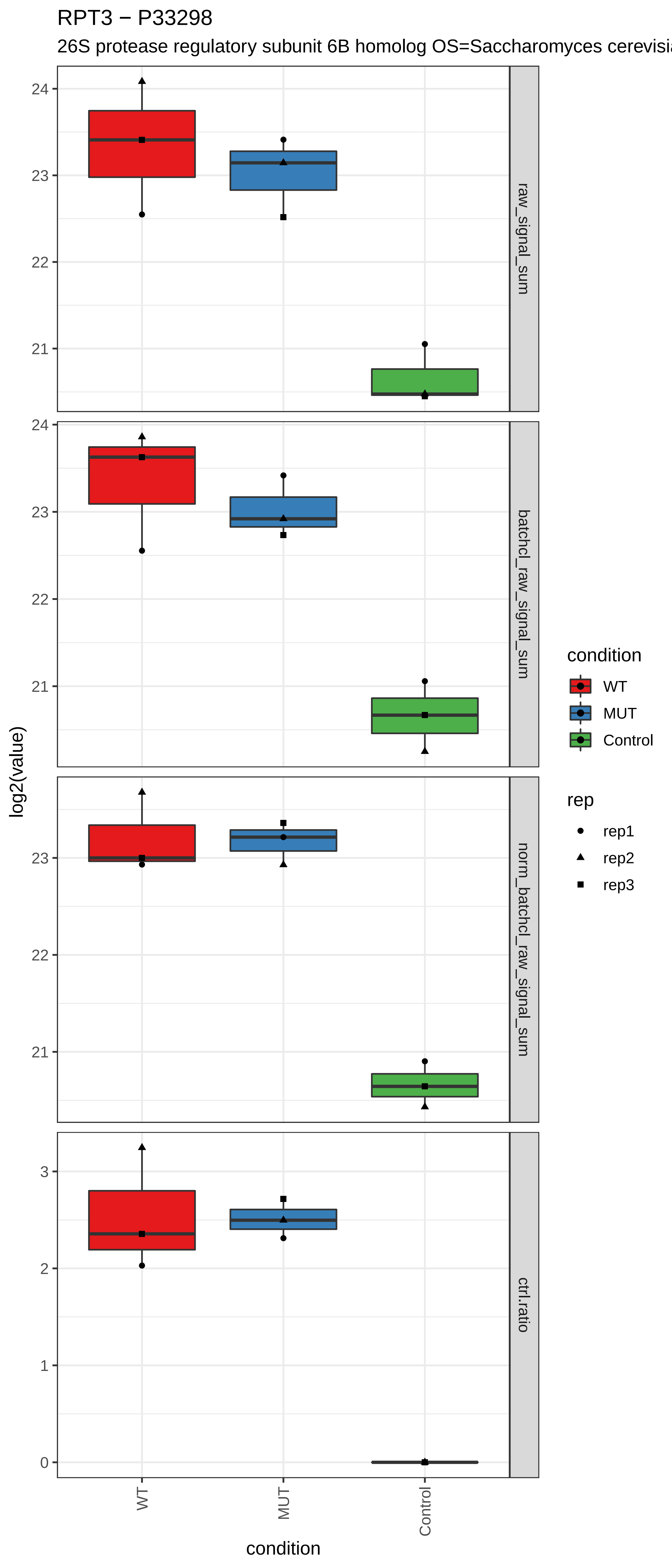

RPT3 − P33298
26S protease regulatory subunit 6B homolog OS=Saccharomyces cerevisia
raw_signal_sum
batchcl_raw_signal_sum
norm_batchcl_raw_signal_sum
ctrl.ratio
log2(value)
condition
WT
MUT
Control
rep
rep1
rep2
rep3

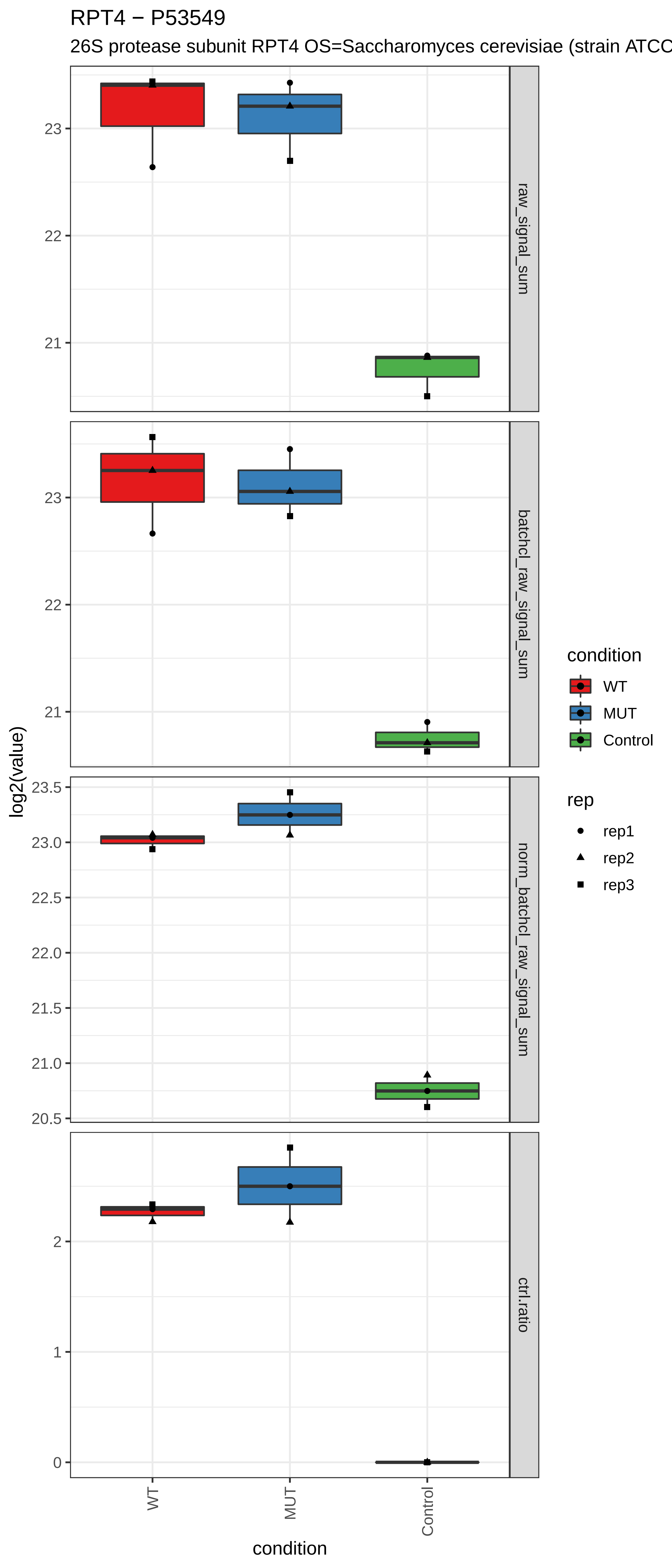

RPT4 − P53549
26S protease subunit RPT4 OS=Saccharomyces cerevisiae (strain ATCC
raw_signal_sum
batchcl_raw_signal_sum
norm_batchcl_raw_signal_sum
ctrl.ratio
log2(value)
condition
WT
MUT
Control
rep
rep1
rep2
rep3

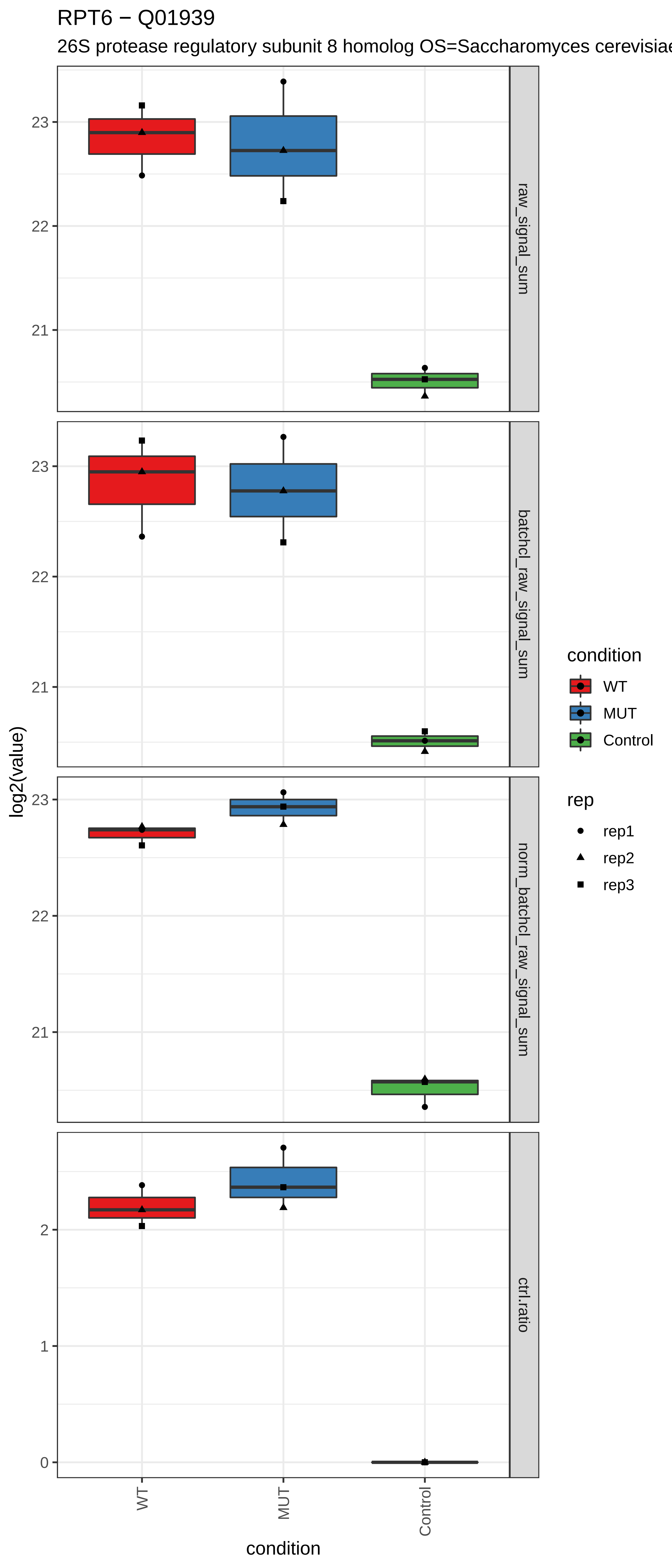

RPT6 − Q01939
26S protease regulatory subunit 8 homolog OS=Saccharomyces cerevisiae
raw_signal_sum
batchcl_raw_signal_sum
norm_batchcl_raw_signal_sum
ctrl.ratio
log2(value)
23
22
21
2
1
0
WT
MUT
Control
condition
WT
MUT
Control
rep
rep1
rep2
rep3

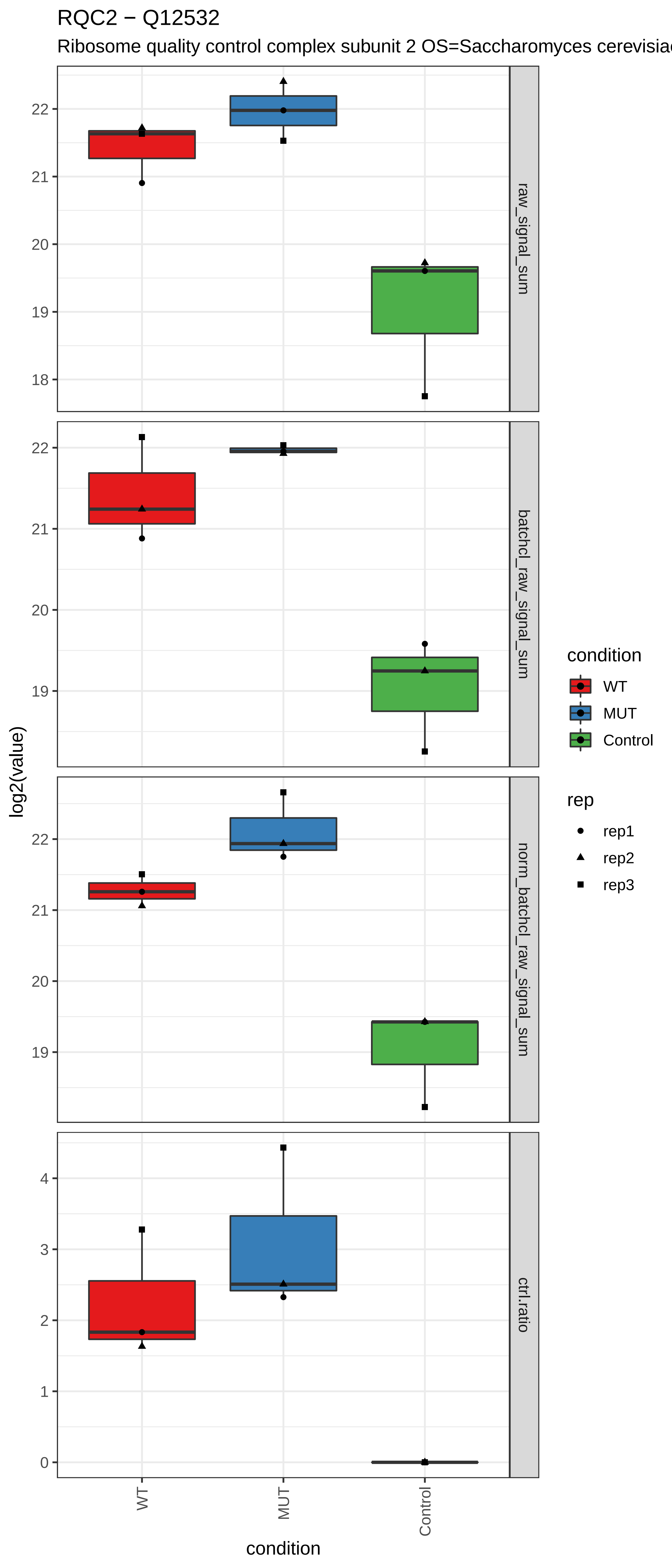

RQC2 − Q12532
Ribosome quality control complex subunit 2 OS=Saccharomyces cerevisiae
raw_signal_sum
batchcl_raw_signal_sum
norm_batchcl_raw_signal_sum
ctrl.ratio
log2(value)
condition
WT
MUT
Control
rep
rep1
rep2
rep3

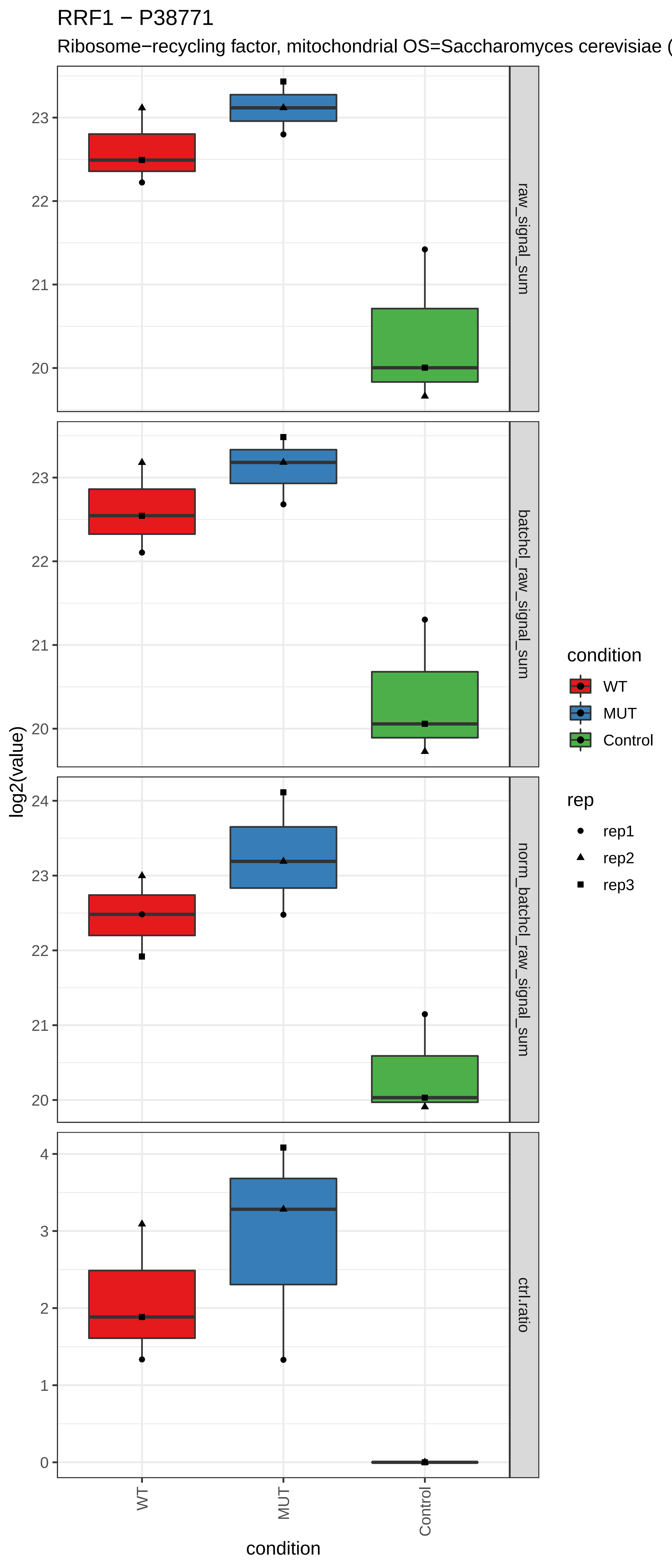
RRF1 − P38771
Ribosome−recycling factor, mitochondrial OS=Saccharomyces cerevisiae (
raw_signal_sum
batchcl_raw_signal_sum
norm_batchcl_raw_signal_sum
ctrl.ratio
log2(value)
condition
WT
MUT
Control
rep
rep1
rep2
rep3
20
21
22
23
24
0
1
2
3
4

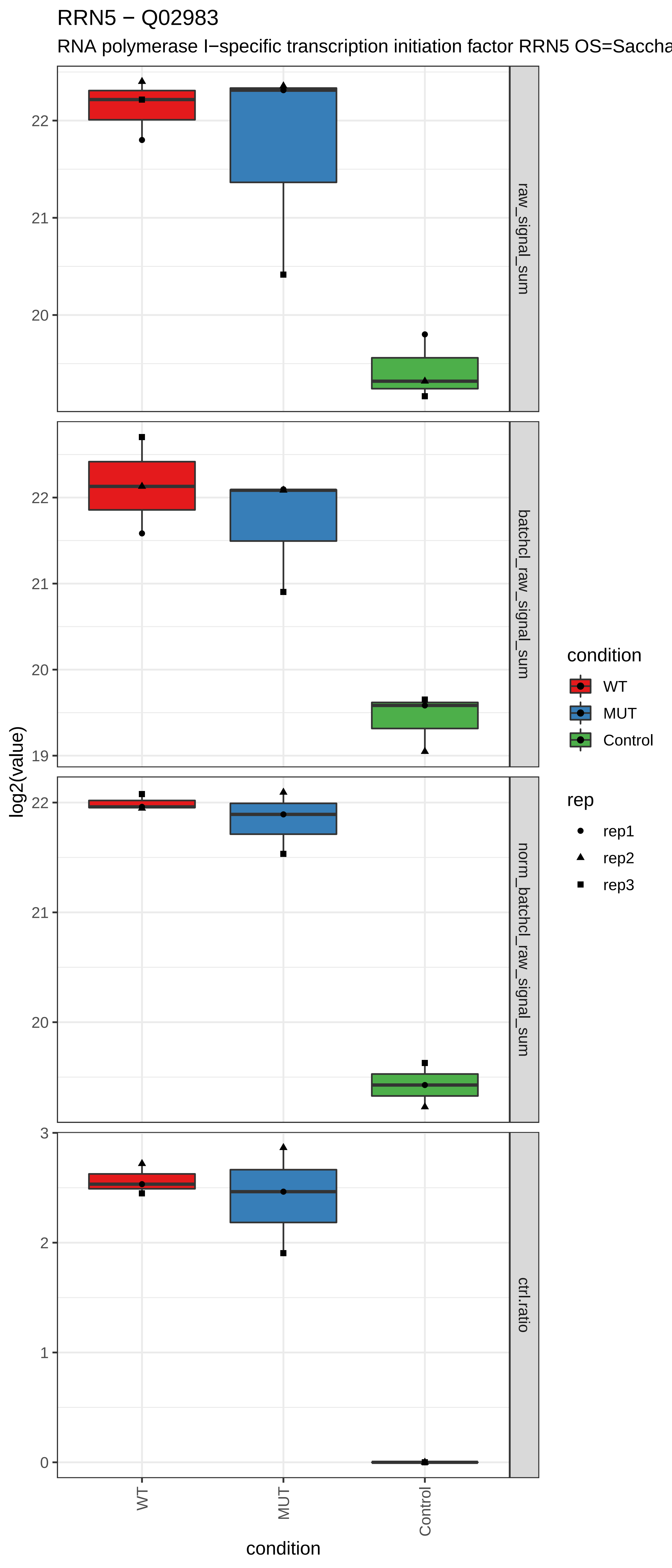
RRN5 − Q02983
RNA polymerase I−specific transcription initiation factor RRN5 OS=Saccha
raw_signal_sum
batchcl_raw_signal_sum
norm_batchcl_raw_signal_sum
ctrl.ratio
log2(value)
condition
WT
MUT
Control
rep
rep1
rep2
rep3

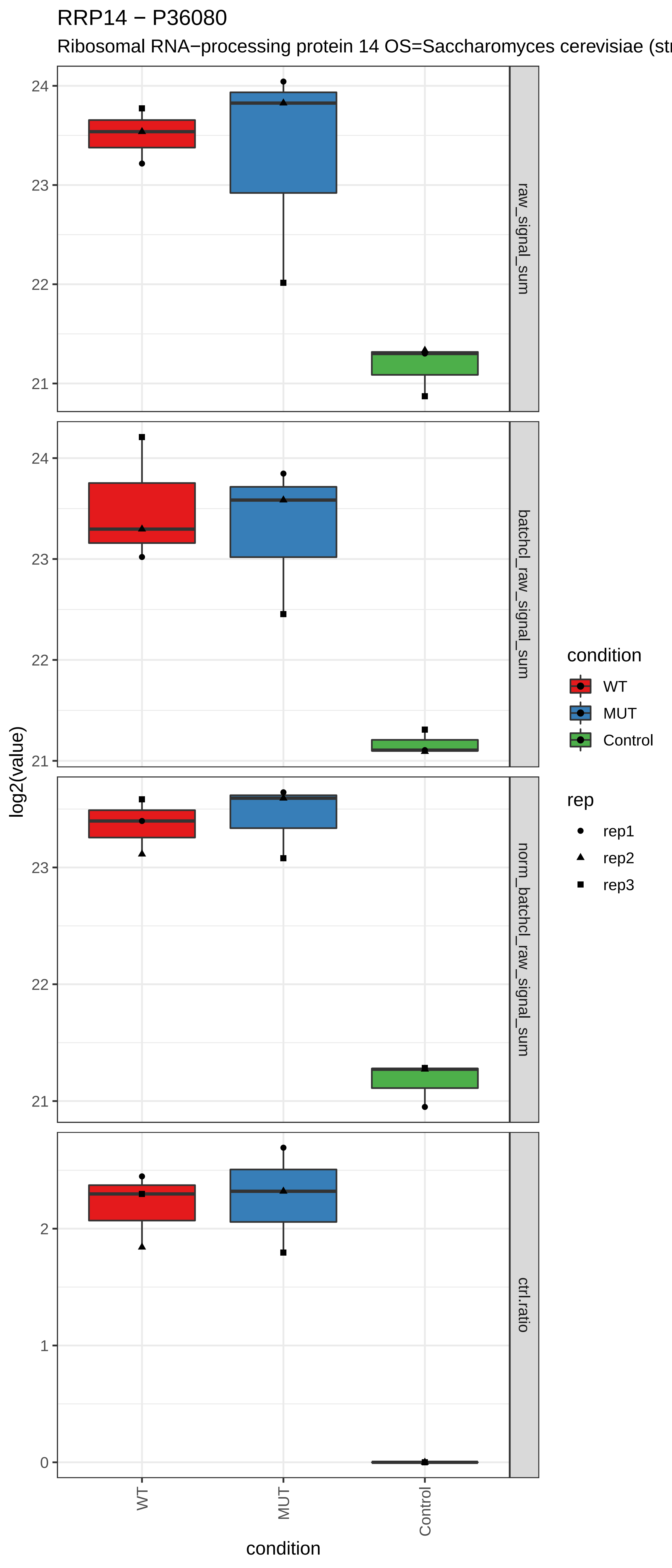
RRP14 − P36080
Ribosomal RNA−processing protein 14 OS=Saccharomyces cerevisiae (str
raw_signal_sum
batchcl_raw_signal_sum
norm_batchcl_raw_signal_sum
ctrl.ratio
log2(value)
condition
WT
MUT
Control
rep
rep1
rep2
rep3
24
23
22
21
2
1
0

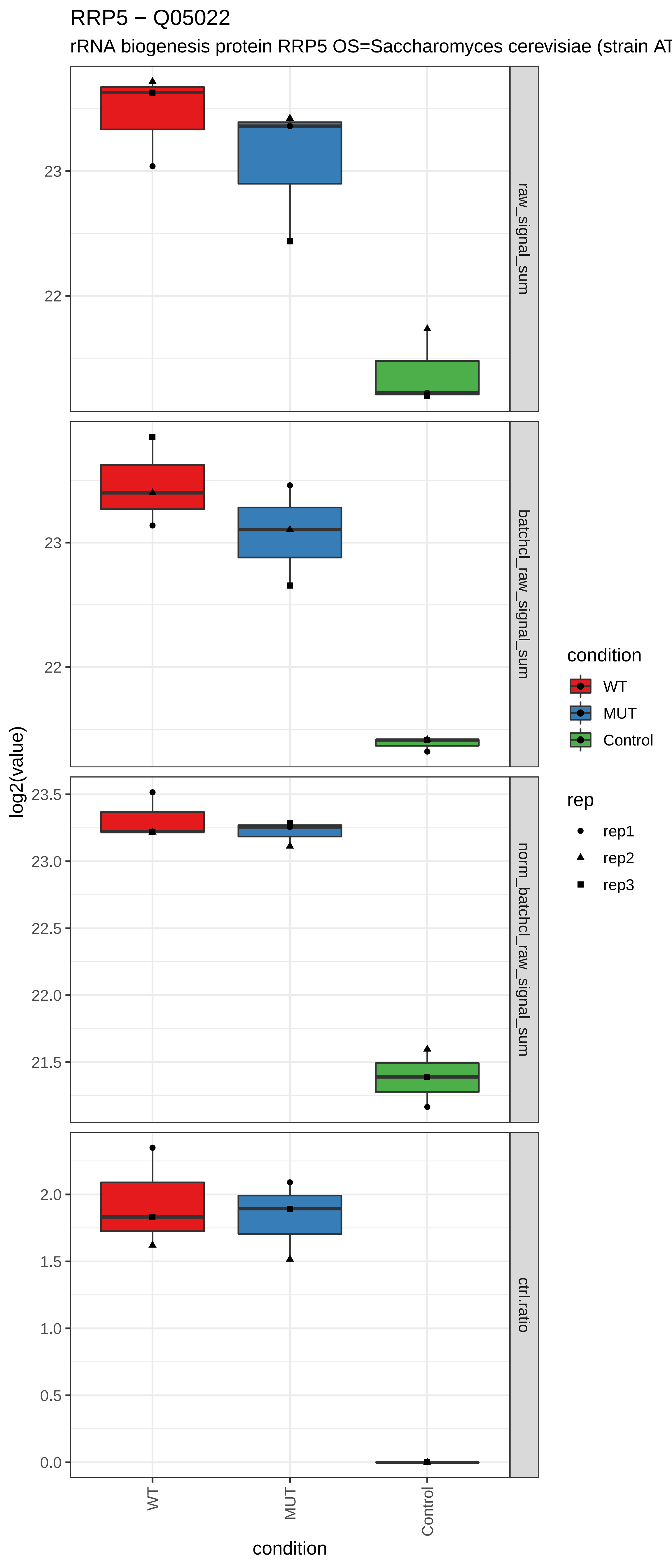

RRP5 − Q05022
rRNA biogenesis protein RRP5 OS=Saccharomyces cerevisiae (strain AT
raw_signal_sum
batchcl_raw_signal_sum
norm_batchcl_raw_signal_sum
ctrl.ratio
log2(value)
condition
WT
MUT
Control
rep
rep1
rep2
rep3

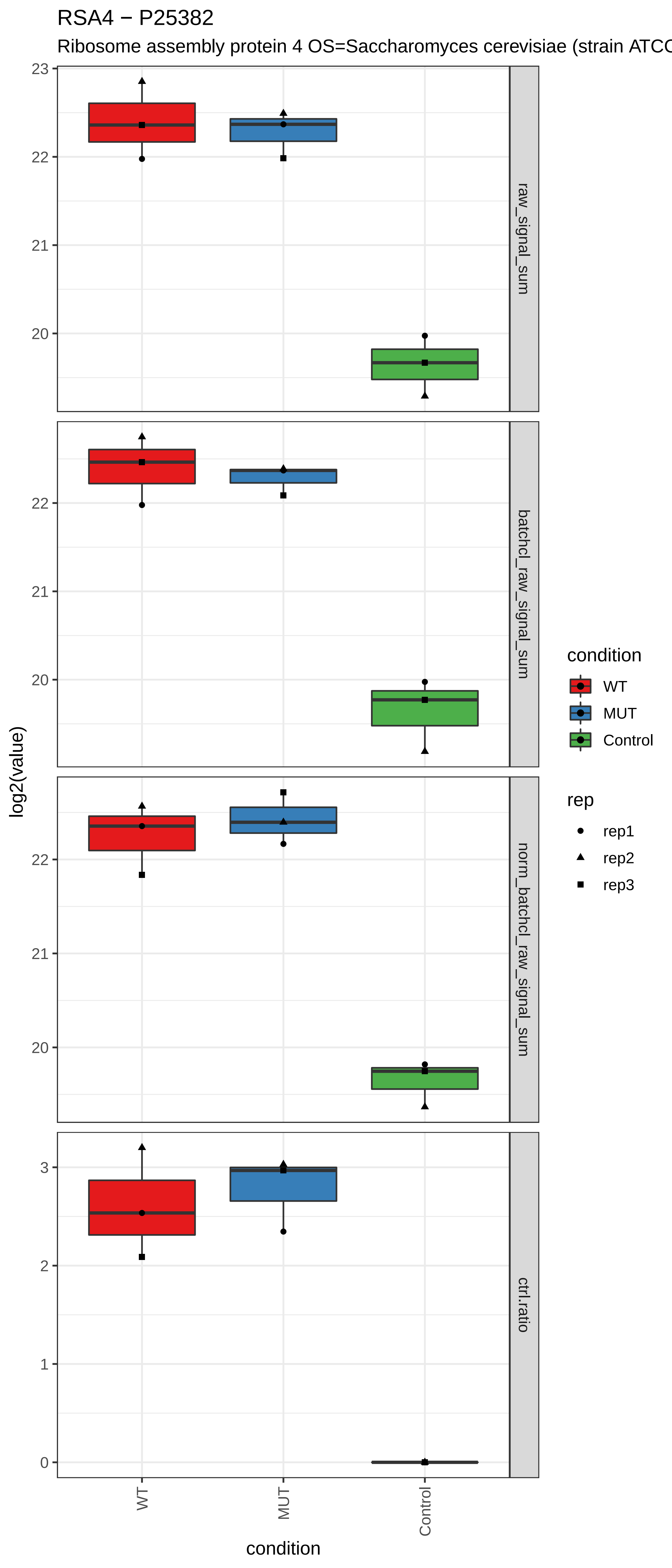

RSA4 − P25382
Ribosome assembly protein 4 OS=Saccharomyces cerevisiae (strain ATCC
raw_signal_sum
batchcl_raw_signal_sum
norm_batchcl_raw_signal_sum
ctrl.ratio
log2(value)
condition
WT
MUT
Control
rep
rep1
rep2
rep3

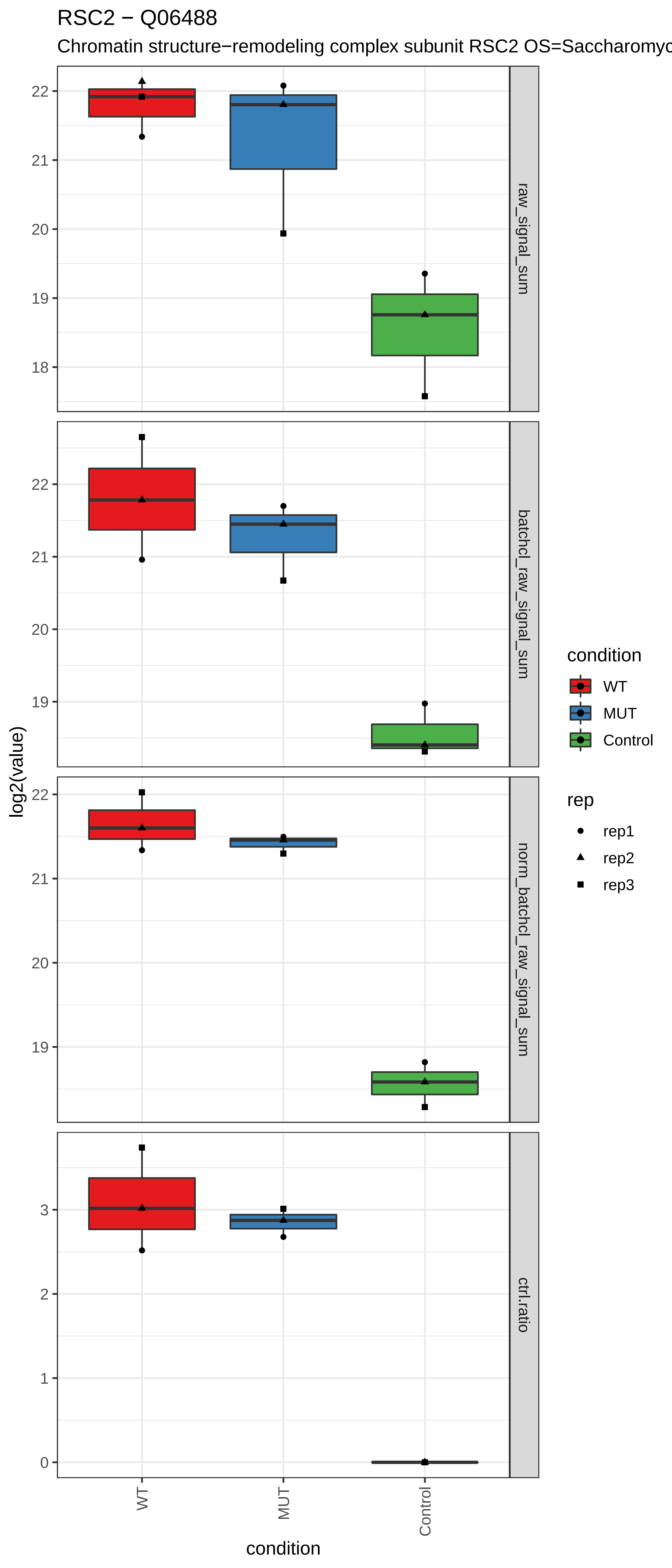
RSC2 − Q06488
Chromatin structure−remodeling complex subunit RSC2 OS=Saccharomyc
raw_signal_sum
batchcl_raw_signal_sum
norm_batchcl_raw_signal_sum
ctrl.ratio
log2(value)
condition
WT
MUT
Control
rep
rep1
rep2
rep3

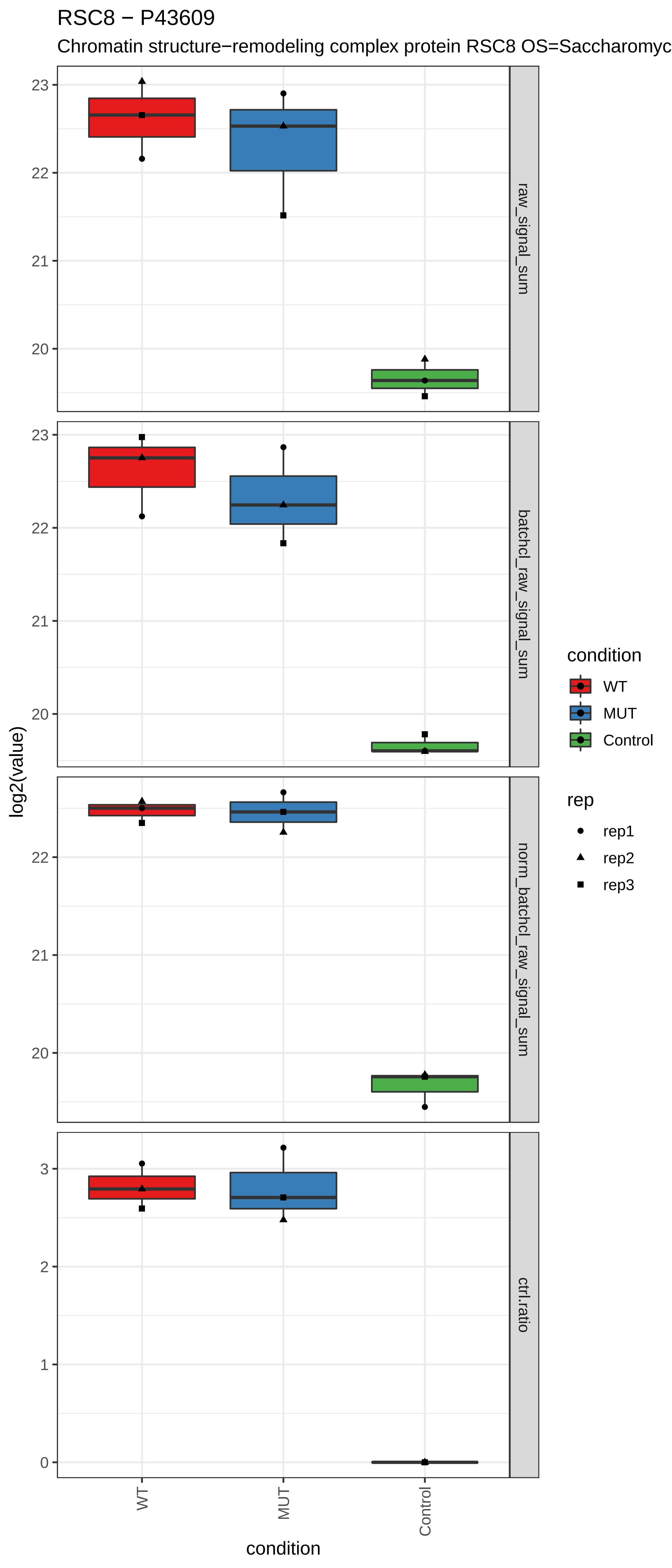
RSC8 − P43609
Chromatin structure−remodeling complex protein RSC8 OS=Saccharomyc
raw_signal_sum
batchcl_raw_signal_sum
norm_batchcl_raw_signal_sum
ctrl.ratio
log2(value)
condition
WT
MUT
Control
rep
rep1
rep2
rep3
23
22
21
20
3
2
1
0

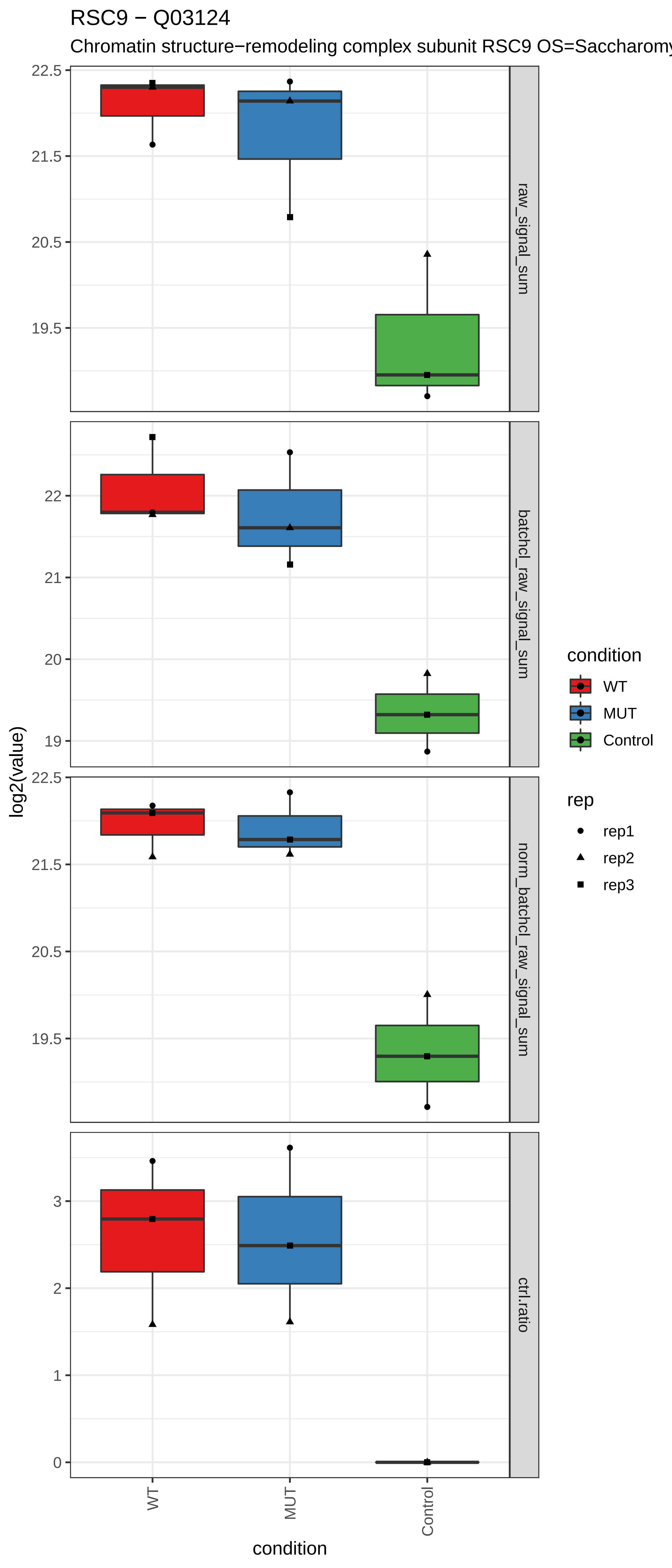

RSC9 − Q03124
Chromatin structure−remodeling complex subunit RSC9 OS=Saccharomy
raw_signal_sum
batchcl_raw_signal_sum
norm_batchcl_raw_signal_sum
ctrl.ratio
22.5
21.5
20.5
19.5
22
21
20
19
3
2
1
0
log2(value)
WT
MUT
Control
condition
condition
WT
MUT
Control
rep
rep1
rep2
rep3

RSF1 − Q05043

Respiration factor 1 OS=Saccharomyces cerevisiae (strain ATCC 204508 /

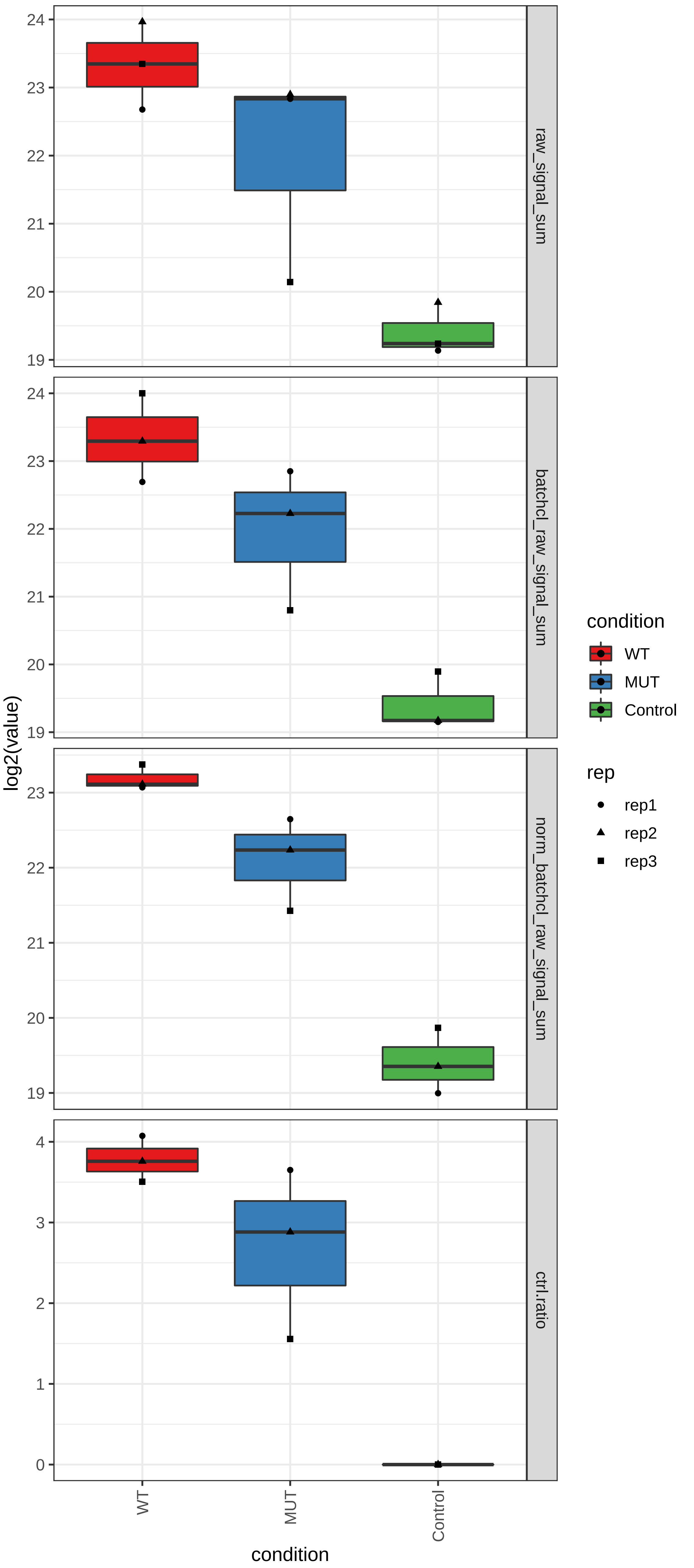

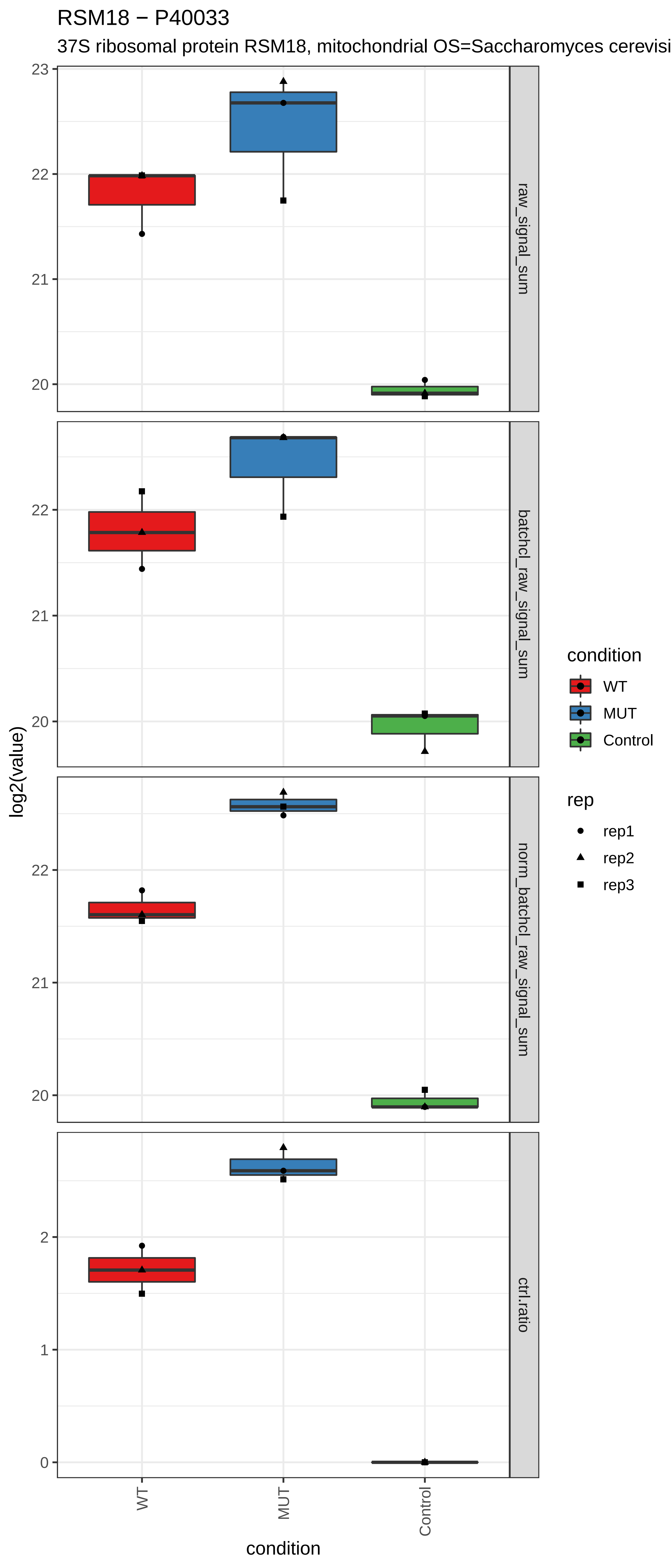
RSM18 − P40033
37S ribosomal protein RSM18, mitochondrial OS=Saccharomyces cerevisi
raw_signal_sum
batchcl_raw_signal_sum
norm_batchcl_raw_signal_sum
ctrl.ratio
log2(value)
condition
WT
MUT
Control
rep
rep1
rep2
rep3

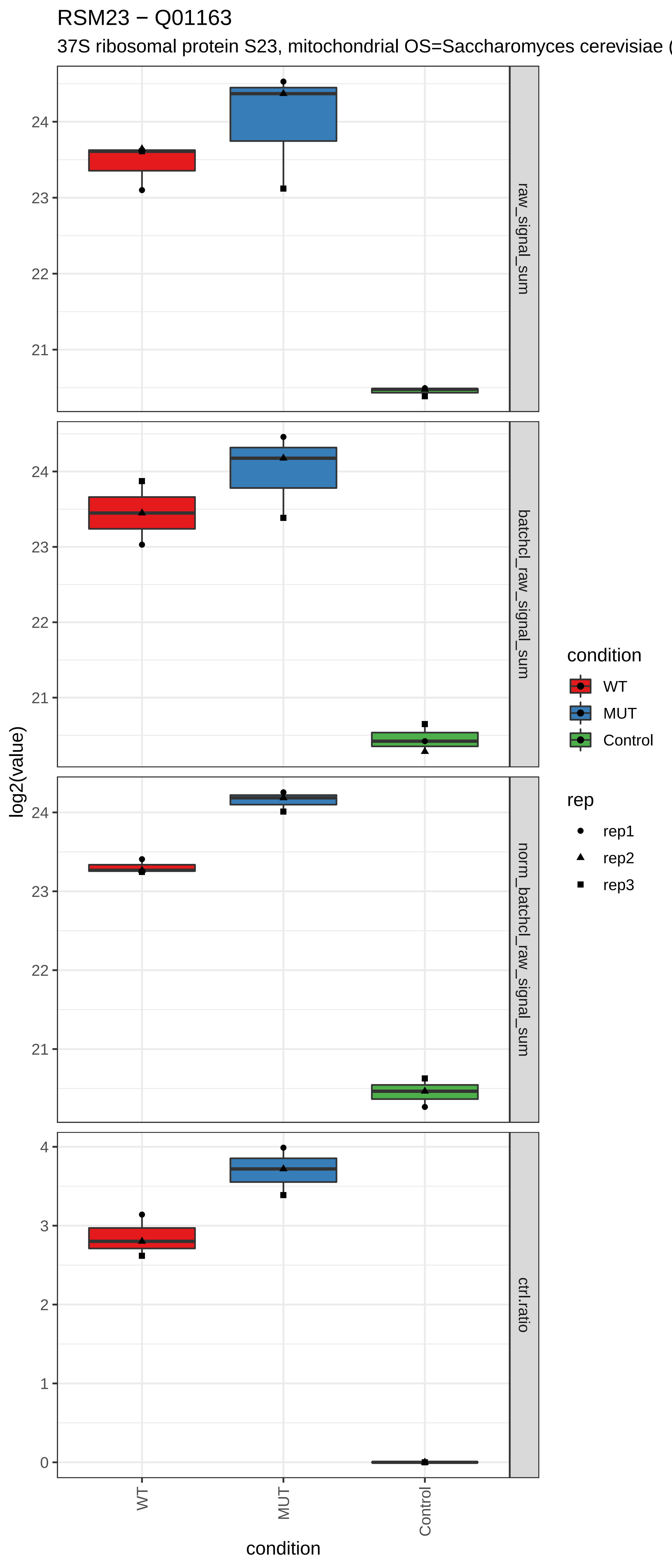

RSM23 − Q01163
37S ribosomal protein S23, mitochondrial OS=Saccharomyces cerevisiae (
raw_signal_sum
batchcl_raw_signal_sum
norm_batchcl_raw_signal_sum
ctrl.ratio
log2(value)
condition
WT
MUT
Control
rep
rep1
rep2
rep3

RSM24 − Q03976

37S ribosomal protein S24, mitochondrial OS=Saccharomyces cerevisiae (

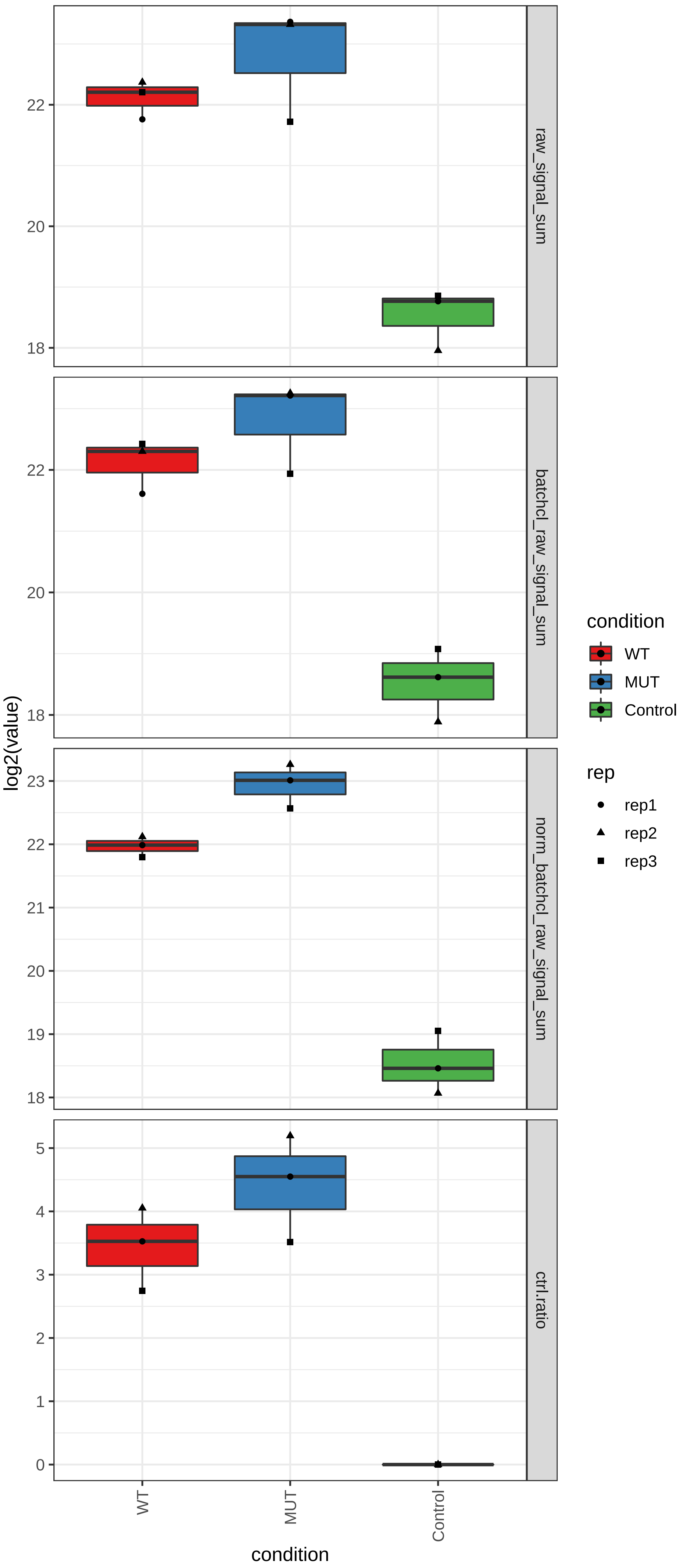

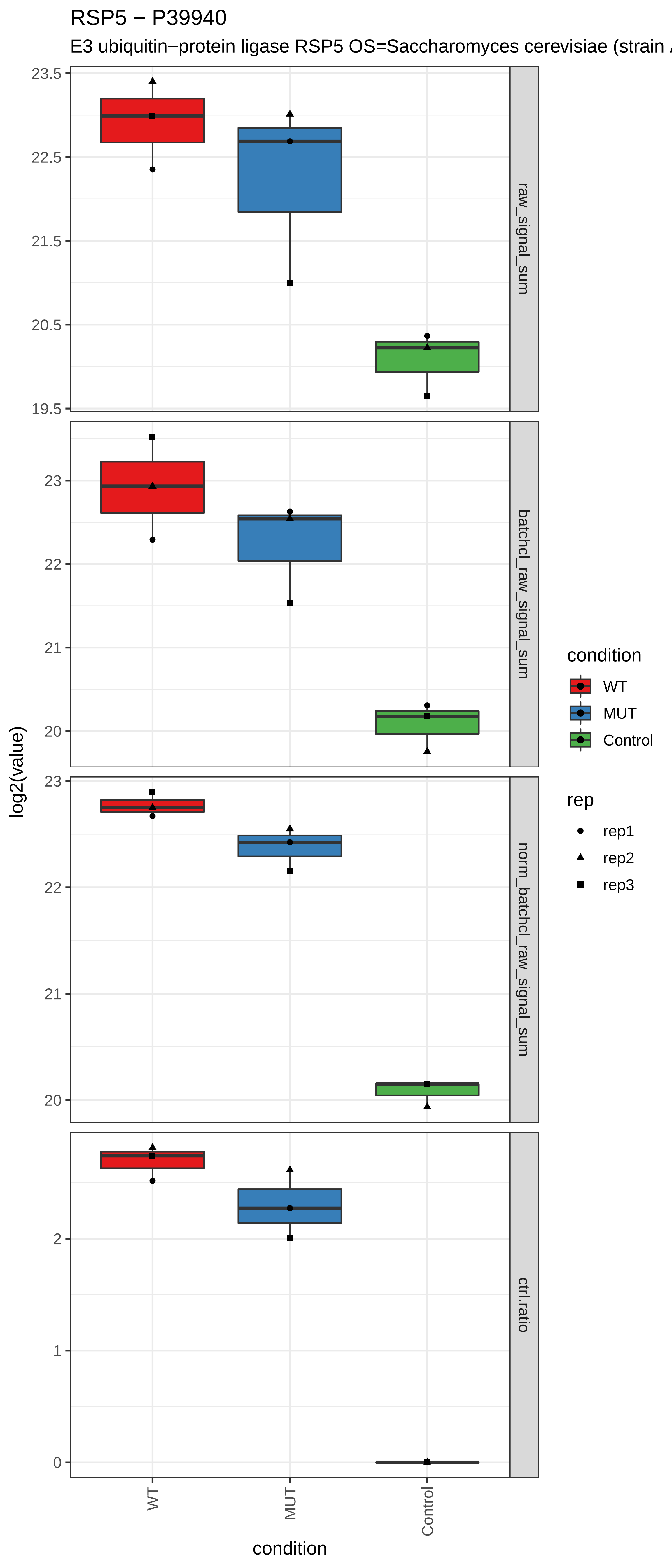

RSP5 − P39940
E3 ubiquitin−protein ligase RSP5 OS=Saccharomyces cerevisiae (strain
log2(value)
raw_signal_sum
batchcl_raw_signal_sum
norm_batchcl_raw_signal_sum
ctrl.ratio
condition
WT
MUT
Control
rep
rep1
rep2
rep3

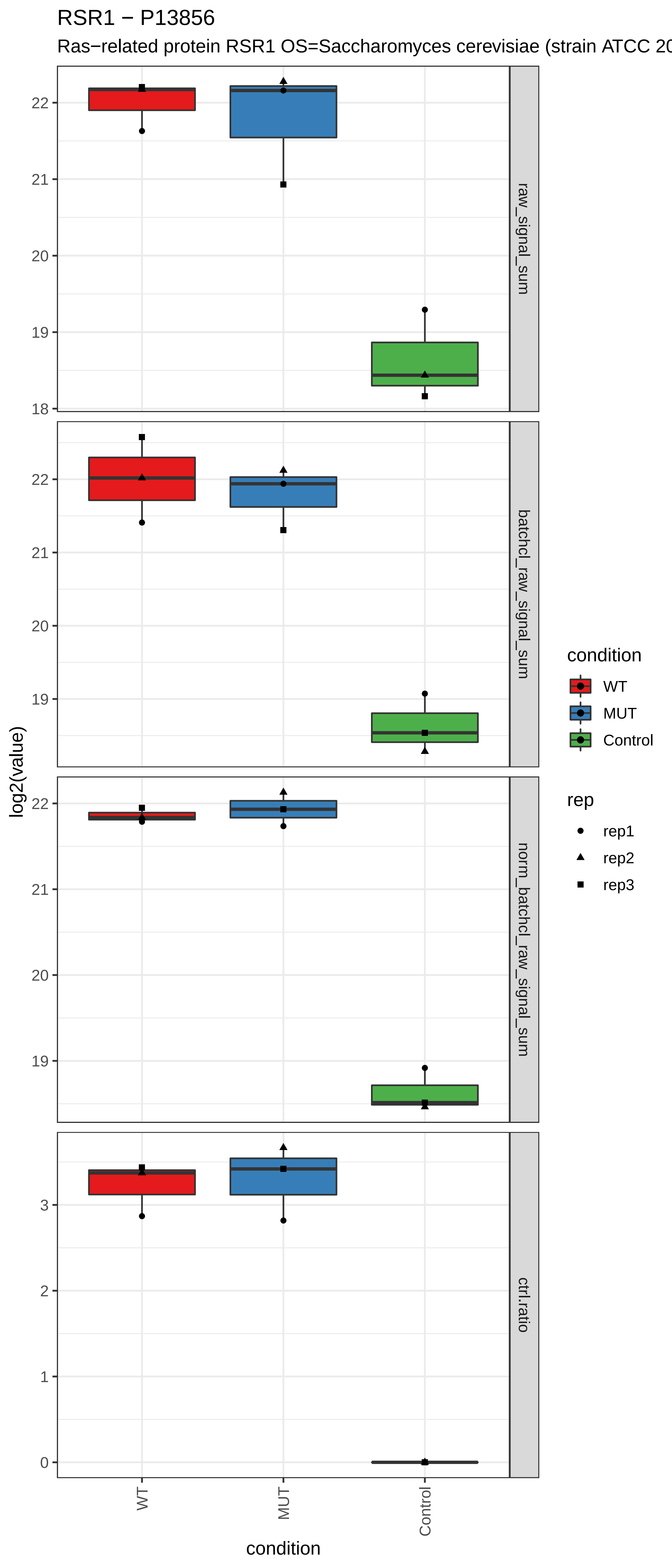
RSR1 − P13856
Ras−related protein RSR1 OS=Saccharomyces cerevisiae (strain ATCC 20
raw_signal_sum
batchcl_raw_signal_sum
norm_batchcl_raw_signal_sum
ctrl.ratio
log2(value)
condition
WT
MUT
Control
rep
rep1
rep2
rep3

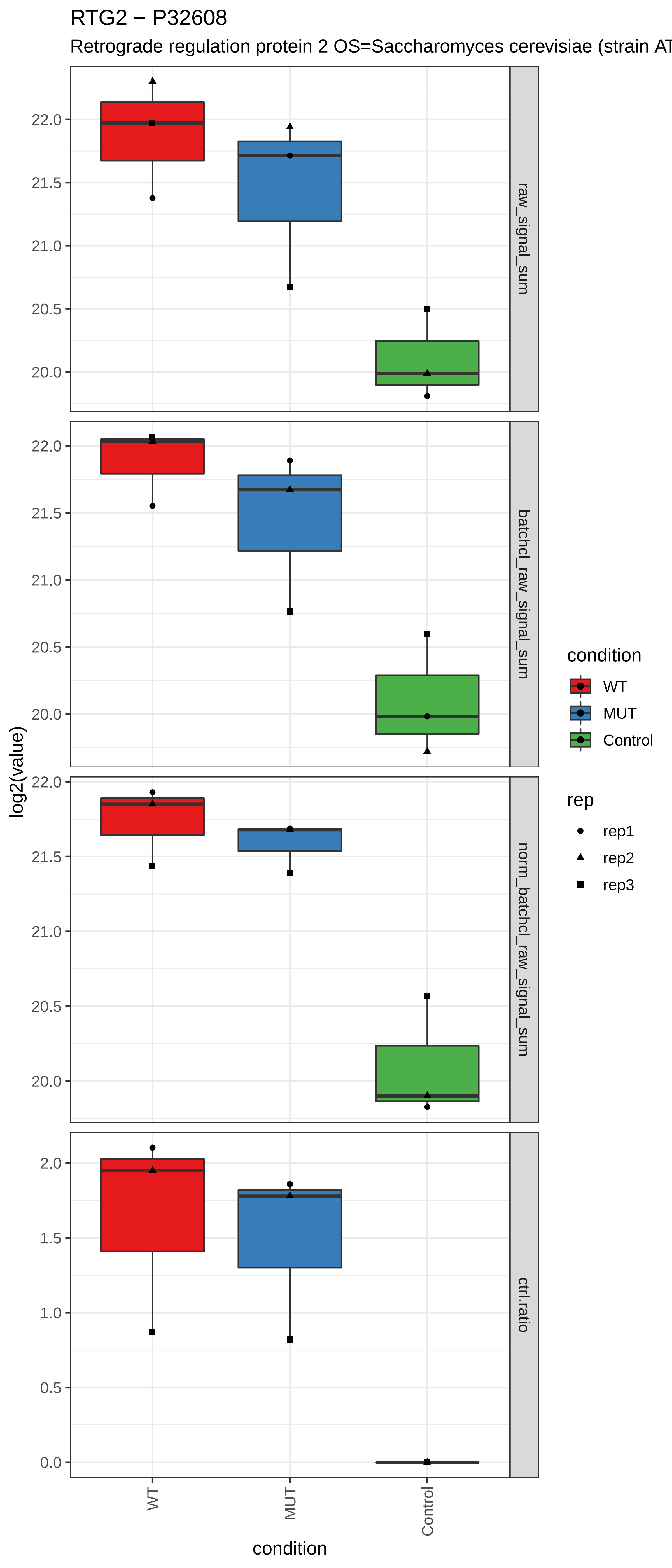

RTG2 − P32608
Retrograde regulation protein 2 OS=Saccharomyces cerevisiae (strain AT
raw_signal_sum
batchcl_raw_signal_sum
norm_batchcl_raw_signal_sum
ctrl.ratio
log2(value)
condition
WT
MUT
Control
rep
rep1
rep2
rep3
22.0
21.5
21.0
20.5
20.0
2.0
1.5
1.0
0.5
0.0

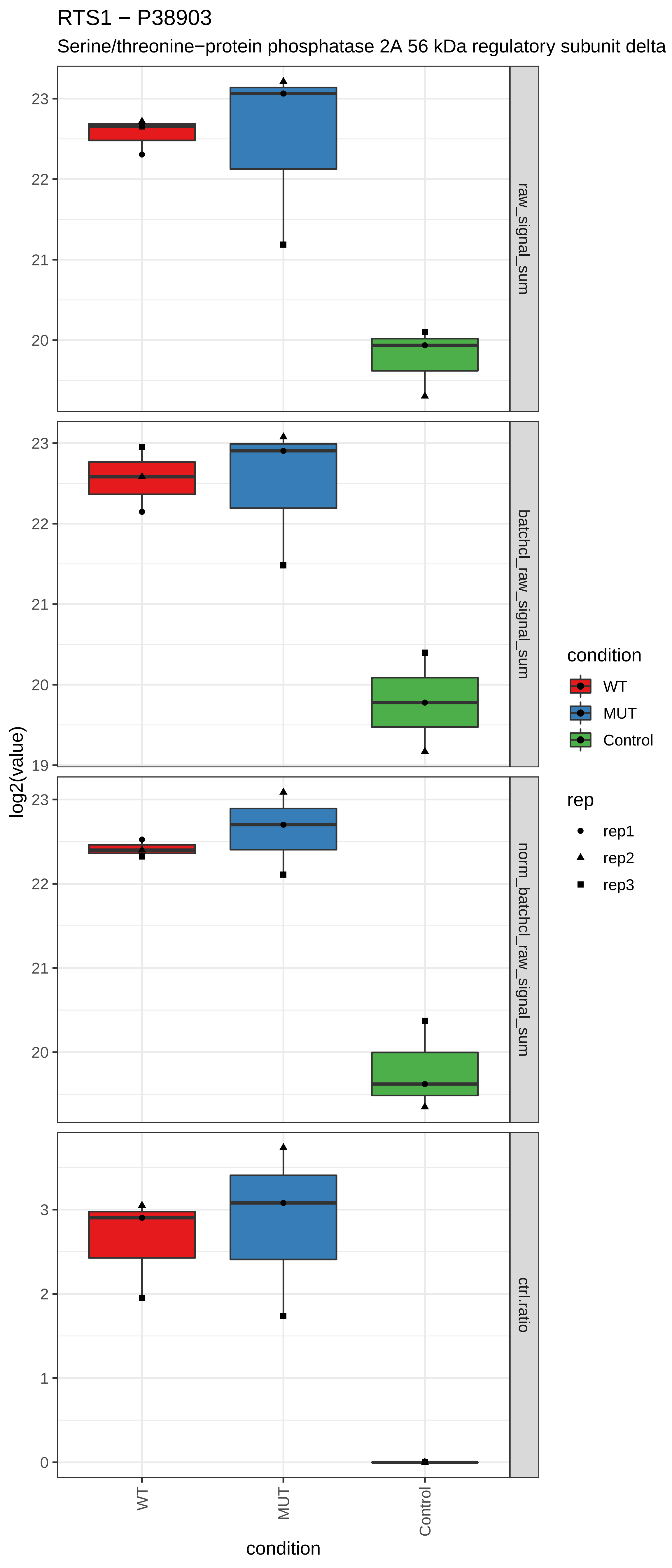

RTS1 − P38903
Serine/threonine−protein phosphatase 2A 56 kDa regulatory subunit delta
raw_signal_sum
batchcl_raw_signal_sum
norm_batchcl_raw_signal_sum
ctrl.ratio
log2(value)
condition
WT
MUT
Control
rep
rep1
rep2
rep3

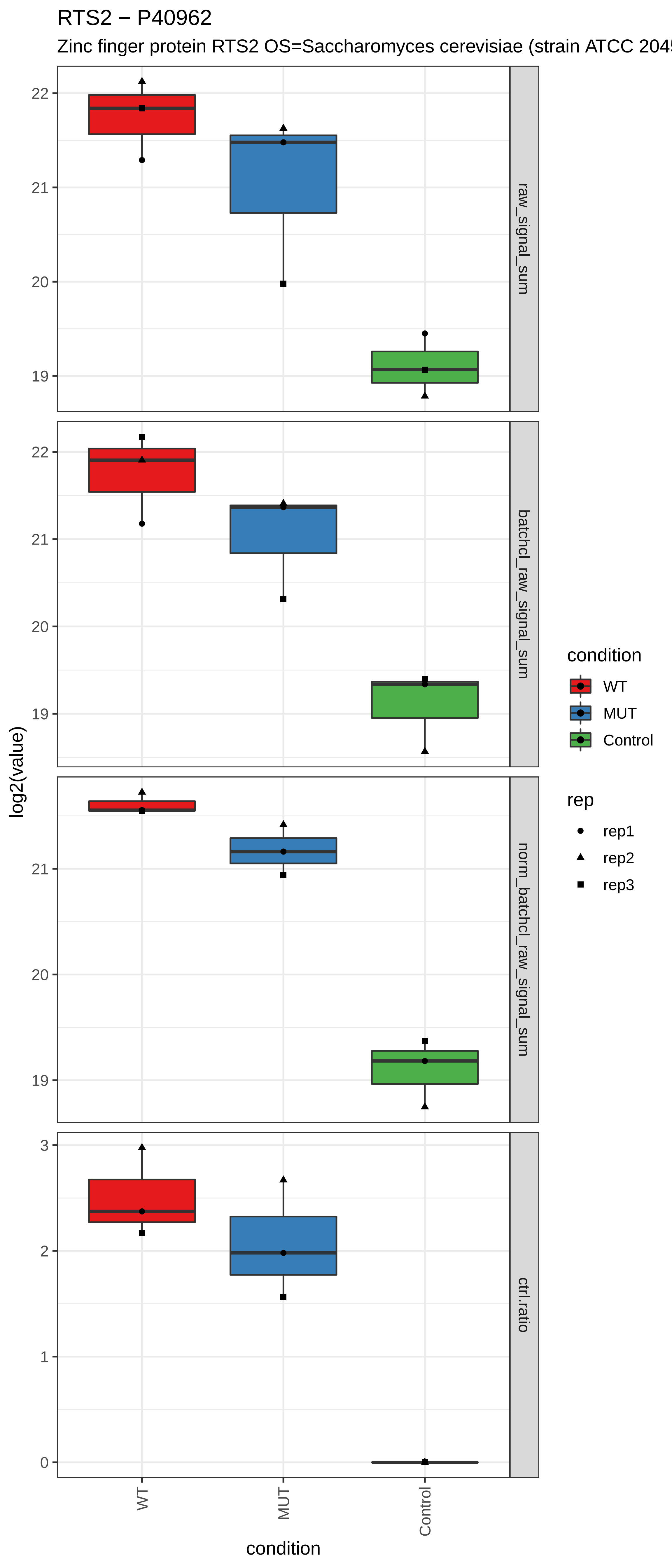
RTS2 − P40962
Zinc finger protein RTS2 OS=Saccharomyces cerevisiae (strain ATCC 204
raw_signal_sum
batchcl_raw_signal_sum
norm_batchcl_raw_signal_sum
ctrl.ratio
log2(value)
condition
WT
MUT
Control
rep
rep1
rep2
rep3

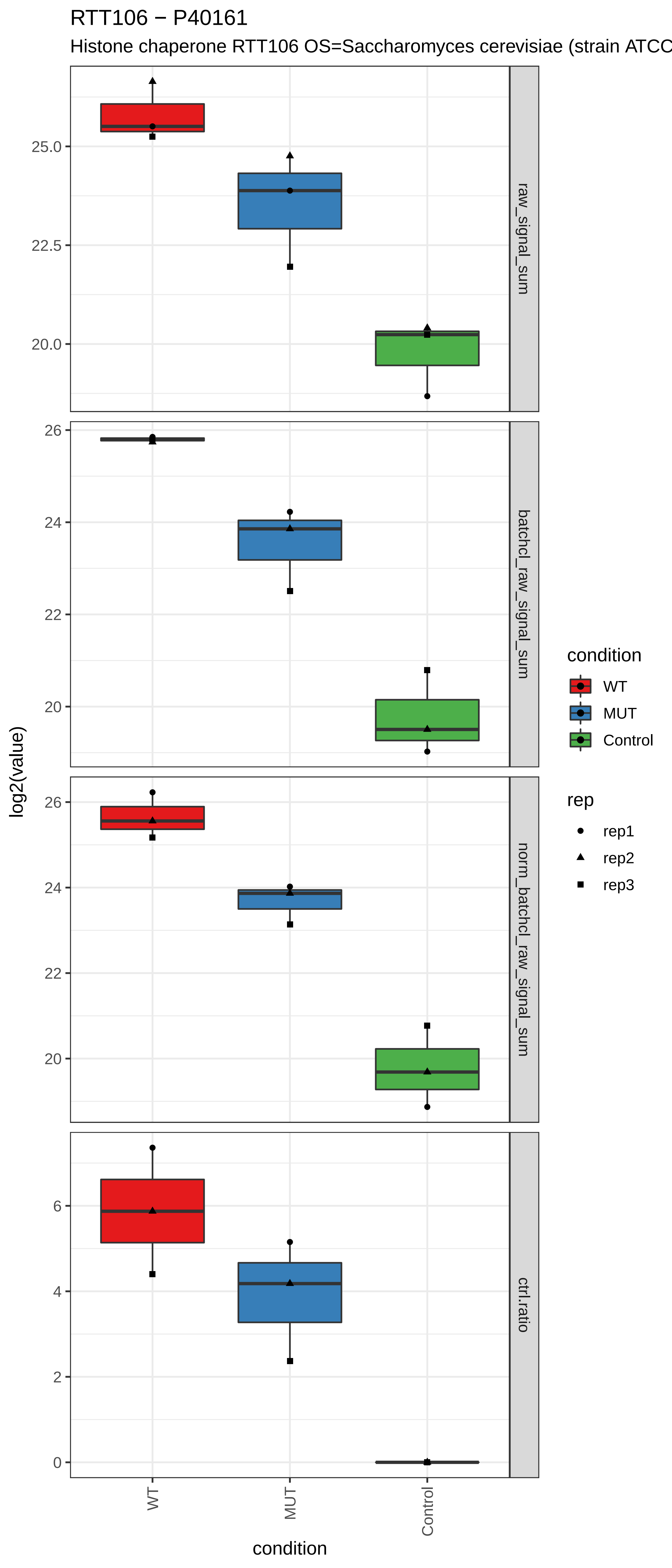

RTT106 − P40161
Histone chaperone RTT106 OS=Saccharomyces cerevisiae (strain ATCC
log2(value)
25.0
22.5
20.0
raw_signal_sum
26
24
22
20
batchcl_raw_signal_sum
26
24
22
20
norm_batchcl_raw_signal_sum
6
4
2
0
ctrl.ratio
WT
MUT
Control
condition
condition
WT
MUT
Control
rep
rep1
rep2
rep3

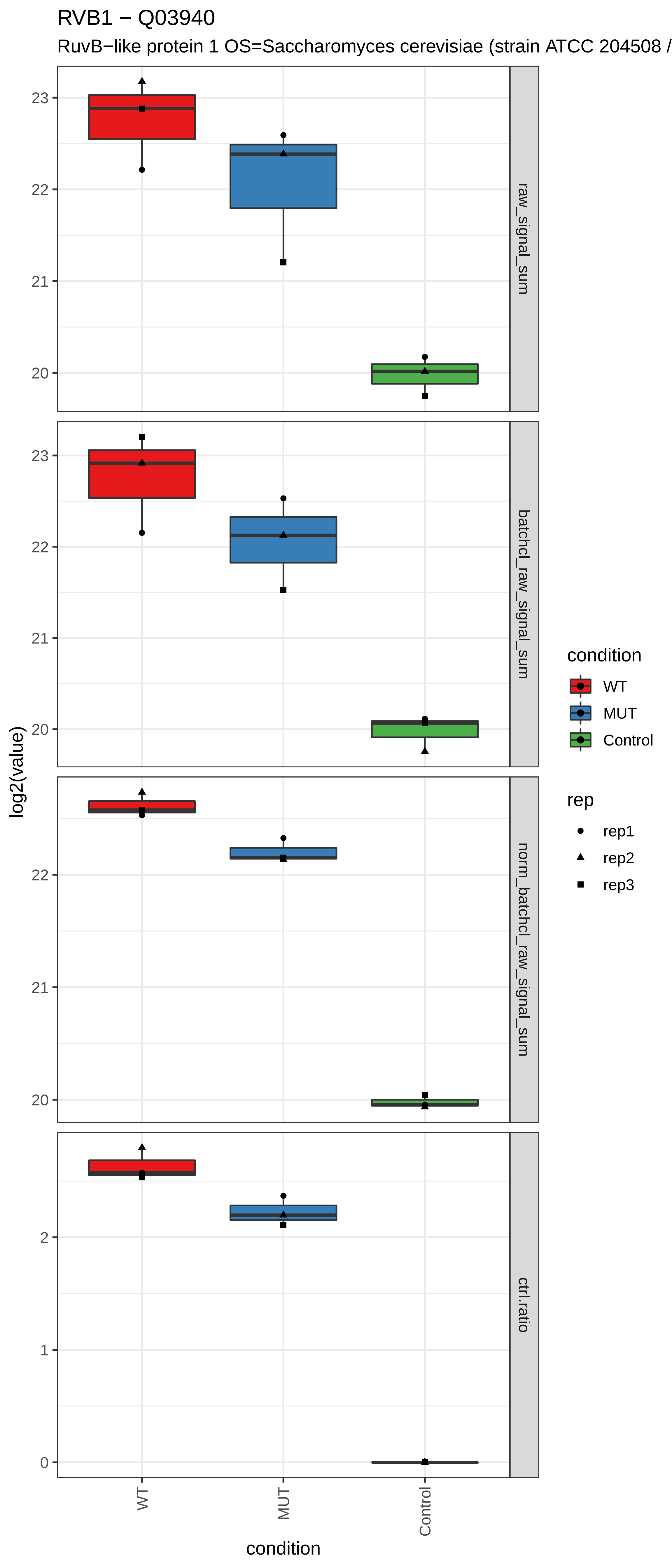
RVB1 − Q03940
RuvB−like protein 1 OS=Saccharomyces cerevisiae (strain ATCC 204508 /
raw_signal_sum
batchcl_raw_signal_sum
norm_batchcl_raw_signal_sum
ctrl.ratio
log2(value)
condition
WT
MUT
Control
rep
rep1
rep2
rep3
23
22
21
20
2
1
0

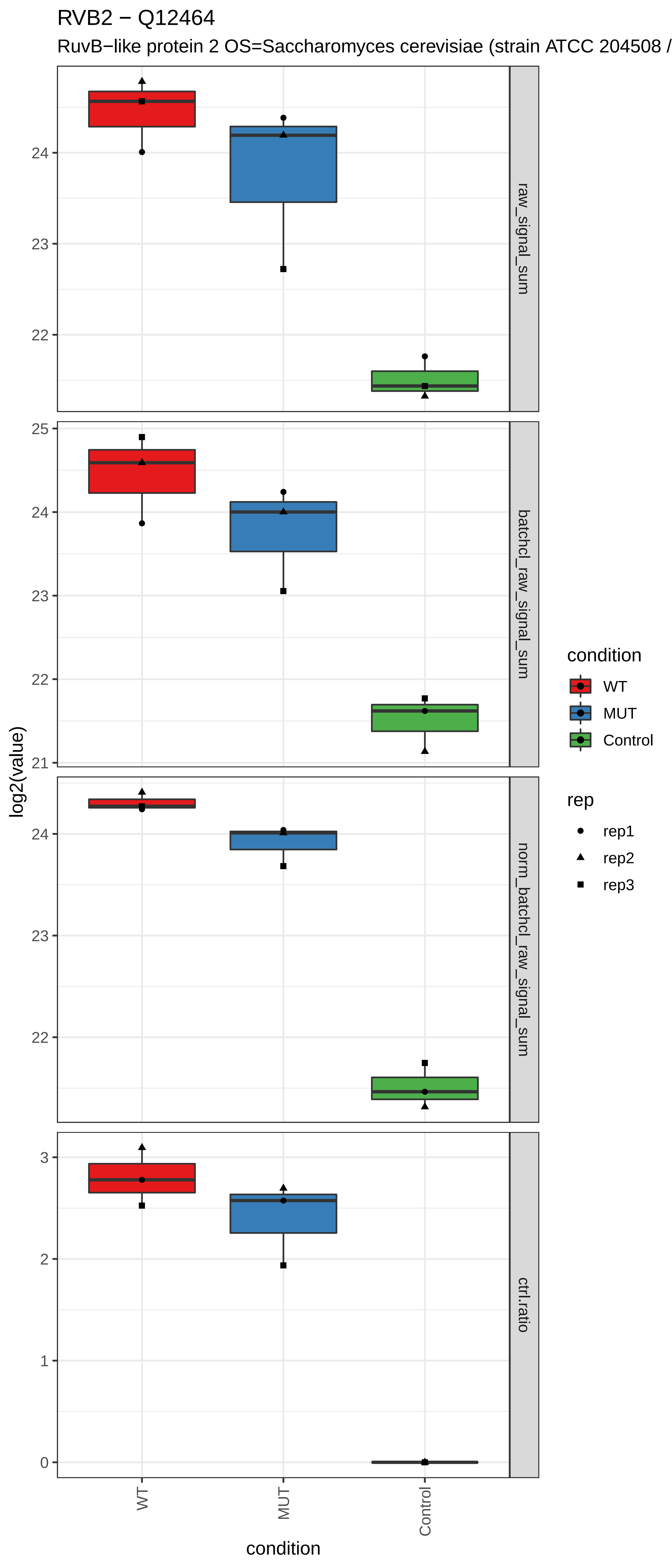

RVB2 − Q12464
RuvB−like protein 2 OS=Saccharomyces cerevisiae (strain ATCC 204508 /
raw_signal_sum
batchcl_raw_signal_sum
norm_batchcl_raw_signal_sum
ctrl.ratio
log2(value)
condition
WT
MUT
Control
rep
rep1
rep2
rep3

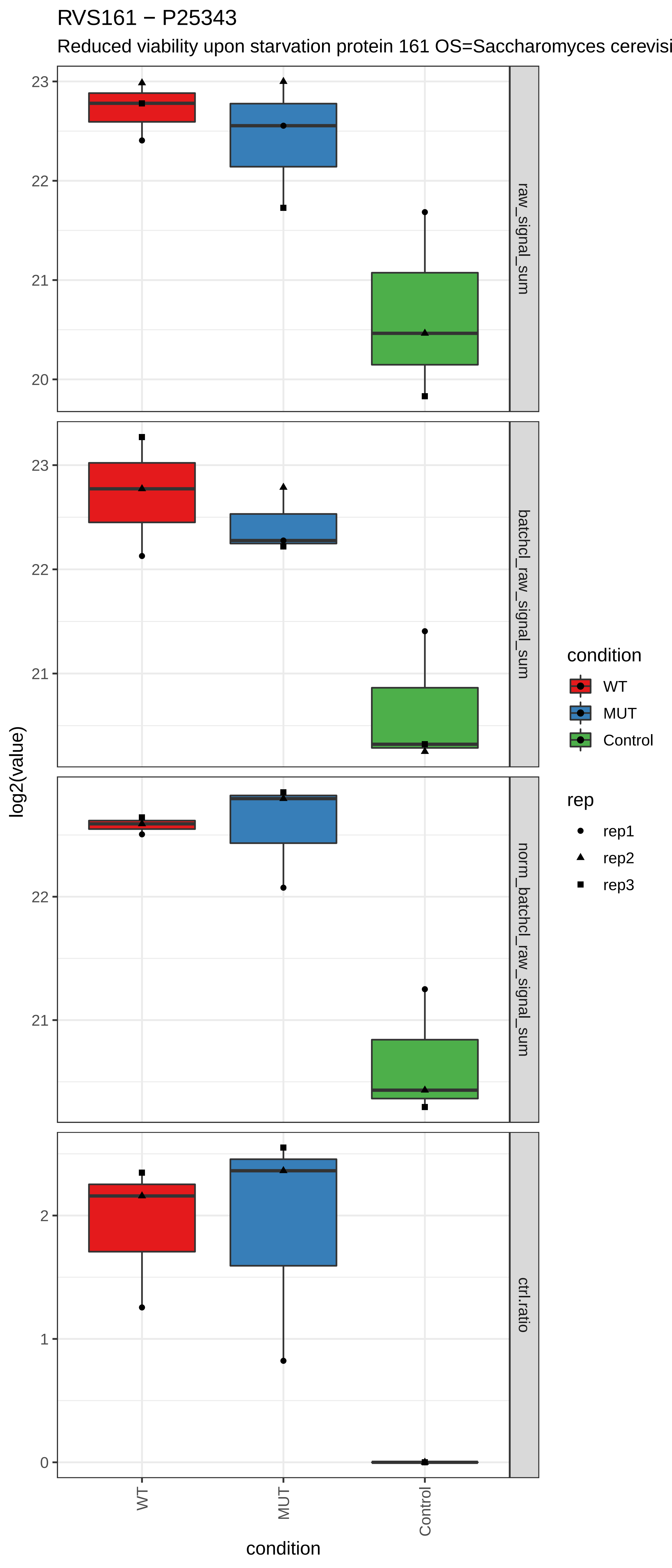

RVS161 − P25343
Reduced viability upon starvation protein 161 OS=Saccharomyces cerevisi
raw_signal_sum
batchcl_raw_signal_sum
norm_batchcl_raw_signal_sum
ctrl.ratio
log2(value)
condition
WT
MUT
Control
rep
rep1
rep2
rep3

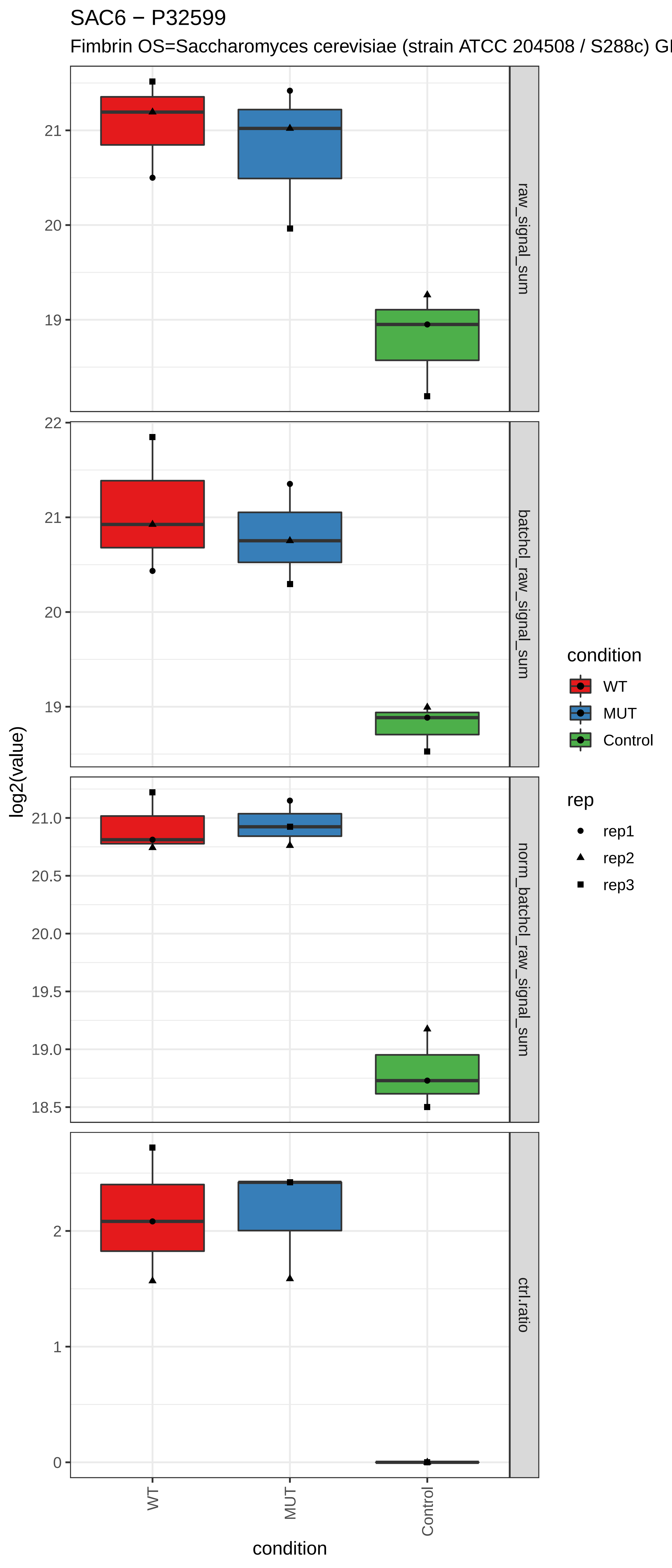

SAC6 − P32599
Fimbrin OS=Saccharomyces cerevisiae (strain ATCC 204508 / S288c) GI
raw_signal_sum
batchcl_raw_signal_sum
norm_batchcl_raw_signal_sum
ctrl.ratio
log2(value)
condition
WT
MUT
Control
rep
rep1
rep2
rep3
21
20
19
22
21.0
20.5
20.0
19.5
19.0
18.5
2
1
0

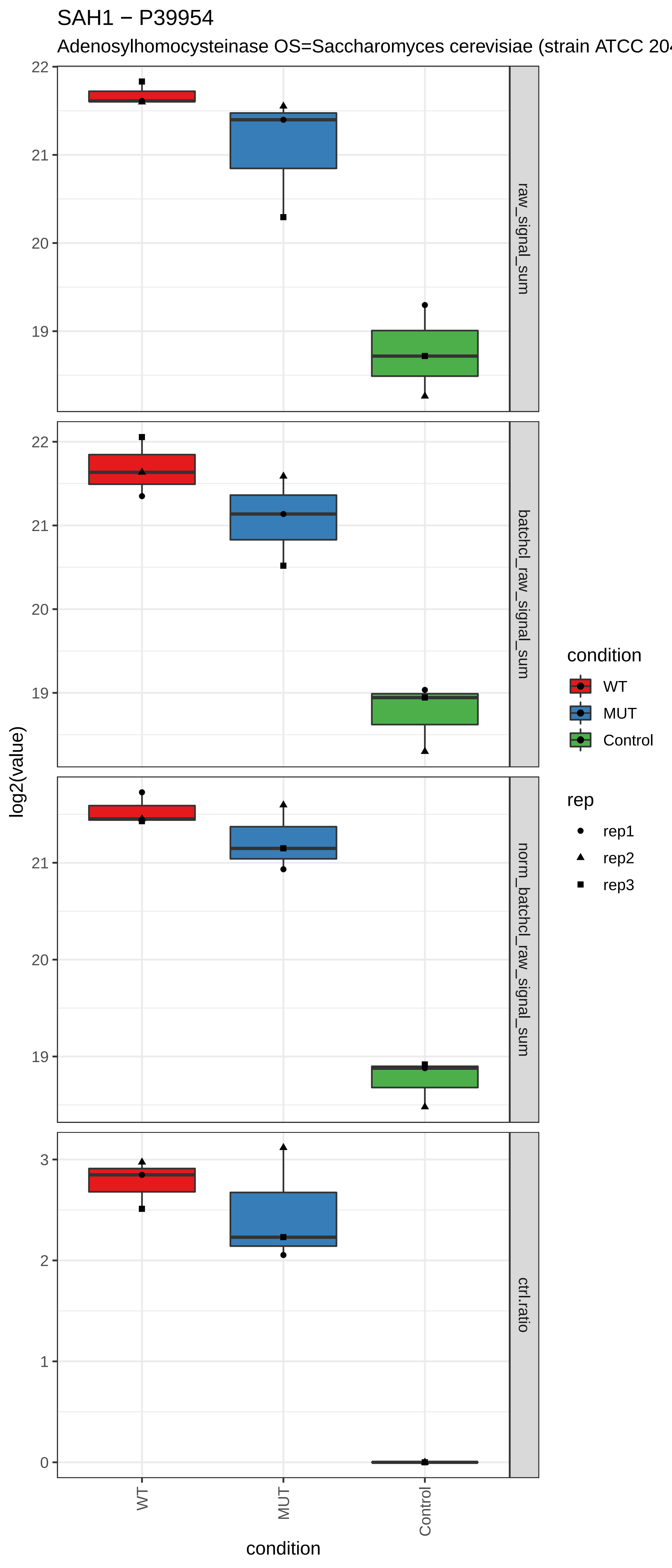

SAH1 − P39954
Adenosylhomocysteinase OS=Saccharomyces cerevisiae (strain ATCC 20
raw_signal_sum
batchcl_raw_signal_sum
norm_batchcl_raw_signal_sum
ctrl.ratio
log2(value)
condition
WT
MUT
Control
rep
rep1
rep2
rep3
22
21
20
19
3
2
1
0

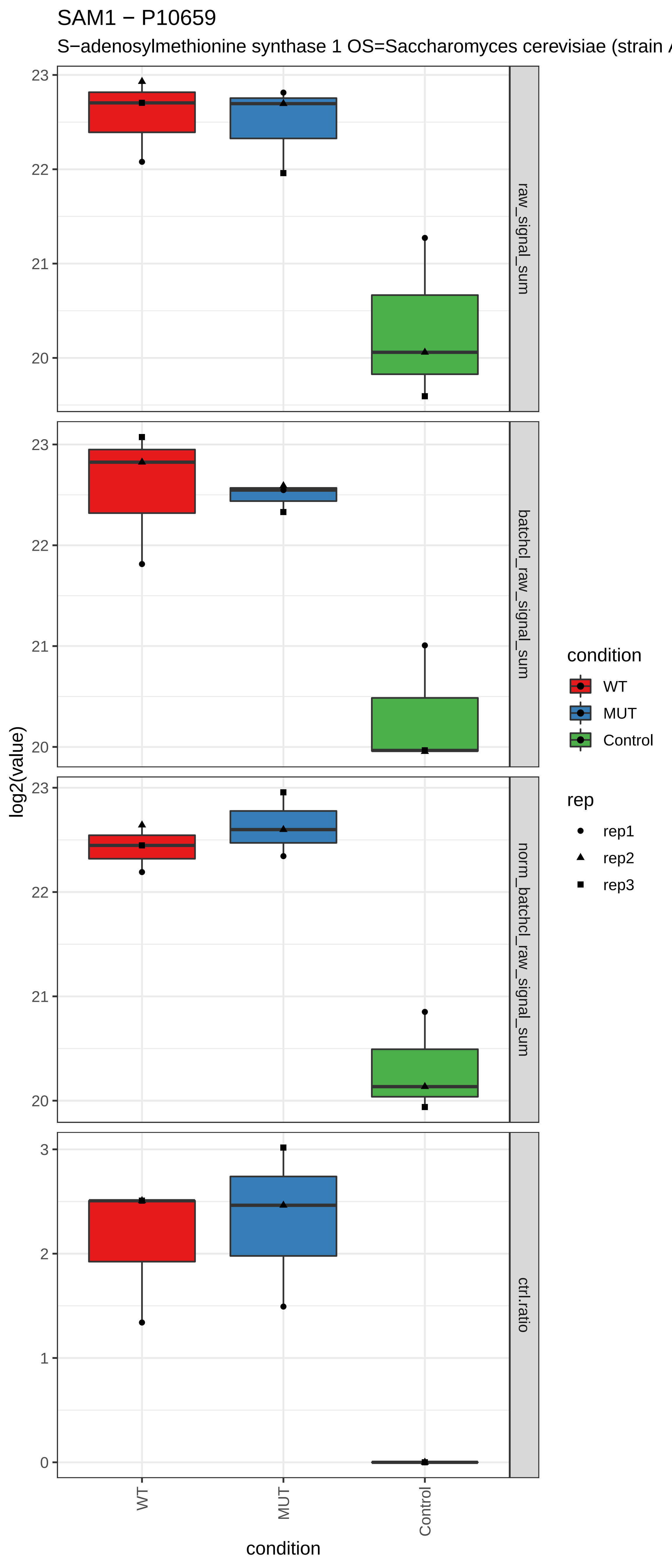

SAM1 − P10659
S−adenosylmethionine synthase 1 OS=Saccharomyces cerevisiae (strain A
raw_signal_sum
batchcl_raw_signal_sum
norm_batchcl_raw_signal_sum
ctrl.ratio
log2(value)
condition
WT
MUT
Control
rep
rep1
rep2
rep3

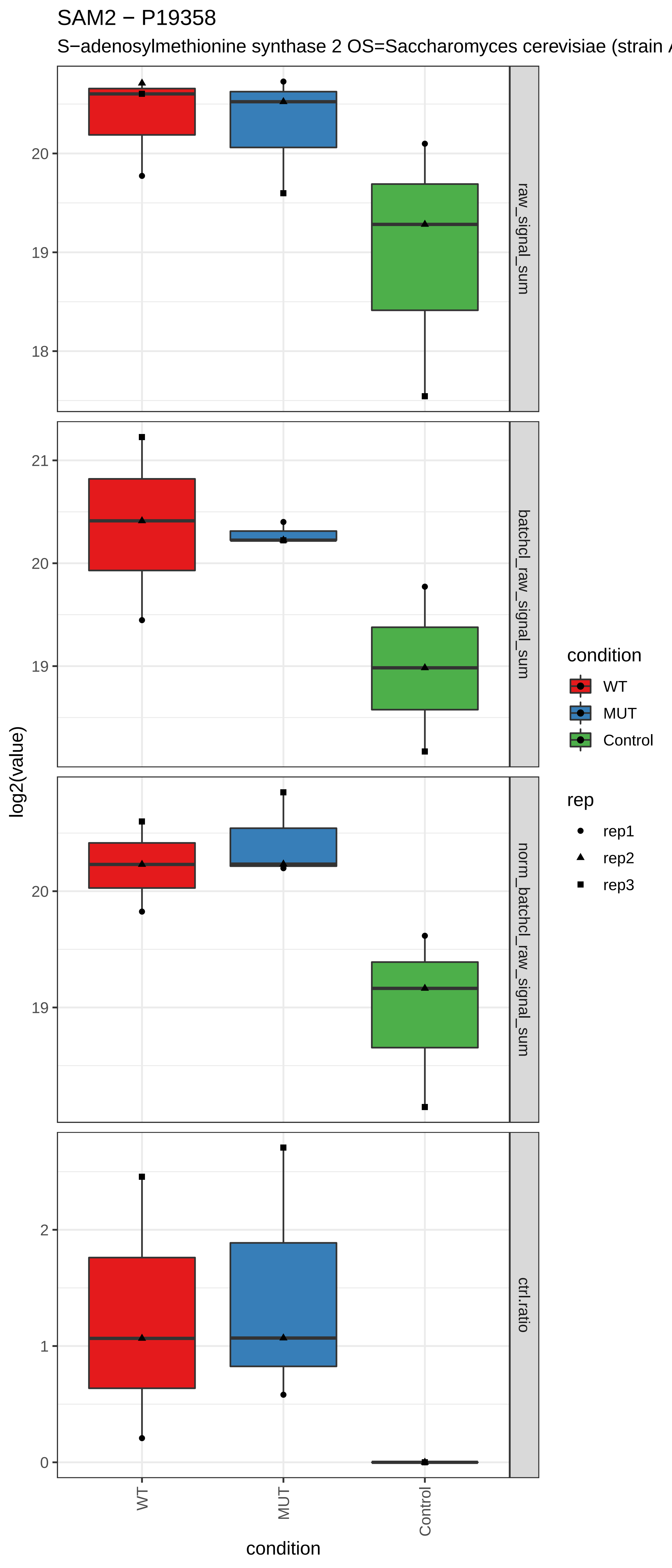

SAM2 − P19358
S−adenosylmethionine synthase 2 OS=Saccharomyces cerevisiae (strain A
raw_signal_sum
batchcl_raw_signal_sum
norm_batchcl_raw_signal_sum
ctrl.ratio
log2(value)
condition
WT
MUT
Control
rep
rep1
rep2
rep3

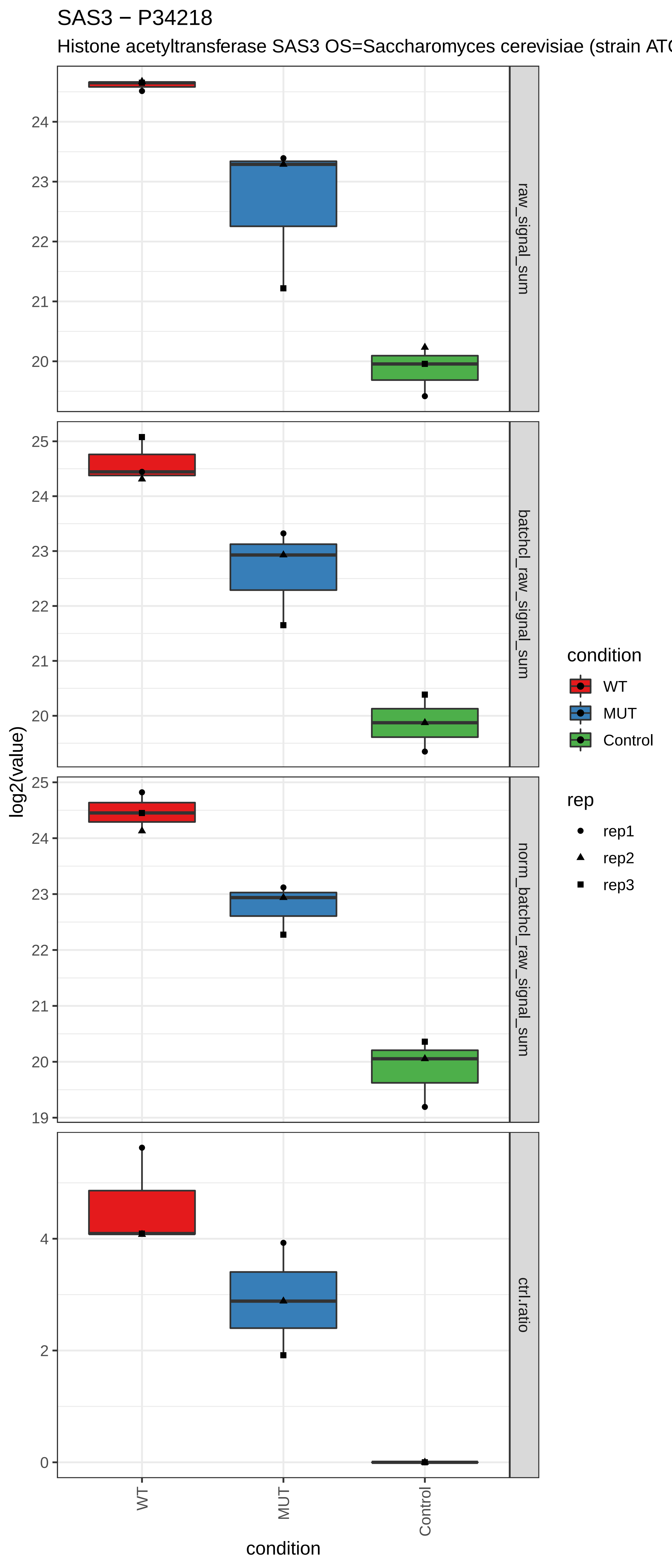

SAS3 − P34218
Histone acetyltransferase SAS3 OS=Saccharomyces cerevisiae (strain ATC
raw_signal_sum
batchcl_raw_signal_sum
norm_batchcl_raw_signal_sum
ctrl.ratio
log2(value)
condition
WT
MUT
Control
rep
rep1
rep2
rep3

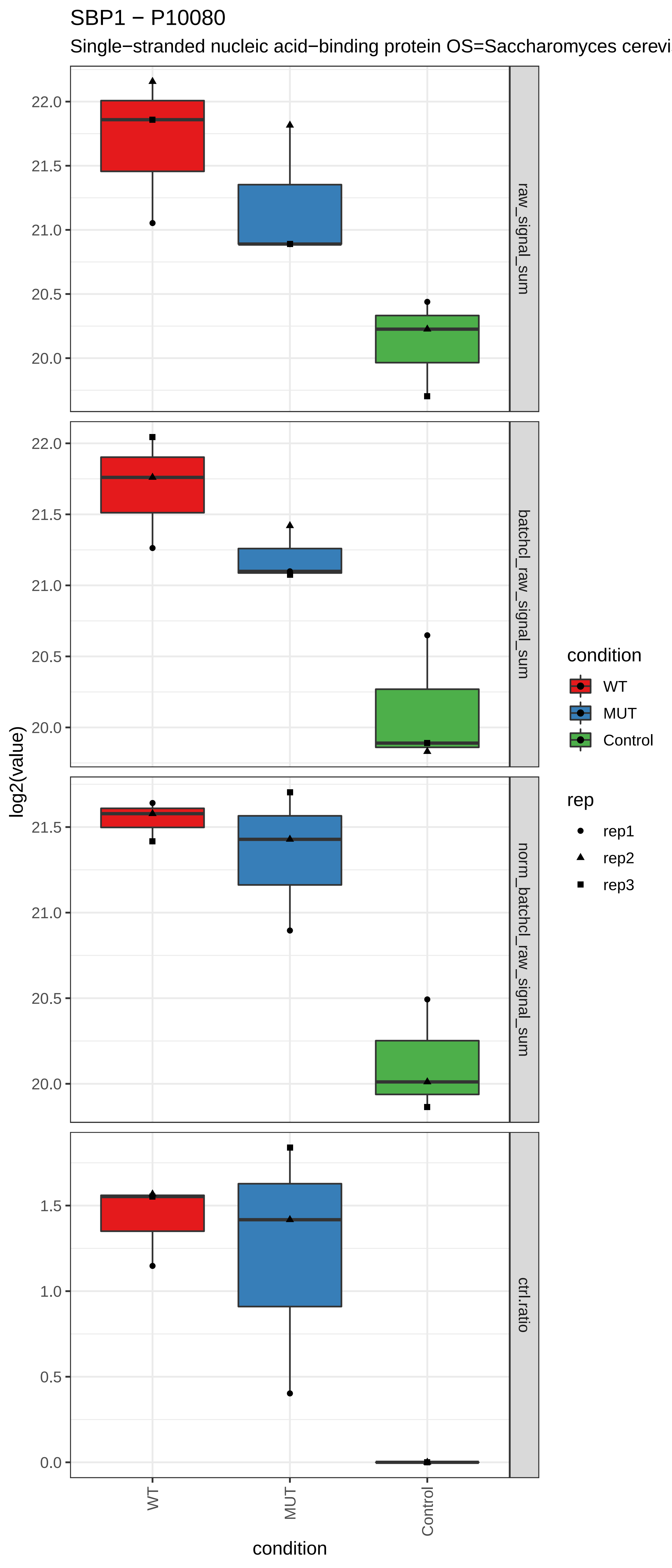

SBP1 − P10080
Single−stranded nucleic acid−binding protein OS=Saccharomyces cerevi
raw_signal_sum
batchcl_raw_signal_sum
norm_batchcl_raw_signal_sum
ctrl.ratio
22.0
21.5
21.0
20.5
20.0
1.5
1.0
0.5
0.0
log2(value)
WT
MUT
Control
condition
rep
rep1
rep2
rep3

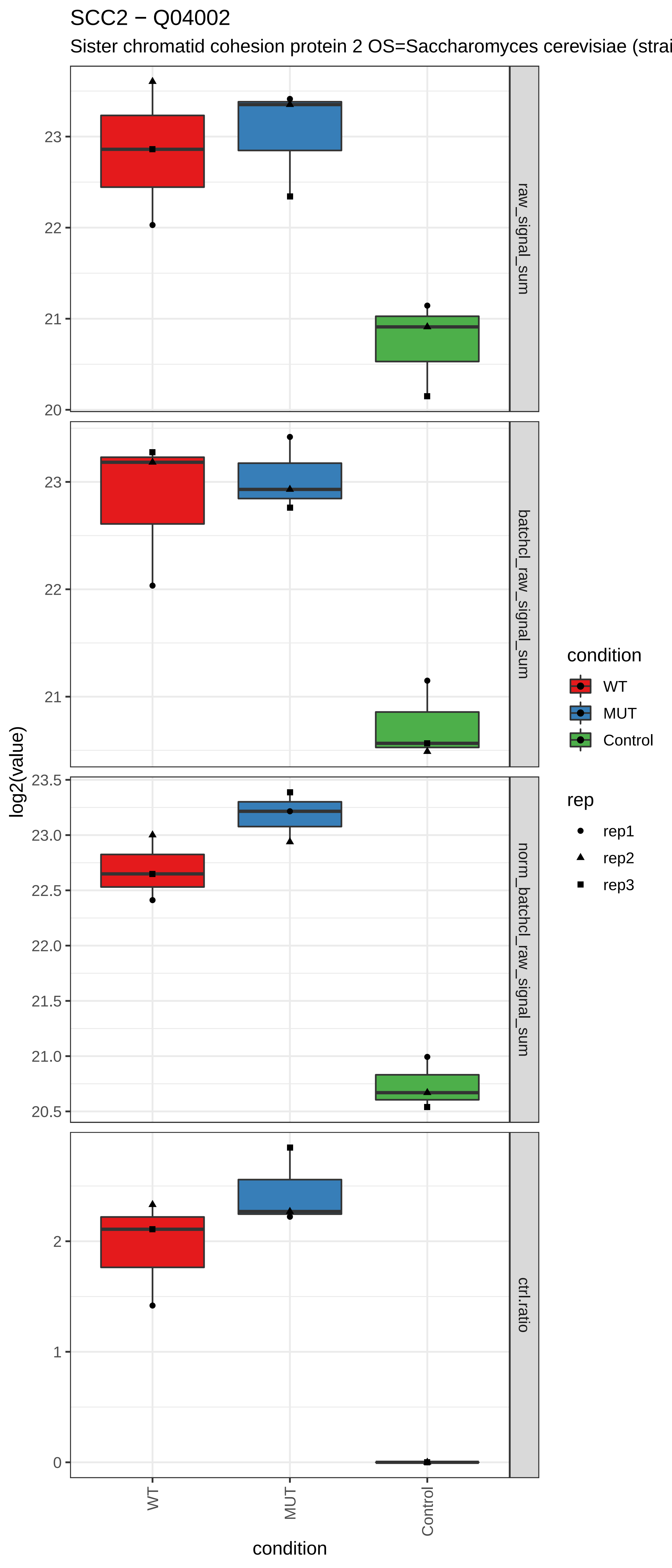

SCC2 − Q04002
Sister chromatid cohesion protein 2 OS=Saccharomyces cerevisiae (strai
raw_signal_sum
batchcl_raw_signal_sum
norm_batchcl_raw_signal_sum
ctrl.ratio
log2(value)
condition
WT
MUT
Control
rep
rep1
rep2
rep3
23
22
21
20
23.5
23.0
22.5
22.0
21.5
21.0
20.5
2
1
0

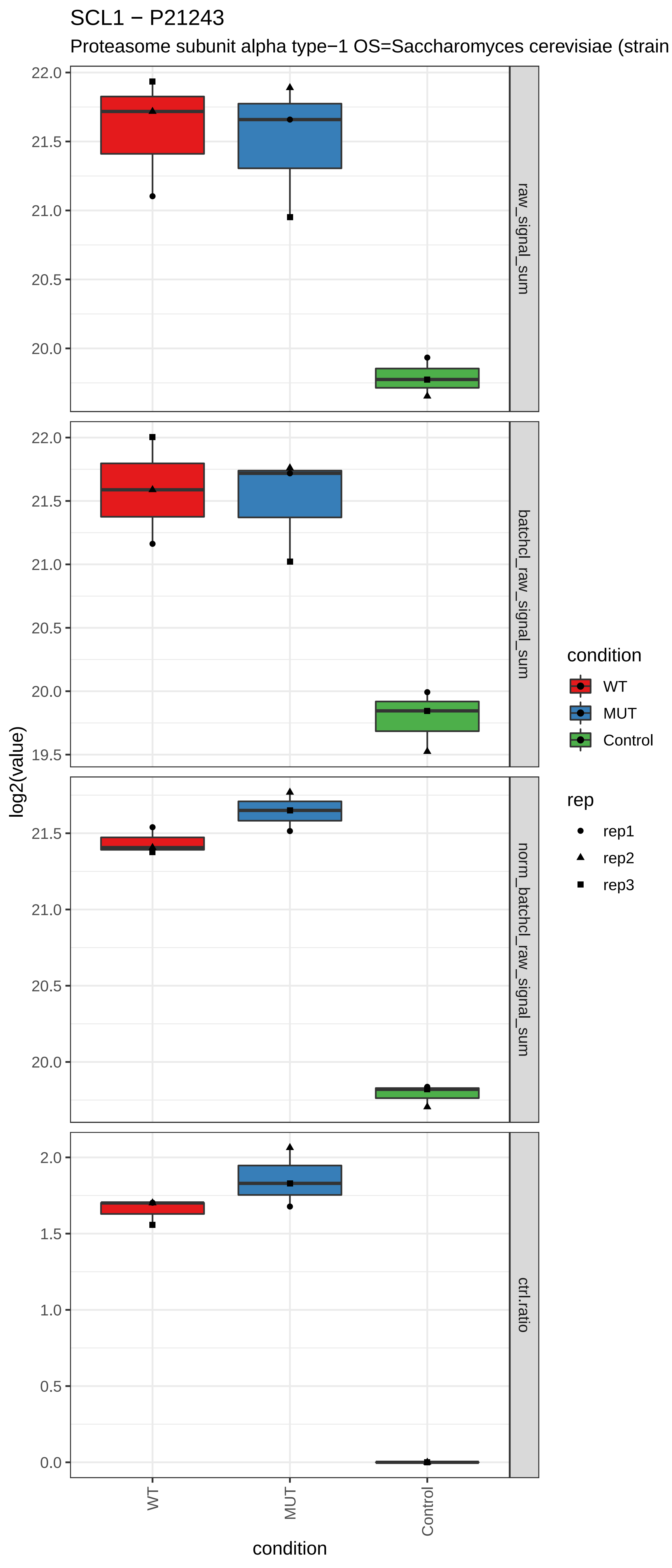

SCL1 − P21243
Proteasome subunit alpha type−1 OS=Saccharomyces cerevisiae (strain
raw_signal_sum
batchcl_raw_signal_sum
norm_batchcl_raw_signal_sum
ctrl.ratio
log2(value)
condition
WT
MUT
Control
rep
rep1
rep2
rep3

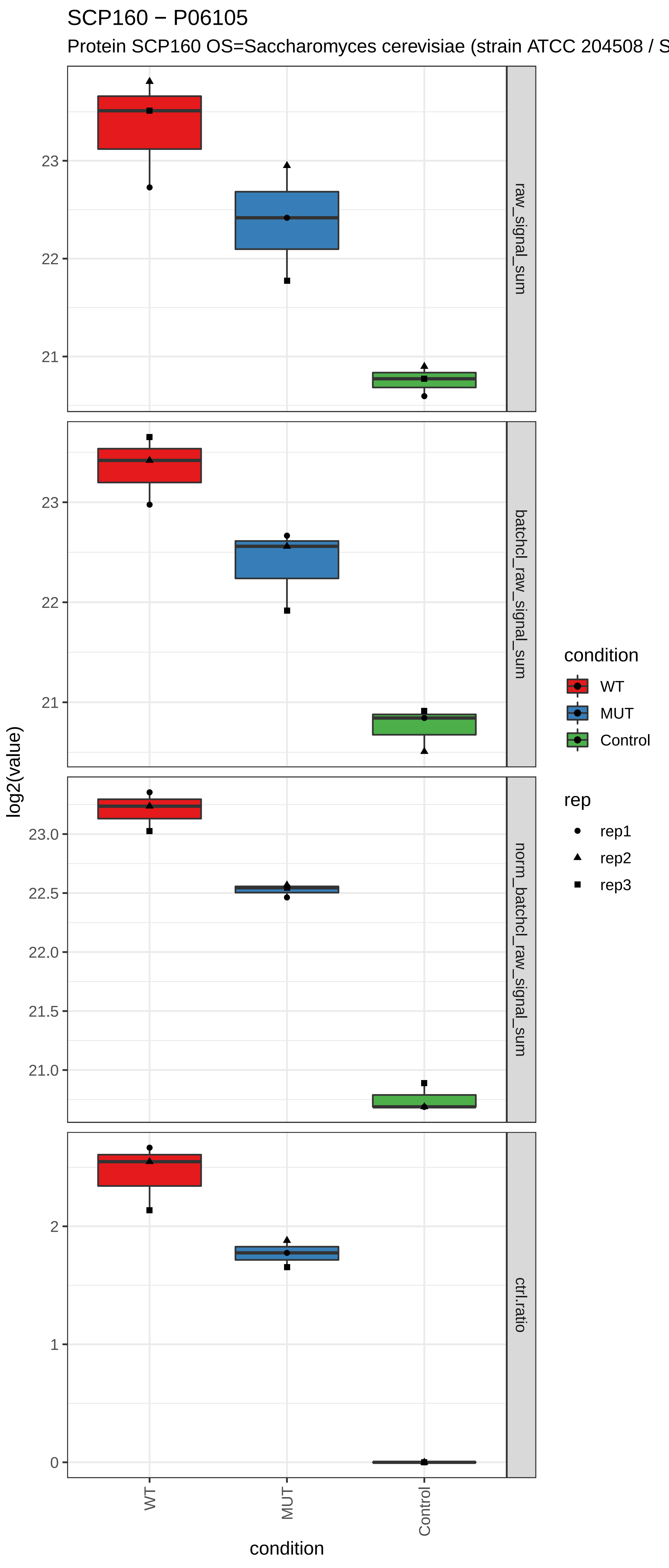

SCP160 − P06105
Protein SCP160 OS=Saccharomyces cerevisiae (strain ATCC 204508 / S
raw_signal_sum
batchcl_raw_signal_sum
norm_batchcl_raw_signal_sum
ctrl.ratio
log2(value)
condition
WT
MUT
Control
rep
rep1
rep2
rep3
23
22
21
23.0
22.5
22.0
21.5
21.0
2
1
0

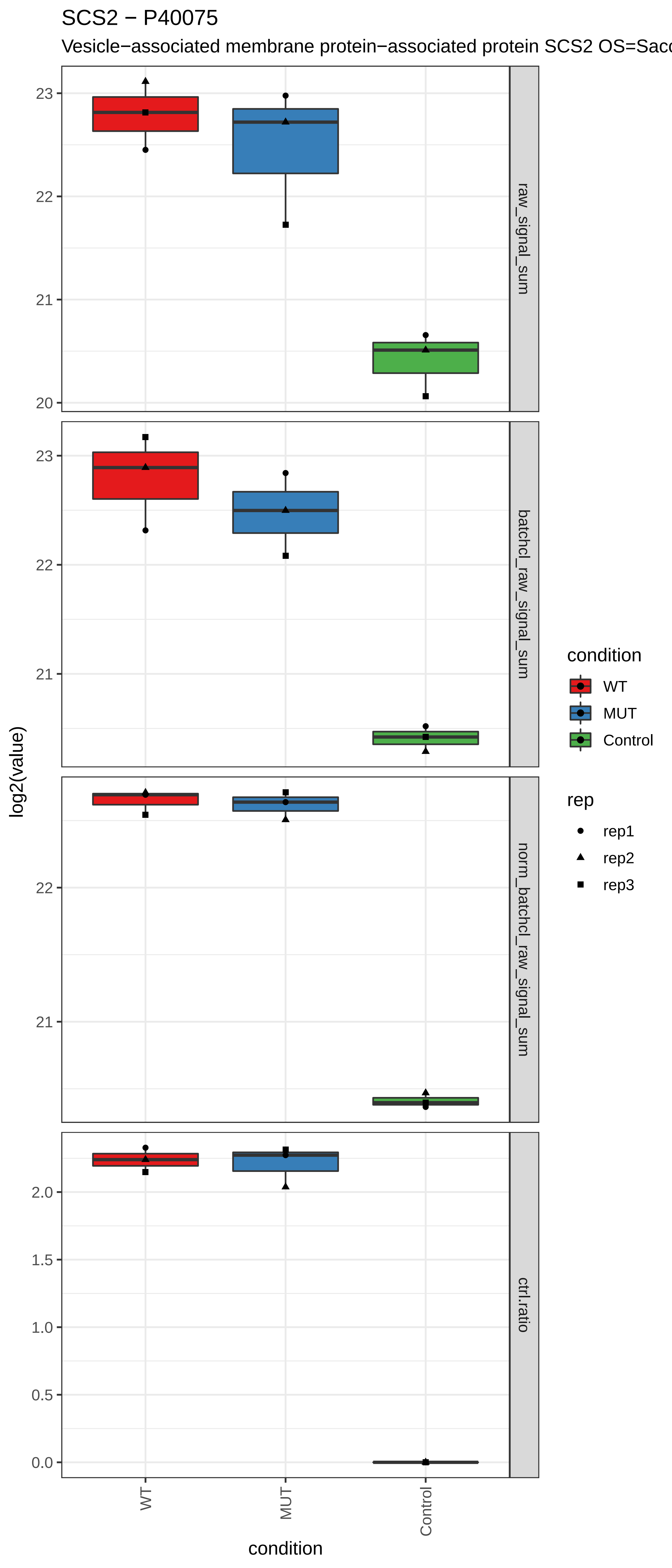
SCS2 − P40075
Vesicle−associated membrane protein−associated protein SCS2 OS=Sacc
raw_signal_sum
batchcl_raw_signal_sum
norm_batchcl_raw_signal_sum
ctrl.ratio
log2(value)
condition
WT
MUT
Control
rep
rep1
rep2
rep3

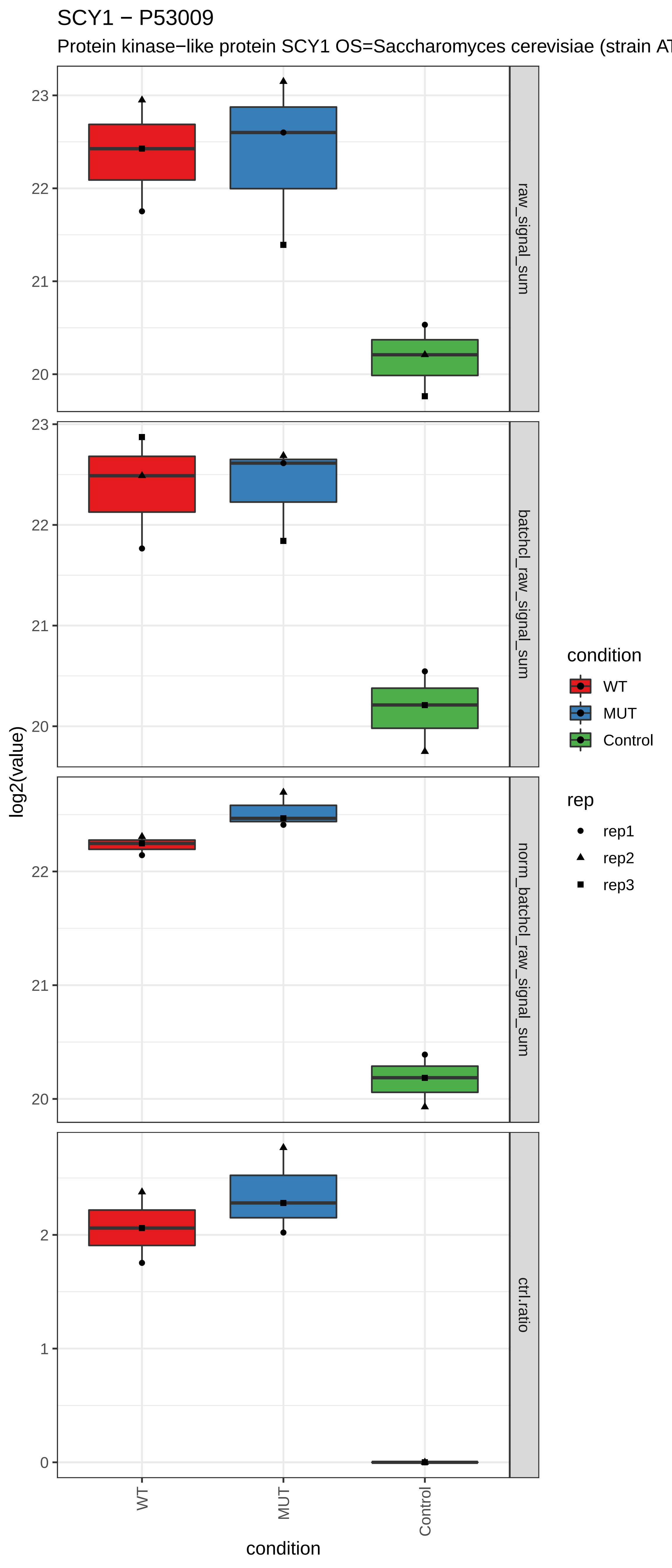
SCY1 − P53009
Protein kinase−like protein SCY1 OS=Saccharomyces cerevisiae (strain AT
raw_signal_sum
batchcl_raw_signal_sum
norm_batchcl_raw_signal_sum
ctrl.ratio
log2(value)
condition
WT
MUT
Control
condition
WT
MUT
Control
rep
rep1
rep2
rep3

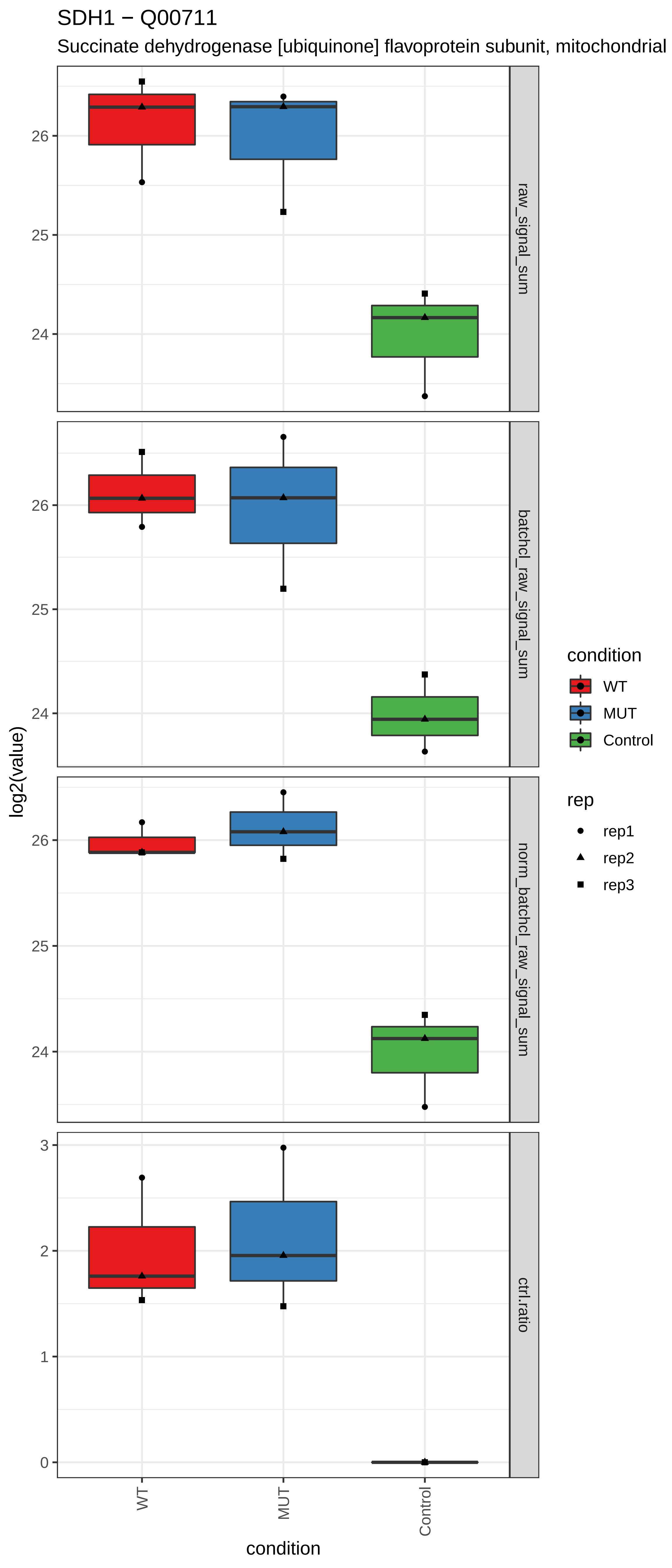
SDH1 − Q00711
Succinate dehydrogenase [ubiquinone] flavoprotein subunit, mitochondrial
raw_signal_sum
batchcl_raw_signal_sum
norm_batchcl_raw_signal_sum
ctrl.ratio
log2(value)
condition
WT
MUT
Control
rep
rep1
rep2
rep3
26
25
24
3
2
1
0

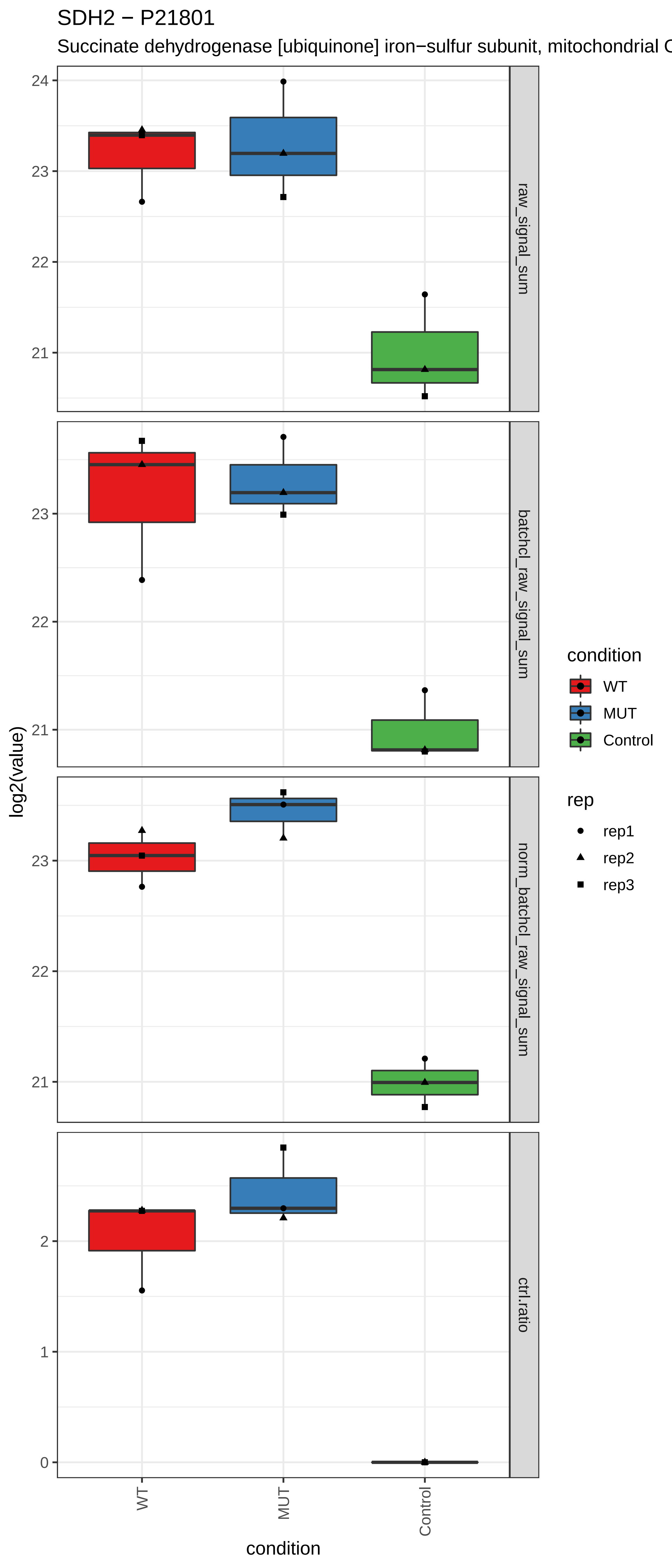

SDH2 − P21801
Succinate dehydrogenase [ubiquinone] iron−sulfur subunit, mitochondrial C
raw_signal_sum
batchcl_raw_signal_sum
norm_batchcl_raw_signal_sum
ctrl.ratio
log2(value)
condition
WT
MUT
Control
condition
WT
MUT
Control
rep
rep1
rep2
rep3
24
23
22
21
0
1
2

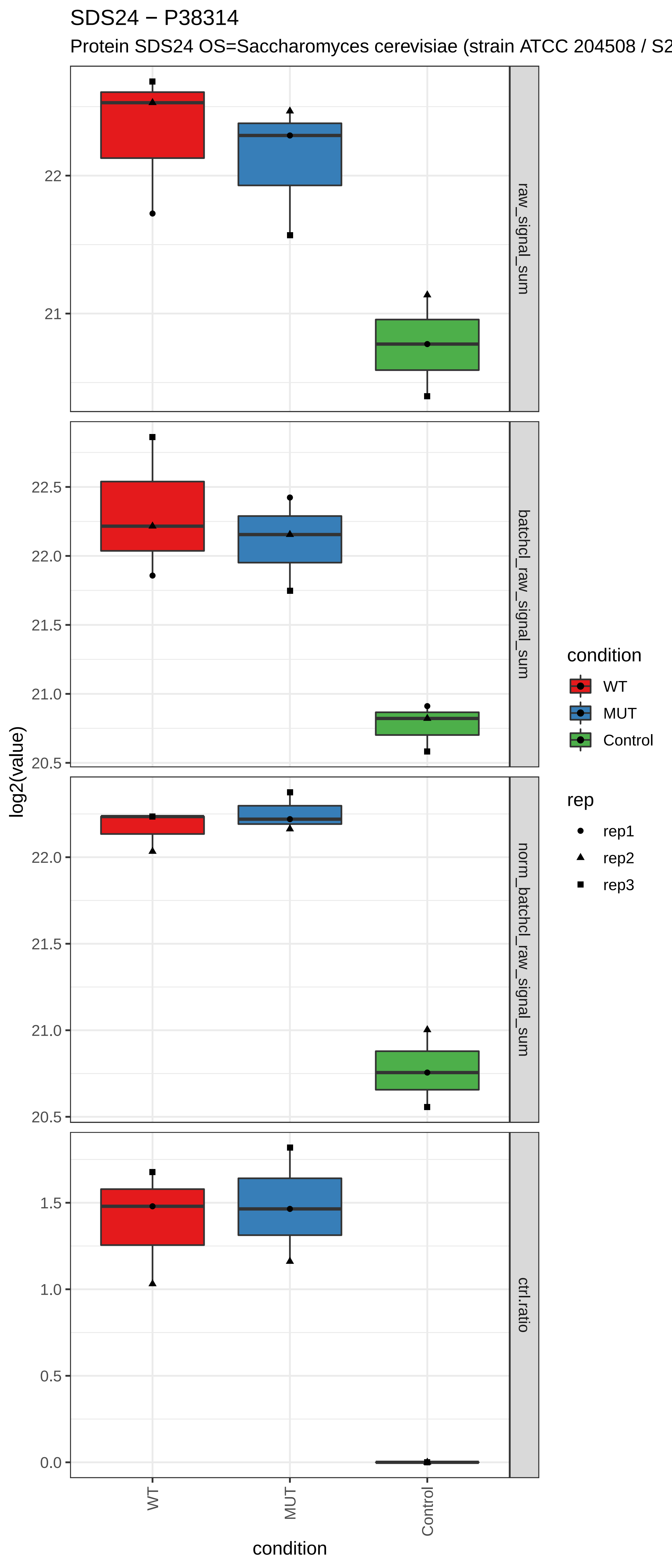

SDS24 − P38314
Protein SDS24 OS=Saccharomyces cerevisiae (strain ATCC 204508 / S2
raw_signal_sum
batchcl_raw_signal_sum
norm_batchcl_raw_signal_sum
ctrl.ratio
log2(value)
condition
WT
MUT
Control
rep
rep1
rep2
rep3
22
21
22.5
22.0
21.5
21.0
20.5
1.5
1.0
0.5
0.0

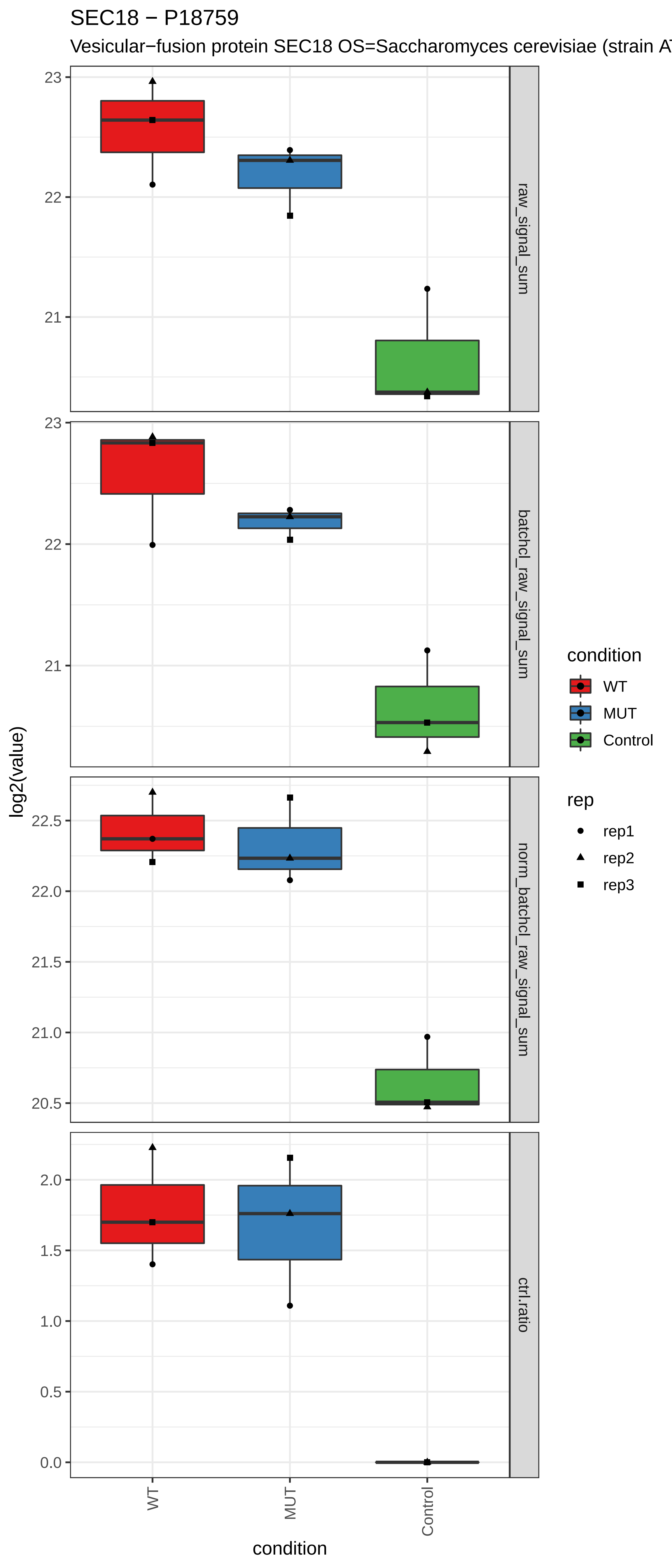

SEC18 − P18759
Vesicular−fusion protein SEC18 OS=Saccharomyces cerevisiae (strain A
raw_signal_sum
batchcl_raw_signal_sum
norm_batchcl_raw_signal_sum
ctrl.ratio
log2(value)
condition
WT
MUT
Control
rep
rep1
rep2
rep3

SEC23 − P15303

Protein transport protein SEC23 OS=Saccharomyces cerevisiae (strain A

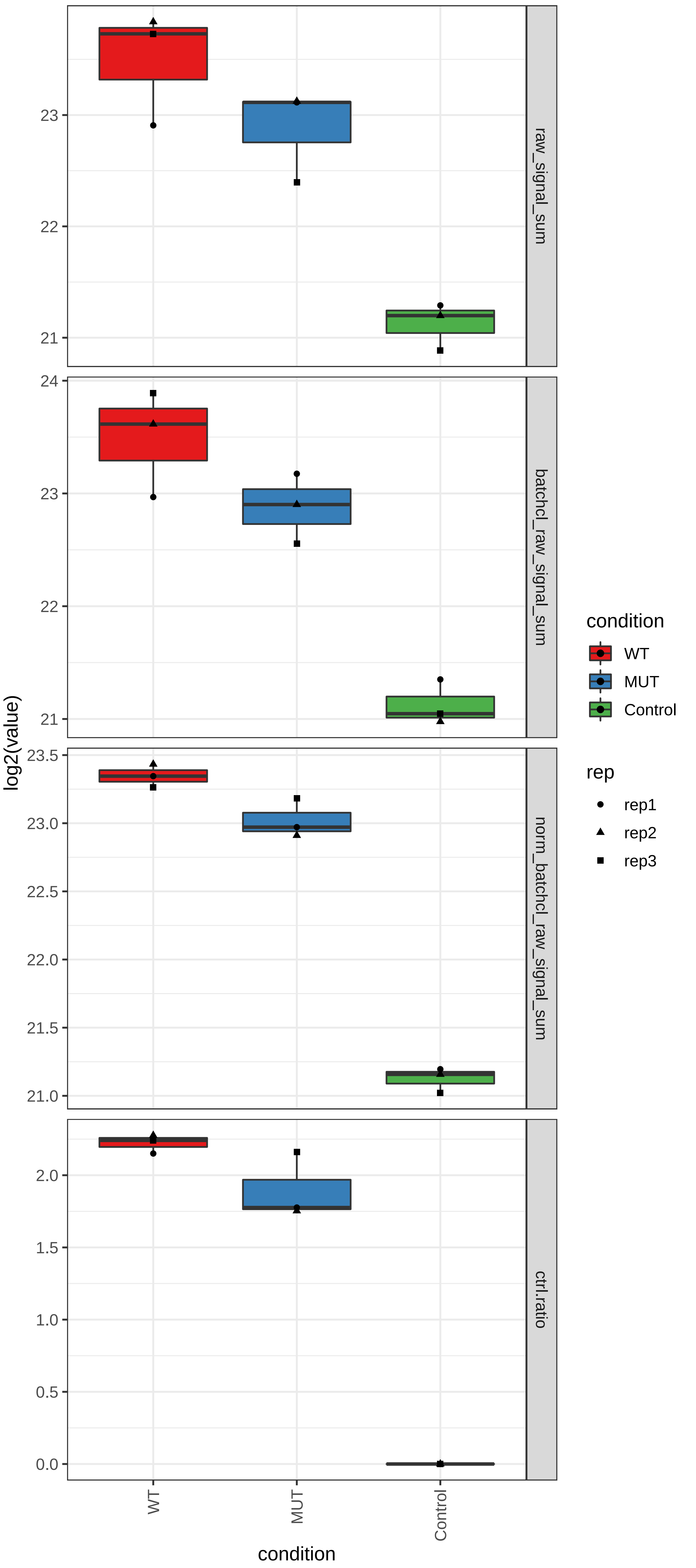

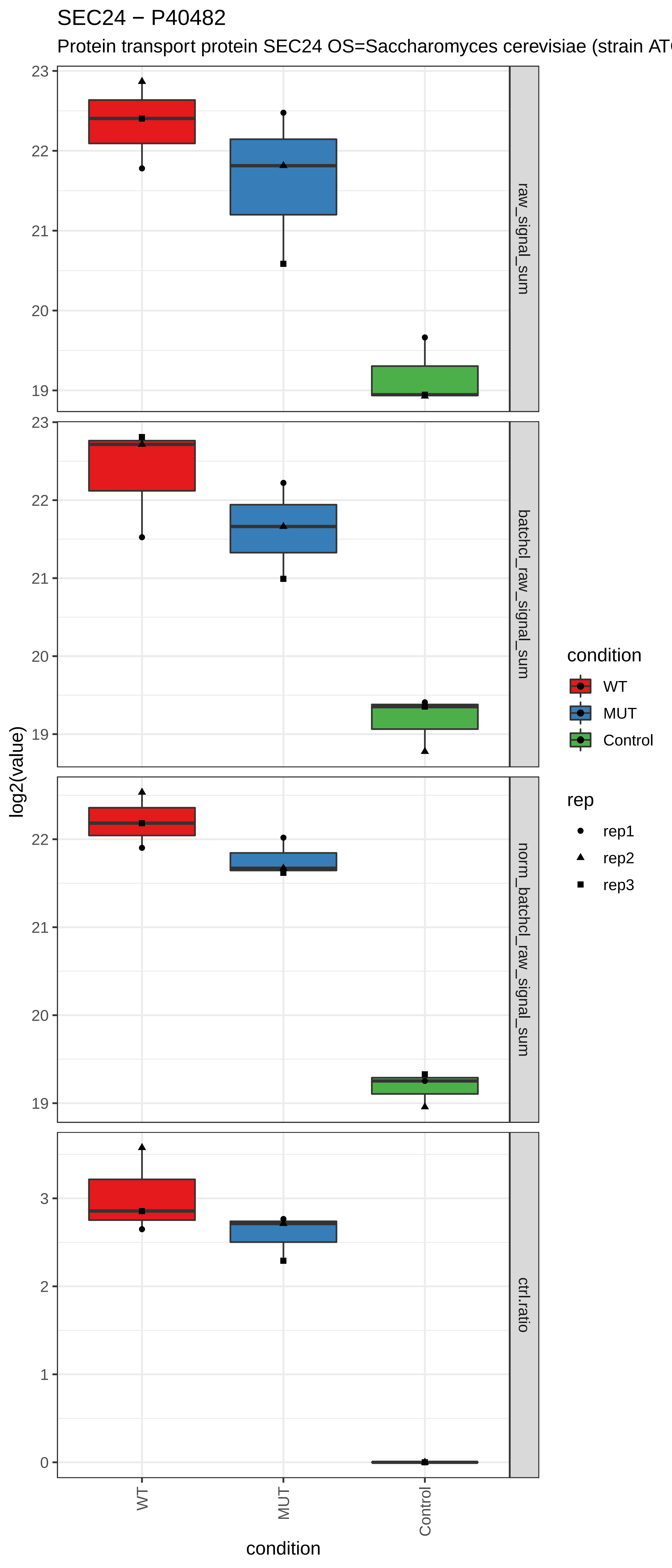

SEC24 − P40482
Protein transport protein SEC24 OS=Saccharomyces cerevisiae (strain ATC
raw_signal_sum
batchcl_raw_signal_sum
norm_batchcl_raw_signal_sum
ctrl.ratio
log2(value)
condition
WT
MUT
Control
rep
rep1
rep2
rep3

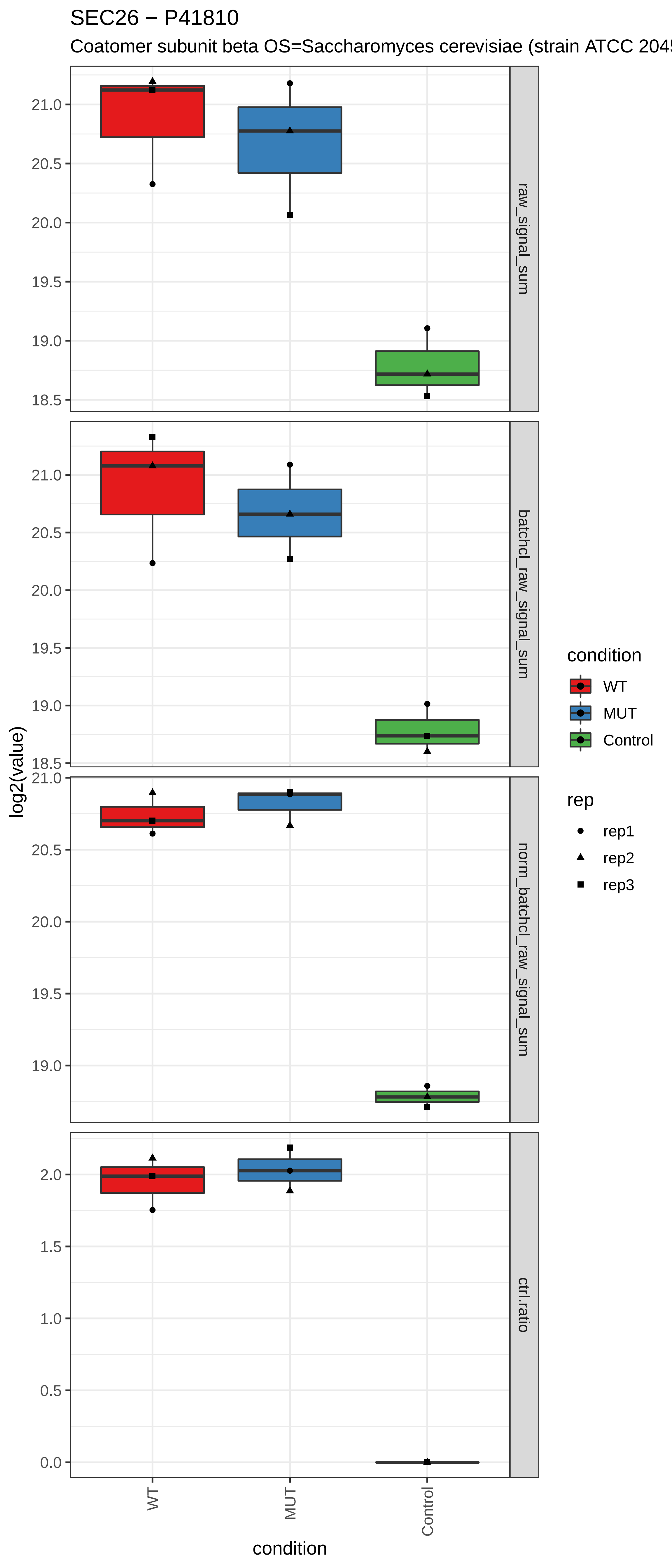

SEC26 − P41810
Coatomer subunit beta OS=Saccharomyces cerevisiae (strain ATCC 204
raw_signal_sum
batchcl_raw_signal_sum
norm_batchcl_raw_signal_sum
ctrl.ratio
log2(value)
condition
WT
MUT
Control
rep
rep1
rep2
rep3
21.0
20.5
20.0
19.5
19.0
18.5
2.0
1.5
1.0
0.5
0.0

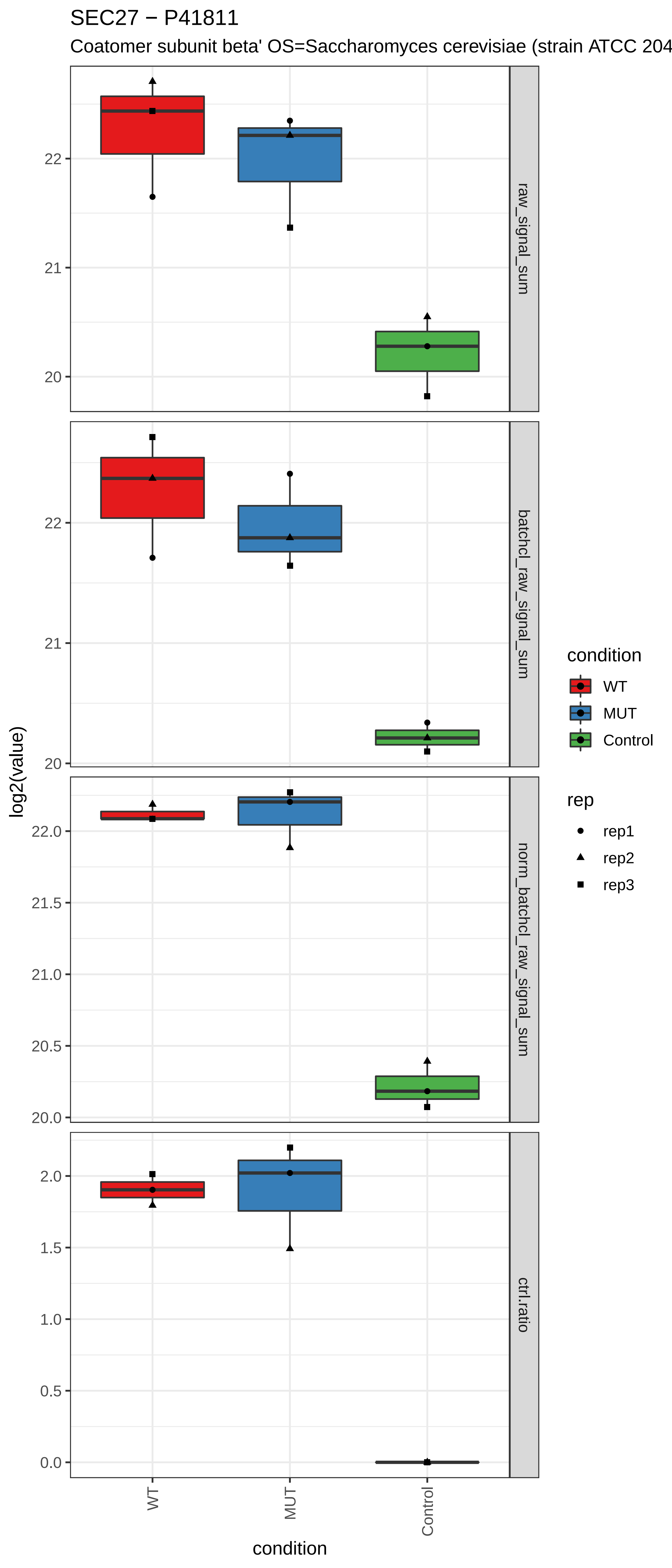

SEC27 − P41811
Coatomer subunit beta' OS=Saccharomyces cerevisiae (strain ATCC 204
raw_signal_sum
batchcl_raw_signal_sum
norm_batchcl_raw_signal_sum
ctrl.ratio
log2(value)
condition
WT
MUT
Control
rep
rep1
rep2
rep3

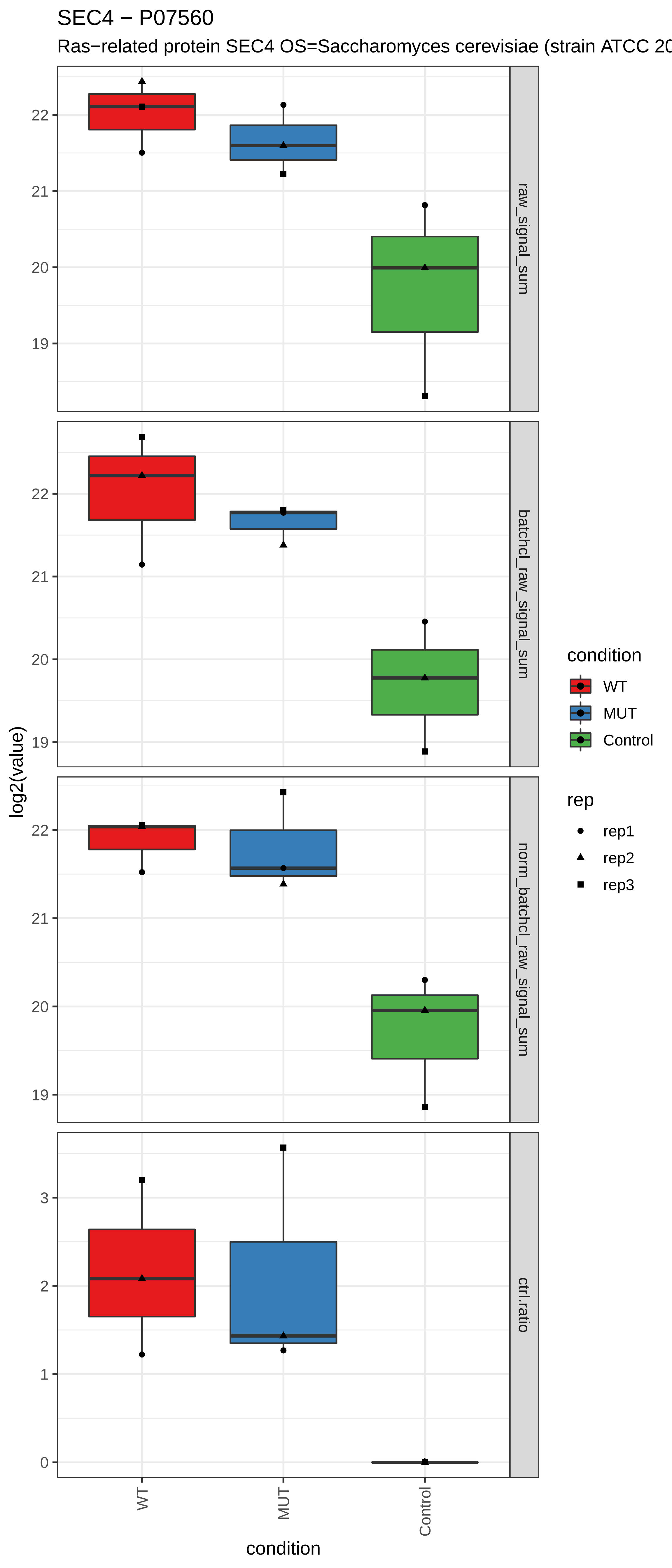

SEC4 − P07560
Ras−related protein SEC4 OS=Saccharomyces cerevisiae (strain ATCC 20
raw_signal_sum
batchcl_raw_signal_sum
norm_batchcl_raw_signal_sum
ctrl.ratio
log2(value)
condition
WT
MUT
Control
rep
rep1
rep2
rep3

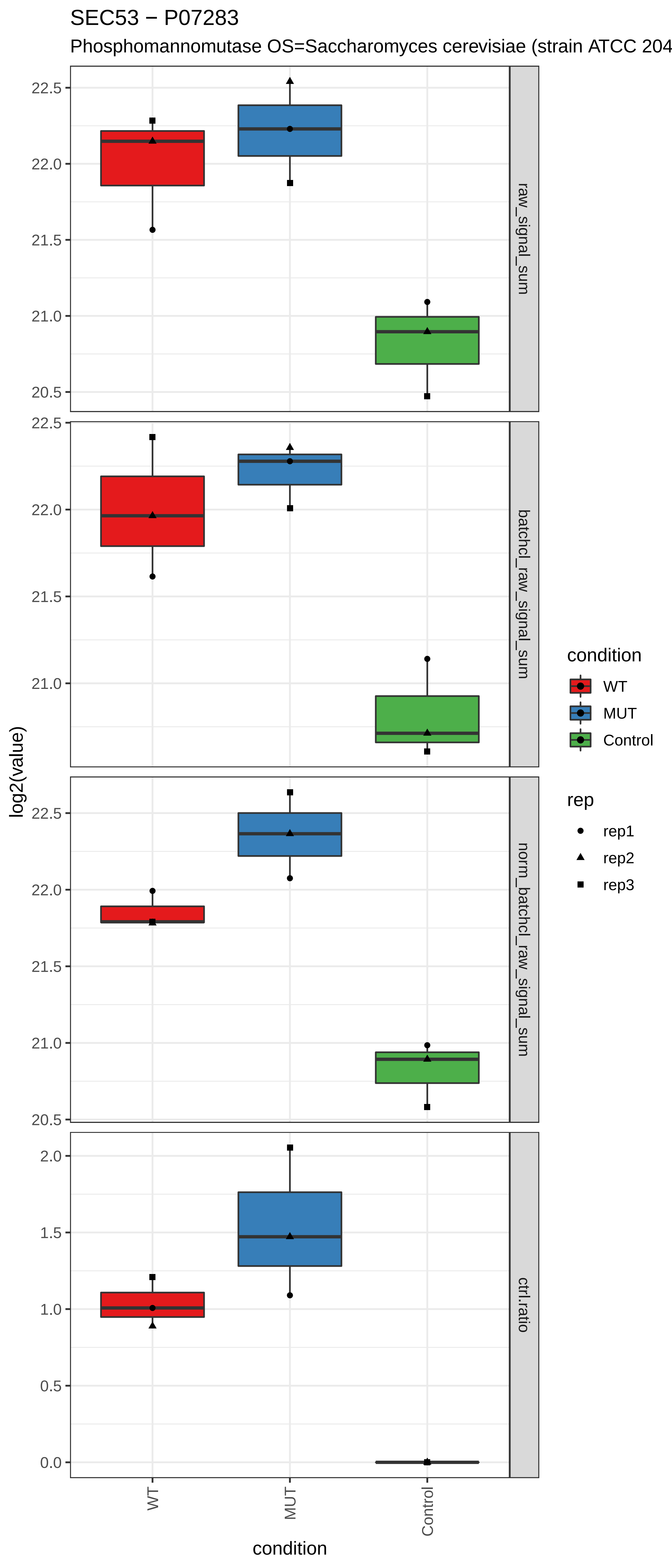

SEC53 − P07283
Phosphomannomutase OS=Saccharomyces cerevisiae (strain ATCC 204
raw_signal_sum
batchcl_raw_signal_sum
norm_batchcl_raw_signal_sum
ctrl.ratio
log2(value)
condition
WT
MUT
Control
rep
rep1
rep2
rep3
22.5
22.0
21.5
21.0
20.5
2.0
1.5
1.0
0.5
0.0

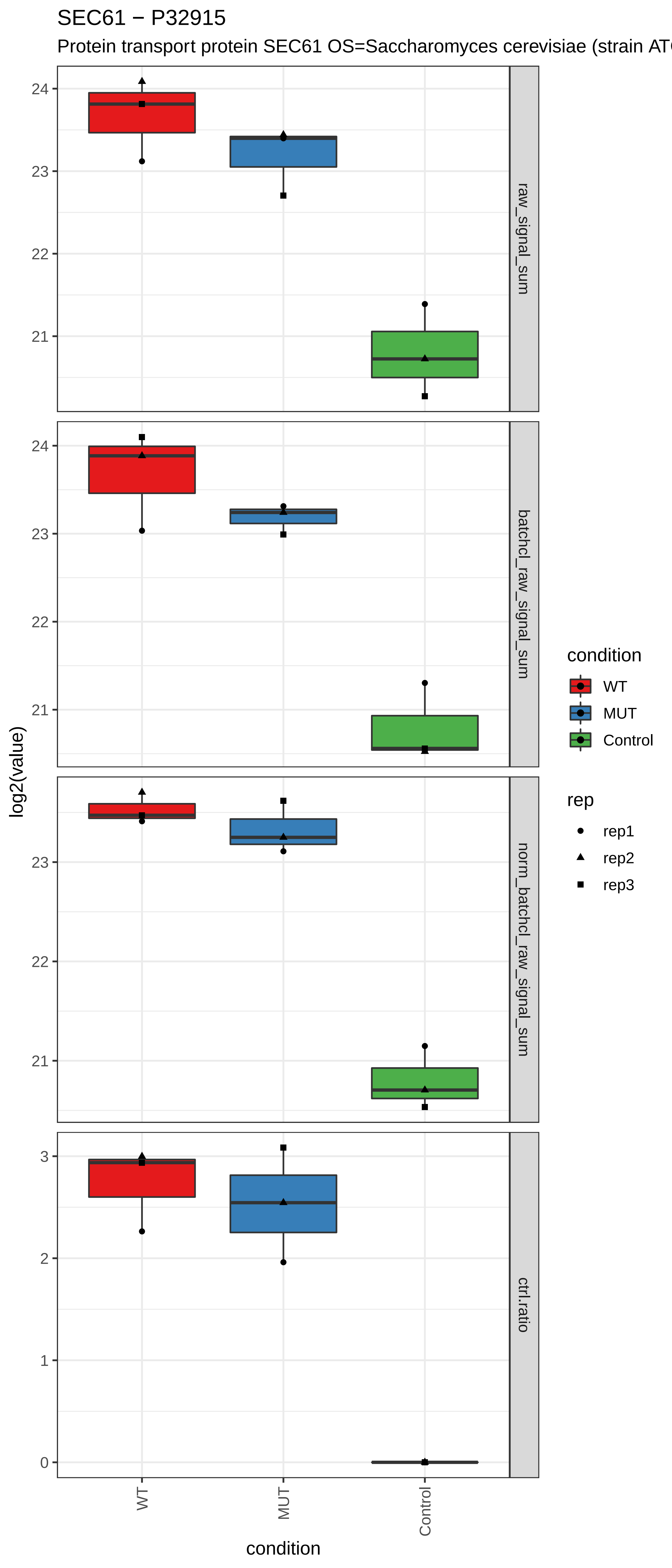

SEC61 − P32915
Protein transport protein SEC61 OS=Saccharomyces cerevisiae (strain AT
log2(value)
raw_signal_sum
batchcl_raw_signal_sum
norm_batchcl_raw_signal_sum
ctrl.ratio
condition
WT
MUT
Control
rep
rep1
rep2
rep3
24
23
22
21
3
2
1
0

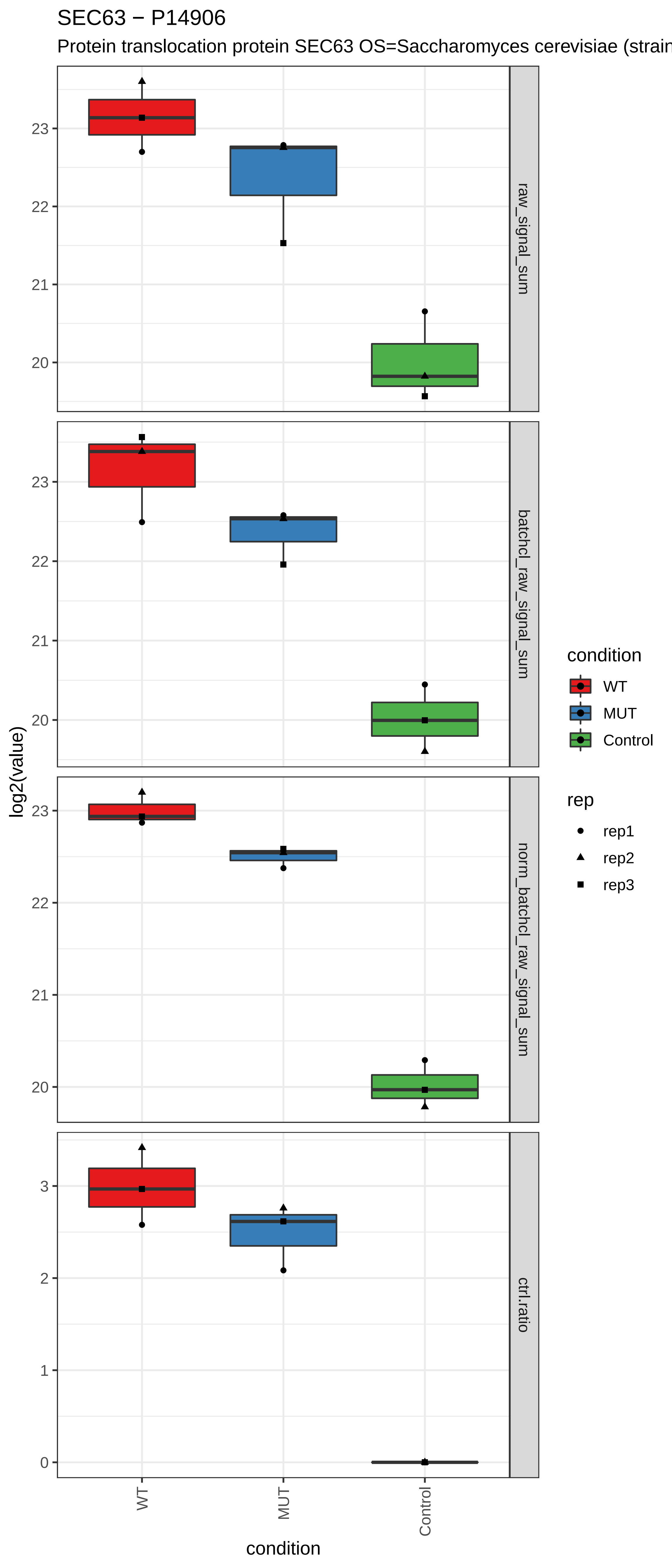
SEC63 − P14906
Protein translocation protein SEC63 OS=Saccharomyces cerevisiae (strain
log2(value)
raw_signal_sum
batchcl_raw_signal_sum
norm_batchcl_raw_signal_sum
ctrl.ratio
23
22
21
20
3
2
1
0
WT
MUT
Control
condition
condition
WT
MUT
Control
rep
rep1
rep2
rep3

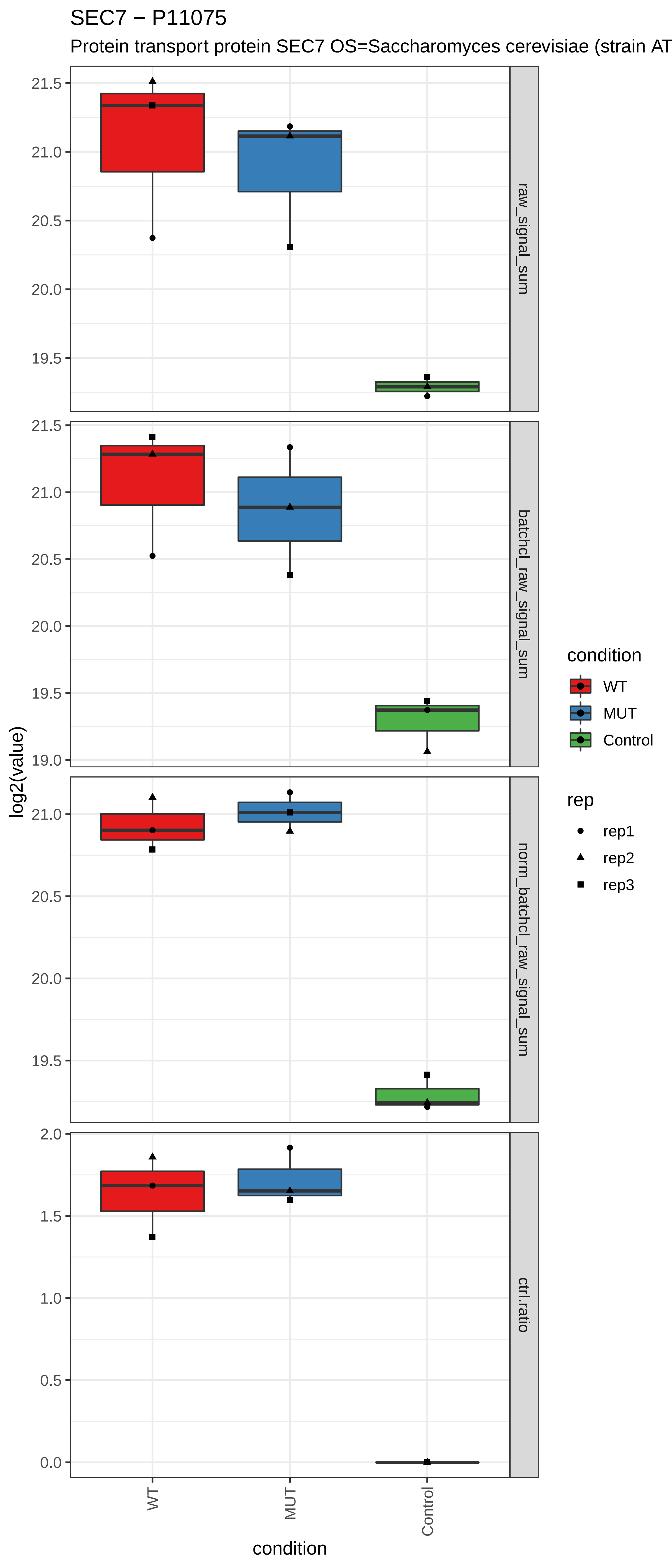

SEC7 − P11075
Protein transport protein SEC7 OS=Saccharomyces cerevisiae (strain AT
raw_signal_sum
batchcl_raw_signal_sum
norm_batchcl_raw_signal_sum
ctrl.ratio
log2(value)
condition
WT
MUT
Control
rep
rep1
rep2
rep3
21.5
21.0
20.5
20.0
19.5
19.0
2.0
1.5
1.0
0.5
0.0

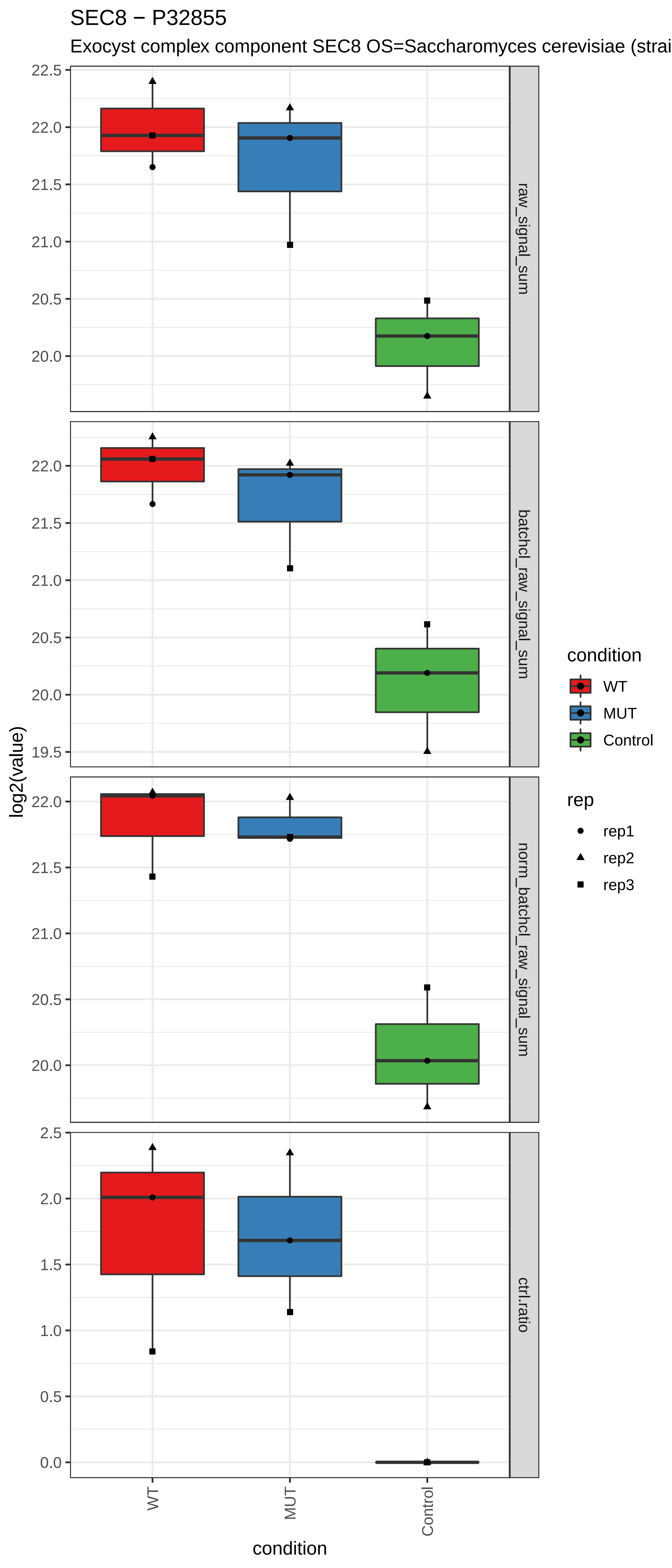

SEC8 − P32855
Exocyst complex component SEC8 OS=Saccharomyces cerevisiae (strai
raw_signal_sum
batchcl_raw_signal_sum
norm_batchcl_raw_signal_sum
ctrl.ratio
log2(value)
condition
WT
MUT
Control
rep
rep1
rep2
rep3
22.5
22.0
21.5
21.0
20.5
20.0
19.5
2.5
2.0
1.5
1.0
0.5
0.0

SEG1 − Q04279

Eisosome protein SEG1 OS=Saccharomyces cerevisiae (strain ATCC 2045

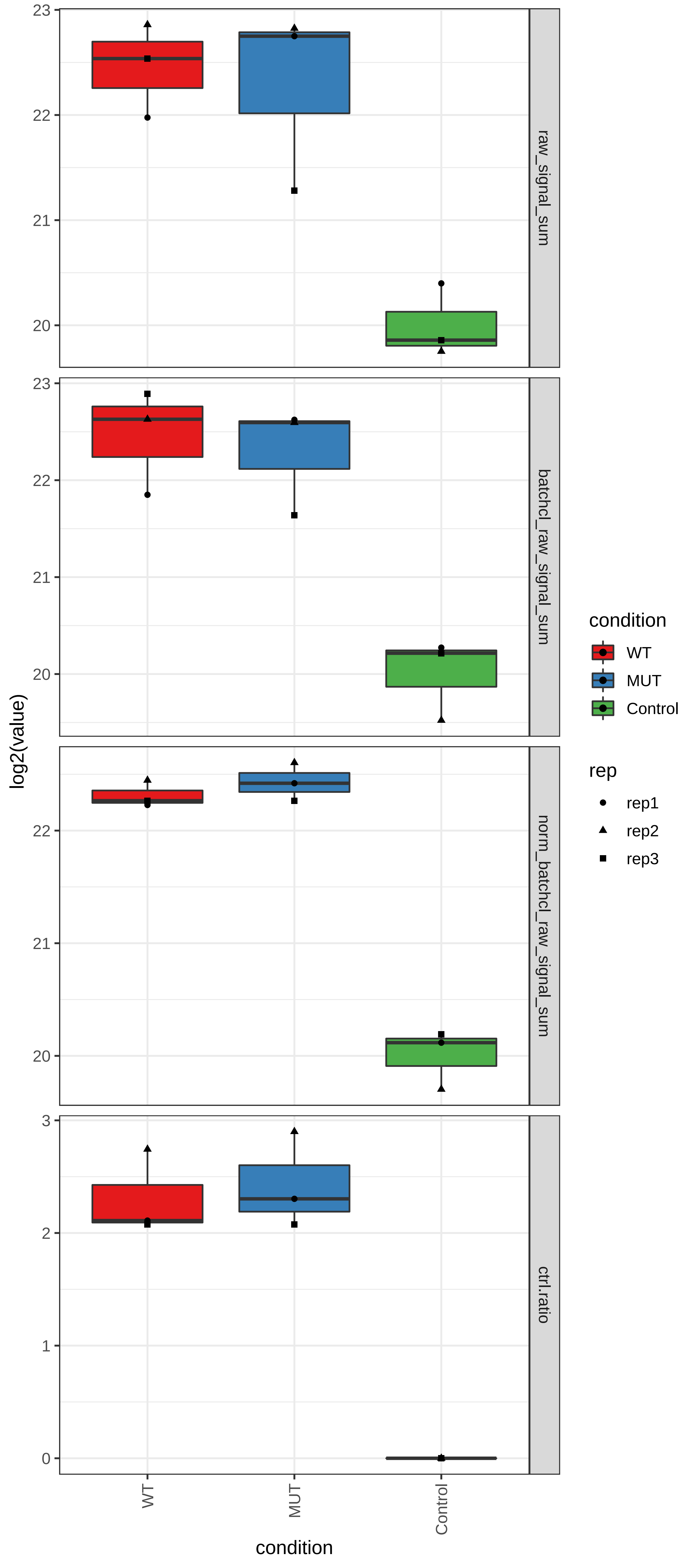

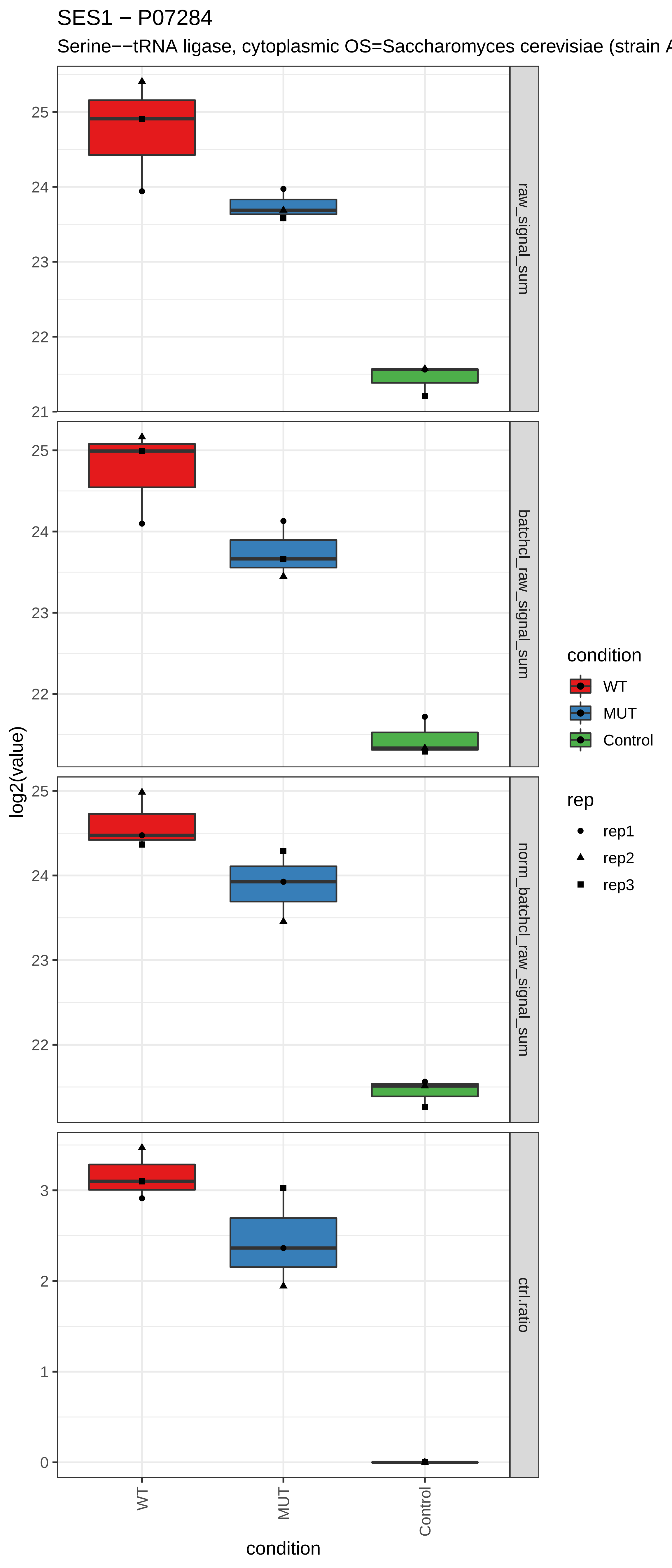
SES1 − P07284
Serine−−tRNA ligase, cytoplasmic OS=Saccharomyces cerevisiae (strain A
raw_signal_sum
batchcl_raw_signal_sum
norm_batchcl_raw_signal_sum
ctrl.ratio
log2(value)
condition
WT
MUT
Control
rep
rep1
rep2
rep3
25
24
23
22
21
3
2
1
0

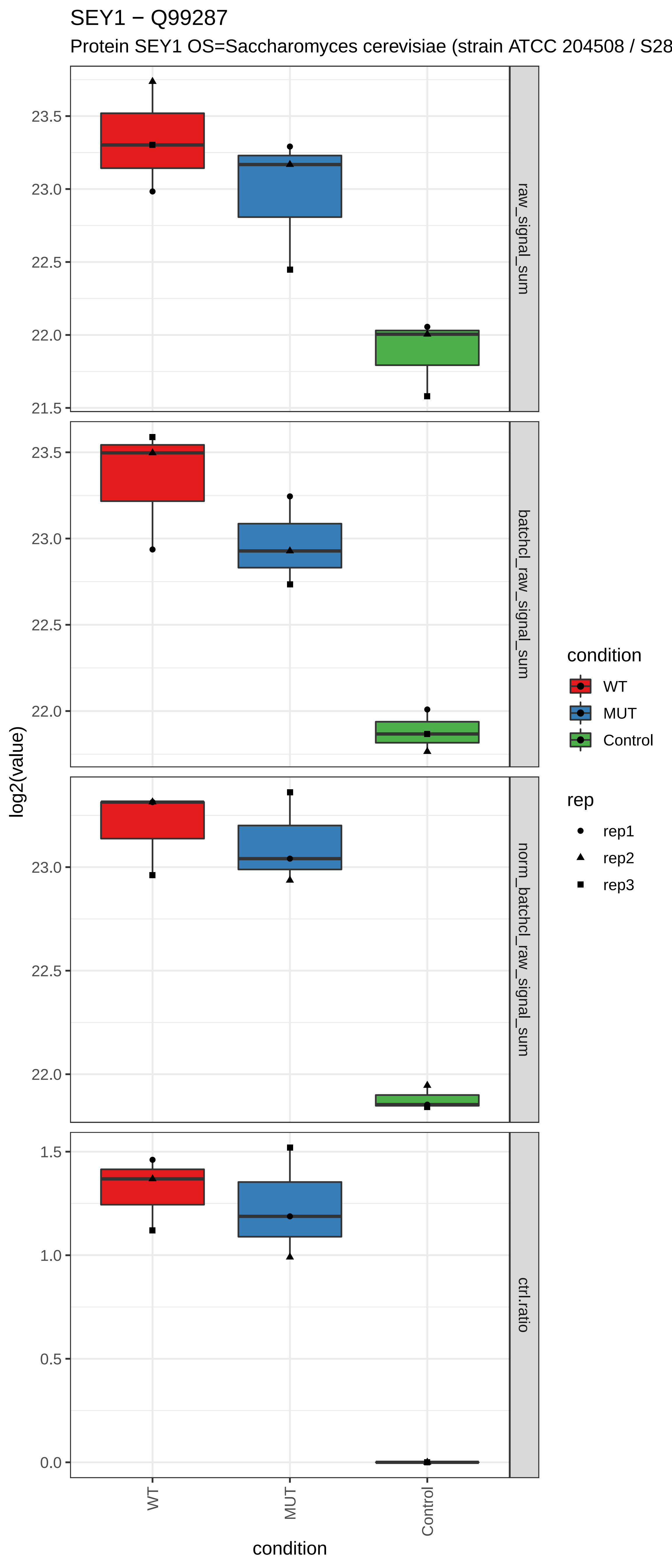

SEY1 − Q99287
Protein SEY1 OS=Saccharomyces cerevisiae (strain ATCC 204508 / S28
raw_signal_sum
batchcl_raw_signal_sum
norm_batchcl_raw_signal_sum
ctrl.ratio
log2(value)
condition
WT
MUT
Control
rep
rep1
rep2
rep3
23.5
23.0
22.5
22.0
21.5
1.5
1.0
0.5
0.0

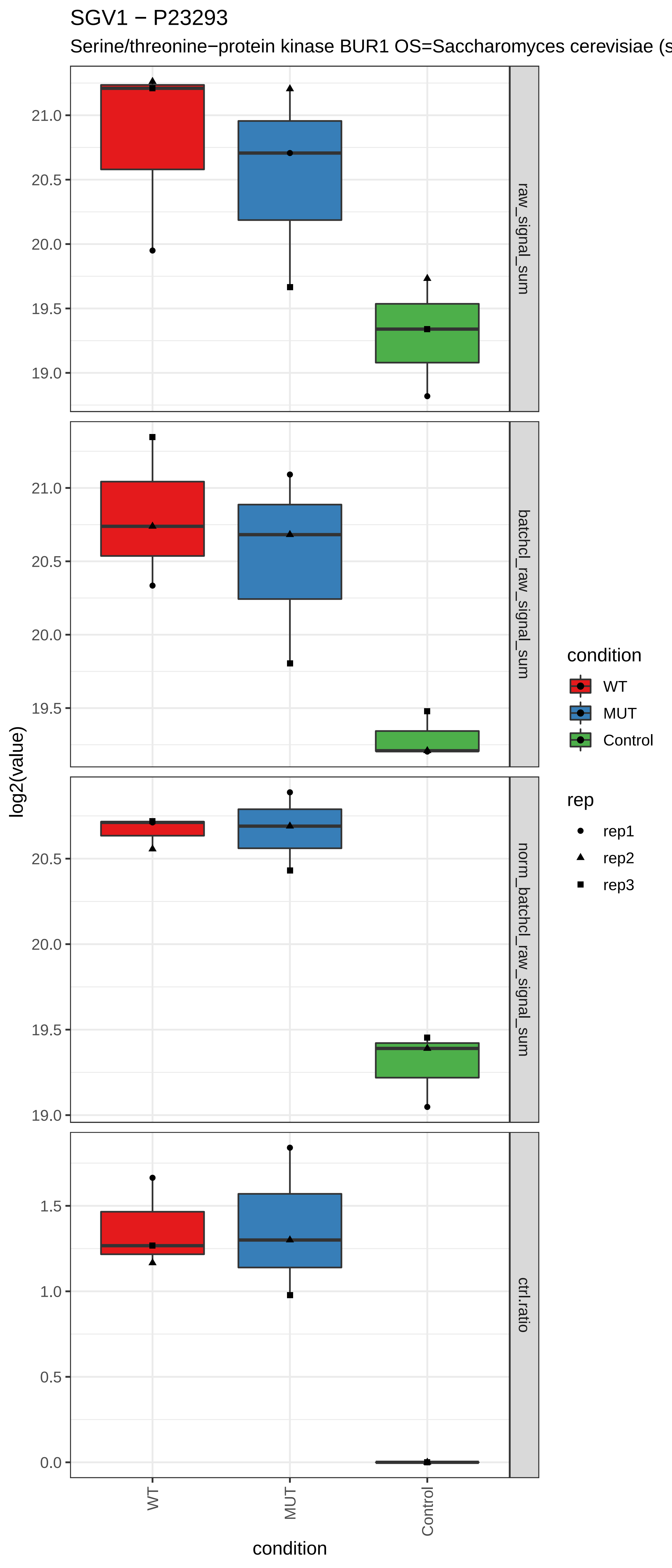
SGV1 − P23293
Serine/threonine−protein kinase BUR1 OS=Saccharomyces cerevisiae (s
raw_signal_sum
batchcl_raw_signal_sum
norm_batchcl_raw_signal_sum
ctrl.ratio
log2(value)
condition
WT
MUT
Control
rep
rep1
rep2
rep3

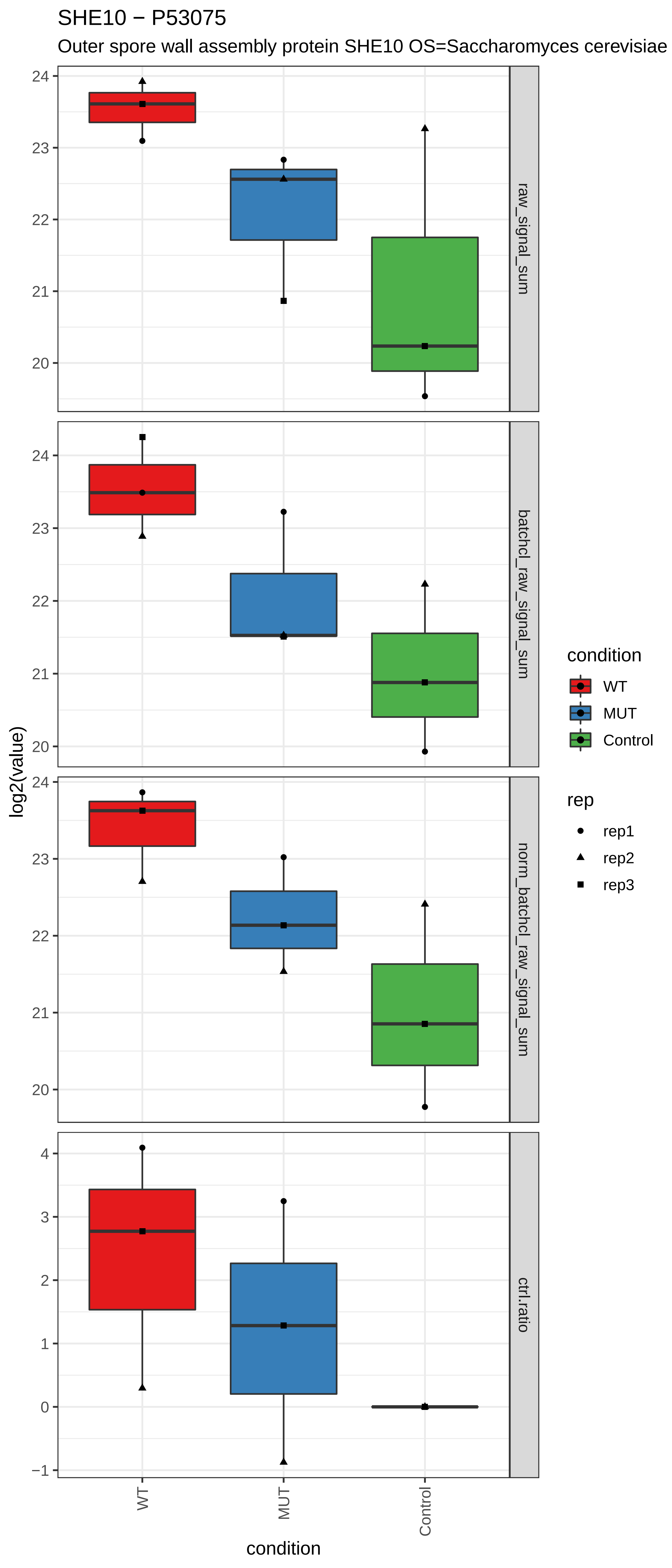

SHE10 − P53075
Outer spore wall assembly protein SHE10 OS=Saccharomyces cerevisiae
raw_signal_sum
batchcl_raw_signal_sum
norm_batchcl_raw_signal_sum
ctrl.ratio
log2(value)
condition
WT
MUT
Control
rep
rep1
rep2
rep3

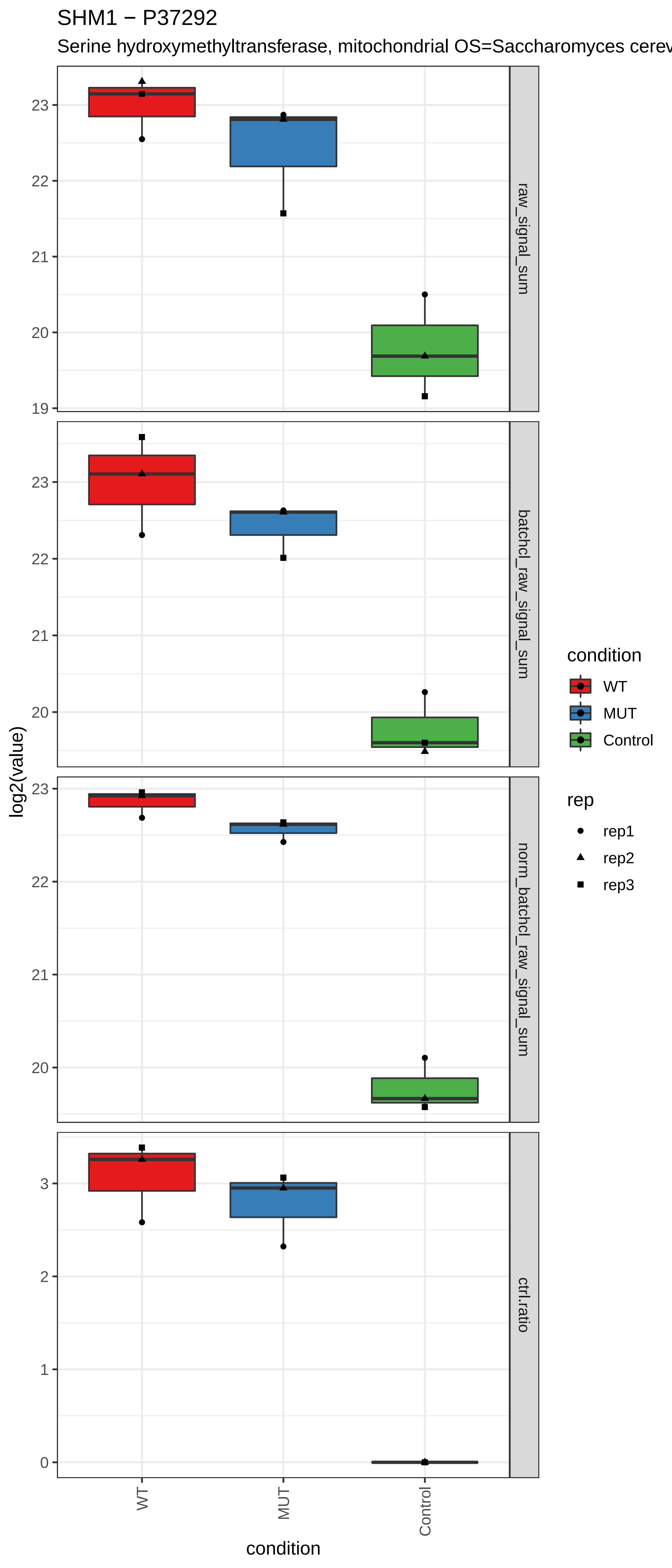

SHM1 − P37292
Serine hydroxymethyltransferase, mitochondrial OS=Saccharomyces cerev
raw_signal_sum
batchcl_raw_signal_sum
norm_batchcl_raw_signal_sum
ctrl.ratio
log2(value)
condition
WT
MUT
Control
rep
rep1
rep2
rep3

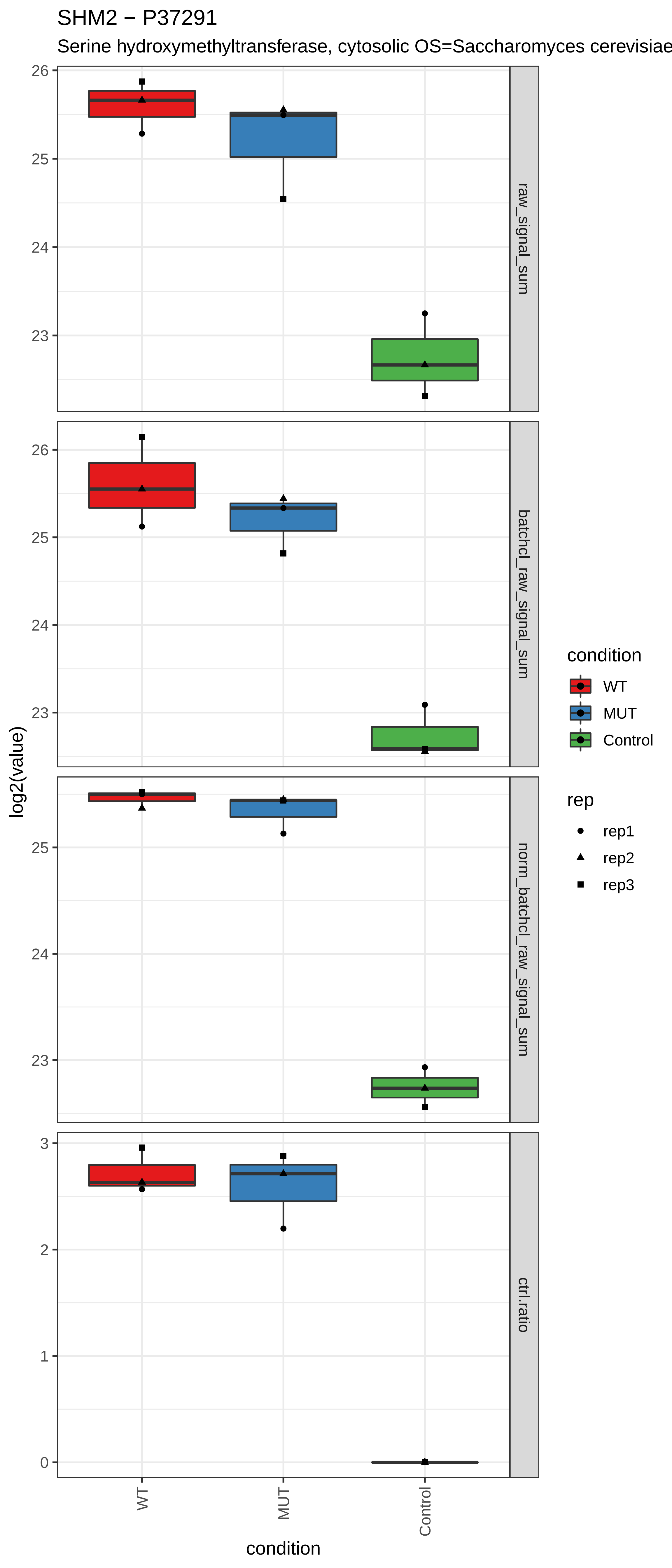

SHM2 − P37291
Serine hydroxymethyltransferase, cytosolic OS=Saccharomyces cerevisiae
log2(value)
raw_signal_sum
batchcl_raw_signal_sum
norm_batchcl_raw_signal_sum
ctrl.ratio
26
25
24
23
3
2
1
0
WT
MUT
Control
condition
condition
WT
MUT
Control
rep
rep1
rep2
rep3

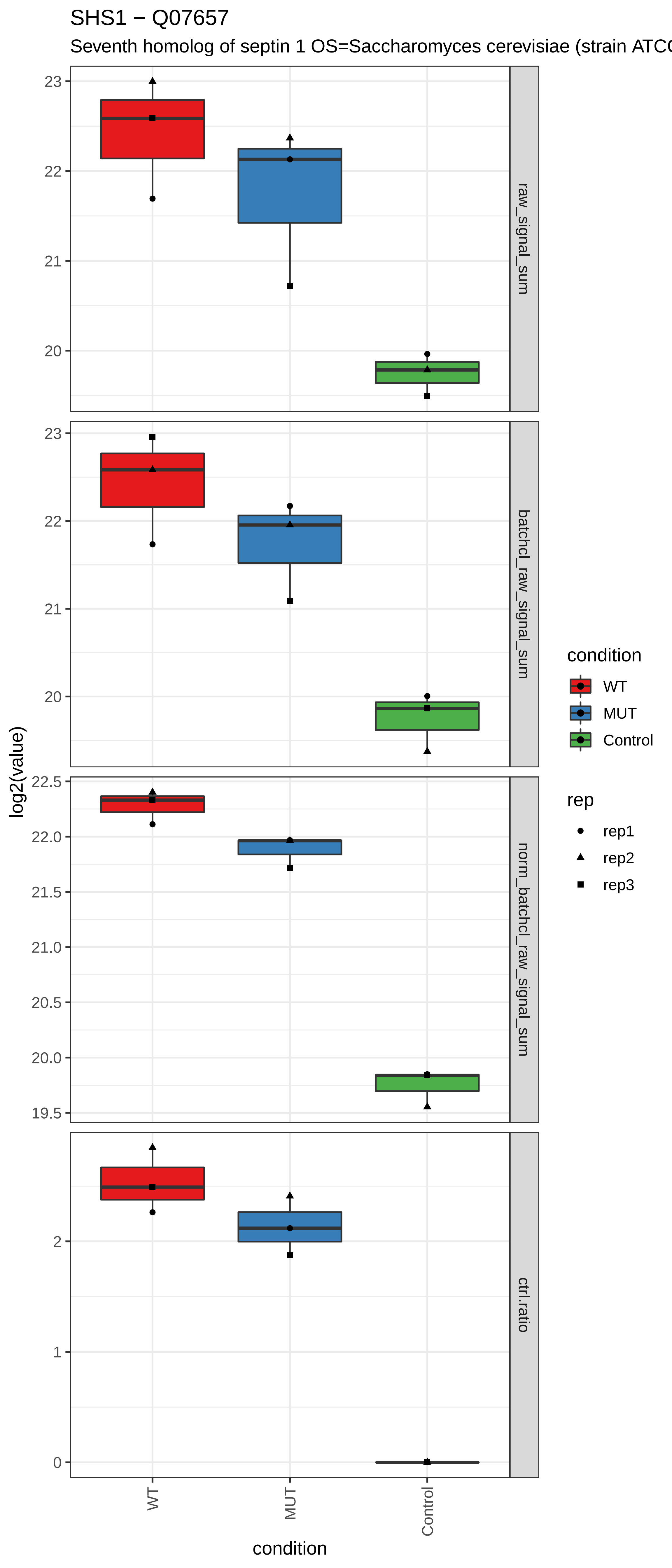

SHS1 − Q07657
Seventh homolog of septin 1 OS=Saccharomyces cerevisiae (strain ATCC
raw_signal_sum
batchcl_raw_signal_sum
norm_batchcl_raw_signal_sum
ctrl.ratio
log2(value)
condition
WT
MUT
Control
rep
rep1
rep2
rep3
23
22
21
20
22.5
22.0
21.5
21.0
20.5
20.0
19.5
2
1
0

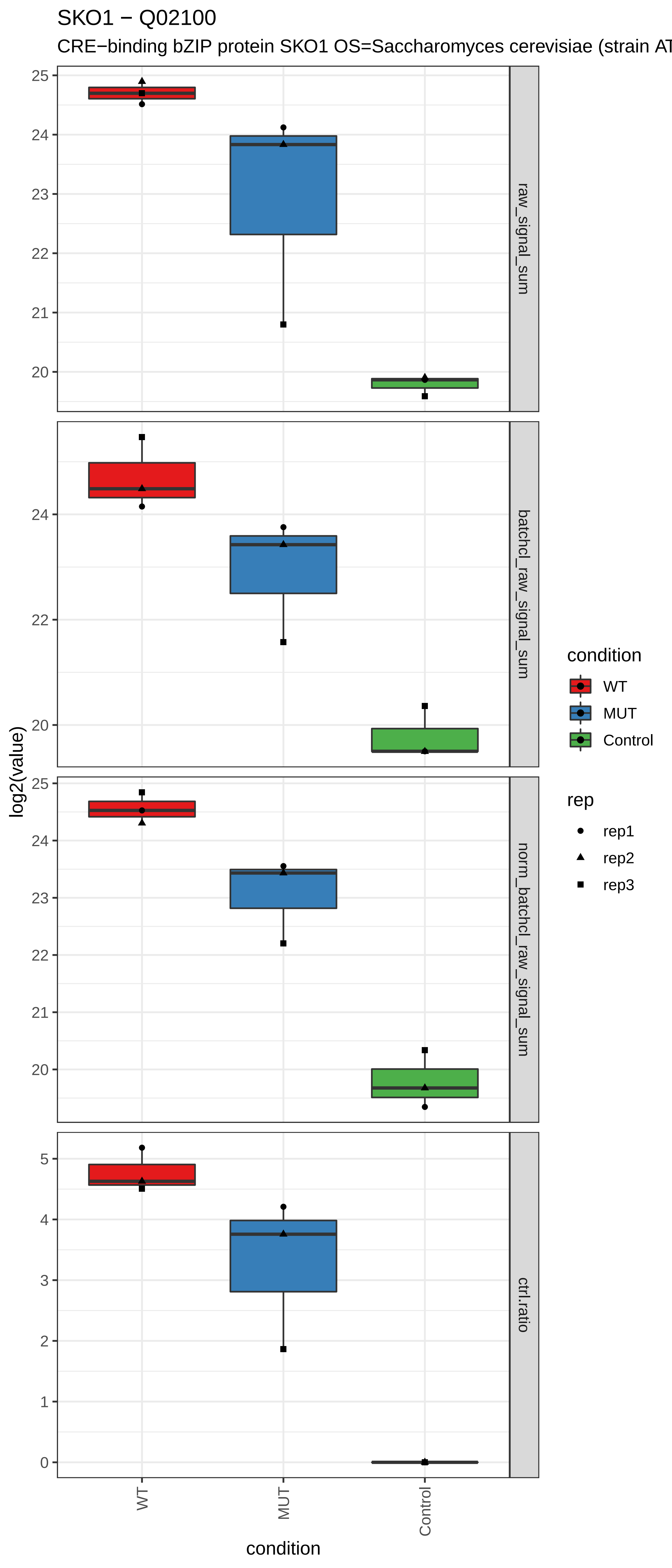

SKO1 − Q02100
CRE−binding bZIP protein SKO1 OS=Saccharomyces cerevisiae (strain AT
raw_signal_sum
batchcl_raw_signal_sum
norm_batchcl_raw_signal_sum
ctrl.ratio
log2(value)
condition
WT
MUT
Control
rep
rep1
rep2
rep3

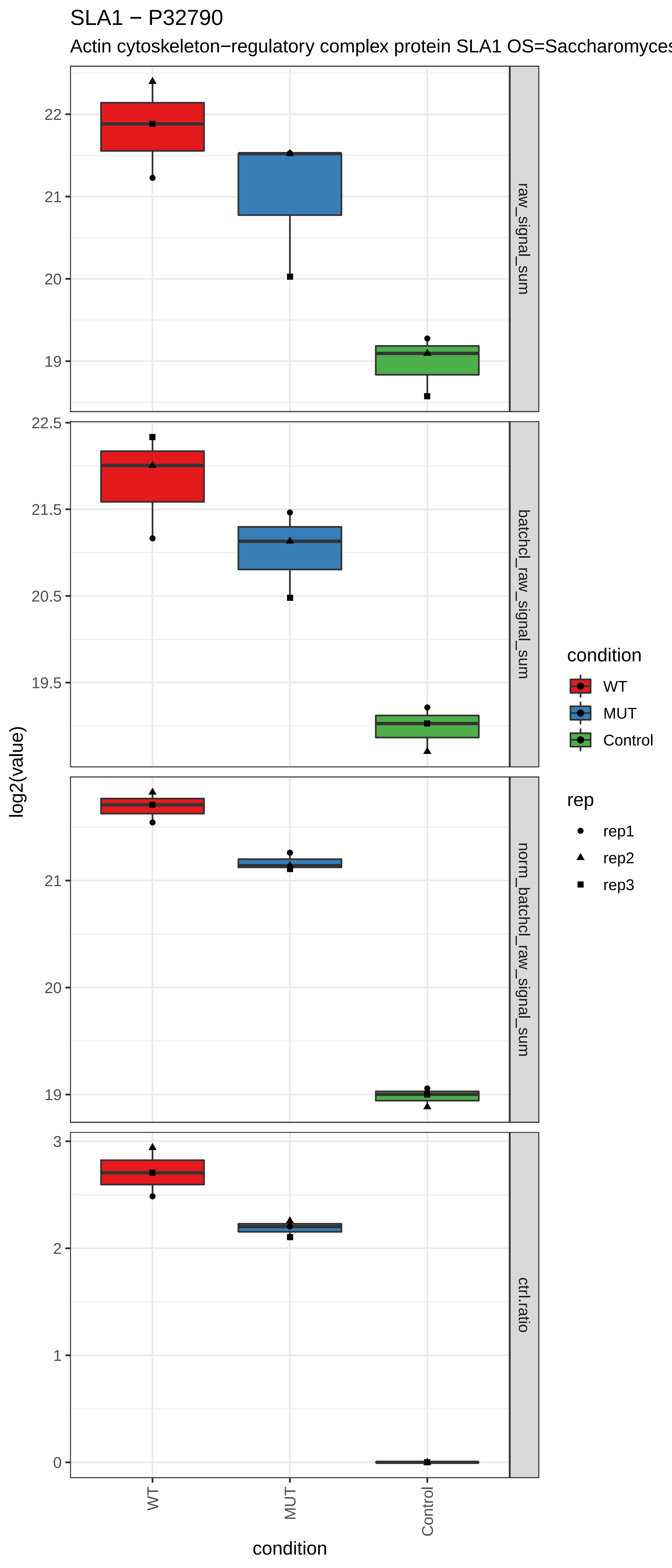

SLA1 − P32790
Actin cytoskeleton−regulatory complex protein SLA1 OS=Saccharomyces
raw_signal_sum
batchcl_raw_signal_sum
norm_batchcl_raw_signal_sum
ctrl.ratio
log2(value)
condition
WT
MUT
Control
rep
rep1
rep2
rep3

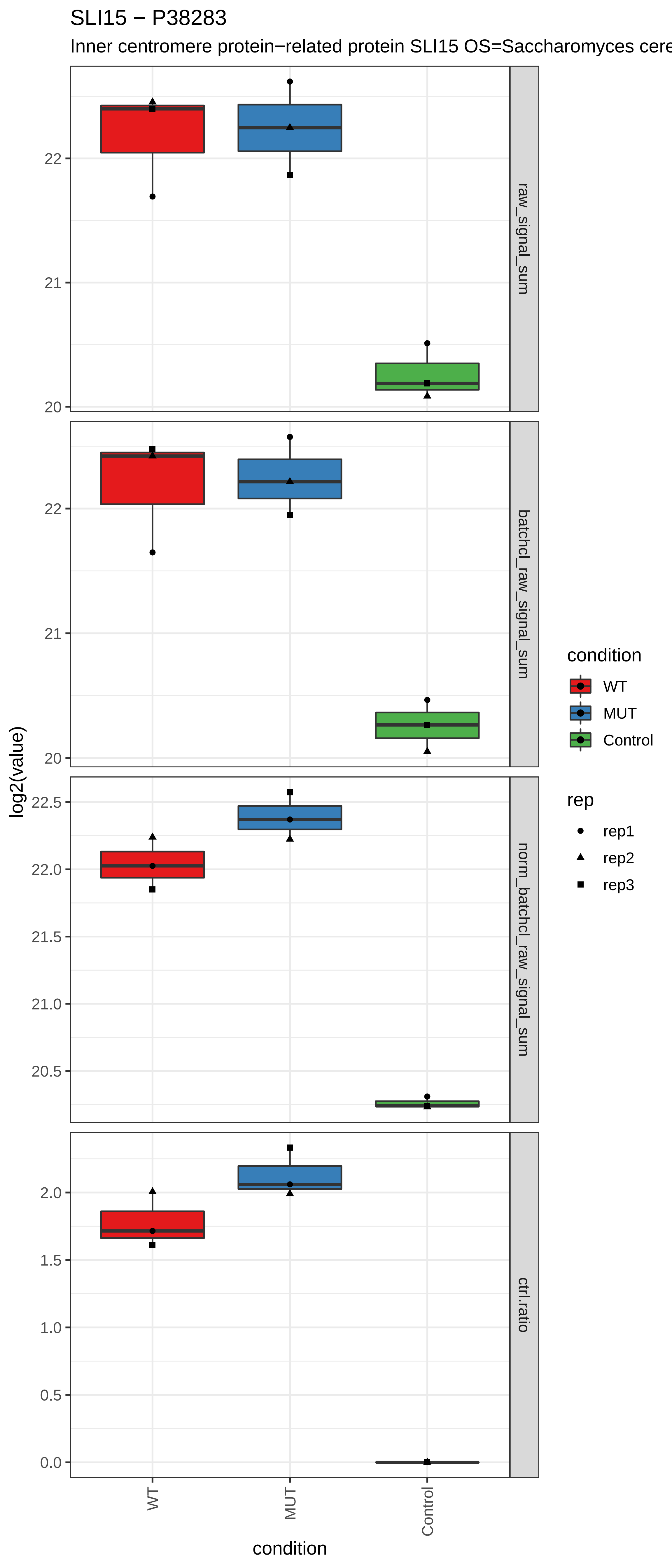

SLI15 − P38283
Inner centromere protein−related protein SLI15 OS=Saccharomyces cere
raw_signal_sum
batchcl_raw_signal_sum
norm_batchcl_raw_signal_sum
ctrl.ratio
log2(value)
condition
WT
MUT
Control
rep
rep1
rep2
rep3
22
21
20
22.5
22.0
21.5
21.0
20.5
2.0
1.5
1.0
0.5
0.0

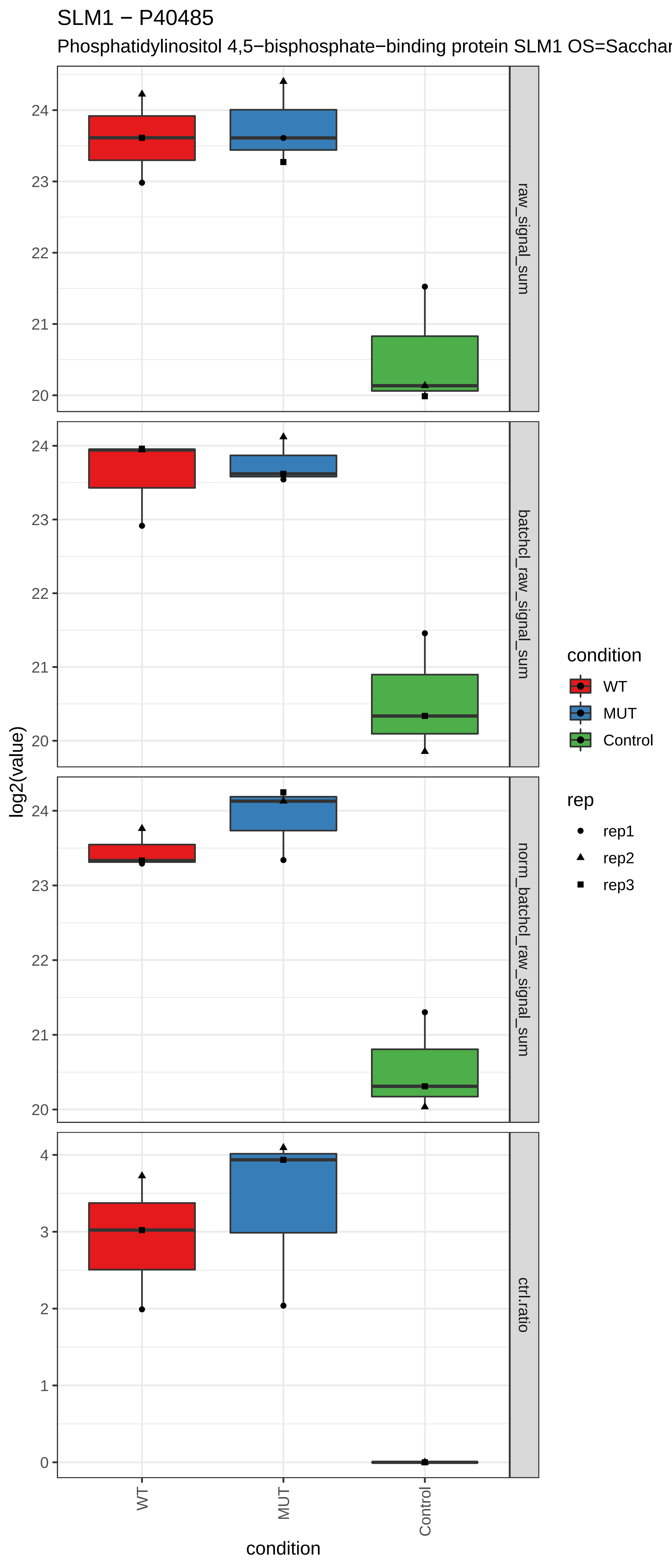

SLM1 − P40485
Phosphatidylinositol 4,5−bisphosphate−binding protein SLM1 OS=Sacchar
raw_signal_sum
batchcl_raw_signal_sum
norm_batchcl_raw_signal_sum
ctrl.ratio
log2(value)
condition
WT
MUT
Control
rep
rep1
rep2
rep3

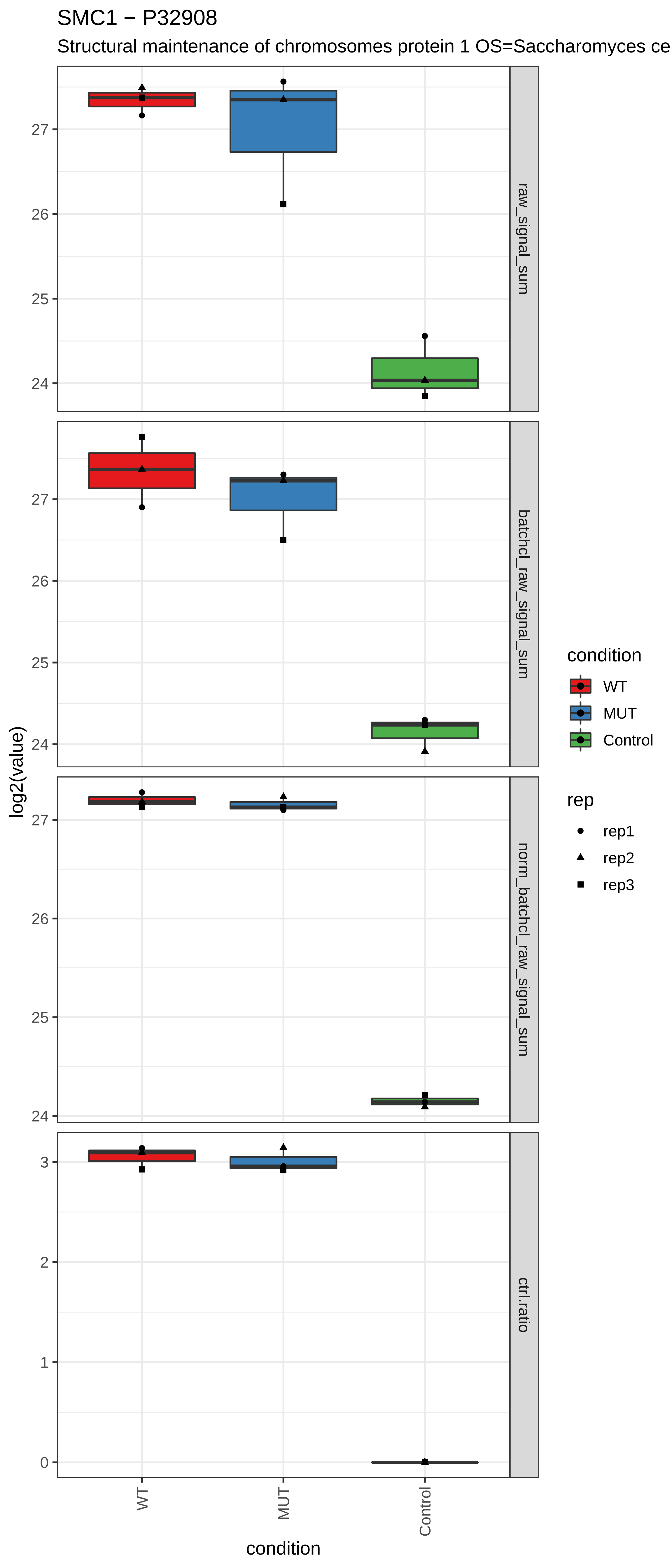
SMC1 − P32908
Structural maintenance of chromosomes protein 1 OS=Saccharomyces ce
raw_signal_sum
batchcl_raw_signal_sum
norm_batchcl_raw_signal_sum
ctrl.ratio
log2(value)
condition
WT
MUT
Control
rep
rep1
rep2
rep3
27
26
25
24
3
2
1
0

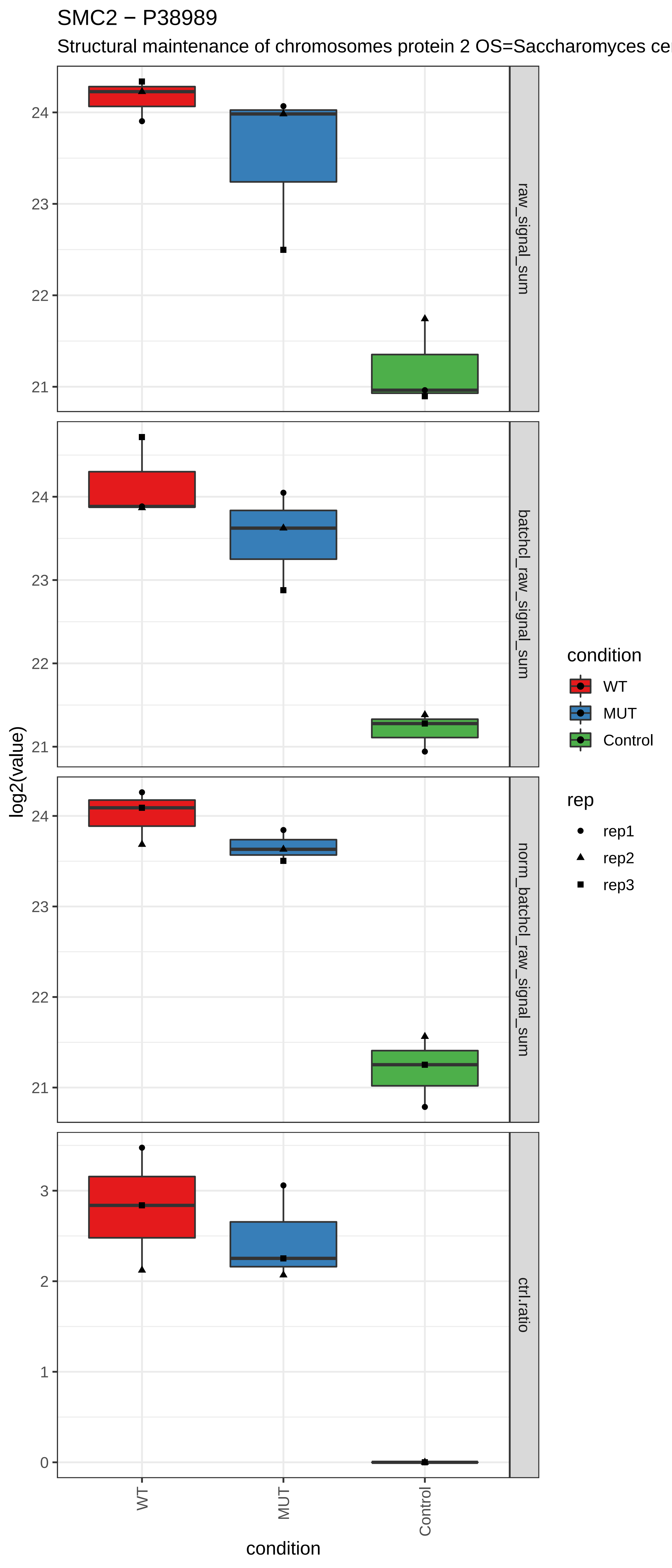
SMC2 − P38989
Structural maintenance of chromosomes protein 2 OS=Saccharomyces ce
raw_signal_sum
batchcl_raw_signal_sum
norm_batchcl_raw_signal_sum
ctrl.ratio
log2(value)
condition
WT
MUT
Control
rep
rep1
rep2
rep3
24
23
22
21
3
2
1
0

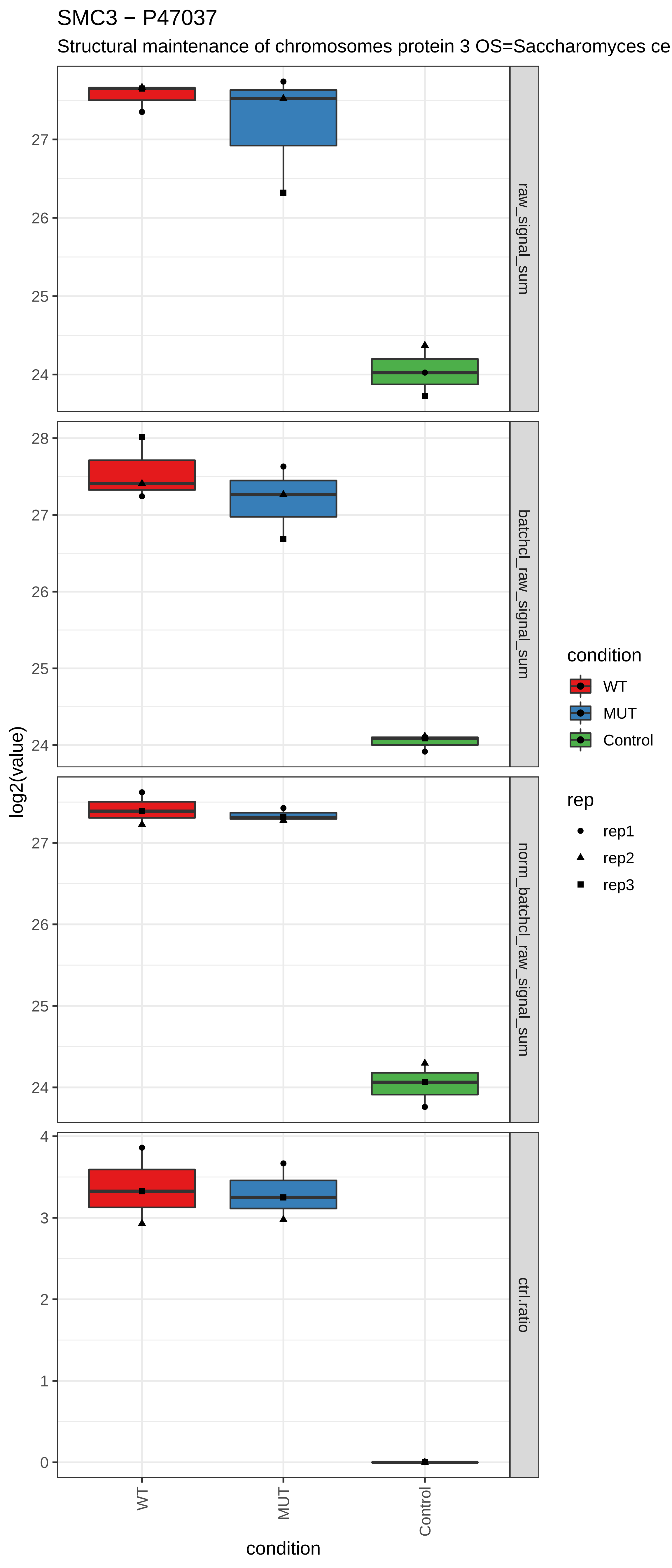

SMC3 − P47037
Structural maintenance of chromosomes protein 3 OS=Saccharomyces ce
raw_signal_sum
batchcl_raw_signal_sum
norm_batchcl_raw_signal_sum
ctrl.ratio
log2(value)
condition
WT
MUT
Control
rep
rep1
rep2
rep3

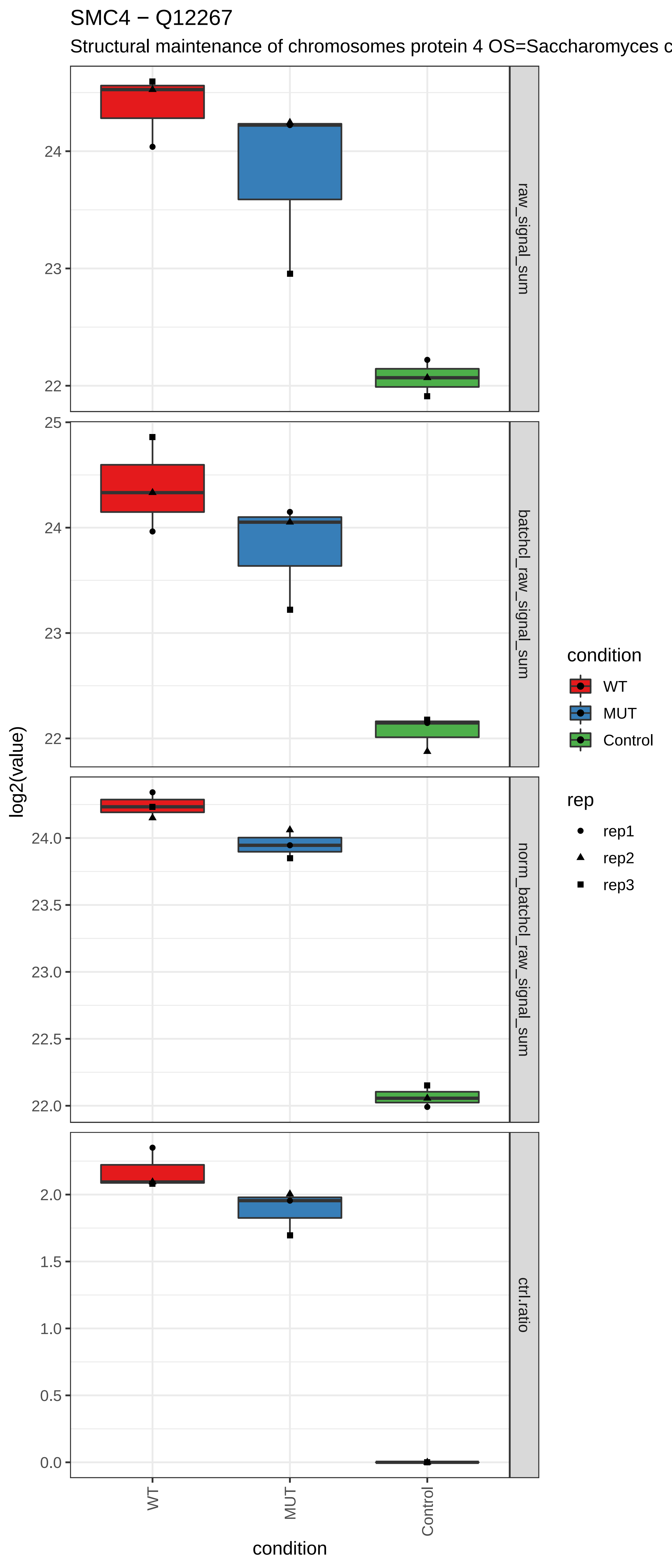

SMC4 − Q12267
Structural maintenance of chromosomes protein 4 OS=Saccharomyces c
raw_signal_sum
batchcl_raw_signal_sum
norm_batchcl_raw_signal_sum
ctrl.ratio
log2(value)
condition
WT
MUT
Control
rep
rep1
rep2
rep3
24
23
22
25
24.0
23.5
23.0
22.5
22.0
2.0
1.5
1.0
0.5
0.0

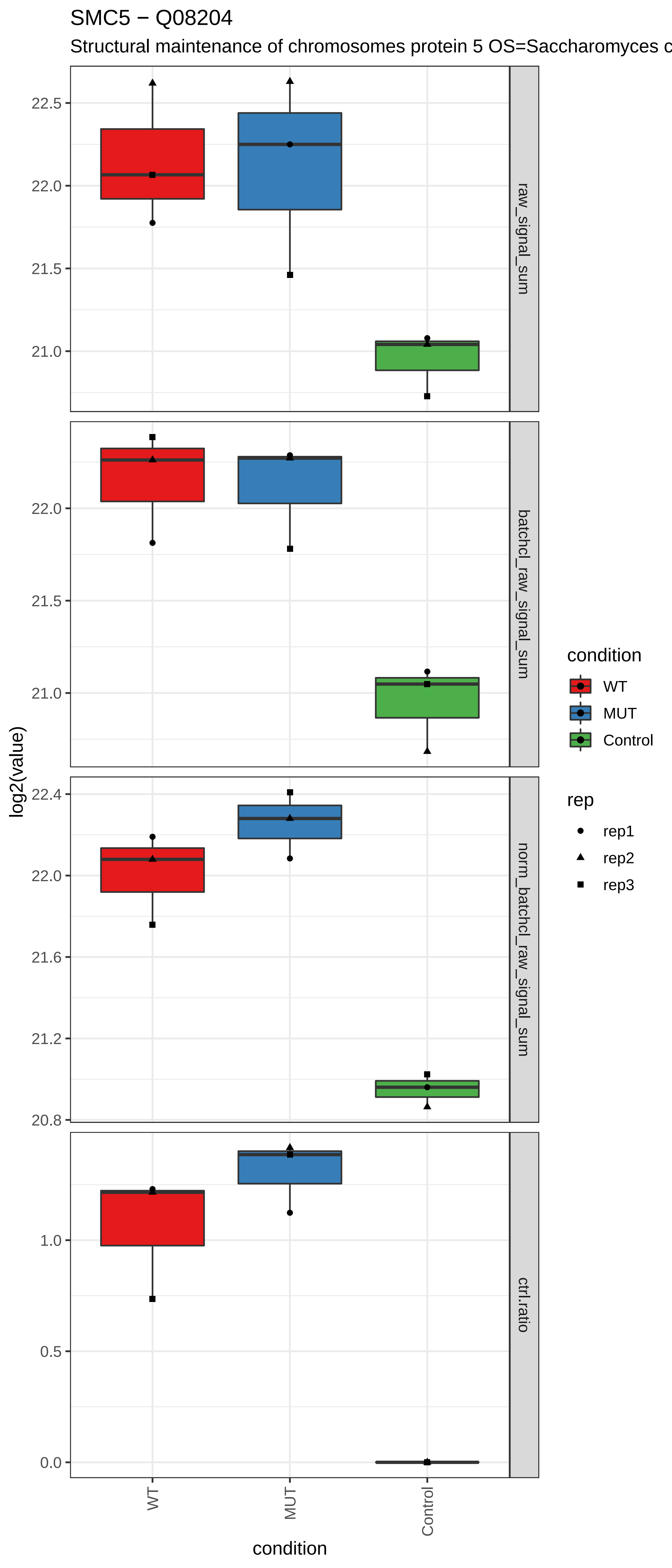

SMC5 − Q08204
Structural maintenance of chromosomes protein 5 OS=Saccharomyces c
raw_signal_sum
batchcl_raw_signal_sum
norm_batchcl_raw_signal_sum
ctrl.ratio
22.5
22.0
21.5
21.0
22.4
21.6
21.2
20.8
1.0
0.5
0.0
log2(value)
WT
MUT
Control
condition
condition
WT
MUT
Control
rep
rep1
rep2
rep3

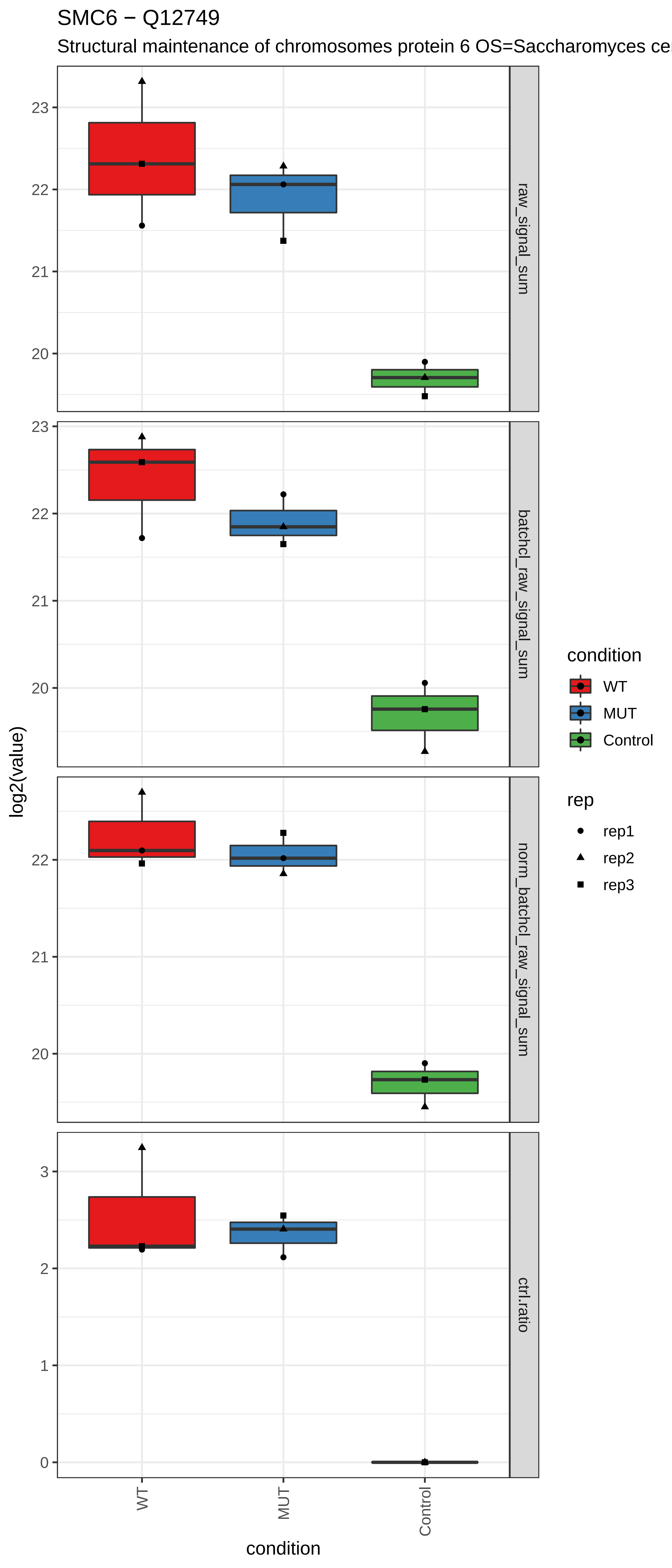
SMC6 − Q12749
Structural maintenance of chromosomes protein 6 OS=Saccharomyces ce
raw_signal_sum
batchcl_raw_signal_sum
norm_batchcl_raw_signal_sum
ctrl.ratio
log2(value)
condition
WT
MUT
Control
rep
rep1
rep2
rep3
23
22
21
20
3
2
1
0

SMY2 − P32909

Protein SMY2 OS=Saccharomyces cerevisiae (strain ATCC 204508 / S28

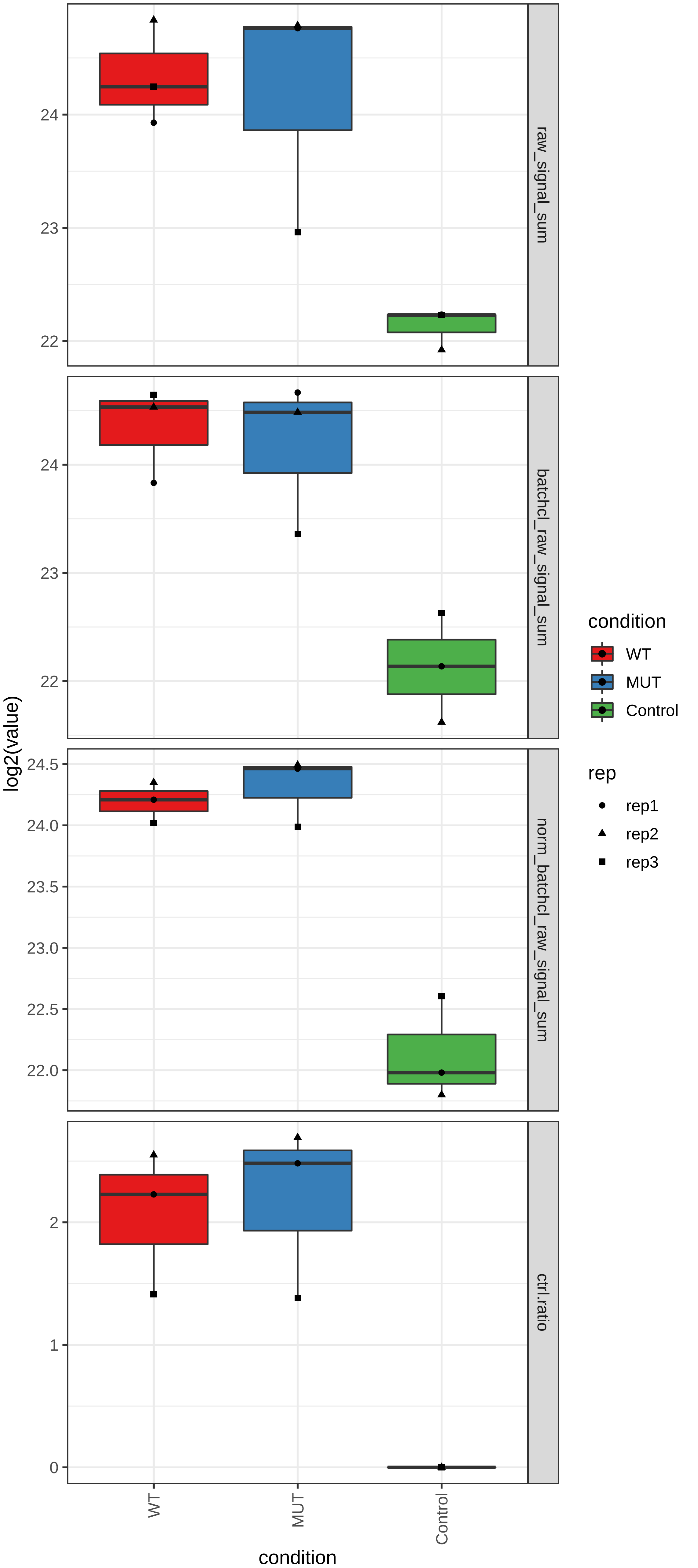

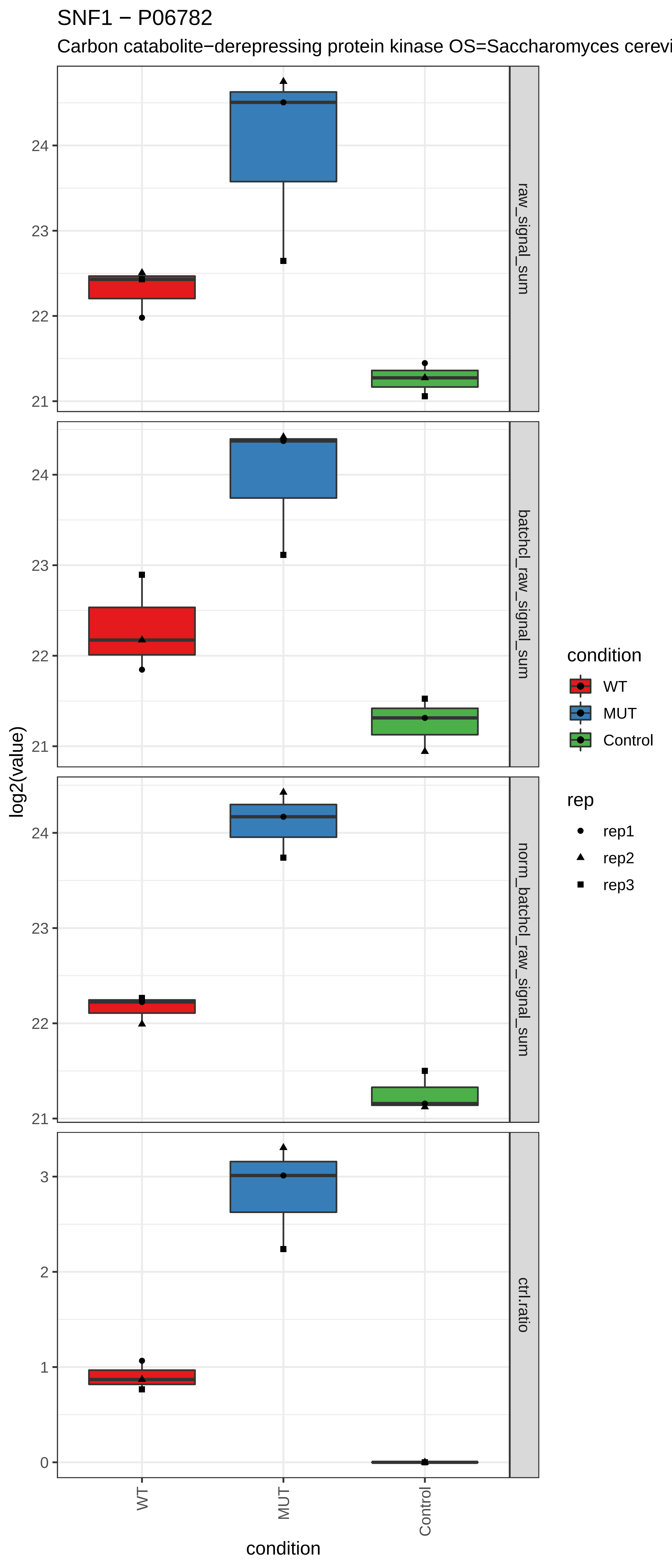

SNF1 − P06782
Carbon catabolite−derepressing protein kinase OS=Saccharomyces cerevi
raw_signal_sum
batchcl_raw_signal_sum
norm_batchcl_raw_signal_sum
ctrl.ratio
log2(value)
condition
WT
MUT
Control
rep
rep1
rep2
rep3

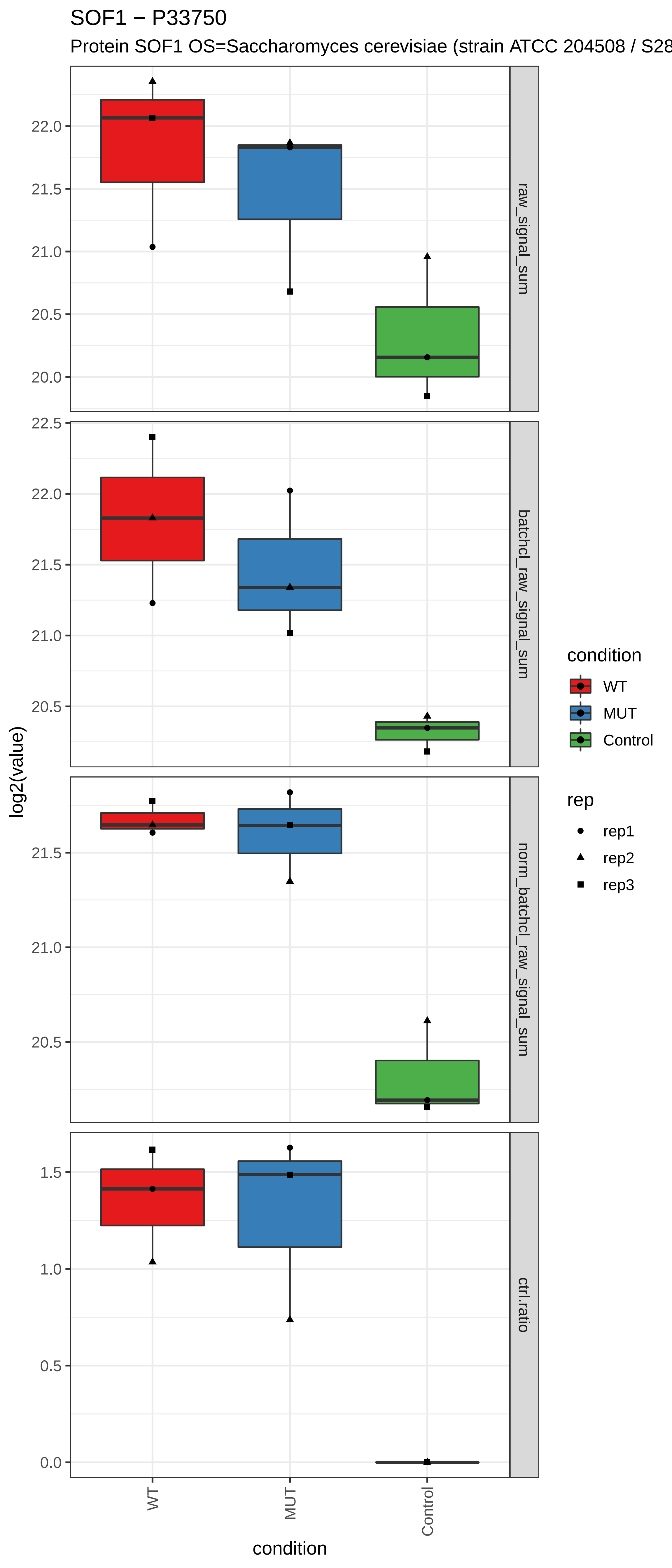

SOF1 − P33750
Protein SOF1 OS=Saccharomyces cerevisiae (strain ATCC 204508 / S28
raw_signal_sum
batchcl_raw_signal_sum
norm_batchcl_raw_signal_sum
ctrl.ratio
log2(value)
condition
WT
MUT
Control
rep
rep1
rep2
rep3
22.5
22.0
21.5
21.0
20.5
20.0
1.5
1.0
0.5
0.0

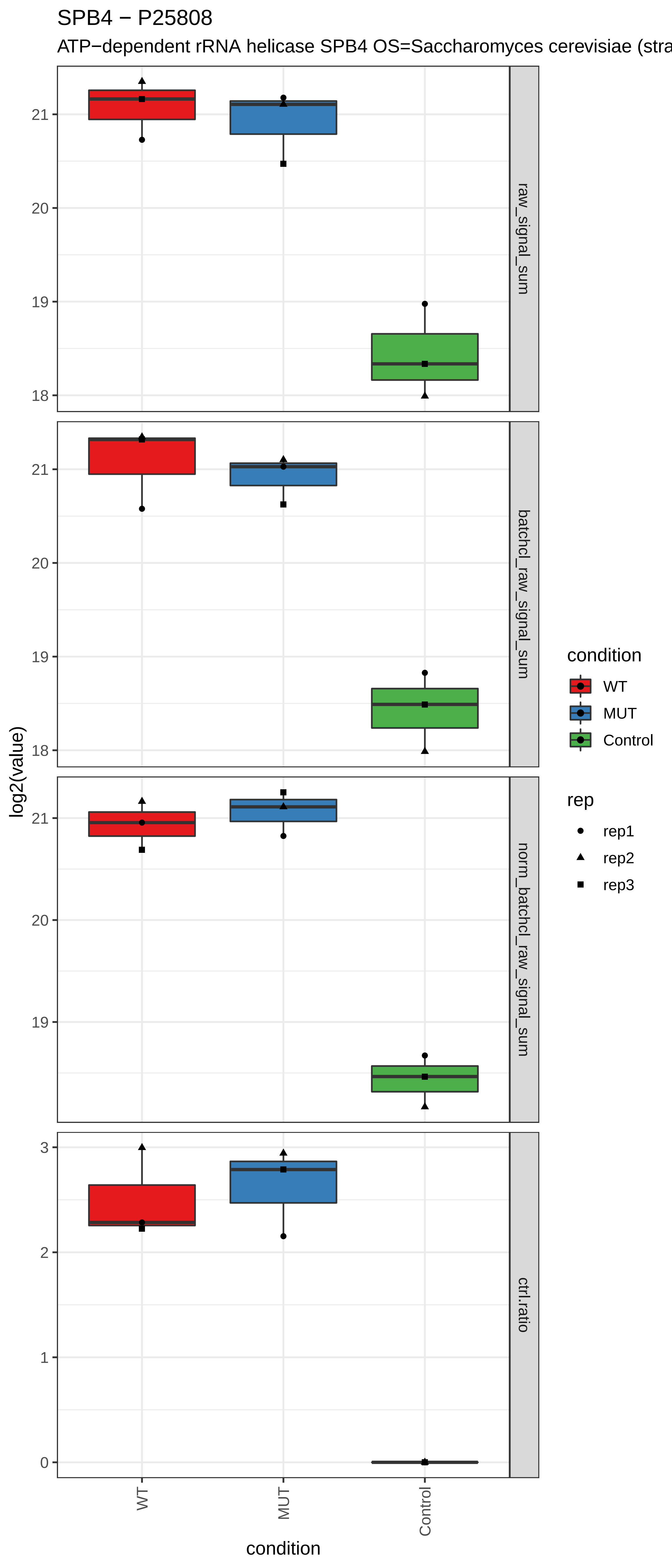

SPB4 − P25808
ATP−dependent rRNA helicase SPB4 OS=Saccharomyces cerevisiae (stra
raw_signal_sum
batchcl_raw_signal_sum
norm_batchcl_raw_signal_sum
ctrl.ratio
log2(value)
condition
WT
MUT
Control
rep
rep1
rep2
rep3

SPF1 − P39986

Manganese−transporting ATPase 1 OS=Saccharomyces cerevisiae (strain

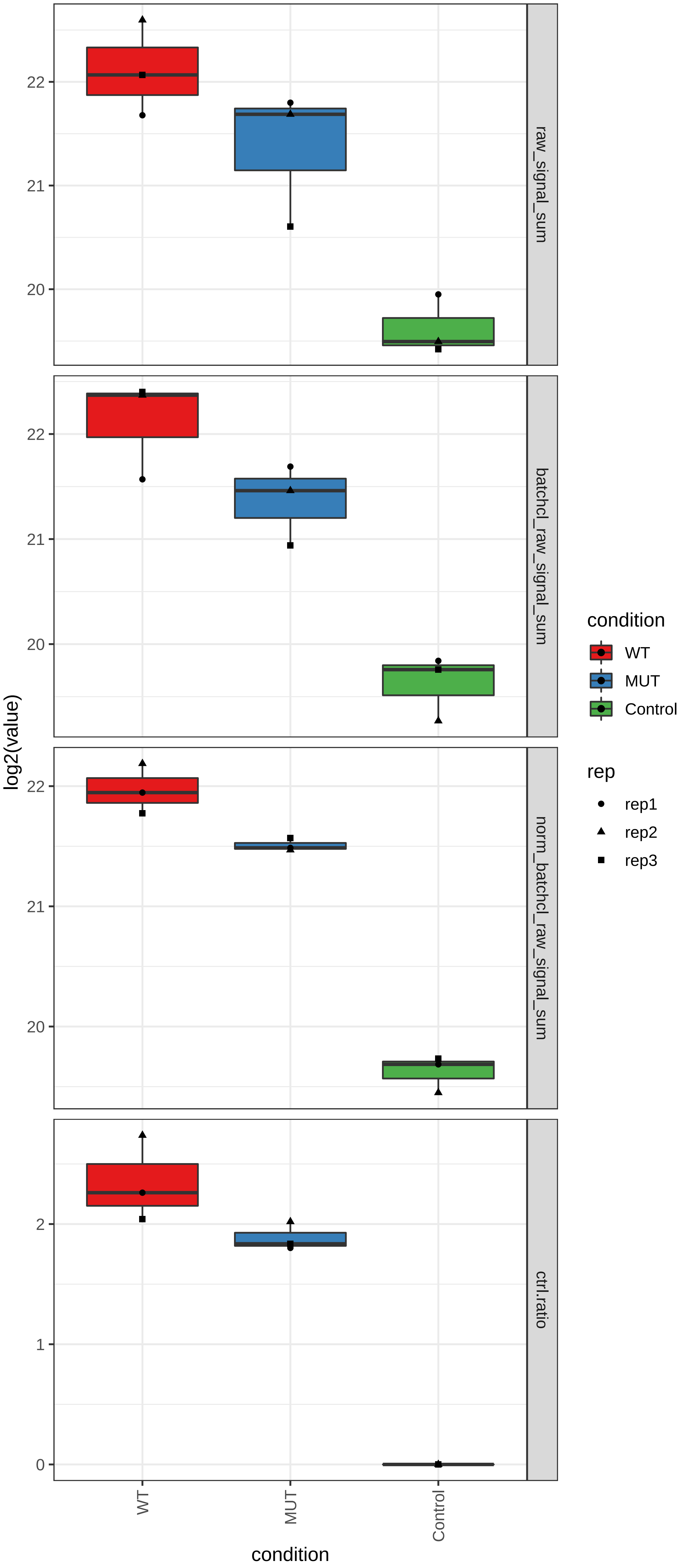

SPO14 − P36126

Phospholipase D1 OS=Saccharomyces cerevisiae (strain ATCC 204508 / S

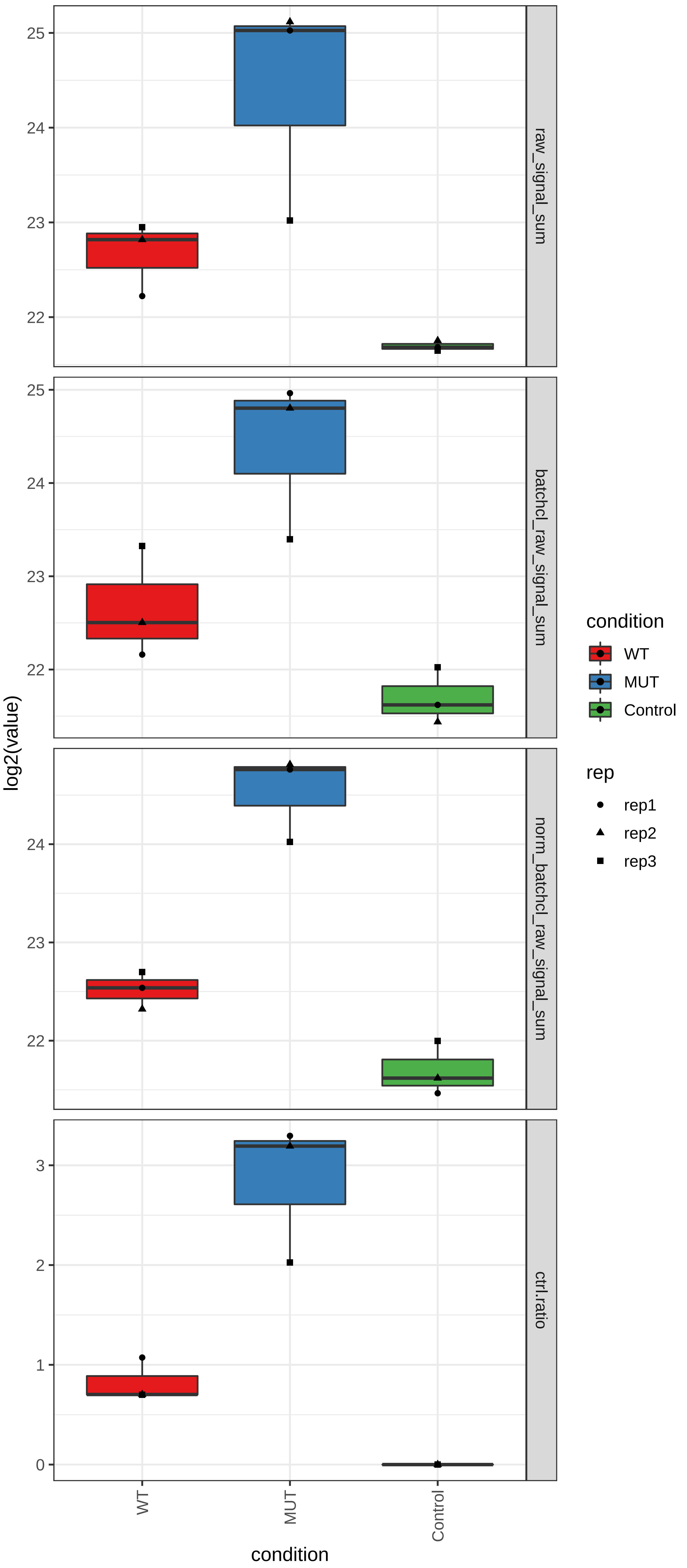

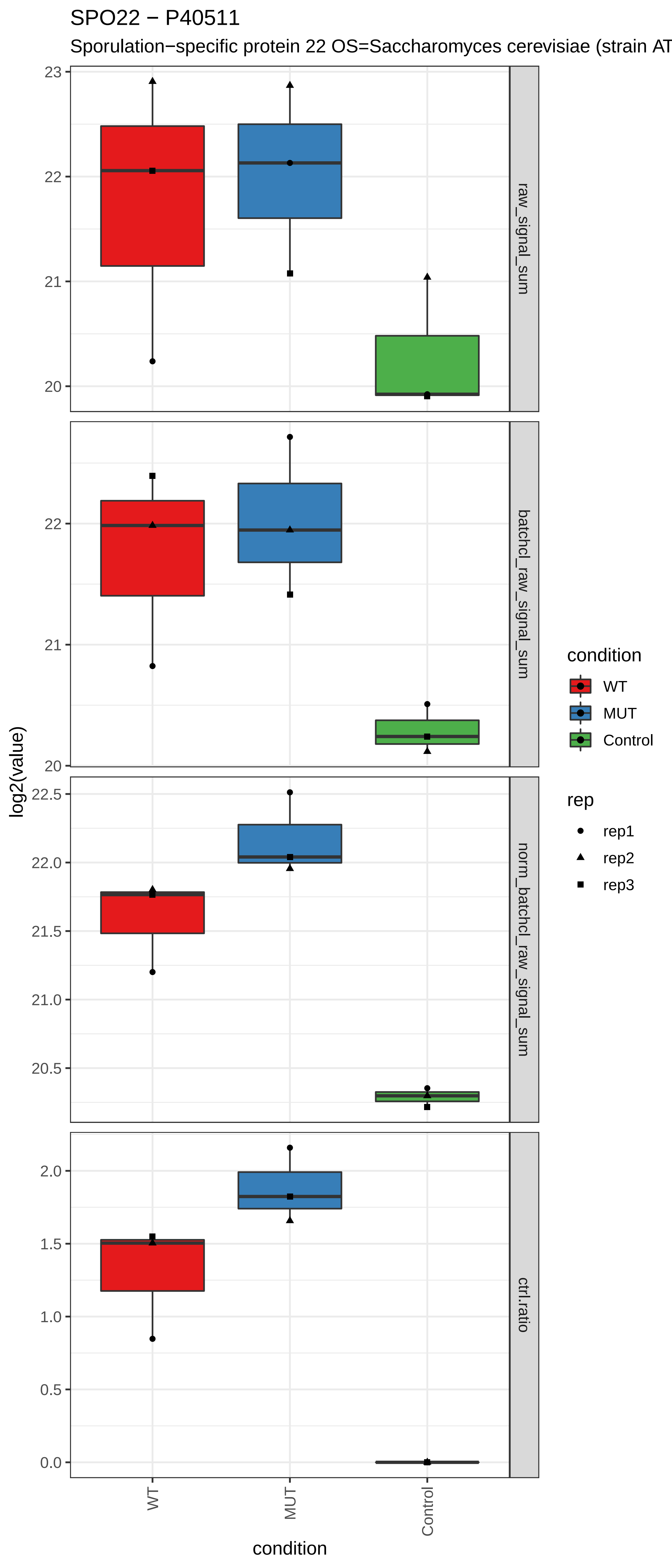
SPO22 − P40511
Sporulation−specific protein 22 OS=Saccharomyces cerevisiae (strain AT
raw_signal_sum
batchcl_raw_signal_sum
norm_batchcl_raw_signal_sum
ctrl.ratio
log2(value)
condition
WT
MUT
Control
rep
rep1
rep2
rep3

SPP1 − Q03012

COMPASS component SPP1 OS=Saccharomyces cerevisiae (strain ATCC

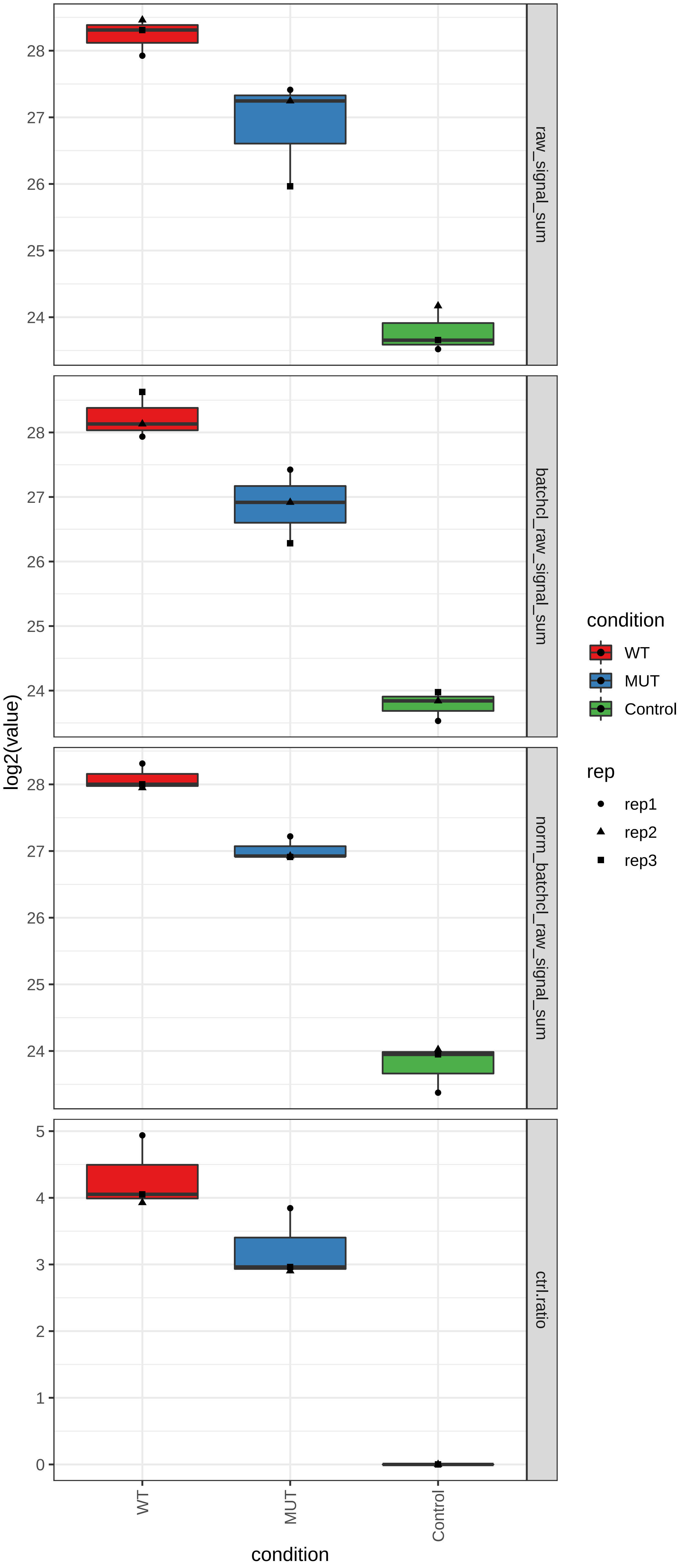

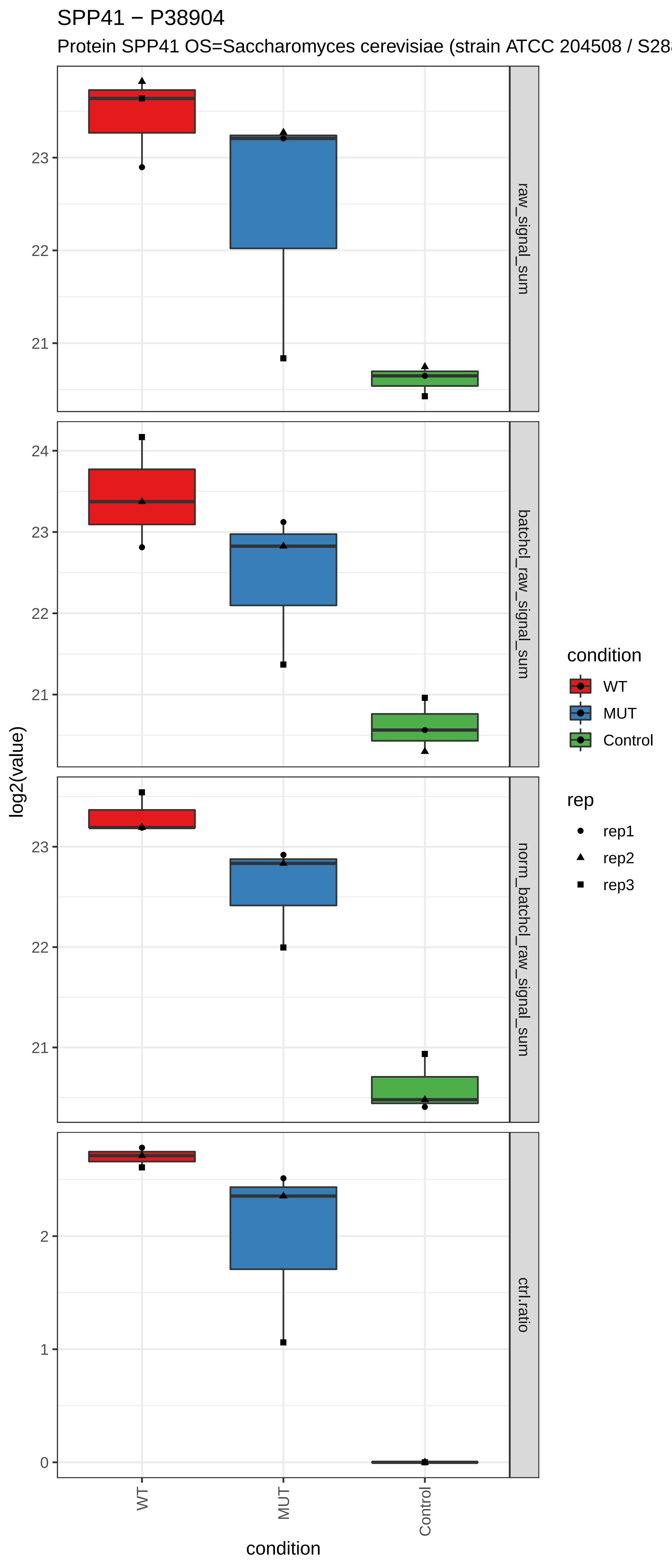
SPP41 − P38904
Protein SPP41 OS=Saccharomyces cerevisiae (strain ATCC 204508 / S28
raw_signal_sum
batchcl_raw_signal_sum
norm_batchcl_raw_signal_sum
ctrl.ratio
log2(value)
condition
WT
MUT
Control
rep
rep1
rep2
rep3
23
22
21
24
2
1
0

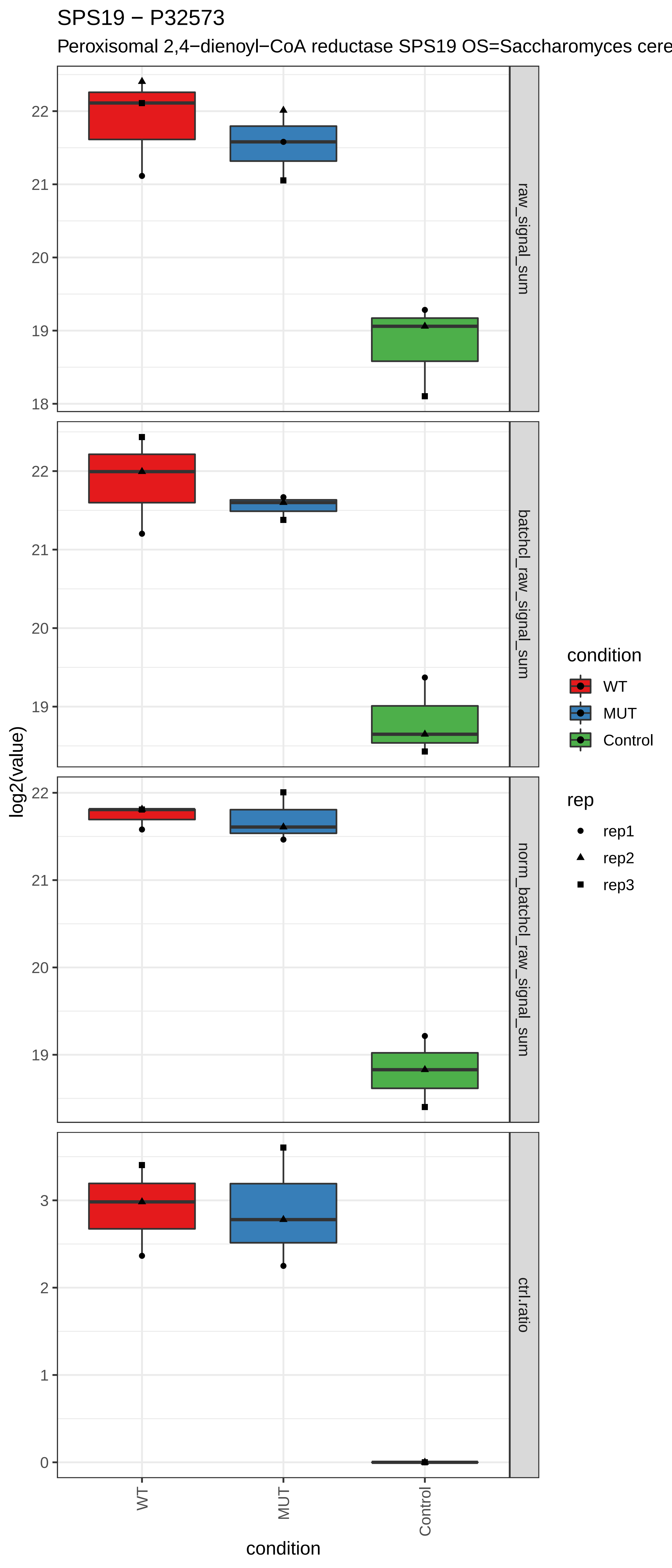

SPS19 − P32573
Peroxisomal 2,4−dienoyl−CoA reductase SPS19 OS=Saccharomyces cere
raw_signal_sum
batchcl_raw_signal_sum
norm_batchcl_raw_signal_sum
ctrl.ratio
log2(value)
condition
WT
MUT
Control
rep
rep1
rep2
rep3

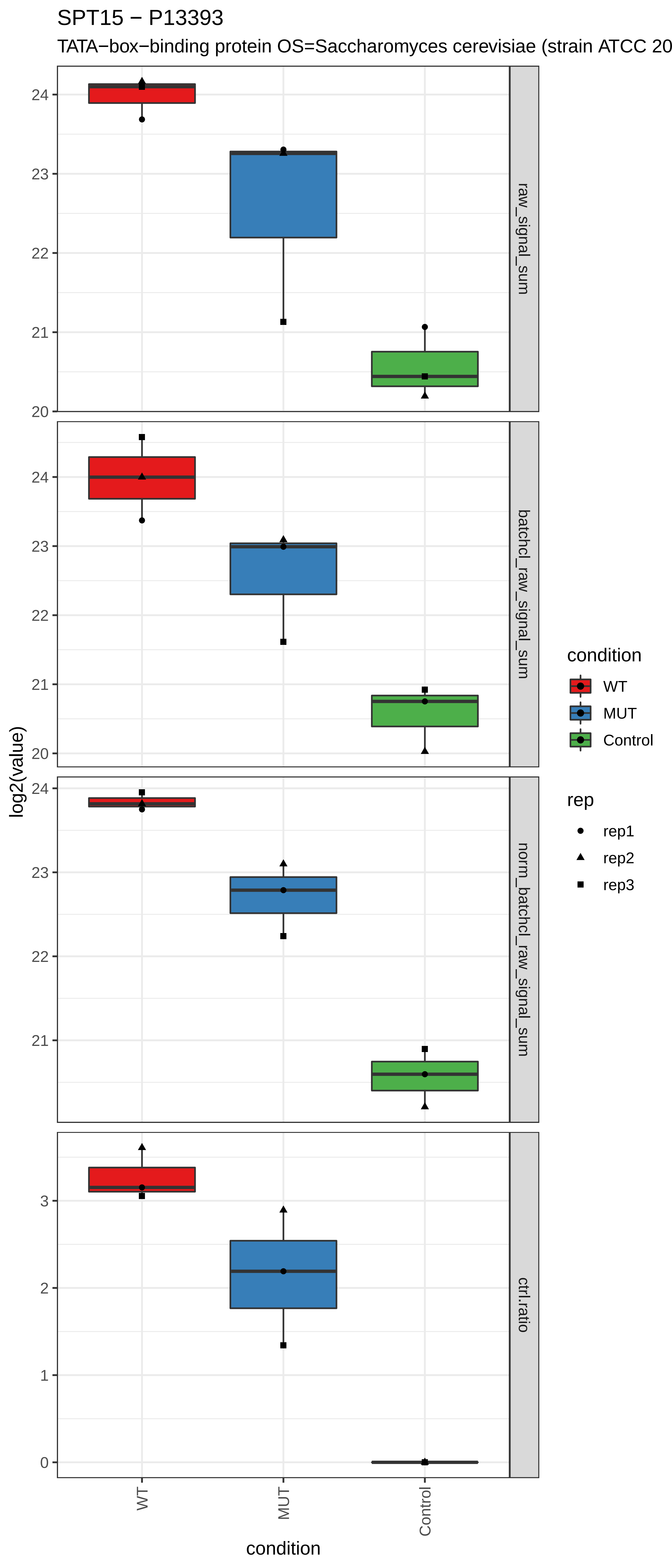
SPT15 − P13393
TATA−box−binding protein OS=Saccharomyces cerevisiae (strain ATCC 20
raw_signal_sum
batchcl_raw_signal_sum
norm_batchcl_raw_signal_sum
ctrl.ratio
log2(value)
condition
WT
MUT
Control
rep
rep1
rep2
rep3

SPT16 − P32558

FACT complex subunit SPT16 OS=Saccharomyces cerevisiae (strain ATCC

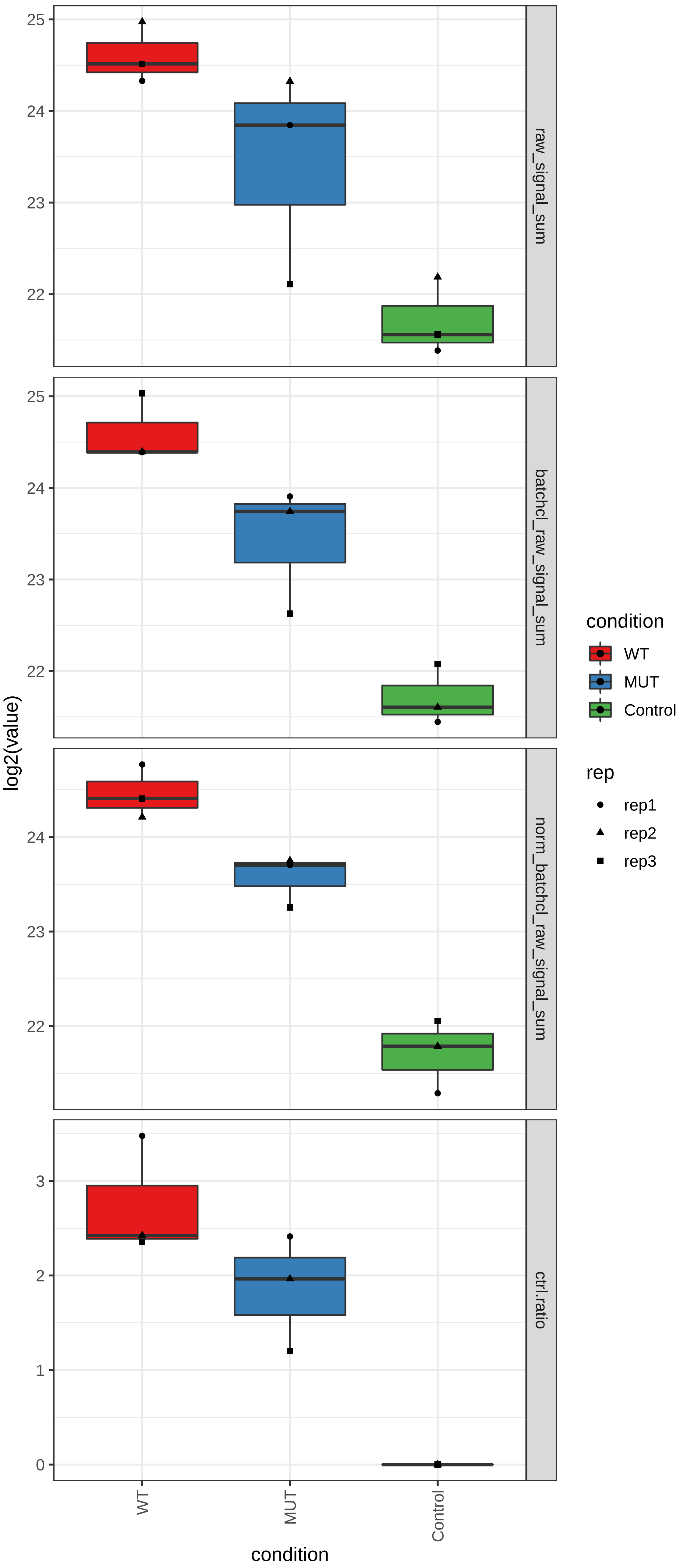

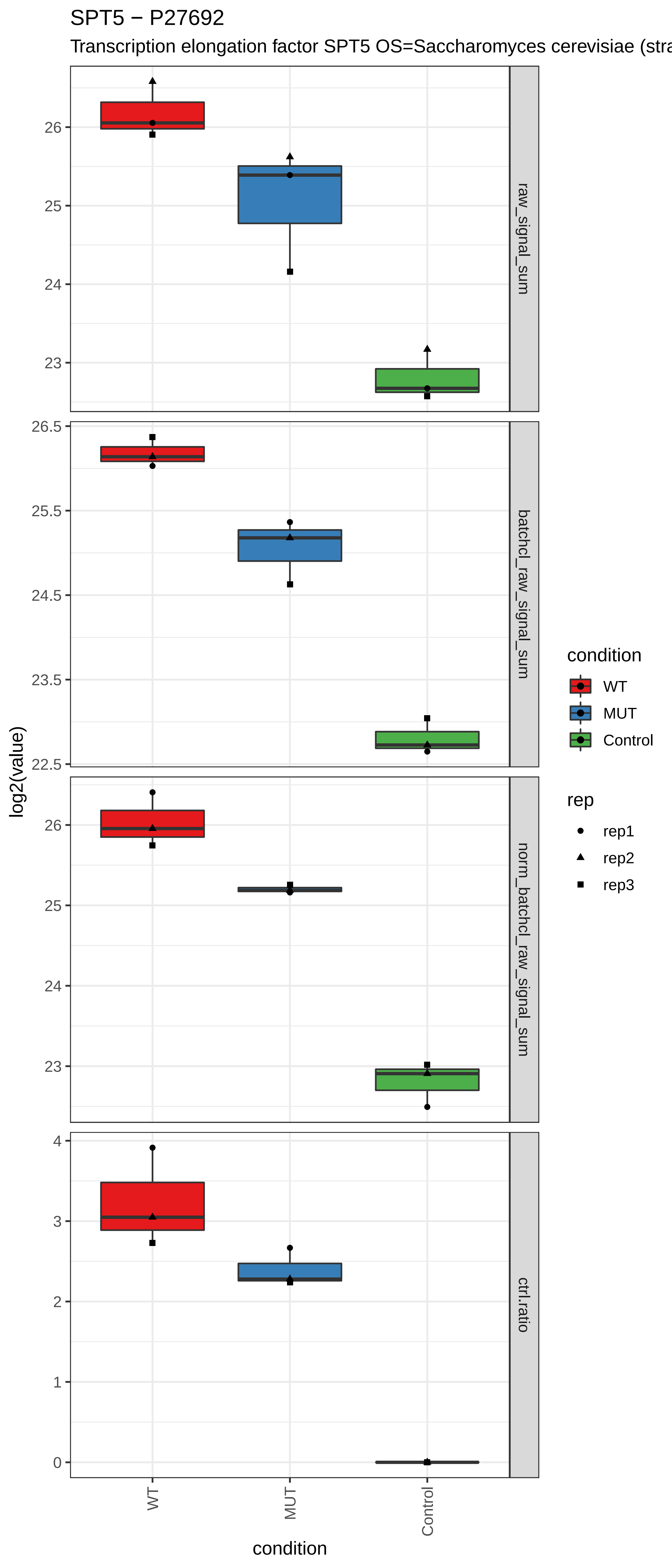
SPT5 − P27692
Transcription elongation factor SPT5 OS=Saccharomyces cerevisiae (stra
raw_signal_sum
batchcl_raw_signal_sum
norm_batchcl_raw_signal_sum
ctrl.ratio
log2(value)
condition
WT
MUT
Control
rep
rep1
rep2
rep3
26
25
24
23
26.5
25.5
24.5
23.5
22.5
4
3
2
1
0

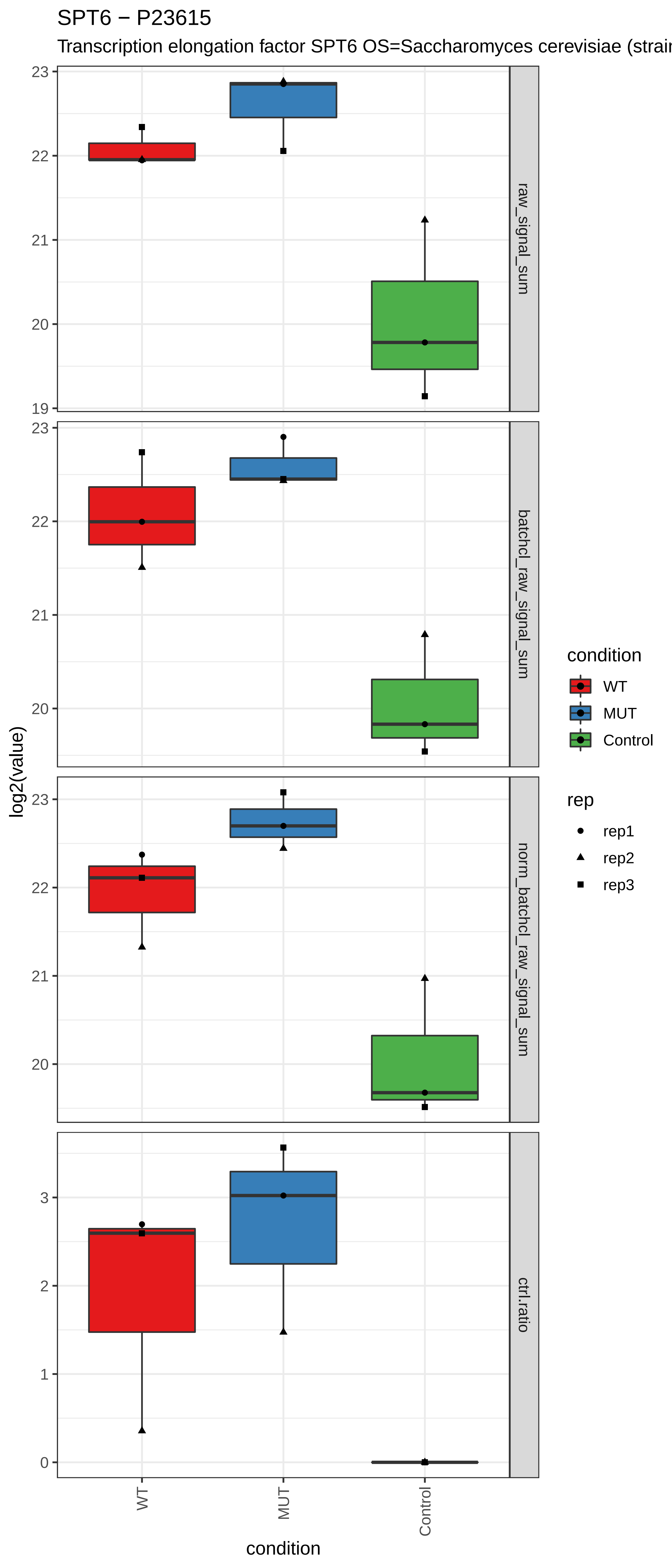

SPT6 − P23615
Transcription elongation factor SPT6 OS=Saccharomyces cerevisiae (strain
raw_signal_sum
batchcl_raw_signal_sum
norm_batchcl_raw_signal_sum
ctrl.ratio
log2(value)
condition
WT
MUT
Control
rep
rep1
rep2
rep3

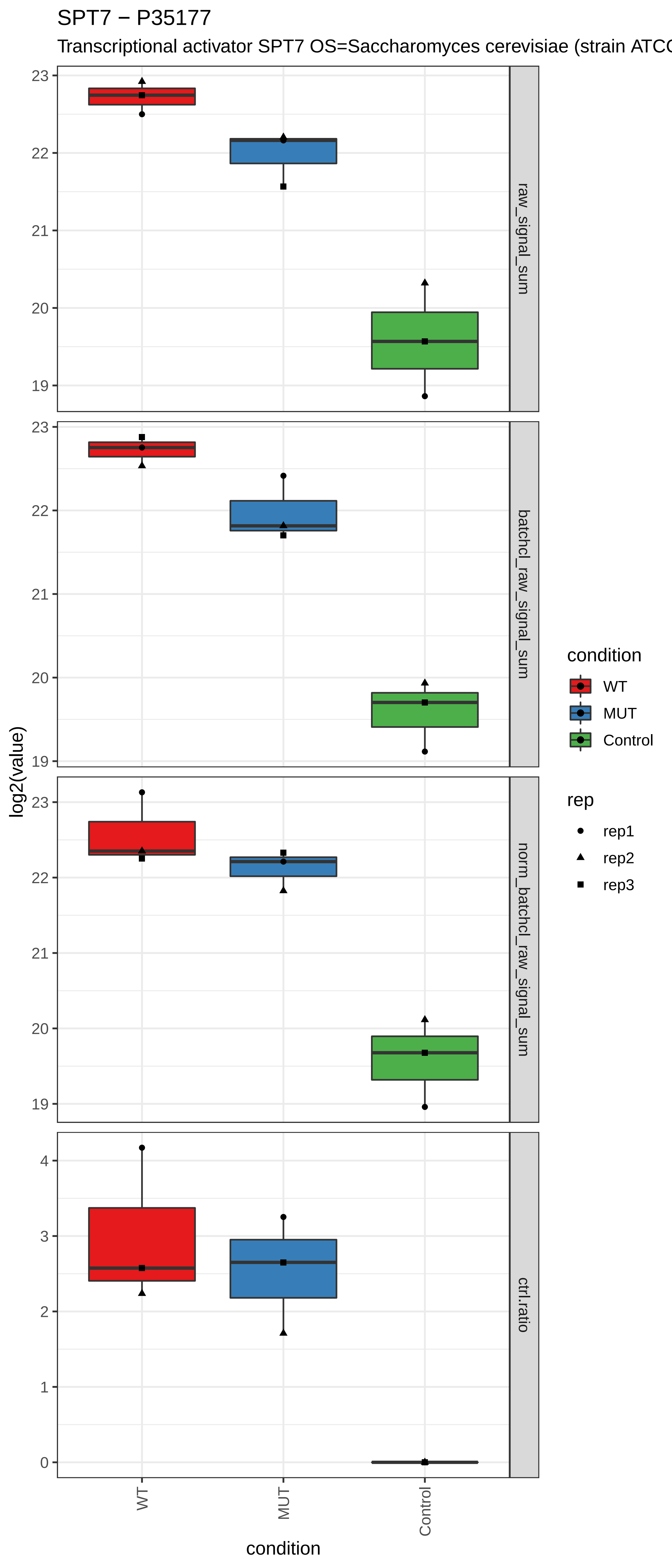

SPT7 − P35177
Transcriptional activator SPT7 OS=Saccharomyces cerevisiae (strain ATCC
raw_signal_sum
batchcl_raw_signal_sum
norm_batchcl_raw_signal_sum
ctrl.ratio
log2(value)
condition
WT
MUT
Control
rep
rep1
rep2
rep3
23
22
21
20
19
4
3
2
1
0

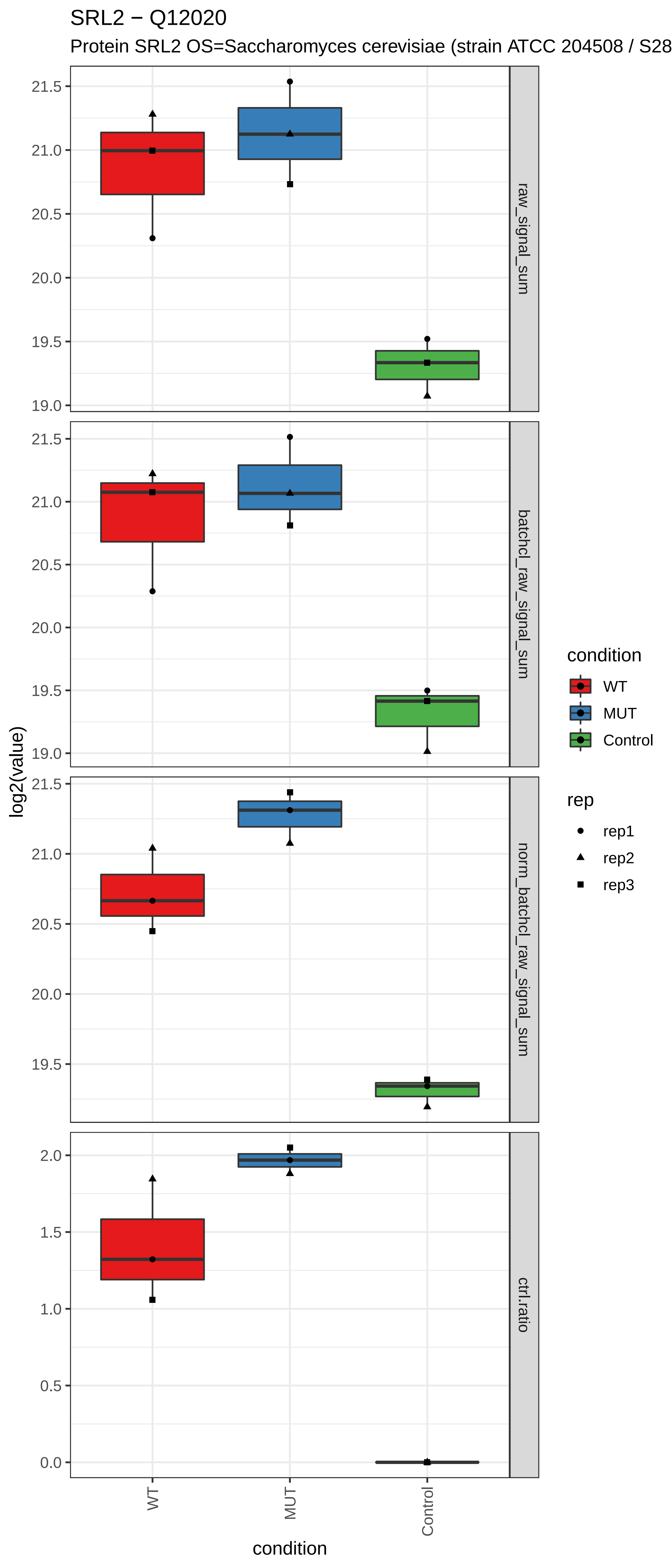
SRL2 − Q12020
Protein SRL2 OS=Saccharomyces cerevisiae (strain ATCC 204508 / S28
raw_signal_sum
batchcl_raw_signal_sum
norm_batchcl_raw_signal_sum
ctrl.ratio
log2(value)
condition
WT
MUT
Control
rep
rep1
rep2
rep3

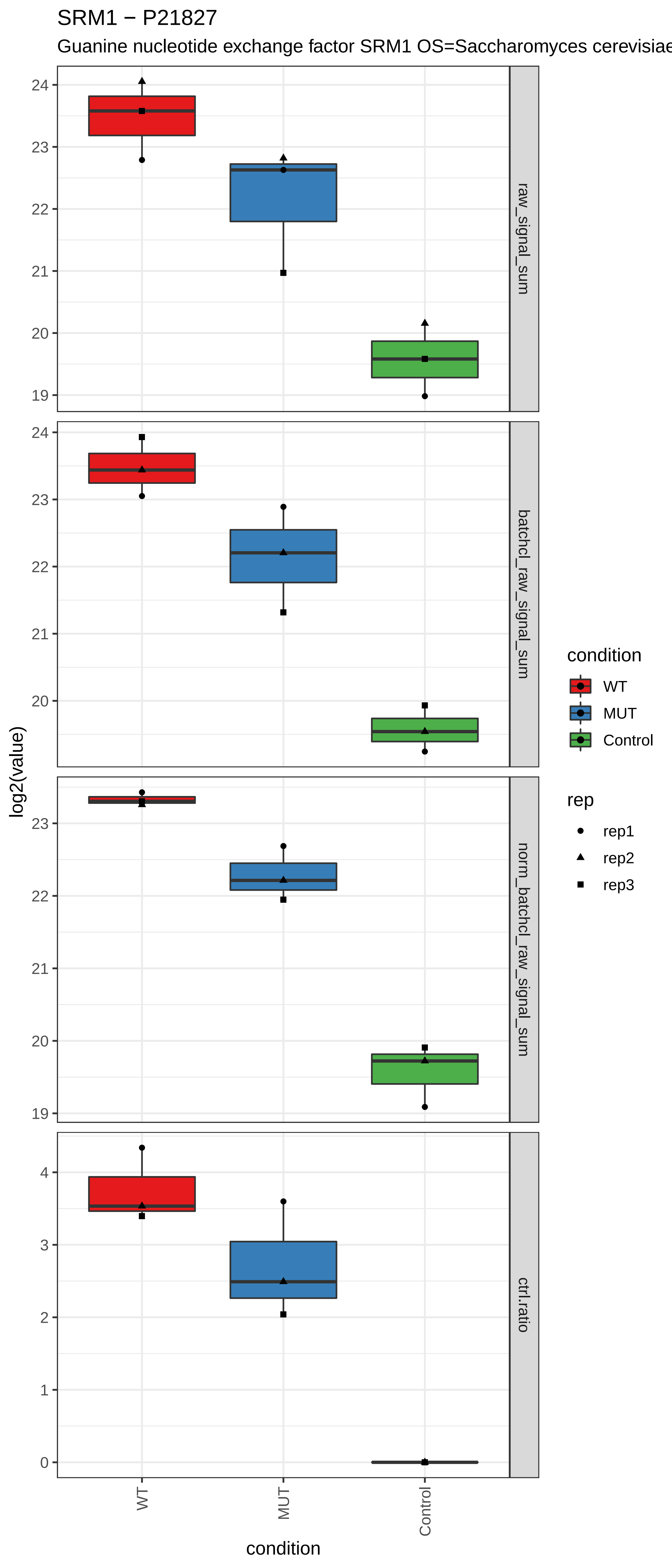

SRM1 − P21827
Guanine nucleotide exchange factor SRM1 OS=Saccharomyces cerevisiae
raw_signal_sum
batchcl_raw_signal_sum
norm_batchcl_raw_signal_sum
ctrl.ratio
log2(value)
condition
WT
MUT
Control
rep
rep1
rep2
rep3

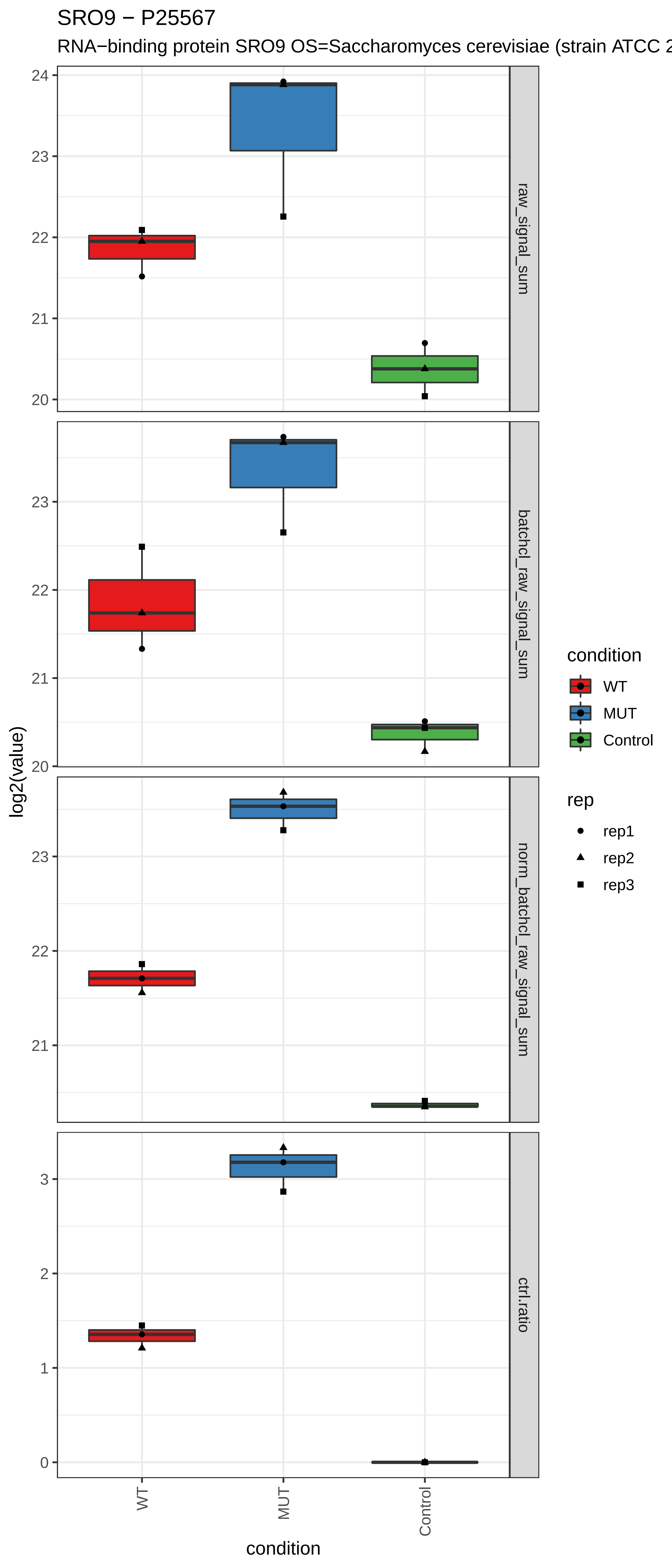

SRO9 − P25567
RNA−binding protein SRO9 OS=Saccharomyces cerevisiae (strain ATCC 2
raw_signal_sum
batchcl_raw_signal_sum
norm_batchcl_raw_signal_sum
ctrl.ratio
log2(value)
condition
WT
MUT
Control
rep
rep1
rep2
rep3

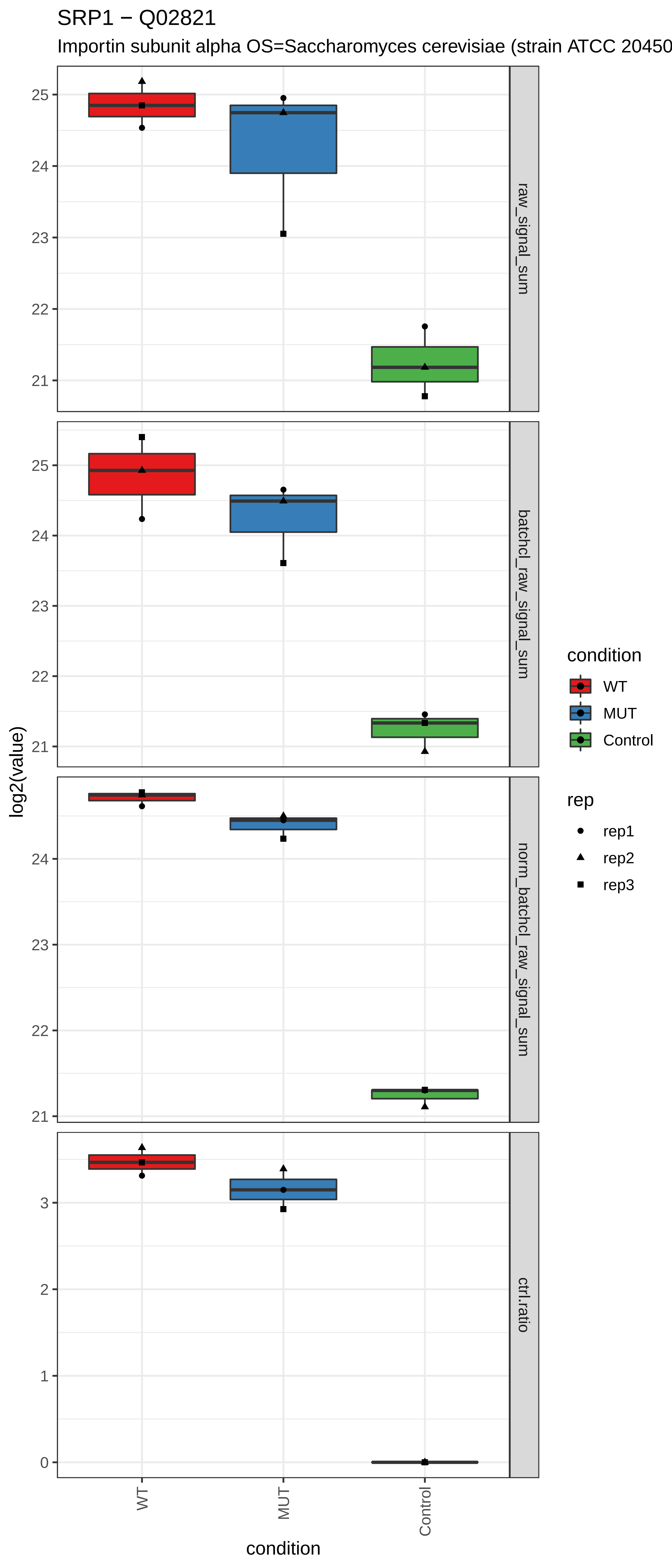
SRP1 − Q02821
Importin subunit alpha OS=Saccharomyces cerevisiae (strain ATCC 20450
raw_signal_sum
batchcl_raw_signal_sum
norm_batchcl_raw_signal_sum
ctrl.ratio
log2(value)
condition
WT
MUT
Control
rep
rep1
rep2
rep3

SRP40 − P32583

Suppressor protein SRP40 OS=Saccharomyces cerevisiae (strain ATCC 20

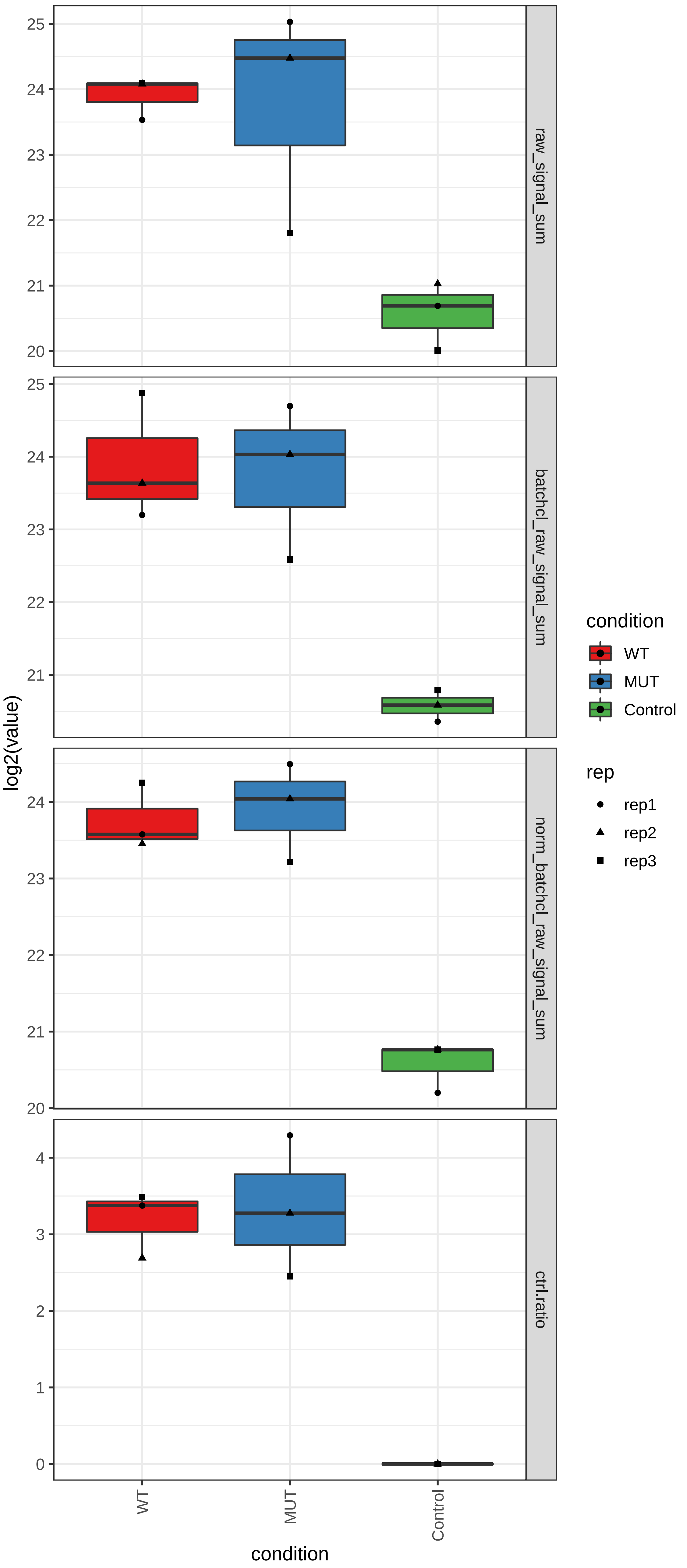

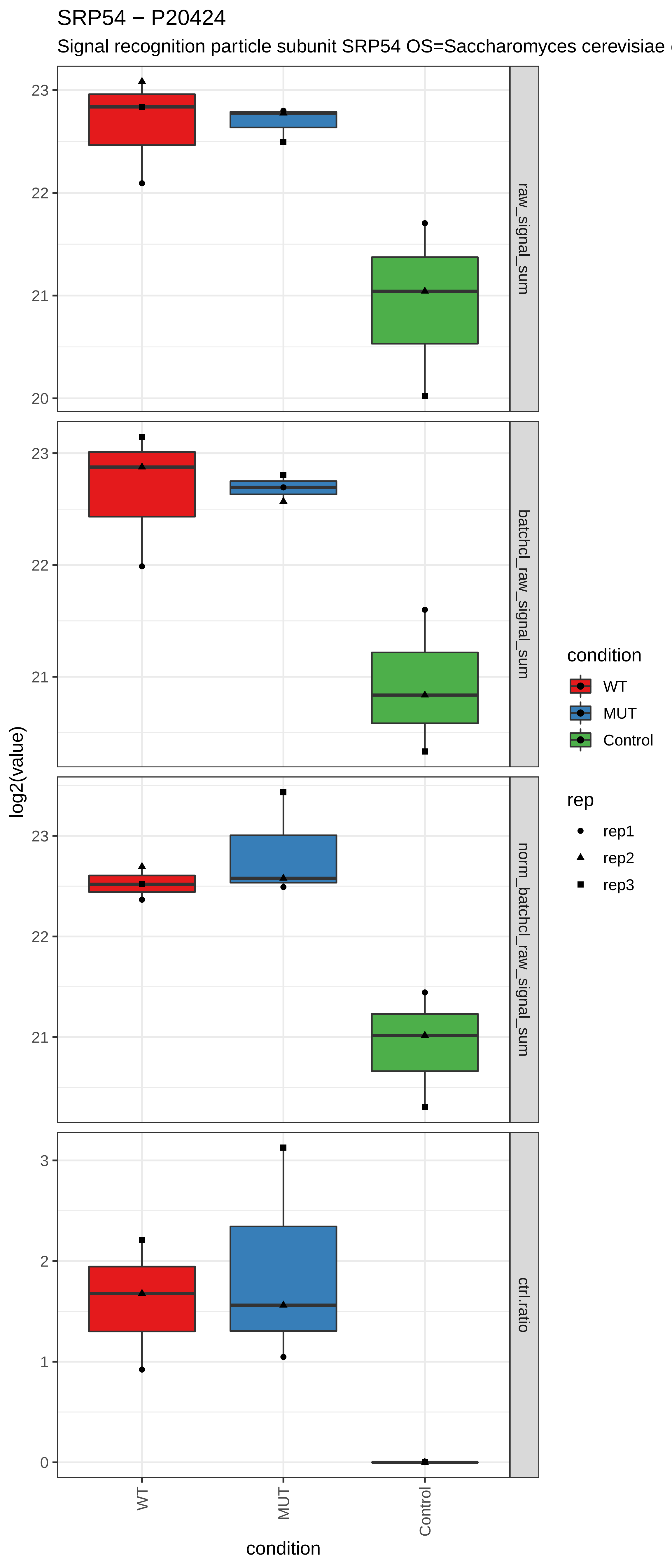
SRP54 − P20424
Signal recognition particle subunit SRP54 OS=Saccharomyces cerevisiae
raw_signal_sum
batchcl_raw_signal_sum
norm_batchcl_raw_signal_sum
ctrl.ratio
log2(value)
condition
WT
MUT
Control
rep
rep1
rep2
rep3

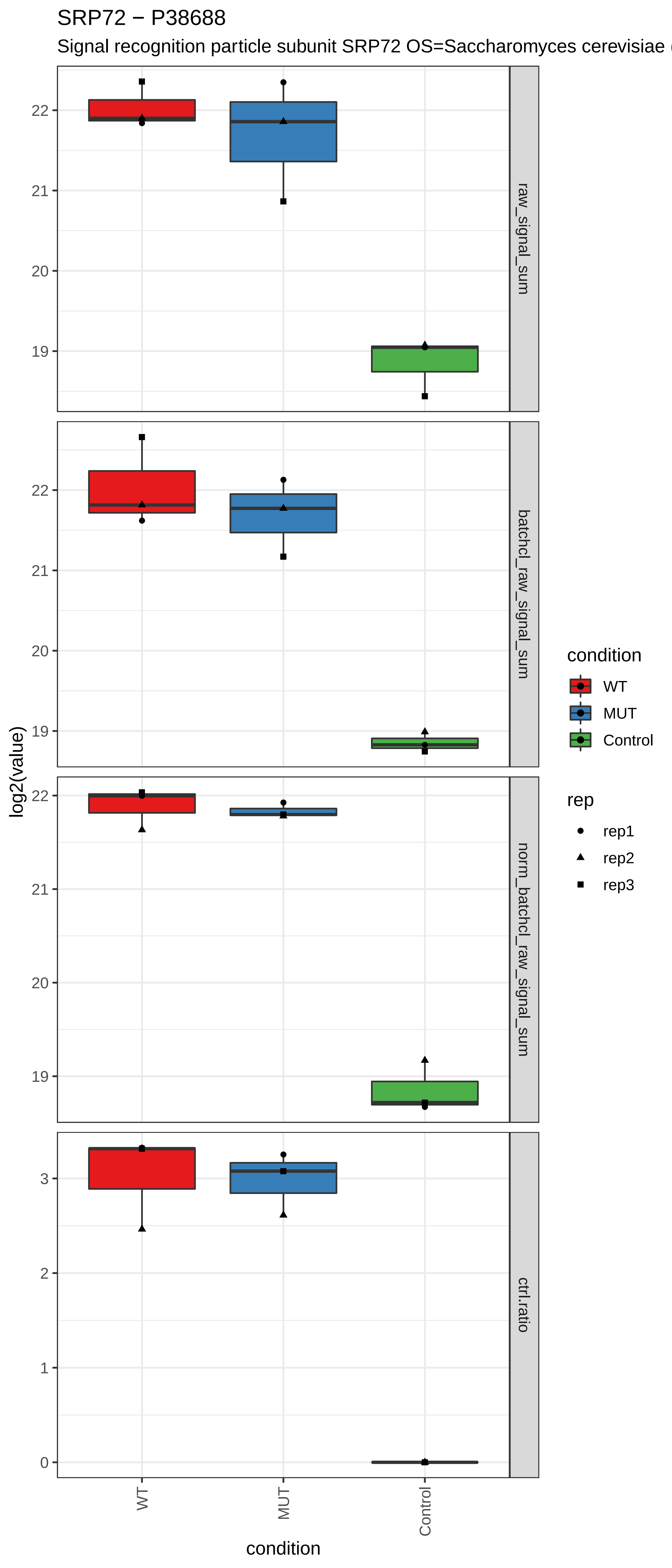

SRP72 − P38688
Signal recognition particle subunit SRP72 OS=Saccharomyces cerevisiae
raw_signal_sum
batchcl_raw_signal_sum
norm_batchcl_raw_signal_sum
ctrl.ratio
log2(value)
condition
WT
MUT
Control
rep
rep1
rep2
rep3
22
21
20
19
3
2
1
0

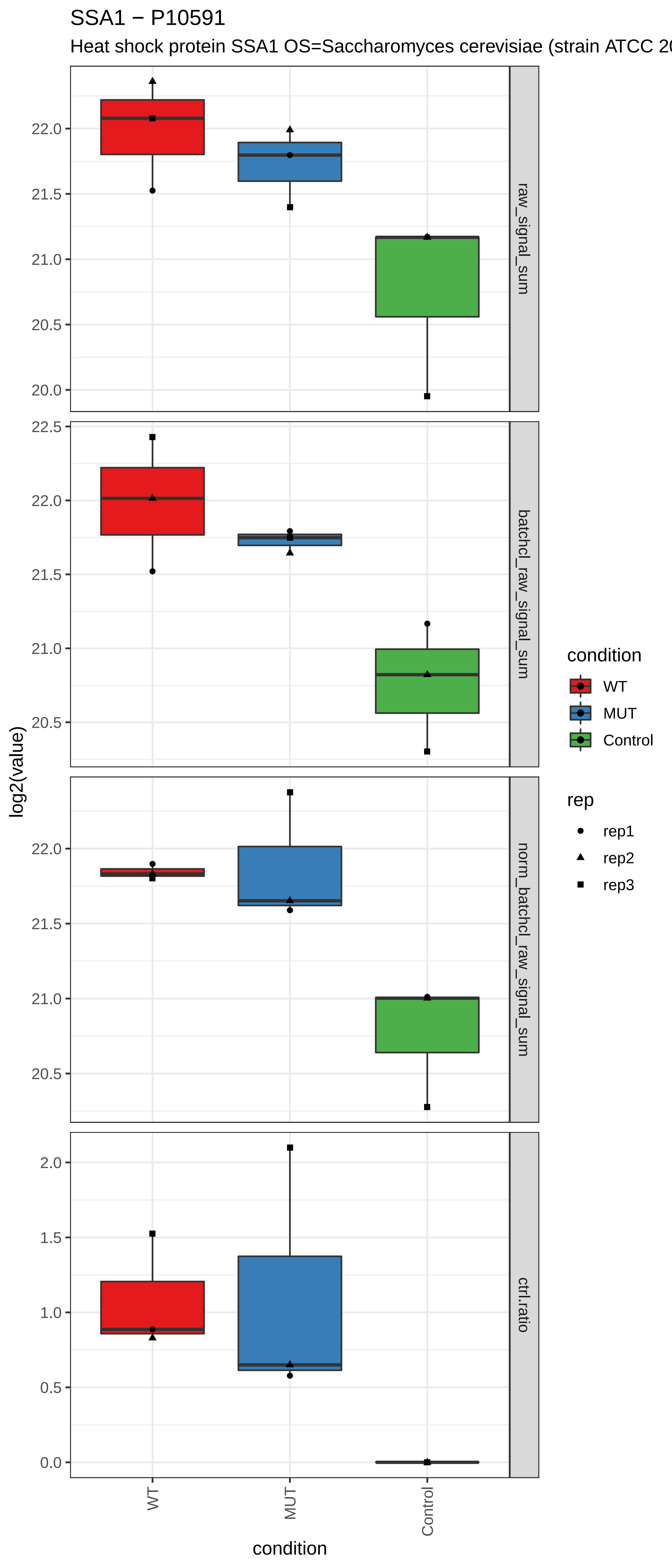

SSA1 − P10591
Heat shock protein SSA1 OS=Saccharomyces cerevisiae (strain ATCC 20
raw_signal_sum
batchcl_raw_signal_sum
norm_batchcl_raw_signal_sum
ctrl.ratio
log2(value)
condition
WT
MUT
Control
rep
rep1
rep2
rep3
22.0
21.5
21.0
20.5
20.0
22.5
2.0
1.5
1.0
0.5
0.0

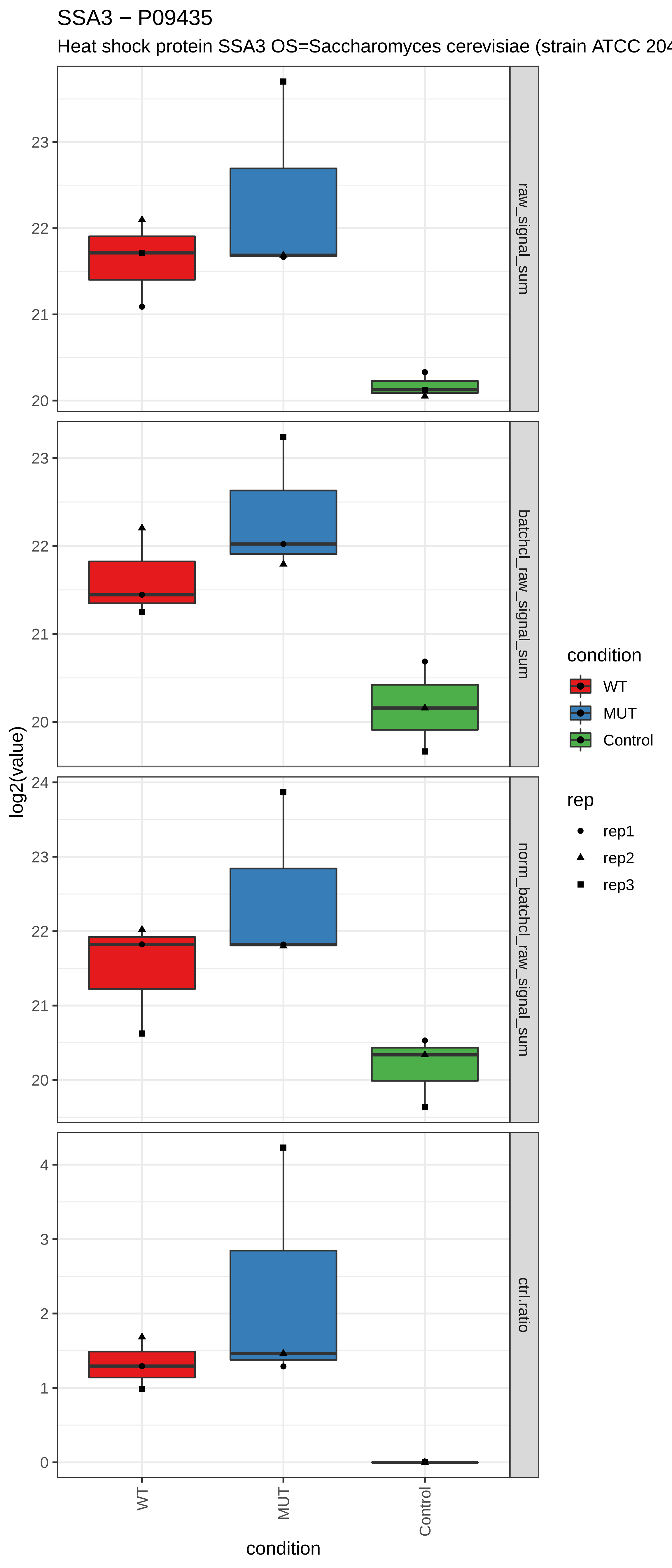

SSA3 − P09435
Heat shock protein SSA3 OS=Saccharomyces cerevisiae (strain ATCC 204
raw_signal_sum
batchcl_raw_signal_sum
norm_batchcl_raw_signal_sum
ctrl.ratio
log2(value)
condition
WT
MUT
Control
rep
rep1
rep2
rep3

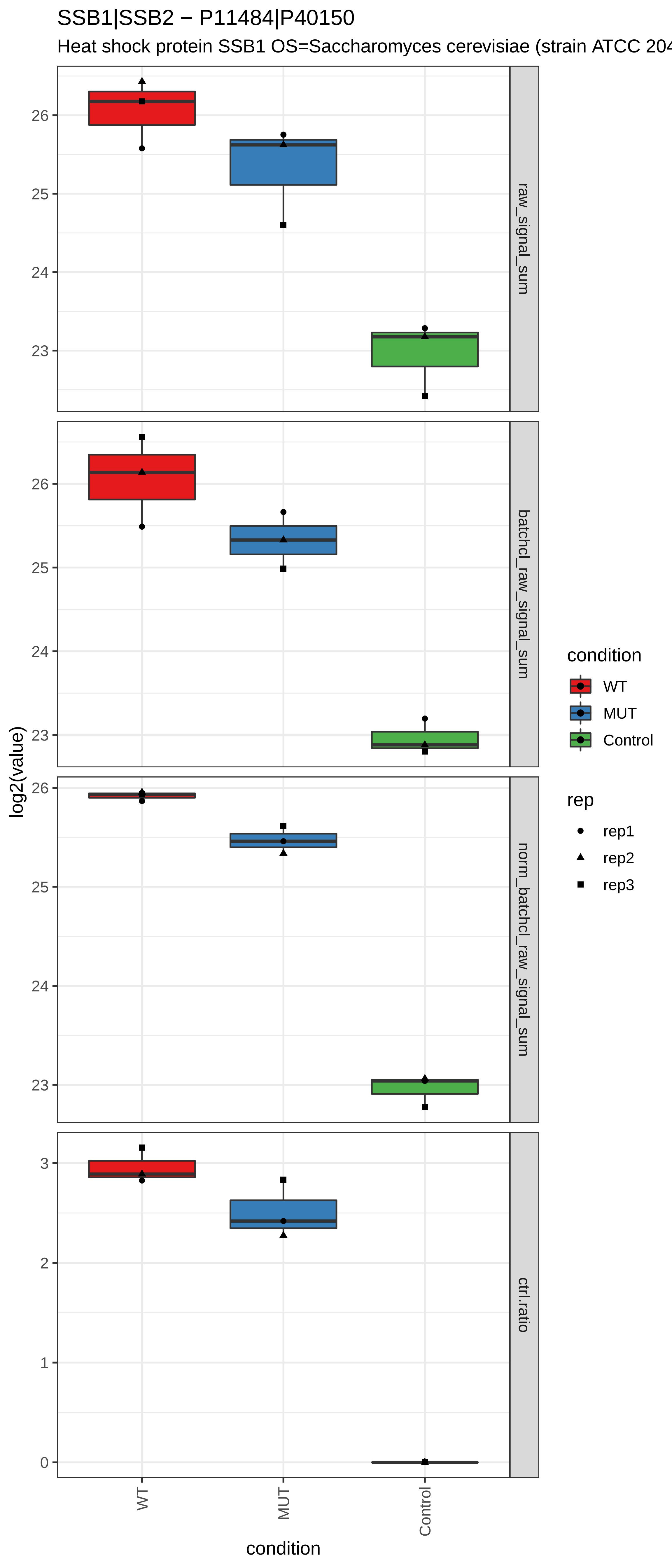

SSB1|SSB2 − P11484|P40150
Heat shock protein SSB1 OS=Saccharomyces cerevisiae (strain ATCC 204
raw_signal_sum
batchcl_raw_signal_sum
norm_batchcl_raw_signal_sum
ctrl.ratio
log2(value)
condition
WT
MUT
Control
rep
rep1
rep2
rep3
26
25
24
23
3
2
1
0

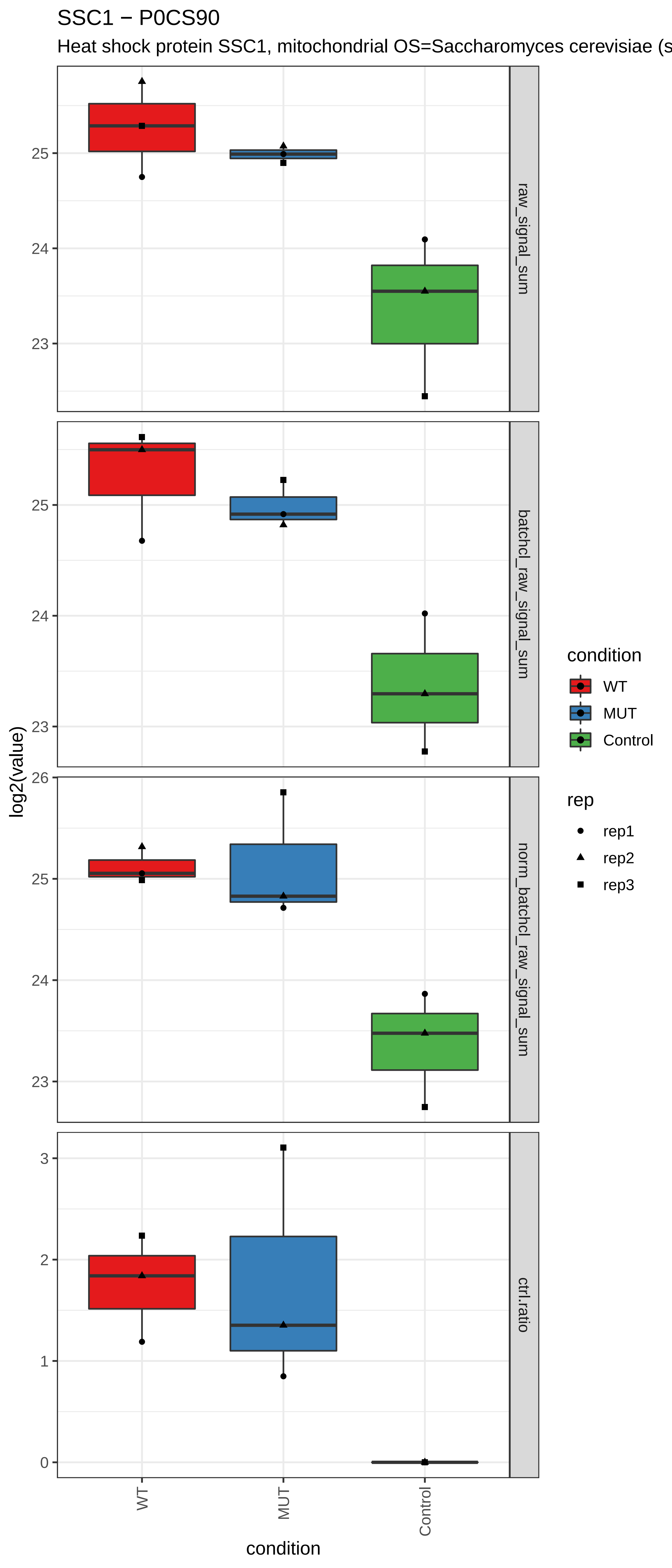

SSC1 − P0CS90
Heat shock protein SSC1, mitochondrial OS=Saccharomyces cerevisiae (s
raw_signal_sum
batchcl_raw_signal_sum
norm_batchcl_raw_signal_sum
ctrl.ratio
log2(value)
condition
WT
MUT
Control
rep
rep1
rep2
rep3

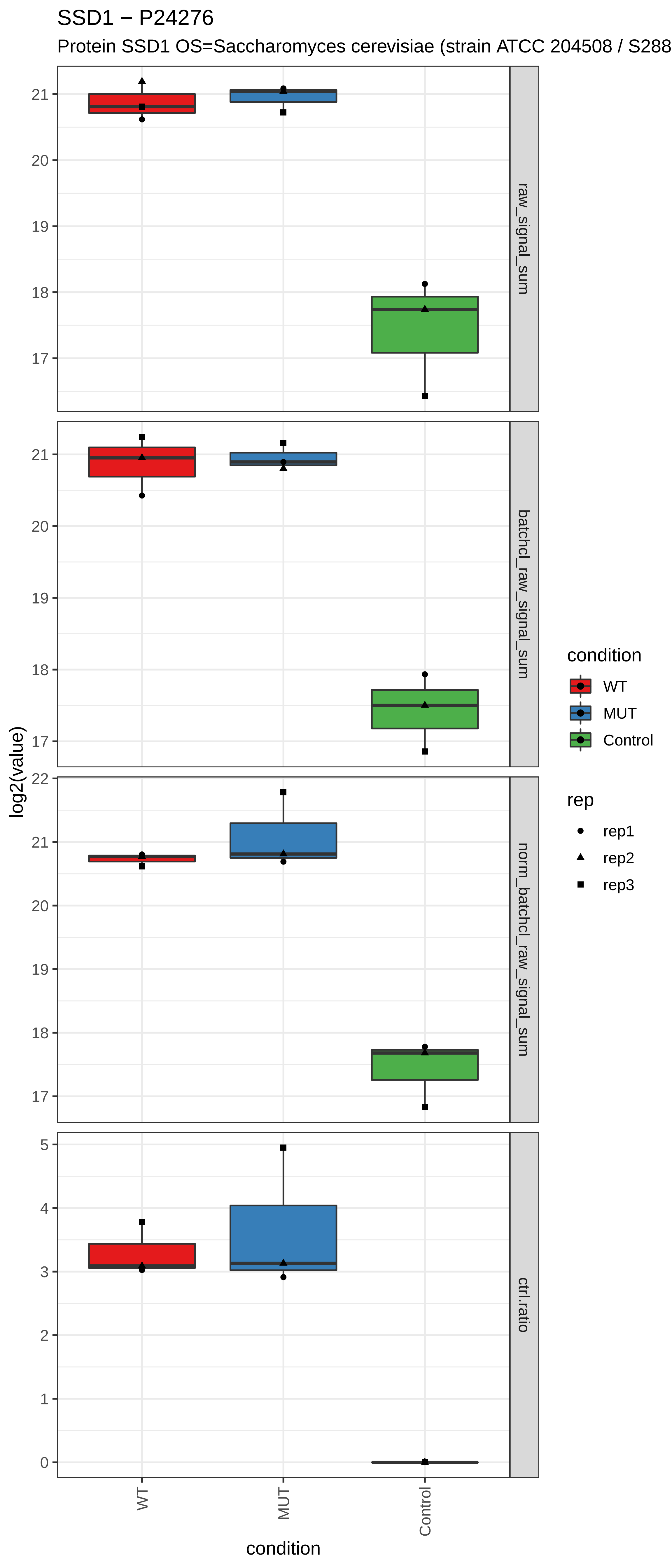

SSD1 − P24276
Protein SSD1 OS=Saccharomyces cerevisiae (strain ATCC 204508 / S288
raw_signal_sum
batchcl_raw_signal_sum
norm_batchcl_raw_signal_sum
ctrl.ratio
log2(value)
condition
WT
MUT
Control
rep
rep1
rep2
rep3

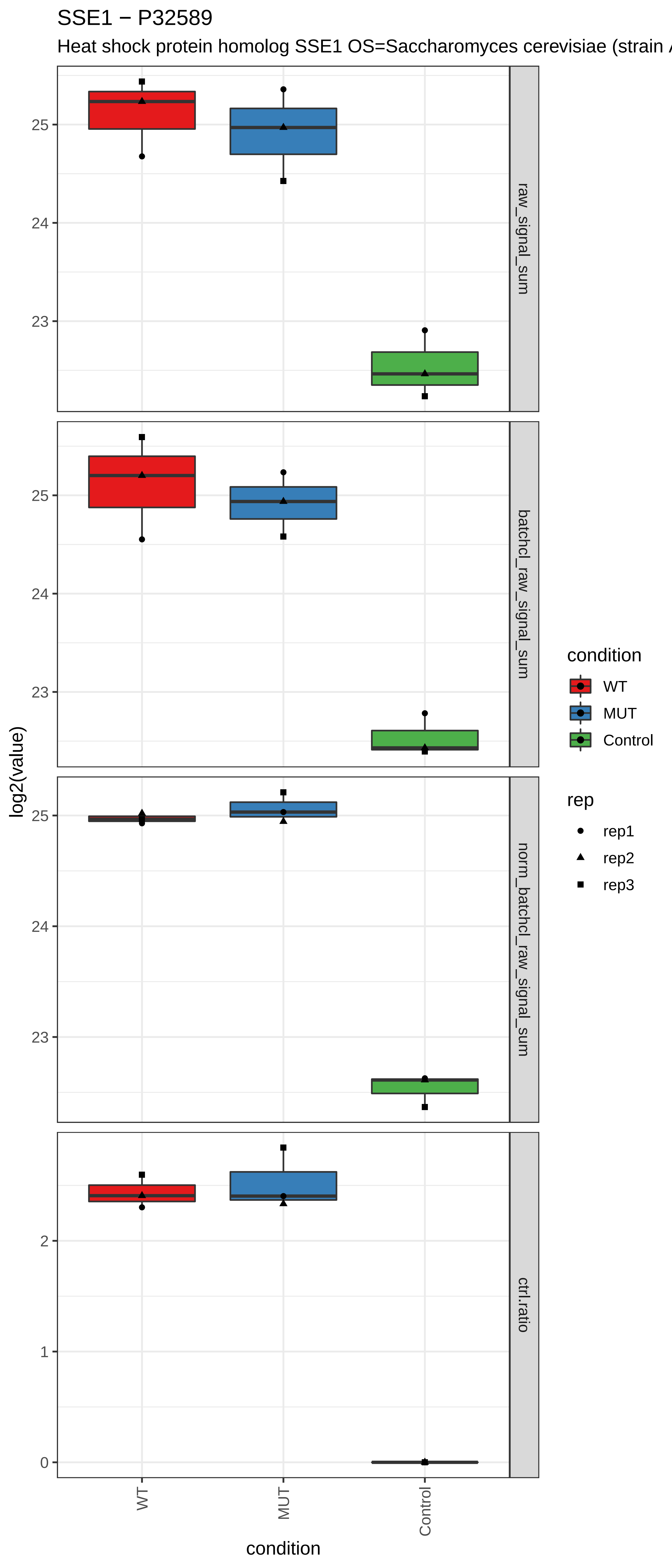
SSE1 − P32589
Heat shock protein homolog SSE1 OS=Saccharomyces cerevisiae (strain
raw_signal_sum
batchcl_raw_signal_sum
norm_batchcl_raw_signal_sum
ctrl.ratio
log2(value)
condition
WT
MUT
Control
rep
rep1
rep2
rep3
25
24
23
2
1
0

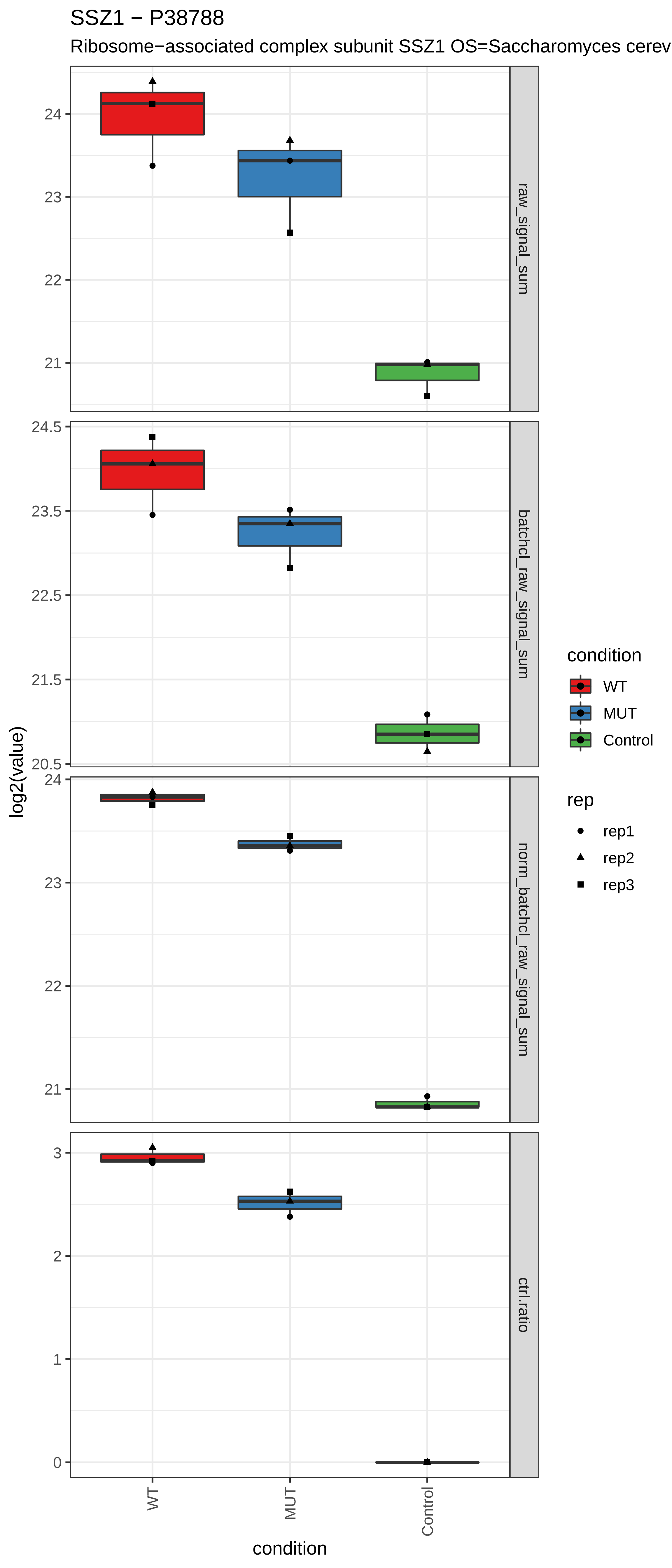
SSZ1 − P38788
Ribosome−associated complex subunit SSZ1 OS=Saccharomyces cerev
raw_signal_sum
batchcl_raw_signal_sum
norm_batchcl_raw_signal_sum
ctrl.ratio
log2(value)
condition
WT
MUT
Control
rep
rep1
rep2
rep3
24
23
22
21
24.5
23.5
22.5
21.5
20.5
3
2
1
0

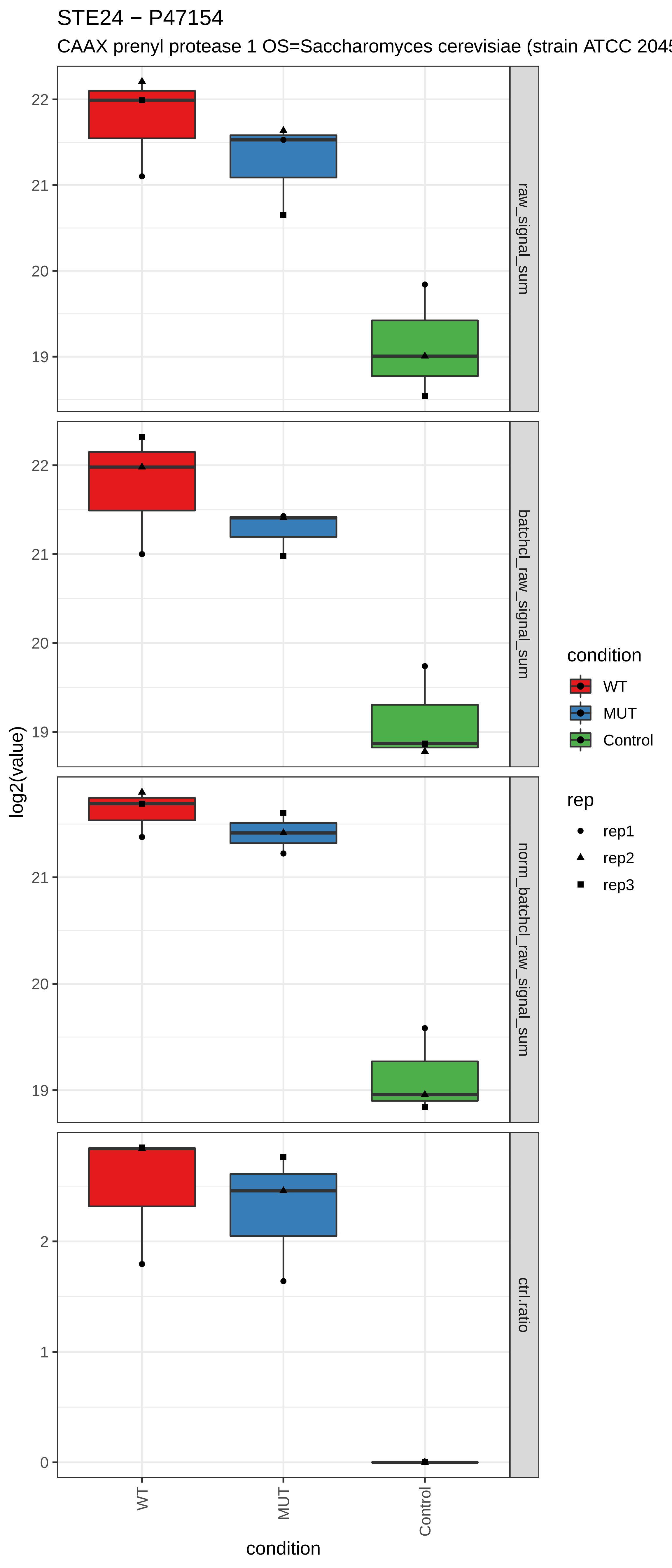

STE24 − P47154
CAAX prenyl protease 1 OS=Saccharomyces cerevisiae (strain ATCC 2045
raw_signal_sum
batchcl_raw_signal_sum
norm_batchcl_raw_signal_sum
ctrl.ratio
log2(value)
condition
WT
MUT
Control
rep
rep1
rep2
rep3
22
21
20
19
2
1
0

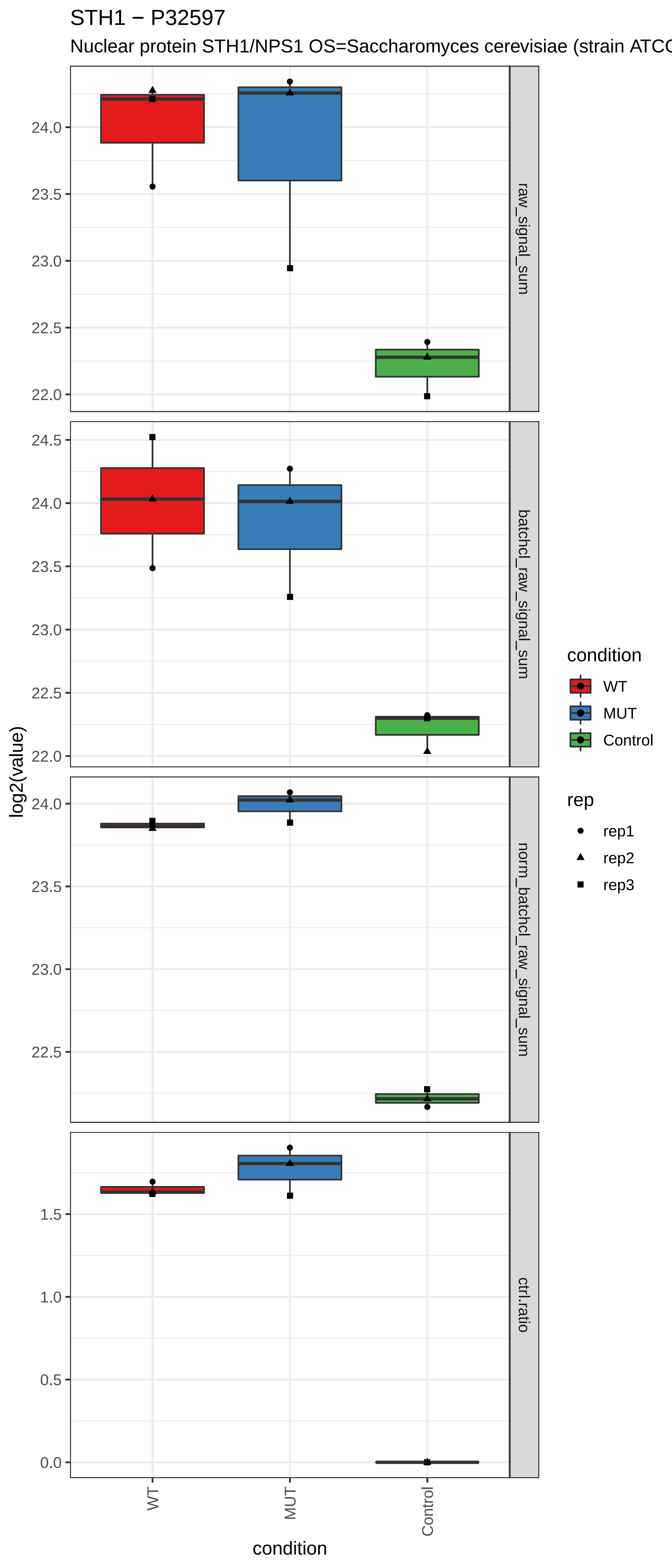

STH1 − P32597
Nuclear protein STH1/NPS1 OS=Saccharomyces cerevisiae (strain ATCC
raw_signal_sum
batchcl_raw_signal_sum
norm_batchcl_raw_signal_sum
ctrl.ratio
log2(value)
condition
WT
MUT
Control
rep
rep1
rep2
rep3

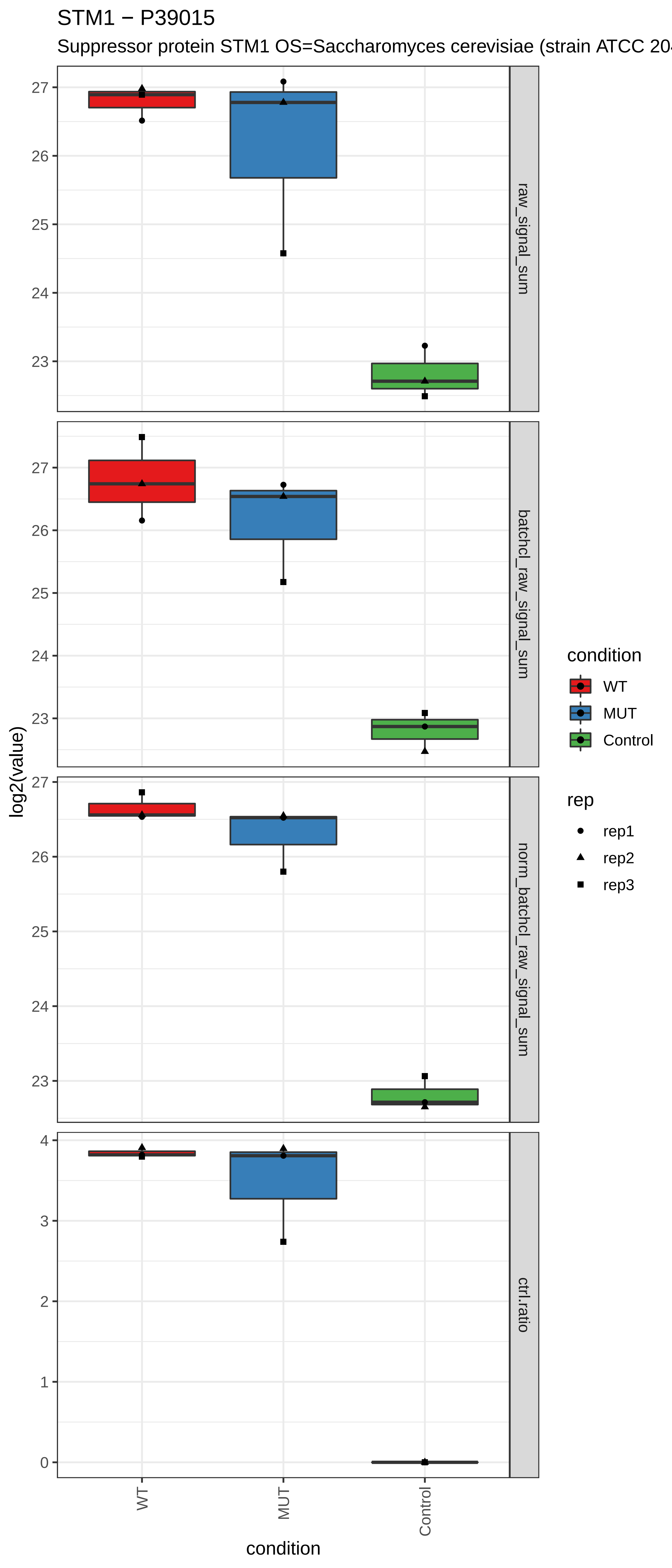

STM1 − P39015
Suppressor protein STM1 OS=Saccharomyces cerevisiae (strain ATCC 20
log2(value)
raw_signal_sum
batchcl_raw_signal_sum
norm_batchcl_raw_signal_sum
ctrl.ratio
WT
MUT
Control
condition
condition
WT
MUT
Control
rep
rep1
rep2
rep3

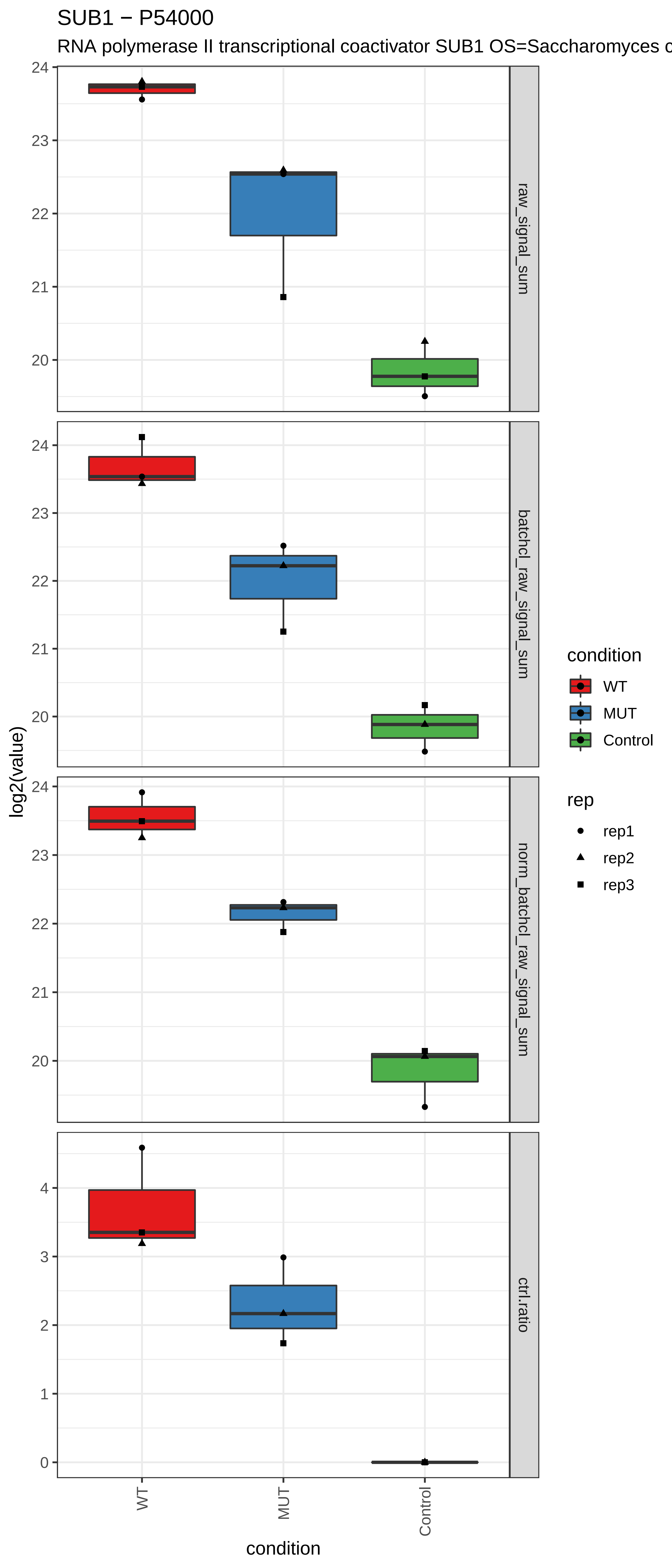

SUB1 − P54000
RNA polymerase II transcriptional coactivator SUB1 OS=Saccharomyces c
raw_signal_sum
batchcl_raw_signal_sum
norm_batchcl_raw_signal_sum
ctrl.ratio
log2(value)
condition
WT
MUT
Control
rep
rep1
rep2
rep3

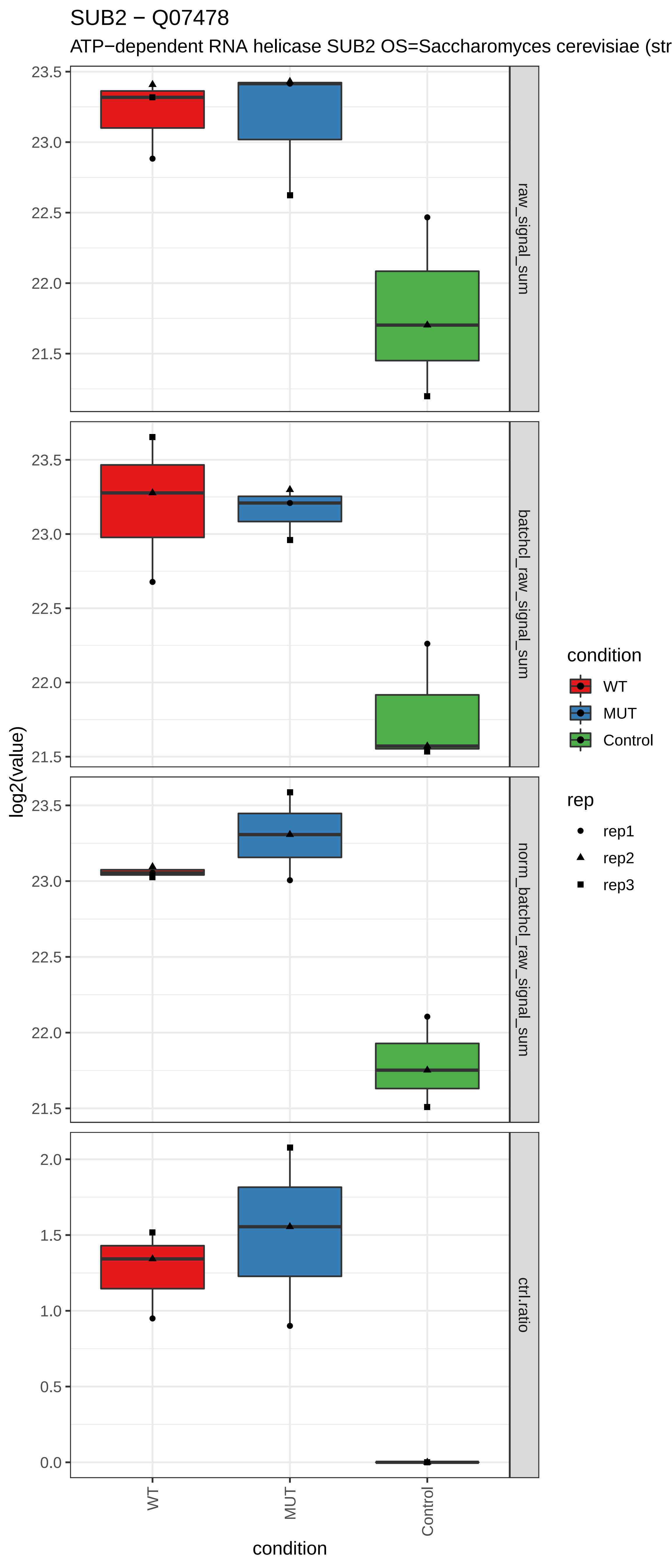

SUB2 − Q07478
ATP−dependent RNA helicase SUB2 OS=Saccharomyces cerevisiae (str
raw_signal_sum
batchcl_raw_signal_sum
norm_batchcl_raw_signal_sum
ctrl.ratio
log2(value)
condition
WT
MUT
Control
rep
rep1
rep2
rep3
23.5
23.0
22.5
22.0
21.5
2.0
1.5
1.0
0.5
0.0

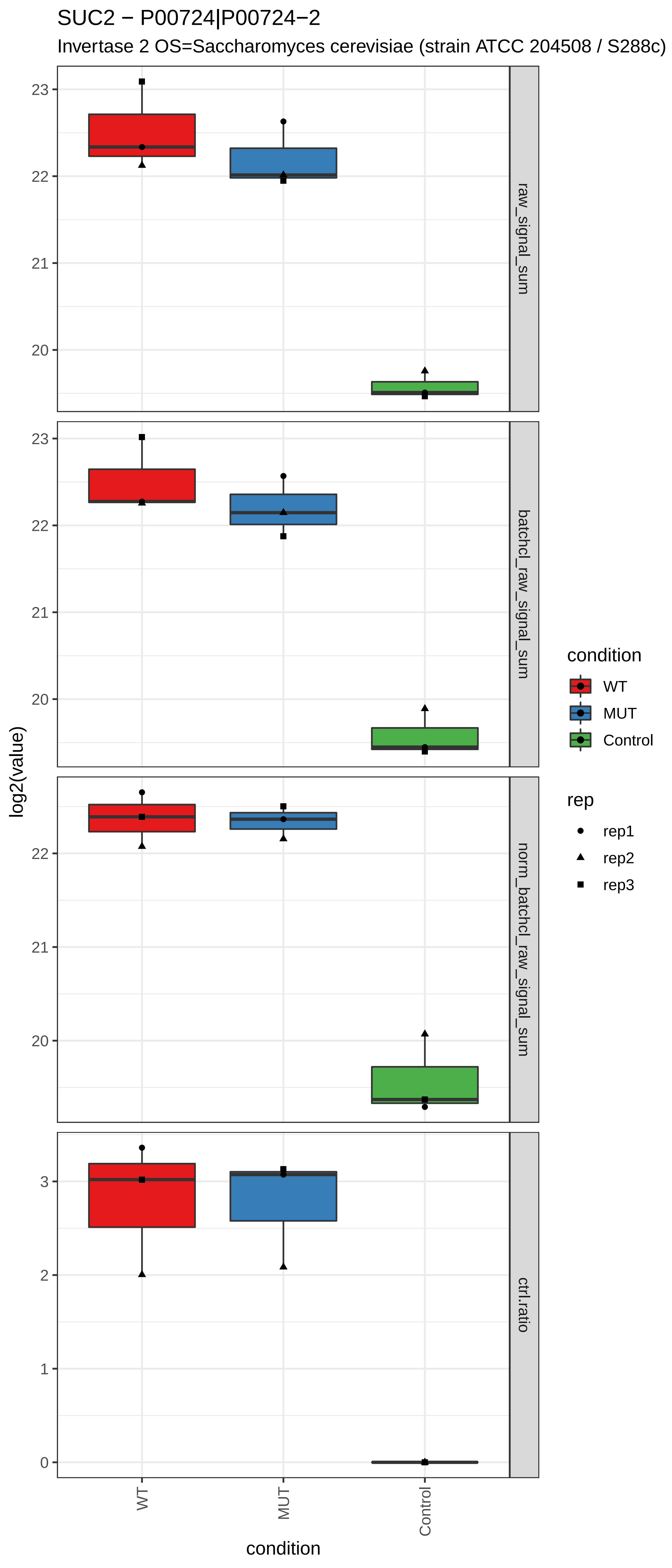

SUC2 − P00724|P00724−2
Invertase 2 OS=Saccharomyces cerevisiae (strain ATCC 204508 / S288c)
raw_signal_sum
batchcl_raw_signal_sum
norm_batchcl_raw_signal_sum
ctrl.ratio
log2(value)
condition
WT
MUT
Control
rep
rep1
rep2
rep3

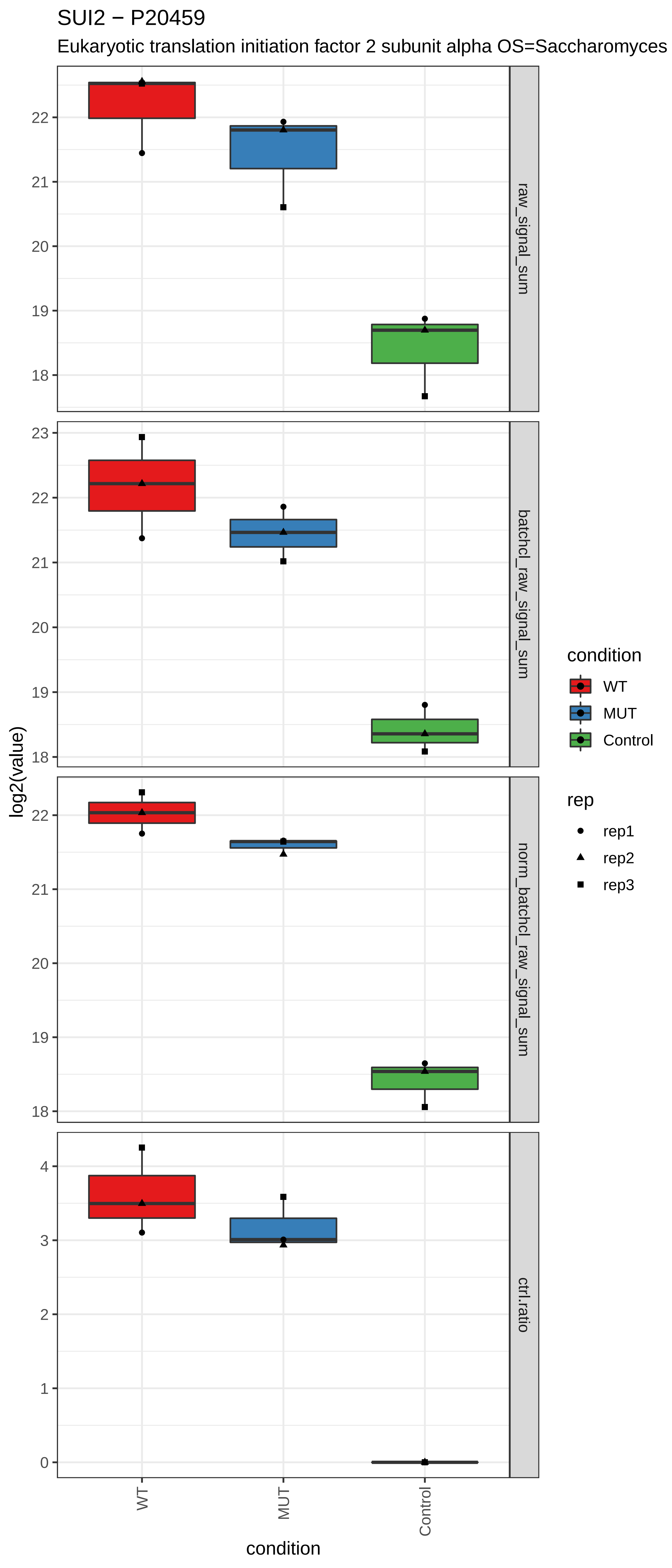

SUI2 − P20459
Eukaryotic translation initiation factor 2 subunit alpha OS=Saccharomyces
raw_signal_sum
batchcl_raw_signal_sum
norm_batchcl_raw_signal_sum
ctrl.ratio
log2(value)
condition
WT
MUT
Control
rep
rep1
rep2
rep3

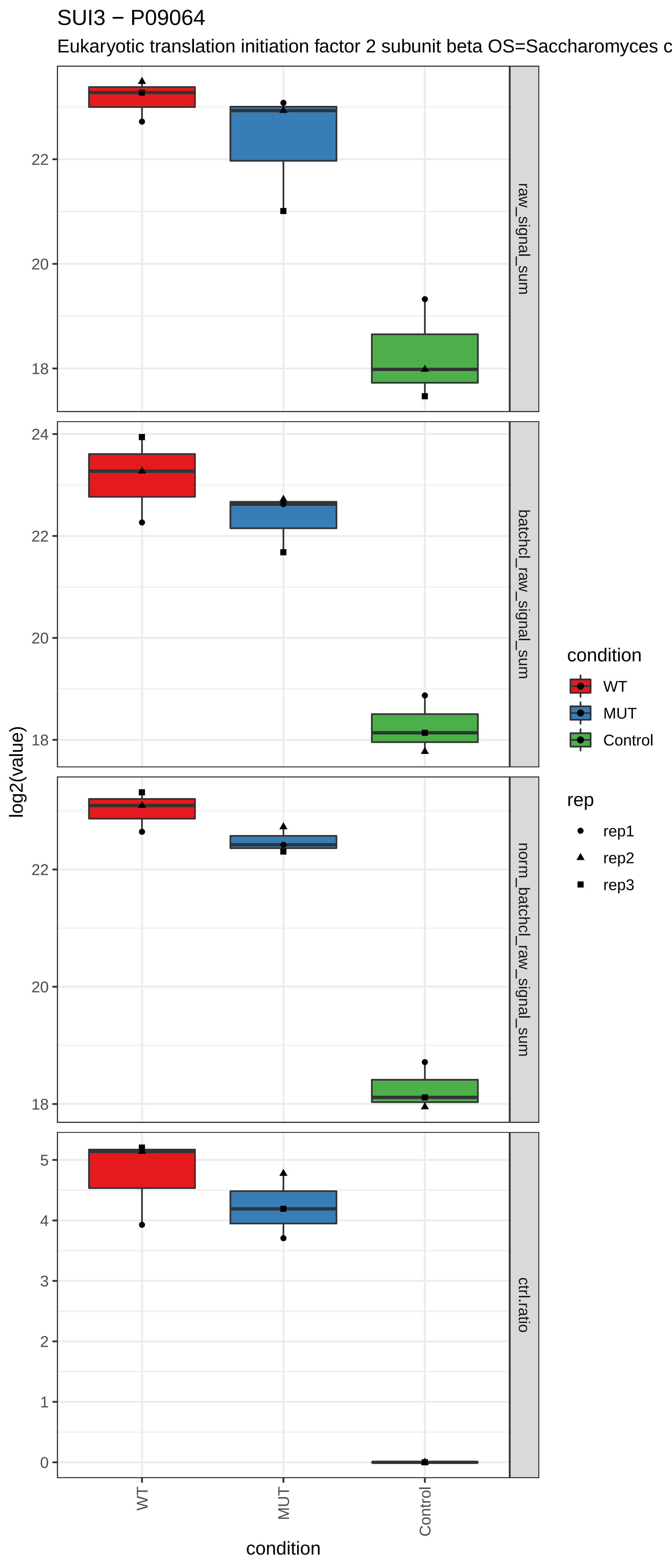
SUI3 − P09064
Eukaryotic translation initiation factor 2 subunit beta OS=Saccharomyces c
raw_signal_sum
batchcl_raw_signal_sum
norm_batchcl_raw_signal_sum
ctrl.ratio
log2(value)
condition
WT
MUT
Control
rep
rep1
rep2
rep3

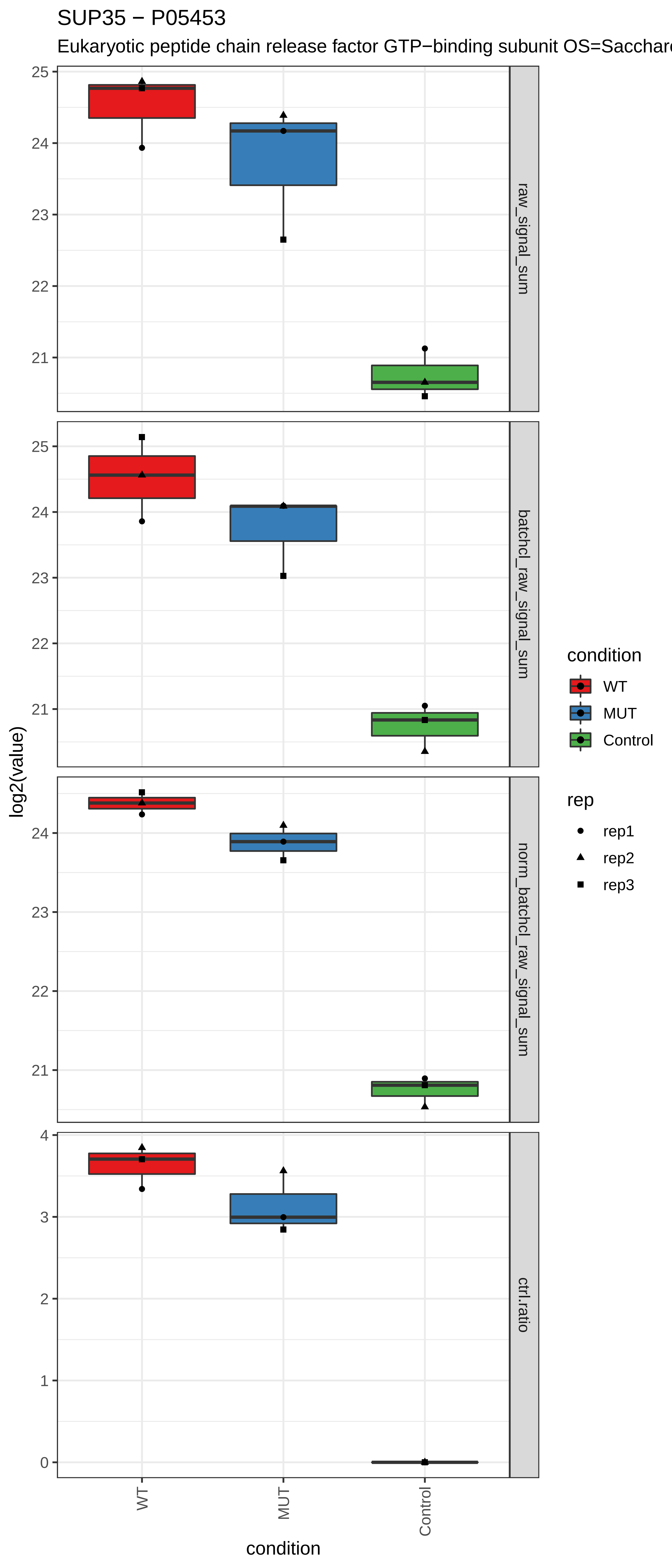
SUP35 − P05453
Eukaryotic peptide chain release factor GTP−binding subunit OS=Sacchar
raw_signal_sum
batchcl_raw_signal_sum
norm_batchcl_raw_signal_sum
ctrl.ratio
log2(value)
condition
WT
MUT
Control
rep
rep1
rep2
rep3

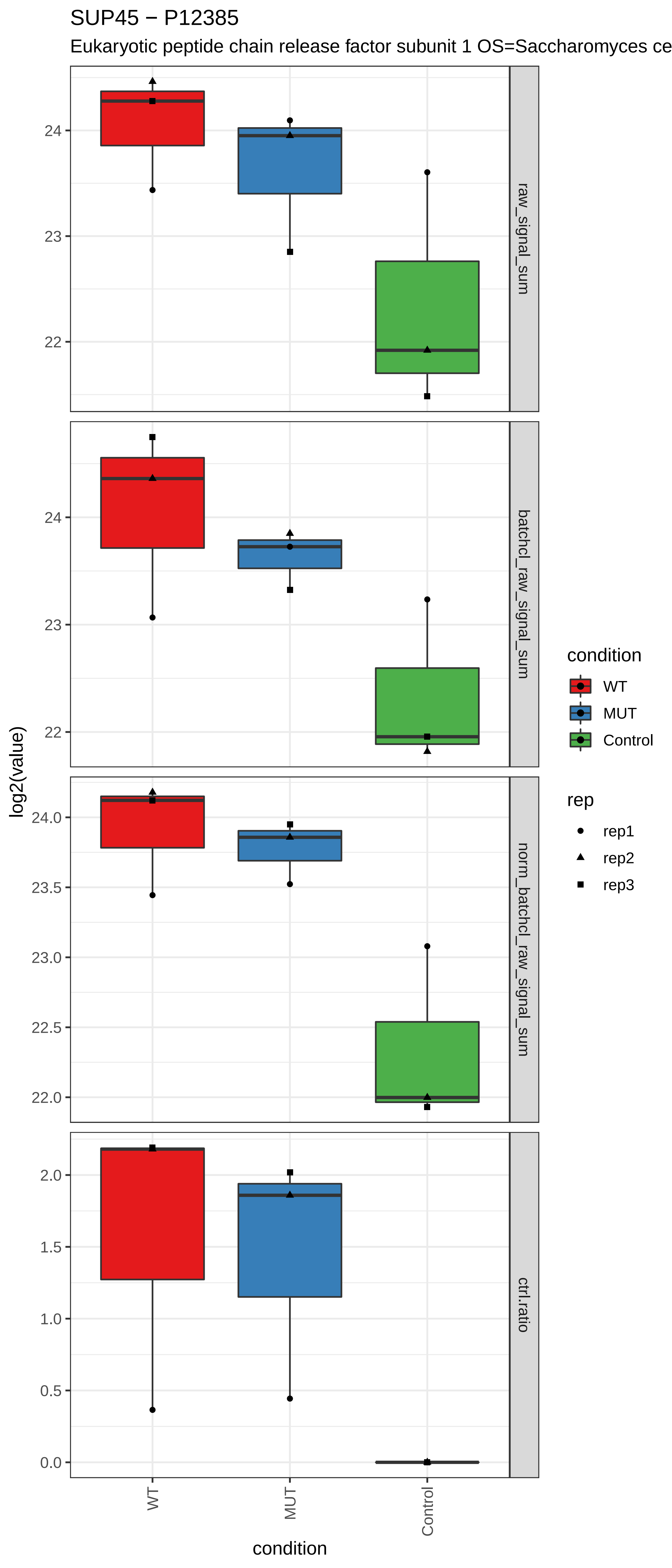
SUP45 − P12385
Eukaryotic peptide chain release factor subunit 1 OS=Saccharomyces ce
raw_signal_sum
batchcl_raw_signal_sum
norm_batchcl_raw_signal_sum
ctrl.ratio
log2(value)
condition
WT
MUT
Control
rep
rep1
rep2
rep3
24
23
22
24.0
23.5
23.0
22.5
22.0
2.0
1.5
1.0
0.5
0.0

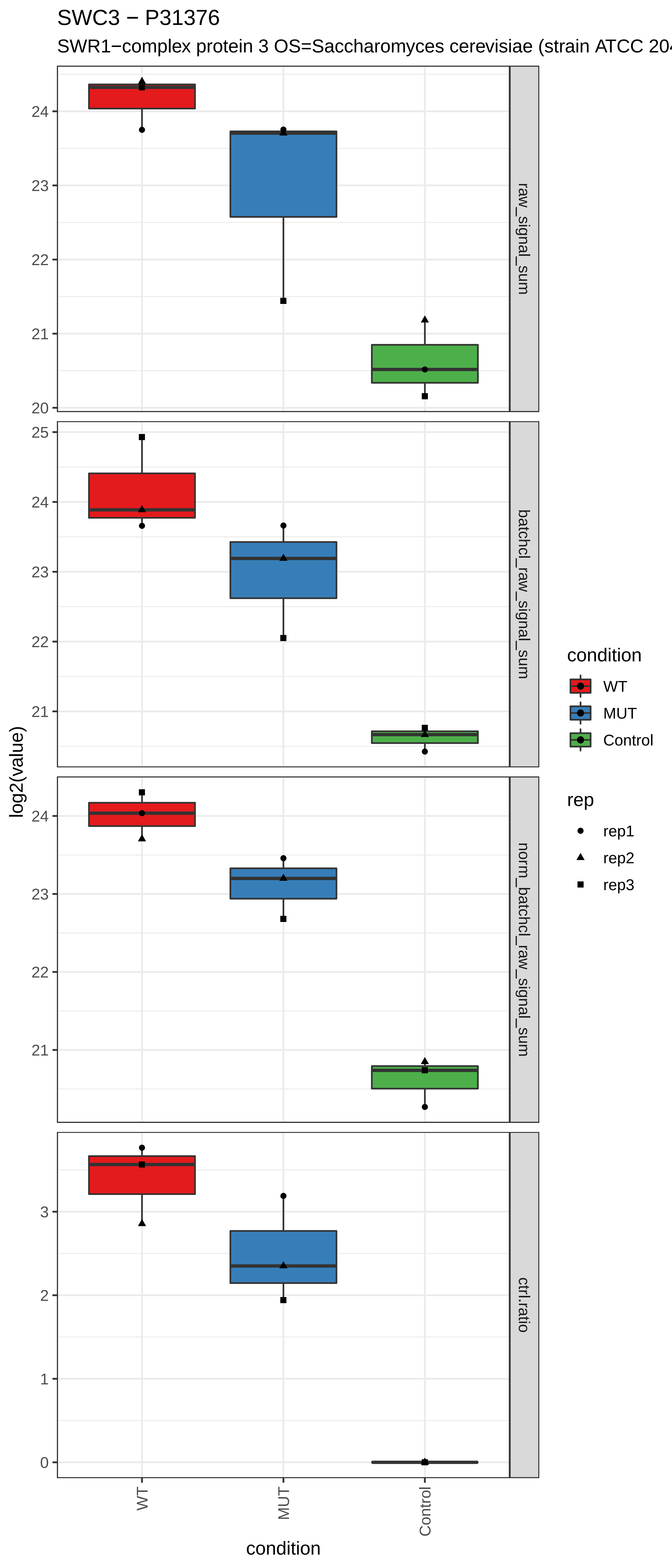

SWC3 − P31376
SWR1−complex protein 3 OS=Saccharomyces cerevisiae (strain ATCC 20
raw_signal_sum
batchcl_raw_signal_sum
norm_batchcl_raw_signal_sum
ctrl.ratio
log2(value)
condition
WT
MUT
Control
rep
rep1
rep2
rep3

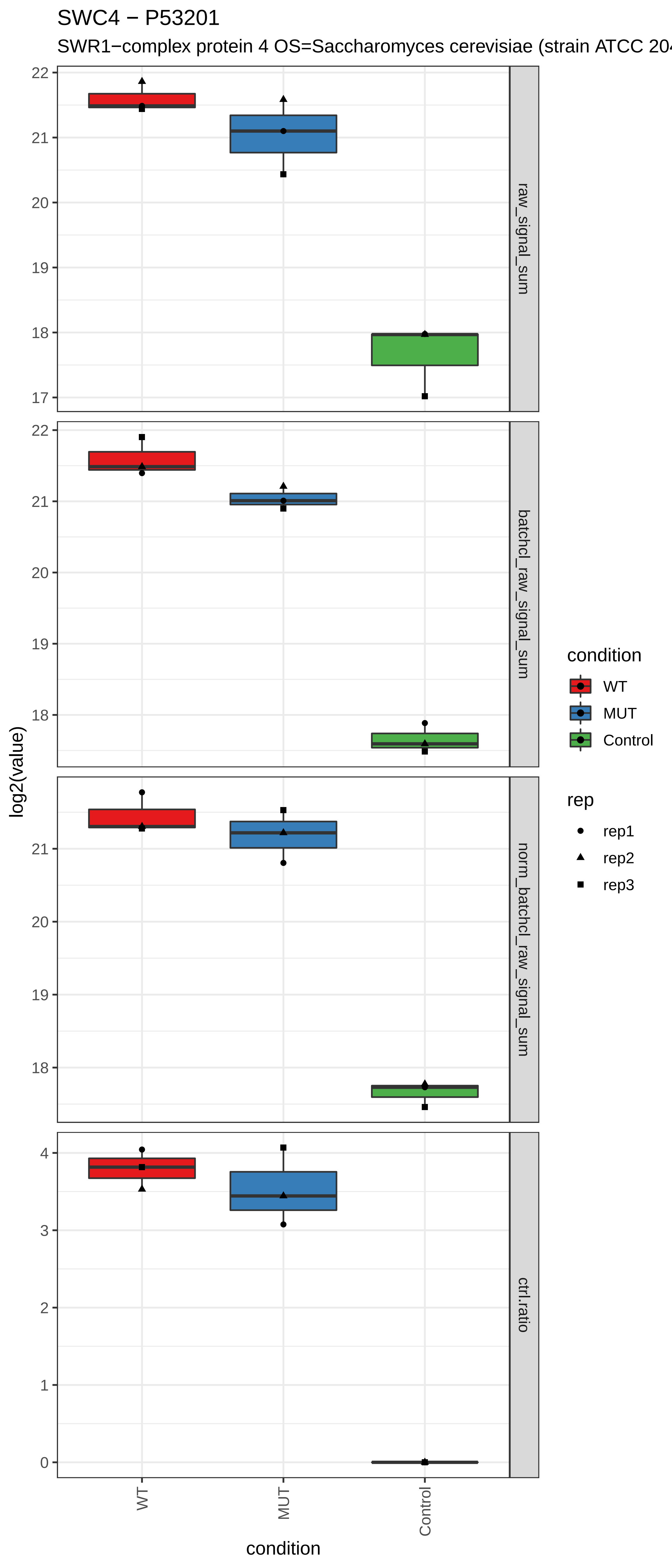

SWC4 − P53201
SWR1−complex protein 4 OS=Saccharomyces cerevisiae (strain ATCC 20
raw_signal_sum
batchcl_raw_signal_sum
norm_batchcl_raw_signal_sum
ctrl.ratio
log2(value)
condition
WT
MUT
Control
rep
rep1
rep2
rep3

SWR1 − Q05471

Helicase SWR1 OS=Saccharomyces cerevisiae (strain ATCC 204508 / S28

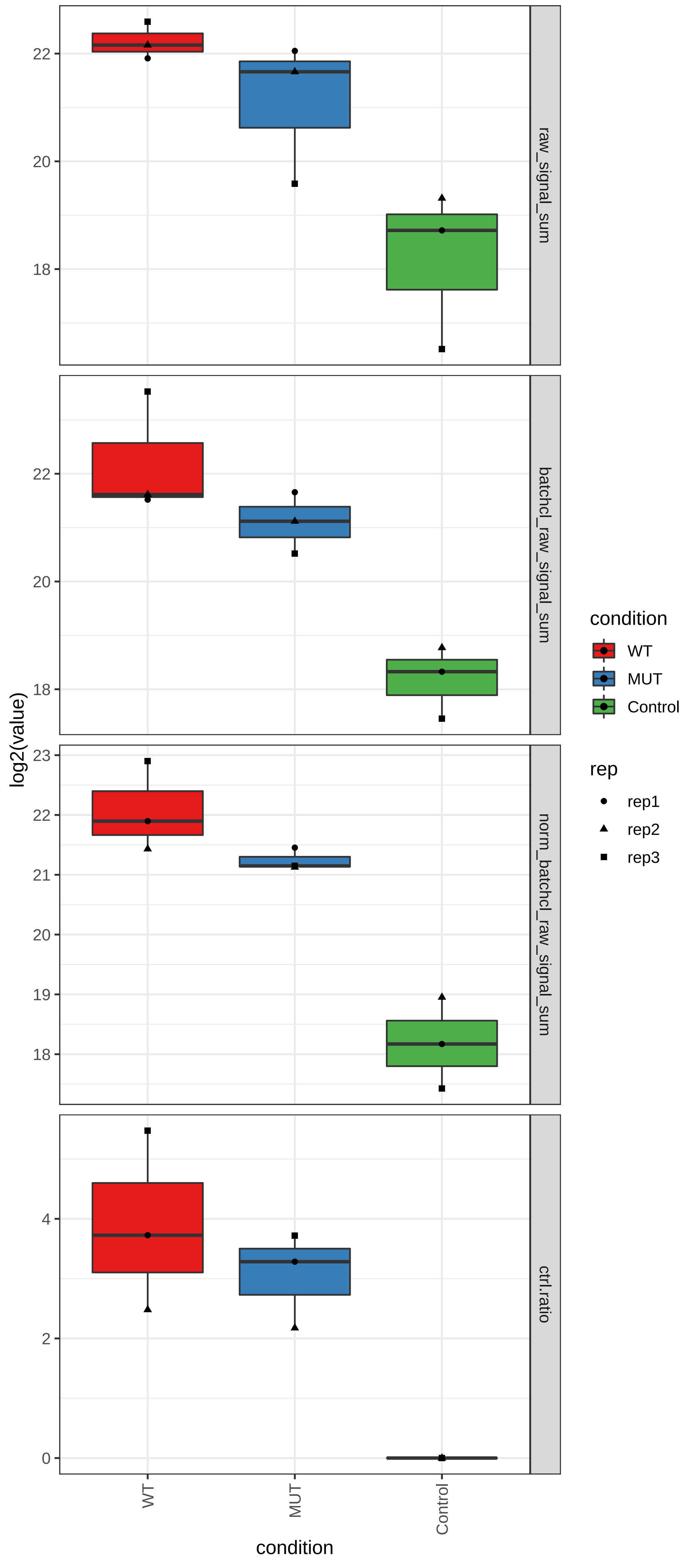

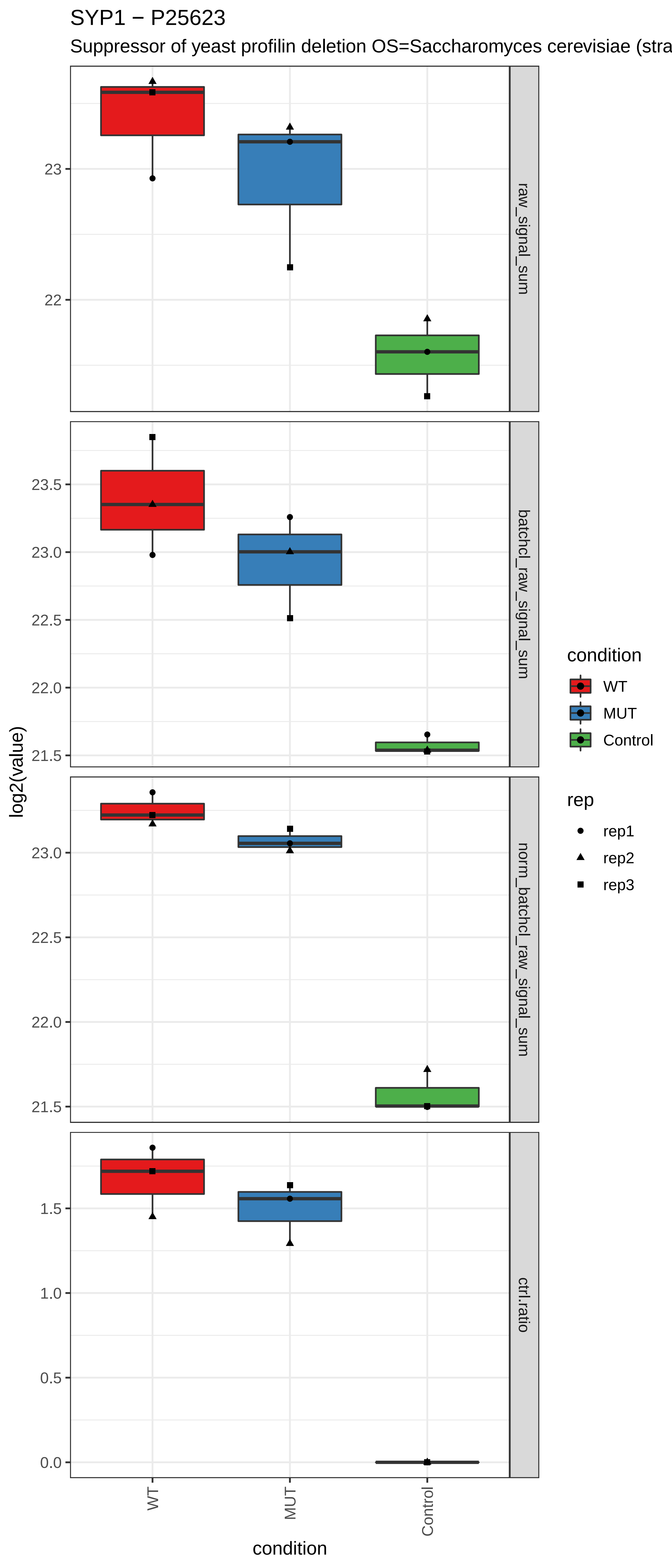
SYP1 − P25623
Suppressor of yeast profilin deletion OS=Saccharomyces cerevisiae (stra
raw_signal_sum
batchcl_raw_signal_sum
norm_batchcl_raw_signal_sum
ctrl.ratio
log2(value)
condition
WT
MUT
Control
rep
rep1
rep2
rep3

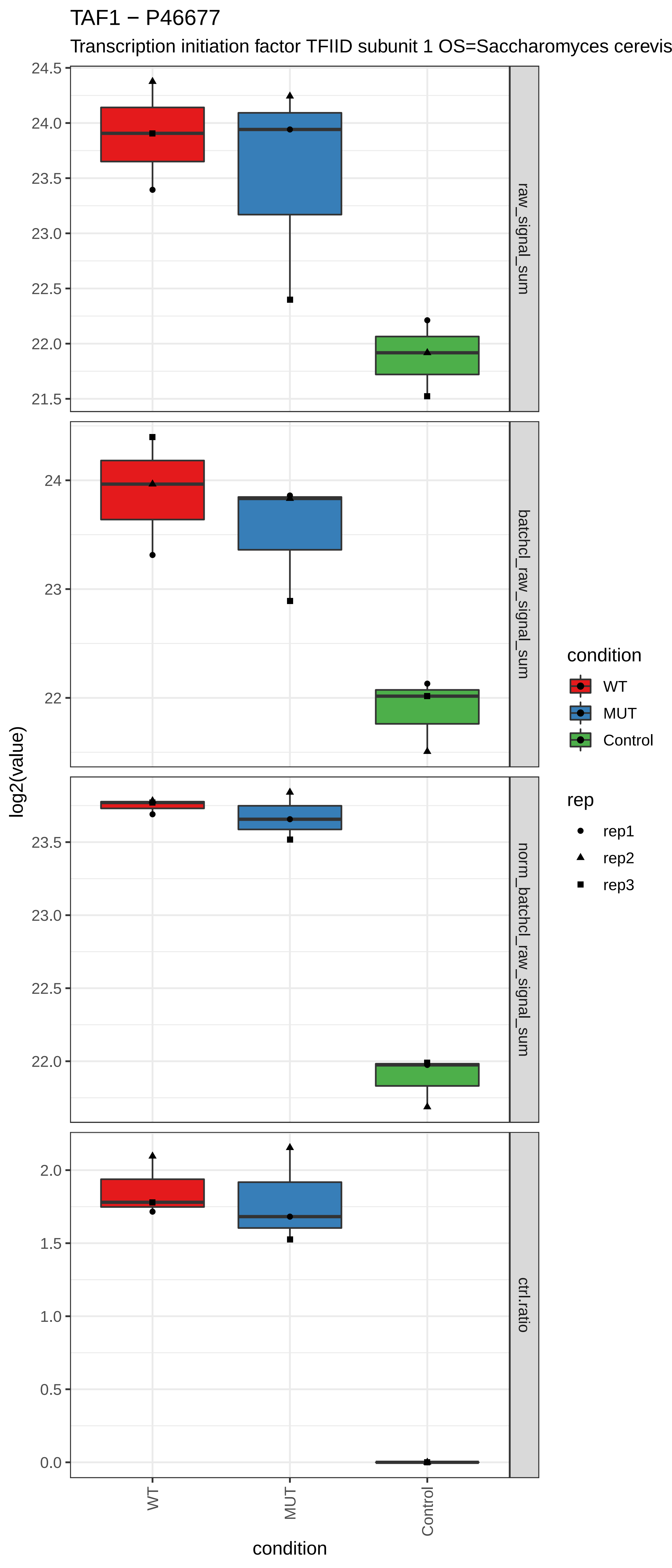

TAF1 − P46677
Transcription initiation factor TFIID subunit 1 OS=Saccharomyces cerevis
log2(value)
raw_signal_sum
batchcl_raw_signal_sum
norm_batchcl_raw_signal_sum
ctrl.ratio
condition
WT
MUT
Control
rep
rep1
rep2
rep3

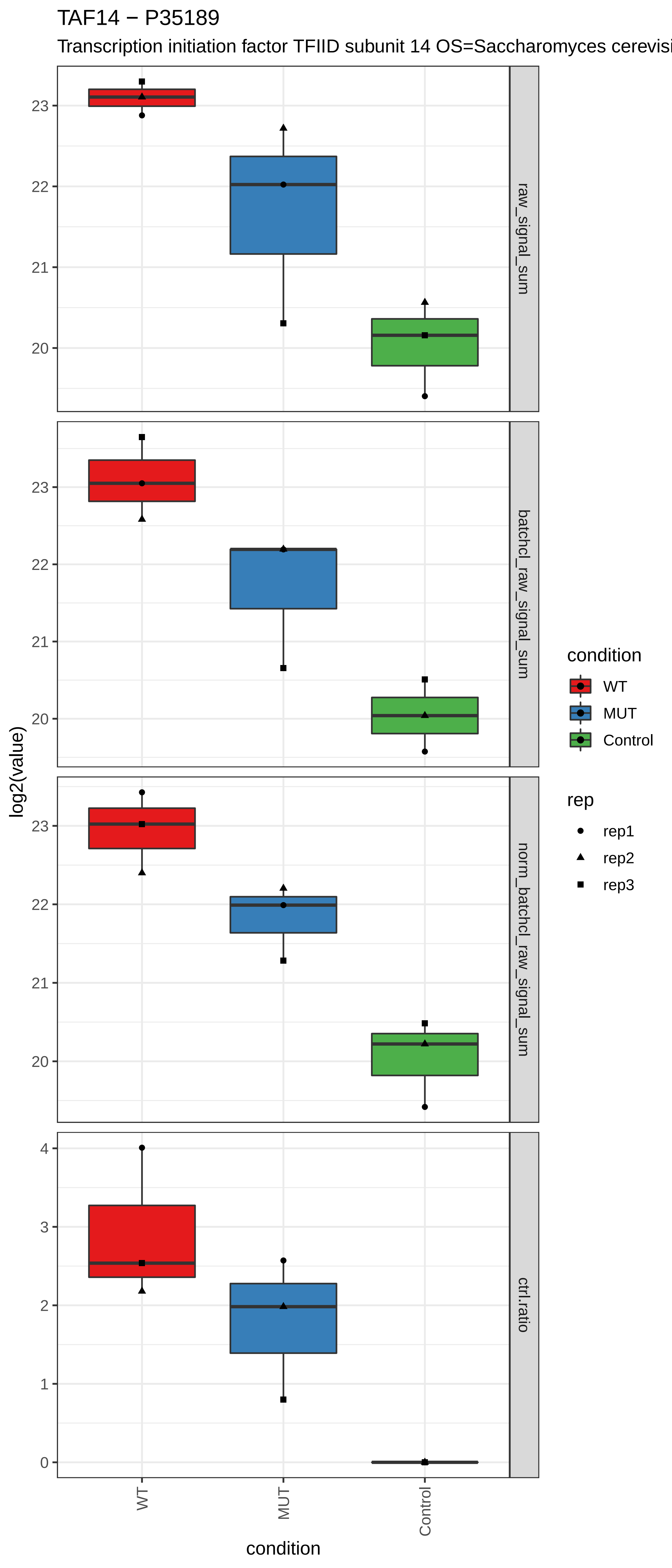

TAF14 − P35189
Transcription initiation factor TFIID subunit 14 OS=Saccharomyces cerevisi
raw_signal_sum
batchcl_raw_signal_sum
norm_batchcl_raw_signal_sum
ctrl.ratio
log2(value)
condition
WT
MUT
Control
rep
rep1
rep2
rep3
23
22
21
20
4
3
2
1
0

TAF5 − P38129

Transcription initiation factor TFIID subunit 5 OS=Saccharomyces cerevisia

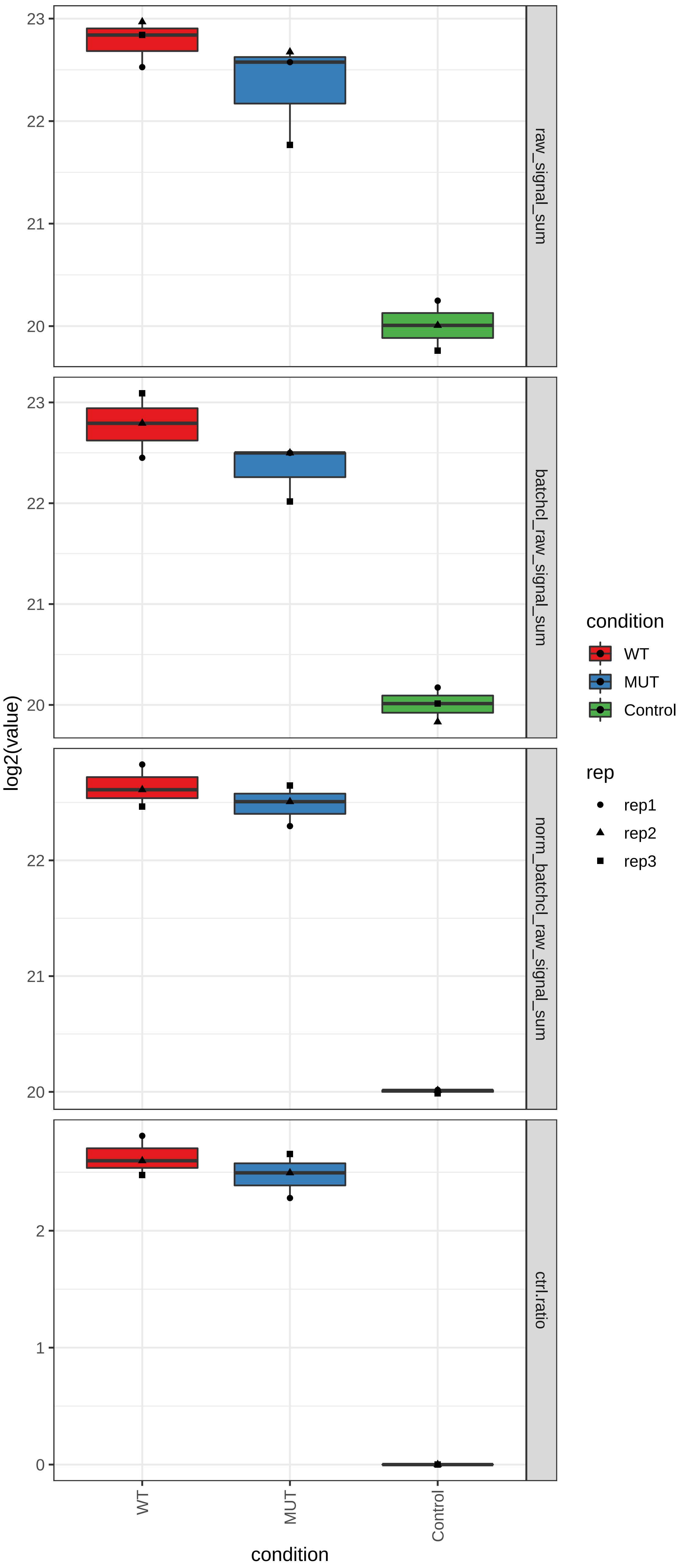

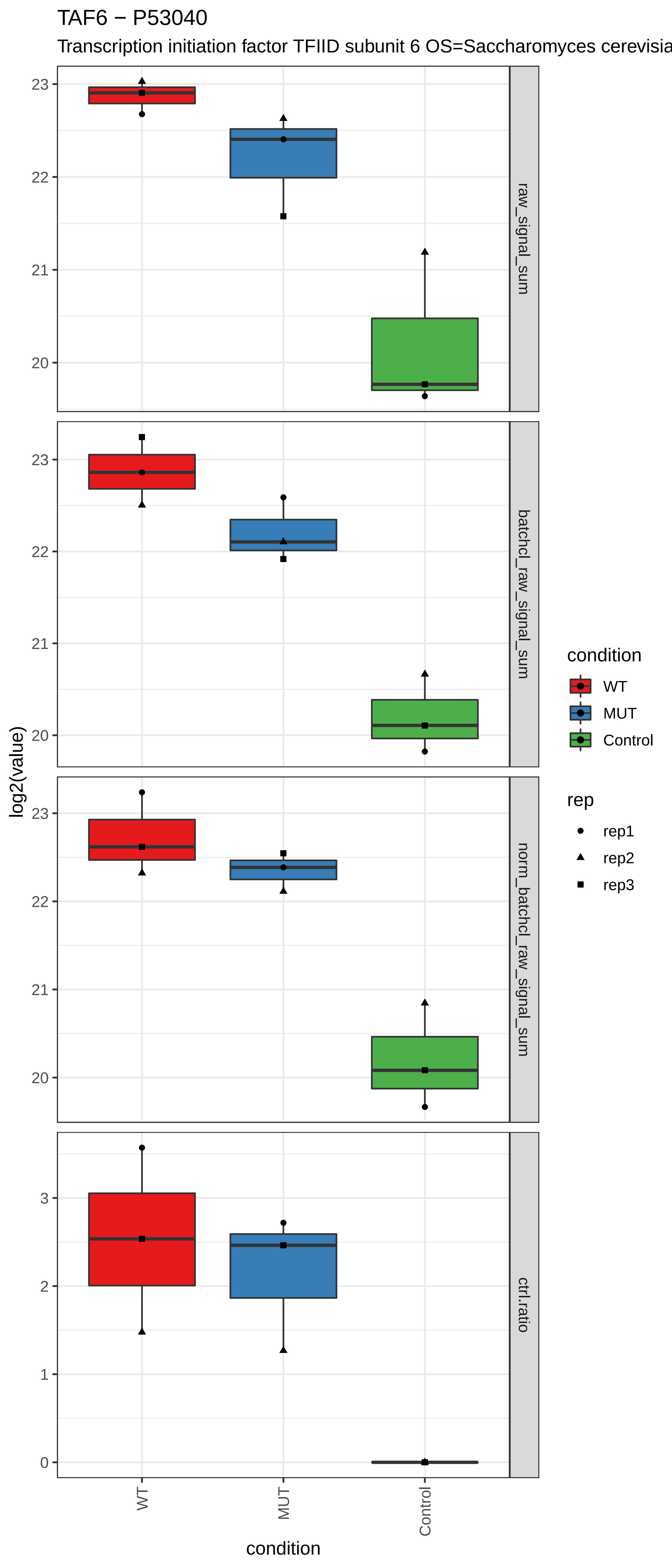

TAF6 − P53040
Transcription initiation factor TFIID subunit 6 OS=Saccharomyces cerevisia
raw_signal_sum
batchcl_raw_signal_sum
norm_batchcl_raw_signal_sum
ctrl.ratio
log2(value)
condition
WT
MUT
Control
rep
rep1
rep2
rep3

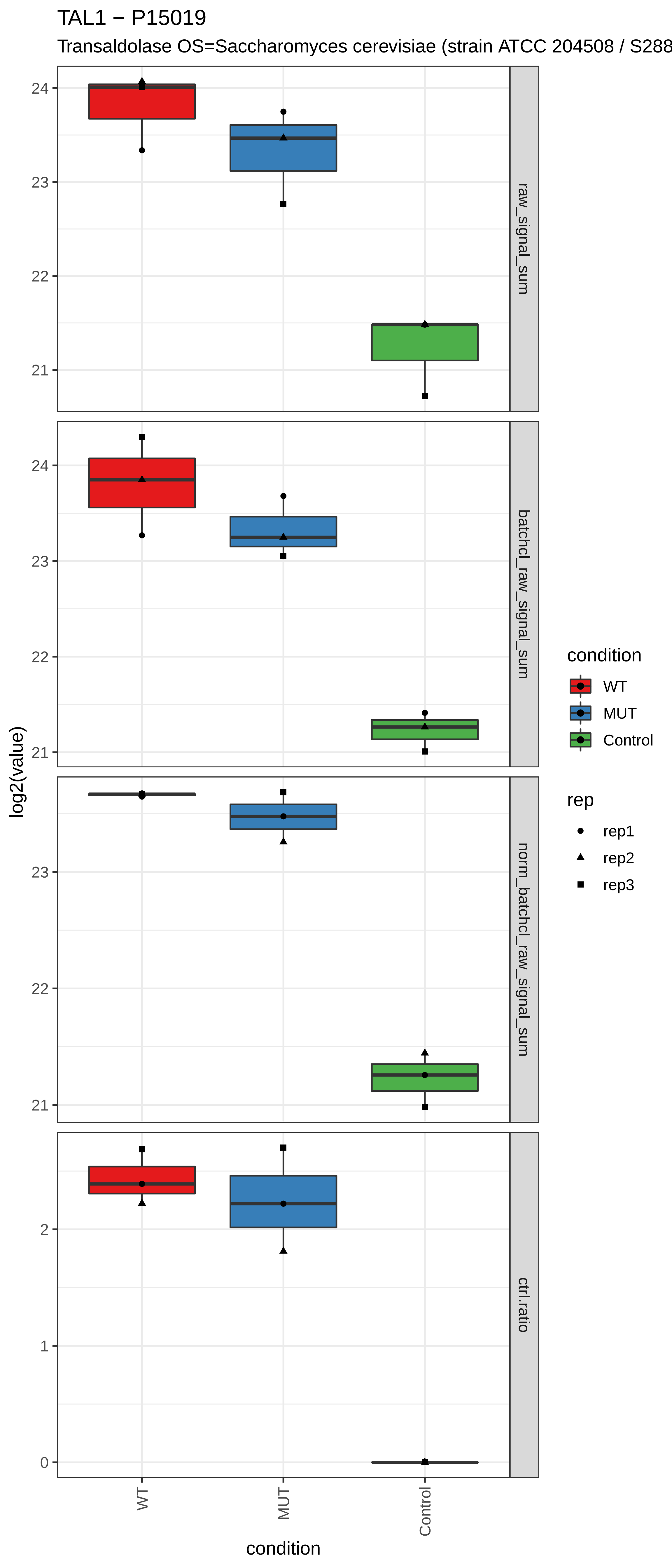

TAL1 − P15019
Transaldolase OS=Saccharomyces cerevisiae (strain ATCC 204508 / S288
raw_signal_sum
batchcl_raw_signal_sum
norm_batchcl_raw_signal_sum
ctrl.ratio
log2(value)
condition
WT
MUT
Control
rep
rep1
rep2
rep3
24
23
22
21
2
1
0

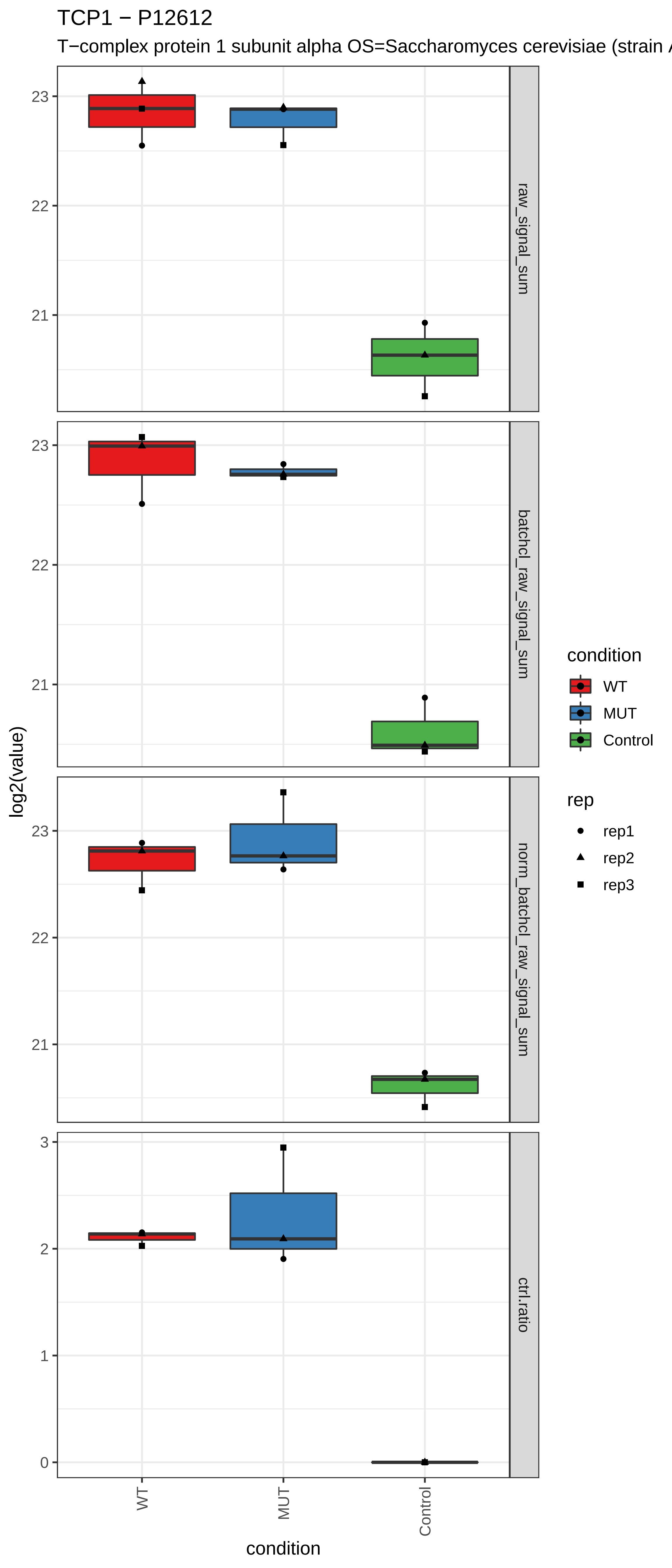
TCP1 − P12612
T−complex protein 1 subunit alpha OS=Saccharomyces cerevisiae (strain
raw_signal_sum
batchcl_raw_signal_sum
norm_batchcl_raw_signal_sum
ctrl.ratio
log2(value)
condition
WT
MUT
Control
rep
rep1
rep2
rep3

TDH1 − P00360

Glyceraldehyde−3−phosphate dehydrogenase 1 OS=Saccharomyces cere

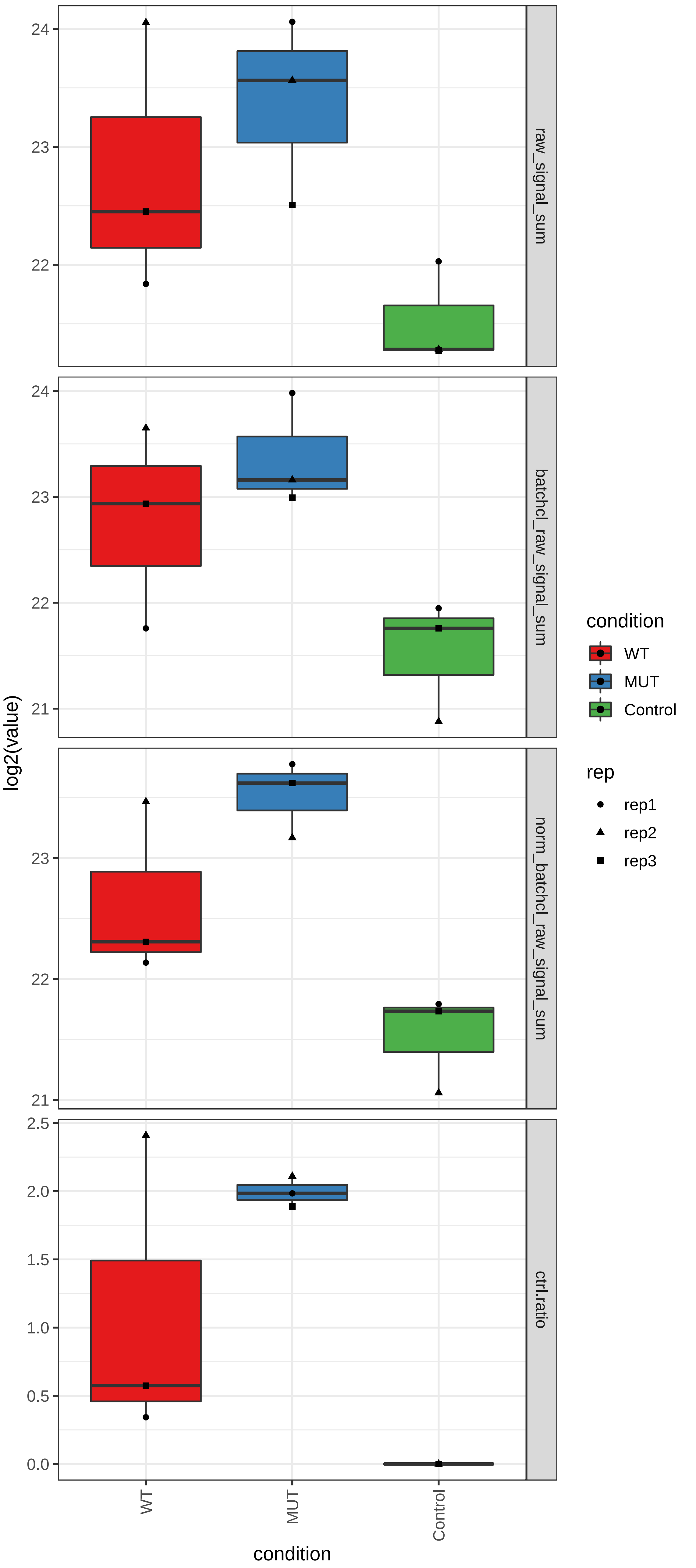

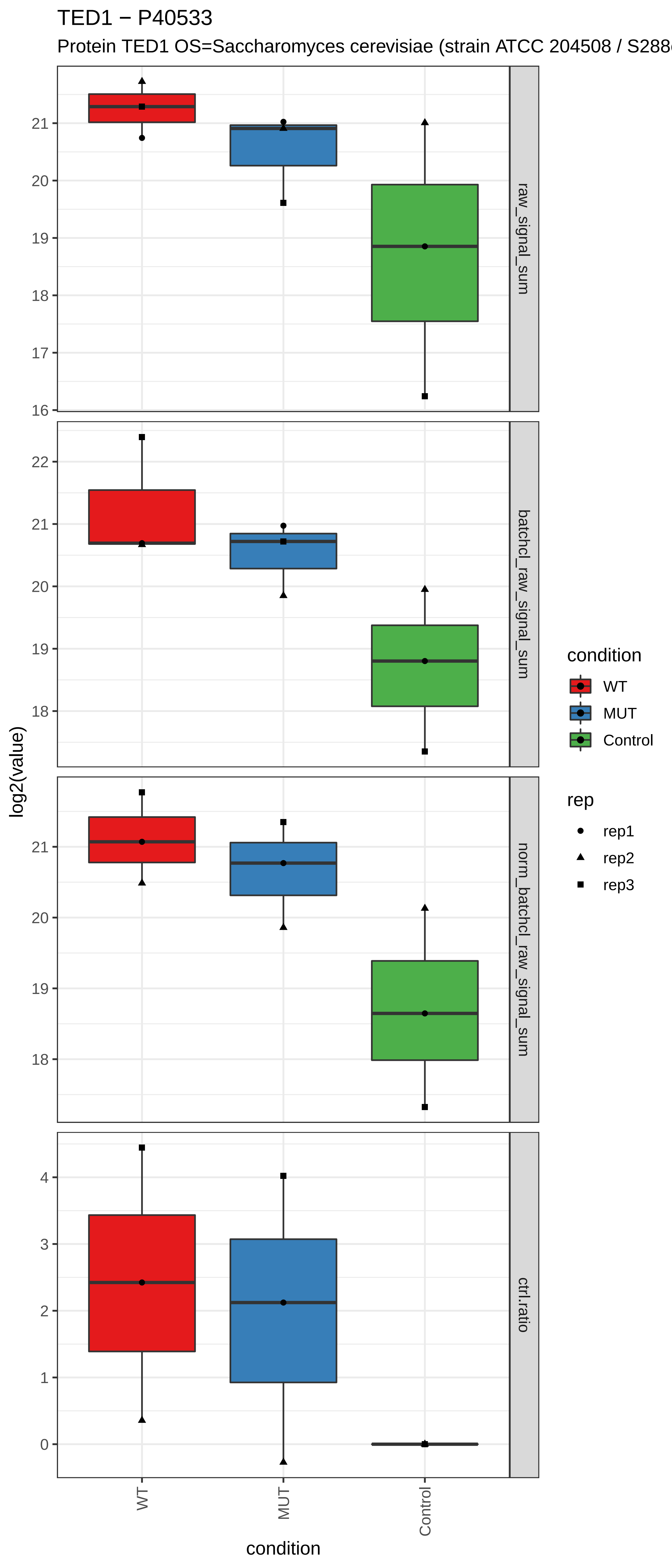
TED1 − P40533
Protein TED1 OS=Saccharomyces cerevisiae (strain ATCC 204508 / S288
raw_signal_sum
batchcl_raw_signal_sum
norm_batchcl_raw_signal_sum
ctrl.ratio
log2(value)
condition
WT
MUT
Control
rep
rep1
rep2
rep3

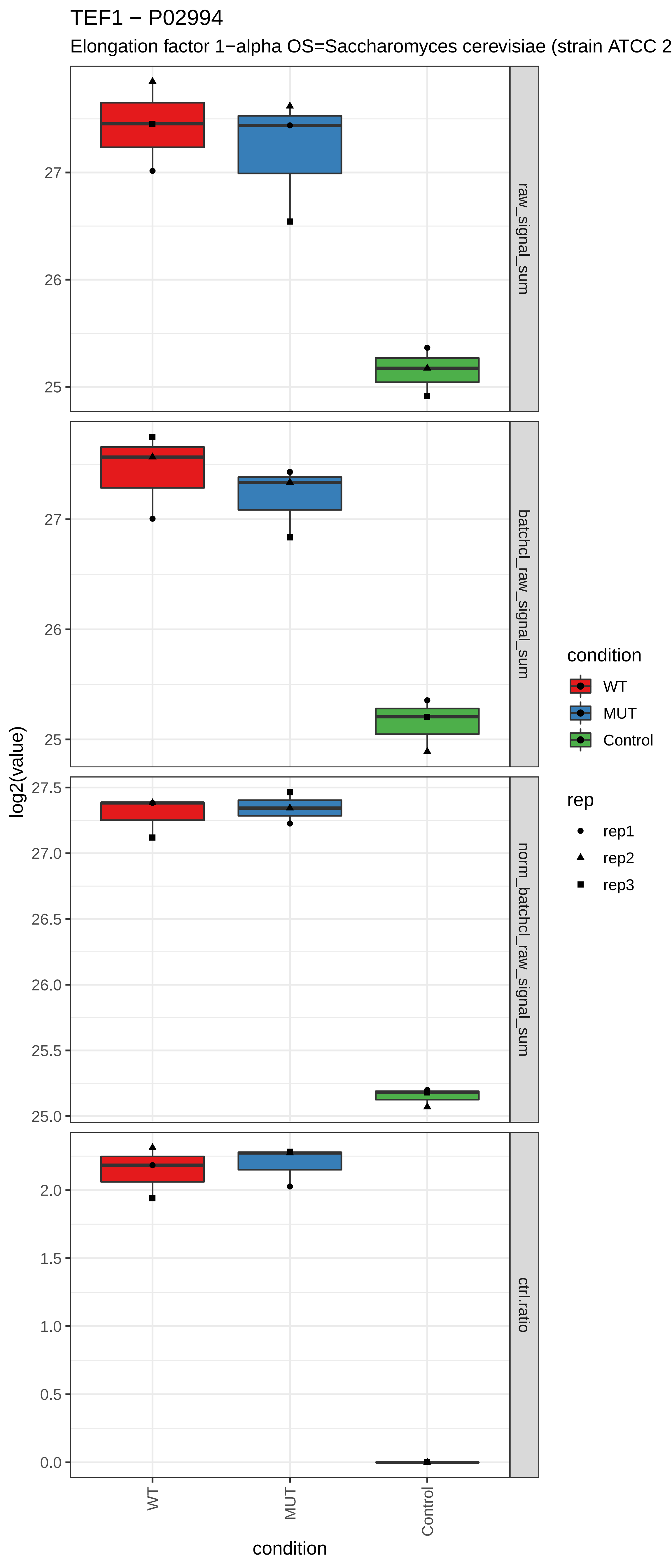

TEF1 − P02994
Elongation factor 1−alpha OS=Saccharomyces cerevisiae (strain ATCC 2
raw_signal_sum
batchcl_raw_signal_sum
norm_batchcl_raw_signal_sum
ctrl.ratio
log2(value)
27
26
25
27.5
27.0
26.5
26.0
25.5
25.0
2.0
1.5
1.0
0.5
0.0
WT
MUT
Control
condition
condition
WT
MUT
Control
rep
rep1
rep2
rep3

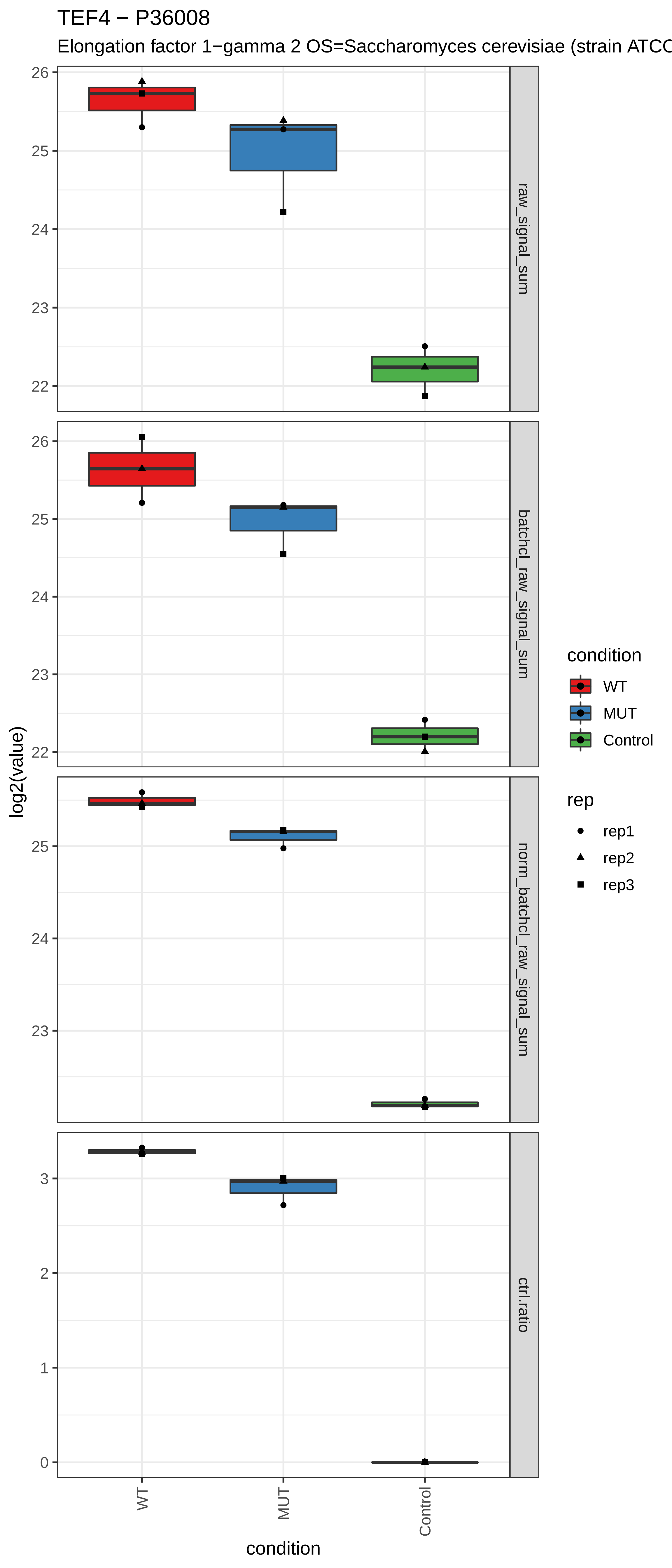

TEF4 − P36008
Elongation factor 1−gamma 2 OS=Saccharomyces cerevisiae (strain ATCC
raw_signal_sum
batchcl_raw_signal_sum
norm_batchcl_raw_signal_sum
ctrl.ratio
log2(value)
condition
WT
MUT
Control
rep
rep1
rep2
rep3
26
25
24
23
22
3
2
1
0

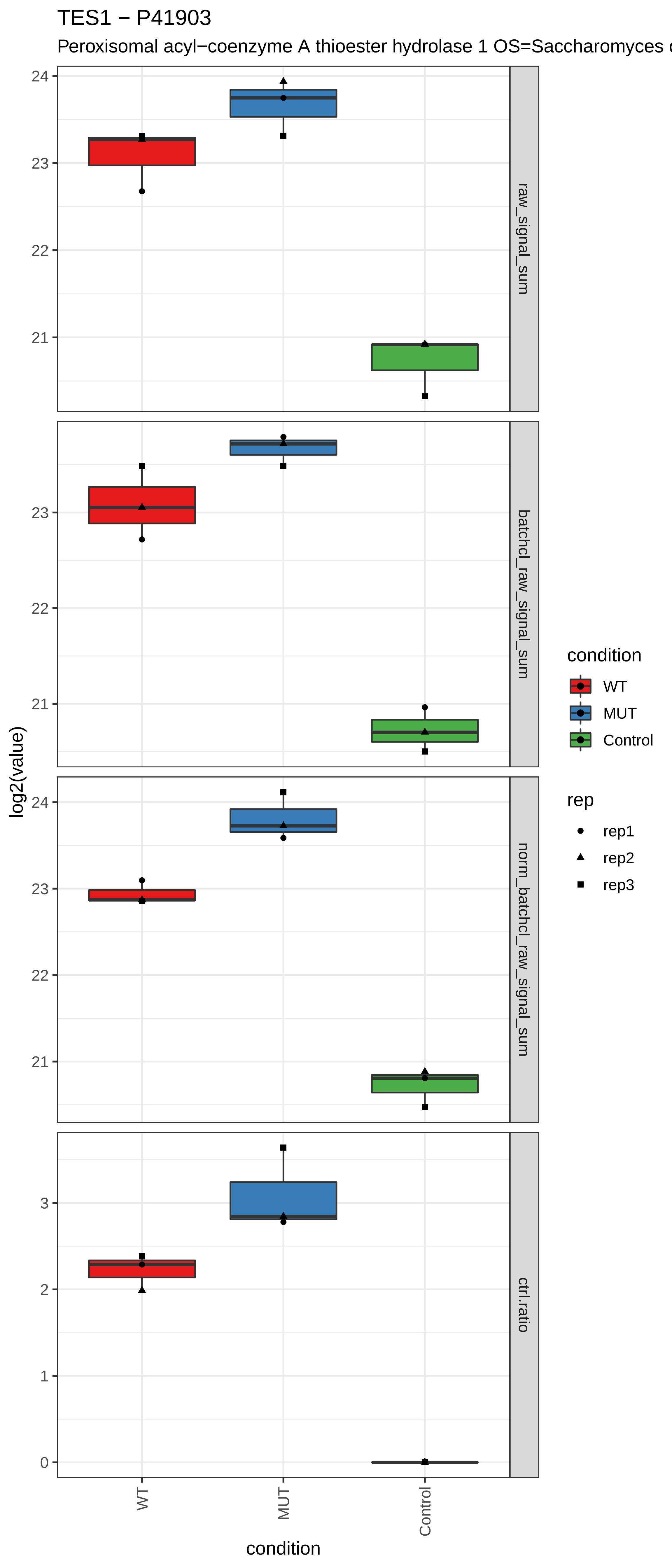
TES1 − P41903
Peroxisomal acyl−coenzyme A thioester hydrolase 1 OS=Saccharomyces c
raw_signal_sum
batchcl_raw_signal_sum
norm_batchcl_raw_signal_sum
ctrl.ratio
log2(value)
condition
WT
MUT
Control
rep
rep1
rep2
rep3
24
23
22
21
3
2
1
0

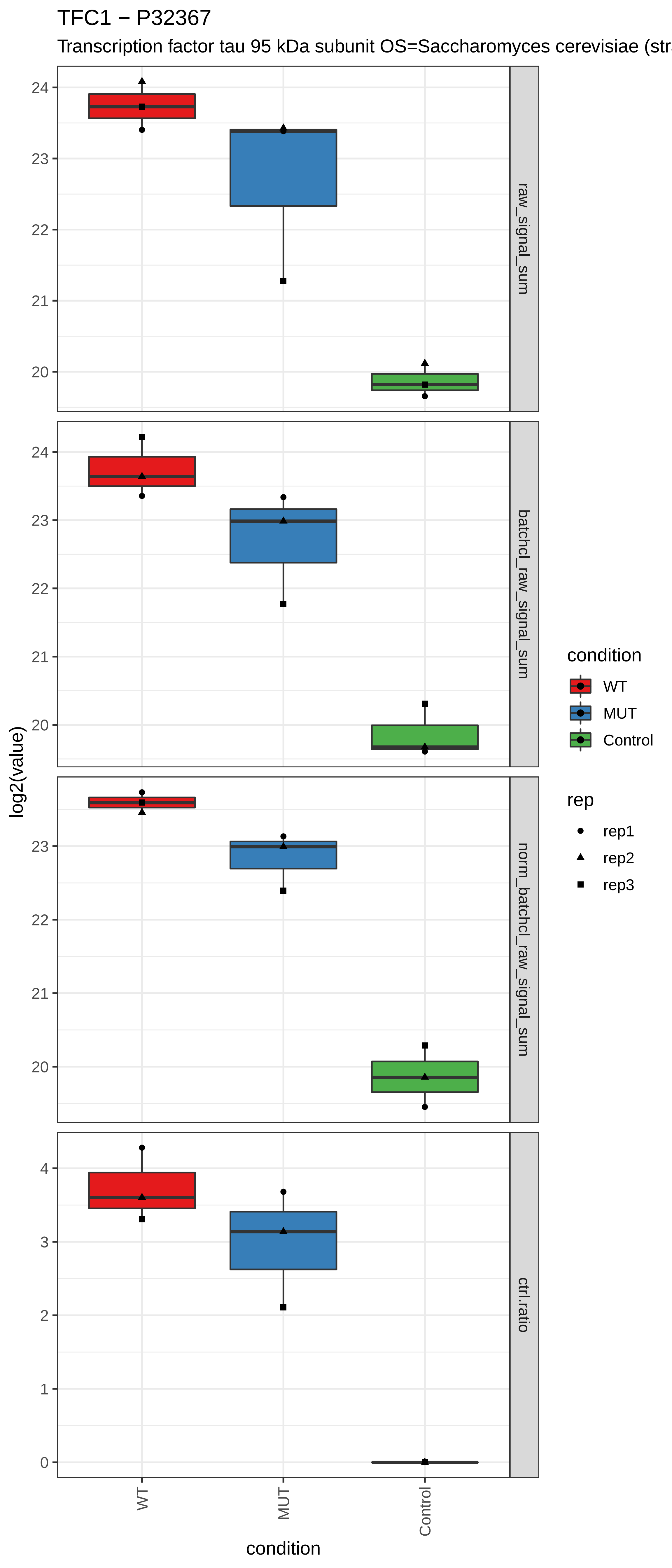
TFC1 − P32367
Transcription factor tau 95 kDa subunit OS=Saccharomyces cerevisiae (str
raw_signal_sum
batchcl_raw_signal_sum
norm_batchcl_raw_signal_sum
ctrl.ratio
log2(value)
condition
WT
MUT
Control
rep
rep1
rep2
rep3

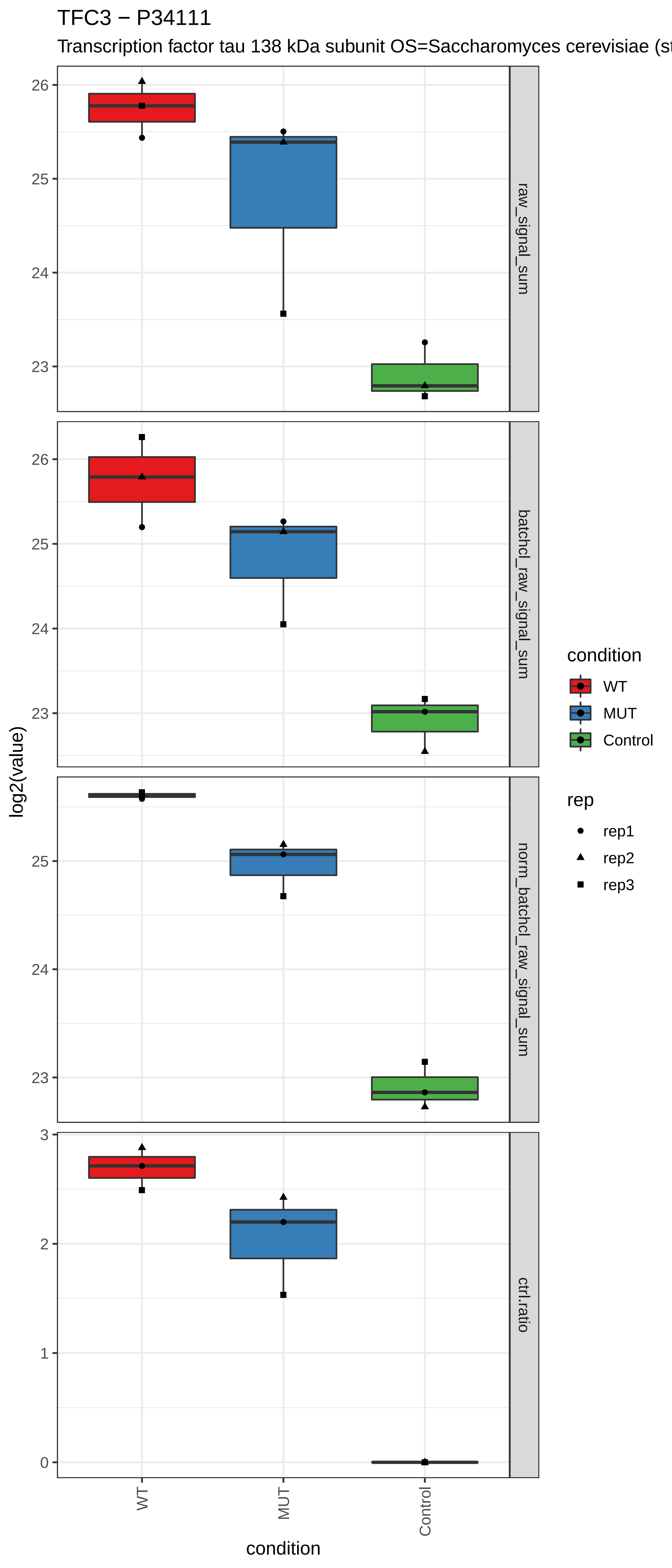
TFC3 − P34111
Transcription factor tau 138 kDa subunit OS=Saccharomyces cerevisiae (st
raw_signal_sum
batchcl_raw_signal_sum
norm_batchcl_raw_signal_sum
ctrl.ratio
log2(value)
condition
WT
MUT
Control
rep
rep1
rep2
rep3
26
25
24
23
3
2
1
0

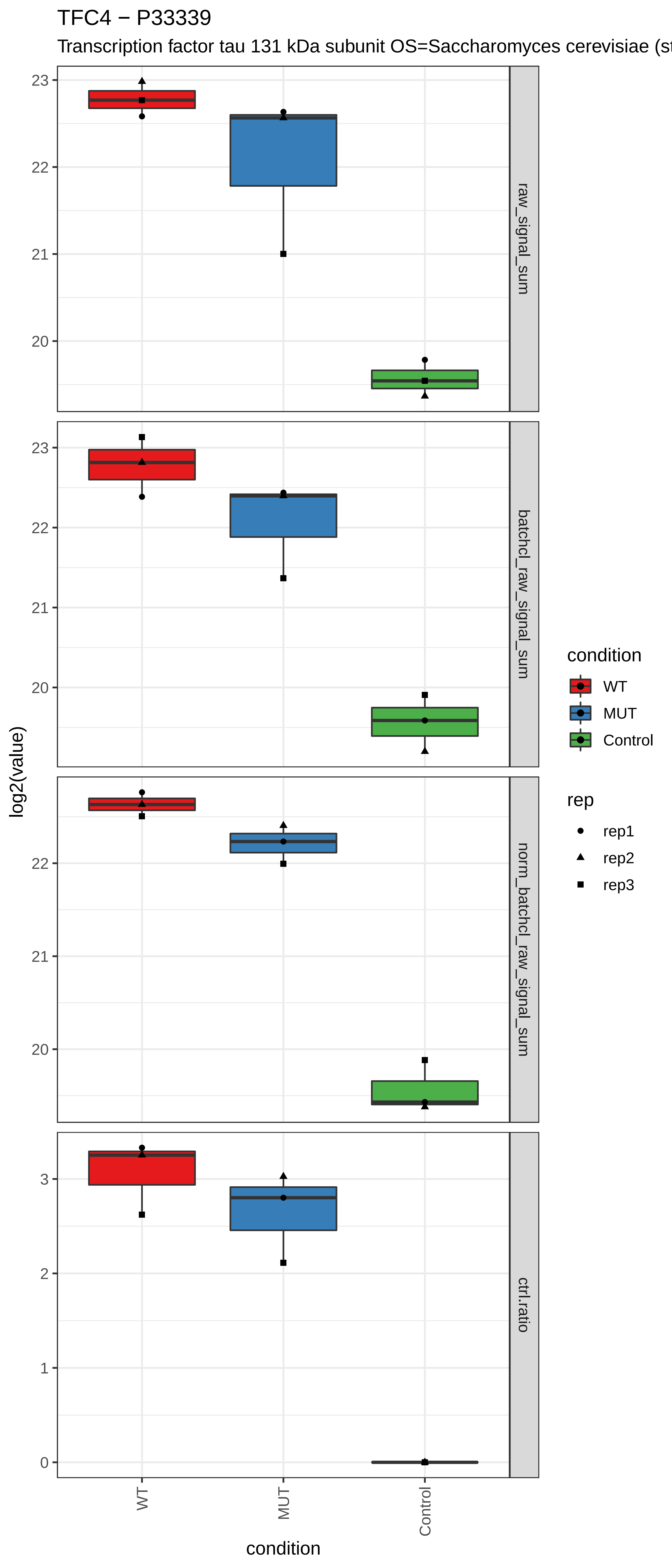

TFC4 − P33339
Transcription factor tau 131 kDa subunit OS=Saccharomyces cerevisiae (st
raw_signal_sum
batchcl_raw_signal_sum
norm_batchcl_raw_signal_sum
ctrl.ratio
log2(value)
condition
WT
MUT
Control
rep
rep1
rep2
rep3

TFC6 − Q06339

Transcription factor tau 91 kDa subunit OS=Saccharomyces cerevisiae (str

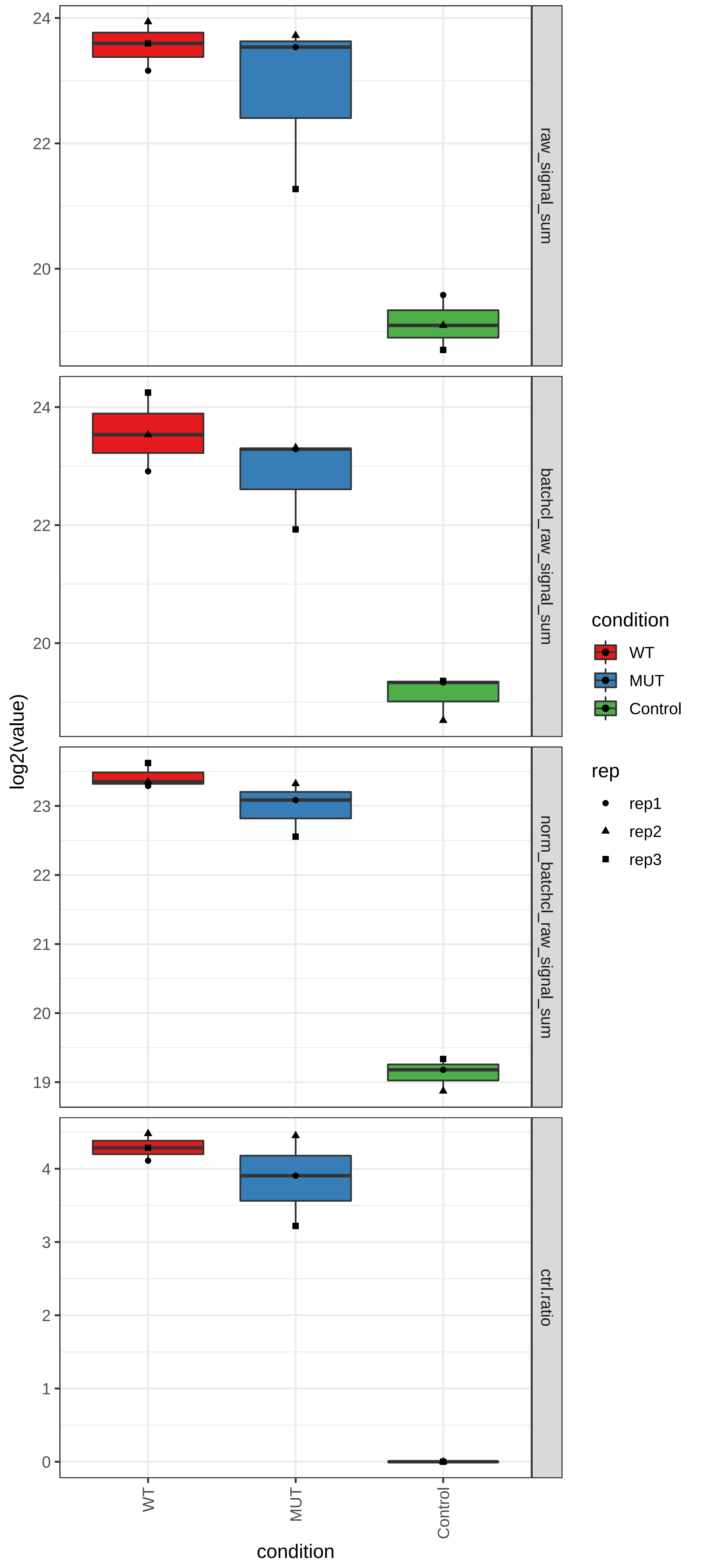

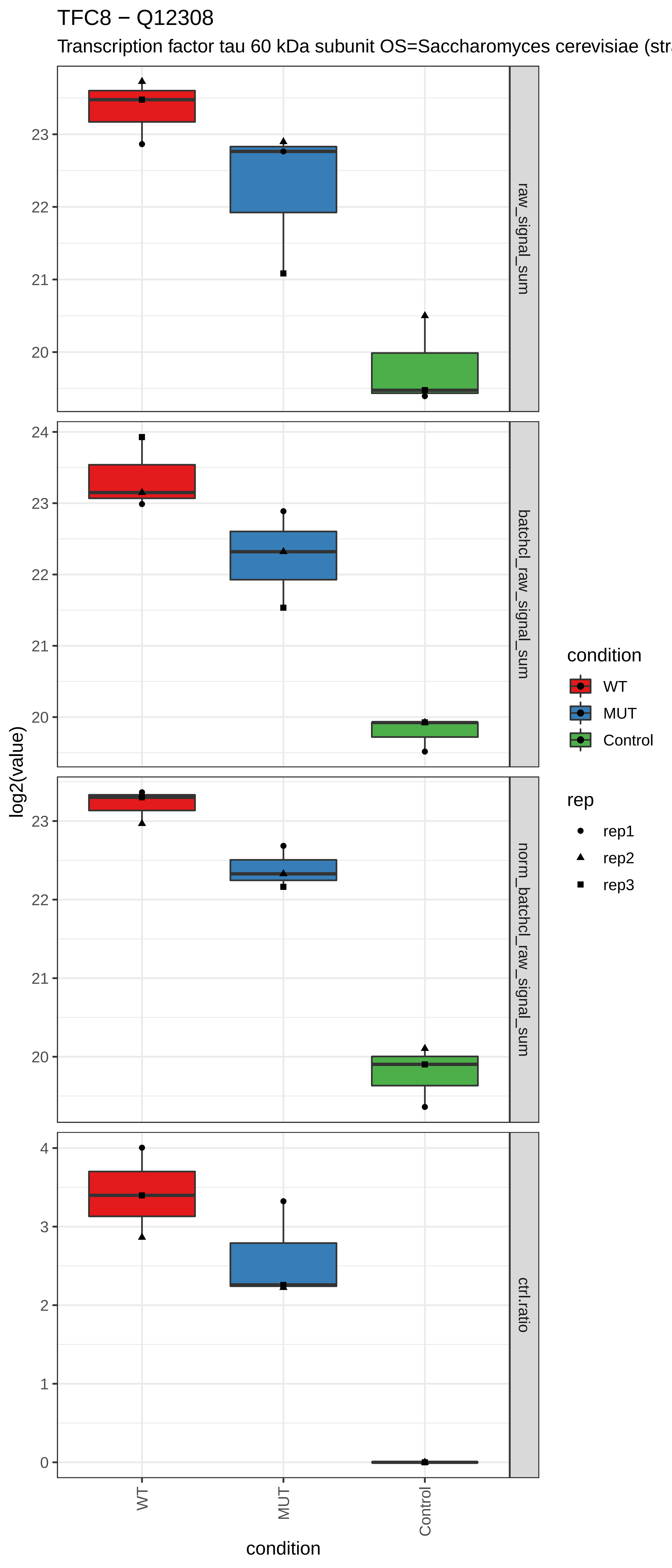

TFC8 − Q12308
Transcription factor tau 60 kDa subunit OS=Saccharomyces cerevisiae (str
raw_signal_sum
batchcl_raw_signal_sum
norm_batchcl_raw_signal_sum
ctrl.ratio
log2(value)
condition
WT
MUT
Control
rep
rep1
rep2
rep3

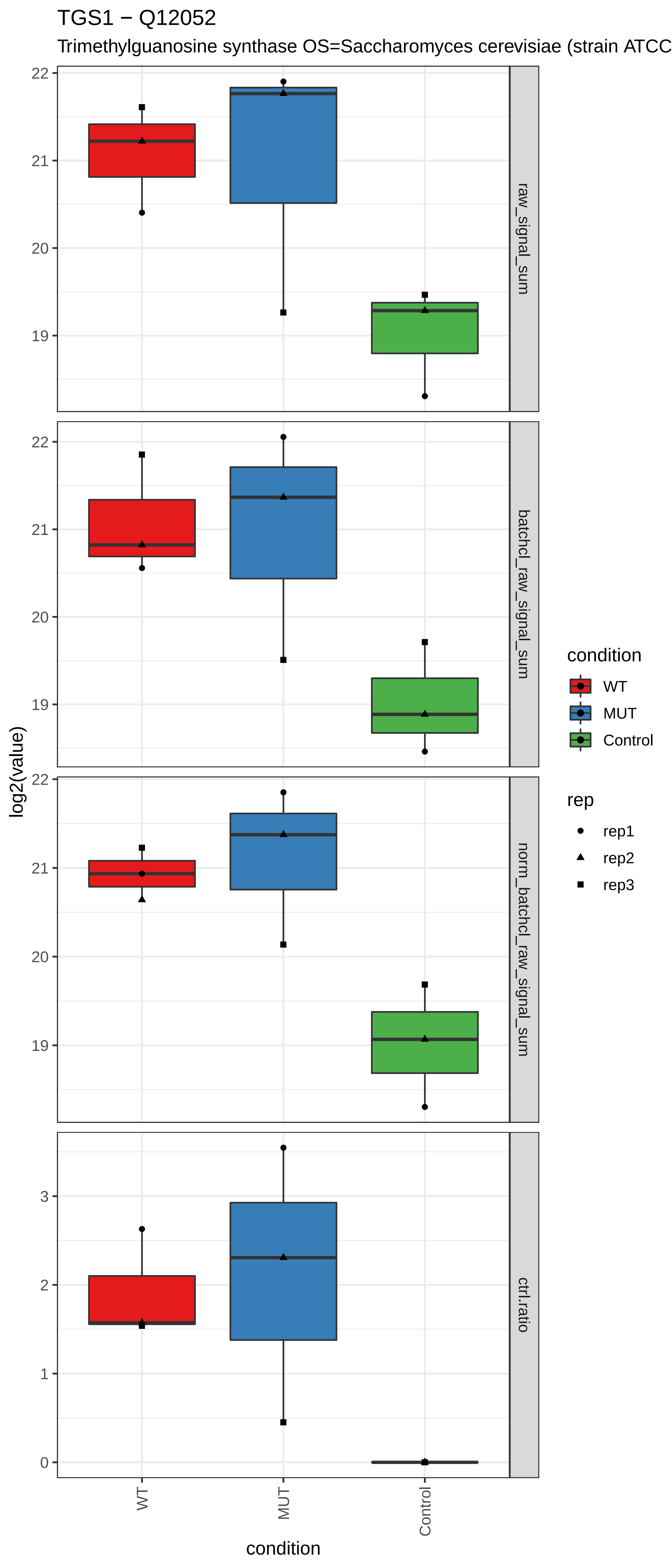

TGS1 − Q12052
Trimethylguanosine synthase OS=Saccharomyces cerevisiae (strain ATCC
raw_signal_sum
batchcl_raw_signal_sum
norm_batchcl_raw_signal_sum
ctrl.ratio
log2(value)
condition
WT
MUT
Control
rep
rep1
rep2
rep3
22
21
20
19
3
2
1
0

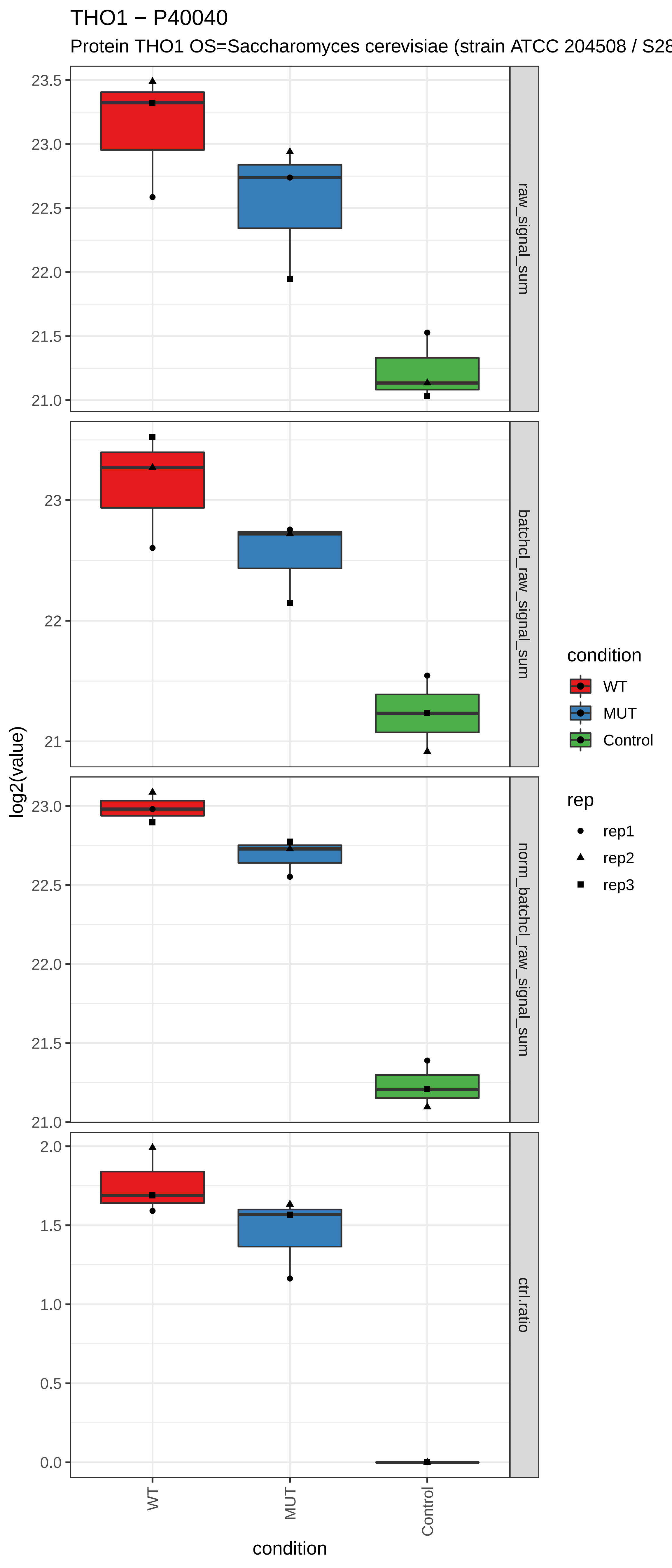

THO1 − P40040
Protein THO1 OS=Saccharomyces cerevisiae (strain ATCC 204508 / S28
raw_signal_sum
batchcl_raw_signal_sum
norm_batchcl_raw_signal_sum
ctrl.ratio
log2(value)
condition
WT
MUT
Control
rep
rep1
rep2
rep3
23.5
23.0
22.5
22.0
21.5
21.0
23
22
21
2.0
1.5
1.0
0.5
0.0

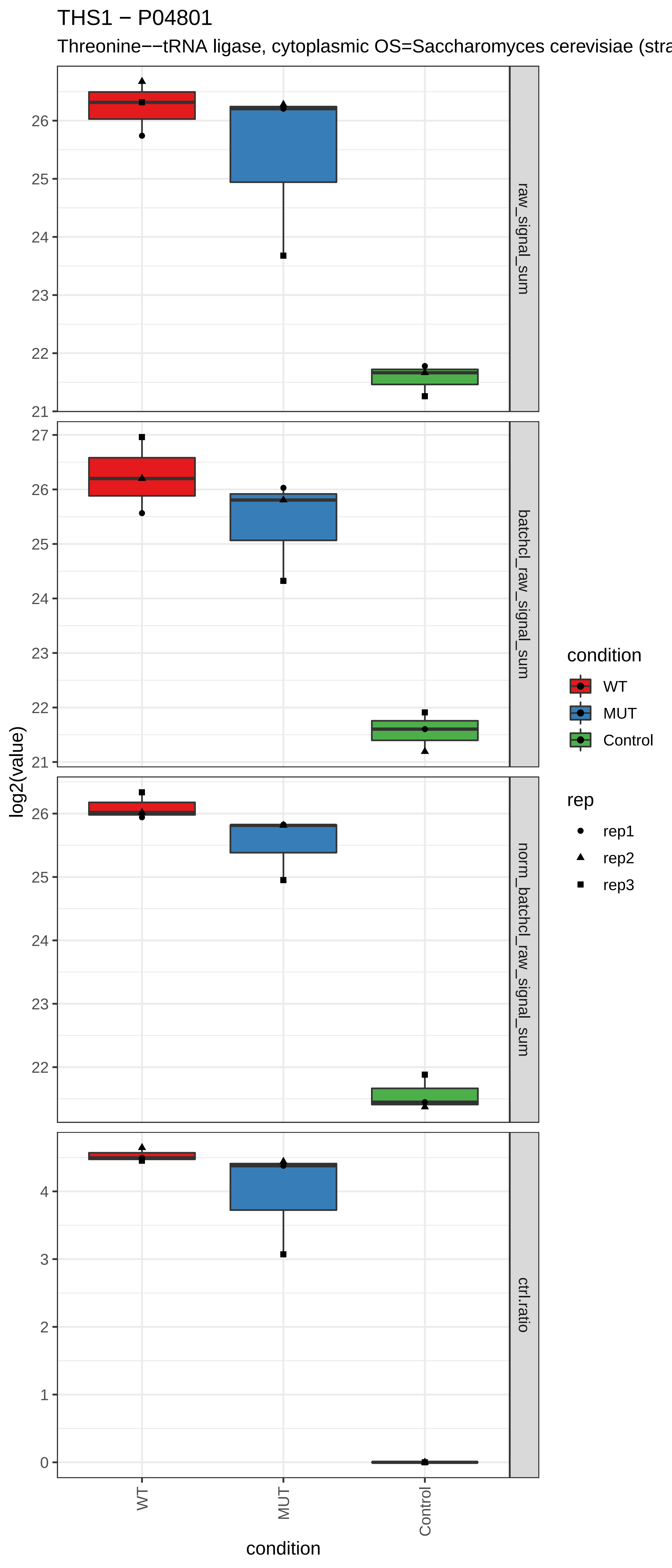

THS1 − P04801
Threonine−−tRNA ligase, cytoplasmic OS=Saccharomyces cerevisiae (stra
raw_signal_sum
batchcl_raw_signal_sum
norm_batchcl_raw_signal_sum
ctrl.ratio
log2(value)
condition
WT
MUT
Control
rep
rep1
rep2
rep3

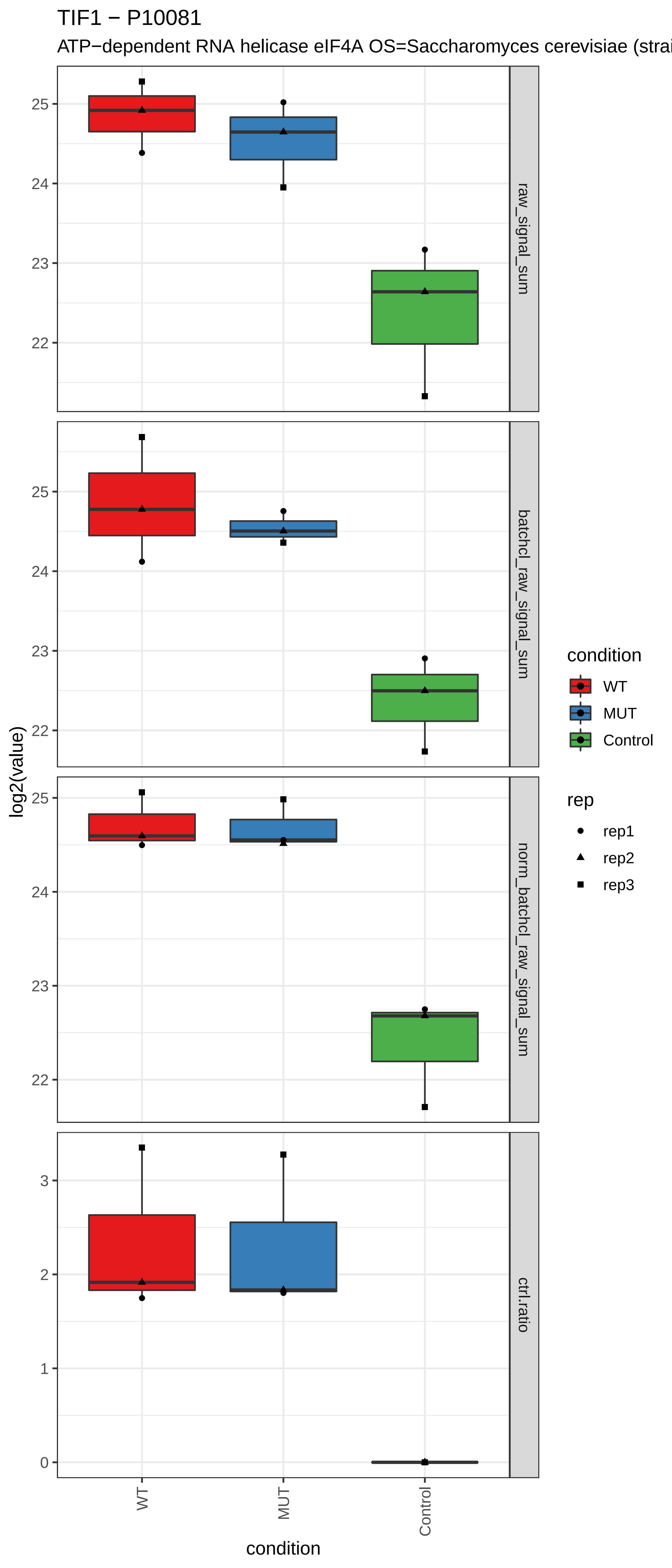

TIF1 − P10081
ATP−dependent RNA helicase eIF4A OS=Saccharomyces cerevisiae (strai
raw_signal_sum
batchcl_raw_signal_sum
norm_batchcl_raw_signal_sum
ctrl.ratio
log2(value)
condition
WT
MUT
Control
rep
rep1
rep2
rep3

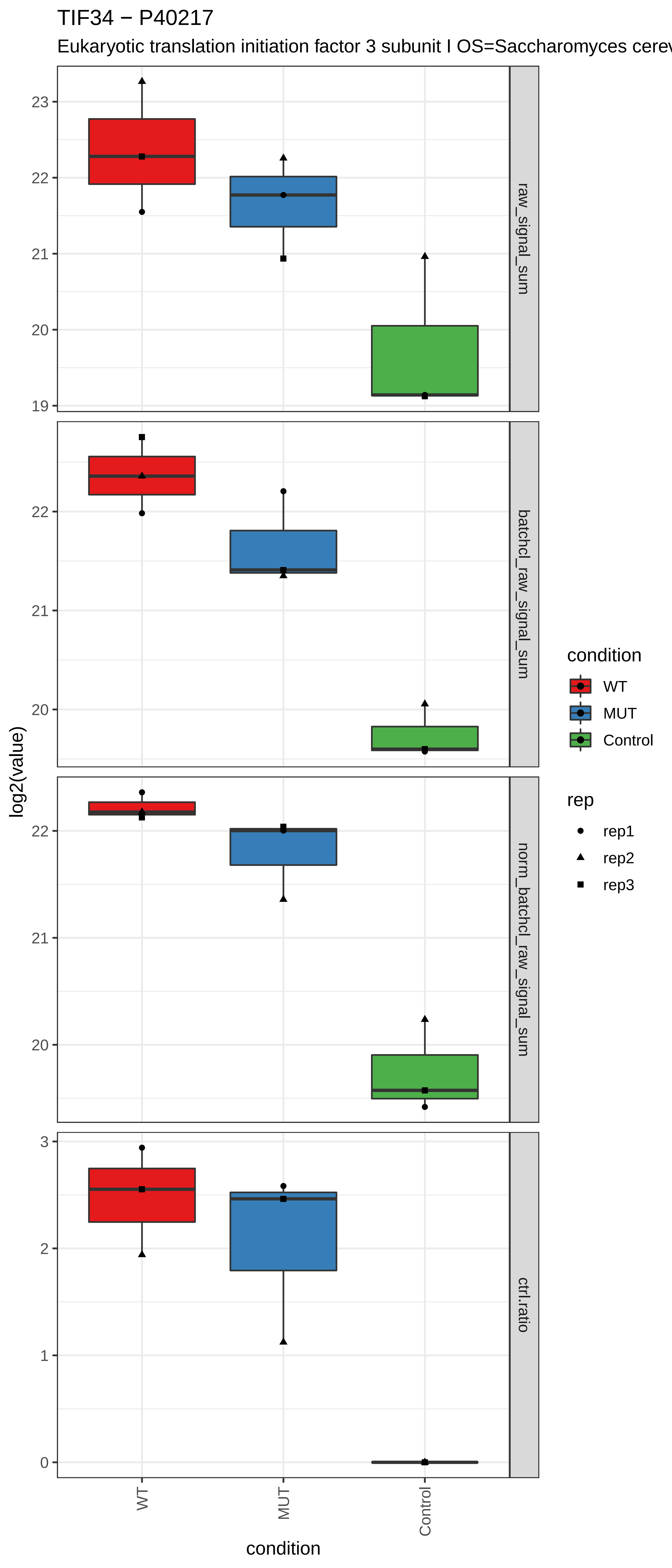

TIF34 − P40217
Eukaryotic translation initiation factor 3 subunit I OS=Saccharomyces cerev
raw_signal_sum
batchcl_raw_signal_sum
norm_batchcl_raw_signal_sum
ctrl.ratio
log2(value)
condition
WT
MUT
Control
rep
rep1
rep2
rep3
23
22
21
20
19
3
2
1
0

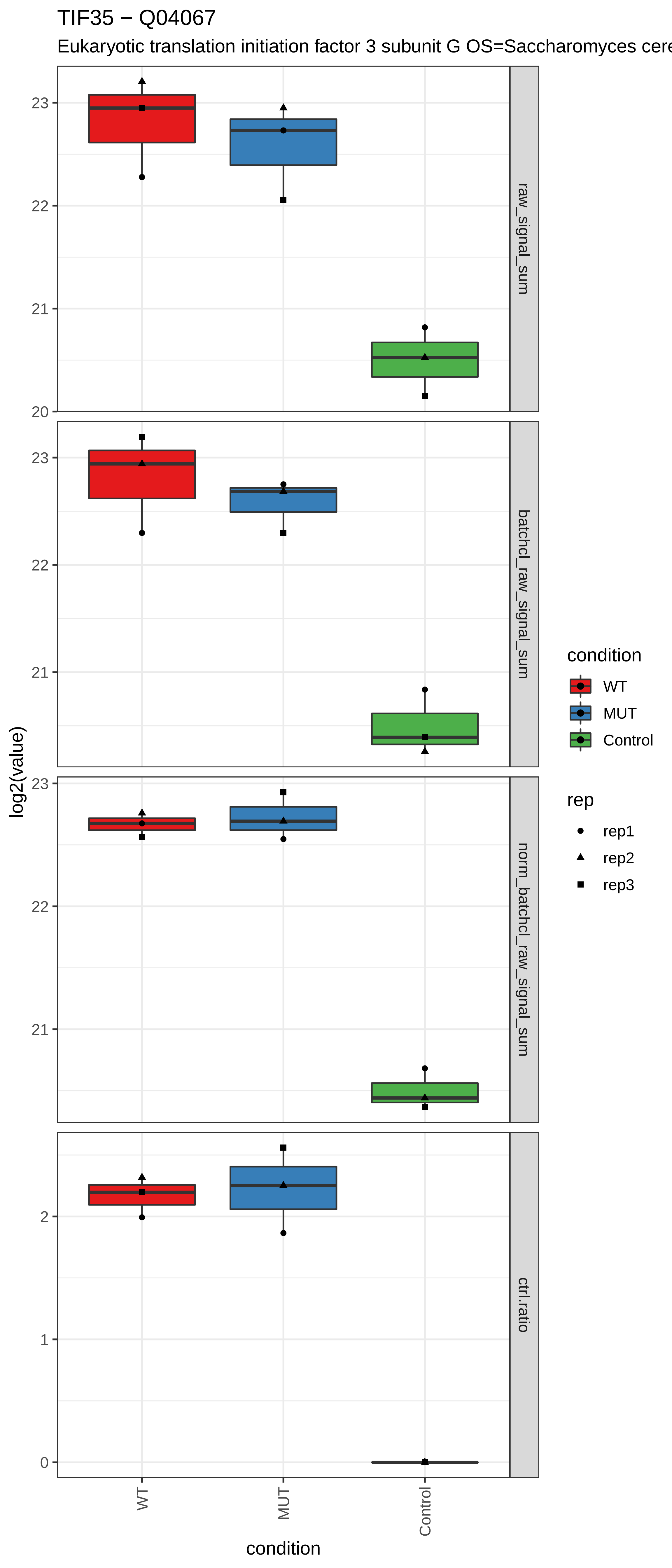

TIF35 − Q04067
Eukaryotic translation initiation factor 3 subunit G OS=Saccharomyces cere
raw_signal_sum
batchcl_raw_signal_sum
norm_batchcl_raw_signal_sum
ctrl.ratio
log2(value)
condition
WT
MUT
Control
rep
rep1
rep2
rep3

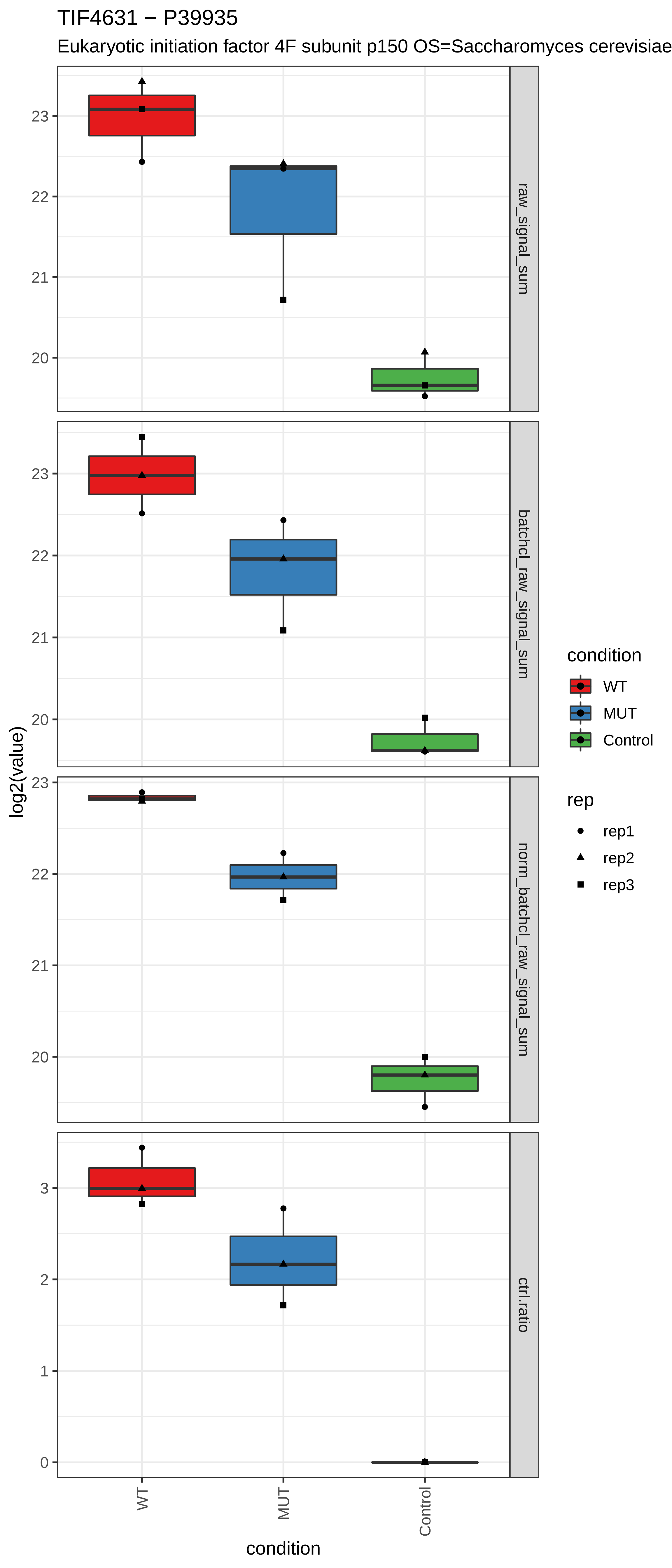
TIF4631 − P39935
Eukaryotic initiation factor 4F subunit p150 OS=Saccharomyces cerevisiae
raw_signal_sum
batchcl_raw_signal_sum
norm_batchcl_raw_signal_sum
ctrl.ratio
log2(value)
condition
WT
MUT
Control
rep
rep1
rep2
rep3
23
22
21
20
3
2
1
0

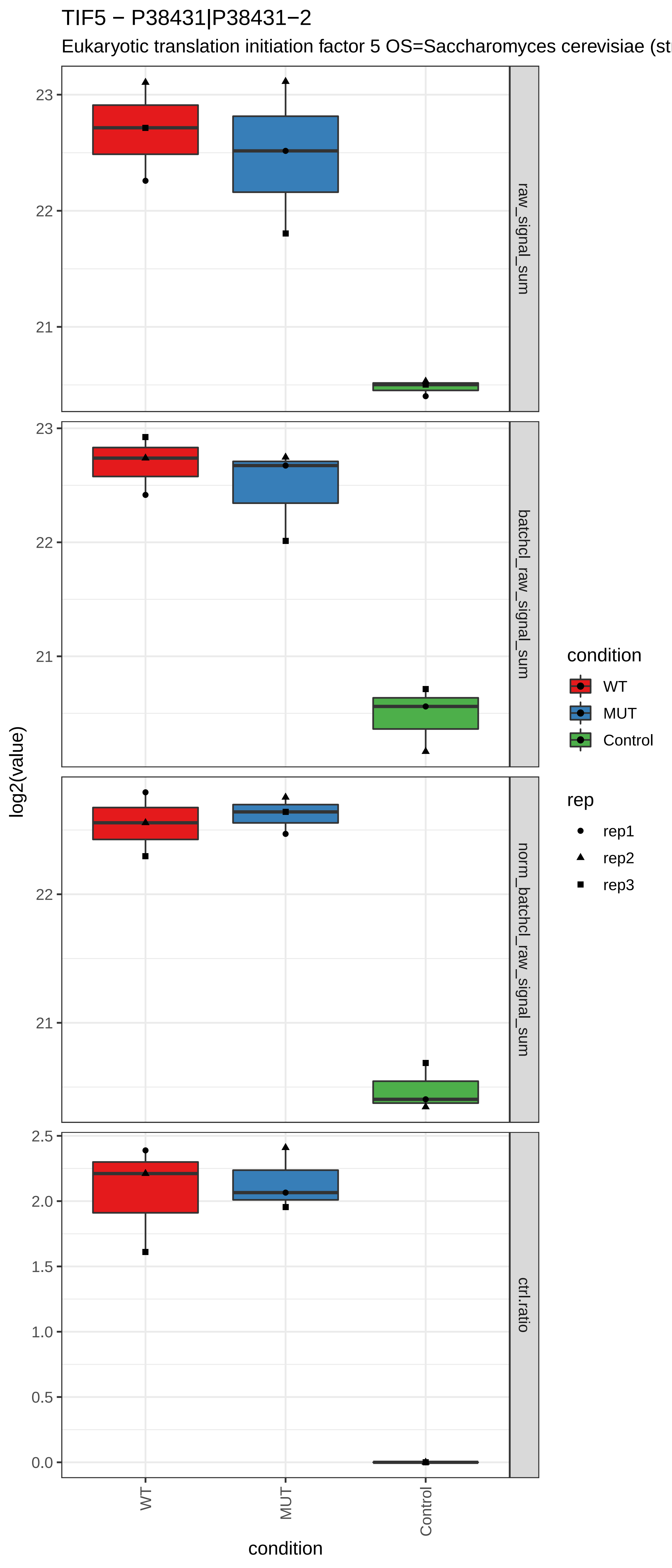
TIF5 − P38431|P38431−2
Eukaryotic translation initiation factor 5 OS=Saccharomyces cerevisiae (st
raw_signal_sum
batchcl_raw_signal_sum
norm_batchcl_raw_signal_sum
ctrl.ratio
log2(value)
condition
WT
MUT
Control
rep
rep1
rep2
rep3
23
22
21
2.5
2.0
1.5
1.0
0.5
0.0

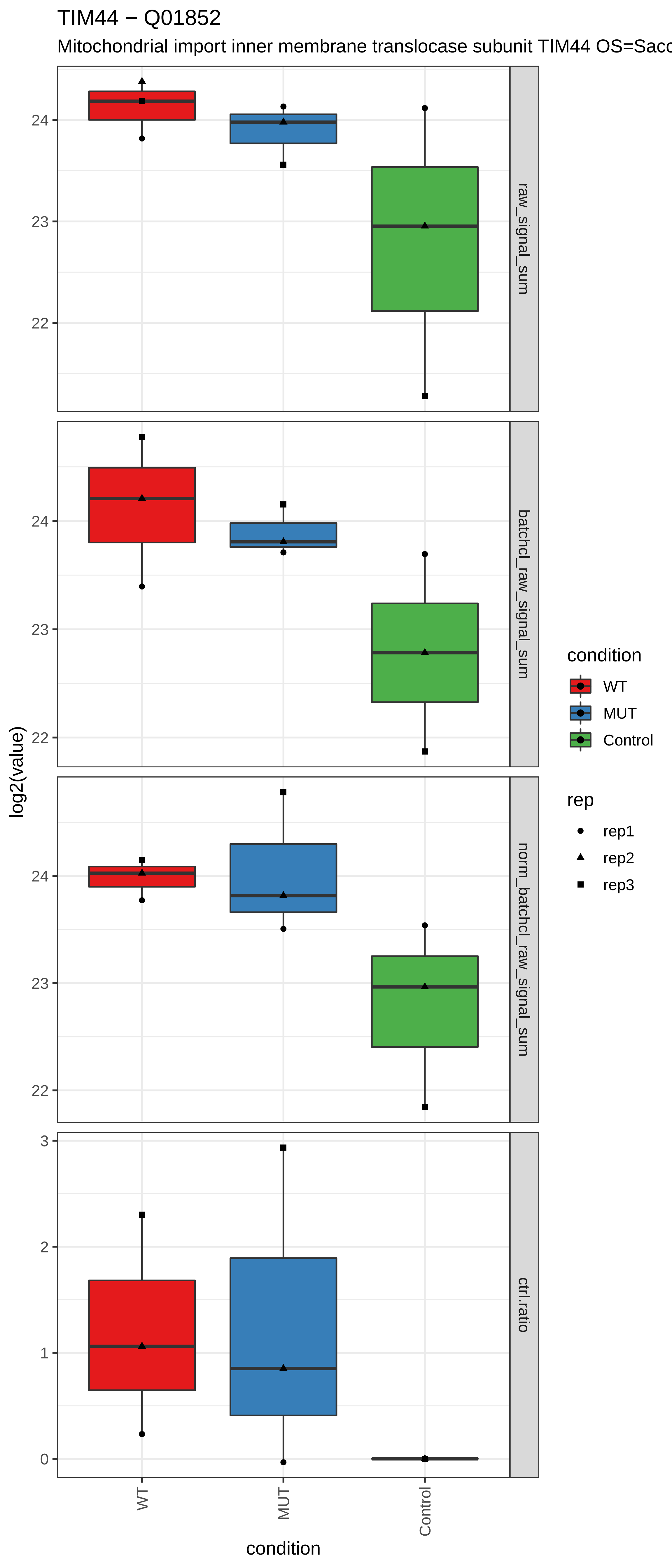

TIM44 − Q01852
Mitochondrial import inner membrane translocase subunit TIM44 OS=Sacc
raw_signal_sum
batchcl_raw_signal_sum
norm_batchcl_raw_signal_sum
ctrl.ratio
log2(value)
condition
WT
MUT
Control
rep
rep1
rep2
rep3

TKL1 − P23254

Transketolase 1 OS=Saccharomyces cerevisiae (strain ATCC 204508 / S28

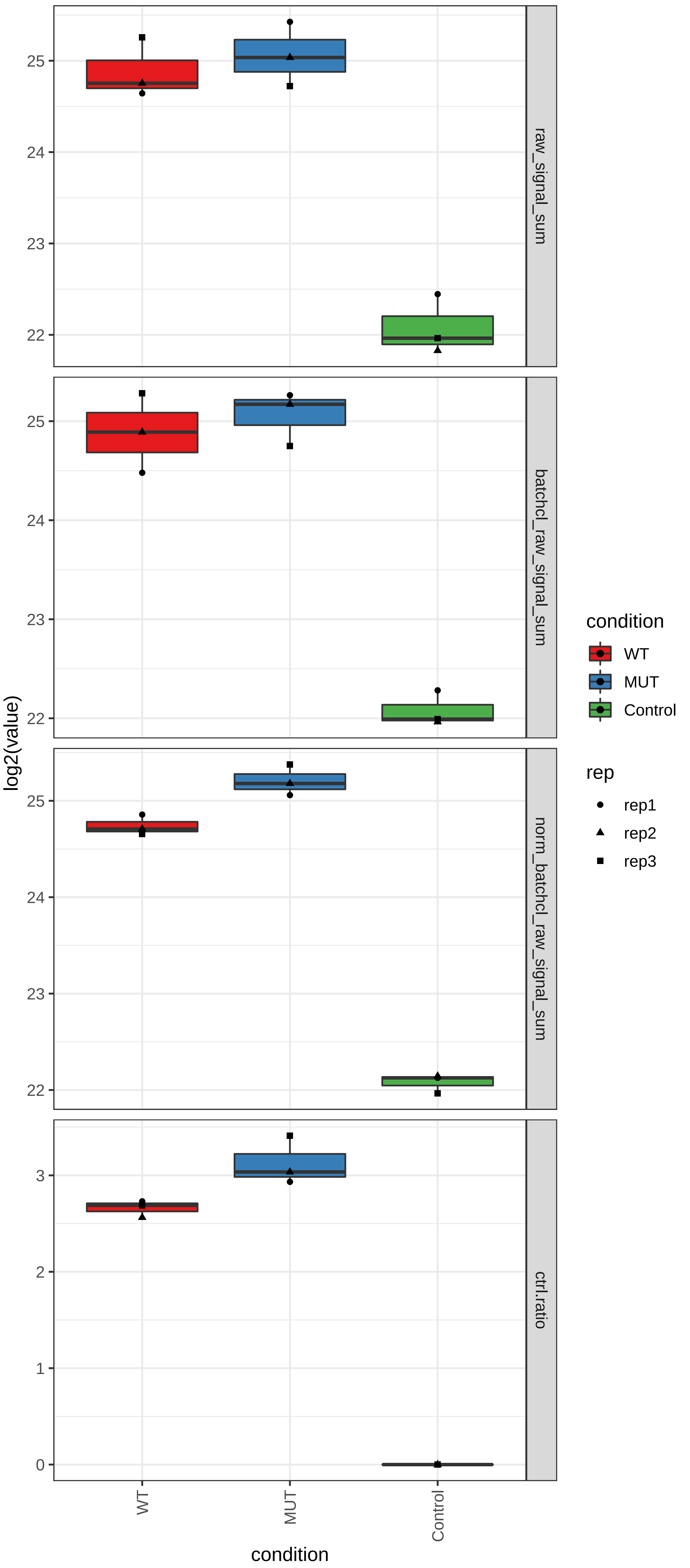

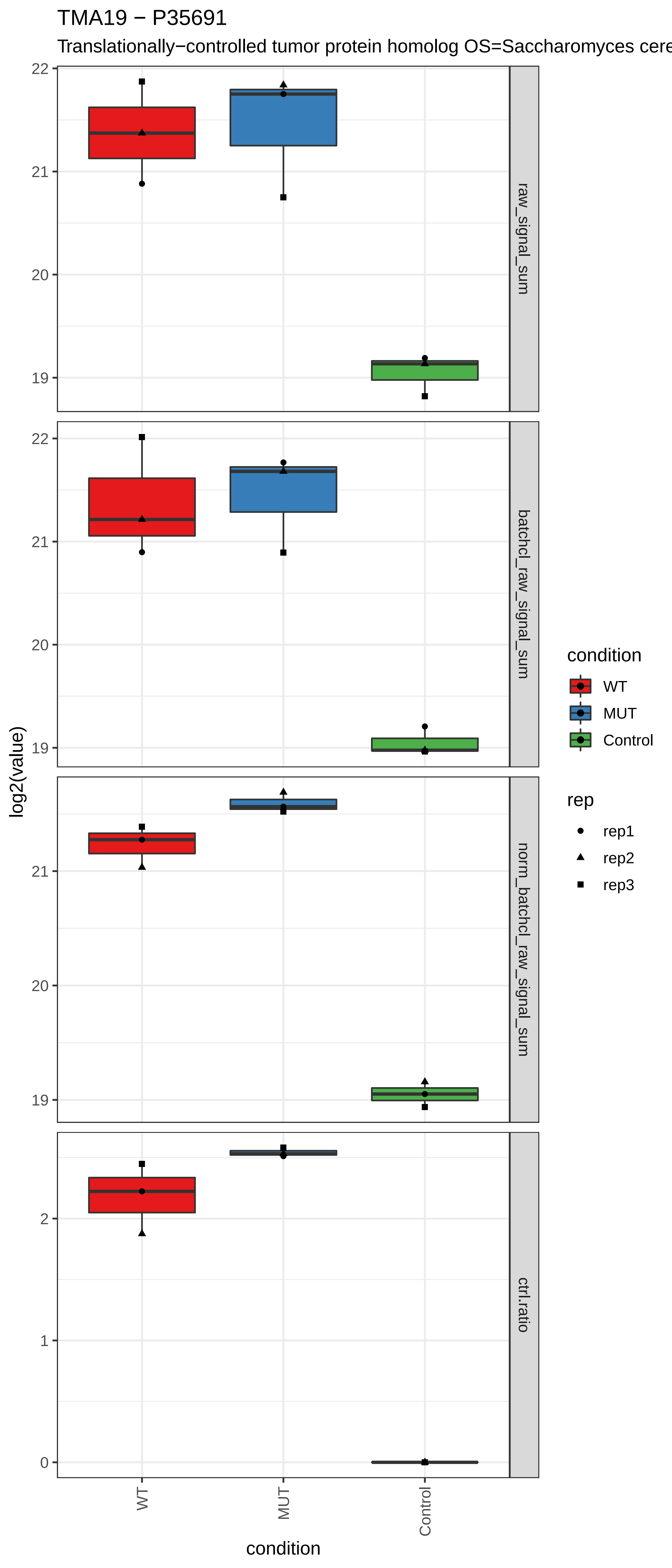

TMA19 − P35691
Translationally−controlled tumor protein homolog OS=Saccharomyces cere
raw_signal_sum
batchcl_raw_signal_sum
norm_batchcl_raw_signal_sum
ctrl.ratio
log2(value)
condition
WT
MUT
Control
rep
rep1
rep2
rep3

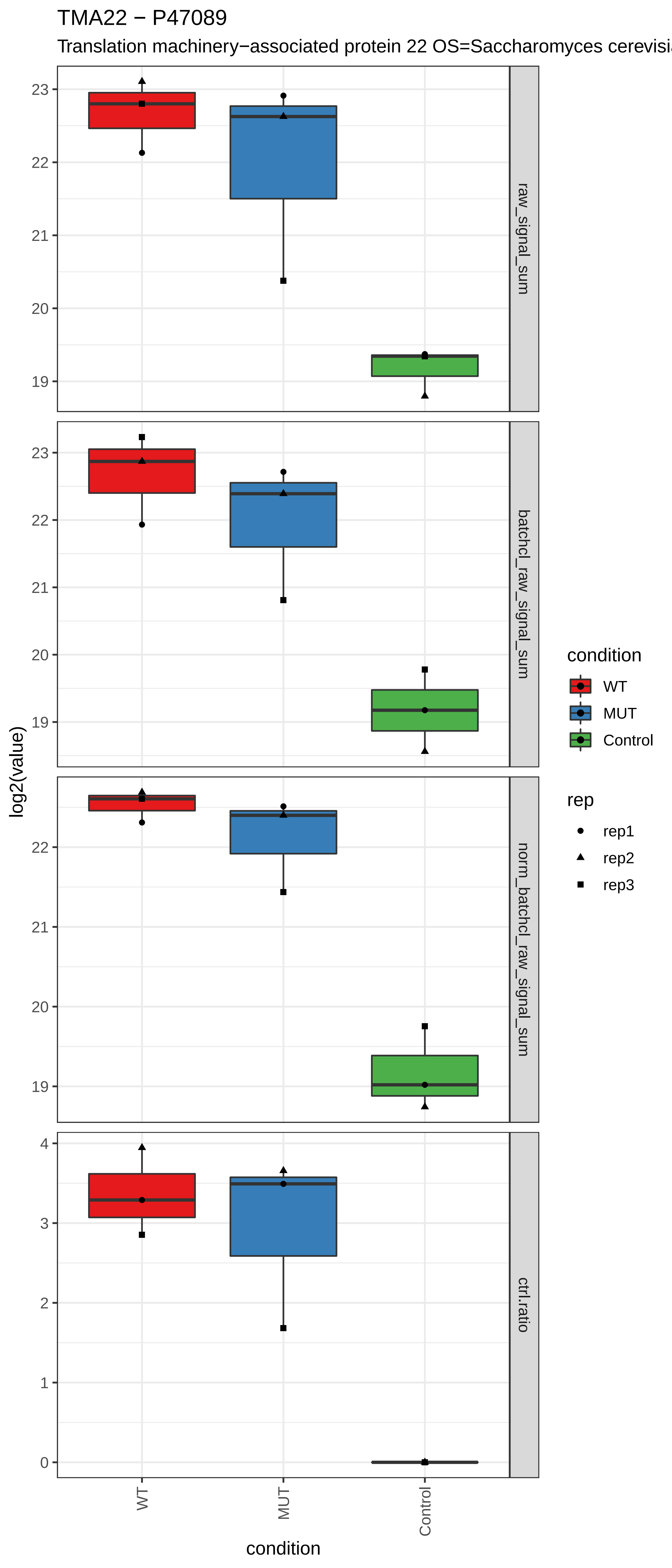

TMA22 − P47089
Translation machinery−associated protein 22 OS=Saccharomyces cerevisi
raw_signal_sum
batchcl_raw_signal_sum
norm_batchcl_raw_signal_sum
ctrl.ratio
log2(value)
condition
WT
MUT
Control
rep
rep1
rep2
rep3

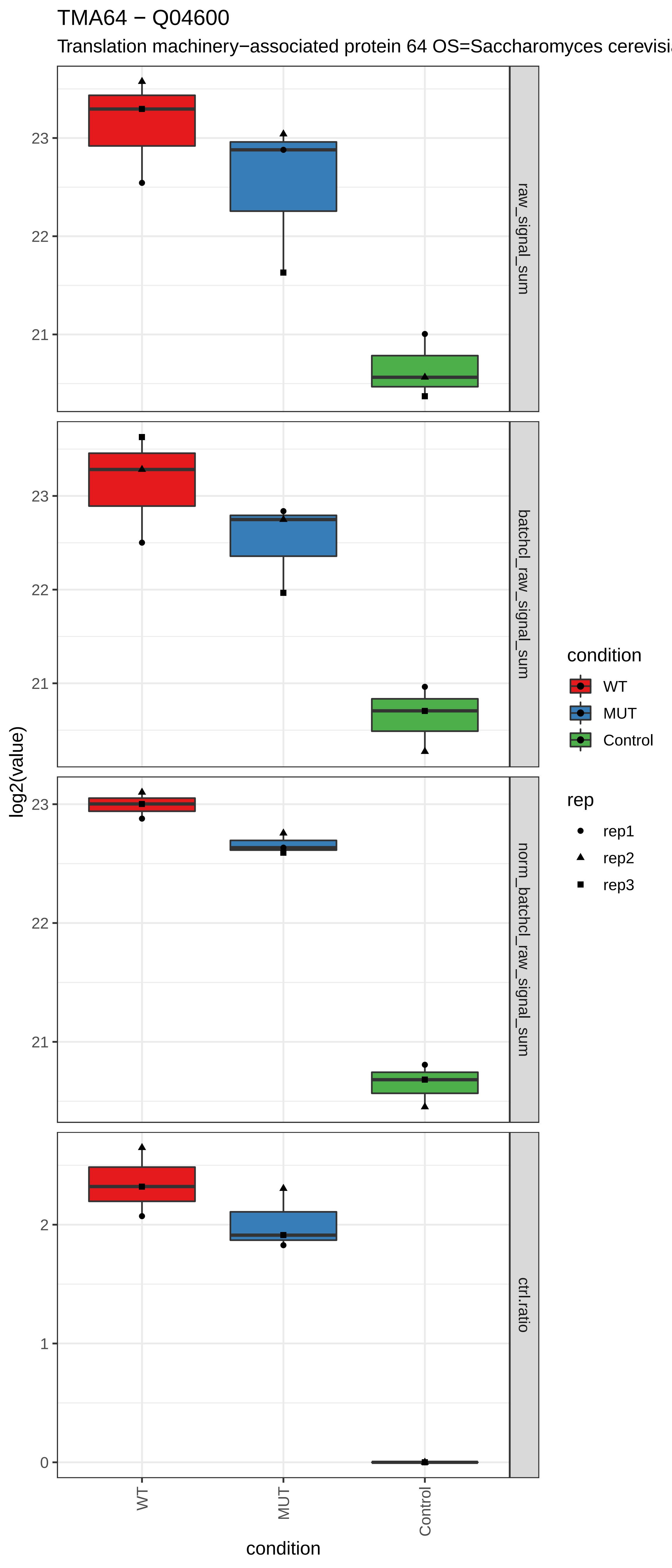

TMA64 − Q04600
Translation machinery−associated protein 64 OS=Saccharomyces cerevisi
raw_signal_sum
batchcl_raw_signal_sum
norm_batchcl_raw_signal_sum
ctrl.ratio
log2(value)
condition
WT
MUT
Control
rep
rep1
rep2
rep3
23
22
21
2
1
0

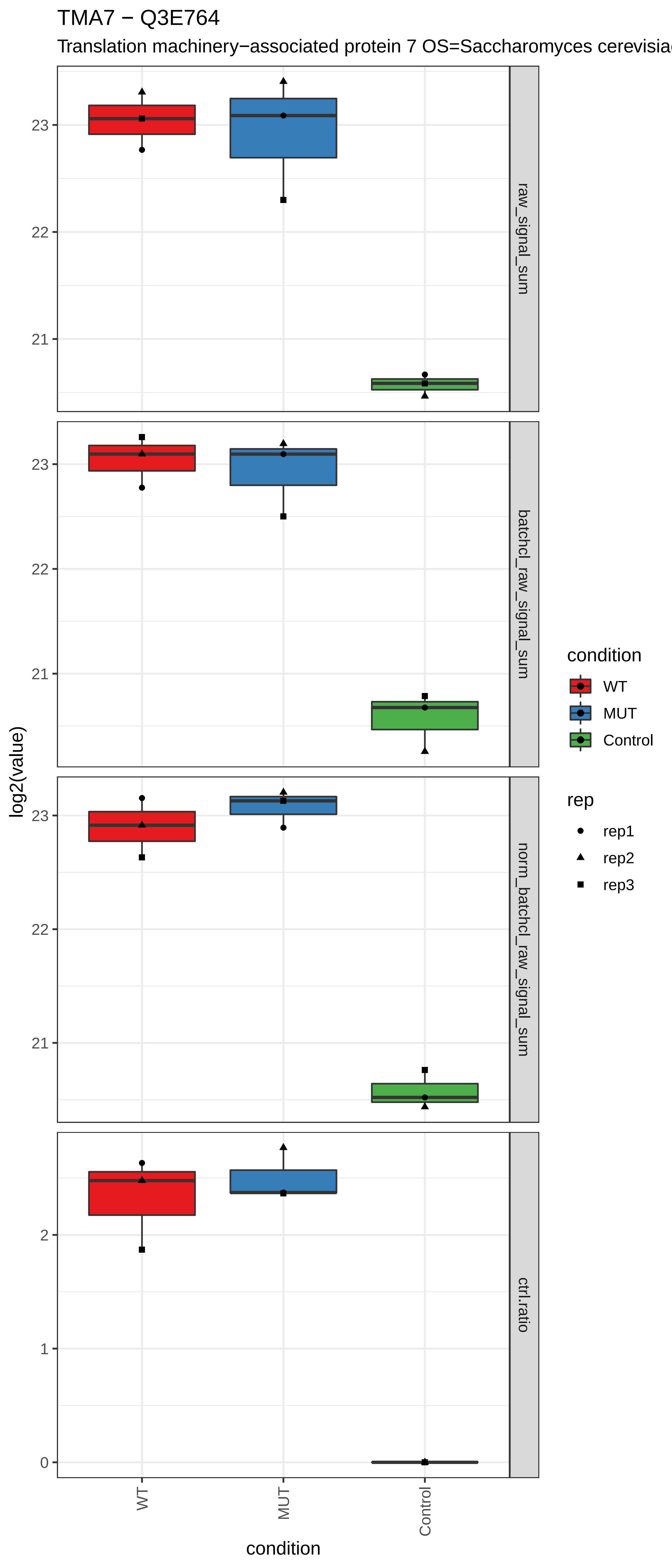
TMA7 − Q3E764
Translation machinery−associated protein 7 OS=Saccharomyces cerevisiae
raw_signal_sum
batchcl_raw_signal_sum
norm_batchcl_raw_signal_sum
ctrl.ratio
log2(value)
condition
WT
MUT
Control
rep
rep1
rep2
rep3

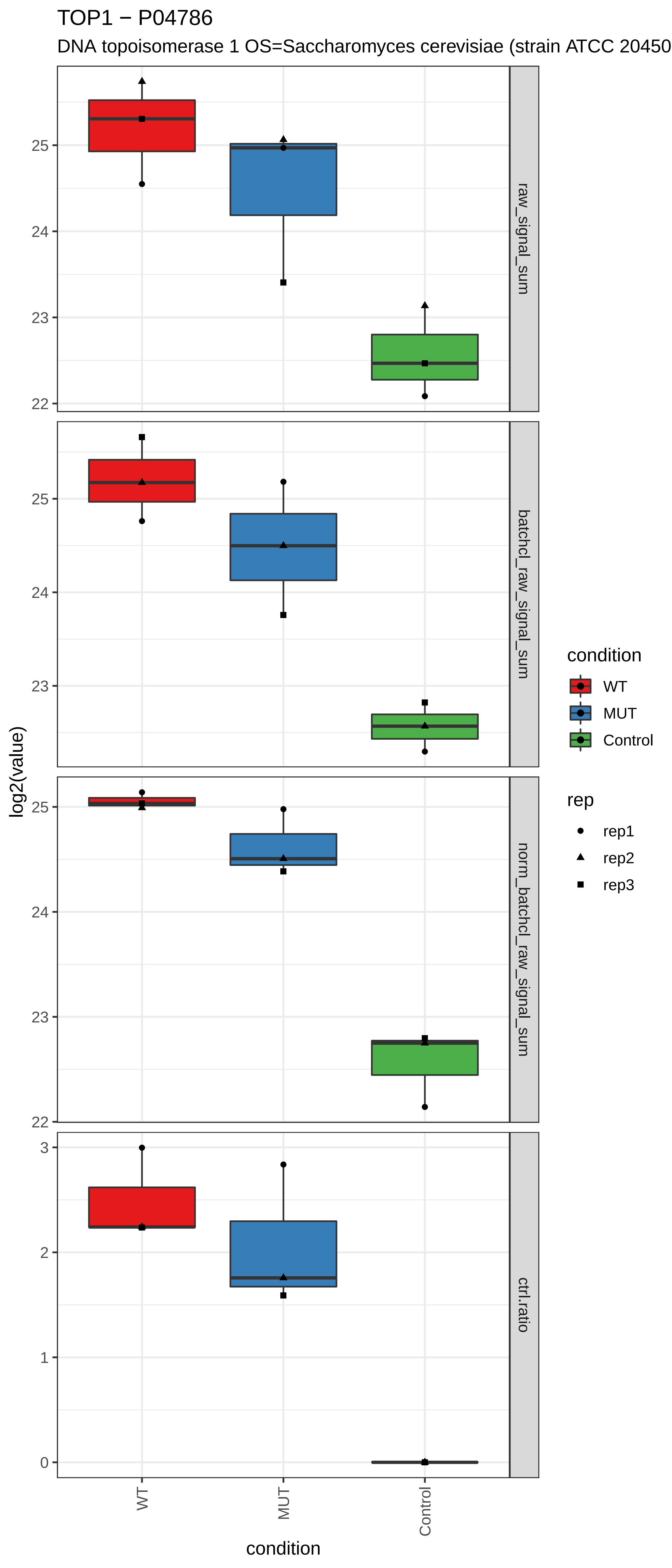

TOP1 − P04786
DNA topoisomerase 1 OS=Saccharomyces cerevisiae (strain ATCC 20450
raw_signal_sum
batchcl_raw_signal_sum
norm_batchcl_raw_signal_sum
ctrl.ratio
log2(value)
condition
WT
MUT
Control
rep
rep1
rep2
rep3

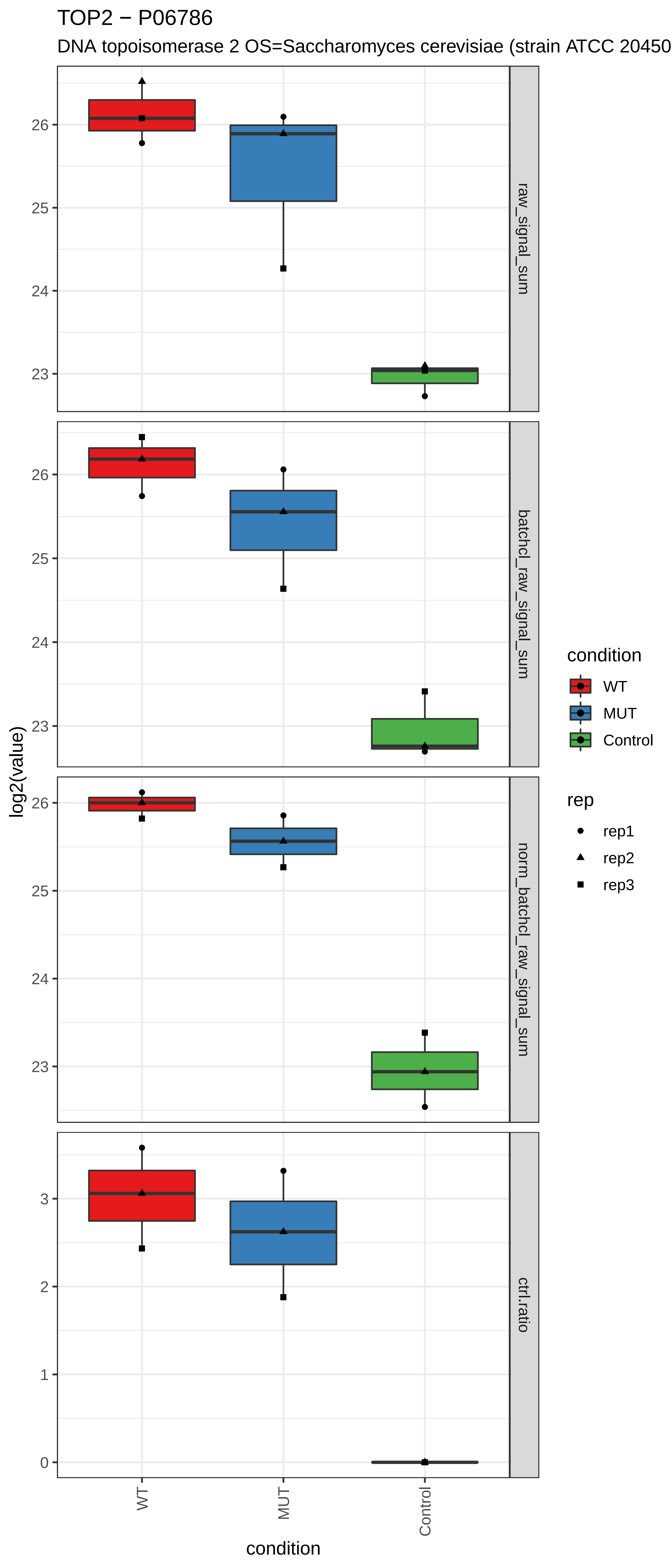

TOP2 − P06786
DNA topoisomerase 2 OS=Saccharomyces cerevisiae (strain ATCC 20450
raw_signal_sum
batchcl_raw_signal_sum
norm_batchcl_raw_signal_sum
ctrl.ratio
log2(value)
condition
WT
MUT
Control
rep
rep1
rep2
rep3

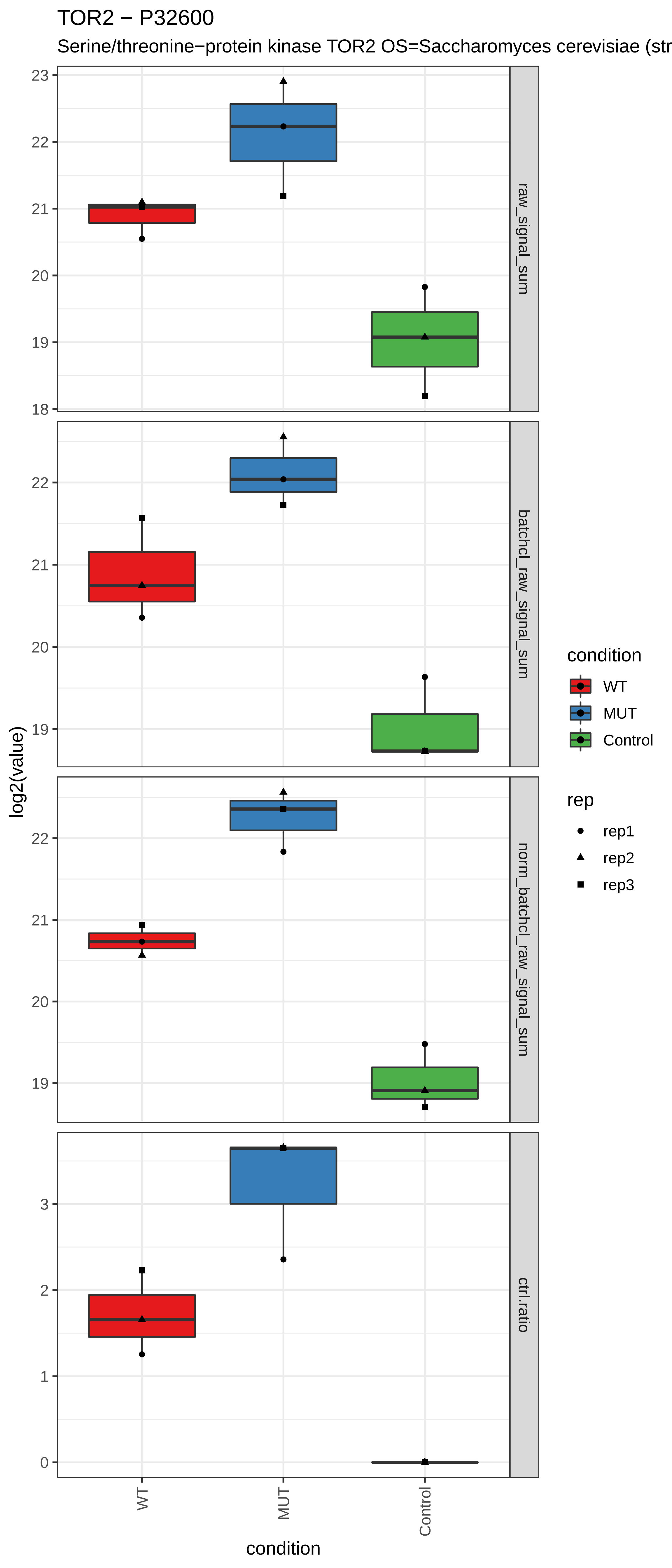

TOR2 − P32600
Serine/threonine−protein kinase TOR2 OS=Saccharomyces cerevisiae (str
raw_signal_sum
batchcl_raw_signal_sum
norm_batchcl_raw_signal_sum
ctrl.ratio
log2(value)
condition
WT
MUT
Control
rep
rep1
rep2
rep3

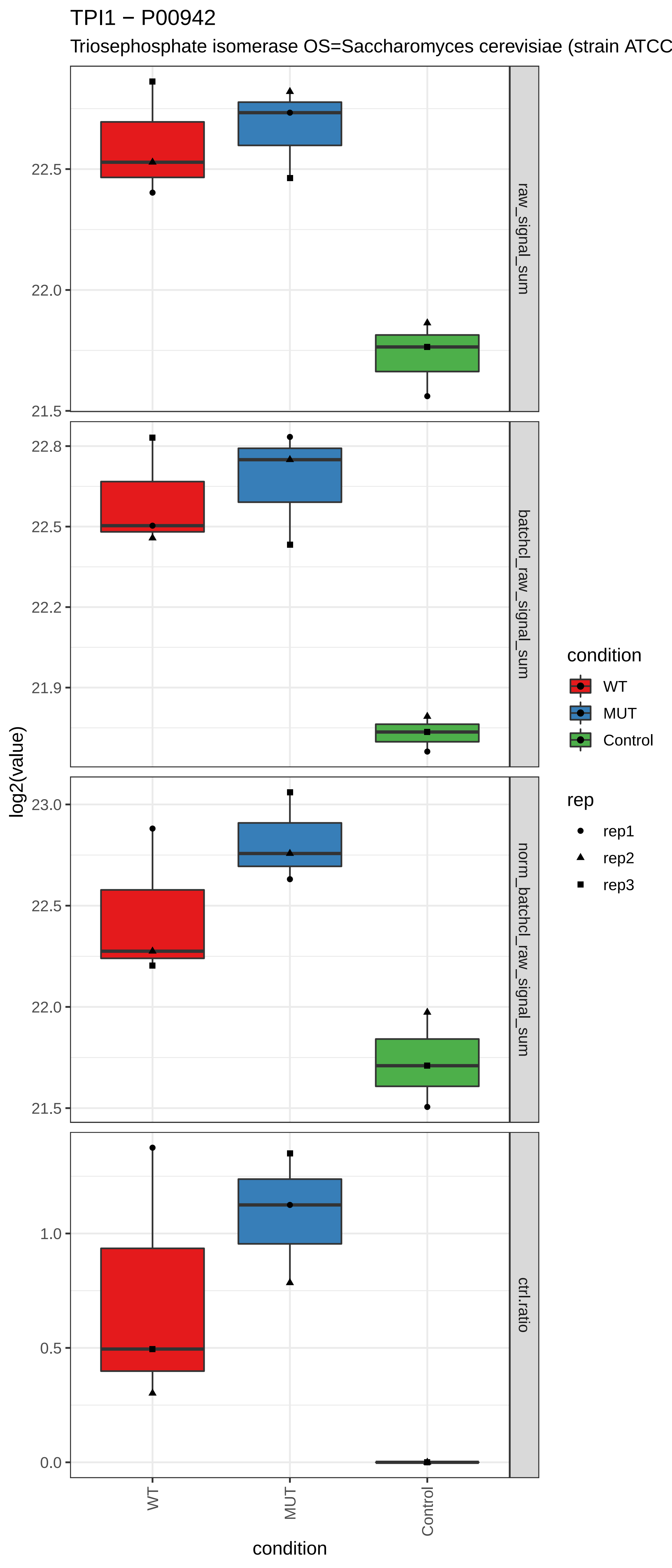
TPI1 − P00942
Triosephosphate isomerase OS=Saccharomyces cerevisiae (strain ATCC
raw_signal_sum
batchcl_raw_signal_sum
norm_batchcl_raw_signal_sum
ctrl.ratio
log2(value)
condition
WT
MUT
Control
rep
rep1
rep2
rep3

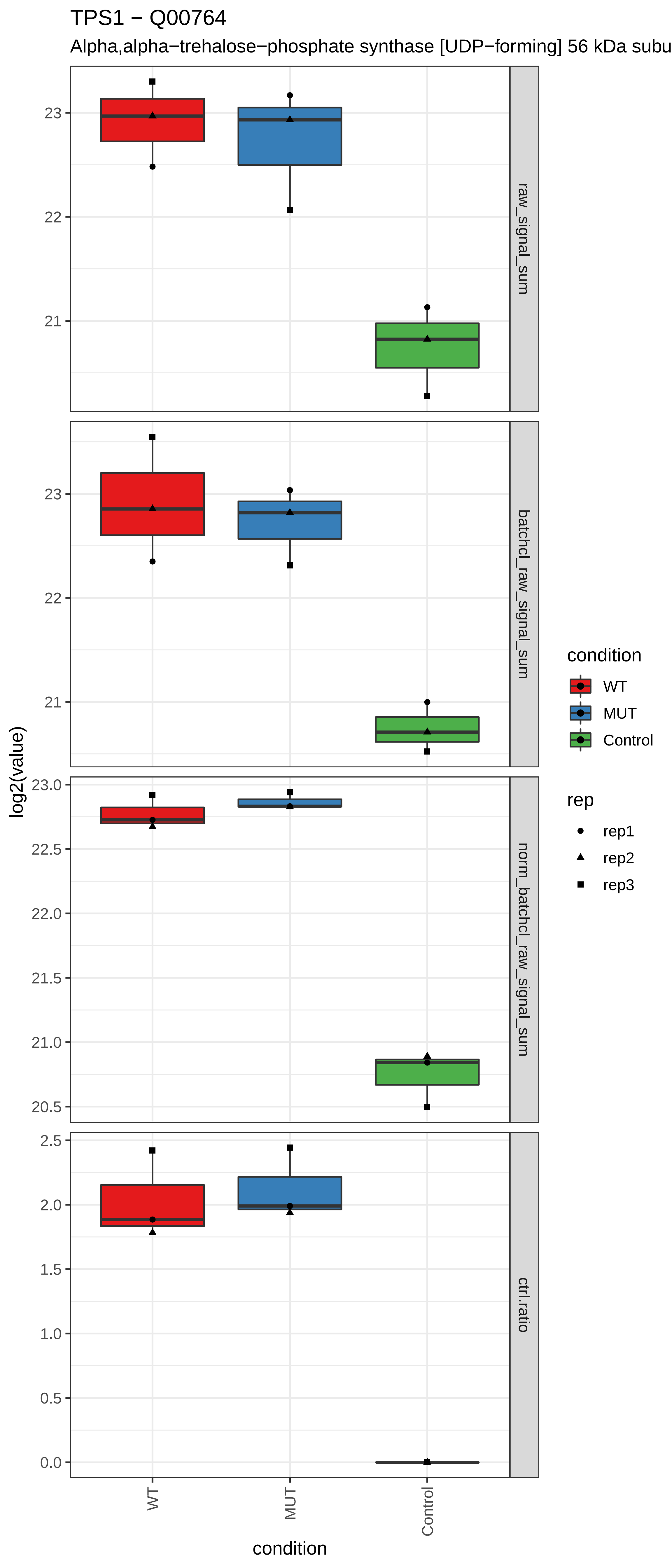

TPS1 − Q00764
Alpha,alpha−trehalose−phosphate synthase [UDP−forming] 56 kDa subu
raw_signal_sum
batchcl_raw_signal_sum
norm_batchcl_raw_signal_sum
ctrl.ratio
log2(value)
condition
WT
MUT
Control
rep
rep1
rep2
rep3

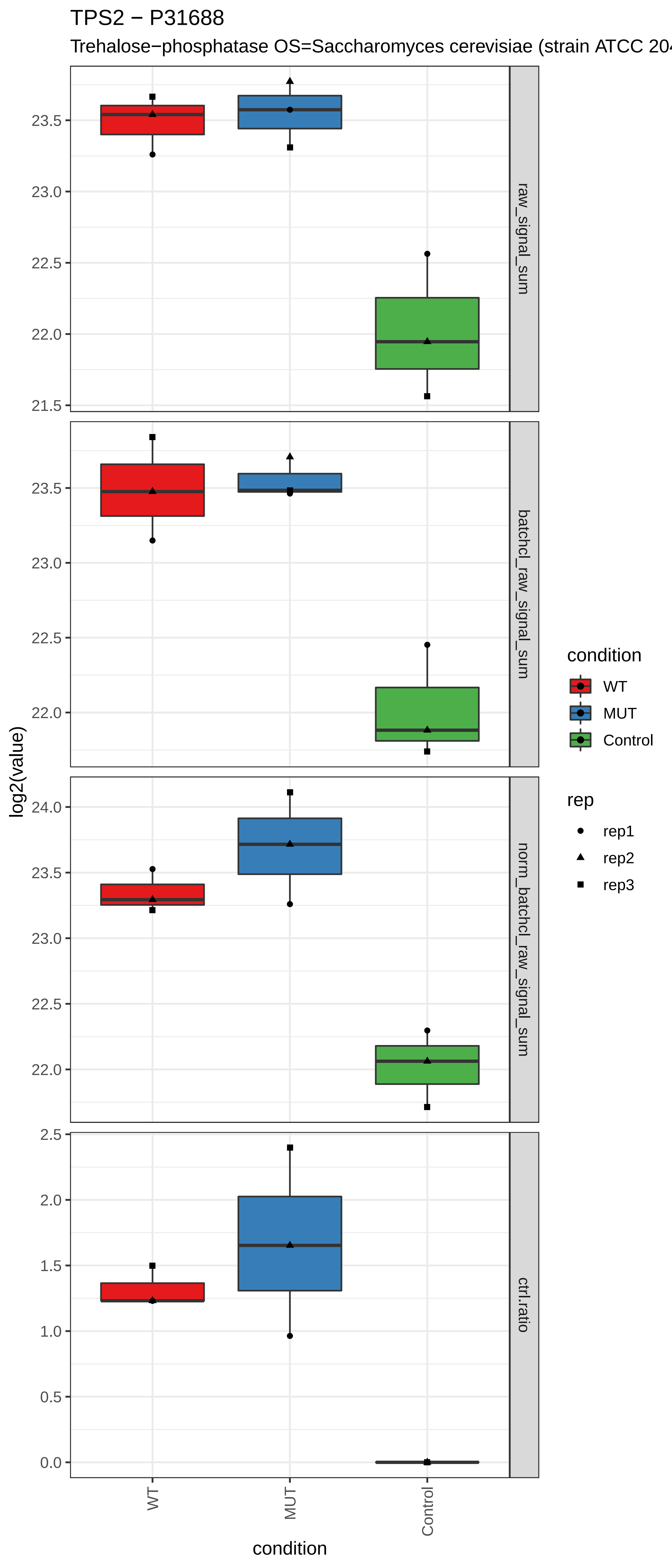

TPS2 − P31688
Trehalose−phosphatase OS=Saccharomyces cerevisiae (strain ATCC 204
raw_signal_sum
batchcl_raw_signal_sum
norm_batchcl_raw_signal_sum
ctrl.ratio
log2(value)
condition
WT
MUT
Control
rep
rep1
rep2
rep3
23.5
23.0
22.5
22.0
21.5
24.0
2.5
2.0
1.5
1.0
0.5
0.0

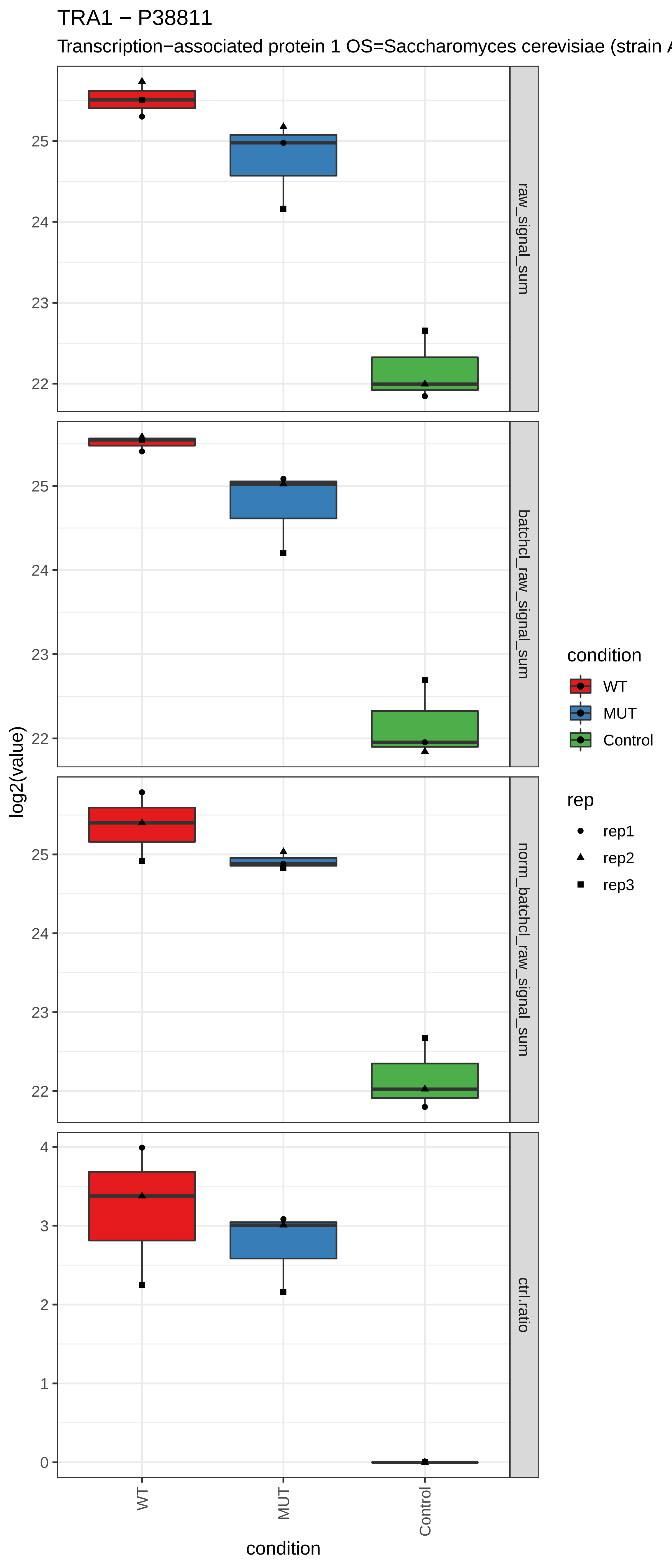

TRA1 − P38811
Transcription−associated protein 1 OS=Saccharomyces cerevisiae (strain A
raw_signal_sum
batchcl_raw_signal_sum
norm_batchcl_raw_signal_sum
ctrl.ratio
log2(value)
condition
WT
MUT
Control
condition
WT
MUT
Control
rep
rep1
rep2
rep3

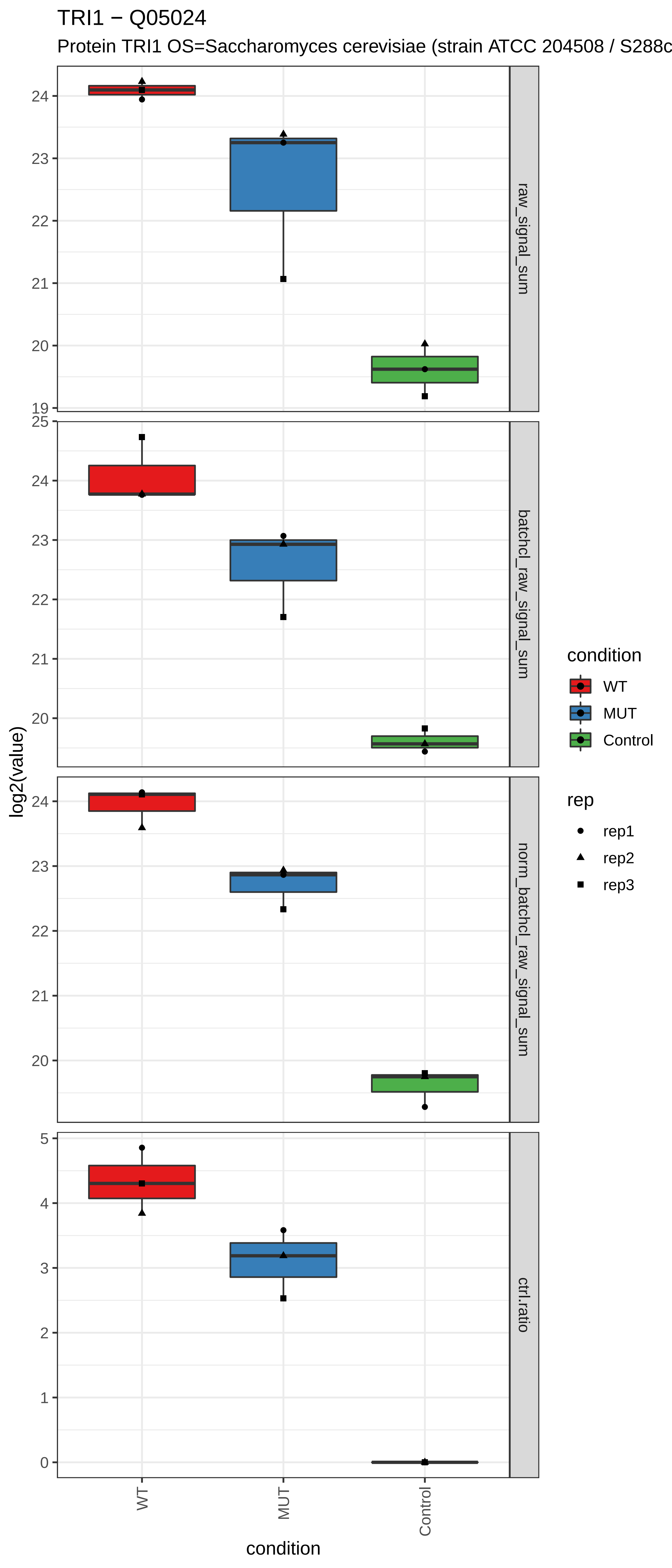

TRI1 − Q05024
Protein TRI1 OS=Saccharomyces cerevisiae (strain ATCC 204508 / S288c
raw_signal_sum
batchcl_raw_signal_sum
norm_batchcl_raw_signal_sum
ctrl.ratio
log2(value)
condition
WT
MUT
Control
rep
rep1
rep2
rep3

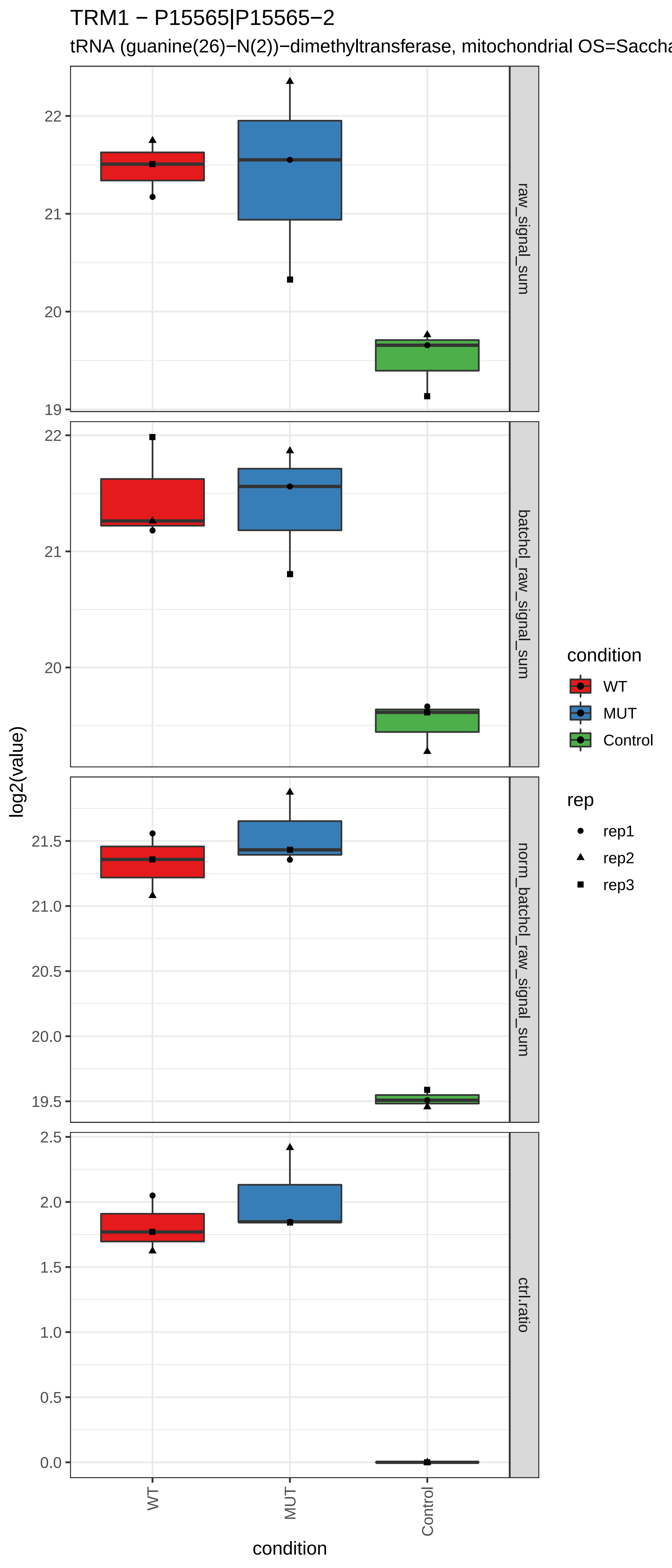
TRM1 − P15565|P15565−2
tRNA (guanine(26)−N(2))−dimethyltransferase, mitochondrial OS=Saccha
raw_signal_sum
batchcl_raw_signal_sum
norm_batchcl_raw_signal_sum
ctrl.ratio
log2(value)
condition
WT
MUT
Control
rep
rep1
rep2
rep3
22
21
20
19
21.5
21.0
20.5
20.0
19.5
2.5
2.0
1.5
1.0
0.5
0.0

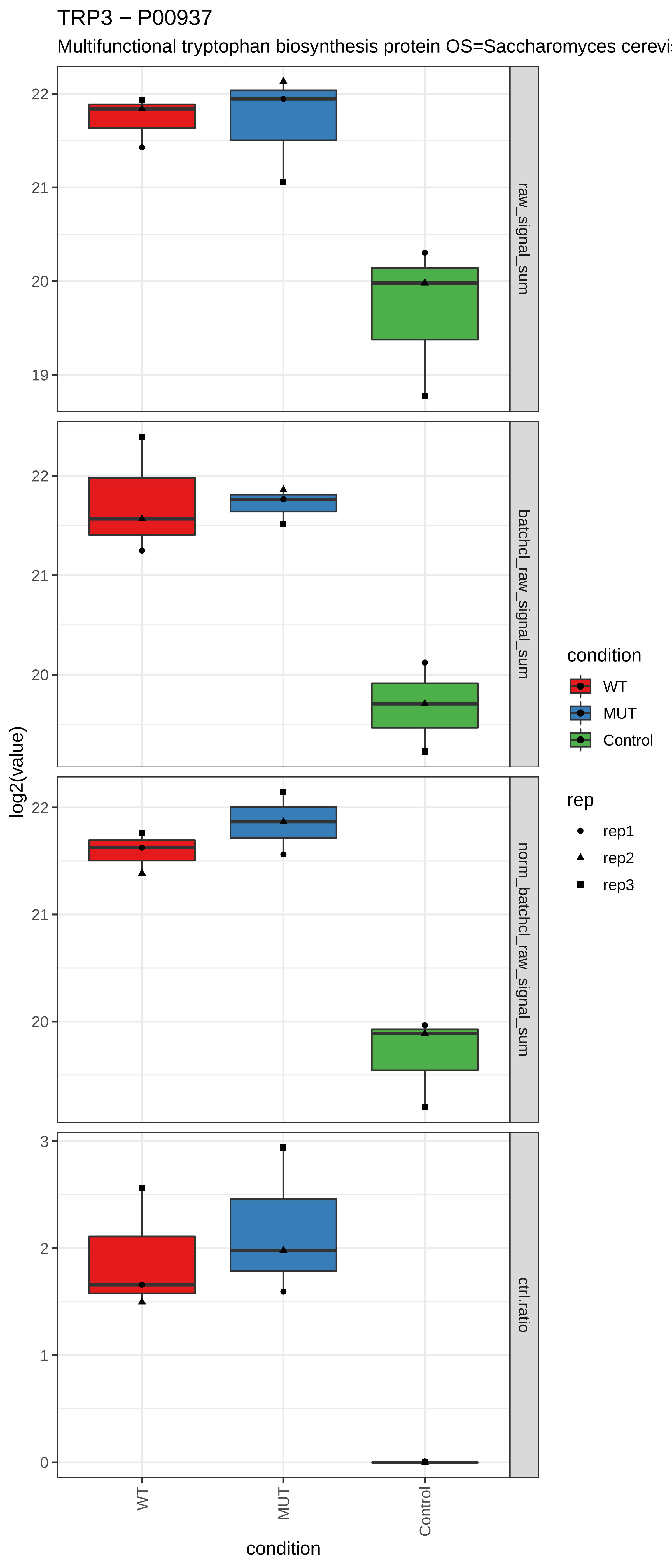

TRP3 − P00937
Multifunctional tryptophan biosynthesis protein OS=Saccharomyces cerevis
raw_signal_sum
batchcl_raw_signal_sum
norm_batchcl_raw_signal_sum
ctrl.ratio
log2(value)
condition
WT
MUT
Control
rep
rep1
rep2
rep3

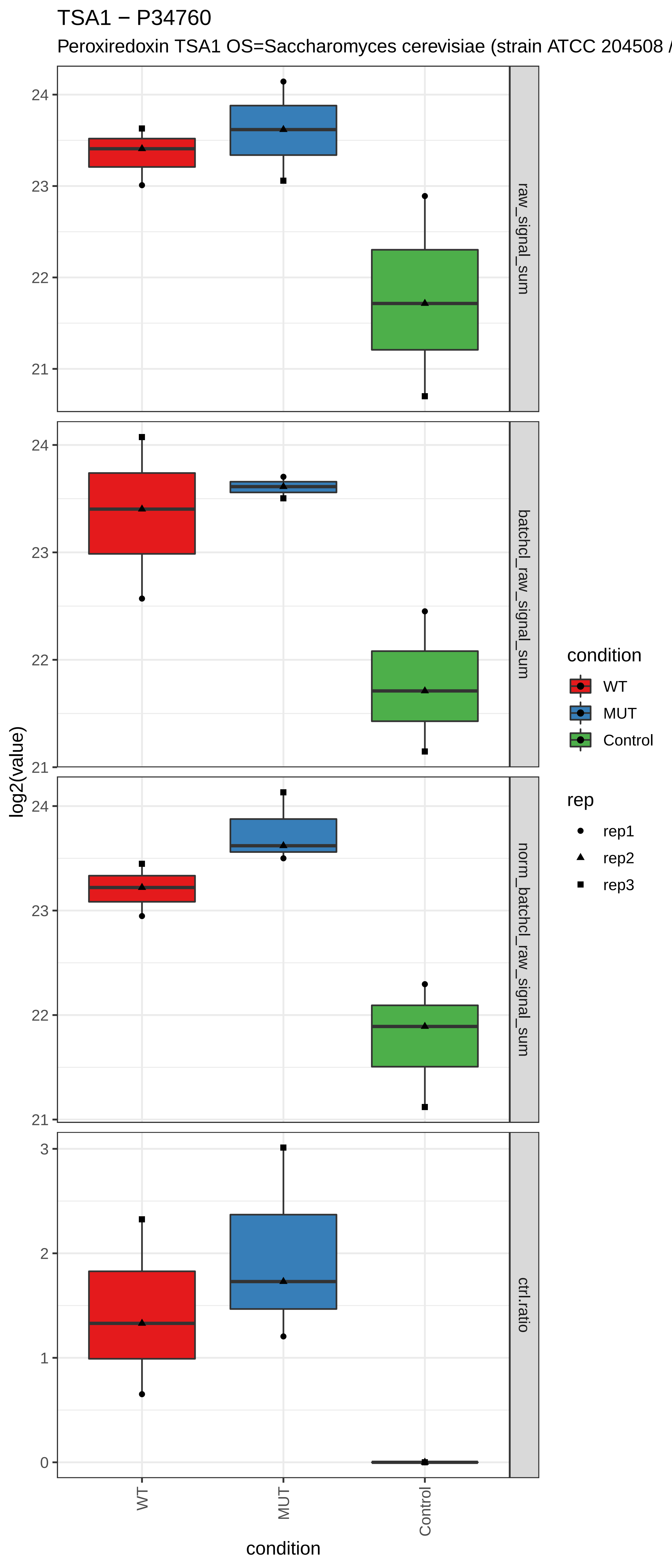

TSA1 − P34760
Peroxiredoxin TSA1 OS=Saccharomyces cerevisiae (strain ATCC 204508 /
raw_signal_sum
batchcl_raw_signal_sum
norm_batchcl_raw_signal_sum
ctrl.ratio
log2(value)
condition
WT
MUT
Control
rep
rep1
rep2
rep3

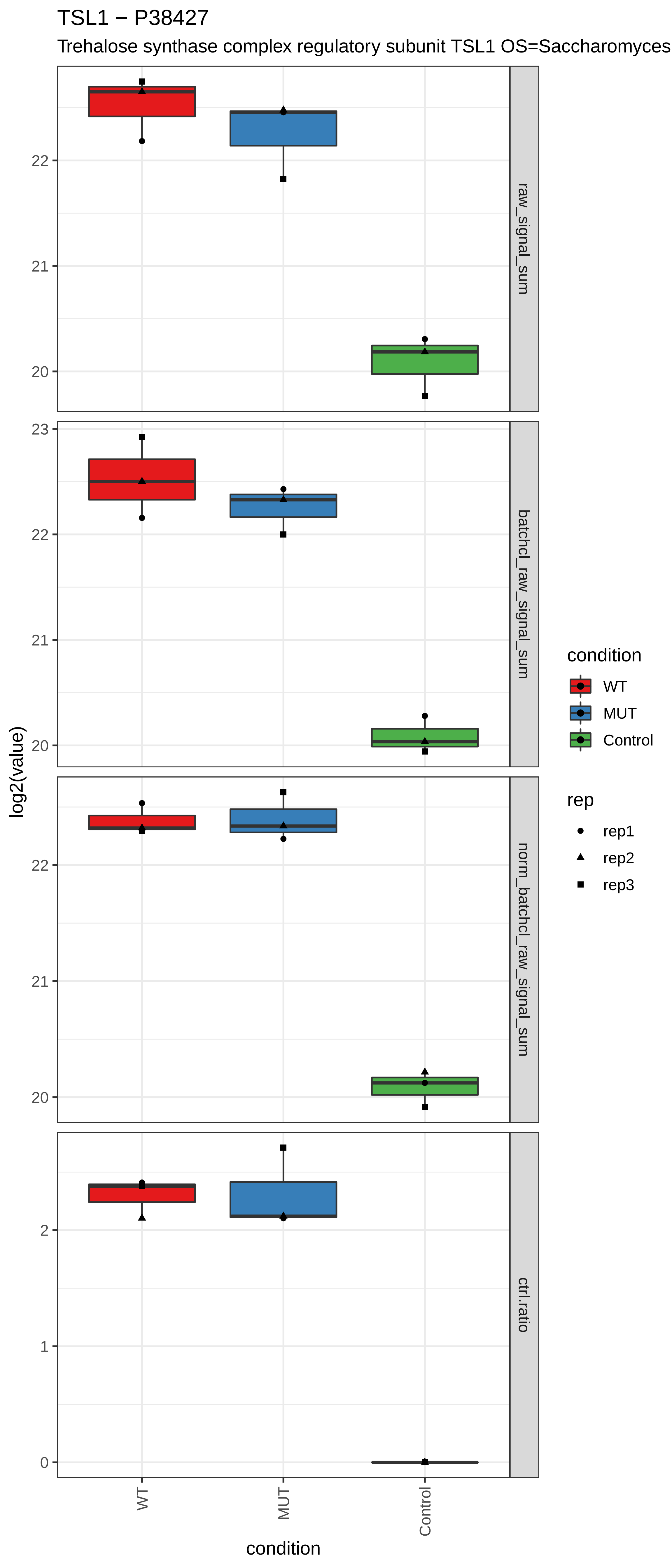

TSL1 − P38427
Trehalose synthase complex regulatory subunit TSL1 OS=Saccharomyces
raw_signal_sum
batchcl_raw_signal_sum
norm_batchcl_raw_signal_sum
ctrl.ratio
log2(value)
condition
WT
MUT
Control
rep
rep1
rep2
rep3

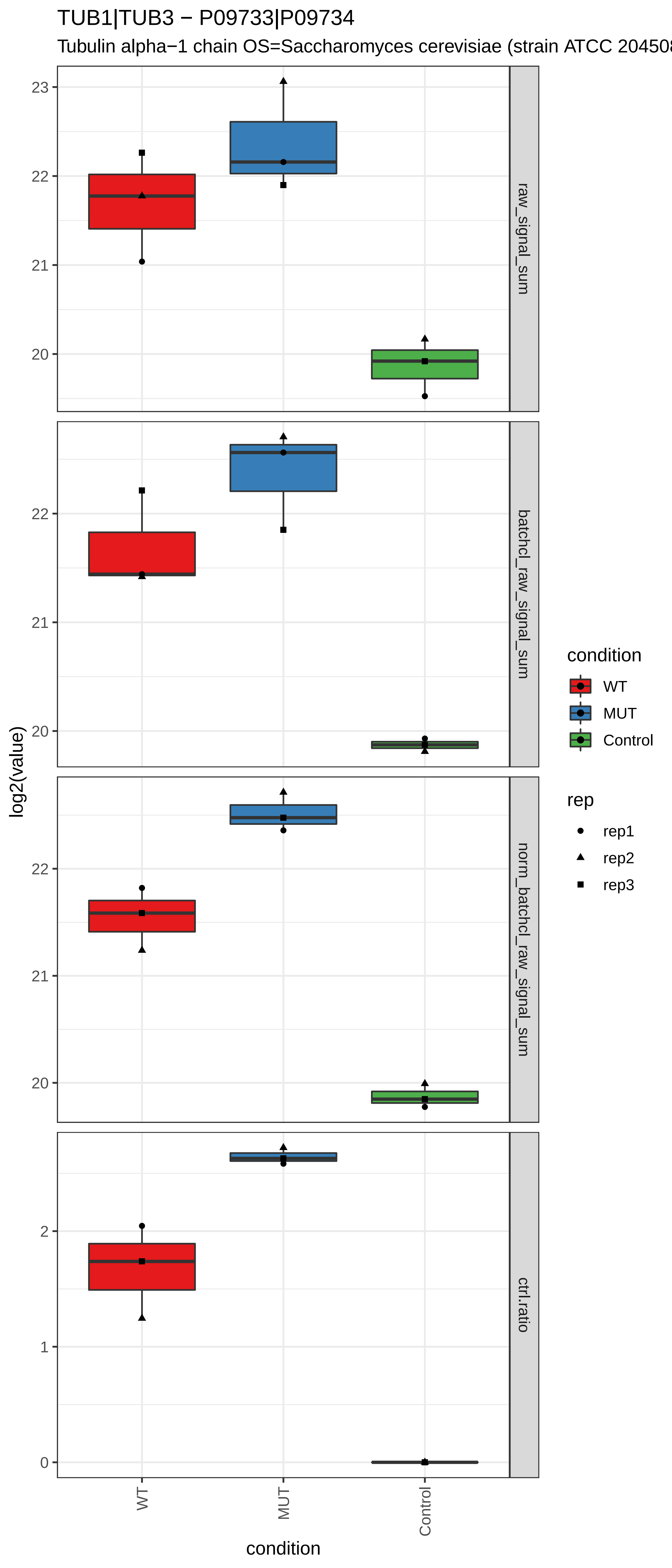

TUB1|TUB3 − P09733|P09734
Tubulin alpha−1 chain OS=Saccharomyces cerevisiae (strain ATCC 204508
raw_signal_sum
batchcl_raw_signal_sum
norm_batchcl_raw_signal_sum
ctrl.ratio
log2(value)
condition
WT
MUT
Control
rep
rep1
rep2
rep3

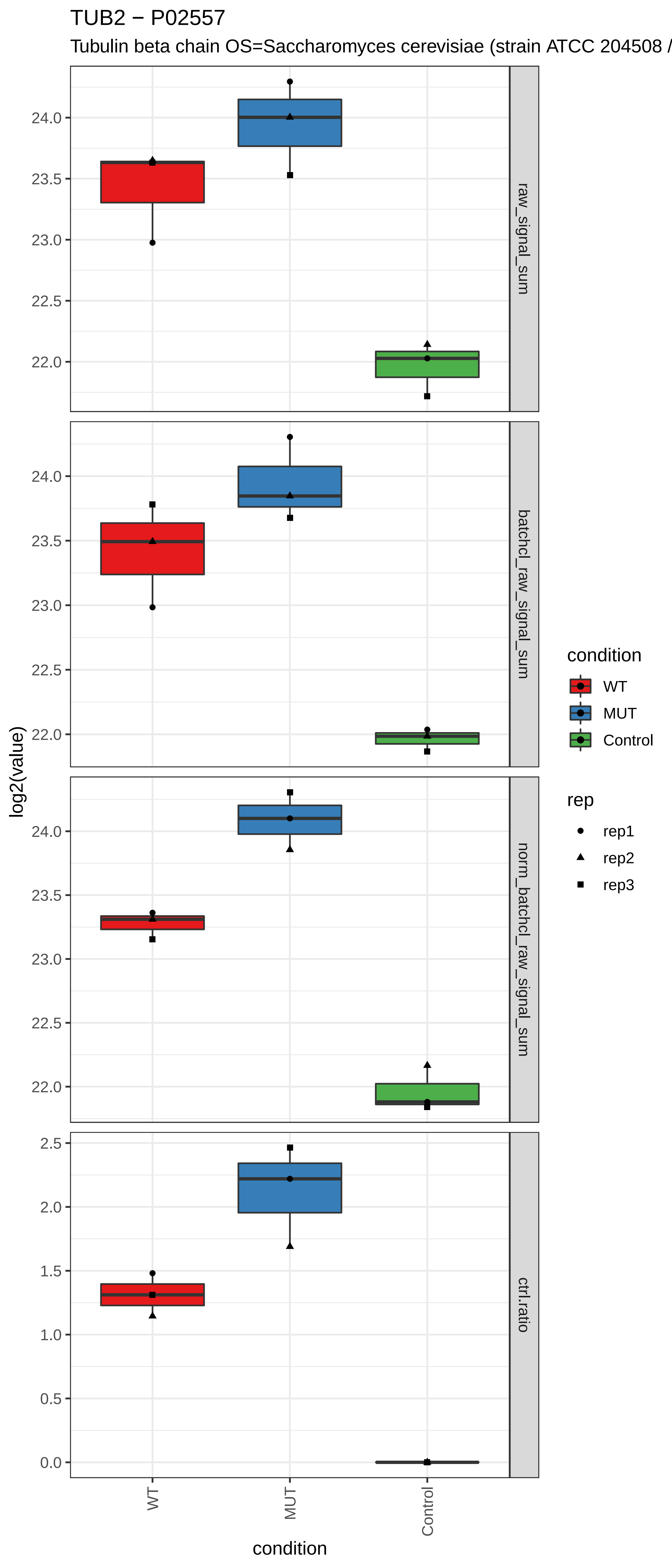

TUB2 − P02557
Tubulin beta chain OS=Saccharomyces cerevisiae (strain ATCC 204508 /
raw_signal_sum
batchcl_raw_signal_sum
norm_batchcl_raw_signal_sum
ctrl.ratio
log2(value)
condition
WT
MUT
Control
rep
rep1
rep2
rep3
24.0
23.5
23.0
22.5
22.0
2.5
2.0
1.5
1.0
0.5
0.0

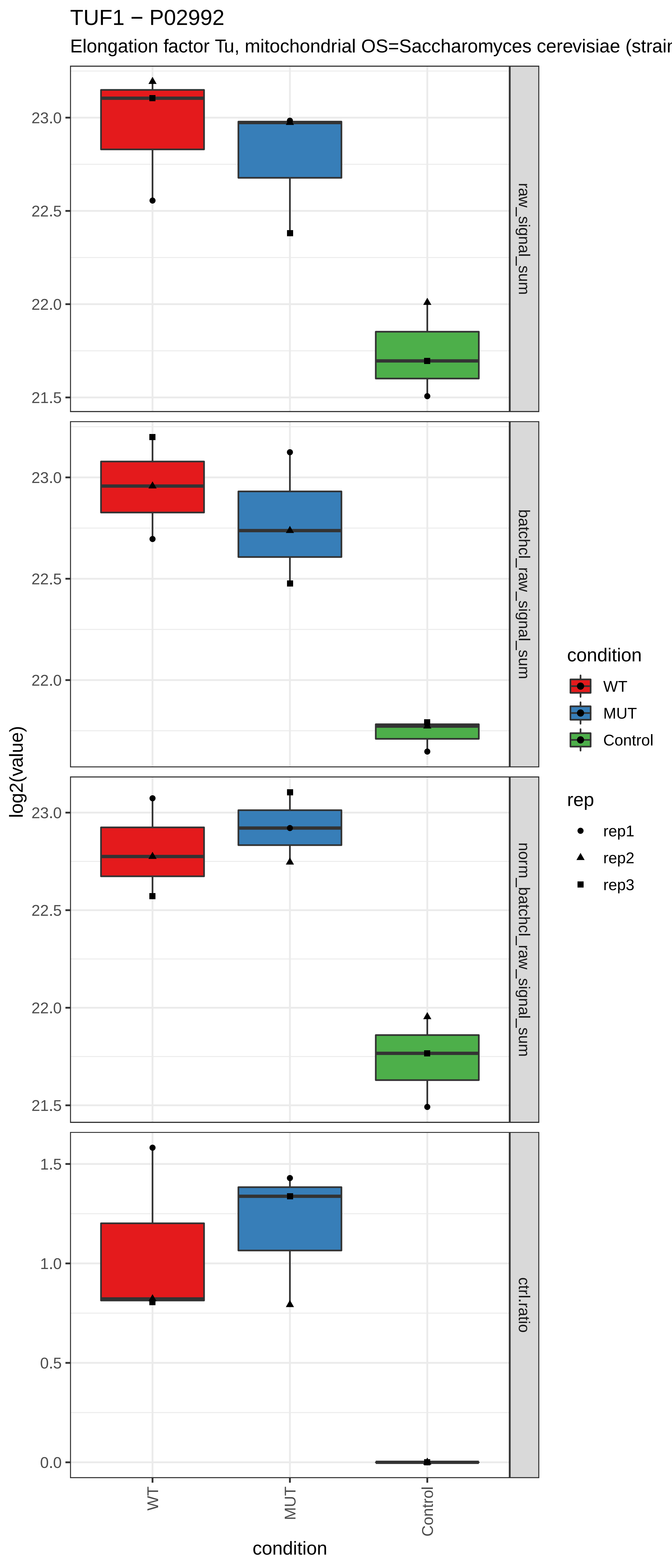

TUF1 − P02992
Elongation factor Tu, mitochondrial OS=Saccharomyces cerevisiae (strain
raw_signal_sum
batchcl_raw_signal_sum
norm_batchcl_raw_signal_sum
ctrl.ratio
log2(value)
condition
WT
MUT
Control
rep
rep1
rep2
rep3
23.0
22.5
22.0
21.5
1.5
1.0
0.5
0.0

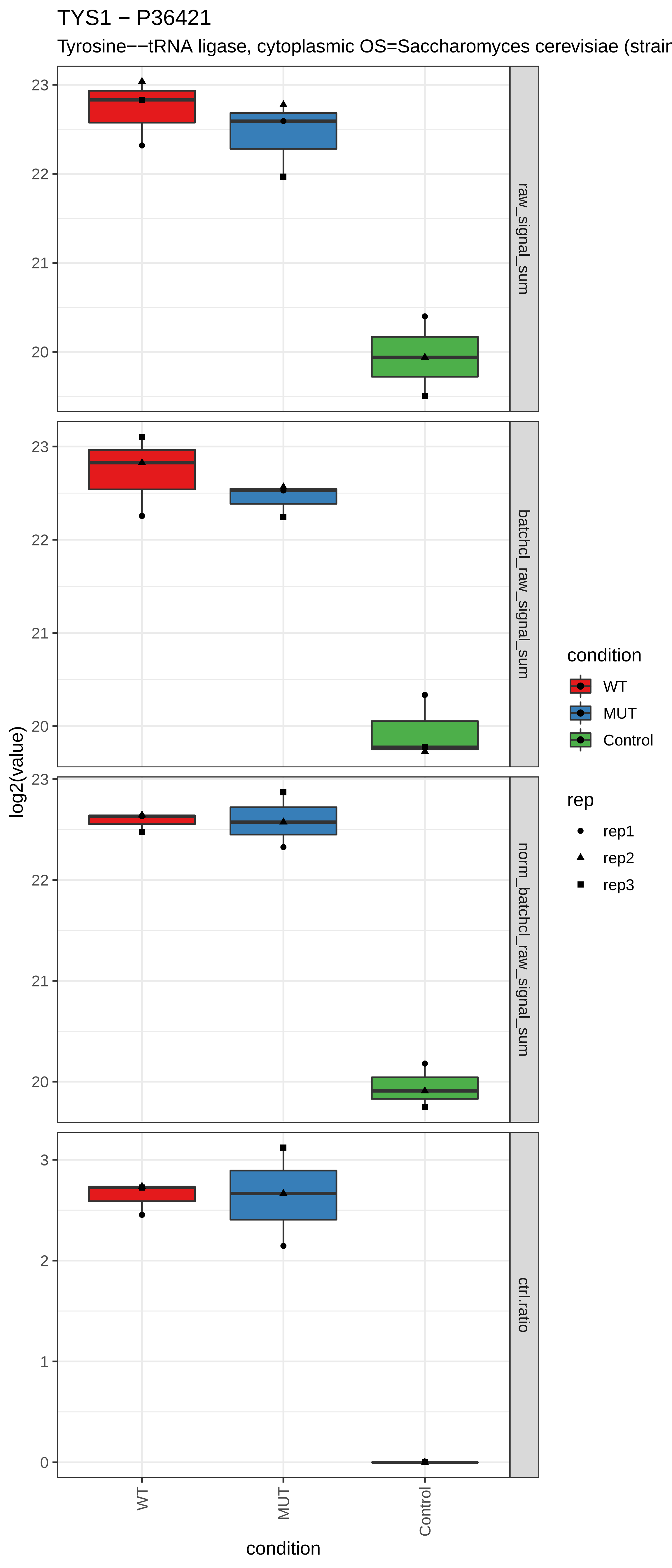

TYS1 − P36421
Tyrosine−−tRNA ligase, cytoplasmic OS=Saccharomyces cerevisiae (strain
raw_signal_sum
batchcl_raw_signal_sum
norm_batchcl_raw_signal_sum
ctrl.ratio
log2(value)
condition
WT
MUT
Control
condition
WT
MUT
Control
rep
rep1
rep2
rep3

UBA1 − P22515

Ubiquitin−activating enzyme E1 1 OS=Saccharomyces cerevisiae (strain A

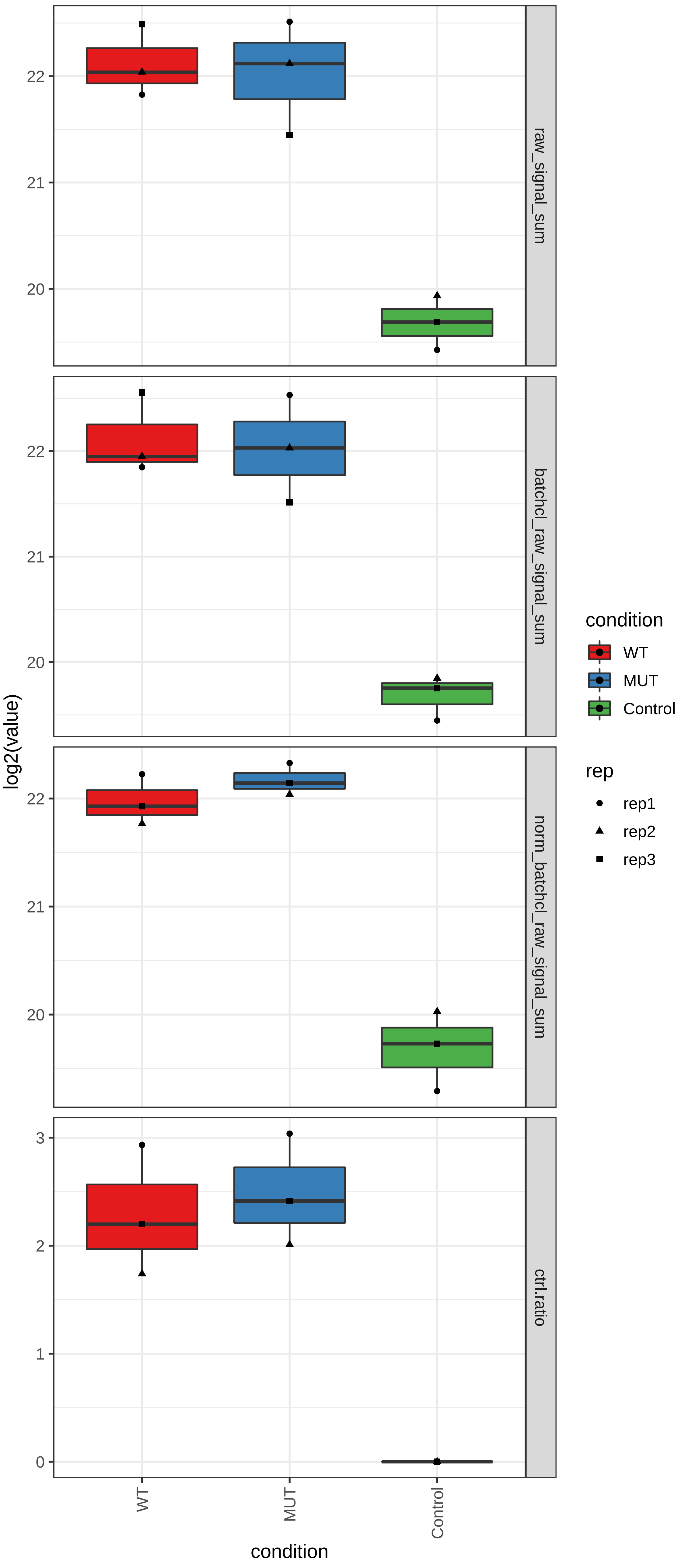

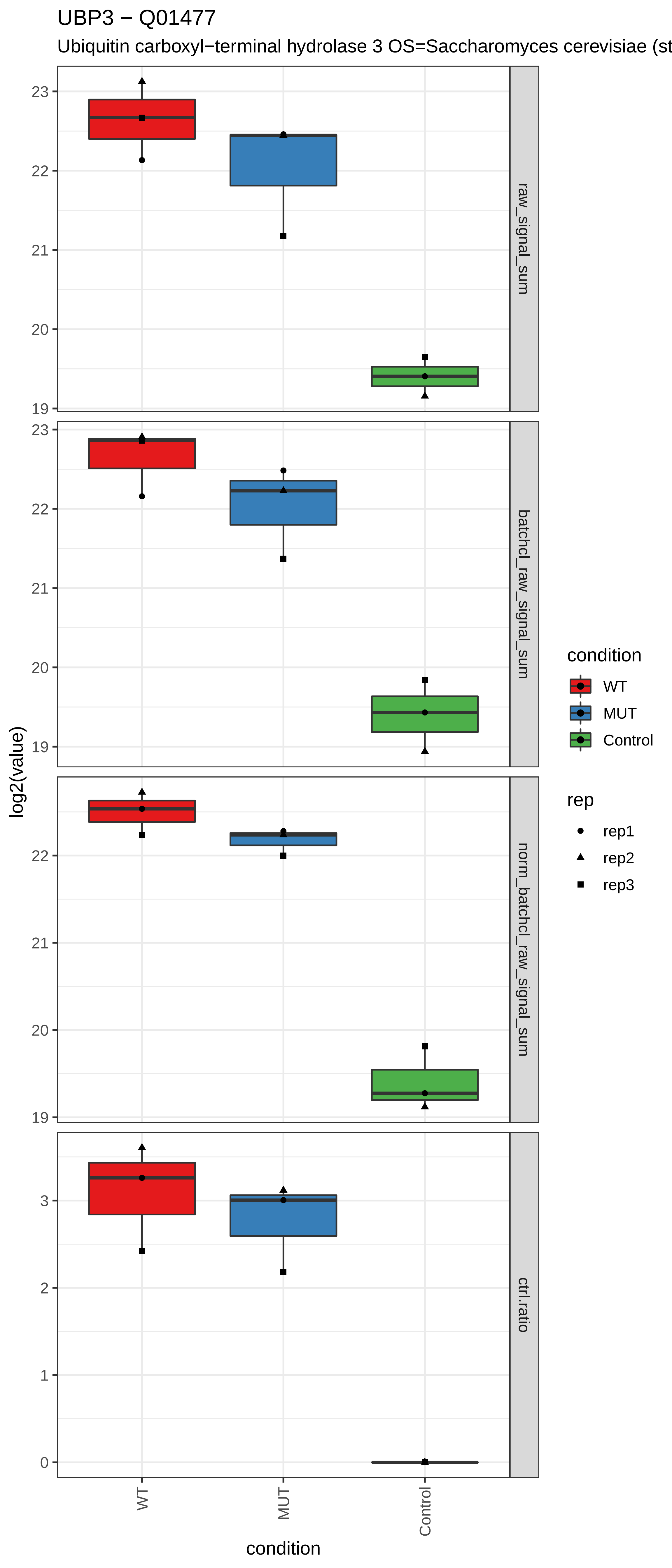

UBP3 − Q01477
Ubiquitin carboxyl−terminal hydrolase 3 OS=Saccharomyces cerevisiae (st
raw_signal_sum
batchcl_raw_signal_sum
norm_batchcl_raw_signal_sum
ctrl.ratio
log2(value)
condition
WT
MUT
Control
rep
rep1
rep2
rep3

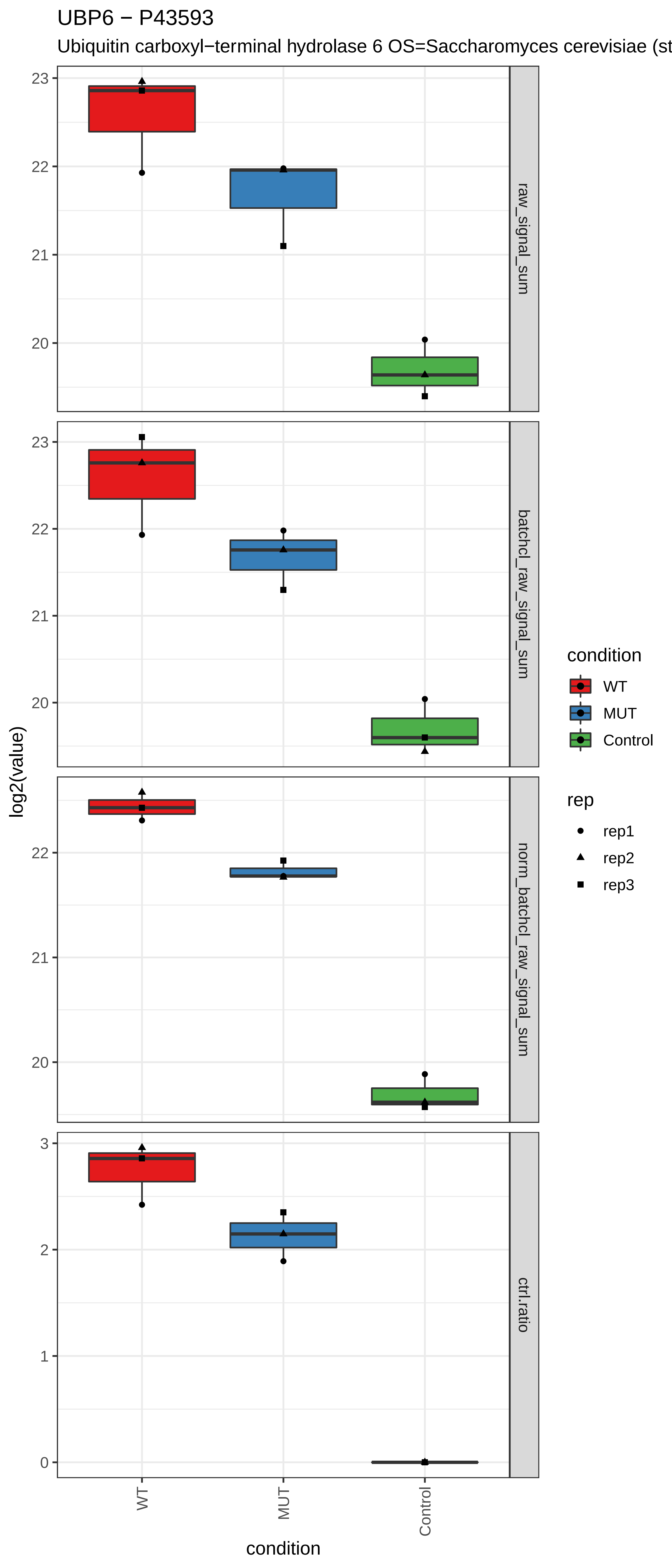

UBP6 − P43593
Ubiquitin carboxyl−terminal hydrolase 6 OS=Saccharomyces cerevisiae (st
raw_signal_sum
batchcl_raw_signal_sum
norm_batchcl_raw_signal_sum
ctrl.ratio
log2(value)
condition
WT
MUT
Control
rep
rep1
rep2
rep3

UBP7 − P40453

Ubiquitin carboxyl−terminal hydrolase 7 OS=Saccharomyces cerevisiae (st

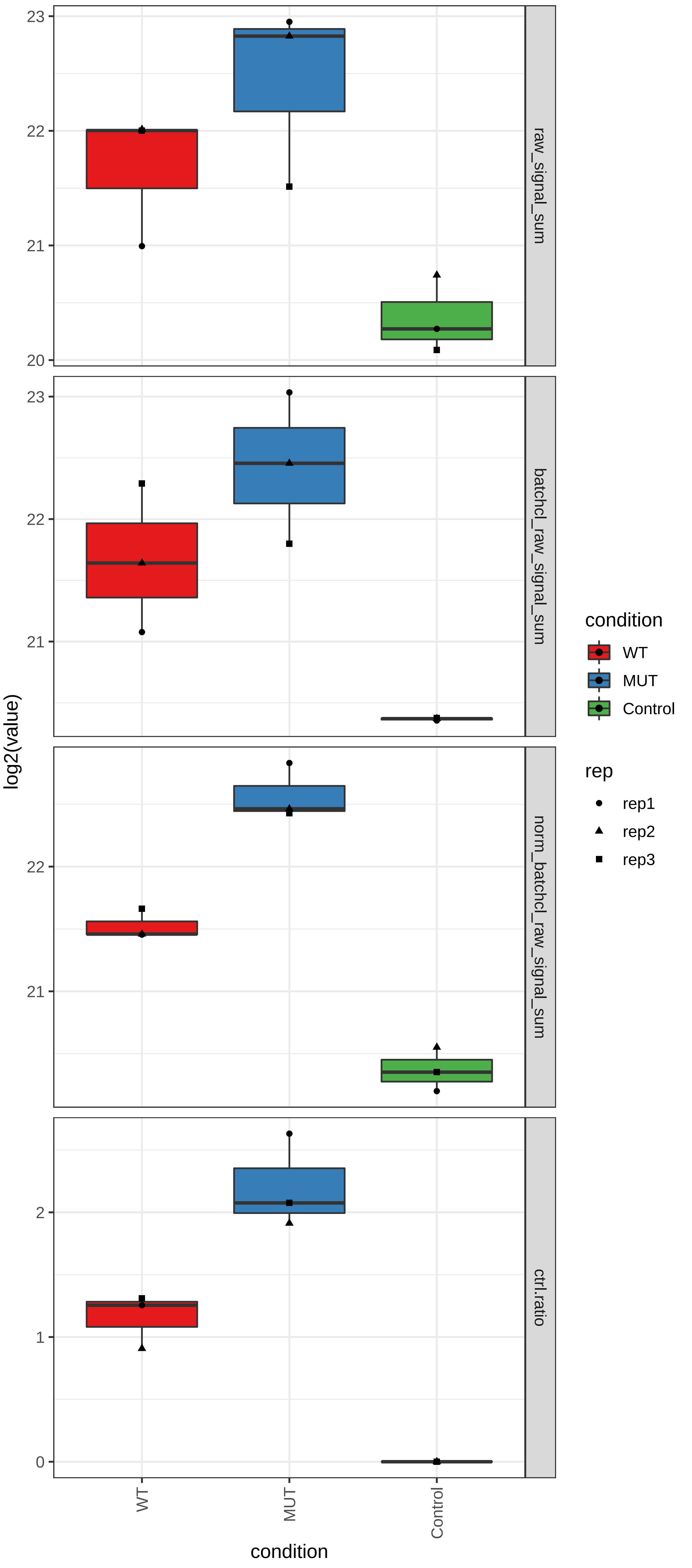

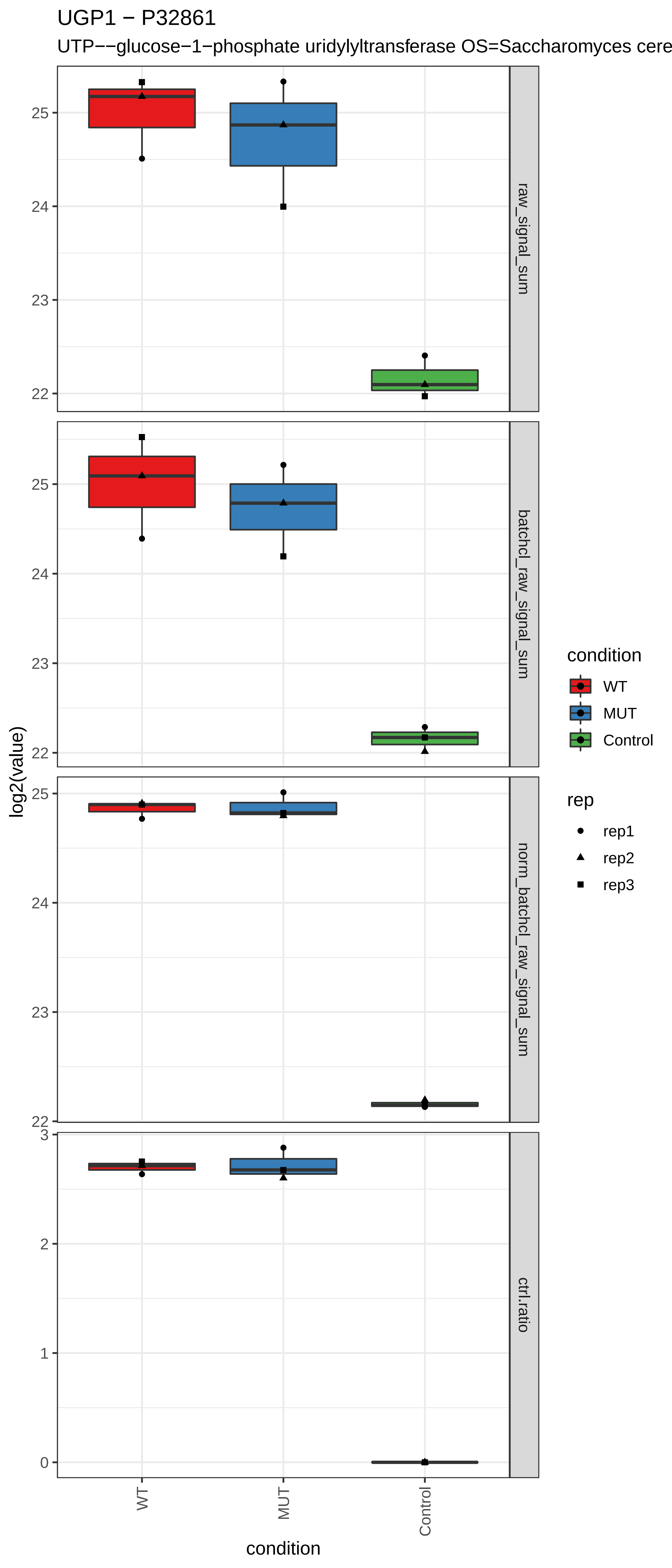
UGP1 − P32861
UTP−−glucose−1−phosphate uridylyltransferase OS=Saccharomyces cere
raw_signal_sum
batchcl_raw_signal_sum
norm_batchcl_raw_signal_sum
ctrl.ratio
log2(value)
condition
WT
MUT
Control
rep
rep1
rep2
rep3
25
24
23
22
3
2
1
0

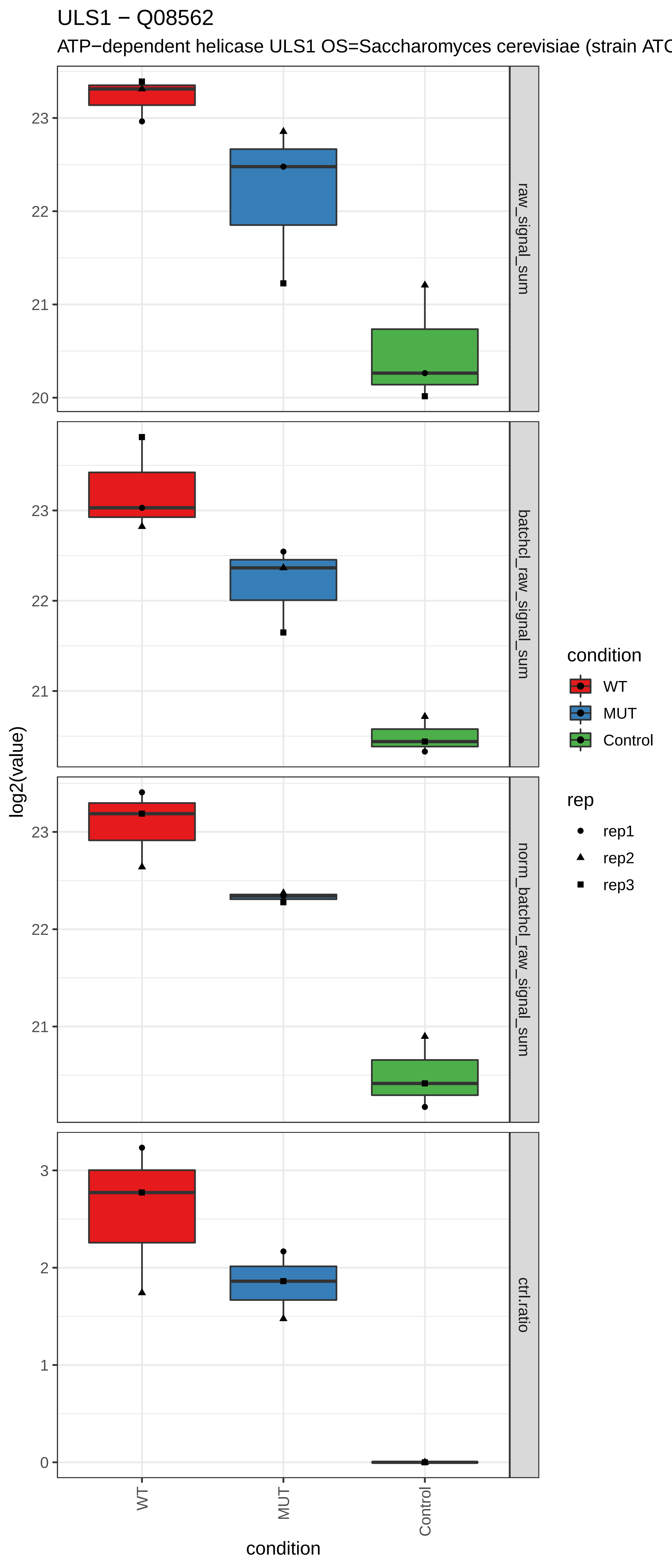

ULS1 − Q08562
ATP−dependent helicase ULS1 OS=Saccharomyces cerevisiae (strain ATC
raw_signal_sum
batchcl_raw_signal_sum
norm_batchcl_raw_signal_sum
ctrl.ratio
log2(value)
condition
WT
MUT
Control
rep
rep1
rep2
rep3

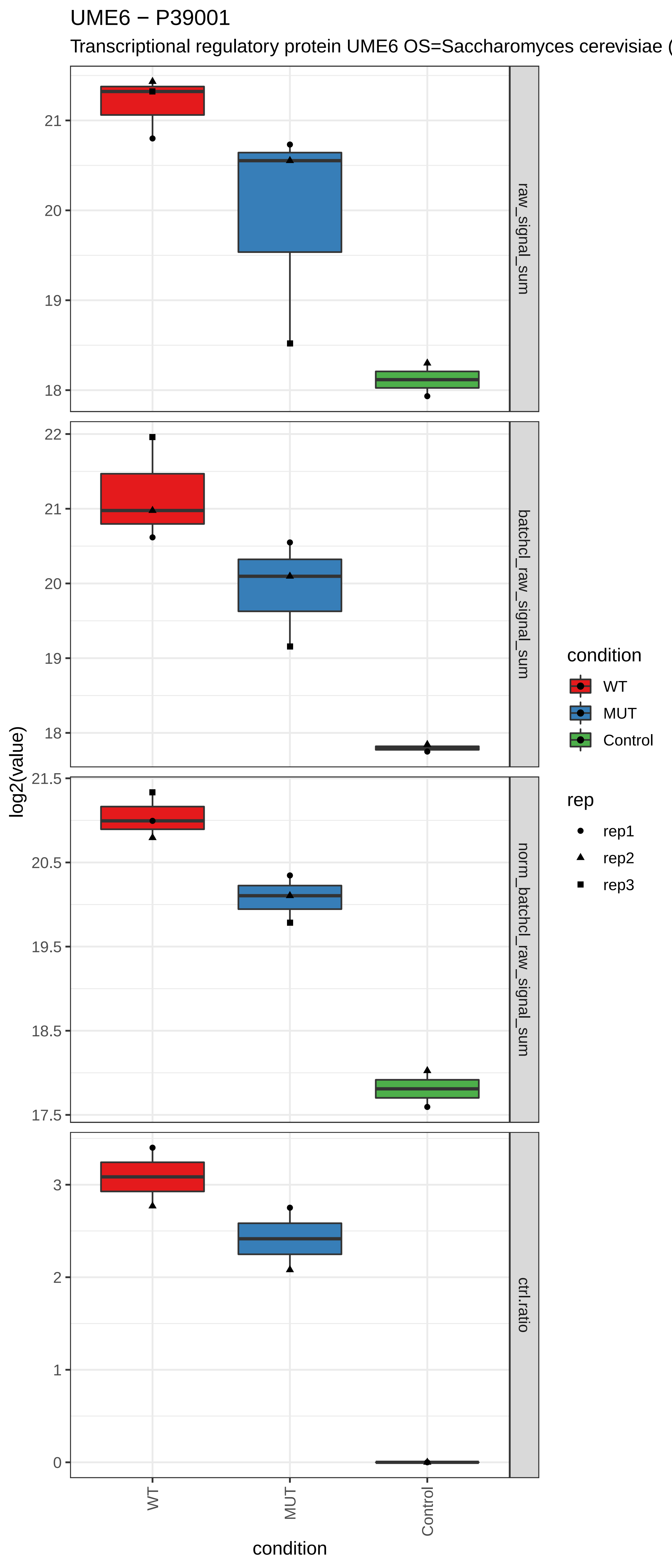

UME6 − P39001
Transcriptional regulatory protein UME6 OS=Saccharomyces cerevisiae (
raw_signal_sum
batchcl_raw_signal_sum
norm_batchcl_raw_signal_sum
ctrl.ratio
log2(value)
condition
WT
MUT
Control
rep
rep1
rep2
rep3
21
20
19
18
22
21.5
20.5
19.5
18.5
17.5
3
2
1
0

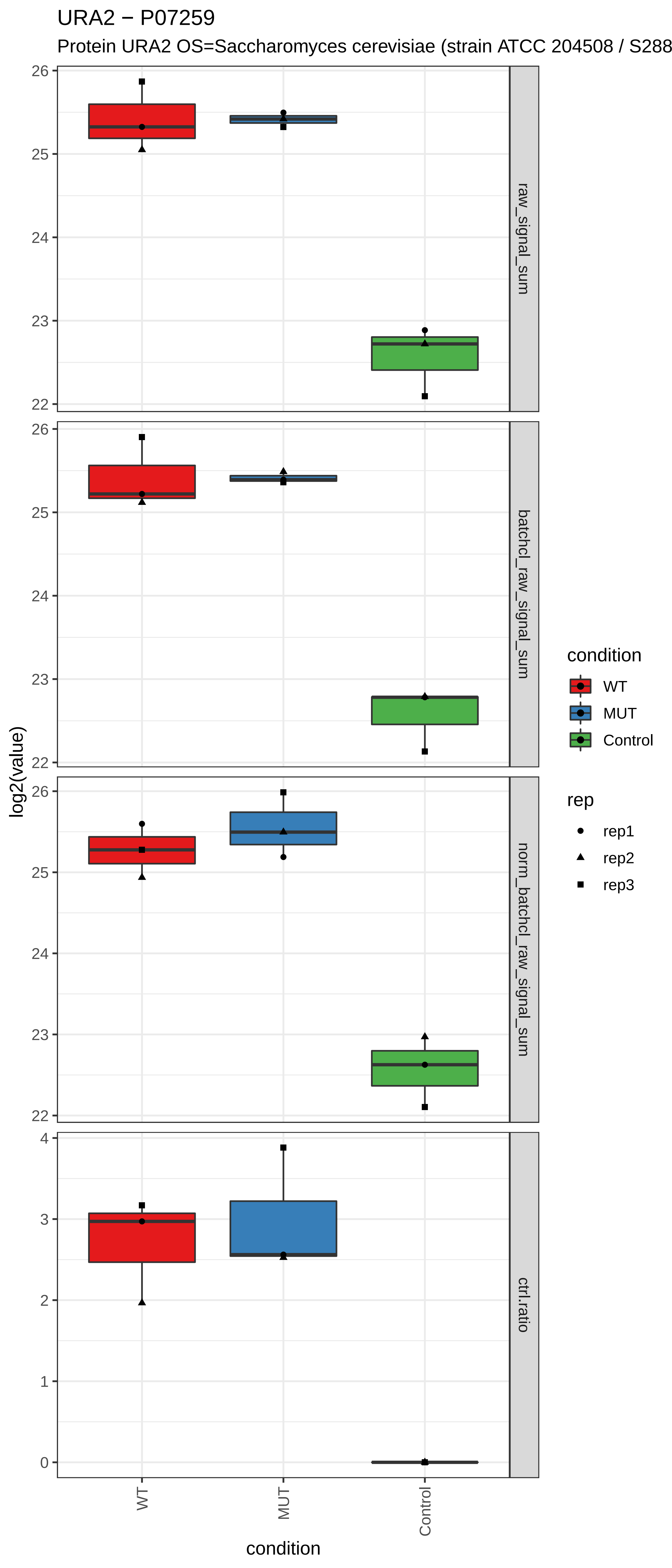

URA2 − P07259
Protein URA2 OS=Saccharomyces cerevisiae (strain ATCC 204508 / S288
raw_signal_sum
batchcl_raw_signal_sum
norm_batchcl_raw_signal_sum
ctrl.ratio
log2(value)
condition
WT
MUT
Control
rep
rep1
rep2
rep3

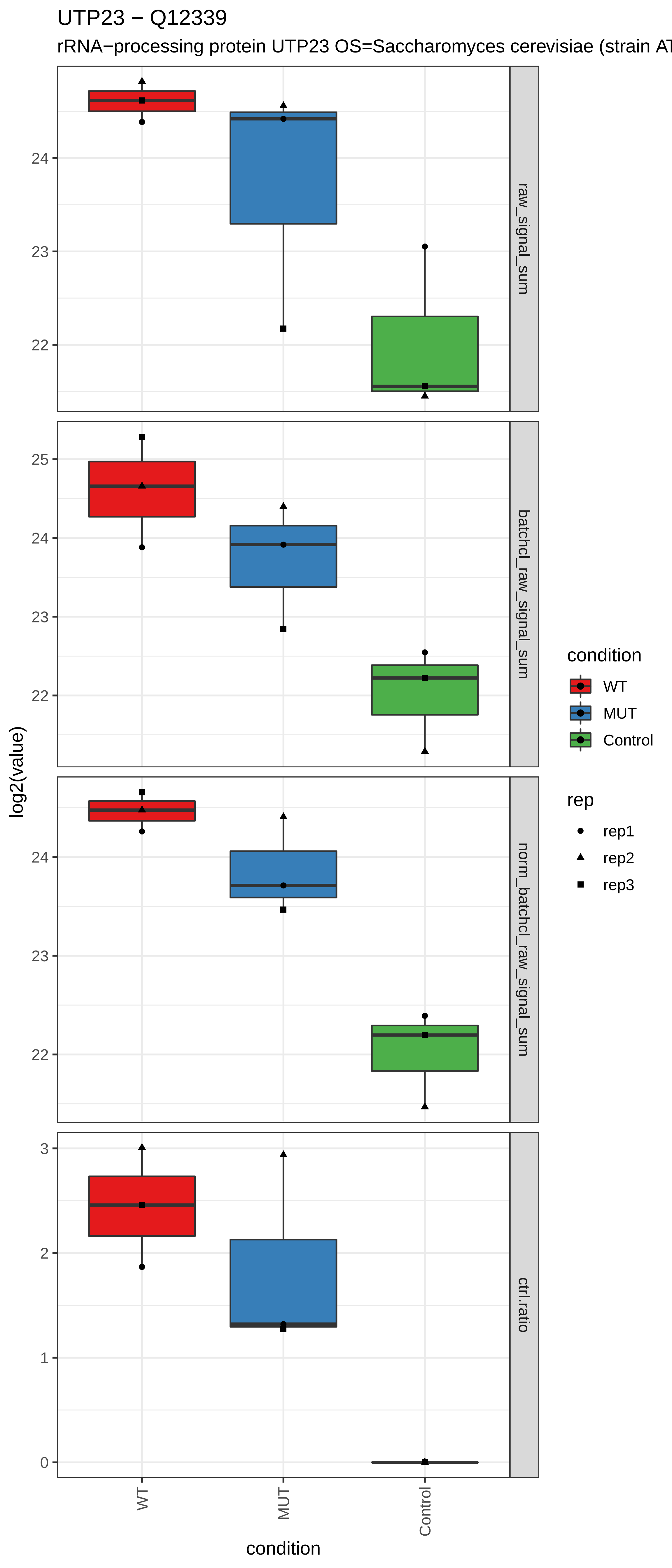

UTP23 − Q12339
rRNA−processing protein UTP23 OS=Saccharomyces cerevisiae (strain AT
raw_signal_sum
batchcl_raw_signal_sum
norm_batchcl_raw_signal_sum
ctrl.ratio
log2(value)
condition
WT
MUT
Control
rep
rep1
rep2
rep3

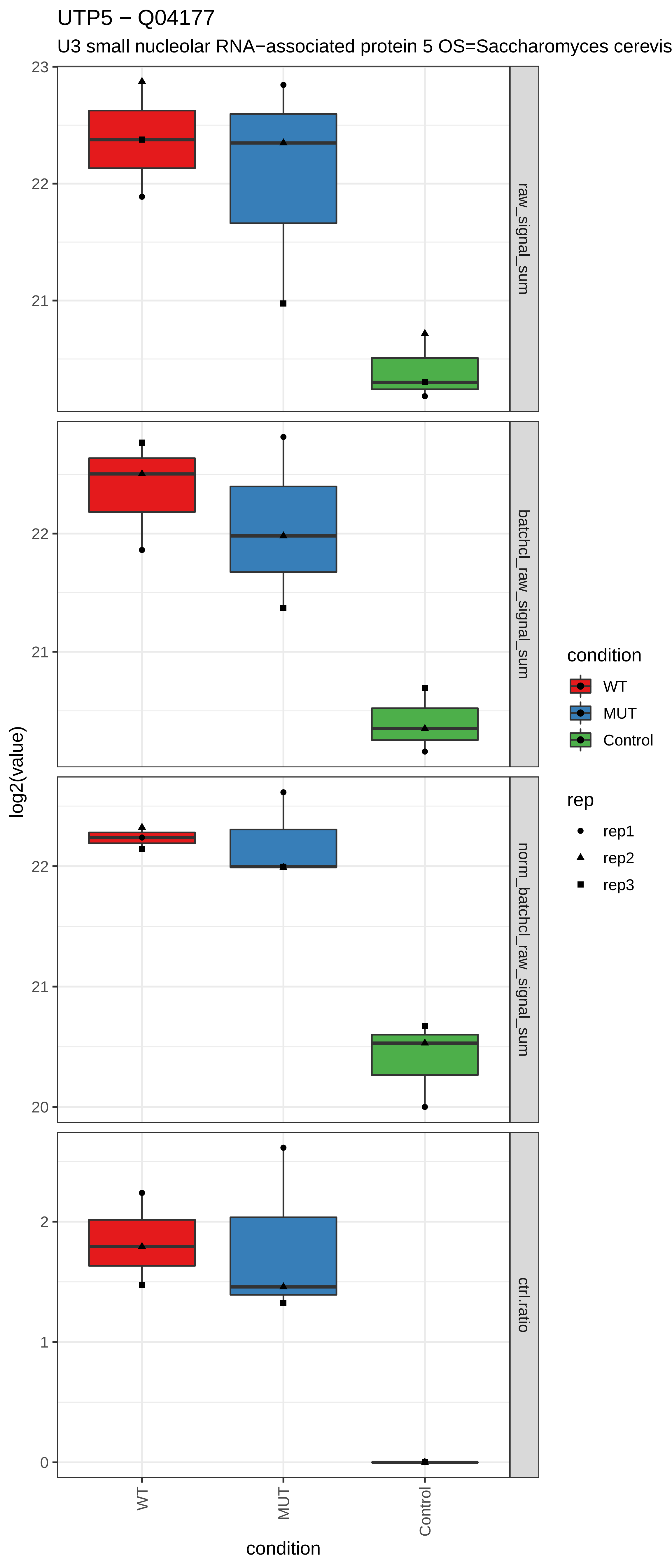

UTP5 − Q04177
U3 small nucleolar RNA−associated protein 5 OS=Saccharomyces cerevis
raw_signal_sum
batchcl_raw_signal_sum
norm_batchcl_raw_signal_sum
ctrl.ratio
log2(value)
condition
WT
MUT
Control
rep
rep1
rep2
rep3
23
22
21
20
2
1
0

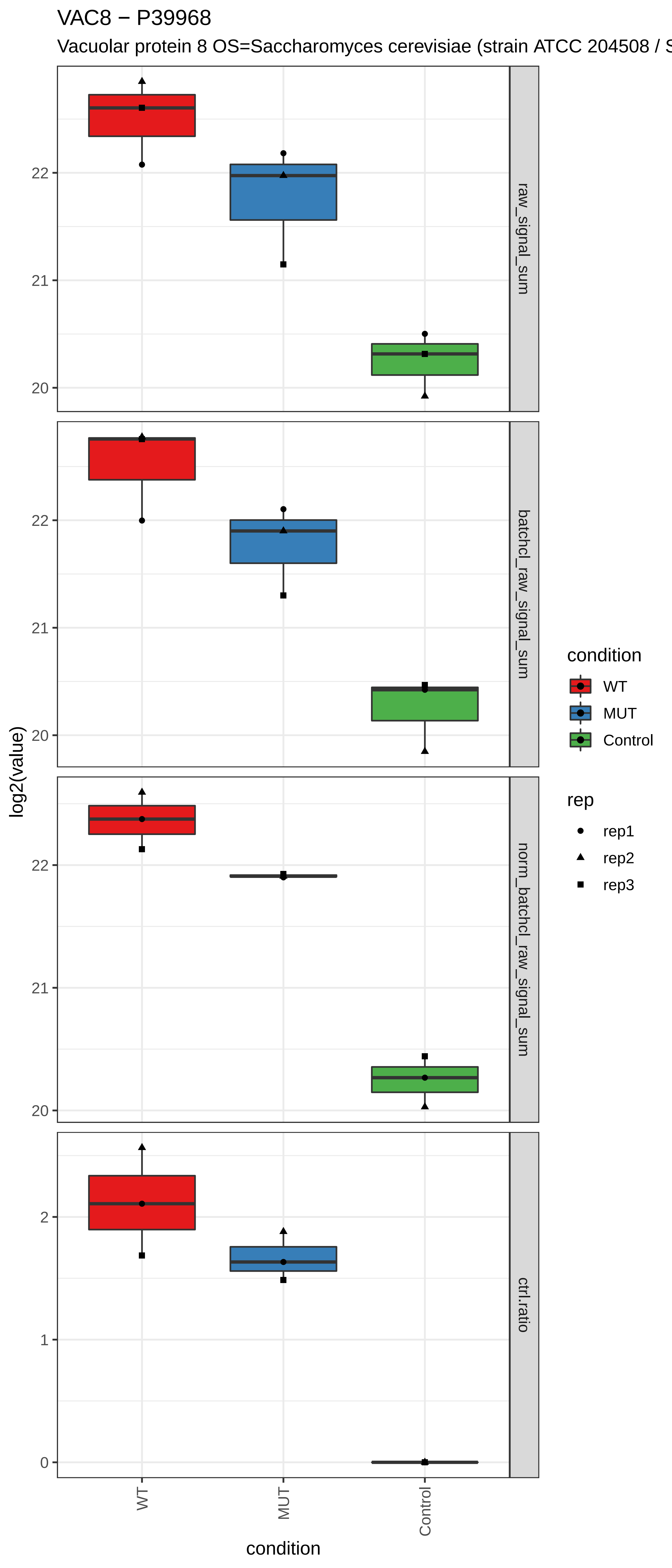
VAC8 − P39968
Vacuolar protein 8 OS=Saccharomyces cerevisiae (strain ATCC 204508 / S
raw_signal_sum
batchcl_raw_signal_sum
norm_batchcl_raw_signal_sum
ctrl.ratio
log2(value)
condition
WT
MUT
Control
rep
rep1
rep2
rep3
22
21
20
2
1
0

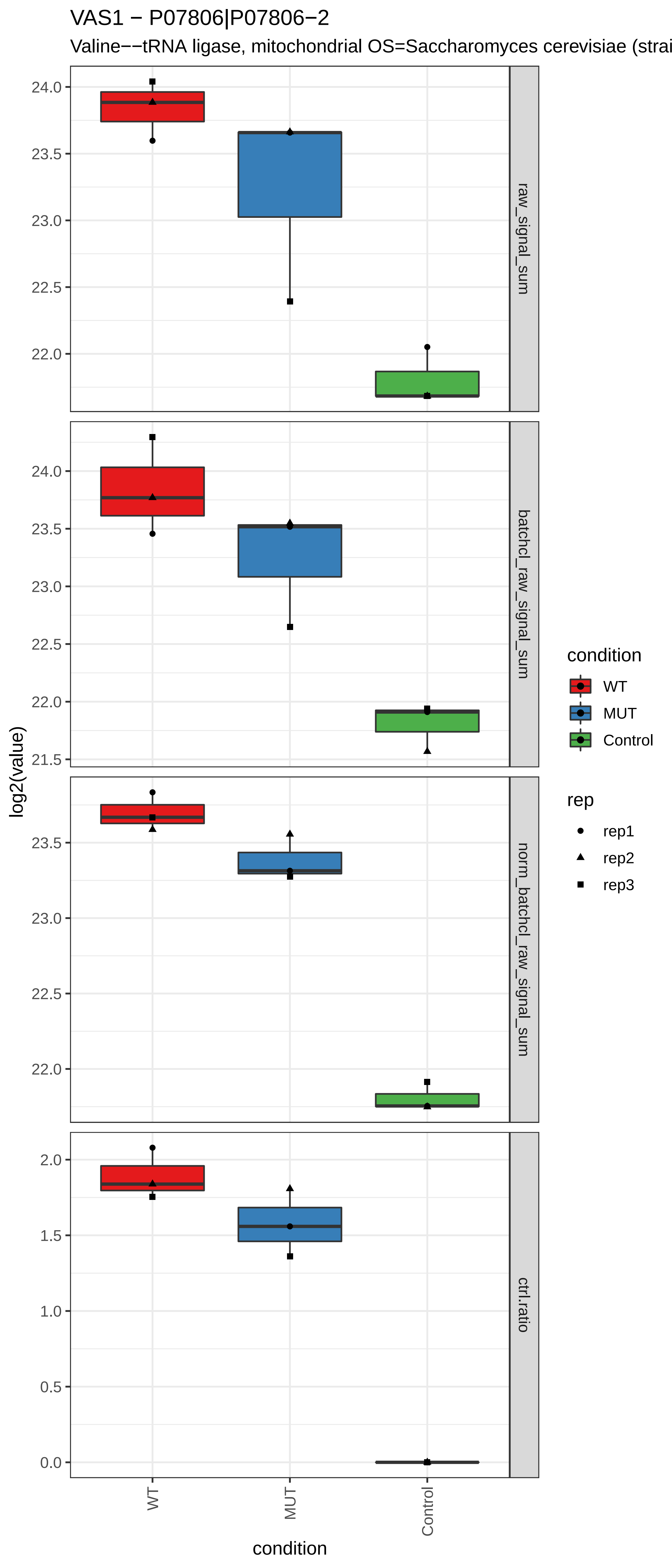

VAS1 − P07806|P07806−2
Valine−−tRNA ligase, mitochondrial OS=Saccharomyces cerevisiae (strai
raw_signal_sum
batchcl_raw_signal_sum
norm_batchcl_raw_signal_sum
ctrl.ratio
log2(value)
condition
WT
MUT
Control
rep
rep1
rep2
rep3

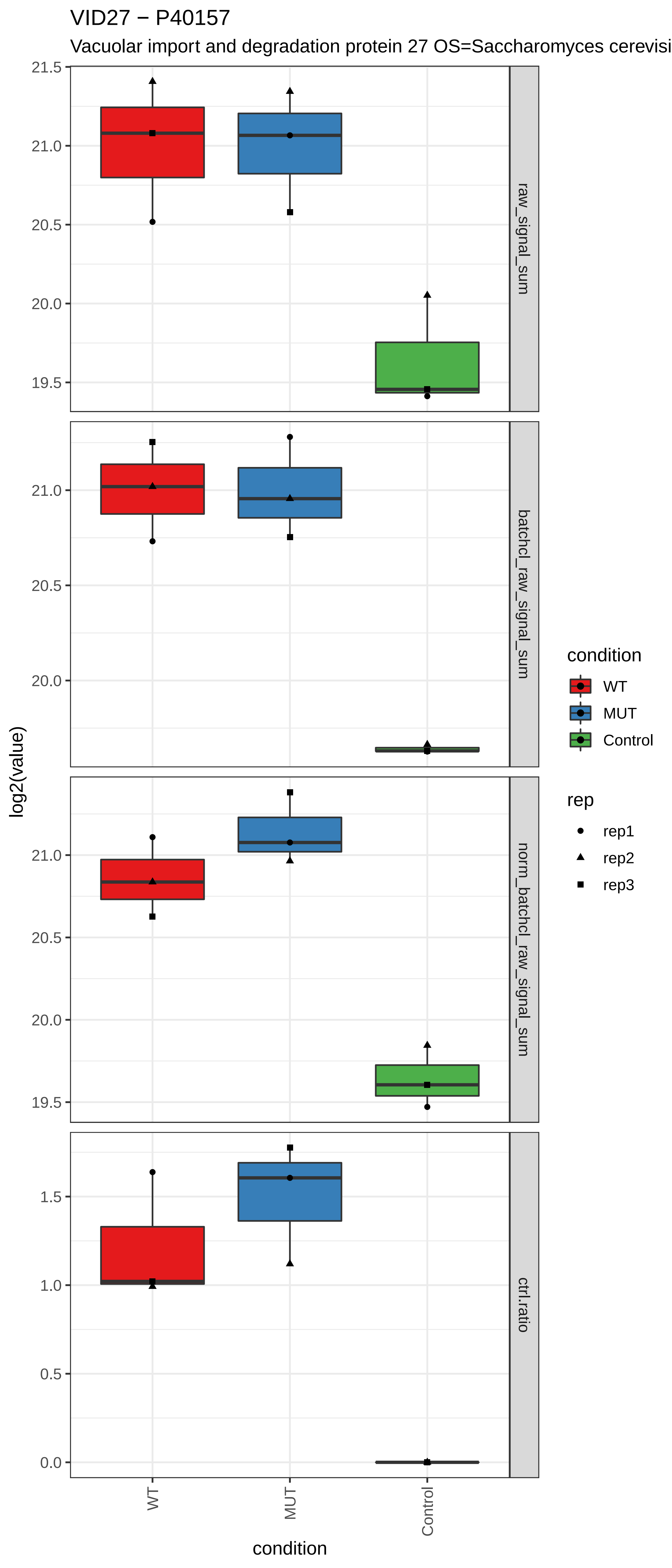

VID27 − P40157
Vacuolar import and degradation protein 27 OS=Saccharomyces cerevisi
raw_signal_sum
batchcl_raw_signal_sum
norm_batchcl_raw_signal_sum
ctrl.ratio
21.5
21.0
20.5
20.0
19.5
1.5
1.0
0.5
0.0
log2(value)
WT
MUT
Control
condition
rep
rep1
rep2
rep3

VMA1 − P17255

V−type proton ATPase catalytic subunit A OS=Saccharomyces cerevisiae (

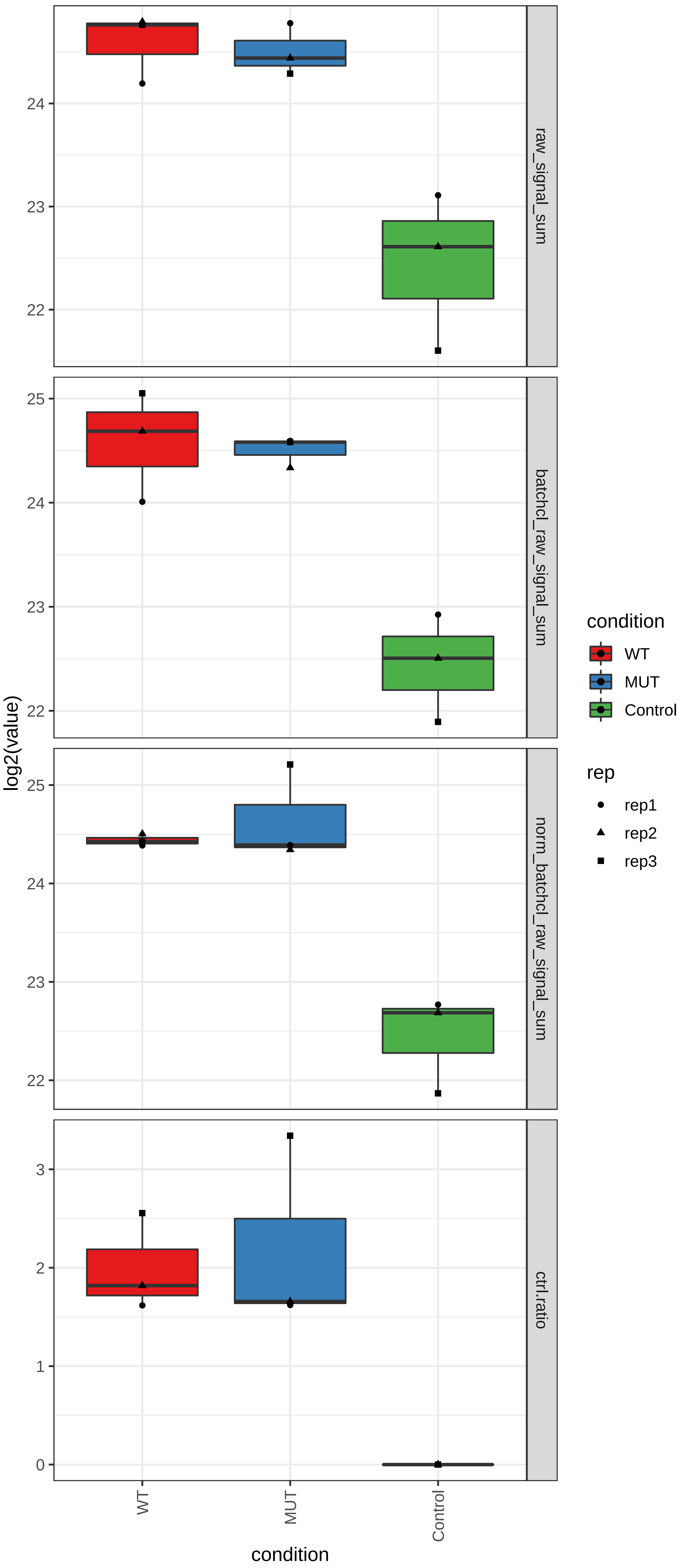

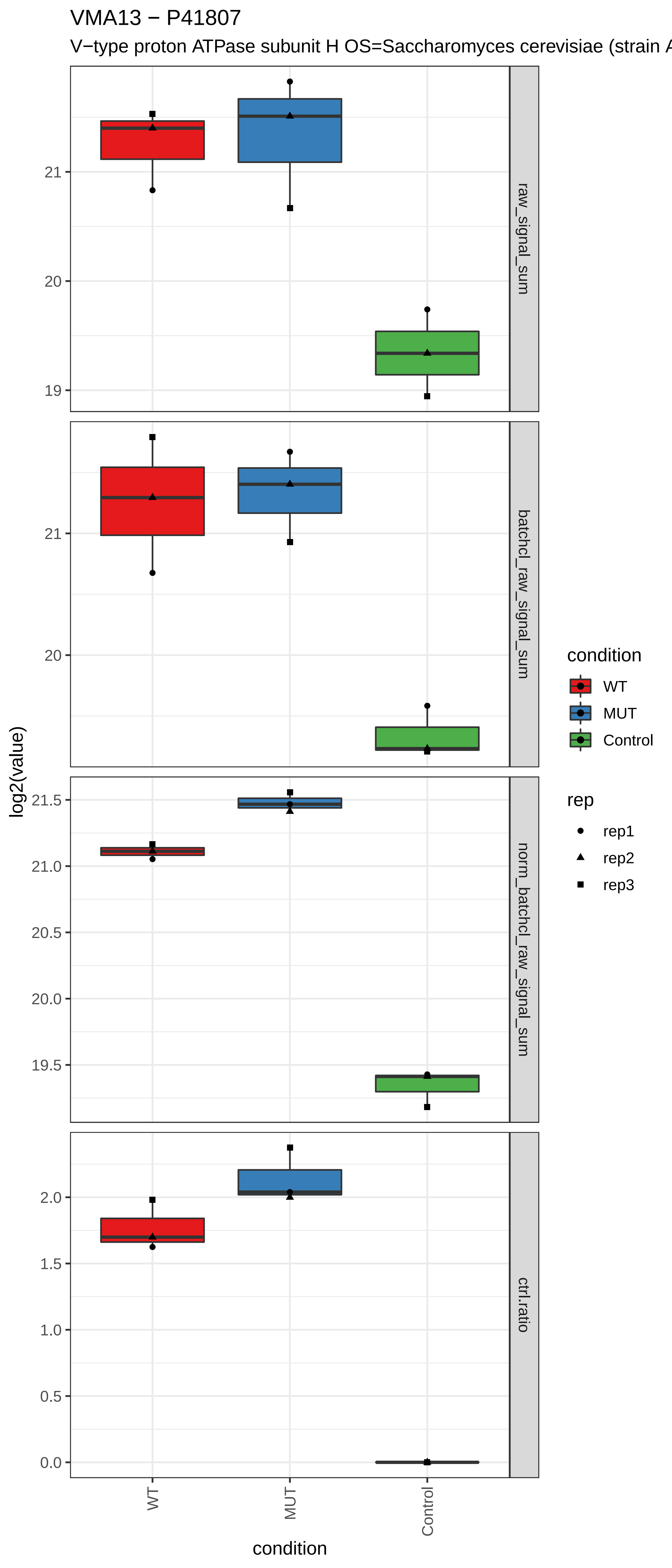

VMA13 − P41807
V−type proton ATPase subunit H OS=Saccharomyces cerevisiae (strain A
raw_signal_sum
batchcl_raw_signal_sum
norm_batchcl_raw_signal_sum
ctrl.ratio
log2(value)
condition
WT
MUT
Control
rep
rep1
rep2
rep3

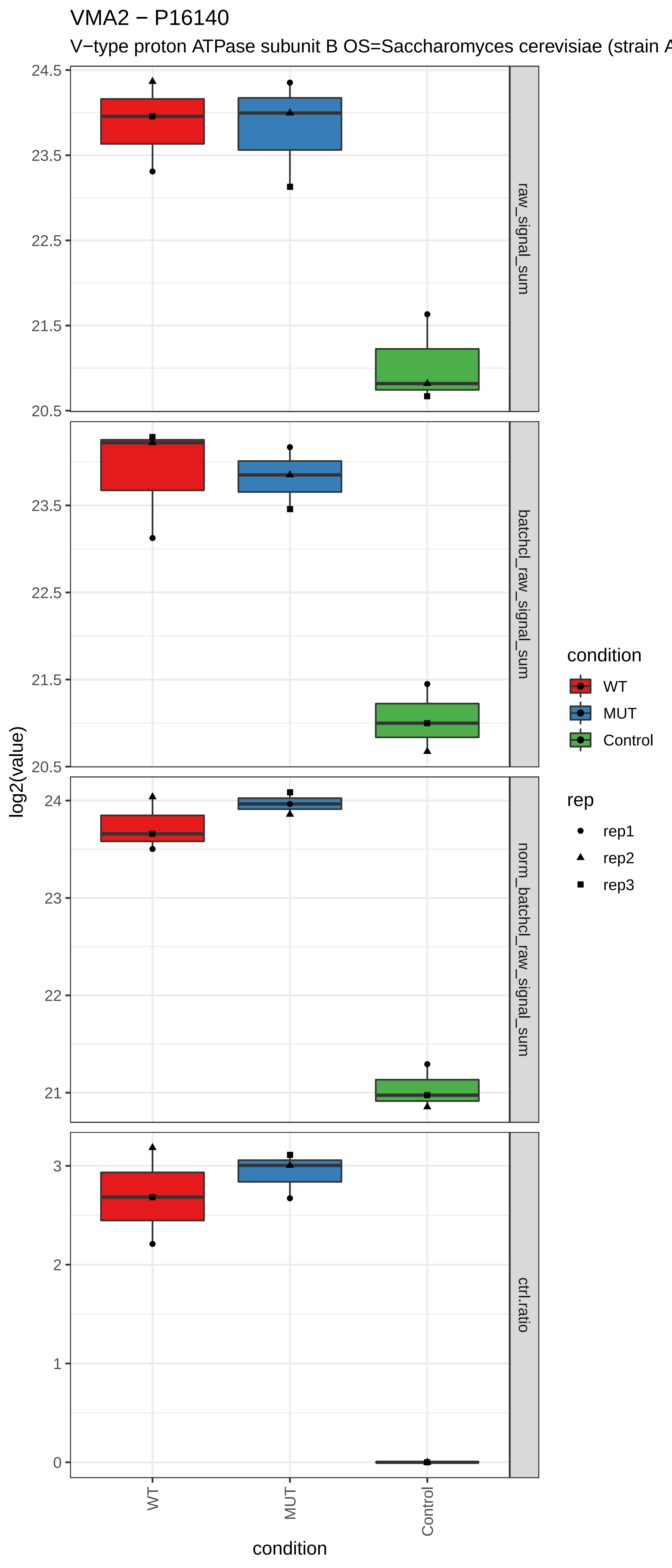
VMA2 − P16140
V−type proton ATPase subunit B OS=Saccharomyces cerevisiae (strain A
raw_signal_sum
batchcl_raw_signal_sum
norm_batchcl_raw_signal_sum
ctrl.ratio
log2(value)
condition
WT
MUT
Control
rep
rep1
rep2
rep3
24.5
23.5
22.5
21.5
20.5
24
23
22
21
3
2
1
0

VPS1 − P21576

Vacuolar protein sorting−associated protein 1 OS=Saccharomyces cerevis

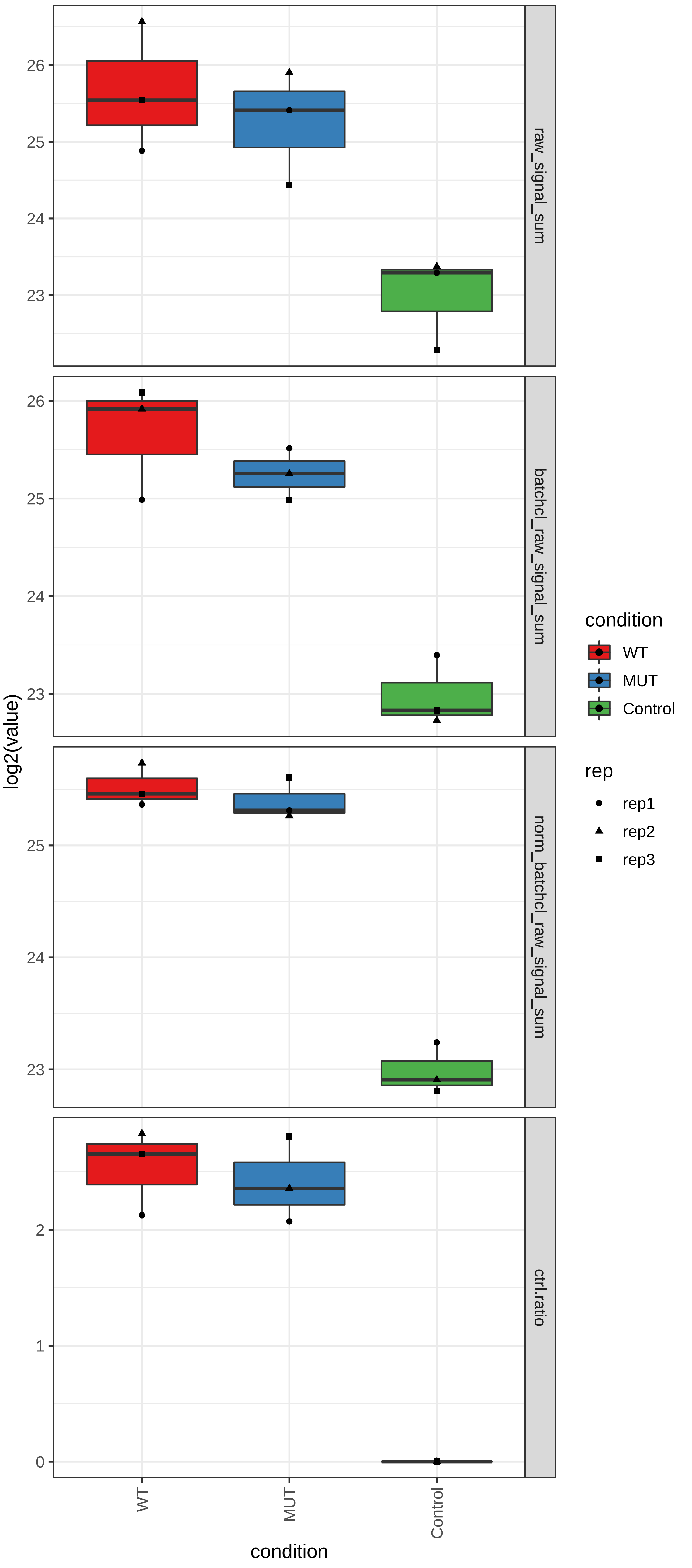

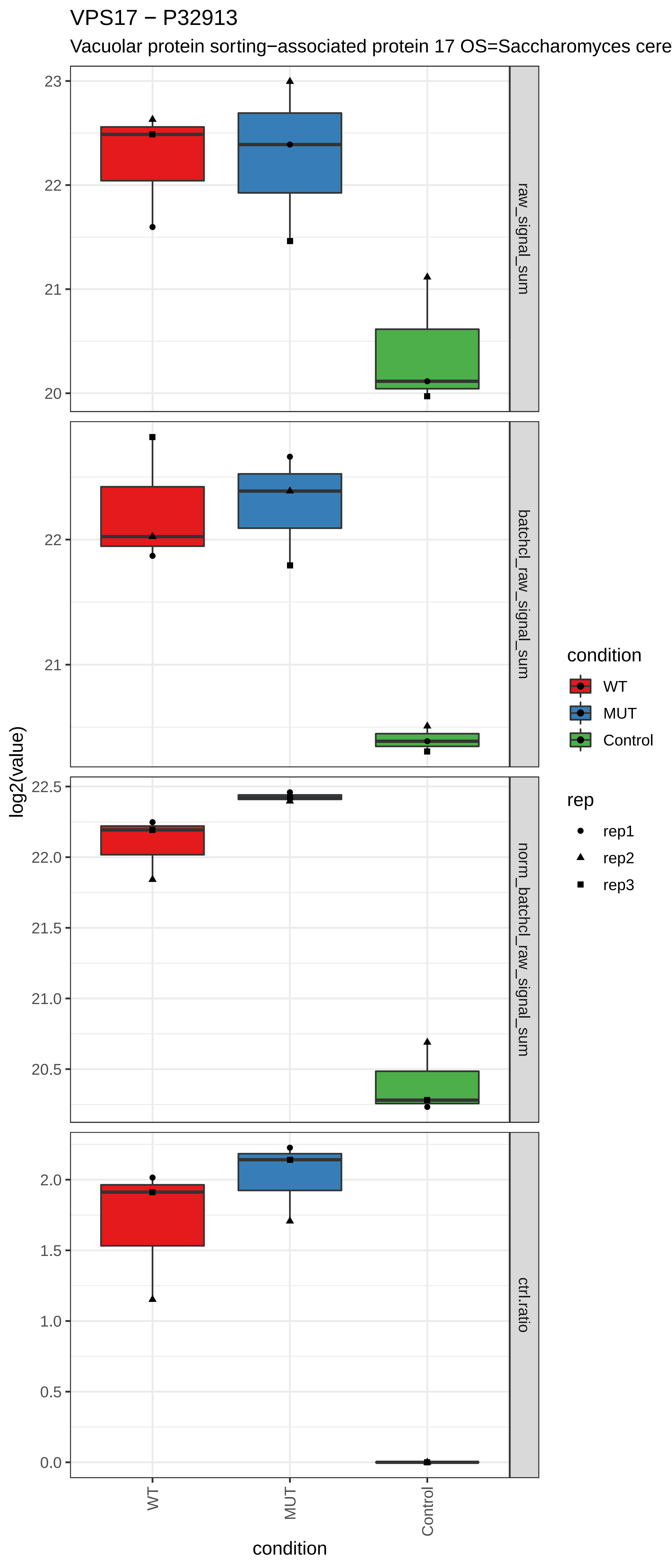
VPS17 − P32913
Vacuolar protein sorting−associated protein 17 OS=Saccharomyces cere
log2(value)
condition
raw_signal_sum
batchcl_raw_signal_sum
norm_batchcl_raw_signal_sum
ctrl.ratio
WT
MUT
Control
rep
rep1
rep2
rep3
23
22
21
20
22.5
22.0
21.5
21.0
20.5
2.0
1.5
1.0
0.5
0.0

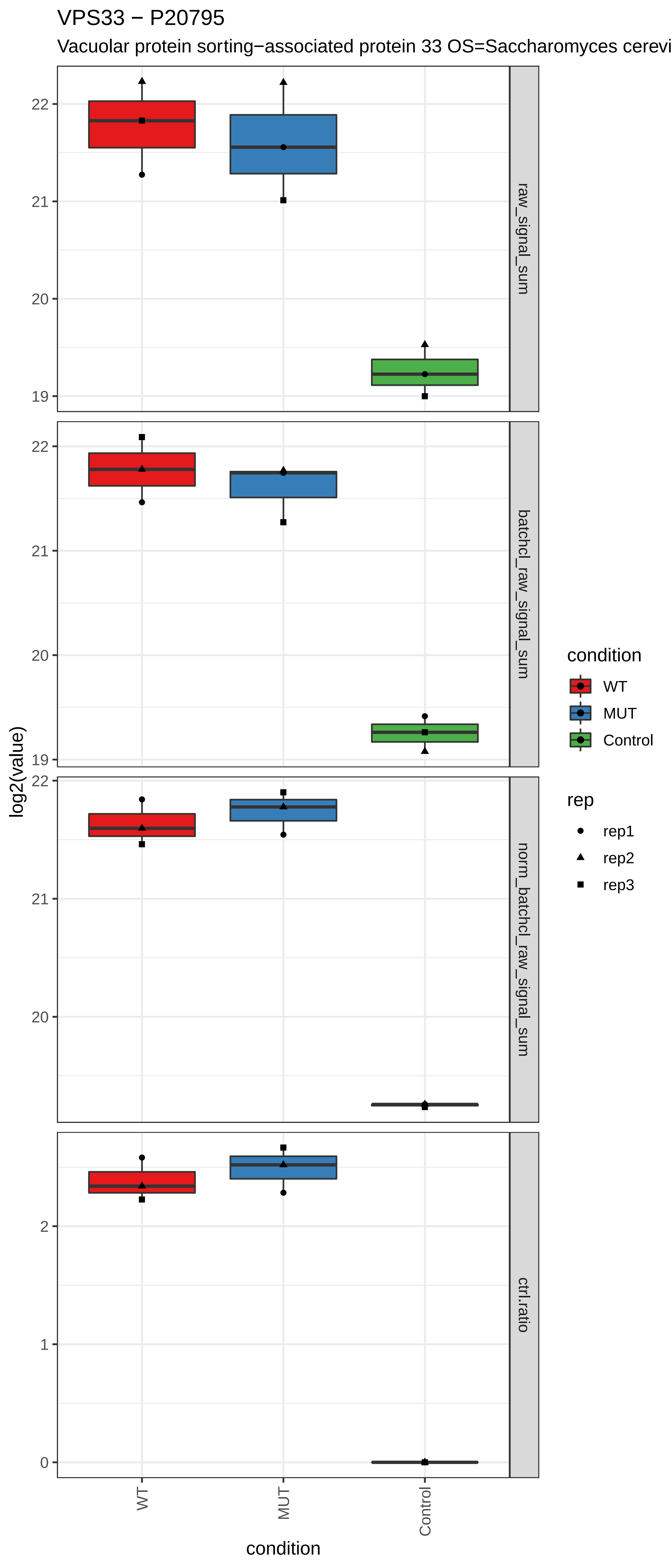
VPS33 − P20795
Vacuolar protein sorting−associated protein 33 OS=Saccharomyces cerevi
raw_signal_sum
batchcl_raw_signal_sum
norm_batchcl_raw_signal_sum
ctrl.ratio
log2(value)
condition
WT
MUT
Control
rep
rep1
rep2
rep3
22
21
20
19
2
1
0

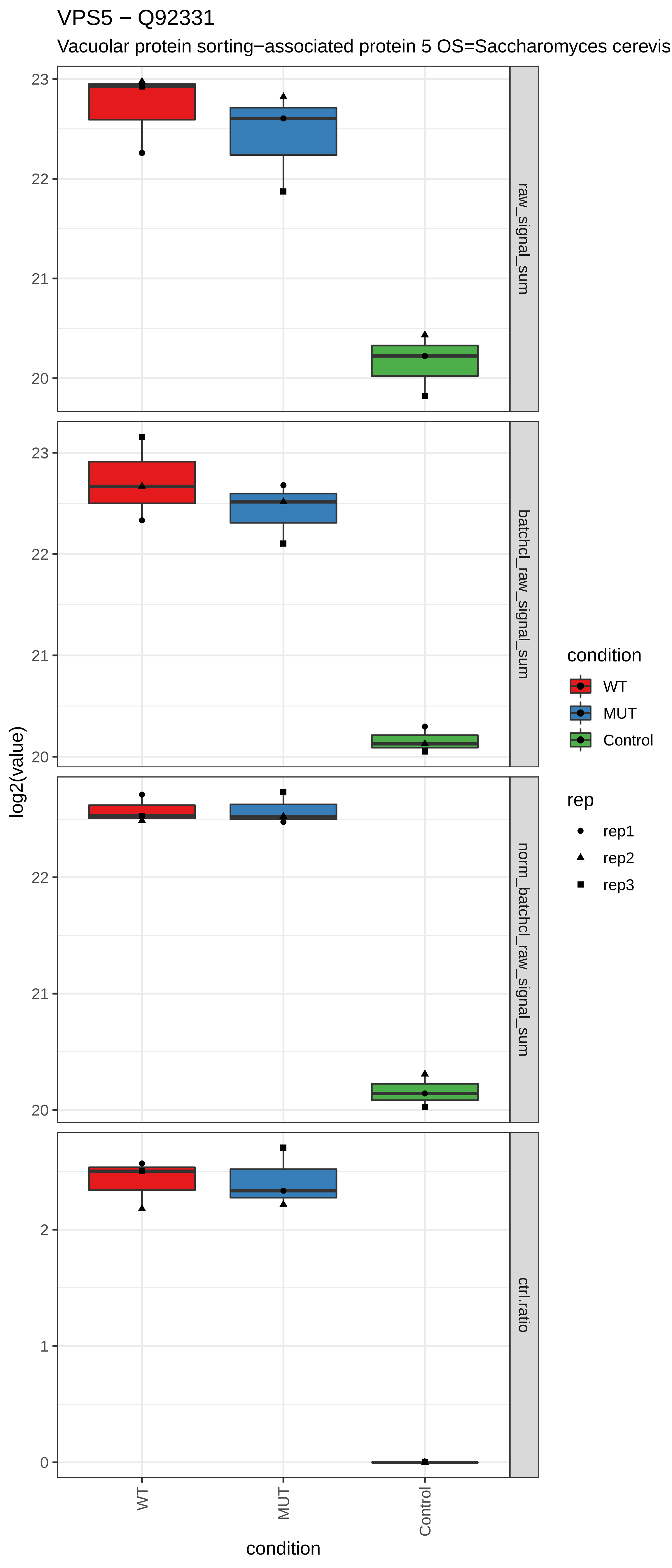
VPS5 − Q92331
Vacuolar protein sorting−associated protein 5 OS=Saccharomyces cerevis
raw_signal_sum
batchcl_raw_signal_sum
norm_batchcl_raw_signal_sum
ctrl.ratio
log2(value)
condition
WT
MUT
Control
rep
rep1
rep2
rep3
23
22
21
20
2
1
0

VPS72 − Q03388

Vacuolar protein sorting−associated protein 72 OS=Saccharomyces cerevi

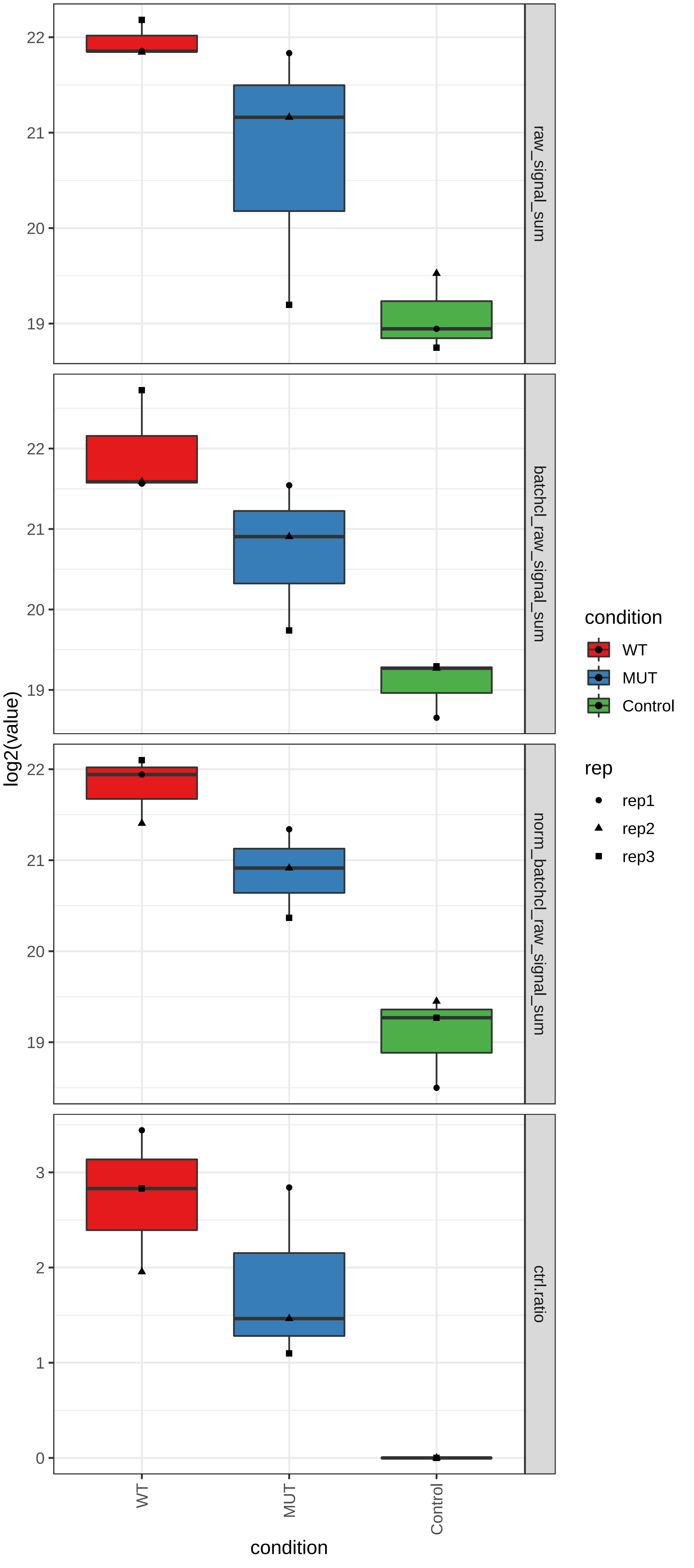

VTC4 − P47075

Vacuolar transporter chaperone 4 OS=Saccharomyces cerevisiae (strain A

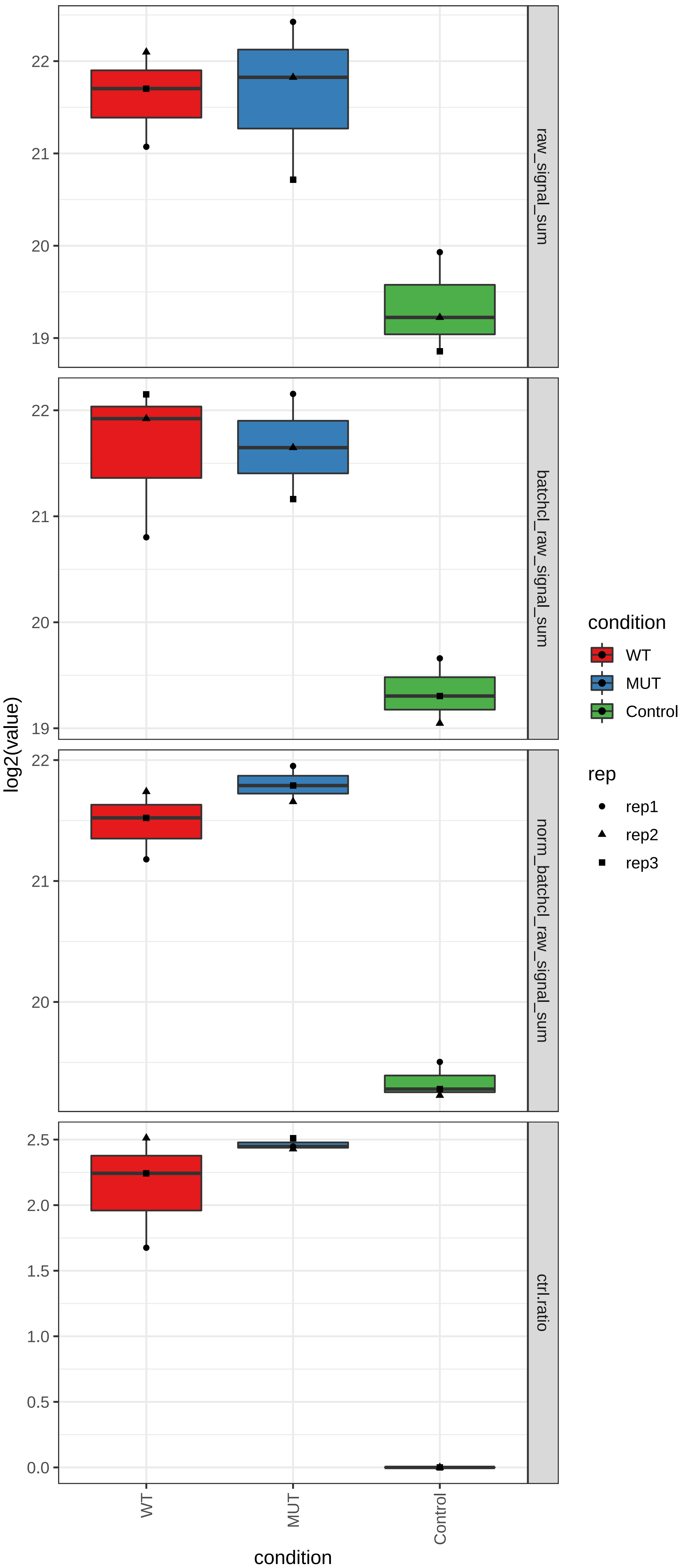

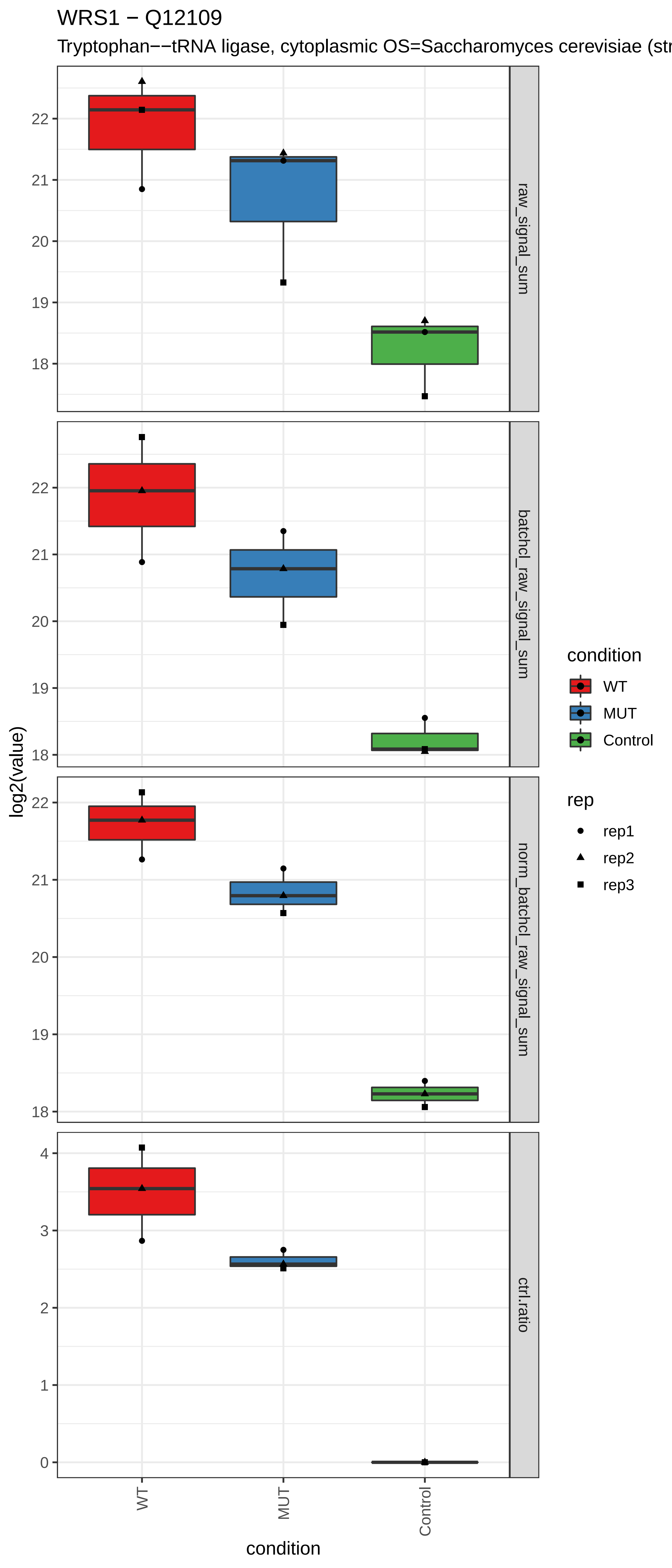

WRS1 − Q12109
Tryptophan−−tRNA ligase, cytoplasmic OS=Saccharomyces cerevisiae (str
raw_signal_sum
batchcl_raw_signal_sum
norm_batchcl_raw_signal_sum
ctrl.ratio
log2(value)
condition
WT
MUT
Control
rep
rep1
rep2
rep3

WTM1 − Q12363

Transcriptional modulator WTM1 OS=Saccharomyces cerevisiae (strain AT

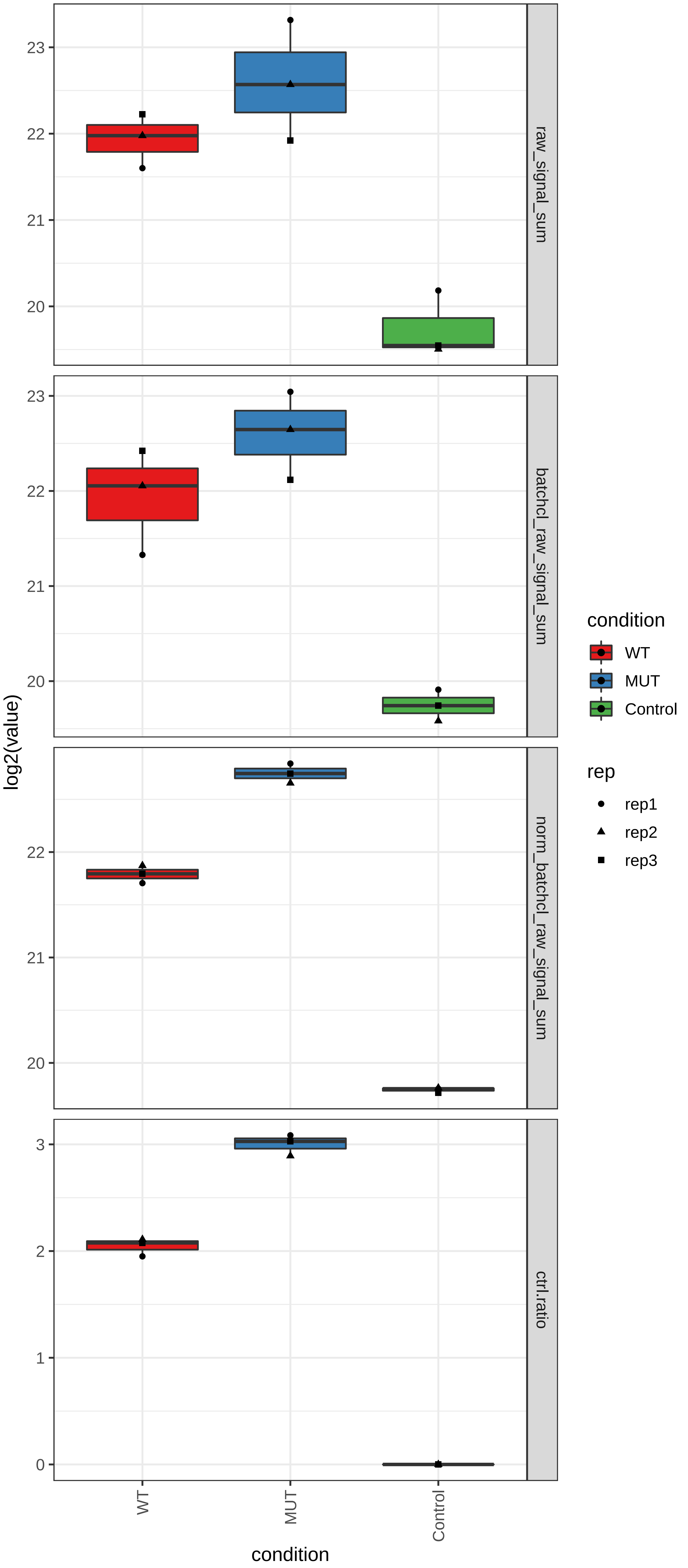

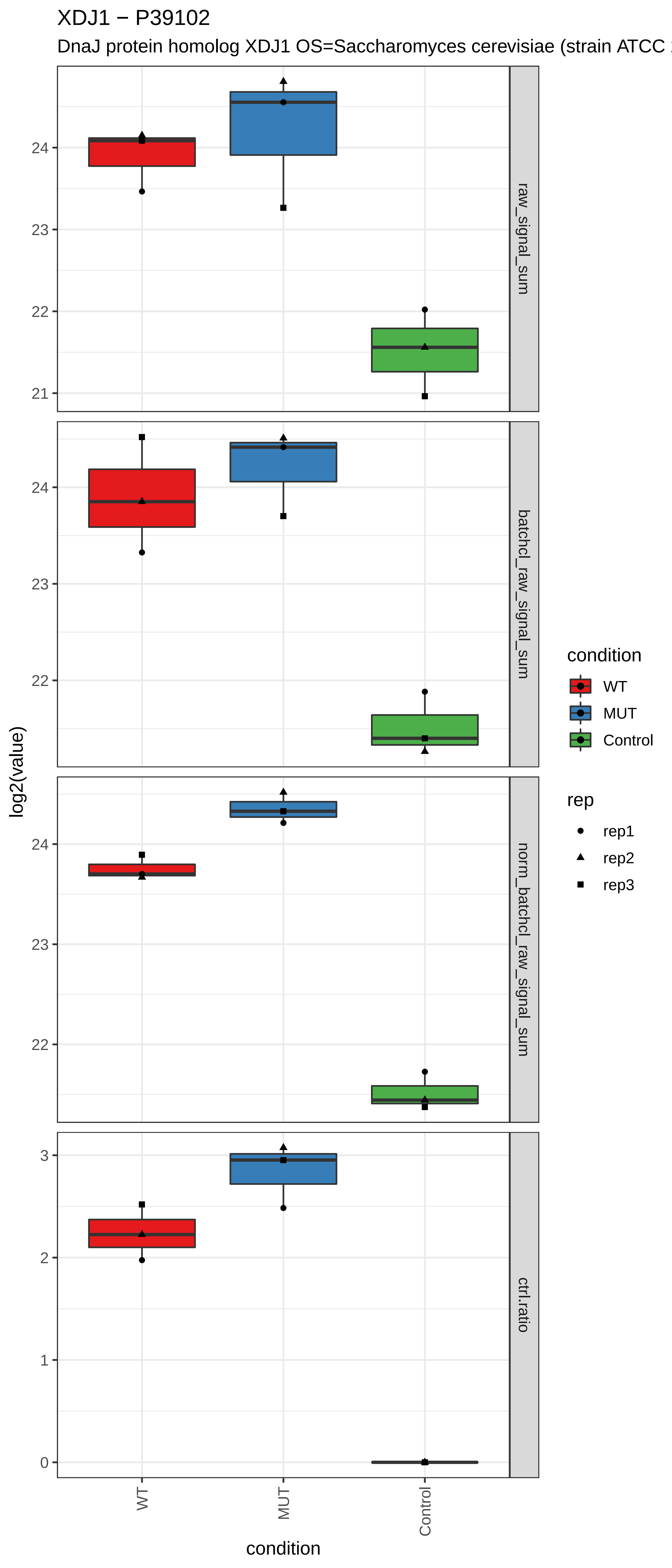
XDJ1 − P39102
DnaJ protein homolog XDJ1 OS=Saccharomyces cerevisiae (strain ATCC
raw_signal_sum
batchcl_raw_signal_sum
norm_batchcl_raw_signal_sum
ctrl.ratio
log2(value)
condition
WT
MUT
Control
rep
rep1
rep2
rep3
24
23
22
21
3
2
1
0

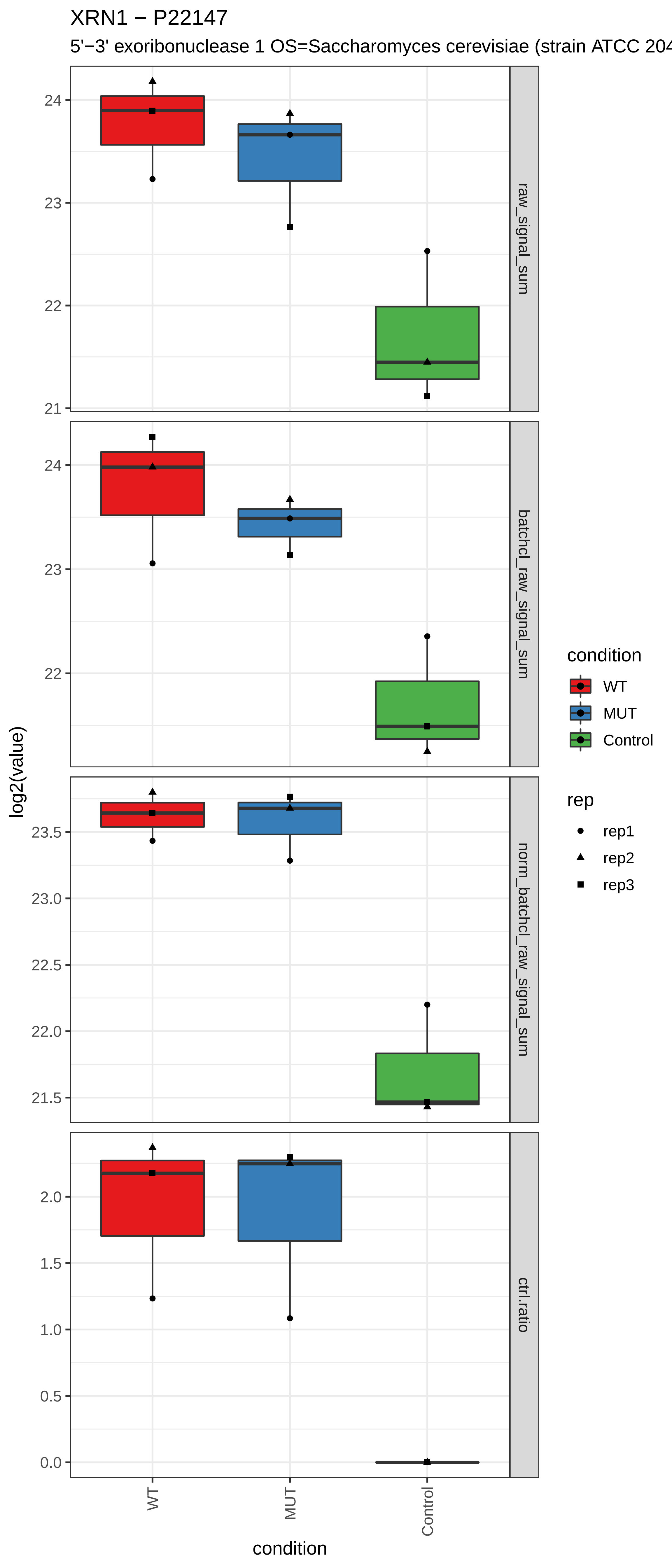
XRN1 − P22147
5'−3' exoribonuclease 1 OS=Saccharomyces cerevisiae (strain ATCC 204
raw_signal_sum
batchcl_raw_signal_sum
norm_batchcl_raw_signal_sum
ctrl.ratio
log2(value)
condition
WT
MUT
Control
rep
rep1
rep2
rep3

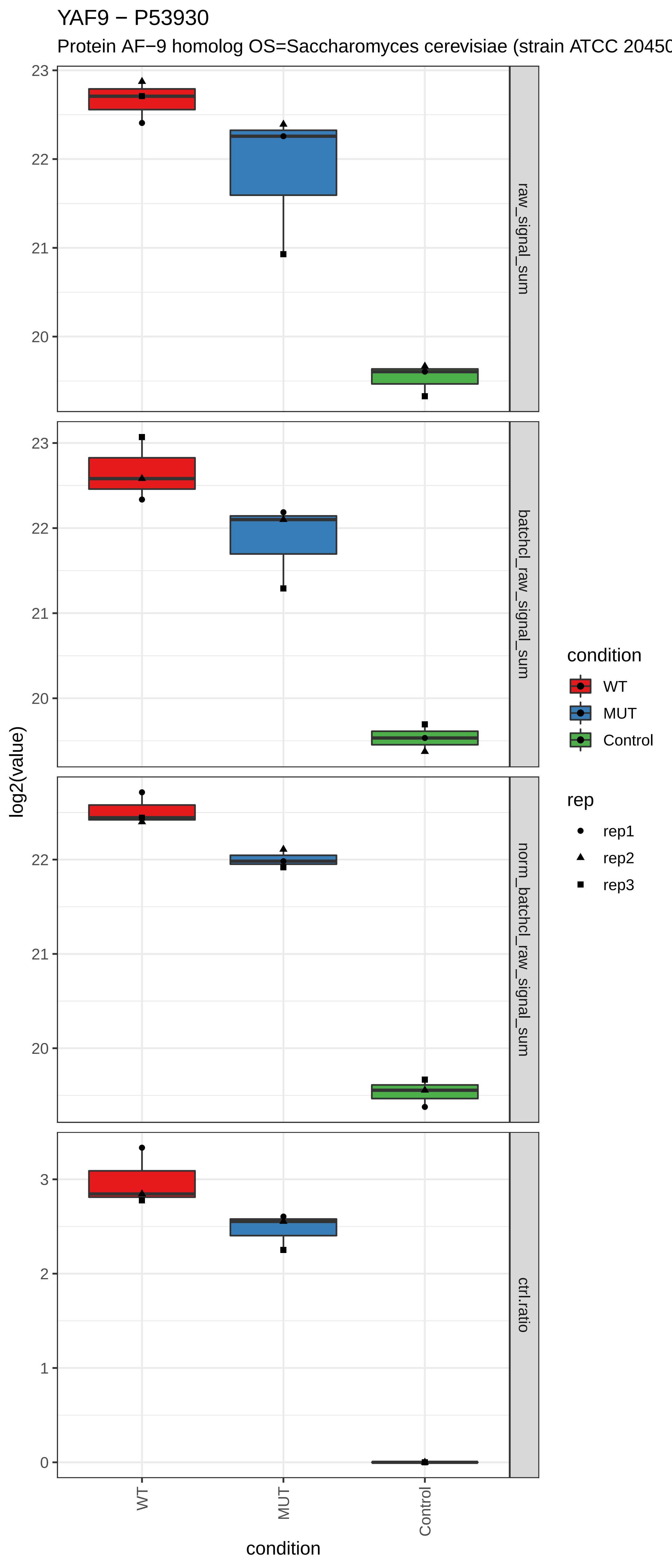

YAF9 − P53930
Protein AF−9 homolog OS=Saccharomyces cerevisiae (strain ATCC 20450
raw_signal_sum
batchcl_raw_signal_sum
norm_batchcl_raw_signal_sum
ctrl.ratio
log2(value)
condition
WT
MUT
Control
rep
rep1
rep2
rep3

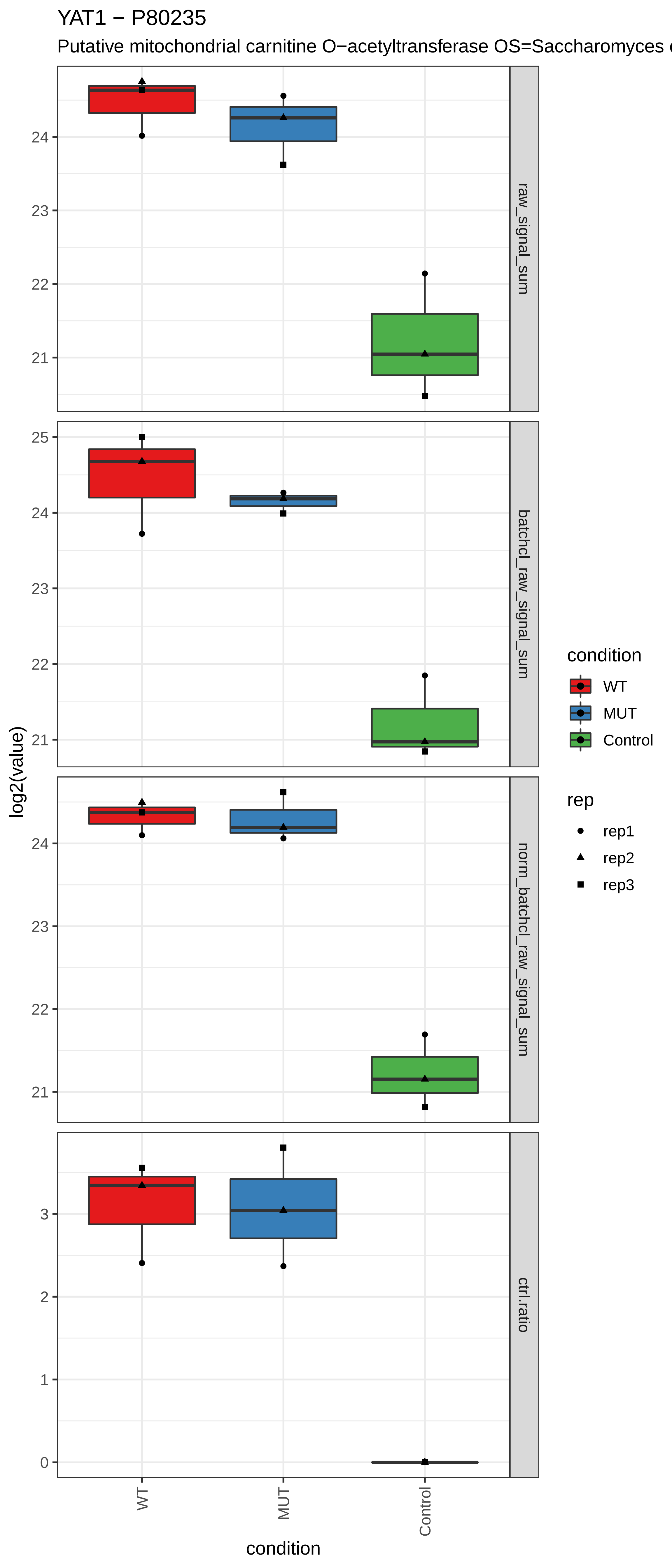

YAT1 − P80235
Putative mitochondrial carnitine O−acetyltransferase OS=Saccharomyces c
raw_signal_sum
batchcl_raw_signal_sum
norm_batchcl_raw_signal_sum
ctrl.ratio
log2(value)
condition
WT
MUT
Control
rep
rep1
rep2
rep3

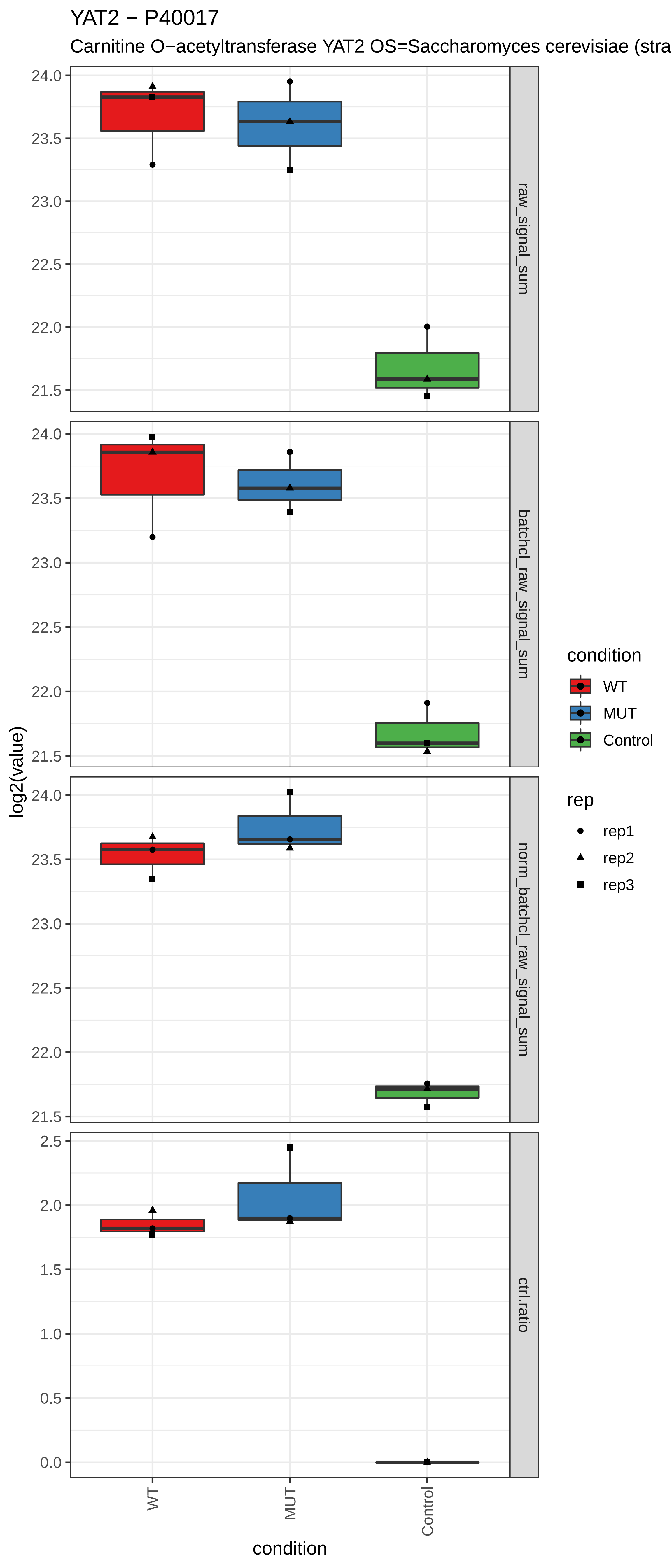

YAT2 − P40017
Carnitine O−acetyltransferase YAT2 OS=Saccharomyces cerevisiae (stra
raw_signal_sum
batchcl_raw_signal_sum
norm_batchcl_raw_signal_sum
ctrl.ratio
log2(value)
condition
WT
MUT
Control
rep
rep1
rep2
rep3
24.0
23.5
23.0
22.5
22.0
21.5
2.5
2.0
1.5
1.0
0.5
0.0

YBT1 − P32386

ATP−dependent bile acid permease OS=Saccharomyces cerevisiae (stra

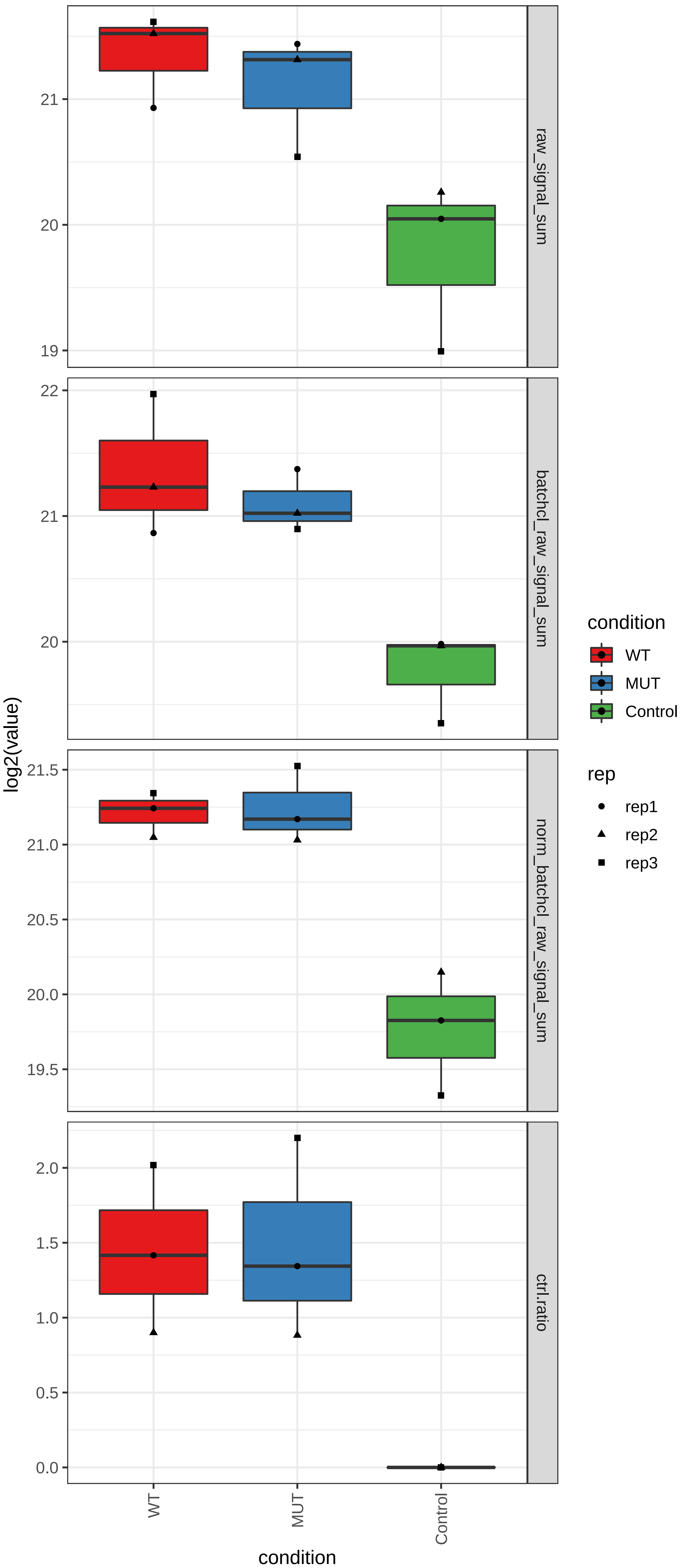

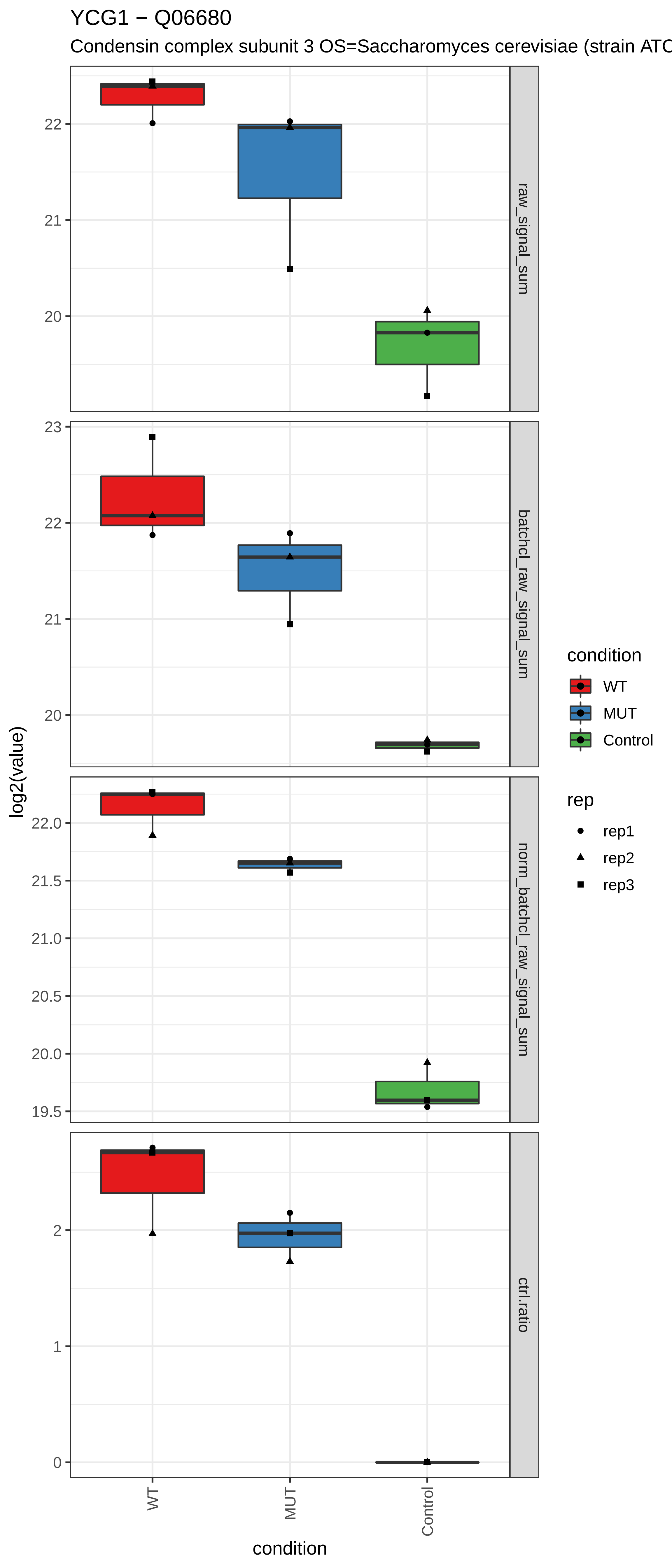

YCG1 − Q06680
Condensin complex subunit 3 OS=Saccharomyces cerevisiae (strain ATC
raw_signal_sum
batchcl_raw_signal_sum
norm_batchcl_raw_signal_sum
ctrl.ratio
log2(value)
condition
WT
MUT
Control
rep
rep1
rep2
rep3
22
21
20
23
22.0
21.5
21.0
20.5
20.0
19.5
2
1
0

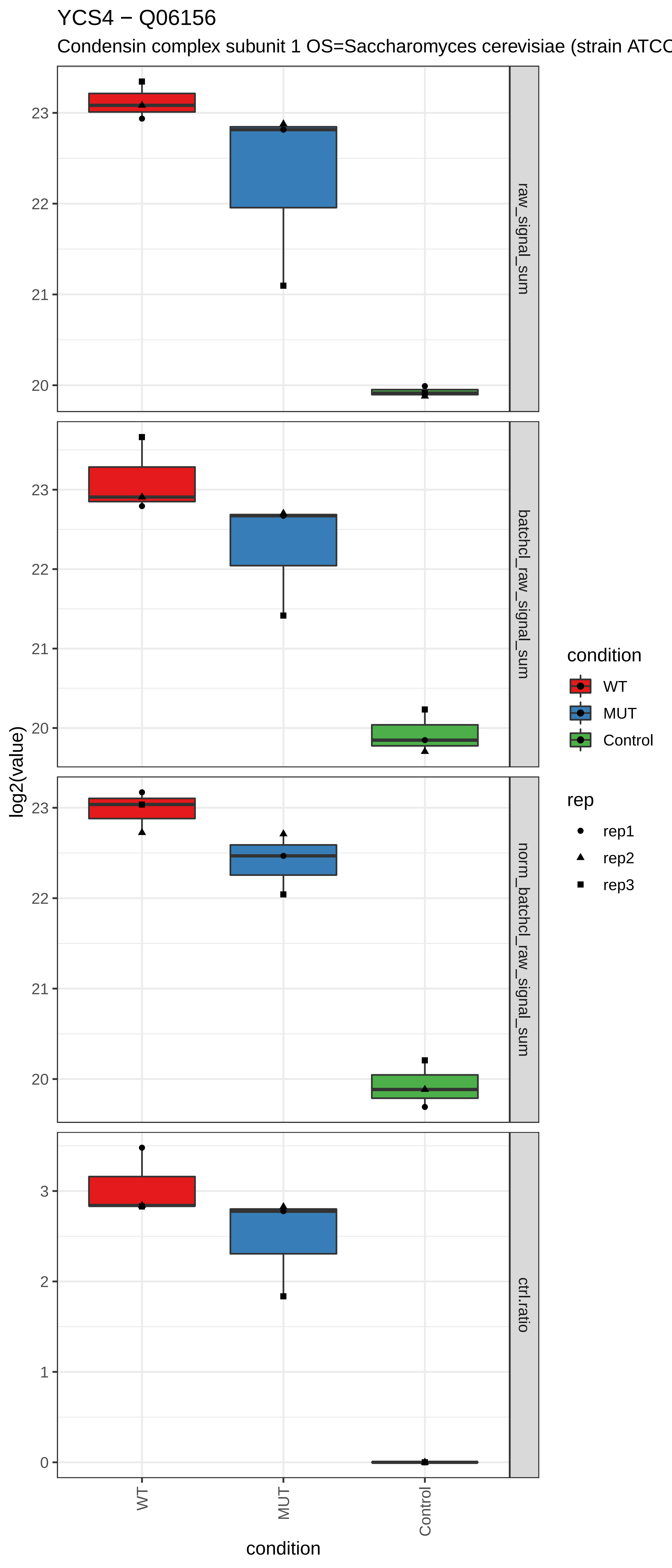

YCS4 − Q06156
Condensin complex subunit 1 OS=Saccharomyces cerevisiae (strain ATCC
raw_signal_sum
batchcl_raw_signal_sum
norm_batchcl_raw_signal_sum
ctrl.ratio
log2(value)
condition
WT
MUT
Control
rep
rep1
rep2
rep3

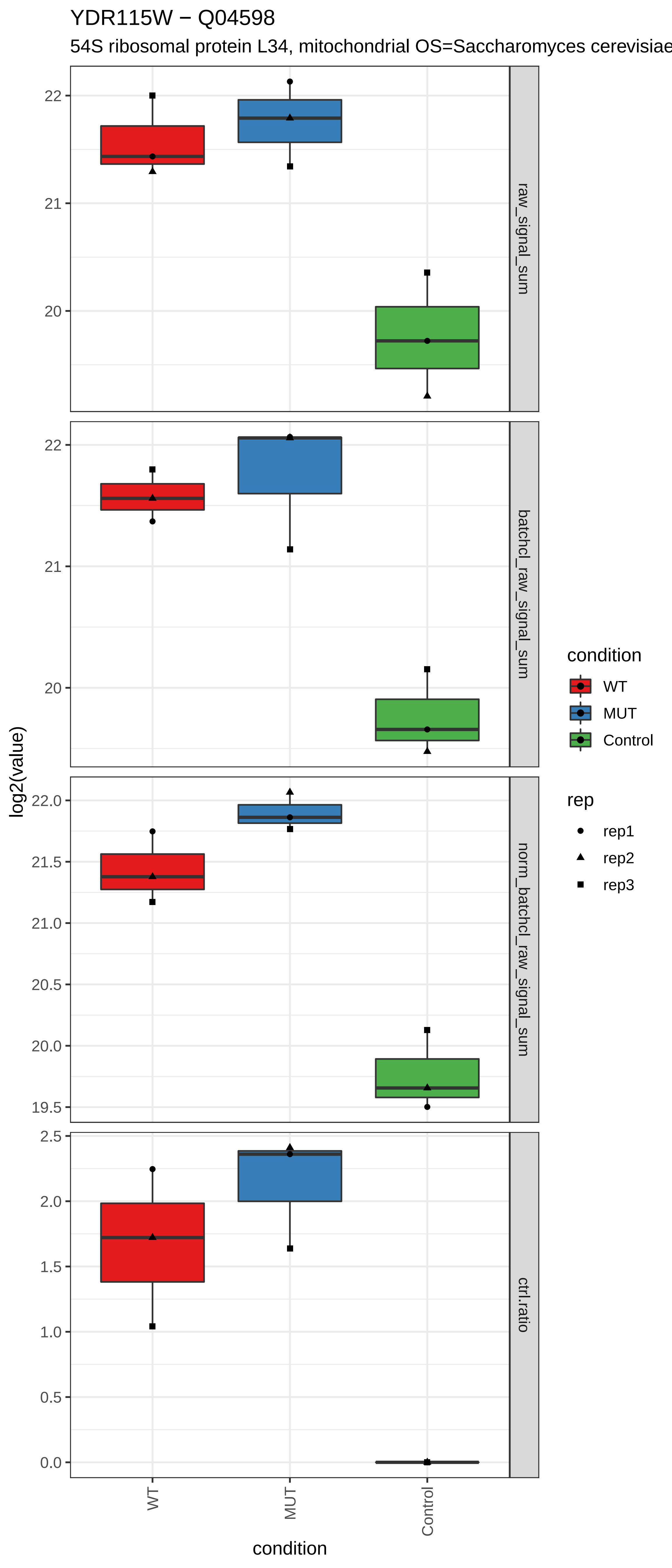
YDR115W − Q04598
54S ribosomal protein L34, mitochondrial OS=Saccharomyces cerevisiae
raw_signal_sum
batchcl_raw_signal_sum
norm_batchcl_raw_signal_sum
ctrl.ratio
log2(value)
condition
WT
MUT
Control
rep
rep1
rep2
rep3

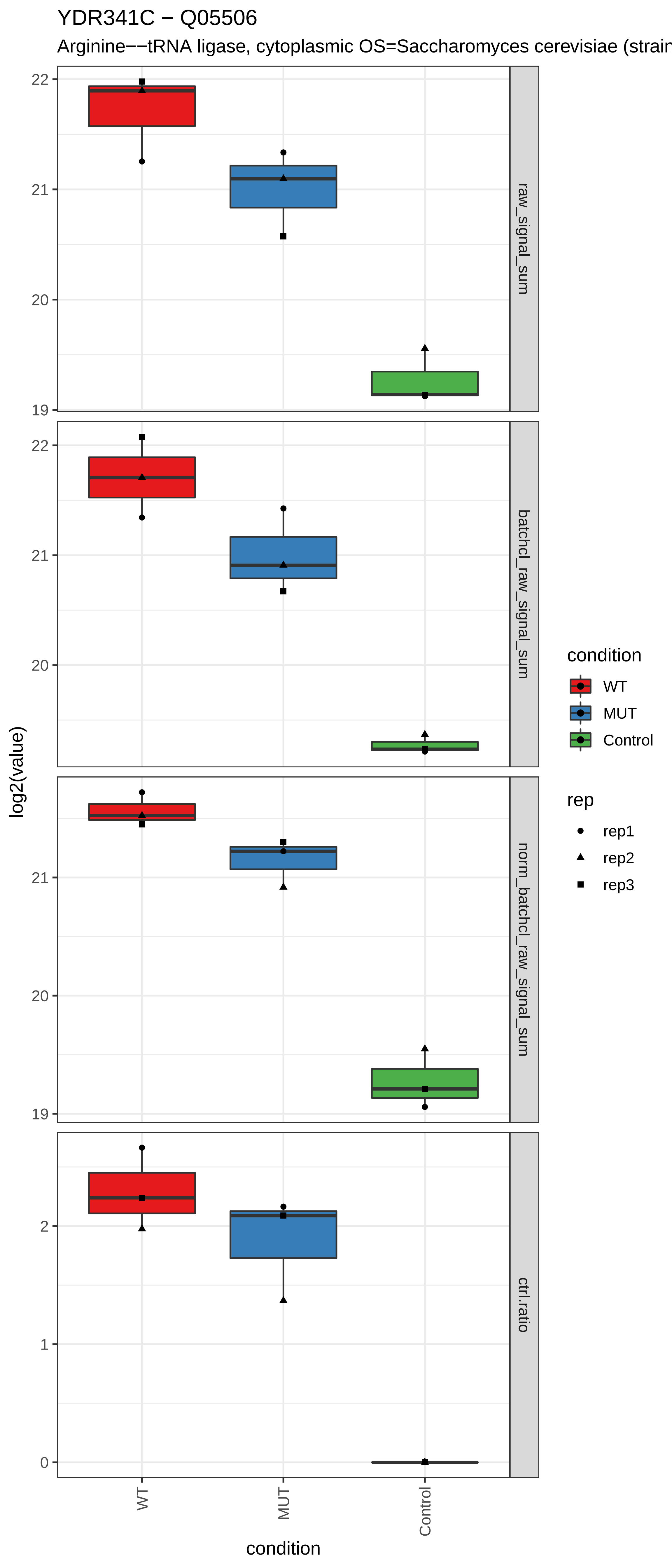

YDR341C − Q05506
Arginine−−tRNA ligase, cytoplasmic OS=Saccharomyces cerevisiae (strain
raw_signal_sum
batchcl_raw_signal_sum
norm_batchcl_raw_signal_sum
ctrl.ratio
log2(value)
condition
WT
MUT
Control
rep
rep1
rep2
rep3
22
21
20
19
2
1
0

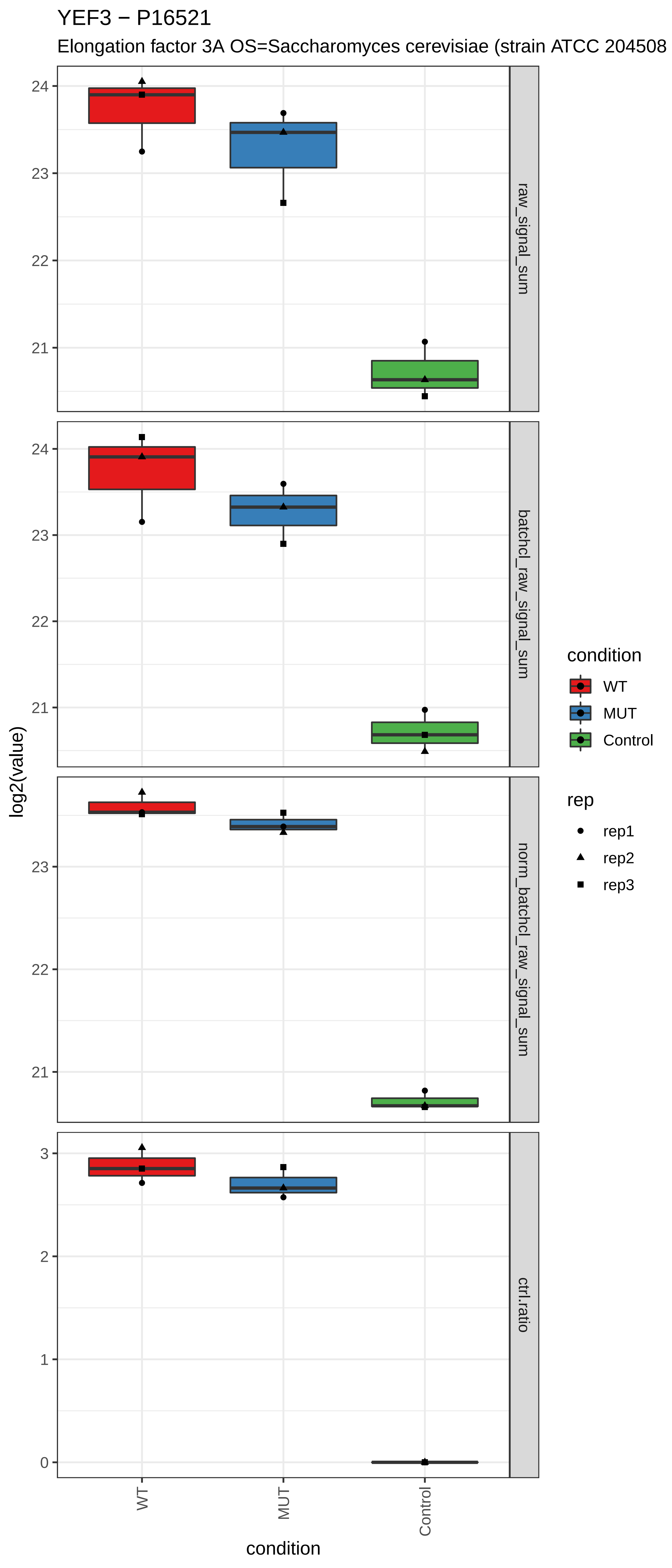

YEF3 − P16521
Elongation factor 3A OS=Saccharomyces cerevisiae (strain ATCC 204508
raw_signal_sum
batchcl_raw_signal_sum
norm_batchcl_raw_signal_sum
ctrl.ratio
log2(value)
condition
WT
MUT
Control
rep
rep1
rep2
rep3
24
23
22
21
3
2
1
0

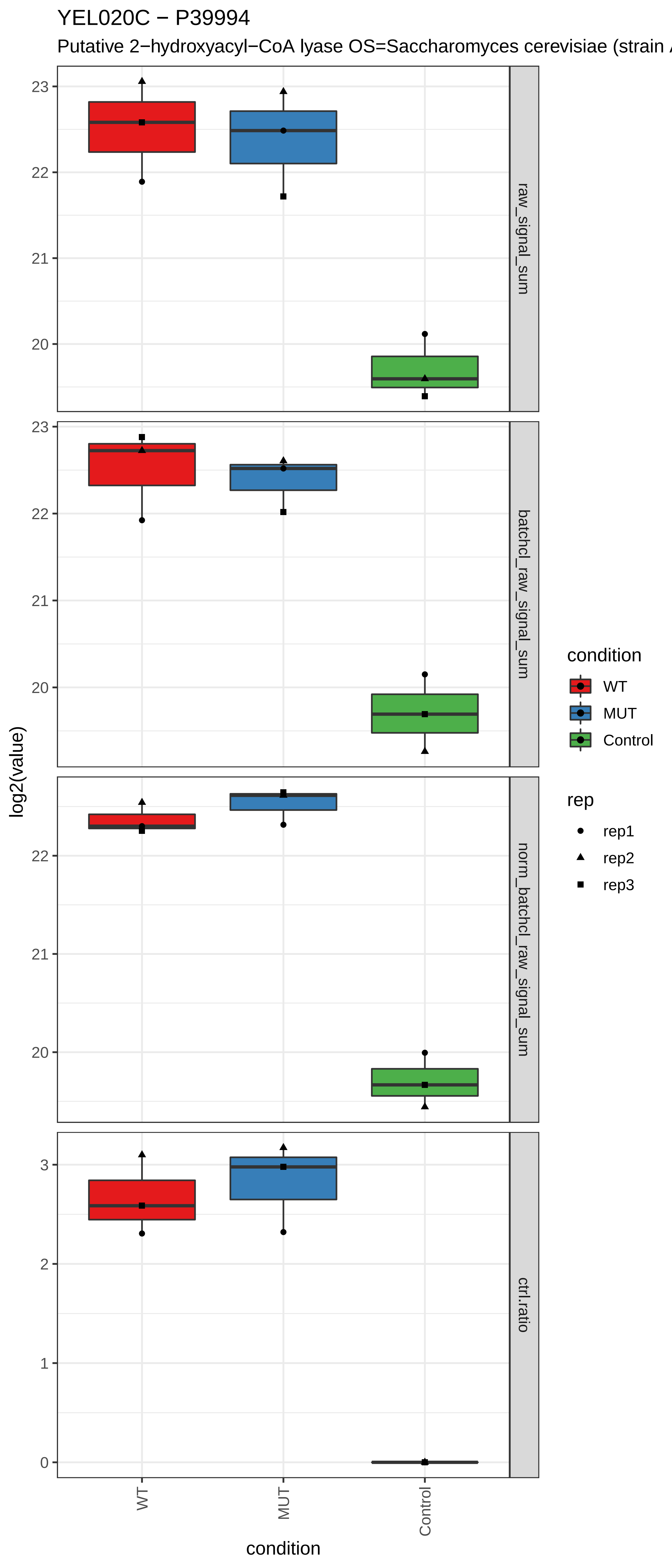

YEL020C − P39994
Putative 2−hydroxyacyl−CoA lyase OS=Saccharomyces cerevisiae (strain
raw_signal_sum
batchcl_raw_signal_sum
norm_batchcl_raw_signal_sum
ctrl.ratio
log2(value)
condition
WT
MUT
Control
rep
rep1
rep2
rep3

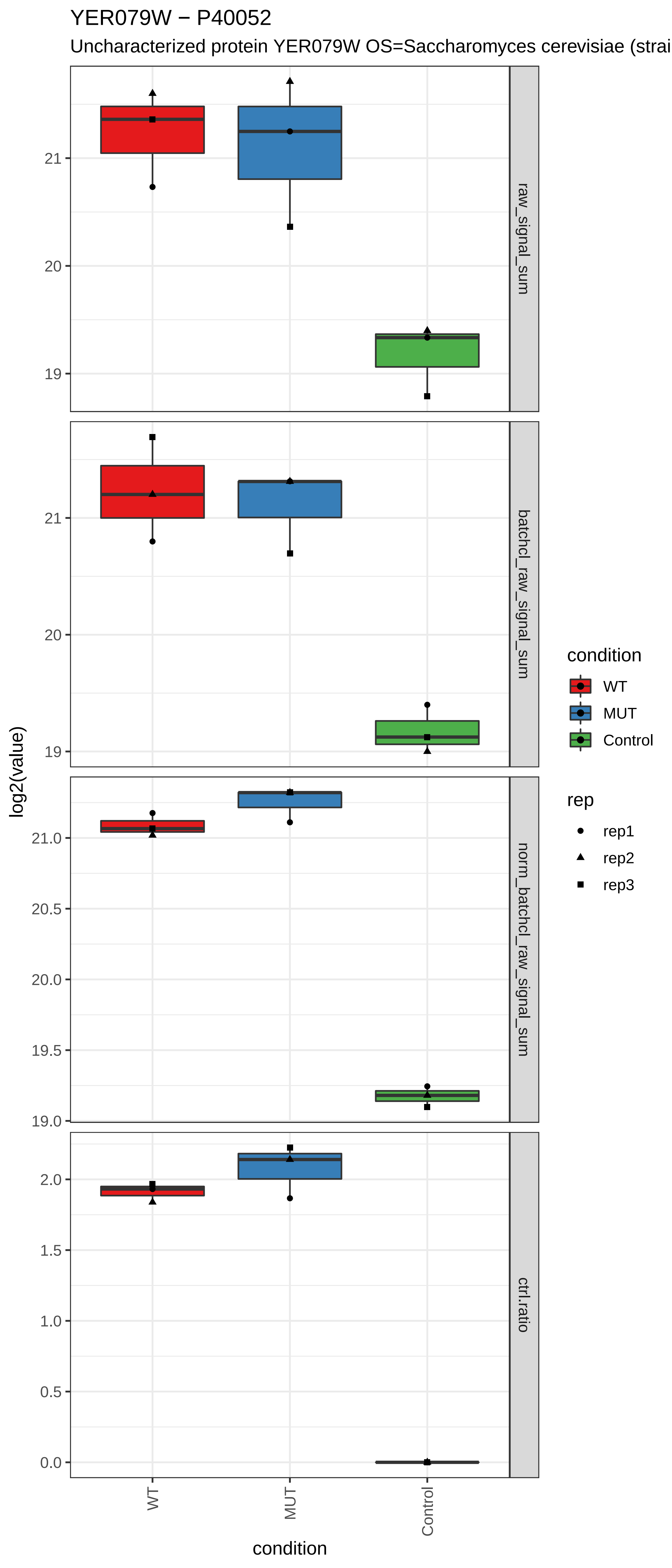

YER079W − P40052
Uncharacterized protein YER079W OS=Saccharomyces cerevisiae (strai
raw_signal_sum
batchcl_raw_signal_sum
norm_batchcl_raw_signal_sum
ctrl.ratio
log2(value)
condition
WT
MUT
Control
rep
rep1
rep2
rep3
21
20
19
21.0
20.5
20.0
19.5
19.0
2.0
1.5
1.0
0.5
0.0

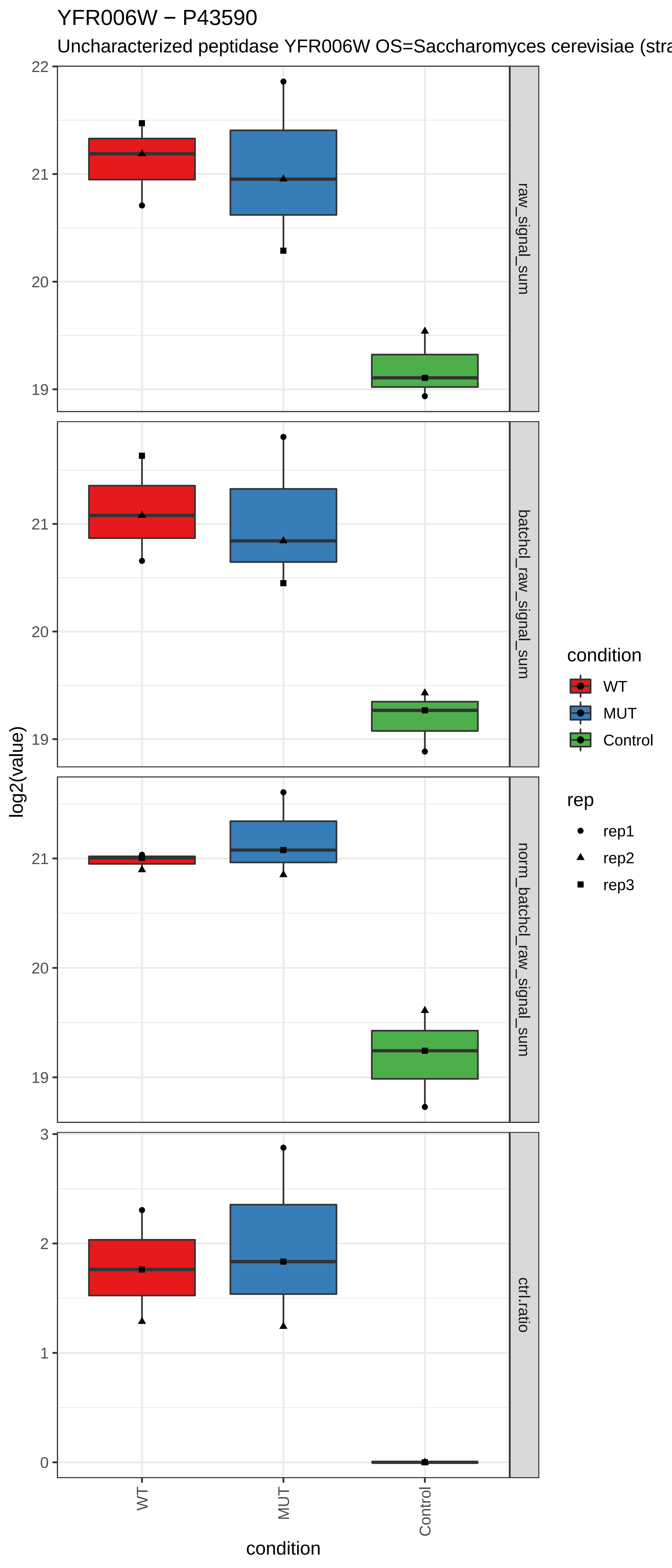
YFR006W − P43590
Uncharacterized peptidase YFR006W OS=Saccharomyces cerevisiae (stra
raw_signal_sum
batchcl_raw_signal_sum
norm_batchcl_raw_signal_sum
ctrl.ratio
log2(value)
condition
WT
MUT
Control
rep
rep1
rep2
rep3

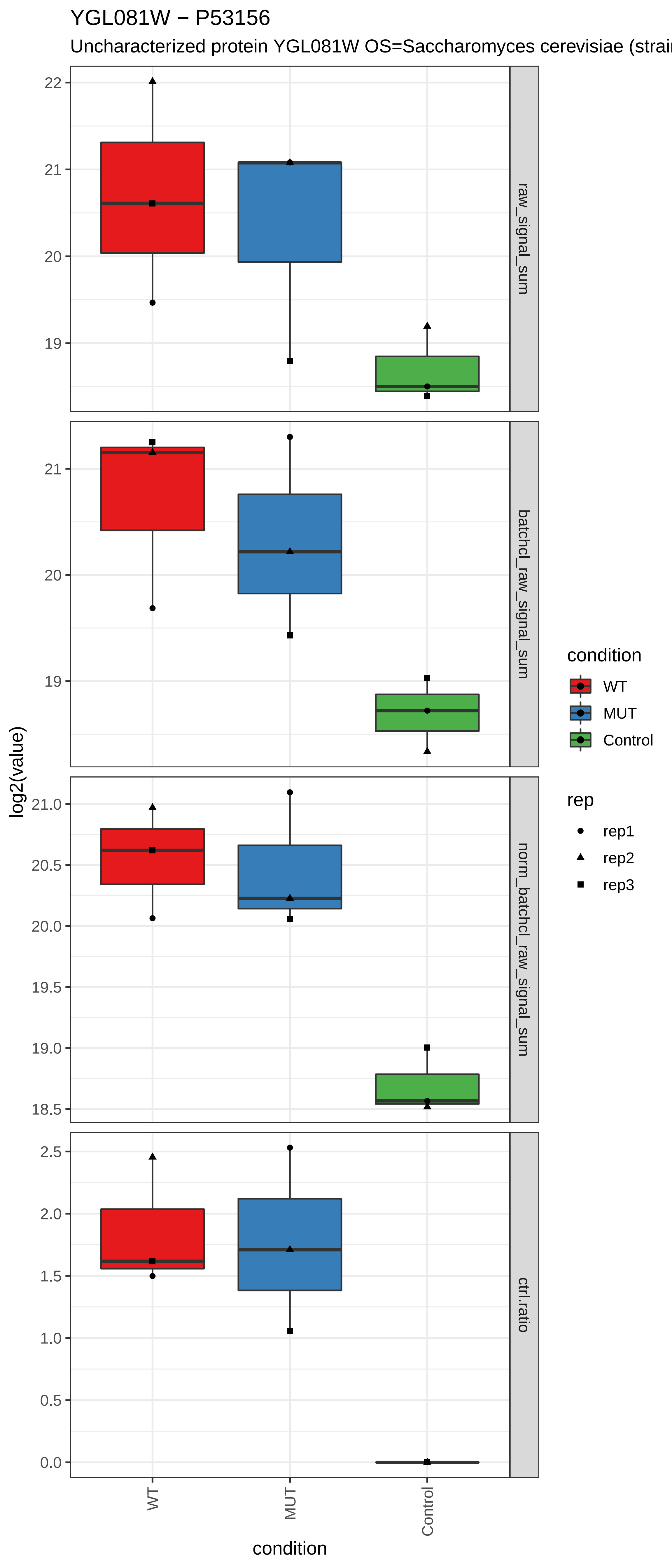

YGL081W − P53156
Uncharacterized protein YGL081W OS=Saccharomyces cerevisiae (strai
raw_signal_sum
batchcl_raw_signal_sum
norm_batchcl_raw_signal_sum
ctrl.ratio
log2(value)
condition
WT
MUT
Control
rep
rep1
rep2
rep3
22
21
20
19
21.0
20.5
20.0
19.5
19.0
18.5
2.5
2.0
1.5
1.0
0.5
0.0

YGL108C − P53139

Uncharacterized protein YGL108C OS=Saccharomyces cerevisiae (strain A

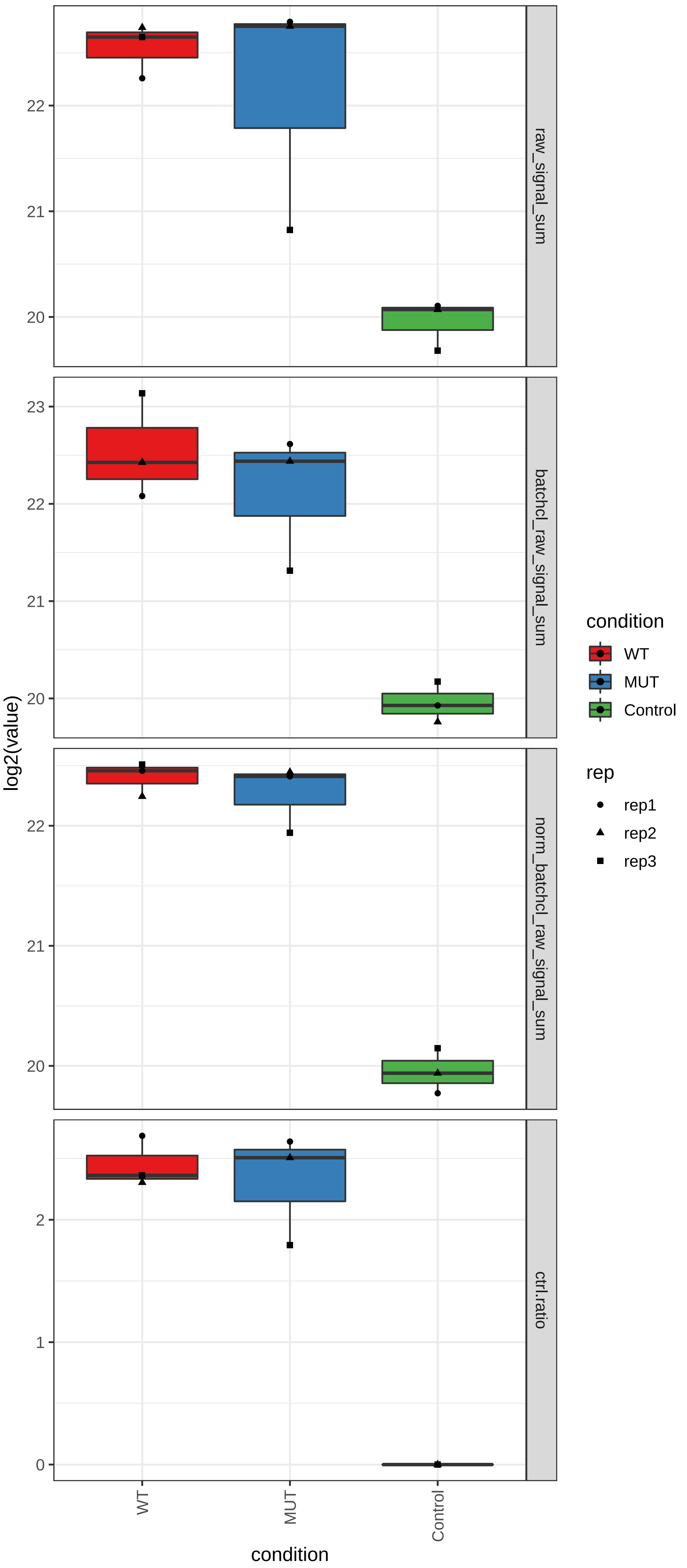

YGR130C − P53278

Uncharacterized protein YGR130C OS=Saccharomyces cerevisiae (strain

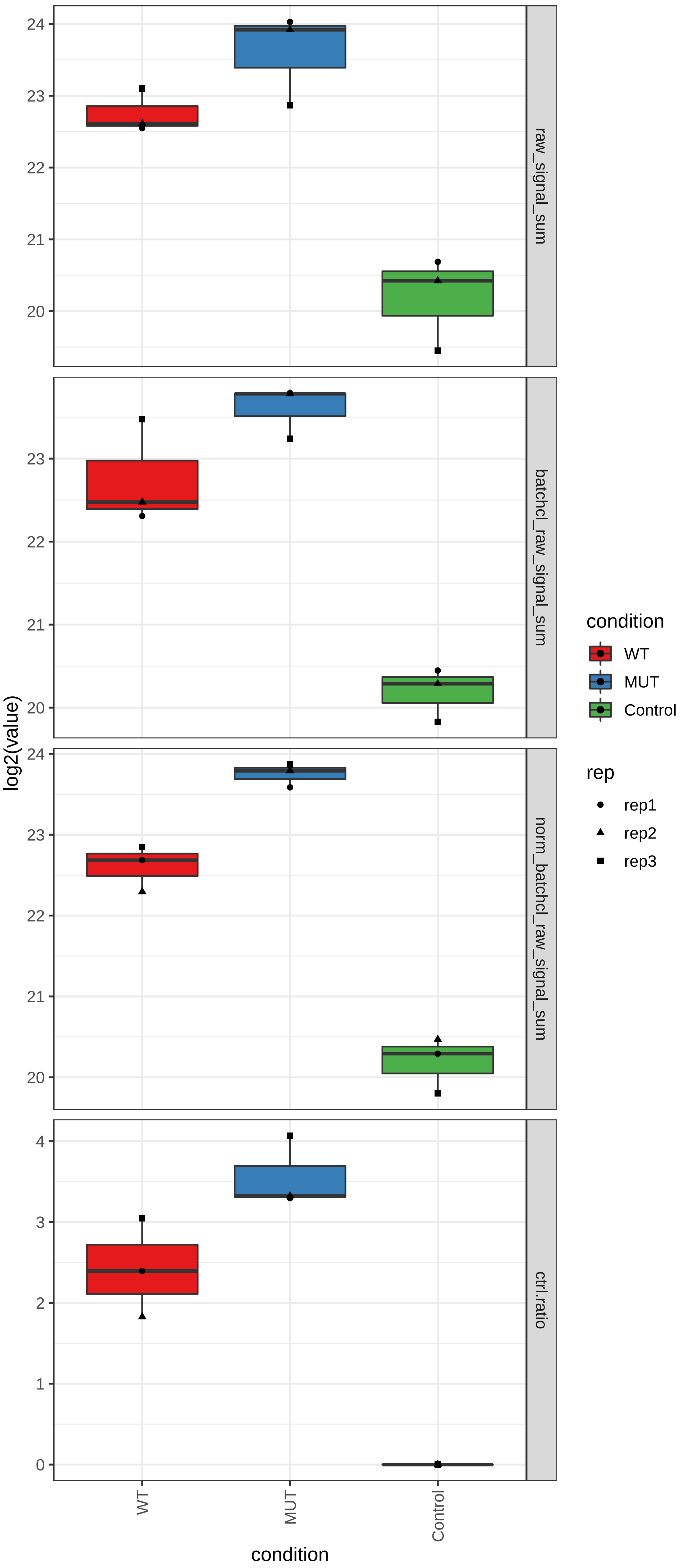

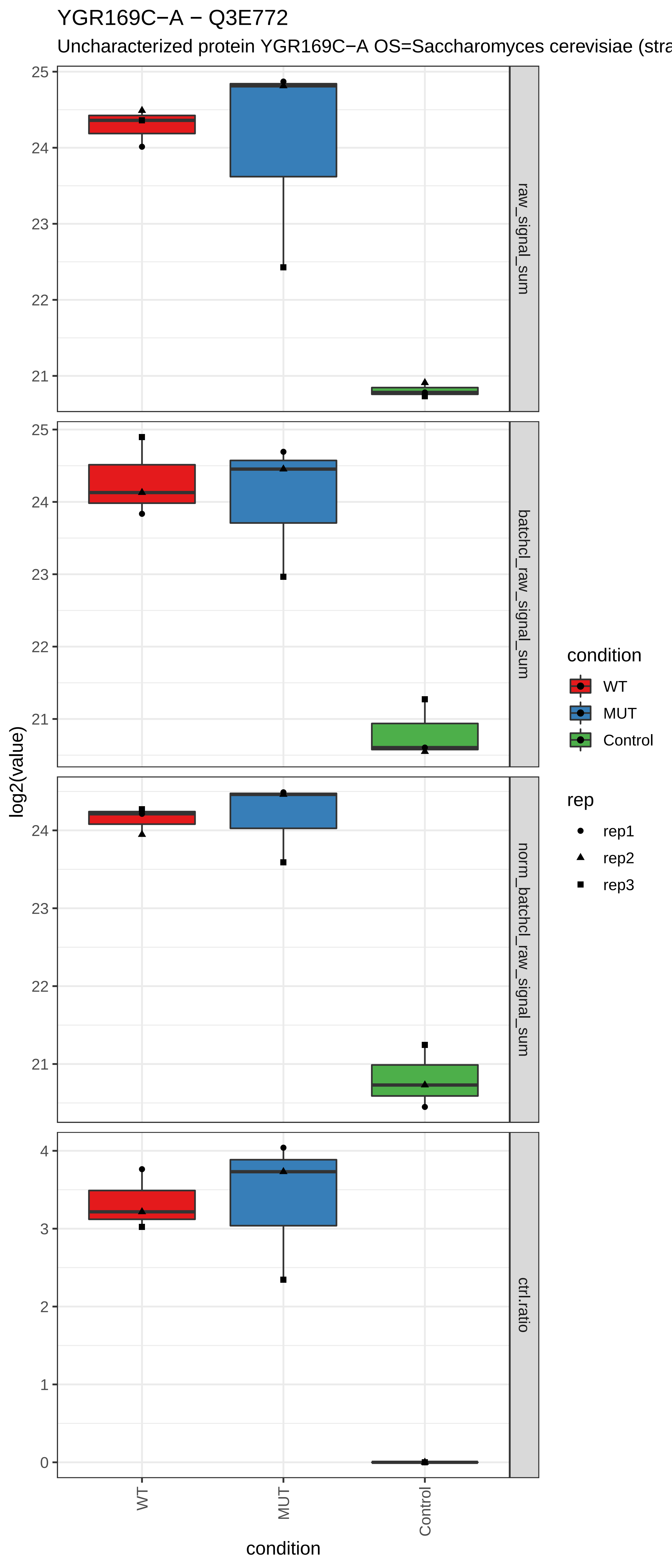
YGR169C−A − Q3E772
Uncharacterized protein YGR169C−A OS=Saccharomyces cerevisiae (stra
raw_signal_sum
batchcl_raw_signal_sum
norm_batchcl_raw_signal_sum
ctrl.ratio
log2(value)
condition
WT
MUT
Control
rep
rep1
rep2
rep3

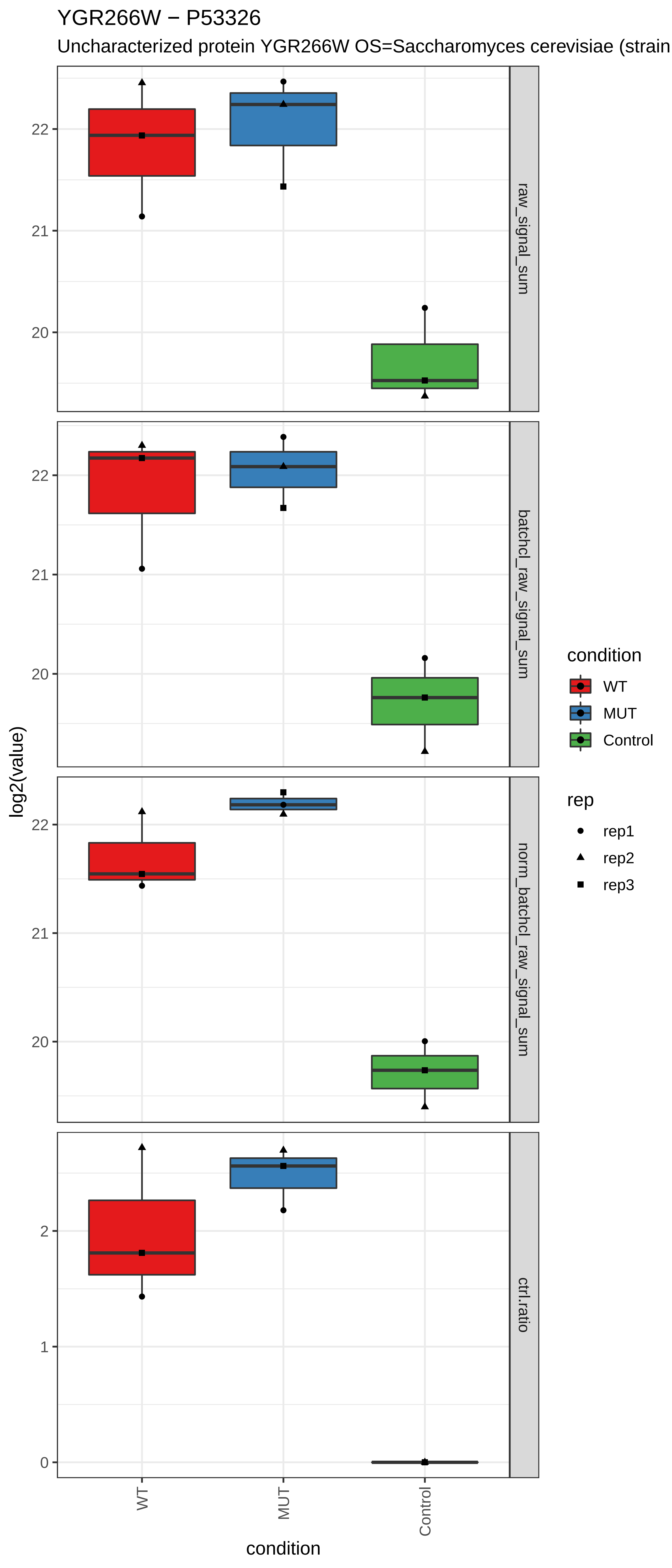

YGR266W − P53326
Uncharacterized protein YGR266W OS=Saccharomyces cerevisiae (strain
raw_signal_sum
batchcl_raw_signal_sum
norm_batchcl_raw_signal_sum
ctrl.ratio
log2(value)
condition
WT
MUT
Control
rep
rep1
rep2
rep3
22
21
20
2
1
0

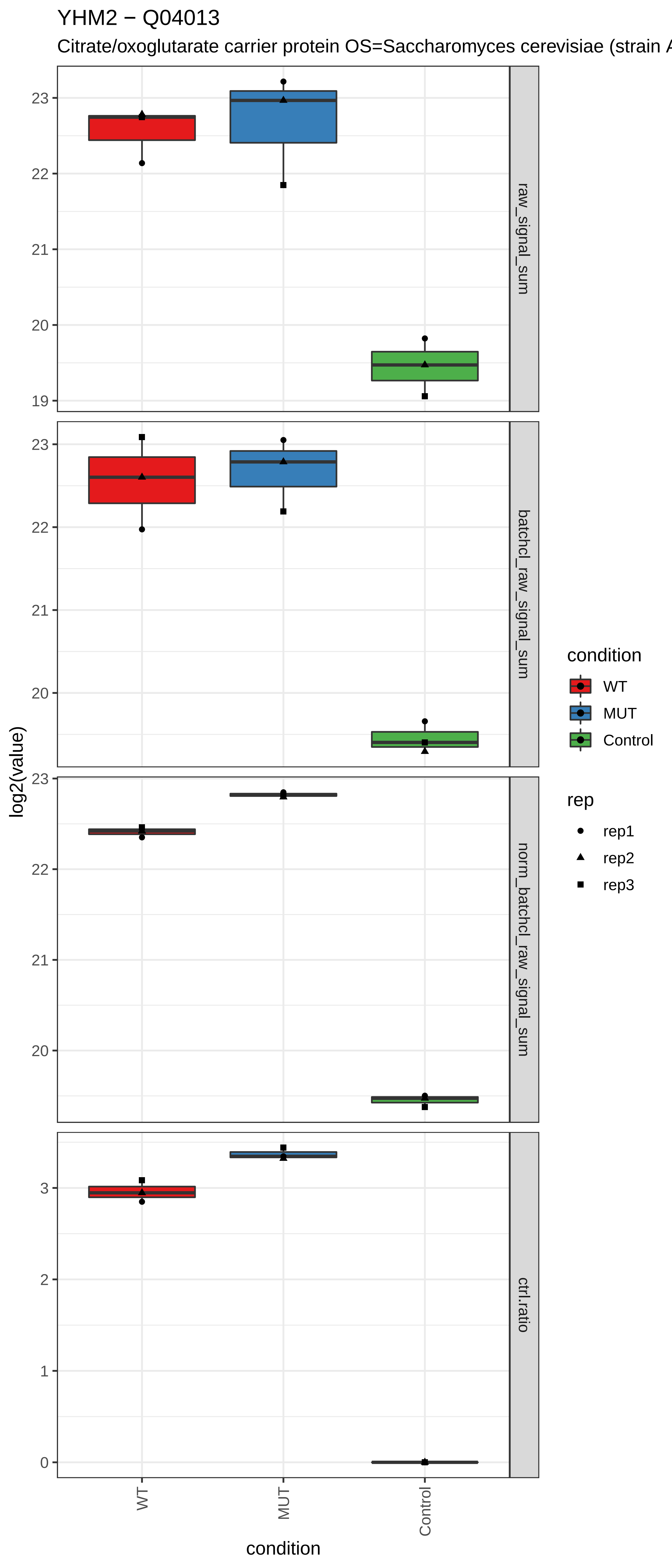
YHM2 − Q04013
Citrate/oxoglutarate carrier protein OS=Saccharomyces cerevisiae (strain A
raw_signal_sum
batchcl_raw_signal_sum
norm_batchcl_raw_signal_sum
ctrl.ratio
log2(value)
condition
WT
MUT
Control
rep
rep1
rep2
rep3
23
22
21
20
19
3
2
1
0

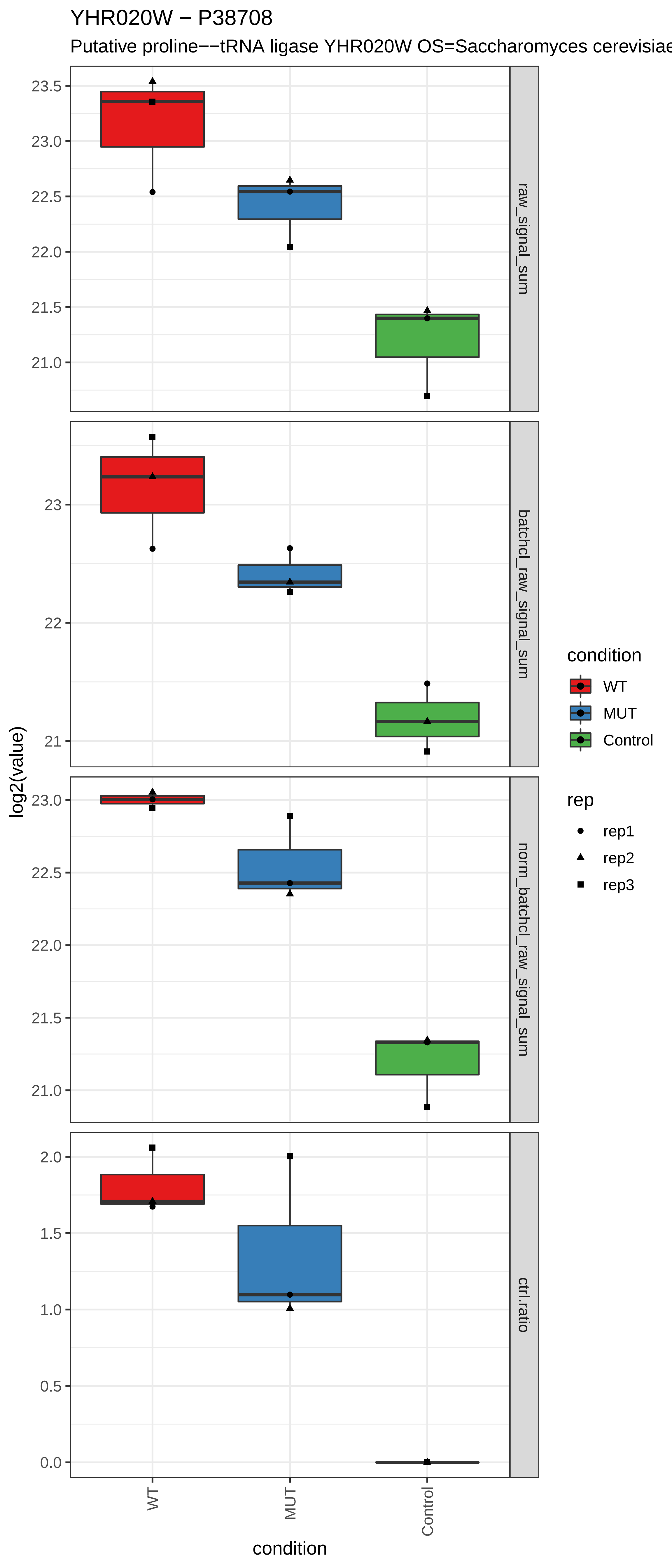
YHR020W − P38708
Putative proline−−tRNA ligase YHR020W OS=Saccharomyces cerevisiae
raw_signal_sum
batchcl_raw_signal_sum
norm_batchcl_raw_signal_sum
ctrl.ratio
log2(value)
condition
WT
MUT
Control
rep
rep1
rep2
rep3
23.5
23.0
22.5
22.0
21.5
21.0
23
22
21
2.0
1.5
1.0
0.5
0.0

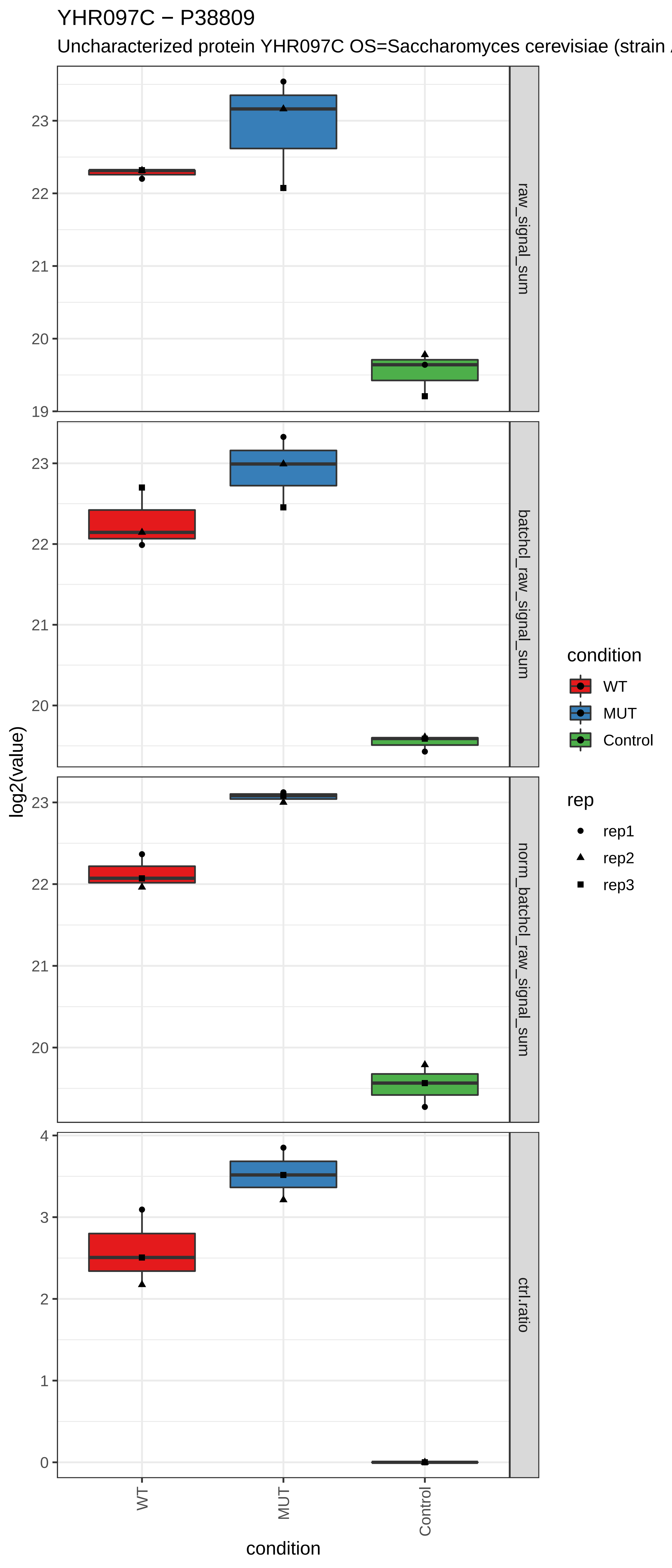

YHR097C − P38809
Uncharacterized protein YHR097C OS=Saccharomyces cerevisiae (strain
raw_signal_sum
batchcl_raw_signal_sum
norm_batchcl_raw_signal_sum
ctrl.ratio
log2(value)
condition
WT
MUT
Control
rep
rep1
rep2
rep3

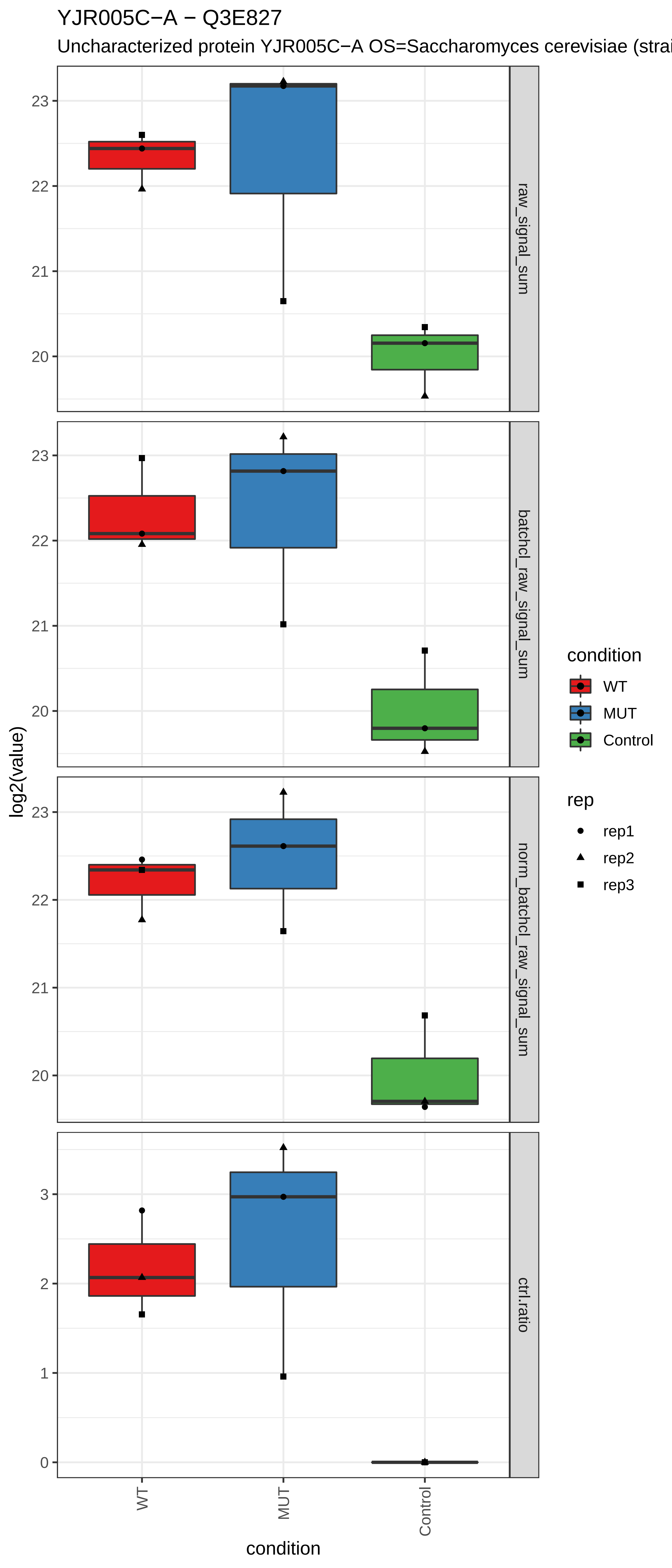

YJR005C−A − Q3E827
Uncharacterized protein YJR005C−A OS=Saccharomyces cerevisiae (strai
raw_signal_sum
batchcl_raw_signal_sum
norm_batchcl_raw_signal_sum
ctrl.ratio
log2(value)
condition
WT
MUT
Control
rep
rep1
rep2
rep3
23
22
21
20
3
2
1
0

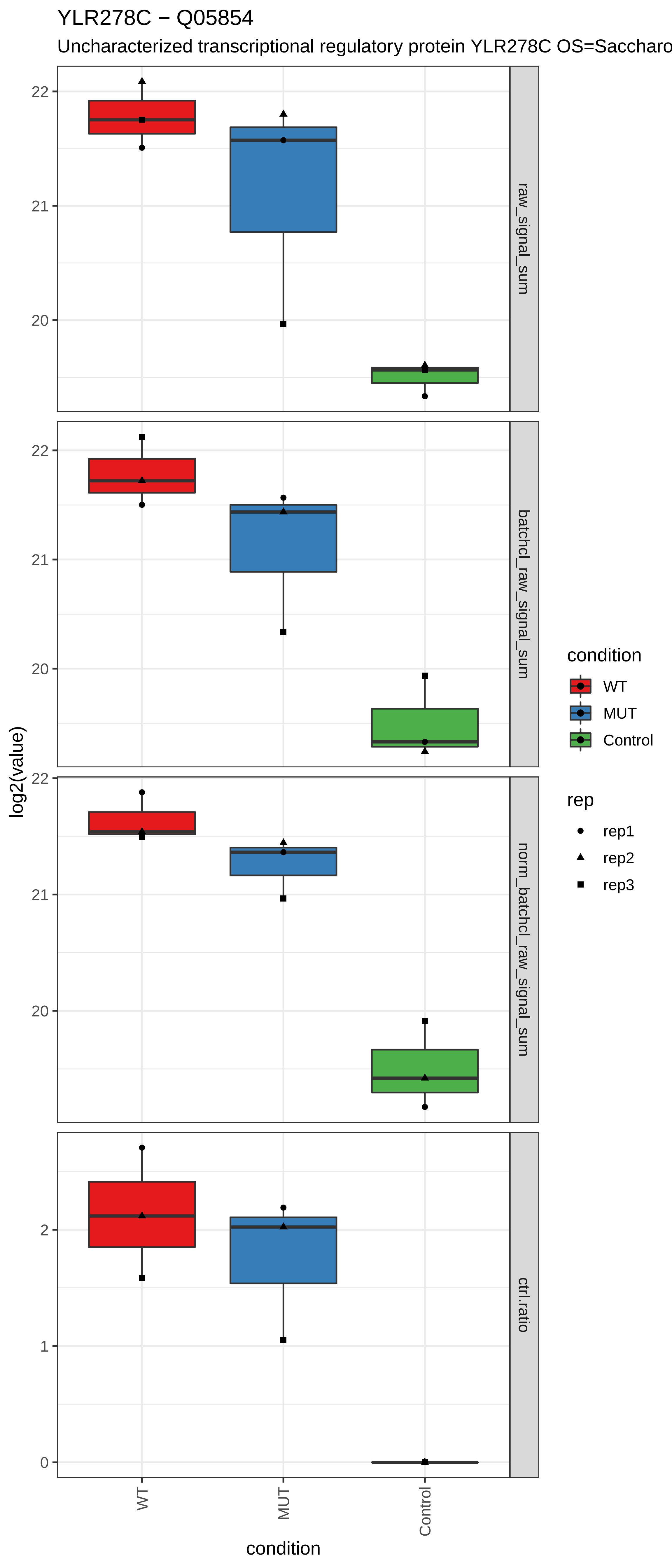
YLR278C − Q05854
Uncharacterized transcriptional regulatory protein YLR278C OS=Saccharo
raw_signal_sum
batchcl_raw_signal_sum
norm_batchcl_raw_signal_sum
ctrl.ratio
log2(value)
condition
WT
MUT
Control
rep
rep1
rep2
rep3
22
21
20
2
1
0

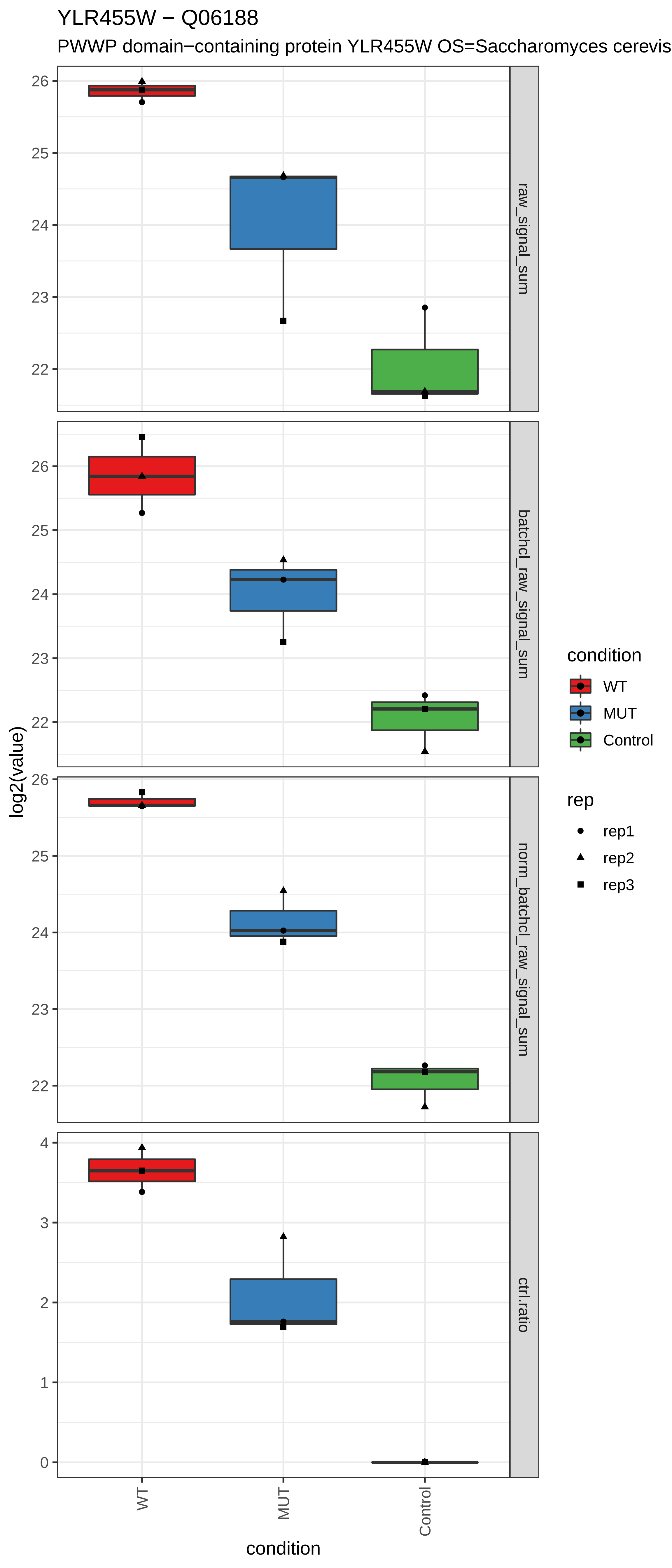

YLR455W − Q06188
PWWP domain−containing protein YLR455W OS=Saccharomyces cerevis
raw_signal_sum
batchcl_raw_signal_sum
norm_batchcl_raw_signal_sum
ctrl.ratio
log2(value)
condition
WT
MUT
Control
rep
rep1
rep2
rep3
26
25
24
23
22
4
3
2
1
0

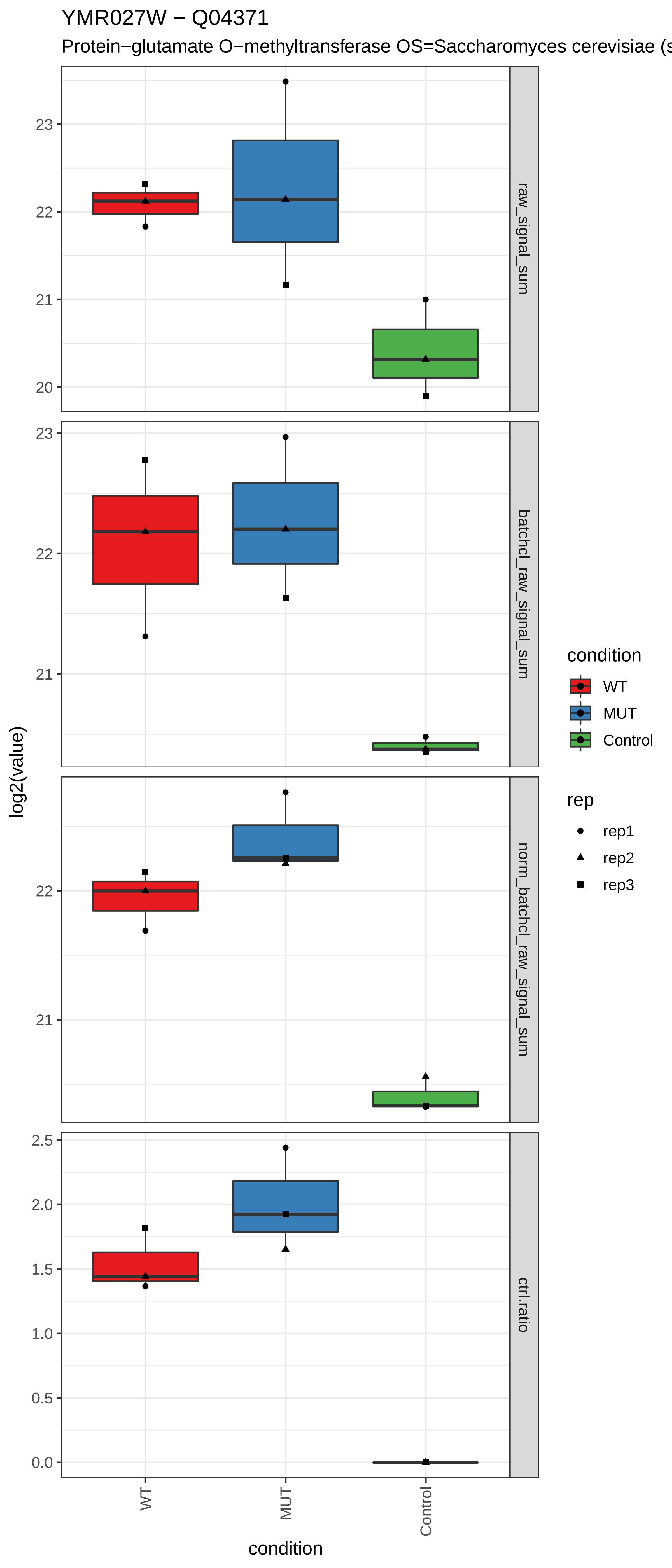
YMR027W − Q04371
Protein−glutamate O−methyltransferase OS=Saccharomyces cerevisiae (s
log2(value)
raw_signal_sum
batchcl_raw_signal_sum
norm_batchcl_raw_signal_sum
ctrl.ratio
23
22
21
20
23
22
21
22
21
2.5
2.0
1.5
1.0
0.5
0.0
WT
MUT
Control
condition
condition
WT
MUT
Control
rep
rep1
rep2
rep3

YNG1 − Q08465

Protein YNG1 OS=Saccharomyces cerevisiae (strain ATCC 204508 / S288

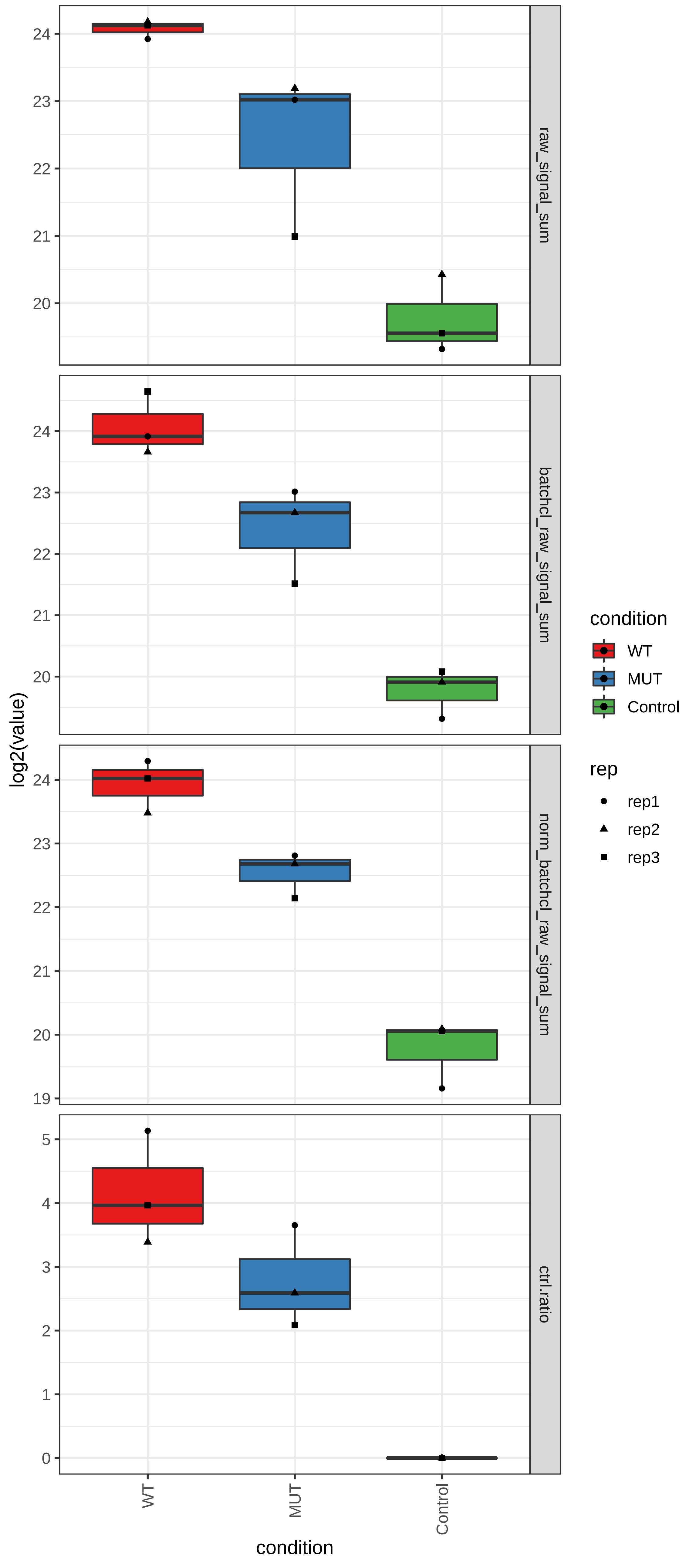

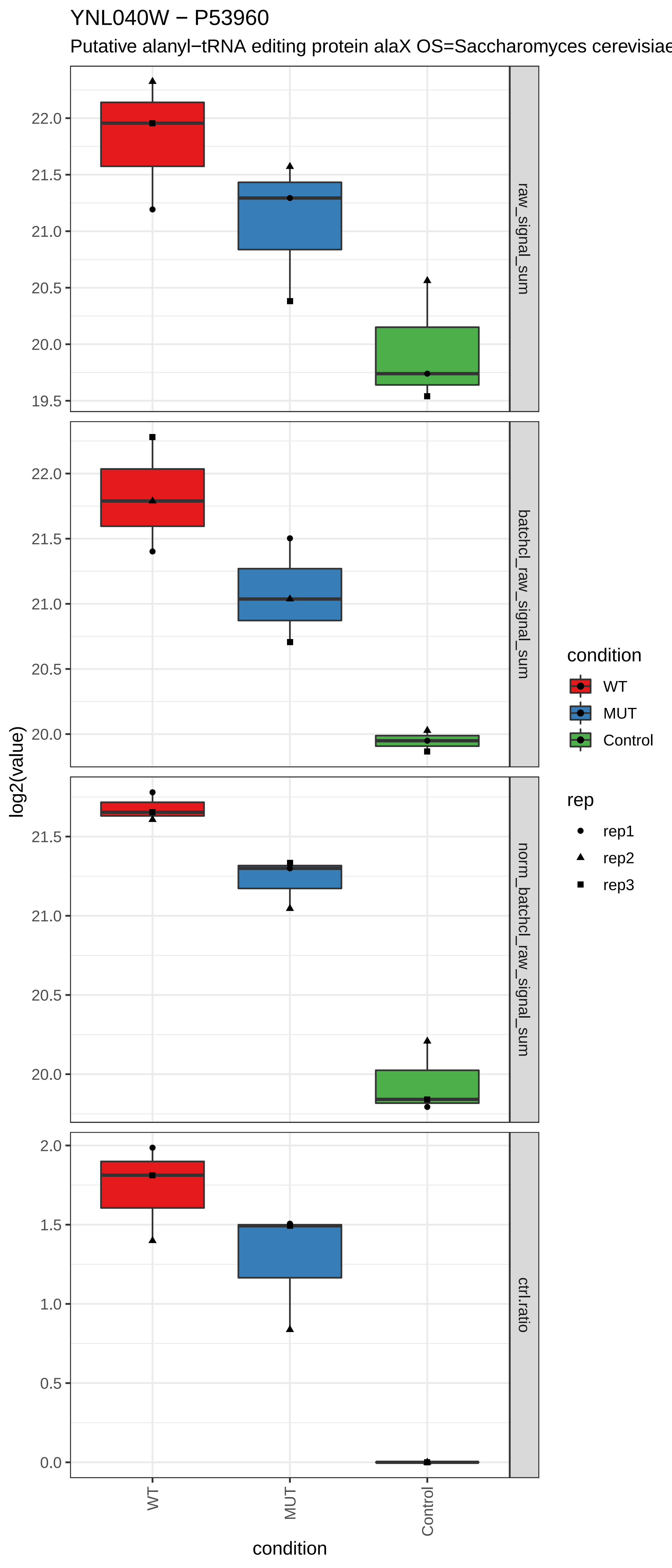
YNL040W − P53960
Putative alanyl−tRNA editing protein alaX OS=Saccharomyces cerevisiae
raw_signal_sum
batchcl_raw_signal_sum
norm_batchcl_raw_signal_sum
ctrl.ratio
log2(value)
condition
WT
MUT
Control
rep
rep1
rep2
rep3
22.0
21.5
21.0
20.5
20.0
19.5
2.0
1.5
1.0
0.5
0.0

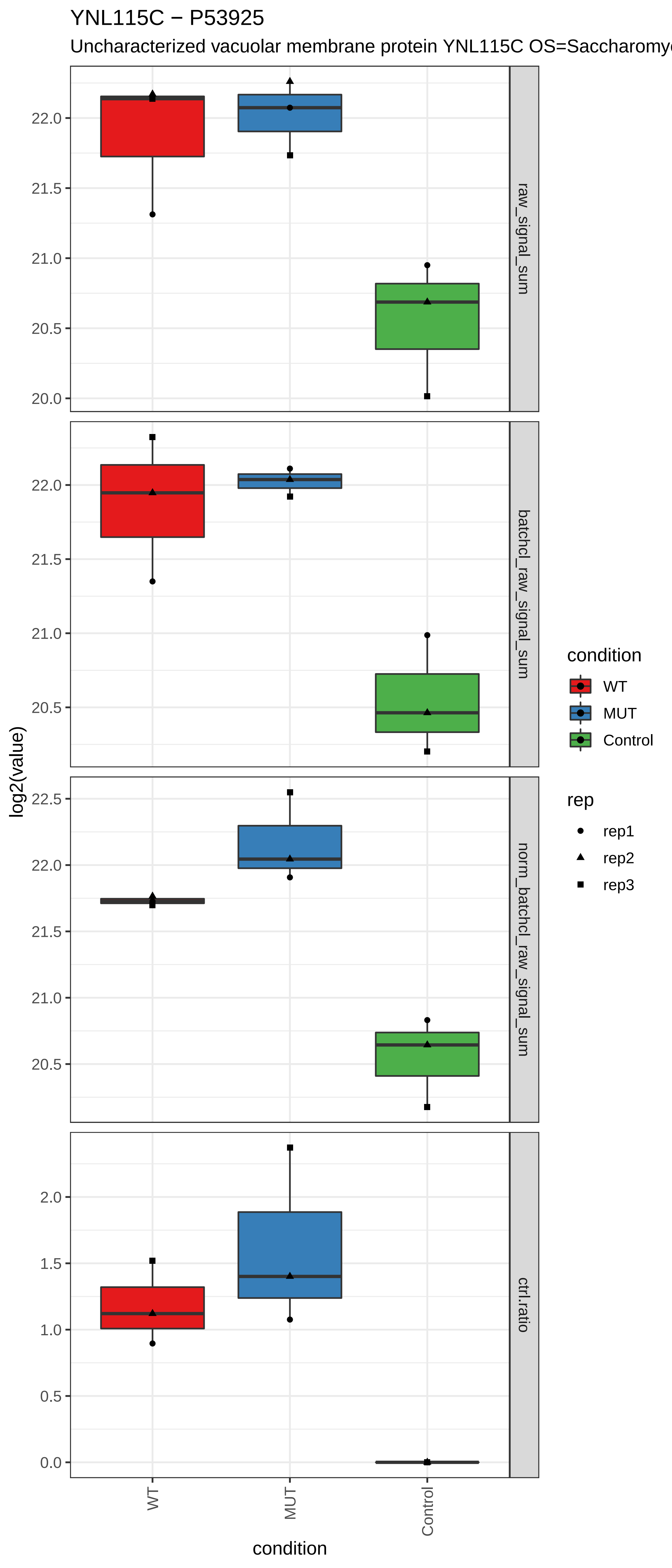

YNL115C − P53925
Uncharacterized vacuolar membrane protein YNL115C OS=Saccharomy
raw_signal_sum
batchcl_raw_signal_sum
norm_batchcl_raw_signal_sum
ctrl.ratio
log2(value)
condition
WT
MUT
Control
rep
rep1
rep2
rep3

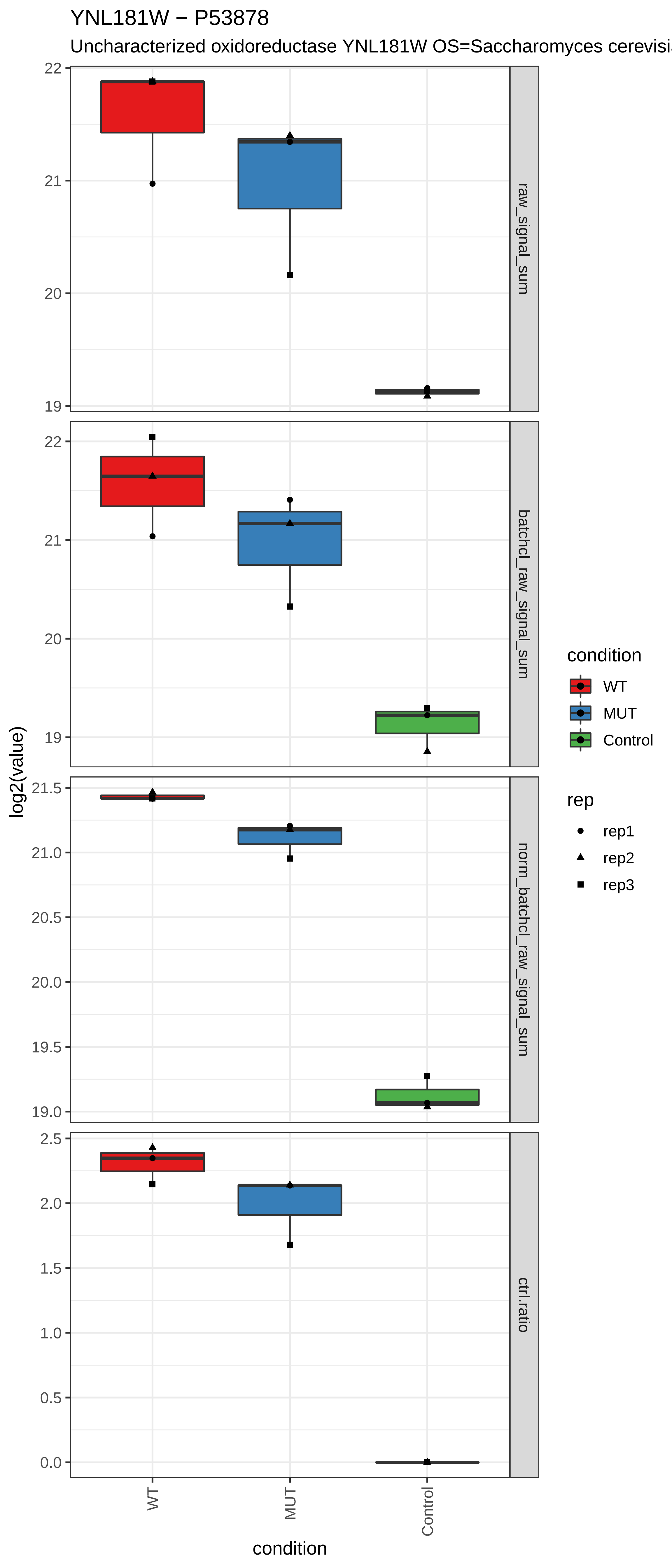

YNL181W − P53878
Uncharacterized oxidoreductase YNL181W OS=Saccharomyces cerevisi
raw_signal_sum
batchcl_raw_signal_sum
norm_batchcl_raw_signal_sum
ctrl.ratio
log2(value)
condition
WT
MUT
Control
rep
rep1
rep2
rep3
22
21
20
19
21.5
21.0
20.5
20.0
19.5
19.0
2.5
2.0
1.5
1.0
0.5
0.0

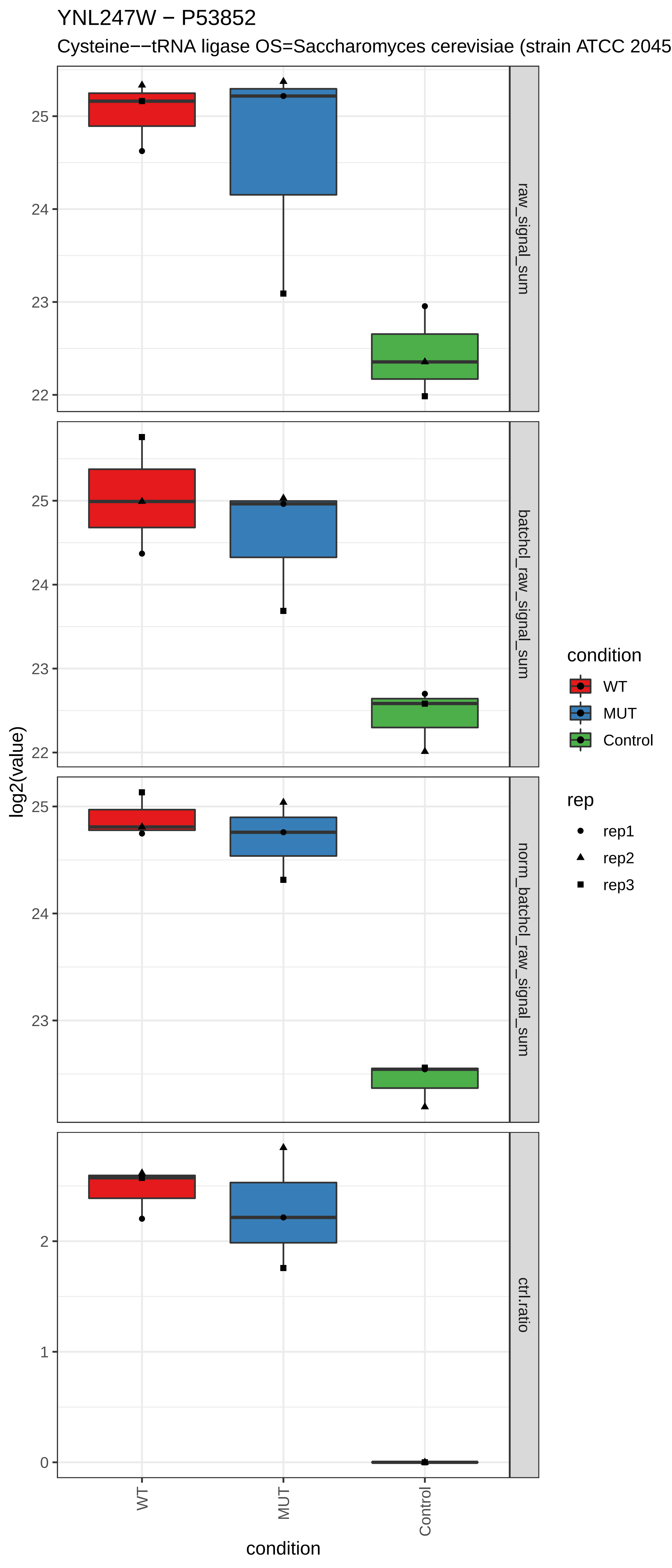

YNL247W − P53852
Cysteine−−tRNA ligase OS=Saccharomyces cerevisiae (strain ATCC 2045
raw_signal_sum
batchcl_raw_signal_sum
norm_batchcl_raw_signal_sum
ctrl.ratio
log2(value)
condition
WT
MUT
Control
rep
rep1
rep2
rep3

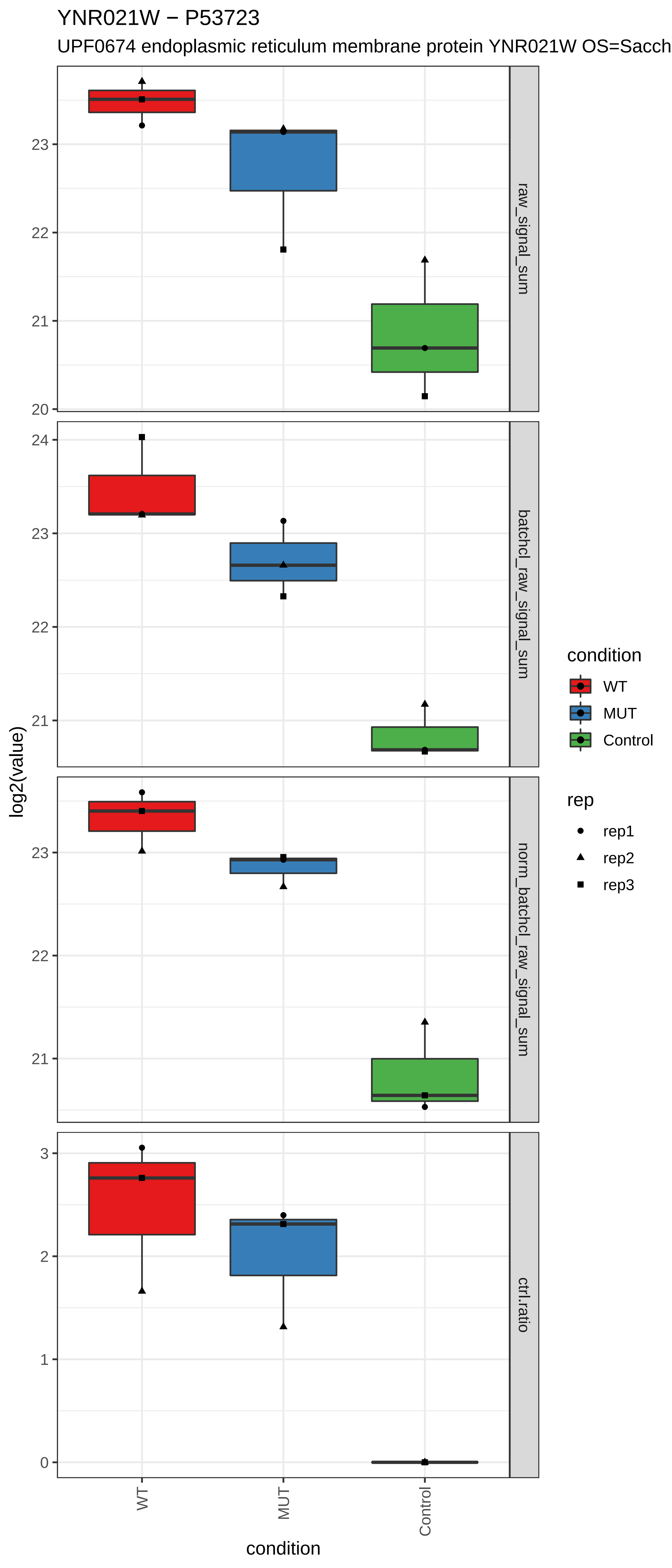
YNR021W − P53723
UPF0674 endoplasmic reticulum membrane protein YNR021W OS=Sacch
raw_signal_sum
batchcl_raw_signal_sum
norm_batchcl_raw_signal_sum
ctrl.ratio
log2(value)
condition
WT
MUT
Control
rep
rep1
rep2
rep3

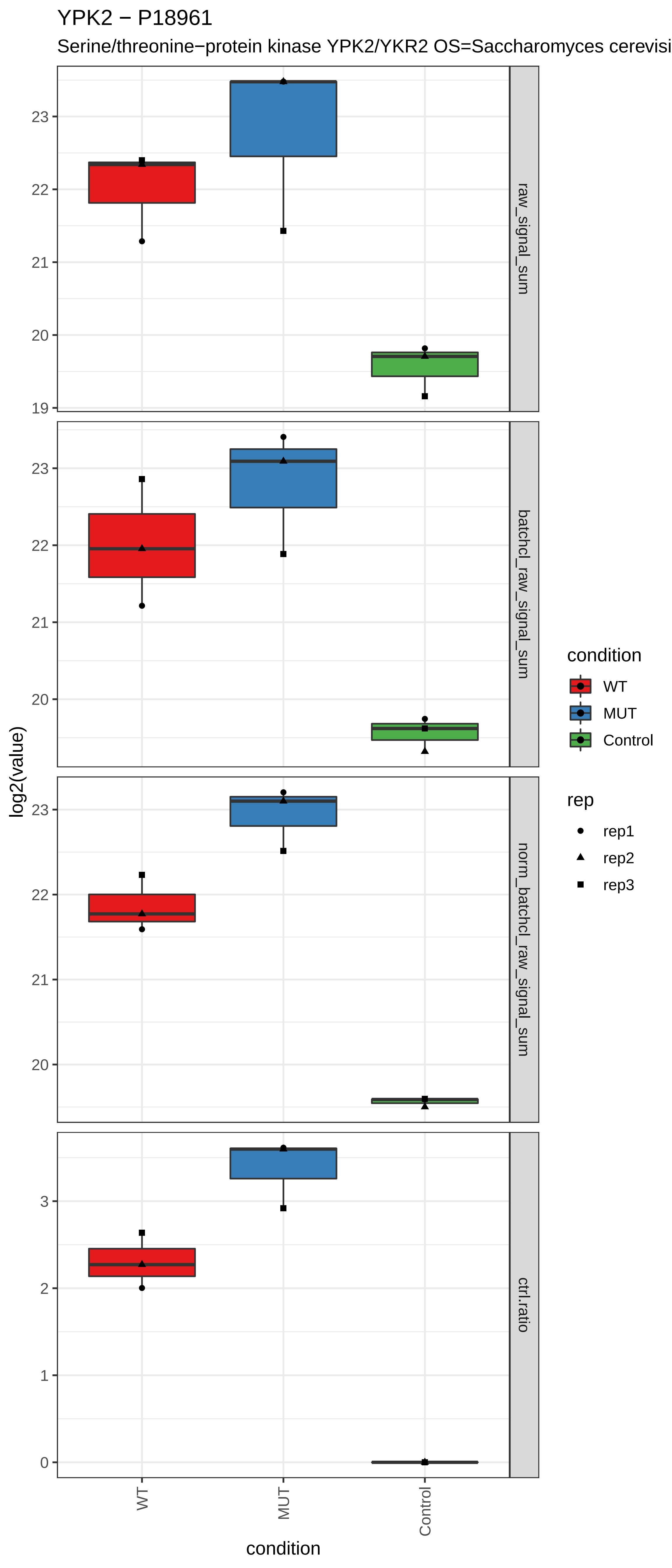
YPK2 − P18961
Serine/threonine−protein kinase YPK2/YKR2 OS=Saccharomyces cerevisi
raw_signal_sum
batchcl_raw_signal_sum
norm_batchcl_raw_signal_sum
ctrl.ratio
log2(value)
condition
WT
MUT
Control
rep
rep1
rep2
rep3

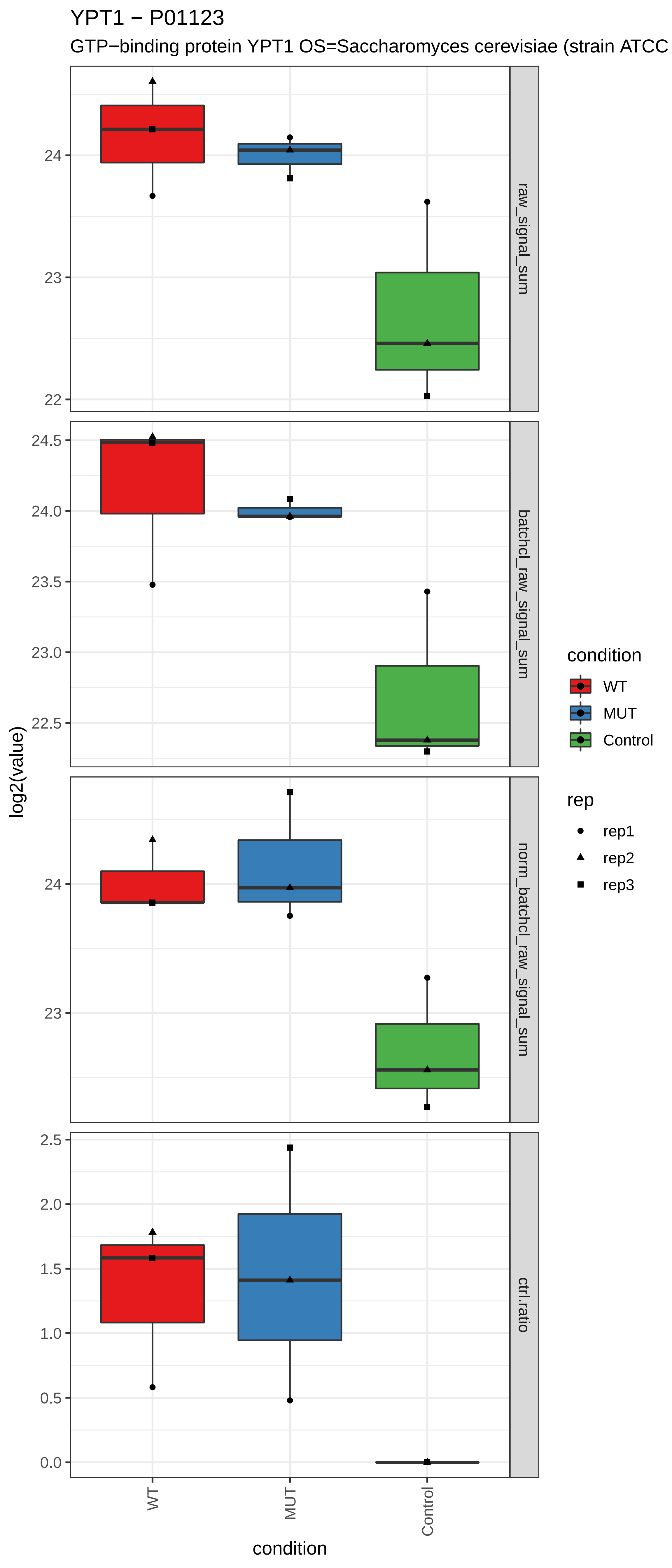
YPT1 − P01123
GTP−binding protein YPT1 OS=Saccharomyces cerevisiae (strain ATCC
raw_signal_sum
batchcl_raw_signal_sum
norm_batchcl_raw_signal_sum
ctrl.ratio
log2(value)
condition
WT
MUT
Control
rep
rep1
rep2
rep3

YPT7 − P32939

GTP−binding protein YPT7 OS=Saccharomyces cerevisiae (strain ATCC

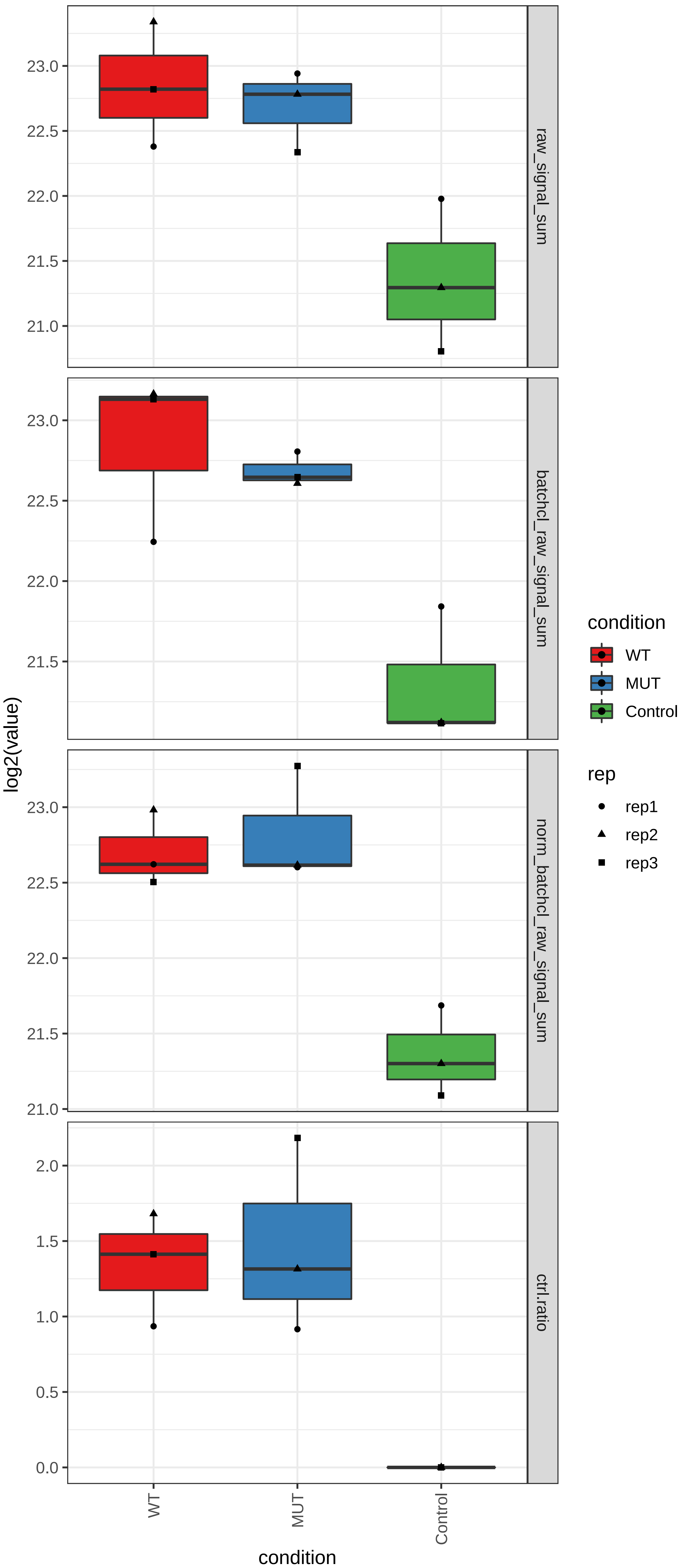

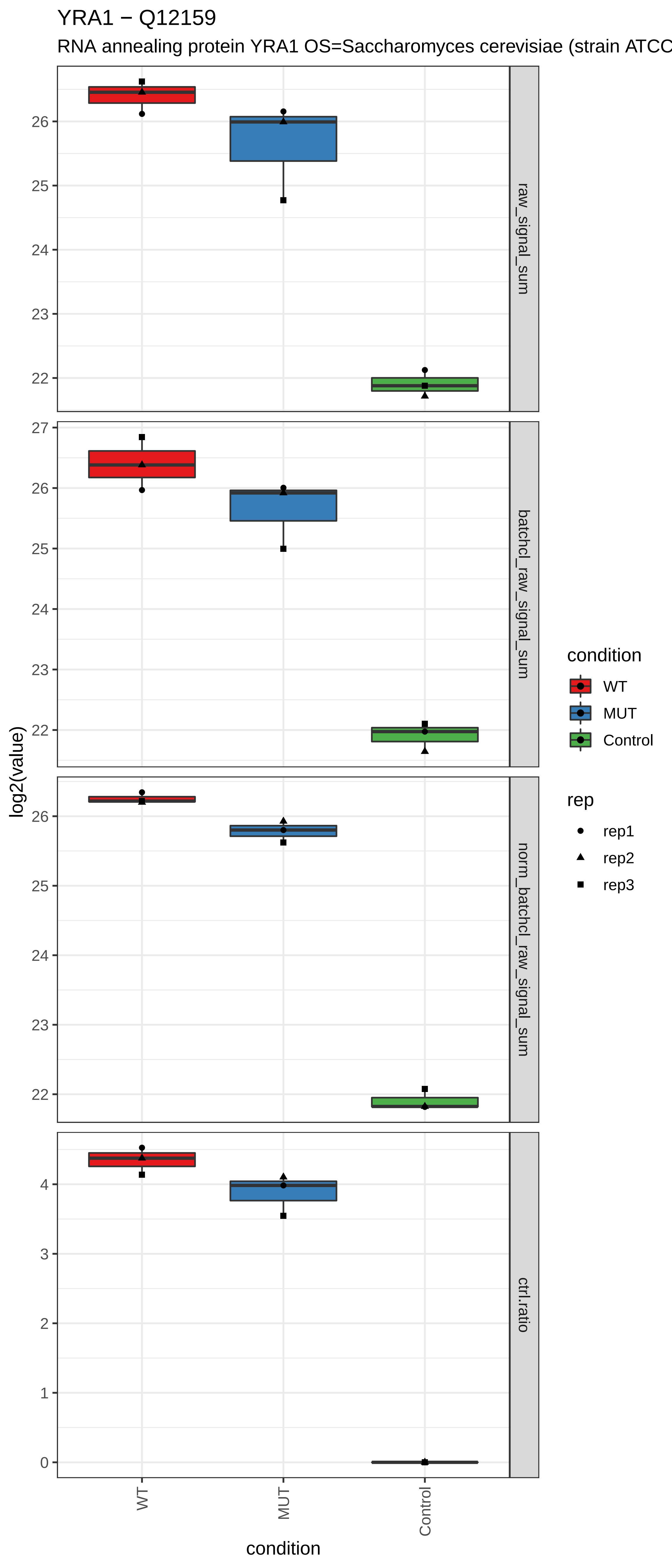

YRA1 − Q12159
RNA annealing protein YRA1 OS=Saccharomyces cerevisiae (strain ATCC
raw_signal_sum
batchcl_raw_signal_sum
norm_batchcl_raw_signal_sum
ctrl.ratio
log2(value)
condition
WT
MUT
Control
rep
rep1
rep2
rep3

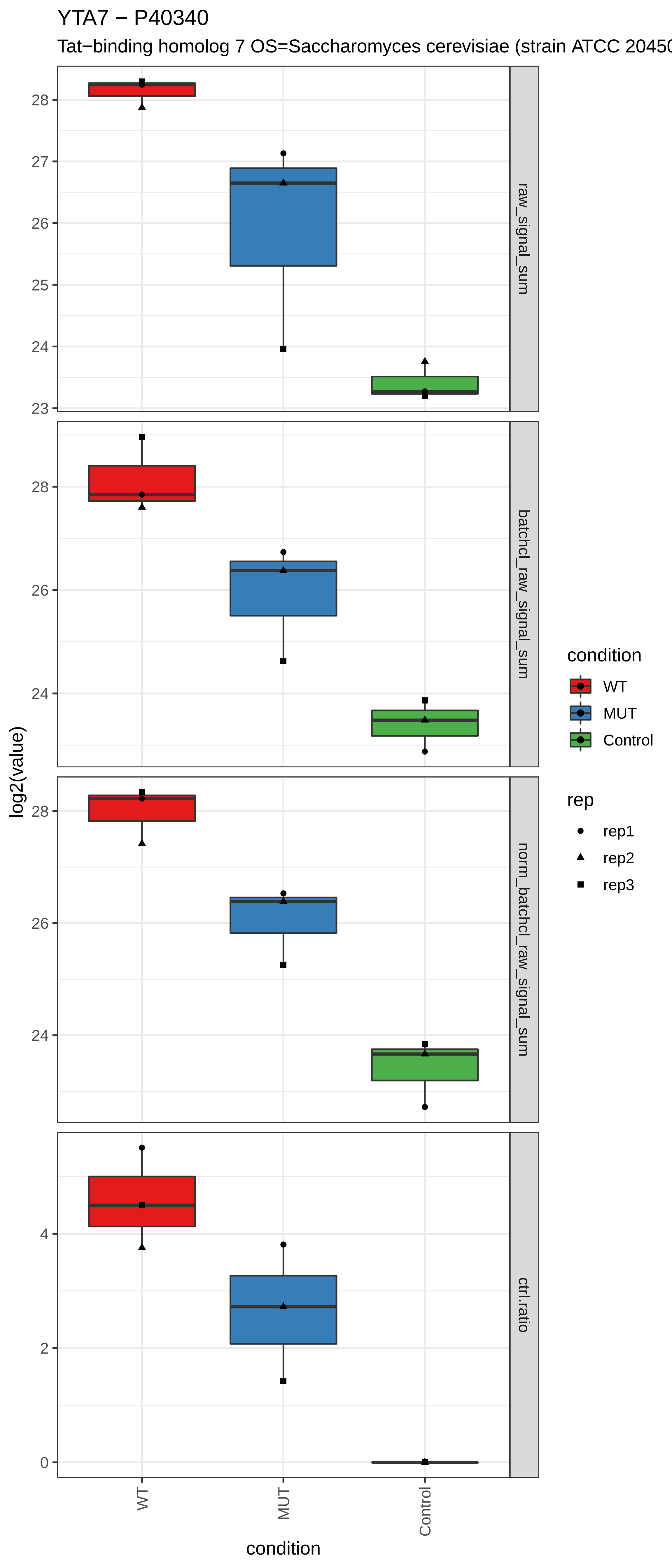

YTA7 − P40340
Tat−binding homolog 7 OS=Saccharomyces cerevisiae (strain ATCC 20450
raw_signal_sum
batchcl_raw_signal_sum
norm_batchcl_raw_signal_sum
ctrl.ratio
log2(value)
condition
WT
MUT
Control
rep
rep1
rep2
rep3
28
27
26
25
24
23
4
2
0

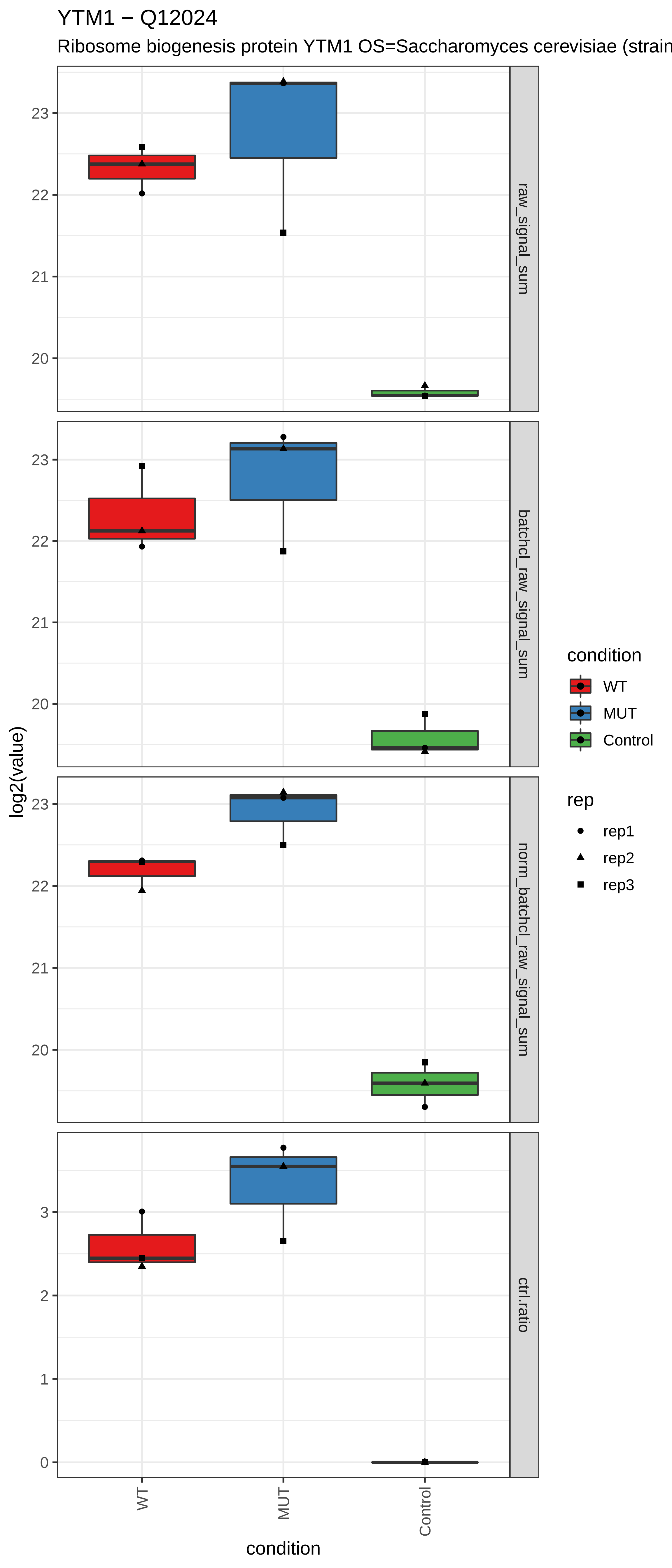

YTM1 − Q12024
Ribosome biogenesis protein YTM1 OS=Saccharomyces cerevisiae (strain
raw_signal_sum
batchcl_raw_signal_sum
norm_batchcl_raw_signal_sum
ctrl.ratio
log2(value)
condition
WT
MUT
Control
rep
rep1
rep2
rep3
23
22
21
20
3
2
1
0

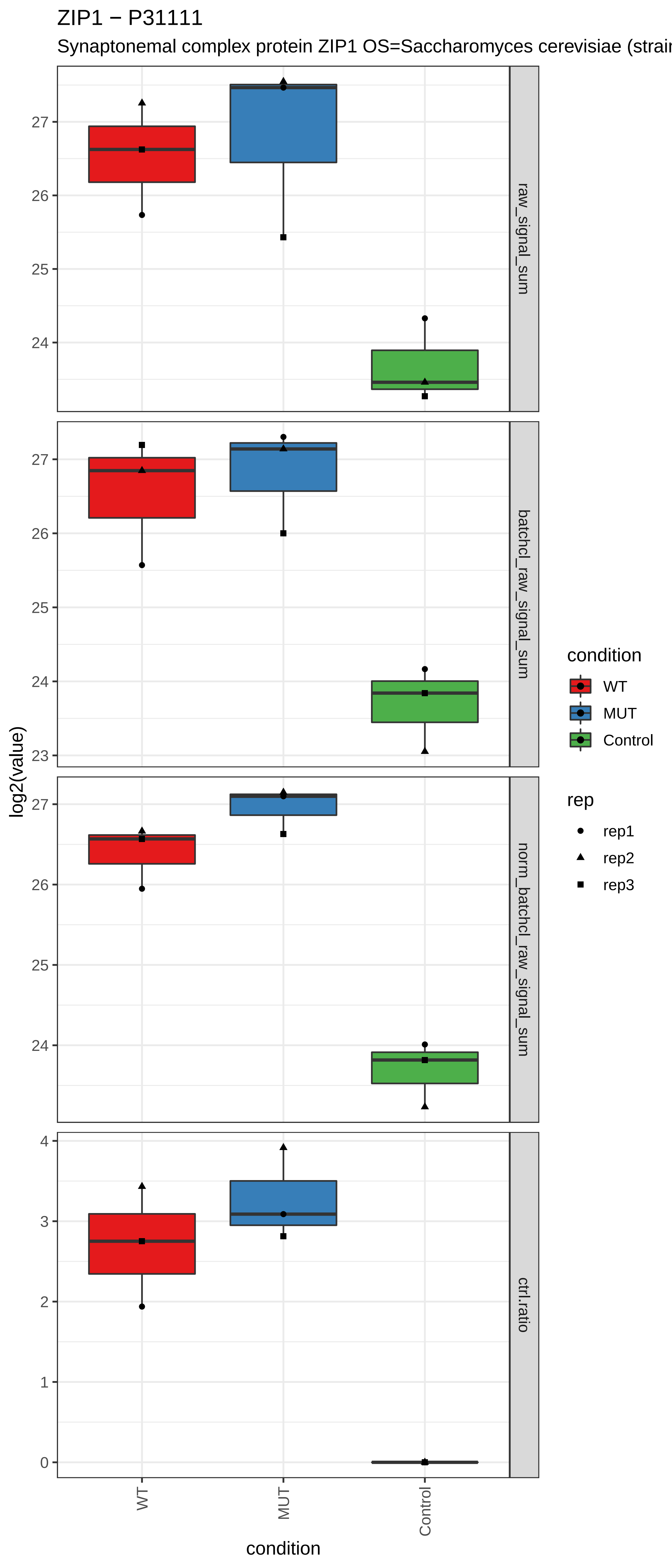

ZIP1 − P31111
Synaptonemal complex protein ZIP1 OS=Saccharomyces cerevisiae (strain
log2(value)
27
26
25
24
23
4
3
2
1
0
raw_signal_sum
batchcl_raw_signal_sum
norm_batchcl_raw_signal_sum
ctrl.ratio
WT
MUT
Control
condition
condition
WT
MUT
Control
rep
rep1
rep2
rep3

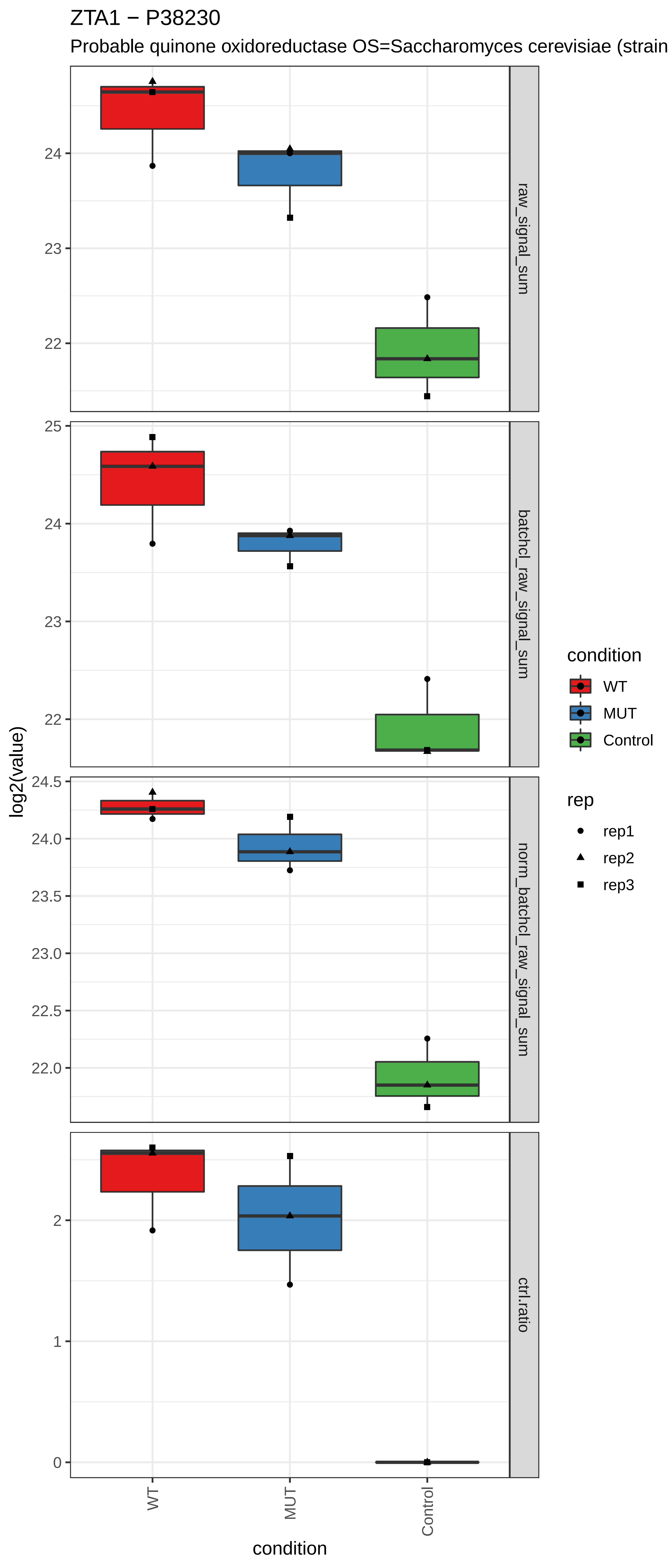

ZTA1 − P38230
Probable quinone oxidoreductase OS=Saccharomyces cerevisiae (strain
log2(value)
raw_signal_sum
batchcl_raw_signal_sum
norm_batchcl_raw_signal_sum
ctrl.ratio
condition
WT
MUT
Control
rep
rep1
rep2
rep3
24
23
22
25
24.5
24.0
23.5
23.0
22.5
22.0
2
1
0

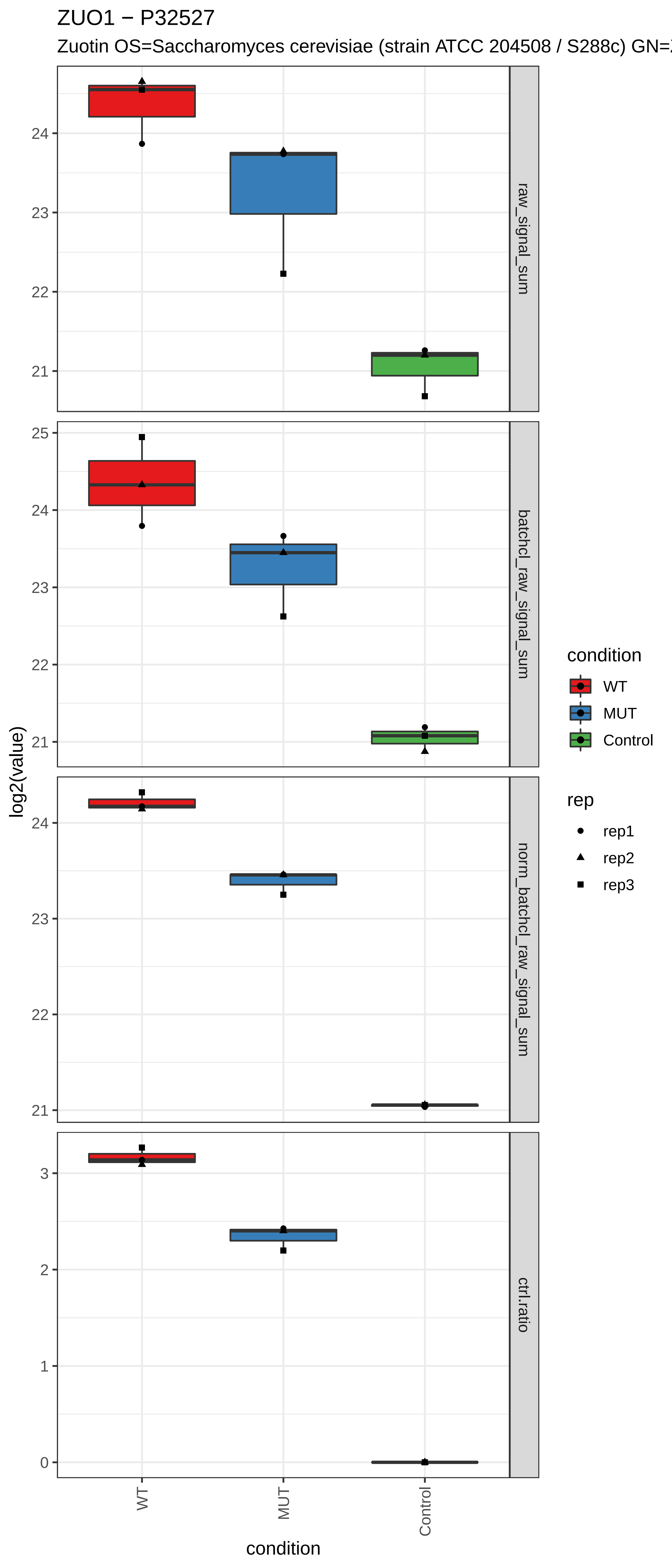
ZUO1 − P32527
Zuotin OS=Saccharomyces cerevisiae (strain ATCC 204508 / S288c) GN=Z
raw_signal_sum
batchcl_raw_signal_sum
norm_batchcl_raw_signal_sum
ctrl.ratio
log2(value)
condition
WT
MUT
Control
condition
WT
MUT
Control
rep
rep1
rep2
rep3
24
23
22
21
25
24
23
22
21
24
23
22
21
3
2
1
0
